# The Construction and Exploration of a Comprehensive MicroRNA Centered Regulatory Network in Foxtail Millet (*Setaria italica* L.)

**DOI:** 10.3389/fpls.2022.848474

**Published:** 2022-05-06

**Authors:** Yang Deng, Haolin Zhang, Hailong Wang, Guofang Xing, Biao Lei, Zheng Kuang, Yongxin Zhao, Congcong Li, Shaojun Dai, Xiaozeng Yang, Jianhua Wei, Jiewei Zhang

**Affiliations:** ^1^Beijing Academy of Agriculture and Forestry Sciences, Beijing, China; ^2^Beijing Key Laboratory of Agricultural Genetic Resources and Biotechnology, Institute of Biotechnology, Beijing, China; ^3^College of Life Science, Shanghai Normal University, Shanghai, China; ^4^College of Agricultural, Shanxi Agricultural University, Jinzhong, China; ^5^Shanxi Key Laboratory of Minor Crop Germplasm Innovation and Molecular Breeding, College of Agriculture, Shanxi Agricultural University, Jinzhong, China

**Keywords:** foxtail millet, expression correlation, regulatory network, microRNA, PARE-seq, sRNA-seq

## Abstract

MicroRNA (miRNA) is an essential endogenous post-transcriptional regulatory factor, and foxtail millet (*Setaria italica* L.) is an ideal C4 model cereal that is a highly valuable crop in semiarid and arid areas. The Research on comprehensive and high confidence identification and annotation of foxtail millet miRNAs needs to be strengthened, and to our knowledge, there is no information on the regulatory network of foxtail millet miRNA. In this study, 136 high confidence miRNAs were identified through high-throughput sequencing of the small RNAs in seven tissues at the shooting and grain filling stages of foxtail millet. A total of 2,417 target genes were obtained by combining computational biology software and degradome sequencing methods. Furthermore, an analysis using transcriptome sequencing revealed the relationships between miRNAs and their target genes and simultaneously explored key regulatory modules in panicles during the grain filling stage. An miRNA regulatory network was constructed to explore the functions of miRNA in more detail. This network, centered on miRNAs and combining upstream transcriptional factors and downstream target genes, is primarily composed of feed forward loop motifs, which greatly enhances our knowledge of the potential functions of miRNAs and uncovers numerous previously unknown regulatory links. This study provides a solid foundation for research on the function and regulatory network of miRNAs in foxtail millet.

## Introduction

Foxtail millet (*Setaria italica*) is well known as an important C4 cereal crop that was domesticated from its wild ancestor green foxtail (*S. viridis*). *S. italica* is evolutionarily close to pearl millet (*Pennisetum glaucum*) and broomcorn millet (*Panicum miliaceum*) among others (Yang et al., 2020). The global production of foxtail millet combined with other millet crops was estimated to be 28.37 million tons in 2019 according to the FAO (Food and Agriculture Organization).^1^ As a cereal crop that is highly drought tolerant and efficient at using water, *S. italica* is still widely cultivated in the semiarid and arid regions in the world, particularly in China and India (Jia et al., 2013). Originating from northern China, it is also one of the earliest domesticated crops. It was estimated to have been domesticated nearly 11,500 years ago (Yang et al., 2012). Foxtail millet has been chosen as the main cereal crop for arid and semiarid regions and emphasized as a strategic reserve crop to manage the increasingly severe drought. EMS-mutagenized mutant ‘*xiaomi’* was selected from an M2 population of the cultivated foxtail millet species ‘Jingu21’, which is widely cultivated in northern China for its good grain quality. ‘*xiaomi’* has the potential to serve as an ideal model plant like *Arabidopsis* and enable basic and applied research to be conducted on a C4 plant (Yang et al., 2020).

MicroRNAs (miRNAs) are non-coding RNAs with 20–24 nucleotide (nt) that perform important regulatory functions in eukaryotes (Fromm et al., 2020). In plants, primary miRNAs are transcribed by RNA polymerase II and then processed by Dicer-like (DCL) into precursors (pre-miRNA) with stem-loop structures (Lee et al., 2004; Carthew and Sontheimer, 2009). The HASTY protein or other mechanisms cleave these pre-miRNAs into mature miRNA:miRNA star (miRNA*) duplexes, which are then transported to the cytoplasm. To target particular mRNAs and down-regulate the production of target mRNAs, mature miRNAs are integrated into the RNA-induced silencing complex (RISC) (Park et al., 2005; Bartel, 2009). Growing evidence suggests that miRNAs have a role in a variety of plant functions, including growth, development, abiotic stress tolerance, and metabolism (Chandra et al., 2017).

With the revolution of high-throughput sequencing technologies and the development of collaborative analytical software during the last decade, the identification of miRNAs and research on their functions ushered in a breakthrough and explosive era of new data (Zhao et al., 2021). These vast and complex data increase the ratio of false-positive miRNA identification in plants and challenge the corresponding research. This problem was partially effectively solved after the release of the newest annotation criteria of plant miRNAs (Axtell and Meyers, 2018), and the annotation software was developed based on this standard (Kuang et al., 2019; Guo et al., 2021). Furthermore, when paired with RNA-seq data, the correlation of expression patterns between miRNAs and prospective targets offers not only a reference for target gene prediction but also the possibility for the identification of novel regulatory miRNA-target pairings. Motif scanning, in combination with methods, such as Chromatin Immunoprecipitation Sequencing (ChIP-Seq) (Johnson et al., 2007) or DNA Affinity Purification Sequencing (DAP-Seq) (Bartlett et al., 2017), has been widely used as high-throughput techniques to detect the regulation between transcriptional factors (TFs) and their targets, which could be used to discover the upstream regulation from TFs to miRNAs. With all these technical advantages, a whole-genome scaled image of the miRNA-based network in Arabidopsis (Gao et al., 2021) and lettuce (*Lactuca sativa*) (Deng et al., 2021) has been constructed. Despite its importance as a crop, there are few case studies of miRNAs in foxtail millet, and no comprehensive identification and annotation of miRNAs and their regulatory networks have been conducted to our knowledge.

In this study, by sequencing 14 small RNA (sRNA) libraries from four tissues under different developmental stages and analyzing them with a pipeline centered by miRDeep-P2 (Kuang et al., 2019), 136 high confidence miRNAs were identified in foxtail millet. The detailed characteristics of these miRNAs were annotated, including their sequences, structure, conservation, clusters, selection after duplication, and patterns of expression in different tissues. A total of 2,417 targets of the 136 miRNAs were identified by five different means, and Gene Ontology (GO) (Mi et al., 2019) and Kyoto Encyclopedia of Genes and Genomes (KEGG) (Ogata et al., 1999) enrichment analyses indicated that these targets primarily function in the lignin catabolic process, regulation of transcription process, and plant hormone signal transduction, protein metabolism, and sphingolipid metabolism pathways. Furthermore, an analysis of transcriptome sequencing revealed the negative correlation of expression between miRNAs and their target genes and simultaneously explored key regulatory modules in panicles during the grain filling stage. The binding of upstream transcriptional factors (TFs) enables the prediction of miRNAs and their targets. This facilitated the construction of an miRNA central regulatory network, where the connection between different feed-forward loops (FFLs) helped to define several new regulatory links. All the findings were double-checked by selecting candidates at random and examining them through real-time quantitative reverse transcription PCR (qRT-PCR) tests. Compared with previous studies (Yi et al., 2013; Han et al., 2014; Khan et al., 2014; Wang et al., 2016), this work established a strong platform for future research on foxtail millet miRNAs by methodically identifying and annotating high confidence miRNAs in foxtail millet and conducting exploratory analyses of their functions using target genes, an expression correlation, and regulatory networks.

## Materials and Methods

### Plant Materials

Yugu1, as the reference genome, is an inbred line that was used in this study. The seeds were deposited in the Beijing Crop Germplasm Resources Infrastructure, part of the Biotechnology Institute of Beijing Academy of Agriculture and Forestry Sciences (BAAFS), Beijing, China. Seeds of Yugu1 were first sown in plastic pots (21 cm/21 cm diameter/height) with a 3 kg mixture of nutrient soil (Pindstrup seedling, pH 5.5, 0–10 mm) from a commercial source (Pindstrup Mosebrug, Denmark). and cultured in a greenhouse [30^°^/25^°^C (day/night), 16-h photoperiod] at 50% humidity. The roots, stems, and leaves were collected from the shooting stage (40 days after germination). The roots, stems, flag leaves and panicles were collected from the grain-filling stage (80 days after germination). After collection, the samples of each tissue were quickly frozen in liquid nitrogen and stored at −80^°^C for the extraction of total RNA and then for the construction of small RNA (sRNA), mRNA, and PARE Libraries.

### Construction and Sequencing of Small RNA, mRNA, and PARE Libraries

Following the manufacturer’s instructions, RNA was extracted from the seven tissues specified using an OminiPlant RNA Kit (Cwbio, China), and its integrity and quality were verified by an Agilent 2100 Bioanalyzer (Agilent Technologies, United States). cDNA libraries of sRNA and mRNA were prepared by a Small RNA Sample Prep Kit (Illumina, United States) and a TruSeq Stranded RNA LT Kit (Illumina, United States) following the manufacturer’s instructions, respectively. Briefly, sRNAs were extracted from 20 μg of total RNA using 15% polyacrylamide gel electrophoresis (PAGE) and the ligation of 5′-RNA and 3′-RNA adaptors. RT-PCR was used to convert and amplify the samples to cDNA. PARE libraries were prepared as previously described (German et al., 2009) but with some modifications. Finally, Illumina HiSeq2500 was used to sequence the verified cDNA libraries. There were two, three, and one biological replicates in each of the sRNA, mRNA and PARE libraries, for a total of 42 libraries.

### Identification of Conserved, Poaceae-Specific, and Foxtail Millet-Specific miRNAs

Clean reads were acquired after sRNA library sequencing by filtering low-quality sequences, such as junk reads, adaptor sequences, polyA tags, and reads lengths, that were less than 18 bp or exceeded 30 bp. For miRNA prediction, the retained clean reads (19–25 nt in length) were utilized. Reads that matched plant non-coding RNAs in the Rfam database (version 13.0) (Kalvari et al., 2018), including tRNA, rRNA small nuclear RNA (snRNA), and small nucleolar RNA (snoRNA) sequences, with no more than one mismatch, were further filtered. The remaining sequences were then mapped to the foxtail millet reference genome (Bennetzen et al., 2012), and potential miRNAs were found using miRDeep-P2 software (version 1.1.4) (Kuang et al., 2019). As specified in the miRDeep-P2 handbook, the neighboring sequences of mapped sRNAs were retrieved as putative miRNA precursor sequences. RNAfold (version 2.1.2) (Tav et al., 2016) was used to estimate the secondary structures of all the candidates. All the identification processes were based on the newly defined plant miRNA annotation criteria (Axtell and Meyers, 2018).

All the predicted mature miRNA sequences with 2 nt contiguous nucleotides were matched with the miRNA dataset in PmiREN2.0 (Guo et al., 2021), with no more than two mismatches, using Bowtie (version 1.2.2) (Langmead, 2010) software to annotate the conservation of miRNAs in foxtail millet. Those that did not match any miRNAs in PmiREN2.0 were classified as foxtail millet-specific miRNAs, while those that only matched miRNAs from other Poaceae species were annotated as Poaceae-specific miRNAs, and the rest were considered as conserved.

### Validation of Reported miRNAs for Unbiased Comparisons

The genomic location and sequence information of candidate miRNAs were collected from former studies. Those candidates without miRNA star sequences were filtered based on the newest plant annotation criteria. The retained miRNAs were aligned into the PmiREN2.0 database for reclassification and reannotation. After the comparison, the miRNA sequences that were not annotated in this study were searched against the 14 sRNA libraries constructed in our research to confirm whether this study was omitted owing to deficiency in the identification process.

### Identification of the Genome-Wide Synteny Analysis of miRNAs

JCVI (MCscanX python version 1.1.18) (Wang et al., 2012) was used to conduct a synteny analysis of miRNAs. The converted Browser Extensible Data (BED) file was modified following the JCVI manuscript, and protein sequences were first used as input files with default settings to produce collinearity blocks over the whole genome. Following that, BEDTools (version 2.26.0; subfunction: intersect) (Quinlan and Hall, 2010) was used to anchor the overlap region between all the miRNA genomic sites and collinearity blocks. Syntenic miRNAs were defined as members of the same miRNA family that followed comparable gene ordering in collinearity blocks. The subfunction “Advanced Circos” of TBtools (version 1.6) (Chen et al., 2020) was used to create the outcome plot.

### Target Gene Prediction and Function Enrichment Analysis

The miRNA targets were predicted using four plant miRNA target prediction toolkits: psRNATarget (Dai et al., 2018), psRobot (version 1.2) (Wu et al., 2012), TargetFinder (version 1.0), and RNAhybrid (version 2.1.2) (Kruger and Rehmsmeier, 2006). The miRNA and mRNA transcript sequences were submitted to the psRNATarget webserver^2^ and utilized with the current default settings (2017 version). Targets with an *E* value less than 3.0 were retained as potential miRNA targets. Under default settings, PsRobot and TargetFinder both utilized the miRNAs and mRNA transcript sequences as input data. Furthermore, considering plant-specific limitations, RNAhybrid was utilized to forecast energetically feasible miRNA-mRNA duplexes. A strict cut-off value of 0.75 was applied for minimum free energy (MFE)/minimum duplex energy (MDE) (Alves et al., 2009).

Under default settings, the CleaveLand4.0 (Addo-Quaye et al., 2009) program was used to identify probable miRNA cleavage sites using clean Degradome-seq (PARE-seq) data. To create density files, the reads were aligned to foxtail millet transcript sequences. To find possible miRNA target sites, the predicted miRNA mature sequences were matched to transcript sequences. To categorize the miRNA target candidates, the density distribution of reads and miRNA-mRNA alignment were combined. To eliminate false positives, all putative miRNA targets were divided into one of five groups, with only data from categories 0 and 1 being kept.

The Gene Ontology (GO) and Kyoto Encyclopedia of Genes and Genomes (KEGG) annotation of all protein sequences were performed using InterProScan (version 5.0) (Jones et al., 2014) and the KEGG webserver,^3^ respectively. A hypergeometric analysis and Fisher test were used for the enrichment analysis of GO and KEGG by the R package “topGO” and in-house R script, respectively. The results plot was generated with the R package “ggplot2.”

### Quantification of the Expression of miRNAs and mRNAs

The expression of mature miRNA was standardized by reads per million to assess the miRNA expression (RPM). The reads that matched to primary miRNAs (pri-miRNAs) and anchored in the genomic locations of mature miRNAs (less than 2-nt shift) were deemed matching mature miRNAs for each sRNA-seq dataset. To compute the RPM for mature miRNAs, the total number of reads was tallied.

FastQC (version 0.11.9) (Brown et al., 2017) was used to test the quality of all raw mRNA reads, and Cutadapt (version 3.2) (Kechin et al., 2017) software was used to cut out the low-quality reads and adapters. HISTAT2 (version 2.1.0) (Kim et al., 2019) was used to align clean reads to the foxtail millet genome. To assemble the transcript and quantify each mRNA-seq dataset, StringTie (version 2.1.5) (Pertea et al., 2015) was utilized. The levels of transcript expression were derived from the StringTie data and standardized by fragments per kilobase of transcript per million mapped reads (FPKM).

### Correlation Analysis Between the Expression of miRNAs and Predicted Targets

Using the R function “cor.test (),” the Pearson correlation coefficient between the expression matrix of miRNAs and their targets was obtained. The raw expression matrix of miRNAs and targets was averaged and transposed using the R function “*t* ()” to generate a matrix with miRNAs and targets as factors, based on biological replicates of seven tissues. The correlation coefficient between each miRNA and target was then calculated using “for loop” of the R language, and significance was determined using a Student’s *t*-test. The R package “Pheatmap” was used to visualize the results.

### Co-expression Network and Trait Association Analyses of the Predicted Targets

A weighted gene co-expression network analysis (WGCNA) was performed using the R package “WGCNA” following the “WGCNA” handbook. After filtering target genes with a variance of 0 and with >10% missing samples, the soft power estimate value was set to eight, and 12 gene modules were produced. The correlation between the module eigengenes and sample tissues was used to estimate module-trait relationships. The modules with an absolute *r* > 0.75 and *P* < 0.01 were retained. The regulatory miRNAs of these genes in the selected modules that integrated the target genes were traced to construct trait highly correlated regulatory networks. Cytoscape (version 3.7.1) (Shannon et al., 2003) software was utilized to visualize the network.

### Network Construction

TF annotations were searched against the TF datasets from PlantTFDB^4^ (Jin et al., 2017) in all the mRNA transcripts, and the default filtered result was maintained. To find the potential binding sites for TFs, the upstream 2,000 nucleotide sequences of all the pre-miRNAs and target transcripts were extracted as candidate promoter sequences and then submitted to the PlantRegMap webserver under default parameters^5^ (Tian et al., 2020). Based on the interactions between the TFs-miRNAs, miRNAs-targets, and the TFs-miRNA targets, an in-house Perl script was used to detect FFL motifs. After that, a genome-wide miRNA network was constructed using all the FFL motifs, and the network topology was visualized by Cytoscape (version 3.7.1).

### Quantitative RT-PCR of miRNAs and Targets

The total RNA of foxtail millet was extracted from separate tissues using the TRIzol reagent (Invitrogen, United States), and the RNA was purified using the RQ1 RNase-free DNase reagent (Promega, United States). It was then reverse transcribed with the Maxima First Strand cDNA Synthesis Kit 222 (Thermo Fisher, United States). Real-time PCR was performed using SYBR Premix Ex Taq™ (TaKaRa, Japan) on a CFX96 Touch System (Bio-Rad, United States). The cycling parameters were as follows: 95°C for 30 s, 40 cycles of 95°C for 1 min, and 60°C for 10 s. The ubiquitin-conjugating enzyme E2 A was used as the endogenous reference. Following the manufacturer’s instructions, total small RNA was isolated using a miRcute miRNA Isolation Kit (TianGen, China). The miRcute Plus miRNA First-Strand cDNA Synthesis Kit was used for reverse miRNA transcription (TianGen). The miRcute miRNA qPCR Detection Kit, SYBR Green (TianGen), and a CFX96 Touch System were used to examine the resulting cDNA (Bio-Rad). U6 snRNA was used as the reference gene. The results of relative levels of gene expression were calculated using the 2^–△△Ct^ method. Each qRT-PCR experiment was performed on three biological replicates. Supplementary Table 19 lists all the primers for qRT-PCR experiments.

## Results

### Comprehensive Identification and Annotation of High Confidence miRNAs

**FIGURE 1:**
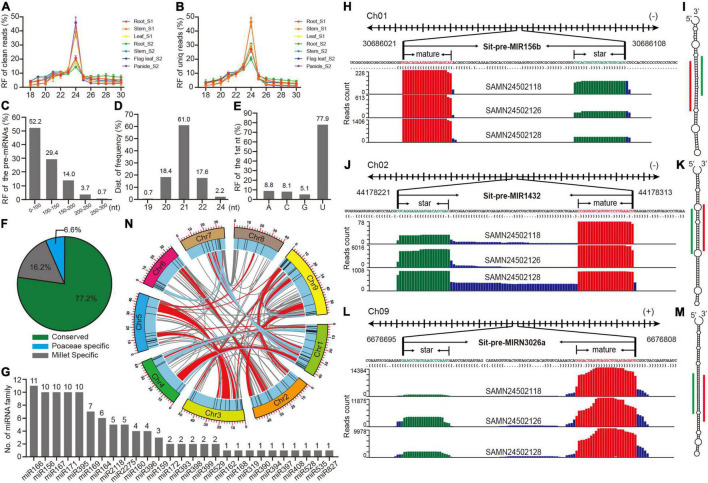
Preliminary analysis of high confidence identified and annotated miRNAs in foxtail millet. **(A,B)** Length distribution of clean and unique reads from sRNA libraries in multiple tissues. **(C)** Length distribution of pre-miRNAs. **(D)** Length distribution of mature miRNAs. **(E)** The proportion of the first nucleotide from mature miRNAs. **(F)** The proportion of three types of miRNAs. **(G)** Member distributions of conserved miRNA families. **(H)** The detailed information and reads mapping to the genome loci of pri-miR156b and pre-miR156b sequences, where mature and star Sit-miR156b are colored with red and green, respectively. **(I)** The secondary structure of pre-miR156b by RNAfold. Mature and star Sti-miR156b are labeled with red and green lines. **(J–M)** The information of Sit-miR1432 and Sit-miRN3026N represented examples of Poaceae-specific and foxtail millet-specific miRNAs, respectively. **(N)** Anchored information of the foxtail millet miRNAs in synteny blocks, while red and blue lines manifest single miRNA and paired miRNAs located in the synteny blocks, respectively.

To comprehensively identify the miRNAs in foxtail millet, 14 small RNA libraries from different tissues at different growth stages (Supplementary Figure 1) were constructed and produced 508.8 million raw reads and 420.2 million clean reads (Supplementary Table 1). Clean and unique sRNA read length distributions both peaked at 24 nt, perhaps indicating the prevalence of short interference RNAs (siRNAs) (Figures 1A,B and Supplementary Tables 2, 3). The results were similar to those of former studies in foxtail millet (Yi et al., 2013). In particular, the 24 nt small RNA from the shooting stem stage and the grain filling stage were more abundant than those of other tissues, suggesting that the activities of siRNAs were potentially higher during stem development and grain filling.

To identify high confidence miRNAs, miRDeep-P2 (Kuang et al., 2019), software that was developed based on the newest strict plant miRNA criteria was utilized (Axtell and Meyers, 2018), and only 19∼25 nt reads were selected as input datasets. A total of 136 miRNA loci that belong to 36 families were annotated in the foxtail millet genome (Supplementary Table 4). A statistical analysis of several basic features of these miRNA loci found that precursor sequences <150 nucleotides comprised > 80% (Figure 1C), and 21 nt mature miRNA was the most prevalent, reaching 61% (Figure 1D), and U was typically the initial nucleic acid of mature miRNAs (Figure 1E). These results were consistent with those of other plant research (Deng et al., 2021; Gao et al., 2021), suggesting that the identification and annotation of these 136 miRNAs were highly confident. The conservation classification of all the miRNAs was searched against the PmiREN2.0 database (Guo et al., 2021). A total of 105, including 27 families, were found to be conserved since their counterparts could be found in other non-Poaceae species, while only nine were considered specific to the Poaceae because their counterparts were only detected in Poaceae species. A total of 22 miRNAs were annotated as specific for foxtail millet since no items could be found in species other than millet (Supplementary Table 4 and Figure 1F). MIR166 had the most members among the conserved families, whereas the other 17 families contained multiple members (Figure 1G). Three examples that are conserved and specific to the Poaceae and foxtail millet were randomly selected to illustrate the detailed information (Figures 1H–M). Standard stem-loop structure and reads signature strongly supported them as high confidence candidates. Furthermore, a comprehensive comparison between this study and previous studies (Yi et al., 2013; Han et al., 2014; Khan et al., 2014; Wang et al., 2016) indicated that most of the conserved families were retrieved by this research, and we also identified 18 high-confident new miRNA families (Supplementary Figure 2 and Supplementary Tables 5, 6).

A cluster analysis of the genomic loci of 136 miRNAs was further searched to preliminarily trace back the origin of these miRNAs in foxtail millet, and 11 clusters were obtained when a cluster region length < 10 kb was defined (Supplementary Table 7). There were two miR395 clusters that only contained members from foxtail millet, indicating that the abundance of miR395 family was owing to segmental duplication after a tandem duplication (Supplementary Table 7 and Figure 1G). In addition, the genome anchored condition of the miR2275 clusters was the same as those of miR395 (Supplementary Table 7 and Figure 1G), which could similarly explain the miR2275 expansion in the foxtail millet genome. Since the foxtail millet genome had experienced multiple whole-genome duplication (WGD) events (Bennetzen et al., 2012), a synteny analysis was performed to identify whether these miRNAs also expanded owing to the WGD events. Many miRNAs were anchored in the synteny block with no member expansion (Supplementary Table 8 and Figure 1N), while members of miR156, miR166, miR167, miR169, miR171, and miR396 were anchored in pairs in the synteny blocks (Supplementary Table 9 and Figure 1N). This demonstrated that the expansion of partial miRNAs could be owing to the WGD events, but only some were retained.

### Exploration of Expression Patterns of miRNAs

**FIGURE 2:**
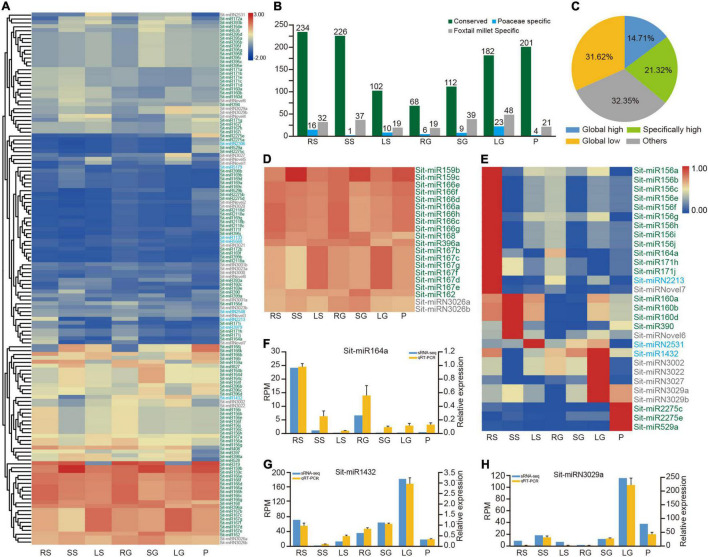
Dynamic expression profiles of miRNAs in foxtail millet. **(A)** The average expression profile of 136 miRNAs in seven tissues. Green, blue, and gray coloring miRNA IDs represent conserved, Poaceae-specific, and foxtail millet-specific miRNAs, respectively. RS, SS, and LS indicate root, stem and leaf tissues at the shooting stage, respectively. RG, SG, LG, and P indicate root, stem, flag leaf, and panicle tissues at the grain filling stage, respectively. **(B)** Average expression values of conserved, Poaceae-specific and foxtail millet-specific types of miRNAs in all samples. **(C)** The pie chart shows the proportion of four expression patterns of miRNAs. **(D)** Global highly expressed (housekeeping) miRNAs in seven tissues. **(E)** Highly expressed miRNAs in only one specific tissue. **(F–H)** qRT-PCR results of Sit-miR164a, Sit-miR1432 and Sit-miRN3029a compared with the sRNA-seq result, respectively. RPM, reads per million; qRT-PCR, real-time quantitative reverse transcription PCR; sRNA, small RNA.

The expression of plant miRNAs was dynamic and spatiotemporally specific. To analyze the expression pattern of miRNAs in foxtail millet, clean reads of 14 libraries were mapped to 136 miRNA sequences, normalized to the RPM value, and averaged for tissues (Figure 2A). An analysis of the expression of three classified miRNAs found that the conserved miRNAs were expressed at much higher levels than those that were specific to the Poaceae and foxtail millet (Figure 2B). Examining the expression profile at the level of tissue found that the expression of conserved miRNAs was higher in the roots and stems at the shooting stage, while relatively few of them were expressed in the leaves. However, the trend was opposite at the grain filling stage. The level of expression of conserved miRNAs in the flag leaves and panicles was higher than those in the roots and stems. This could be owing to the early involvement of the conserved miRNA in the development of roots and stems at the seedling stage. For example, the accumulation of conserved miRNAs in rice (*Oryza sativa* L.) directly regulated the development of rice crowns and roots (Zhu et al., 2019), and Osa-miR396d affected signaling by gibberellin and brassinosteroid to regulate the architecture of rice (Tang et al., 2018). During the latter stage, conserved miRNAs primarily regulated the processes of photosynthesis and grain filling, which are critical for the construction of source-sink system.

To analyze the profile of expression in more detail, two cutoff values of 100 and 10 were established to dissect the matrix into four patterns of expression (Figure 2C and Supplementary Table 10). The miRNAs represented by the miR166 family comprised 14.71%, demonstrating the global highly expressive pattern in the whole tissues with RPM ≥ 100 (Figure 2D and Supplementary Table 10). Most of these housekeeping miRNAs were primarily conserved miRNAs and contained only the two miRNAs Sit-miRN3026a and Sit-miRN3026b that are specific to foxtail millet. In addition, 29 miRNAs were highly expressed in only one specific tissue (Figure 2E and Supplementary Table 10). Except for Sit-miR156d, the members of miR156 were specifically highly expressed in roots at the shooting stage. Sit-miR2275c and Sit-miR2275e, two conserved miRNAs, were expressed at high levels in panicles, which could trigger the production and accumulation of 24 nt phased small interfering RNAs (phasiRNA) and further regulate the development of anther in grass plants (Xia et al., 2019). Compared with their levels of expression in other tissues, Sit-miR159a and Sit-miR319, members of the miR159/319 superfamily, were both poorly expressed in the leaves at the two stages, while the other two members of miR159 were housekeeping miRNAs (Figure 2D and Supplementary Table 10). Consistent with previous studies, Sit-miR408 was only poorly expressed in panicles, which had been proven to reduce crop production by regulating the expression of uclacyanin (*UCL*) in panicles during the grain filling stage in rice (Zhang et al., 2017). All these miRNAs could be candidate targets for genetic breeding improvement in foxtail millet. Finally, eight miRNAs were randomly selected and examined using qRT-PCR experiments (Figures 2F–H and Supplementary Figure 3), and the results were consistent with the profile of expression (Figure 2A and Supplementary Table 10), which further demonstrated the high confidence of the identification and annotation of miRNAs.

### Functional Analysis of Predicted Targets of miRNAs

**FIGURE 3:**
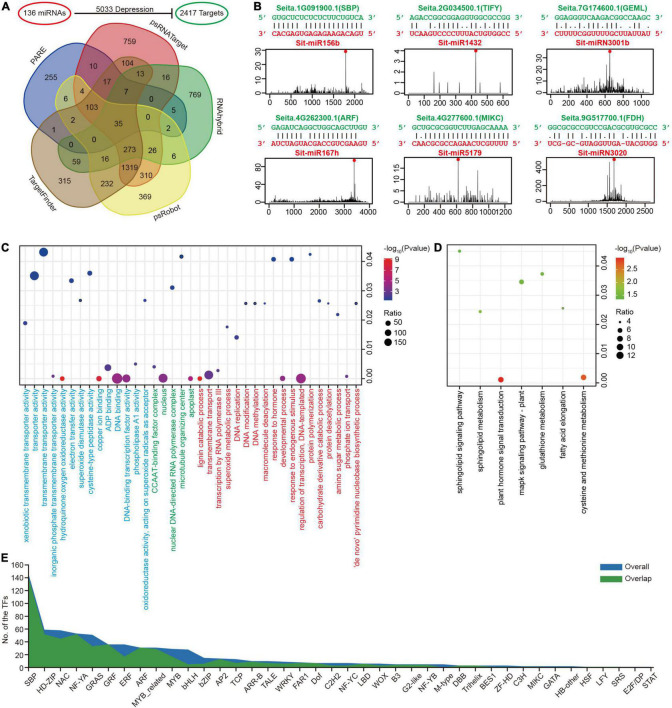
Prediction of miRNA target genes in foxtail millet. **(A)** Candidate miRNA target genes predicted through five methods. **(B)** Six miRNA-target examples represented conserved (Sit-miR156b, Sit-miR167h, and their targets), Poaceae-specific (Sit-miR1432, Sit-miR5179, and their targets), and foxtail millet-specific (Sit-miRN3001b, SitmiRN3020, and their targets) examples. **(C)** Bubble chart of the GO enrichment analysis for miRNA targets. Terms of BP, CC, and MF are labeled red, green, and blue, respectively. **(D)** Bubble chart of the KEGG enrichment analysis for miRNA targets. **(E)** The numbers of transcriptional factor families accounted in the miRNA targets. The “overall” and “overlap” are the overall target genes dataset and overlap target genes dataset, respectively.

The major function of miRNAs is to dampen the expression of their targets to regulate various biological processes. Four types of computational software, psRNATarget, TargetFinder, psRobot, and RNAhybrid, were used to predict the targets of 136 miRNAs using whole-genome transcripts as input data. Combined with the results of degradome (PARE-seq) data, 2,417 targets inside 5,033 miRNA-targeted regulatory pairs were obtained as an overall dataset (Figure 3A and Supplementary Table 11). All the results of PARE-seq and the overlap results predicted by at least two types of computational software were stablished as an overlap dataset for subsequent analysis. Almost all the conserved regulatory links that had been researched or published in model plants such as Arabidopsis, rice, and maize (*Zea mays* L.) were discovered among the regulatory pairings between these miRNAs and the target genes (Supplementary Table 11), showing that the predictions were valid. For example, miR156 regulates the SPL genes; miR159 regulates the MYB genes, and miR160 regulates the ARF genes. There were also many new regulatory pairs uncovered. The conserved miR159a and miR171 target genes, for example, may be TCP, and ARF, respectively, and these predictions were convincingly verified by the PARE-seq data (Supplementary Data 1). Furthermore, the regulatory pairs between Poaceae or foxtail millet-specific miRNAs and their target genes were discovered. For example, Sit-miR5179 and Sit-miRNovel8 could possibly depress TFs, such as MIKC and C2H2, according to the PARE-seq data (Supplementary Table 11 and Supplementary Data 1). Six examples are shown in Figure 3B: Sit-miR156b and Sit-miR167h represented conserved miRNAs; Sit-miR1432 and Sit-miR5179 were specific to the Poaceae, while Sit-miRN3001b and Sit-miRN3020 were specific to foxtail millet. All these regulatory pairs were clearly supported by the PARE-seq results.

GO and KEGG enrichment analyses were performed on all miRNA target genes to have a better understanding of their general status at the genome-wide level. In GO, a biological process (BP) analysis indicated significant enrichment in the regulation of transcription, phosphate ion transport, DNA methylation, superoxide metabolic process and protein deacetylation terms (Figure 3C and Supplementary Table 12), which directly or indirectly affects the ion balance, gene transcription and the response to biological and abiotic stresses. The KEGG enrichment analysis was primarily focused on plant hormone signal transduction and the cysteine and methionine metabolism pathways (Figure 3D and Supplementary Table 13). Moreover, the results of GO and KEGG enrichment in the overlap dataset were similar to the overall condition (Supplementary Figure 4). These were closely related to the miRNAs as regulatory factors, which could regulate the TFs, its large class of target genes. The TFs that can be regulated by miRNAs were studied in more detail, and 40/35 families with different constrictions were identified (Figure 3E). Interestingly, in this study, sphingolipid metabolism and sphingolipid signaling pathways were both detected in the GO enrichment (Supplementary Table 12). In addition, the gene *Seita.5G438700.1* (SPT) in these two pathways was regulated by the only global highly expressed foxtail millet-specific miRNA family miR3026 (Figure 2D). Simultaneously, *Seita.5G438700.1* was globally expressed at lower levels in seven tissues (Supplementary Table 10).

The functions of tissue-specific highly expressed miRNAs were explored in more details. In addition to the 29 miRNAs that were highly expressed in one specific tissue (Figure 2E), candidates that were larger than 100 RPM in specific tissues after filtering out house-keeping miRNAs were also considered as tissue-specific highly expressed miRNAs. Their targets were then extracted and GO enrichment analyses were followed, which resulted in many new discoveries. For example, in roots, miRNA-target pairs that regulate nitrogen biosynthesis process and potassium ion transport process were enriched, suggesting their crucial roles in root structure construction, and the transportation of nutrients from soil to root. We also found that stem-specific miRNAs are involved in shoot development and the transition between different developmental stages. Moreover, panicle-specific highly expressed miRNAs were found to be able to regulate seed development and further impact the final grain yield (Supplementary Table 14). In general, miRNAs regulate important physiological processes, such as plant growth and development, nutritional metabolism, biotic and abiotic stresses, by regulating many functional genes in plants.

### Correlation of the Expression Between miRNAs and Their Targets

**FIGURE 4:**
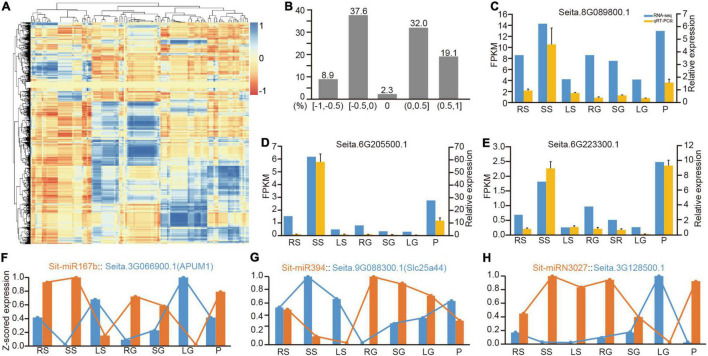
Correlation analysis of the expression between miRNAs and their target genes. **(A)** Correlation matrix of expression between all miRNAs and their targets. **(B)** Distribution of the correlation of miRNA-target expression in divided sections of *r* (correlation value). **(C–E)** The comparison between quantitative results from RNA-Seq and their relative expression from qRT-PCR. **(F–H)** Three representative examples of the negative correlation of conserved miRNA, Poaceae-specific miRNA and foxtail millet-specific miRNA with their target transcripts, respectively. qRT-PCR, real-time quantitative reverse transcription PCR.

The regulatory relationship between miRNAs and targets will cause a negative correlation between their levels of expression. Therefore, identifying the expression patterns of the miRNAs and their target genes in different tissues was a powerful method to corroborate their regulatory patterns. Transcripts in seven tissues were examined by high-throughput sequencing. A correlation analysis and principal component analysis showed that expression levels of the transcripts were consistent between the same samples, indicating the reliability of these data (Supplementary Figure 5). A correlation matrix analysis of the dynamic expression patterns of all miRNAs and their target genes in seven tissues was preliminarily conducted to explore these miRNA-target regulatory pairings (Figure 4A and Supplementary Table 15). A statistical analysis revealed that negative regulatory relationships existed in more than 46% of the miRNA-target combinations (*R* < 0, Figure 4B). Despite the fact that these miRNAs may depress their target genes only in a single tissue, the comprehensive matrix validly supported negative regulation of miRNA and their target genes (Figures 4A,B). Furthermore, nine transcripts were randomly selected for qRT-PCR experiments, and the results were highly consistent with the results of RNA-Seq (Figures 4C–E and Supplementary Figure 6).

Among the predicted regulatory pairs, many pairs exhibited a strict negative correlation. For example, the expression patterns of miR156 and miR172 and their target genes in specific tissues had been genetically verified for their function in developmental transition (Wu et al., 2009). MiR167b was a good example. Sti-MiR167b and its target *Seita.3G066900.1* (APUM1) had reverse patterns of expression in seven tissues (Figure 4F). Sti-miR394 is another representative conserved miRNA. Its target *Seita.9G088300.1* (Slc25a44) also had an opposite pattern of expression (Figure 4G). It also revealed many new regulatory relationships among the Poaceae-specific and foxtail millet-specific miRNAs with their target genes (Supplementary Table 15). Figure 4H lists a representative example of foxtail millet-specific miRNA, Sit-miRN3027 and its target gene *Seita.3G128500.1*, and presented a strong negative correlation between them. Simultaneously, six comparative miRNA-mRNA regulatory pairings were randomly selected for qRT-PCR experiments, and the results exhibited their negative regulatory relationship (Supplementary Figure 7).

### Specific Target Genes and miRNA-Target Regulatory Modules at the Grain Filling Stage

**FIGURE 5:**
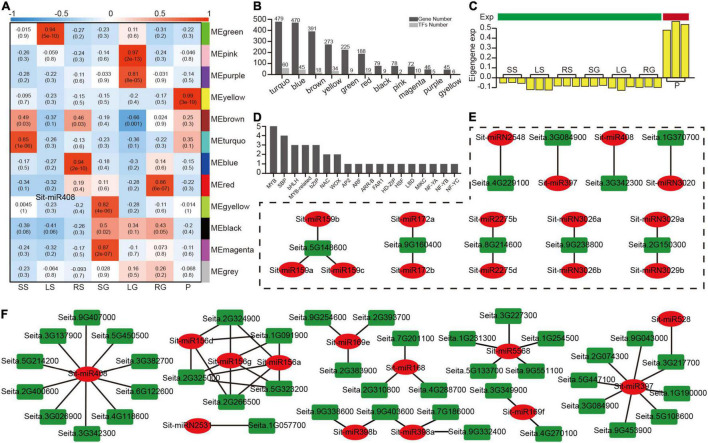
A miRNA modules analysis based on a WGCNA analysis of 2,350 target genes. **(A)** A heatmap of 12 modules correlated with seven tissues. **(B)** The number of target genes and transcription factors in 11 modules (except for the non-sense gray module). **(C)** The bar plot of eigengene expression from the panicle-related yellow module. **(D)** The number of detailed transcription factors in the yellow module. **(E)** The regulatory modules between the nine highest-connectivity target genes in the yellow module and their relevant miRNAs. **(F)** The regulatory modules between the target genes and their relevant miRNAs with opposite patterns of expression.

In most cases, cluster genes exhibit a co-expression pattern when performing different biological functions, which could be related to their response to common environmental factors or their own cascade control. For this reason, after filtering 67 genes with 0 expression variance in seven tissues, 2,350 targets were retained and iterated through the R package “WGCNA” for clustering analysis (Supplementary Figure 8). This constructed a gene co-expression matrix that contained 12 modules. After associating the tissue phenotype, nine modules were highly correlated with the tissues (*R* ≥ 0.75, *P* < 0.01) (Figure 5A). Transcription factors comprised a large proportion in each module (Figure 5B). Among them, the yellow module was the only module that was related to panicle at grain filling stage (Figures 5A,C). Further analysis found that this module contained 34 transcription factors in 18 TF families, including SBP, AP2, and NAC (Figure 5D). A GO enrichment analysis of genes in the yellow module (Supplementary Table 16) found that glucosamine-containing compound metabolic process, aminoglycan metabolic process and amino sugar metabolic process were enriched, and all these terms were related to sugar accumulation in the source-sink system during the grain filling process.

Hub genes normally play an important role in the co-expression network. Based on the evaluation value KME (eigengene connectivity), the top nine hub genes and the genes associated with them in the yellow module were extracted to construct the regulatory network (Supplementary Figure 9). The level of regulation of the miRNA was traced back to further analyze the regulatory relationship of these nine genes (Figure 5E), and these nine genes were only regulated by a few specific miRNAs. For example, *Seita.3G084900* (Laccase25) was only regulated by Sit-miR397, but the relationship of expression between these two nodes does not show a high negative correlation (Supplementary Table 15), which could be owing to the importance of these hub genes and additional simultaneous regulation by multiple TFs. To co-locate the miRNAs that were closely related to the yellow module, an analysis of the expression level between the miRNAs and targets identified the target gene, which was relatively highly expressed in panicles, while the miRNA was expressed in an opposite manner (Supplementary Tables 11, 15). Based on this, a new regulatory network was constructed (Figure 5F). Among them, miR408 occupied the most regulatory relationship pairs, indicating that miR408 may play an important regulatory role during grain filling stage, which was consistent with the previously reported miR408 regulation of seed size and other traits. This part of the regulatory relationship laid a solid foundation for subsequent genetic experiments.

### Exploration of the Regulatory Network of Foxtail Millet miRNAs

**FIGURE 6:**
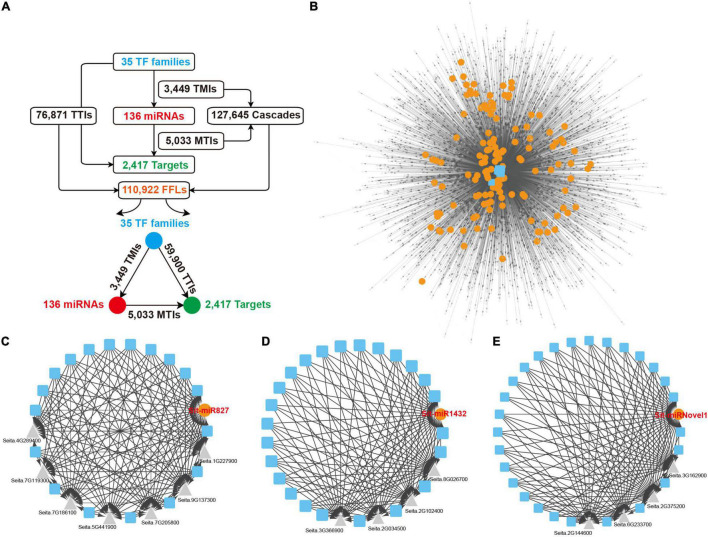
FFLs regulatory network centered by miRNAs. **(A)** Detailed information of all interactions and miRNA-centered FFLs regulatory network from the overall dataset. **(B)** miRNA-centered FFLs regulatory network. Blue squares, orange dots, and gray triangles represent TFs, miRNAs, and targets, respectively. **(C–E)** Three examples of local FFL networks centered by Sit-miR827, Sit-miR1432, and Sit-miRNovel1. Blue squares, orange dots, and gray triangles represent TFs, miRNAs, and target genes, respectively. FFLs, feed forward loops; TFs, transcriptional factors.

As important transcriptional regulatory factors, miRNAs and TFs cooperate to change the gene expression for multiple biological processes. To construct a comprehensive whole genome-wide regulatory network monitored by TFs and miRNAs, the upstream 2,000 nucleotides of the miRNAs and their target genes were extracted to predict the binding site of TFs, and 35 TF families were retained under the default parameter, including WRKY, ERF, and MYB. Every two nodes from the TFs, miRNAs and target genes formed direct regulatory relationships. The TFs regulated miRNAs and formed 3,449 TF and miRNA interactions (TMIs). The genes targeted by the miRNAs formed 5,033 miRNAs and Target interactions (MTIs), and the target genes regulated by the TFs formed 76,871 TFs and Targets interactions (TTIs) (Figure 6A). As for the three nodes together, TFs to miRNAs and then to target genes formed directed acyclic 127,645 cascade motifs, and 110,922 FFL motifs were extracted (Figure 6A and Supplementary Table 17). A comprehensive regulatory network, including TFs, miRNAs, and targets, was constructed based on these FFLs interactions (Figure 6B). To the overlap dataset, 62,328 FFLs were extracted from 71,548 cascades motifs to construct the FFLs network (Supplementary Figures 10A,B and Supplementary Table 18).

The construction of network further unveiled the regulatory relationships between the miRNAs, TFs and their target genes. In this network, any specific miRNA that occupies a central position linked by other elements can present an independent module core. As a conserved miRNA, Sit-miR827 integrated multiple TF families that regulated the expression of seven downstream target genes, including flowering-promoting factor *Seita.5G441900.1* and SPX domain-containing membrane protein encoding gene *Seita.7G205800.1* (Figure 6C). Sit-miR1432, a Poaceae-specific miRNA that highly expressed in flag leaves (Figure 2E), was triggered by various TF families and modulated four target genes expression (Figure 6D). In these four genes, *Seita.8G026700.1* encodes the BTB/POZ domain contained protein that includes an ankyrin repeat, which is a key positive factor for disease resistance, and the encoding protein TIFY5 of *Seita.2G034500.1* had been reported as an endogenous repressor of the salicylic acid signal. Figure 6E showed a foxtail millet-specific miRNA, Sit-miRNovel1, which had a similar regulatory module with the former two examples. Based on the detection of these specific modules, except for those conserved regulatory modules, increasing numbers of novel regulatory relationships were explored and could be chosen as high confidence candidates for further genetic studies and potential breeding targets.

## Discussion

The precise systematic identification and annotation of miRNAs is the premise for exploring their functions in a specific species. There have been several studies on the identification of miRNAs in foxtail millet (Yi et al., 2013; Han et al., 2014; Khan et al., 2014; Wang et al., 2016) with or without sRNA-seq datasets based on the 2008 criteria for plant miRNA annotations (Meyers et al., 2008). It has become clear that many miRNA annotations are questionable following the study of extensive data sets of plant small RNAs by NGS. In this study, the identification and annotation of all the miRNAs were analyzed by updating the criteria for plant miRNAs that were delineated in 2018. To acquire more precise information at the shooting and grain filling stages, we collected the different tissues of these stages. Moreover, the functions of many miRNAs have been elucidated in plants over the past several years. For example, the overexpression of *Ath-miR408* enhances photosynthesis, growth, and seed yield in Arabidopsis, rice, and tobacco (*Nicotiana tabacum*) (Pan et al., 2018), while miR858 controls flavonoid biosynthesis and development in Arabidopsis (Sharma et al., 2016). The combination of these methods and research results provides a strong foundation to precisely identify and mine the functions of miRNA in foxtail millet. Finally, 136 high confidence miRNAs were identified, annotated and classified.

It is well known that the function of miRNAs is primarily to repress the expression of their target genes. It is critical to accurately predict miRNA targets to mine miRNA functions. Four computational prediction software packages, including psRNATarget, TargetFinder, psRobot and RNAhybrid, extra with PARE data, were used to obtain 5,033 potential miRNA-targets regulatory pairs as an overall dataset. GO and KEGG pathway analyses demonstrated that these miRNA target genes were enriched in plant growth, development, nutritional metabolism, and biotic and abiotic stresses. In general, these results suggest that these miRNAs are involved in almost all biological activities (Mi et al., 2019). According to the analysis of TFs, many conservative types of regulation were also found in foxtail millet, such as miR156-SPL and miR159-MYB, which coincided with previous data (Jia et al., 2013; Yi et al., 2015). Simultaneously, a functional analysis of these tissue-specific highly expressed miRNAs revealed their regulatory roles in nutrient absorption, ion transport, carbohydrate metabolism, flower development, reproductive regulation, and seed development, further demonstrating their regulatory function in growth and development. Interestingly, *Seita.5G438700* was regulated by the only global highly expressed foxtail millet-specific miRNA family *Sit-miR3026*, and its expression pattern was globally low. In addition, expression patterns between the miRNAs and their target genes in different tissues and developmental stages were correlated. Despite the high degree of complication among them, many negative correlations were observed between miRNAs and their target genes in general.

Grain filling is a highly complex physiological process. During this stage, numerous storage compounds are synthesized and transported into the endosperm, which primarily determine the yield and grain quality (Wang et al., 2020). The panicle structure is an important agronomic trait that helps to determine crop yield (Xing and Zhang, 2010). The molecular mechanisms of panicle development have been the focus of intensive study (Huang et al., 2017). TFs have extensive and conserved functions in the regulation of panicle development. In this trial, the results of a WGCNA analysis showed that nine modules were highly correlated with different tissues, and only one module was related to the grain filling stage. It contained 34 transcription factors in 18 TF families, including MYB, SBP, AP2 and bZIP. A GO analysis showed that these genes focused on metabolic processes, such as glucosamine-containing, aminoglycan, and amino sugar metabolites. A new miRNA and target gene regulatory network in panicles was constructed and miR408 occupies the largest number of regulatory relationship pairs, indicating that miR408 may play an important regulatory role during the grain filling stage. It has been reported that this miRNA positively regulates the yield of grain and photosynthesis via a cytoplasm-localized phytocyanin protein, OsUCL8 (Zhang et al., 2017). In a recent study, genetic experiments indicated that *Lsa-MIR408* served as a hub gene to increase the fresh weight and achene size of lettuce (Deng et al., 2021).

The construction of a gene regulatory network that integrates multiple regulatory relationships within the appropriate biological process is a desired approach to promote plant biology (Gaudinier and Brady, 2016). The regulatory networks in plants that have been reported to date are primarily focused on transcriptional regulation. MiRNAs are known to have a higher propensity to interact with TFs in plants (Gao et al., 2021). The FFL is a prominent and versatile network motif (Gaudinier and Brady, 2016). In plants, many FFLs have been identified in gene regulatory networks, which involve developmental processes and stress responses (Taylor-Teeples et al., 2015; Gao et al., 2021). In this study, a complex regulatory network centered on miRNAs and the combination of upstream TFs and downstream target genes was constructed in foxtail millet. Based on the reconstructed hub miRNA in an integrated gene regulatory network of Arabidopsis, the *HY5-MIR858A-MYBL2* FFL module was identified and confirmed to play an important role in the regulation of accumulation of anthocyanins in seedlings in response to light (Gao et al., 2021). The rice miR1432-OsACOT module was found to regulate grain size by controlling rice grain filling. The suppressed expression of rice miR1432 significantly increased grain weight and led to an increase of up to 17.14% in grain yield in a field trial (Zhao et al., 2019). Interestingly, Sit-miR1432 was identified as a classic miRNA specific to the Poaceae in our study, and it was highly expressed in the flag leaves and poorly expressed in the panicle. Simultaneously, Sit-miR1432 is triggered by various TF families and modulates the expression of the four target genes *Seita.8G026700.1*, *Seita.3G366900.1*, *Seita.2G102400.1*, and *Seita.2G034500.1*, excluding the homologous genes of *OsACOT*. This raises the possibility that there is a novel module in foxtail millet that regulates grain filling. Based on the regulatory network centered on miRNAs, increasing numbers of novel regulatory relationships were explored and validated by experiments, which could be exploited in crop breeding.

## Conclusion

In conclusion, as a C4 model crop and an important economic crop, research on the variation in morphology, nitrogen and water utilization, and nutritional balance of foxtail millet is still limited. In this study, a comprehensive miRNA centered regulatory network was constructed after high confident identification and annotation of the miRNAs in foxtail millet. This study sets the groundwork and offers tools to explore the roles of miRNAs in foxtail millet, as well as providing innovative crop breeding tactics.

## Data Availability Statement

The sequencing data for this study can be found in the NCBI Sequence Read Archive (SRA) under accession number PRJNA793125. All annotated miRNA loci were deposited into PmiREN2.0 database (https://www.pmiren.com/).

## Author Contributions

JZ, JW, and XY initiated, designed the research, and revised the manuscript. YD, GX, CL, ZK, and SD analyzed the data. HZ, HW, YZ, BL, and GX performed the experiments. JZ, YD, and HZ wrote the manuscript. All authors contributed to the article and approved the submitted version.

## Conflict of Interest

The authors declare that the research was conducted in the absence of any commercial or financial relationships that could be construed as a potential conflict of interest.

## Publisher’s Note

All claims expressed in this article are solely those of the authors and do not necessarily represent those of their affiliated organizations, or those of the publisher, the editors and the reviewers. Any product that may be evaluated in this article, or claim that may be made by its manufacturer, is not guaranteed or endorsed by the publisher.

## Footnotes

1
http://www.fao.org/

2
https://www.zhaolab.org/psRNATarget/

3
https://www.kegg.jp/ghostkoala/

4
http://planttfdb.gao-lab.org/prediction.php

5
http://plantregmap.gao-lab.org/

## References

[B1] Addo-Quaye C. Miller W. Axtell M. J. (2009). CleaveLand: a pipeline for using degradome data to find cleaved small RNA targets. *Bioinform.* 25 130–131. 10.1093/bioinformatics/btn604 19017659 PMC3202307

[B2] Alves L. Jr. Niemeier S. Hauenschild A. Rehmsmeier M. Merkle T. (2009). Comprehensive prediction of novel microRNA targets in Arabidopsis thaliana. *Nucleic. Acids. Res.* 37 4010–4021. 10.1093/nar/gkp272 19417064 PMC2709567

[B3] Axtell M. J. Meyers B. C. (2018). Revisiting Criteria for Plant MicroRNA Annotation in the Era of Big Data. *Plant Cell.* 30 272–284. 10.1105/tpc.17.00851 29343505 PMC5868703

[B4] Bartel D. P. (2009). MicroRNAs: target recognition and regulatory functions. *Cell* 136 215–233. 10.1016/j.cell.2009.01.002 19167326 PMC3794896

[B5] Bartlett A. O’Malley R. C. Huang S. C. Galli M. Nery J. R. Gallavotti A. (2017). Mapping genome-wide transcription-factor binding sites using DAP-seq. *Nat. Protoc.* 12 1659–1672. 10.1038/nprot.2017.055 28726847 PMC5576341

[B6] Bennetzen J. L. Schmutz J. Wang H. Percifield R. Hawkins J. Pontaroli A. C. (2012). Reference genome sequence of the model plant Setaria. *Nat. Biotechnol.* 30 555–561. 10.1038/nbt.2196 22580951

[B7] Brown J. Pirrung M. McCue L. A. (2017). FQC Dashboard: integrates FastQC results into a web-based, interactive, and extensible FASTQ quality control tool. *Bioinform.* 33 3137–3139. 10.1093/bioinformatics/btx373 28605449 PMC5870778

[B8] Carthew R. W. Sontheimer E. J. (2009). Origins and Mechanisms of miRNAs and siRNAs. *Cell* 136 642–655. 10.1016/j.cell.2009.01.035 19239886 PMC2675692

[B9] Chandra S. Vimal D. Sharma D. Rai V. Gupta S. C. Chowdhuri D. K. (2017). Role of miRNAs in development and disease: Lessons learnt from small organisms. *Life Sci.* 185 8–14. 10.1016/j.lfs.2017.07.017 28728902

[B10] Chen C. Chen H. Zhang Y. Thomas H. R. Frank M. H. He Y. (2020). TBtools: An Integrative Toolkit Developed for Interactive Analyses of Big Biological Data. *Mol. Plant.* 13 1194–1202. 10.1016/j.molp.2020.06.009 32585190

[B11] Dai X. Zhuang Z. Zhao P. X. (2018). psRNATarget: a plant small RNA target analysis server. *Nucleic. Acids. Res.* 46 W49–W54. 10.1093/nar/gky316 29718424 PMC6030838

[B12] Deng Y. Qin Y. Yang P. Du J. Kuang Z. Zhao Y. (2021). Comprehensive Annotation and Functional Exploration of MicroRNAs in Lettuce. *Front. Plant. Sci.* 12:781836. 10.3389/fpls.2021.781836 35003165 PMC8739914

[B13] Yi F. Xie S. Liu Y. Qi X. Yu J. (2013). Genome-wide characterization of microRNA in foxtail millet (Setaria italica). *BMC. Plant Biol.* 13:212. 10.1186/1471-2229-13-212 24330712 PMC3878754

[B14] Fromm B. Keller A. Yang X. Friedlander M. R. Peterson K. J. Griffiths-Jones S. (2020). Quo vadis microRNAs? *Trends Genet* 36 461–463. 10.1016/j.tig.2020.03.007 32544447

[B15] Gao Z. Li J. Li L. Yang Y. Li J. Fu C. (2021). Structural and Functional Analyses of Hub MicroRNAs in an Integrated Gene Regulatory Network of Arabidopsis. *Genomics Proteomics Bioinform.* 10.1016/j.gpb.2020.02.004 [Epub ahead of print]. 33662619 PMC9880815

[B16] Gaudinier A. Brady S. M. (2016). Mapping Transcriptional Networks in Plants: Data-Driven Discovery of Novel Biological Mechanisms. *Annu. Rev. Plant Biol.* 67 575–594. 10.1146/annurev-arplant-043015-112205 27128468

[B17] German M. A. Luo S. Schroth G. Meyers B. C. Green P. J. (2009). Construction of Parallel Analysis of RNA Ends (PARE) libraries for the study of cleaved miRNA targets and the RNA degradome. *Nat. Protoc.* 4 356–362. 10.1038/nprot.2009.8 19247285

[B18] Guo Z. Kuang Z. Zhao Y. Deng Y. He H. Wan M. (2021). PmiREN2.0: from data annotation to functional exploration of plant microRNAs. *Nucleic. Acids. Res.* 50 D1475–D1482. 10.1093/nar/gkab811 34554254 PMC8728213

[B19] Han J. Xie H. Sun Q. Wang J. Lu M. Wang W. (2014). Bioinformatic identification and experimental validation of miRNAs from foxtail millet (Setaria italica). *Gene.* 546 367–377. 10.1016/j.gene.2014.05.050 24862217

[B20] Huang P. Jiang H. Zhu C. Barry K. Jenkins J. Sandor L. (2017). Sparse panicle1 is required for inflorescence development in Setaria viridis and maize. *Nat Plants.* 3 17054. 10.1038/nplants.2017.54 28418381

[B21] Jia G. Huang X. Zhi H. Zhao Y. Zhao Q. Li W. (2013). A haplotype map of genomic variations and genome-wide association studies of agronomic traits in foxtail millet (Setaria italica). *Nat. Genet.* 45 957–961. 10.1038/ng.2673 23793027

[B22] Jin J. Tian F. Yang D. C. Meng Y. Q. Kong L. Luo J. (2017). PlantTFDB 4.0: toward a central hub for transcription factors and regulatory interactions in plants. *Nucleic. Acids. Res.* 45 D1040–D1045. 10.1093/nar/gkw982 27924042 PMC5210657

[B23] Johnson D. S. Mortazavi A. Myers R. M. Wold B. (2007). Genome-Wide Mapping of in Vivo Protein-DNA Interactions. *Sci.* 316 1497–1502. 10.1126/science.1141319 17540862

[B24] Jones P. Binns D. Chang H. Y. Fraser M. Li W. McAnulla C. (2014). InterProScan 5: genome-scale protein function classification. *Bioinform.* 30 1236–1240. 10.1093/bioinformatics/btu031 24451626 PMC3998142

[B25] Kalvari I. Argasinska J. Quinones-Olvera N. Nawrocki E. P. Rivas E. Eddy S. R. (2018). Rfam 13.0: shifting to a genome-centric resource for non-coding RNA families. *Nucleic. Acids. Res.* 46 D335–D342. 10.1093/nar/gkx1038 29112718 PMC5753348

[B26] Kechin A. Boyarskikh U. Kel A. Filipenko M. (2017). cutPrimers: A New Tool for Accurate Cutting of Primers from Reads of Targeted Next Generation Sequencing. *J. Comput. Biol.* 24 1138–1143. 10.1089/cmb.2017.0096 28715235

[B27] Khan Y. Yadav A. Bonthala V. S. Muthamilarasan M. Yadav C. B. Prasad M. (2014). Comprehensive genome-wide identification and expression profiling of foxtail millet [Setaria italica (L.)] miRNAs in response to abiotic stress and development of miRNA database. *Plant Cell, Tissue and Organ Culture (PCTOC)* 118 279–292. 10.1007/s11240-014-0480-x

[B28] Kim D. Paggi J. M. Park C. Bennett C. Salzberg S. L. (2019). Graph-based genome alignment and genotyping with HISAT2 and HISAT-genotype. *Nat. Biotechnol.* 37 907–915. 10.1038/s41587-019-0201-4 31375807 PMC7605509

[B29] Kruger J. Rehmsmeier M. (2006). RNAhybrid: microRNA target prediction easy, fast and flexible. *Nucleic. Acids. Res.* 34 W451–W454. 10.1093/nar/gkl243 16845047 PMC1538877

[B30] Kuang Z. Wang Y. Li L. Yang X. (2019). miRDeep-P2: accurate and fast analysis of the microRNA transcriptome in plants. *Bioinform.* 35 2521–2522. 10.1093/bioinformatics/bty972 30521000

[B31] Langmead B. (2010). Aligning short sequencing reads with Bowtie. *Curr. Protoc. Bioinform. Chapter.* 11 17. 10.1002/0471250953.bi1107s32 21154709 PMC3010897

[B32] Lee Y. Kim M. Han J. Yeom K. H. Lee S. Baek S. H. (2004). MicroRNA genes are transcribed by RNA polymerase II. *EMBO J.* 23 4051–4060. 10.1038/sj.emboj.7600385 15372072 PMC524334

[B33] Park MY Wu G Gonzalez-Sulser A Vaucheret H Poethig RS. (2005). Nuclear processing and export of microRNAs in Arabidopsis. *Proc. Natl. Acad. Sci. U S A.* 102 3691–3696. 10.1073/pnas.0405570102 15738428 PMC553294

[B34] Meyers B. C. Axtell M. J. Bartel B. Bartel D. P. Baulcombe D. Bowman J. L. (2008). Criteria for annotation of plant MicroRNAs. *Plant Cell* 20 3186–3190. 10.1105/tpc.108.064311 19074682 PMC2630443

[B35] Mi H. Muruganujan A. Ebert D. Huang X. Thomas P. D. (2019). PANTHER version 14: more genomes, a new PANTHER GO-slim and improvements in enrichment analysis tools. *Nucleic. Acids. Res.* 47 D419–D426. 10.1093/nar/gky1038 30407594 PMC6323939

[B36] Ogata H. Goto S. Sato K. Fujibuchi W. Bono H. Kanehisa M. (1999). KEGG: Kyoto Encyclopedia of Genes and Genomes. *Nucleic. Acids. Res.* 27 29–34. 9847135 10.1093/nar/27.1.29 PMC148090

[B37] Pan J. Huang D. Guo Z. Kuang Z. Zhang H. Xie X. (2018). Overexpression of microRNA408 enhances photosynthesis, growth, and seed yield in diverse plants. *J. Integr. Plant. Biol.* 60 323–340. 10.1111/jipb.12634 29330900

[B38] Pertea M. Pertea G. M. Antonescu C. M. Chang T. C. Mendell J. T. Salzberg S. L. (2015). StringTie enables improved reconstruction of a transcriptome from RNA-seq reads. *Nat. Biotechnol.* 33 290–295. 10.1038/nbt.3122 25690850 PMC4643835

[B39] Quinlan A. R. Hall I. M. (2010). BEDTools: a flexible suite of utilities for comparing genomic features. *Bioinform.* 26 841–842. 10.1093/bioinformatics/btq033 20110278 PMC2832824

[B40] Shannon P. Markiel A. Ozier O. Baliga N. S. Wang J. T. Ramage D. (2003). Cytoscape: a software environment for integrated models of biomolecular interaction networks. *Genome. Res.* 13 2498–2504. 10.1101/gr.1239303 14597658 PMC403769

[B41] Sharma D. Tiwari M. Pandey A. Bhatia C. Sharma A. Trivedi P. K. (2016). MicroRNA858 Is a Potential Regulator of Phenylpropanoid Pathway and Plant Development. *Plant Physiol.* 171 944–959. 10.1104/pp.15.01831 27208307 PMC4902582

[B42] Tang Y. Liu H. Guo S. Wang B. Li Z. Chong K. (2018). OsmiR396d Affects Gibberellin and Brassinosteroid Signaling to Regulate Plant Architecture in Rice. *Plant Physiol.* 176 946–959. 10.1104/pp.17.00964 29180380 PMC5761777

[B43] Tav C. Tempel S. Poligny L. Tahi F. (2016). miRNAFold: a web server for fast miRNA precursor prediction in genomes. *Nucleic. Acids. Res.* 44 W181–W184. 10.1093/nar/gkw459 27242364 PMC4987958

[B44] Taylor-Teeples M. Lin L. de Lucas M. Turco G. Toal T. W. Gaudinier A. (2015). An Arabidopsis gene regulatory network for secondary cell wall synthesis. *Nat.* 517 571–575. 10.1038/nature14099 25533953 PMC4333722

[B45] Tian F. Yang D. C. Meng Y. Q. Jin J. Gao G. (2020). PlantRegMap: charting functional regulatory maps in plants. *Nucleic. Acids. Res.* 48 D1104–D1113. 10.1093/nar/gkz1020 31701126 PMC7145545

[B46] Wang G. Li H. Wang K. Yang J. Duan M. Zhang J. (2020). Regulation of gene expression involved in the remobilization of rice straw carbon reserves results from moderate soil drying during grain filling. *Plant J.* 101 604–618. 10.1111/tpj.14565 31621135

[B47] Wang Y. Li L. Tang S. Liu J. Zhang H. Zhi H. (2016). Combined small RNA and degradome sequencing to identify miRNAs and their targets in response to drought in foxtail millet. *BMC. Genet.* 17:57. 10.1186/s12863-016-0364-7 27068810 PMC4828802

[B48] Wang Y. Tang H. Debarry J. D. Tan X. Li J. Wang X. (2012). MCScanX: a toolkit for detection and evolutionary analysis of gene synteny and collinearity. *Nucleic. Acids. Res.* 40 e49. 10.1093/nar/gkr1293 22217600 PMC3326336

[B49] Wu G. Park M. Y. Conway S. R. Wang J. W. Weigel D. Poethig R. S. (2009). The sequential action of miR156 and miR172 regulates developmental timing in Arabidopsis. *Cell* 138 750–759. 10.1016/j.cell.2009.06.031 19703400 PMC2732587

[B50] Wu H. J. Ma Y. K. Chen T. Wang M. Wang X. J. (2012). PsRobot: a web-based plant small RNA meta-analysis toolbox. *Nucleic. Acids. Res.* 40 W22–W28. 10.1093/nar/gks554 22693224 PMC3394341

[B51] Xia R. Chen C. Pokhrel S. Ma W. Huang K. Patel P. (2019). 24-nt reproductive phasiRNAs are broadly present in angiosperms. *Nat. Commun.* 10 627. 10.1038/s41467-019-08543-0 30733503 PMC6367383

[B52] Xing Y. Zhang Q. (2010). Genetic and molecular bases of rice yield. *Annu. Rev. Plant. Biol.* 61 421–442. 10.1146/annurev-arplant-042809-112209 20192739

[B53] Yang X. Wan Z. Perry L. Lu H. Wang Q. Zhao C. (2012). Early millet use in northern China. *Proc. Natl. Acad. Sci. U S A.* 109 3726–3730. 10.1073/pnas.1115430109 22355109 PMC3309722

[B54] Yang Z. Zhang H. Li X. Shen H. Gao J. Hou S. (2020). A mini foxtail millet with an Arabidopsis-like life cycle as a C4 model system. *Nat. Plants.* 6 1167–1178. 10.1038/s41477-020-0747-7 32868891

[B55] Yi F. Chen J. Yu J. (2015). Global analysis of uncapped mRNA changes under drought stress and microRNA-dependent endonucleolytic cleavages in foxtail millet. *BMC. Plant. Biol.* 15:241. 10.1186/s12870-015-0632-0 26444665 PMC4594888

[B56] Zhang J. P. Yu Y. Feng Y. Z. Zhou Y. F. Zhang F. Yang Y. W. (2017). MiR408 Regulates Grain Yield and Photosynthesis via a Phytocyanin Protein. *Plant Physiol.* 175 1175–1185. 10.1104/pp.17.01169 28904074 PMC5664482

[B57] Zhao Y. Kuang Z. Wang Y. Li L. Yang X. (2021). MicroRNA annotation in plants: current status and challenges. *Brief. Bioinform.* 22 bbab075. 10.1093/bib/bbab075 33754625

[B58] Zhao Y. F. Peng T. Sun H. Z. Teotia S. Wen H. L. Du Y. X. (2019). miR1432-OsACOT (Acyl-CoA thioesterase) module determines grain yield via enhancing grain filling rate in rice. *Plant. Biotechnol. J.* 17 712–723. 30183128 10.1111/pbi.13009 PMC6419572

[B59] Zhu J. Li Y. Lin J. Wu Y. Guo H. Shao Y. (2019). CRD1, an Xpo1 domain protein, regulates miRNA accumulation and crown root development in rice. *Plant J.* 100 328–342. 10.1111/tpj.14445 31257621

