# Supplementary figures and images for: The Construction and Exploration of a Comprehensive MicroRNA Centered Regulatory Network in Foxtail Millet (Setaria italica L.) (part 2 of 14)
Source: Front Plant Sci. 2022 May 6;13:848474. doi: 10.3389/fpls.2022.848474 (PMC9121102; doi:10.3389/fpls.2022.848474)

**T=Seita.9G254200.1\_Q=Sit-miR160b\_S=262**

category=2\_p=0.999997819533941

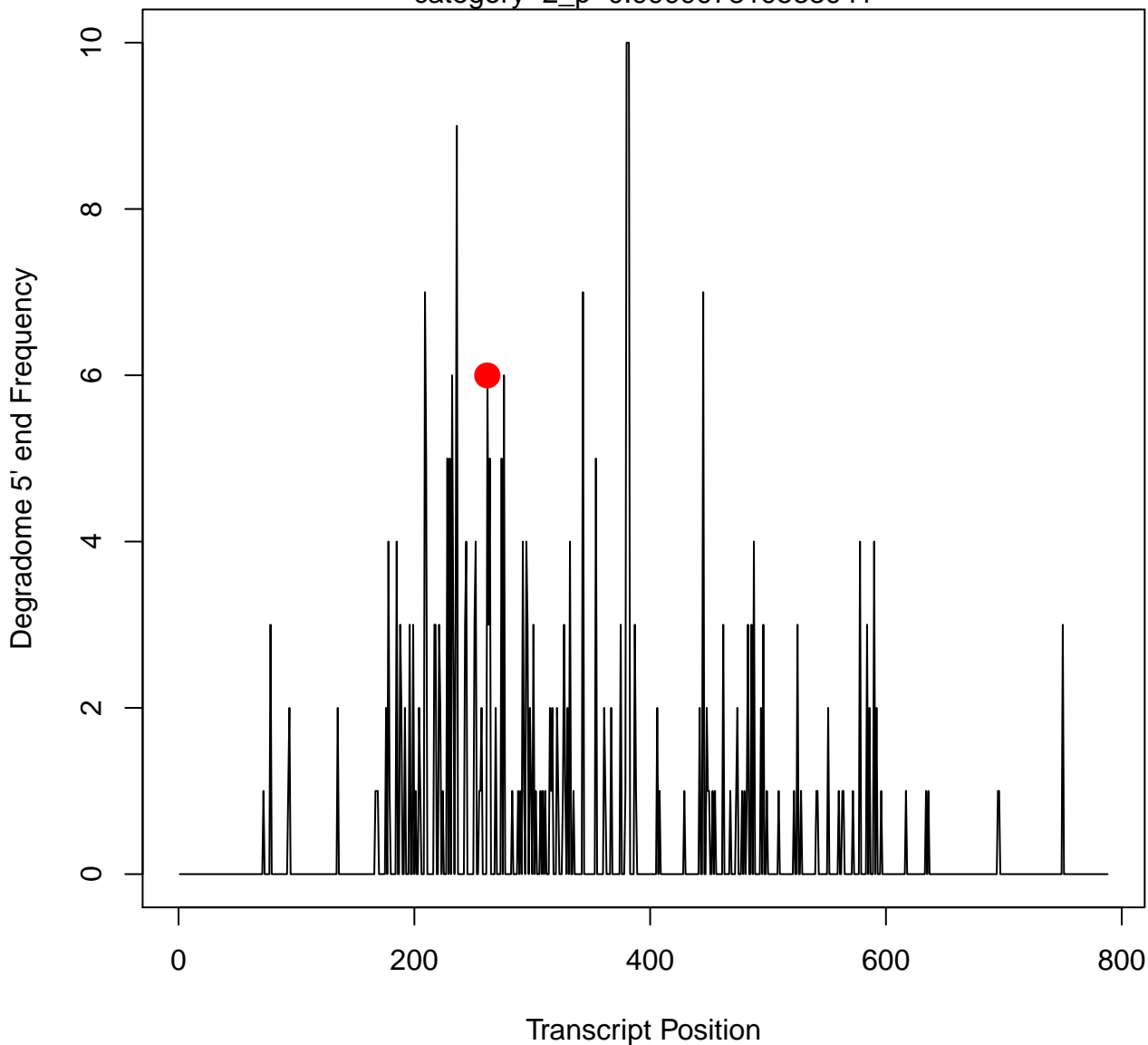

Supplement: Supplementary file 1 [file Data_Sheet_1.zip › Sit-miR160b_Seita.9G254200.1_262_TPlot.pdf]

**T=Seita.9G259700.1\_Q=Sit-miR160b\_S=239**

category=2\_p=0.999999997876074

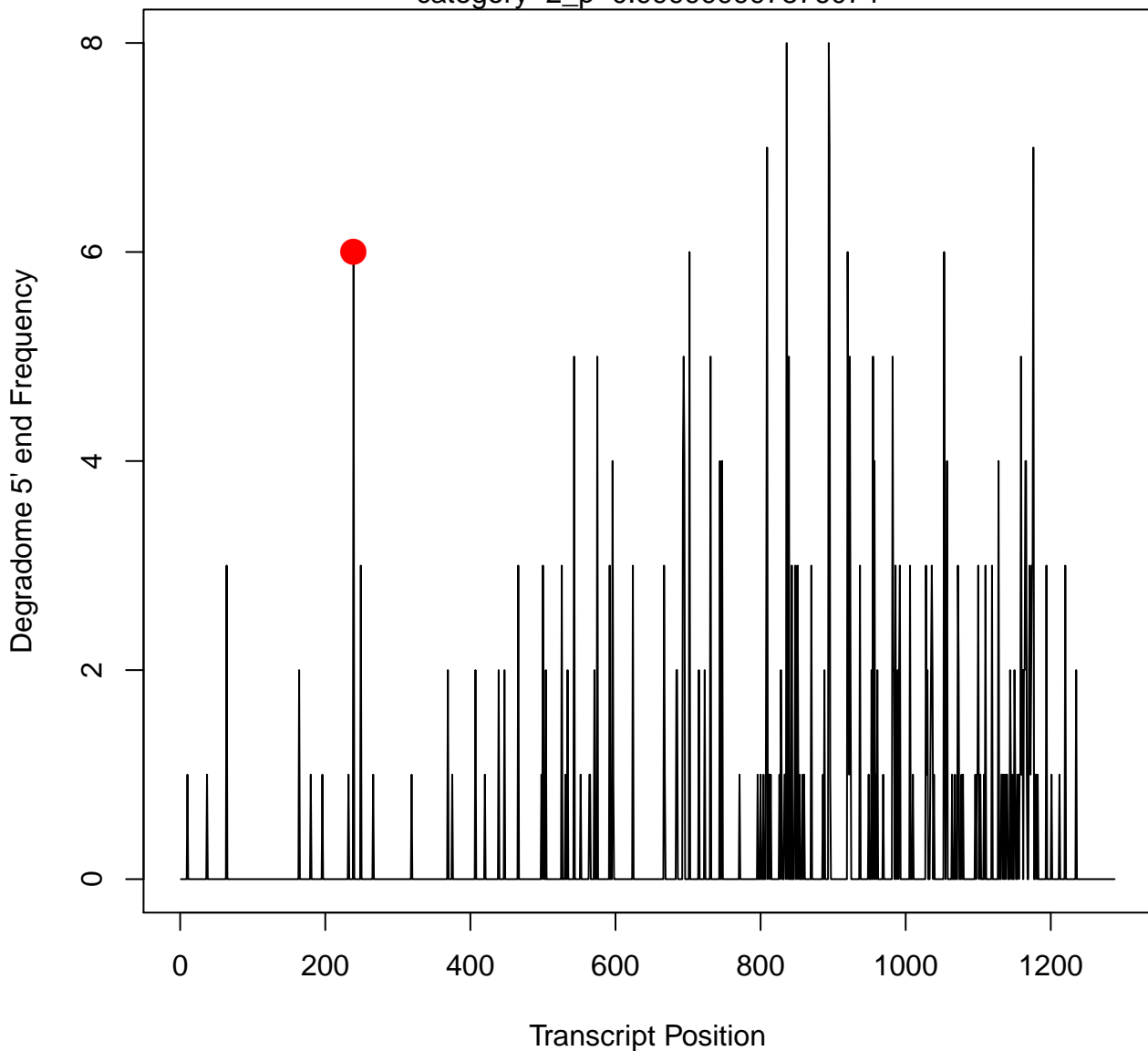

Supplement: Supplementary file 1 [file Data_Sheet_1.zip › Sit-miR160b_Seita.9G259700.1_239_TPlot.pdf]

**T=Seita.1G141900.1\_Q=Sit-miR160c\_S=1482**

category=2\_p=0.999999999245889

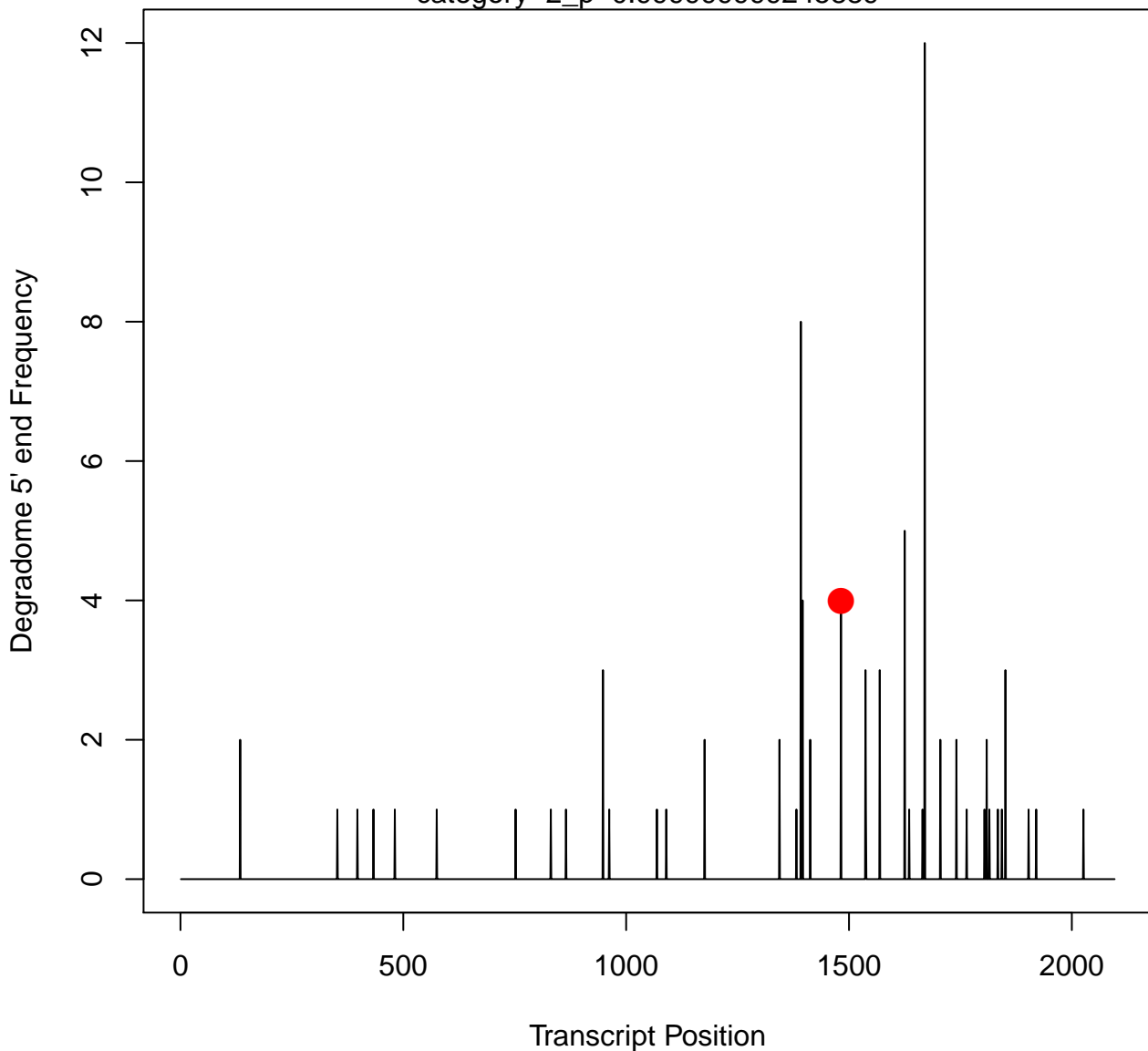

Supplement: Supplementary file 1 [file Data_Sheet_1.zip › Sit-miR160c_Seita.1G141900.1_1482_TPlot.pdf]

**T=Seita.1G335300.1\_Q=Sit-miR160c\_S=1303**

category=2\_p=0.999999985468724

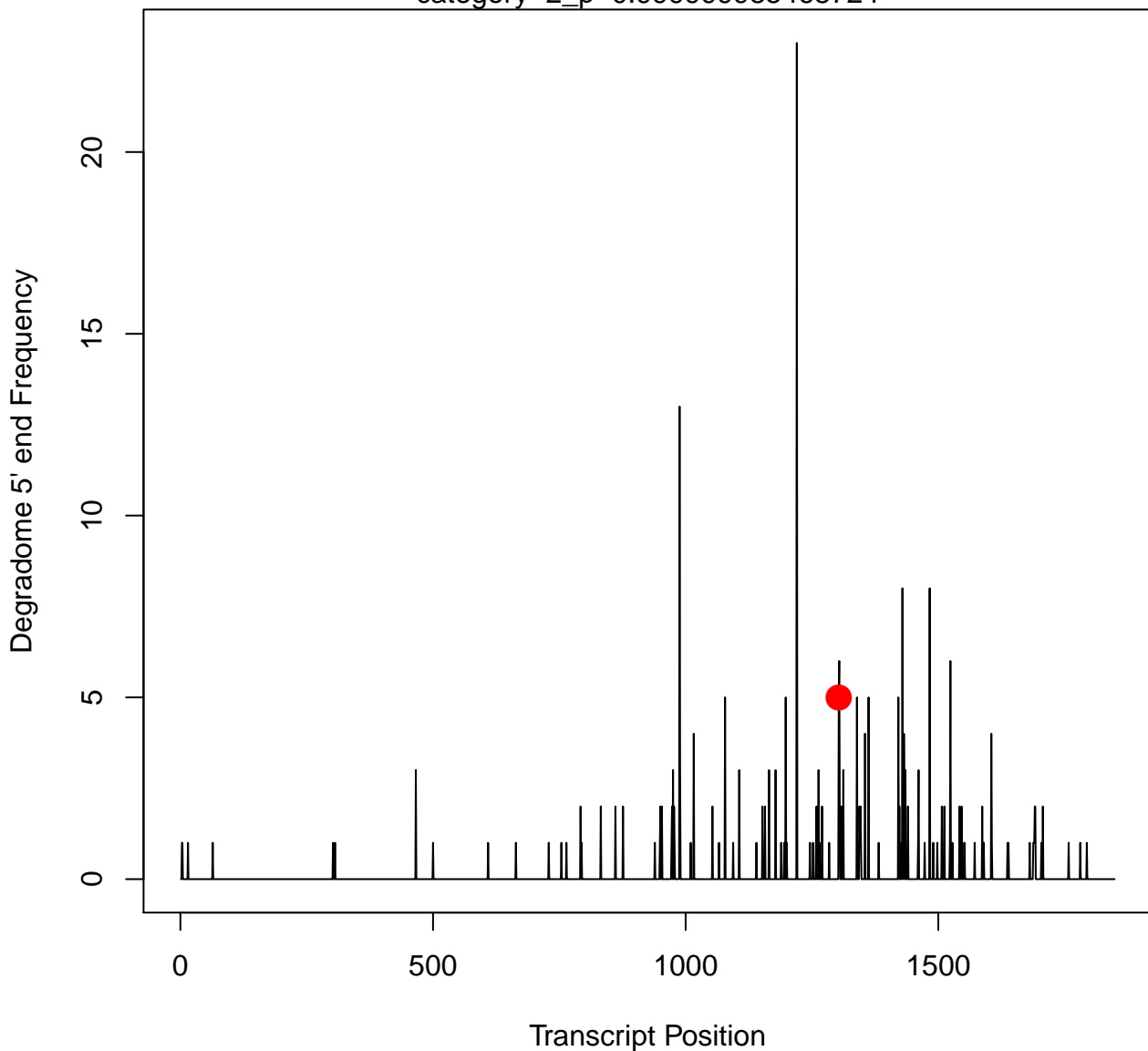

Supplement: Supplementary file 1 [file Data_Sheet_1.zip › Sit-miR160c_Seita.1G335300.1_1303_TPlot.pdf]

**T=Seita.2G038000.1\_Q=Sit-miR160c\_S=734**

category=2\_p=0.99999992739762

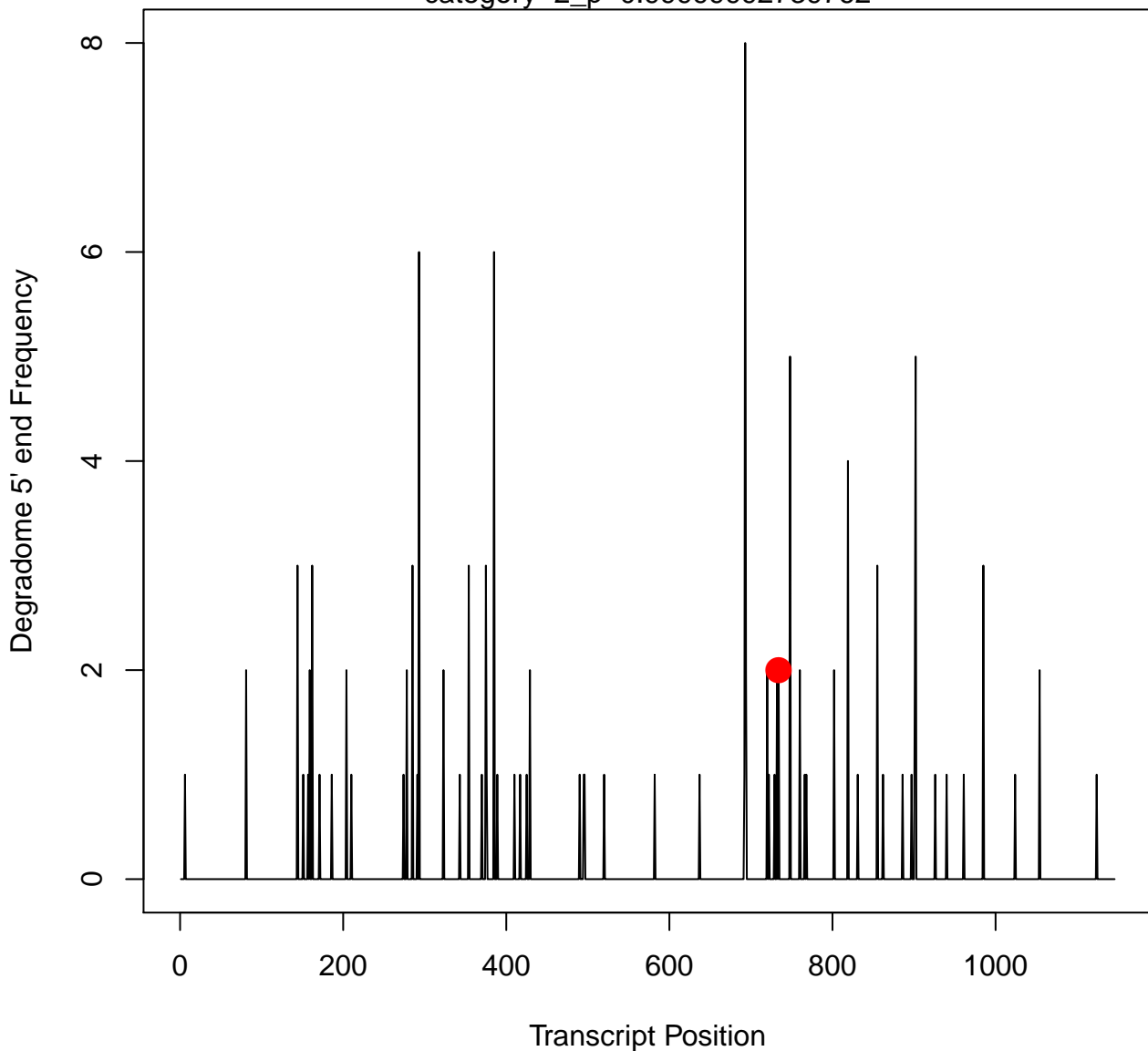

Supplement: Supplementary file 1 [file Data_Sheet_1.zip › Sit-miR160c_Seita.2G038000.1_734_TPlot.pdf]

**T=Seita.2G137400.1\_Q=Sit-miR160c\_S=817**

category=2\_p=0.999999999653137

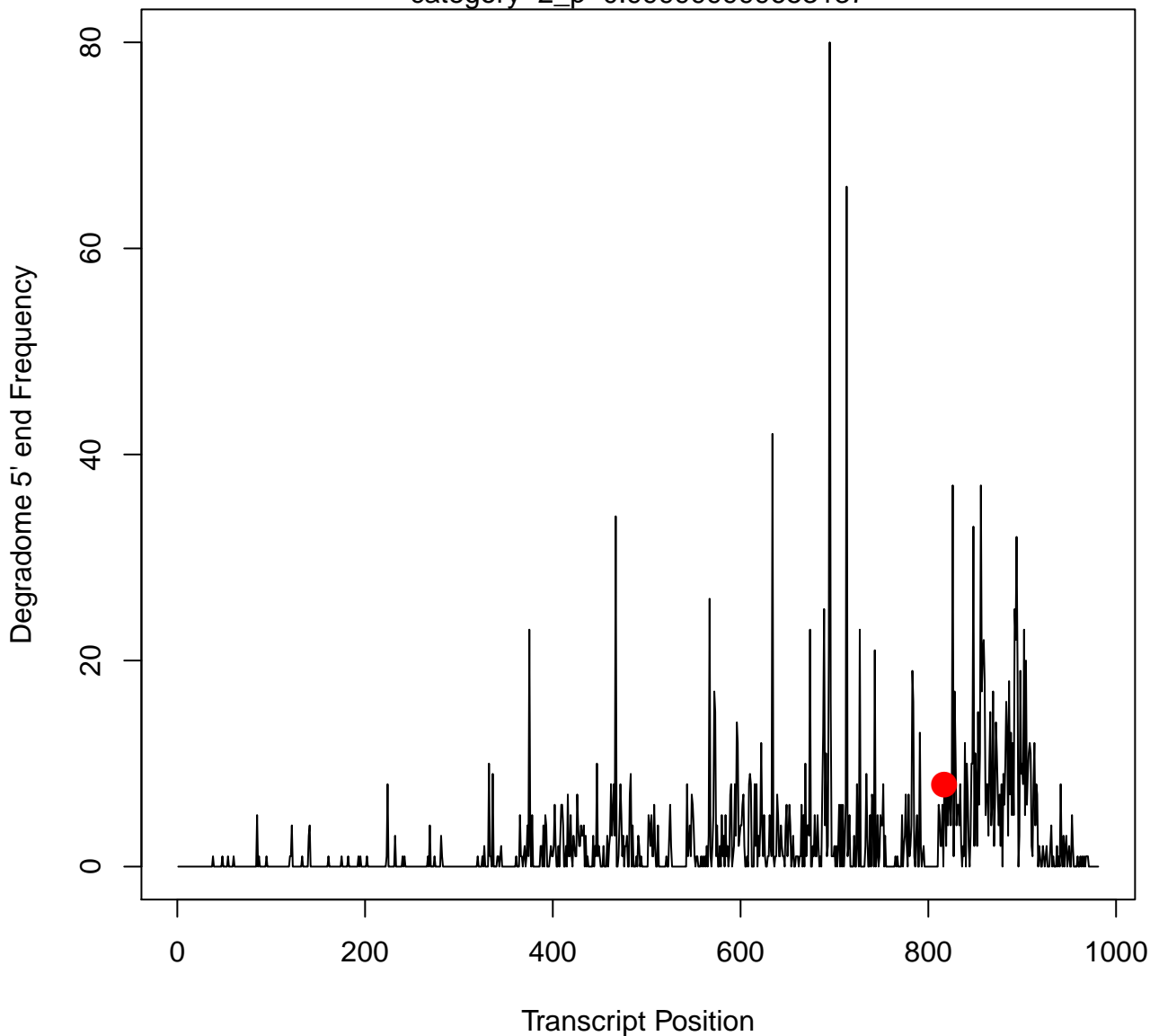

Supplement: Supplementary file 1 [file Data_Sheet_1.zip › Sit-miR160c_Seita.2G137400.1_817_TPlot.pdf]

**T=Seita.2G444000.1\_Q=Sit-miR160c\_S=581**

category=2\_p=0.999999991015112

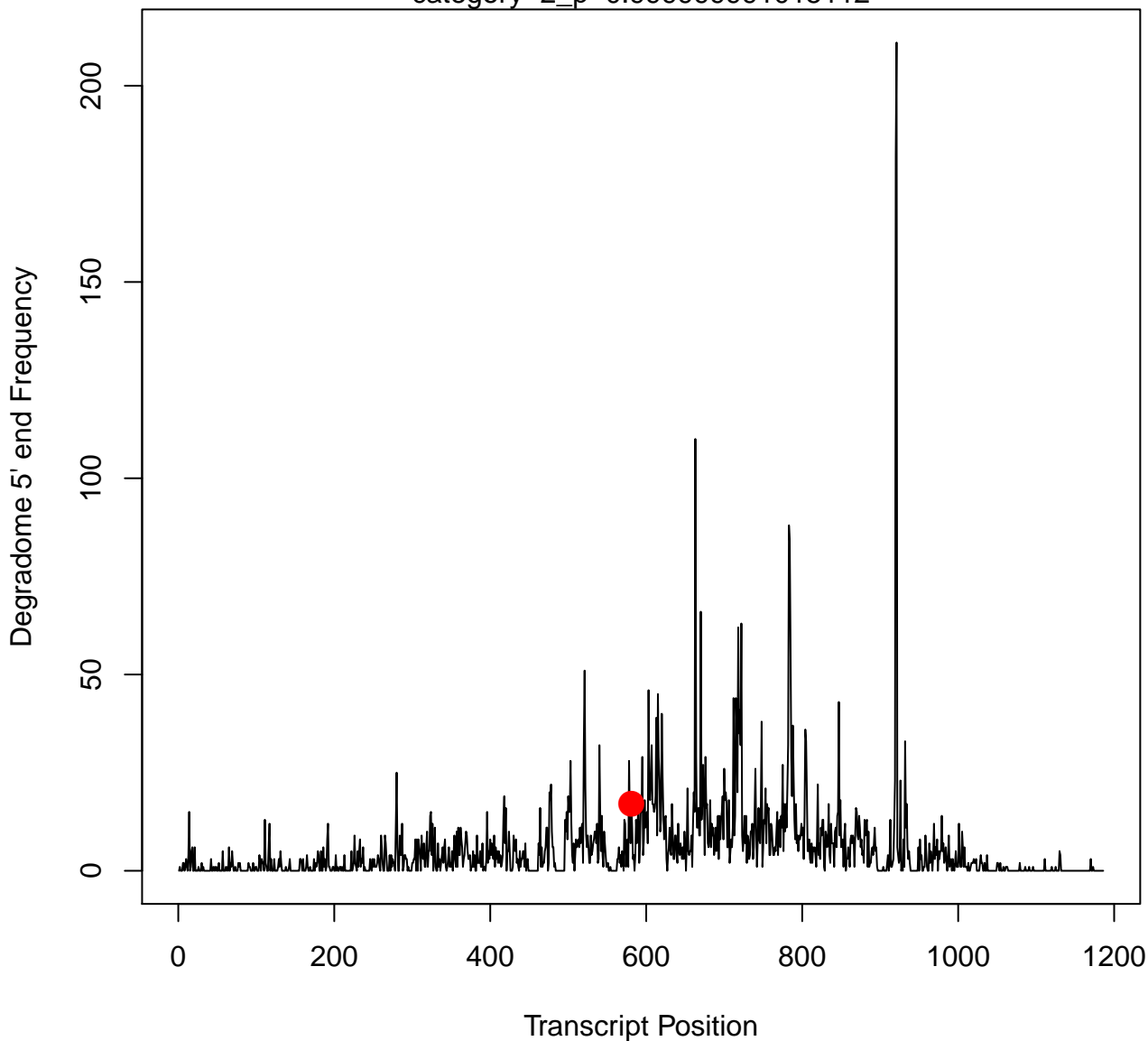

Supplement: Supplementary file 1 [file Data_Sheet_1.zip › Sit-miR160c_Seita.2G444000.1_581_TPlot.pdf]

**T=Seita.3G003300.1\_Q=Sit-miR160c\_S=1363**

category=0\_p=0.000437760109239571

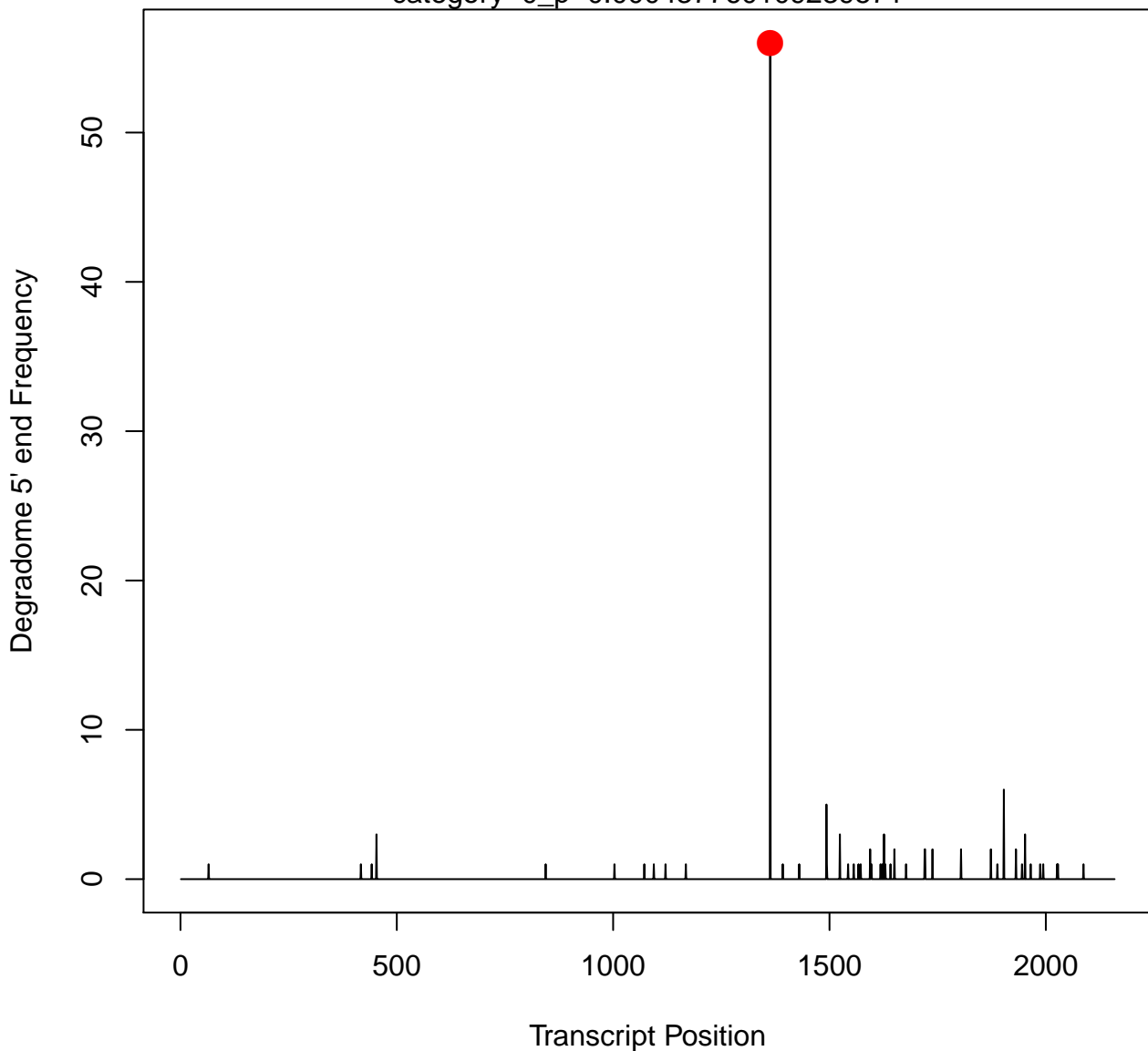

Supplement: Supplementary file 1 [file Data_Sheet_1.zip › Sit-miR160c_Seita.3G003300.1_1363_TPlot.pdf]

**T=Seita.3G014300.1\_Q=Sit-miR160c\_S=744**

category=2\_p=0.999999884731267

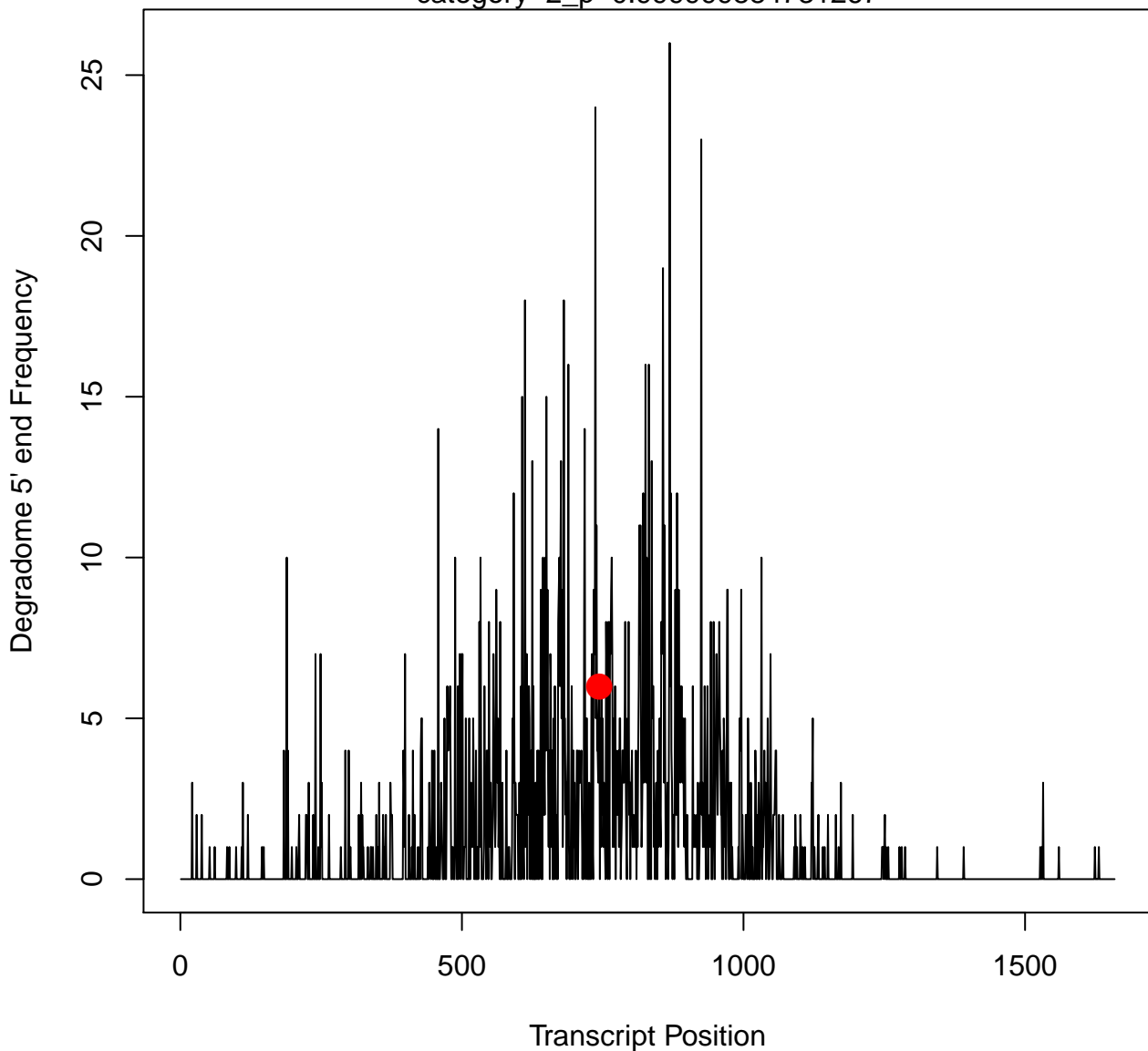

Supplement: Supplementary file 1 [file Data_Sheet_1.zip › Sit-miR160c_Seita.3G014300.1_744_TPlot.pdf]

**T=Seita.4G216400.1\_Q=Sit-miR160c\_S=1036**

category=2\_p=0.999999999903159

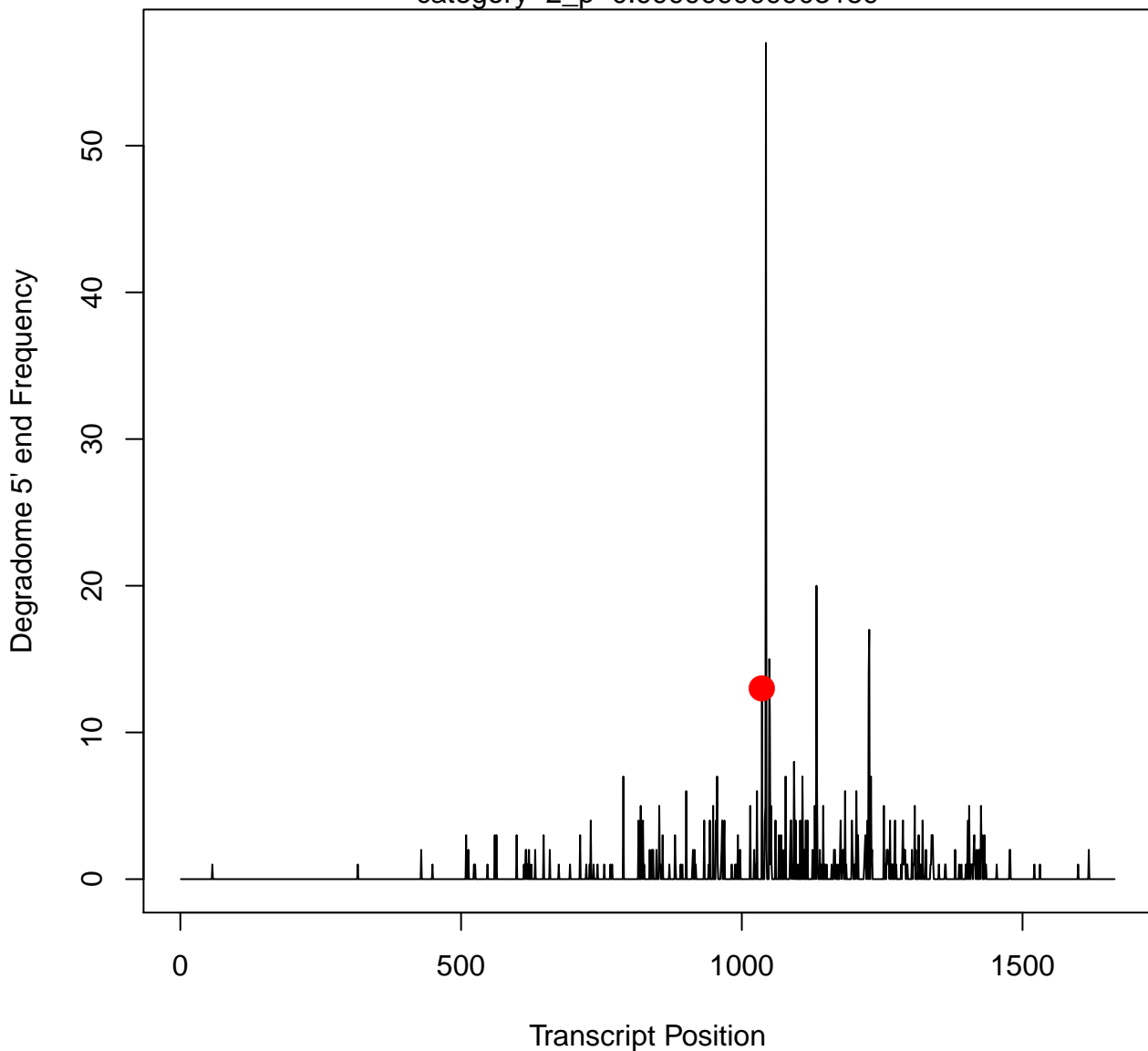

Supplement: Supplementary file 1 [file Data_Sheet_1.zip › Sit-miR160c_Seita.4G216400.1_1036_TPlot.pdf]

**T=Seita.5G158700.1\_Q=Sit-miR160c\_S=2845**

category=2\_p=0.999712537021004

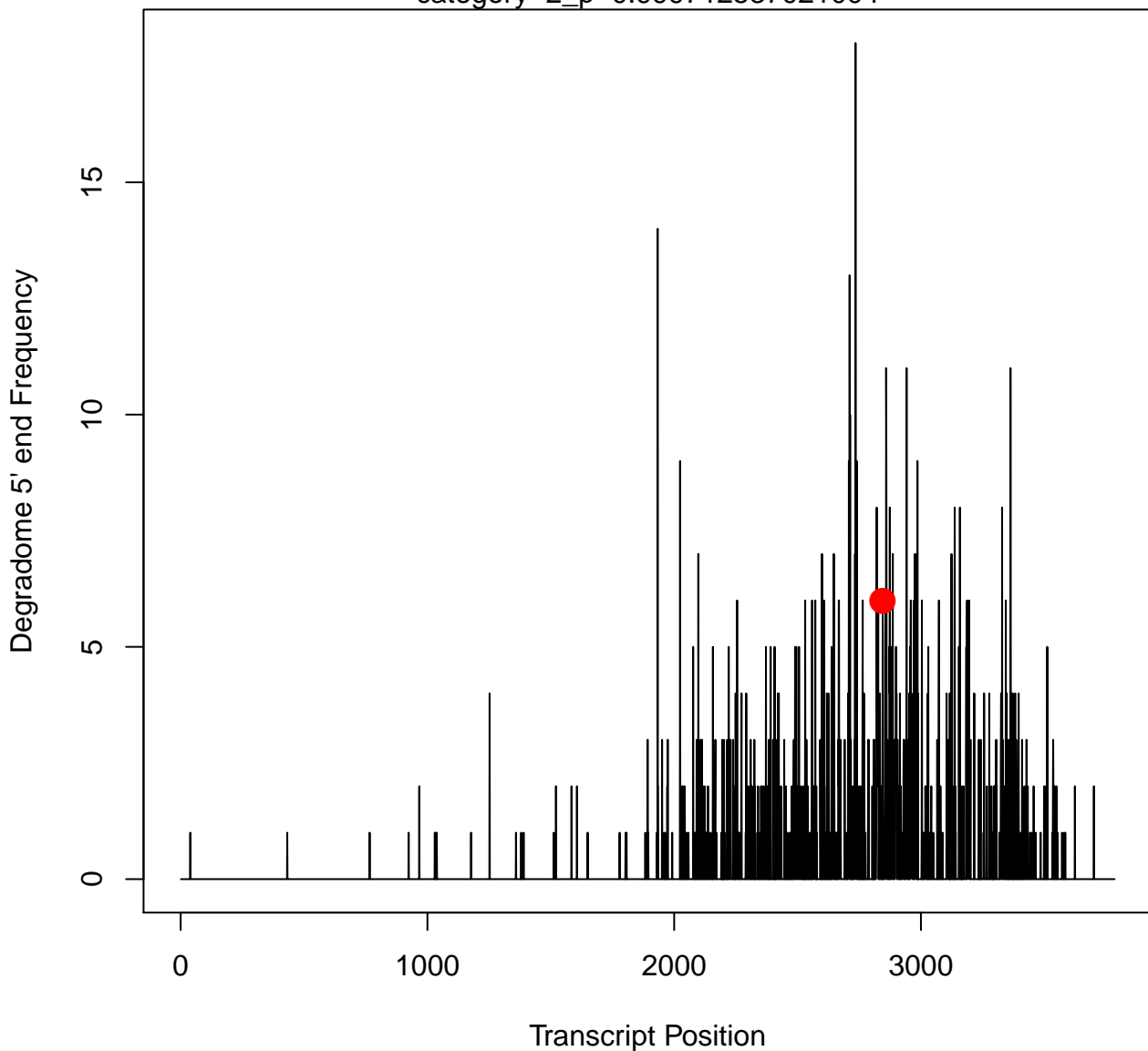

Supplement: Supplementary file 1 [file Data_Sheet_1.zip › Sit-miR160c_Seita.5G158700.1_2845_TPlot.pdf]

**T=Seita.6G055900.1\_Q=Sit-miR160c\_S=1207**

category=2\_p=0.999999890951363

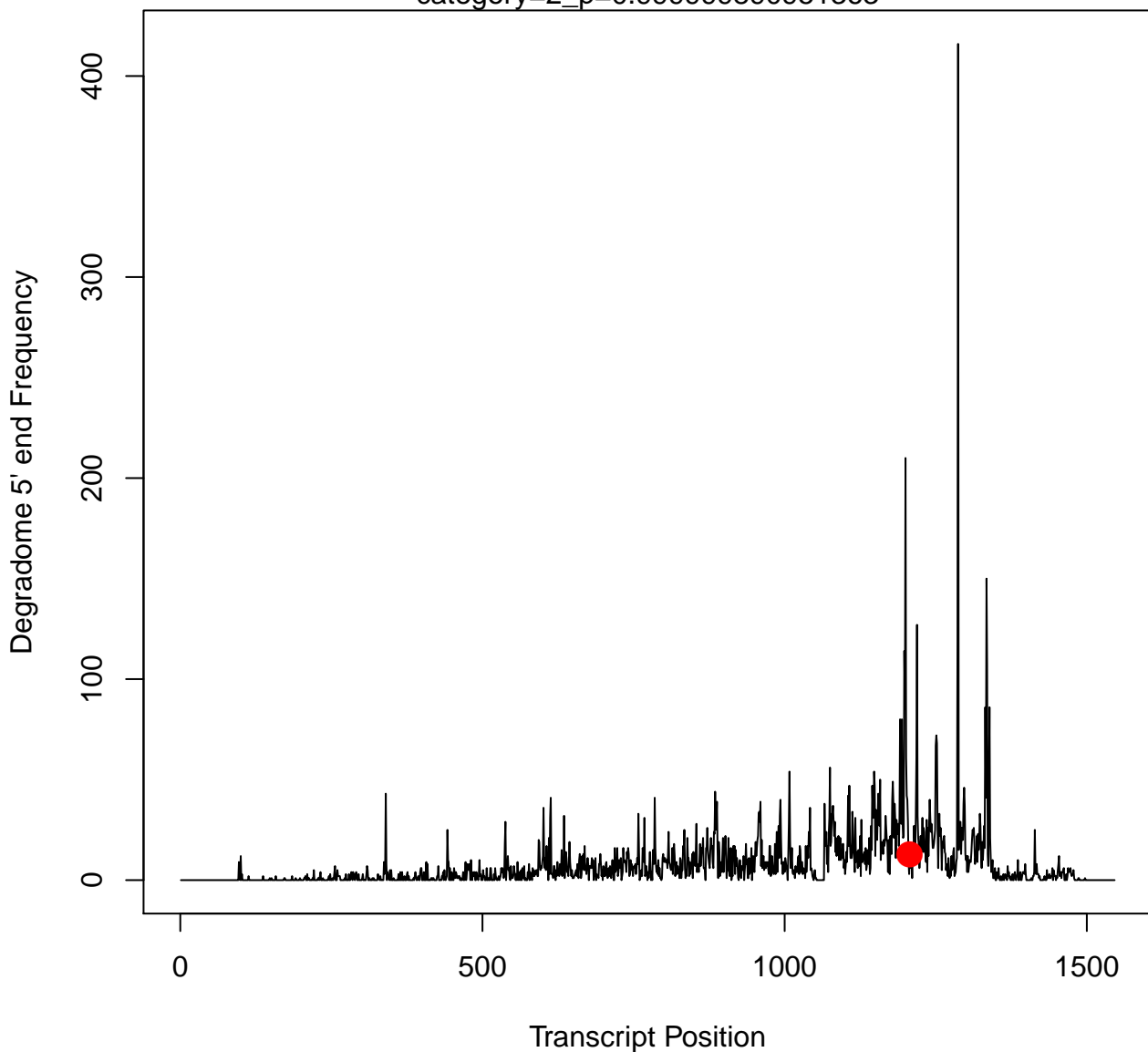

Supplement: Supplementary file 1 [file Data_Sheet_1.zip › Sit-miR160c_Seita.6G055900.1_1207_TPlot.pdf]

**T=Seita.7G155700.1\_Q=Sit-miR160c\_S=1461**

category=2\_p=0.99556304482646

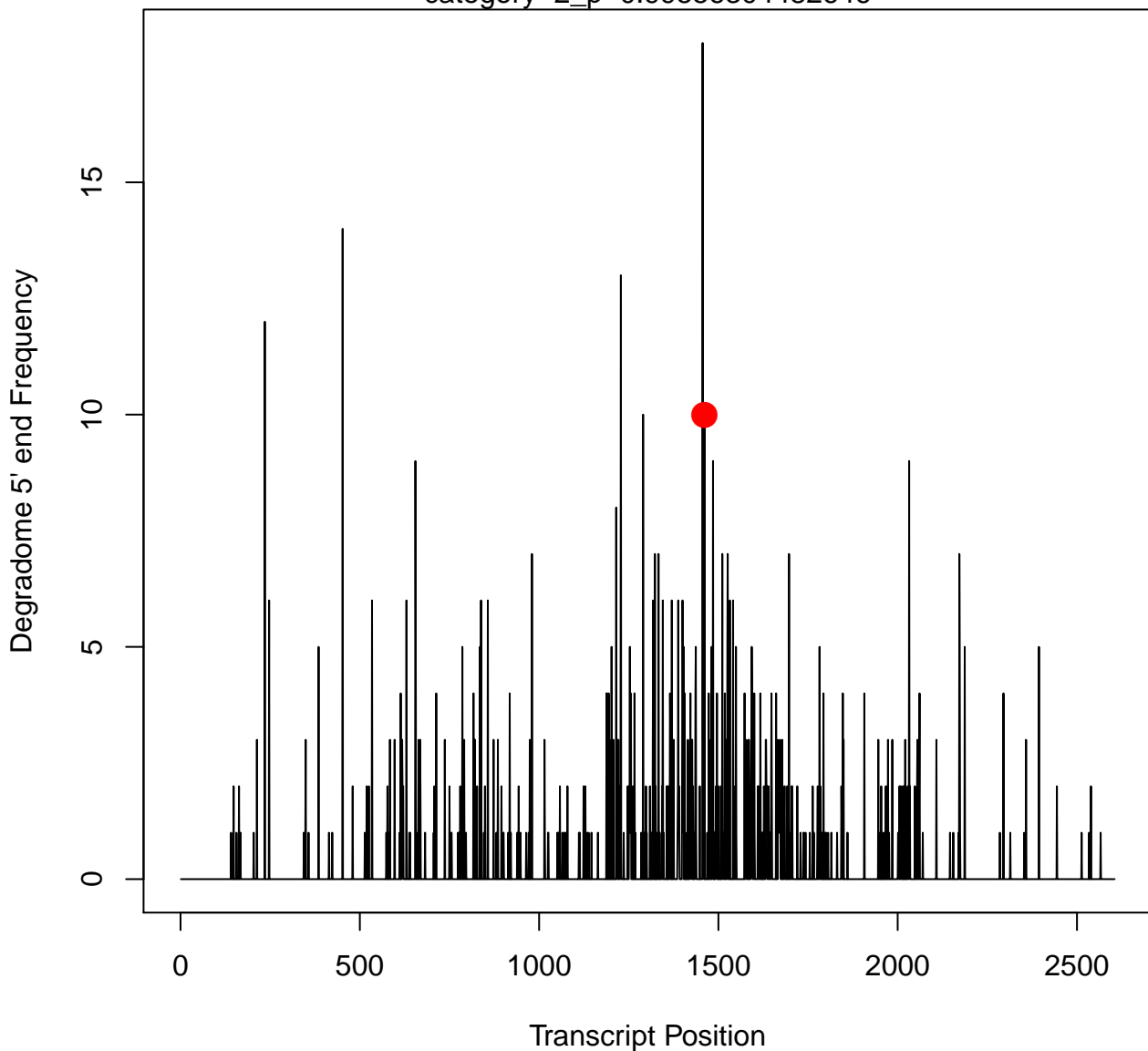

Supplement: Supplementary file 1 [file Data_Sheet_1.zip › Sit-miR160c_Seita.7G155700.1_1461_TPlot.pdf]

**T=Seita.7G174600.1\_Q=Sit-miR160c\_S=223**

category=2\_p=0.999988695345656

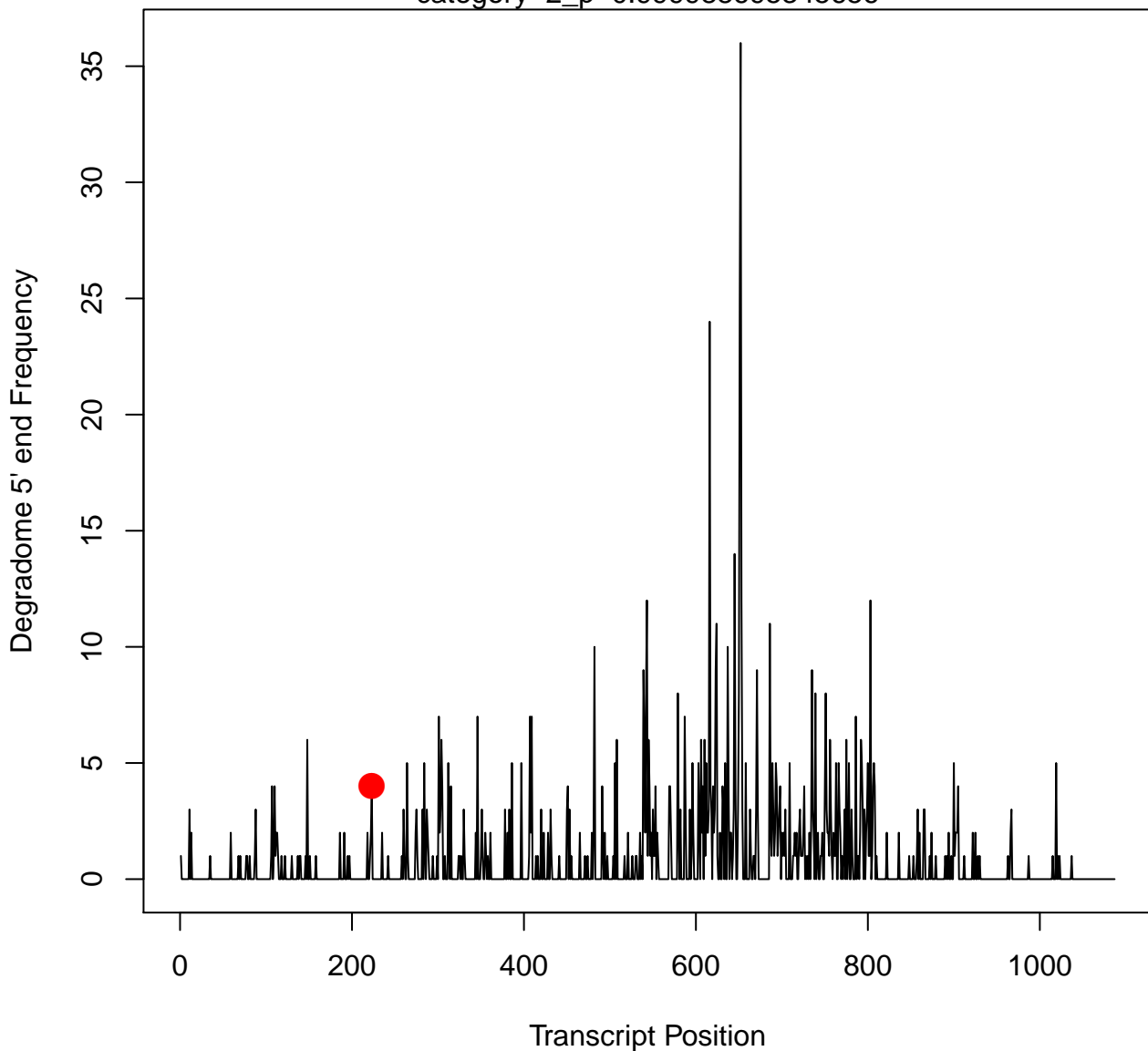

Supplement: Supplementary file 1 [file Data_Sheet_1.zip › Sit-miR160c_Seita.7G174600.1_223_TPlot.pdf]

**T=Seita.9G038800.1\_Q=Sit-miR160c\_S=969**

category=2\_p=0.999997277834982

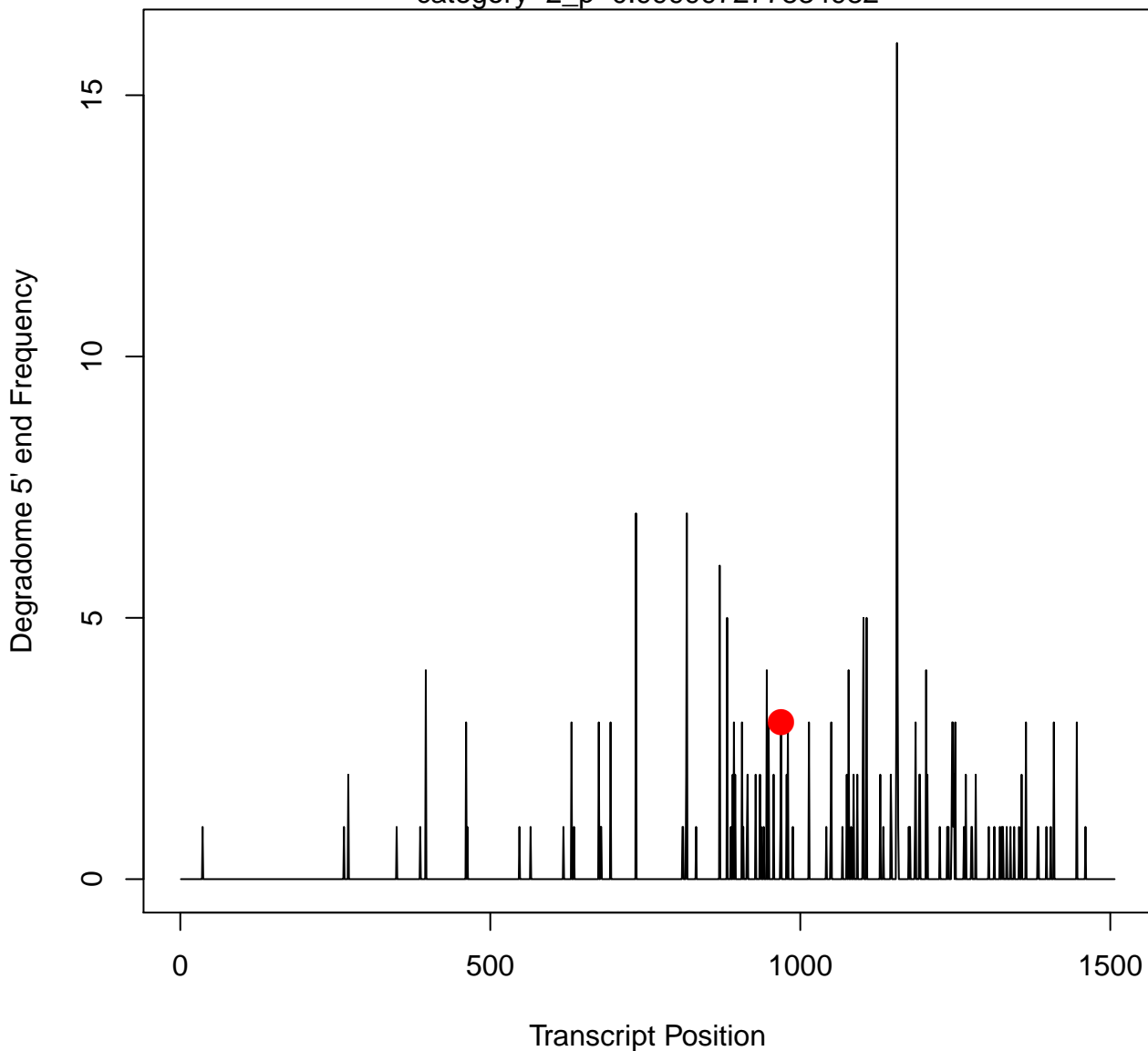

Supplement: Supplementary file 1 [file Data_Sheet_1.zip › Sit-miR160c_Seita.9G038800.1_969_TPlot.pdf]

**T=Seita.9G113800.1\_Q=Sit-miR160c\_S=1086**

category=2\_p=0.999460965673923

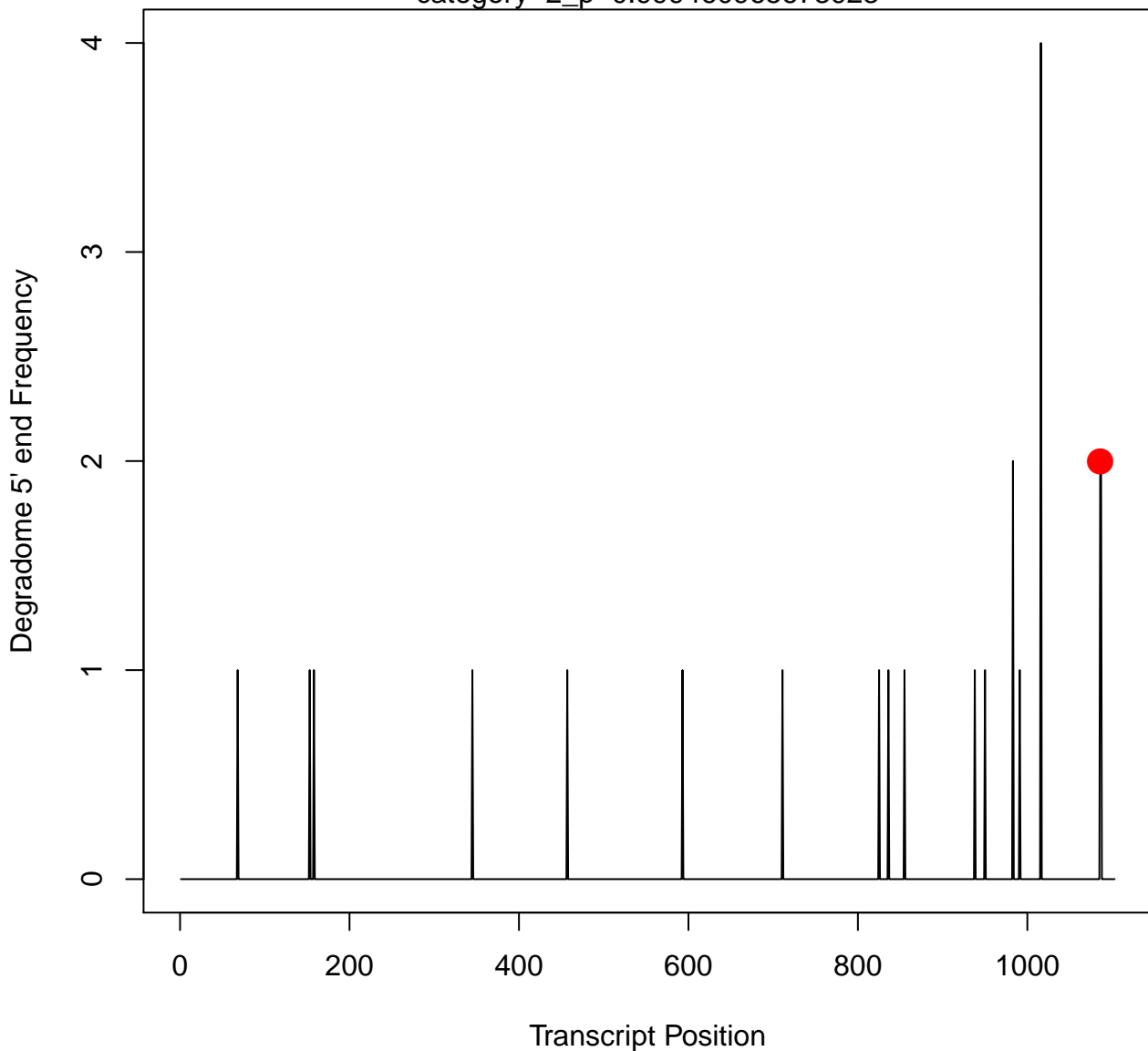

Supplement: Supplementary file 1 [file Data_Sheet_1.zip › Sit-miR160c_Seita.9G113800.1_1086_TPlot.pdf]

**T=Seita.9G182200.1\_Q=Sit-miR160c\_S=215**

category=2\_p=0.999999999633352

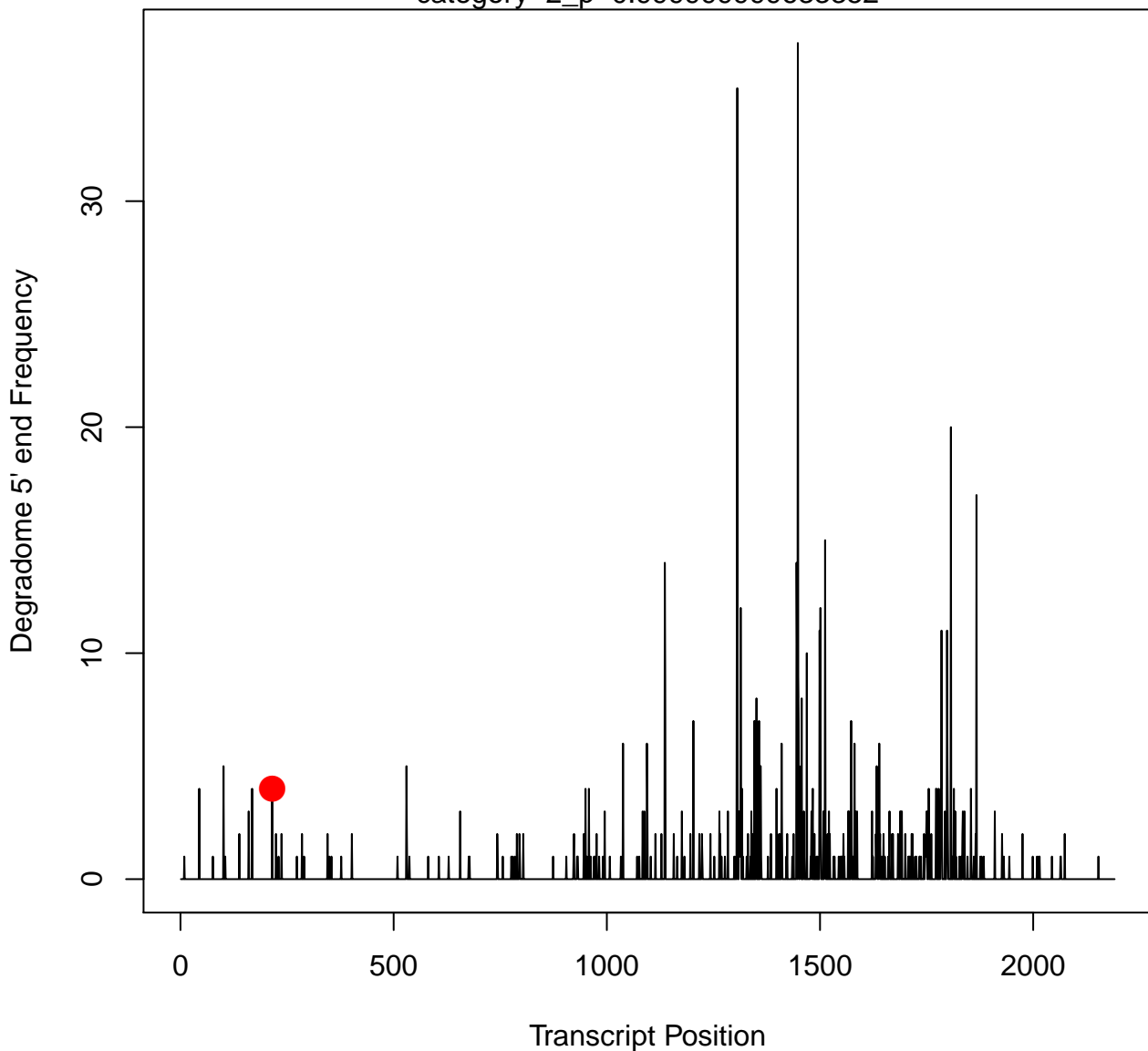

Supplement: Supplementary file 1 [file Data_Sheet_1.zip › Sit-miR160c_Seita.9G182200.1_215_TPlot.pdf]

**T=Seita.9G204900.1\_Q=Sit-miR160c\_S=1104**

category=2\_p=0.999728049011065

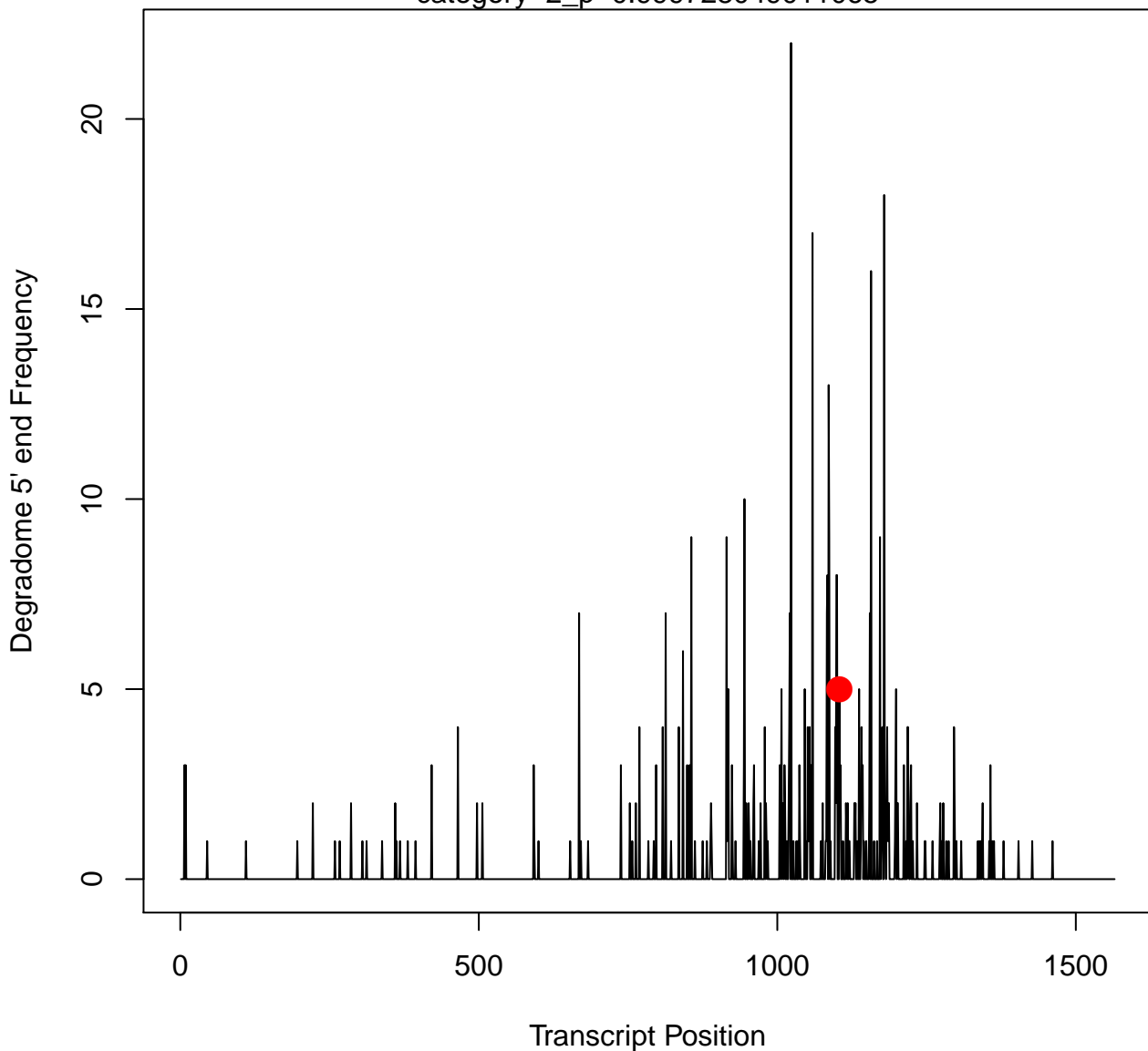

Supplement: Supplementary file 1 [file Data_Sheet_1.zip › Sit-miR160c_Seita.9G204900.1_1104_TPlot.pdf]

**T=Seita.9G236900.1\_Q=Sit-miR160c\_S=1291**

category=2\_p=0.984965354623466

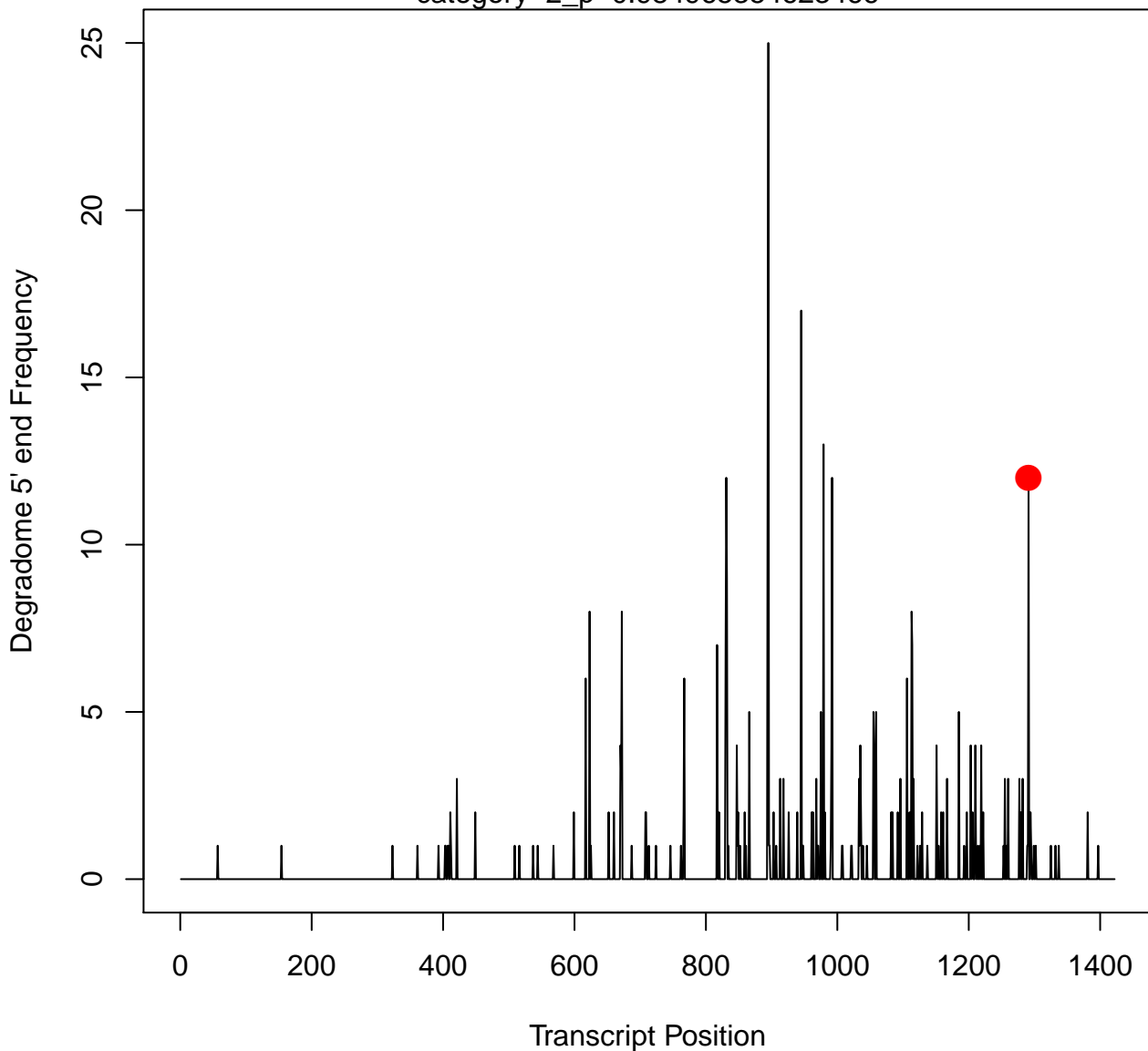

Supplement: Supplementary file 1 [file Data_Sheet_1.zip › Sit-miR160c_Seita.9G236900.1_1291_TPlot.pdf]

**T=Seita.9G259100.1\_Q=Sit-miR160c\_S=1247**

category=2\_p=0.999999704019383

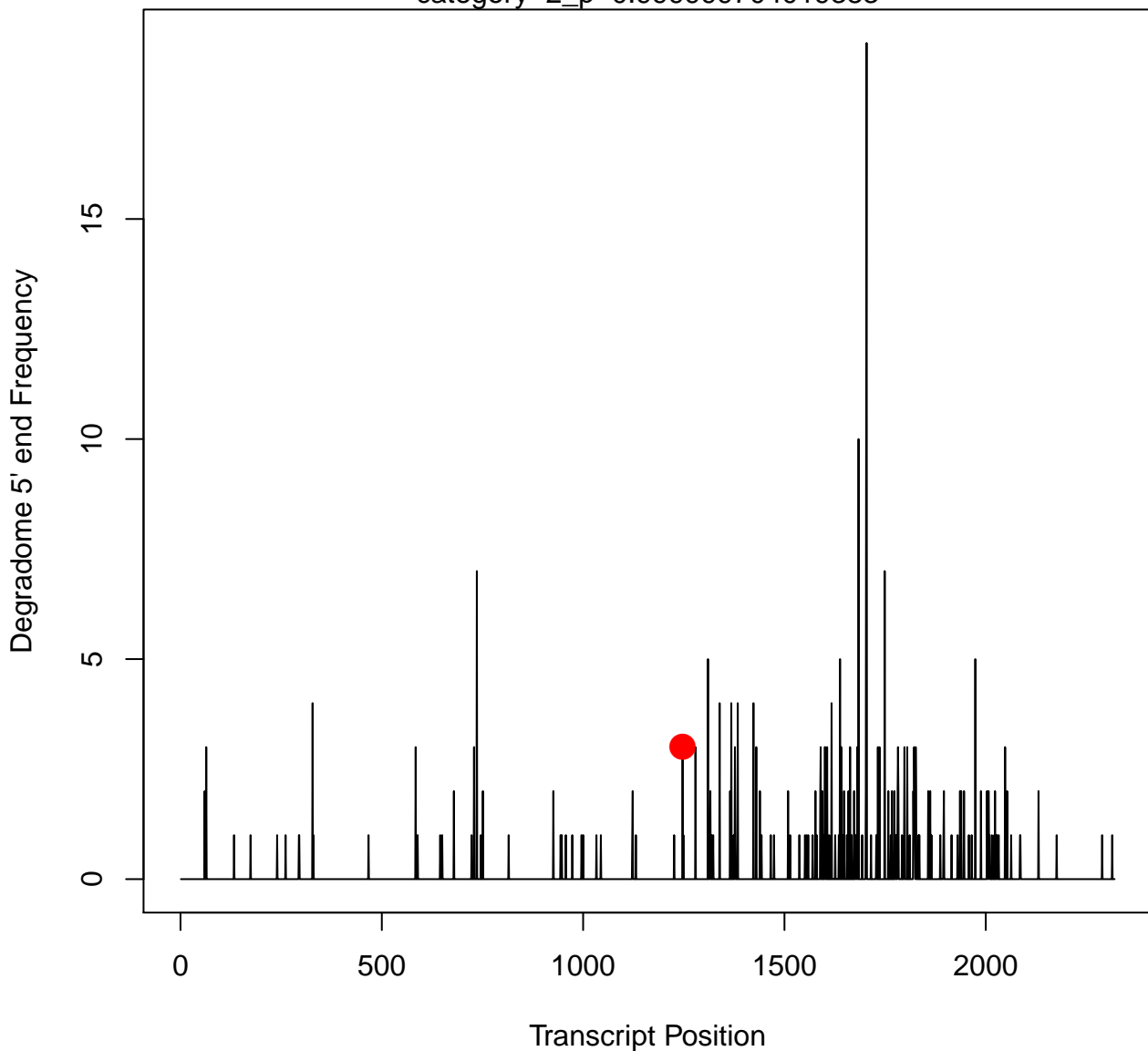

Supplement: Supplementary file 1 [file Data_Sheet_1.zip › Sit-miR160c_Seita.9G259100.1_1247_TPlot.pdf]

**T=Seita.9G409200.1\_Q=Sit-miR160c\_S=875**

category=2\_p=0.99999999926616

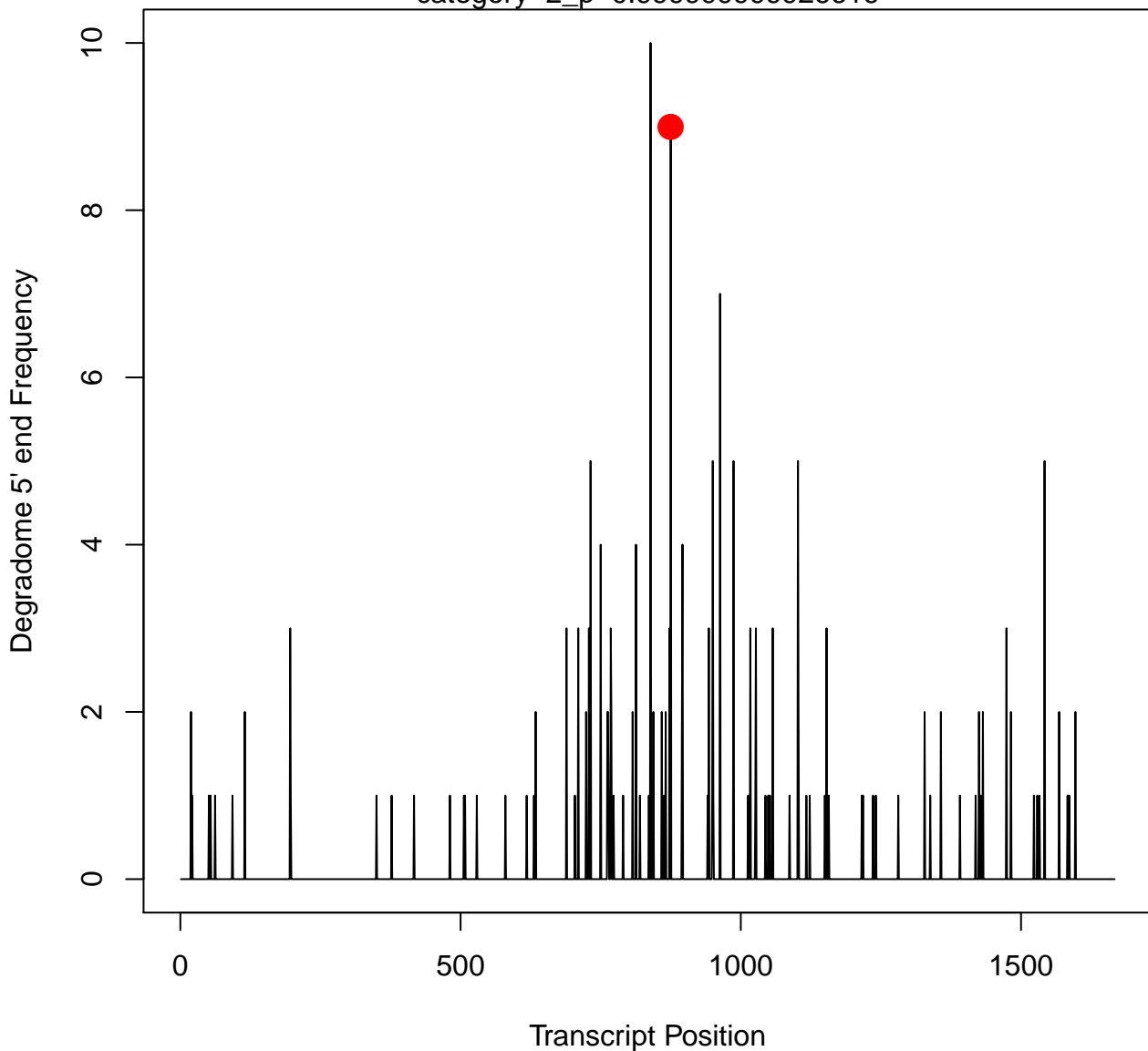

Supplement: Supplementary file 1 [file Data_Sheet_1.zip › Sit-miR160c_Seita.9G409200.1_875_TPlot.pdf]

**T=Seita.9G427100.1\_Q=Sit-miR160c\_S=386**

category=2\_p=0.999999991808555

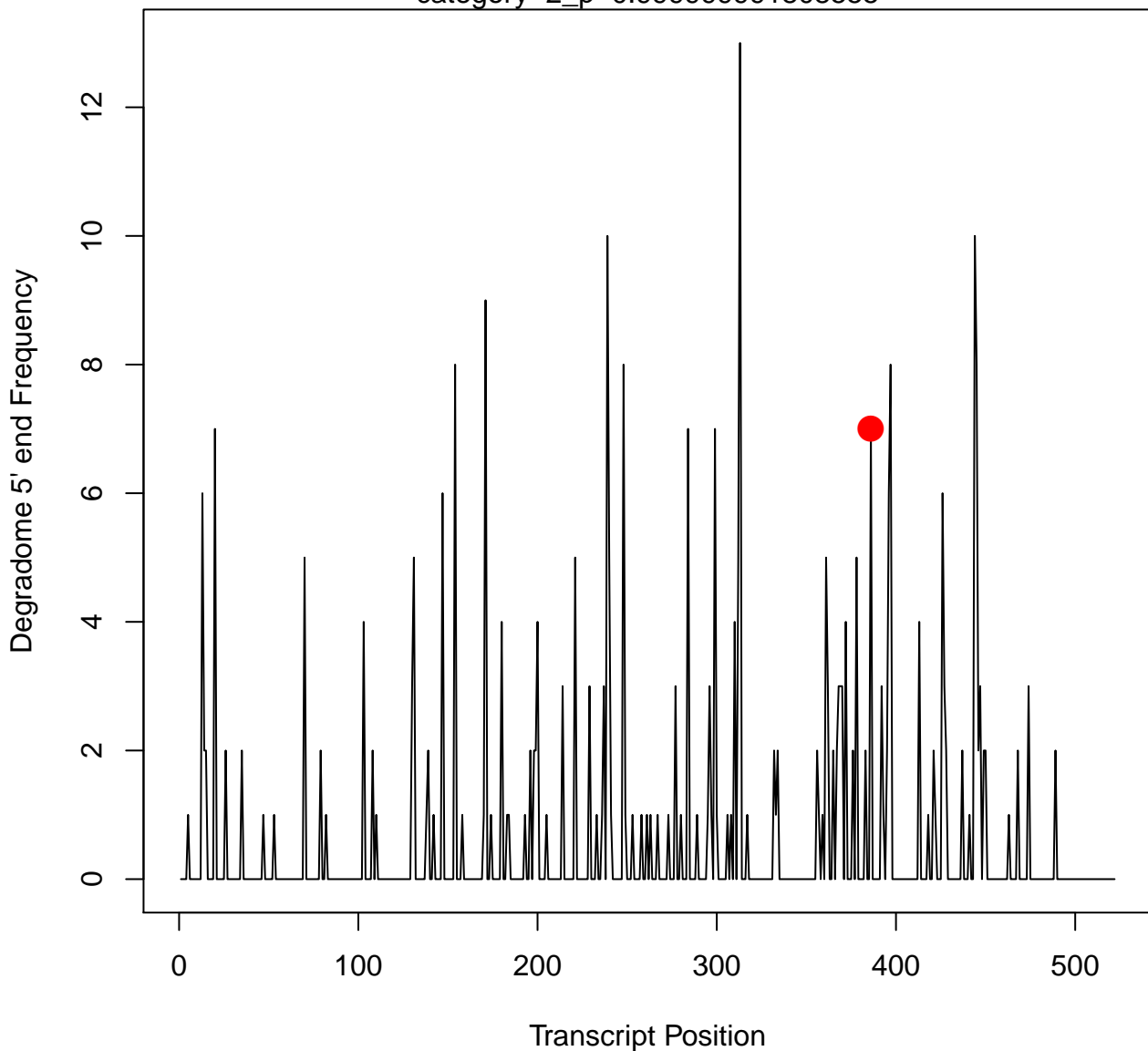

Supplement: Supplementary file 1 [file Data_Sheet_1.zip › Sit-miR160c_Seita.9G427100.1_386_TPlot.pdf]

**T=Seita.J025900.1\_Q=Sit-miR160c\_S=1332**

category=2\_p=0.999924073878556

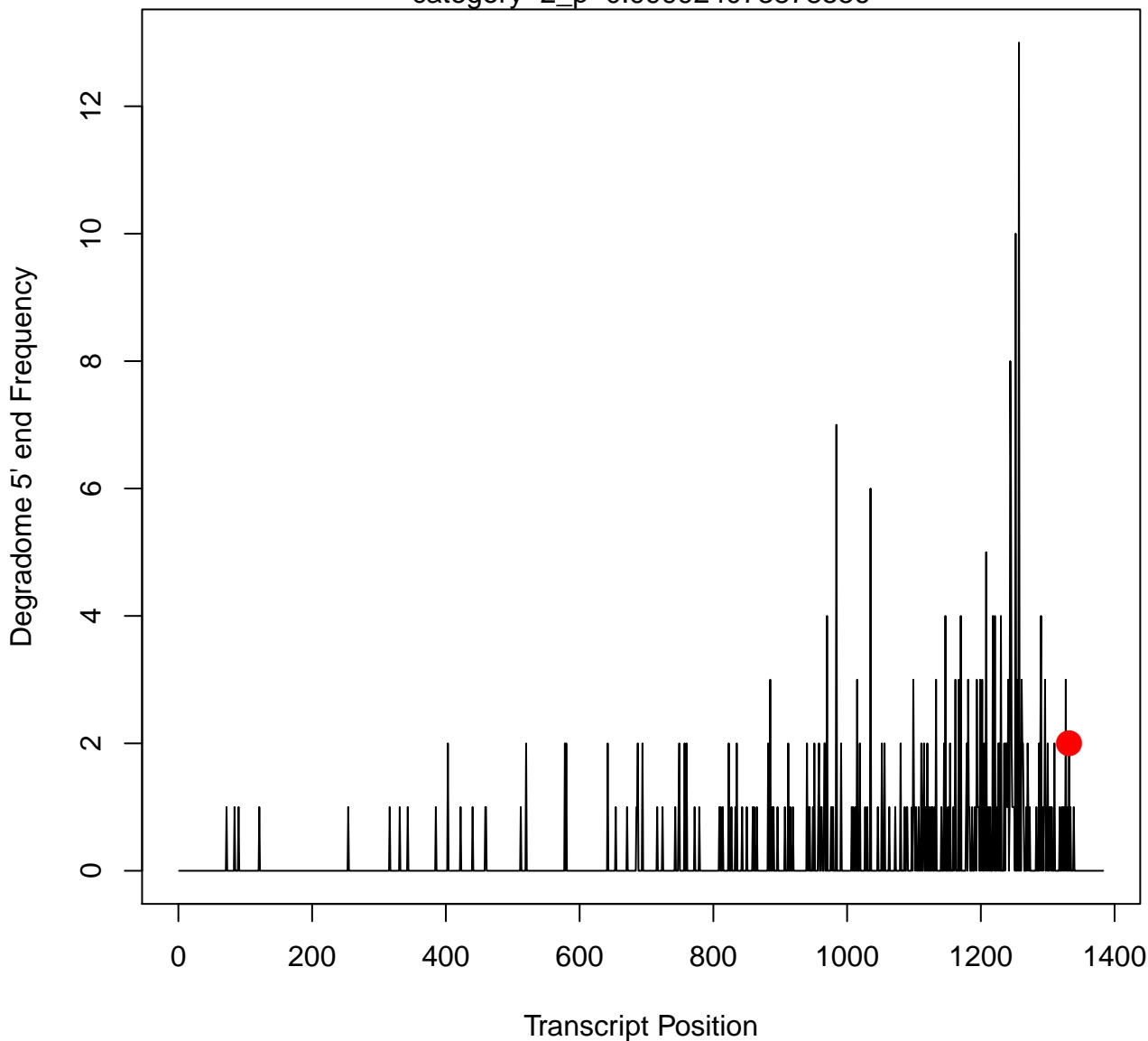

Supplement: Supplementary file 1 [file Data_Sheet_1.zip › Sit-miR160c_Seita.J025900.1_1332_TPlot.pdf]

**T=Seita.1G241500.1\_Q=Sit-miR160d\_S=1660**

category=2\_p=0.088308580092527

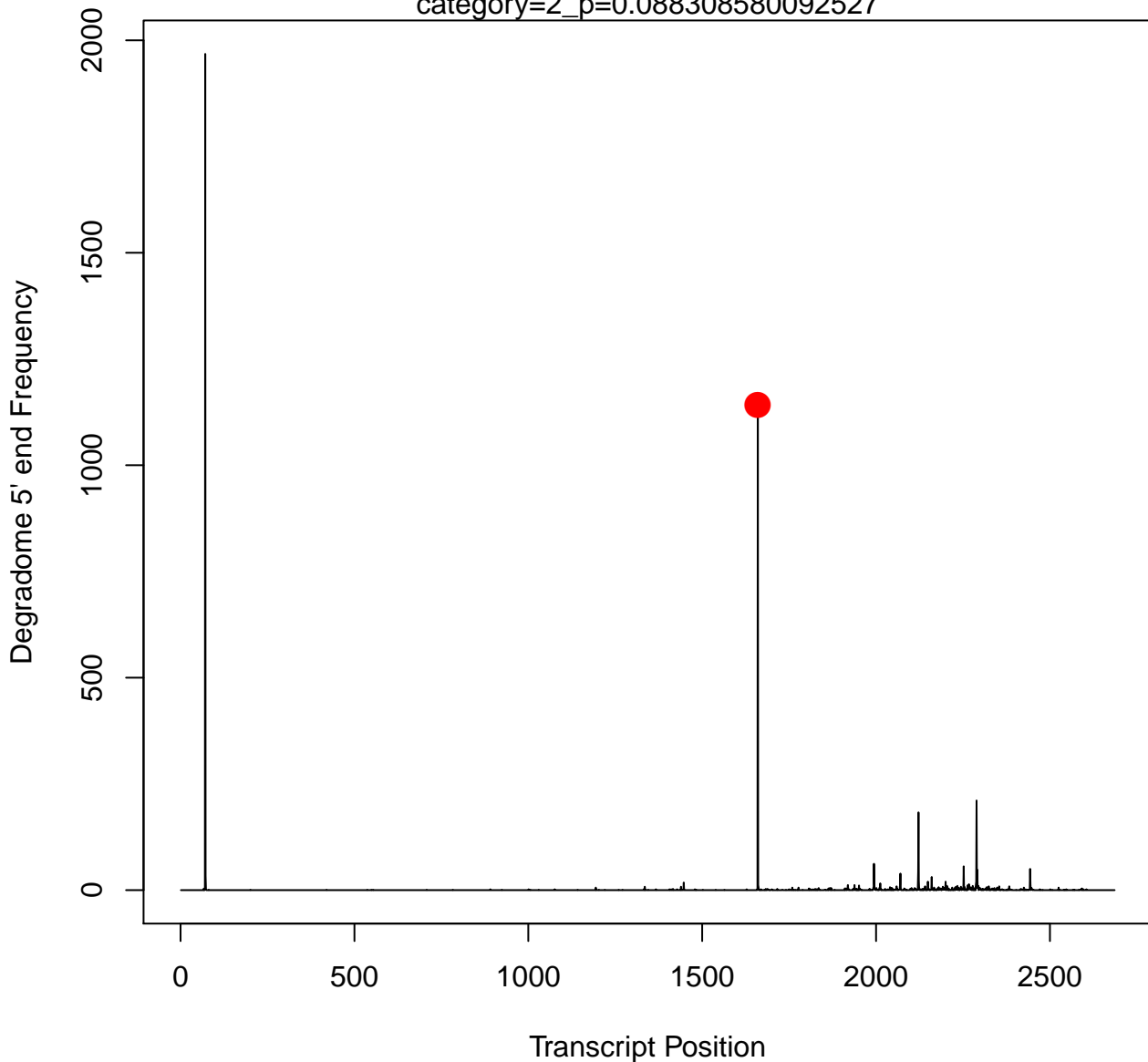

Supplement: Supplementary file 1 [file Data_Sheet_1.zip › Sit-miR160d_Seita.1G241500.1_1660_TPlot.pdf]

**T=Seita.1G320200.1\_Q=Sit-miR160d\_S=569**

category=2\_p=1

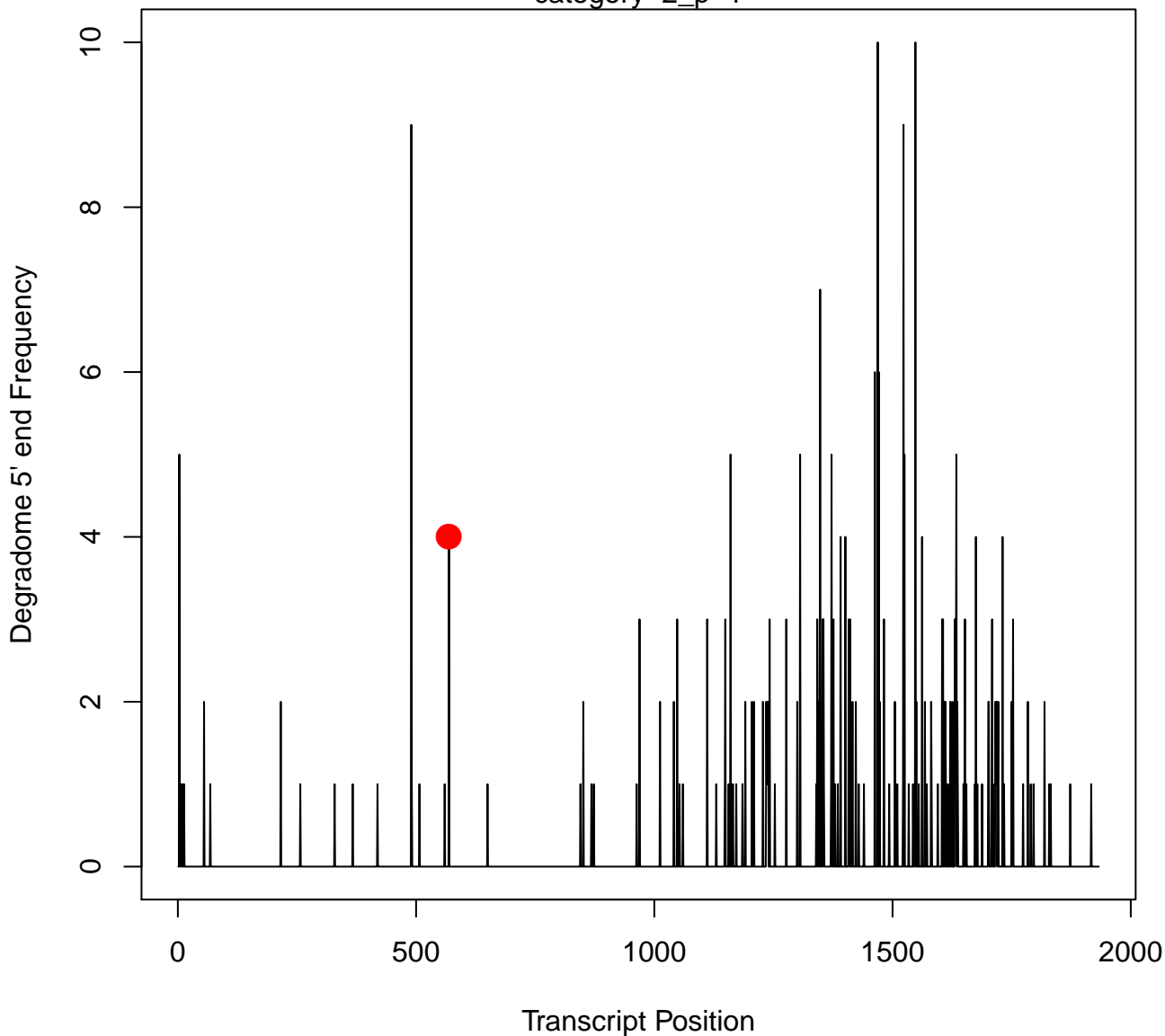

Supplement: Supplementary file 1 [file Data_Sheet_1.zip › Sit-miR160d_Seita.1G320200.1_569_TPlot.pdf]

**T=Seita.2G412300.1\_Q=Sit-miR160d\_S=1580**

category=2\_p=1

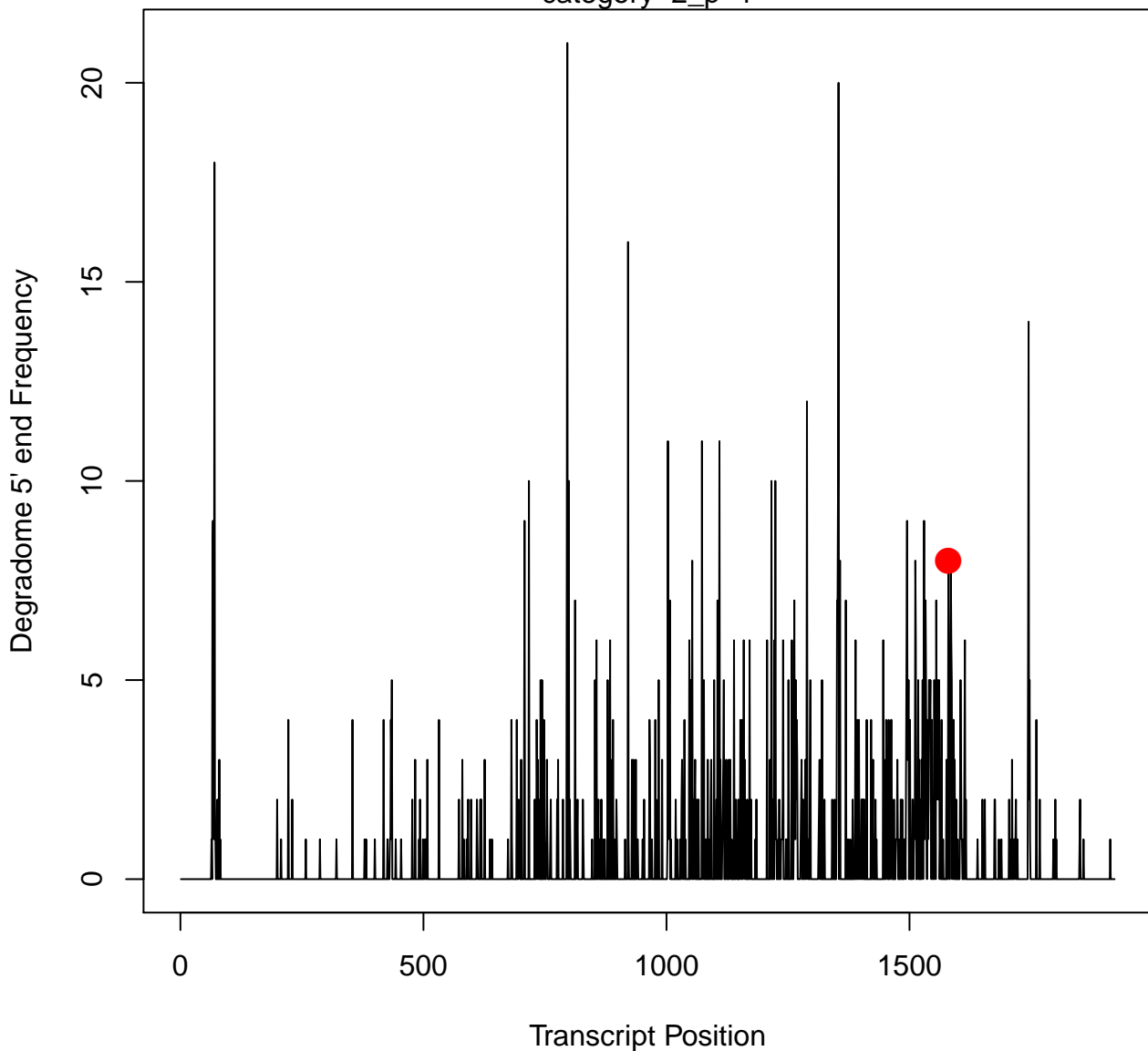

Supplement: Supplementary file 1 [file Data_Sheet_1.zip › Sit-miR160d_Seita.2G412300.1_1580_TPlot.pdf]

**T=Seita.5G273800.1\_Q=Sit-miR160d\_S=477**

category=2\_p=0.999999999417884

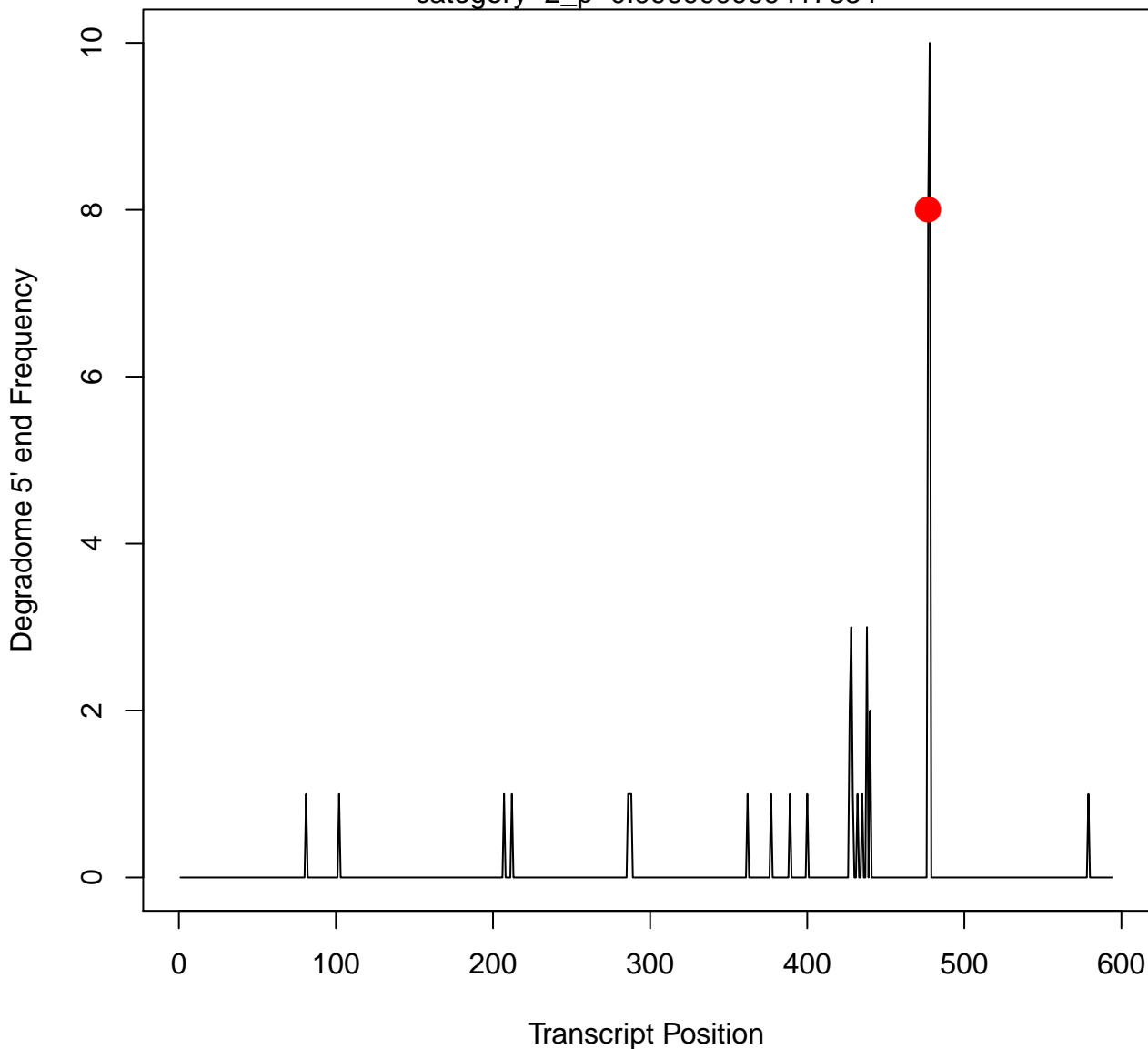

Supplement: Supplementary file 1 [file Data_Sheet_1.zip › Sit-miR160d_Seita.5G273800.1_477_TPlot.pdf]

**T=Seita.6G024600.1\_Q=Sit-miR160d\_S=310**

category=2\_p=0.168818754867096

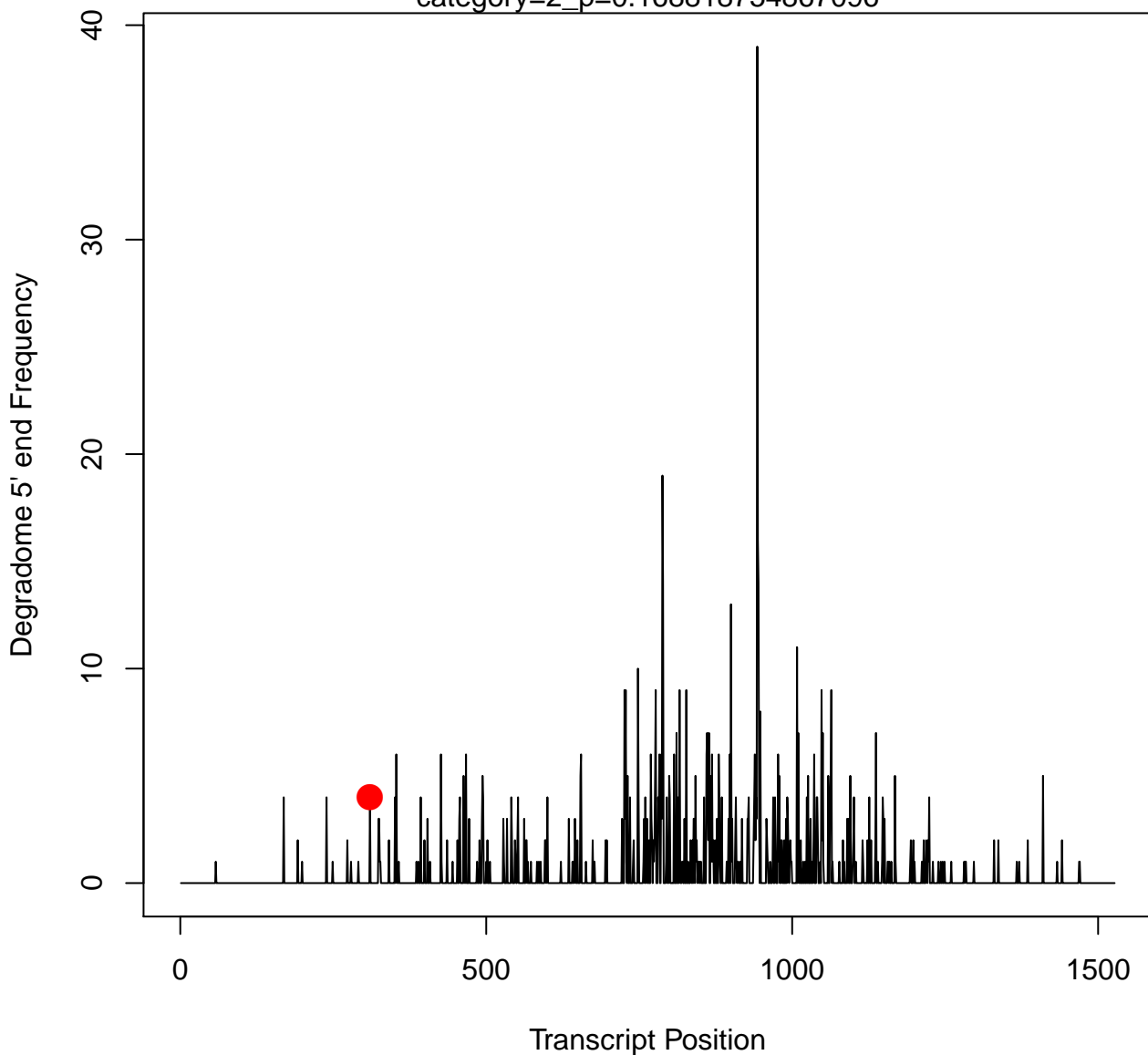

Supplement: Supplementary file 1 [file Data_Sheet_1.zip › Sit-miR160d_Seita.6G024600.1_310_TPlot.pdf]

**T=Seita.7G072600.1\_Q=Sit-miR160d\_S=258**

category=2\_p=0.999999999995665

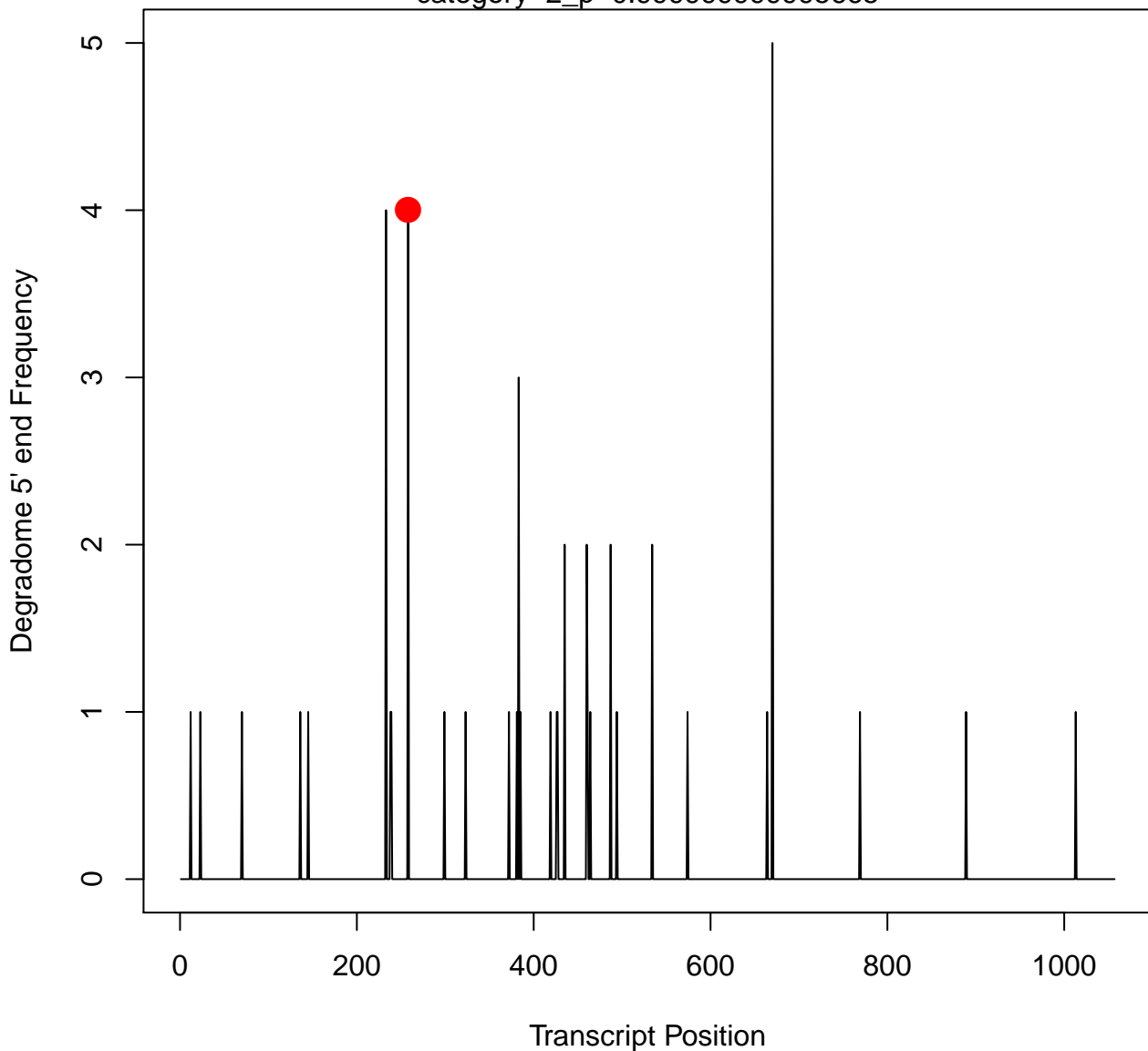

Supplement: Supplementary file 1 [file Data_Sheet_1.zip › Sit-miR160d_Seita.7G072600.1_258_TPlot.pdf]

**T=Seita.7G253500.1\_Q=Sit-miR160d\_S=53**

category=2\_p=0.999499395537929

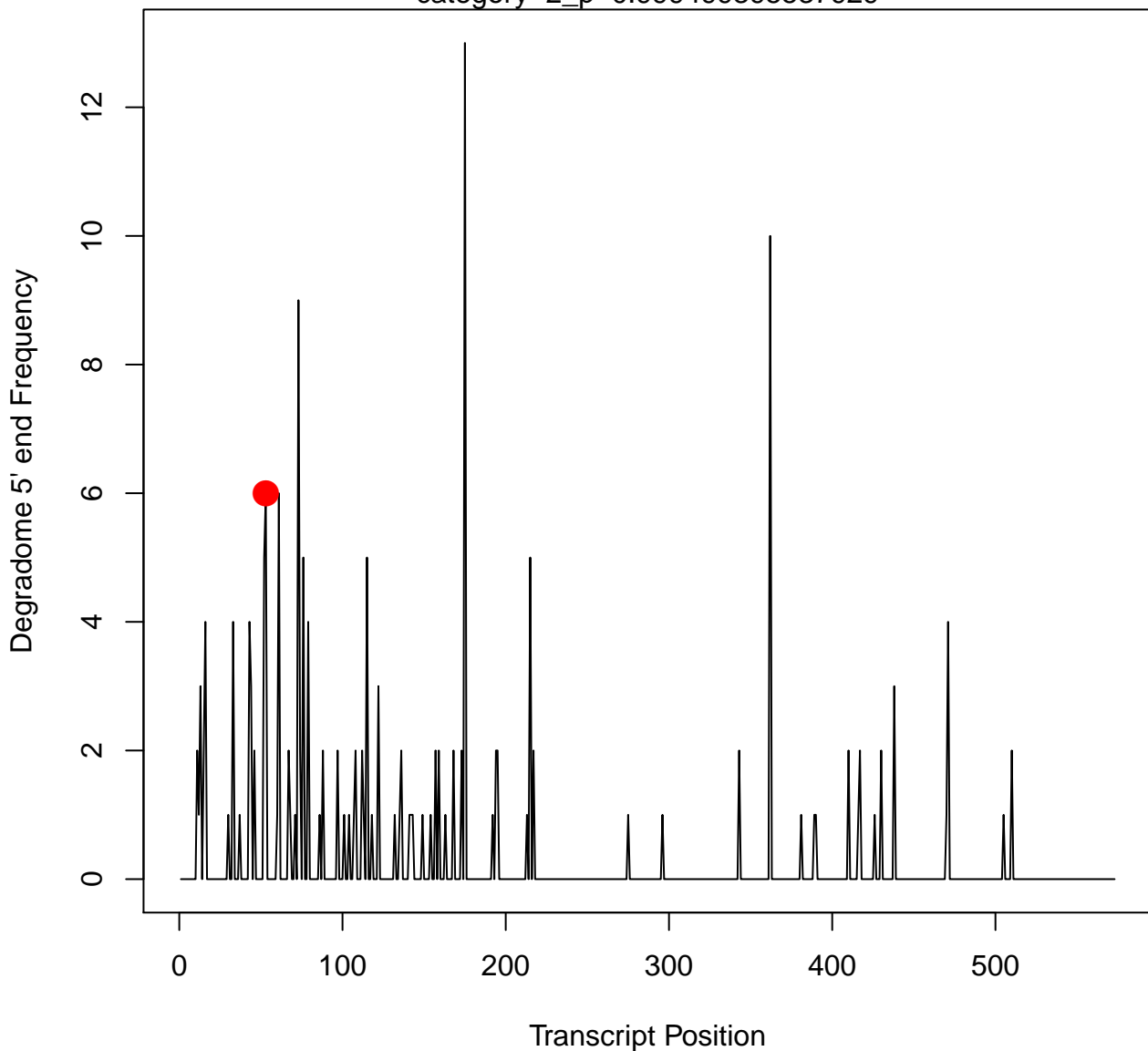

Supplement: Supplementary file 1 [file Data_Sheet_1.zip › Sit-miR160d_Seita.7G253500.1_53_TPlot.pdf]

**T=Seita.9G060800.1\_Q=Sit-miR160d\_S=2103**

category=2\_p=0.999999999999083

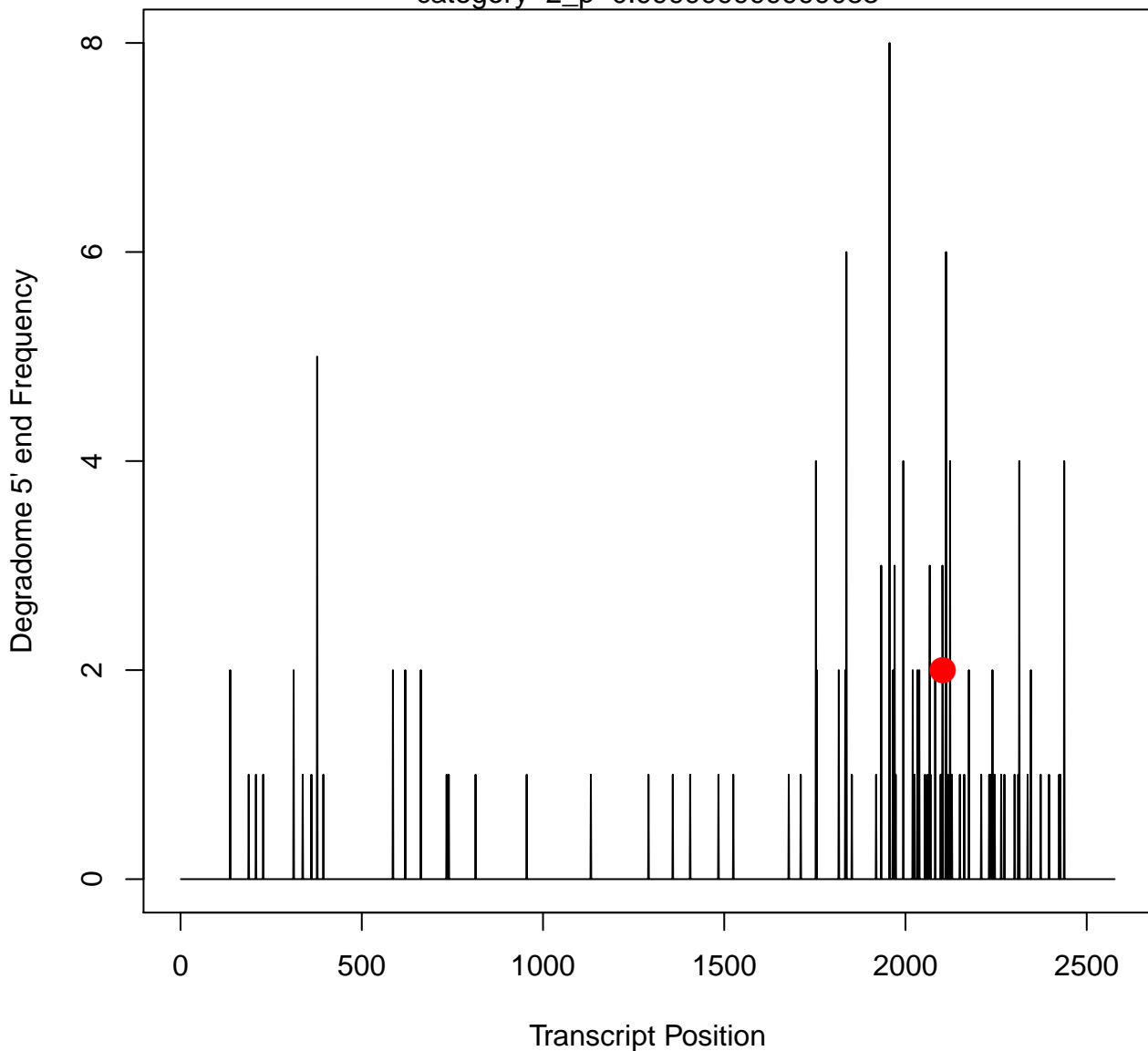

Supplement: Supplementary file 1 [file Data_Sheet_1.zip › Sit-miR160d_Seita.9G060800.1_2103_TPlot.pdf]

**T=Seita.9G356100.1\_Q=Sit-miR160d\_S=700**

category=2\_p=0.664103995728674

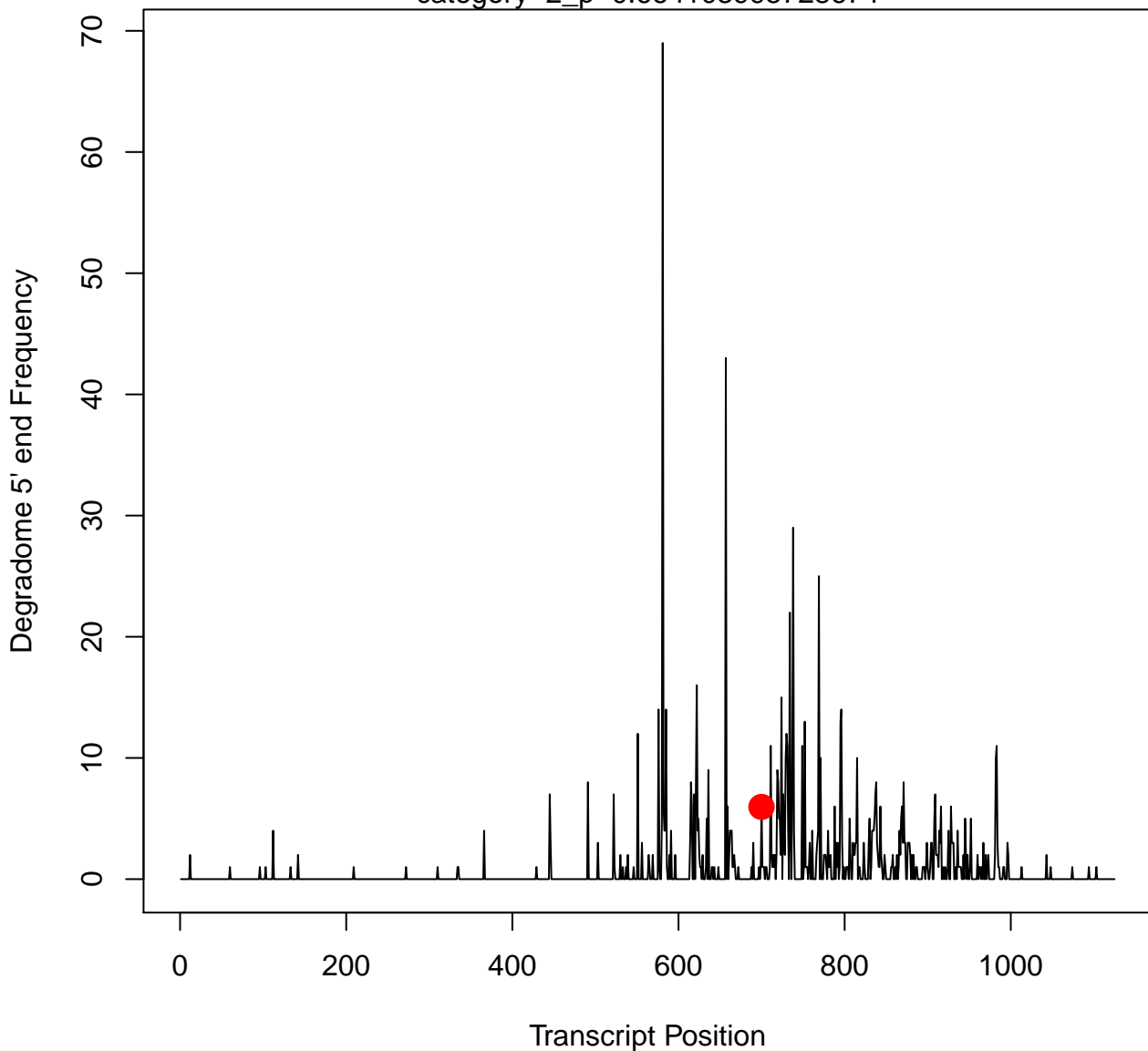

Supplement: Supplementary file 1 [file Data_Sheet_1.zip › Sit-miR160d_Seita.9G356100.1_700_TPlot.pdf]

**T=Seita.2G035900.1\_Q=Sit-miR162\_S=1041**

category=2\_p=0.848331113290476

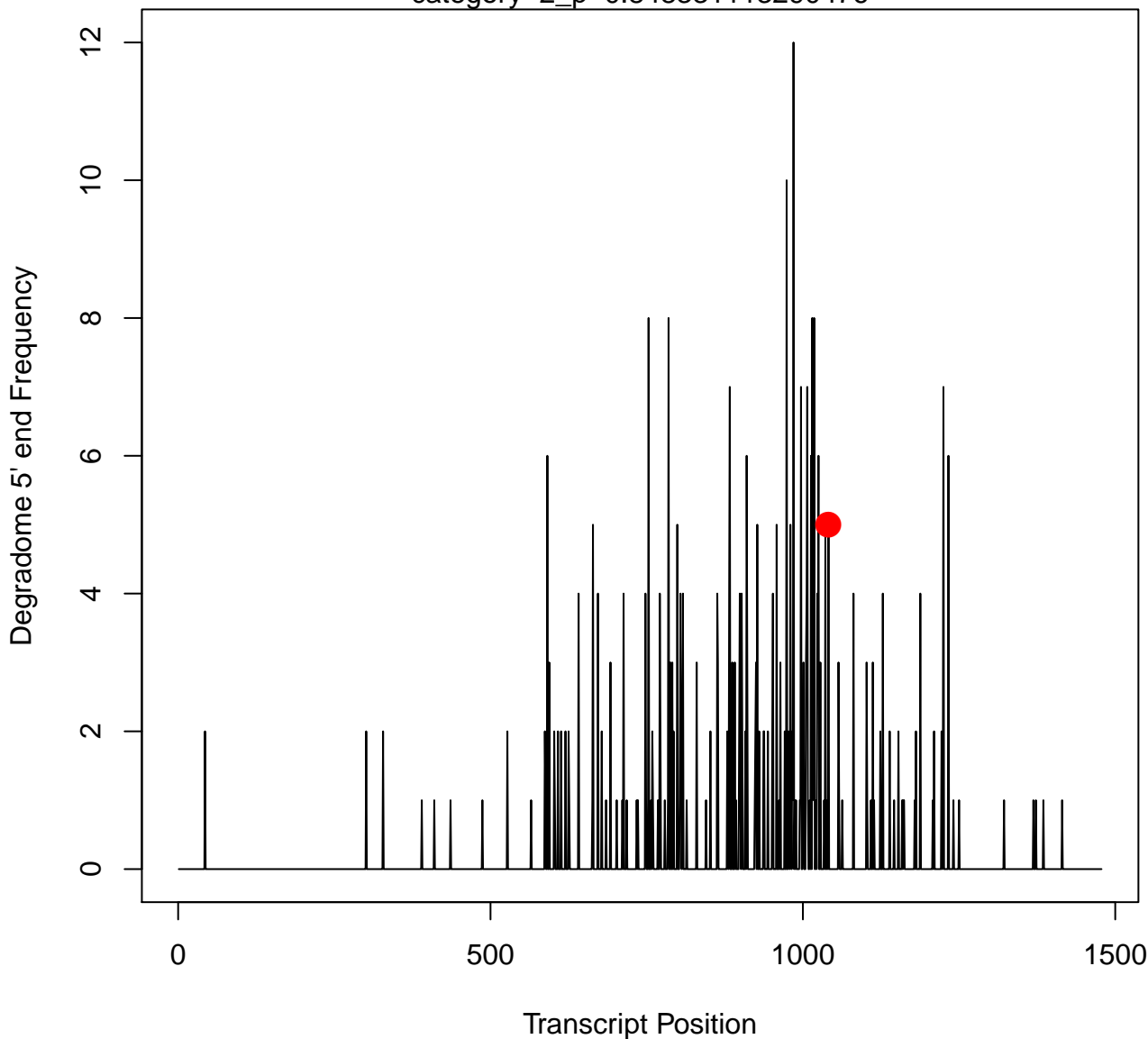

Supplement: Supplementary file 1 [file Data_Sheet_1.zip › Sit-miR162_Seita.2G035900.1_1041_TPlot.pdf]

**T=Seita.2G057700.1\_Q=Sit-miR162\_S=1013**

category=2\_p=0.817526096025816

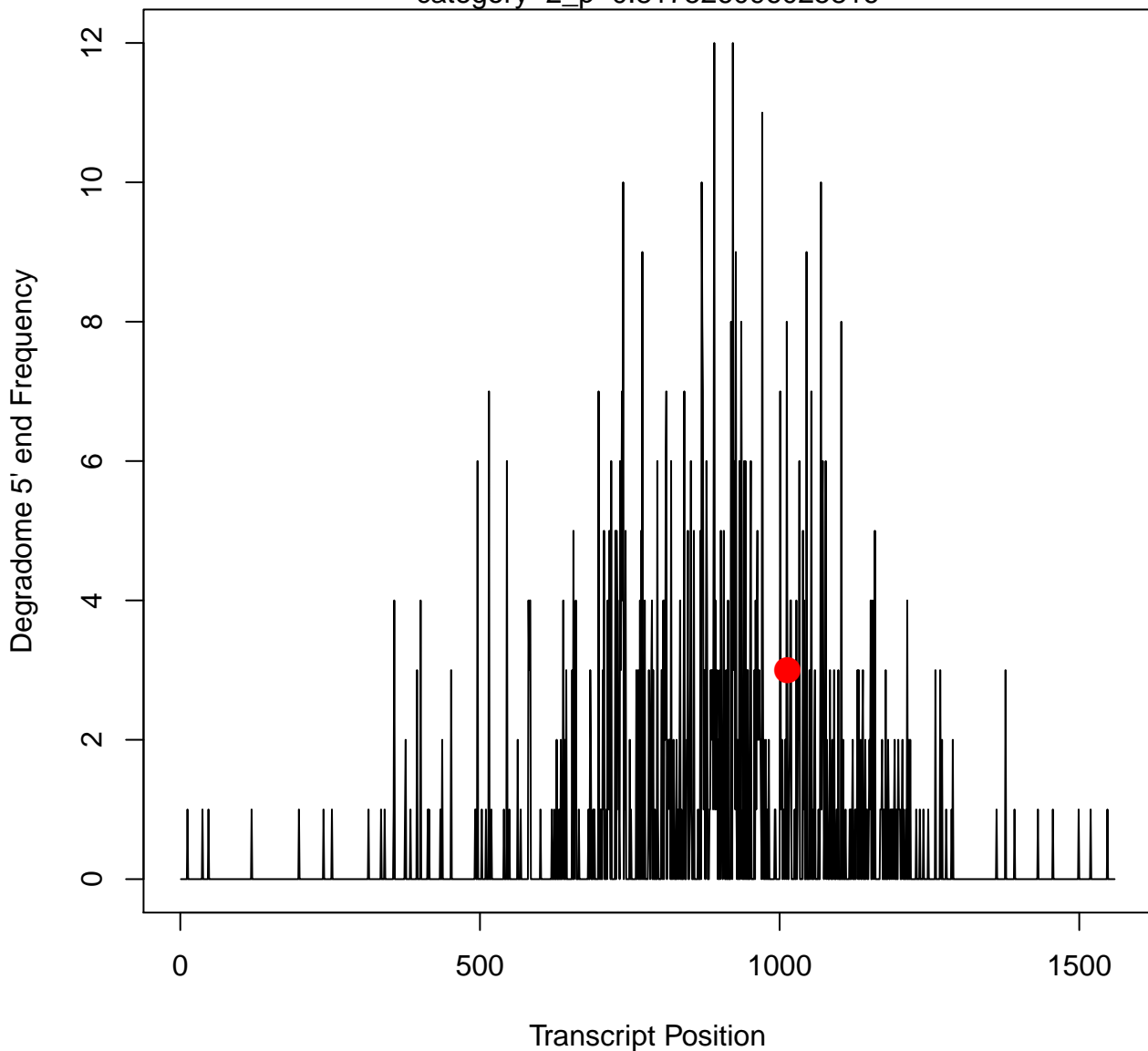

Supplement: Supplementary file 1 [file Data_Sheet_1.zip › Sit-miR162_Seita.2G057700.1_1013_TPlot.pdf]

**T=Seita.5G064400.1\_Q=Sit-miR162\_S=878**

category=2\_p=0.036306017792474

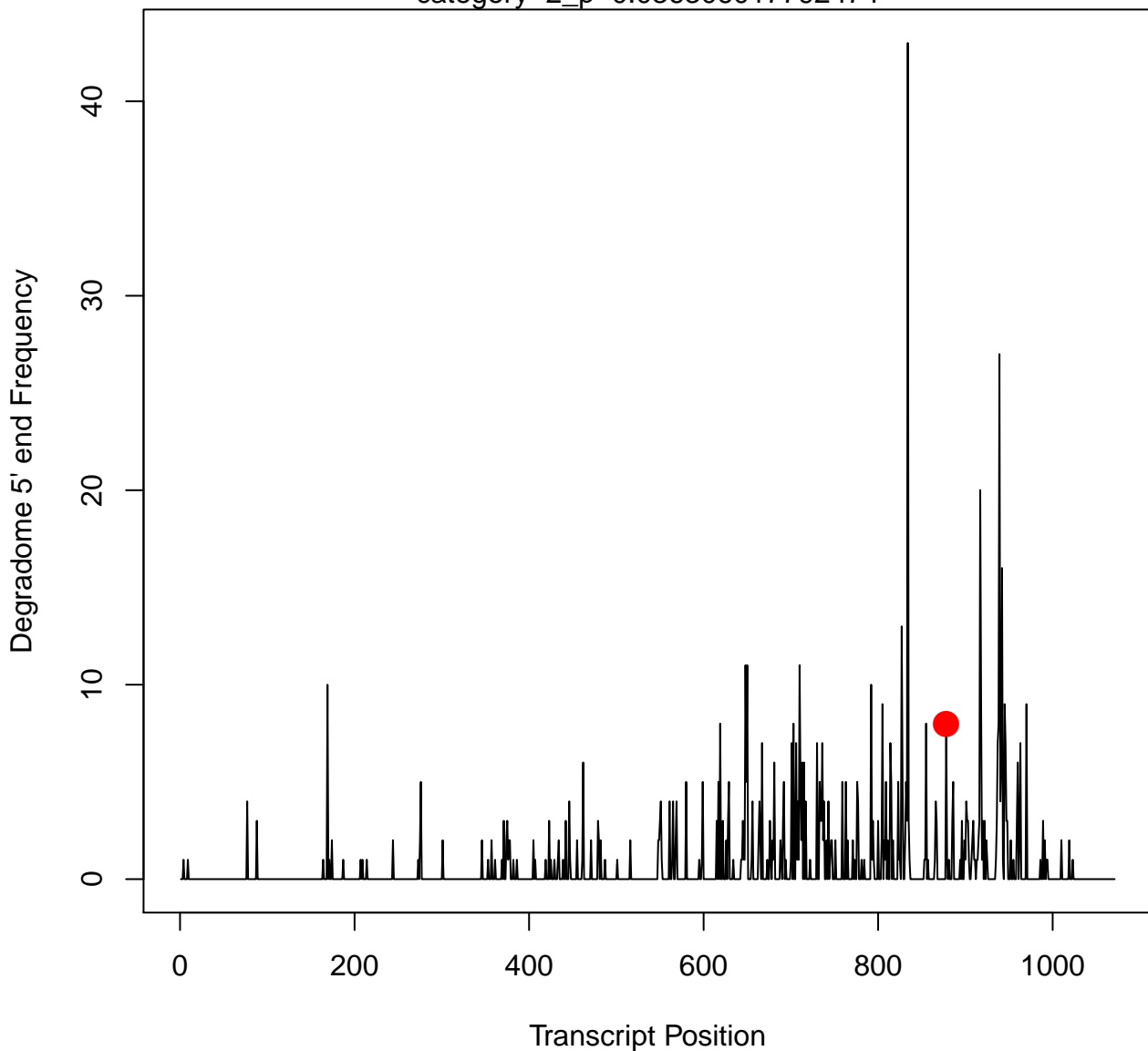

Supplement: Supplementary file 1 [file Data_Sheet_1.zip › Sit-miR162_Seita.5G064400.1_878_TPlot.pdf]

**T=Seita.5G414500.1\_Q=Sit-miR162\_S=1422**

category=2\_p=0.814120630696337

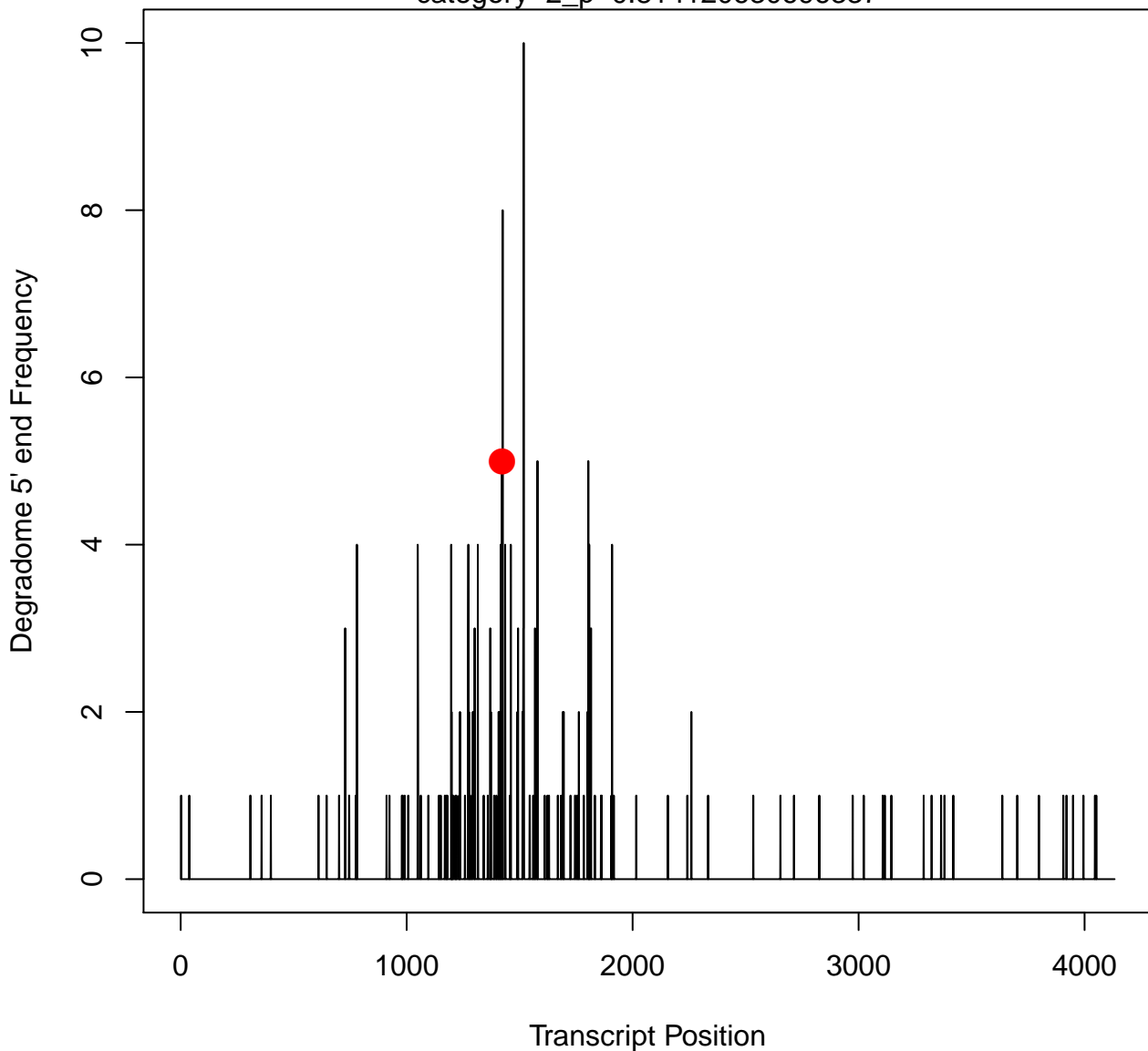

Supplement: Supplementary file 1 [file Data_Sheet_1.zip › Sit-miR162_Seita.5G414500.1_1422_TPlot.pdf]

**T=Seita.9G332000.1\_Q=Sit-miR162\_S=1088**

category=2\_p=0.780464363166656

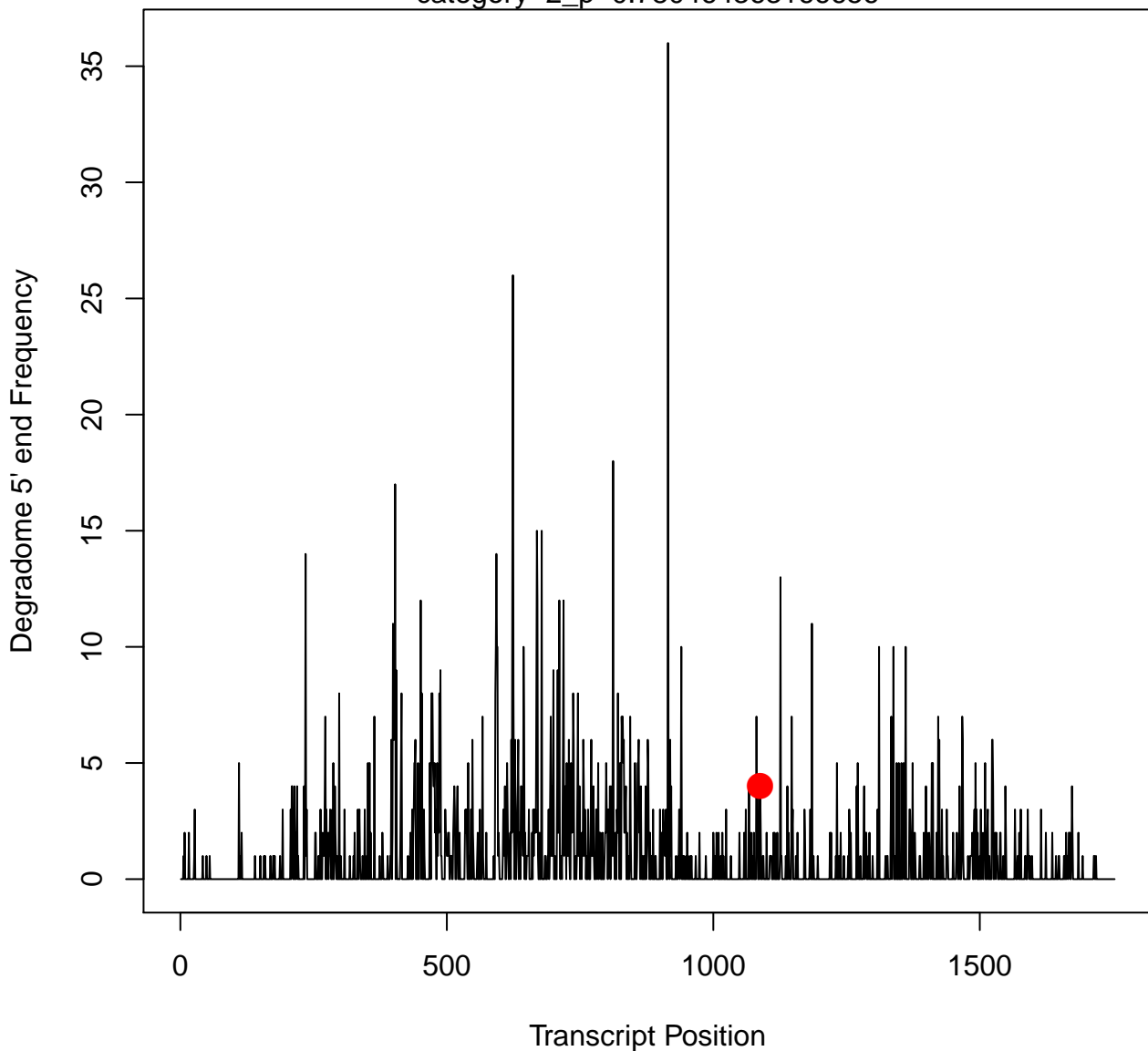

Supplement: Supplementary file 1 [file Data_Sheet_1.zip › Sit-miR162_Seita.9G332000.1_1088_TPlot.pdf]

**T=Seita.9G562200.1\_Q=Sit-miR162\_S=3530**

category=2\_p=0.0183208354011348

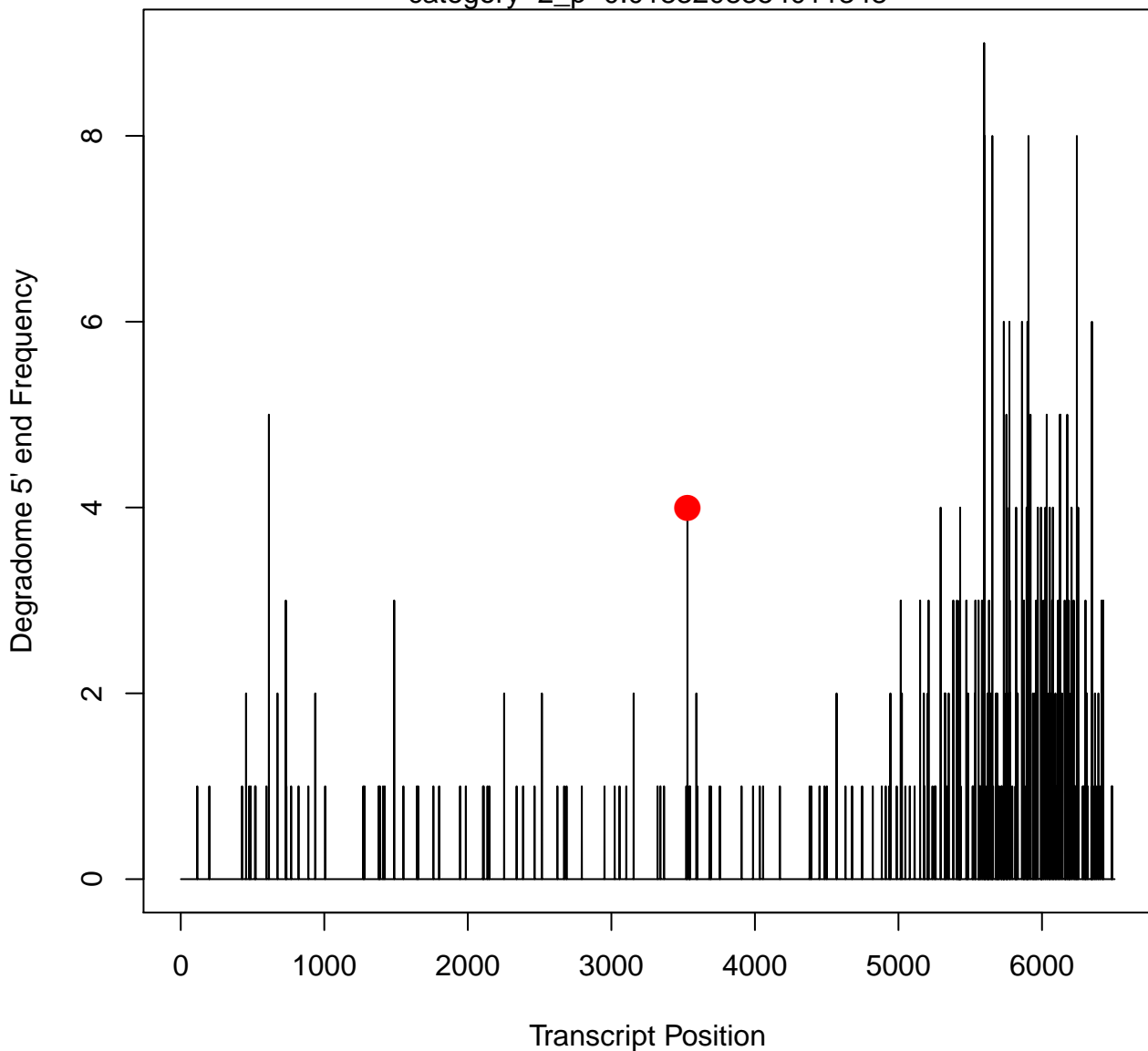

Supplement: Supplementary file 1 [file Data_Sheet_1.zip › Sit-miR162_Seita.9G562200.1_3530_TPlot.pdf]

**T=Seita.1G209000.1\_Q=Sit-miR164a\_S=999**

category=0\_p=0.00218688504577869

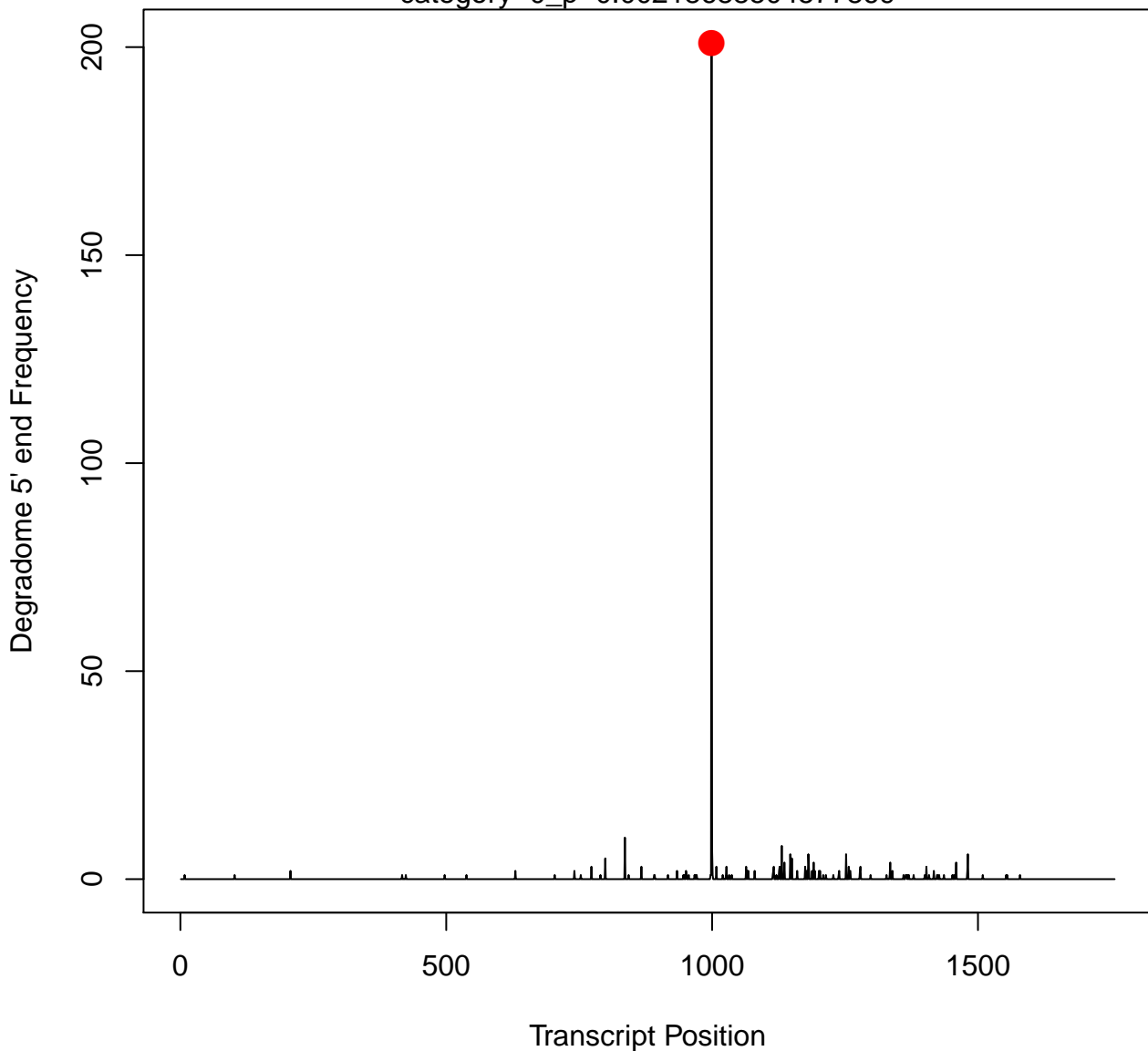

Supplement: Supplementary file 1 [file Data_Sheet_1.zip › Sit-miR164a_Seita.1G209000.1_999_TPlot.pdf]

**T=Seita.7G124900.1\_Q=Sit-miR164a\_S=859**

category=0\_p=0.0017498909690008

Degradsome 5' end Frequency

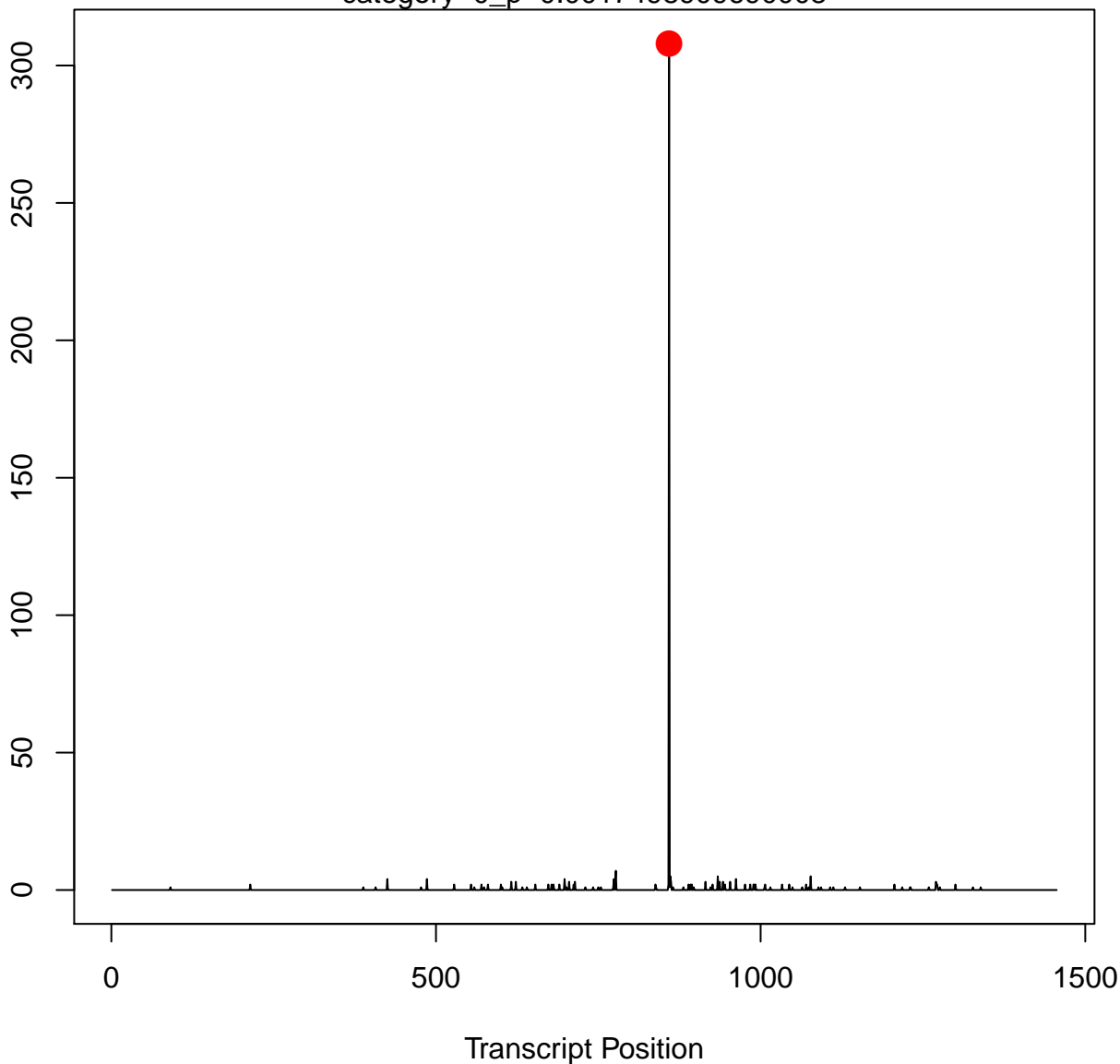

Supplement: Supplementary file 1 [file Data_Sheet_1.zip › Sit-miR164a_Seita.7G124900.1_859_TPlot.pdf]

**T=Seita.1G369500.1\_Q=Sit-miR164b\_S=3079**

category=2\_p=0.99999901544271

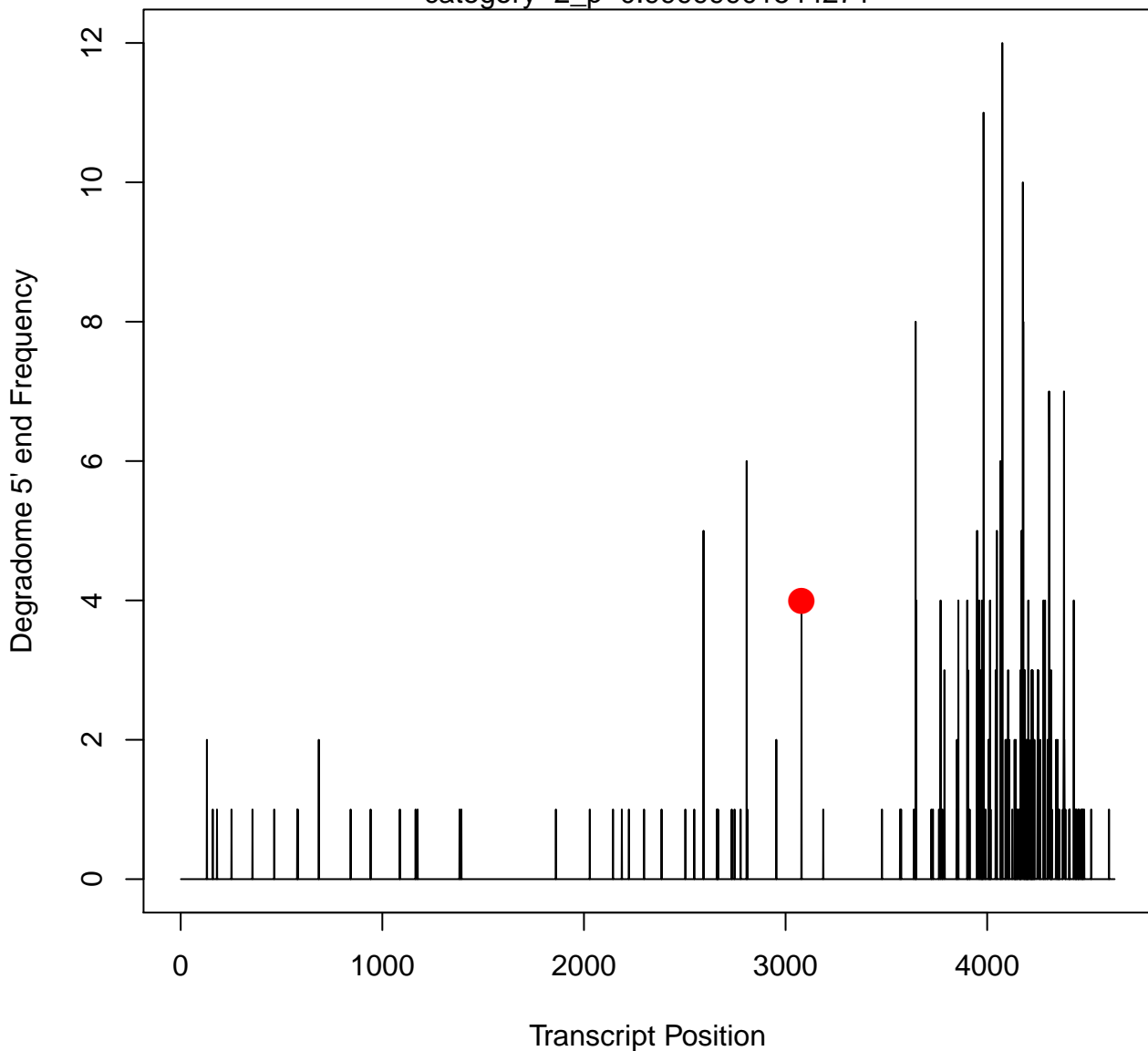

Supplement: Supplementary file 1 [file Data_Sheet_1.zip › Sit-miR164b_Seita.1G369500.1_3079_TPlot.pdf]

**T=Seita.3G386200.1\_Q=Sit-miR164b\_S=790**

category=0\_p=0.000437760109239571

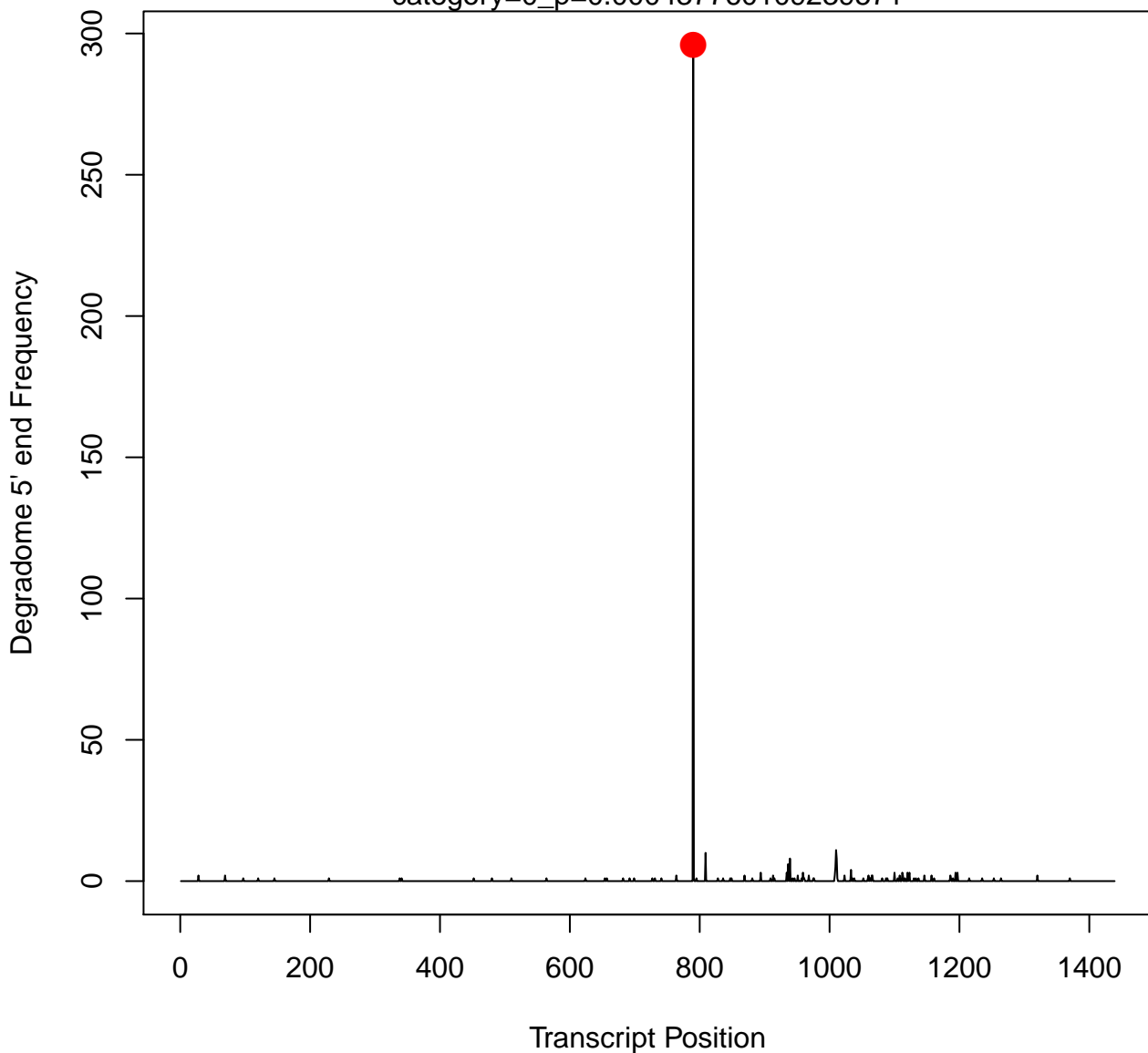

Supplement: Supplementary file 1 [file Data_Sheet_1.zip › Sit-miR164b_Seita.3G386200.1_790_TPlot.pdf]

**T=Seita.4G263400.1\_Q=Sit-miR164b\_S=1216**

category=0\_p=0.0017498909690008

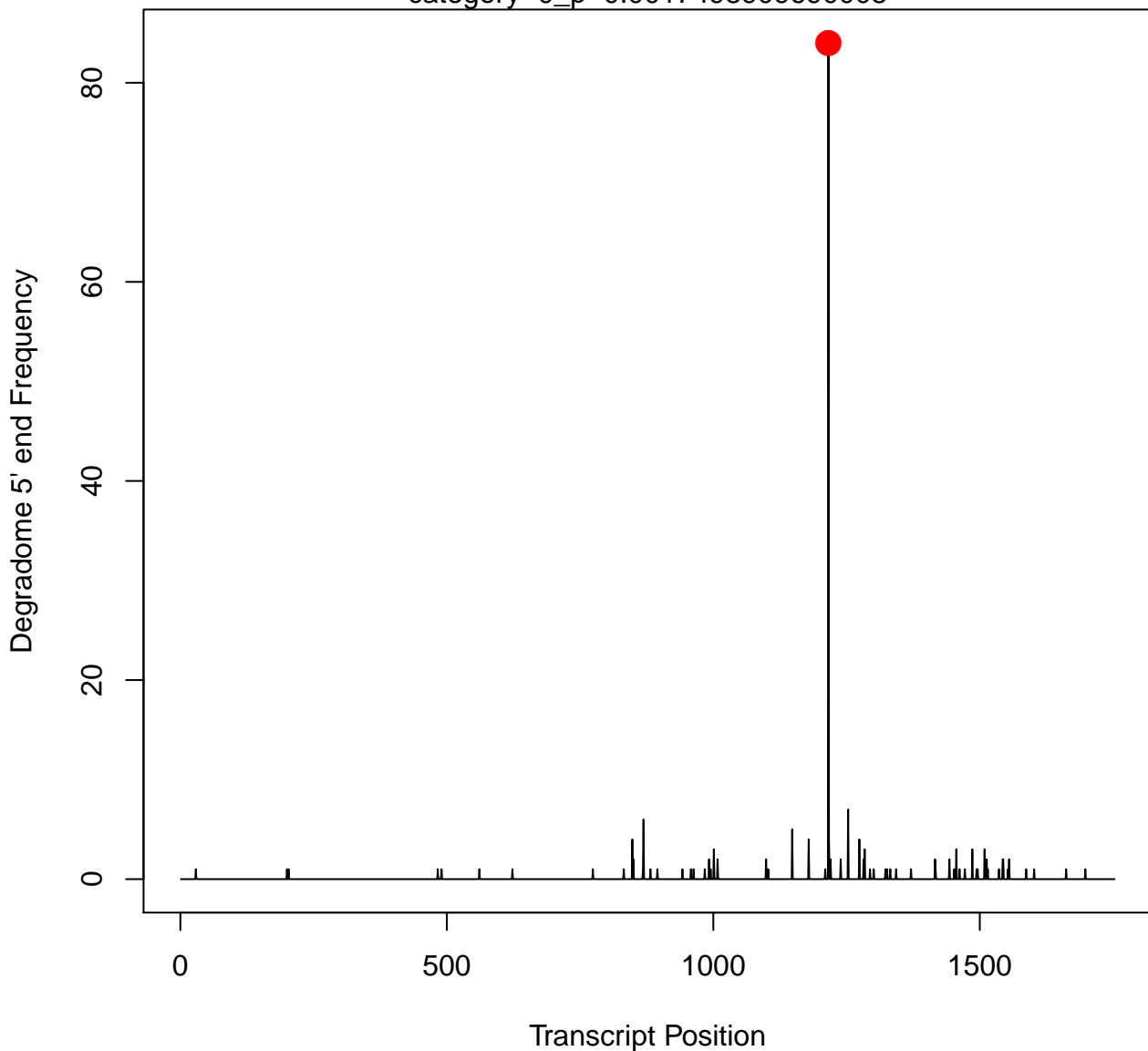

Supplement: Supplementary file 1 [file Data_Sheet_1.zip › Sit-miR164b_Seita.4G263400.1_1216_TPlot.pdf]

**T=Seita.2G381900.1\_Q=Sit-miR164c\_S=1305**

category=1\_p=0.0667291732820193

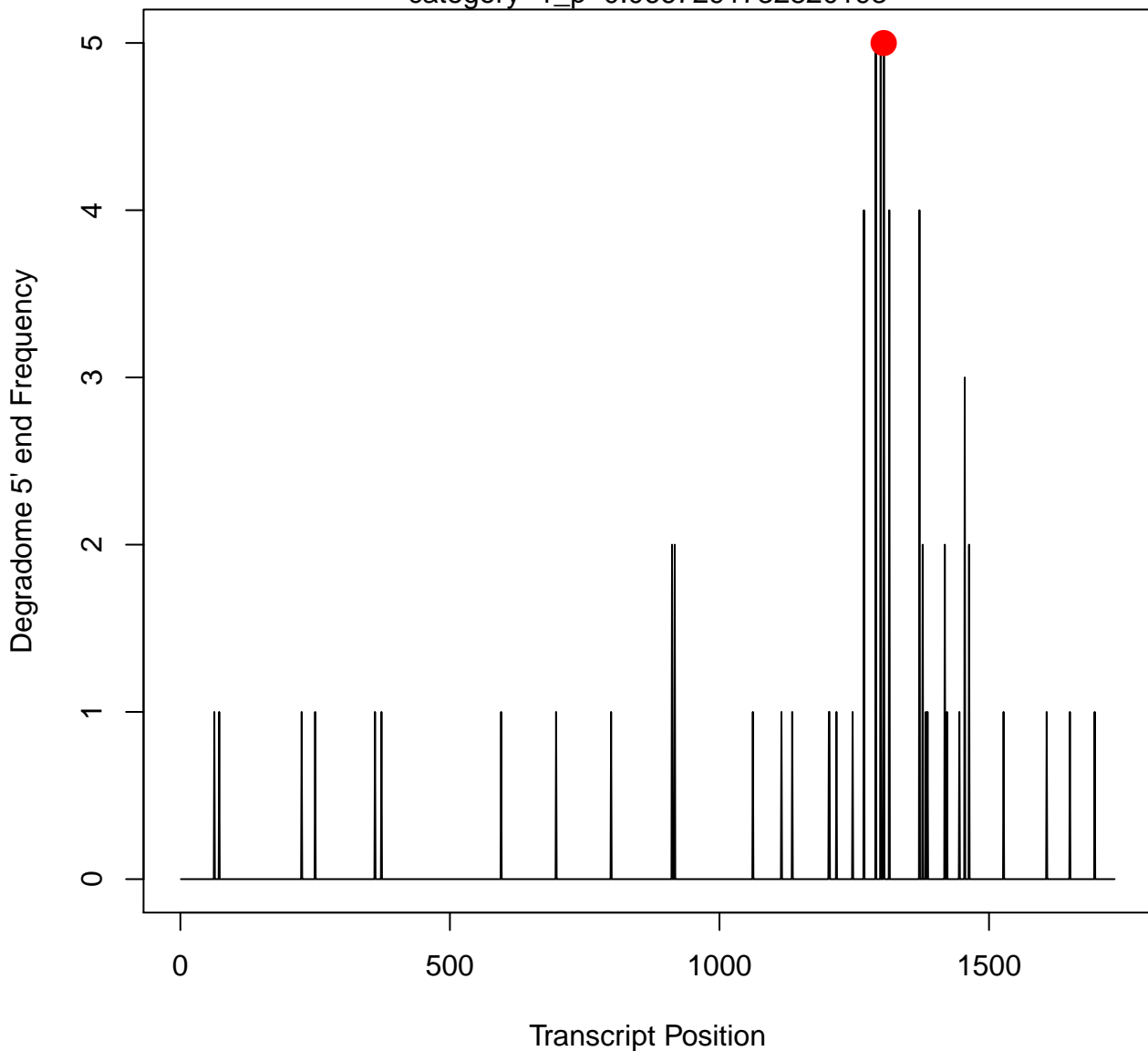

Supplement: Supplementary file 1 [file Data_Sheet_1.zip › Sit-miR164c_Seita.2G381900.1_1305_TPlot.pdf]

**T=Seita.6G004200.1\_Q=Sit-miR164c\_S=2284**

category=2\_p=0.436288650110905

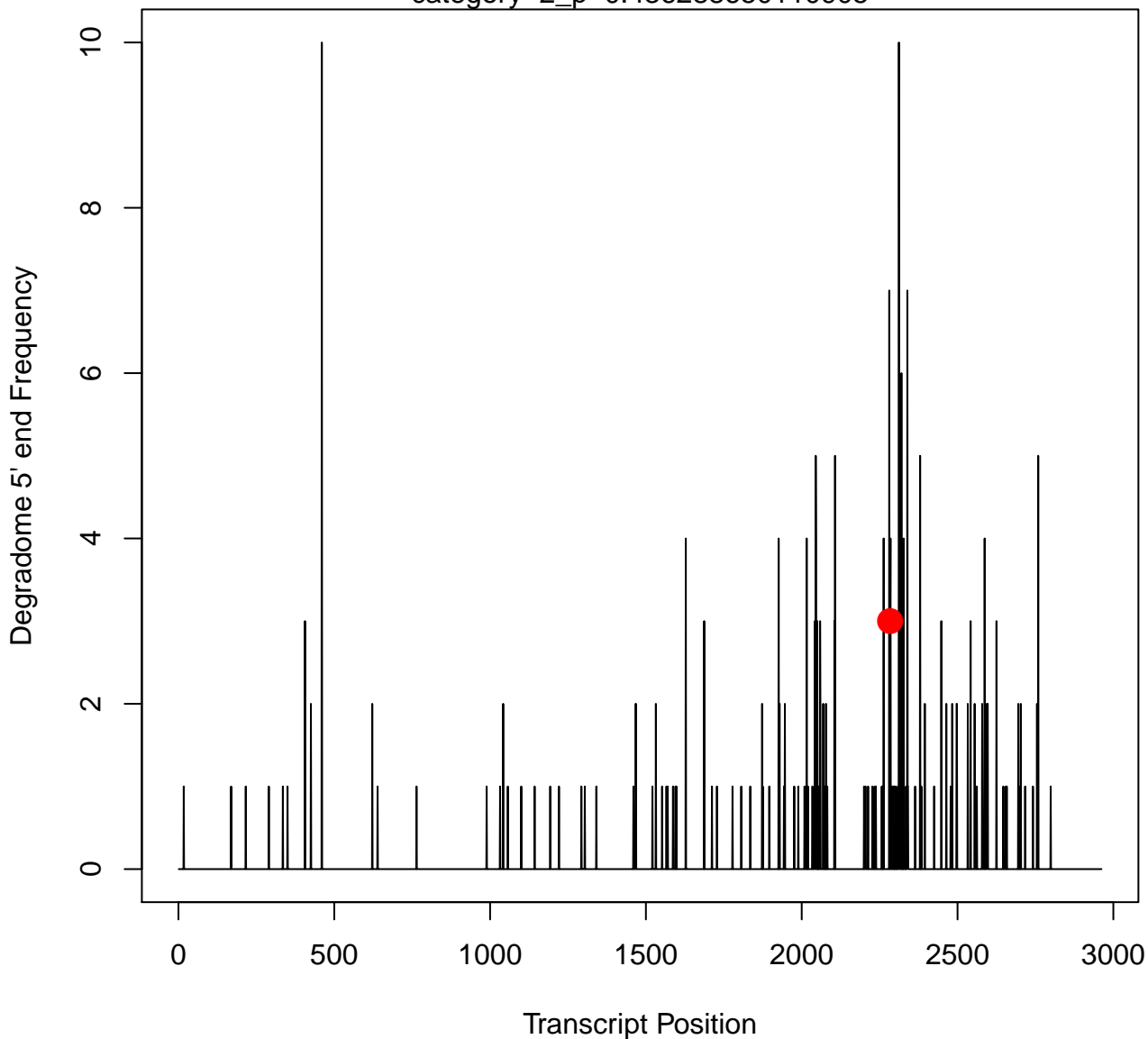

Supplement: Supplementary file 1 [file Data_Sheet_1.zip › Sit-miR164c_Seita.6G004200.1_2284_TPlot.pdf]

**T=Seita.3G001500.1\_Q=Sit-miR164d\_S=686**

category=2\_p=0.991205506730575

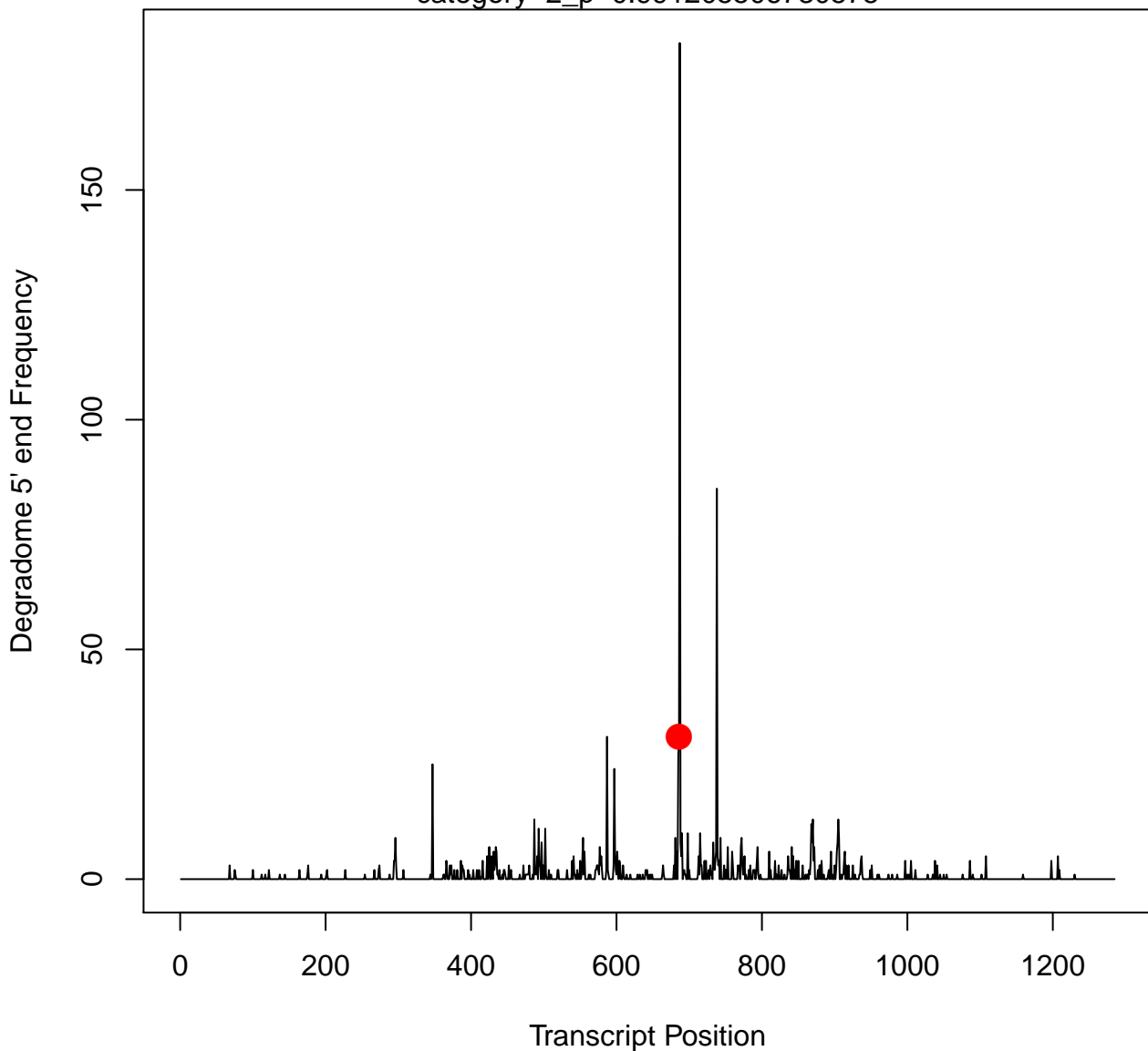

Supplement: Supplementary file 1 [file Data_Sheet_1.zip › Sit-miR164d_Seita.3G001500.1_686_TPlot.pdf]

**T=Seita.3G120800.1\_Q=Sit-miR164e\_S=2100**

category=2\_p=0.99988166275595

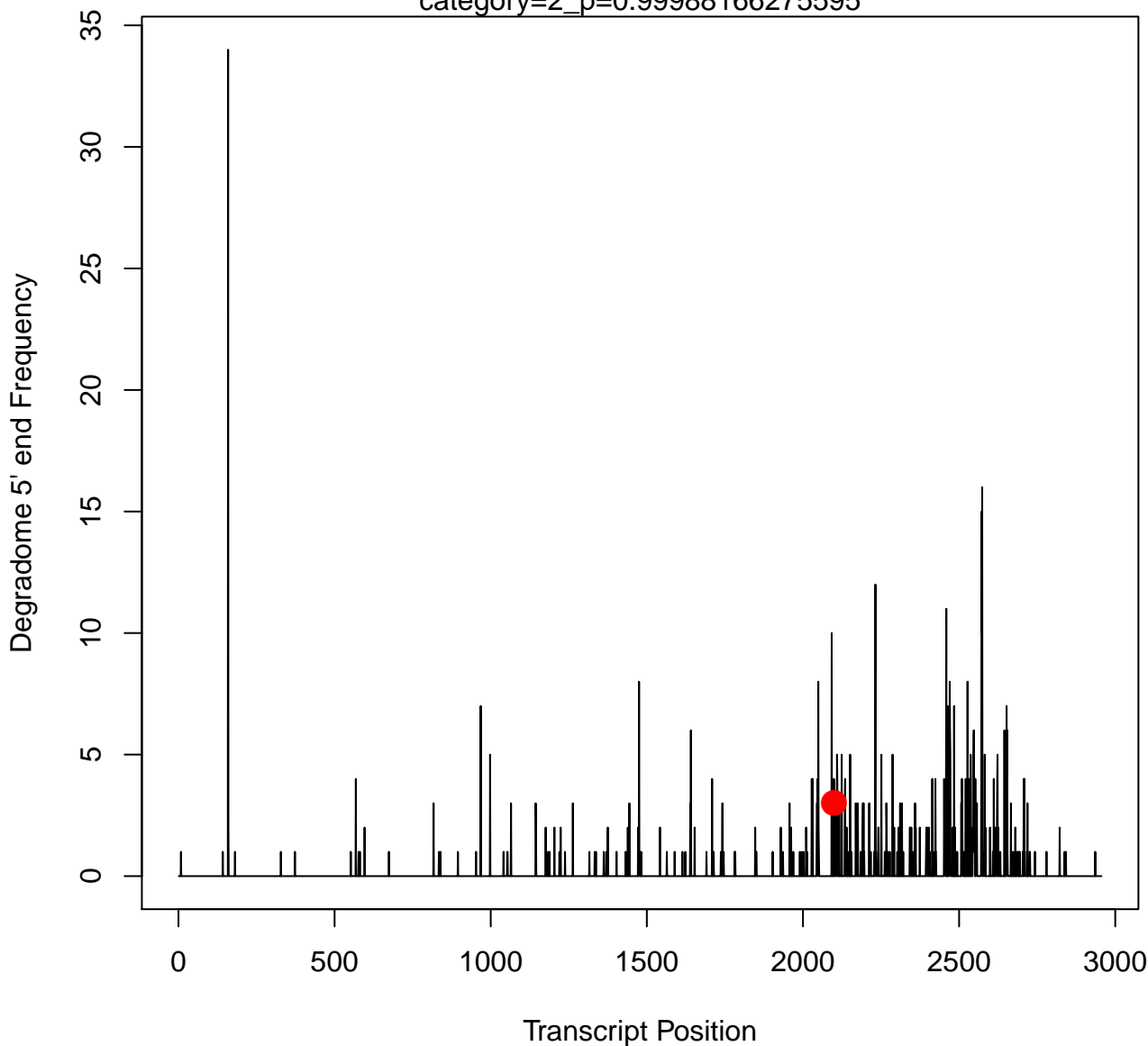

Supplement: Supplementary file 1 [file Data_Sheet_1.zip › Sit-miR164e_Seita.3G120800.1_2100_TPlot.pdf]

**T=Seita.7G028400.1\_Q=Sit-miR164e\_S=641**

category=2\_p=0.807117847848473

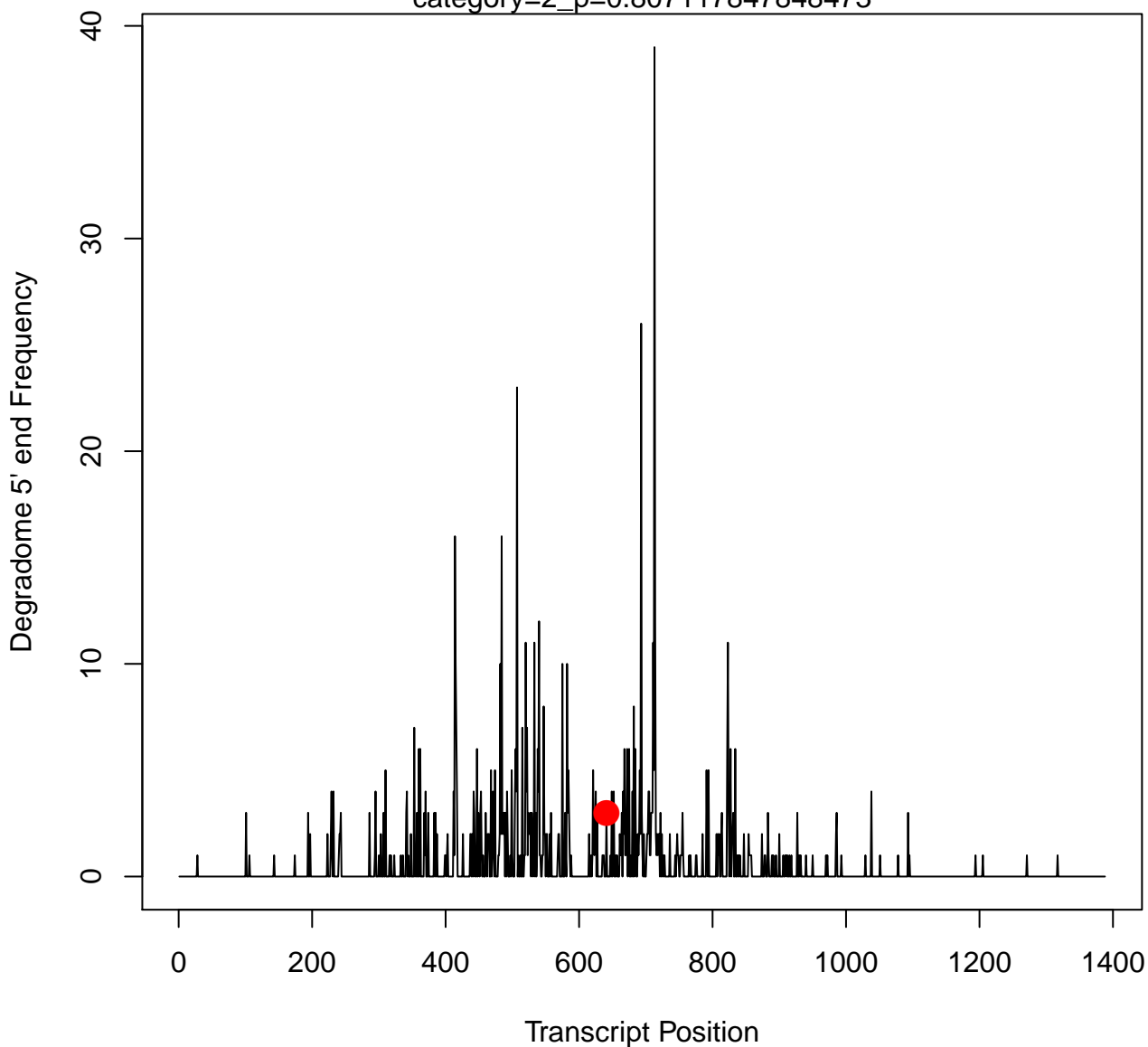

Supplement: Supplementary file 1 [file Data_Sheet_1.zip › Sit-miR164e_Seita.7G028400.1_641_TPlot.pdf]

**T=Seita.8G179000.1\_Q=Sit-miR164e\_S=122**

category=2\_p=0.137504995902408

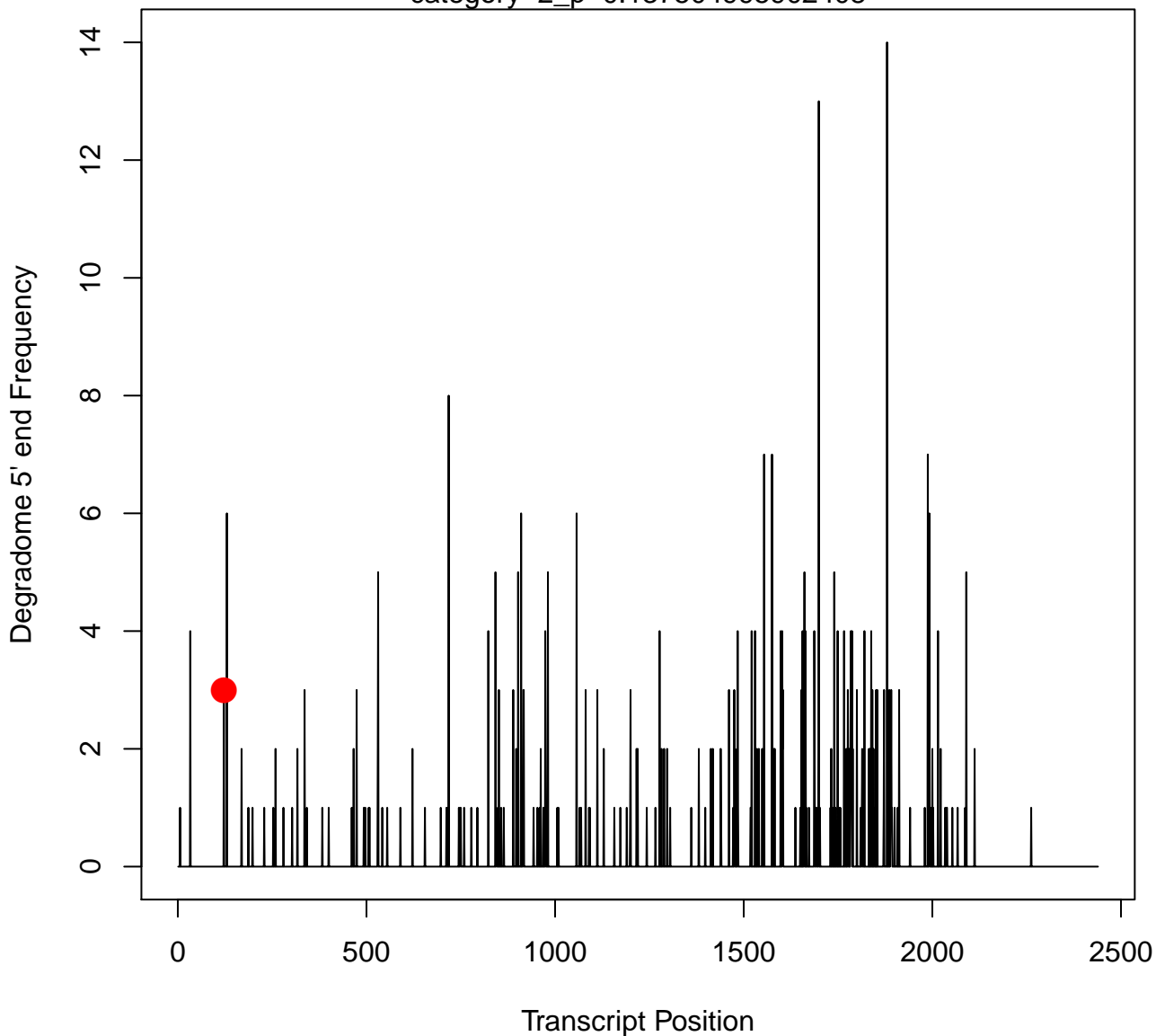

Supplement: Supplementary file 1 [file Data_Sheet_1.zip › Sit-miR164e_Seita.8G179000.1_122_TPlot.pdf]

**T=Seita.9G089800.1\_Q=Sit-miR164e\_S=1145**

category=2\_p=0.710291374419638

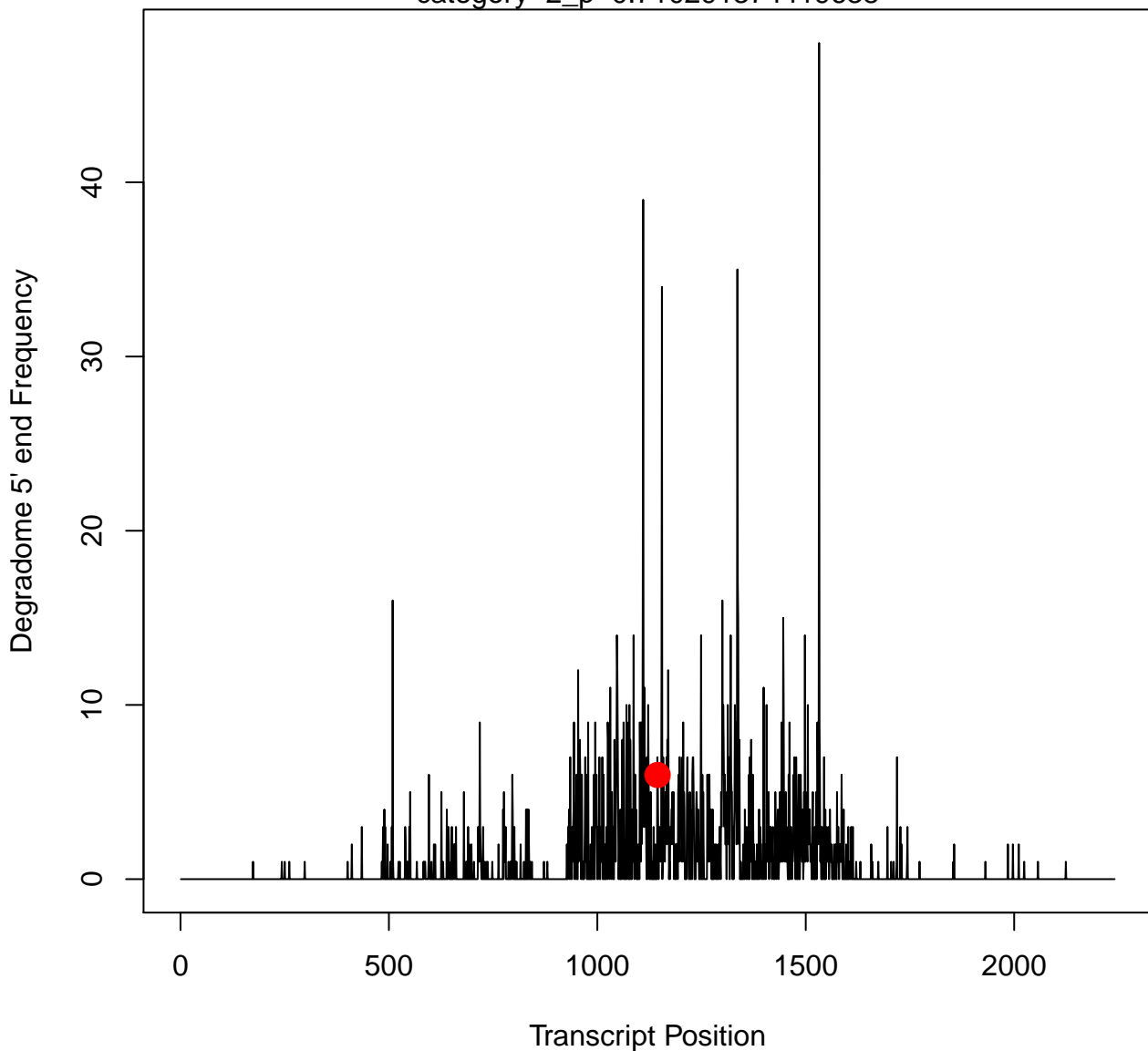

Supplement: Supplementary file 1 [file Data_Sheet_1.zip › Sit-miR164e_Seita.9G089800.1_1145_TPlot.pdf]

**T=Seita.9G141600.1\_Q=Sit-miR164e\_S=638**

category=2\_p=0.9999923331259

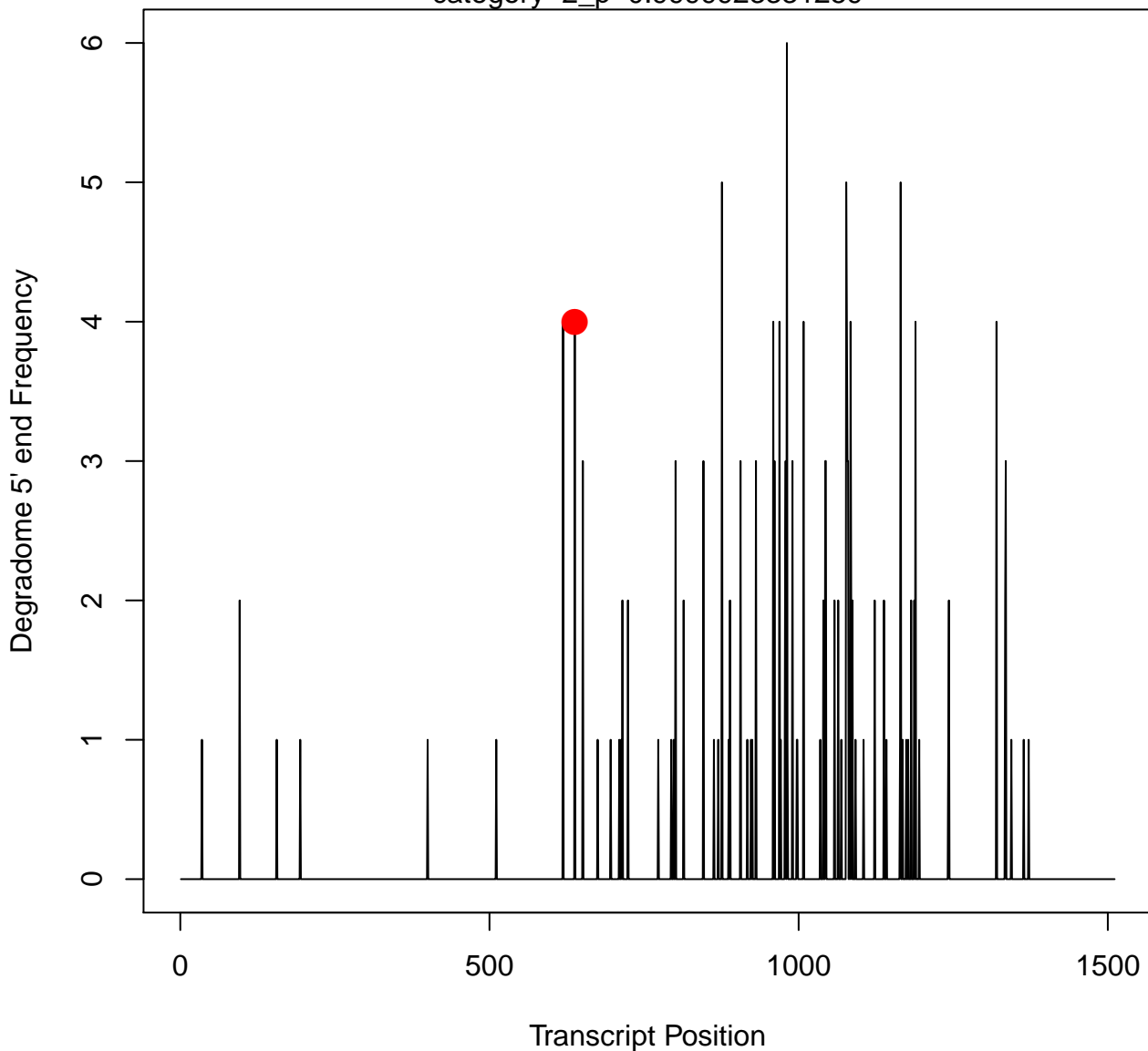

Supplement: Supplementary file 1 [file Data_Sheet_1.zip › Sit-miR164e_Seita.9G141600.1_638_TPlot.pdf]

**T=Seita.9G161900.1\_Q=Sit-miR164e\_S=2120**

category=2\_p=0.999999413336525

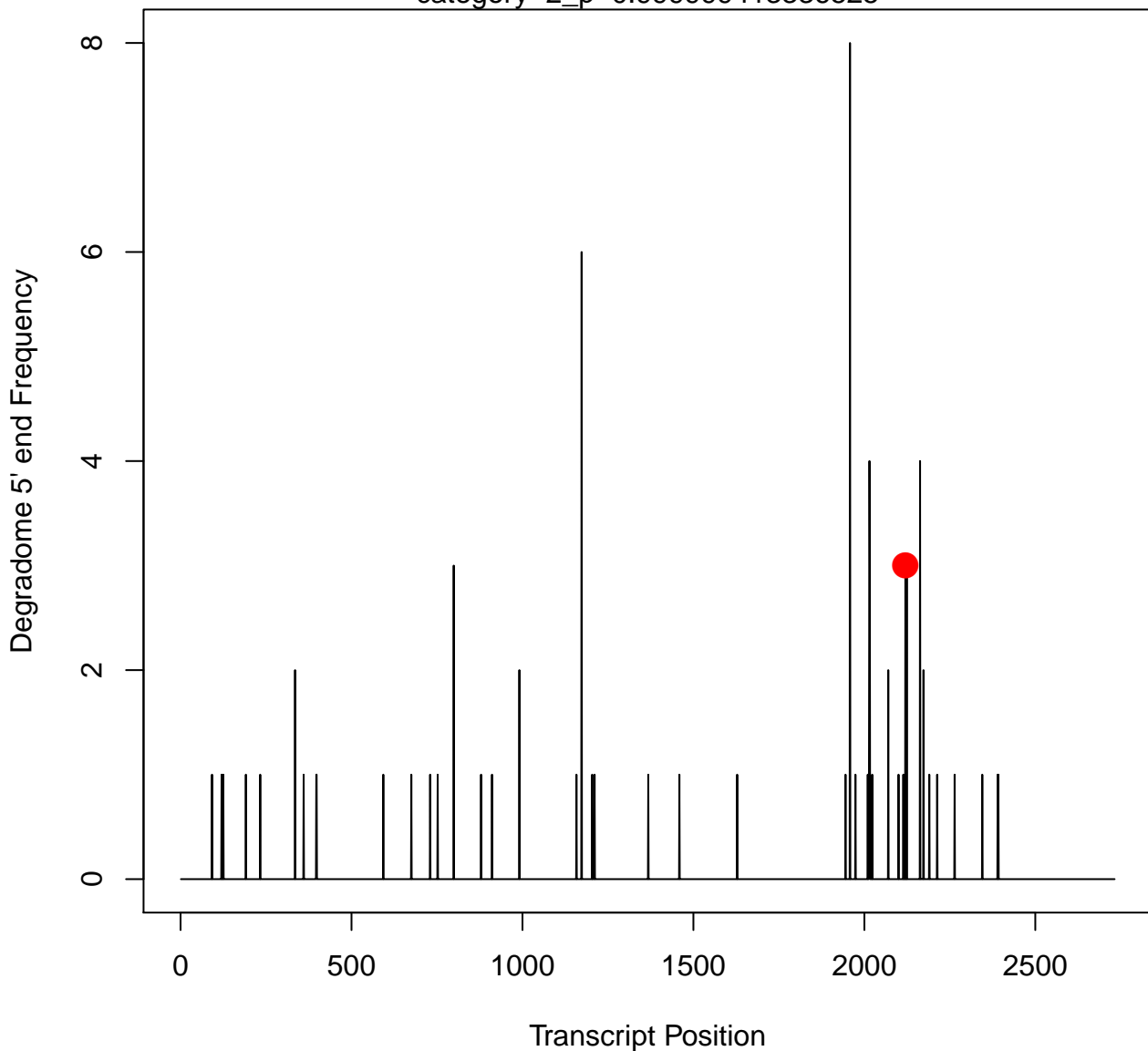

Supplement: Supplementary file 1 [file Data_Sheet_1.zip › Sit-miR164e_Seita.9G161900.1_2120_TPlot.pdf]

**T=Seita.9G171900.1\_Q=Sit-miR164e\_S=2520**

category=2\_p=0.95268709323487

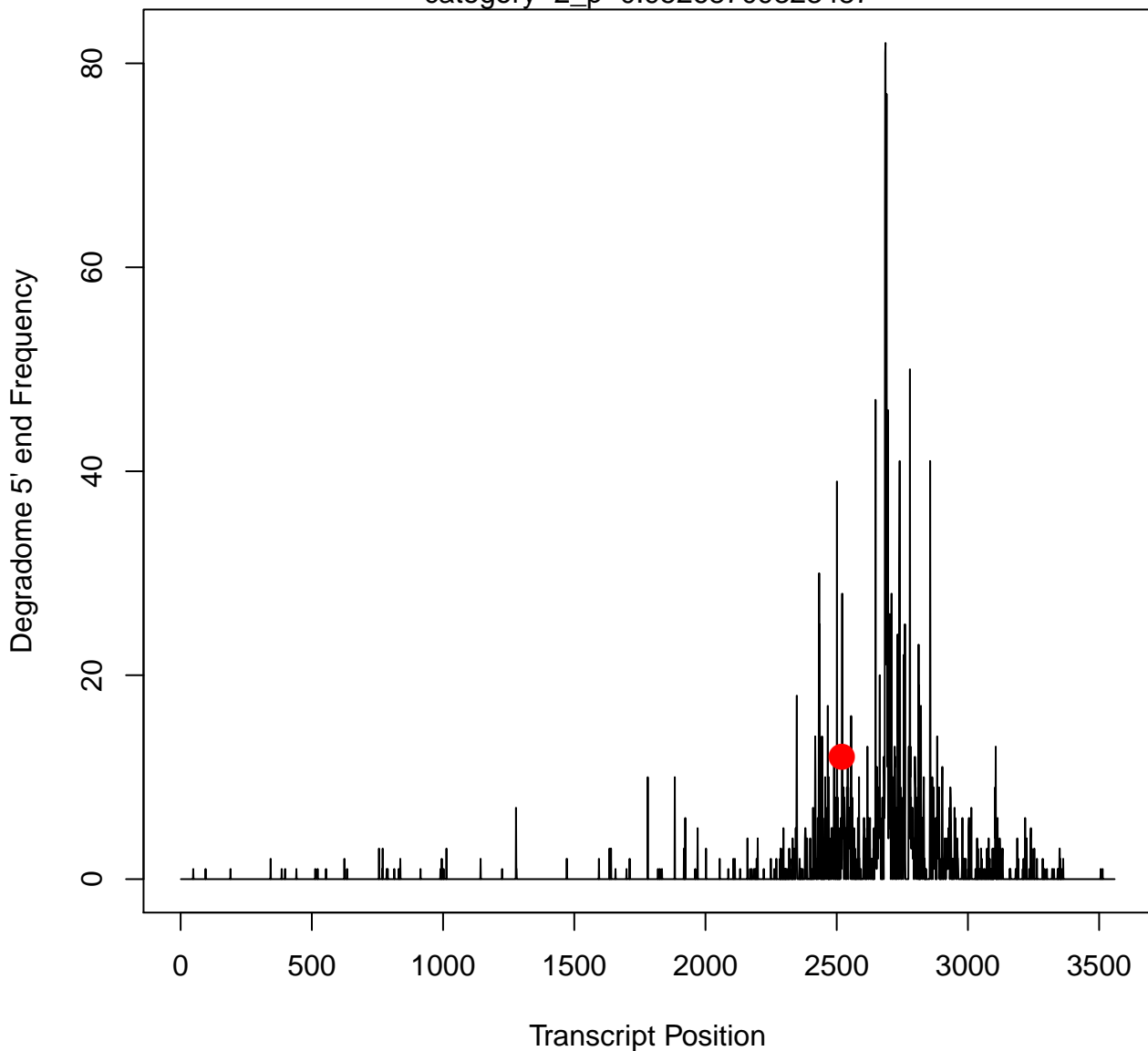

Supplement: Supplementary file 1 [file Data_Sheet_1.zip › Sit-miR164e_Seita.9G171900.1_2520_TPlot.pdf]

**T=Seita.4G157500.1\_Q=Sit-miR164f\_S=1077**

category=0\_p=0.00087532858456596

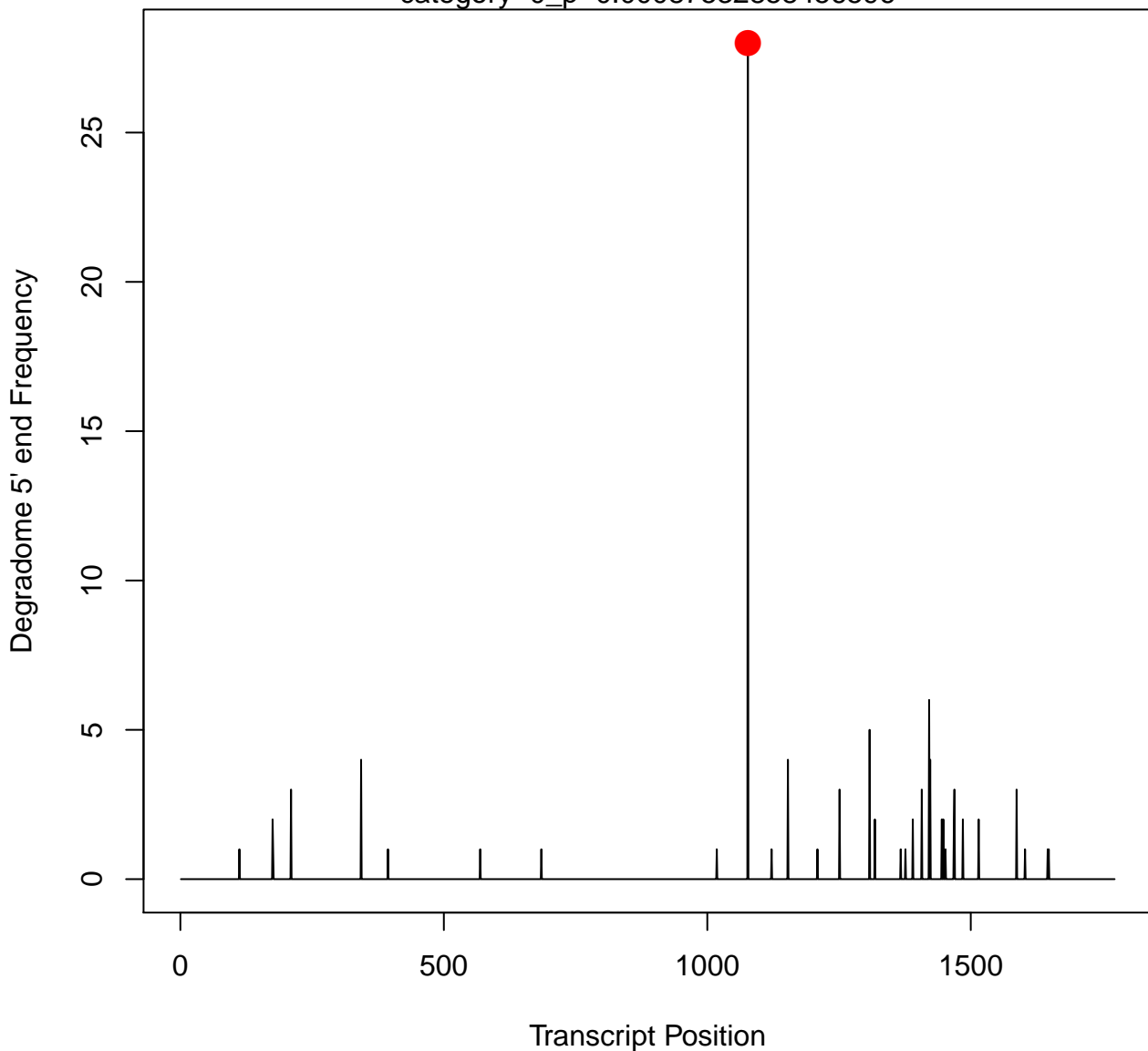

Supplement: Supplementary file 1 [file Data_Sheet_1.zip › Sit-miR164f_Seita.4G157500.1_1077_TPlot.pdf]

**T=Seita.5G087200.1\_Q=Sit-miR164f\_S=748**

category=2\_p=0.105011528533279

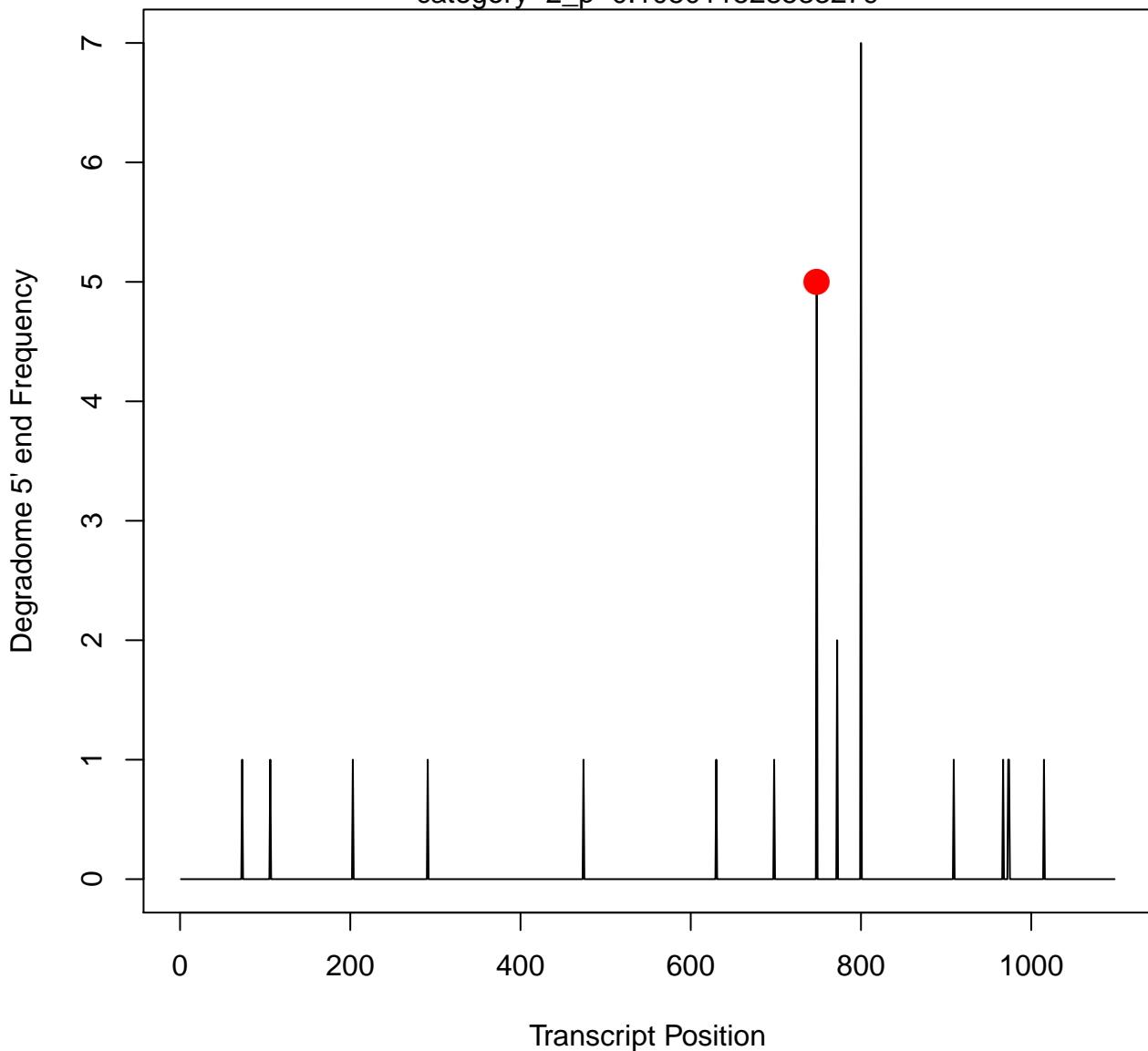

Supplement: Supplementary file 1 [file Data_Sheet_1.zip › Sit-miR164f_Seita.5G087200.1_748_TPlot.pdf]

**T=Seita.9G391300.1\_Q=Sit-miR164f\_S=1593**

category=2\_p=0.495484840614317

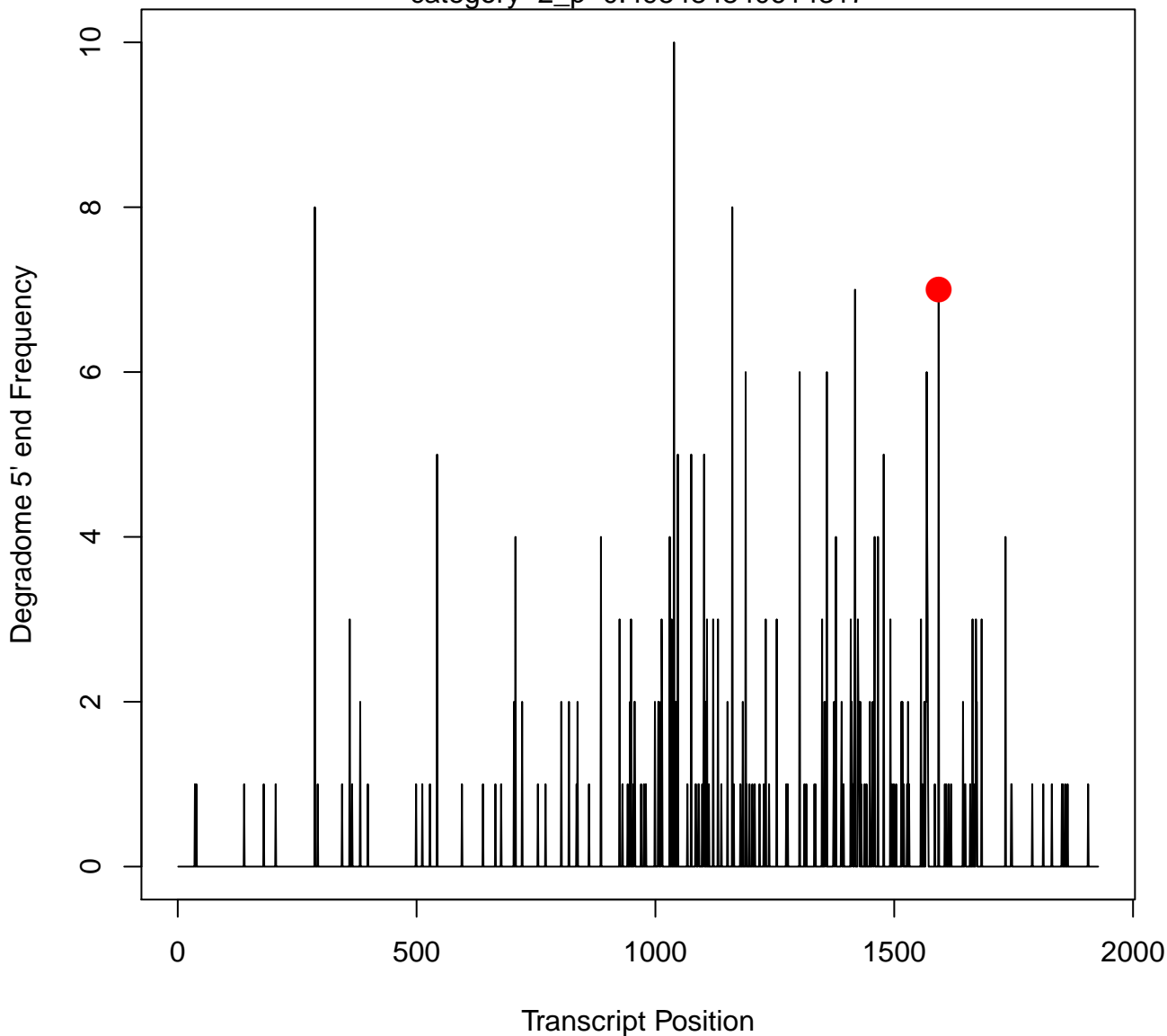

Supplement: Supplementary file 1 [file Data_Sheet_1.zip › Sit-miR164f_Seita.9G391300.1_1593_TPlot.pdf]

**T=Seita.3G105500.1\_Q=Sit-miR166a\_S=844**

category=0\_p=0.0039329492043878

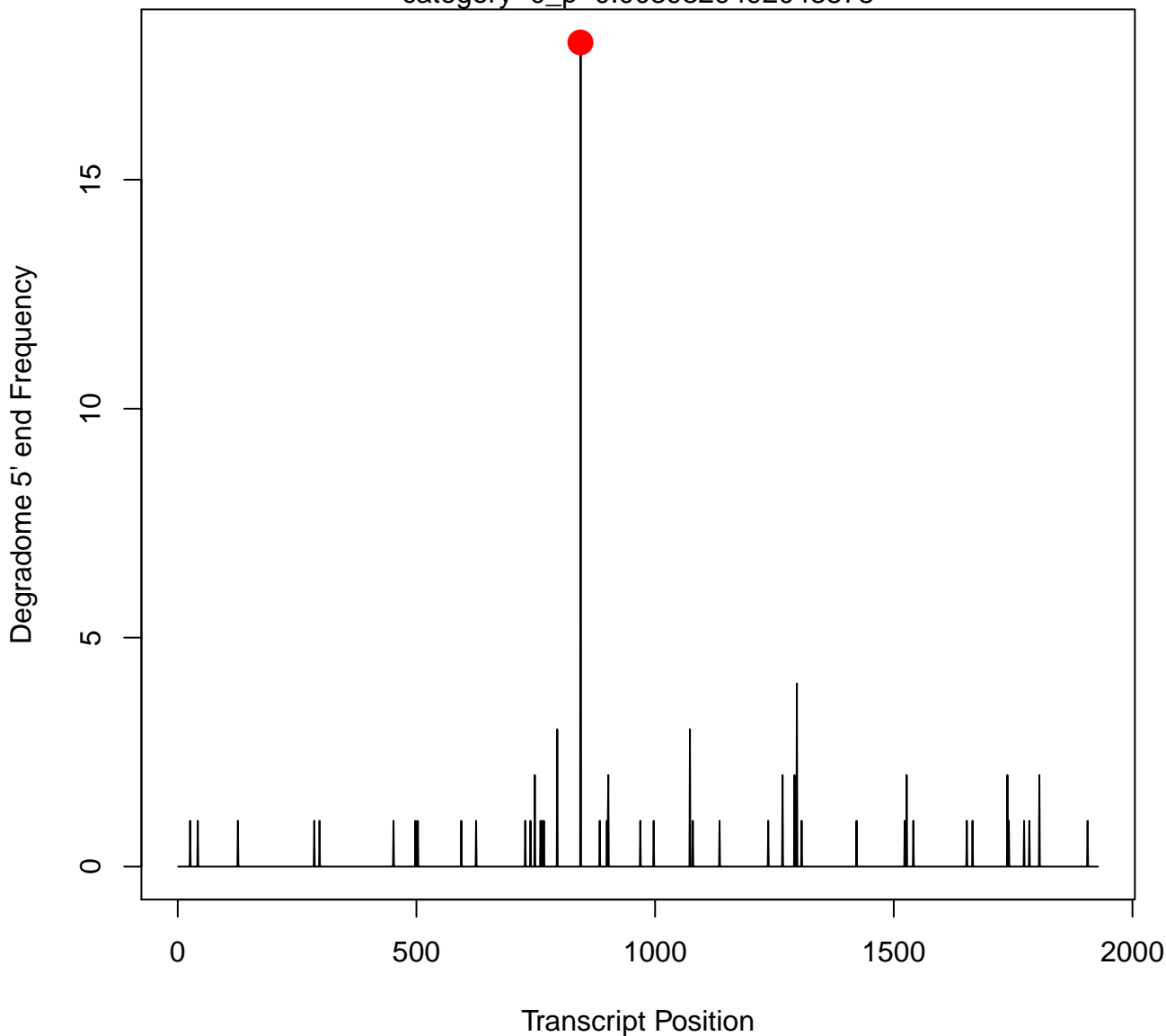

Supplement: Supplementary file 1 [file Data_Sheet_1.zip › Sit-miR166a_Seita.3G105500.1_844_TPlot.pdf]

**T=Seita.3G395000.1\_Q=Sit-miR166a\_S=774**

category=2\_p=0.0539616966175622

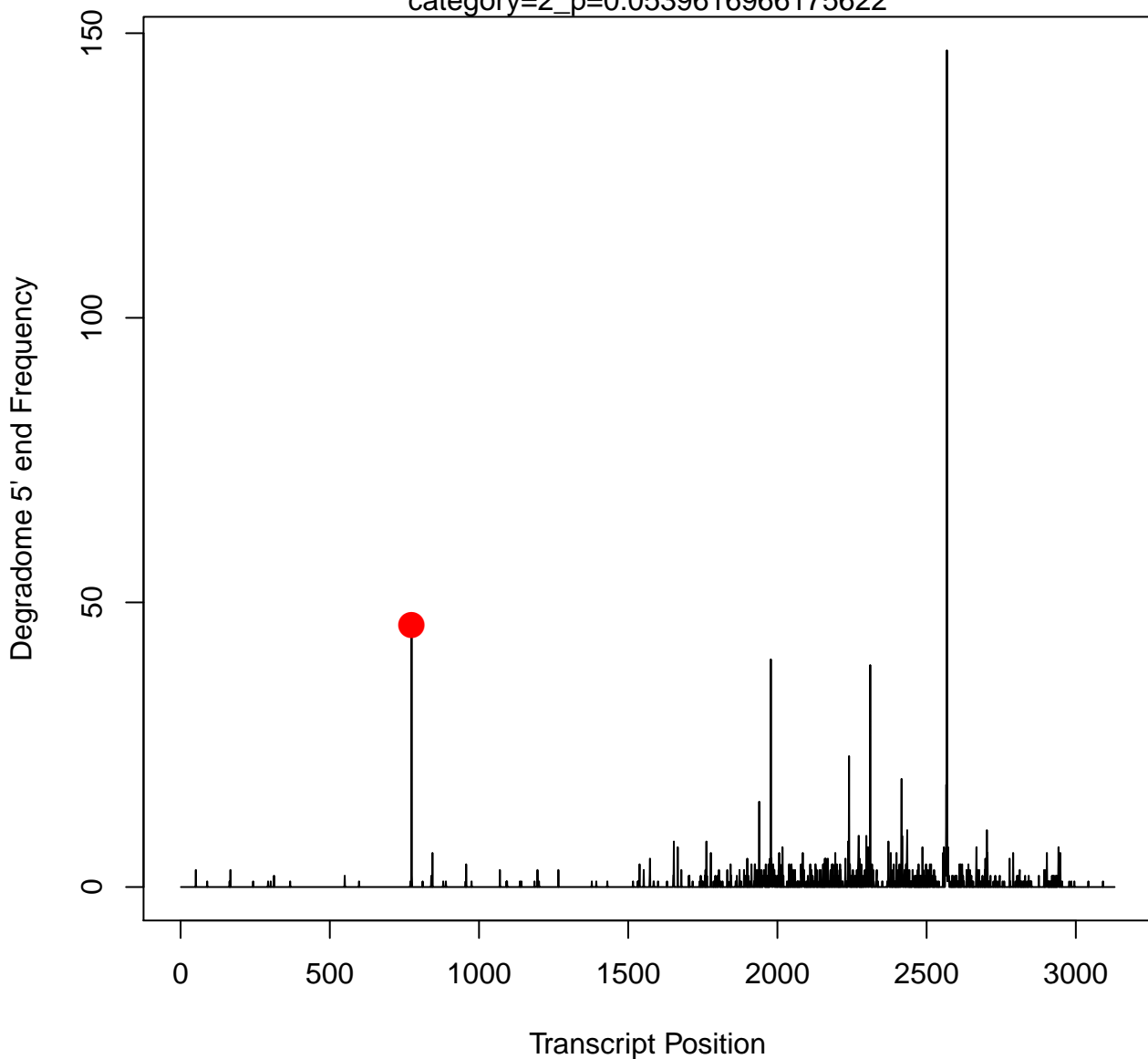

Supplement: Supplementary file 1 [file Data_Sheet_1.zip › Sit-miR166a_Seita.3G395000.1_774_TPlot.pdf]

**T=Seita.5G261100.1\_Q=Sit-miR166b\_S=1617**

category=2\_p=0.988815762905034

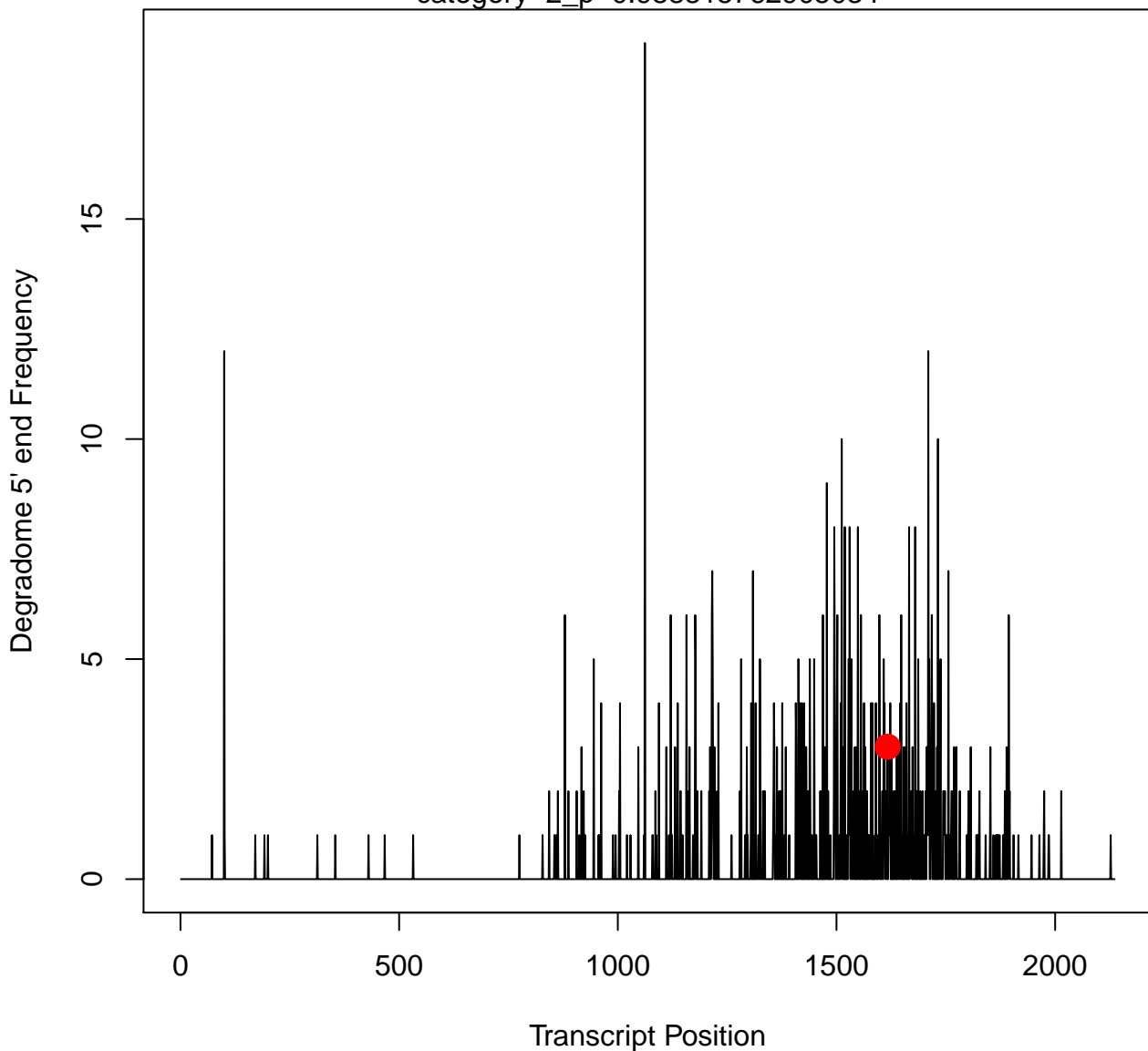

Supplement: Supplementary file 1 [file Data_Sheet_1.zip › Sit-miR166b_Seita.5G261100.1_1617_TPlot.pdf]

**T=Seita.8G184200.1\_Q=Sit-miR166b\_S=481**

category=2\_p=0.513801776967546

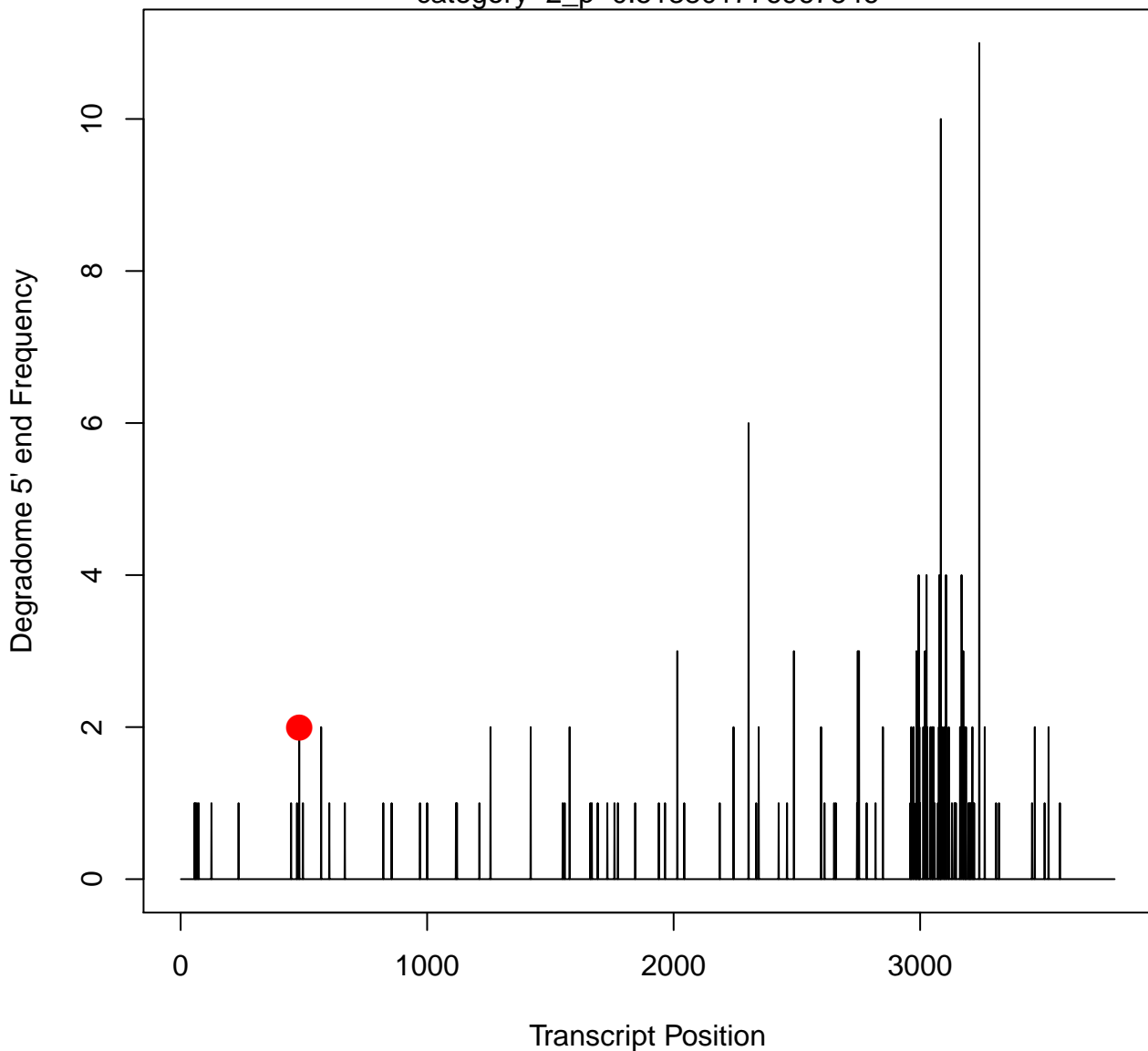

Supplement: Supplementary file 1 [file Data_Sheet_1.zip › Sit-miR166b_Seita.8G184200.1_481_TPlot.pdf]

**T=Seita.6G225800.1\_Q=Sit-miR166d\_S=732**

category=2\_p=0.917606599907781

Degradome 5' end Frequency

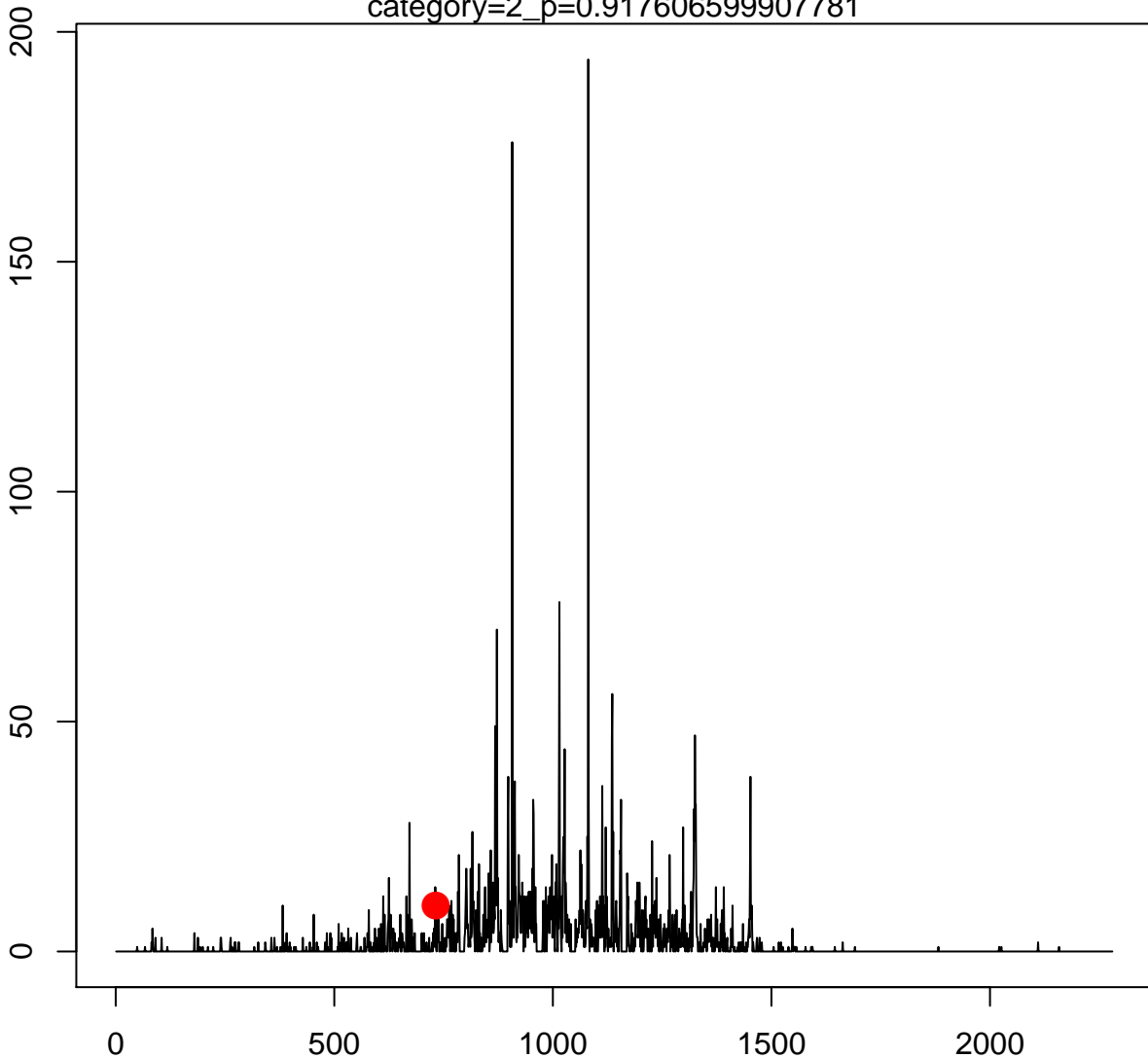

Transcript Position

Supplement: Supplementary file 1 [file Data_Sheet_1.zip › Sit-miR166d_Seita.6G225800.1_732_TPlot.pdf]

**T=Seita.7G313100.1\_Q=Sit-miR166i\_S=1430**

category=2\_p=0.955240177955825

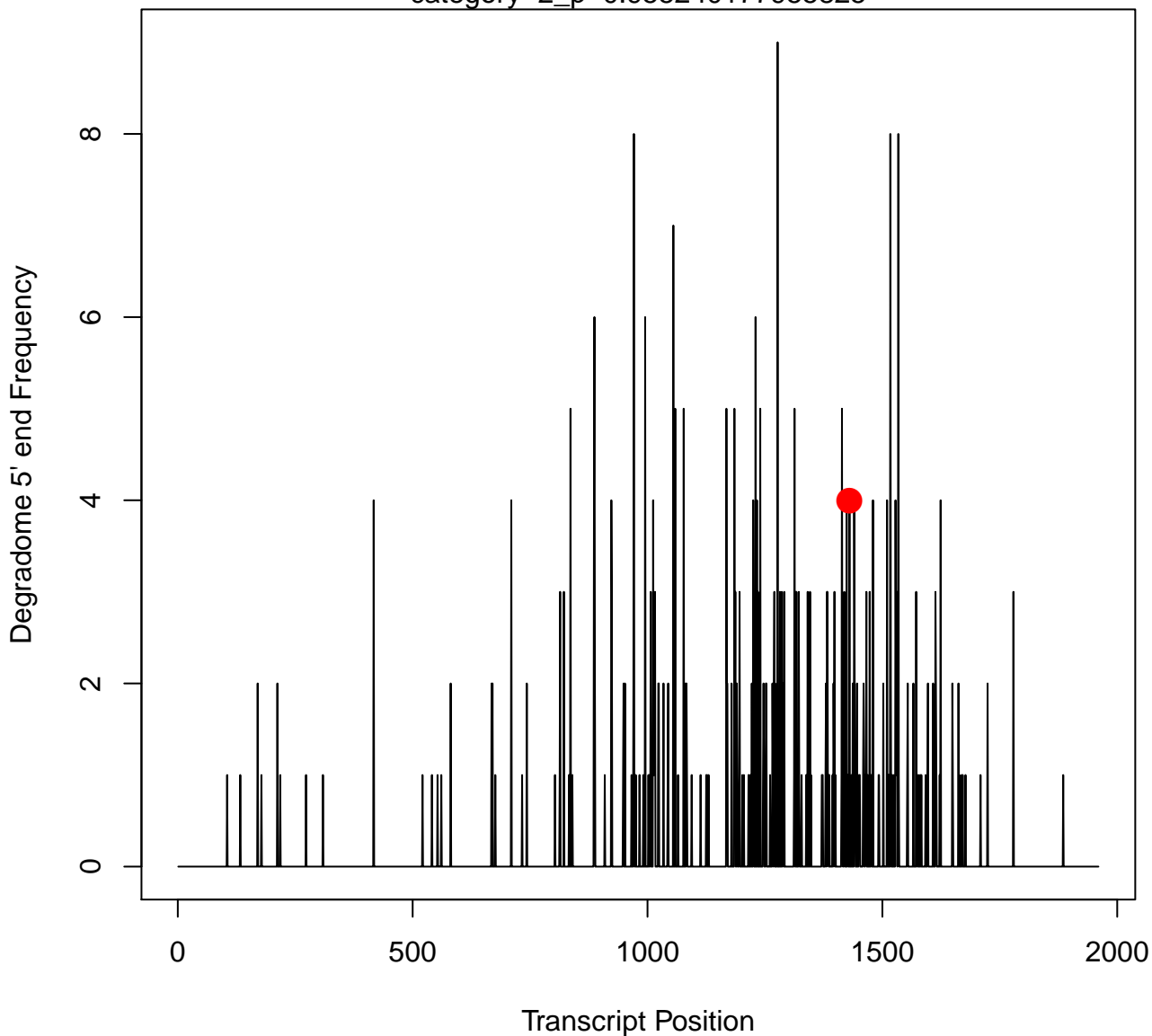

Supplement: Supplementary file 1 [file Data_Sheet_1.zip › Sit-miR166i_Seita.7G313100.1_1430_TPlot.pdf]

**T=Seita.9G219700.1\_Q=Sit-miR166i\_S=1105**

category=0\_p=0.00131270550986873

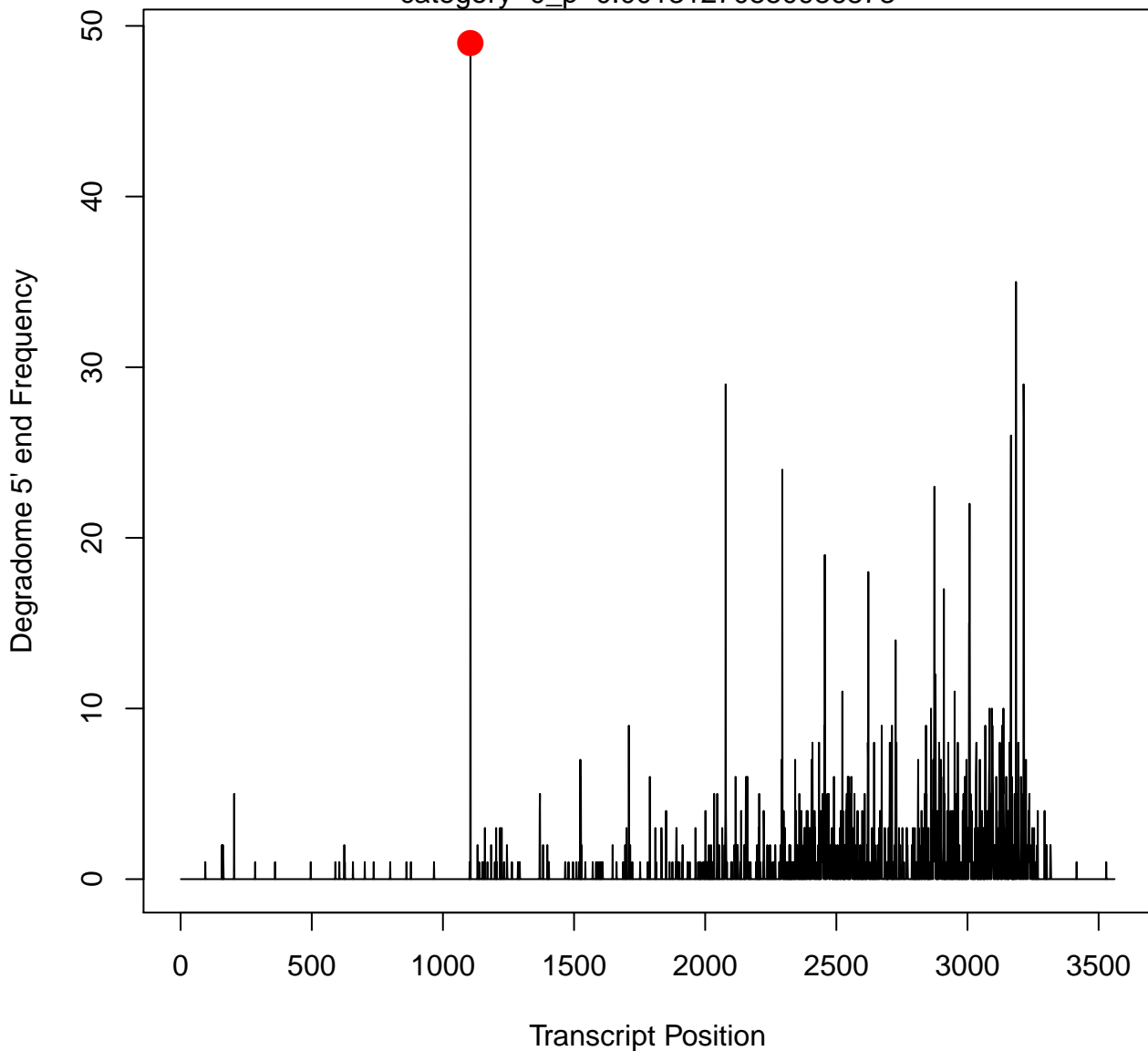

Supplement: Supplementary file 1 [file Data_Sheet_1.zip › Sit-miR166i_Seita.9G219700.1_1105_TPlot.pdf]

**T=Seita.9G250400.1\_Q=Sit-miR166i\_S=2289**

category=2\_p=0.631568316936211

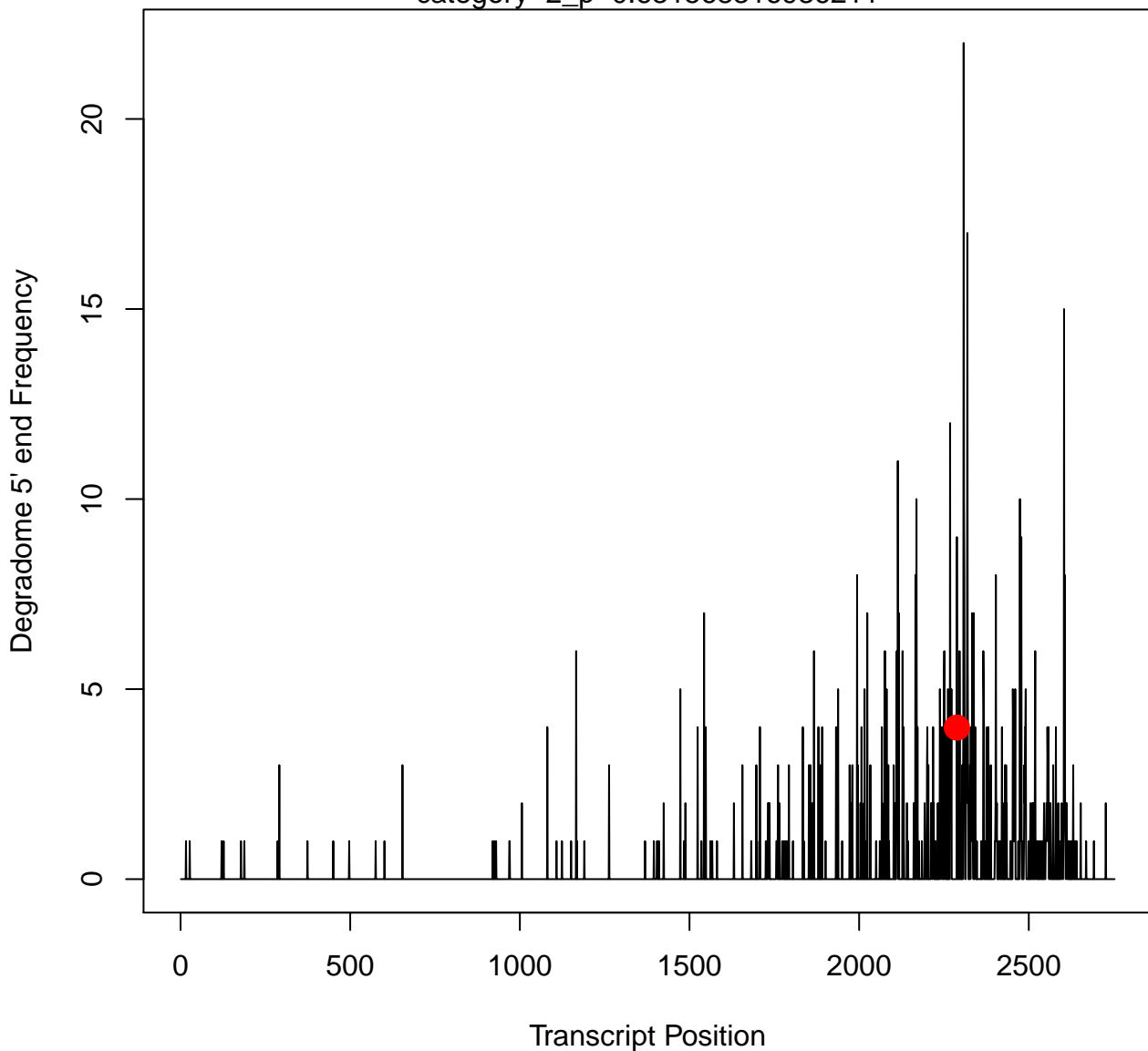

Supplement: Supplementary file 1 [file Data_Sheet_1.zip › Sit-miR166i_Seita.9G250400.1_2289_TPlot.pdf]

**T=Seita.2G145300.1\_Q=Sit-miR166j\_S=940**

category=2\_p=0.504727979806799

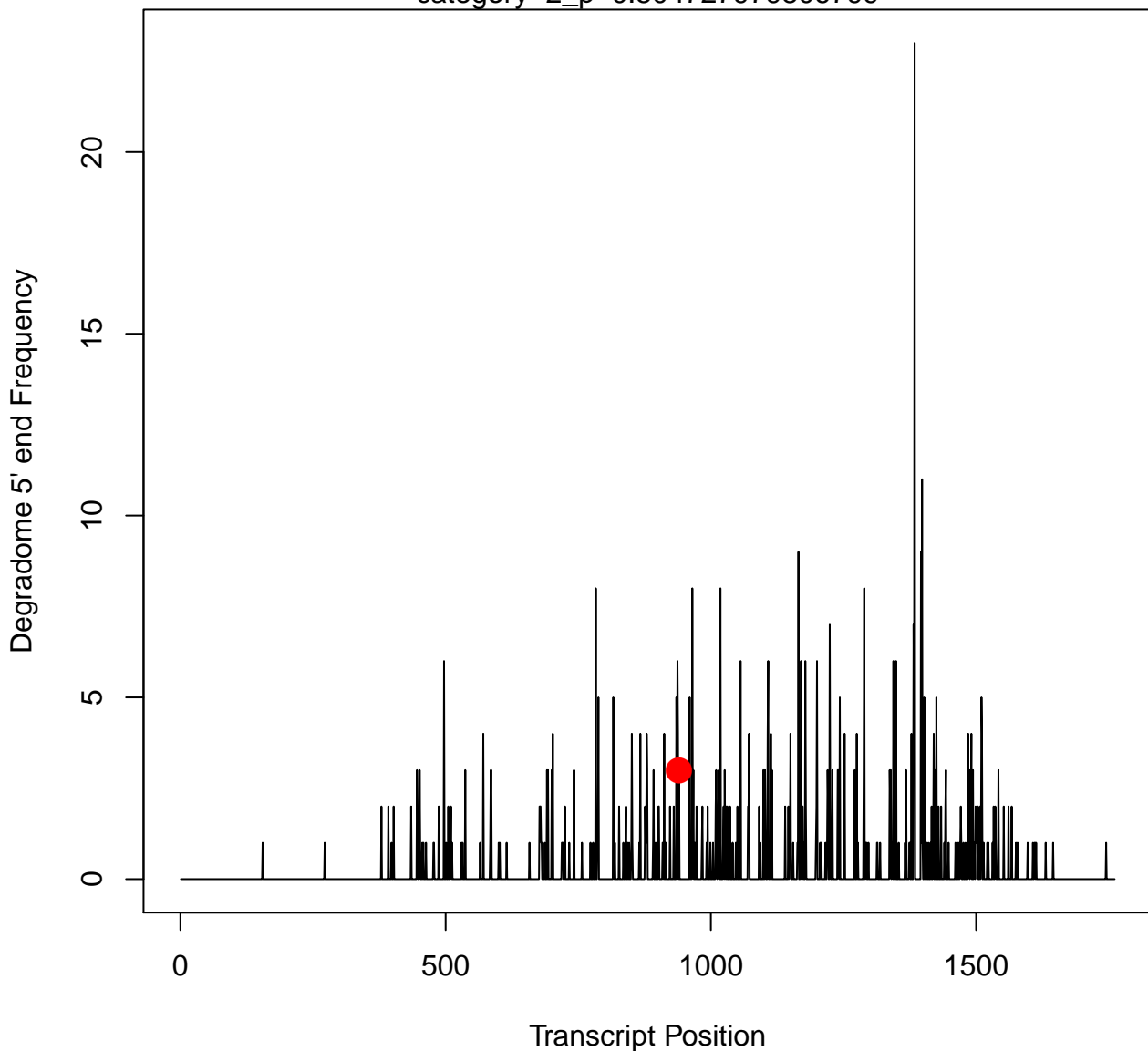

Supplement: Supplementary file 1 [file Data_Sheet_1.zip › Sit-miR166j_Seita.2G145300.1_940_TPlot.pdf]

**T=Seita.5G141300.1\_Q=Sit-miR166j\_S=1222**

category=2\_p=0.0712939086570006

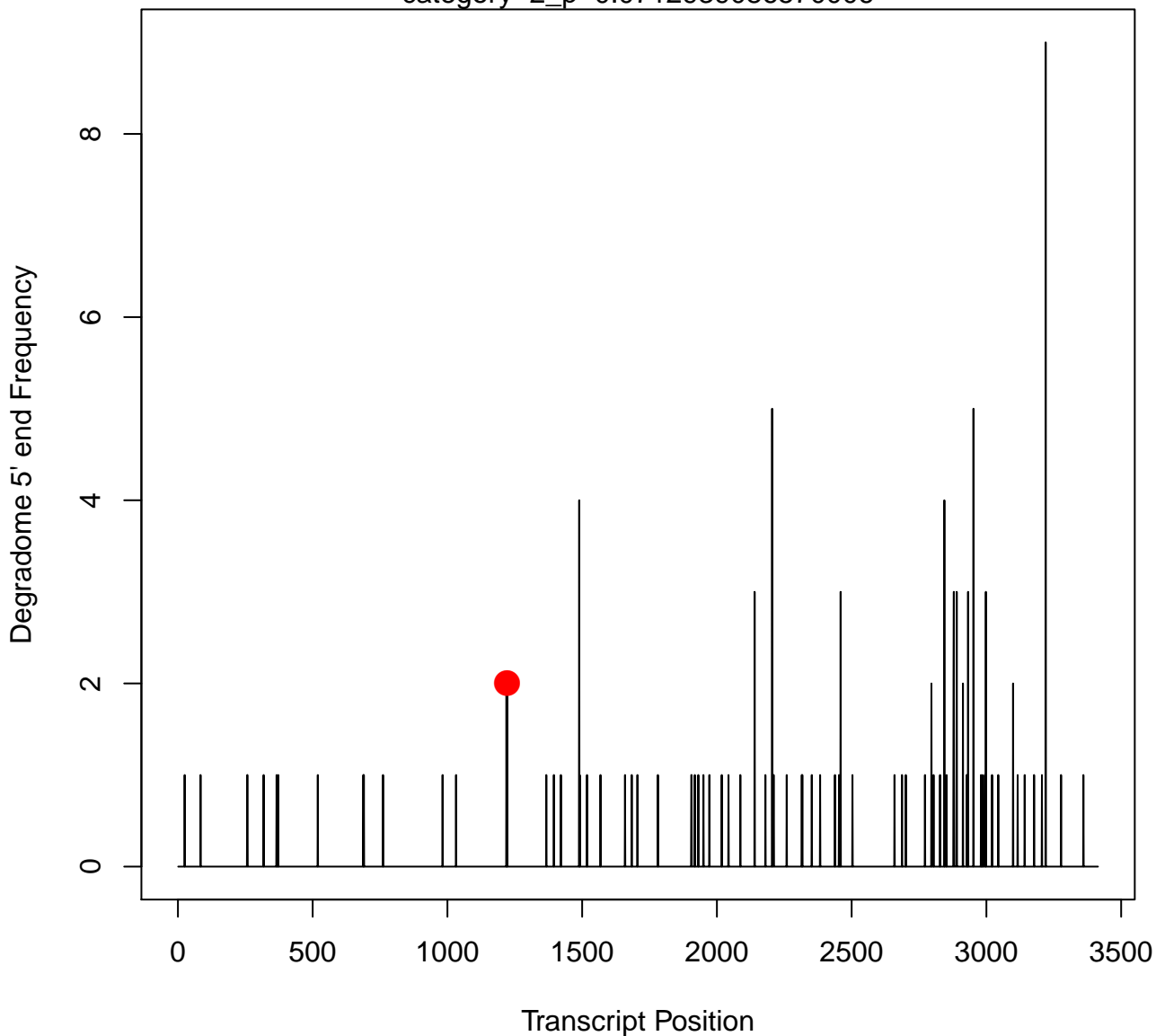

Supplement: Supplementary file 1 [file Data_Sheet_1.zip › Sit-miR166j_Seita.5G141300.1_1222_TPlot.pdf]

**T=Seita.9G572600.1\_Q=Sit-miR166j\_S=1138**

category=0\_p=0.00131270550986873

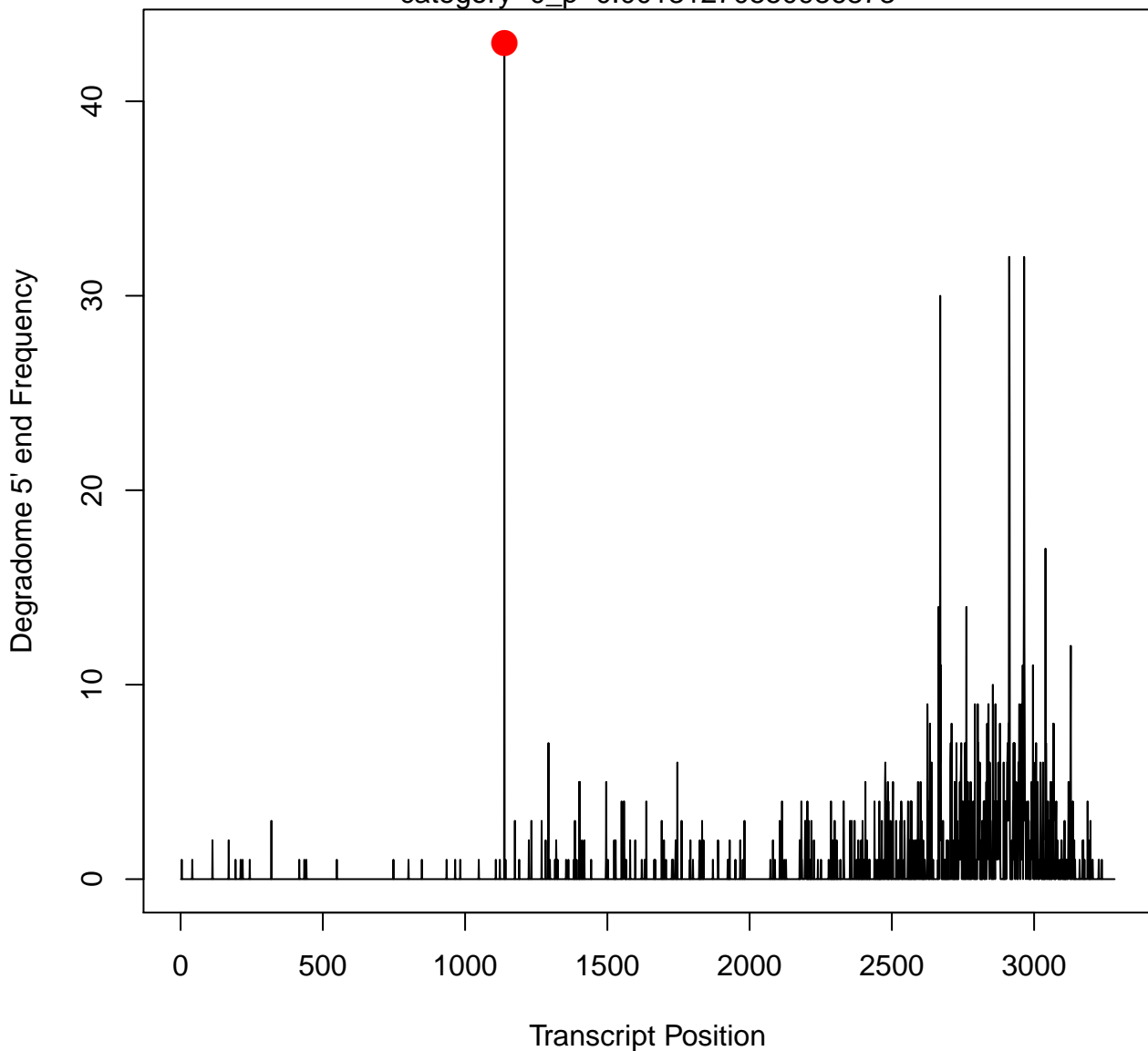

Supplement: Supplementary file 1 [file Data_Sheet_1.zip › Sit-miR166j_Seita.9G572600.1_1138_TPlot.pdf]

**T=Seita.3G185800.1\_Q=Sit-miR166k\_S=1028**

category=2\_p=0.767942126604787

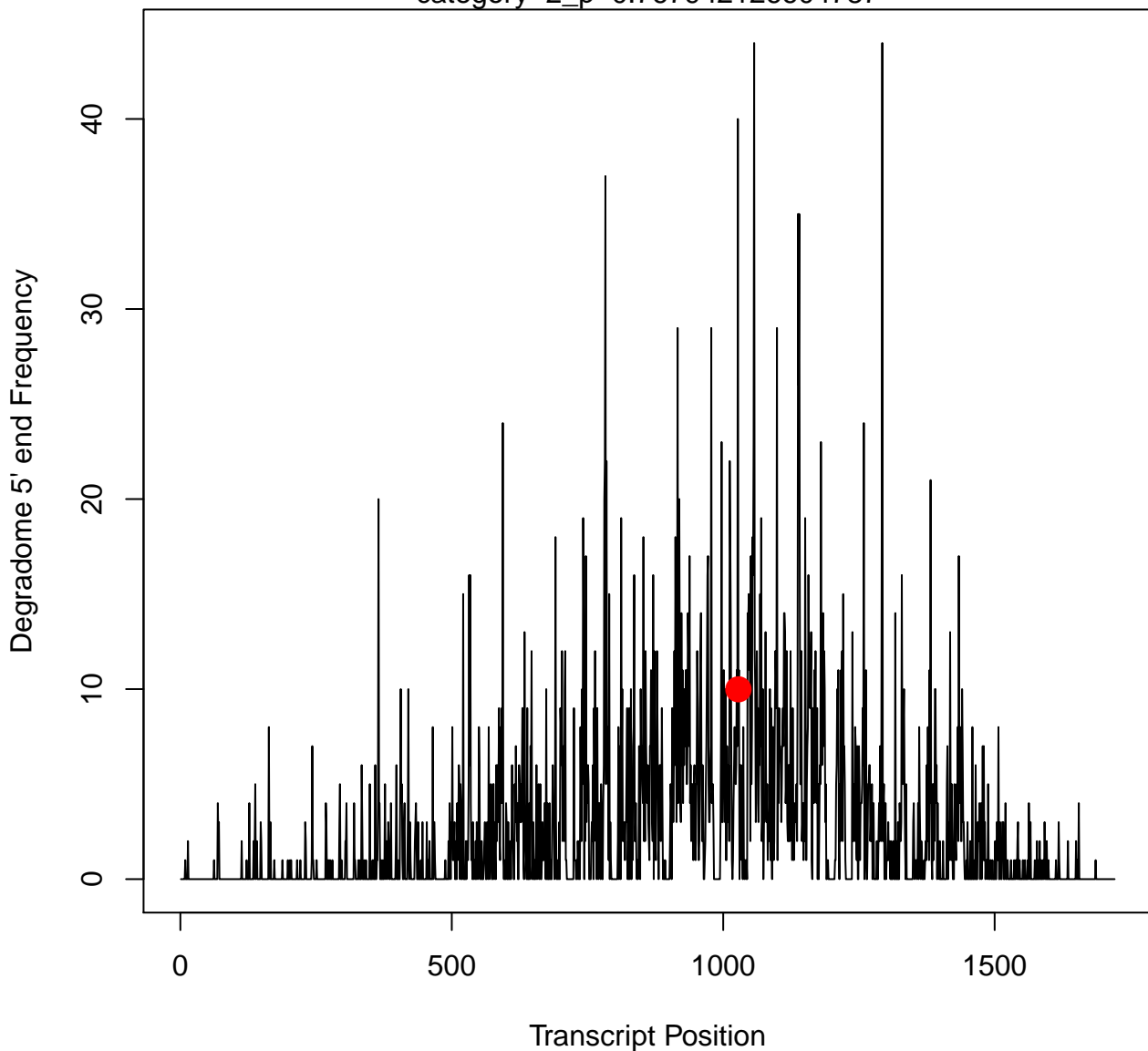

Supplement: Supplementary file 1 [file Data_Sheet_1.zip › Sit-miR166k_Seita.3G185800.1_1028_TPlot.pdf]

**T=Seita.1G101200.1\_Q=Sit-miR167a\_S=317**

category=2\_p=0.998365285137734

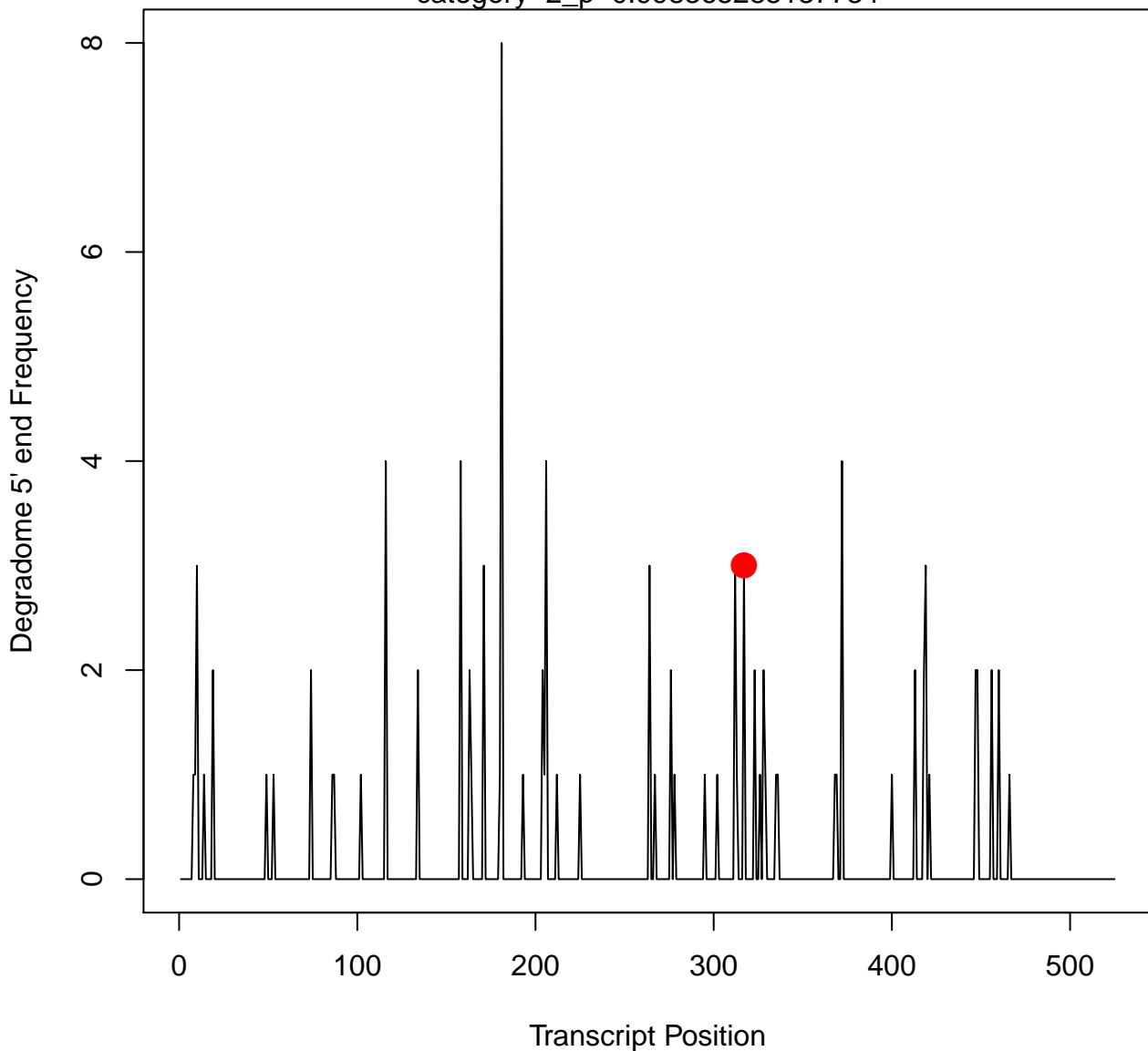

Supplement: Supplementary file 1 [file Data_Sheet_1.zip › Sit-miR167a_Seita.1G101200.1_317_TPlot.pdf]

**T=Seita.7G160400.1\_Q=Sit-miR167a\_S=808**

category=2\_p=0.994252076071229

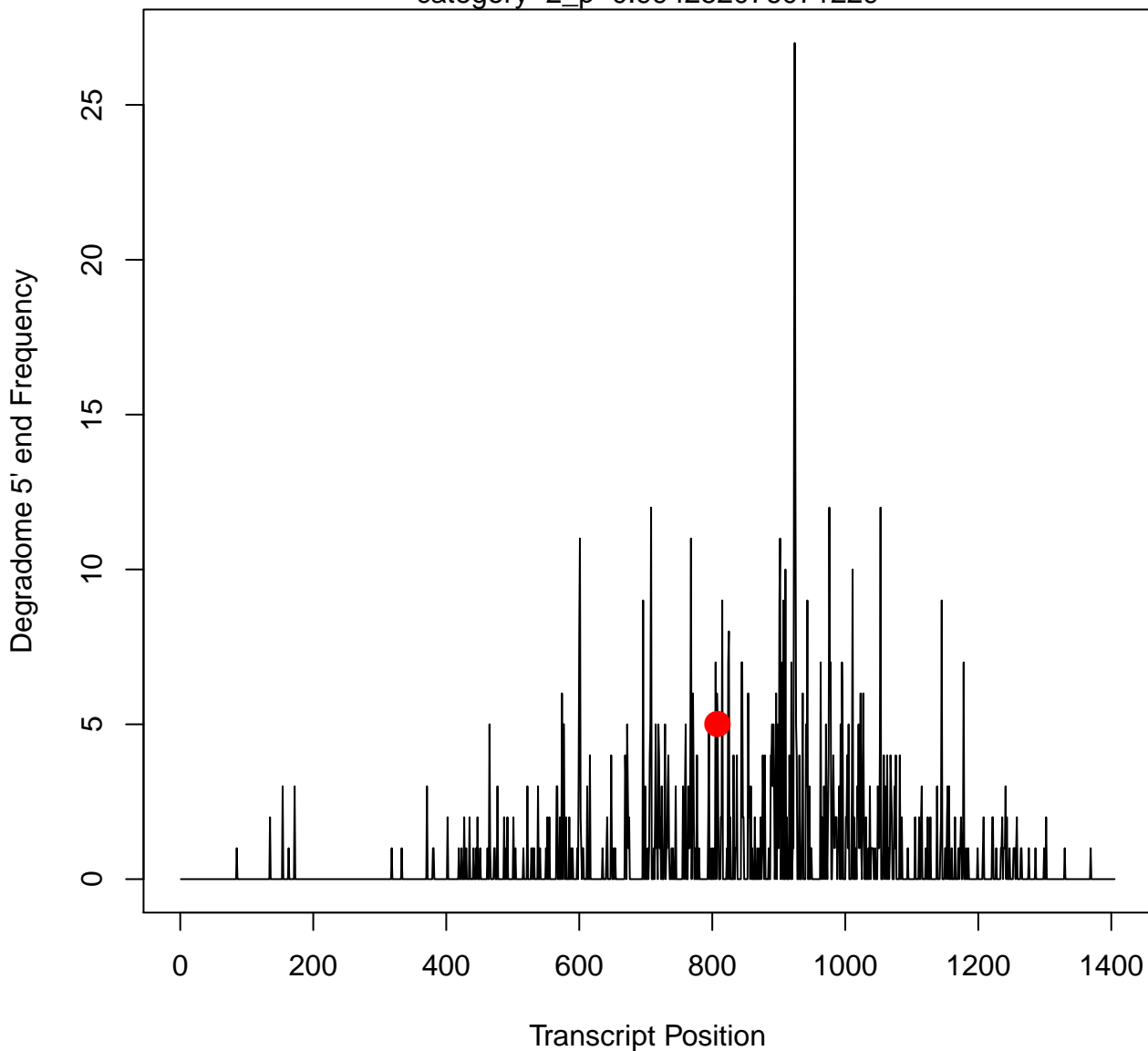

Supplement: Supplementary file 1 [file Data_Sheet_1.zip › Sit-miR167a_Seita.7G160400.1_808_TPlot.pdf]

**T=Seita.9G410200.1\_Q=Sit-miR167b\_S=401**

category=2\_p=0.83668795959958

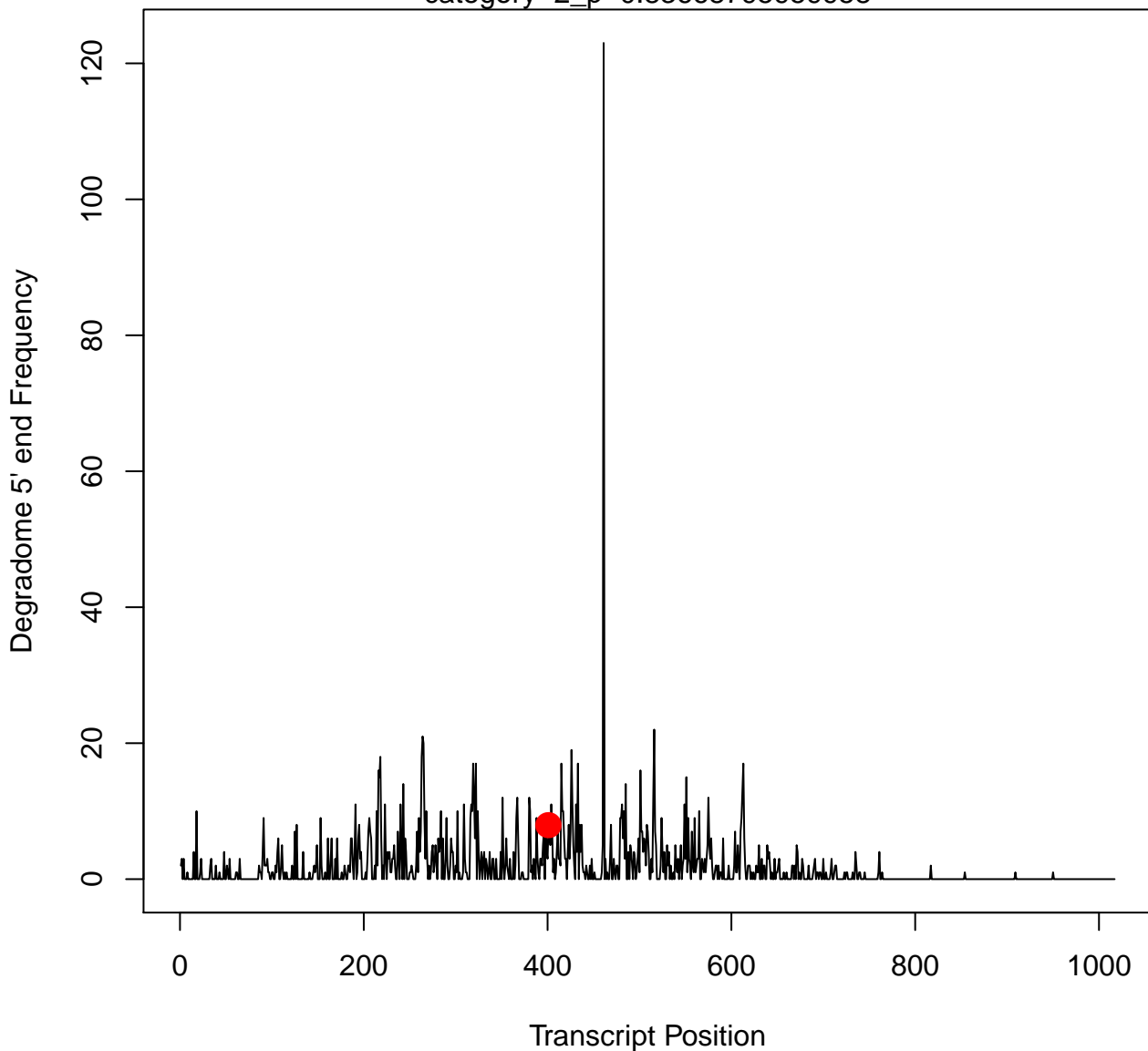

Supplement: Supplementary file 1 [file Data_Sheet_1.zip › Sit-miR167b_Seita.9G410200.1_401_TPlot.pdf]

**T=Seita.1G023300.1\_Q=Sit-miR167c\_S=1378**

category=2\_p=0.94307750921694

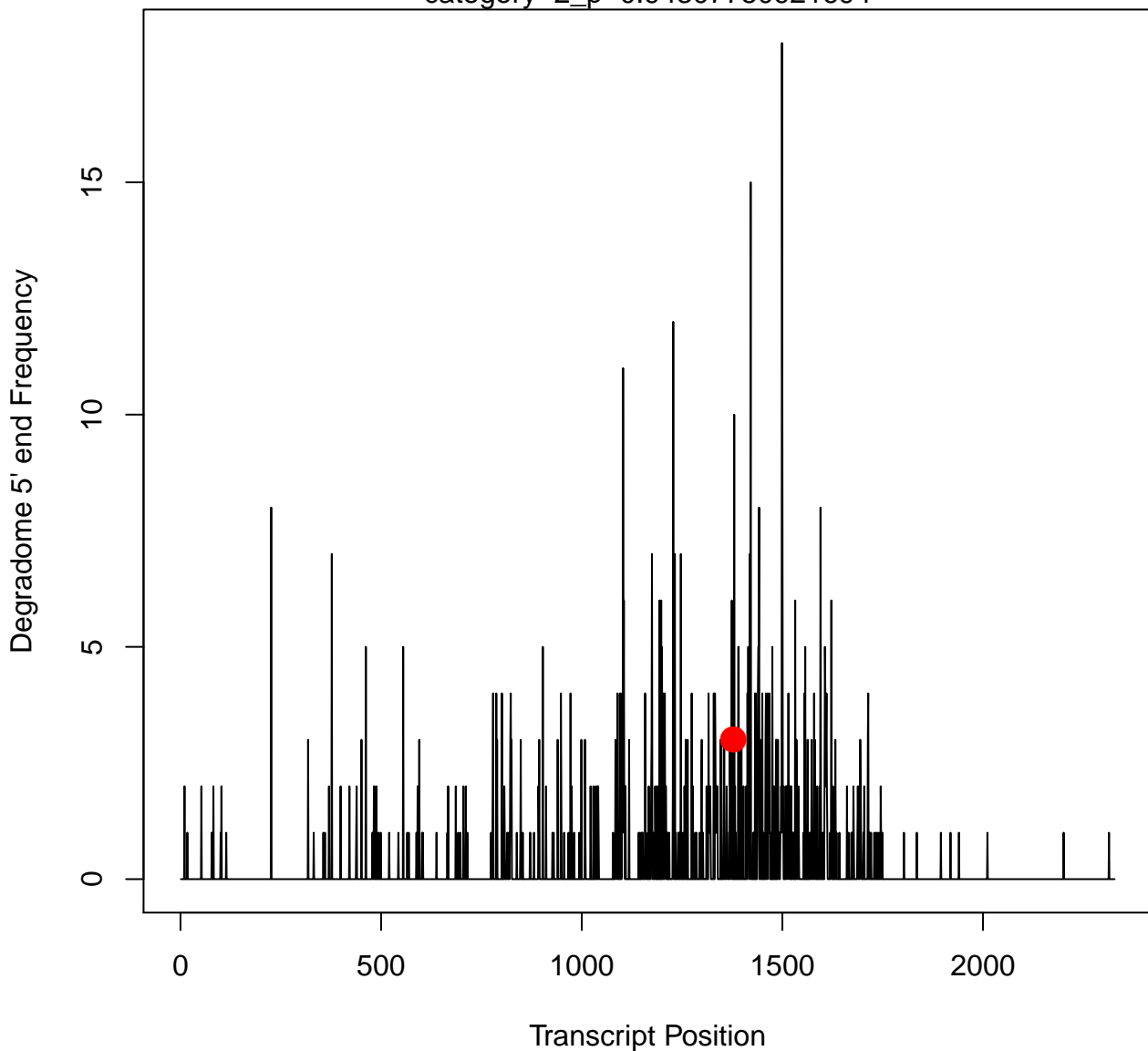

Supplement: Supplementary file 1 [file Data_Sheet_1.zip › Sit-miR167c_Seita.1G023300.1_1378_TPlot.pdf]

**T=Seita.3G138800.1\_Q=Sit-miR167d\_S=1329**

category=2\_p=0.269731193979571

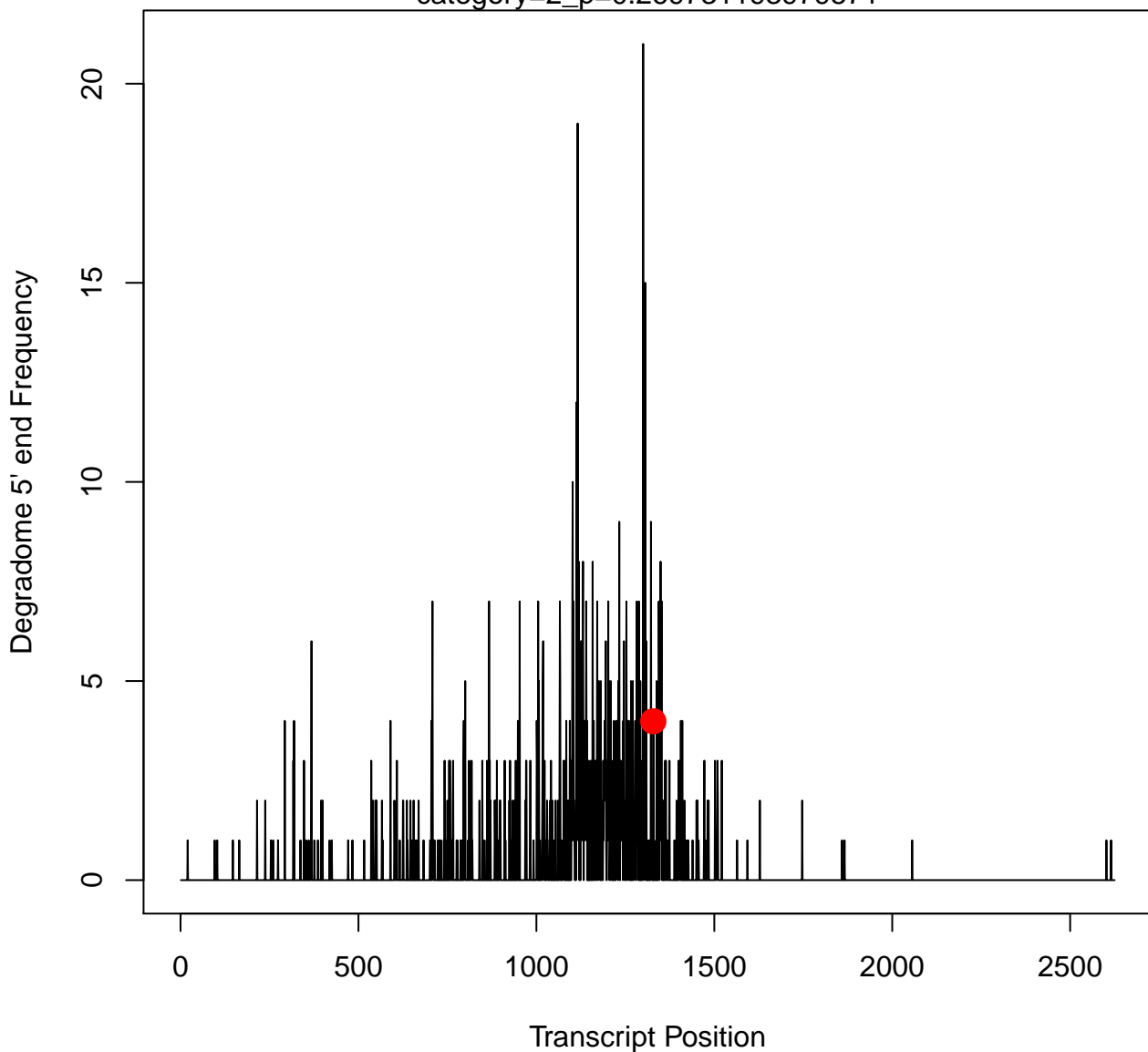

Supplement: Supplementary file 1 [file Data_Sheet_1.zip › Sit-miR167d_Seita.3G138800.1_1329_TPlot.pdf]

**T=Seita.3G394000.1\_Q=Sit-miR167h\_S=3277**

category=0\_p=0.0039329492043878

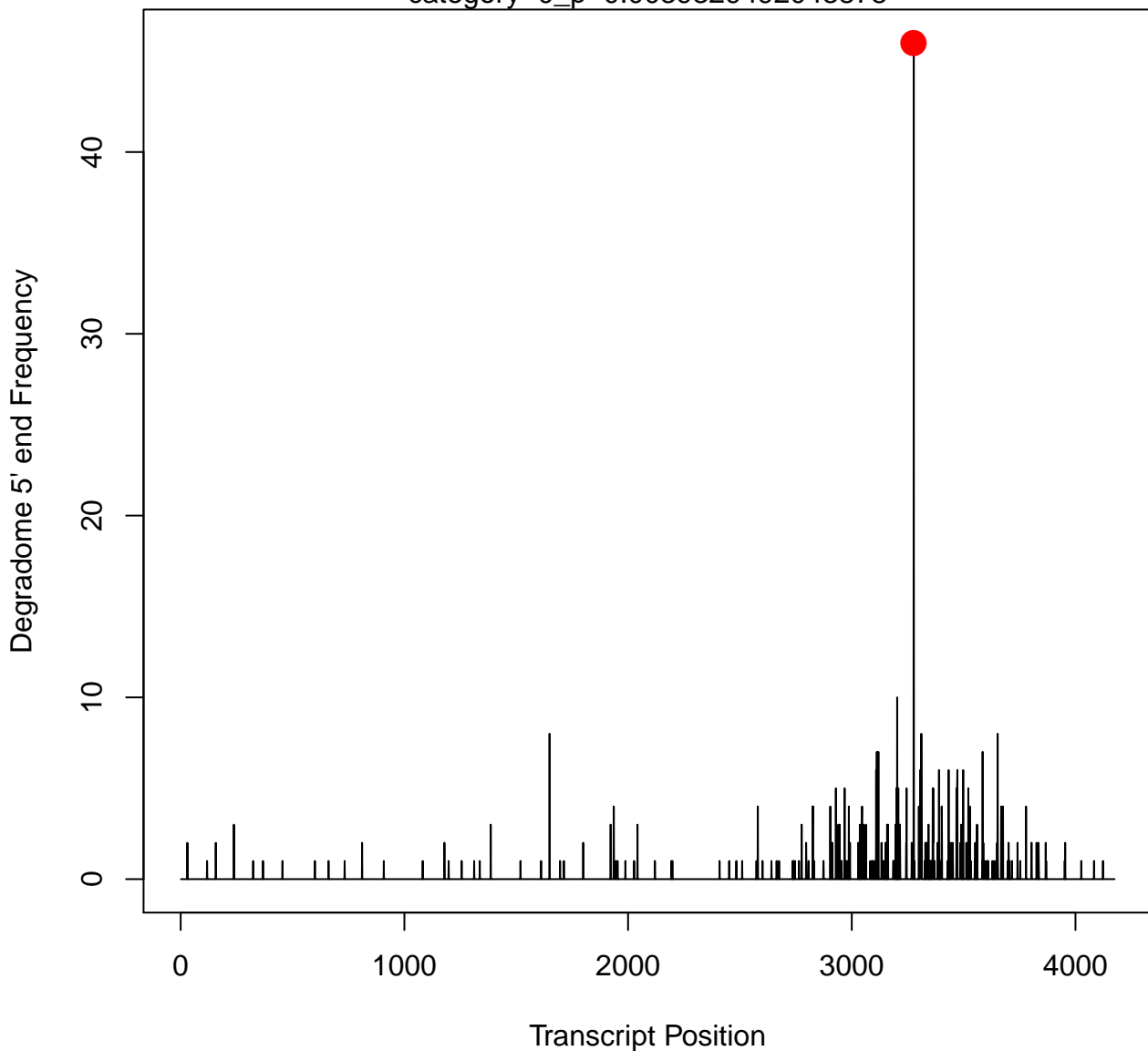

Supplement: Supplementary file 1 [file Data_Sheet_1.zip › Sit-miR167h_Seita.3G394000.1_3277_TPlot.pdf]

**T=Seita.4G262300.1\_Q=Sit-miR167h\_S=3389**

category=0\_p=0.00262368782398181

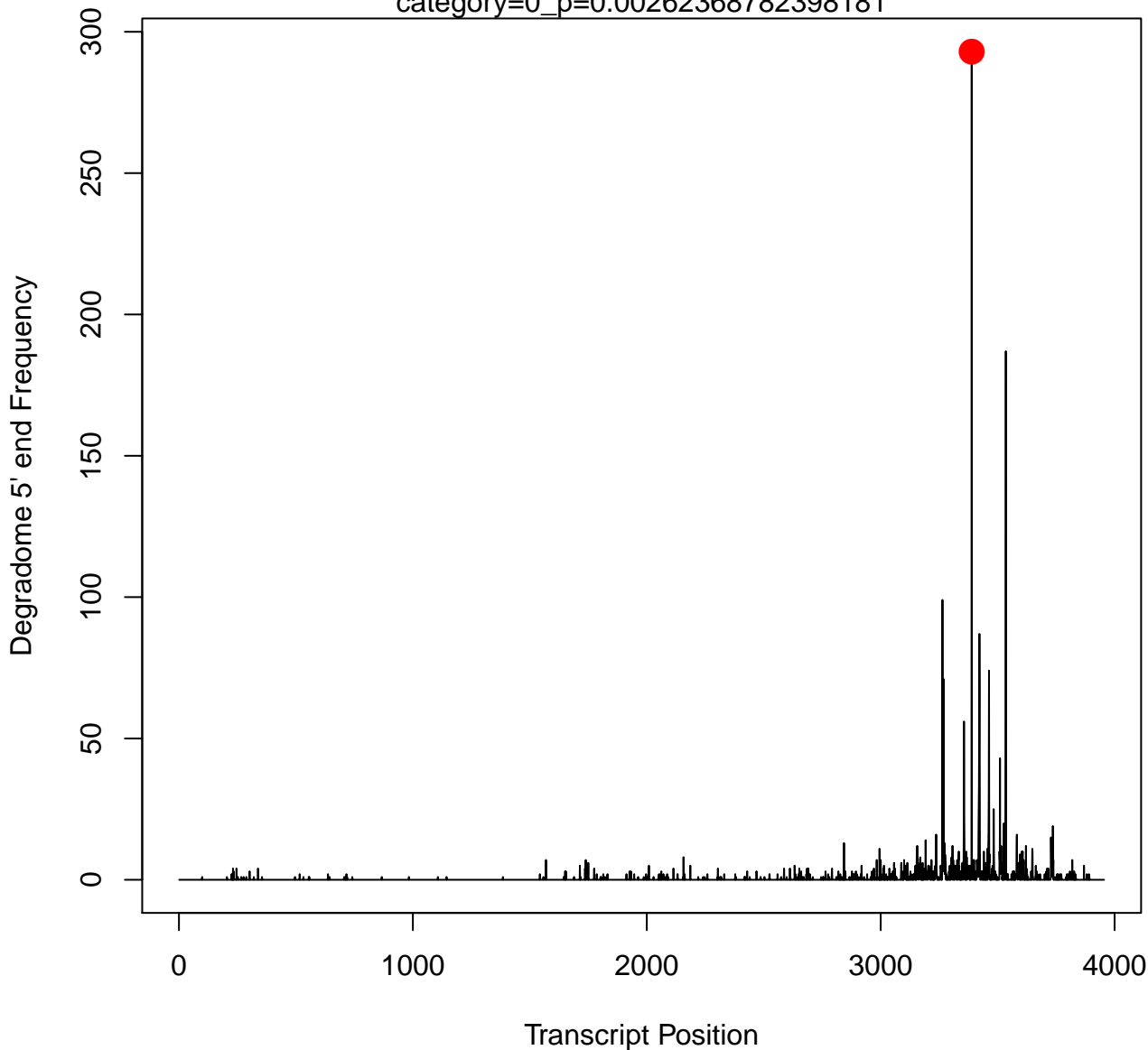

Supplement: Supplementary file 1 [file Data_Sheet_1.zip › Sit-miR167h_Seita.4G262300.1_3389_TPlot.pdf]

**T=Seita.5G251600.1\_Q=Sit-miR167h\_S=910**

category=2\_p=0.969077128084175

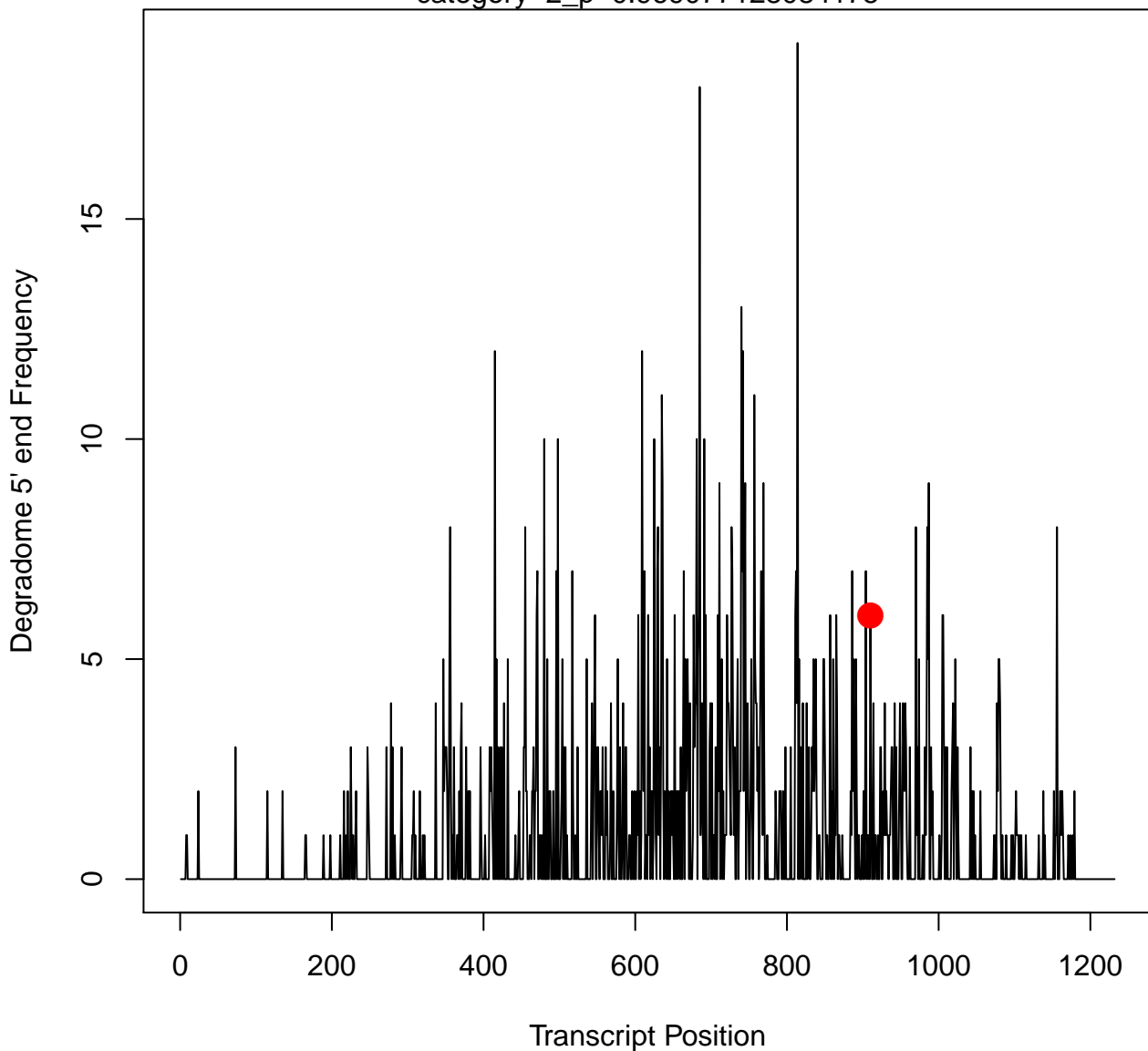

Supplement: Supplementary file 1 [file Data_Sheet_1.zip › Sit-miR167h_Seita.5G251600.1_910_TPlot.pdf]

**T=Seita.9G111400.1\_Q=Sit-miR167h\_S=3265**

category=2\_p=0.381686239905268

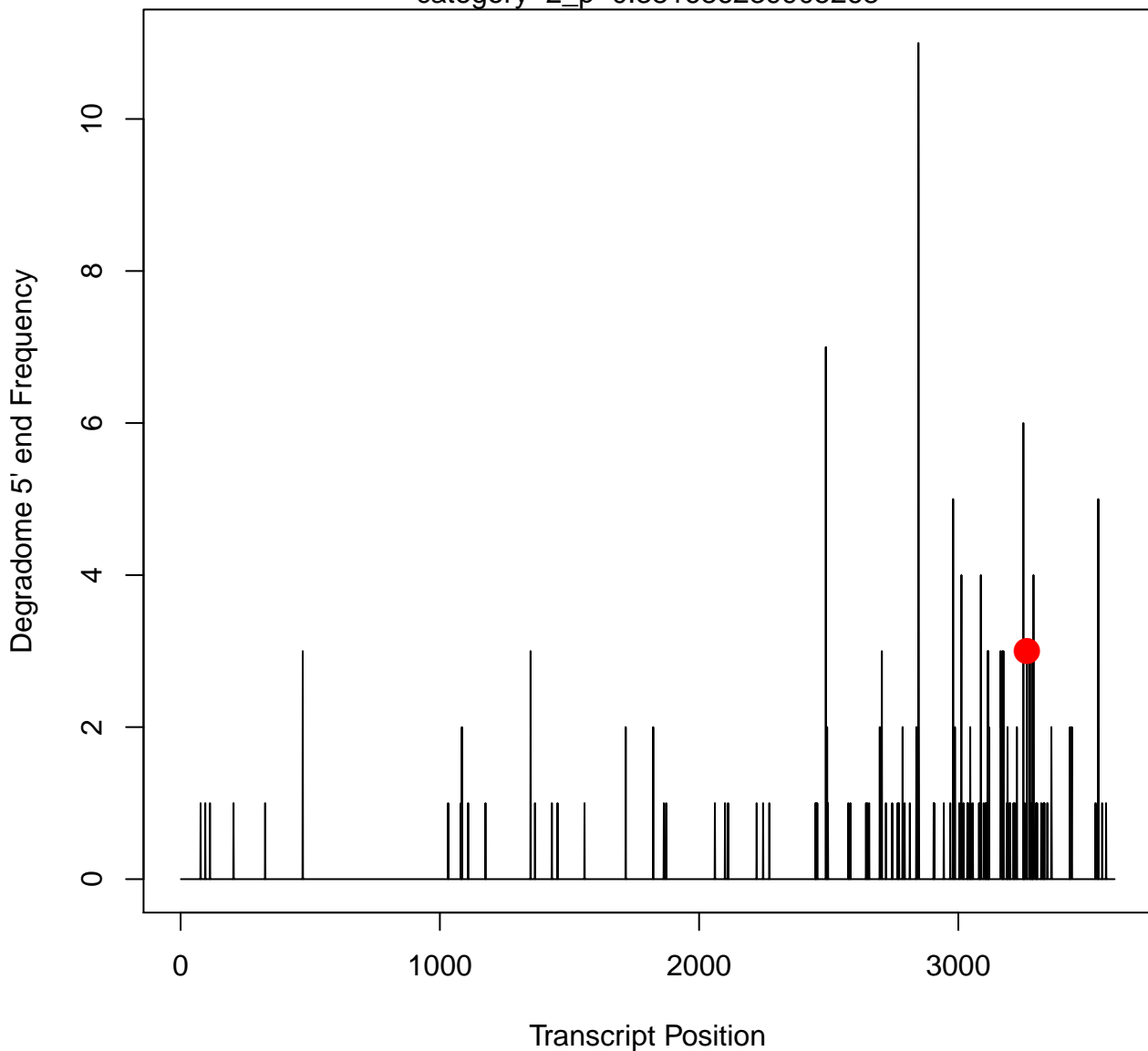

Supplement: Supplementary file 1 [file Data_Sheet_1.zip › Sit-miR167h_Seita.9G111400.1_3265_TPlot.pdf]

**T=Seita.7G303100.1\_Q=Sit-miR167i\_S=1350**

category=2\_p=0.999551964777682

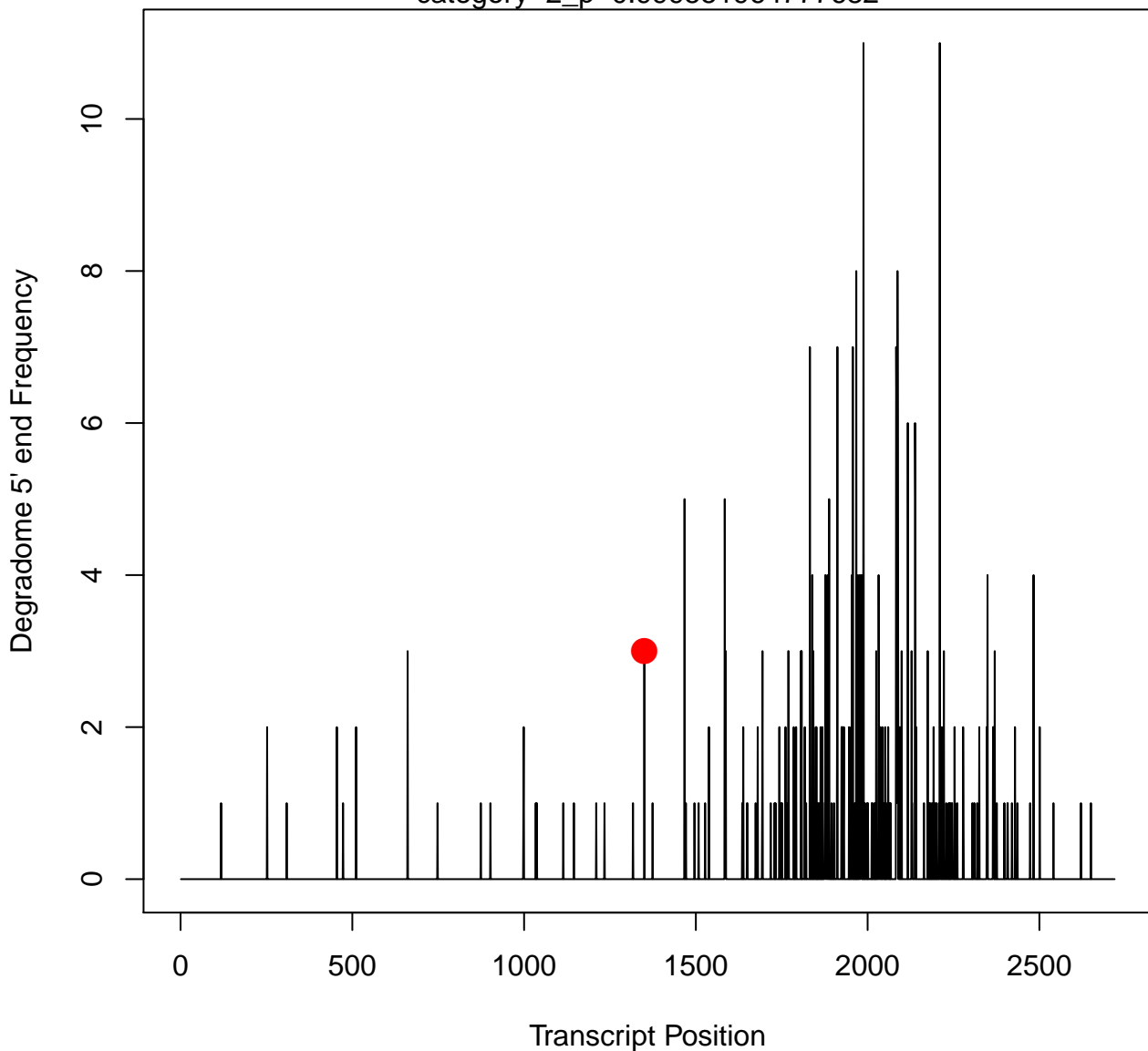

Supplement: Supplementary file 1 [file Data_Sheet_1.zip › Sit-miR167i_Seita.7G303100.1_1350_TPlot.pdf]

**T=Seita.1G077200.1\_Q=Sit-miR167j\_S=3508**

category=1\_p=0.00164853358359274

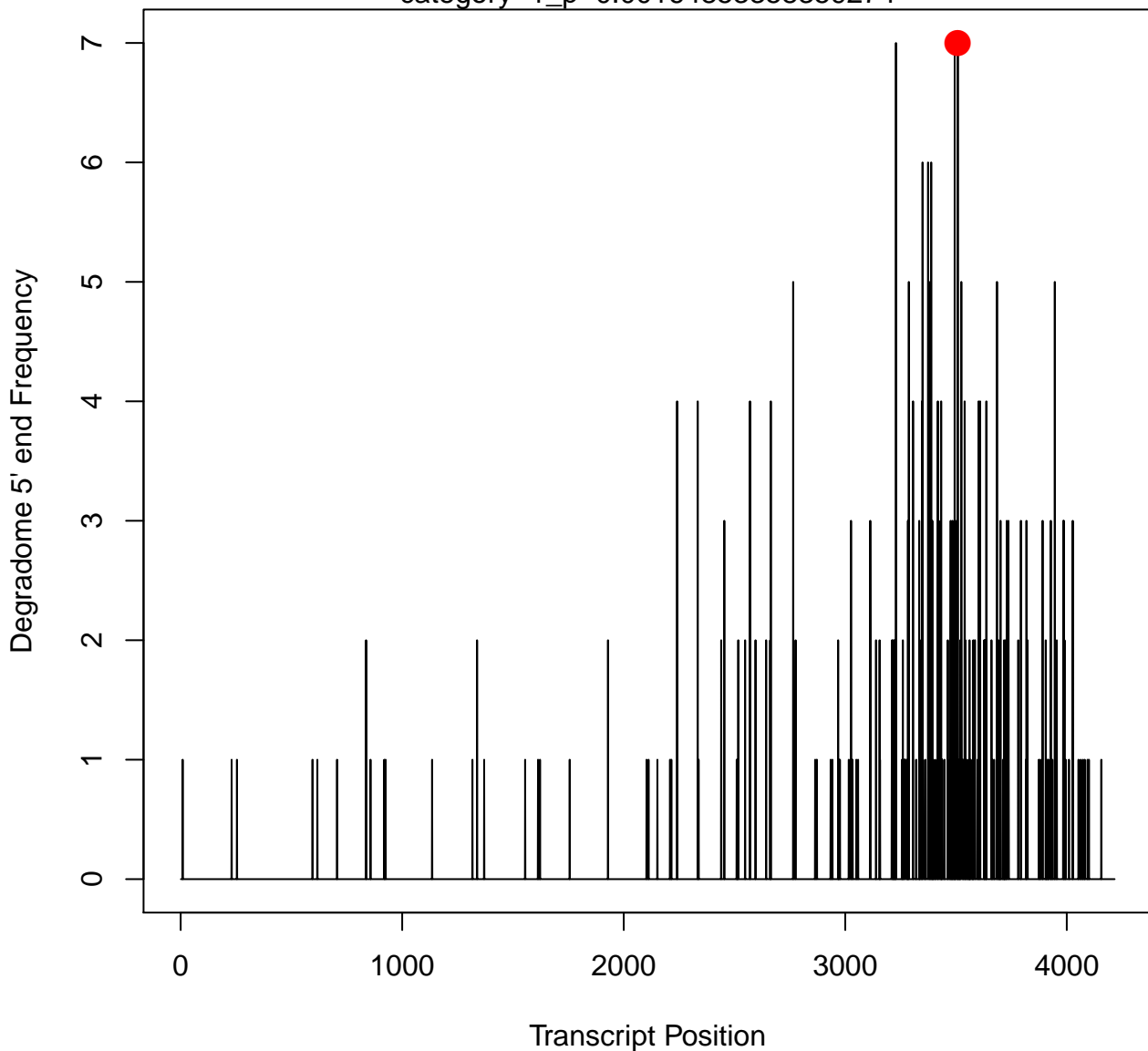

Supplement: Supplementary file 1 [file Data_Sheet_1.zip › Sit-miR167j_Seita.1G077200.1_3508_TPlot.pdf]

**T=Seita.1G238800.1\_Q=Sit-miR167j\_S=507**

category=2\_p=0.999943518738044

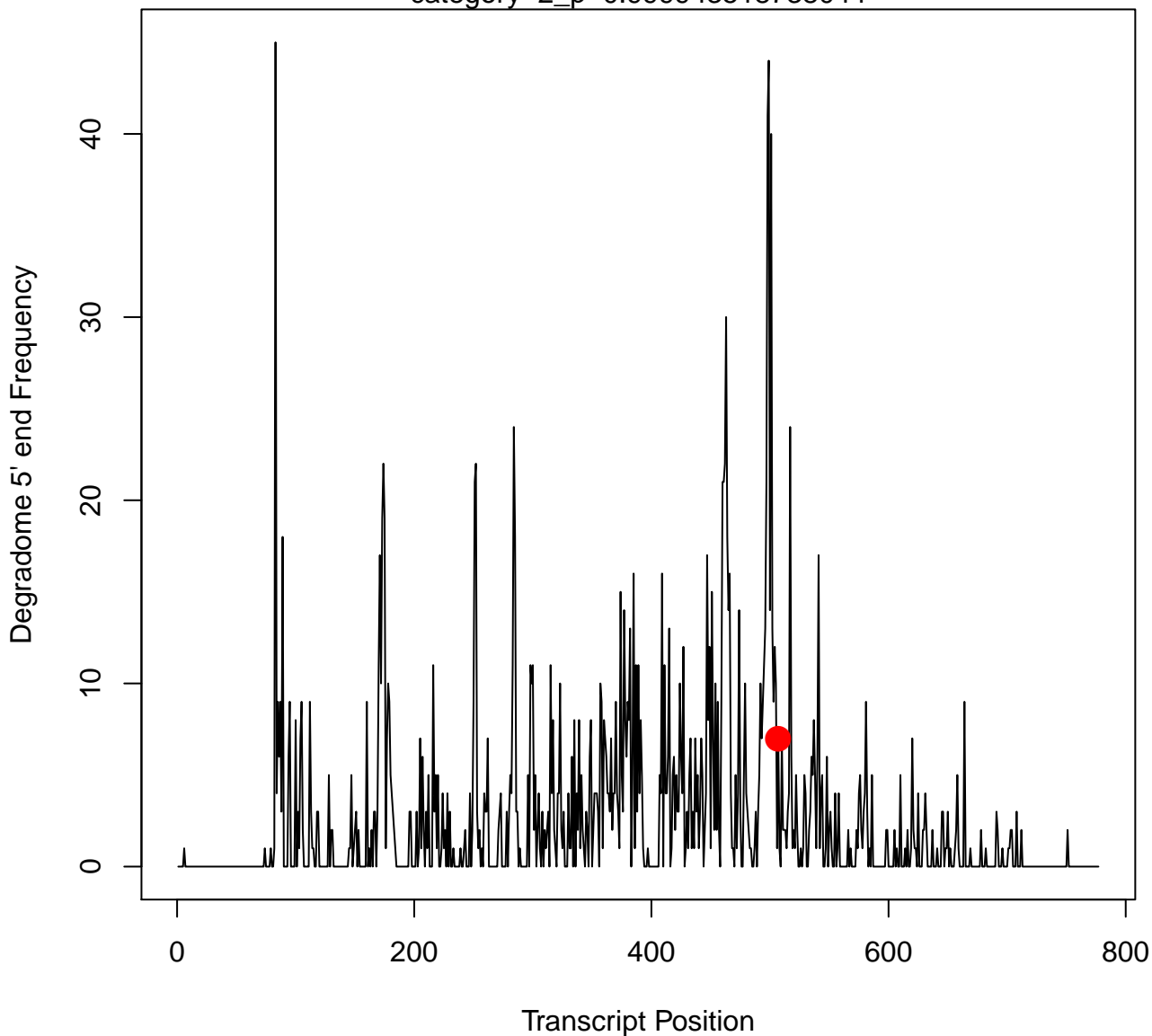

Supplement: Supplementary file 1 [file Data_Sheet_1.zip › Sit-miR167j_Seita.1G238800.1_507_TPlot.pdf]

**T=Seita.3G020000.1\_Q=Sit-miR167j\_S=2739**

category=0\_p=0.00131270550986873

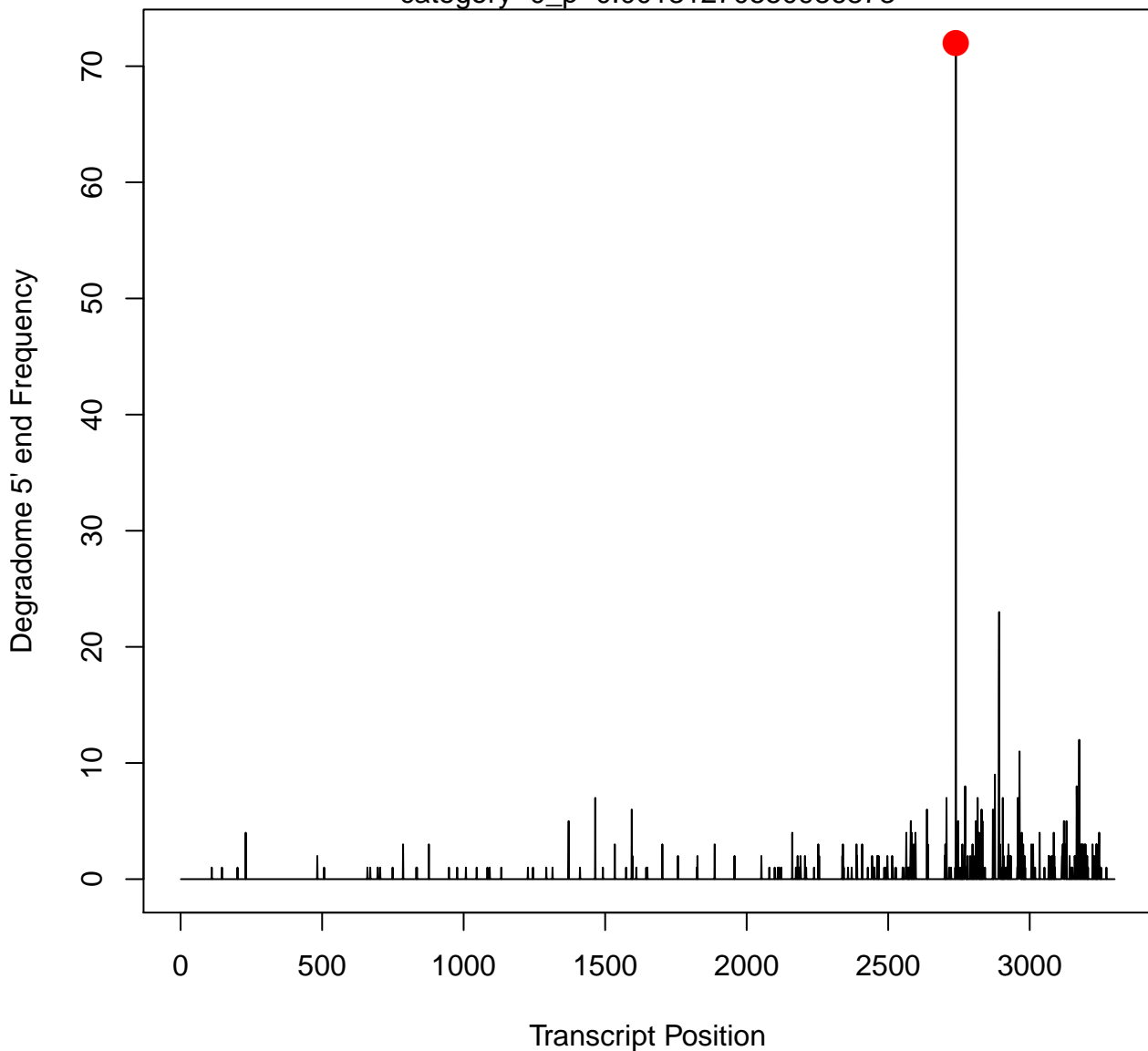

Supplement: Supplementary file 1 [file Data_Sheet_1.zip › Sit-miR167j_Seita.3G020000.1_2739_TPlot.pdf]

**T=Seita.3G353500.1\_Q=Sit-miR167j\_S=725**

category=0\_p=0.0627476173676701

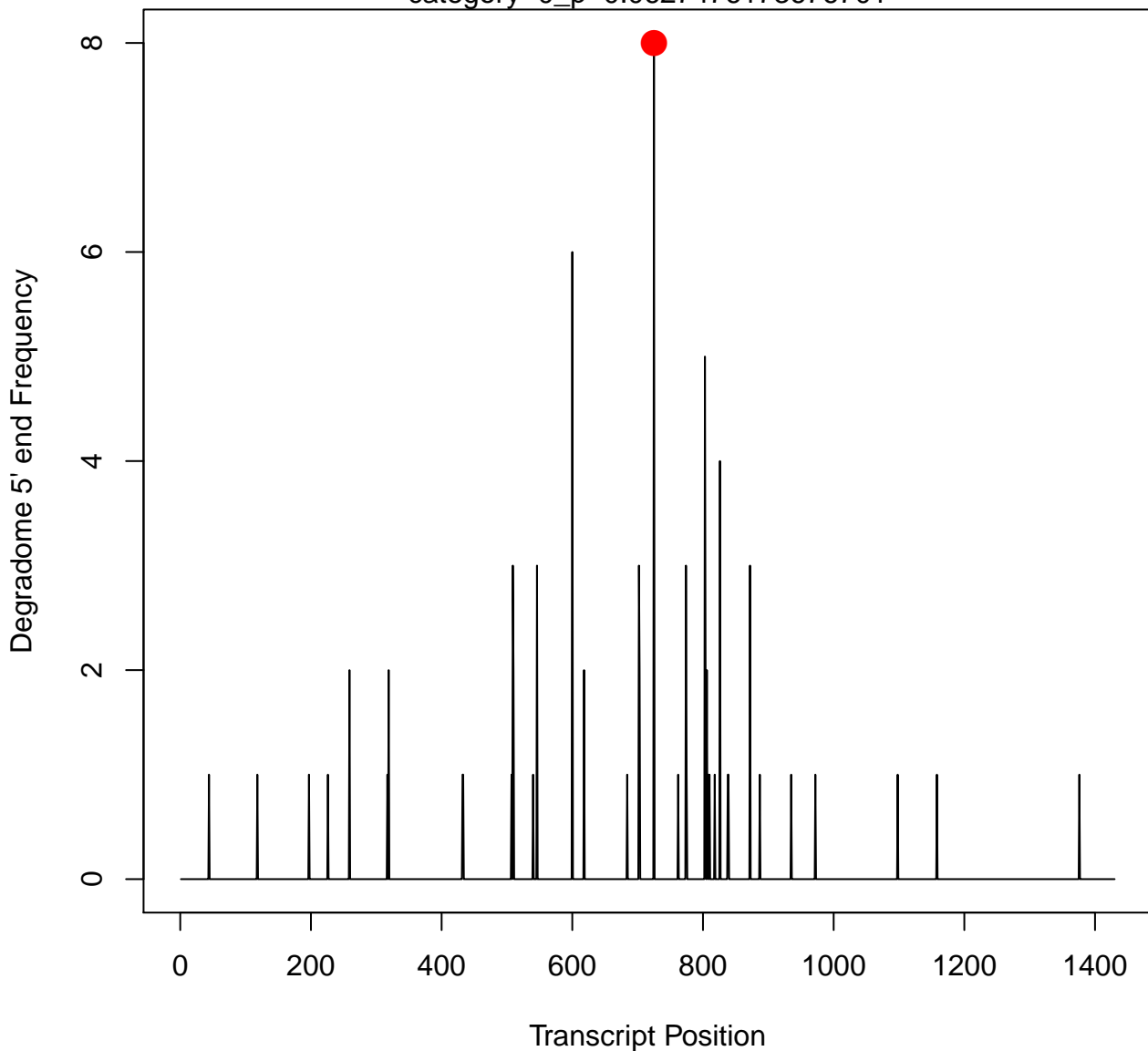

Supplement: Supplementary file 1 [file Data_Sheet_1.zip › Sit-miR167j_Seita.3G353500.1_725_TPlot.pdf]

**T=Seita.7G045700.1\_Q=Sit-miR167j\_S=613**

category=2\_p=0.999957983722973

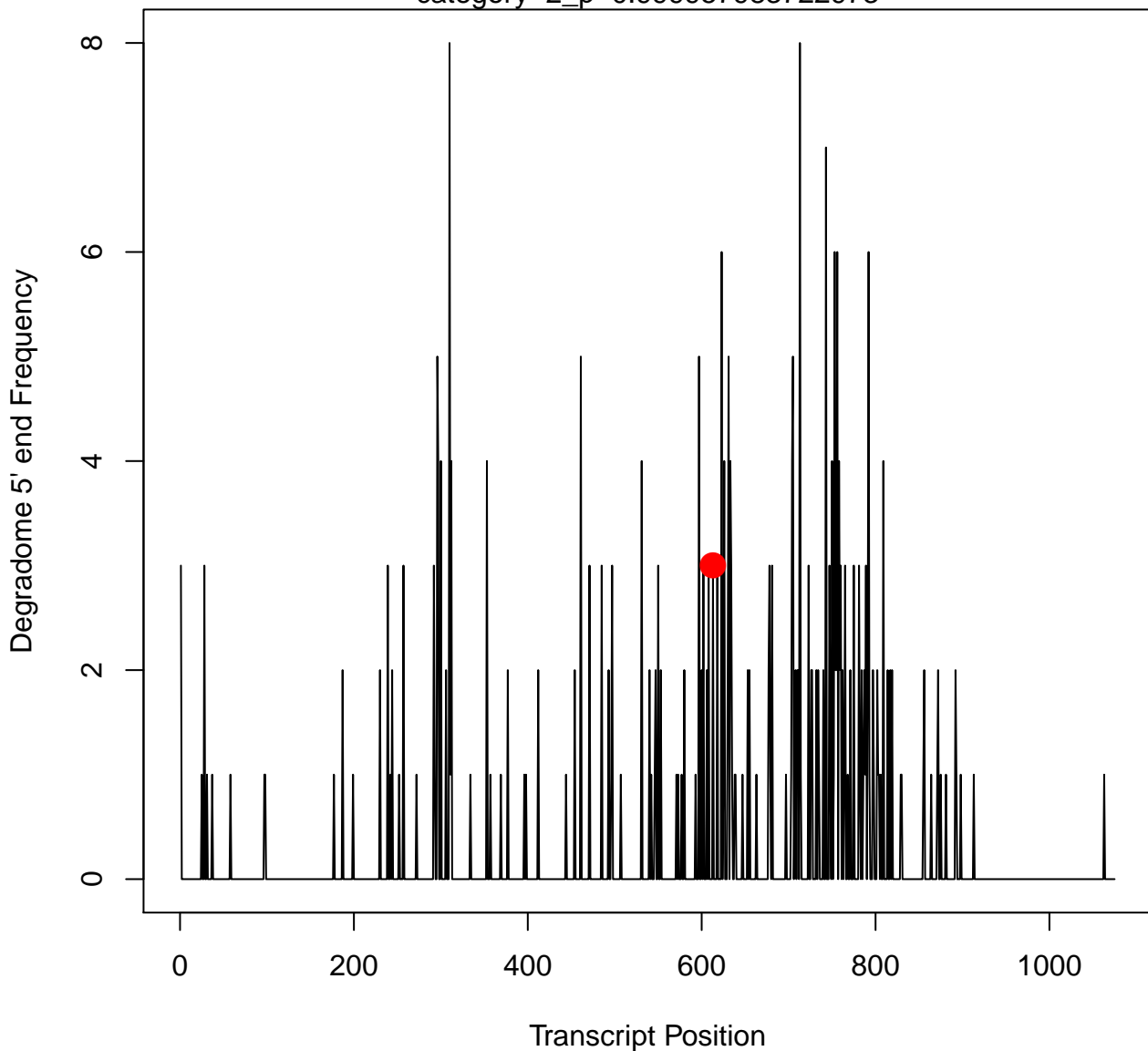

Supplement: Supplementary file 1 [file Data_Sheet_1.zip › Sit-miR167j_Seita.7G045700.1_613_TPlot.pdf]

**T=Seita.7G235100.1\_Q=Sit-miR167j\_S=1010**

category=2\_p=0.088308580092527

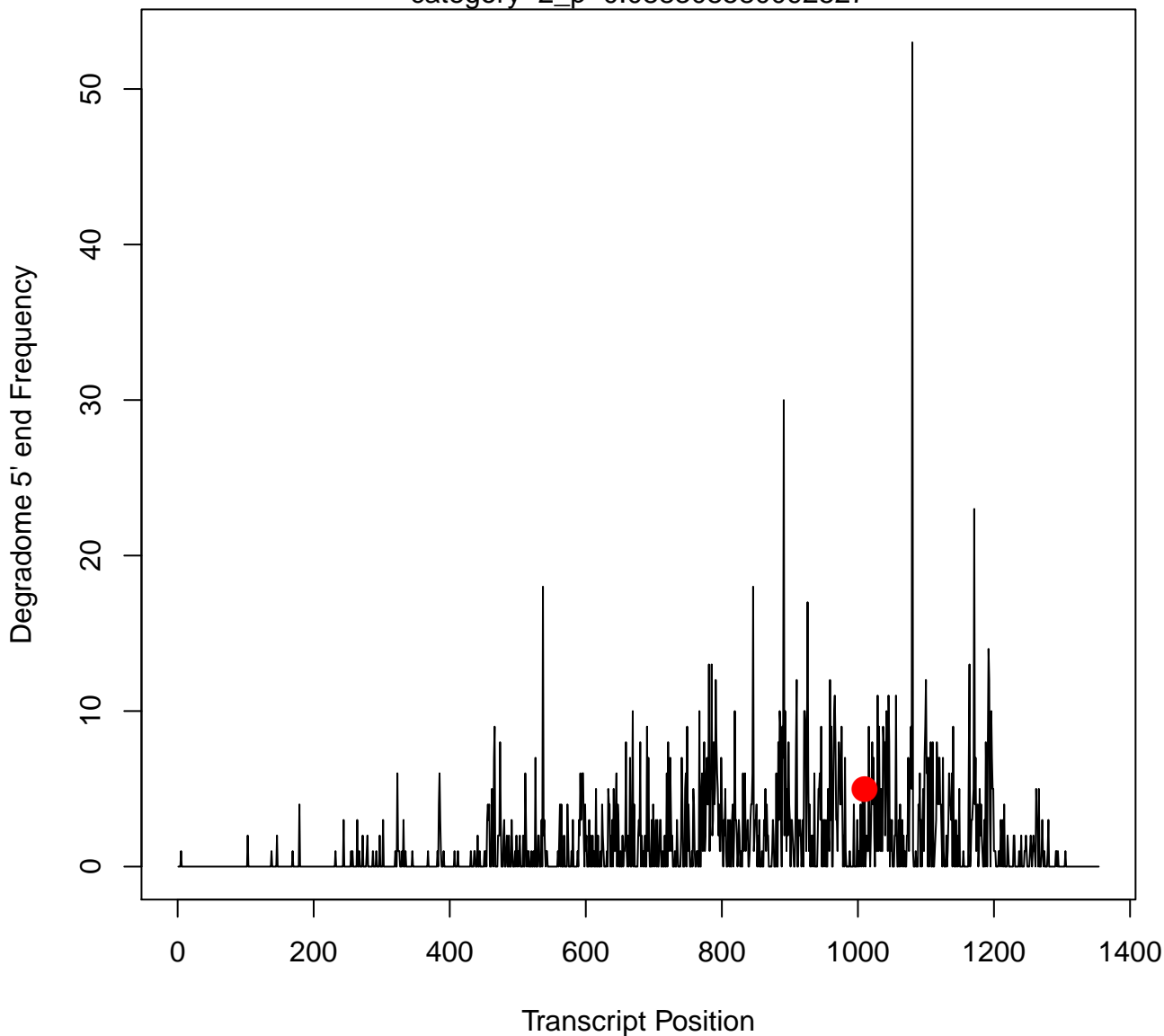

Supplement: Supplementary file 1 [file Data_Sheet_1.zip › Sit-miR167j_Seita.7G235100.1_1010_TPlot.pdf]

**T=Seita.1G218400.1\_Q=Sit-miR168\_S=725**

category=2\_p=0.971281740489954

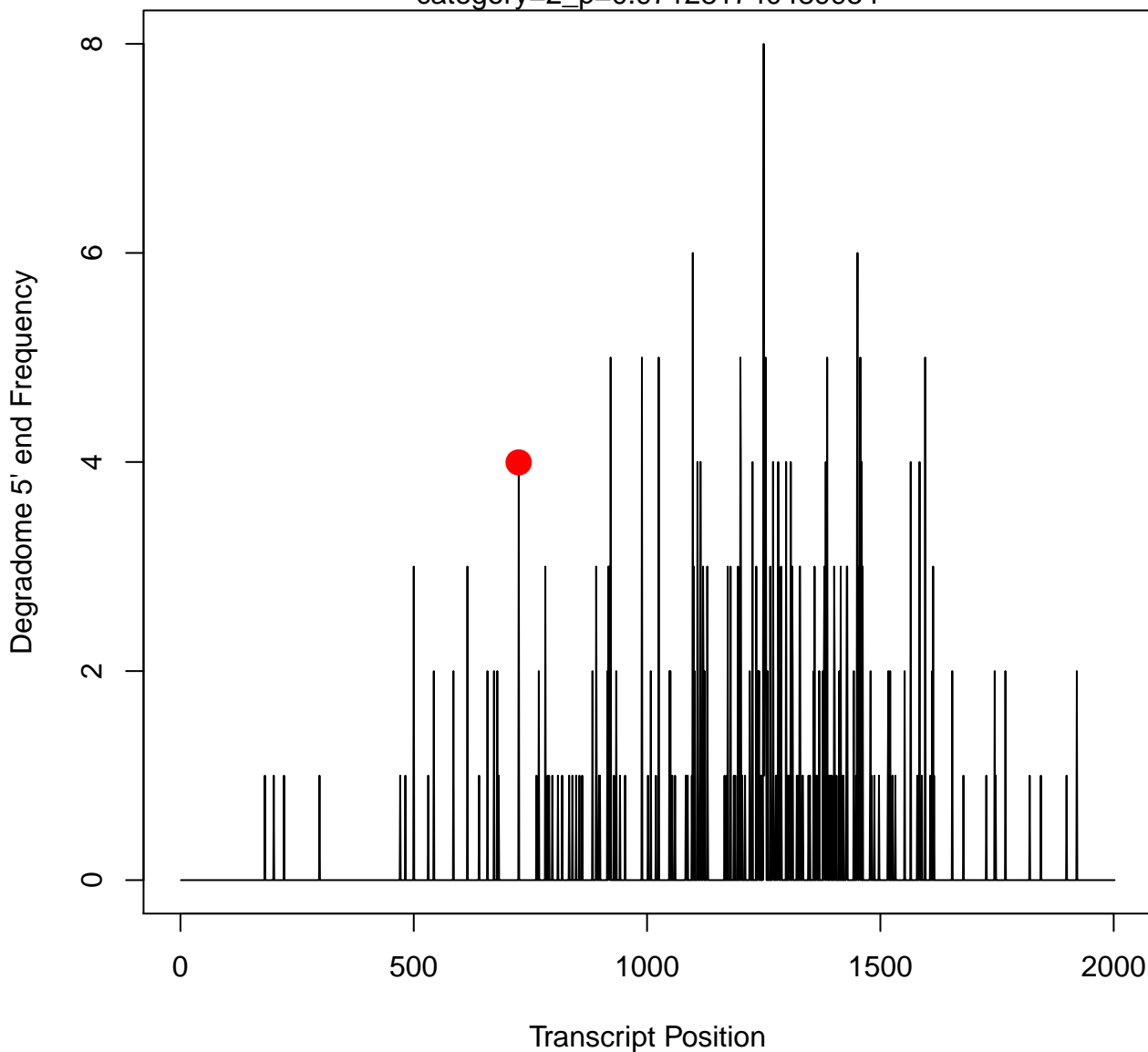

Supplement: Supplementary file 1 [file Data_Sheet_1.zip › Sit-miR168_Seita.1G218400.1_725_TPlot.pdf]

**T=Seita.1G378700.1\_Q=Sit-miR168\_S=527**

category=2\_p=0.425768244638829

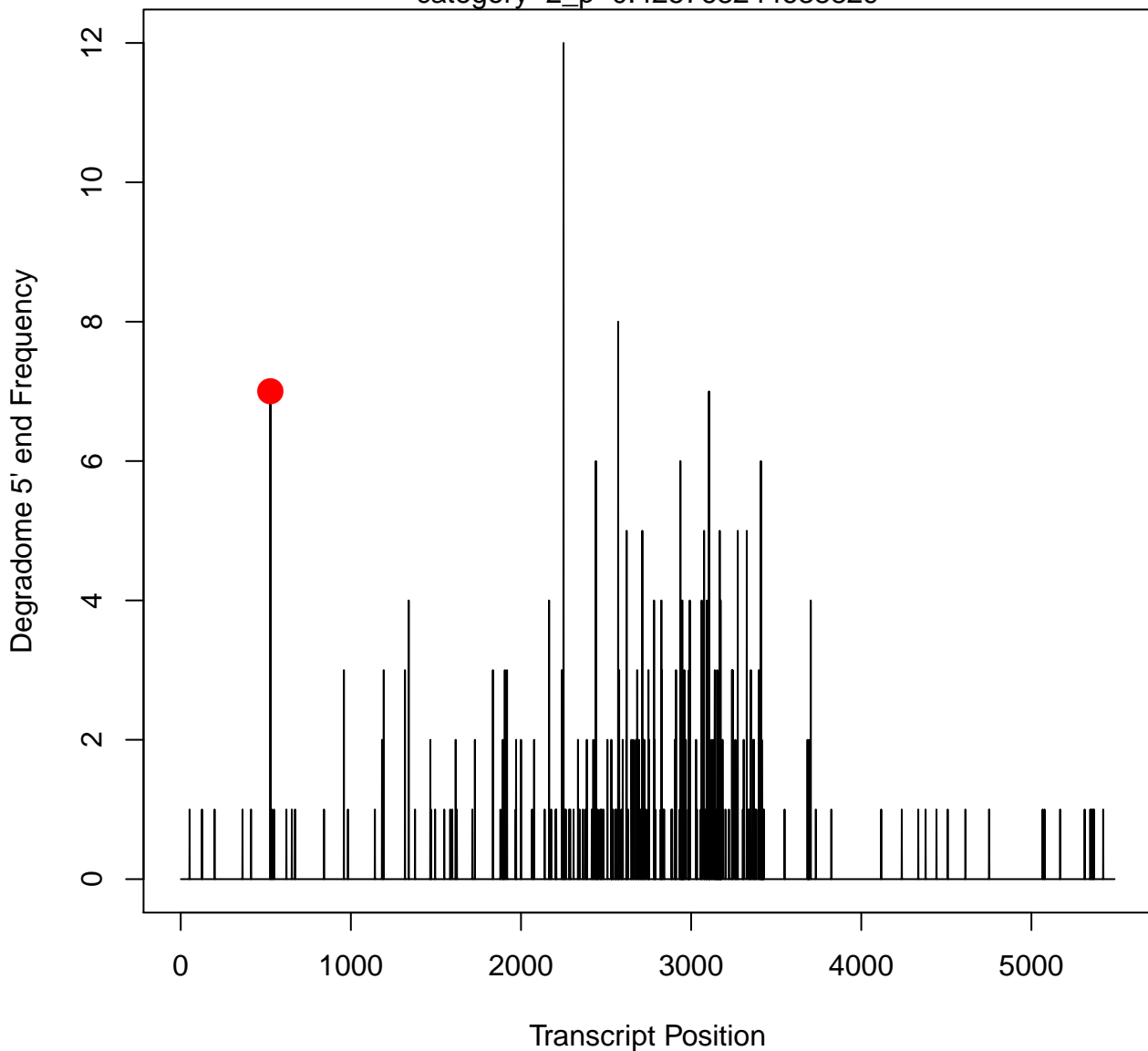

Supplement: Supplementary file 1 [file Data_Sheet_1.zip › Sit-miR168_Seita.1G378700.1_527_TPlot.pdf]

**T=Seita.2G191500.1\_Q=Sit-miR168\_S=704**

category=2\_p=0.882923285017311

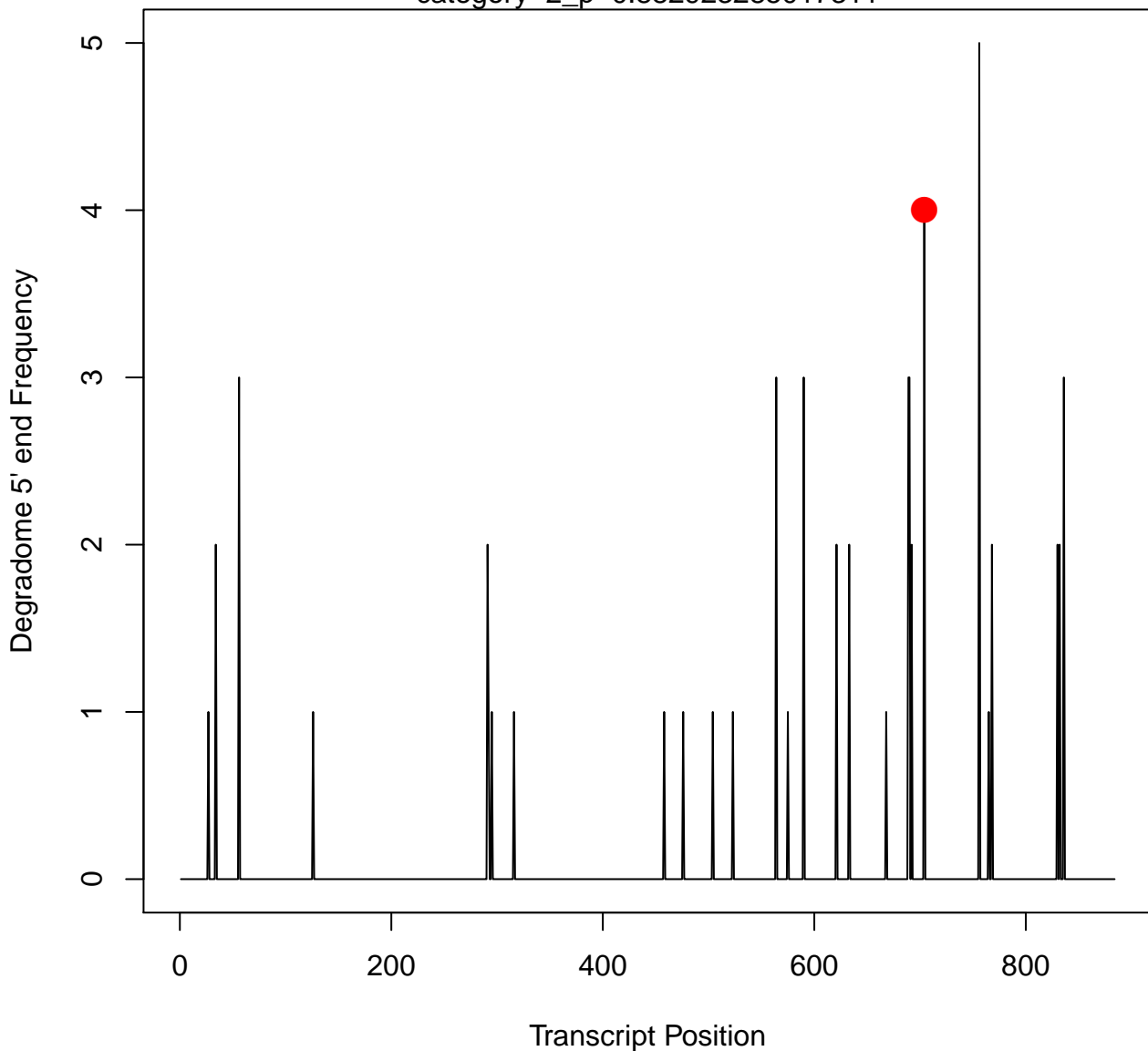

Supplement: Supplementary file 1 [file Data_Sheet_1.zip › Sit-miR168_Seita.2G191500.1_704_TPlot.pdf]

**T=Seita.2G261600.1\_Q=Sit-miR168\_S=131**

category=2\_p=0.985776649596027

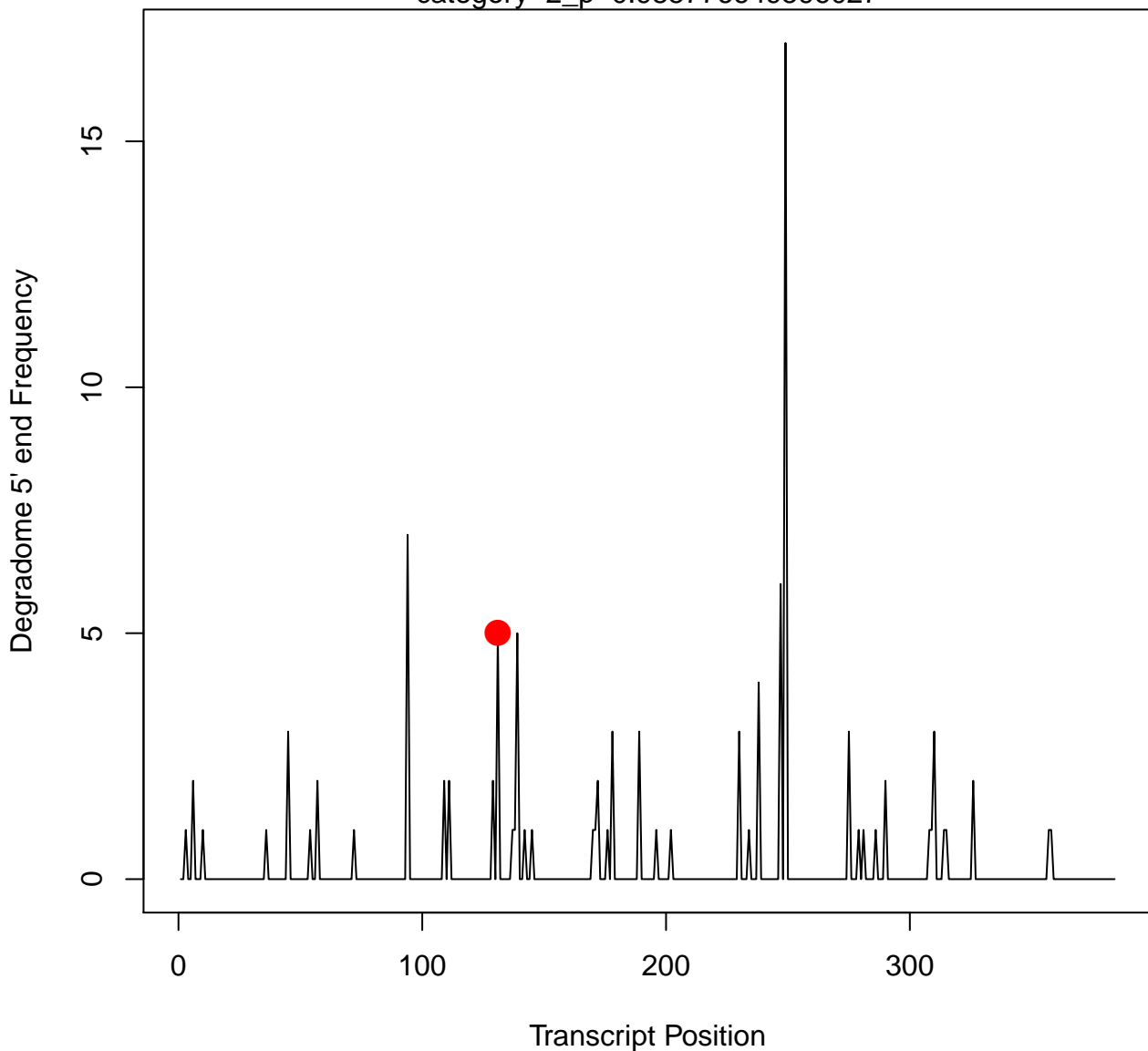

Supplement: Supplementary file 1 [file Data_Sheet_1.zip › Sit-miR168_Seita.2G261600.1_131_TPlot.pdf]

**T=Seita.4G158600.1\_Q=Sit-miR168\_S=436**

category=2\_p=0.961394877102602

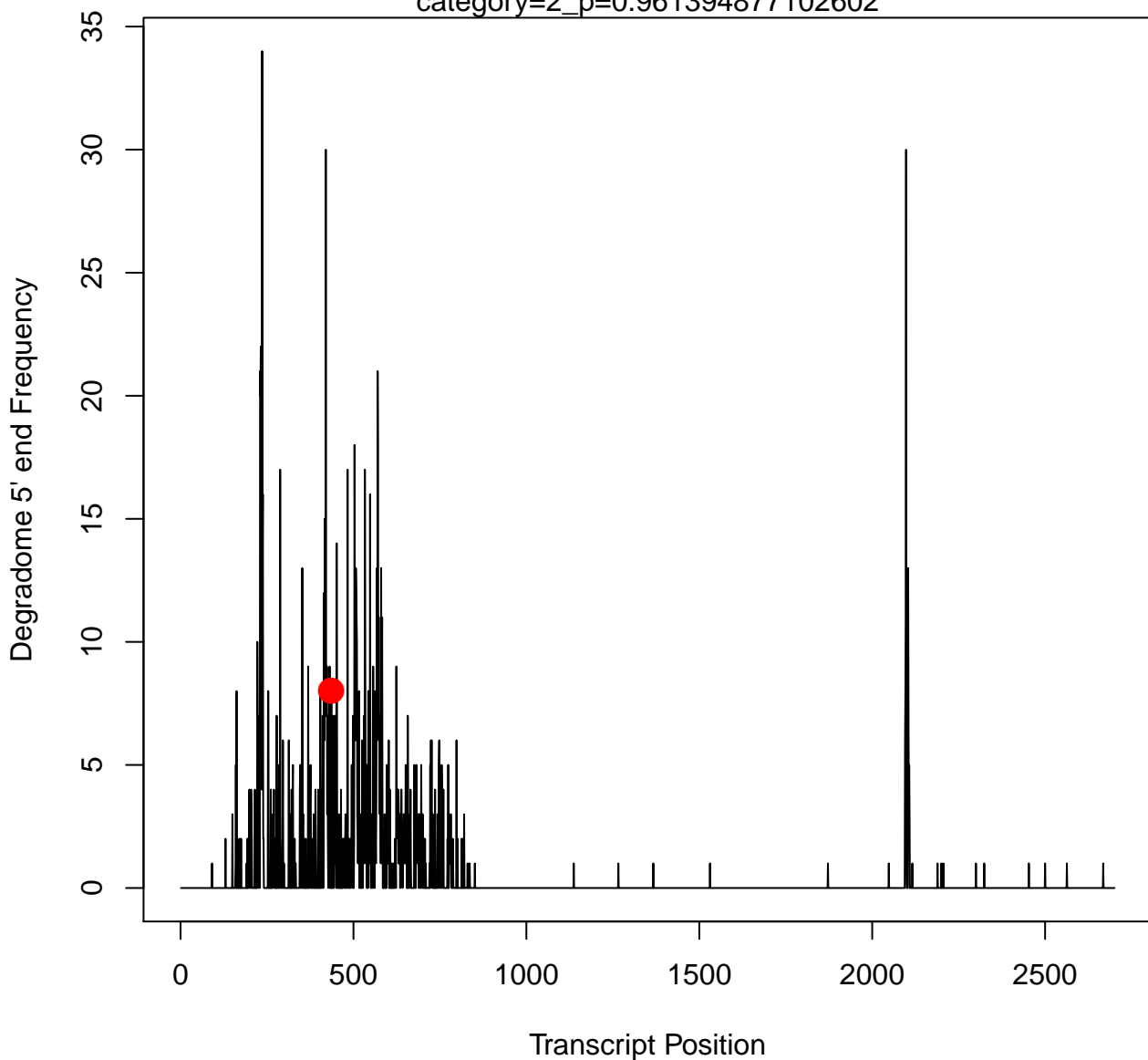

Supplement: Supplementary file 1 [file Data_Sheet_1.zip › Sit-miR168_Seita.4G158600.1_436_TPlot.pdf]

**T=Seita.5G022800.1\_Q=Sit-miR168\_S=2856**

category=2\_p=0.977831712512693

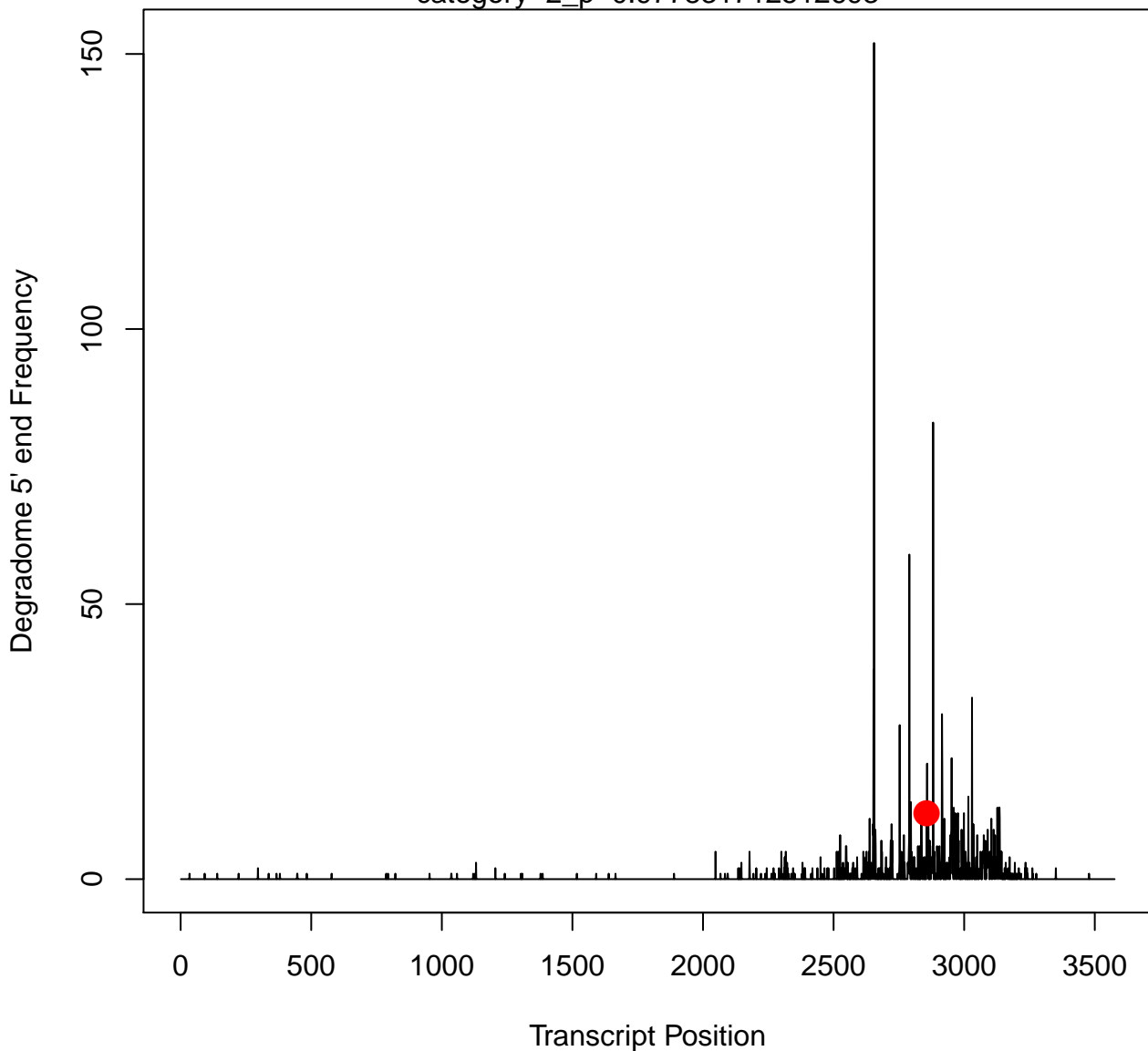

Supplement: Supplementary file 1 [file Data_Sheet_1.zip › Sit-miR168_Seita.5G022800.1_2856_TPlot.pdf]

**T=Seita.5G261900.1\_Q=Sit-miR168\_S=537**

category=2\_p=0.992553760811476

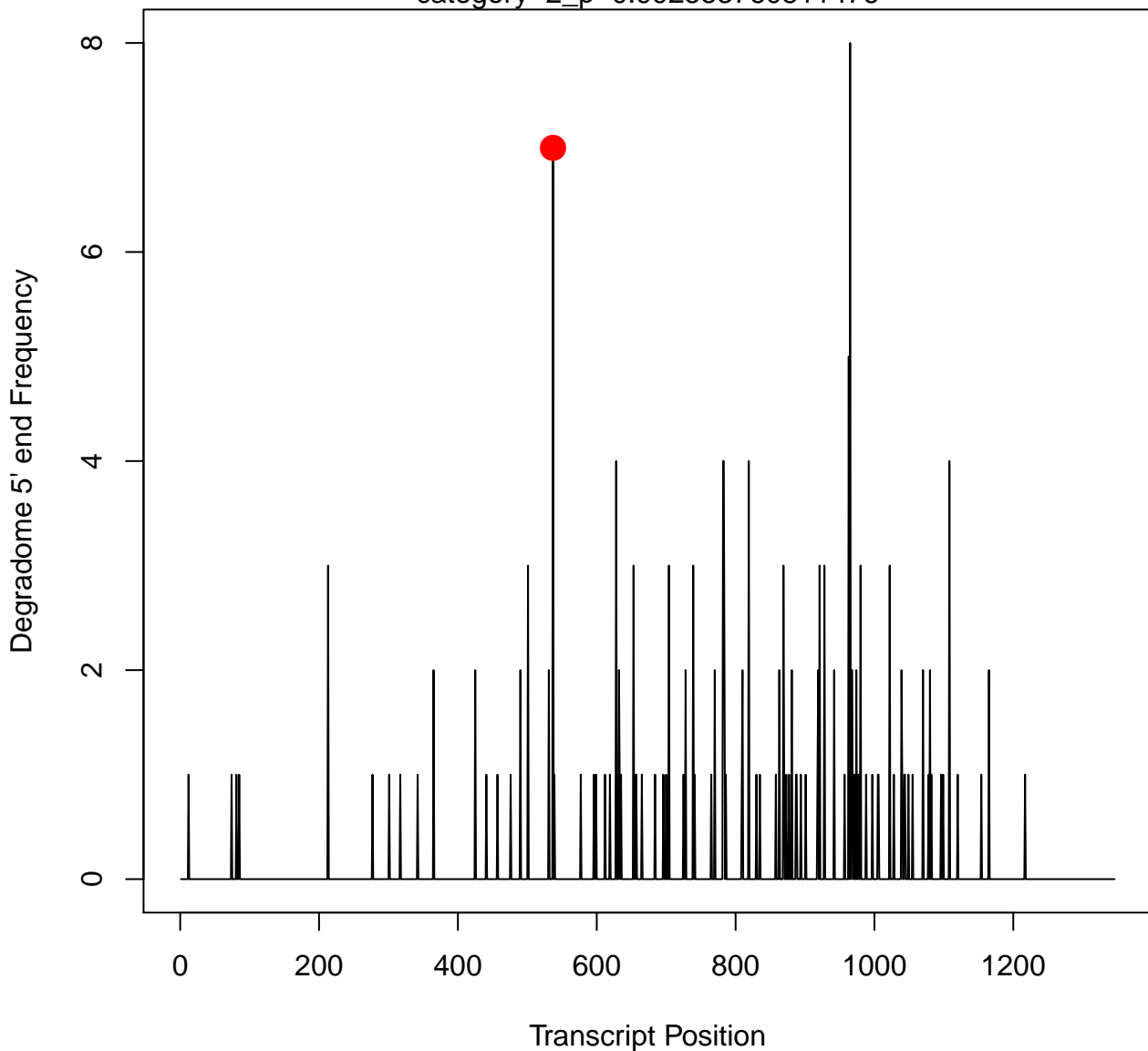

Supplement: Supplementary file 1 [file Data_Sheet_1.zip › Sit-miR168_Seita.5G261900.1_537_TPlot.pdf]

**T=Seita.5G435400.1\_Q=Sit-miR168\_S=595**

category=0\_p=0.00524049191016585

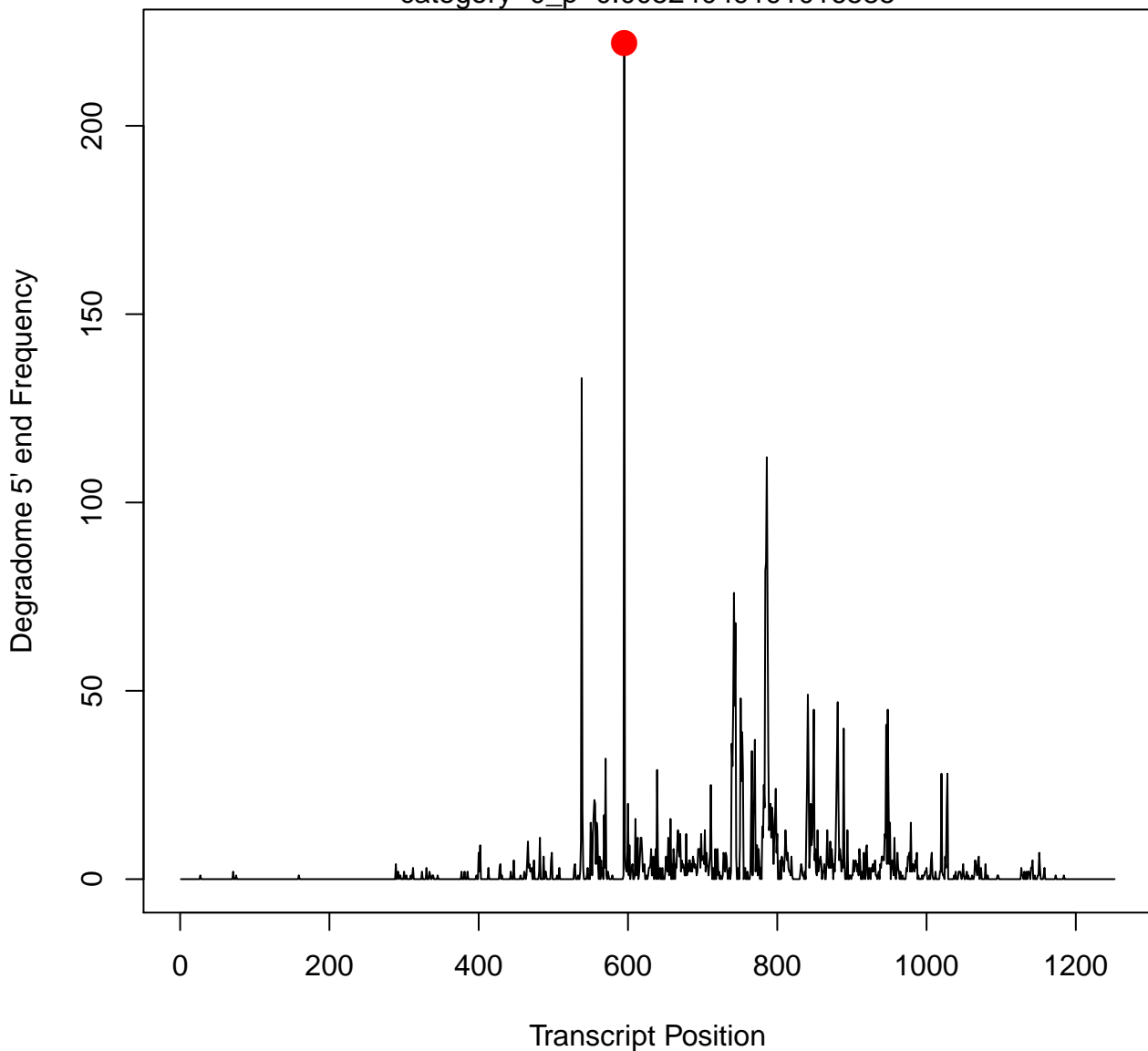

Supplement: Supplementary file 1 [file Data_Sheet_1.zip › Sit-miR168_Seita.5G435400.1_595_TPlot.pdf]

**T=Seita.7G114700.1\_Q=Sit-miR168\_S=402**

category=0\_p=0.0898437857076038

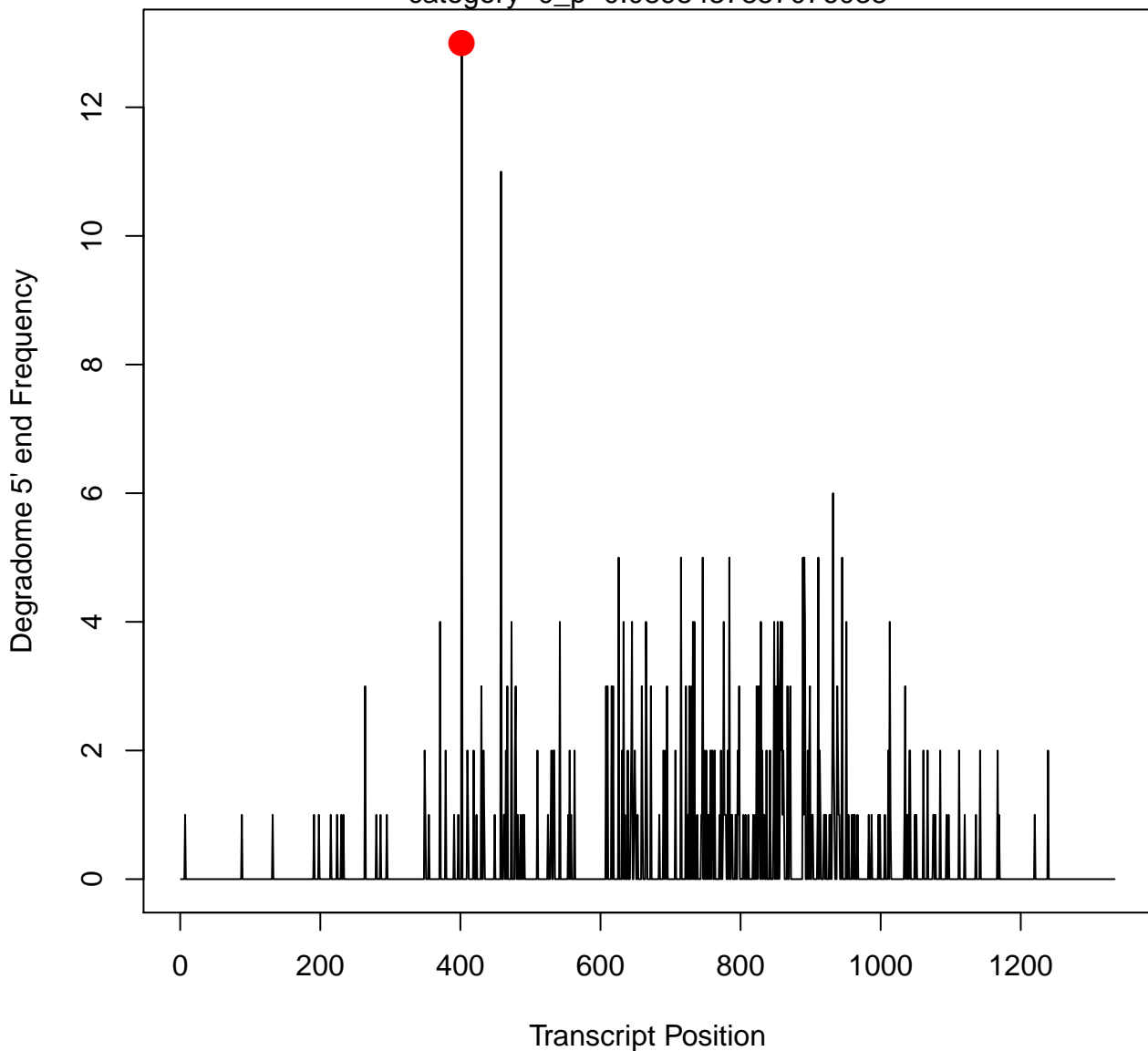

Supplement: Supplementary file 1 [file Data_Sheet_1.zip › Sit-miR168_Seita.7G114700.1_402_TPlot.pdf]

**T=Seita.7G115200.1\_Q=Sit-miR168\_S=418**

category=2\_p=0.98551120272601

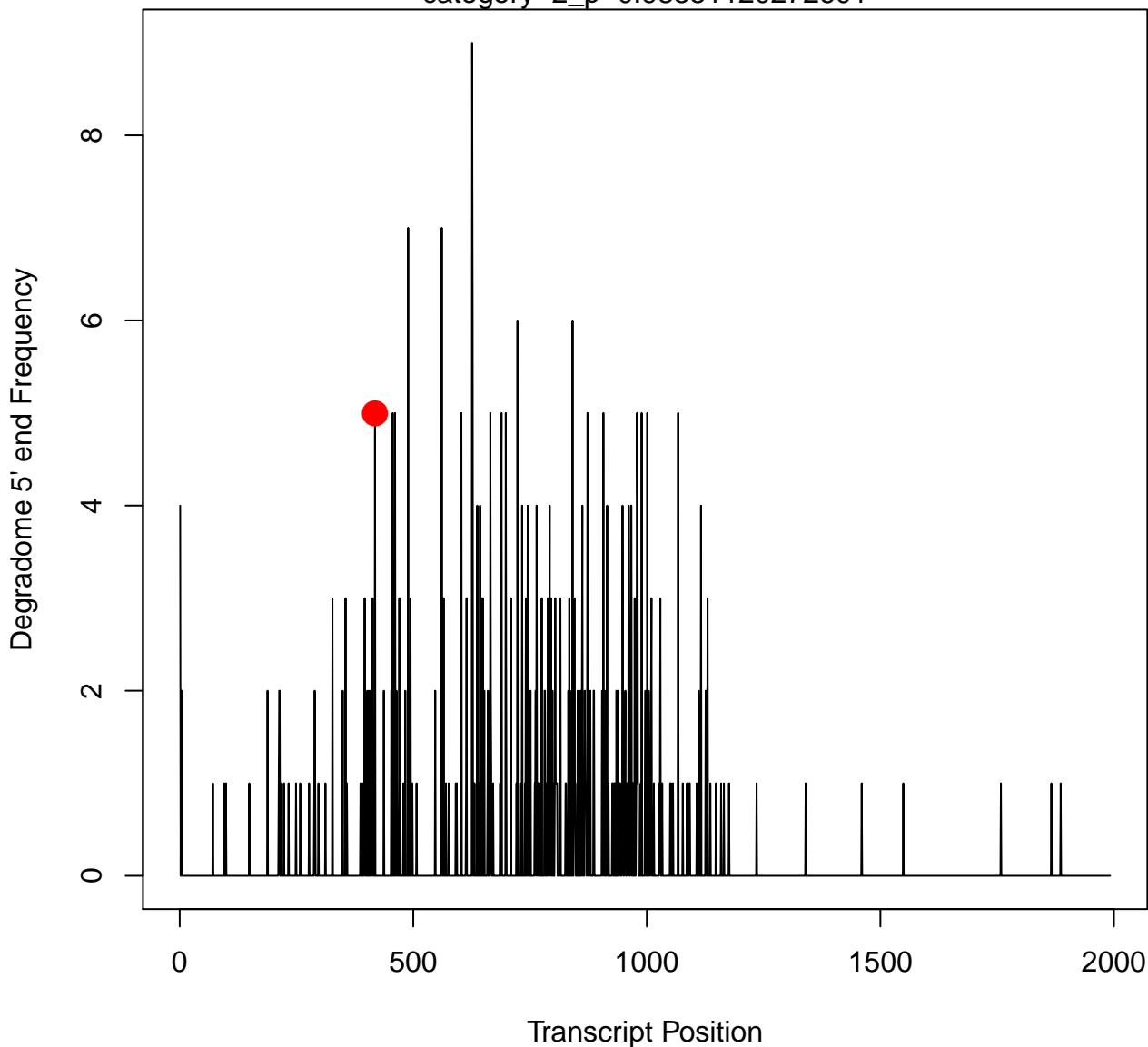

Supplement: Supplementary file 1 [file Data_Sheet_1.zip › Sit-miR168_Seita.7G115200.1_418_TPlot.pdf]

**T=Seita.7G200400.1\_Q=Sit-miR168\_S=1236**

category=2\_p=0.999159869681432

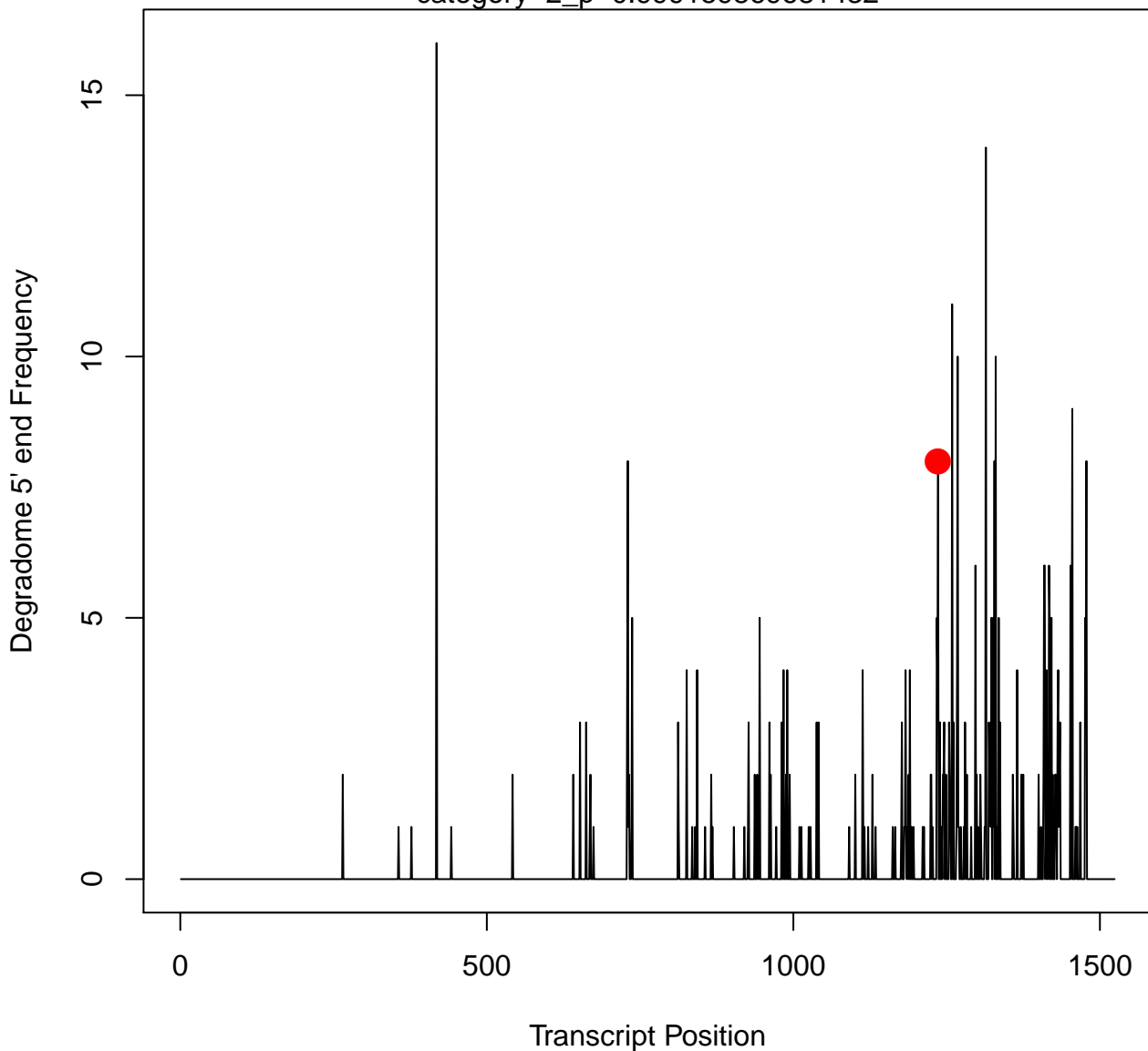

Supplement: Supplementary file 1 [file Data_Sheet_1.zip › Sit-miR168_Seita.7G200400.1_1236_TPlot.pdf]

**T=Seita.7G201100.1\_Q=Sit-miR168\_S=664**

category=2\_p=0.415051499441964

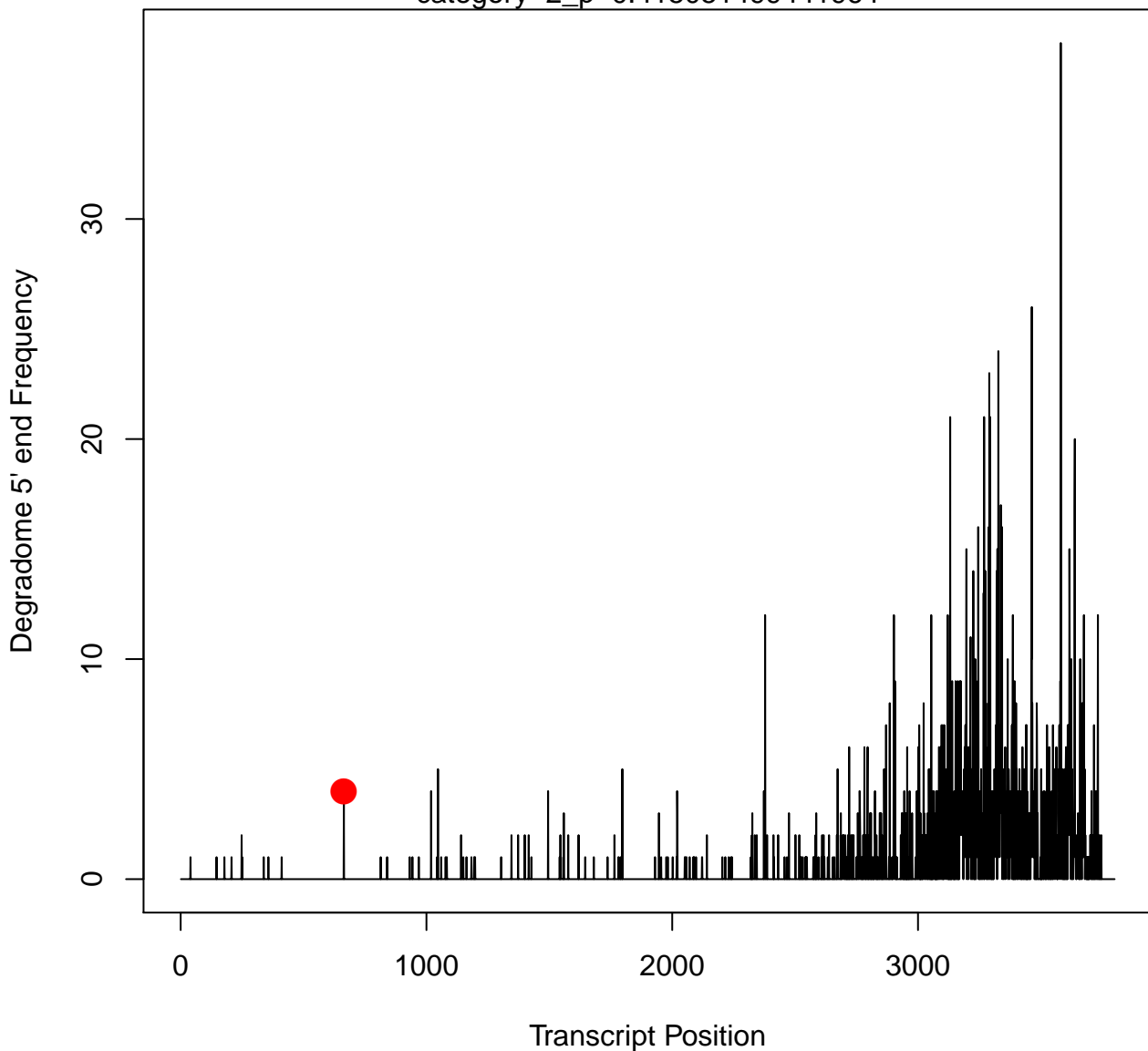

Supplement: Supplementary file 1 [file Data_Sheet_1.zip › Sit-miR168_Seita.7G201100.1_664_TPlot.pdf]

**T=Seita.8G219700.1\_Q=Sit-miR168\_S=355**

category=2\_p=0.39301426453022

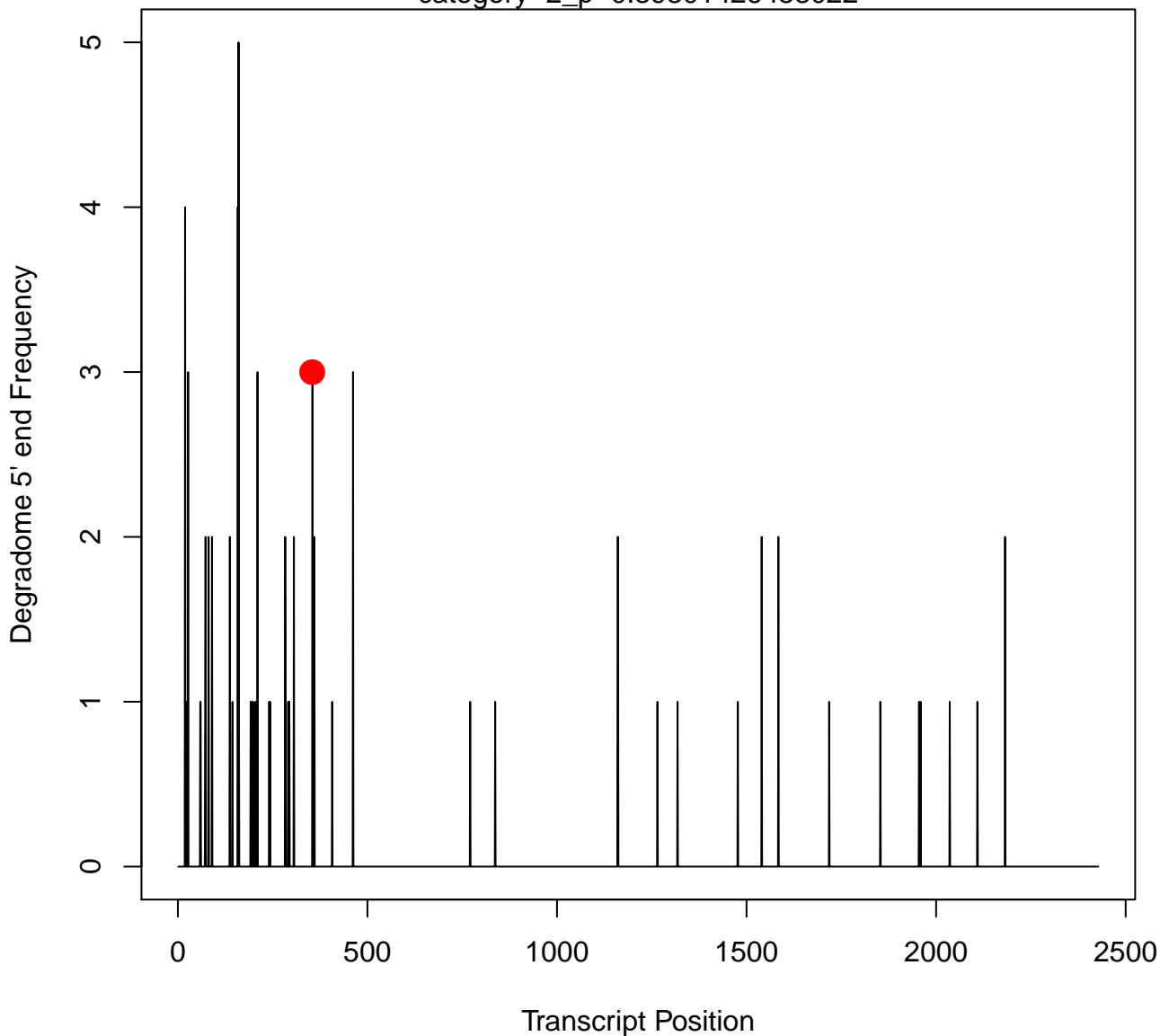

Supplement: Supplementary file 1 [file Data_Sheet_1.zip › Sit-miR168_Seita.8G219700.1_355_TPlot.pdf]

**T=Seita.9G245400.1\_Q=Sit-miR168\_S=343**

category=2\_p=0.997761488853434

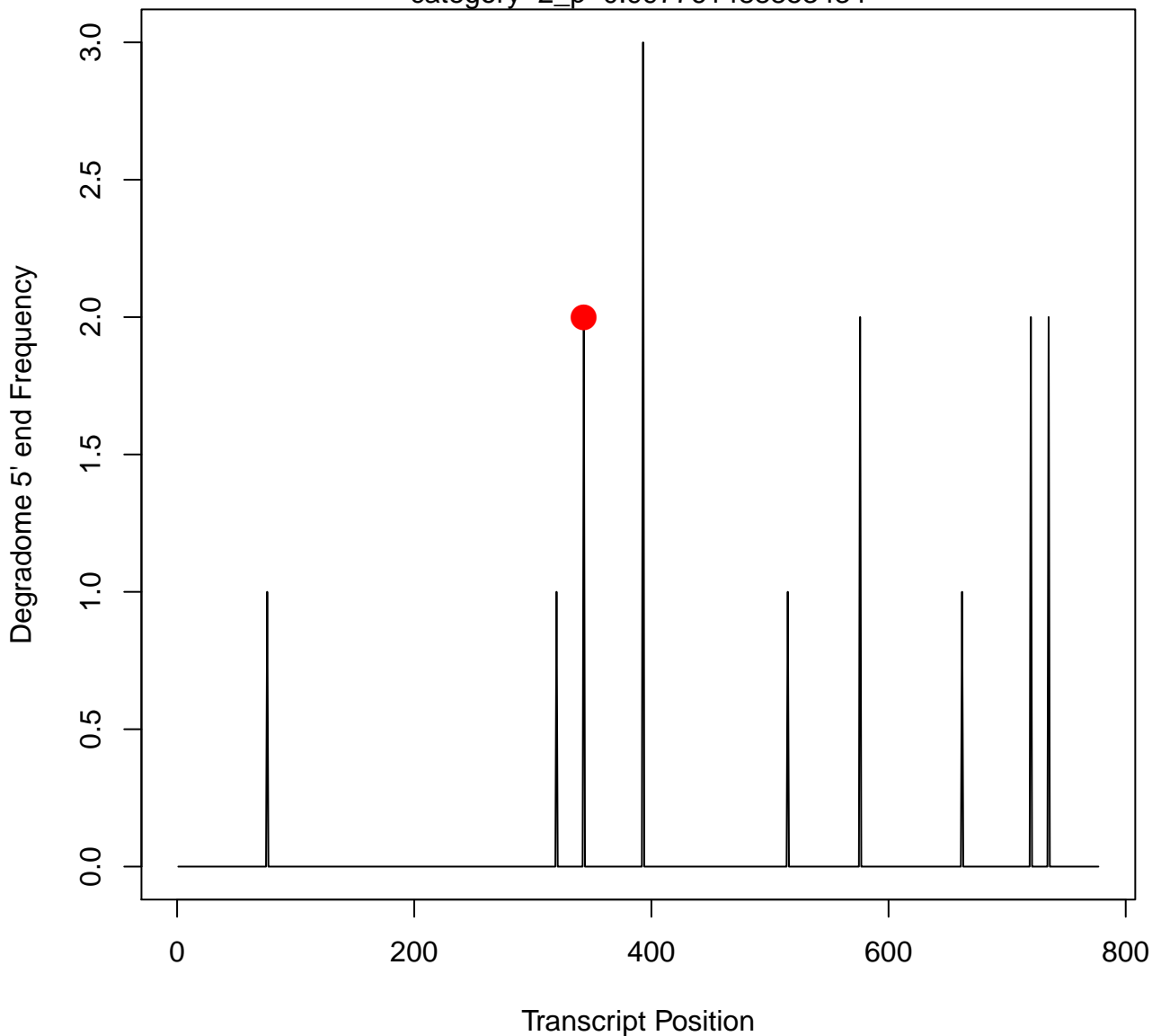

Supplement: Supplementary file 1 [file Data_Sheet_1.zip › Sit-miR168_Seita.9G245400.1_343_TPlot.pdf]

**T=Seita.9G280700.1\_Q=Sit-miR168\_S=533**

category=2\_p=0.99308463230802

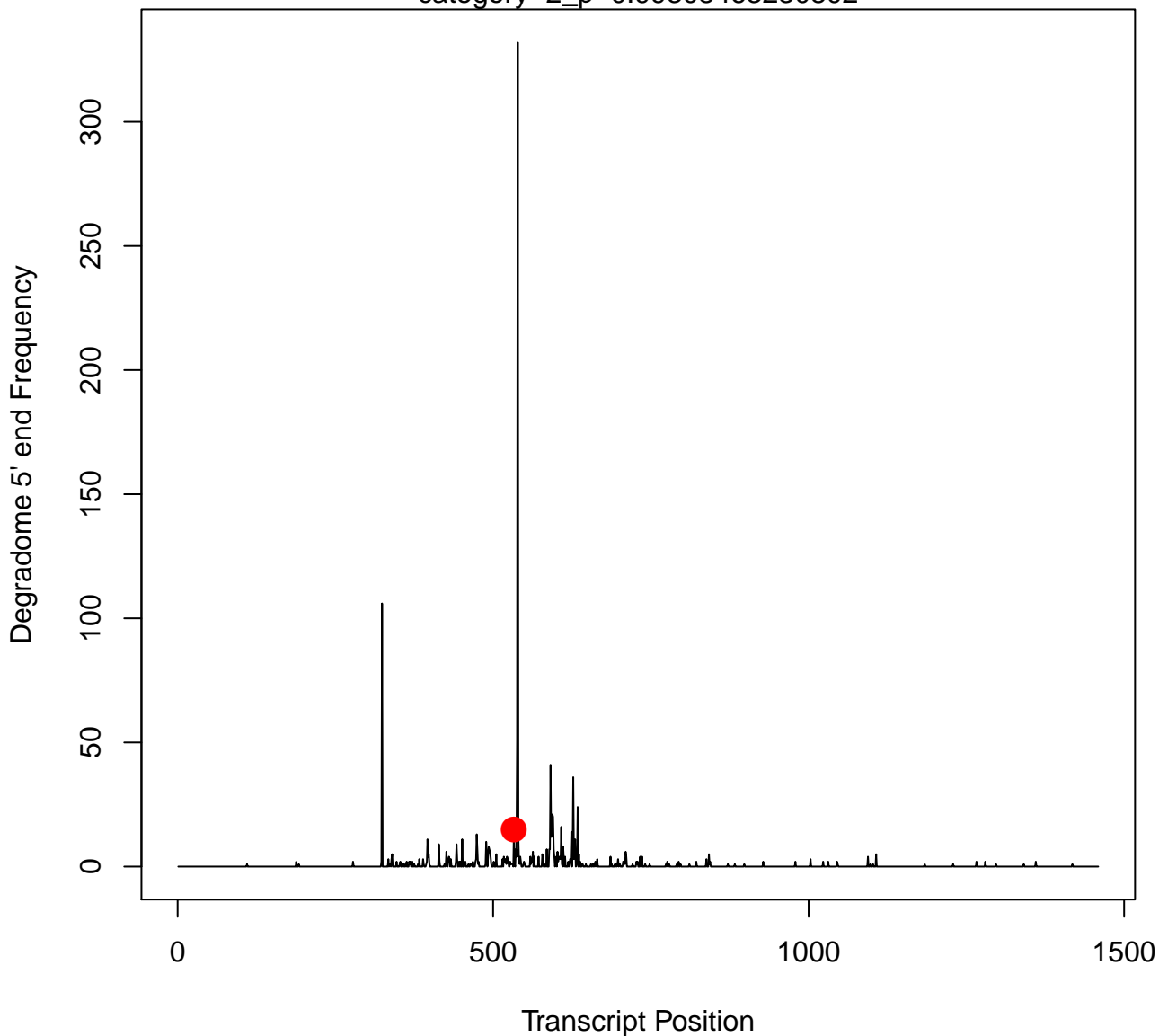

Supplement: Supplementary file 1 [file Data_Sheet_1.zip › Sit-miR168_Seita.9G280700.1_533_TPlot.pdf]

**T=Seita.5G243500.1\_Q=Sit-miR169a\_S=1691**

category=2\_p=0.999790074753553

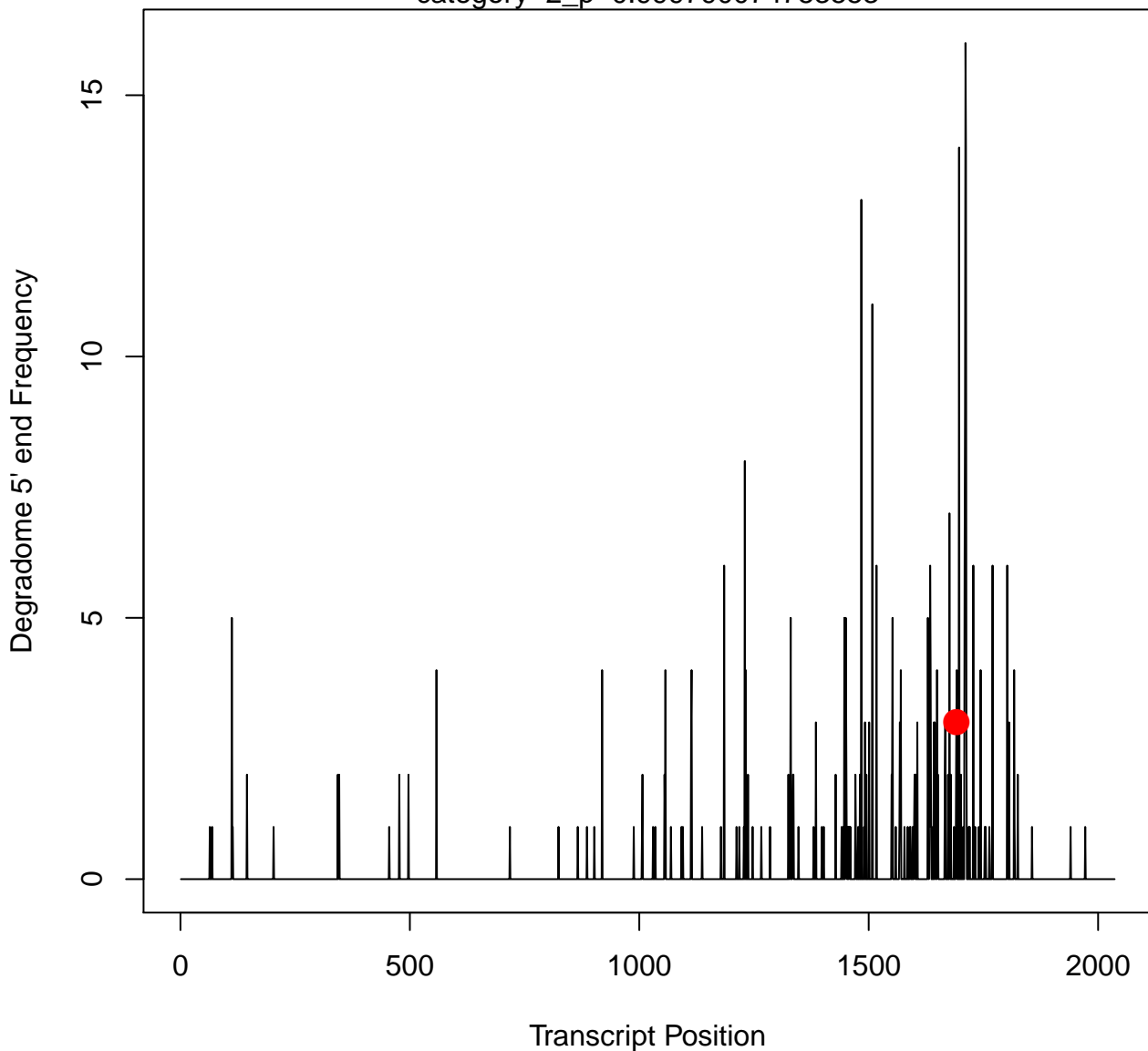

Supplement: Supplementary file 1 [file Data_Sheet_1.zip › Sit-miR169a_Seita.5G243500.1_1691_TPlot.pdf]
