# Supplementary figures and images for: The Construction and Exploration of a Comprehensive MicroRNA Centered Regulatory Network in Foxtail Millet (Setaria italica L.) (part 1 of 14)
Source: Front Plant Sci. 2022 May 6;13:848474. doi: 10.3389/fpls.2022.848474 (PMC9121102; doi:10.3389/fpls.2022.848474)

**T=Seita.2G293100.1\_Q=Sit-miR1133\_S=567**

category=2\_p=0.999999968986467

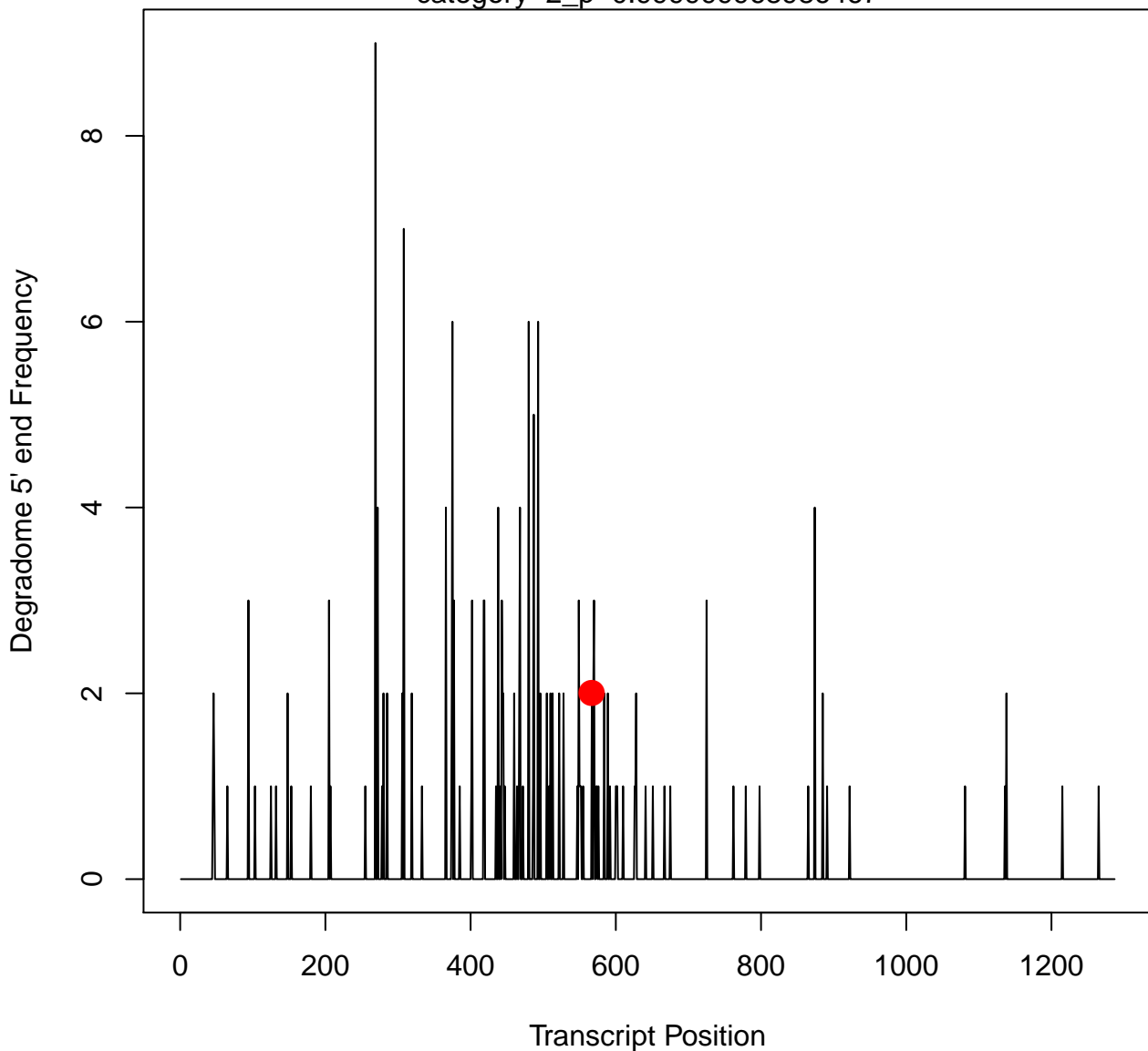

Supplement: Supplementary file 1 [file Data_Sheet_1.zip › Sit-miR1133_Seita.2G293100.1_567_TPlot.pdf]

**T=Seita.3G163400.1\_Q=Sit-miR1133\_S=876**

category=2\_p=0.99992546490853

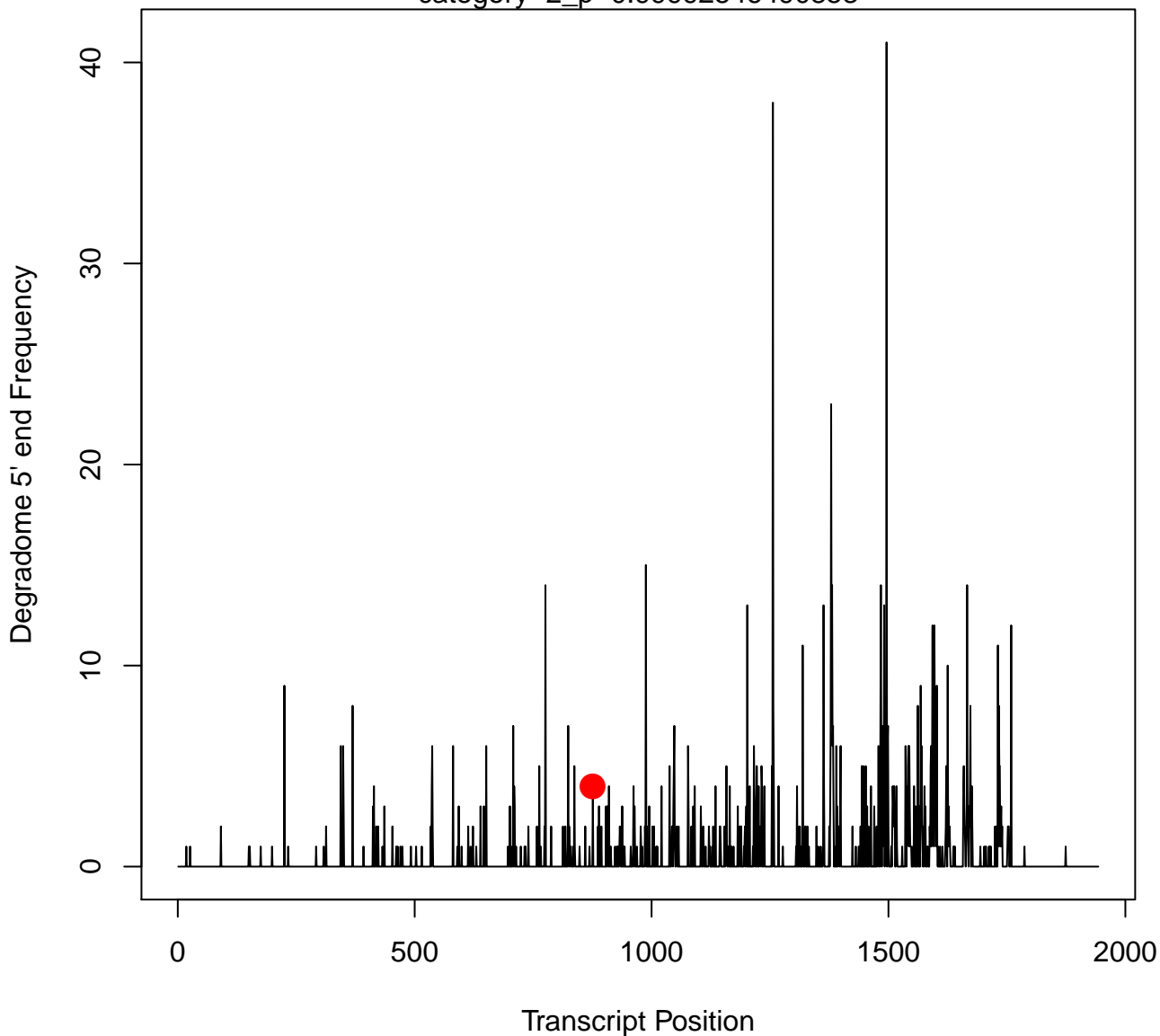

Supplement: Supplementary file 1 [file Data_Sheet_1.zip › Sit-miR1133_Seita.3G163400.1_876_TPlot.pdf]

**T=Seita.4G191500.1\_Q=Sit-miR1133\_S=1058**

category=2\_p=0.99999973510076

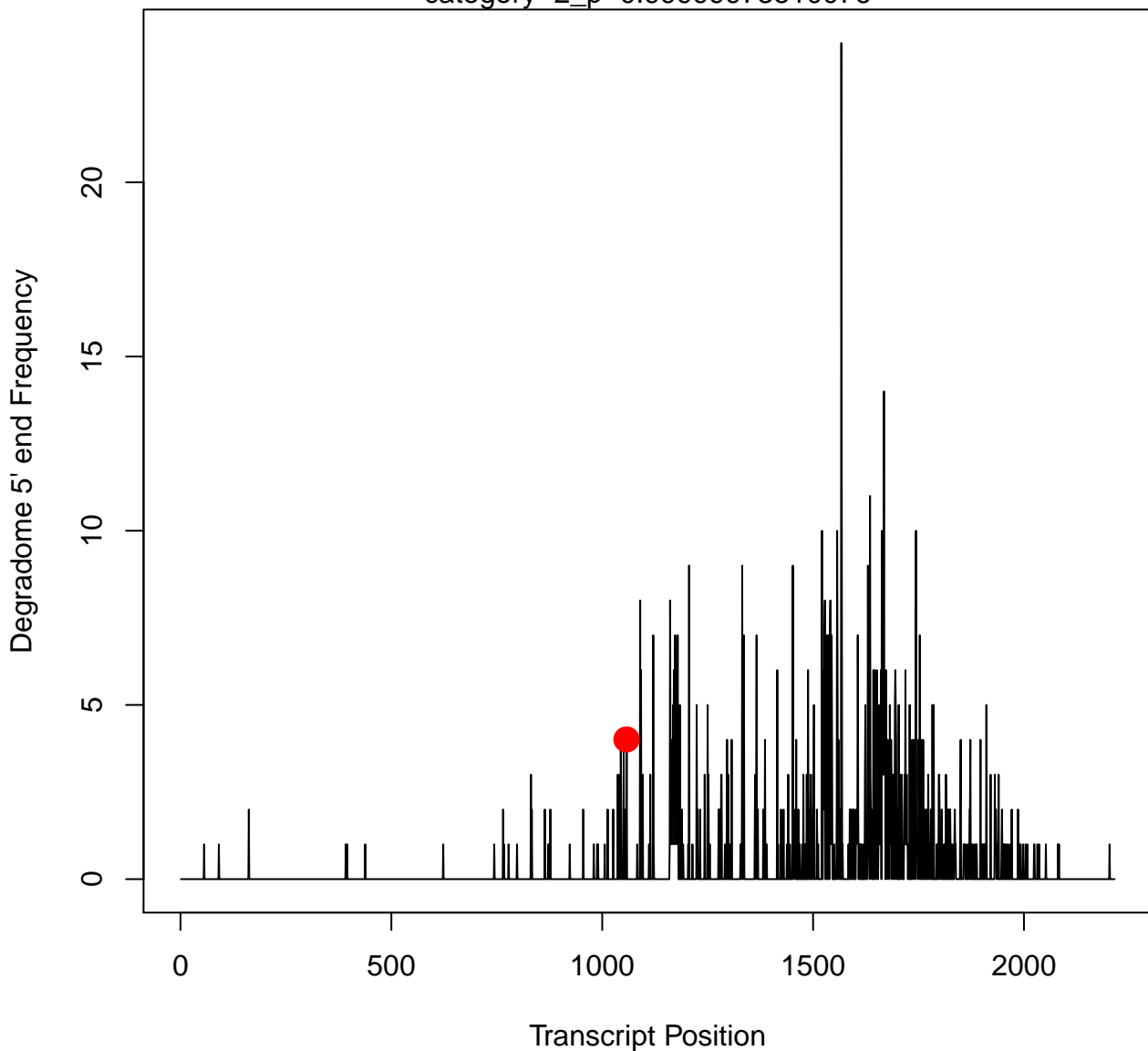

Supplement: Supplementary file 1 [file Data_Sheet_1.zip › Sit-miR1133_Seita.4G191500.1_1058_TPlot.pdf]

**T=Seita.5G069500.1\_Q=Sit-miR1133\_S=1445**

category=2\_p=0.999560173157244

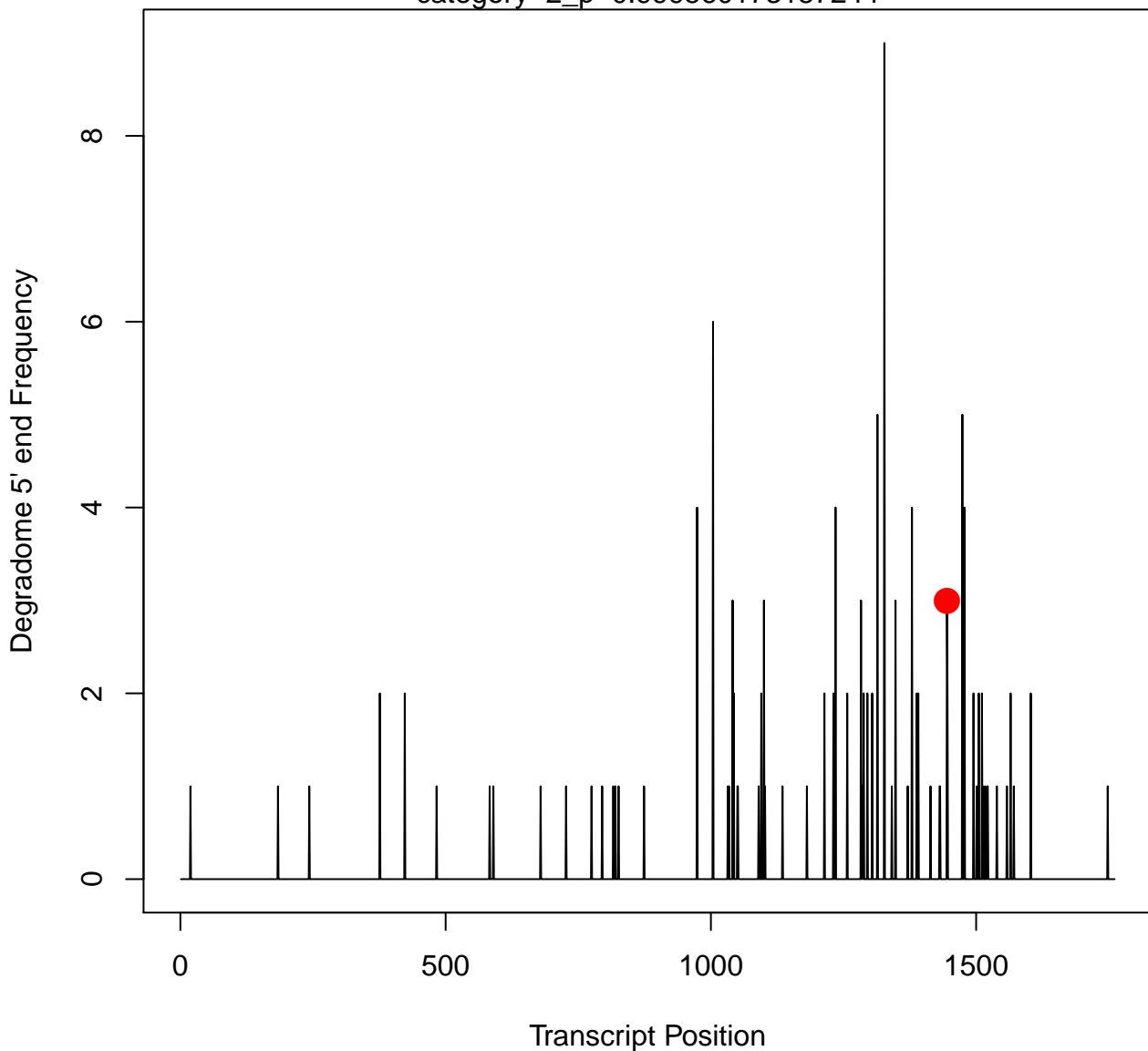

Supplement: Supplementary file 1 [file Data_Sheet_1.zip › Sit-miR1133_Seita.5G069500.1_1445_TPlot.pdf]

**T=Seita.7G236300.1\_Q=Sit-miR1133\_S=2052**

category=2\_p=0.987957183441327

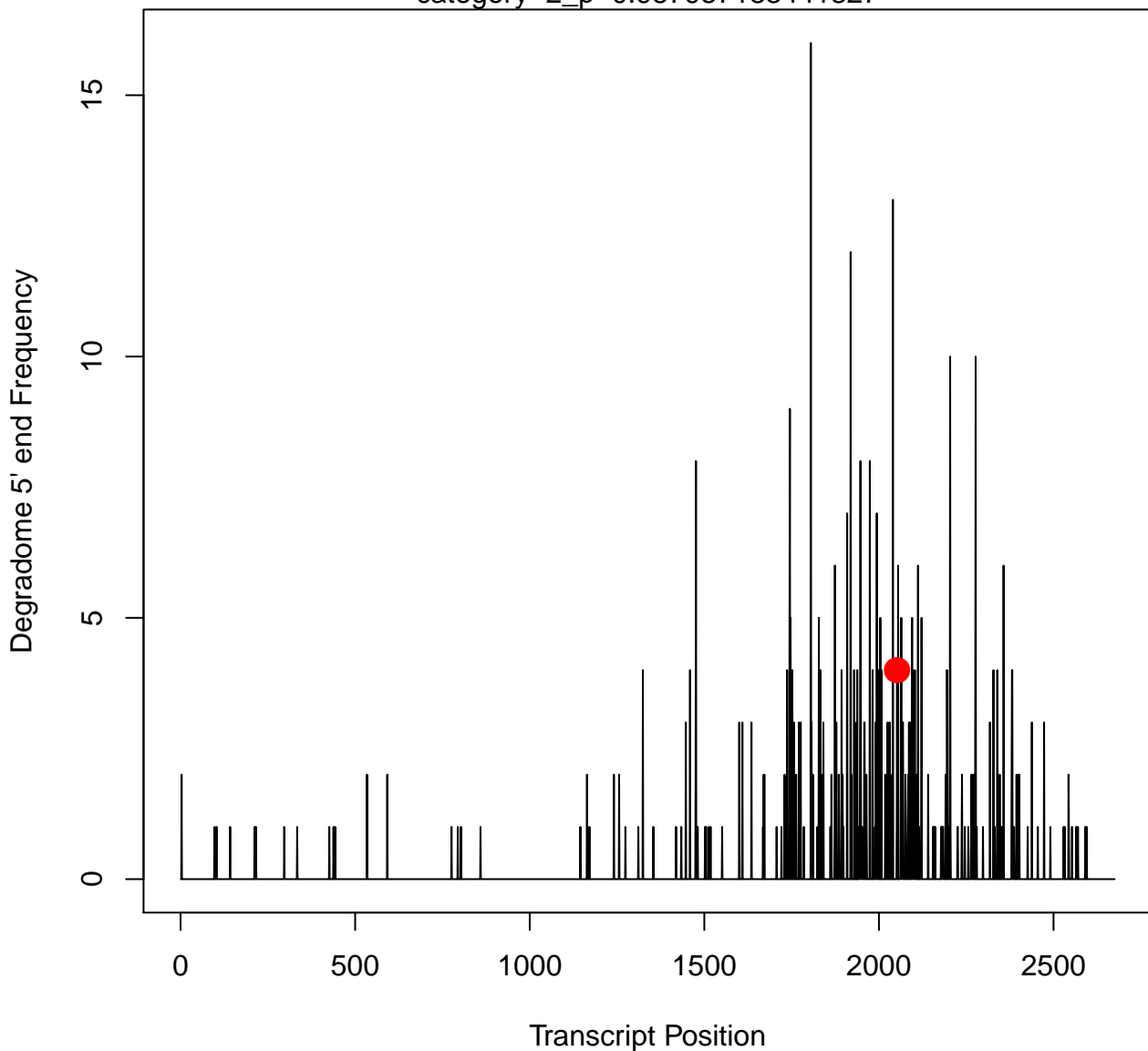

Supplement: Supplementary file 1 [file Data_Sheet_1.zip › Sit-miR1133_Seita.7G236300.1_2052_TPlot.pdf]

**T=Seita.9G053500.1\_Q=Sit-miR1133\_S=782**

category=2\_p=0.999999973250983

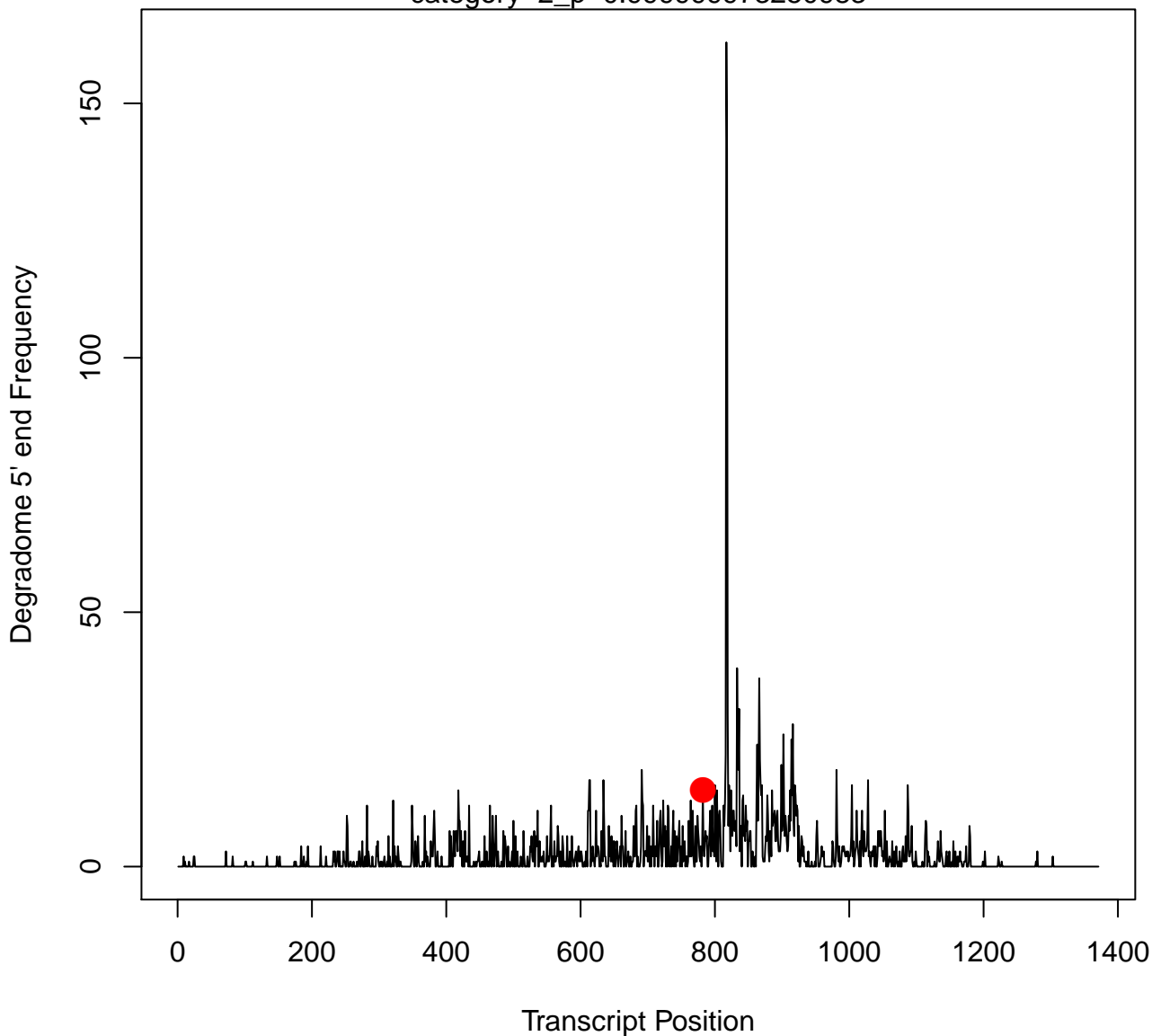

Supplement: Supplementary file 1 [file Data_Sheet_1.zip › Sit-miR1133_Seita.9G053500.1_782_TPlot.pdf]

**T=Seita.2G034500.1\_Q=Sit-miR1432\_S=427**

category=0\_p=0.200130377624318

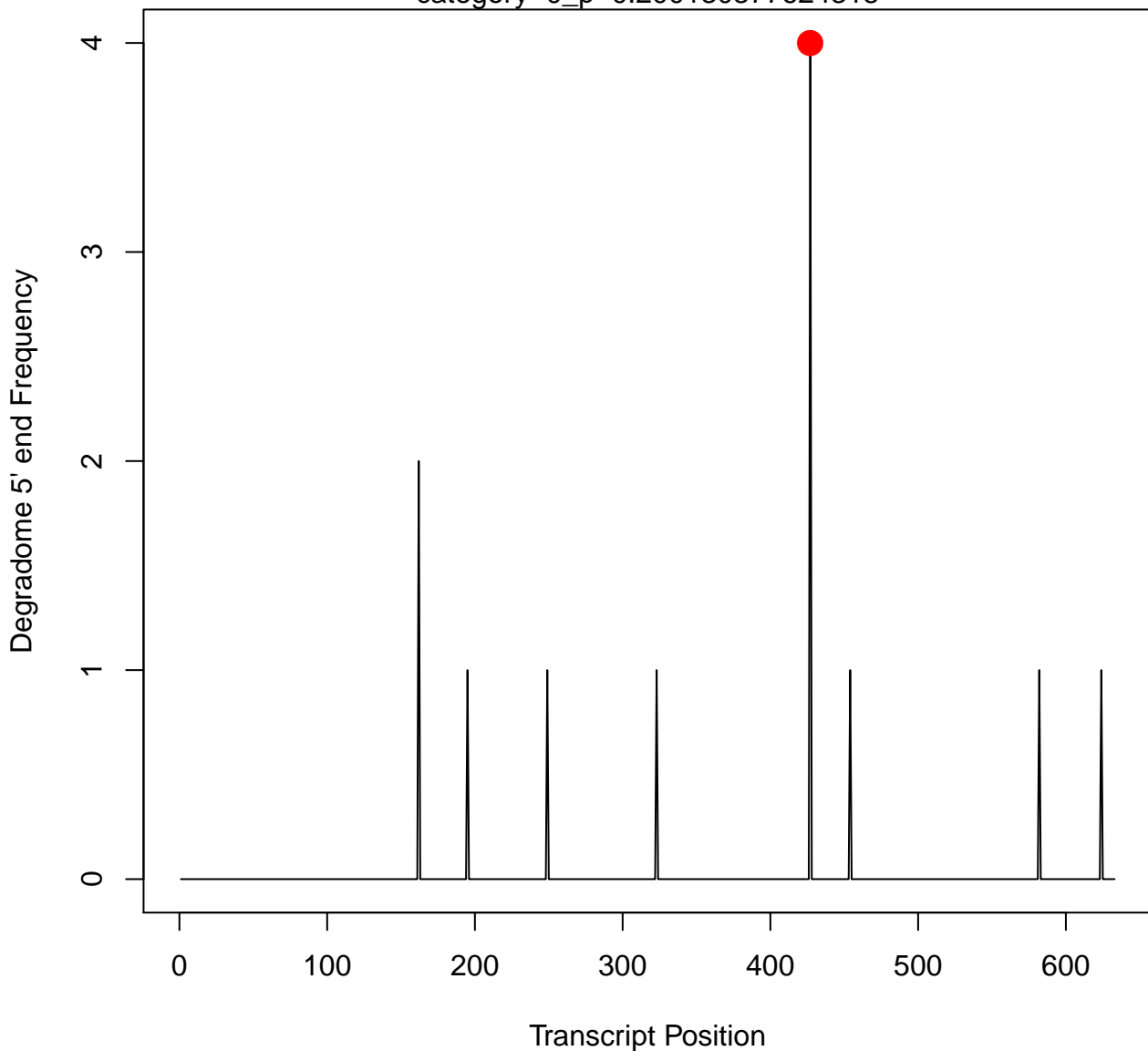

Supplement: Supplementary file 1 [file Data_Sheet_1.zip › Sit-miR1432_Seita.2G034500.1_427_TPlot.pdf]

**T=Seita.2G394300.1\_Q=Sit-miR1432\_S=1038**

category=2\_p=0.99998736893859

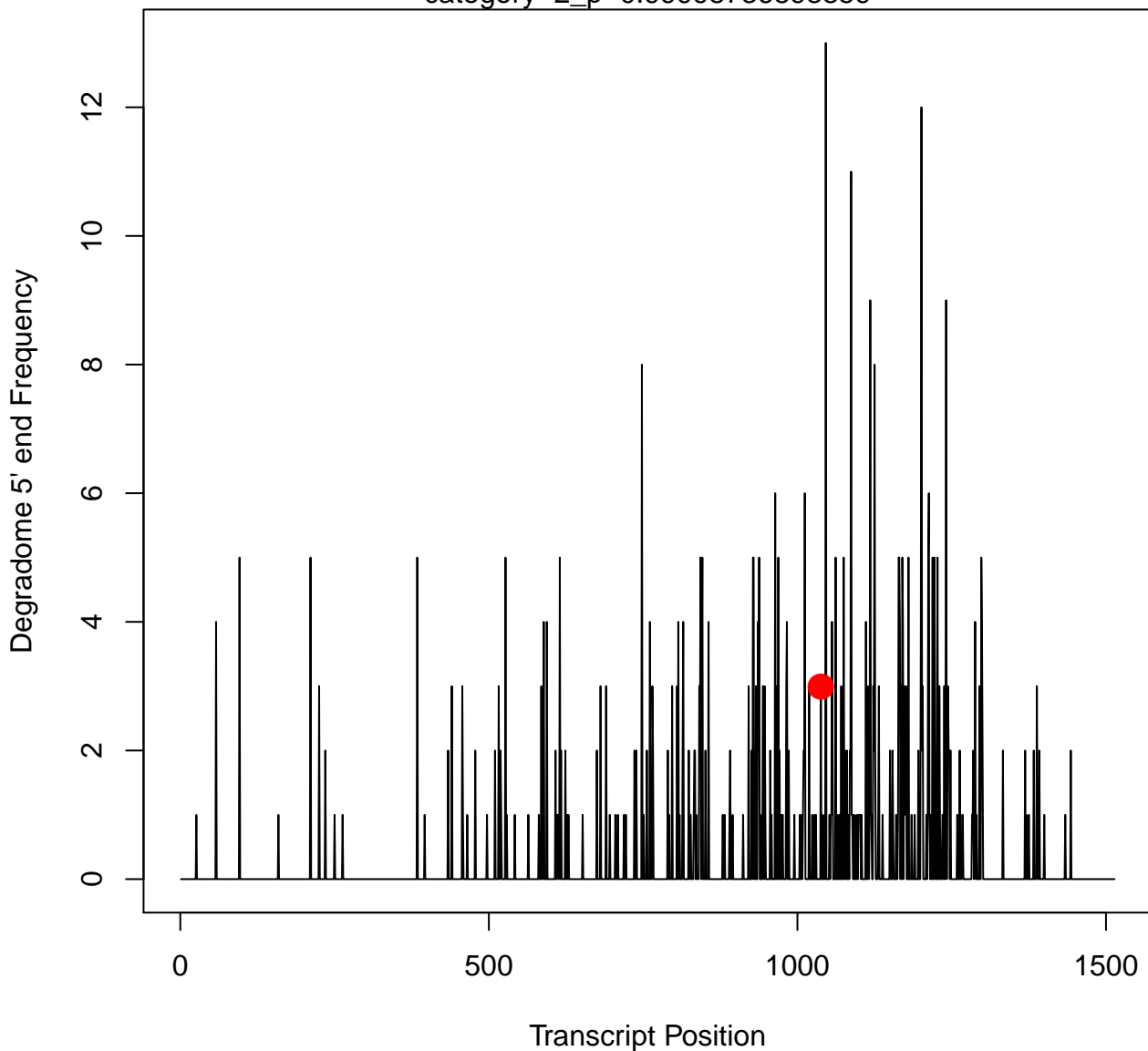

Supplement: Supplementary file 1 [file Data_Sheet_1.zip › Sit-miR1432_Seita.2G394300.1_1038_TPlot.pdf]

**T=Seita.2G415400.1\_Q=Sit-miR1432\_S=600**

category=2\_p=0.999996473528167

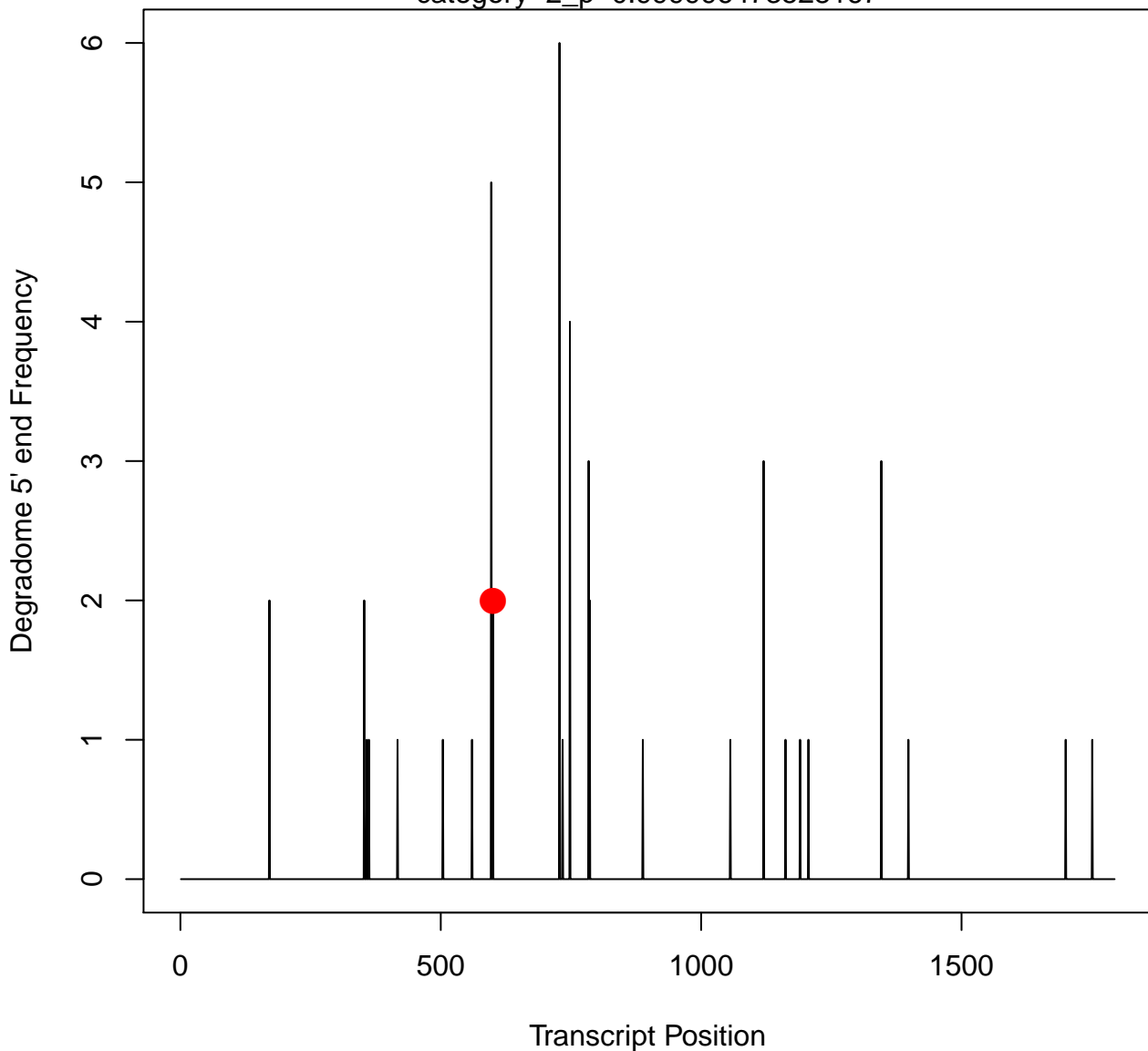

Supplement: Supplementary file 1 [file Data_Sheet_1.zip › Sit-miR1432_Seita.2G415400.1_600_TPlot.pdf]

**T=Seita.3G130300.1\_Q=Sit-miR1432\_S=1130**

category=2\_p=0.522709334584048

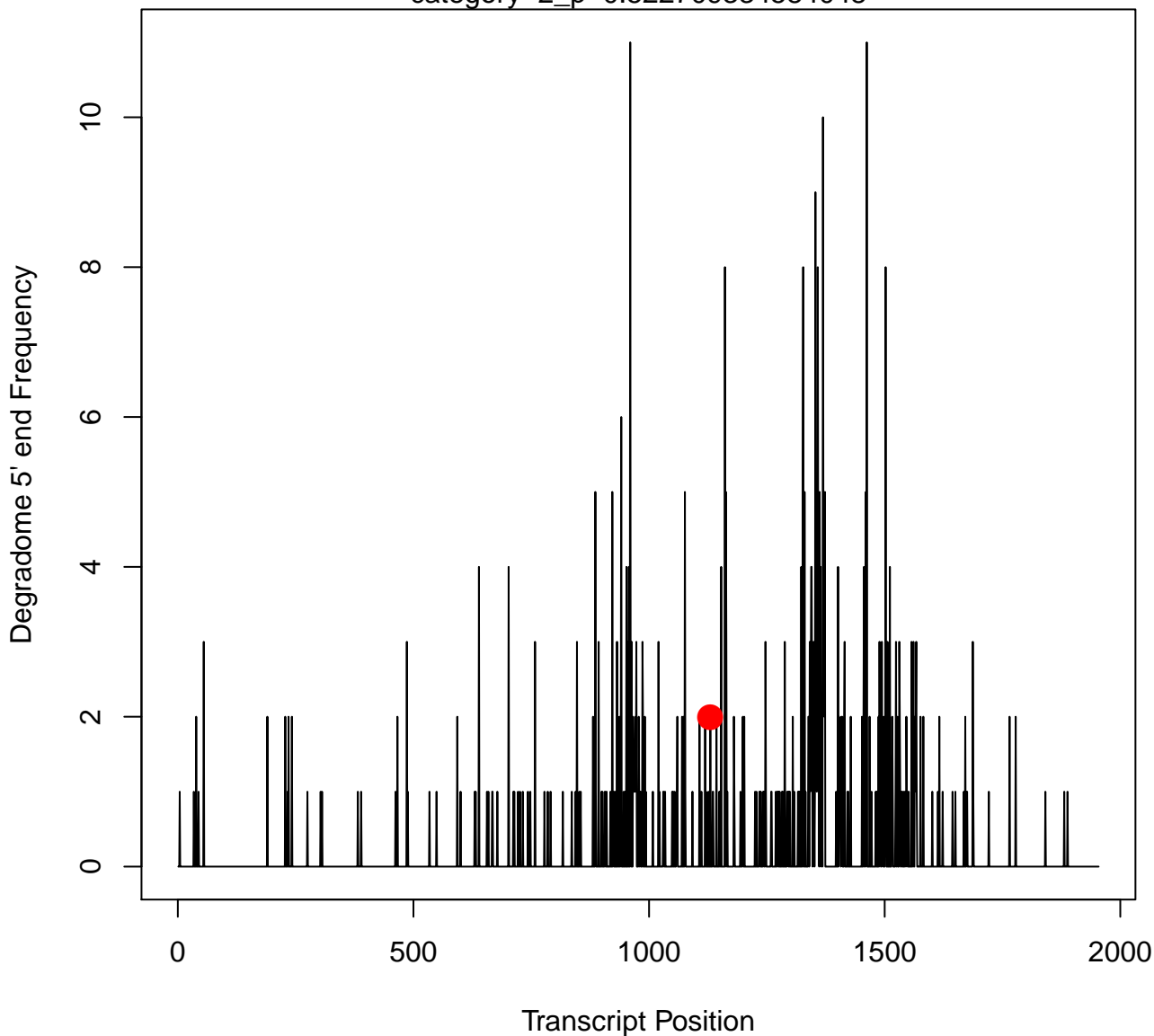

Supplement: Supplementary file 1 [file Data_Sheet_1.zip › Sit-miR1432_Seita.3G130300.1_1130_TPlot.pdf]

**T=Seita.3G207900.1\_Q=Sit-miR1432\_S=480**

category=2\_p=0.987503484735801

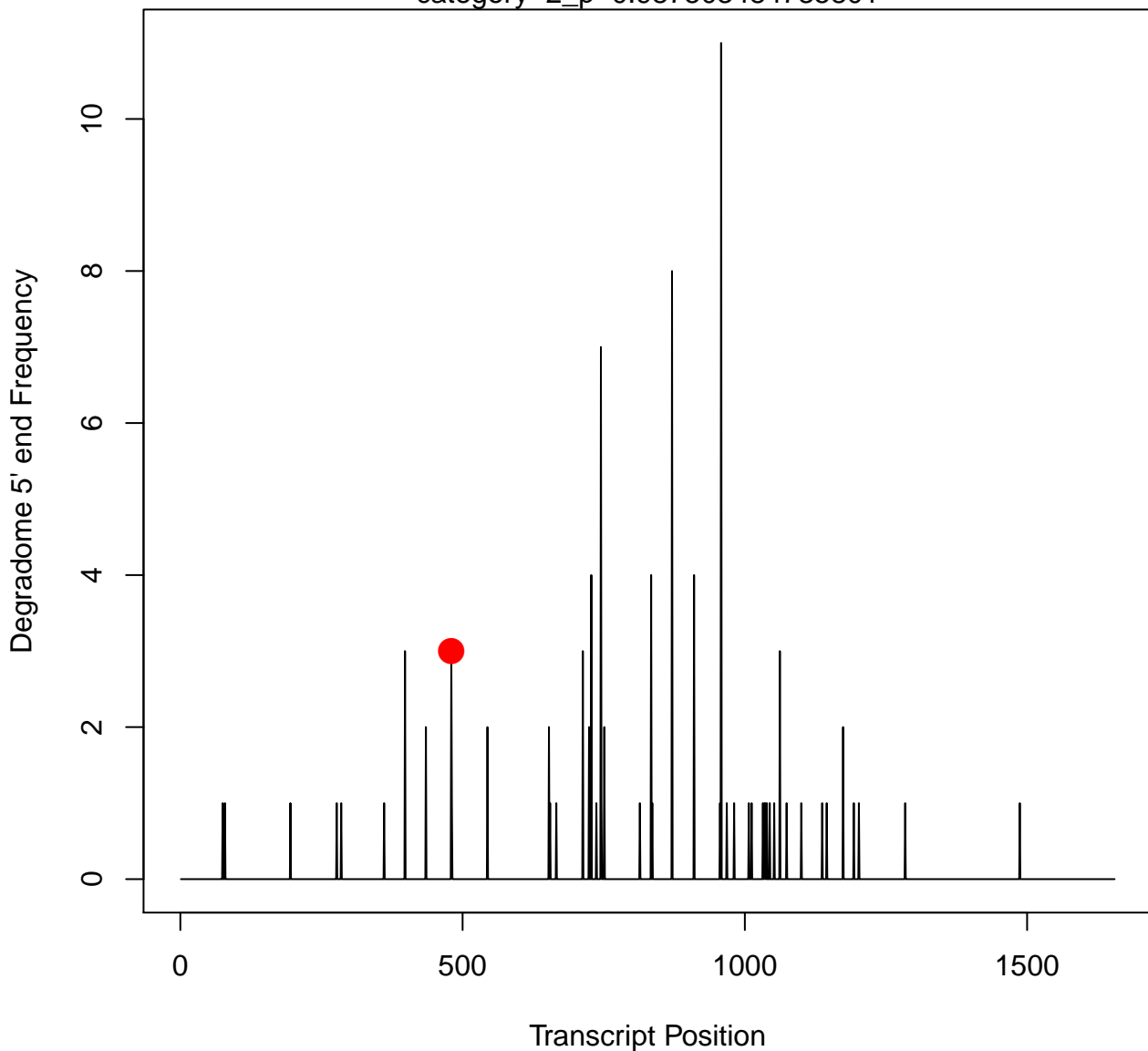

Supplement: Supplementary file 1 [file Data_Sheet_1.zip › Sit-miR1432_Seita.3G207900.1_480_TPlot.pdf]

**T=Seita.3G376600.1\_Q=Sit-miR1432\_S=1585**

category=2\_p=0.999969878964968

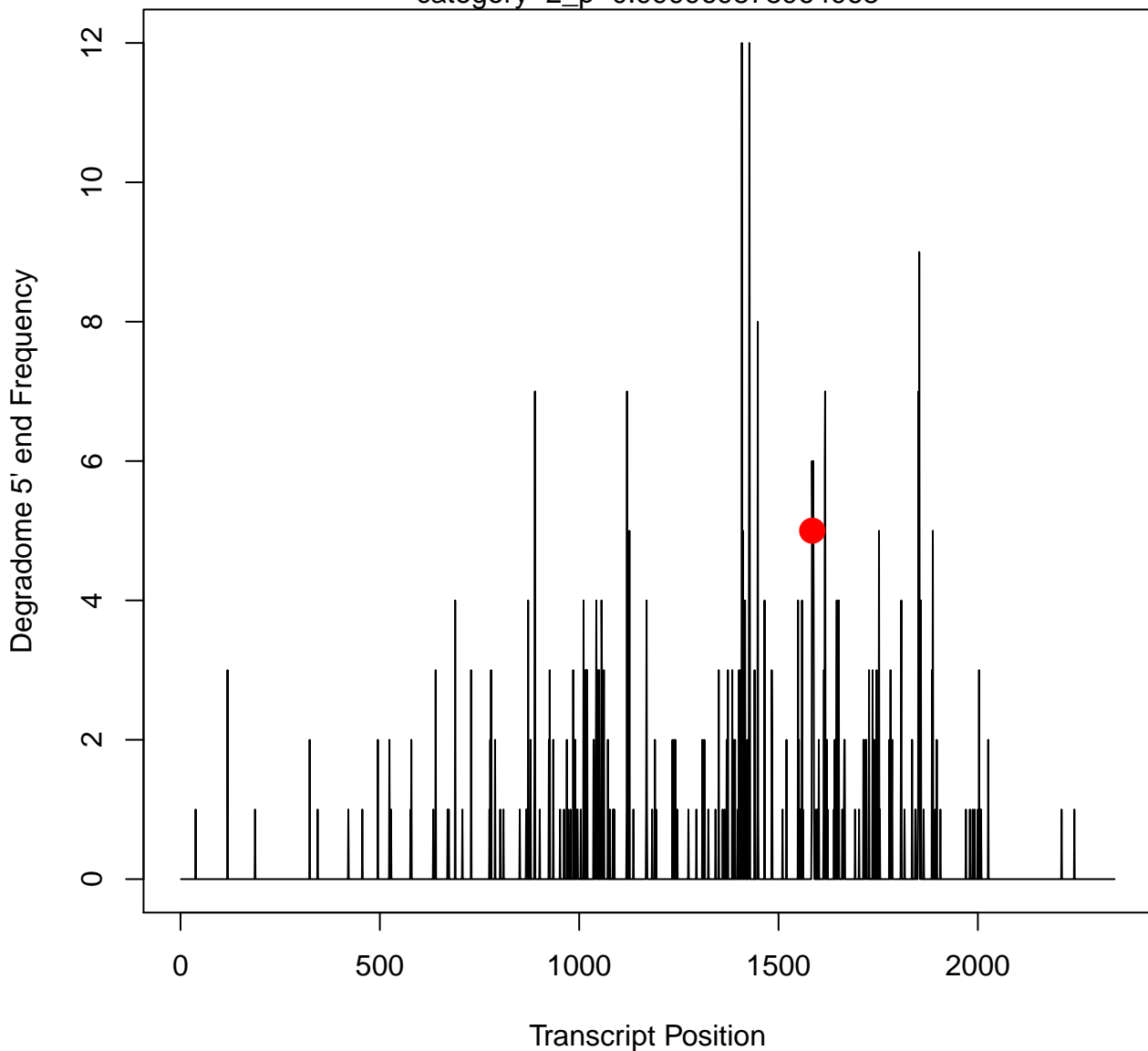

Supplement: Supplementary file 1 [file Data_Sheet_1.zip › Sit-miR1432_Seita.3G376600.1_1585_TPlot.pdf]

**T=Seita.4G002100.1\_Q=Sit-miR1432\_S=488**

category=2\_p=0.999993744188698

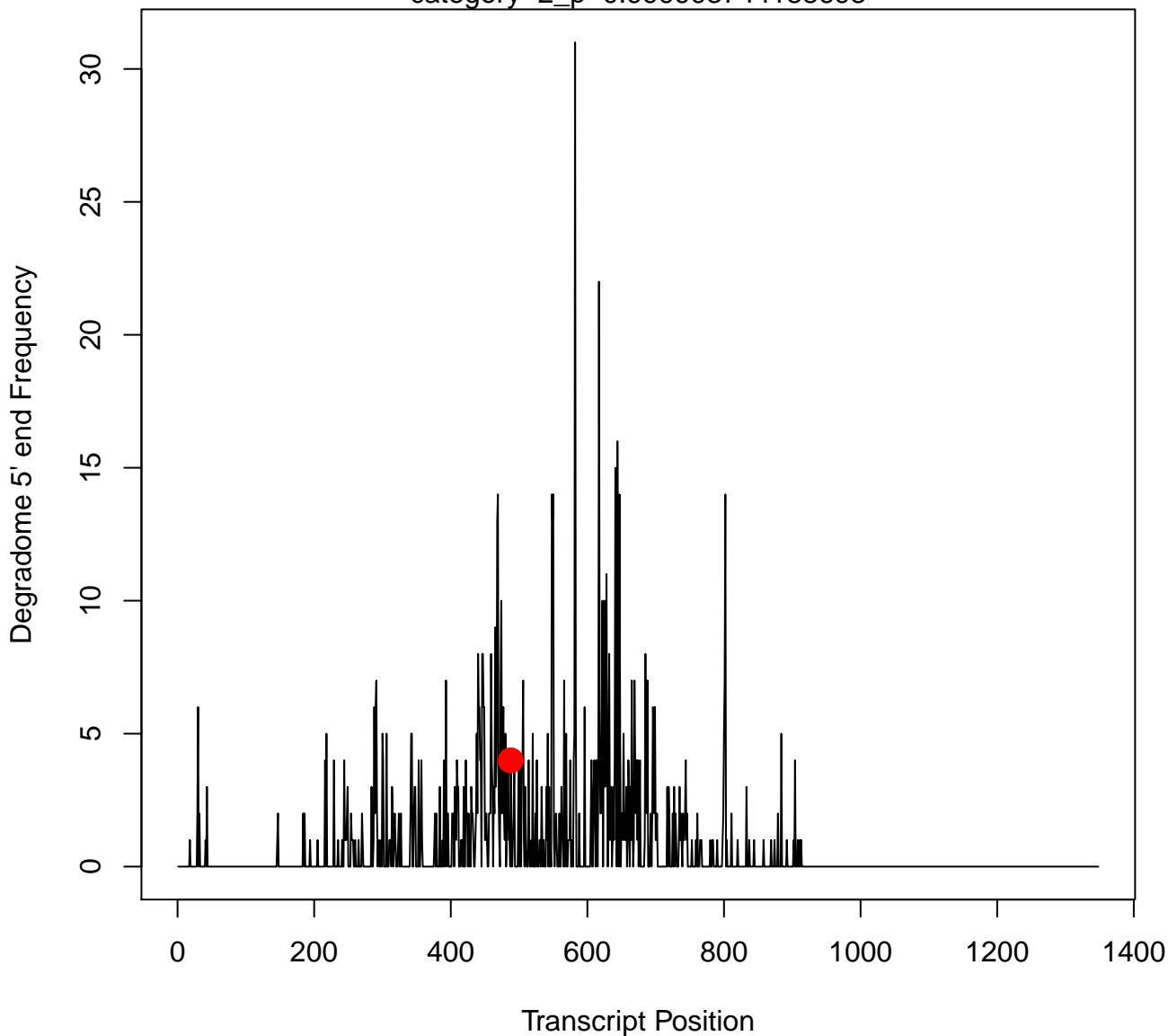

Supplement: Supplementary file 1 [file Data_Sheet_1.zip › Sit-miR1432_Seita.4G002100.1_488_TPlot.pdf]

**T=Seita.4G249500.1\_Q=Sit-miR1432\_S=991**

category=2\_p=0.999981375749571

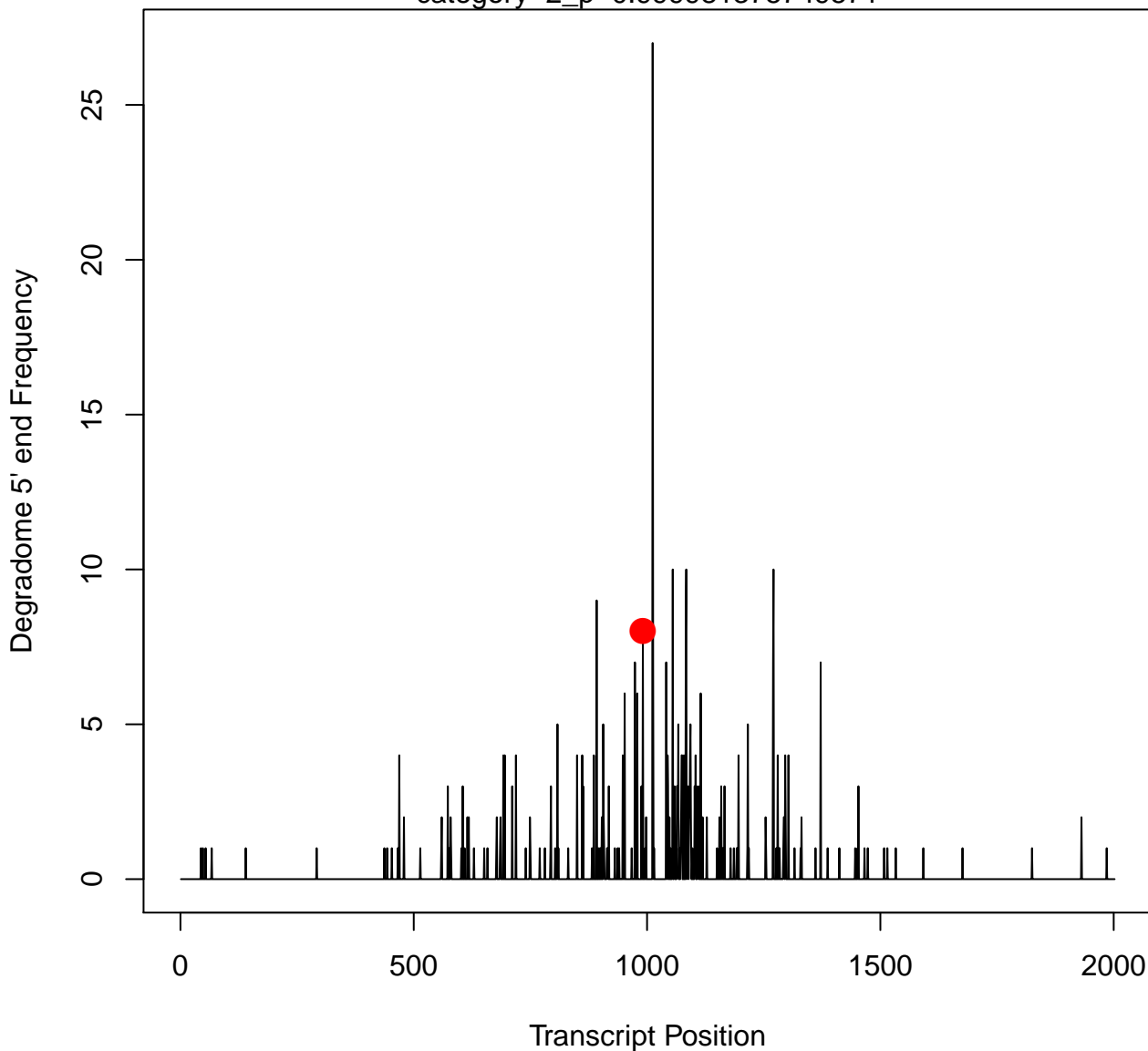

Supplement: Supplementary file 1 [file Data_Sheet_1.zip › Sit-miR1432_Seita.4G249500.1_991_TPlot.pdf]

**T=Seita.5G027200.1\_Q=Sit-miR1432\_S=1158**

category=2\_p=0.994759667071858

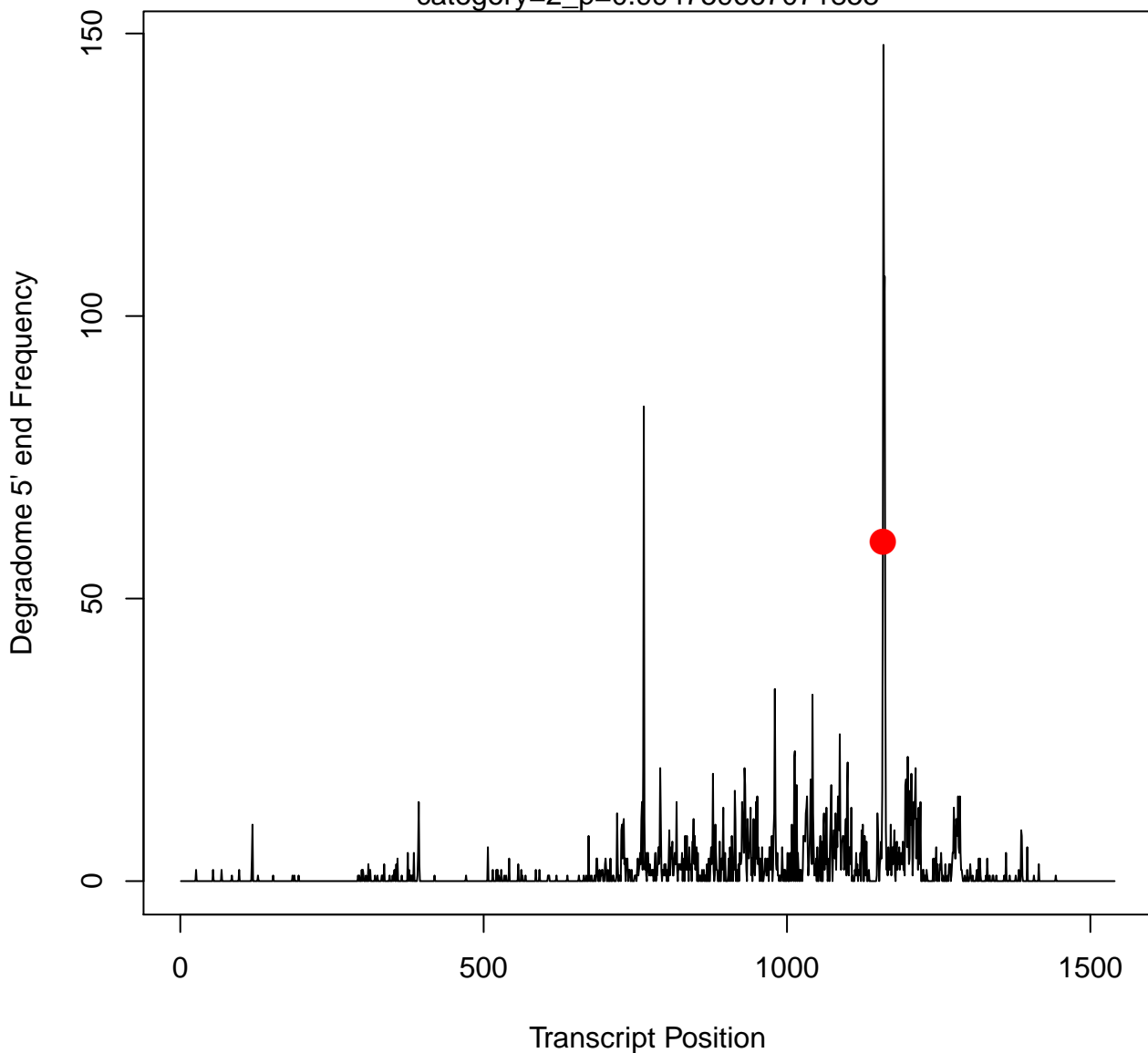

Supplement: Supplementary file 1 [file Data_Sheet_1.zip › Sit-miR1432_Seita.5G027200.1_1158_TPlot.pdf]

**T=Seita.5G220500.1\_Q=Sit-miR1432\_S=2566**

category=2\_p=0.999992044285592

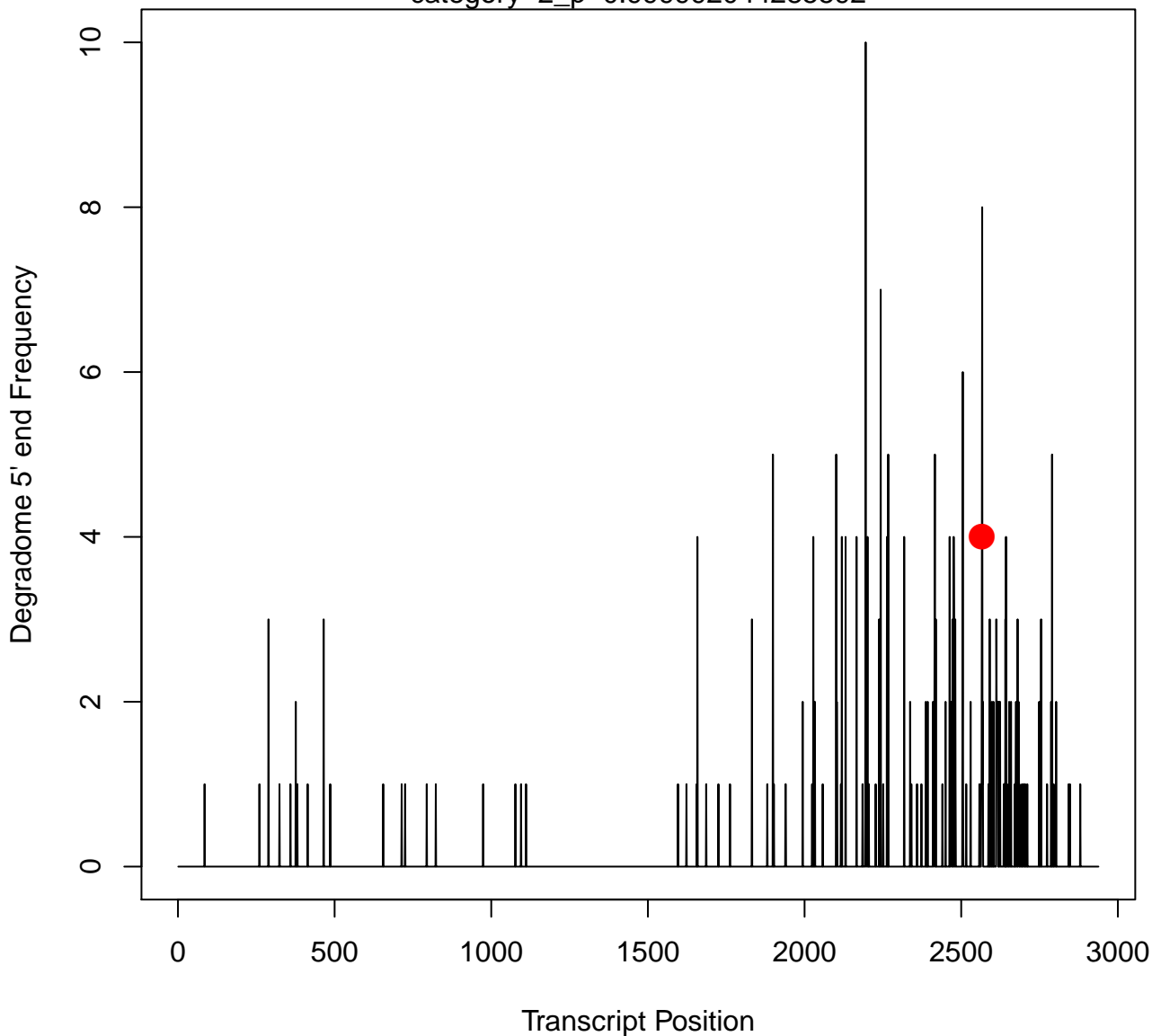

Supplement: Supplementary file 1 [file Data_Sheet_1.zip › Sit-miR1432_Seita.5G220500.1_2566_TPlot.pdf]

**T=Seita.5G407500.1\_Q=Sit-miR1432\_S=990**

category=2\_p=0.99989408953083

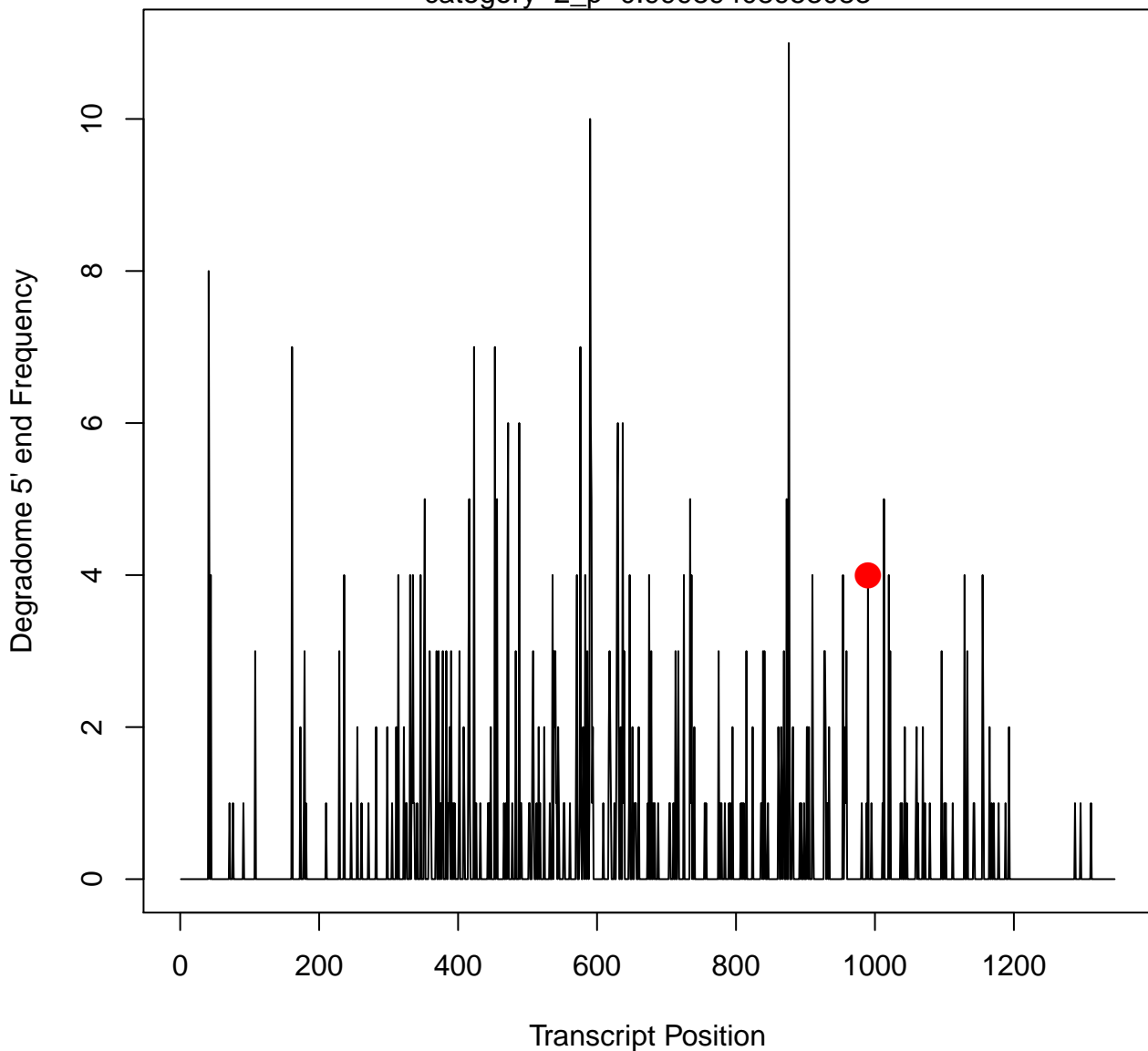

Supplement: Supplementary file 1 [file Data_Sheet_1.zip › Sit-miR1432_Seita.5G407500.1_990_TPlot.pdf]

**T=Seita.9G090200.1\_Q=Sit-miR1432\_S=4065**

category=2\_p=0.99950856702988

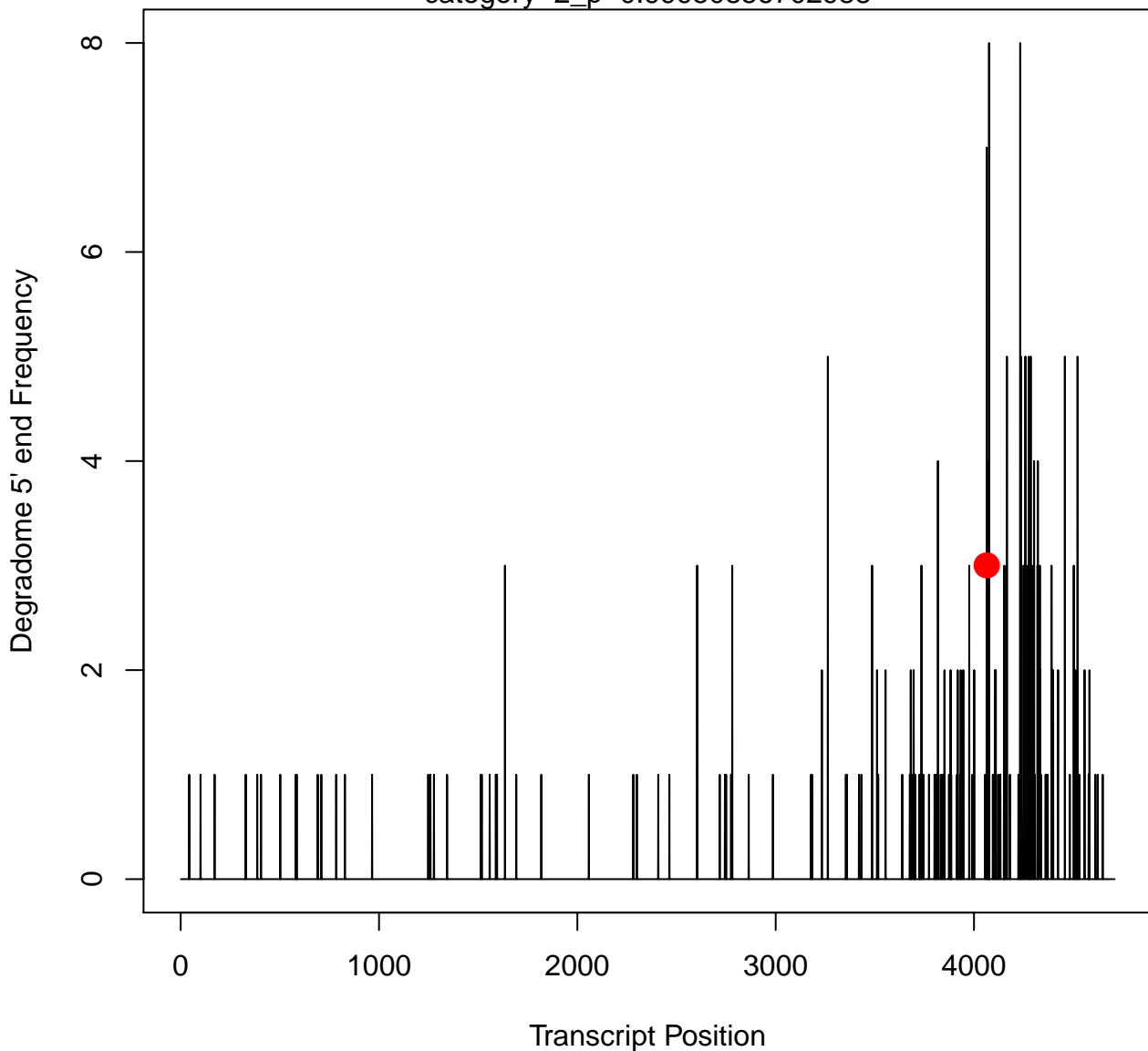

Supplement: Supplementary file 1 [file Data_Sheet_1.zip › Sit-miR1432_Seita.9G090200.1_4065_TPlot.pdf]

**T=Seita.9G259600.1\_Q=Sit-miR1432\_S=856**

category=2\_p=0.999908652749459

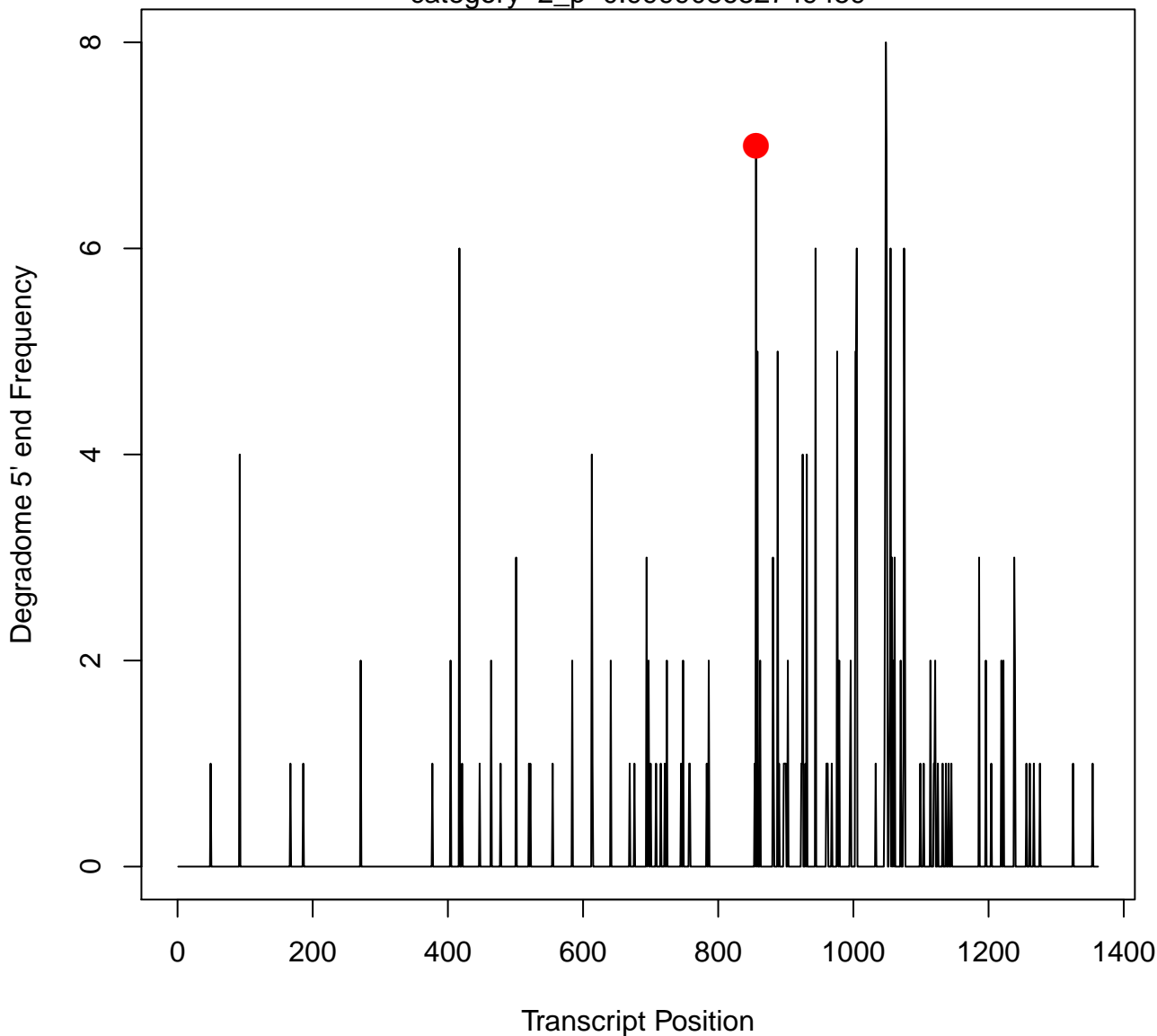

Supplement: Supplementary file 1 [file Data_Sheet_1.zip › Sit-miR1432_Seita.9G259600.1_856_TPlot.pdf]

**T=Seita.9G410000.1\_Q=Sit-miR1432\_S=1012**

category=2\_p=0.989990236737855

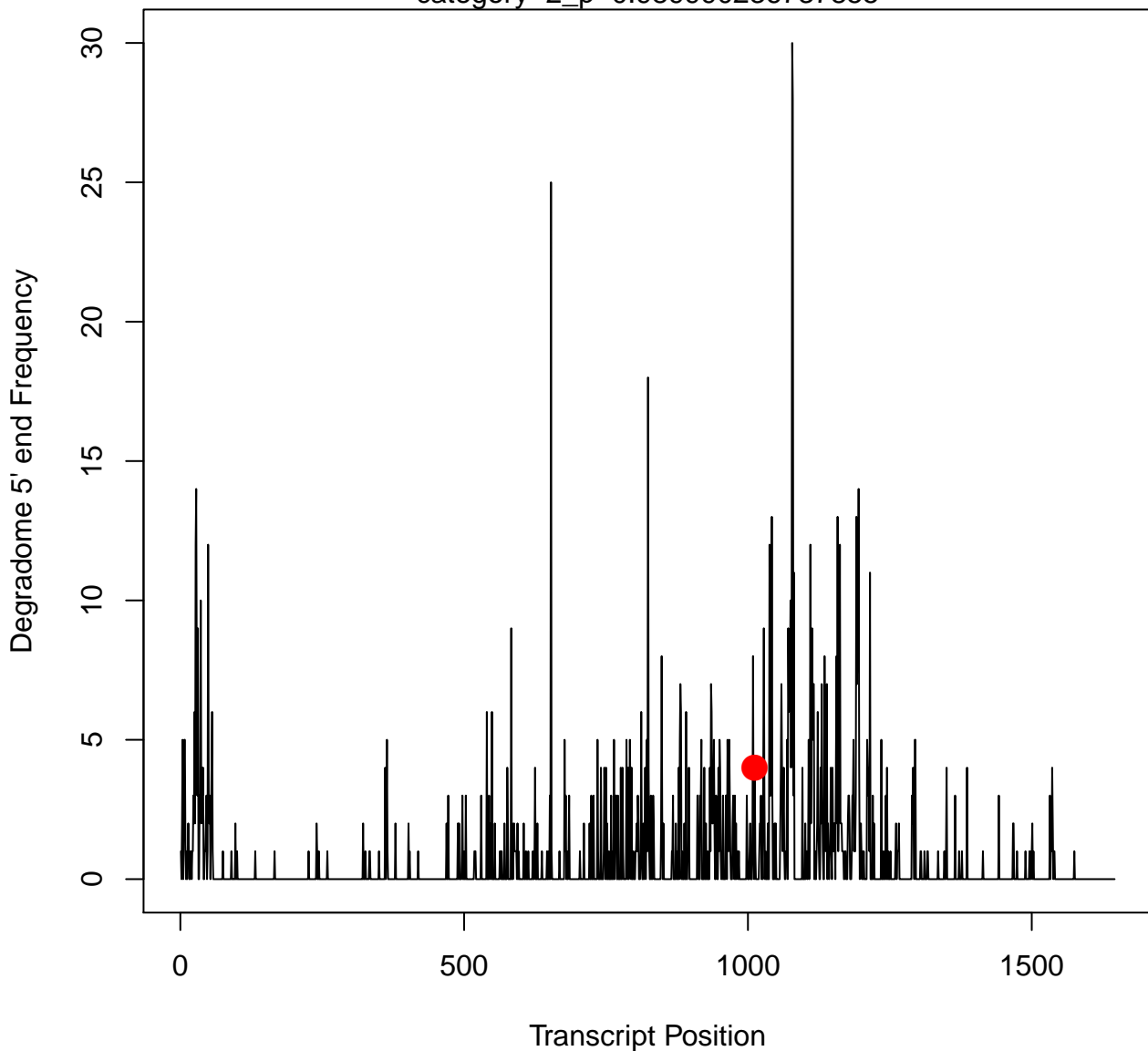

Supplement: Supplementary file 1 [file Data_Sheet_1.zip › Sit-miR1432_Seita.9G410000.1_1012_TPlot.pdf]

**T=Seita.2G324900.1\_Q=Sit-miR156a\_S=833**

category=0\_p=0.00436898762535398

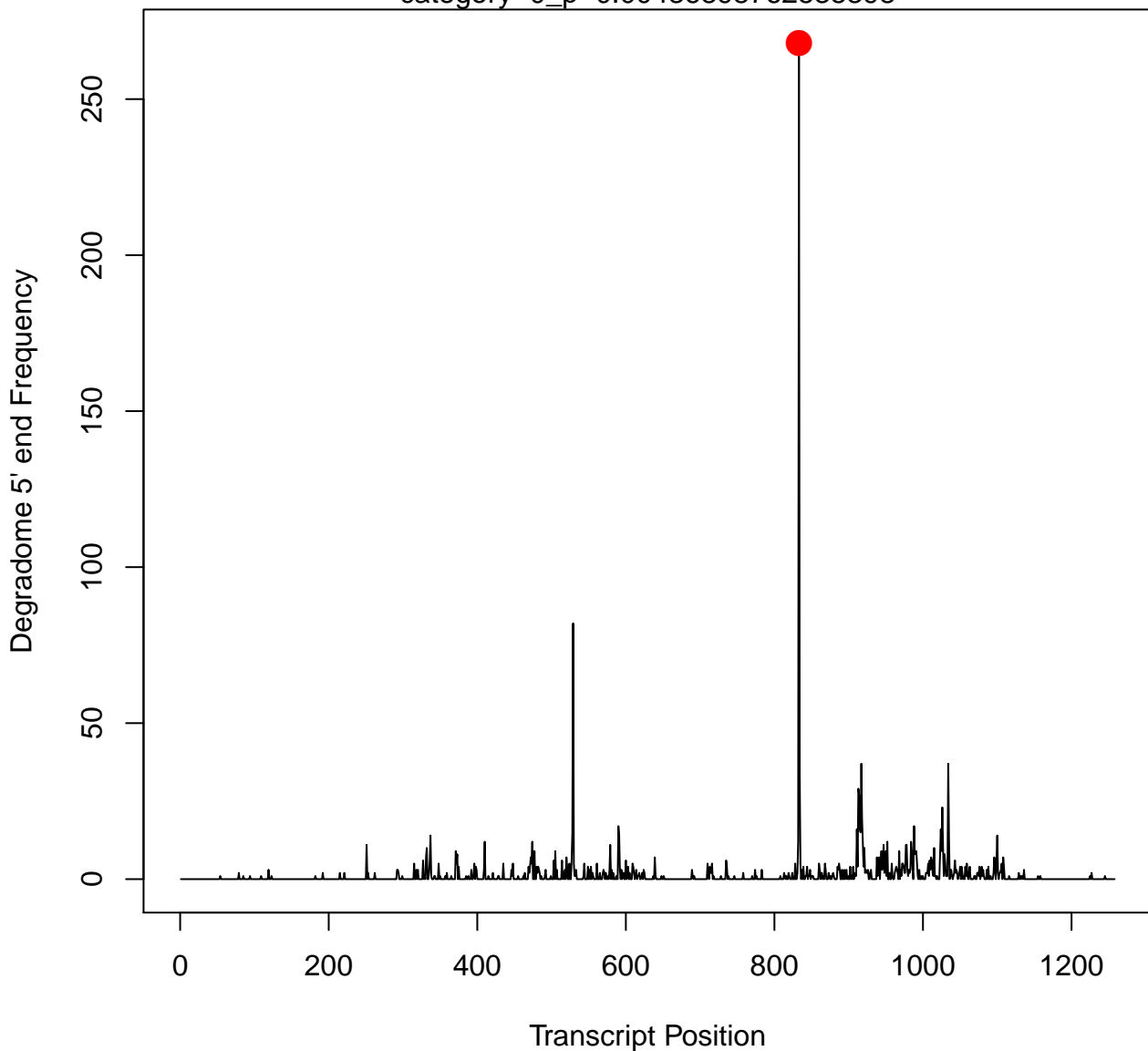

Supplement: Supplementary file 1 [file Data_Sheet_1.zip › Sit-miR156a_Seita.2G324900.1_833_TPlot.pdf]

**T=Seita.4G270400.1\_Q=Sit-miR156a\_S=2084**

category=0\_p=0.000437760109239571

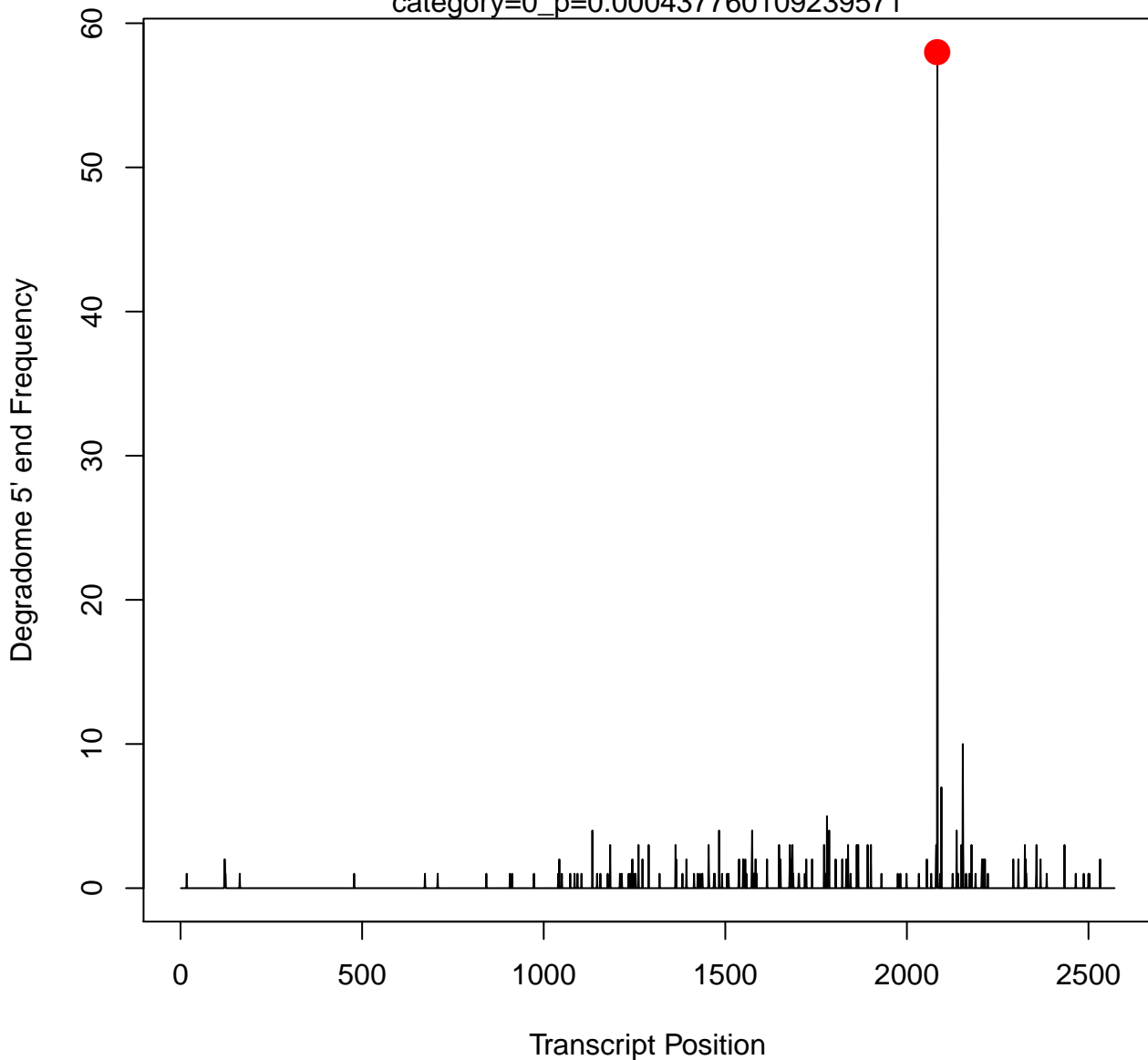

Supplement: Supplementary file 1 [file Data_Sheet_1.zip › Sit-miR156a_Seita.4G270400.1_2084_TPlot.pdf]

**T=Seita.1G069300.1\_Q=Sit-miR156c\_S=873**

category=0\_p=0.000437760109239571

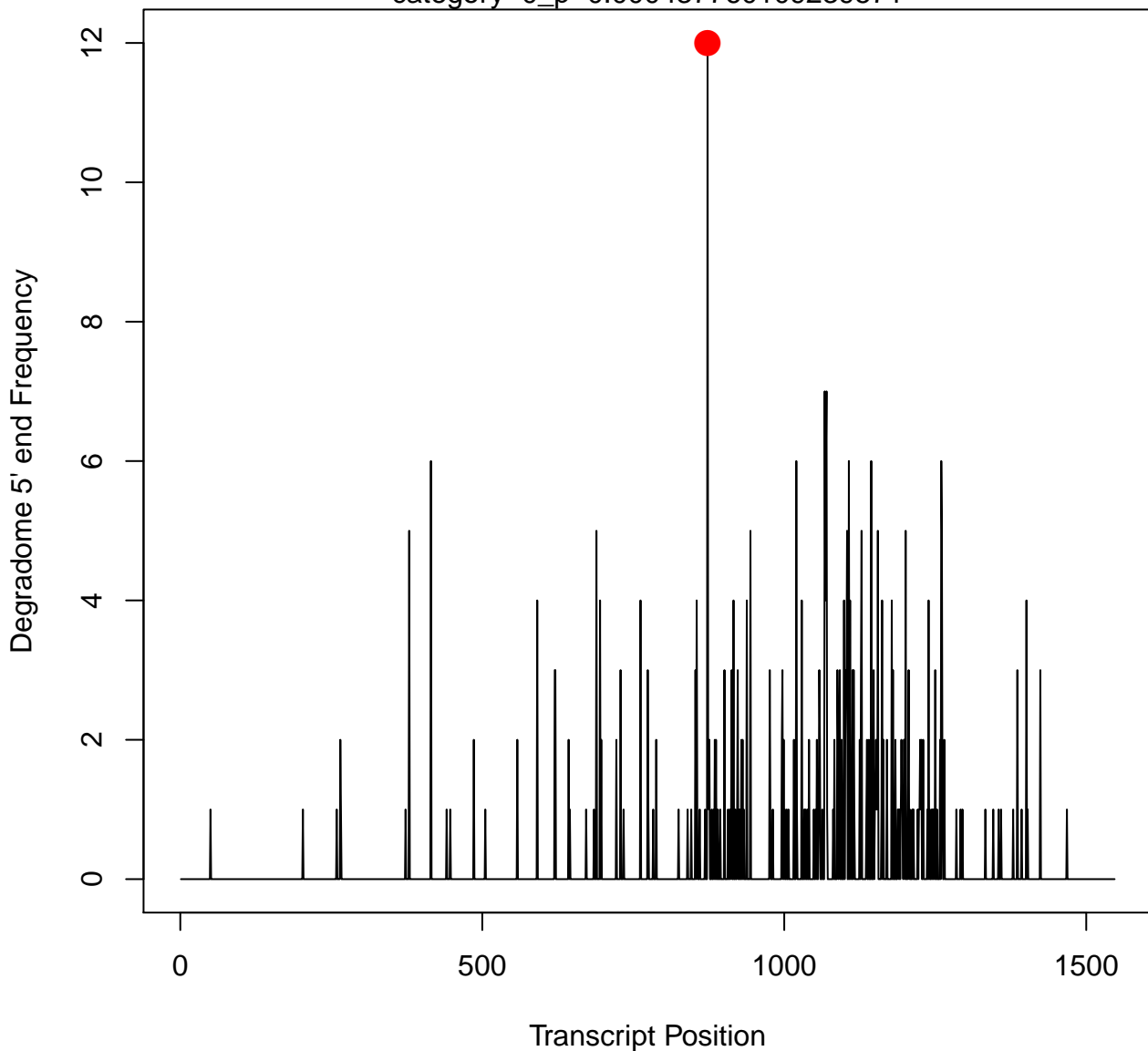

Supplement: Supplementary file 1 [file Data_Sheet_1.zip › Sit-miR156c_Seita.1G069300.1_873_TPlot.pdf]

**T=Seita.1G091900.1\_Q=Sit-miR156d\_S=1787**

category=2\_p=0.0183208354011348

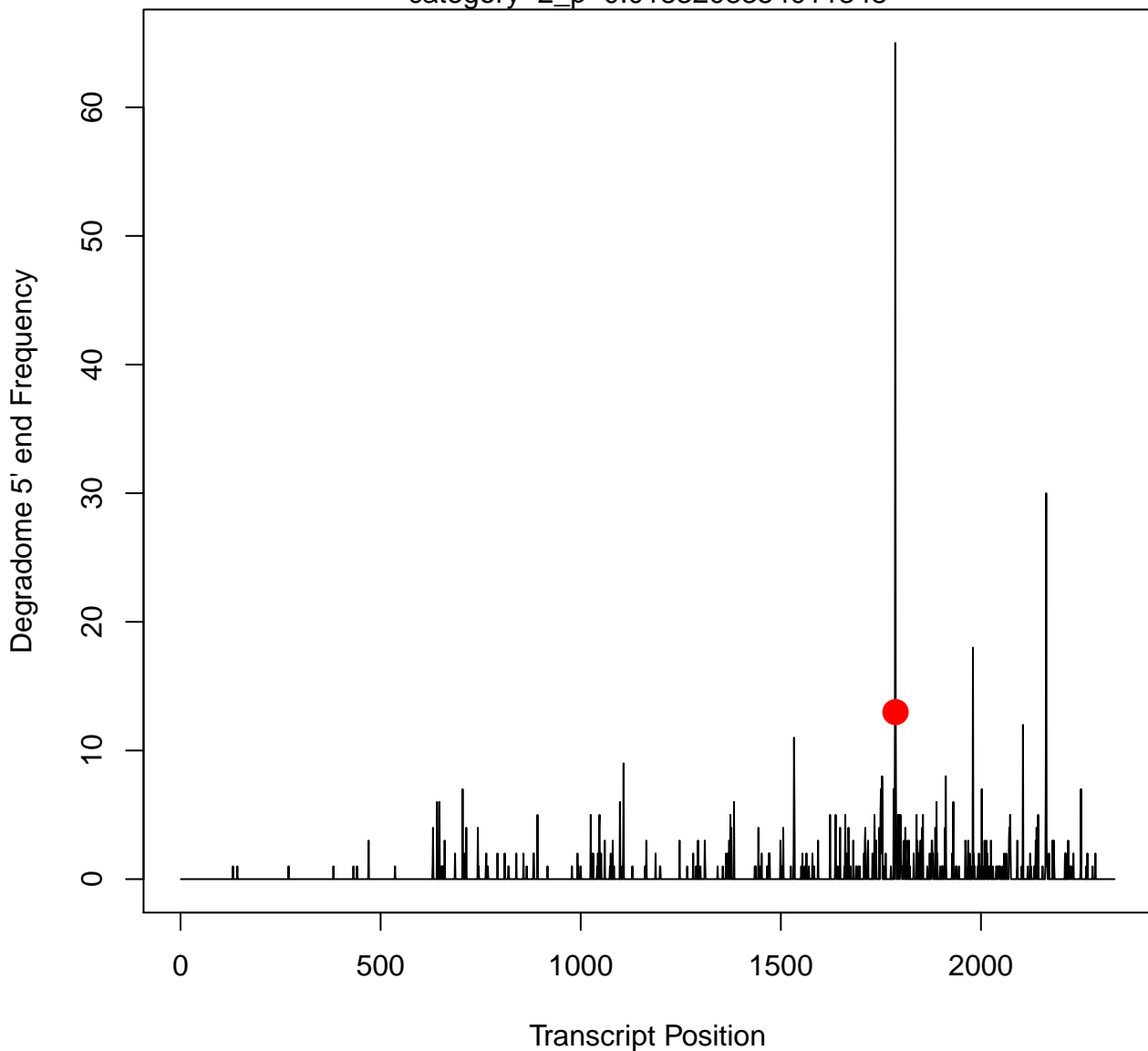

Supplement: Supplementary file 1 [file Data_Sheet_1.zip › Sit-miR156d_Seita.1G091900.1_1787_TPlot.pdf]

T=Seita.1G134200.1\_Q=Sit-miR156d\_S=60

category=2\_p=0.969643660930676

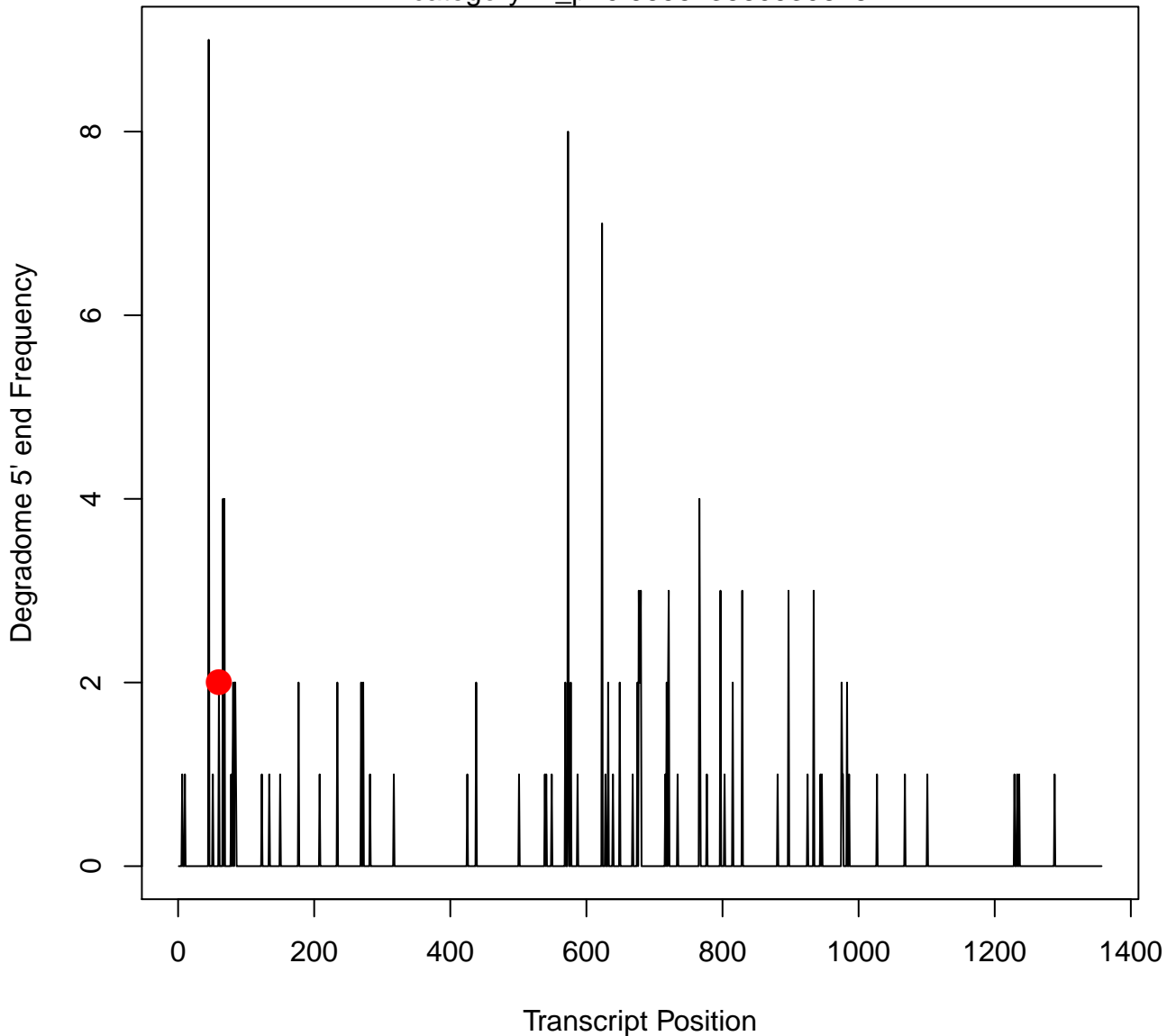

Supplement: Supplementary file 1 [file Data_Sheet_1.zip › Sit-miR156d_Seita.1G134200.1_60_TPlot.pdf]

**T=Seita.2G254300.1\_Q=Sit-miR156d\_S=1267**

category=2\_p=0.0539616966175622

Degradome 5' end Frequency

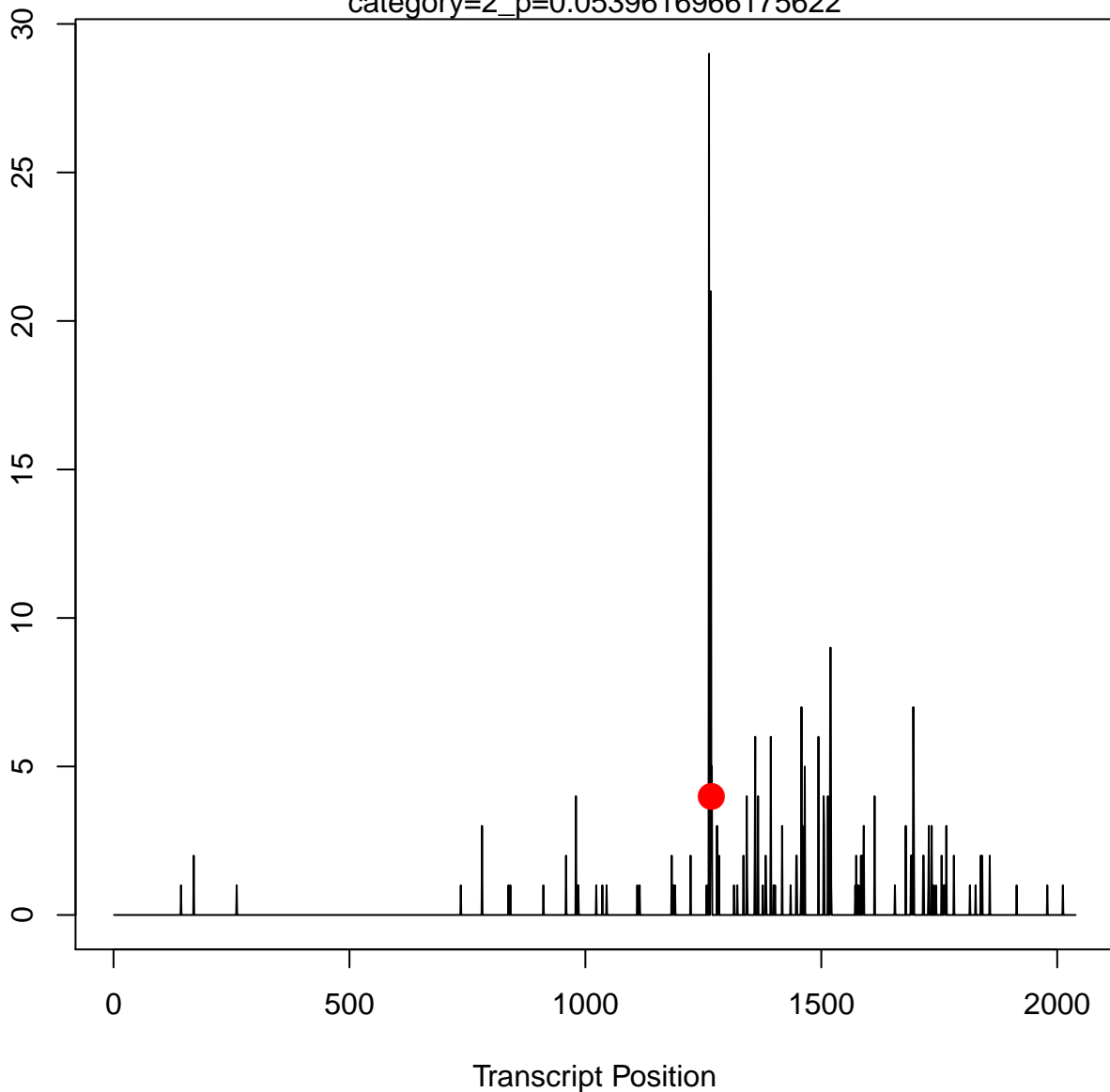

Supplement: Supplementary file 1 [file Data_Sheet_1.zip › Sit-miR156d_Seita.2G254300.1_1267_TPlot.pdf]

**T=Seita.2G266500.1\_Q=Sit-miR156d\_S=1897**

category=2\_p=0.153306624906782

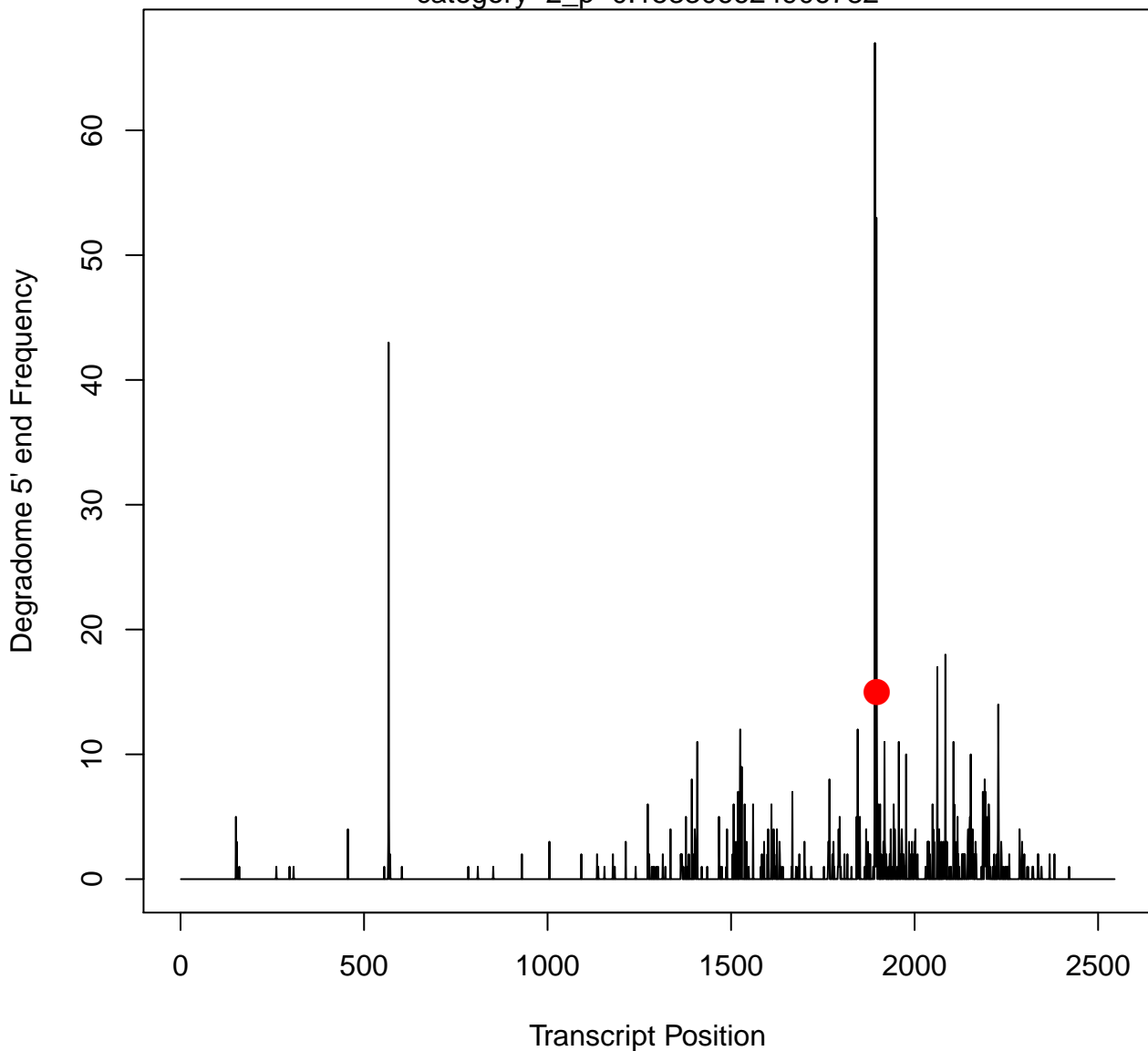

Supplement: Supplementary file 1 [file Data_Sheet_1.zip › Sit-miR156d_Seita.2G266500.1_1897_TPlot.pdf]

**T=Seita.2G324900.1\_Q=Sit-miR156d\_S=834**

category=2\_p=0.184046689647686

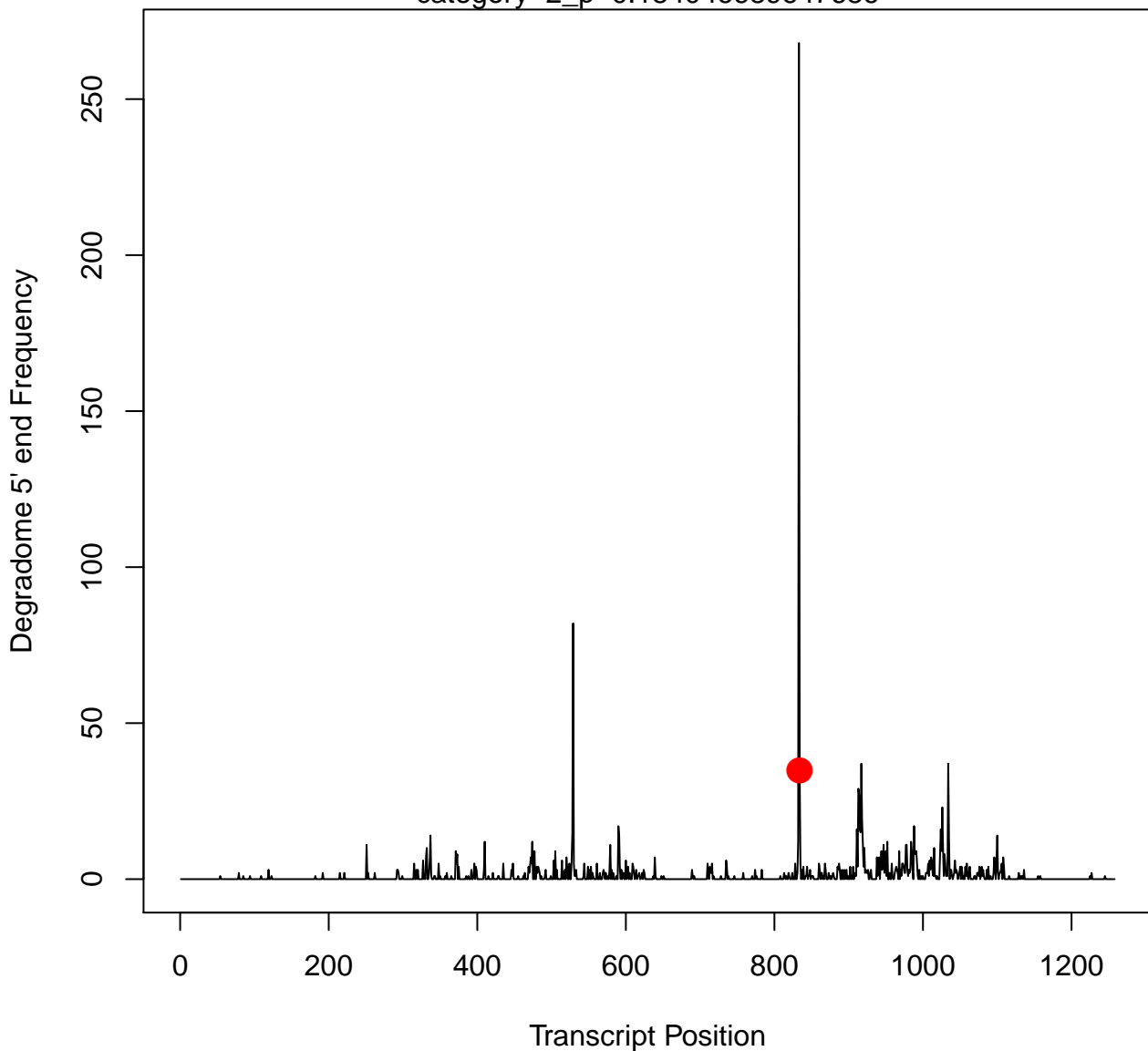

Supplement: Supplementary file 1 [file Data_Sheet_1.zip › Sit-miR156d_Seita.2G324900.1_834_TPlot.pdf]

**T=Seita.2G325000.1\_Q=Sit-miR156d\_S=666**

category=2\_p=0.198995635941662

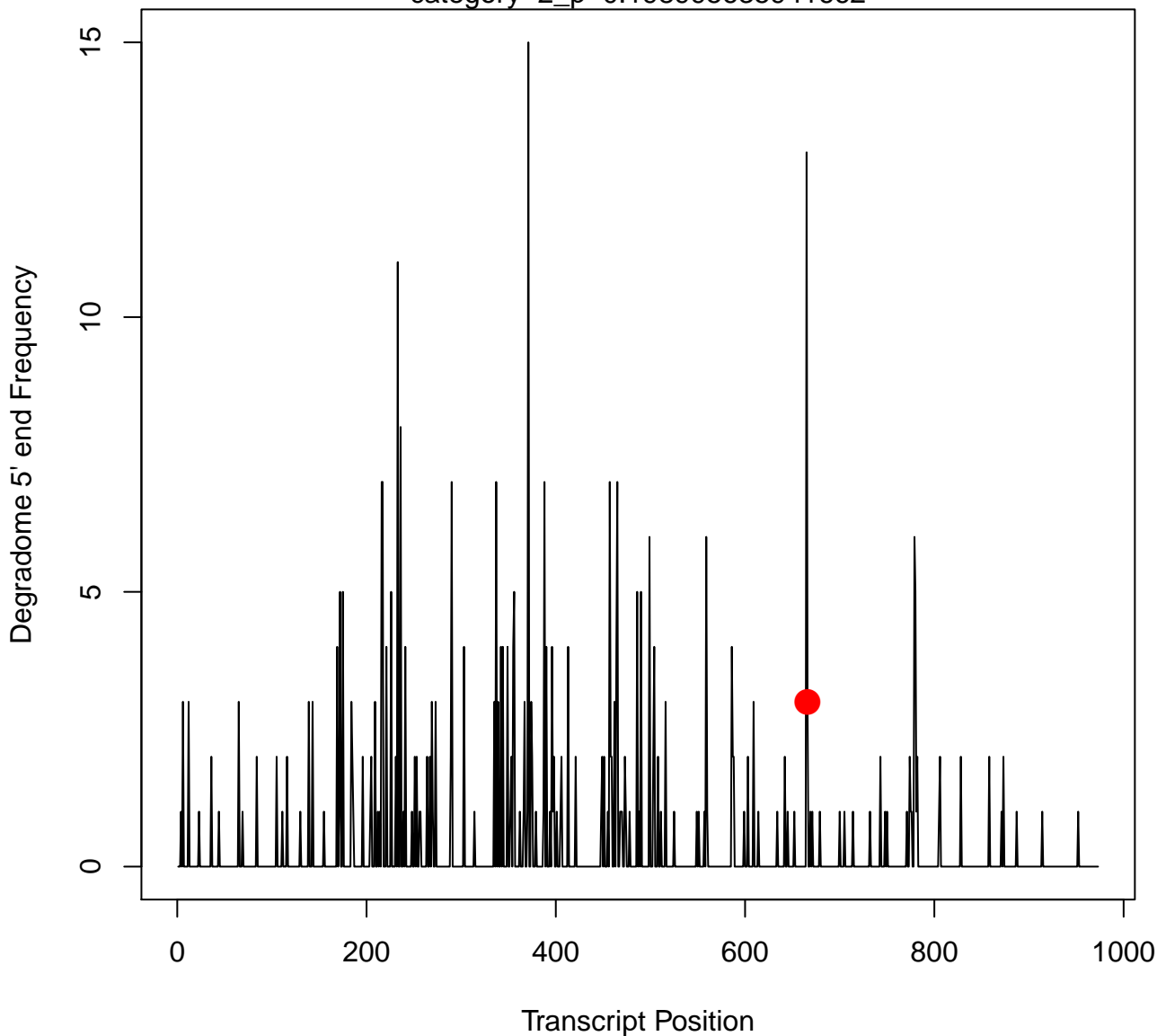

Supplement: Supplementary file 1 [file Data_Sheet_1.zip › Sit-miR156d_Seita.2G325000.1_666_TPlot.pdf]

**T=Seita.7G162400.1\_Q=Sit-miR156d\_S=1617**

category=2\_p=0.283110328573254

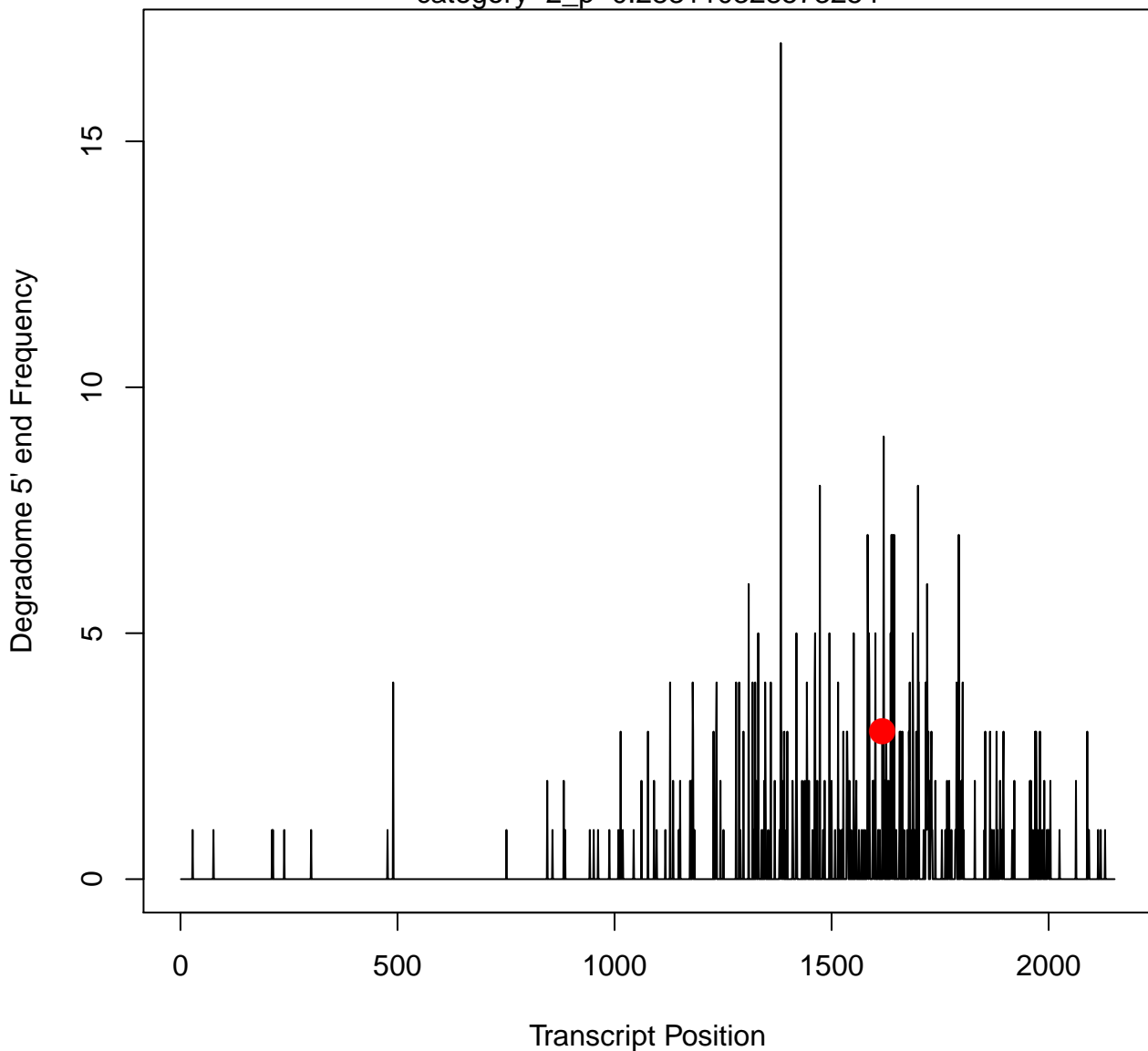

Supplement: Supplementary file 1 [file Data_Sheet_1.zip › Sit-miR156d_Seita.7G162400.1_1617_TPlot.pdf]

**T=Seita.9G485400.1\_Q=Sit-miR156d\_S=1791**

category=2\_p=0.976567241085221

Degradome 5' end Frequency

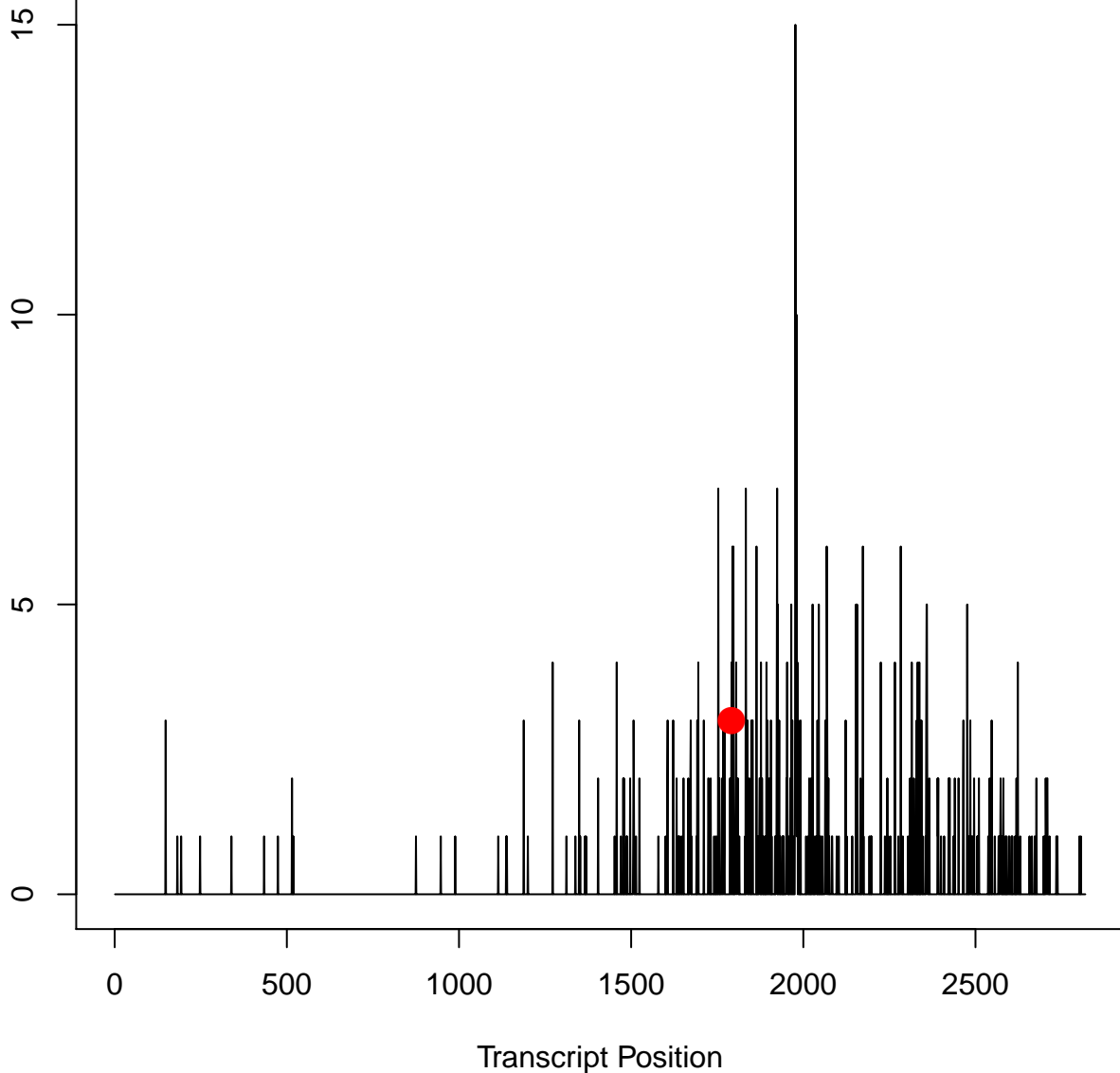

Supplement: Supplementary file 1 [file Data_Sheet_1.zip › Sit-miR156d_Seita.9G485400.1_1791_TPlot.pdf]

**T=Seita.2G254300.1\_Q=Sit-miR156e\_S=1266**

category=2\_p=0.088308580092527

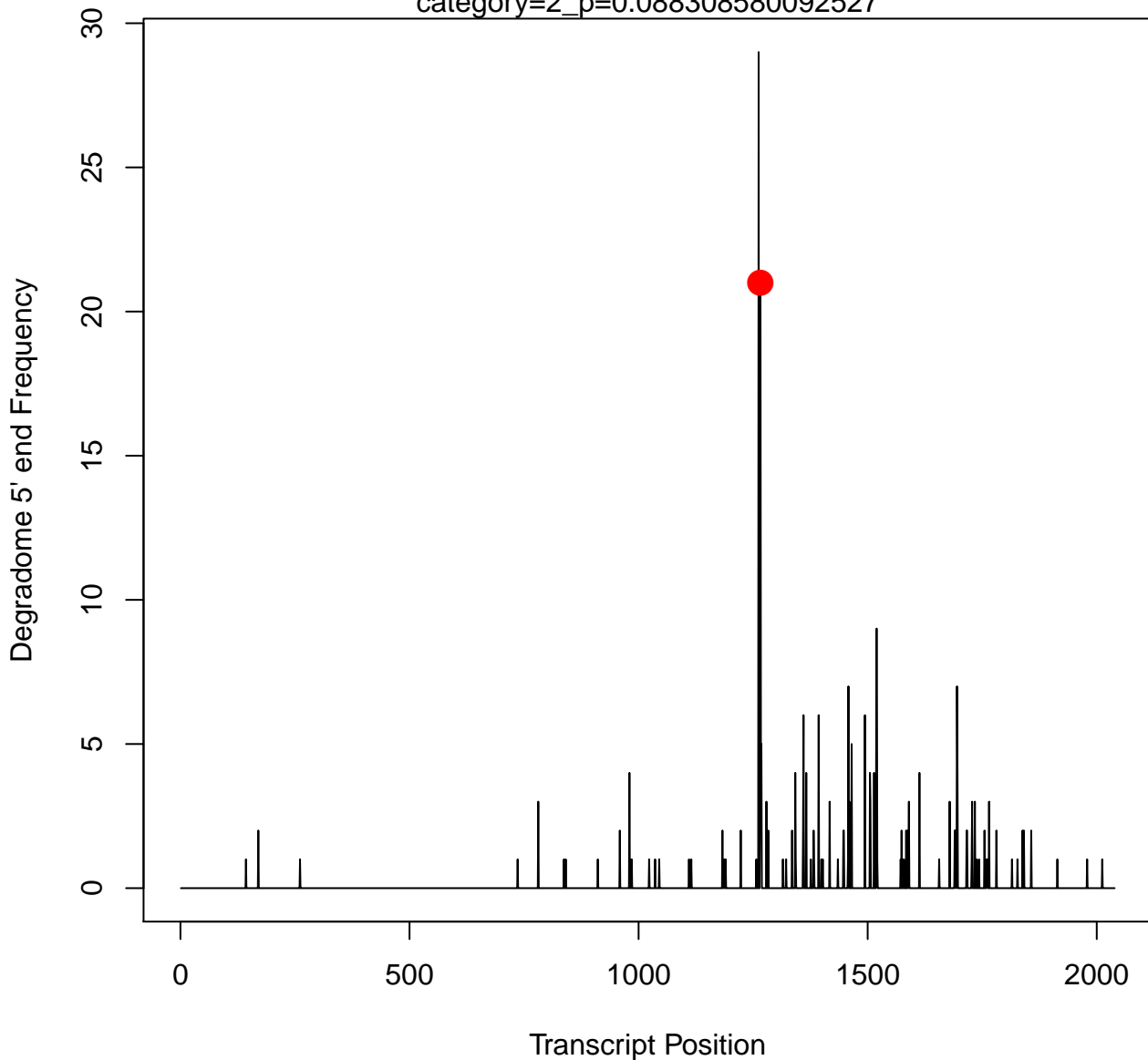

Supplement: Supplementary file 1 [file Data_Sheet_1.zip › Sit-miR156e_Seita.2G254300.1_1266_TPlot.pdf]

**T=Seita.1G091900.1\_Q=Sit-miR156f\_S=1786**

category=0\_p=0.000437760109239571

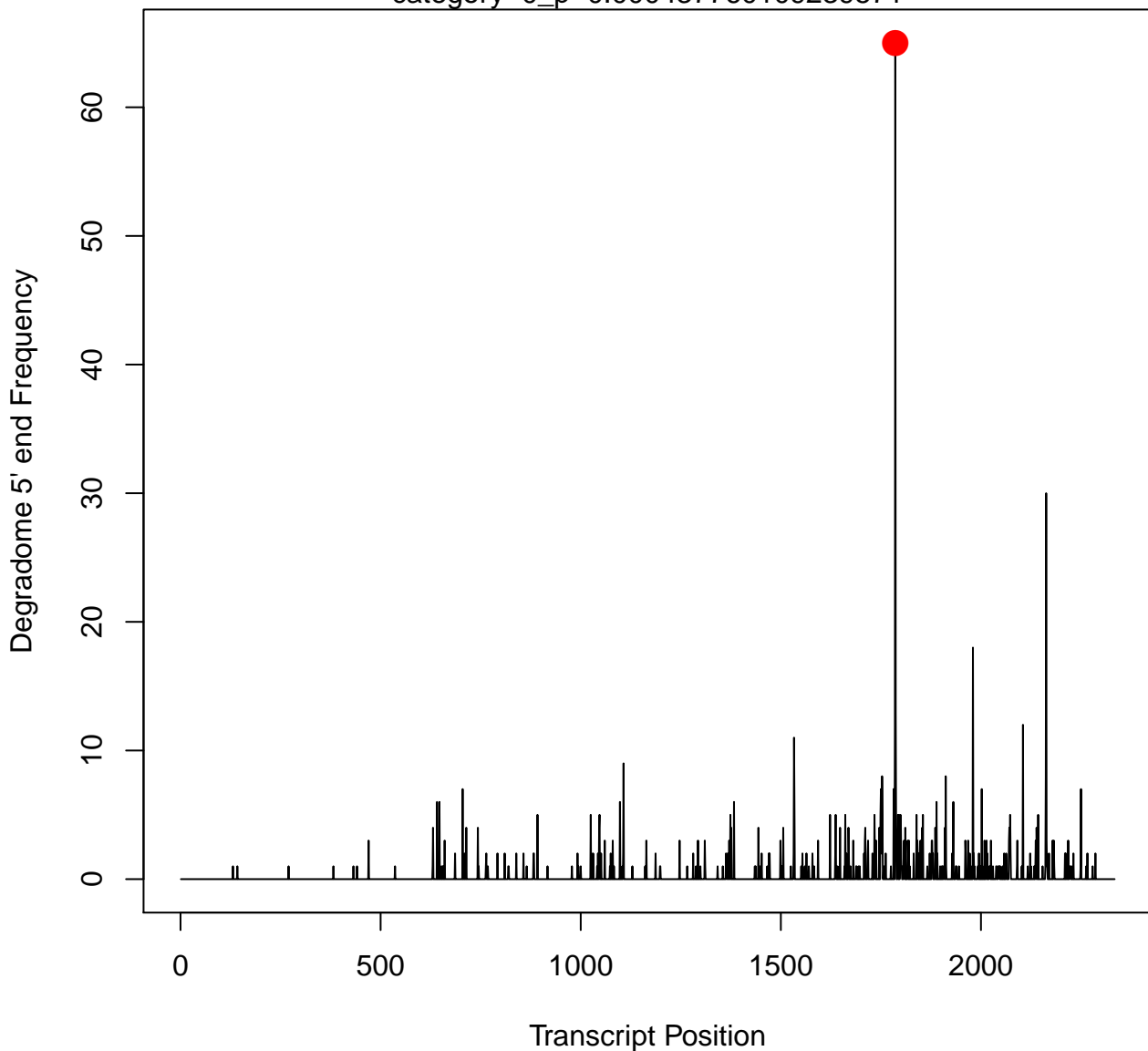

Supplement: Supplementary file 1 [file Data_Sheet_1.zip › Sit-miR156f_Seita.1G091900.1_1786_TPlot.pdf]

**T=Seita.7G318000.1\_Q=Sit-miR156f\_S=1016**

category=2\_p=0.971281740489954

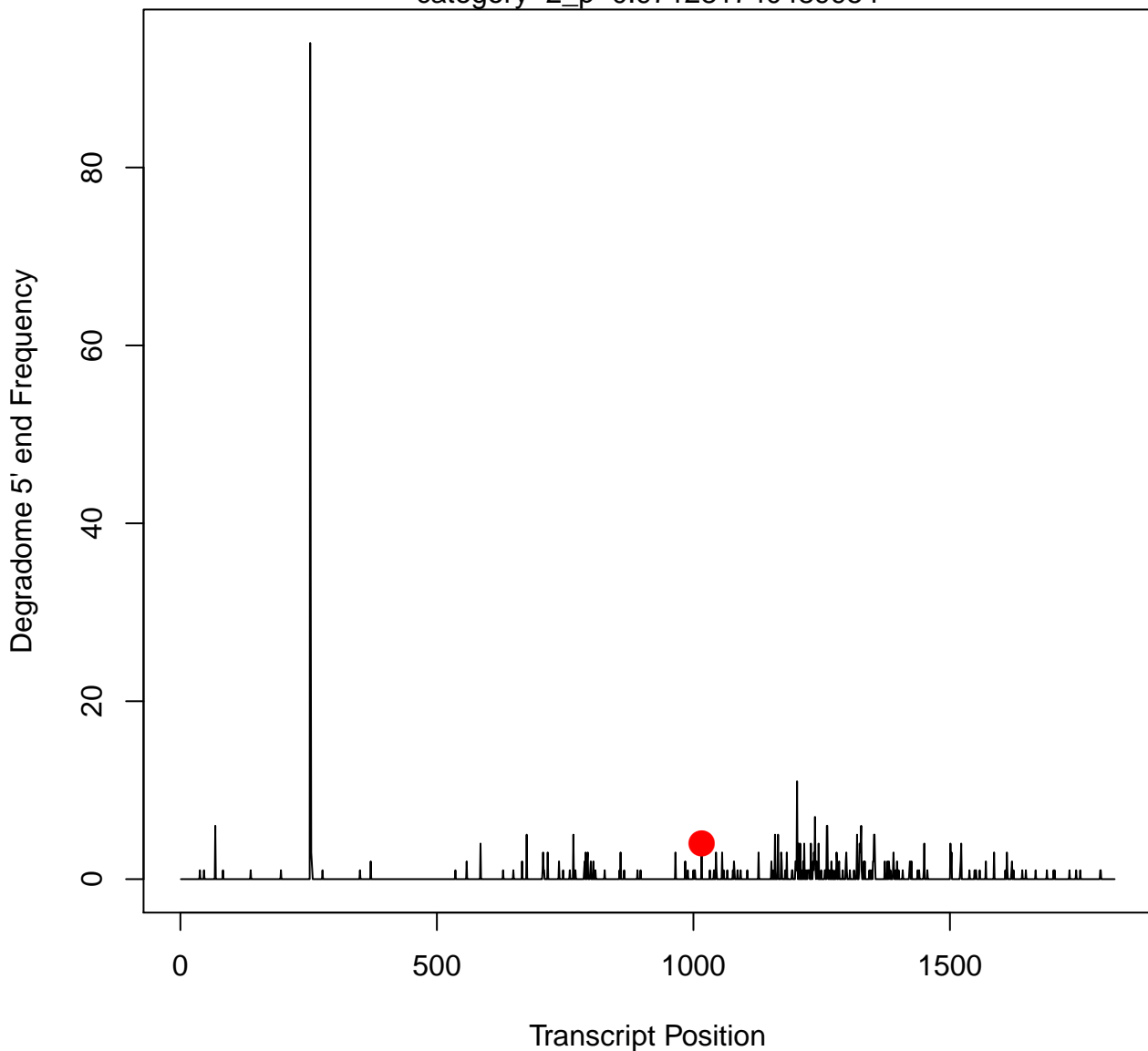

Supplement: Supplementary file 1 [file Data_Sheet_1.zip › Sit-miR156f_Seita.7G318000.1_1016_TPlot.pdf]

**T=Seita.8G124900.1\_Q=Sit-miR156f\_S=1295**

category=0\_p=0.00654631819742957

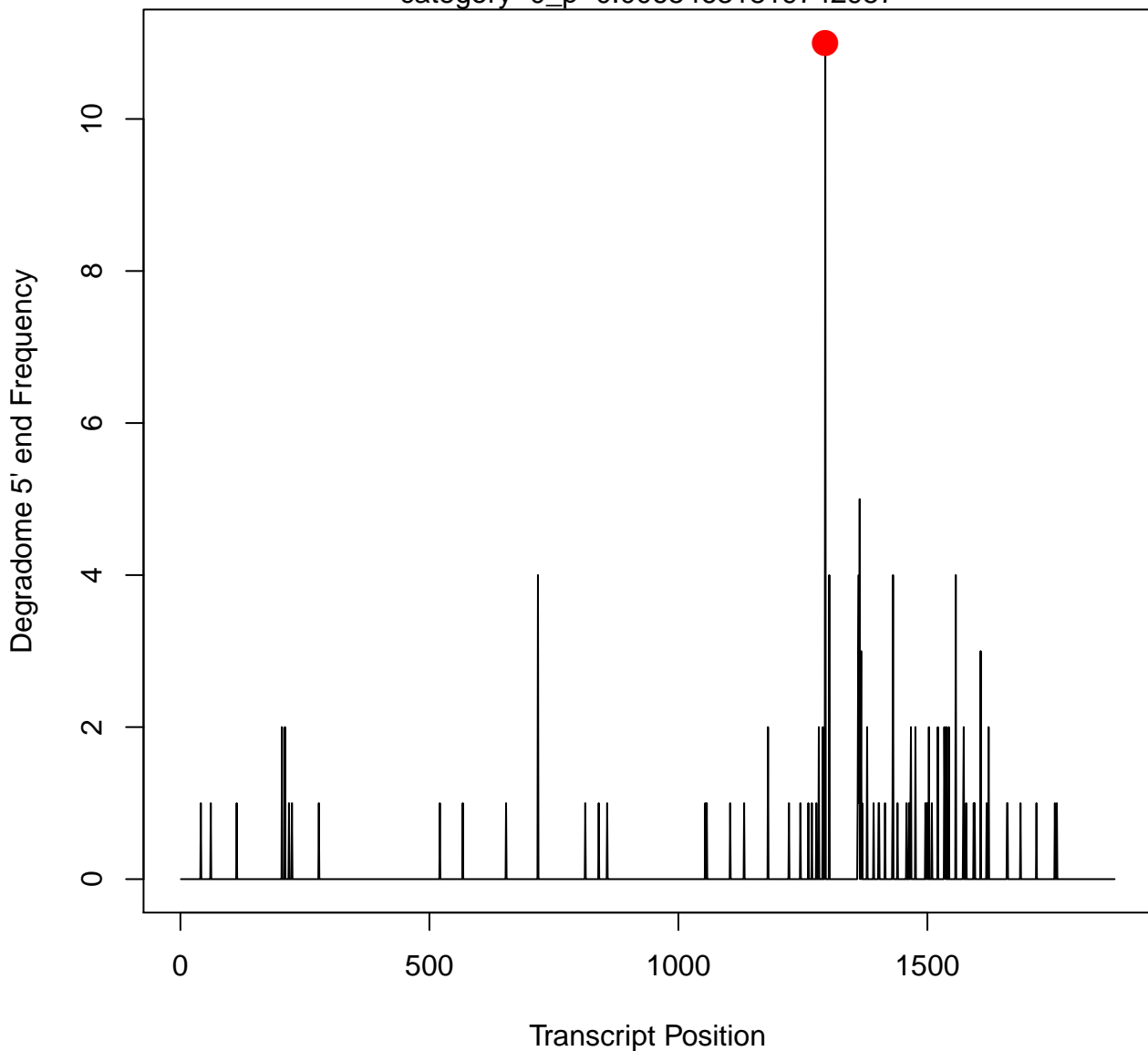

Supplement: Supplementary file 1 [file Data_Sheet_1.zip › Sit-miR156f_Seita.8G124900.1_1295_TPlot.pdf]

**T=Seita.3G202800.1\_Q=Sit-miR156g\_S=1107**

category=2\_p=0.960674399244102

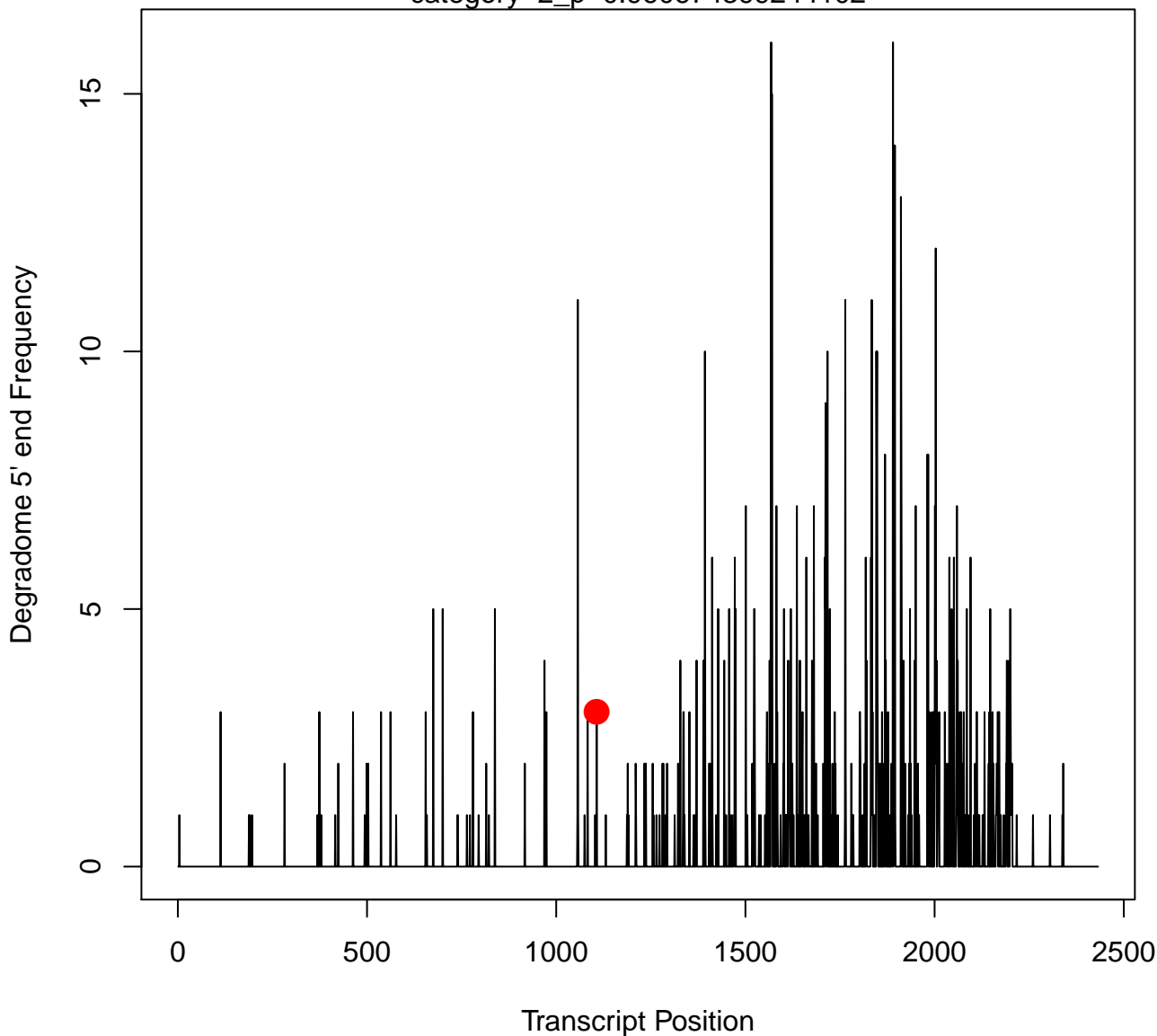

Supplement: Supplementary file 1 [file Data_Sheet_1.zip › Sit-miR156g_Seita.3G202800.1_1107_TPlot.pdf]

**T=Seita.2G325000.1\_Q=Sit-miR156h\_S=665**

category=2\_p=0.184046689647686

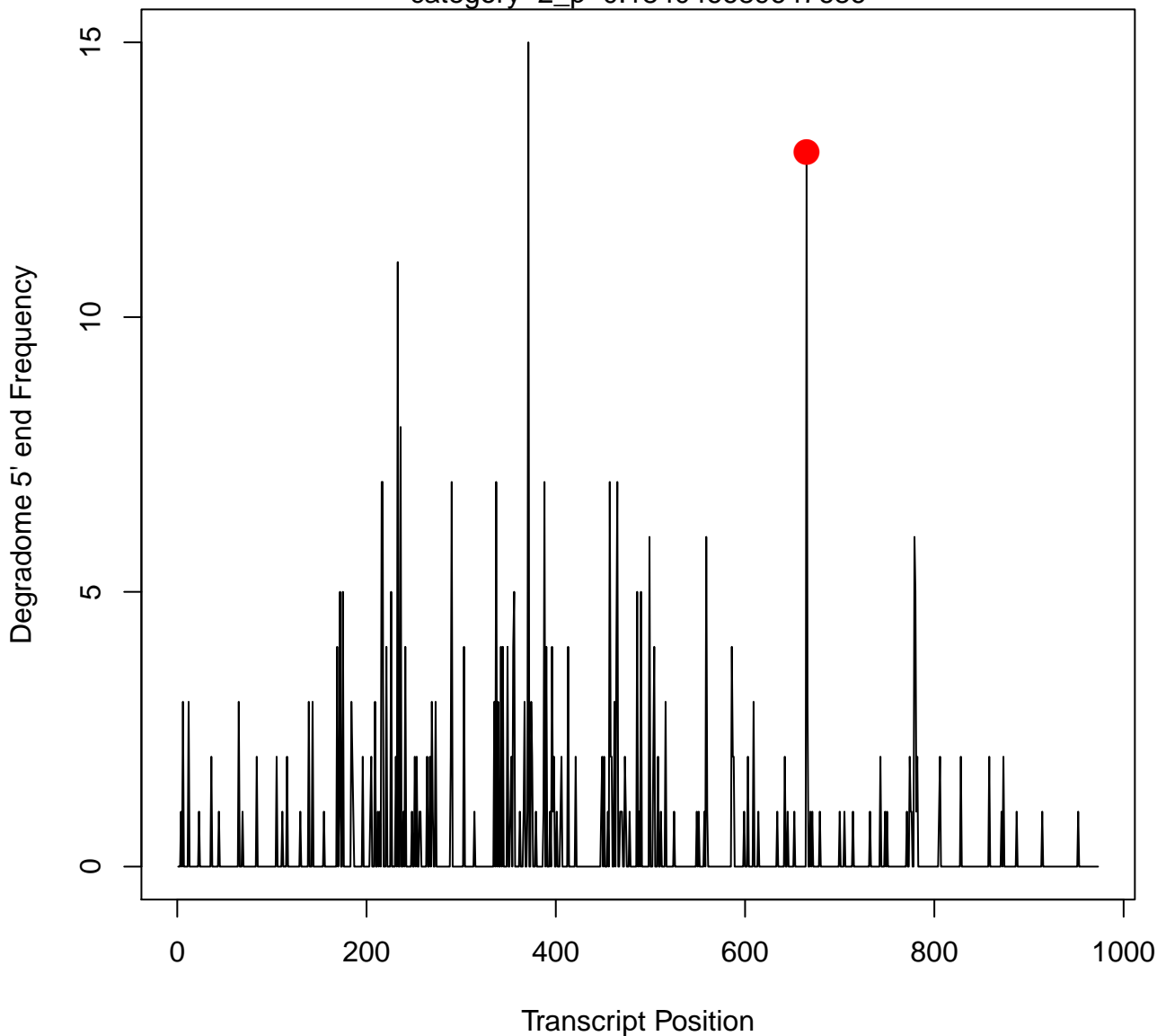

Supplement: Supplementary file 1 [file Data_Sheet_1.zip › Sit-miR156h_Seita.2G325000.1_665_TPlot.pdf]

**T=Seita.2G266500.1\_Q=Sit-miR156j\_S=1896**

category=2\_p=0.153306624906782

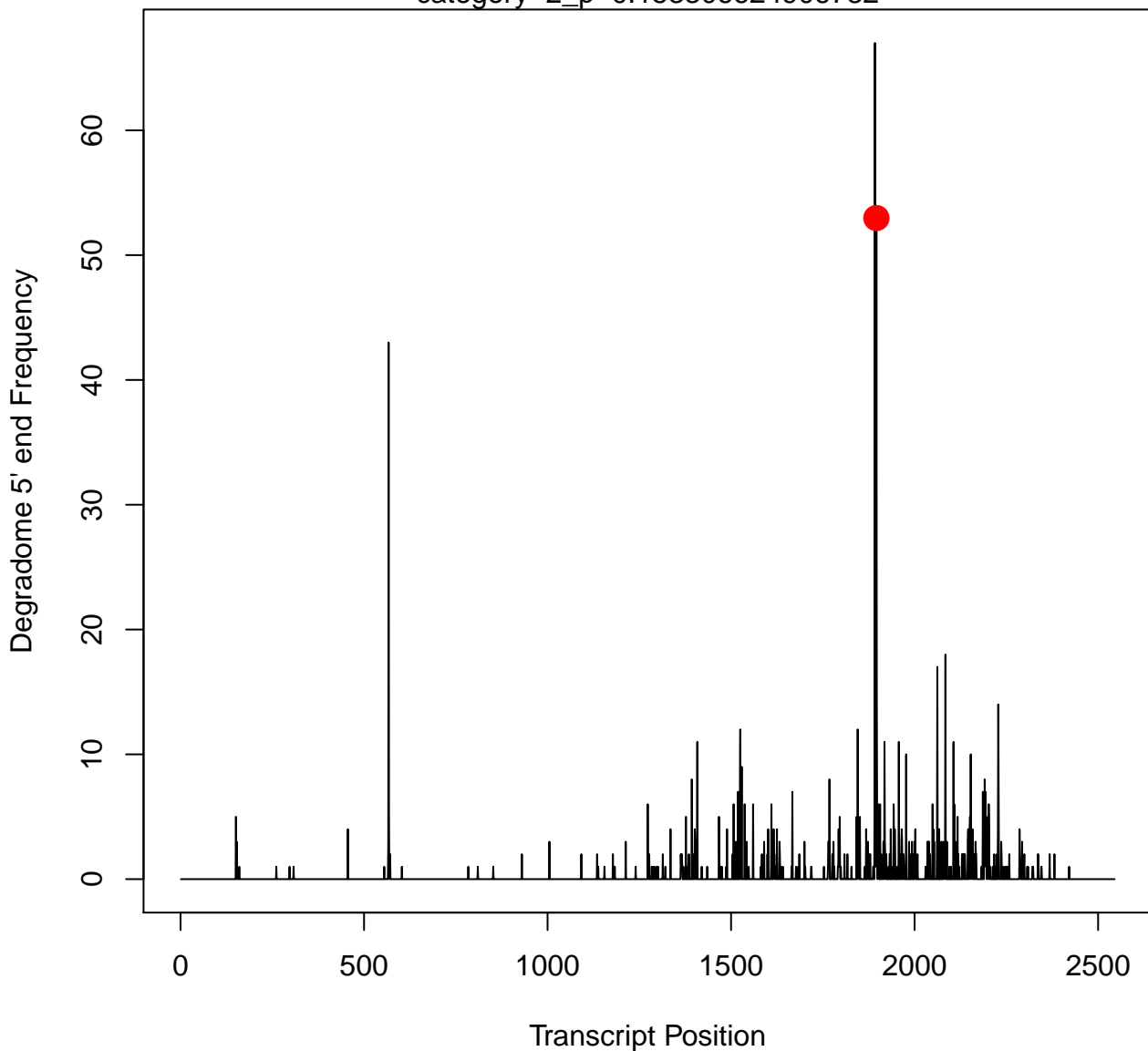

Supplement: Supplementary file 1 [file Data_Sheet_1.zip › Sit-miR156j_Seita.2G266500.1_1896_TPlot.pdf]

**T=Seita.6G223300.1\_Q=Sit-miR156j\_S=981**

category=2\_p=0.0712939086570006

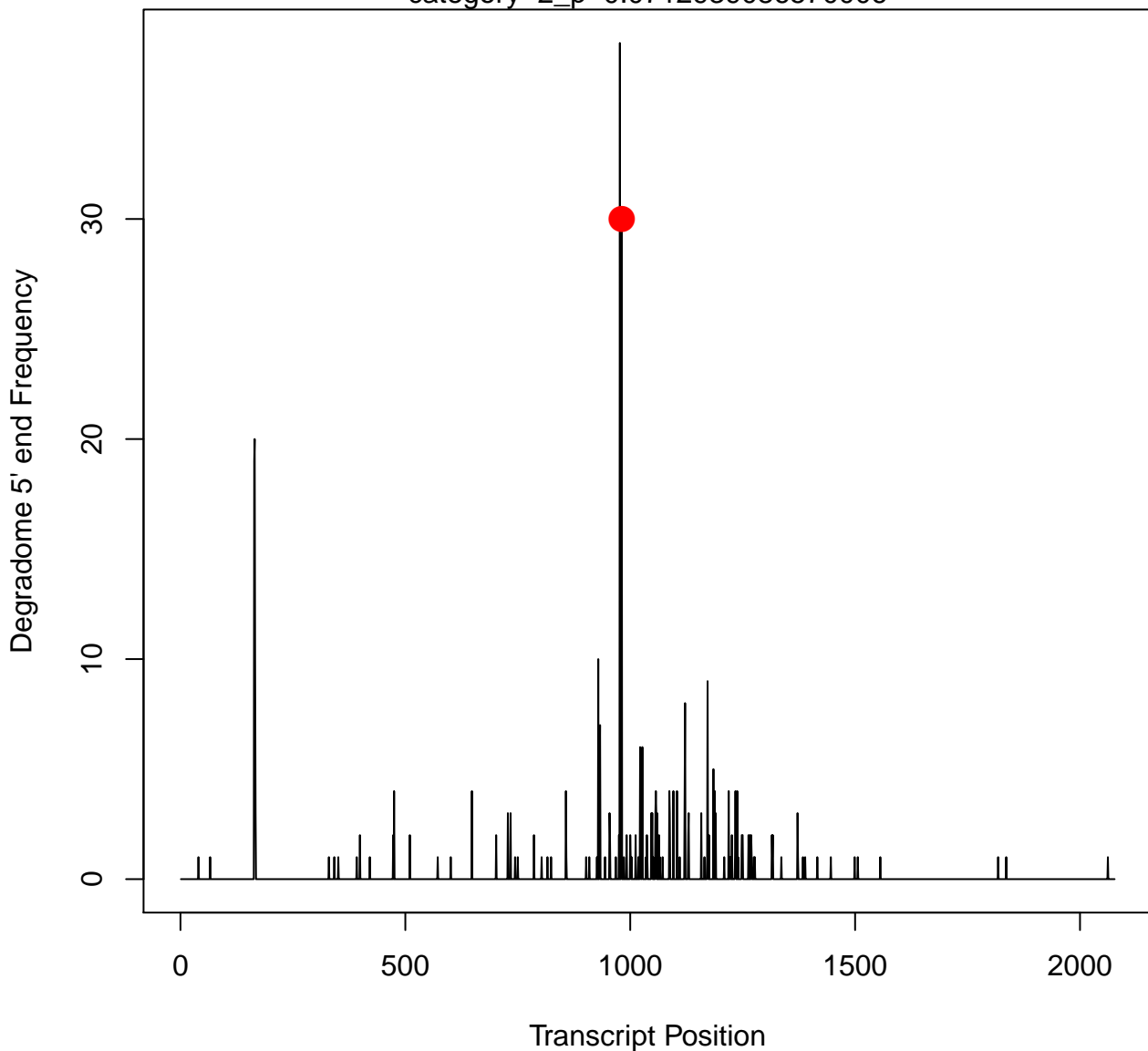

Supplement: Supplementary file 1 [file Data_Sheet_1.zip › Sit-miR156j_Seita.6G223300.1_981_TPlot.pdf]

**T=Seita.1G186900.1\_Q=Sit-miR159a\_S=885**

category=0\_p=0.0126175454617572

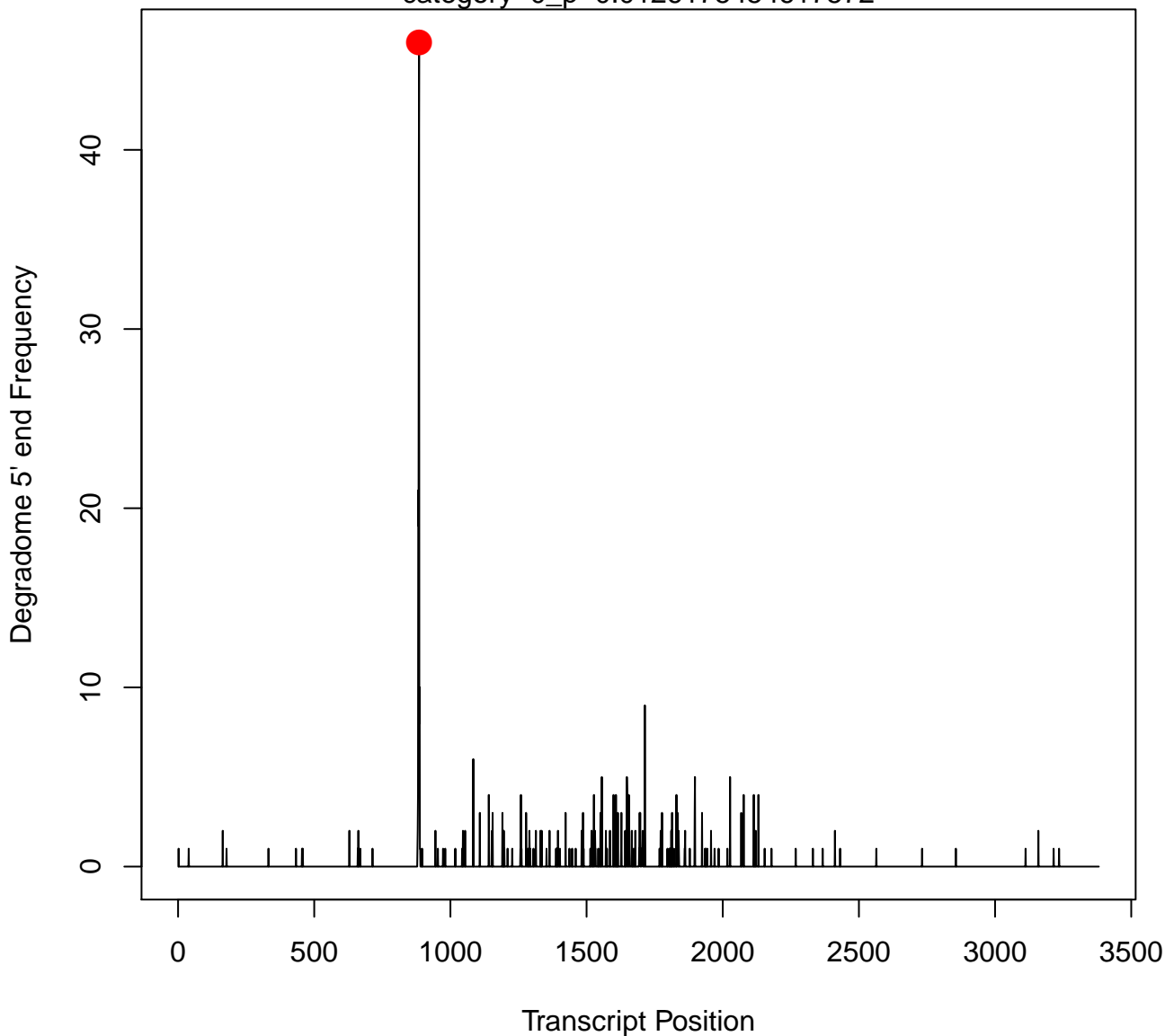

Supplement: Supplementary file 1 [file Data_Sheet_1.zip › Sit-miR159a_Seita.1G186900.1_885_TPlot.pdf]

**T=Seita.1G226400.1\_Q=Sit-miR159a\_S=1975**

category=2\_p=0.999999896835813

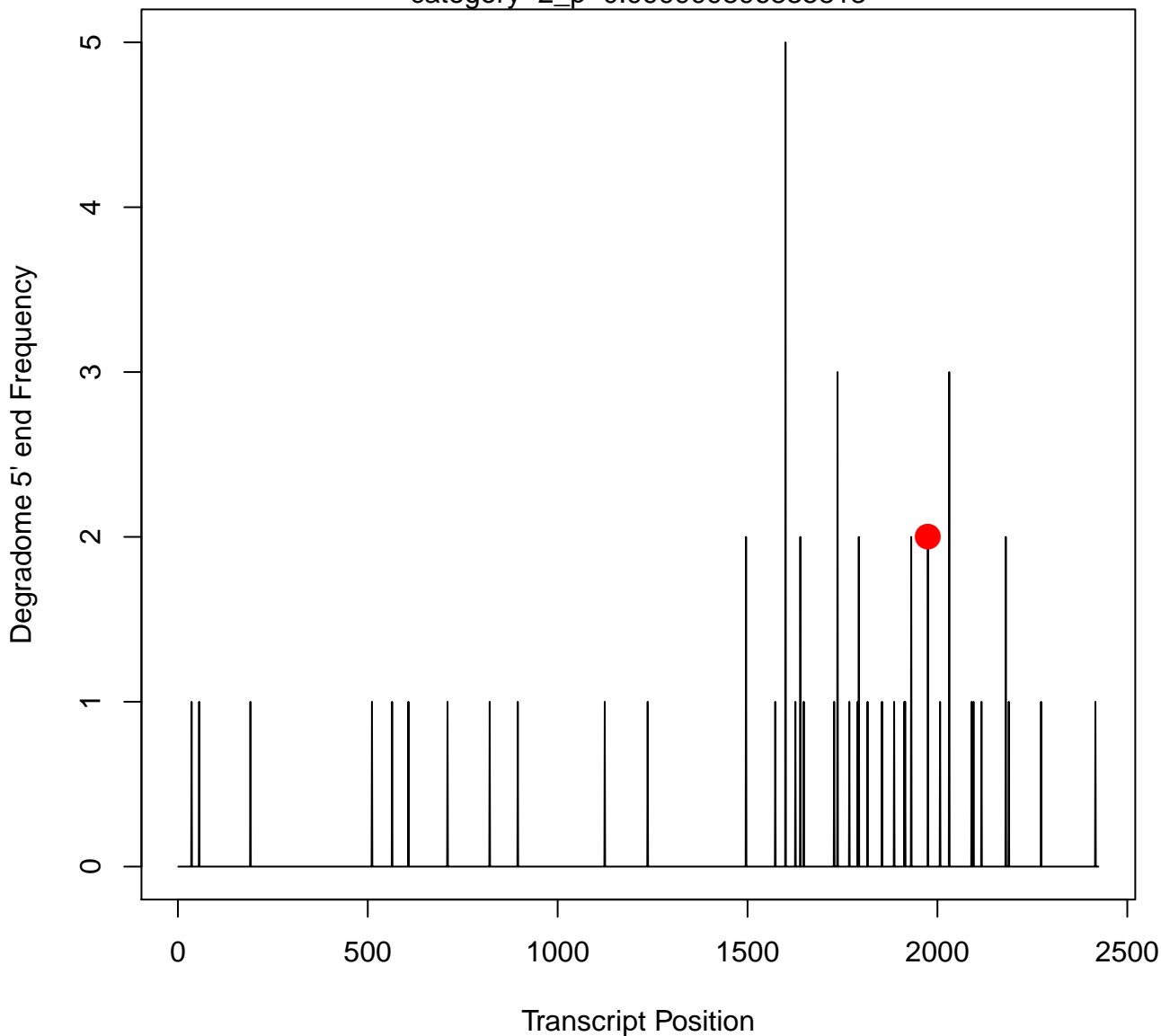

Supplement: Supplementary file 1 [file Data_Sheet_1.zip › Sit-miR159a_Seita.1G226400.1_1975_TPlot.pdf]

**T=Seita.2G109700.1\_Q=Sit-miR159a\_S=1844**

category=2\_p=0.999999898725867

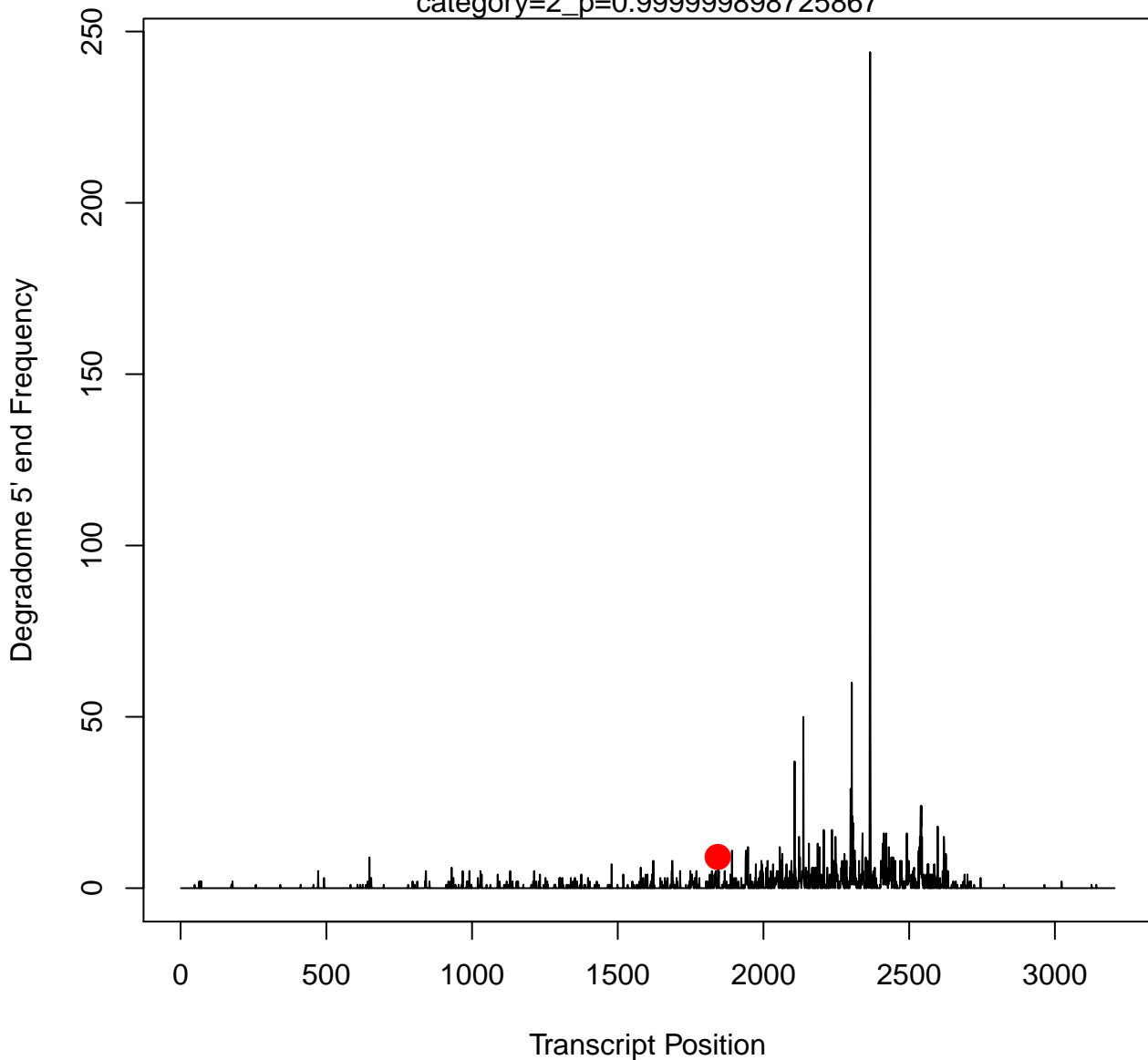

Supplement: Supplementary file 1 [file Data_Sheet_1.zip › Sit-miR159a_Seita.2G109700.1_1844_TPlot.pdf]

**T=Seita.2G388600.1\_Q=Sit-miR159a\_S=2451**

category=2\_p=0.999805041144898

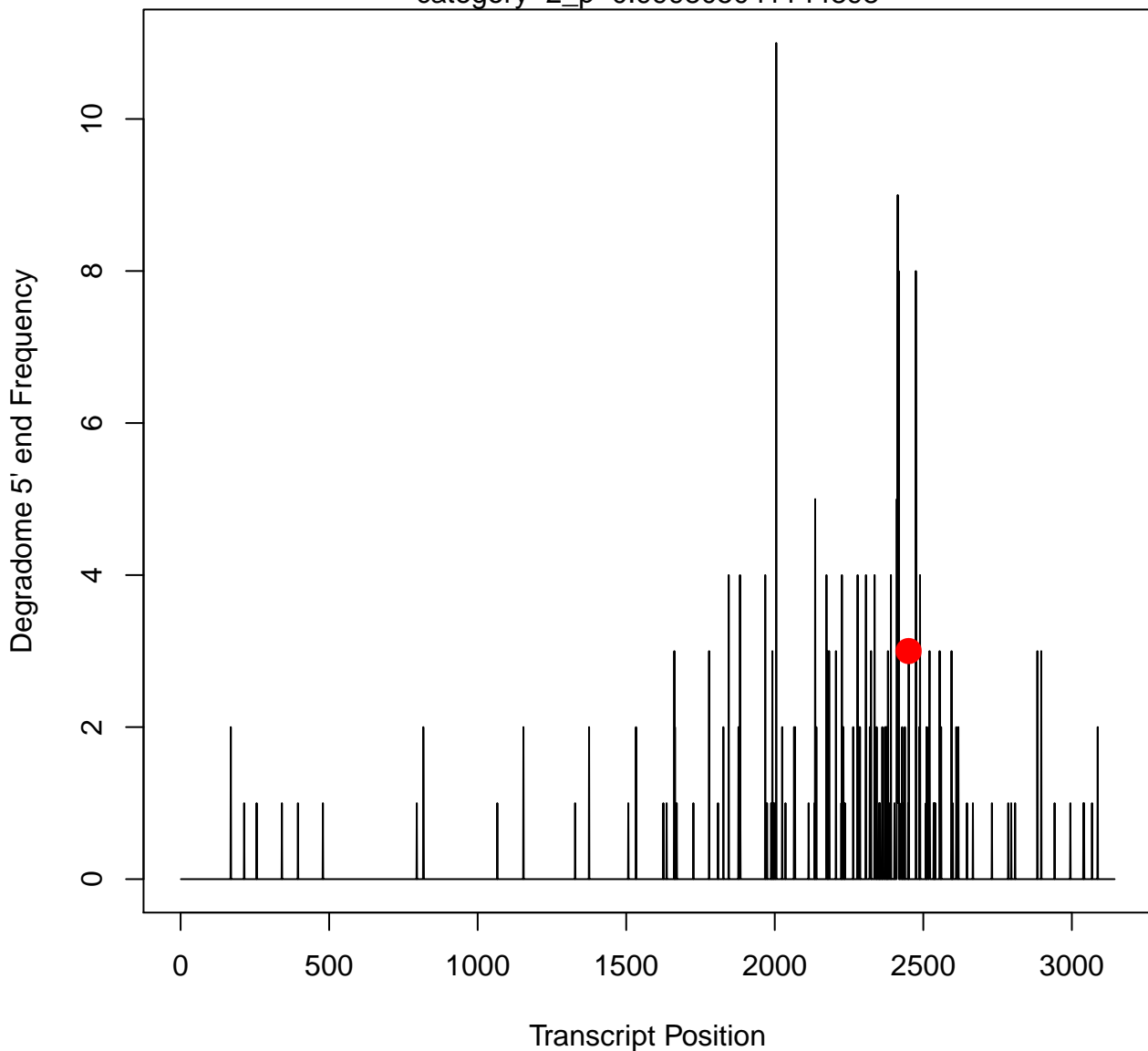

Supplement: Supplementary file 1 [file Data_Sheet_1.zip › Sit-miR159a_Seita.2G388600.1_2451_TPlot.pdf]

**T=Seita.3G001200.1\_Q=Sit-miR159a\_S=1489**

category=2\_p=0.889240943192188

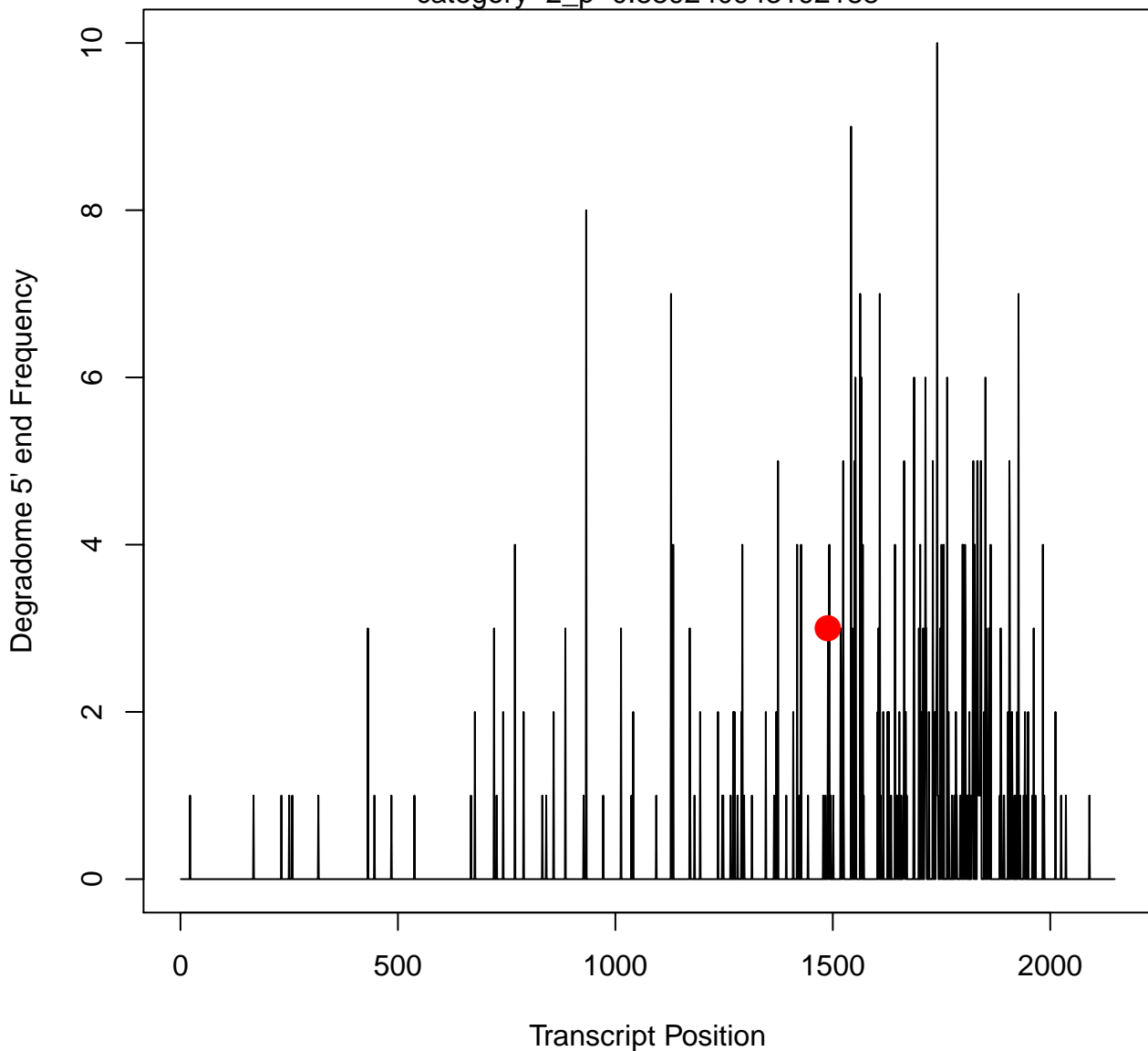

Supplement: Supplementary file 1 [file Data_Sheet_1.zip › Sit-miR159a_Seita.3G001200.1_1489_TPlot.pdf]

**T=Seita.4G020800.1\_Q=Sit-miR159a\_S=284**

category=2\_p=0.999999998757613

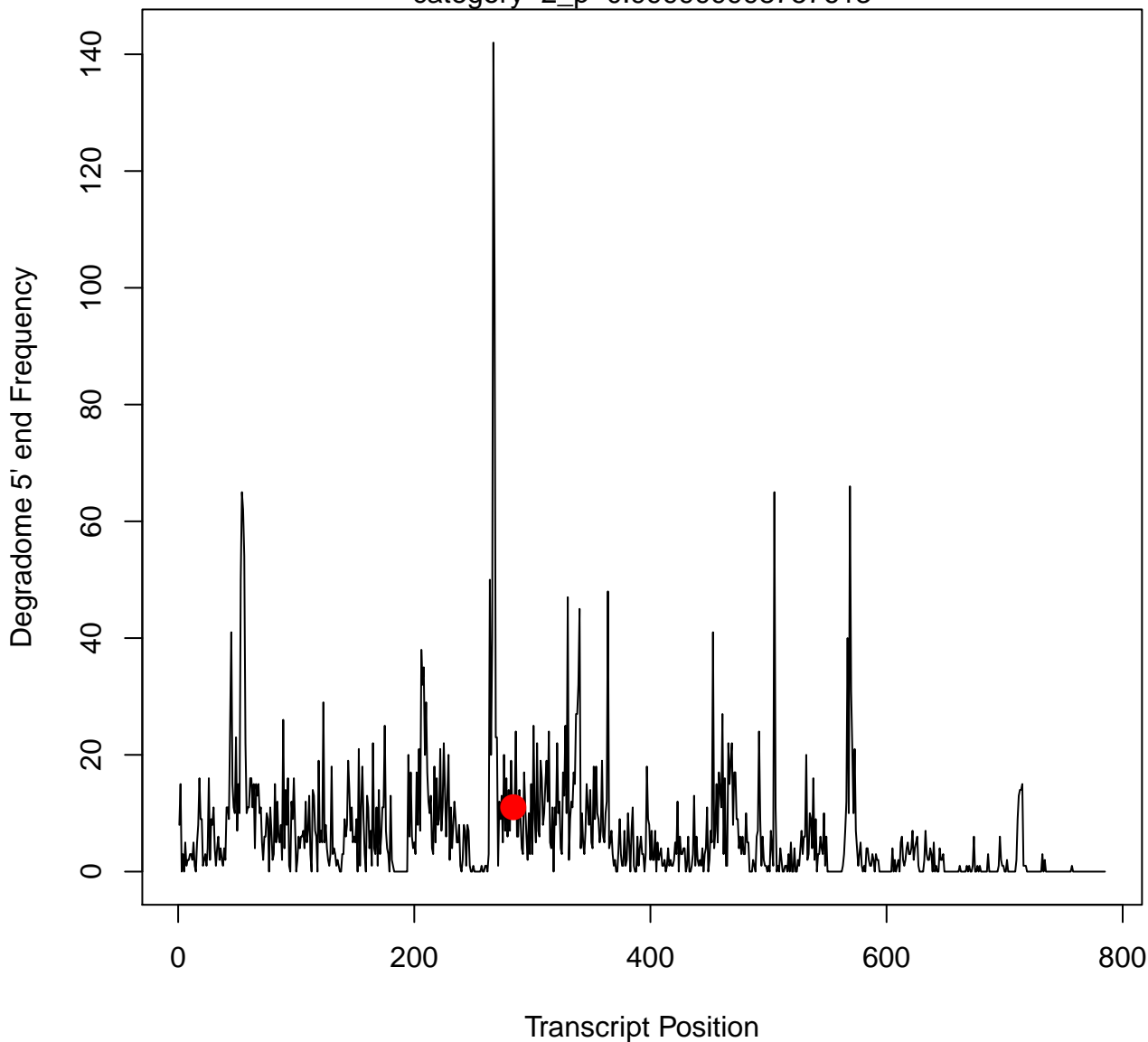

Supplement: Supplementary file 1 [file Data_Sheet_1.zip › Sit-miR159a_Seita.4G020800.1_284_TPlot.pdf]

**T=Seita.5G046700.1\_Q=Sit-miR159a\_S=479**

category=2\_p=0.999808612953992

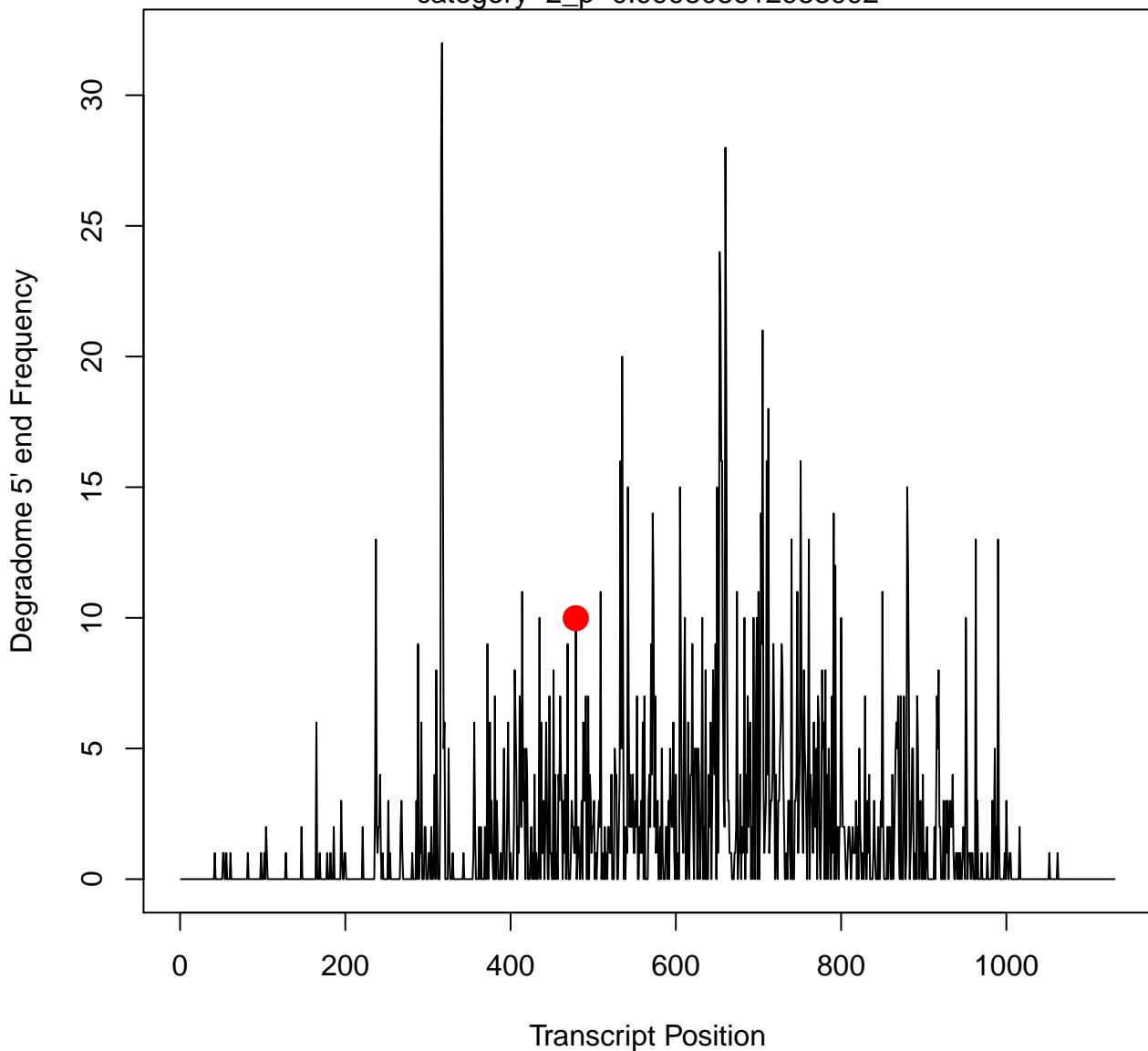

Supplement: Supplementary file 1 [file Data_Sheet_1.zip › Sit-miR159a_Seita.5G046700.1_479_TPlot.pdf]

**T=Seita.5G149100.1\_Q=Sit-miR159a\_S=1902**

category=0\_p=0.0108867050395873

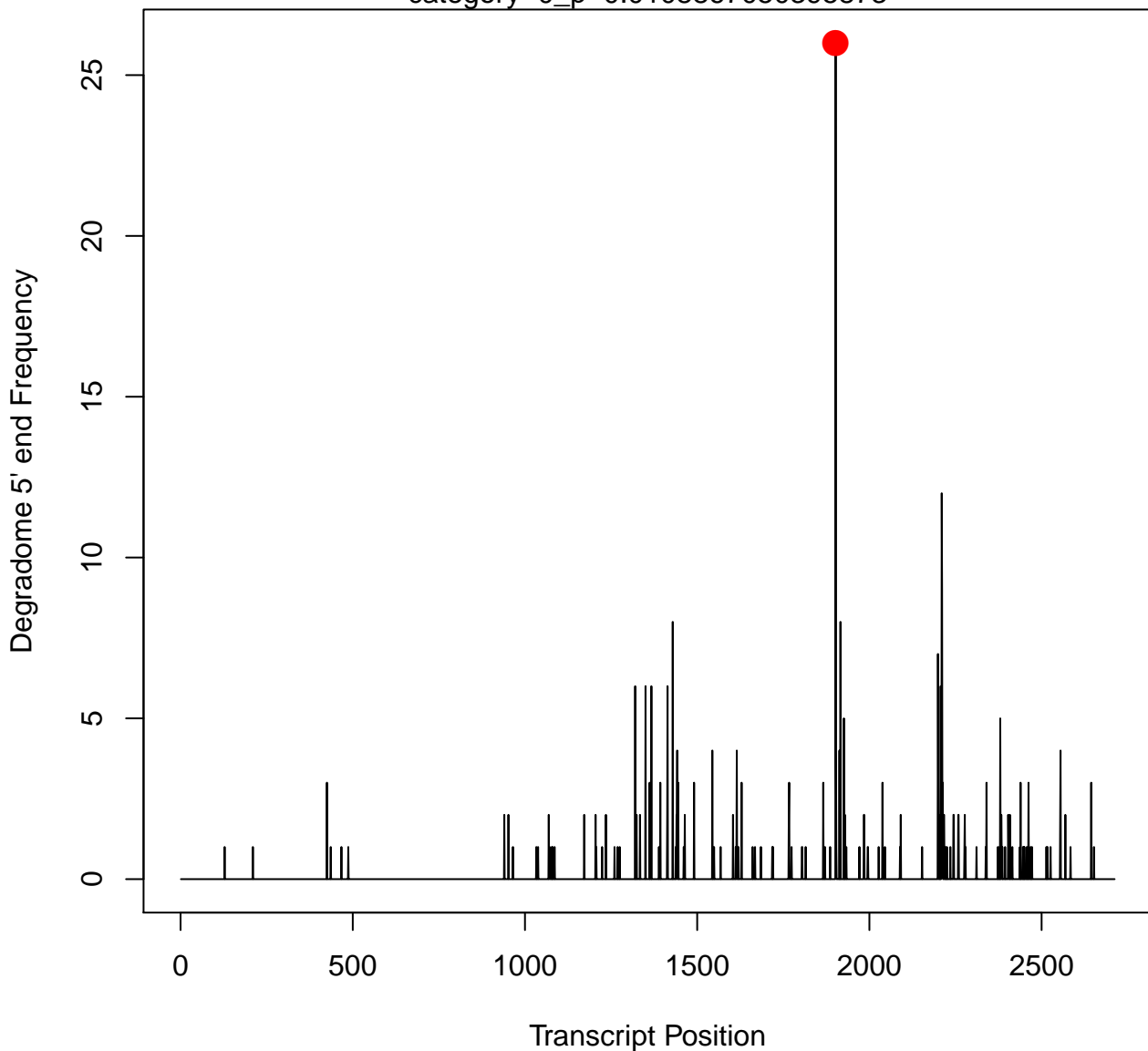

Supplement: Supplementary file 1 [file Data_Sheet_1.zip › Sit-miR159a_Seita.5G149100.1_1902_TPlot.pdf]

**T=Seita.5G301800.1\_Q=Sit-miR159a\_S=1762**

category=2\_p=0.999994895550059

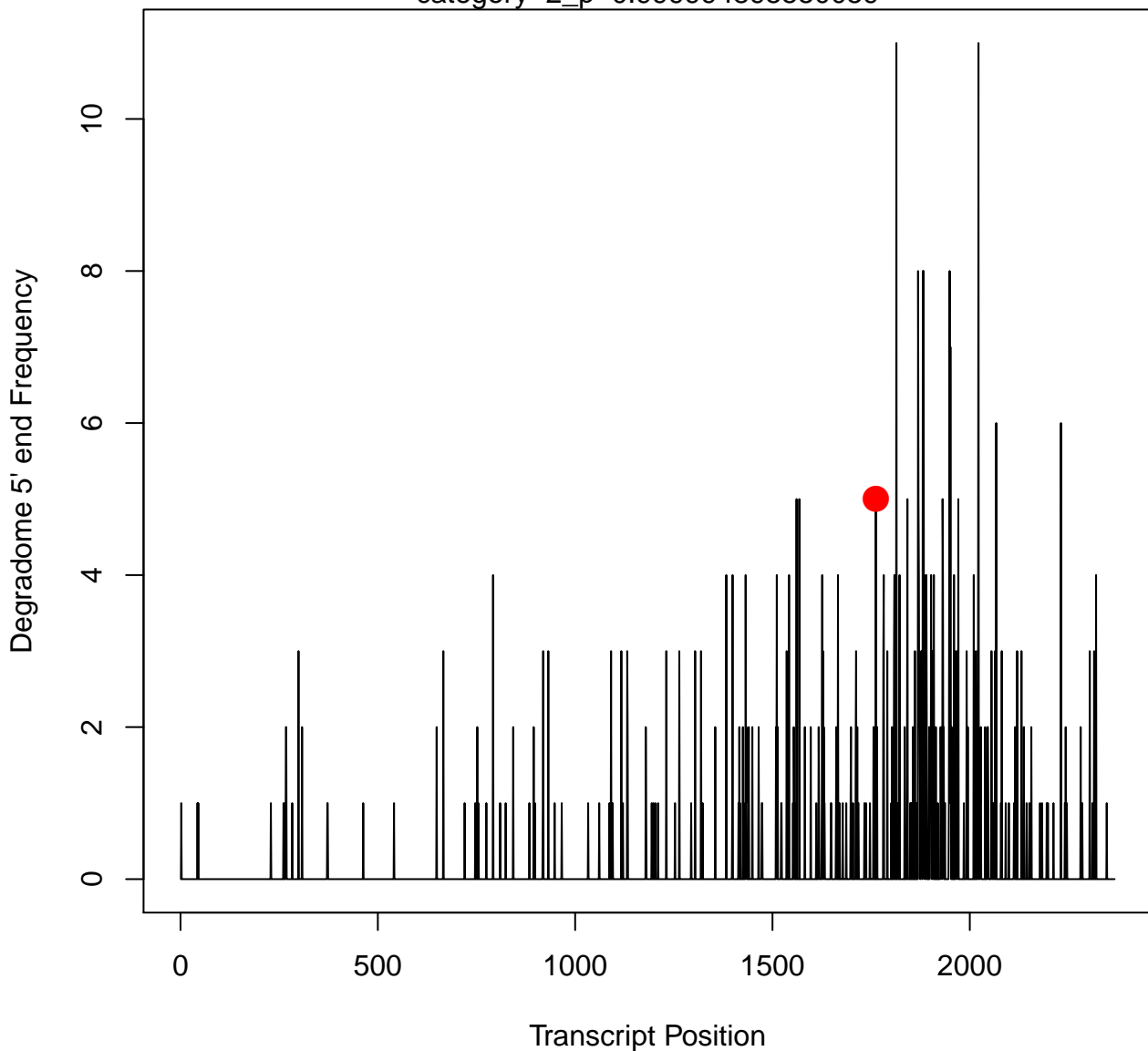

Supplement: Supplementary file 1 [file Data_Sheet_1.zip › Sit-miR159a_Seita.5G301800.1_1762_TPlot.pdf]

**T=Seita.5G401200.1\_Q=Sit-miR159a\_S=510**

category=2\_p=0.917606599907781

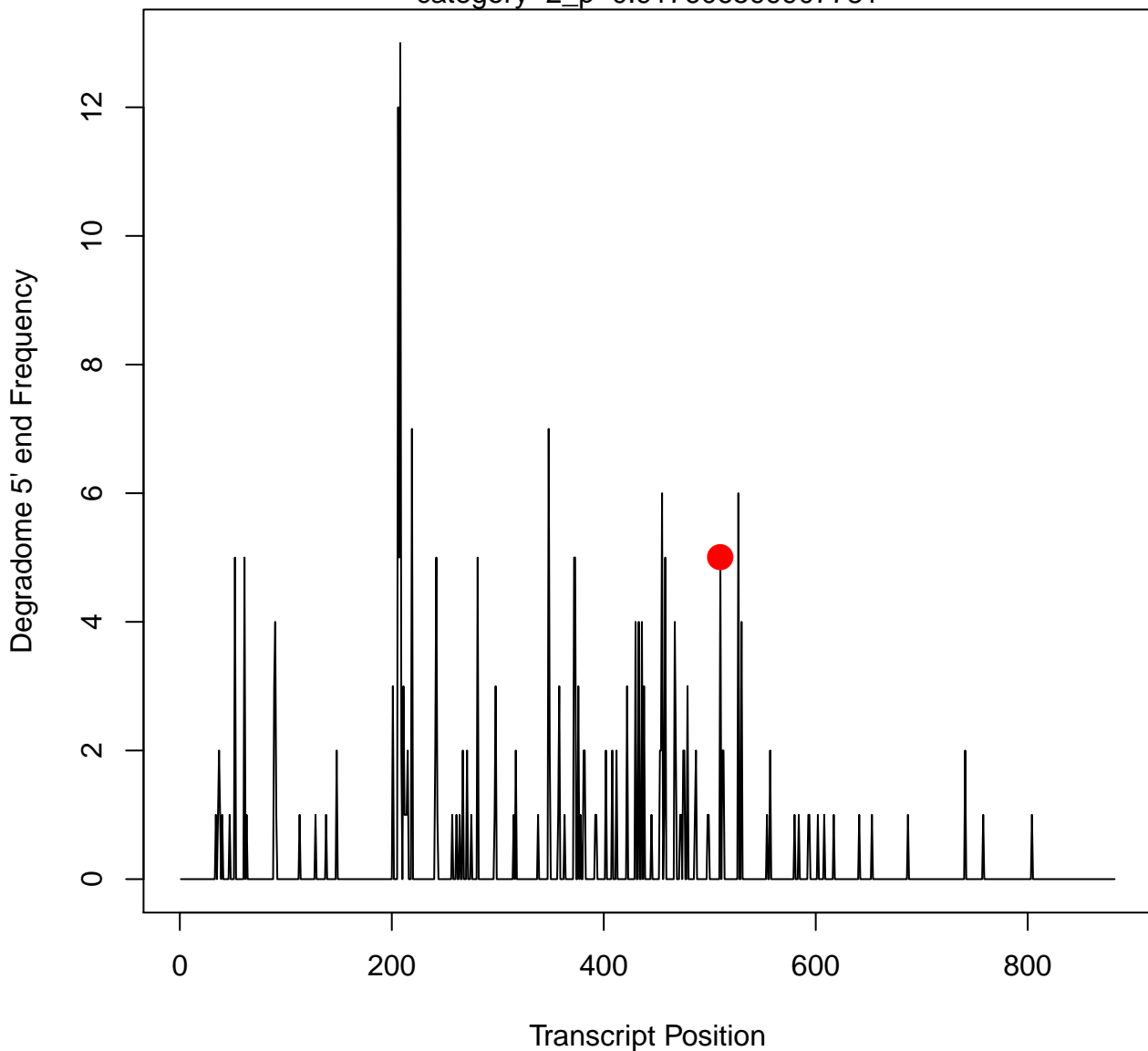

Supplement: Supplementary file 1 [file Data_Sheet_1.zip › Sit-miR159a_Seita.5G401200.1_510_TPlot.pdf]

**T=Seita.6G063700.1\_Q=Sit-miR159a\_S=883**

category=2\_p=0.99999999839053

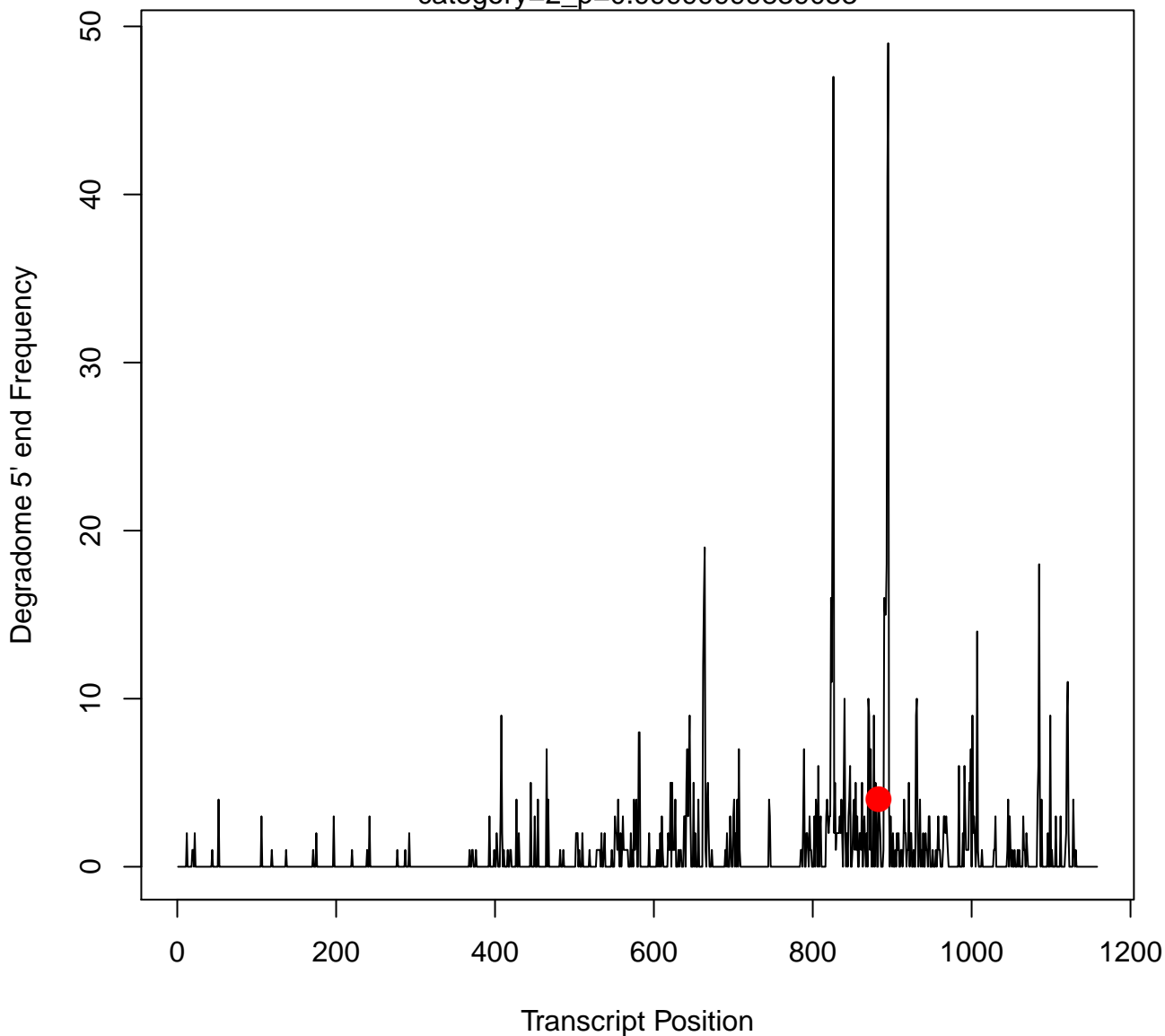

Supplement: Supplementary file 1 [file Data_Sheet_1.zip › Sit-miR159a_Seita.6G063700.1_883_TPlot.pdf]

**T=Seita.7G237800.1\_Q=Sit-miR159a\_S=2992**

category=2\_p=0.999999999859826

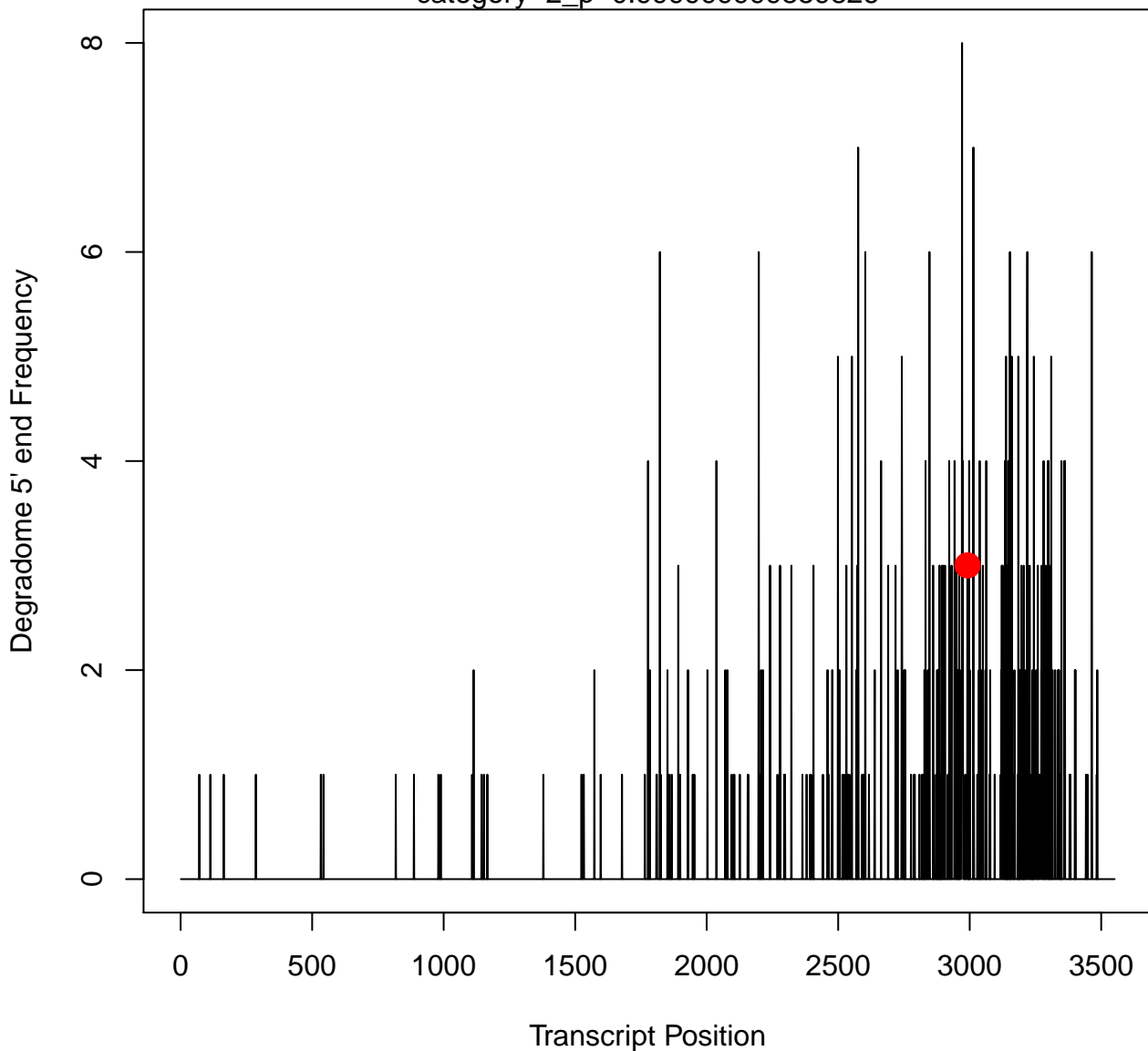

Supplement: Supplementary file 1 [file Data_Sheet_1.zip › Sit-miR159a_Seita.7G237800.1_2992_TPlot.pdf]

**T=Seita.7G280400.1\_Q=Sit-miR159a\_S=2378**

category=2\_p=0.495484840614317

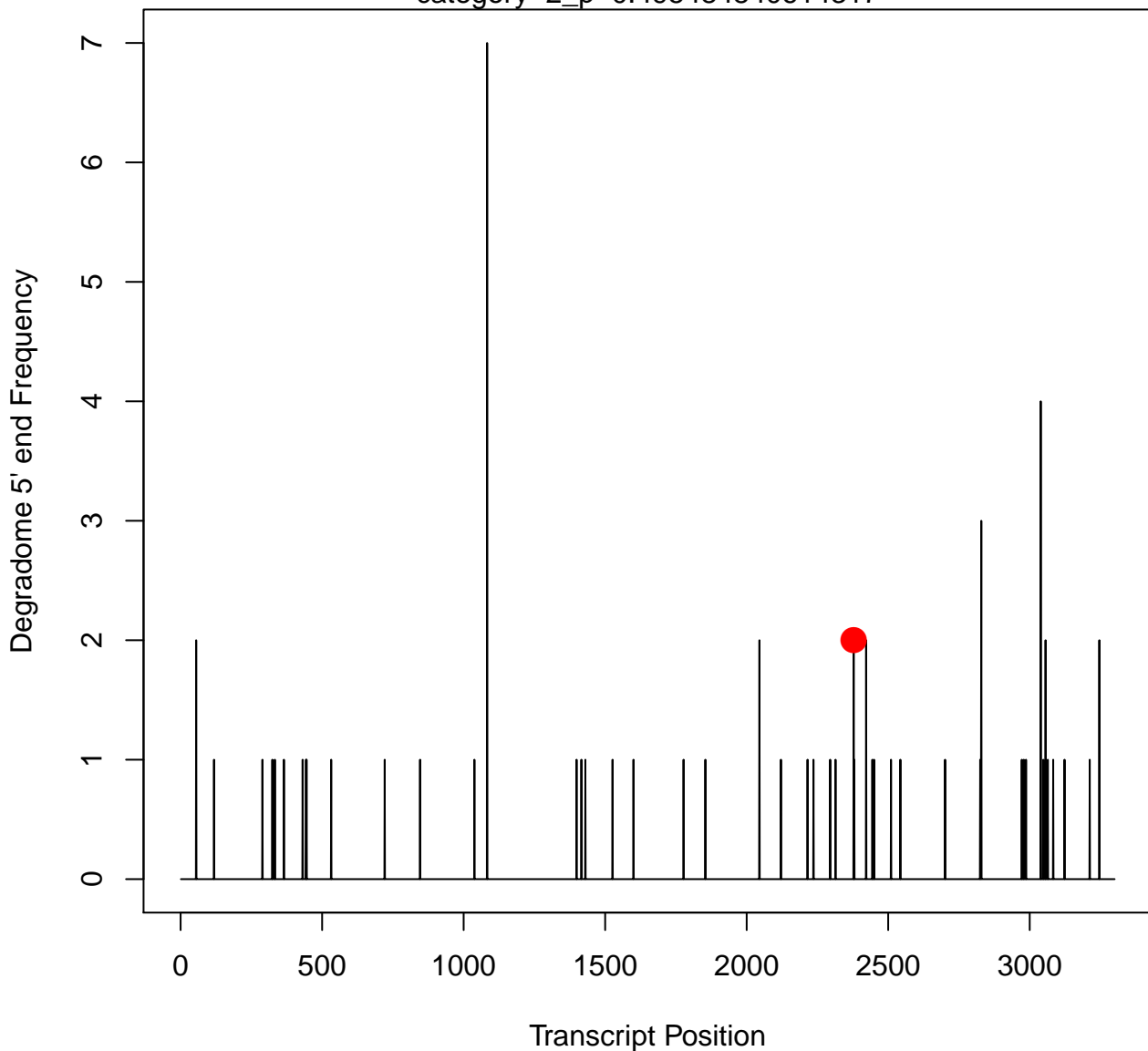

Supplement: Supplementary file 1 [file Data_Sheet_1.zip › Sit-miR159a_Seita.7G280400.1_2378_TPlot.pdf]

**T=Seita.8G215200.1\_Q=Sit-miR159a\_S=2970**

category=2\_p=0.999999999683768

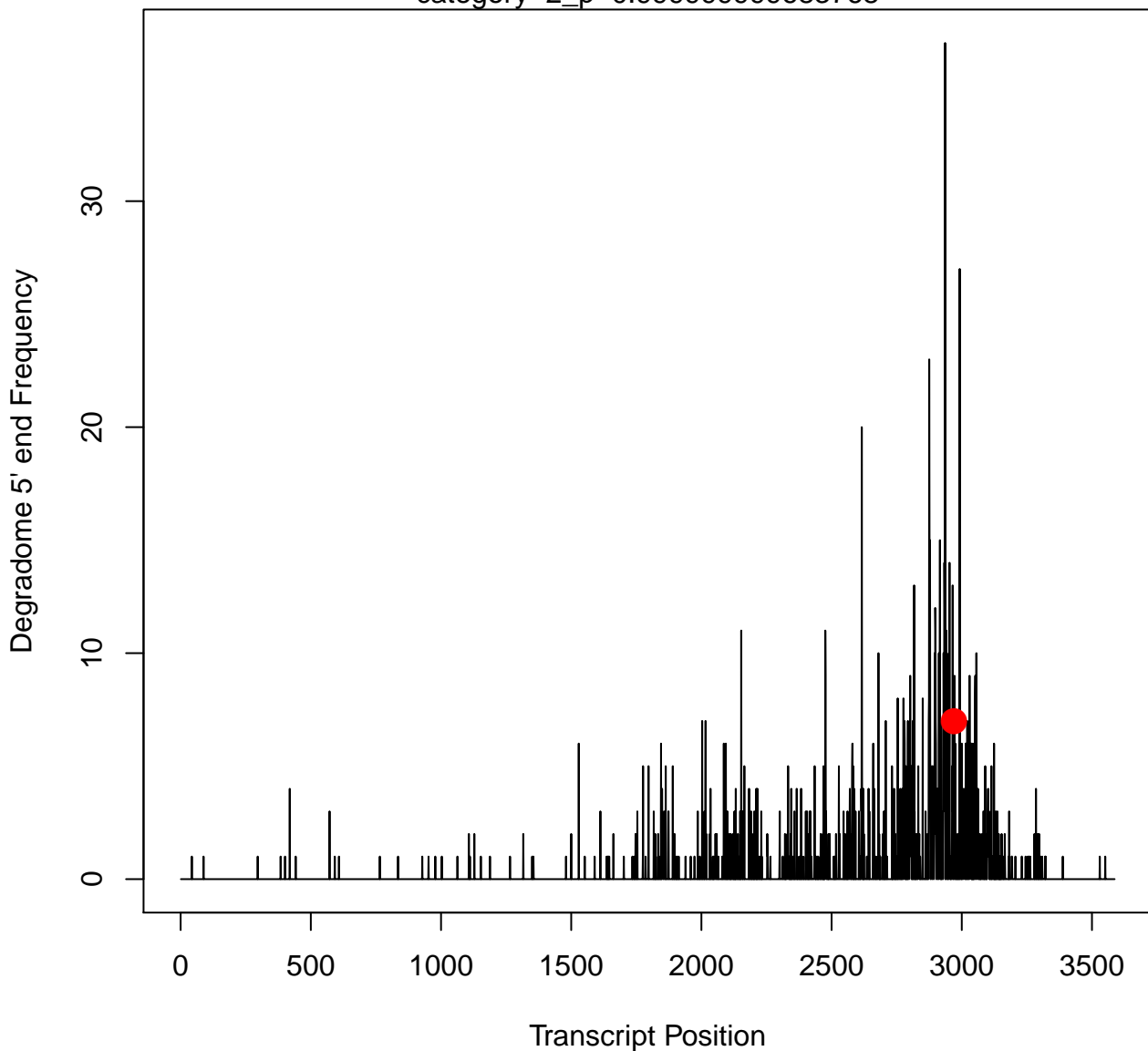

Supplement: Supplementary file 1 [file Data_Sheet_1.zip › Sit-miR159a_Seita.8G215200.1_2970_TPlot.pdf]

**T=Seita.9G007800.1\_Q=Sit-miR159a\_S=735**

category=2\_p=0.999999882580035

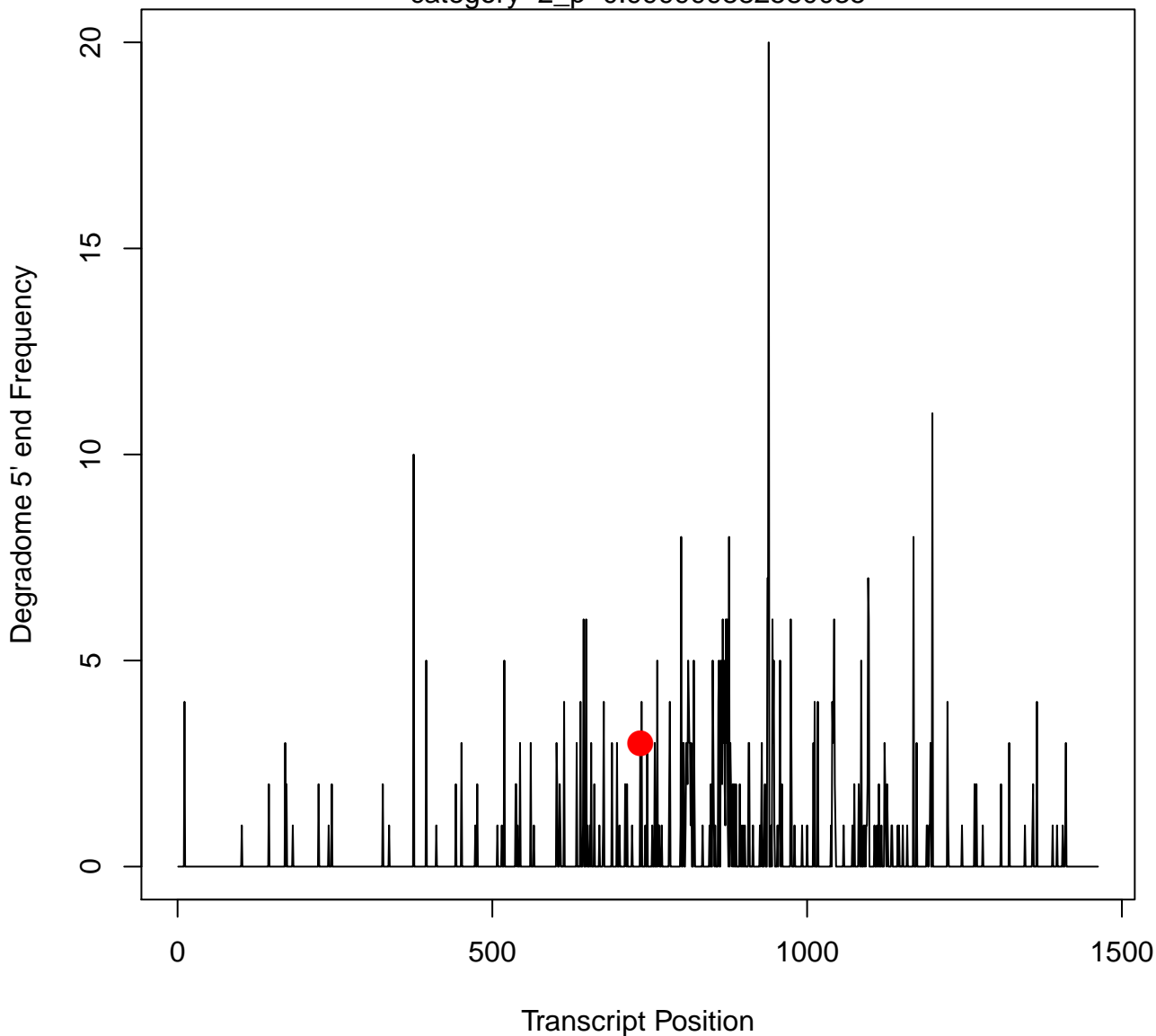

Supplement: Supplementary file 1 [file Data_Sheet_1.zip › Sit-miR159a_Seita.9G007800.1_735_TPlot.pdf]

**T=Seita.9G134700.1\_Q=Sit-miR159a\_S=4274**

category=0\_p=0.426294571892068

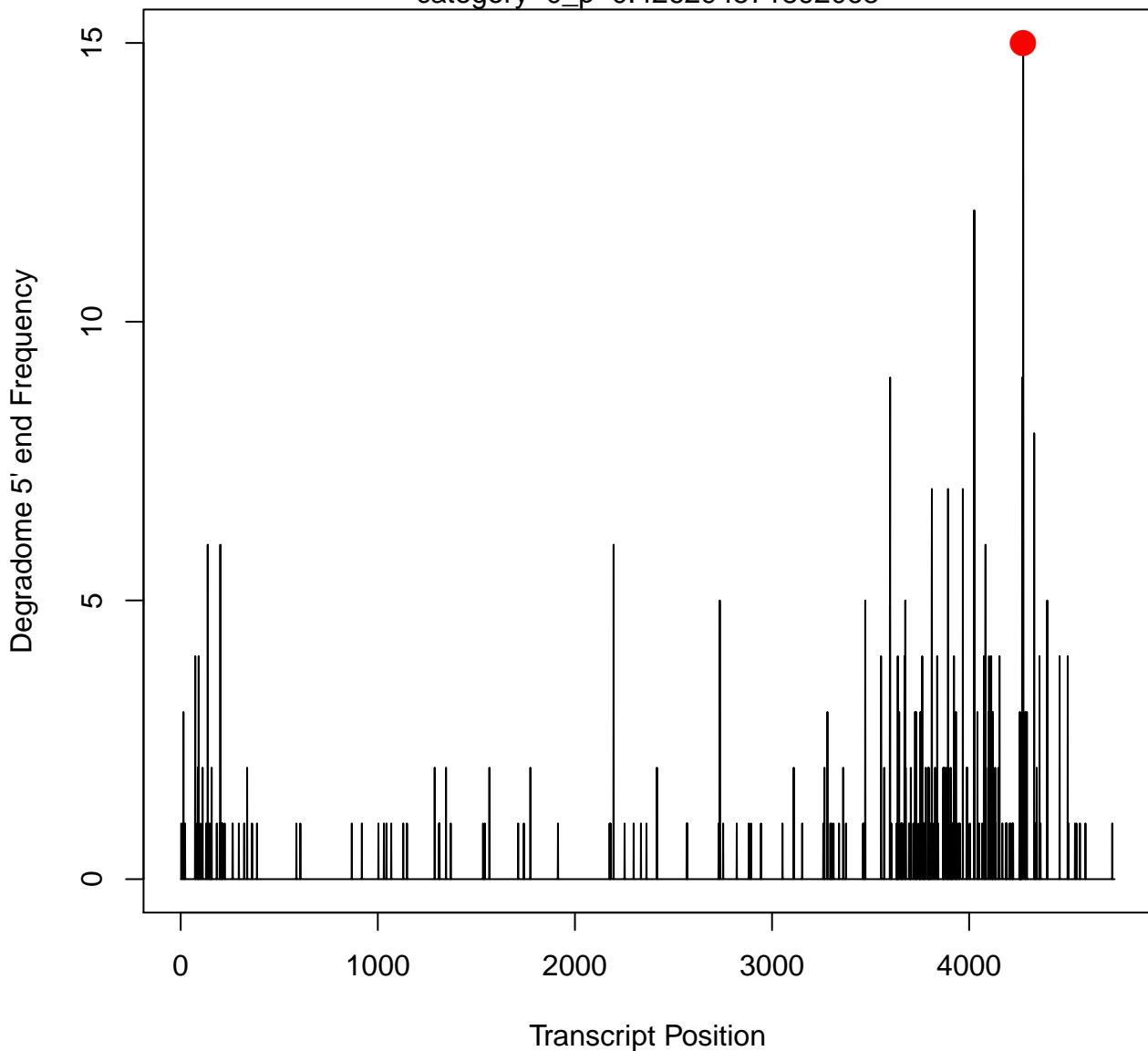

Supplement: Supplementary file 1 [file Data_Sheet_1.zip › Sit-miR159a_Seita.9G134700.1_4274_TPlot.pdf]

**T=Seita.9G225800.1\_Q=Sit-miR159a\_S=3112**

category=2\_p=0.99999999988349

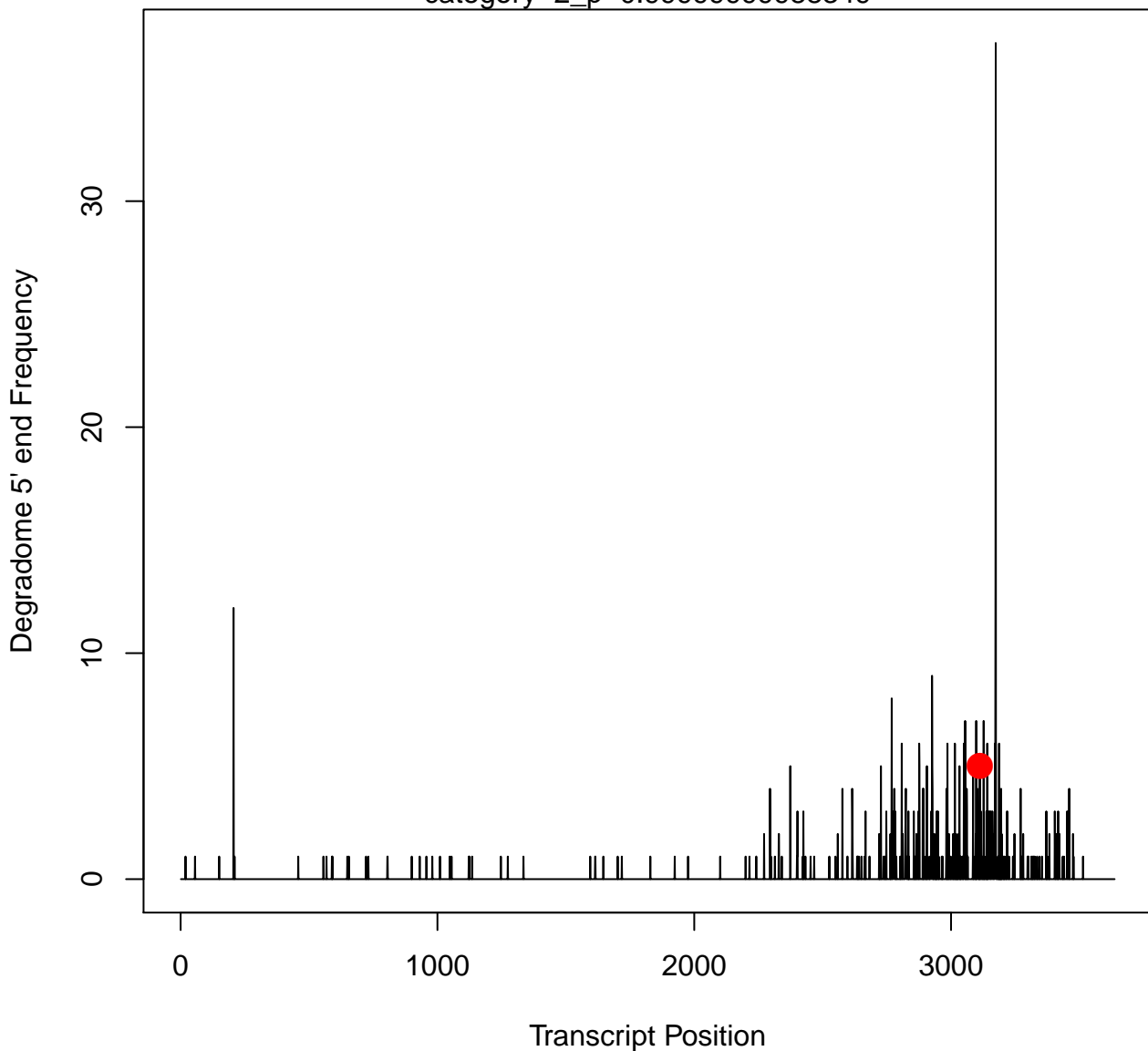

Supplement: Supplementary file 1 [file Data_Sheet_1.zip › Sit-miR159a_Seita.9G225800.1_3112_TPlot.pdf]

**T=Seita.9G294600.1\_Q=Sit-miR159a\_S=1202**

category=2\_p=0.99998736893859

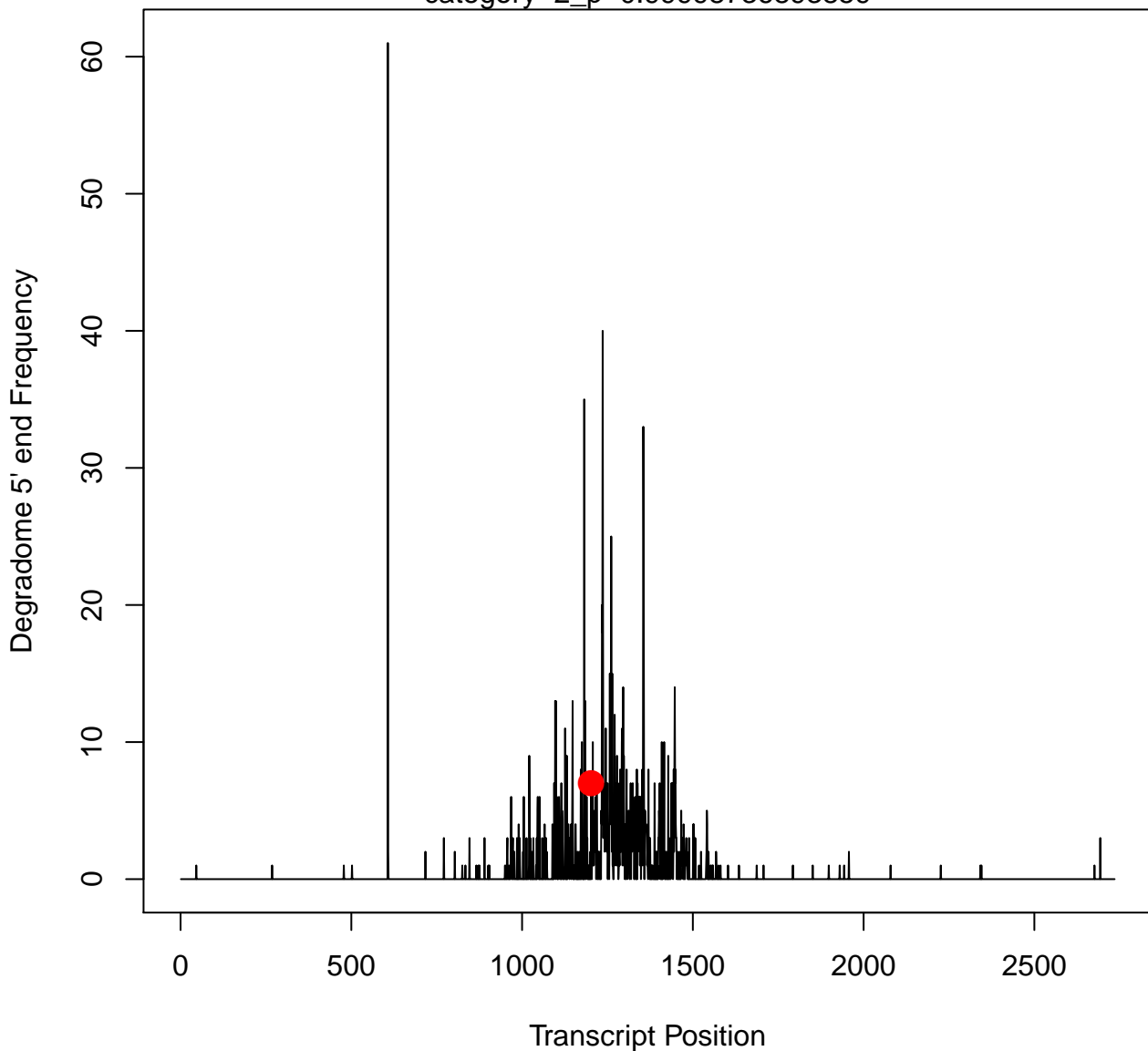

Supplement: Supplementary file 1 [file Data_Sheet_1.zip › Sit-miR159a_Seita.9G294600.1_1202_TPlot.pdf]

**T=Seita.9G411900.1\_Q=Sit-miR159a\_S=738**

category=2\_p=0.990703871885544

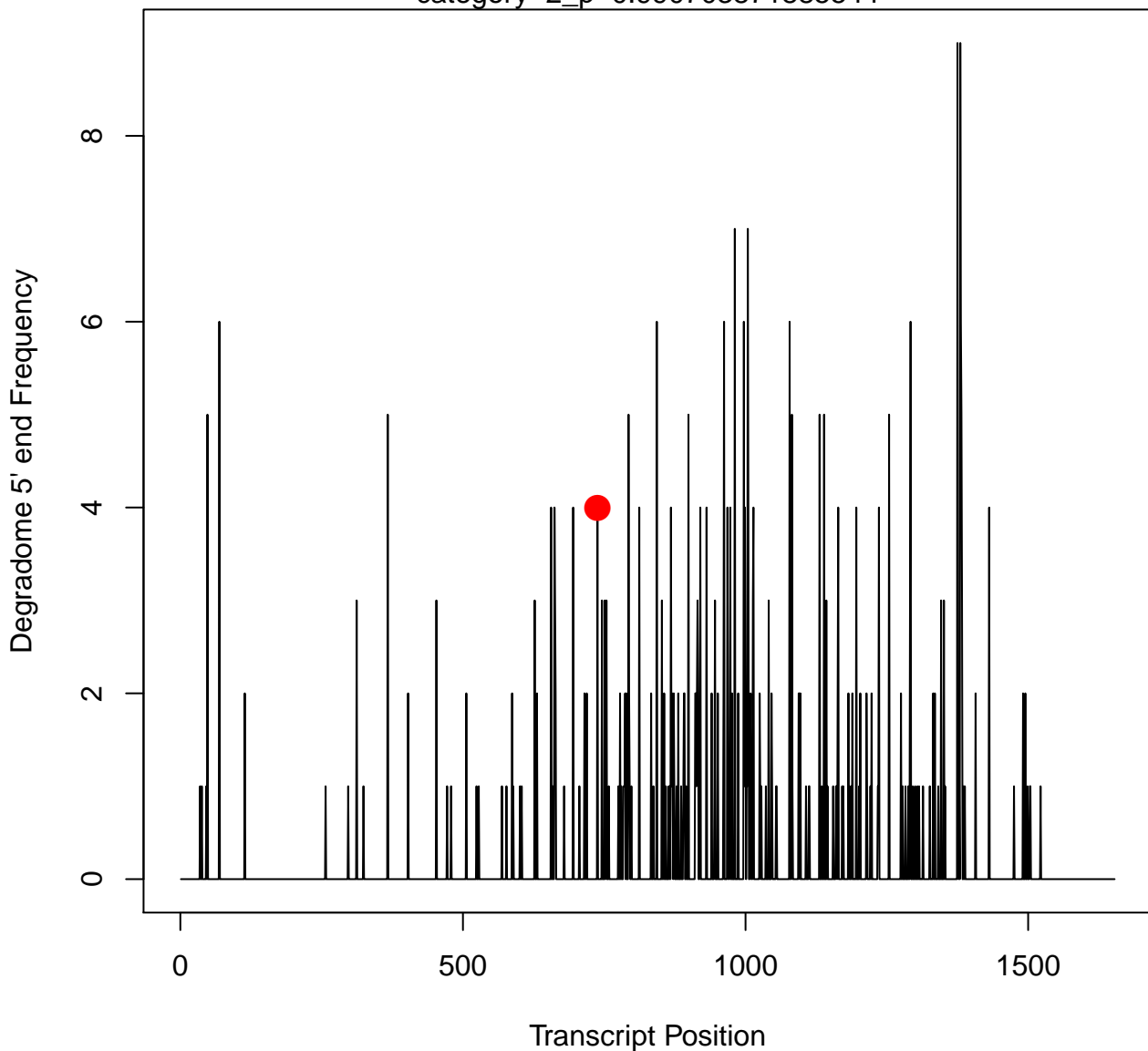

Supplement: Supplementary file 1 [file Data_Sheet_1.zip › Sit-miR159a_Seita.9G411900.1_738_TPlot.pdf]

**T=Seita.9G444100.1\_Q=Sit-miR159a\_S=1241**

category=2\_p=0.999999986504715

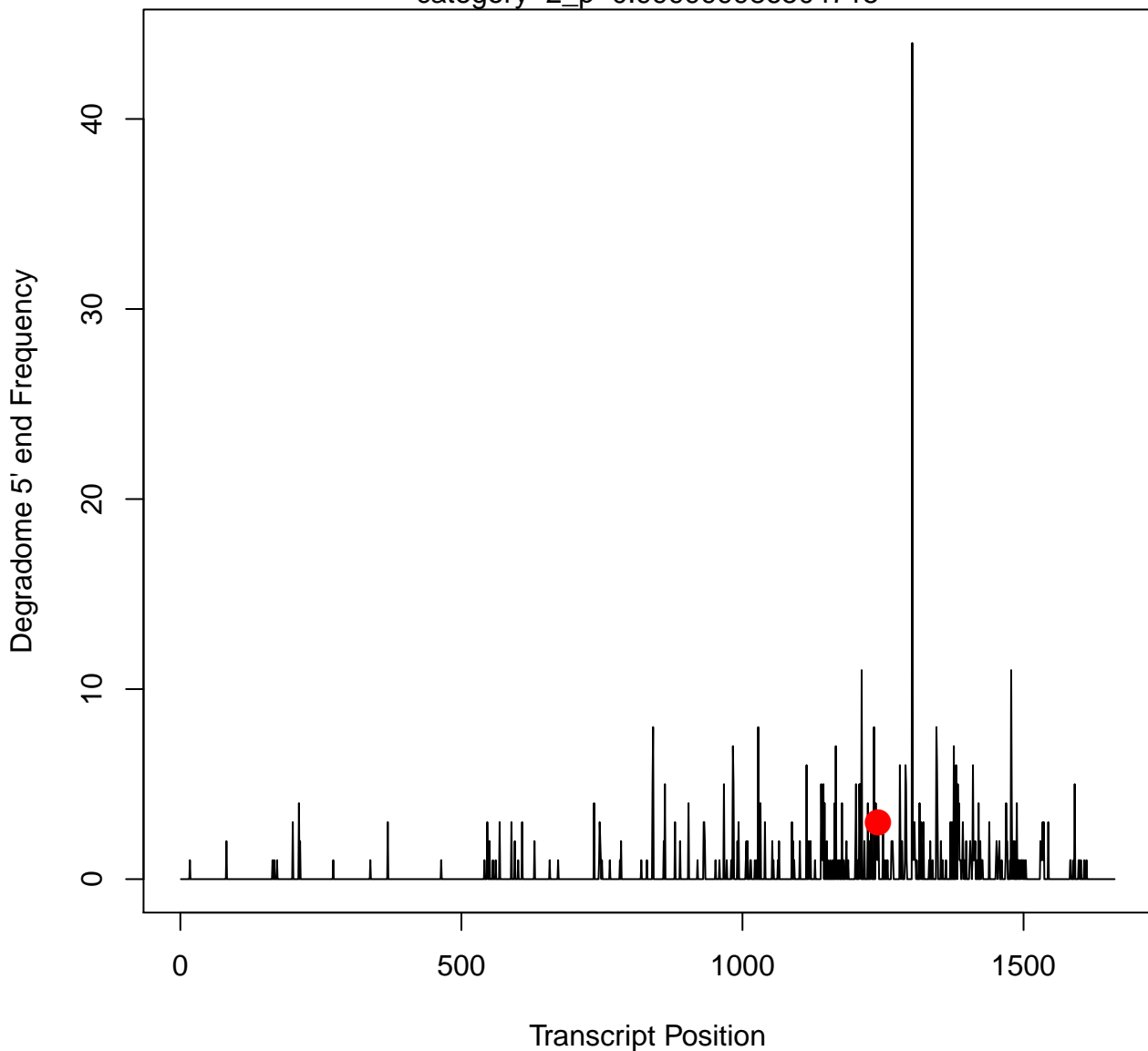

Supplement: Supplementary file 1 [file Data_Sheet_1.zip › Sit-miR159a_Seita.9G444100.1_1241_TPlot.pdf]

**T=Seita.1G077900.1\_Q=Sit-miR159b\_S=859**

category=0\_p=0.000437760109239571

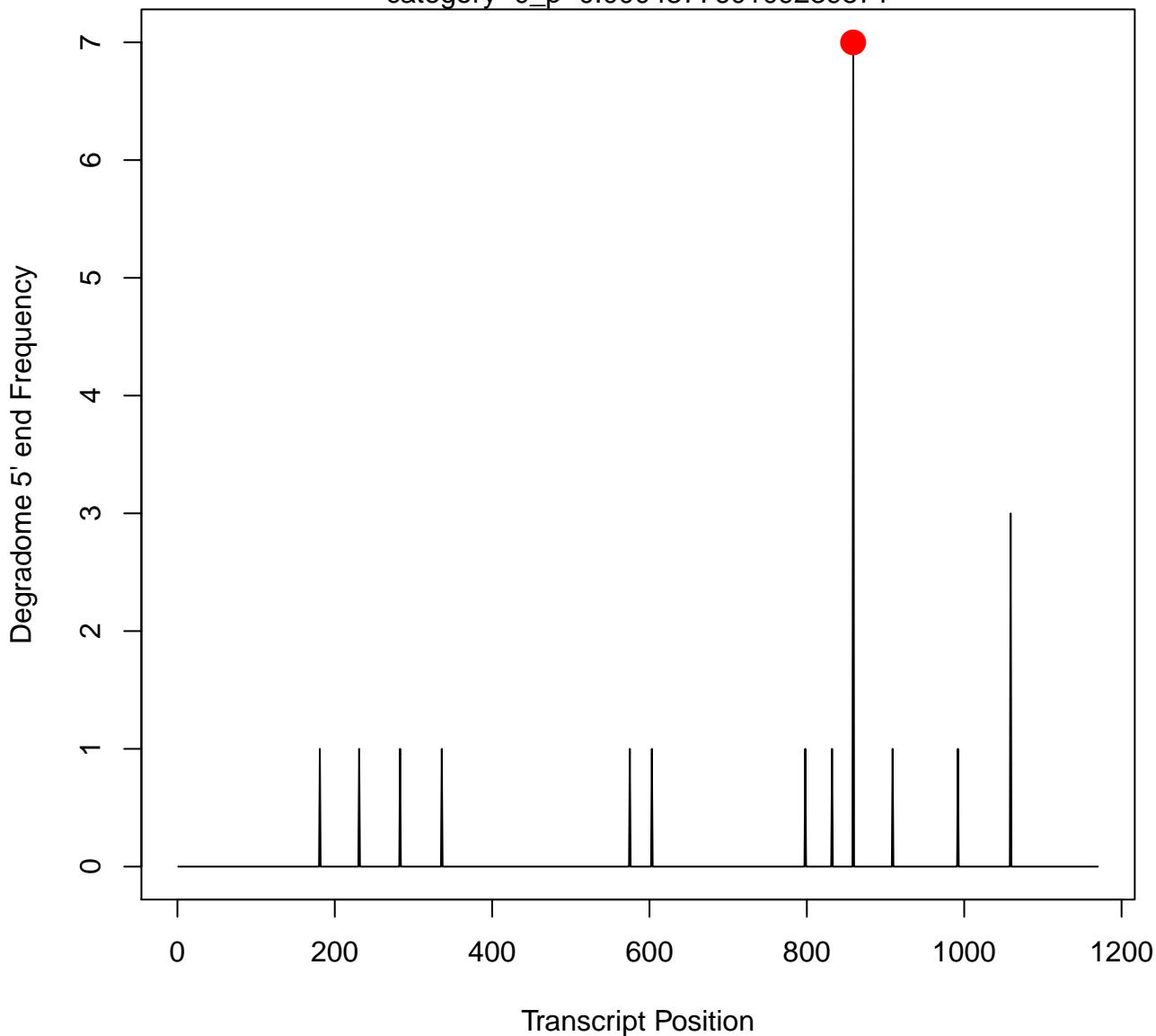

Supplement: Supplementary file 1 [file Data_Sheet_1.zip › Sit-miR159b_Seita.1G077900.1_859_TPlot.pdf]

**T=Seita.1G216500.1\_Q=Sit-miR159b\_S=2157**

category=2\_p=0.982243104978967

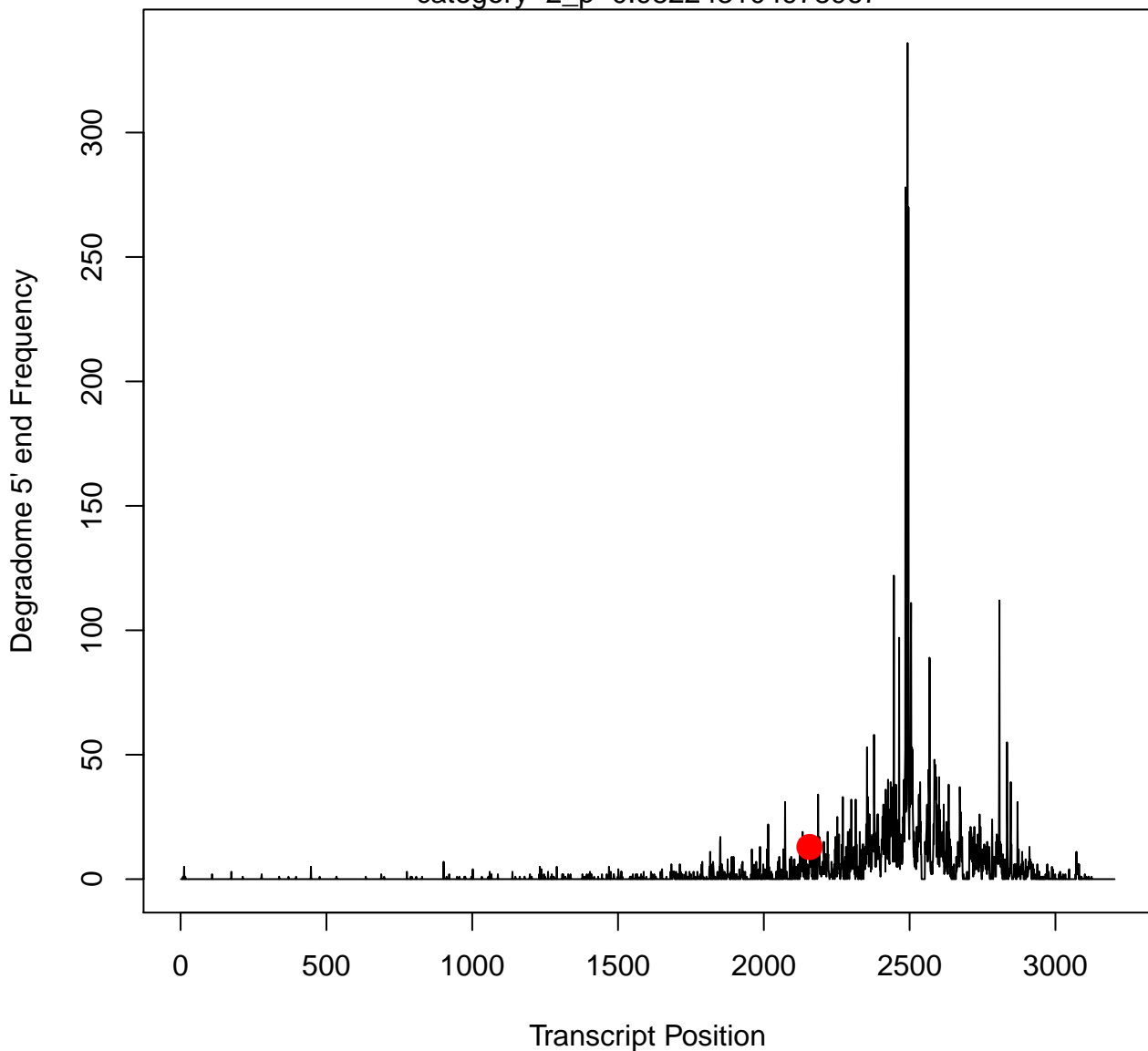

Supplement: Supplementary file 1 [file Data_Sheet_1.zip › Sit-miR159b_Seita.1G216500.1_2157_TPlot.pdf]

**T=Seita.4G083900.1\_Q=Sit-miR159b\_S=4244**

category=2\_p=0.456754764409798

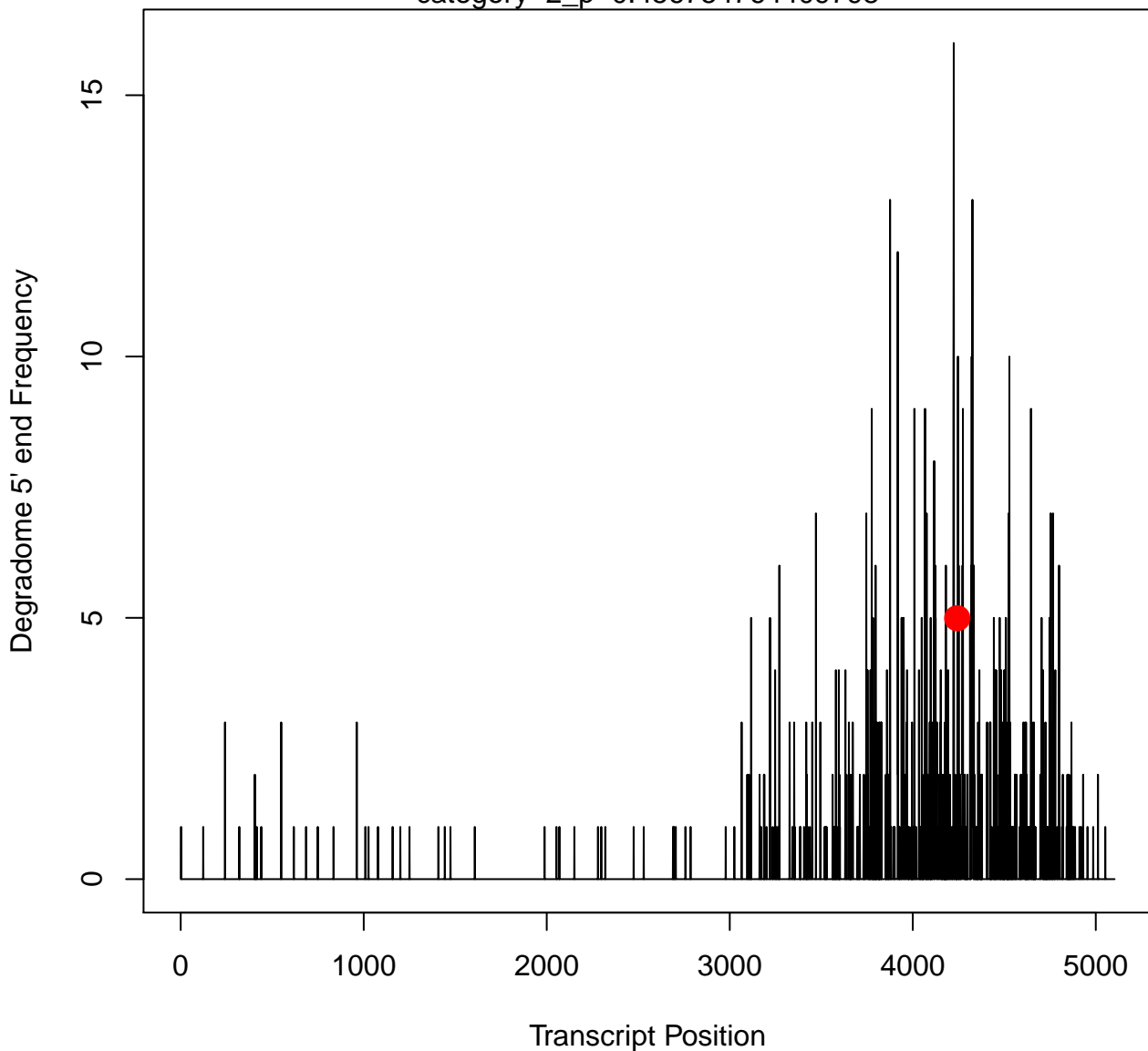

Supplement: Supplementary file 1 [file Data_Sheet_1.zip › Sit-miR159b_Seita.4G083900.1_4244_TPlot.pdf]

**T=Seita.4G221900.1\_Q=Sit-miR159b\_S=1159**

category=2\_p=0.036306017792474

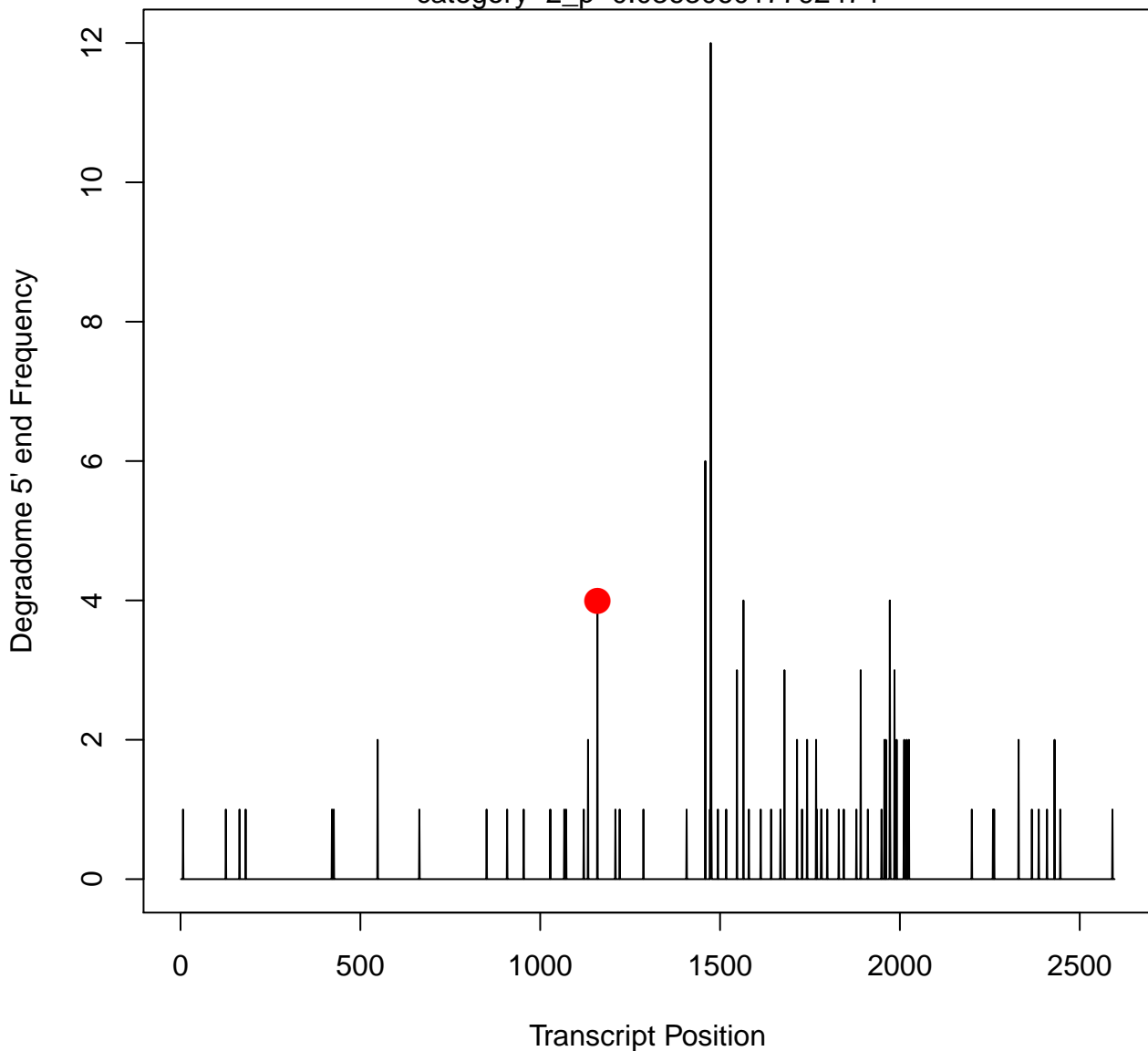

Supplement: Supplementary file 1 [file Data_Sheet_1.zip › Sit-miR159b_Seita.4G221900.1_1159_TPlot.pdf]

**T=Seita.5G148600.1\_Q=Sit-miR159b\_S=1382**

category=0\_p=0.0082847538233366

Degradome 5' end Frequency

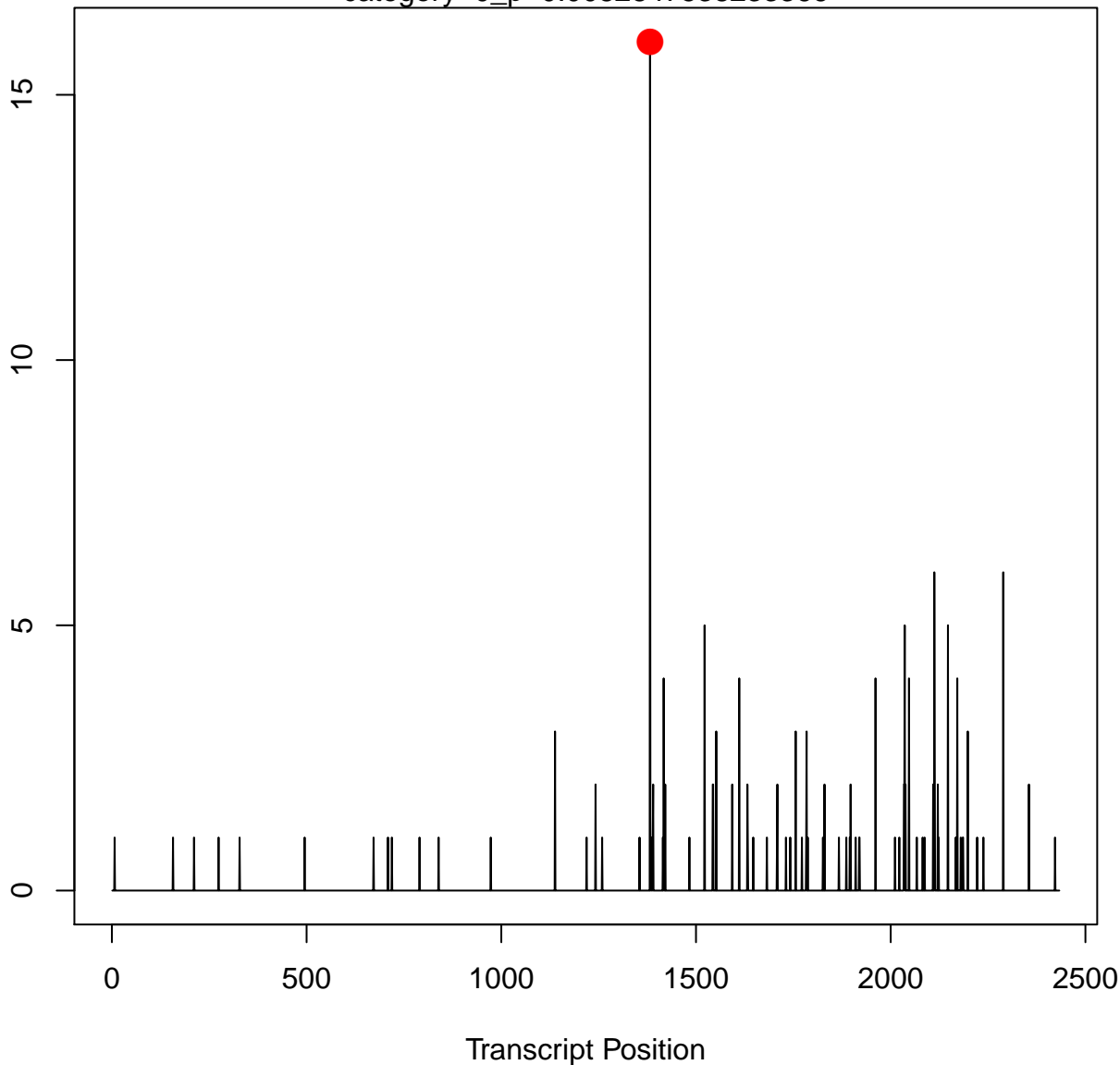

Supplement: Supplementary file 1 [file Data_Sheet_1.zip › Sit-miR159b_Seita.5G148600.1_1382_TPlot.pdf]

**T=Seita.5G149100.1\_Q=Sit-miR159b\_S=1903**

category=2\_p=0.725924543380696

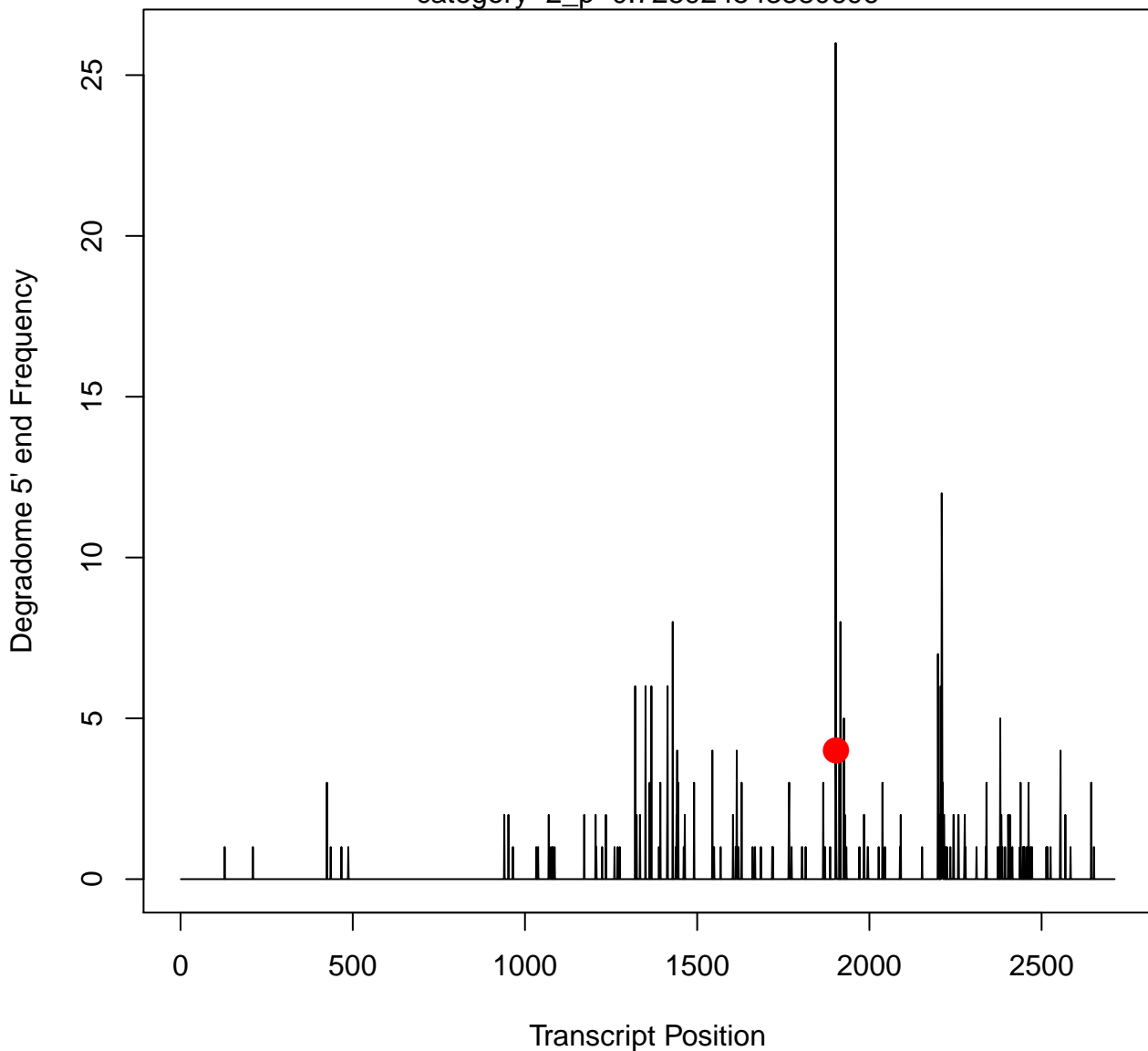

Supplement: Supplementary file 1 [file Data_Sheet_1.zip › Sit-miR159b_Seita.5G149100.1_1903_TPlot.pdf]

**T=Seita.5G355300.1\_Q=Sit-miR159b\_S=1278**

category=0\_p=0.0017498909690008

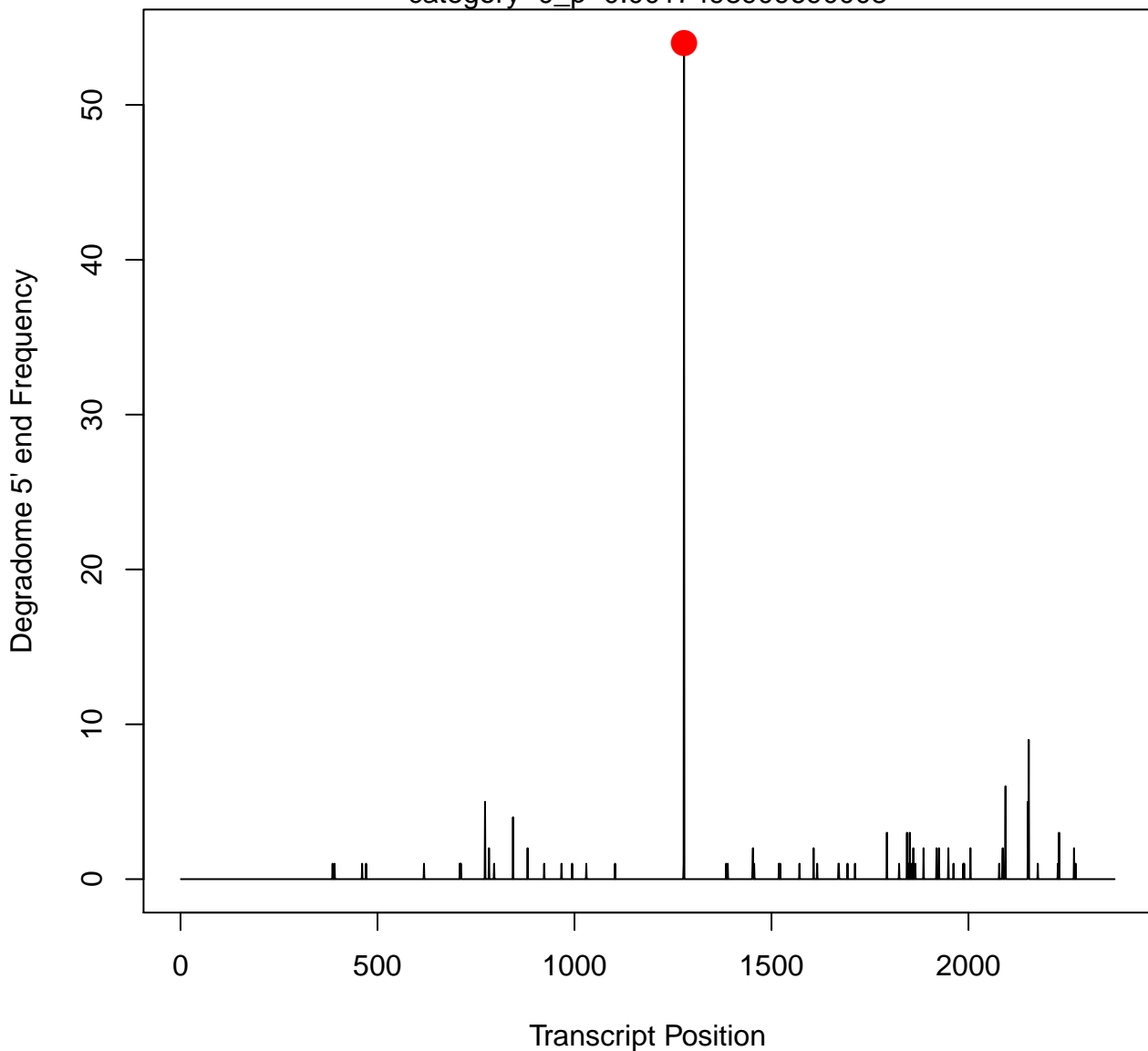

Supplement: Supplementary file 1 [file Data_Sheet_1.zip › Sit-miR159b_Seita.5G355300.1_1278_TPlot.pdf]

**T=Seita.7G264300.1\_Q=Sit-miR159b\_S=1536**

category=2\_p=0.999690469372737

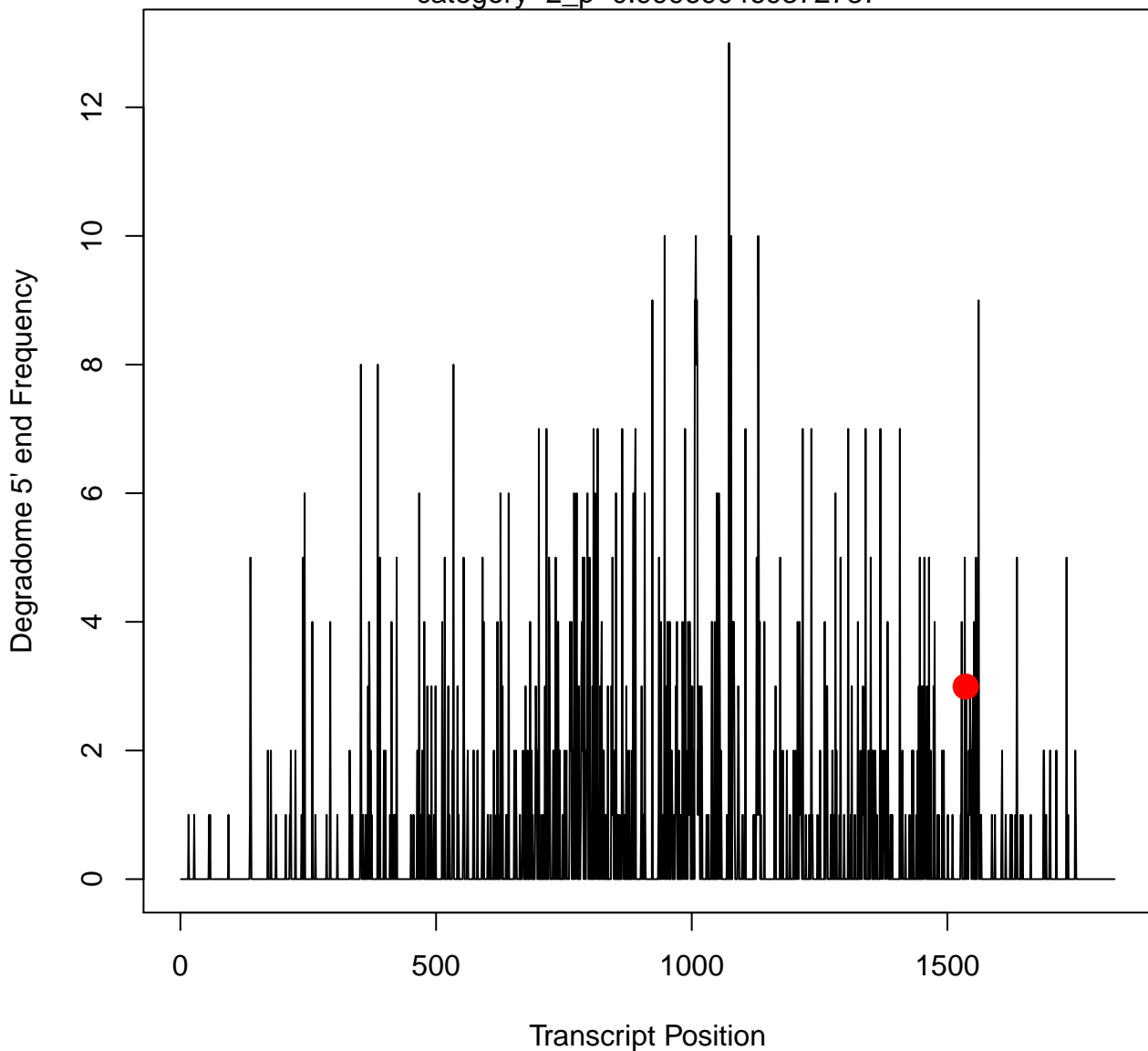

Supplement: Supplementary file 1 [file Data_Sheet_1.zip › Sit-miR159b_Seita.7G264300.1_1536_TPlot.pdf]

**T=Seita.8G230000.1\_Q=Sit-miR159b\_S=678**

category=2\_p=0.92488264407892

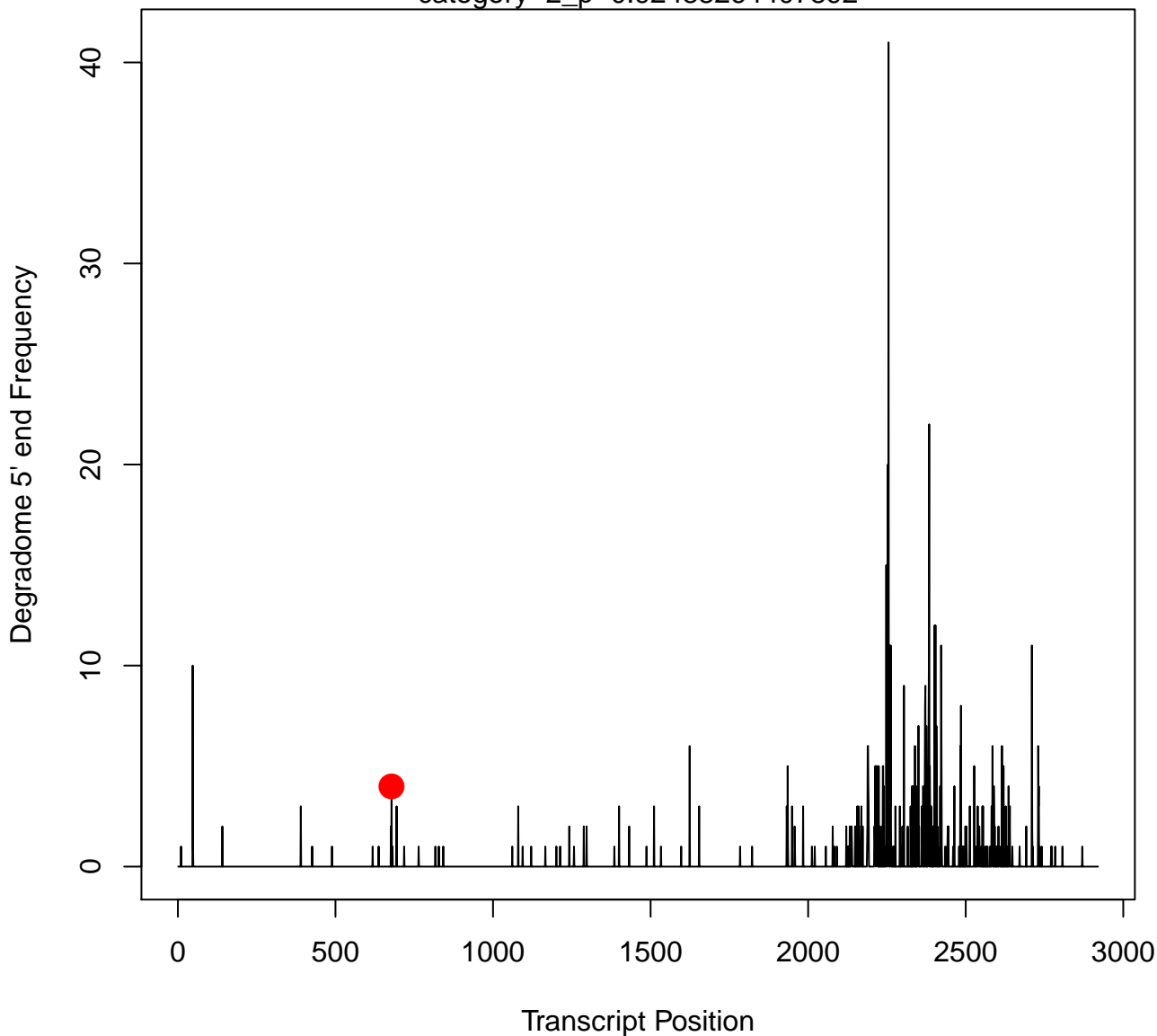

Supplement: Supplementary file 1 [file Data_Sheet_1.zip › Sit-miR159b_Seita.8G230000.1_678_TPlot.pdf]

**T=Seita.1G186900.1\_Q=Sit-miR159c\_S=886**

category=2\_p=0.283110328573254

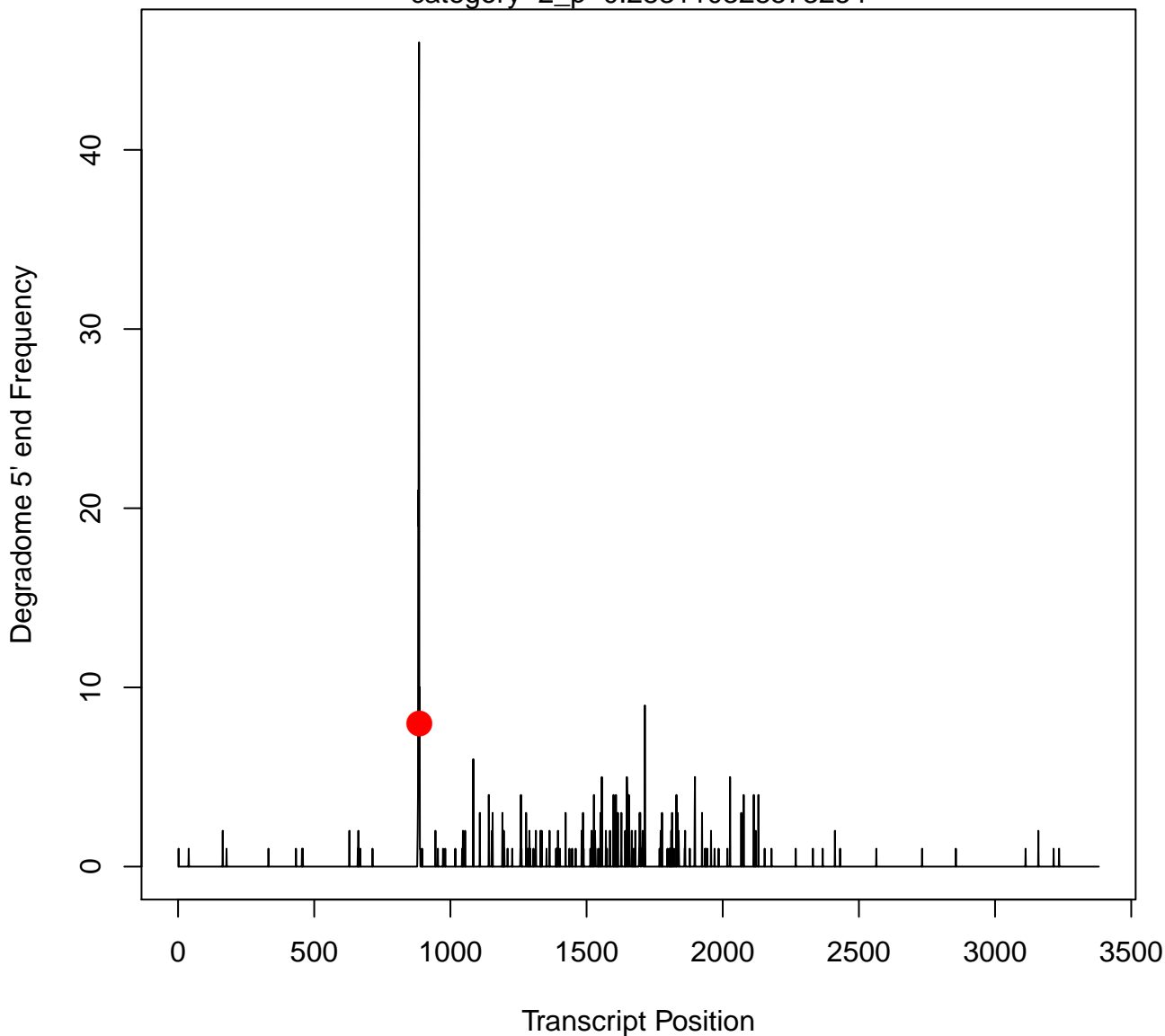

Supplement: Supplementary file 1 [file Data_Sheet_1.zip › Sit-miR159c_Seita.1G186900.1_886_TPlot.pdf]

**T=Seita.2G032900.1\_Q=Sit-miR159c\_S=1116**

category=2\_p=0.772193620706798

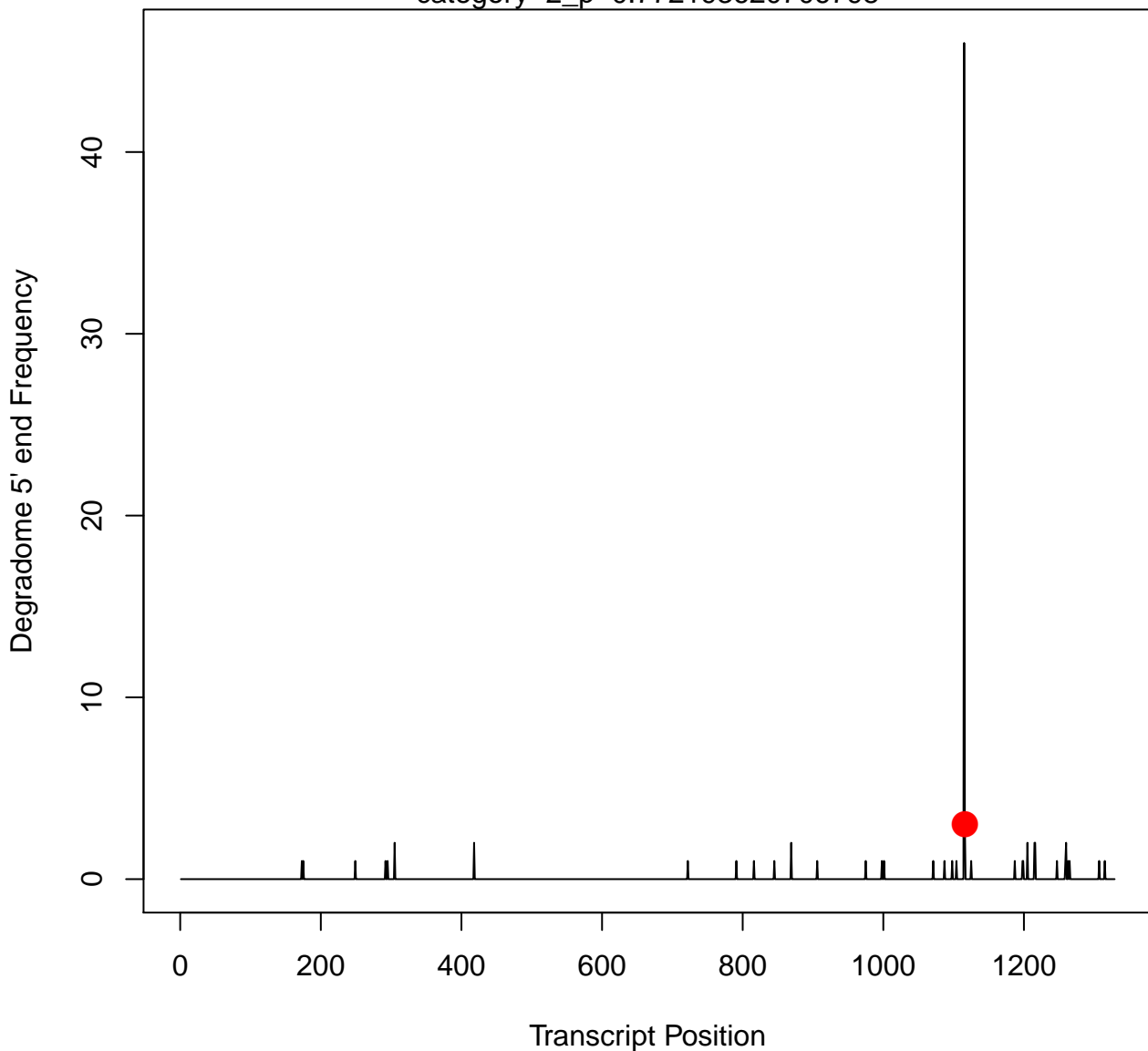

Supplement: Supplementary file 1 [file Data_Sheet_1.zip › Sit-miR159c_Seita.2G032900.1_1116_TPlot.pdf]

**T=Seita.3G188300.1\_Q=Sit-miR159c\_S=759**

category=0\_p=0.00915283052606486

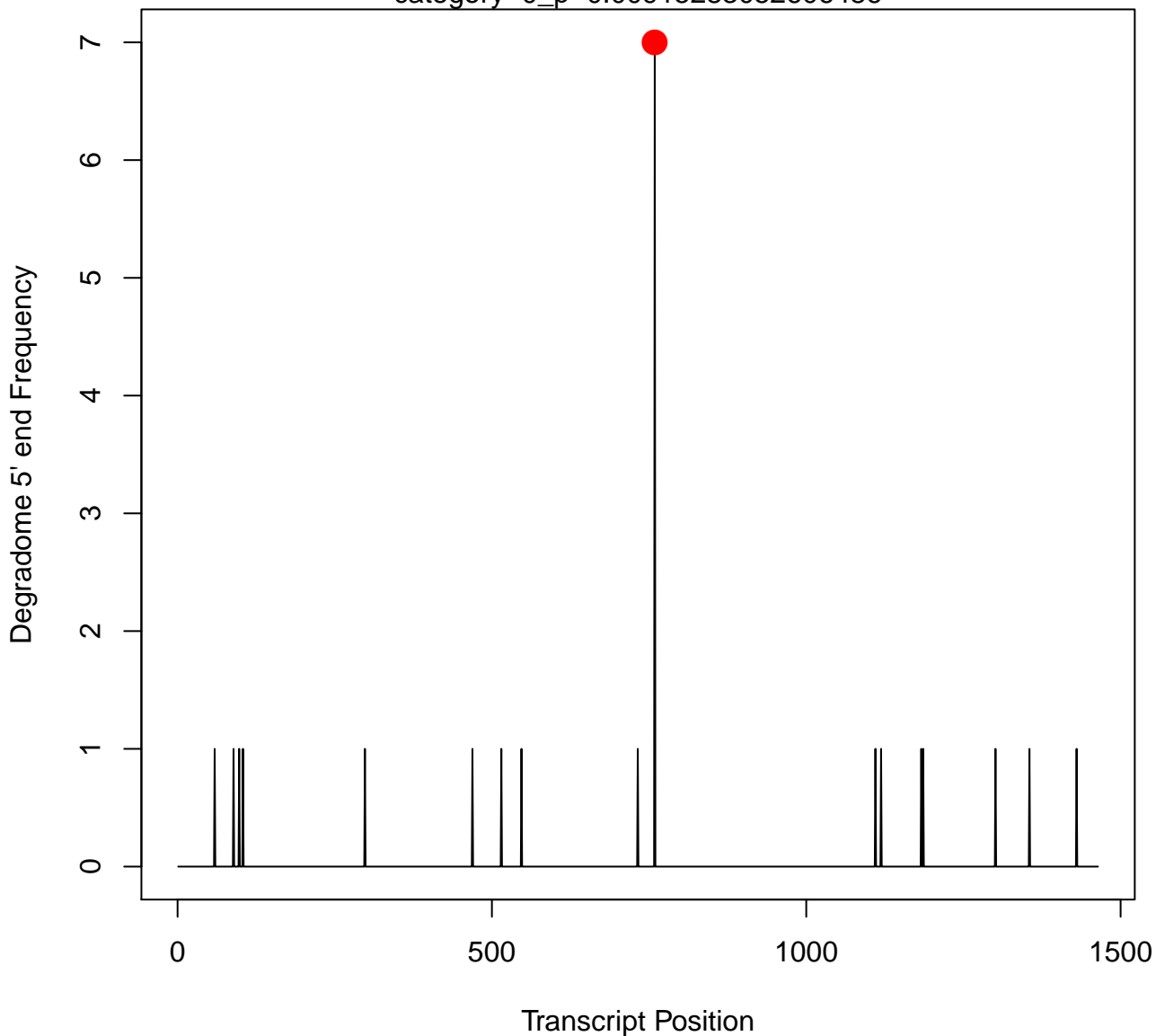

Supplement: Supplementary file 1 [file Data_Sheet_1.zip › Sit-miR159c_Seita.3G188300.1_759_TPlot.pdf]

**T=Seita.5G370400.1\_Q=Sit-miR159c\_S=899**

category=2\_p=0.999678808176685

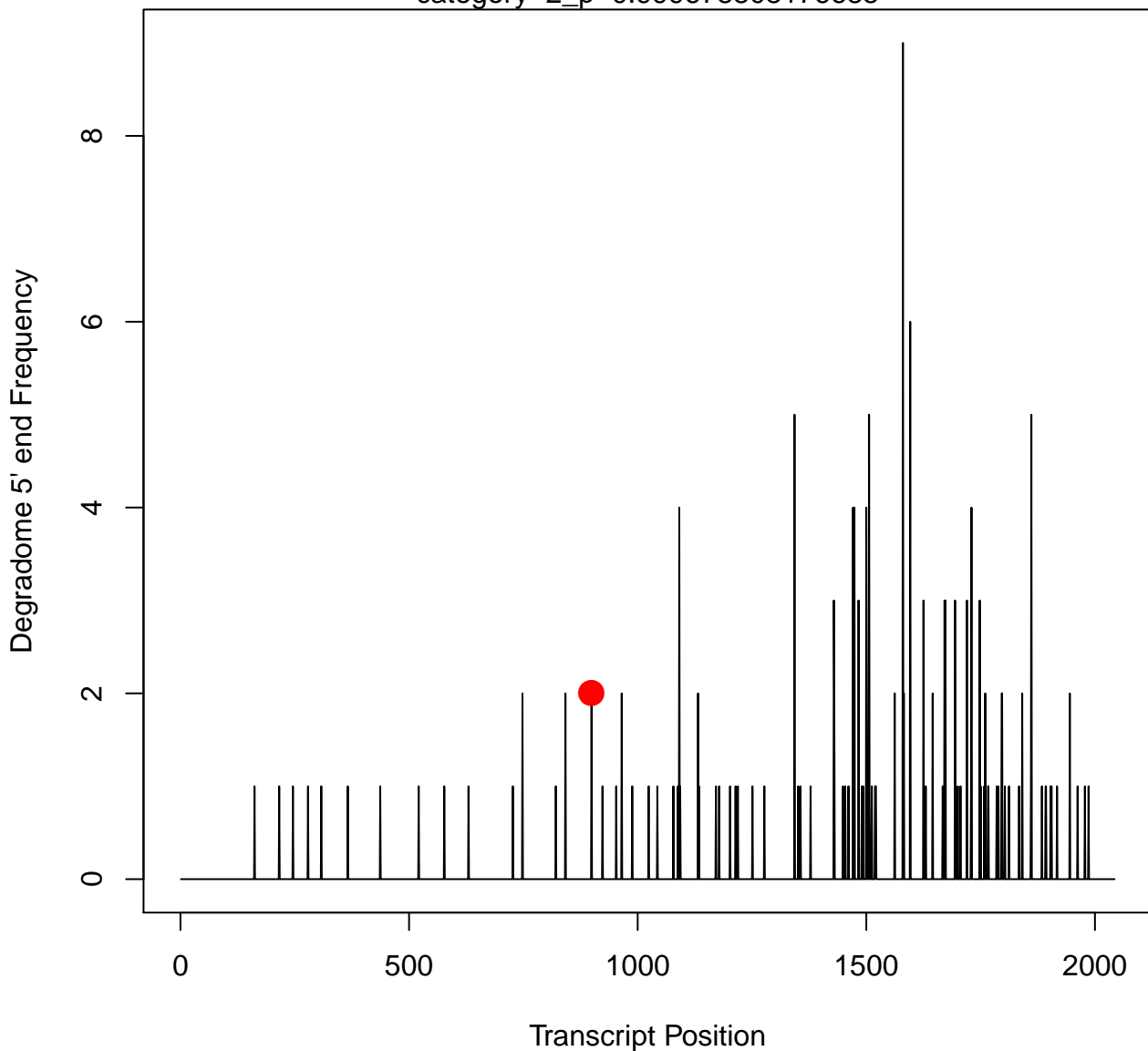

Supplement: Supplementary file 1 [file Data_Sheet_1.zip › Sit-miR159c_Seita.5G370400.1_899_TPlot.pdf]

**T=Seita.6G026800.1\_Q=Sit-miR159c\_S=1459**

category=2\_p=0.99978615697061

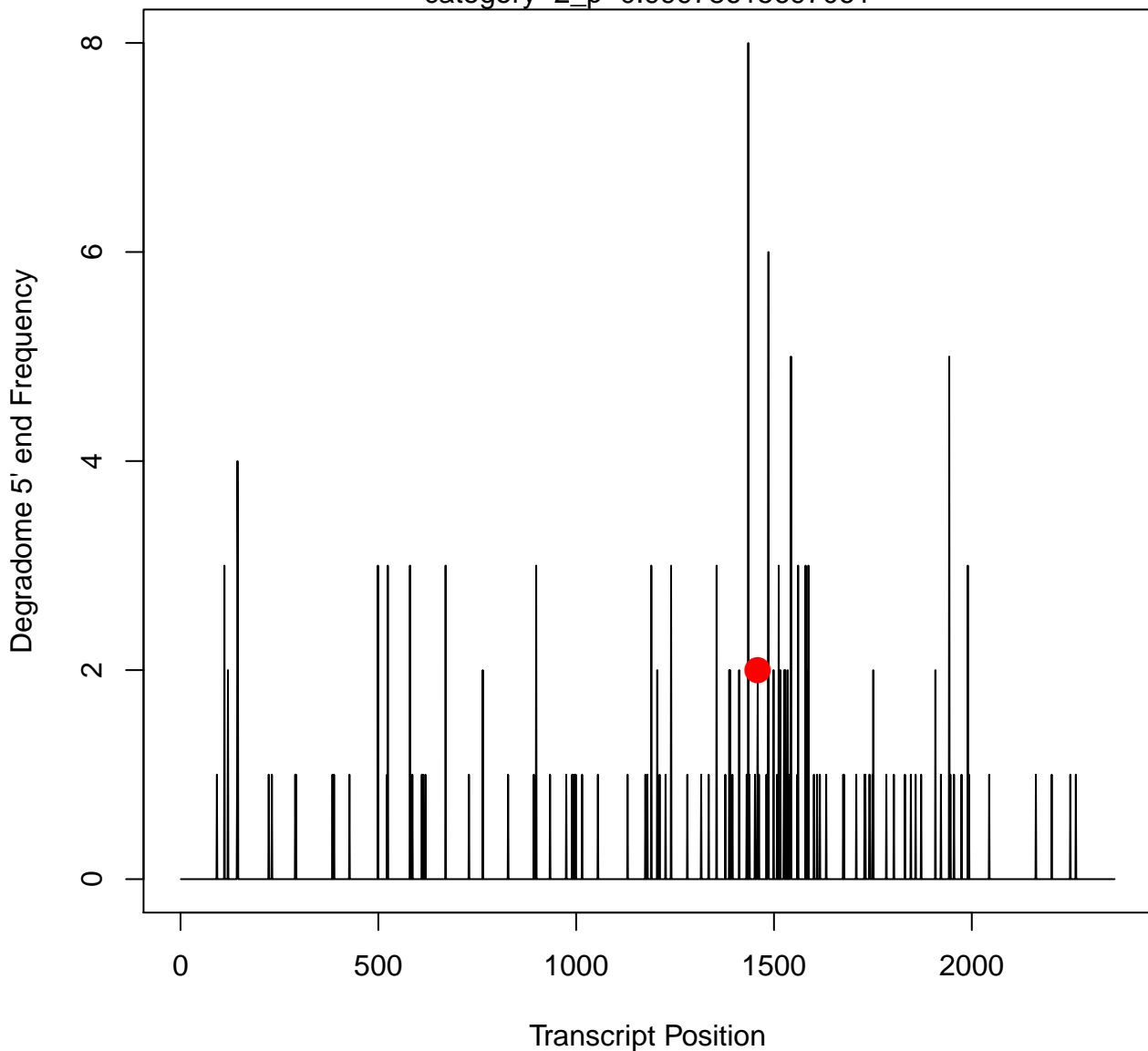

Supplement: Supplementary file 1 [file Data_Sheet_1.zip › Sit-miR159c_Seita.6G026800.1_1459_TPlot.pdf]

**T=Seita.6G100100.1\_Q=Sit-miR159c\_S=1109**

category=2\_p=0.710291374419638

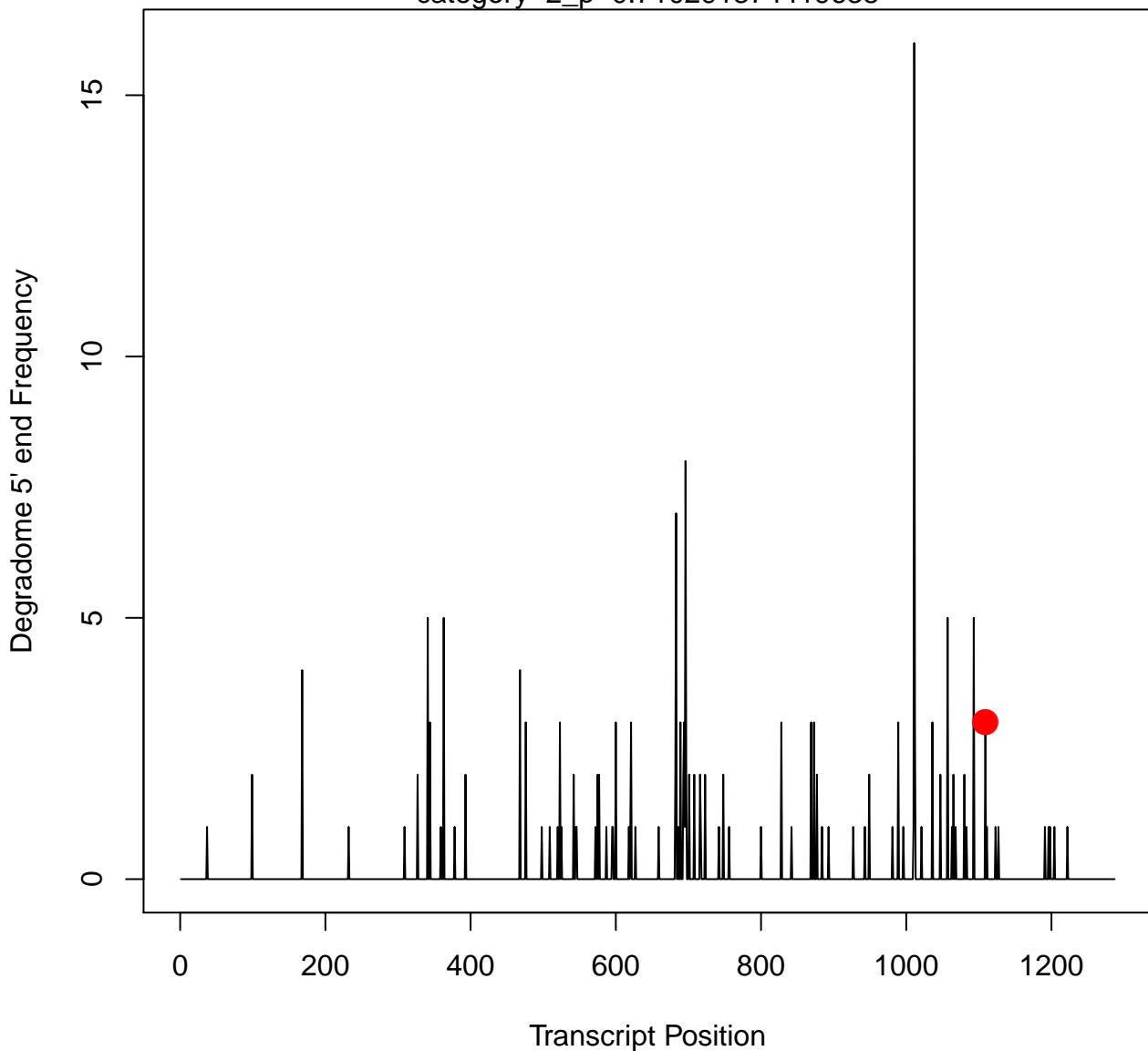

Supplement: Supplementary file 1 [file Data_Sheet_1.zip › Sit-miR159c_Seita.6G100100.1_1109_TPlot.pdf]

**T=Seita.2G369900.1\_Q=Sit-miR160a\_S=2167**

category=0\_p=0.57792667736209

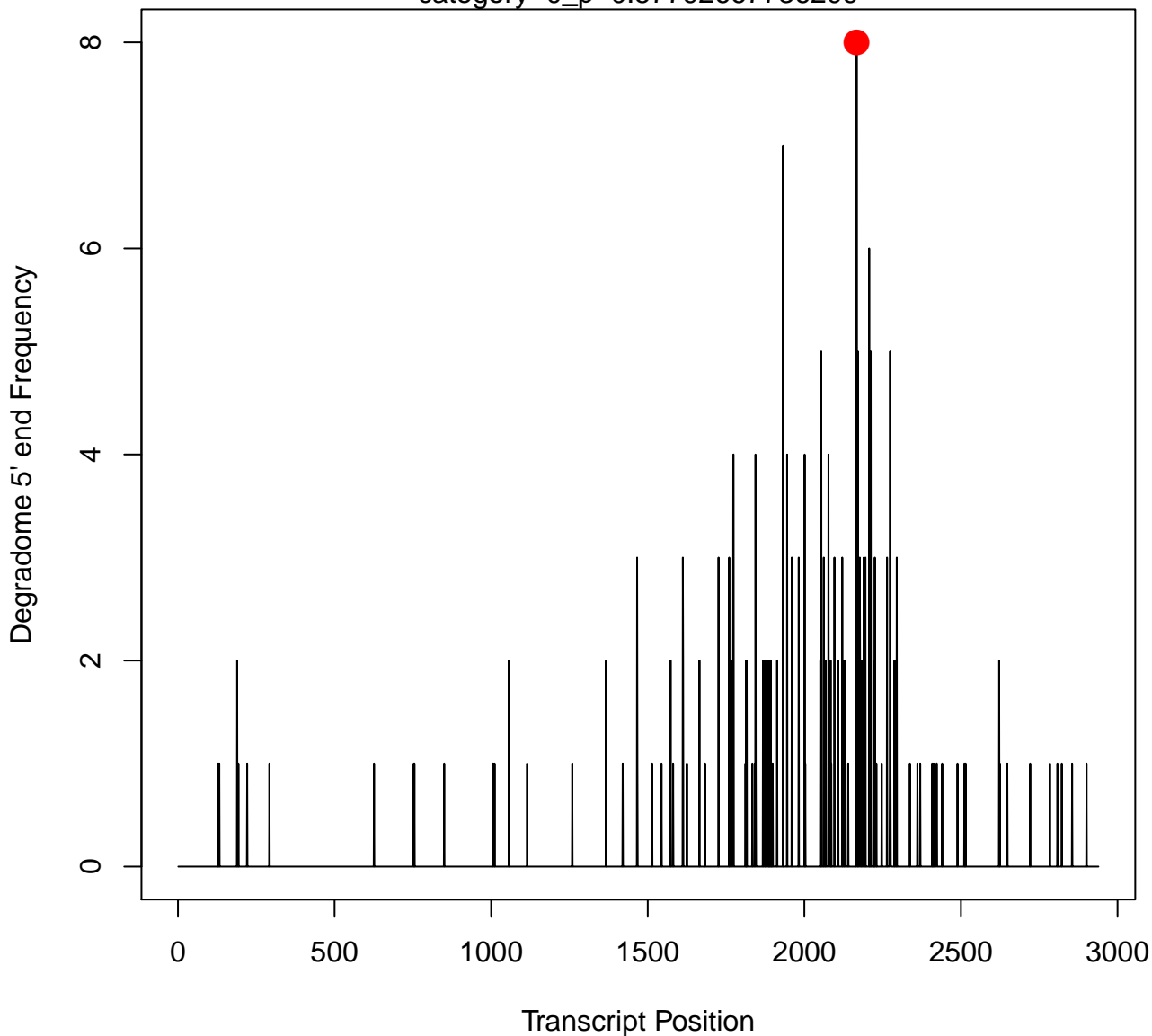

Supplement: Supplementary file 1 [file Data_Sheet_1.zip › Sit-miR160a_Seita.2G369900.1_2167_TPlot.pdf]

**T=Seita.2G429500.1\_Q=Sit-miR160a\_S=228**

category=2\_p=0.999999999995418

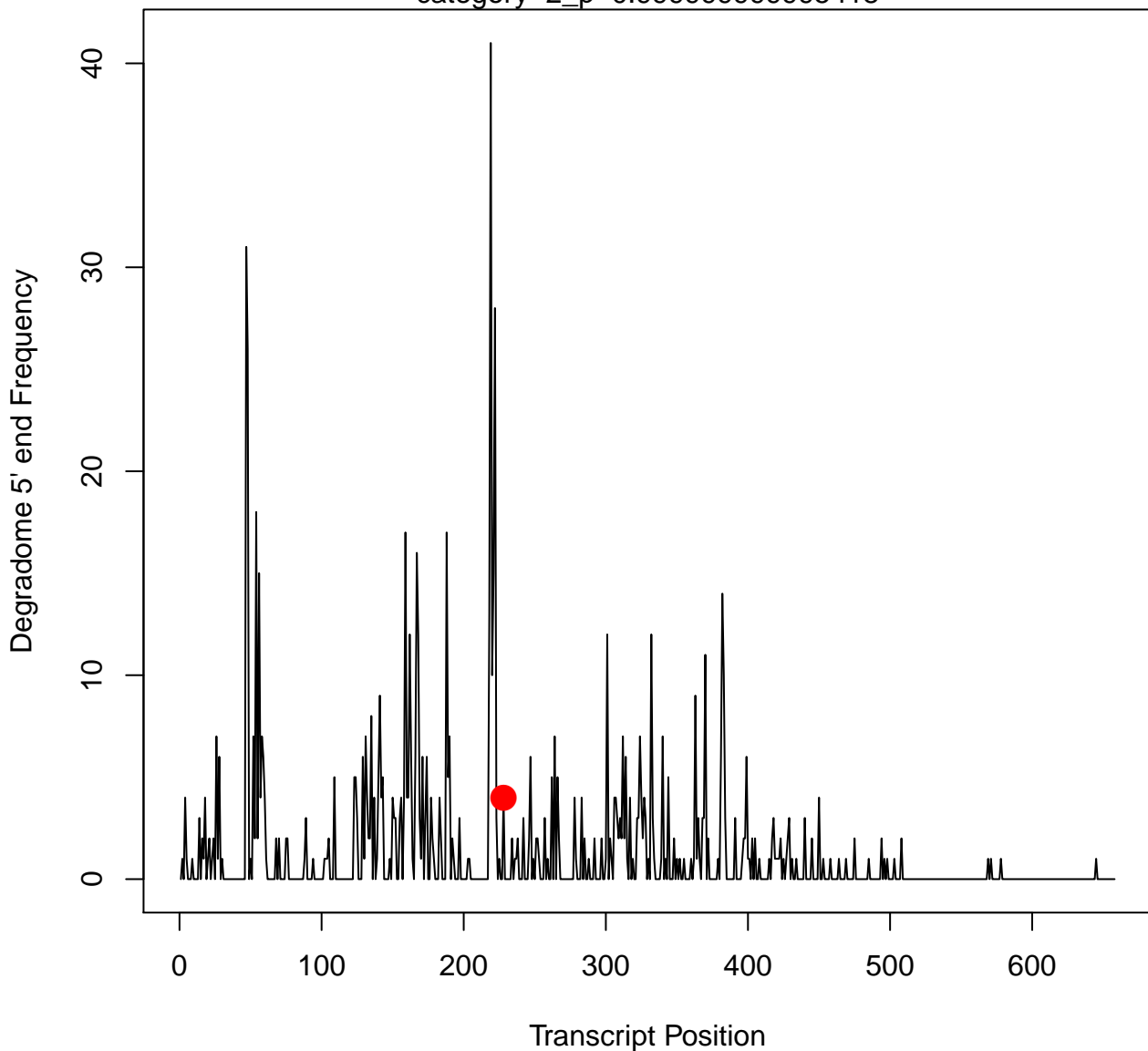

Supplement: Supplementary file 1 [file Data_Sheet_1.zip › Sit-miR160a_Seita.2G429500.1_228_TPlot.pdf]

**T=Seita.4G006200.1\_Q=Sit-miR160a\_S=649**

category=2\_p=0.97283142649702

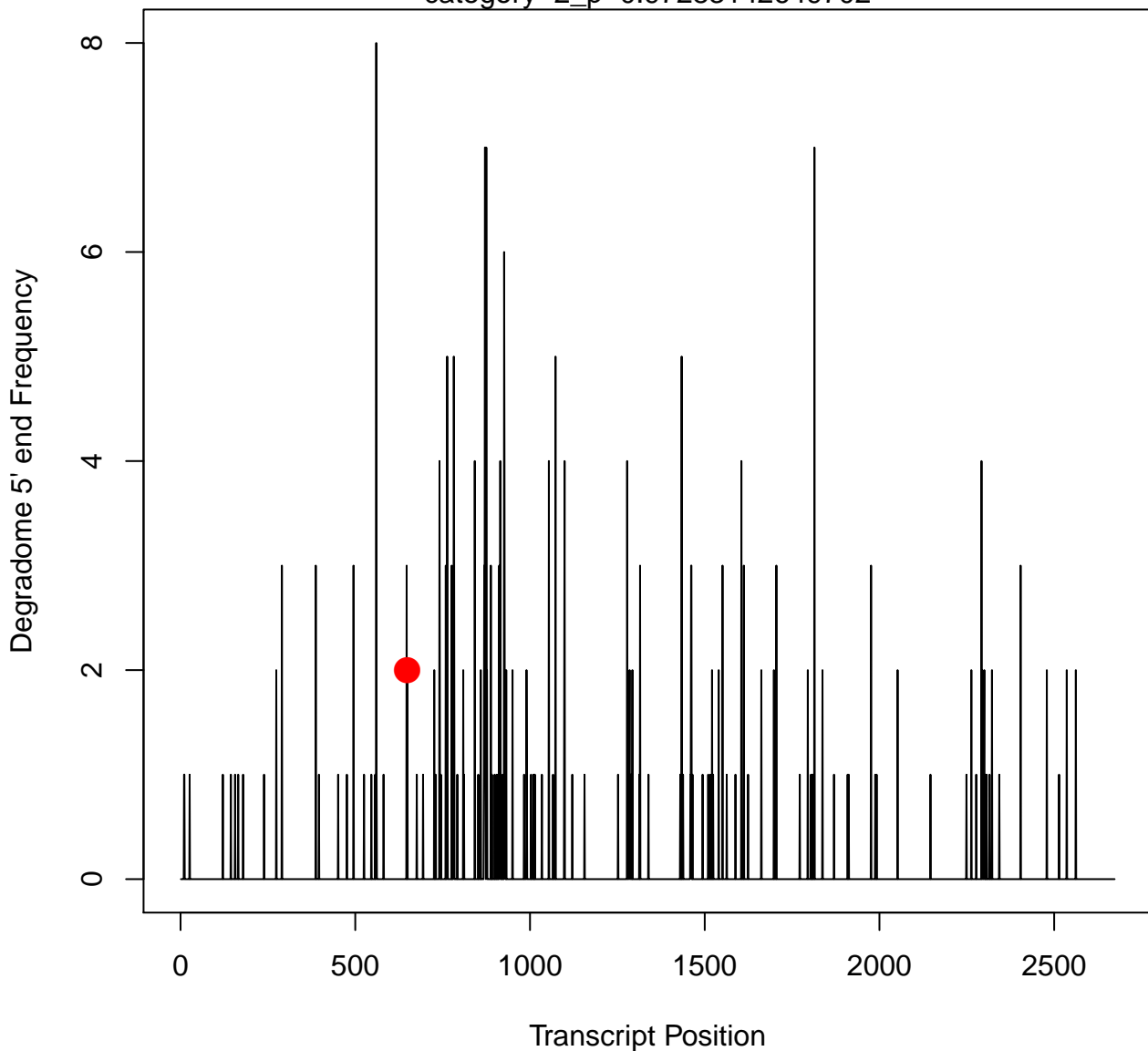

Supplement: Supplementary file 1 [file Data_Sheet_1.zip › Sit-miR160a_Seita.4G006200.1_649_TPlot.pdf]

**T=Seita.4G043900.1\_Q=Sit-miR160a\_S=308**

category=2\_p=0.999998119358917

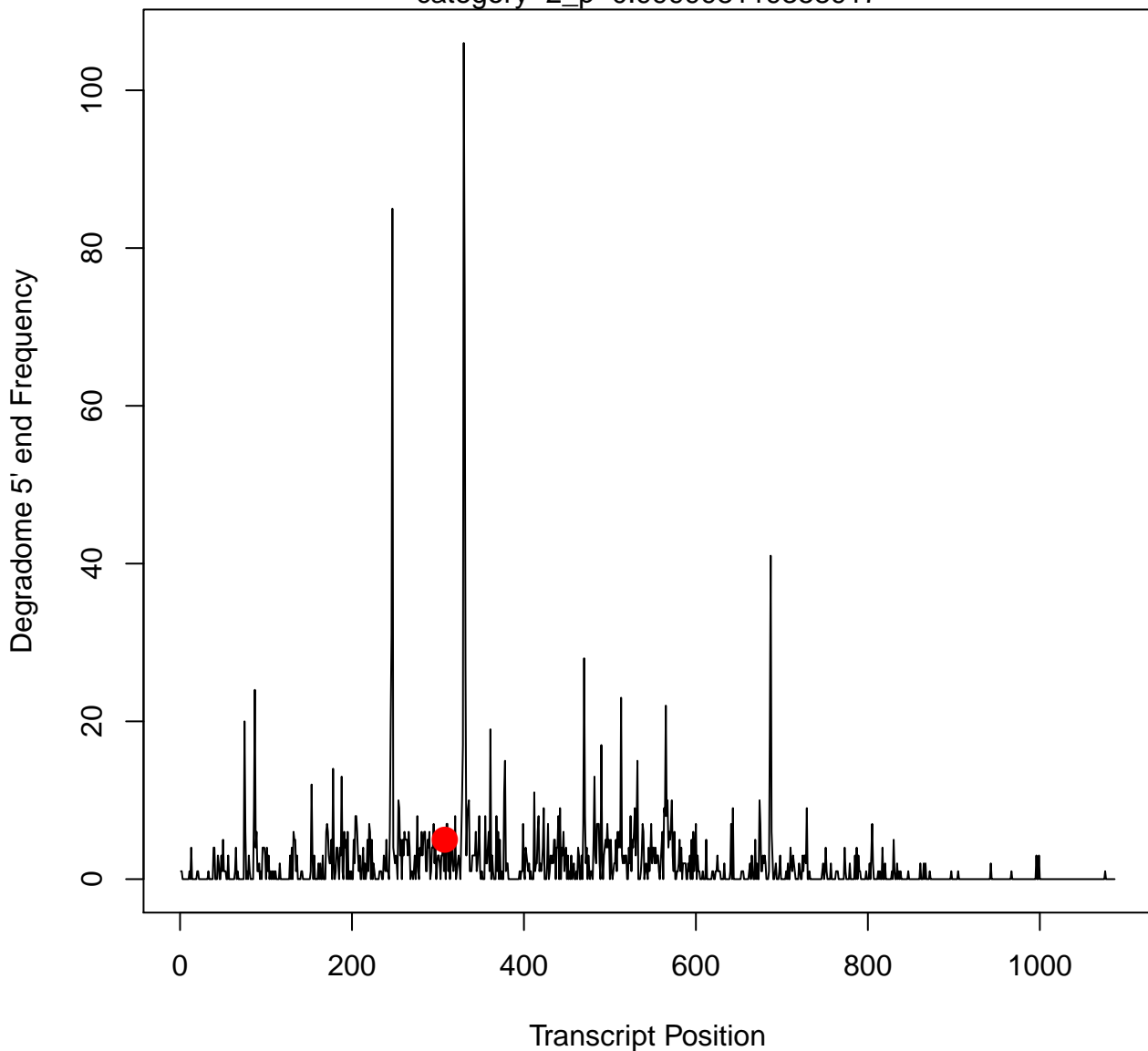

Supplement: Supplementary file 1 [file Data_Sheet_1.zip › Sit-miR160a_Seita.4G043900.1_308_TPlot.pdf]

**T=Seita.4G257800.1\_Q=Sit-miR160a\_S=1974**

category=0\_p=0.000437760109239571

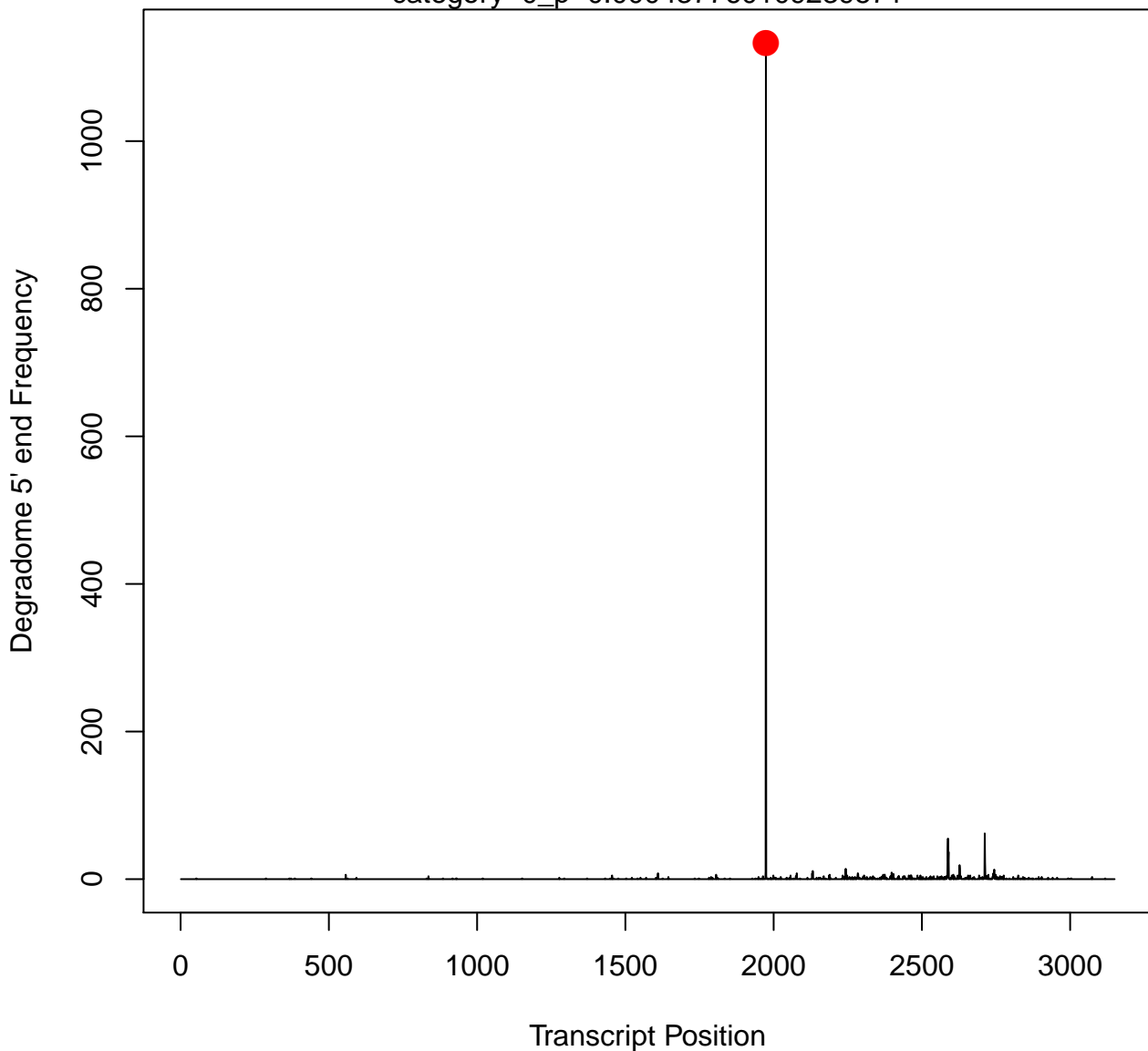

Supplement: Supplementary file 1 [file Data_Sheet_1.zip › Sit-miR160a_Seita.4G257800.1_1974_TPlot.pdf]

**T=Seita.5G079500.1\_Q=Sit-miR160a\_S=3803**

category=2\_p=0.999999999999749

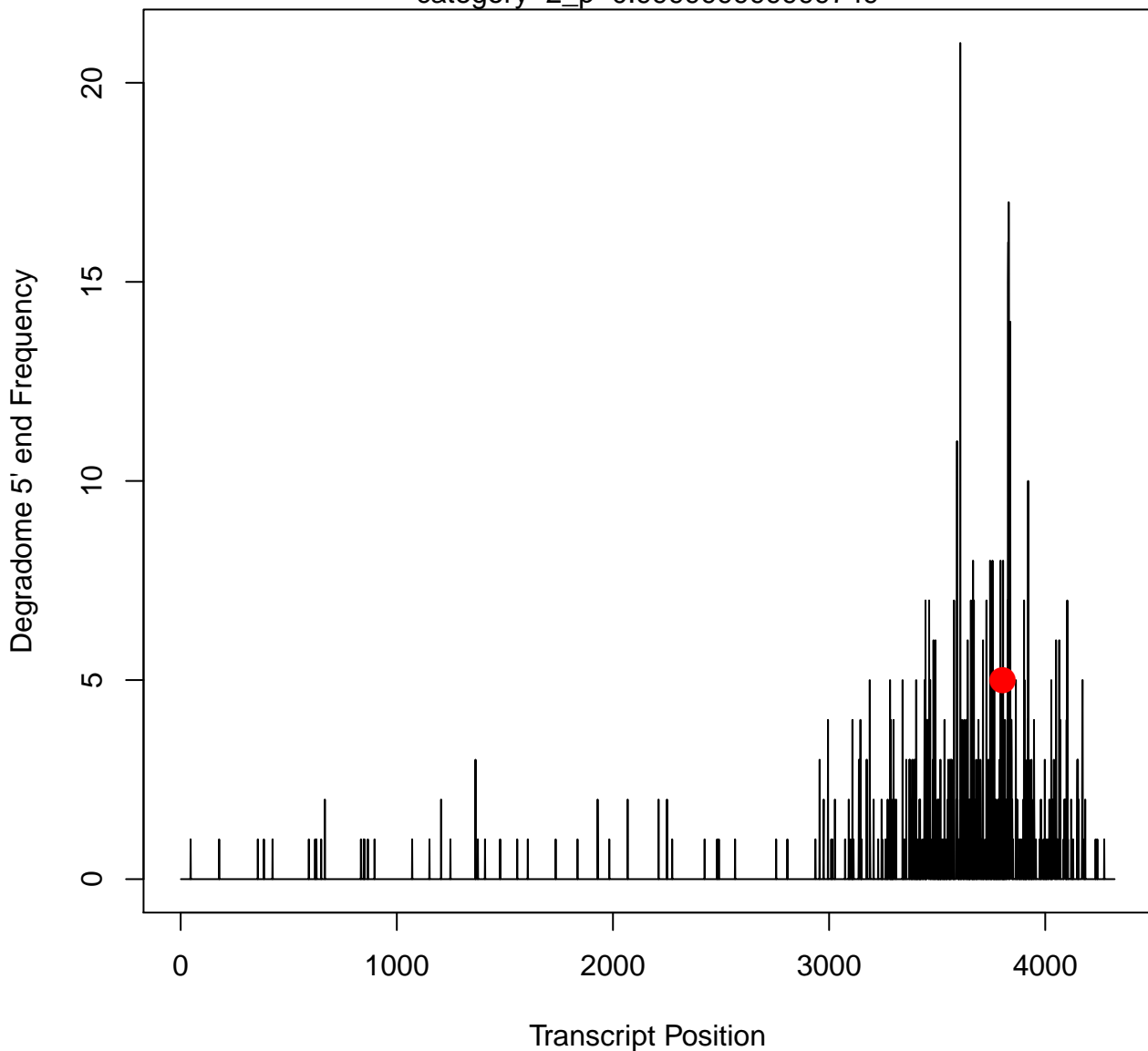

Supplement: Supplementary file 1 [file Data_Sheet_1.zip › Sit-miR160a_Seita.5G079500.1_3803_TPlot.pdf]

**T=Seita.5G267100.1\_Q=Sit-miR160a\_S=1283**

category=2\_p=0.9999999999999999

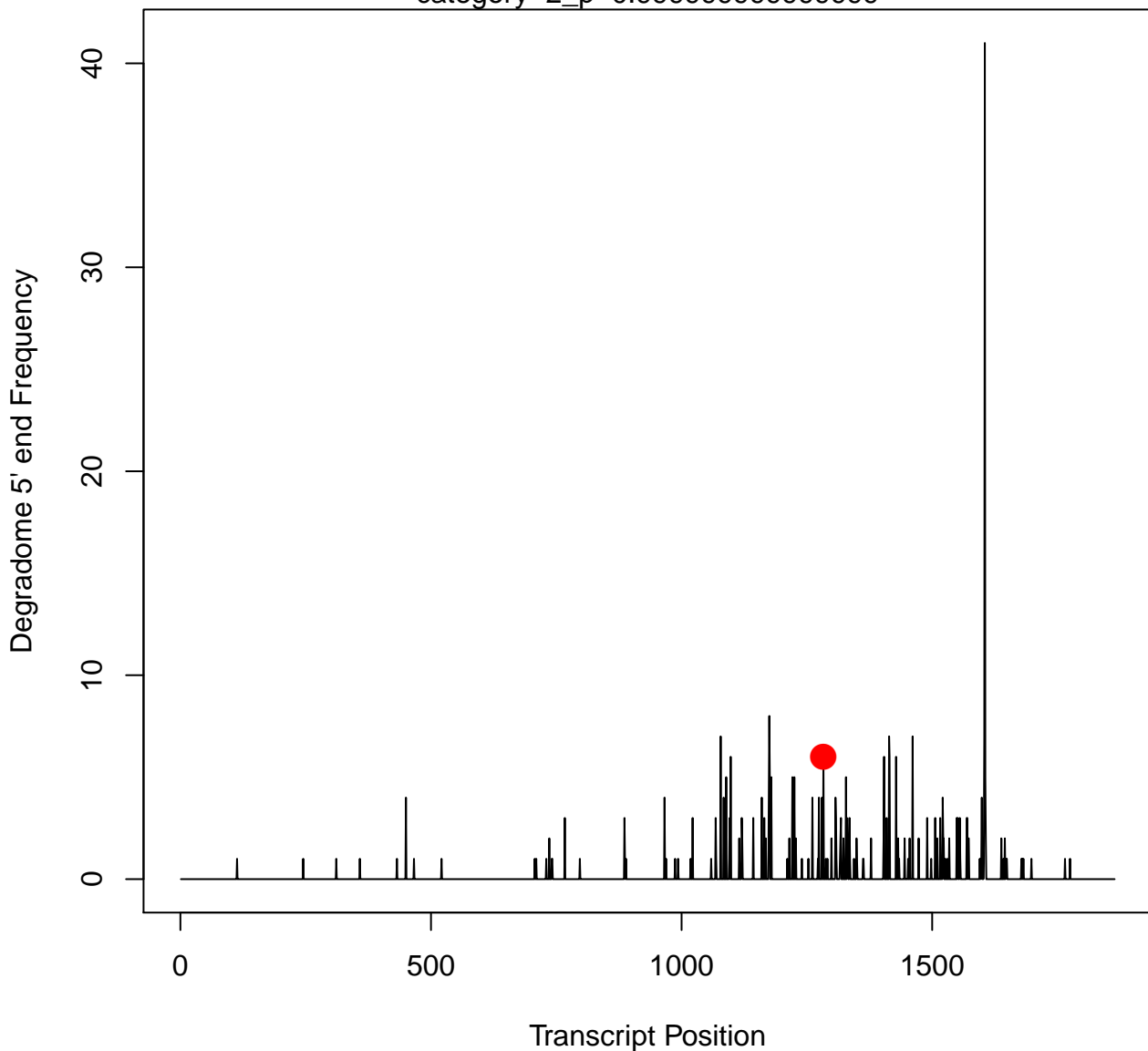

Supplement: Supplementary file 1 [file Data_Sheet_1.zip › Sit-miR160a_Seita.5G267100.1_1283_TPlot.pdf]

**T=Seita.6G044900.1\_Q=Sit-miR160a\_S=839**

category=2\_p=0.999999999997772

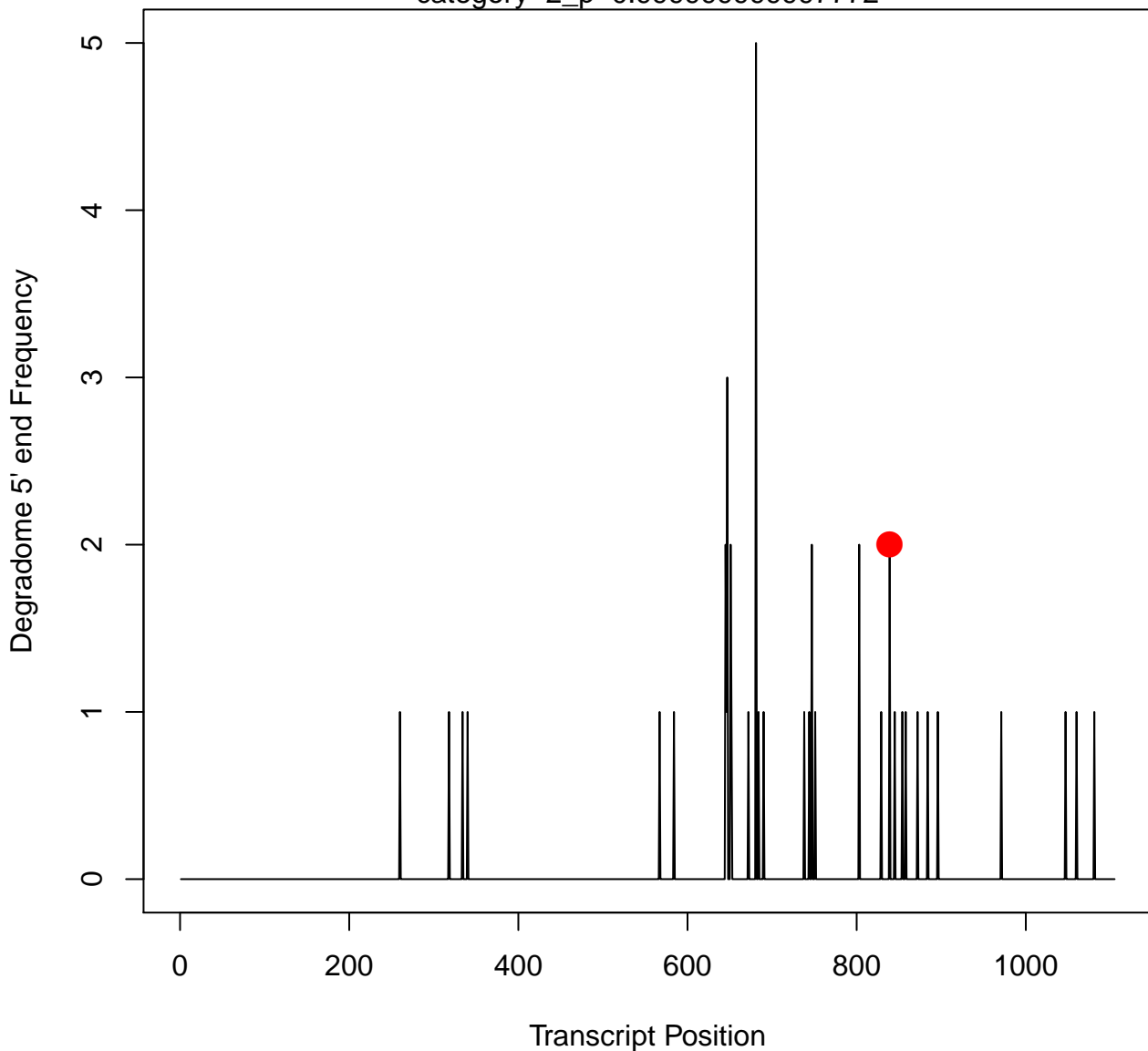

Supplement: Supplementary file 1 [file Data_Sheet_1.zip › Sit-miR160a_Seita.6G044900.1_839_TPlot.pdf]

**T=Seita.6G252500.1\_Q=Sit-miR160a\_S=1030**

category=2\_p=0.999999999996715

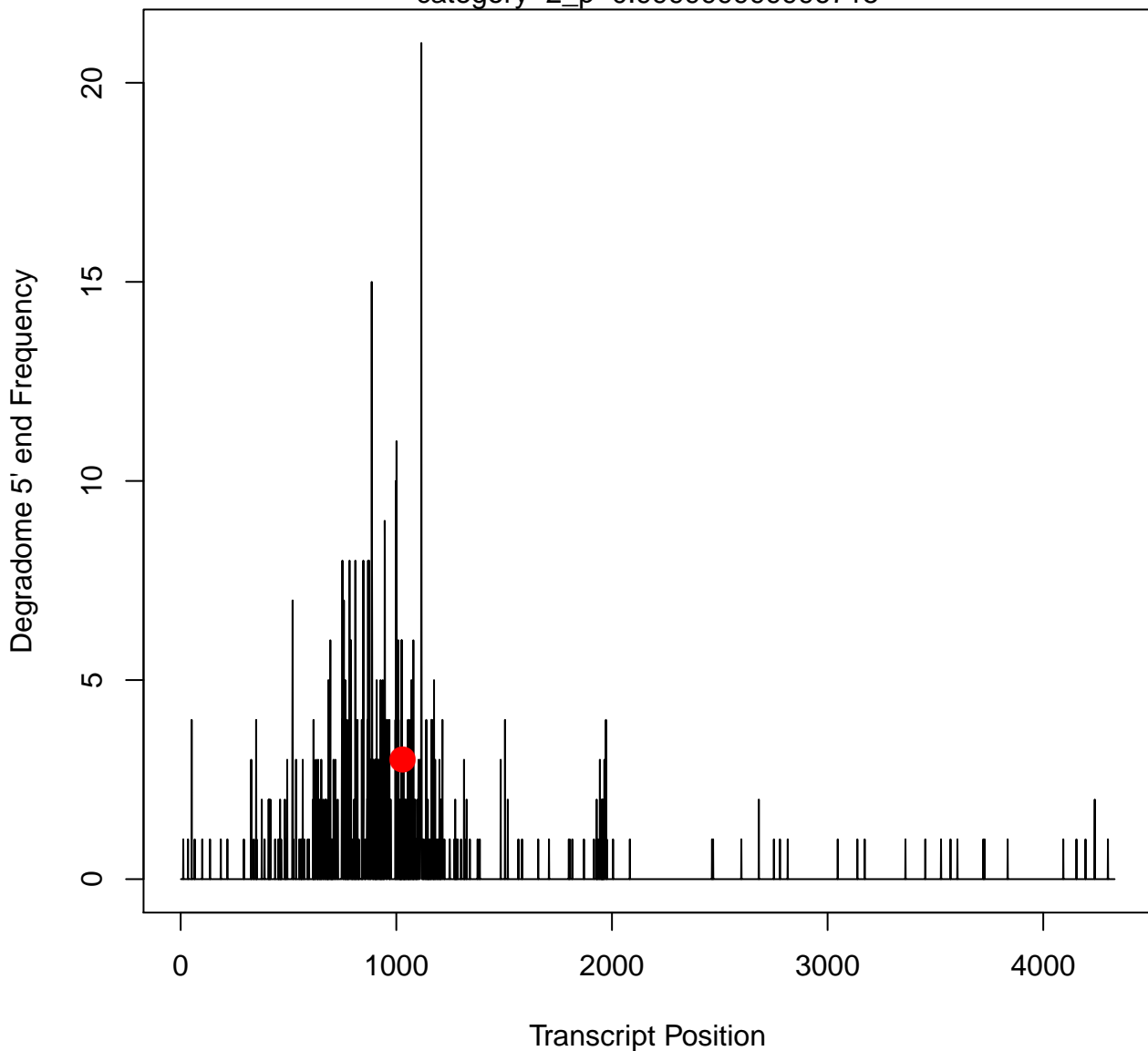

Supplement: Supplementary file 1 [file Data_Sheet_1.zip › Sit-miR160a_Seita.6G252500.1_1030_TPlot.pdf]

**T=Seita.7G078300.1\_Q=Sit-miR160a\_S=2649**

category=2\_p=0.999994503697146

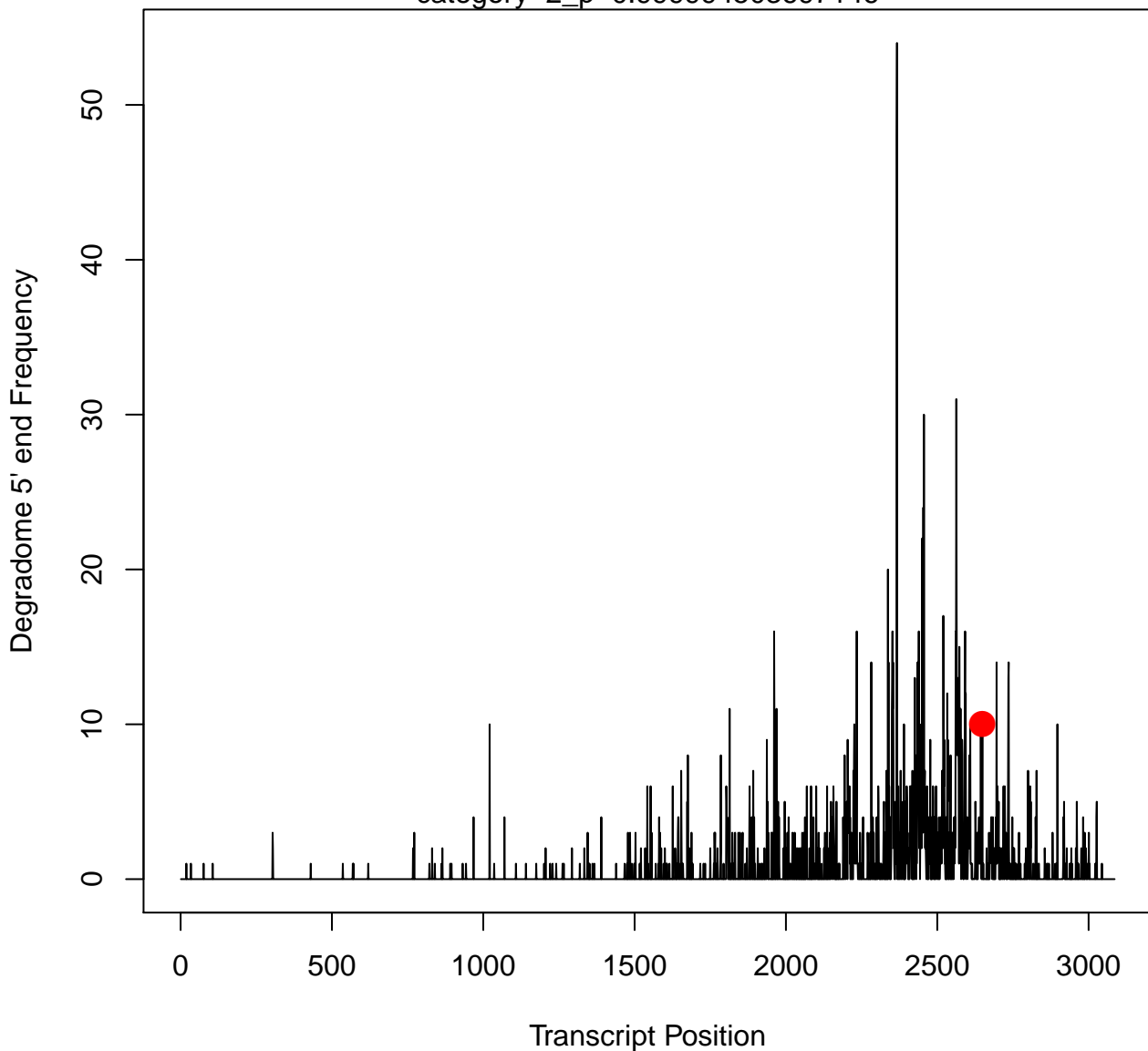

Supplement: Supplementary file 1 [file Data_Sheet_1.zip › Sit-miR160a_Seita.7G078300.1_2649_TPlot.pdf]

**T=Seita.7G169600.1\_Q=Sit-miR160a\_S=1802**

category=0\_p=0.00087532858456596

Degradsome 5' end Frequency

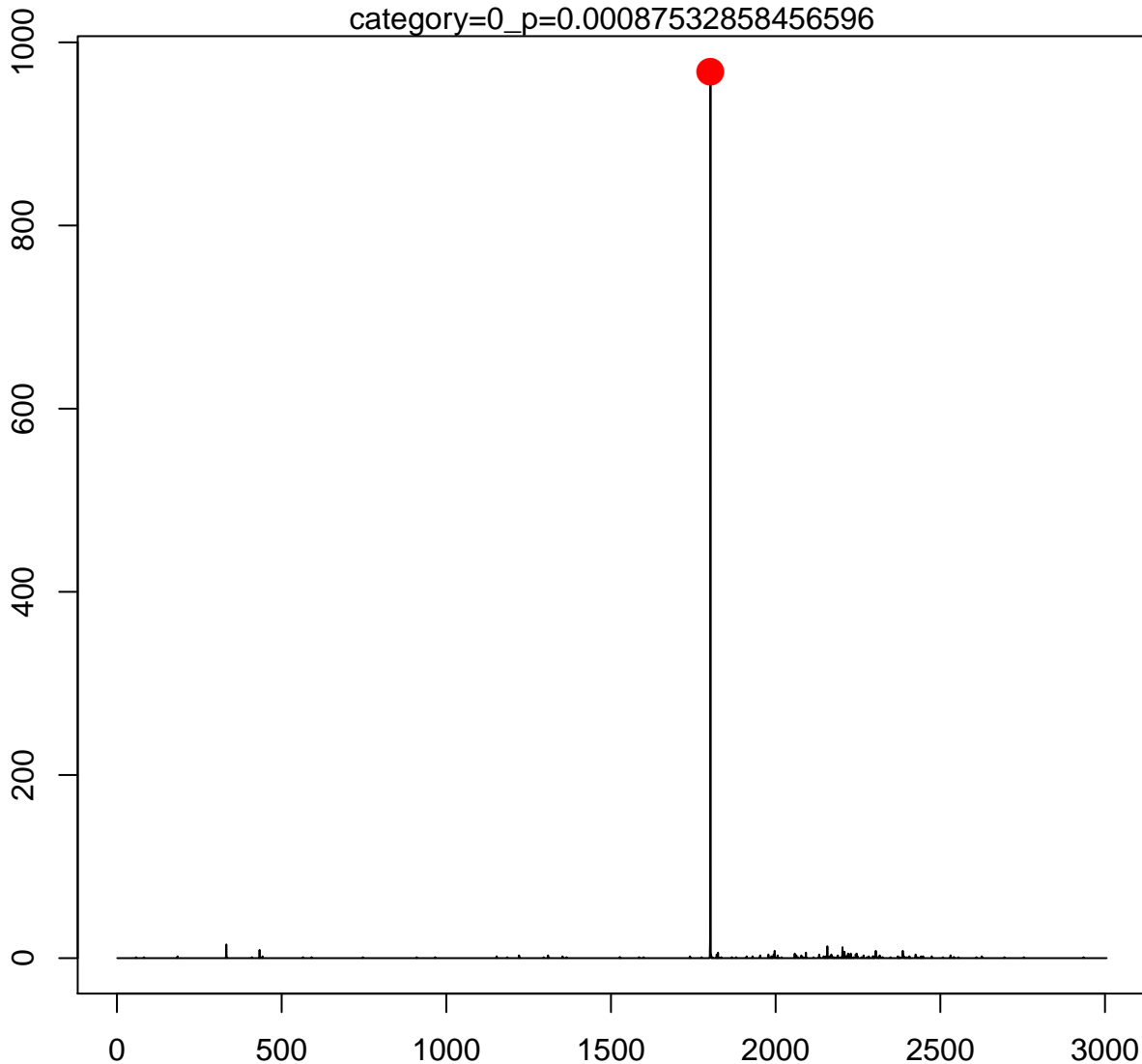

Transcript Position

Supplement: Supplementary file 1 [file Data_Sheet_1.zip › Sit-miR160a_Seita.7G169600.1_1802_TPlot.pdf]

**T=Seita.7G295800.1\_Q=Sit-miR160a\_S=368**

category=2\_p=0.999999999999252

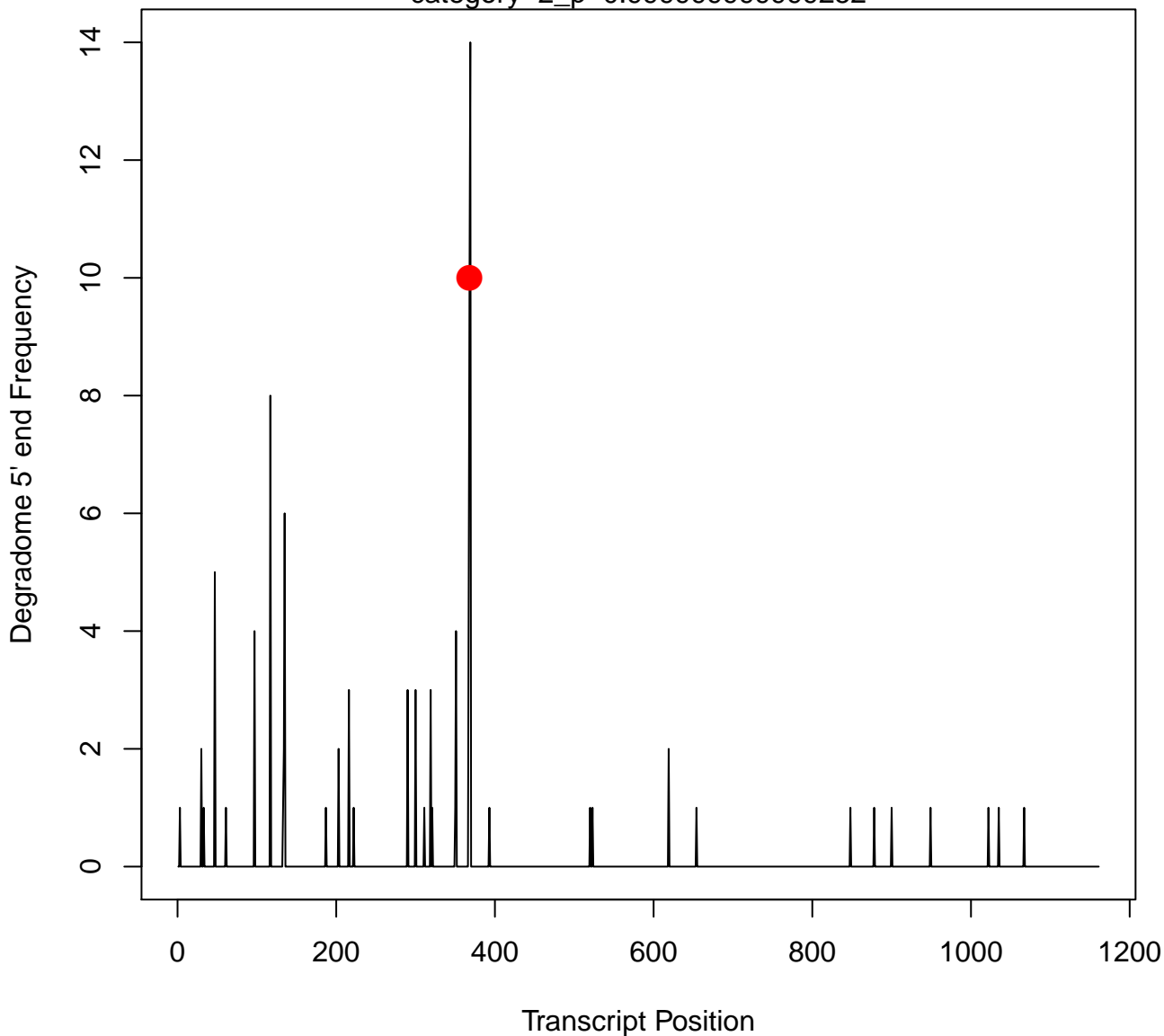

Supplement: Supplementary file 1 [file Data_Sheet_1.zip › Sit-miR160a_Seita.7G295800.1_368_TPlot.pdf]

**T=Seita.8G171500.1\_Q=Sit-miR160a\_S=190**

category=1\_p=0.348054246735839

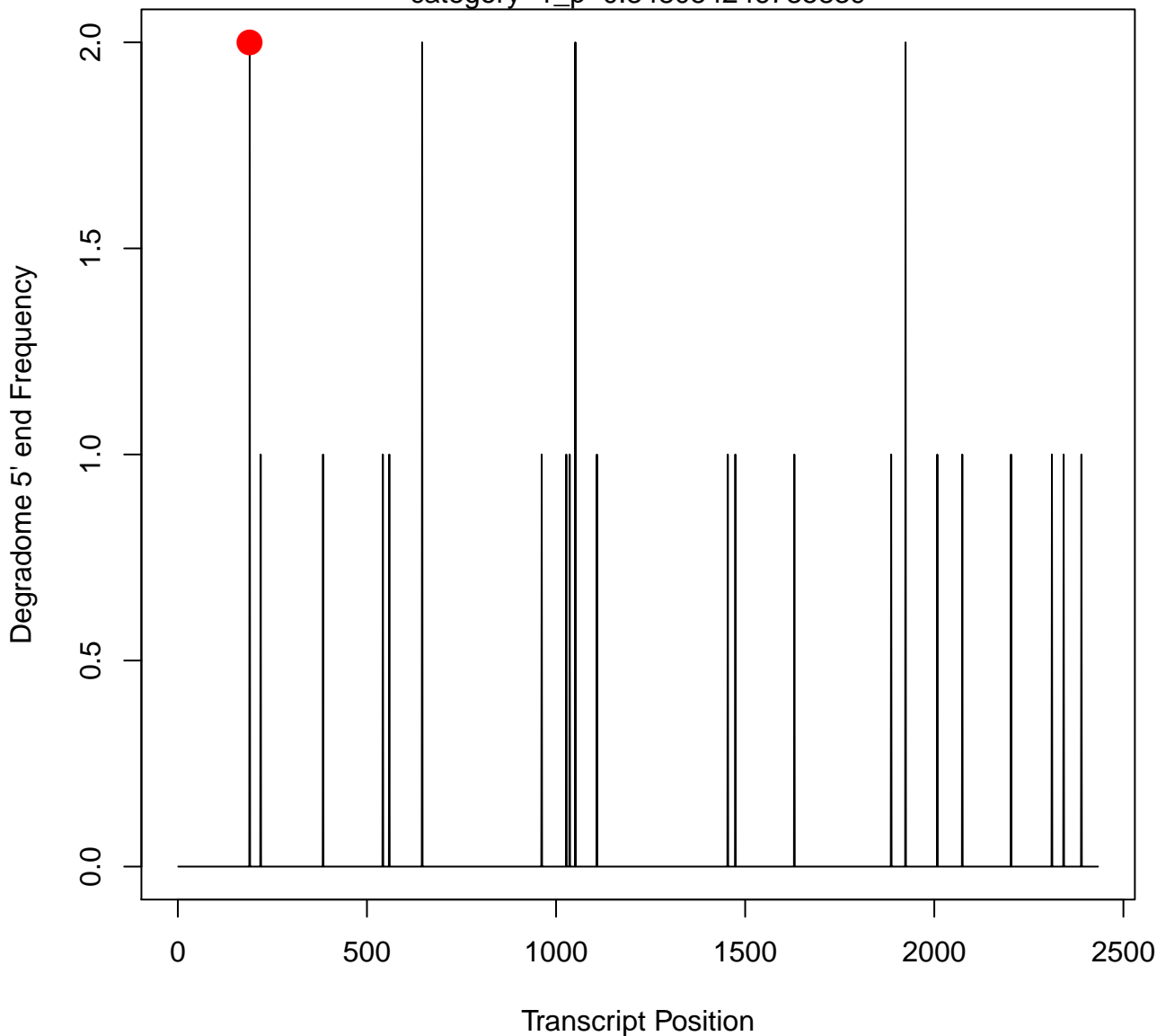

Supplement: Supplementary file 1 [file Data_Sheet_1.zip › Sit-miR160a_Seita.8G171500.1_190_TPlot.pdf]

**T=Seita.9G161900.1\_Q=Sit-miR160a\_S=2014**

category=2\_p=0.996699359552699

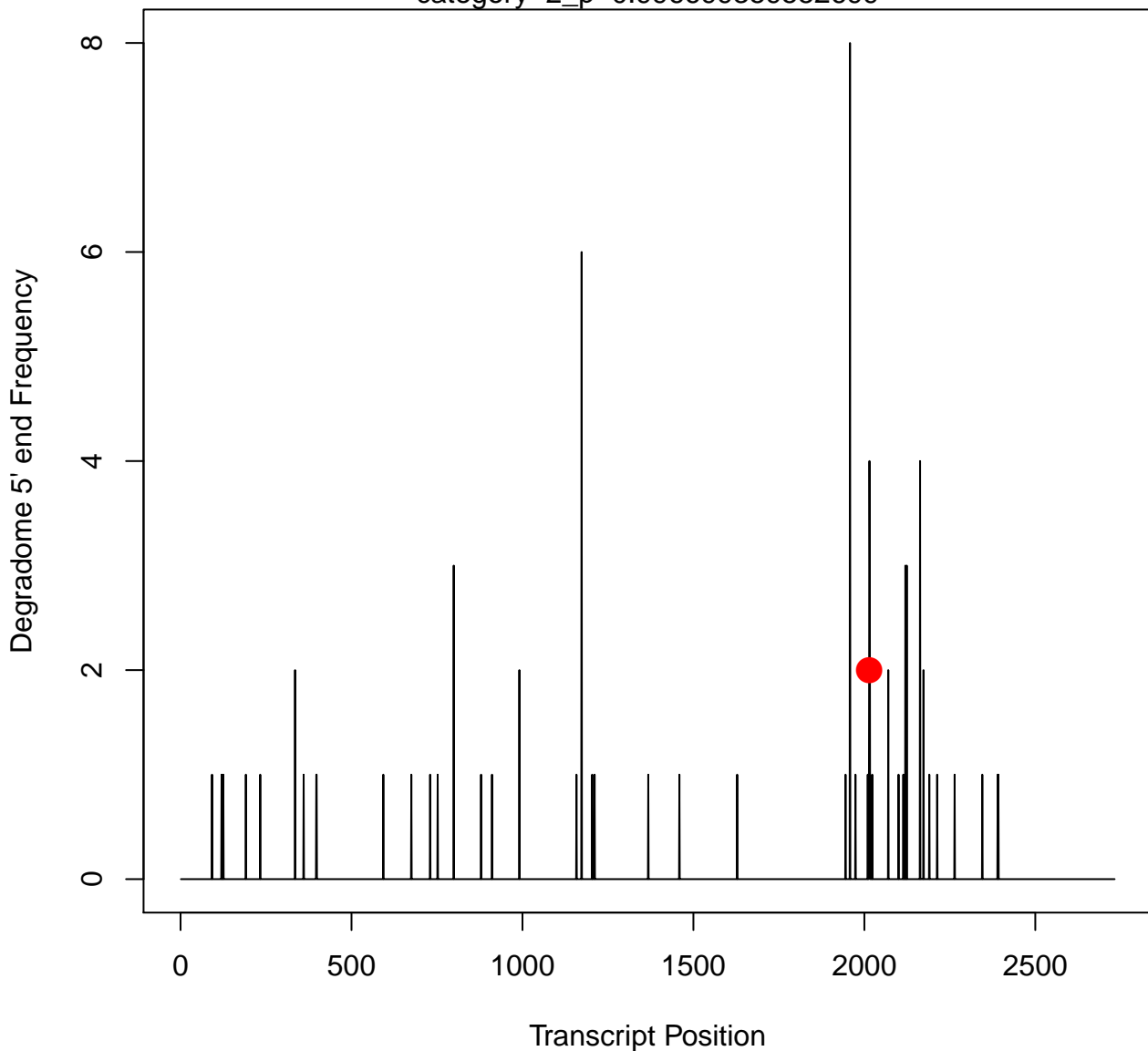

Supplement: Supplementary file 1 [file Data_Sheet_1.zip › Sit-miR160a_Seita.9G161900.1_2014_TPlot.pdf]

**T=Seita.2G068600.1\_Q=Sit-miR160b\_S=3061**

category=2\_p=0.999999998298726

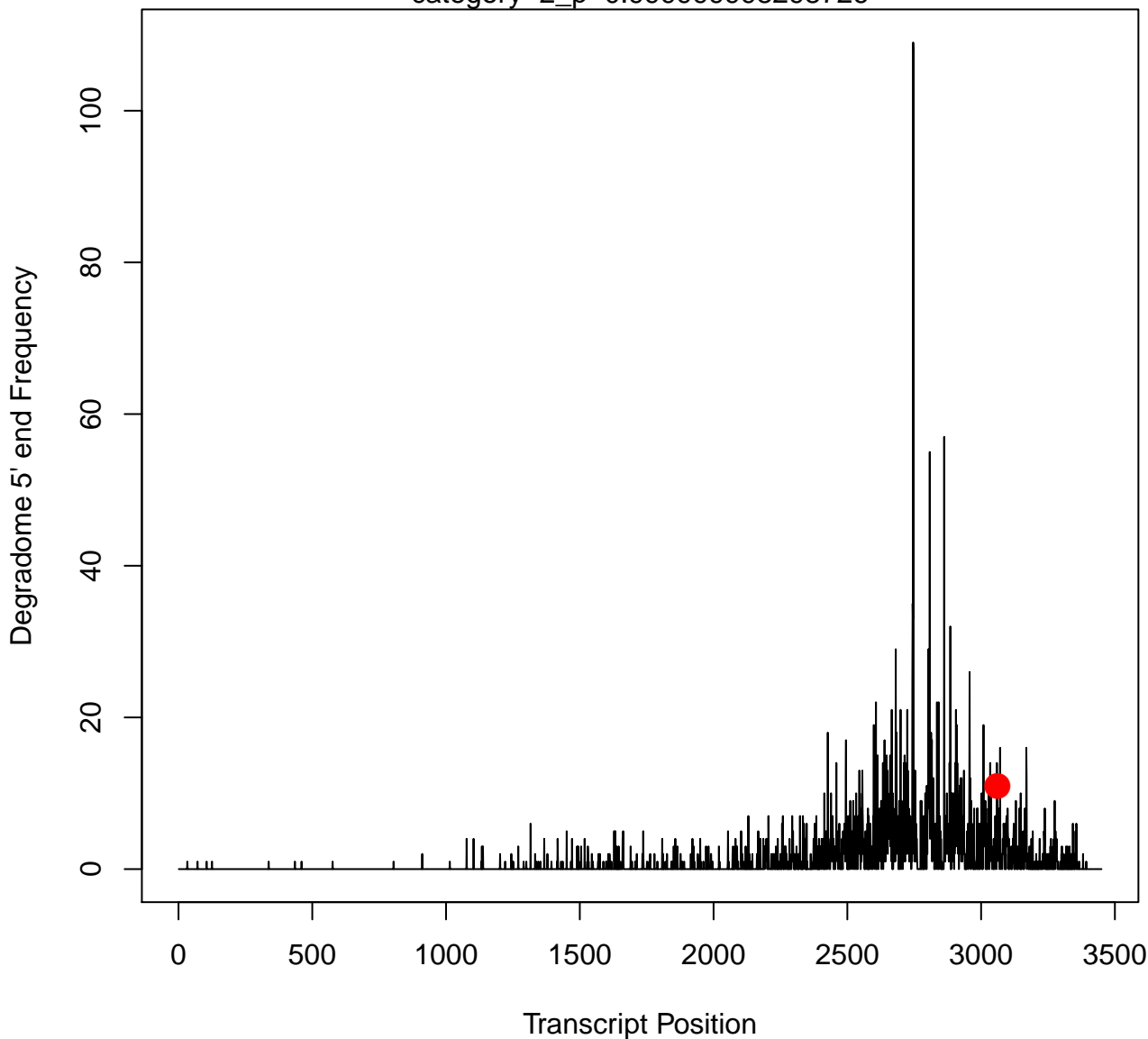

Supplement: Supplementary file 1 [file Data_Sheet_1.zip › Sit-miR160b_Seita.2G068600.1_3061_TPlot.pdf]

**T=Seita.2G374100.1\_Q=Sit-miR160b\_S=1439**

category=2\_p=0.92488264407892

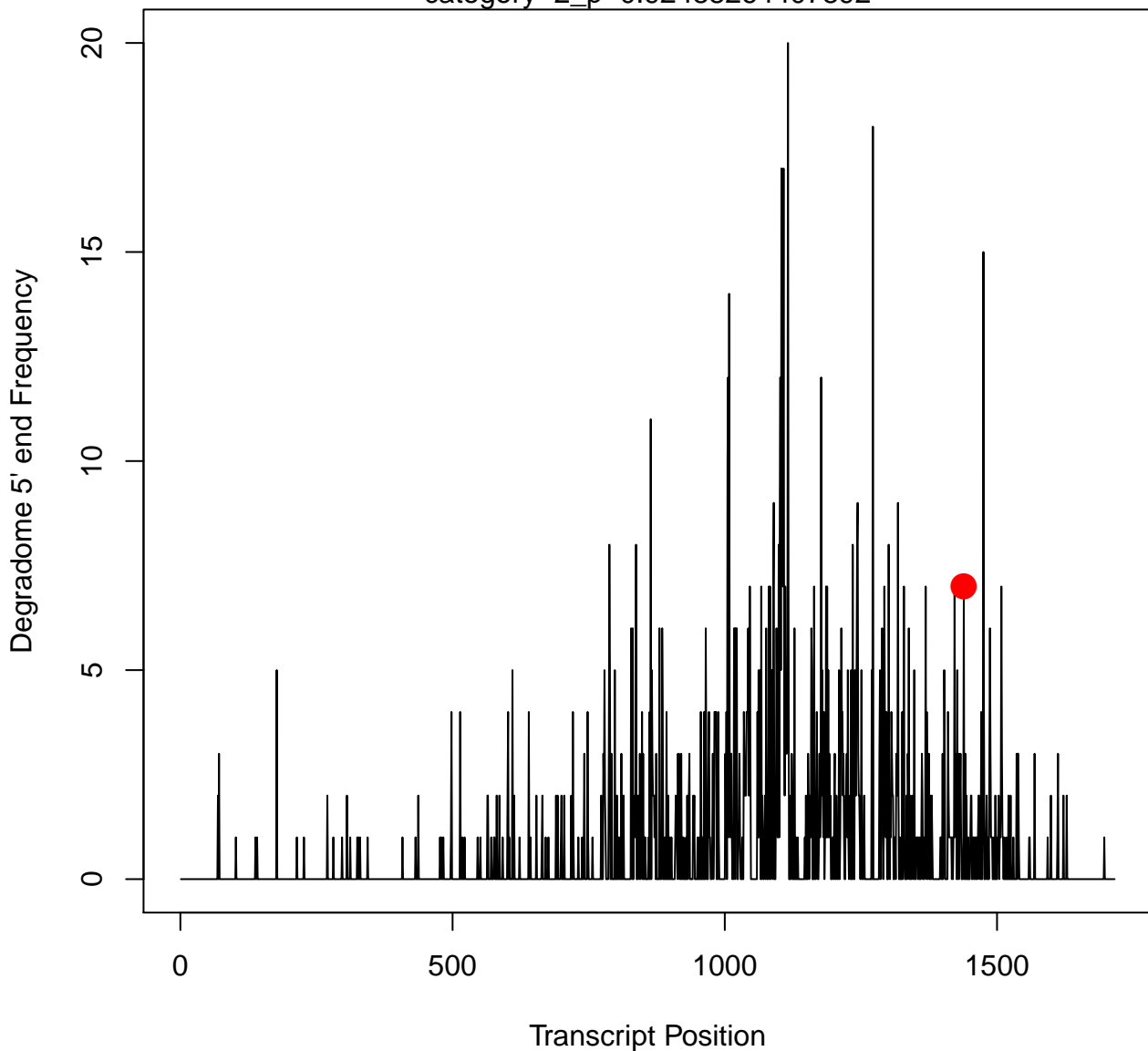

Supplement: Supplementary file 1 [file Data_Sheet_1.zip › Sit-miR160b_Seita.2G374100.1_1439_TPlot.pdf]

**T=Seita.4G006100.1\_Q=Sit-miR160b\_S=1393**

category=2\_p=0.999999999999318

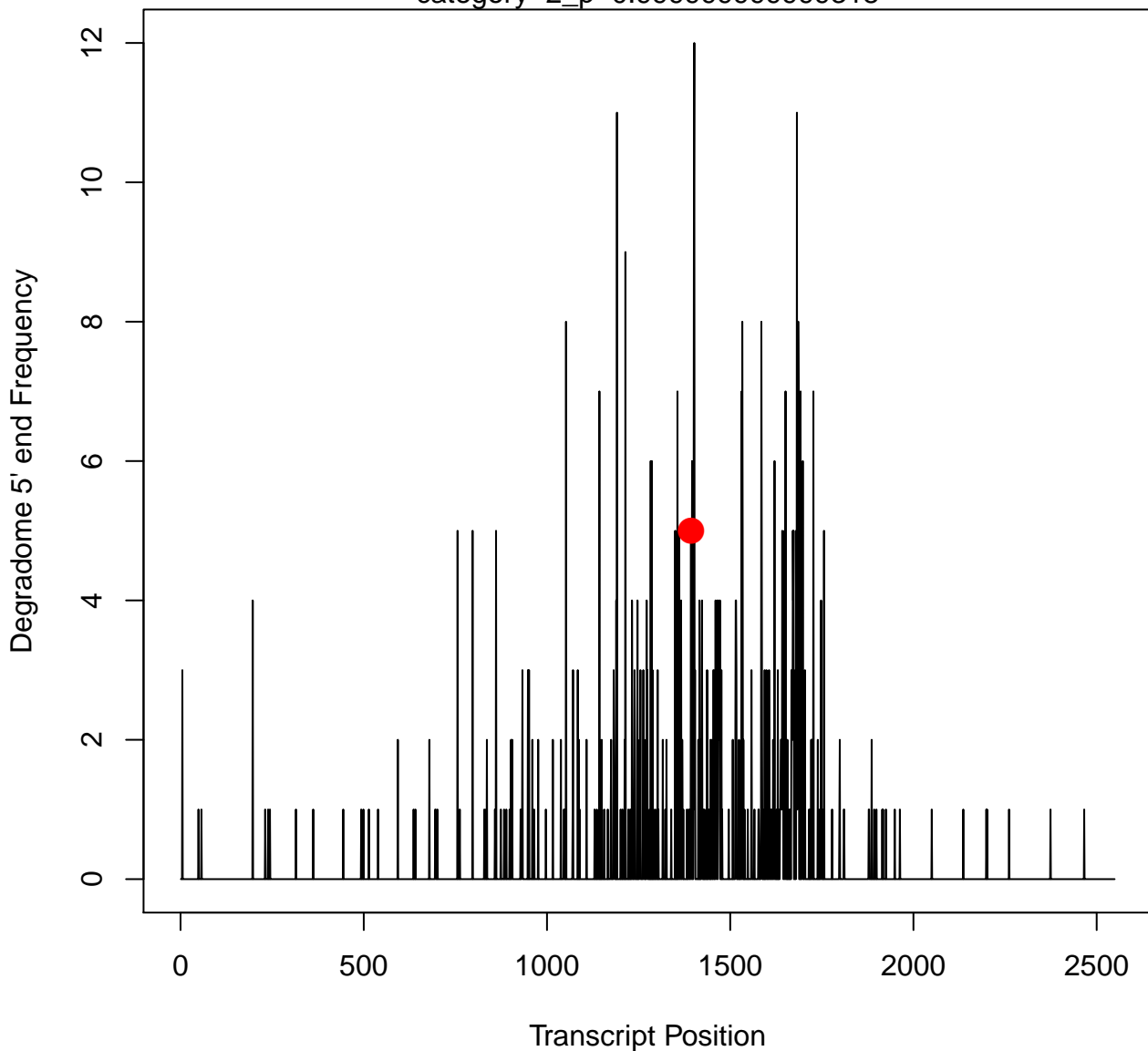

Supplement: Supplementary file 1 [file Data_Sheet_1.zip › Sit-miR160b_Seita.4G006100.1_1393_TPlot.pdf]

**T=Seita.4G090500.1\_Q=Sit-miR160b\_S=945**

category=2\_p=0.999999999895725

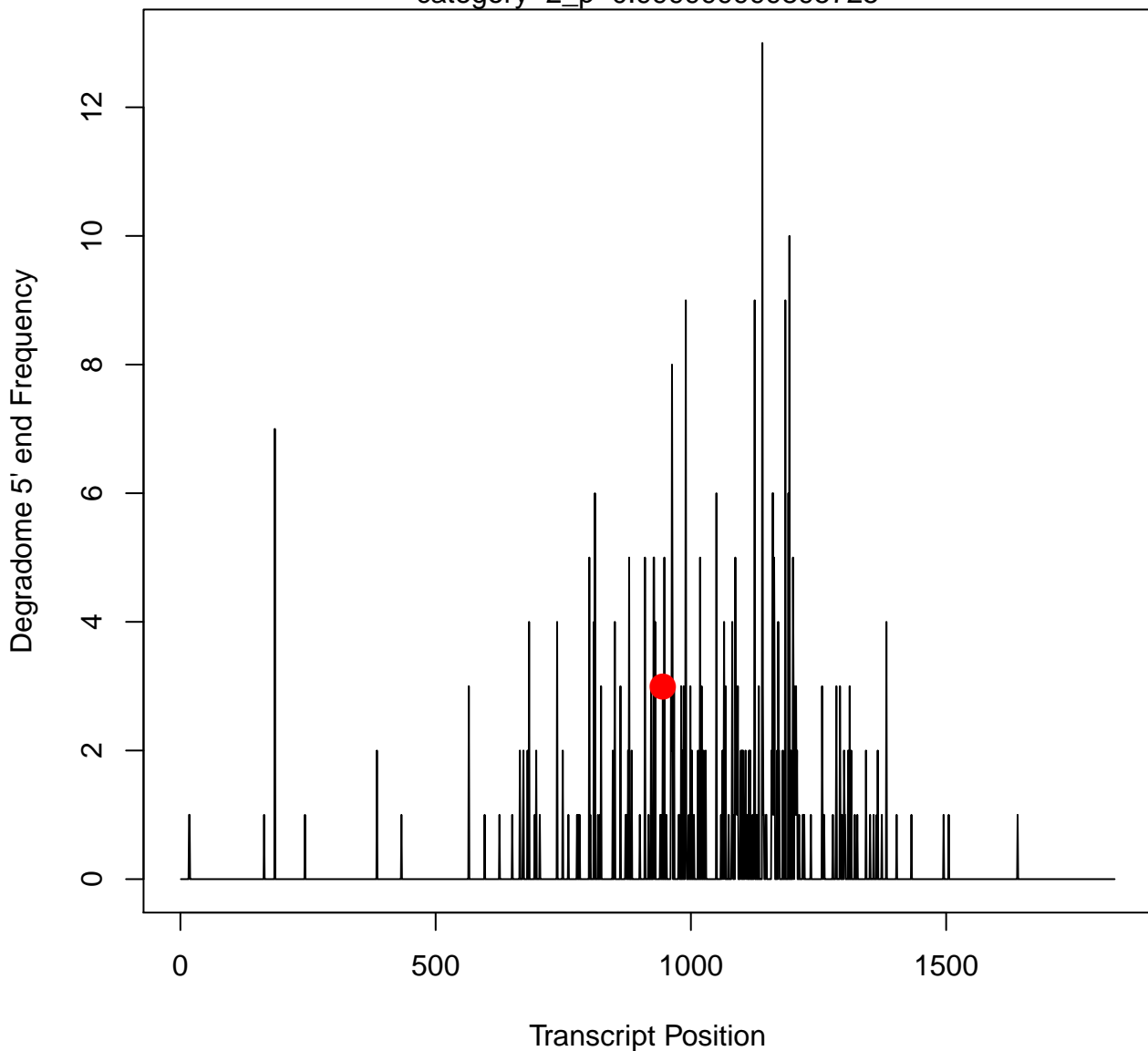

Supplement: Supplementary file 1 [file Data_Sheet_1.zip › Sit-miR160b_Seita.4G090500.1_945_TPlot.pdf]

**T=Seita.4G258300.1\_Q=Sit-miR160b\_S=212**

category=2\_p=0.999999999914917

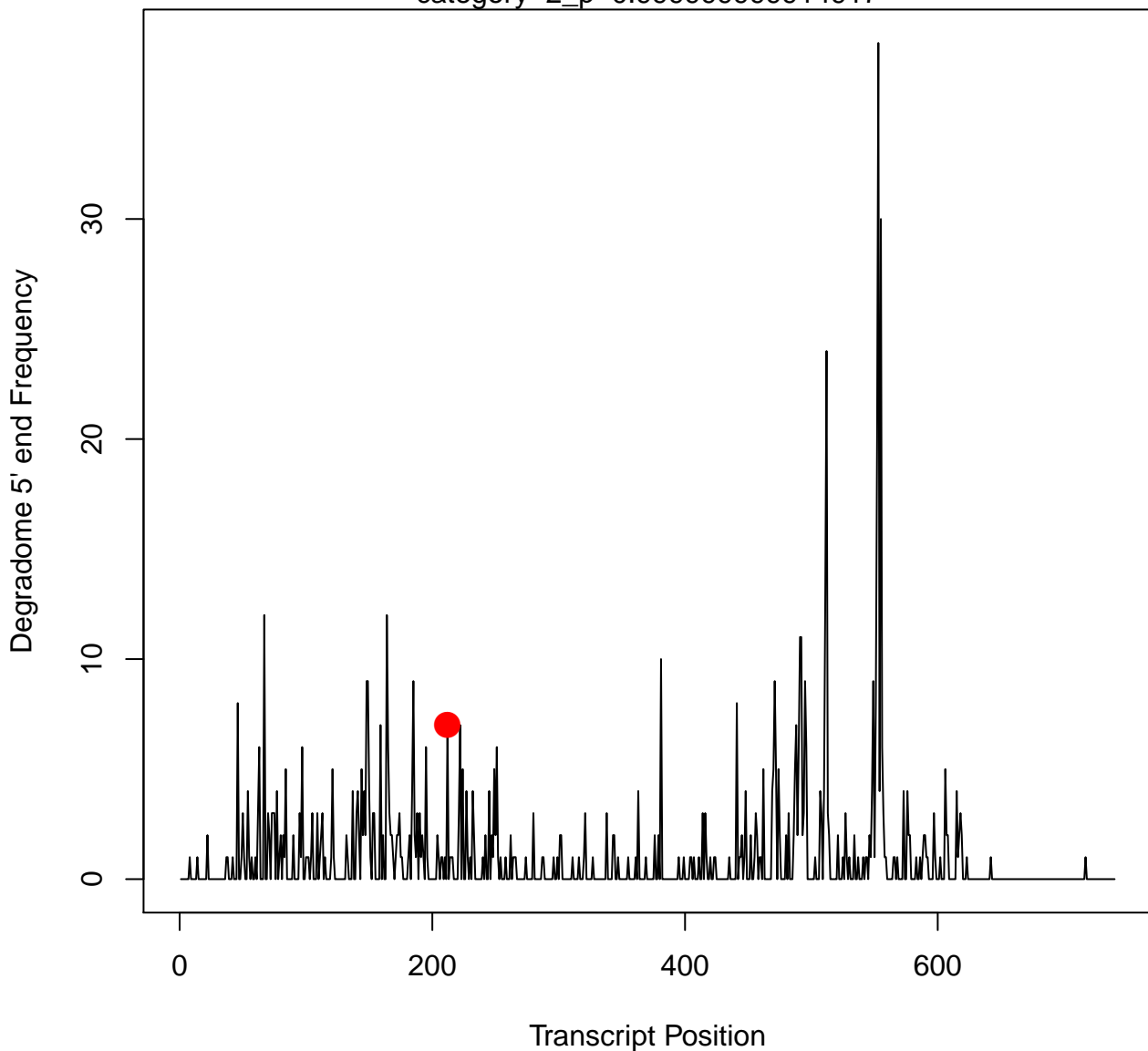

Supplement: Supplementary file 1 [file Data_Sheet_1.zip › Sit-miR160b_Seita.4G258300.1_212_TPlot.pdf]

**T=Seita.5G260400.1\_Q=Sit-miR160b\_S=1308**

category=2\_p=0.999999999997511

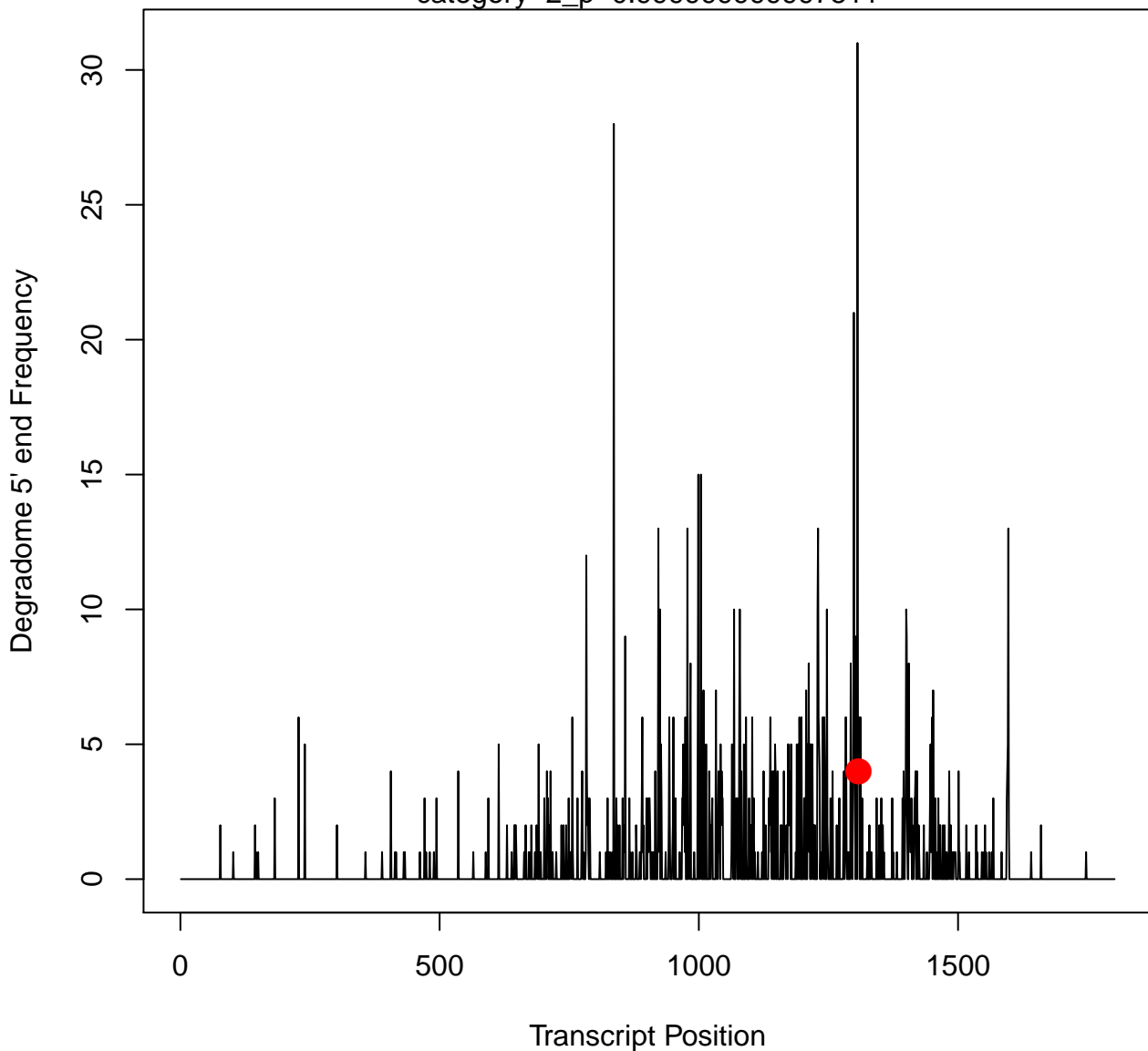

Supplement: Supplementary file 1 [file Data_Sheet_1.zip › Sit-miR160b_Seita.5G260400.1_1308_TPlot.pdf]

**T=Seita.7G182800.1\_Q=Sit-miR160b\_S=514**

category=2\_p=0.999999999999132

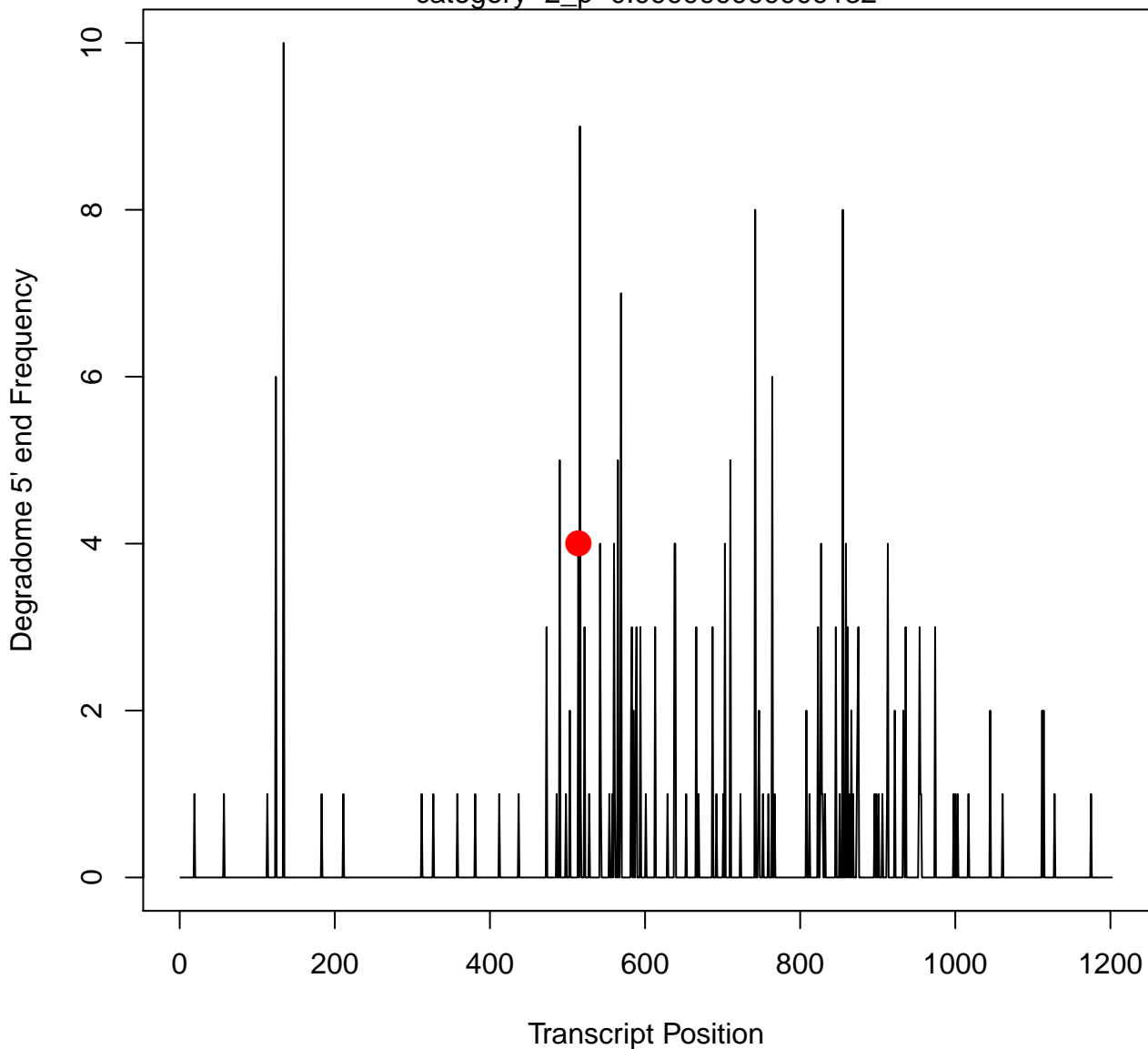

Supplement: Supplementary file 1 [file Data_Sheet_1.zip › Sit-miR160b_Seita.7G182800.1_514_TPlot.pdf]

**T=Seita.7G262800.1\_Q=Sit-miR160b\_S=1155**

category=2\_p=0.99999999999998

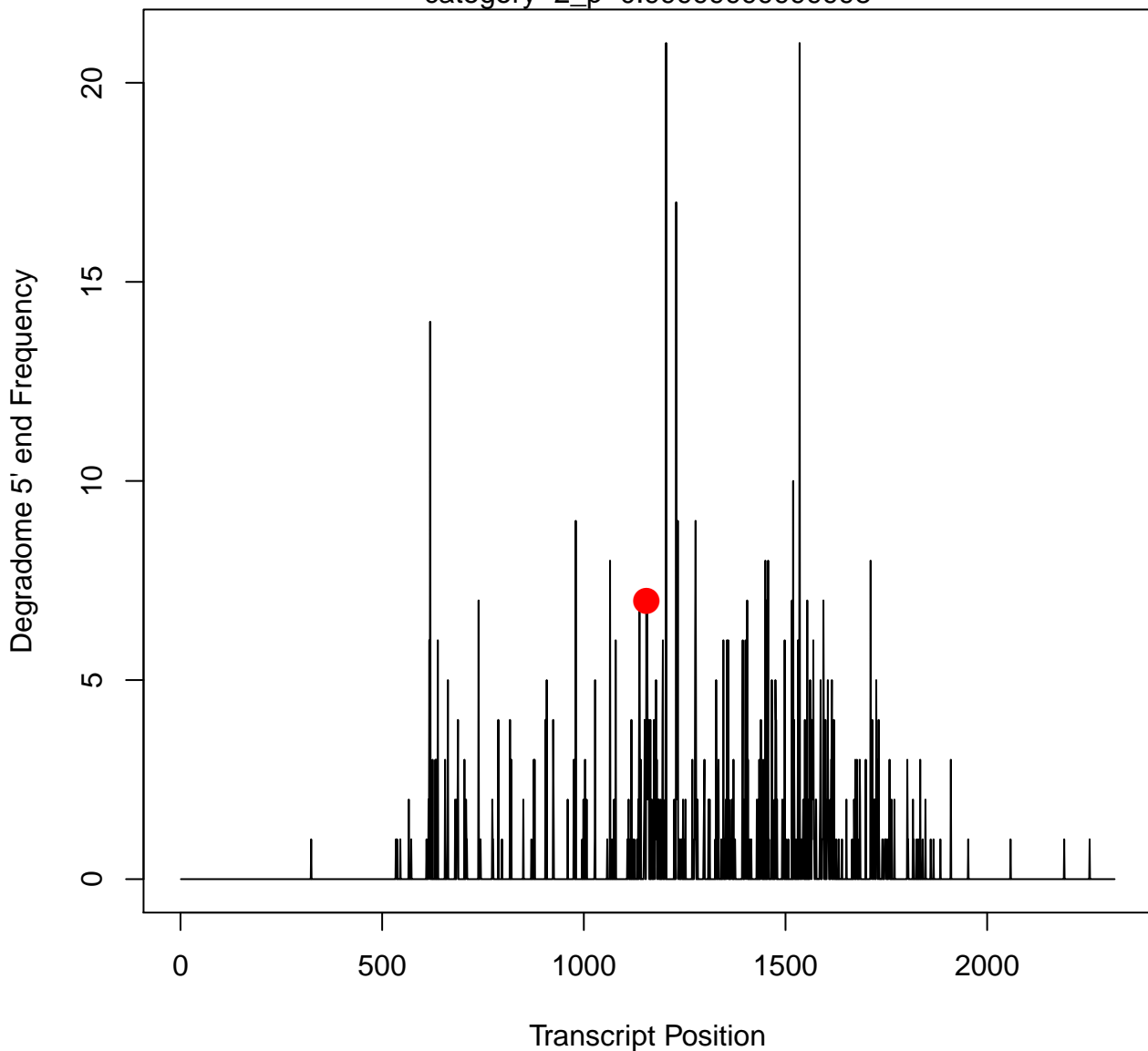

Supplement: Supplementary file 1 [file Data_Sheet_1.zip › Sit-miR160b_Seita.7G262800.1_1155_TPlot.pdf]

**T=Seita.9G127600.1\_Q=Sit-miR160b\_S=2497**

category=2\_p=0.999999976929106

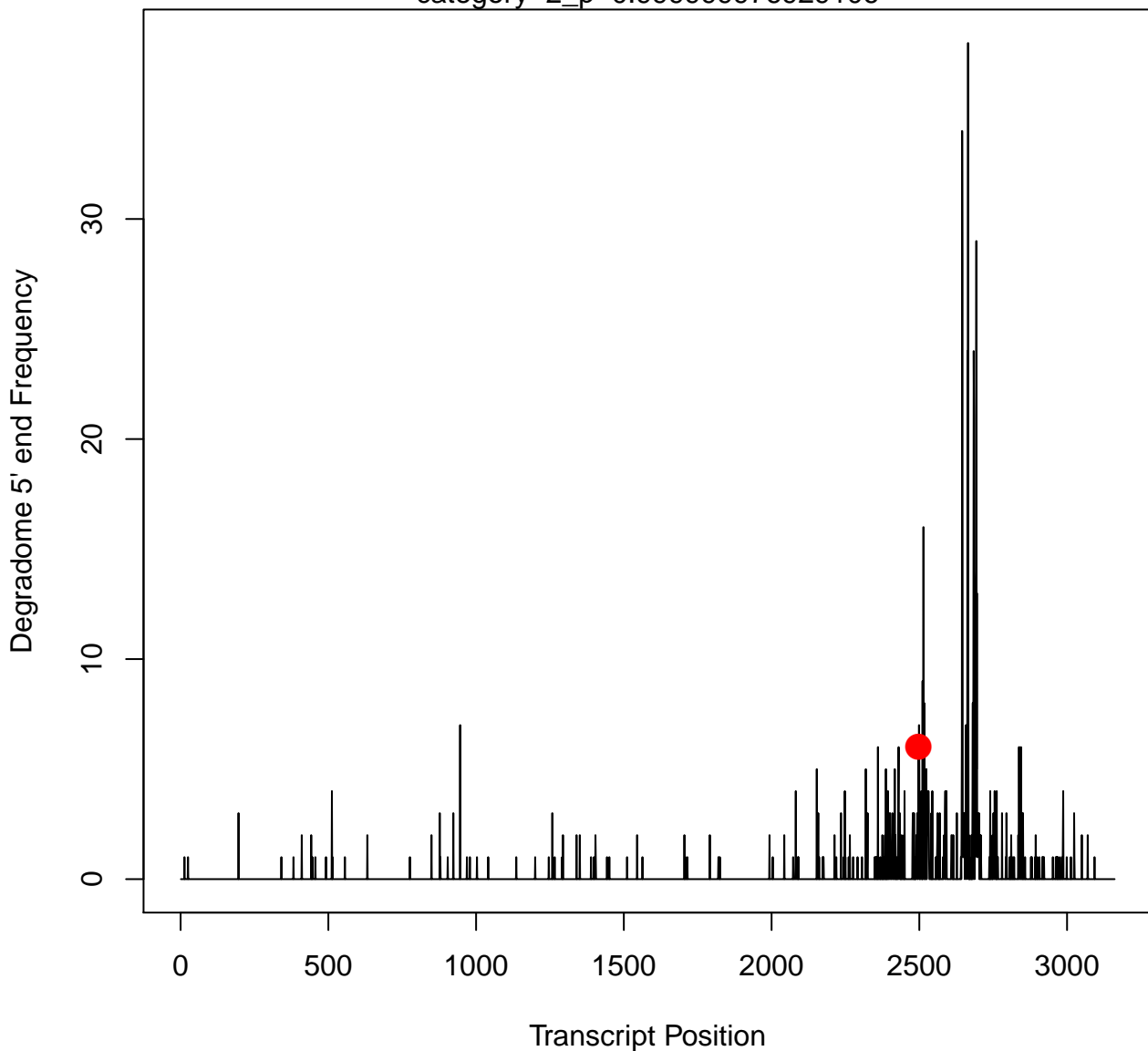

Supplement: Supplementary file 1 [file Data_Sheet_1.zip › Sit-miR160b_Seita.9G127600.1_2497_TPlot.pdf]

**T=Seita.9G127700.1\_Q=Sit-miR160b\_S=2456**

category=2\_p=0.999999979351816

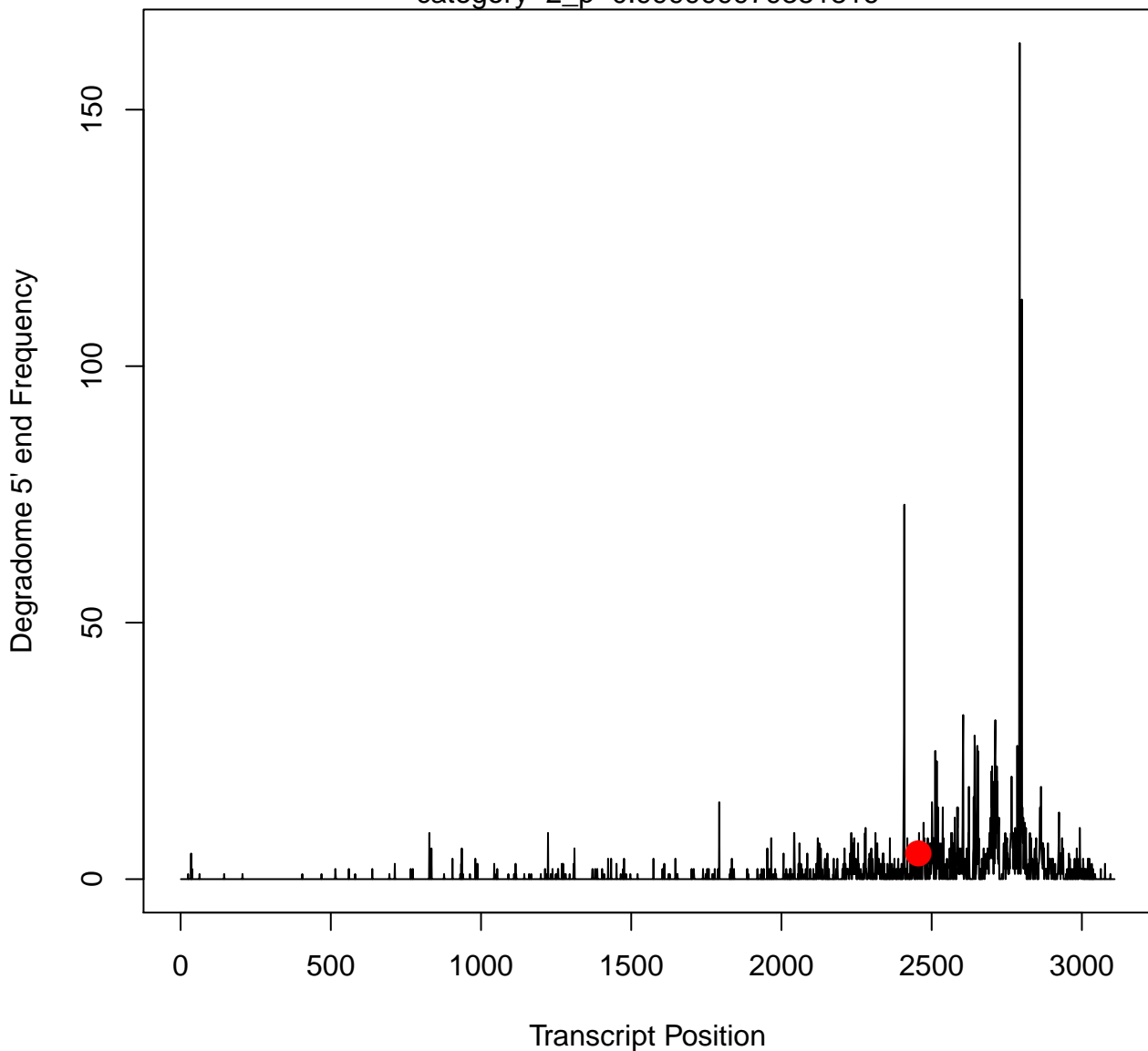

Supplement: Supplementary file 1 [file Data_Sheet_1.zip › Sit-miR160b_Seita.9G127700.1_2456_TPlot.pdf]

**T=Seita.9G127800.1\_Q=Sit-miR160b\_S=2313**

category=2\_p=0.999999999995246

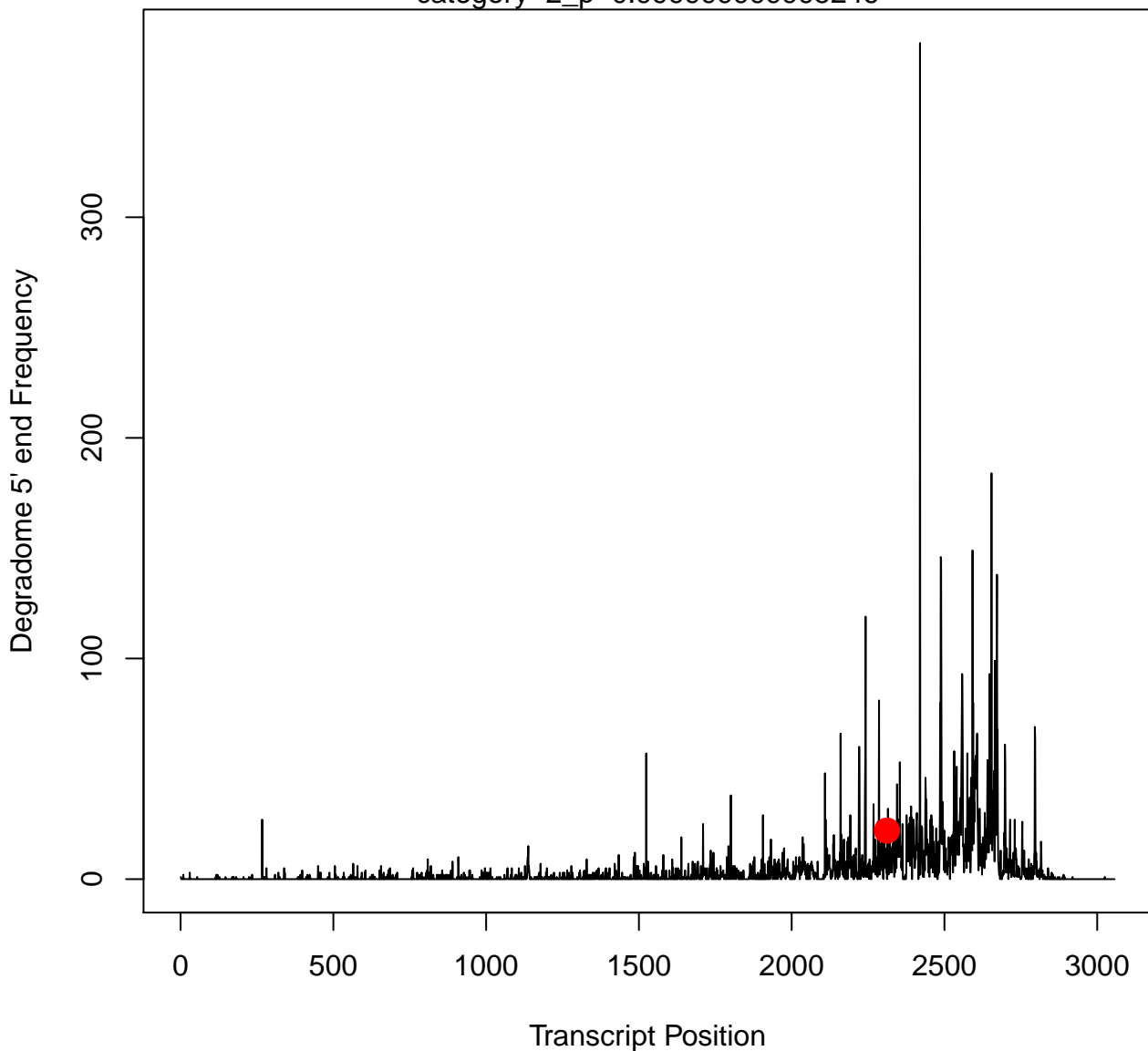

Supplement: Supplementary file 1 [file Data_Sheet_1.zip › Sit-miR160b_Seita.9G127800.1_2313_TPlot.pdf]

**T=Seita.9G219800.1\_Q=Sit-miR160b\_S=1729**

category=0\_p=0.000437760109239571

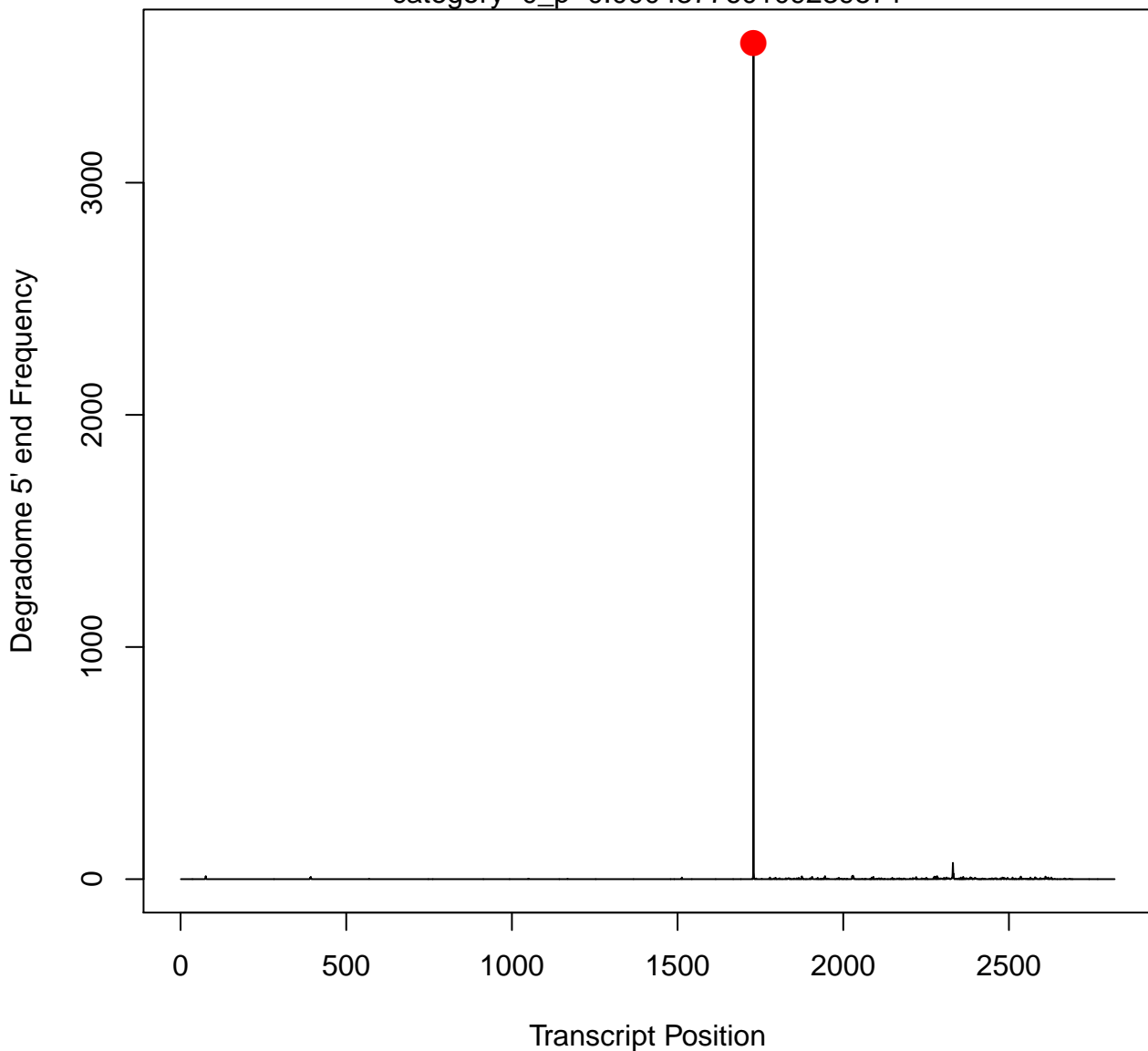

Supplement: Supplementary file 1 [file Data_Sheet_1.zip › Sit-miR160b_Seita.9G219800.1_1729_TPlot.pdf]
