# Supplementary figures and images for: The Construction and Exploration of a Comprehensive MicroRNA Centered Regulatory Network in Foxtail Millet (Setaria italica L.) (part 4 of 14)
Source: Front Plant Sci. 2022 May 6;13:848474. doi: 10.3389/fpls.2022.848474 (PMC9121102; doi:10.3389/fpls.2022.848474)

**T=Seita.6G026800.1\_Q=Sit-miR159b\_S=1459**

category=2\_p=0.999995945846198

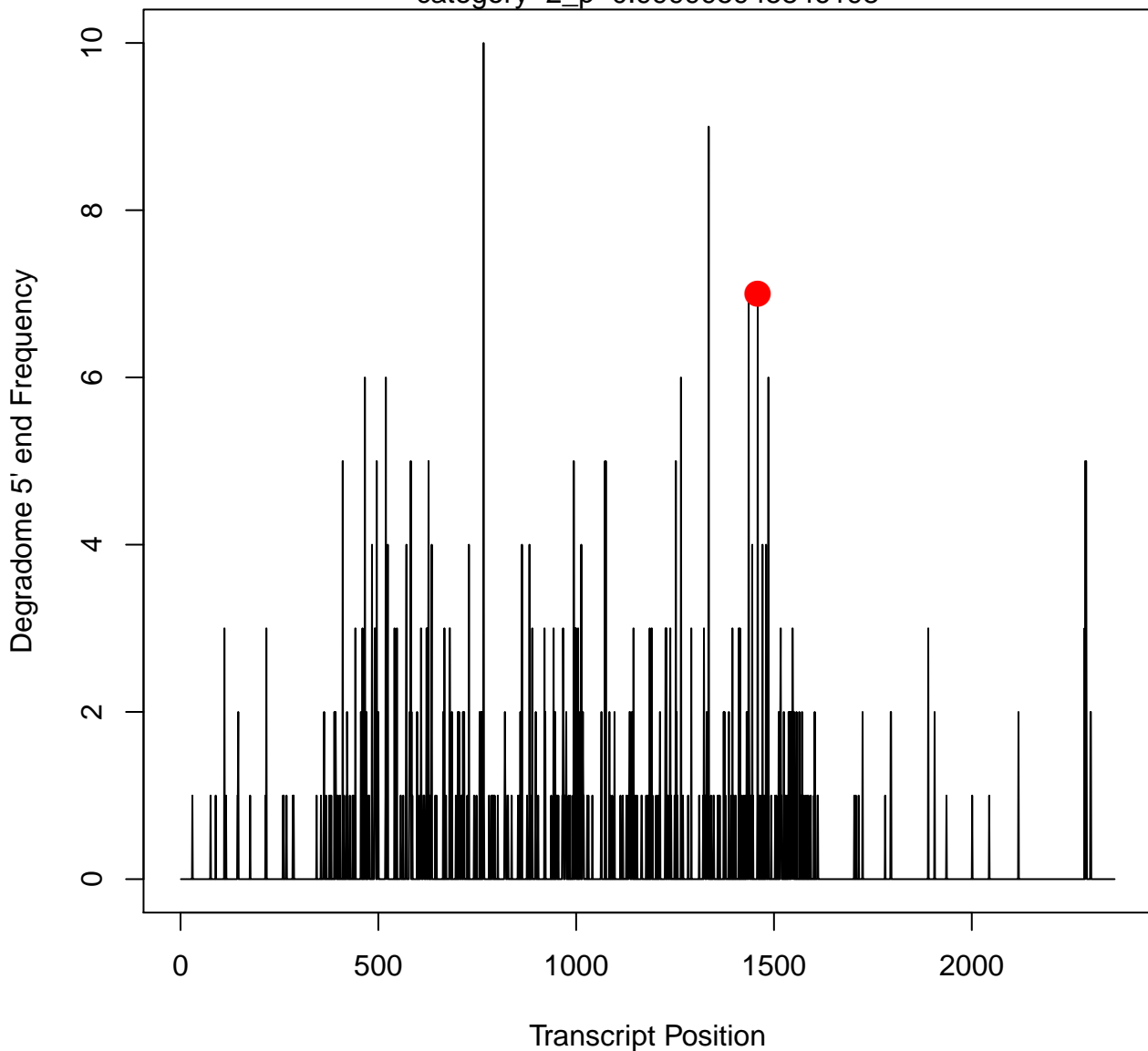

Supplement: Supplementary file 2 [file Data_Sheet_2.zip › Sit-miR159b_Seita.6G026800.1_1459_TPlot.pdf]

**T=Seita.6G091300.1\_Q=Sit-miR159b\_S=1708**

category=2\_p=0.994347140371361

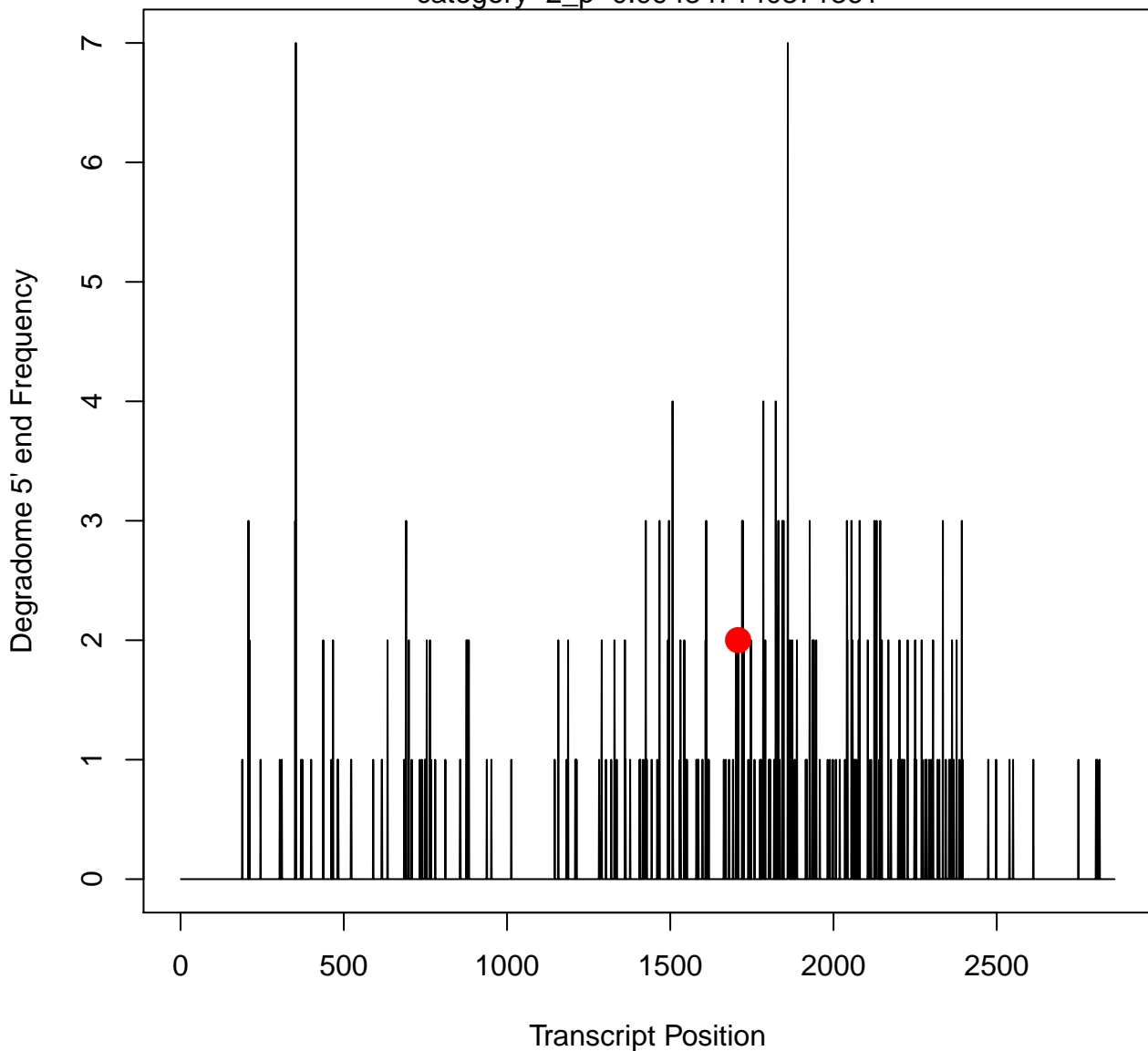

Supplement: Supplementary file 2 [file Data_Sheet_2.zip › Sit-miR159b_Seita.6G091300.1_1708_TPlot.pdf]

**T=Seita.7G067100.1\_Q=Sit-miR159b\_S=590**

category=2\_p=0.0813460690731977

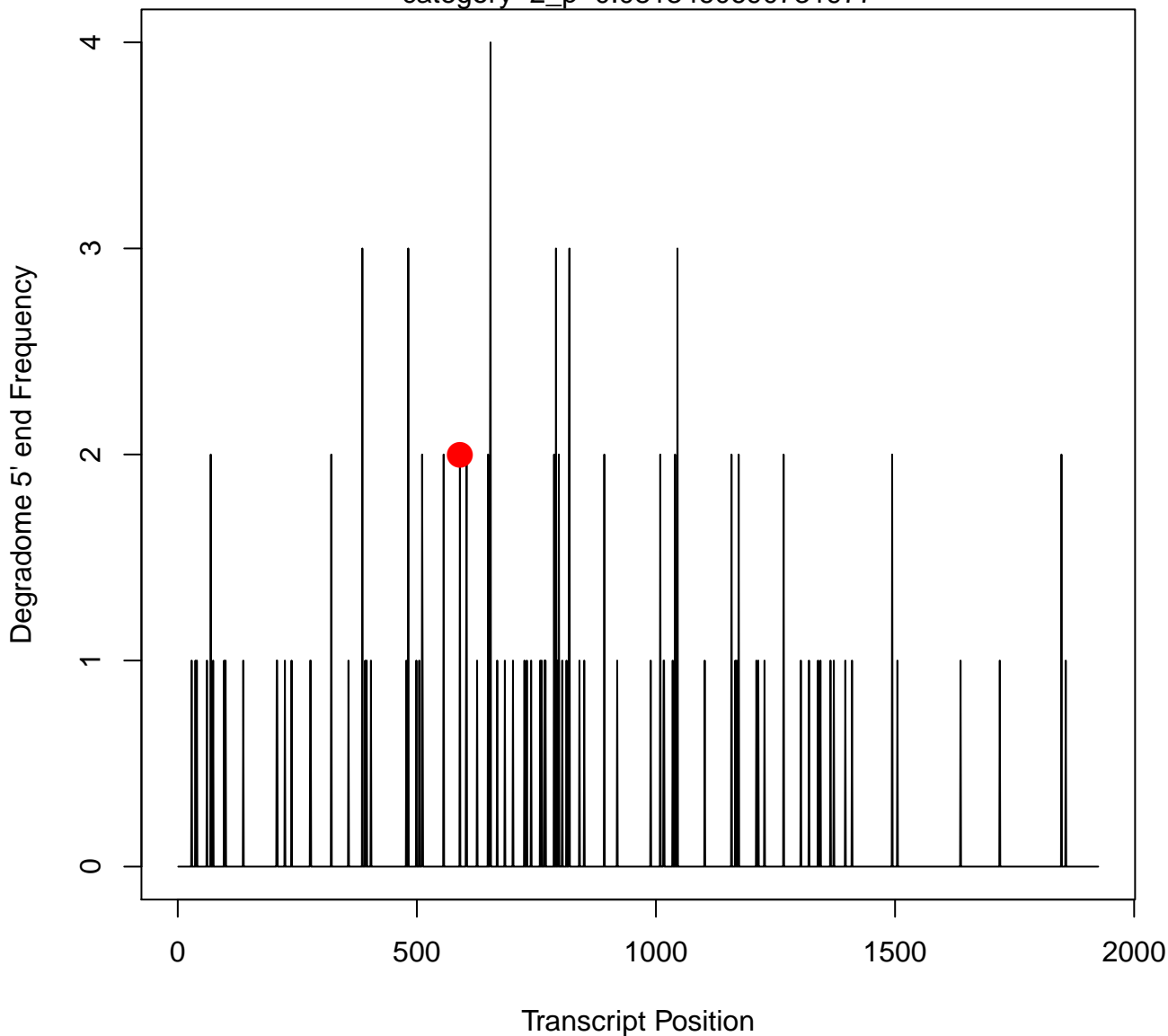

Supplement: Supplementary file 2 [file Data_Sheet_2.zip › Sit-miR159b_Seita.7G067100.1_590_TPlot.pdf]

**T=Seita.7G326700.1\_Q=Sit-miR159b\_S=134**

category=2\_p=0.988536020867649

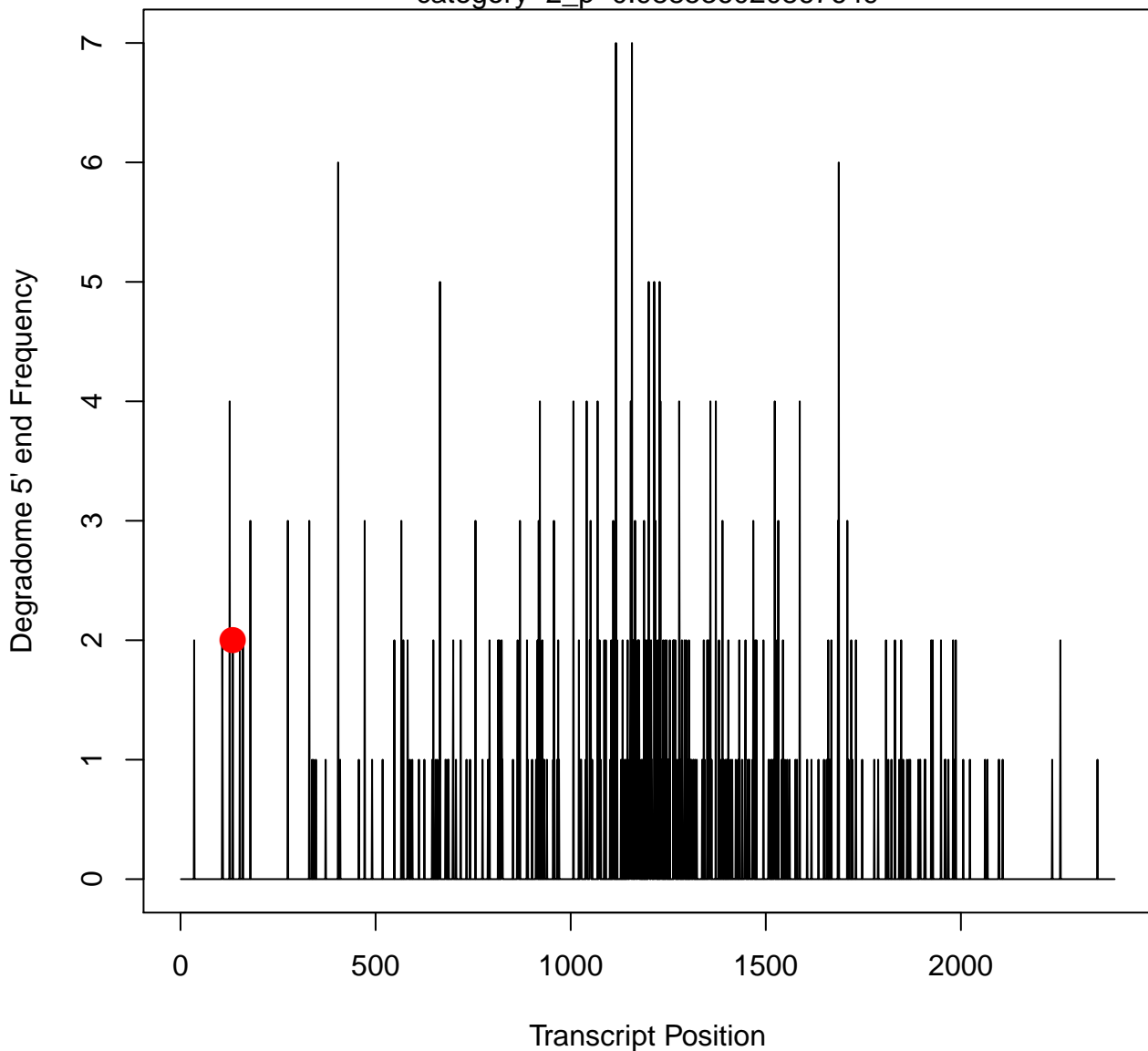

Supplement: Supplementary file 2 [file Data_Sheet_2.zip › Sit-miR159b_Seita.7G326700.1_134_TPlot.pdf]

**T=Seita.9G063000.1\_Q=Sit-miR159b\_S=224**

category=0\_p=0.116756774743294

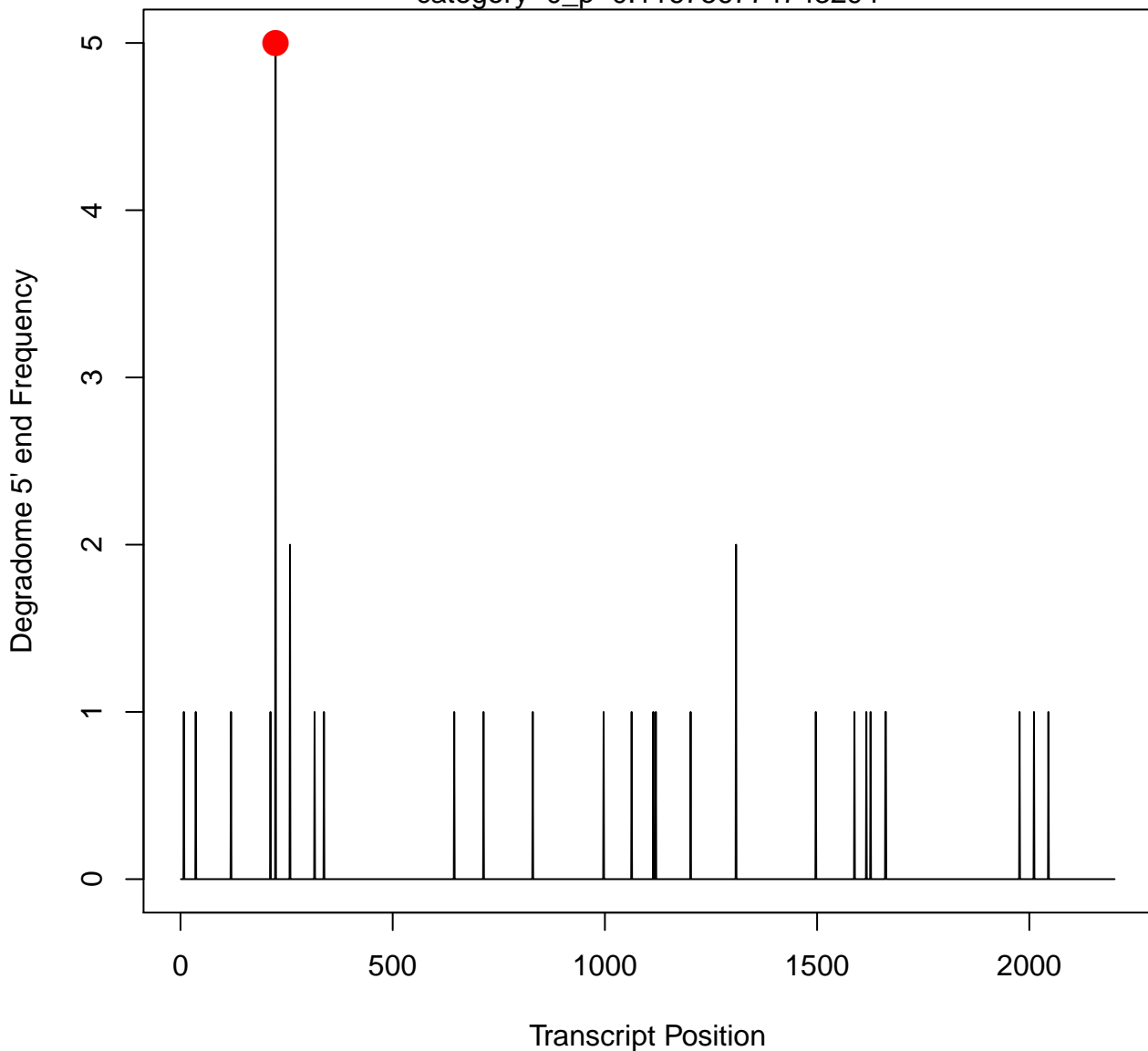

Supplement: Supplementary file 2 [file Data_Sheet_2.zip › Sit-miR159b_Seita.9G063000.1_224_TPlot.pdf]

**T=Seita.9G064600.1\_Q=Sit-miR159b\_S=1006**

category=2\_p=0.895915752674564

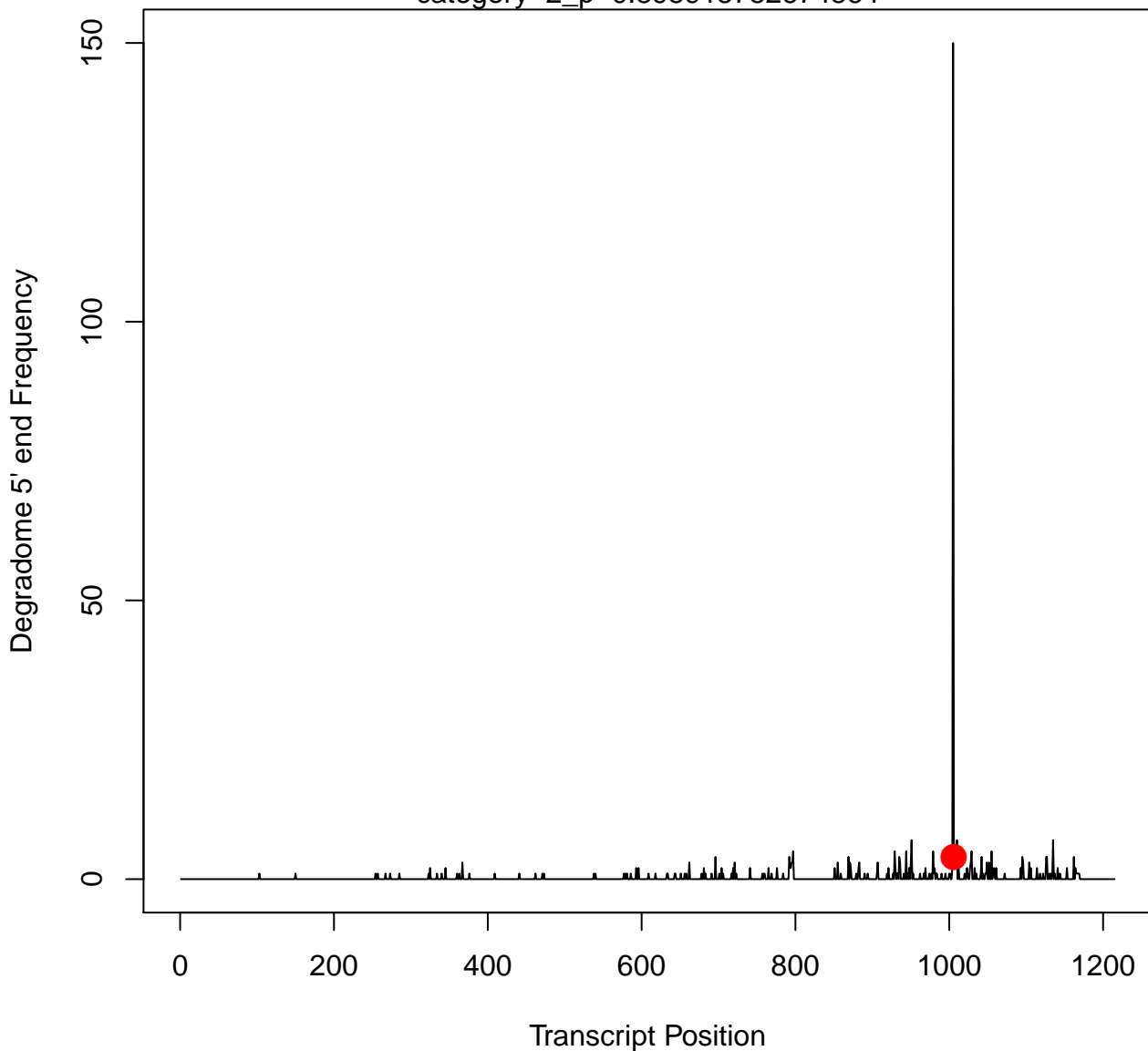

Supplement: Supplementary file 2 [file Data_Sheet_2.zip › Sit-miR159b_Seita.9G064600.1_1006_TPlot.pdf]

**T=Seita.9G181600.1\_Q=Sit-miR159b\_S=1283**

category=2\_p=0.179608432013757

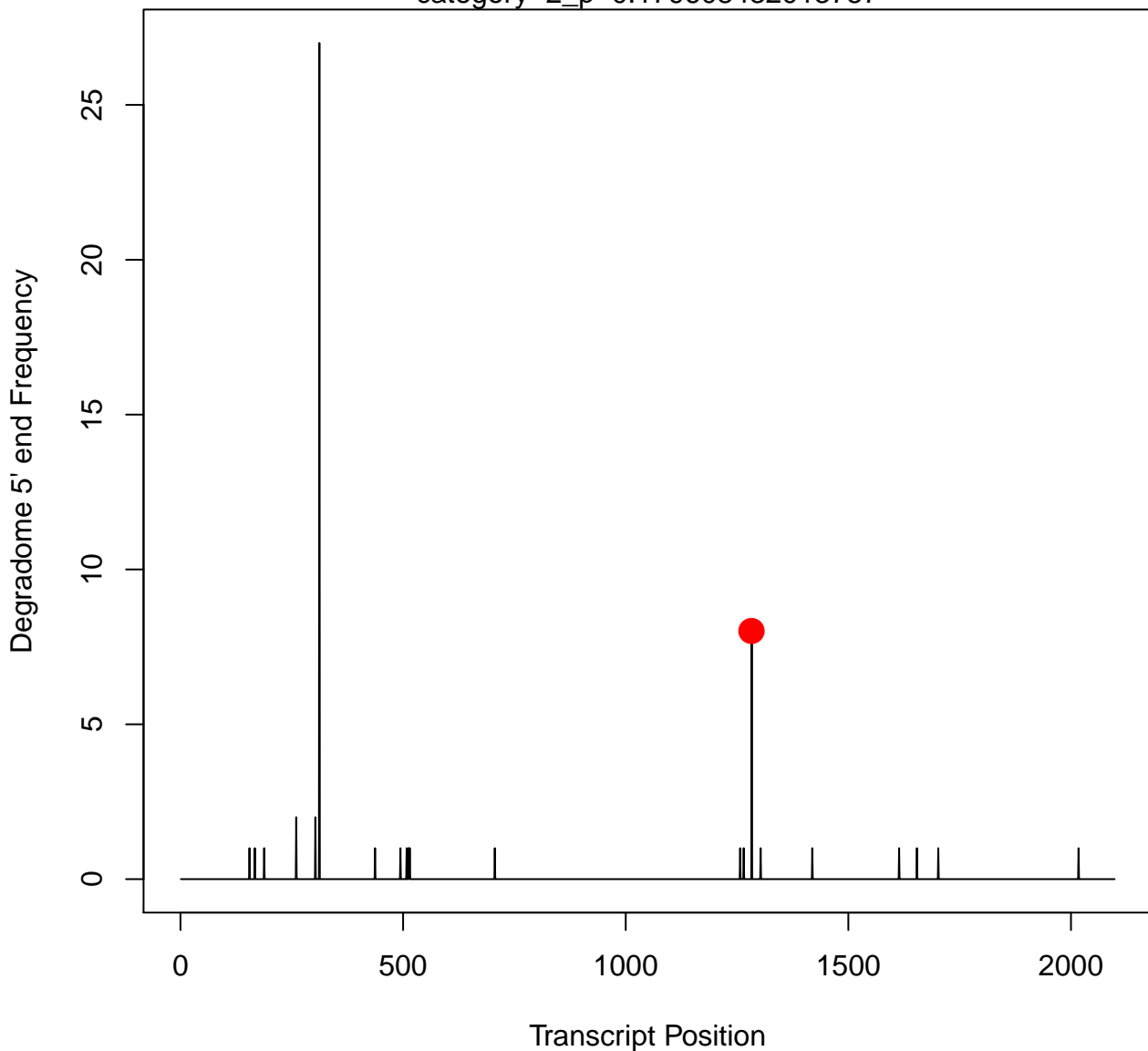

Supplement: Supplementary file 2 [file Data_Sheet_2.zip › Sit-miR159b_Seita.9G181600.1_1283_TPlot.pdf]

**T=Seita.9G188600.1\_Q=Sit-miR159b\_S=4508**

category=2\_p=0.727732139623031

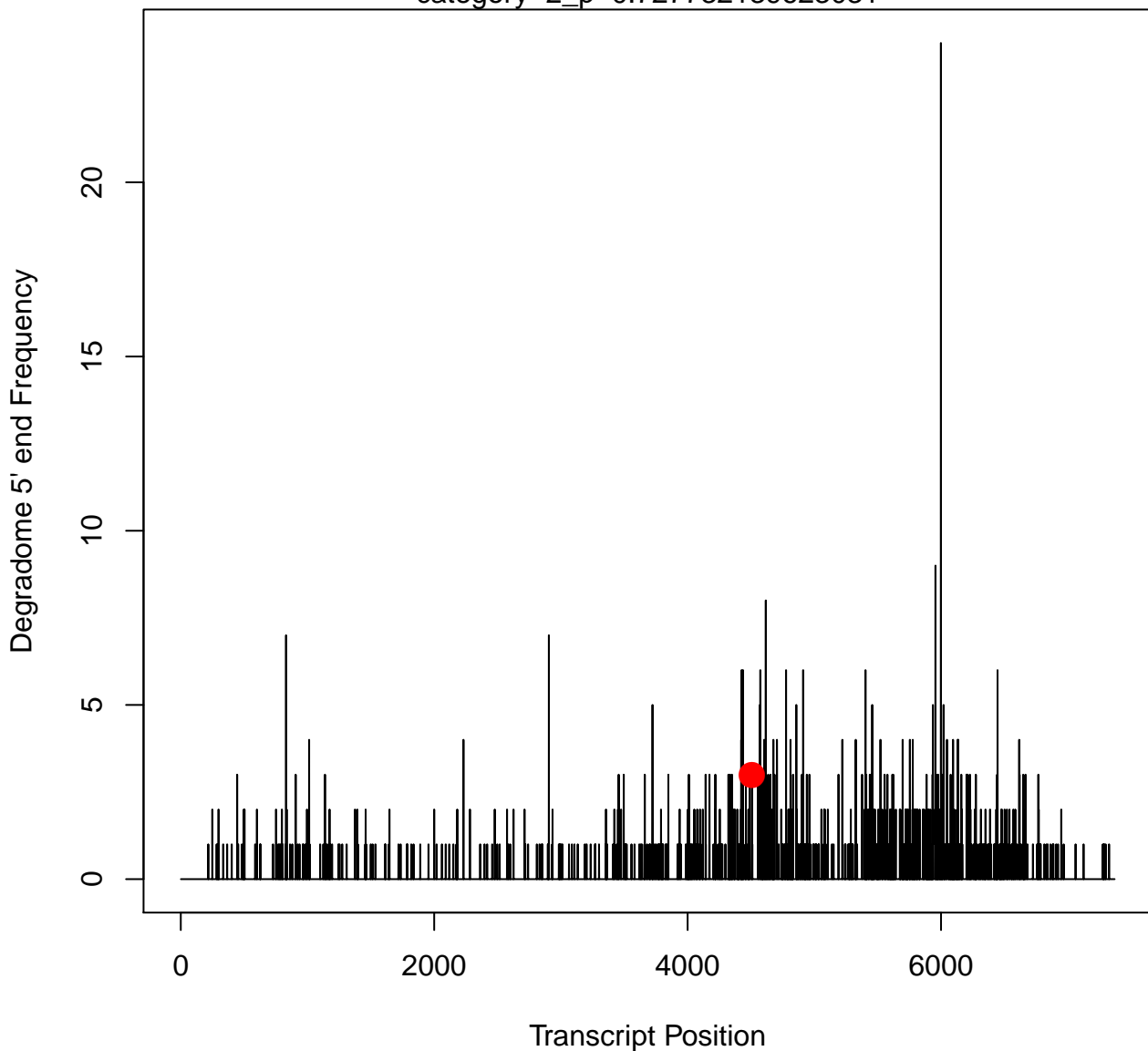

Supplement: Supplementary file 2 [file Data_Sheet_2.zip › Sit-miR159b_Seita.9G188600.1_4508_TPlot.pdf]

**T=Seita.9G439200.1\_Q=Sit-miR159b\_S=131**

category=2\_p=0.999965215604152

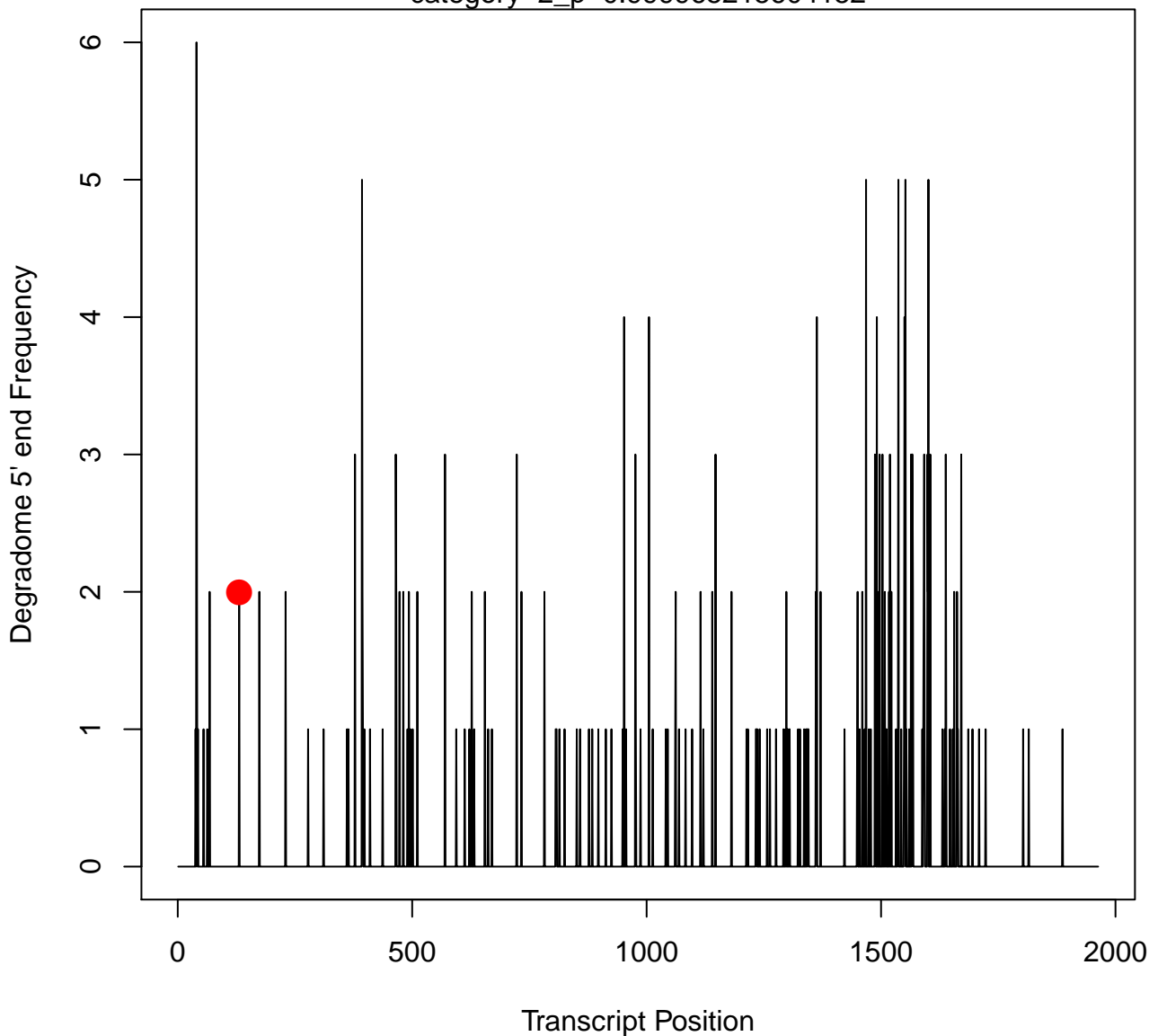

Supplement: Supplementary file 2 [file Data_Sheet_2.zip › Sit-miR159b_Seita.9G439200.1_131_TPlot.pdf]

**T=Seita.9G534000.1\_Q=Sit-miR159b\_S=2152**

category=2\_p=0.999999753444801

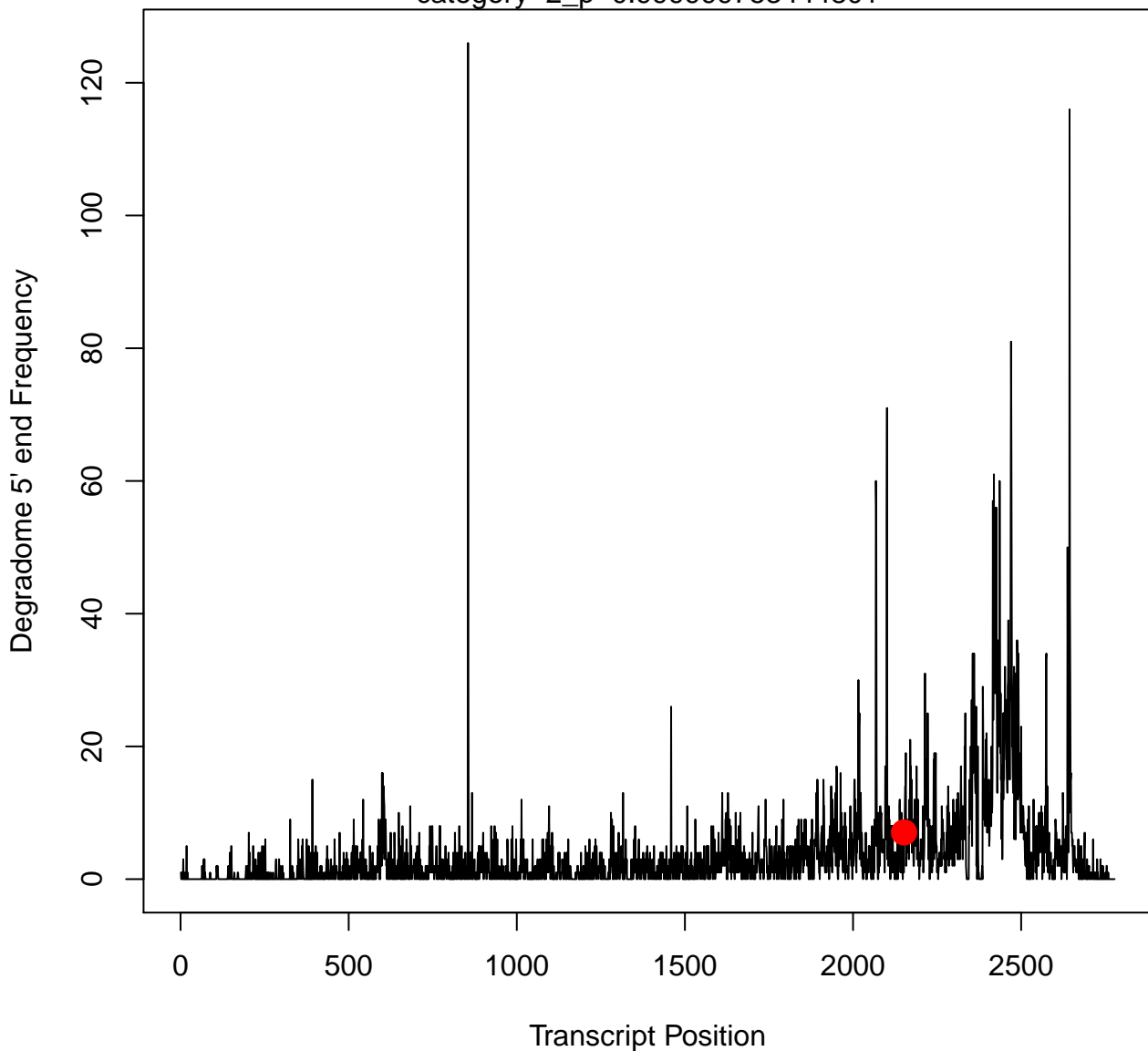

Supplement: Supplementary file 2 [file Data_Sheet_2.zip › Sit-miR159b_Seita.9G534000.1_2152_TPlot.pdf]

**T=Seita.4G221900.1\_Q=Sit-miR159c\_S=1159**

category=2\_p=0.0549938711306051

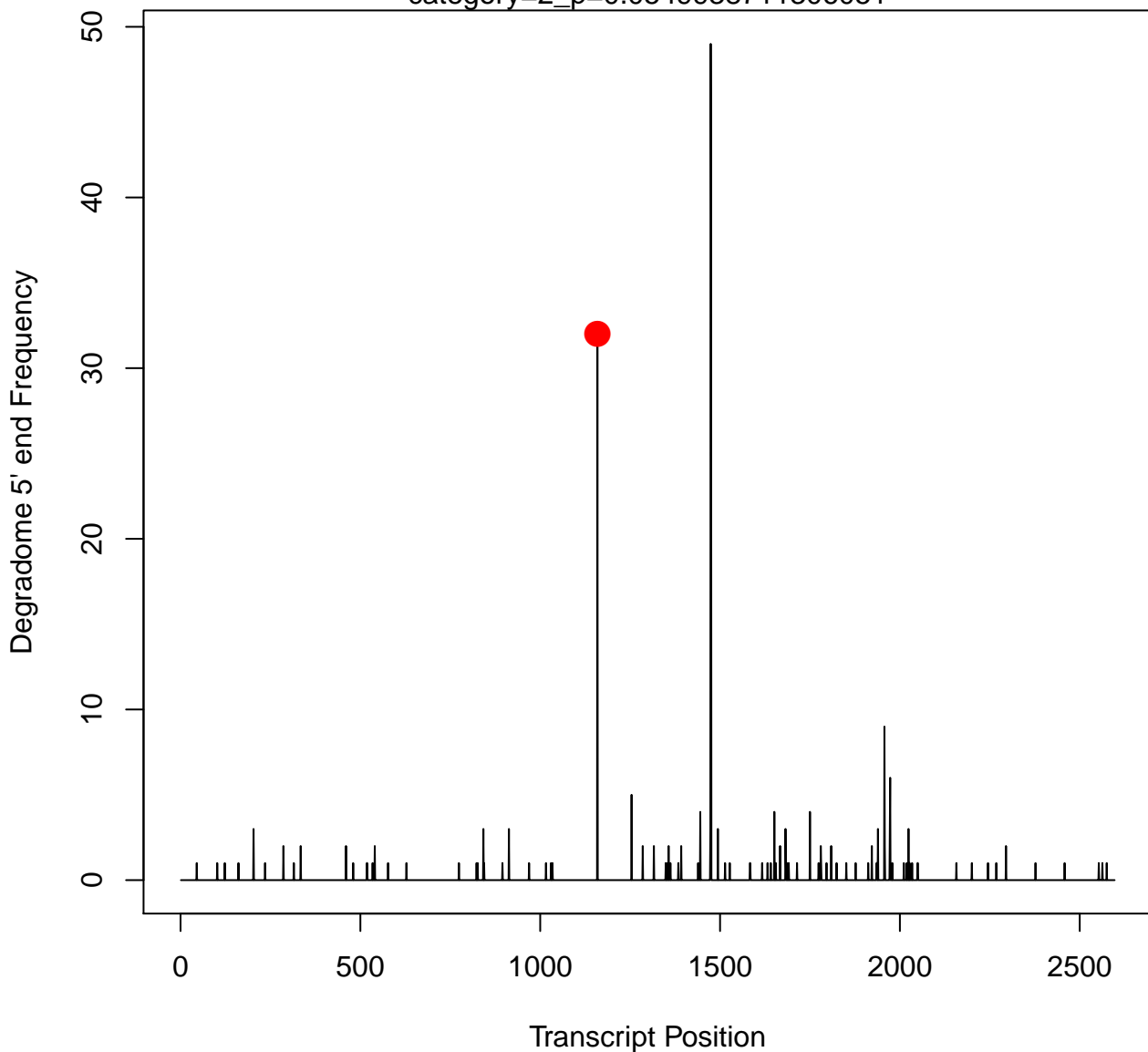

Supplement: Supplementary file 2 [file Data_Sheet_2.zip › Sit-miR159c_Seita.4G221900.1_1159_TPlot.pdf]

**T=Seita.4G232800.1\_Q=Sit-miR159c\_S=47**

category=2\_p=0.999996766754208

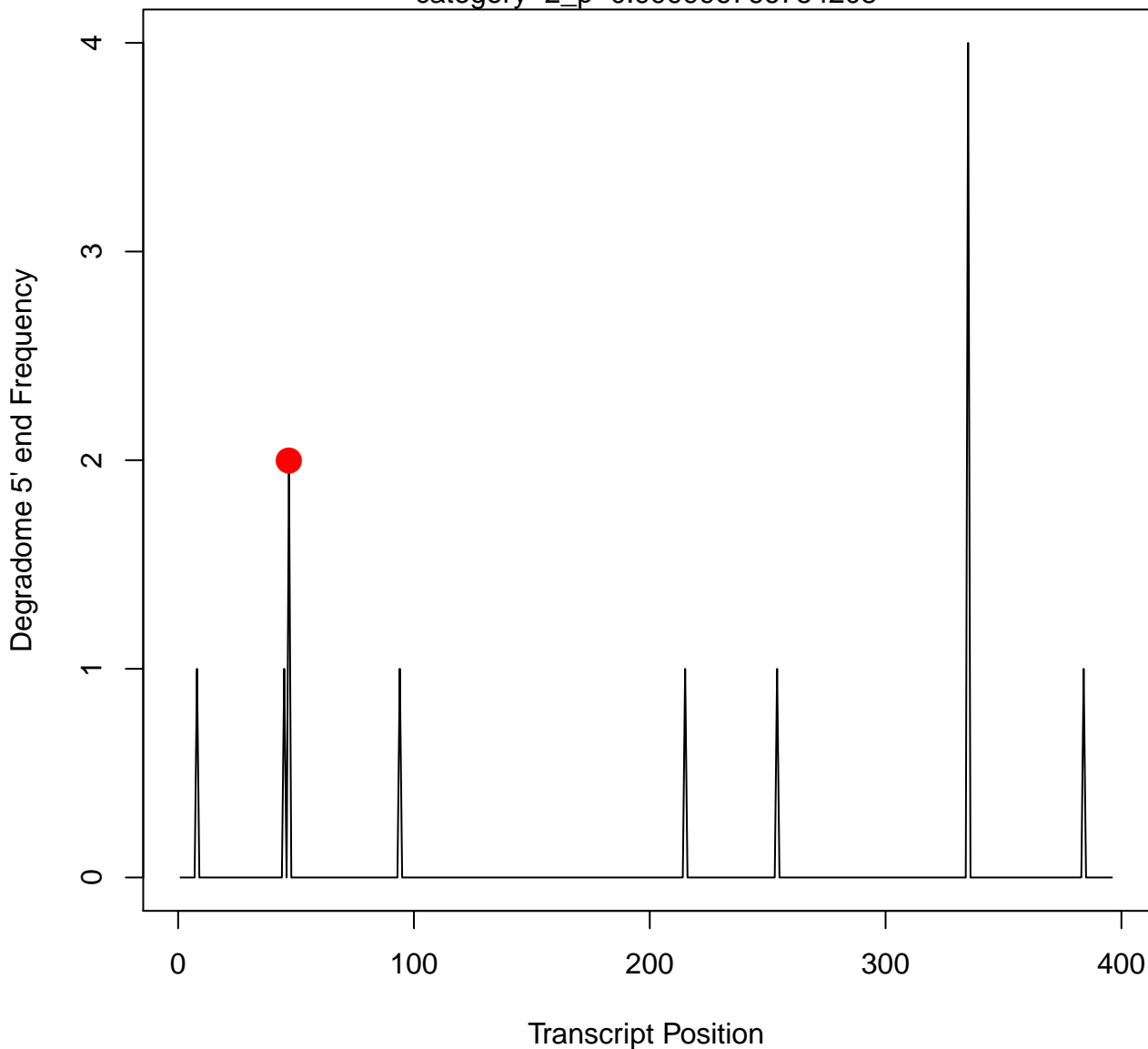

Supplement: Supplementary file 2 [file Data_Sheet_2.zip › Sit-miR159c_Seita.4G232800.1_47_TPlot.pdf]

**T=Seita.5G002500.1\_Q=Sit-miR159c\_S=441**

category=0\_p=0.0160636672179344

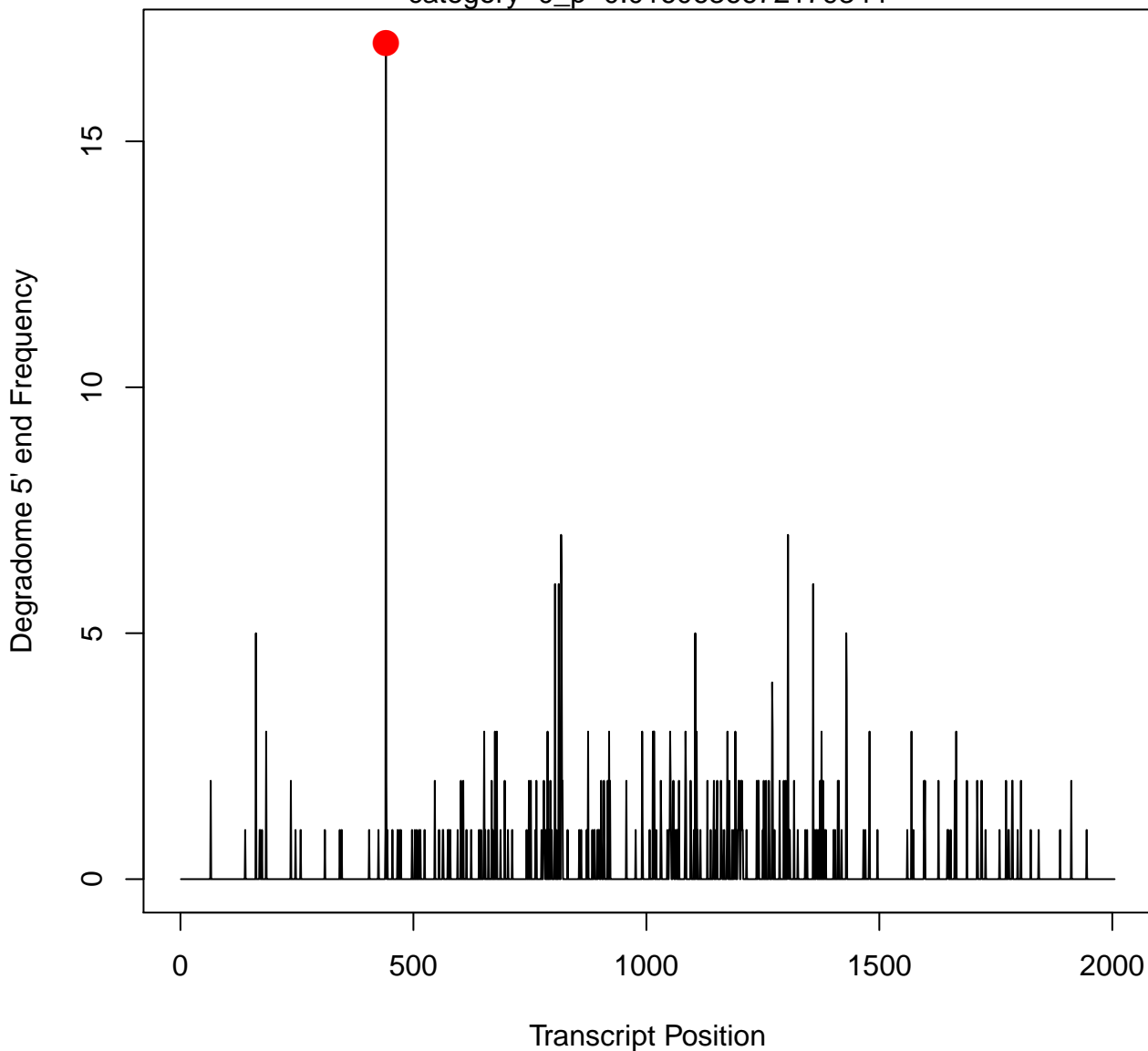

Supplement: Supplementary file 2 [file Data_Sheet_2.zip › Sit-miR159c_Seita.5G002500.1_441_TPlot.pdf]

**T=Seita.5G148600.1\_Q=Sit-miR159c\_S=1382**

category=0\_p=0.00729912729569815

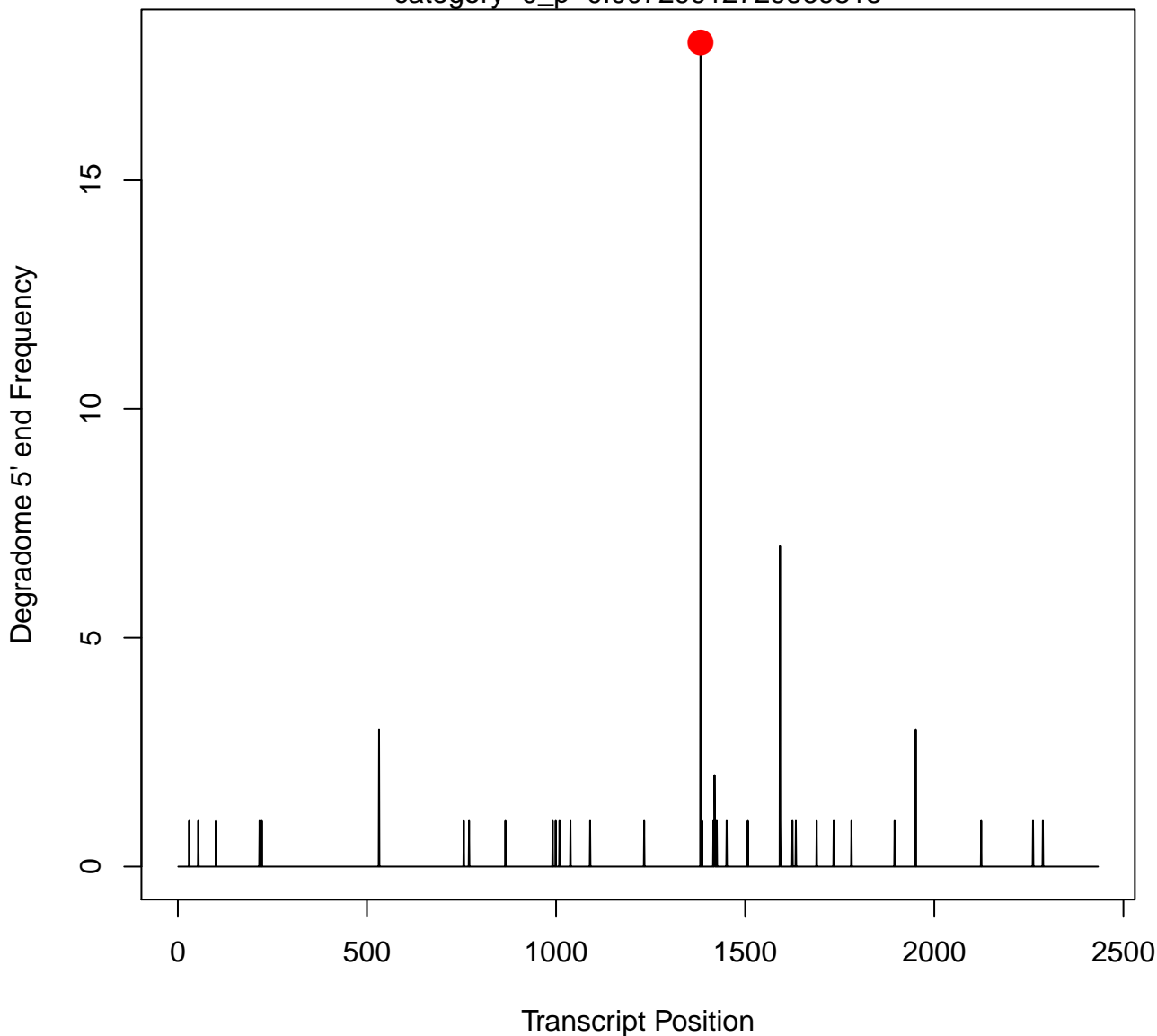

Supplement: Supplementary file 2 [file Data_Sheet_2.zip › Sit-miR159c_Seita.5G148600.1_1382_TPlot.pdf]

**T=Seita.6G235700.1\_Q=Sit-miR159c\_S=1043**

category=2\_p=0.999999859957319

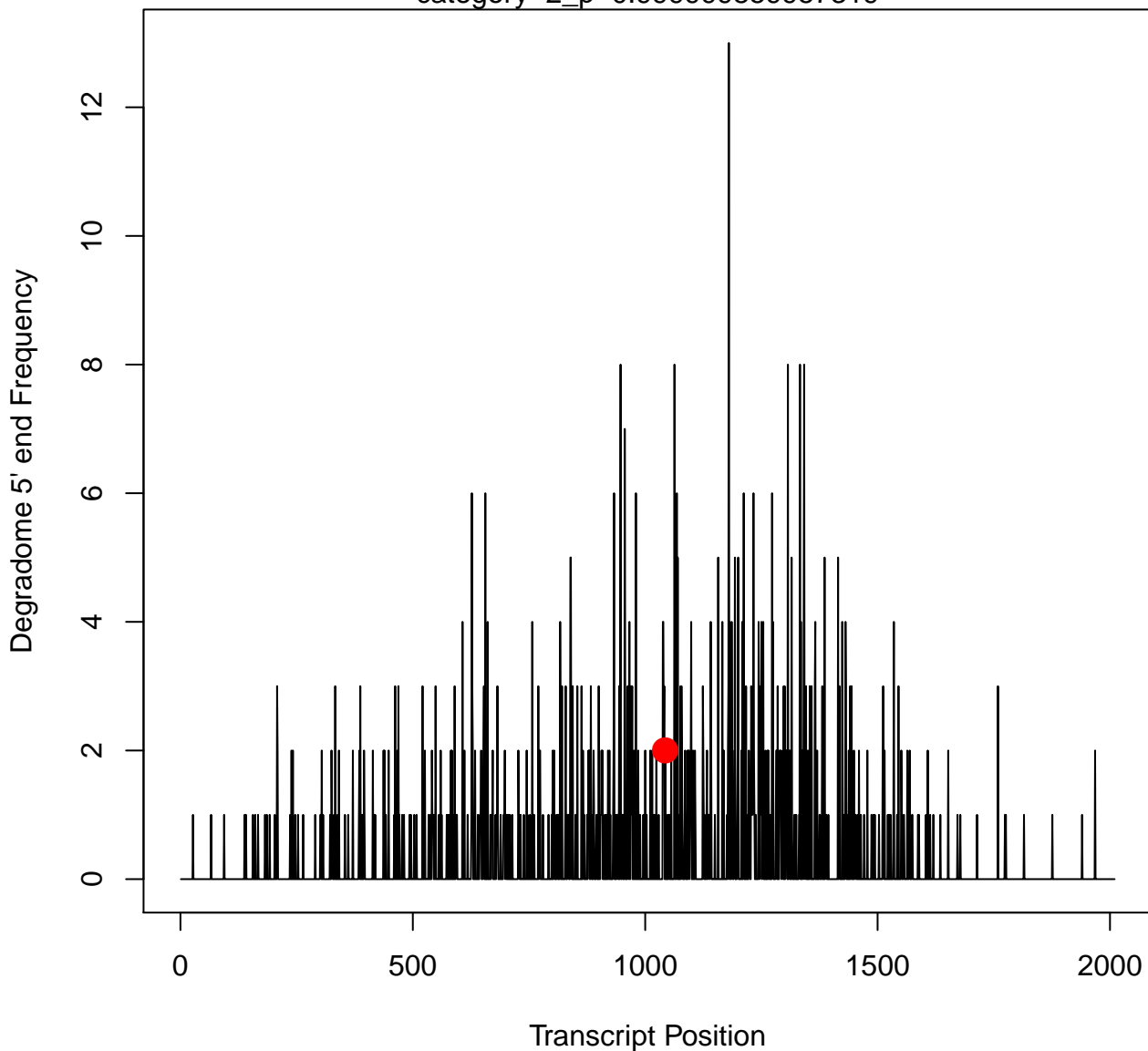

Supplement: Supplementary file 2 [file Data_Sheet_2.zip › Sit-miR159c_Seita.6G235700.1_1043_TPlot.pdf]

**T=Seita.7G006100.1\_Q=Sit-miR159c\_S=784**

category=2\_p=0.901639748358705

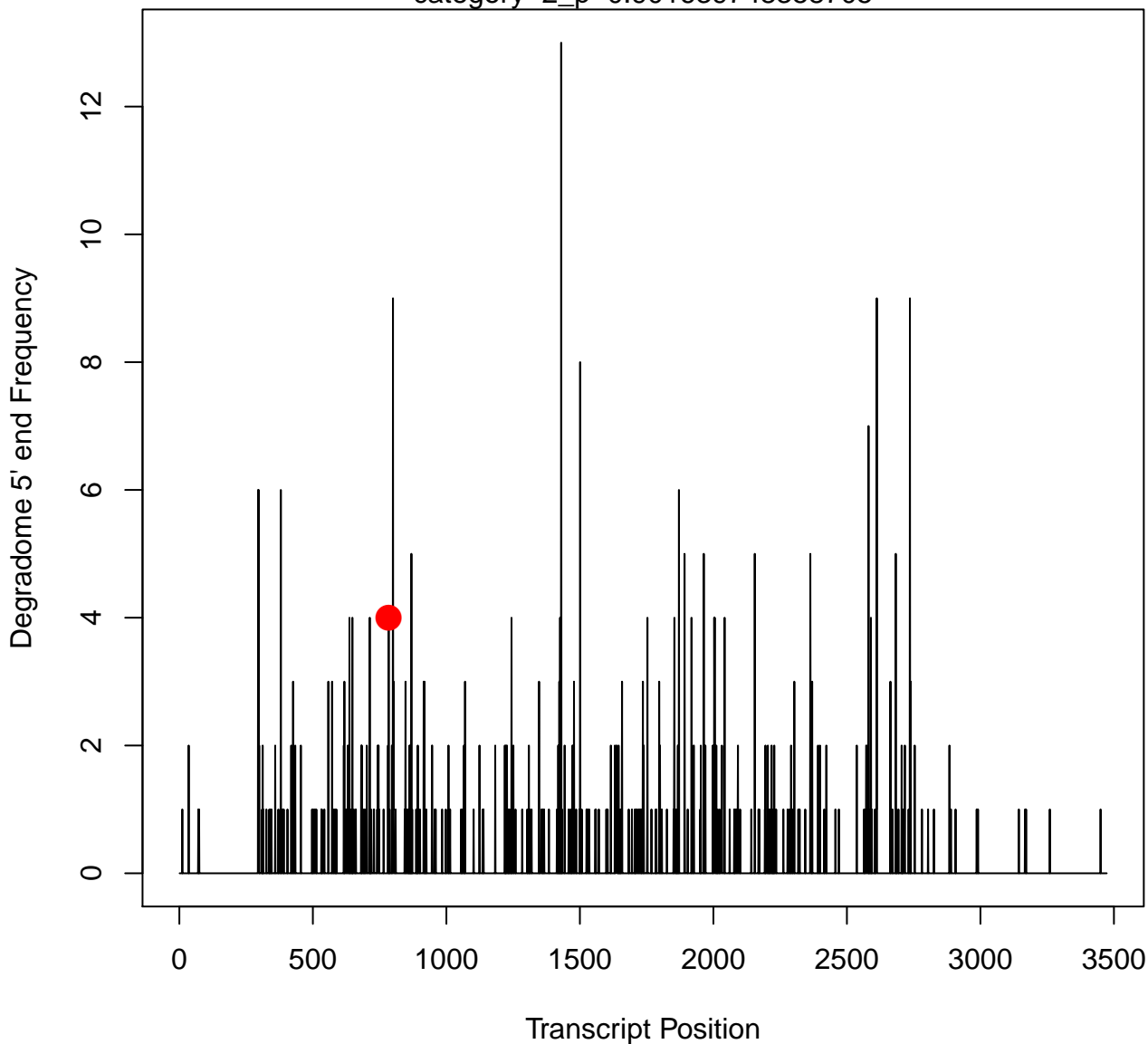

Supplement: Supplementary file 2 [file Data_Sheet_2.zip › Sit-miR159c_Seita.7G006100.1_784_TPlot.pdf]

**T=Seita.7G320500.1\_Q=Sit-miR159c\_S=341**

category=2\_p=0.999839804768021

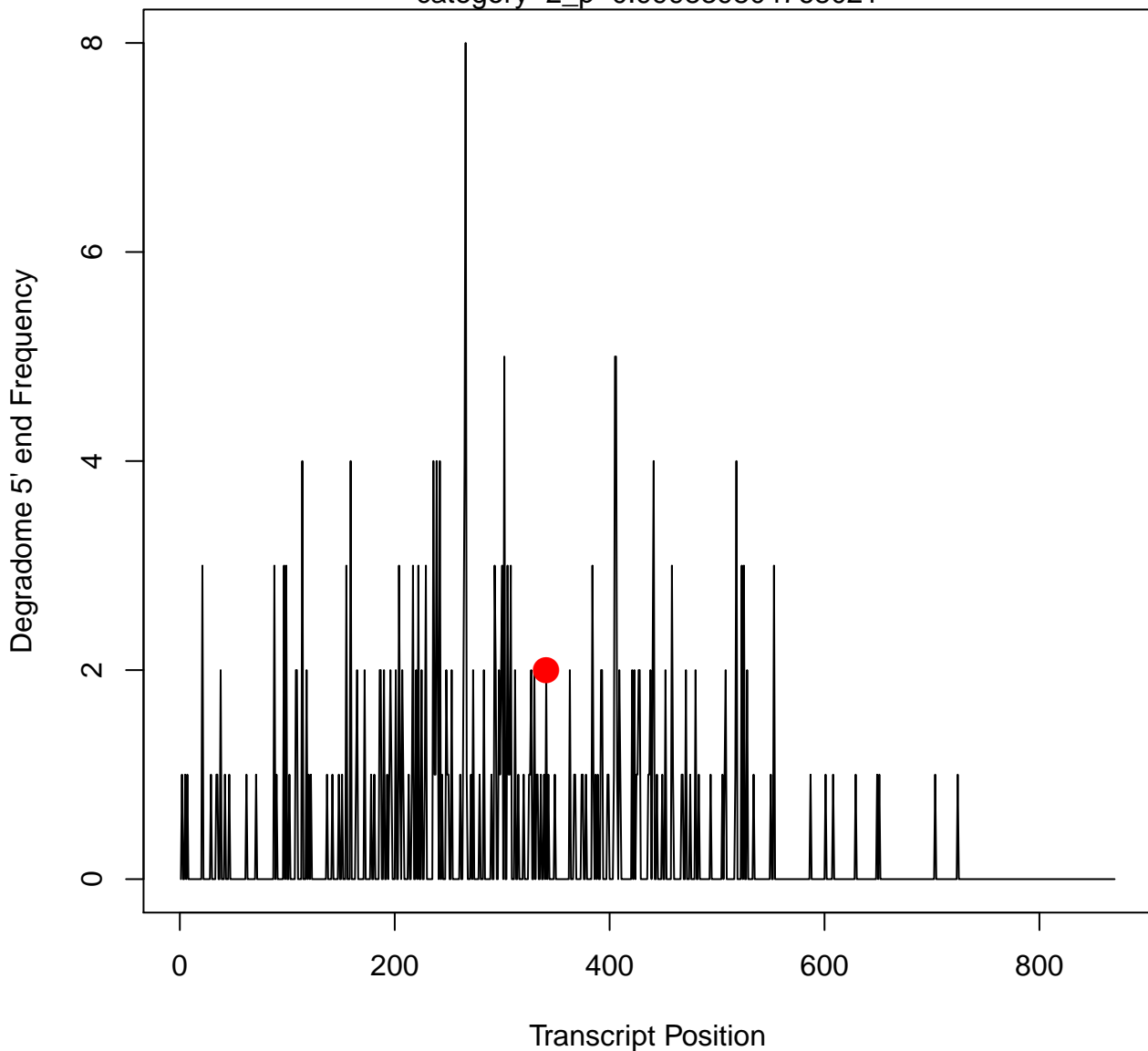

Supplement: Supplementary file 2 [file Data_Sheet_2.zip › Sit-miR159c_Seita.7G320500.1_341_TPlot.pdf]

**T=Seita.8G035200.1\_Q=Sit-miR159c\_S=59**

category=0\_p=0.0616381393818213

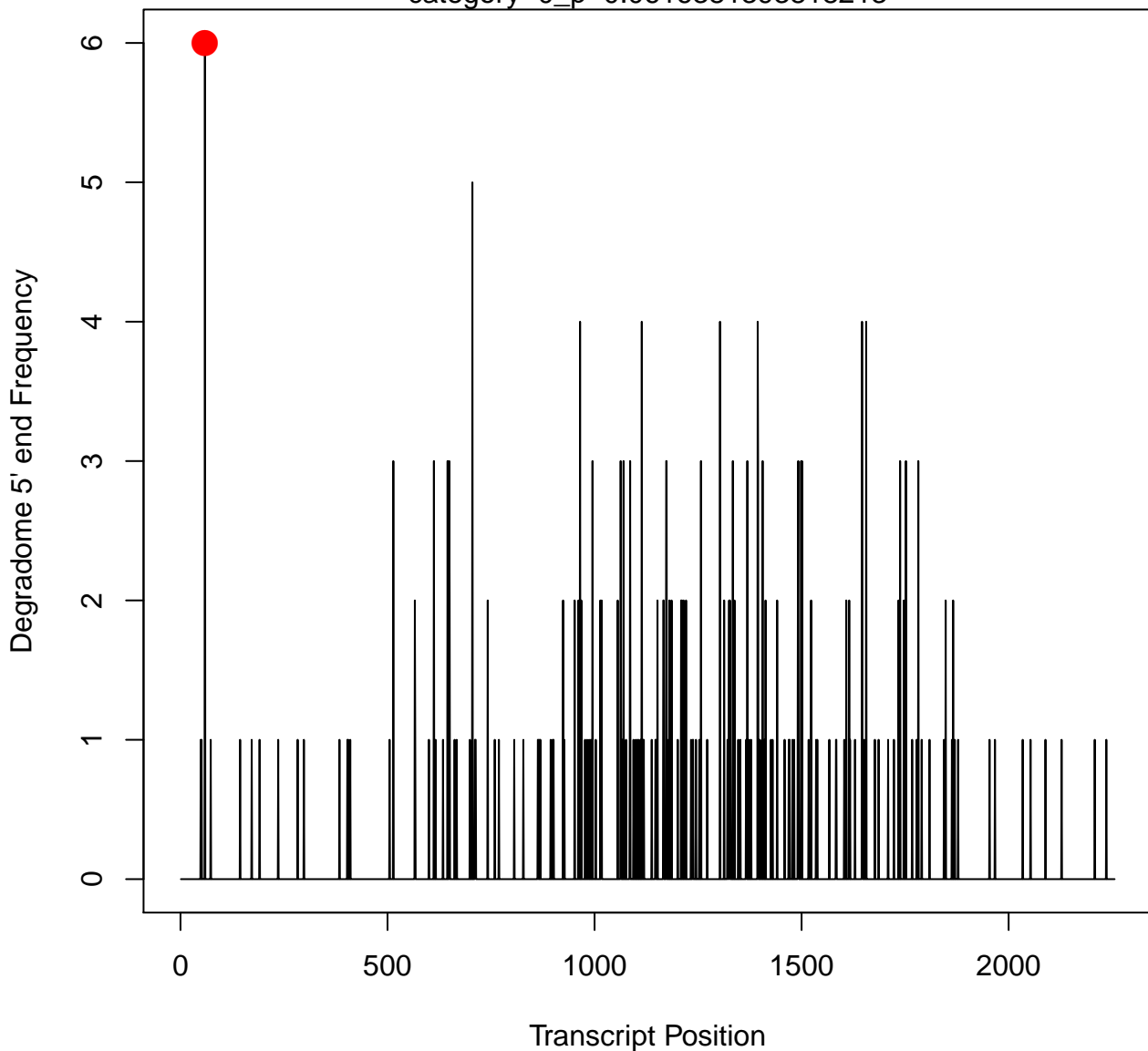

Supplement: Supplementary file 2 [file Data_Sheet_2.zip › Sit-miR159c_Seita.8G035200.1_59_TPlot.pdf]

**T=Seita.9G090700.1\_Q=Sit-miR159c\_S=433**

category=2\_p=0.999998763974734

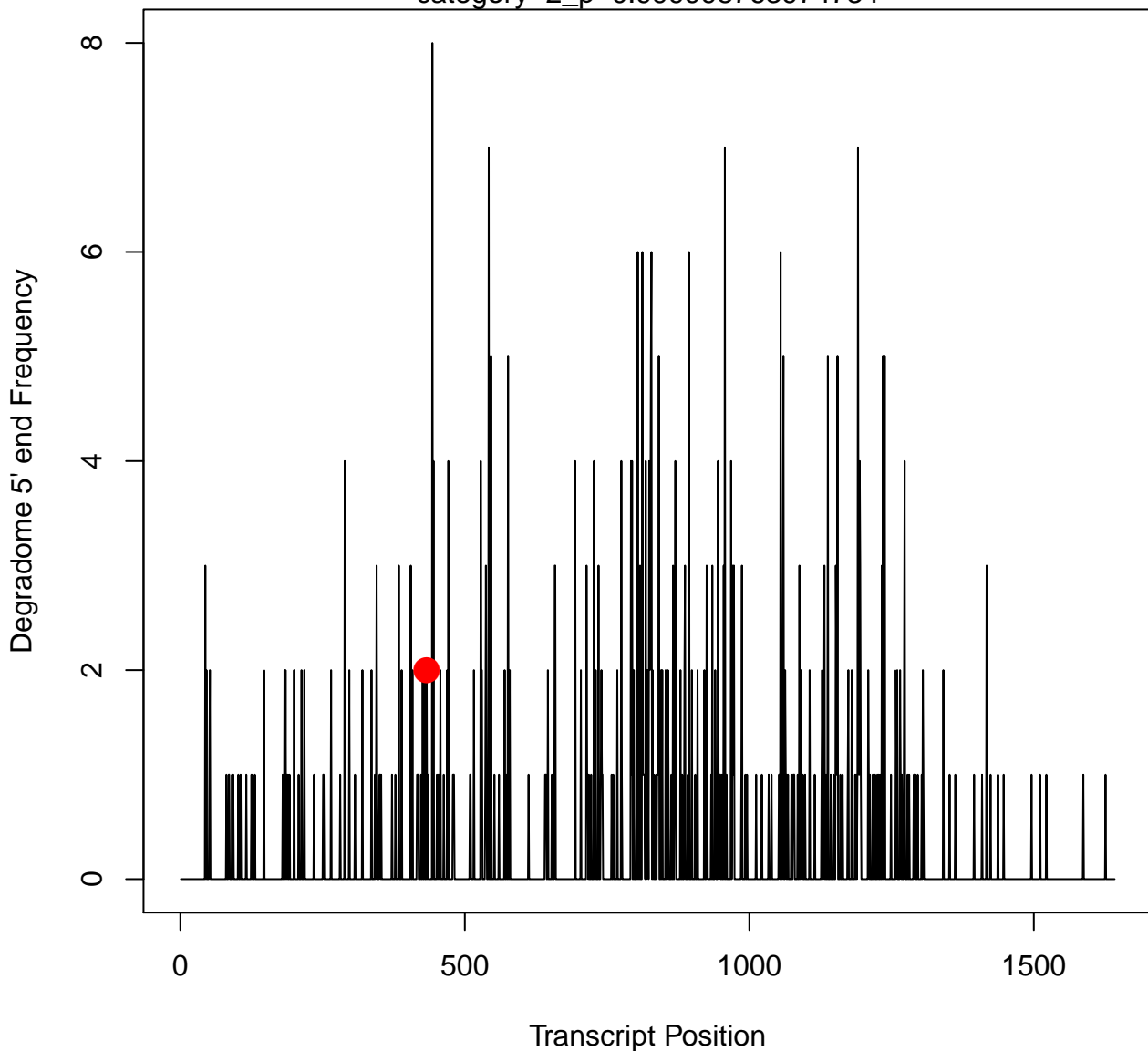

Supplement: Supplementary file 2 [file Data_Sheet_2.zip › Sit-miR159c_Seita.9G090700.1_433_TPlot.pdf]

**T=Seita.9G091300.1\_Q=Sit-miR159c\_S=2347**

category=2\_p=0.950107515284326

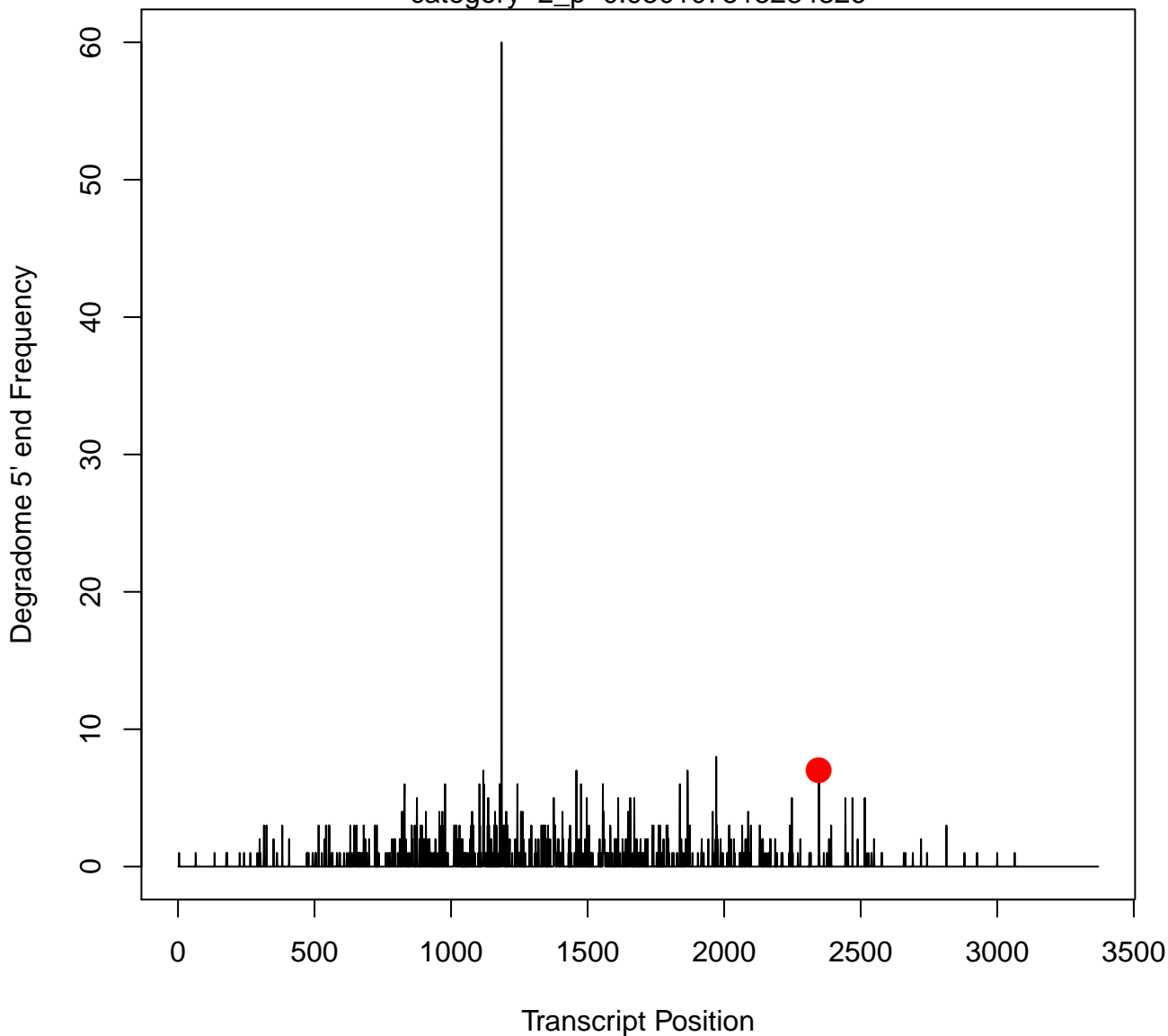

Supplement: Supplementary file 2 [file Data_Sheet_2.zip › Sit-miR159c_Seita.9G091300.1_2347_TPlot.pdf]

**T=Seita.3G102500.1\_Q=Sit-miR160a\_S=316**

category=2\_p=0.999942127420742

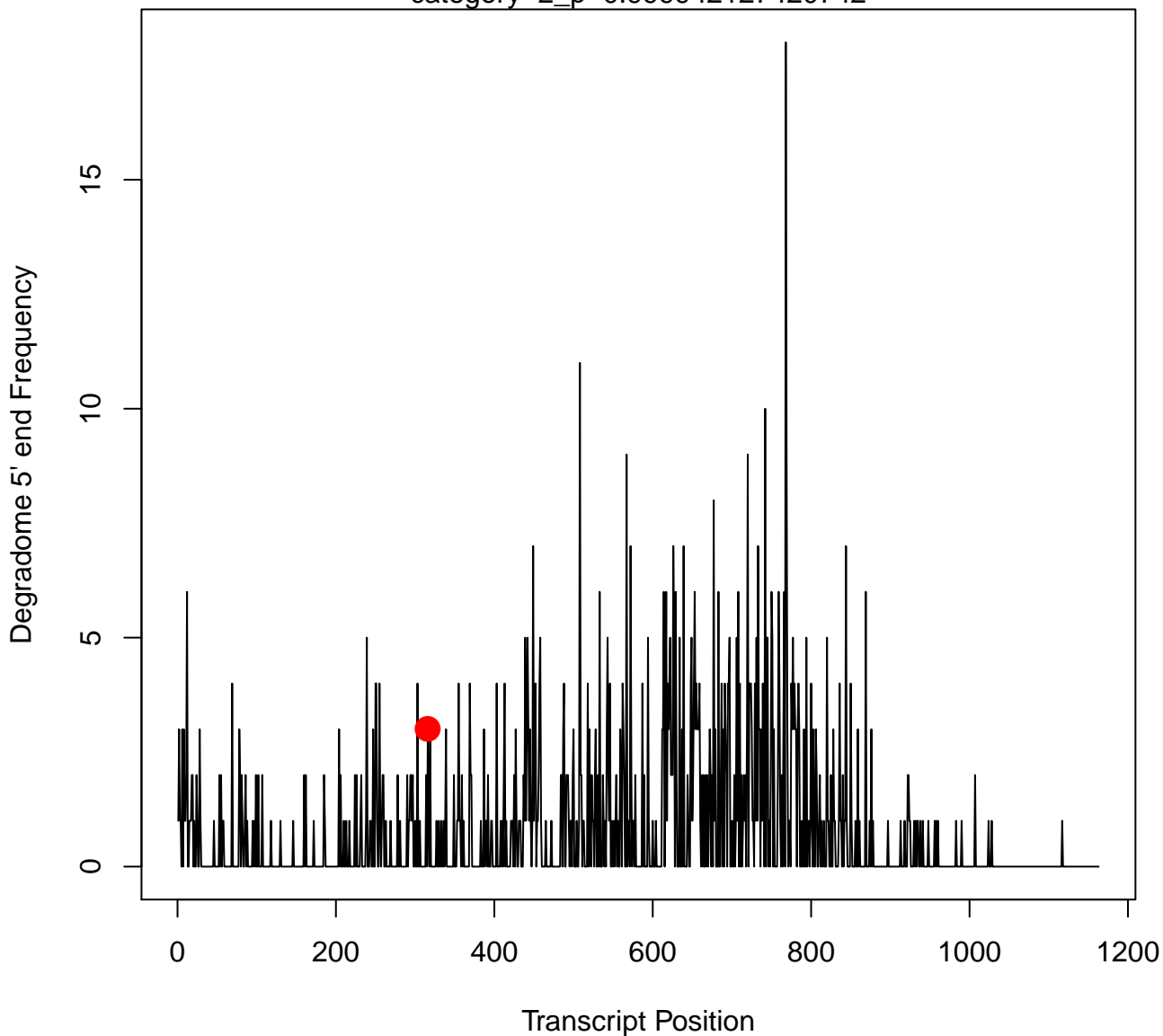

Supplement: Supplementary file 2 [file Data_Sheet_2.zip › Sit-miR160a_Seita.3G102500.1_316_TPlot.pdf]

**T=Seita.3G327800.1\_Q=Sit-miR160a\_S=1619**

category=2\_p=0.999999999999686

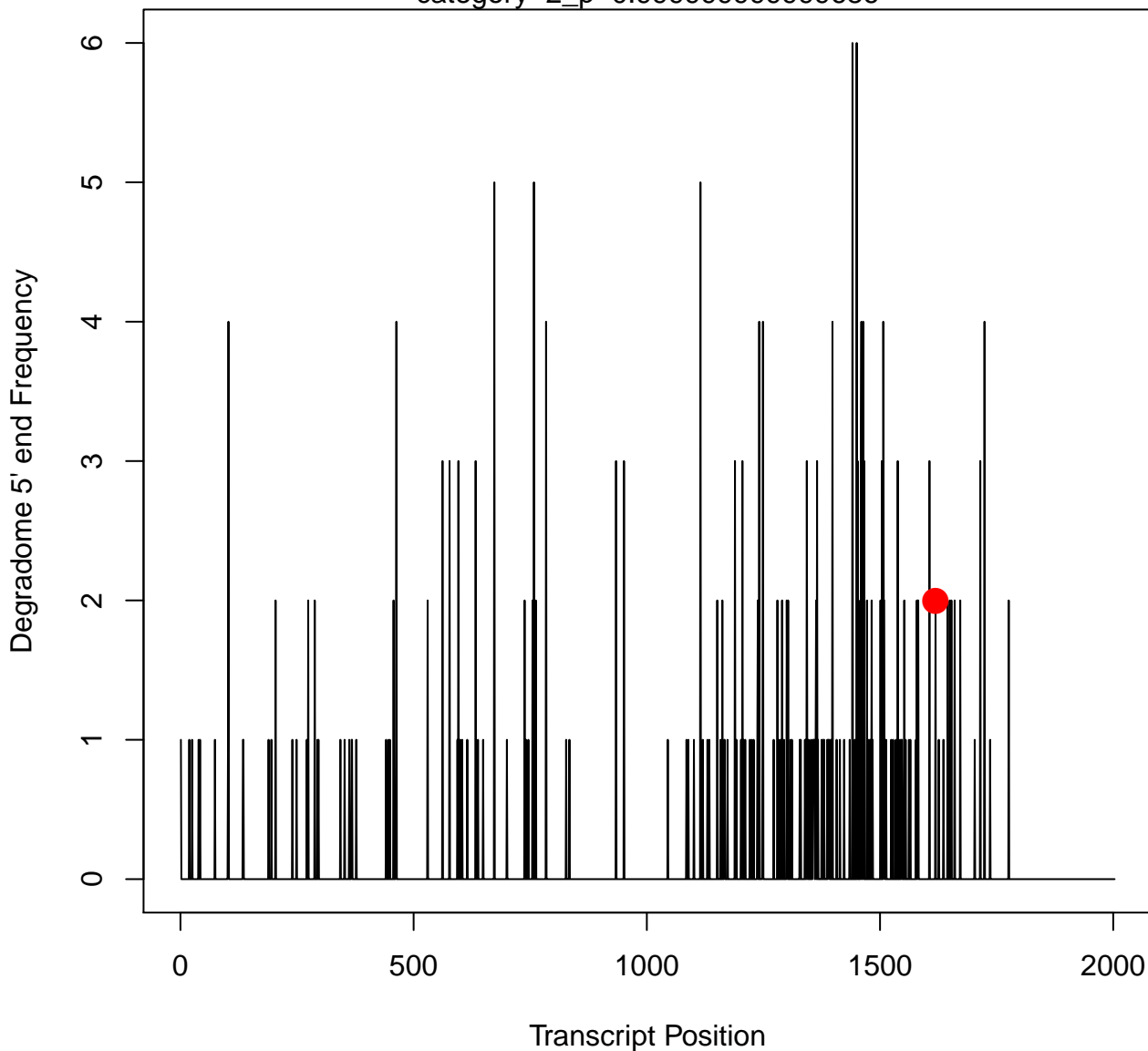

Supplement: Supplementary file 2 [file Data_Sheet_2.zip › Sit-miR160a_Seita.3G327800.1_1619_TPlot.pdf]

**T=Seita.3G383600.1\_Q=Sit-miR160a\_S=182**

category=2\_p=1

Degradome 5' end Frequency

5  
4  
3  
2  
1  
0

0 200 400 600 800 1000 1200

Transcript Position

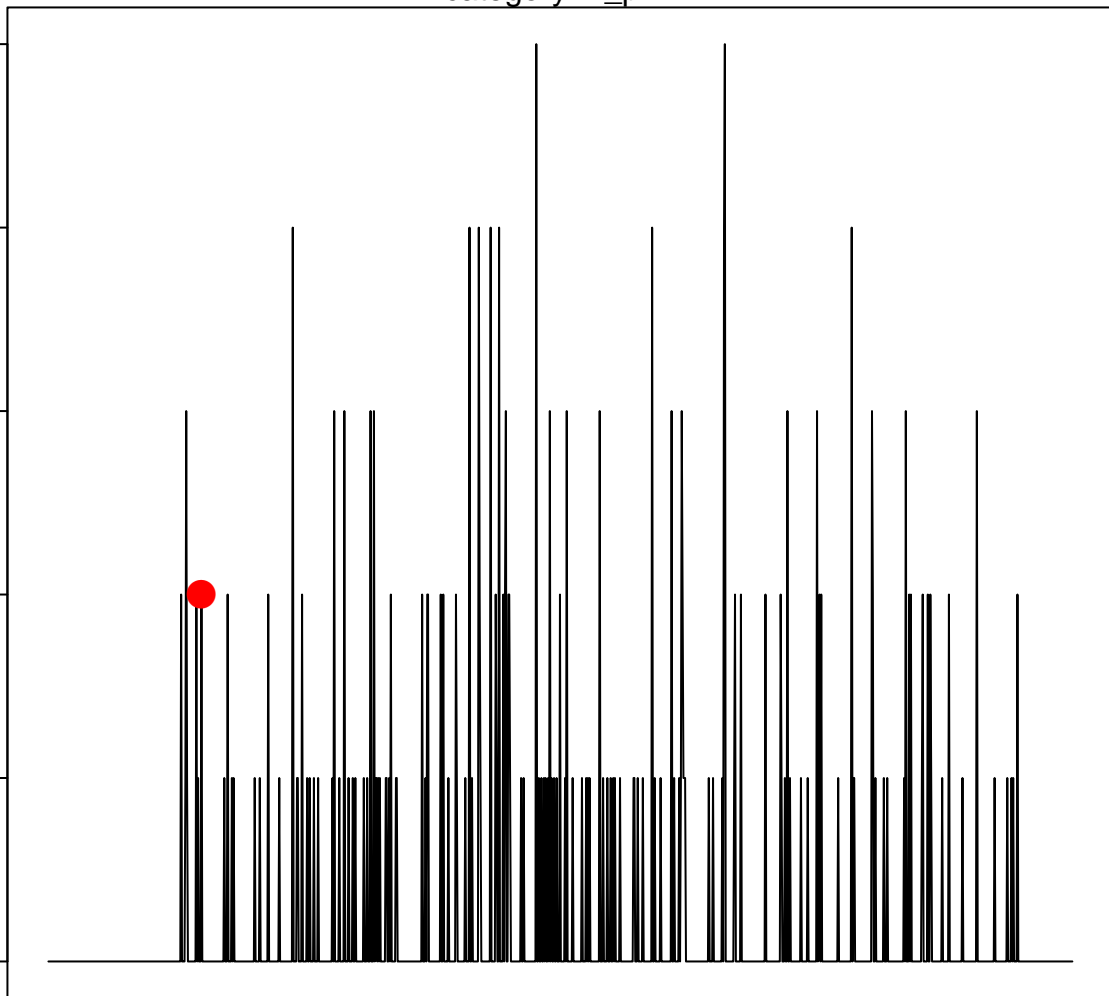

Supplement: Supplementary file 2 [file Data_Sheet_2.zip › Sit-miR160a_Seita.3G383600.1_182_TPlot.pdf]

**T=Seita.5G140500.1\_Q=Sit-miR160a\_S=1934**

category=2\_p=1

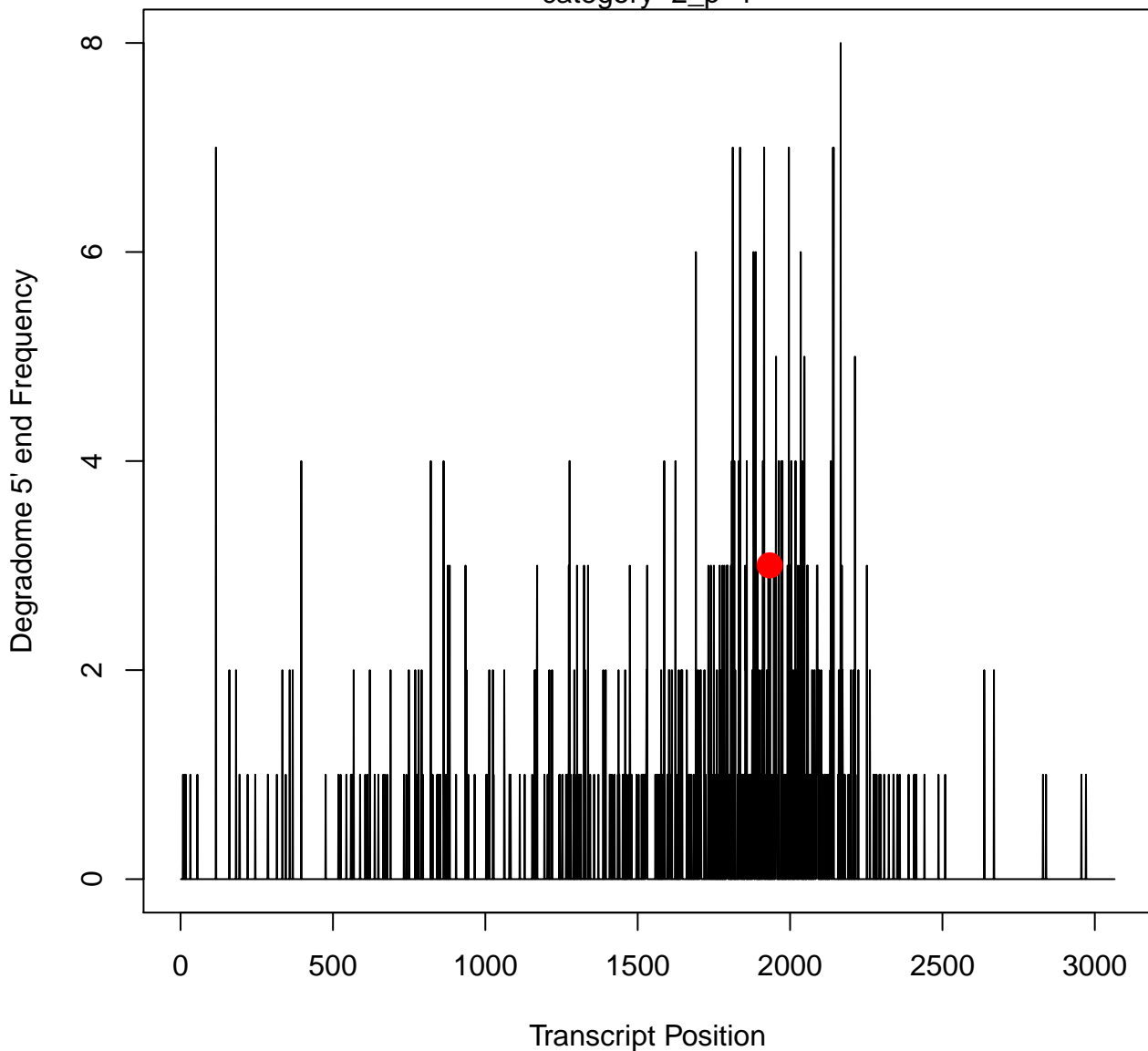

Supplement: Supplementary file 2 [file Data_Sheet_2.zip › Sit-miR160a_Seita.5G140500.1_1934_TPlot.pdf]

**T=Seita.5G273800.1\_Q=Sit-miR160a\_S=477**

category=0\_p=0.360132463759389

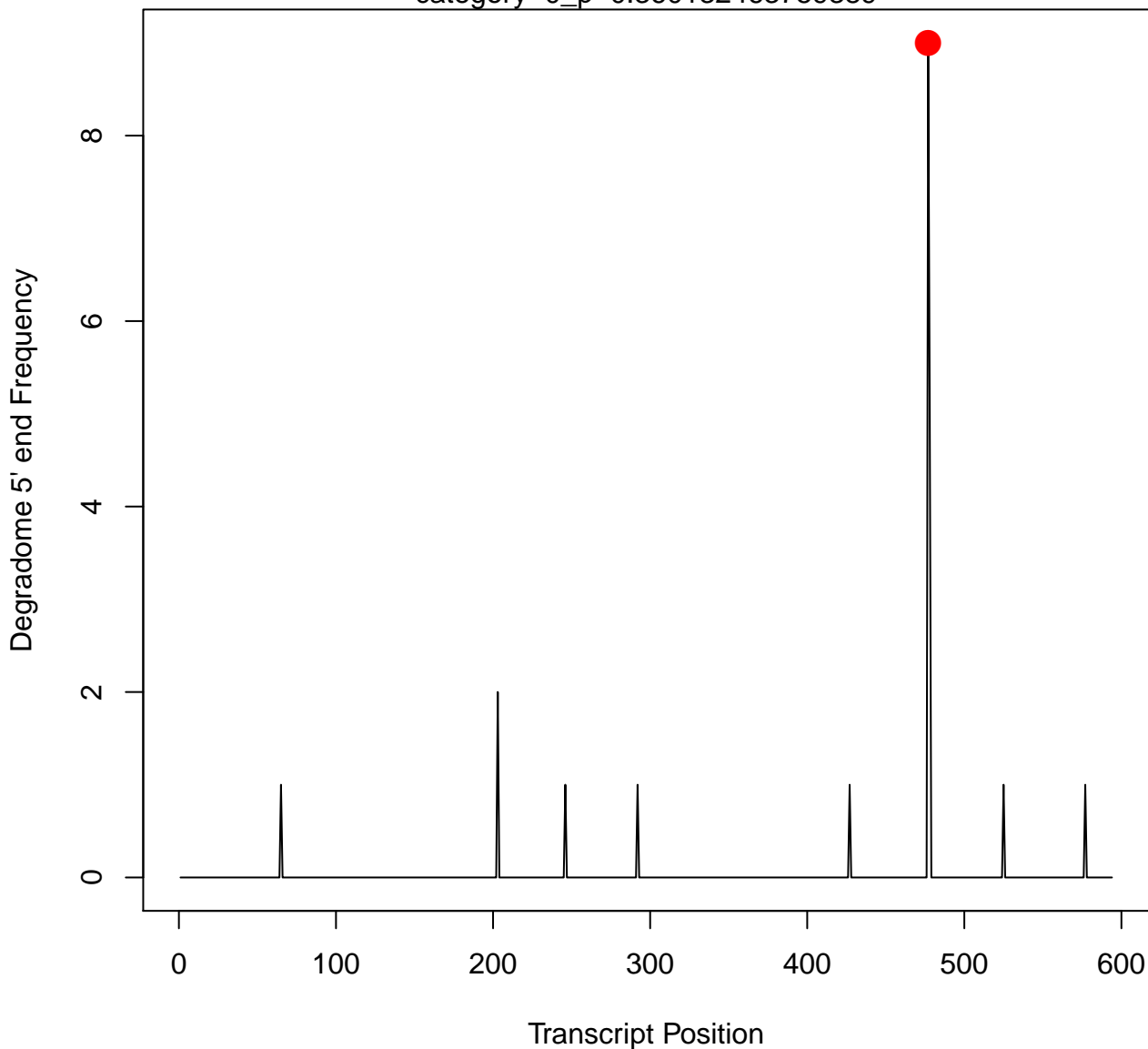

Supplement: Supplementary file 2 [file Data_Sheet_2.zip › Sit-miR160a_Seita.5G273800.1_477_TPlot.pdf]

**T=Seita.5G390300.1\_Q=Sit-miR160a\_S=756**

category=2\_p=1

Degradome 5' end Frequency

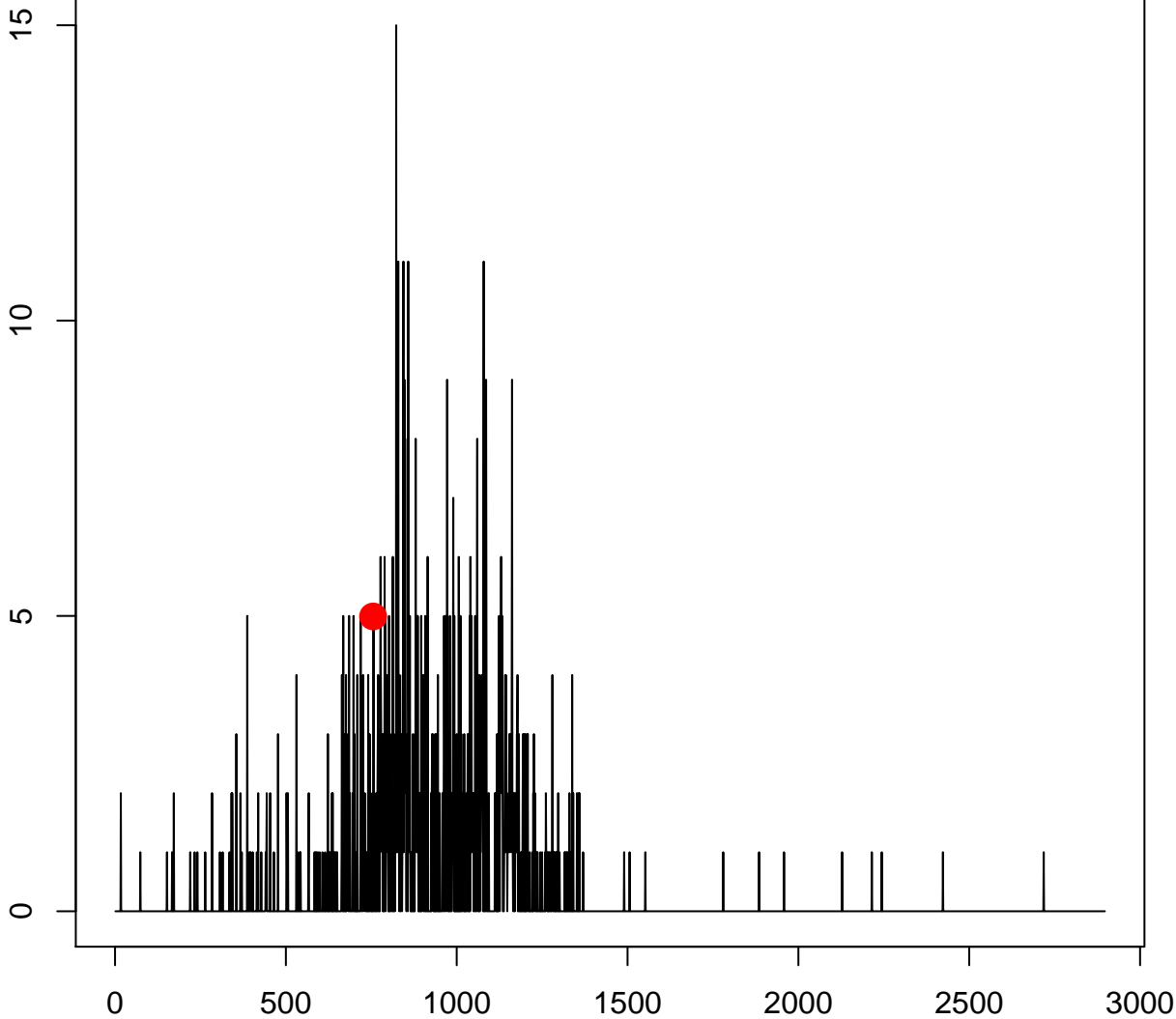

Transcript Position

Supplement: Supplementary file 2 [file Data_Sheet_2.zip › Sit-miR160a_Seita.5G390300.1_756_TPlot.pdf]

**T=Seita.6G173700.1\_Q=Sit-miR160a\_S=1111**

category=2\_p=1

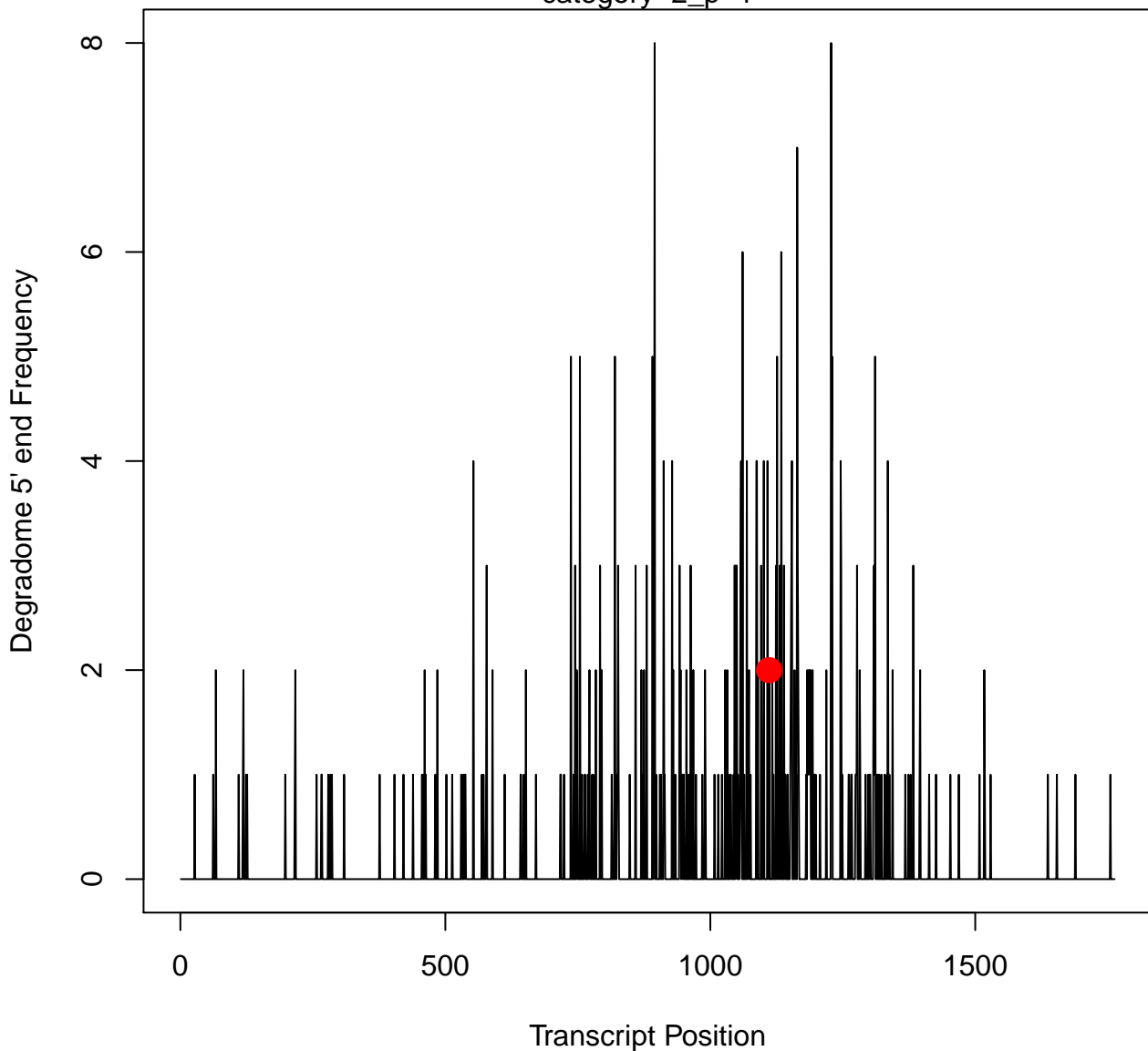

Supplement: Supplementary file 2 [file Data_Sheet_2.zip › Sit-miR160a_Seita.6G173700.1_1111_TPlot.pdf]

**T=Seita.7G207700.1\_Q=Sit-miR160a\_S=3709**

category=2\_p=1

Degradome 5' end Frequency

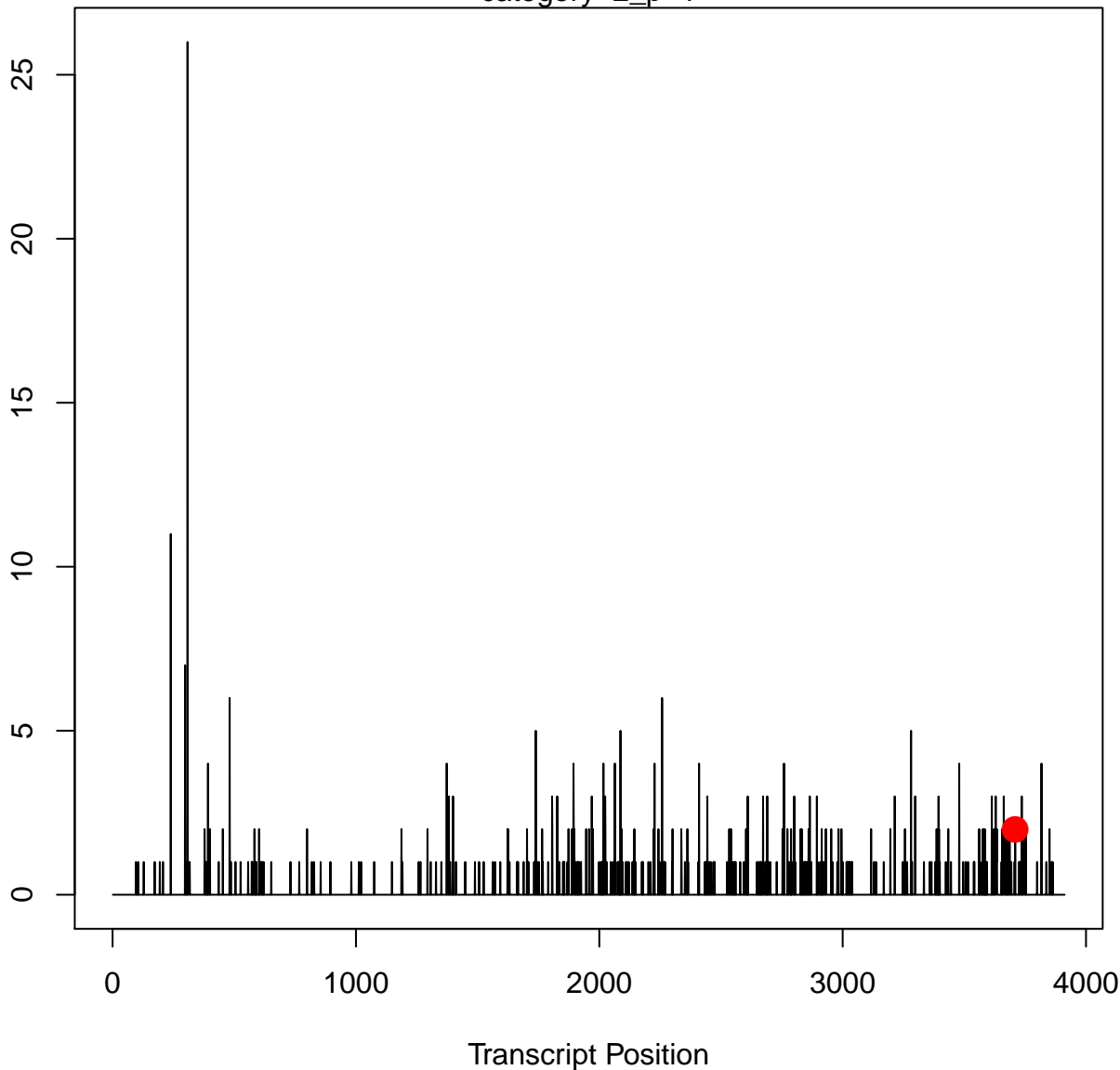

Supplement: Supplementary file 2 [file Data_Sheet_2.zip › Sit-miR160a_Seita.7G207700.1_3709_TPlot.pdf]

**T=Seita.7G295800.1\_Q=Sit-miR160a\_S=368**

category=1\_p=0.355098926341321

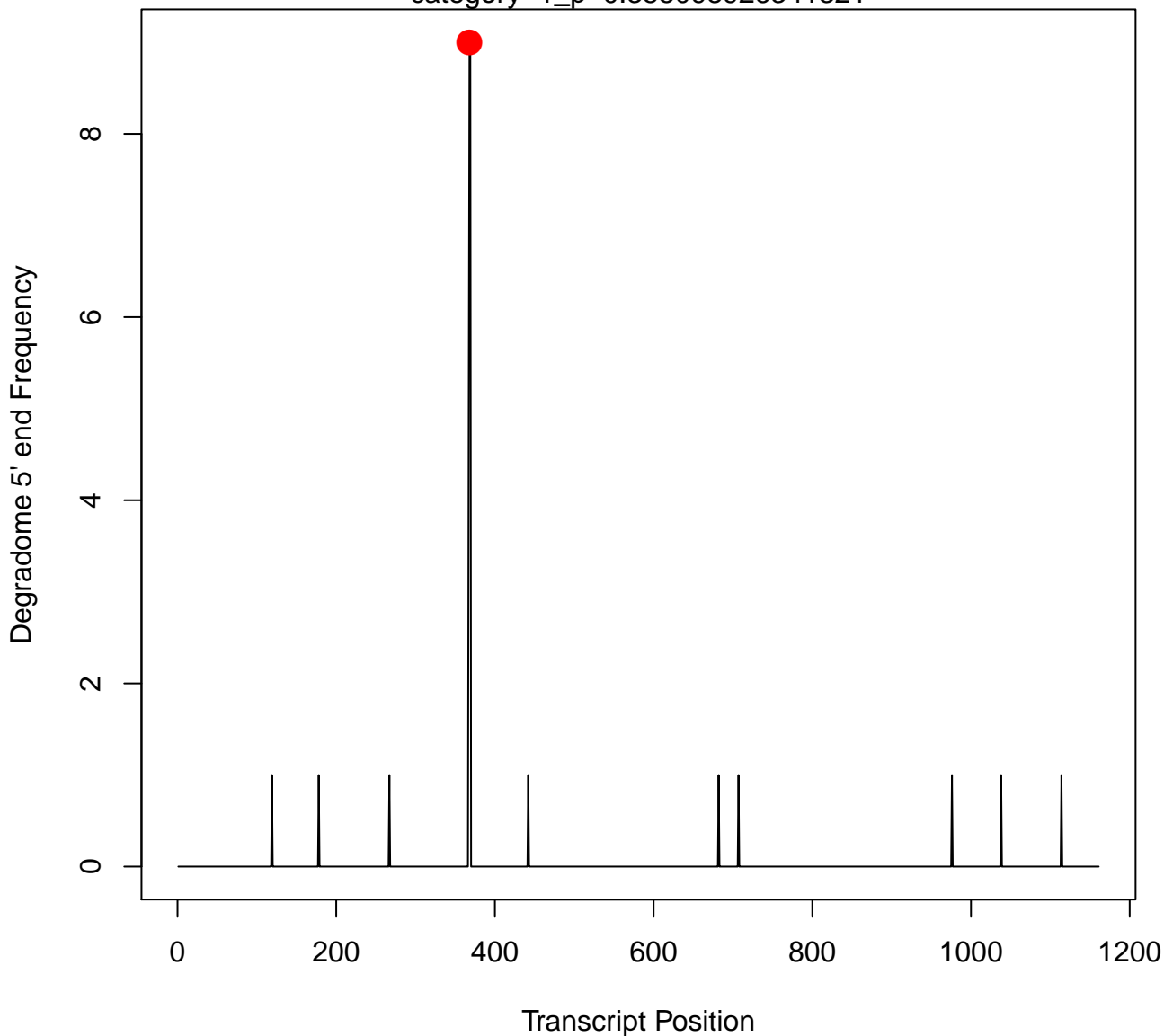

Supplement: Supplementary file 2 [file Data_Sheet_2.zip › Sit-miR160a_Seita.7G295800.1_368_TPlot.pdf]

**T=Seita.8G239900.1\_Q=Sit-miR160a\_S=5654**

category=2\_p=1

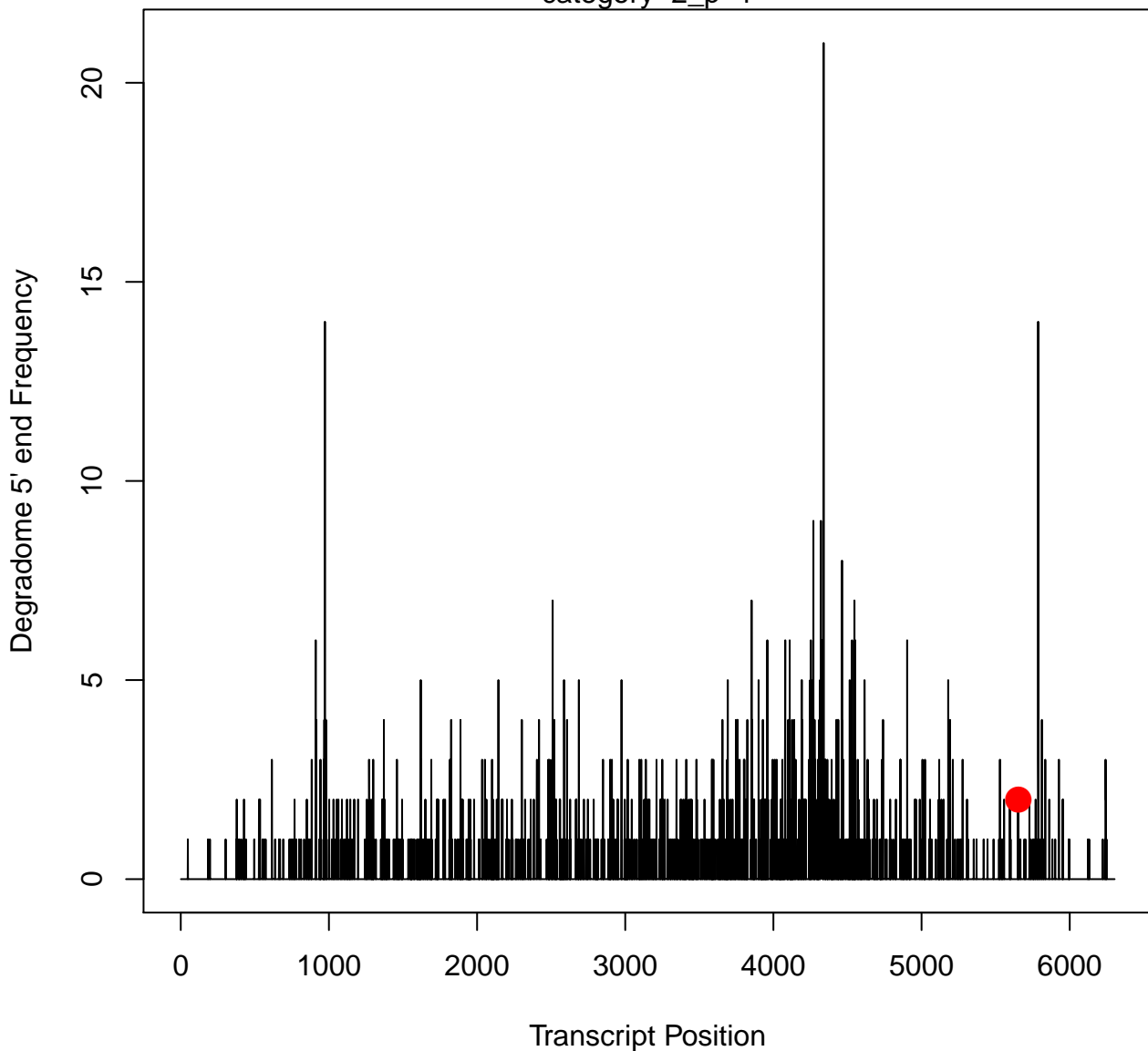

Supplement: Supplementary file 2 [file Data_Sheet_2.zip › Sit-miR160a_Seita.8G239900.1_5654_TPlot.pdf]

**T=Seita.9G219800.1\_Q=Sit-miR160a\_S=1729**

category=0\_p=0.000385499163724878

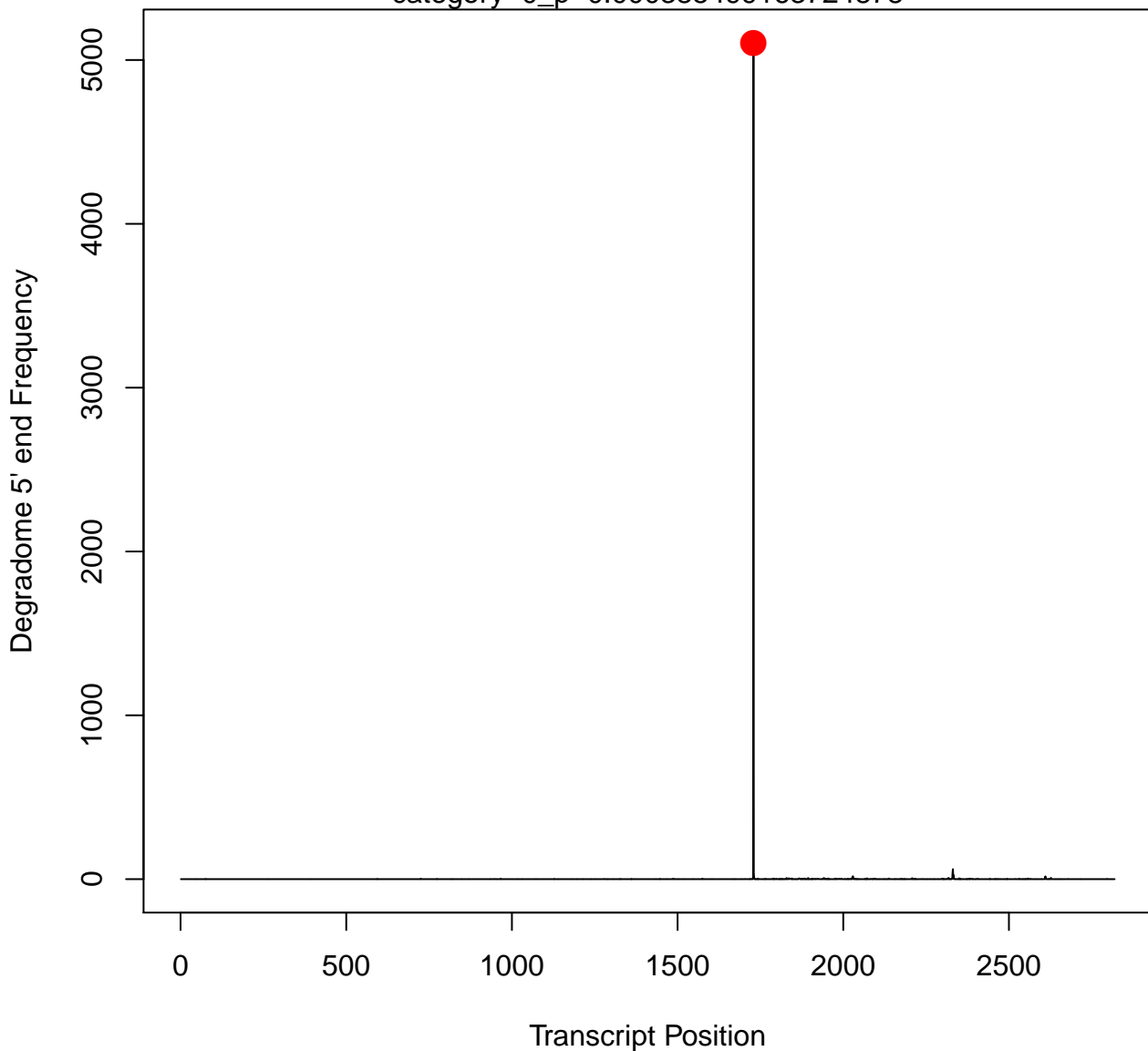

Supplement: Supplementary file 2 [file Data_Sheet_2.zip › Sit-miR160a_Seita.9G219800.1_1729_TPlot.pdf]

**T=Seita.9G474800.1\_Q=Sit-miR160a\_S=1972**

category=2\_p=0.287790518747054

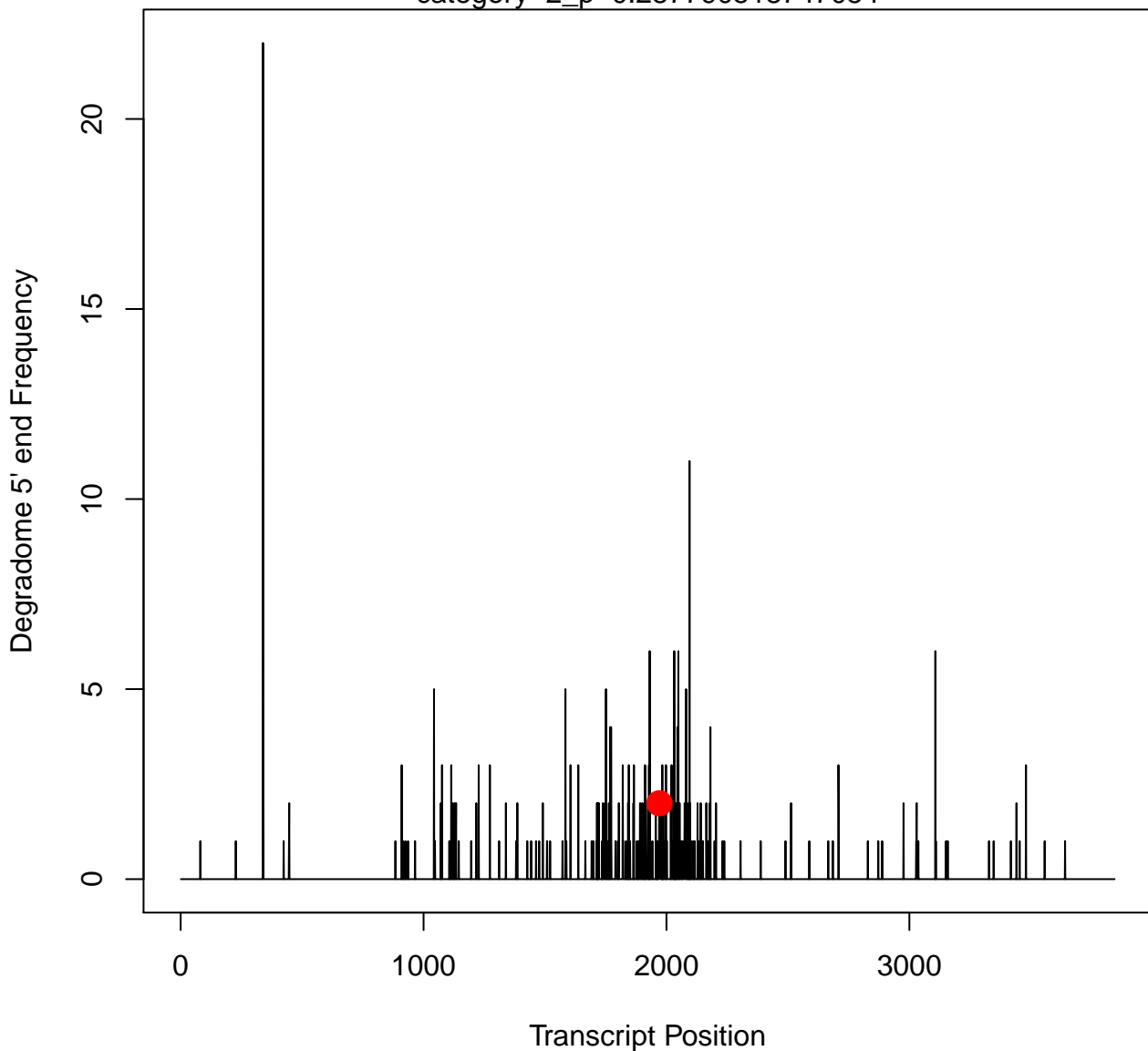

Supplement: Supplementary file 2 [file Data_Sheet_2.zip › Sit-miR160a_Seita.9G474800.1_1972_TPlot.pdf]

**T=Seita.1G264900.1\_Q=Sit-miR160b\_S=256**

category=2\_p=0.999999997986937

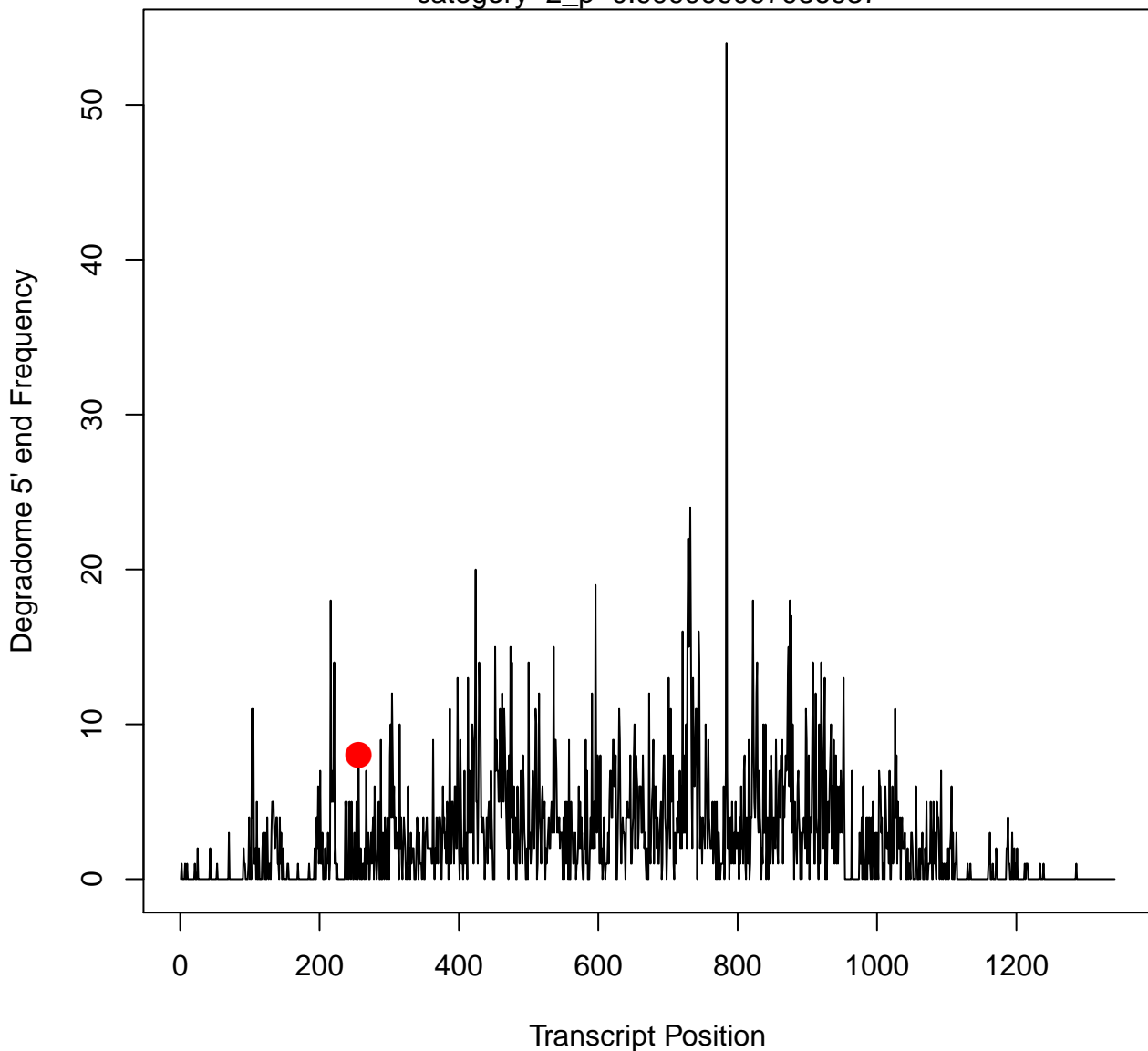

Supplement: Supplementary file 2 [file Data_Sheet_2.zip › Sit-miR160b_Seita.1G264900.1_256_TPlot.pdf]

**T=Seita.2G058900.1\_Q=Sit-miR160b\_S=570**

category=2\_p=0.99999999768116

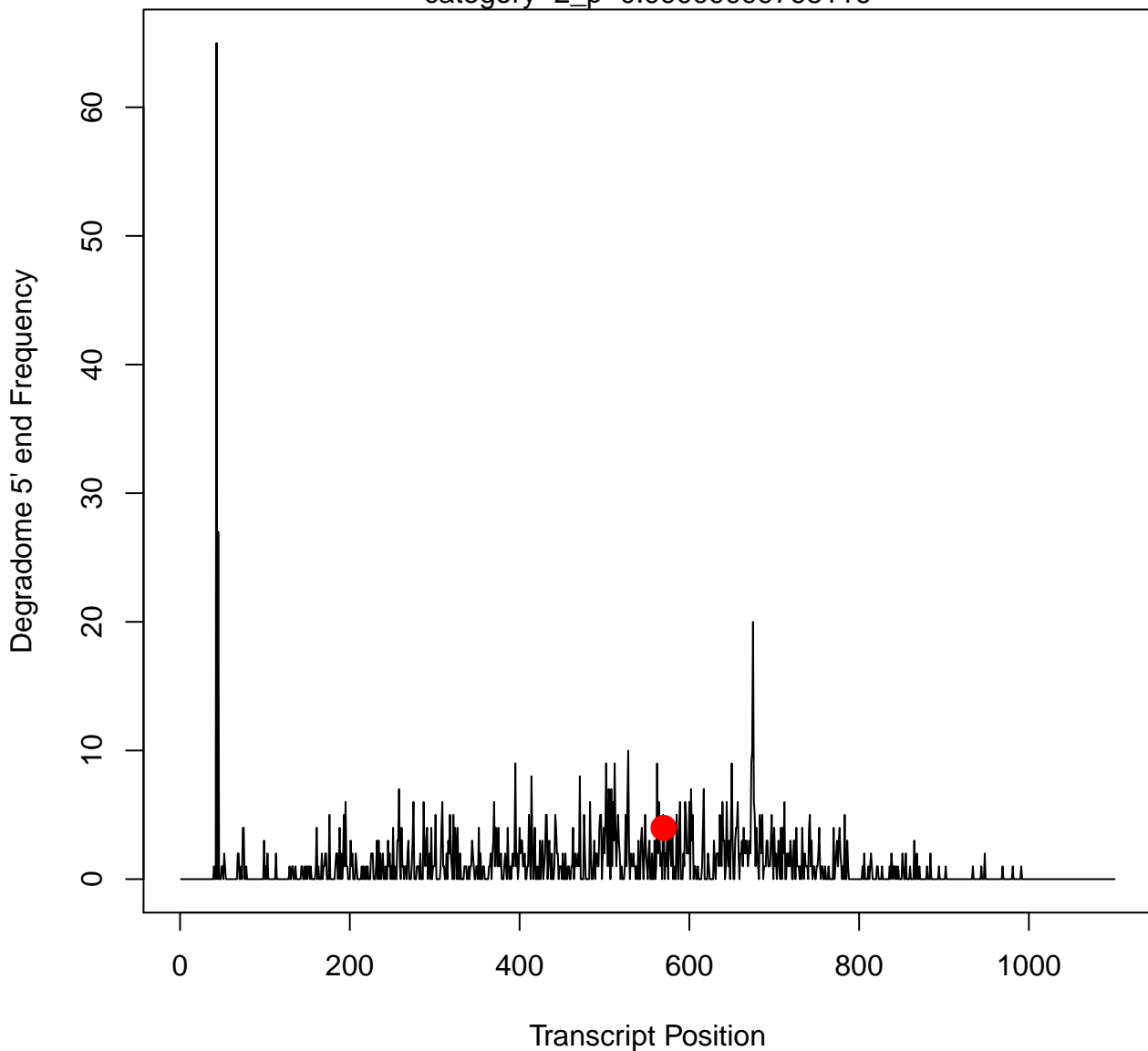

Supplement: Supplementary file 2 [file Data_Sheet_2.zip › Sit-miR160b_Seita.2G058900.1_570_TPlot.pdf]

**T=Seita.3G003300.1\_Q=Sit-miR160b\_S=1363**

category=0\_p=0.000385499163724878

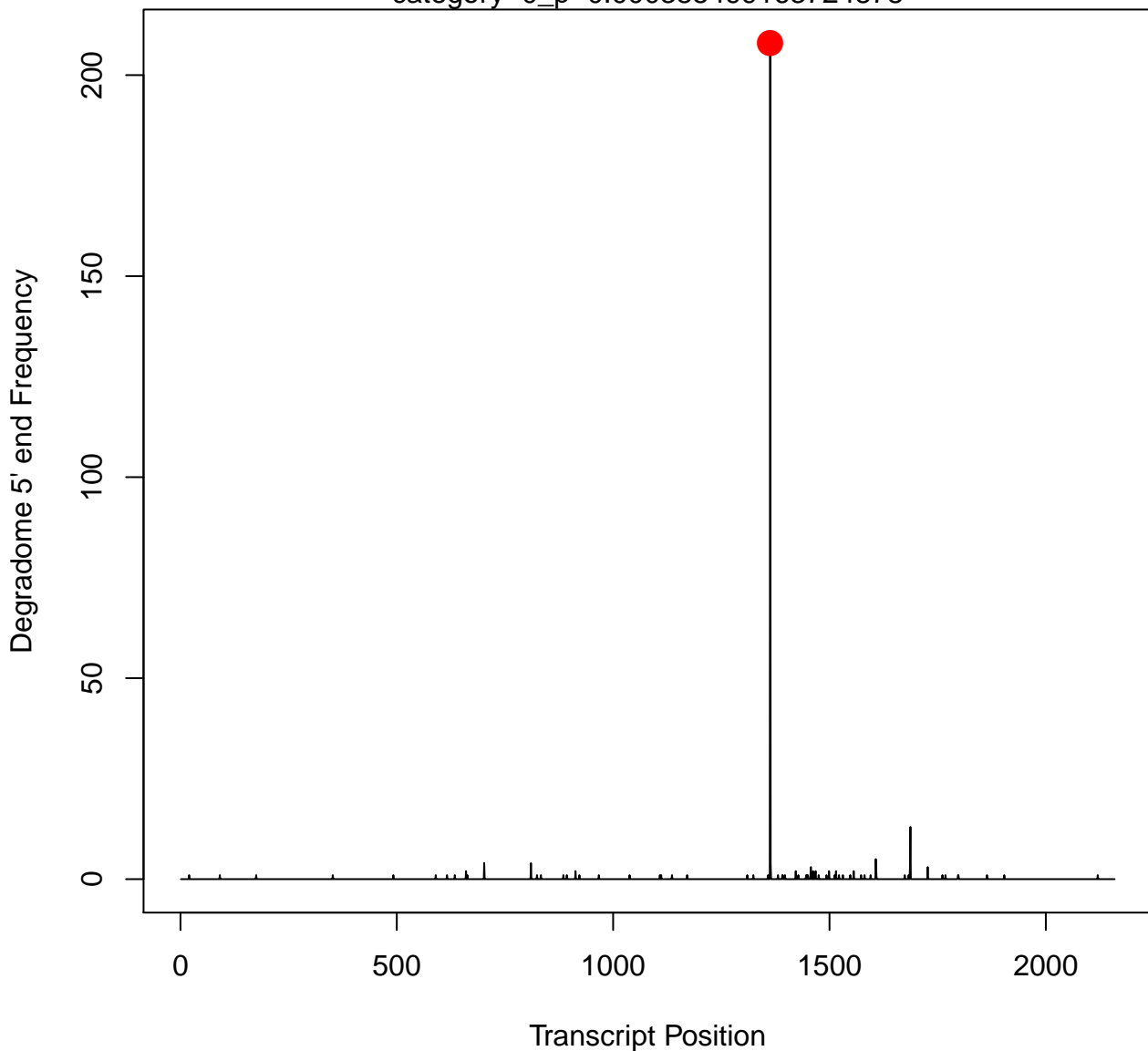

Supplement: Supplementary file 2 [file Data_Sheet_2.zip › Sit-miR160b_Seita.3G003300.1_1363_TPlot.pdf]

**T=Seita.5G267100.1\_Q=Sit-miR160b\_S=1283**

category=0\_p=0.519522654758098

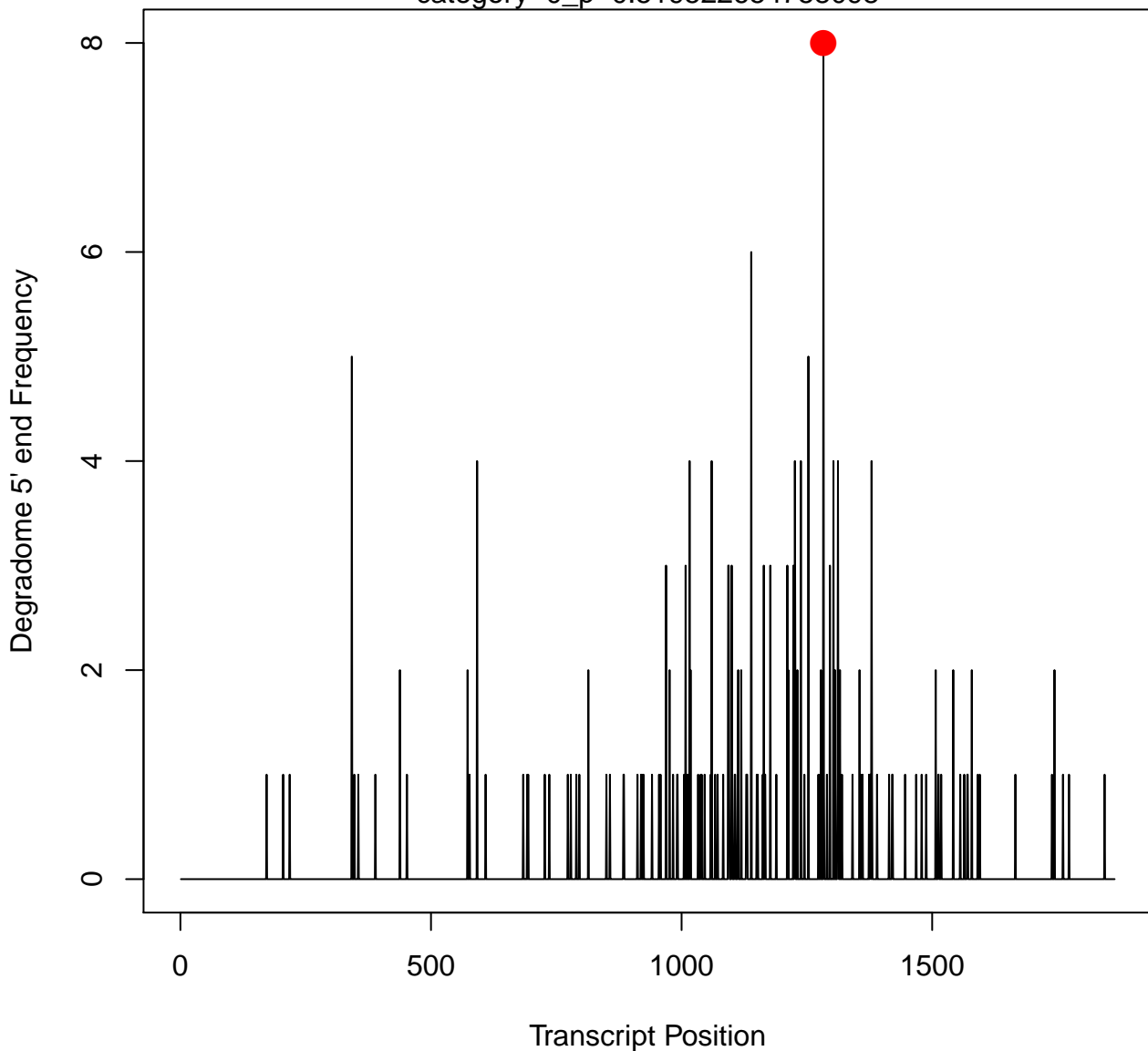

Supplement: Supplementary file 2 [file Data_Sheet_2.zip › Sit-miR160b_Seita.5G267100.1_1283_TPlot.pdf]

**T=Seita.8G079000.1\_Q=Sit-miR160b\_S=2497**

category=2\_p=0.984788842571649

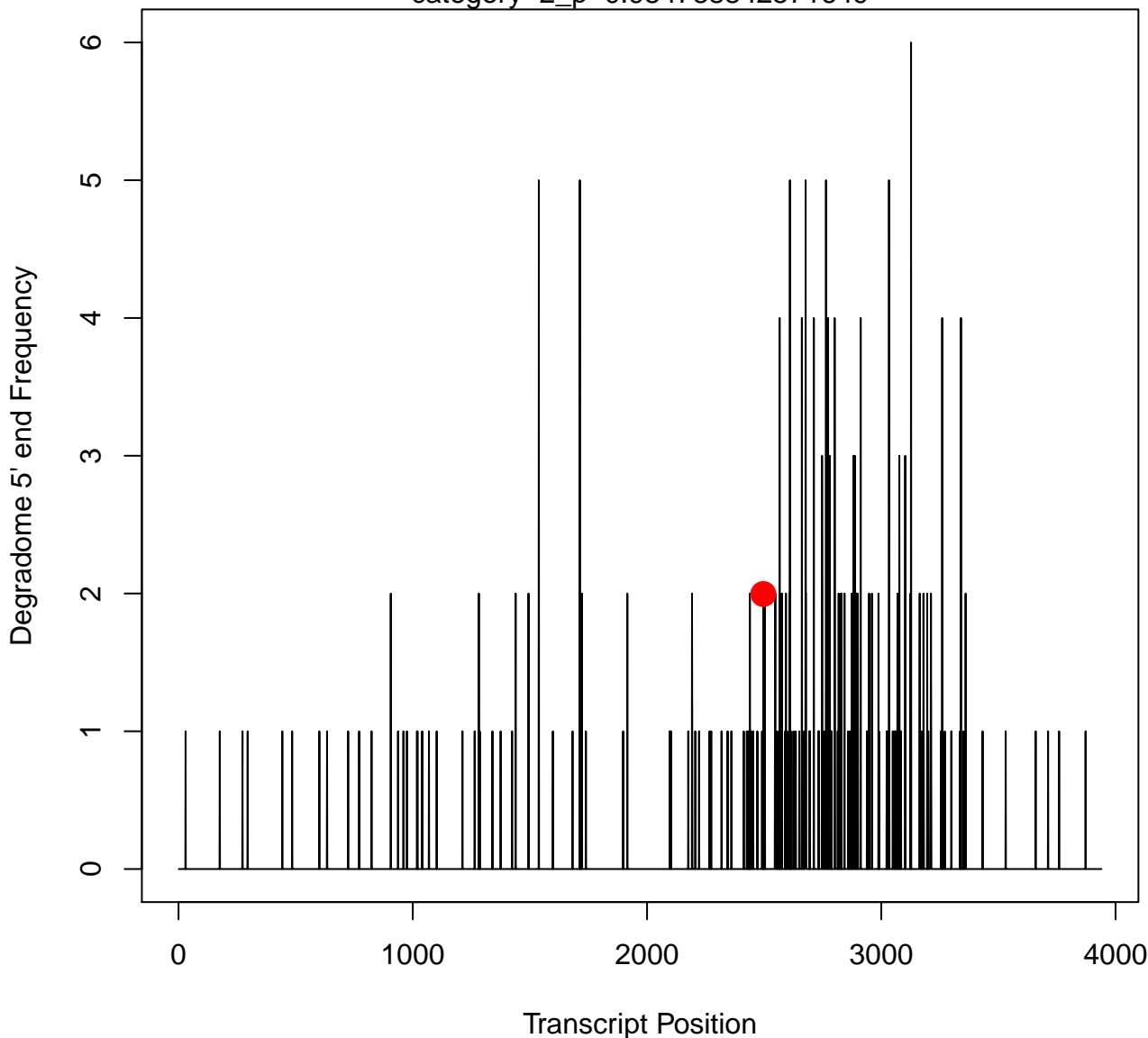

Supplement: Supplementary file 2 [file Data_Sheet_2.zip › Sit-miR160b_Seita.8G079000.1_2497_TPlot.pdf]

**T=Seita.2G374100.1\_Q=Sit-miR160c\_S=780**

category=2\_p=0.999733474995515

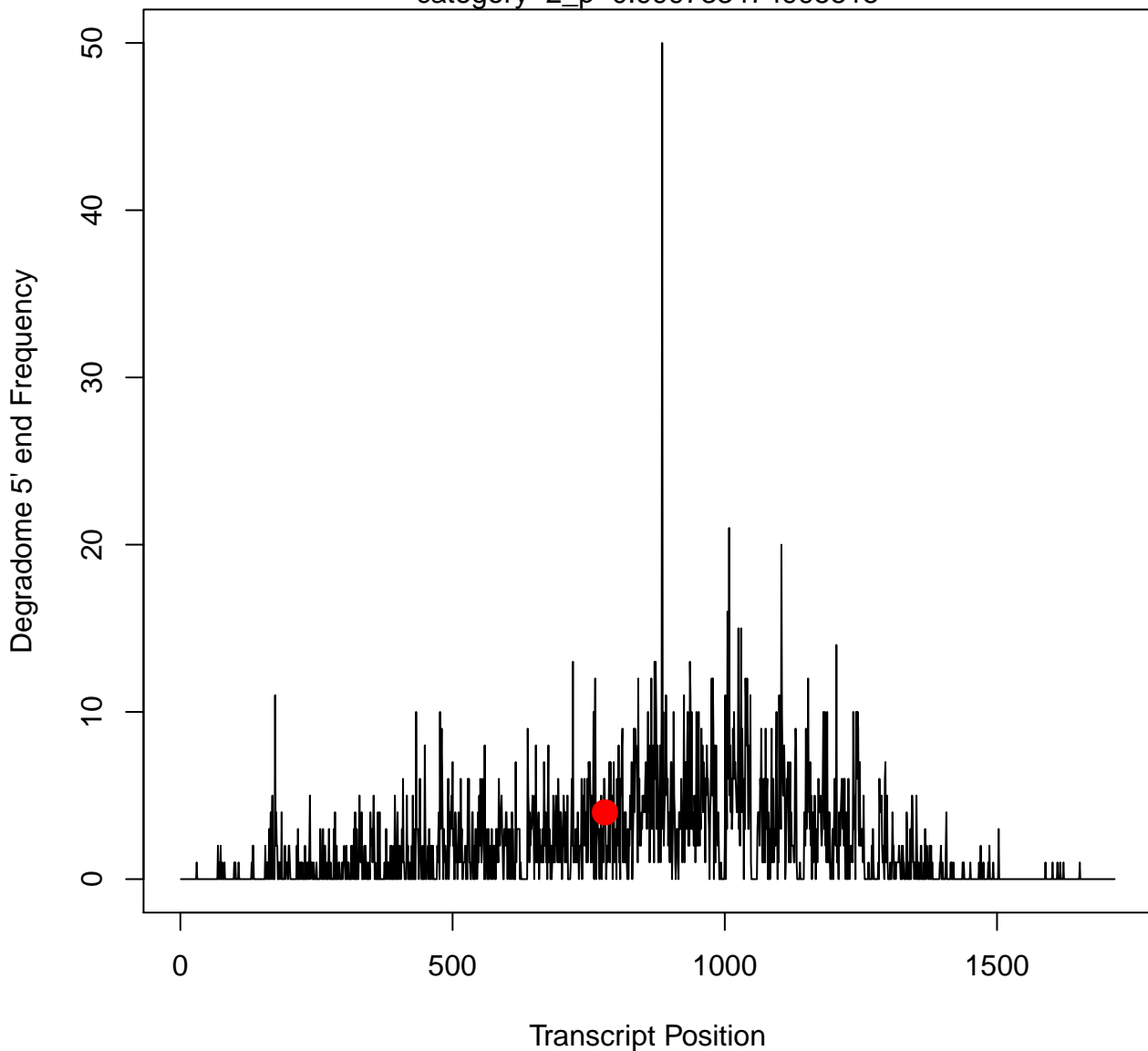

Supplement: Supplementary file 2 [file Data_Sheet_2.zip › Sit-miR160c_Seita.2G374100.1_780_TPlot.pdf]

**T=Seita.2G444000.1\_Q=Sit-miR160c\_S=581**

category=2\_p=0.999999999999268

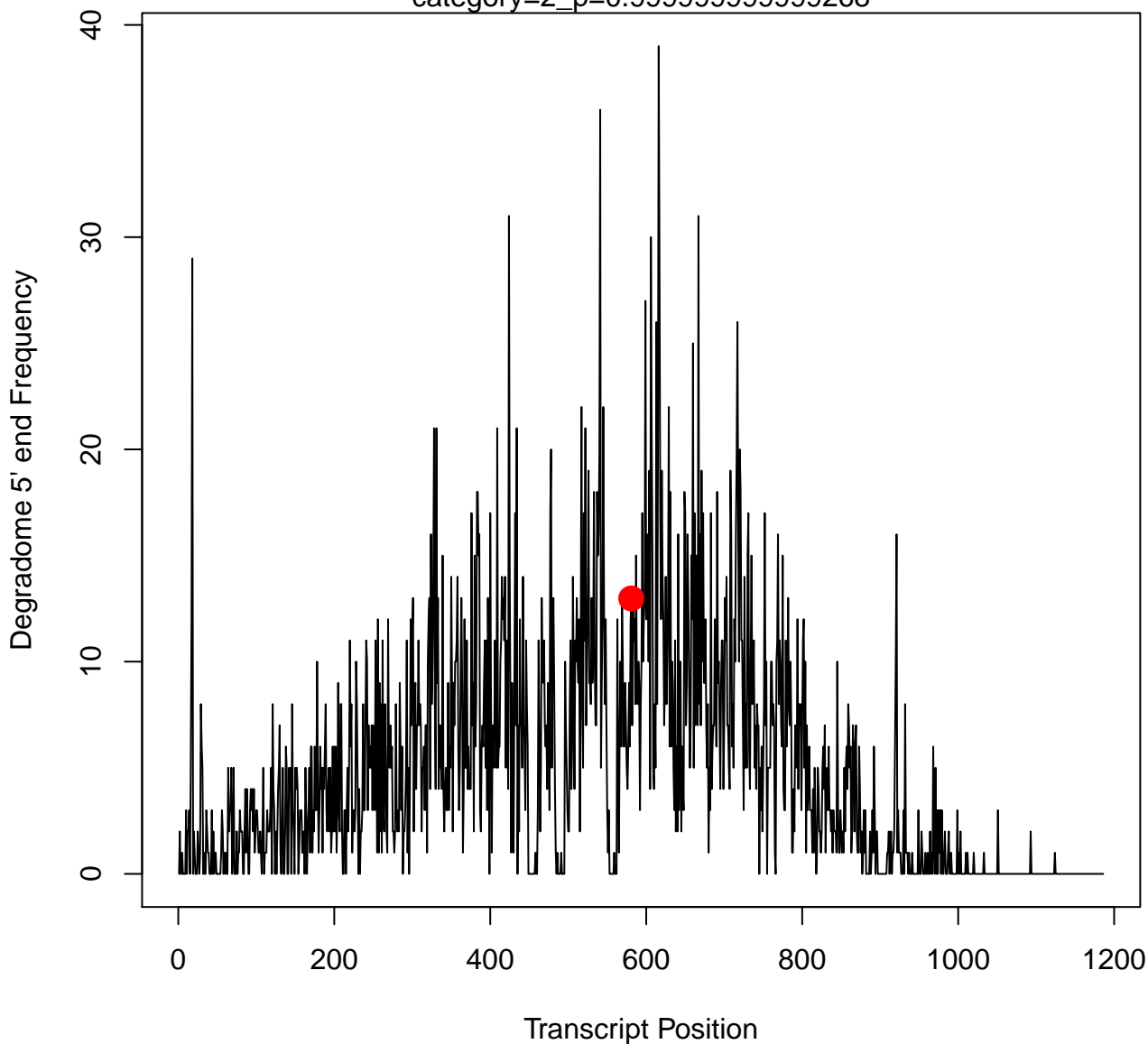

Supplement: Supplementary file 2 [file Data_Sheet_2.zip › Sit-miR160c_Seita.2G444000.1_581_TPlot.pdf]

**T=Seita.3G014300.1\_Q=Sit-miR160c\_S=744**

category=2\_p=0.99999999997626

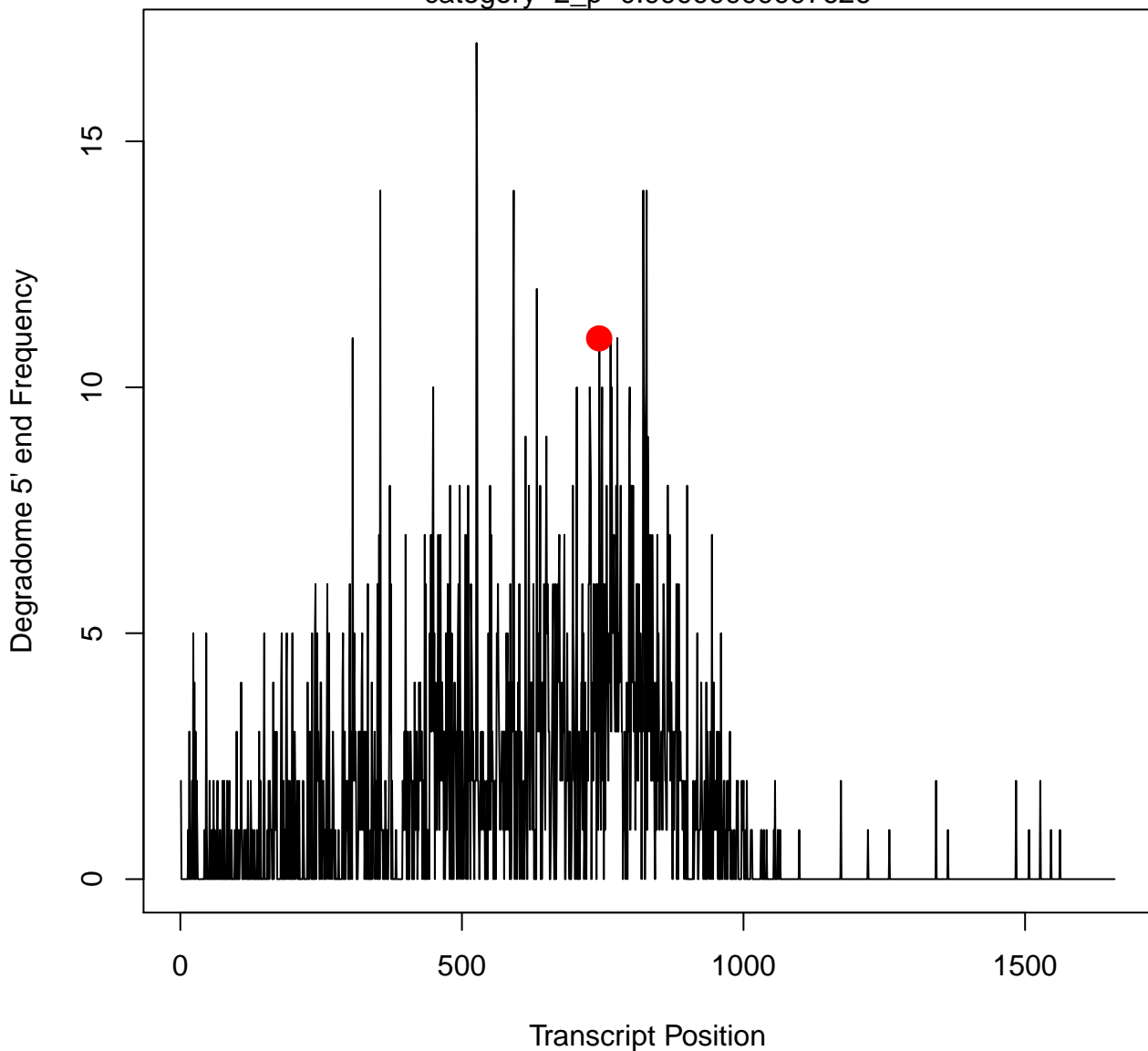

Supplement: Supplementary file 2 [file Data_Sheet_2.zip › Sit-miR160c_Seita.3G014300.1_744_TPlot.pdf]

**T=Seita.3G020200.1\_Q=Sit-miR160c\_S=1771**

category=2\_p=0.999999999999972

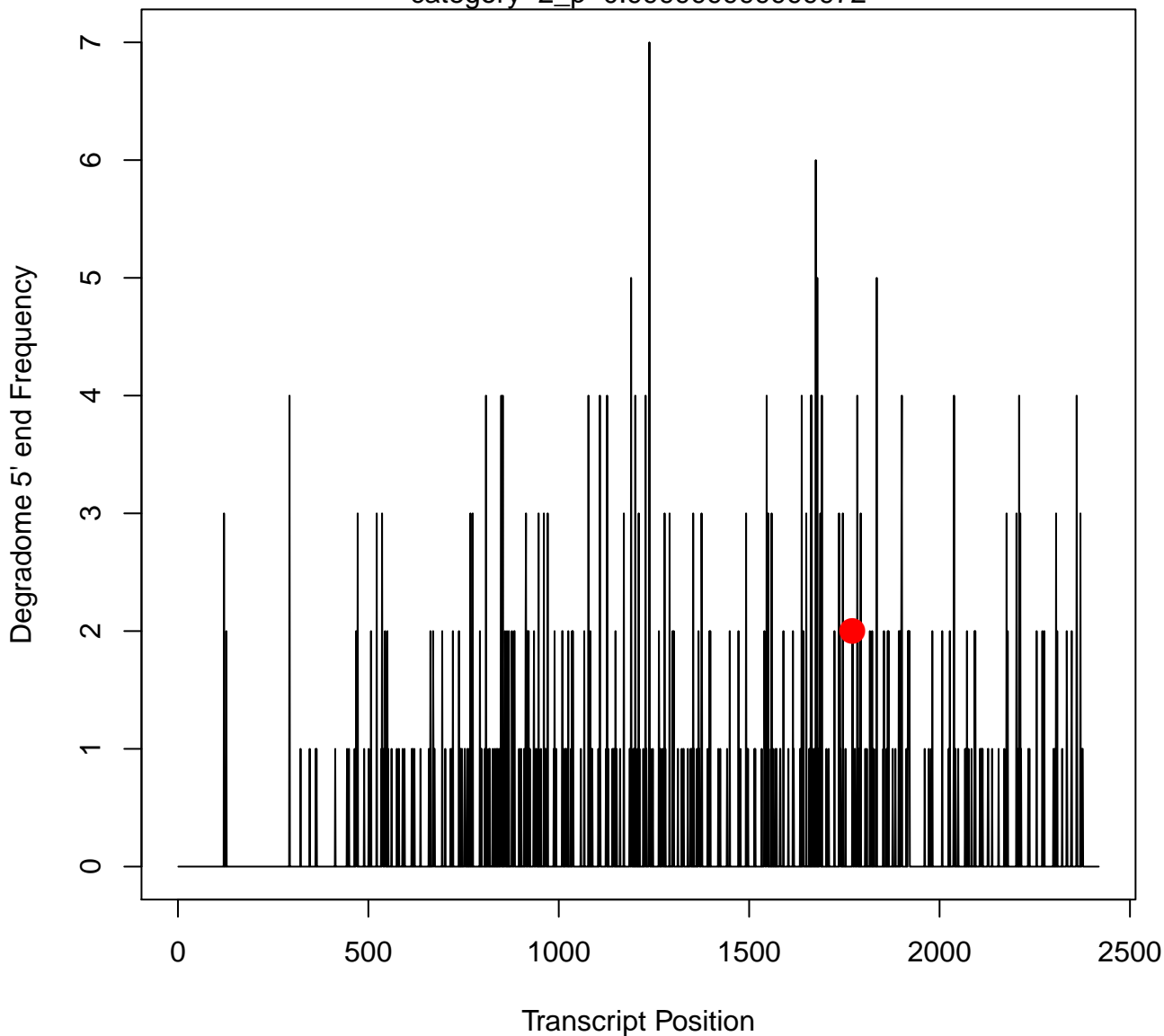

Supplement: Supplementary file 2 [file Data_Sheet_2.zip › Sit-miR160c_Seita.3G020200.1_1771_TPlot.pdf]

**T=Seita.3G386700.1\_Q=Sit-miR160c\_S=363**

category=2\_p=0.999999894456002

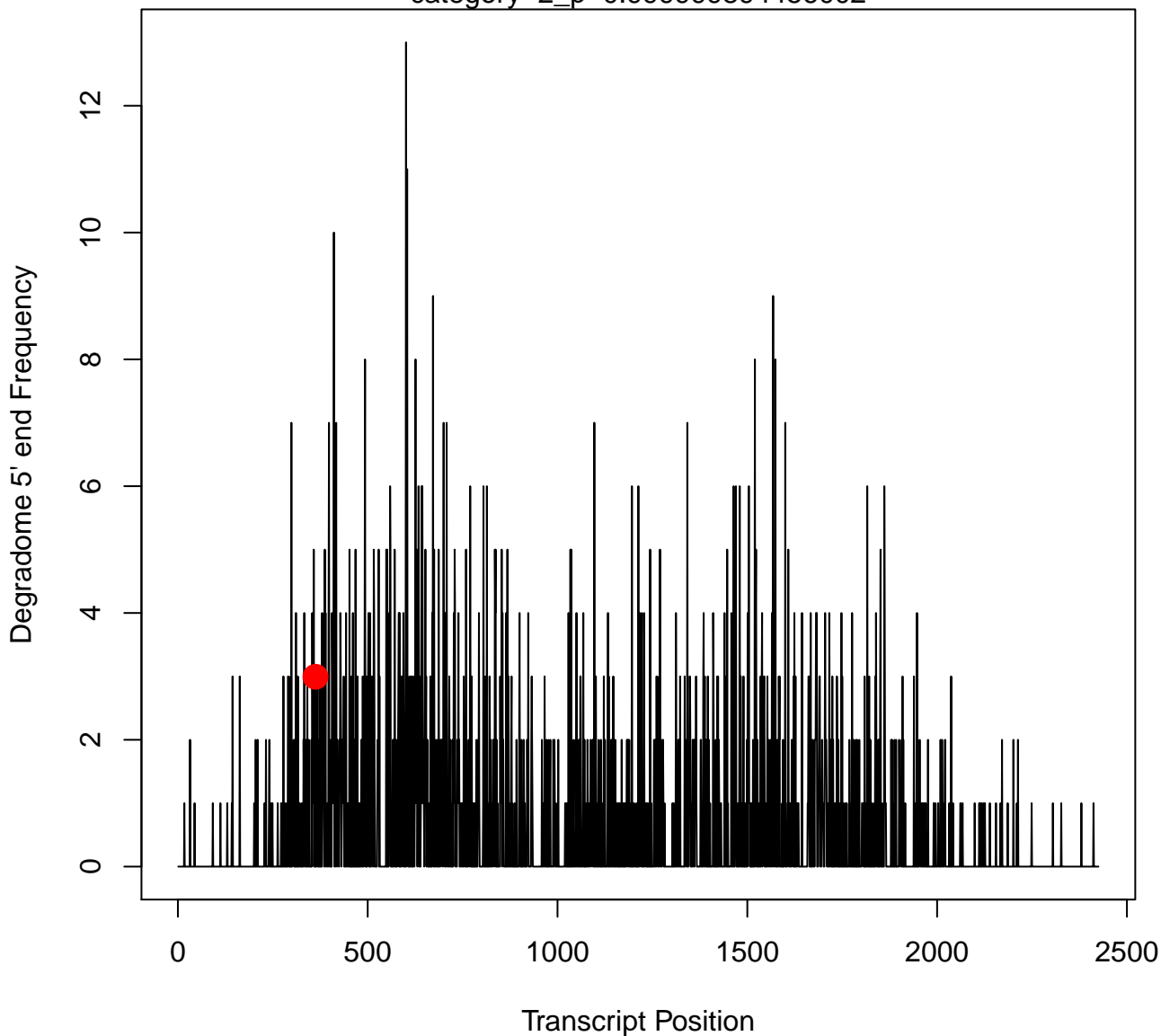

Supplement: Supplementary file 2 [file Data_Sheet_2.zip › Sit-miR160c_Seita.3G386700.1_363_TPlot.pdf]

**T=Seita.4G053700.1\_Q=Sit-miR160c\_S=1847**

category=2\_p=0.999999999994062

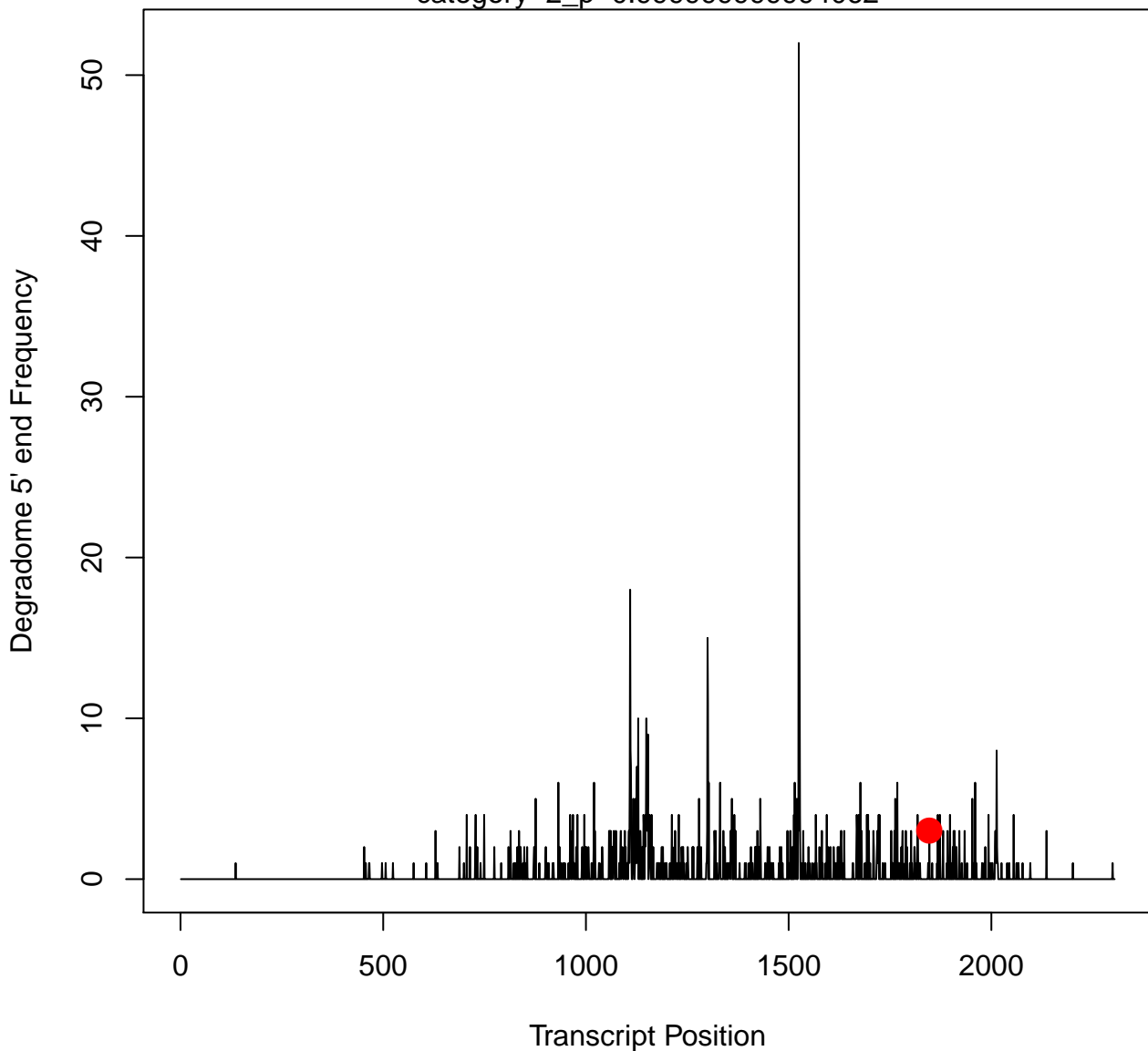

Supplement: Supplementary file 2 [file Data_Sheet_2.zip › Sit-miR160c_Seita.4G053700.1_1847_TPlot.pdf]

**T=Seita.4G216400.1\_Q=Sit-miR160c\_S=1036**

category=2\_p=1

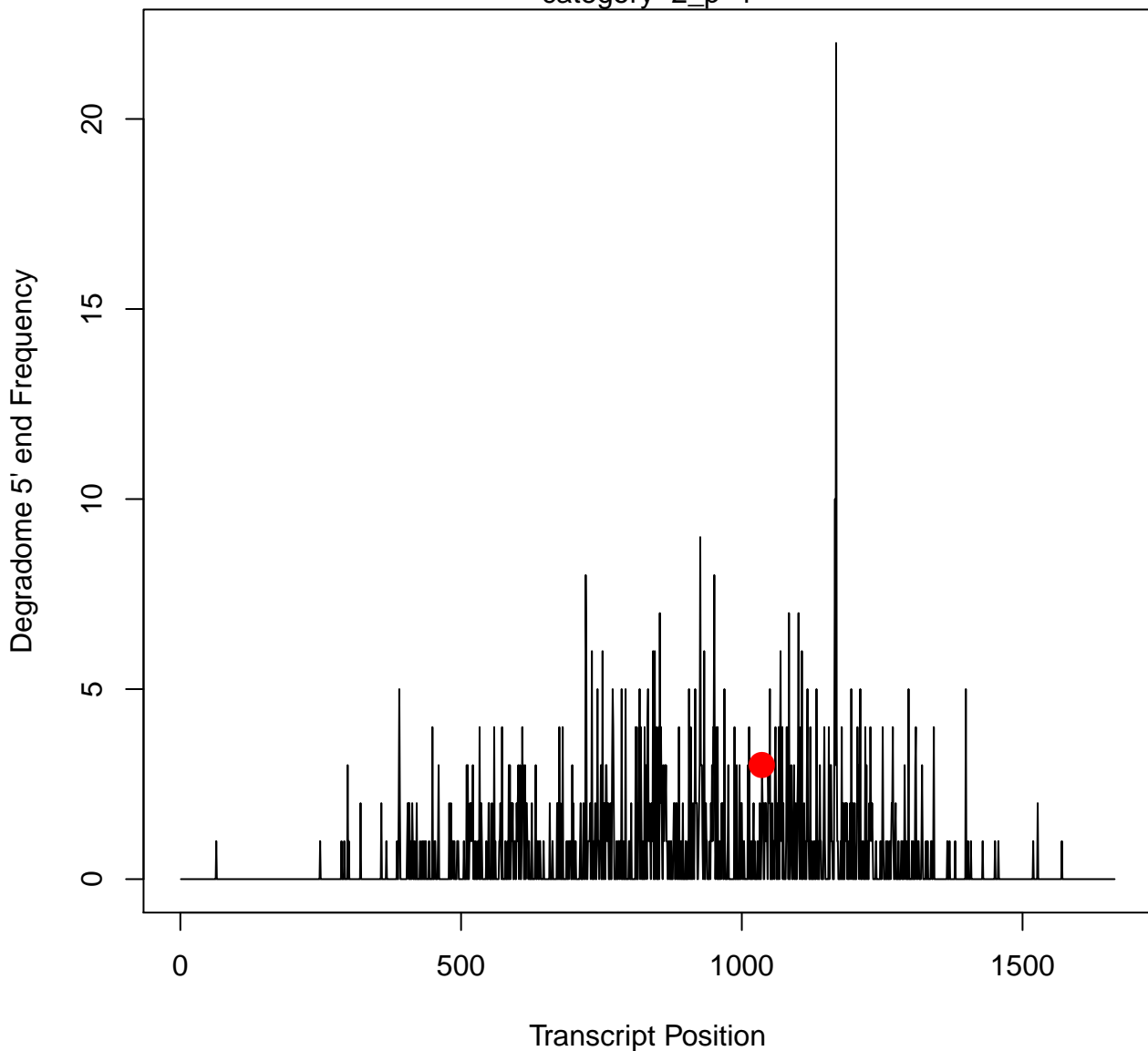

Supplement: Supplementary file 2 [file Data_Sheet_2.zip › Sit-miR160c_Seita.4G216400.1_1036_TPlot.pdf]

**T=Seita.4G222400.1\_Q=Sit-miR160c\_S=1446**

category=2\_p=0.719921955277745

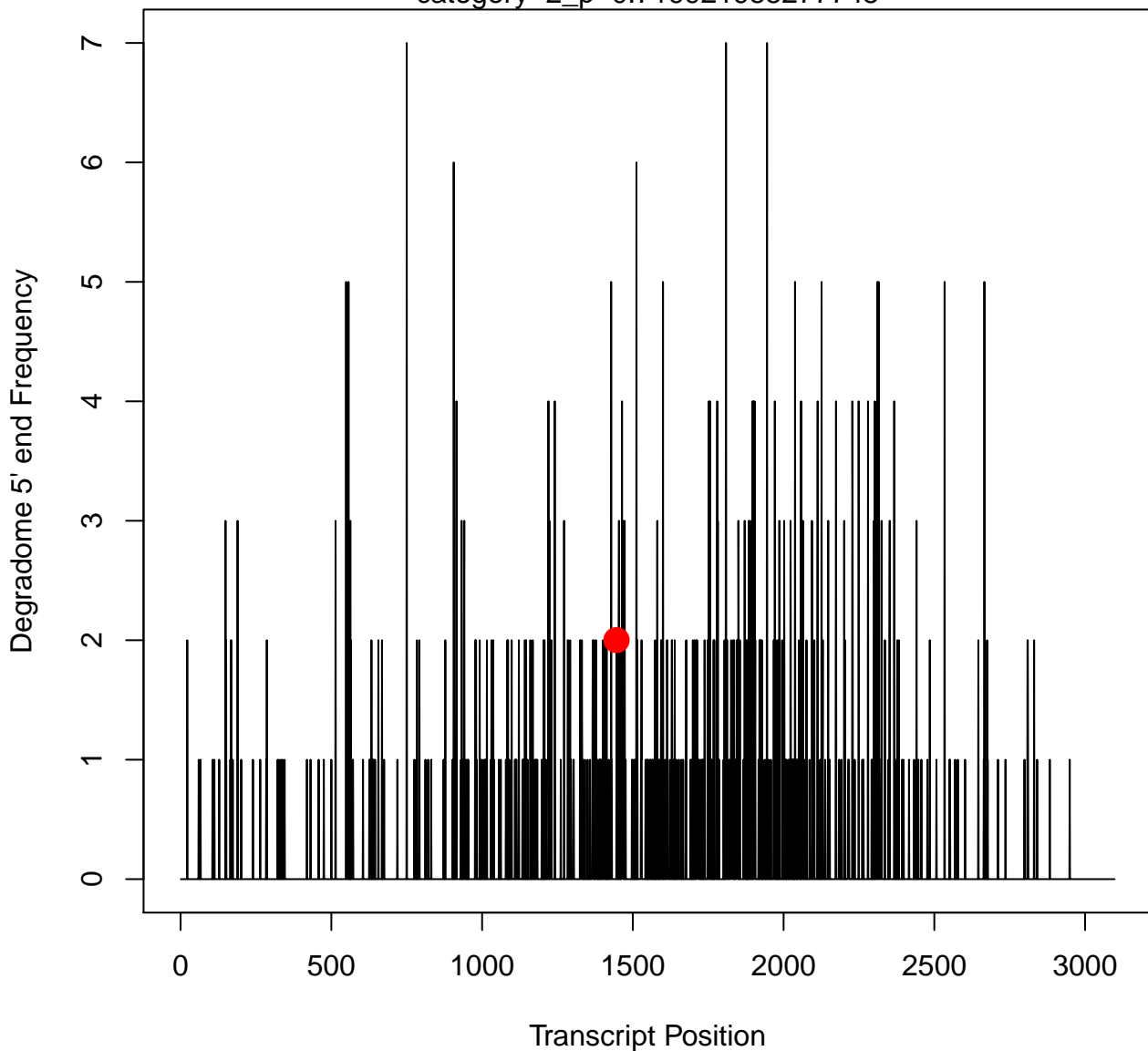

Supplement: Supplementary file 2 [file Data_Sheet_2.zip › Sit-miR160c_Seita.4G222400.1_1446_TPlot.pdf]

**T=Seita.4G265400.1\_Q=Sit-miR160c\_S=870**

category=2\_p=1

Degradsome 5' end Frequency

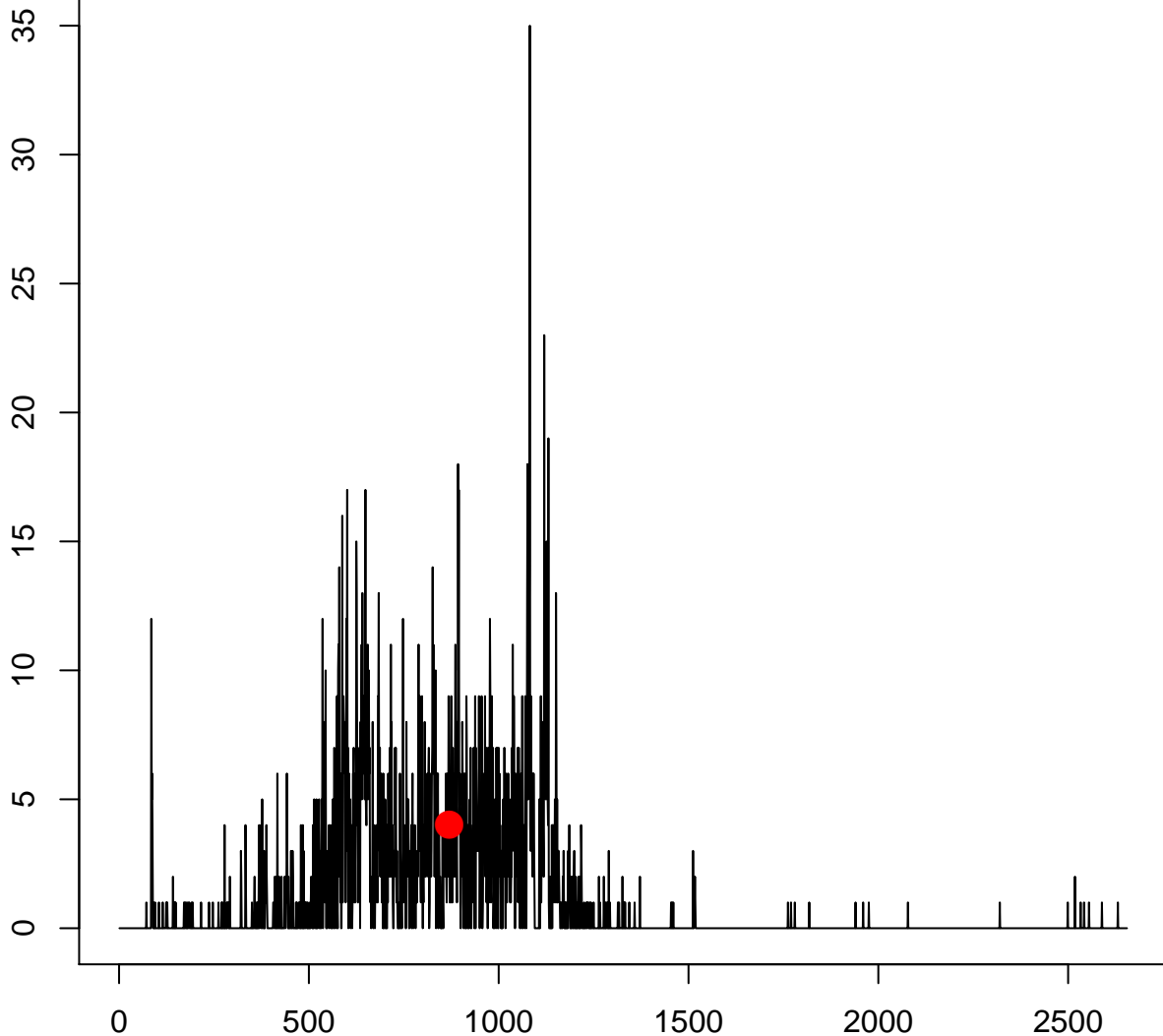

Transcript Position

Supplement: Supplementary file 2 [file Data_Sheet_2.zip › Sit-miR160c_Seita.4G265400.1_870_TPlot.pdf]

**T=Seita.7G090000.1\_Q=Sit-miR160c\_S=838**

category=2\_p=0.999999999955772

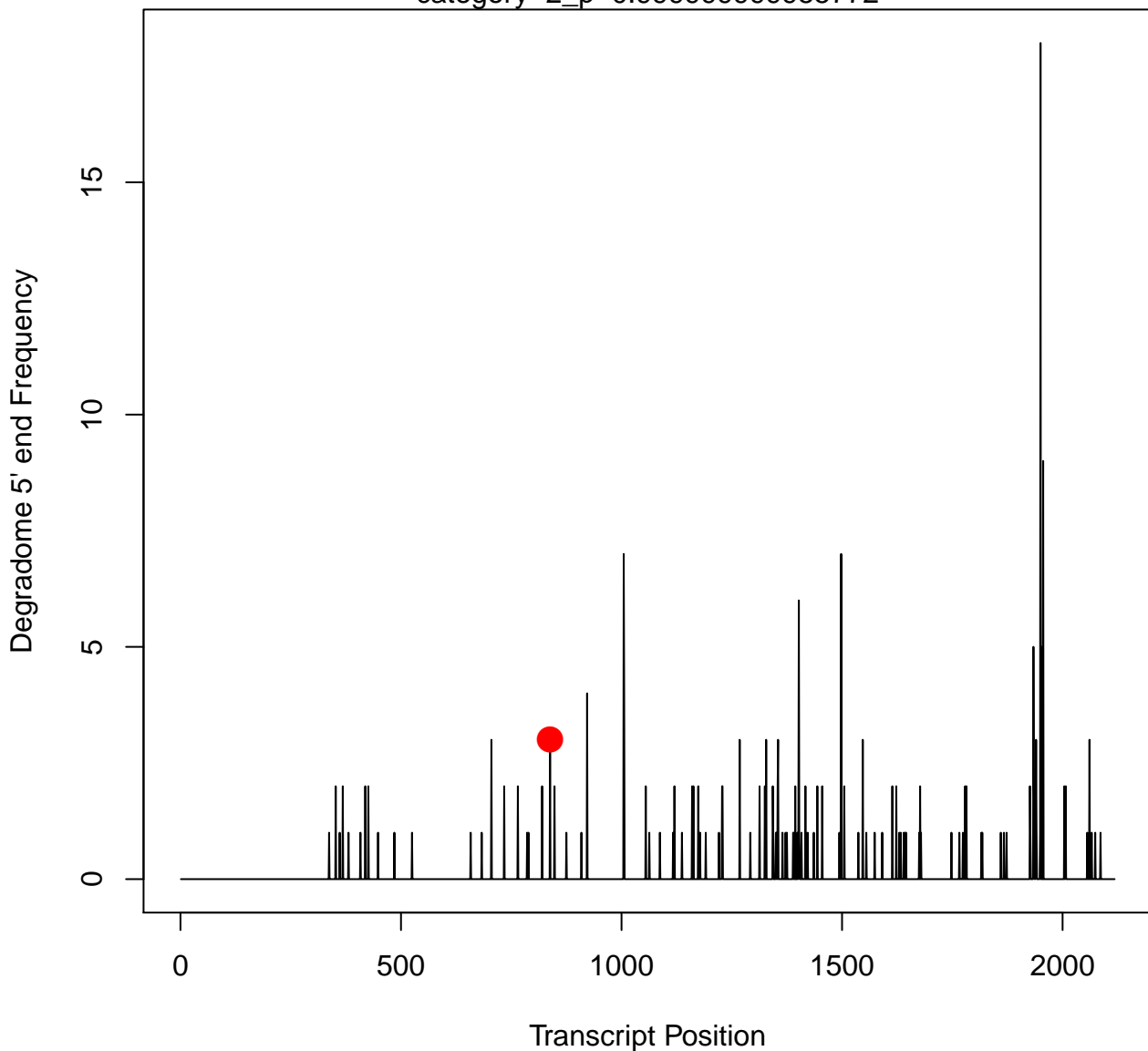

Supplement: Supplementary file 2 [file Data_Sheet_2.zip › Sit-miR160c_Seita.7G090000.1_838_TPlot.pdf]

**T=Seita.7G155700.1\_Q=Sit-miR160c\_S=1461**

category=2\_p=0.999787442487049

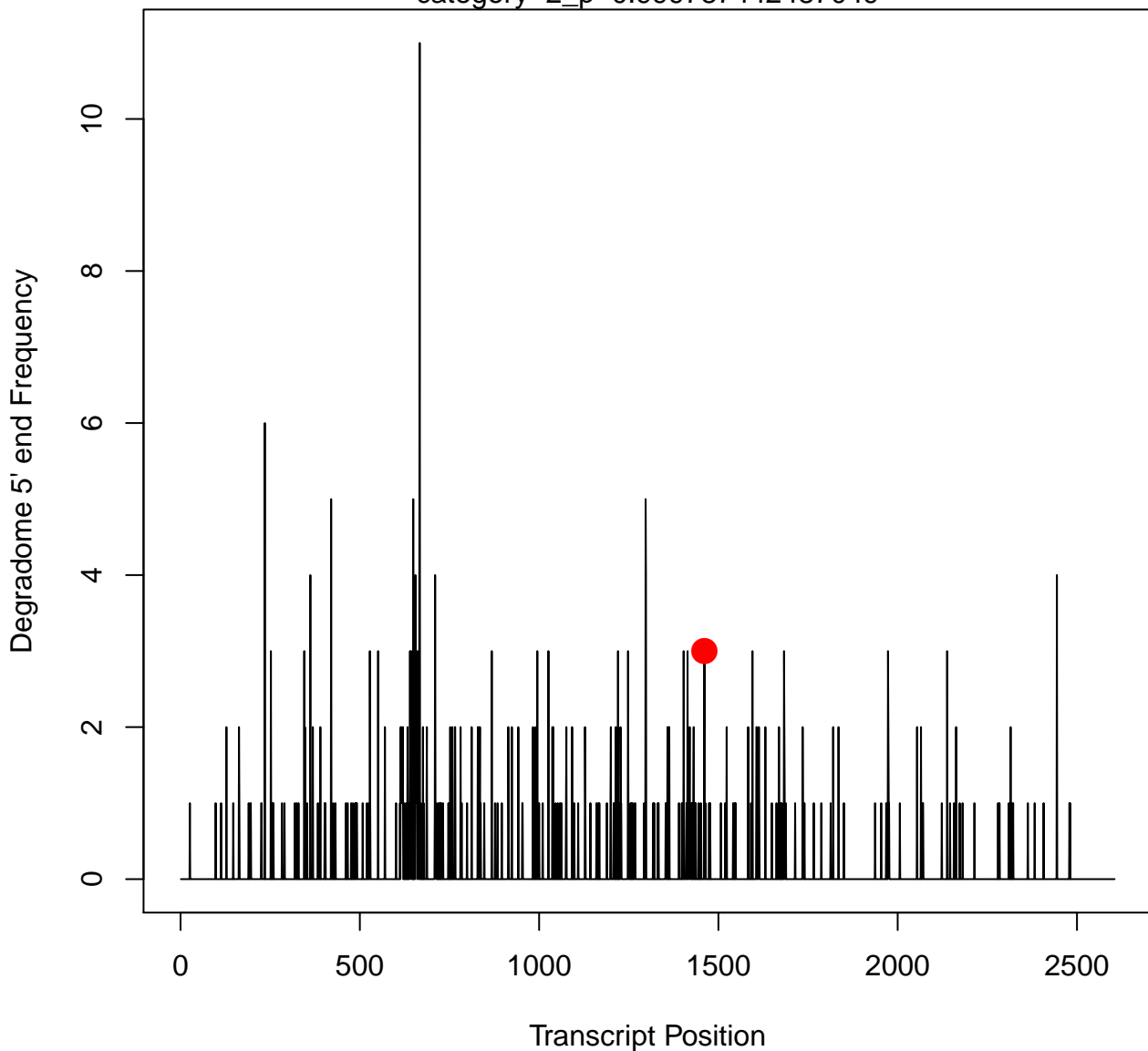

Supplement: Supplementary file 2 [file Data_Sheet_2.zip › Sit-miR160c_Seita.7G155700.1_1461_TPlot.pdf]

**T=Seita.7G194100.1\_Q=Sit-miR160c\_S=435**

category=2\_p=0.9999999999999998

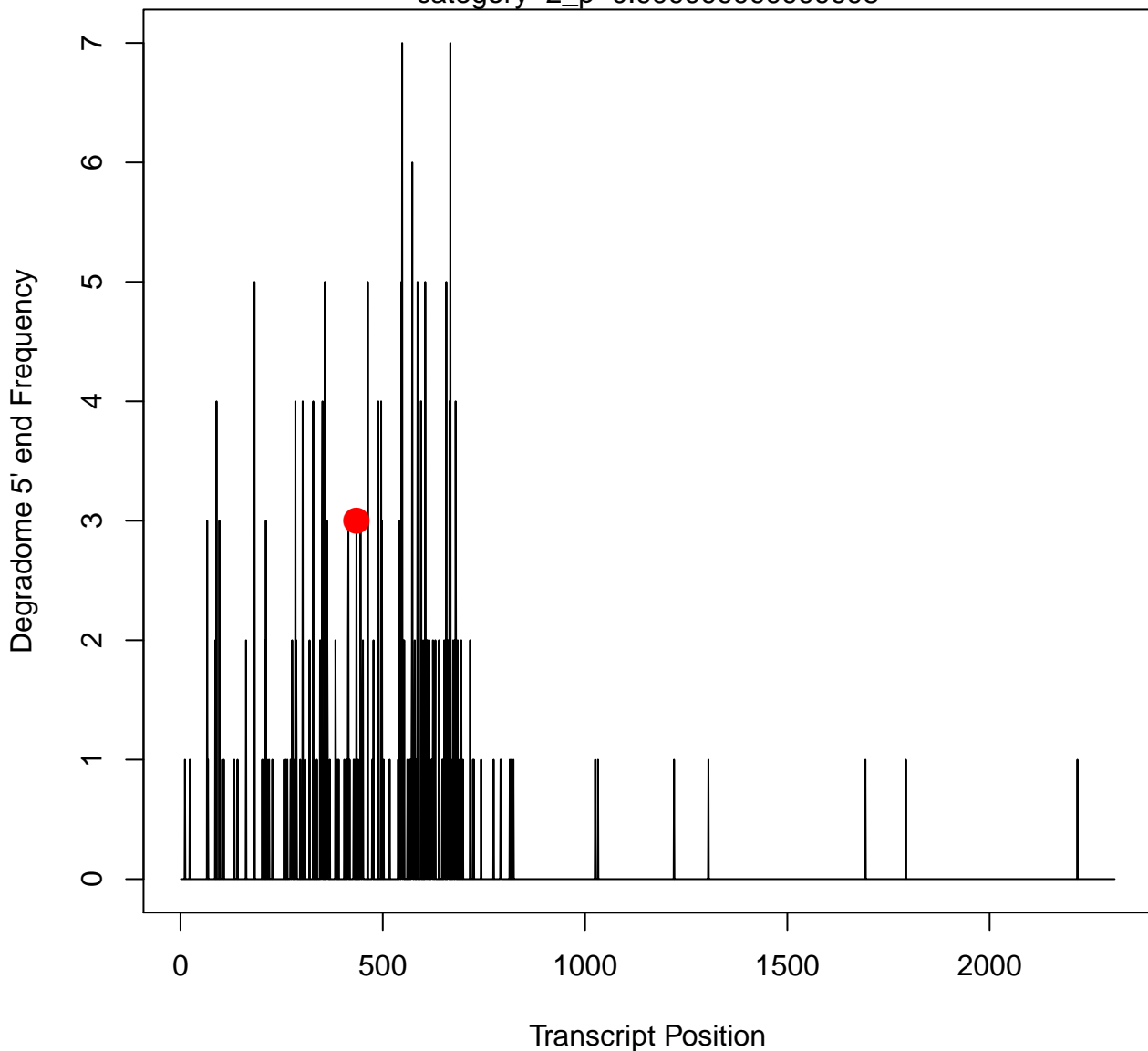

Supplement: Supplementary file 2 [file Data_Sheet_2.zip › Sit-miR160c_Seita.7G194100.1_435_TPlot.pdf]

**T=Seita.9G045100.1\_Q=Sit-miR160c\_S=835**

category=2\_p=0.998934411209794

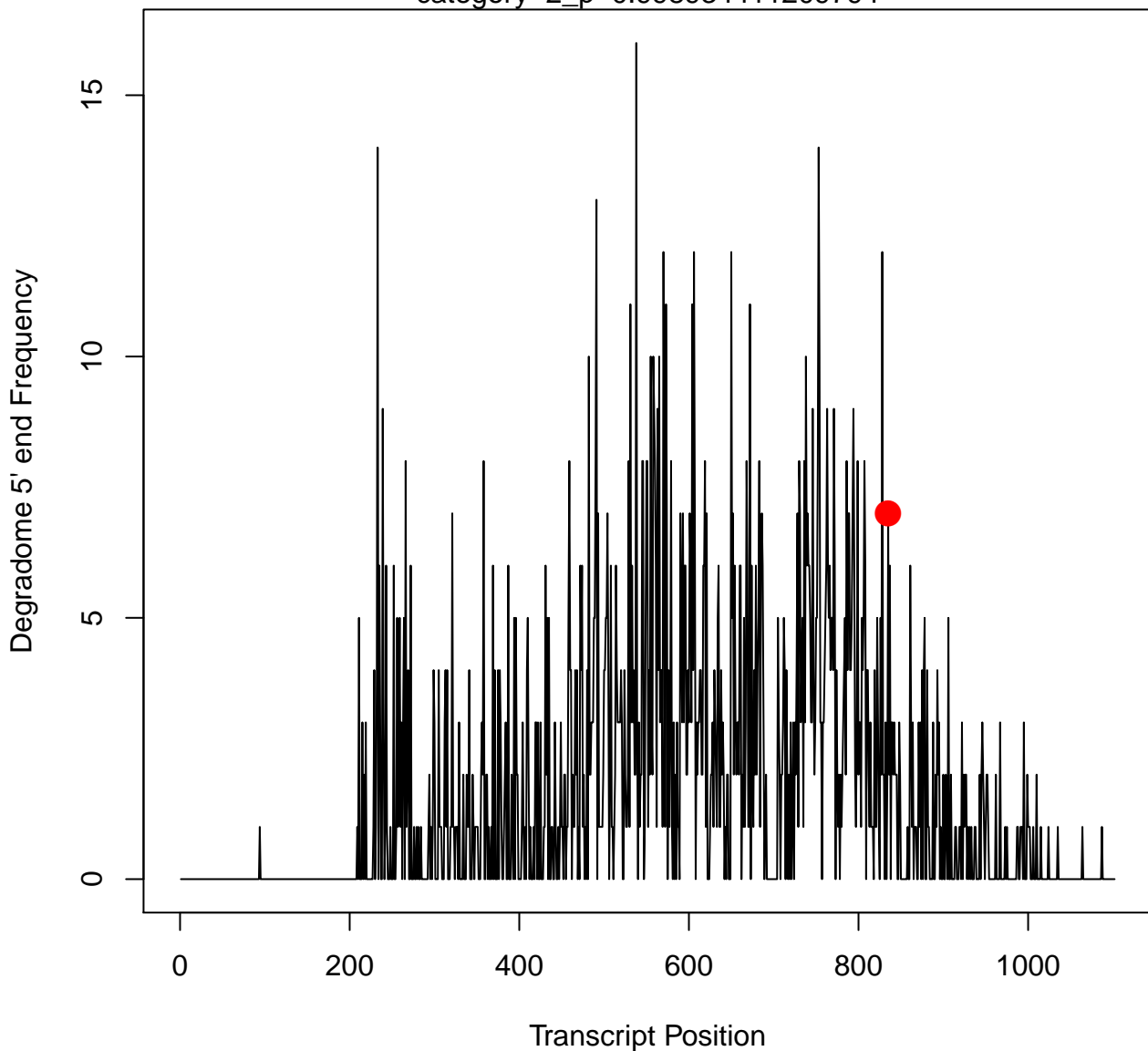

Supplement: Supplementary file 2 [file Data_Sheet_2.zip › Sit-miR160c_Seita.9G045100.1_835_TPlot.pdf]

**T=Seita.9G050200.1\_Q=Sit-miR160c\_S=1386**

category=2\_p=0.945689575762944

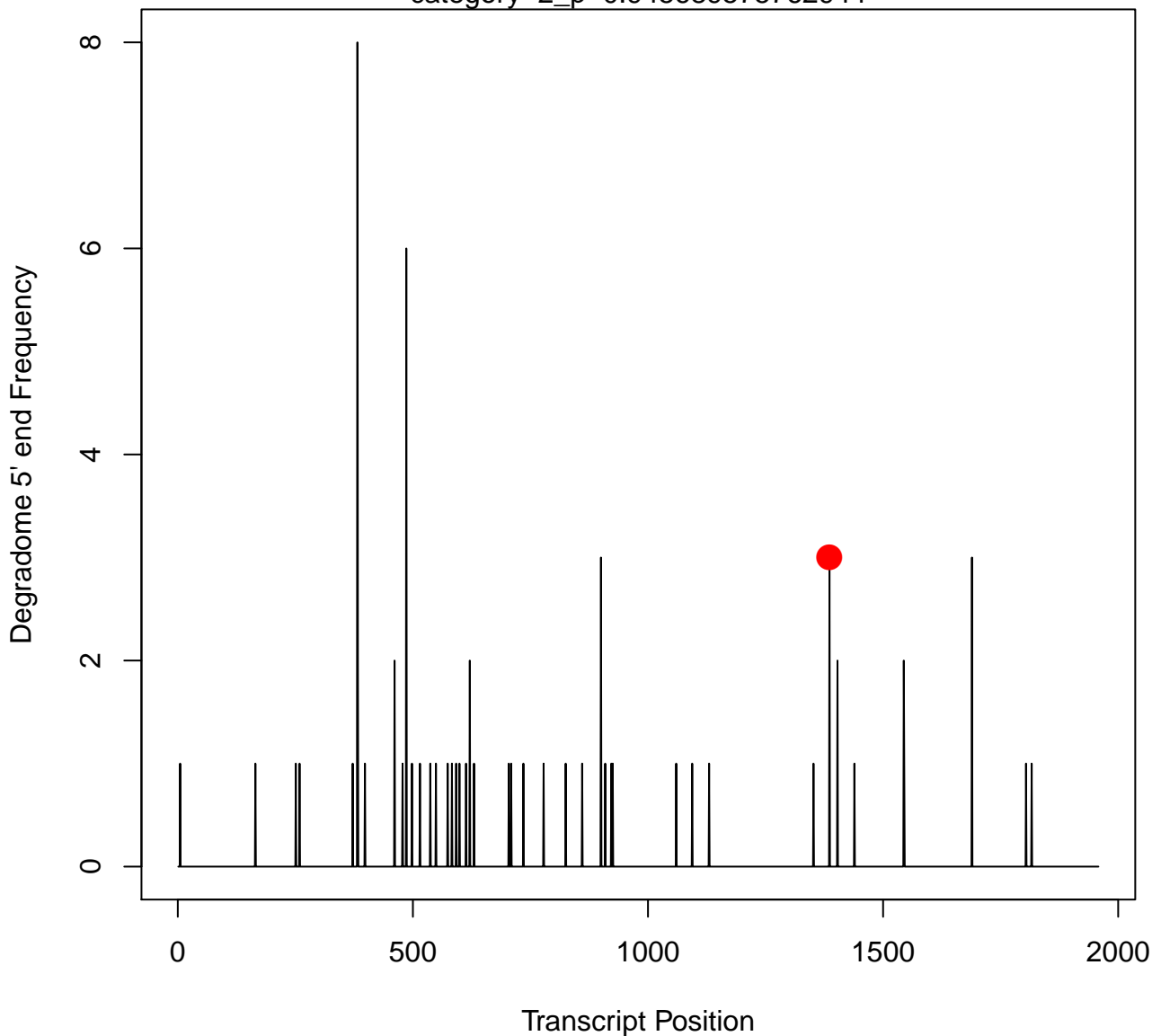

Supplement: Supplementary file 2 [file Data_Sheet_2.zip › Sit-miR160c_Seita.9G050200.1_1386_TPlot.pdf]

**T=Seita.J025900.1\_Q=Sit-miR160c\_S=1332**

category=2\_p=0.99999965381646

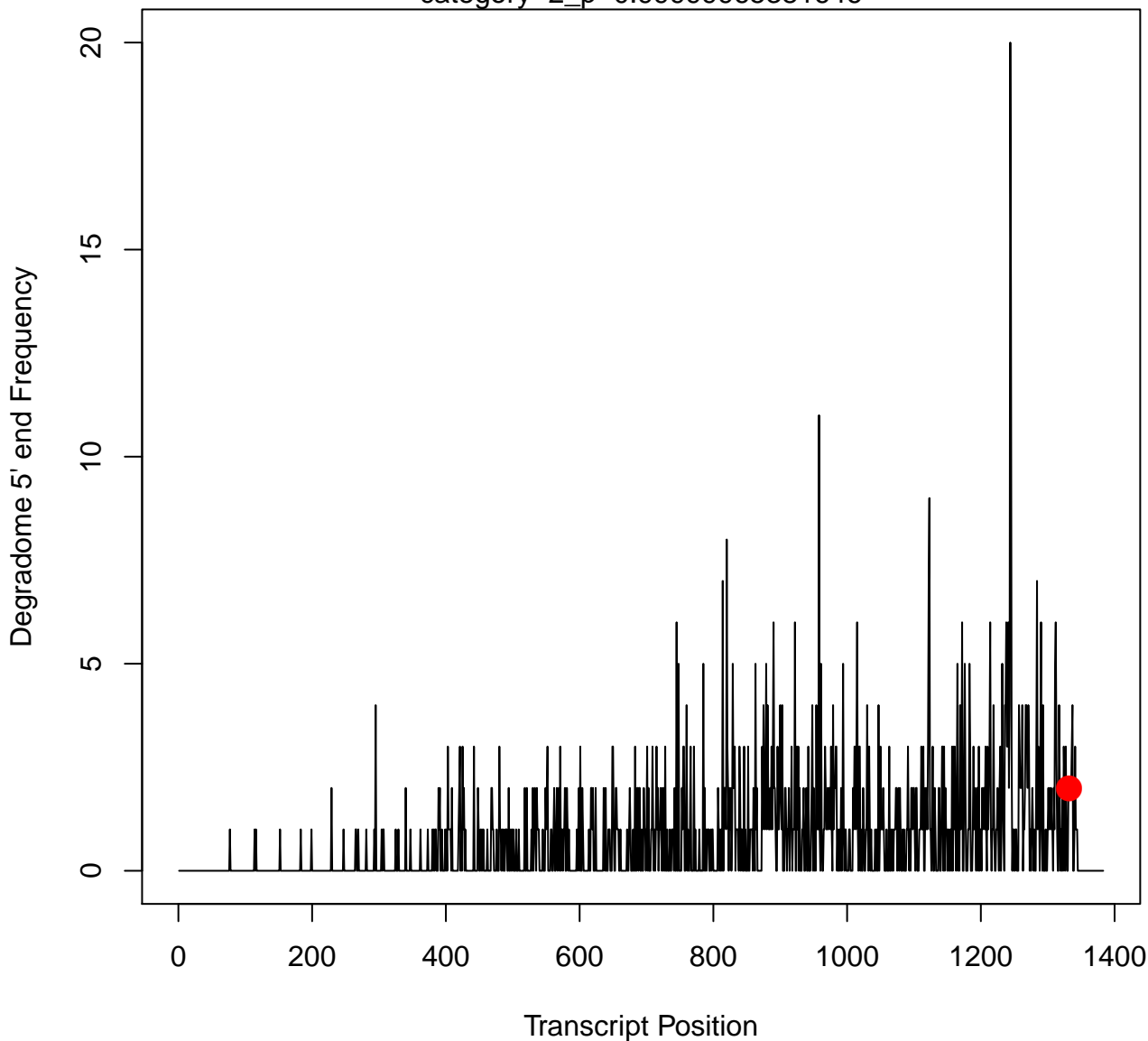

Supplement: Supplementary file 2 [file Data_Sheet_2.zip › Sit-miR160c_Seita.J025900.1_1332_TPlot.pdf]

**T=Seita.1G241500.1\_Q=Sit-miR160d\_S=1660**

category=2\_p=0.13186640496421

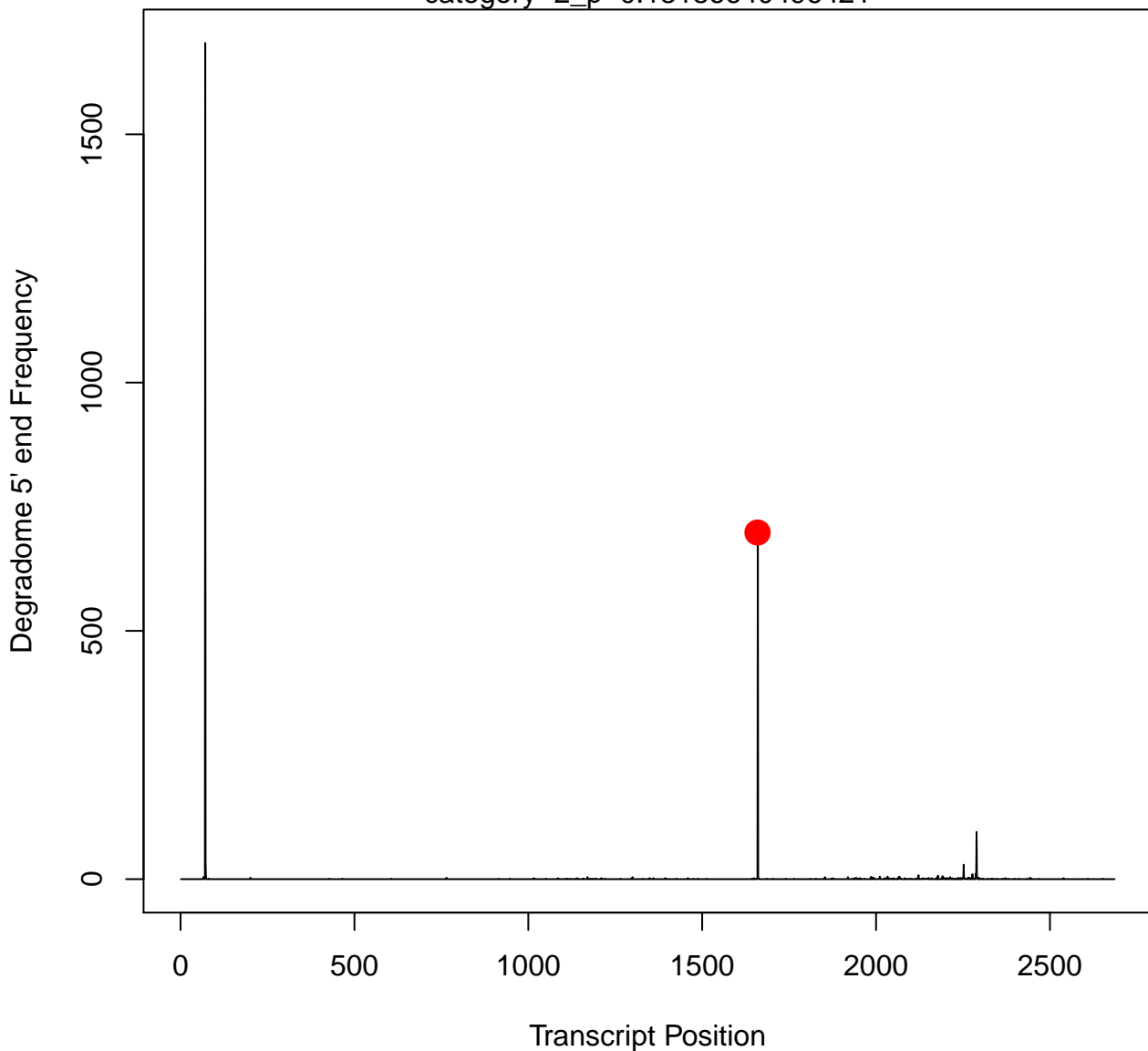

Supplement: Supplementary file 2 [file Data_Sheet_2.zip › Sit-miR160d_Seita.1G241500.1_1660_TPlot.pdf]

**T=Seita.4G043900.1\_Q=Sit-miR160d\_S=308**

category=2\_p=0.999999998482843

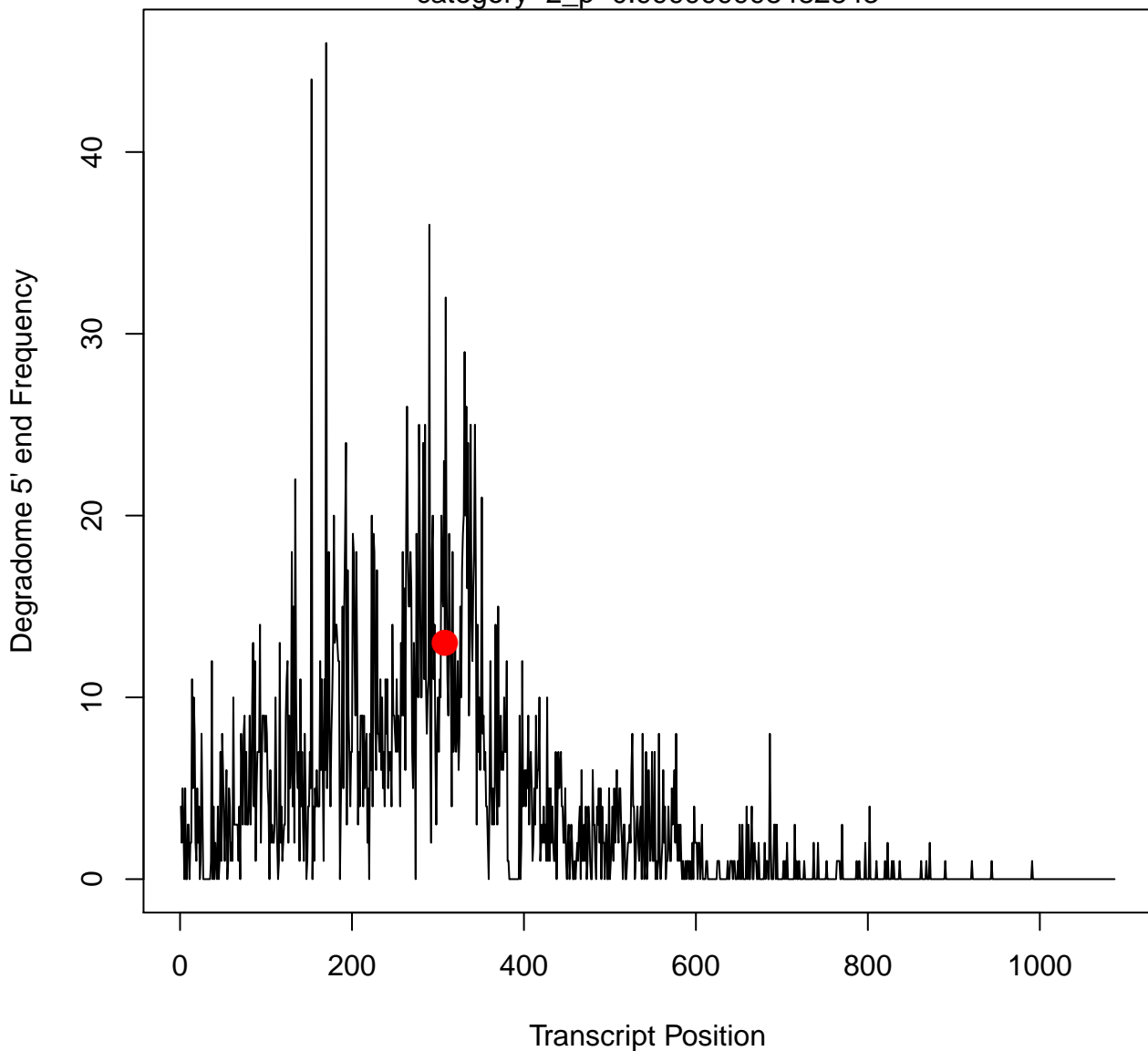

Supplement: Supplementary file 2 [file Data_Sheet_2.zip › Sit-miR160d_Seita.4G043900.1_308_TPlot.pdf]

**T=Seita.4G174800.1\_Q=Sit-miR160d\_S=455**

category=2\_p=0.999999999994062

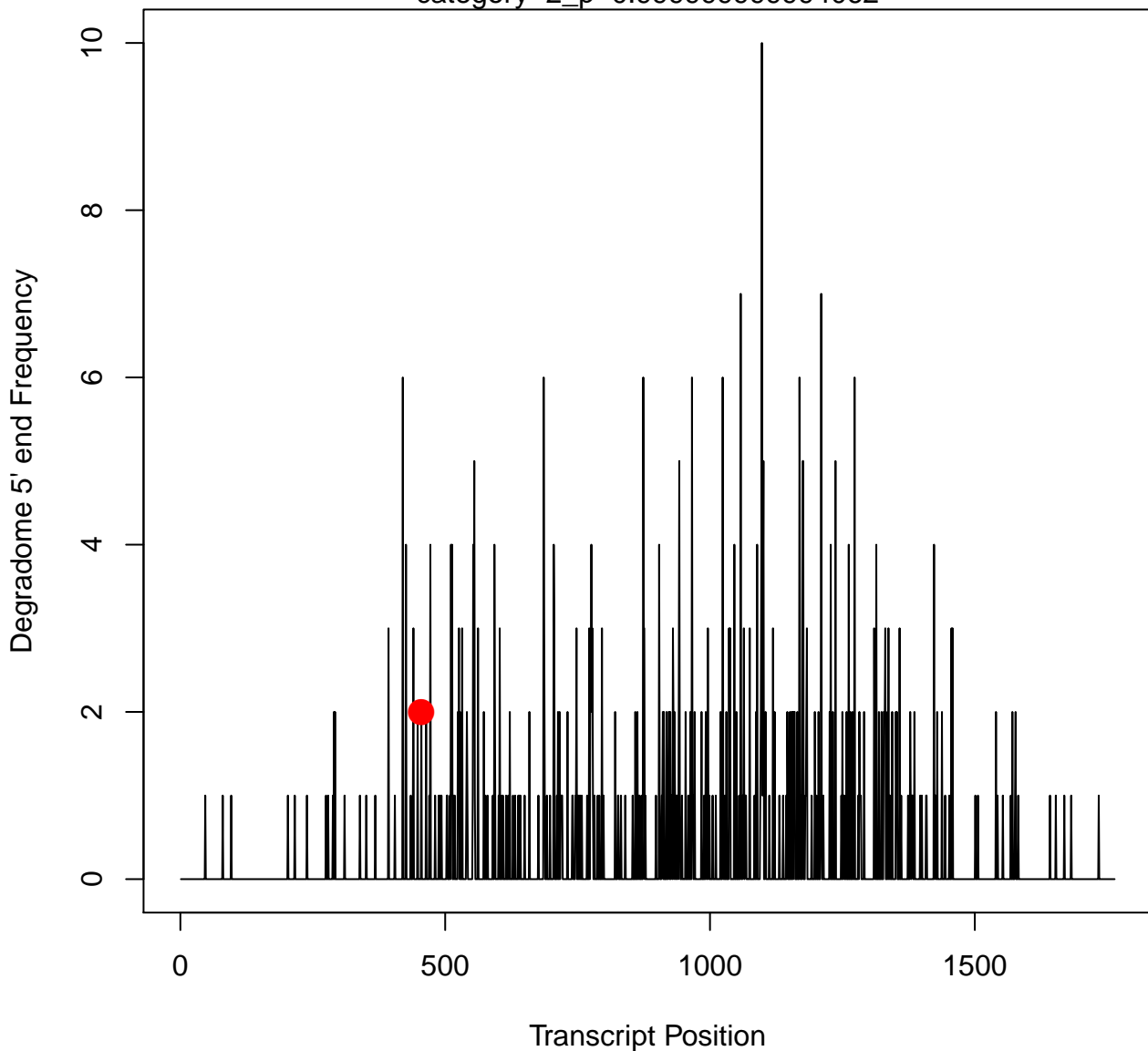

Supplement: Supplementary file 2 [file Data_Sheet_2.zip › Sit-miR160d_Seita.4G174800.1_455_TPlot.pdf]

**T=Seita.4G257800.1\_Q=Sit-miR160d\_S=1974**

category=0\_p=0.000770849717844468

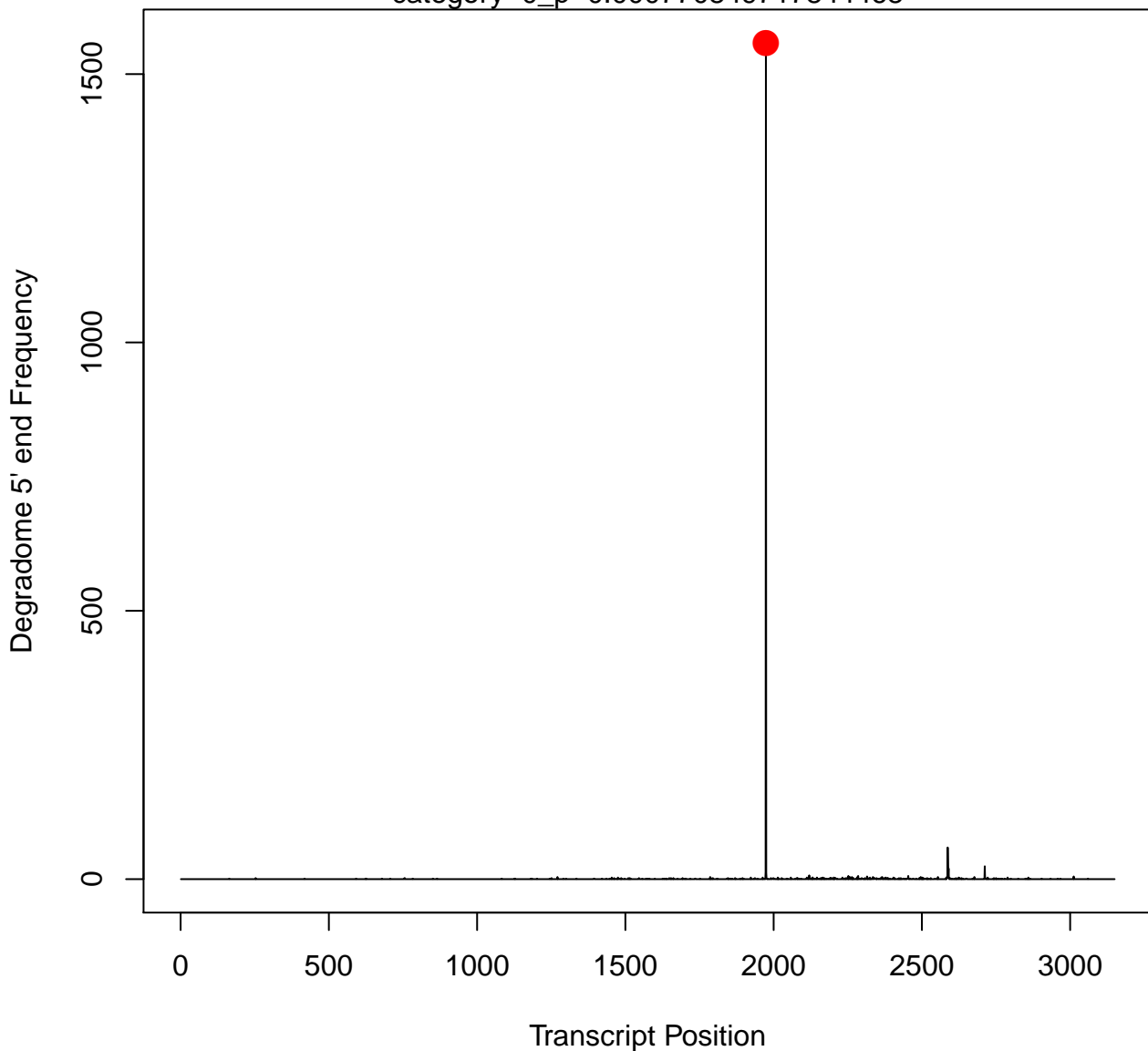

Supplement: Supplementary file 2 [file Data_Sheet_2.zip › Sit-miR160d_Seita.4G257800.1_1974_TPlot.pdf]

**T=Seita.4G290000.1\_Q=Sit-miR160d\_S=1772**

category=2\_p=0.99999991817437

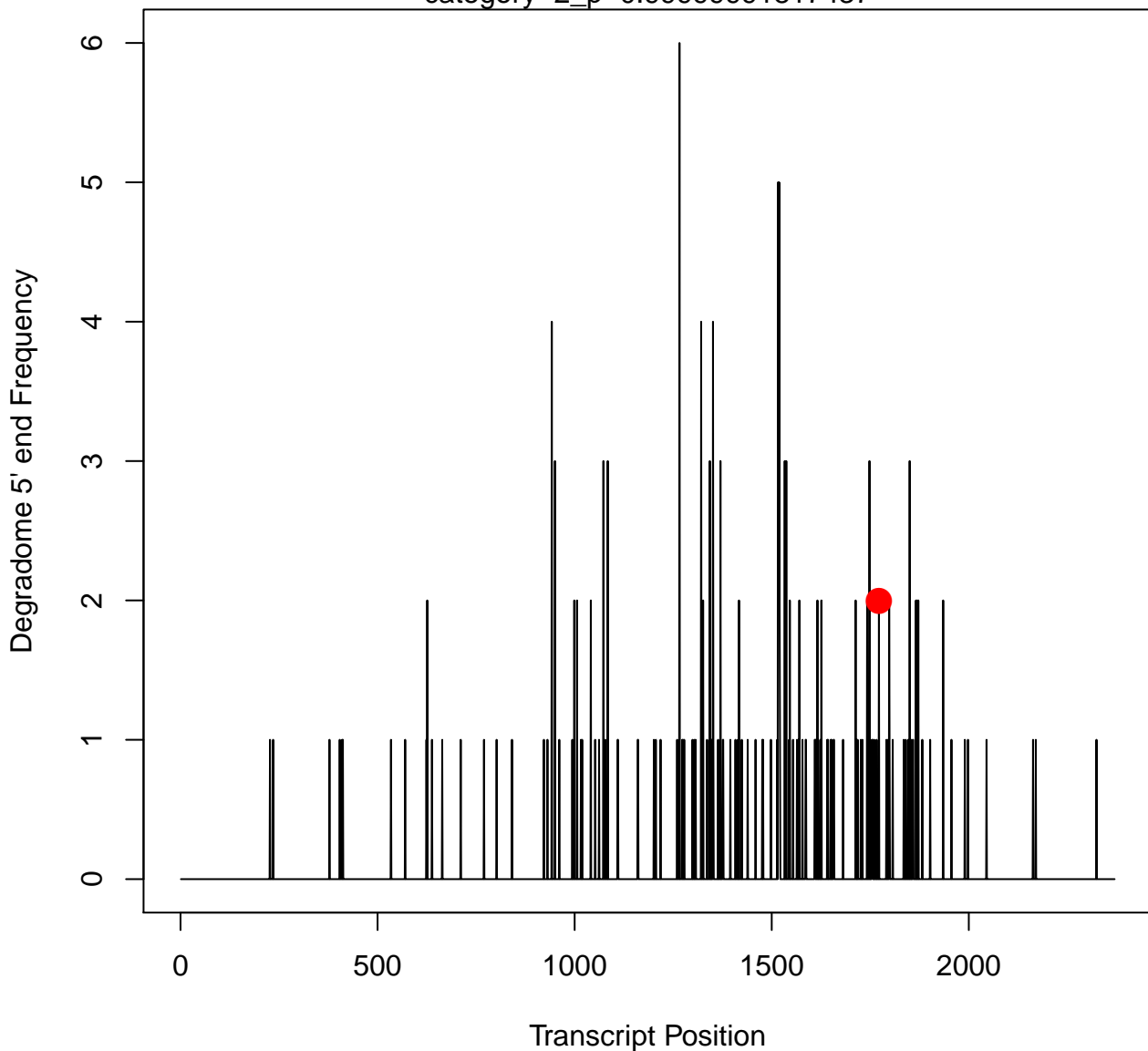

Supplement: Supplementary file 2 [file Data_Sheet_2.zip › Sit-miR160d_Seita.4G290000.1_1772_TPlot.pdf]

**T=Seita.6G173400.1\_Q=Sit-miR160d\_S=505**

category=2\_p=0.99999999877575

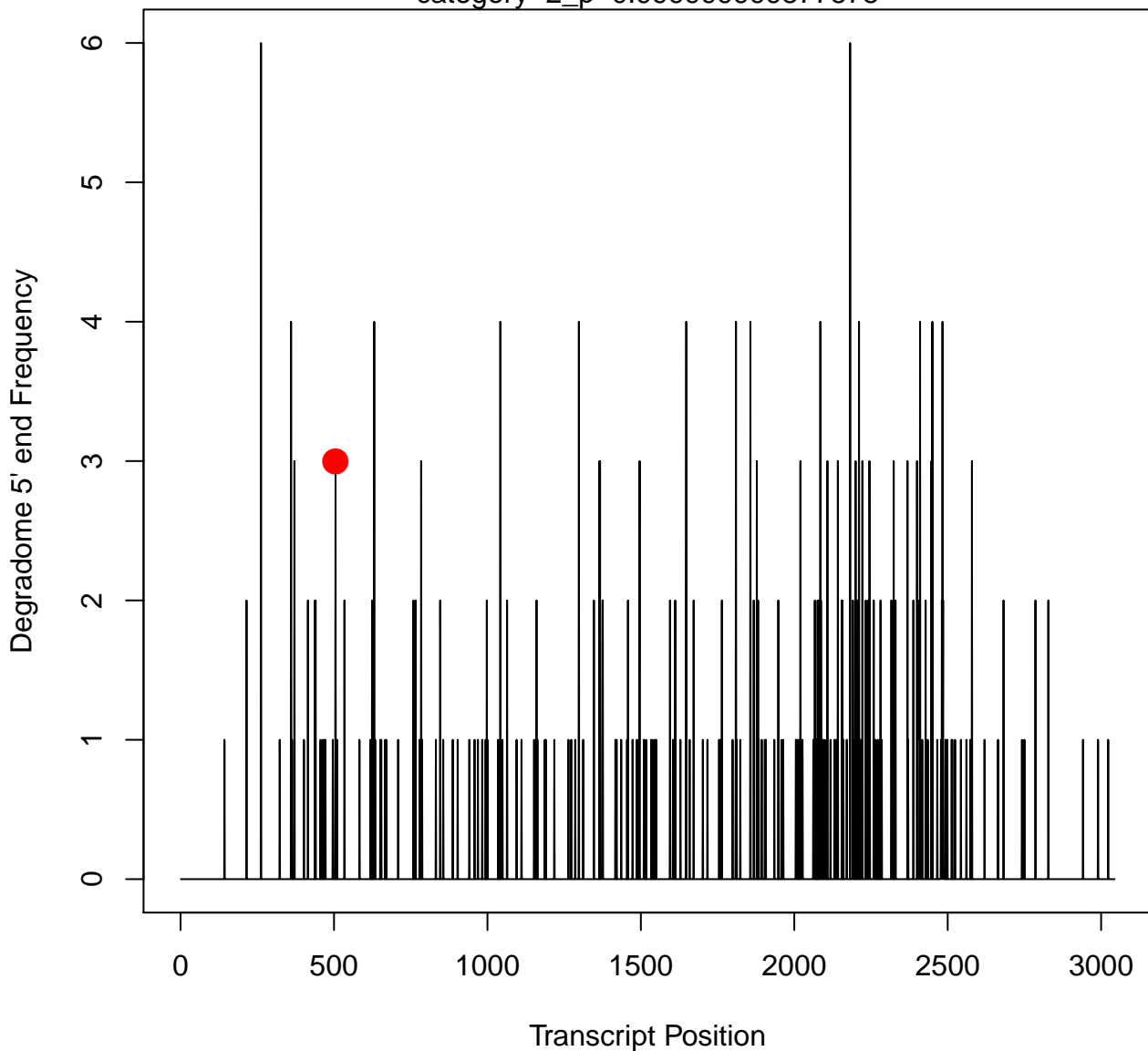

Supplement: Supplementary file 2 [file Data_Sheet_2.zip › Sit-miR160d_Seita.6G173400.1_505_TPlot.pdf]

**T=Seita.7G169600.1\_Q=Sit-miR160d\_S=1802**

category=0\_p=0.000385499163724878

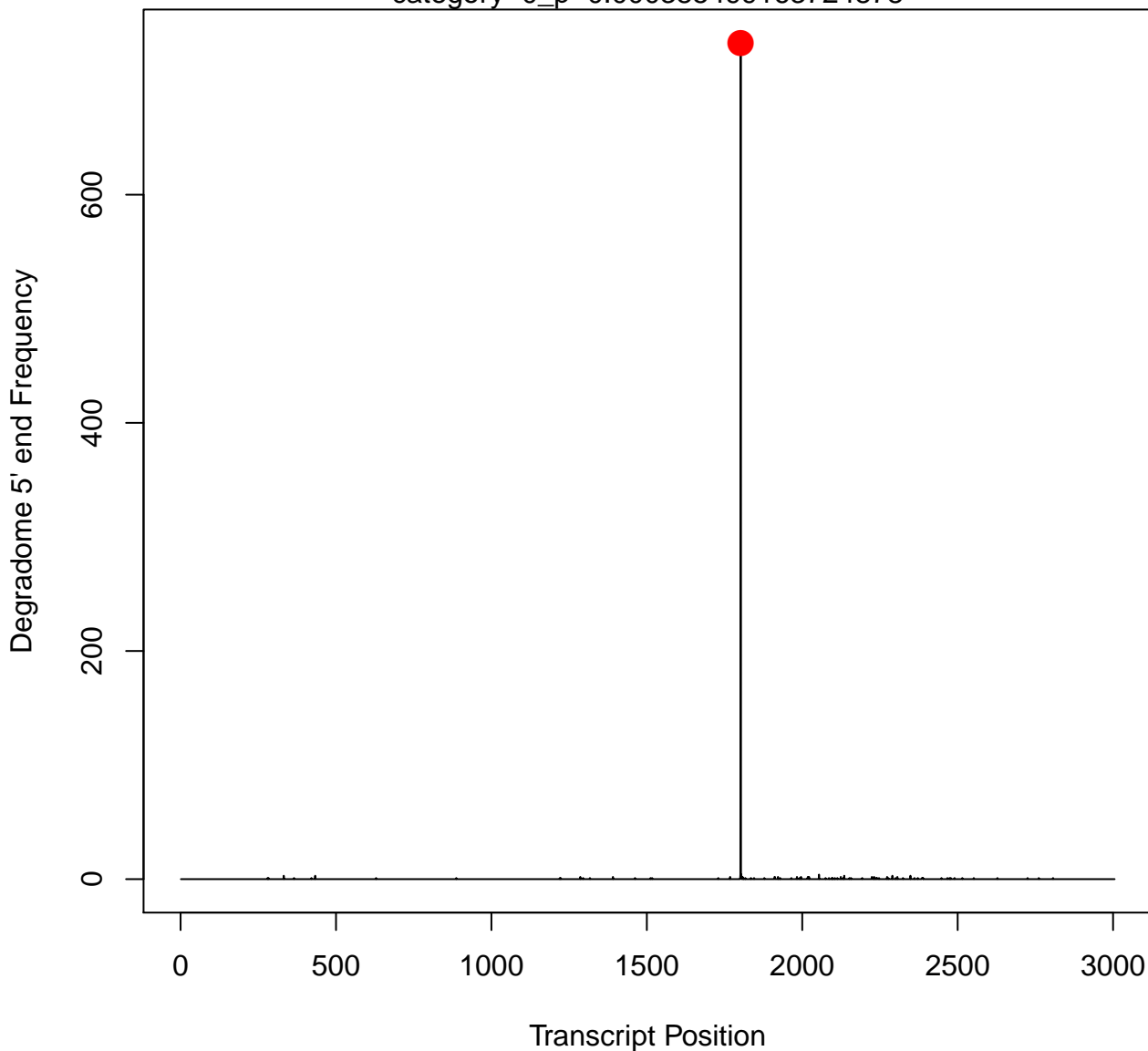

Supplement: Supplementary file 2 [file Data_Sheet_2.zip › Sit-miR160d_Seita.7G169600.1_1802_TPlot.pdf]

**T=Seita.8G049300.1\_Q=Sit-miR160d\_S=1249**

category=2\_p=1

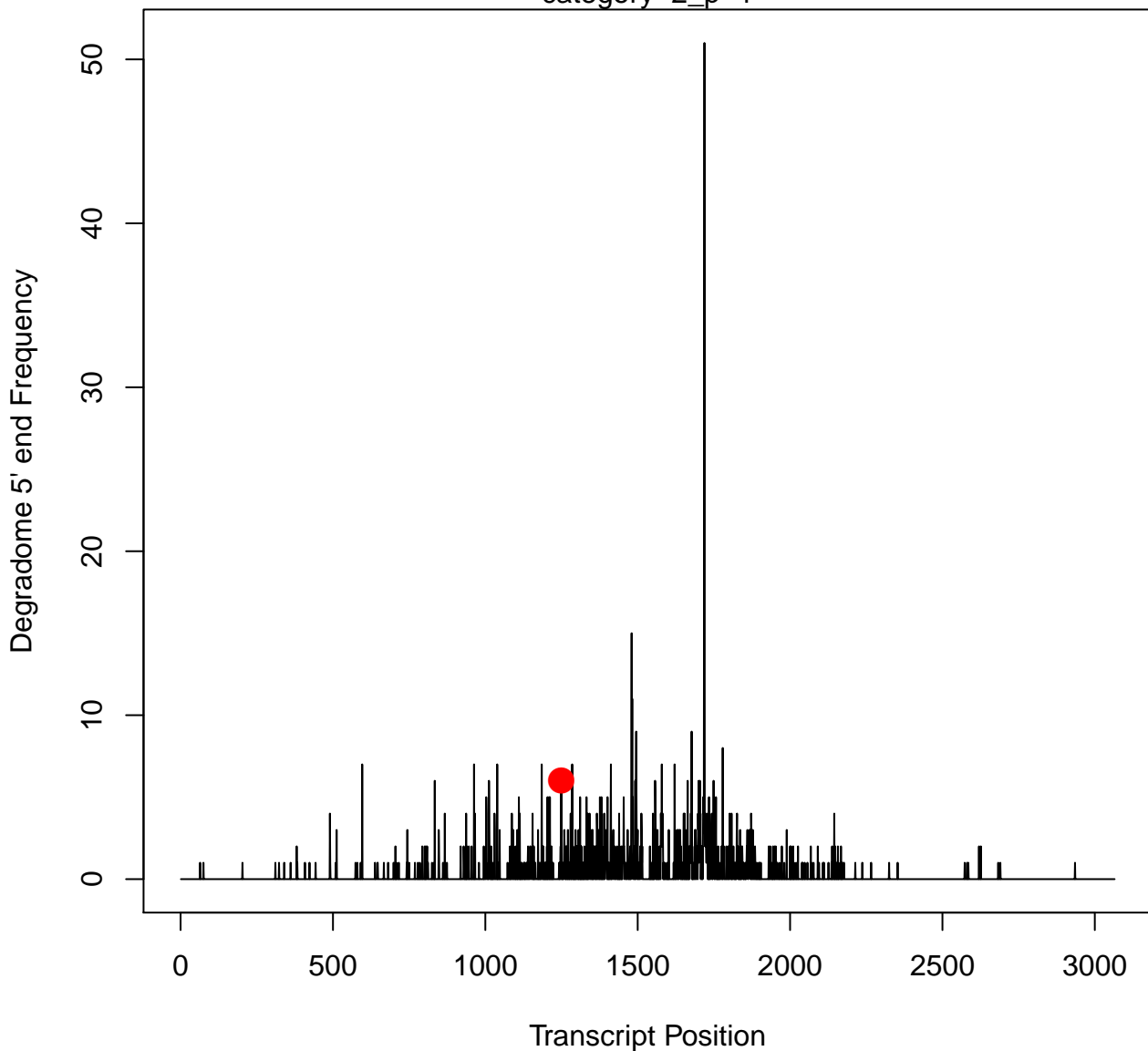

Supplement: Supplementary file 2 [file Data_Sheet_2.zip › Sit-miR160d_Seita.8G049300.1_1249_TPlot.pdf]

**T=Seita.9G237700.1\_Q=Sit-miR160d\_S=613**

category=2\_p=0.999999513930172

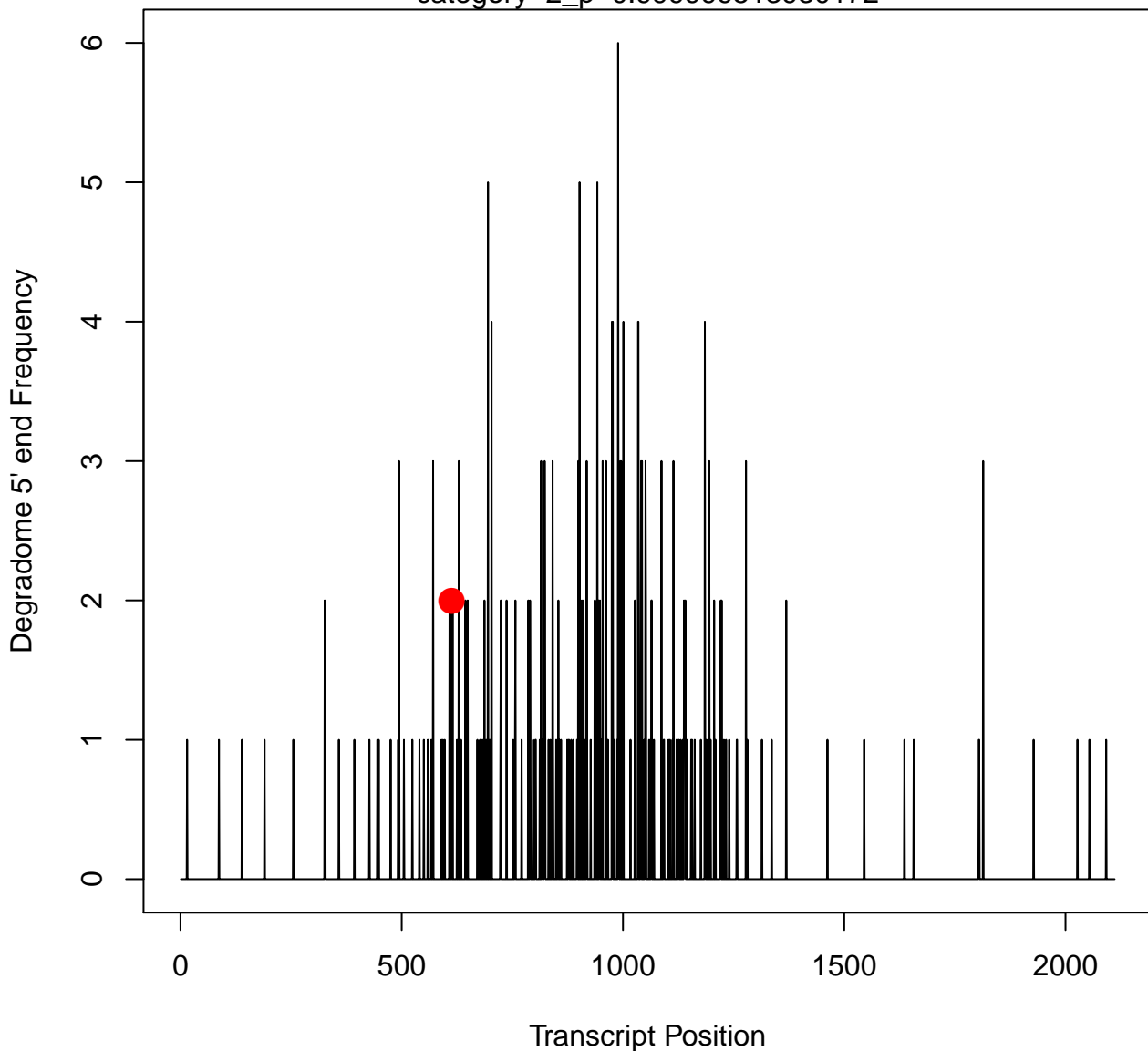

Supplement: Supplementary file 2 [file Data_Sheet_2.zip › Sit-miR160d_Seita.9G237700.1_613_TPlot.pdf]

**T=Seita.2G133300.1\_Q=Sit-miR162\_S=1963**

category=2\_p=0.986026210434499

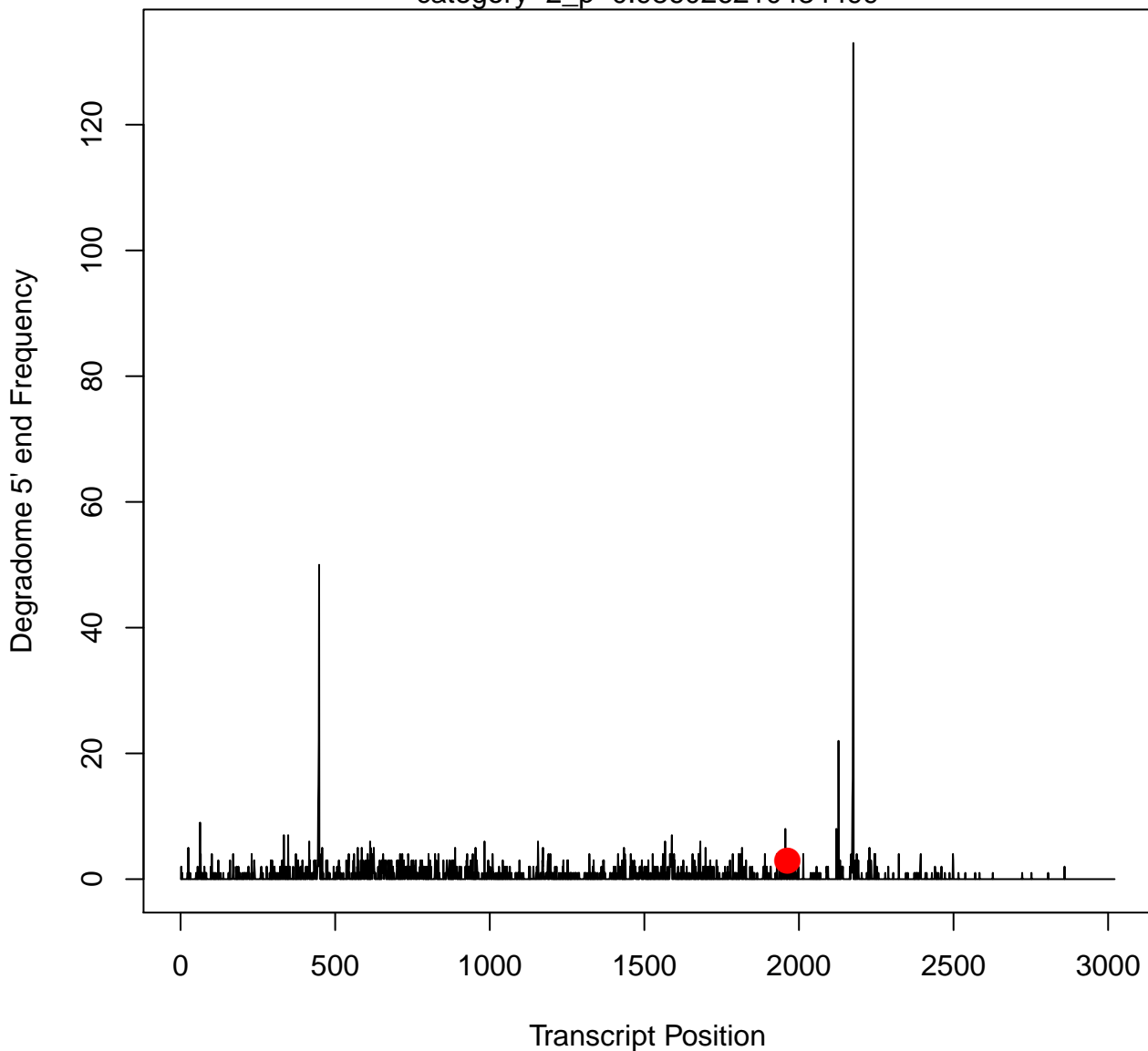

Supplement: Supplementary file 2 [file Data_Sheet_2.zip › Sit-miR162_Seita.2G133300.1_1963_TPlot.pdf]

**T=Seita.2G271300.1\_Q=Sit-miR162\_S=793**

category=2\_p=0.995617478913254

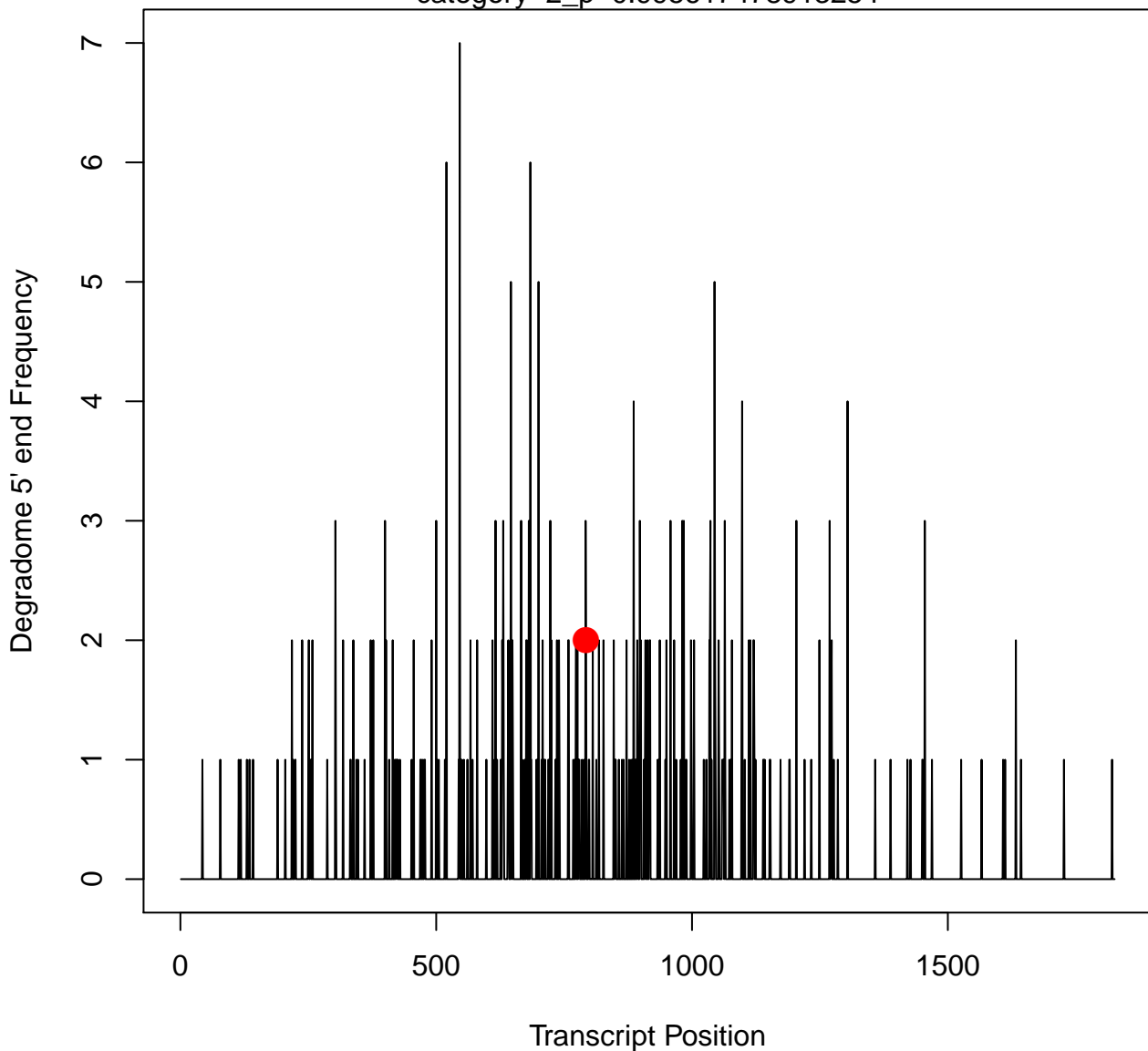

Supplement: Supplementary file 2 [file Data_Sheet_2.zip › Sit-miR162_Seita.2G271300.1_793_TPlot.pdf]

**T=Seita.5G064400.1\_Q=Sit-miR162\_S=878**

category=0\_p=0.000770849717844468

Degradome 5' end Frequency

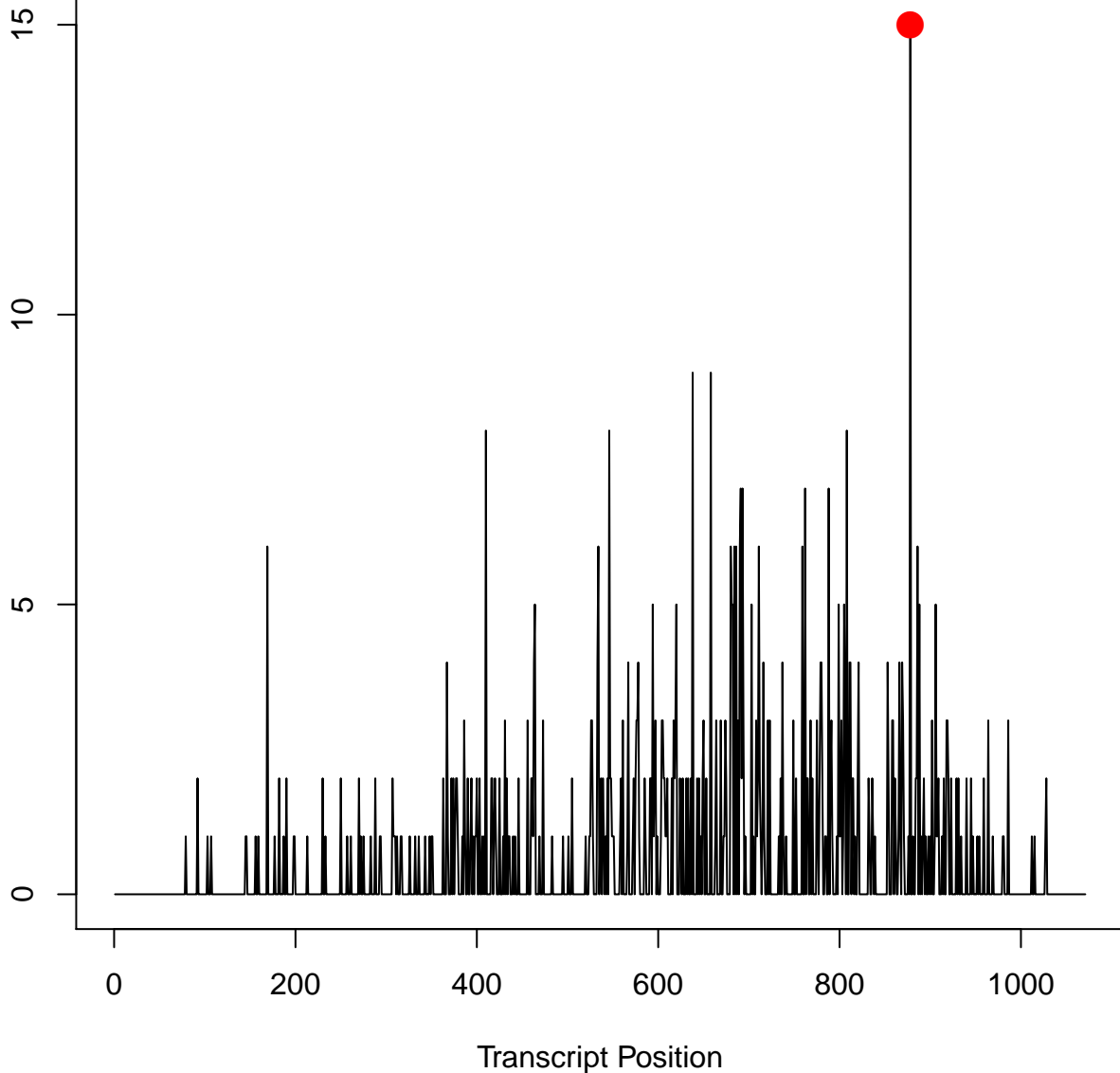

Supplement: Supplementary file 2 [file Data_Sheet_2.zip › Sit-miR162_Seita.5G064400.1_878_TPlot.pdf]

**T=Seita.5G177000.1\_Q=Sit-miR162\_S=1193**

category=2\_p=0.398948581621448

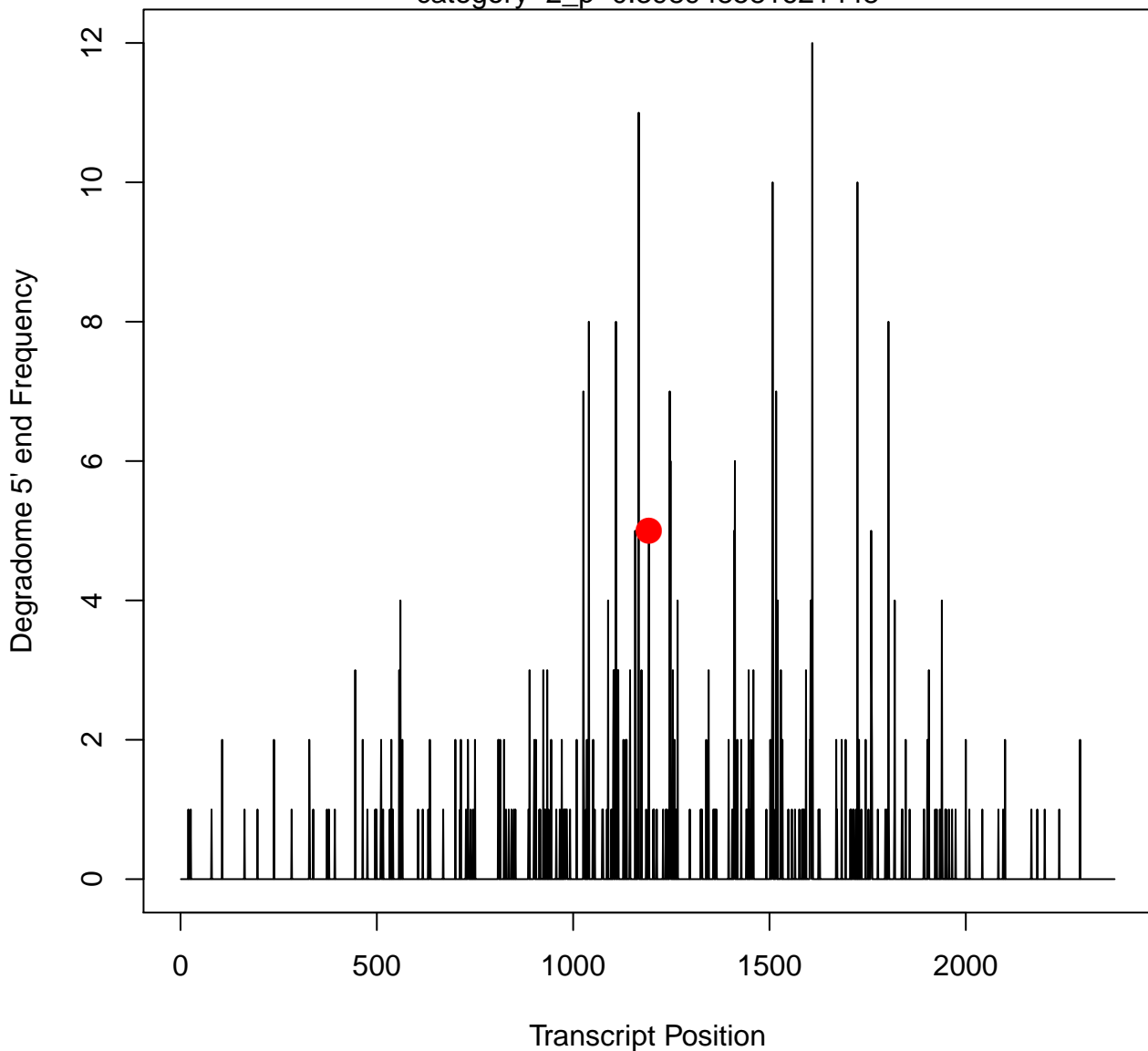

Supplement: Supplementary file 2 [file Data_Sheet_2.zip › Sit-miR162_Seita.5G177000.1_1193_TPlot.pdf]

**T=Seita.9G562200.1\_Q=Sit-miR162\_S=3530**

category=0\_p=0.000385499163724878

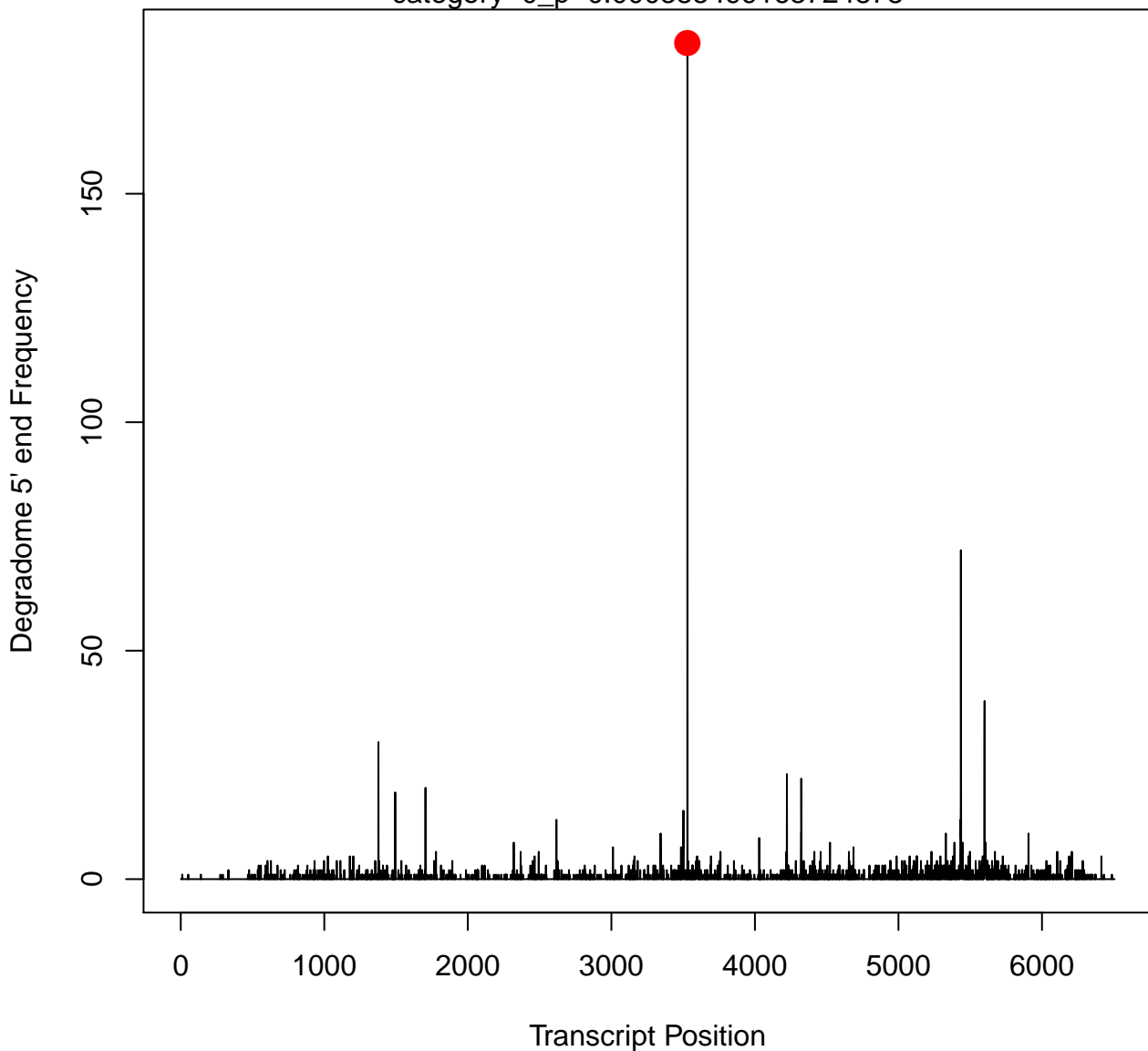

Supplement: Supplementary file 2 [file Data_Sheet_2.zip › Sit-miR162_Seita.9G562200.1_3530_TPlot.pdf]

**T=Seita.1G209000.1\_Q=Sit-miR164a\_S=999**

category=0\_p=0.00192601029535011

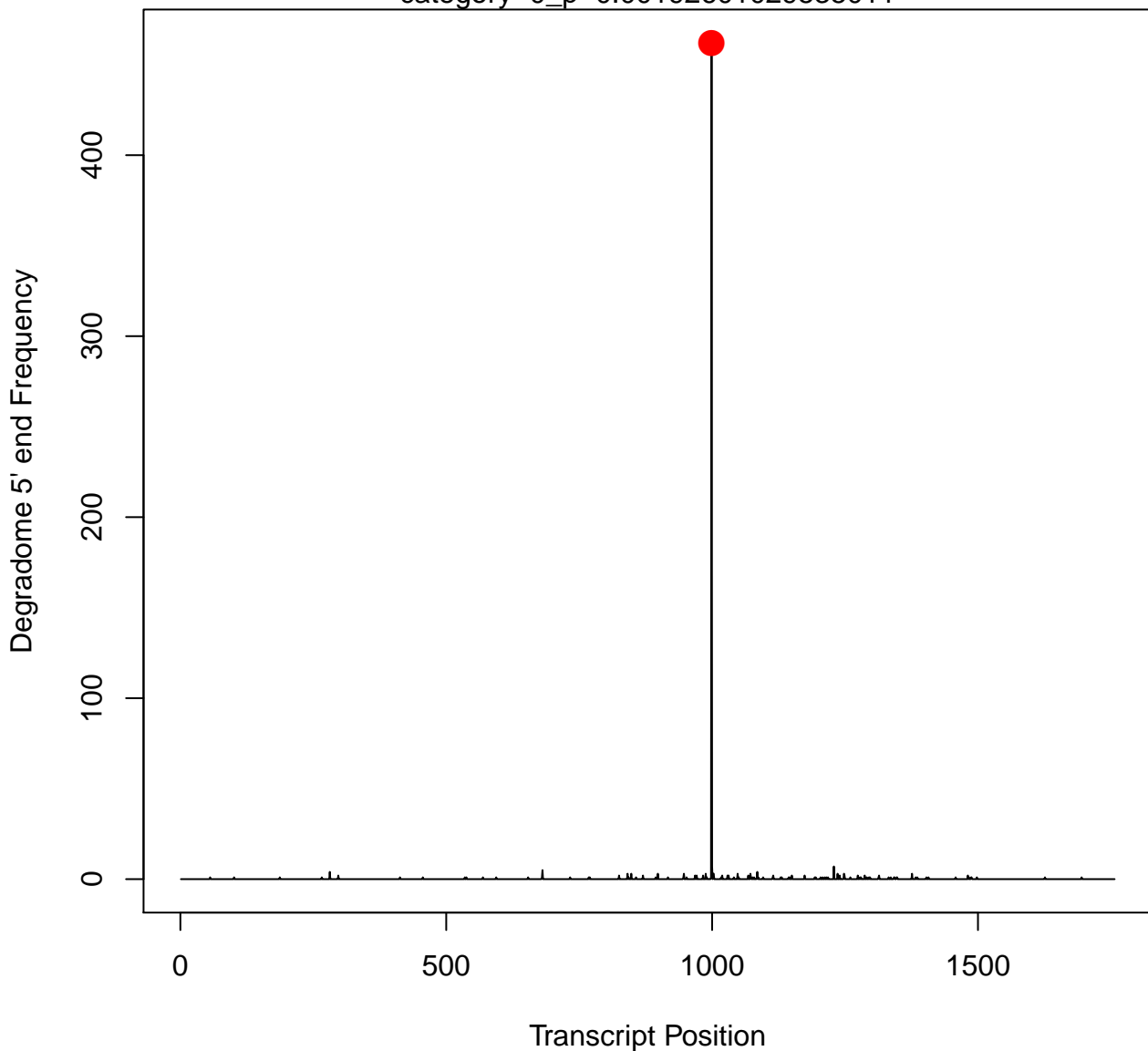

Supplement: Supplementary file 2 [file Data_Sheet_2.zip › Sit-miR164a_Seita.1G209000.1_999_TPlot.pdf]

**T=Seita.1G348900.1\_Q=Sit-miR164a\_S=673**

category=2\_p=0.90704895936184

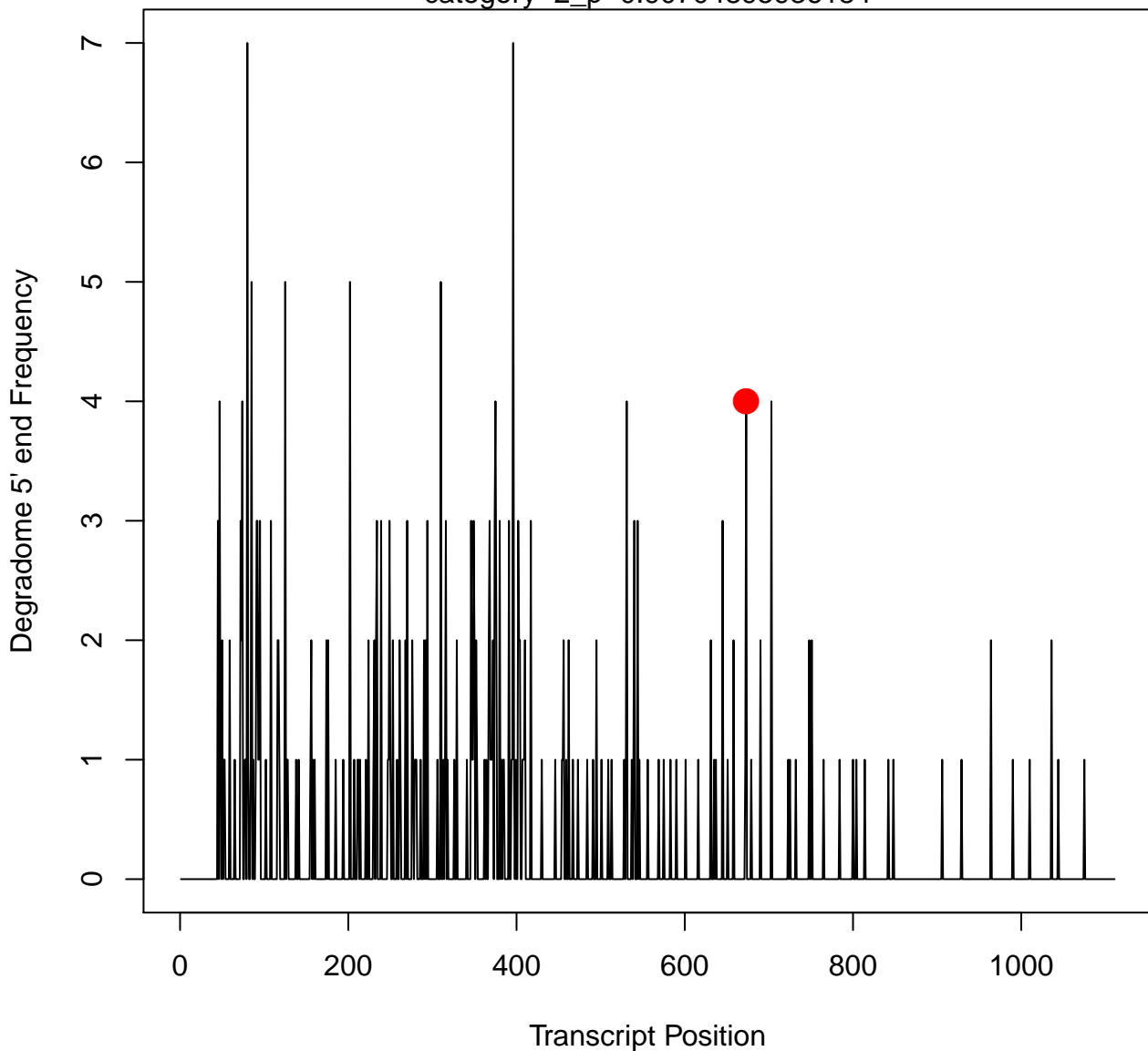

Supplement: Supplementary file 2 [file Data_Sheet_2.zip › Sit-miR164a_Seita.1G348900.1_673_TPlot.pdf]

**T=Seita.2G188000.1\_Q=Sit-miR164a\_S=584**

category=2\_p=0.606747660492345

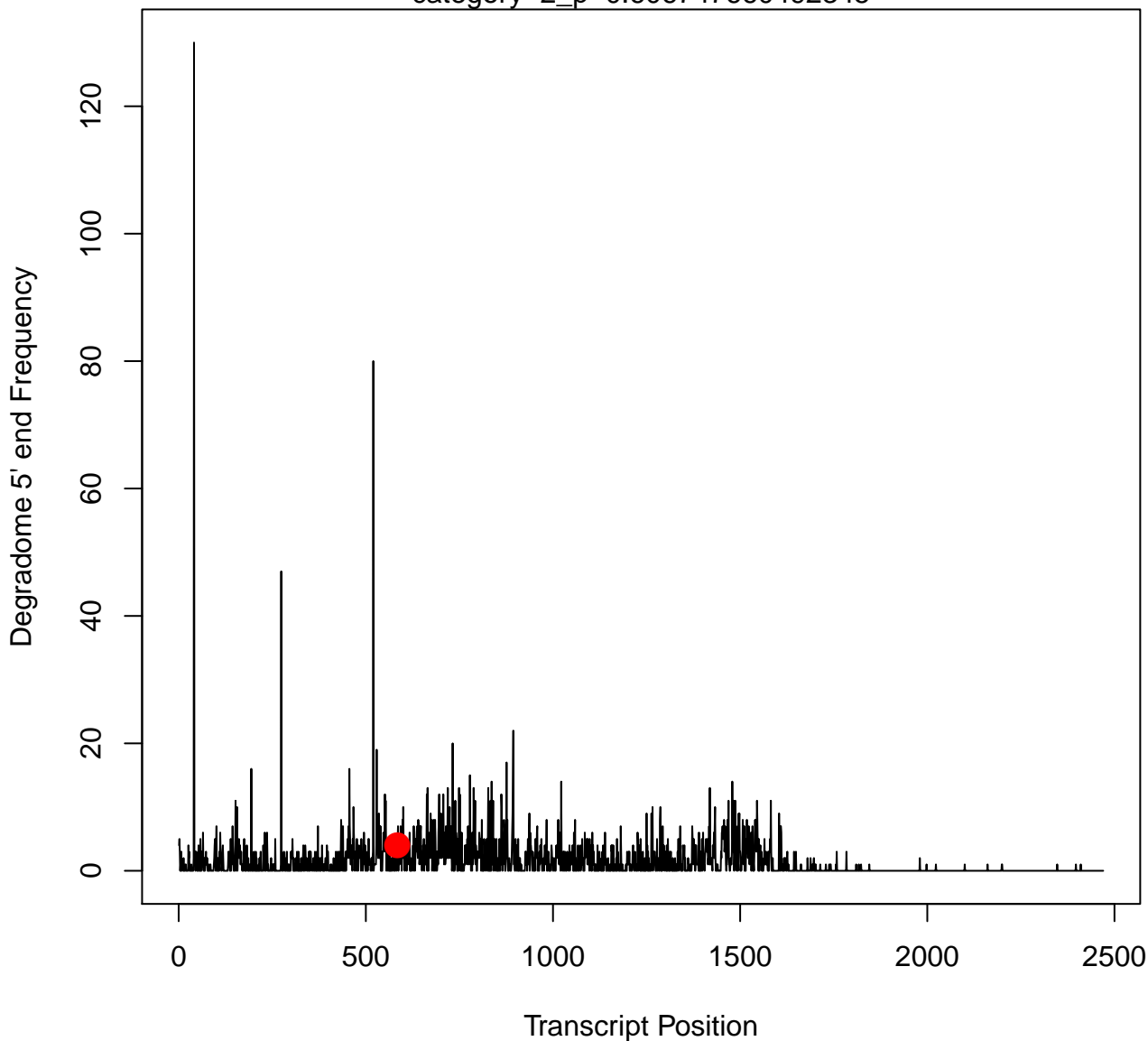

Supplement: Supplementary file 2 [file Data_Sheet_2.zip › Sit-miR164a_Seita.2G188000.1_584_TPlot.pdf]

**T=Seita.6G032700.1\_Q=Sit-miR164a\_S=727**

category=0\_p=0.000385499163724878

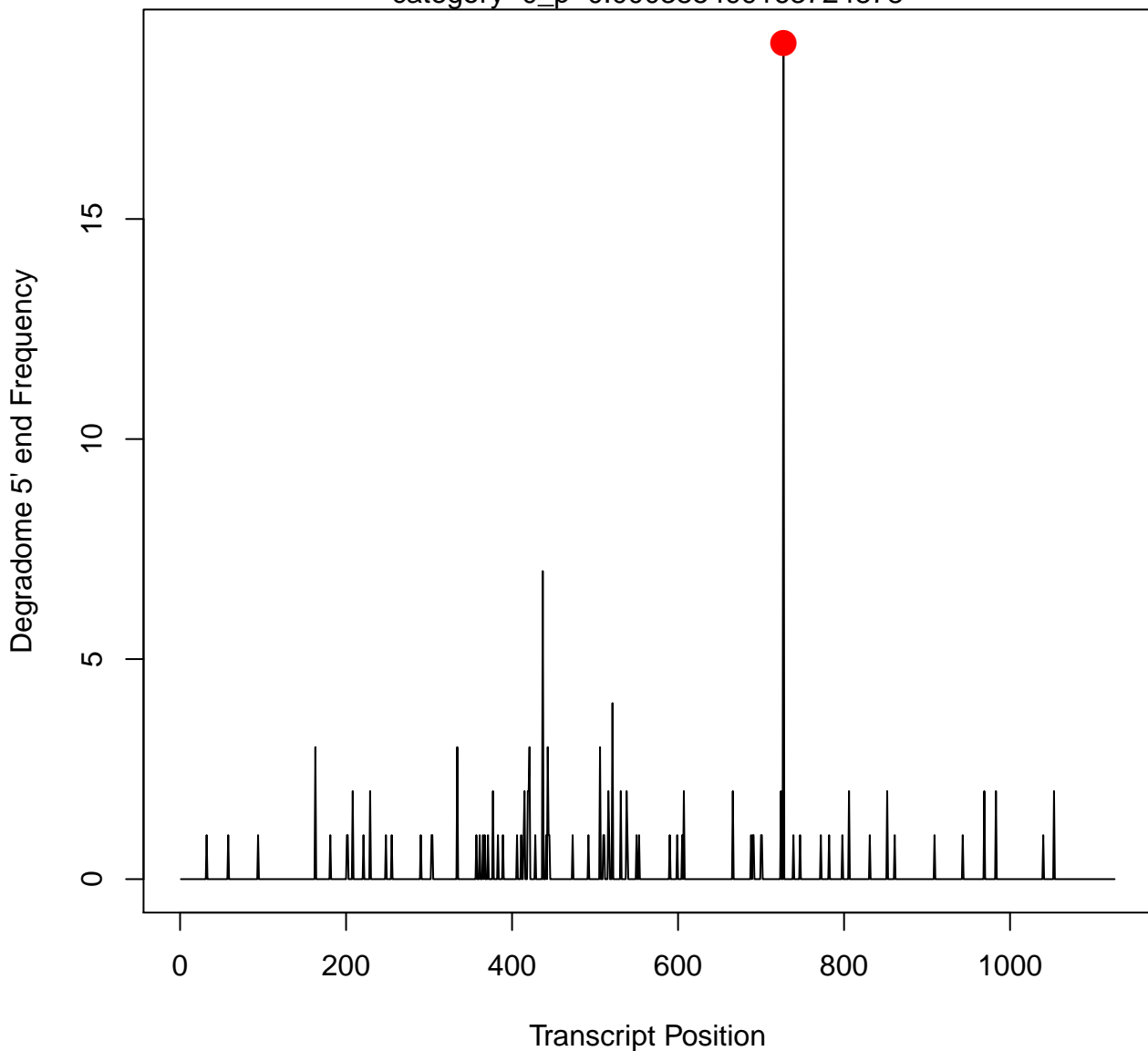

Supplement: Supplementary file 2 [file Data_Sheet_2.zip › Sit-miR164a_Seita.6G032700.1_727_TPlot.pdf]

**T=Seita.7G124900.1\_Q=Sit-miR164a\_S=859**

category=0\_p=0.00154110522640127

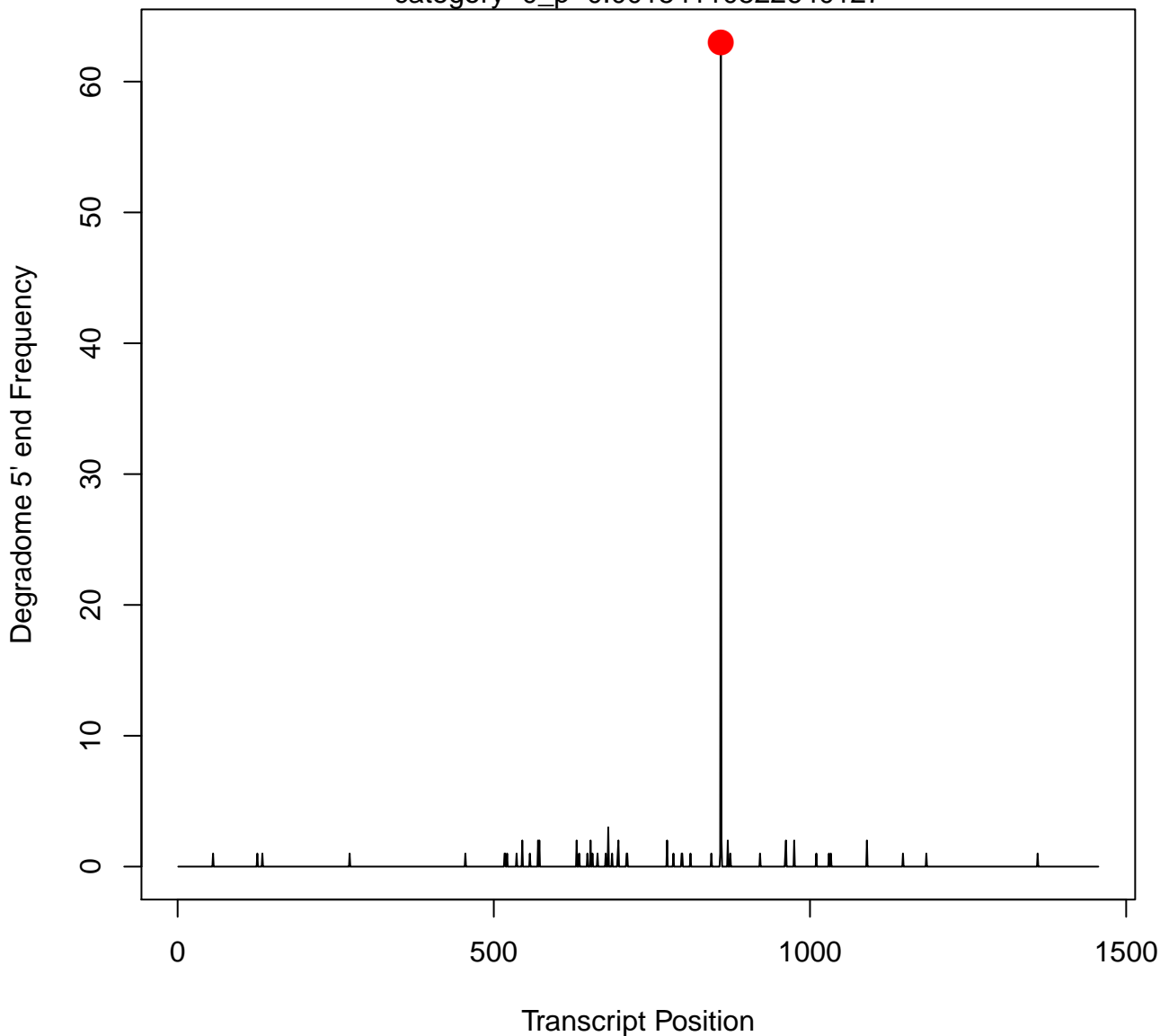

Supplement: Supplementary file 2 [file Data_Sheet_2.zip › Sit-miR164a_Seita.7G124900.1_859_TPlot.pdf]

**T=Seita.7G238600.1\_Q=Sit-miR164a\_S=2269**

category=2\_p=0.995617478913254

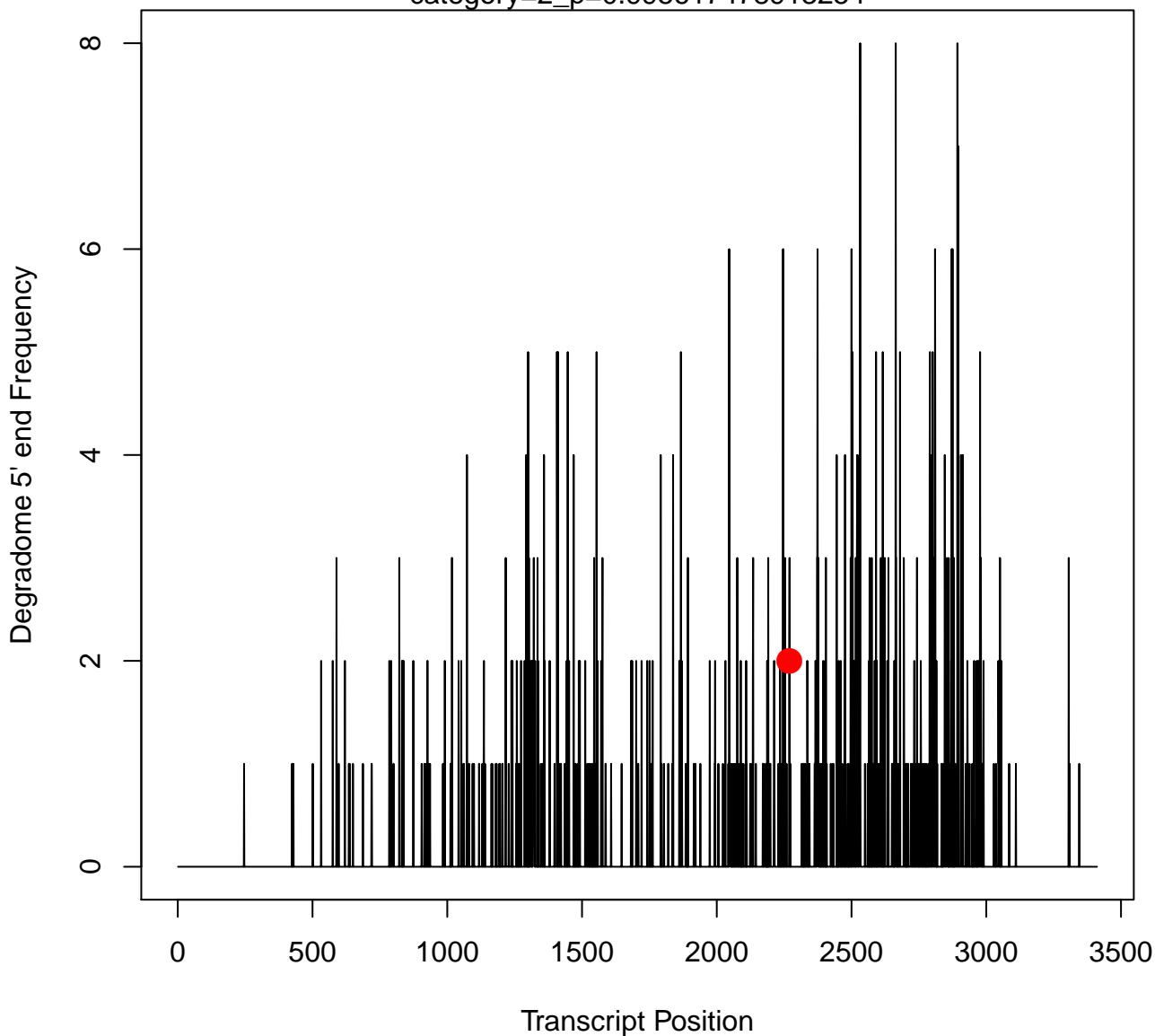

Supplement: Supplementary file 2 [file Data_Sheet_2.zip › Sit-miR164a_Seita.7G238600.1_2269_TPlot.pdf]

**T=Seita.7G280600.1\_Q=Sit-miR164a\_S=215**

category=2\_p=0.703623038871356

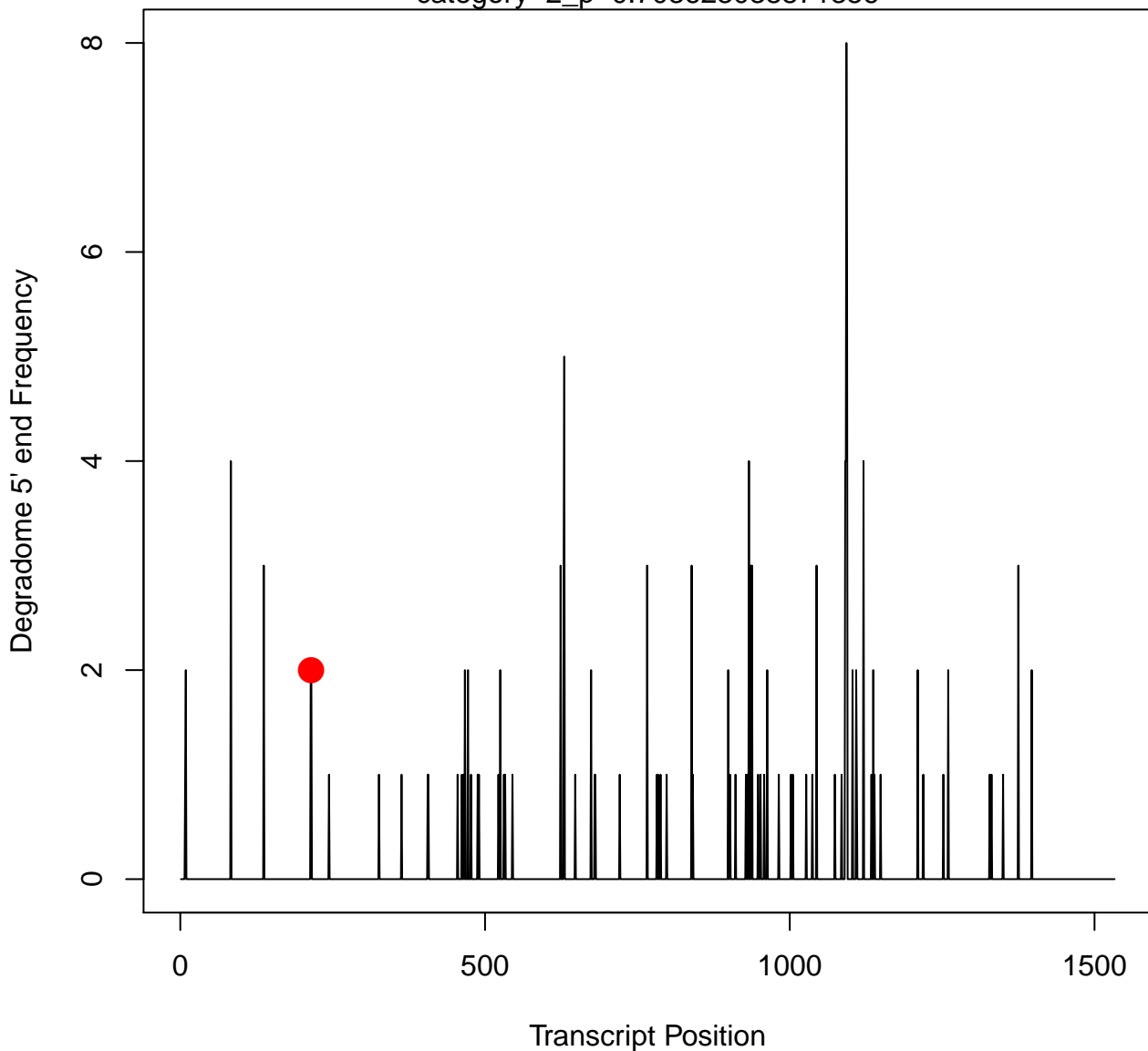

Supplement: Supplementary file 2 [file Data_Sheet_2.zip › Sit-miR164a_Seita.7G280600.1_215_TPlot.pdf]

**T=Seita.2G174200.1\_Q=Sit-miR164b\_S=201**

category=0\_p=0.00115605171964761

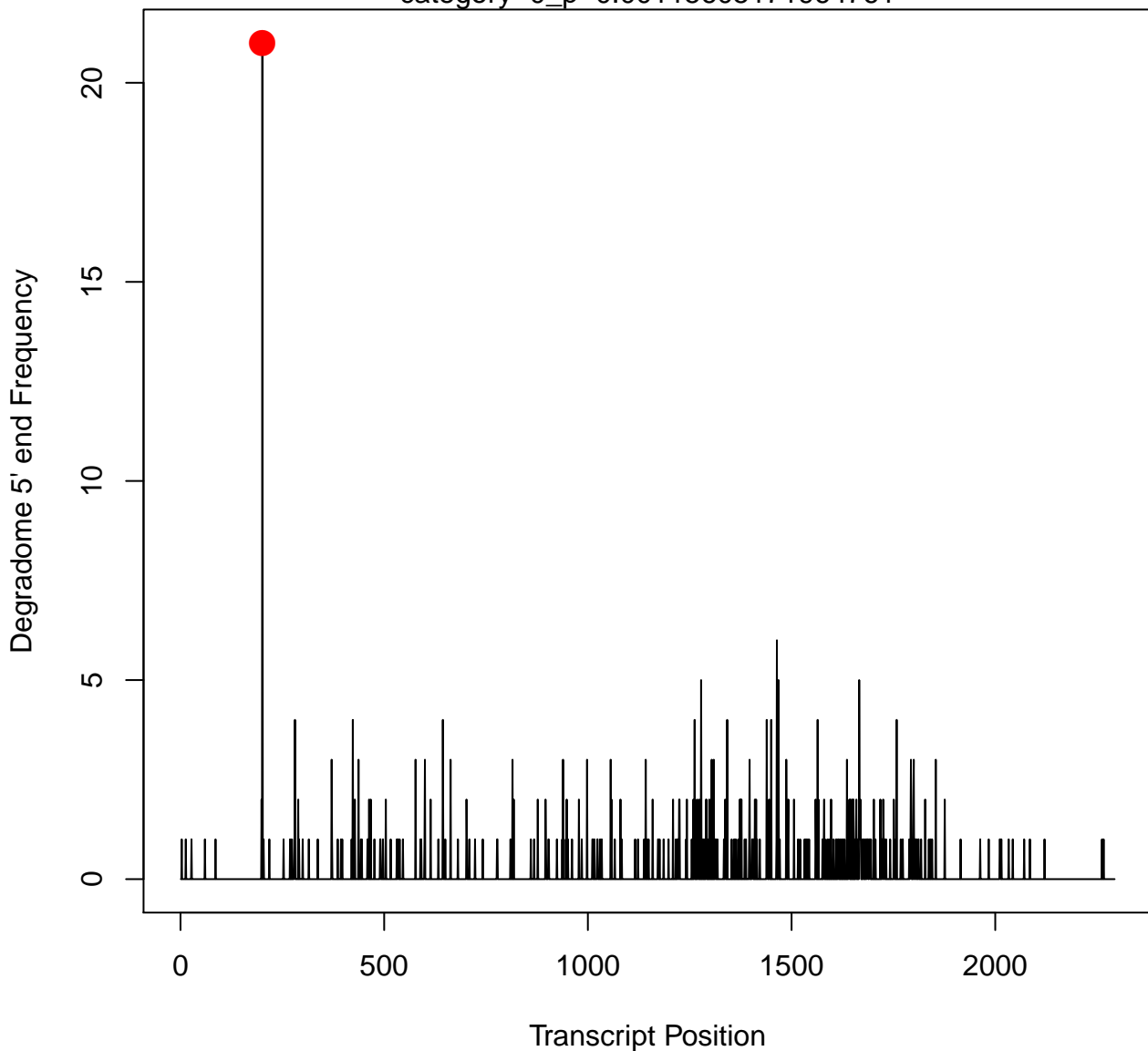

Supplement: Supplementary file 2 [file Data_Sheet_2.zip › Sit-miR164b_Seita.2G174200.1_201_TPlot.pdf]

**T=Seita.3G220200.1\_Q=Sit-miR164b\_S=319**

category=2\_p=0.999999834057915

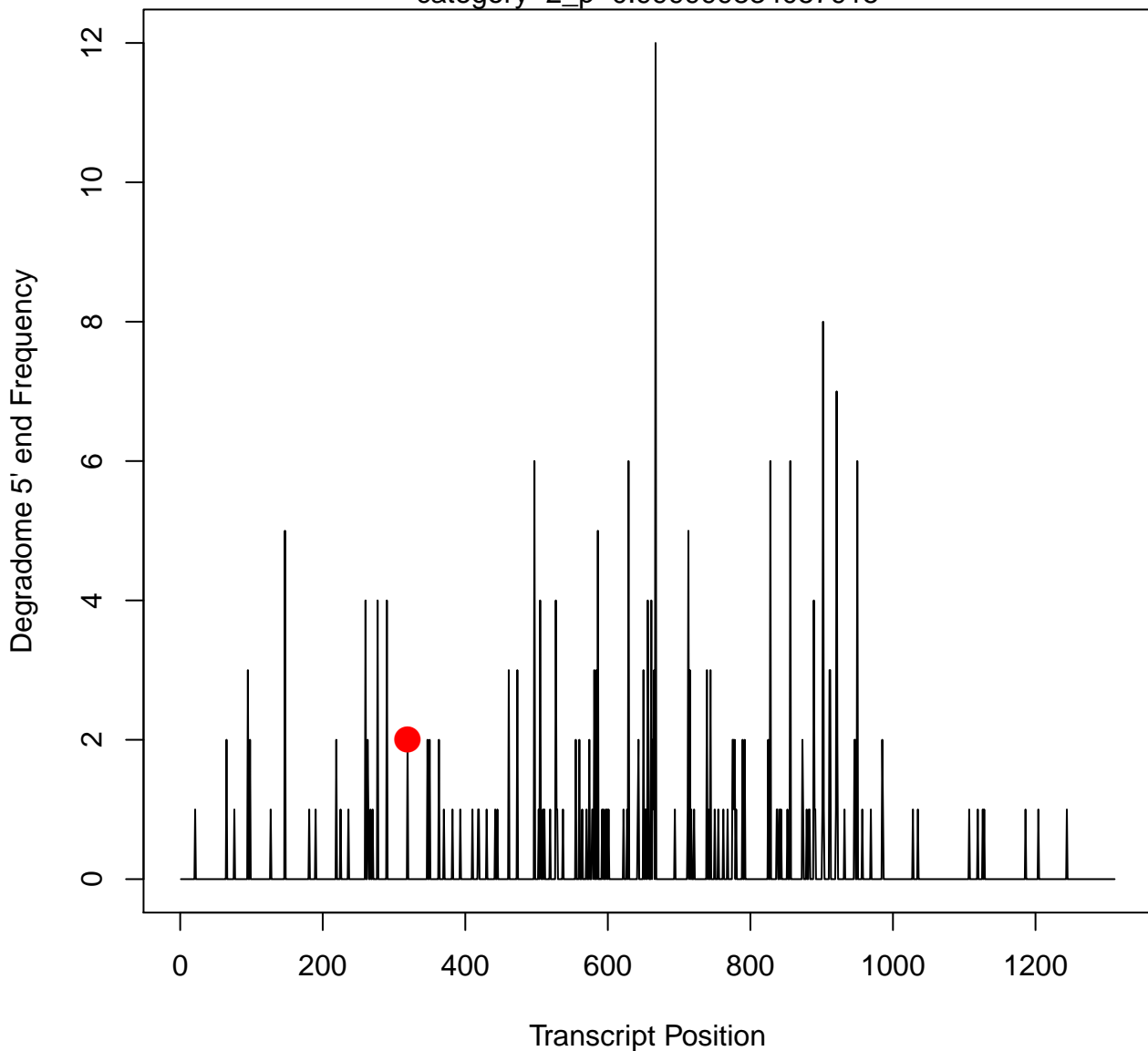

Supplement: Supplementary file 2 [file Data_Sheet_2.zip › Sit-miR164b_Seita.3G220200.1_319_TPlot.pdf]

**T=Seita.3G386200.1\_Q=Sit-miR164b\_S=790**

category=0\_p=0.000385499163724878

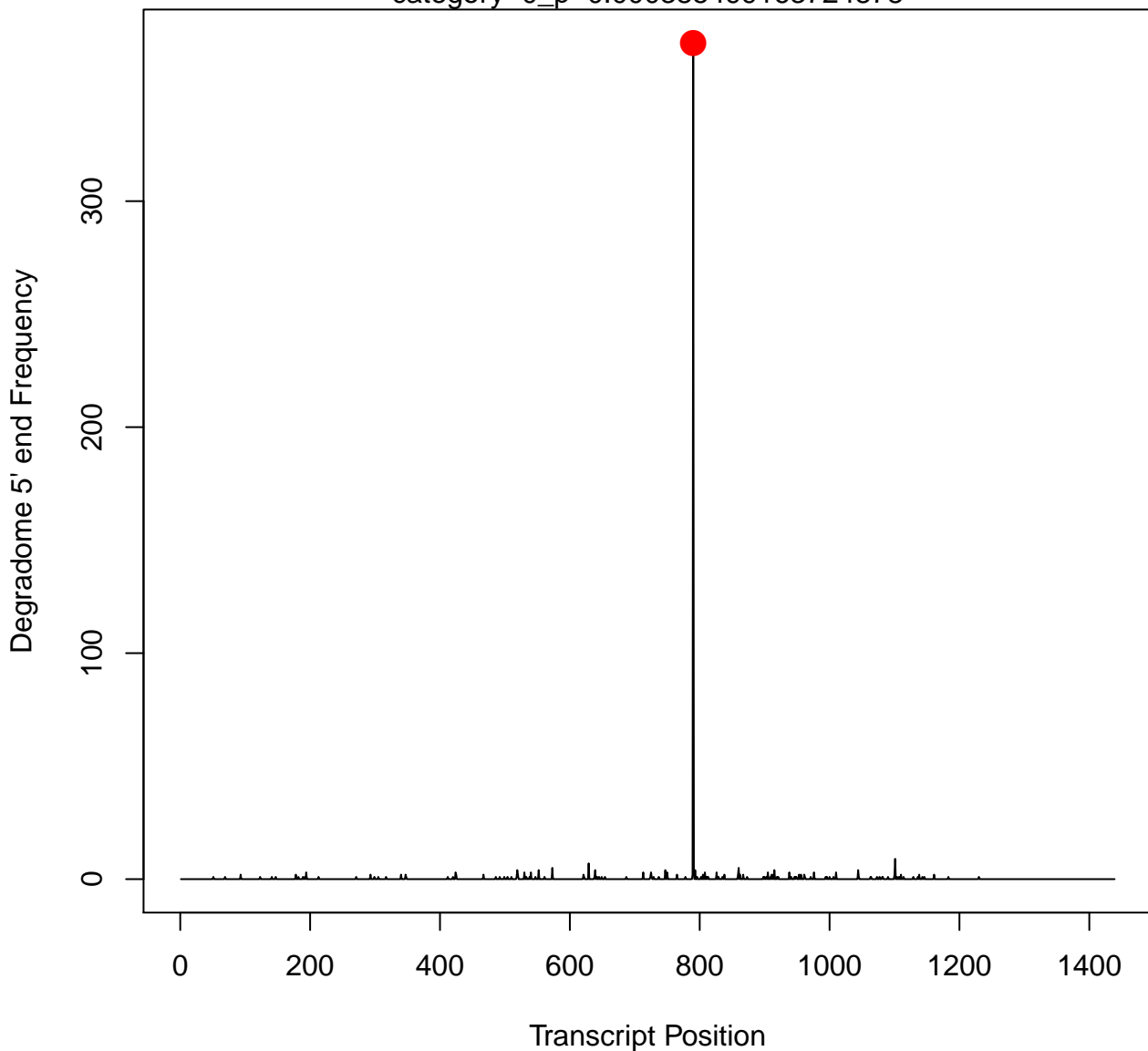

Supplement: Supplementary file 2 [file Data_Sheet_2.zip › Sit-miR164b_Seita.3G386200.1_790_TPlot.pdf]

**T=Seita.4G157500.1\_Q=Sit-miR164b\_S=1077**

category=0\_p=0.000770849717844468

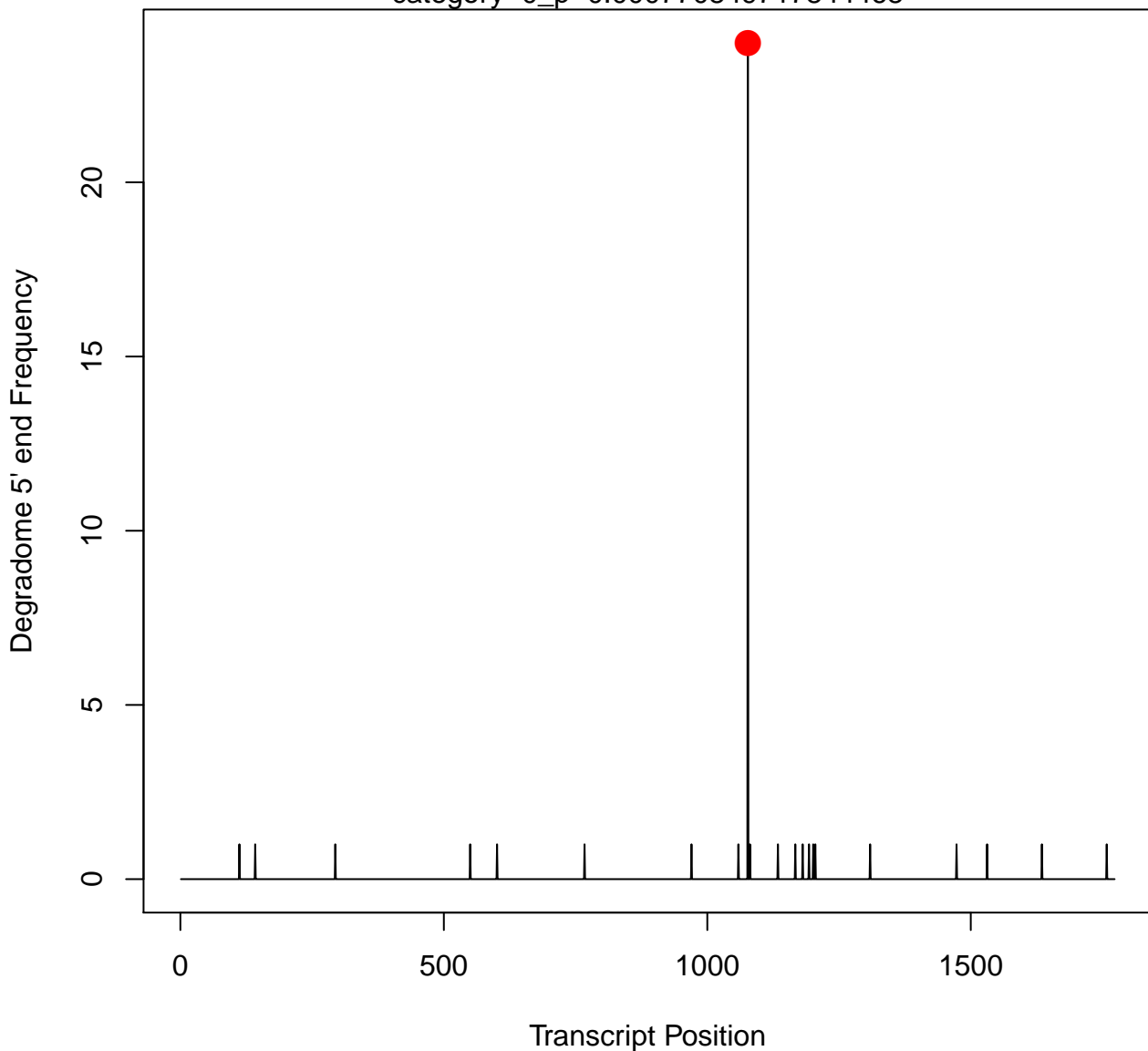

Supplement: Supplementary file 2 [file Data_Sheet_2.zip › Sit-miR164b_Seita.4G157500.1_1077_TPlot.pdf]

**T=Seita.4G263400.1\_Q=Sit-miR164b\_S=1216**

category=0\_p=0.00154110522640127

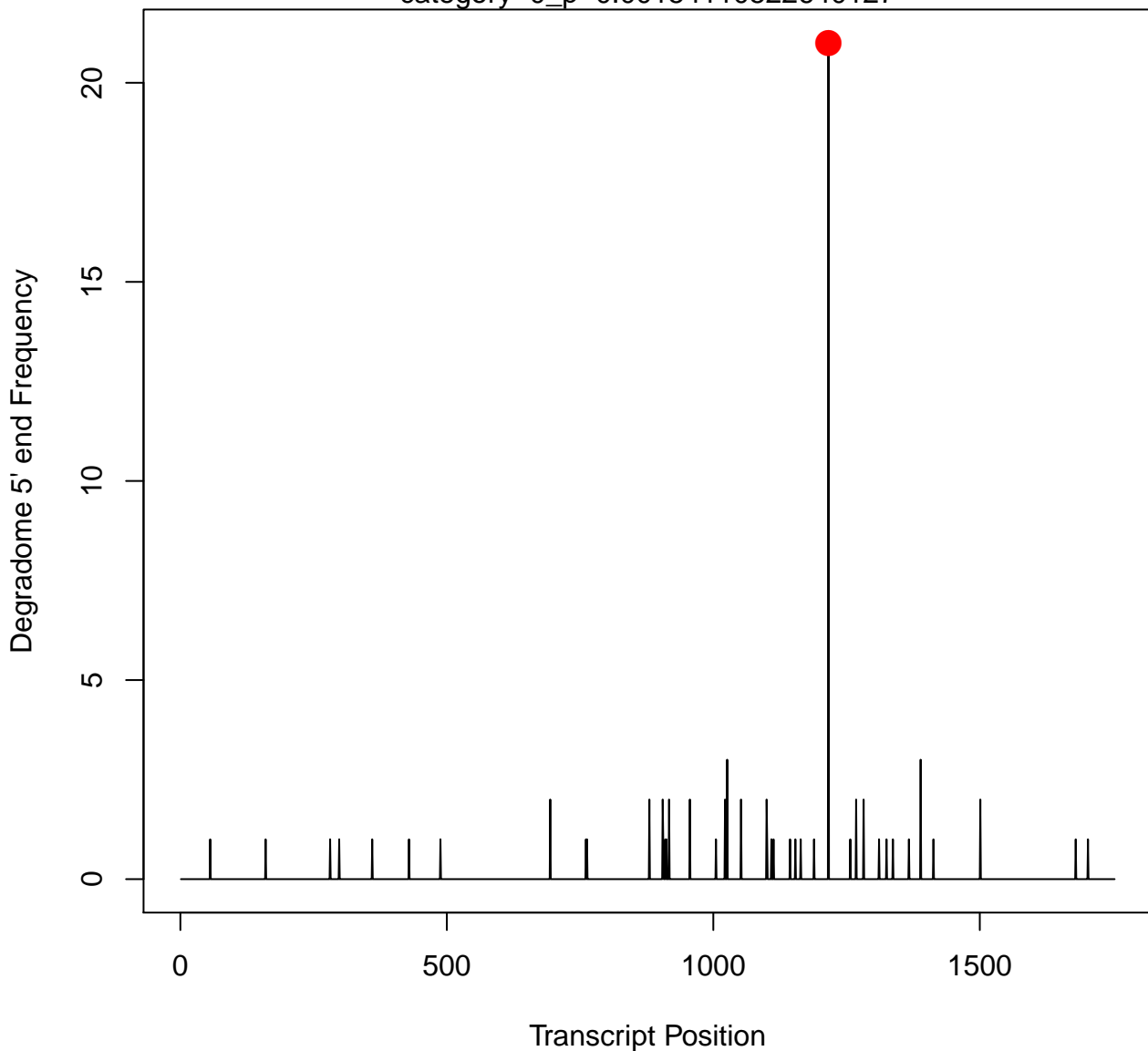

Supplement: Supplementary file 2 [file Data_Sheet_2.zip › Sit-miR164b_Seita.4G263400.1_1216_TPlot.pdf]

**T=Seita.3G291200.1\_Q=Sit-miR164c\_S=34**

category=2\_p=0.999999984133049

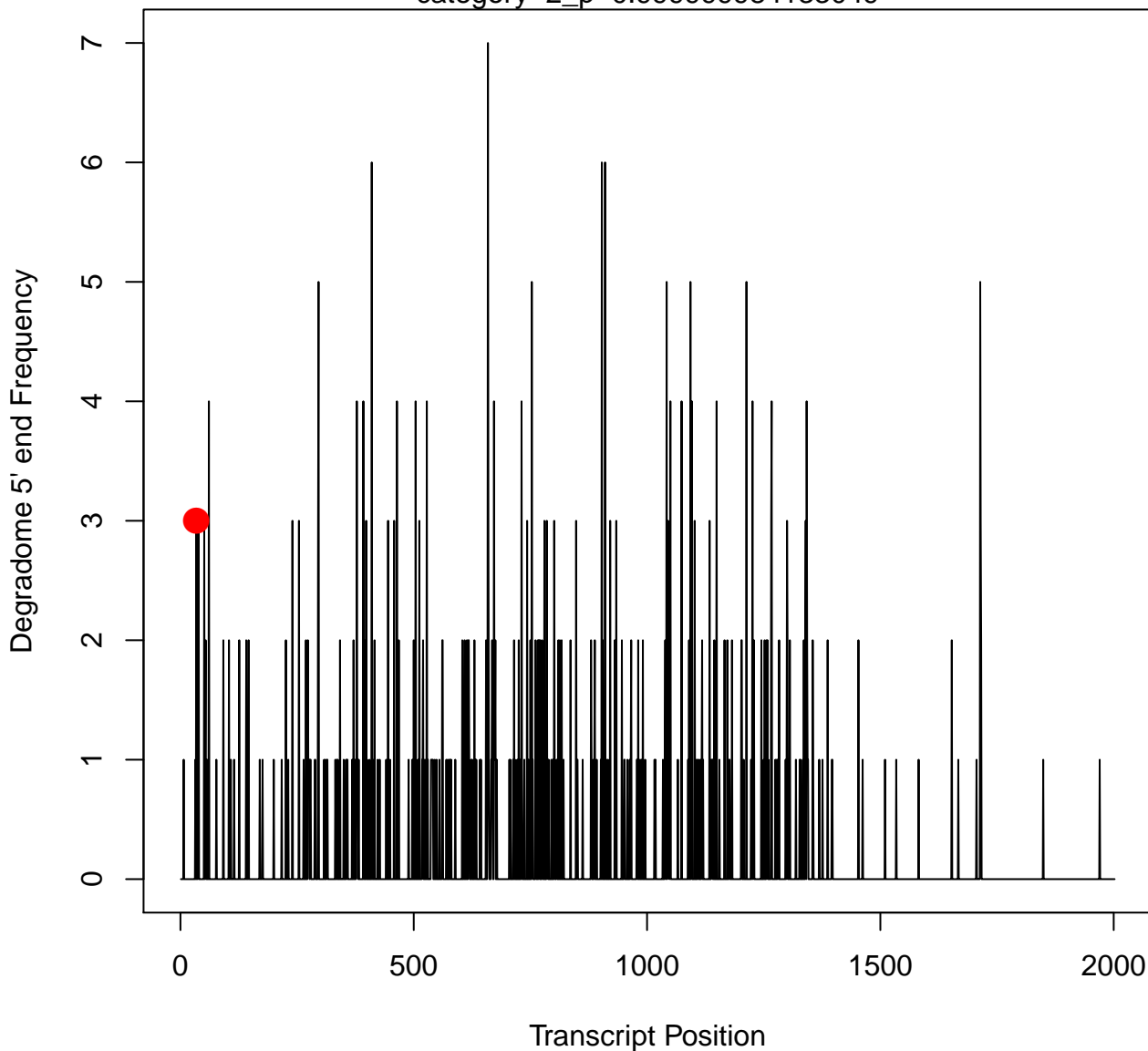

Supplement: Supplementary file 2 [file Data_Sheet_2.zip › Sit-miR164c_Seita.3G291200.1_34_TPlot.pdf]

**T=Seita.1G229400.1\_Q=Sit-miR164d\_S=1272**

category=2\_p=0.999787442487049

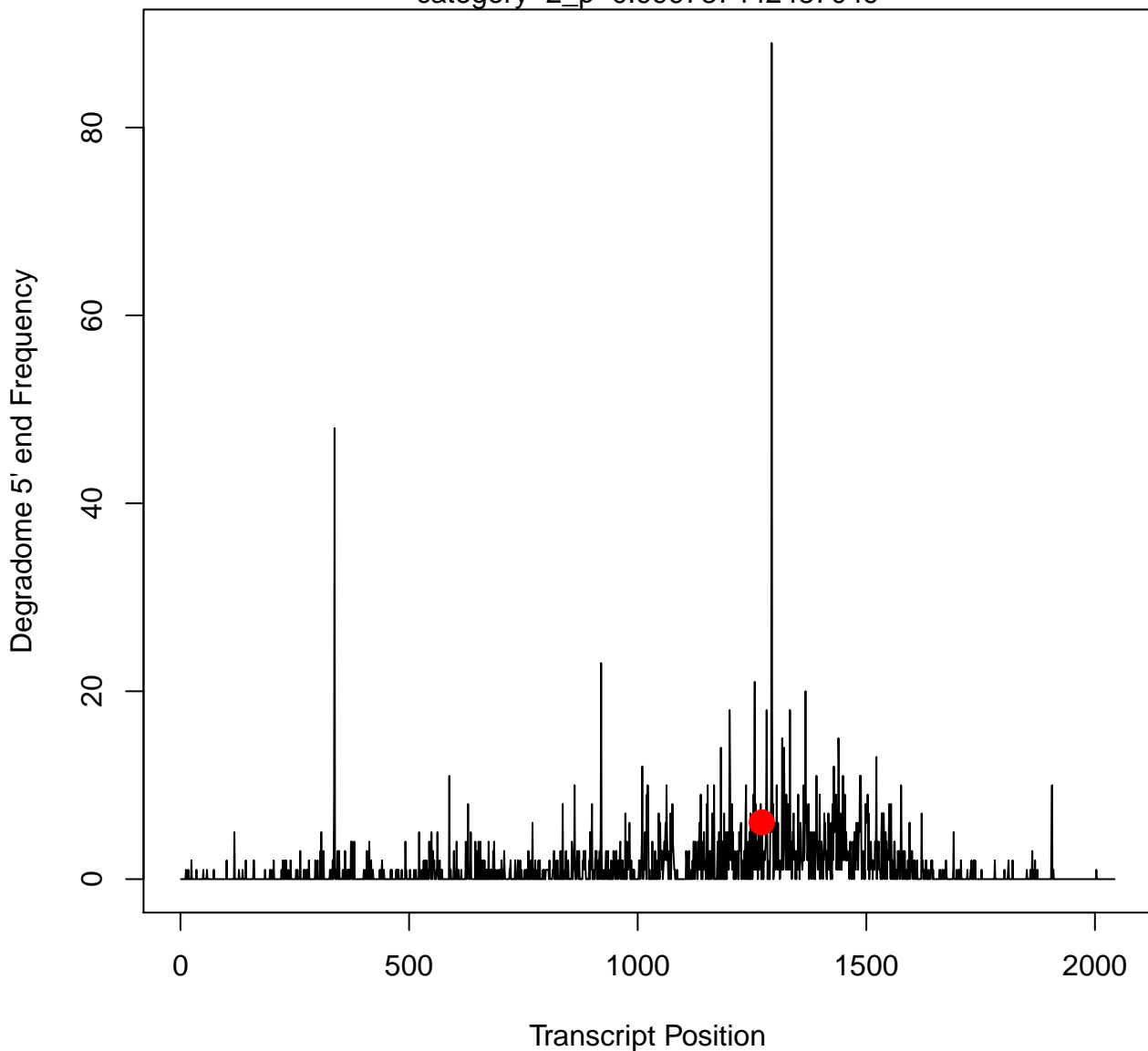

Supplement: Supplementary file 2 [file Data_Sheet_2.zip › Sit-miR164d_Seita.1G229400.1_1272_TPlot.pdf]

**T=Seita.3G001500.1\_Q=Sit-miR164d\_S=686**

category=2\_p=0.99932225449741

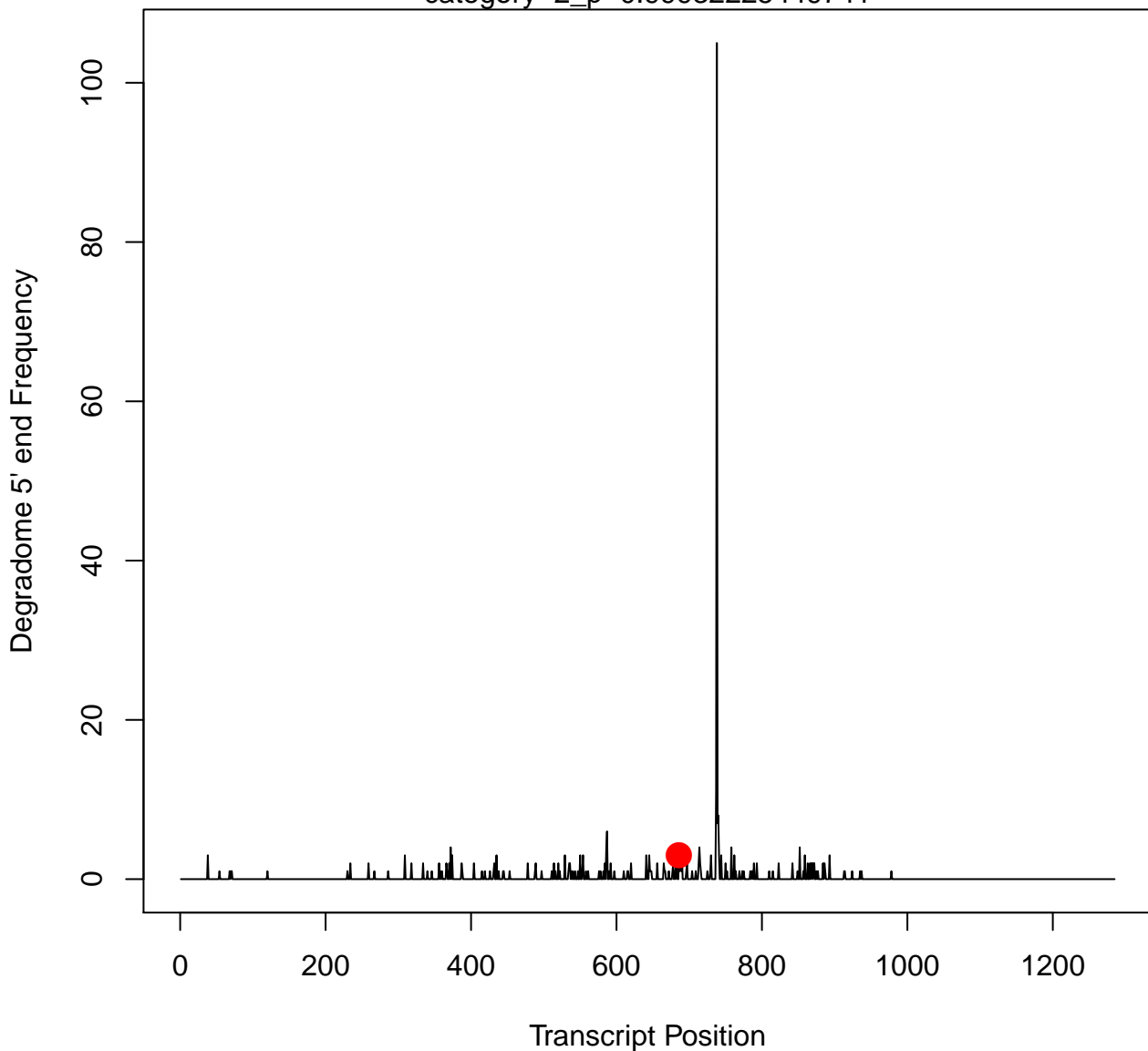

Supplement: Supplementary file 2 [file Data_Sheet_2.zip › Sit-miR164d_Seita.3G001500.1_686_TPlot.pdf]

**T=Seita.1G081100.1\_Q=Sit-miR164e\_S=494**

category=2\_p=0.999875804631945

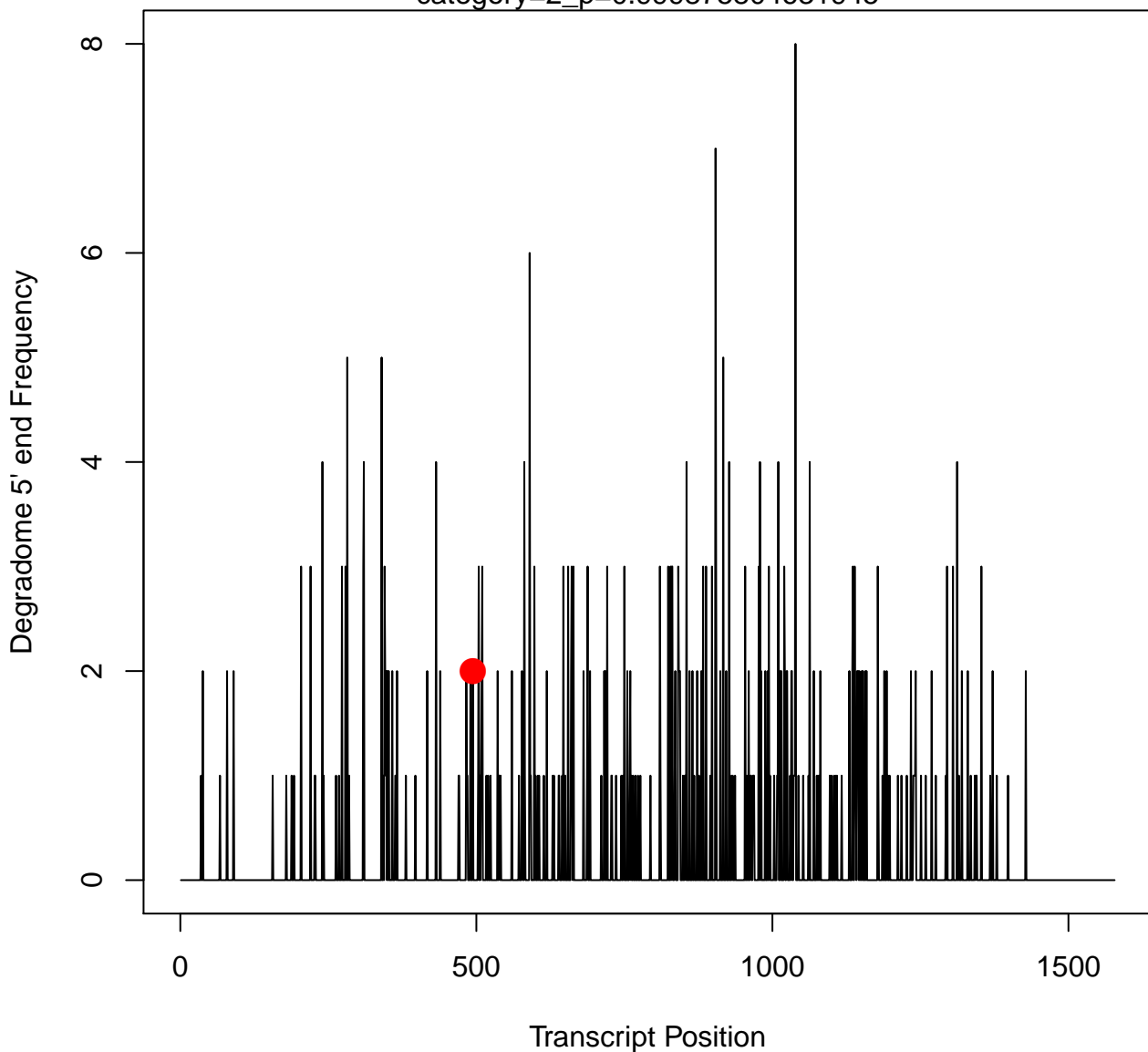

Supplement: Supplementary file 2 [file Data_Sheet_2.zip › Sit-miR164e_Seita.1G081100.1_494_TPlot.pdf]

**T=Seita.3G181300.1\_Q=Sit-miR164e\_S=1067**

category=2\_p=0.999999999951856

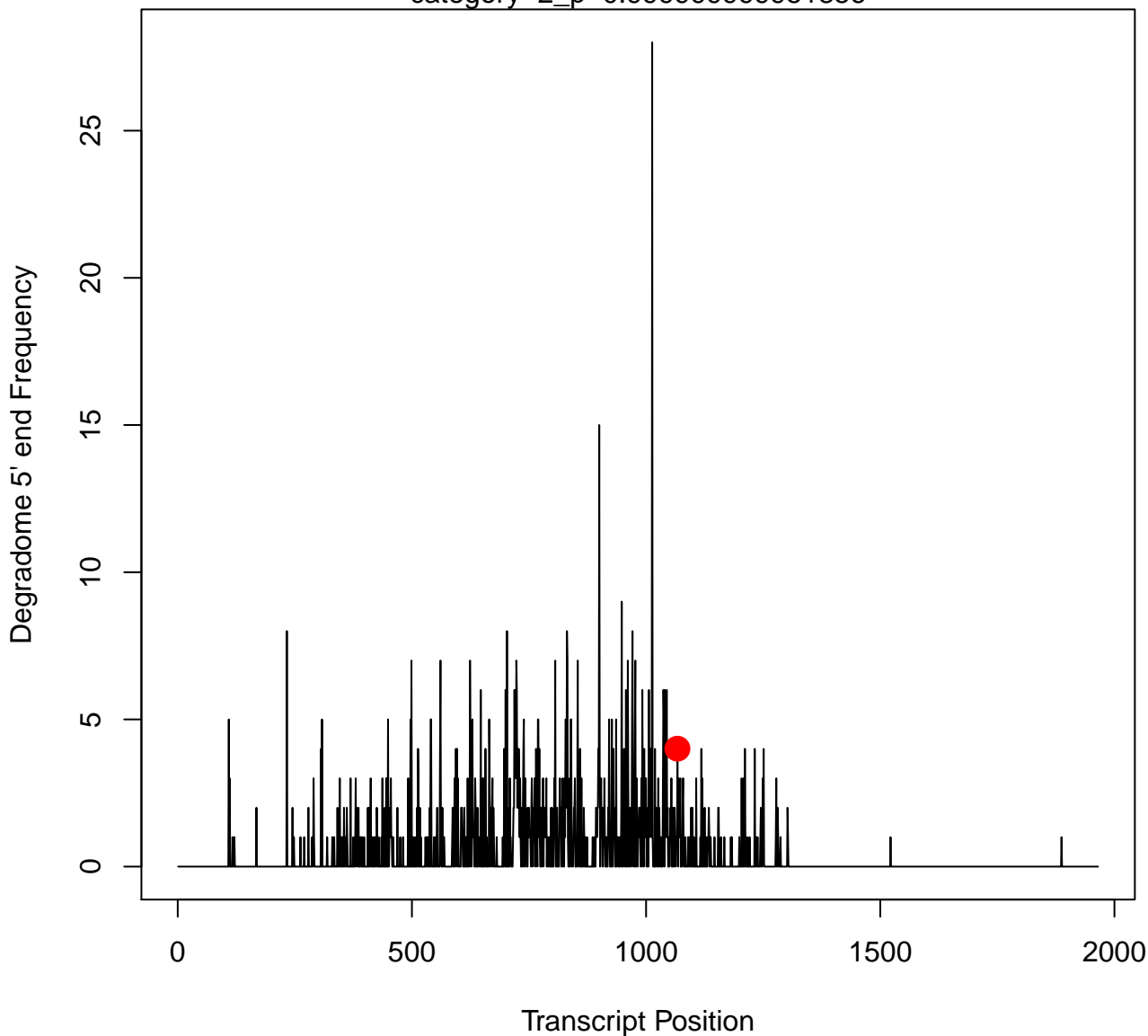

Supplement: Supplementary file 2 [file Data_Sheet_2.zip › Sit-miR164e_Seita.3G181300.1_1067_TPlot.pdf]

**T=Seita.3G394000.1\_Q=Sit-miR164e\_S=431**

category=2\_p=0.995739688769505

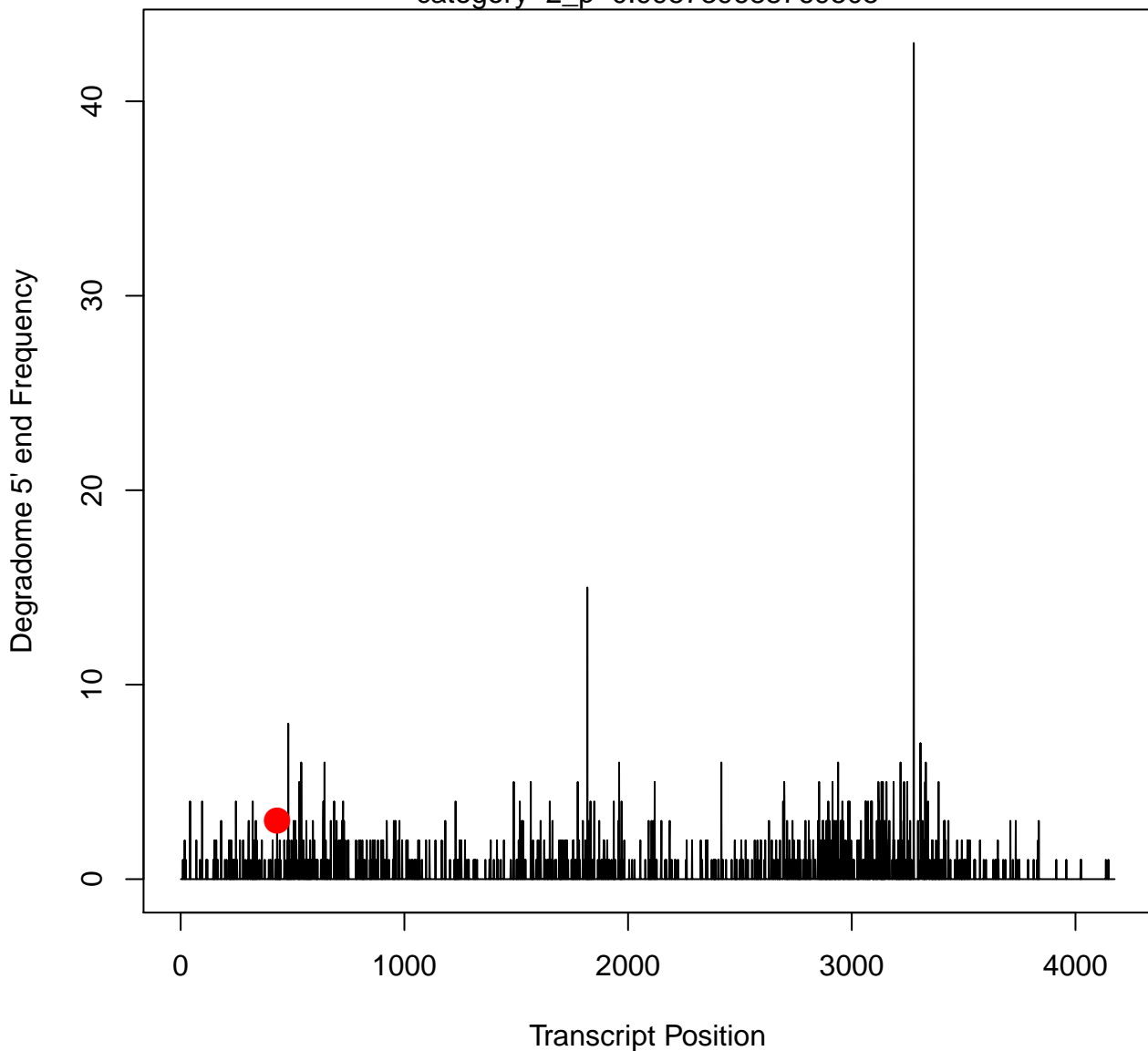

Supplement: Supplementary file 2 [file Data_Sheet_2.zip › Sit-miR164e_Seita.3G394000.1_431_TPlot.pdf]

**T=Seita.4G186700.1\_Q=Sit-miR164e\_S=664**

category=2\_p=0.99999999976922

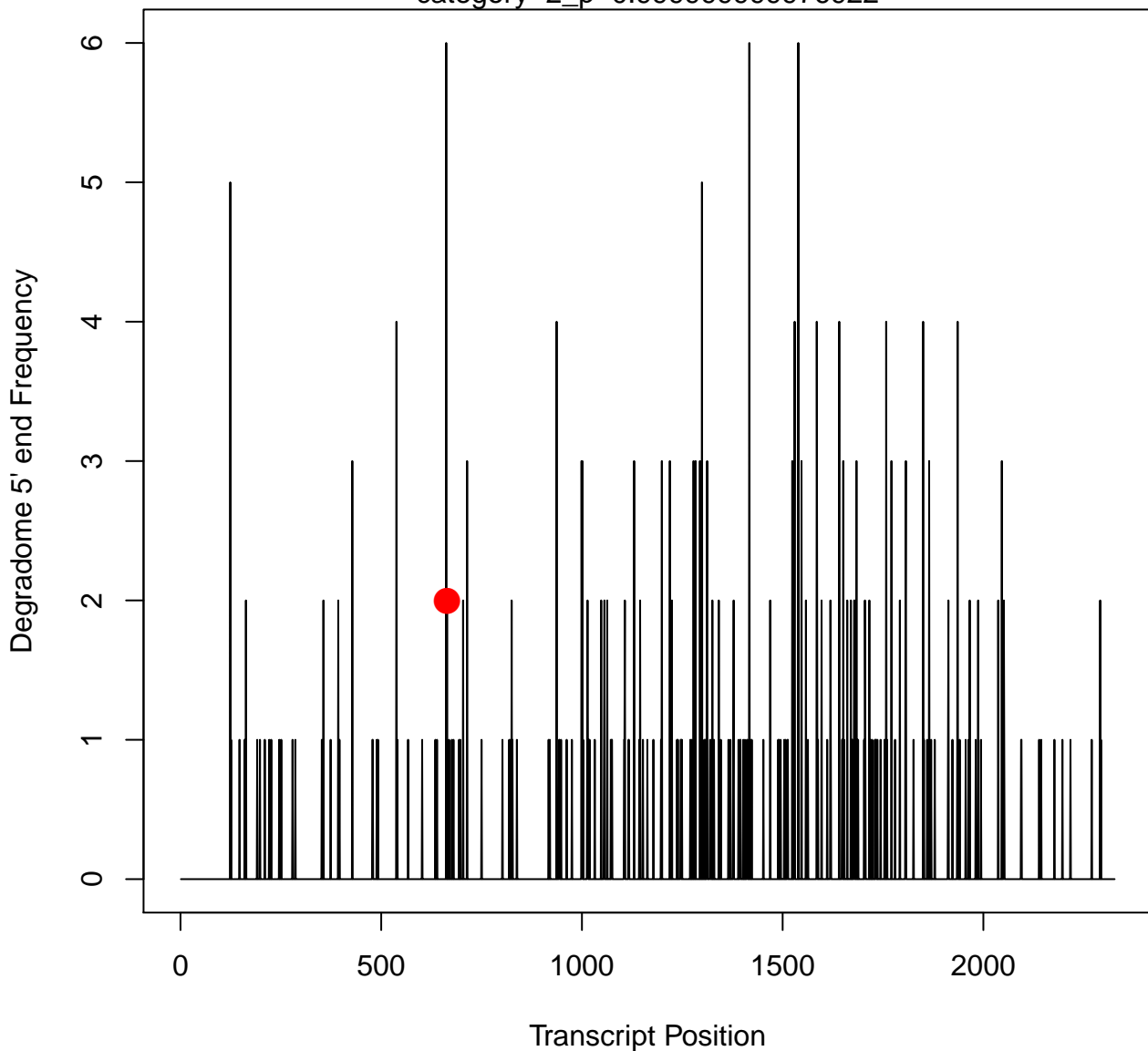

Supplement: Supplementary file 2 [file Data_Sheet_2.zip › Sit-miR164e_Seita.4G186700.1_664_TPlot.pdf]

**T=Seita.5G156700.1\_Q=Sit-miR164e\_S=145**

category=2\_p=0.999998056653031

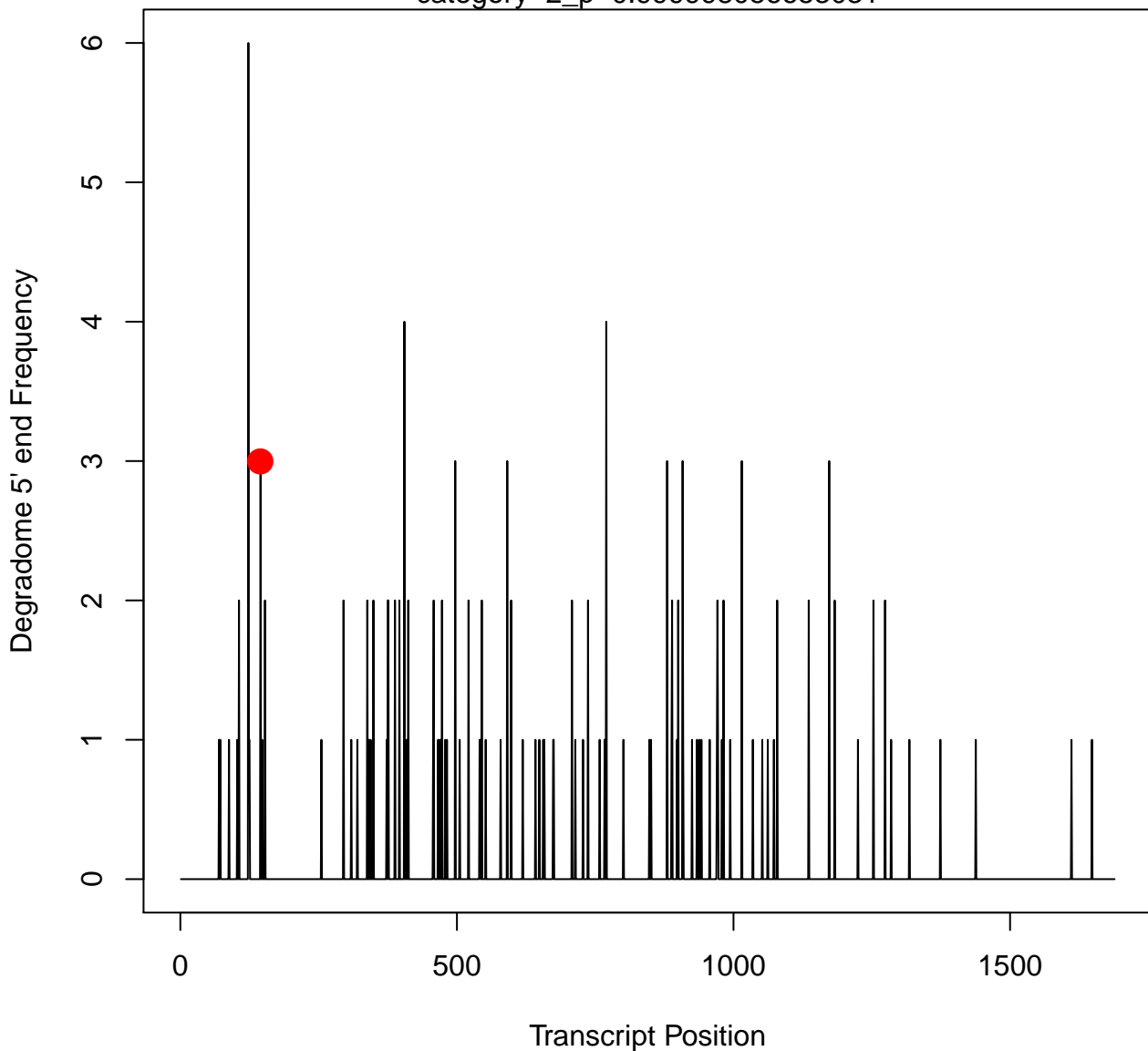

Supplement: Supplementary file 2 [file Data_Sheet_2.zip › Sit-miR164e_Seita.5G156700.1_145_TPlot.pdf]

**T=Seita.5G431200.1\_Q=Sit-miR164e\_S=572**

category=0\_p=0.0543740177473359

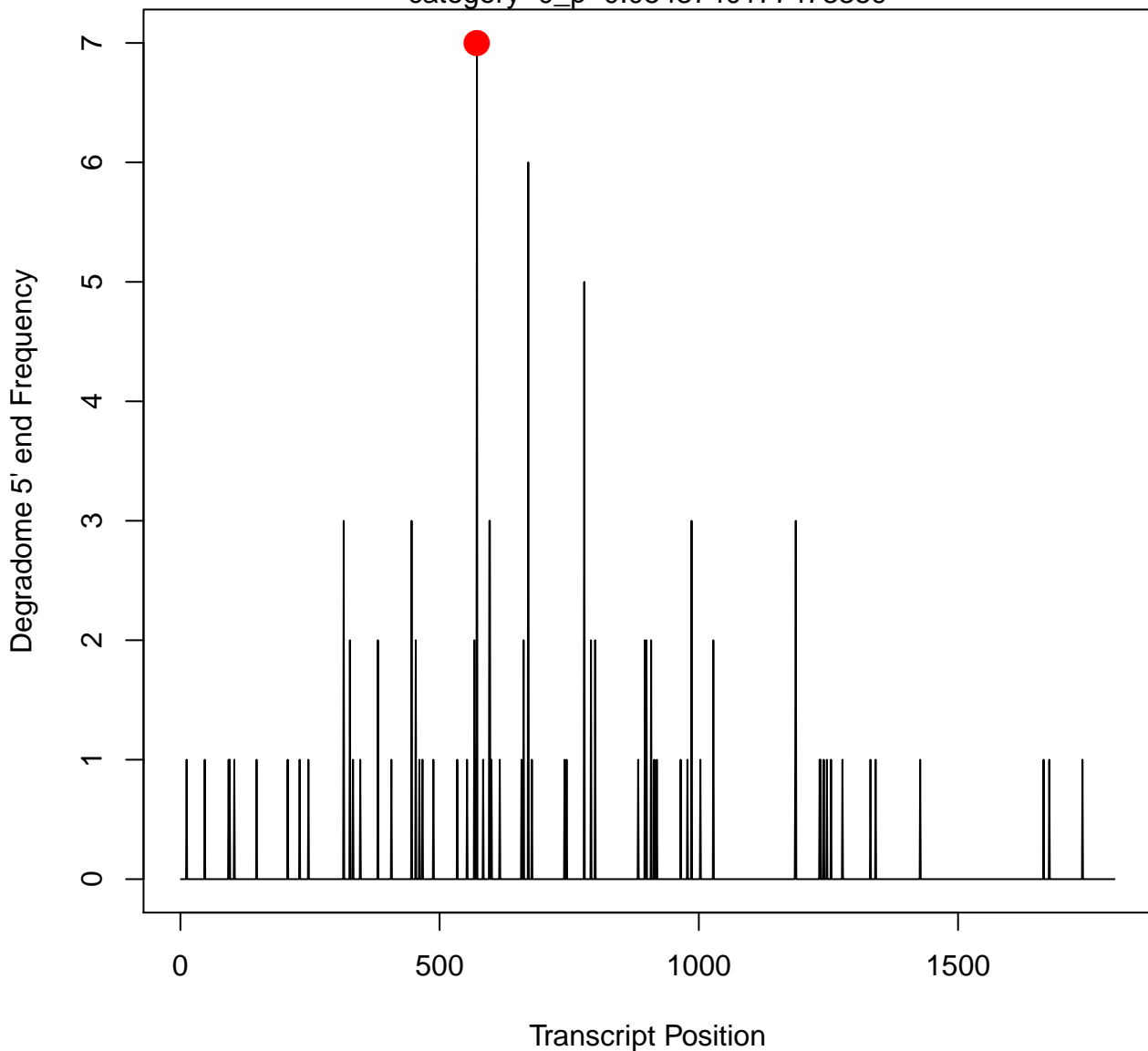

Supplement: Supplementary file 2 [file Data_Sheet_2.zip › Sit-miR164e_Seita.5G431200.1_572_TPlot.pdf]

**T=Seita.7G031200.1\_Q=Sit-miR164e\_S=548**

category=2\_p=0.976751091274025

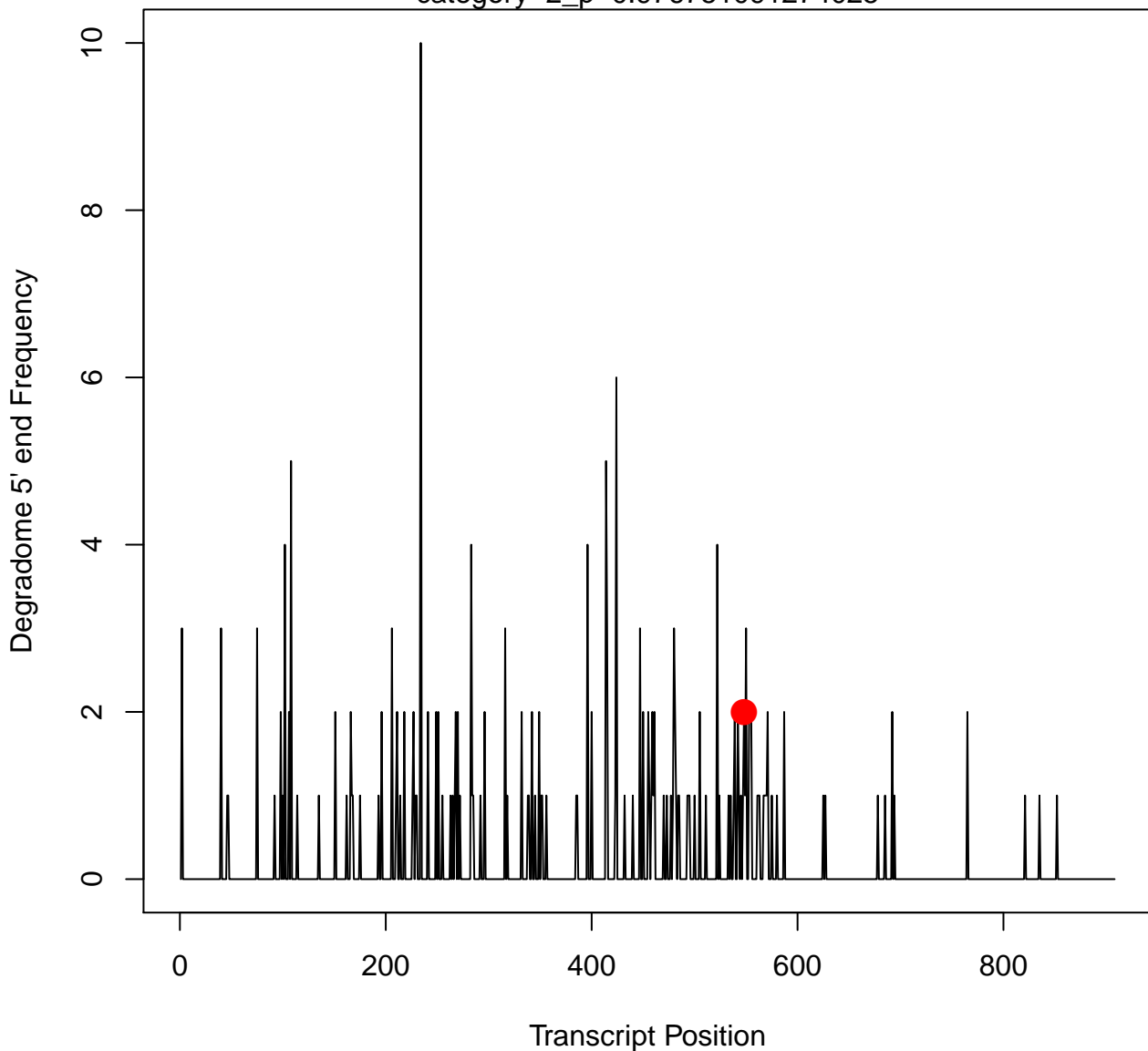

Supplement: Supplementary file 2 [file Data_Sheet_2.zip › Sit-miR164e_Seita.7G031200.1_548_TPlot.pdf]

**T=Seita.7G205800.1\_Q=Sit-miR164e\_S=1612**

category=2\_p=0.950107515284326

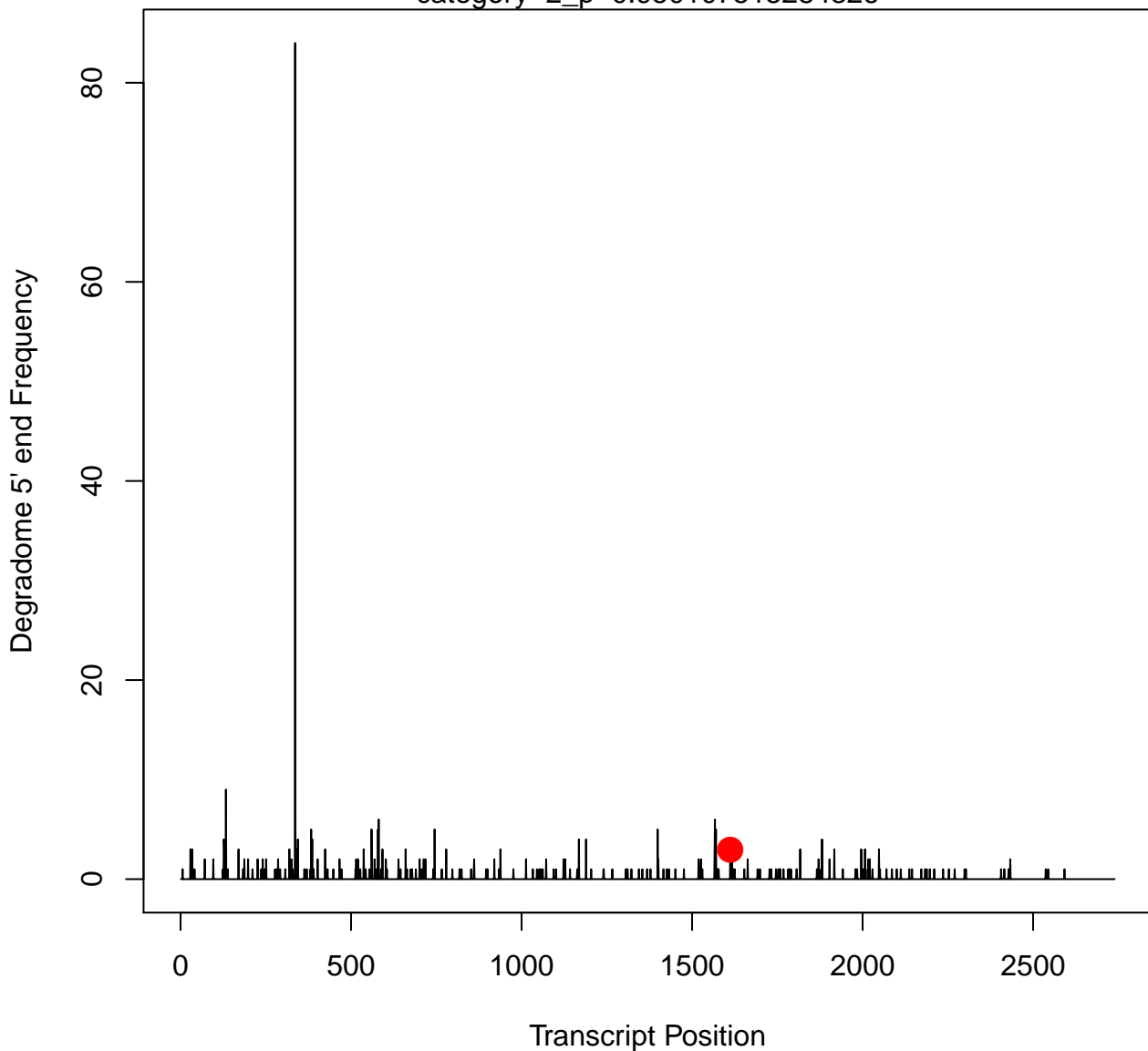

Supplement: Supplementary file 2 [file Data_Sheet_2.zip › Sit-miR164e_Seita.7G205800.1_1612_TPlot.pdf]

**T=Seita.7G333700.1\_Q=Sit-miR164e\_S=768**

category=2\_p=0.999991778190573

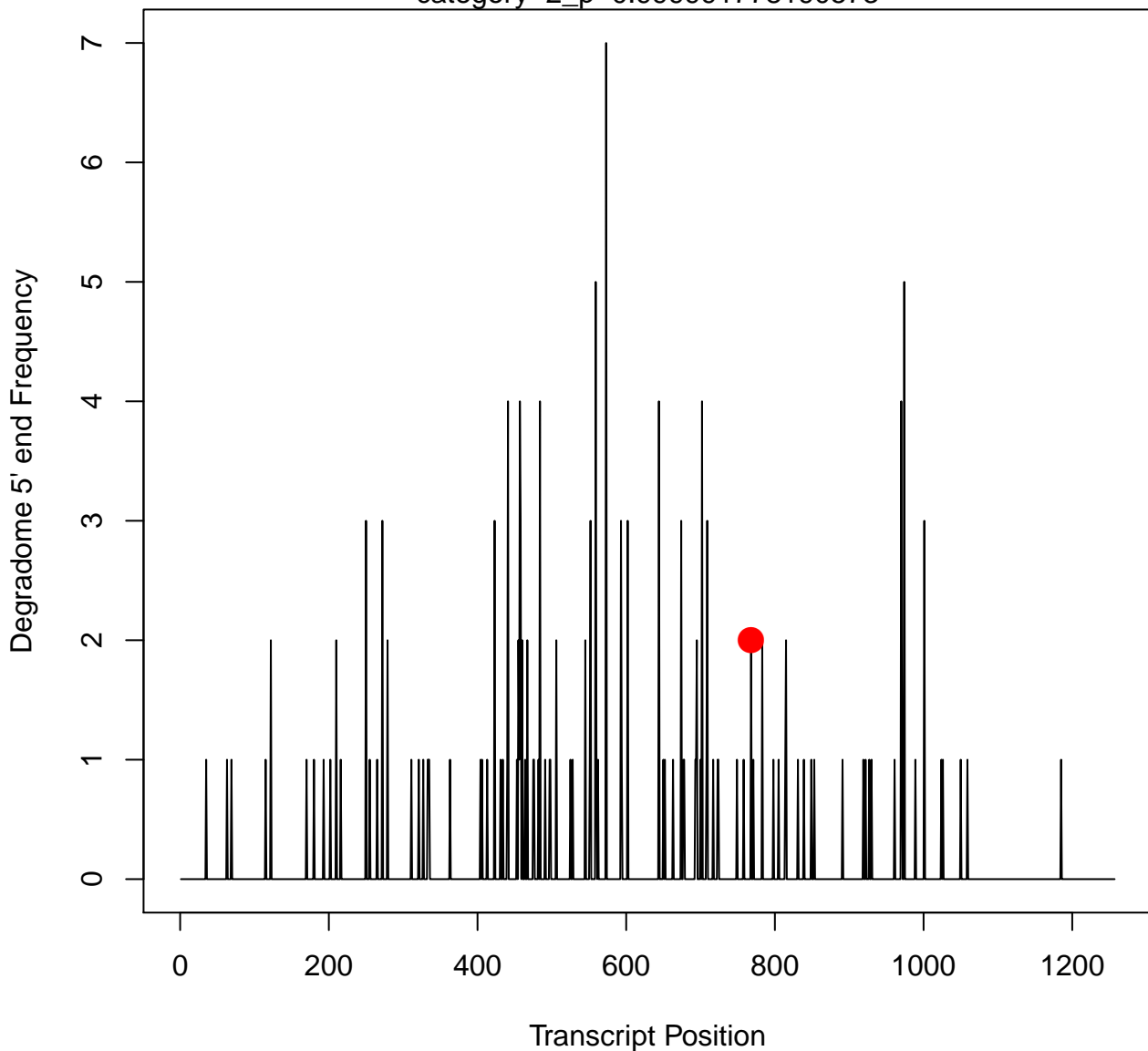

Supplement: Supplementary file 2 [file Data_Sheet_2.zip › Sit-miR164e_Seita.7G333700.1_768_TPlot.pdf]

**T=Seita.8G030100.1\_Q=Sit-miR164e\_S=133**

category=2\_p=0.999976588629351

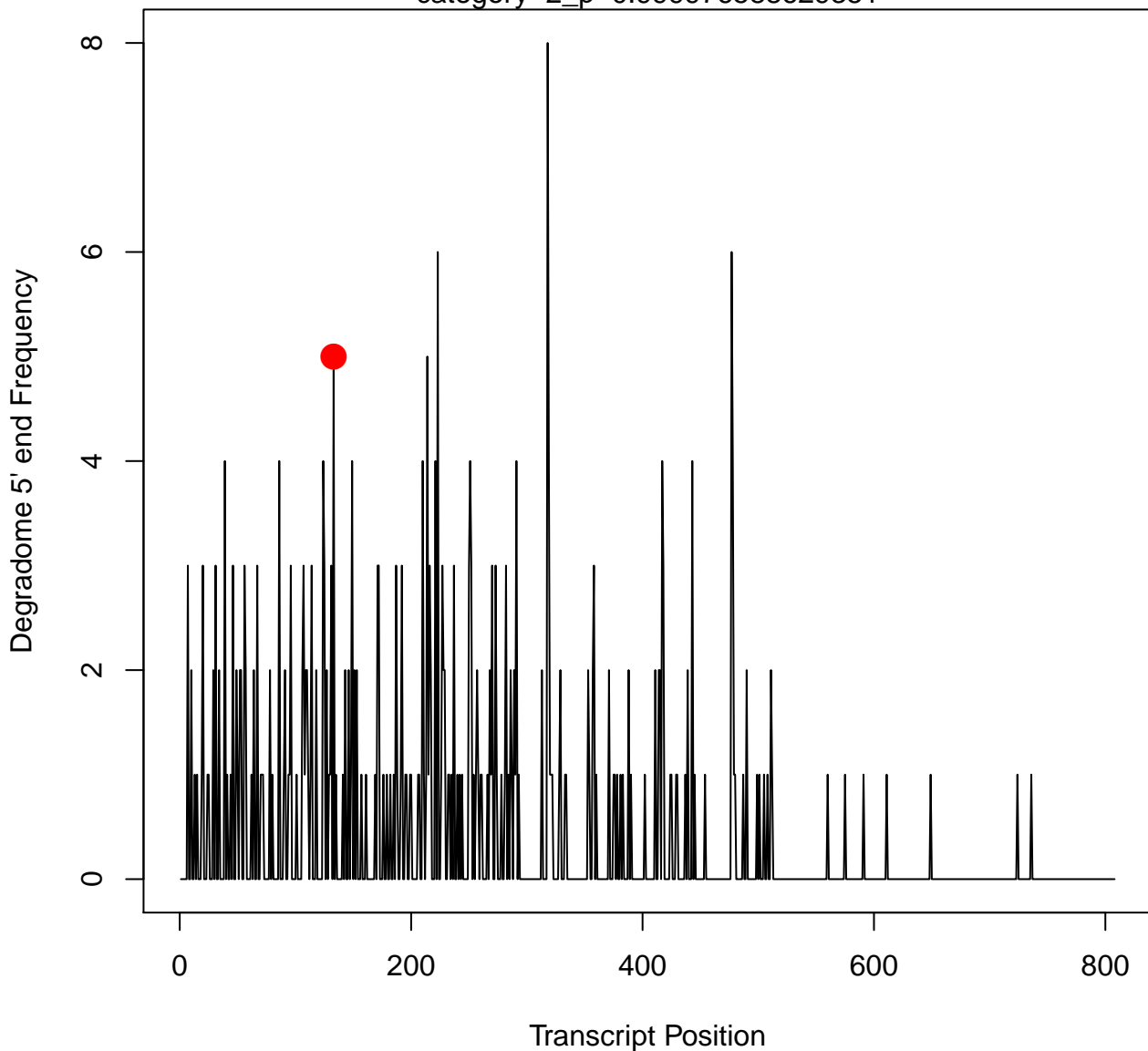

Supplement: Supplementary file 2 [file Data_Sheet_2.zip › Sit-miR164e_Seita.8G030100.1_133_TPlot.pdf]

**T=Seita.9G089800.1\_Q=Sit-miR164e\_S=1145**

category=2\_p=0.853857256797077

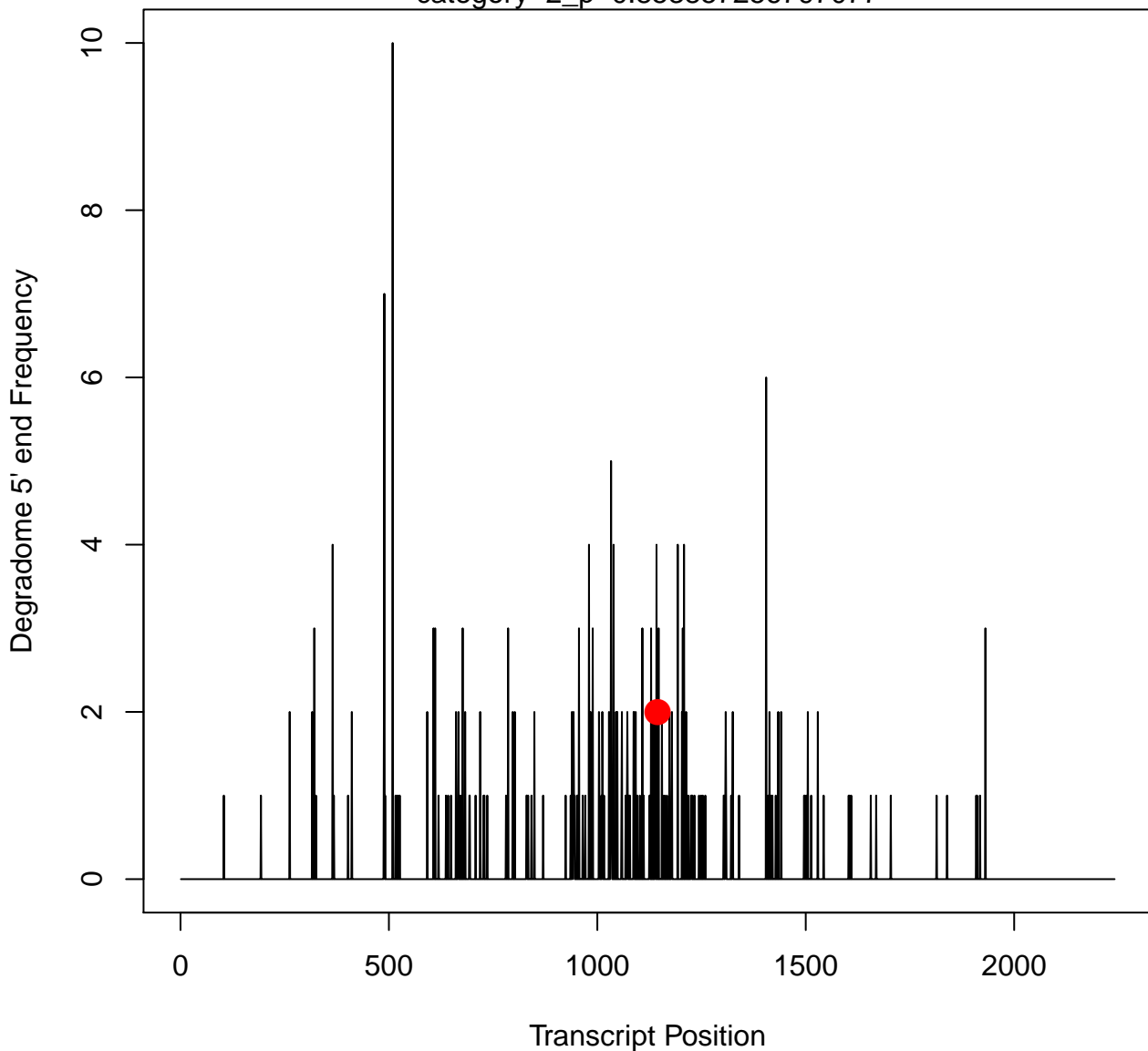

Supplement: Supplementary file 2 [file Data_Sheet_2.zip › Sit-miR164e_Seita.9G089800.1_1145_TPlot.pdf]

**T=Seita.9G171900.1\_Q=Sit-miR164e\_S=2520**

category=2\_p=0.990325260897061

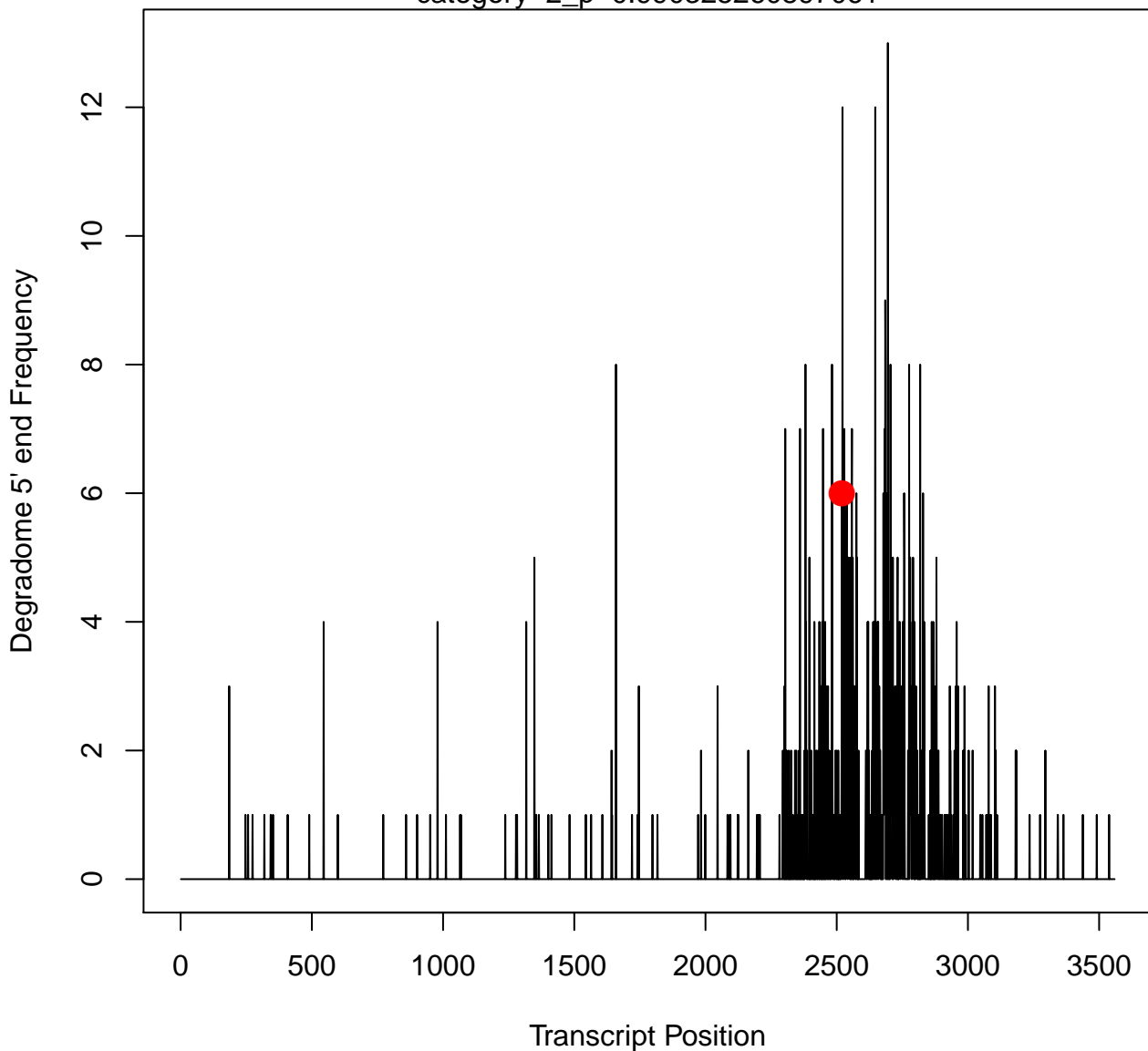

Supplement: Supplementary file 2 [file Data_Sheet_2.zip › Sit-miR164e_Seita.9G171900.1_2520_TPlot.pdf]

**T=Seita.9G403000.1\_Q=Sit-miR164f\_S=1037**

category=2\_p=0.999999941723011

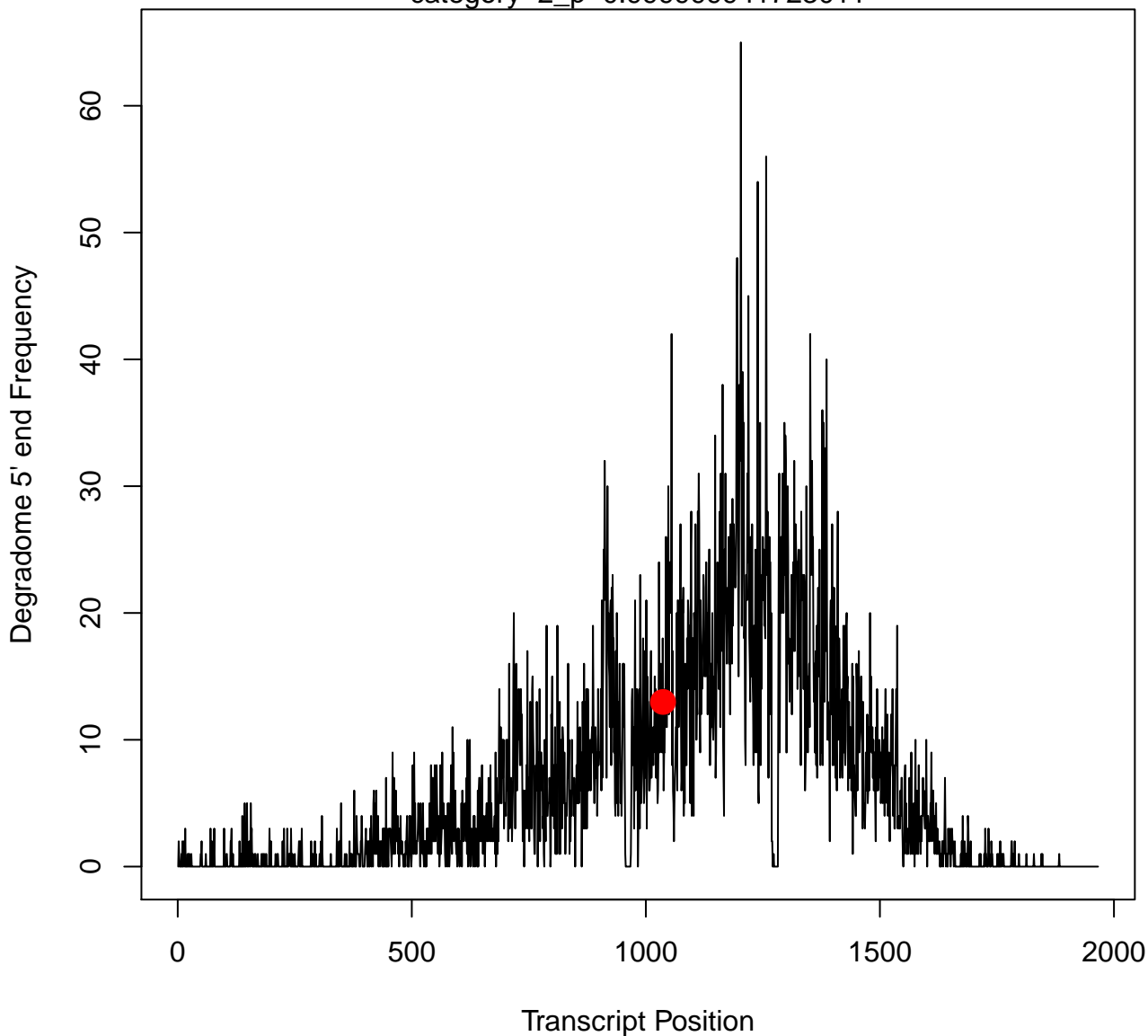

Supplement: Supplementary file 2 [file Data_Sheet_2.zip › Sit-miR164f_Seita.9G403000.1_1037_TPlot.pdf]

**T=Seita.9G484900.1\_Q=Sit-miR164f\_S=1768**

category=2\_p=0.999998000906833

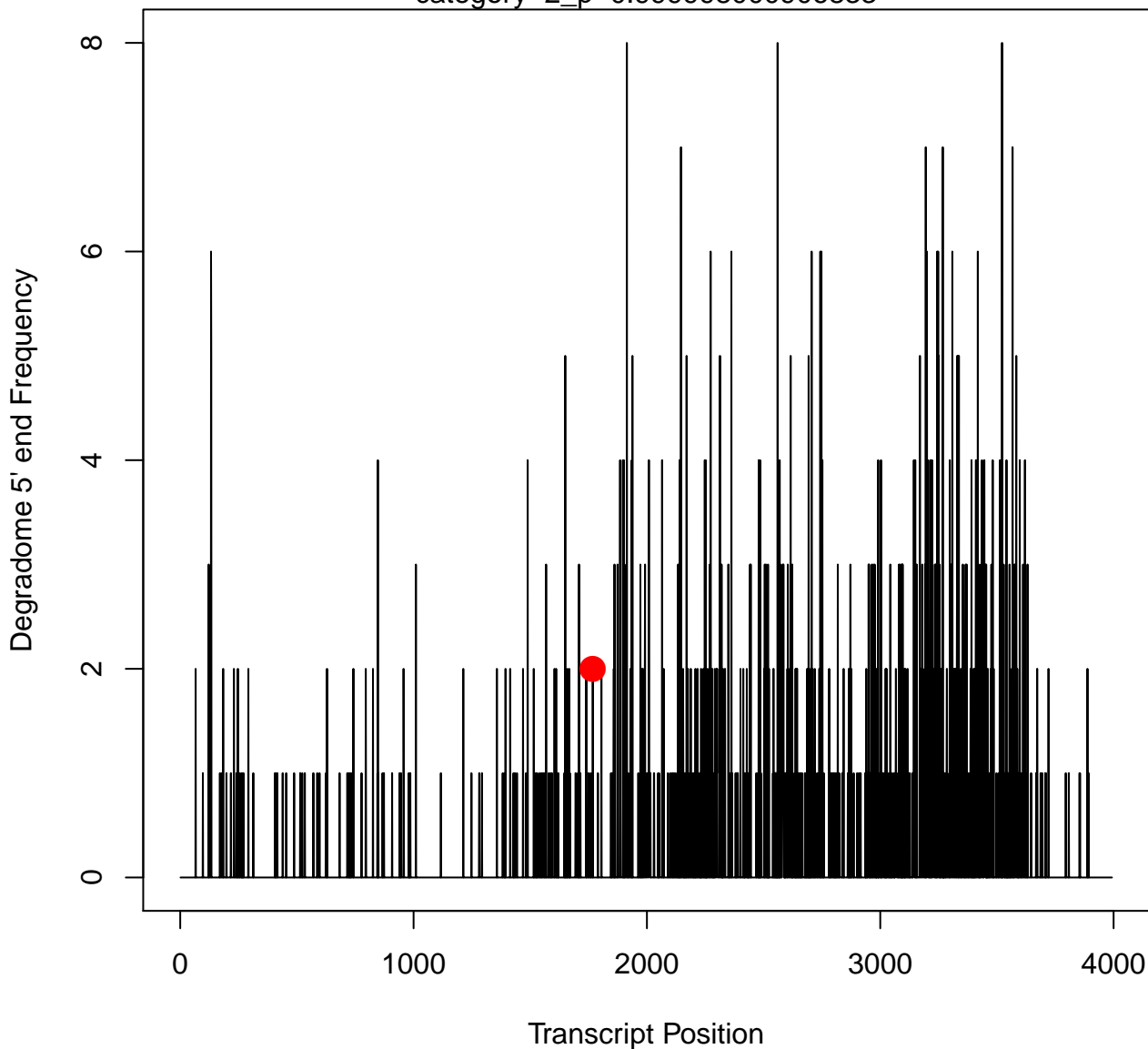

Supplement: Supplementary file 2 [file Data_Sheet_2.zip › Sit-miR164f_Seita.9G484900.1_1768_TPlot.pdf]

**T=Seita.3G395000.1\_Q=Sit-miR166a\_S=774**

category=0\_p=0.00115605171964761

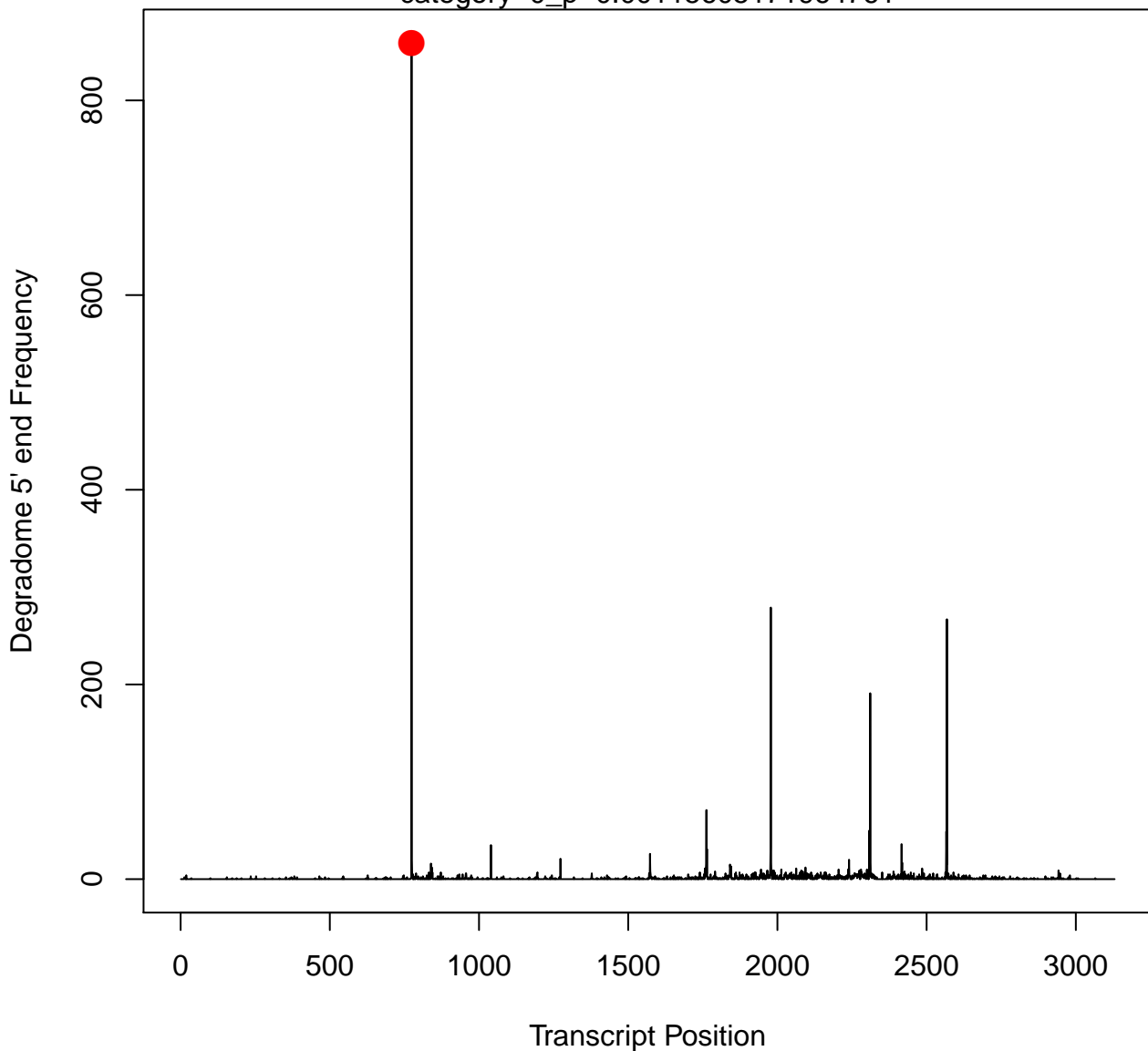

Supplement: Supplementary file 2 [file Data_Sheet_2.zip › Sit-miR166a_Seita.3G395000.1_774_TPlot.pdf]

**T=Seita.1G244300.1\_Q=Sit-miR166b\_S=481**

category=2\_p=0.999740907243265

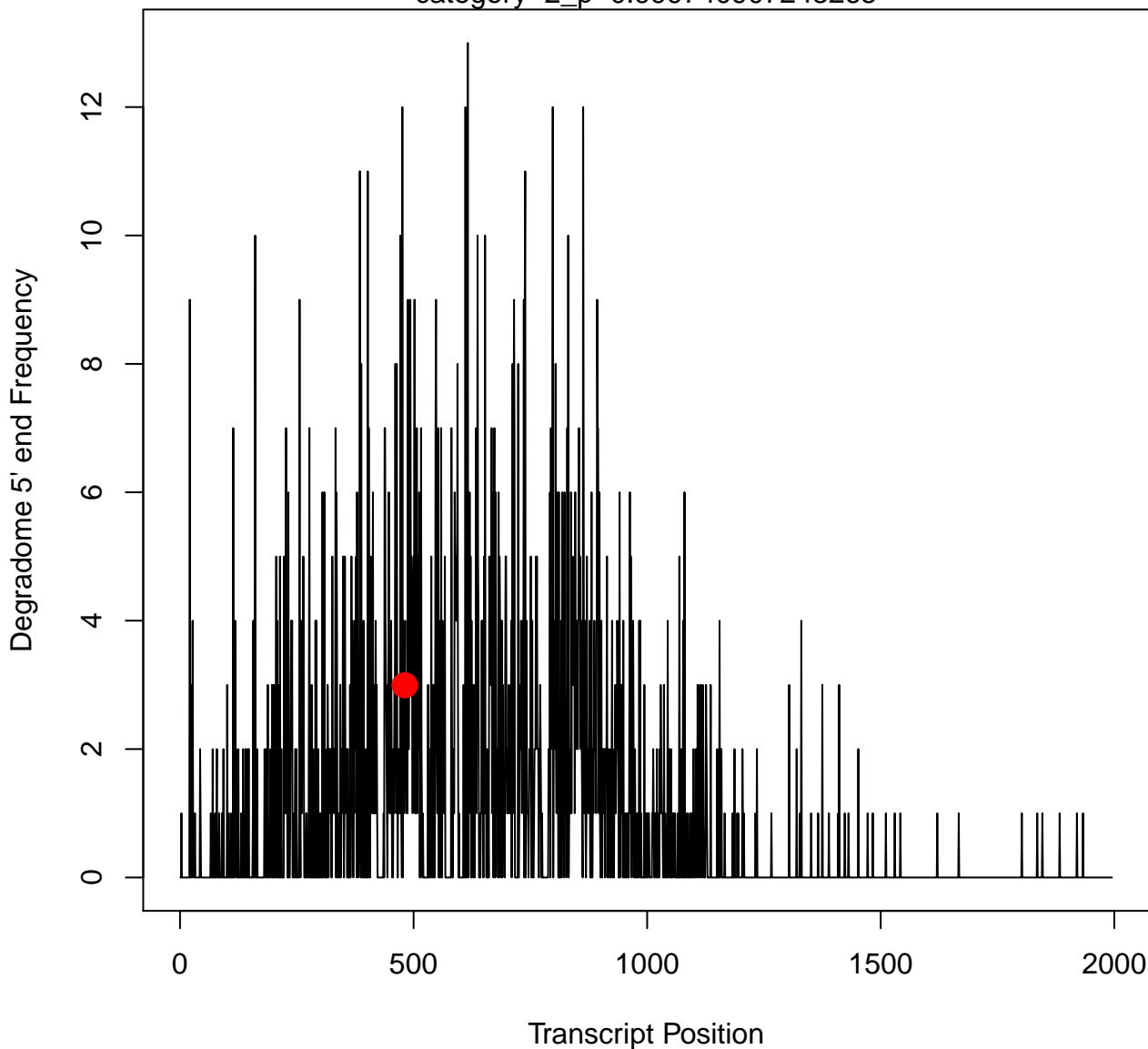

Supplement: Supplementary file 2 [file Data_Sheet_2.zip › Sit-miR166b_Seita.1G244300.1_481_TPlot.pdf]

**T=Seita.1G338400.1\_Q=Sit-miR166b\_S=1948**

category=2\_p=0.998227125404544

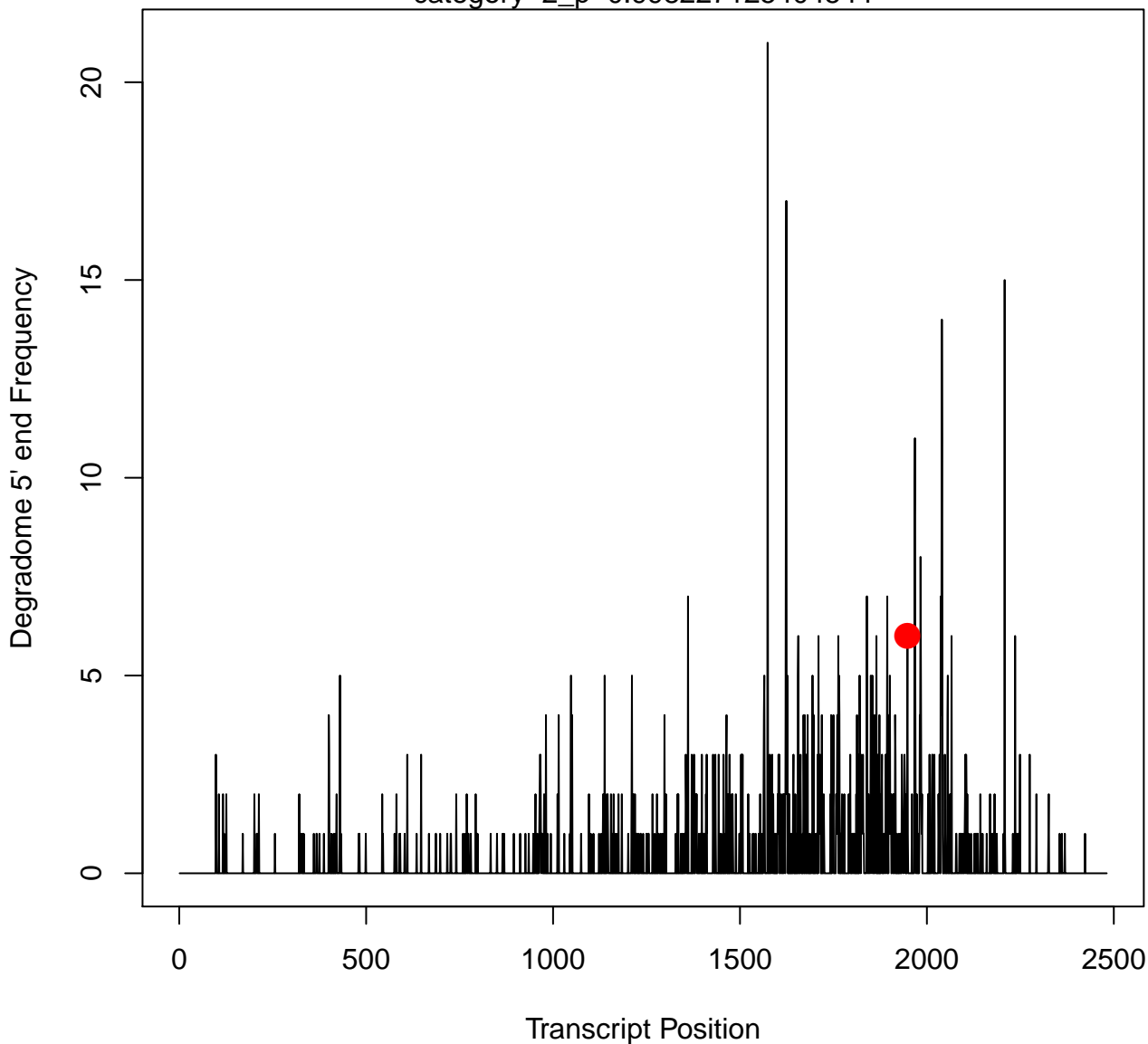

Supplement: Supplementary file 2 [file Data_Sheet_2.zip › Sit-miR166b_Seita.1G338400.1_1948_TPlot.pdf]

**T=Seita.2G382900.1\_Q=Sit-miR166b\_S=1099**

category=2\_p=0.892930026934013

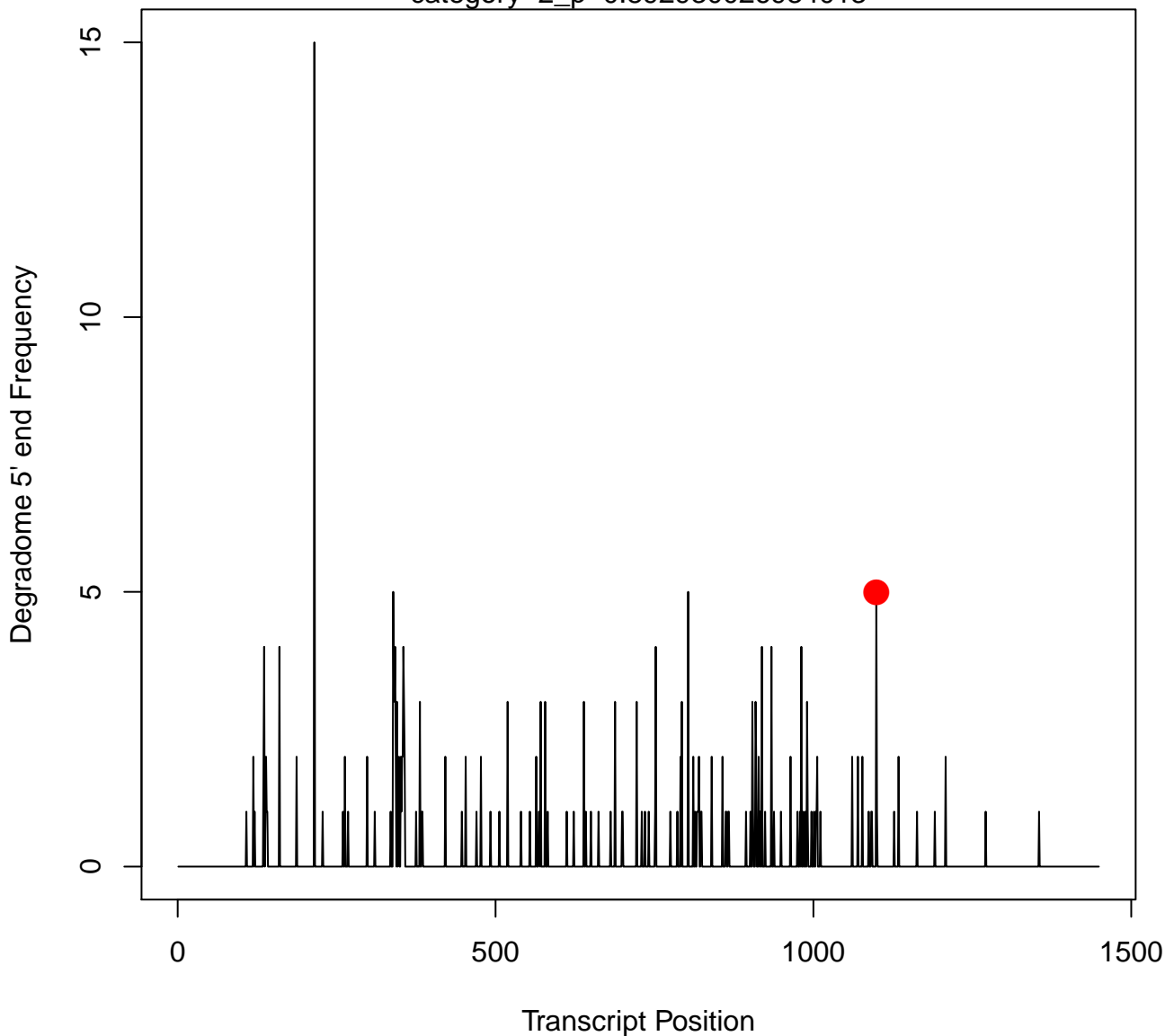

Supplement: Supplementary file 2 [file Data_Sheet_2.zip › Sit-miR166b_Seita.2G382900.1_1099_TPlot.pdf]

**T=Seita.3G035500.1\_Q=Sit-miR166b\_S=2169**

category=2\_p=0.999615042401972

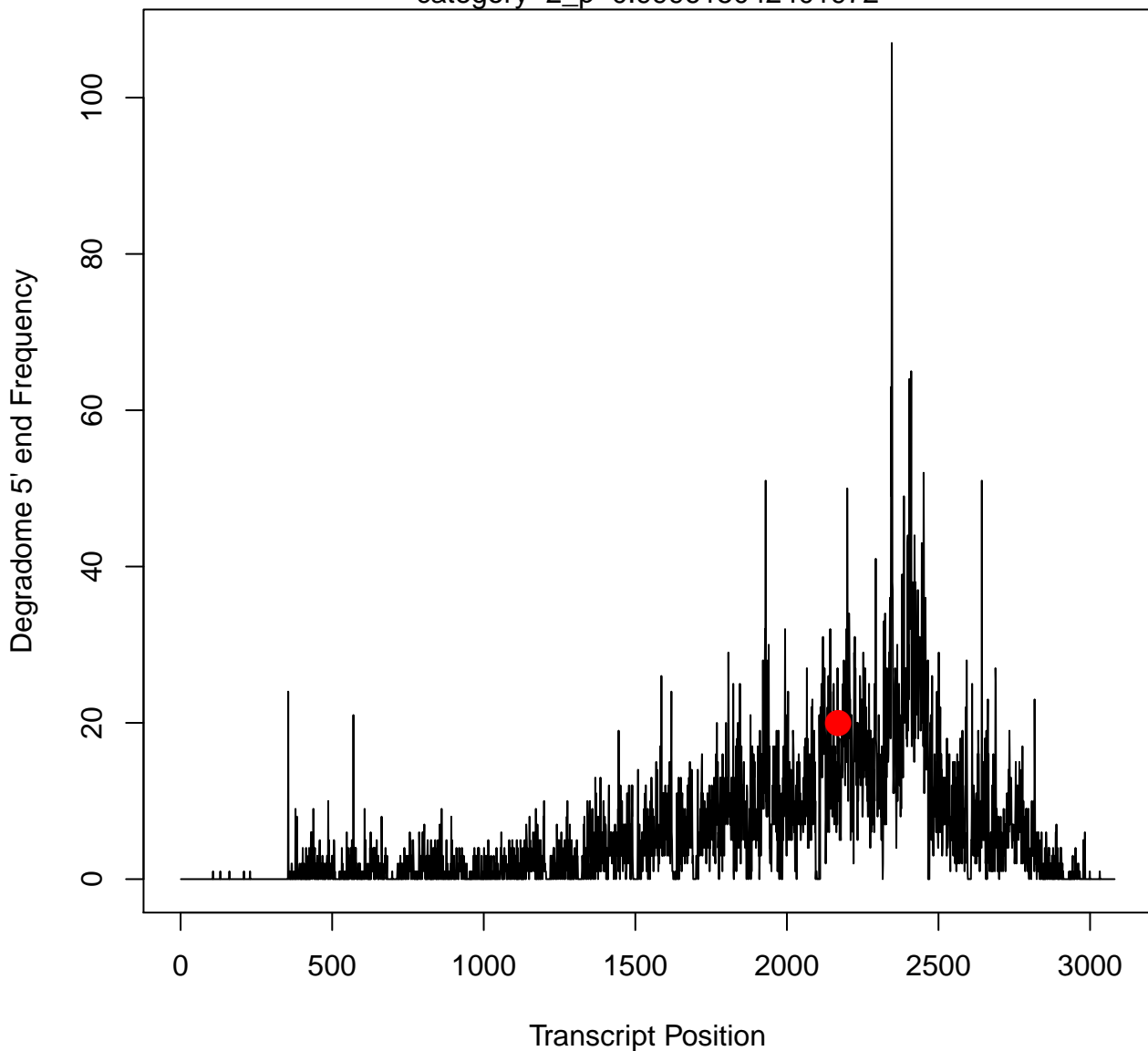

Supplement: Supplementary file 2 [file Data_Sheet_2.zip › Sit-miR166b_Seita.3G035500.1_2169_TPlot.pdf]
