# Supplementary figures and images for: The Construction and Exploration of a Comprehensive MicroRNA Centered Regulatory Network in Foxtail Millet (Setaria italica L.) (part 3 of 14)
Source: Front Plant Sci. 2022 May 6;13:848474. doi: 10.3389/fpls.2022.848474 (PMC9121102; doi:10.3389/fpls.2022.848474)

**T=Seita.1G096400.1\_Q=Sit-miR1133\_S=100**

category=2\_p=0.999999999842088

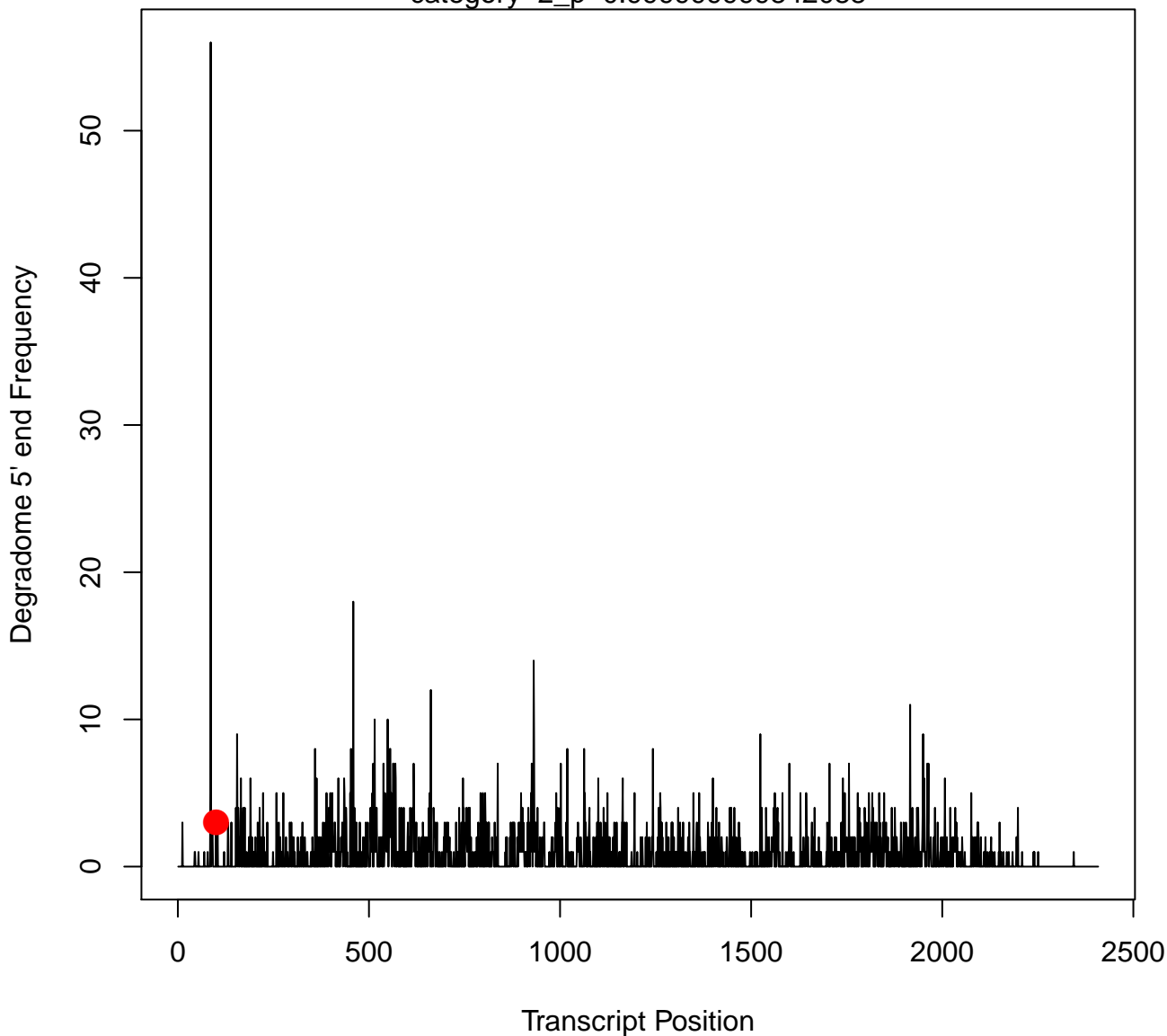

Supplement: Supplementary file 2 [file Data_Sheet_2.zip › Sit-miR1133_Seita.1G096400.1_100_TPlot.pdf]

**T=Seita.2G199300.1\_Q=Sit-miR1133\_S=450**

category=2\_p=0.999999829297757

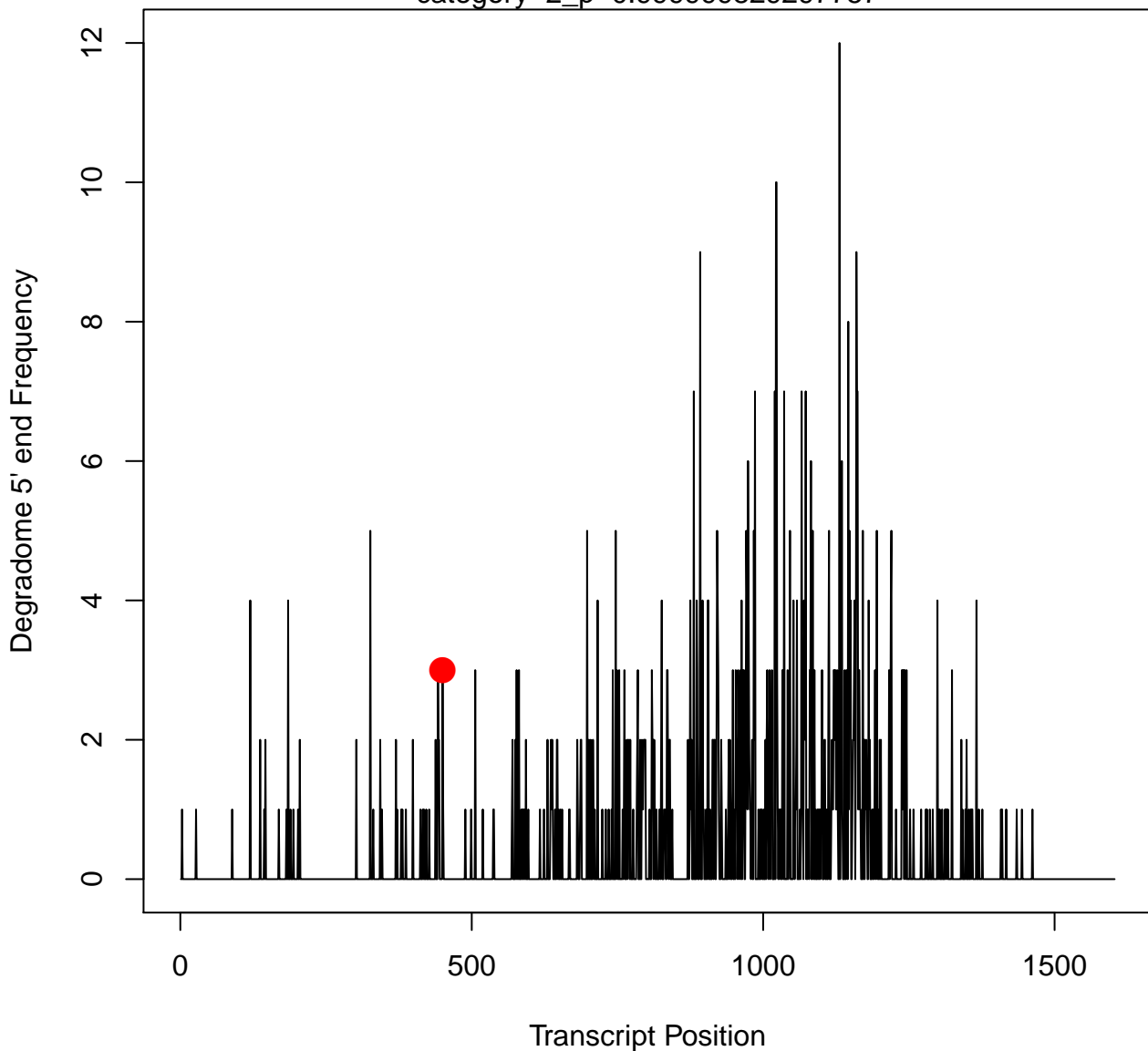

Supplement: Supplementary file 2 [file Data_Sheet_2.zip › Sit-miR1133_Seita.2G199300.1_450_TPlot.pdf]

**T=Seita.2G311900.1\_Q=Sit-miR1133\_S=126**

category=2\_p=0.999999999999629

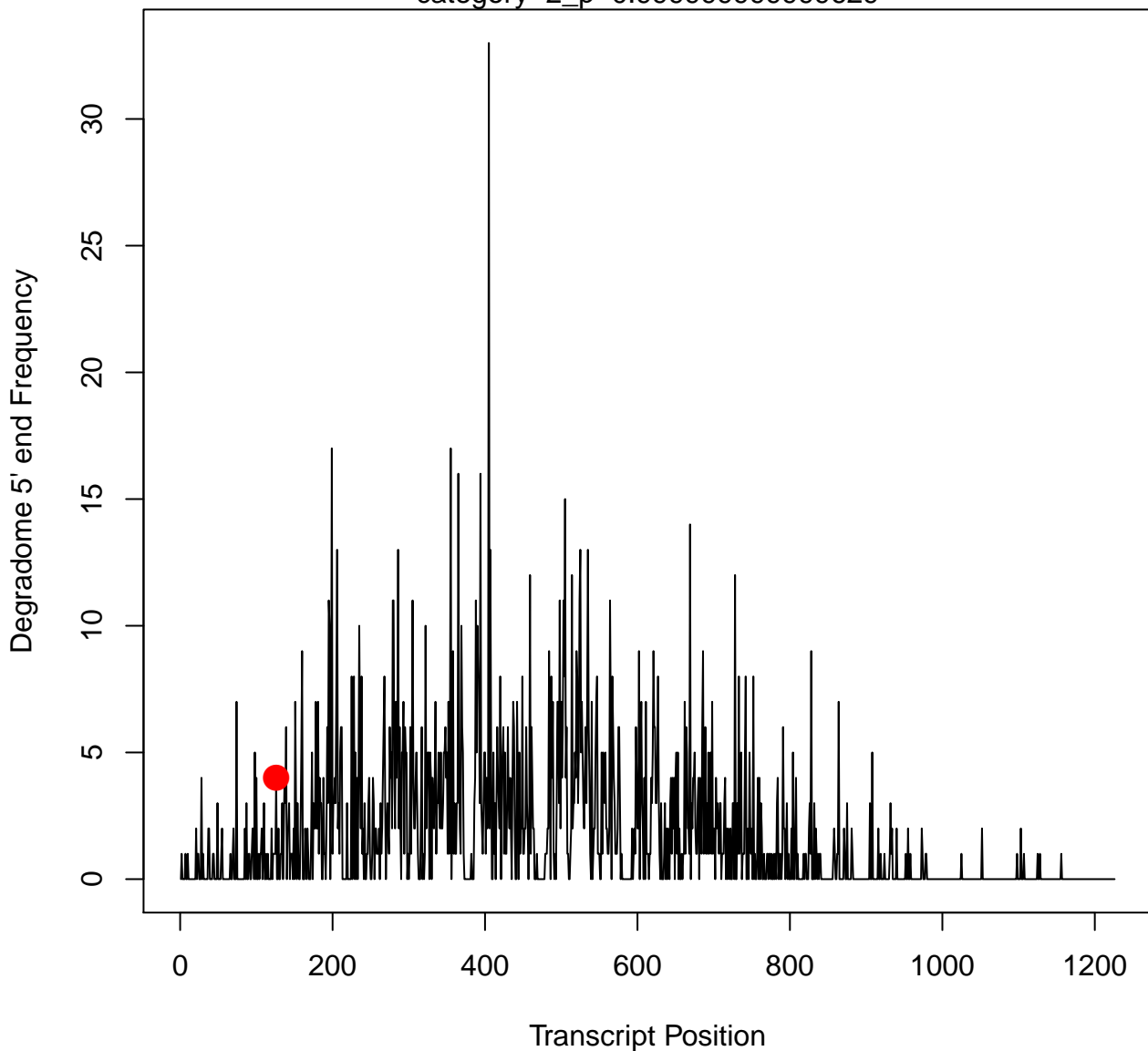

Supplement: Supplementary file 2 [file Data_Sheet_2.zip › Sit-miR1133_Seita.2G311900.1_126_TPlot.pdf]

**T=Seita.3G120600.1\_Q=Sit-miR1133\_S=1175**

category=2\_p=0.520652874976587

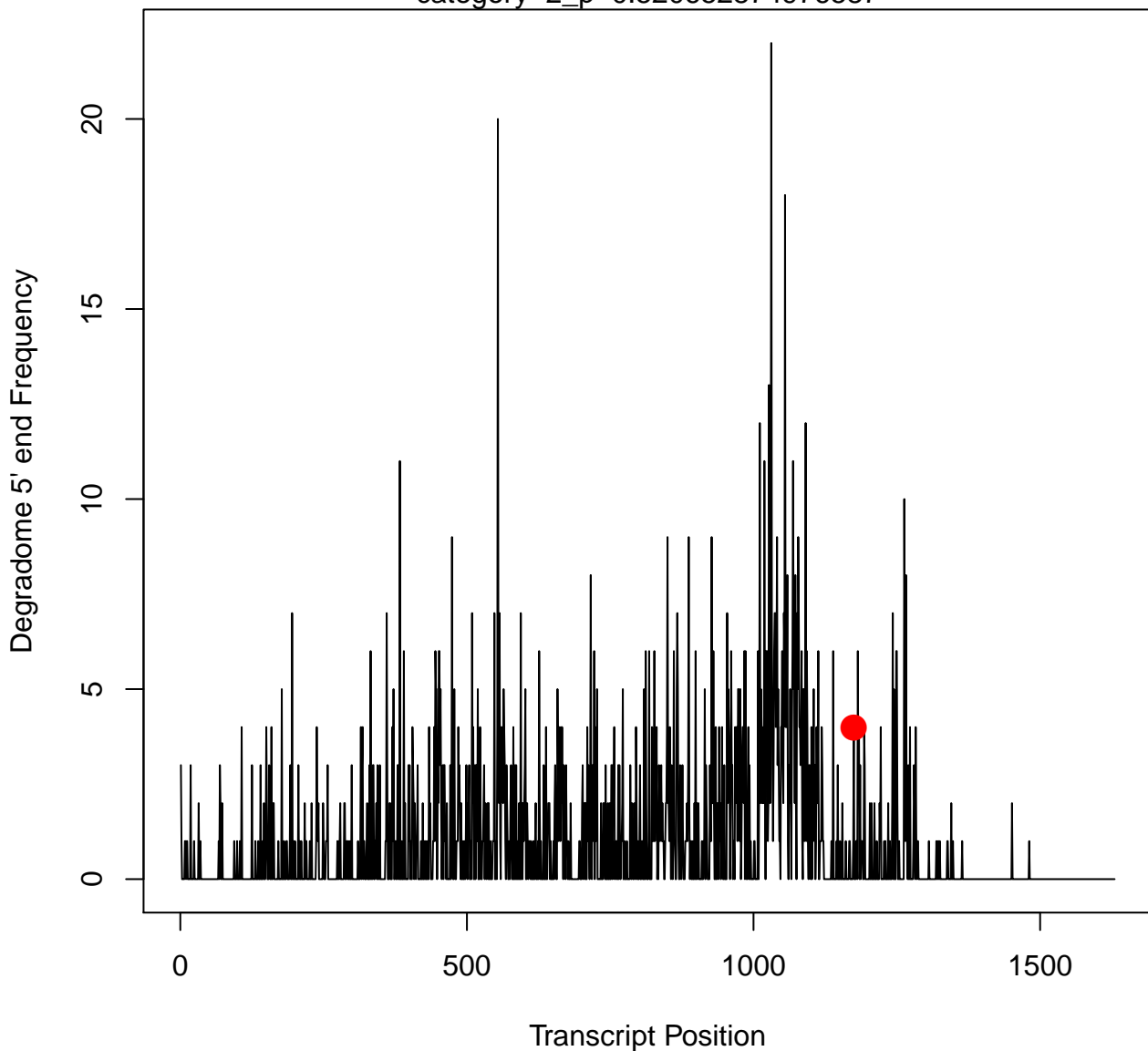

Supplement: Supplementary file 2 [file Data_Sheet_2.zip › Sit-miR1133_Seita.3G120600.1_1175_TPlot.pdf]

**T=Seita.3G158600.1\_Q=Sit-miR1133\_S=1059**

category=2\_p=0.999929457369485

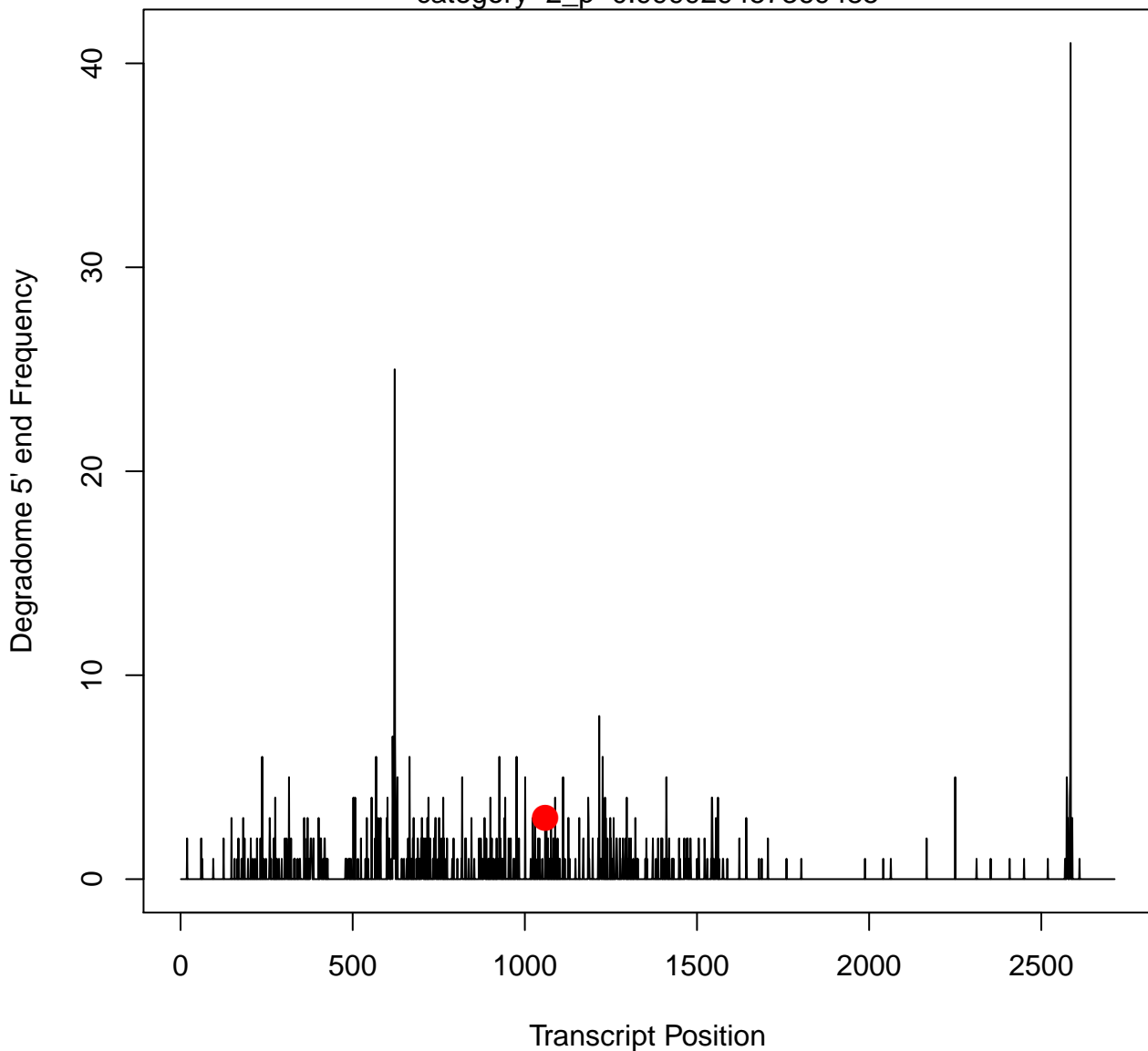

Supplement: Supplementary file 2 [file Data_Sheet_2.zip › Sit-miR1133_Seita.3G158600.1_1059_TPlot.pdf]

**T=Seita.3G335400.1\_Q=Sit-miR1133\_S=726**

category=2\_p=0.999999963967747

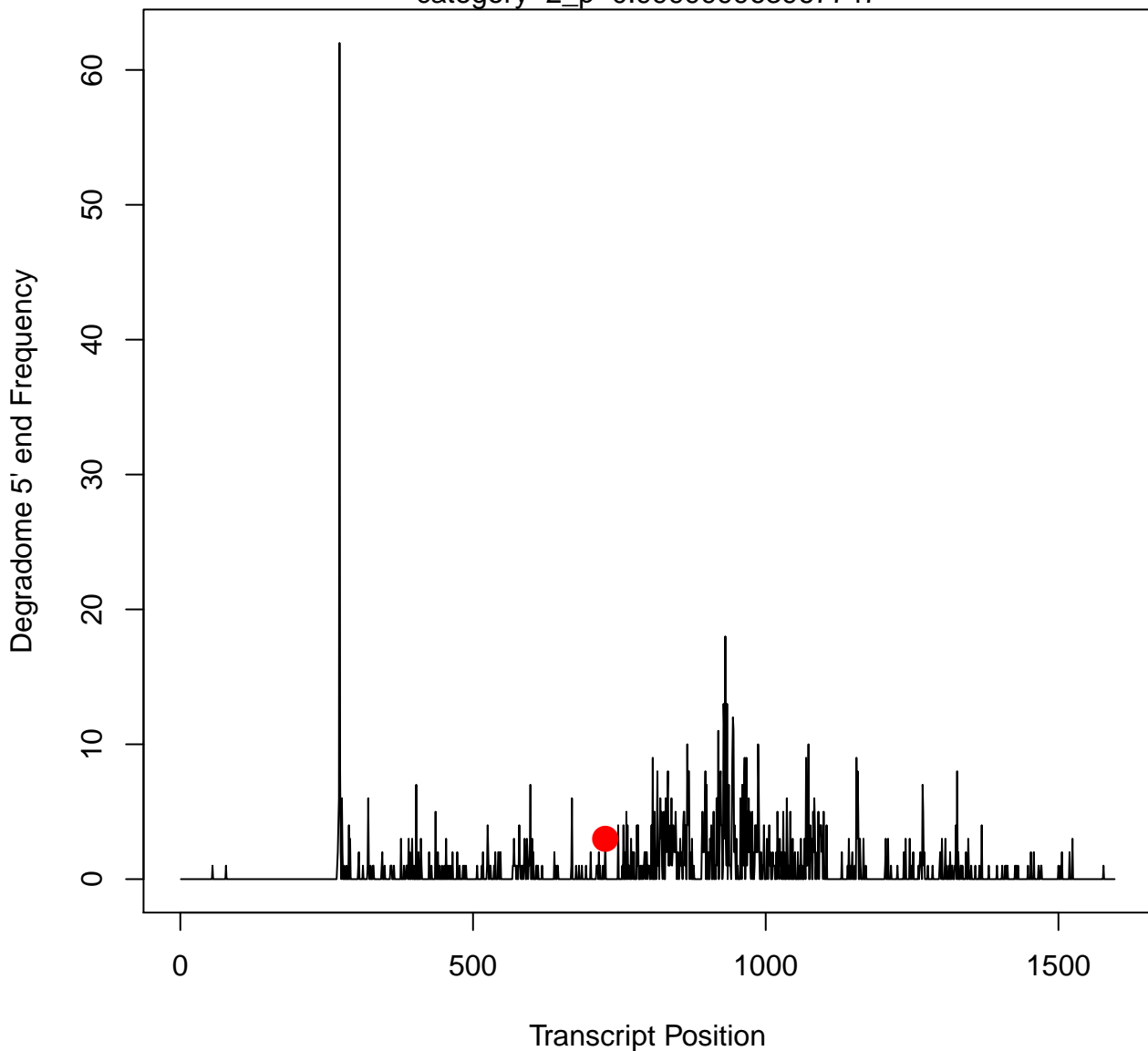

Supplement: Supplementary file 2 [file Data_Sheet_2.zip › Sit-miR1133_Seita.3G335400.1_726_TPlot.pdf]

**T=Seita.4G239100.1\_Q=Sit-miR1133\_S=41**

category=2\_p=0.999999928964422

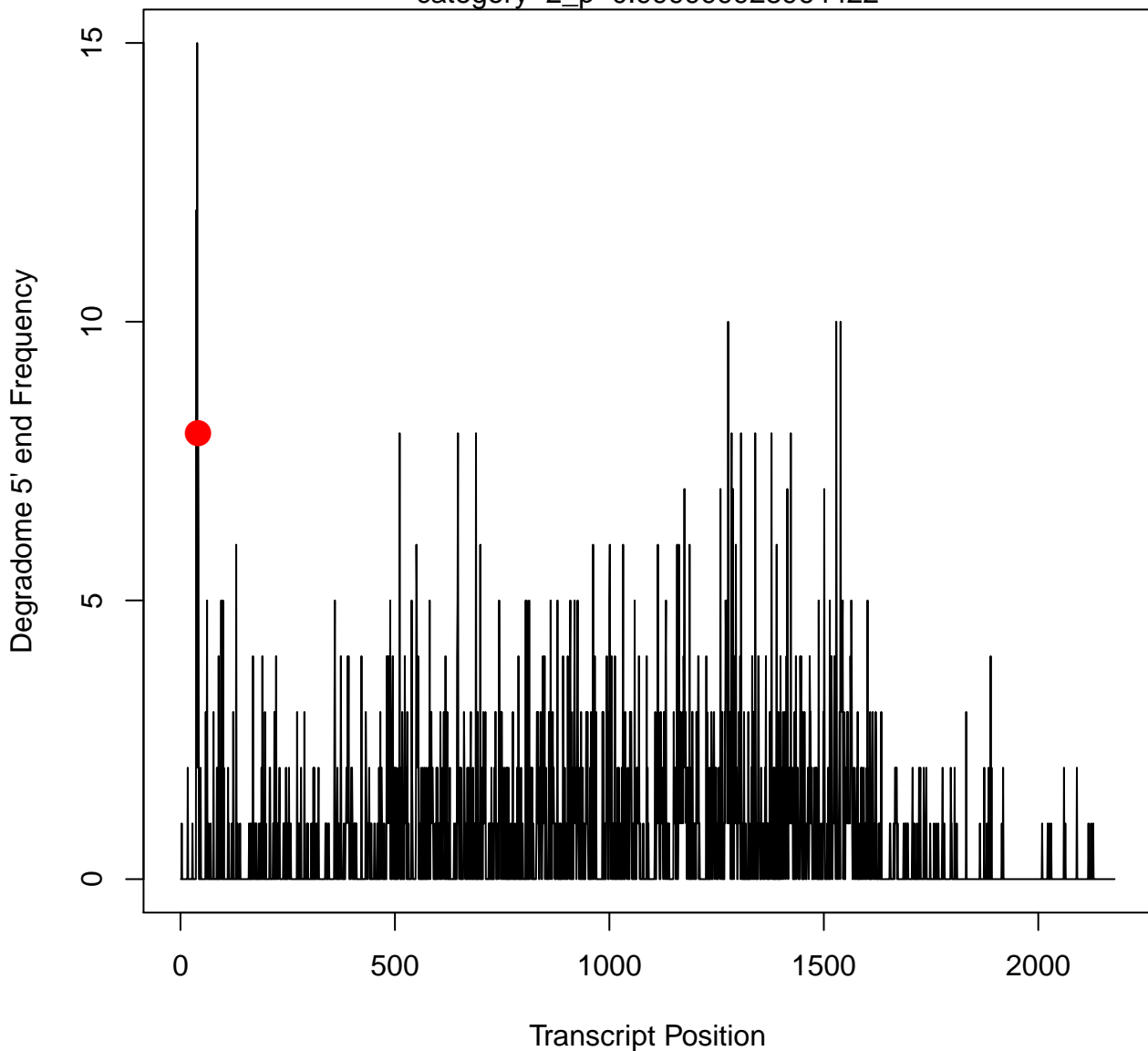

Supplement: Supplementary file 2 [file Data_Sheet_2.zip › Sit-miR1133_Seita.4G239100.1_41_TPlot.pdf]

**T=Seita.9G029000.1\_Q=Sit-miR1133\_S=111**

category=2\_p=0.999994307638541

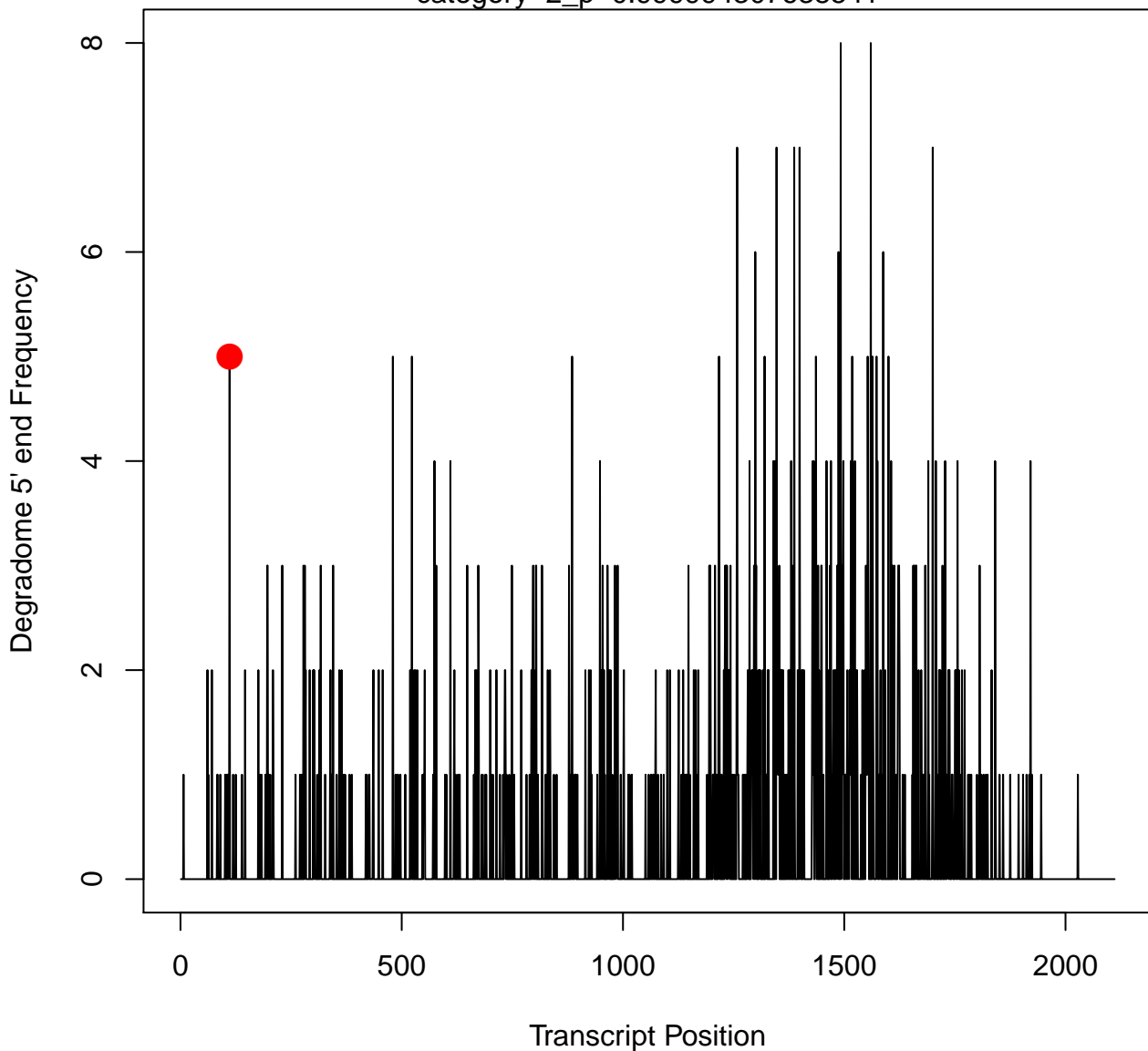

Supplement: Supplementary file 2 [file Data_Sheet_2.zip › Sit-miR1133_Seita.9G029000.1_111_TPlot.pdf]

**T=Seita.9G053500.1\_Q=Sit-miR1133\_S=782**

category=2\_p=0.999999999997529

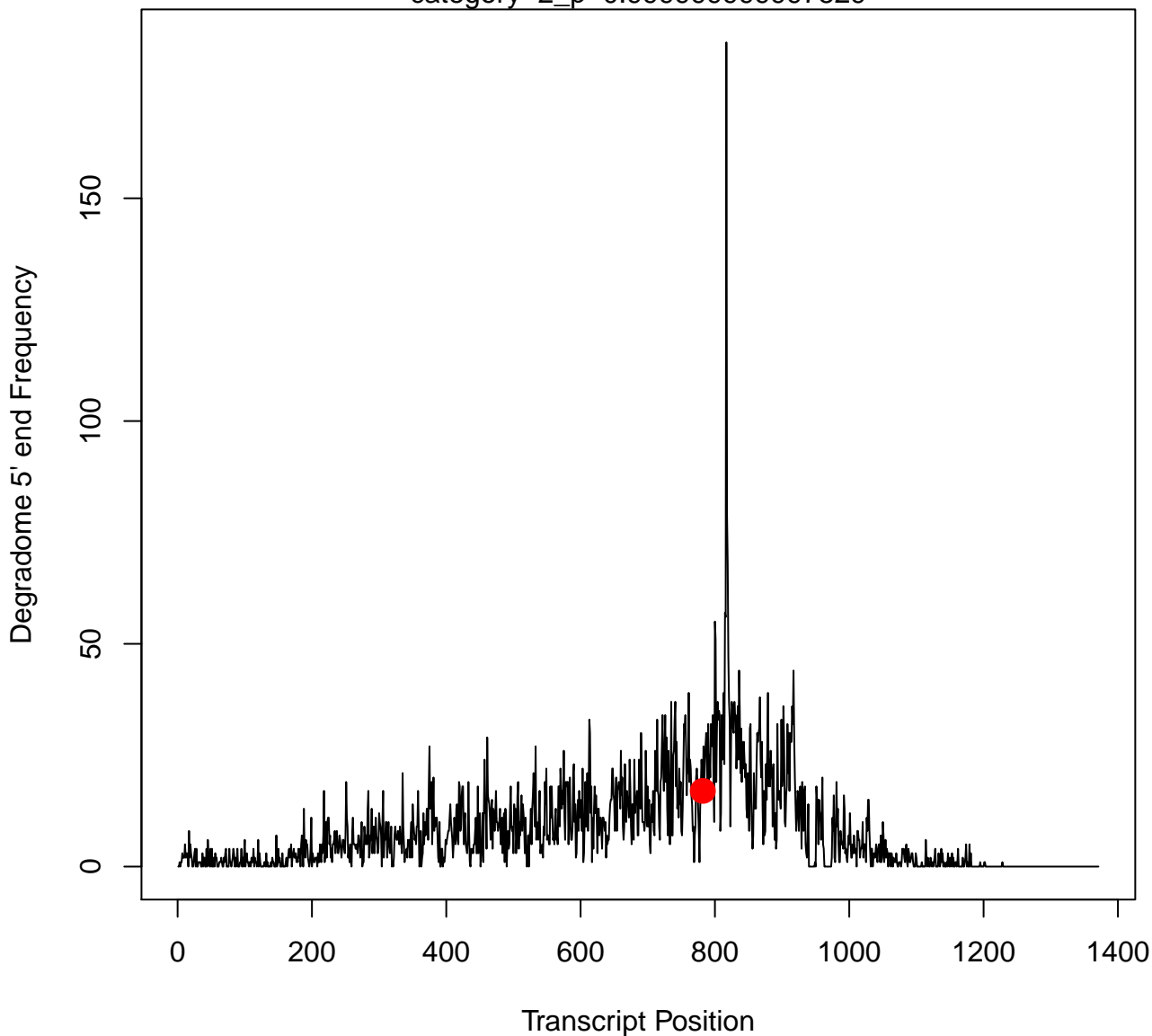

Supplement: Supplementary file 2 [file Data_Sheet_2.zip › Sit-miR1133_Seita.9G053500.1_782_TPlot.pdf]

**T=Seita.9G214500.1\_Q=Sit-miR1133\_S=1282**

category=2\_p=0.999999455711181

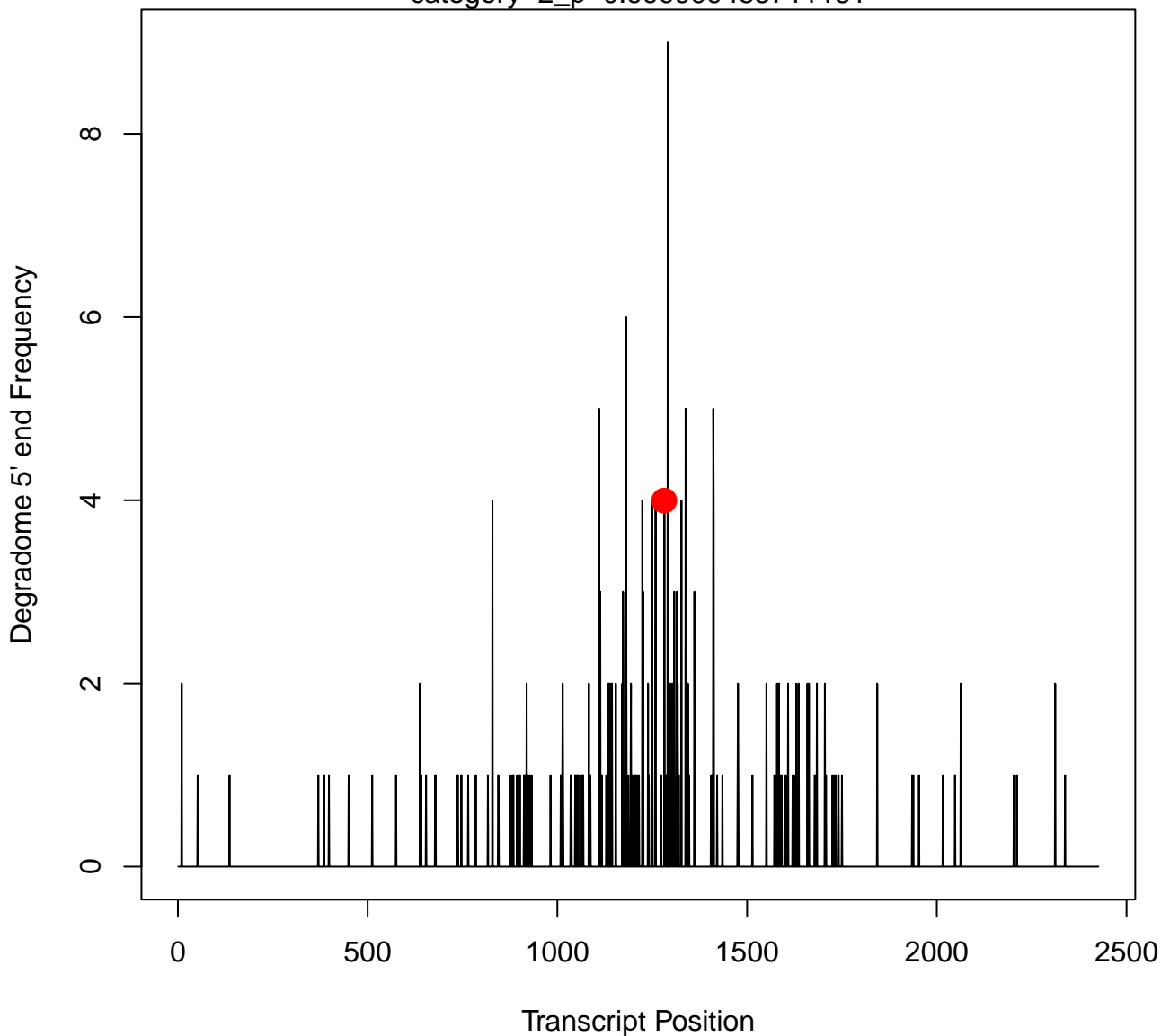

Supplement: Supplementary file 2 [file Data_Sheet_2.zip › Sit-miR1133_Seita.9G214500.1_1282_TPlot.pdf]

**T=Seita.9G281900.1\_Q=Sit-miR1133\_S=22**

category=2\_p=0.876666478894216

Degradsome 5' end Frequency

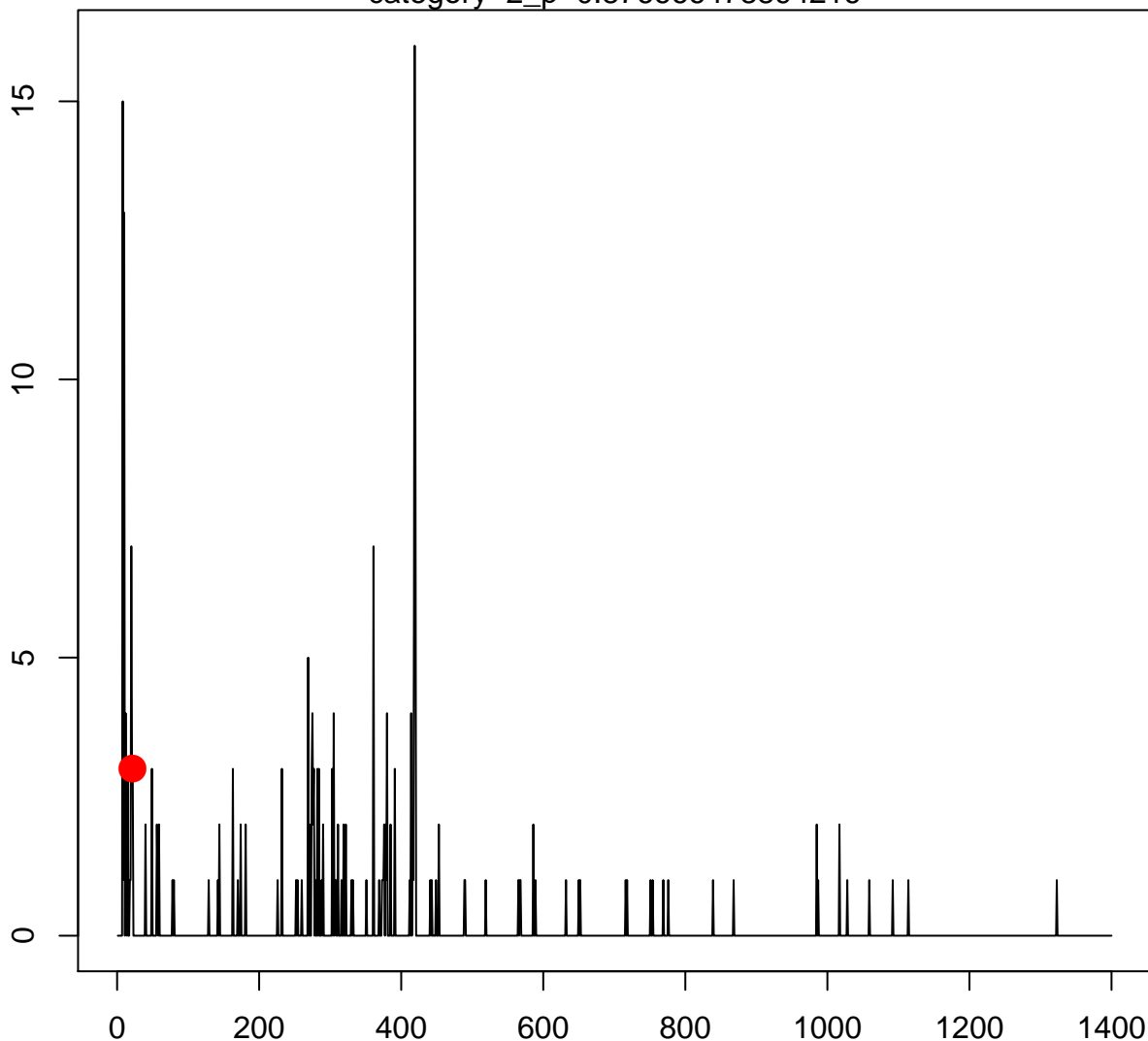

Transcript Position

Supplement: Supplementary file 2 [file Data_Sheet_2.zip › Sit-miR1133_Seita.9G281900.1_22_TPlot.pdf]

**T=Seita.9G305400.1\_Q=Sit-miR1133\_S=381**

category=2\_p=0.999999986982887

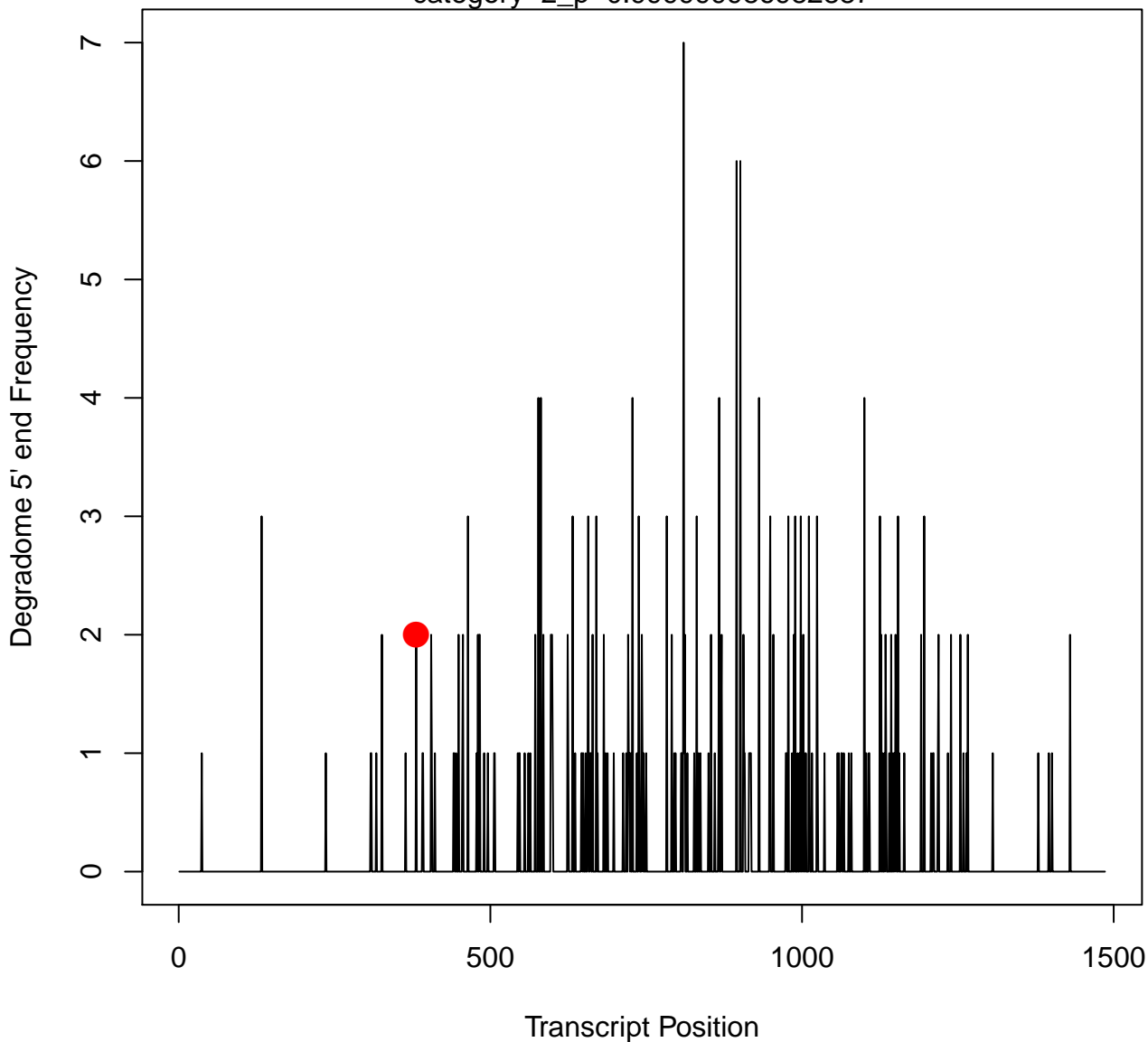

Supplement: Supplementary file 2 [file Data_Sheet_2.zip › Sit-miR1133_Seita.9G305400.1_381_TPlot.pdf]

**T=Seita.1G177000.1\_Q=Sit-miR1432\_S=764**

category=2\_p=0.999999317518454

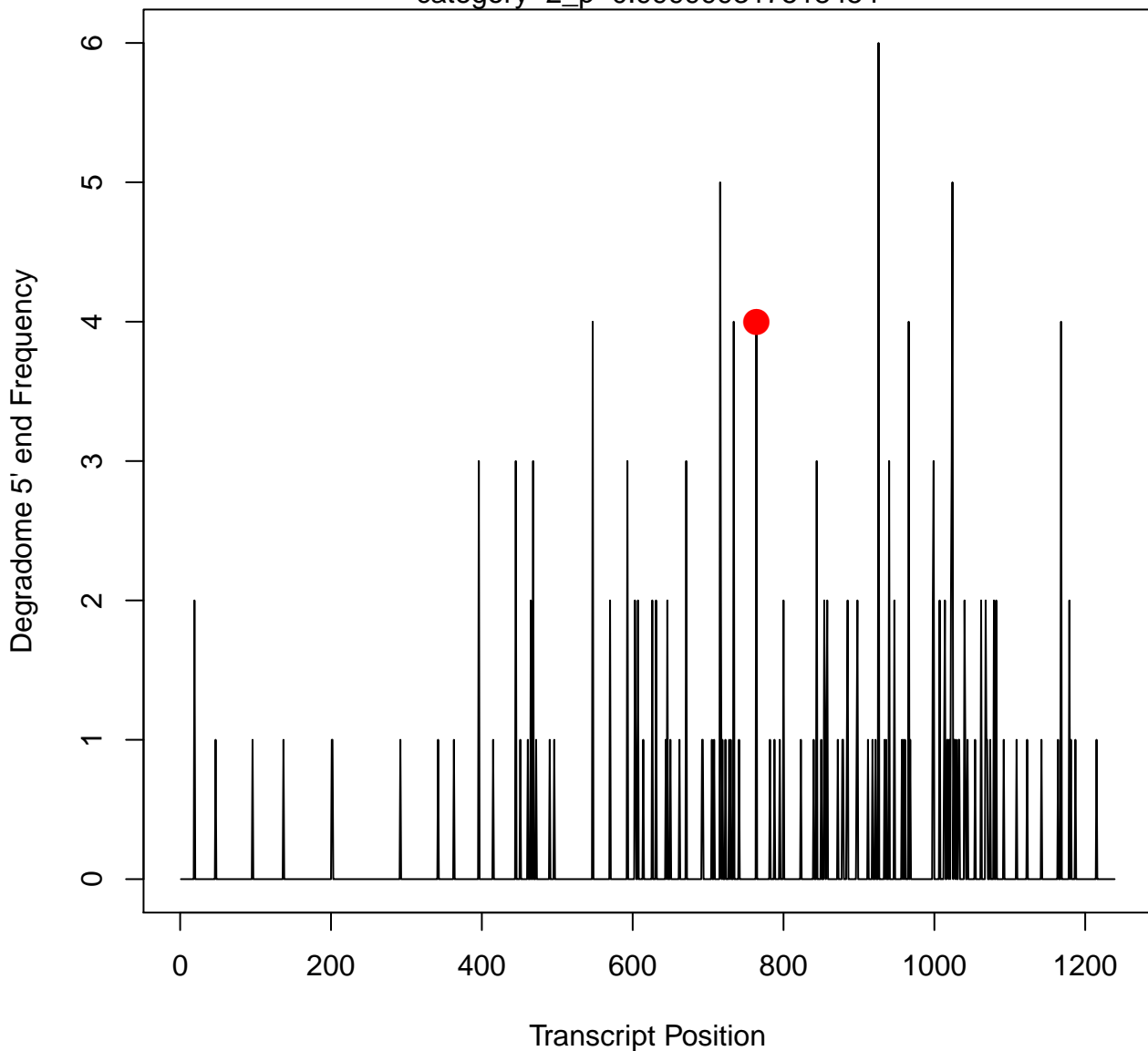

Supplement: Supplementary file 2 [file Data_Sheet_2.zip › Sit-miR1432_Seita.1G177000.1_764_TPlot.pdf]

**T=Seita.1G302400.1\_Q=Sit-miR1432\_S=858**

category=2\_p=0.999872242005354

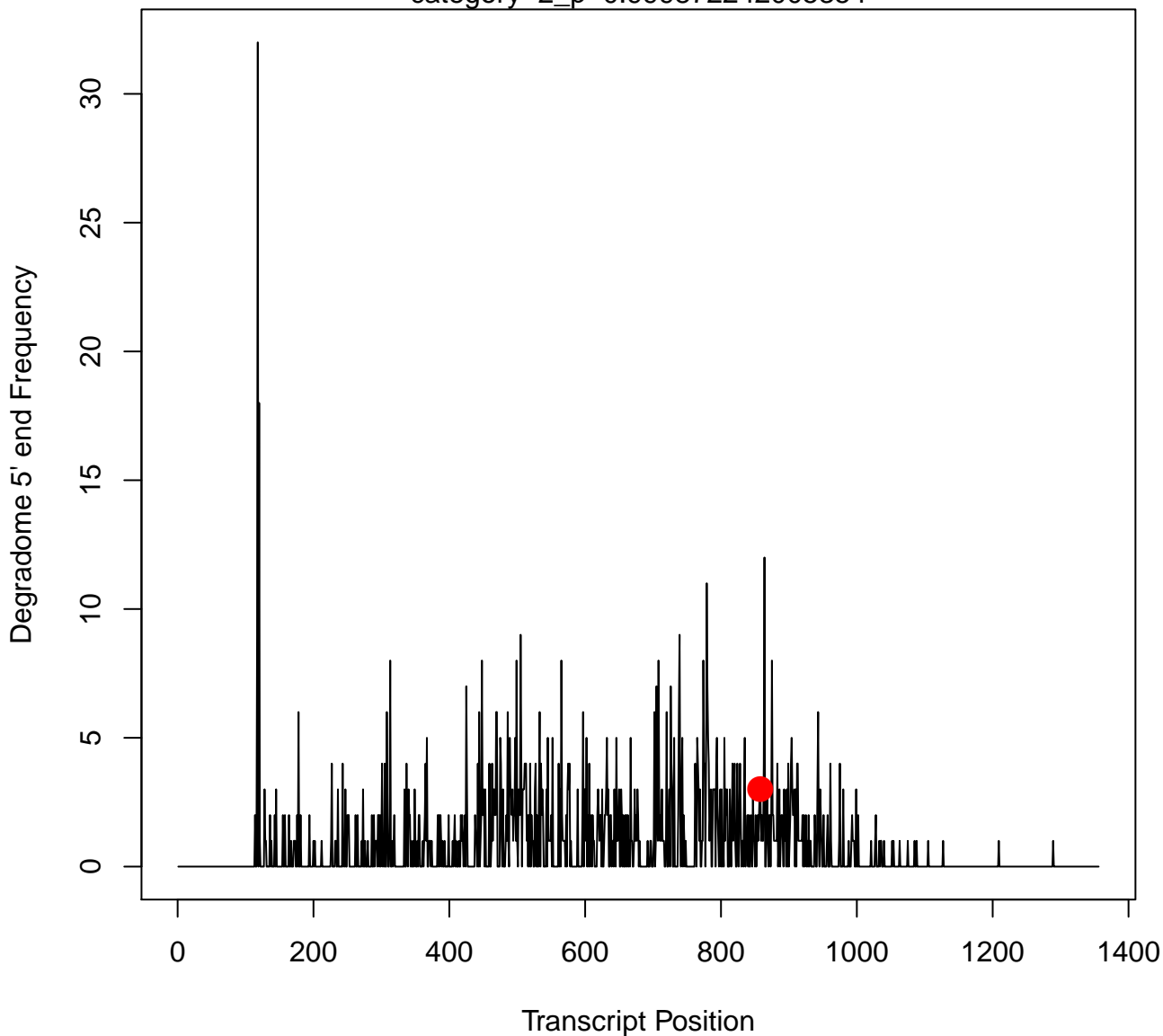

Supplement: Supplementary file 2 [file Data_Sheet_2.zip › Sit-miR1432_Seita.1G302400.1_858_TPlot.pdf]

**T=Seita.1G373400.1\_Q=Sit-miR1432\_S=943**

category=2\_p=0.999996578598071

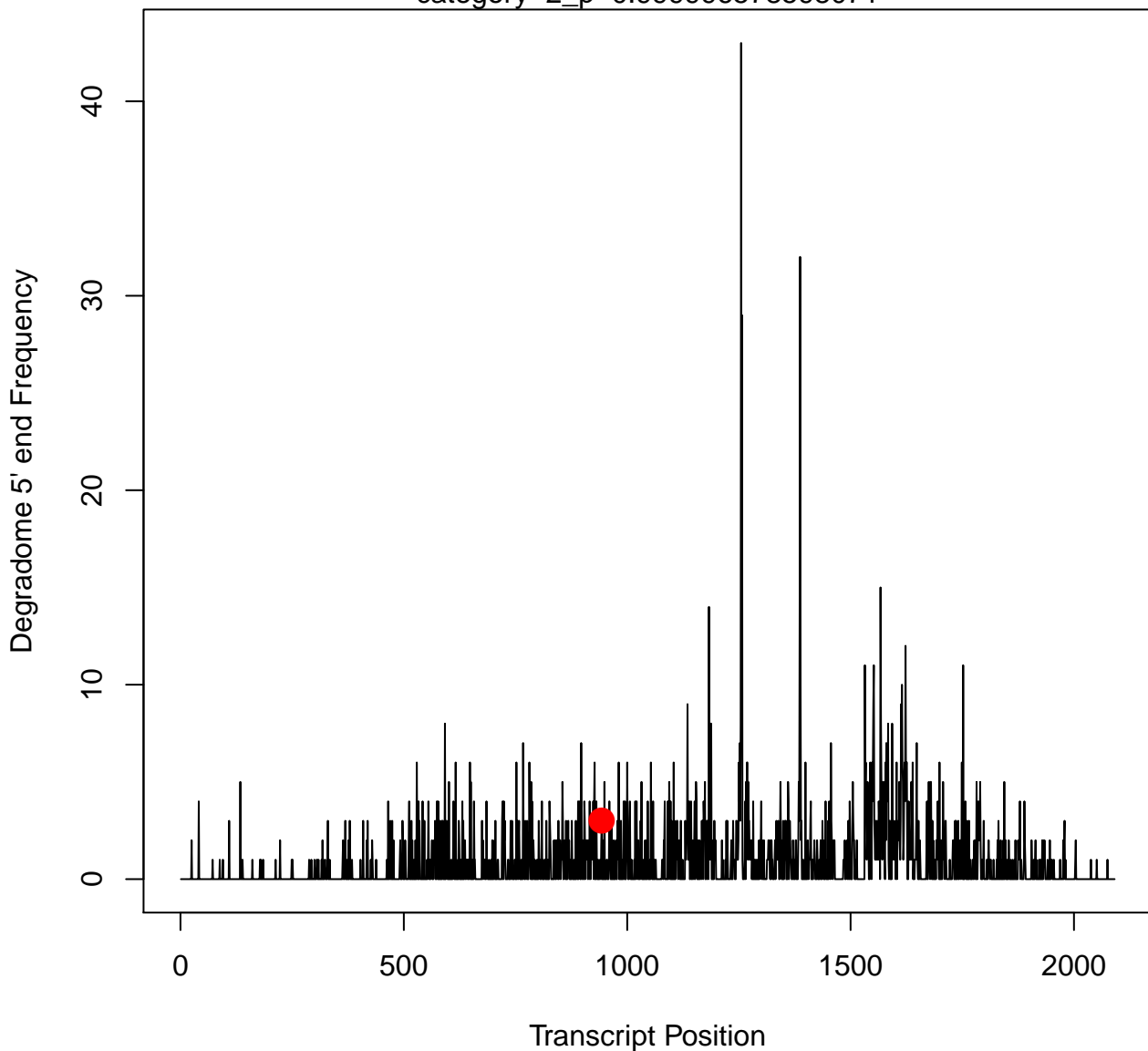

Supplement: Supplementary file 2 [file Data_Sheet_2.zip › Sit-miR1432_Seita.1G373400.1_943_TPlot.pdf]

**T=Seita.2G158400.1\_Q=Sit-miR1432\_S=994**

category=2\_p=0.695121268963937

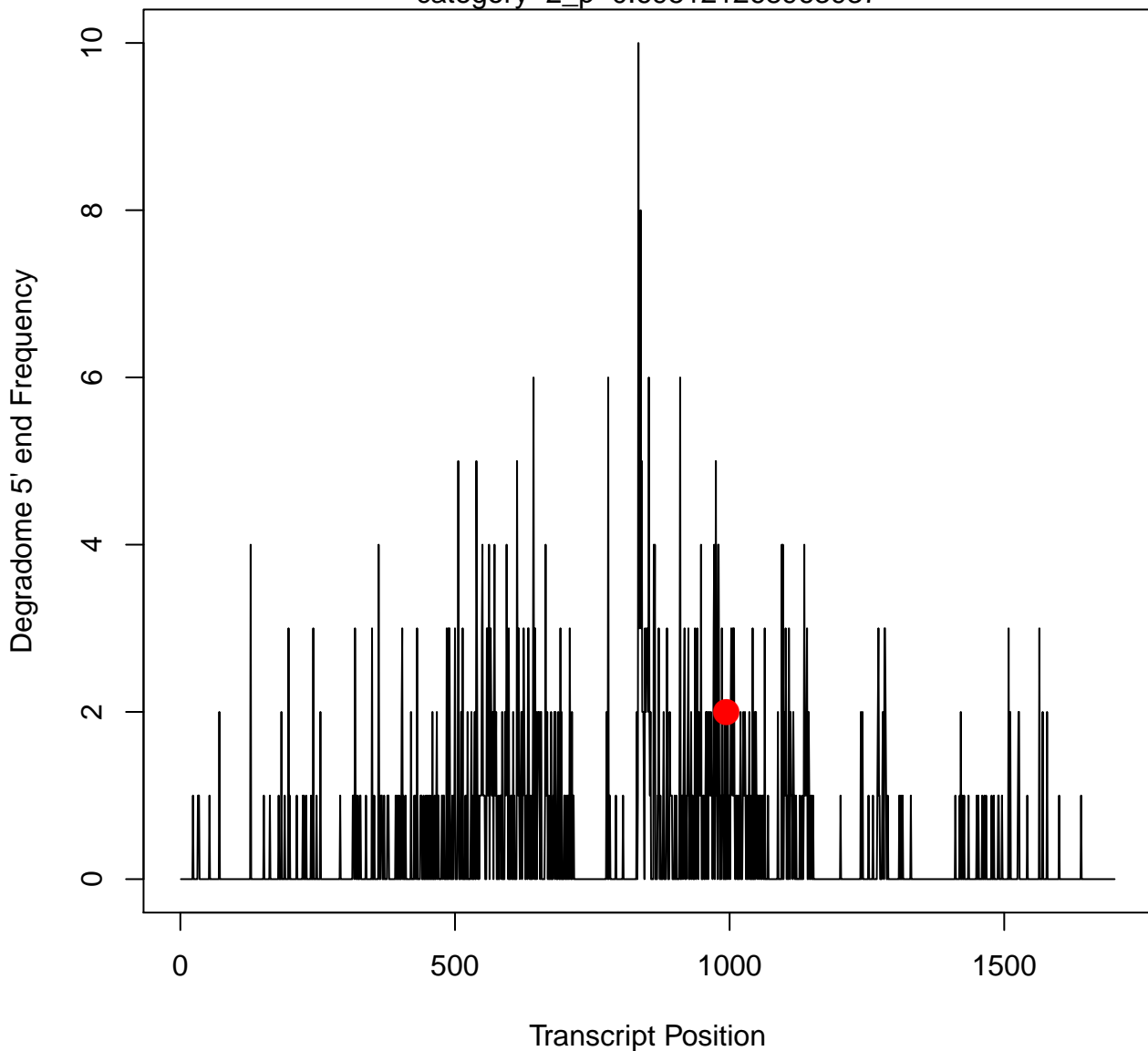

Supplement: Supplementary file 2 [file Data_Sheet_2.zip › Sit-miR1432_Seita.2G158400.1_994_TPlot.pdf]

**T=Seita.5G071400.1\_Q=Sit-miR1432\_S=905**

category=2\_p=0.994951789549666

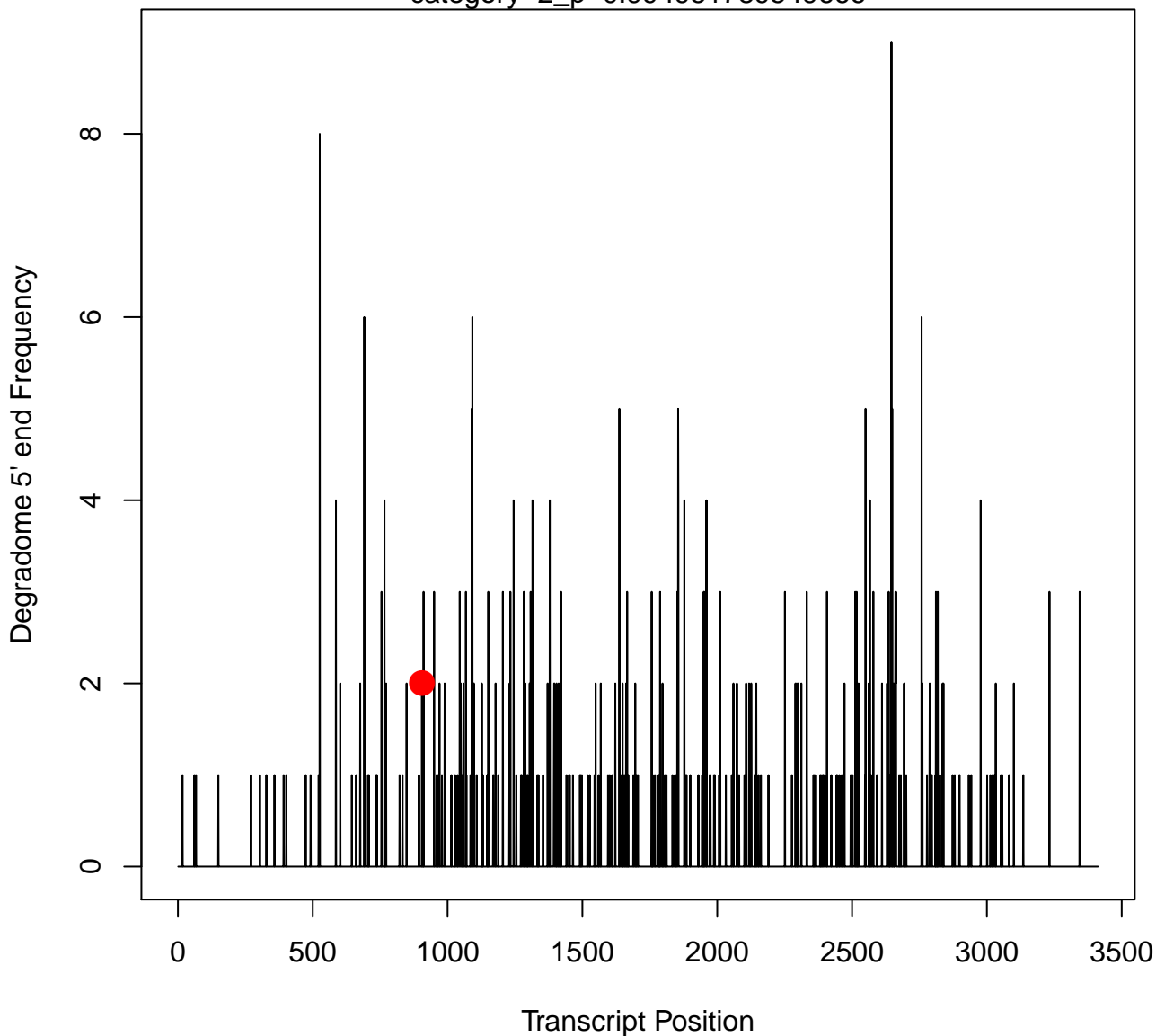

Supplement: Supplementary file 2 [file Data_Sheet_2.zip › Sit-miR1432_Seita.5G071400.1_905_TPlot.pdf]

**T=Seita.9G140000.1\_Q=Sit-miR1432\_S=1349**

category=2\_p=0.997899251116714

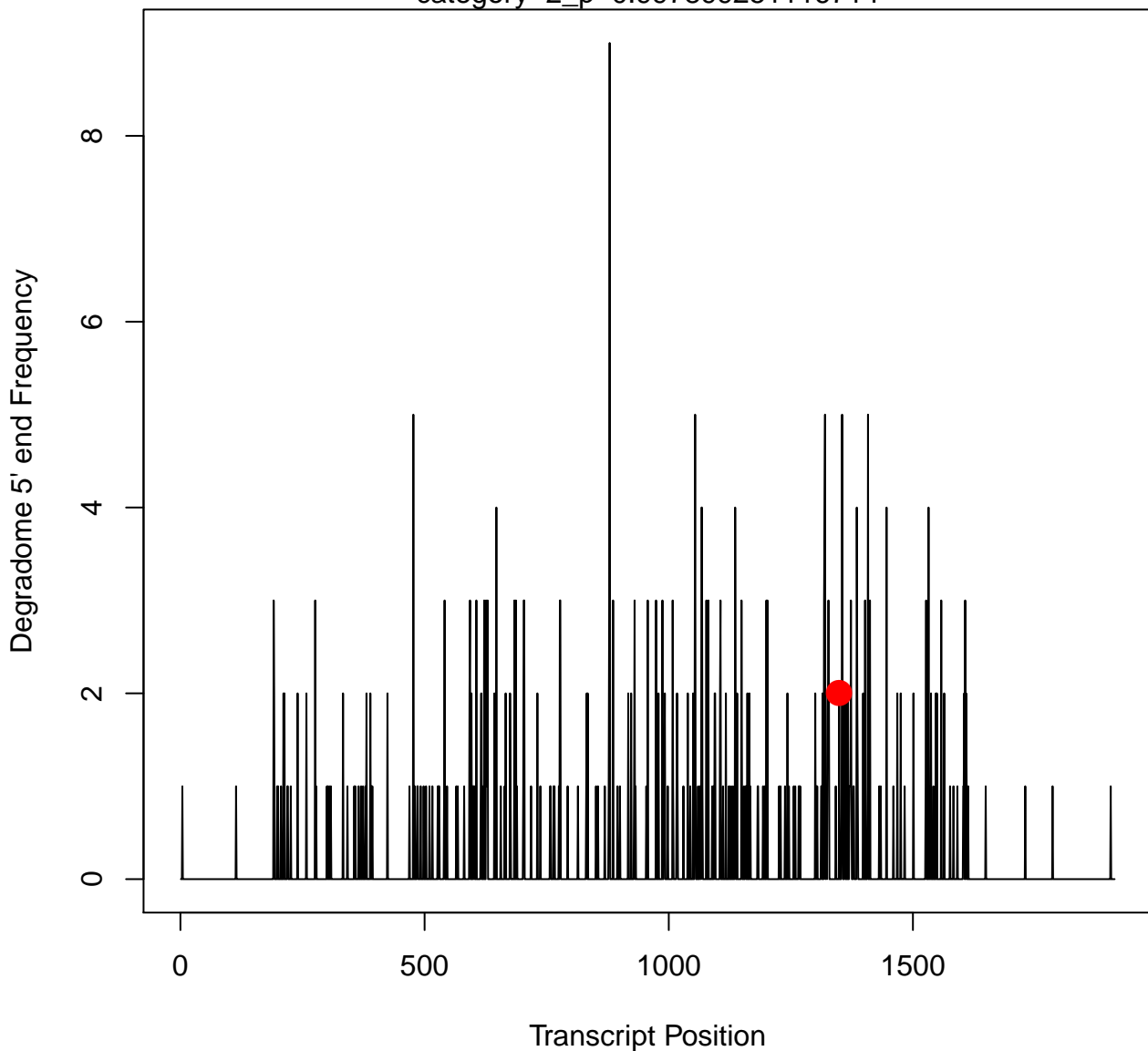

Supplement: Supplementary file 2 [file Data_Sheet_2.zip › Sit-miR1432_Seita.9G140000.1_1349_TPlot.pdf]

**T=Seita.9G455700.1\_Q=Sit-miR1432\_S=274**

category=2\_p=0.999999954819304

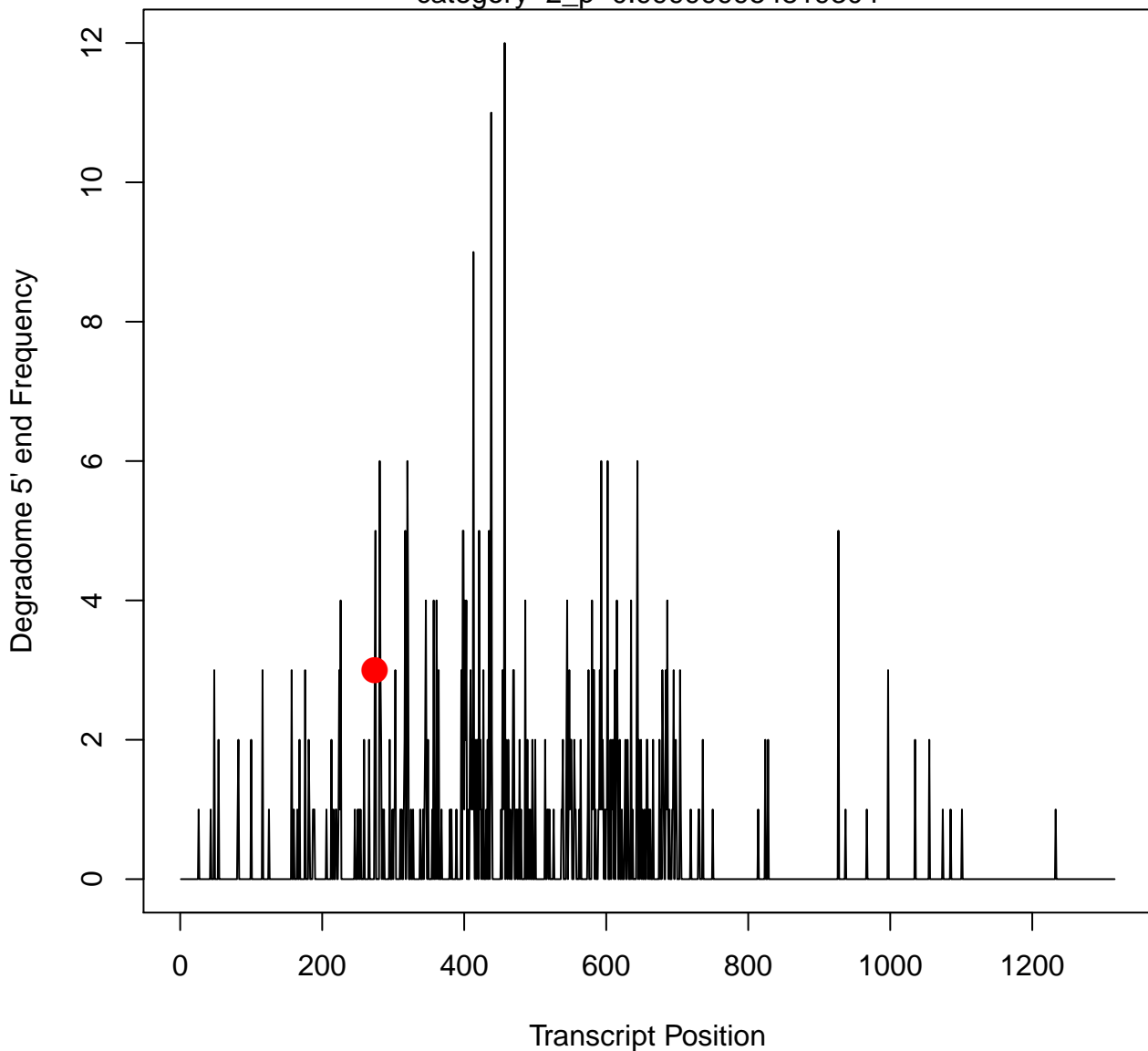

Supplement: Supplementary file 2 [file Data_Sheet_2.zip › Sit-miR1432_Seita.9G455700.1_274_TPlot.pdf]

**T=Seita.9G540200.1\_Q=Sit-miR1432\_S=1028**

category=2\_p=0.999997631195828

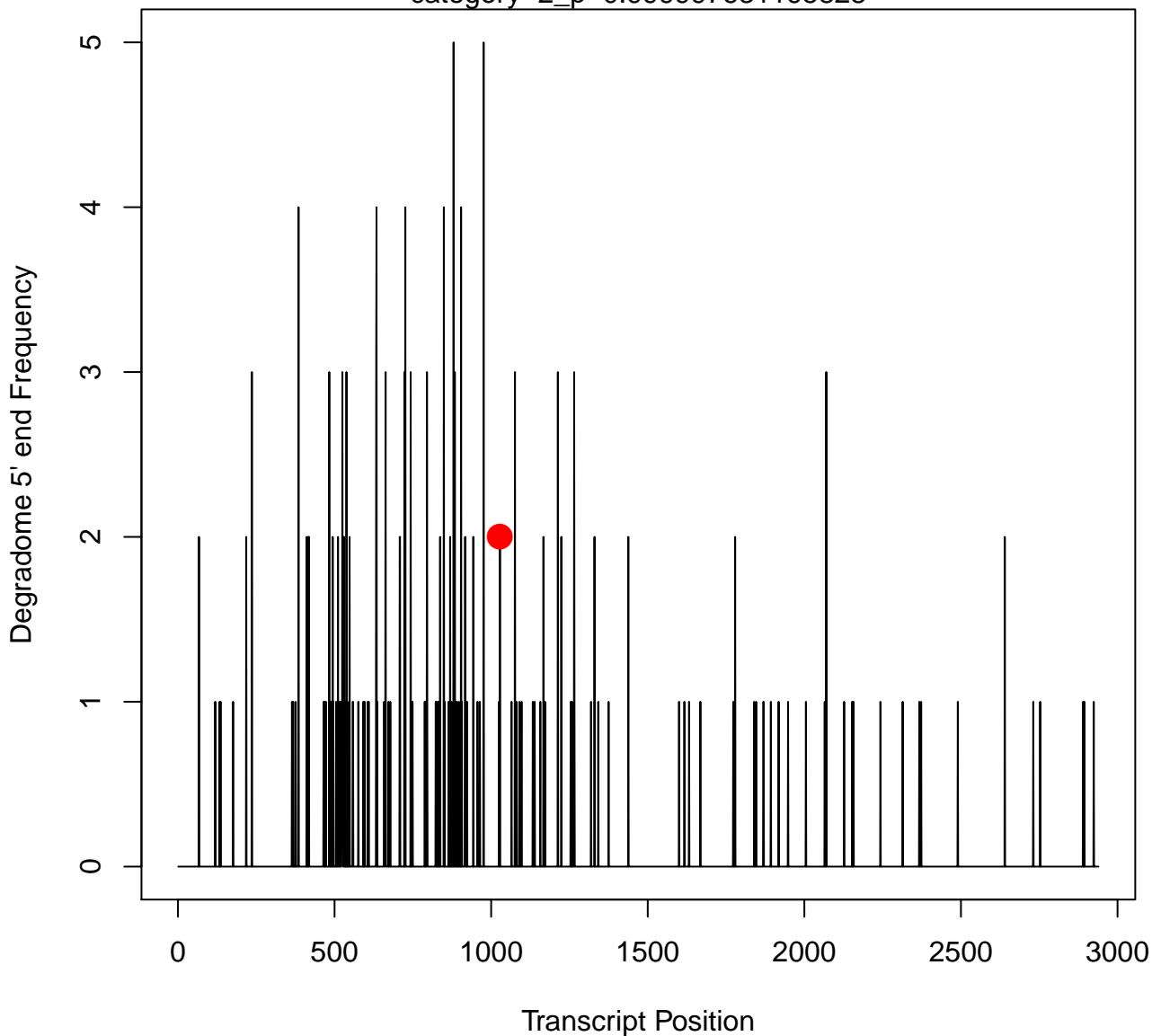

Supplement: Supplementary file 2 [file Data_Sheet_2.zip › Sit-miR1432_Seita.9G540200.1_1028_TPlot.pdf]

**T=Seita.4G270400.1\_Q=Sit-miR156a\_S=2084**

category=0\_p=0.000385499163724878

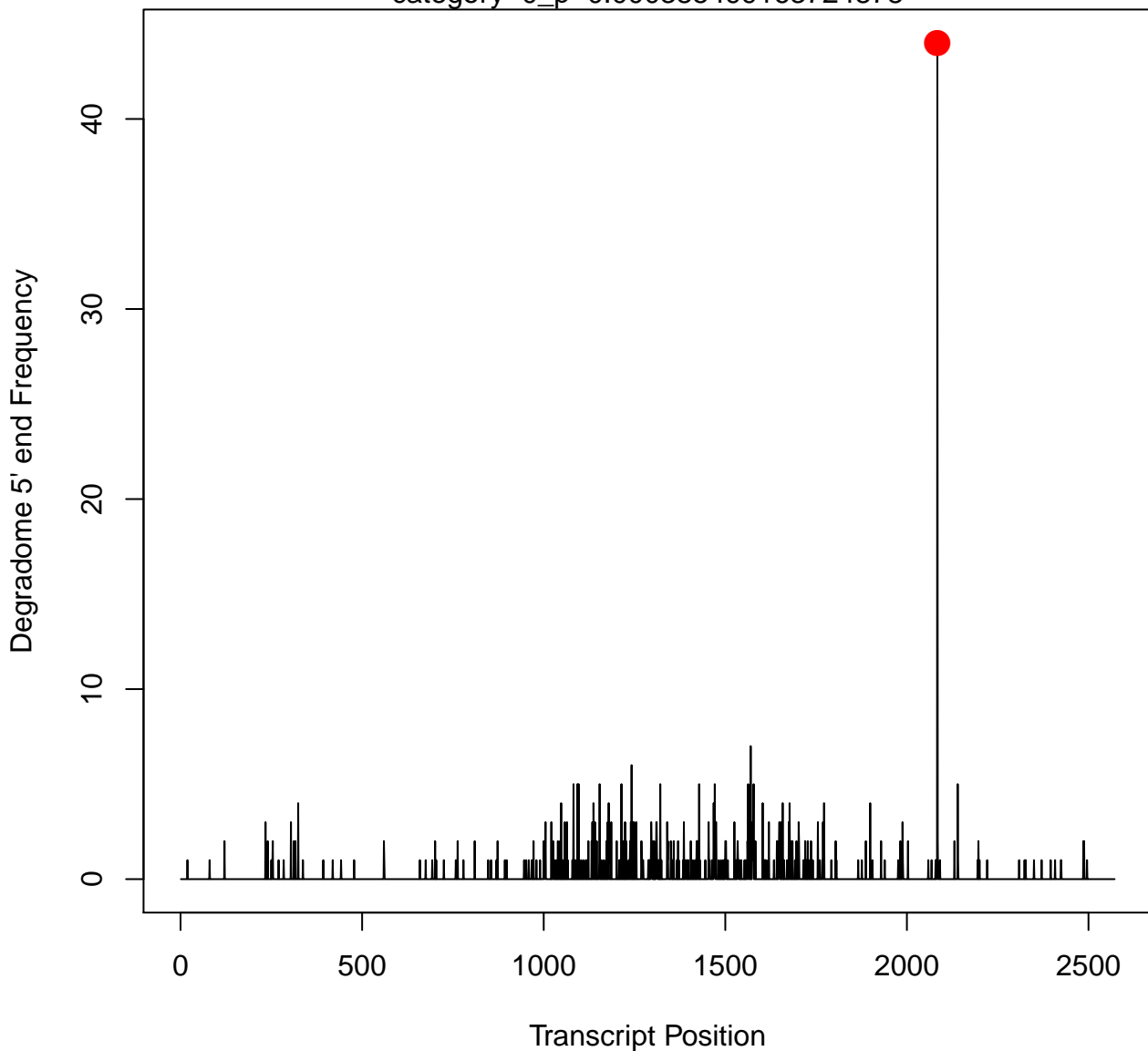

Supplement: Supplementary file 2 [file Data_Sheet_2.zip › Sit-miR156a_Seita.4G270400.1_2084_TPlot.pdf]

**T=Seita.5G001600.1\_Q=Sit-miR156a\_S=1748**

category=2\_p=0.968266926754577

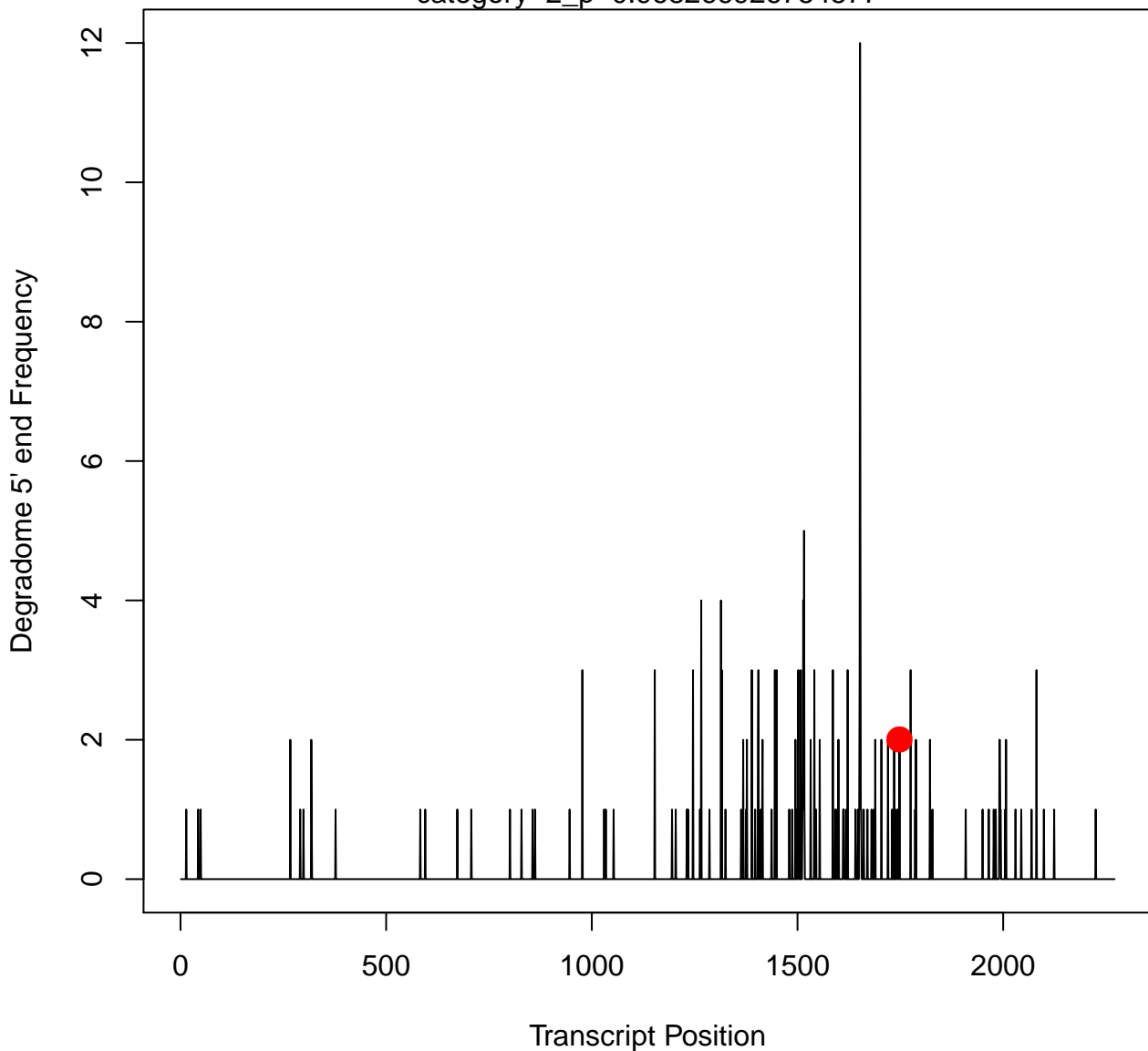

Supplement: Supplementary file 2 [file Data_Sheet_2.zip › Sit-miR156a_Seita.5G001600.1_1748_TPlot.pdf]

**T=Seita.6G223300.1\_Q=Sit-miR156a\_S=981**

category=0\_p=0.00192601029535011

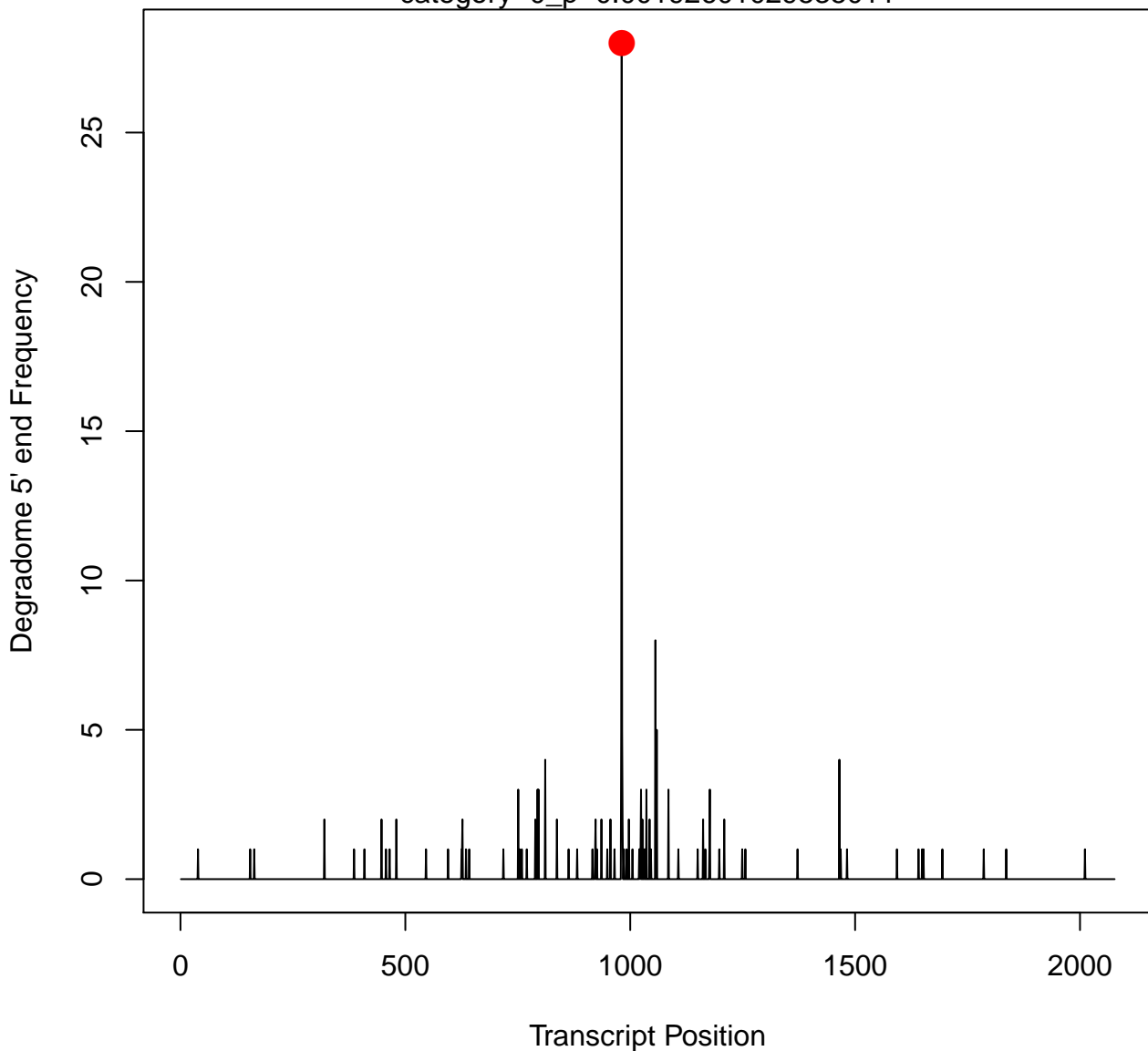

Supplement: Supplementary file 2 [file Data_Sheet_2.zip › Sit-miR156a_Seita.6G223300.1_981_TPlot.pdf]

**T=Seita.9G520200.1\_Q=Sit-miR156a\_S=1152**

category=2\_p=0.998070138780479

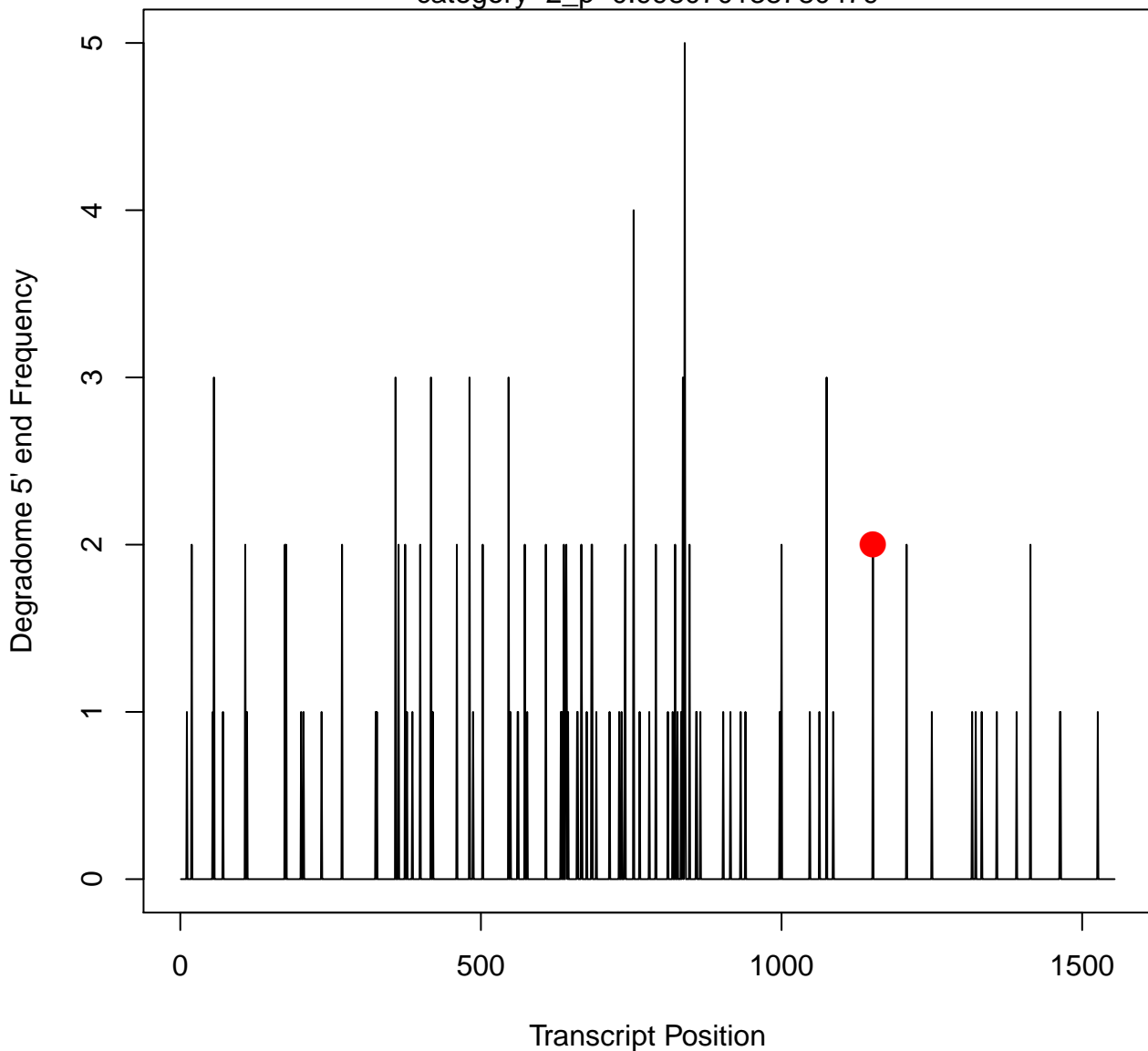

Supplement: Supplementary file 2 [file Data_Sheet_2.zip › Sit-miR156a_Seita.9G520200.1_1152_TPlot.pdf]

**T=Seita.1G091900.1\_Q=Sit-miR156b\_S=1786**

category=0\_p=0.000385499163724878

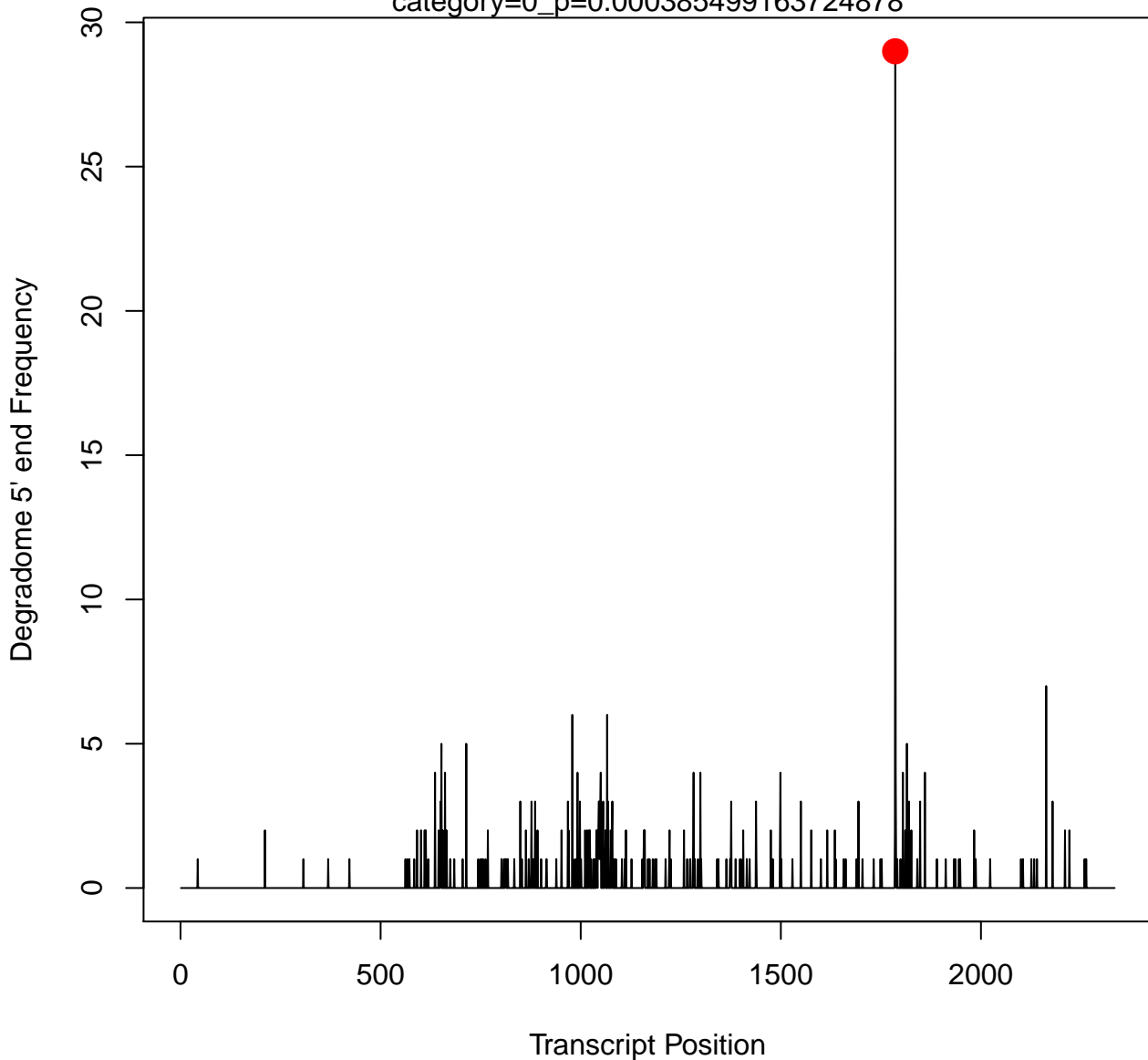

Supplement: Supplementary file 2 [file Data_Sheet_2.zip › Sit-miR156b_Seita.1G091900.1_1786_TPlot.pdf]

**T=Seita.1G134200.1\_Q=Sit-miR156b\_S=59**

category=2\_p=0.987868883828228

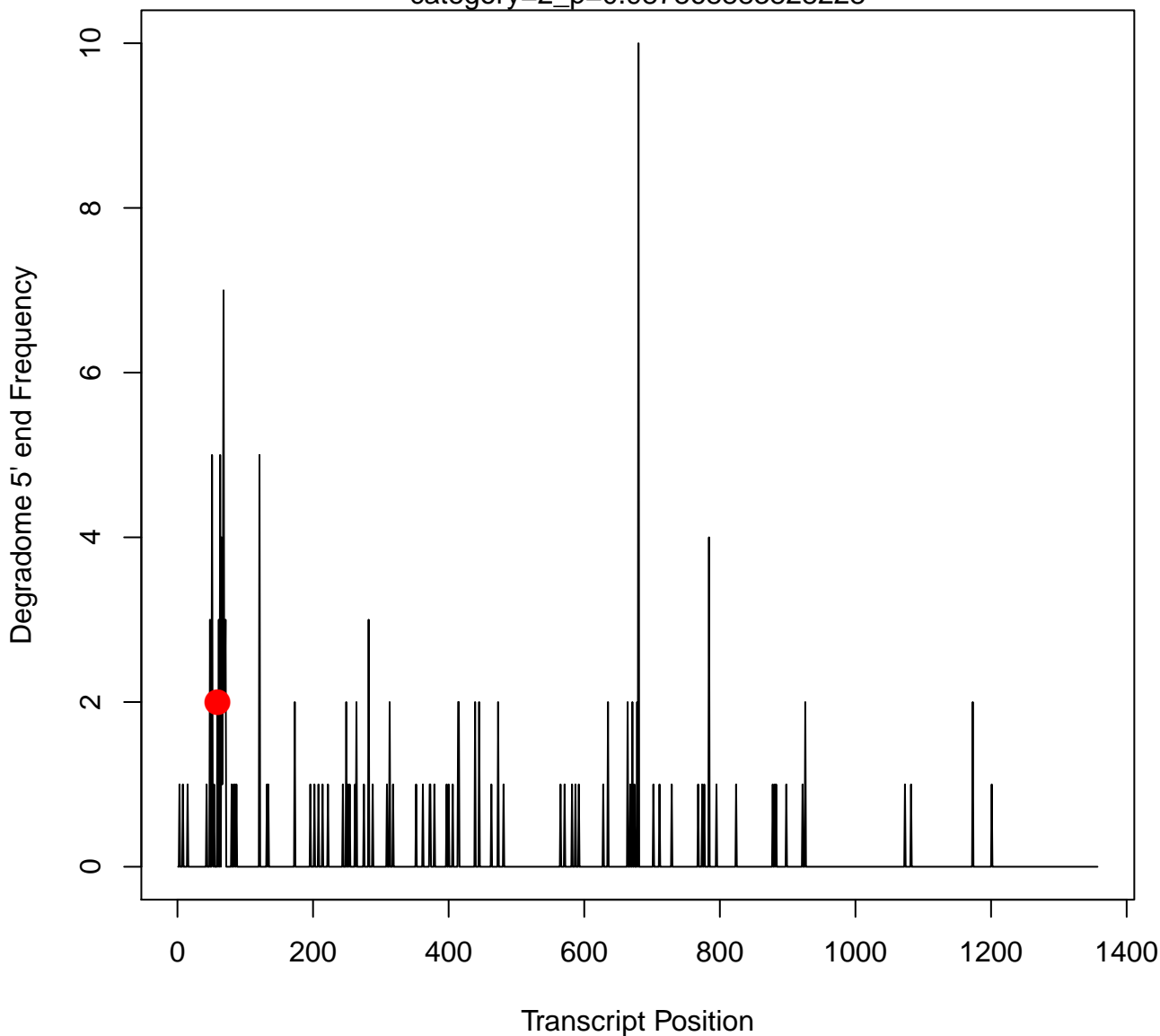

Supplement: Supplementary file 2 [file Data_Sheet_2.zip › Sit-miR156b_Seita.1G134200.1_59_TPlot.pdf]

**T=Seita.6G205500.1\_Q=Sit-miR156b\_S=1434**

category=0\_p=0.00154110522640127

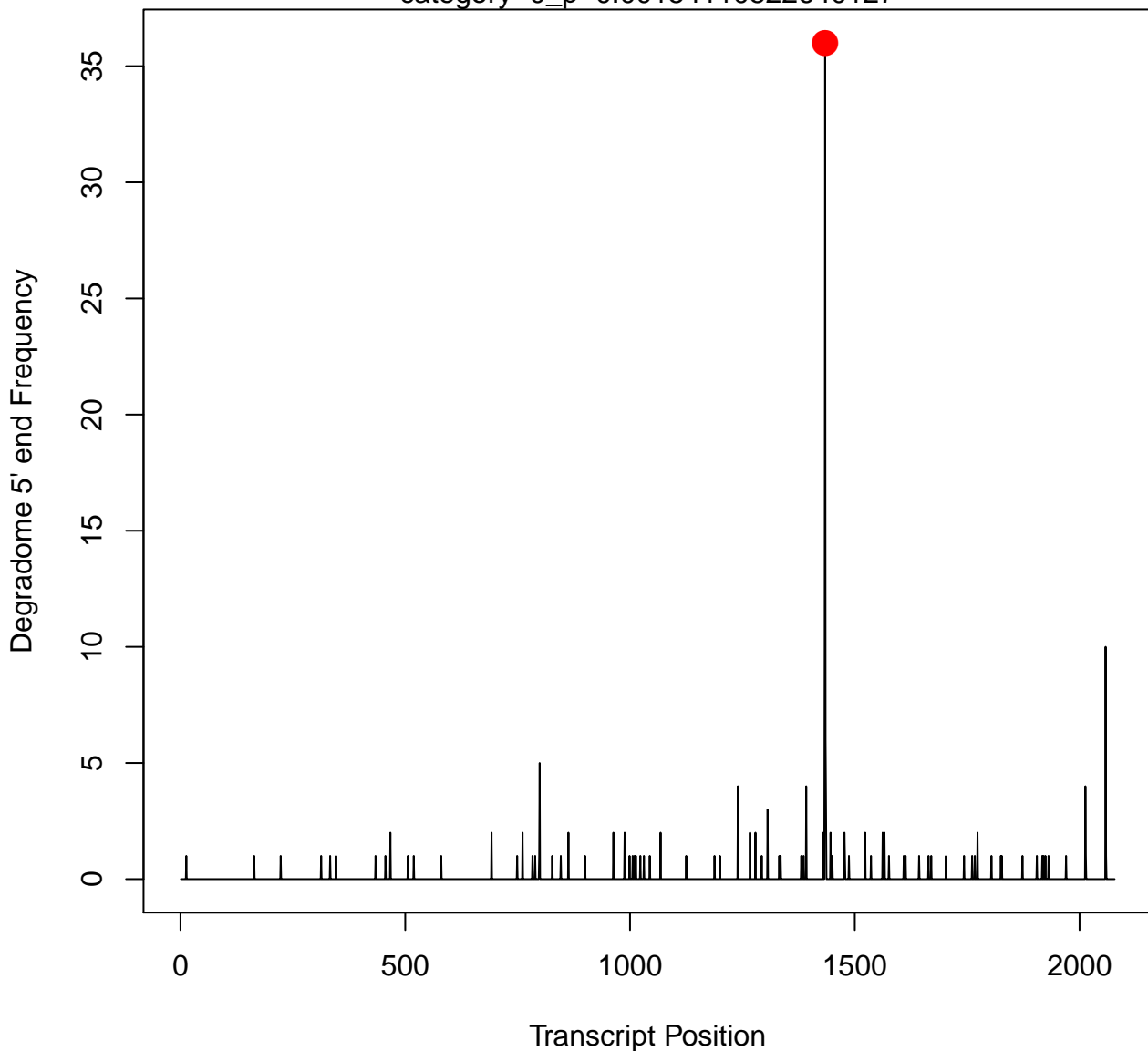

Supplement: Supplementary file 2 [file Data_Sheet_2.zip › Sit-miR156b_Seita.6G205500.1_1434_TPlot.pdf]

**T=Seita.2G254300.1\_Q=Sit-miR156c\_S=1266**

category=0\_p=0.00154110522640127

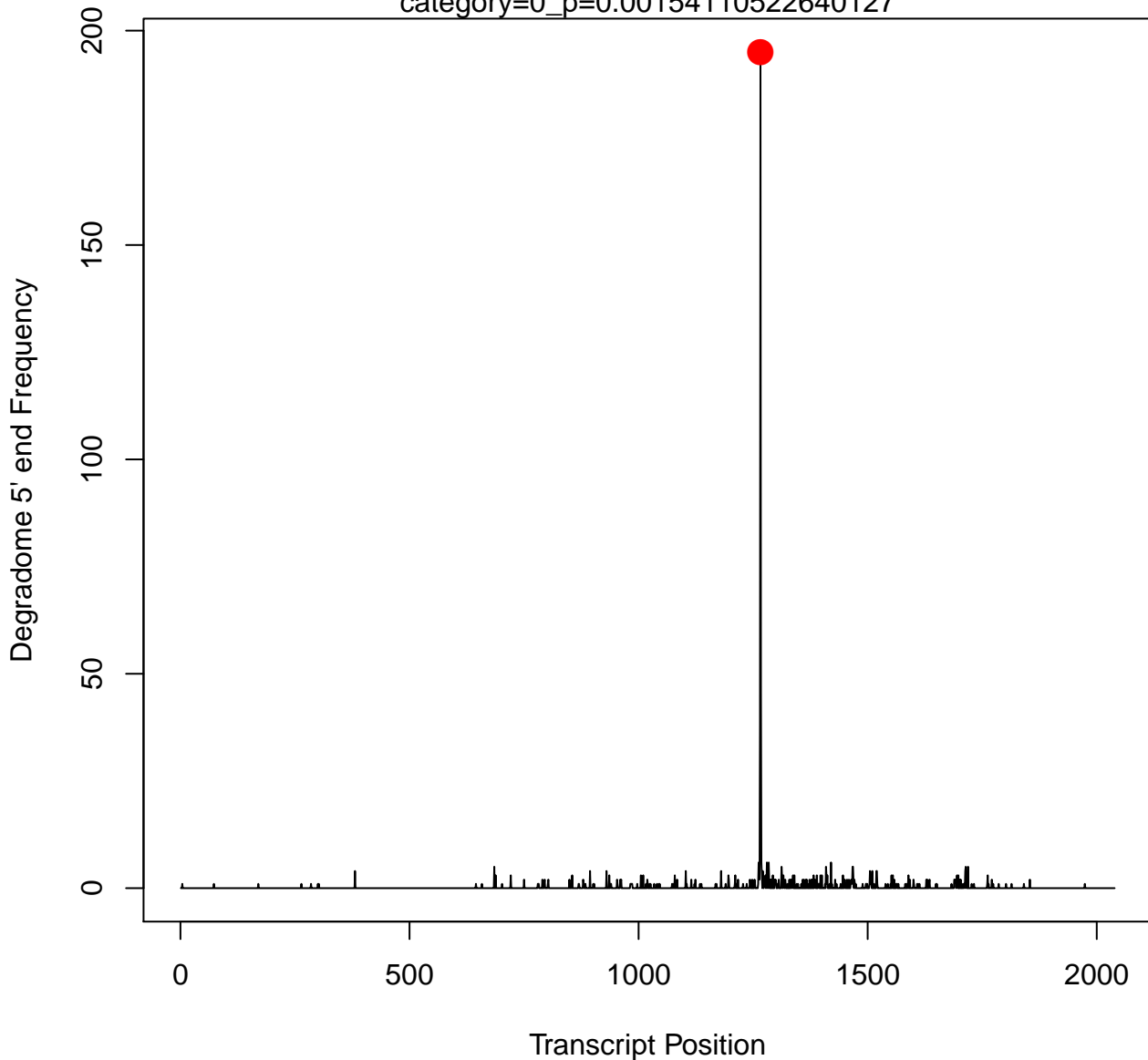

Supplement: Supplementary file 2 [file Data_Sheet_2.zip › Sit-miR156c_Seita.2G254300.1_1266_TPlot.pdf]

**T=Seita.1G069300.1\_Q=Sit-miR156d\_S=874**

category=2\_p=0.0813460690731977

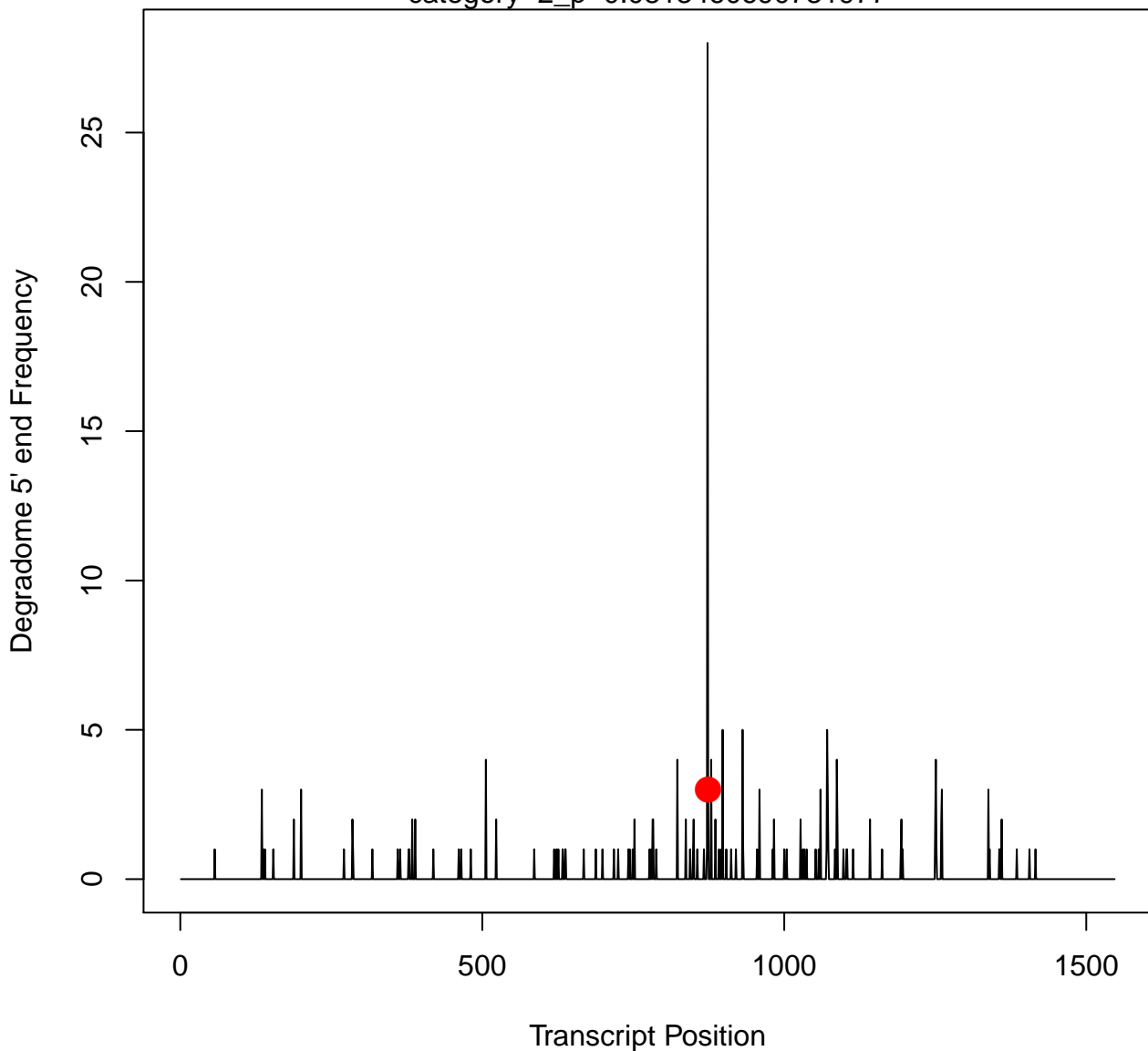

Supplement: Supplementary file 2 [file Data_Sheet_2.zip › Sit-miR156d_Seita.1G069300.1_874_TPlot.pdf]

**T=Seita.1G091900.1\_Q=Sit-miR156d\_S=1787**

category=2\_p=0.0278857428936683

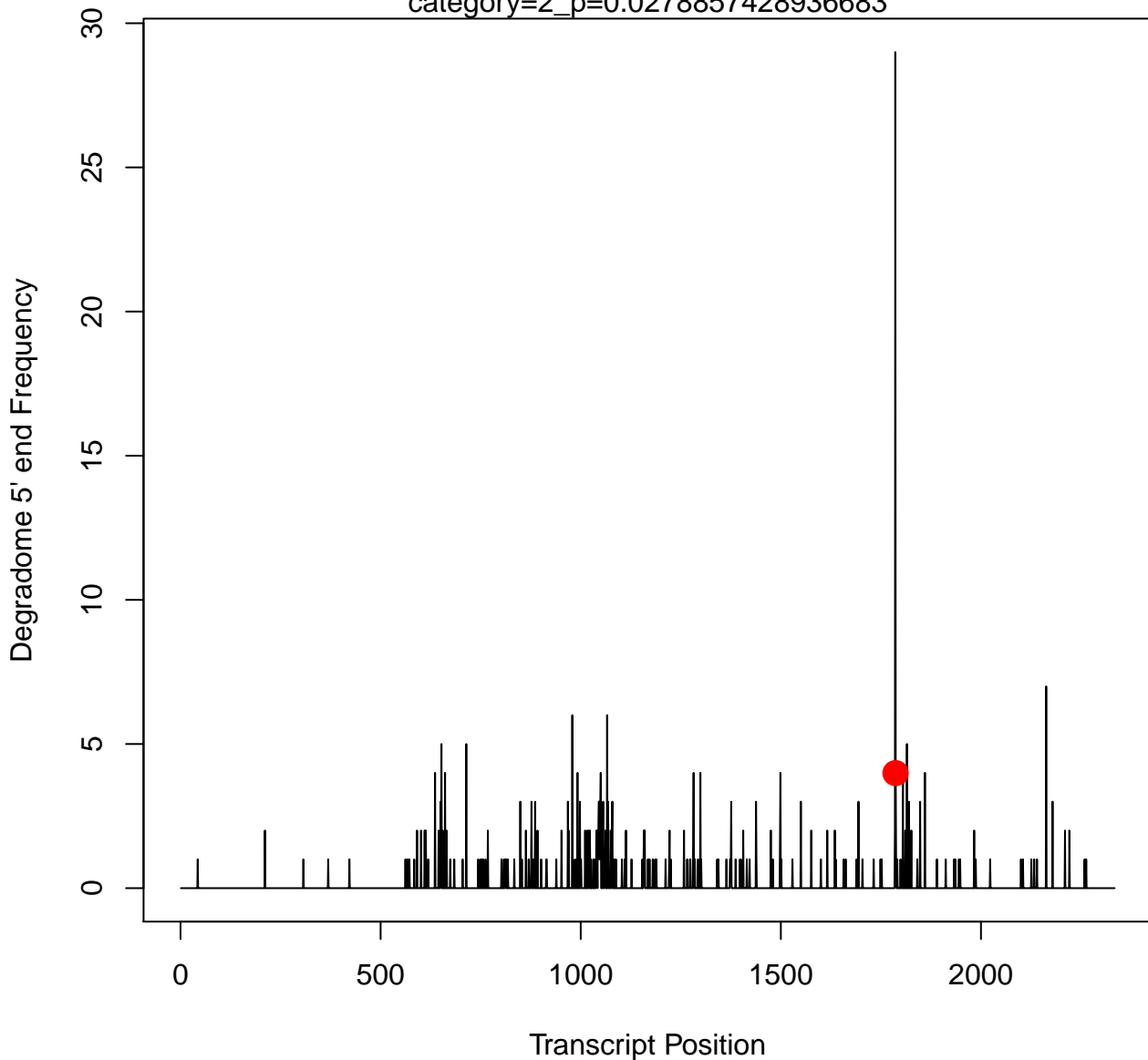

Supplement: Supplementary file 2 [file Data_Sheet_2.zip › Sit-miR156d_Seita.1G091900.1_1787_TPlot.pdf]

**T=Seita.2G254300.1\_Q=Sit-miR156d\_S=1267**

category=2\_p=0.10696341639928

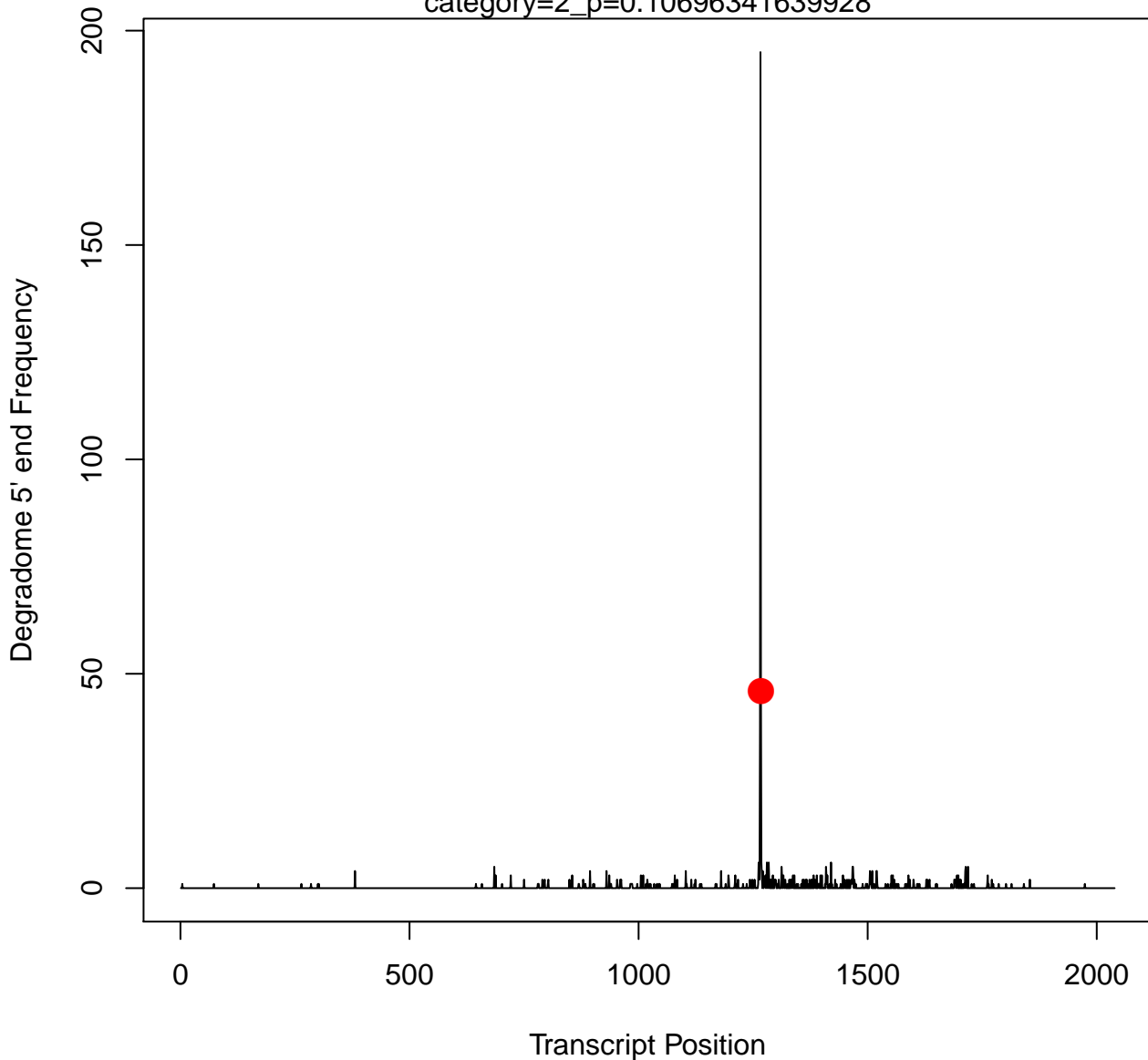

Supplement: Supplementary file 2 [file Data_Sheet_2.zip › Sit-miR156d_Seita.2G254300.1_1267_TPlot.pdf]

**T=Seita.2G266500.1\_Q=Sit-miR156d\_S=1897**

category=2\_p=0.224724940180228

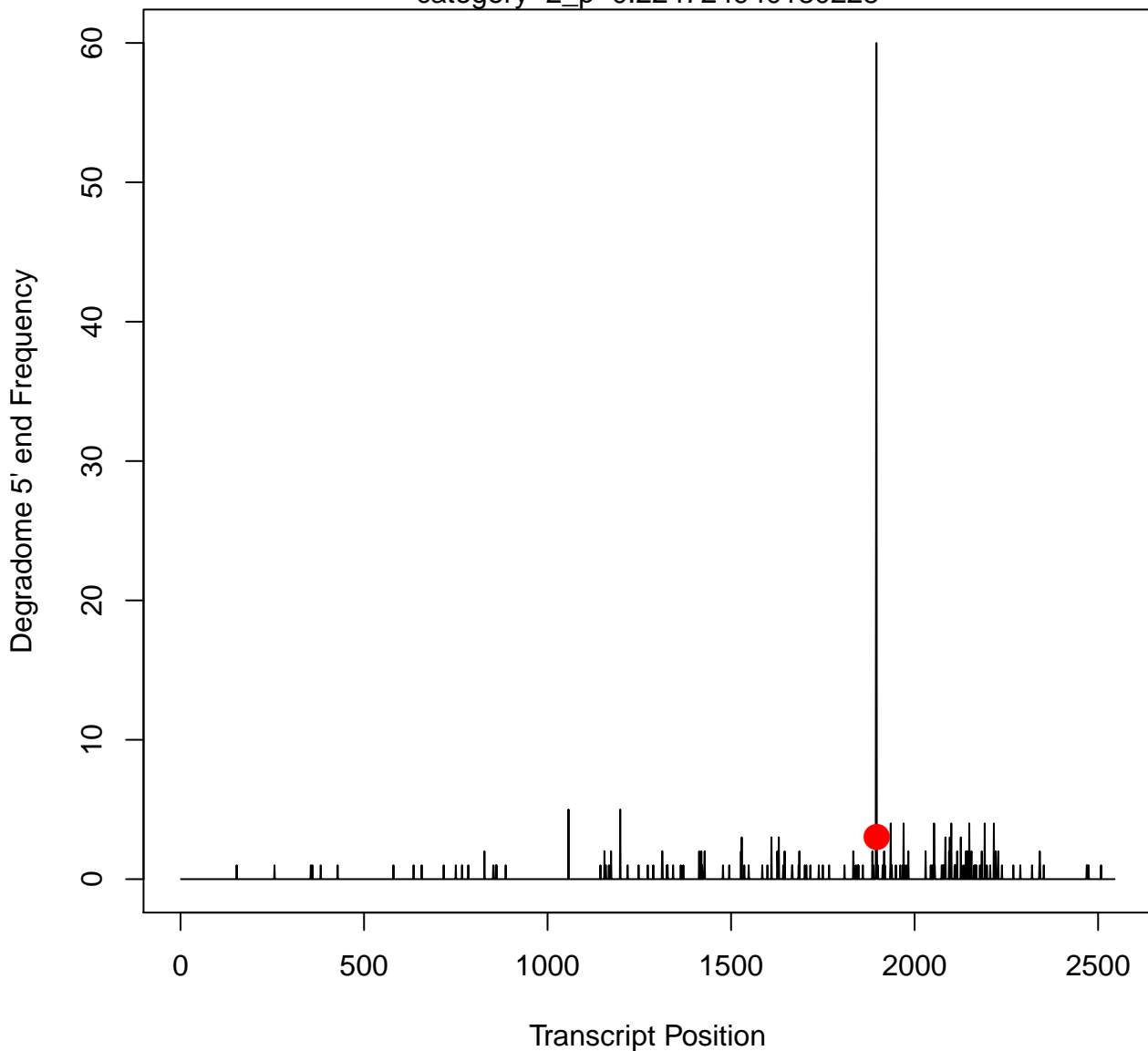

Supplement: Supplementary file 2 [file Data_Sheet_2.zip › Sit-miR156d_Seita.2G266500.1_1897_TPlot.pdf]

**T=Seita.2G324900.1\_Q=Sit-miR156d\_S=834**

category=2\_p=0.267360316910728

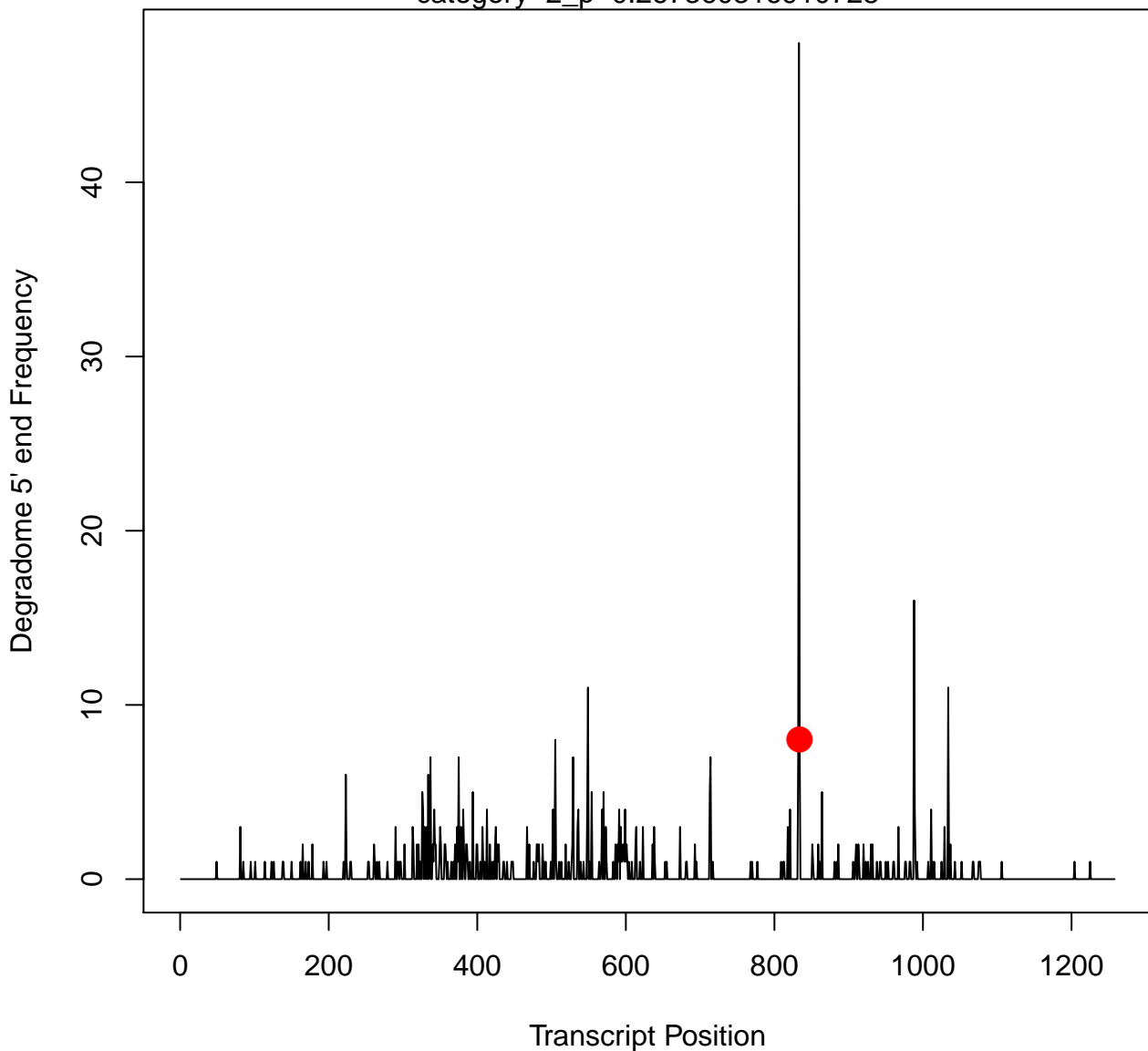

Supplement: Supplementary file 2 [file Data_Sheet_2.zip › Sit-miR156d_Seita.2G324900.1_834_TPlot.pdf]

**T=Seita.2G381400.1\_Q=Sit-miR156d\_S=644**

category=2\_p=0.992911902209651

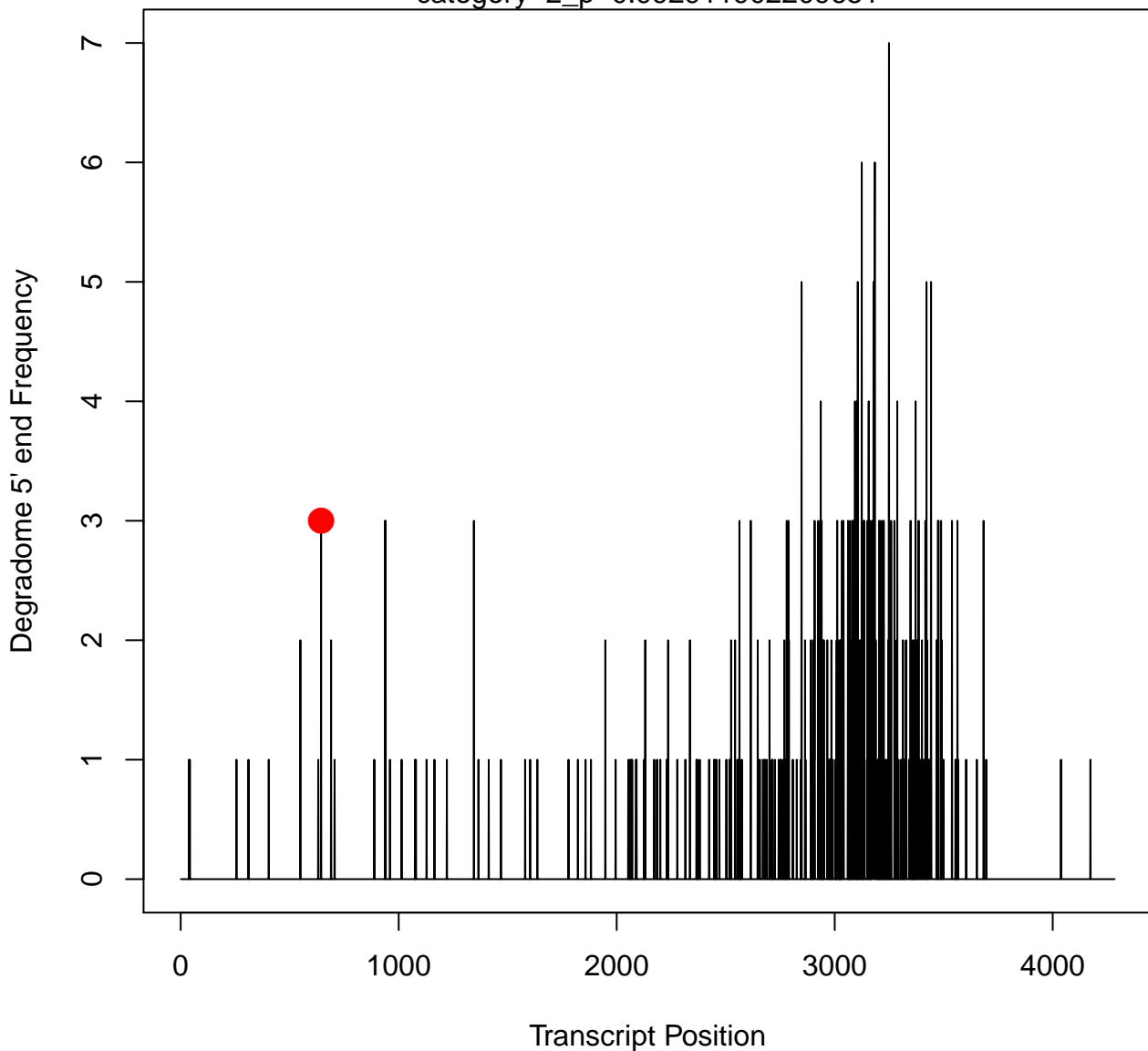

Supplement: Supplementary file 2 [file Data_Sheet_2.zip › Sit-miR156d_Seita.2G381400.1_644_TPlot.pdf]

**T=Seita.2G444600.1\_Q=Sit-miR156d\_S=147**

category=2\_p=0.831658693962997

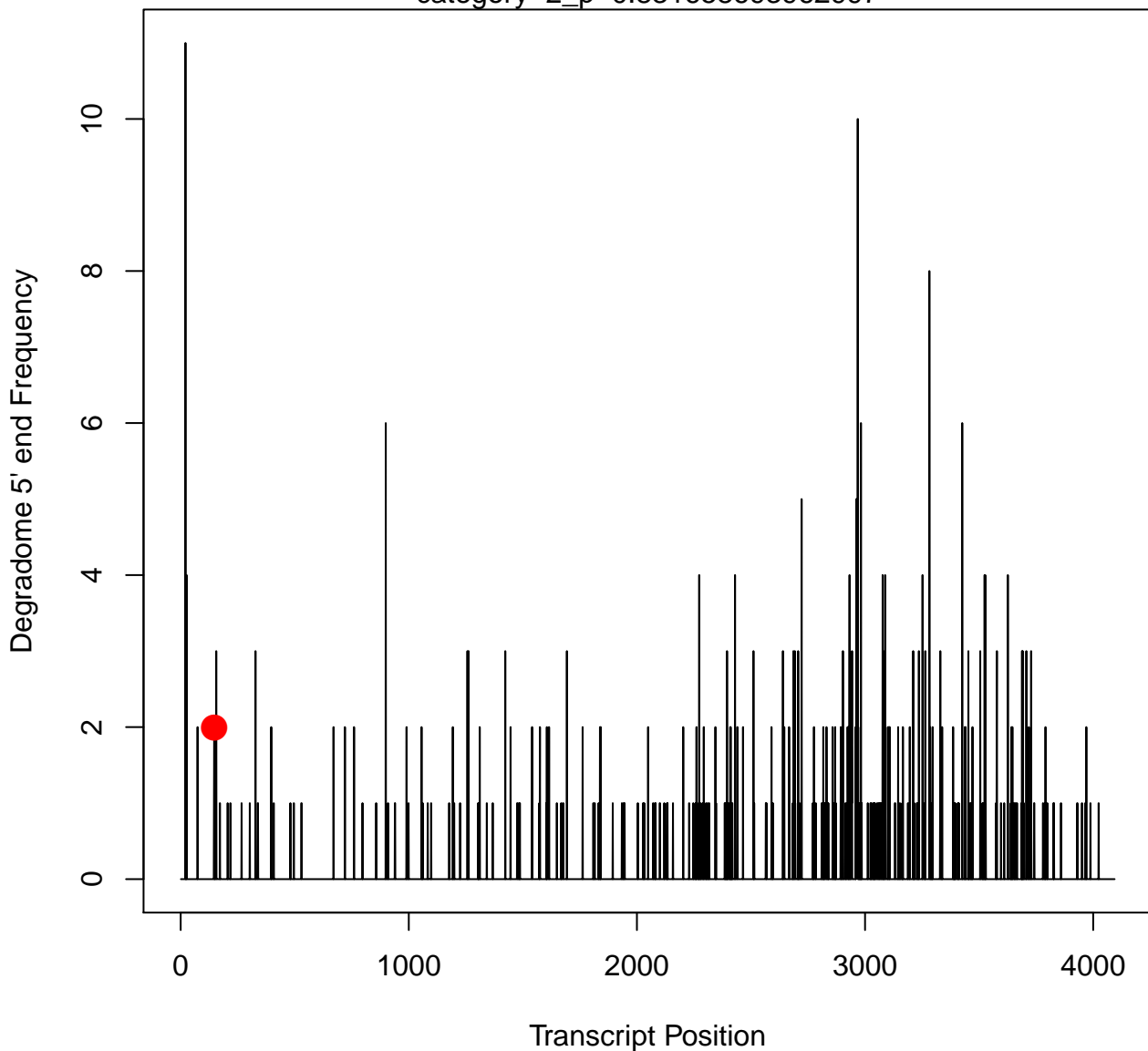

Supplement: Supplementary file 2 [file Data_Sheet_2.zip › Sit-miR156d_Seita.2G444600.1_147_TPlot.pdf]

**T=Seita.3G125200.1\_Q=Sit-miR156d\_S=8985**

category=2\_p=0.520652874976587

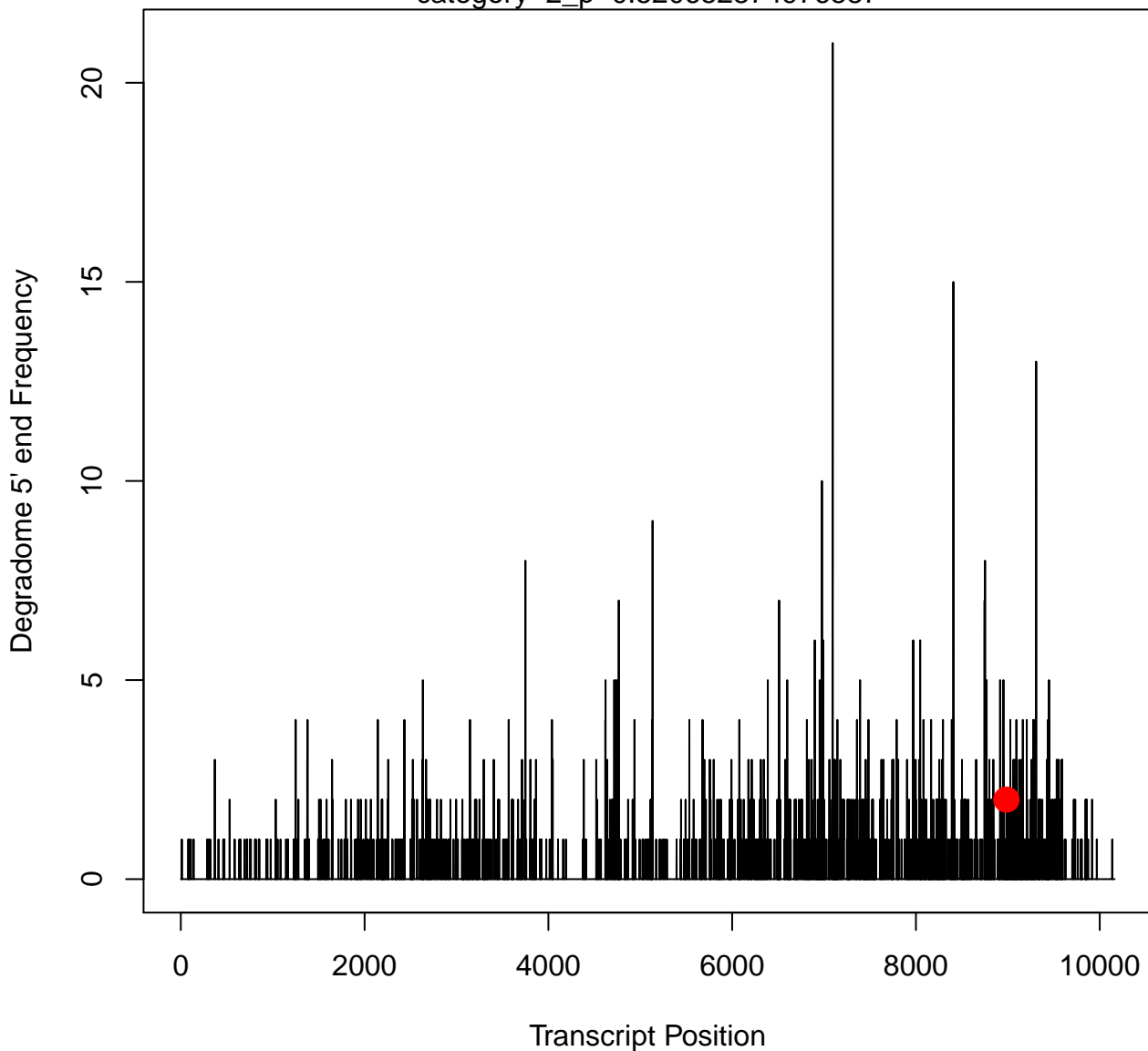

Supplement: Supplementary file 2 [file Data_Sheet_2.zip › Sit-miR156d_Seita.3G125200.1_8985_TPlot.pdf]

**T=Seita.3G205200.1\_Q=Sit-miR156d\_S=48**

category=2\_p=0.994504774561579

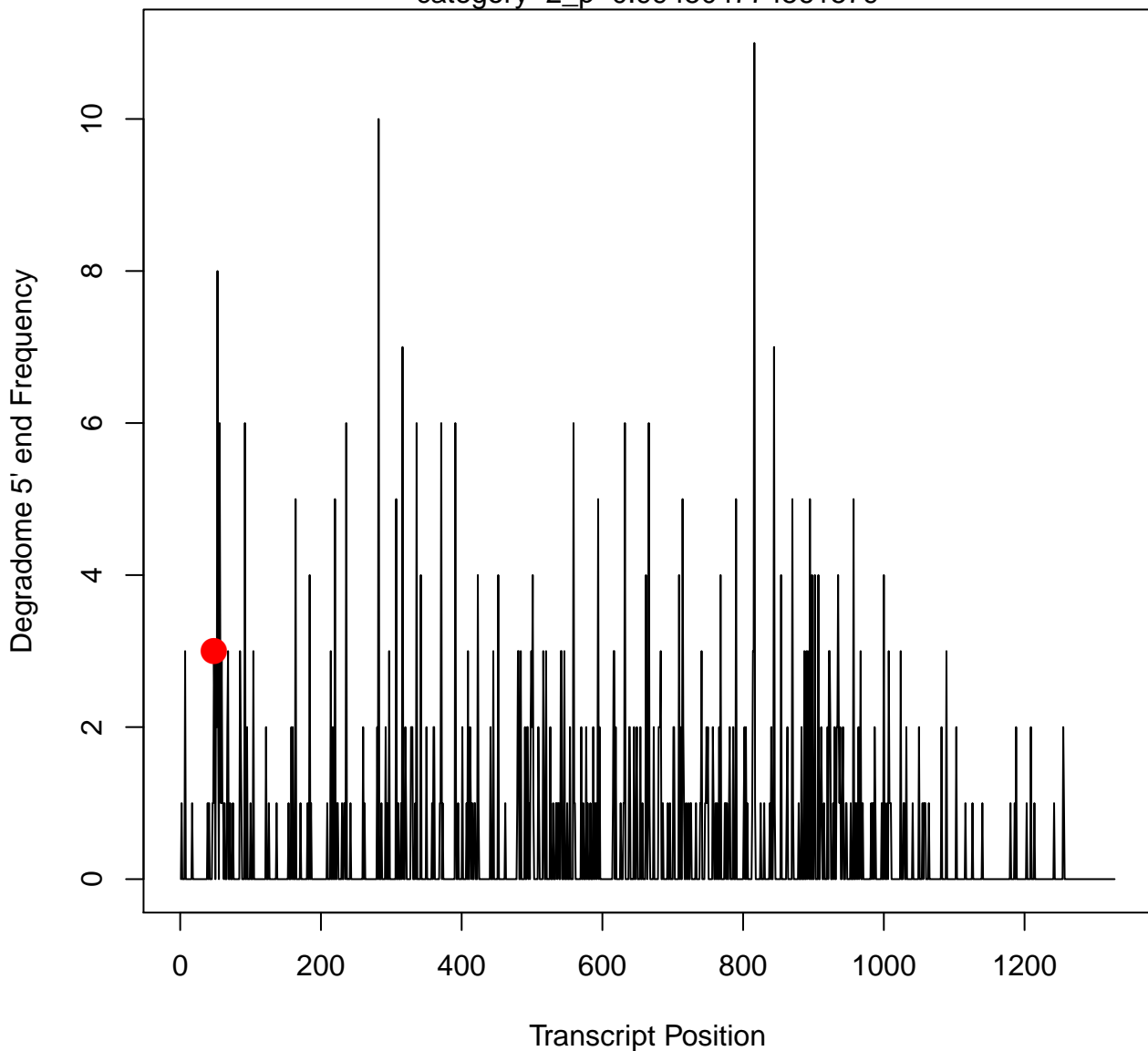

Supplement: Supplementary file 2 [file Data_Sheet_2.zip › Sit-miR156d_Seita.3G205200.1_48_TPlot.pdf]

**T=Seita.3G214700.1\_Q=Sit-miR156d\_S=3012**

category=2\_p=0.970012051295214

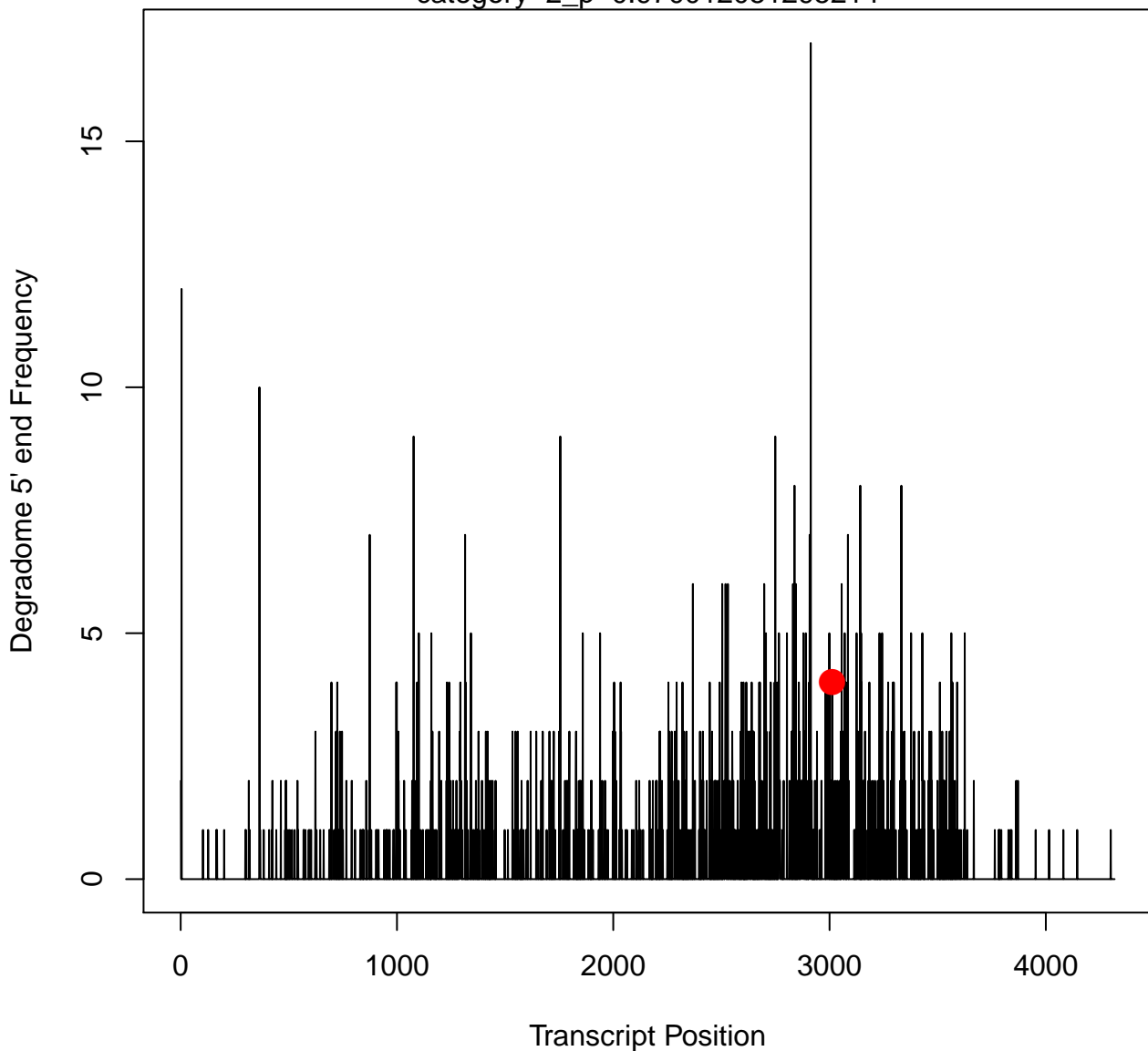

Supplement: Supplementary file 2 [file Data_Sheet_2.zip › Sit-miR156d_Seita.3G214700.1_3012_TPlot.pdf]

**T=Seita.3G222000.1\_Q=Sit-miR156d\_S=2038**

category=2\_p=0.892930026934013

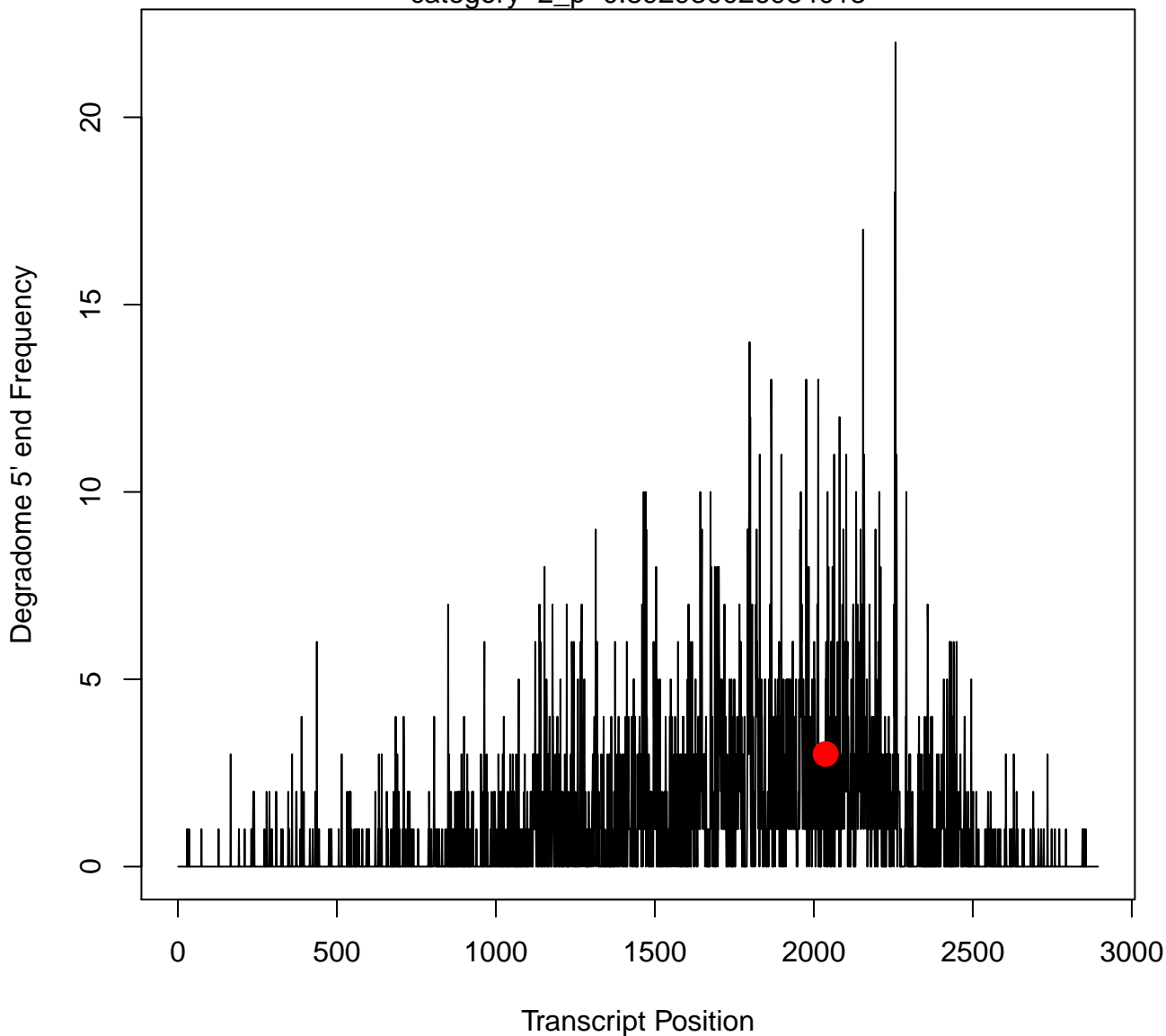

Supplement: Supplementary file 2 [file Data_Sheet_2.zip › Sit-miR156d_Seita.3G222000.1_2038_TPlot.pdf]

**T=Seita.4G270400.1\_Q=Sit-miR156d\_S=2085**

category=2\_p=0.13186640496421

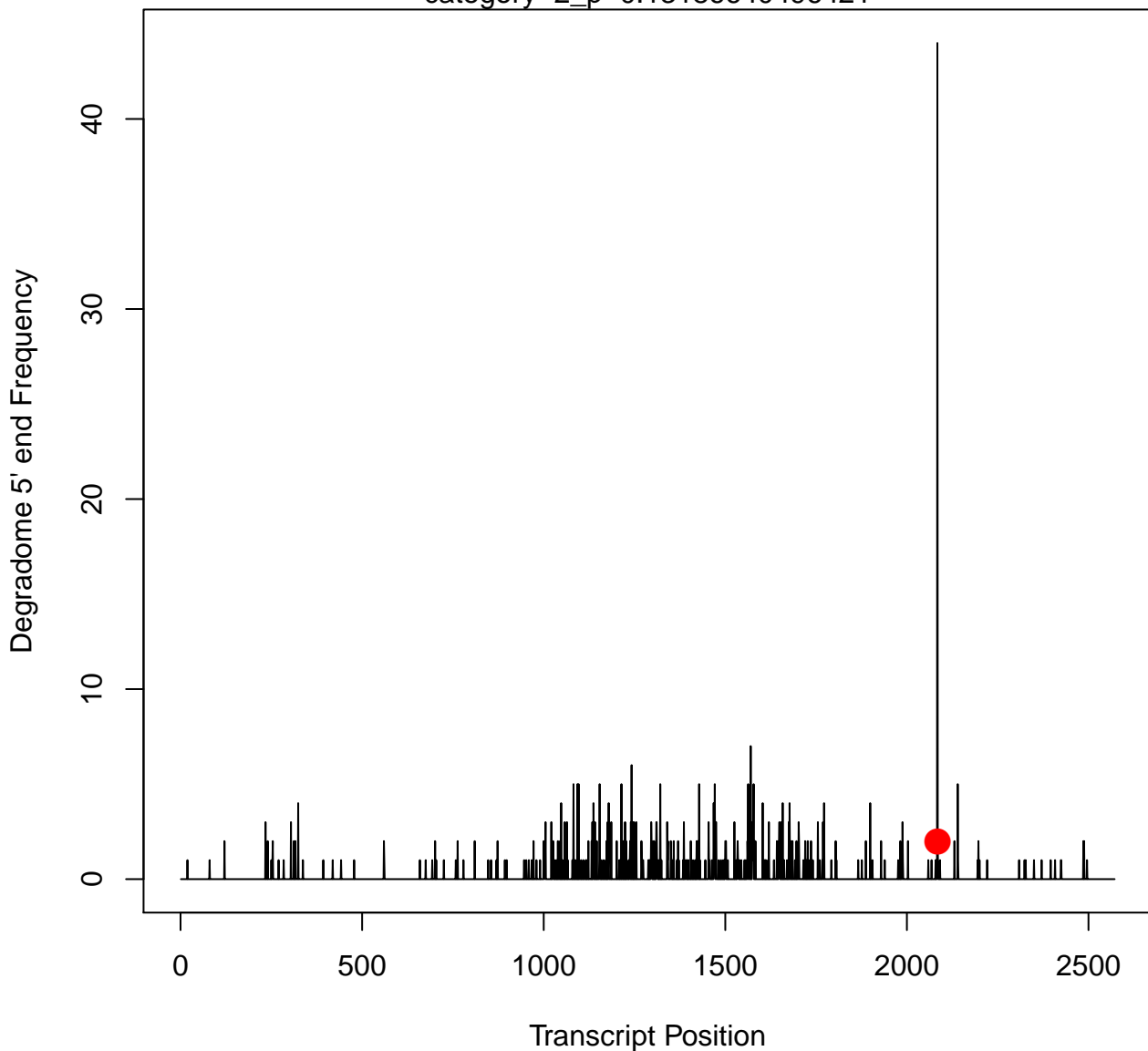

Supplement: Supplementary file 2 [file Data_Sheet_2.zip › Sit-miR156d_Seita.4G270400.1_2085_TPlot.pdf]

**T=Seita.6G176000.1\_Q=Sit-miR156d\_S=189**

category=2\_p=0.83635301634153

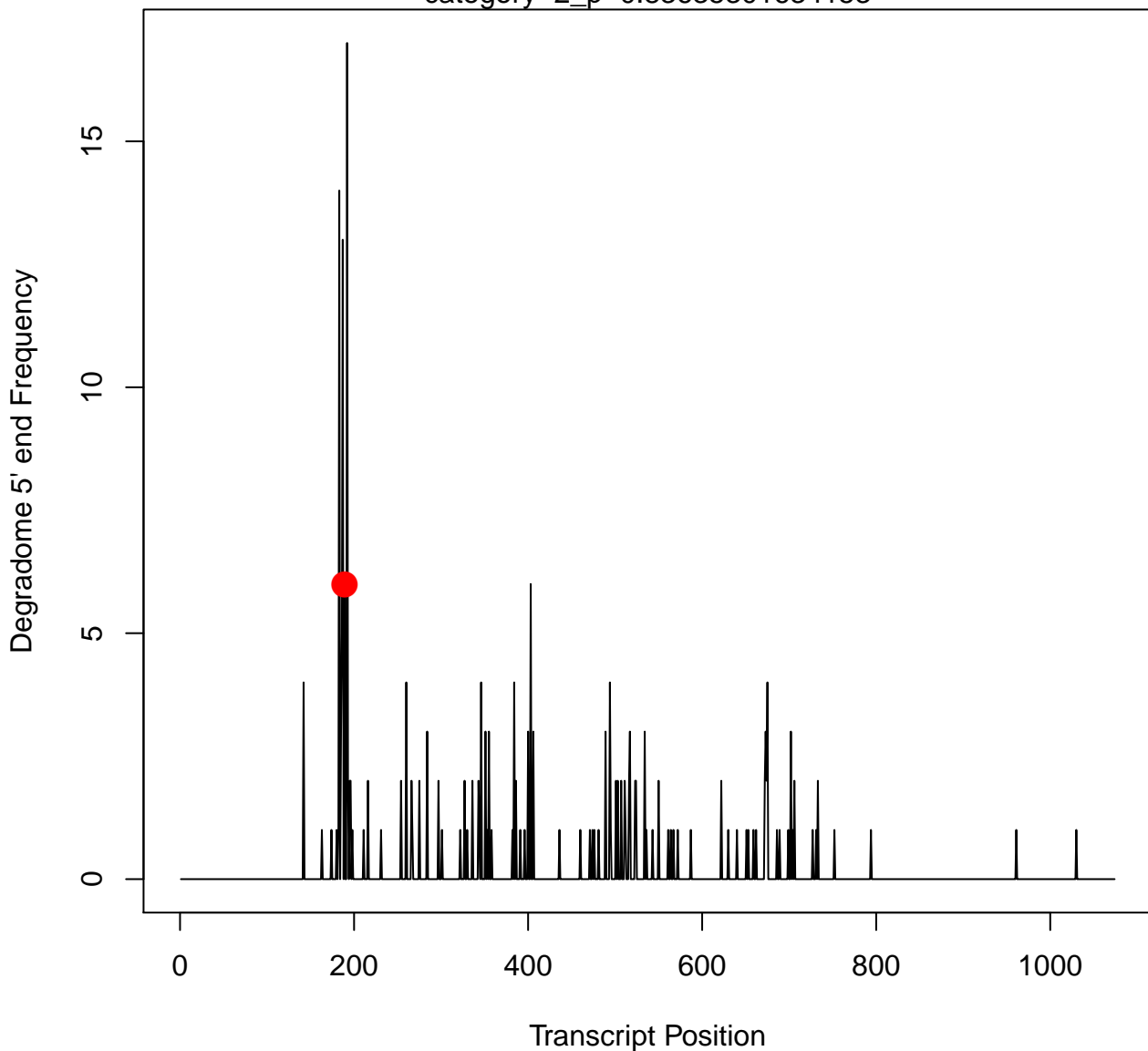

Supplement: Supplementary file 2 [file Data_Sheet_2.zip › Sit-miR156d_Seita.6G176000.1_189_TPlot.pdf]

**T=Seita.6G205500.1\_Q=Sit-miR156d\_S=1435**

category=2\_p=0.0549938711306051

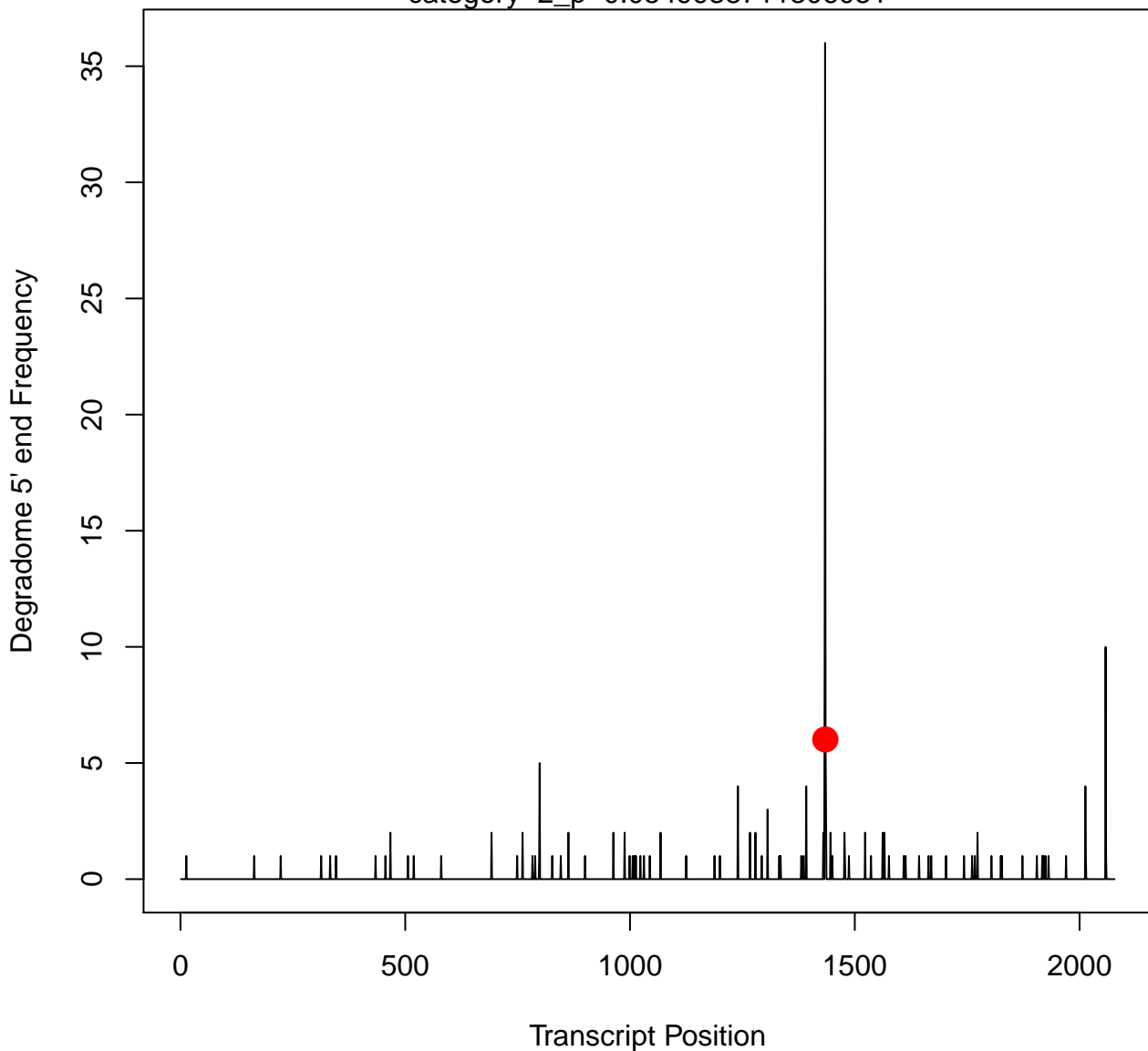

Supplement: Supplementary file 2 [file Data_Sheet_2.zip › Sit-miR156d_Seita.6G205500.1_1435_TPlot.pdf]

**T=Seita.6G223300.1\_Q=Sit-miR156d\_S=982**

category=2\_p=0.156074955192734

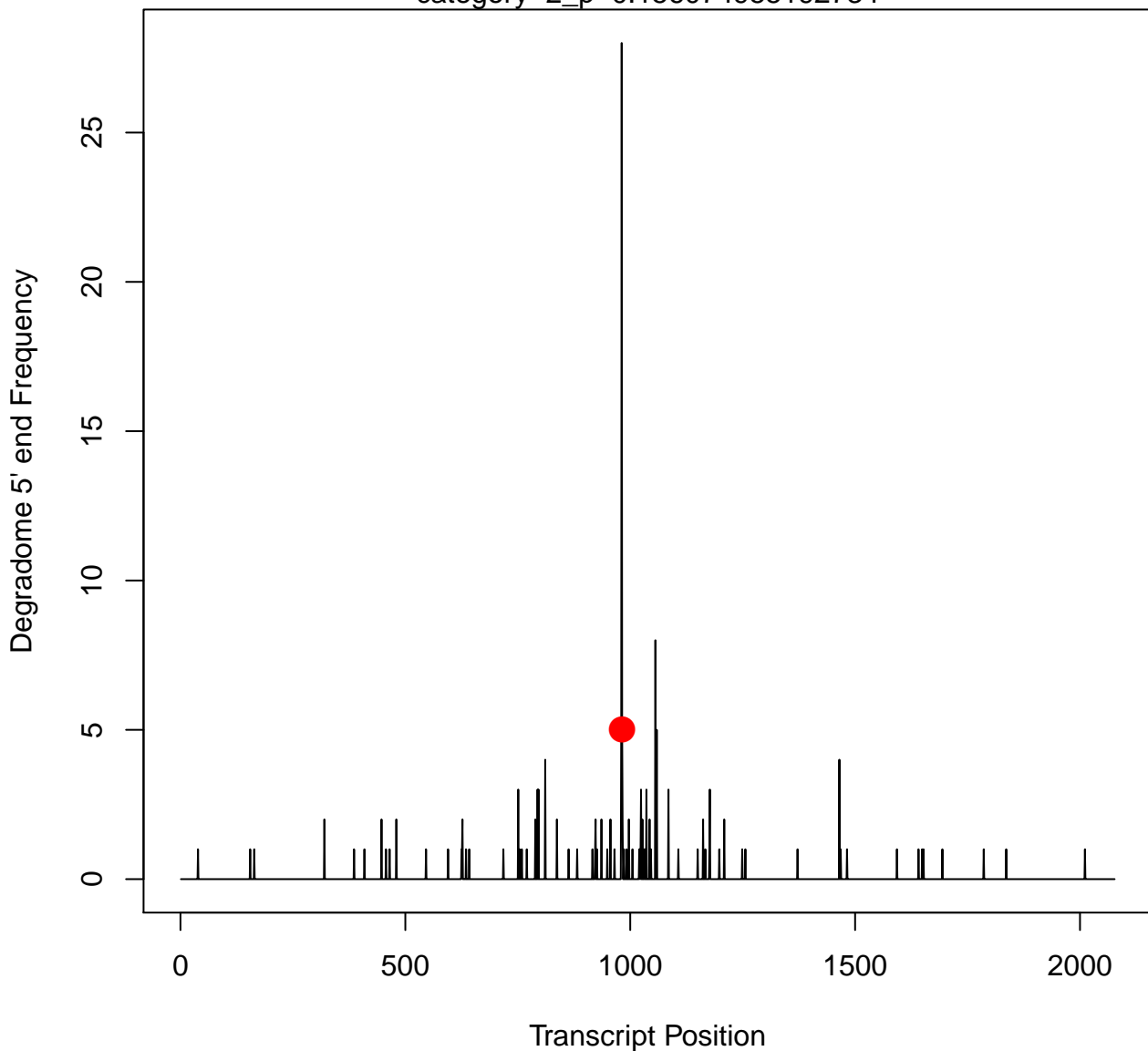

Supplement: Supplementary file 2 [file Data_Sheet_2.zip › Sit-miR156d_Seita.6G223300.1_982_TPlot.pdf]

**T=Seita.9G485400.1\_Q=Sit-miR156d\_S=1791**

category=2\_p=0.996301480695499

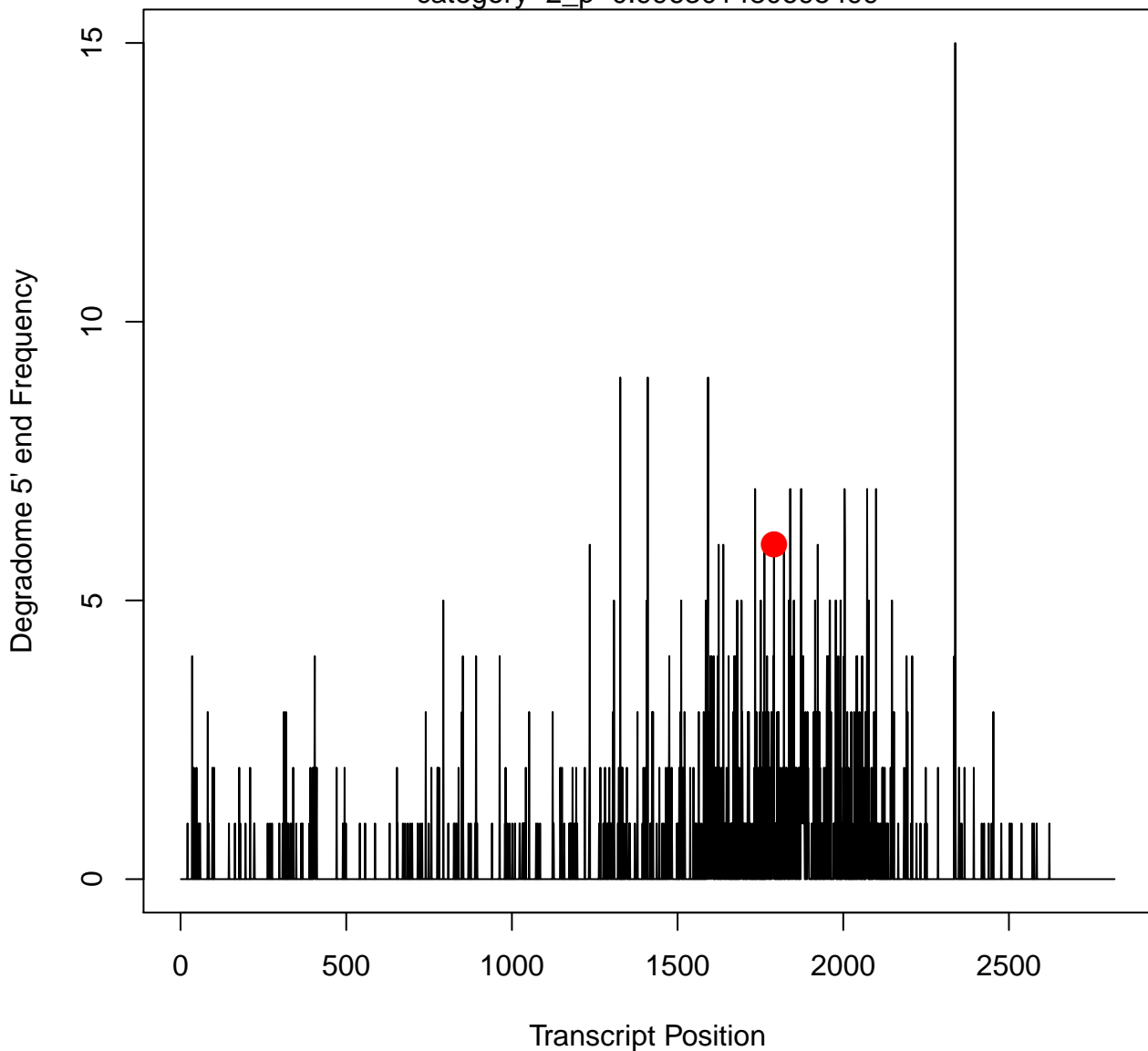

Supplement: Supplementary file 2 [file Data_Sheet_2.zip › Sit-miR156d_Seita.9G485400.1_1791_TPlot.pdf]

**T=Seita.9G556900.1\_Q=Sit-miR156d\_S=786**

category=2\_p=0.937440015514182

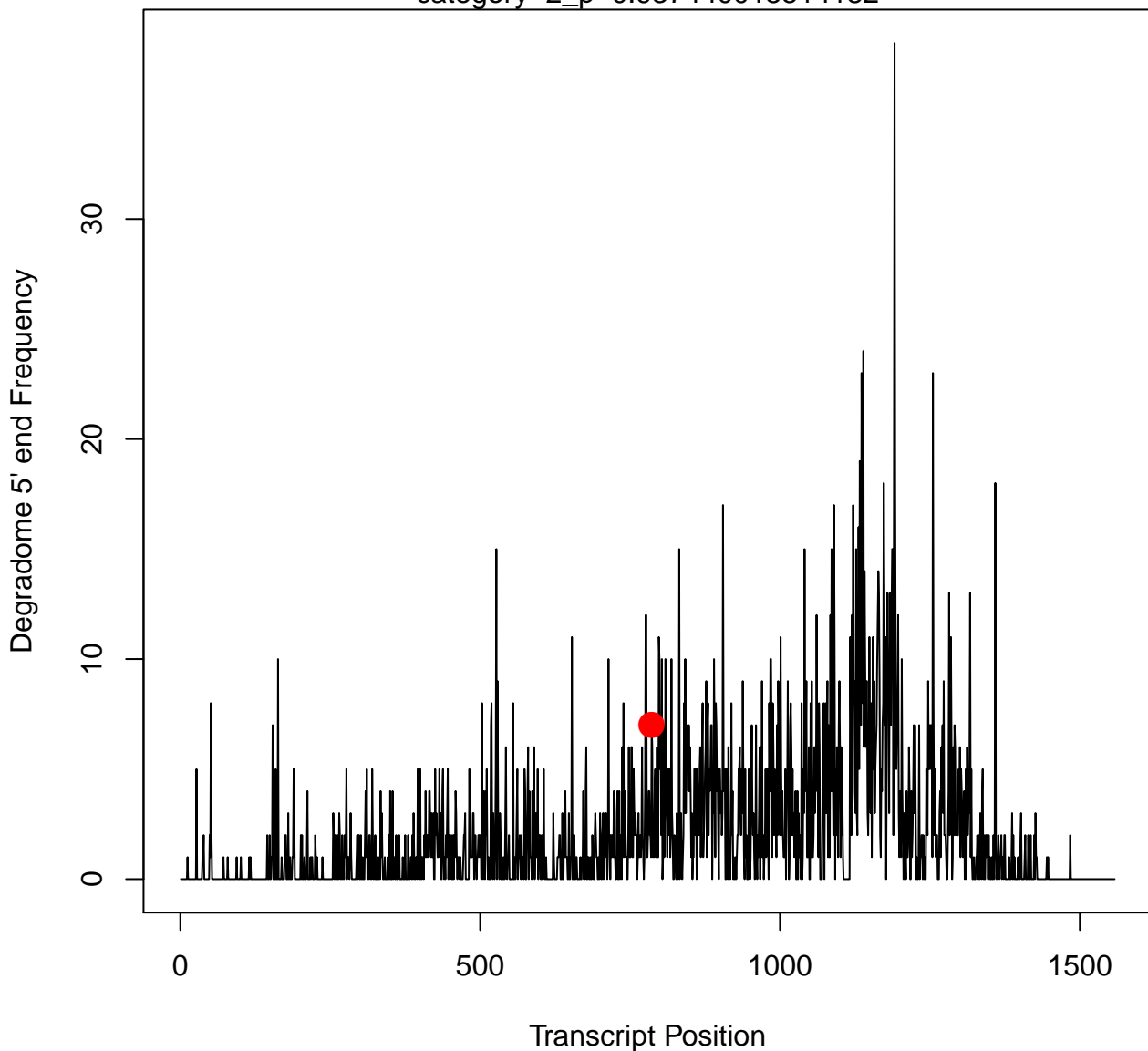

Supplement: Supplementary file 2 [file Data_Sheet_2.zip › Sit-miR156d_Seita.9G556900.1_786_TPlot.pdf]

**T=Seita.2G325000.1\_Q=Sit-miR156e\_S=665**

category=2\_p=0.246344061170235

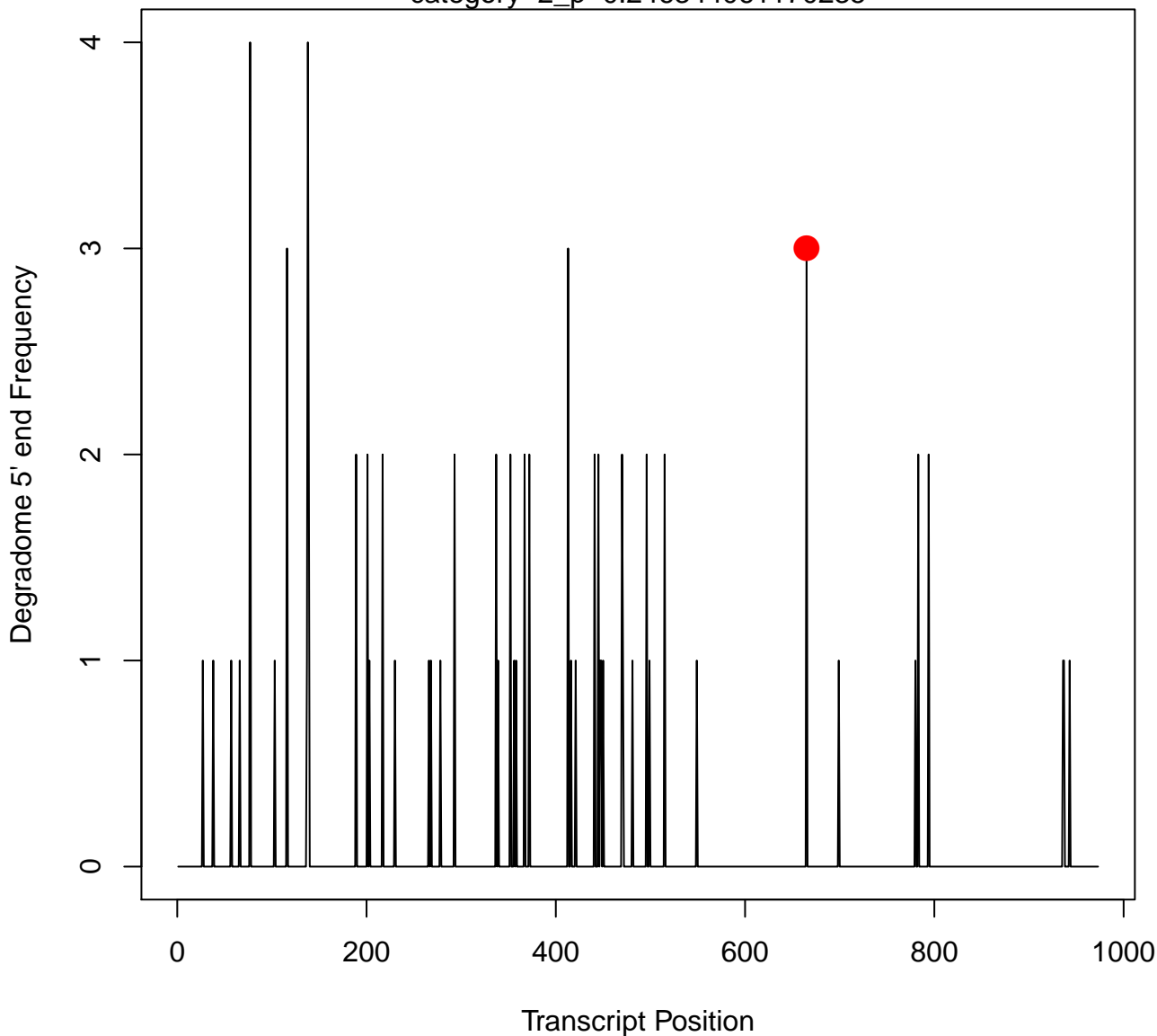

Supplement: Supplementary file 2 [file Data_Sheet_2.zip › Sit-miR156e_Seita.2G325000.1_665_TPlot.pdf]

**T=Seita.5G432500.1\_Q=Sit-miR156e\_S=2375**

category=2\_p=0.10696341639928

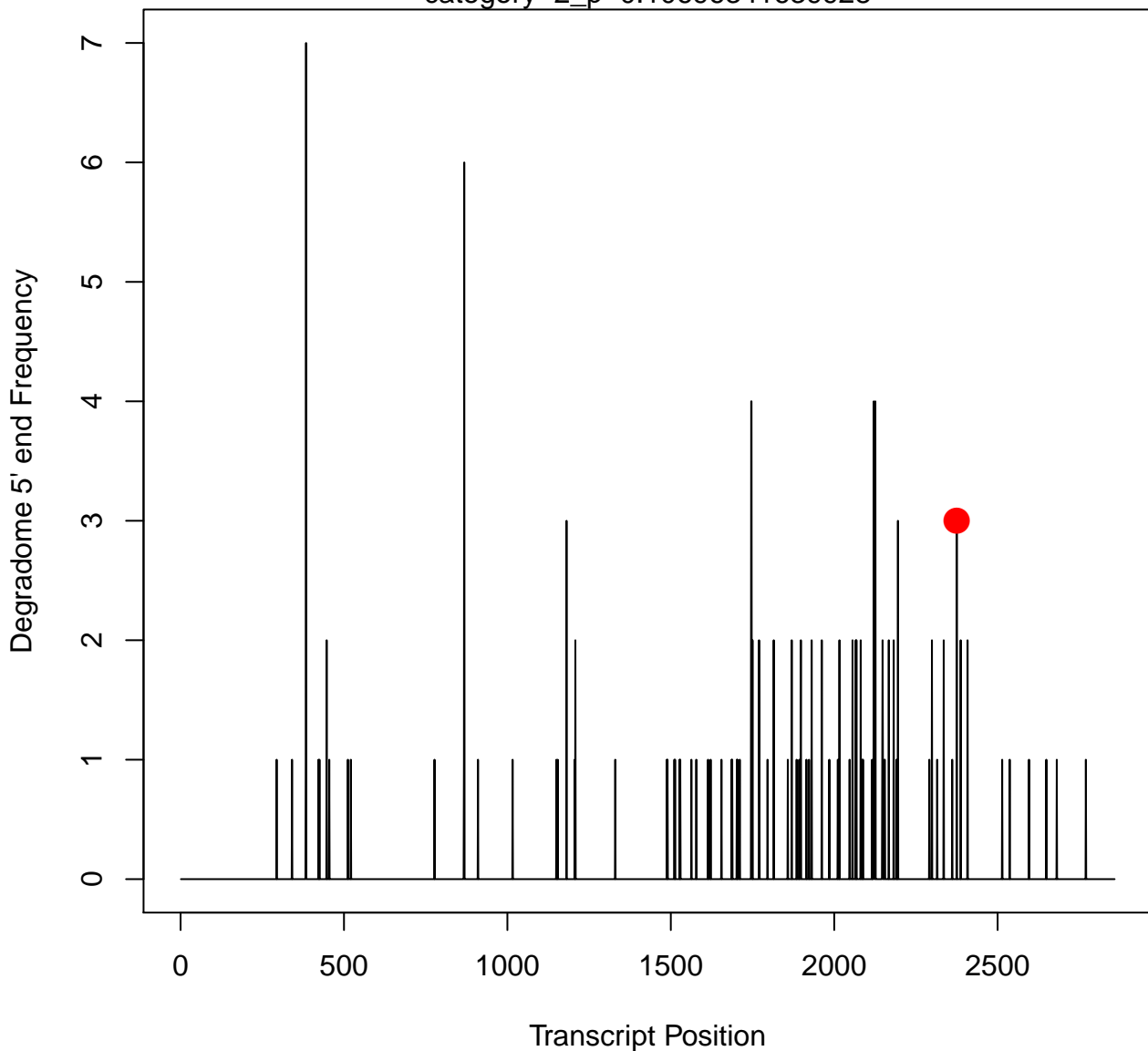

Supplement: Supplementary file 2 [file Data_Sheet_2.zip › Sit-miR156e_Seita.5G432500.1_2375_TPlot.pdf]

**T=Seita.2G324900.1\_Q=Sit-miR156g\_S=833**

category=0\_p=0.00384831107504247

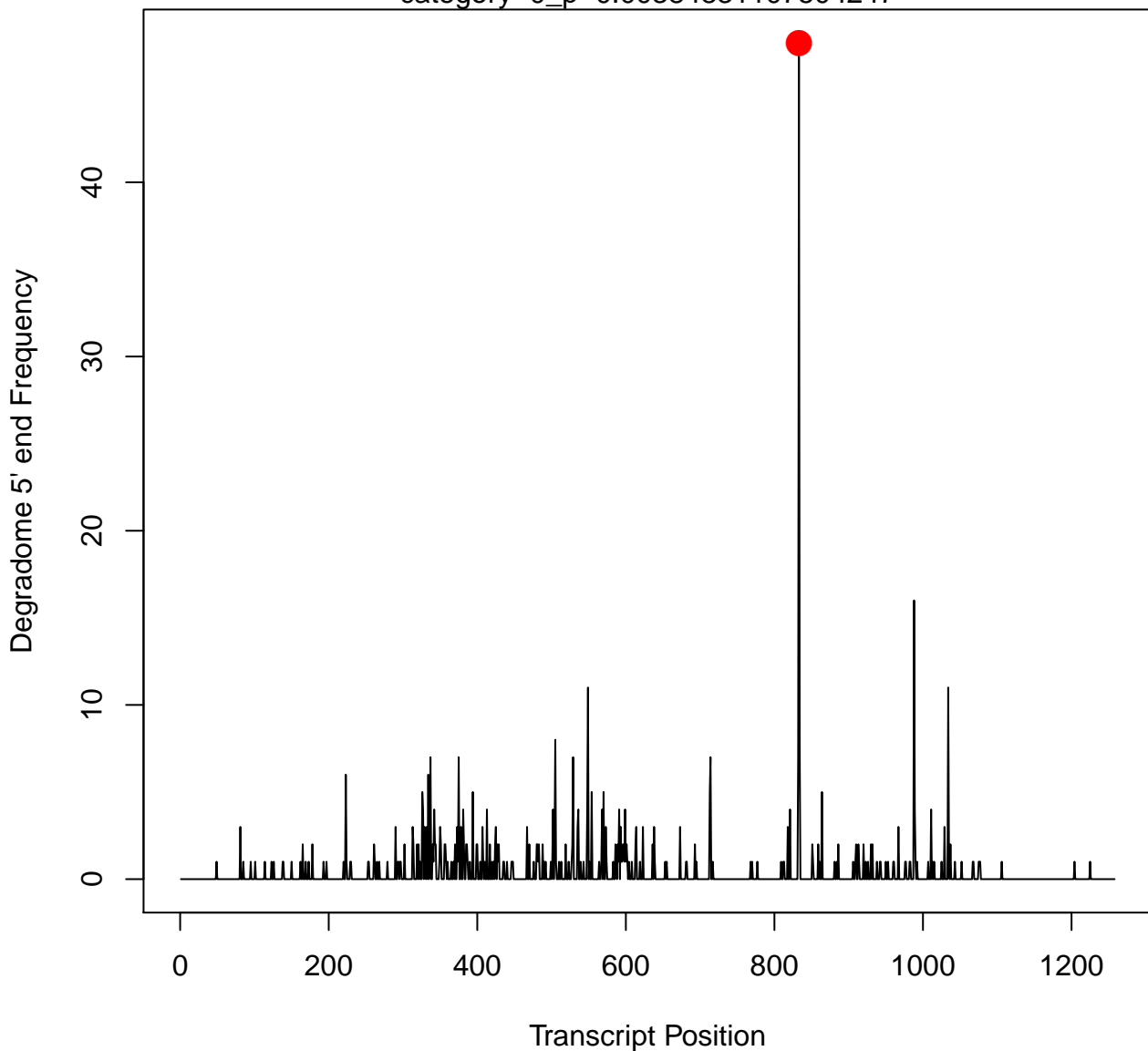

Supplement: Supplementary file 2 [file Data_Sheet_2.zip › Sit-miR156g_Seita.2G324900.1_833_TPlot.pdf]

**T=Seita.9G207500.1\_Q=Sit-miR156g\_S=1162**

category=2\_p=0.97539813974139

Degradome 5' end Frequency

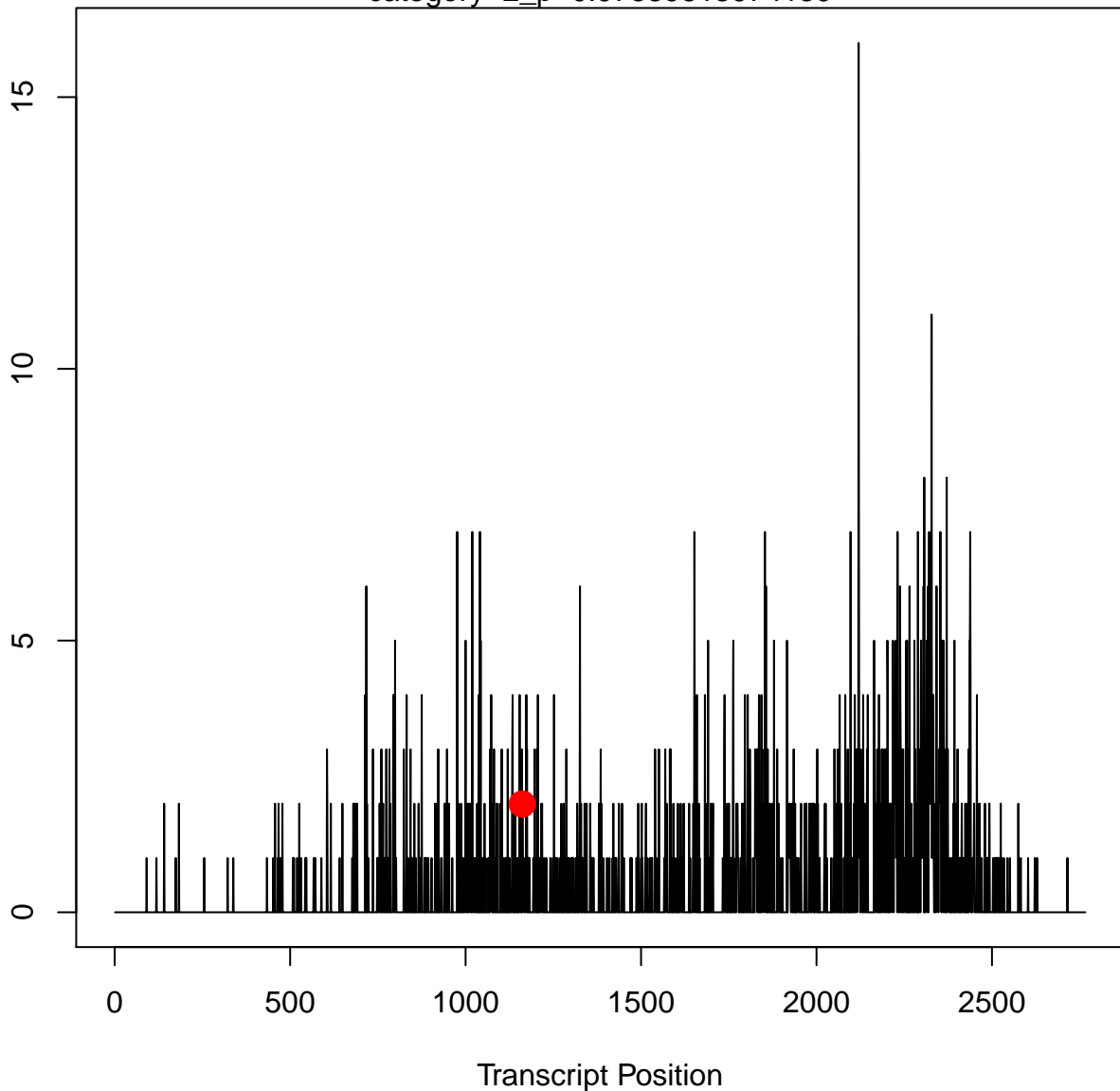

Supplement: Supplementary file 2 [file Data_Sheet_2.zip › Sit-miR156g_Seita.9G207500.1_1162_TPlot.pdf]

**T=Seita.8G124900.1\_Q=Sit-miR156i\_S=1295**

category=0\_p=0.00576690948364234

Degradome 5' end Frequency

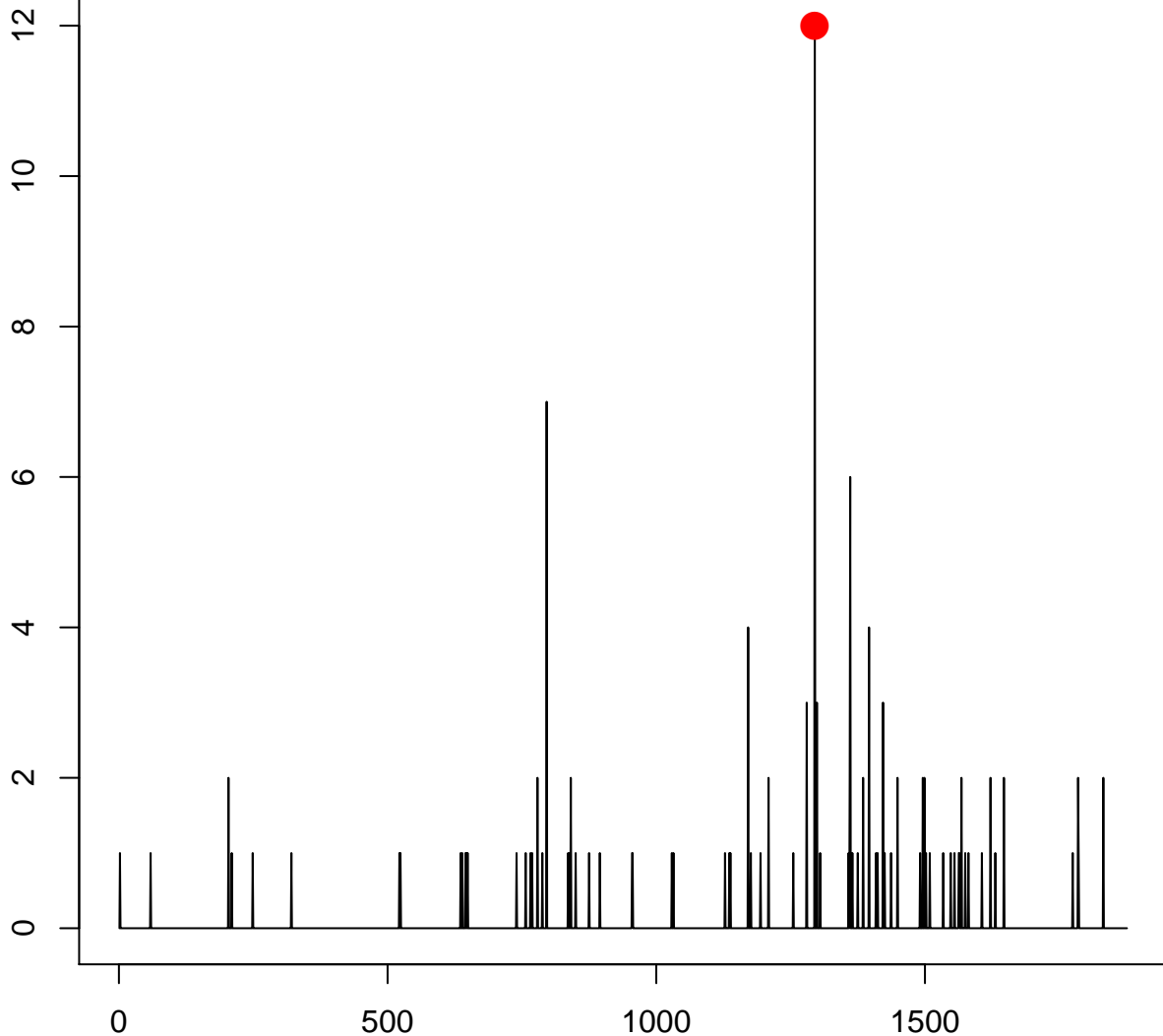

Transcript Position

Supplement: Supplementary file 2 [file Data_Sheet_2.zip › Sit-miR156i_Seita.8G124900.1_1295_TPlot.pdf]

**T=Seita.1G069300.1\_Q=Sit-miR156j\_S=873**

category=0\_p=0.000385499163724878

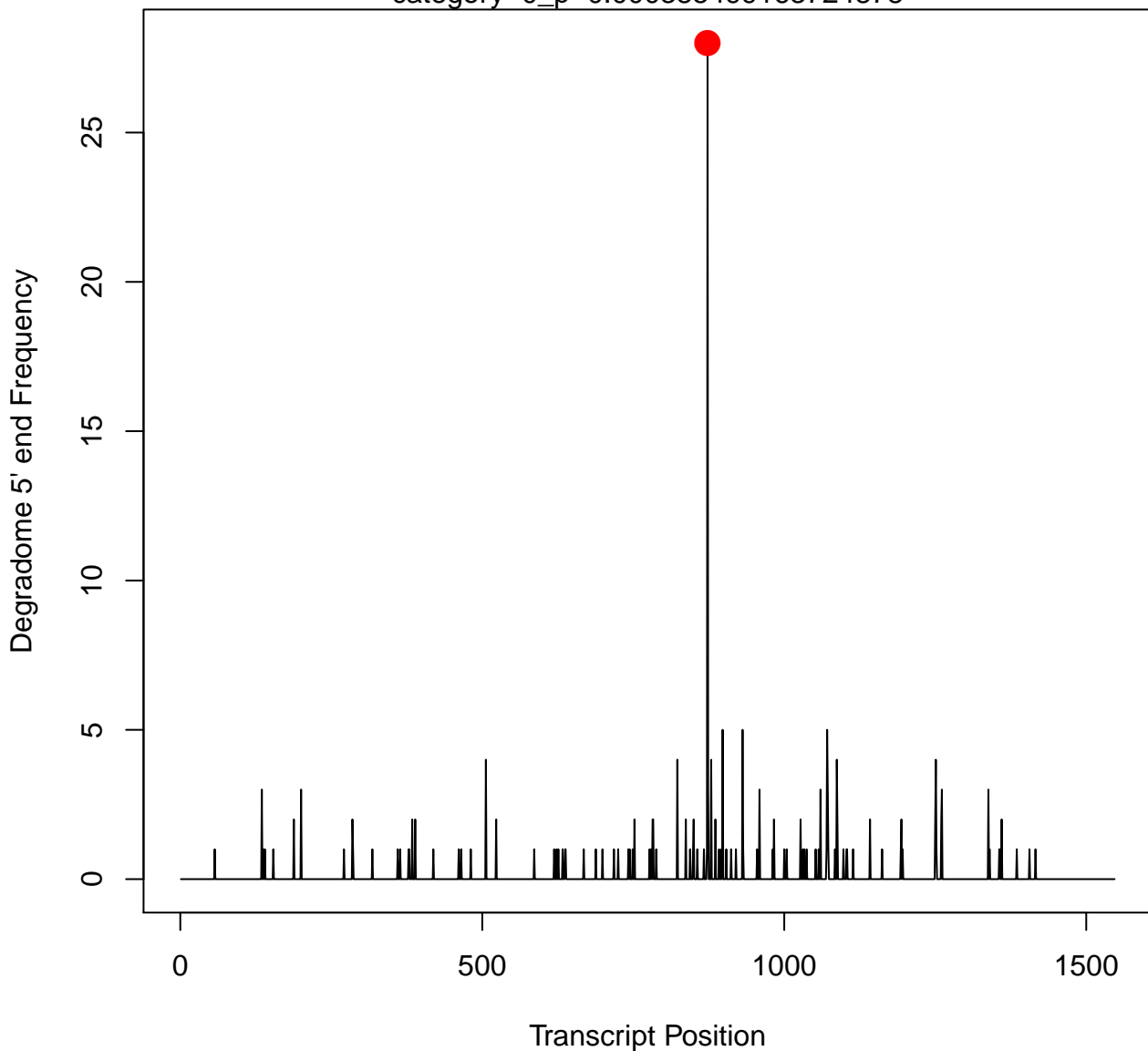

Supplement: Supplementary file 2 [file Data_Sheet_2.zip › Sit-miR156j_Seita.1G069300.1_873_TPlot.pdf]

**T=Seita.2G266500.1\_Q=Sit-miR156j\_S=1896**

category=0\_p=0.00346414733721923

Degradome 5' end Frequency

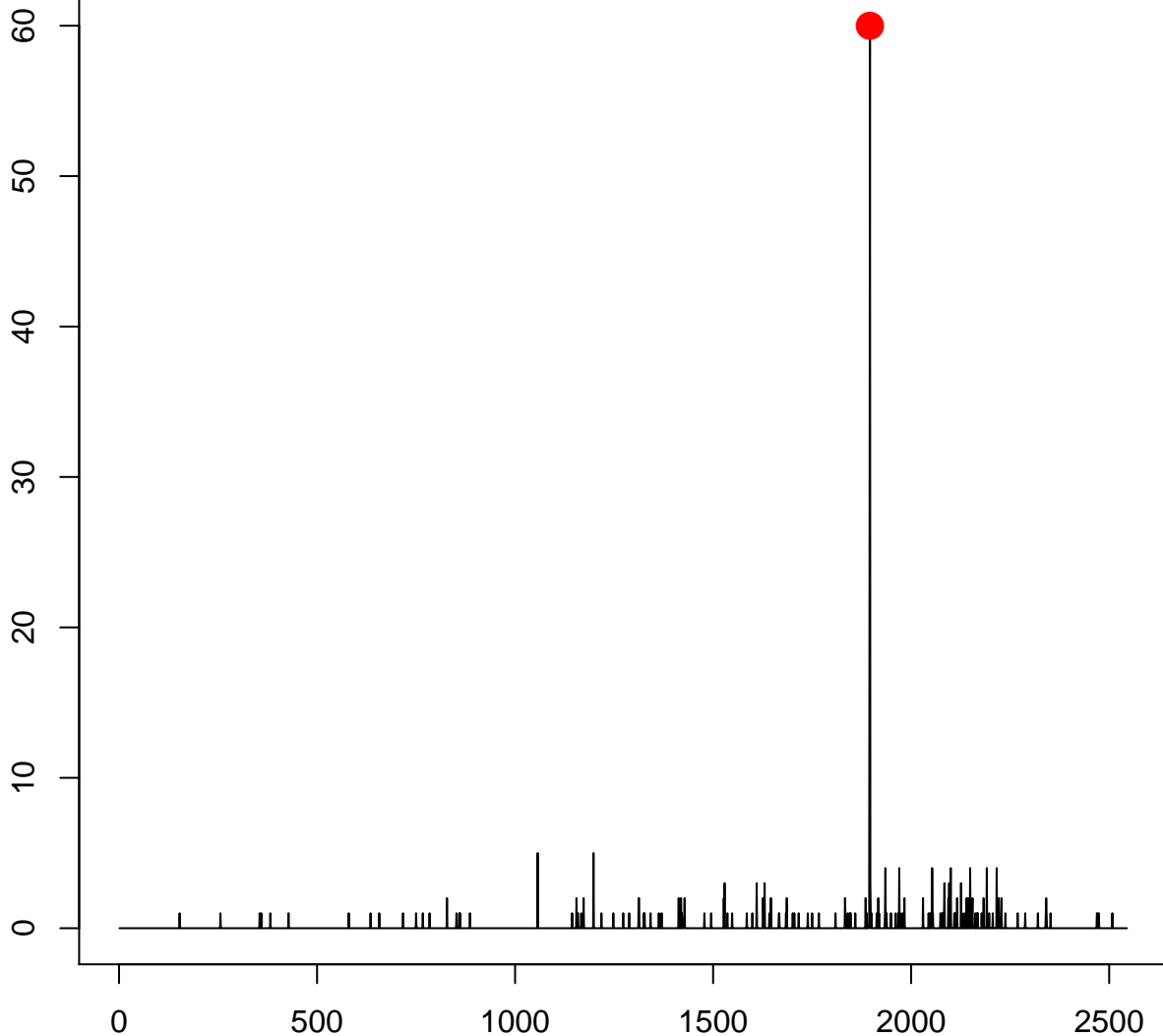

Transcript Position

Supplement: Supplementary file 2 [file Data_Sheet_2.zip › Sit-miR156j_Seita.2G266500.1_1896_TPlot.pdf]

**T=Seita.6G058700.1\_Q=Sit-miR156j\_S=606**

category=2\_p=0.98752089470647

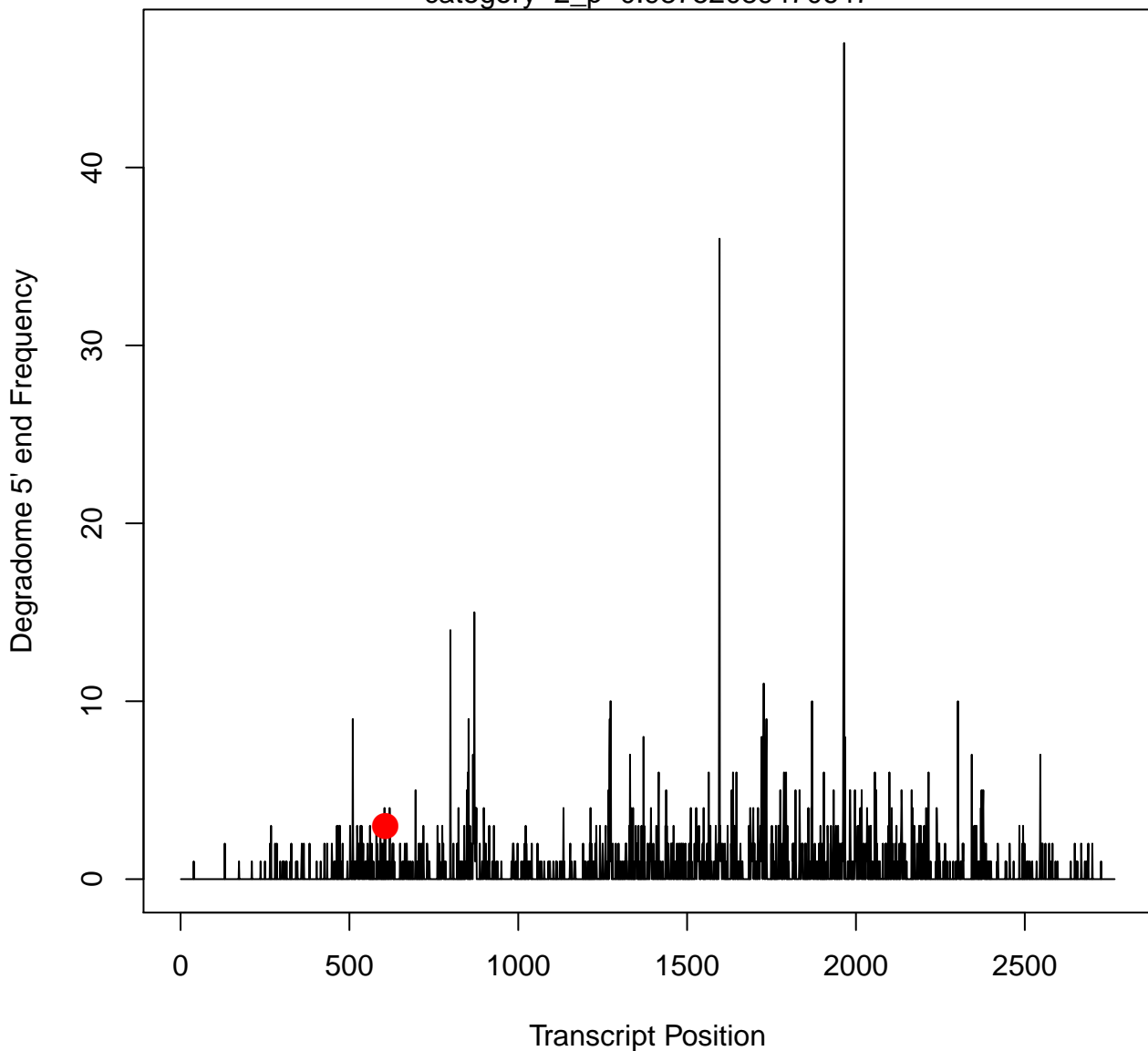

Supplement: Supplementary file 2 [file Data_Sheet_2.zip › Sit-miR156j_Seita.6G058700.1_606_TPlot.pdf]

**T=Seita.1G019300.1\_Q=Sit-miR159a\_S=990**

category=2\_p=0.999835209461431

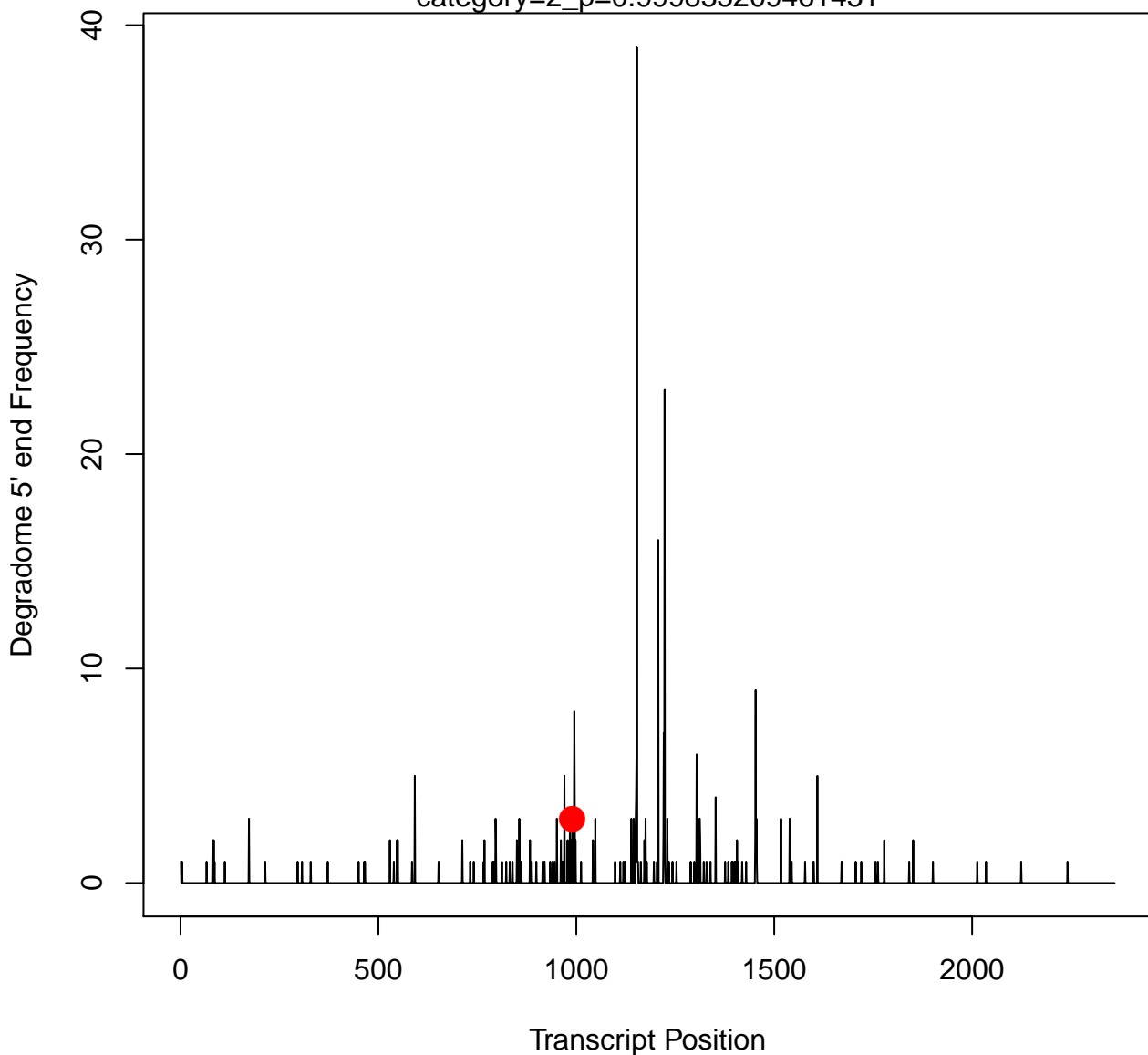

Supplement: Supplementary file 2 [file Data_Sheet_2.zip › Sit-miR159a_Seita.1G019300.1_990_TPlot.pdf]

**T=Seita.1G093400.1\_Q=Sit-miR159a\_S=3602**

category=2\_p=0.711887730608996

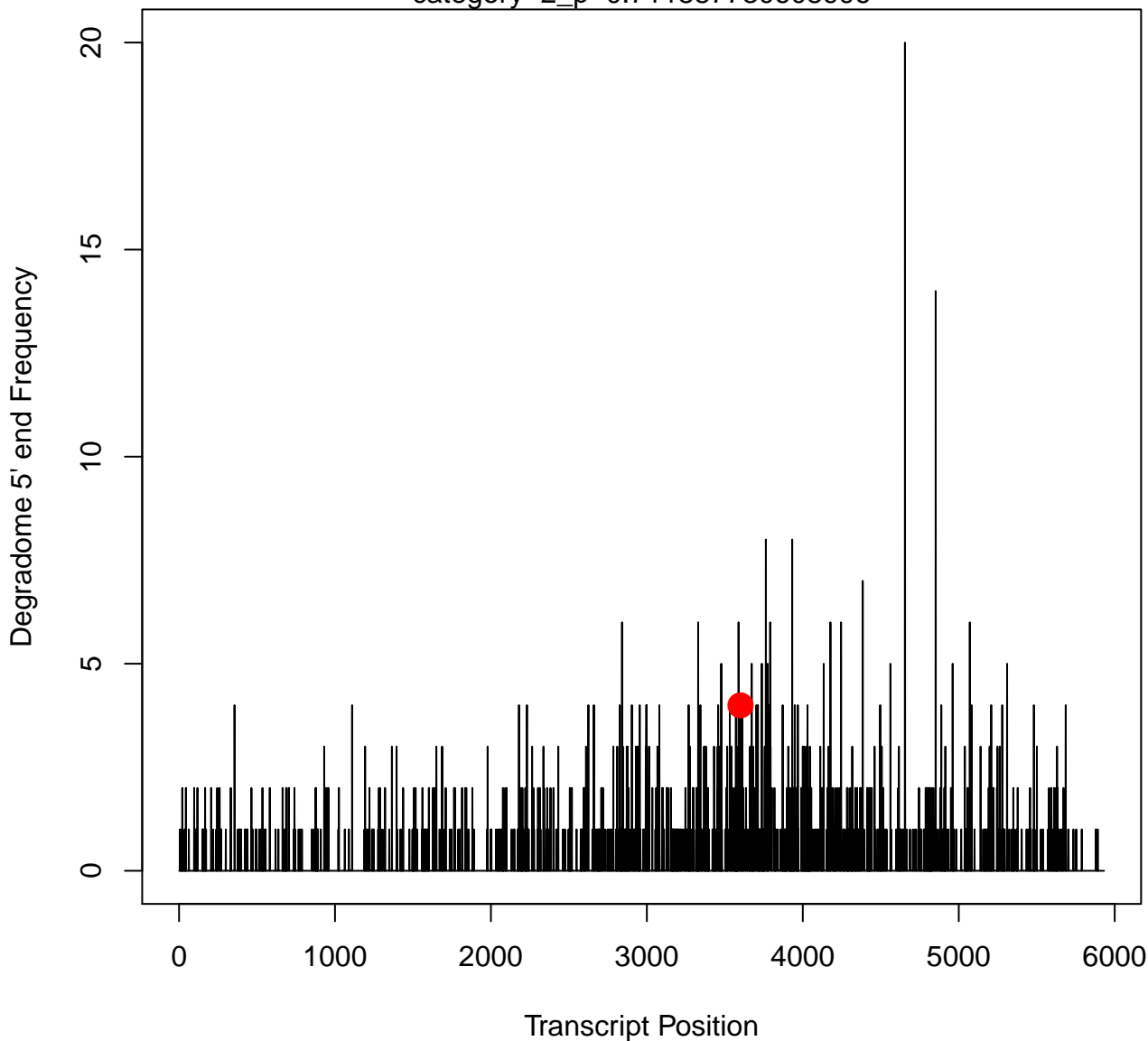

Supplement: Supplementary file 2 [file Data_Sheet_2.zip › Sit-miR159a_Seita.1G093400.1_3602_TPlot.pdf]

**T=Seita.1G134200.1\_Q=Sit-miR159a\_S=61**

category=2\_p=0.999999993934283

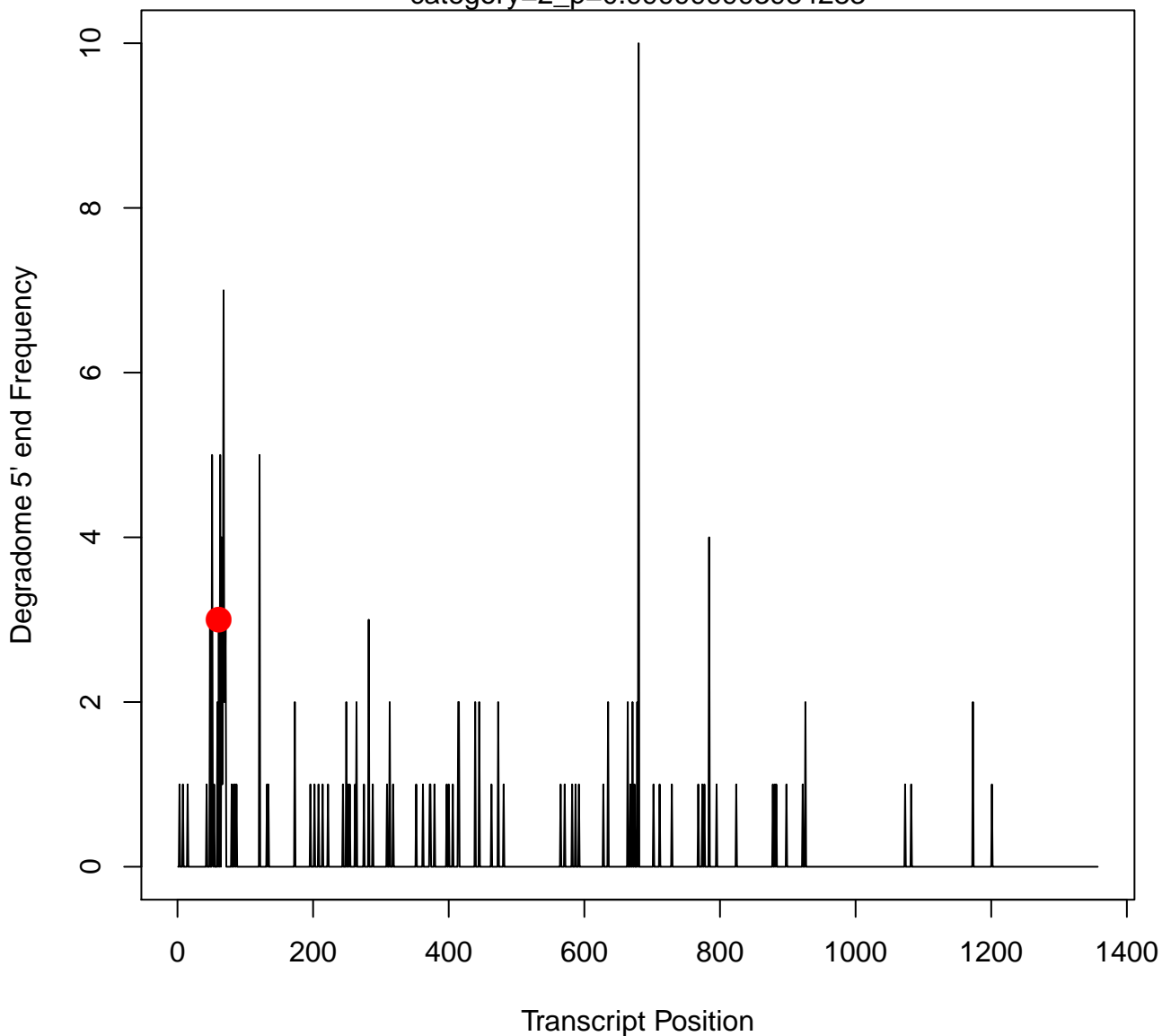

Supplement: Supplementary file 2 [file Data_Sheet_2.zip › Sit-miR159a_Seita.1G134200.1_61_TPlot.pdf]

**T=Seita.2G109700.1\_Q=Sit-miR159a\_S=1844**

category=1\_p=0.223335330947717

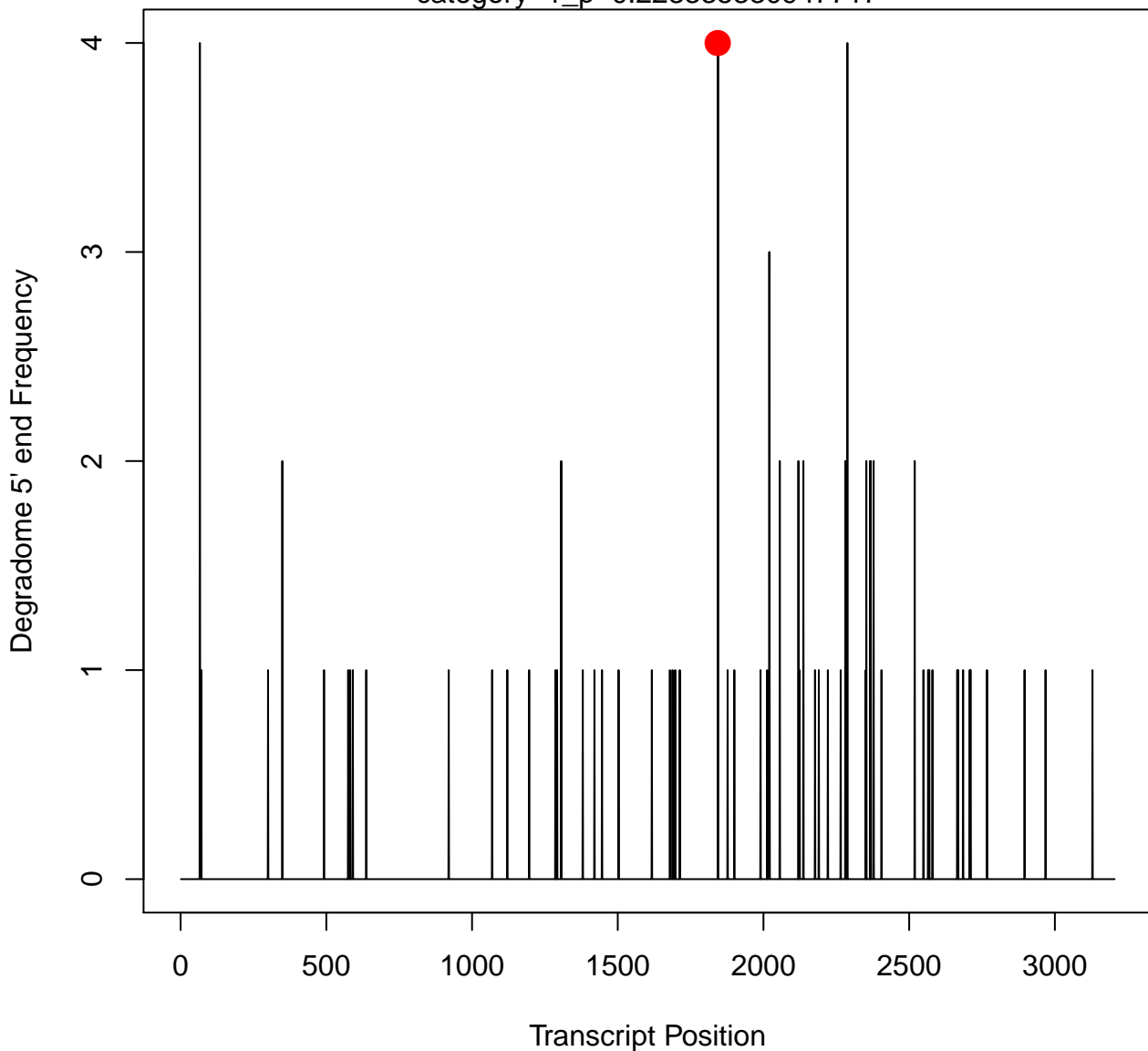

Supplement: Supplementary file 2 [file Data_Sheet_2.zip › Sit-miR159a_Seita.2G109700.1_1844_TPlot.pdf]

**T=Seita.2G132500.1\_Q=Sit-miR159a\_S=265**

category=2\_p=0.999998264520062

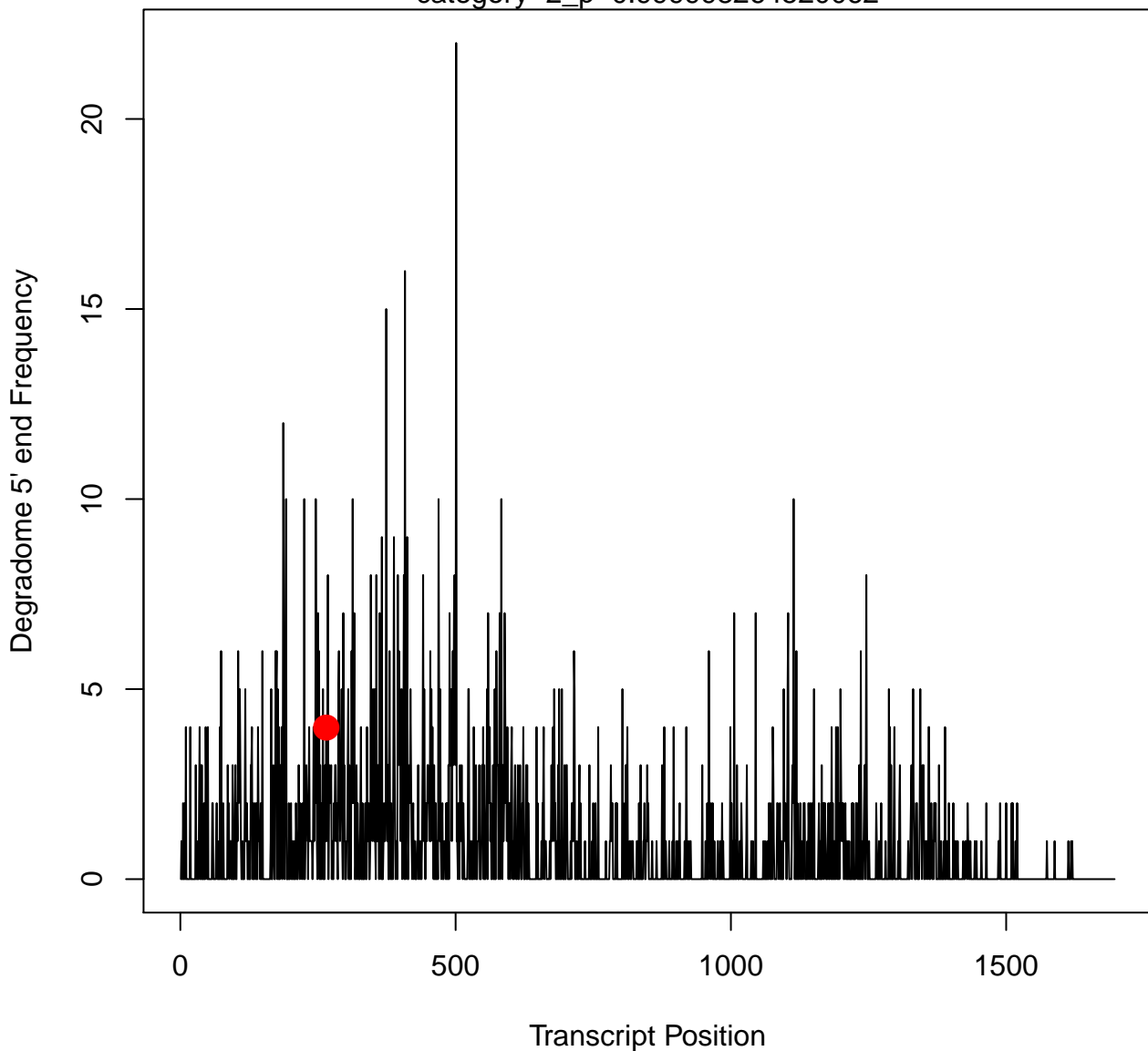

Supplement: Supplementary file 2 [file Data_Sheet_2.zip › Sit-miR159a_Seita.2G132500.1_265_TPlot.pdf]

**T=Seita.2G141700.1\_Q=Sit-miR159a\_S=625**

category=2\_p=0.99999546026011

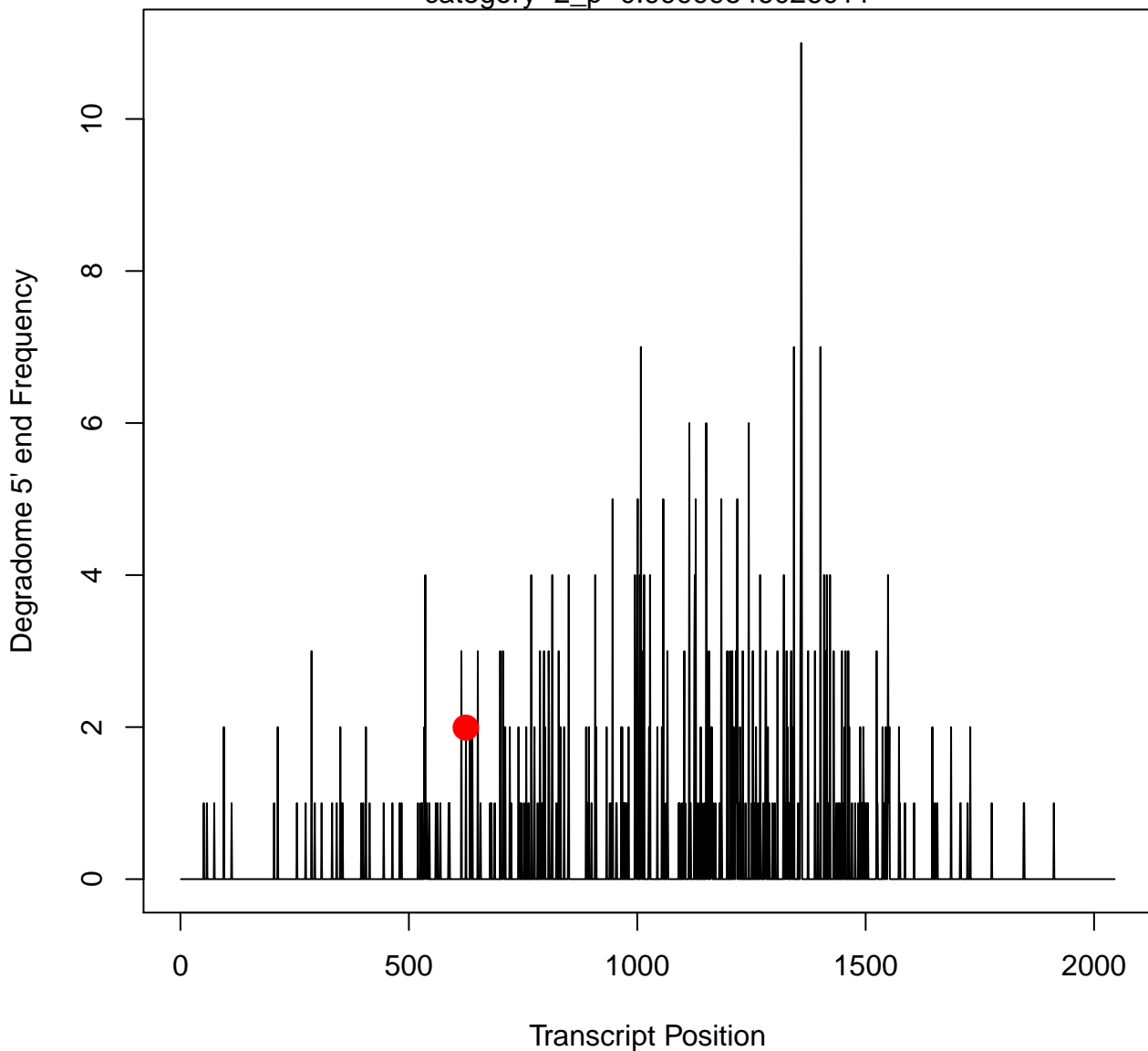

Supplement: Supplementary file 2 [file Data_Sheet_2.zip › Sit-miR159a_Seita.2G141700.1_625_TPlot.pdf]

**T=Seita.2G142600.1\_Q=Sit-miR159a\_S=96**

category=0\_p=0.0795552481614903

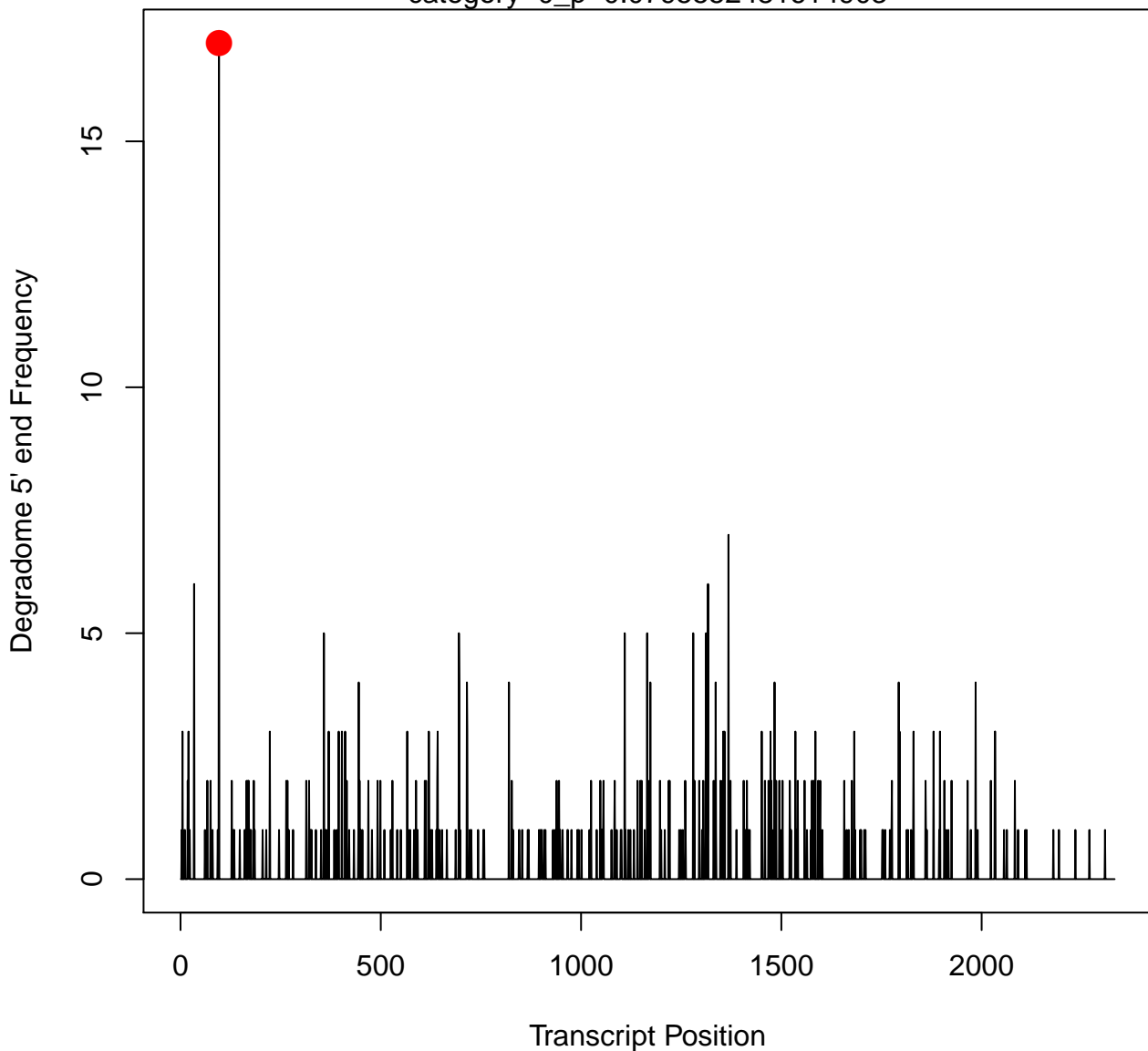

Supplement: Supplementary file 2 [file Data_Sheet_2.zip › Sit-miR159a_Seita.2G142600.1_96_TPlot.pdf]

**T=Seita.2G193900.1\_Q=Sit-miR159a\_S=1942**

category=2\_p=0.970012051295214

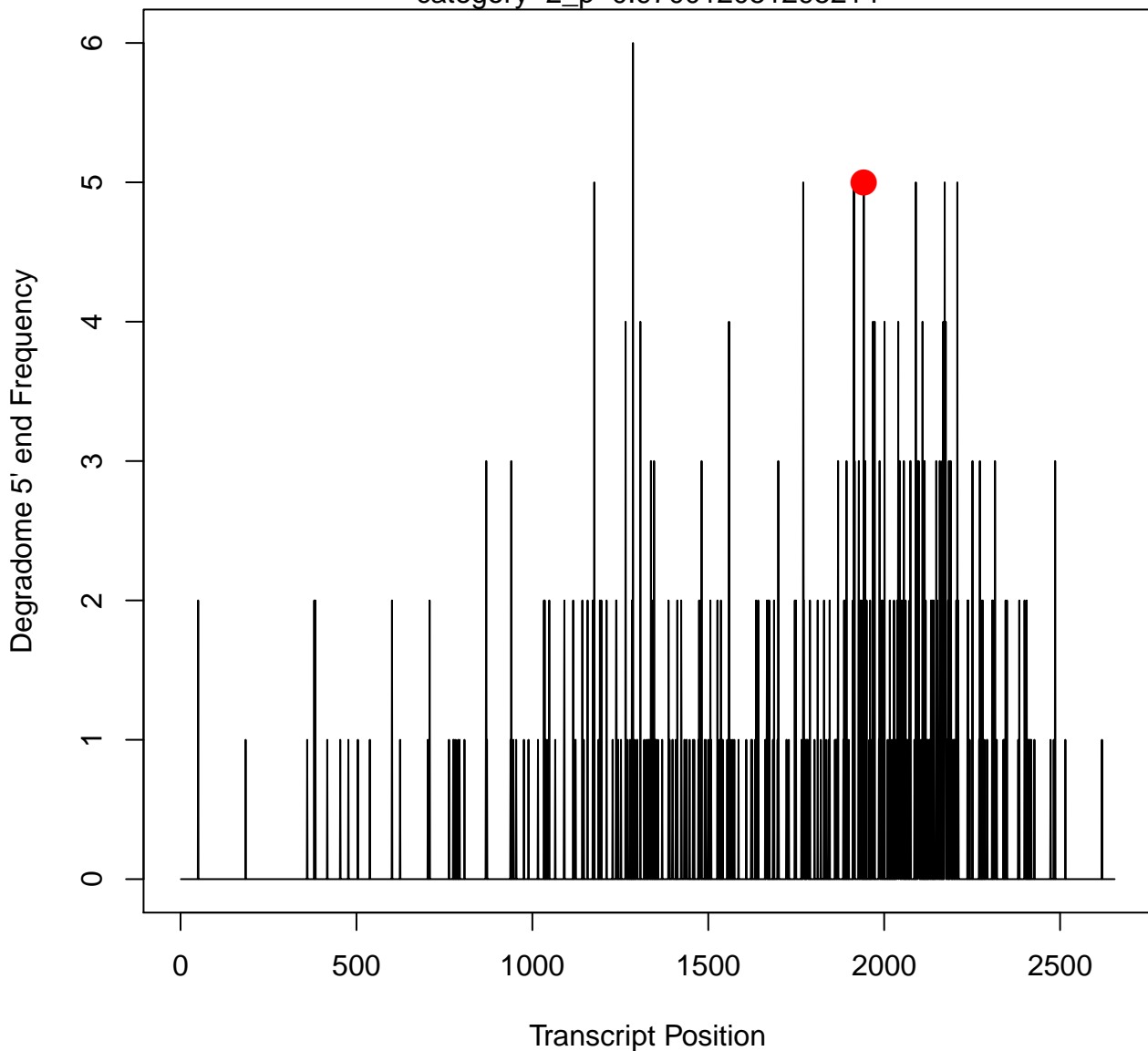

Supplement: Supplementary file 2 [file Data_Sheet_2.zip › Sit-miR159a_Seita.2G193900.1_1942_TPlot.pdf]

**T=Seita.2G258400.1\_Q=Sit-miR159a\_S=480**

category=2\_p=0.999999999999972

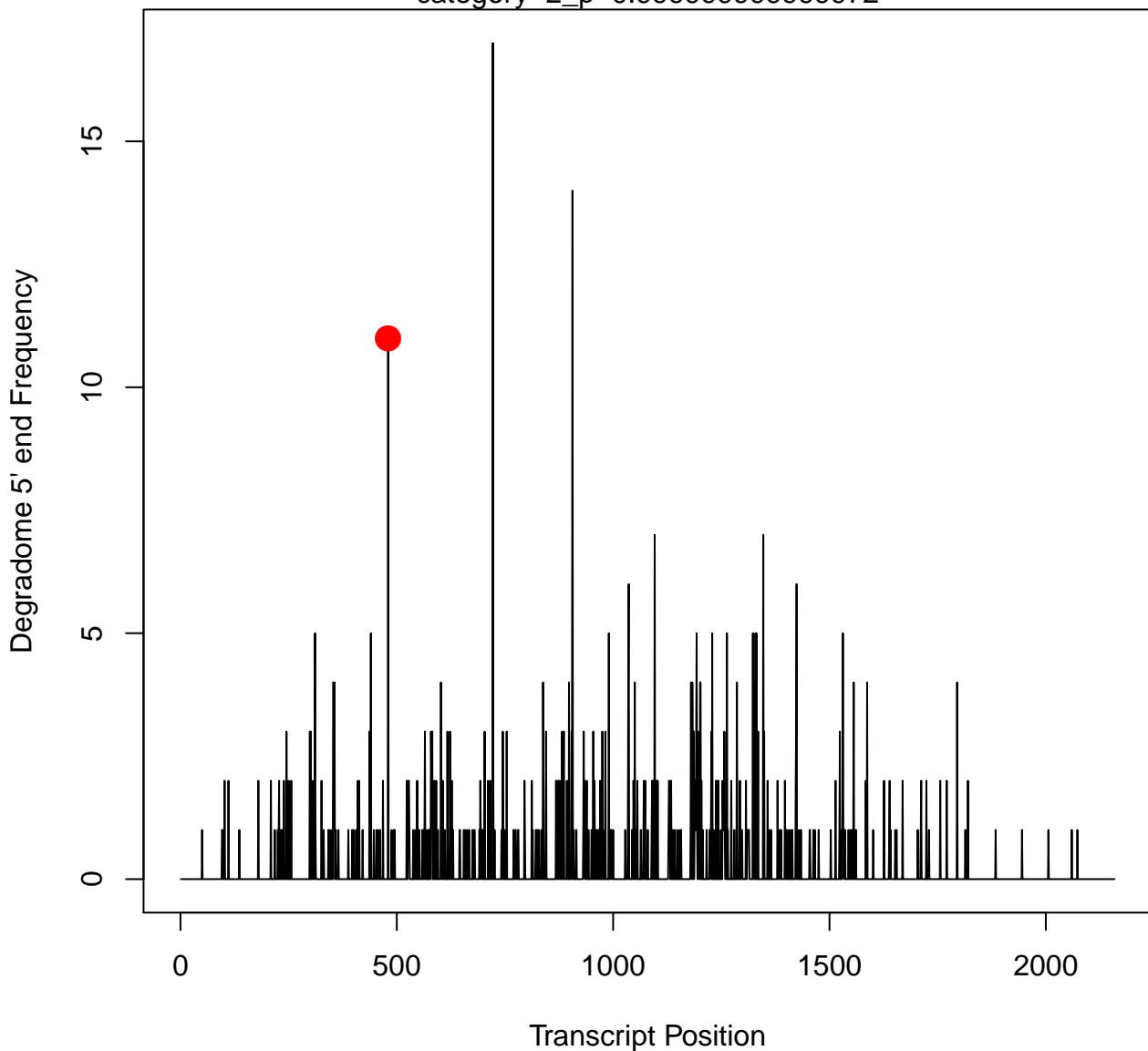

Supplement: Supplementary file 2 [file Data_Sheet_2.zip › Sit-miR159a_Seita.2G258400.1_480_TPlot.pdf]

**T=Seita.2G269400.1\_Q=Sit-miR159a\_S=445**

category=2\_p=0.999665805376488

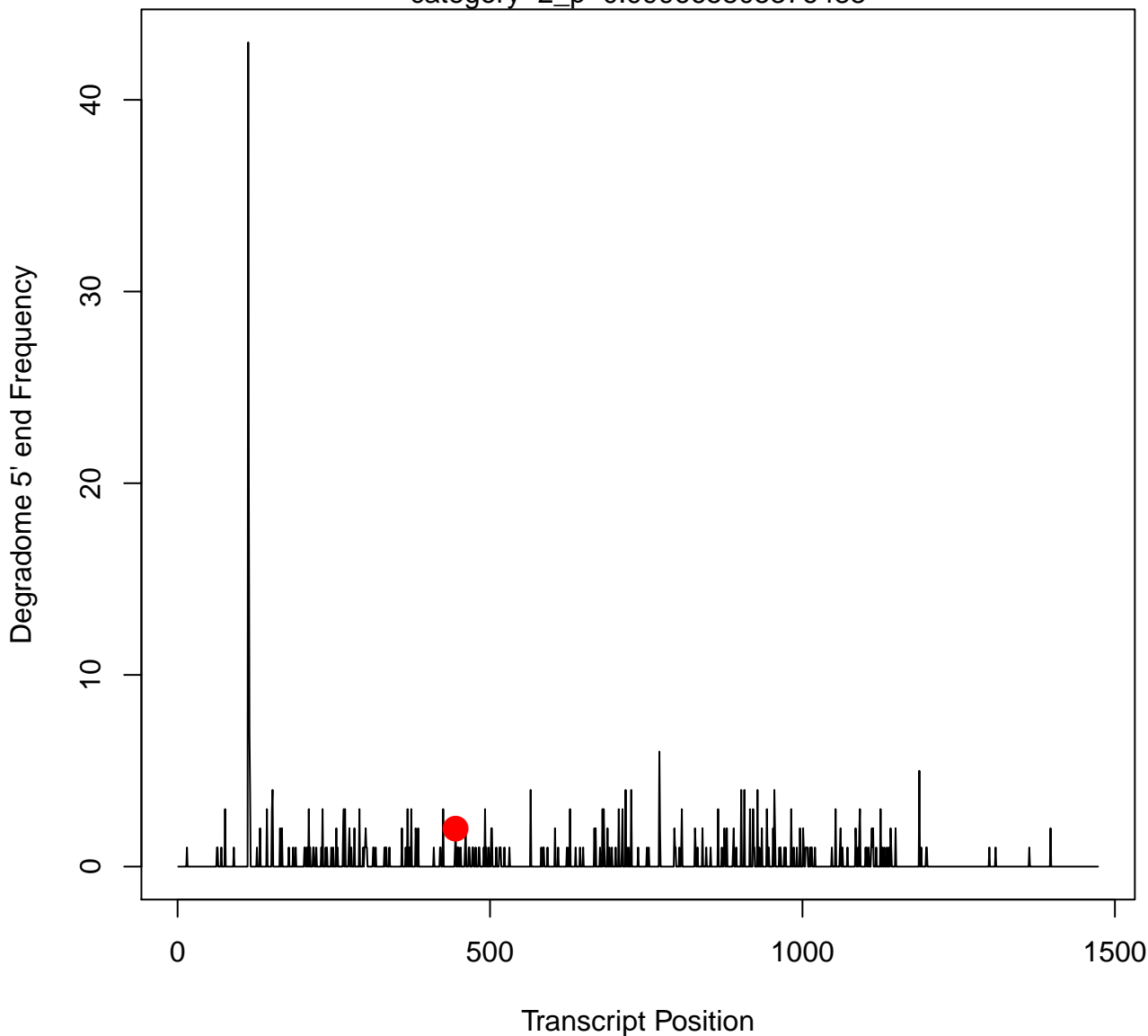

Supplement: Supplementary file 2 [file Data_Sheet_2.zip › Sit-miR159a_Seita.2G269400.1_445_TPlot.pdf]

**T=Seita.2G434400.1\_Q=Sit-miR159a\_S=362**

category=2\_p=0.595466956036382

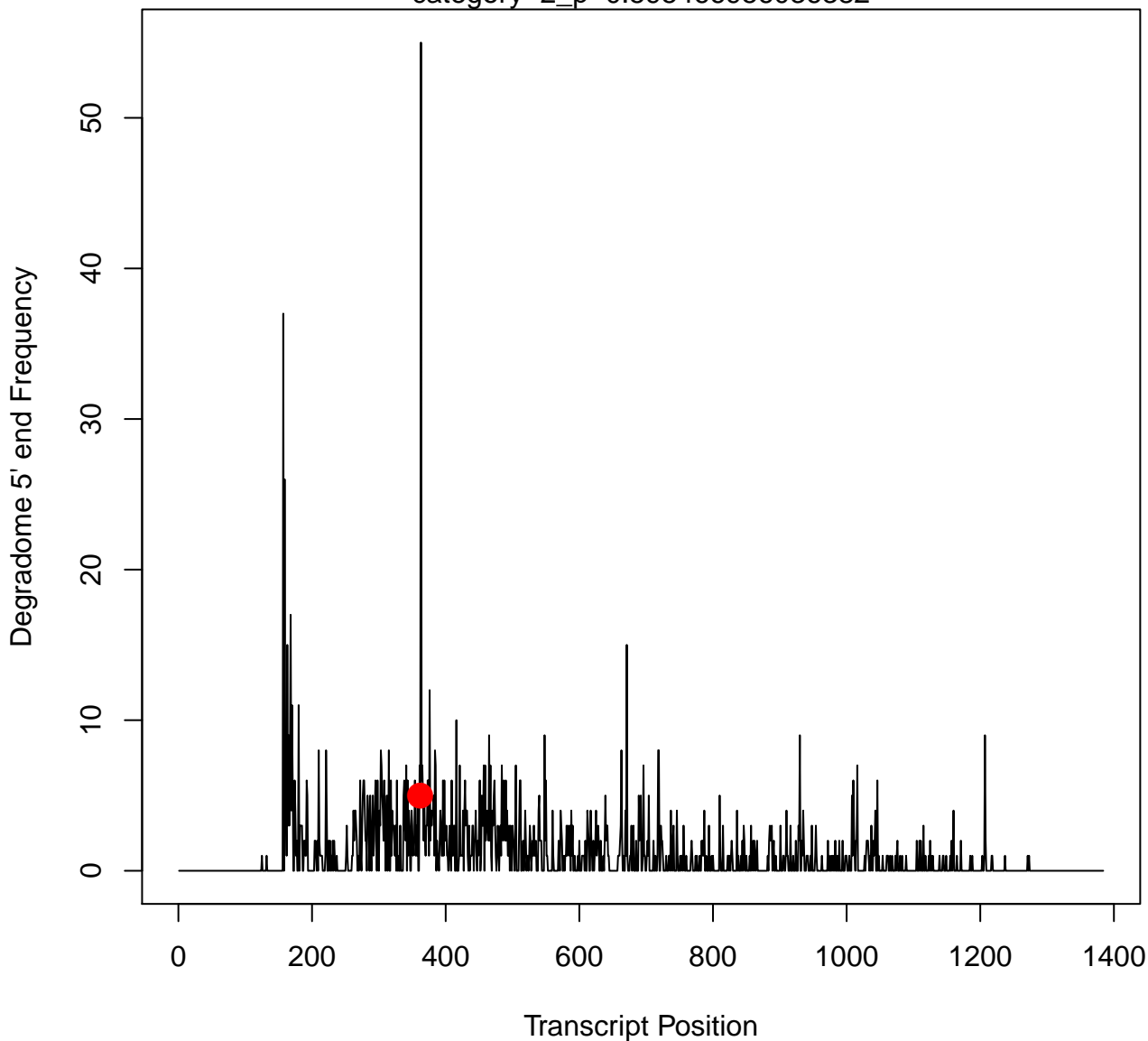

Supplement: Supplementary file 2 [file Data_Sheet_2.zip › Sit-miR159a_Seita.2G434400.1_362_TPlot.pdf]

**T=Seita.3G069700.1\_Q=Sit-miR159a\_S=4218**

category=2\_p=0.999999999999899

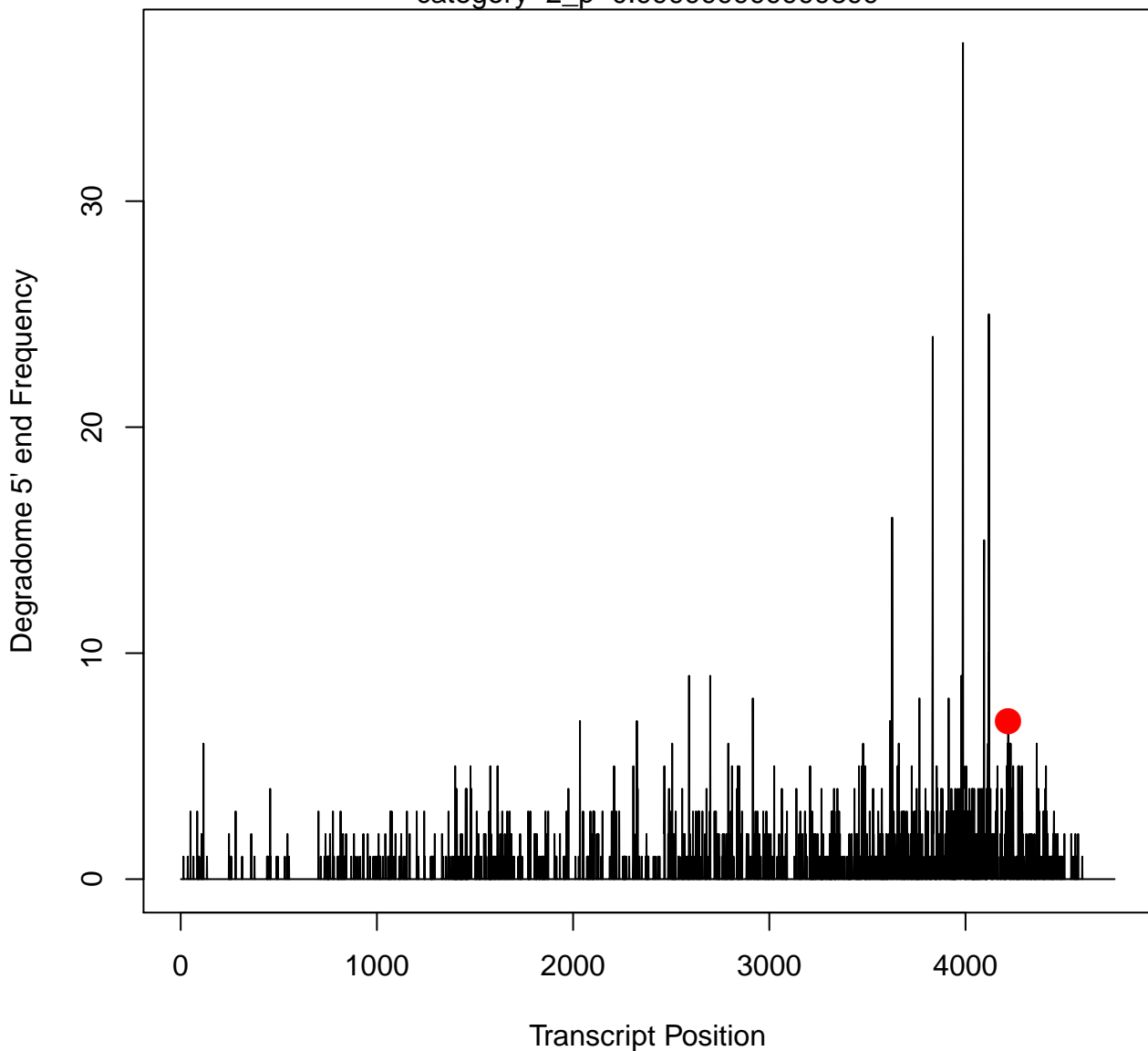

Supplement: Supplementary file 2 [file Data_Sheet_2.zip › Sit-miR159a_Seita.3G069700.1_4218_TPlot.pdf]

**T=Seita.3G096800.1\_Q=Sit-miR159a\_S=1199**

category=2\_p=0.99999999999996

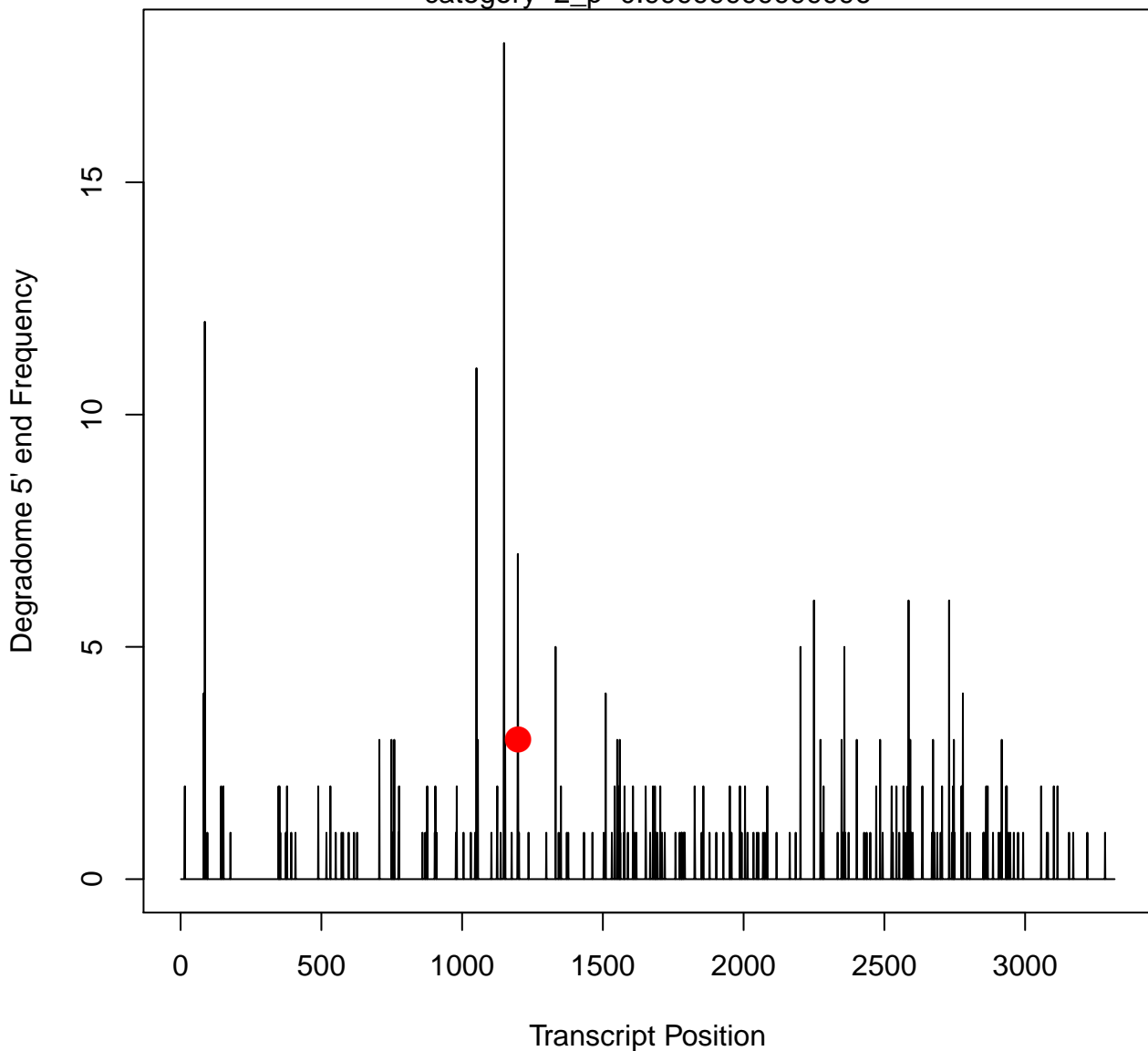

Supplement: Supplementary file 2 [file Data_Sheet_2.zip › Sit-miR159a_Seita.3G096800.1_1199_TPlot.pdf]

**T=Seita.3G103400.1\_Q=Sit-miR159a\_S=1570**

category=2\_p=0.99999999950475

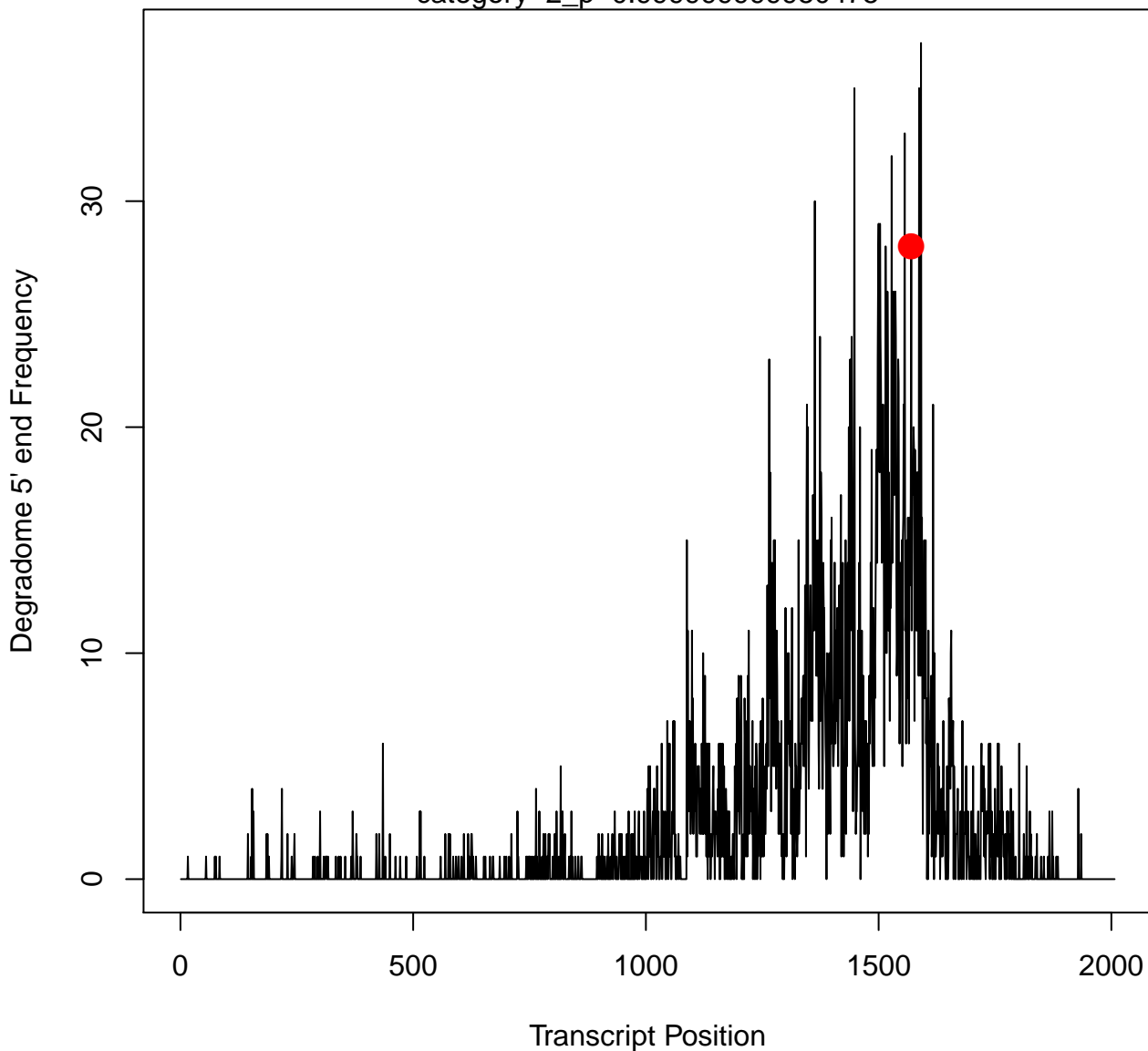

Supplement: Supplementary file 2 [file Data_Sheet_2.zip › Sit-miR159a_Seita.3G103400.1_1570_TPlot.pdf]

**T=Seita.3G125200.1\_Q=Sit-miR159a\_S=6511**

category=2\_p=0.999999999999382

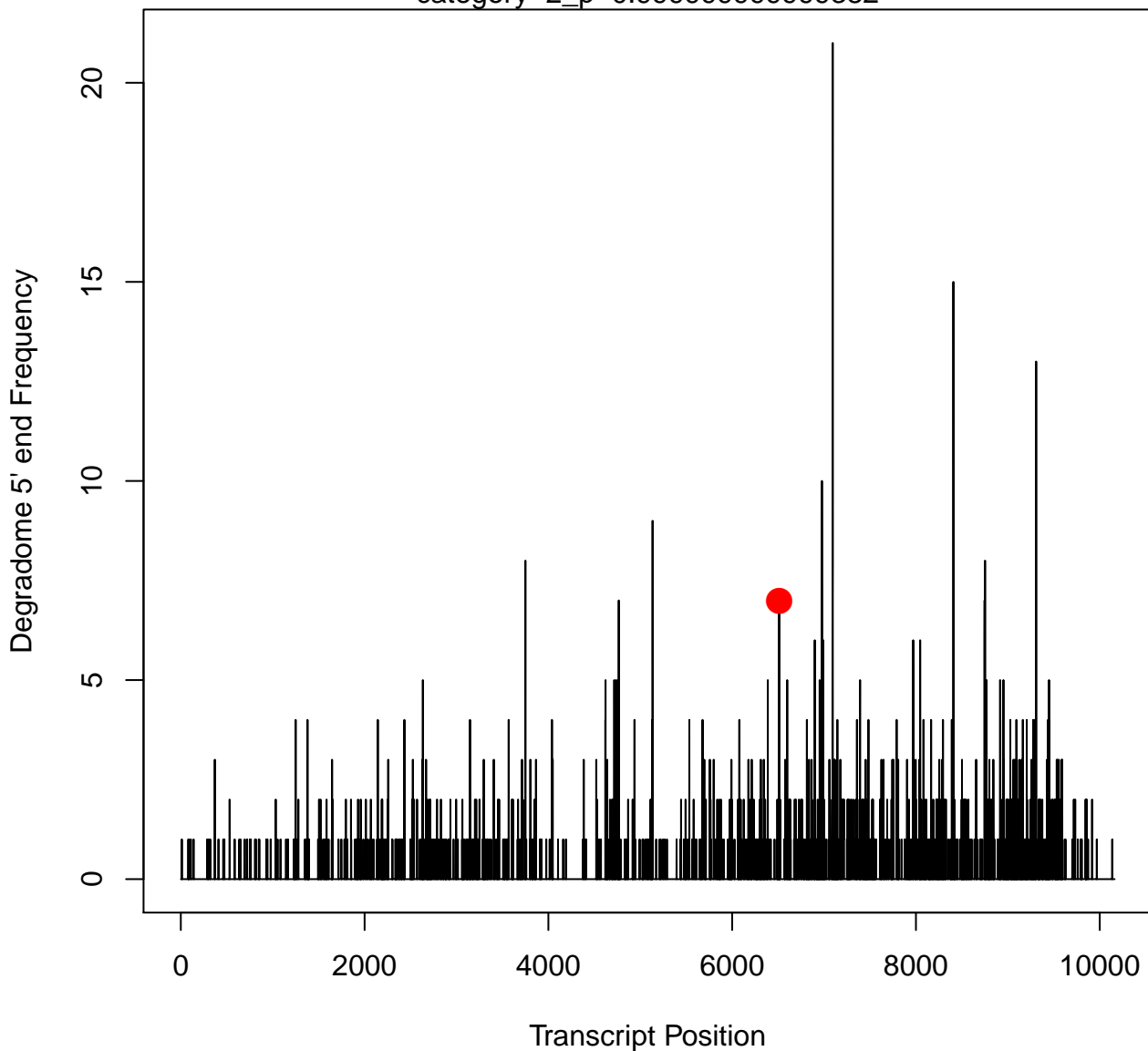

Supplement: Supplementary file 2 [file Data_Sheet_2.zip › Sit-miR159a_Seita.3G125200.1_6511_TPlot.pdf]

**T=Seita.3G182700.1\_Q=Sit-miR159a\_S=1027**

category=2\_p=0.999999999998472

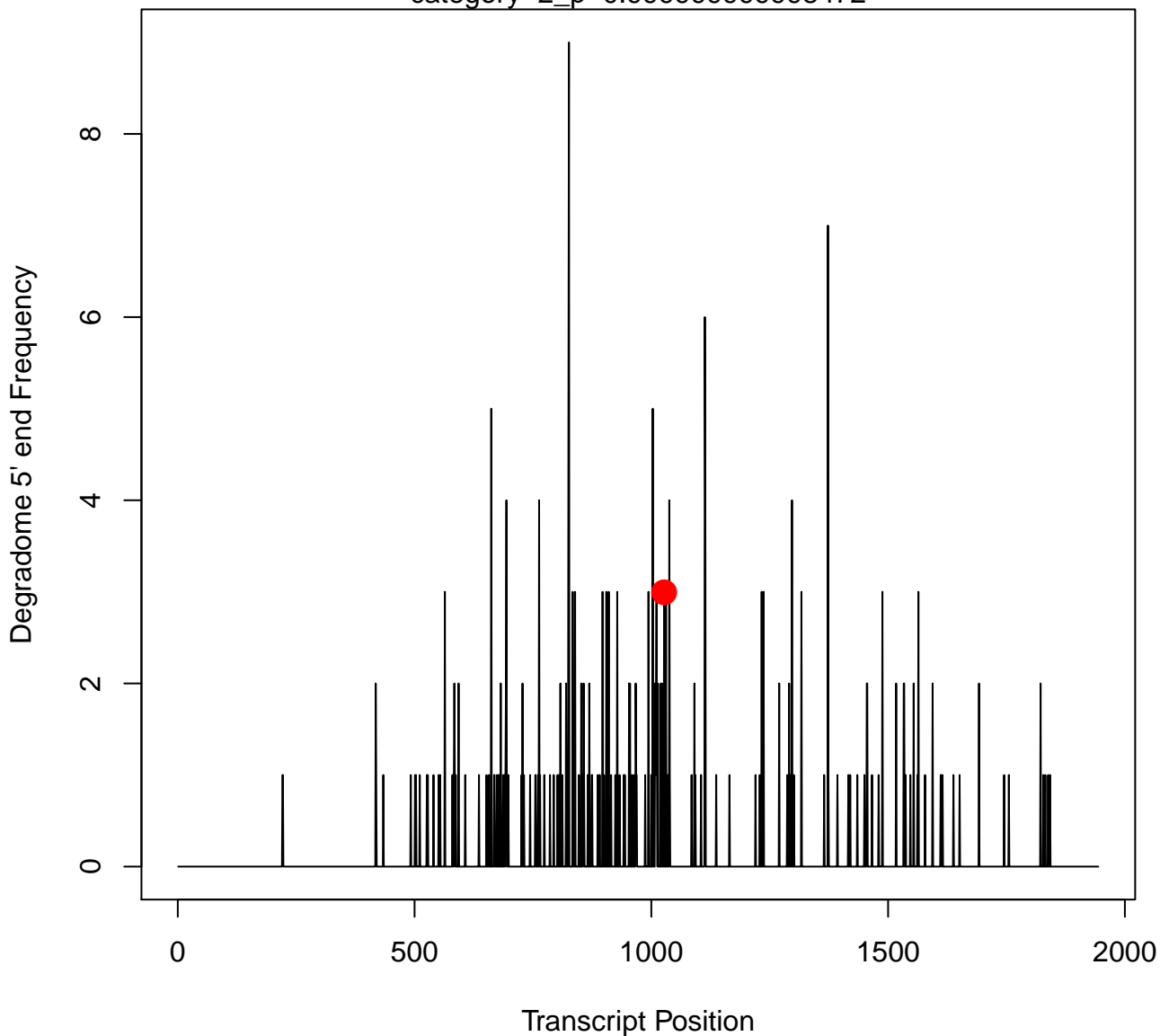

Supplement: Supplementary file 2 [file Data_Sheet_2.zip › Sit-miR159a_Seita.3G182700.1_1027_TPlot.pdf]

**T=Seita.4G020800.1\_Q=Sit-miR159a\_S=284**

category=2\_p=0.999999999999992

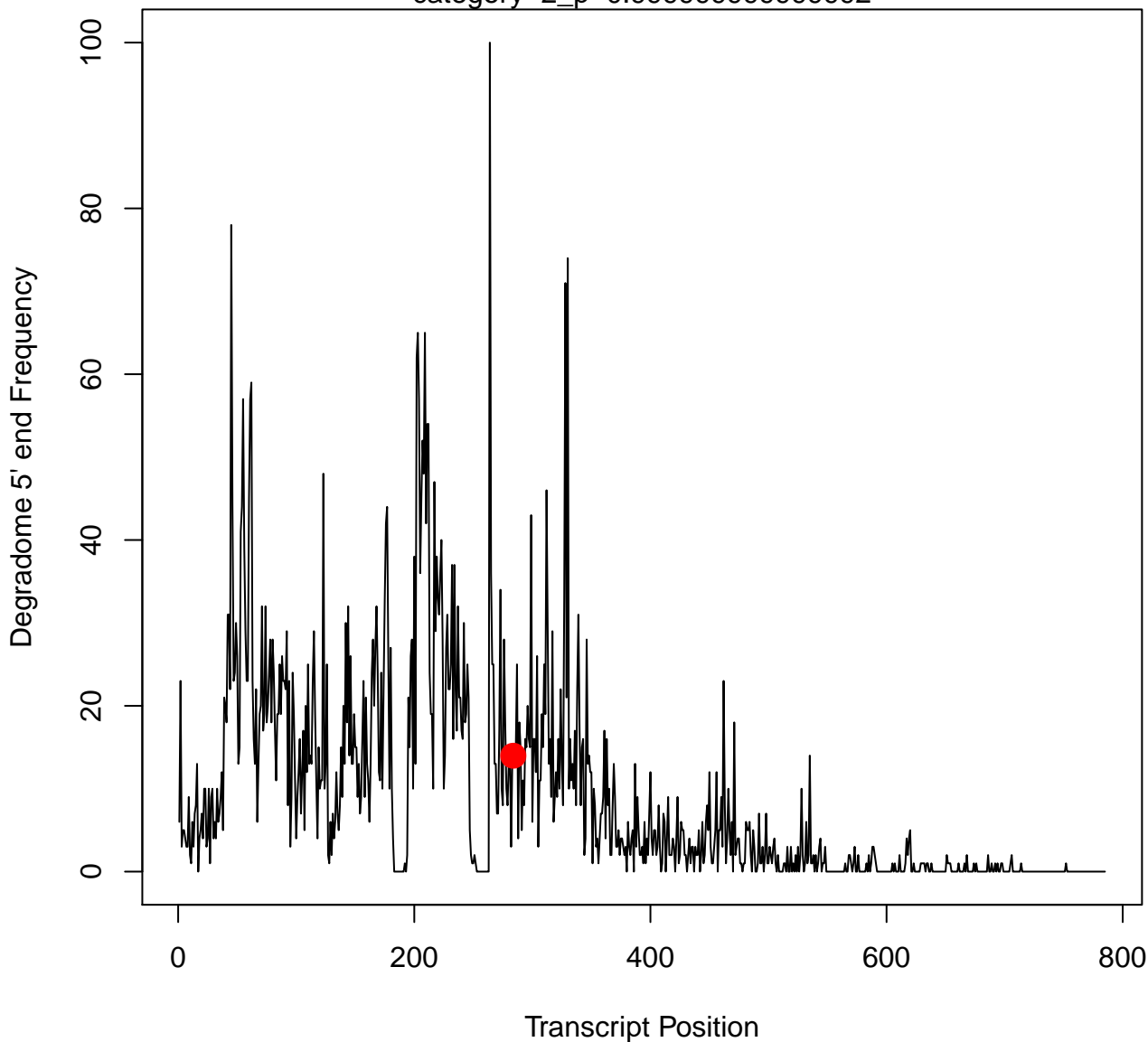

Supplement: Supplementary file 2 [file Data_Sheet_2.zip › Sit-miR159a_Seita.4G020800.1_284_TPlot.pdf]

**T=Seita.4G083900.1\_Q=Sit-miR159a\_S=4243**

category=2\_p=0.770226333731788

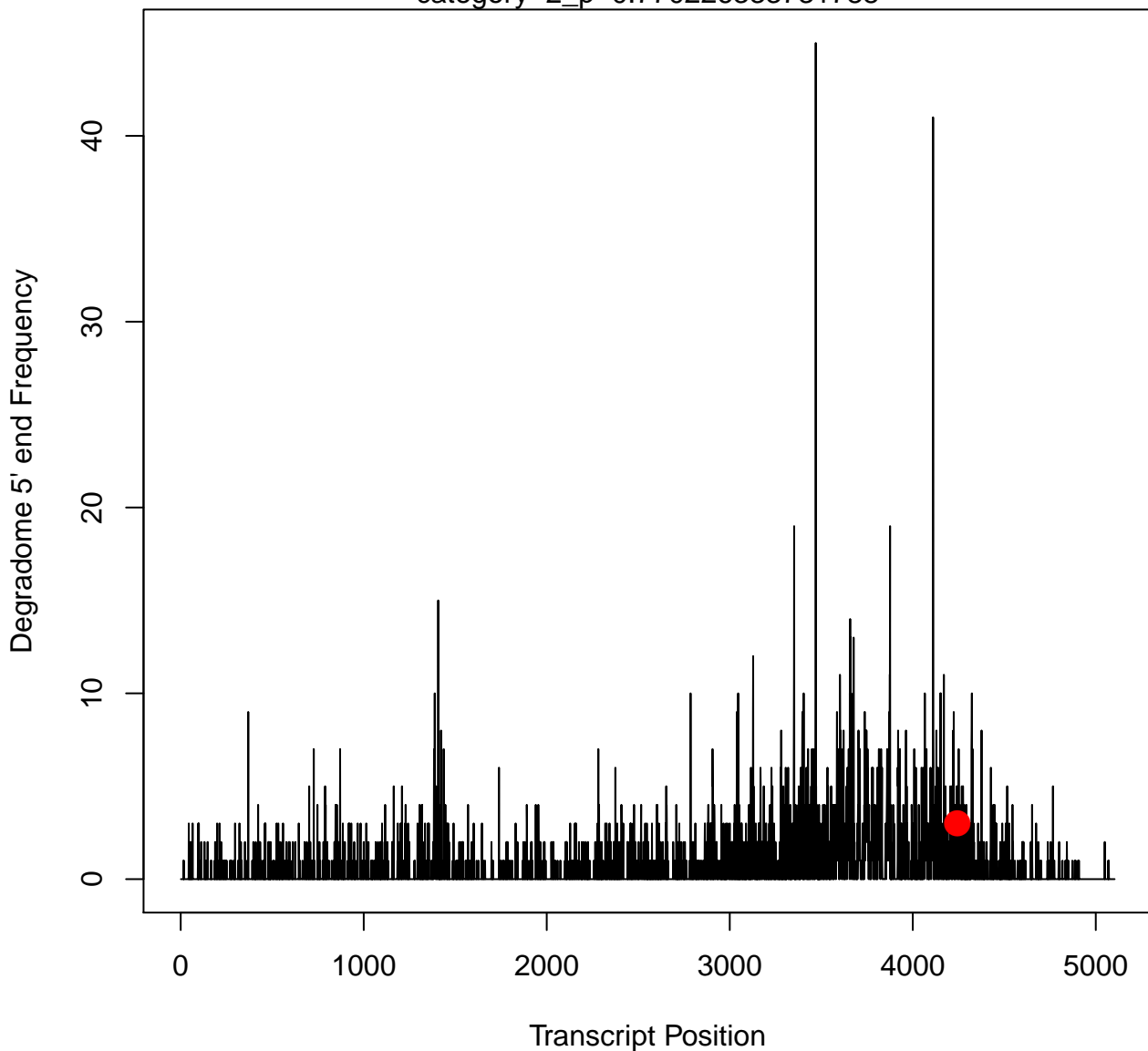

Supplement: Supplementary file 2 [file Data_Sheet_2.zip › Sit-miR159a_Seita.4G083900.1_4243_TPlot.pdf]

**T=Seita.4G265500.1\_Q=Sit-miR159a\_S=936**

category=2\_p=0.999074926572728

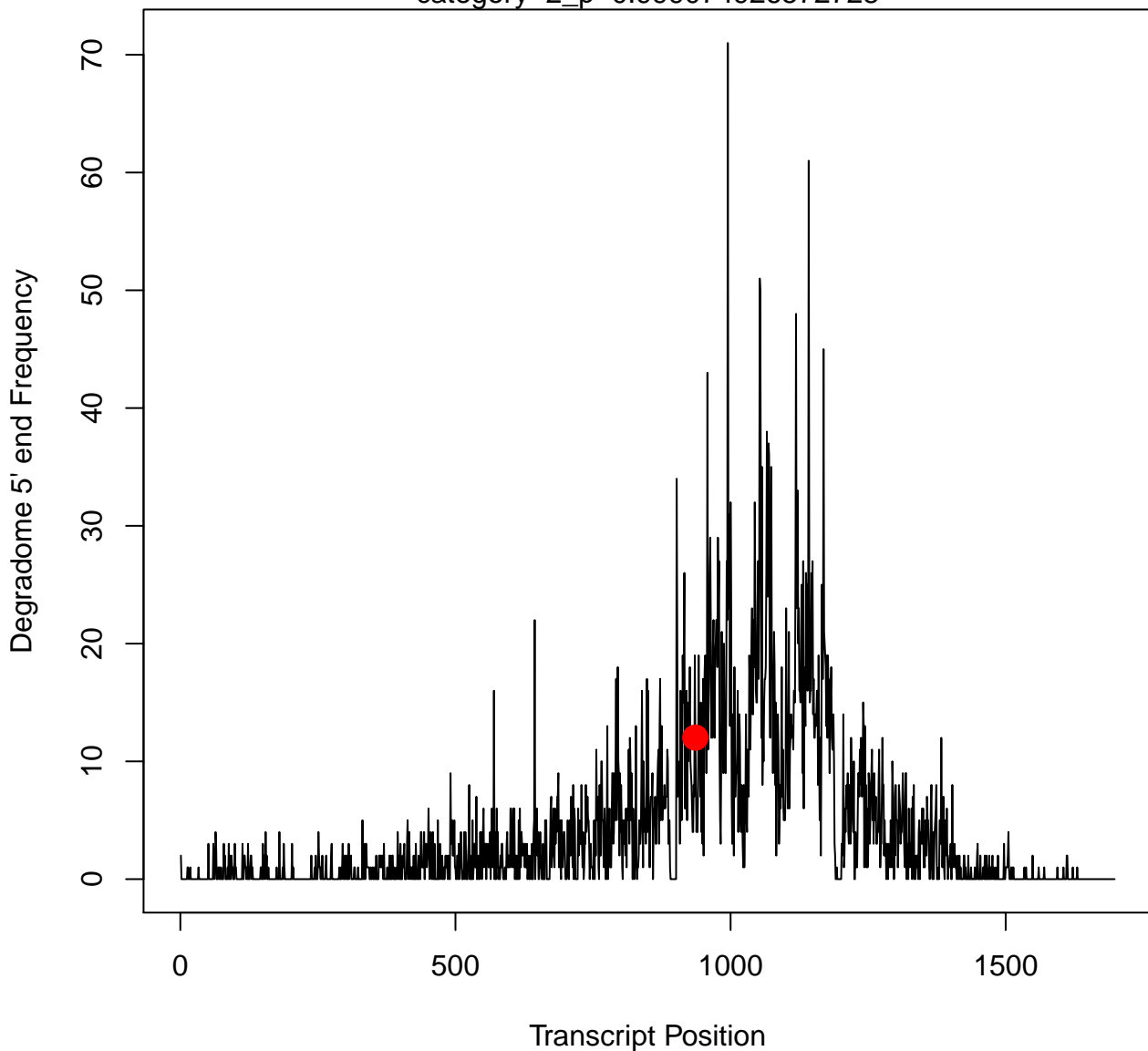

Supplement: Supplementary file 2 [file Data_Sheet_2.zip › Sit-miR159a_Seita.4G265500.1_936_TPlot.pdf]

**T=Seita.5G046700.1\_Q=Sit-miR159a\_S=479**

category=2\_p=0.999998163525204

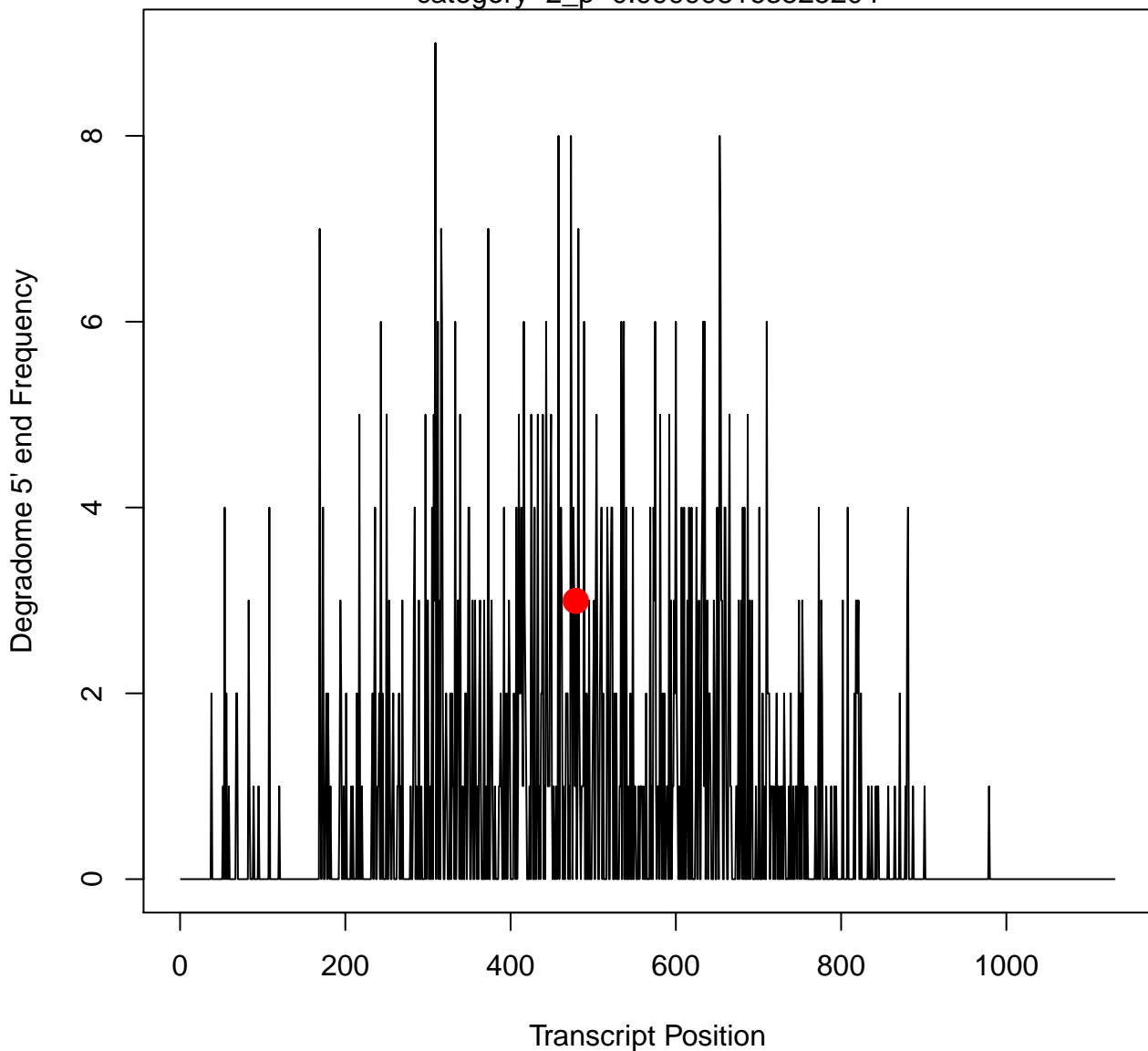

Supplement: Supplementary file 2 [file Data_Sheet_2.zip › Sit-miR159a_Seita.5G046700.1_479_TPlot.pdf]

**T=Seita.5G149100.1\_Q=Sit-miR159a\_S=1902**

category=0\_p=0.00959302769705017

Degradome 5' end Frequency

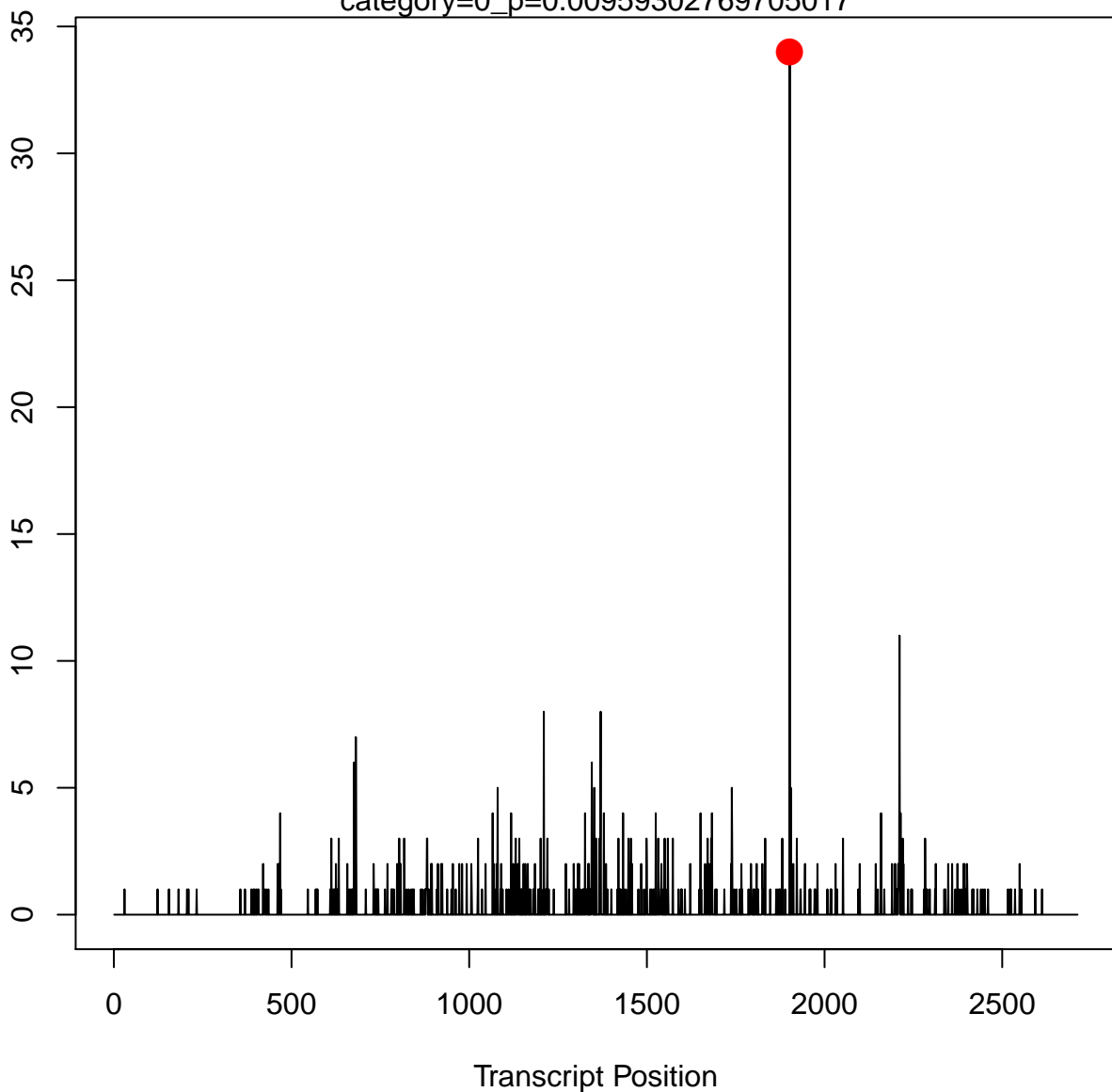

Supplement: Supplementary file 2 [file Data_Sheet_2.zip › Sit-miR159a_Seita.5G149100.1_1902_TPlot.pdf]

**T=Seita.5G210200.1\_Q=Sit-miR159a\_S=1203**

category=2\_p=0.999999999974158

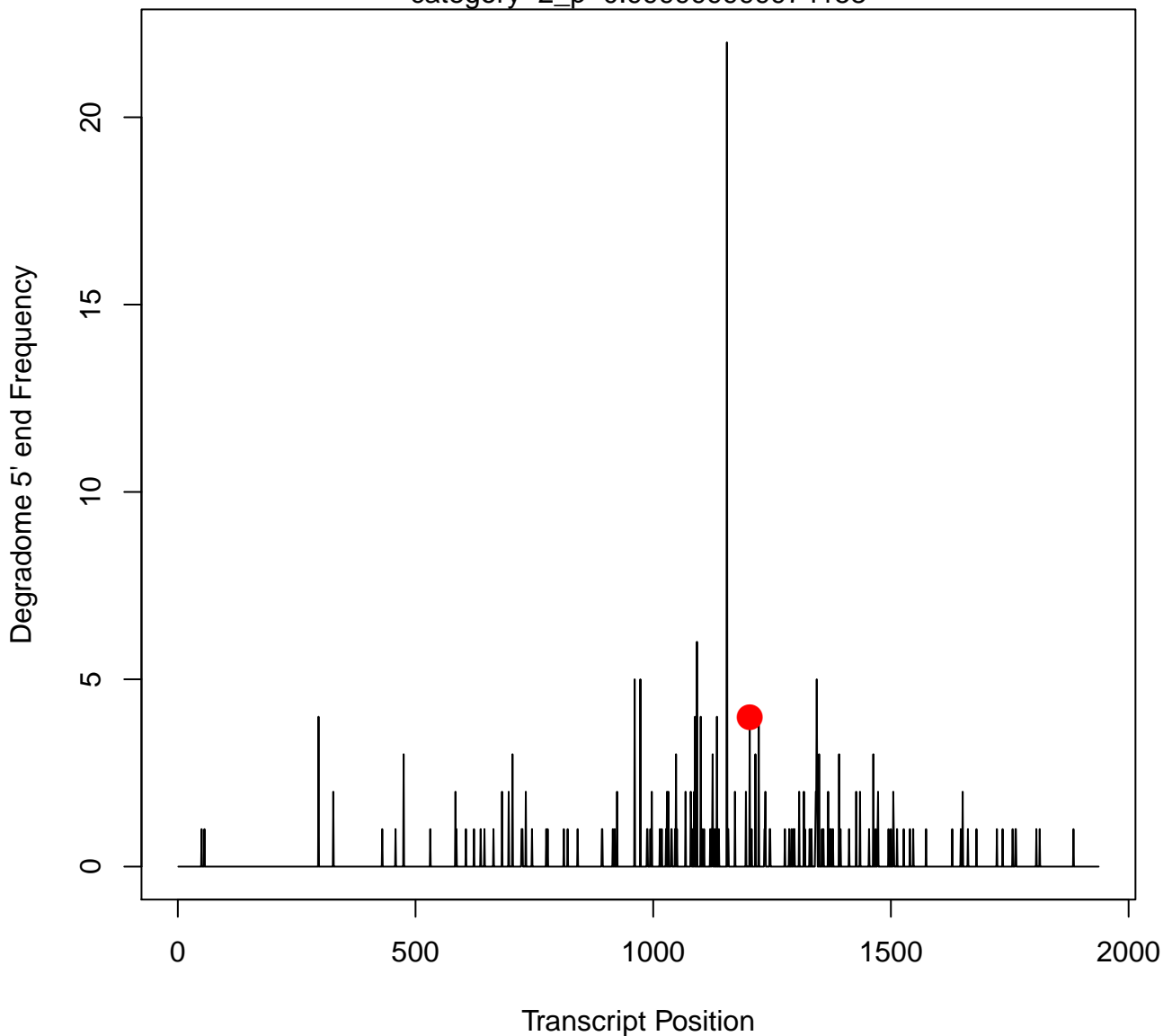

Supplement: Supplementary file 2 [file Data_Sheet_2.zip › Sit-miR159a_Seita.5G210200.1_1203_TPlot.pdf]

**T=Seita.5G395000.1\_Q=Sit-miR159a\_S=3104**

category=2\_p=0.995229410184612

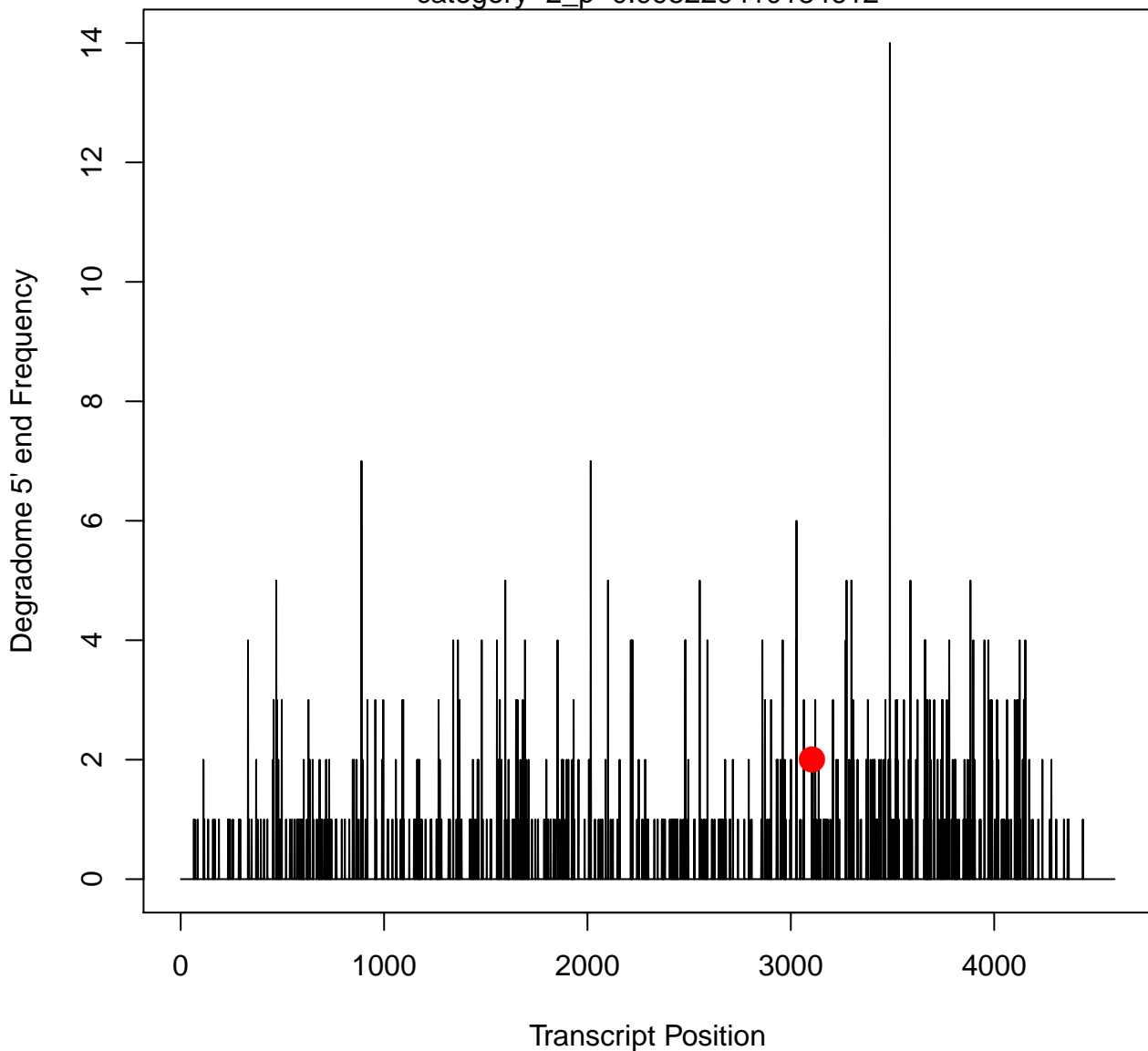

Supplement: Supplementary file 2 [file Data_Sheet_2.zip › Sit-miR159a_Seita.5G395000.1_3104_TPlot.pdf]

**T=Seita.6G009100.1\_Q=Sit-miR159a\_S=509**

category=2\_p=0.999999998150692

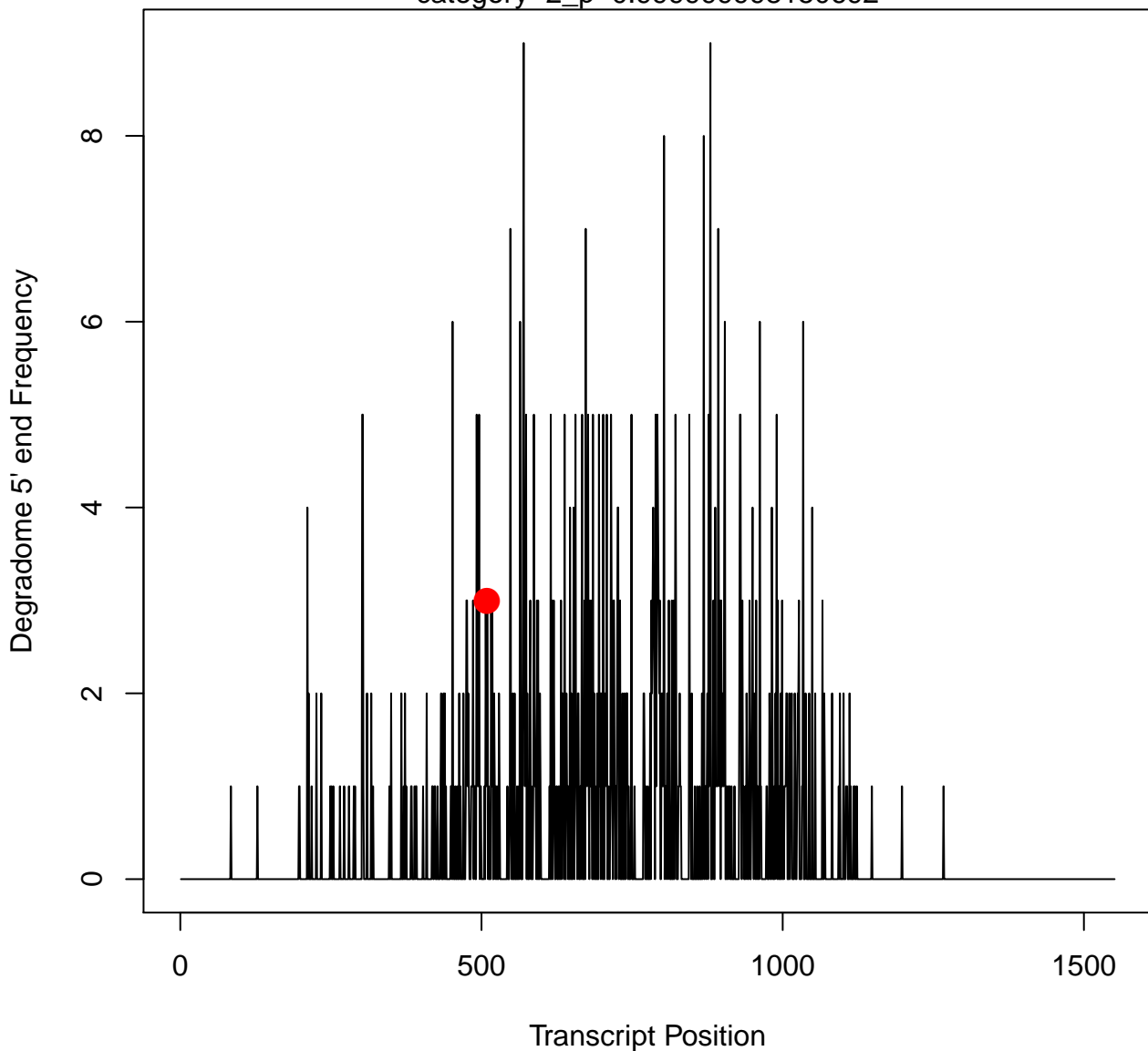

Supplement: Supplementary file 2 [file Data_Sheet_2.zip › Sit-miR159a_Seita.6G009100.1_509_TPlot.pdf]

**T=Seita.6G063700.1\_Q=Sit-miR159a\_S=883**

category=2\_p=0.999999999999962

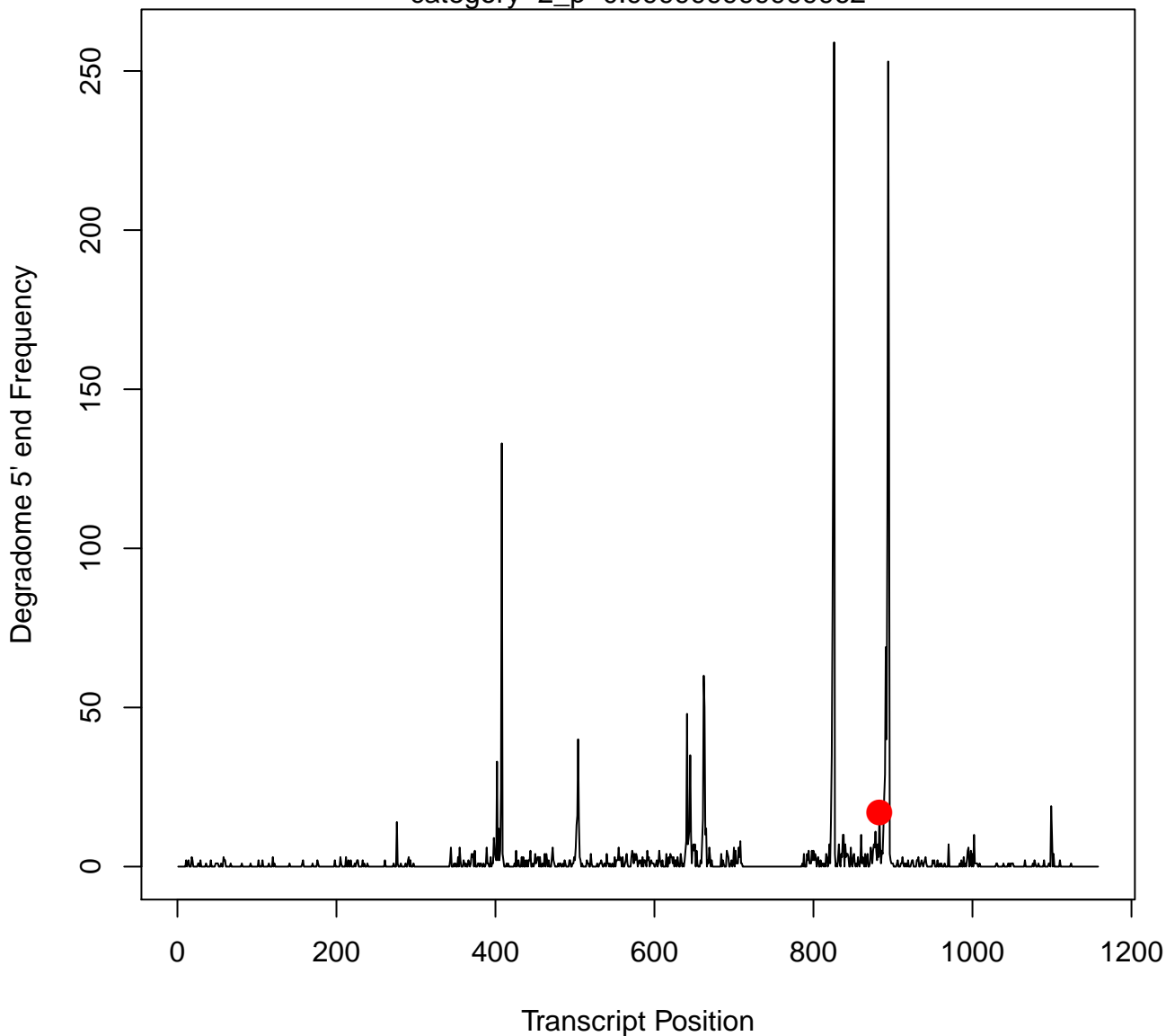

Supplement: Supplementary file 2 [file Data_Sheet_2.zip › Sit-miR159a_Seita.6G063700.1_883_TPlot.pdf]

**T=Seita.7G075500.1\_Q=Sit-miR159a\_S=599**

category=2\_p=0.9999999999999836

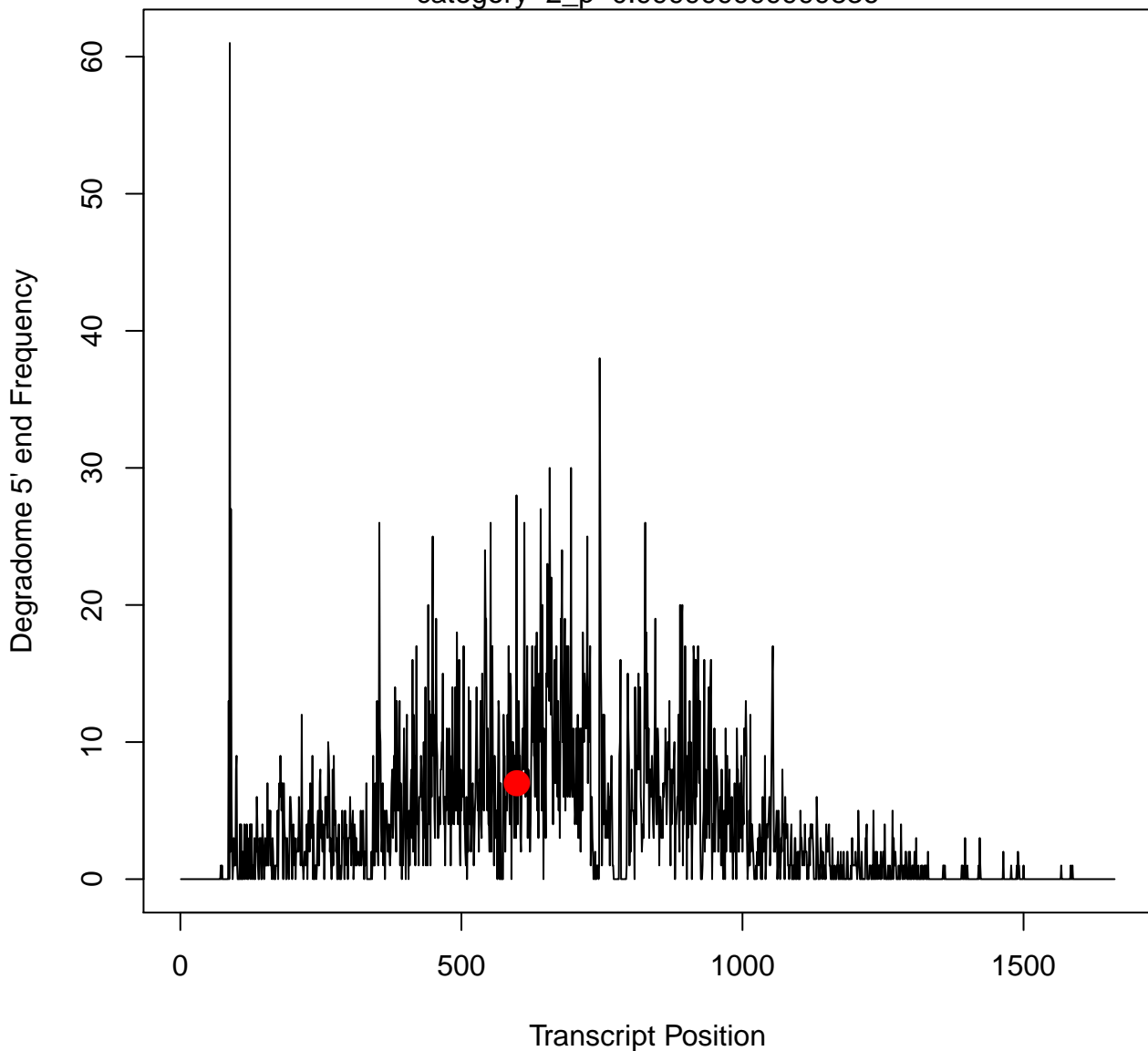

Supplement: Supplementary file 2 [file Data_Sheet_2.zip › Sit-miR159a_Seita.7G075500.1_599_TPlot.pdf]

**T=Seita.7G219300.1\_Q=Sit-miR159a\_S=2404**

category=2\_p=0.999999888313954

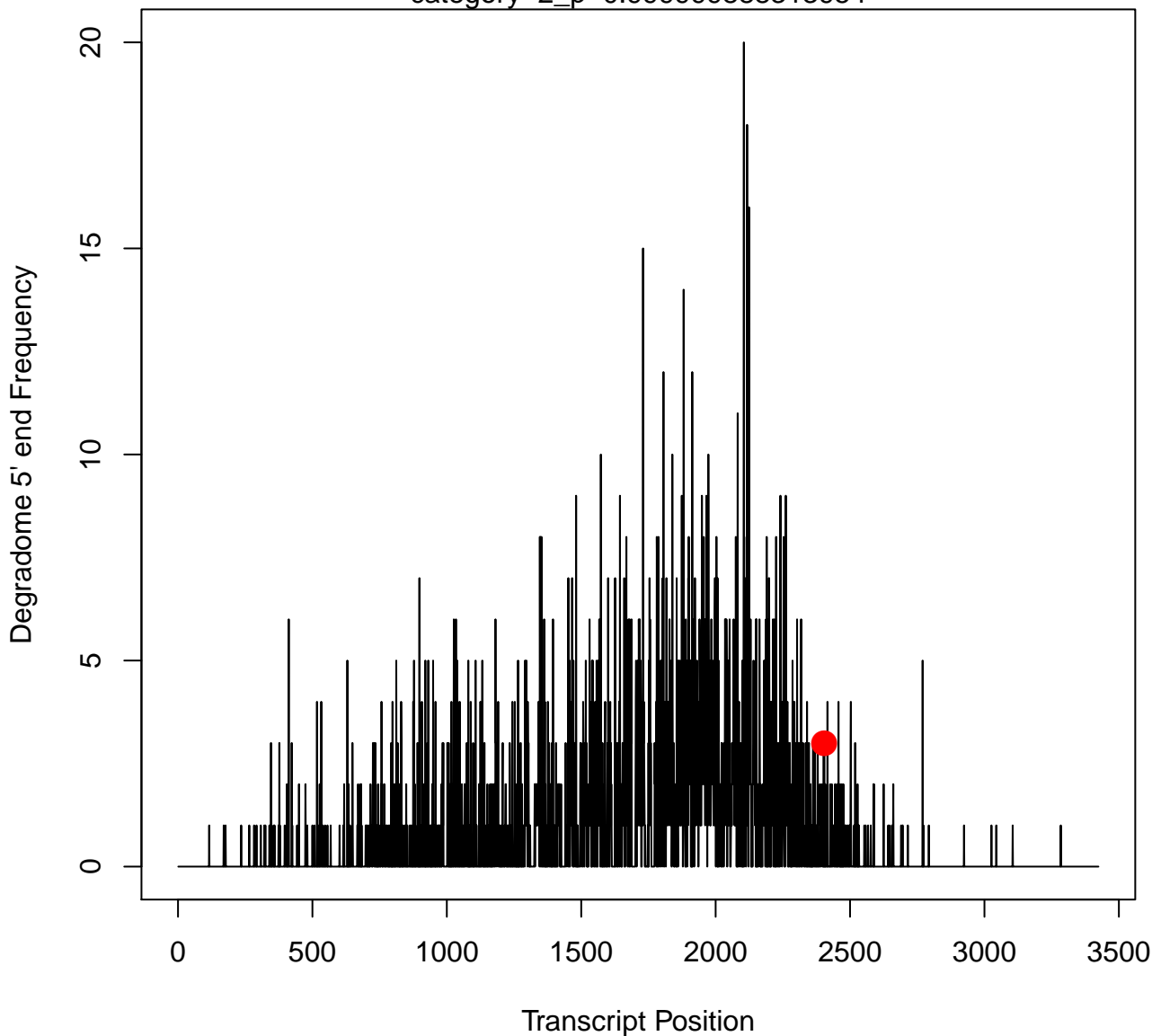

Supplement: Supplementary file 2 [file Data_Sheet_2.zip › Sit-miR159a_Seita.7G219300.1_2404_TPlot.pdf]

**T=Seita.7G254600.1\_Q=Sit-miR159a\_S=1602**

category=2\_p=0.9999999999999988

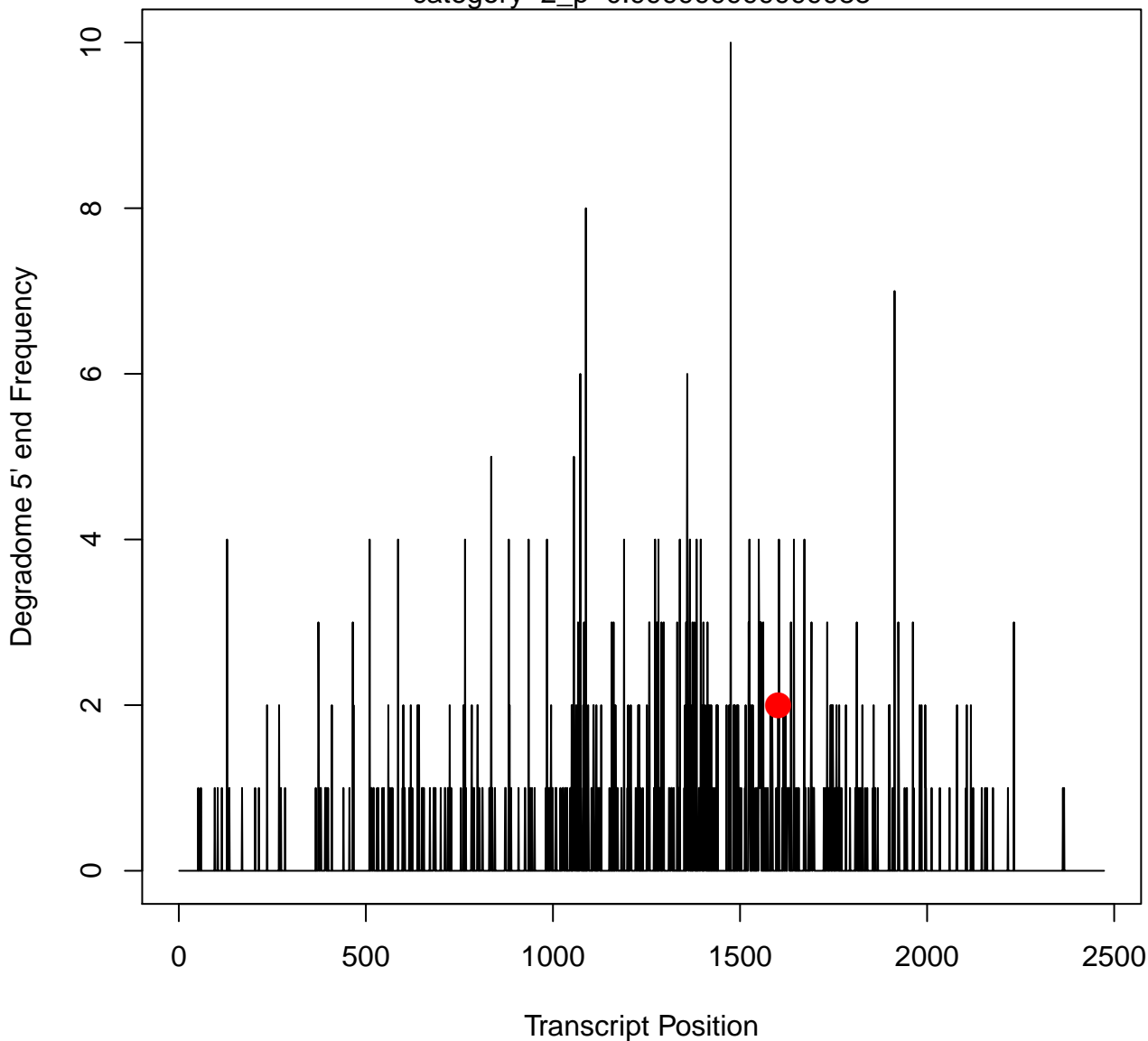

Supplement: Supplementary file 2 [file Data_Sheet_2.zip › Sit-miR159a_Seita.7G254600.1_1602_TPlot.pdf]

**T=Seita.7G311500.1\_Q=Sit-miR159a\_S=313**

category=2\_p=0.999885907436933

Degradome 5' end Frequency

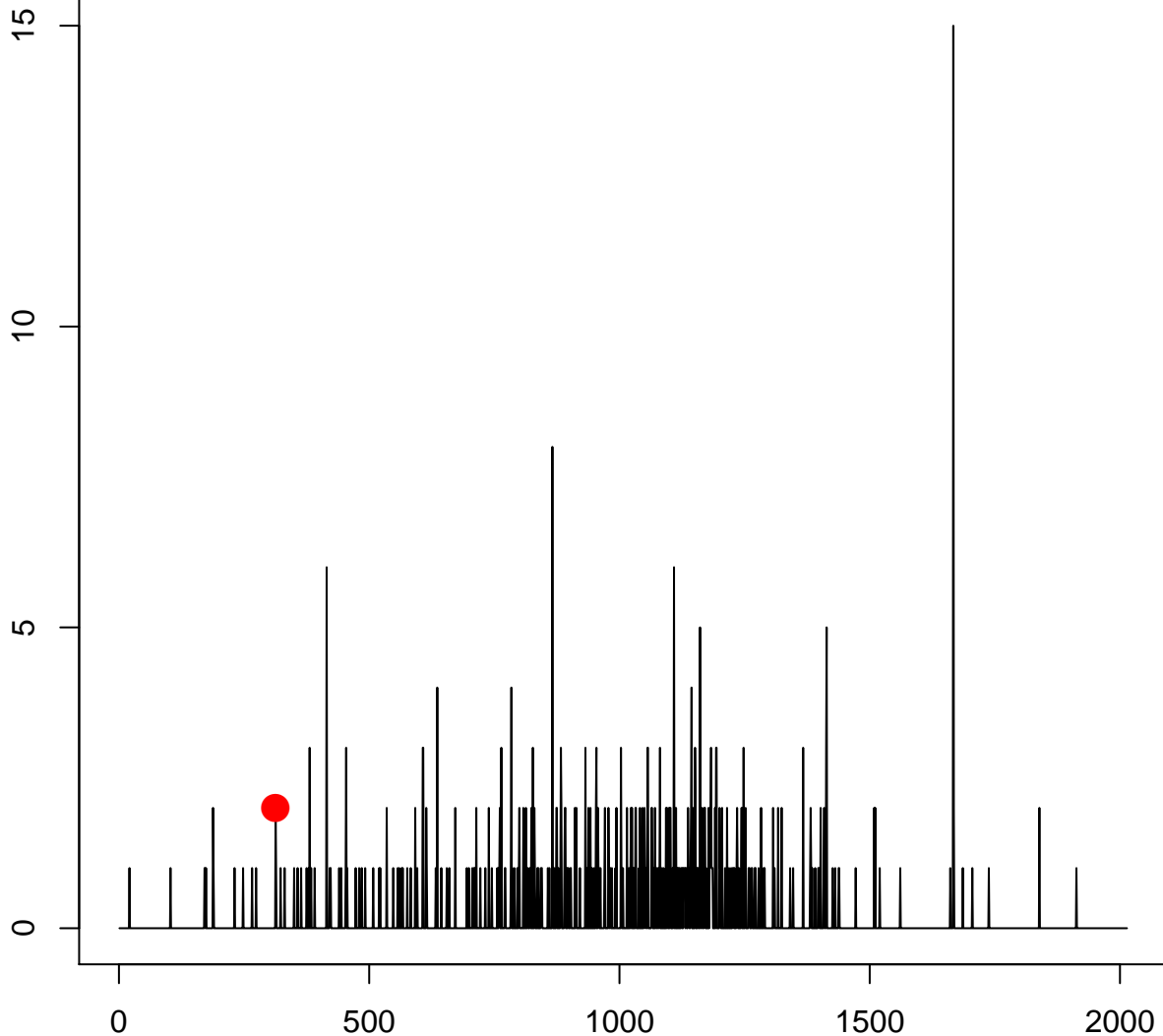

Transcript Position

Supplement: Supplementary file 2 [file Data_Sheet_2.zip › Sit-miR159a_Seita.7G311500.1_313_TPlot.pdf]

**T=Seita.9G120100.1\_Q=Sit-miR159a\_S=599**

category=2\_p=0.999999999999607

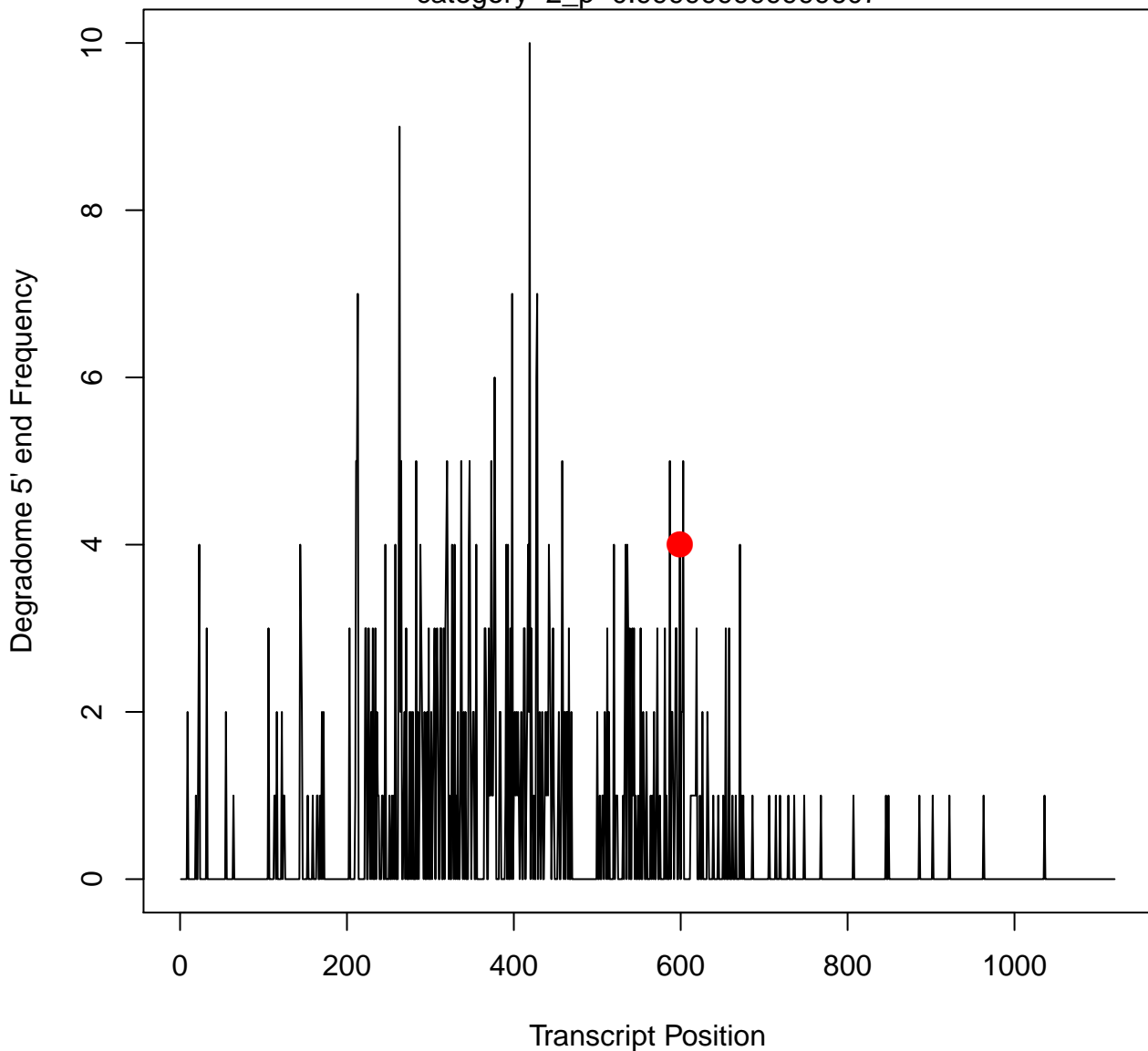

Supplement: Supplementary file 2 [file Data_Sheet_2.zip › Sit-miR159a_Seita.9G120100.1_599_TPlot.pdf]

**T=Seita.9G134700.1\_Q=Sit-miR159a\_S=4274**

category=2\_p=1

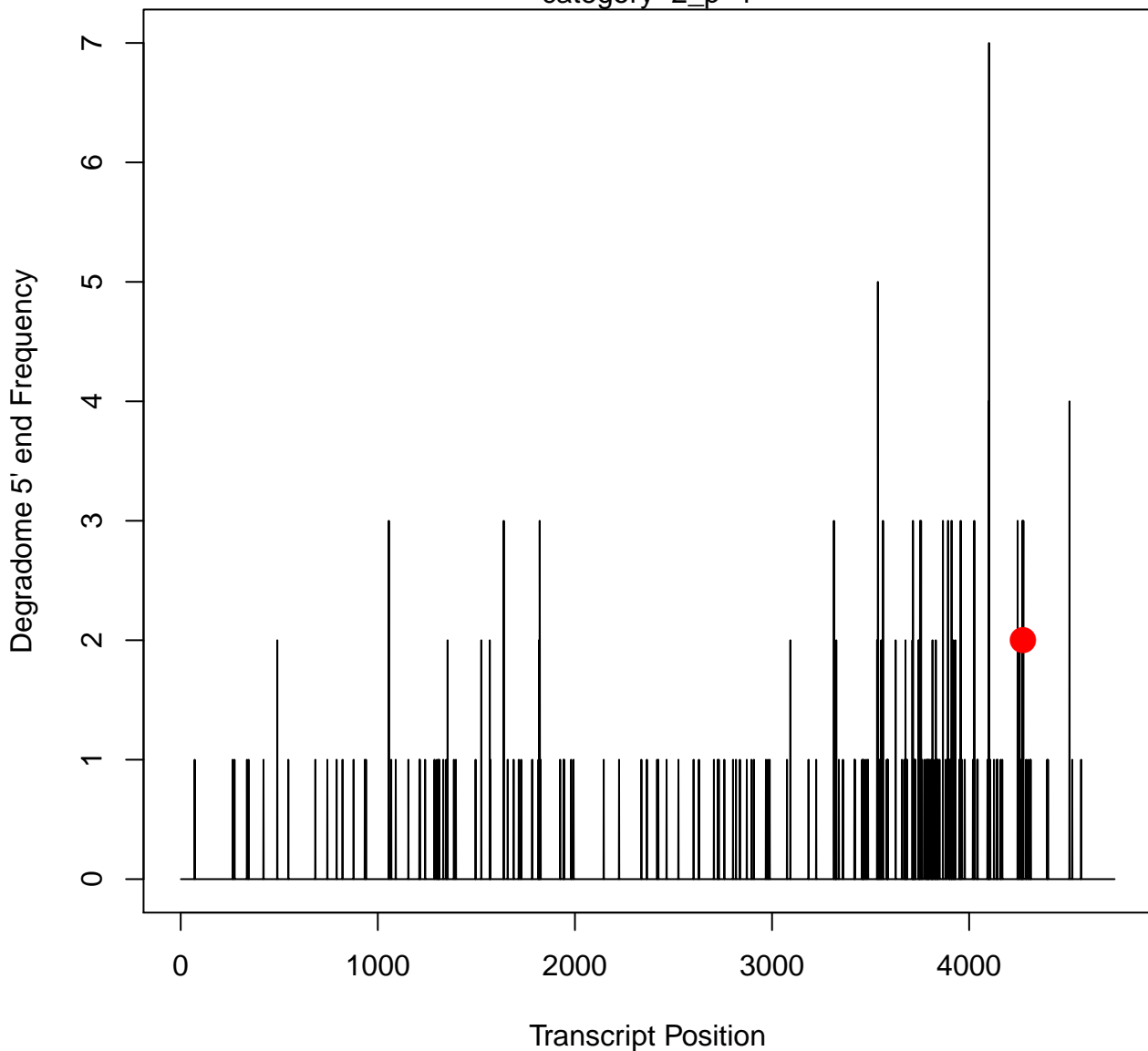

Supplement: Supplementary file 2 [file Data_Sheet_2.zip › Sit-miR159a_Seita.9G134700.1_4274_TPlot.pdf]

**T=Seita.9G224900.1\_Q=Sit-miR159a\_S=950**

category=2\_p=0.999999988699407

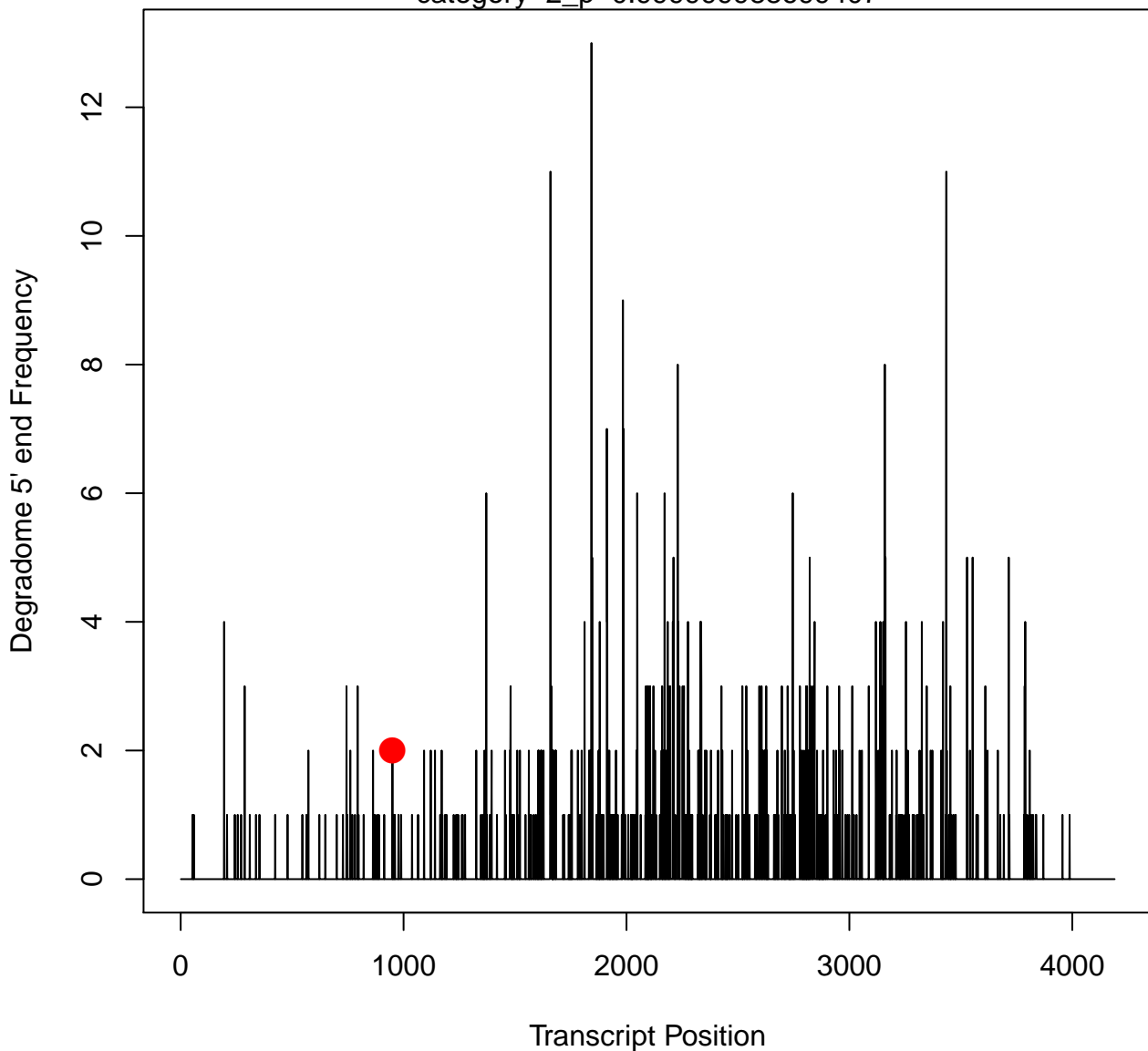

Supplement: Supplementary file 2 [file Data_Sheet_2.zip › Sit-miR159a_Seita.9G224900.1_950_TPlot.pdf]

**T=Seita.9G361700.1\_Q=Sit-miR159a\_S=214**

category=2\_p=0.999999999641397

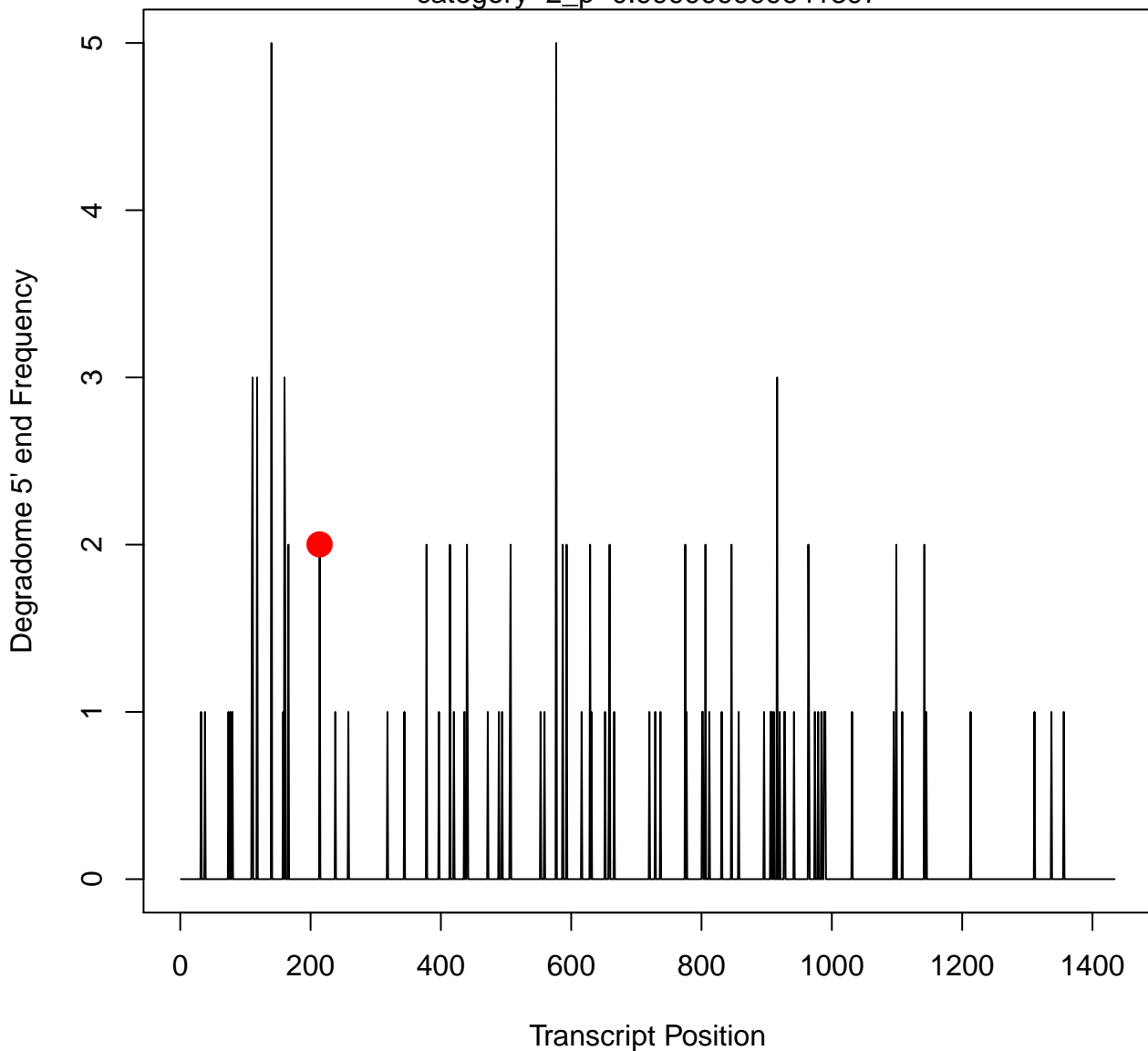

Supplement: Supplementary file 2 [file Data_Sheet_2.zip › Sit-miR159a_Seita.9G361700.1_214_TPlot.pdf]

**T=Seita.9G371700.1\_Q=Sit-miR159a\_S=1867**

category=2\_p=0.999999973601341

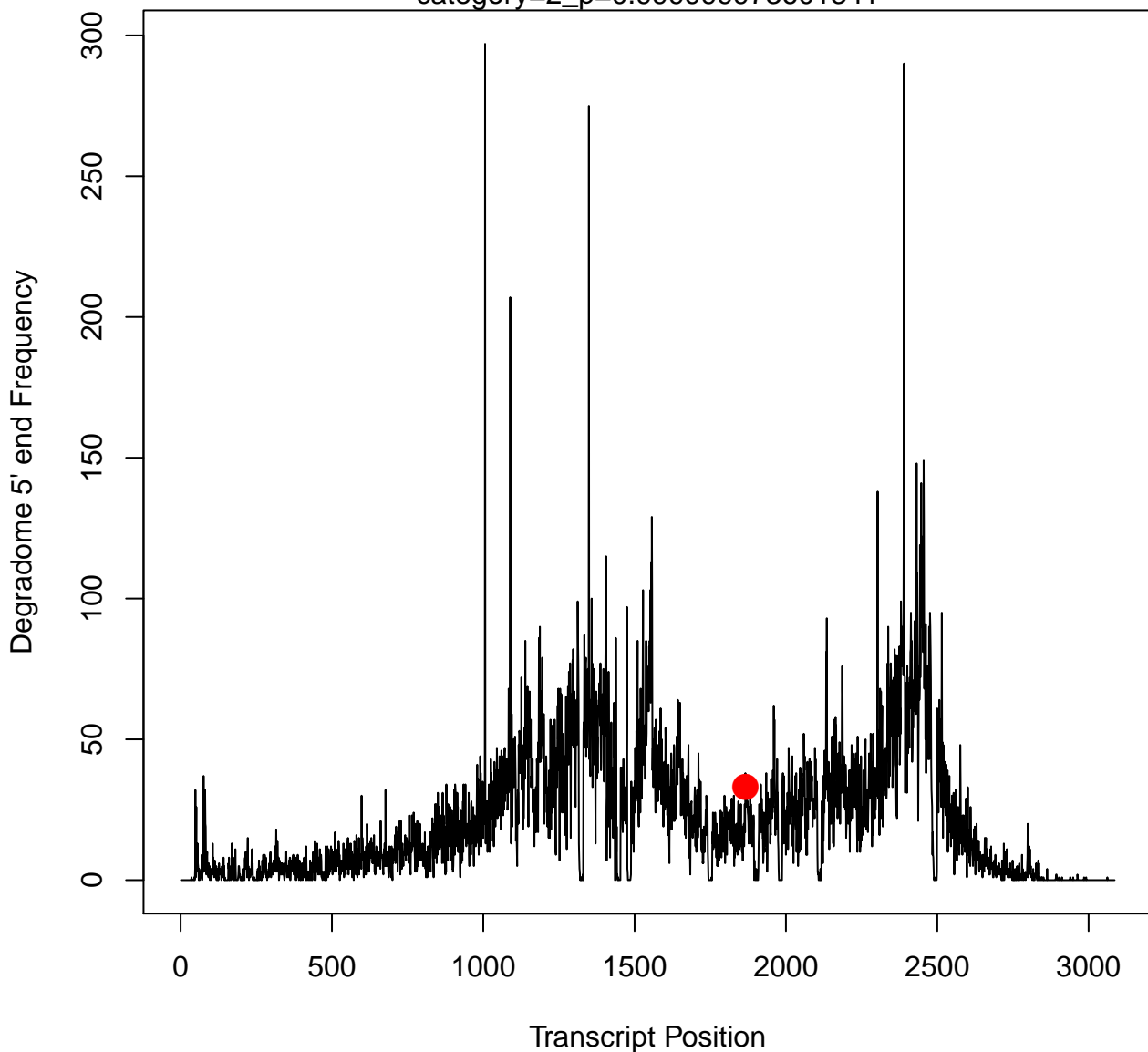

Supplement: Supplementary file 2 [file Data_Sheet_2.zip › Sit-miR159a_Seita.9G371700.1_1867_TPlot.pdf]

**T=Seita.9G459700.1\_Q=Sit-miR159a\_S=93**

category=2\_p=0.997580154834119

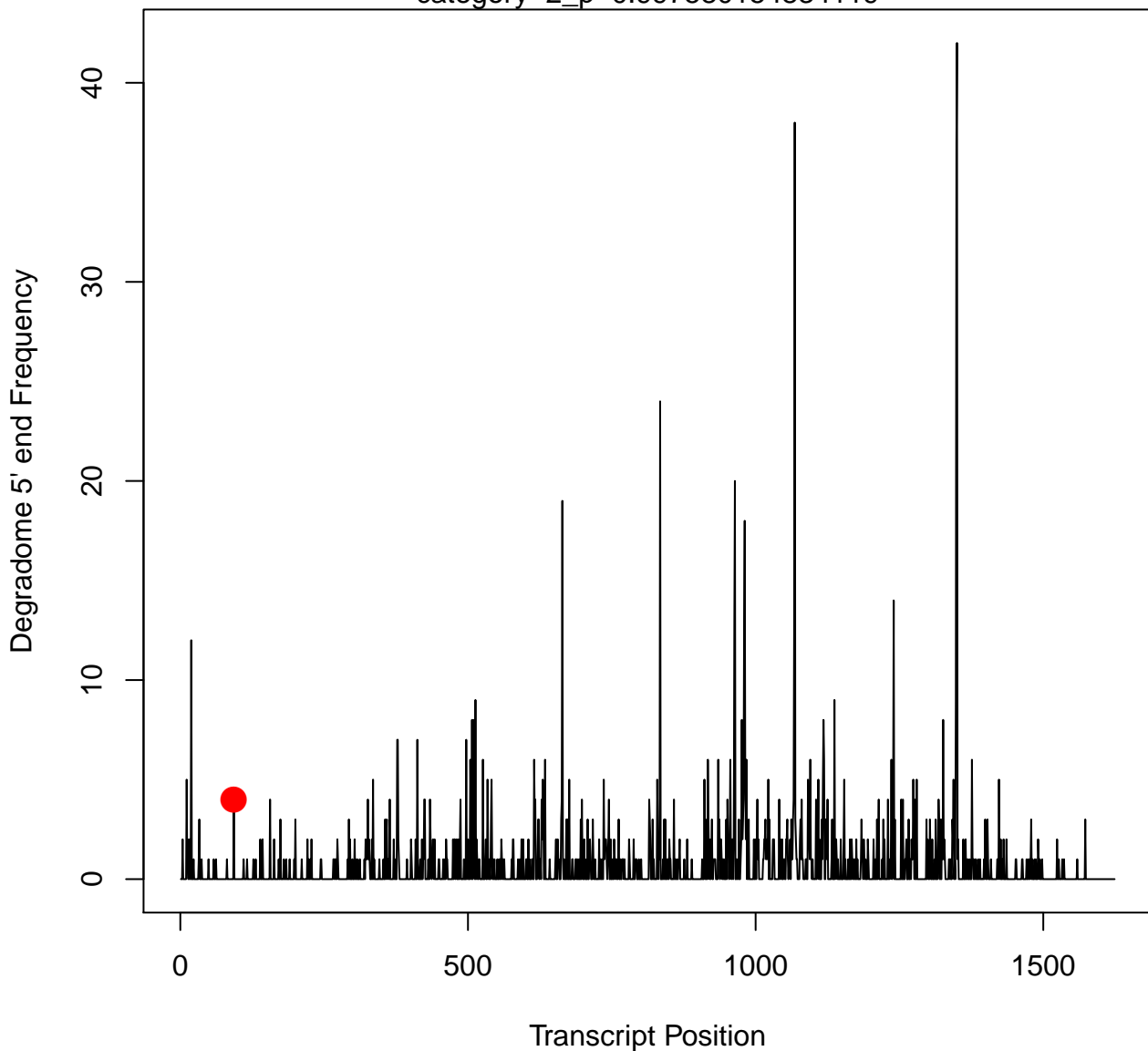

Supplement: Supplementary file 2 [file Data_Sheet_2.zip › Sit-miR159a_Seita.9G459700.1_93_TPlot.pdf]

**T=Seita.9G517700.1\_Q=Sit-miR159a\_S=1633**

category=2\_p=0.999999999924305

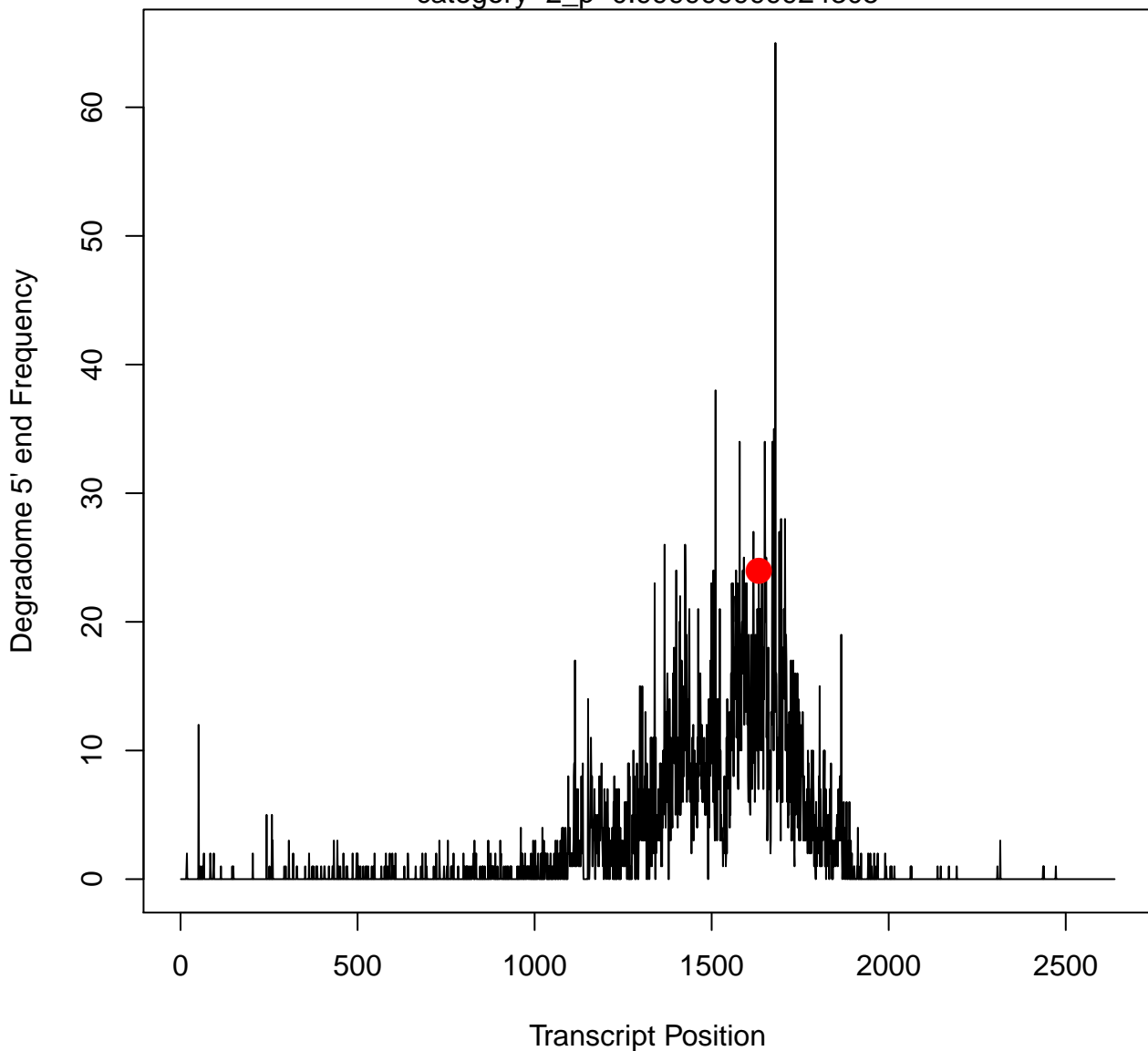

Supplement: Supplementary file 2 [file Data_Sheet_2.zip › Sit-miR159a_Seita.9G517700.1_1633_TPlot.pdf]

**T=Seita.9G552500.1\_Q=Sit-miR159a\_S=789**

category=2\_p=0.478207071361667

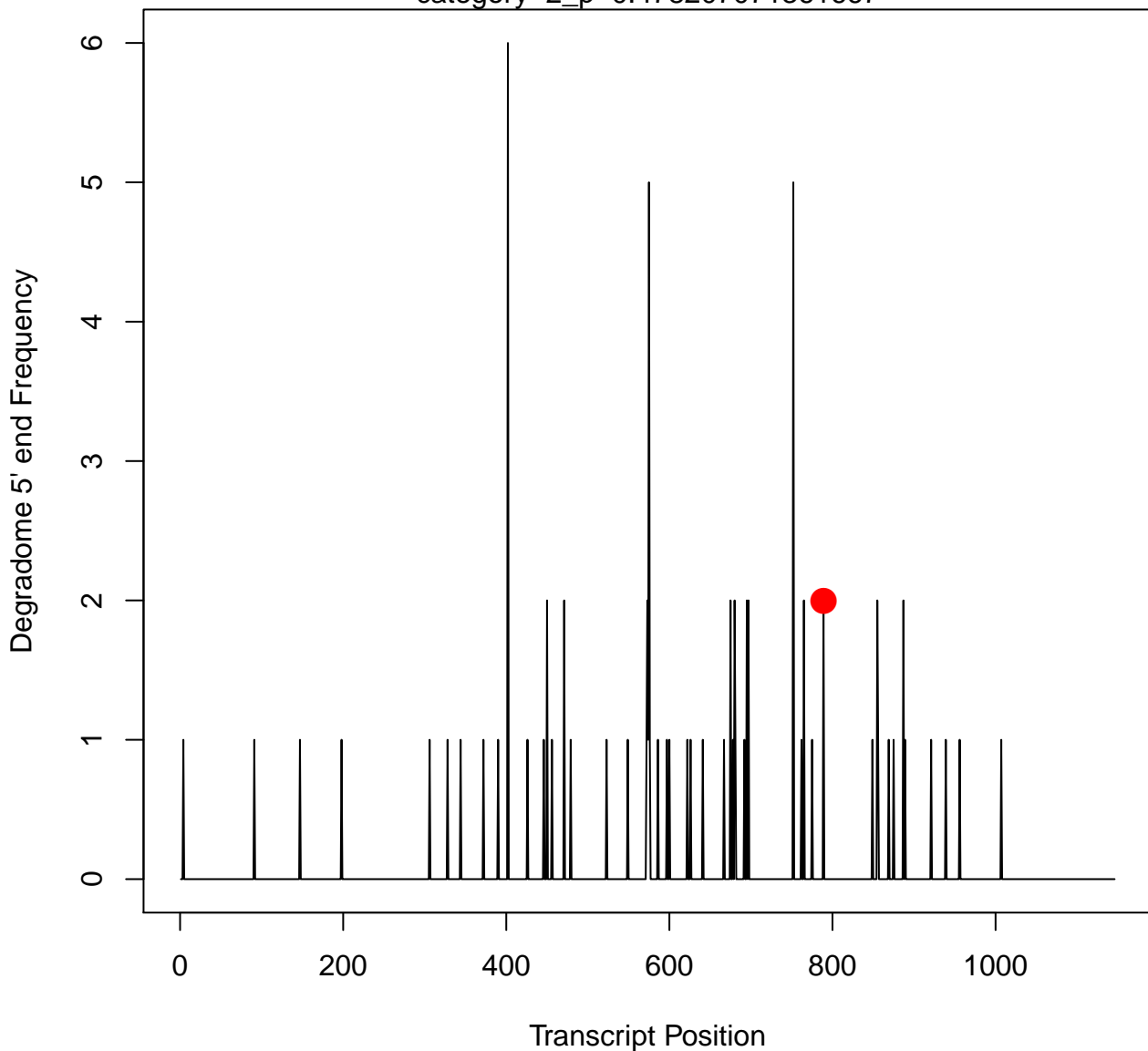

Supplement: Supplementary file 2 [file Data_Sheet_2.zip › Sit-miR159a_Seita.9G552500.1_789_TPlot.pdf]

**T=Seita.9G569300.1\_Q=Sit-miR159a\_S=1398**

category=2\_p=0.999982355881474

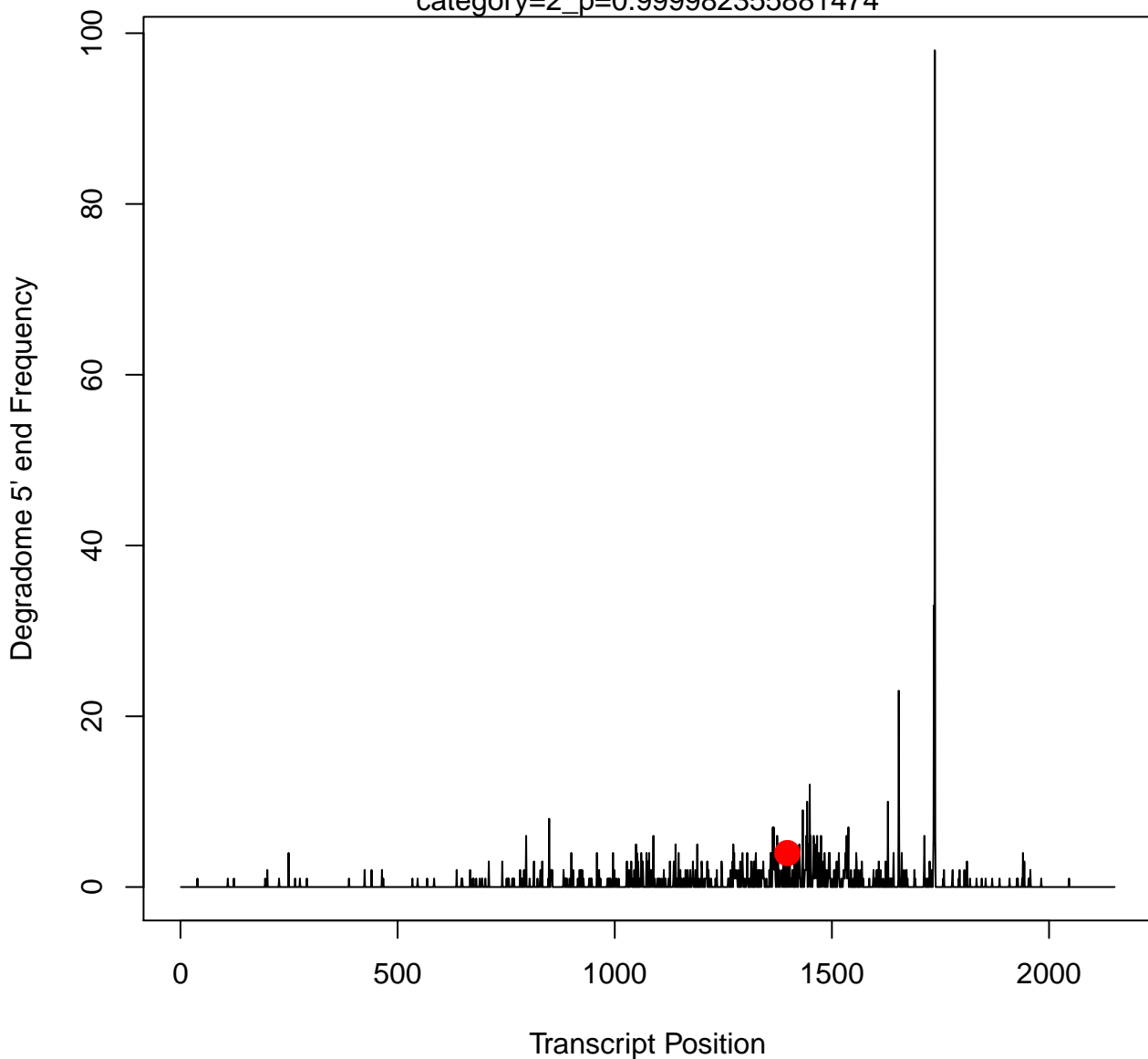

Supplement: Supplementary file 2 [file Data_Sheet_2.zip › Sit-miR159a_Seita.9G569300.1_1398_TPlot.pdf]

**T=Seita.1G093400.1\_Q=Sit-miR159b\_S=3603**

category=2\_p=0.996878726930228

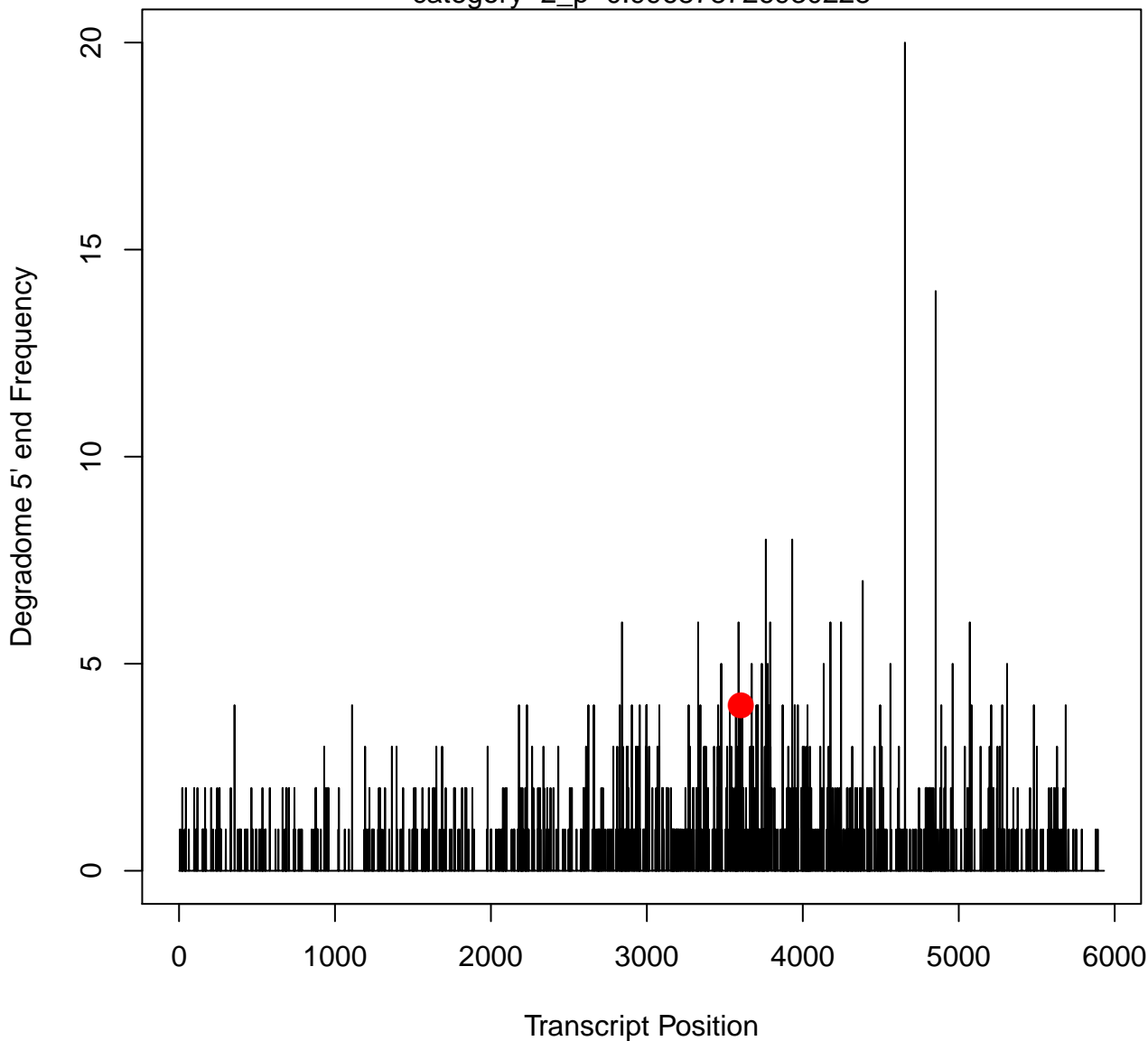

Supplement: Supplementary file 2 [file Data_Sheet_2.zip › Sit-miR159b_Seita.1G093400.1_3603_TPlot.pdf]

**T=Seita.1G348600.1\_Q=Sit-miR159b\_S=4876**

category=2\_p=0.998123954394267

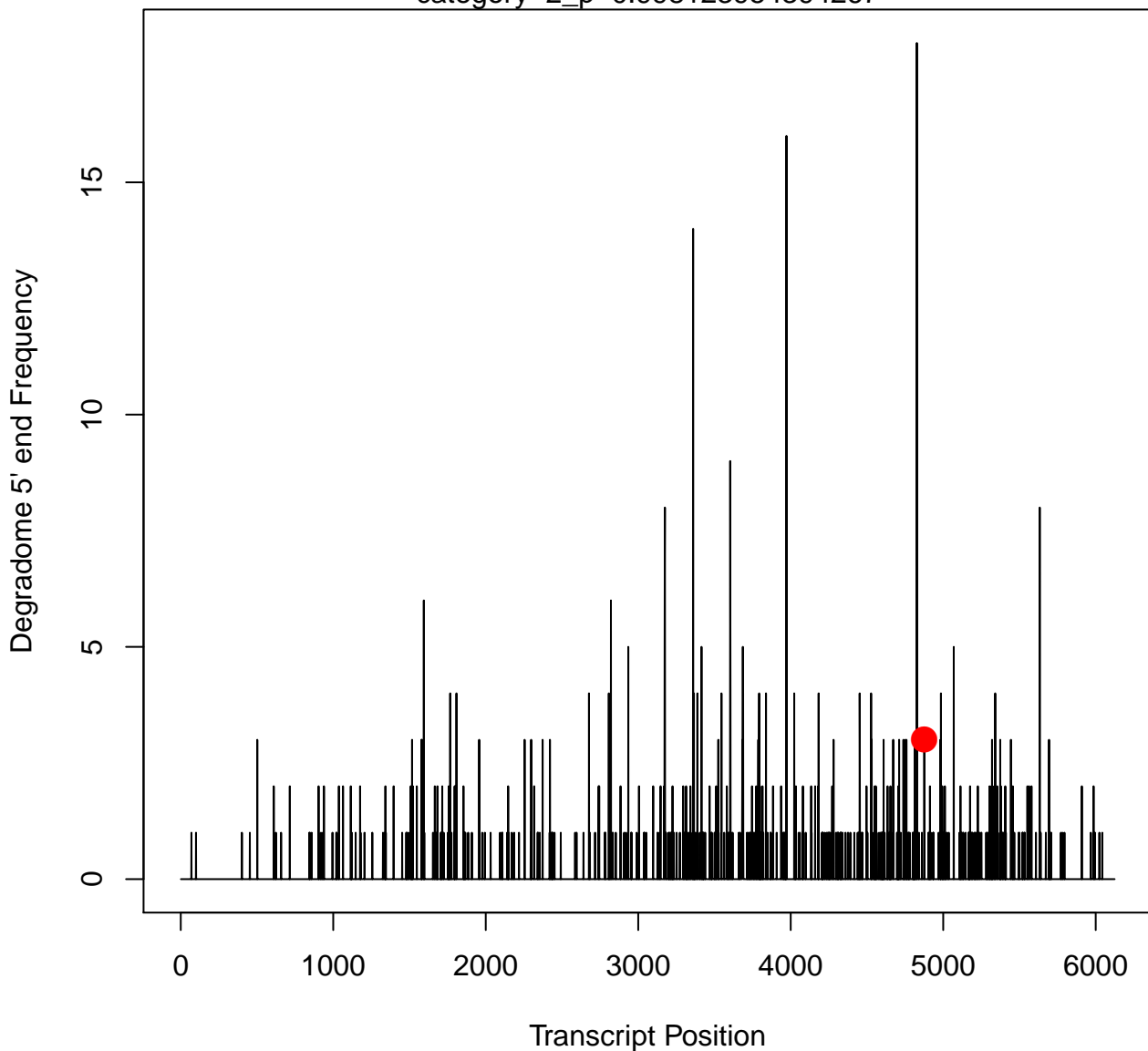

Supplement: Supplementary file 2 [file Data_Sheet_2.zip › Sit-miR159b_Seita.1G348600.1_4876_TPlot.pdf]

**T=Seita.2G434400.1\_Q=Sit-miR159b\_S=363**

category=0\_p=0.0141649387404315

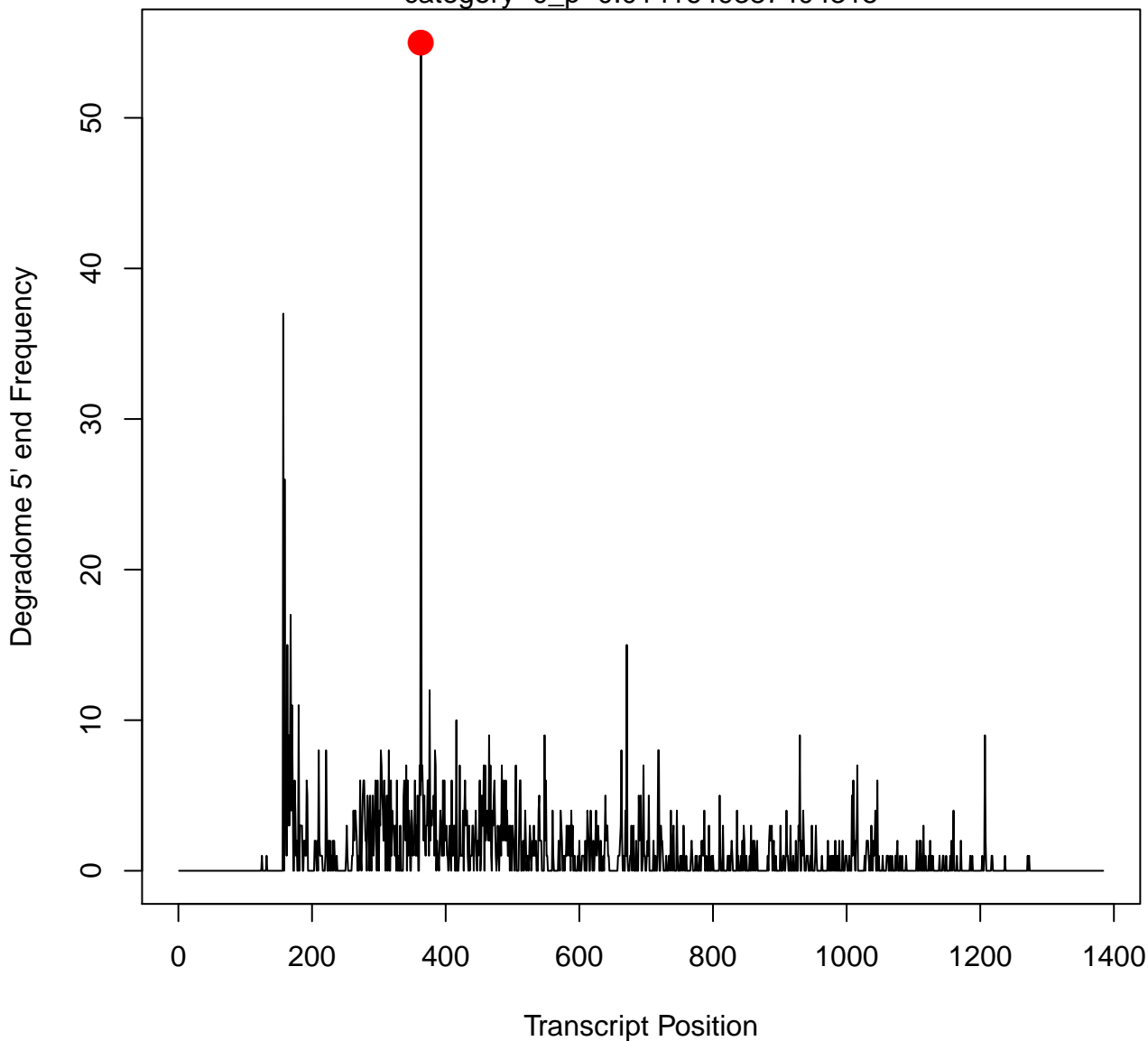

Supplement: Supplementary file 2 [file Data_Sheet_2.zip › Sit-miR159b_Seita.2G434400.1_363_TPlot.pdf]

**T=Seita.3G097700.1\_Q=Sit-miR159b\_S=3459**

category=0\_p=0.030748788501557

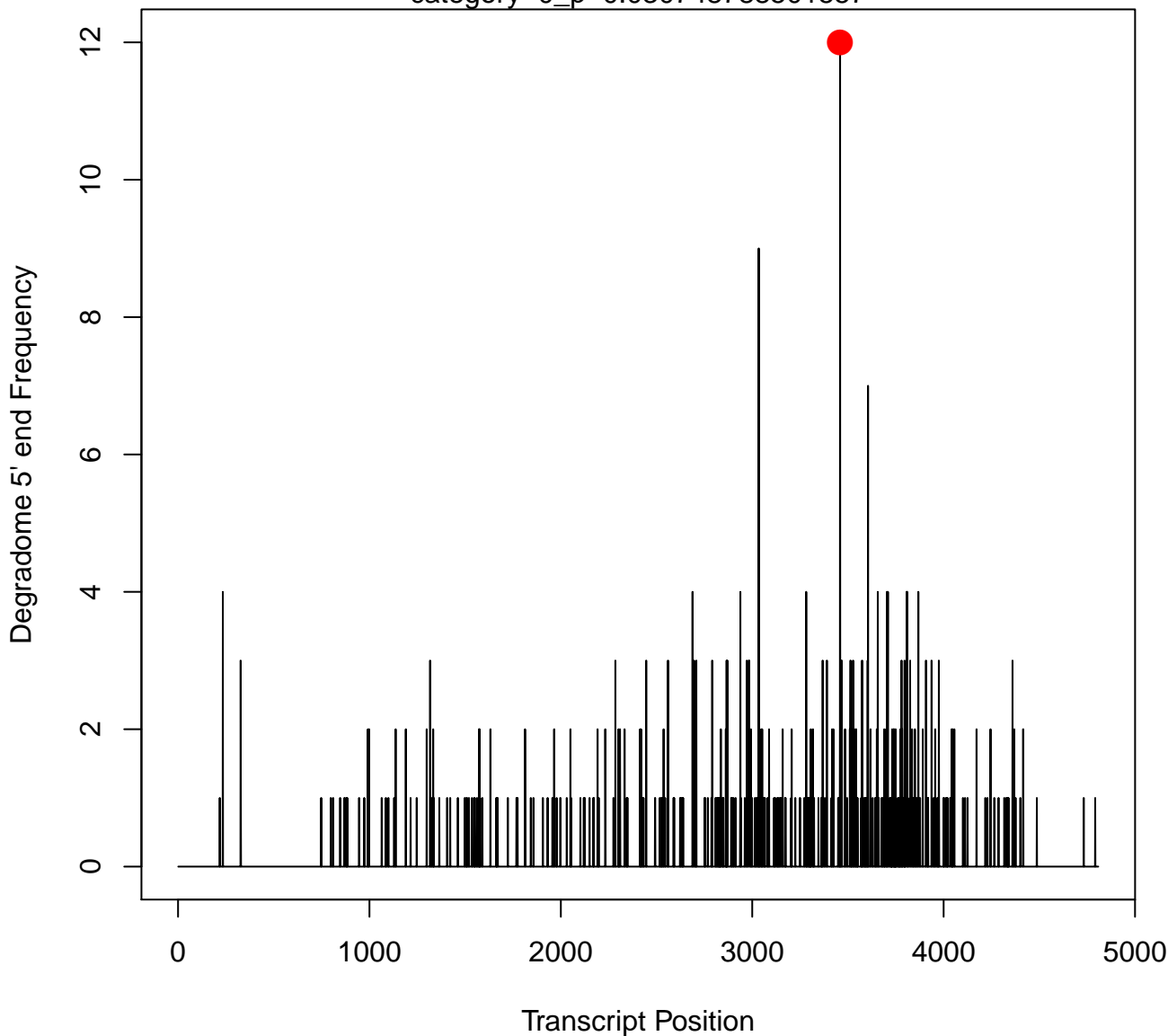

Supplement: Supplementary file 2 [file Data_Sheet_2.zip › Sit-miR159b_Seita.3G097700.1_3459_TPlot.pdf]

**T=Seita.3G188300.1\_Q=Sit-miR159b\_S=759**

category=0\_p=0.00806435048332621

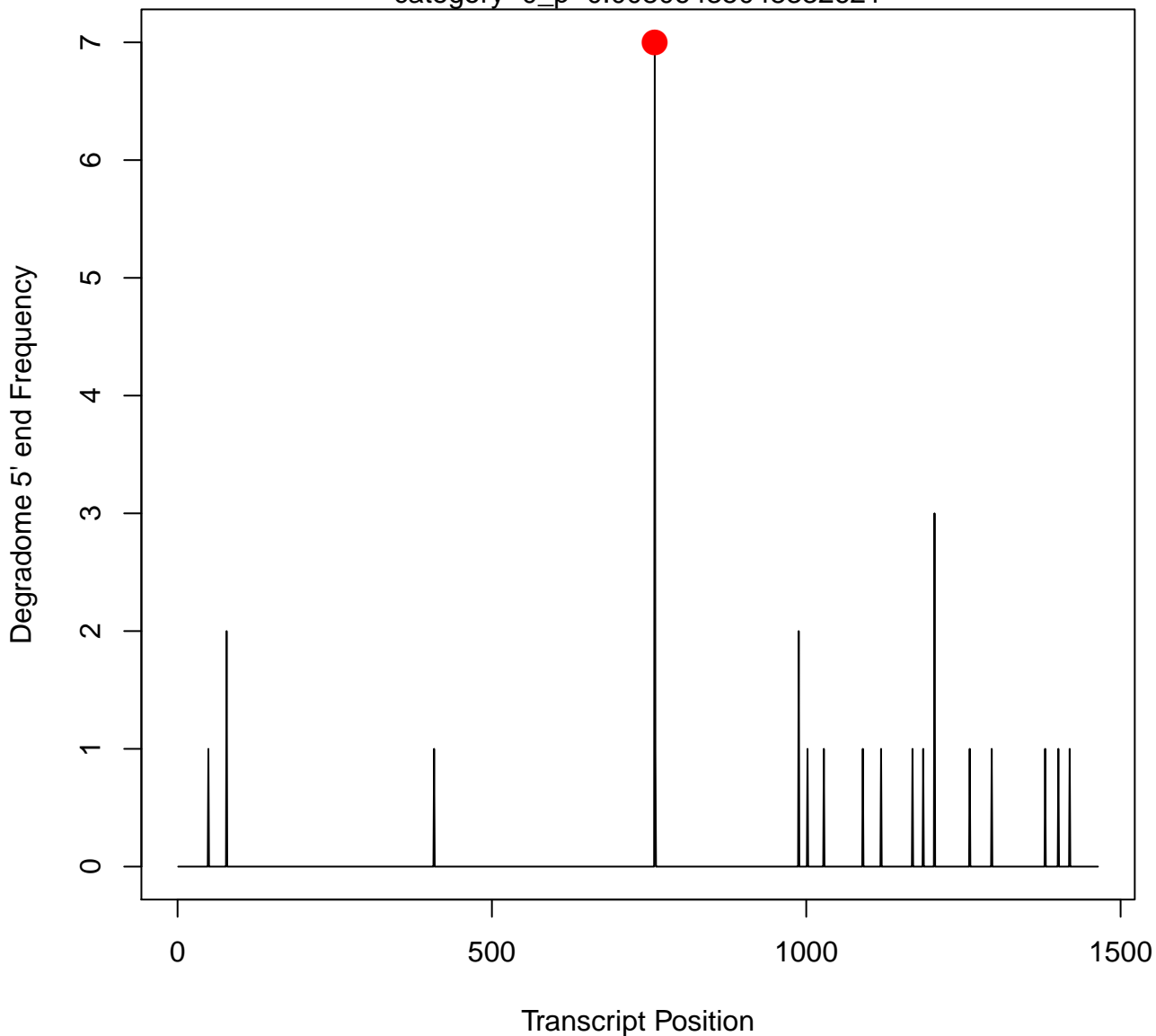

Supplement: Supplementary file 2 [file Data_Sheet_2.zip › Sit-miR159b_Seita.3G188300.1_759_TPlot.pdf]

**T=Seita.4G083900.1\_Q=Sit-miR159b\_S=4244**

category=2\_p=0.606747660492345

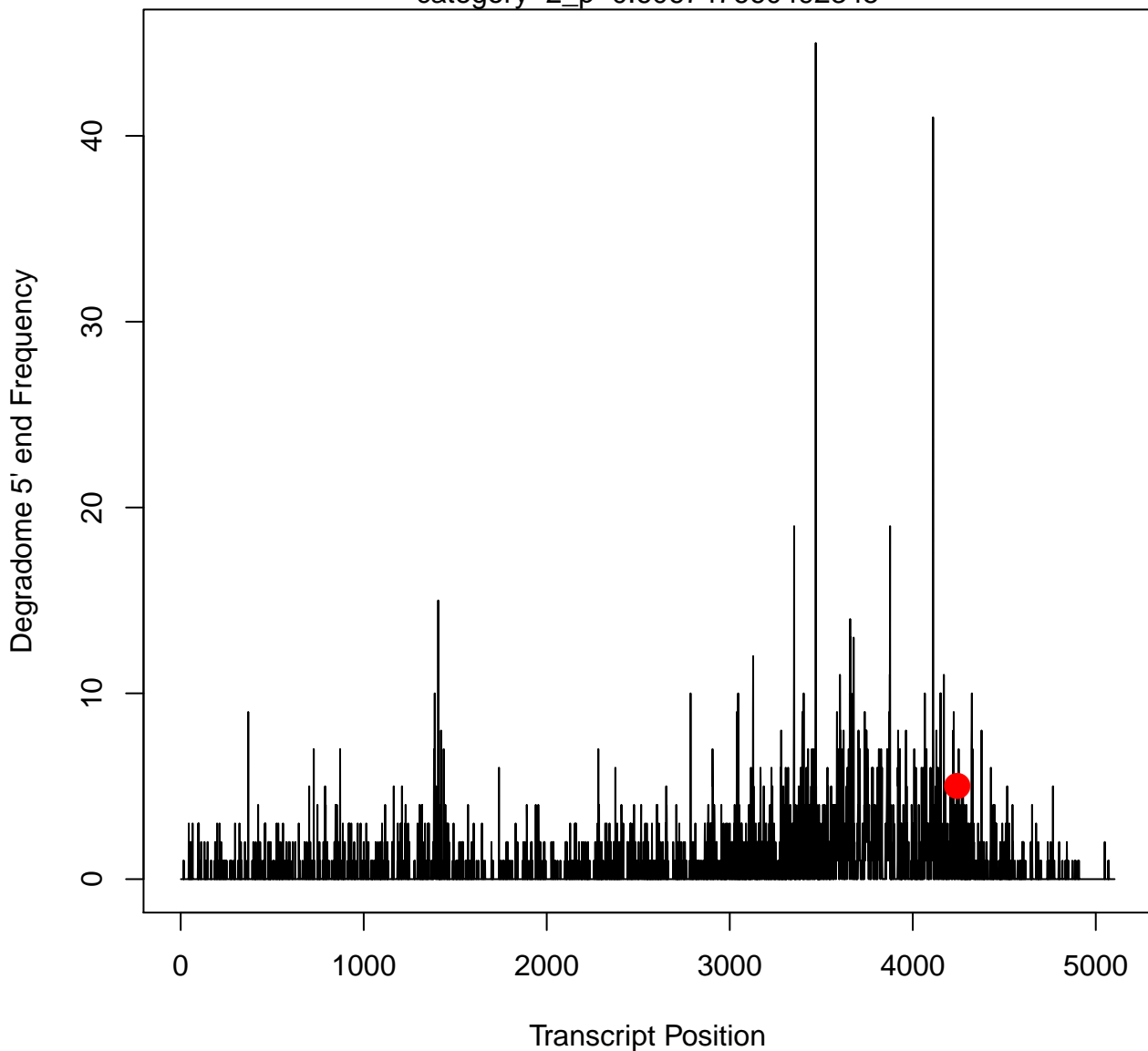

Supplement: Supplementary file 2 [file Data_Sheet_2.zip › Sit-miR159b_Seita.4G083900.1_4244_TPlot.pdf]

**T=Seita.4G265500.1\_Q=Sit-miR159b\_S=937**

category=2\_p=0.998903844087856

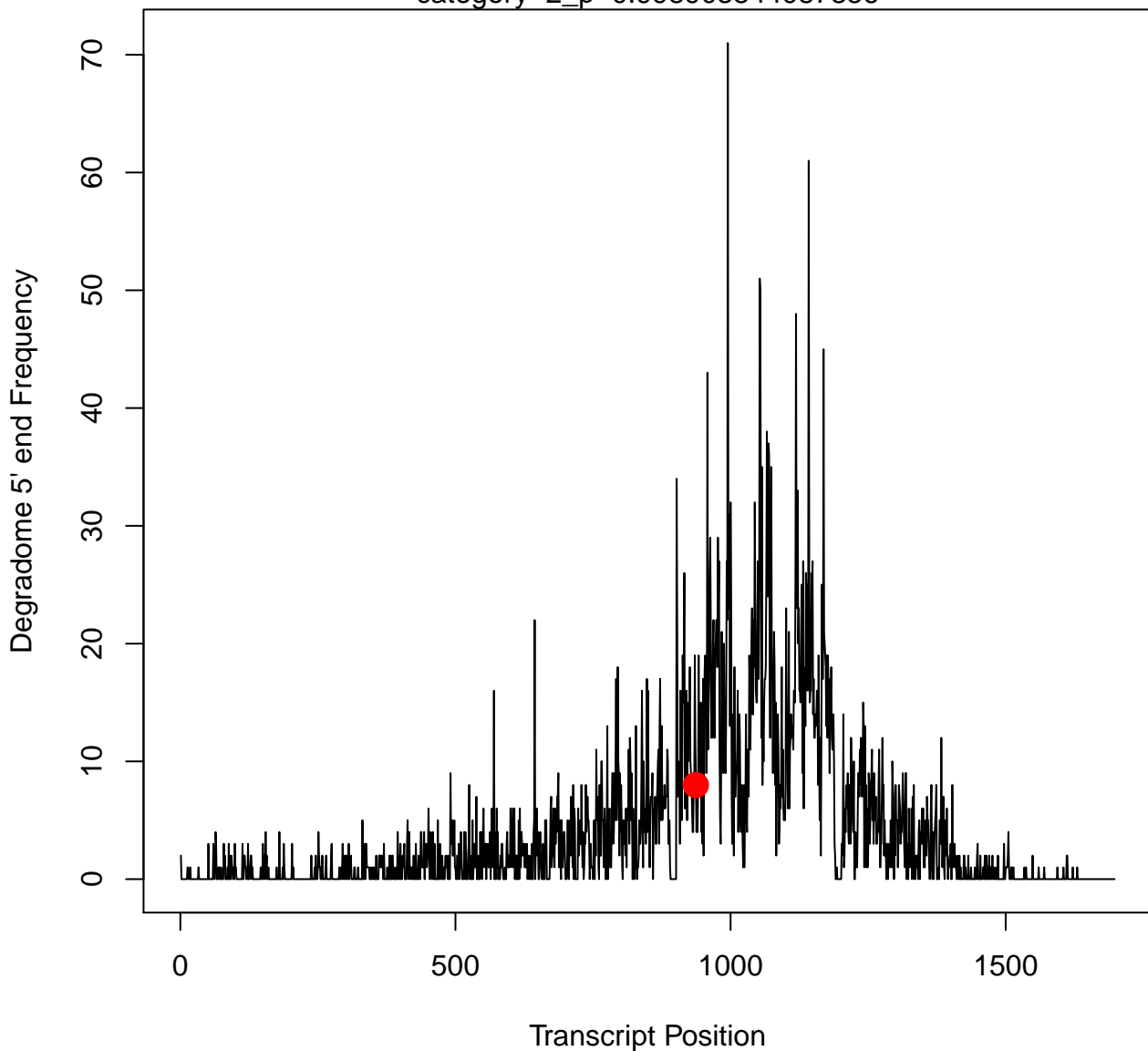

Supplement: Supplementary file 2 [file Data_Sheet_2.zip › Sit-miR159b_Seita.4G265500.1_937_TPlot.pdf]

**T=Seita.5G331000.1\_Q=Sit-miR159b\_S=2699**

category=2\_p=0.986026210434499

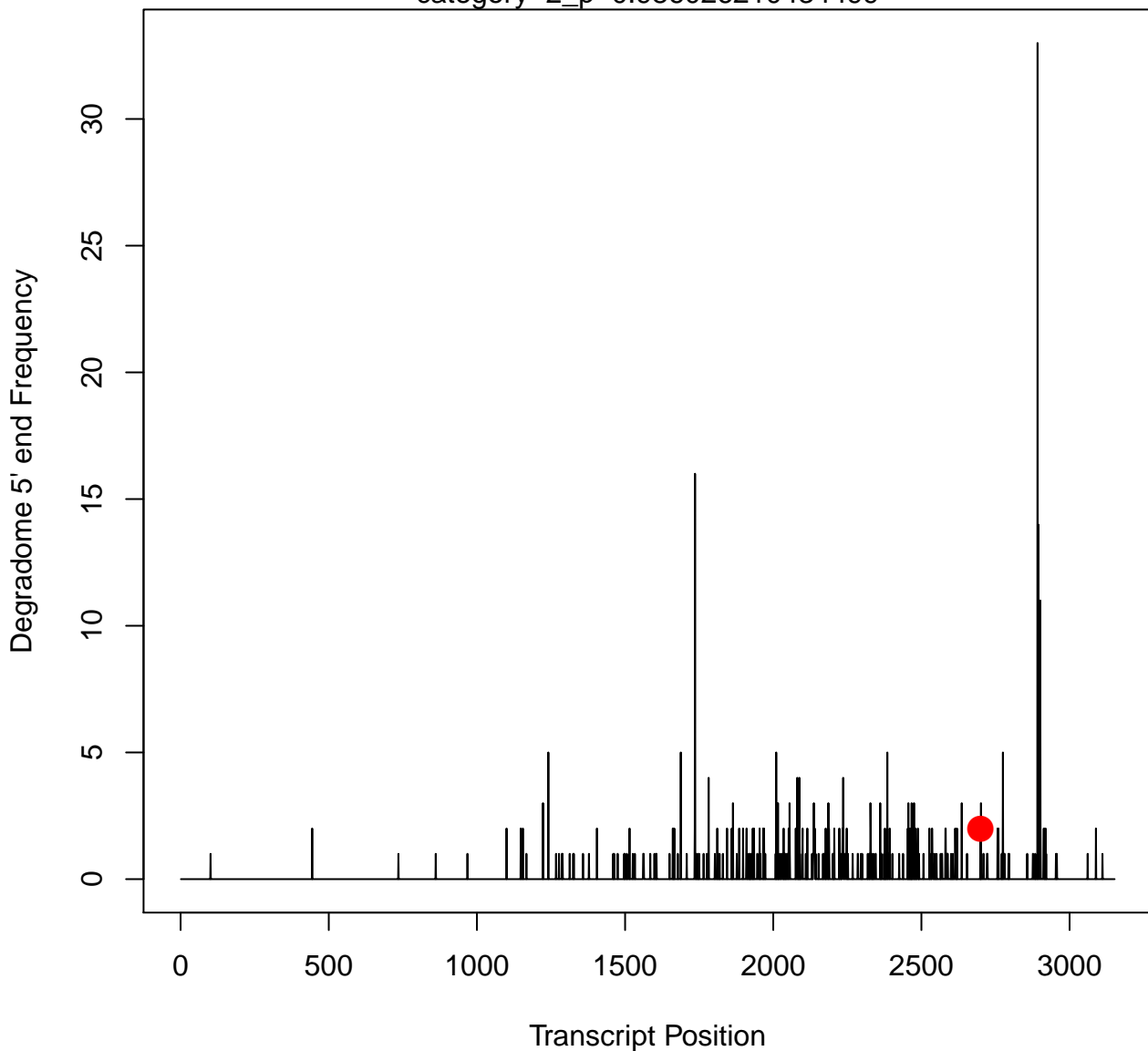

Supplement: Supplementary file 2 [file Data_Sheet_2.zip › Sit-miR159b_Seita.5G331000.1_2699_TPlot.pdf]

**T=Seita.5G355300.1\_Q=Sit-miR159b\_S=1278**

category=0\_p=0.00154110522640127

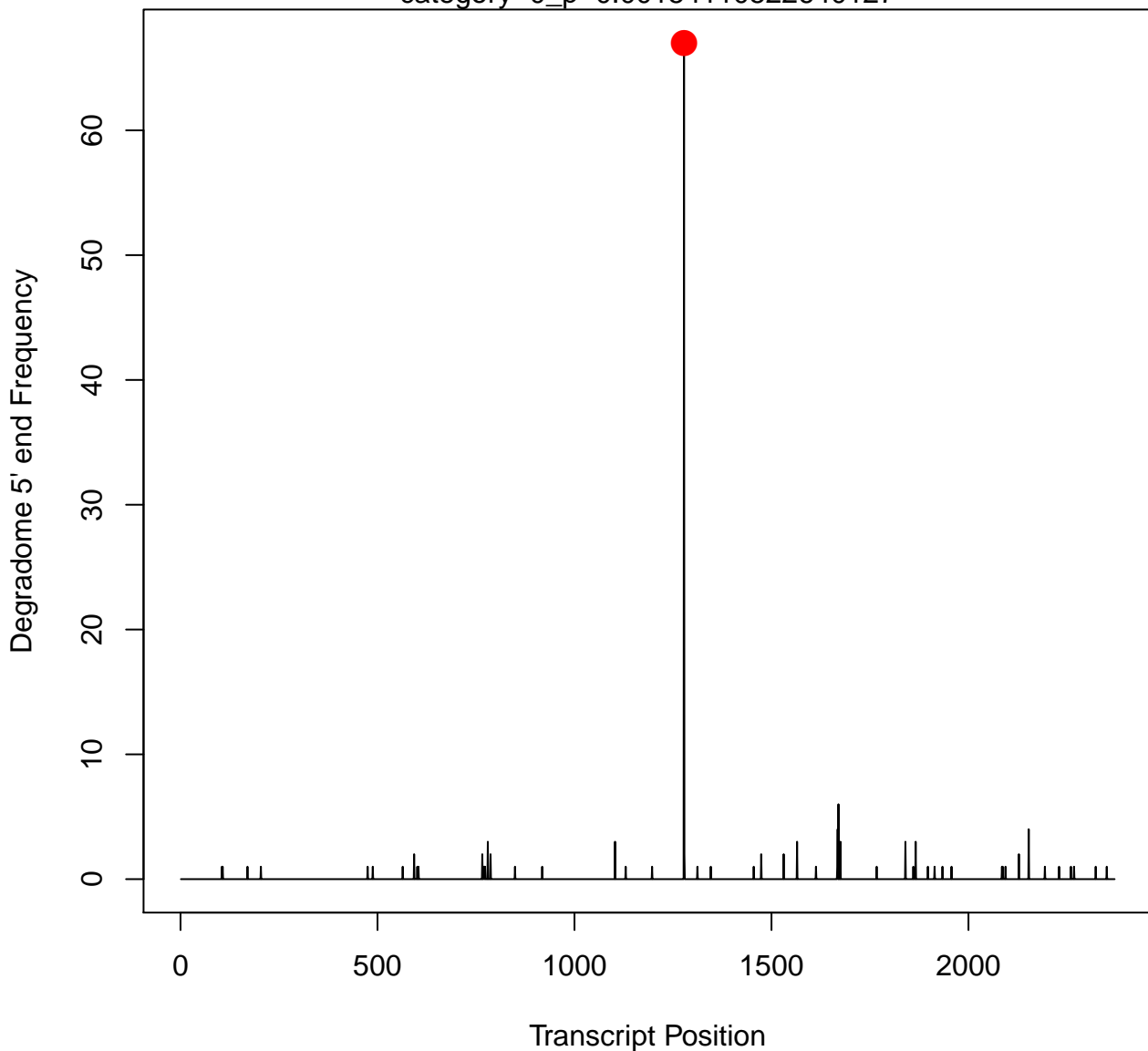

Supplement: Supplementary file 2 [file Data_Sheet_2.zip › Sit-miR159b_Seita.5G355300.1_1278_TPlot.pdf]
