# Supplementary figures and images for: The Construction and Exploration of a Comprehensive MicroRNA Centered Regulatory Network in Foxtail Millet (Setaria italica L.) (part 6 of 14)
Source: Front Plant Sci. 2022 May 6;13:848474. doi: 10.3389/fpls.2022.848474 (PMC9121102; doi:10.3389/fpls.2022.848474)

**T=Seita.2G396200.1\_Q=Sit-miR160c\_S=1590**

category=2\_p=0.998512779037902

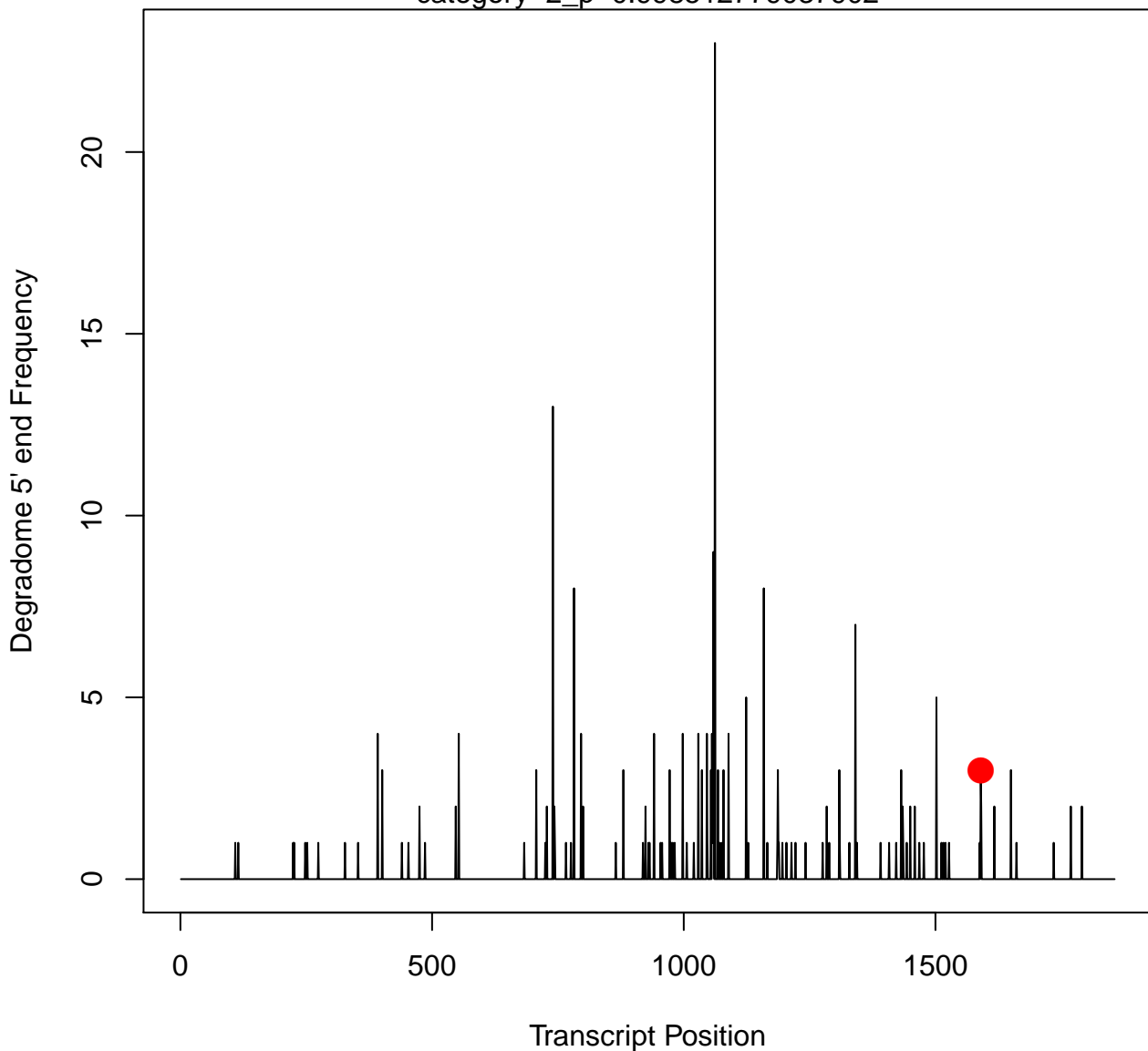

Supplement: Supplementary file 3 [file Data_Sheet_3.zip › Sit-miR160c_Seita.2G396200.1_1590_TPlot.pdf]

**T=Seita.2G444000.1\_Q=Sit-miR160c\_S=581**

category=2\_p=0.999999942364628

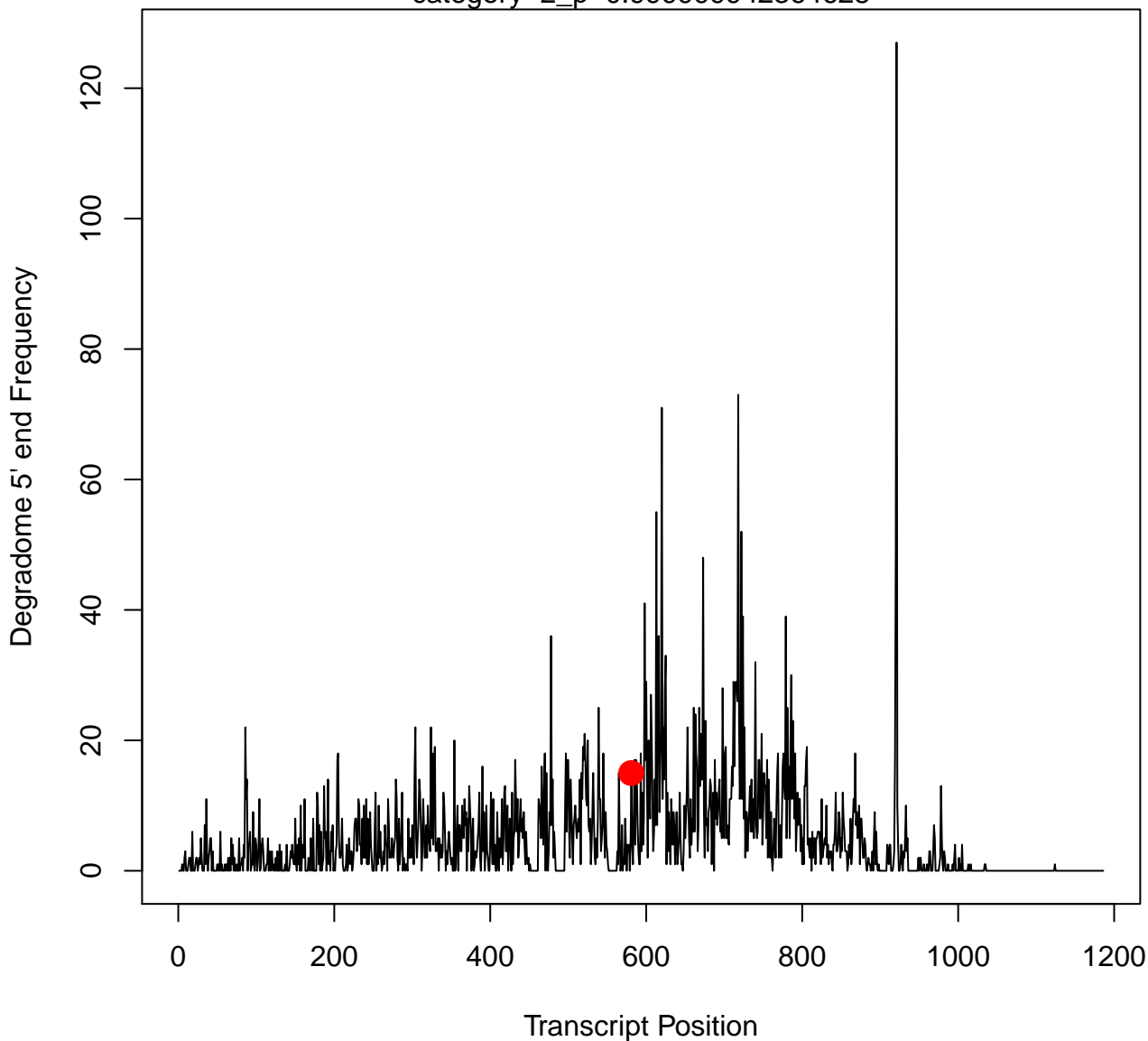

Supplement: Supplementary file 3 [file Data_Sheet_3.zip › Sit-miR160c_Seita.2G444000.1_581_TPlot.pdf]

**T=Seita.3G003300.1\_Q=Sit-miR160c\_S=1363**

category=0\_p=0.000401294755915105

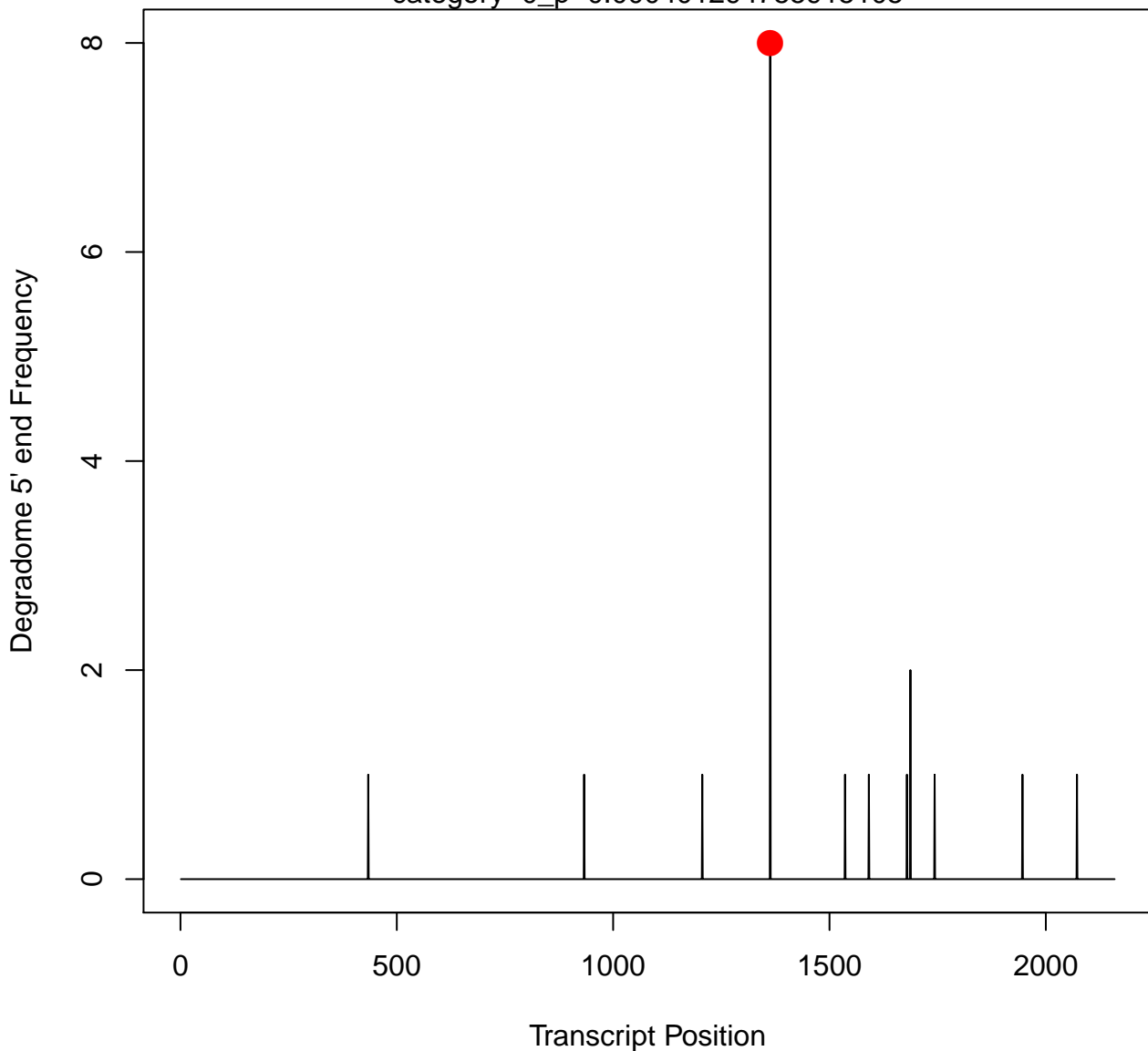

Supplement: Supplementary file 3 [file Data_Sheet_3.zip › Sit-miR160c_Seita.3G003300.1_1363_TPlot.pdf]

**T=Seita.3G122800.1\_Q=Sit-miR160c\_S=280**

category=2\_p=0.999935378303417

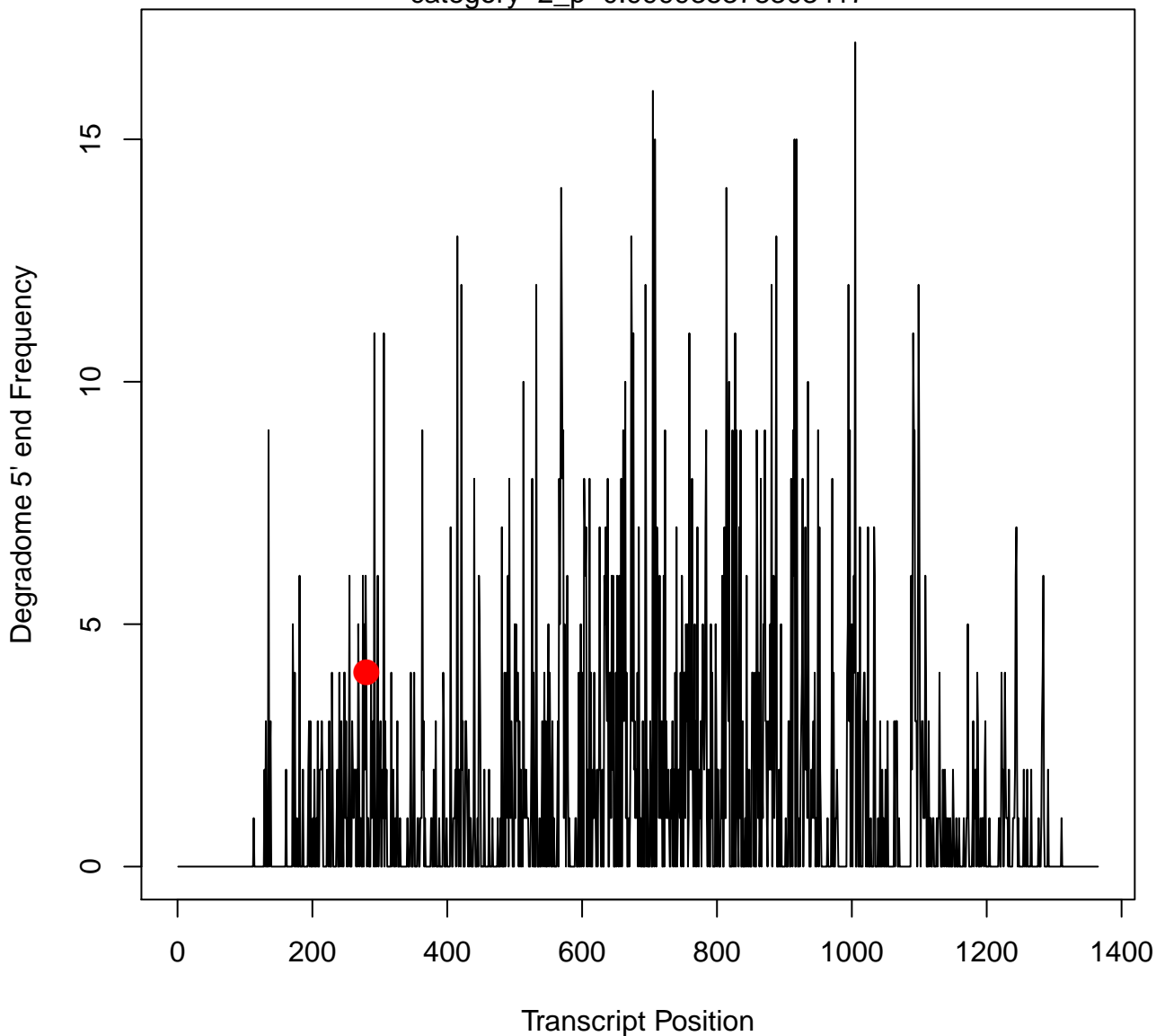

Supplement: Supplementary file 3 [file Data_Sheet_3.zip › Sit-miR160c_Seita.3G122800.1_280_TPlot.pdf]

**T=Seita.4G143100.1\_Q=Sit-miR160c\_S=300**

category=2\_p=0.999996554858103

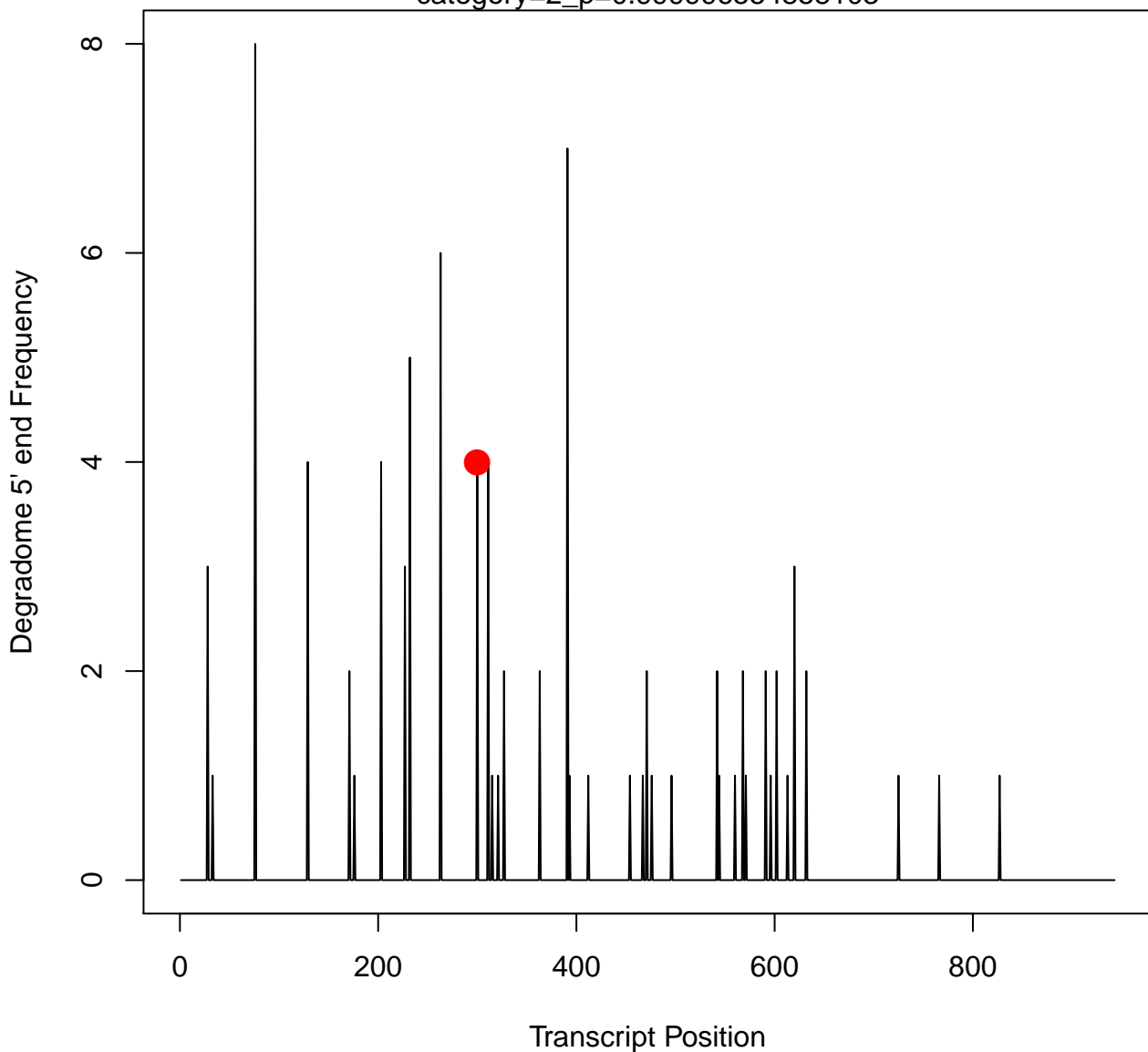

Supplement: Supplementary file 3 [file Data_Sheet_3.zip › Sit-miR160c_Seita.4G143100.1_300_TPlot.pdf]

**T=Seita.5G070500.1\_Q=Sit-miR160c\_S=709**

category=2\_p=0.851762438849464

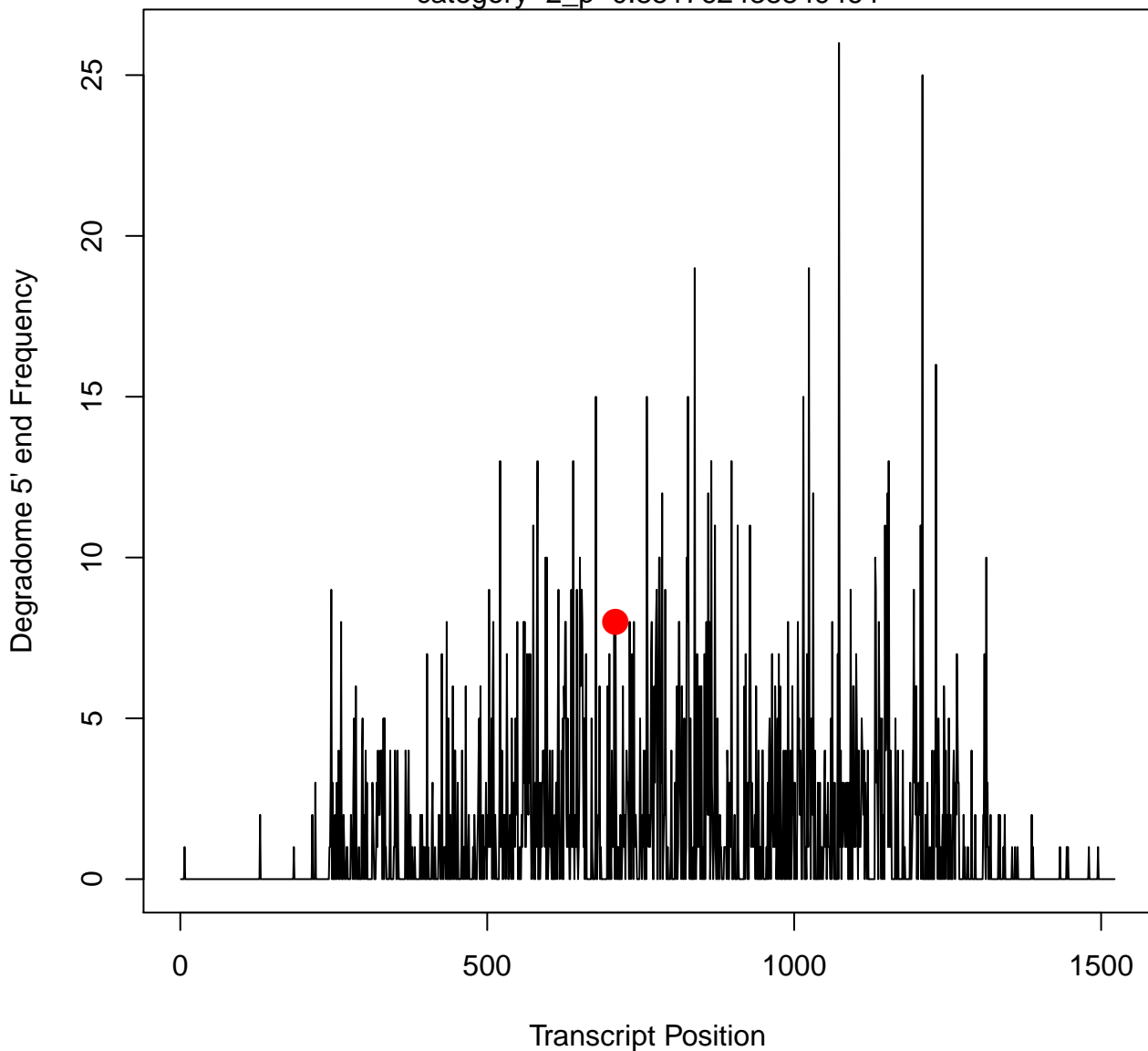

Supplement: Supplementary file 3 [file Data_Sheet_3.zip › Sit-miR160c_Seita.5G070500.1_709_TPlot.pdf]

**T=Seita.6G093900.1\_Q=Sit-miR160c\_S=1130**

category=2\_p=0.999999983948071

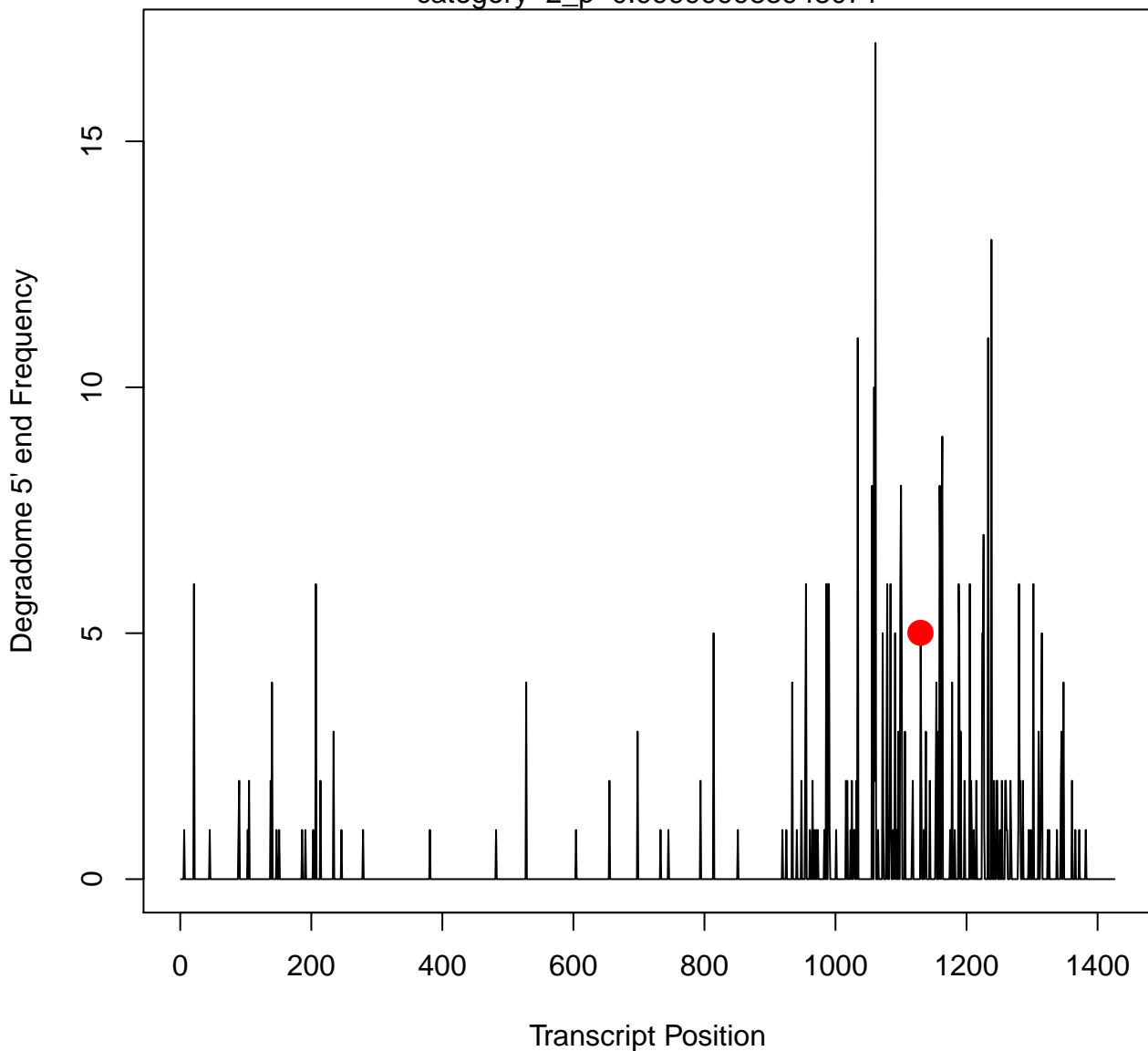

Supplement: Supplementary file 3 [file Data_Sheet_3.zip › Sit-miR160c_Seita.6G093900.1_1130_TPlot.pdf]

**T=Seita.6G170500.1\_Q=Sit-miR160c\_S=2580**

category=2\_p=0.0972093558831681

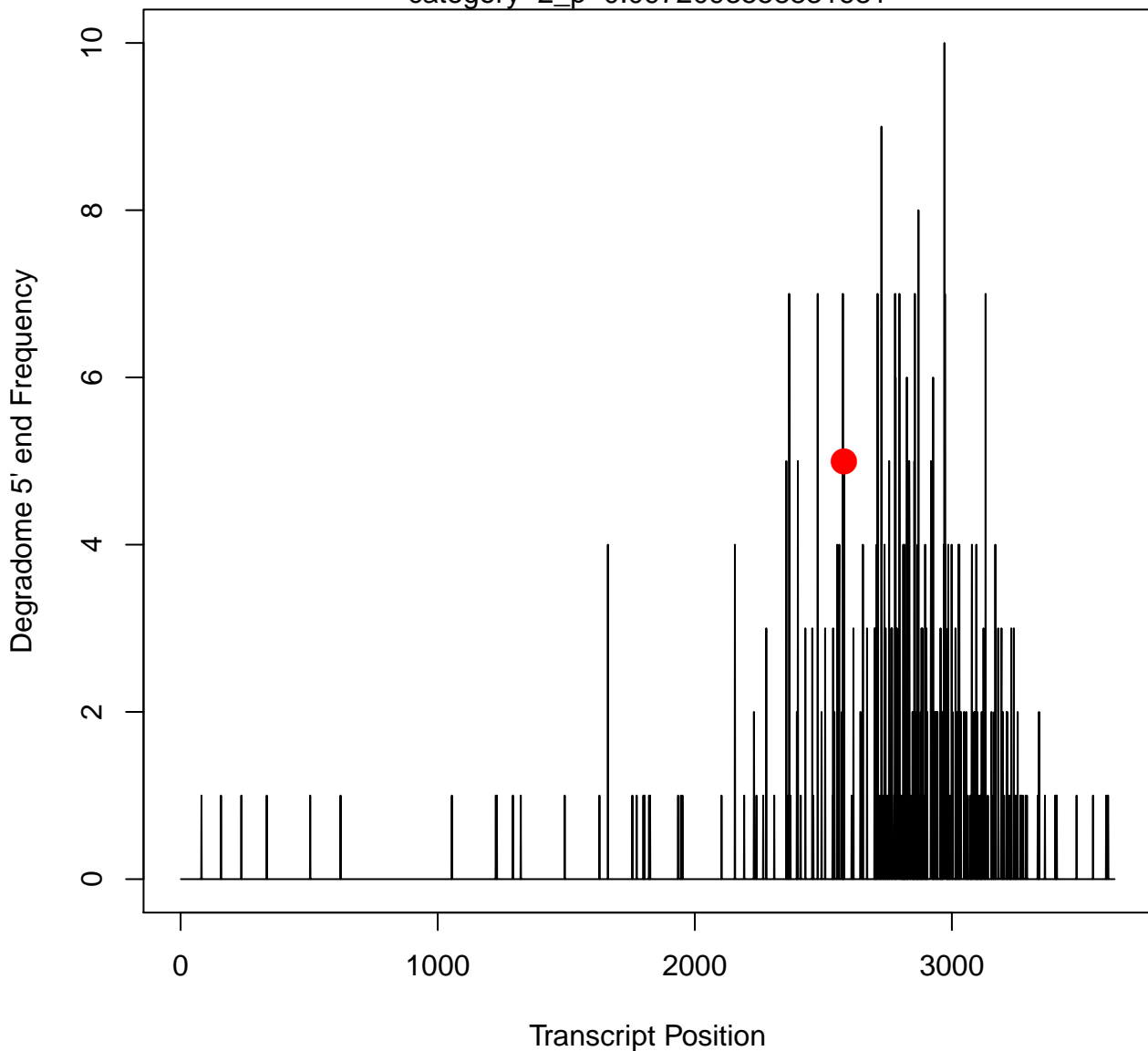

Supplement: Supplementary file 3 [file Data_Sheet_3.zip › Sit-miR160c_Seita.6G170500.1_2580_TPlot.pdf]

**T=Seita.7G133400.1\_Q=Sit-miR160c\_S=493**

category=2\_p=0.999999999227421

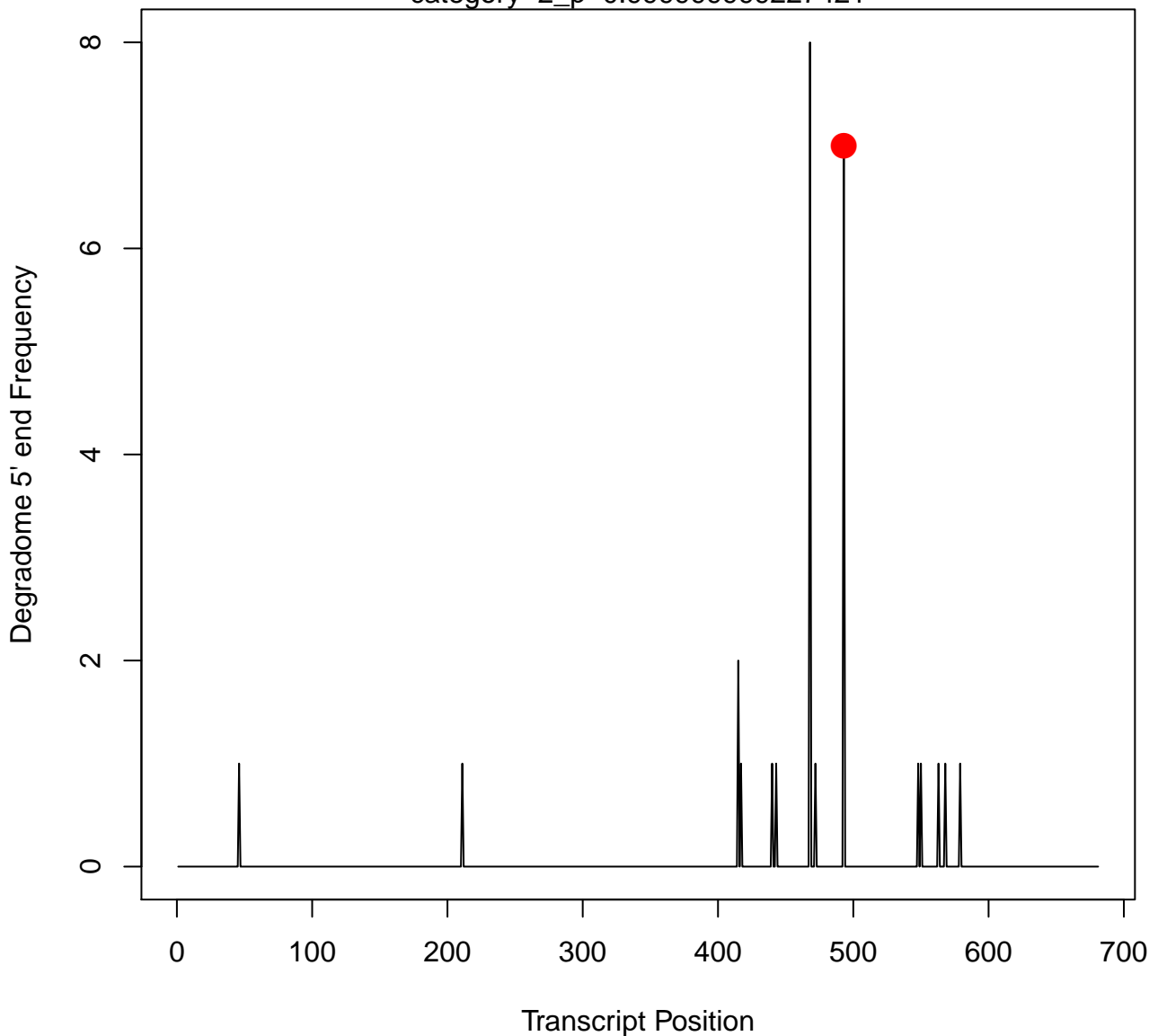

Supplement: Supplementary file 3 [file Data_Sheet_3.zip › Sit-miR160c_Seita.7G133400.1_493_TPlot.pdf]

**T=Seita.7G155700.1\_Q=Sit-miR160c\_S=1461**

category=0\_p=0.113801190999216

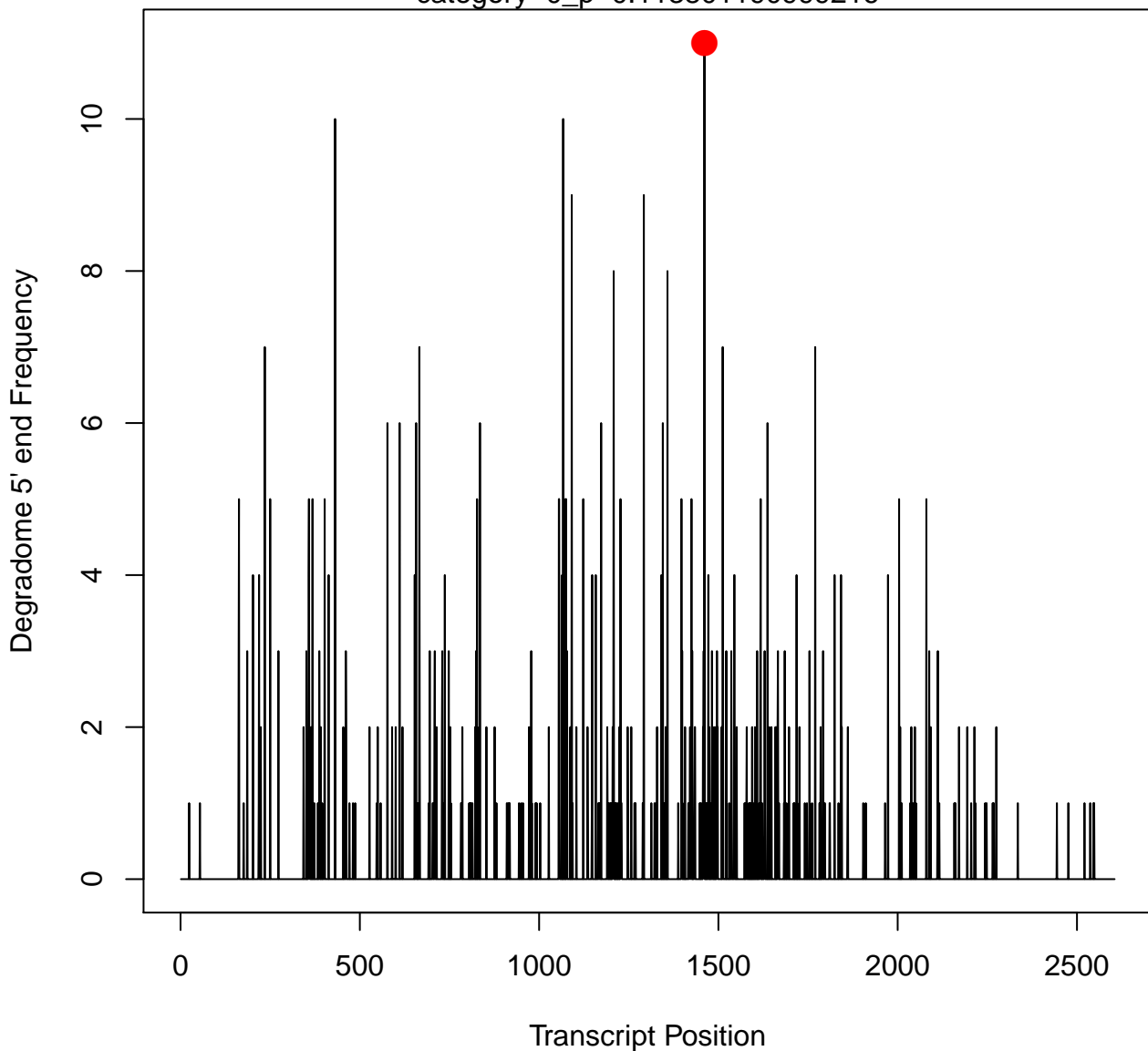

Supplement: Supplementary file 3 [file Data_Sheet_3.zip › Sit-miR160c_Seita.7G155700.1_1461_TPlot.pdf]

**T=Seita.9G038800.1\_Q=Sit-miR160c\_S=969**

category=2\_p=0.999993187704953

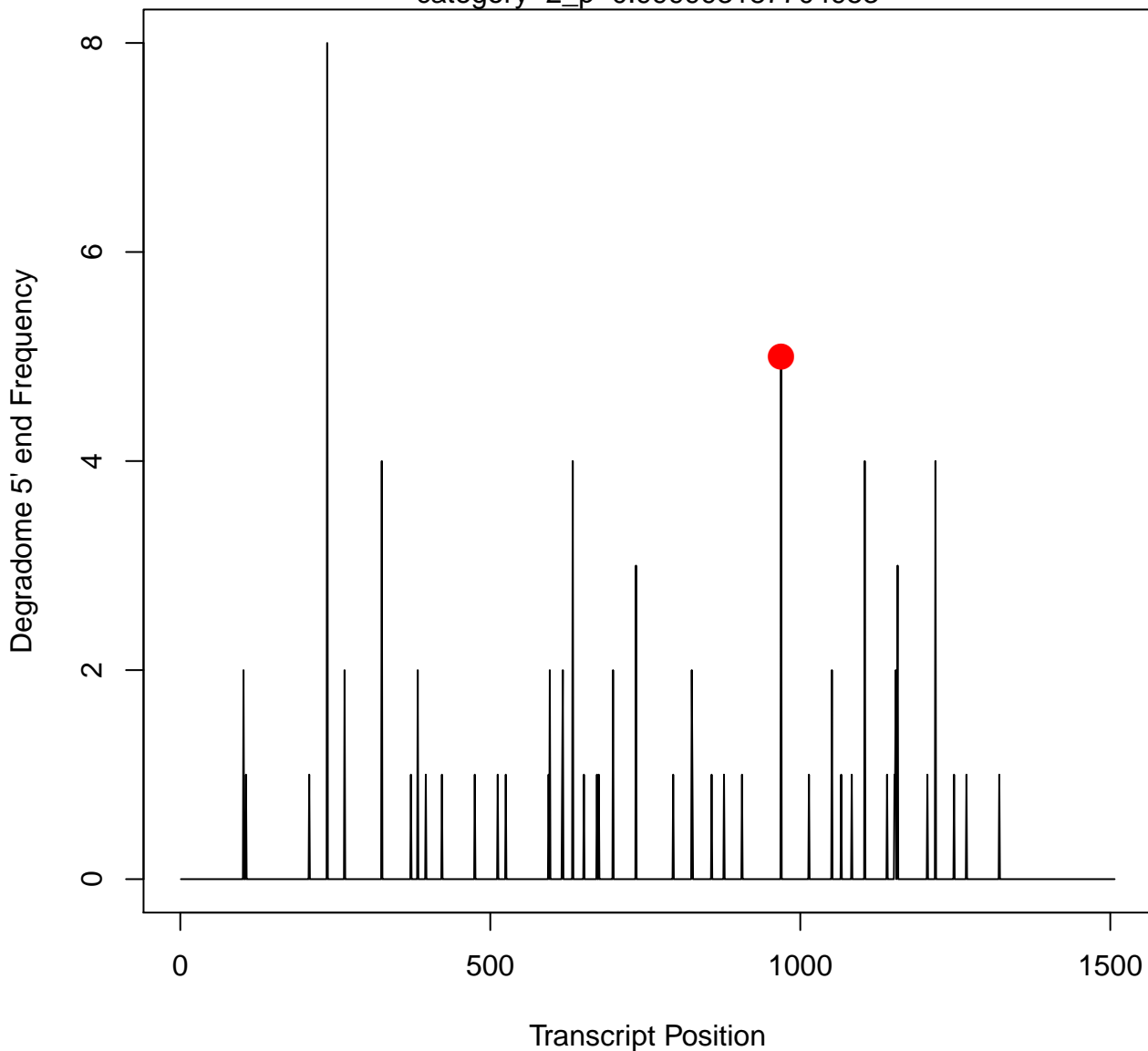

Supplement: Supplementary file 3 [file Data_Sheet_3.zip › Sit-miR160c_Seita.9G038800.1_969_TPlot.pdf]

**T=Seita.9G314200.1\_Q=Sit-miR160c\_S=341**

category=2\_p=0.999999932809399

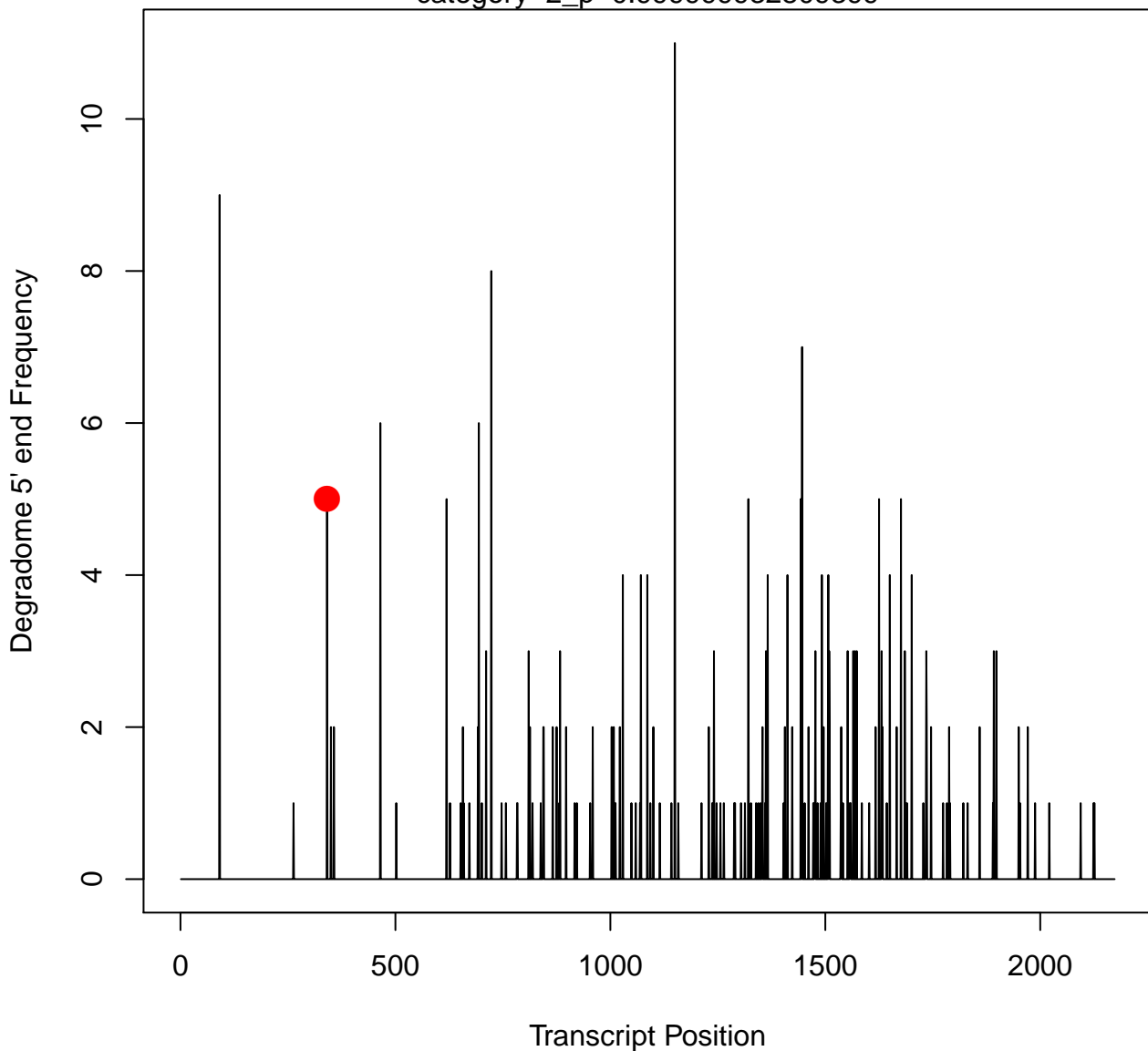

Supplement: Supplementary file 3 [file Data_Sheet_3.zip › Sit-miR160c_Seita.9G314200.1_341_TPlot.pdf]

**T=Seita.9G409200.1\_Q=Sit-miR160c\_S=875**

category=2\_p=0.999999999495468

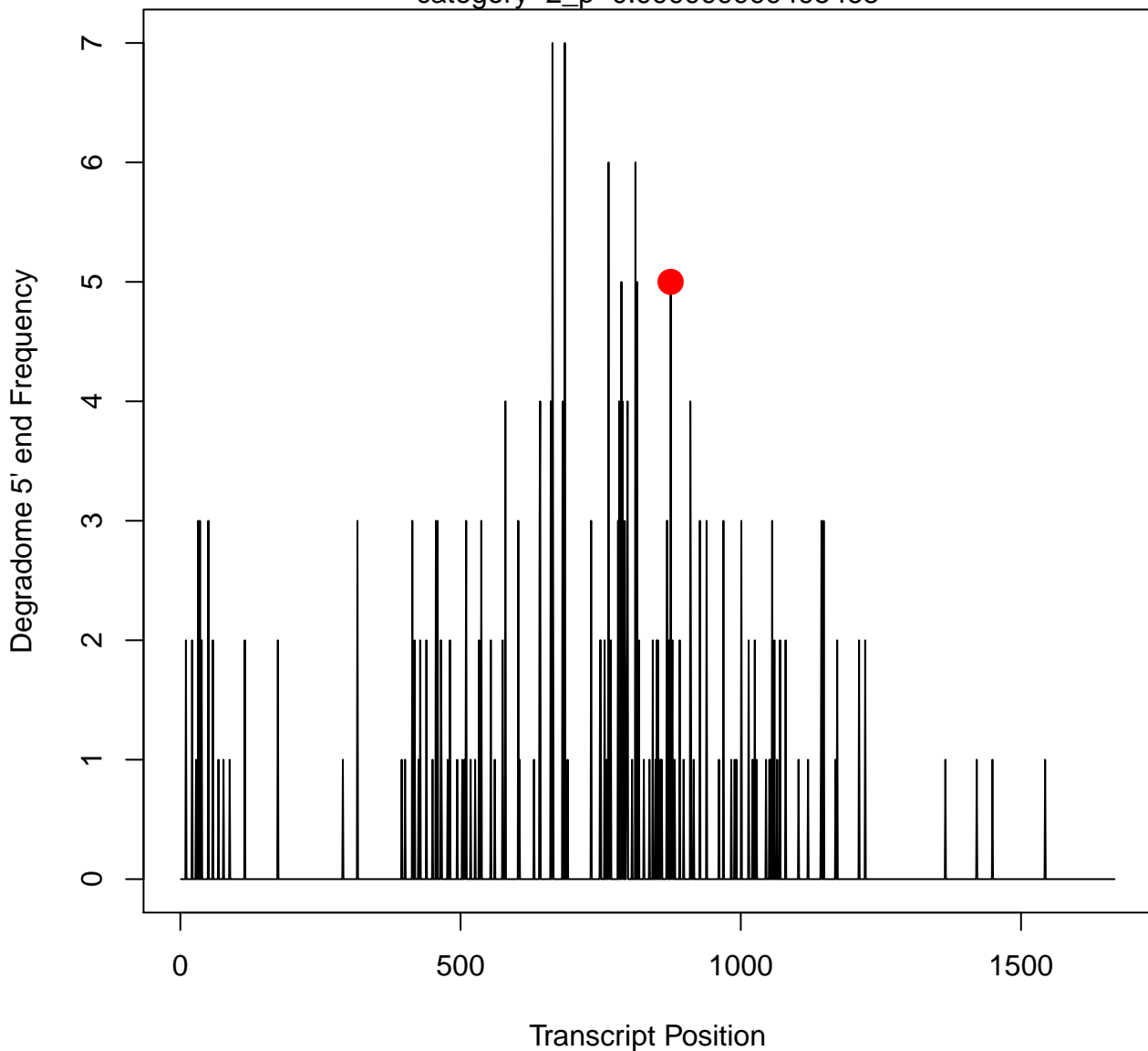

Supplement: Supplementary file 3 [file Data_Sheet_3.zip › Sit-miR160c_Seita.9G409200.1_875_TPlot.pdf]

**T=Seita.1G031300.1\_Q=Sit-miR160d\_S=282**

category=2\_p=0.970135491386263

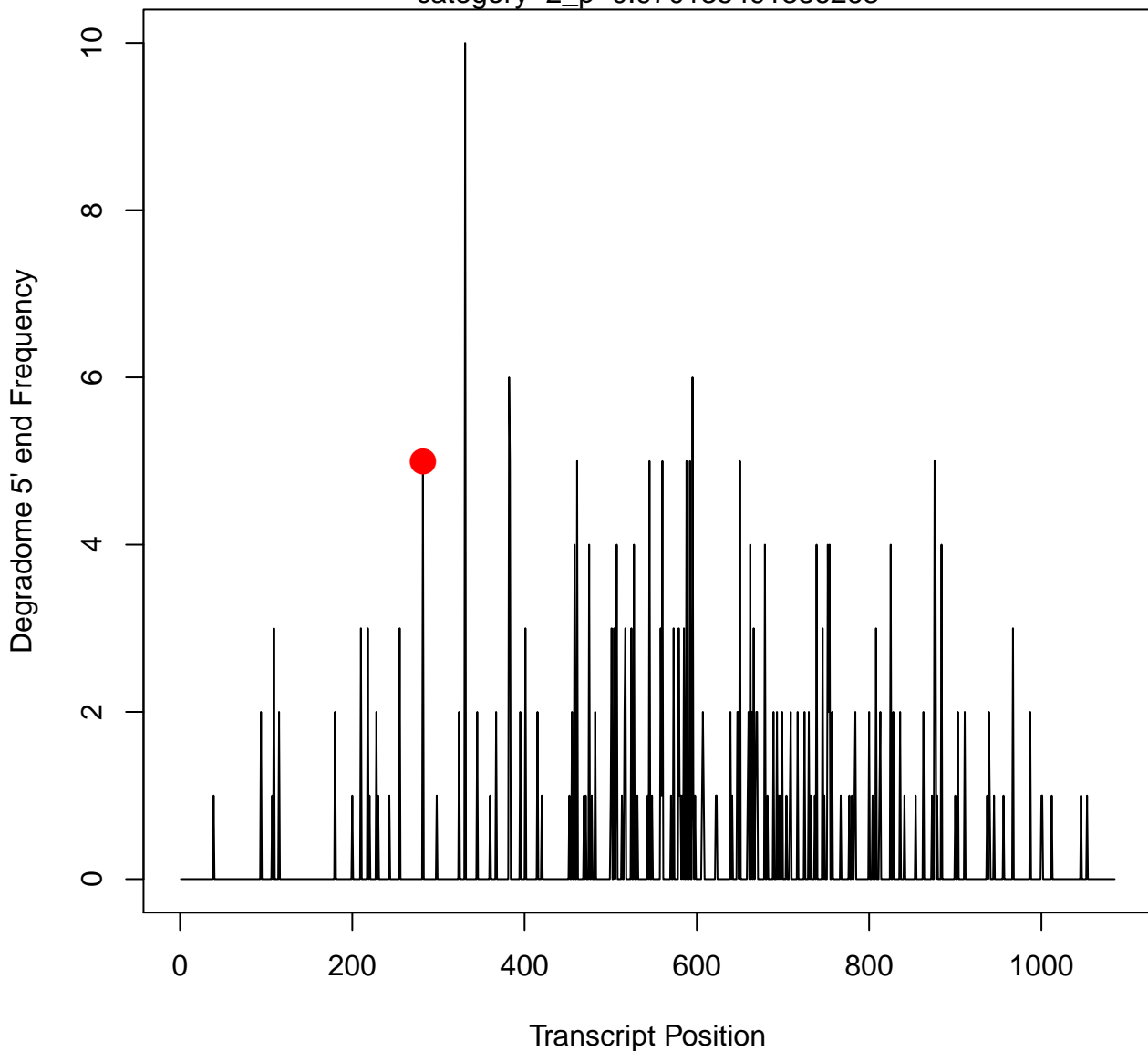

Supplement: Supplementary file 3 [file Data_Sheet_3.zip › Sit-miR160d_Seita.1G031300.1_282_TPlot.pdf]

**T=Seita.1G031700.1\_Q=Sit-miR160d\_S=1282**

category=2\_p=0.824215796674898

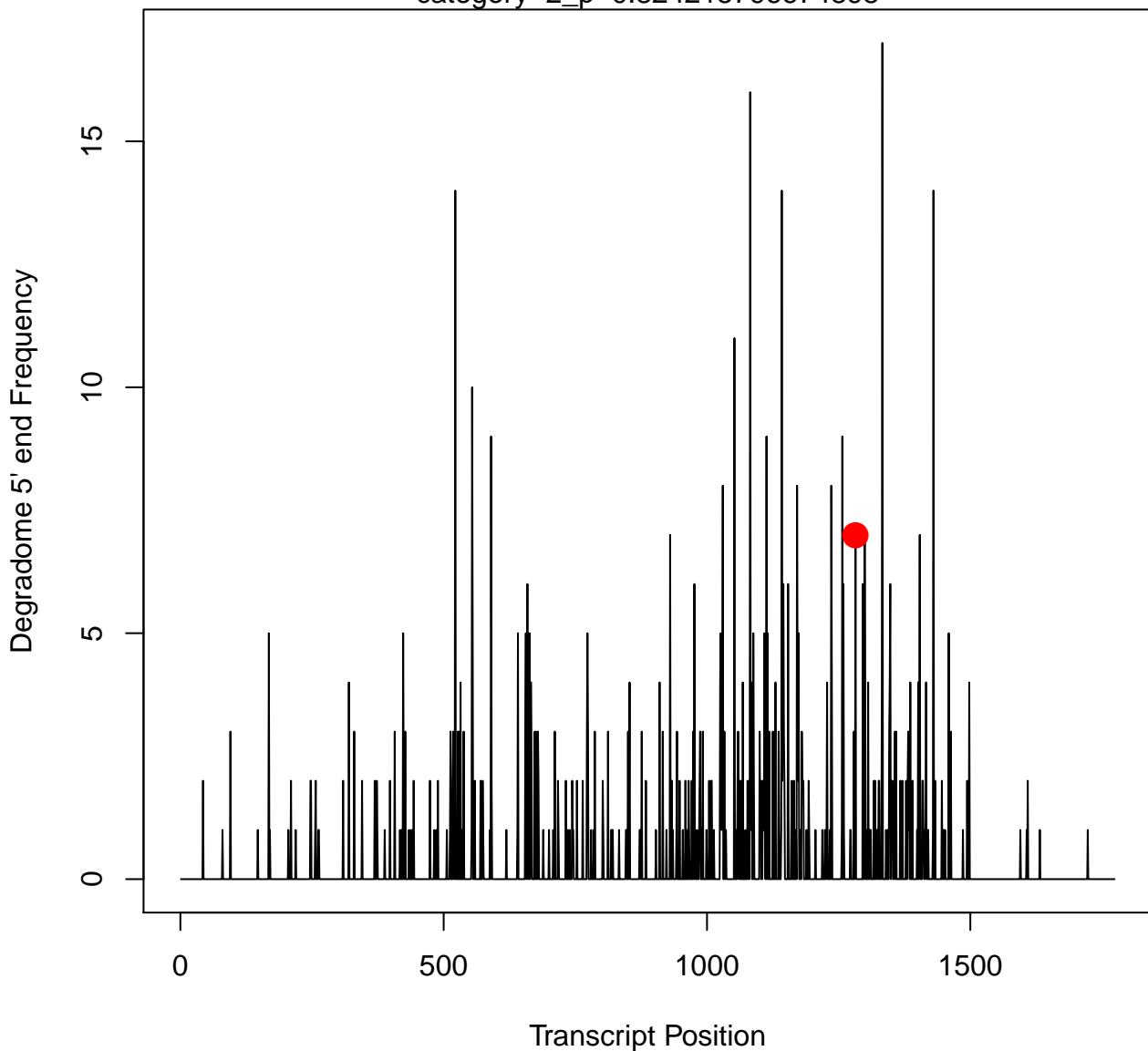

Supplement: Supplementary file 3 [file Data_Sheet_3.zip › Sit-miR160d_Seita.1G031700.1_1282_TPlot.pdf]

**T=Seita.2G439900.1\_Q=Sit-miR160d\_S=610**

category=2\_p=0.99999999999827

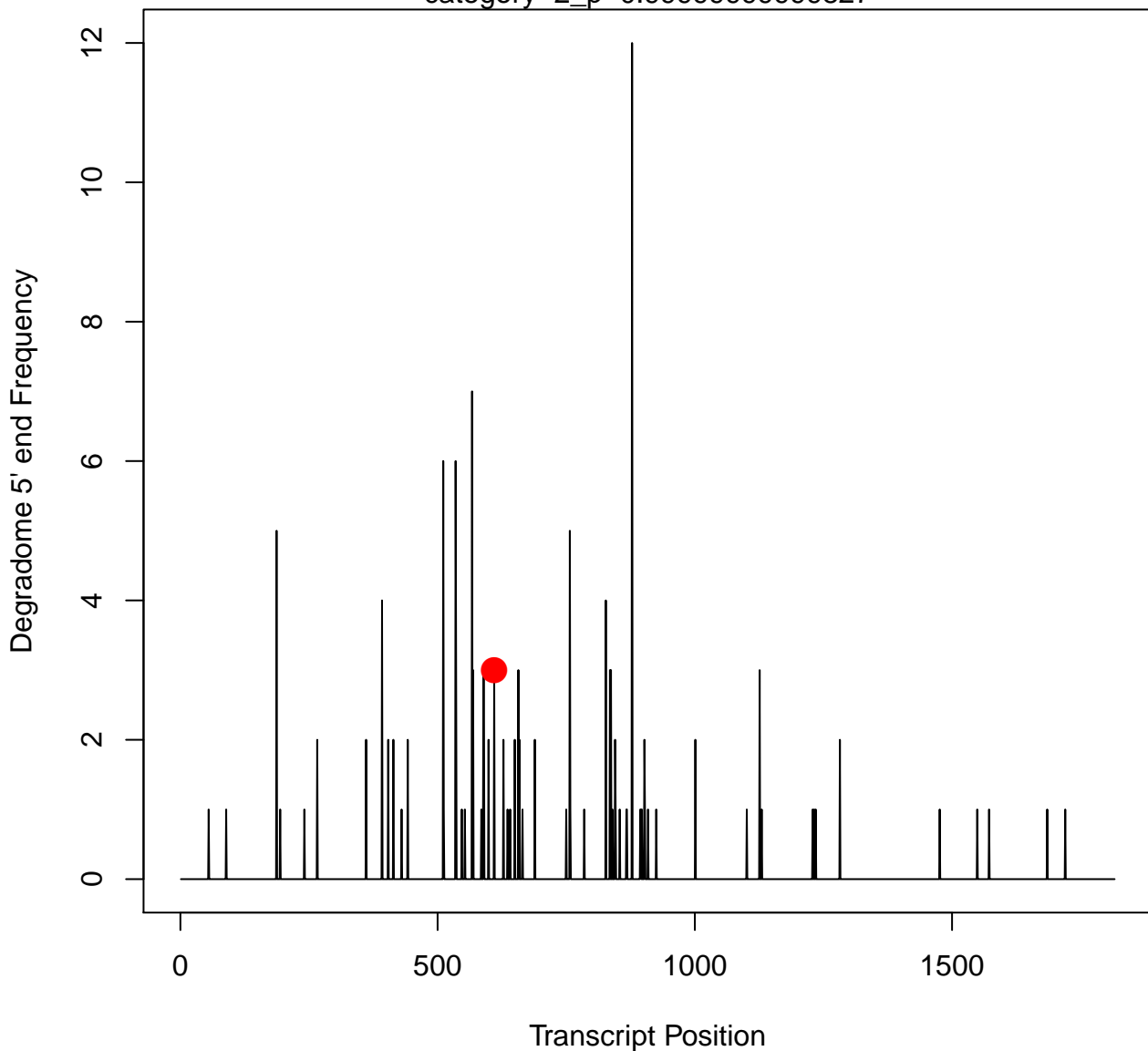

Supplement: Supplementary file 3 [file Data_Sheet_3.zip › Sit-miR160d_Seita.2G439900.1_610_TPlot.pdf]

**T=Seita.5G273800.1\_Q=Sit-miR160d\_S=477**

category=2\_p=0.99999999722601

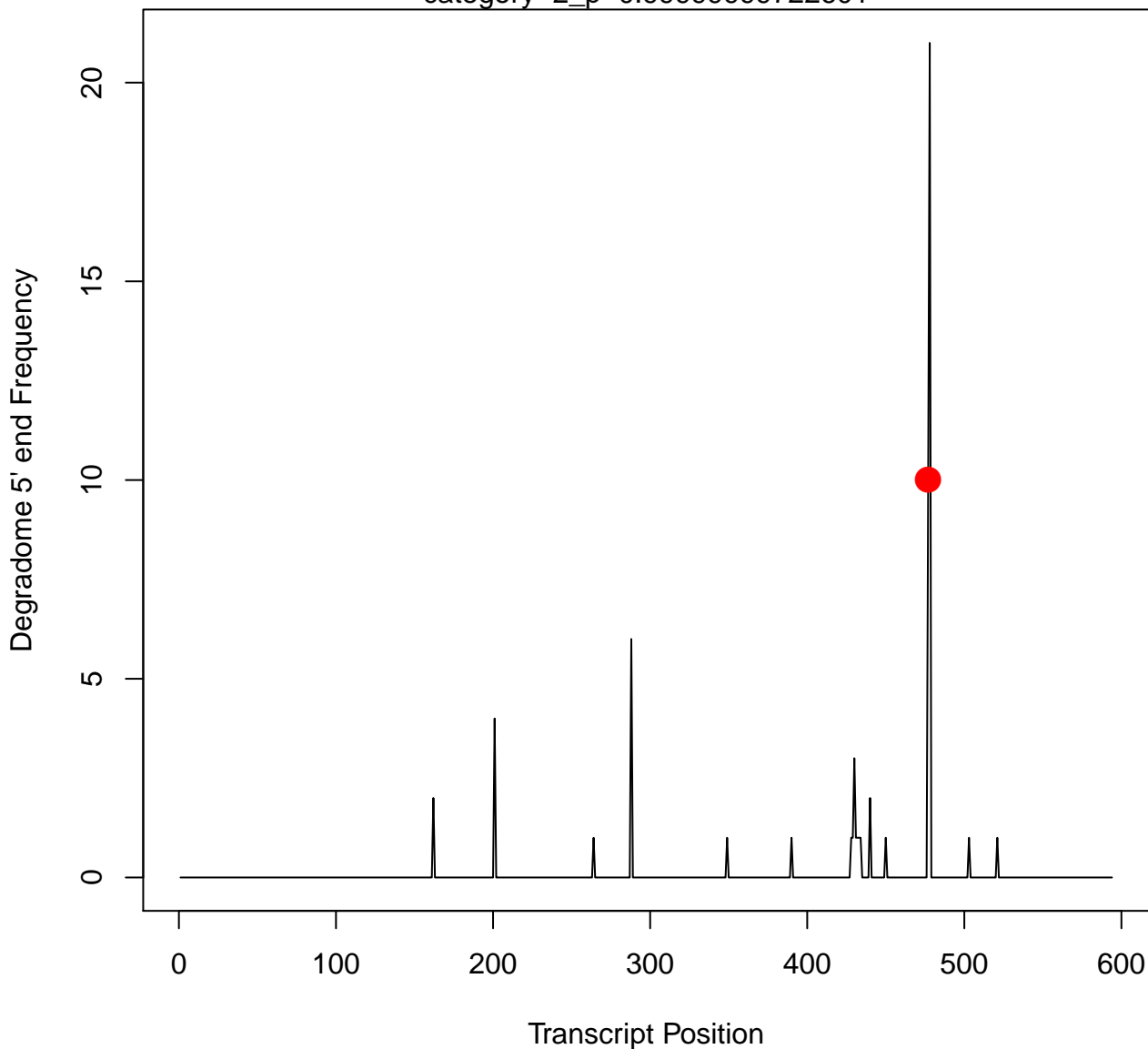

Supplement: Supplementary file 3 [file Data_Sheet_3.zip › Sit-miR160d_Seita.5G273800.1_477_TPlot.pdf]

**T=Seita.6G053700.1\_Q=Sit-miR160d\_S=1004**

category=2\_p=0.999999999968104

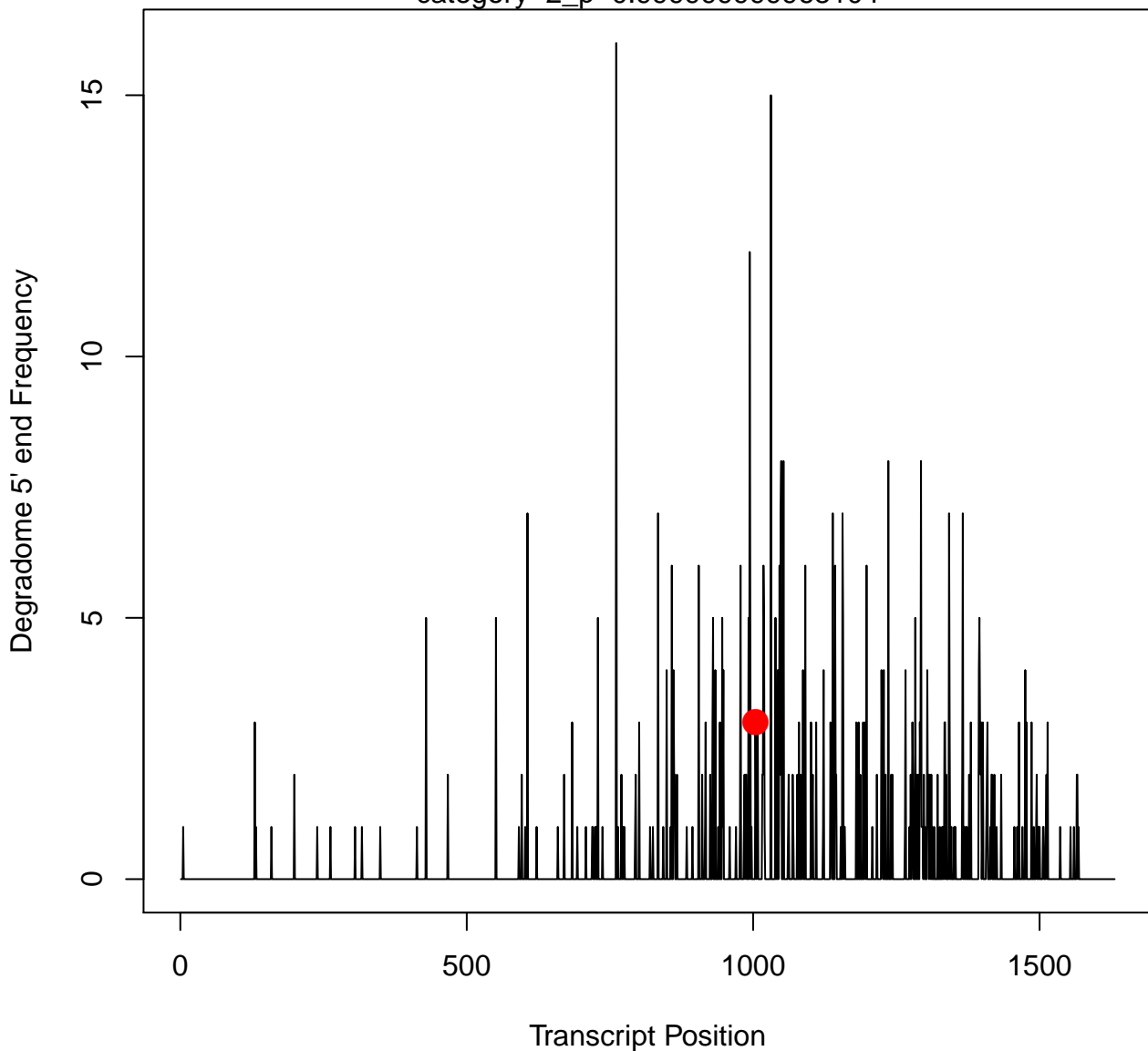

Supplement: Supplementary file 3 [file Data_Sheet_3.zip › Sit-miR160d_Seita.6G053700.1_1004_TPlot.pdf]

**T=Seita.6G192200.1\_Q=Sit-miR160d\_S=88**

category=2\_p=0.999994447768716

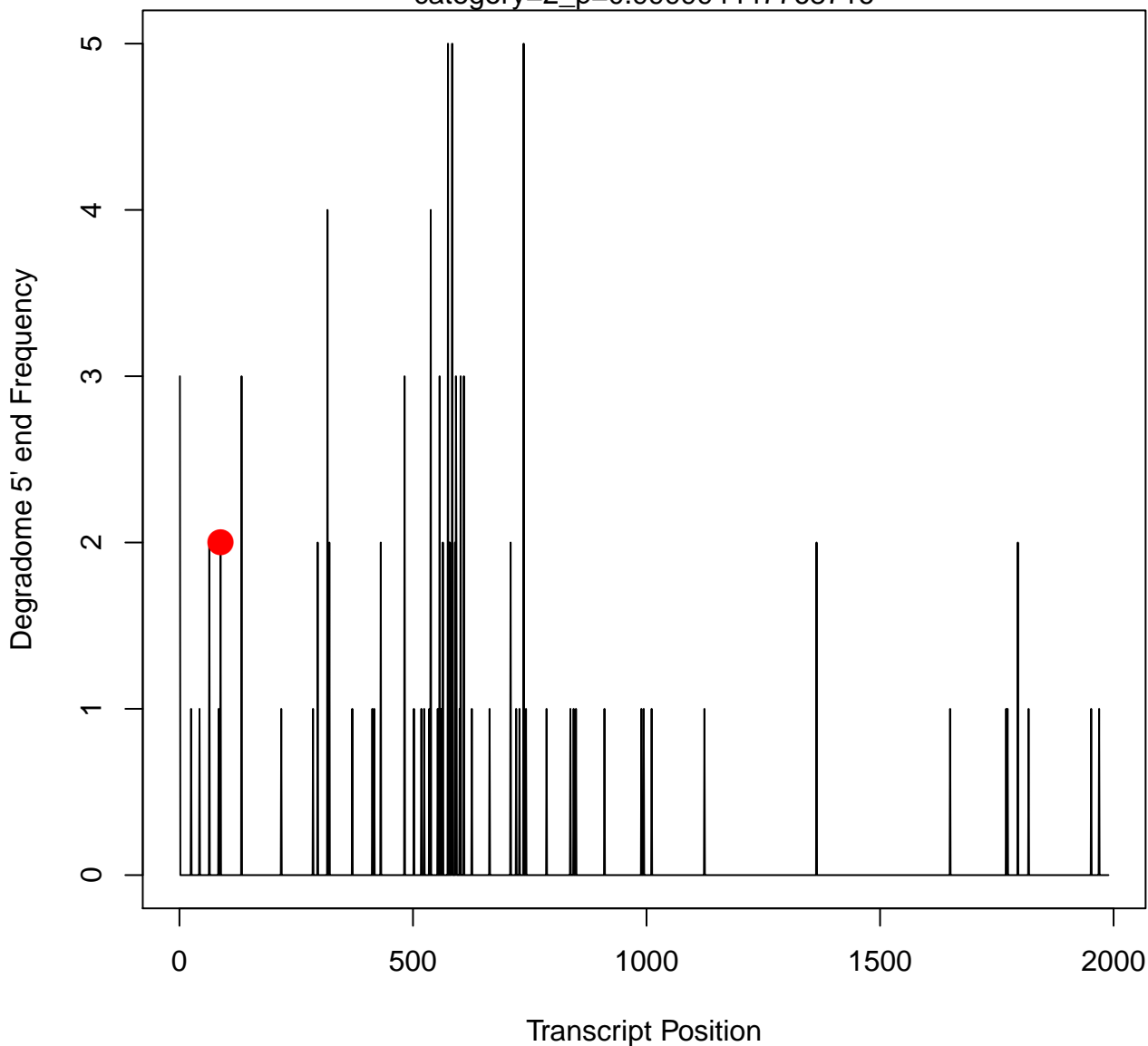

Supplement: Supplementary file 3 [file Data_Sheet_3.zip › Sit-miR160d_Seita.6G192200.1_88_TPlot.pdf]

**T=Seita.9G219800.1\_Q=Sit-miR160d\_S=1729**

category=0\_p=0.000401294755915105

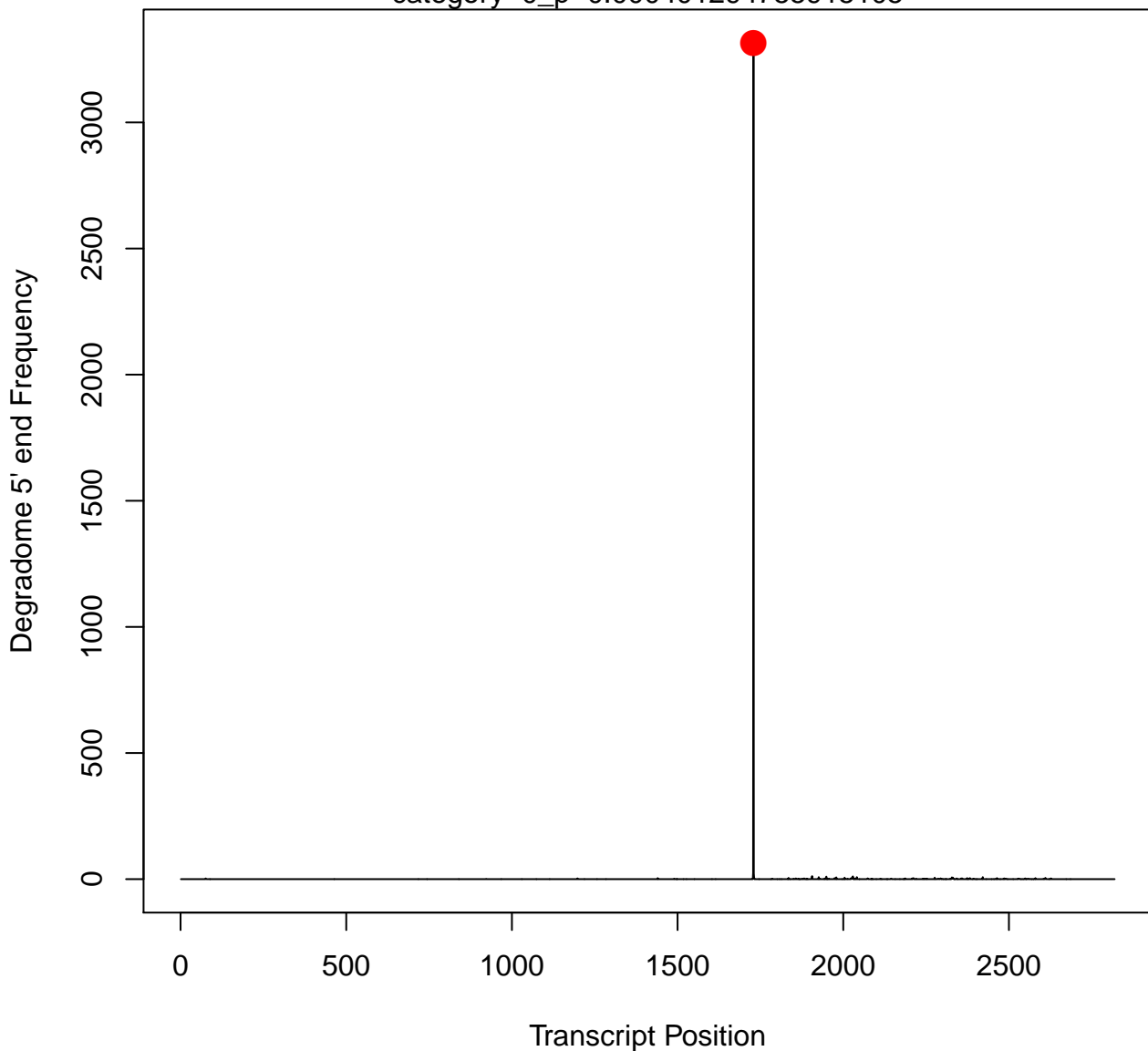

Supplement: Supplementary file 3 [file Data_Sheet_3.zip › Sit-miR160d_Seita.9G219800.1_1729_TPlot.pdf]

**T=Seita.9G254200.1\_Q=Sit-miR160d\_S=262**

category=2\_p=0.999994541599596

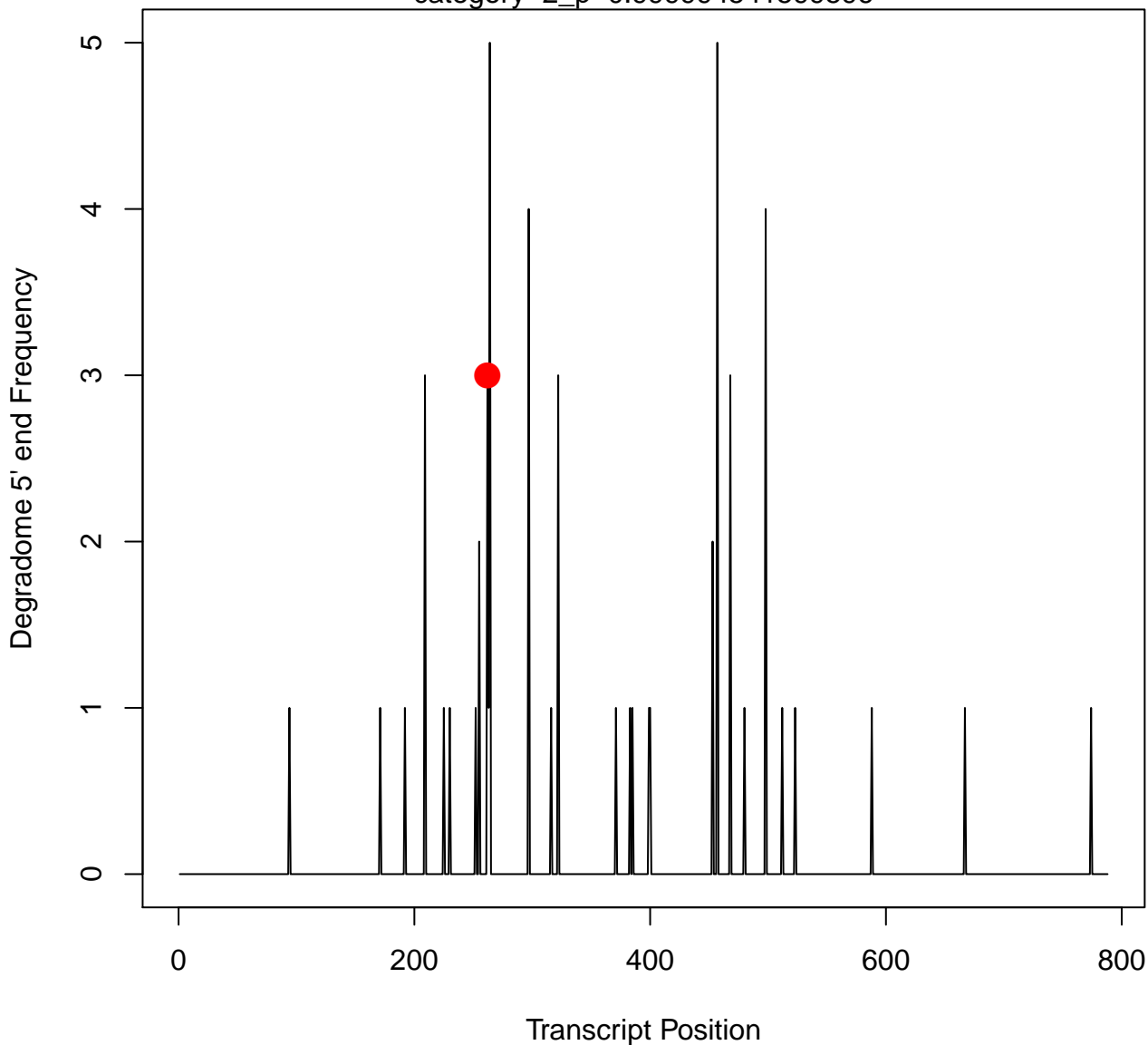

Supplement: Supplementary file 3 [file Data_Sheet_3.zip › Sit-miR160d_Seita.9G254200.1_262_TPlot.pdf]

**T=Seita.2G116400.1\_Q=Sit-miR162\_S=761**

category=2\_p=0.300875254921719

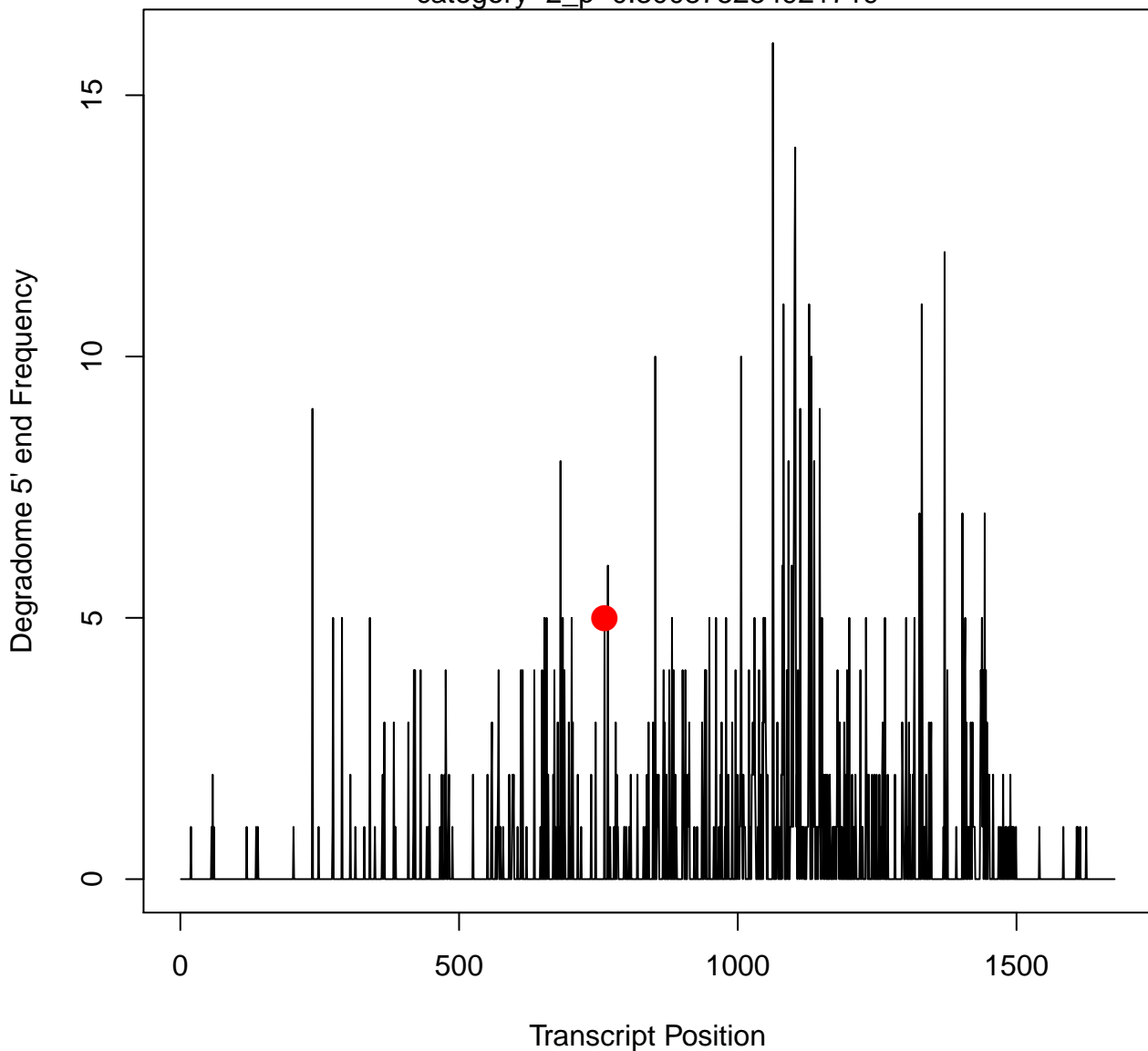

Supplement: Supplementary file 3 [file Data_Sheet_3.zip › Sit-miR162_Seita.2G116400.1_761_TPlot.pdf]

**T=Seita.3G186400.1\_Q=Sit-miR162\_S=4897**

category=2\_p=0.957283004465257

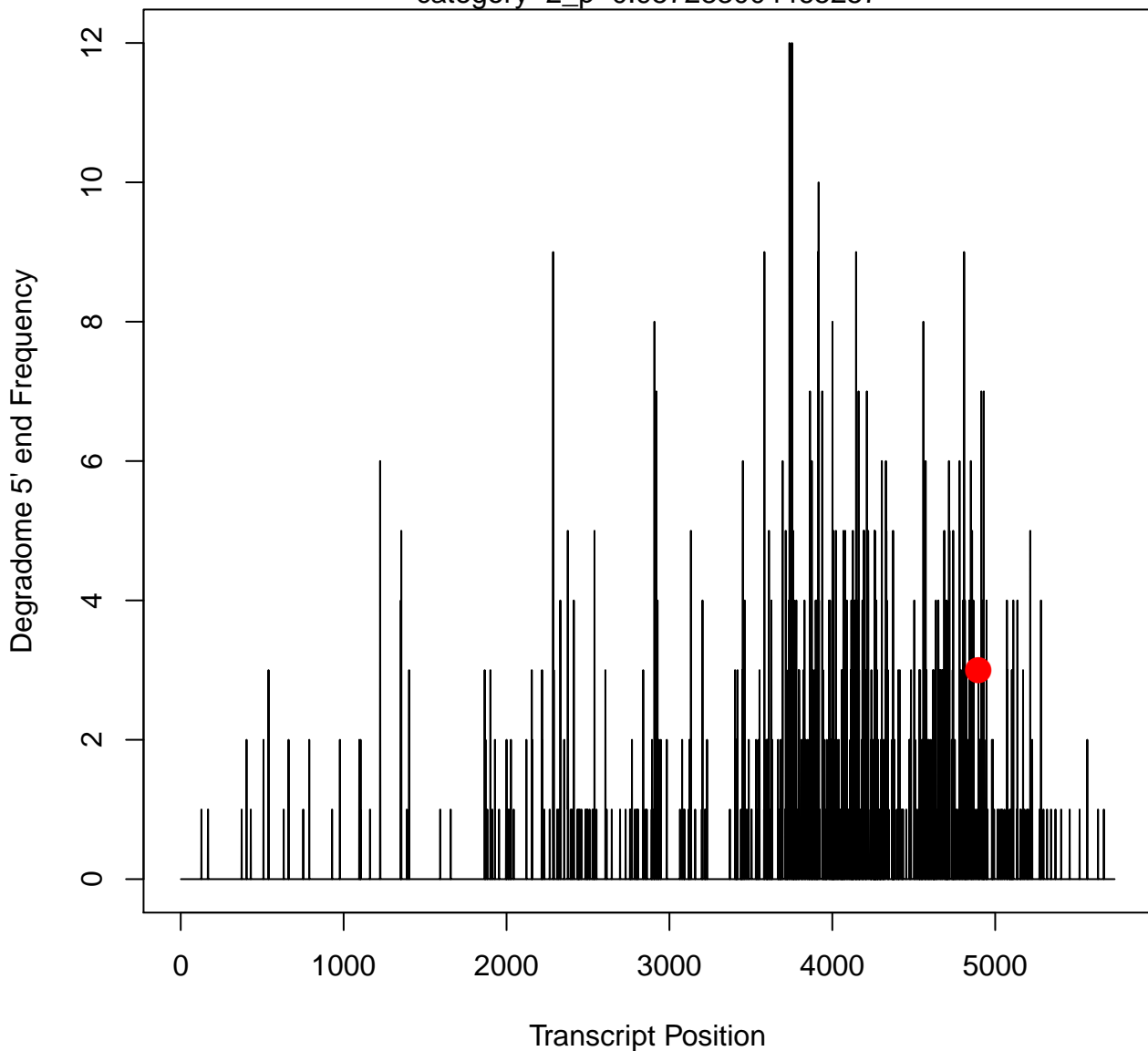

Supplement: Supplementary file 3 [file Data_Sheet_3.zip › Sit-miR162_Seita.3G186400.1_4897_TPlot.pdf]

**T=Seita.5G064400.1\_Q=Sit-miR162\_S=878**

category=2\_p=0.033513742332527

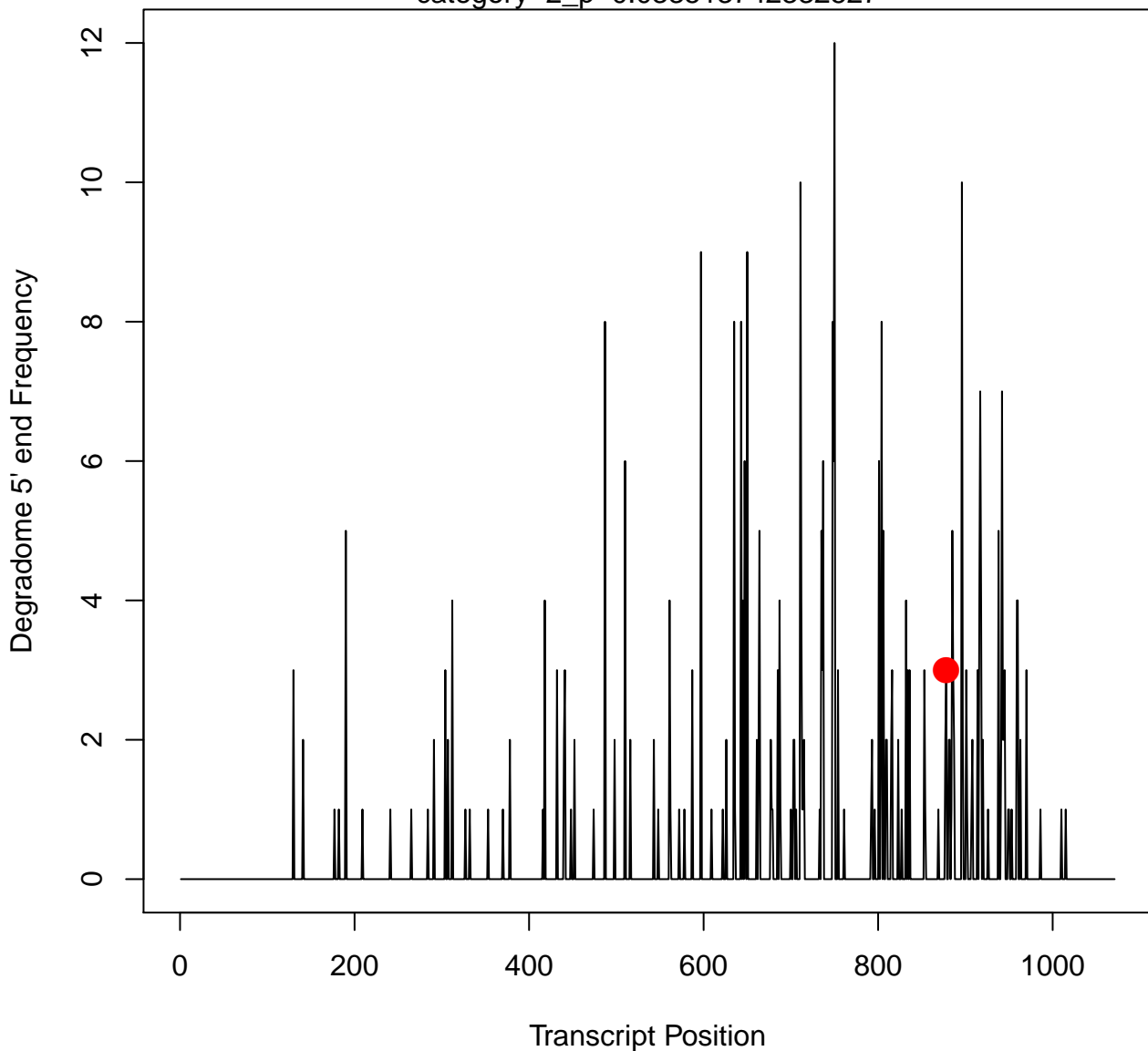

Supplement: Supplementary file 3 [file Data_Sheet_3.zip › Sit-miR162_Seita.5G064400.1_878_TPlot.pdf]

**T=Seita.7G320600.1\_Q=Sit-miR162\_S=1126**

category=2\_p=0.870657576479375

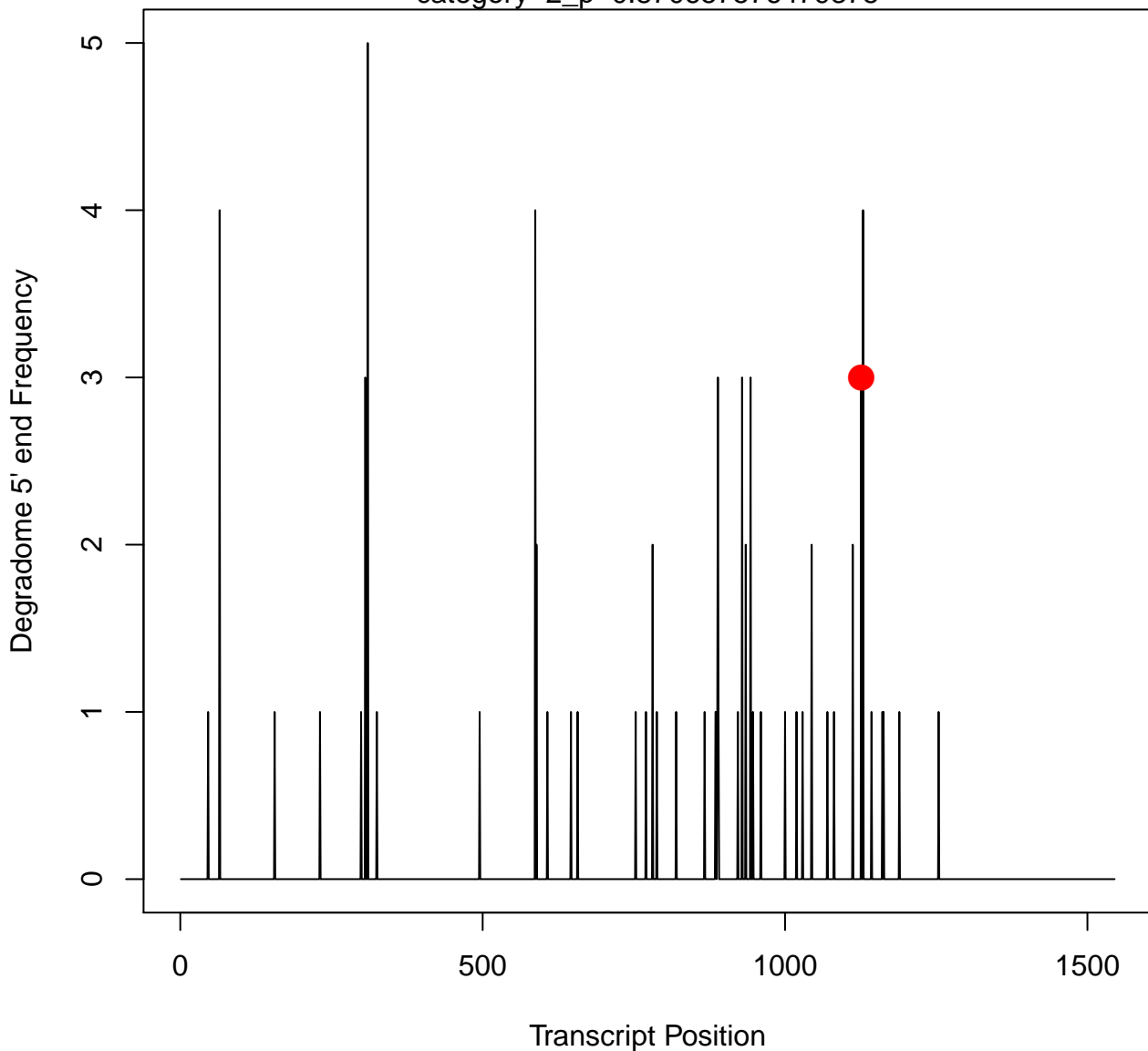

Supplement: Supplementary file 3 [file Data_Sheet_3.zip › Sit-miR162_Seita.7G320600.1_1126_TPlot.pdf]

**T=Seita.9G178200.1\_Q=Sit-miR162\_S=3612**

category=2\_p=0.950201057002698

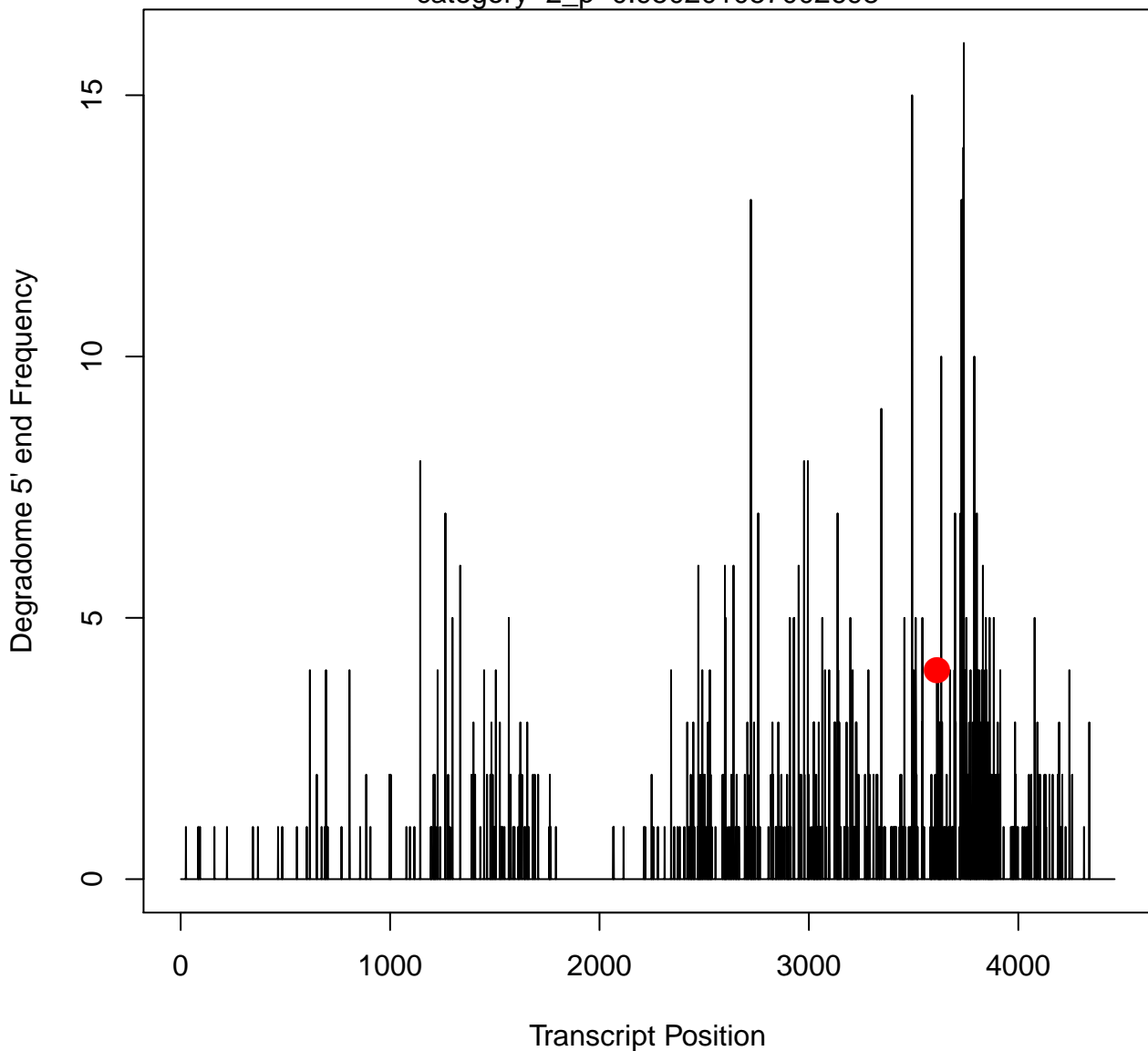

Supplement: Supplementary file 3 [file Data_Sheet_3.zip › Sit-miR162_Seita.9G178200.1_3612_TPlot.pdf]

**T=Seita.9G562200.1\_Q=Sit-miR162\_S=3530**

category=0\_p=0.000401294755915105

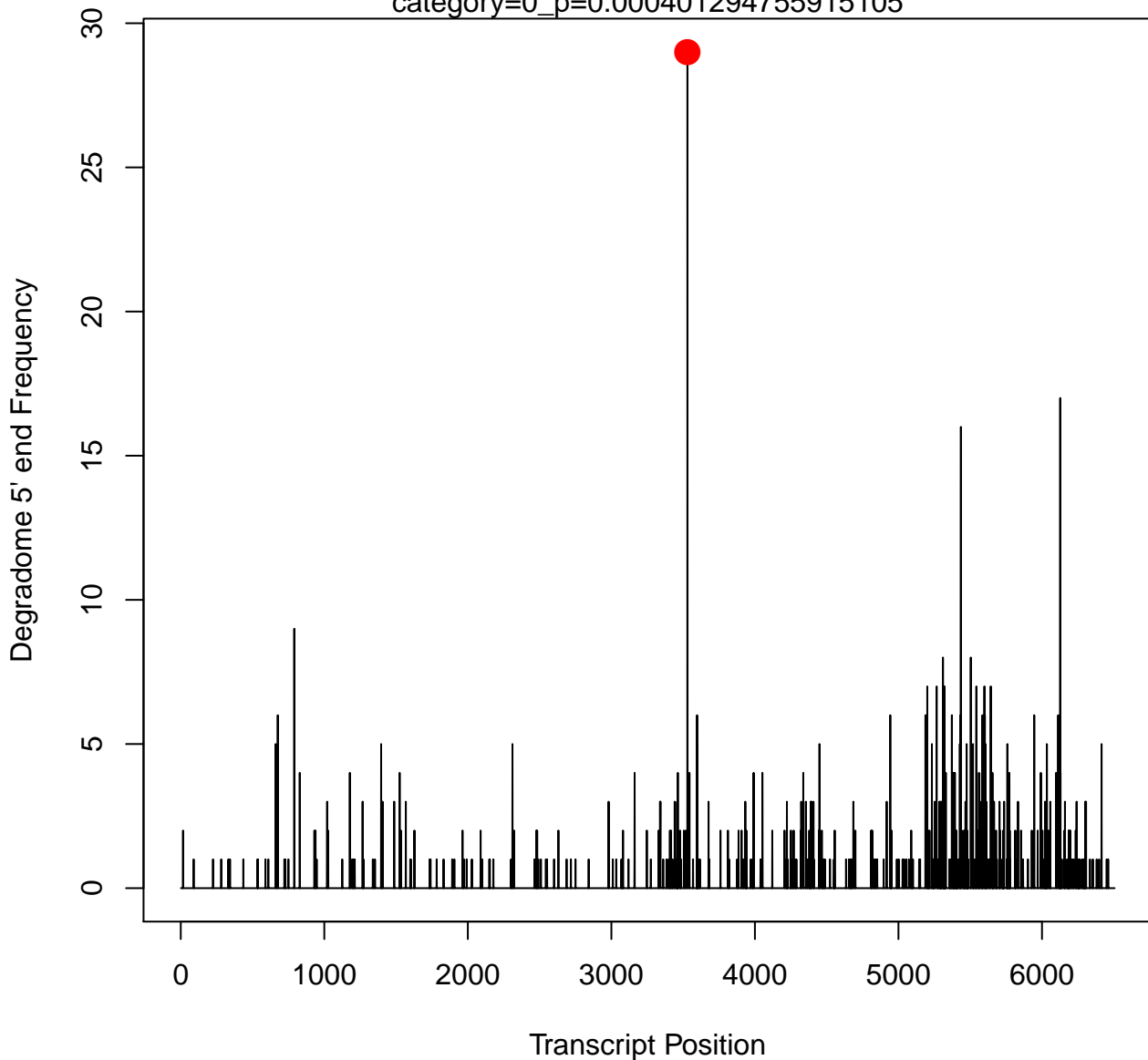

Supplement: Supplementary file 3 [file Data_Sheet_3.zip › Sit-miR162_Seita.9G562200.1_3530_TPlot.pdf]

**T=Seita.1G209000.1\_Q=Sit-miR164a\_S=999**

category=0\_p=0.00160421305724157

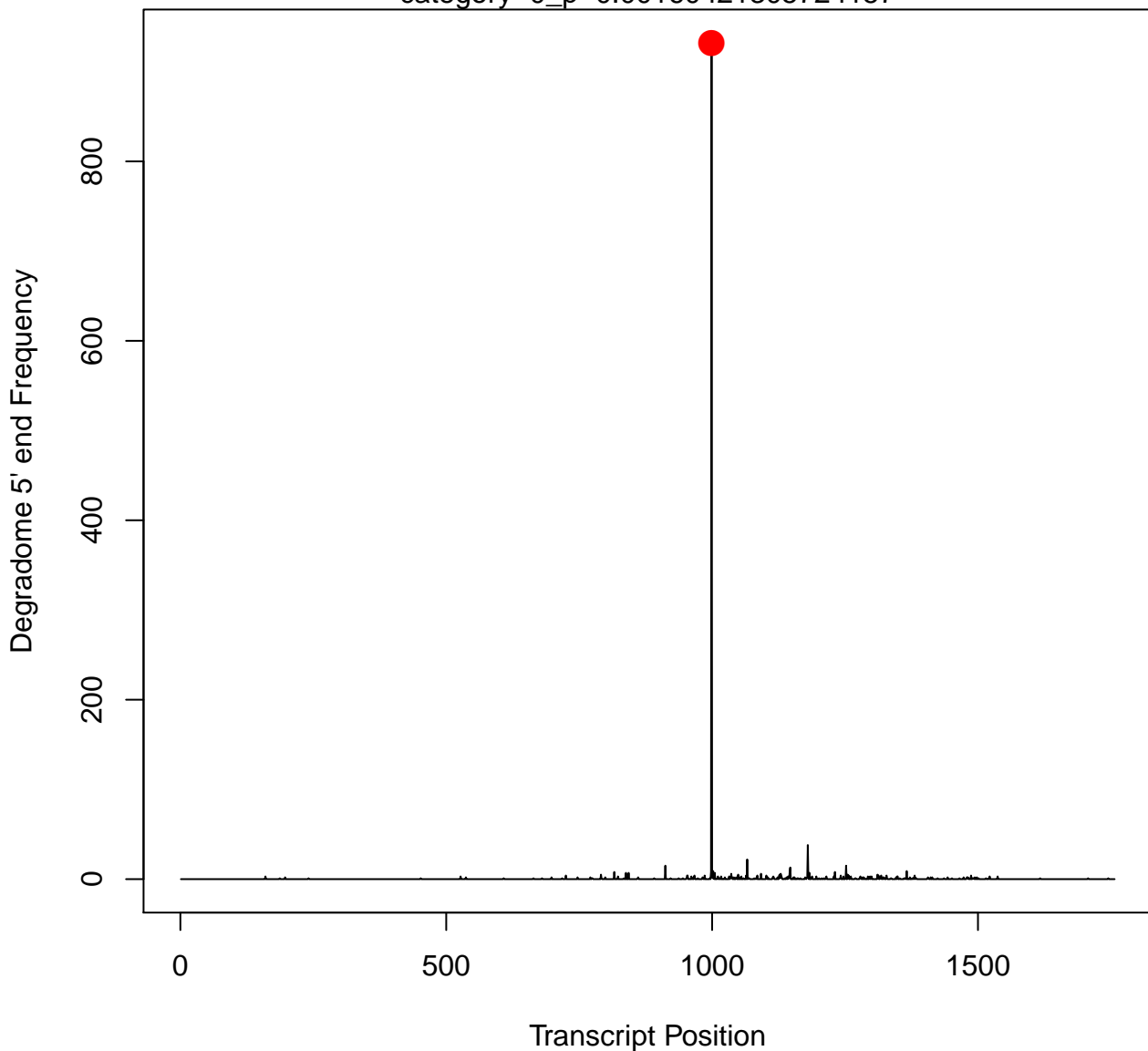

Supplement: Supplementary file 3 [file Data_Sheet_3.zip › Sit-miR164a_Seita.1G209000.1_999_TPlot.pdf]

**T=Seita.5G306600.1\_Q=Sit-miR164a\_S=101**

category=2\_p=0.93458841708386

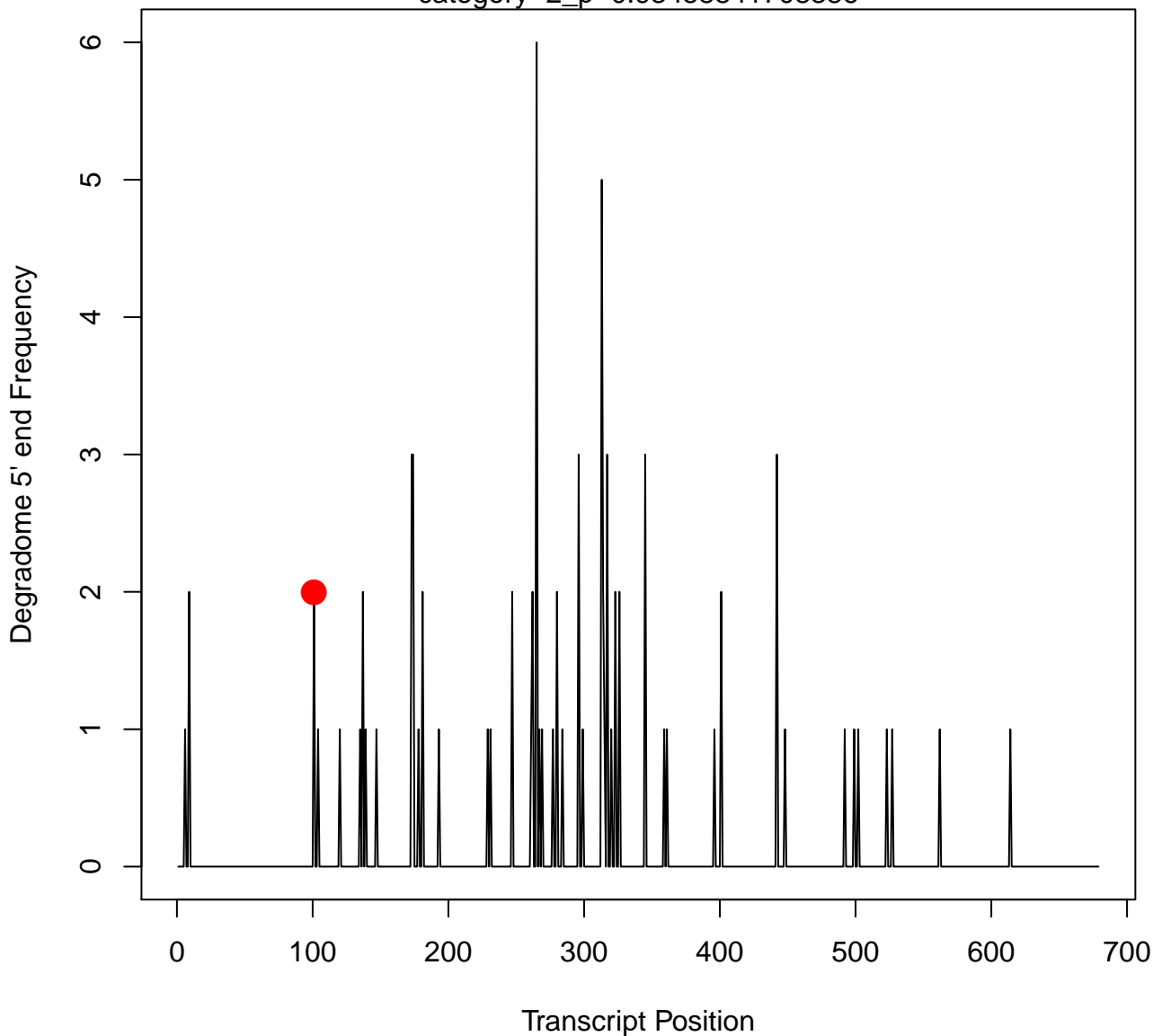

Supplement: Supplementary file 3 [file Data_Sheet_3.zip › Sit-miR164a_Seita.5G306600.1_101_TPlot.pdf]

**T=Seita.7G124900.1\_Q=Sit-miR164a\_S=859**

category=0\_p=0.00200486405086941

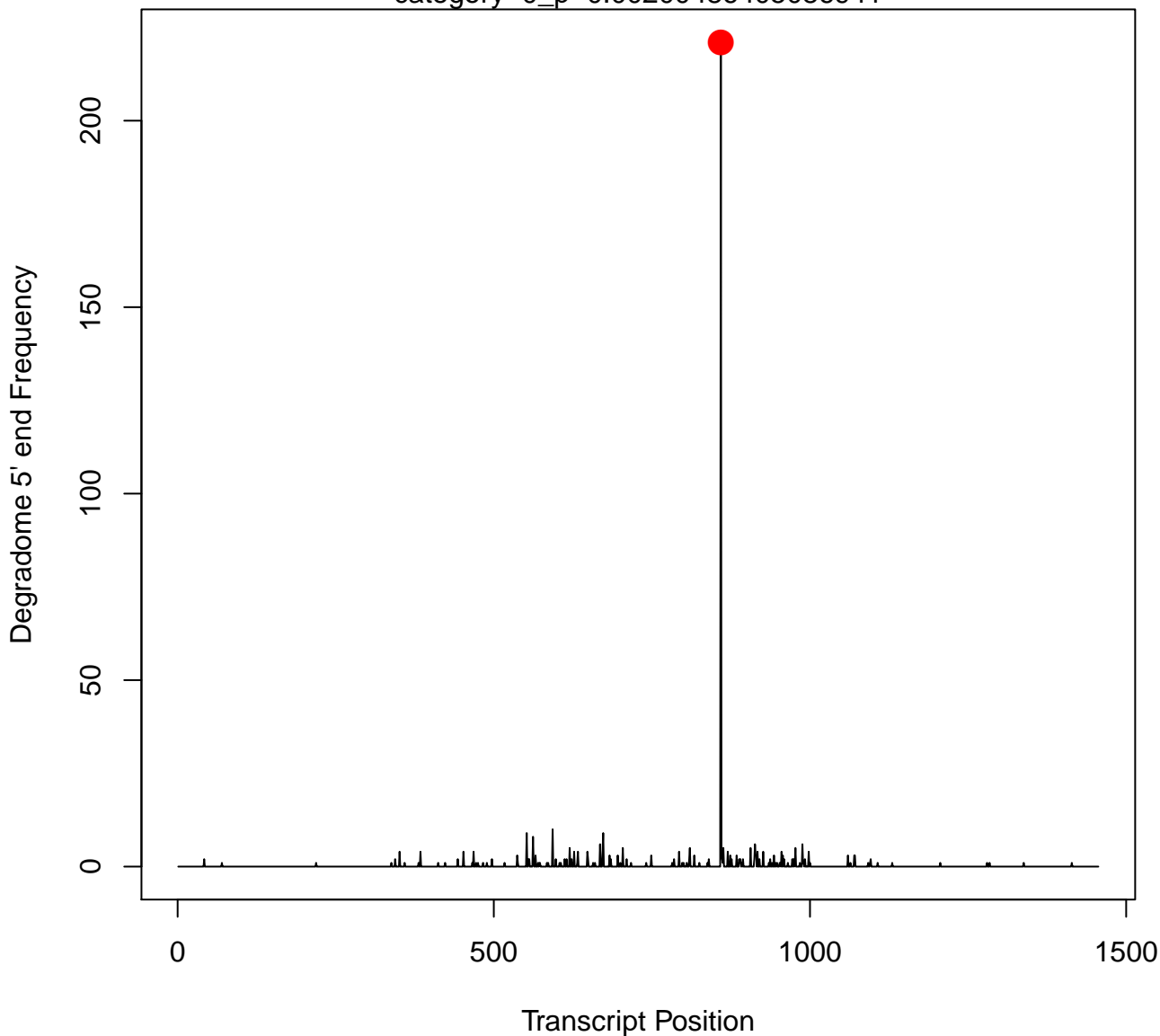

Supplement: Supplementary file 3 [file Data_Sheet_3.zip › Sit-miR164a_Seita.7G124900.1_859_TPlot.pdf]

**T=Seita.7G150700.1\_Q=Sit-miR164a\_S=225**

category=0\_p=0.0670819586851021

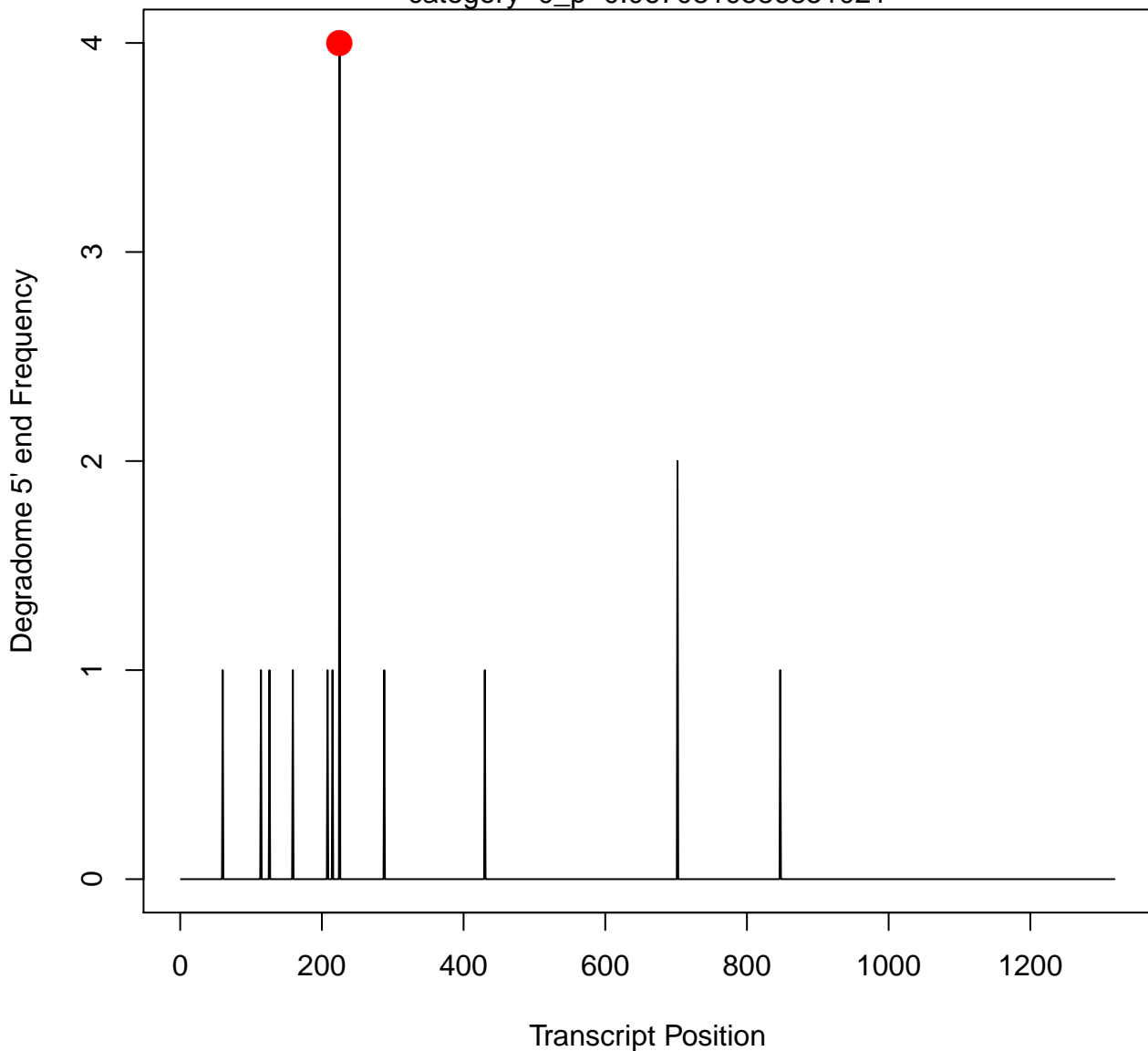

Supplement: Supplementary file 3 [file Data_Sheet_3.zip › Sit-miR164a_Seita.7G150700.1_225_TPlot.pdf]

**T=Seita.8G106600.1\_Q=Sit-miR164a\_S=485**

category=2\_p=0.212283790049424

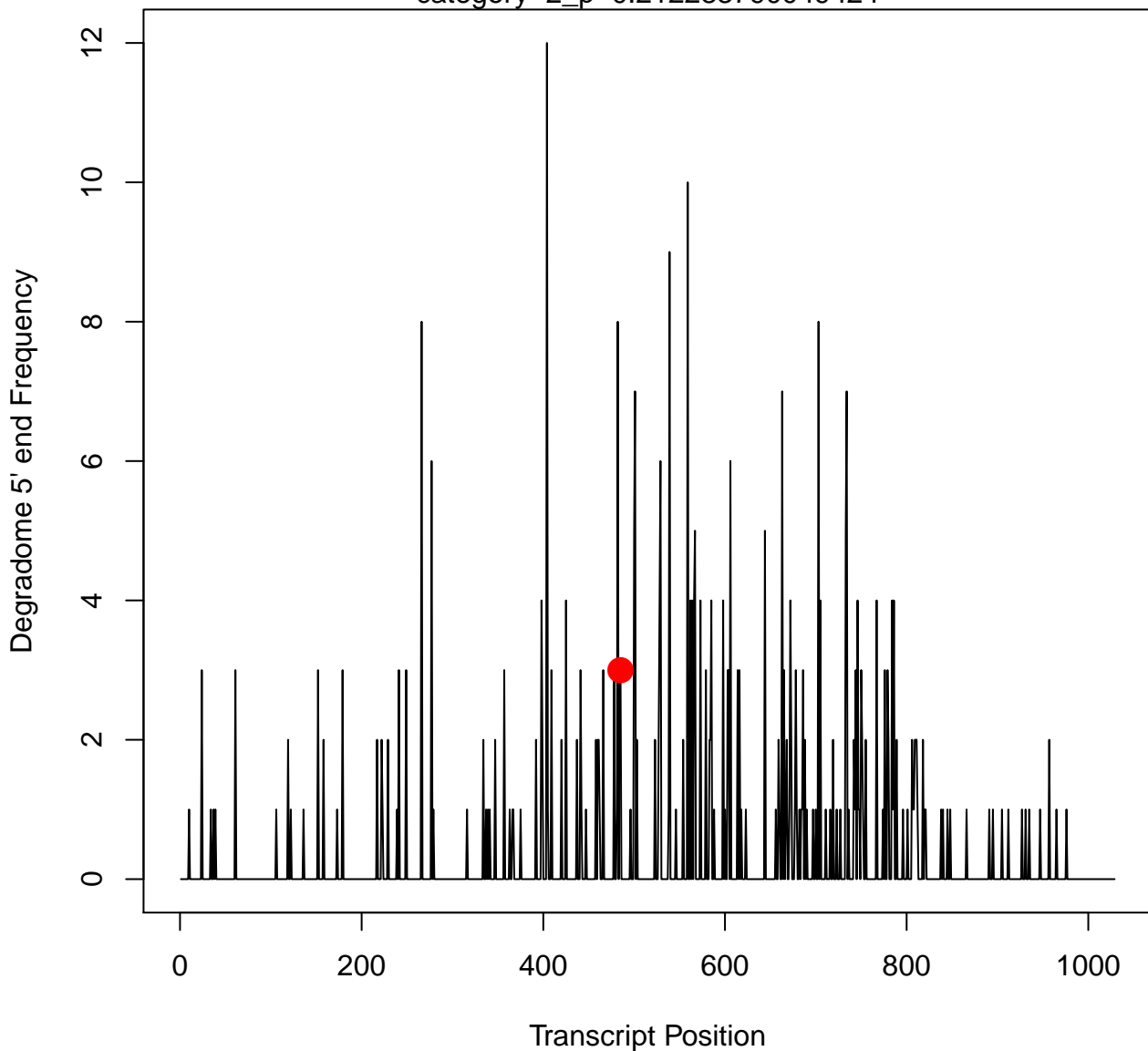

Supplement: Supplementary file 3 [file Data_Sheet_3.zip › Sit-miR164a_Seita.8G106600.1_485_TPlot.pdf]

**T=Seita.1G369500.1\_Q=Sit-miR164c\_S=3079**

category=2\_p=0.999997332068109

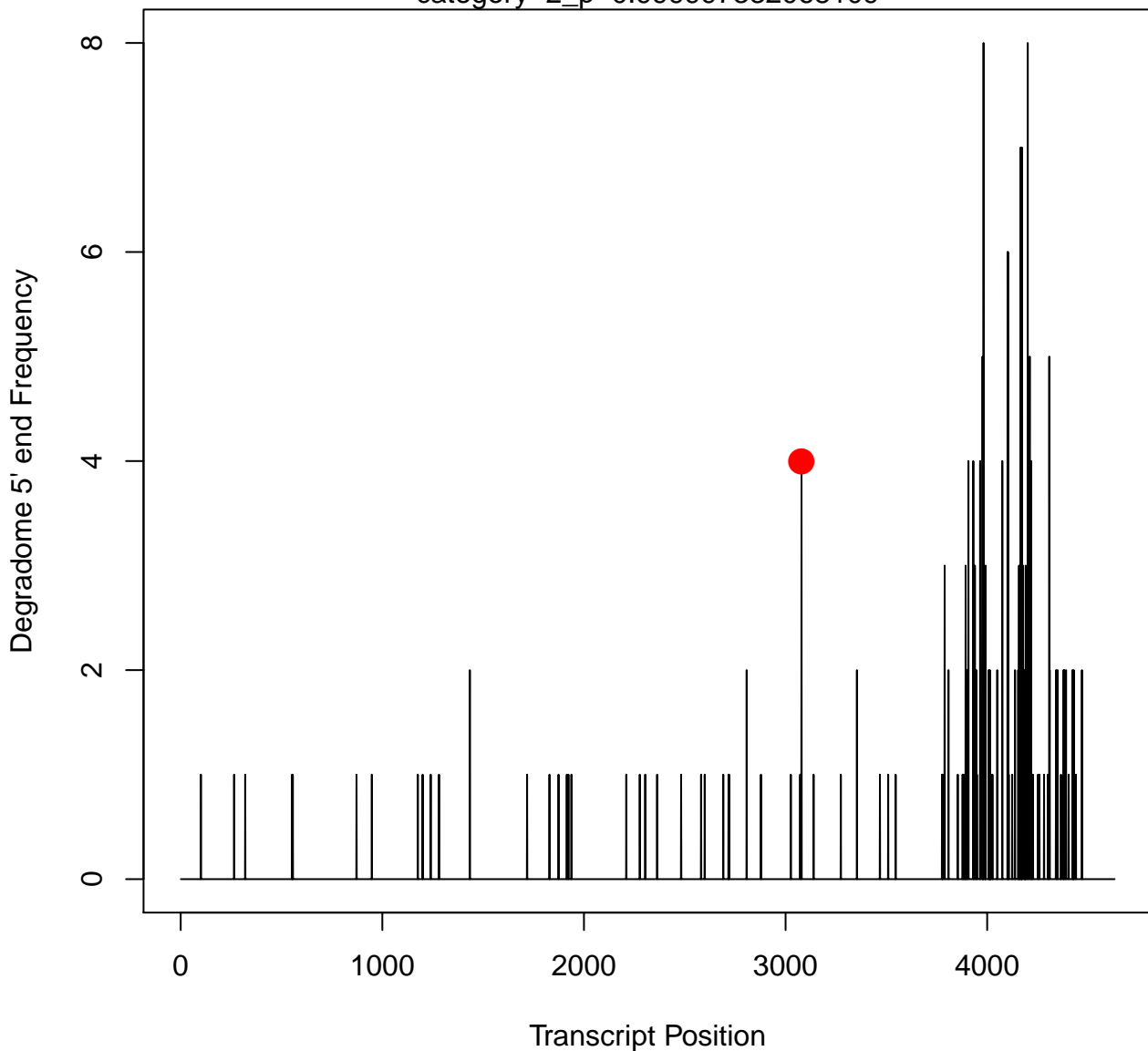

Supplement: Supplementary file 3 [file Data_Sheet_3.zip › Sit-miR164c_Seita.1G369500.1_3079_TPlot.pdf]

**T=Seita.3G302600.1\_Q=Sit-miR164c\_S=10996**

category=2\_p=0.999996836292513

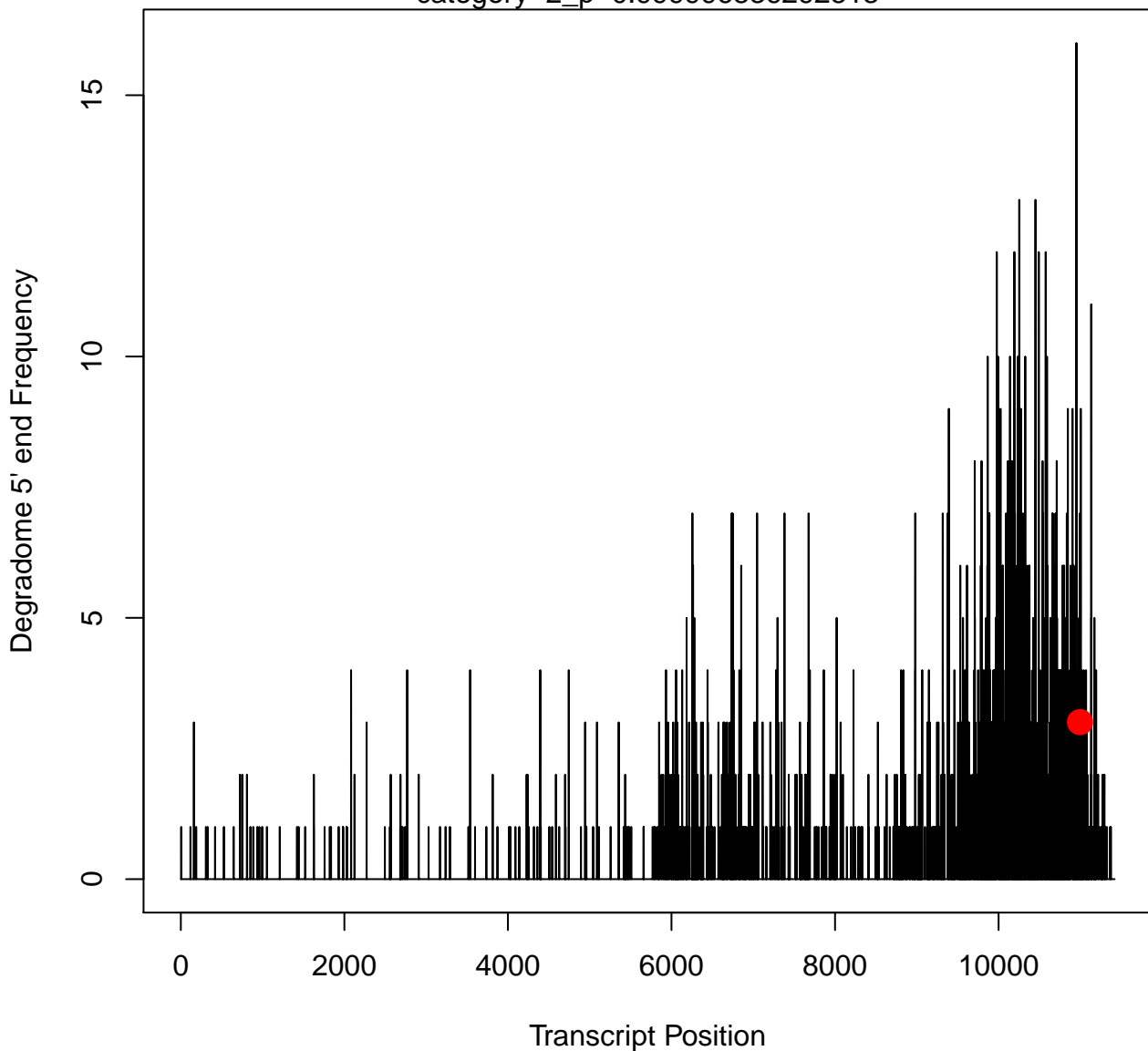

Supplement: Supplementary file 3 [file Data_Sheet_3.zip › Sit-miR164c_Seita.3G302600.1_10996_TPlot.pdf]

**T=Seita.7G090700.1\_Q=Sit-miR164c\_S=1294**

category=2\_p=0.998960247023998

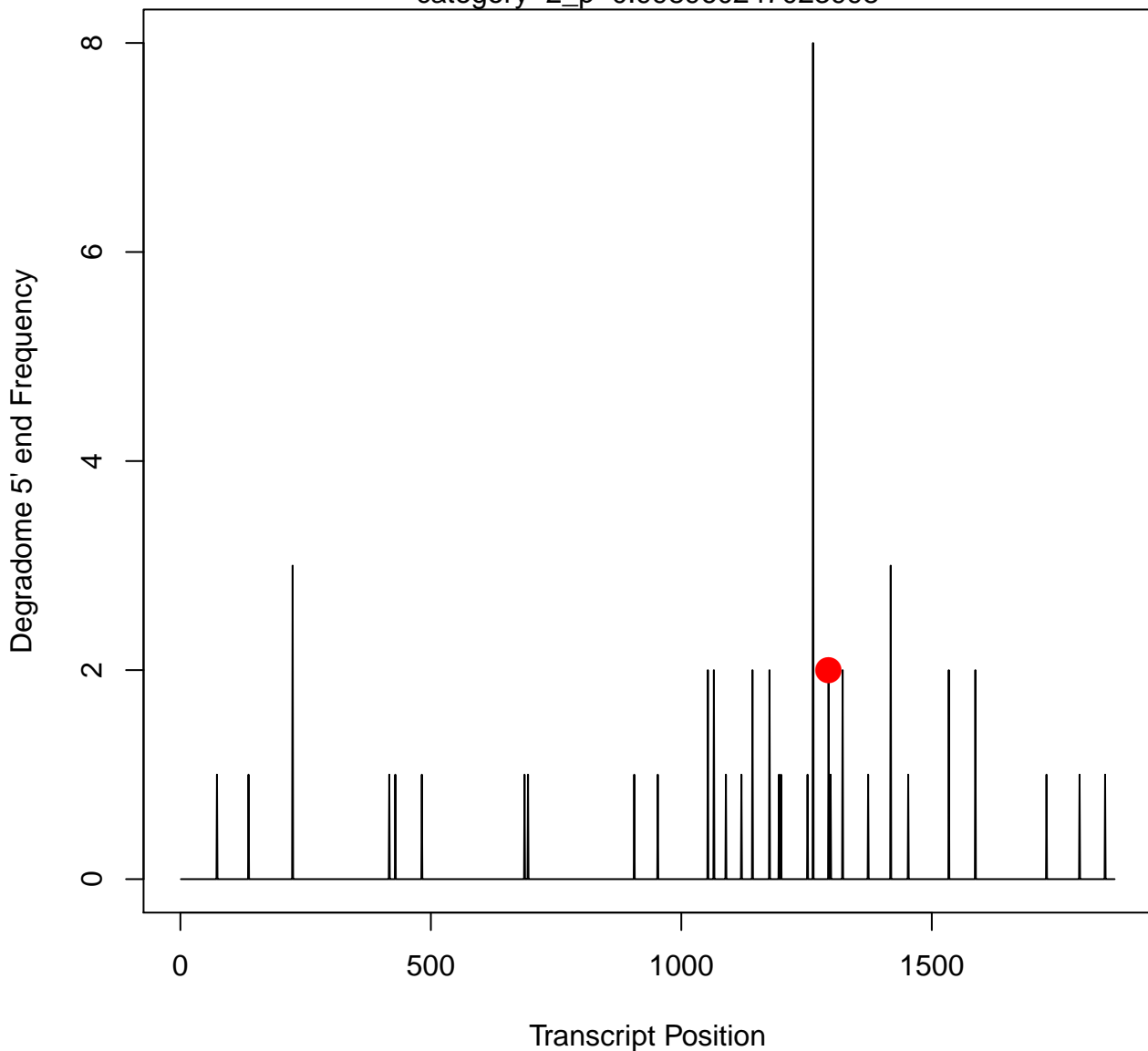

Supplement: Supplementary file 3 [file Data_Sheet_3.zip › Sit-miR164c_Seita.7G090700.1_1294_TPlot.pdf]

**T=Seita.9G564500.1\_Q=Sit-miR164c\_S=1007**

category=2\_p=0.824215796674898

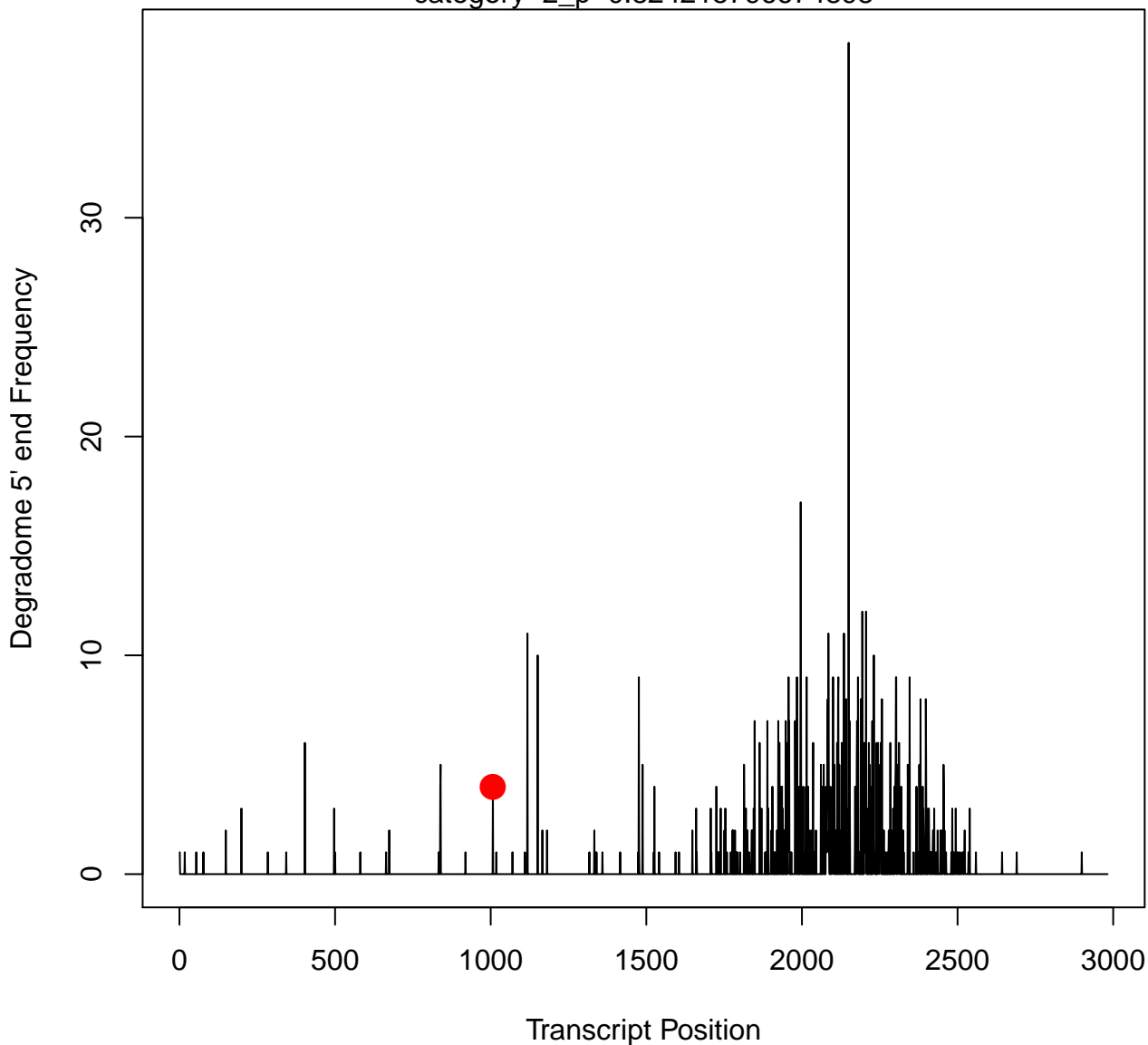

Supplement: Supplementary file 3 [file Data_Sheet_3.zip › Sit-miR164c_Seita.9G564500.1_1007_TPlot.pdf]

**T=Seita.6G097500.1\_Q=Sit-miR164d\_S=1205**

category=2\_p=0.999365739626422

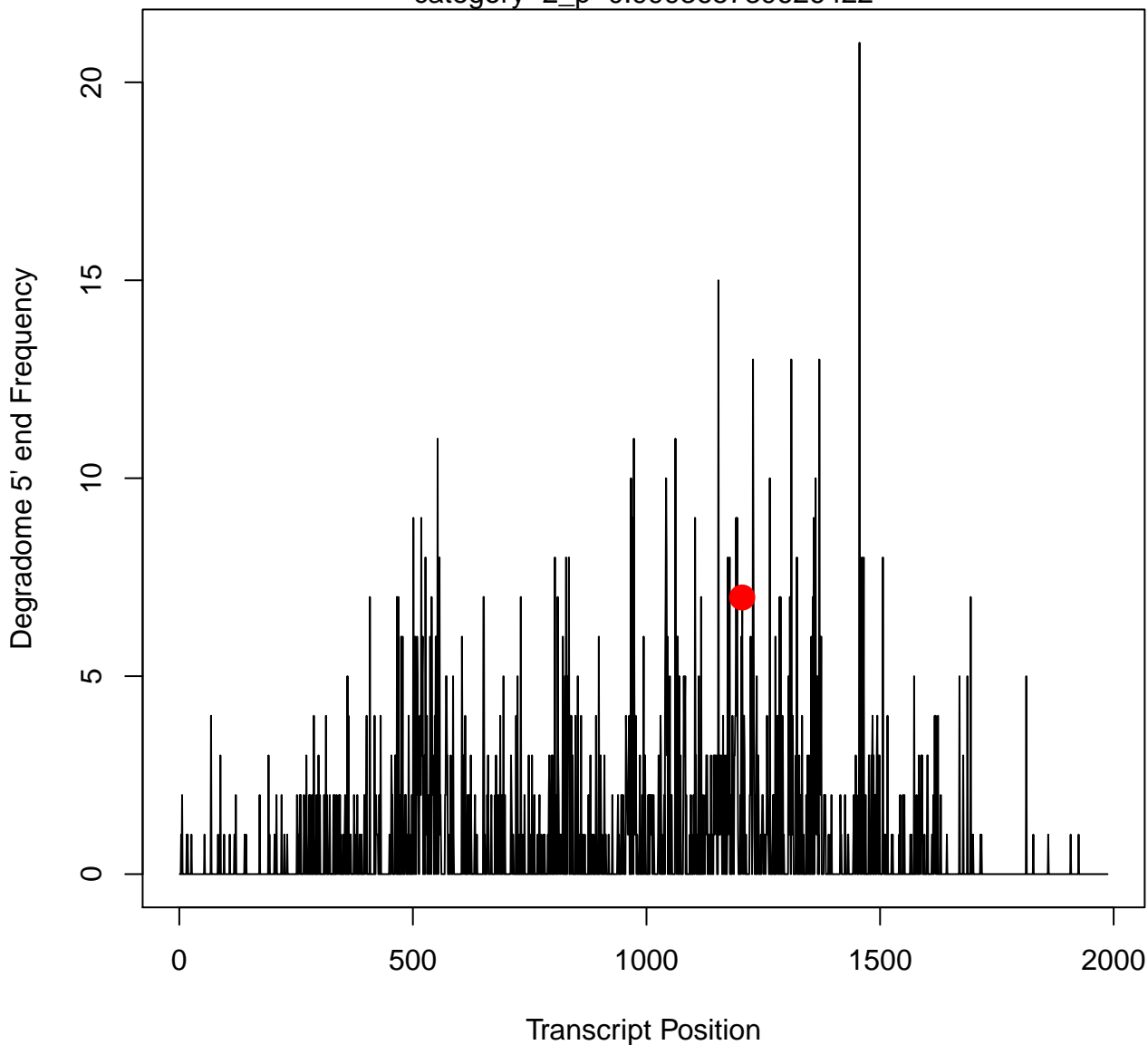

Supplement: Supplementary file 3 [file Data_Sheet_3.zip › Sit-miR164d_Seita.6G097500.1_1205_TPlot.pdf]

**T=Seita.3G244600.1\_Q=Sit-miR164e\_S=300**

category=2\_p=0.999905978789086

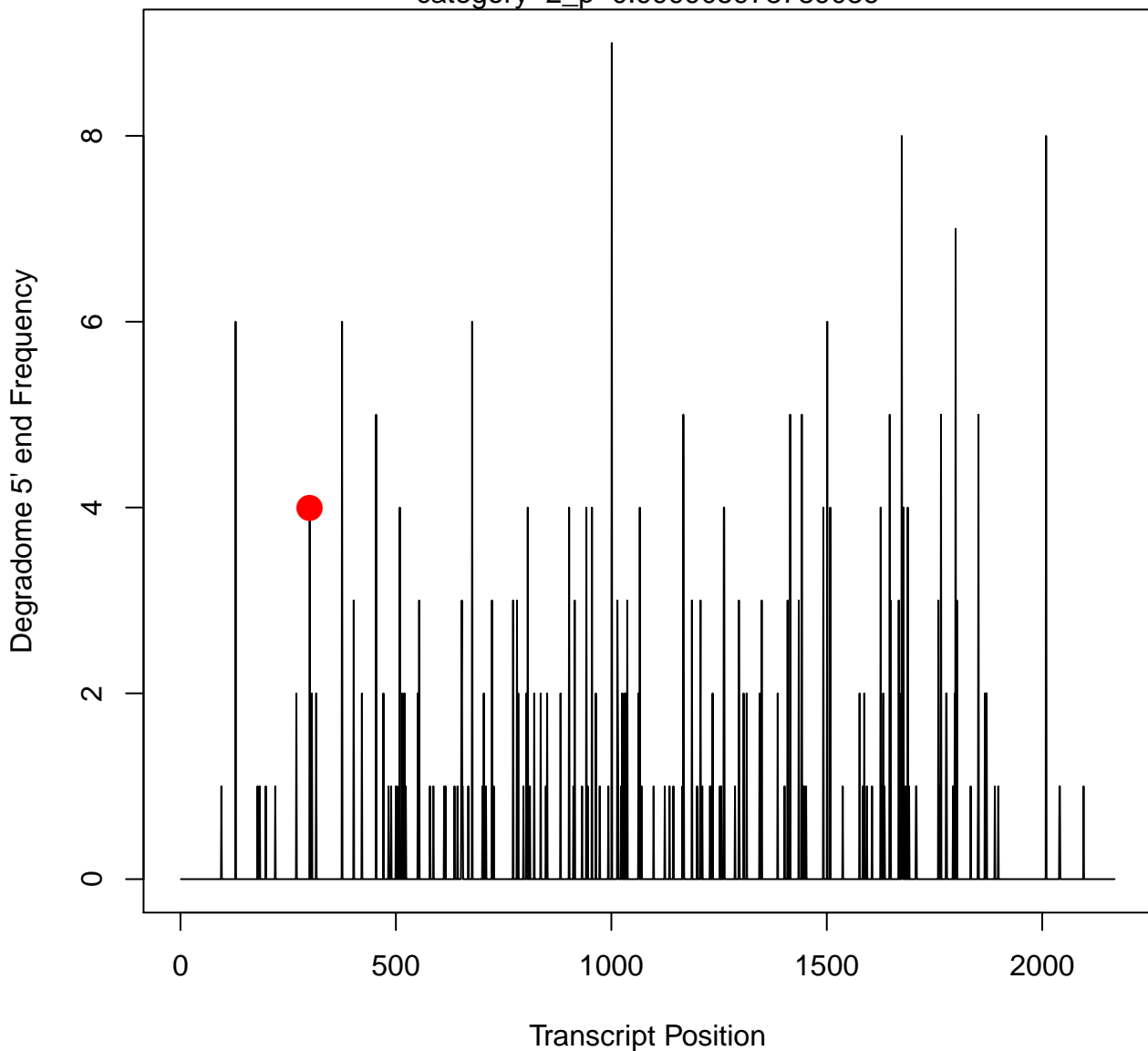

Supplement: Supplementary file 3 [file Data_Sheet_3.zip › Sit-miR164e_Seita.3G244600.1_300_TPlot.pdf]

**T=Seita.4G090500.1\_Q=Sit-miR164e\_S=744**

category=2\_p=0.999606443450437

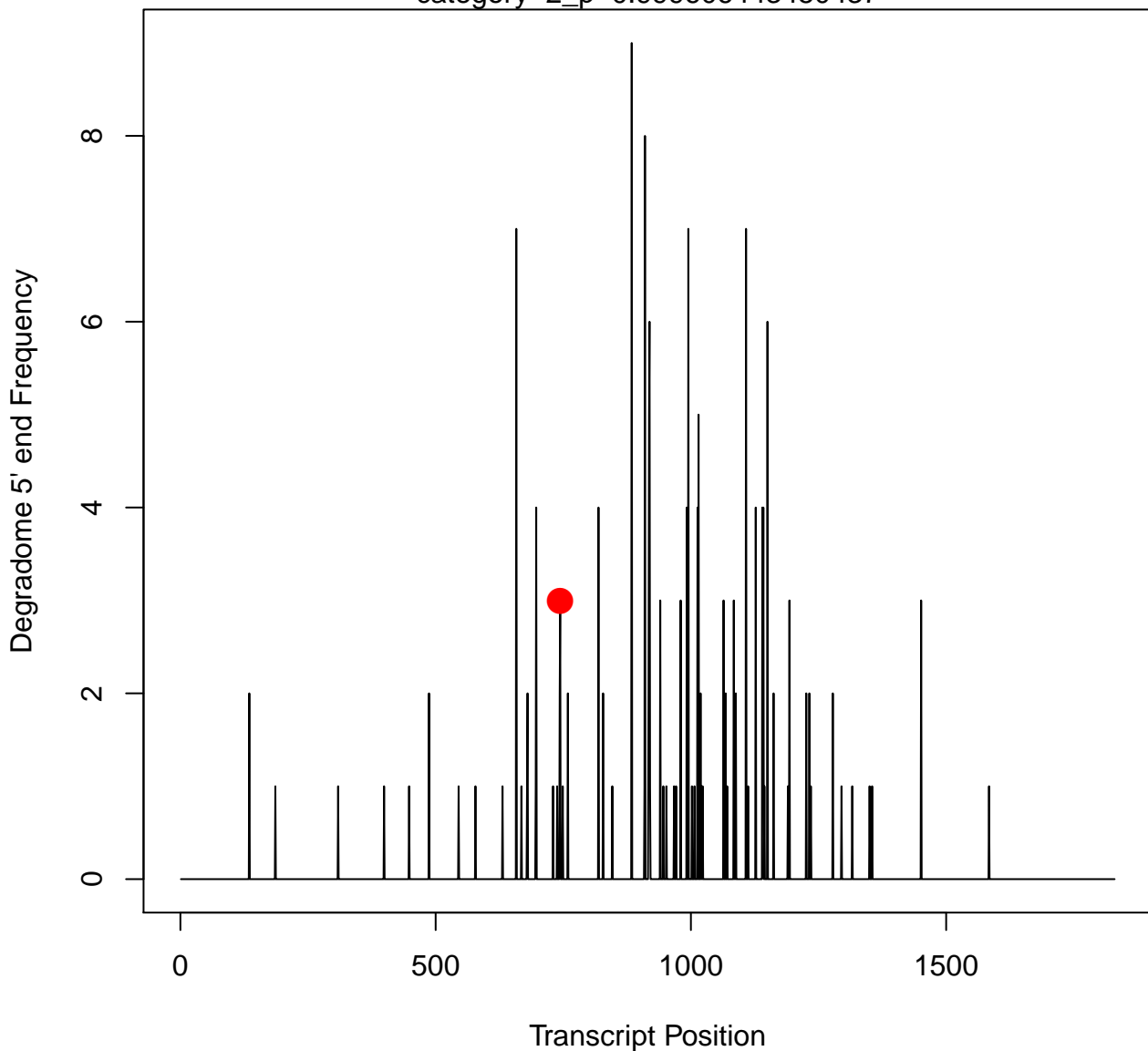

Supplement: Supplementary file 3 [file Data_Sheet_3.zip › Sit-miR164e_Seita.4G090500.1_744_TPlot.pdf]

**T=Seita.5G099100.1\_Q=Sit-miR164e\_S=291**

category=2\_p=0.998206095826271

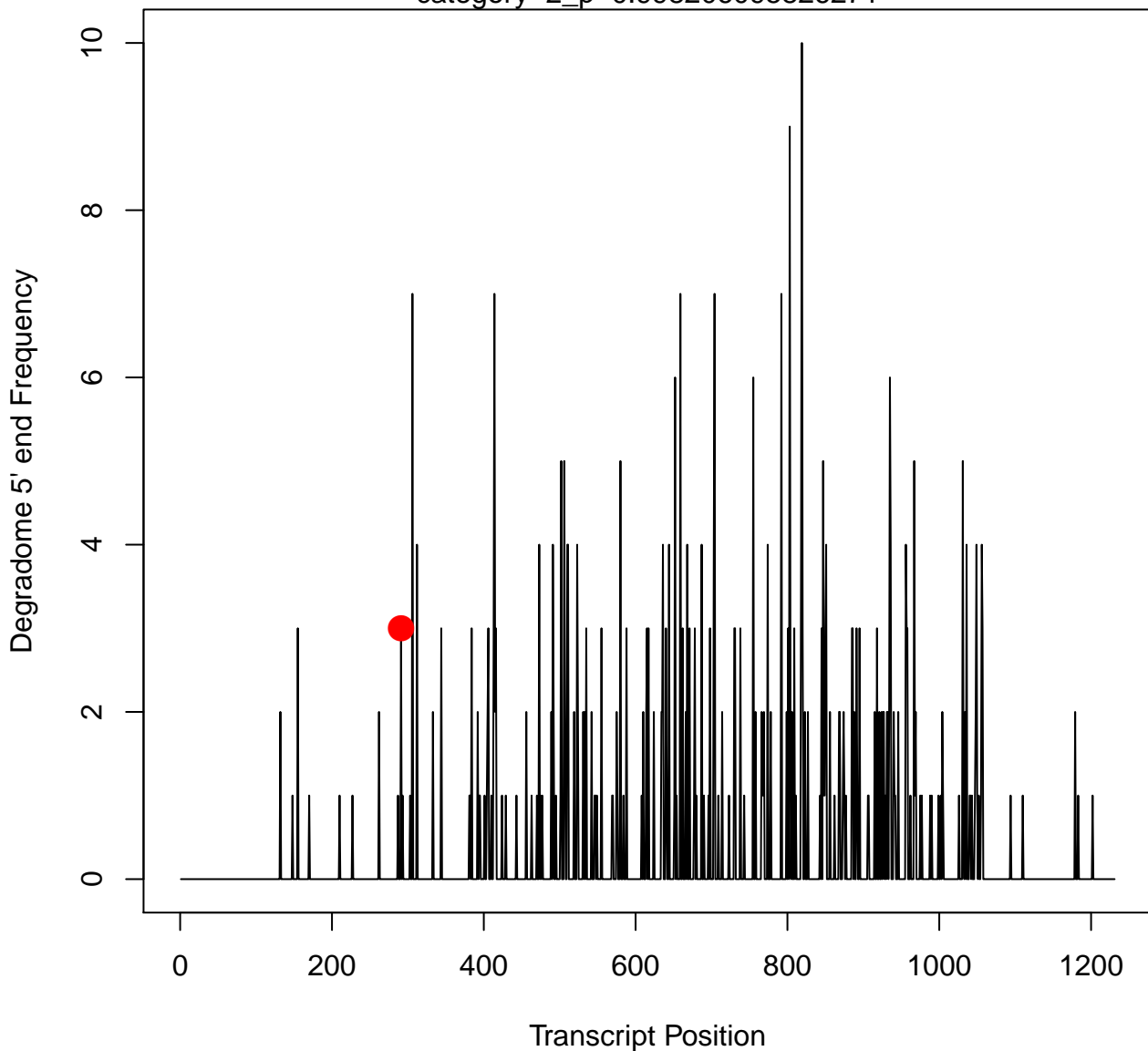

Supplement: Supplementary file 3 [file Data_Sheet_3.zip › Sit-miR164e_Seita.5G099100.1_291_TPlot.pdf]

**T=Seita.5G130400.1\_Q=Sit-miR164e\_S=380**

category=2\_p=0.769105079363422

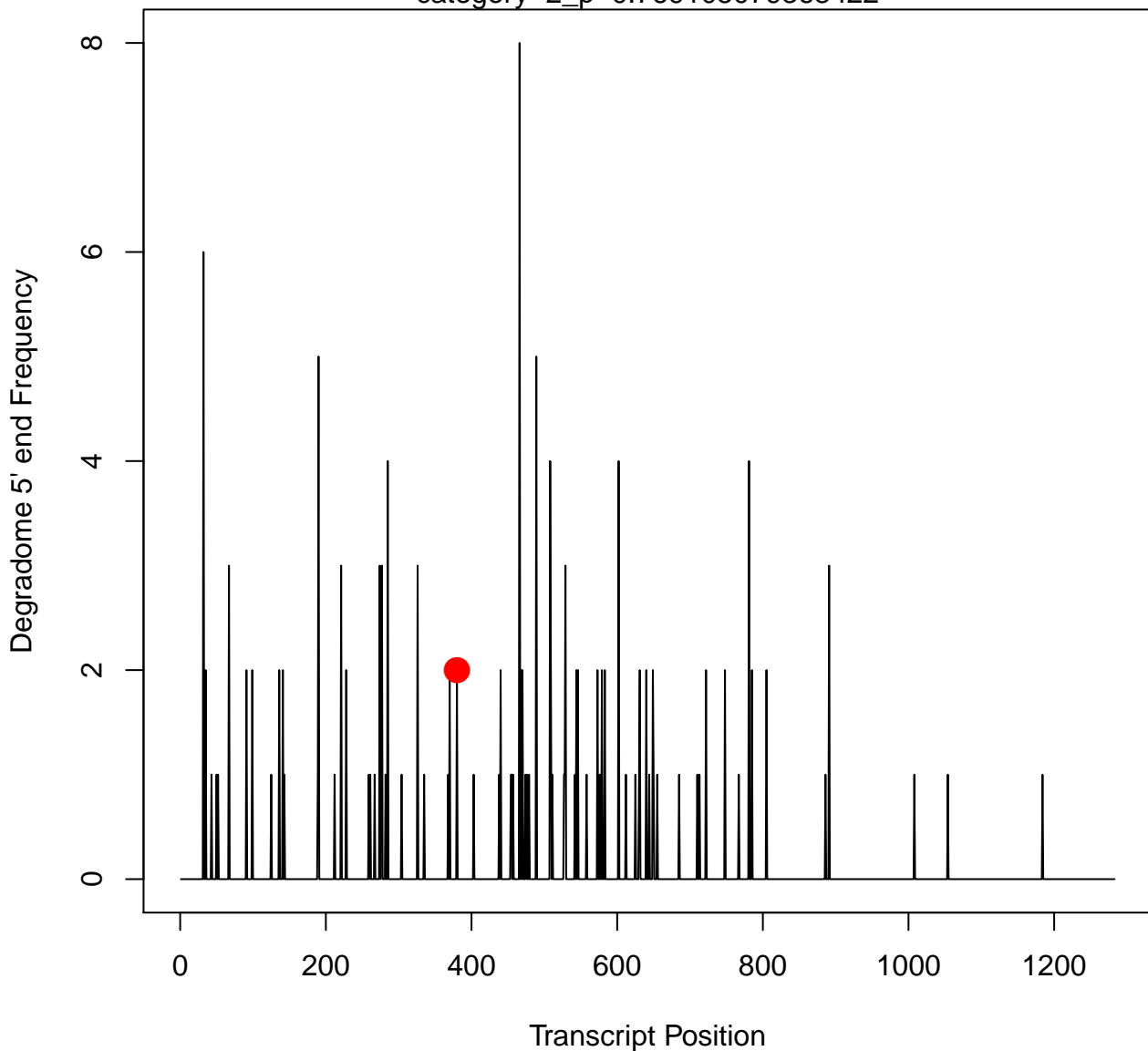

Supplement: Supplementary file 3 [file Data_Sheet_3.zip › Sit-miR164e_Seita.5G130400.1_380_TPlot.pdf]

**T=Seita.2G174200.1\_Q=Sit-miR164f\_S=201**

category=0\_p=0.00120340121992535

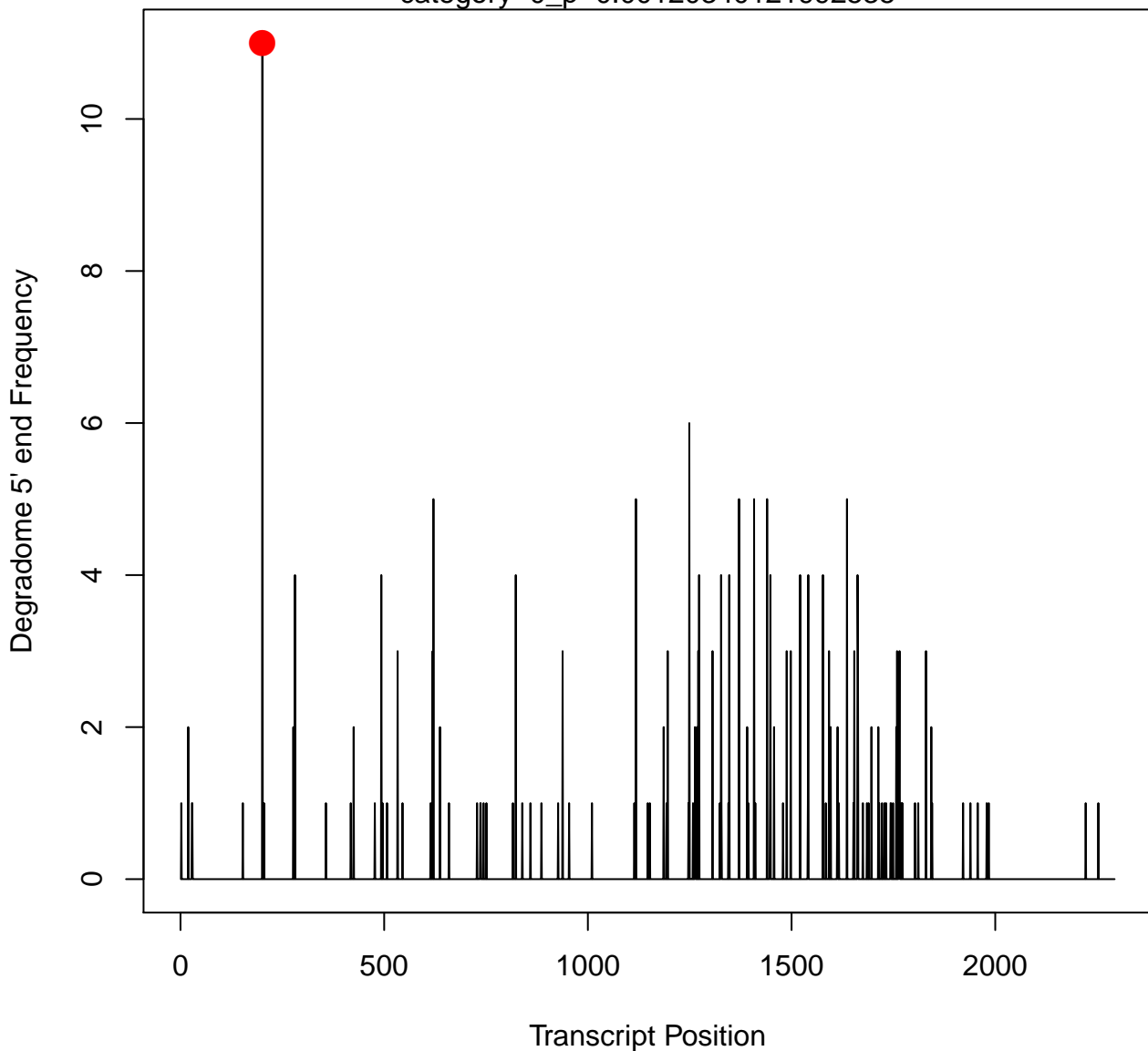

Supplement: Supplementary file 3 [file Data_Sheet_3.zip › Sit-miR164f_Seita.2G174200.1_201_TPlot.pdf]

**T=Seita.3G386200.1\_Q=Sit-miR164f\_S=790**

category=0\_p=0.000401294755915105

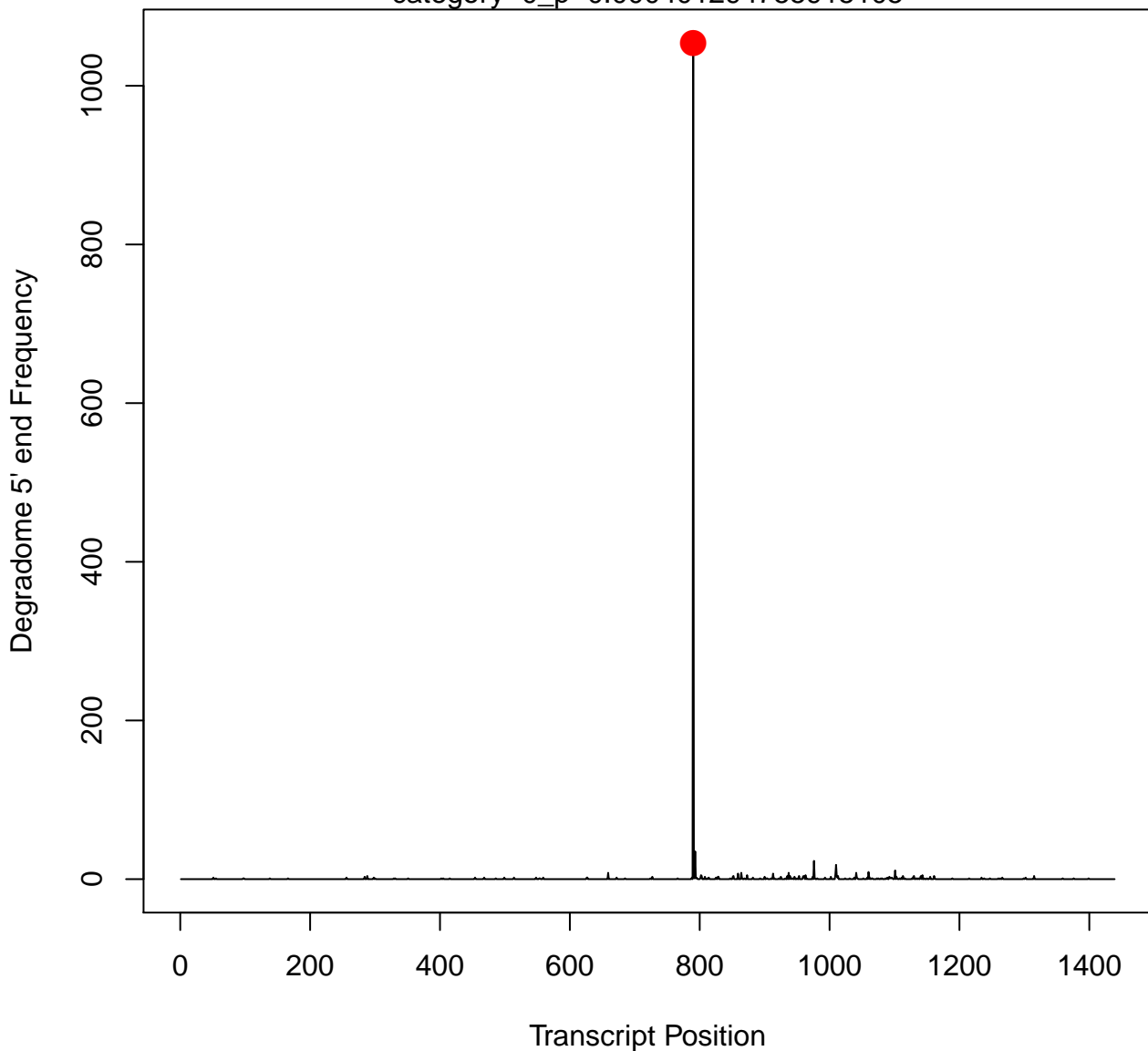

Supplement: Supplementary file 3 [file Data_Sheet_3.zip › Sit-miR164f_Seita.3G386200.1_790_TPlot.pdf]

**T=Seita.4G263400.1\_Q=Sit-miR164f\_S=1216**

category=0\_p=0.00160421305724157

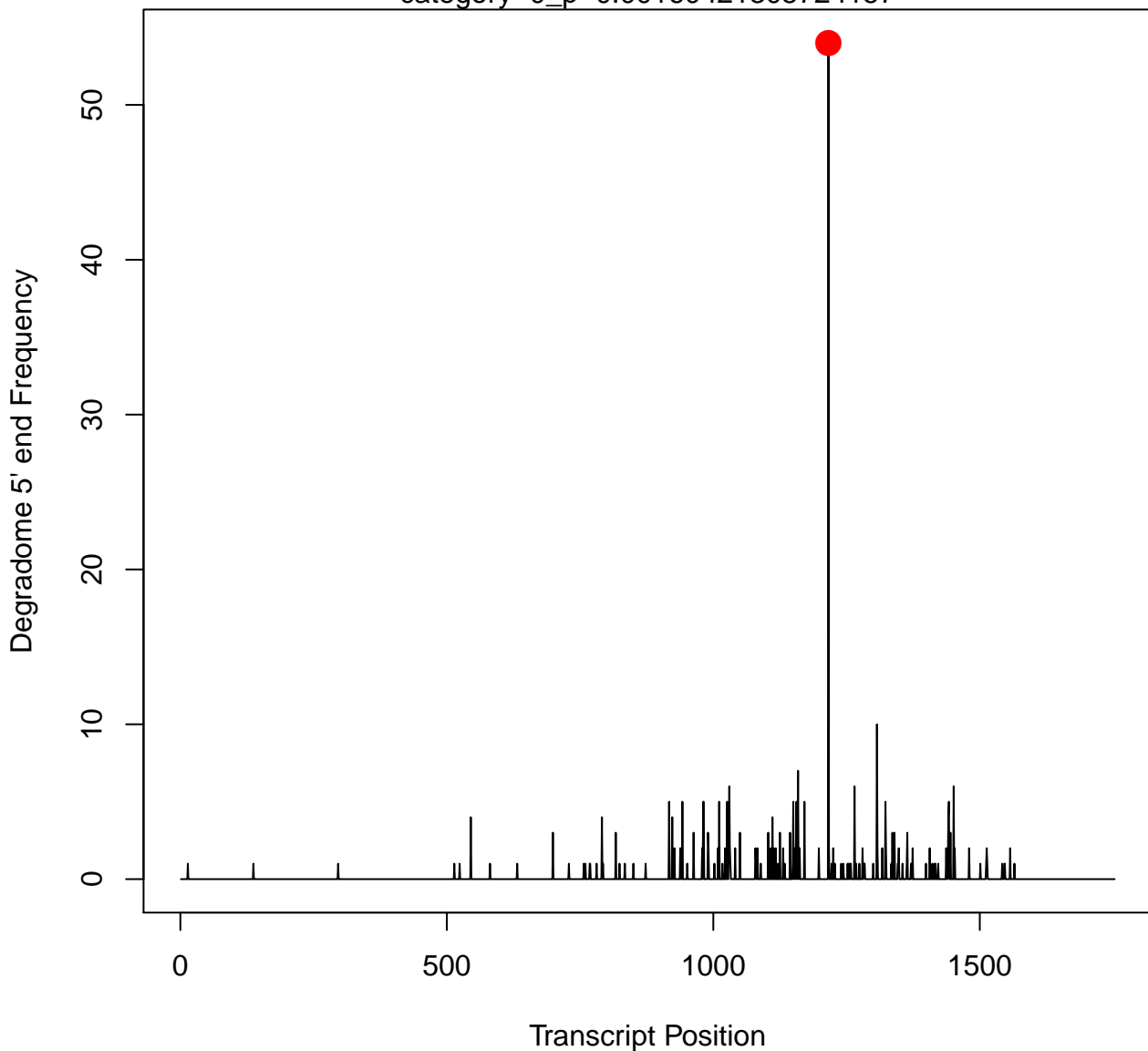

Supplement: Supplementary file 3 [file Data_Sheet_3.zip › Sit-miR164f_Seita.4G263400.1_1216_TPlot.pdf]

**T=Seita.1G338400.1\_Q=Sit-miR166b\_S=1948**

category=2\_p=0.977647882033304

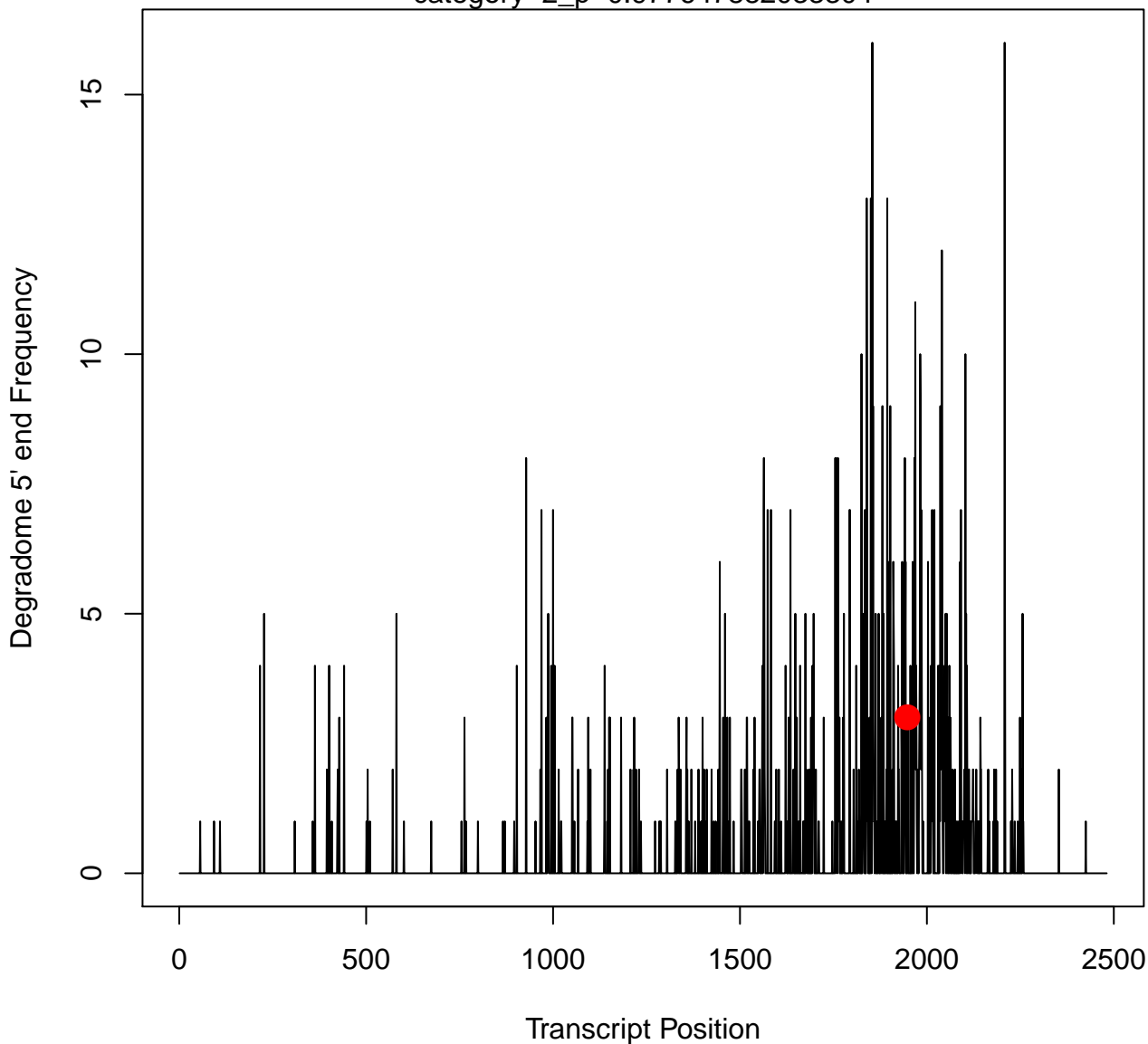

Supplement: Supplementary file 3 [file Data_Sheet_3.zip › Sit-miR166b_Seita.1G338400.1_1948_TPlot.pdf]

**T=Seita.3G035500.1\_Q=Sit-miR166b\_S=2169**

category=2\_p=0.990628510922407

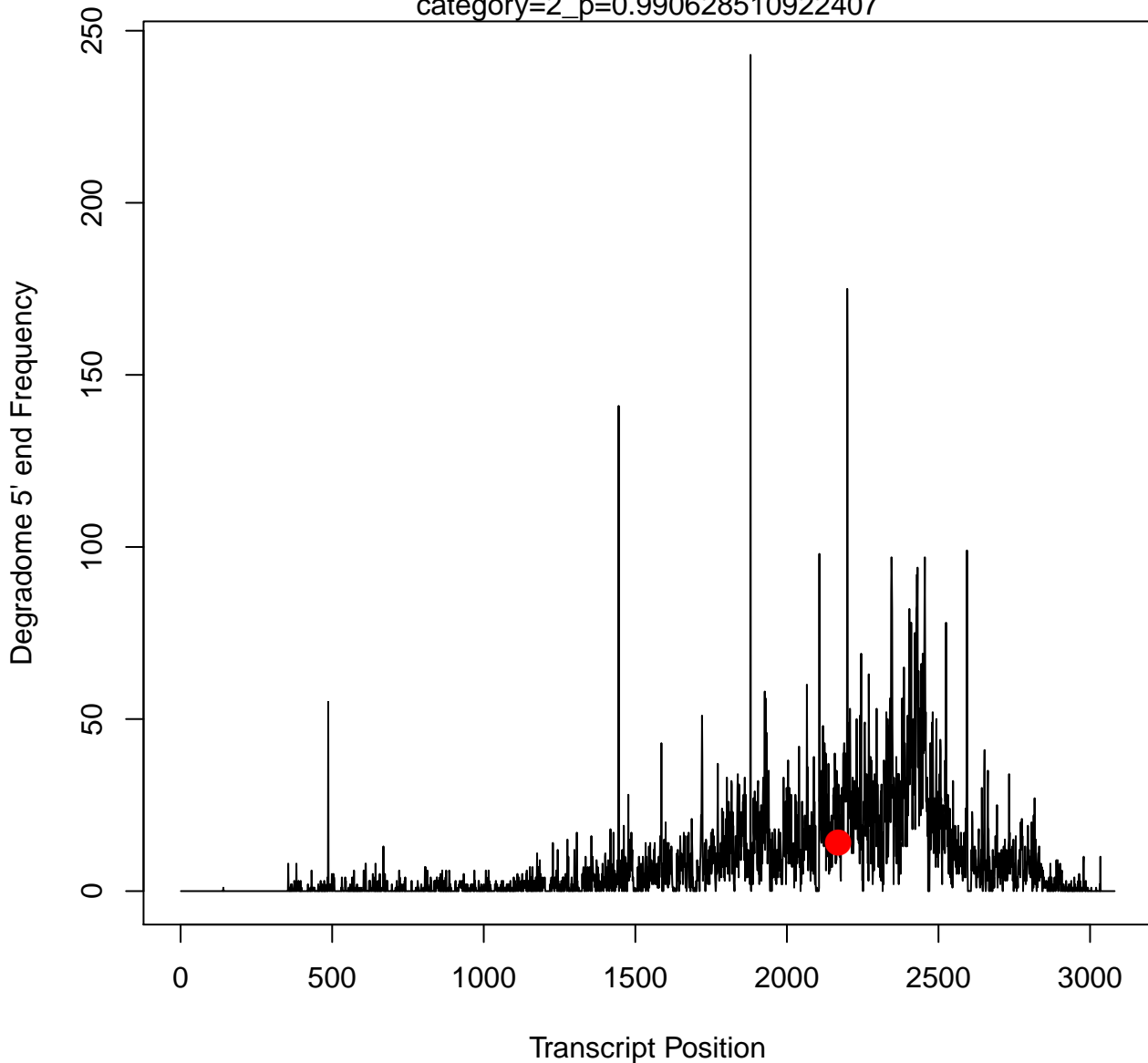

Supplement: Supplementary file 3 [file Data_Sheet_3.zip › Sit-miR166b_Seita.3G035500.1_2169_TPlot.pdf]

**T=Seita.3G256700.1\_Q=Sit-miR166b\_S=308**

category=2\_p=0.896363452849605

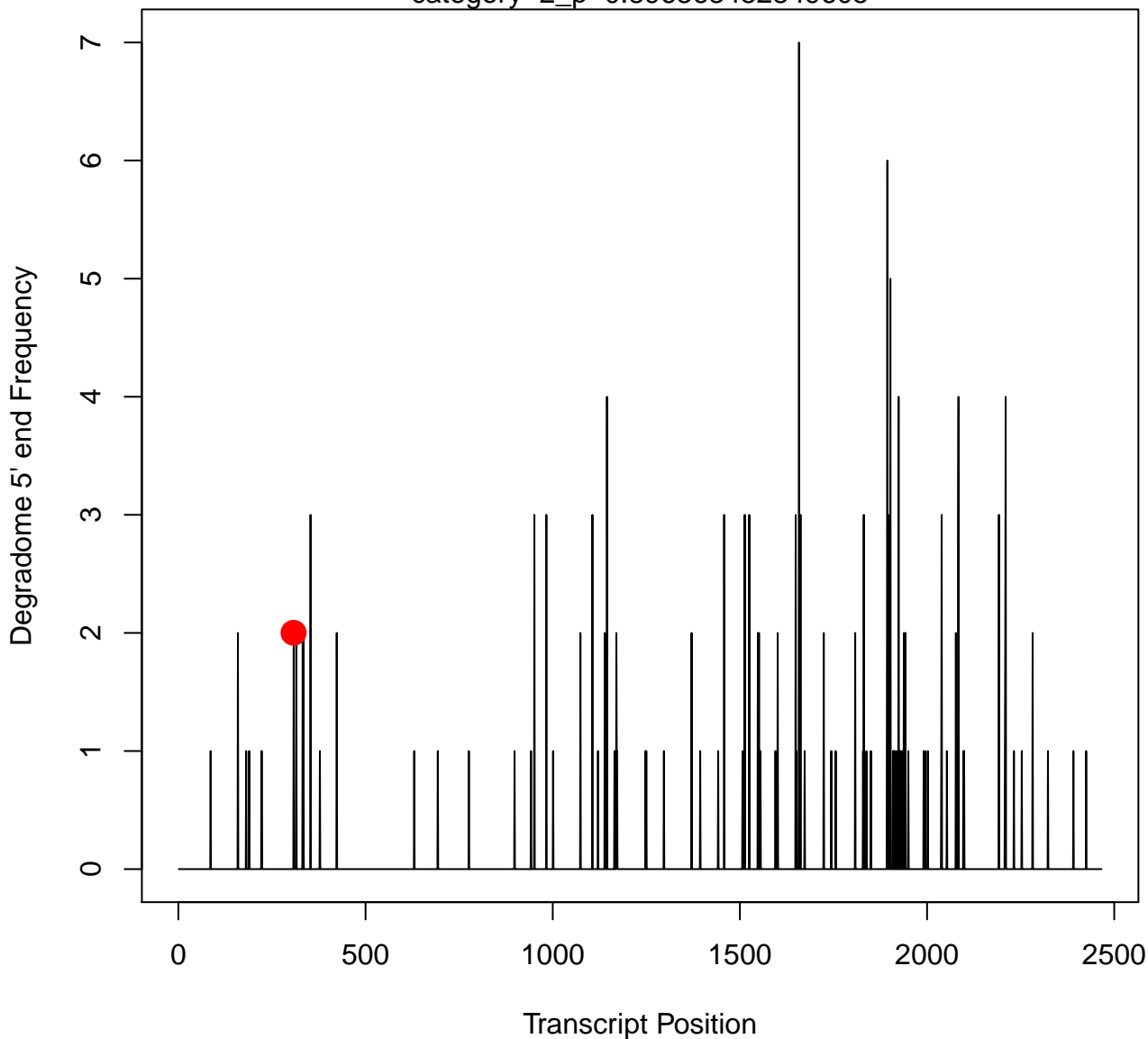

Supplement: Supplementary file 3 [file Data_Sheet_3.zip › Sit-miR166b_Seita.3G256700.1_308_TPlot.pdf]

**T=Seita.3G395000.1\_Q=Sit-miR166b\_S=776**

category=2\_p=0.065904313739923

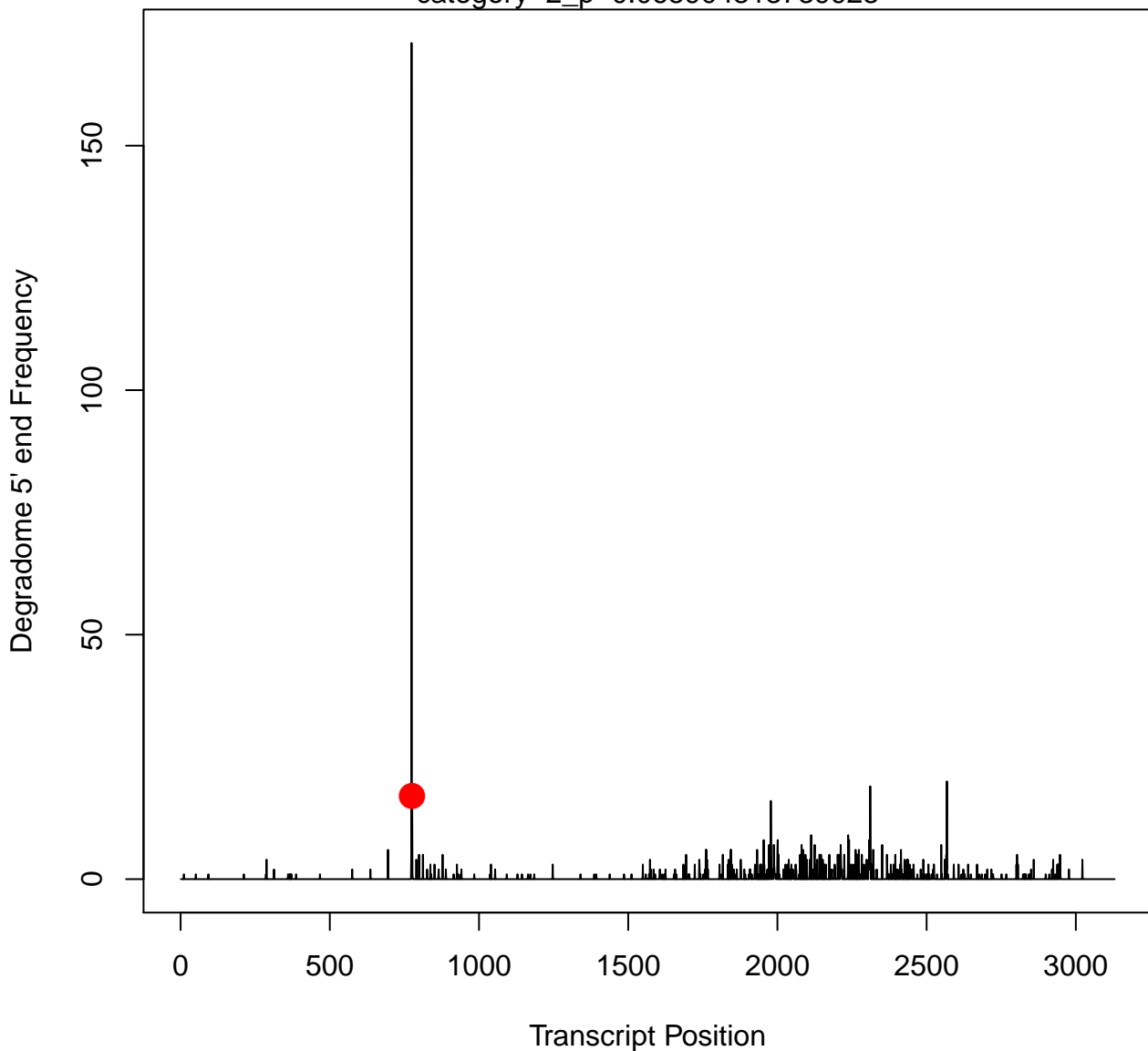

Supplement: Supplementary file 3 [file Data_Sheet_3.zip › Sit-miR166b_Seita.3G395000.1_776_TPlot.pdf]

**T=Seita.5G141300.1\_Q=Sit-miR166b\_S=1224**

category=2\_p=0.081690223146084

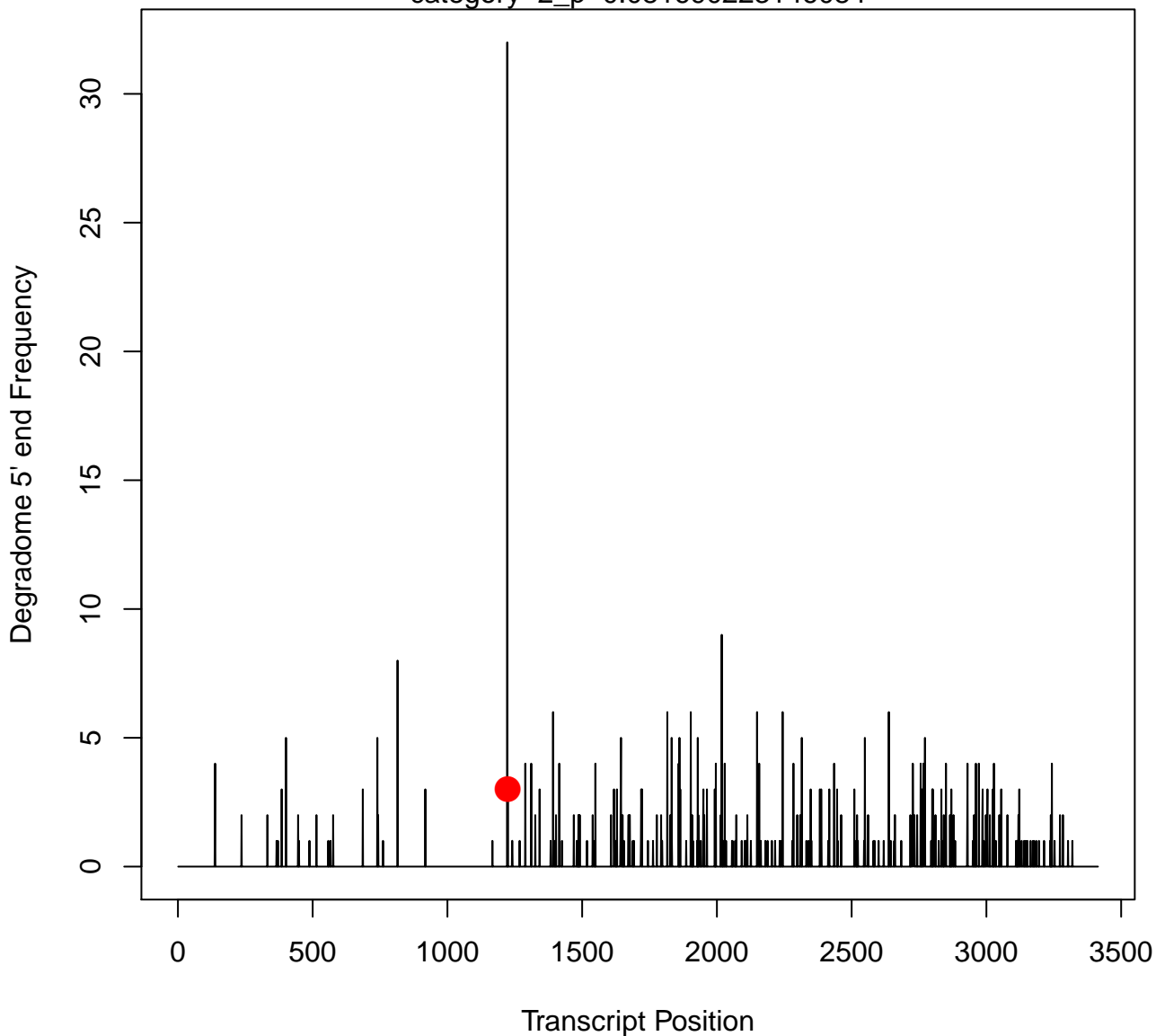

Supplement: Supplementary file 3 [file Data_Sheet_3.zip › Sit-miR166b_Seita.5G141300.1_1224_TPlot.pdf]

**T=Seita.7G154500.1\_Q=Sit-miR166b\_S=406**

category=2\_p=0.621490336025439

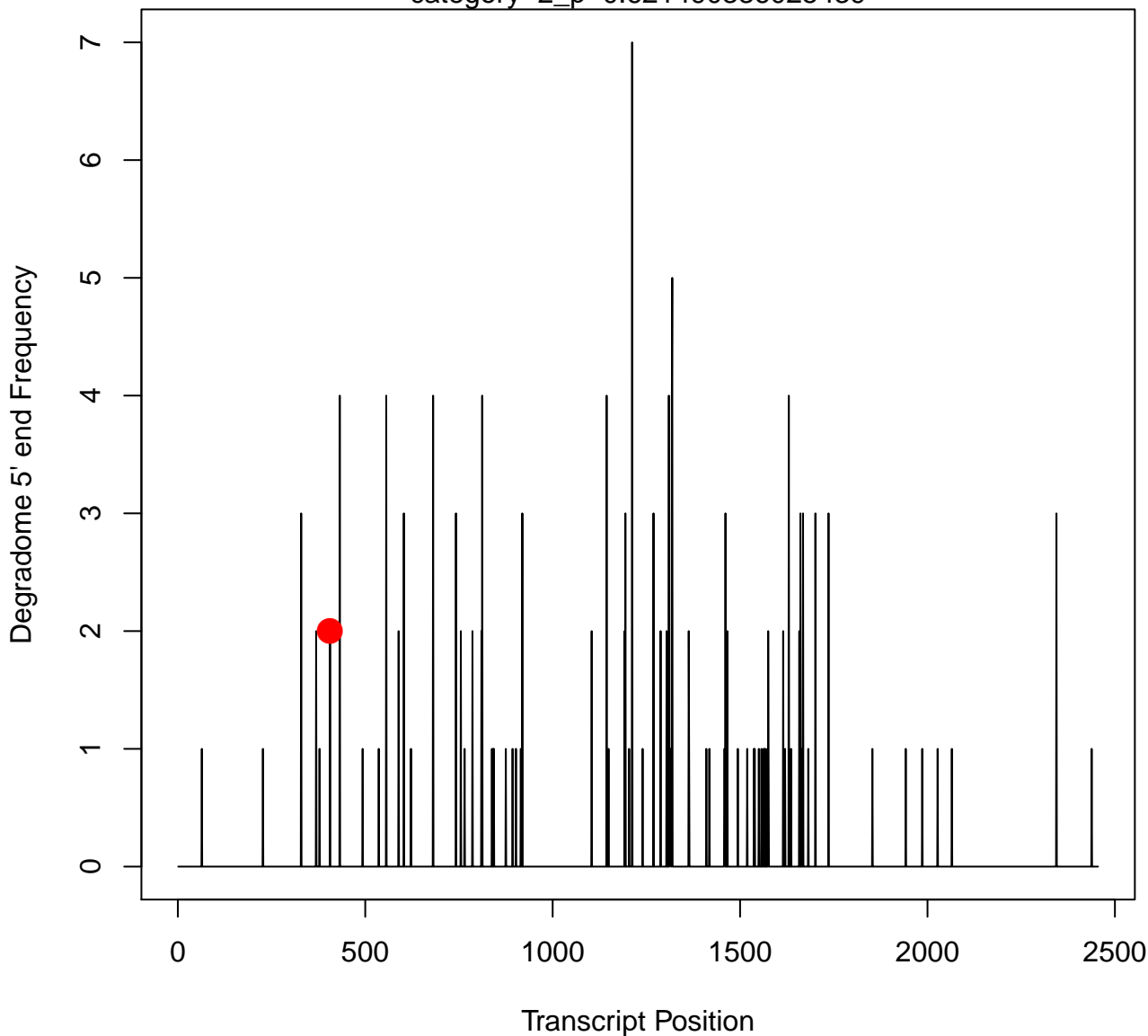

Supplement: Supplementary file 3 [file Data_Sheet_3.zip › Sit-miR166b_Seita.7G154500.1_406_TPlot.pdf]

**T=Seita.7G243800.1\_Q=Sit-miR166b\_S=1926**

category=2\_p=0.78432205065401

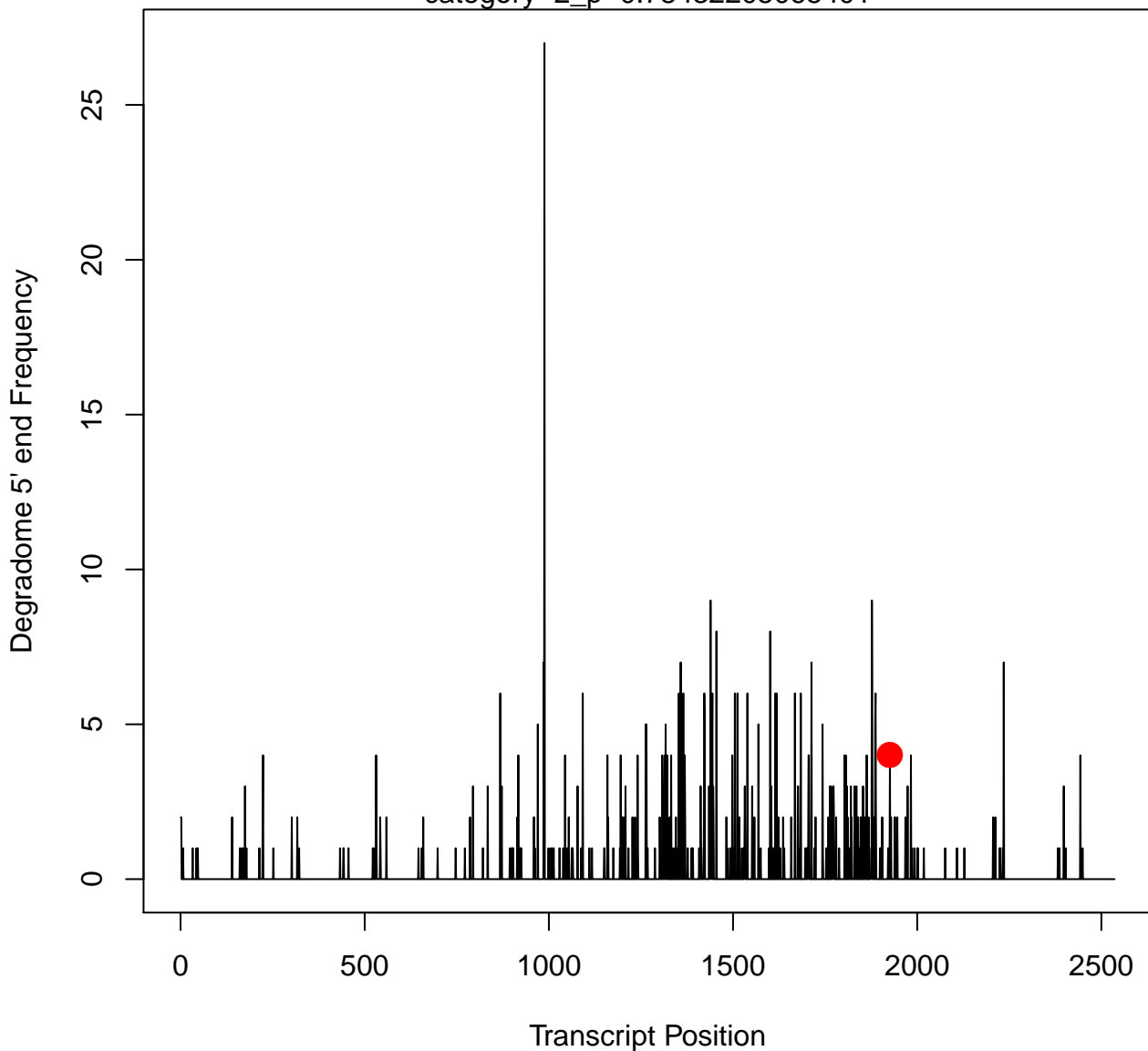

Supplement: Supplementary file 3 [file Data_Sheet_3.zip › Sit-miR166b_Seita.7G243800.1_1926_TPlot.pdf]

**T=Seita.9G572600.1\_Q=Sit-miR166b\_S=1140**

category=2\_p=0.0972093558831681

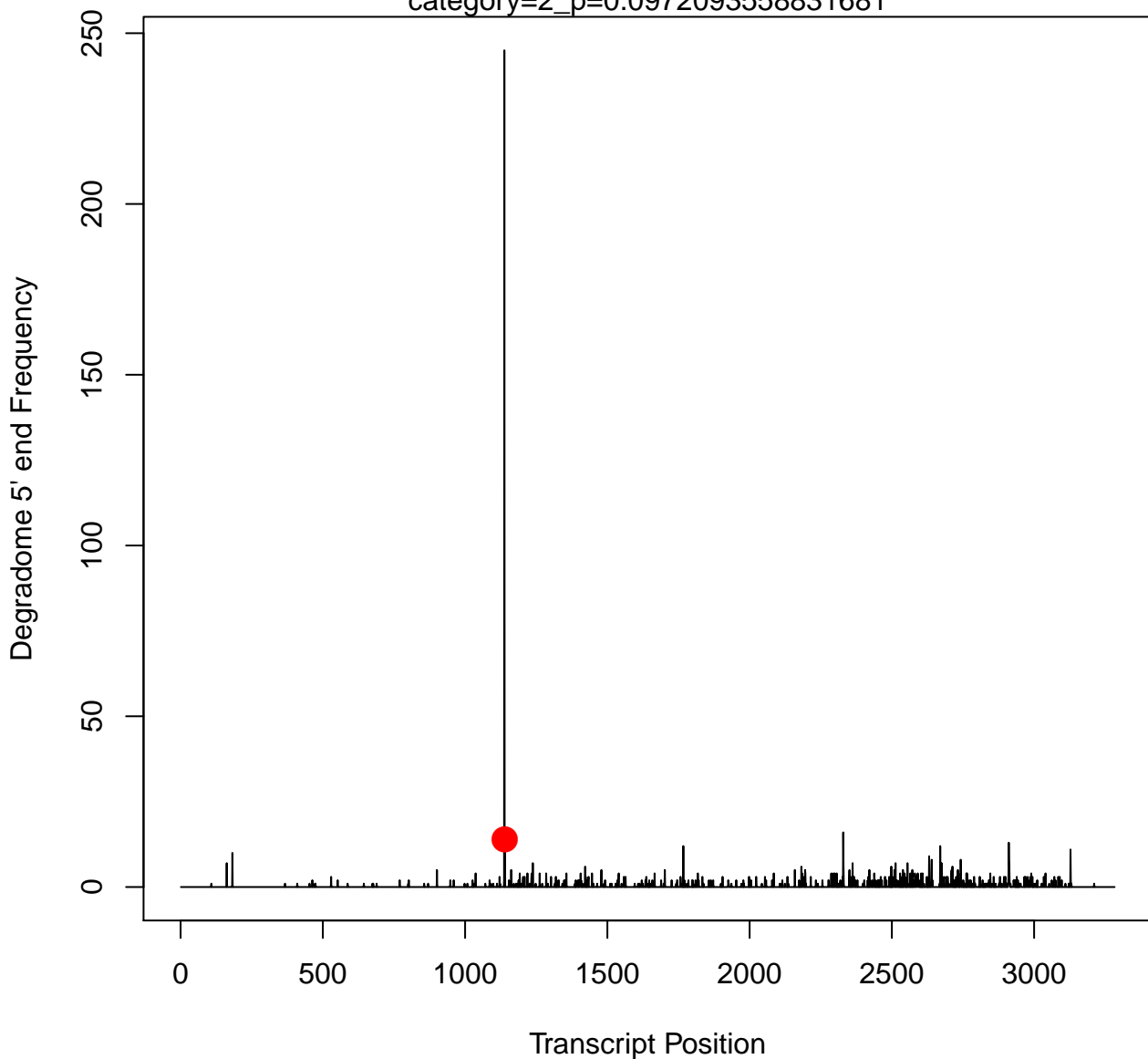

Supplement: Supplementary file 3 [file Data_Sheet_3.zip › Sit-miR166b_Seita.9G572600.1_1140_TPlot.pdf]

**T=Seita.3G164700.1\_Q=Sit-miR166e\_S=458**

category=2\_p=0.919743436554781

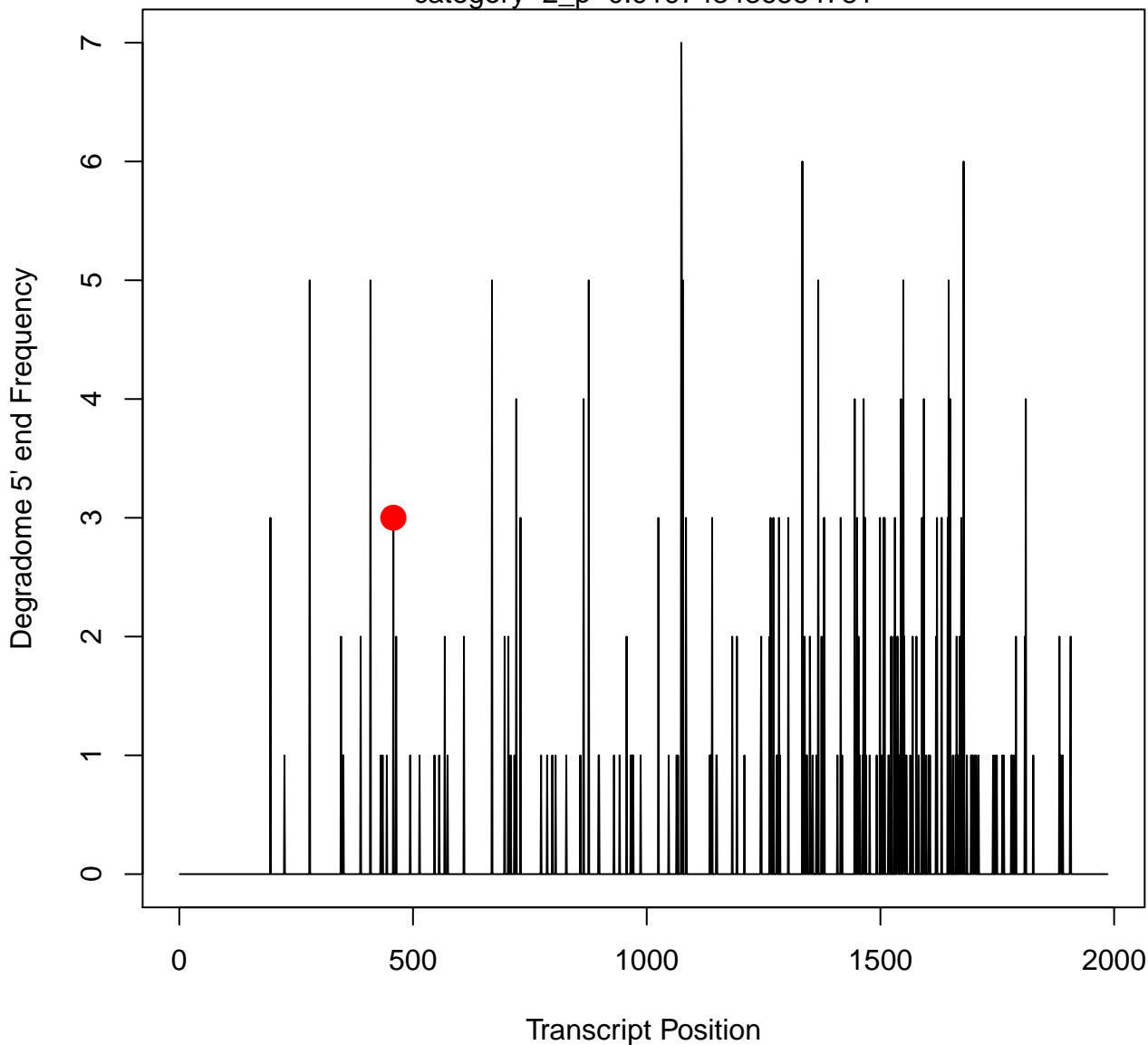

Supplement: Supplementary file 3 [file Data_Sheet_3.zip › Sit-miR166e_Seita.3G164700.1_458_TPlot.pdf]

**T=Seita.4G049700.1\_Q=Sit-miR166e\_S=680**

category=2\_p=0.830106983171264

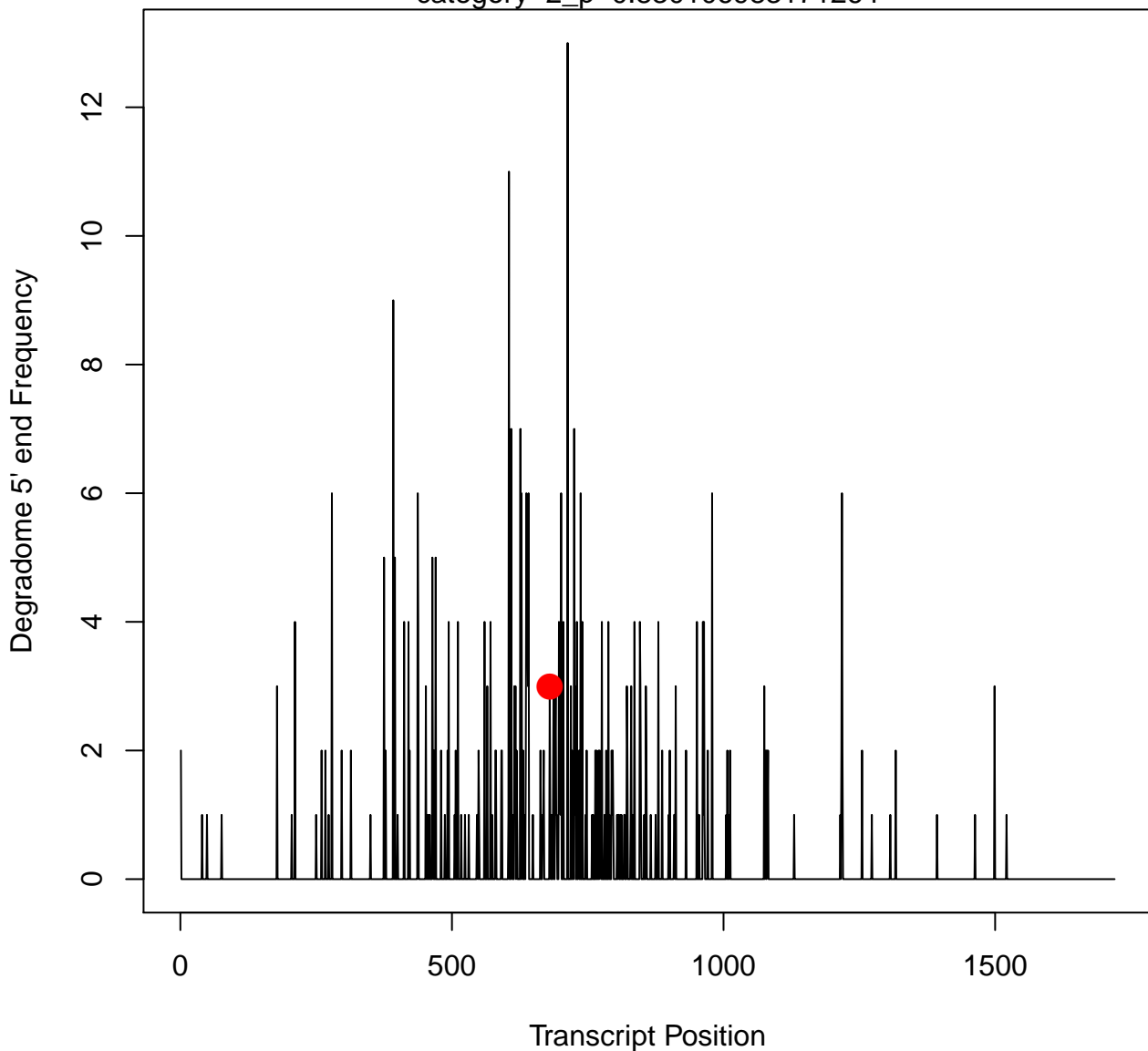

Supplement: Supplementary file 3 [file Data_Sheet_3.zip › Sit-miR166e_Seita.4G049700.1_680_TPlot.pdf]

**T=Seita.4G154400.1\_Q=Sit-miR166e\_S=568**

category=2\_p=0.962087223499423

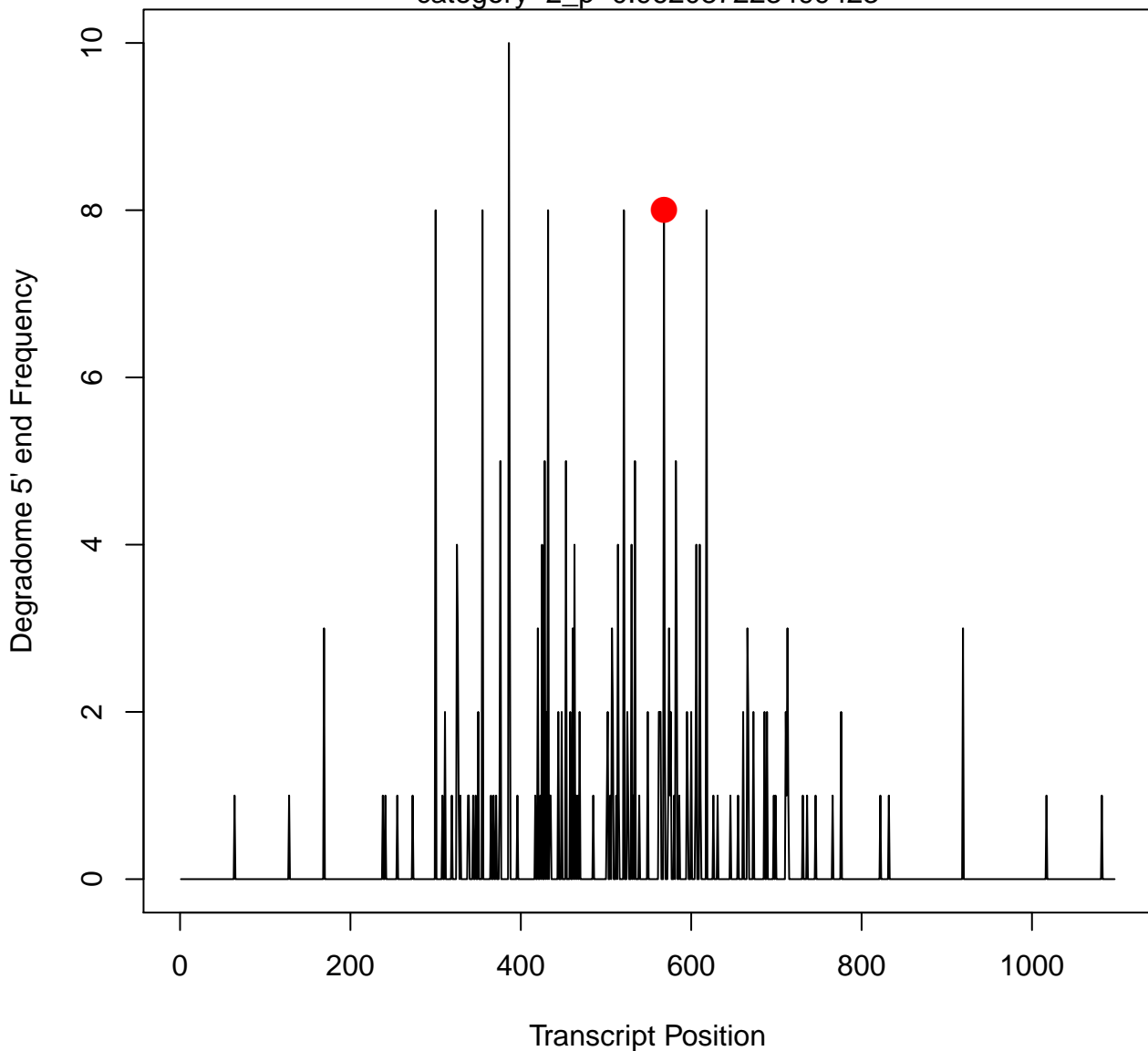

Supplement: Supplementary file 3 [file Data_Sheet_3.zip › Sit-miR166e_Seita.4G154400.1_568_TPlot.pdf]

**T=Seita.6G192900.1\_Q=Sit-miR166e\_S=173**

category=2\_p=0.994085060225356

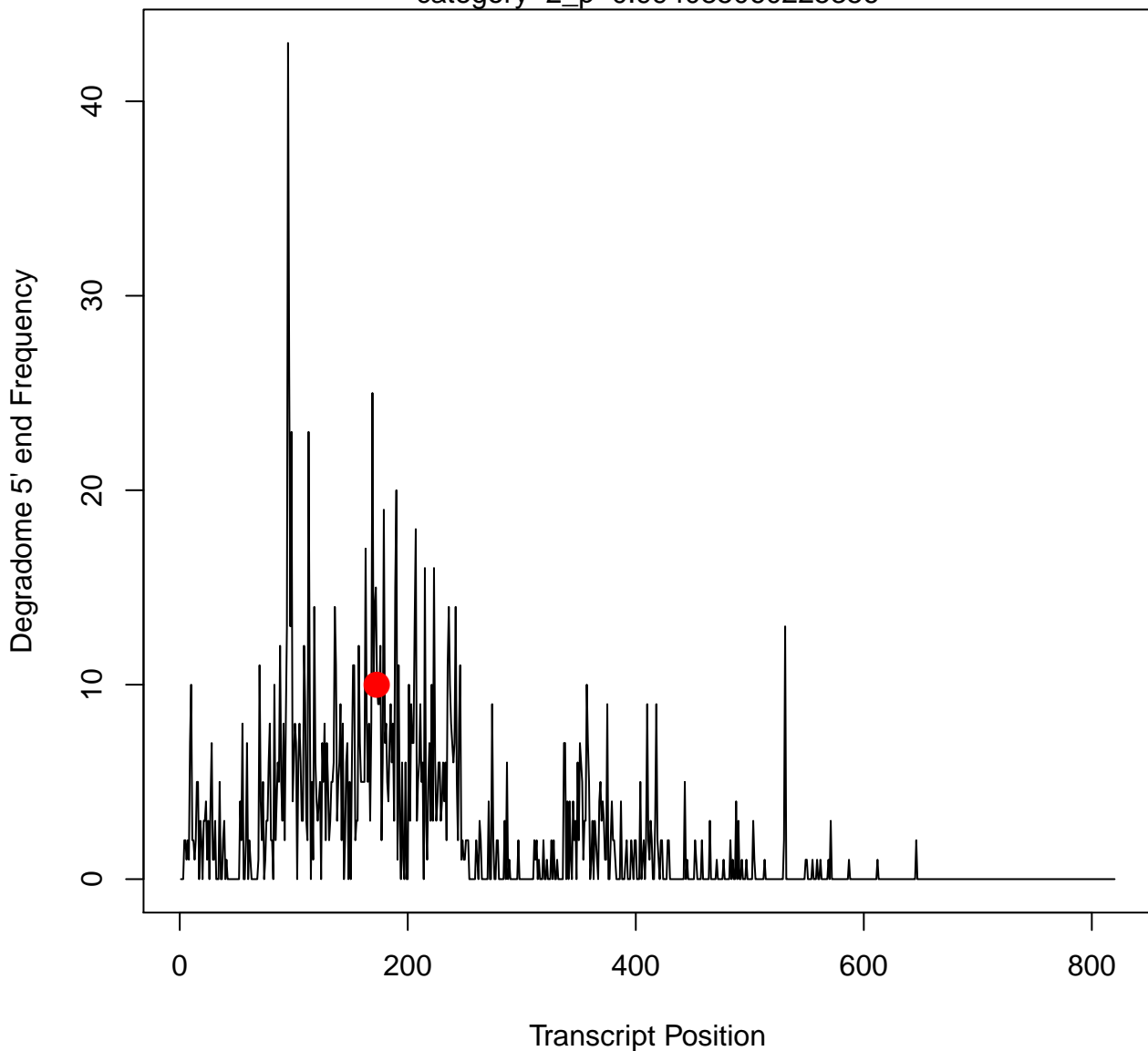

Supplement: Supplementary file 3 [file Data_Sheet_3.zip › Sit-miR166e_Seita.6G192900.1_173_TPlot.pdf]

**T=Seita.9G340800.1\_Q=Sit-miR166g\_S=727**

category=2\_p=0.995721324820405

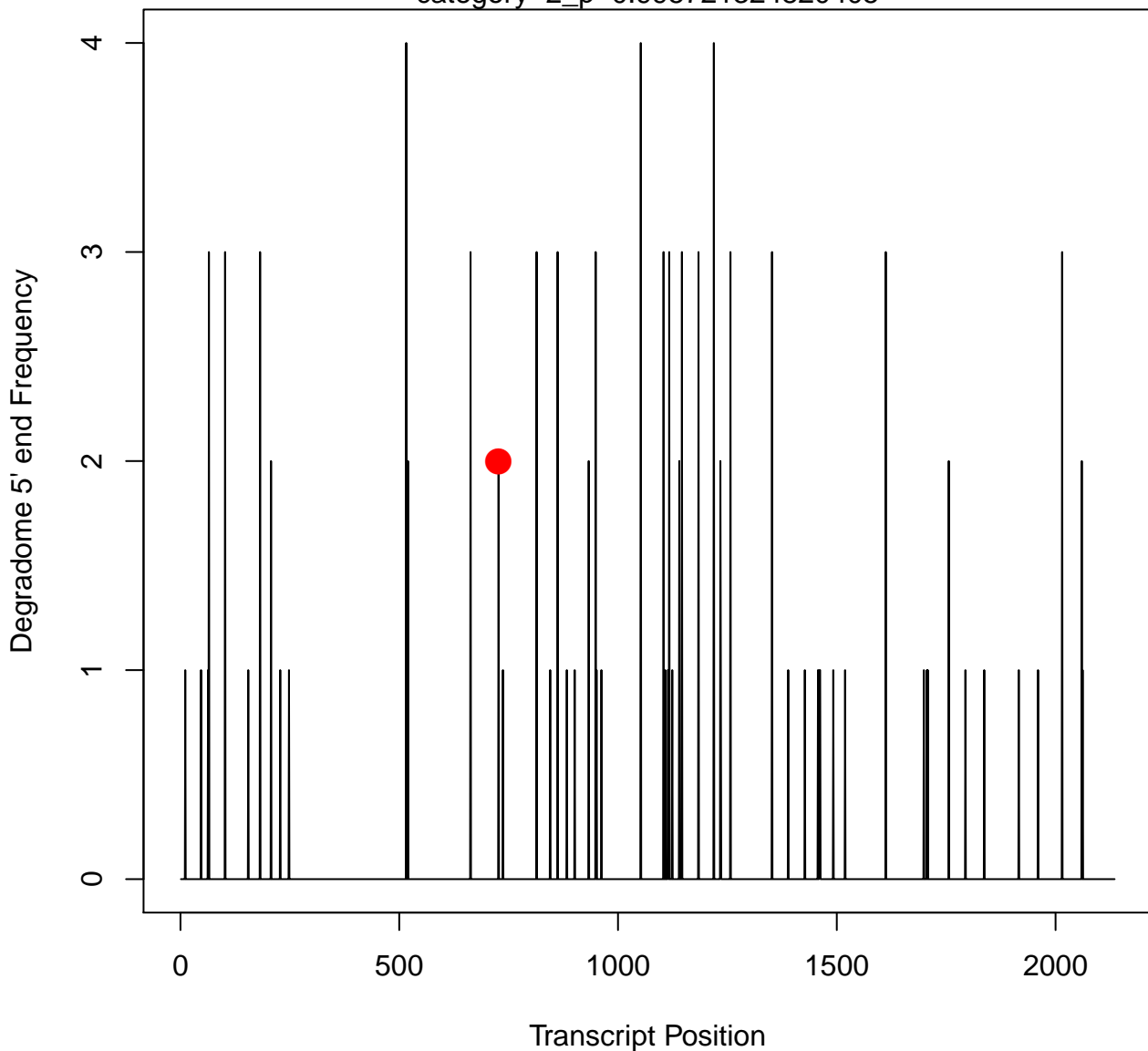

Supplement: Supplementary file 3 [file Data_Sheet_3.zip › Sit-miR166g_Seita.9G340800.1_727_TPlot.pdf]

**T=Seita.4G263300.1\_Q=Sit-miR166h\_S=2995**

category=2\_p=0.198742807429545

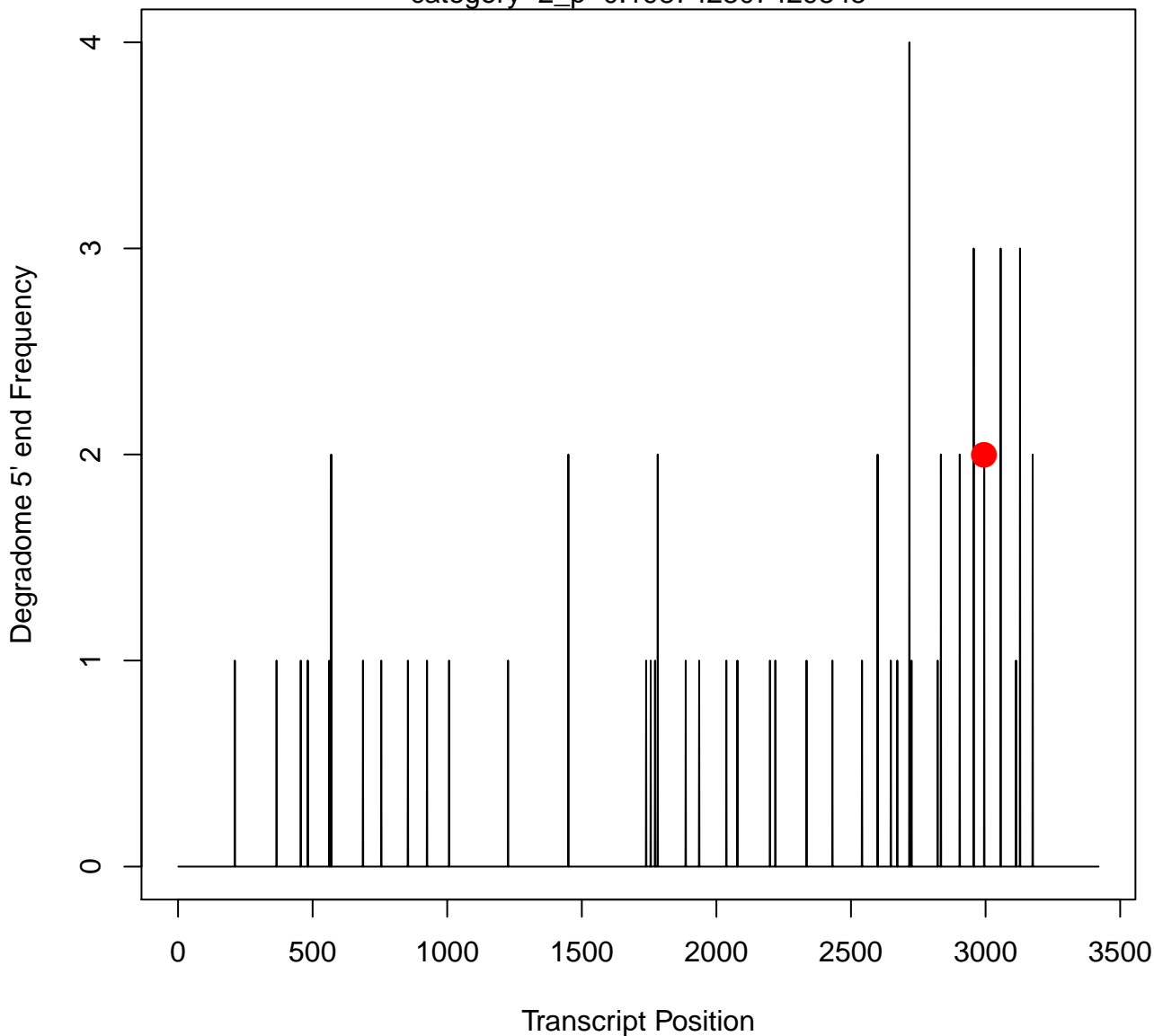

Supplement: Supplementary file 3 [file Data_Sheet_3.zip › Sit-miR166h_Seita.4G263300.1_2995_TPlot.pdf]

**T=Seita.5G128100.1\_Q=Sit-miR166h\_S=780**

category=2\_p=0.184969052895116

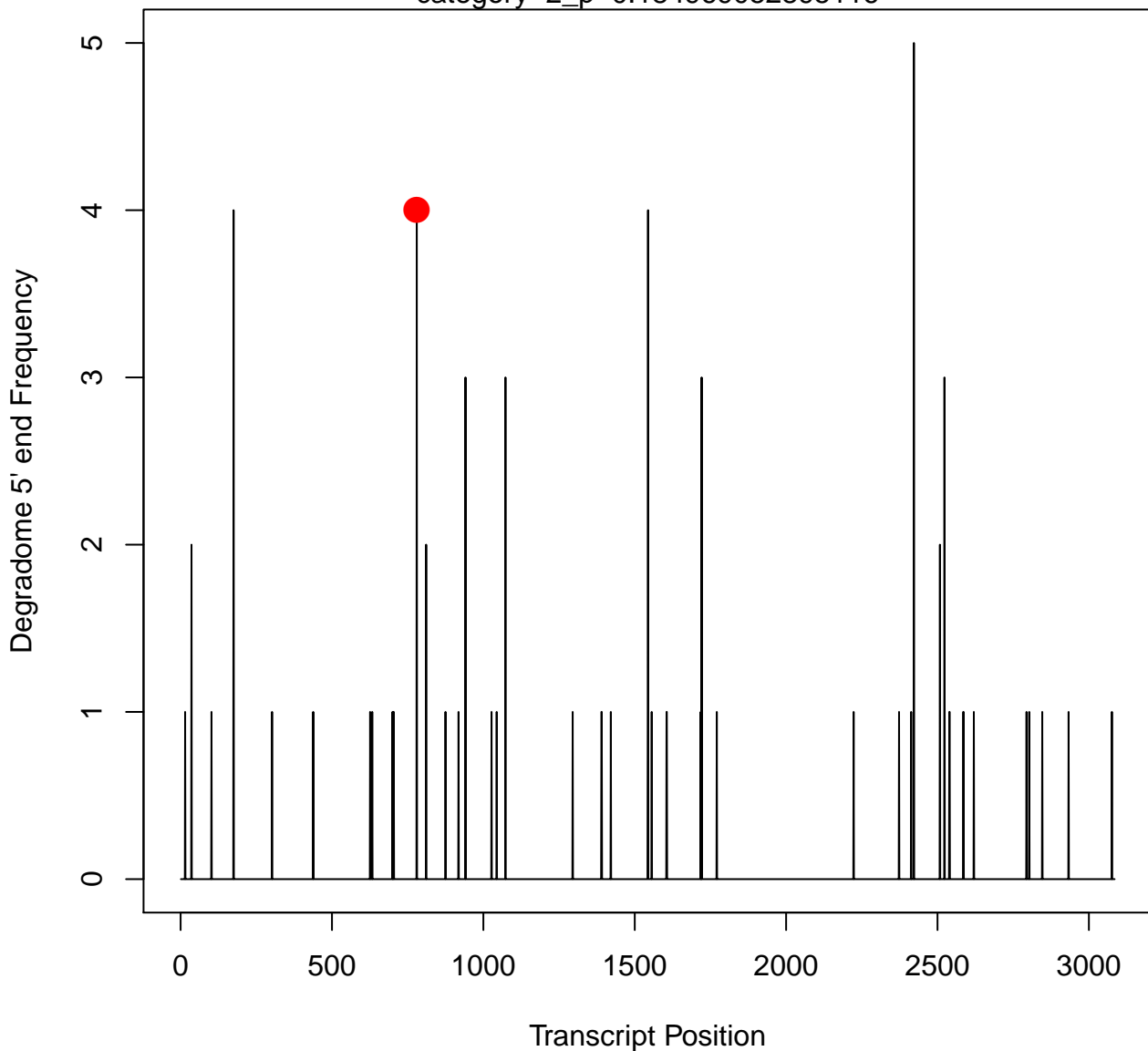

Supplement: Supplementary file 3 [file Data_Sheet_3.zip › Sit-miR166h_Seita.5G128100.1_780_TPlot.pdf]

**T=Seita.1G004100.1\_Q=Sit-miR166i\_S=2969**

category=2\_p=0.997253034532636

Degradome 5' end Frequency

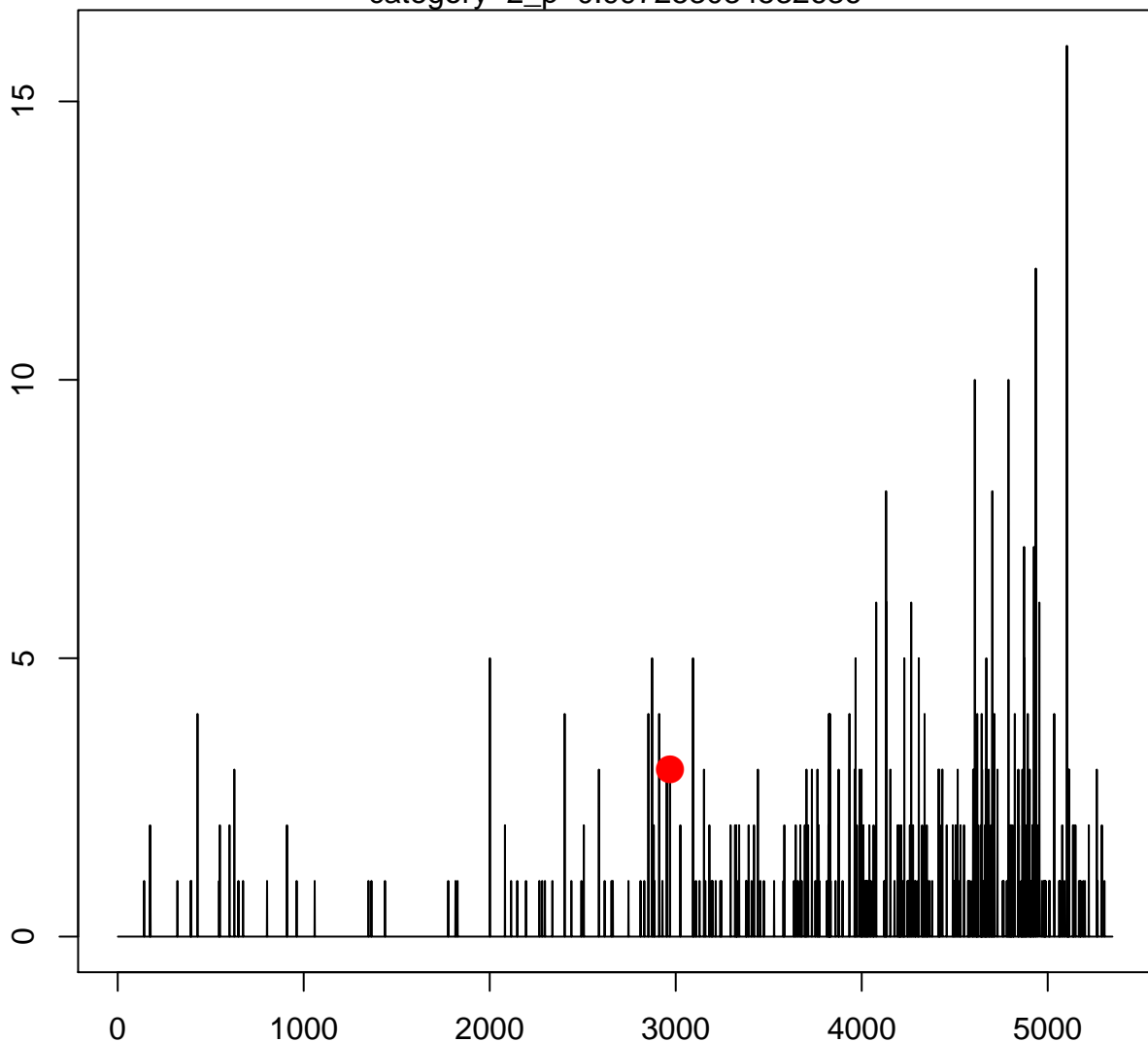

Transcript Position

Supplement: Supplementary file 3 [file Data_Sheet_3.zip › Sit-miR166i_Seita.1G004100.1_2969_TPlot.pdf]

**T=Seita.1G080100.1\_Q=Sit-miR166i\_S=2661**

category=2\_p=0.841303665854567

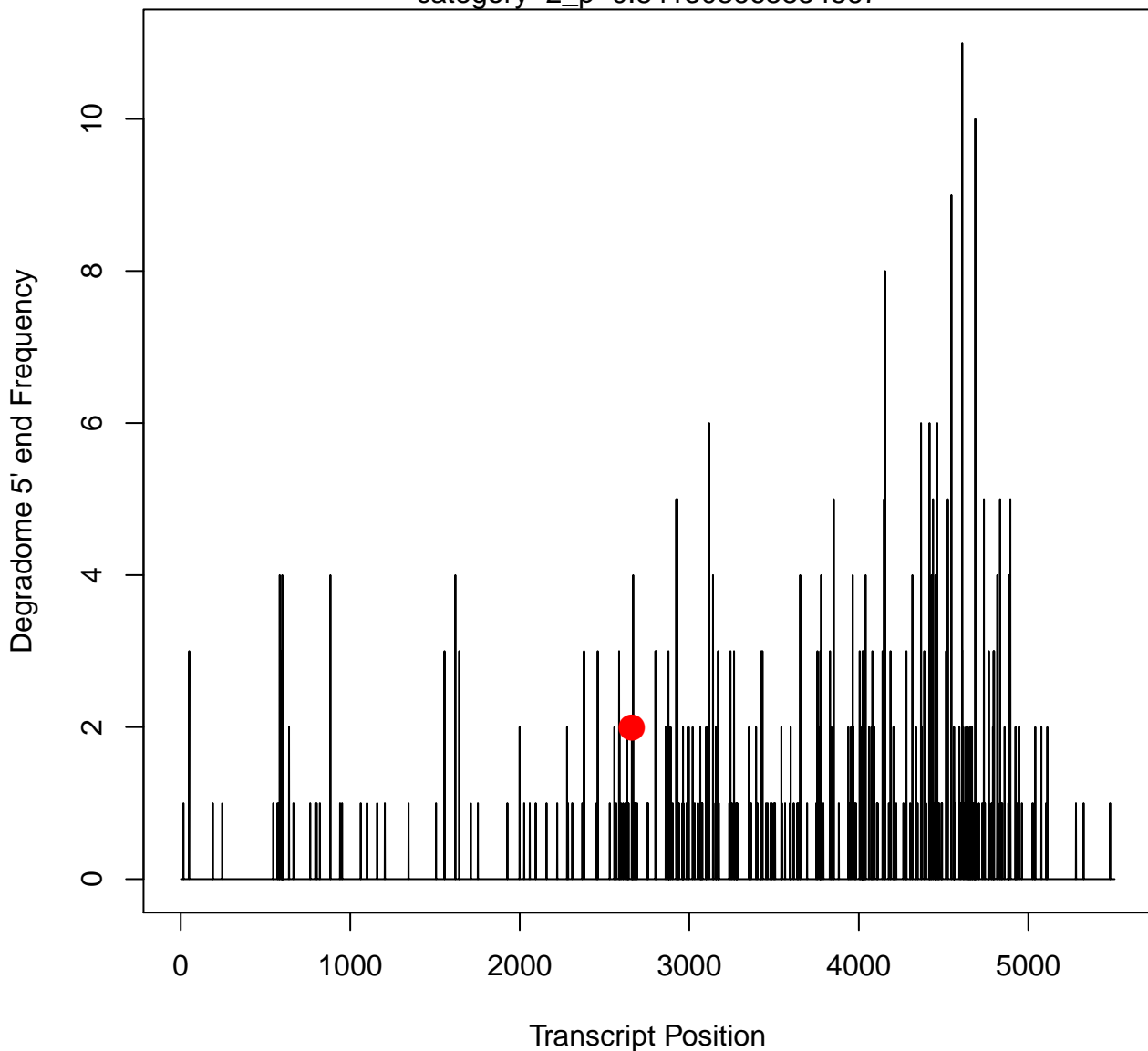

Supplement: Supplementary file 3 [file Data_Sheet_3.zip › Sit-miR166i_Seita.1G080100.1_2661_TPlot.pdf]

**T=Seita.3G001200.1\_Q=Sit-miR166i\_S=1817**

category=2\_p=0.992230646872684

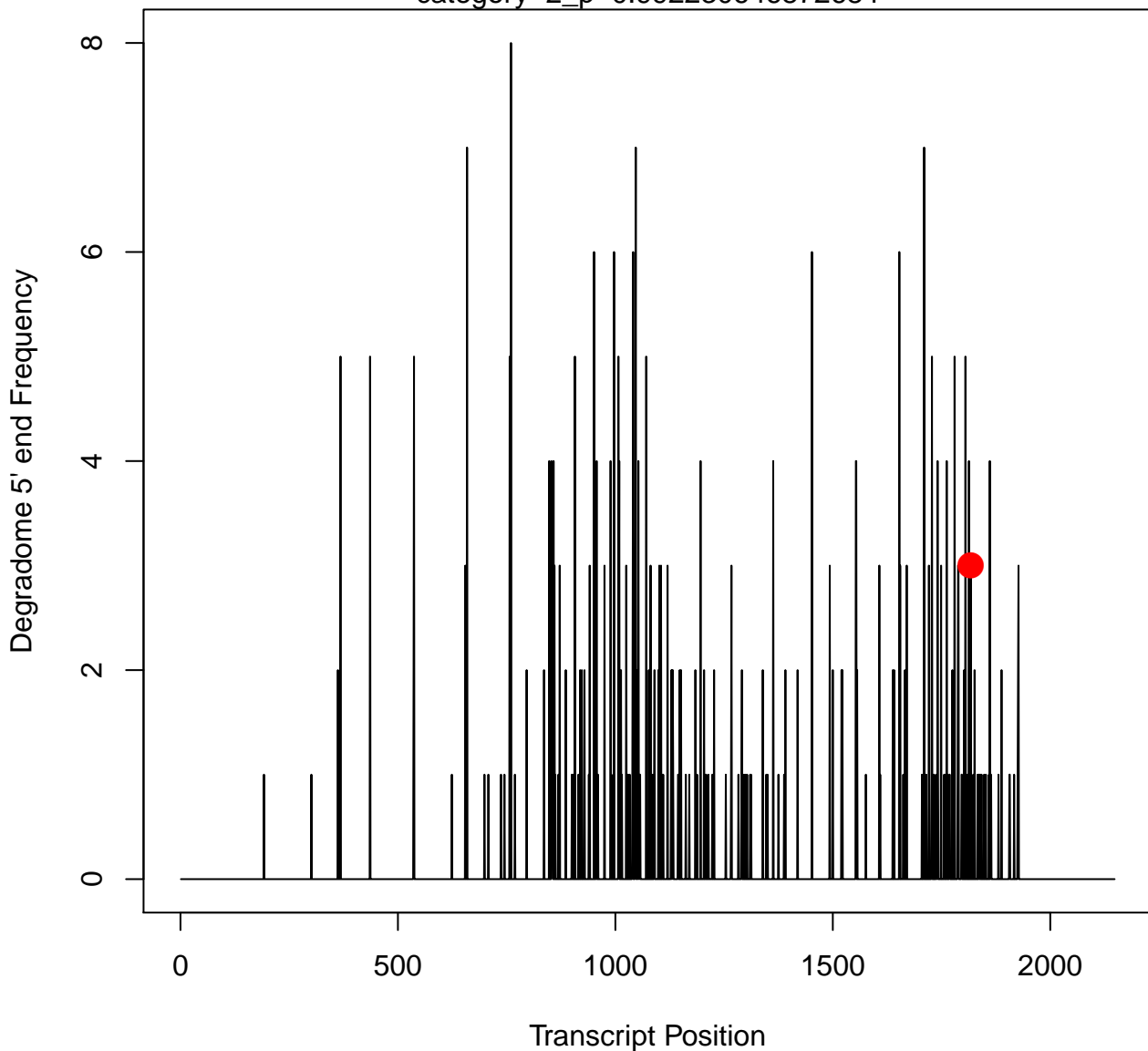

Supplement: Supplementary file 3 [file Data_Sheet_3.zip › Sit-miR166i_Seita.3G001200.1_1817_TPlot.pdf]

**T=Seita.3G395000.1\_Q=Sit-miR166i\_S=774**

category=0\_p=0.00120340121992535

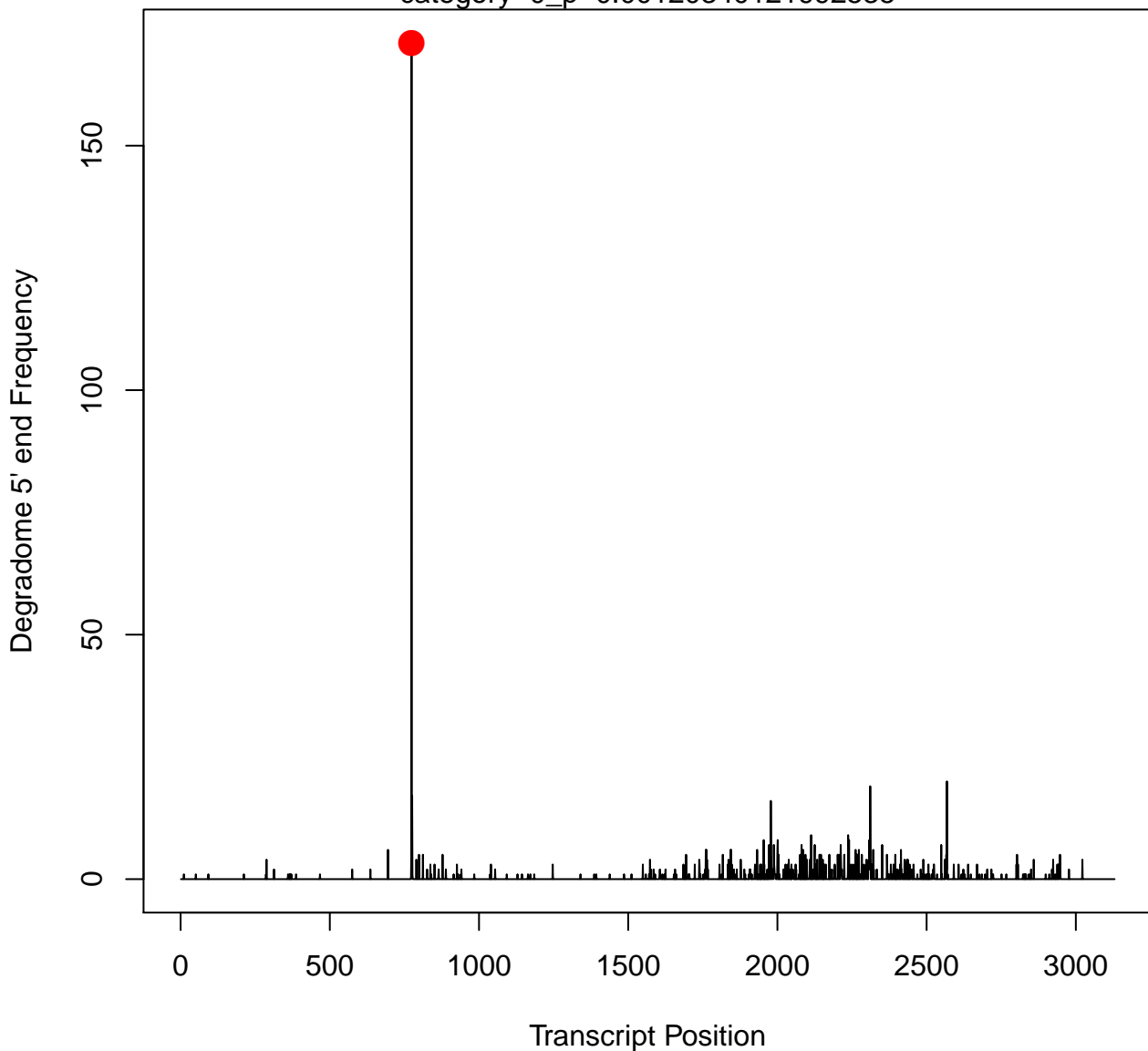

Supplement: Supplementary file 3 [file Data_Sheet_3.zip › Sit-miR166i_Seita.3G395000.1_774_TPlot.pdf]

**T=Seita.4G058600.1\_Q=Sit-miR166i\_S=731**

category=2\_p=0.996137252078647

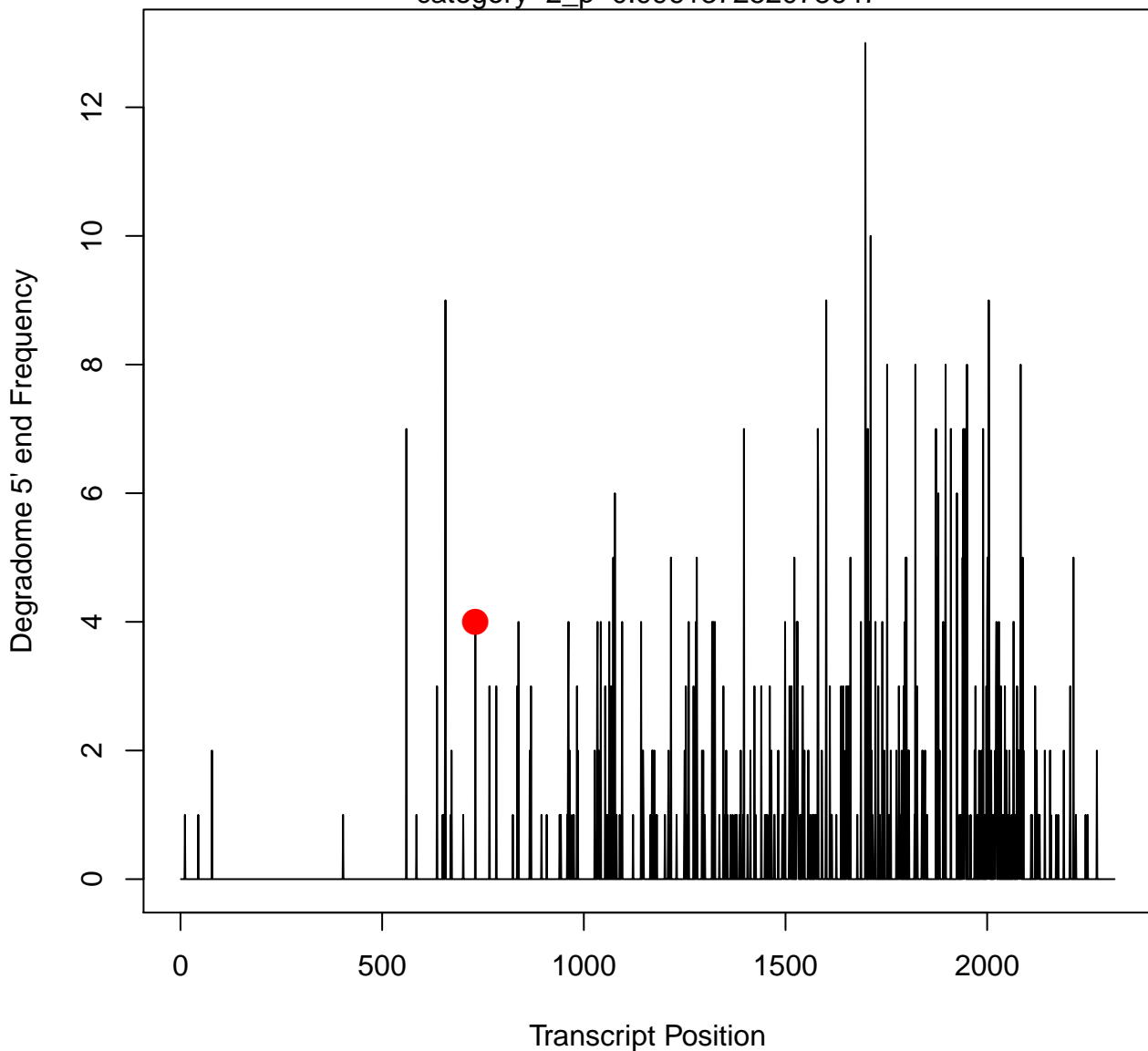

Supplement: Supplementary file 3 [file Data_Sheet_3.zip › Sit-miR166i_Seita.4G058600.1_731_TPlot.pdf]

**T=Seita.5G141300.1\_Q=Sit-miR166i\_S=1222**

category=0\_p=0.000802428474349082

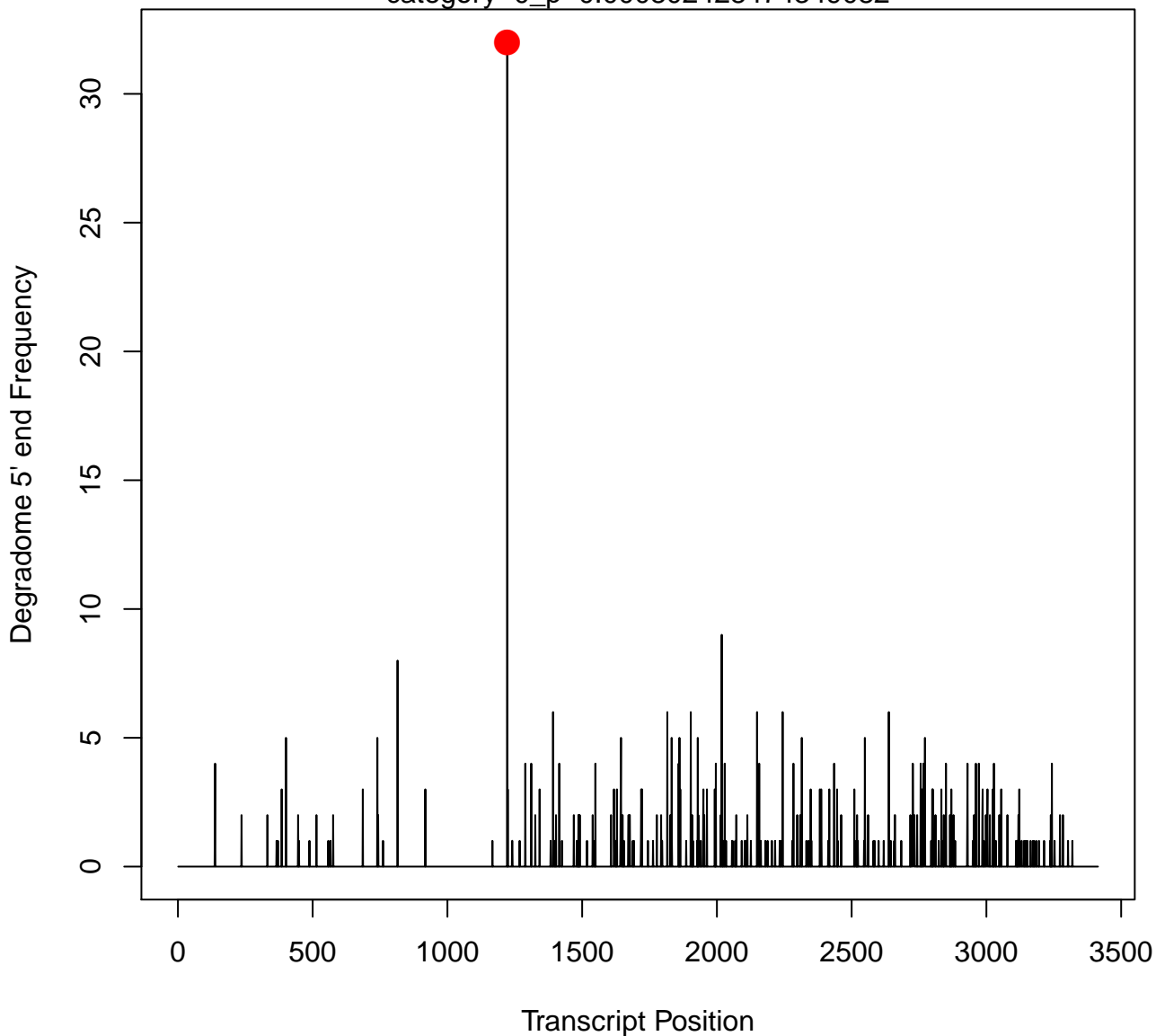

Supplement: Supplementary file 3 [file Data_Sheet_3.zip › Sit-miR166i_Seita.5G141300.1_1222_TPlot.pdf]

**T=Seita.5G261100.1\_Q=Sit-miR166i\_S=1615**

category=2\_p=0.87499232513391

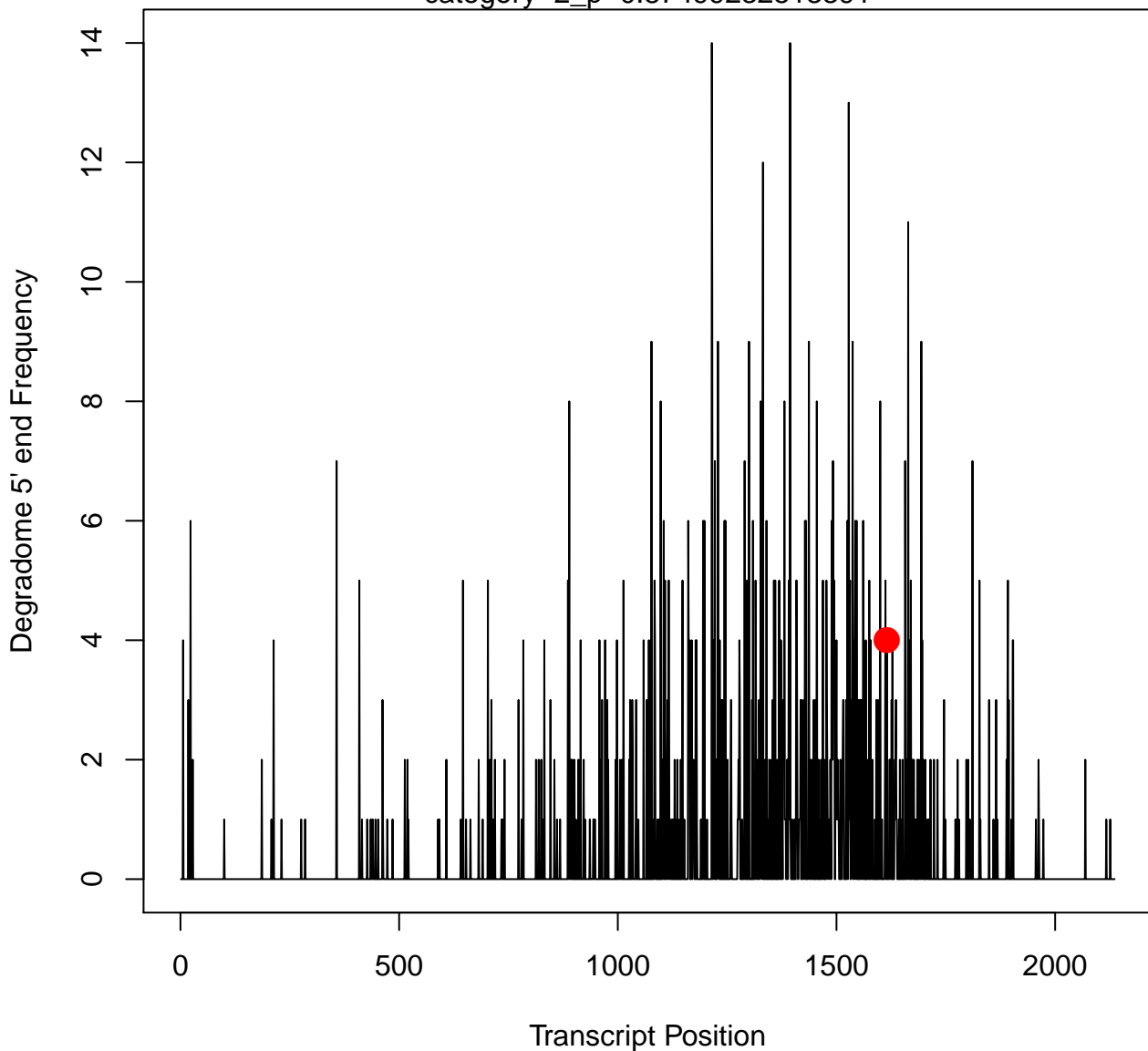

Supplement: Supplementary file 3 [file Data_Sheet_3.zip › Sit-miR166i_Seita.5G261100.1_1615_TPlot.pdf]

**T=Seita.7G217400.1\_Q=Sit-miR166i\_S=595**

category=2\_p=0.990786886000845

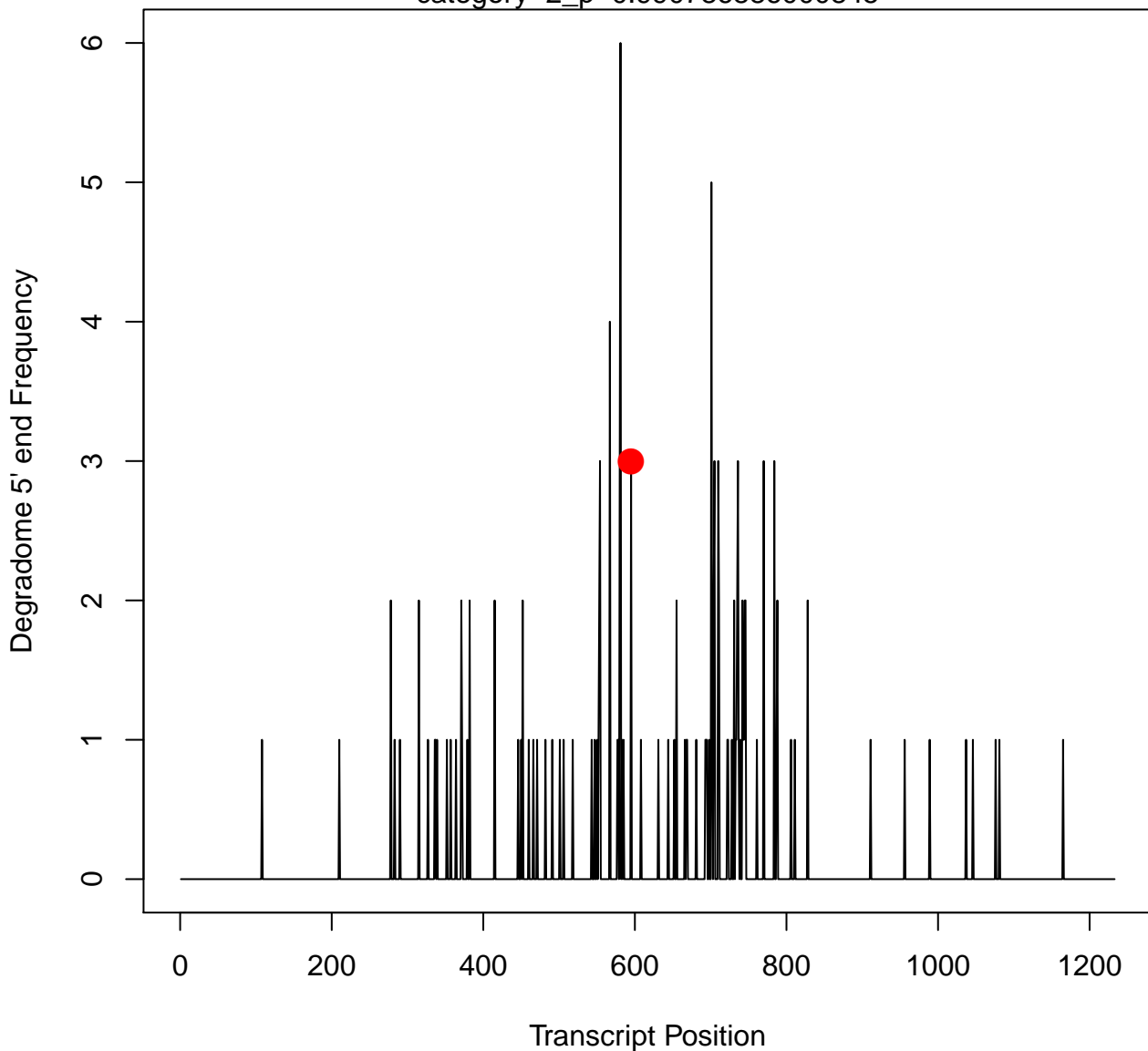

Supplement: Supplementary file 3 [file Data_Sheet_3.zip › Sit-miR166i_Seita.7G217400.1_595_TPlot.pdf]

**T=Seita.7G240200.1\_Q=Sit-miR166i\_S=1983**

category=2\_p=0.899836701387036

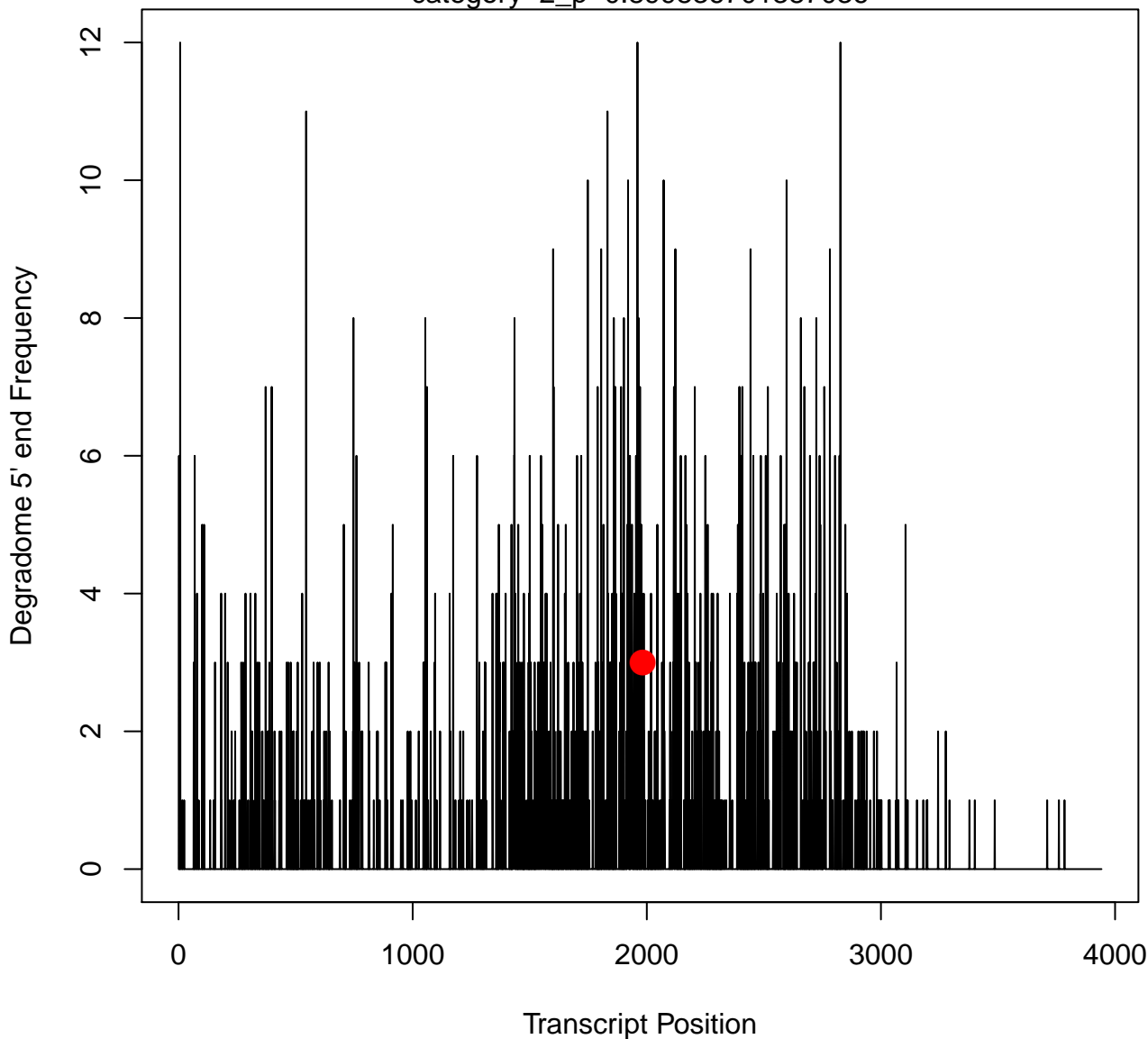

Supplement: Supplementary file 3 [file Data_Sheet_3.zip › Sit-miR166i_Seita.7G240200.1_1983_TPlot.pdf]

**T=Seita.3G185800.1\_Q=Sit-miR166j\_S=1028**

category=2\_p=0.739846610978819

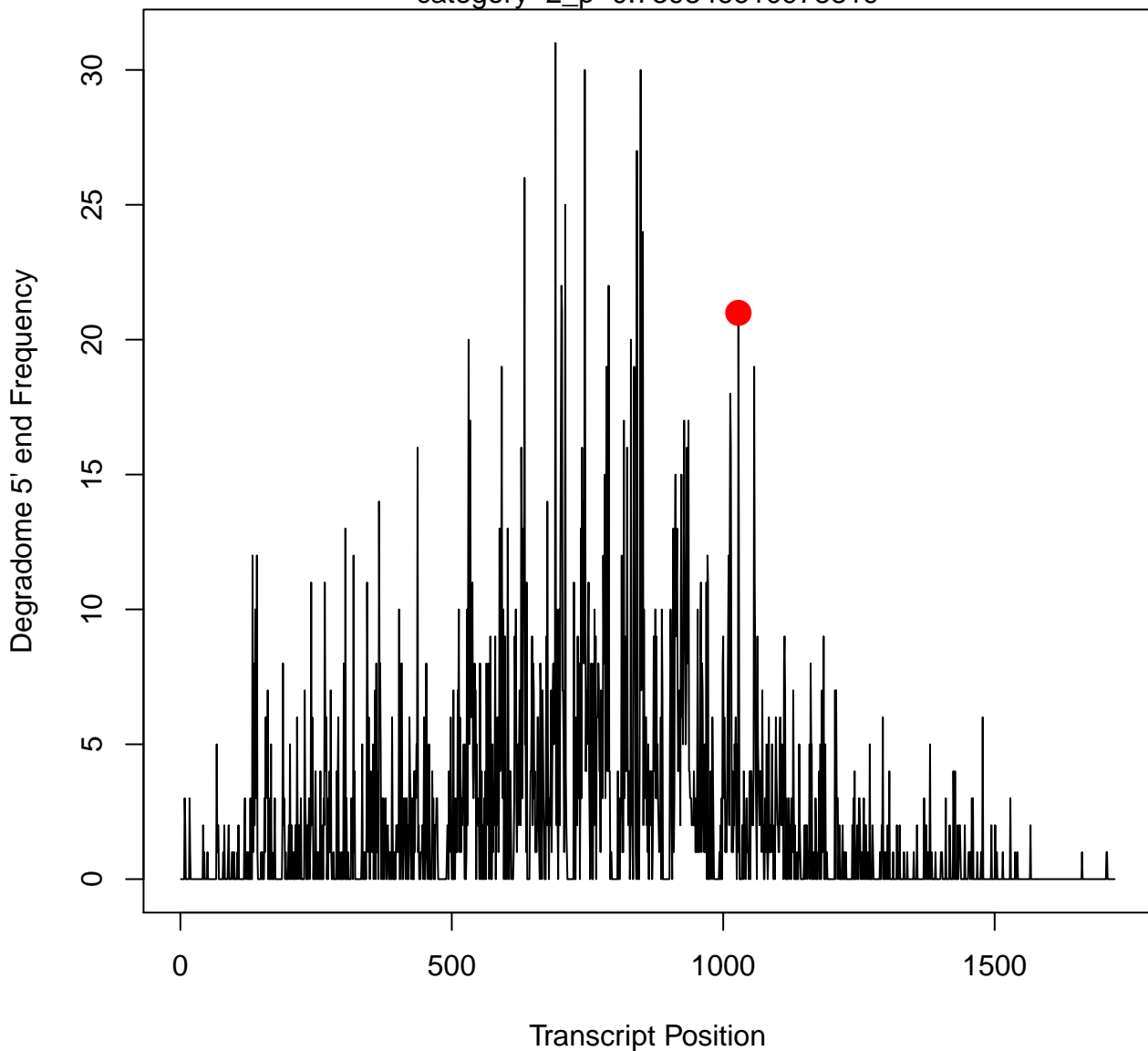

Supplement: Supplementary file 3 [file Data_Sheet_3.zip › Sit-miR166j_Seita.3G185800.1_1028_TPlot.pdf]

**T=Seita.9G219700.1\_Q=Sit-miR166j\_S=1105**

category=0\_p=0.000802428474349082

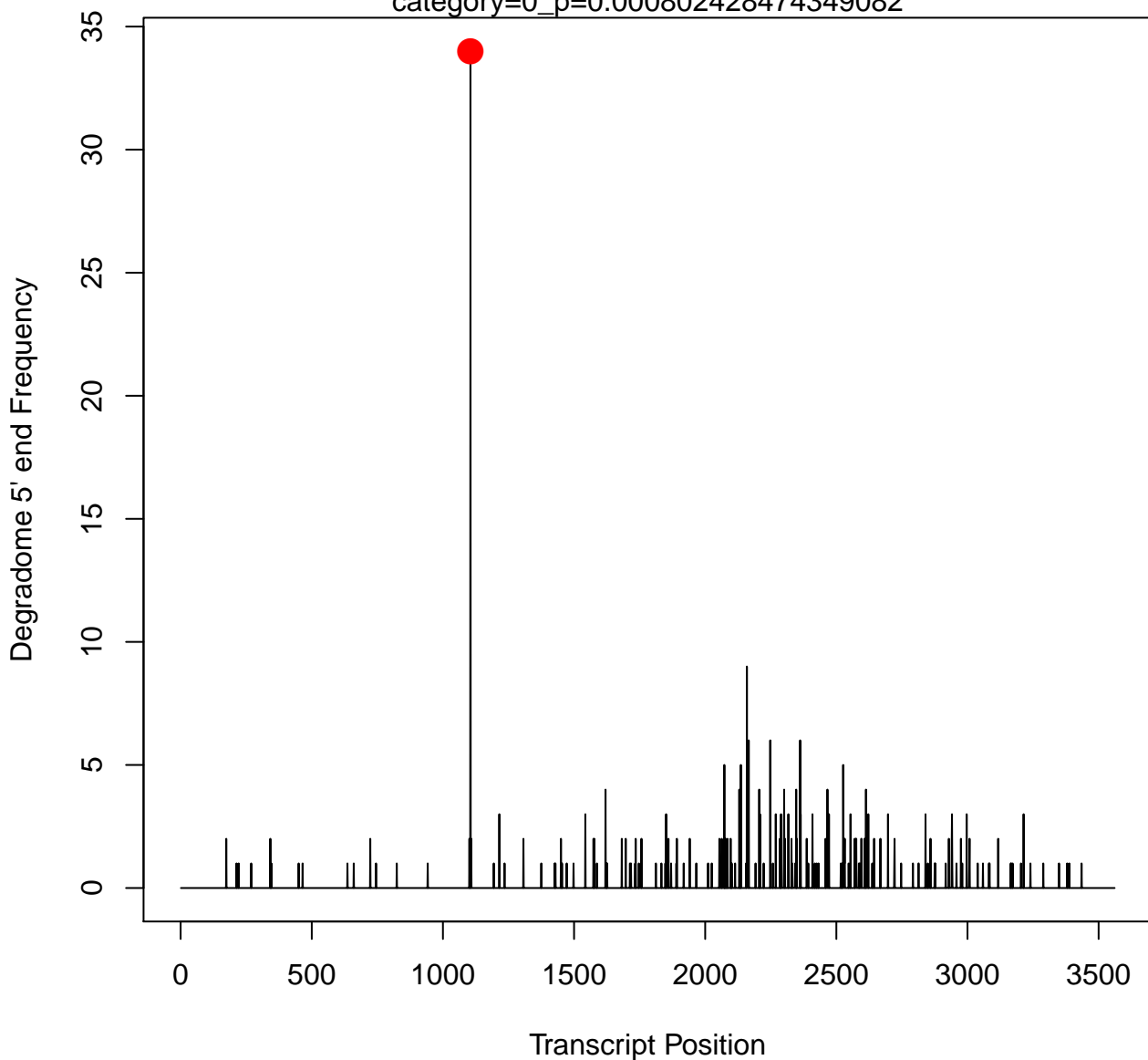

Supplement: Supplementary file 3 [file Data_Sheet_3.zip › Sit-miR166j_Seita.9G219700.1_1105_TPlot.pdf]

**T=Seita.9G560400.1\_Q=Sit-miR166j\_S=917**

category=2\_p=0.982690428974579

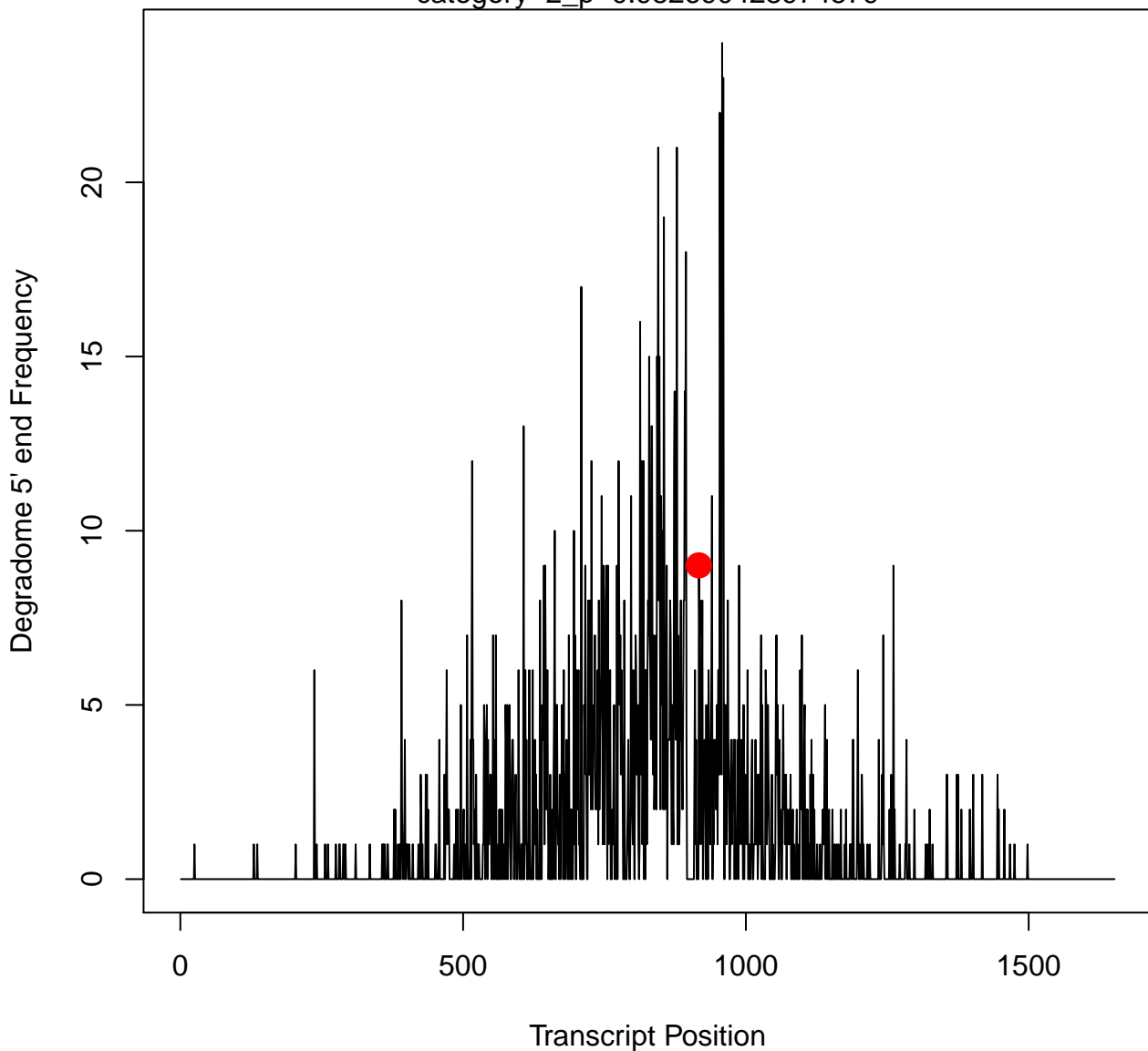

Supplement: Supplementary file 3 [file Data_Sheet_3.zip › Sit-miR166j_Seita.9G560400.1_917_TPlot.pdf]

**T=Seita.9G158800.1\_Q=Sit-miR166k\_S=868**

category=2\_p=0.049847041726001

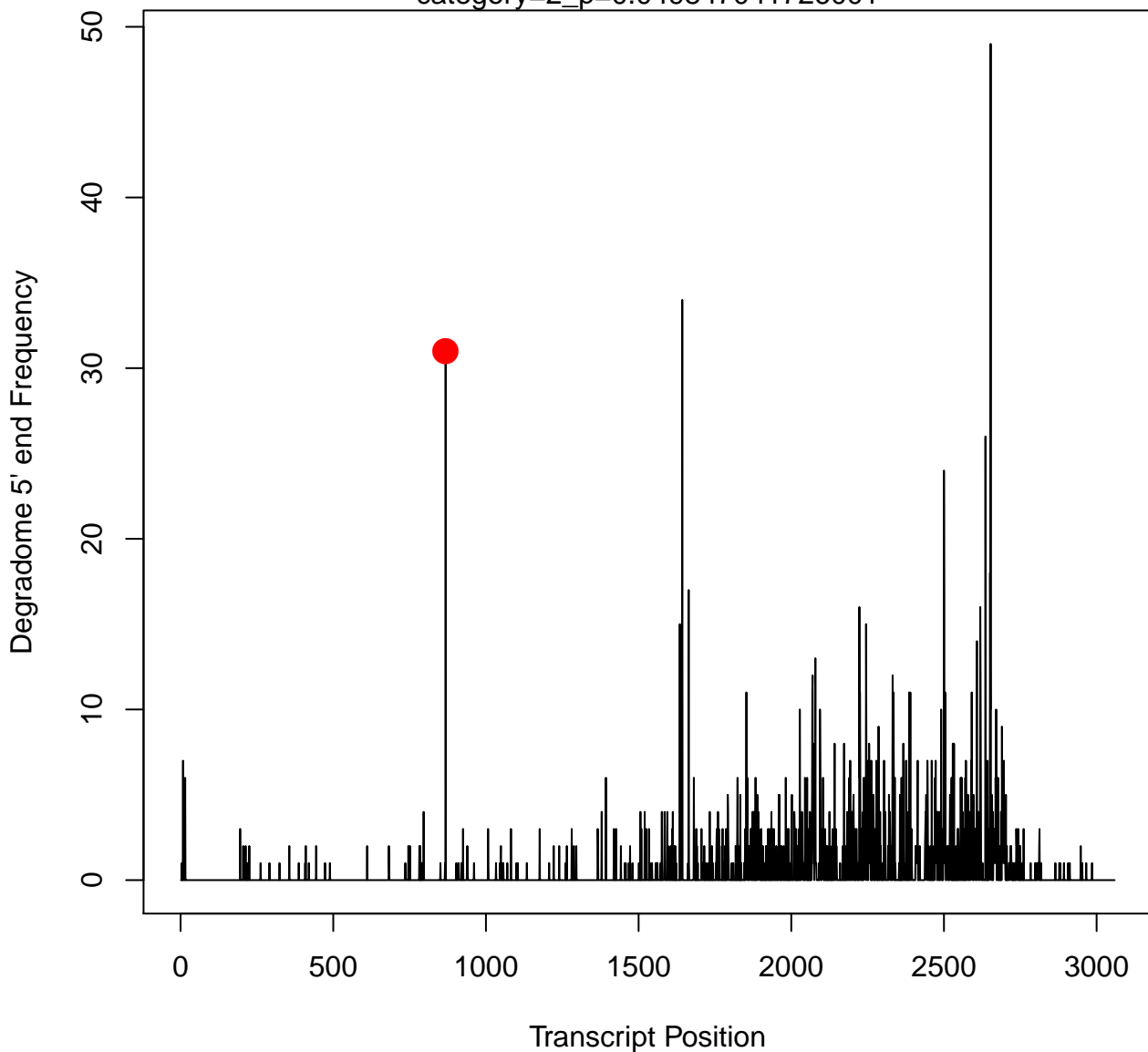

Supplement: Supplementary file 3 [file Data_Sheet_3.zip › Sit-miR166k_Seita.9G158800.1_868_TPlot.pdf]

**T=Seita.9G572600.1\_Q=Sit-miR166k\_S=1138**

category=0\_p=0.000802428474349082

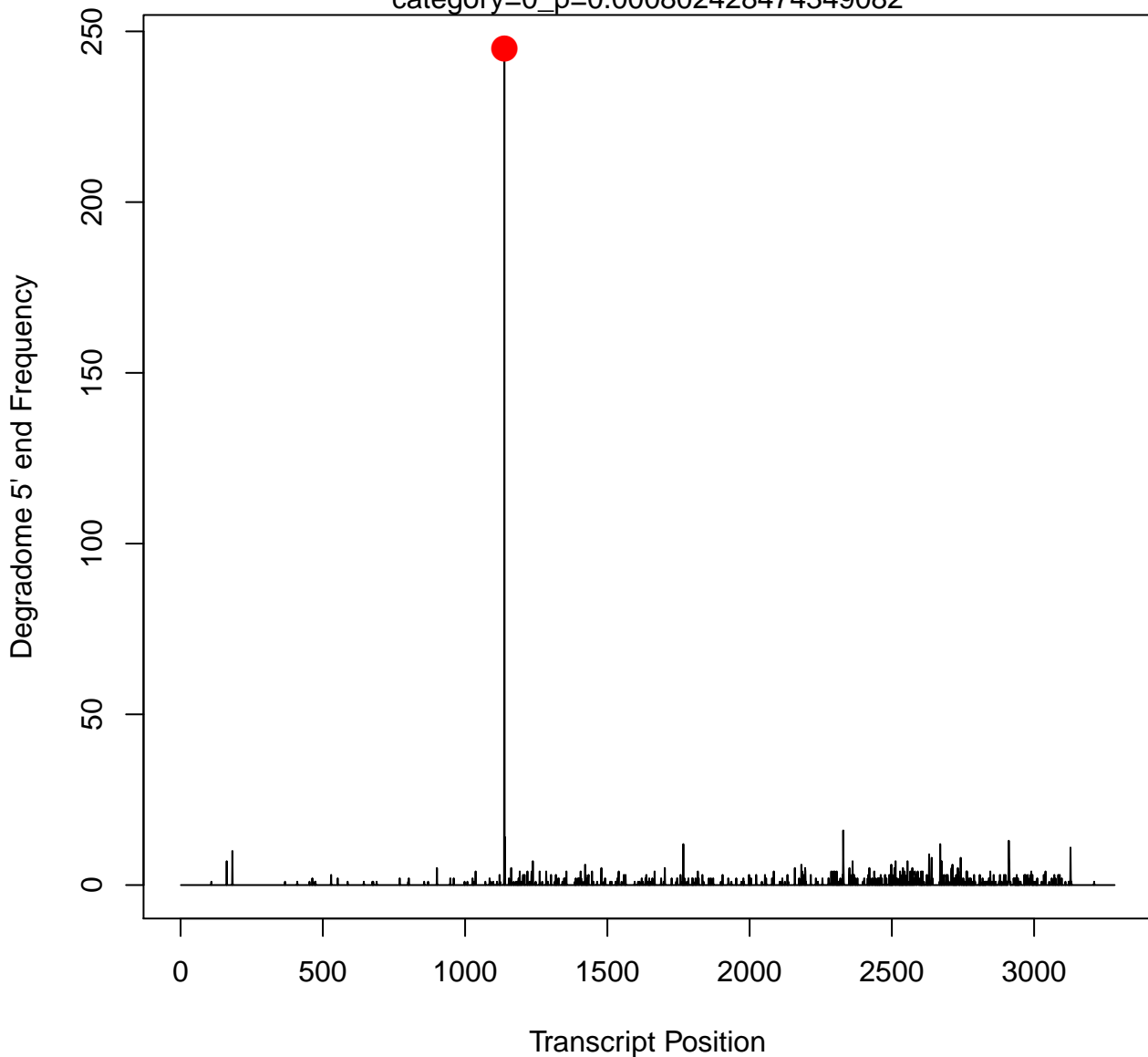

Supplement: Supplementary file 3 [file Data_Sheet_3.zip › Sit-miR166k_Seita.9G572600.1_1138_TPlot.pdf]

**T=Seita.6G093200.1\_Q=Sit-miR167a\_S=329**

category=2\_p=0.706880577395931

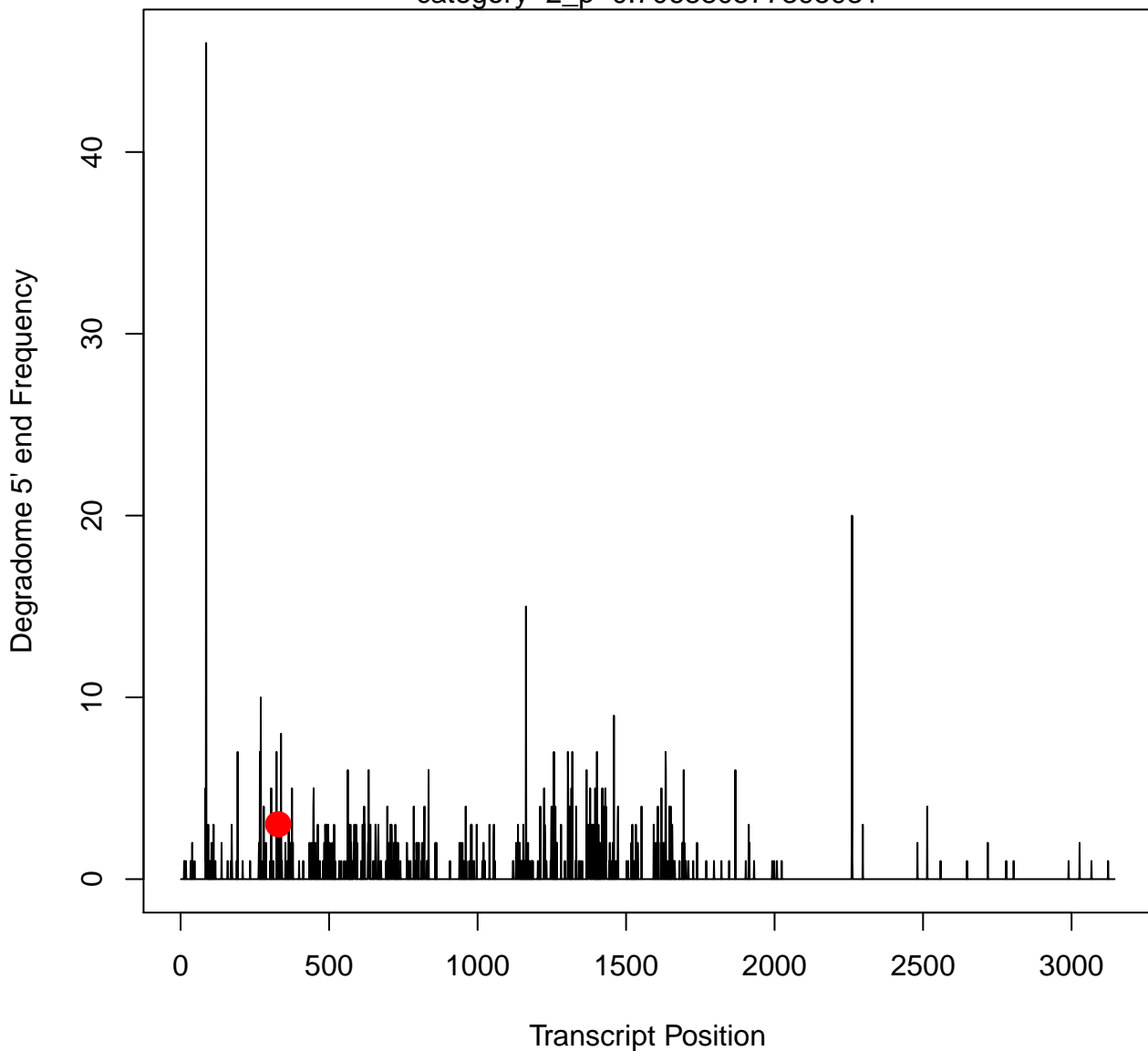

Supplement: Supplementary file 3 [file Data_Sheet_3.zip › Sit-miR167a_Seita.6G093200.1_329_TPlot.pdf]

**T=Seita.2G293900.1\_Q=Sit-miR167g\_S=1829**

category=2\_p=0.711834199084241

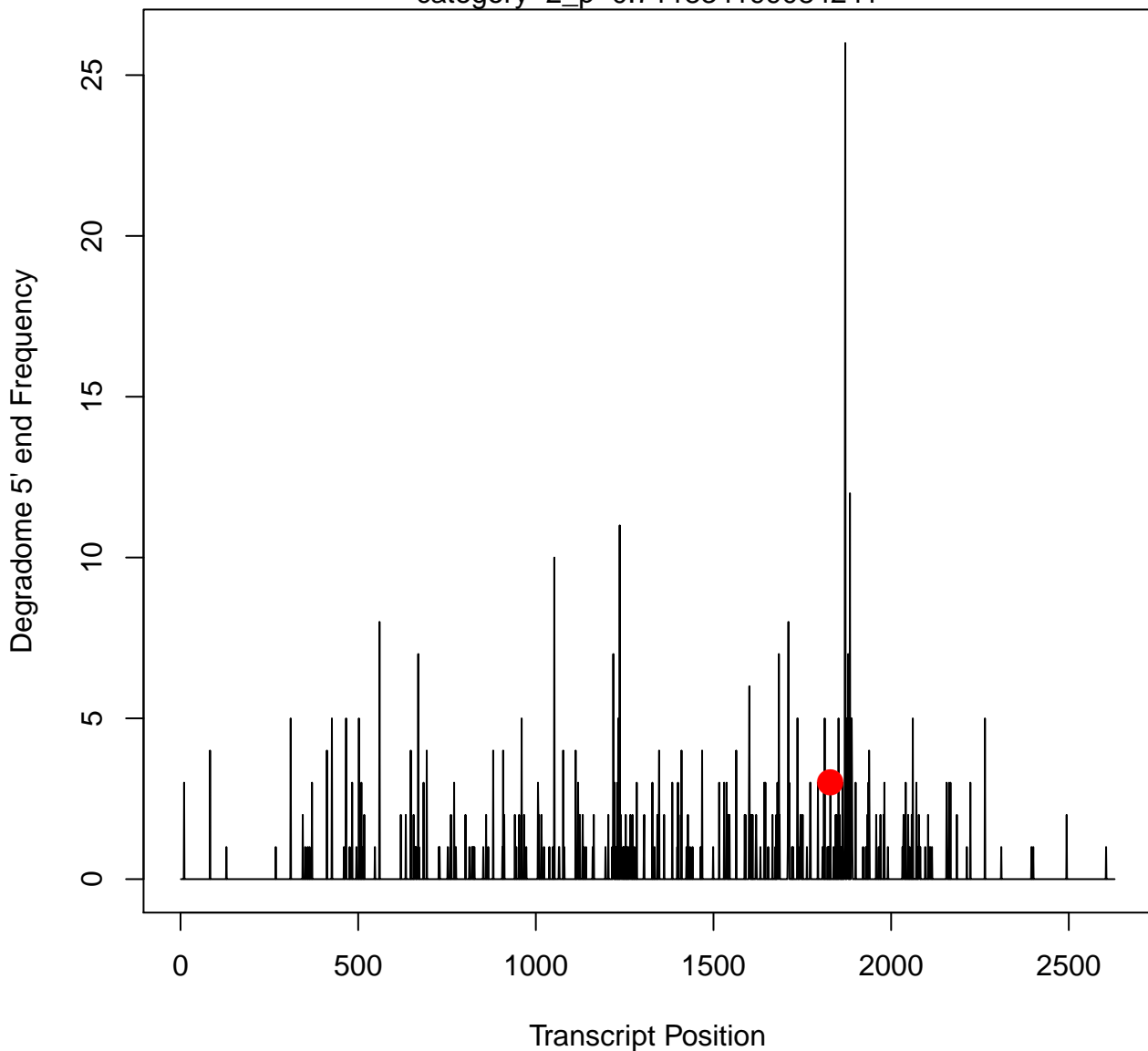

Supplement: Supplementary file 3 [file Data_Sheet_3.zip › Sit-miR167g_Seita.2G293900.1_1829_TPlot.pdf]

**T=Seita.3G161400.1\_Q=Sit-miR167h\_S=640**

category=2\_p=0.926299613131558

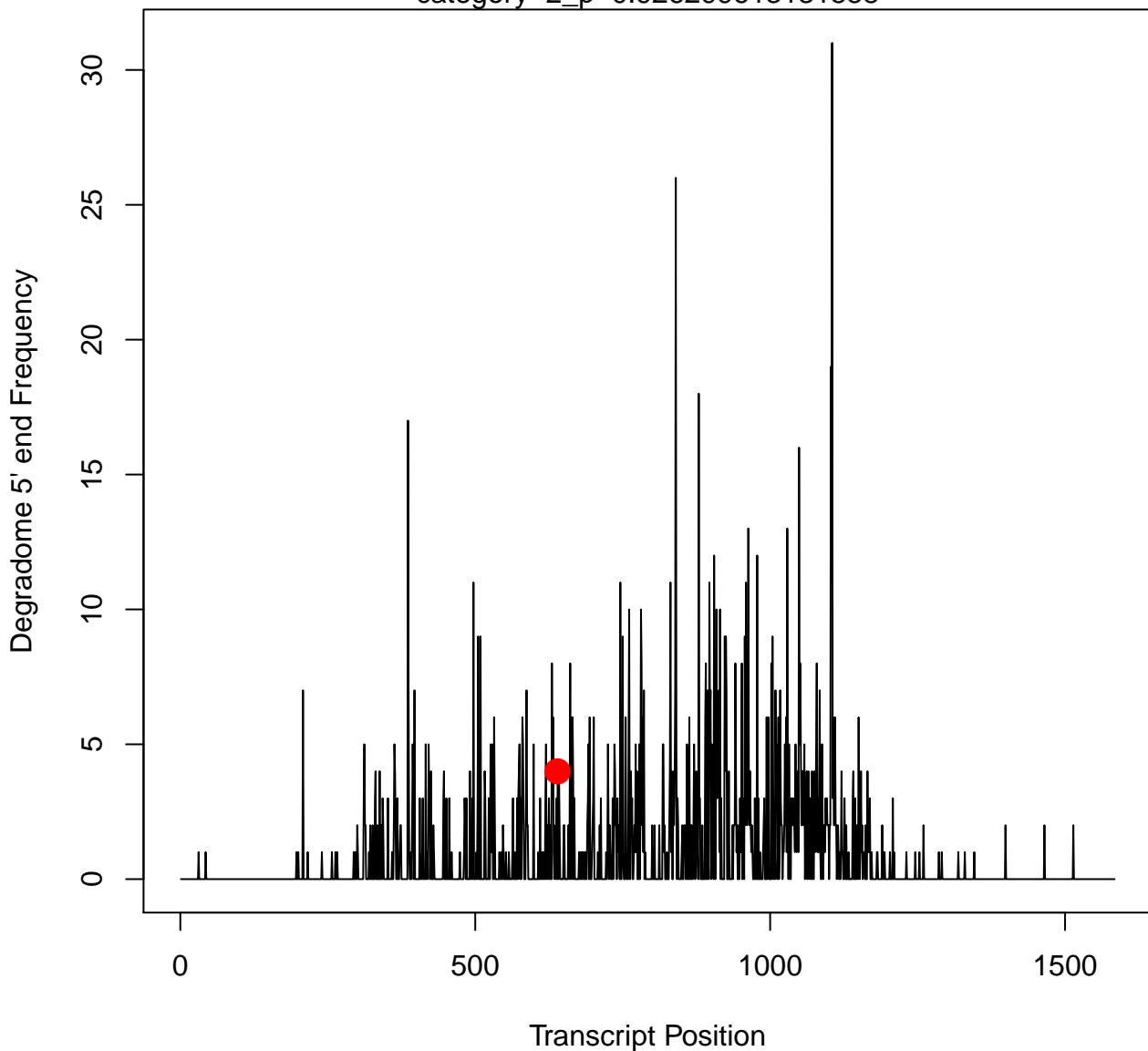

Supplement: Supplementary file 3 [file Data_Sheet_3.zip › Sit-miR167h_Seita.3G161400.1_640_TPlot.pdf]

**T=Seita.3G394000.1\_Q=Sit-miR167h\_S=3277**

category=0\_p=0.00360586087902259

Degradome 5' end Frequency

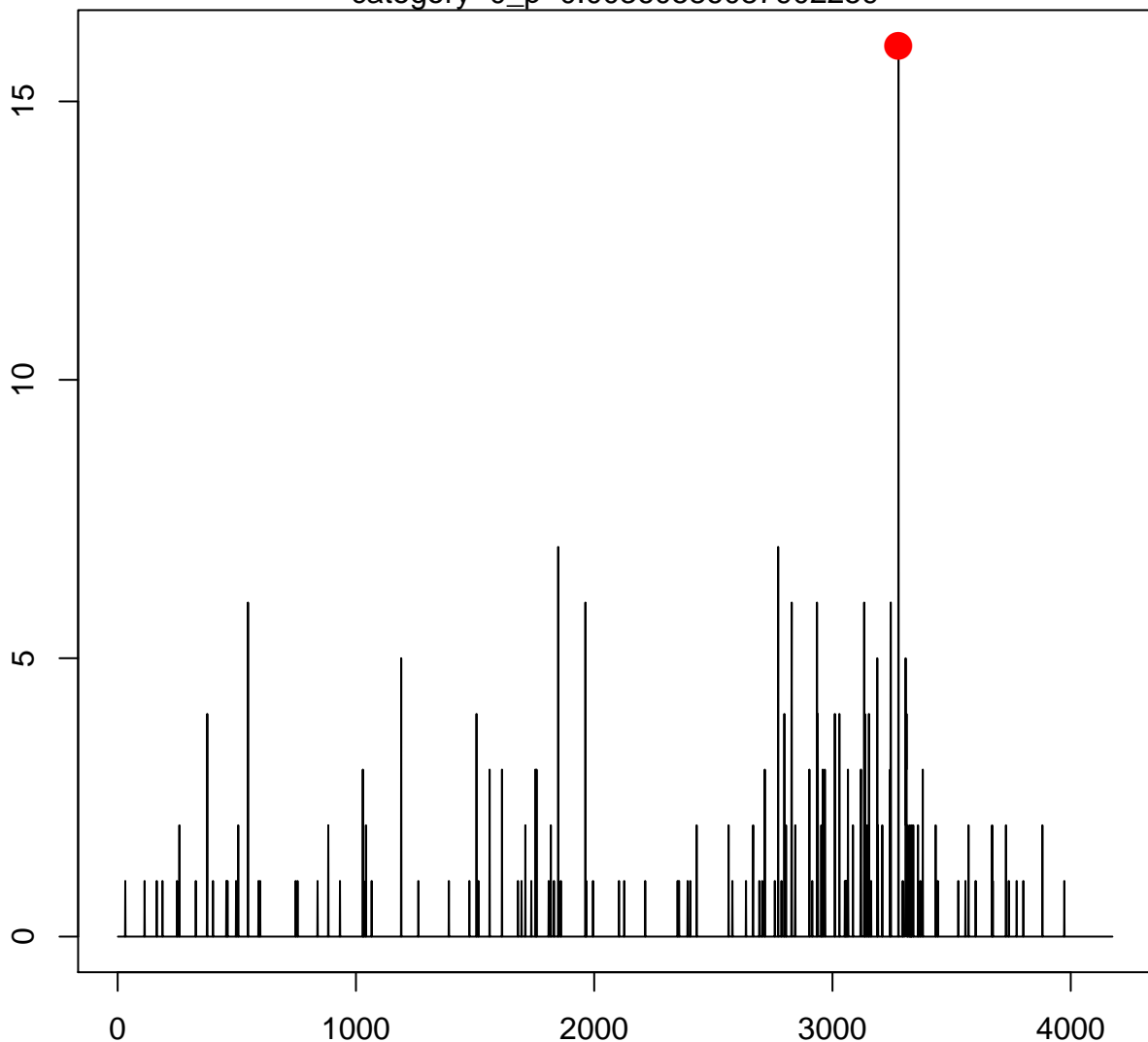

Transcript Position

Supplement: Supplementary file 3 [file Data_Sheet_3.zip › Sit-miR167h_Seita.3G394000.1_3277_TPlot.pdf]

**T=Seita.4G253100.1\_Q=Sit-miR167h\_S=819**

category=2\_p=0.999571433780318

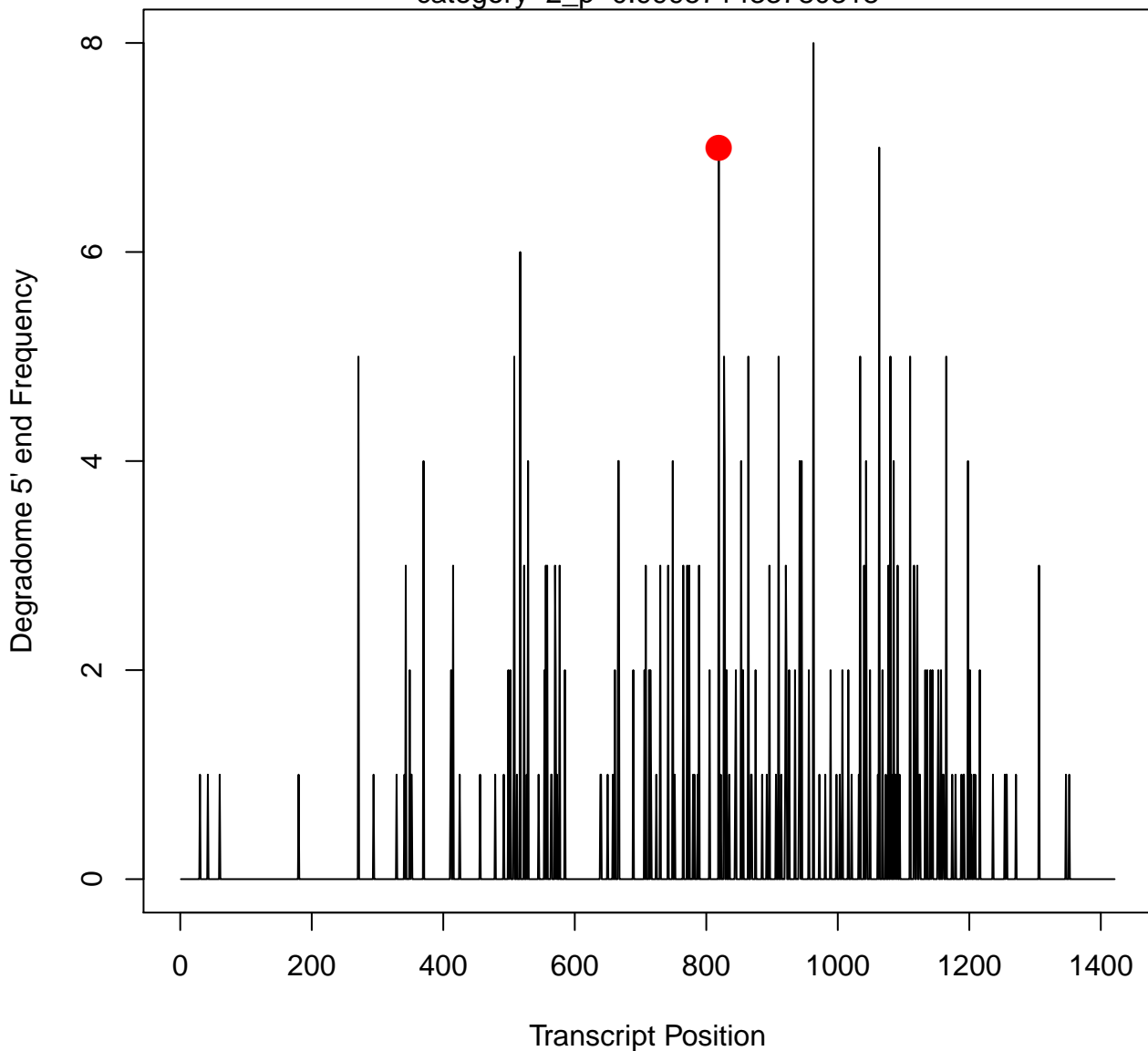

Supplement: Supplementary file 3 [file Data_Sheet_3.zip › Sit-miR167h_Seita.4G253100.1_819_TPlot.pdf]

**T=Seita.6G097500.1\_Q=Sit-miR167h\_S=1476**

category=2\_p=0.955801755900972

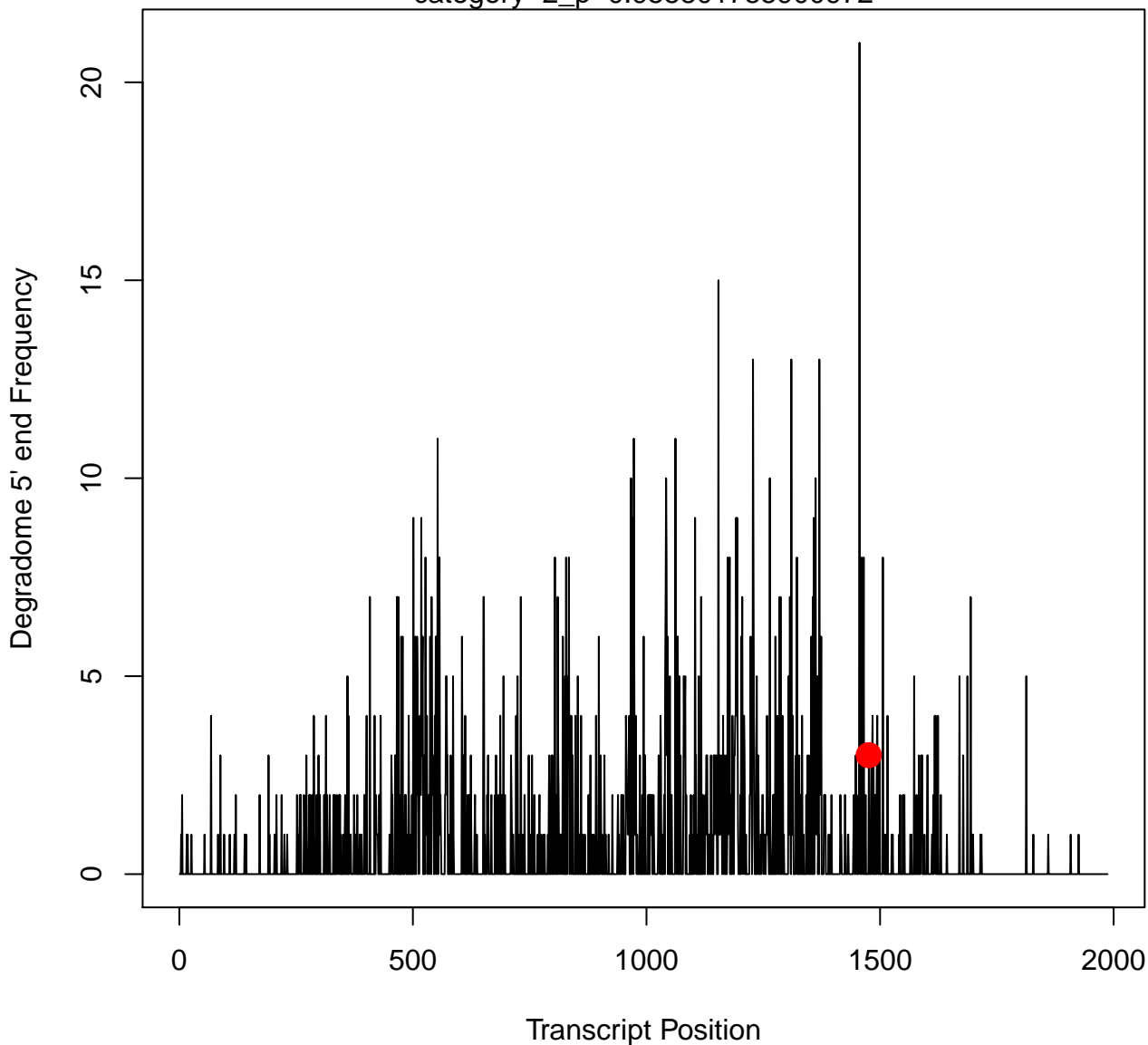

Supplement: Supplementary file 3 [file Data_Sheet_3.zip › Sit-miR167h_Seita.6G097500.1_1476_TPlot.pdf]

**T=Seita.9G357300.1\_Q=Sit-miR167h\_S=251**

category=2\_p=0.901529428139749

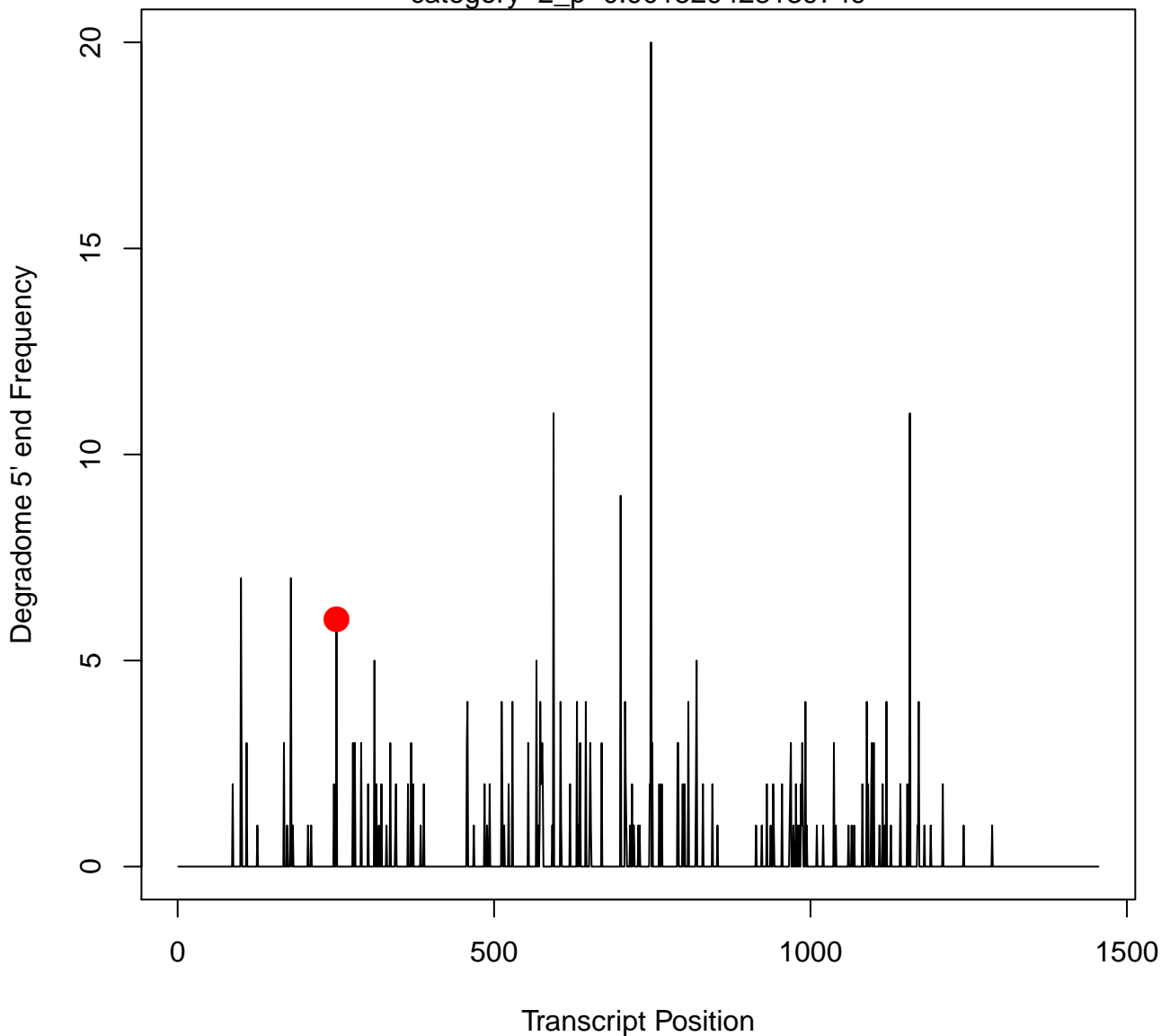

Supplement: Supplementary file 3 [file Data_Sheet_3.zip › Sit-miR167h_Seita.9G357300.1_251_TPlot.pdf]

**T=Seita.2G366700.1\_Q=Sit-miR167i\_S=960**

category=2\_p=0.995340706058631

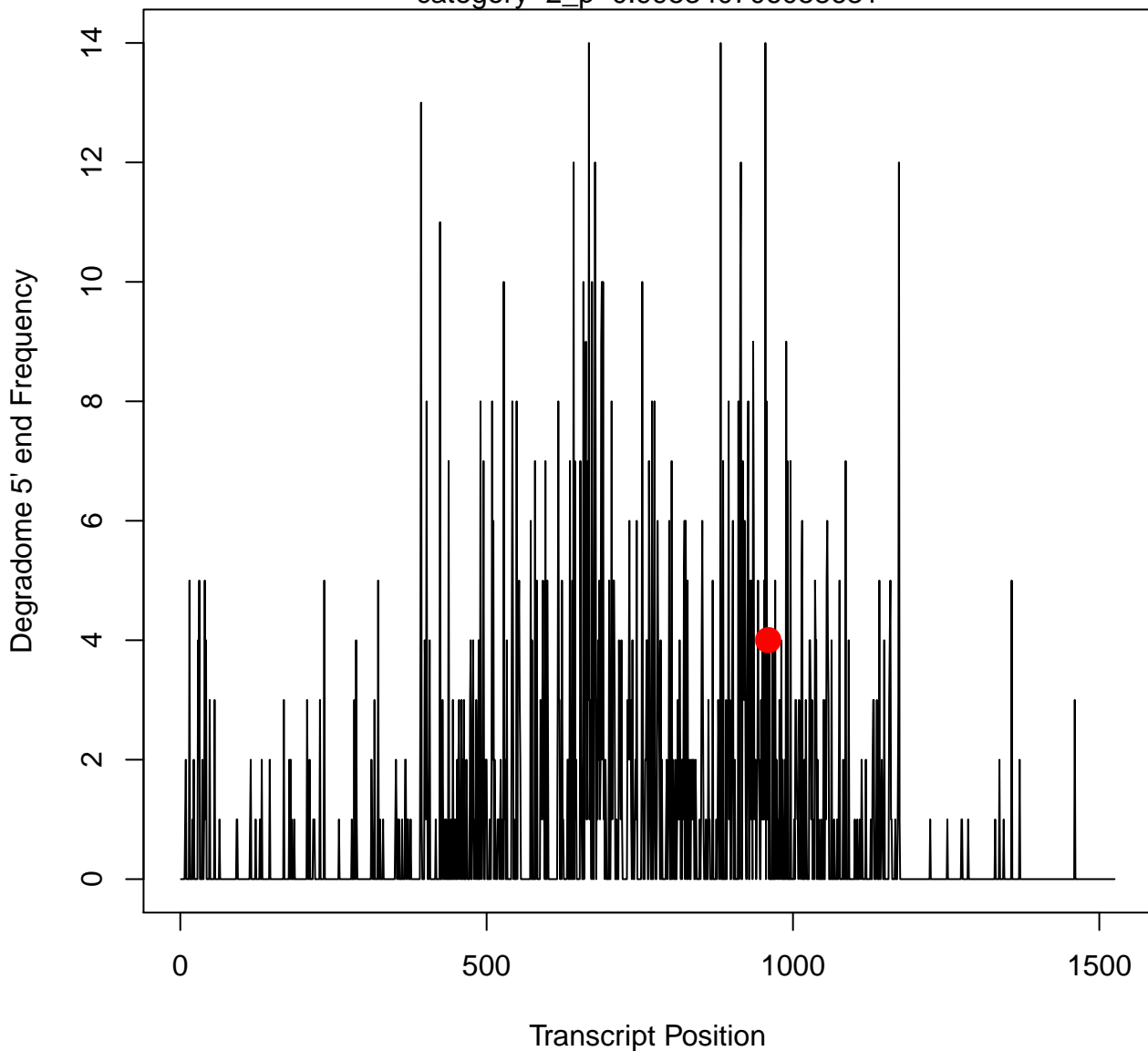

Supplement: Supplementary file 3 [file Data_Sheet_3.zip › Sit-miR167i_Seita.2G366700.1_960_TPlot.pdf]

**T=Seita.4G262300.1\_Q=Sit-miR167i\_S=3389**

category=0\_p=0.00240535426535449

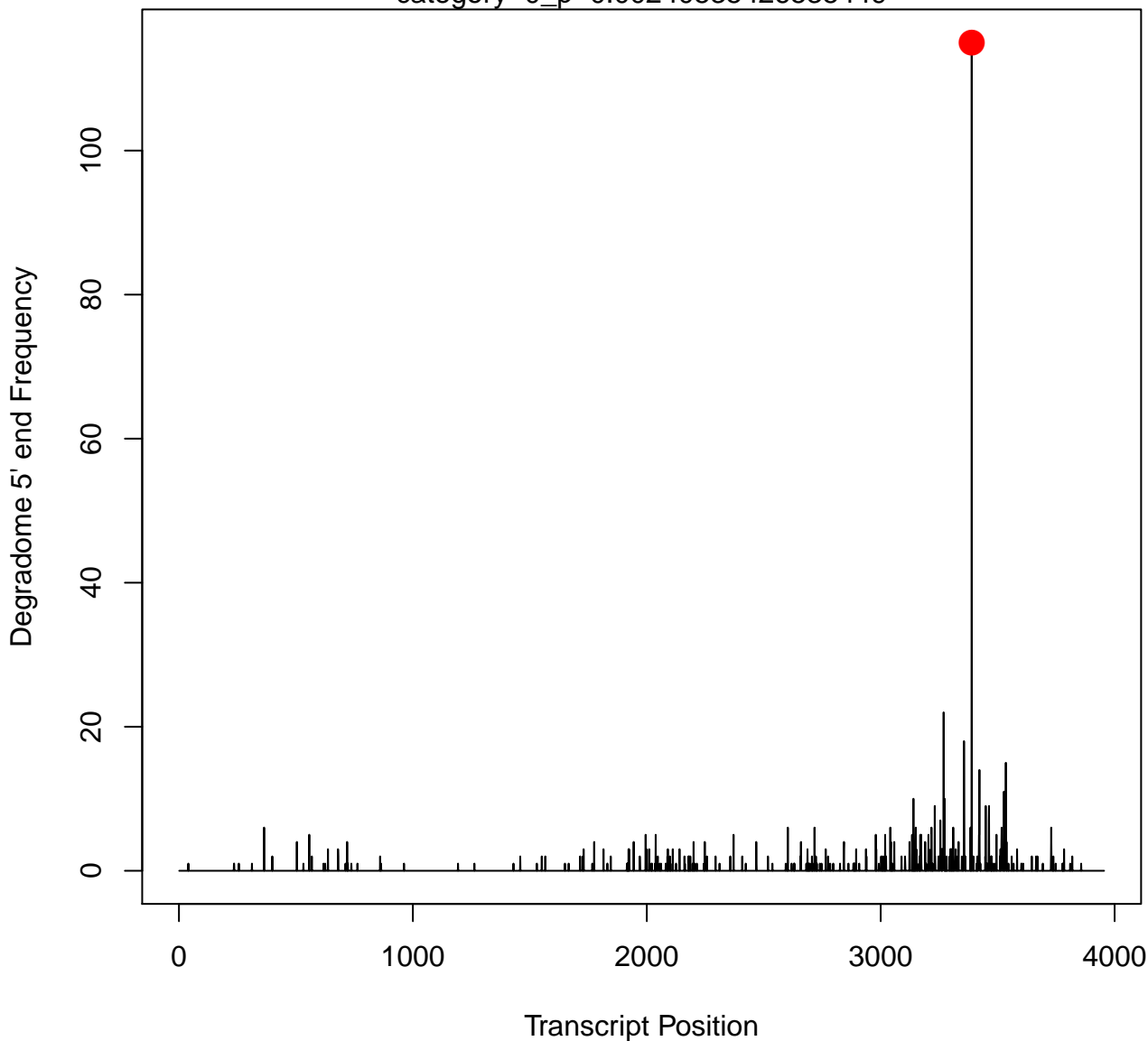

Supplement: Supplementary file 3 [file Data_Sheet_3.zip › Sit-miR167i_Seita.4G262300.1_3389_TPlot.pdf]

**T=Seita.9G385200.1\_Q=Sit-miR167i\_S=387**

category=2\_p=0.999564066650305

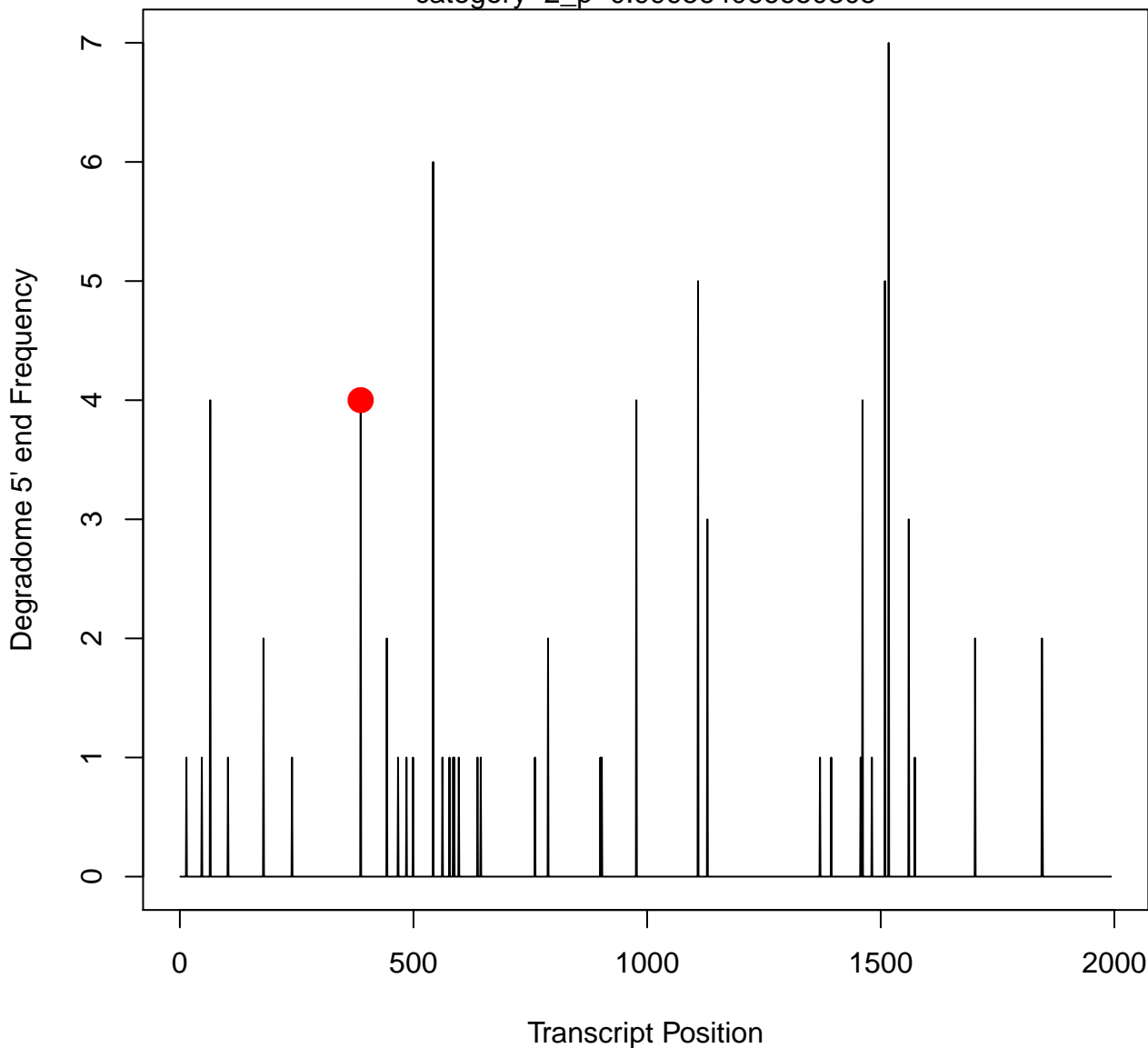

Supplement: Supplementary file 3 [file Data_Sheet_3.zip › Sit-miR167i_Seita.9G385200.1_387_TPlot.pdf]

**T=Seita.9G448300.1\_Q=Sit-miR167i\_S=2577**

category=2\_p=0.896363452849605

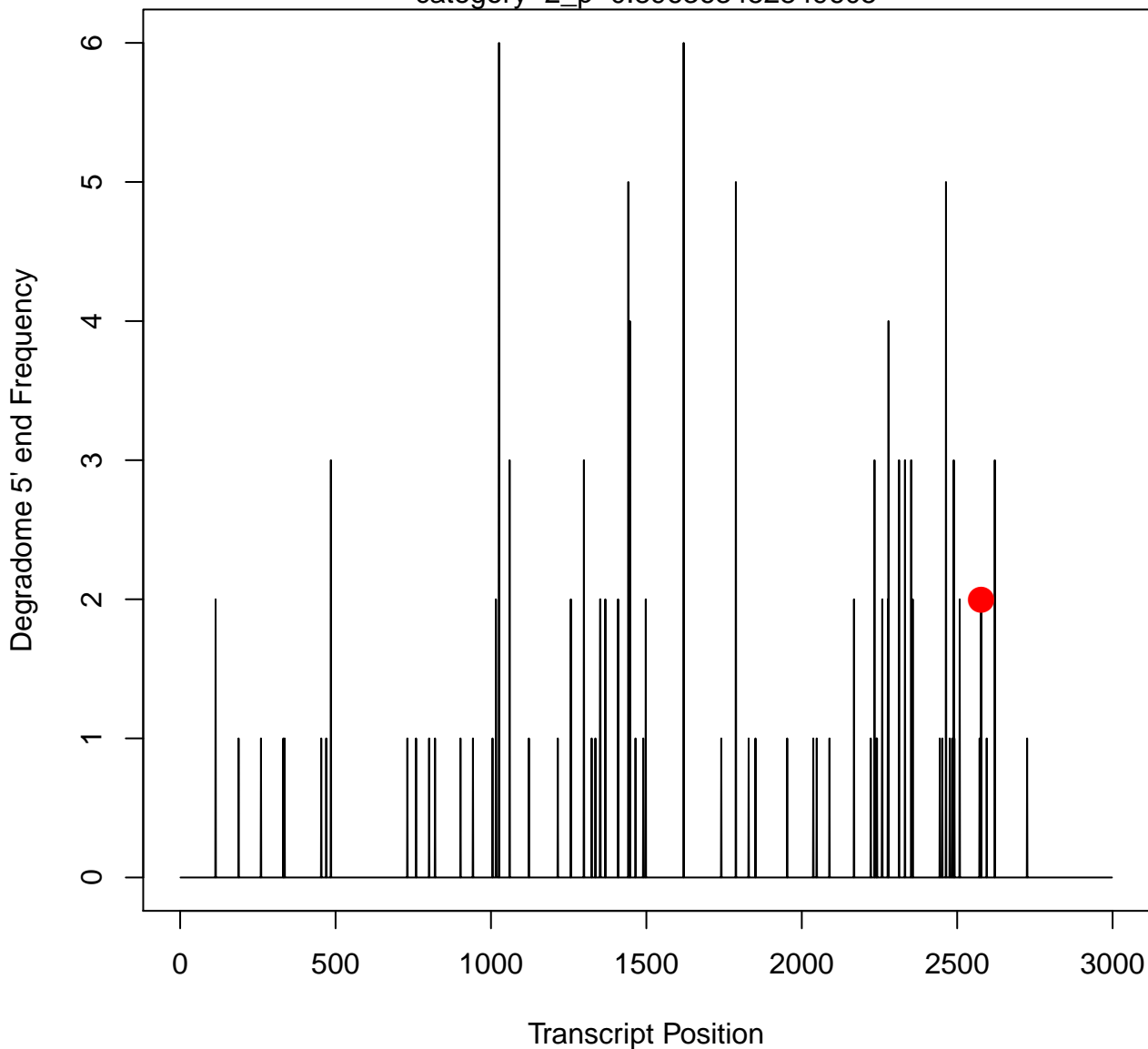

Supplement: Supplementary file 3 [file Data_Sheet_3.zip › Sit-miR167i_Seita.9G448300.1_2577_TPlot.pdf]

**T=Seita.1G077200.1\_Q=Sit-miR167j\_S=3508**

category=0\_p=0.00240535426535449

Degradome 5' end Frequency

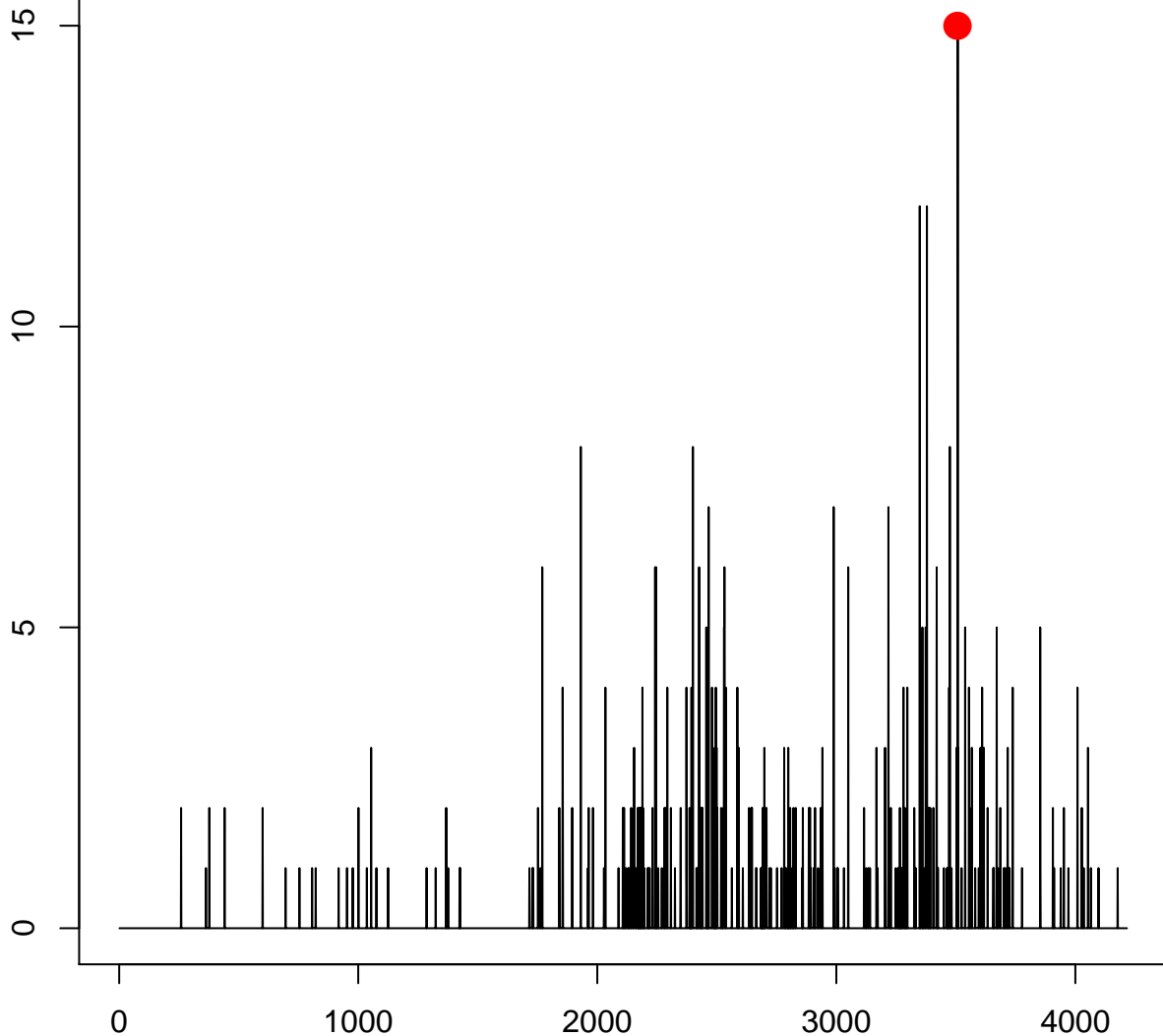

Transcript Position

Supplement: Supplementary file 3 [file Data_Sheet_3.zip › Sit-miR167j_Seita.1G077200.1_3508_TPlot.pdf]

**T=Seita.3G020000.1\_Q=Sit-miR167j\_S=2739**

category=0\_p=0.00120340121992535

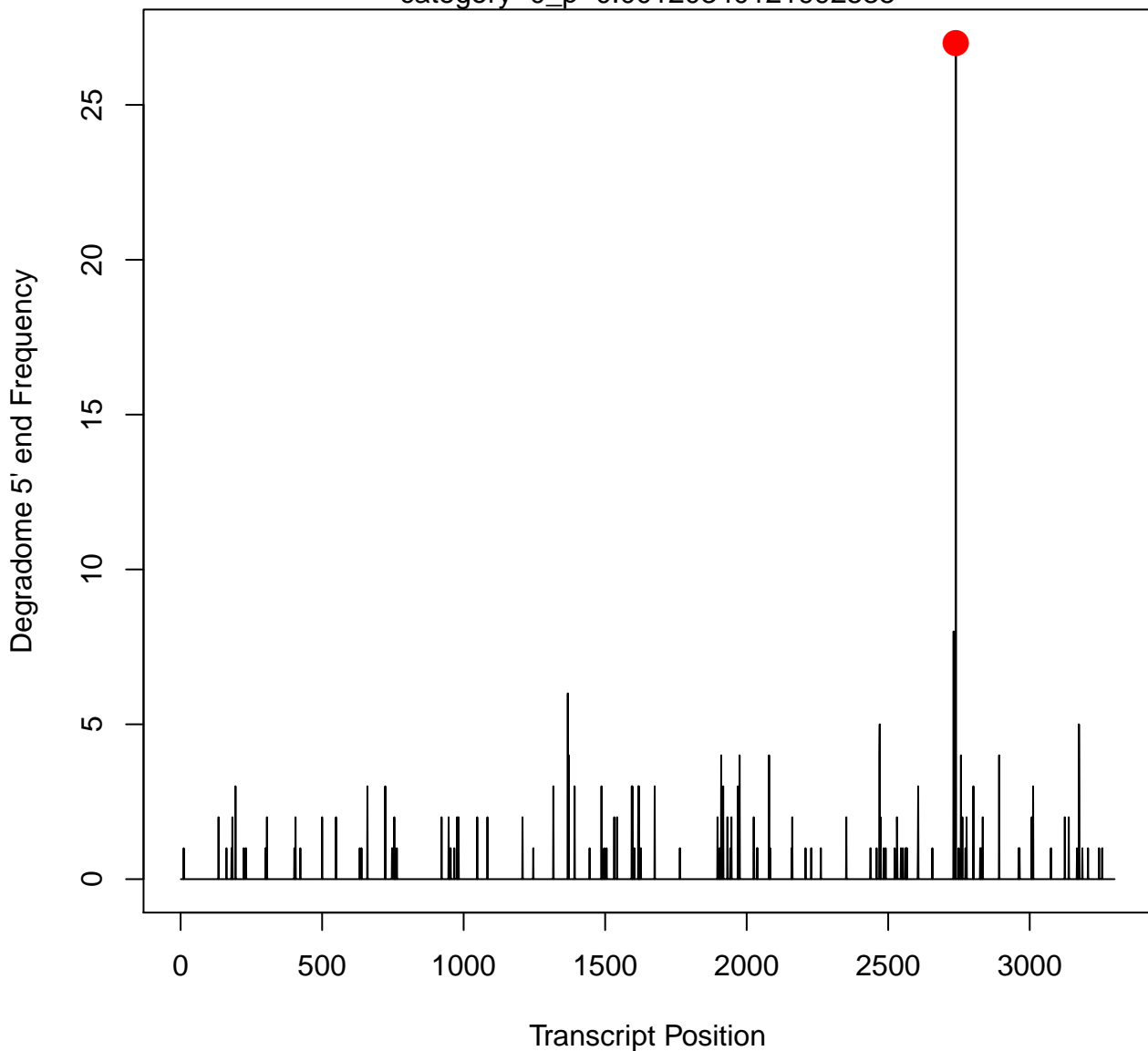

Supplement: Supplementary file 3 [file Data_Sheet_3.zip › Sit-miR167j_Seita.3G020000.1_2739_TPlot.pdf]

**T=Seita.3G255600.1\_Q=Sit-miR167j\_S=1750**

category=2\_p=0.982392874350906

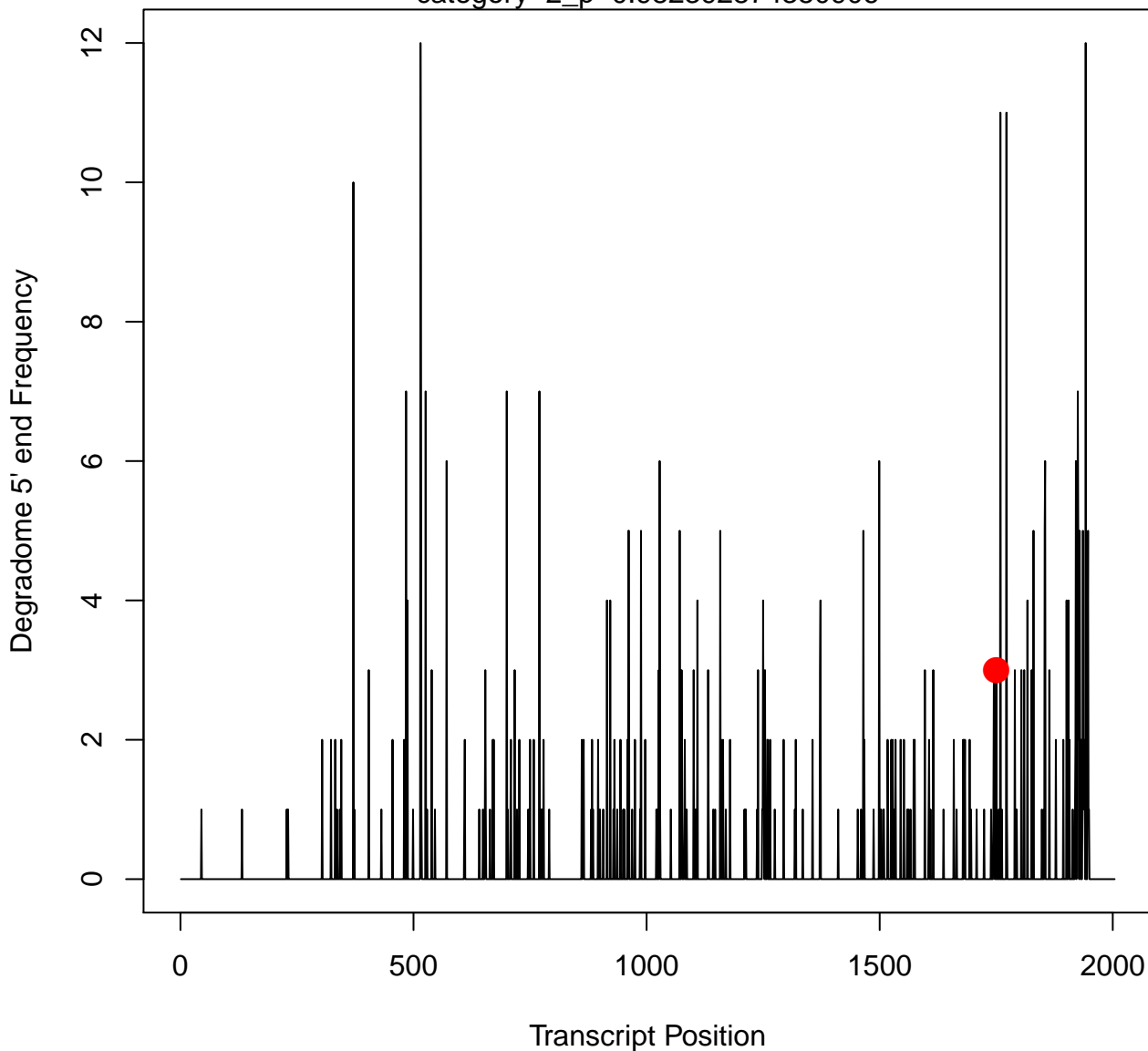

Supplement: Supplementary file 3 [file Data_Sheet_3.zip › Sit-miR167j_Seita.3G255600.1_1750_TPlot.pdf]

**T=Seita.7G235100.1\_Q=Sit-miR167j\_S=1010**

category=2\_p=0.081690223146084

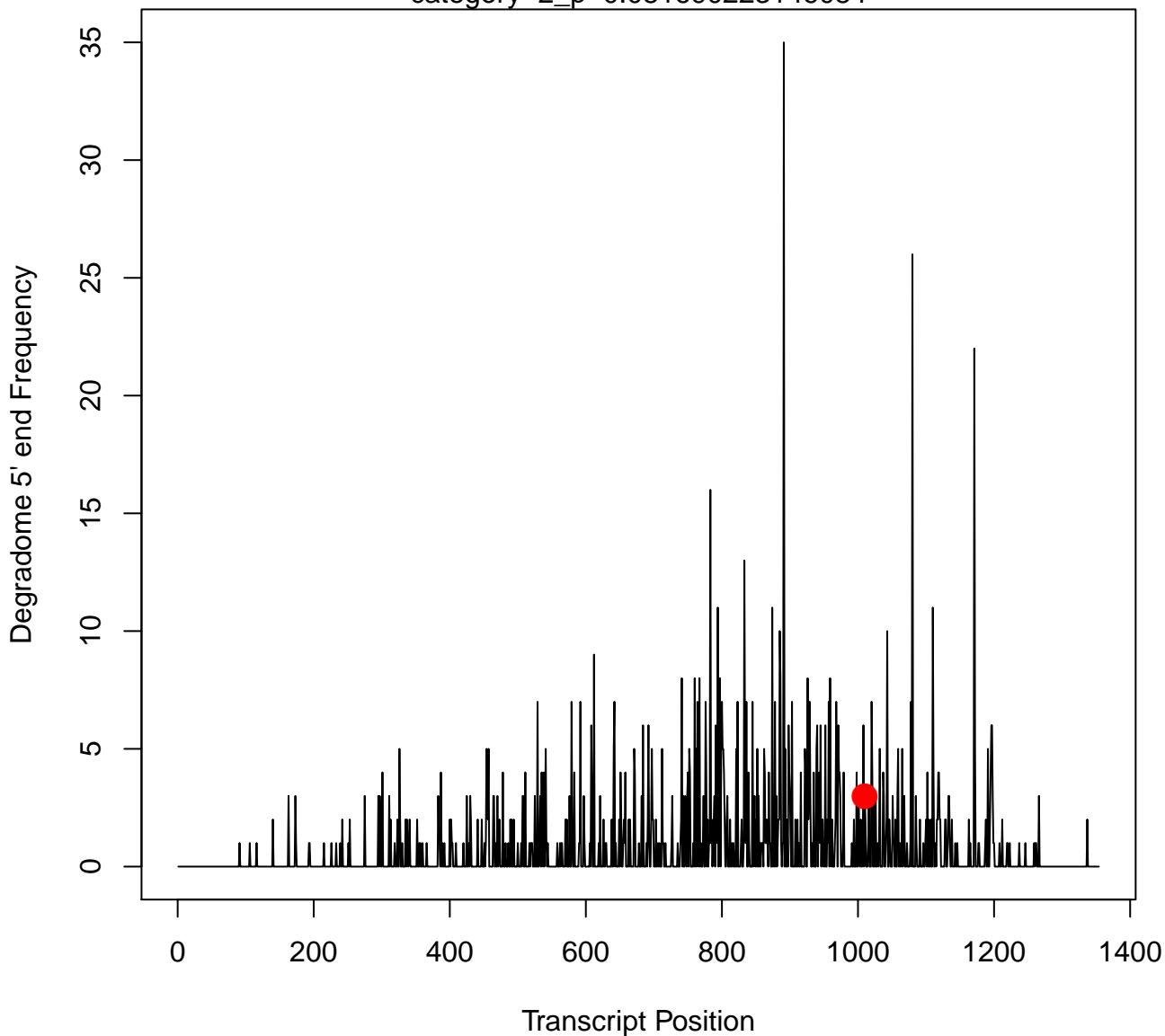

Supplement: Supplementary file 3 [file Data_Sheet_3.zip › Sit-miR167j_Seita.7G235100.1_1010_TPlot.pdf]

**T=Seita.1G268300.1\_Q=Sit-miR168\_S=631**

category=2\_p=0.978025625464141

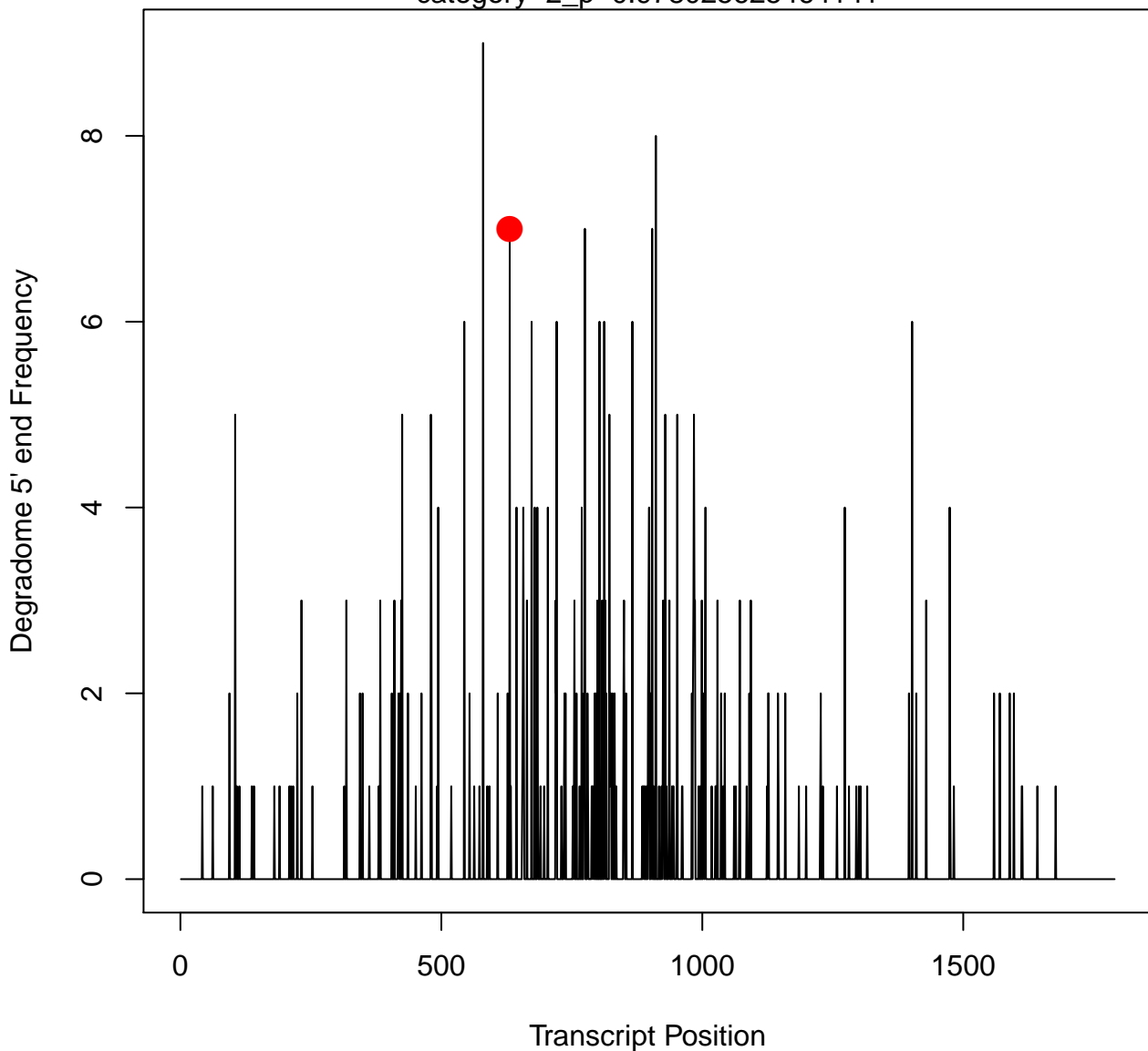

Supplement: Supplementary file 3 [file Data_Sheet_3.zip › Sit-miR168_Seita.1G268300.1_631_TPlot.pdf]

**T=Seita.1G361000.1\_Q=Sit-miR168\_S=1045**

category=0\_p=0.0175054801675408

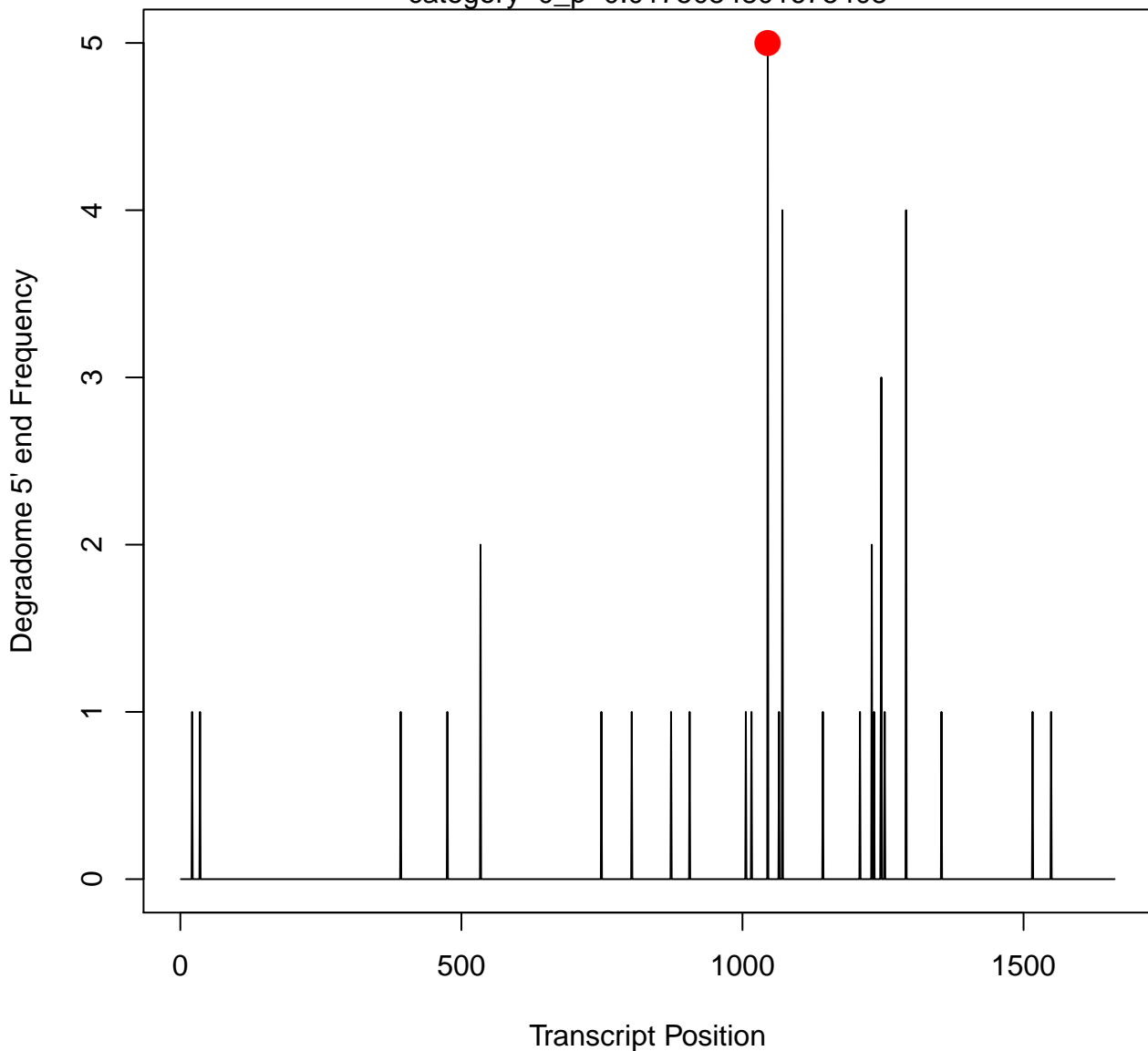

Supplement: Supplementary file 3 [file Data_Sheet_3.zip › Sit-miR168_Seita.1G361000.1_1045_TPlot.pdf]

**T=Seita.1G378700.1\_Q=Sit-miR168\_S=527**

category=0\_p=0.0111755921743733

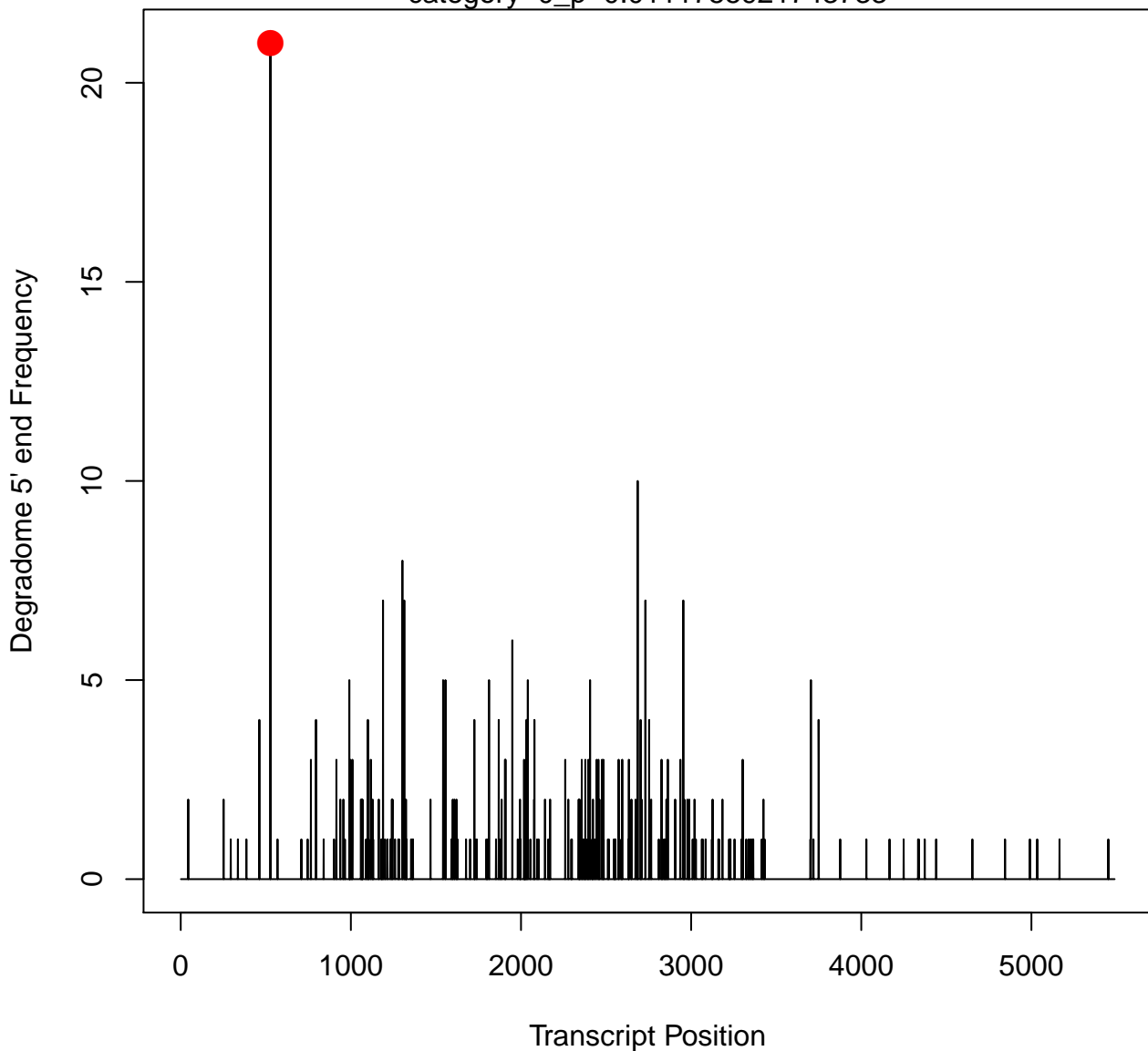

Supplement: Supplementary file 3 [file Data_Sheet_3.zip › Sit-miR168_Seita.1G378700.1_527_TPlot.pdf]

**T=Seita.3G070600.1\_Q=Sit-miR168\_S=747**

category=0\_p=0.0103814941065016

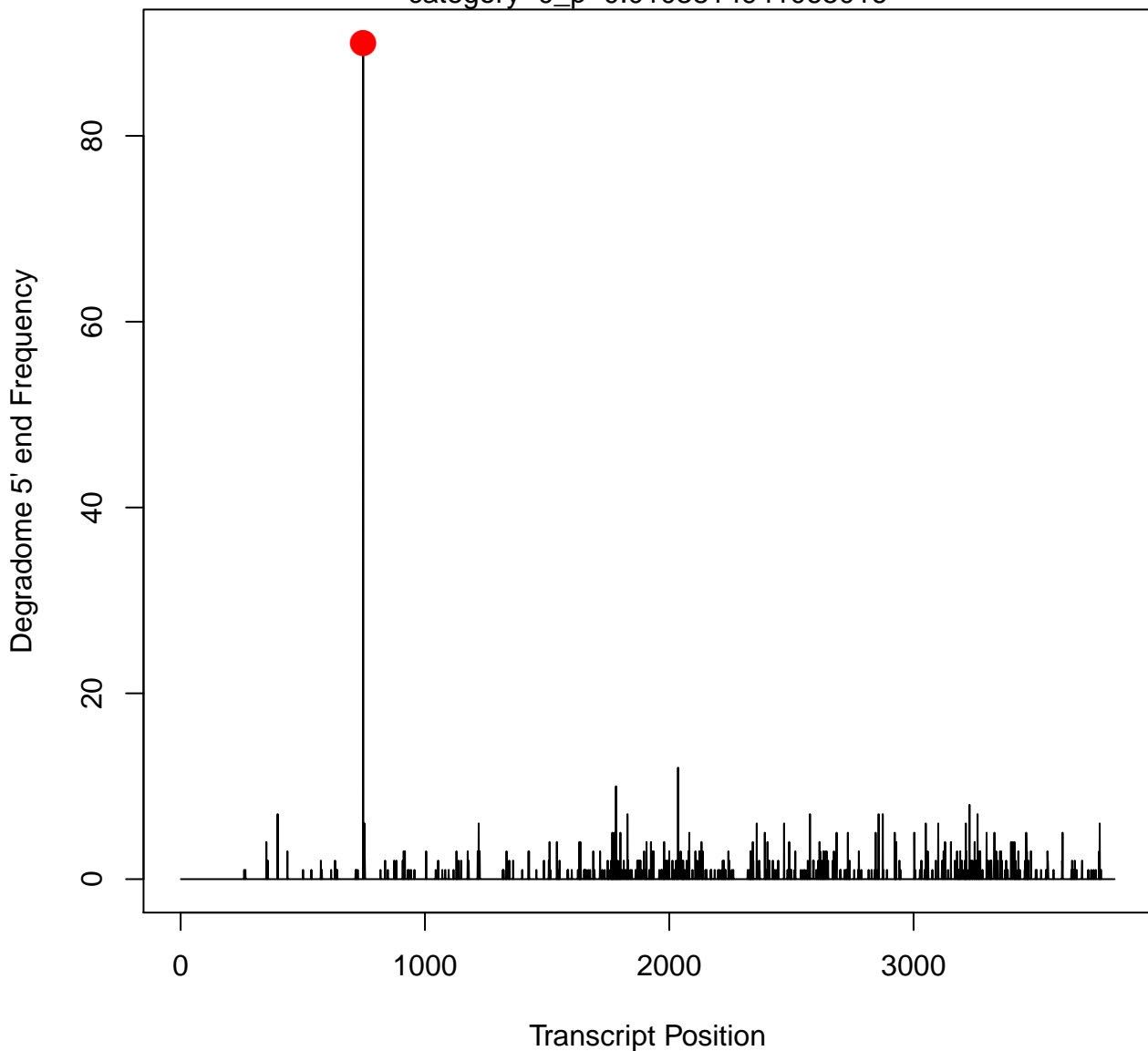

Supplement: Supplementary file 3 [file Data_Sheet_3.zip › Sit-miR168_Seita.3G070600.1_747_TPlot.pdf]

**T=Seita.4G288700.1\_Q=Sit-miR168\_S=343**

category=2\_p=0.368836721072763

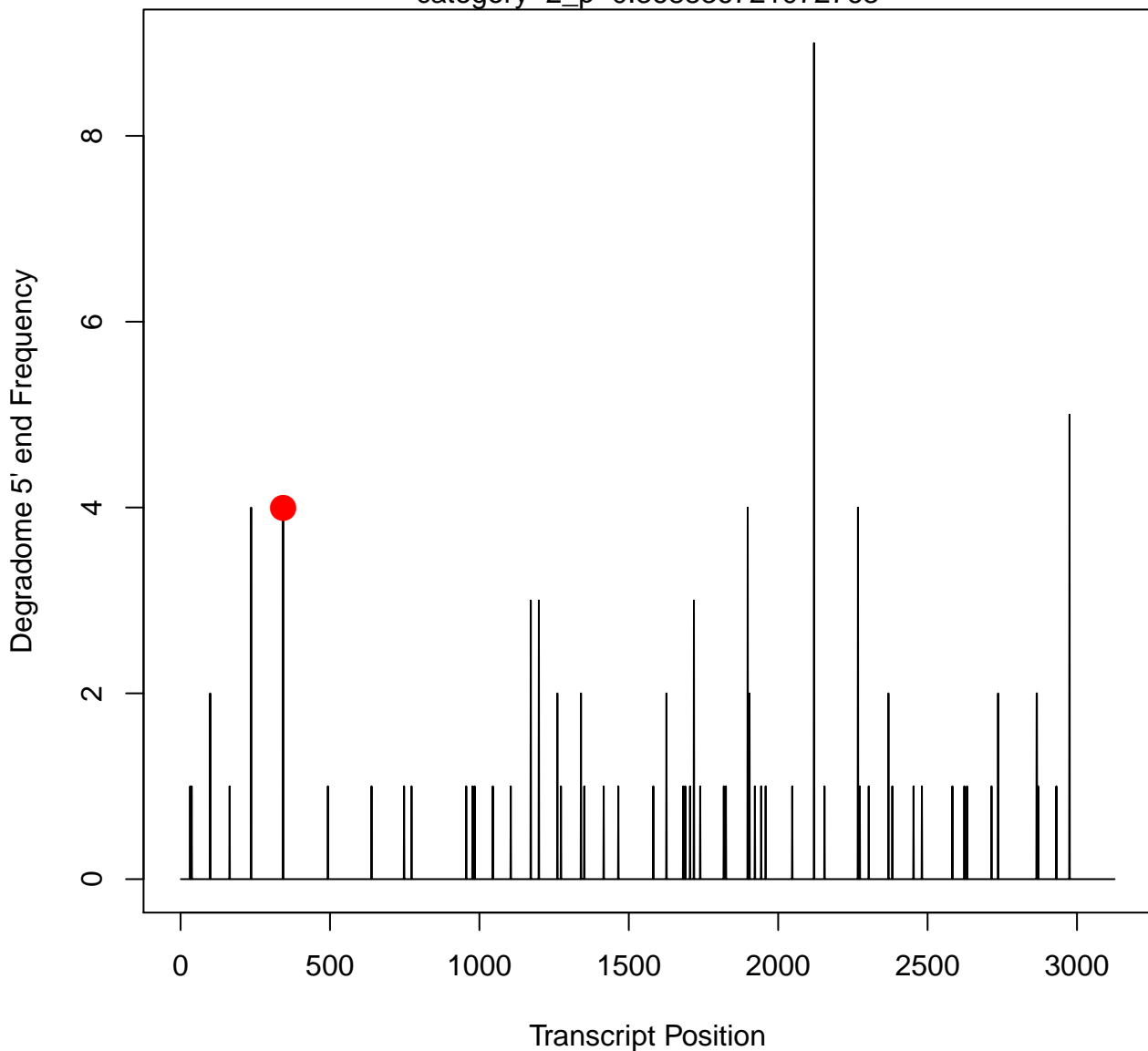

Supplement: Supplementary file 3 [file Data_Sheet_3.zip › Sit-miR168_Seita.4G288700.1_343_TPlot.pdf]

**T=Seita.5G017300.1\_Q=Sit-miR168\_S=2602**

category=2\_p=0.998266216268518

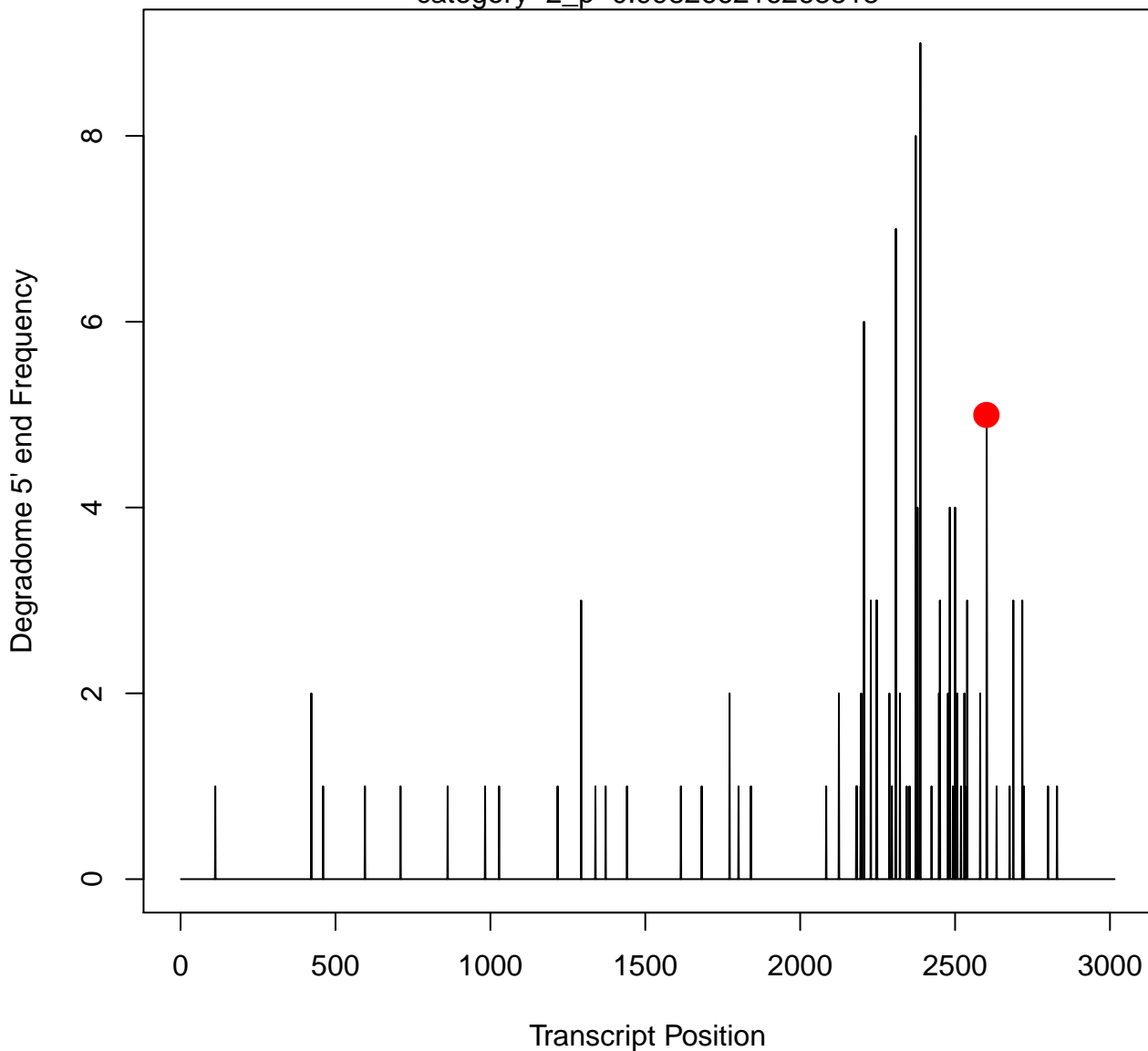

Supplement: Supplementary file 3 [file Data_Sheet_3.zip › Sit-miR168_Seita.5G017300.1_2602_TPlot.pdf]

**T=Seita.5G022800.1\_Q=Sit-miR168\_S=2856**

category=2\_p=0.971624148793258

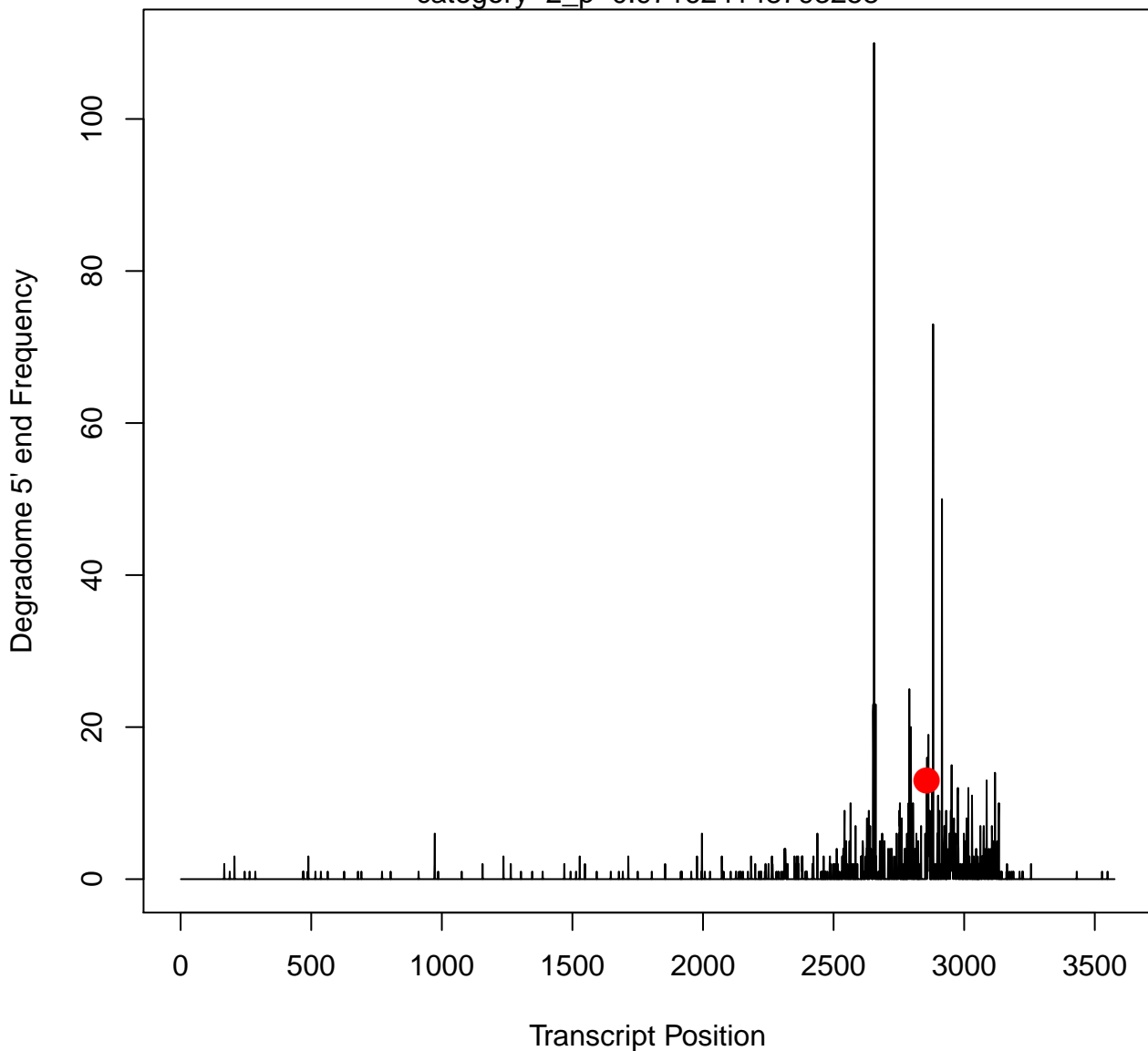

Supplement: Supplementary file 3 [file Data_Sheet_3.zip › Sit-miR168_Seita.5G022800.1_2856_TPlot.pdf]

**T=Seita.5G261900.1\_Q=Sit-miR168\_S=537**

category=0\_p=0.0972870857494557

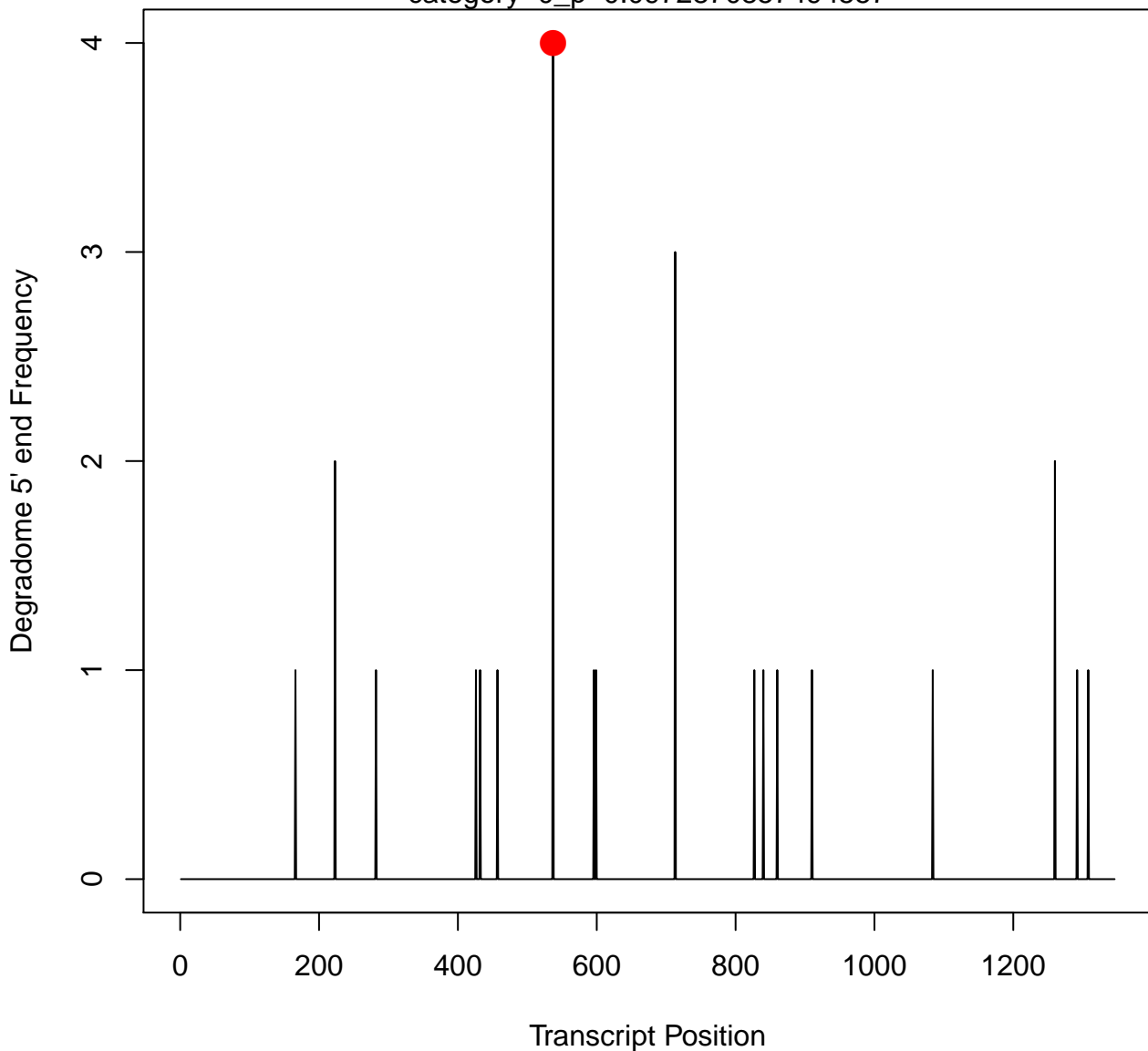

Supplement: Supplementary file 3 [file Data_Sheet_3.zip › Sit-miR168_Seita.5G261900.1_537_TPlot.pdf]

**T=Seita.5G307700.1\_Q=Sit-miR168\_S=524**

category=2\_p=0.99557295807814

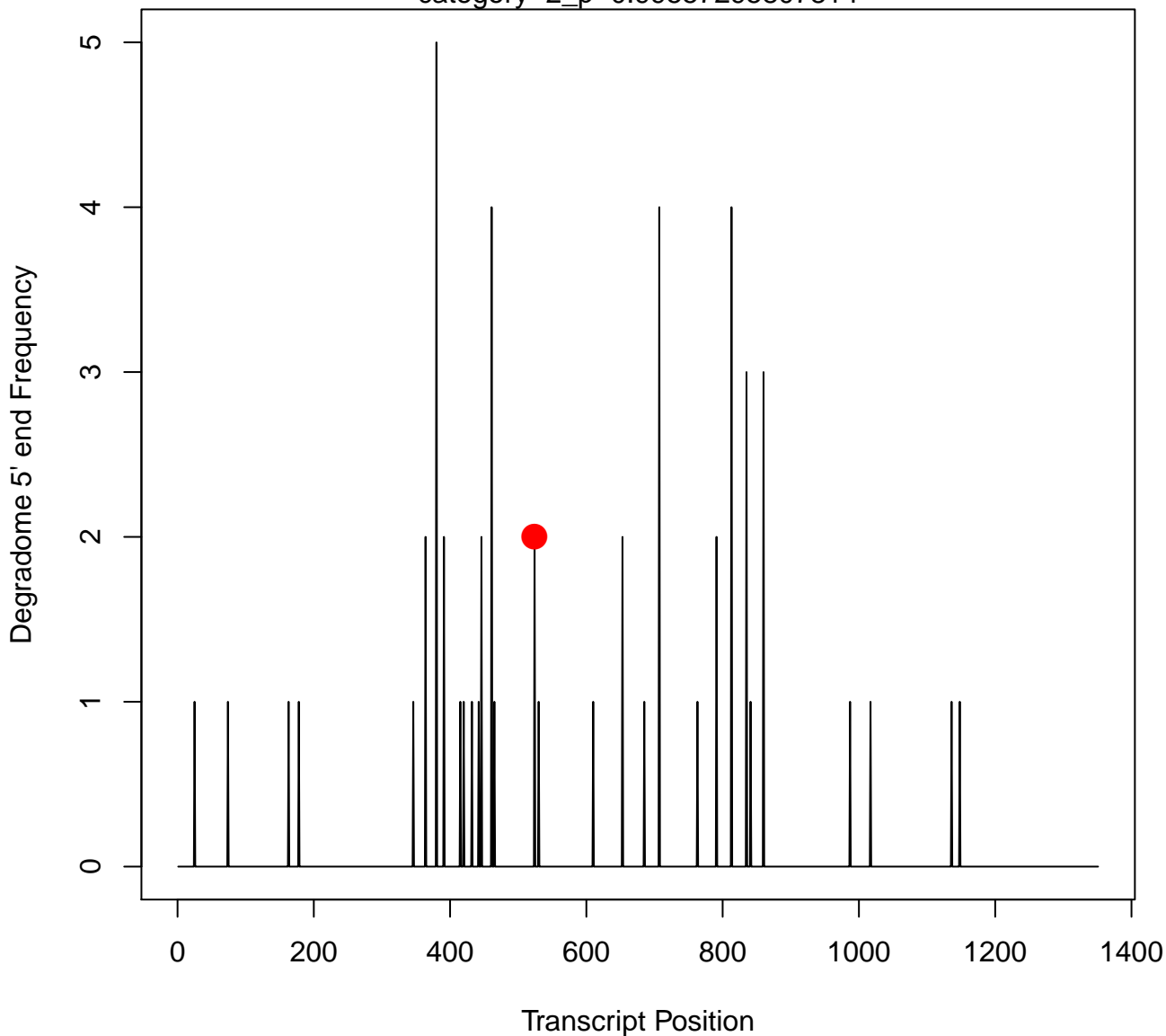

Supplement: Supplementary file 3 [file Data_Sheet_3.zip › Sit-miR168_Seita.5G307700.1_524_TPlot.pdf]

**T=Seita.5G333700.1\_Q=Sit-miR168\_S=911**

category=1\_p=0.0828820166471202

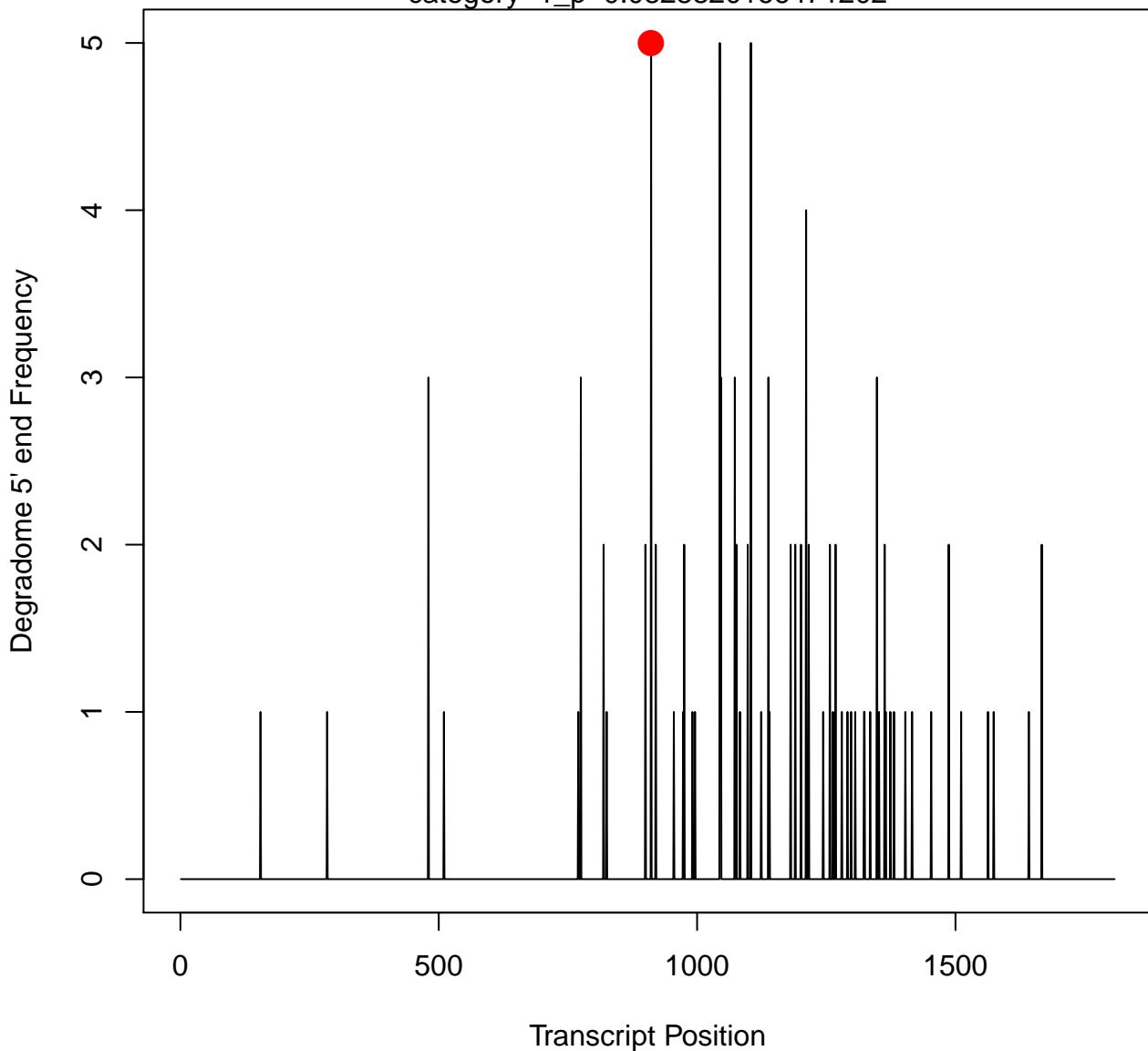

Supplement: Supplementary file 3 [file Data_Sheet_3.zip › Sit-miR168_Seita.5G333700.1_911_TPlot.pdf]

**T=Seita.5G435400.1\_Q=Sit-miR168\_S=595**

category=0\_p=0.00440539590792766

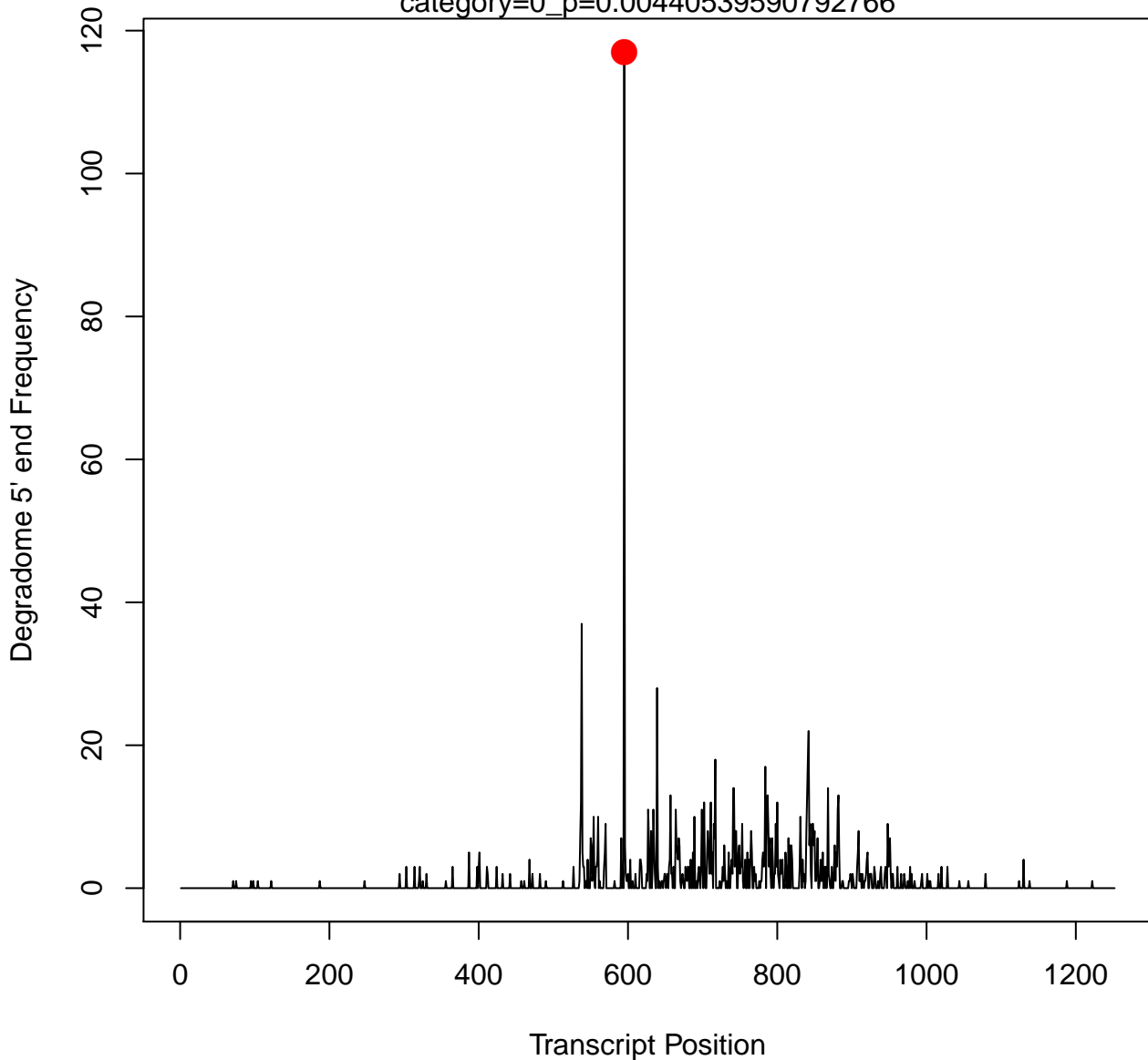

Supplement: Supplementary file 3 [file Data_Sheet_3.zip › Sit-miR168_Seita.5G435400.1_595_TPlot.pdf]

**T=Seita.6G158900.1\_Q=Sit-miR168\_S=1249**

category=2\_p=0.991823050110343

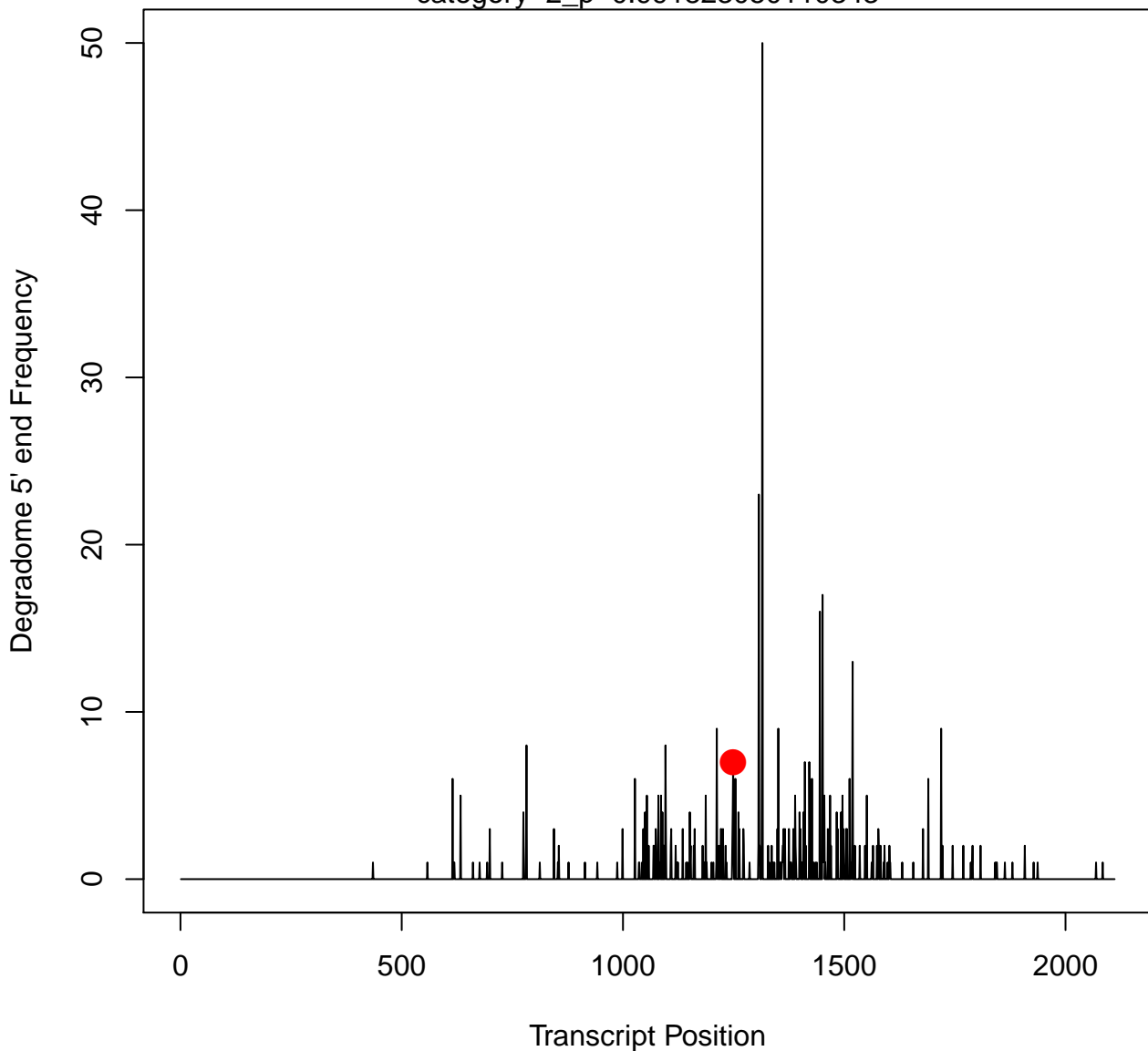

Supplement: Supplementary file 3 [file Data_Sheet_3.zip › Sit-miR168_Seita.6G158900.1_1249_TPlot.pdf]
