# Supplementary figures and images for: The Construction and Exploration of a Comprehensive MicroRNA Centered Regulatory Network in Foxtail Millet (Setaria italica L.) (part 5 of 14)
Source: Front Plant Sci. 2022 May 6;13:848474. doi: 10.3389/fpls.2022.848474 (PMC9121102; doi:10.3389/fpls.2022.848474)

**T=Seita.1G177900.1\_Q=Sit-miR1133\_S=1890**

category=0\_p=0.0194752481105291

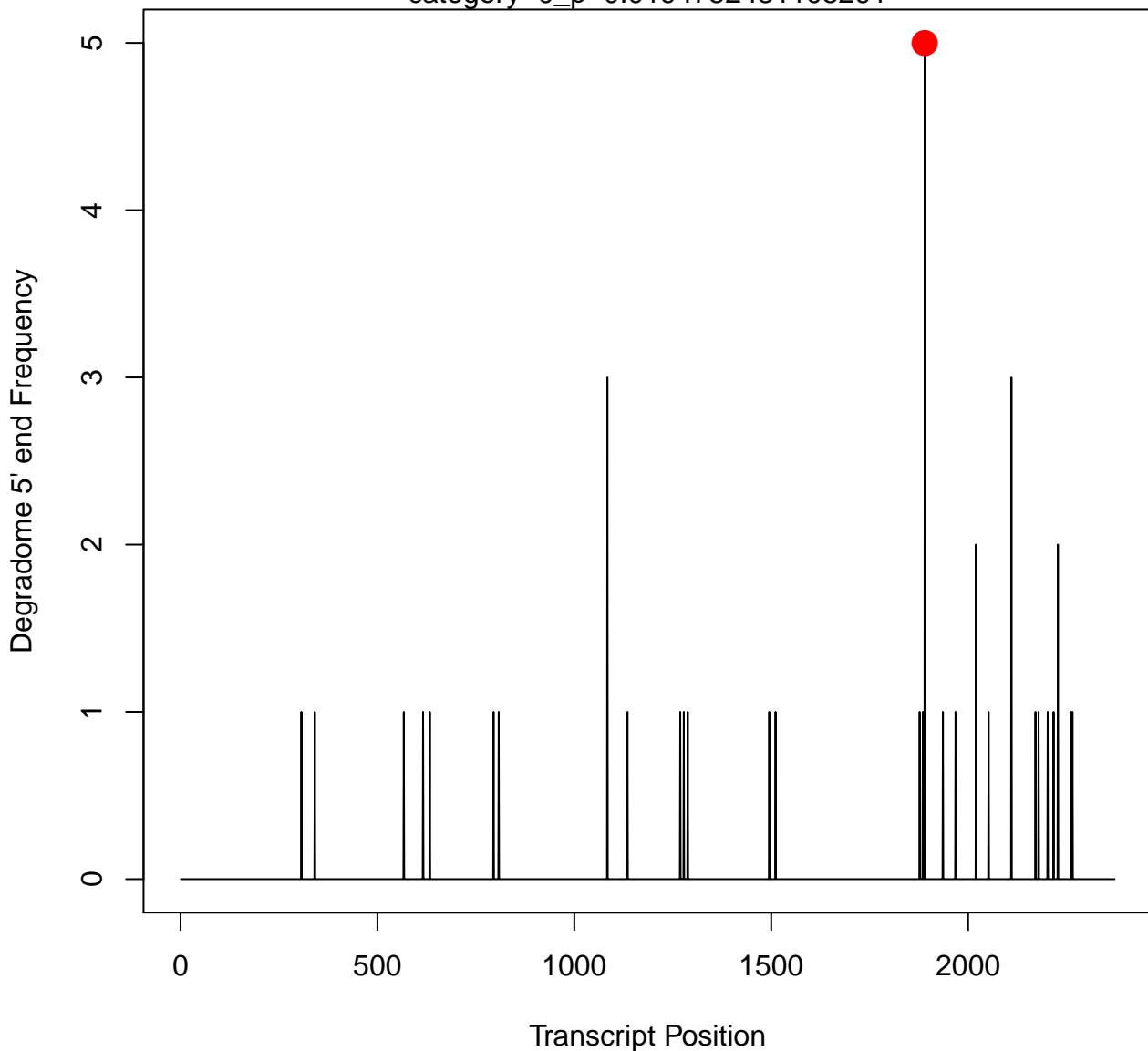

Supplement: Supplementary file 3 [file Data_Sheet_3.zip › Sit-miR1133_Seita.1G177900.1_1890_TPlot.pdf]

**T=Seita.1G300800.1\_Q=Sit-miR1133\_S=71**

category=2\_p=0.999999654923223

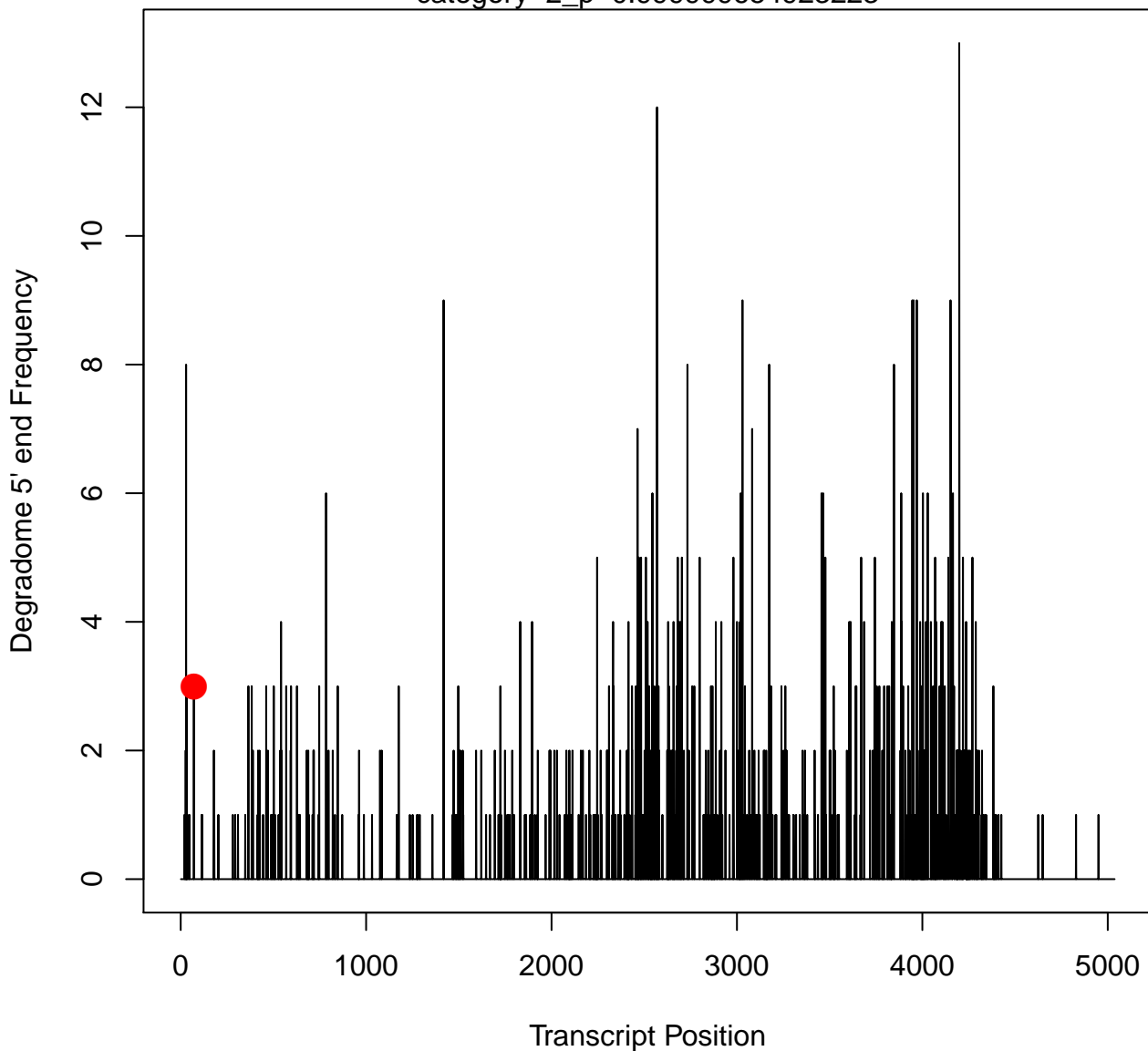

Supplement: Supplementary file 3 [file Data_Sheet_3.zip › Sit-miR1133_Seita.1G300800.1_71_TPlot.pdf]

**T=Seita.2G387500.1\_Q=Sit-miR1133\_S=21**

category=2\_p=0.956548691667319

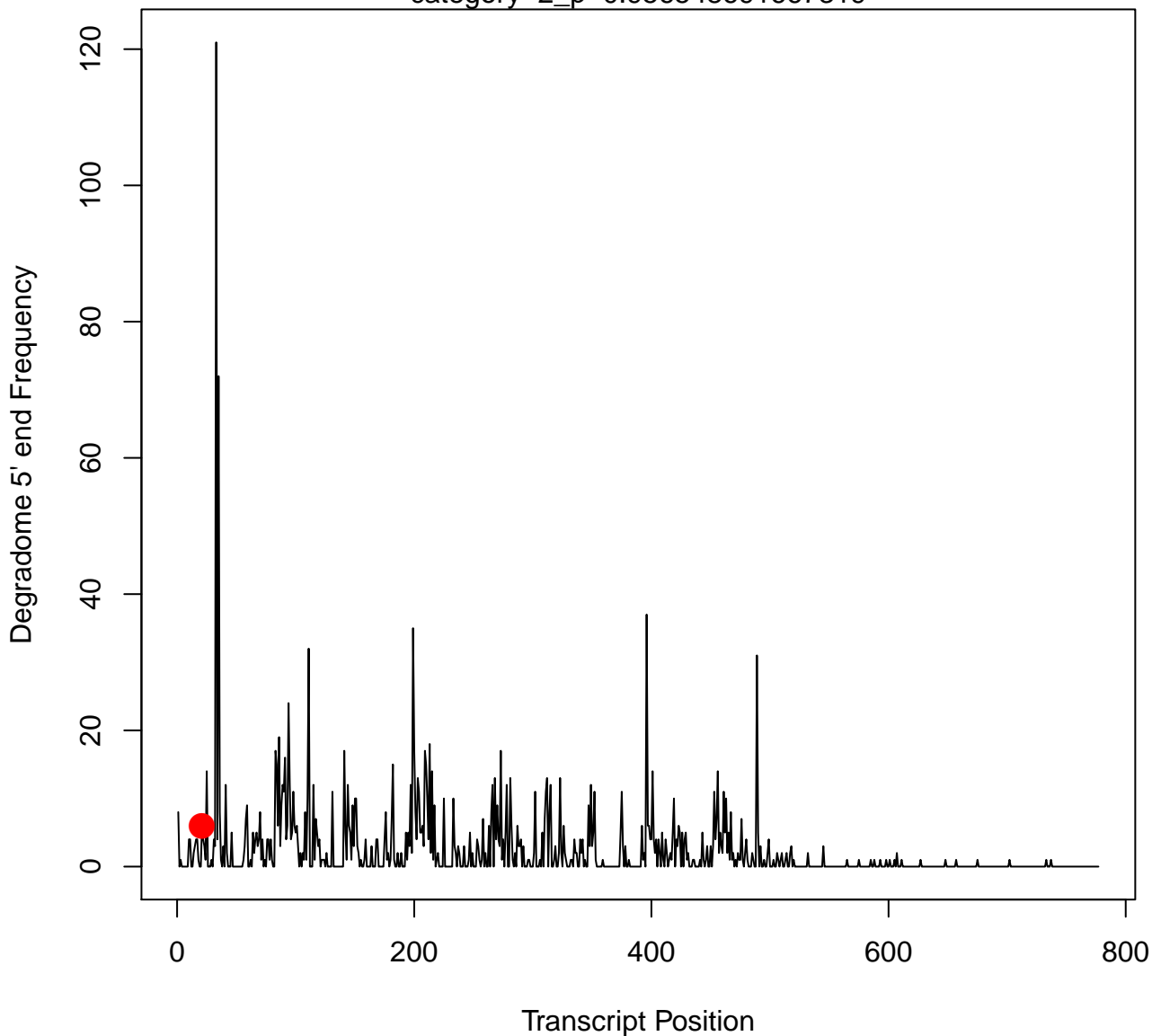

Supplement: Supplementary file 3 [file Data_Sheet_3.zip › Sit-miR1133_Seita.2G387500.1_21_TPlot.pdf]

**T=Seita.2G393200.1\_Q=Sit-miR1133\_S=44**

category=2\_p=0.999446585782814

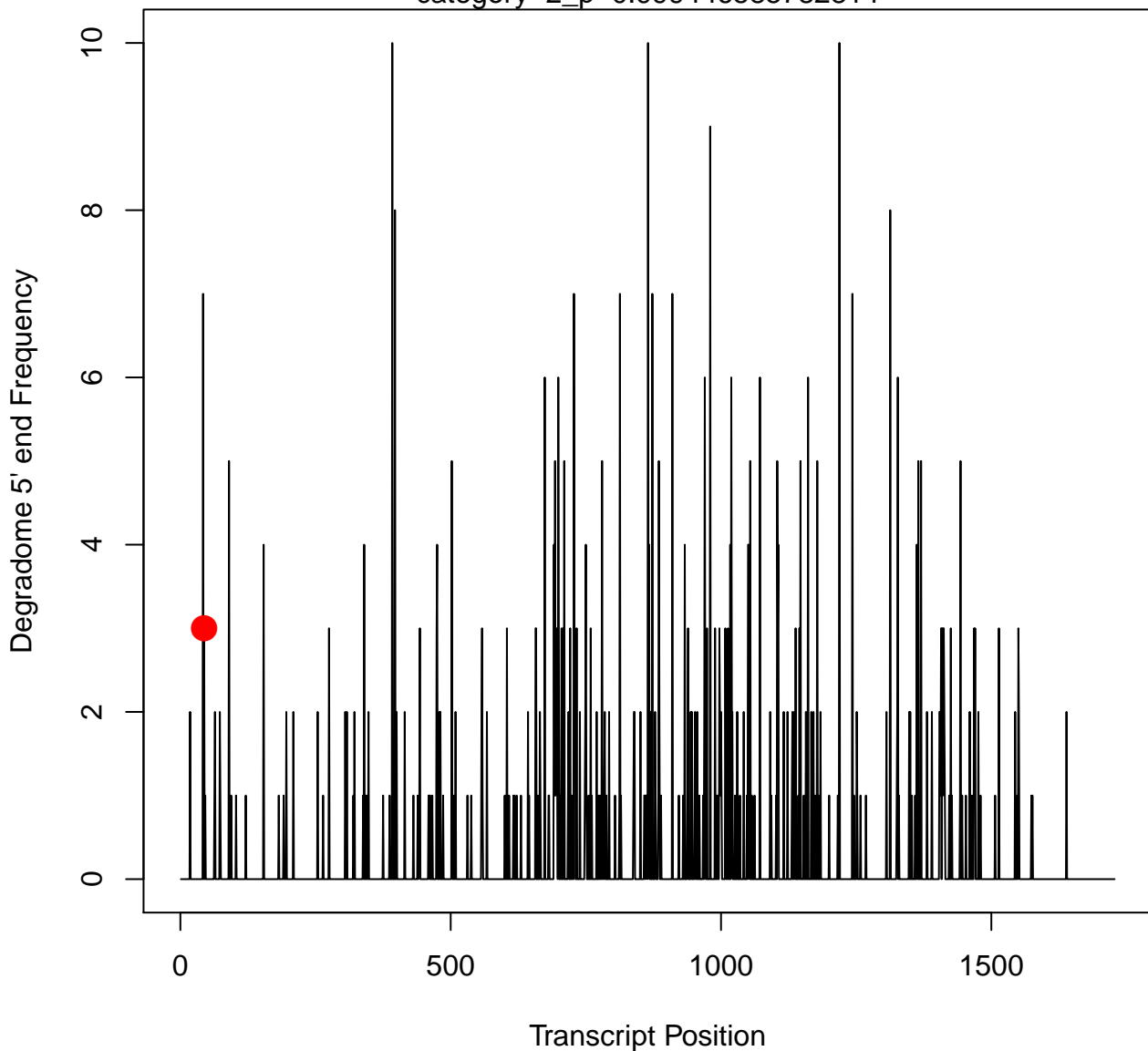

Supplement: Supplementary file 3 [file Data_Sheet_3.zip › Sit-miR1133_Seita.2G393200.1_44_TPlot.pdf]

**T=Seita.4G129400.1\_Q=Sit-miR1133\_S=457**

category=2\_p=0.999999546737002

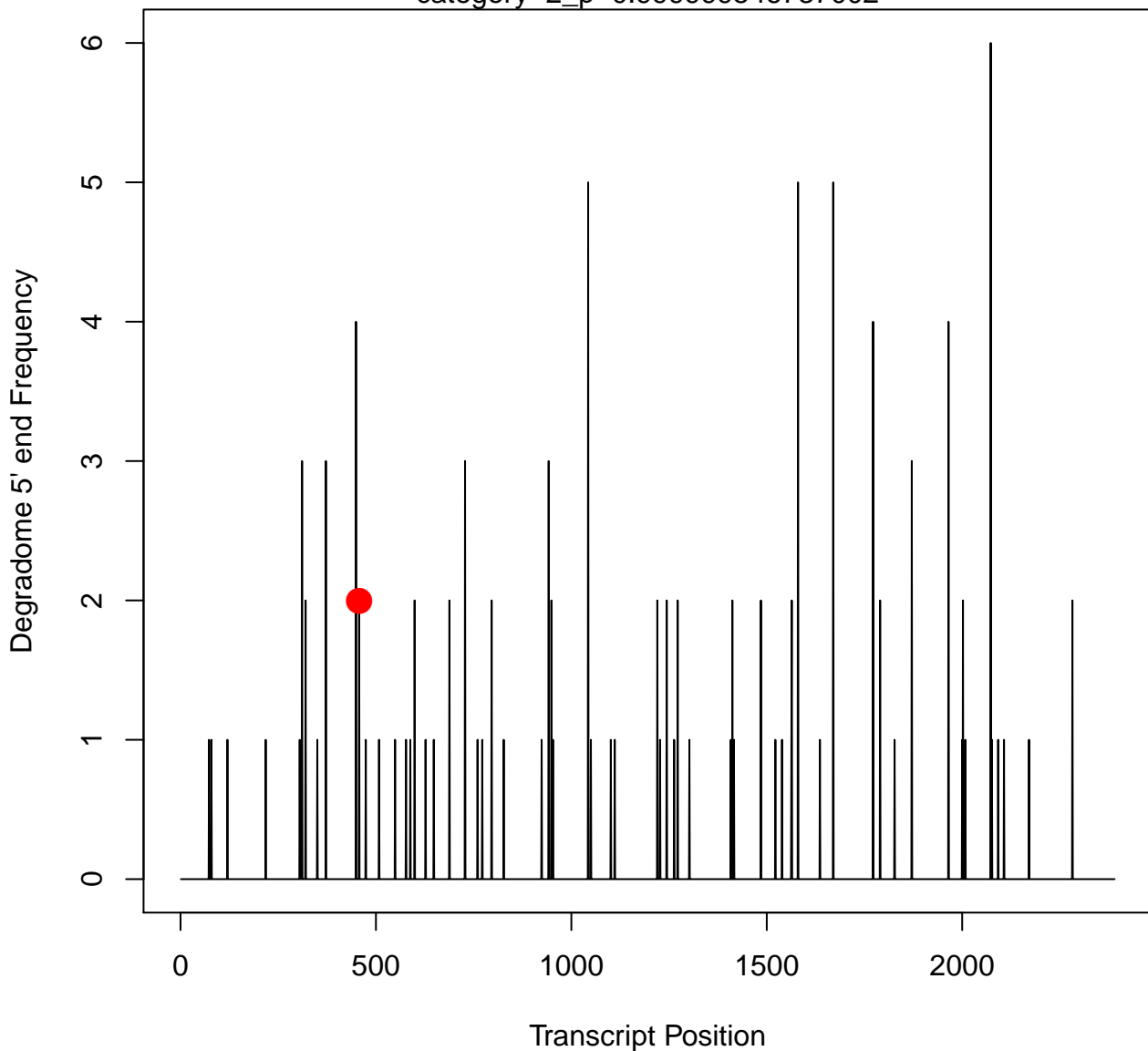

Supplement: Supplementary file 3 [file Data_Sheet_3.zip › Sit-miR1133_Seita.4G129400.1_457_TPlot.pdf]

**T=Seita.4G167500.1\_Q=Sit-miR1133\_S=1023**

category=2\_p=0.999928420063938

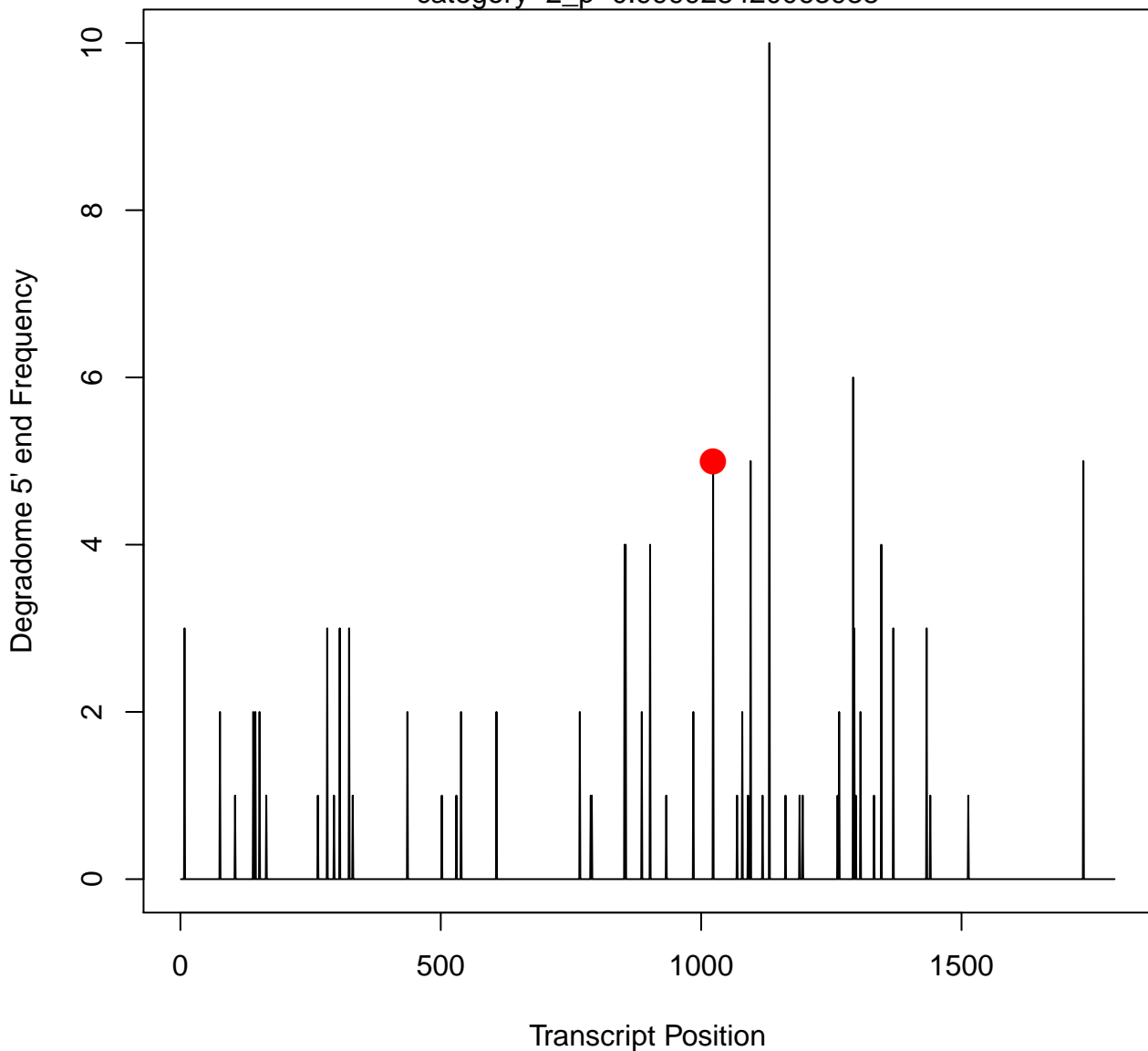

Supplement: Supplementary file 3 [file Data_Sheet_3.zip › Sit-miR1133_Seita.4G167500.1_1023_TPlot.pdf]

**T=Seita.4G191500.1\_Q=Sit-miR1133\_S=1058**

category=2\_p=0.999998781921009

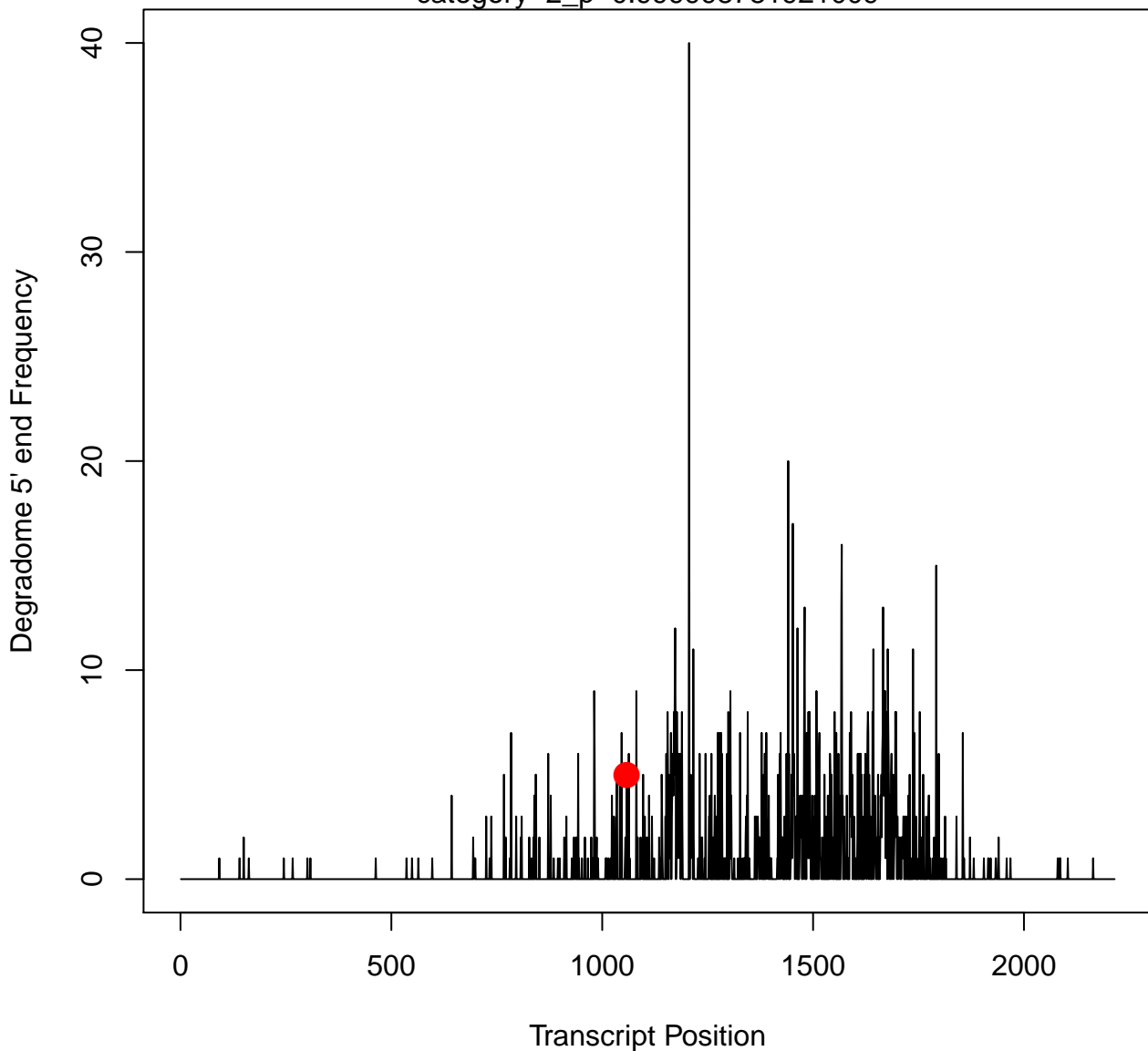

Supplement: Supplementary file 3 [file Data_Sheet_3.zip › Sit-miR1133_Seita.4G191500.1_1058_TPlot.pdf]

**T=Seita.4G239100.1\_Q=Sit-miR1133\_S=41**

category=2\_p=0.999952451206138

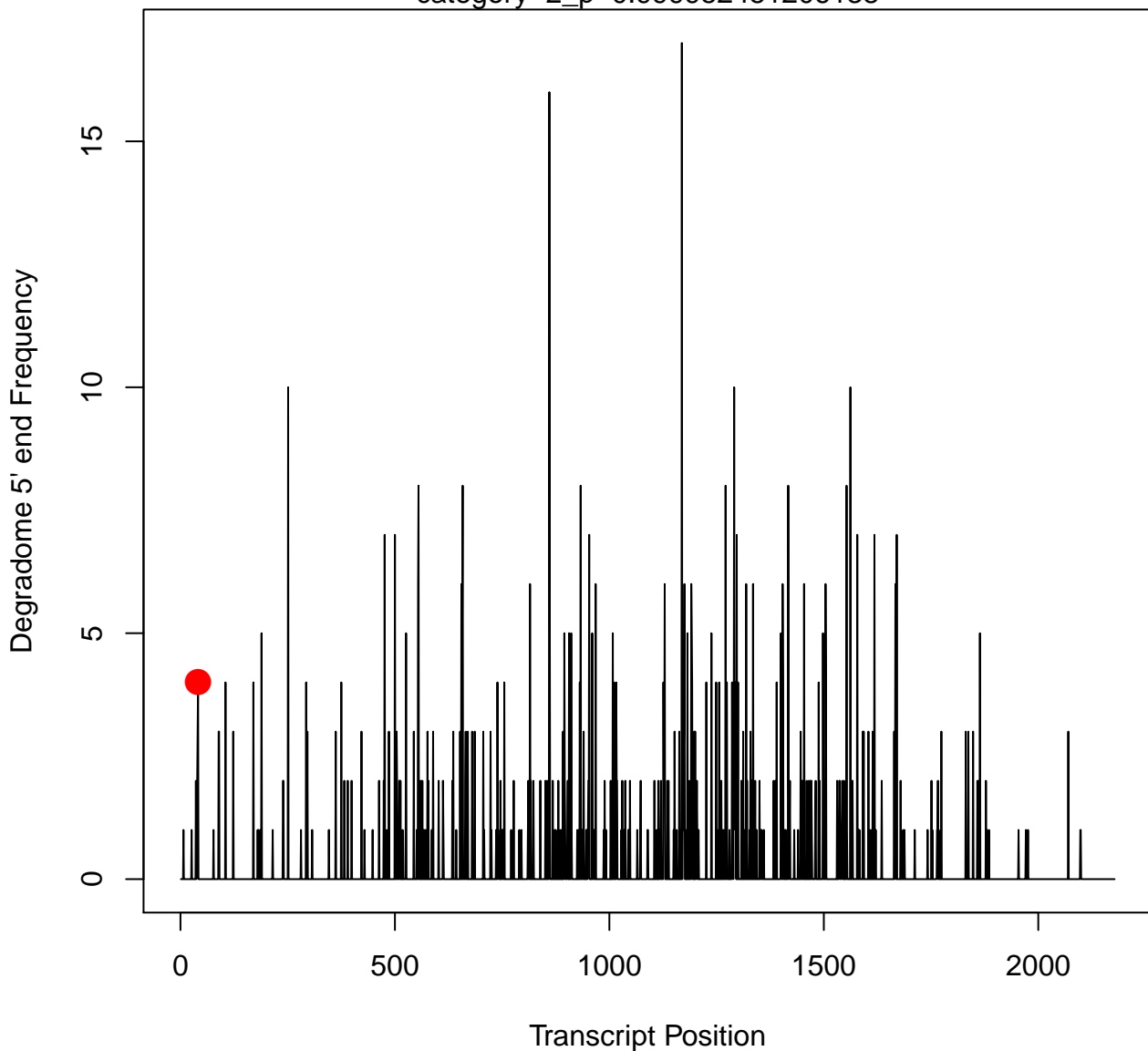

Supplement: Supplementary file 3 [file Data_Sheet_3.zip › Sit-miR1133_Seita.4G239100.1_41_TPlot.pdf]

**T=Seita.9G074400.1\_Q=Sit-miR1133\_S=151**

category=2\_p=0.983553259883742

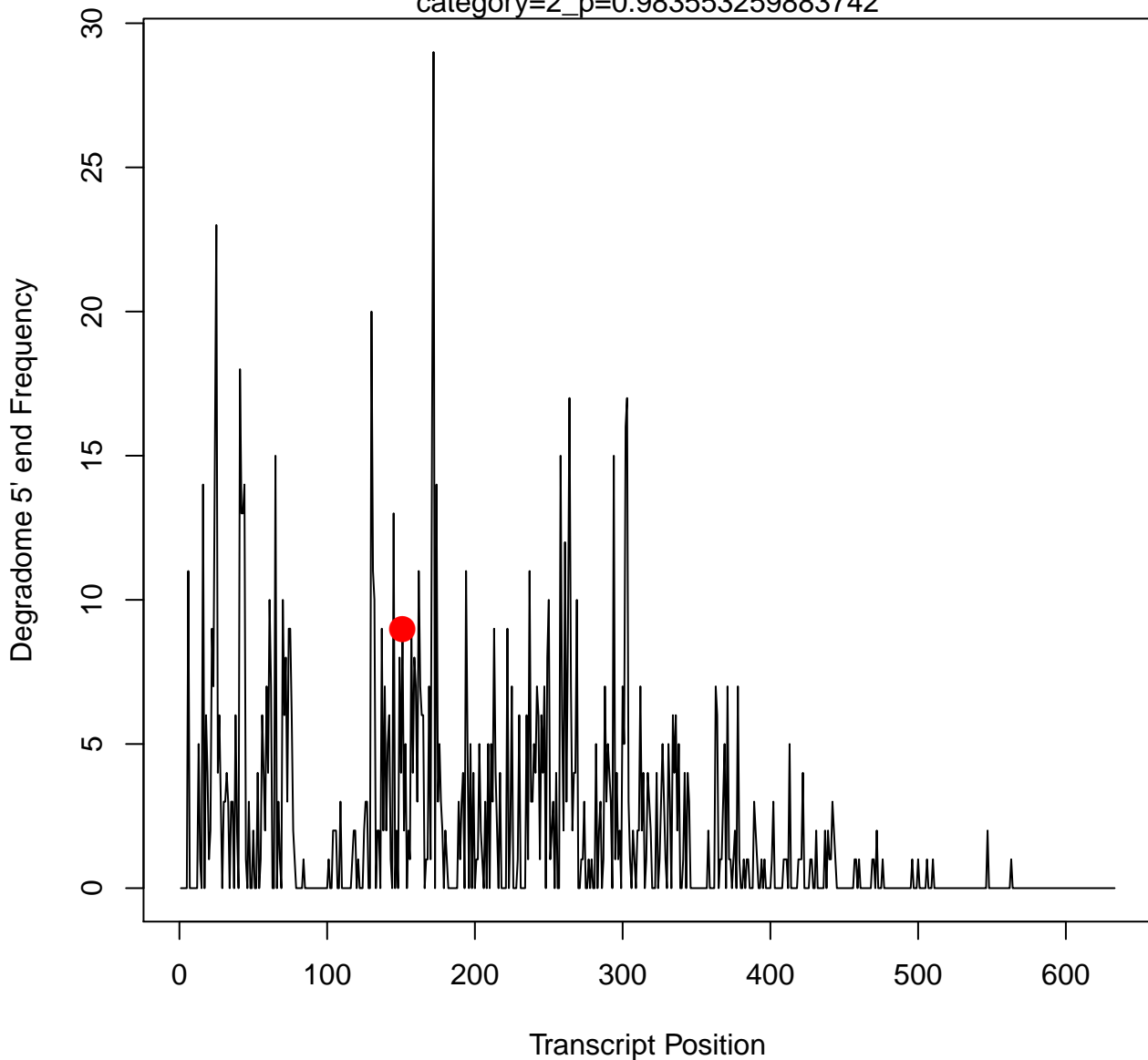

Supplement: Supplementary file 3 [file Data_Sheet_3.zip › Sit-miR1133_Seita.9G074400.1_151_TPlot.pdf]

**T=Seita.9G435700.1\_Q=Sit-miR1133\_S=163**

category=2\_p=0.999985579231063

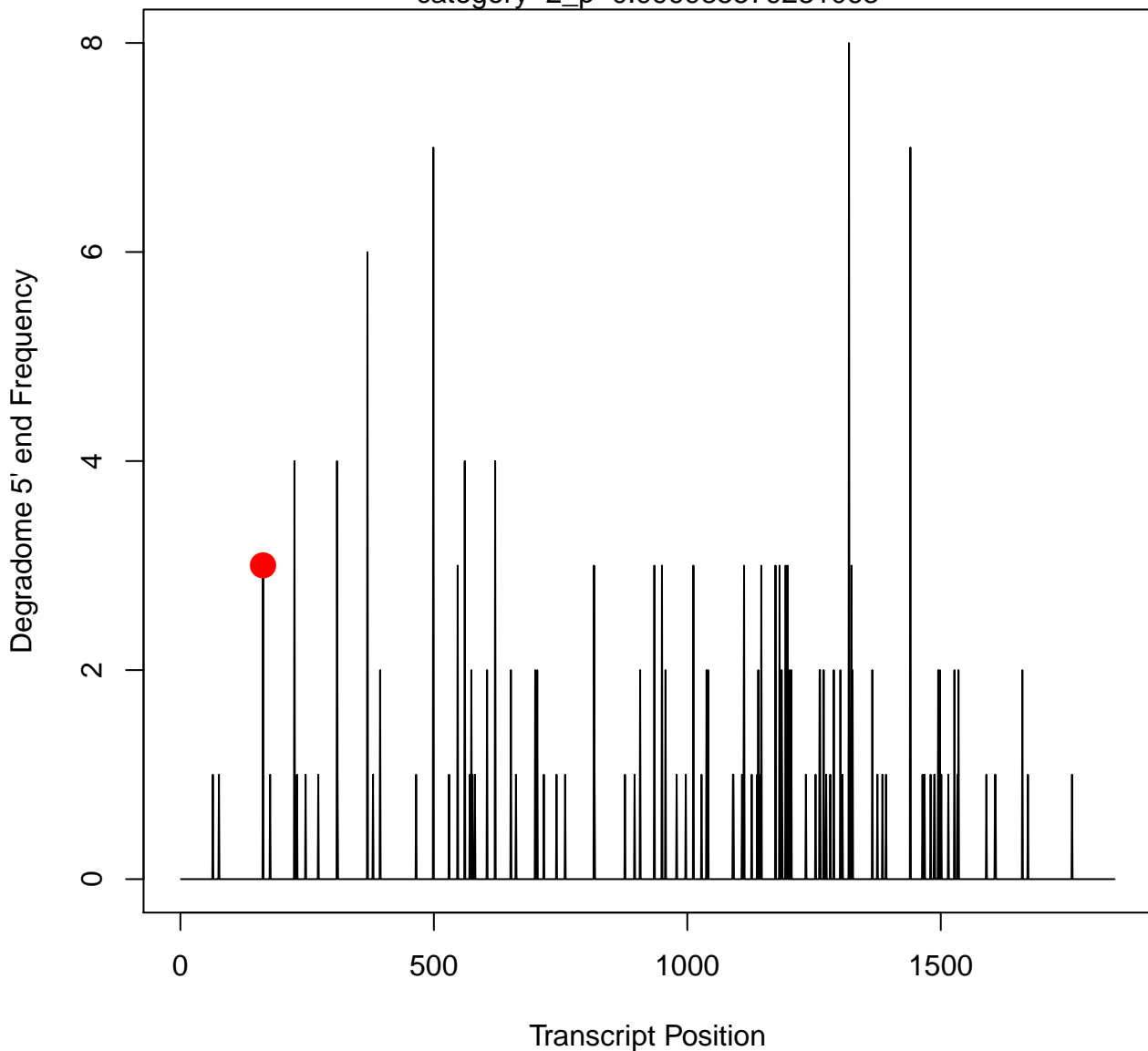

Supplement: Supplementary file 3 [file Data_Sheet_3.zip › Sit-miR1133_Seita.9G435700.1_163_TPlot.pdf]

**T=Seita.2G424800.1\_Q=Sit-miR1432\_S=450**

category=2\_p=0.894581922057144

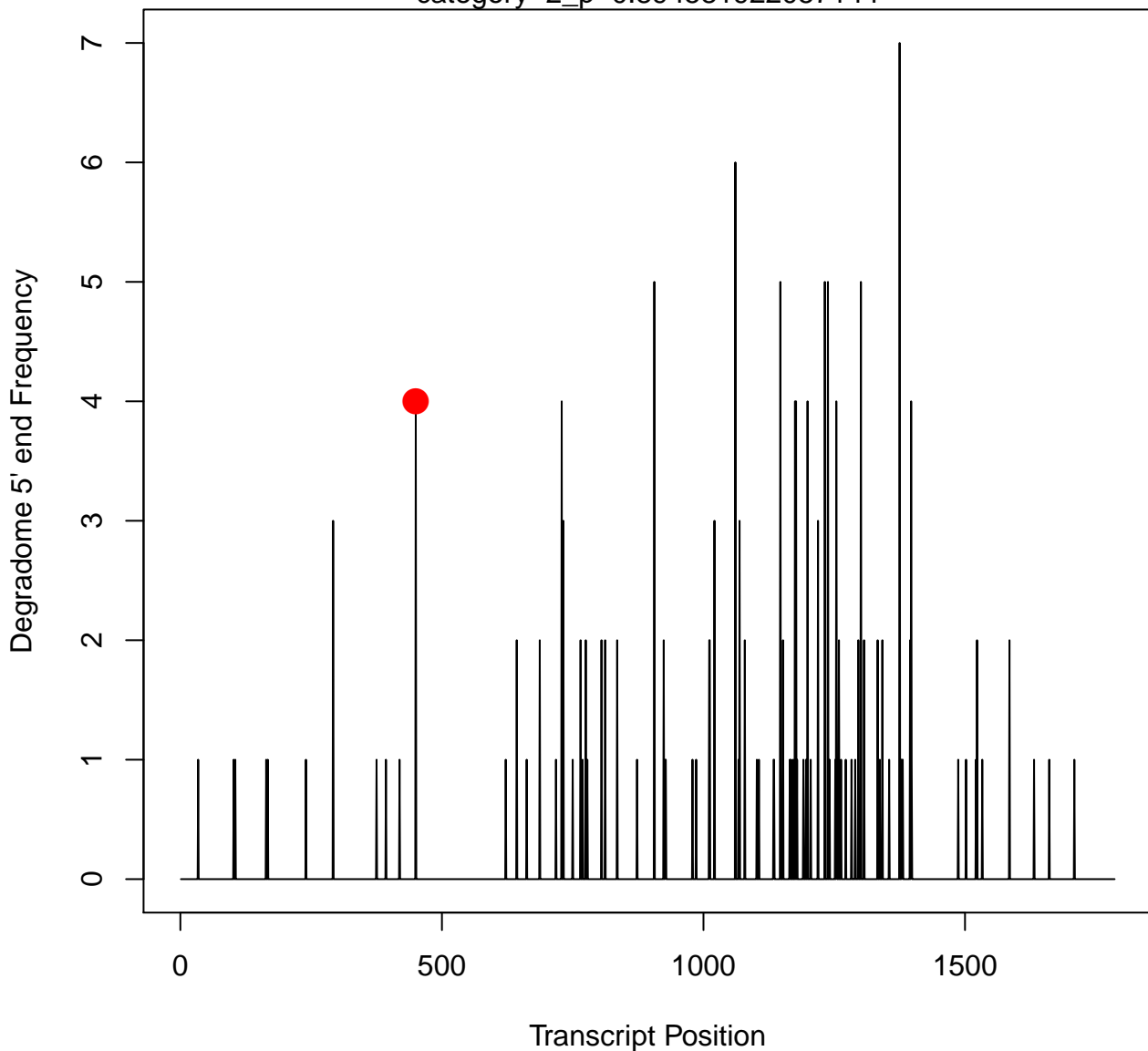

Supplement: Supplementary file 3 [file Data_Sheet_3.zip › Sit-miR1432_Seita.2G424800.1_450_TPlot.pdf]

**T=Seita.3G228000.1\_Q=Sit-miR1432\_S=704**

category=2\_p=0.998680041158392

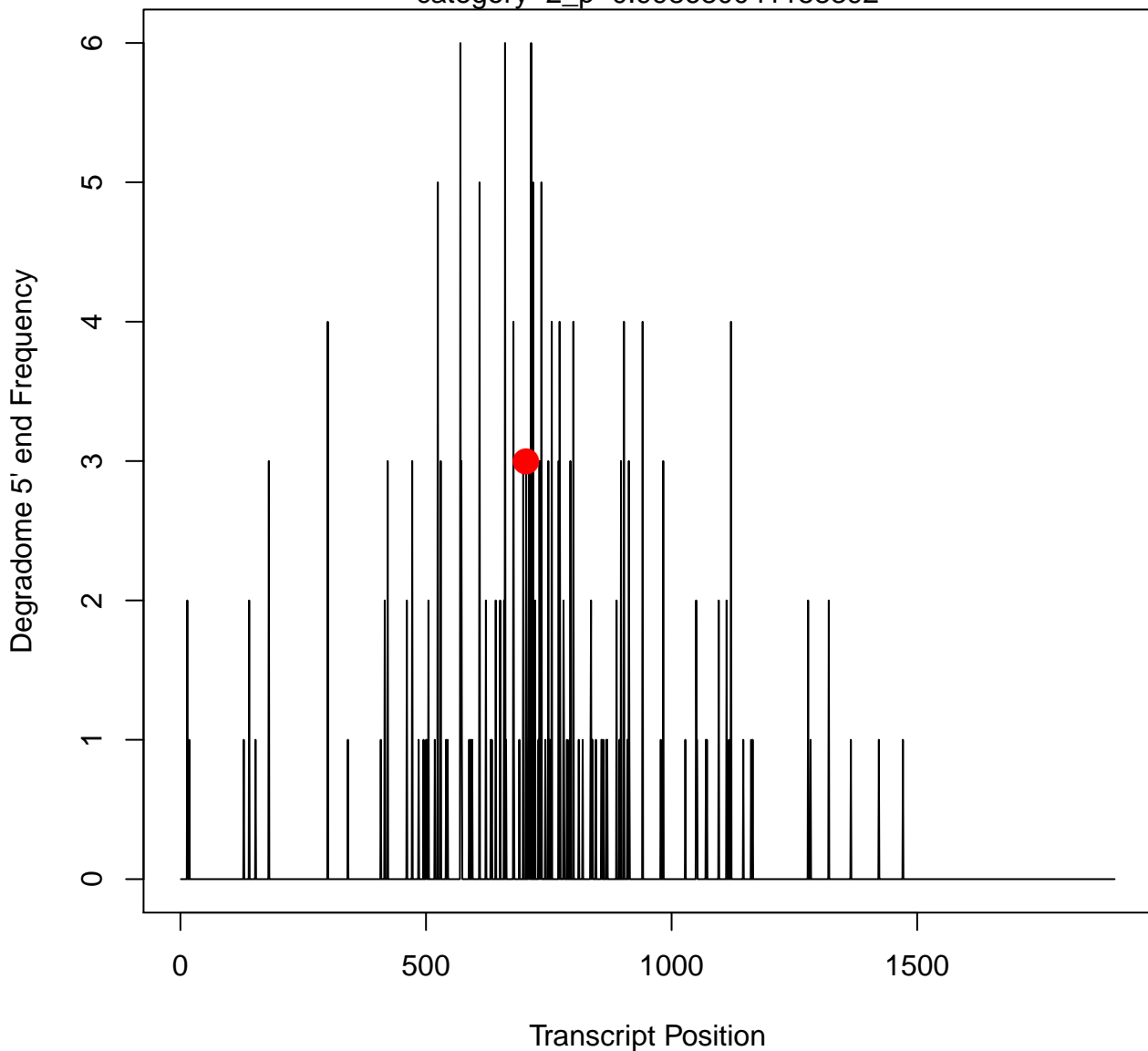

Supplement: Supplementary file 3 [file Data_Sheet_3.zip › Sit-miR1432_Seita.3G228000.1_704_TPlot.pdf]

**T=Seita.5G126800.1\_Q=Sit-miR1432\_S=988**

category=2\_p=0.335724555261316

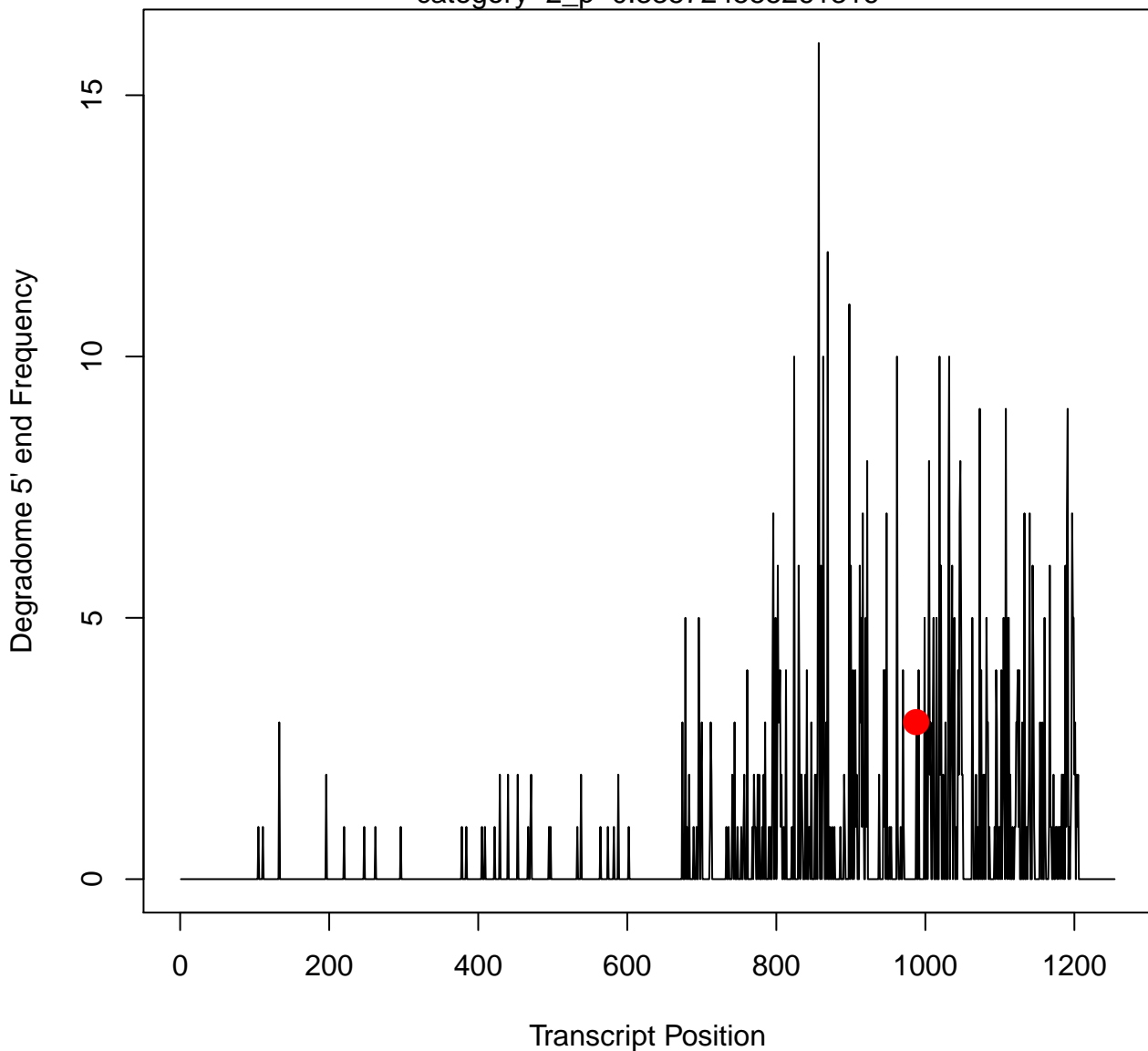

Supplement: Supplementary file 3 [file Data_Sheet_3.zip › Sit-miR1432_Seita.5G126800.1_988_TPlot.pdf]

**T=Seita.5G220500.1\_Q=Sit-miR1432\_S=2566**

category=2\_p=0.999987839068718

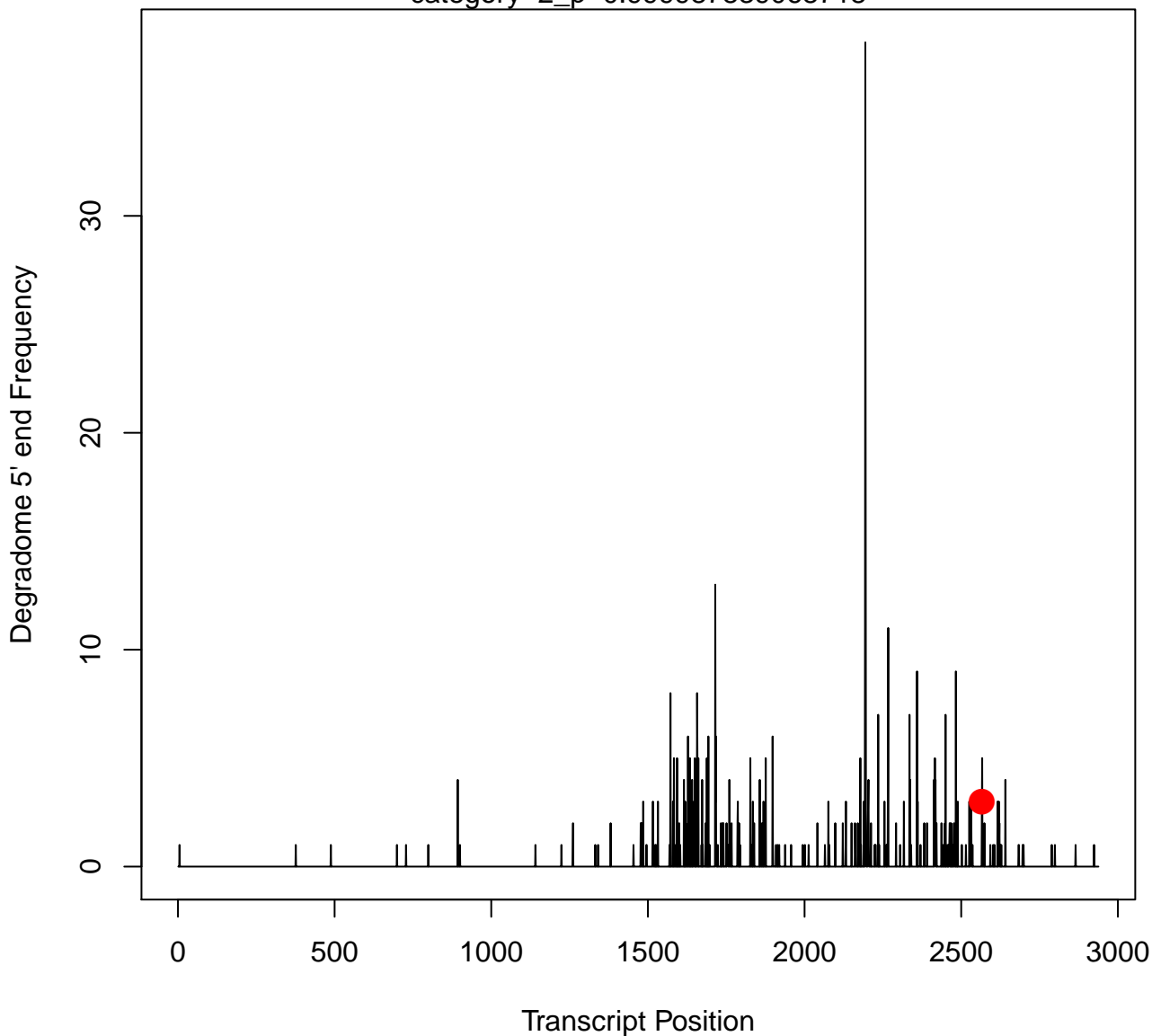

Supplement: Supplementary file 3 [file Data_Sheet_3.zip › Sit-miR1432_Seita.5G220500.1_2566_TPlot.pdf]

**T=Seita.5G458200.1\_Q=Sit-miR1432\_S=2965**

category=2\_p=0.999972440556944

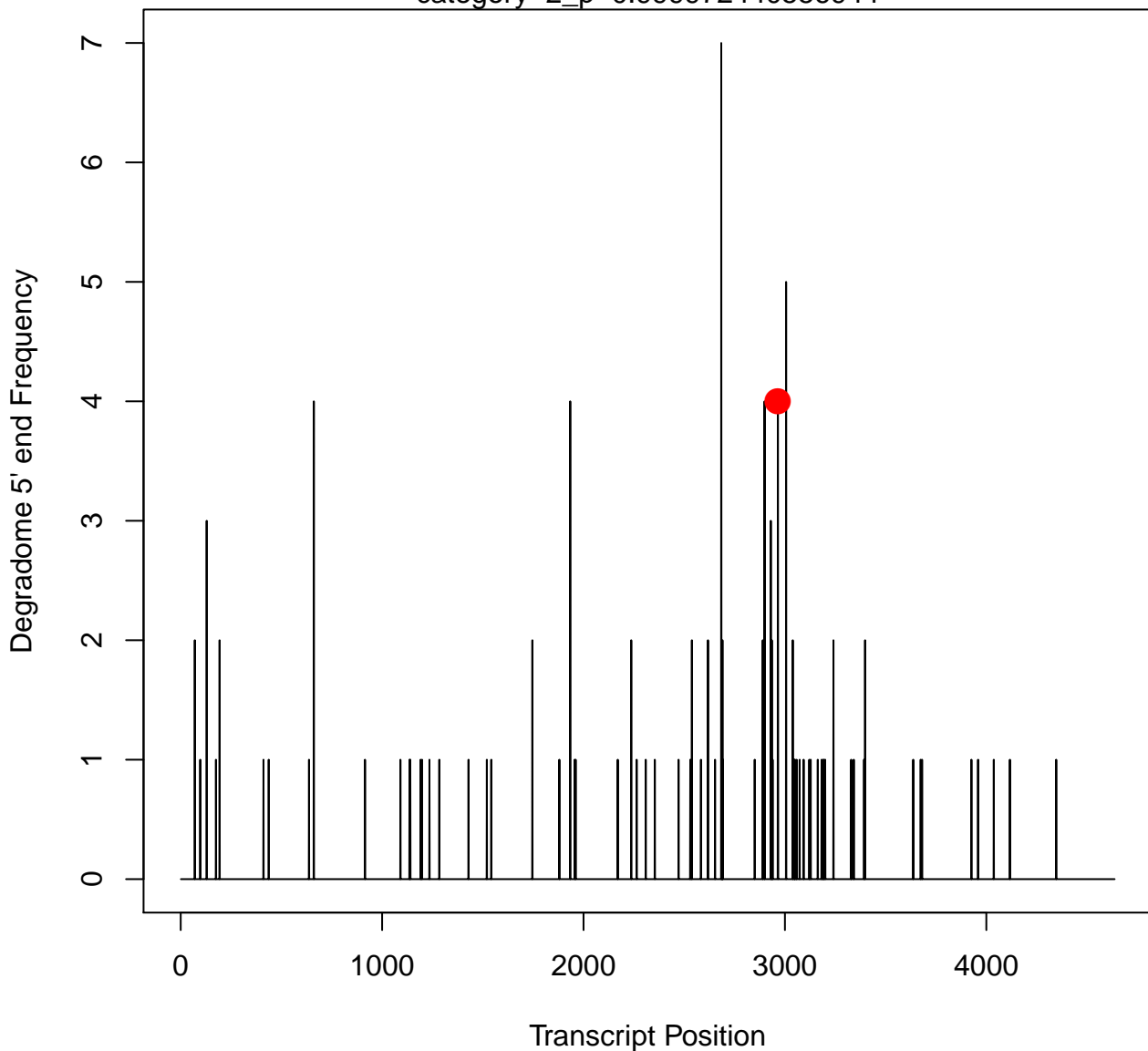

Supplement: Supplementary file 3 [file Data_Sheet_3.zip › Sit-miR1432_Seita.5G458200.1_2965_TPlot.pdf]

**T=Seita.6G140900.1\_Q=Sit-miR1432\_S=1664**

category=2\_p=0.999890391239735

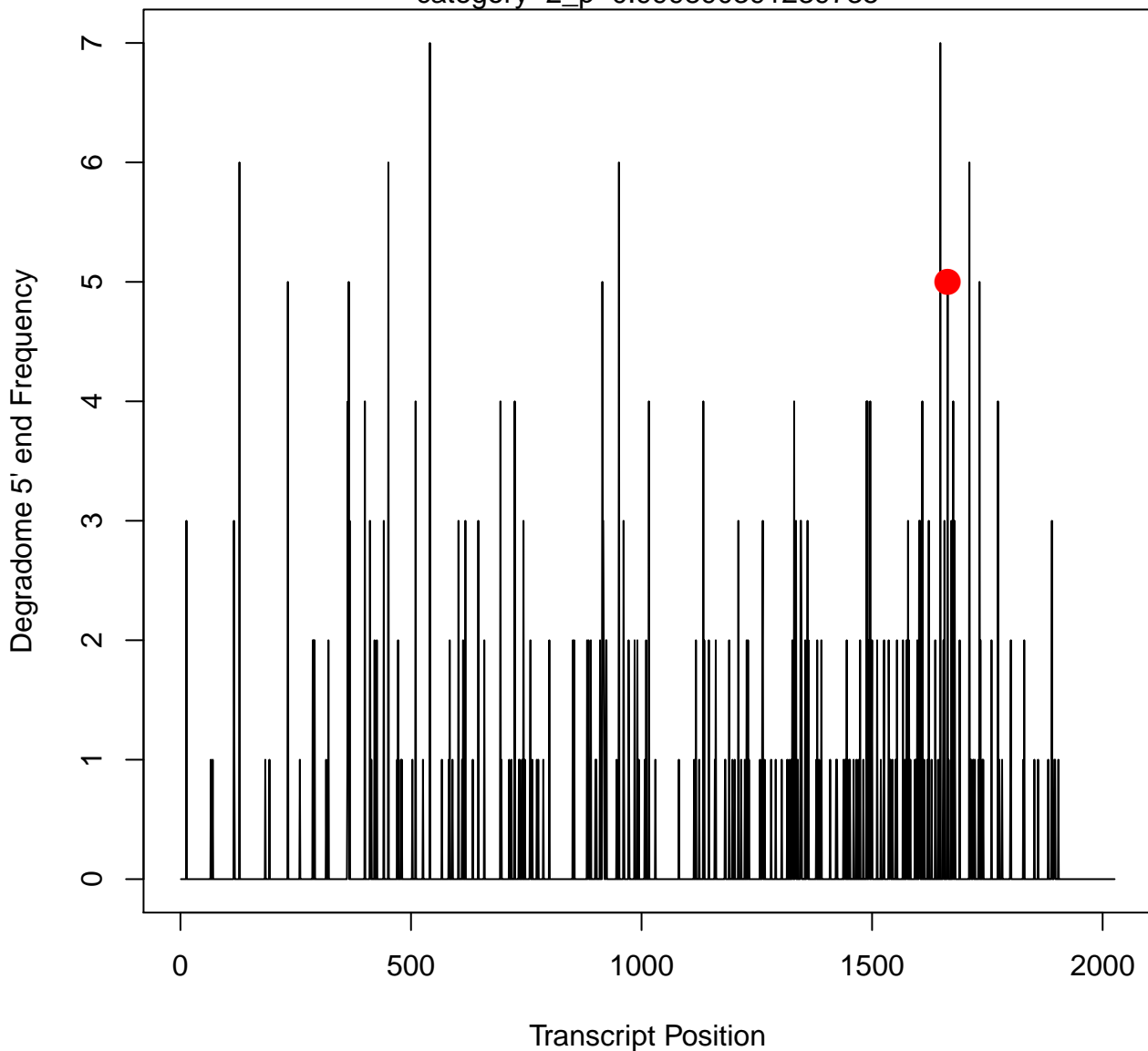

Supplement: Supplementary file 3 [file Data_Sheet_3.zip › Sit-miR1432_Seita.6G140900.1_1664_TPlot.pdf]

**T=Seita.7G153300.1\_Q=Sit-miR1432\_S=300**

category=2\_p=0.996266707217178

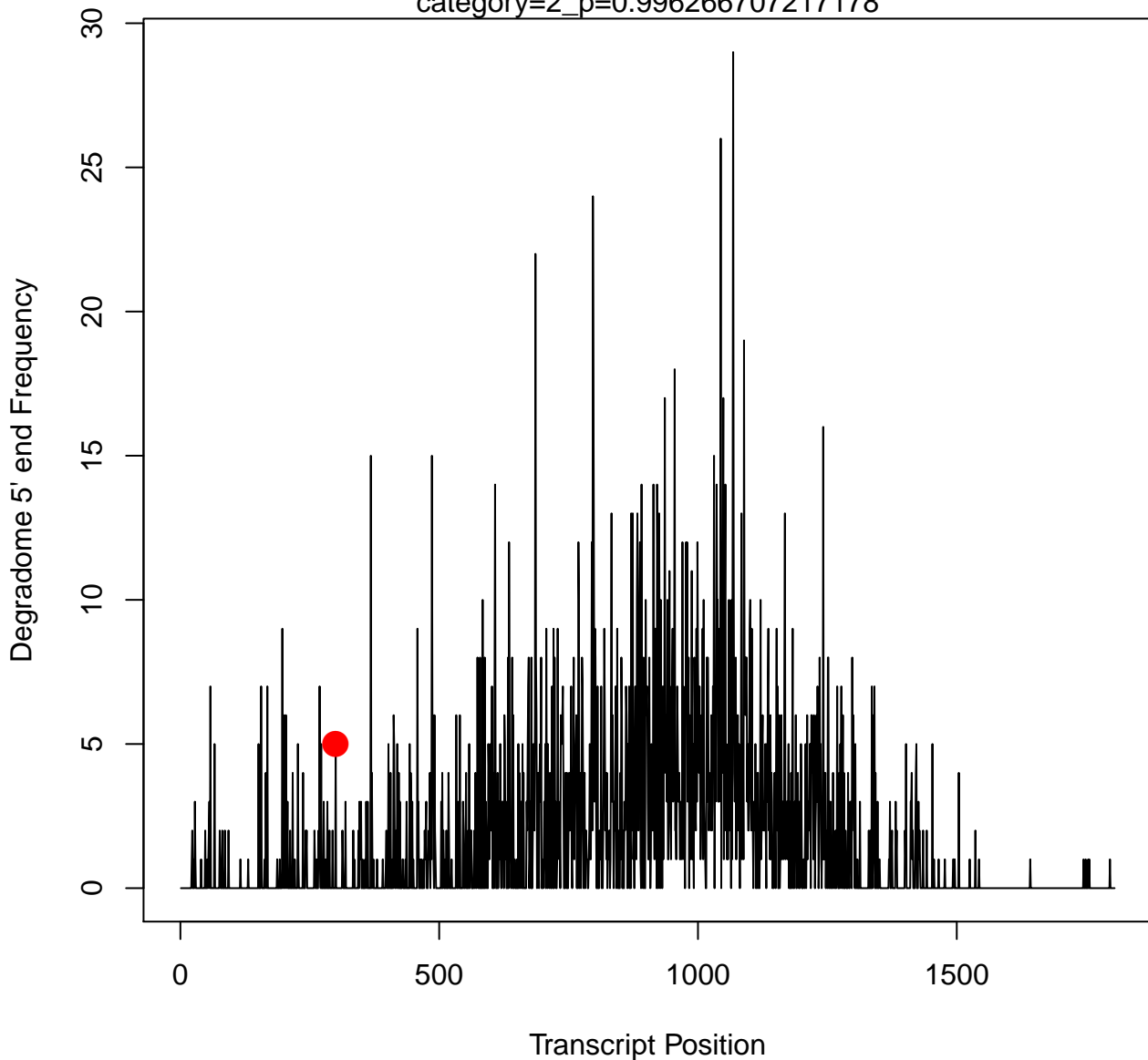

Supplement: Supplementary file 3 [file Data_Sheet_3.zip › Sit-miR1432_Seita.7G153300.1_300_TPlot.pdf]

**T=Seita.7G267000.1\_Q=Sit-miR1432\_S=450**

category=2\_p=0.999061320741073

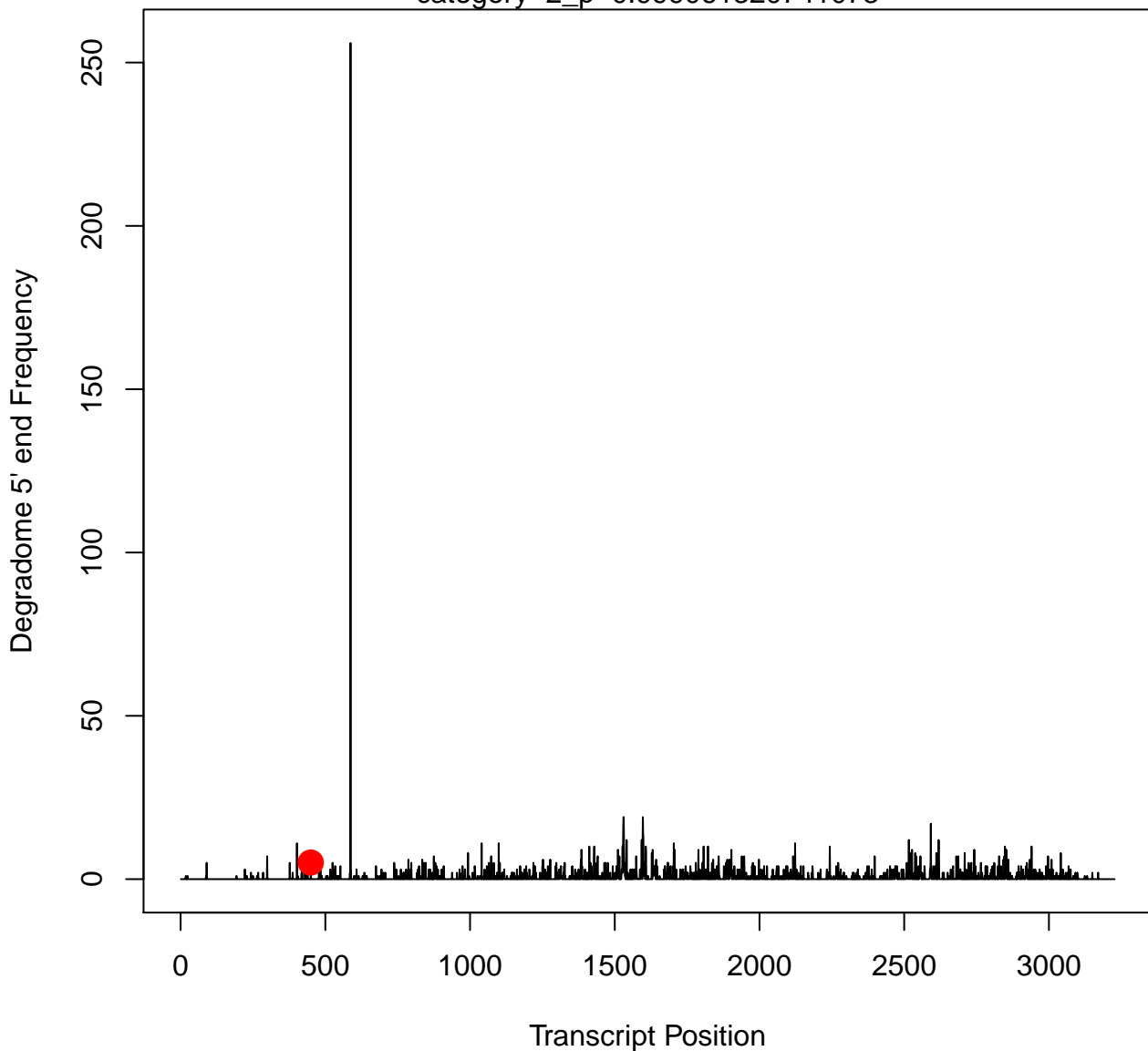

Supplement: Supplementary file 3 [file Data_Sheet_3.zip › Sit-miR1432_Seita.7G267000.1_450_TPlot.pdf]

**T=Seita.7G286000.1\_Q=Sit-miR1432\_S=736**

category=2\_p=0.940947034926436

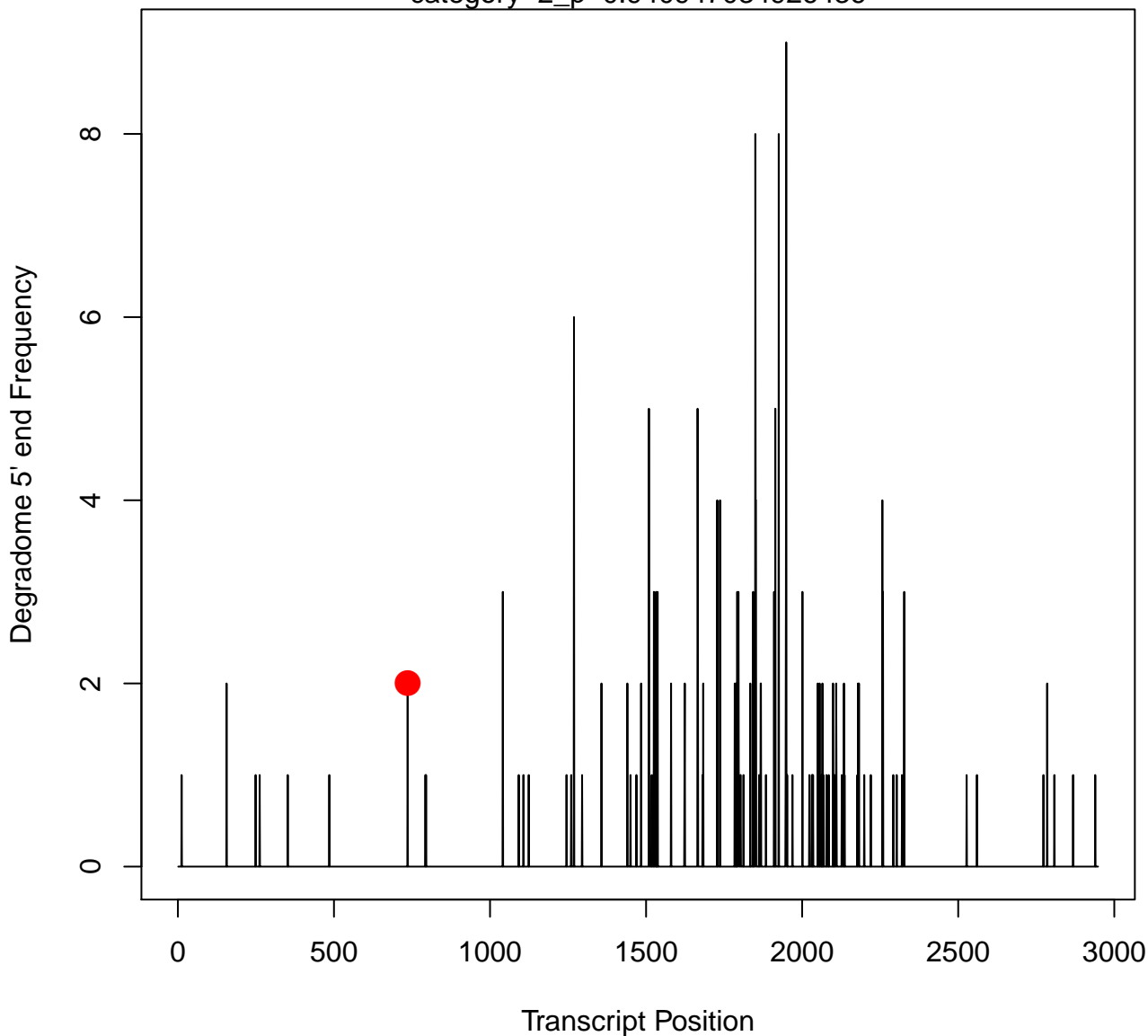

Supplement: Supplementary file 3 [file Data_Sheet_3.zip › Sit-miR1432_Seita.7G286000.1_736_TPlot.pdf]

**T=Seita.8G026700.1\_Q=Sit-miR1432\_S=1262**

category=1\_p=0.0425808924130384

Degradome 5' end Frequency

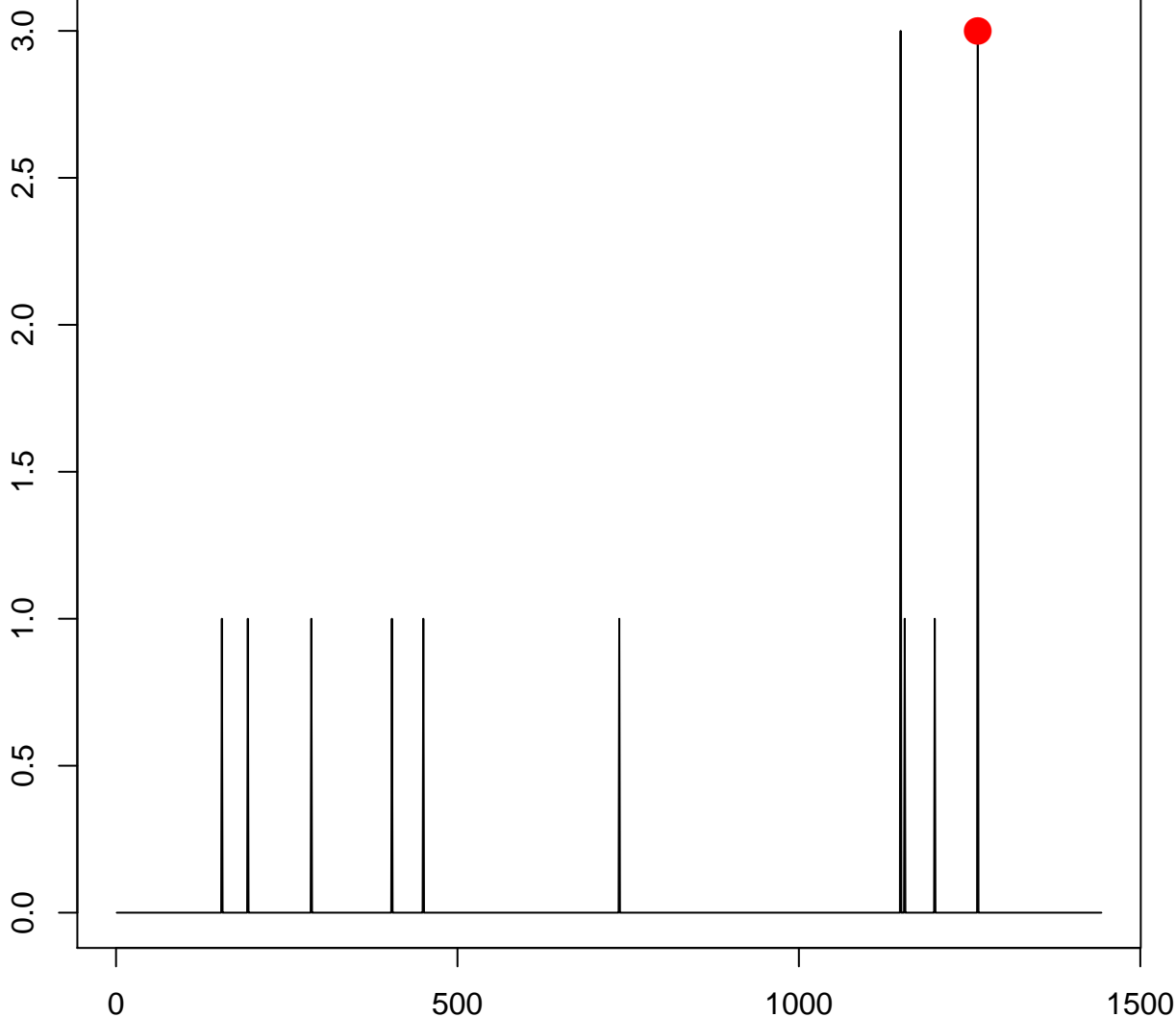

Transcript Position

Supplement: Supplementary file 3 [file Data_Sheet_3.zip › Sit-miR1432_Seita.8G026700.1_1262_TPlot.pdf]

**T=Seita.9G235000.1\_Q=Sit-miR1432\_S=170**

category=2\_p=0.887144240688095

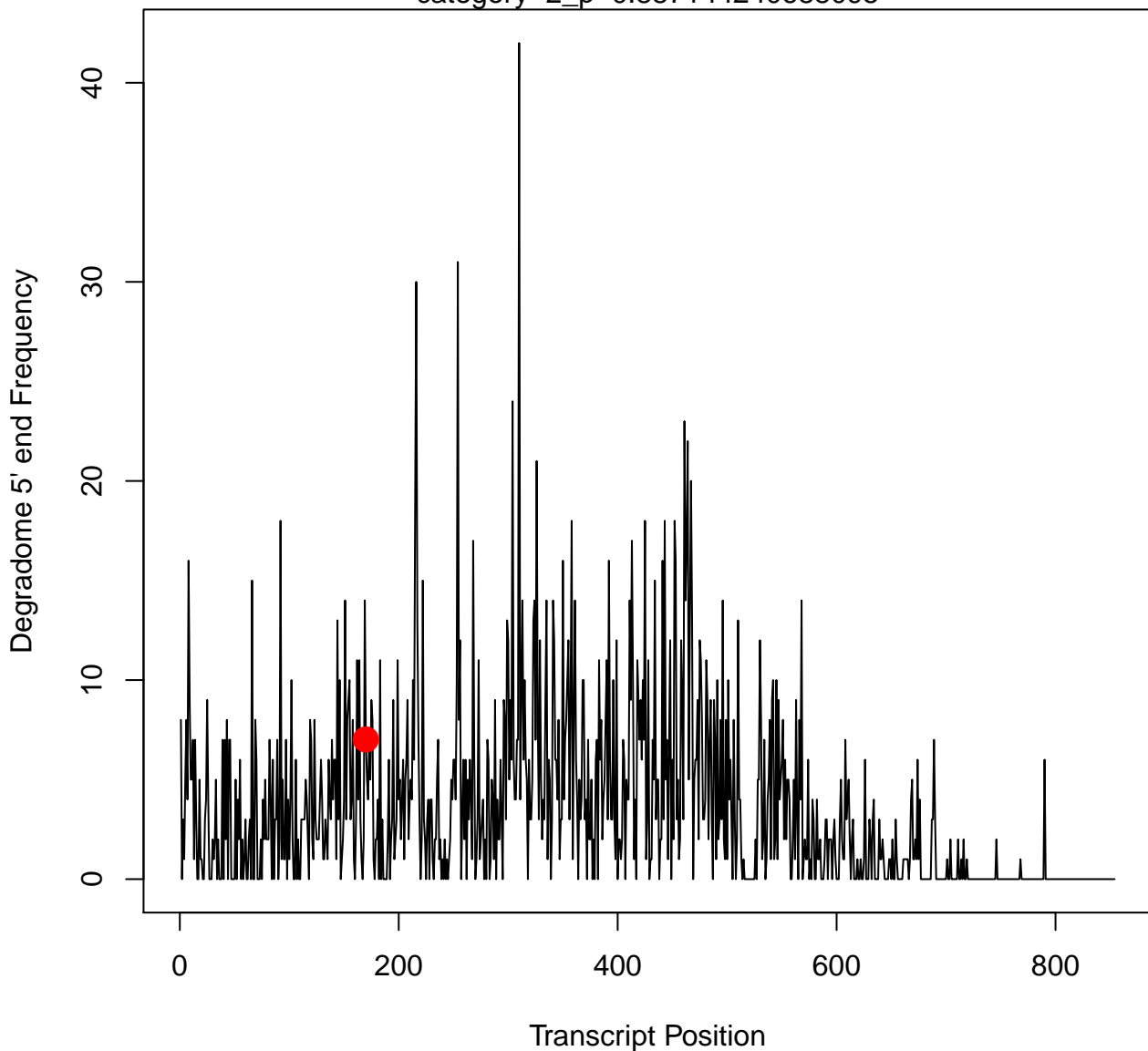

Supplement: Supplementary file 3 [file Data_Sheet_3.zip › Sit-miR1432_Seita.9G235000.1_170_TPlot.pdf]

**T=Seita.9G380800.1\_Q=Sit-miR1432\_S=232**

category=2\_p=0.805287965188136

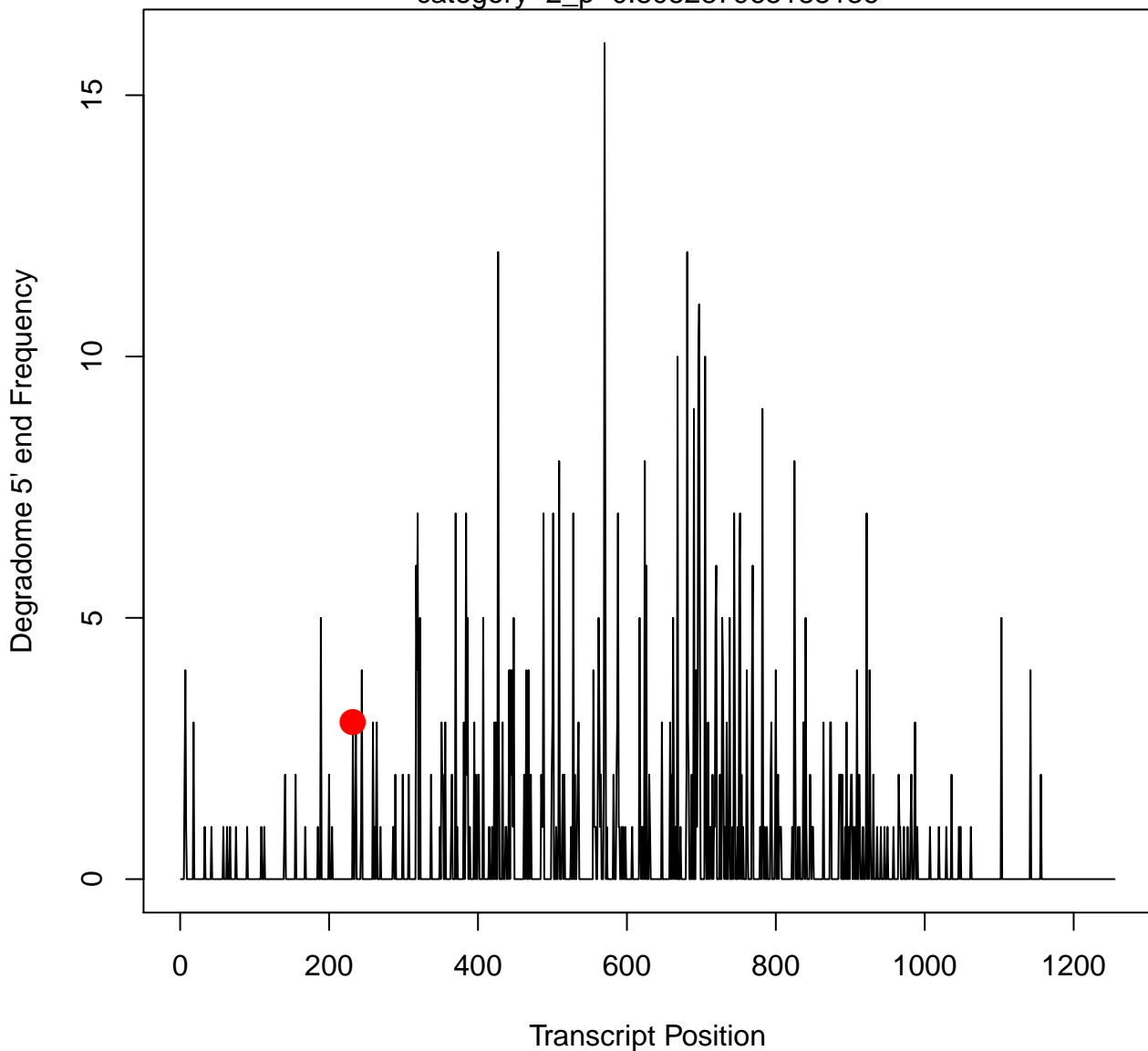

Supplement: Supplementary file 3 [file Data_Sheet_3.zip › Sit-miR1432_Seita.9G380800.1_232_TPlot.pdf]

**T=Seita.2G266500.1\_Q=Sit-miR156a\_S=1896**

category=0\_p=0.00360586087902259

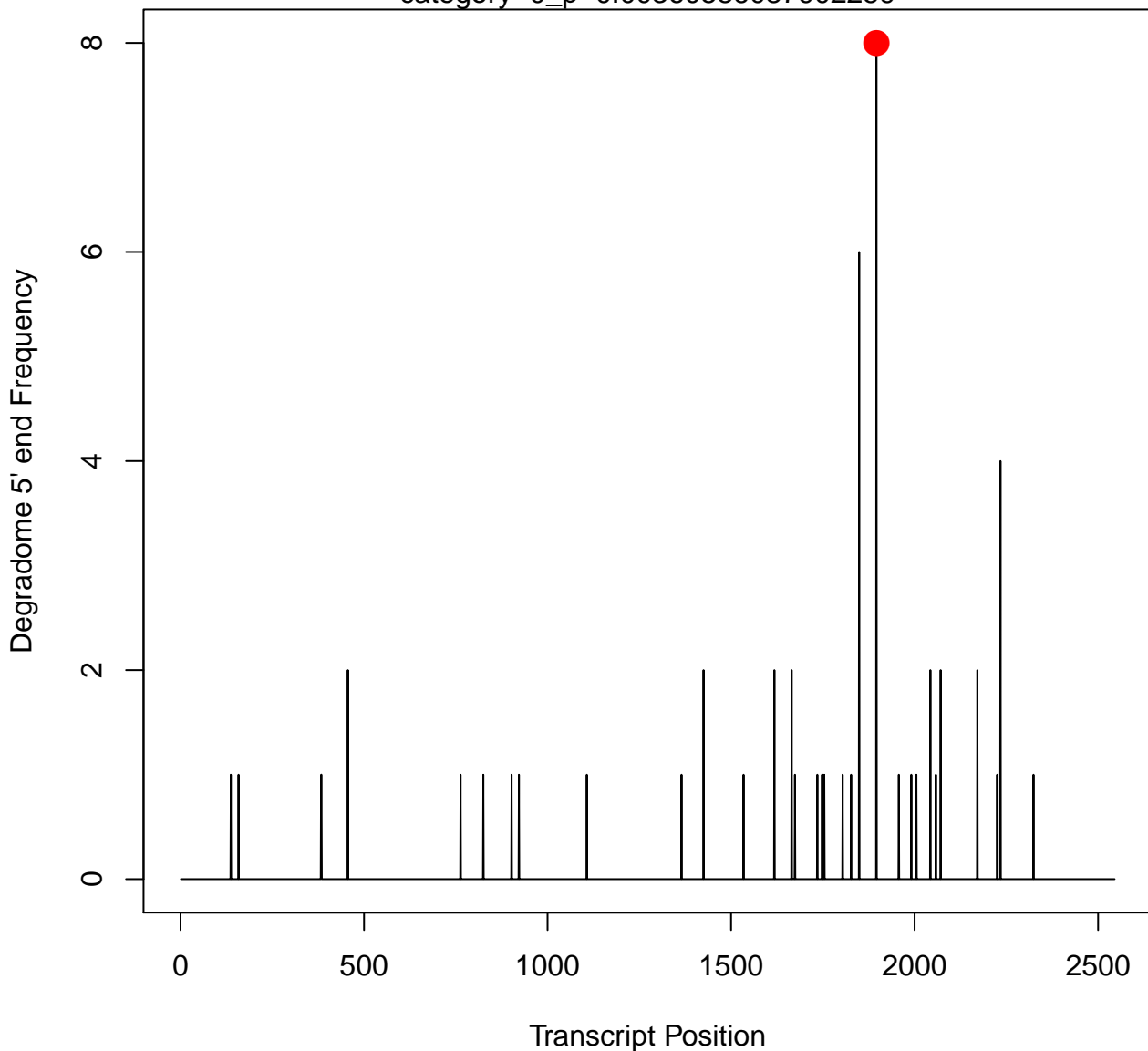

Supplement: Supplementary file 3 [file Data_Sheet_3.zip › Sit-miR156a_Seita.2G266500.1_1896_TPlot.pdf]

**T=Seita.2G324900.1\_Q=Sit-miR156c\_S=833**

category=0\_p=0.00400570862187632

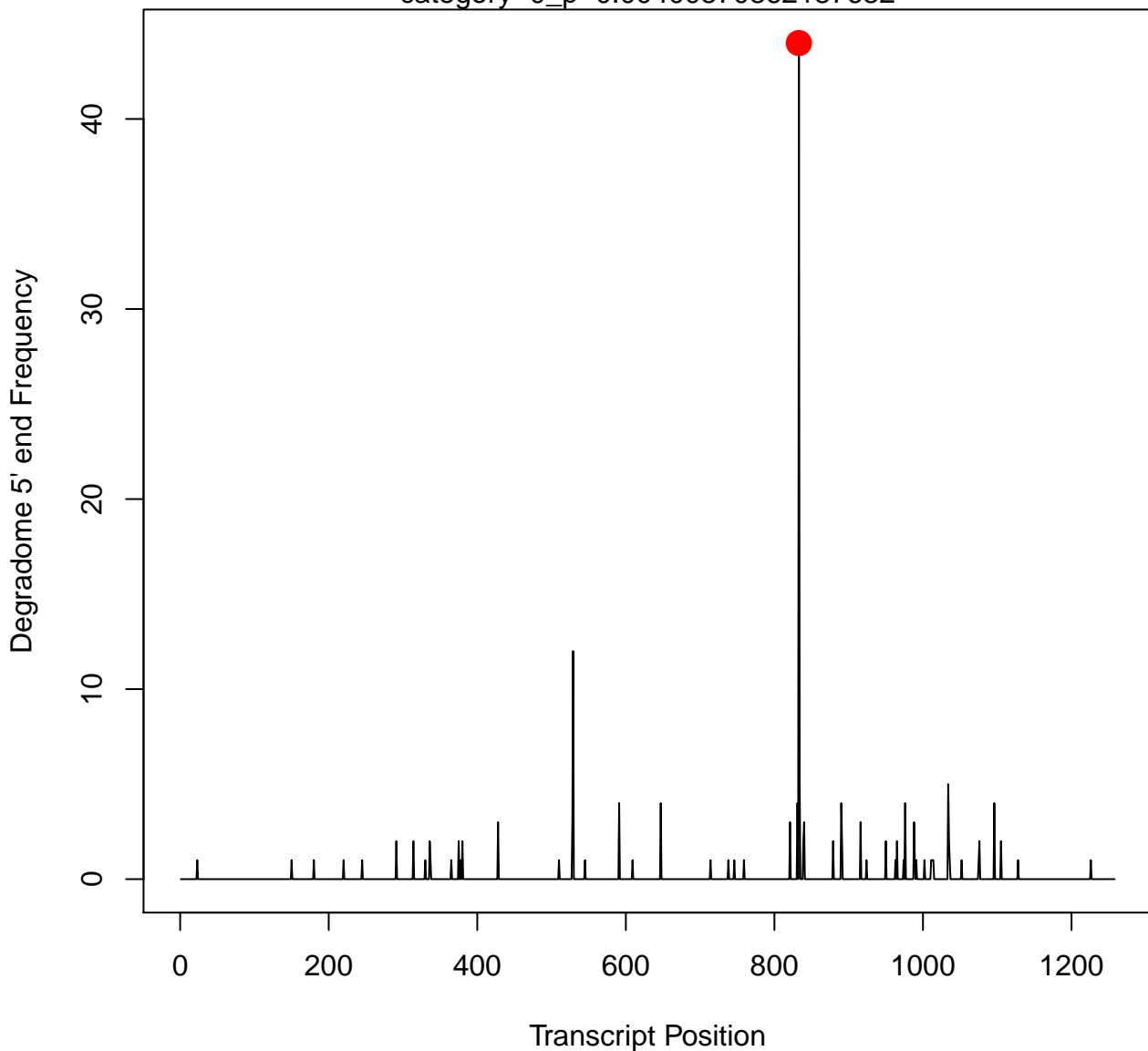

Supplement: Supplementary file 3 [file Data_Sheet_3.zip › Sit-miR156c_Seita.2G324900.1_833_TPlot.pdf]

**T=Seita.6G205500.1\_Q=Sit-miR156c\_S=1434**

category=0\_p=0.00200486405086941

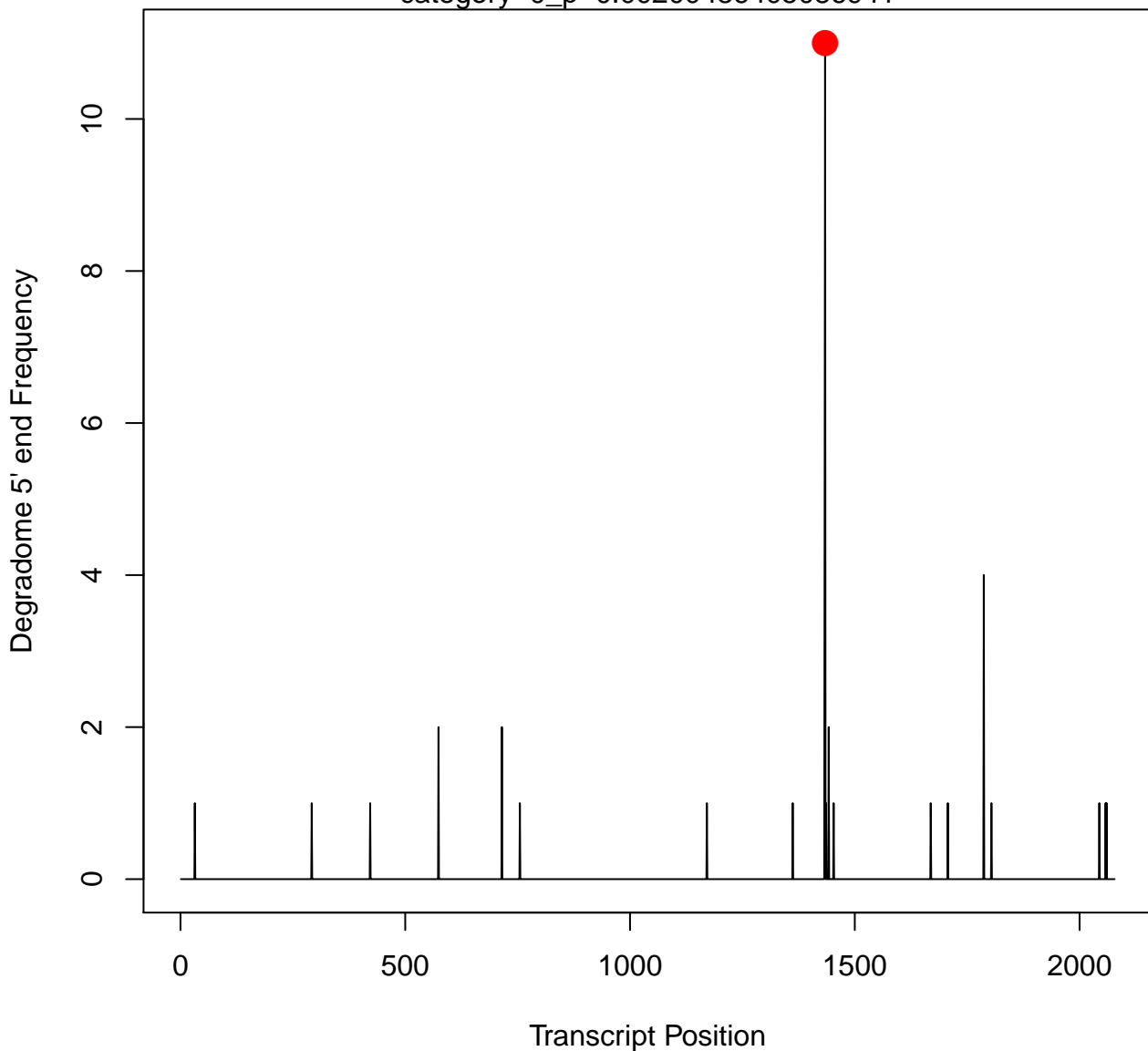

Supplement: Supplementary file 3 [file Data_Sheet_3.zip › Sit-miR156c_Seita.6G205500.1_1434_TPlot.pdf]

**T=Seita.6G208700.1\_Q=Sit-miR156c\_S=163**

category=2\_p=0.225595934523435

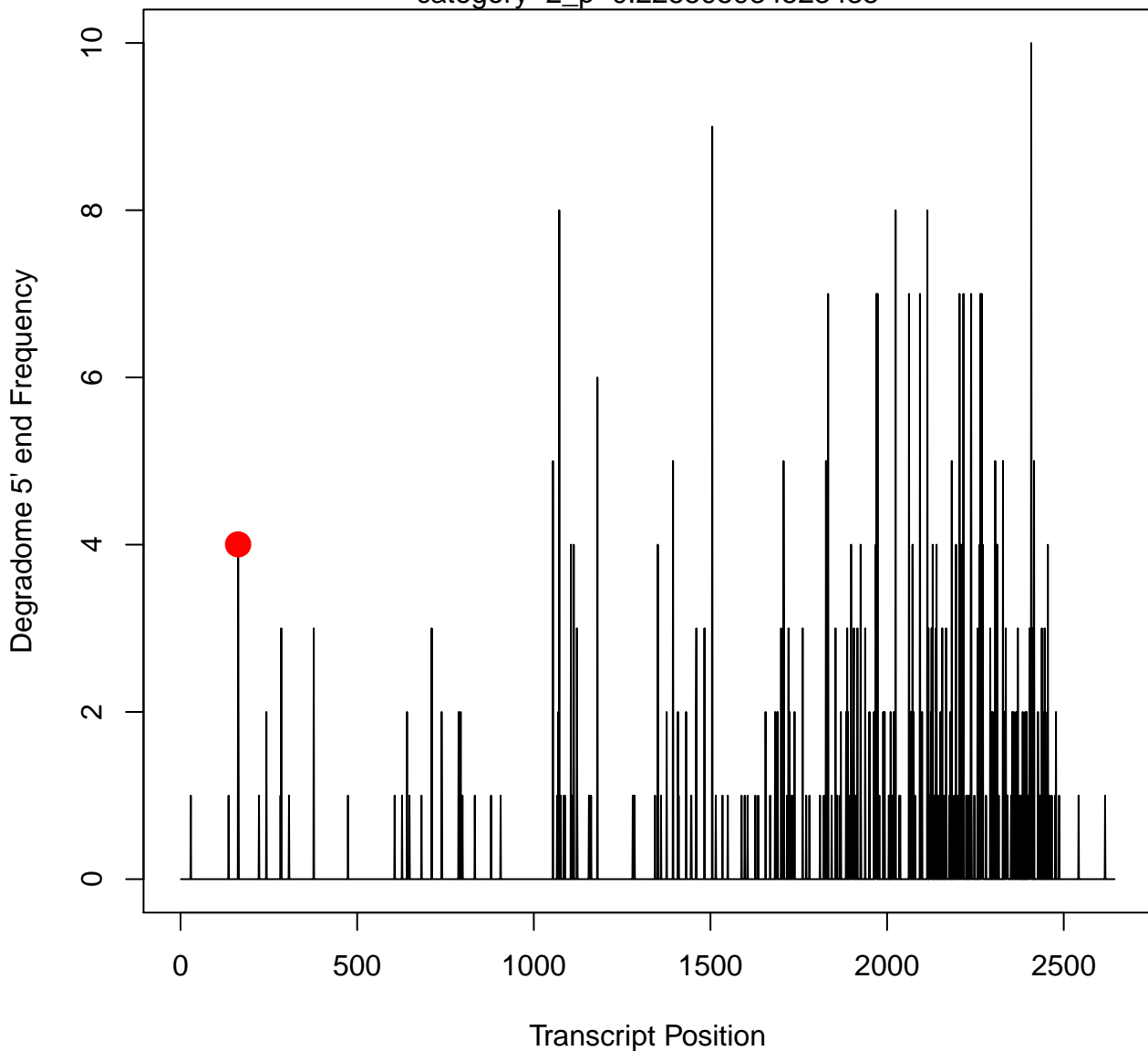

Supplement: Supplementary file 3 [file Data_Sheet_3.zip › Sit-miR156c_Seita.6G208700.1_163_TPlot.pdf]

**T=Seita.1G091900.1\_Q=Sit-miR156d\_S=1787**

category=2\_p=0.0168996705994484

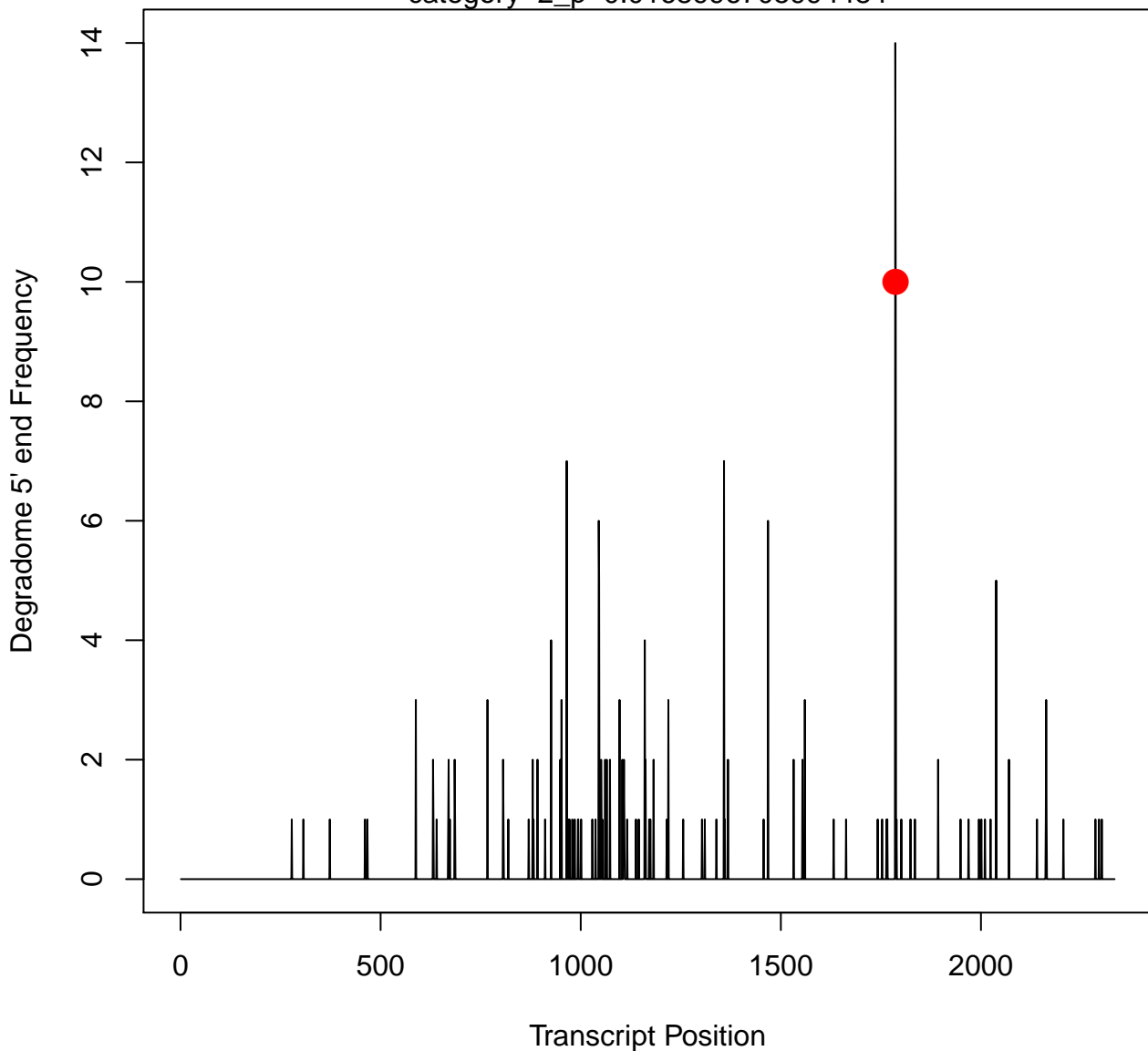

Supplement: Supplementary file 3 [file Data_Sheet_3.zip › Sit-miR156d_Seita.1G091900.1_1787_TPlot.pdf]

**T=Seita.1G227400.1\_Q=Sit-miR156d\_S=2070**

category=2\_p=0.945771084820733

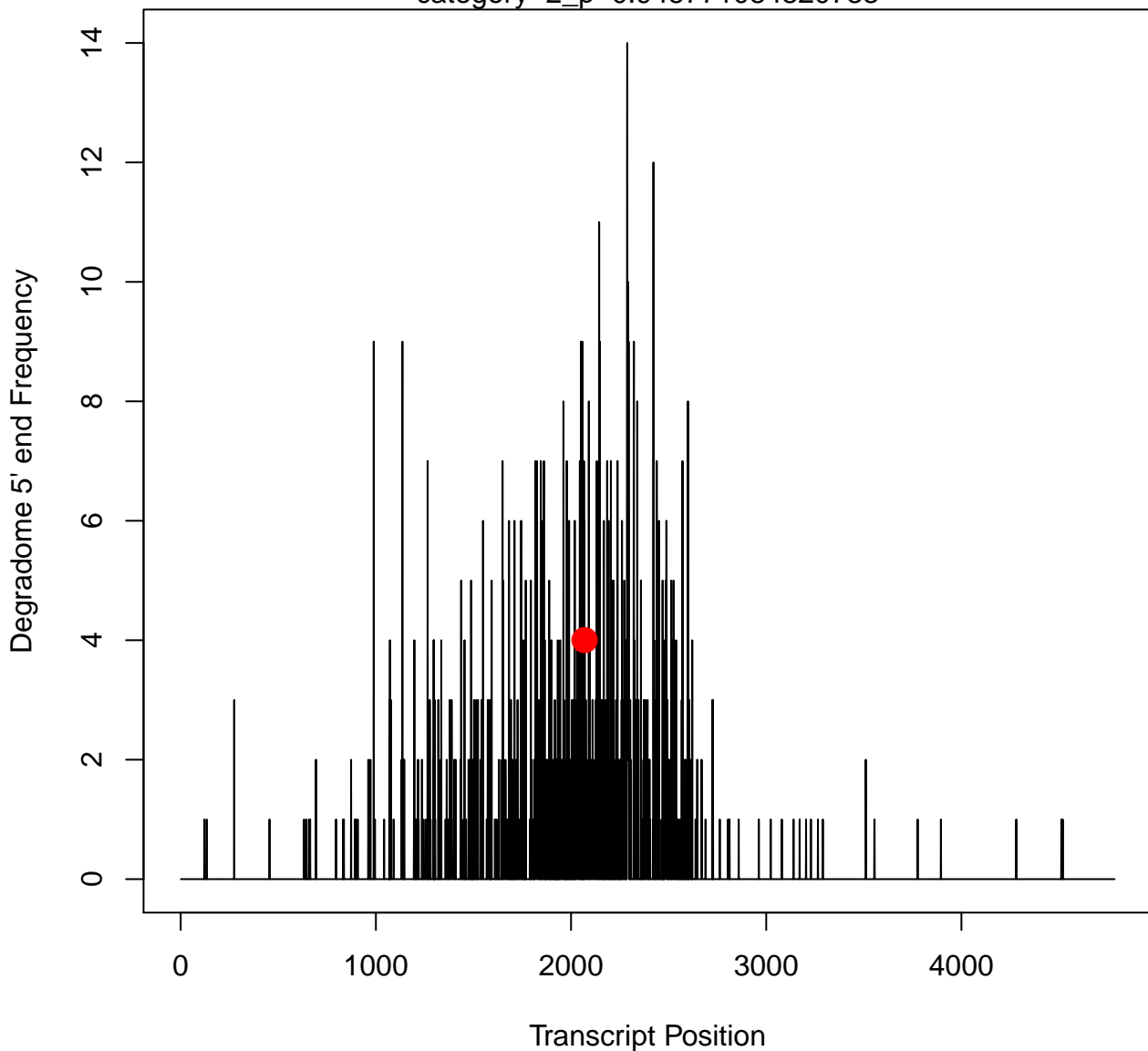

Supplement: Supplementary file 3 [file Data_Sheet_3.zip › Sit-miR156d_Seita.1G227400.1_2070_TPlot.pdf]

**T=Seita.2G324900.1\_Q=Sit-miR156d\_S=834**

category=2\_p=0.184969052895116

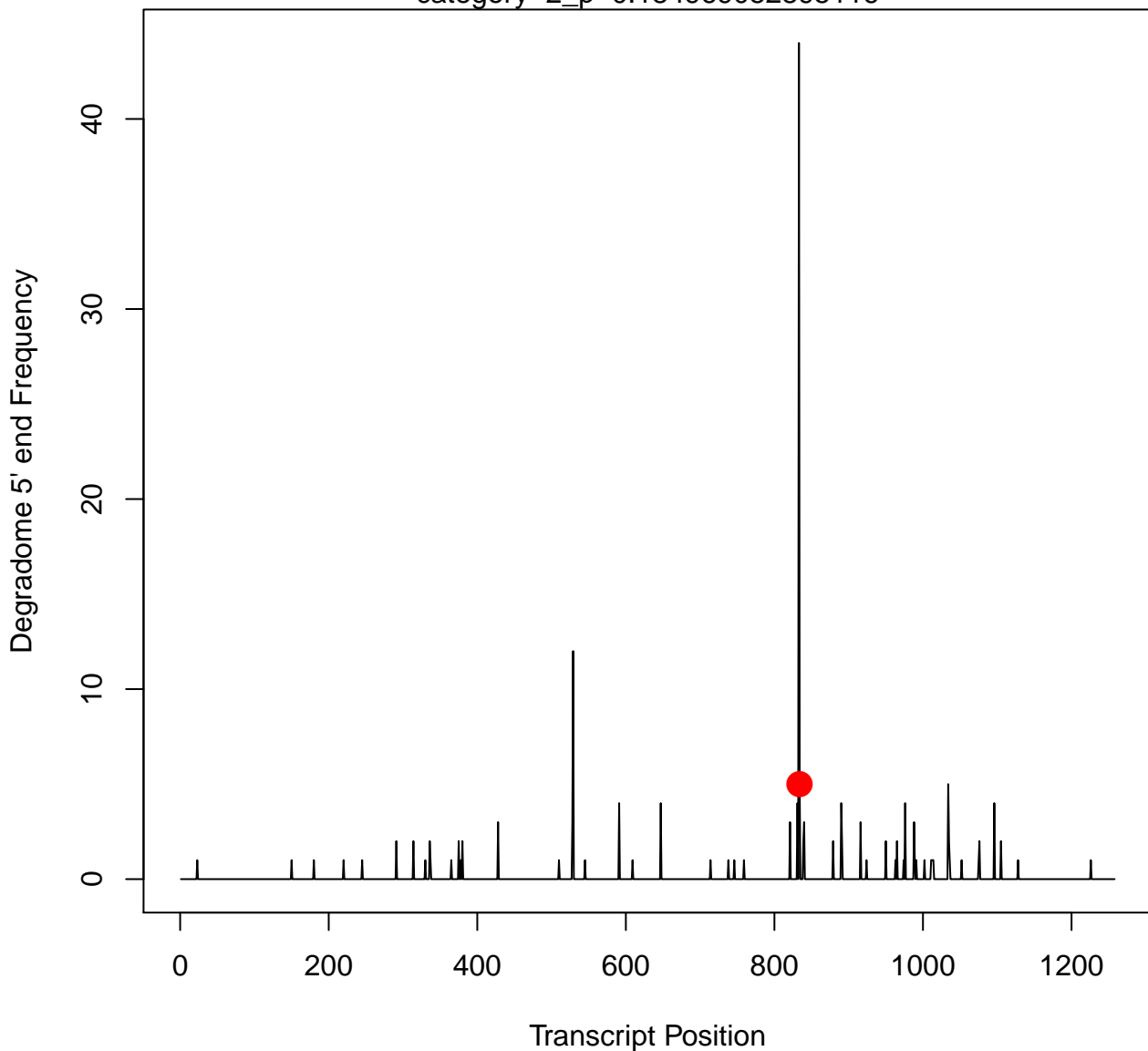

Supplement: Supplementary file 3 [file Data_Sheet_3.zip › Sit-miR156d_Seita.2G324900.1_834_TPlot.pdf]

**T=Seita.6G223300.1\_Q=Sit-miR156d\_S=982**

category=2\_p=0.0972093558831681

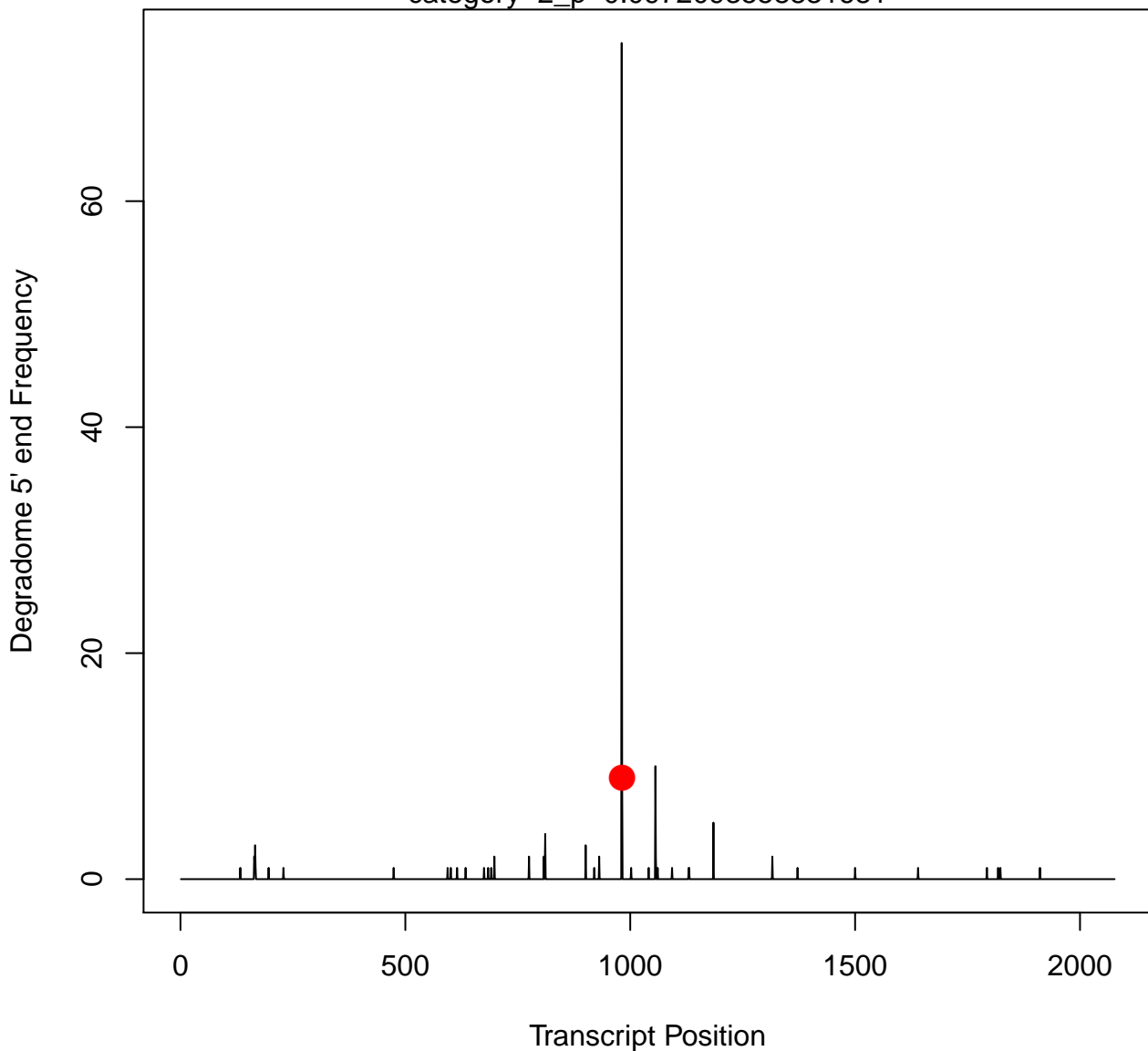

Supplement: Supplementary file 3 [file Data_Sheet_3.zip › Sit-miR156d_Seita.6G223300.1_982_TPlot.pdf]

**T=Seita.9G485100.1\_Q=Sit-miR156d\_S=3193**

category=2\_p=0.940947034926436

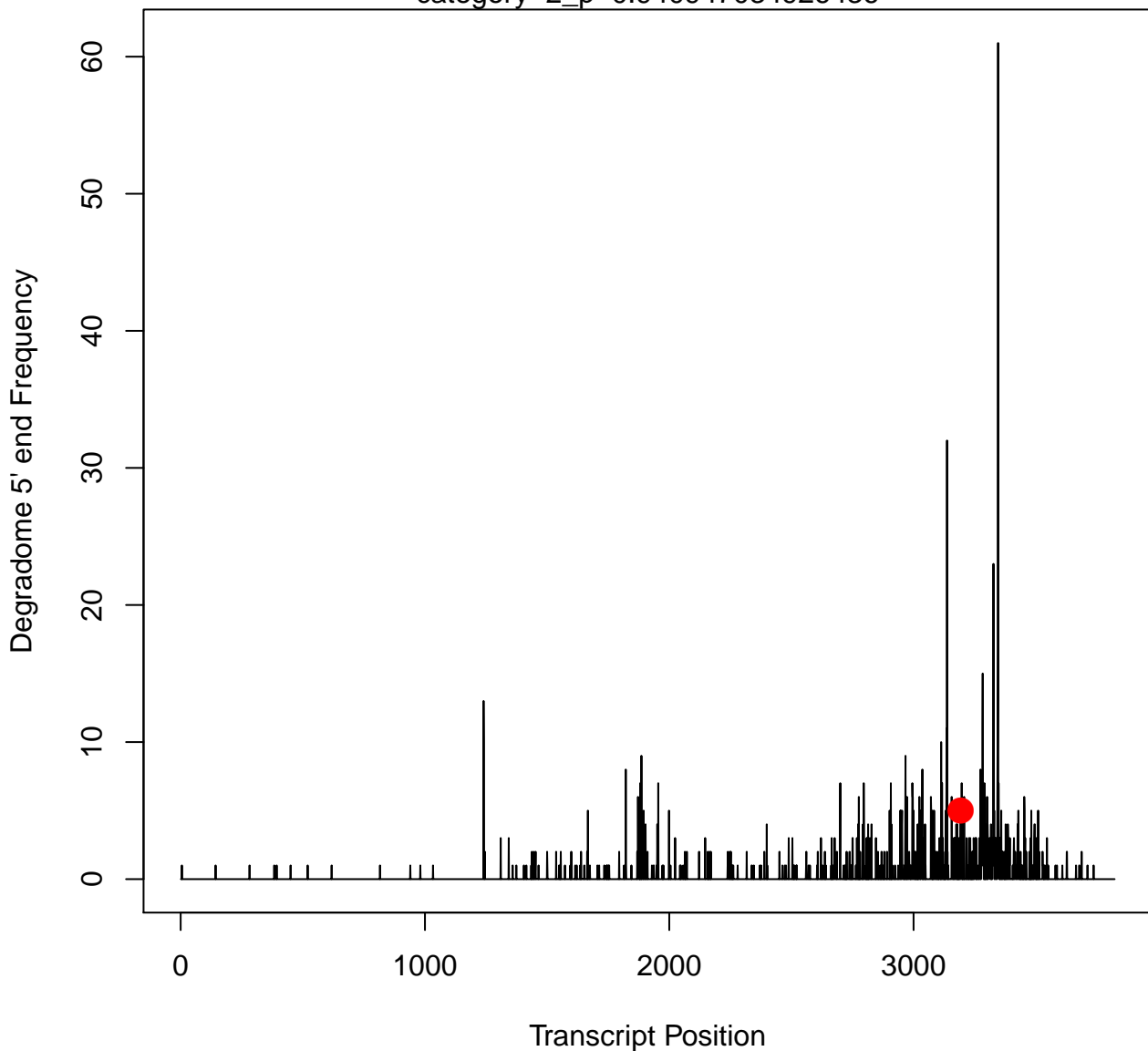

Supplement: Supplementary file 3 [file Data_Sheet_3.zip › Sit-miR156d_Seita.9G485100.1_3193_TPlot.pdf]

**T=Seita.6G223300.1\_Q=Sit-miR156e\_S=981**

category=0\_p=0.00160421305724157

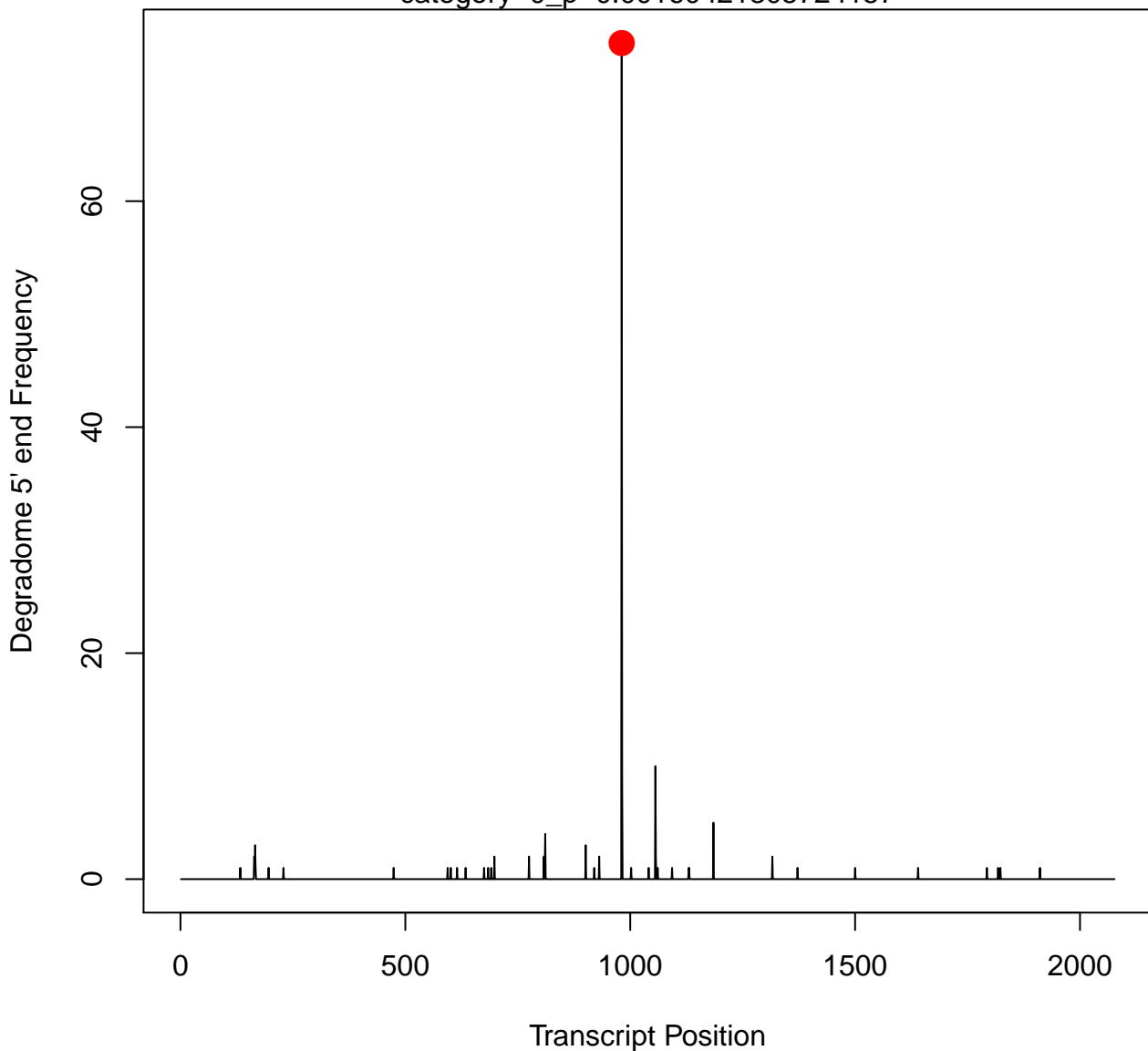

Supplement: Supplementary file 3 [file Data_Sheet_3.zip › Sit-miR156e_Seita.6G223300.1_981_TPlot.pdf]

**T=Seita.1G091900.1\_Q=Sit-miR156i\_S=1786**

category=0\_p=0.000401294755915105

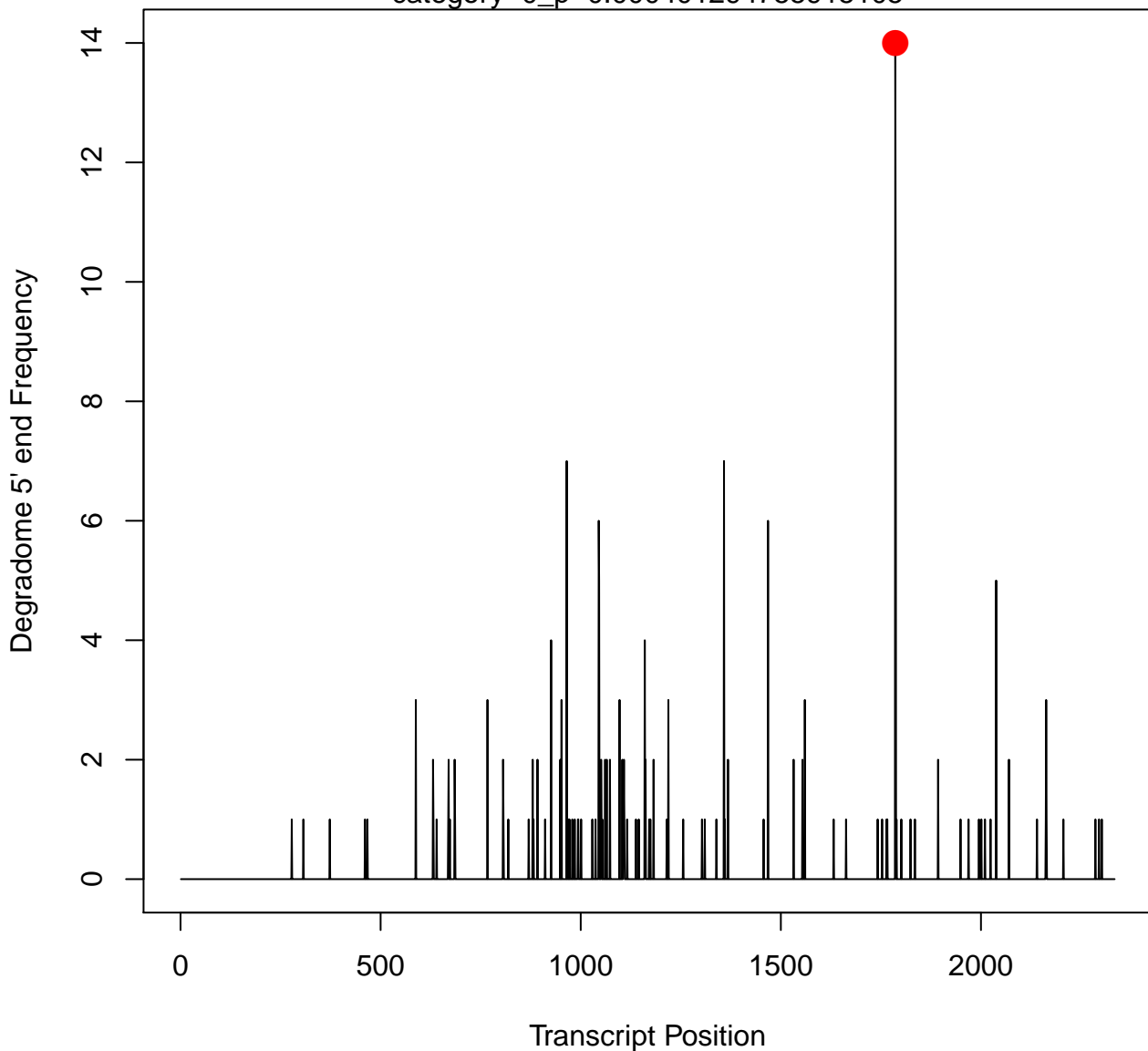

Supplement: Supplementary file 3 [file Data_Sheet_3.zip › Sit-miR156i_Seita.1G091900.1_1786_TPlot.pdf]

**T=Seita.1G134200.1\_Q=Sit-miR156i\_S=59**

category=2\_p=0.93346398026738

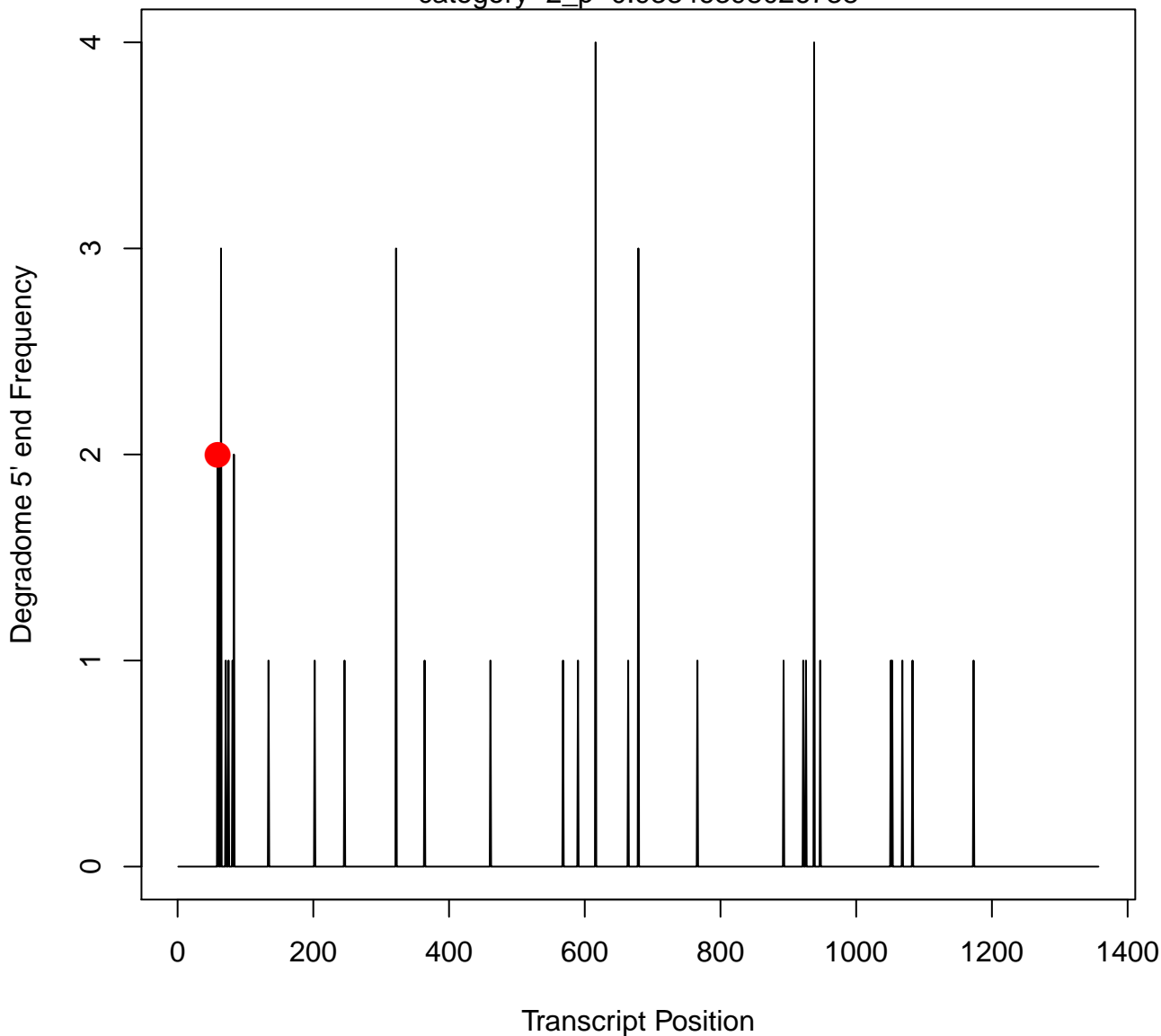

Supplement: Supplementary file 3 [file Data_Sheet_3.zip › Sit-miR156i_Seita.1G134200.1_59_TPlot.pdf]

**T=Seita.1G069300.1\_Q=Sit-miR156j\_S=873**

category=0\_p=0.000802428474349082

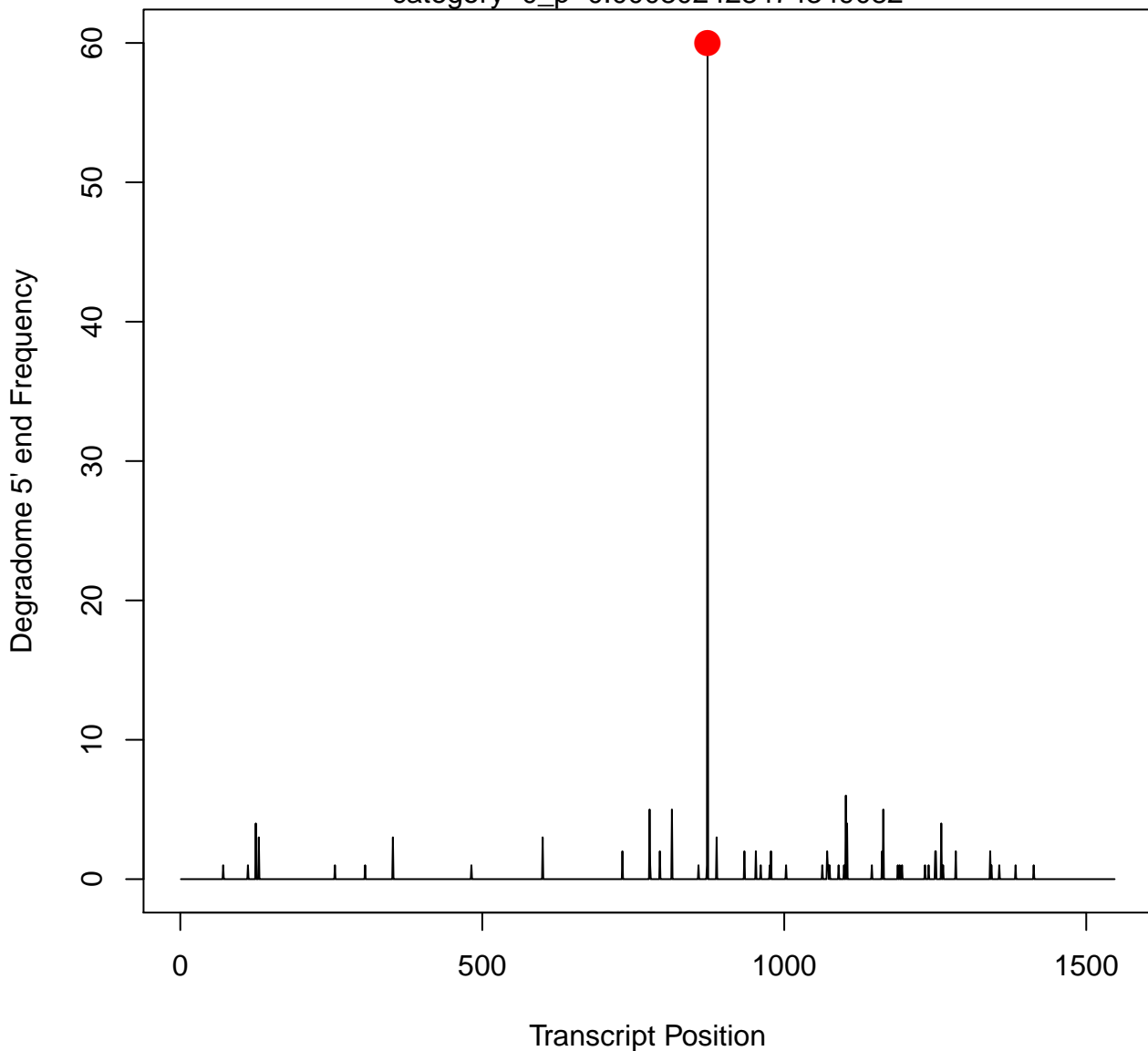

Supplement: Supplementary file 3 [file Data_Sheet_3.zip › Sit-miR156j_Seita.1G069300.1_873_TPlot.pdf]

**T=Seita.2G254300.1\_Q=Sit-miR156j\_S=1266**

category=0\_p=0.00240535426535449

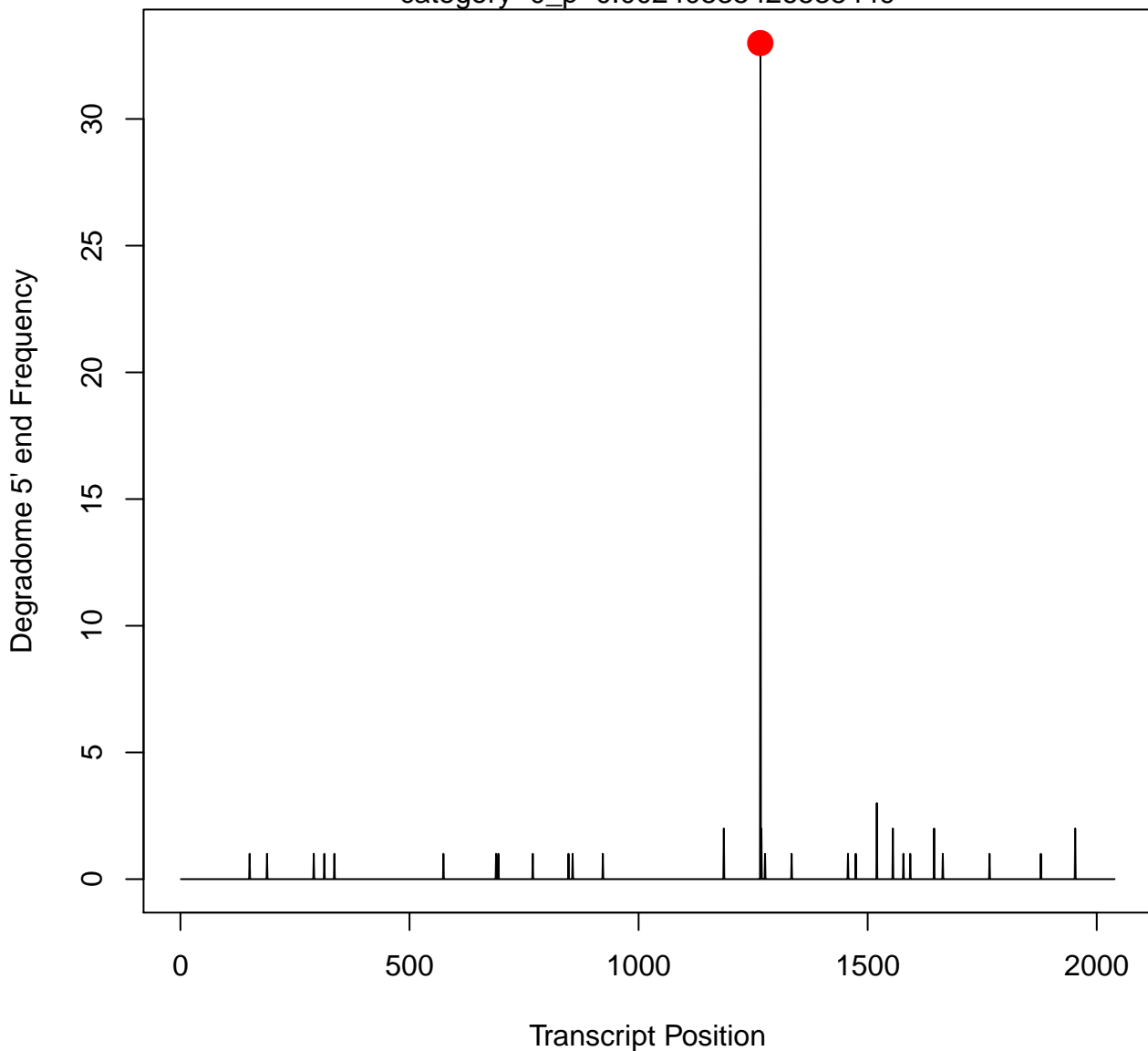

Supplement: Supplementary file 3 [file Data_Sheet_3.zip › Sit-miR156j_Seita.2G254300.1_1266_TPlot.pdf]

**T=Seita.3G205200.1\_Q=Sit-miR156j\_S=47**

category=2\_p=0.93115678655049

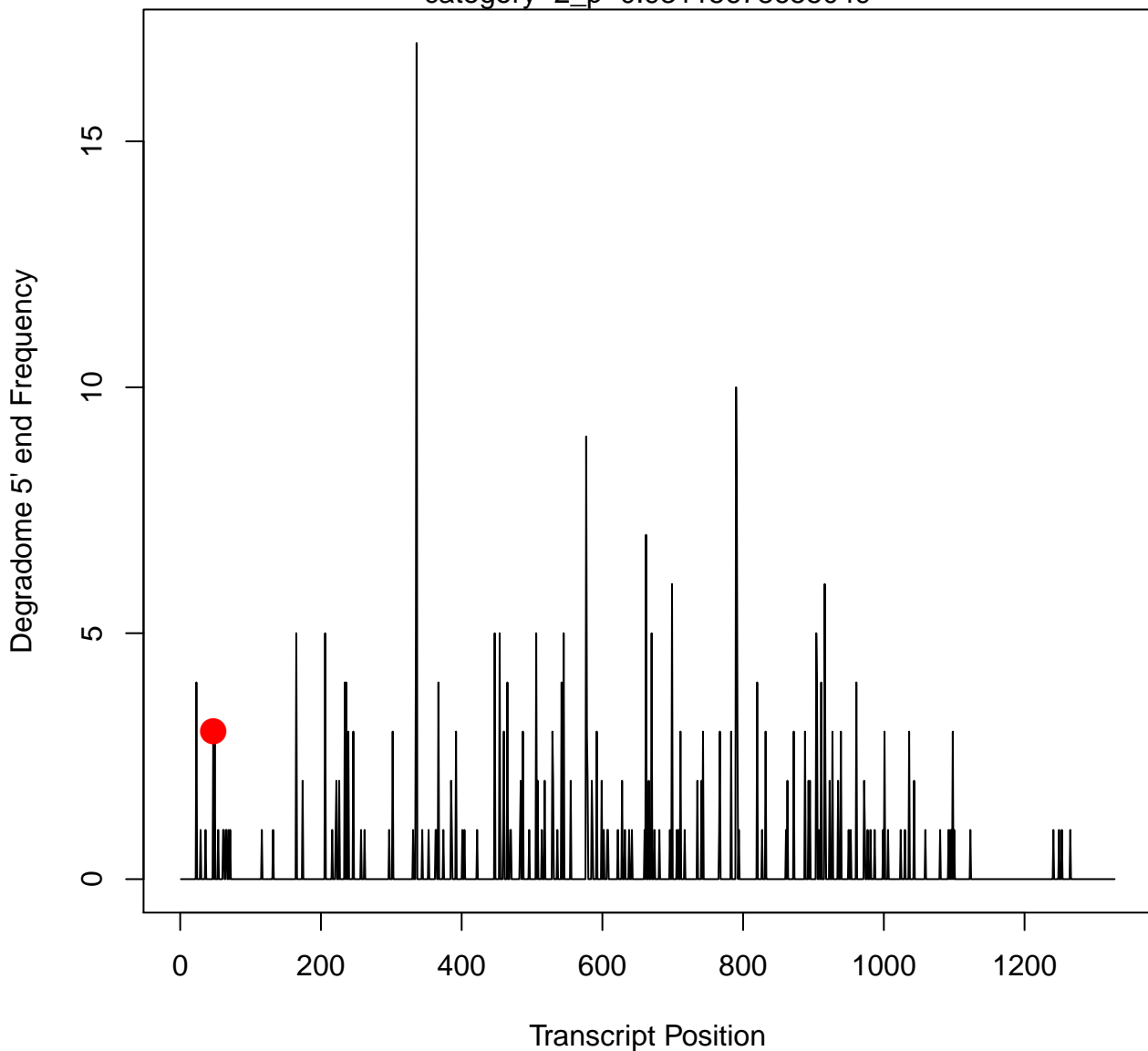

Supplement: Supplementary file 3 [file Data_Sheet_3.zip › Sit-miR156j_Seita.3G205200.1_47_TPlot.pdf]

**T=Seita.4G270400.1\_Q=Sit-miR156j\_S=2084**

category=0\_p=0.000401294755915105

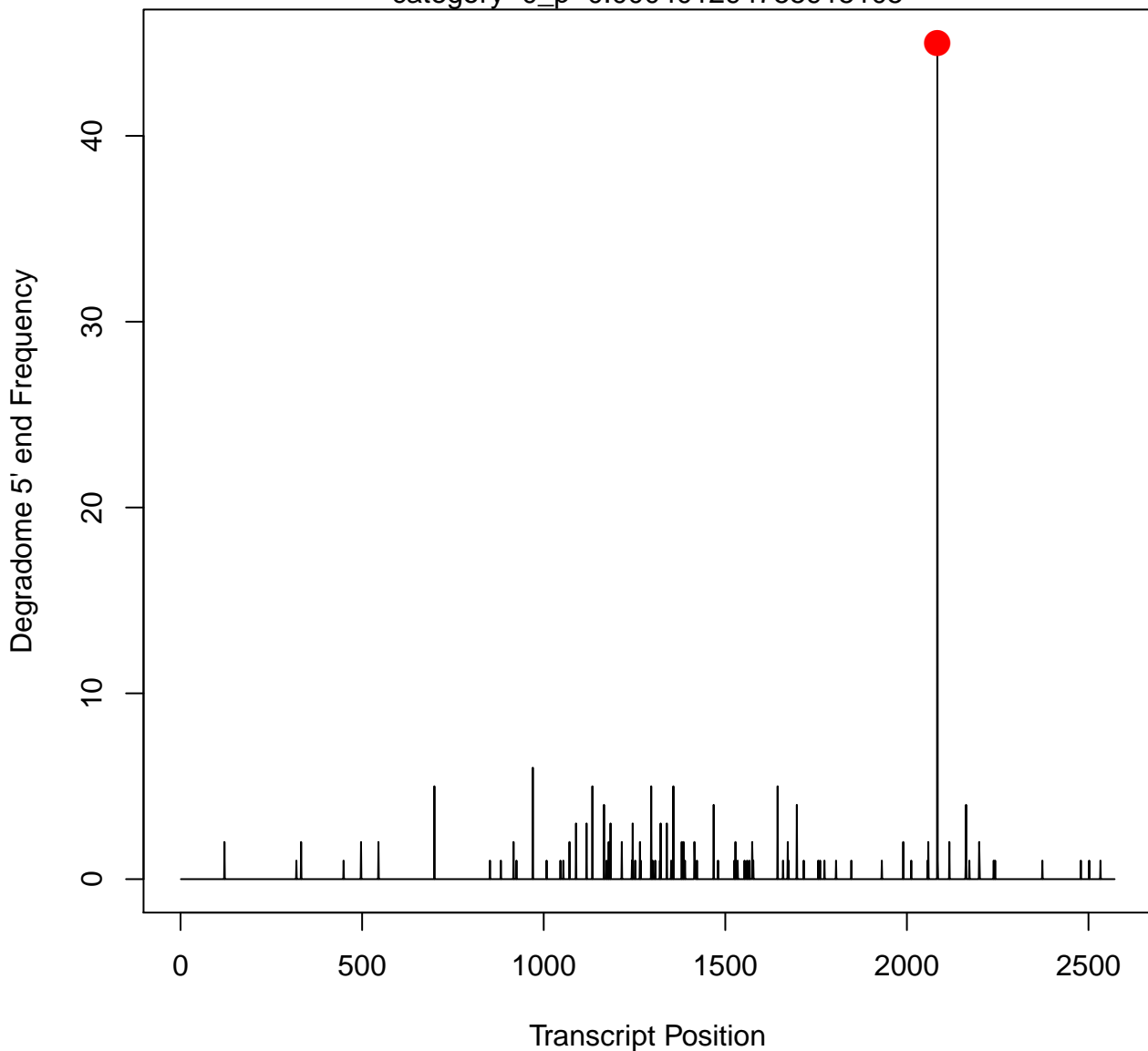

Supplement: Supplementary file 3 [file Data_Sheet_3.zip › Sit-miR156j_Seita.4G270400.1_2084_TPlot.pdf]

**T=Seita.5G432500.1\_Q=Sit-miR156j\_S=2375**

category=2\_p=0.065904313739923

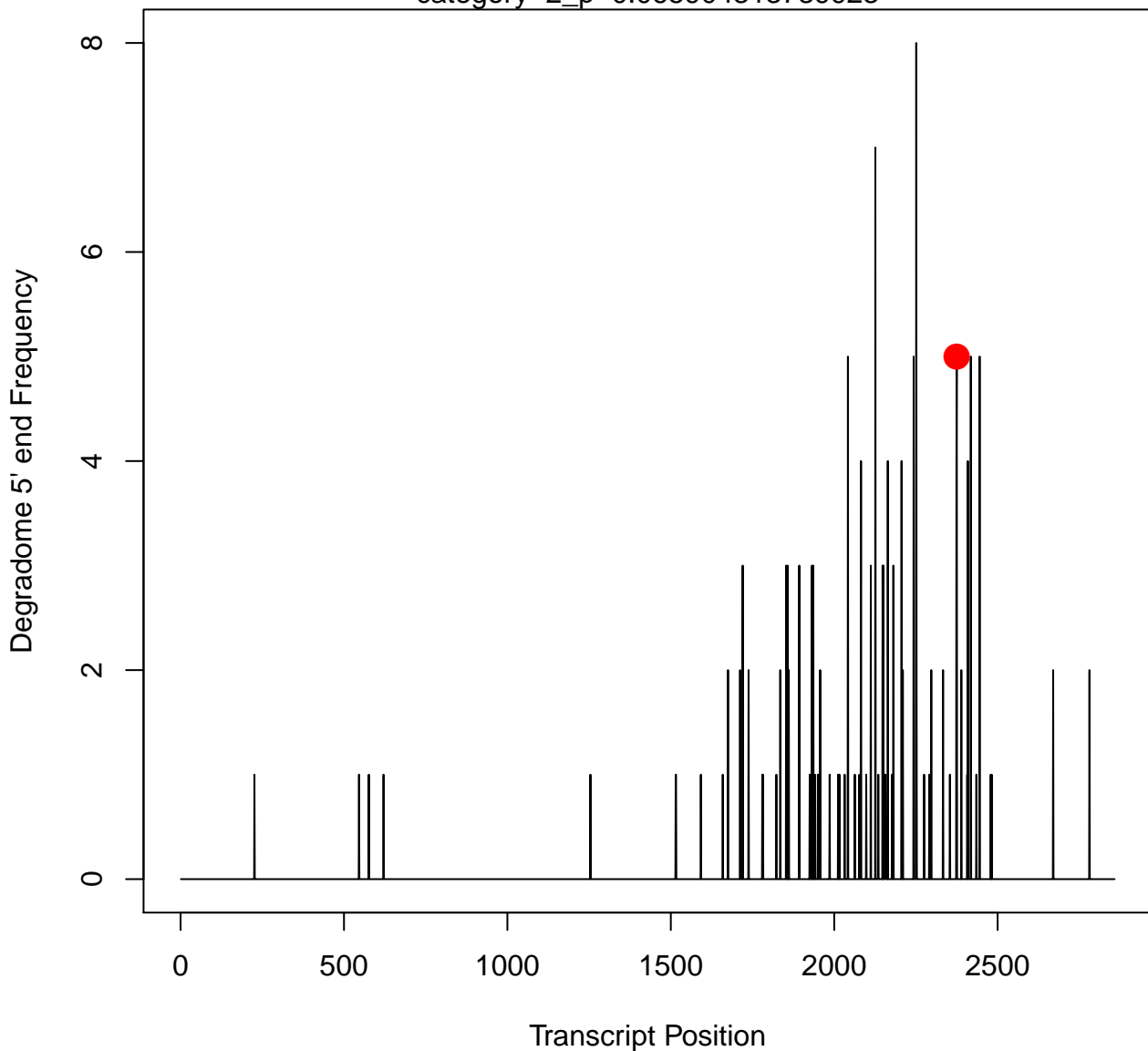

Supplement: Supplementary file 3 [file Data_Sheet_3.zip › Sit-miR156j_Seita.5G432500.1_2375_TPlot.pdf]

**T=Seita.8G124900.1\_Q=Sit-miR156j\_S=1295**

category=0\_p=0.00600254177153148

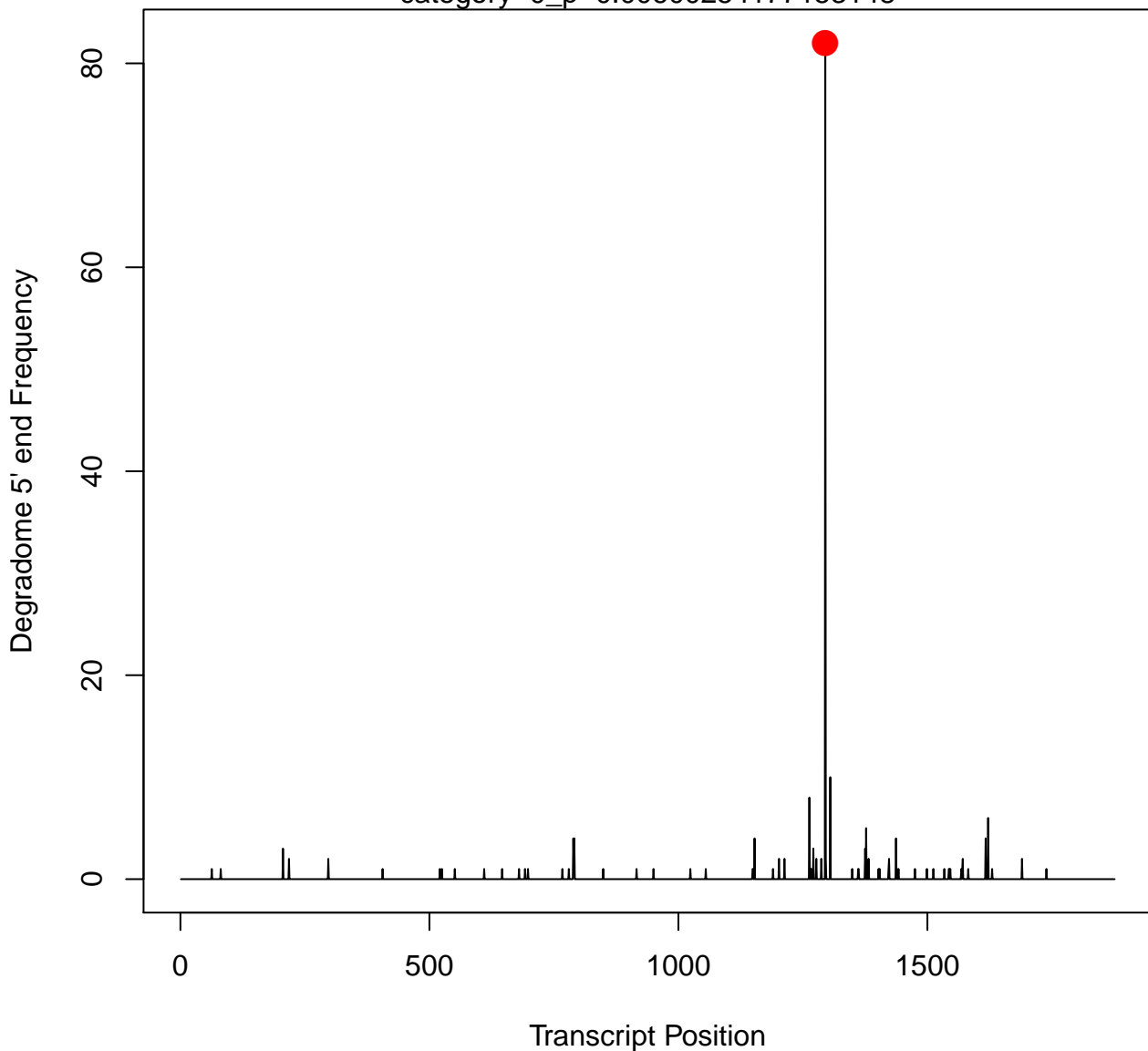

Supplement: Supplementary file 3 [file Data_Sheet_3.zip › Sit-miR156j_Seita.8G124900.1_1295_TPlot.pdf]

**T=Seita.1G134200.1\_Q=Sit-miR159a\_S=61**

category=2\_p=0.999988246627036

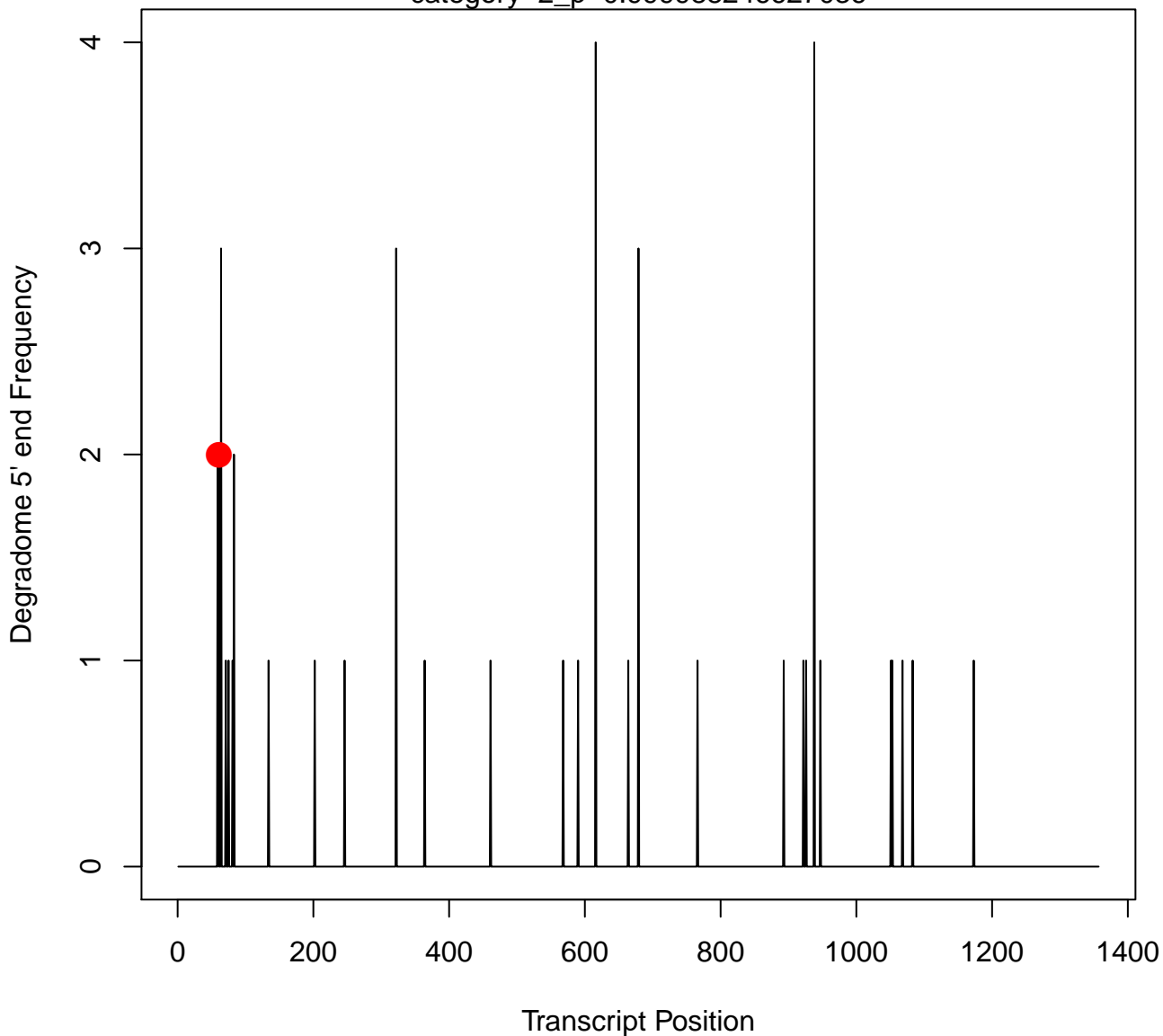

Supplement: Supplementary file 3 [file Data_Sheet_3.zip › Sit-miR159a_Seita.1G134200.1_61_TPlot.pdf]

**T=Seita.1G226400.1\_Q=Sit-miR159a\_S=1975**

category=2\_p=0.999999654923223

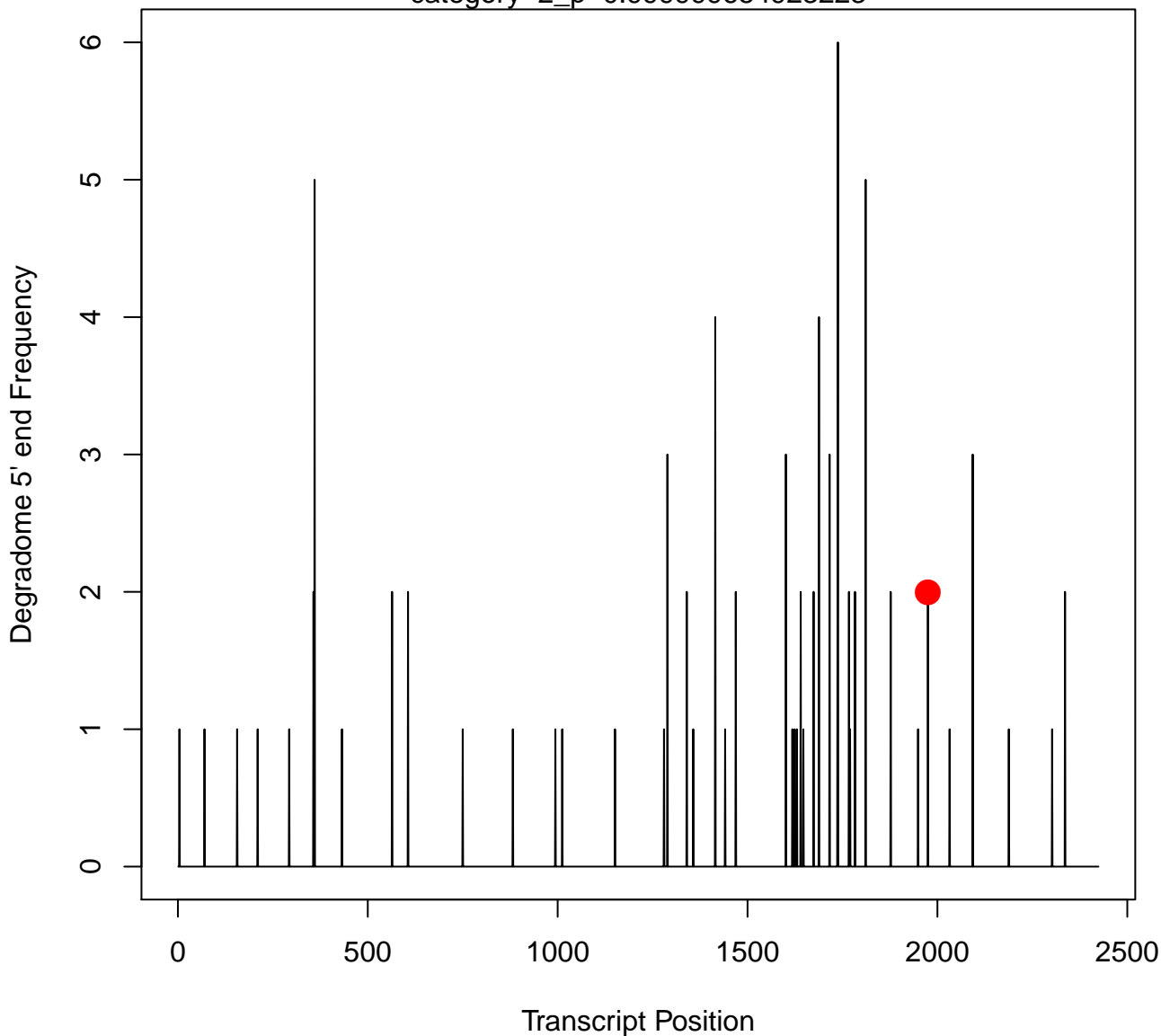

Supplement: Supplementary file 3 [file Data_Sheet_3.zip › Sit-miR159a_Seita.1G226400.1_1975_TPlot.pdf]

**T=Seita.1G230100.1\_Q=Sit-miR159a\_S=1456**

category=2\_p=0.96077256536262

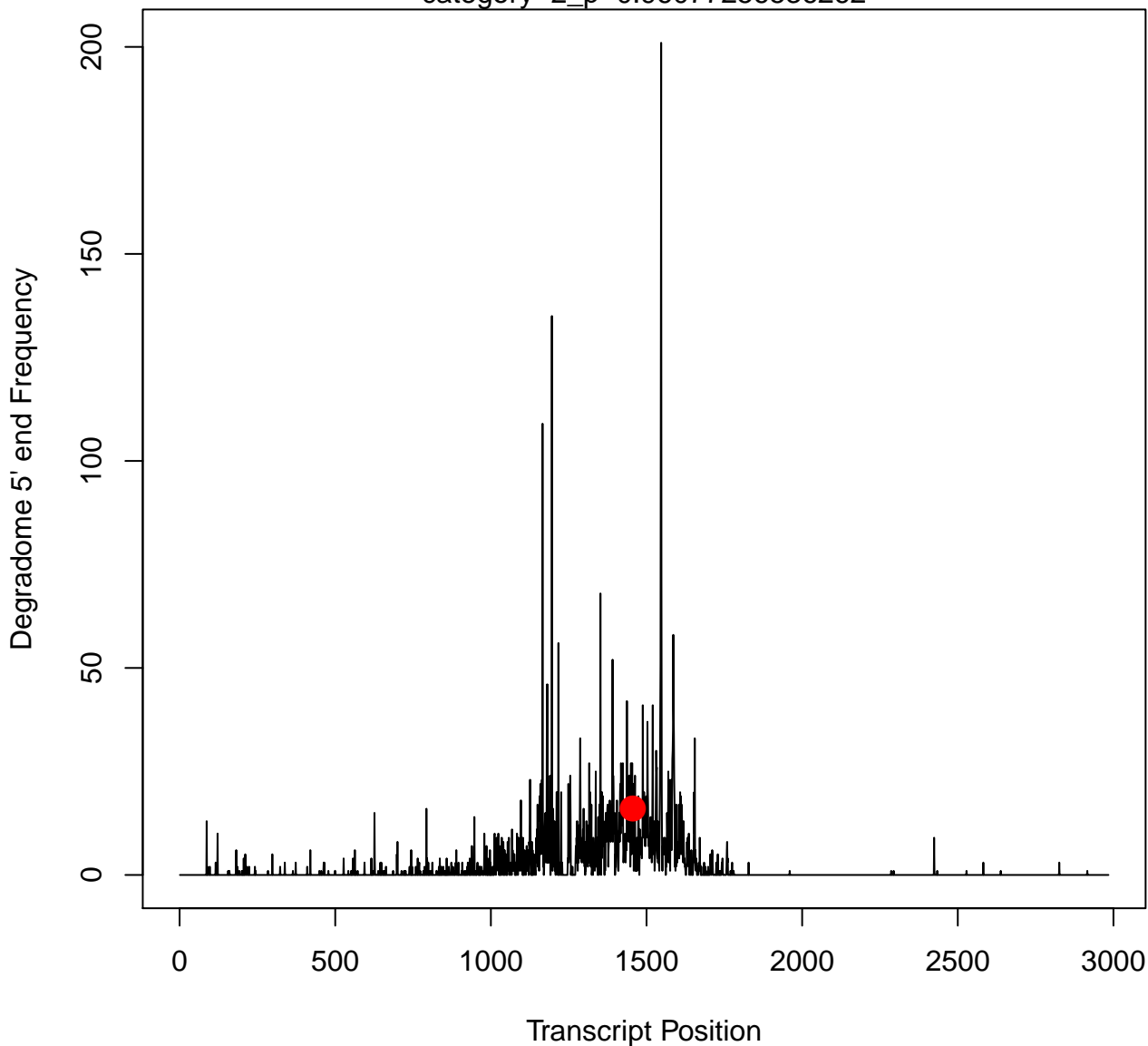

Supplement: Supplementary file 3 [file Data_Sheet_3.zip › Sit-miR159a_Seita.1G230100.1_1456_TPlot.pdf]

**T=Seita.1G247600.1\_Q=Sit-miR159a\_S=1943**

category=2\_p=0.999988445255167

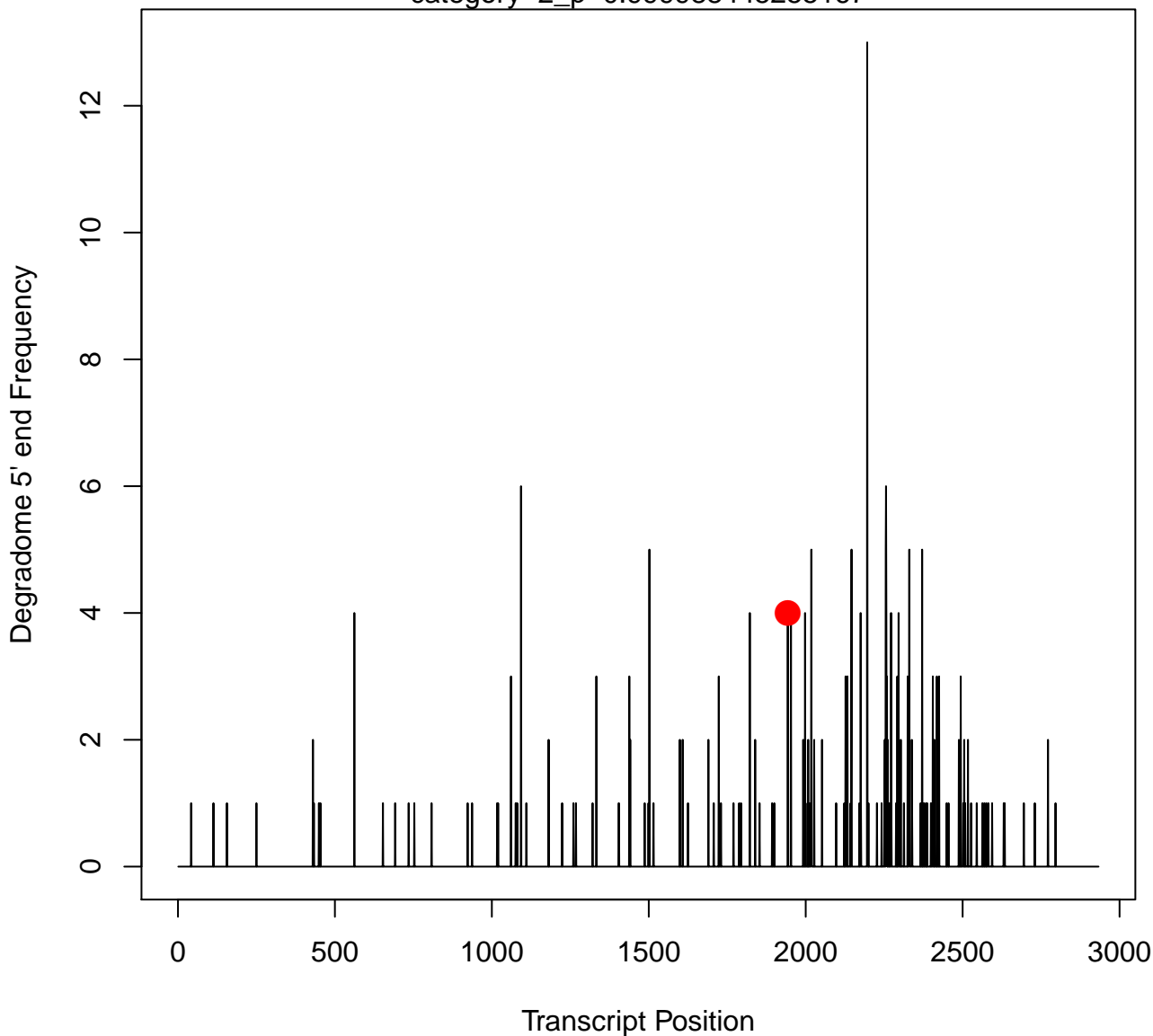

Supplement: Supplementary file 3 [file Data_Sheet_3.zip › Sit-miR159a_Seita.1G247600.1_1943_TPlot.pdf]

**T=Seita.2G258400.1\_Q=Sit-miR159a\_S=480**

category=2\_p=0.999999996356327

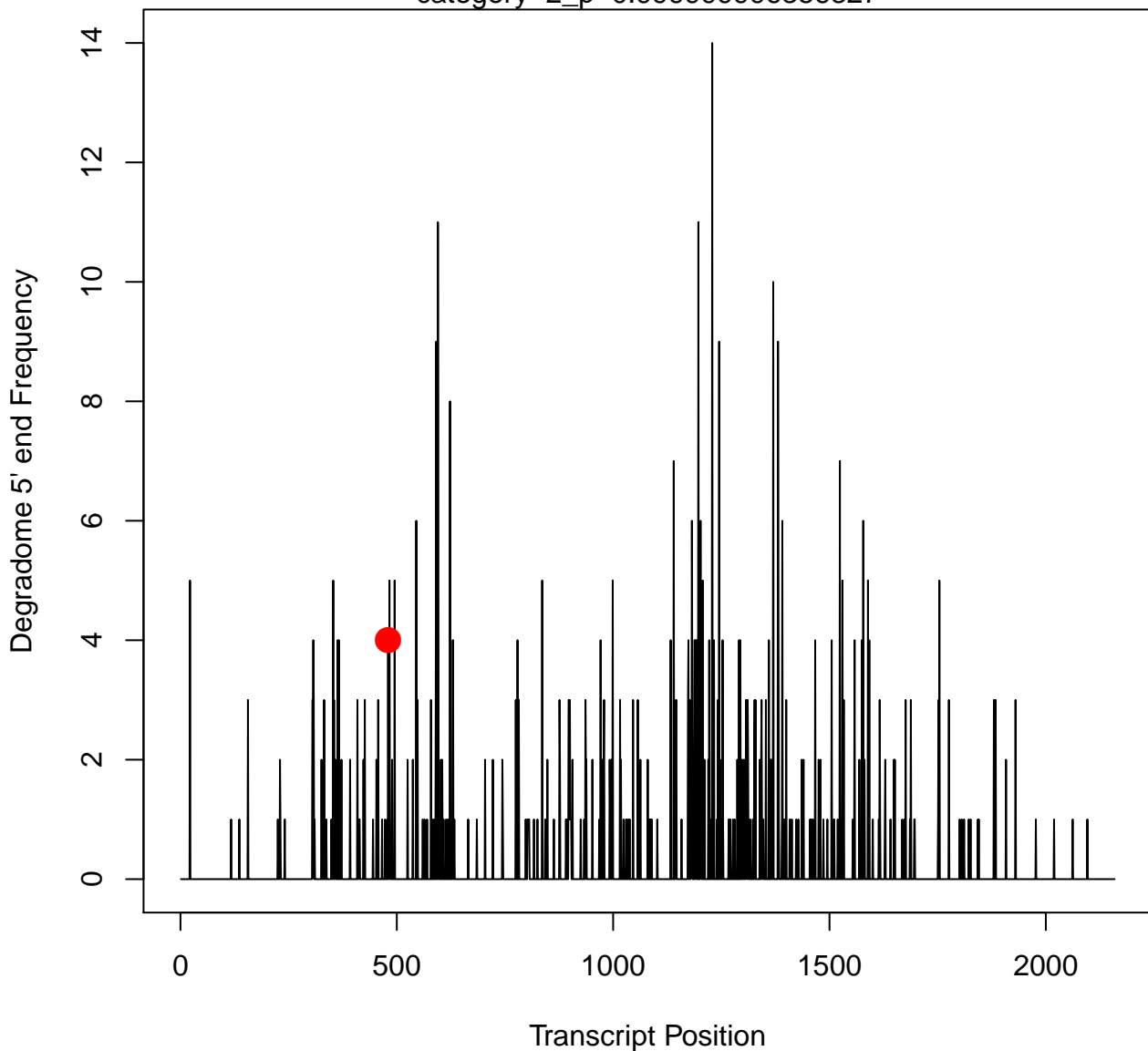

Supplement: Supplementary file 3 [file Data_Sheet_3.zip › Sit-miR159a_Seita.2G258400.1_480_TPlot.pdf]

**T=Seita.2G434400.1\_Q=Sit-miR159a\_S=362**

category=2\_p=0.420396590169943

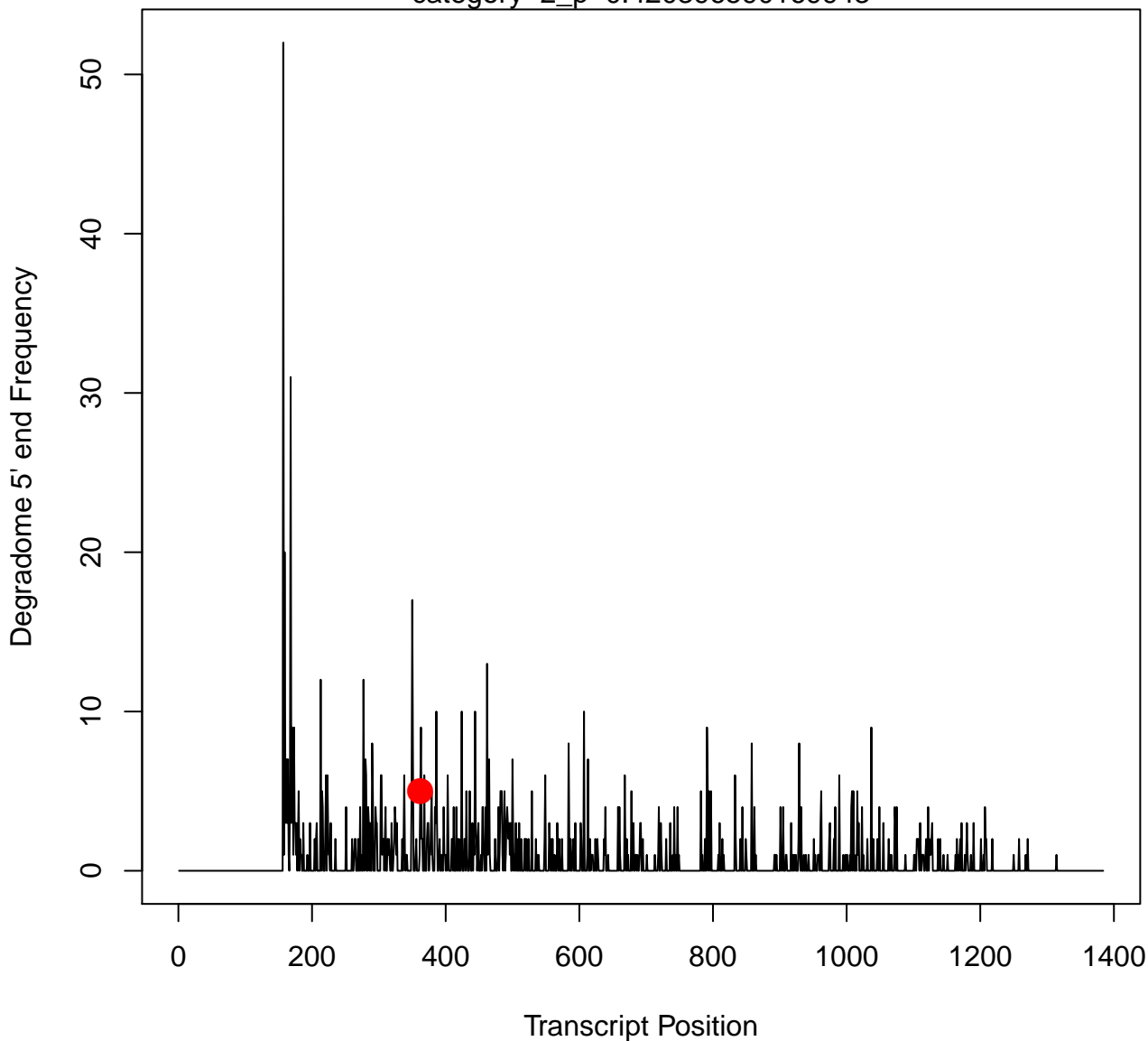

Supplement: Supplementary file 3 [file Data_Sheet_3.zip › Sit-miR159a_Seita.2G434400.1_362_TPlot.pdf]

**T=Seita.4G020800.1\_Q=Sit-miR159a\_S=284**

category=2\_p=0.999999996874496

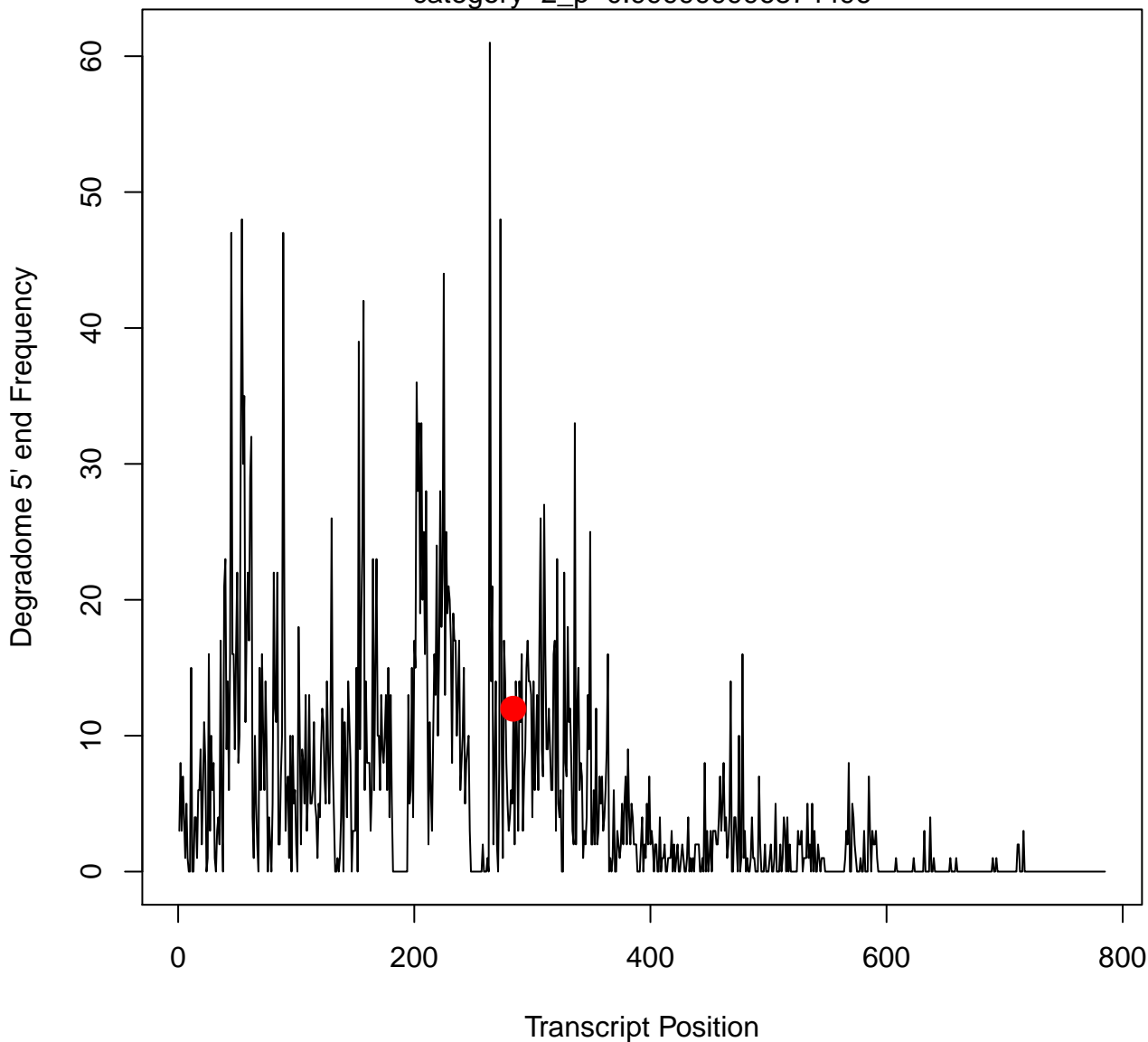

Supplement: Supplementary file 3 [file Data_Sheet_3.zip › Sit-miR159a_Seita.4G020800.1_284_TPlot.pdf]

**T=Seita.4G274900.1\_Q=Sit-miR159a\_S=1312**

category=2\_p=0.999999947072874

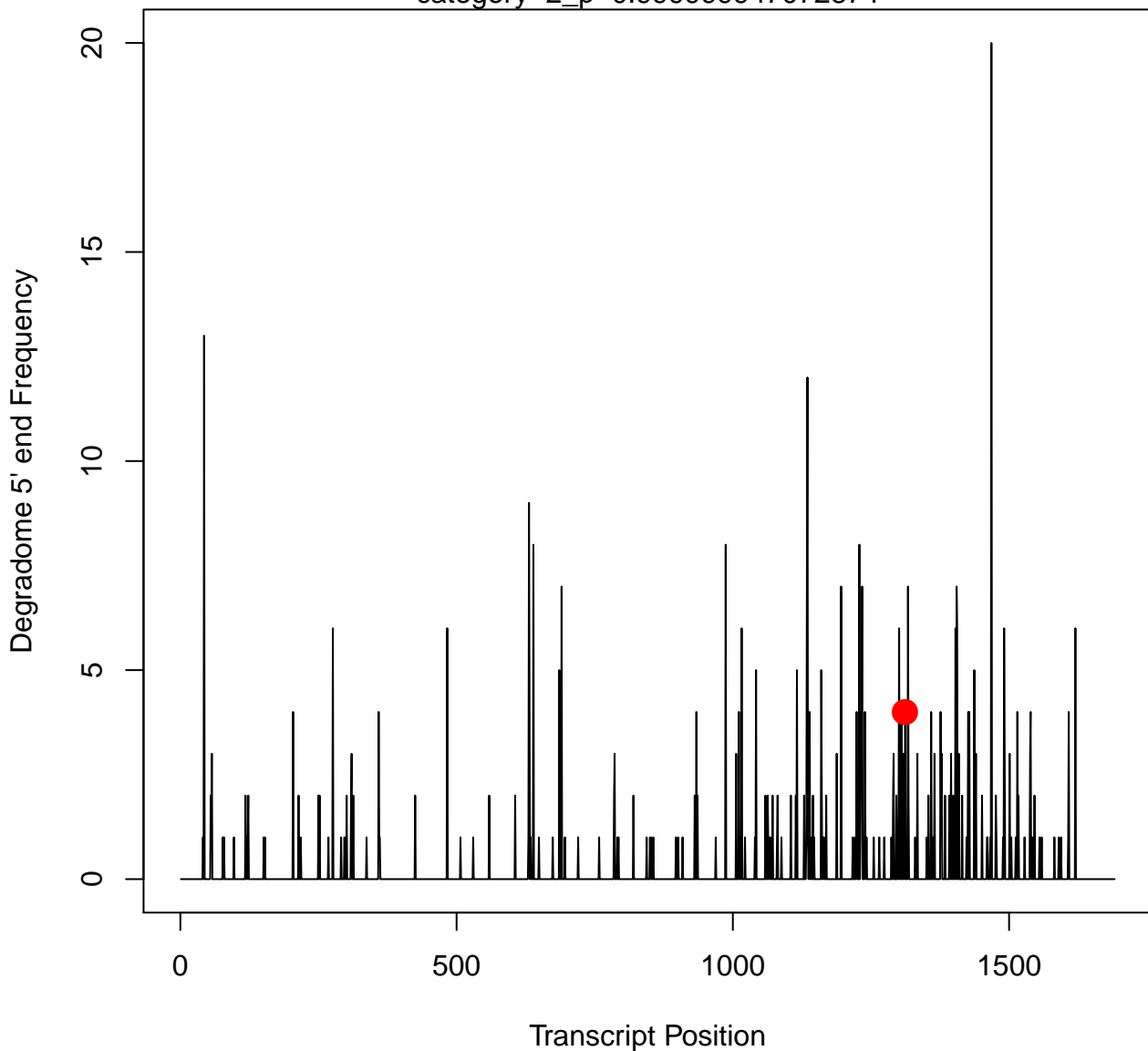

Supplement: Supplementary file 3 [file Data_Sheet_3.zip › Sit-miR159a_Seita.4G274900.1_1312_TPlot.pdf]

**T=Seita.5G081000.1\_Q=Sit-miR159a\_S=274**

category=2\_p=0.999999920323525

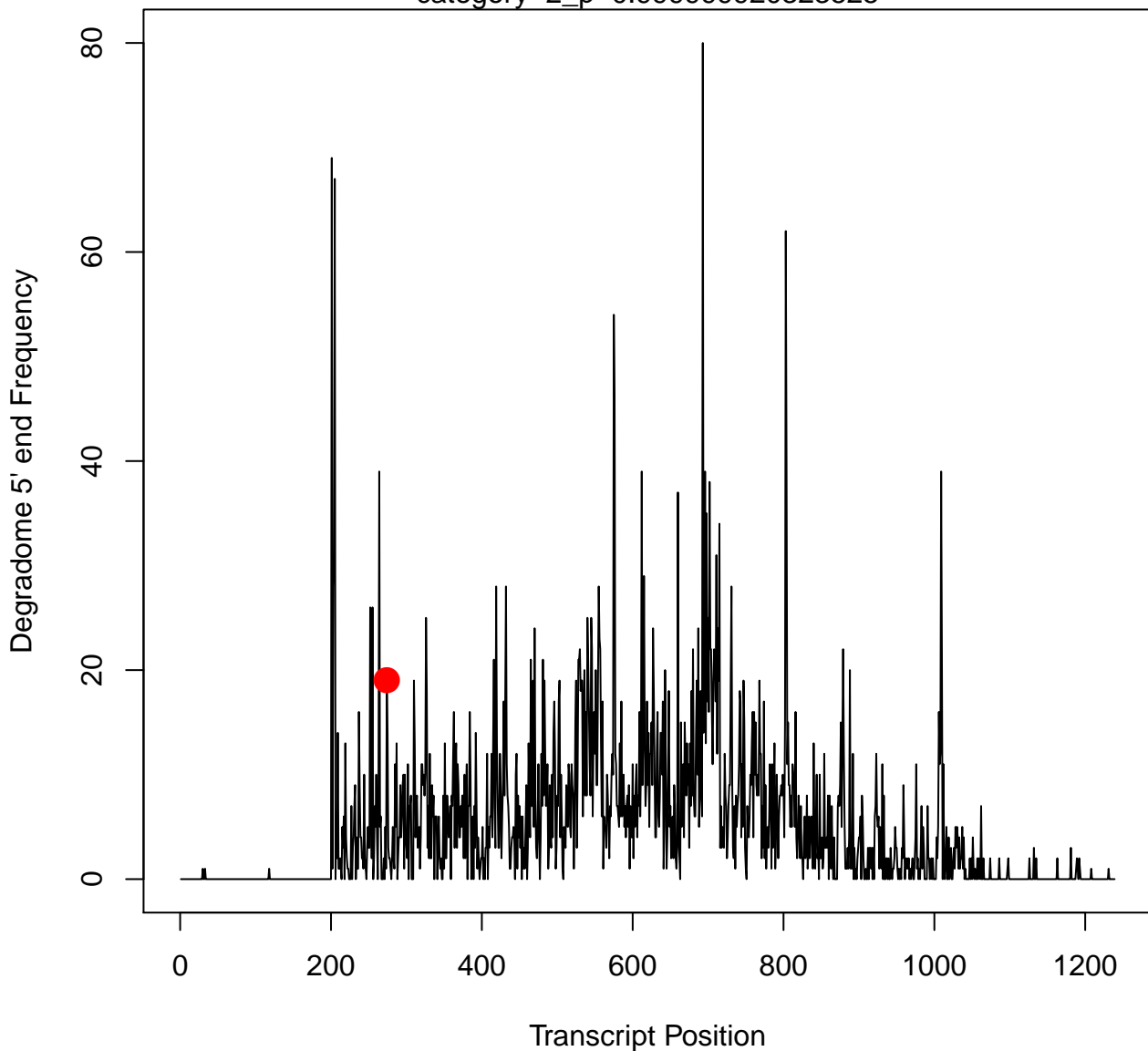

Supplement: Supplementary file 3 [file Data_Sheet_3.zip › Sit-miR159a_Seita.5G081000.1_274_TPlot.pdf]

**T=Seita.5G149100.1\_Q=Sit-miR159a\_S=1902**

category=0\_p=0.0103814941065016

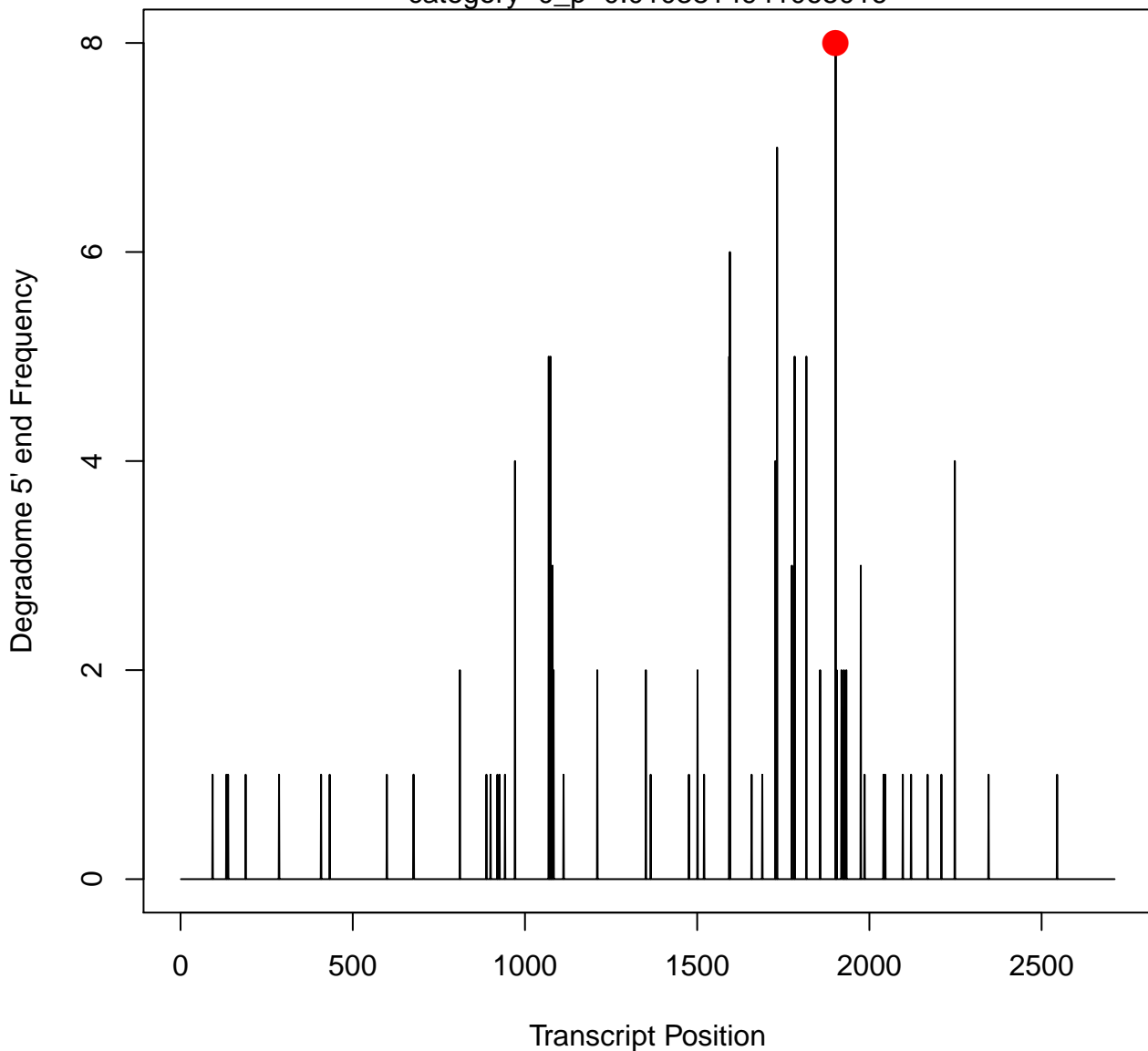

Supplement: Supplementary file 3 [file Data_Sheet_3.zip › Sit-miR159a_Seita.5G149100.1_1902_TPlot.pdf]

**T=Seita.5G166700.1\_Q=Sit-miR159a\_S=1718**

category=2\_p=0.999999994420524

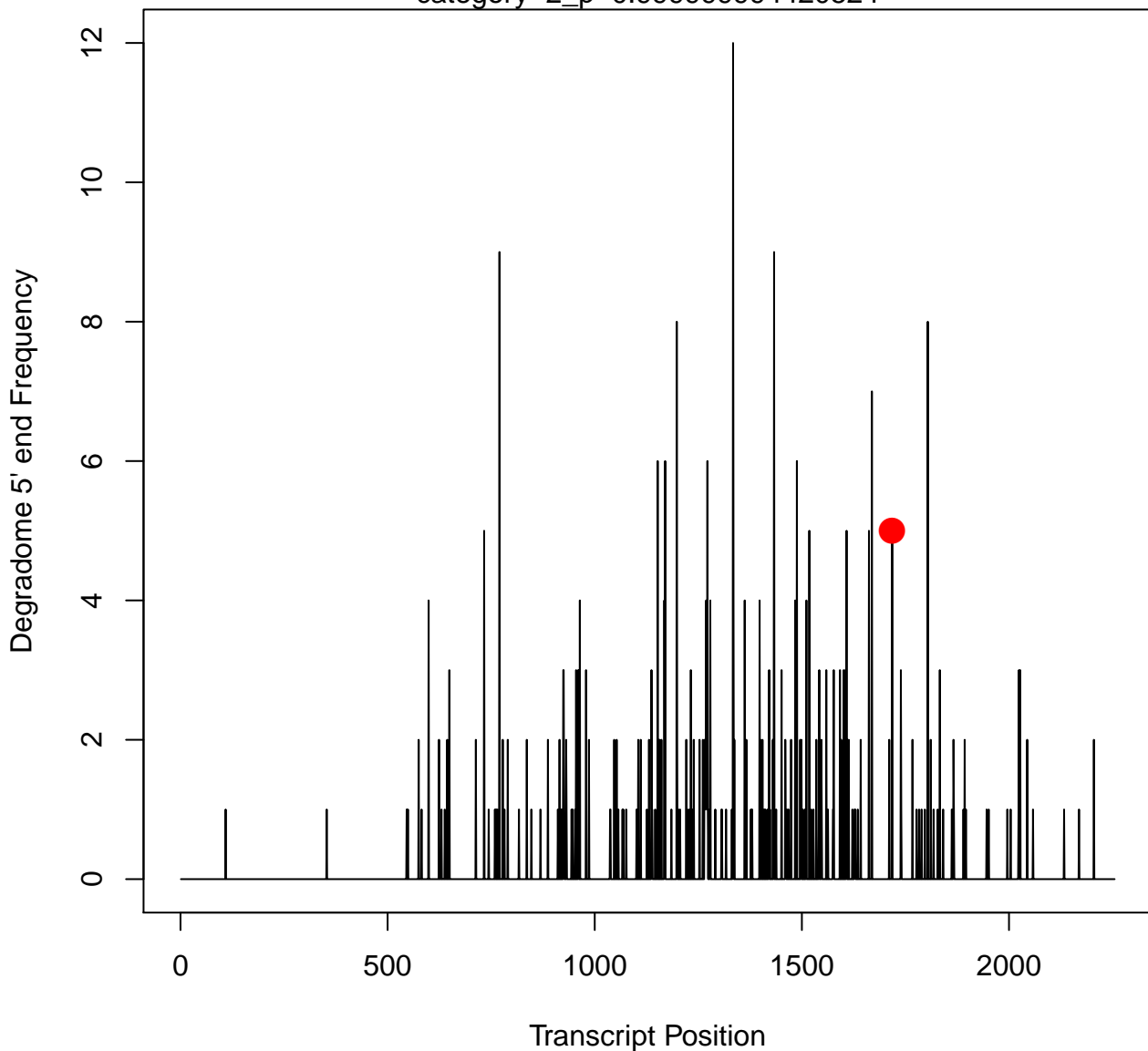

Supplement: Supplementary file 3 [file Data_Sheet_3.zip › Sit-miR159a_Seita.5G166700.1_1718_TPlot.pdf]

**T=Seita.5G263700.1\_Q=Sit-miR159a\_S=7179**

category=2\_p=0.999981378237048

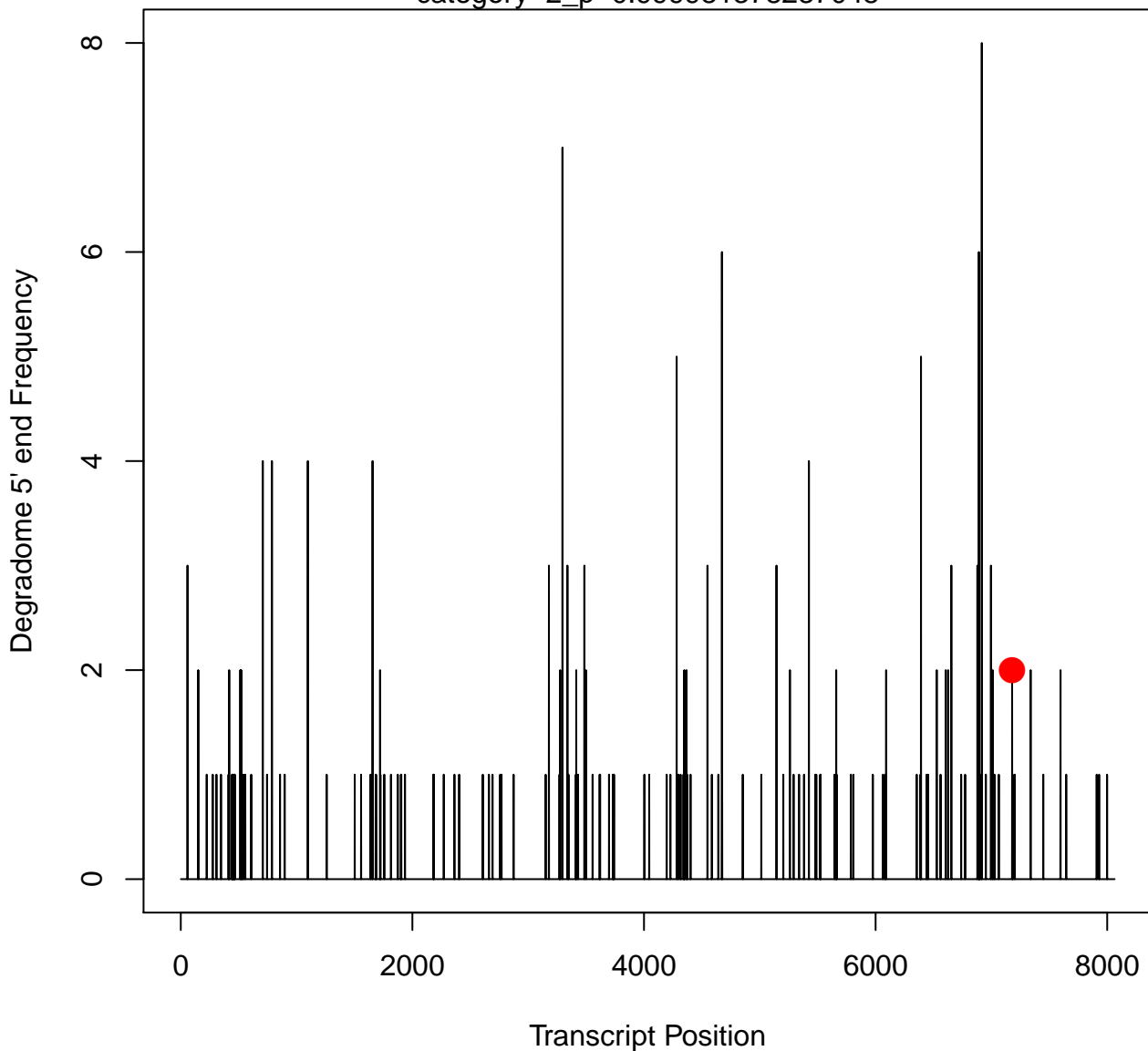

Supplement: Supplementary file 3 [file Data_Sheet_3.zip › Sit-miR159a_Seita.5G263700.1_7179_TPlot.pdf]

**T=Seita.5G301800.1\_Q=Sit-miR159a\_S=1762**

category=2\_p=0.999977917798028

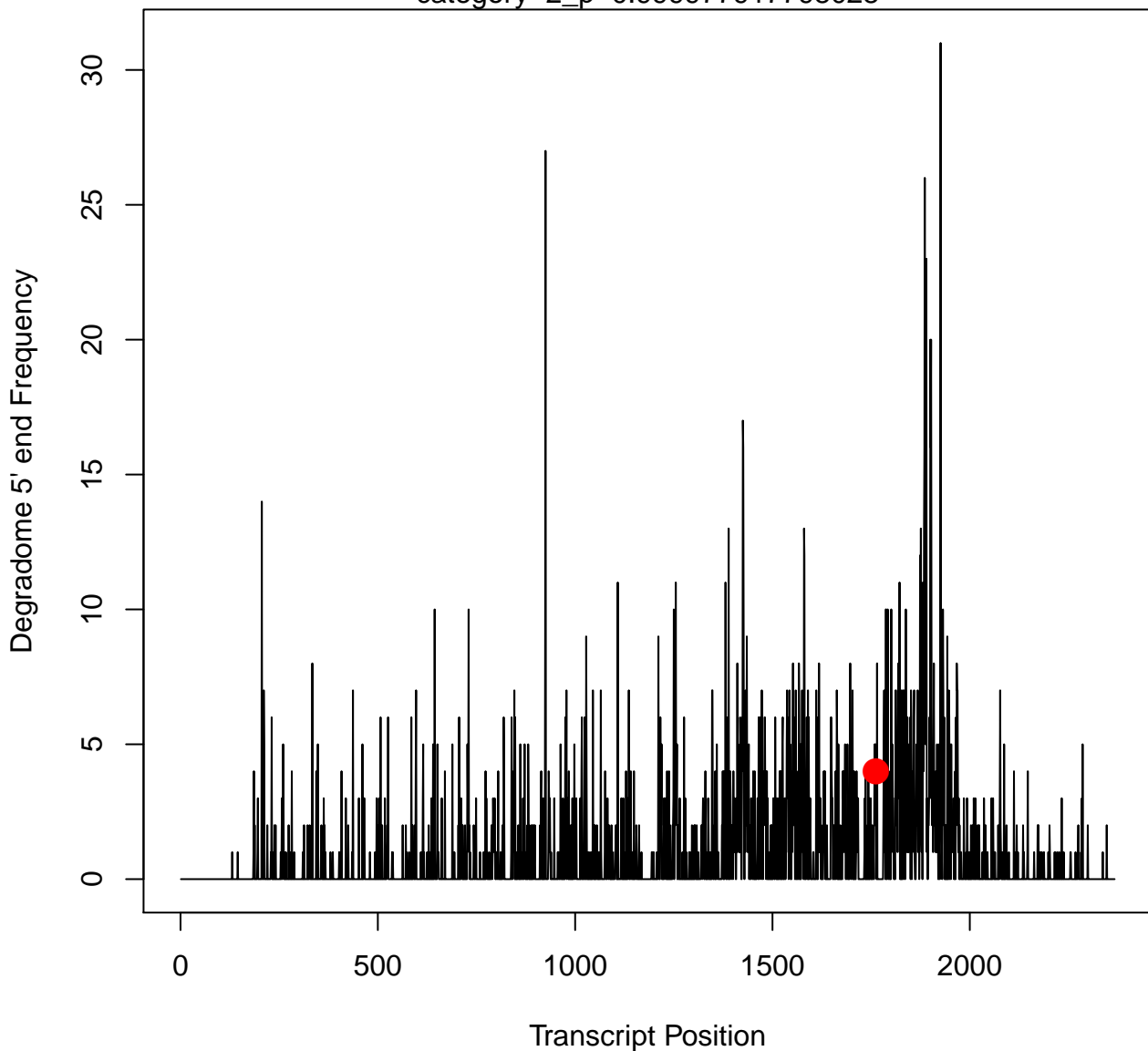

Supplement: Supplementary file 3 [file Data_Sheet_3.zip › Sit-miR159a_Seita.5G301800.1_1762_TPlot.pdf]

**T=Seita.7G320500.1\_Q=Sit-miR159a\_S=340**

category=2\_p=0.99840784944843

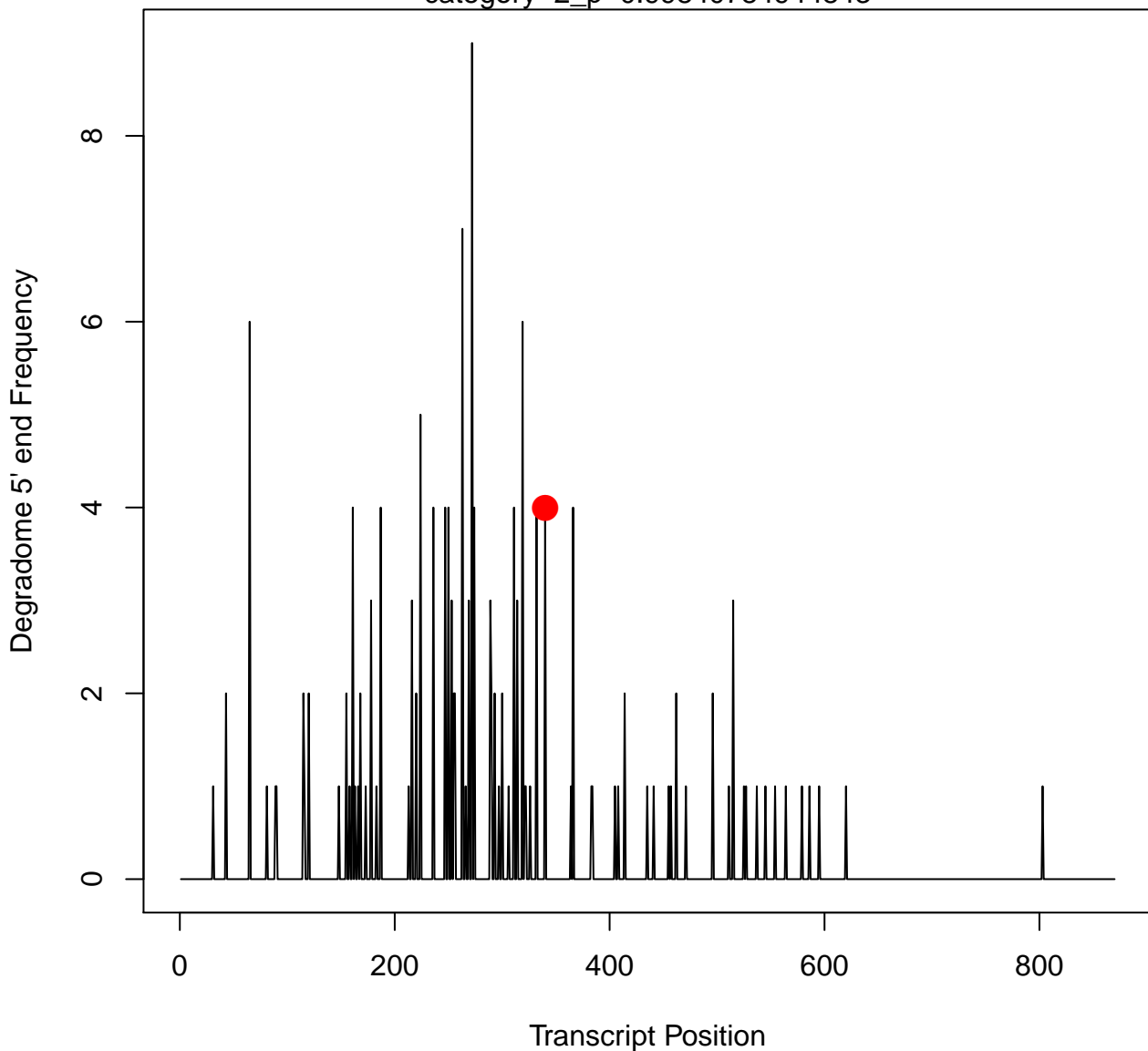

Supplement: Supplementary file 3 [file Data_Sheet_3.zip › Sit-miR159a_Seita.7G320500.1_340_TPlot.pdf]

**T=Seita.8G014200.1\_Q=Sit-miR159a\_S=446**

category=2\_p=0.999517126863758

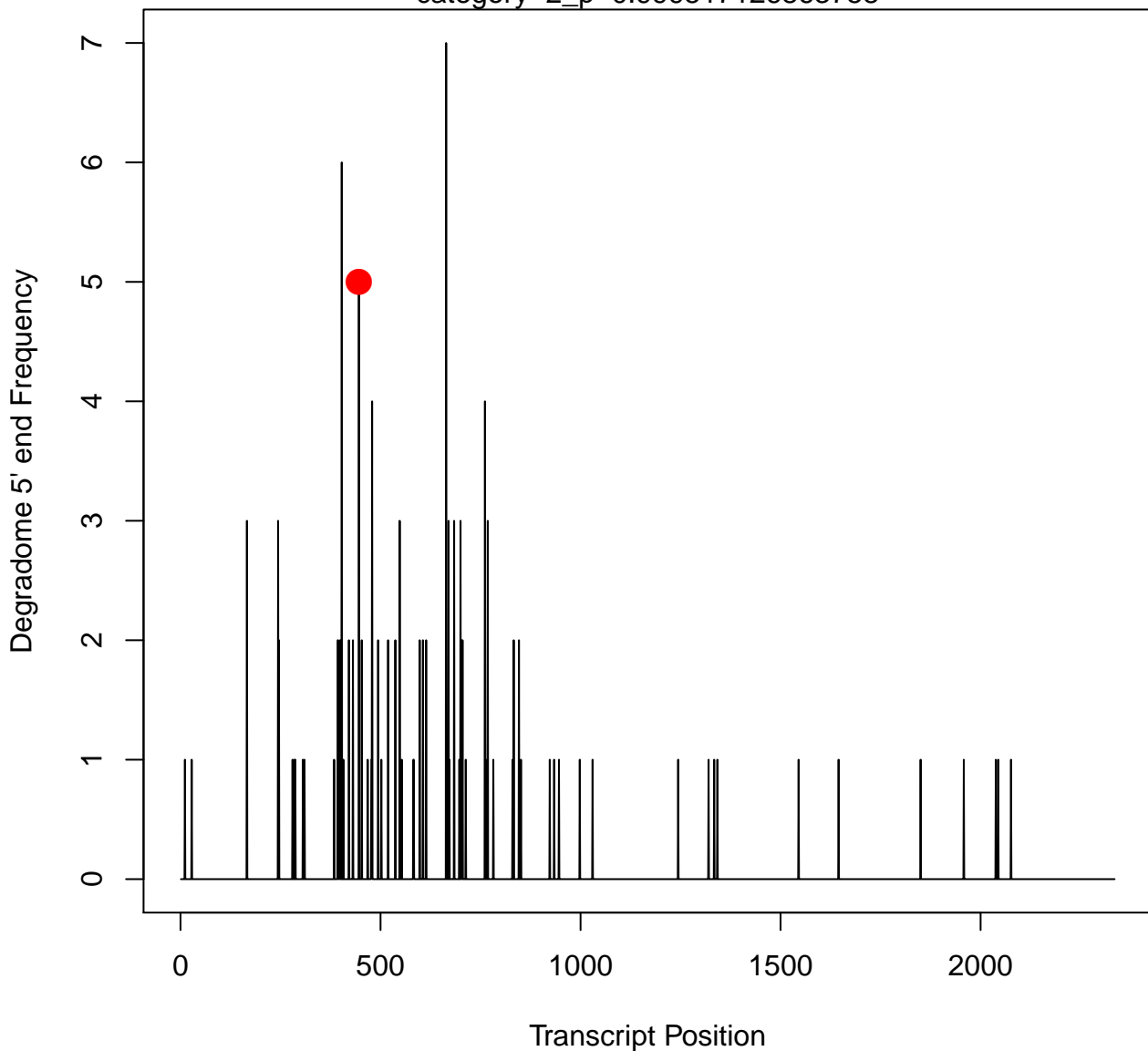

Supplement: Supplementary file 3 [file Data_Sheet_3.zip › Sit-miR159a_Seita.8G014200.1_446_TPlot.pdf]

**T=Seita.8G199800.1\_Q=Sit-miR159a\_S=3572**

category=2\_p=0.992097090302011

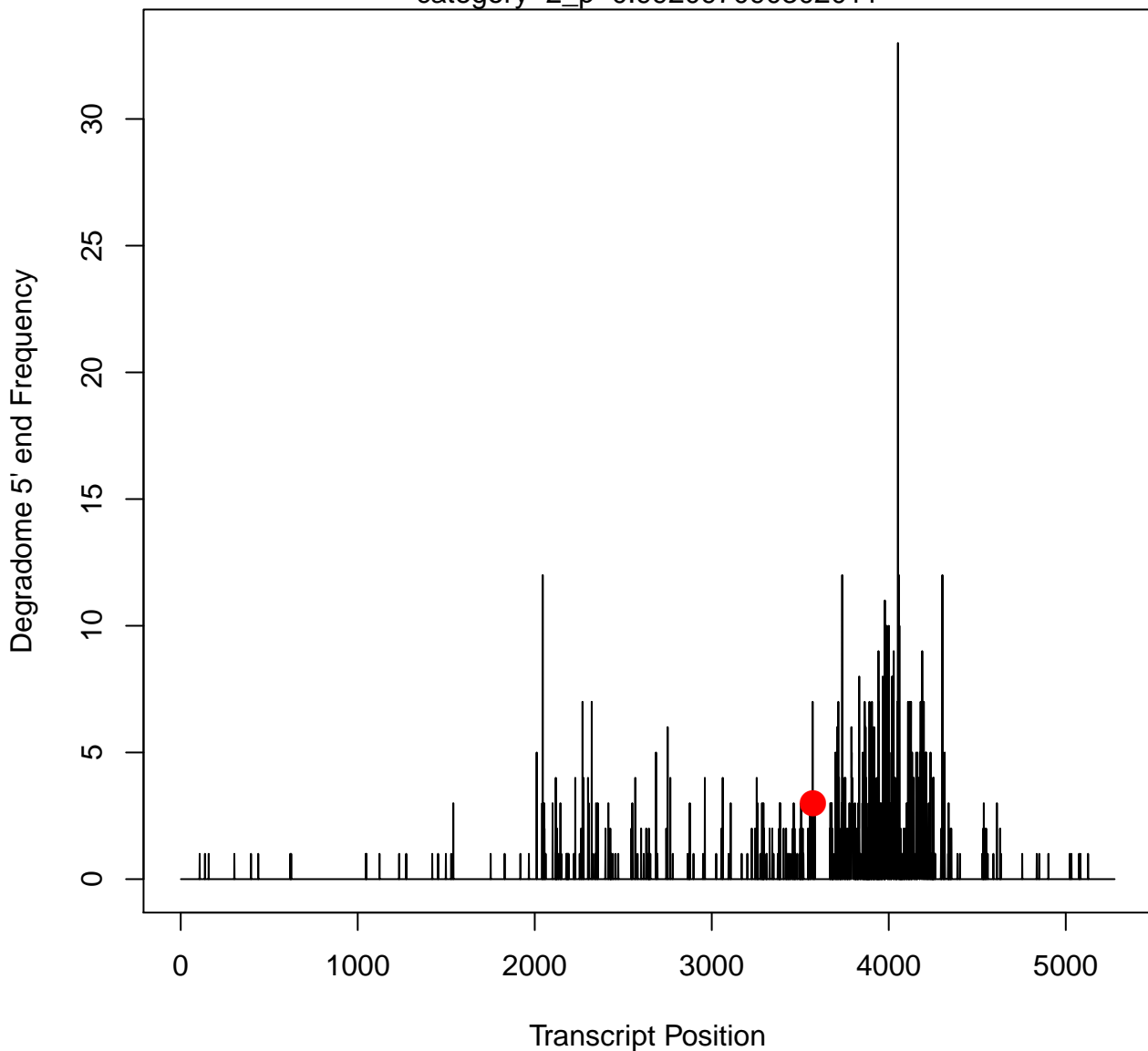

Supplement: Supplementary file 3 [file Data_Sheet_3.zip › Sit-miR159a_Seita.8G199800.1_3572_TPlot.pdf]

**T=Seita.9G503600.1\_Q=Sit-miR159a\_S=368**

category=2\_p=0.999999999647269

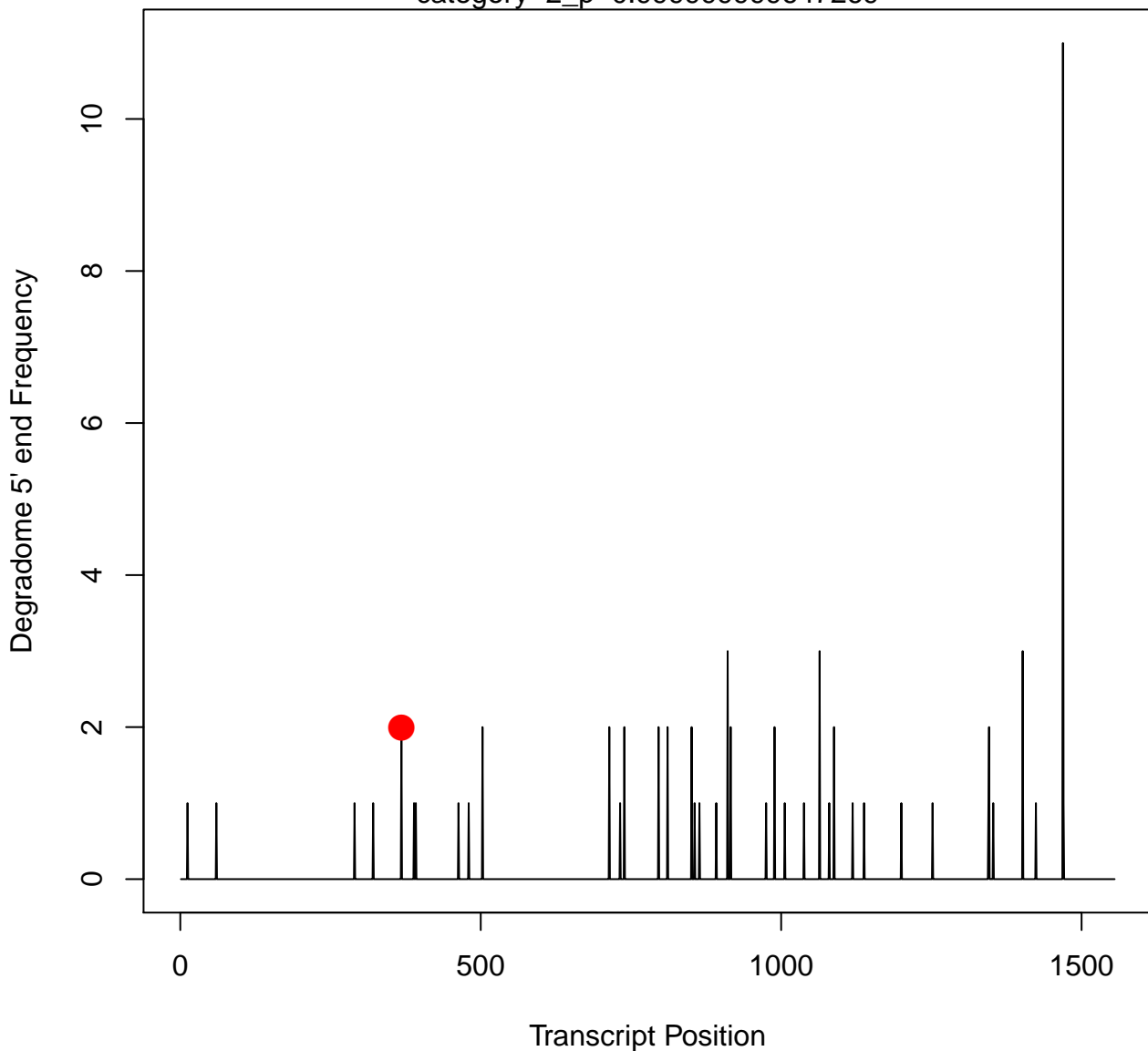

Supplement: Supplementary file 3 [file Data_Sheet_3.zip › Sit-miR159a_Seita.9G503600.1_368_TPlot.pdf]

**T=Seita.9G569300.1\_Q=Sit-miR159a\_S=1398**

category=2\_p=0.998977818506797

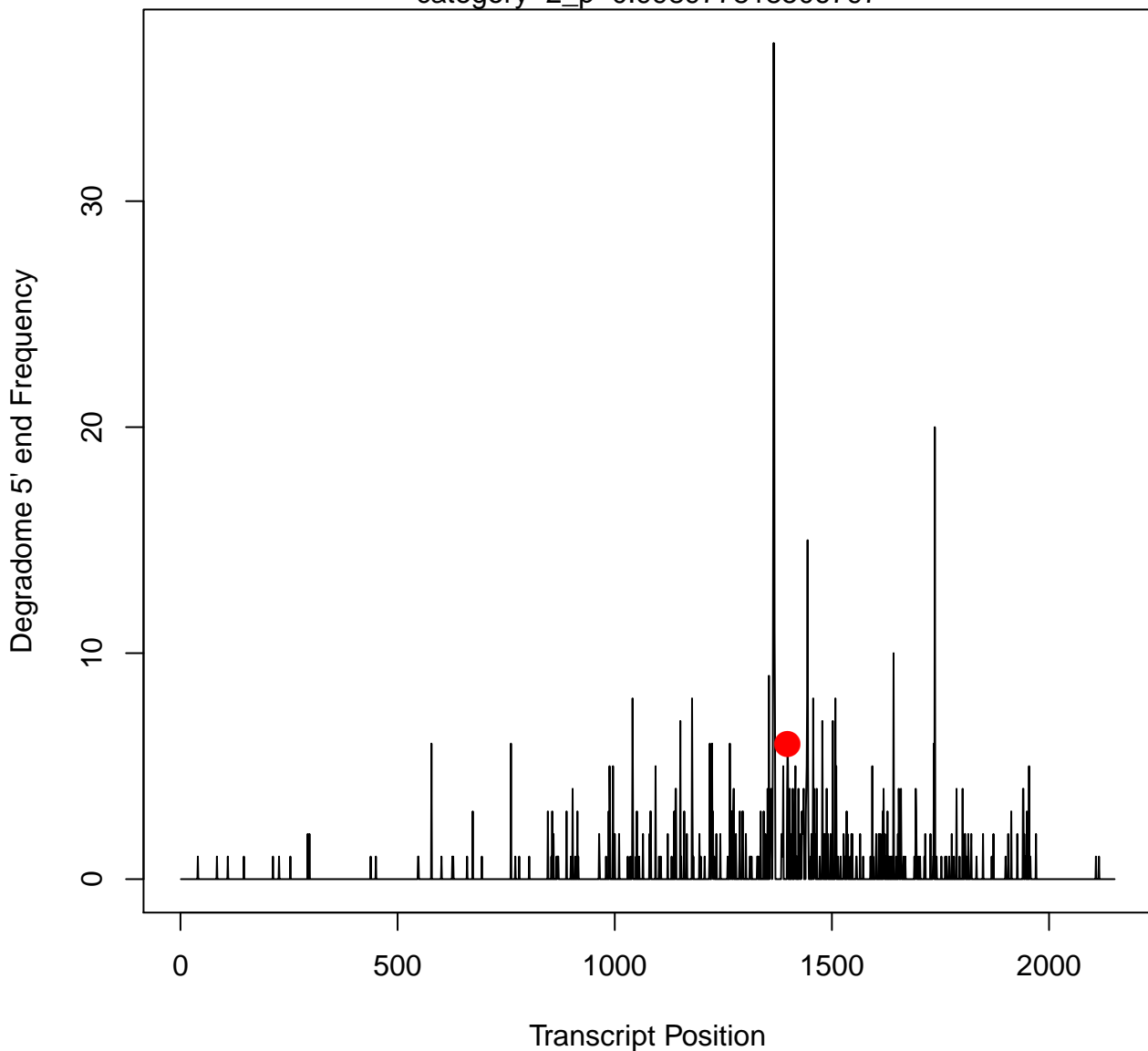

Supplement: Supplementary file 3 [file Data_Sheet_3.zip › Sit-miR159a_Seita.9G569300.1_1398_TPlot.pdf]

**T=Seita.2G434400.1\_Q=Sit-miR159b\_S=363**

category=2\_p=0.467744522055191

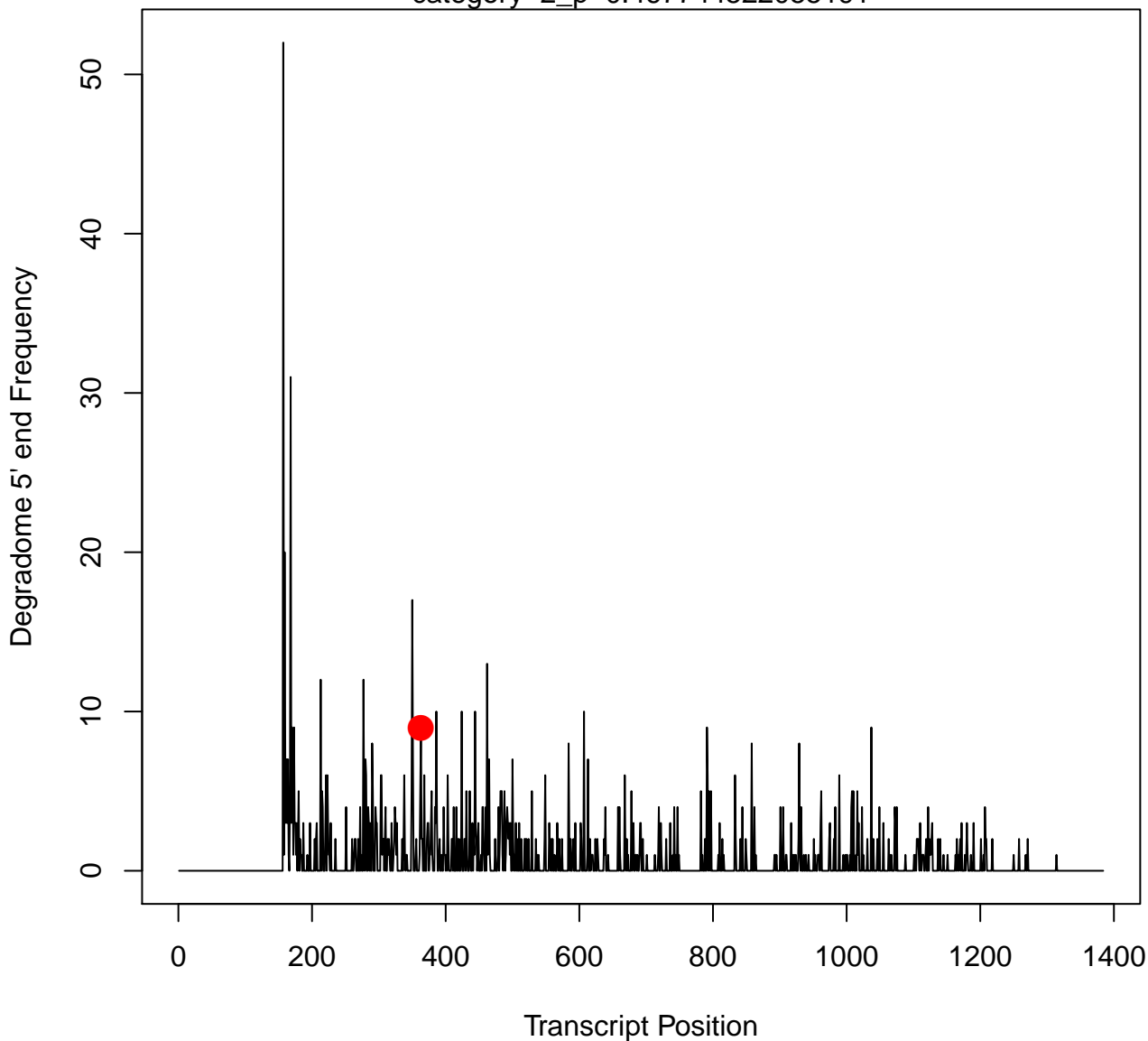

Supplement: Supplementary file 3 [file Data_Sheet_3.zip › Sit-miR159b_Seita.2G434400.1_363_TPlot.pdf]

**T=Seita.4G069500.1\_Q=Sit-miR159b\_S=1936**

category=2\_p=0.993879954600783

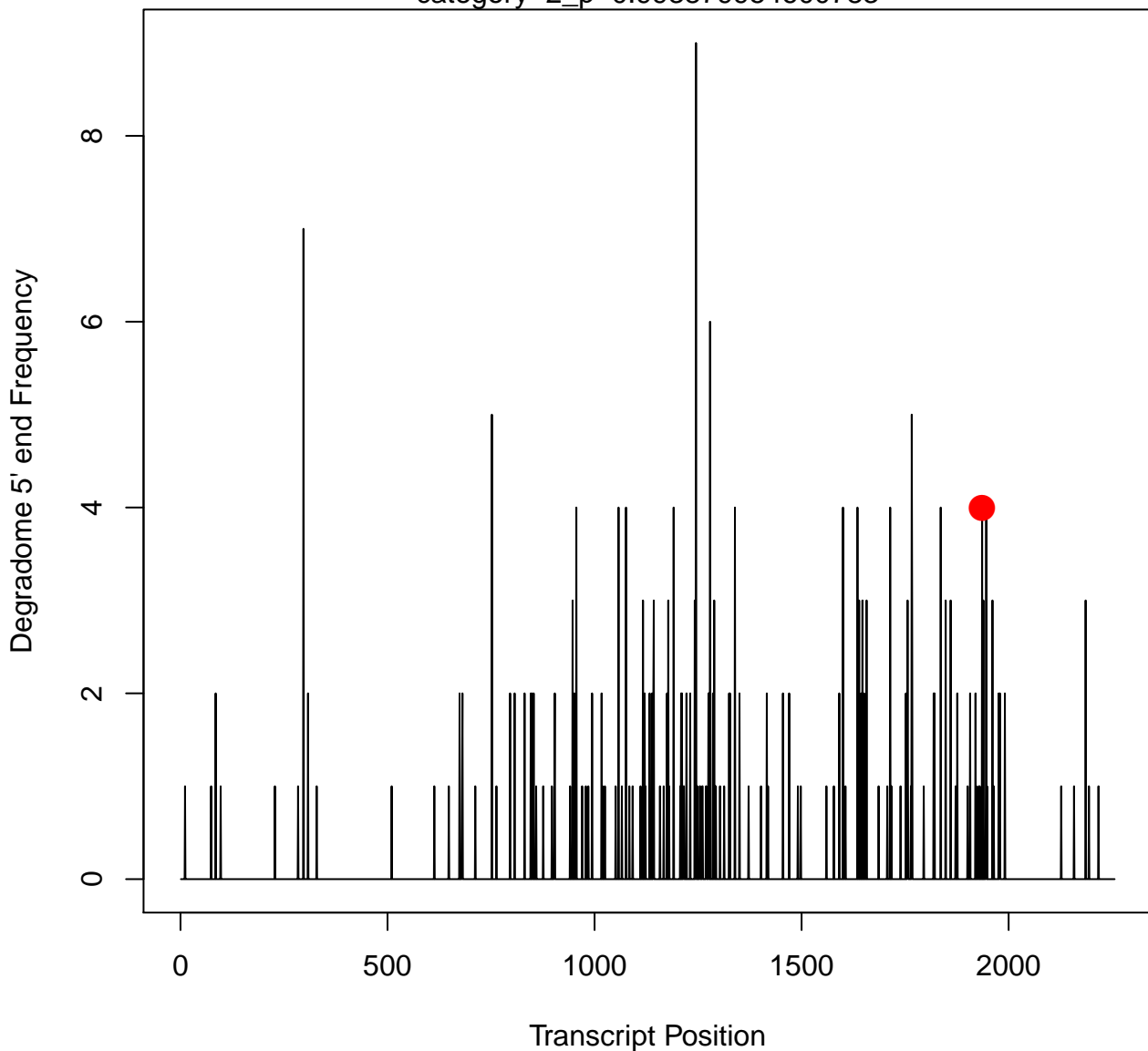

Supplement: Supplementary file 3 [file Data_Sheet_3.zip › Sit-miR159b_Seita.4G069500.1_1936_TPlot.pdf]

**T=Seita.4G131700.1\_Q=Sit-miR159b\_S=1283**

category=2\_p=0.981782332123804

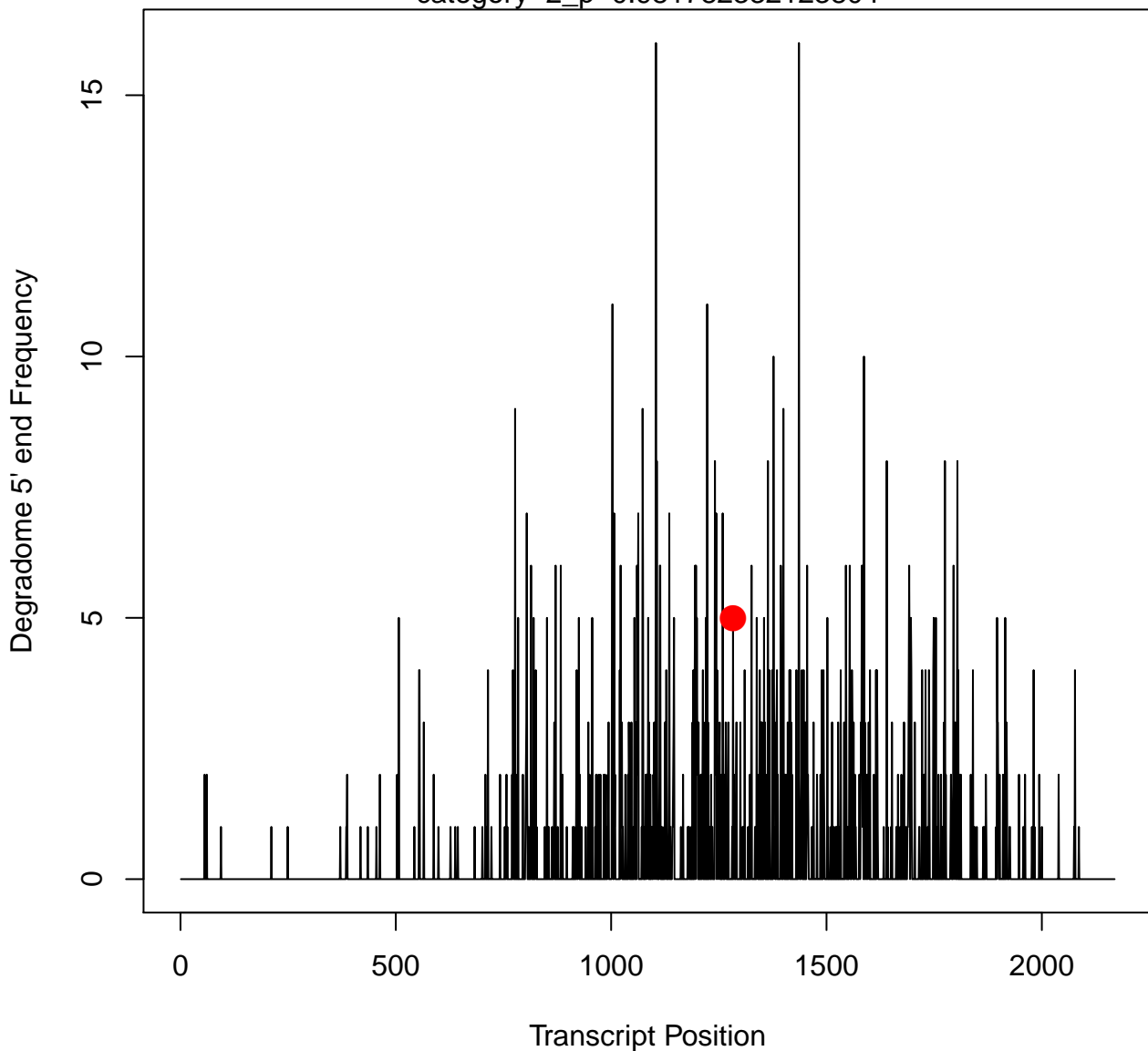

Supplement: Supplementary file 3 [file Data_Sheet_3.zip › Sit-miR159b_Seita.4G131700.1_1283_TPlot.pdf]

**T=Seita.4G265500.1\_Q=Sit-miR159b\_S=937**

category=2\_p=0.983553259883742

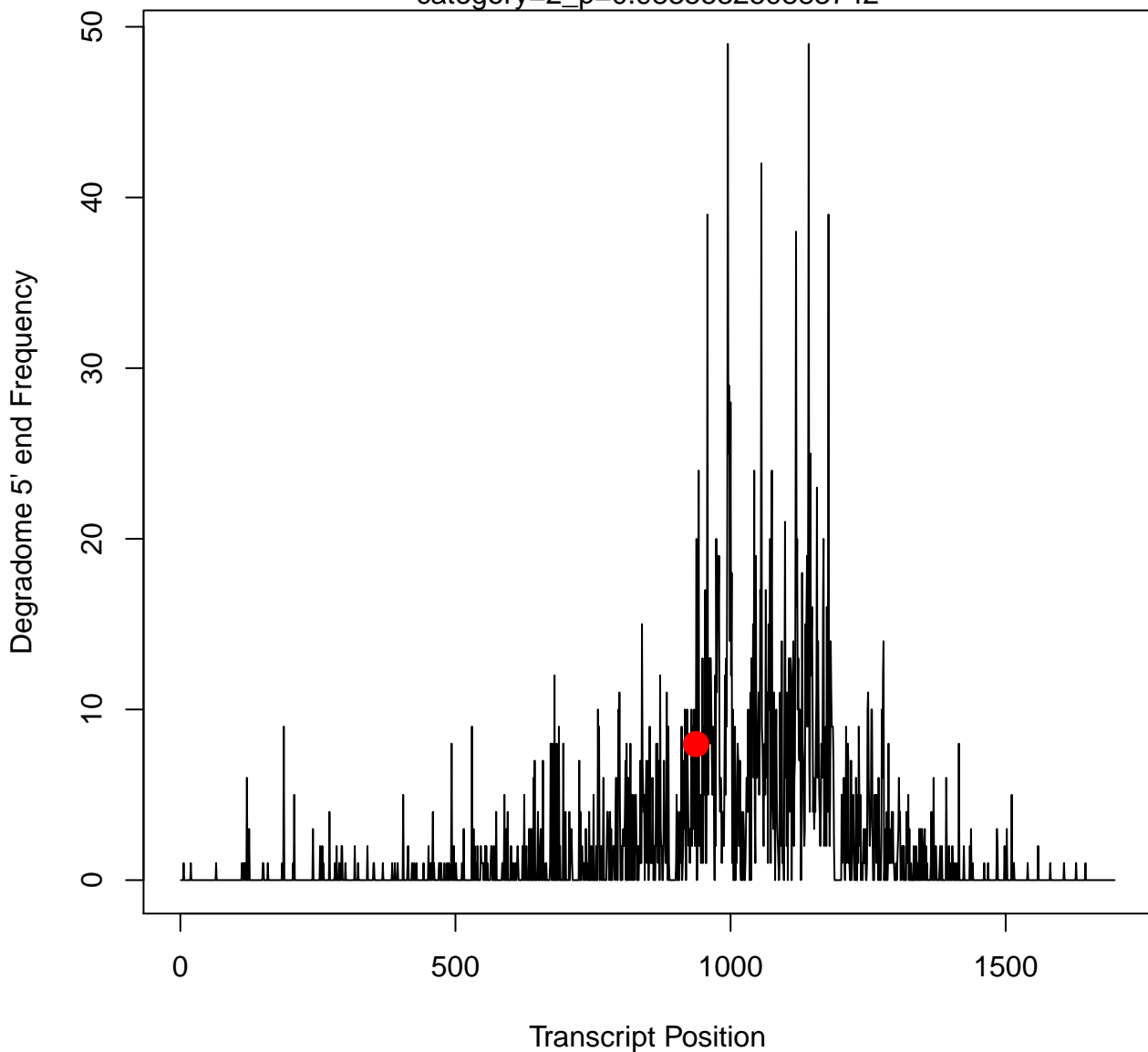

Supplement: Supplementary file 3 [file Data_Sheet_3.zip › Sit-miR159b_Seita.4G265500.1_937_TPlot.pdf]

**T=Seita.5G306500.1\_Q=Sit-miR159b\_S=3489**

category=2\_p=0.960098238741005

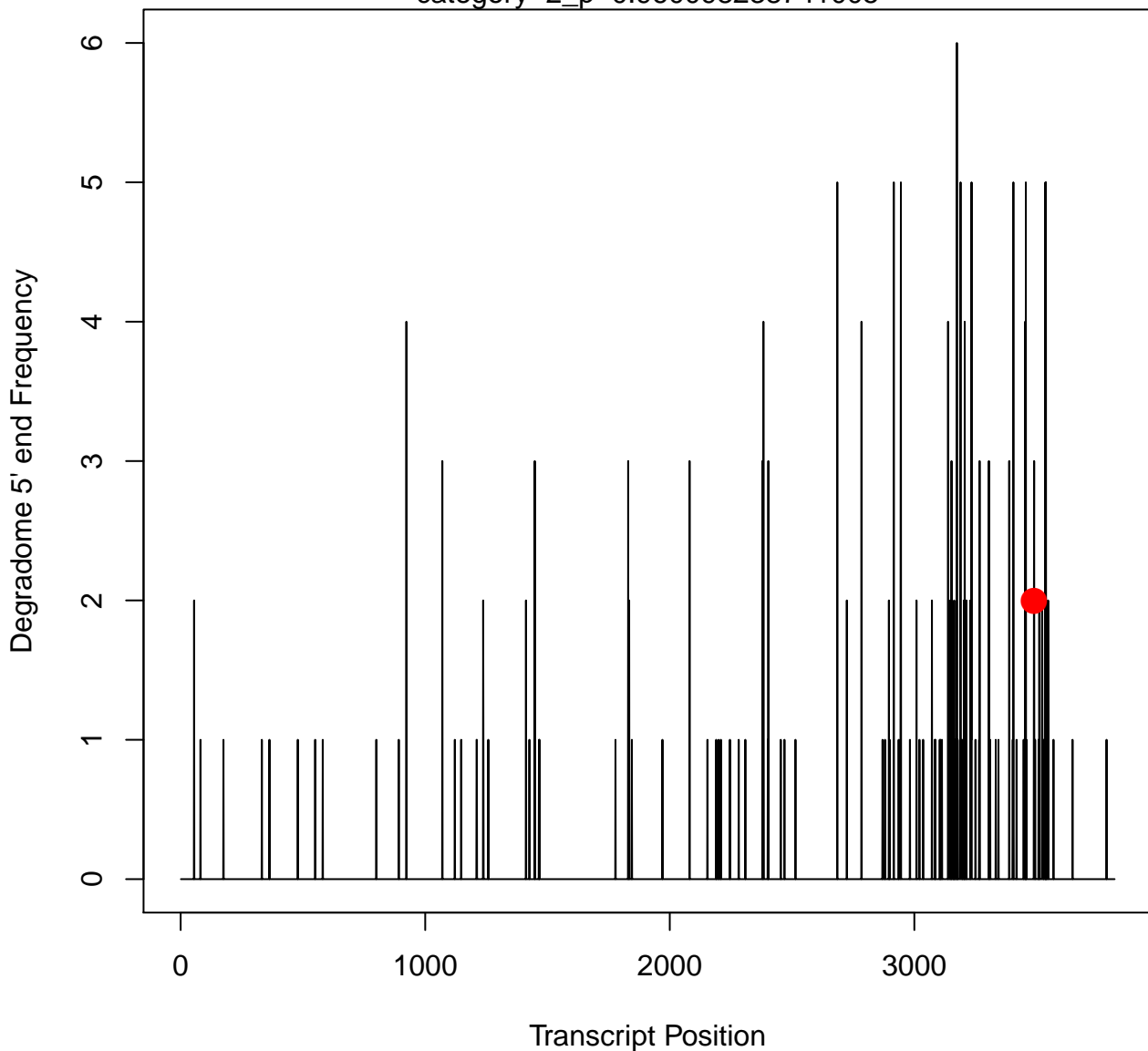

Supplement: Supplementary file 3 [file Data_Sheet_3.zip › Sit-miR159b_Seita.5G306500.1_3489_TPlot.pdf]

**T=Seita.5G317100.1\_Q=Sit-miR159b\_S=838**

category=2\_p=0.999965013487462

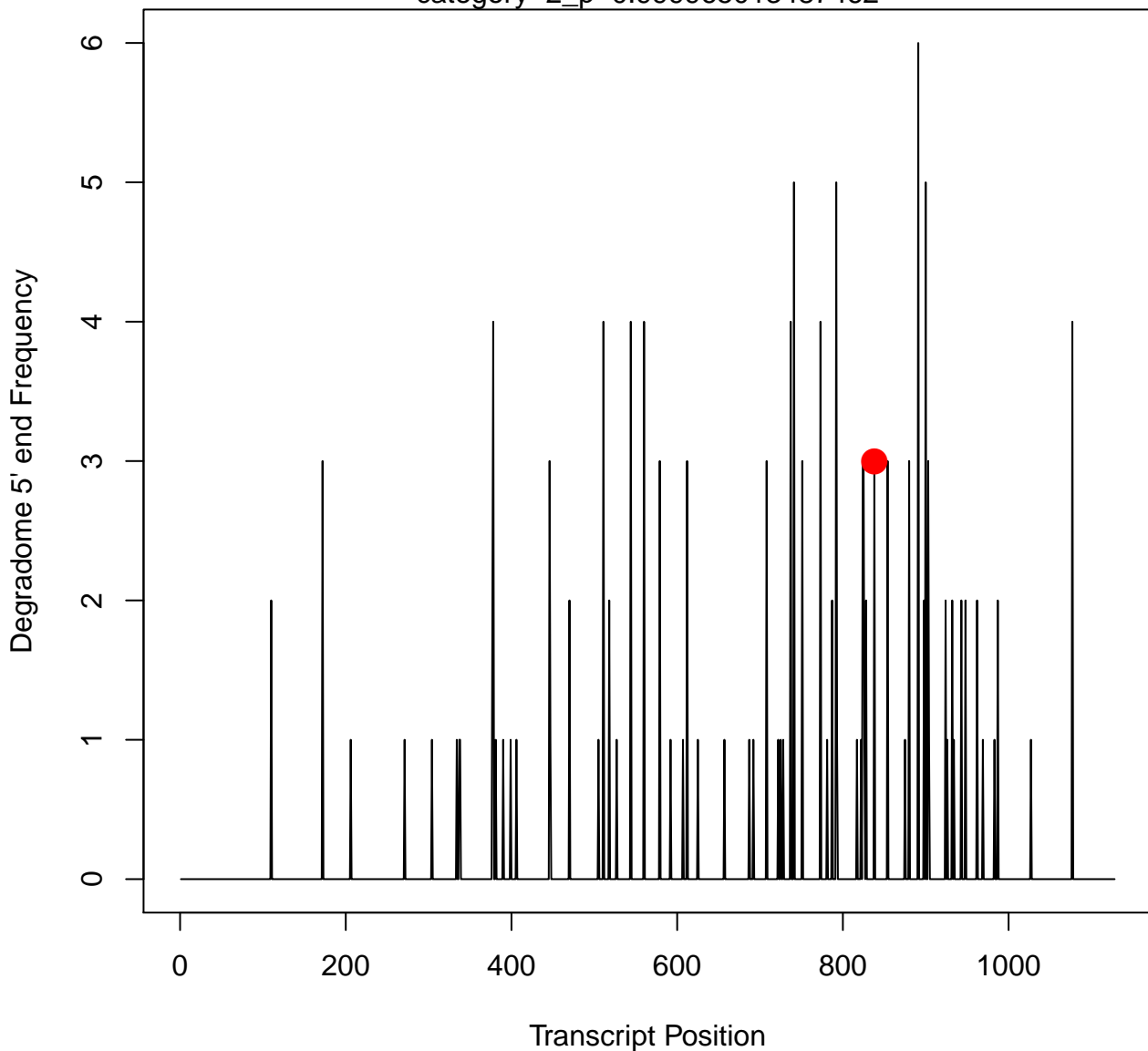

Supplement: Supplementary file 3 [file Data_Sheet_3.zip › Sit-miR159b_Seita.5G317100.1_838_TPlot.pdf]

**T=Seita.4G085800.1\_Q=Sit-miR159c\_S=367**

category=2\_p=0.87499232513391

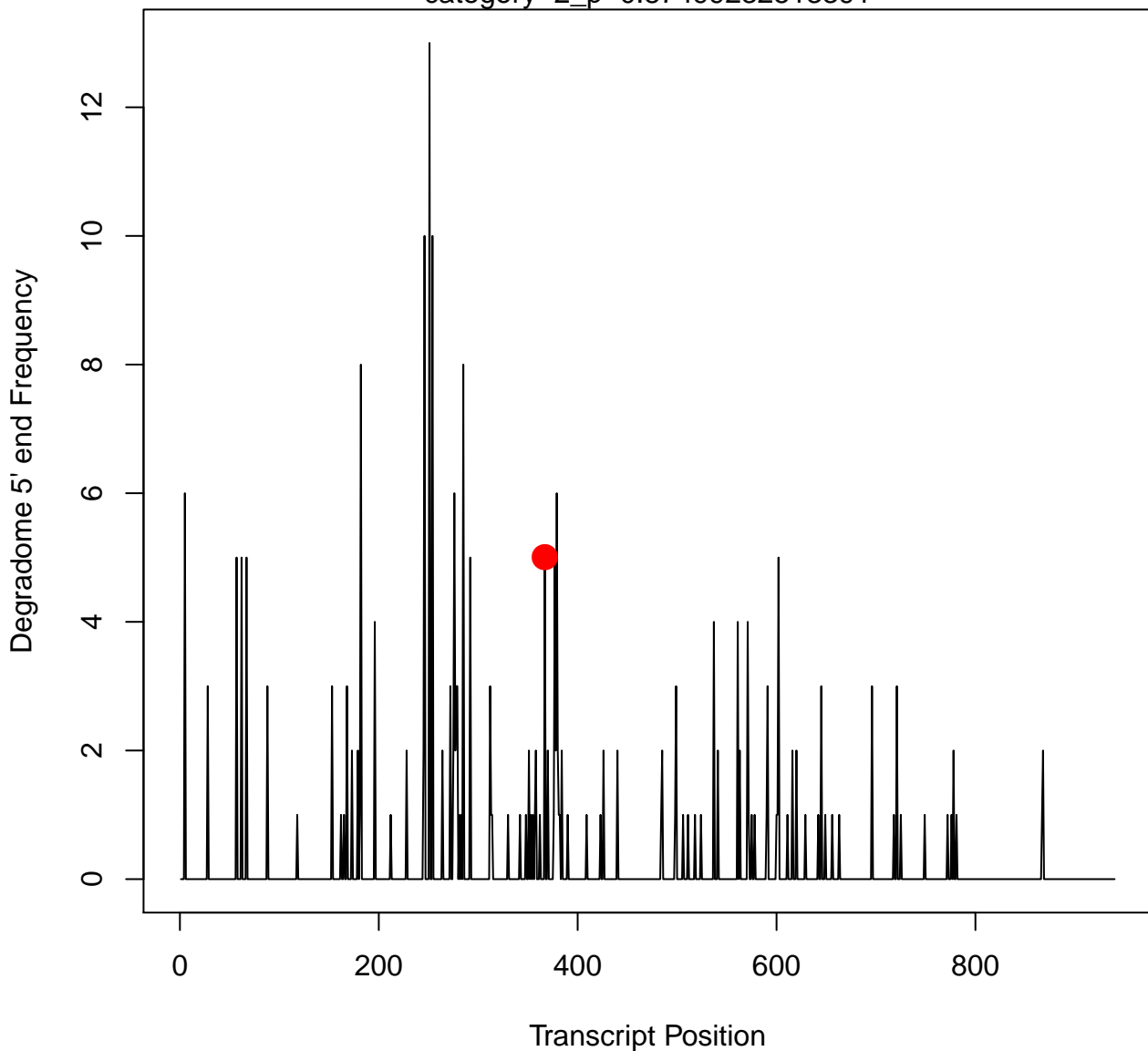

Supplement: Supplementary file 3 [file Data_Sheet_3.zip › Sit-miR159c_Seita.4G085800.1_367_TPlot.pdf]

**T=Seita.4G221900.1\_Q=Sit-miR159c\_S=1159**

category=0\_p=0.000802428474349082

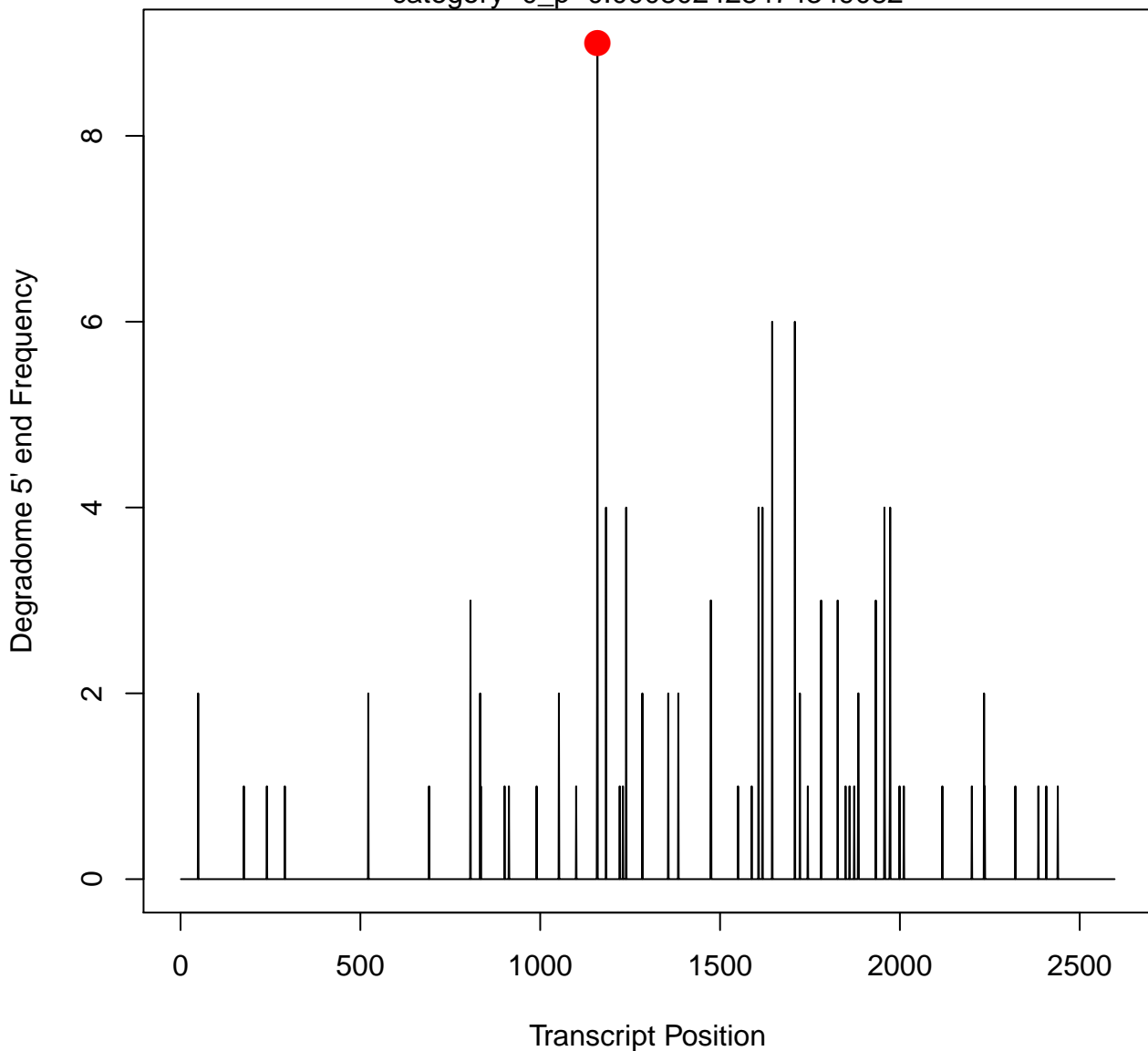

Supplement: Supplementary file 3 [file Data_Sheet_3.zip › Sit-miR159c_Seita.4G221900.1_1159_TPlot.pdf]

**T=Seita.5G221400.1\_Q=Sit-miR159c\_S=193**

category=2\_p=0.999260587131799

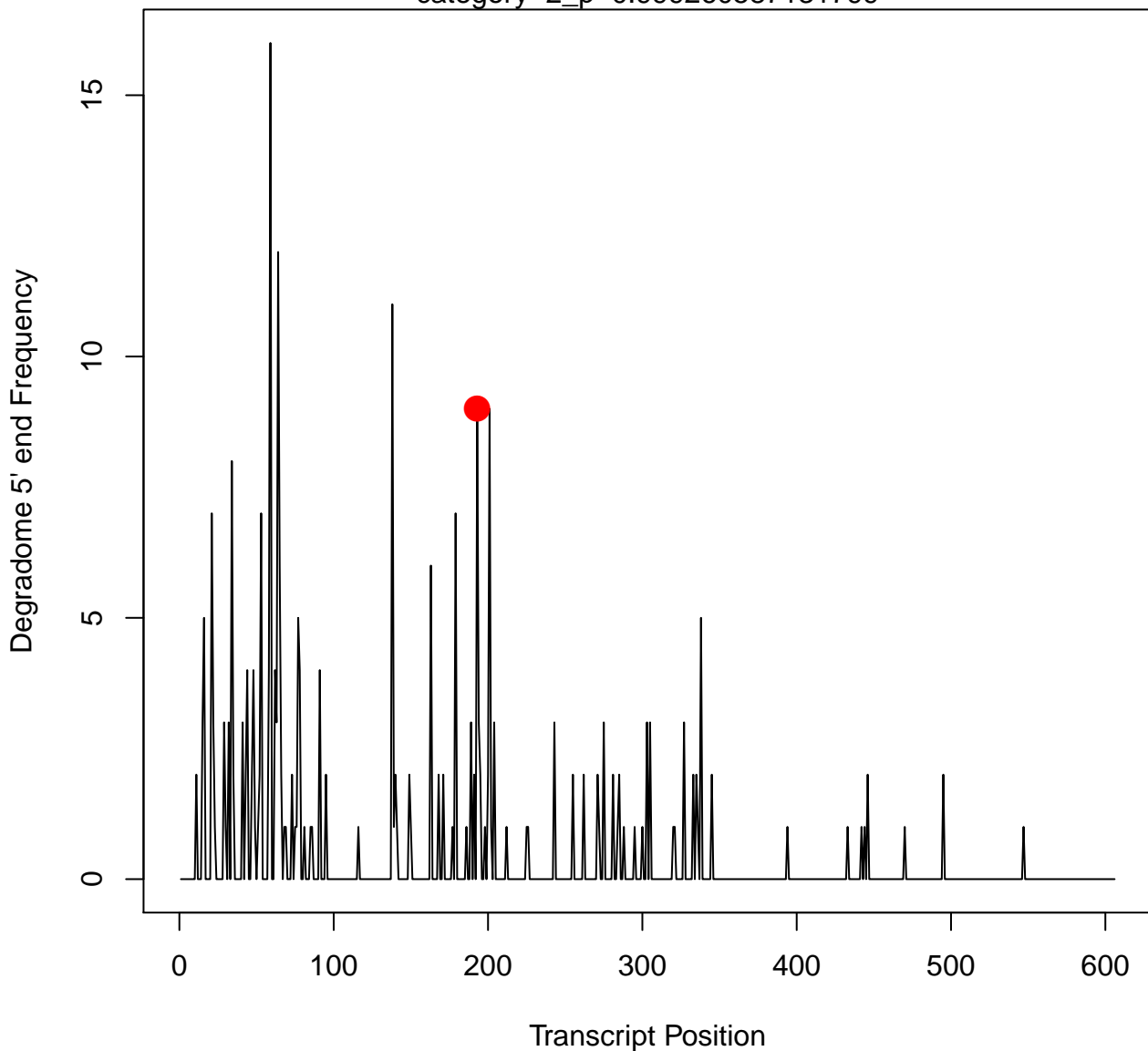

Supplement: Supplementary file 3 [file Data_Sheet_3.zip › Sit-miR159c_Seita.5G221400.1_193_TPlot.pdf]

**T=Seita.5G355300.1\_Q=Sit-miR159c\_S=1278**

category=0\_p=0.00160421305724157

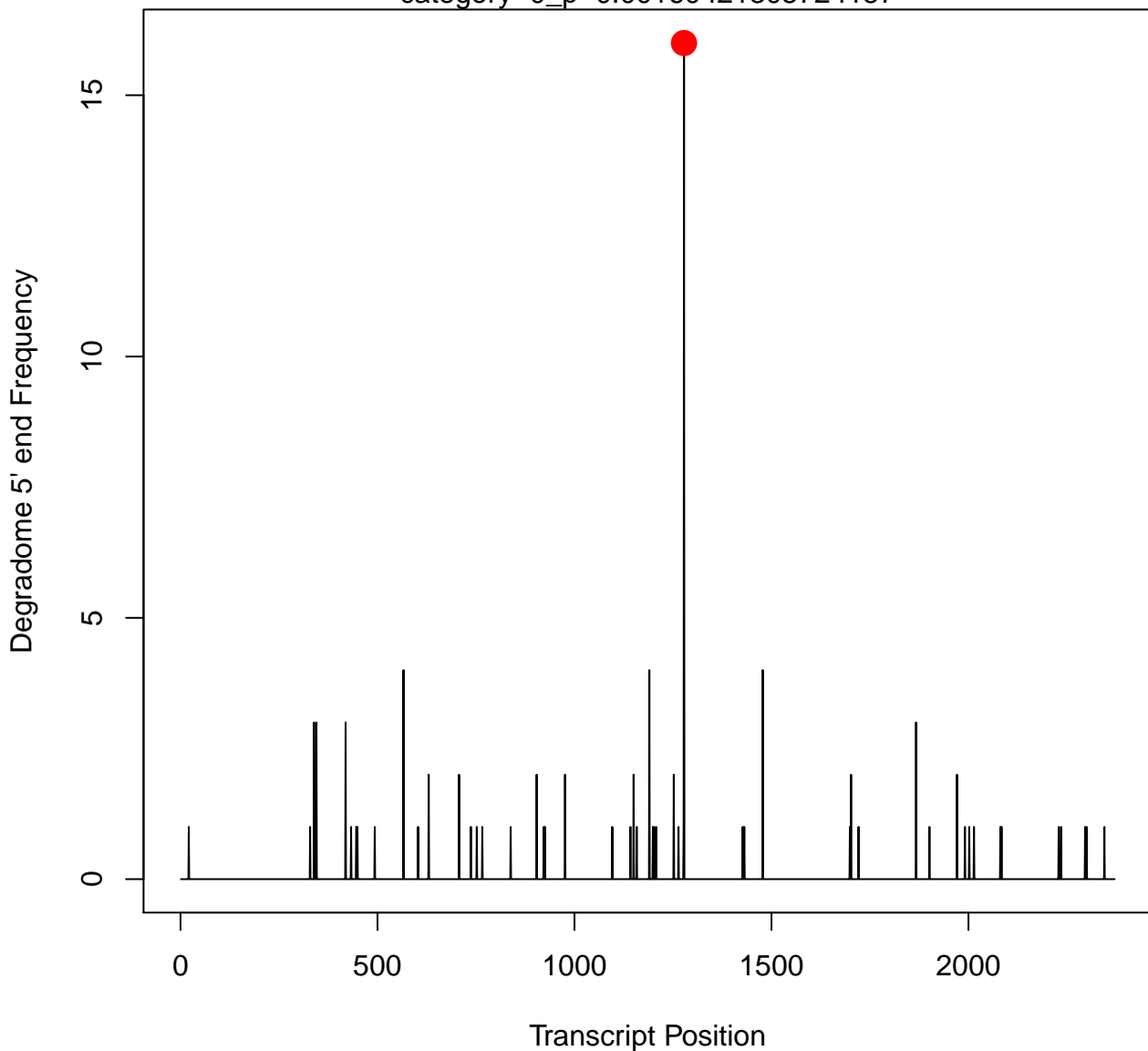

Supplement: Supplementary file 3 [file Data_Sheet_3.zip › Sit-miR159c_Seita.5G355300.1_1278_TPlot.pdf]

**T=Seita.6G015200.1\_Q=Sit-miR159c\_S=270**

category=2\_p=0.980161740258707

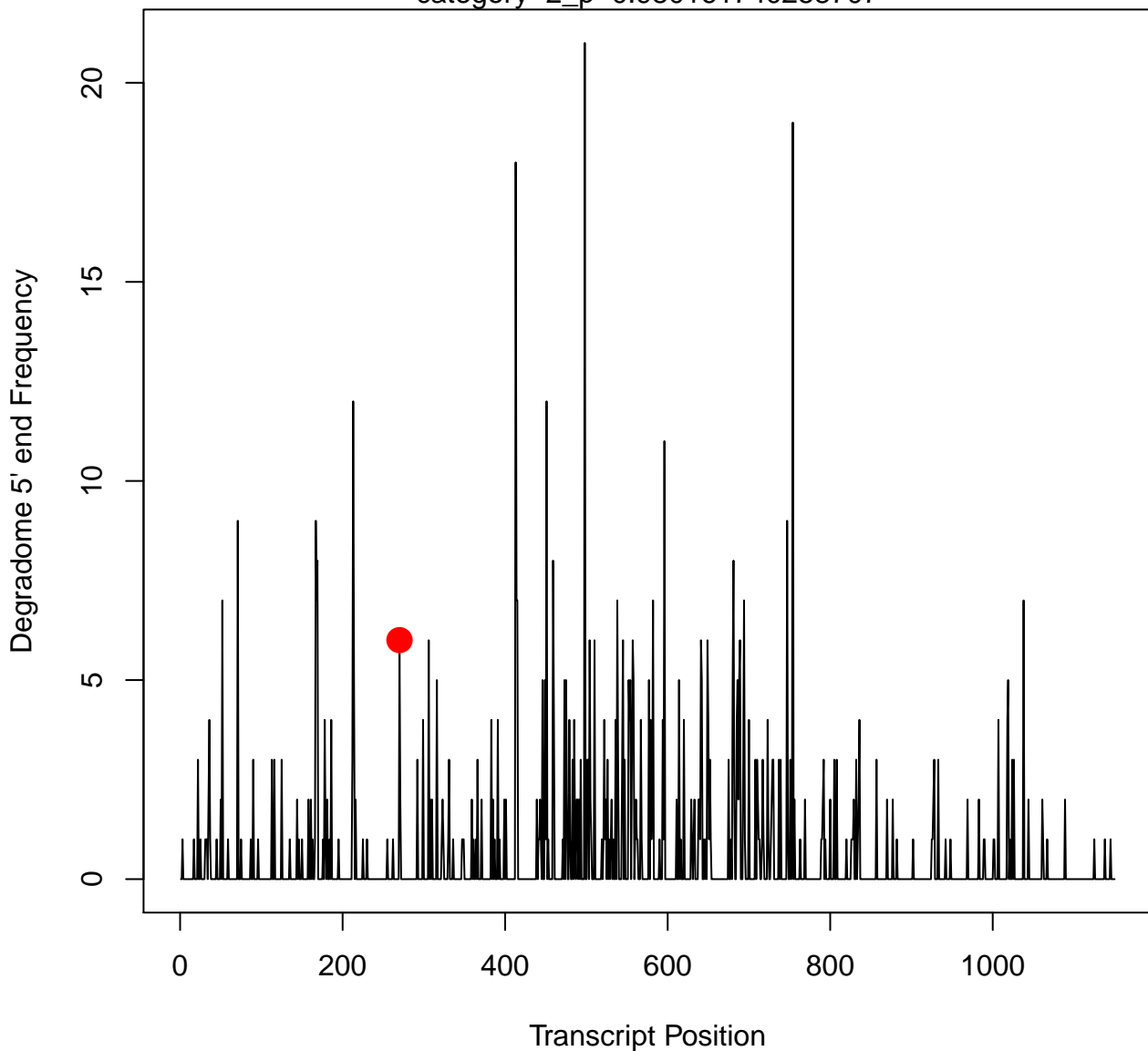

Supplement: Supplementary file 3 [file Data_Sheet_3.zip › Sit-miR159c_Seita.6G015200.1_270_TPlot.pdf]

**T=Seita.9G001800.1\_Q=Sit-miR159c\_S=1099**

category=2\_p=0.744243117558607

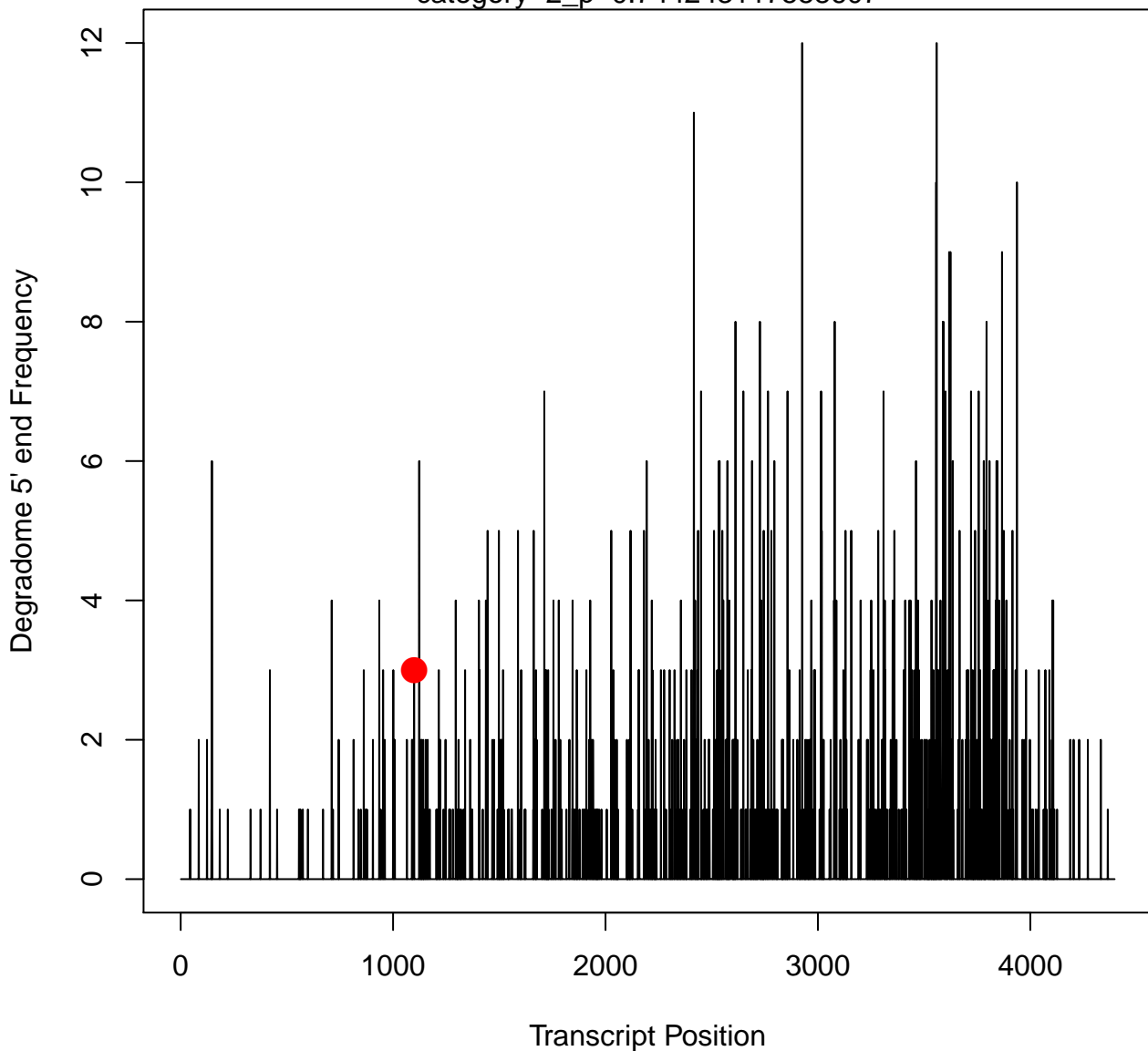

Supplement: Supplementary file 3 [file Data_Sheet_3.zip › Sit-miR159c_Seita.9G001800.1_1099_TPlot.pdf]

**T=Seita.9G016500.1\_Q=Sit-miR159c\_S=1336**

category=2\_p=0.999738570048009

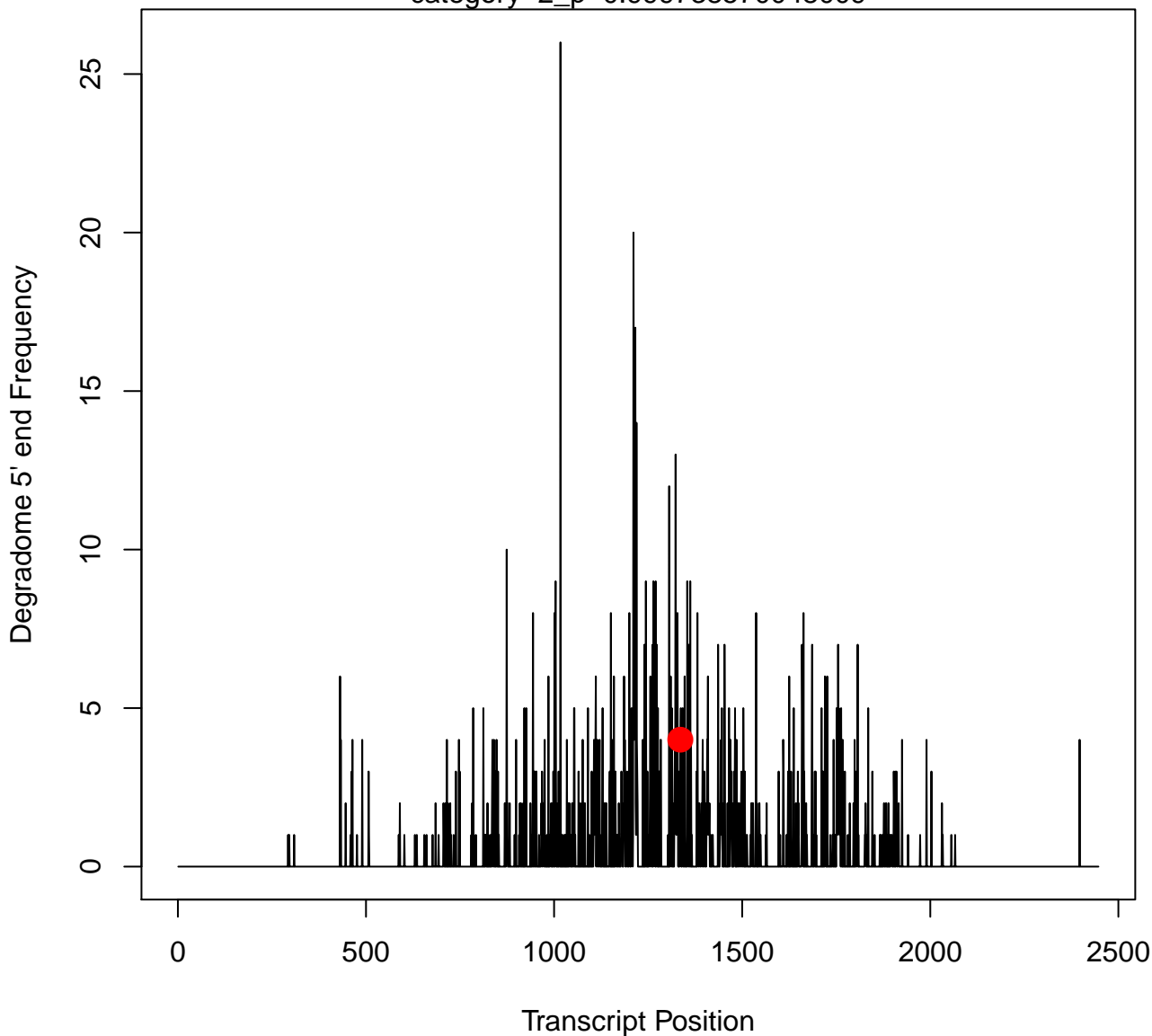

Supplement: Supplementary file 3 [file Data_Sheet_3.zip › Sit-miR159c_Seita.9G016500.1_1336_TPlot.pdf]

**T=Seita.9G181600.1\_Q=Sit-miR159c\_S=1283**

category=0\_p=0.00280568376521684

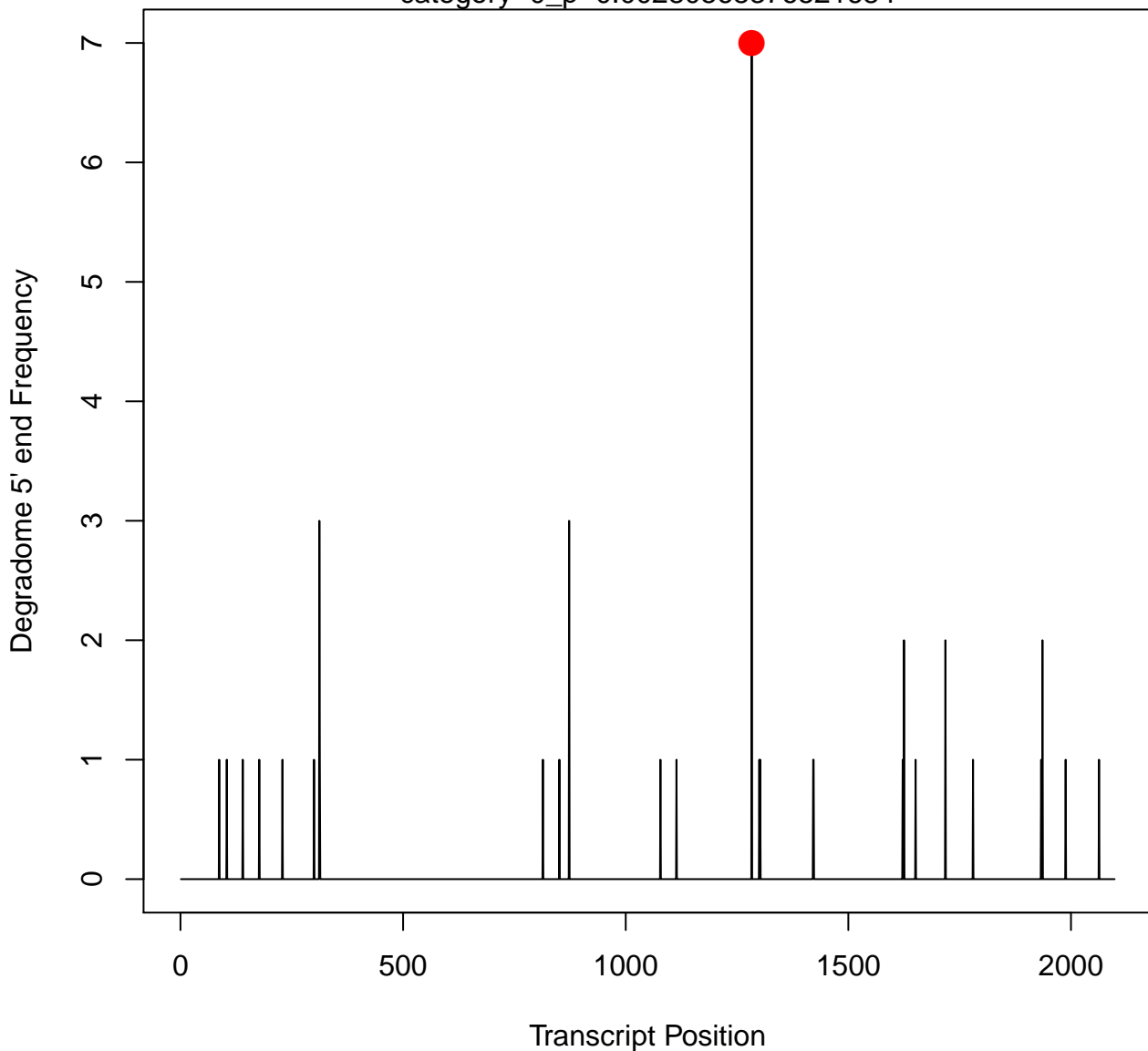

Supplement: Supplementary file 3 [file Data_Sheet_3.zip › Sit-miR159c_Seita.9G181600.1_1283_TPlot.pdf]

**T=Seita.1G109500.1\_Q=Sit-miR160a\_S=471**

category=2\_p=0.999999996927316

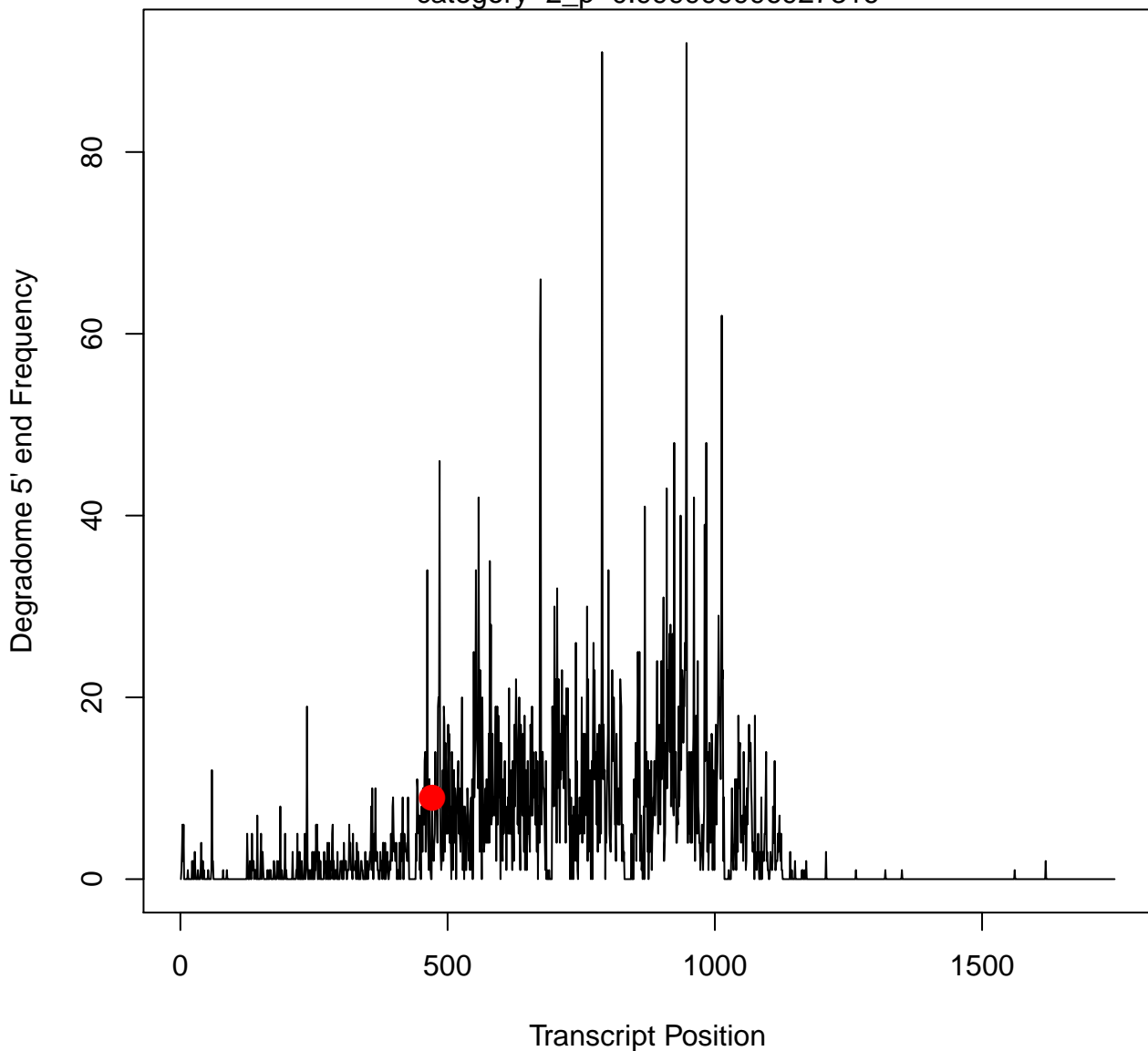

Supplement: Supplementary file 3 [file Data_Sheet_3.zip › Sit-miR160a_Seita.1G109500.1_471_TPlot.pdf]

**T=Seita.2G369900.1\_Q=Sit-miR160a\_S=2167**

category=2\_p=0.999999999999995

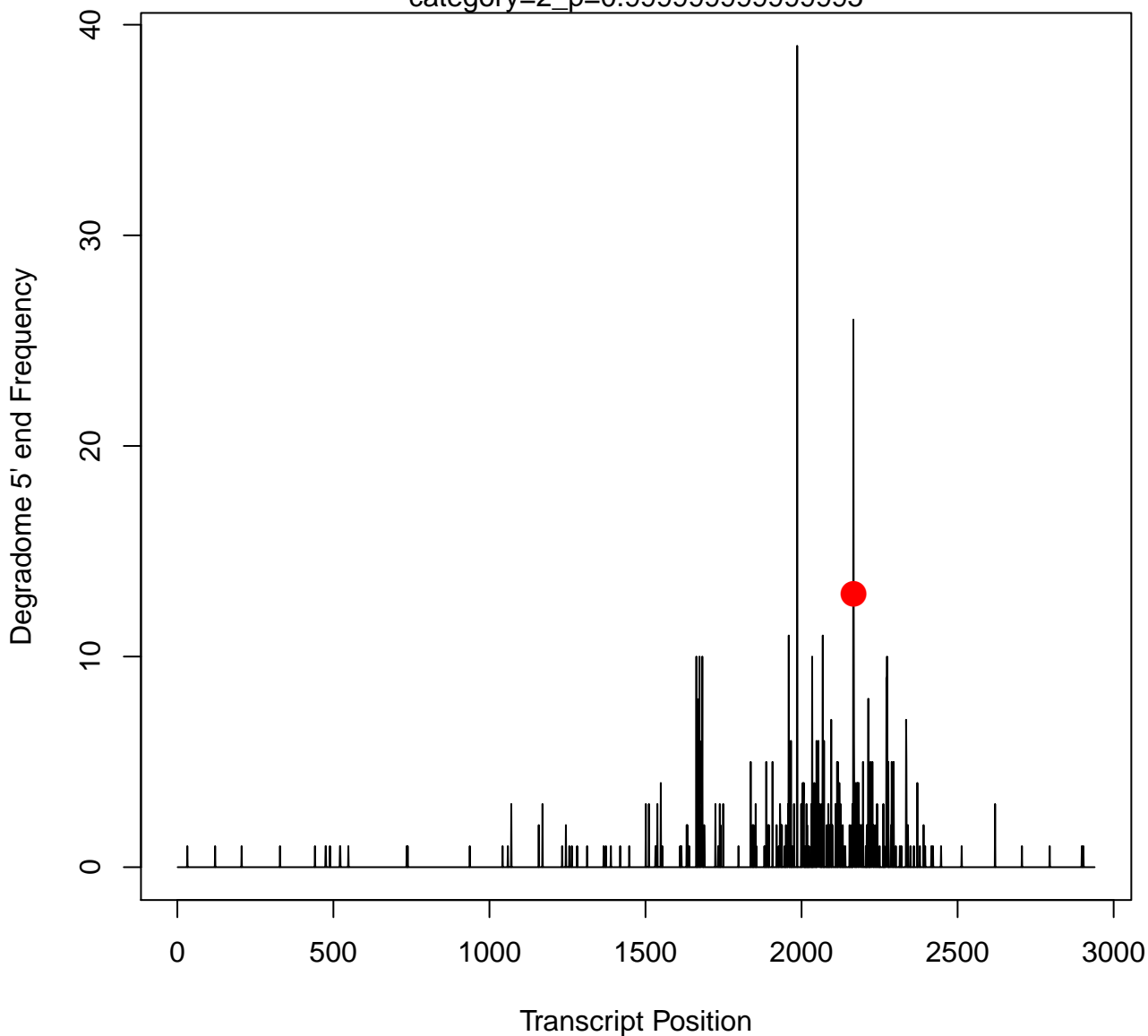

Supplement: Supplementary file 3 [file Data_Sheet_3.zip › Sit-miR160a_Seita.2G369900.1_2167_TPlot.pdf]

**T=Seita.5G353200.1\_Q=Sit-miR160a\_S=2118**

category=2\_p=0.999999999431534

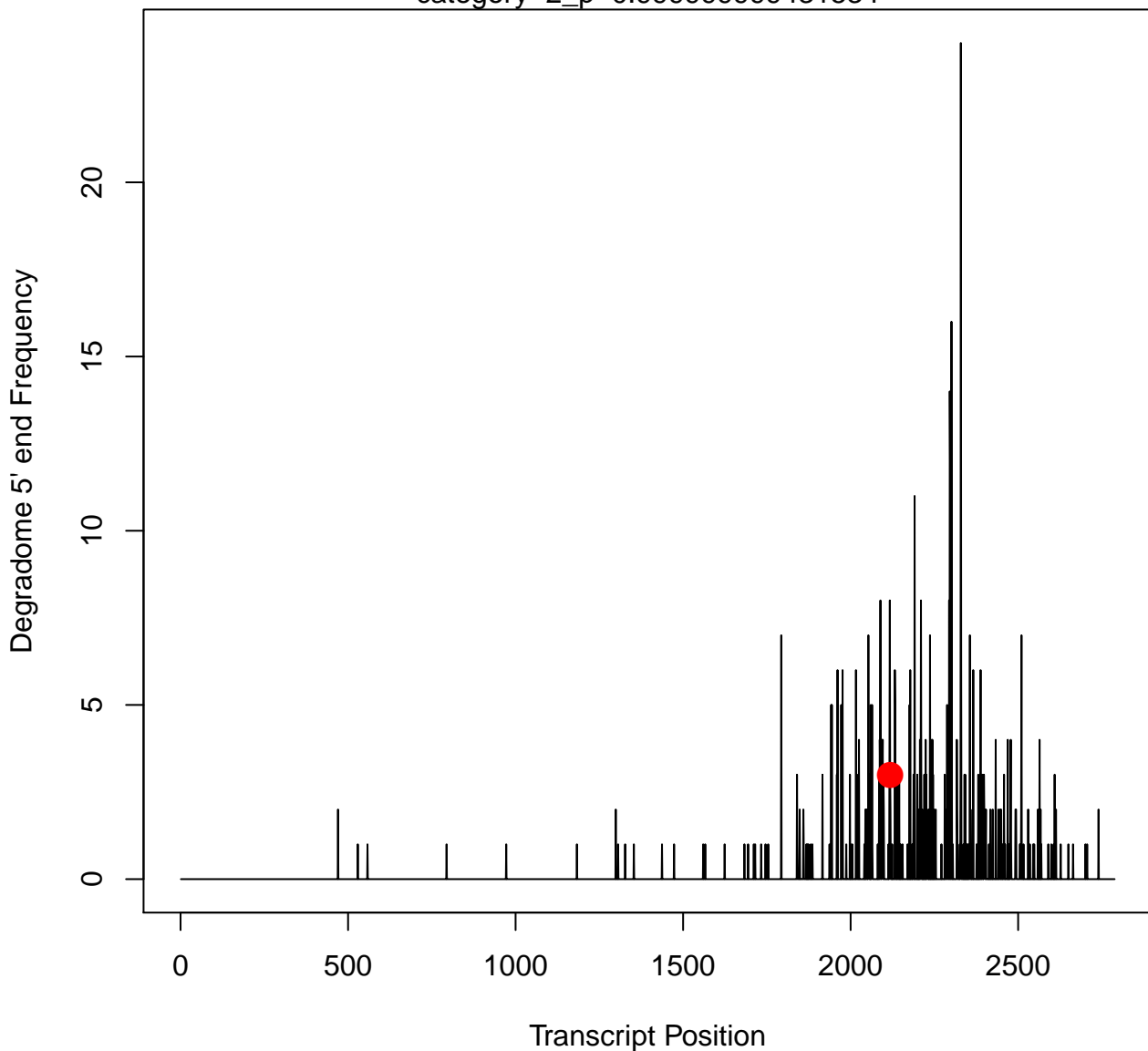

Supplement: Supplementary file 3 [file Data_Sheet_3.zip › Sit-miR160a_Seita.5G353200.1_2118_TPlot.pdf]

**T=Seita.6G024600.1\_Q=Sit-miR160a\_S=310**

category=2\_p=0.156707153734511

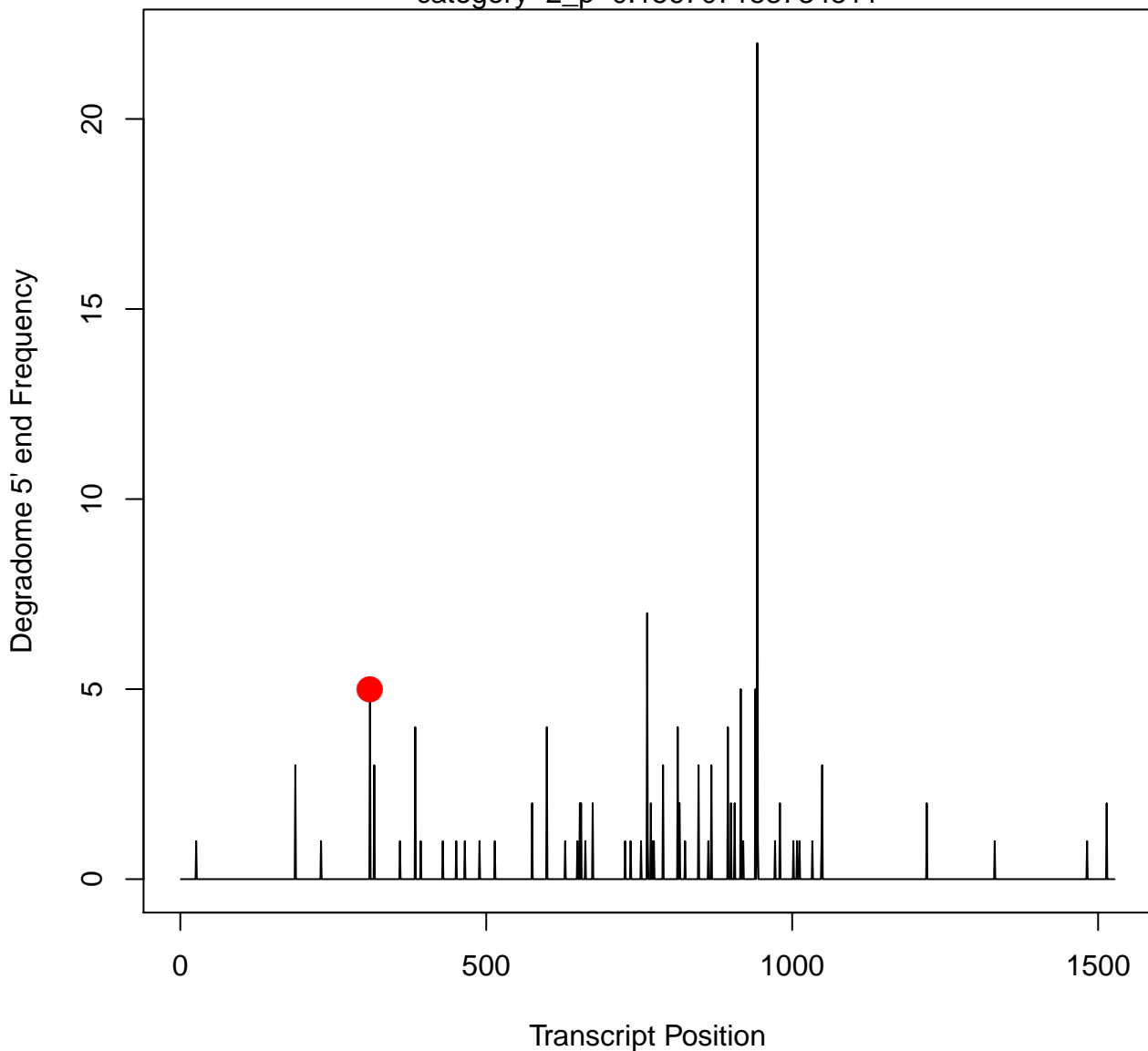

Supplement: Supplementary file 3 [file Data_Sheet_3.zip › Sit-miR160a_Seita.6G024600.1_310_TPlot.pdf]

**T=Seita.6G173700.1\_Q=Sit-miR160a\_S=1111**

category=2\_p=0.999999999964669

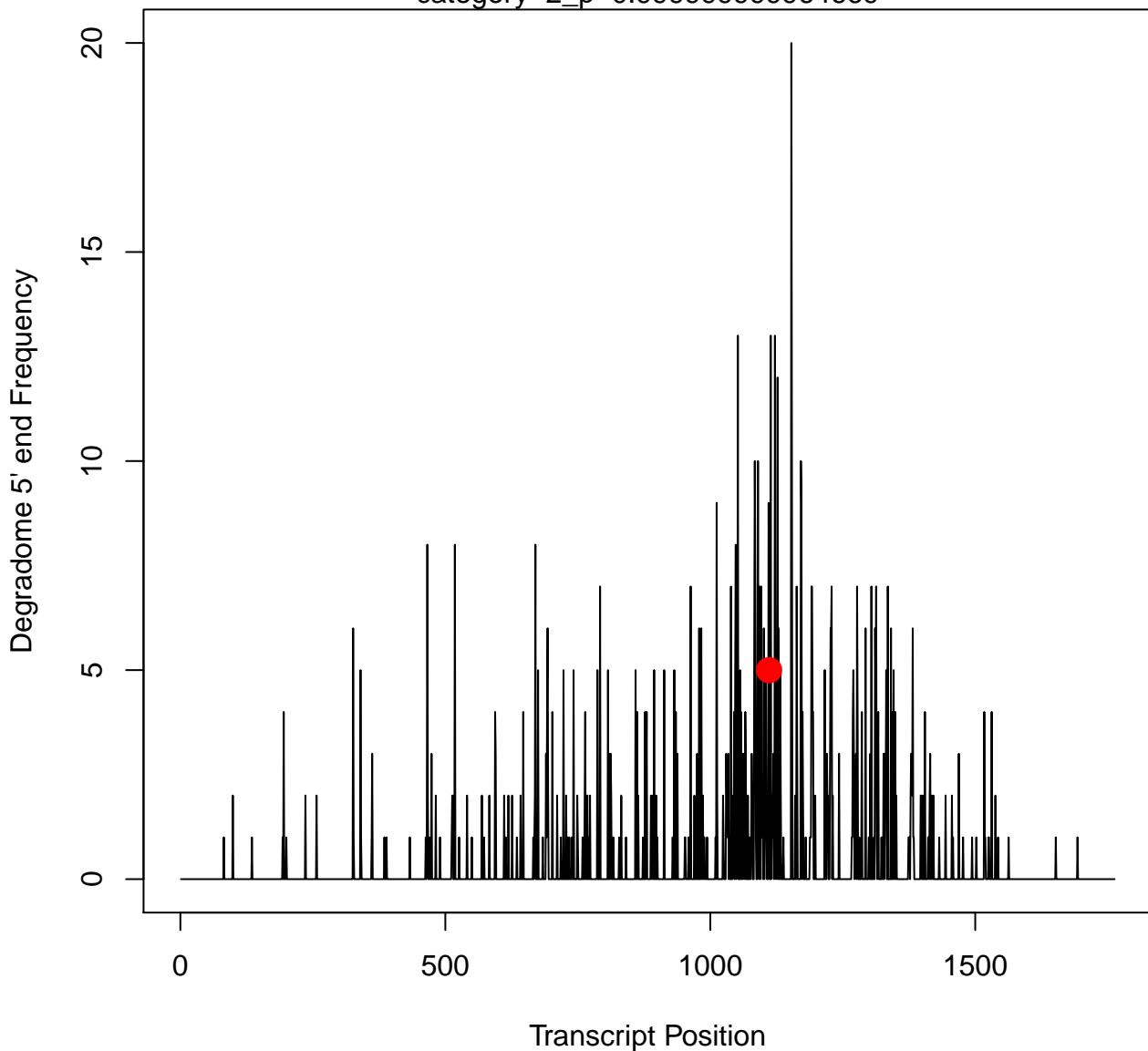

Supplement: Supplementary file 3 [file Data_Sheet_3.zip › Sit-miR160a_Seita.6G173700.1_1111_TPlot.pdf]

**T=Seita.9G127600.1\_Q=Sit-miR160a\_S=2497**

category=2\_p=0.999999926832314

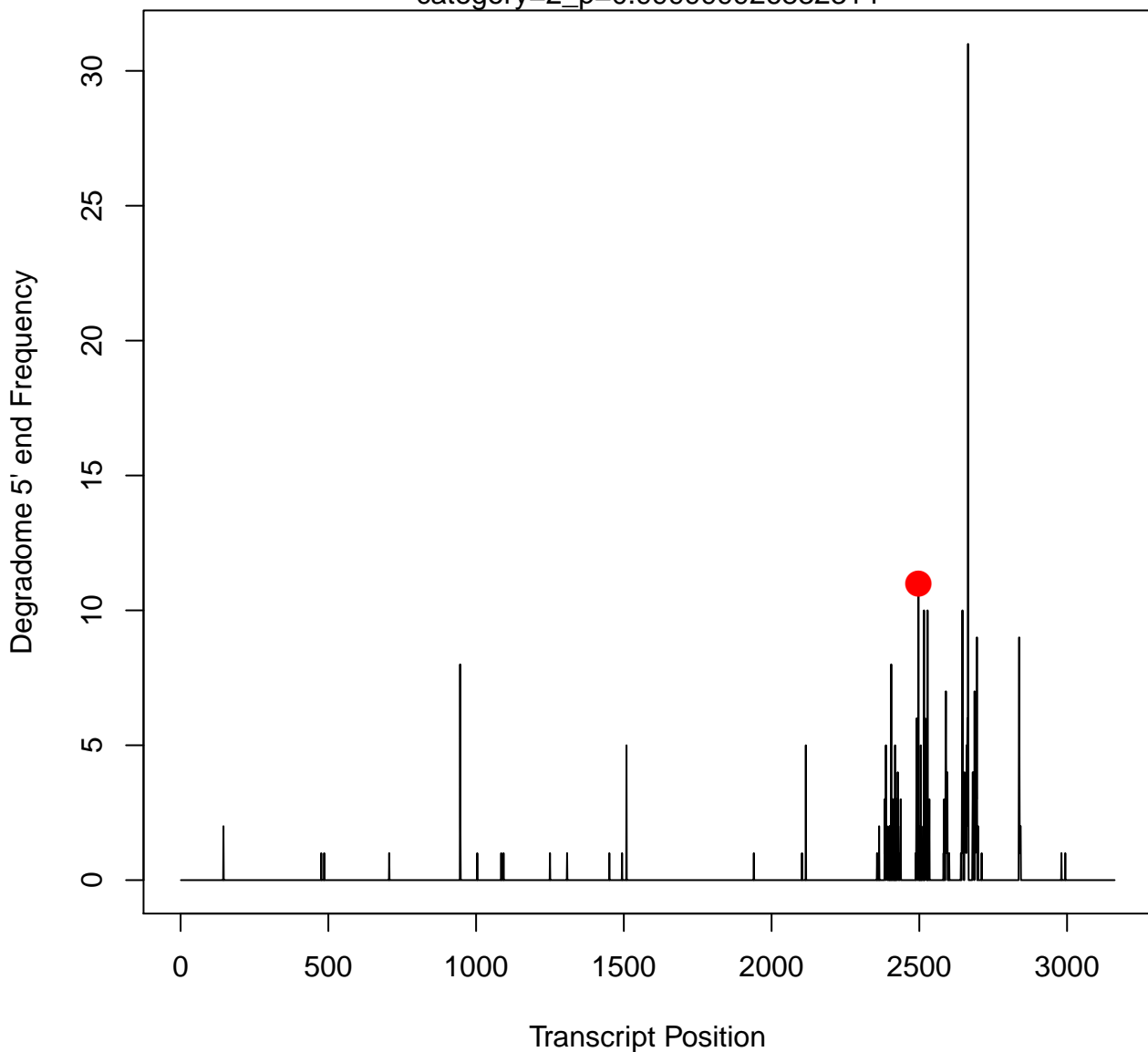

Supplement: Supplementary file 3 [file Data_Sheet_3.zip › Sit-miR160a_Seita.9G127600.1_2497_TPlot.pdf]

**T=Seita.9G127700.1\_Q=Sit-miR160a\_S=2456**

category=2\_p=0.999999910227107

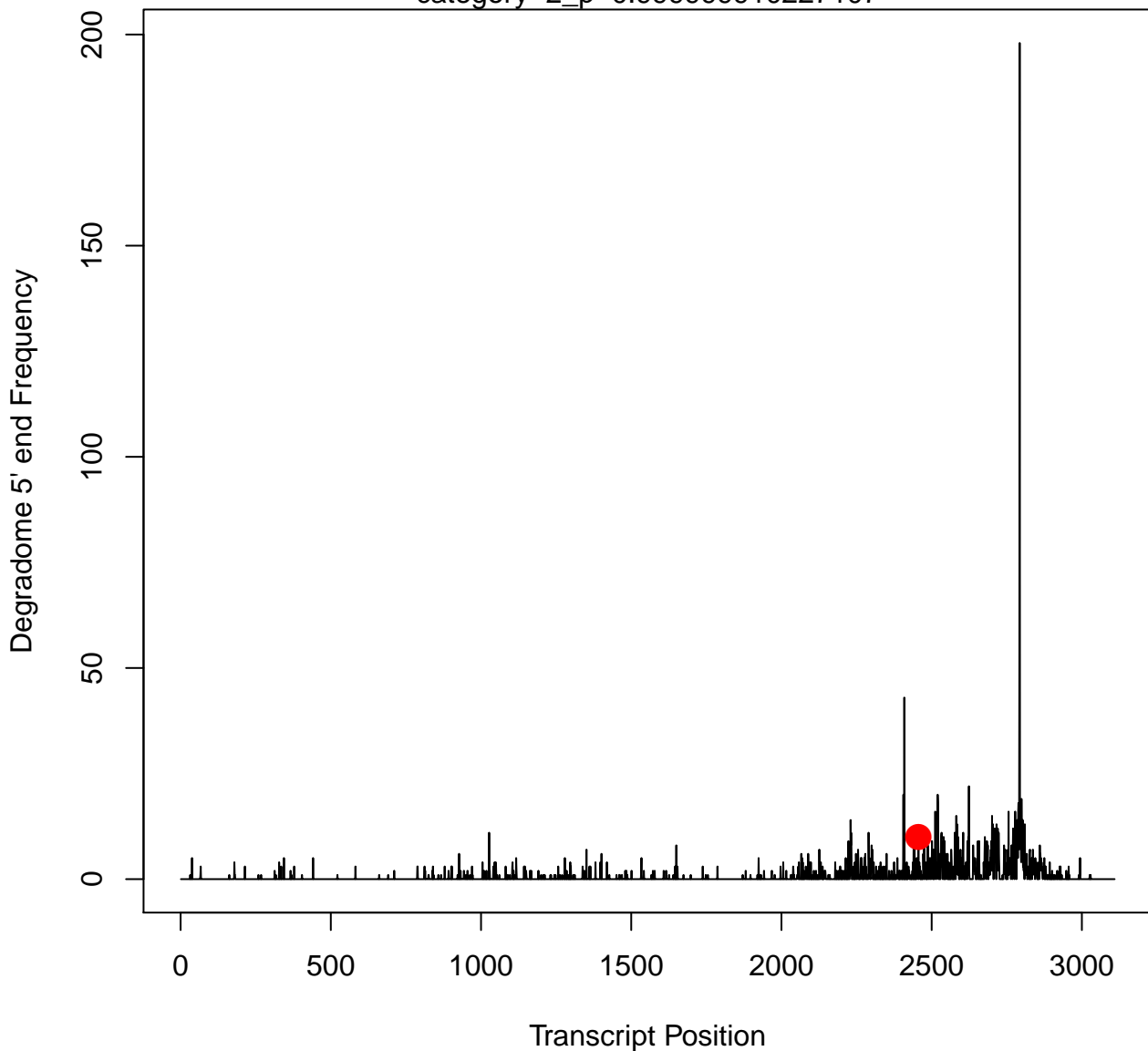

Supplement: Supplementary file 3 [file Data_Sheet_3.zip › Sit-miR160a_Seita.9G127700.1_2456_TPlot.pdf]

**T=Seita.1G241500.1\_Q=Sit-miR160b\_S=1660**

category=2\_p=0.081690223146084

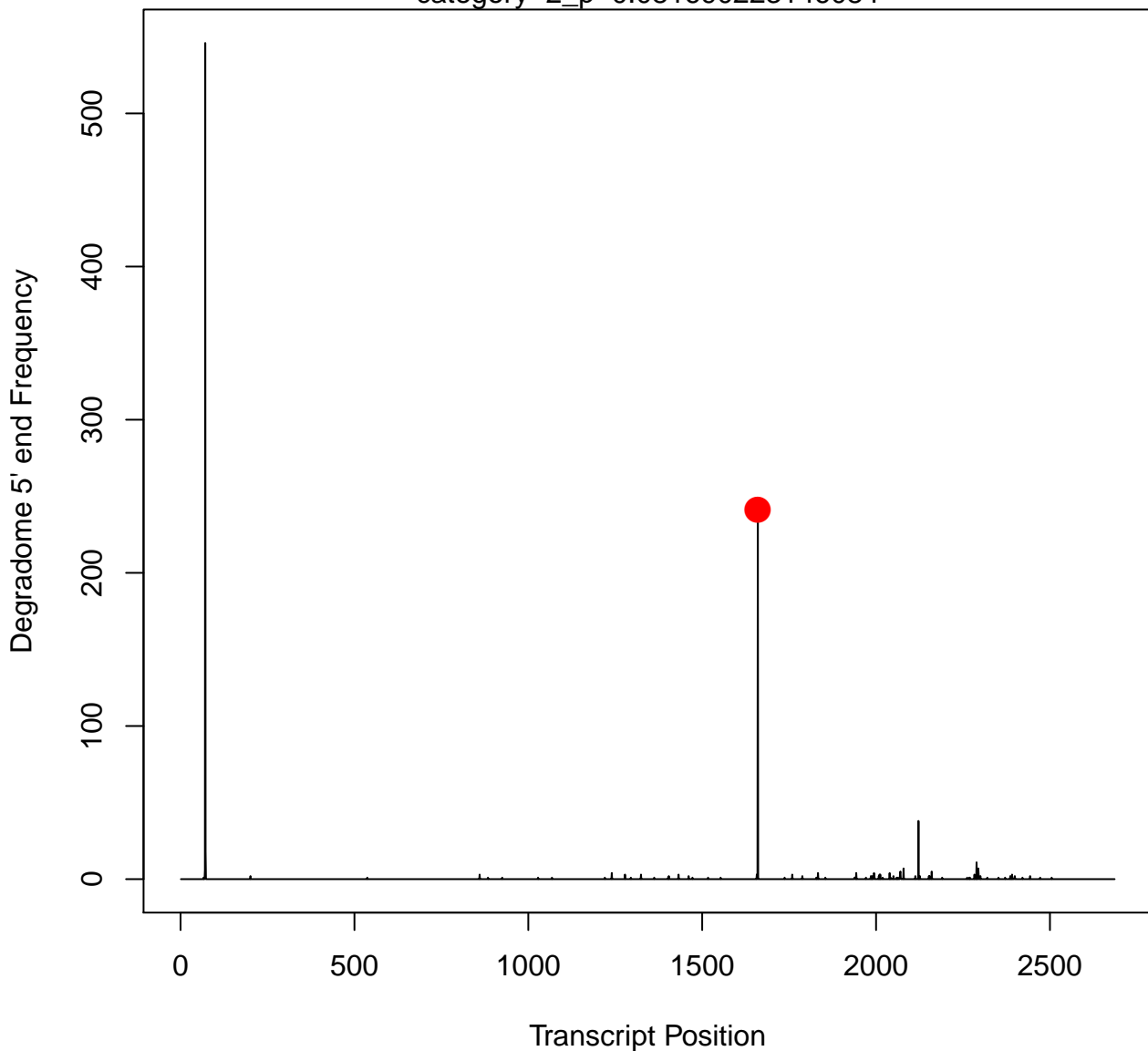

Supplement: Supplementary file 3 [file Data_Sheet_3.zip › Sit-miR160b_Seita.1G241500.1_1660_TPlot.pdf]

**T=Seita.1G264900.1\_Q=Sit-miR160b\_S=256**

category=2\_p=0.999994352324866

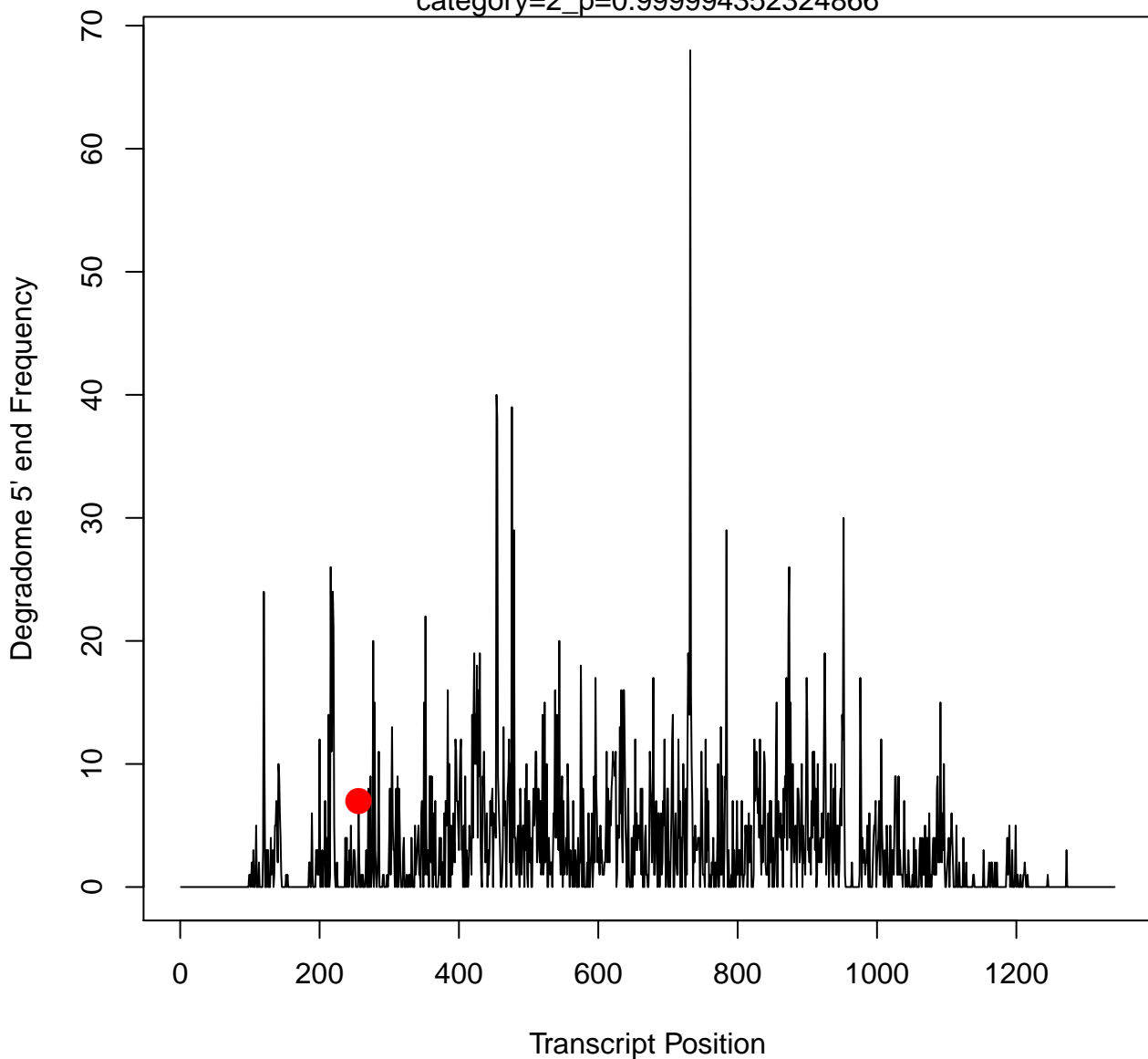

Supplement: Supplementary file 3 [file Data_Sheet_3.zip › Sit-miR160b_Seita.1G264900.1_256_TPlot.pdf]

**T=Seita.1G336800.1\_Q=Sit-miR160b\_S=900**

category=2\_p=0.977647882033304

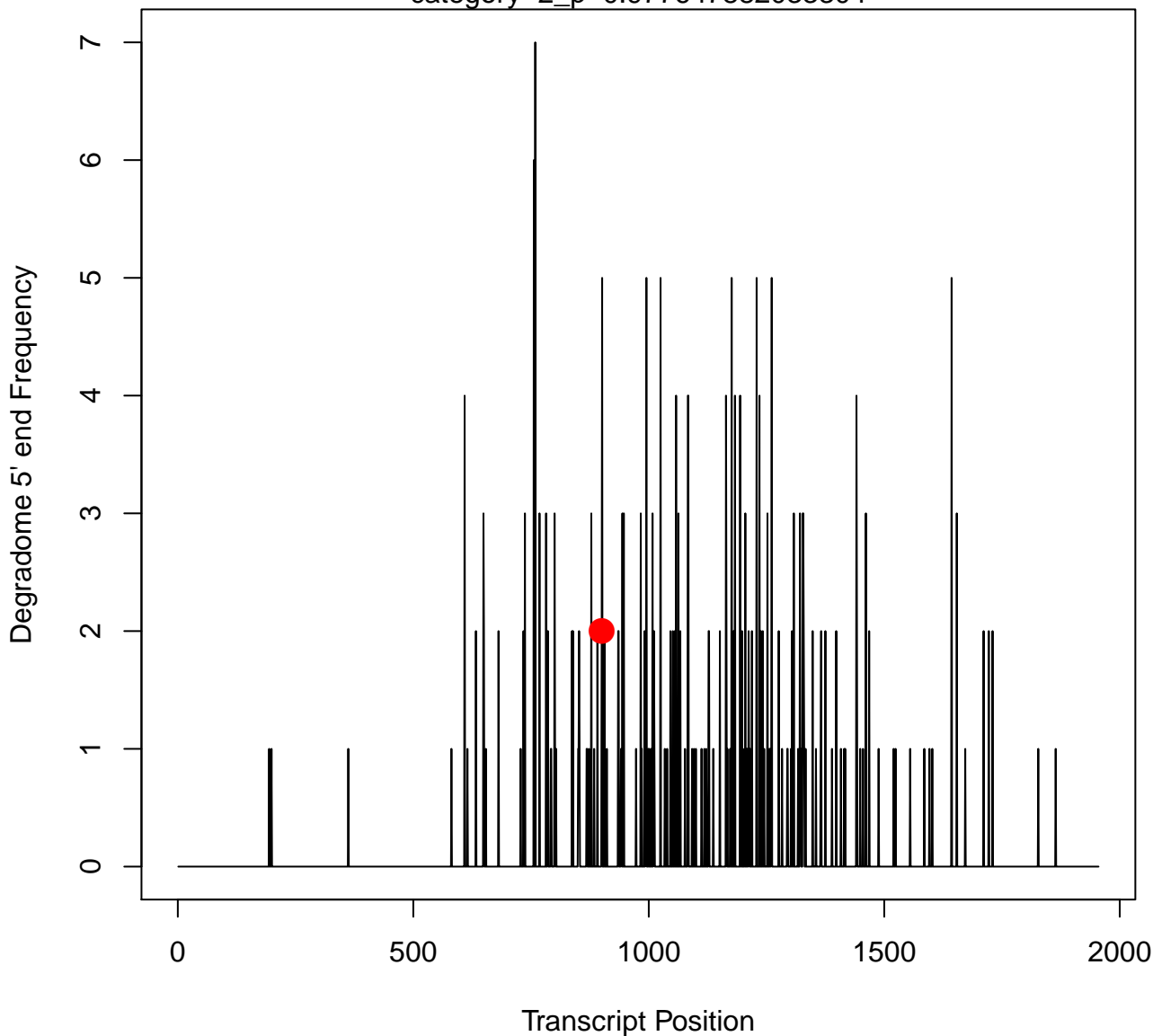

Supplement: Supplementary file 3 [file Data_Sheet_3.zip › Sit-miR160b_Seita.1G336800.1_900_TPlot.pdf]

**T=Seita.3G383600.1\_Q=Sit-miR160b\_S=182**

category=2\_p=0.999999999999997

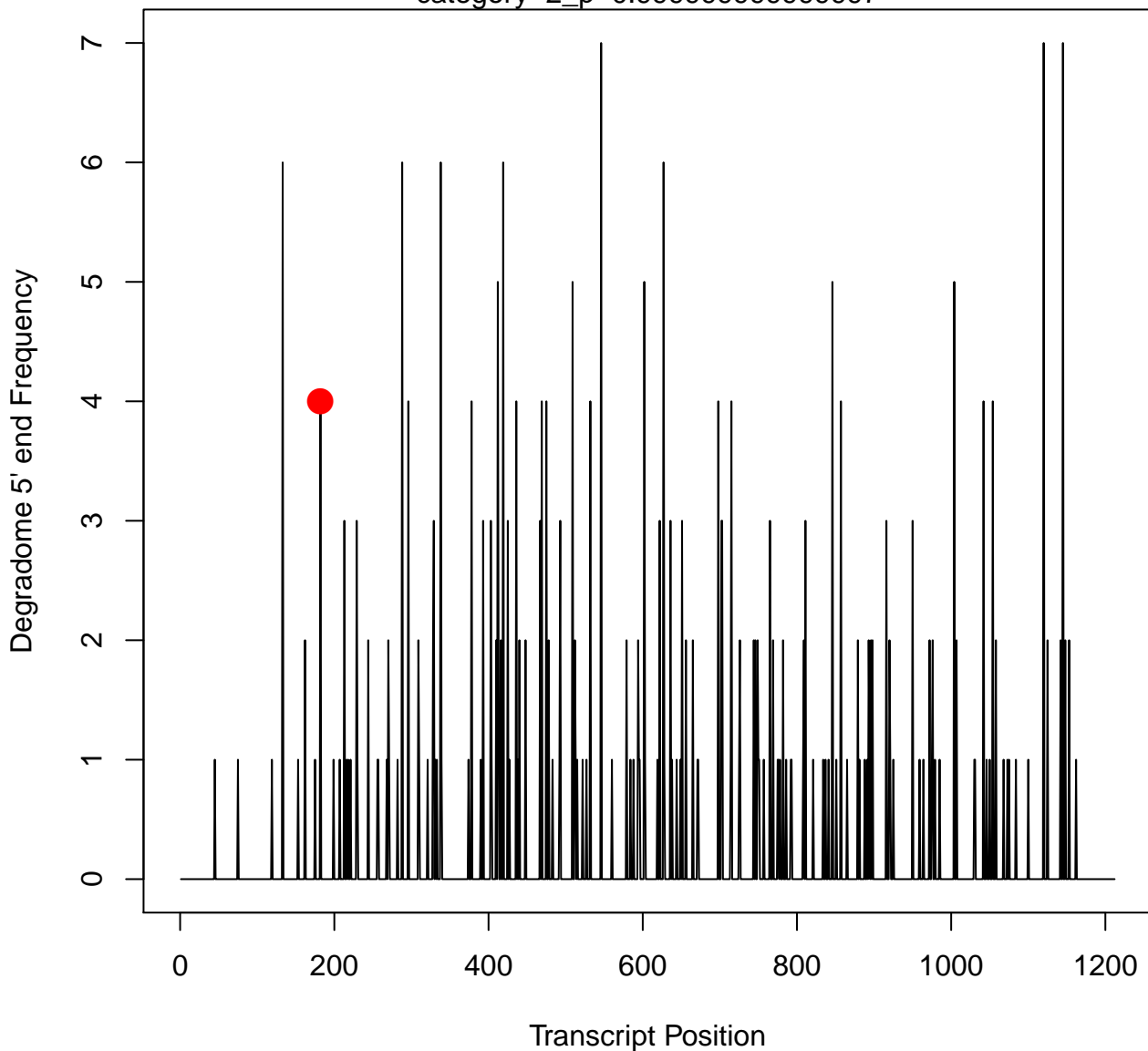

Supplement: Supplementary file 3 [file Data_Sheet_3.zip › Sit-miR160b_Seita.3G383600.1_182_TPlot.pdf]

**T=Seita.4G043900.1\_Q=Sit-miR160b\_S=308**

category=2\_p=0.999994156486873

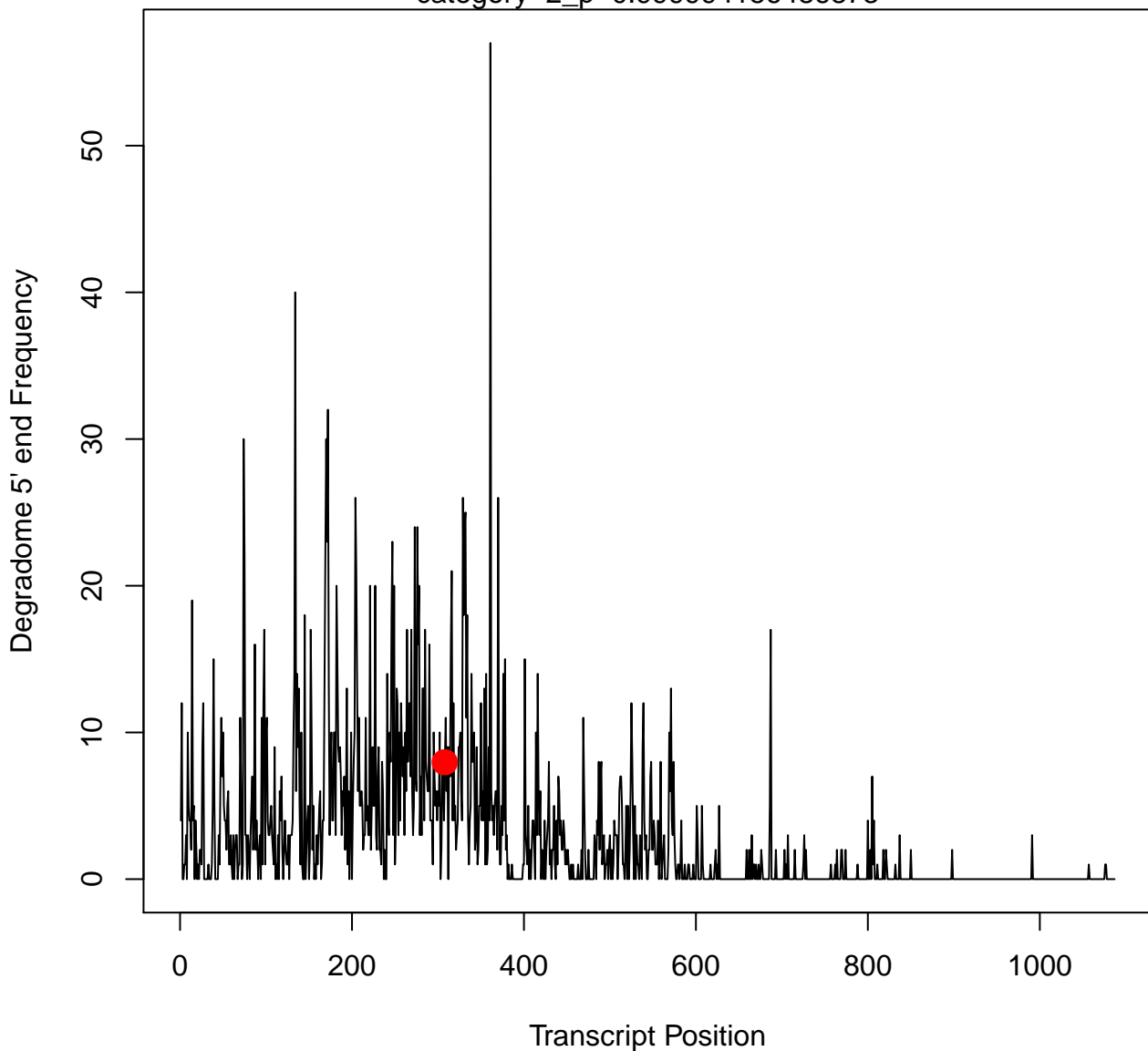

Supplement: Supplementary file 3 [file Data_Sheet_3.zip › Sit-miR160b_Seita.4G043900.1_308_TPlot.pdf]

**T=Seita.4G110300.1\_Q=Sit-miR160b\_S=2568**

category=2\_p=0.980497000313601

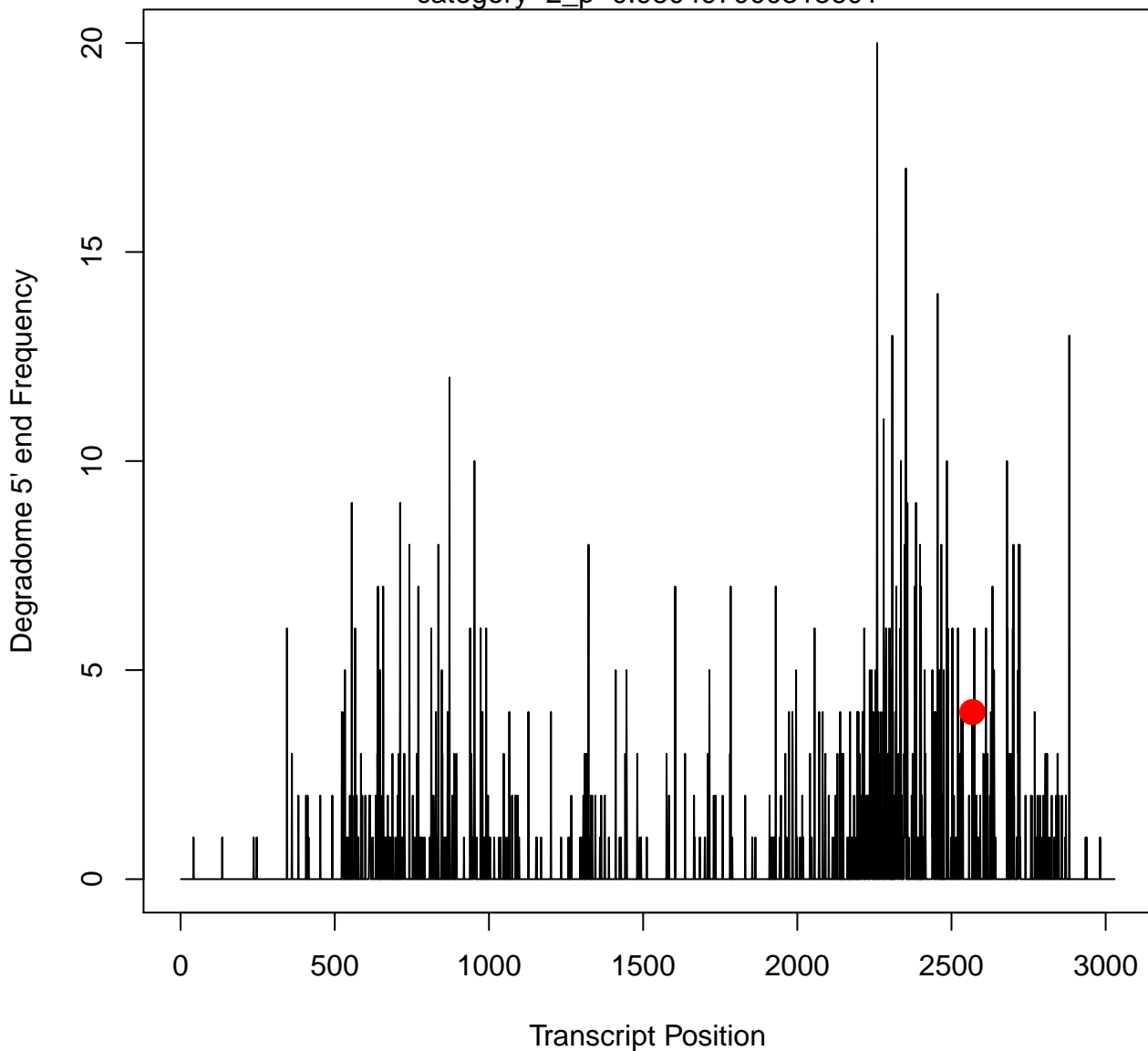

Supplement: Supplementary file 3 [file Data_Sheet_3.zip › Sit-miR160b_Seita.4G110300.1_2568_TPlot.pdf]

**T=Seita.4G179100.1\_Q=Sit-miR160b\_S=221**

category=2\_p=0.999999999391427

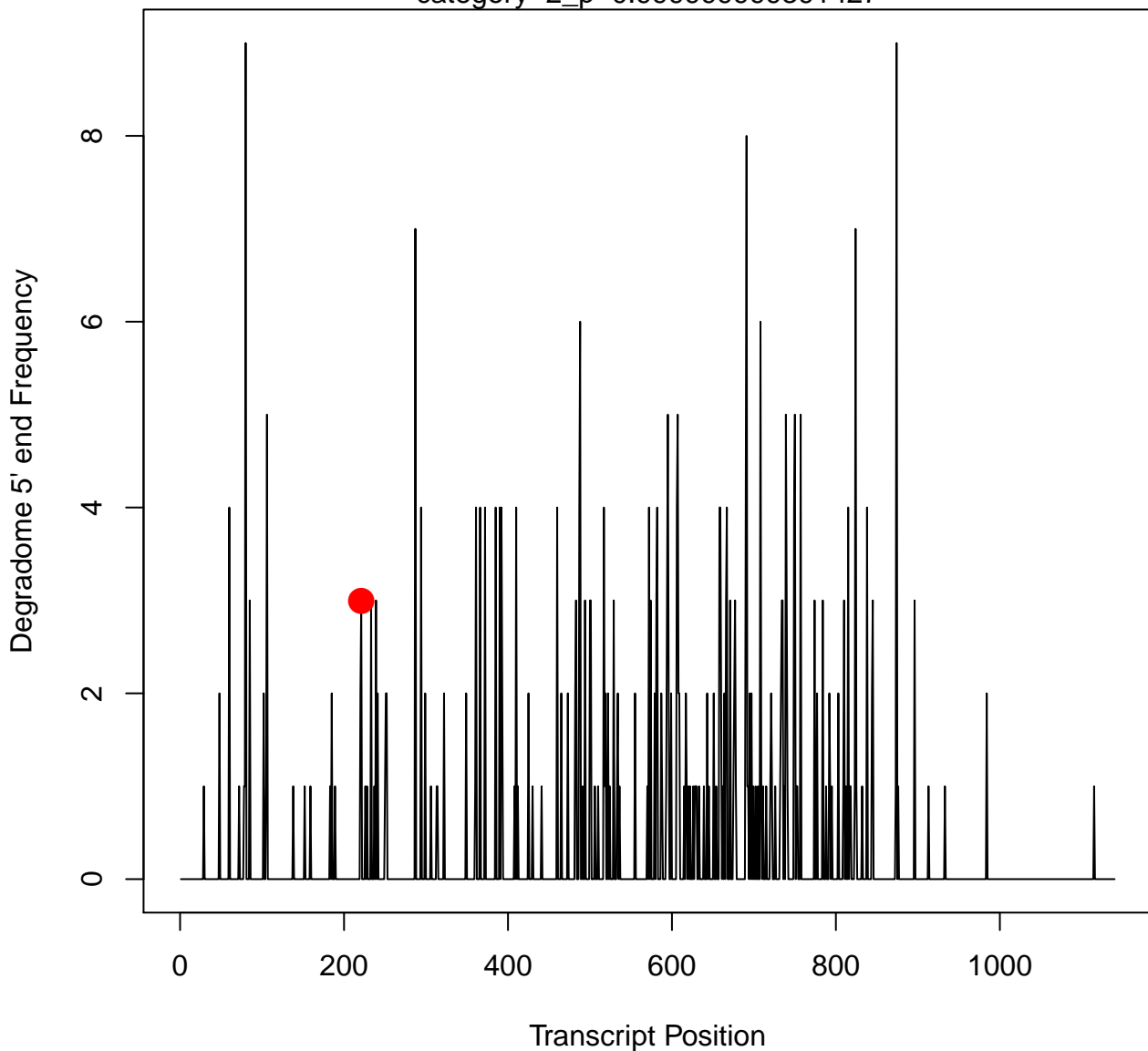

Supplement: Supplementary file 3 [file Data_Sheet_3.zip › Sit-miR160b_Seita.4G179100.1_221_TPlot.pdf]

**T=Seita.4G257800.1\_Q=Sit-miR160b\_S=1974**

category=0\_p=0.000802428474349082

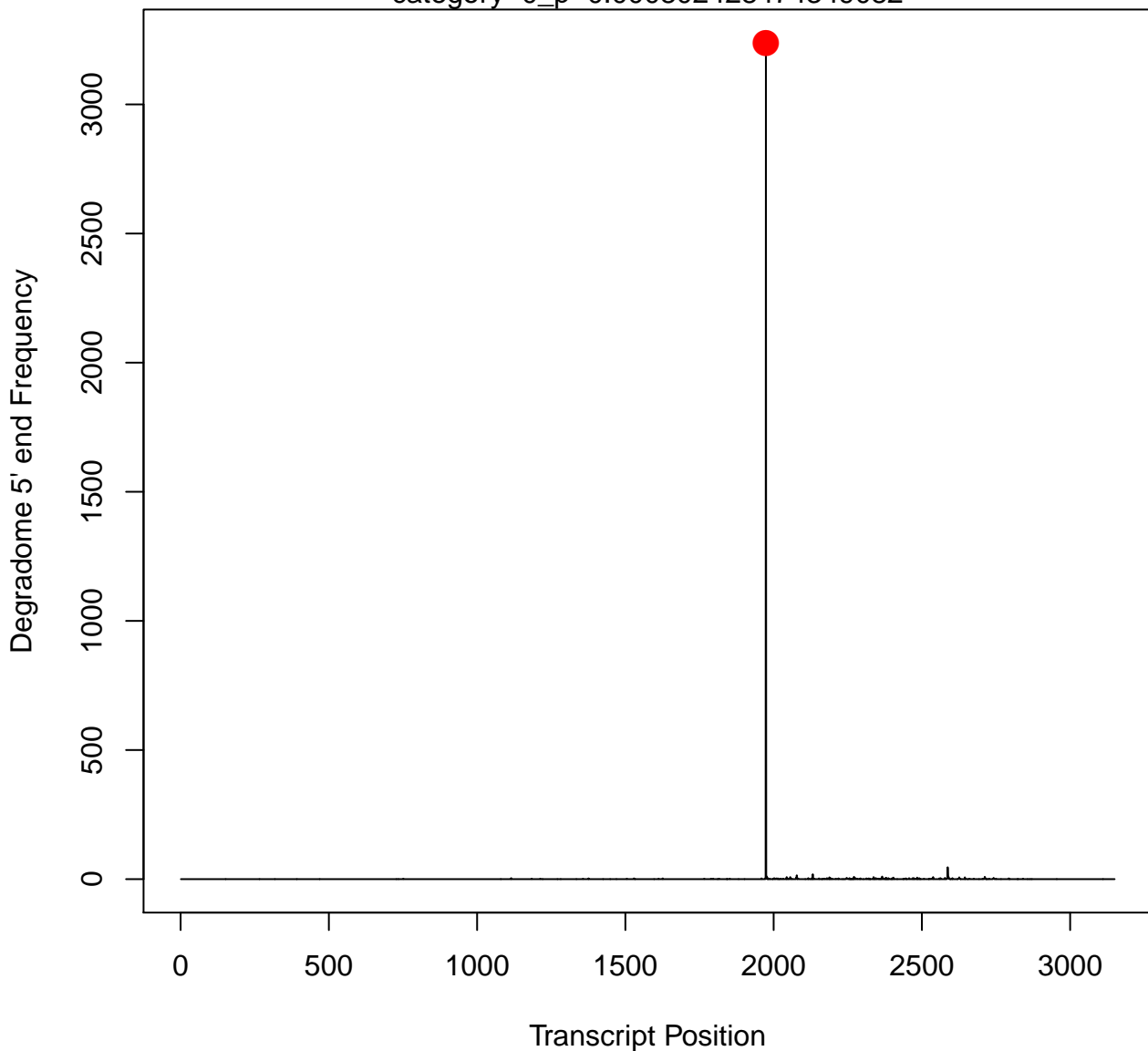

Supplement: Supplementary file 3 [file Data_Sheet_3.zip › Sit-miR160b_Seita.4G257800.1_1974_TPlot.pdf]

**T=Seita.4G282900.1\_Q=Sit-miR160b\_S=353**

category=2\_p=0.870657576479375

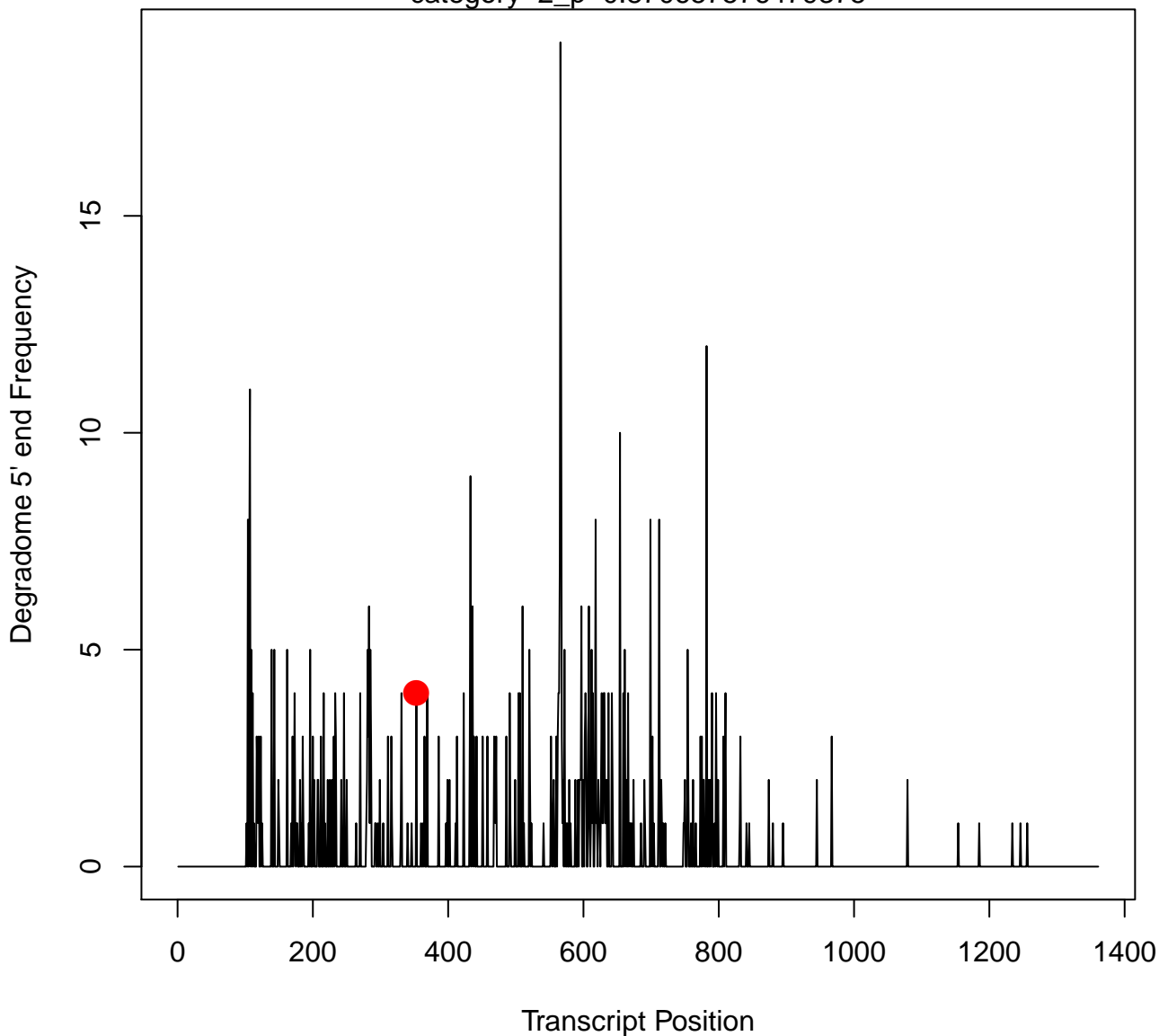

Supplement: Supplementary file 3 [file Data_Sheet_3.zip › Sit-miR160b_Seita.4G282900.1_353_TPlot.pdf]

**T=Seita.7G078300.1\_Q=Sit-miR160b\_S=2649**

category=2\_p=0.999987630020133

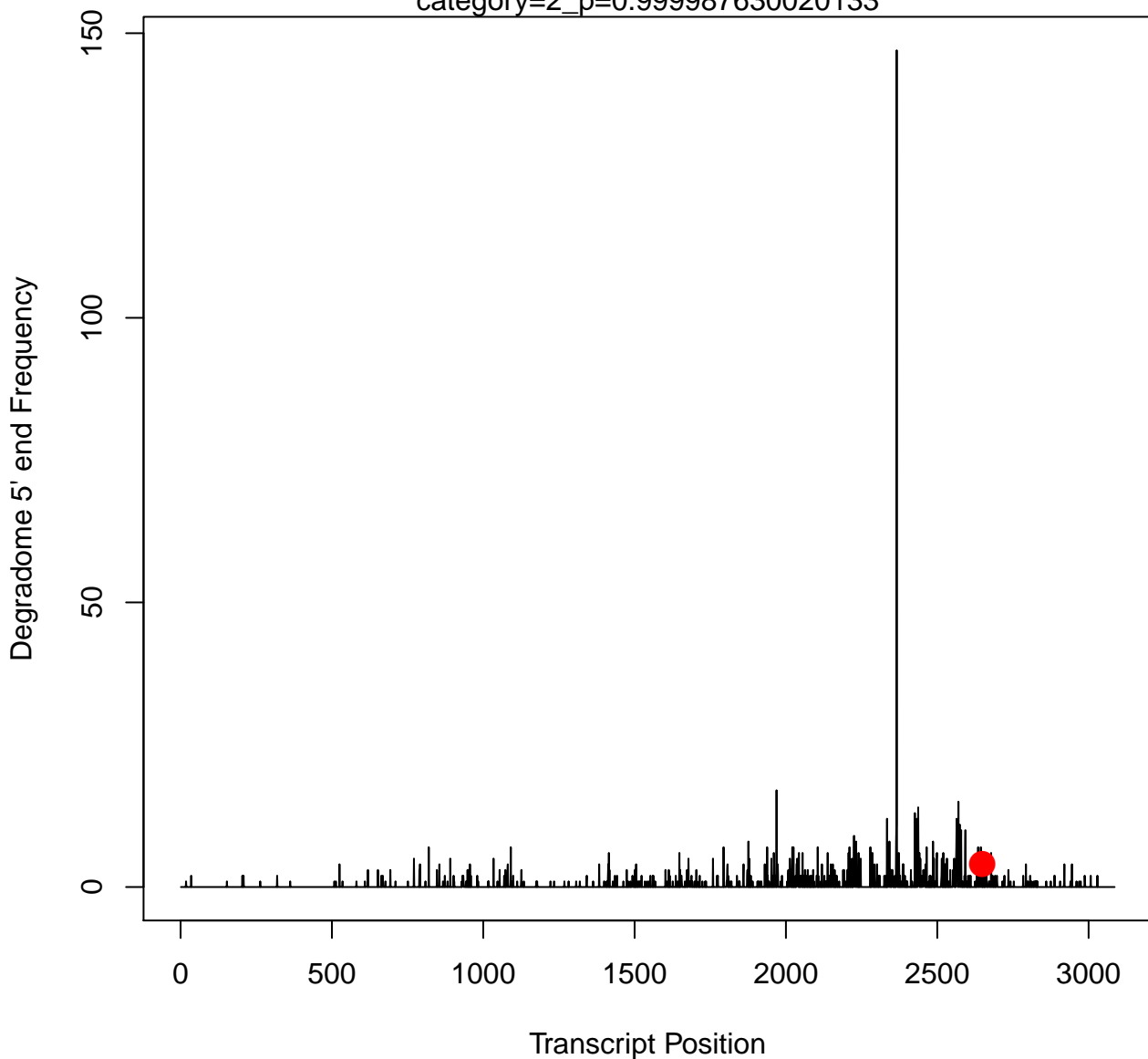

Supplement: Supplementary file 3 [file Data_Sheet_3.zip › Sit-miR160b_Seita.7G078300.1_2649_TPlot.pdf]

**T=Seita.7G138100.1\_Q=Sit-miR160b\_S=1082**

category=2\_p=0.999999822486431

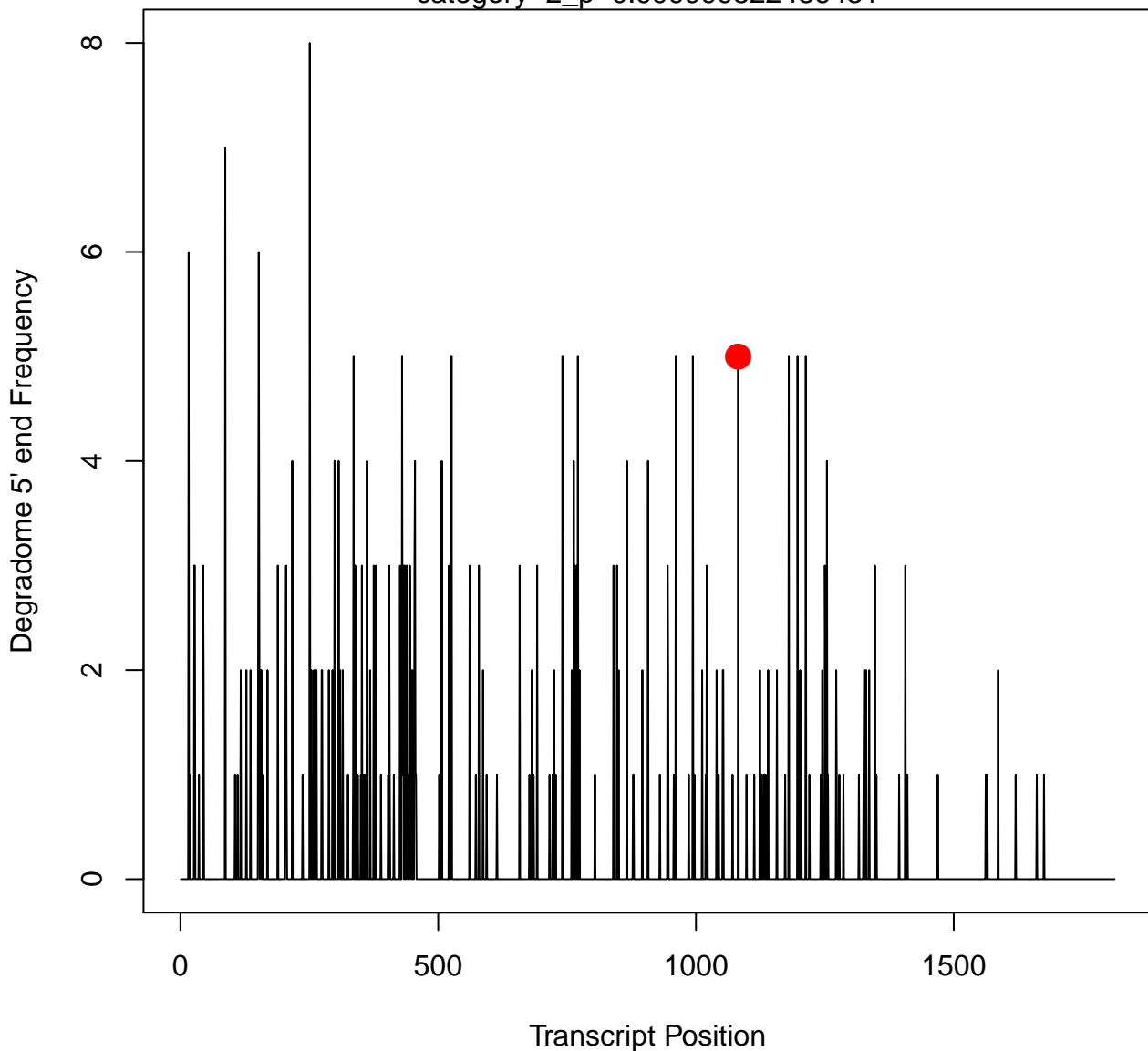

Supplement: Supplementary file 3 [file Data_Sheet_3.zip › Sit-miR160b_Seita.7G138100.1_1082_TPlot.pdf]

**T=Seita.7G169600.1\_Q=Sit-miR160b\_S=1802**

category=0\_p=0.000401294755915105

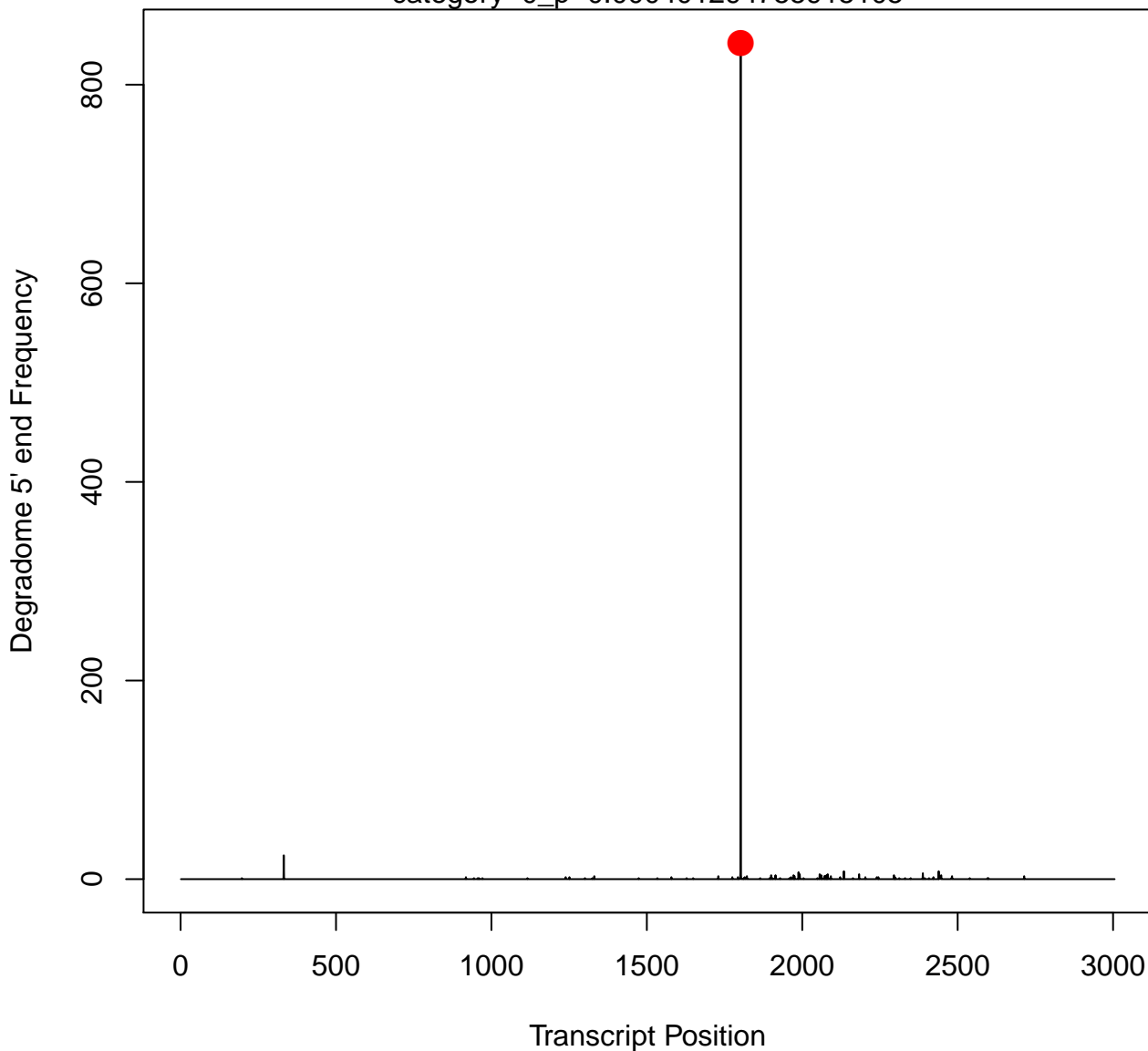

Supplement: Supplementary file 3 [file Data_Sheet_3.zip › Sit-miR160b_Seita.7G169600.1_1802_TPlot.pdf]

**T=Seita.7G214300.1\_Q=Sit-miR160b\_S=1011**

category=2\_p=0.999999975420039

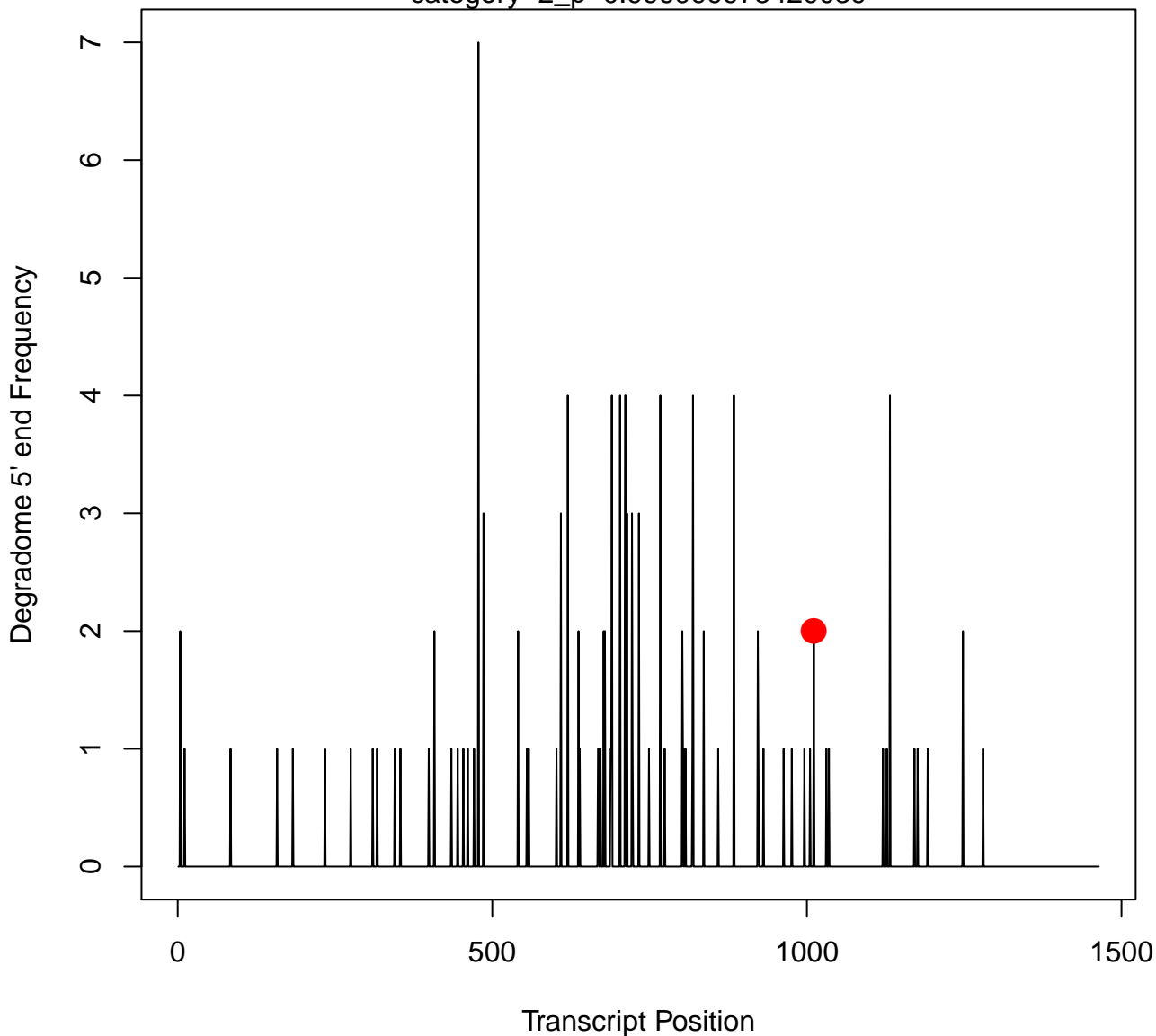

Supplement: Supplementary file 3 [file Data_Sheet_3.zip › Sit-miR160b_Seita.7G214300.1_1011_TPlot.pdf]

**T=Seita.7G295800.1\_Q=Sit-miR160b\_S=368**

category=2\_p=0.99999999999238

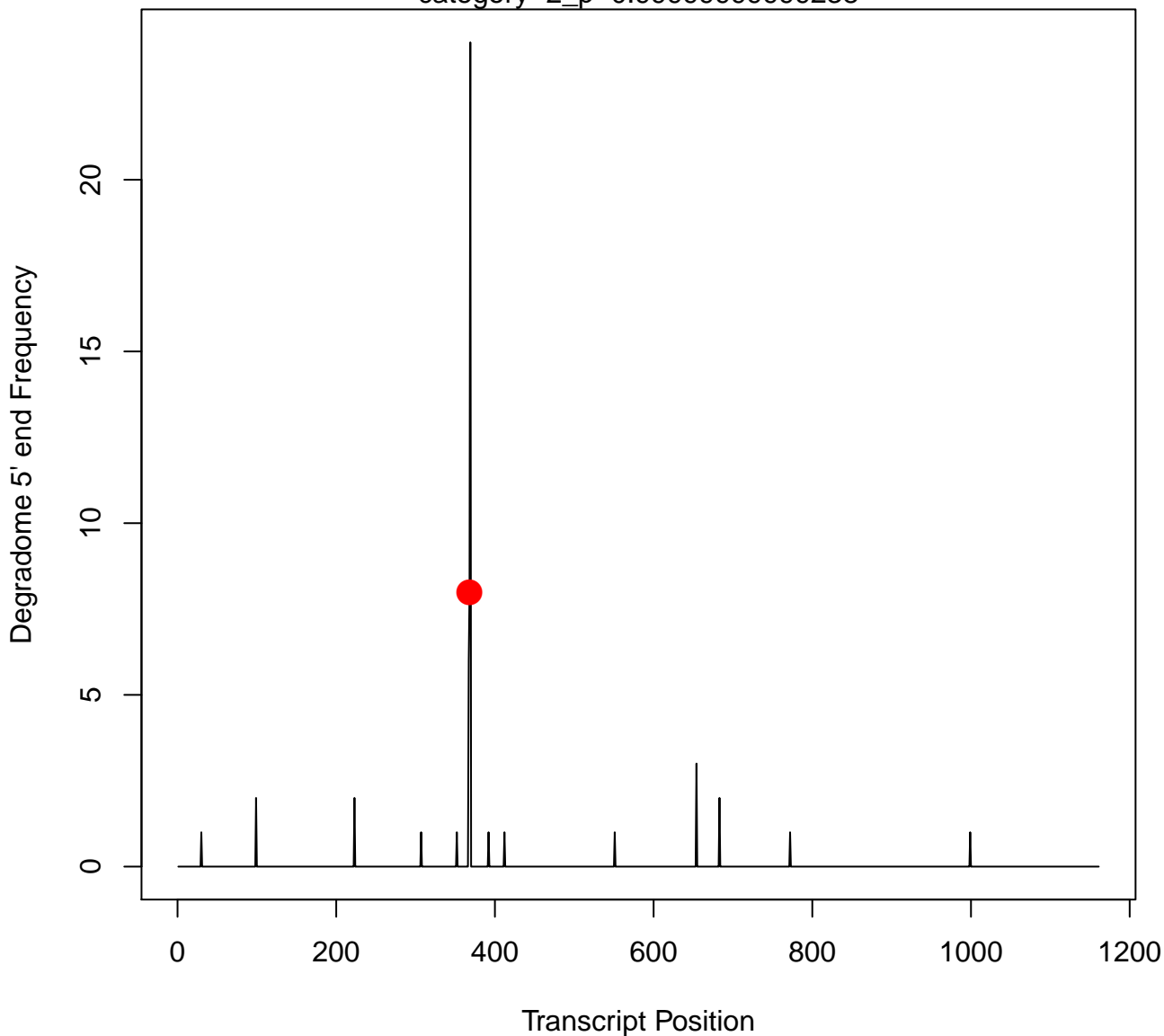

Supplement: Supplementary file 3 [file Data_Sheet_3.zip › Sit-miR160b_Seita.7G295800.1_368_TPlot.pdf]

**T=Seita.8G063600.1\_Q=Sit-miR160b\_S=1651**

category=2\_p=0.99999999984957

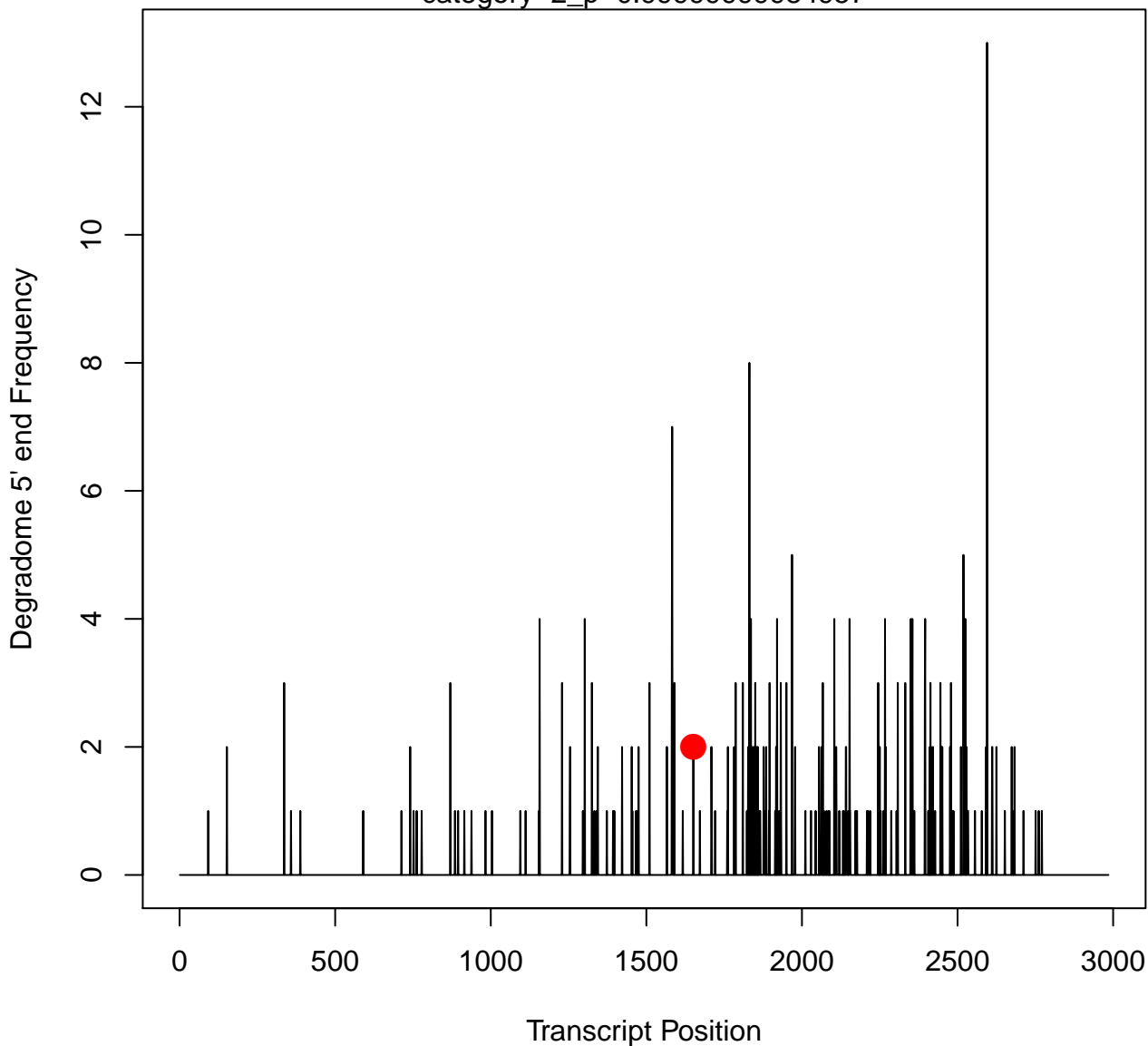

Supplement: Supplementary file 3 [file Data_Sheet_3.zip › Sit-miR160b_Seita.8G063600.1_1651_TPlot.pdf]

**T=Seita.8G178900.1\_Q=Sit-miR160b\_S=612**

category=2\_p=0.999998822743394

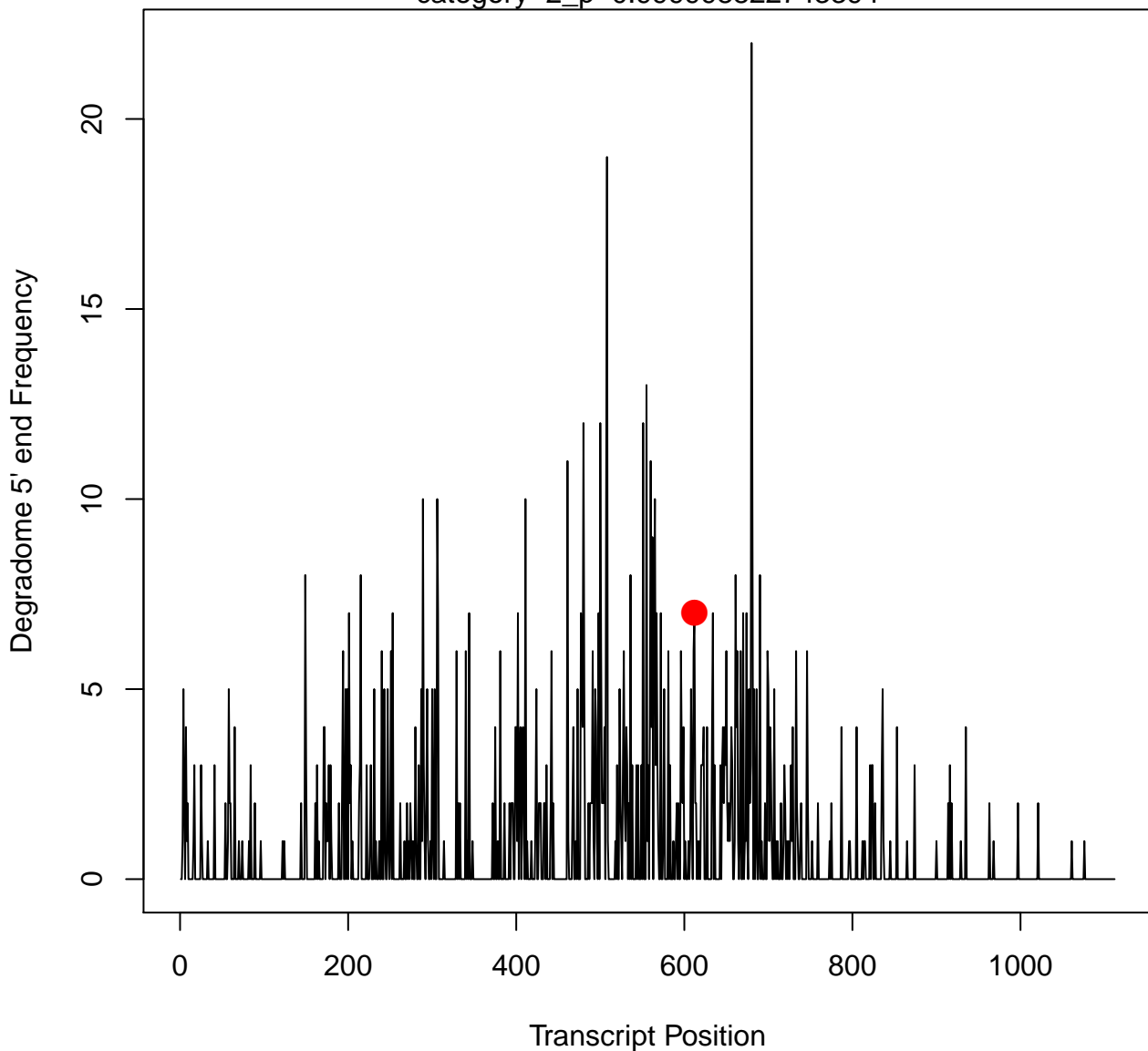

Supplement: Supplementary file 3 [file Data_Sheet_3.zip › Sit-miR160b_Seita.8G178900.1_612_TPlot.pdf]

**T=Seita.8G230000.1\_Q=Sit-miR160b\_S=1637**

category=2\_p=0.999999999999971

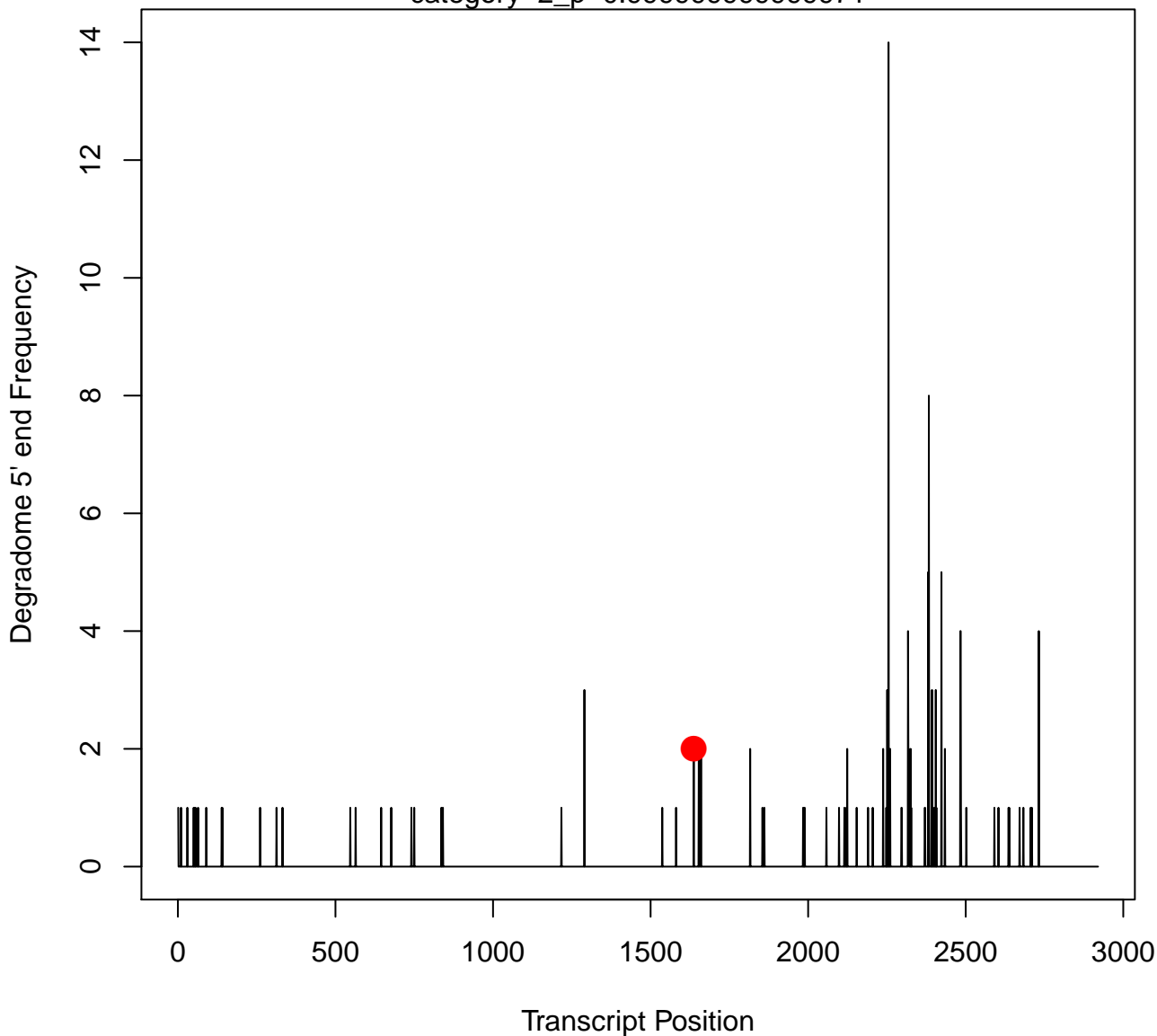

Supplement: Supplementary file 3 [file Data_Sheet_3.zip › Sit-miR160b_Seita.8G230000.1_1637_TPlot.pdf]

**T=Seita.1G154600.1\_Q=Sit-miR160c\_S=222**

category=2\_p=0.984104451694208

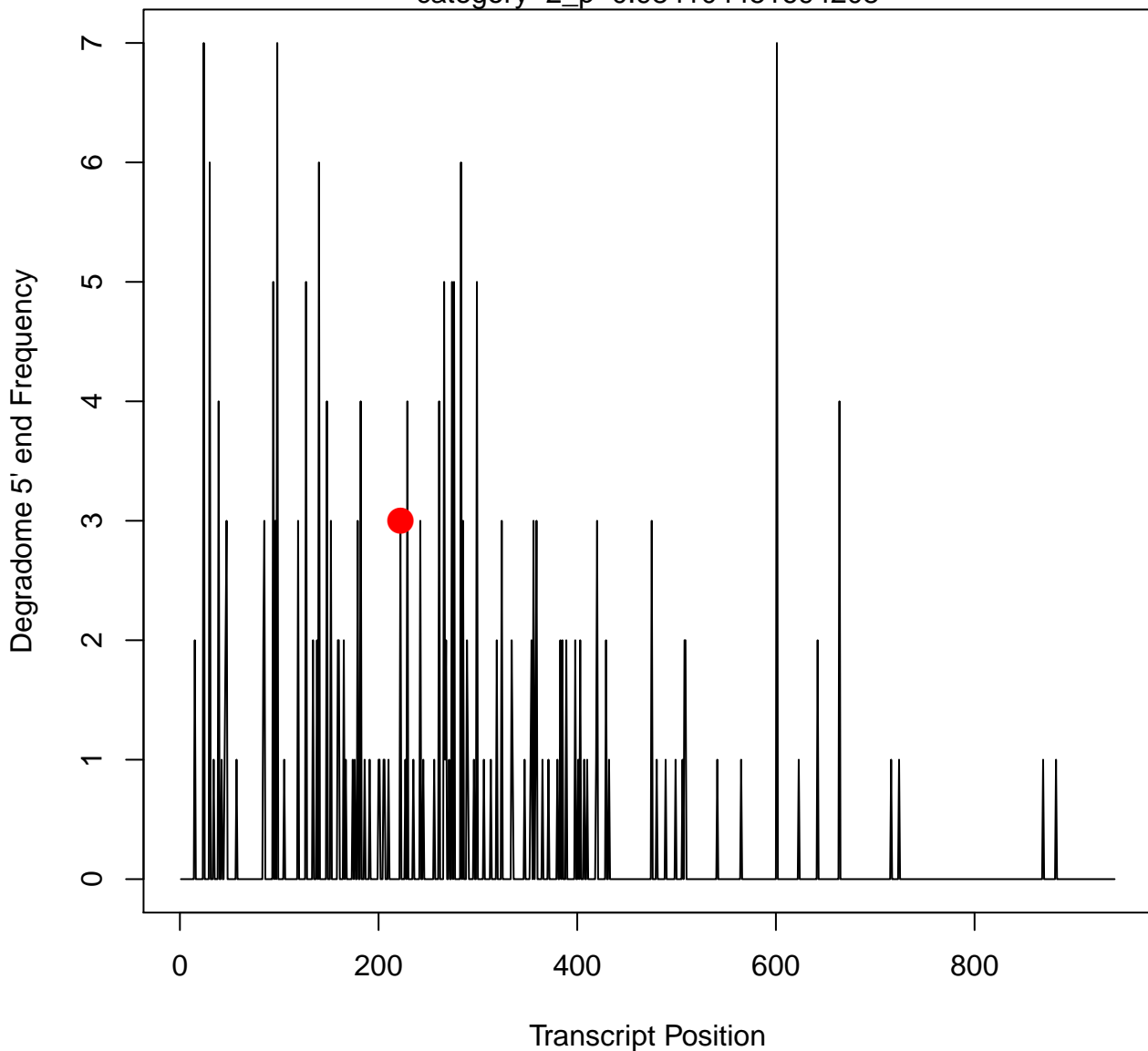

Supplement: Supplementary file 3 [file Data_Sheet_3.zip › Sit-miR160c_Seita.1G154600.1_222_TPlot.pdf]

**T=Seita.1G325400.1\_Q=Sit-miR160c\_S=578**

category=2\_p=0.999863204023275

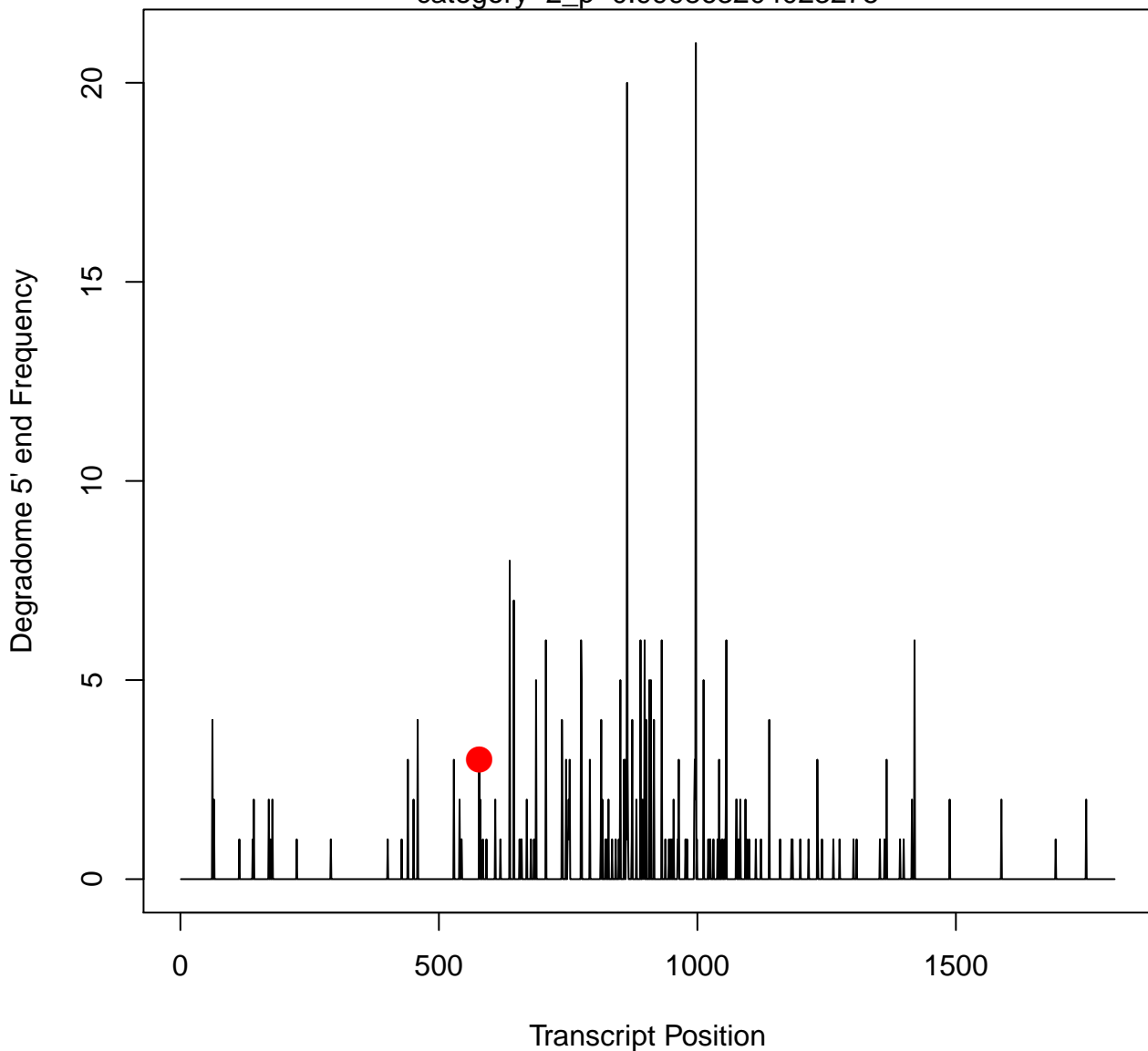

Supplement: Supplementary file 3 [file Data_Sheet_3.zip › Sit-miR160c_Seita.1G325400.1_578_TPlot.pdf]

**T=Seita.2G056700.1\_Q=Sit-miR160c\_S=406**

category=2\_p=0.999999979622193

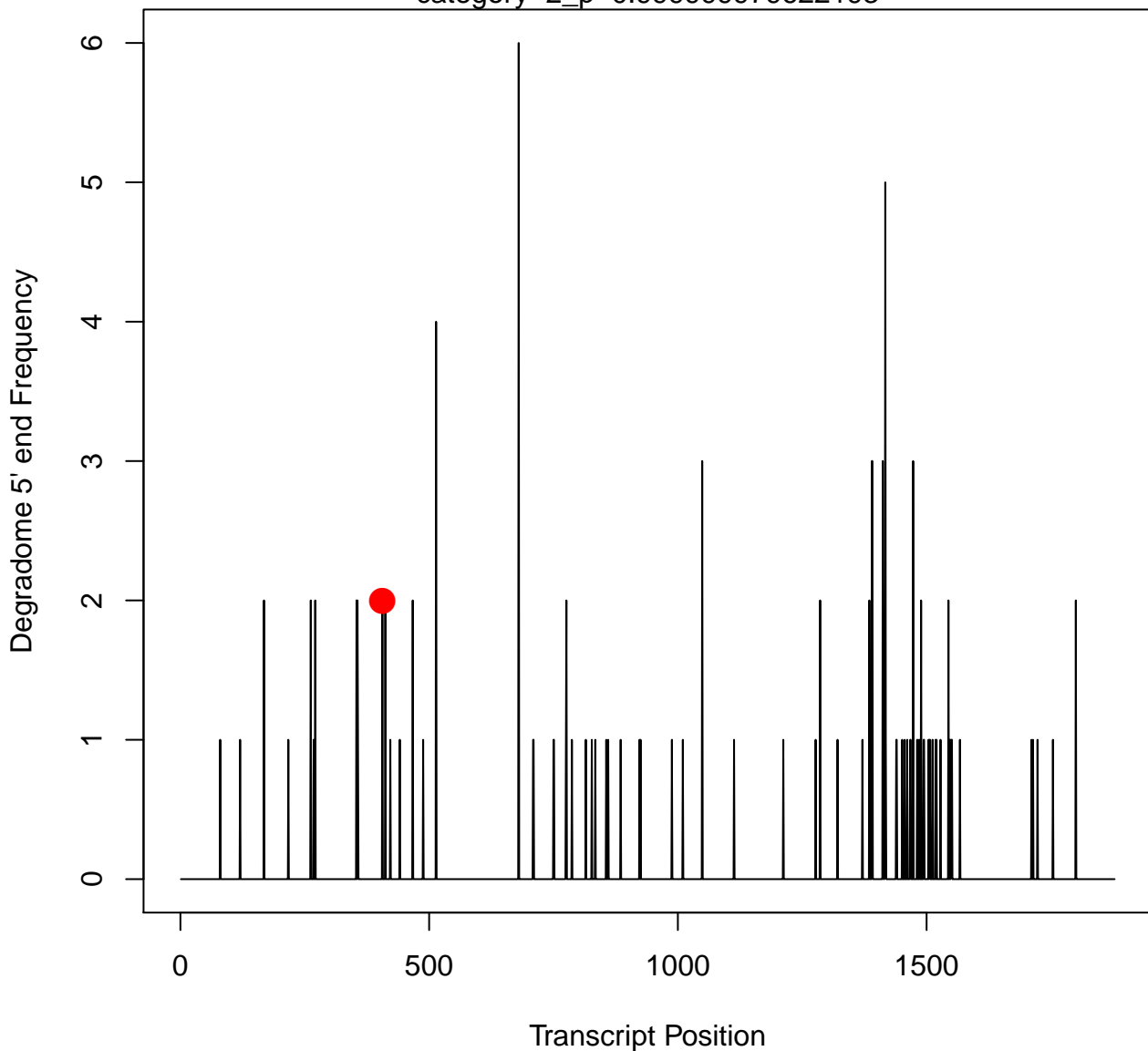

Supplement: Supplementary file 3 [file Data_Sheet_3.zip › Sit-miR160c_Seita.2G056700.1_406_TPlot.pdf]

**T=Seita.2G137400.1\_Q=Sit-miR160c\_S=817**

category=2\_p=0.999999998597126

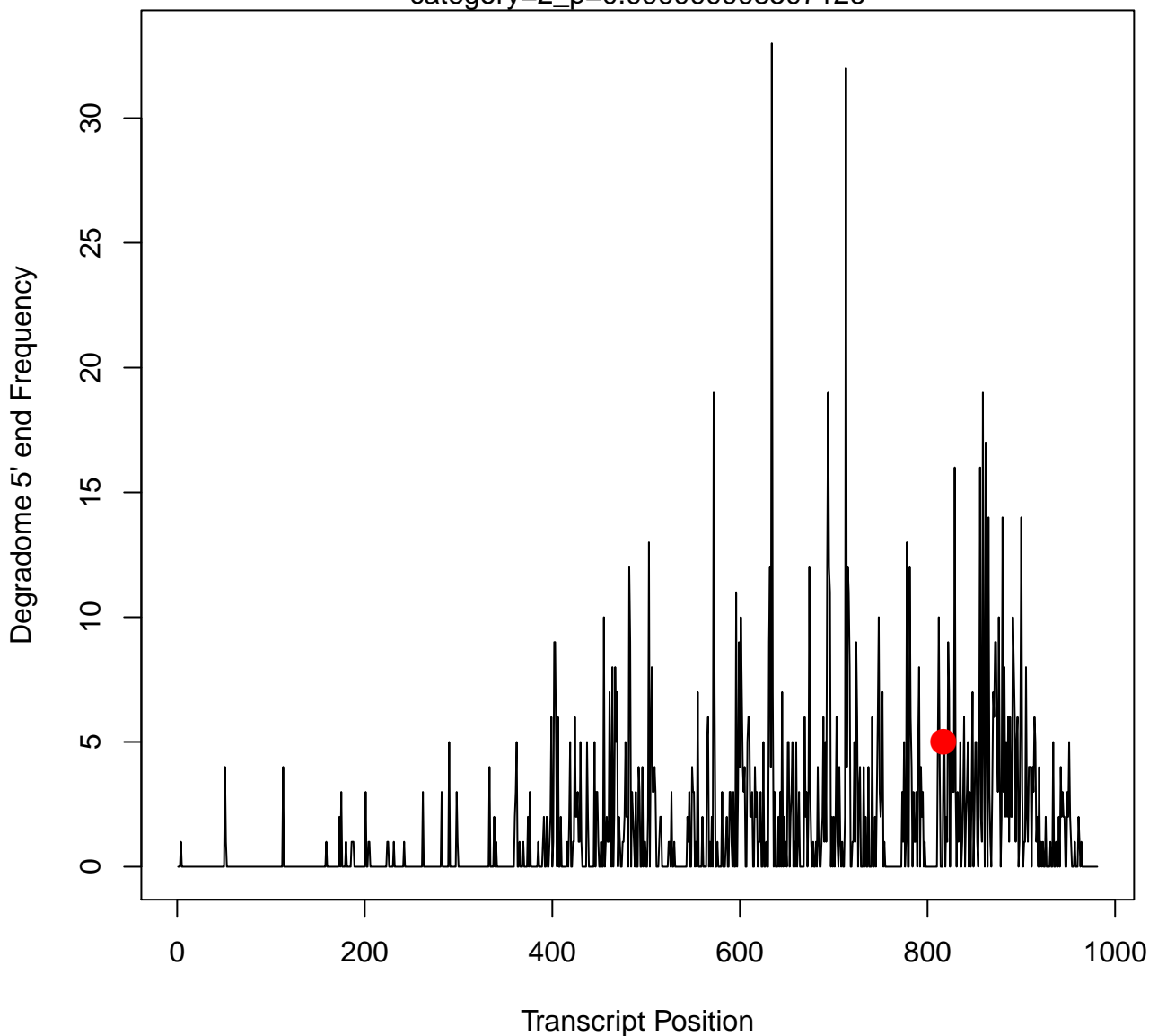

Supplement: Supplementary file 3 [file Data_Sheet_3.zip › Sit-miR160c_Seita.2G137400.1_817_TPlot.pdf]
