# Supplementary figures and images for: The Construction and Exploration of a Comprehensive MicroRNA Centered Regulatory Network in Foxtail Millet (Setaria italica L.) (part 8 of 14)
Source: Front Plant Sci. 2022 May 6;13:848474. doi: 10.3389/fpls.2022.848474 (PMC9121102; doi:10.3389/fpls.2022.848474)

**T=Seita.1G241500.1\_Q=Sit-miR160d\_S=1660**

category=0\_p=0.00190818165876372

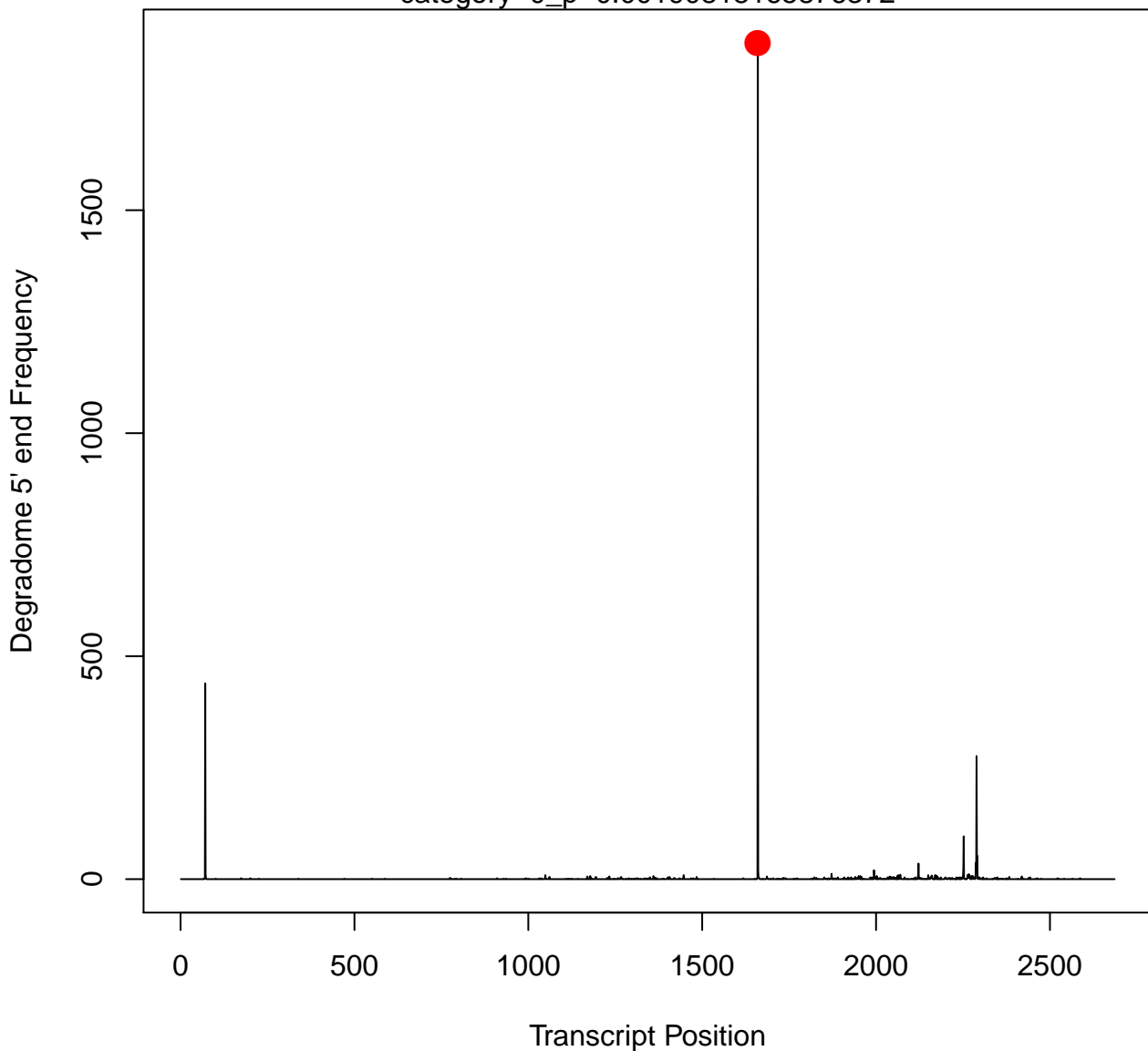

Supplement: Supplementary file 4 [file Data_Sheet_4.zip › Sit-miR160d_Seita.1G241500.1_1660_TPlot.pdf]

**T=Seita.4G123000.1\_Q=Sit-miR160d\_S=2617**

category=2\_p=0.999999860952524

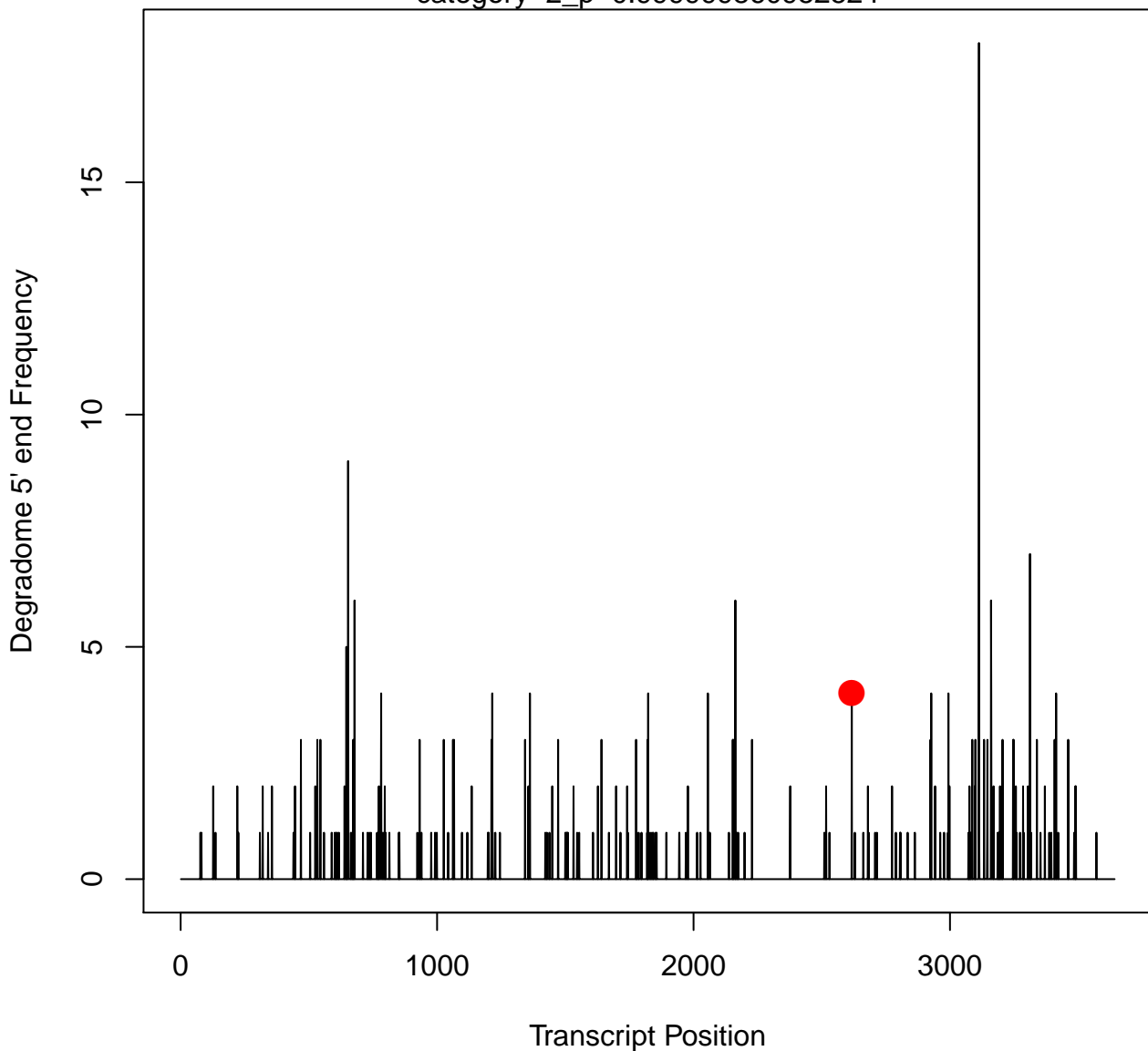

Supplement: Supplementary file 4 [file Data_Sheet_4.zip › Sit-miR160d_Seita.4G123000.1_2617_TPlot.pdf]

**T=Seita.4G257800.1\_Q=Sit-miR160d\_S=1974**

category=0\_p=0.000763710047598232

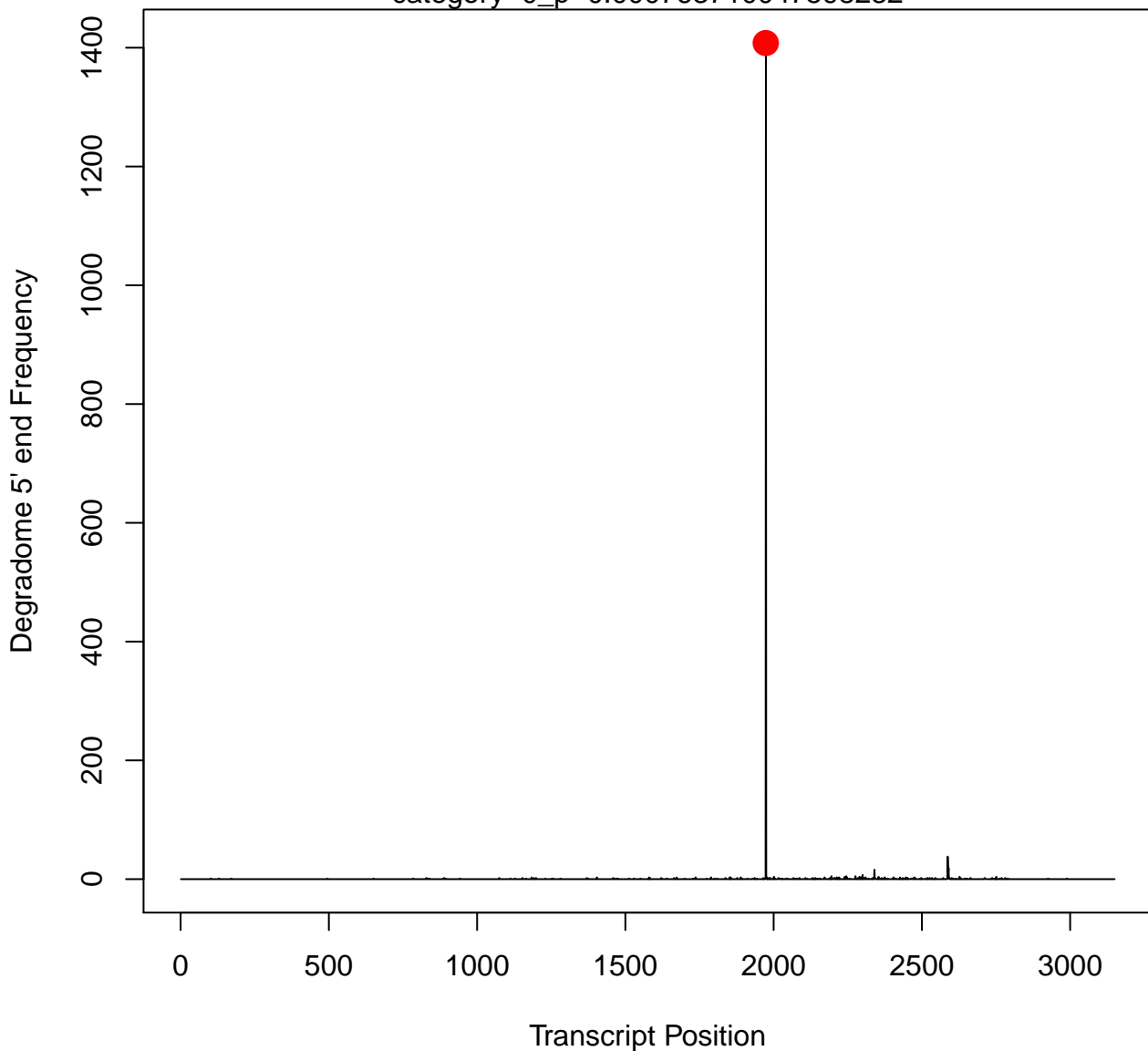

Supplement: Supplementary file 4 [file Data_Sheet_4.zip › Sit-miR160d_Seita.4G257800.1_1974_TPlot.pdf]

**T=Seita.5G273800.1\_Q=Sit-miR160d\_S=477**

category=2\_p=0.999999998553017

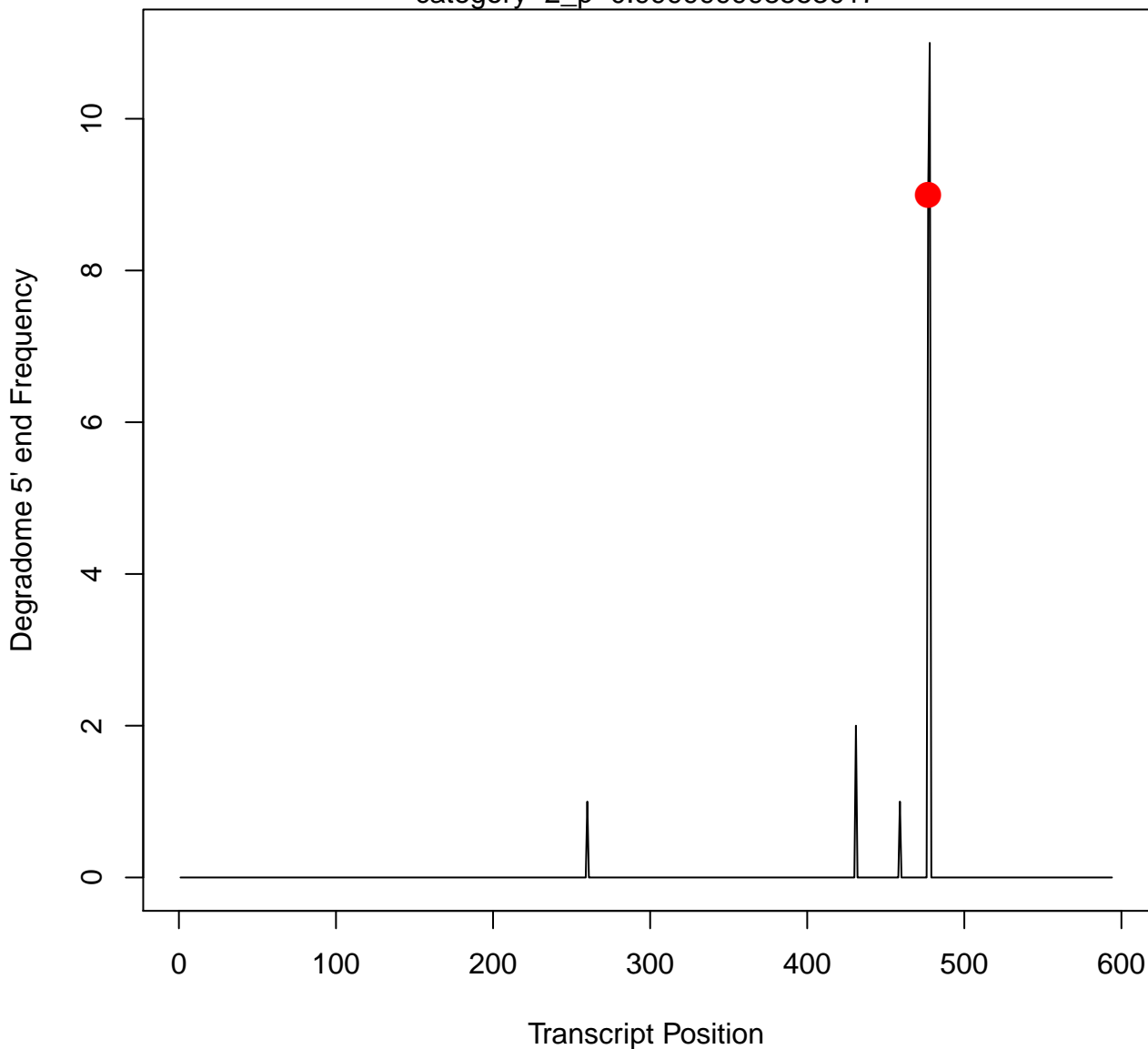

Supplement: Supplementary file 4 [file Data_Sheet_4.zip › Sit-miR160d_Seita.5G273800.1_477_TPlot.pdf]

**T=Seita.6G024600.1\_Q=Sit-miR160d\_S=310**

category=2\_p=0.158802963625695

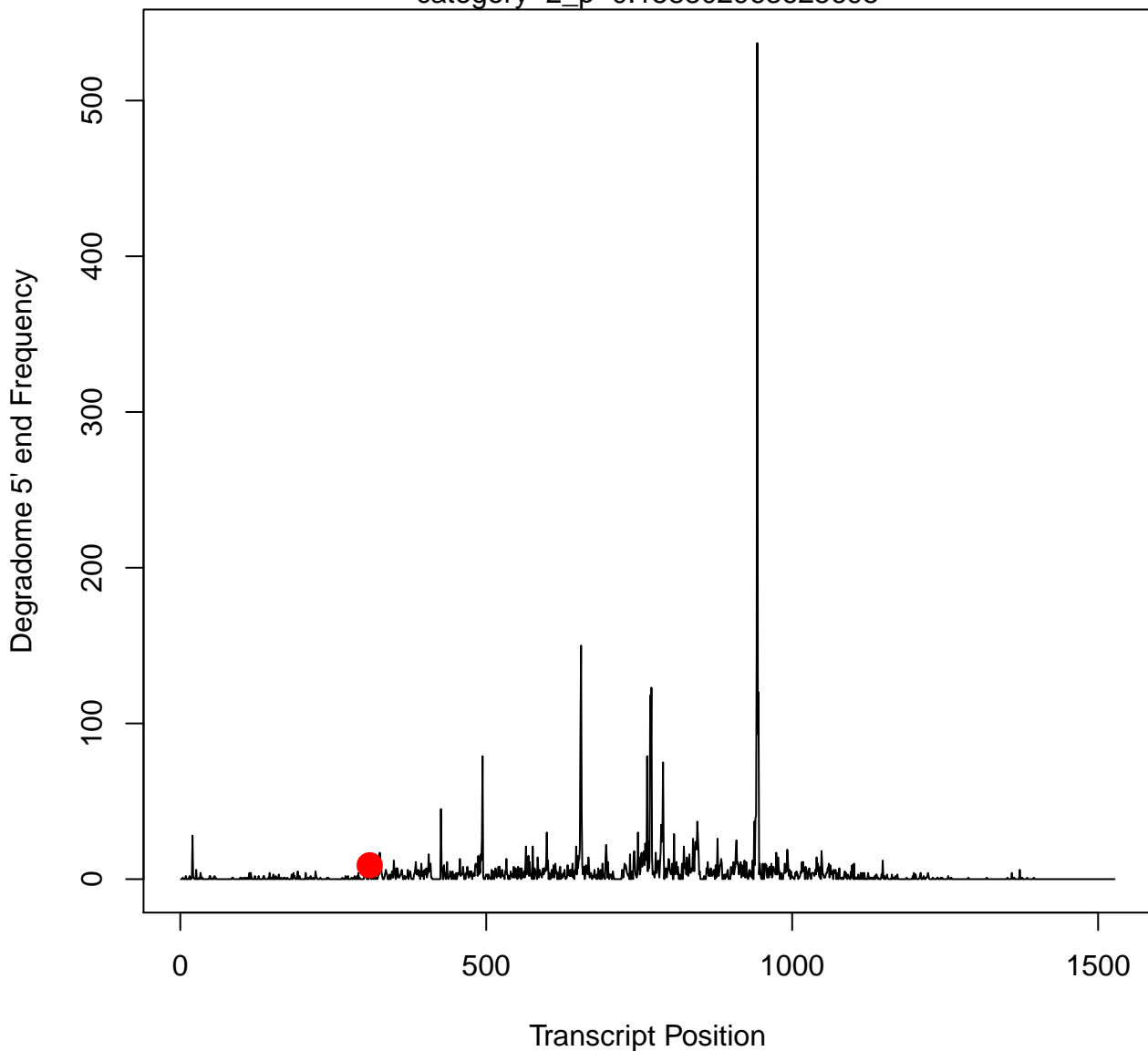

Supplement: Supplementary file 4 [file Data_Sheet_4.zip › Sit-miR160d_Seita.6G024600.1_310_TPlot.pdf]

**T=Seita.7G169600.1\_Q=Sit-miR160d\_S=1802**

category=0\_p=0.000381927958281736

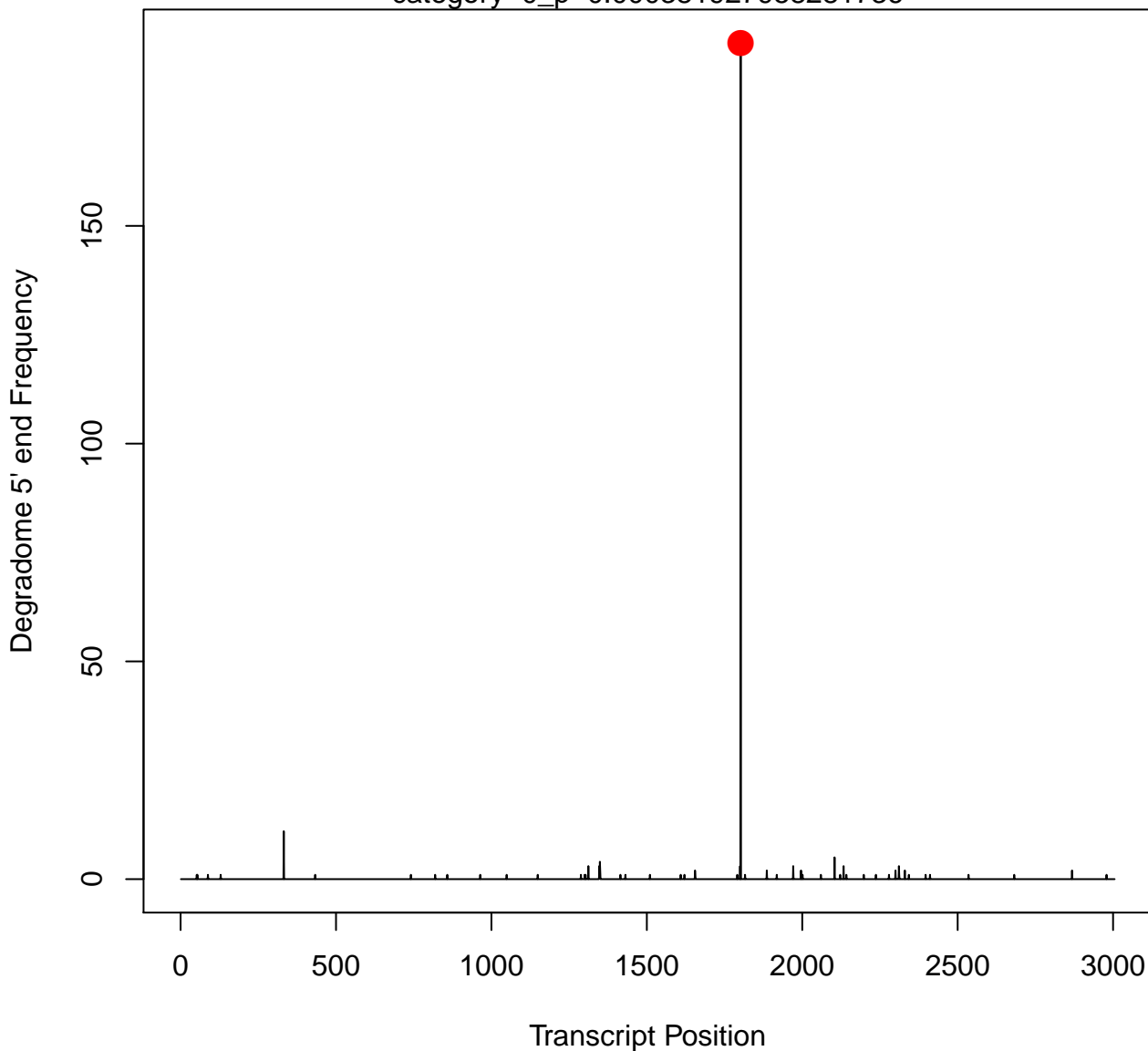

Supplement: Supplementary file 4 [file Data_Sheet_4.zip › Sit-miR160d_Seita.7G169600.1_1802_TPlot.pdf]

**T=Seita.7G295800.1\_Q=Sit-miR160d\_S=368**

category=2\_p=0.999999999996353

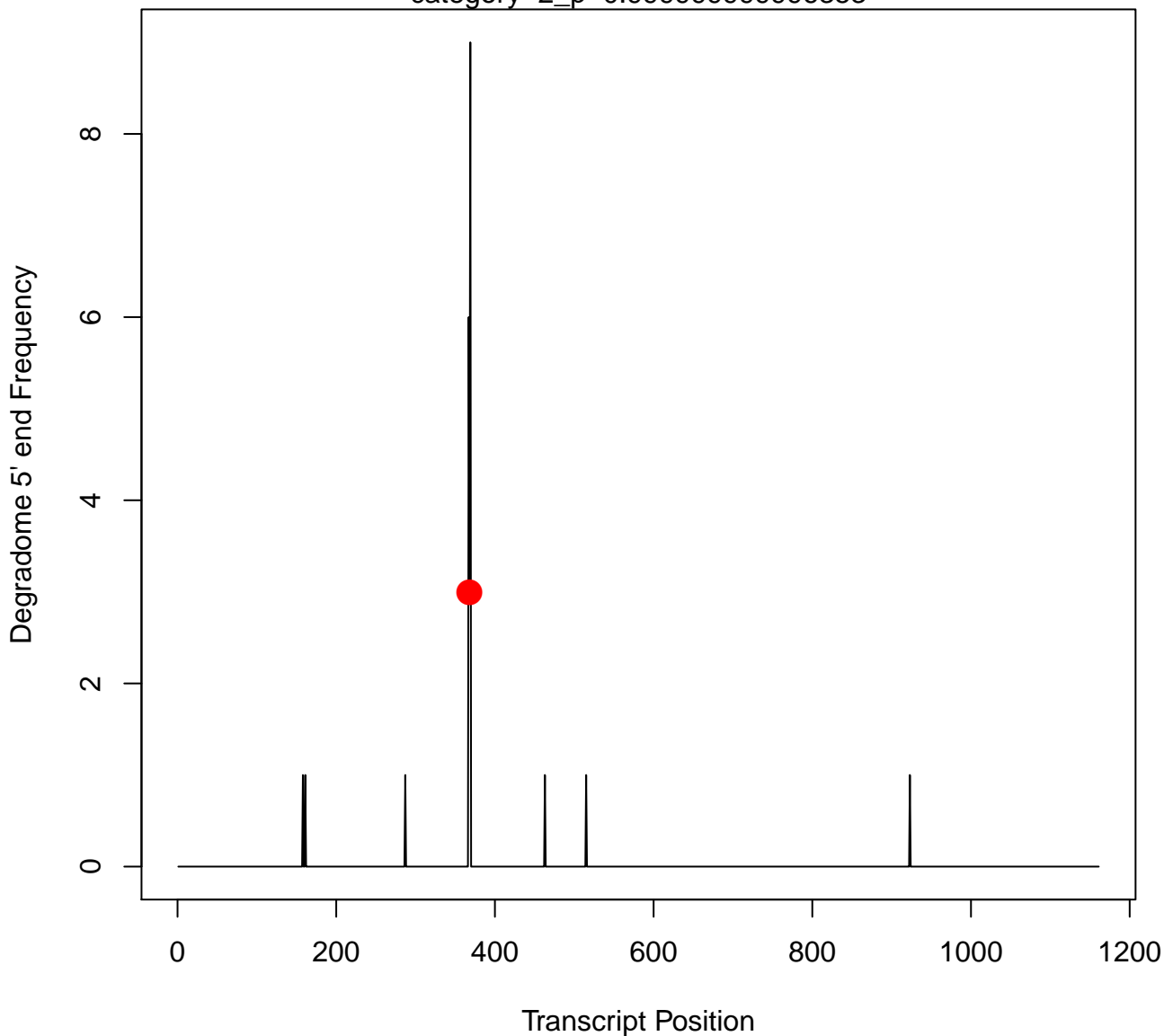

Supplement: Supplementary file 4 [file Data_Sheet_4.zip › Sit-miR160d_Seita.7G295800.1_368_TPlot.pdf]

**T=Seita.9G142000.1\_Q=Sit-miR160d\_S=849**

category=2\_p=0.999999999999976

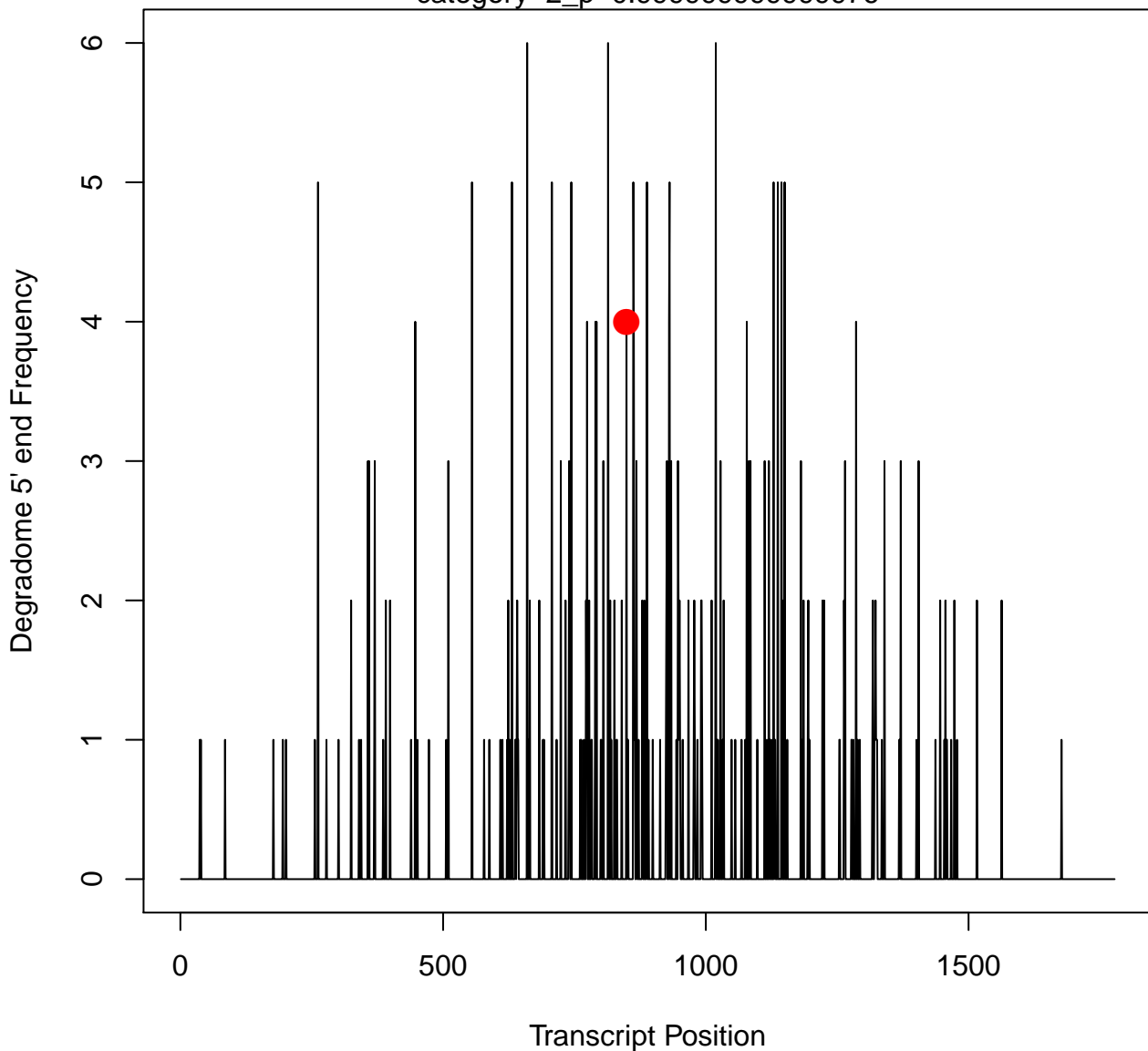

Supplement: Supplementary file 4 [file Data_Sheet_4.zip › Sit-miR160d_Seita.9G142000.1_849_TPlot.pdf]

**T=Seita.9G295400.1\_Q=Sit-miR160d\_S=103**

category=2\_p=0.999999999966639

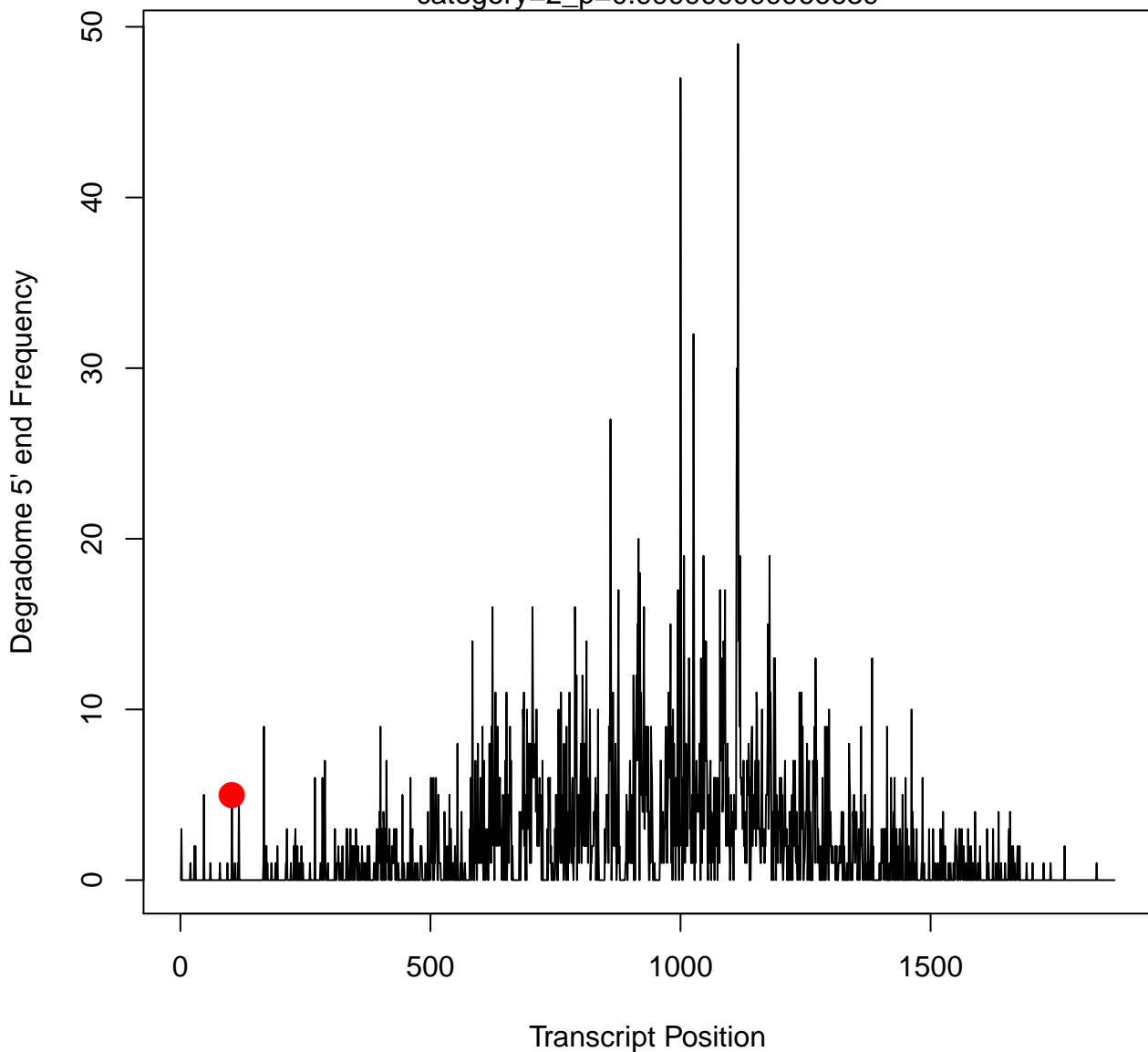

Supplement: Supplementary file 4 [file Data_Sheet_4.zip › Sit-miR160d_Seita.9G295400.1_103_TPlot.pdf]

**T=Seita.9G458800.1\_Q=Sit-miR162\_S=769**

category=2\_p=0.853322044825994

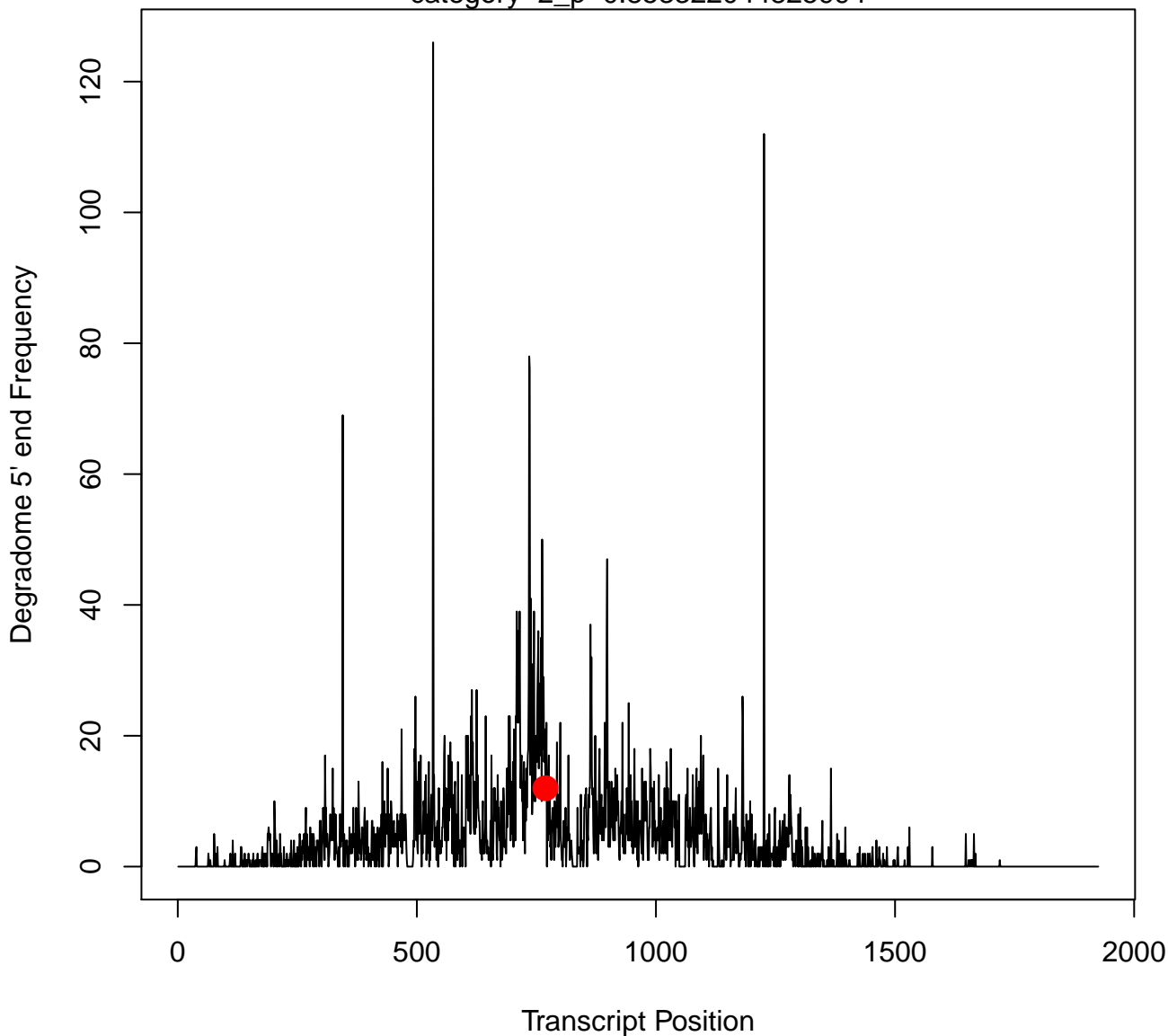

Supplement: Supplementary file 4 [file Data_Sheet_4.zip › Sit-miR162_Seita.9G458800.1_769_TPlot.pdf]

**T=Seita.9G562200.1\_Q=Sit-miR162\_S=3530**

category=0\_p=0.000381927958281736

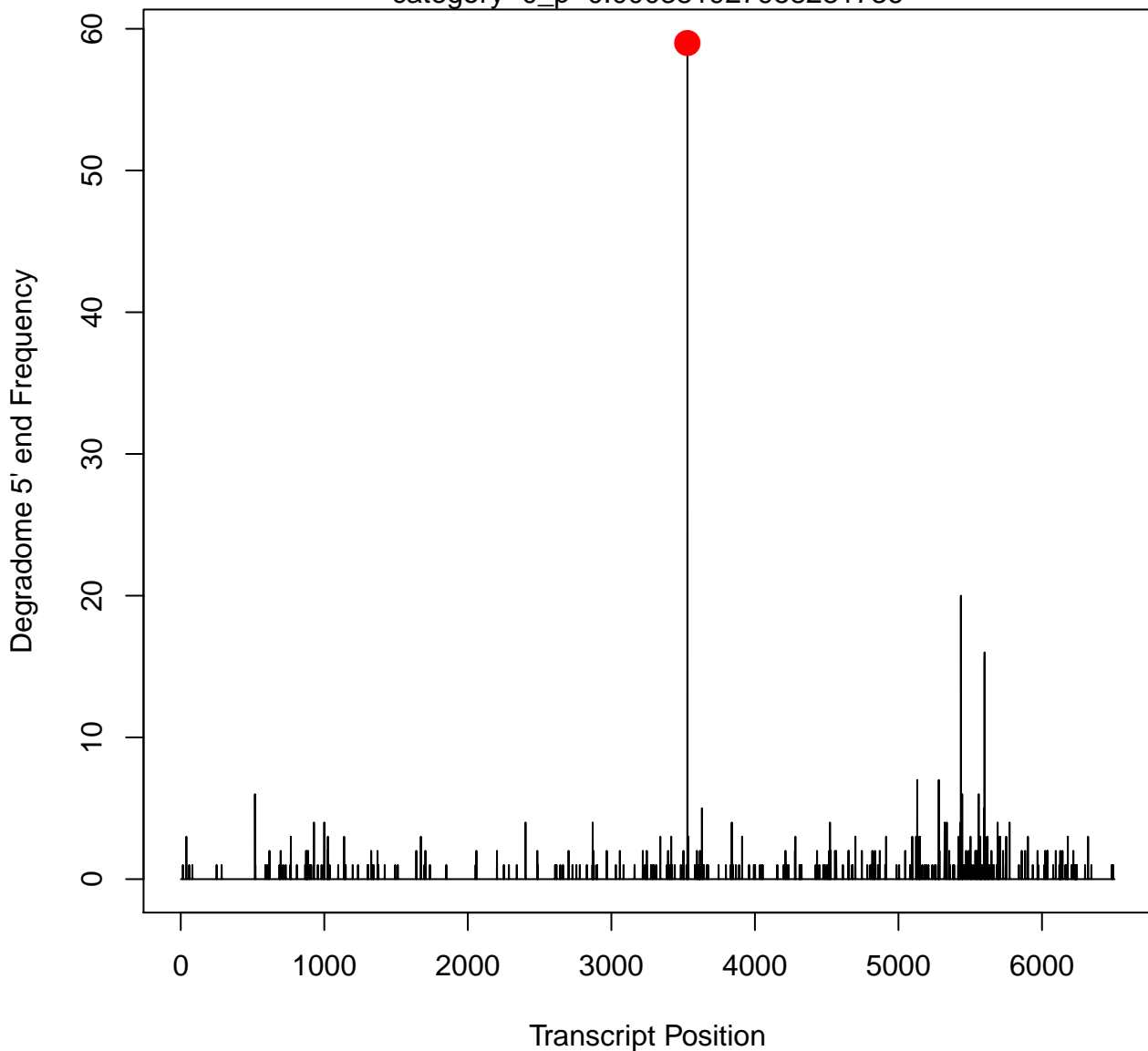

Supplement: Supplementary file 4 [file Data_Sheet_4.zip › Sit-miR162_Seita.9G562200.1_3530_TPlot.pdf]

**T=Seita.J001500.1\_Q=Sit-miR162\_S=1487**

category=2\_p=0.785419707886666

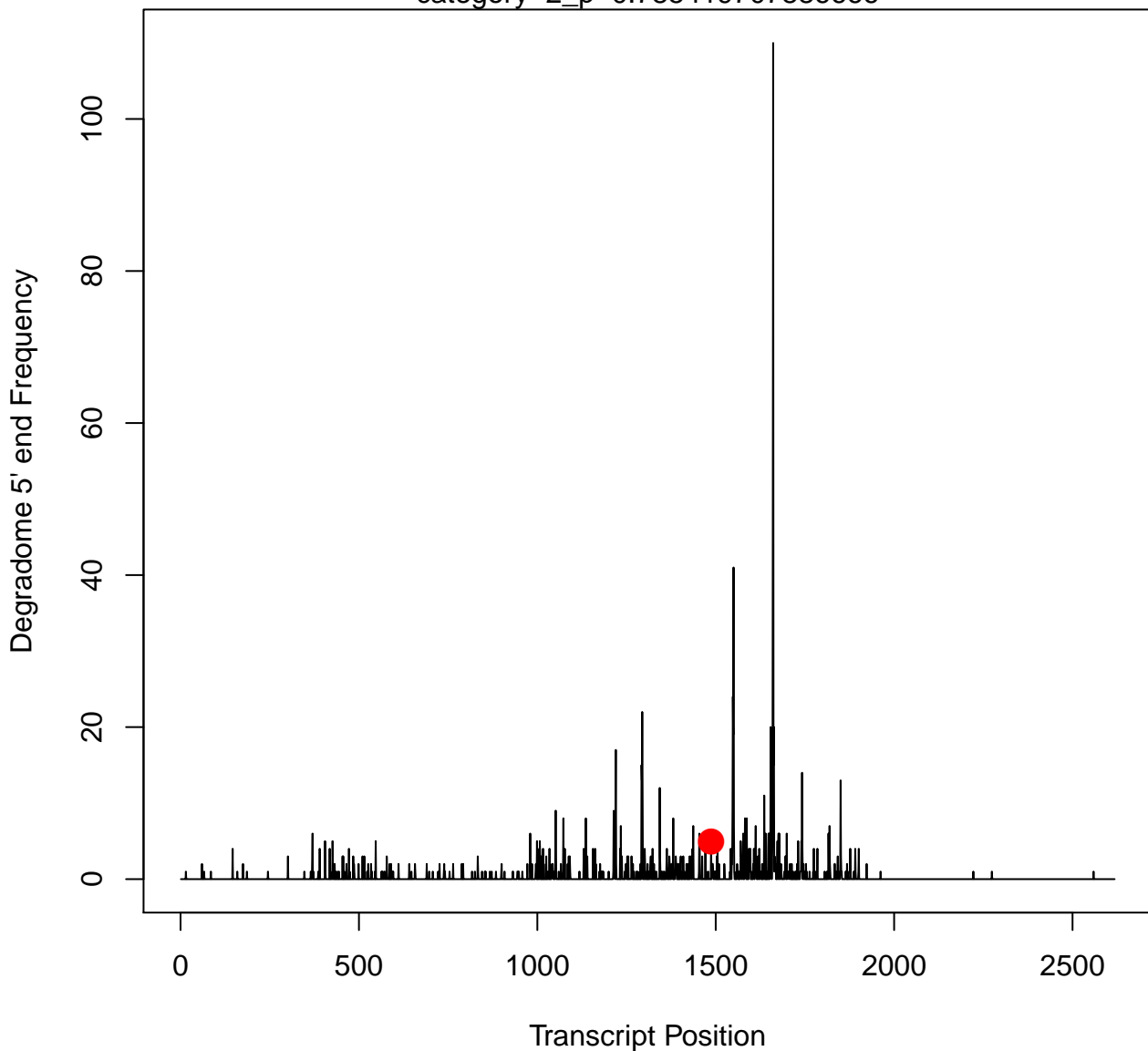

Supplement: Supplementary file 4 [file Data_Sheet_4.zip › Sit-miR162_Seita.J001500.1_1487_TPlot.pdf]

**T=Seita.1G209000.1\_Q=Sit-miR164a\_S=999**

category=0\_p=0.00190818165876372

Degradome 5' end Frequency

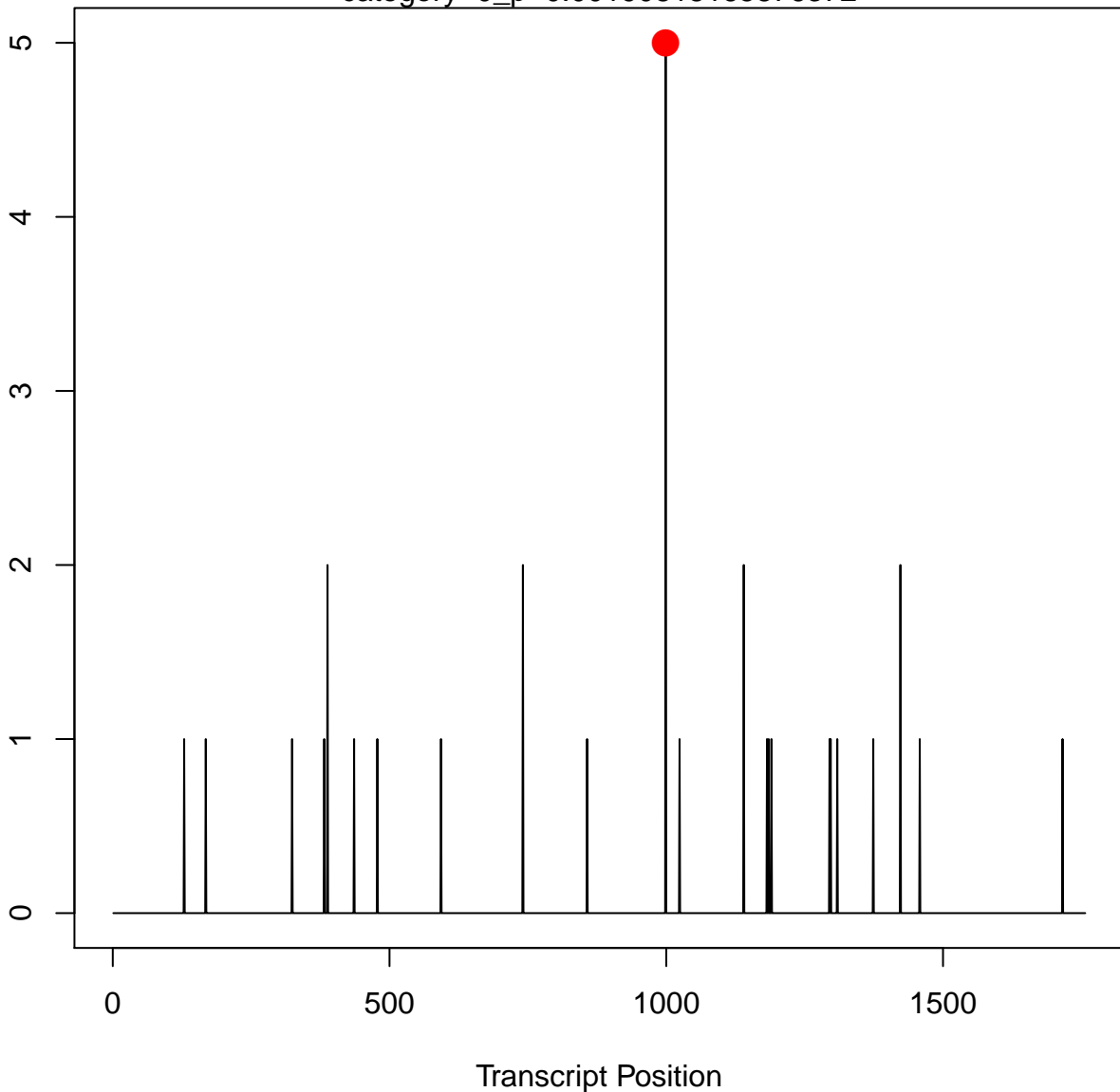

Supplement: Supplementary file 4 [file Data_Sheet_4.zip › Sit-miR164a_Seita.1G209000.1_999_TPlot.pdf]

**T=Seita.1G267400.1\_Q=Sit-miR164a\_S=640**

category=2\_p=0.620311616542702

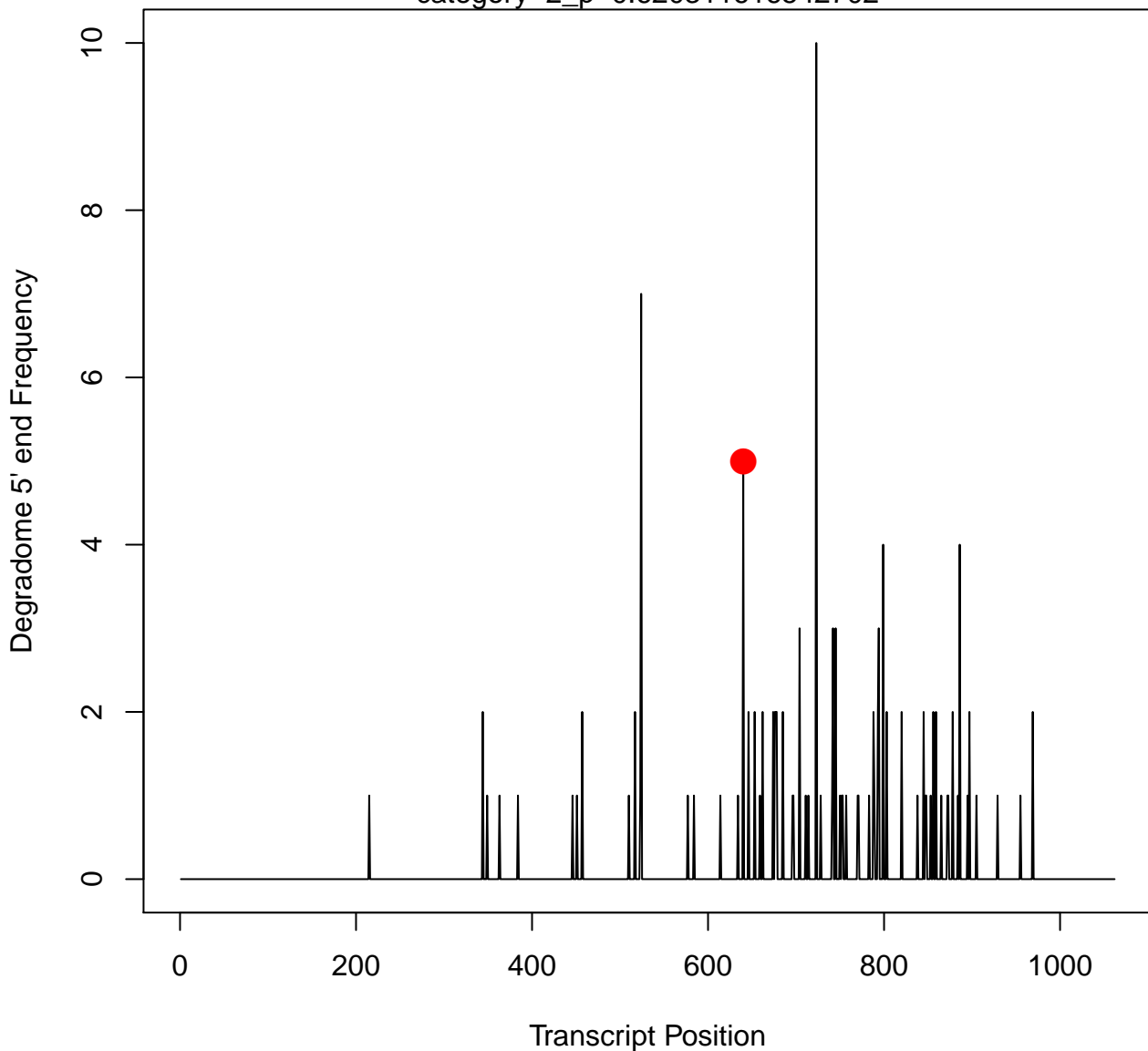

Supplement: Supplementary file 4 [file Data_Sheet_4.zip › Sit-miR164a_Seita.1G267400.1_640_TPlot.pdf]

**T=Seita.7G124900.1\_Q=Sit-miR164a\_S=859**

category=0\_p=0.00152683684215971

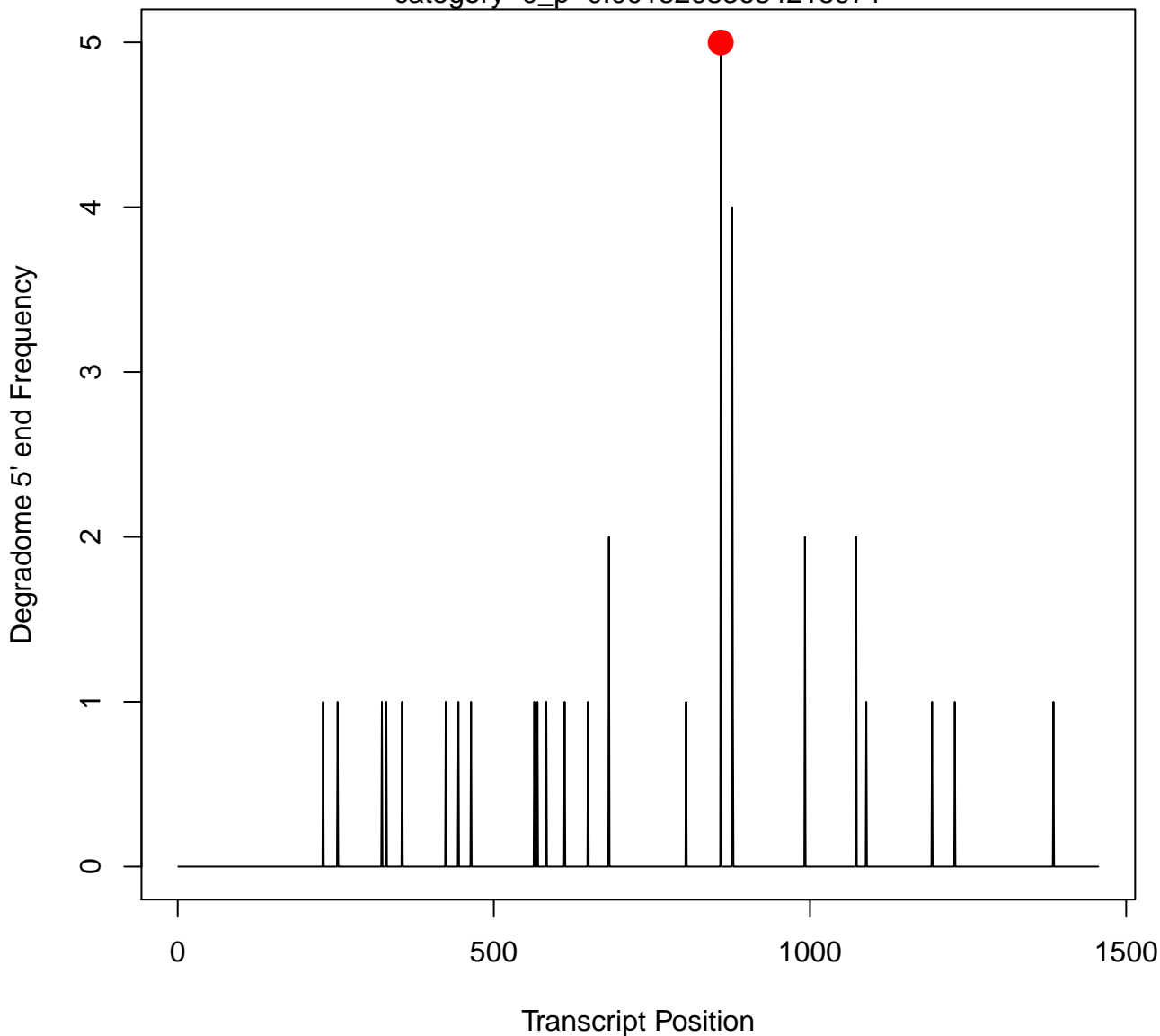

Supplement: Supplementary file 4 [file Data_Sheet_4.zip › Sit-miR164a_Seita.7G124900.1_859_TPlot.pdf]

**T=Seita.2G174200.1\_Q=Sit-miR164b\_S=201**

category=0\_p=0.00114534632366081

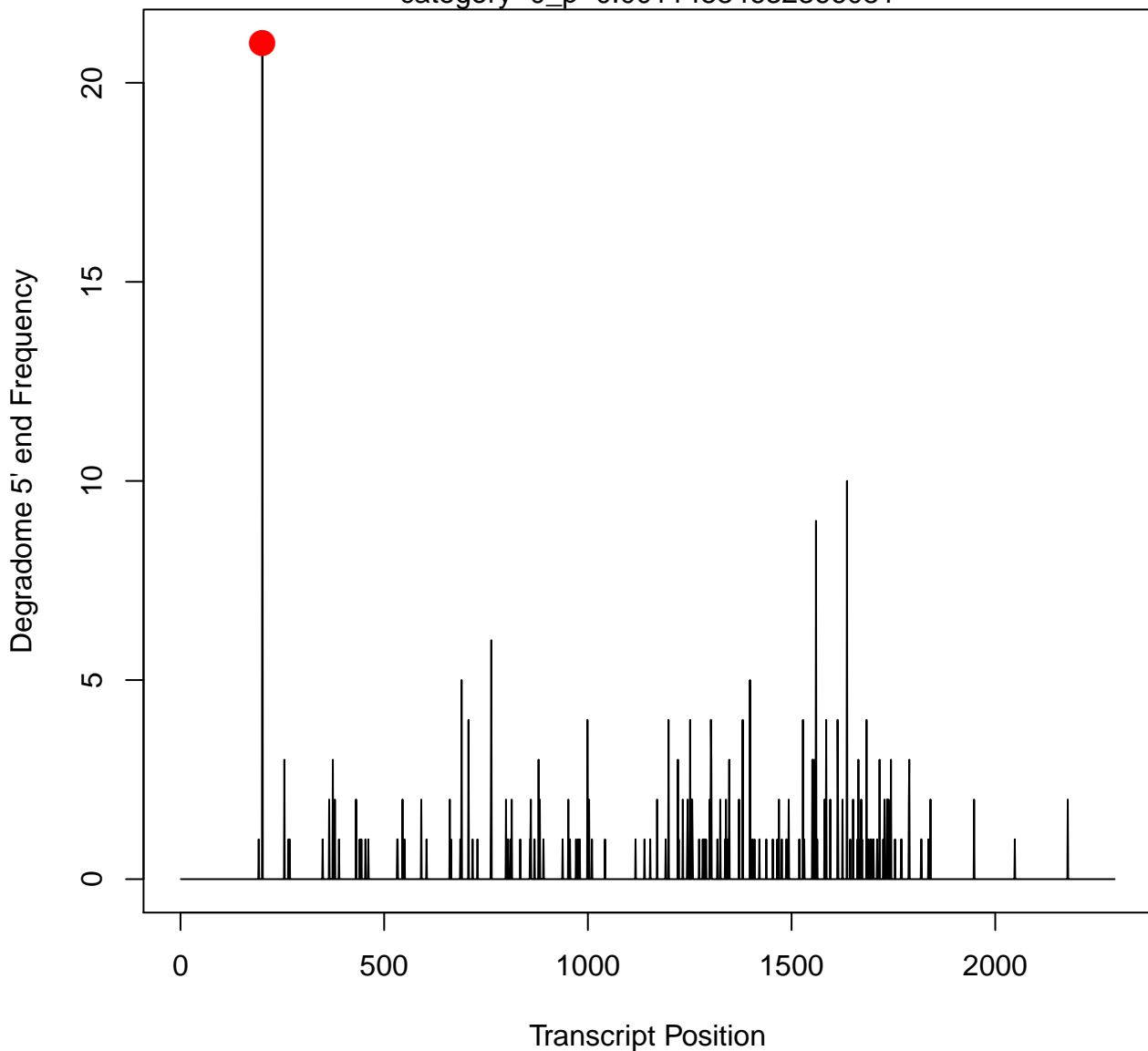

Supplement: Supplementary file 4 [file Data_Sheet_4.zip › Sit-miR164b_Seita.2G174200.1_201_TPlot.pdf]

**T=Seita.2G386600.1\_Q=Sit-miR164b\_S=180**

category=2\_p=0.999999052021991

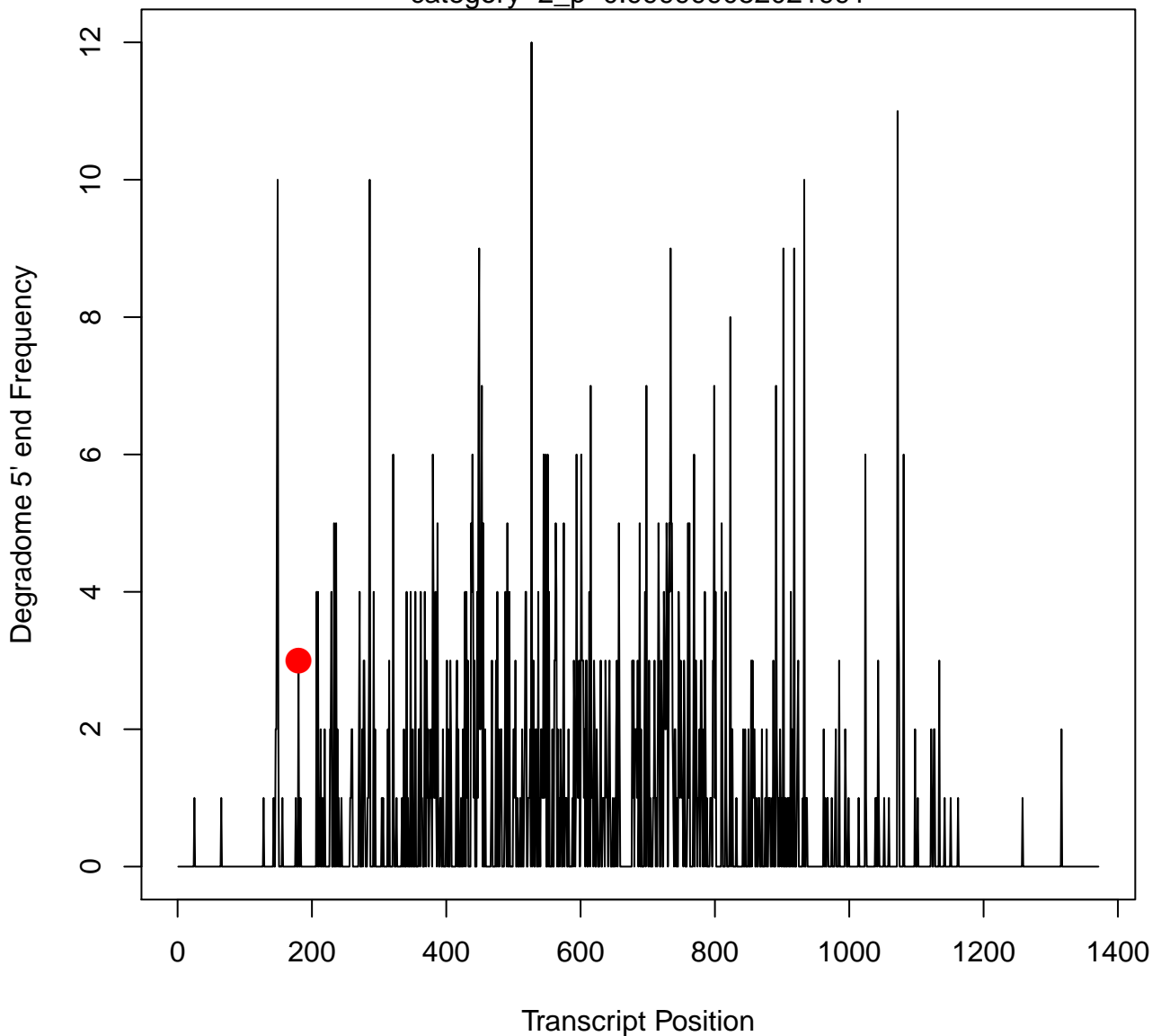

Supplement: Supplementary file 4 [file Data_Sheet_4.zip › Sit-miR164b_Seita.2G386600.1_180_TPlot.pdf]

**T=Seita.5G086100.1\_Q=Sit-miR164b\_S=1397**

category=2\_p=0.9997910756962

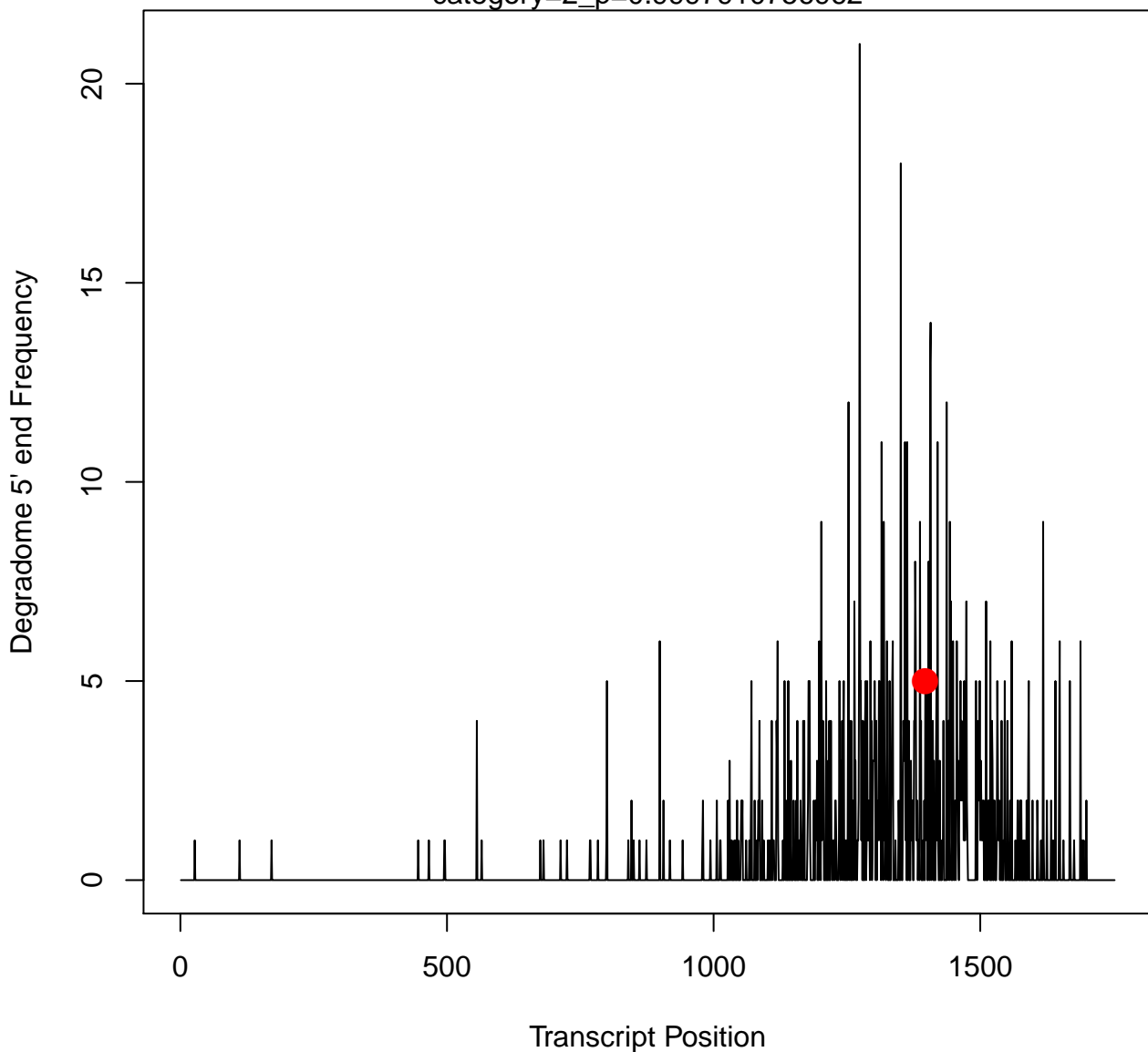

Supplement: Supplementary file 4 [file Data_Sheet_4.zip › Sit-miR164b_Seita.5G086100.1_1397_TPlot.pdf]

**T=Seita.2G271300.1\_Q=Sit-miR164e\_S=1443**

category=2\_p=0.999998073728258

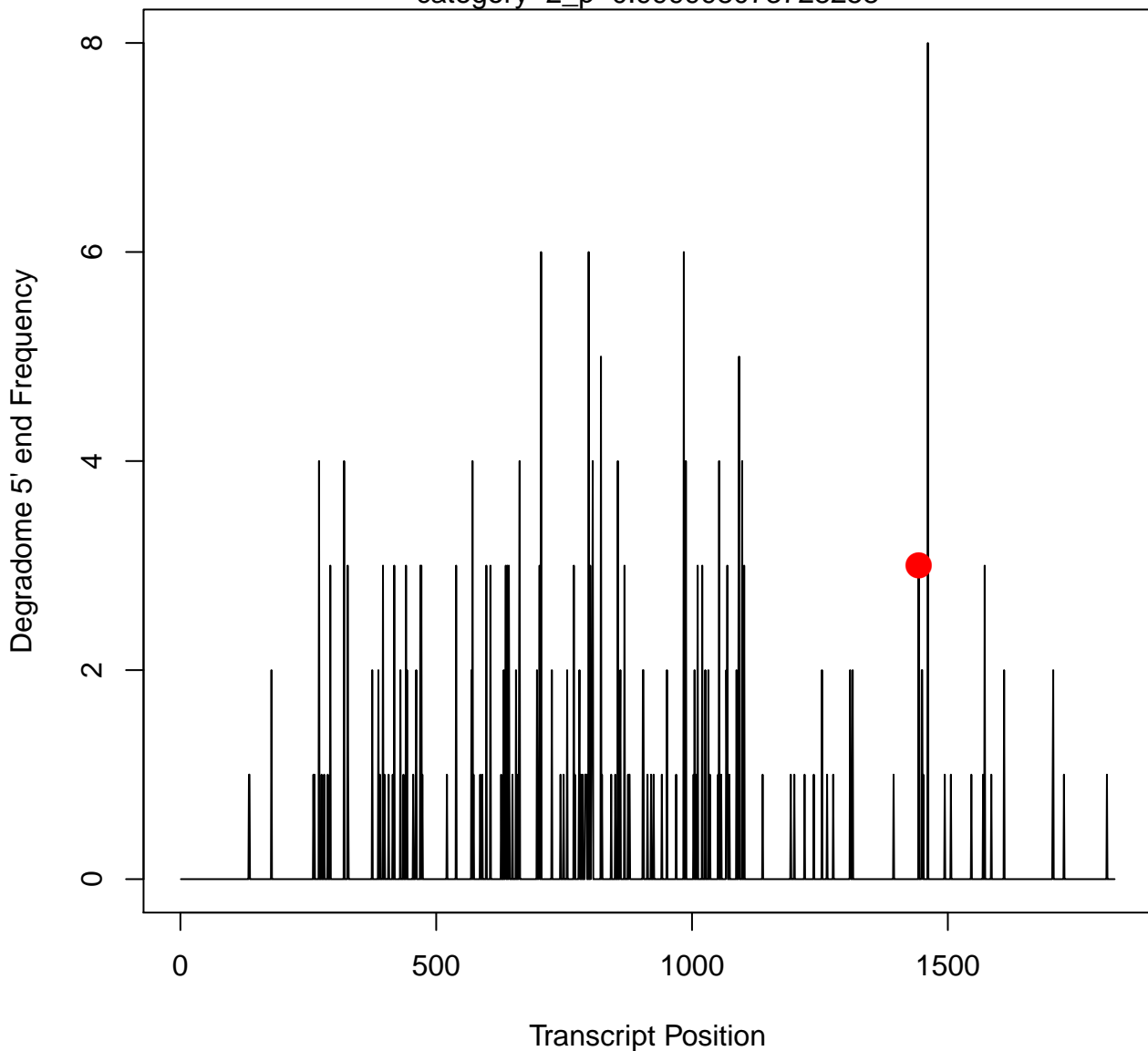

Supplement: Supplementary file 4 [file Data_Sheet_4.zip › Sit-miR164e_Seita.2G271300.1_1443_TPlot.pdf]

**T=Seita.3G120800.1\_Q=Sit-miR164e\_S=2100**

category=2\_p=0.999704747559745

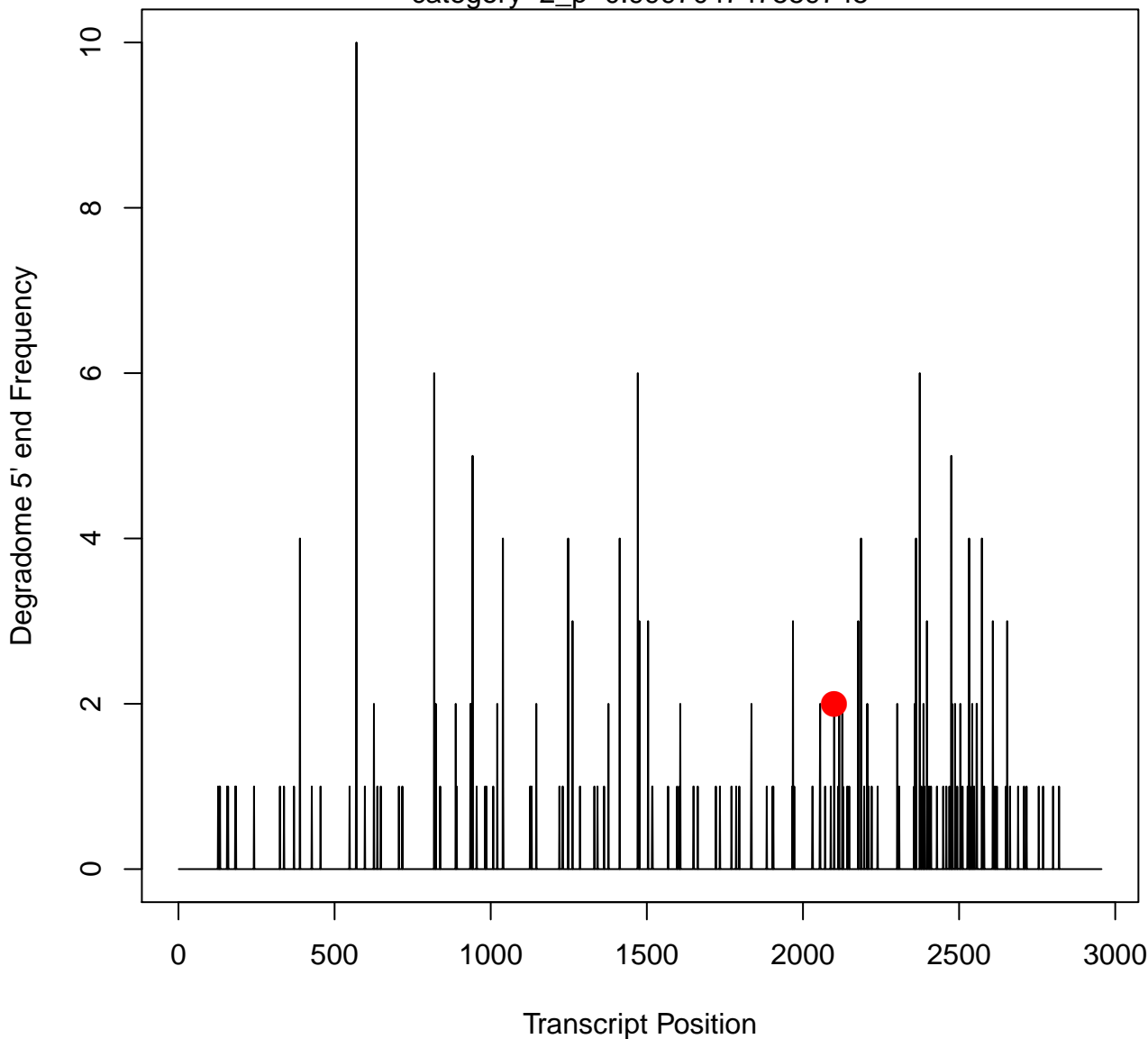

Supplement: Supplementary file 4 [file Data_Sheet_4.zip › Sit-miR164e_Seita.3G120800.1_2100_TPlot.pdf]

**T=Seita.3G324800.1\_Q=Sit-miR164e\_S=887**

category=2\_p=0.947123648956648

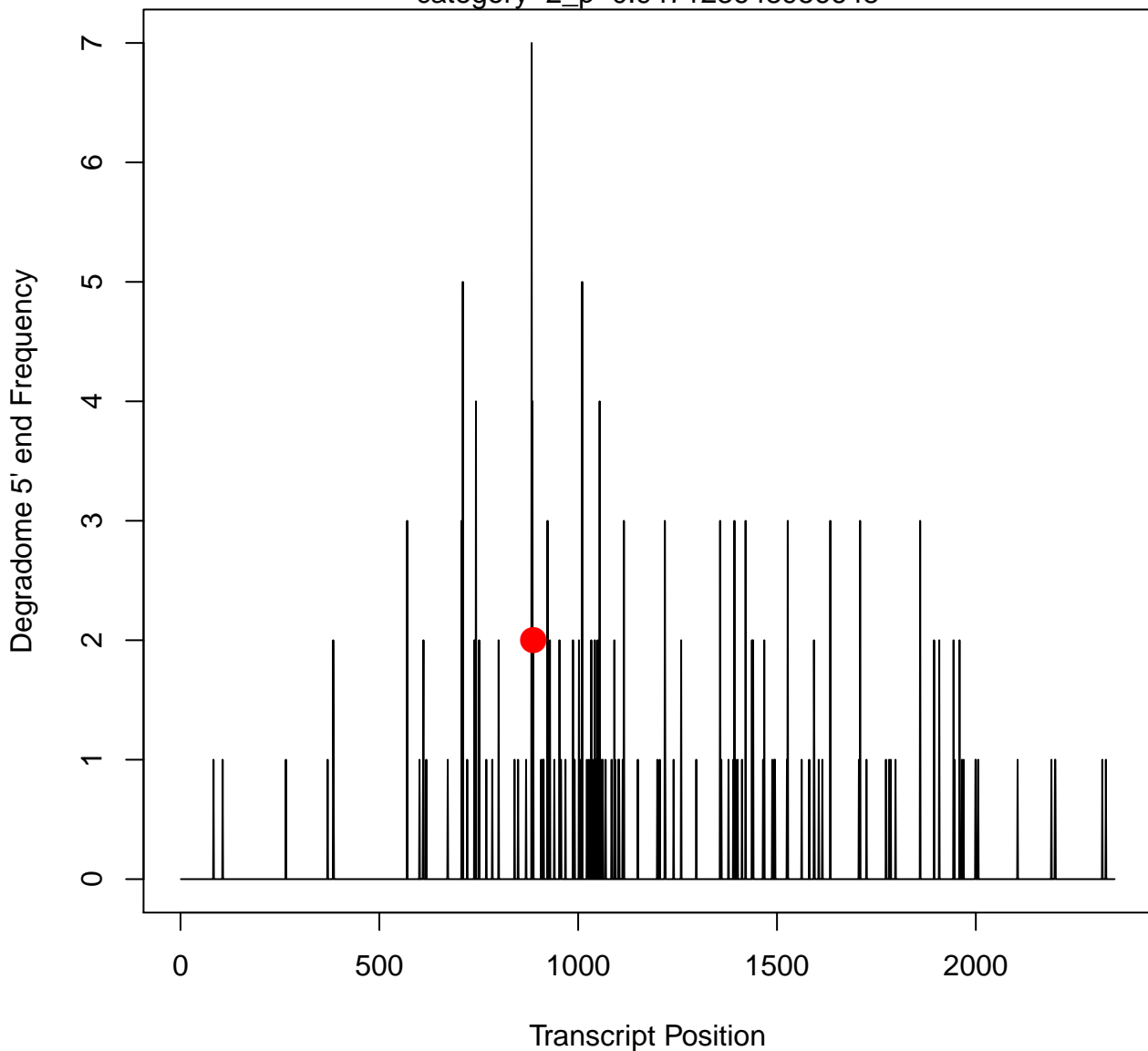

Supplement: Supplementary file 4 [file Data_Sheet_4.zip › Sit-miR164e_Seita.3G324800.1_887_TPlot.pdf]

**T=Seita.4G090500.1\_Q=Sit-miR164e\_S=744**

category=2\_p=0.999642886730072

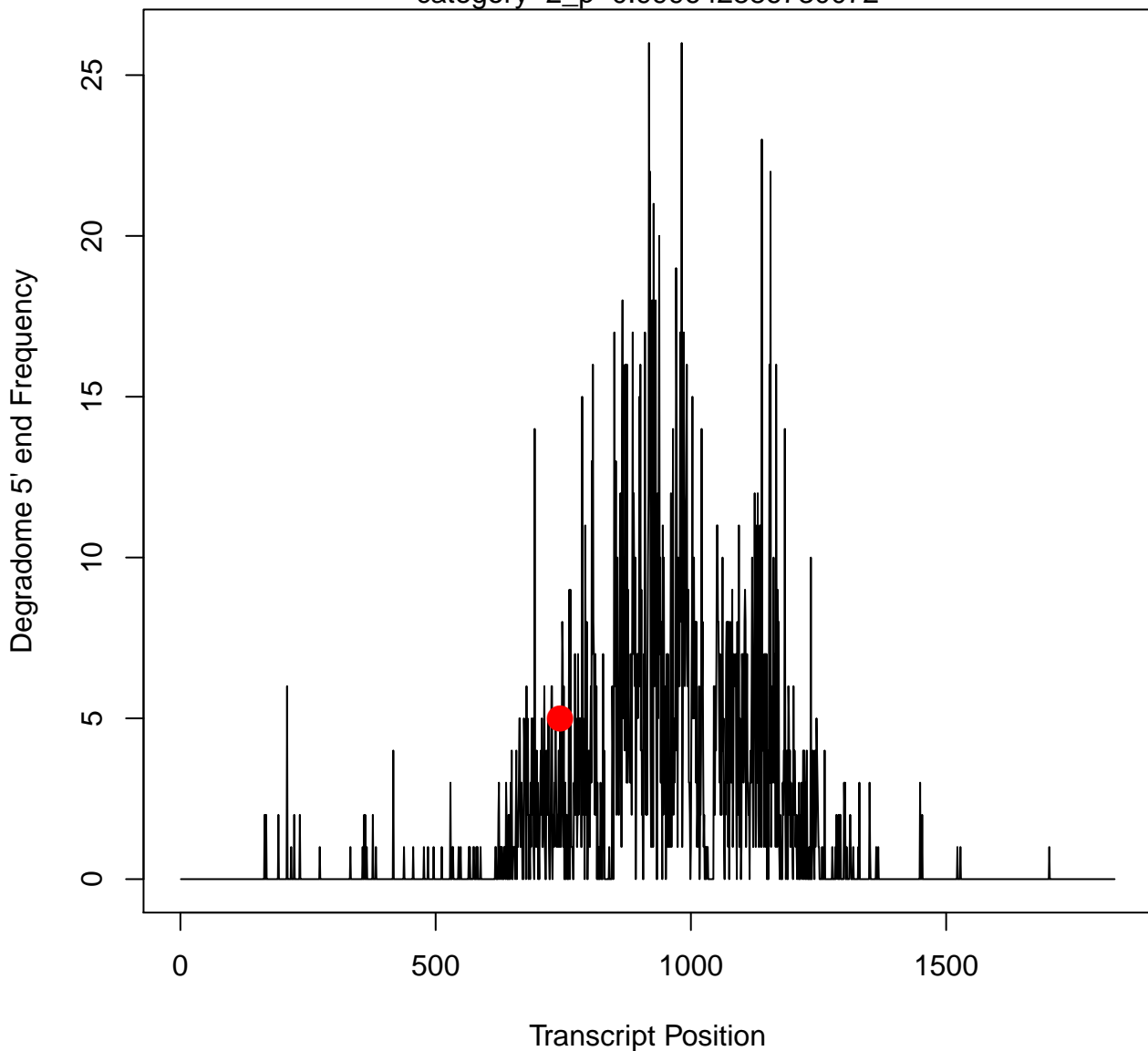

Supplement: Supplementary file 4 [file Data_Sheet_4.zip › Sit-miR164e_Seita.4G090500.1_744_TPlot.pdf]

**T=Seita.7G286800.1\_Q=Sit-miR164e\_S=1119**

category=2\_p=0.999998322603468

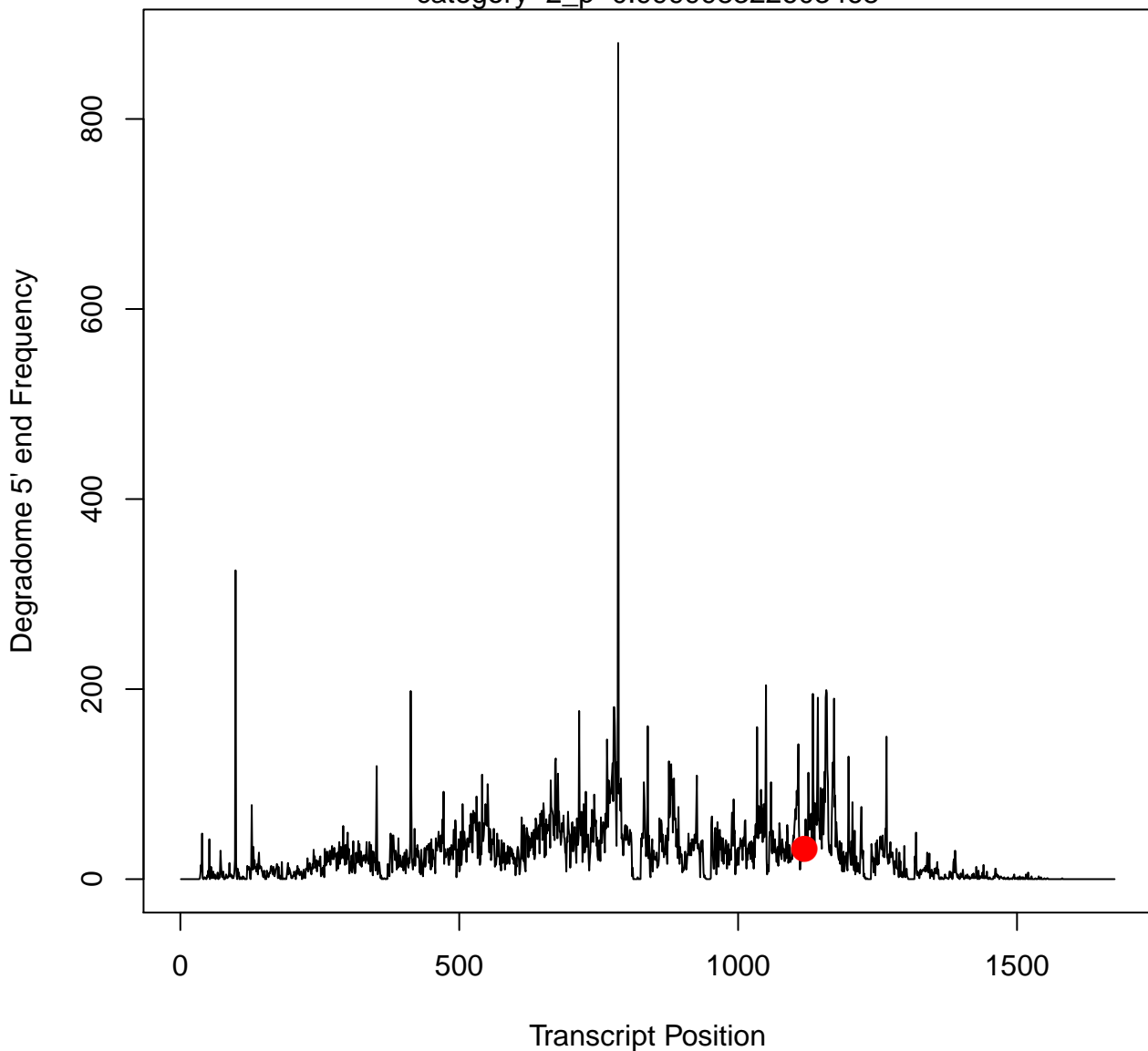

Supplement: Supplementary file 4 [file Data_Sheet_4.zip › Sit-miR164e_Seita.7G286800.1_1119_TPlot.pdf]

**T=Seita.9G108000.1\_Q=Sit-miR164e\_S=364**

category=2\_p=0.999871263226975

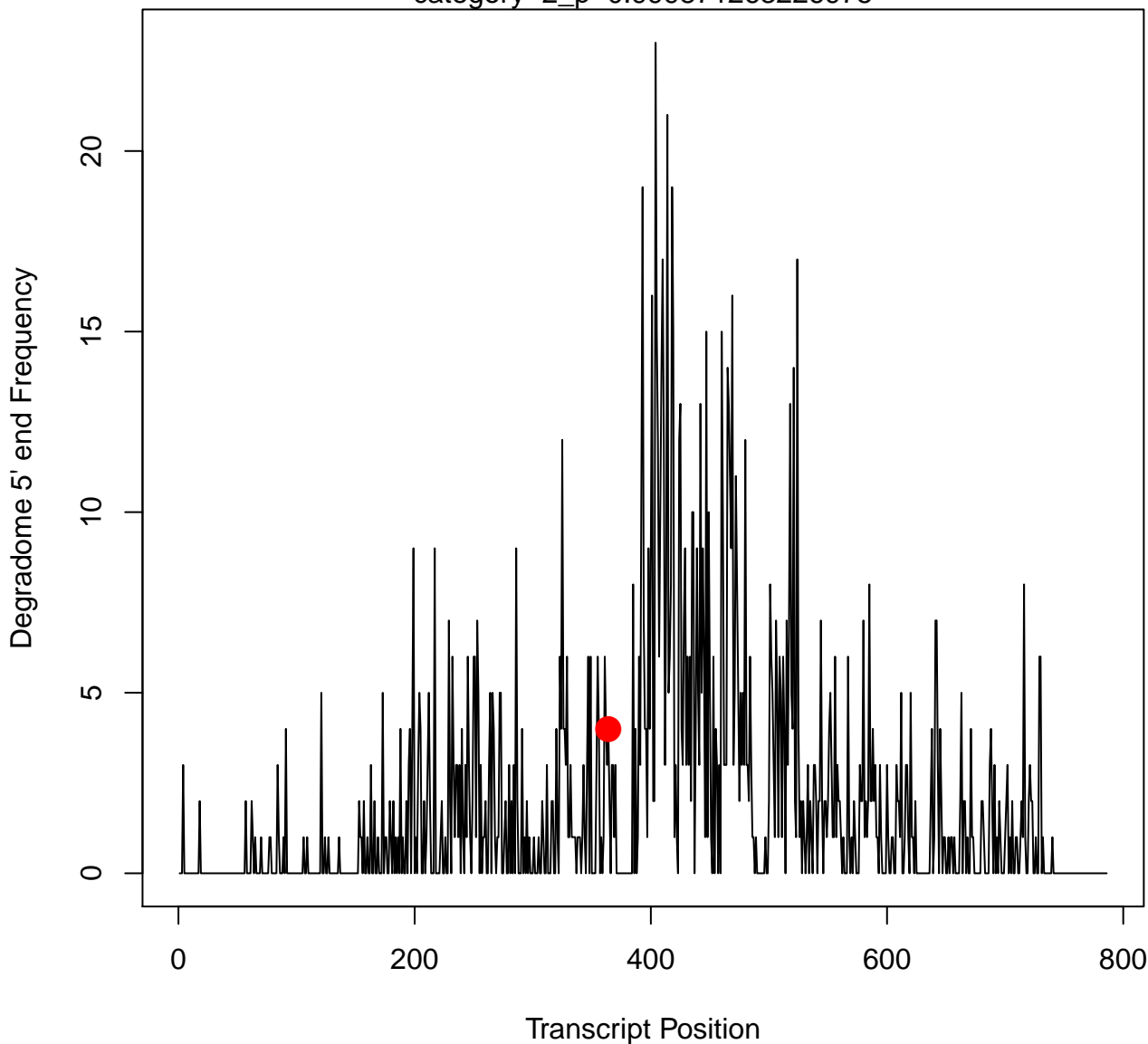

Supplement: Supplementary file 4 [file Data_Sheet_4.zip › Sit-miR164e_Seita.9G108000.1_364_TPlot.pdf]

**T=Seita.J003600.1\_Q=Sit-miR164e\_S=116**

category=2\_p=0.999965412004266

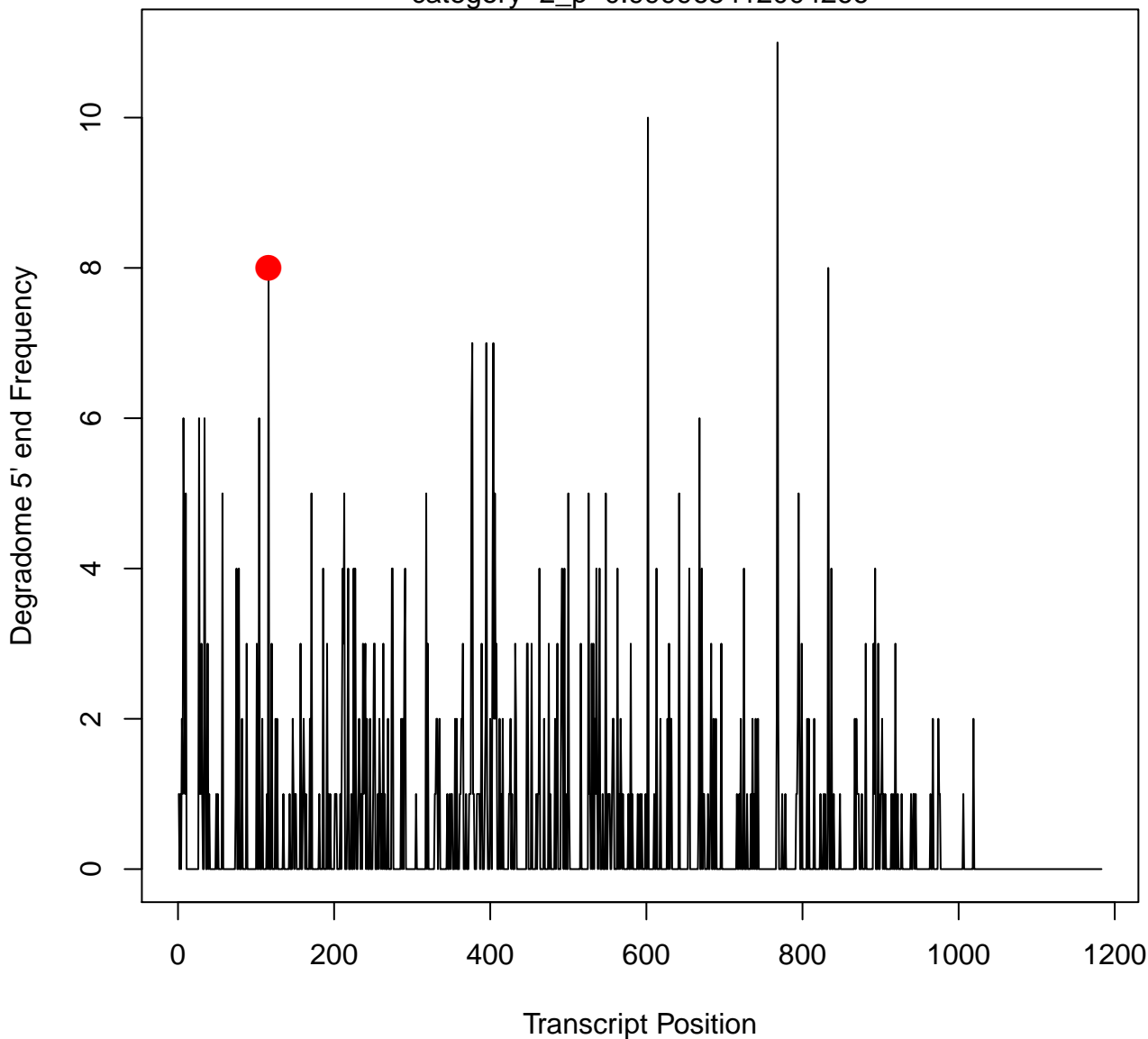

Supplement: Supplementary file 4 [file Data_Sheet_4.zip › Sit-miR164e_Seita.J003600.1_116_TPlot.pdf]

**T=Seita.3G001500.1\_Q=Sit-miR164f\_S=686**

category=2\_p=0.987193722876198

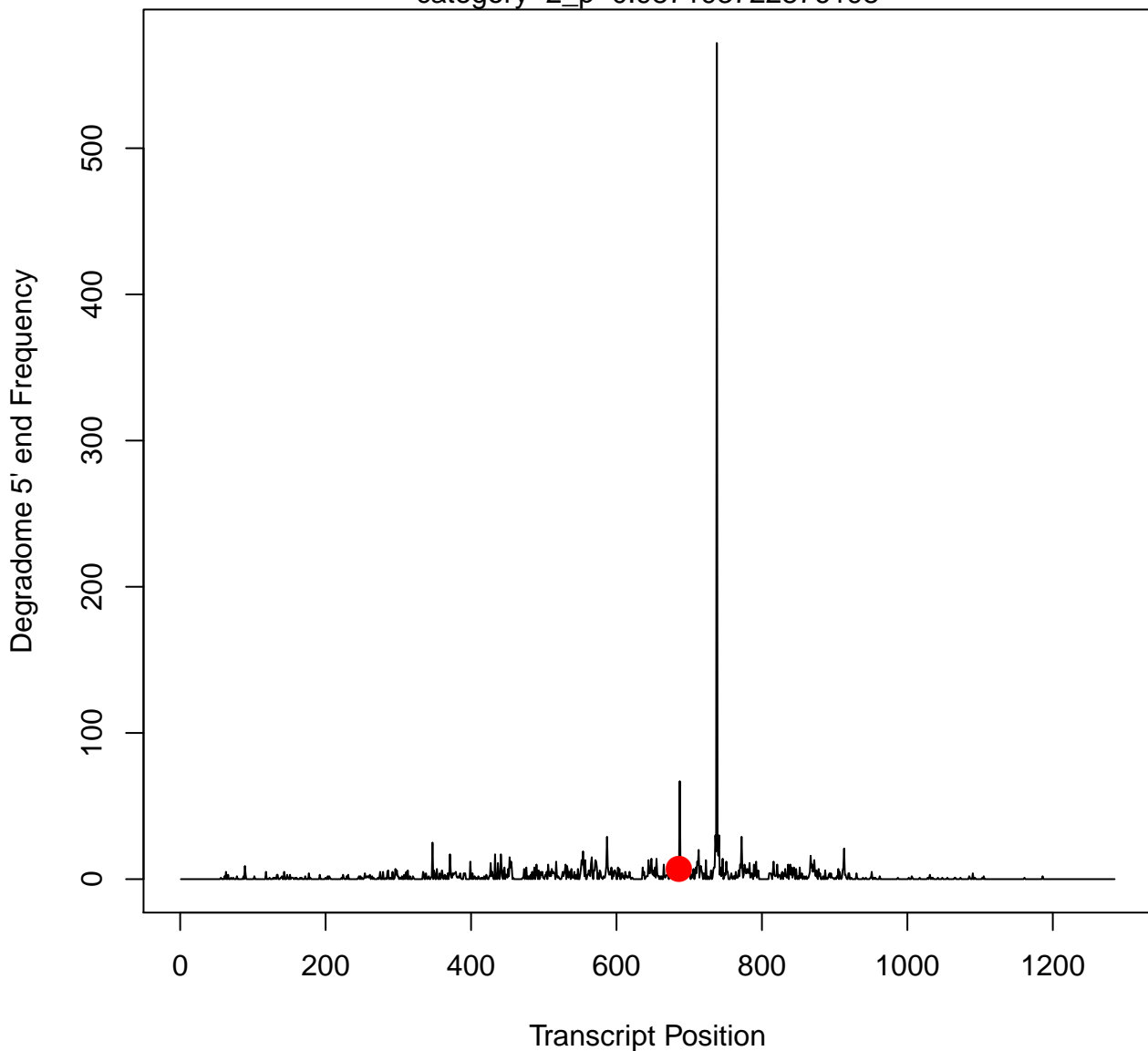

Supplement: Supplementary file 4 [file Data_Sheet_4.zip › Sit-miR164f_Seita.3G001500.1_686_TPlot.pdf]

**T=Seita.3G386200.1\_Q=Sit-miR164f\_S=790**

category=0\_p=0.000381927958281736

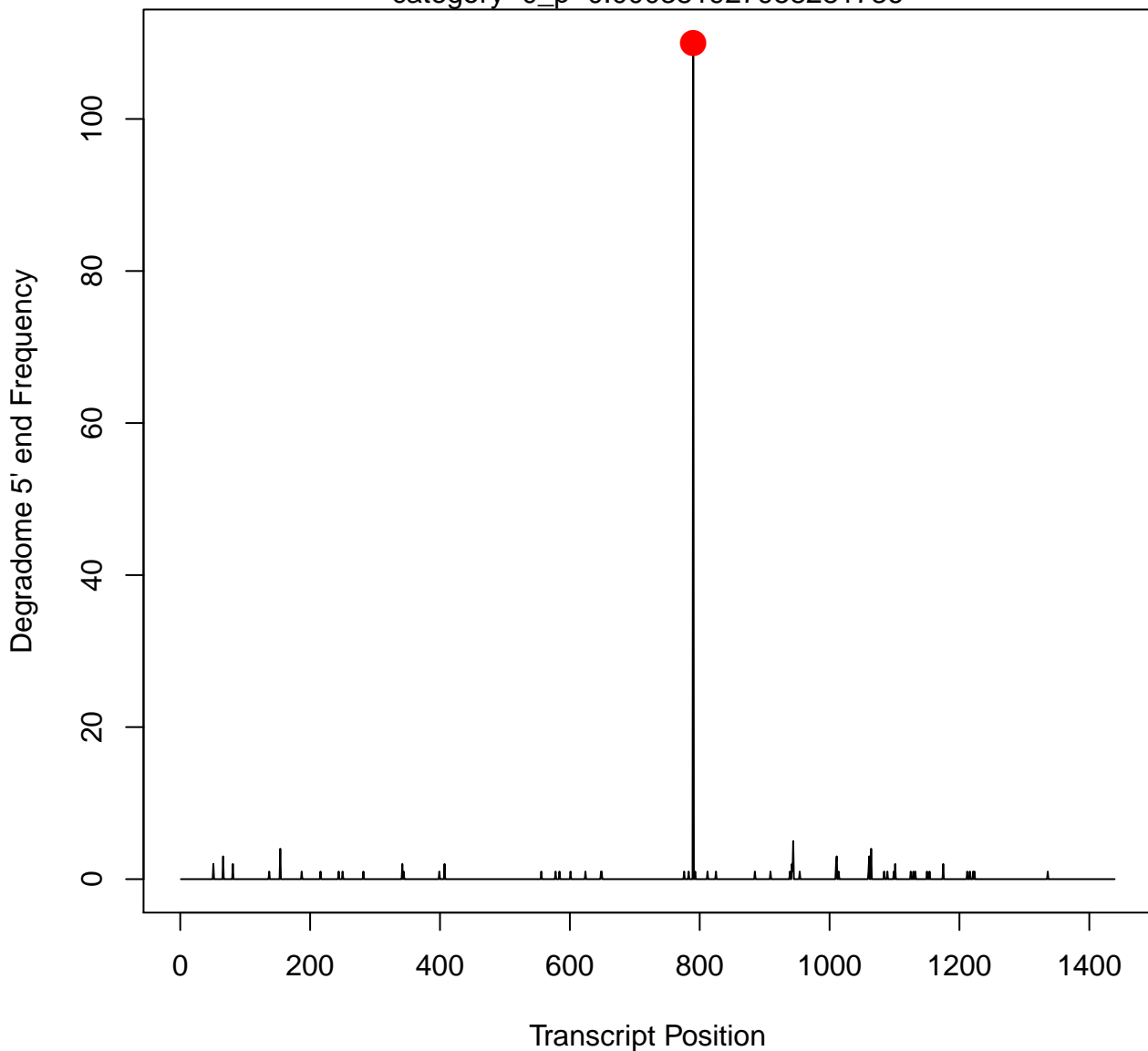

Supplement: Supplementary file 4 [file Data_Sheet_4.zip › Sit-miR164f_Seita.3G386200.1_790_TPlot.pdf]

**T=Seita.4G263400.1\_Q=Sit-miR164f\_S=1216**

category=0\_p=0.00152683684215971

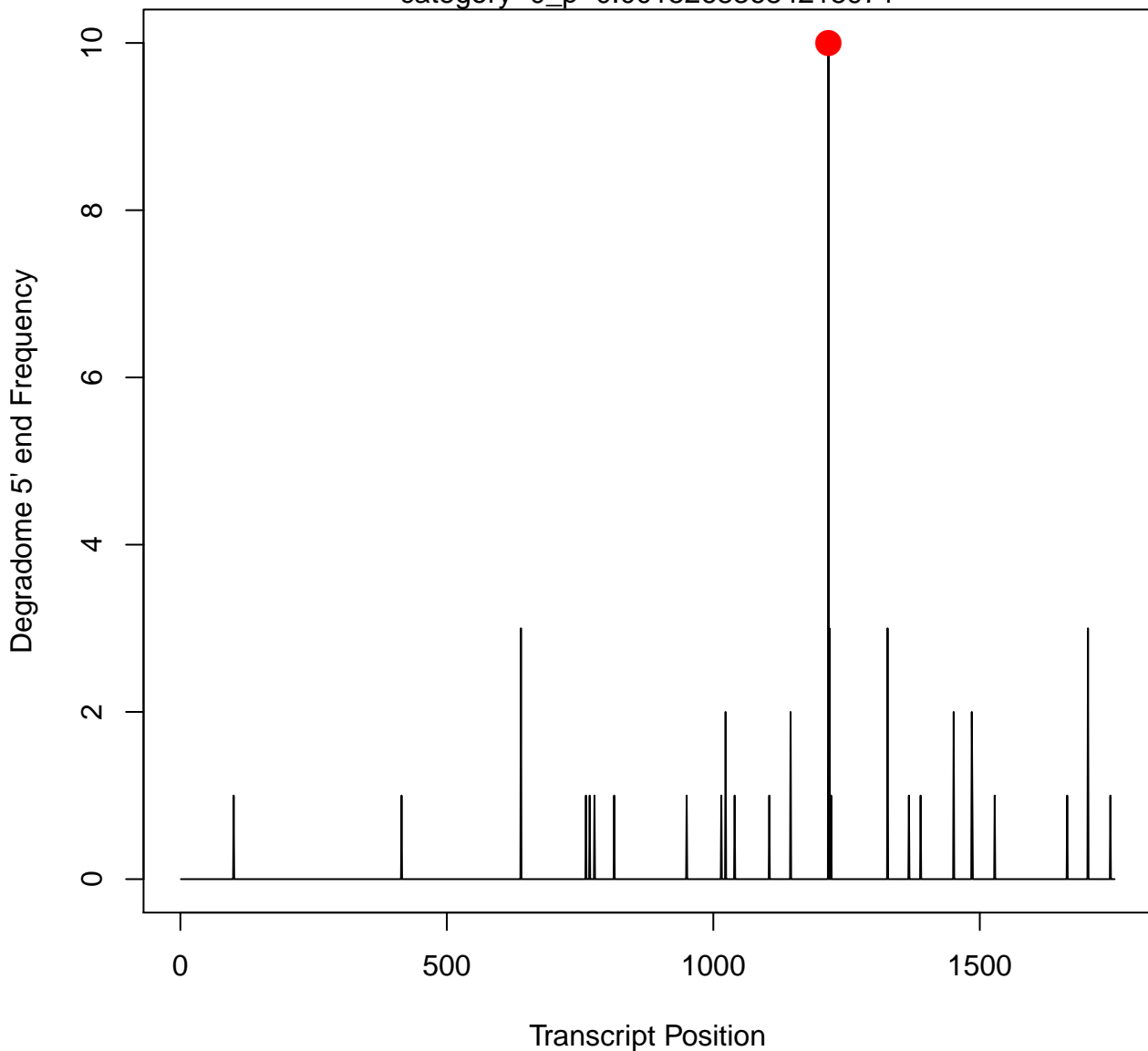

Supplement: Supplementary file 4 [file Data_Sheet_4.zip › Sit-miR164f_Seita.4G263400.1_1216_TPlot.pdf]

**T=Seita.6G004200.1\_Q=Sit-miR164f\_S=2284**

category=2\_p=0.41496348247752

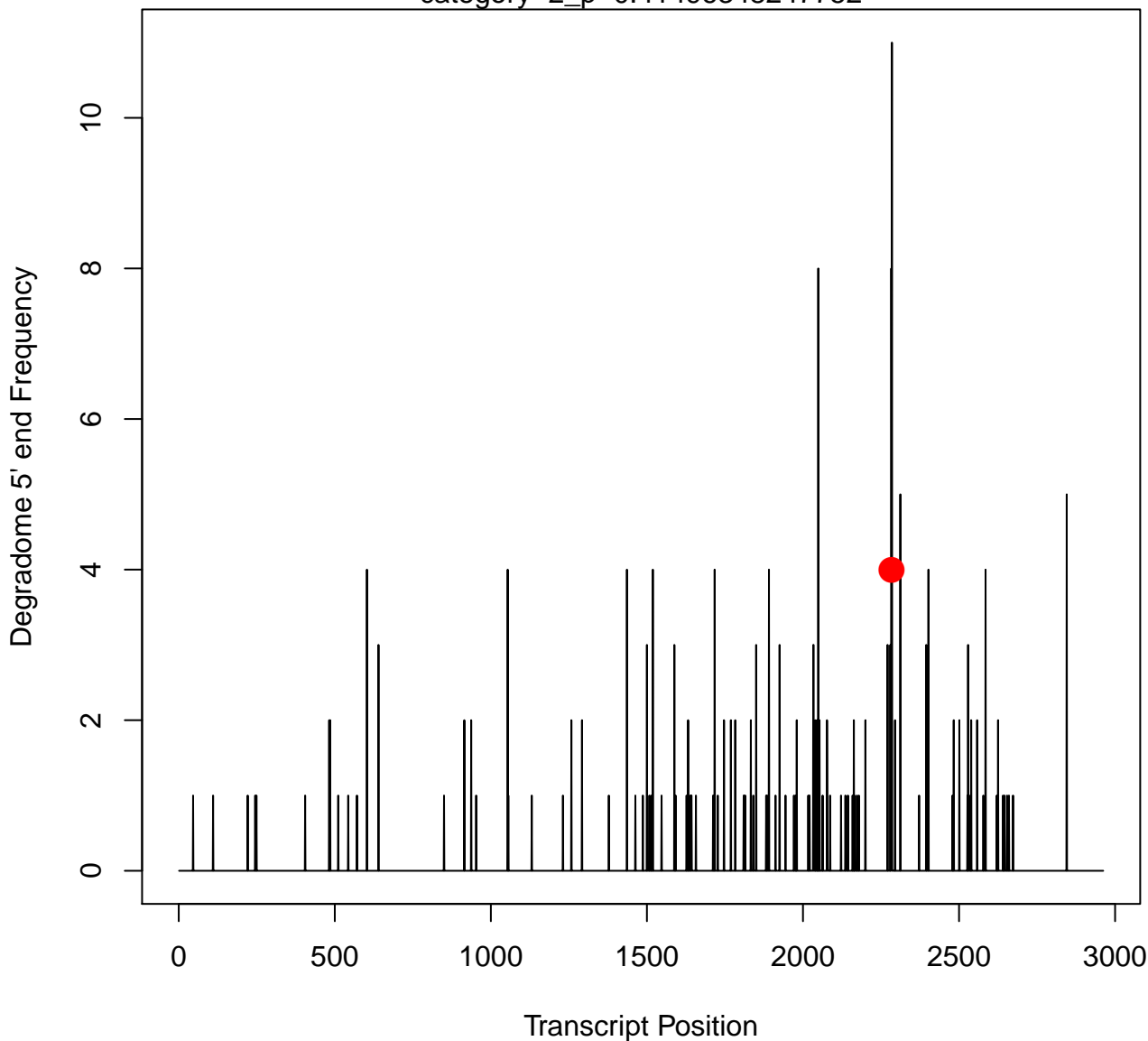

Supplement: Supplementary file 4 [file Data_Sheet_4.zip › Sit-miR164f_Seita.6G004200.1_2284_TPlot.pdf]

**T=Seita.3G395000.1\_Q=Sit-miR166a\_S=774**

category=0\_p=0.00114534632366081

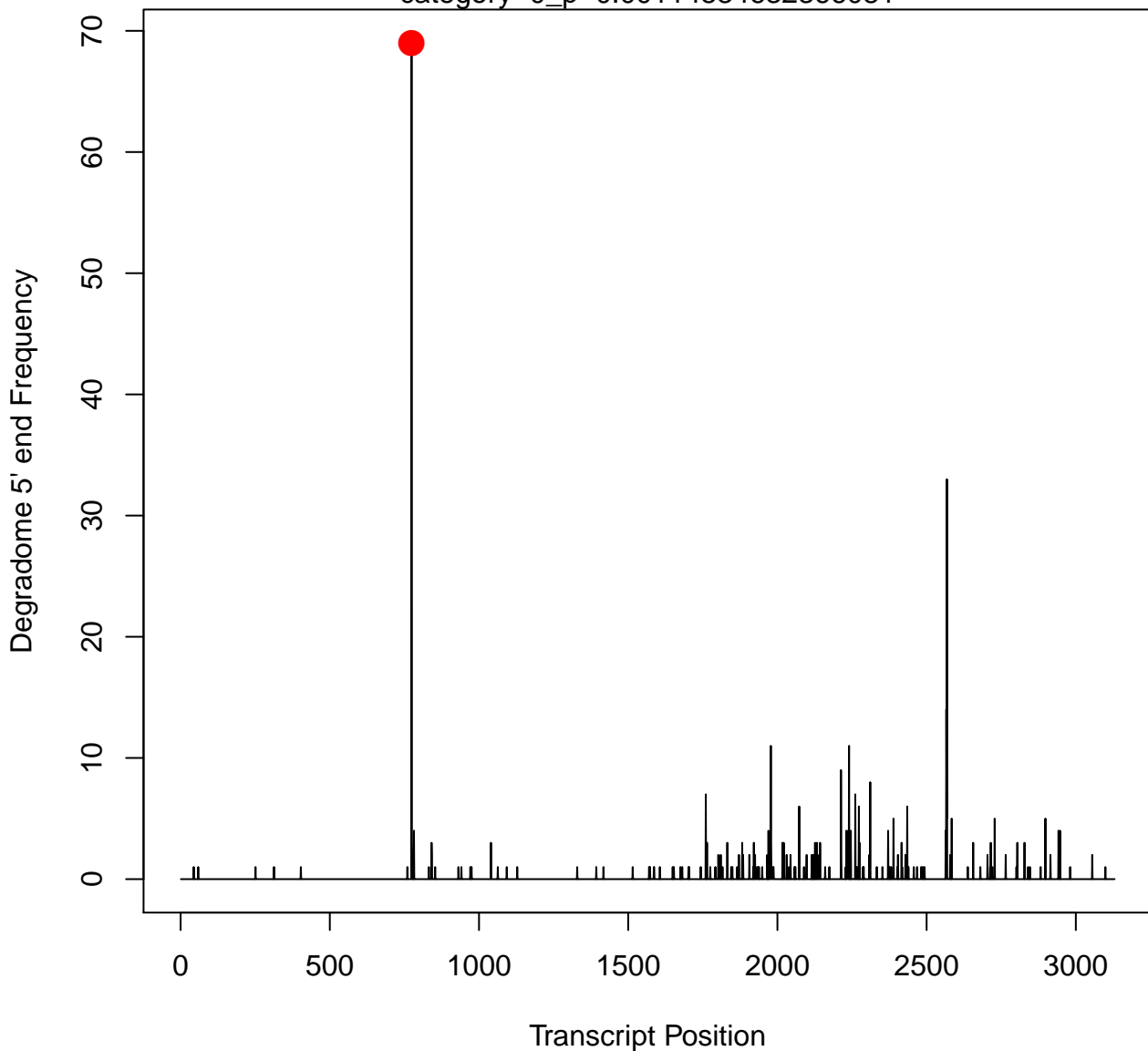

Supplement: Supplementary file 4 [file Data_Sheet_4.zip › Sit-miR166a_Seita.3G395000.1_774_TPlot.pdf]

**T=Seita.3G079300.1\_Q=Sit-miR166b\_S=1192**

category=2\_p=0.707063214852899

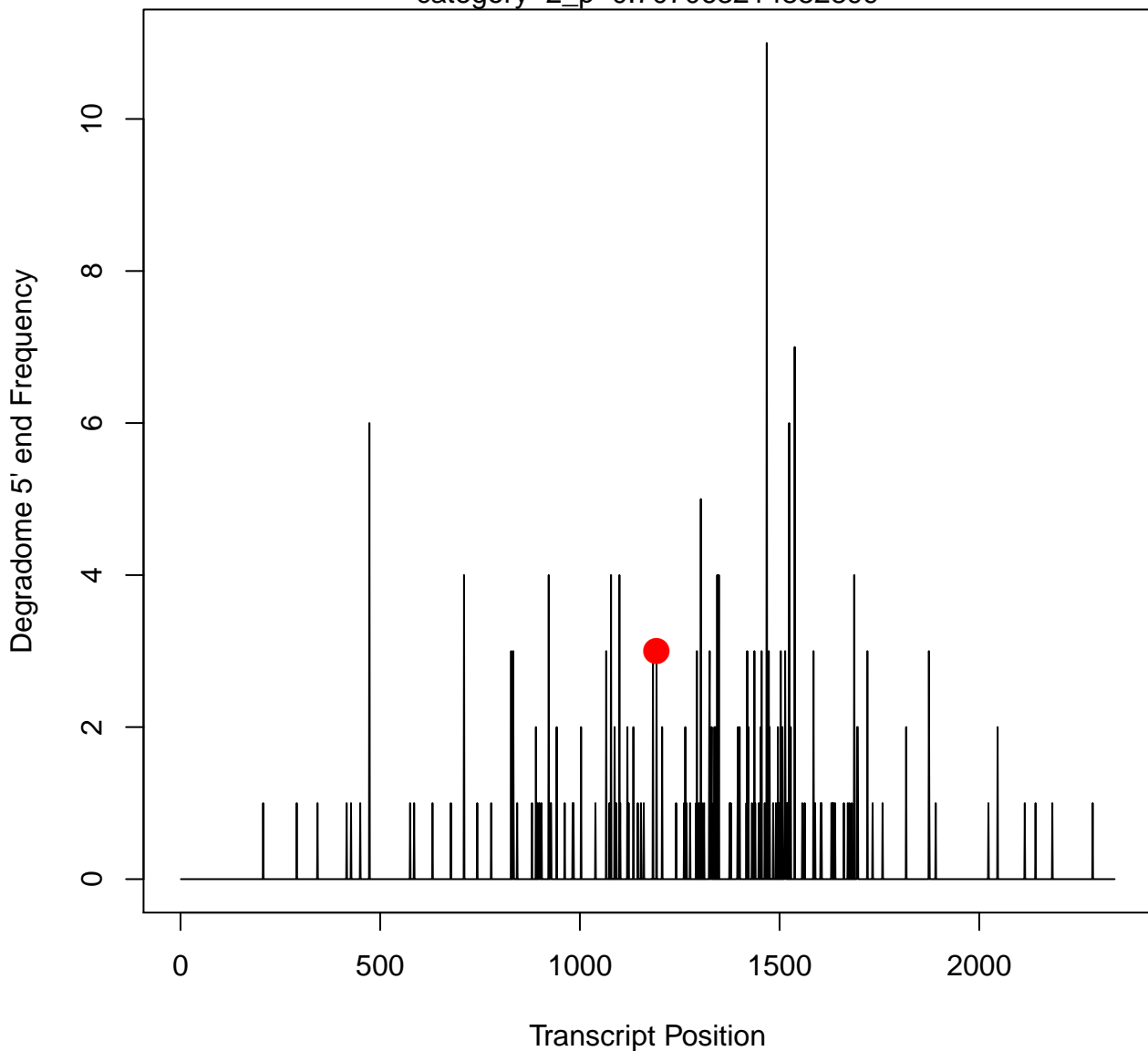

Supplement: Supplementary file 4 [file Data_Sheet_4.zip › Sit-miR166b_Seita.3G079300.1_1192_TPlot.pdf]

**T=Seita.4G034100.1\_Q=Sit-miR166b\_S=359**

category=2\_p=0.888775305592319

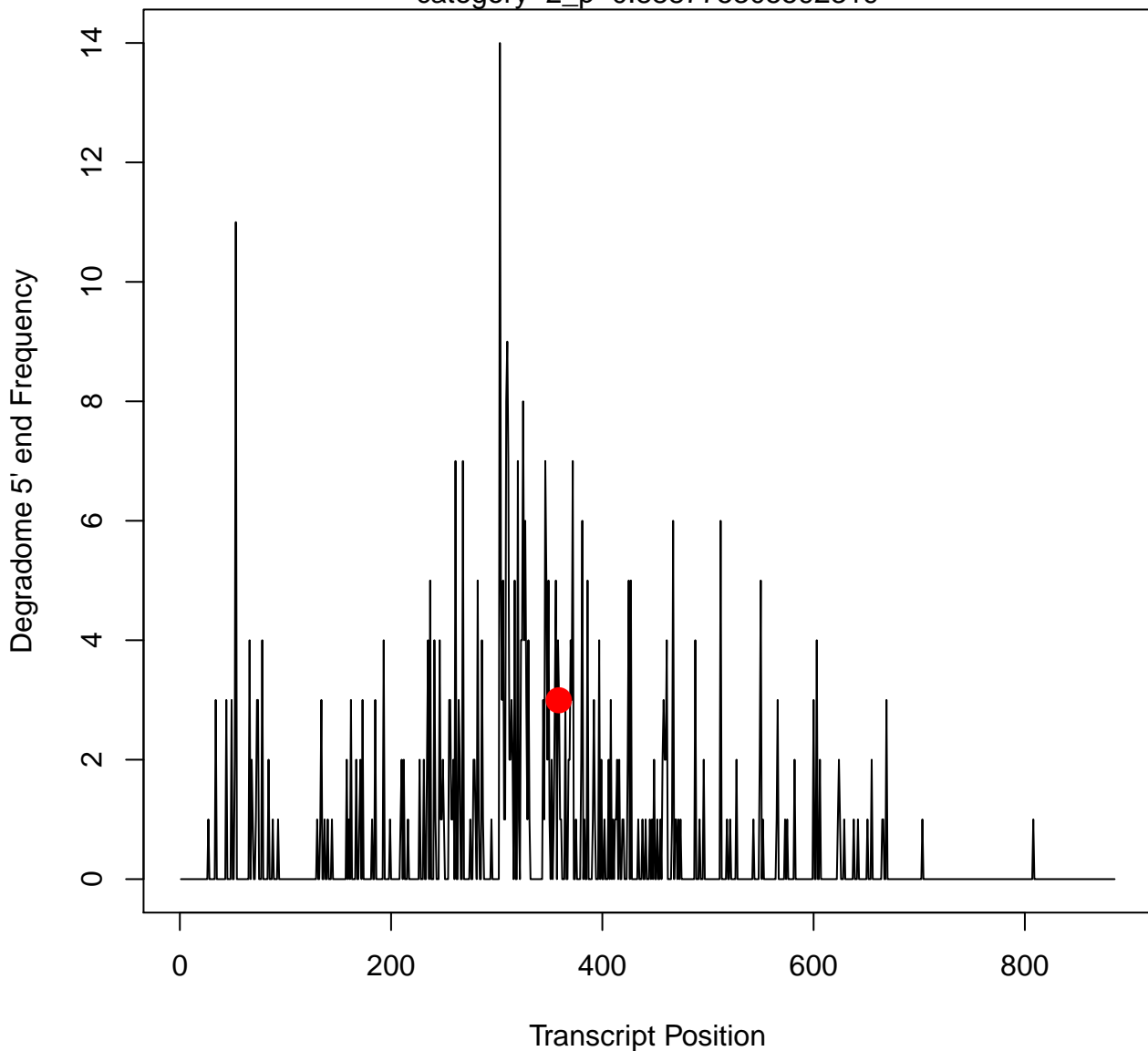

Supplement: Supplementary file 4 [file Data_Sheet_4.zip › Sit-miR166b_Seita.4G034100.1_359_TPlot.pdf]

**T=Seita.5G435000.1\_Q=Sit-miR166b\_S=2929**

category=2\_p=0.989772018125449

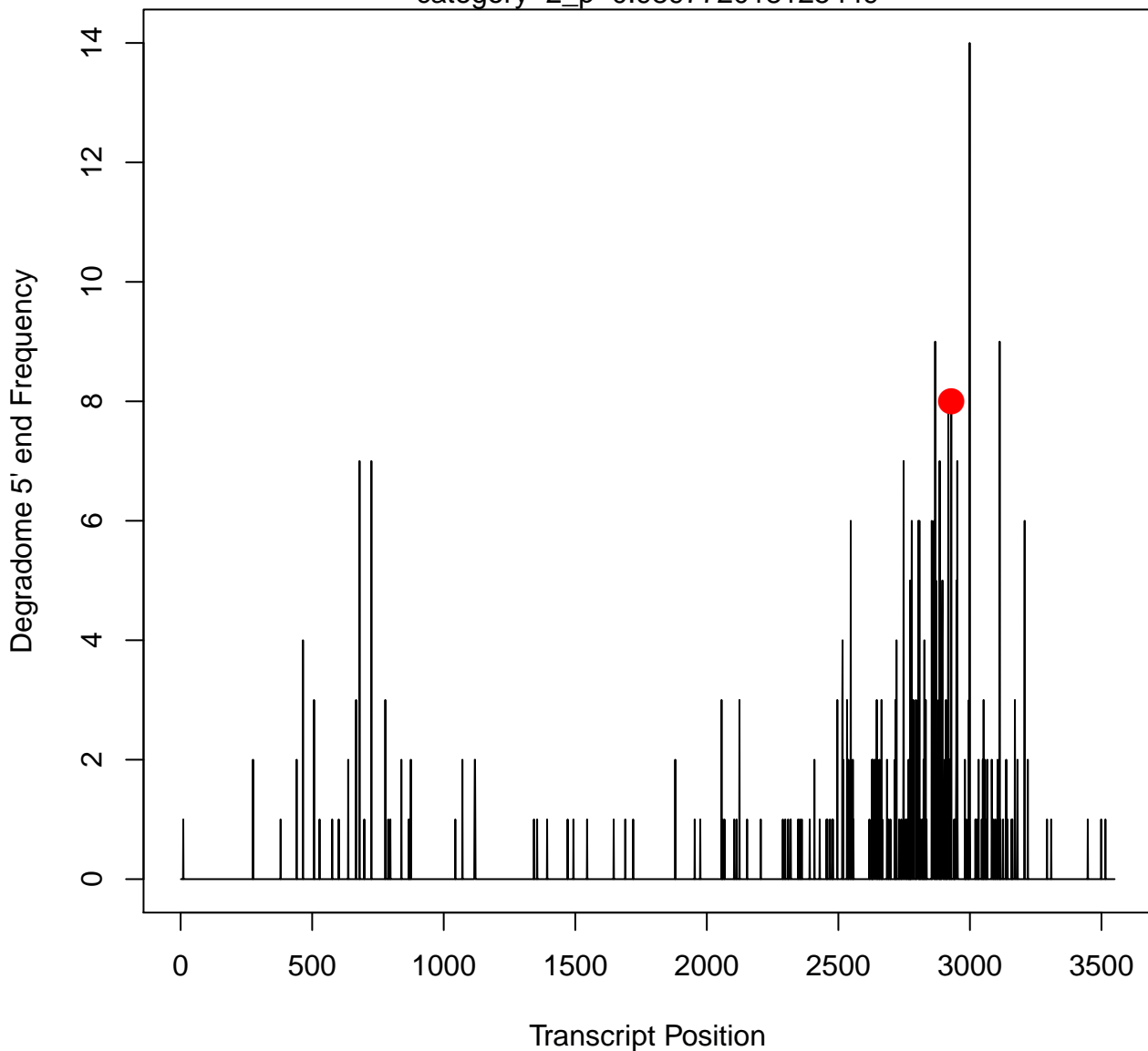

Supplement: Supplementary file 4 [file Data_Sheet_4.zip › Sit-miR166b_Seita.5G435000.1_2929_TPlot.pdf]

**T=Seita.8G157800.1\_Q=Sit-miR166c\_S=635**

category=2\_p=0.890682171916642

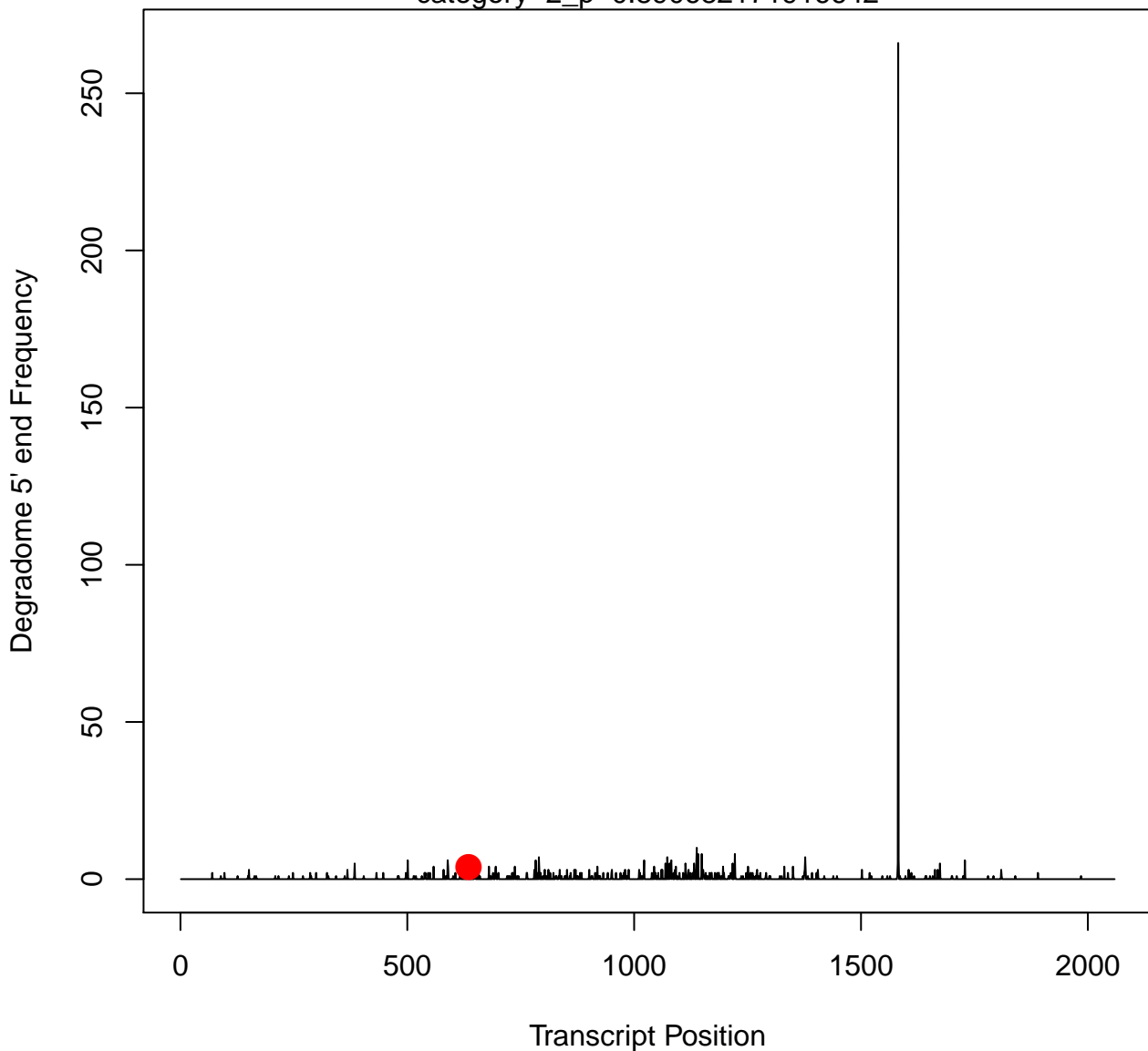

Supplement: Supplementary file 4 [file Data_Sheet_4.zip › Sit-miR166c_Seita.8G157800.1_635_TPlot.pdf]

**T=Seita.3G105500.1\_Q=Sit-miR166h\_S=844**

category=2\_p=0.144129690940846

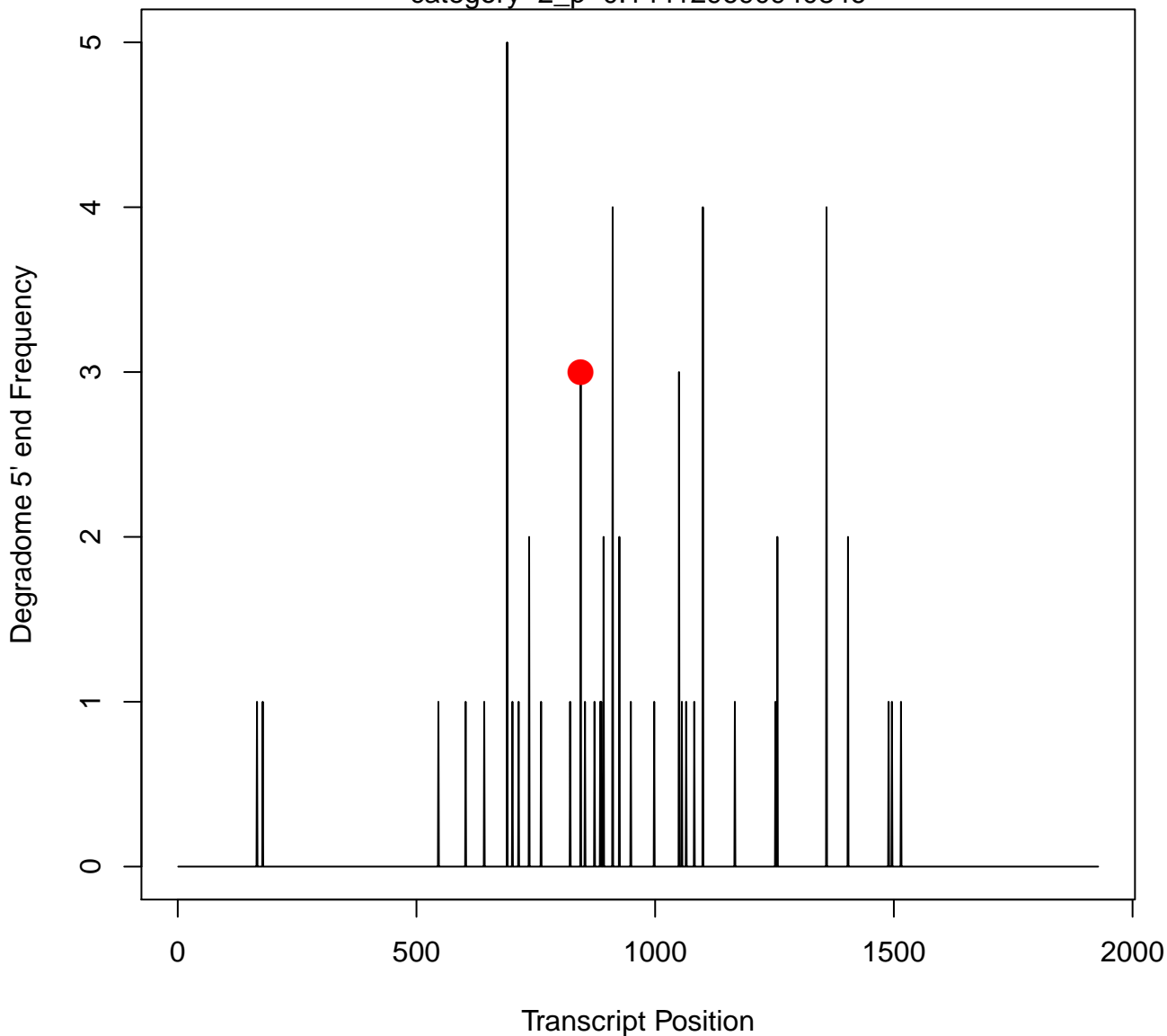

Supplement: Supplementary file 4 [file Data_Sheet_4.zip › Sit-miR166h_Seita.3G105500.1_844_TPlot.pdf]

**T=Seita.9G158800.1\_Q=Sit-miR166h\_S=868**

category=2\_p=0.0505560746234568

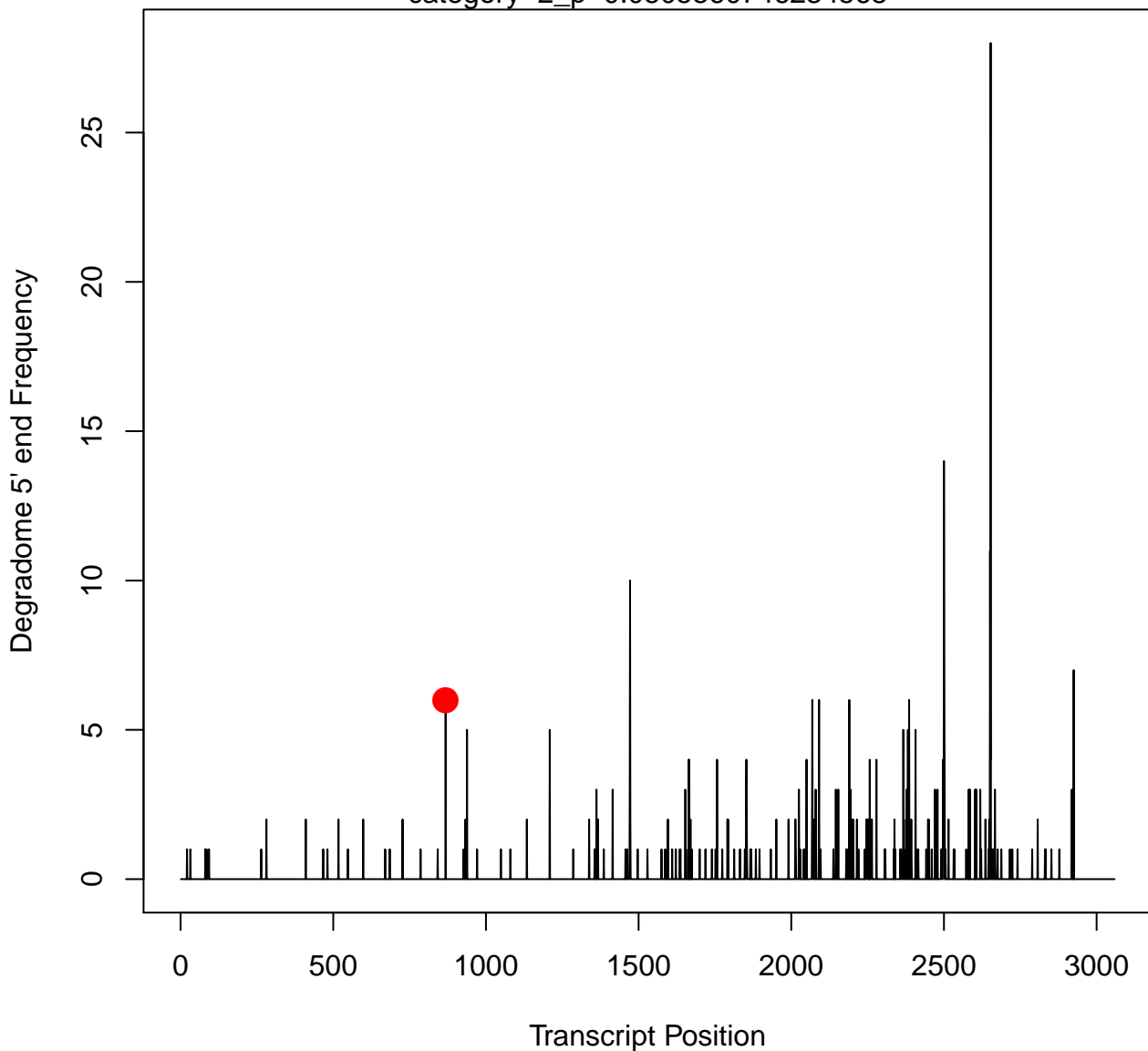

Supplement: Supplementary file 4 [file Data_Sheet_4.zip › Sit-miR166h_Seita.9G158800.1_868_TPlot.pdf]

**T=Seita.3G317100.1\_Q=Sit-miR166i\_S=874**

category=2\_p=0.999122316827556

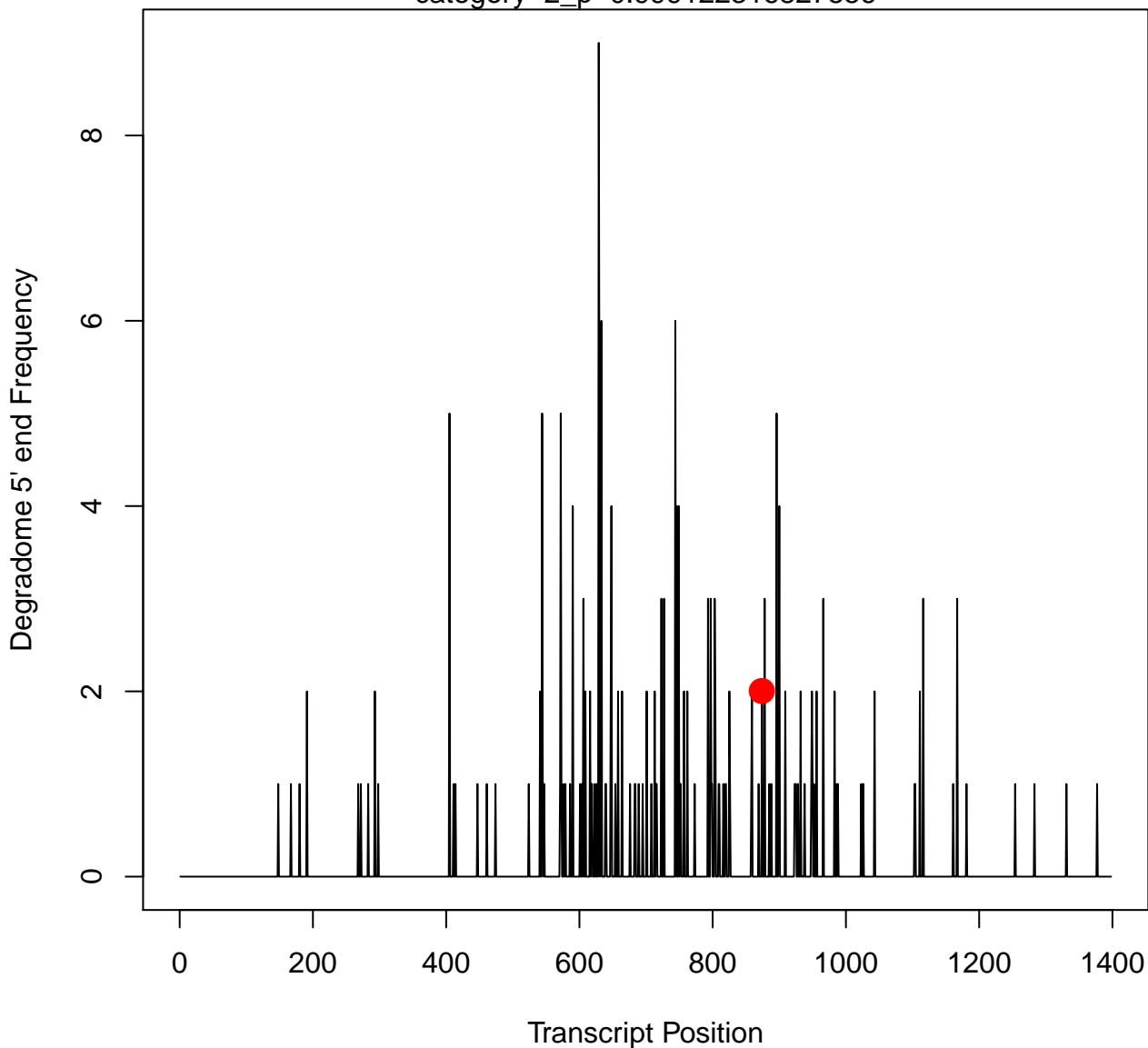

Supplement: Supplementary file 4 [file Data_Sheet_4.zip › Sit-miR166i_Seita.3G317100.1_874_TPlot.pdf]

**T=Seita.9G028300.1\_Q=Sit-miR166i\_S=150**

category=2\_p=0.980938454614104

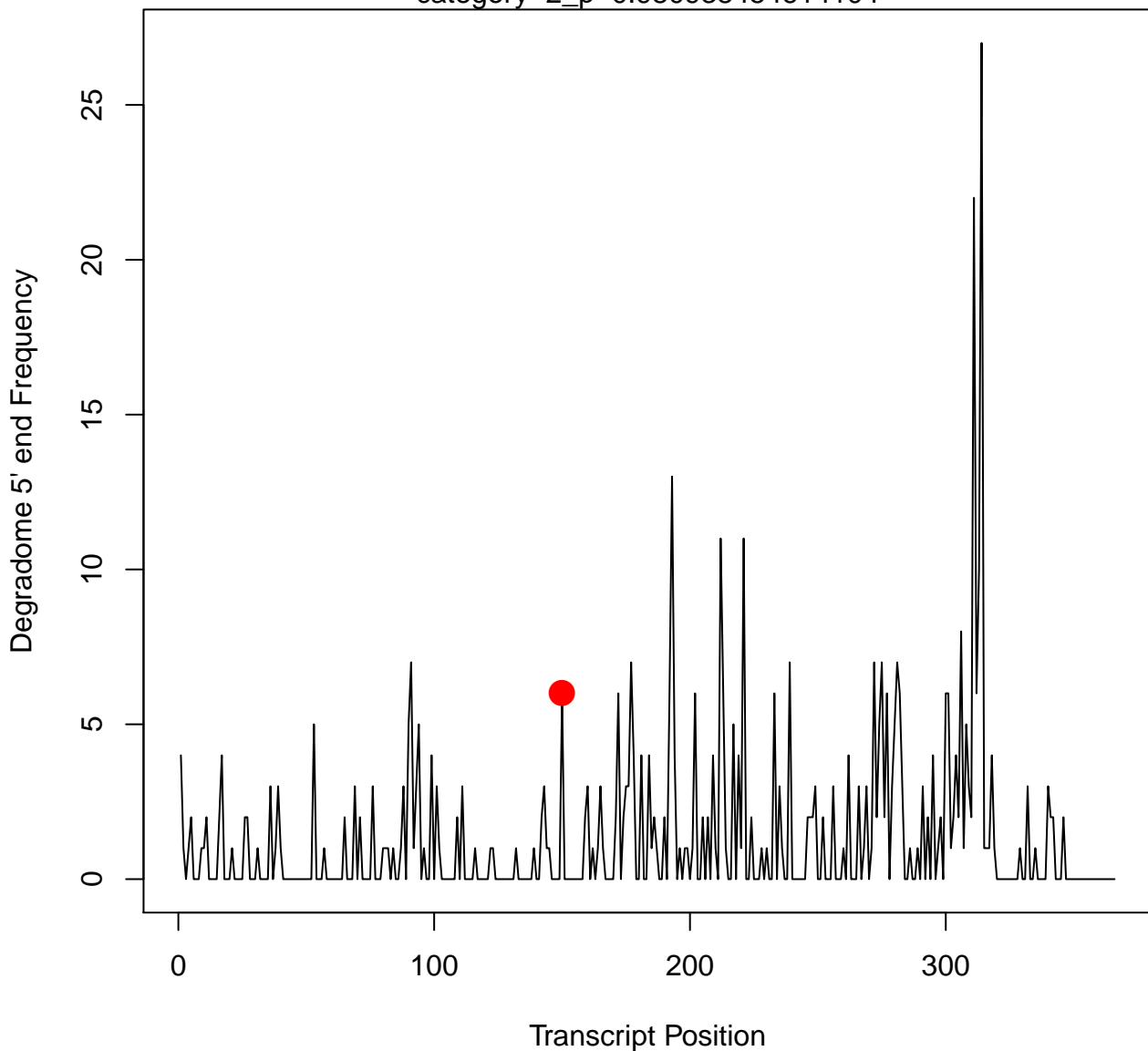

Supplement: Supplementary file 4 [file Data_Sheet_4.zip › Sit-miR166i_Seita.9G028300.1_150_TPlot.pdf]

**T=Seita.9G273900.1\_Q=Sit-miR166i\_S=216**

category=2\_p=0.976133372583784

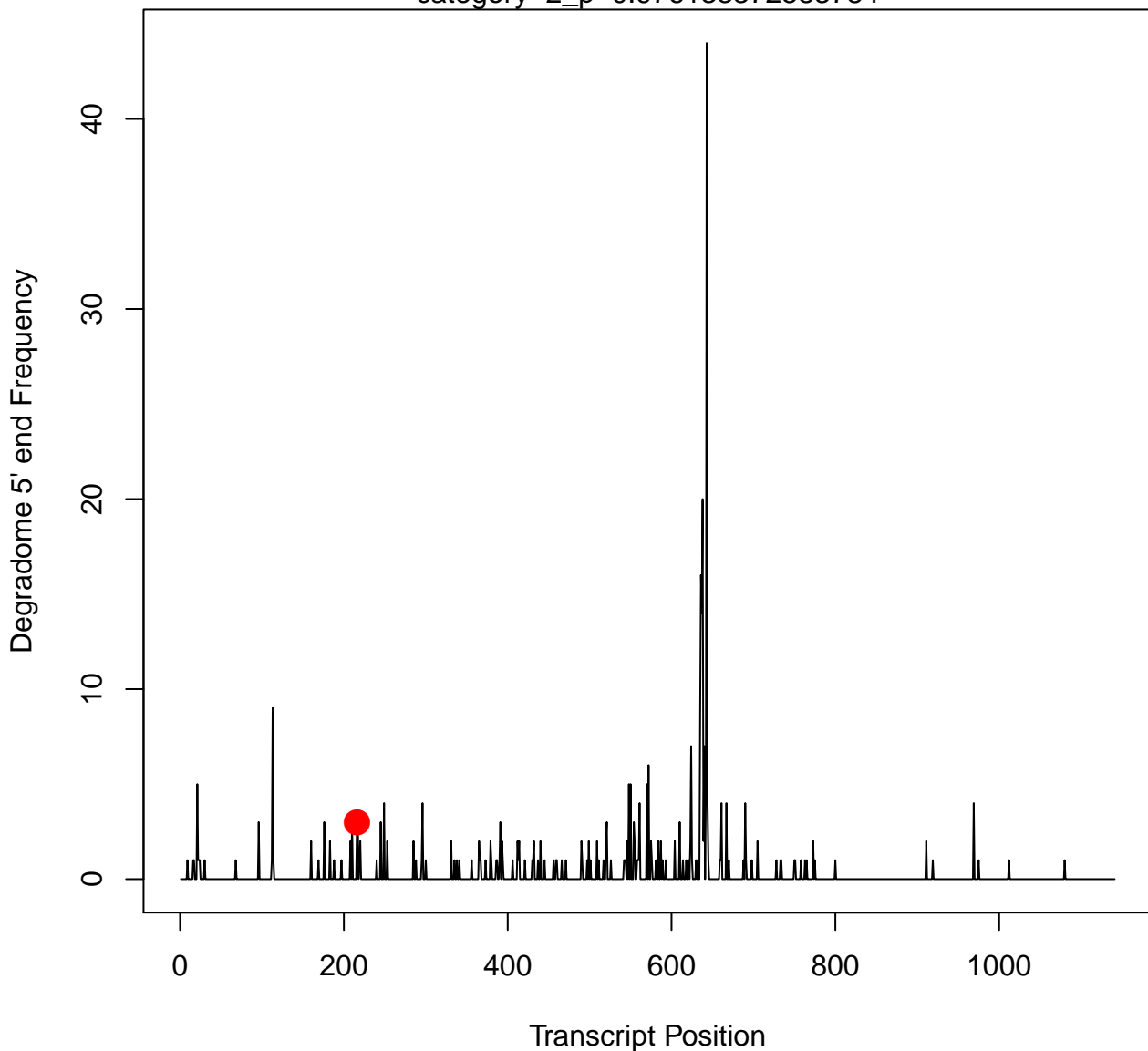

Supplement: Supplementary file 4 [file Data_Sheet_4.zip › Sit-miR166i_Seita.9G273900.1_216_TPlot.pdf]

**T=Seita.3G185800.1\_Q=Sit-miR166j\_S=1028**

category=2\_p=0.744910784471841

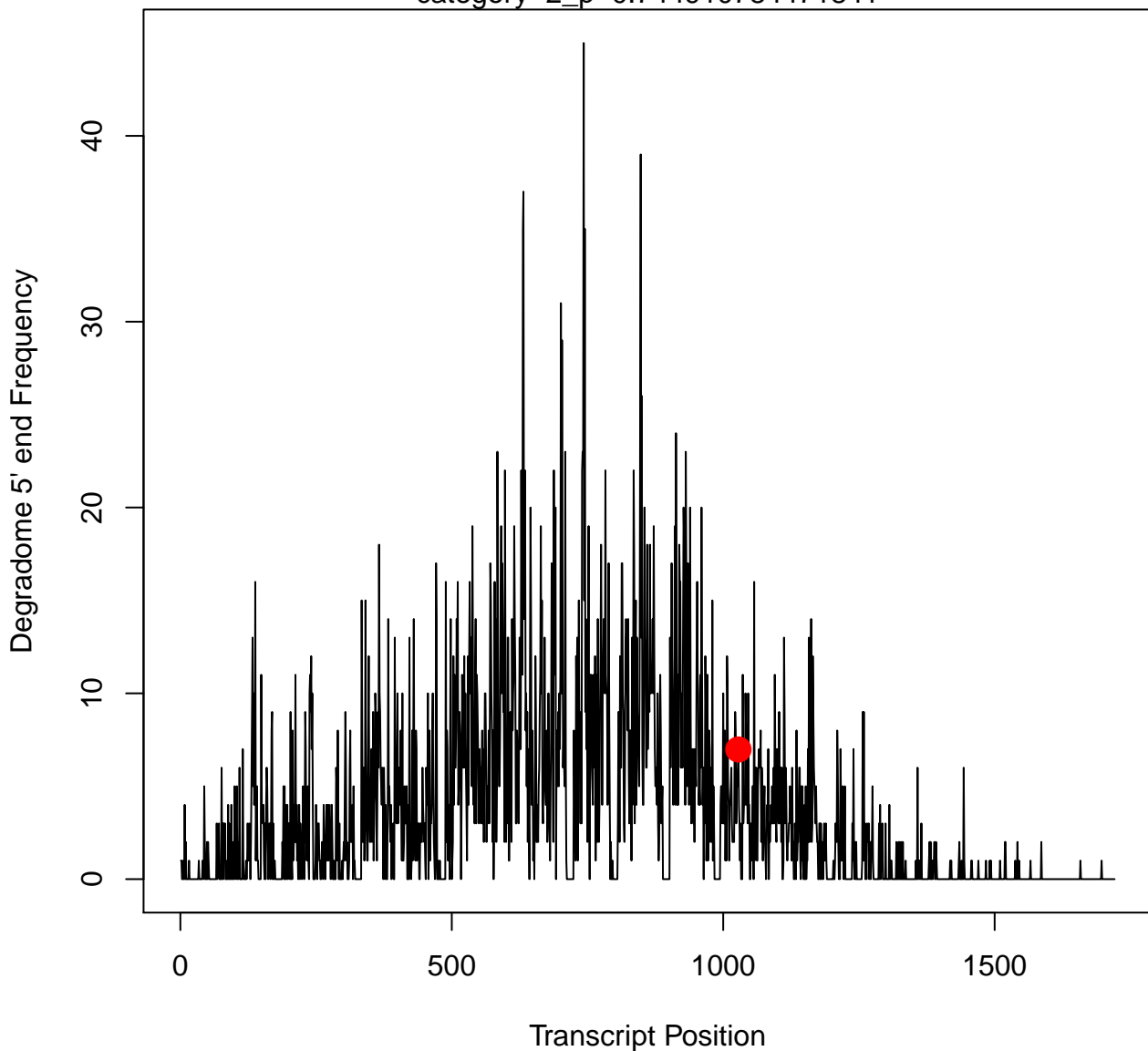

Supplement: Supplementary file 4 [file Data_Sheet_4.zip › Sit-miR166j_Seita.3G185800.1_1028_TPlot.pdf]

**T=Seita.5G141300.1\_Q=Sit-miR166j\_S=1222**

category=0\_p=0.00152683684215971

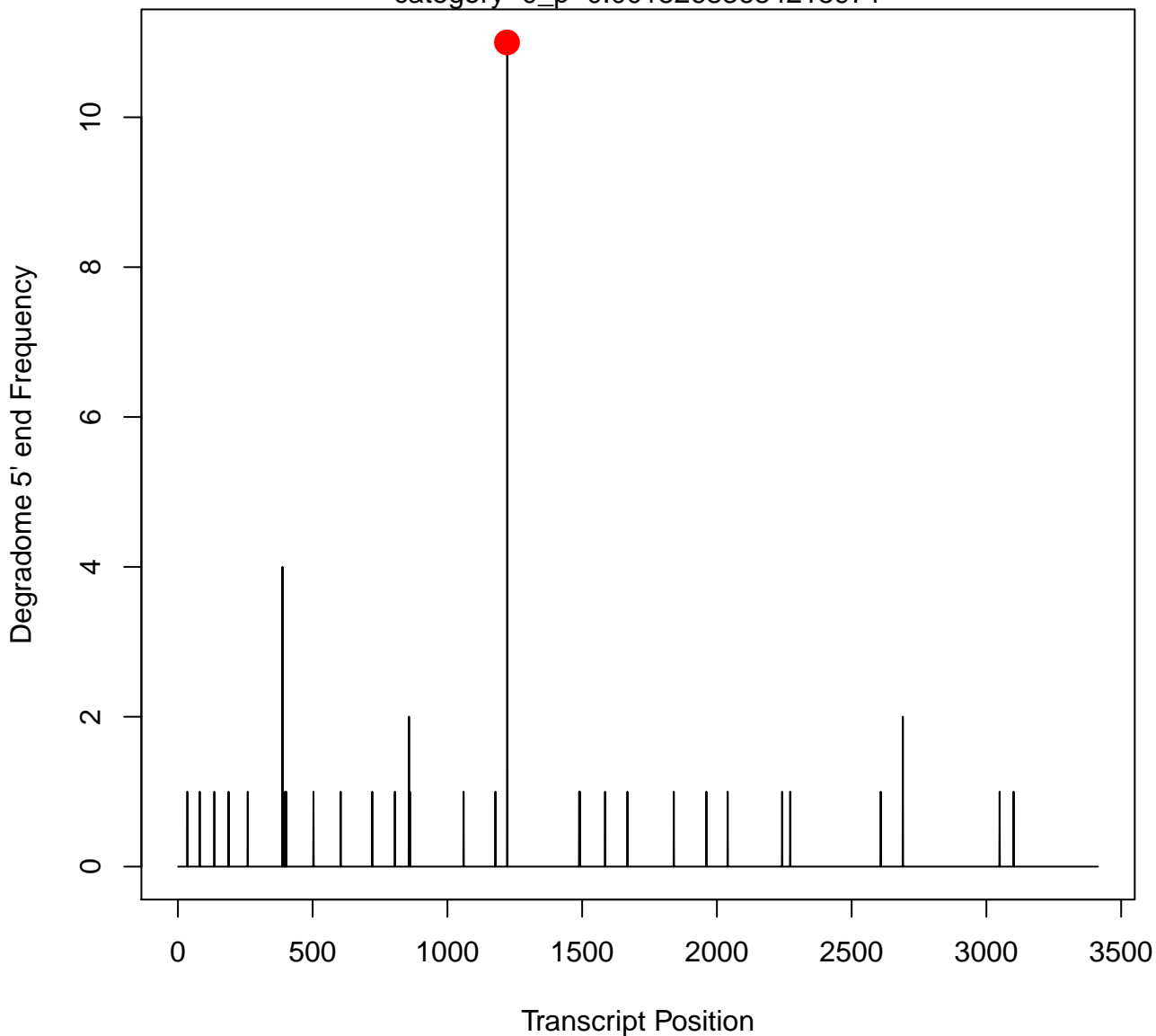

Supplement: Supplementary file 4 [file Data_Sheet_4.zip › Sit-miR166j_Seita.5G141300.1_1222_TPlot.pdf]

**T=Seita.6G050300.1\_Q=Sit-miR166j\_S=683**

category=2\_p=0.979923463749229

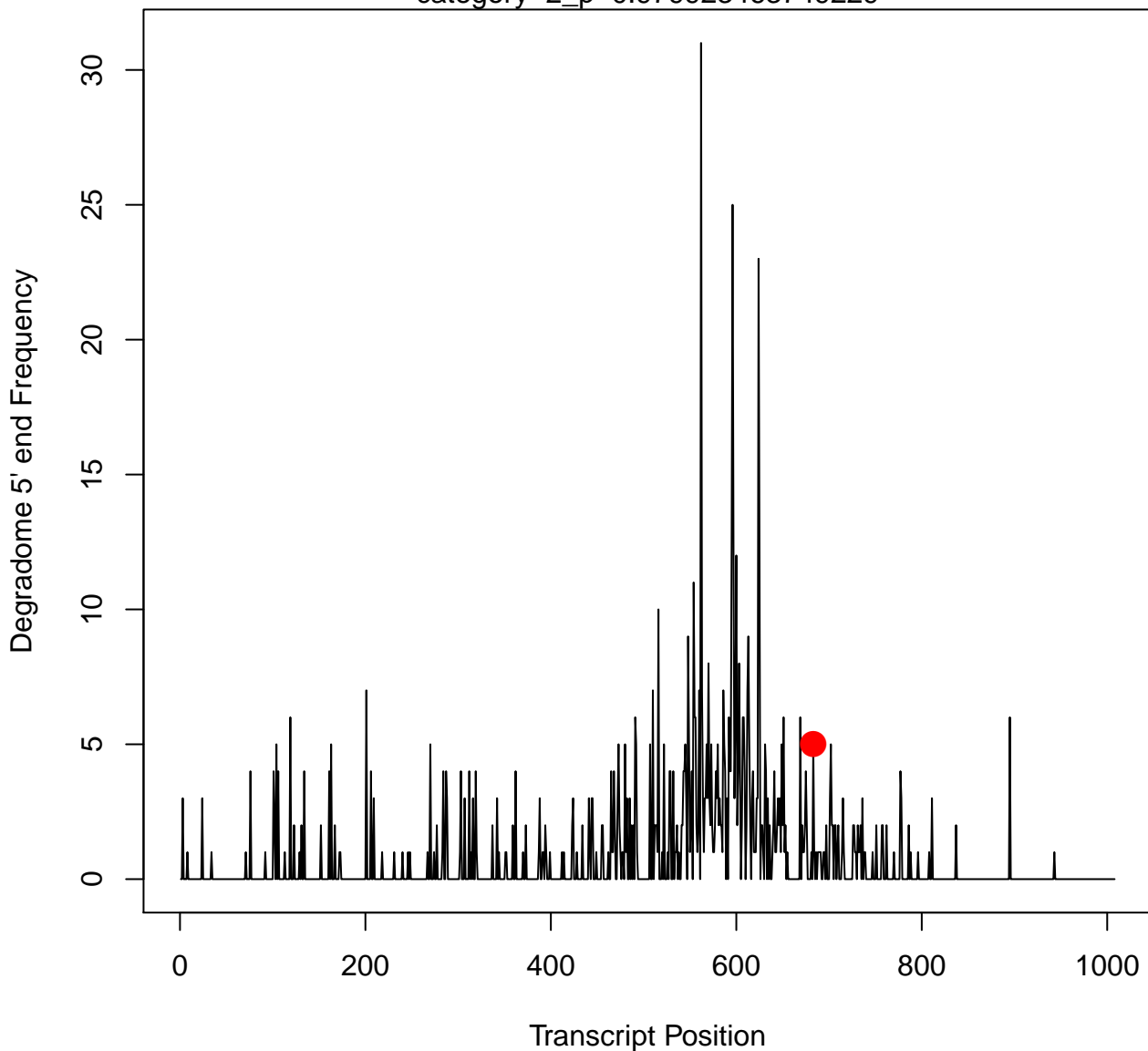

Supplement: Supplementary file 4 [file Data_Sheet_4.zip › Sit-miR166j_Seita.6G050300.1_683_TPlot.pdf]

**T=Seita.9G572600.1\_Q=Sit-miR166j\_S=1138**

category=0\_p=0.00114534632366081

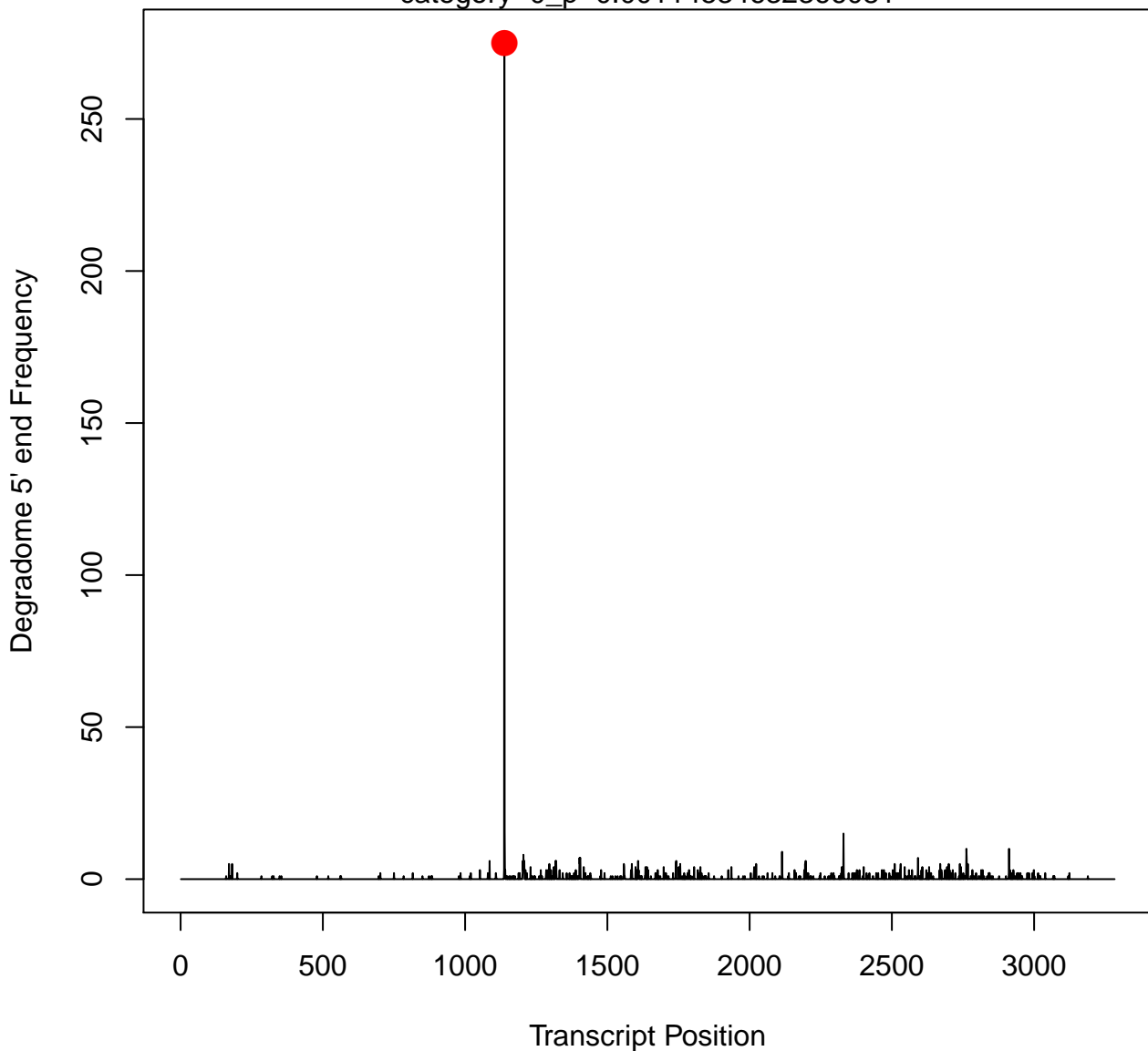

Supplement: Supplementary file 4 [file Data_Sheet_4.zip › Sit-miR166j_Seita.9G572600.1_1138_TPlot.pdf]

**T=Seita.9G219700.1\_Q=Sit-miR166k\_S=1105**

category=0\_p=0.000763710047598232

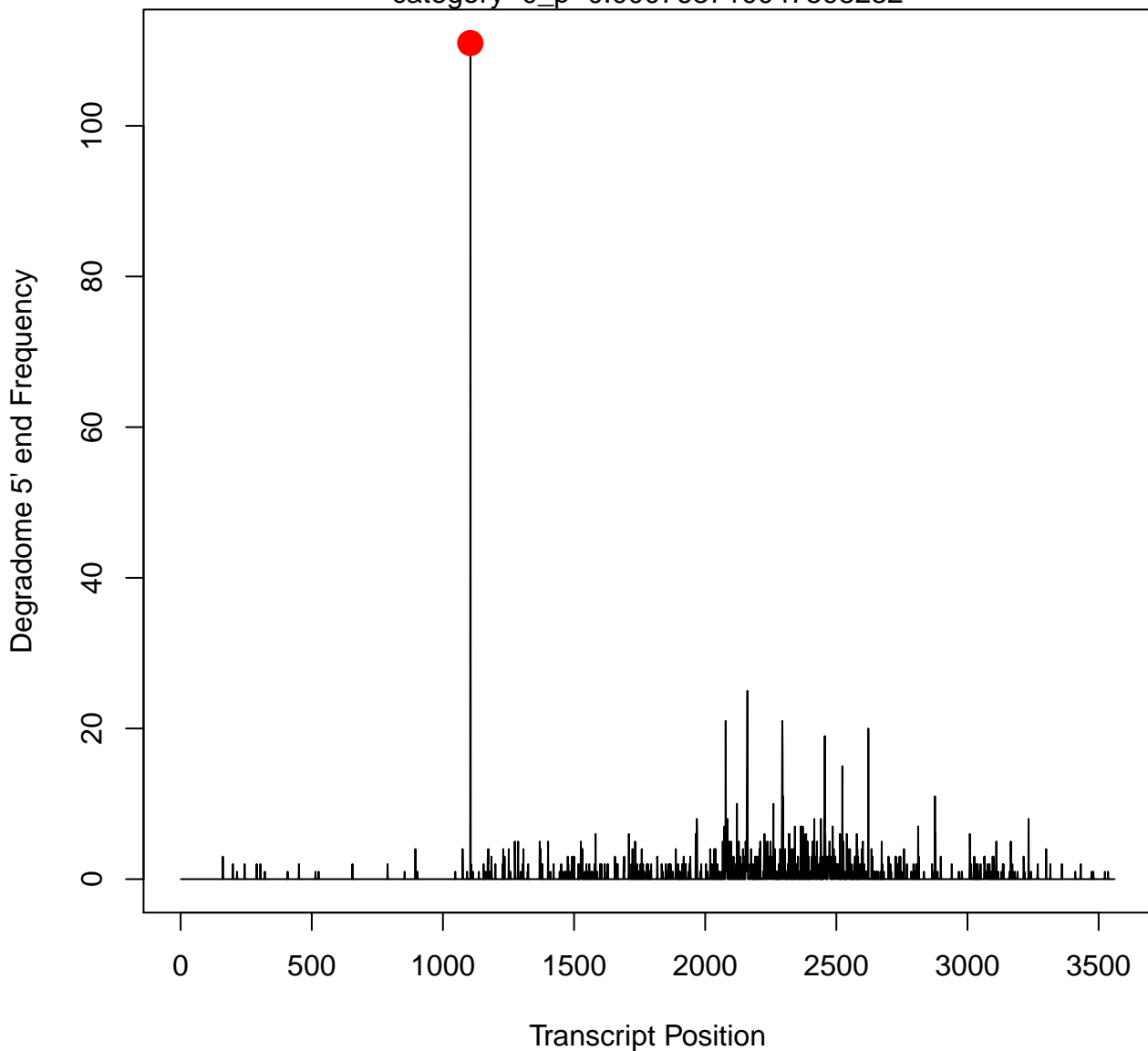

Supplement: Supplementary file 4 [file Data_Sheet_4.zip › Sit-miR166k_Seita.9G219700.1_1105_TPlot.pdf]

**T=Seita.9G560400.1\_Q=Sit-miR166k\_S=917**

category=2\_p=0.98060595789675

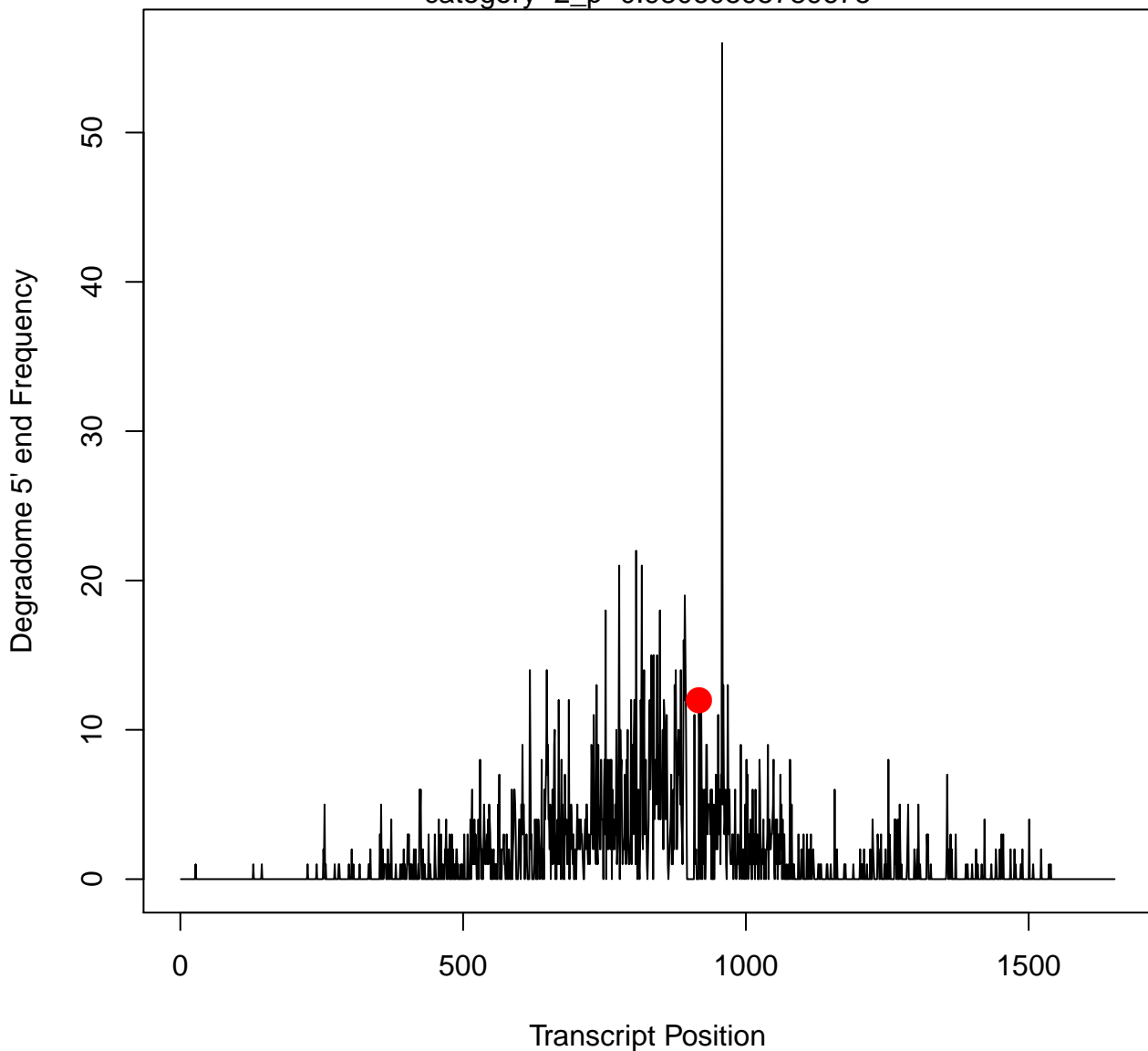

Supplement: Supplementary file 4 [file Data_Sheet_4.zip › Sit-miR166k_Seita.9G560400.1_917_TPlot.pdf]

**T=Seita.1G035000.1\_Q=Sit-miR167a\_S=1306**

category=2\_p=0.99441617217472

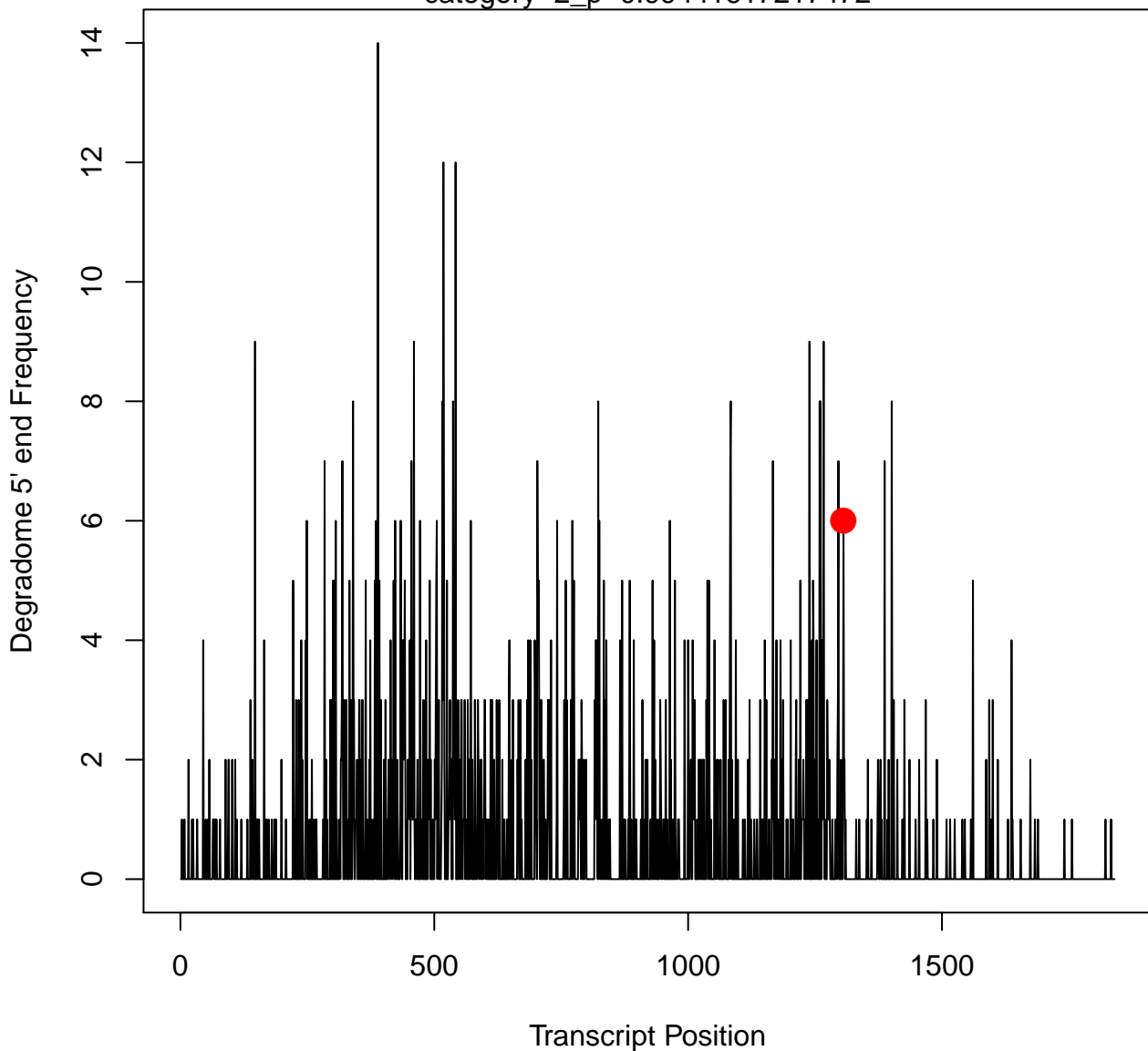

Supplement: Supplementary file 4 [file Data_Sheet_4.zip › Sit-miR167a_Seita.1G035000.1_1306_TPlot.pdf]

**T=Seita.7G048200.1\_Q=Sit-miR167a\_S=569**

category=2\_p=0.651761986217039

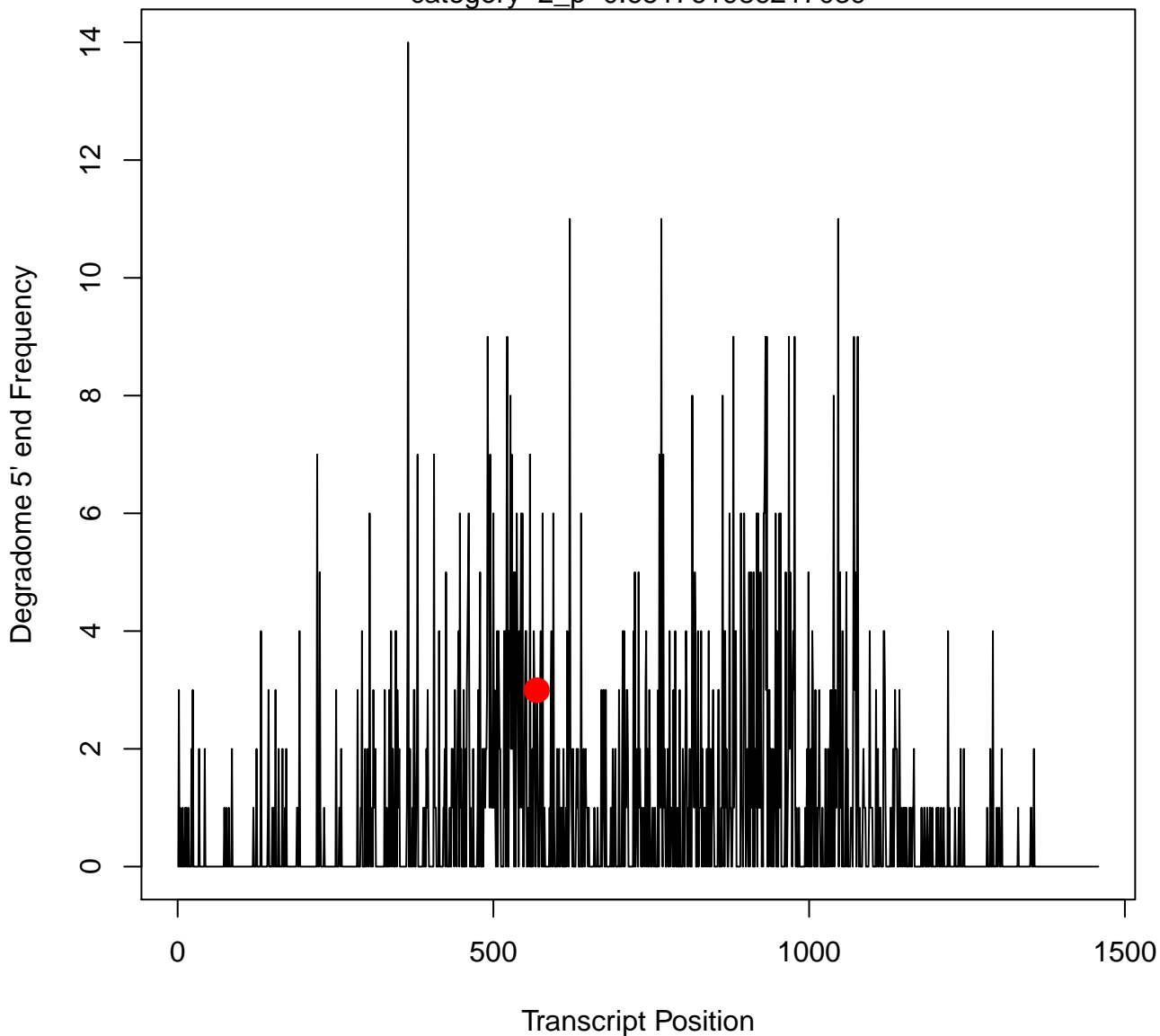

Supplement: Supplementary file 4 [file Data_Sheet_4.zip › Sit-miR167a_Seita.7G048200.1_569_TPlot.pdf]

**T=Seita.7G160400.1\_Q=Sit-miR167a\_S=808**

category=2\_p=0.991831223687038

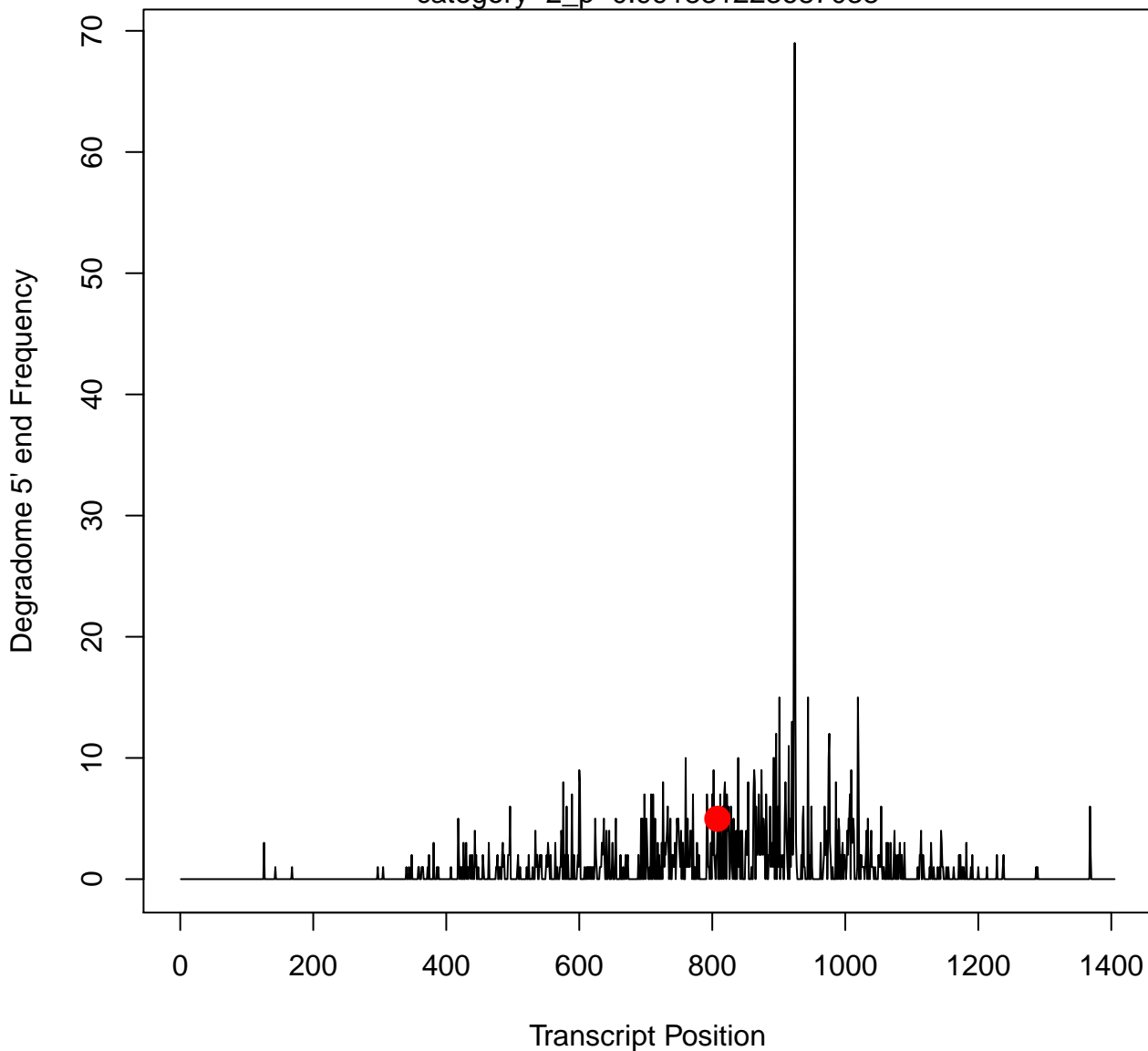

Supplement: Supplementary file 4 [file Data_Sheet_4.zip › Sit-miR167a_Seita.7G160400.1_808_TPlot.pdf]

**T=Seita.2G221100.1\_Q=Sit-miR167h\_S=2599**

category=2\_p=0.998088835943204

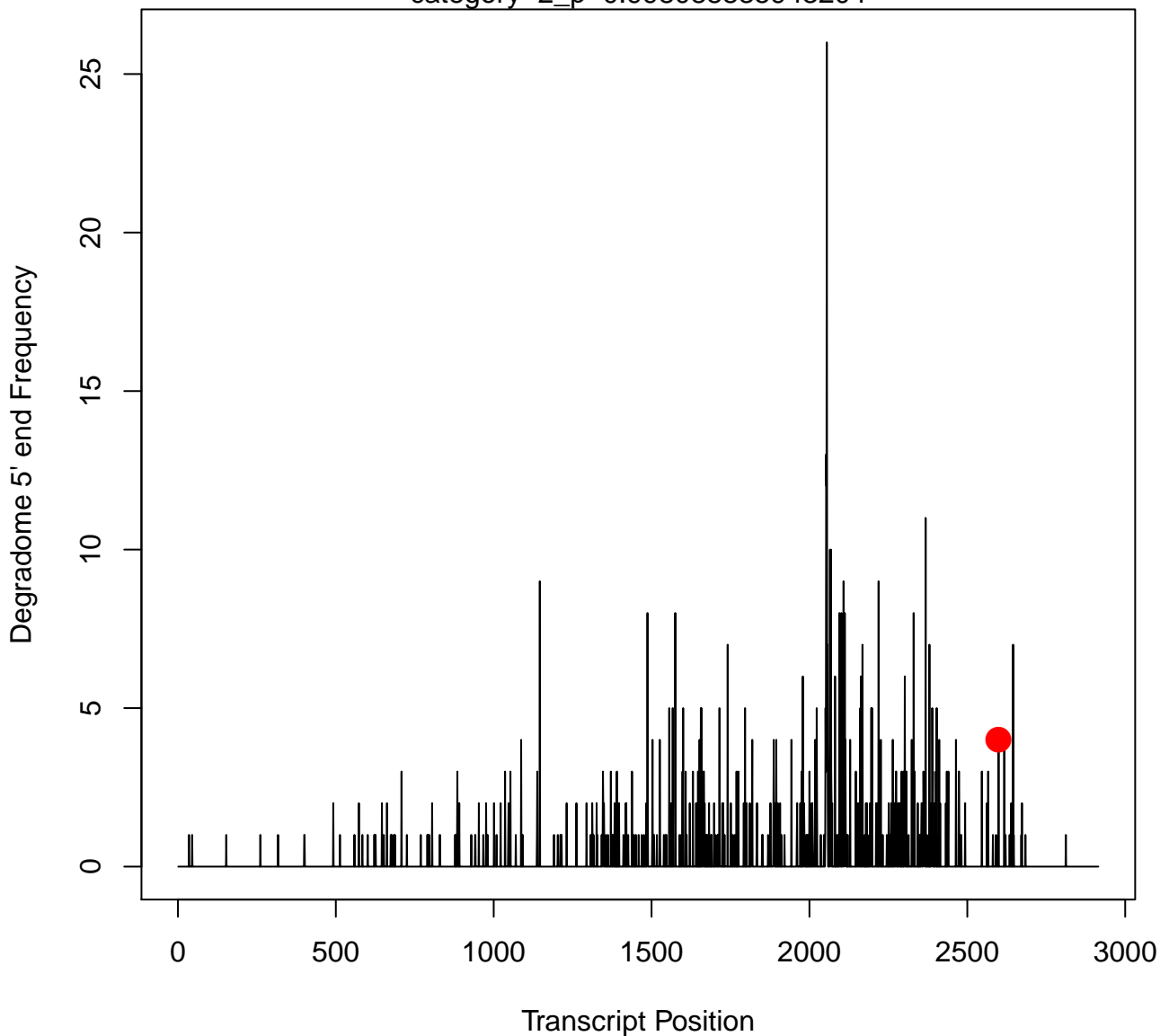

Supplement: Supplementary file 4 [file Data_Sheet_4.zip › Sit-miR167h_Seita.2G221100.1_2599_TPlot.pdf]

**T=Seita.3G394000.1\_Q=Sit-miR167h\_S=3277**

category=0\_p=0.00343210501886526

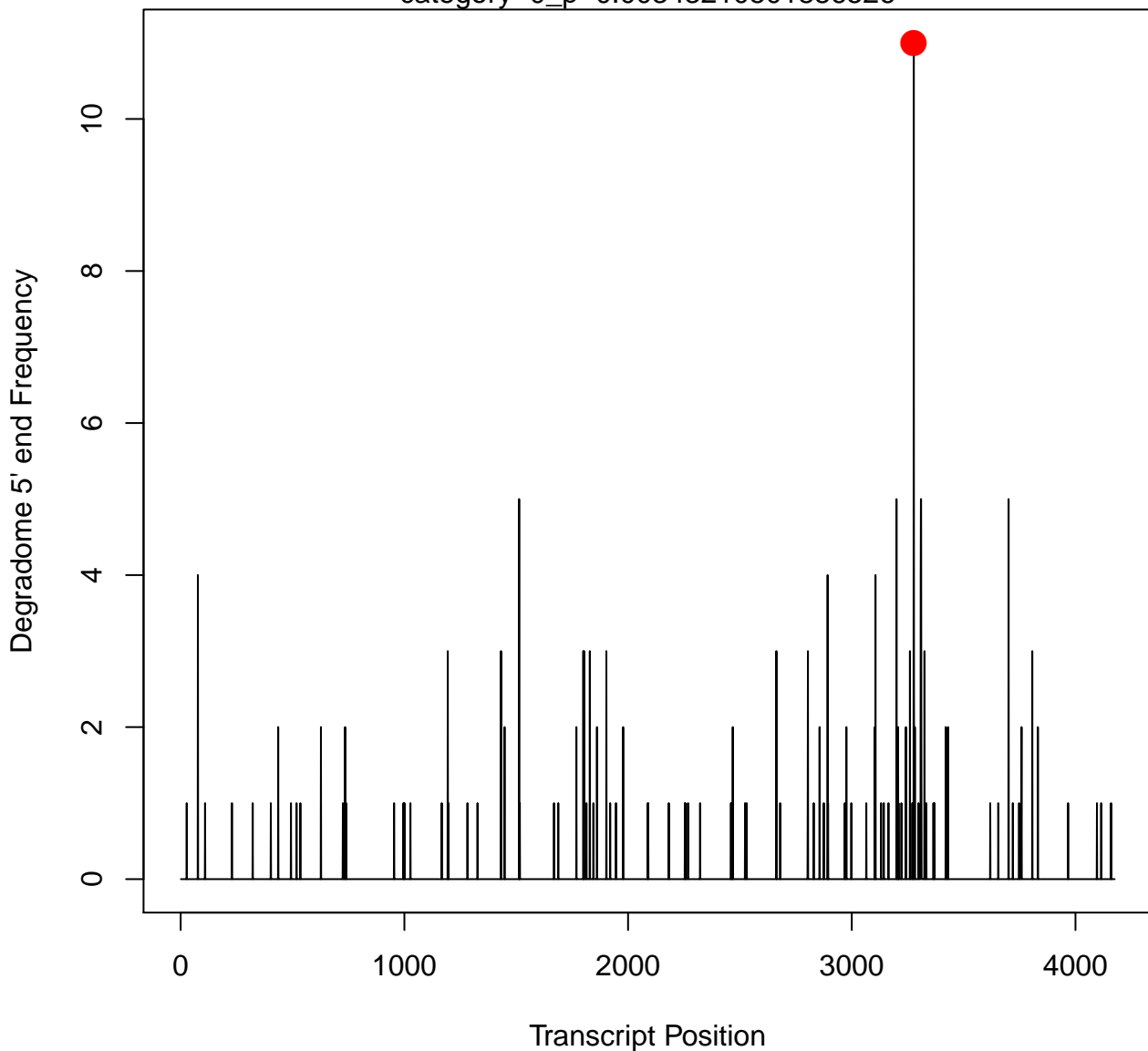

Supplement: Supplementary file 4 [file Data_Sheet_4.zip › Sit-miR167h_Seita.3G394000.1_3277_TPlot.pdf]

**T=Seita.4G262300.1\_Q=Sit-miR167h\_S=3389**

category=0\_p=0.00267043440885661

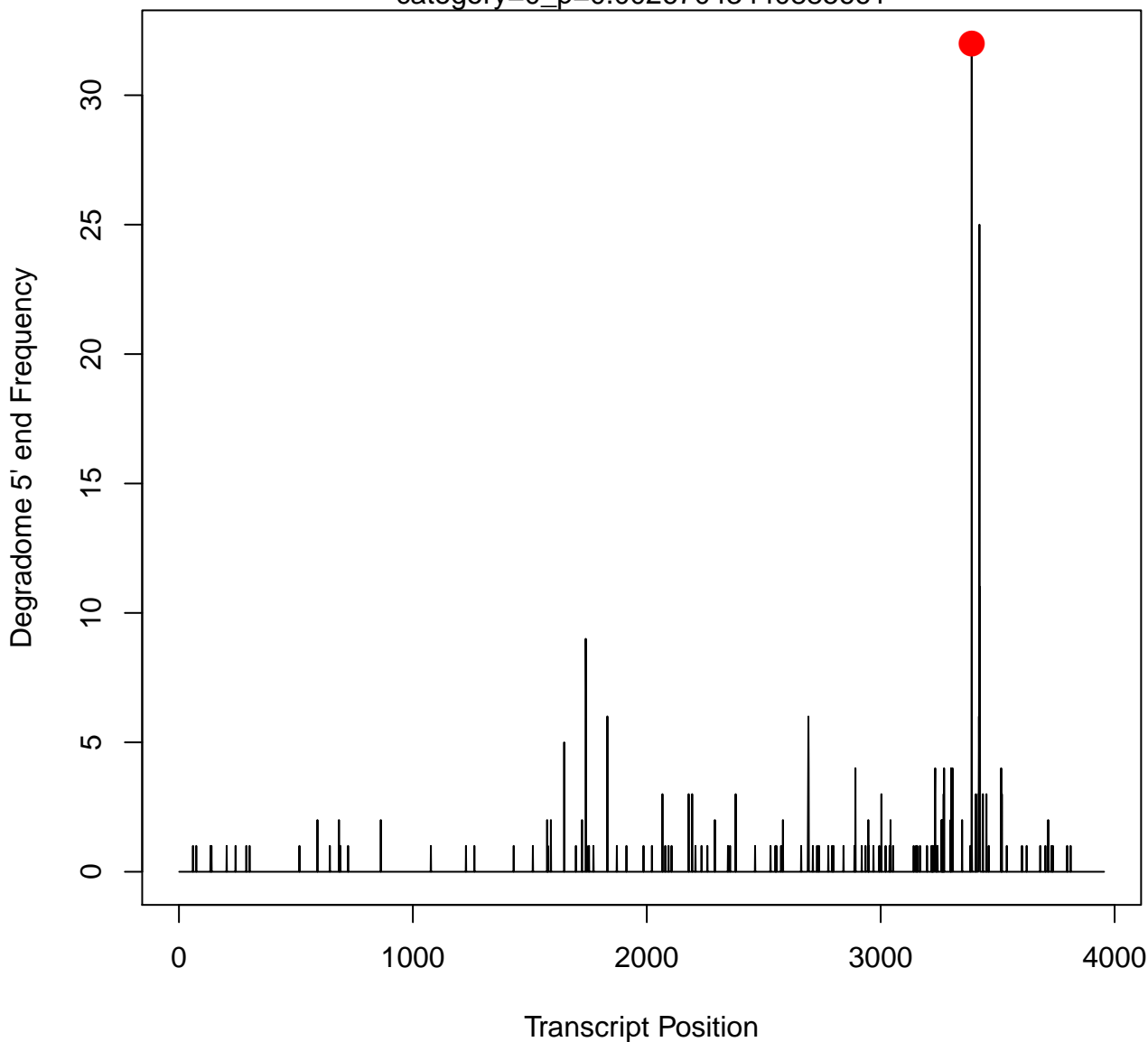

Supplement: Supplementary file 4 [file Data_Sheet_4.zip › Sit-miR167h_Seita.4G262300.1_3389_TPlot.pdf]

**T=Seita.9G047600.1\_Q=Sit-miR167h\_S=2323**

category=2\_p=0.999933271093549

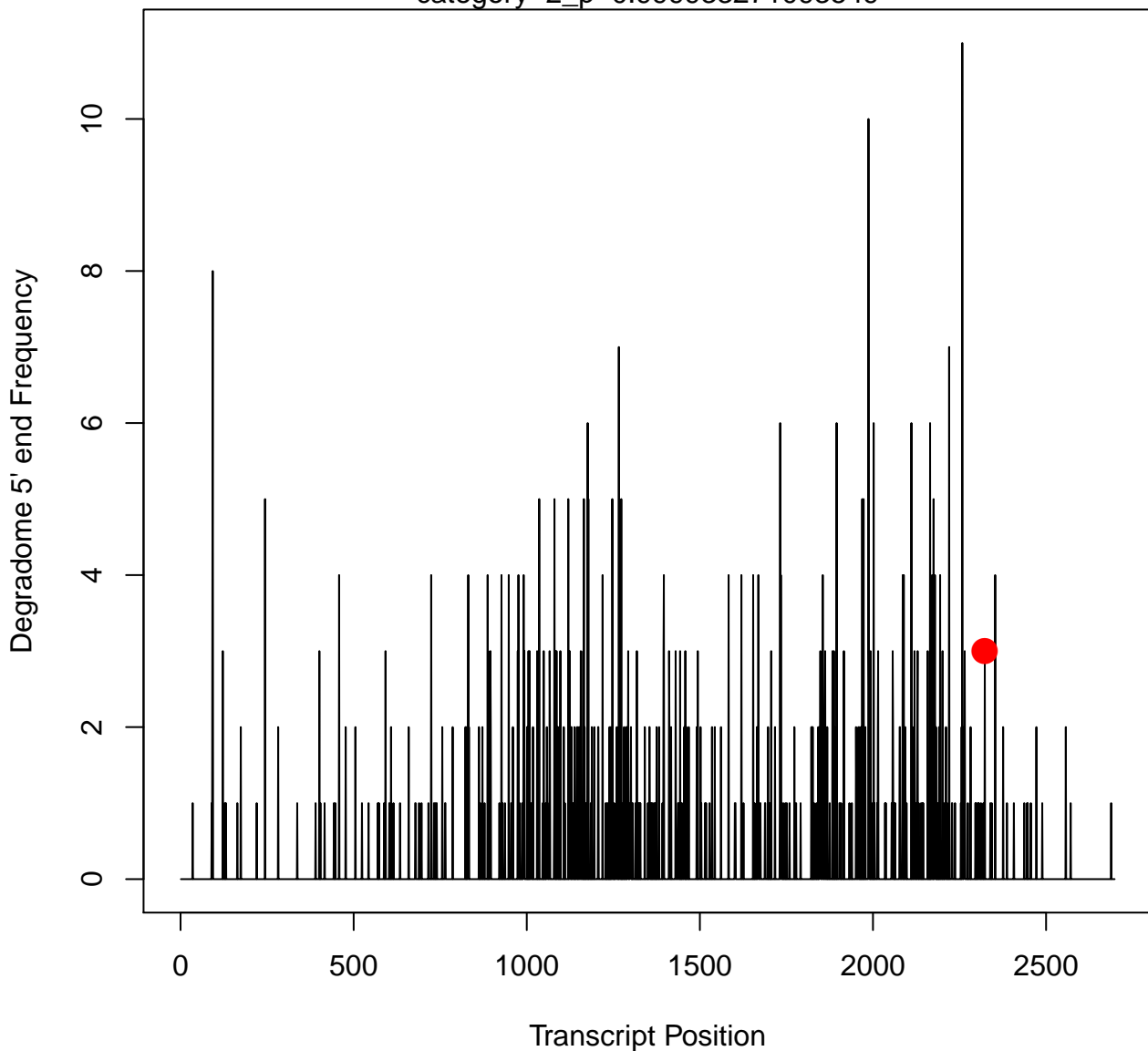

Supplement: Supplementary file 4 [file Data_Sheet_4.zip › Sit-miR167h_Seita.9G047600.1_2323_TPlot.pdf]

**T=Seita.9G320900.1\_Q=Sit-miR167h\_S=1109**

category=2\_p=0.806567933060178

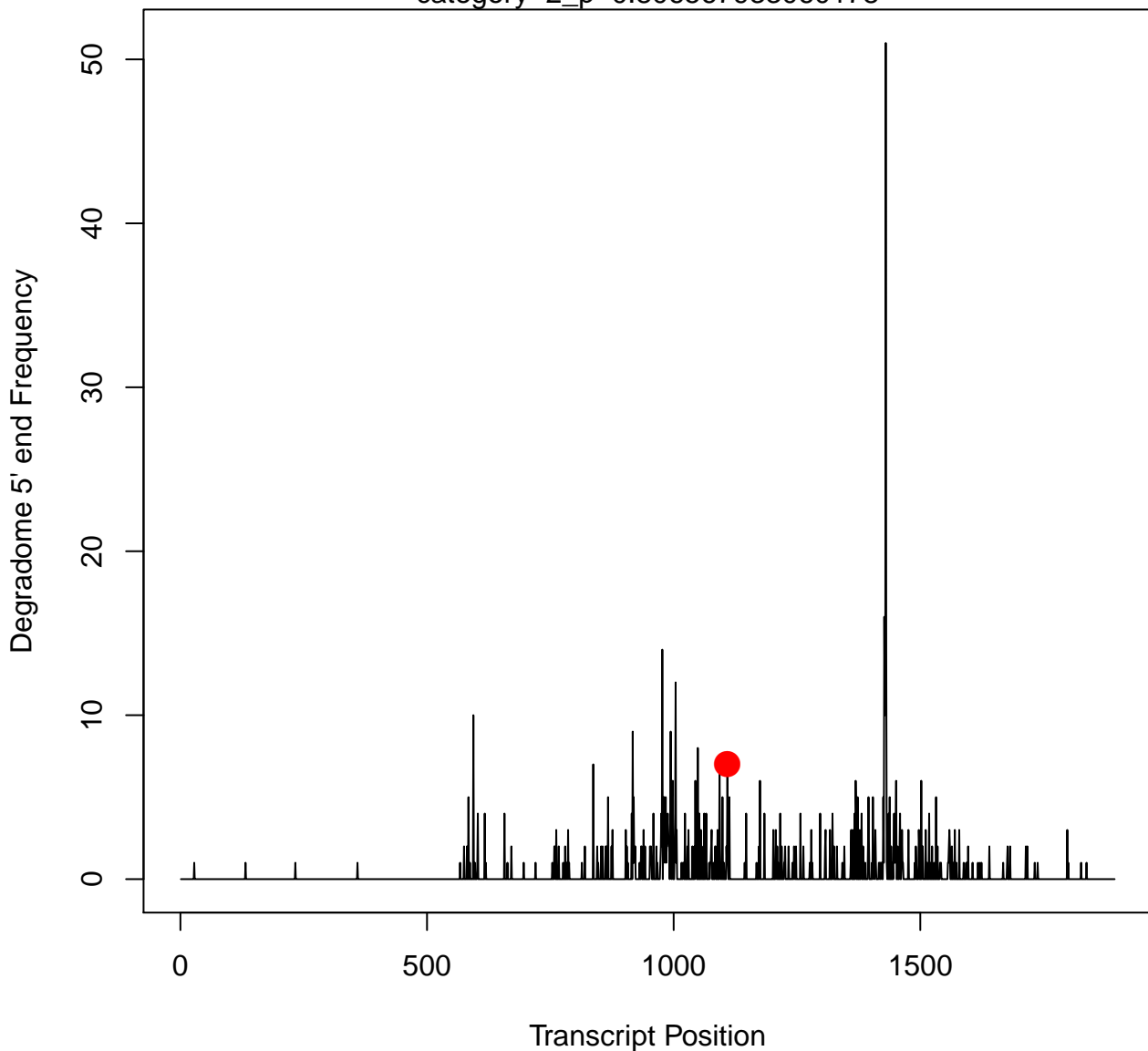

Supplement: Supplementary file 4 [file Data_Sheet_4.zip › Sit-miR167h_Seita.9G320900.1_1109_TPlot.pdf]

**T=Seita.1G377700.1\_Q=Sit-miR167j\_S=3379**

category=2\_p=0.998525500702474

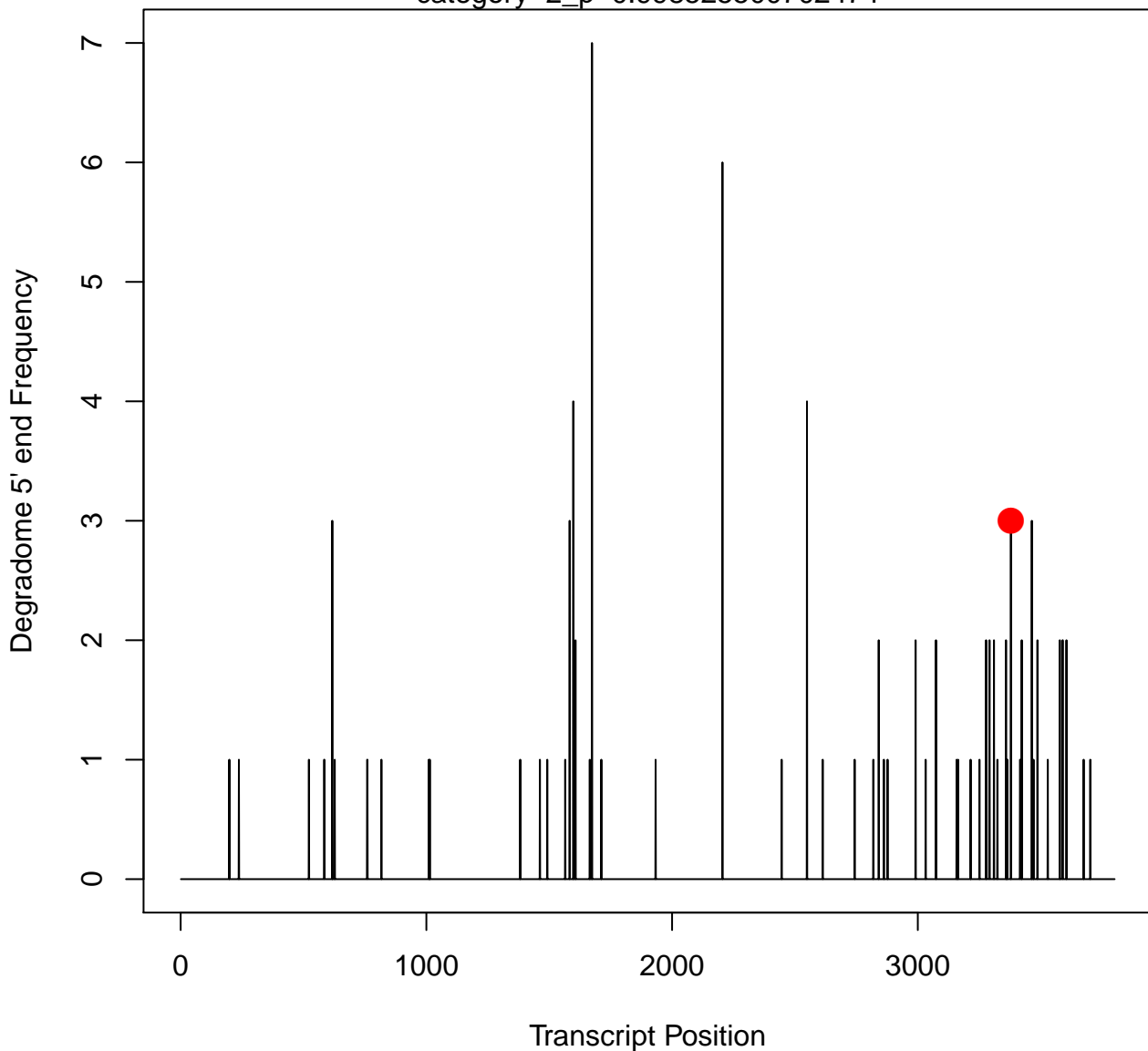

Supplement: Supplementary file 4 [file Data_Sheet_4.zip › Sit-miR167j_Seita.1G377700.1_3379_TPlot.pdf]

**T=Seita.3G020000.1\_Q=Sit-miR167j\_S=2739**

category=2\_p=0.0505560746234568

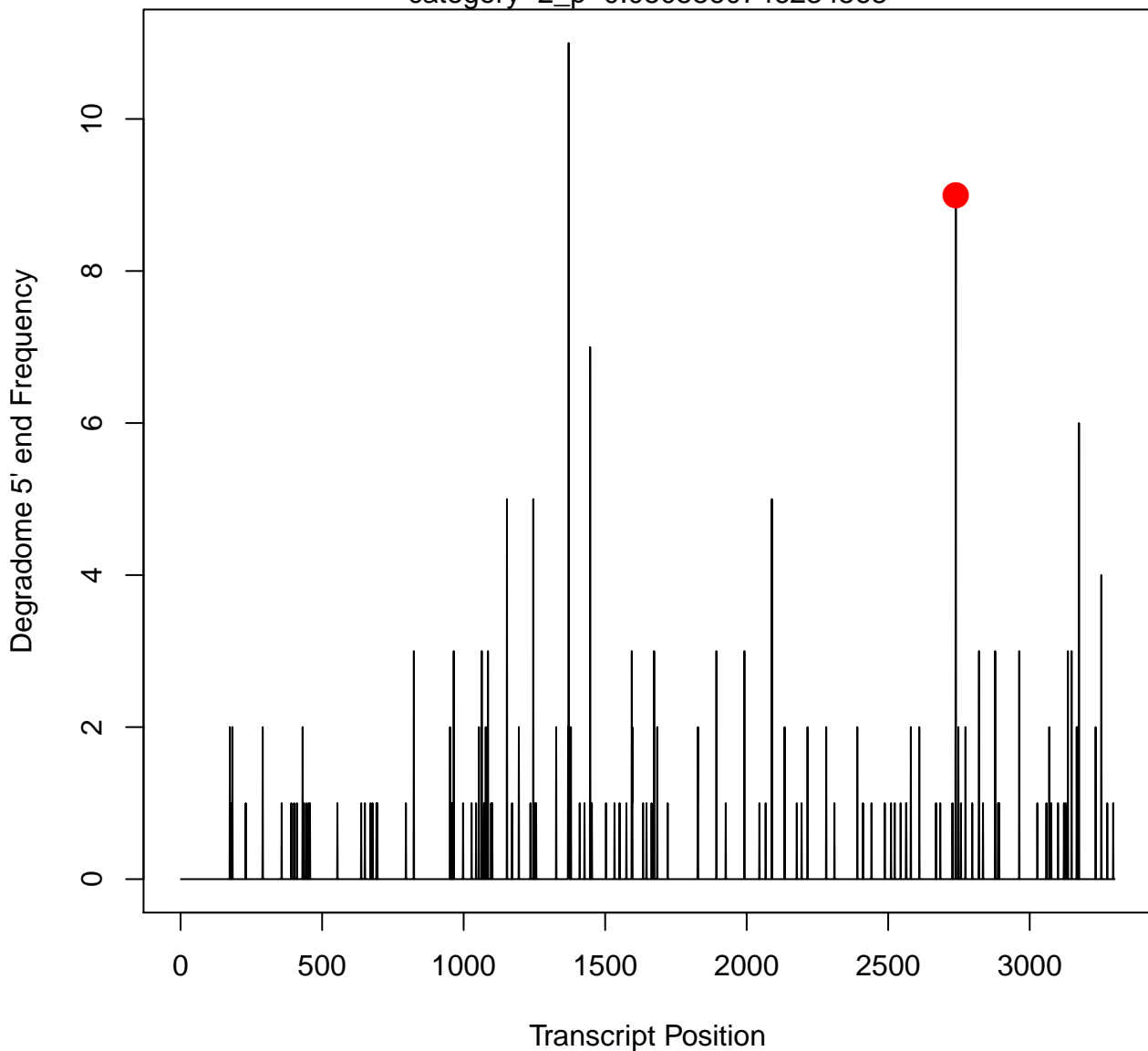

Supplement: Supplementary file 4 [file Data_Sheet_4.zip › Sit-miR167j_Seita.3G020000.1_2739_TPlot.pdf]

**T=Seita.7G045700.1\_Q=Sit-miR167j\_S=613**

category=2\_p=0.999895388386774

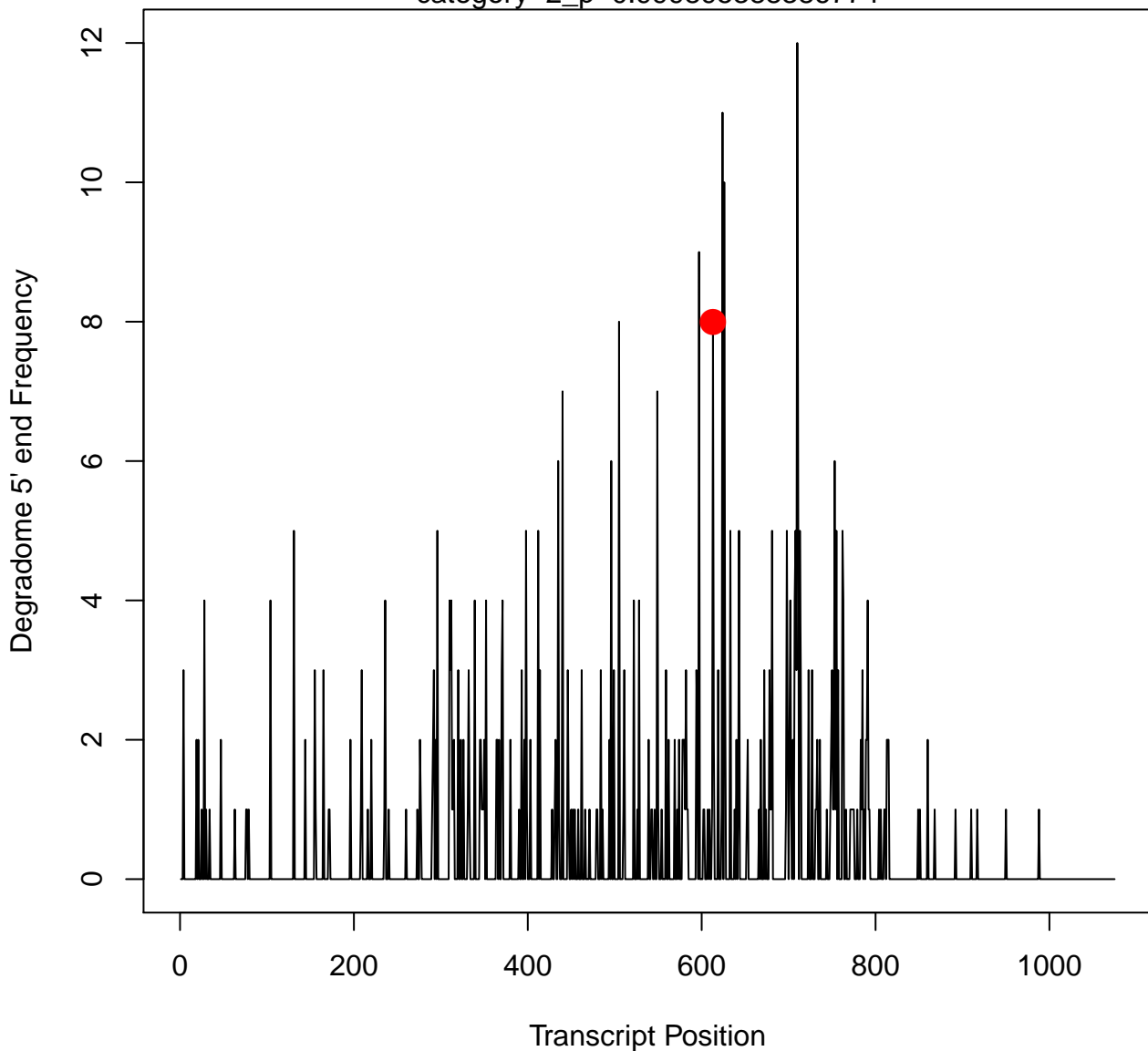

Supplement: Supplementary file 4 [file Data_Sheet_4.zip › Sit-miR167j_Seita.7G045700.1_613_TPlot.pdf]

**T=Seita.8G184000.1\_Q=Sit-miR167j\_S=281**

category=2\_p=0.989412086052848

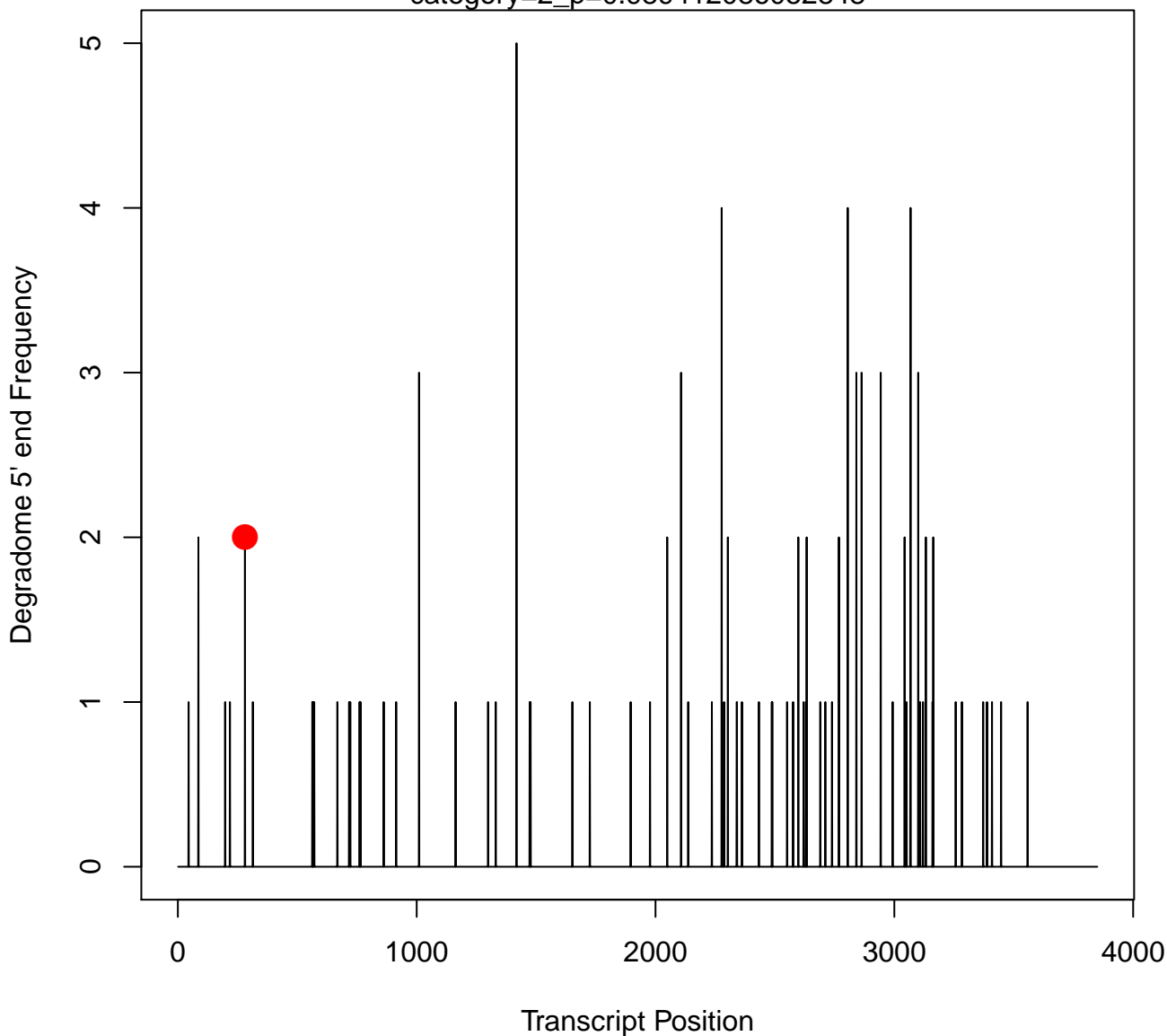

Supplement: Supplementary file 4 [file Data_Sheet_4.zip › Sit-miR167j_Seita.8G184000.1_281_TPlot.pdf]

**T=Seita.1G378700.1\_Q=Sit-miR168\_S=527**

category=0\_p=0.0121496192234285

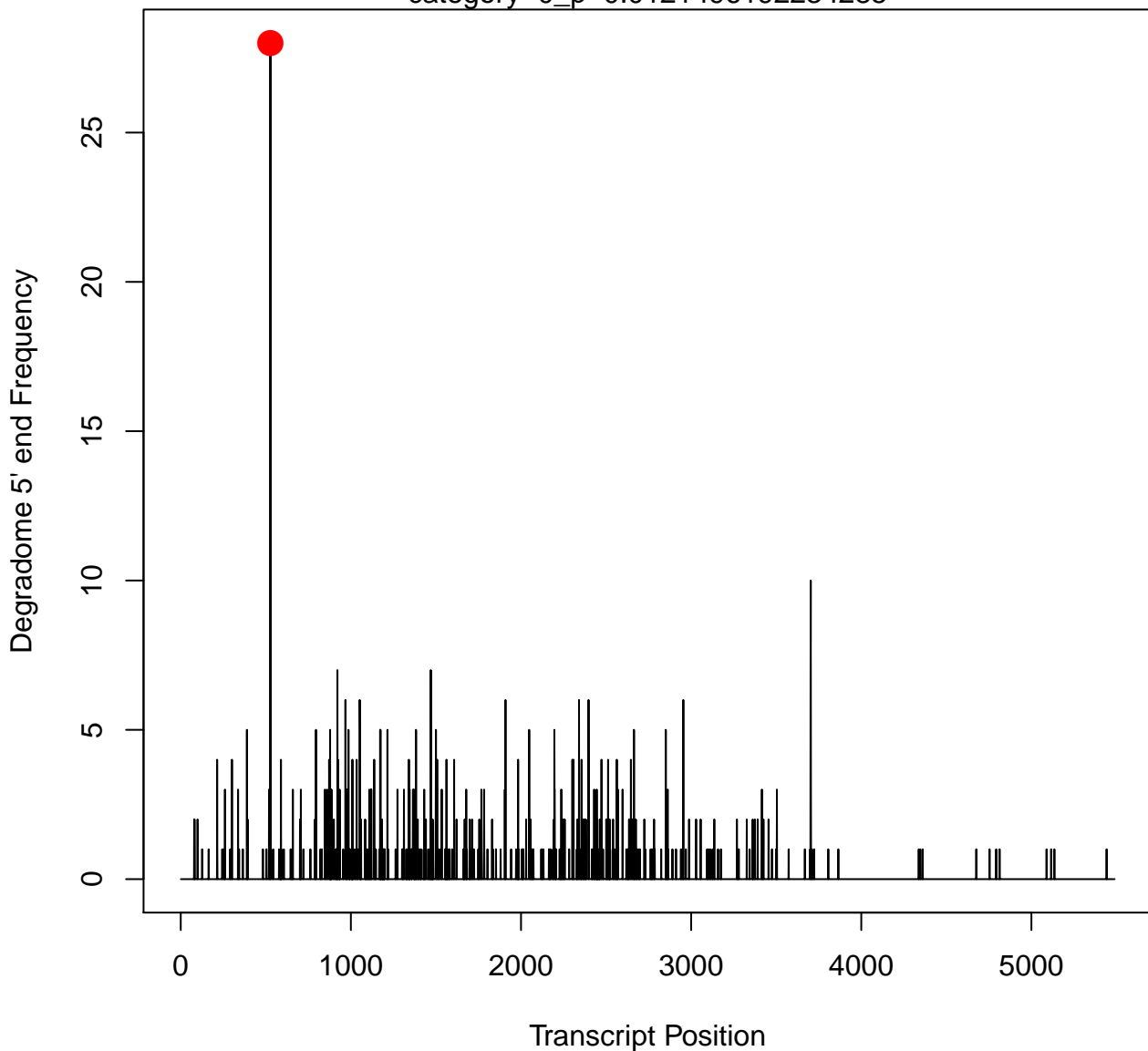

Supplement: Supplementary file 4 [file Data_Sheet_4.zip › Sit-miR168_Seita.1G378700.1_527_TPlot.pdf]

**T=Seita.3G070600.1\_Q=Sit-miR168\_S=747**

category=0\_p=0.011772187392642

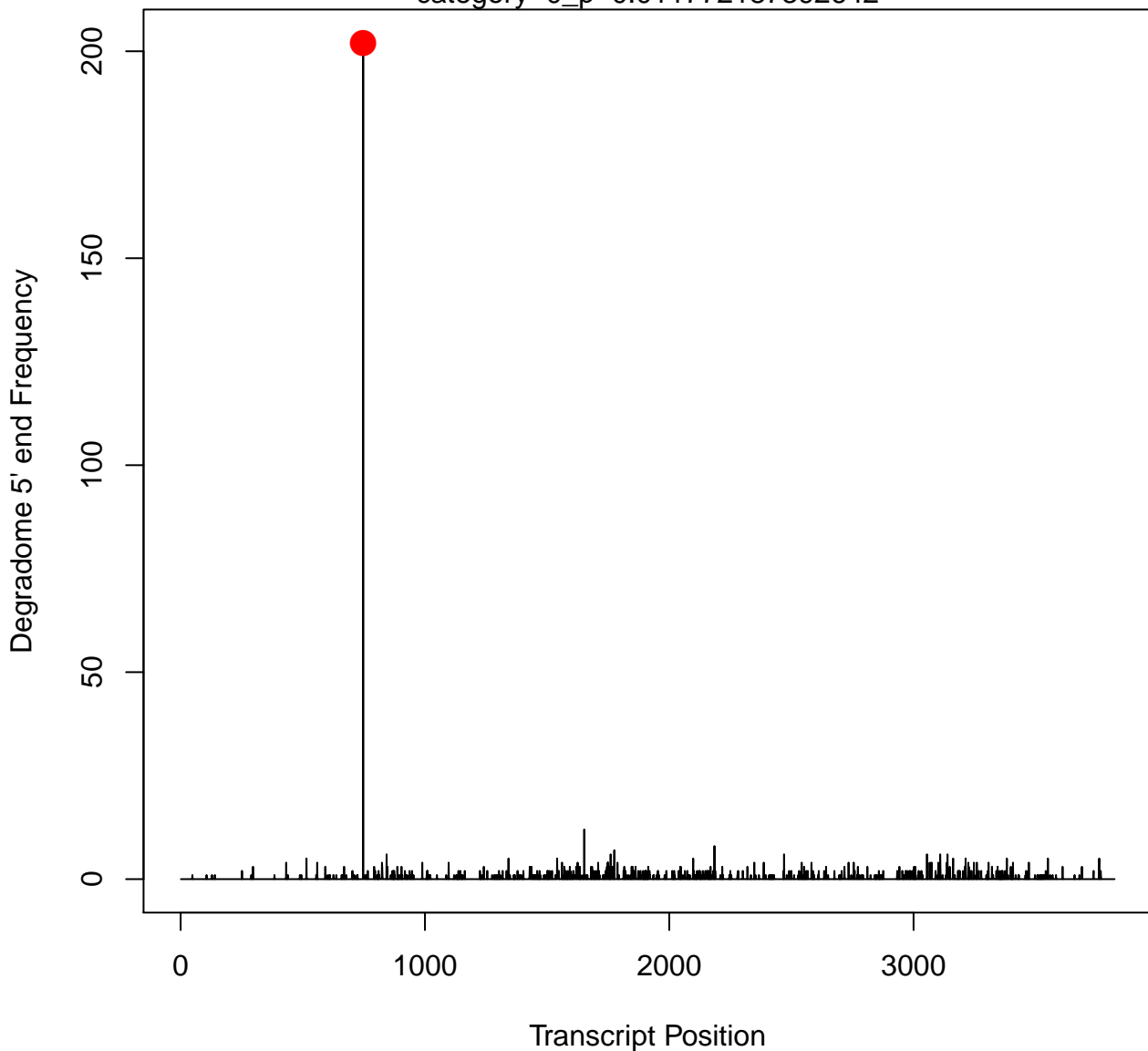

Supplement: Supplementary file 4 [file Data_Sheet_4.zip › Sit-miR168_Seita.3G070600.1_747_TPlot.pdf]

**T=Seita.3G298400.1\_Q=Sit-miR168\_S=1403**

category=2\_p=0.942348234666639

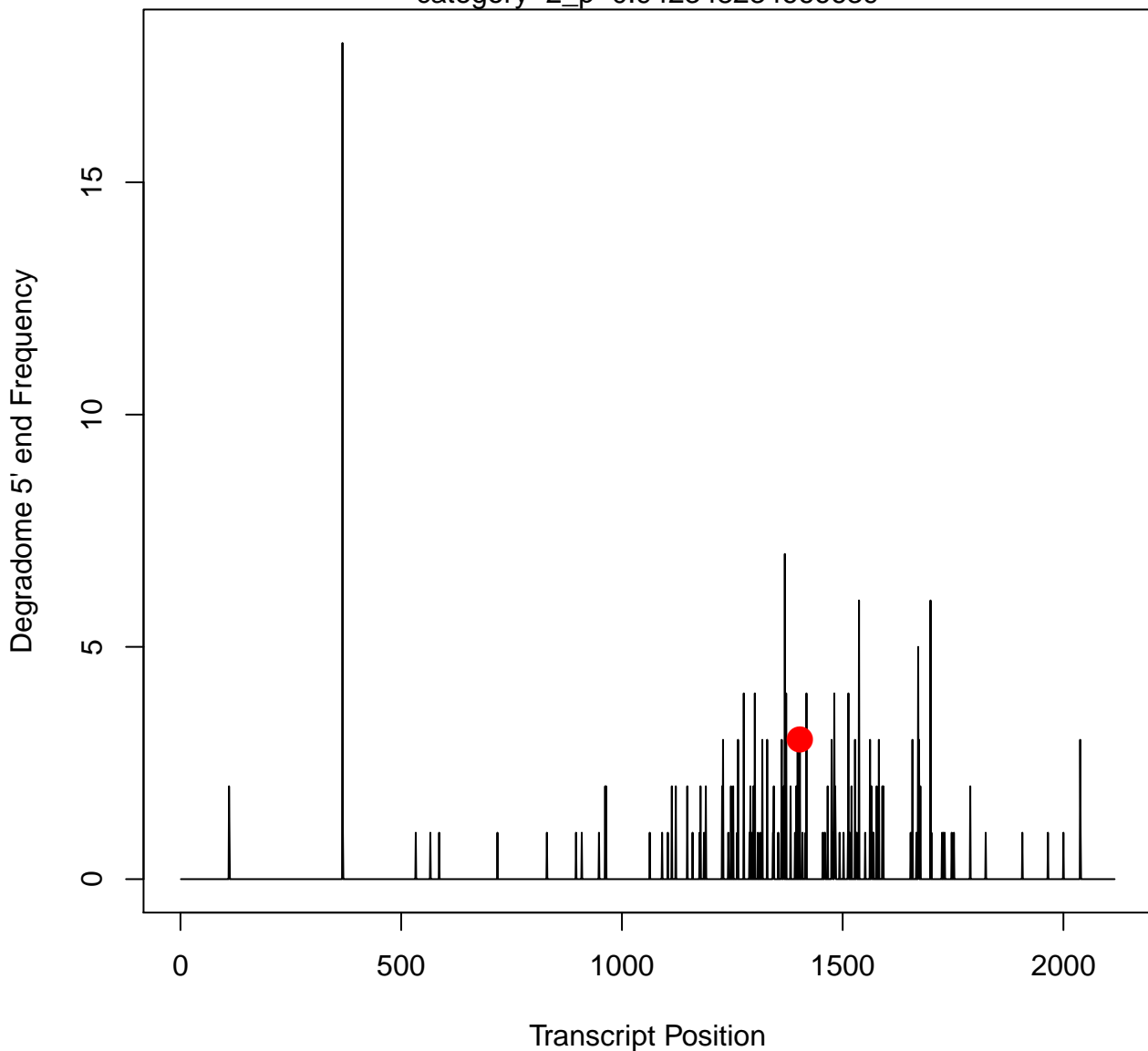

Supplement: Supplementary file 4 [file Data_Sheet_4.zip › Sit-miR168_Seita.3G298400.1_1403_TPlot.pdf]

**T=Seita.4G158600.1\_Q=Sit-miR168\_S=436**

category=2\_p=0.954744701090606

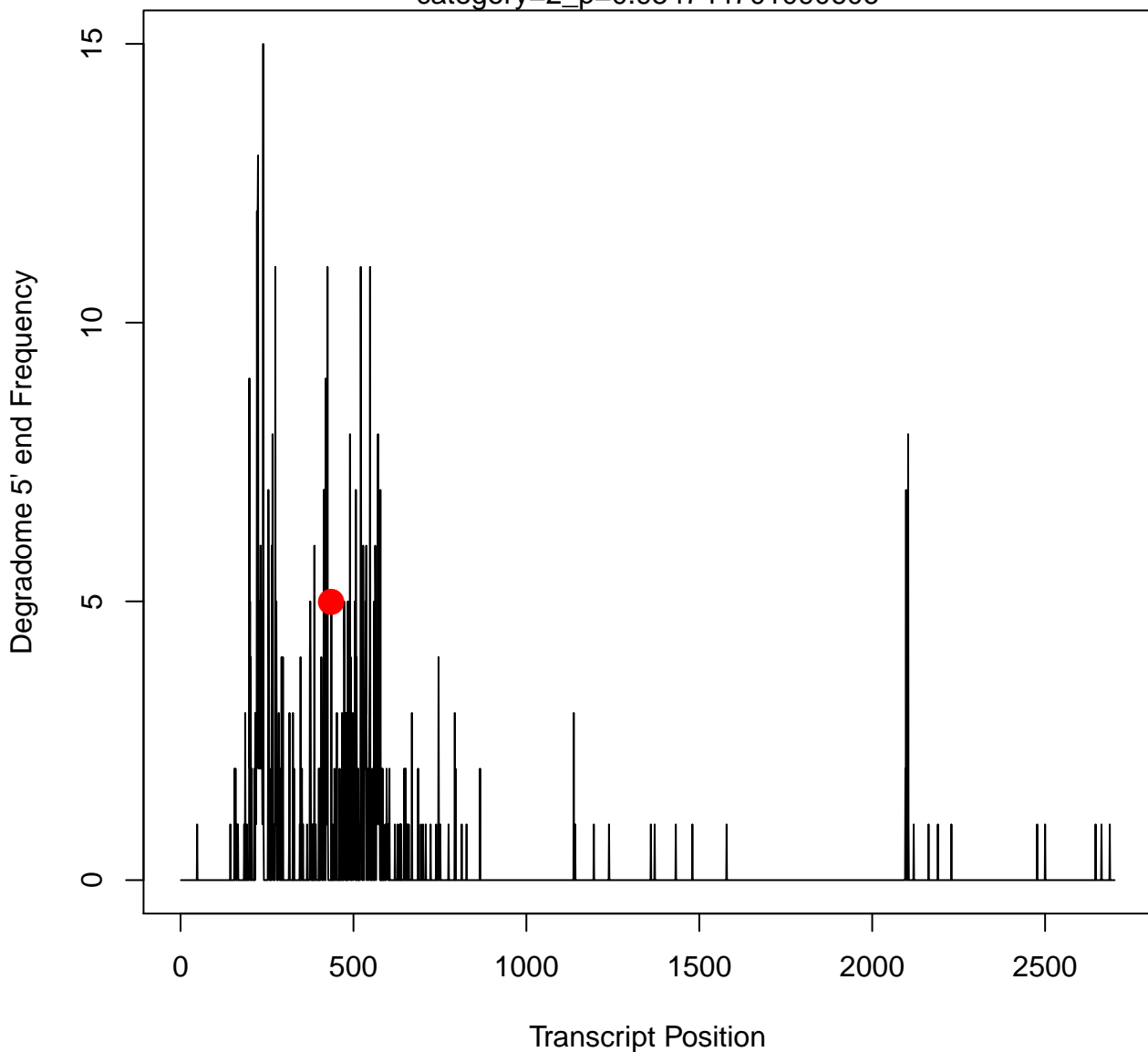

Supplement: Supplementary file 4 [file Data_Sheet_4.zip › Sit-miR168_Seita.4G158600.1_436_TPlot.pdf]

**T=Seita.4G288700.1\_Q=Sit-miR168\_S=343**

category=2\_p=0.362127163578296

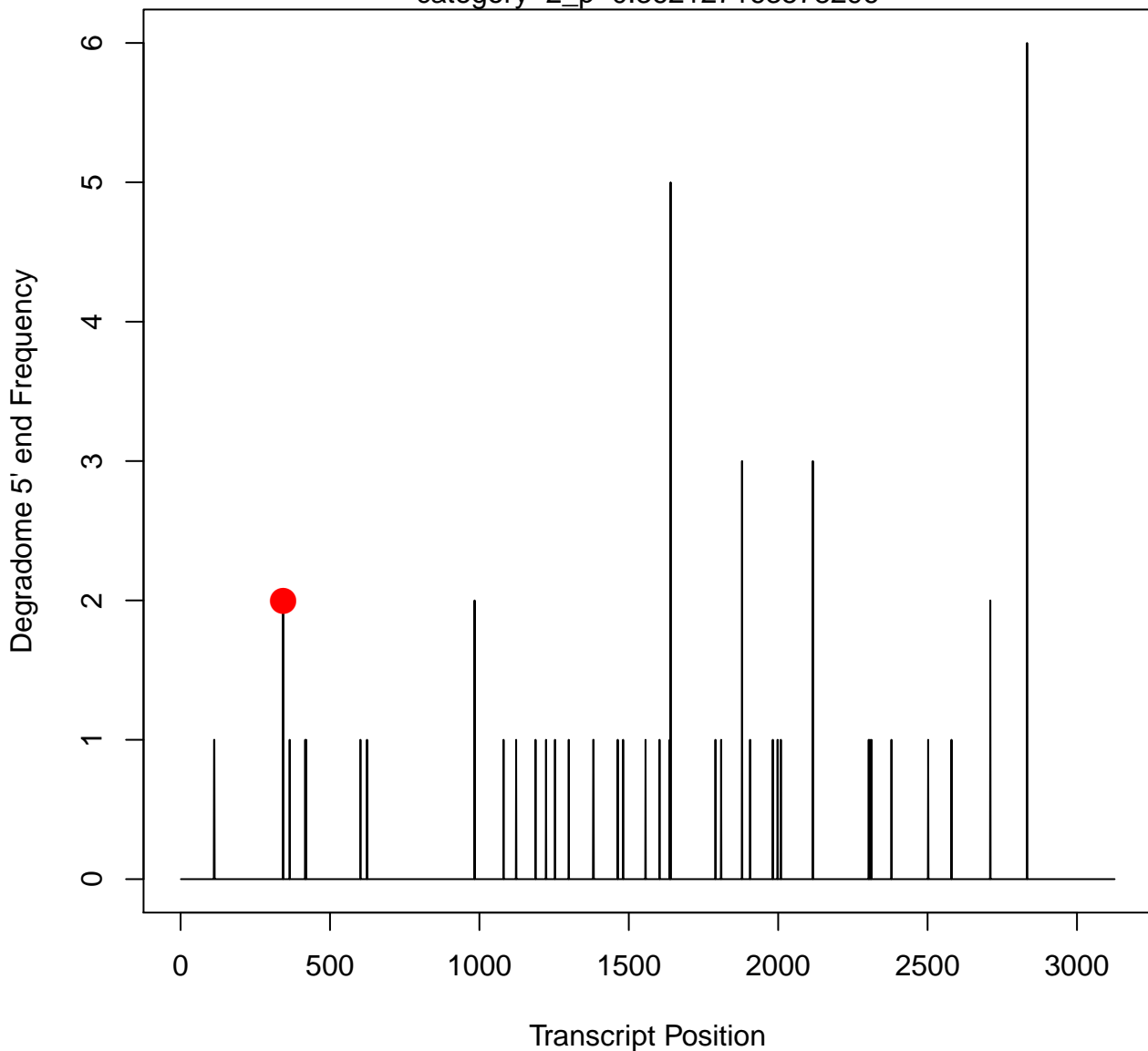

Supplement: Supplementary file 4 [file Data_Sheet_4.zip › Sit-miR168_Seita.4G288700.1_343_TPlot.pdf]

**T=Seita.5G261900.1\_Q=Sit-miR168\_S=537**

category=0\_p=0.096275066058769

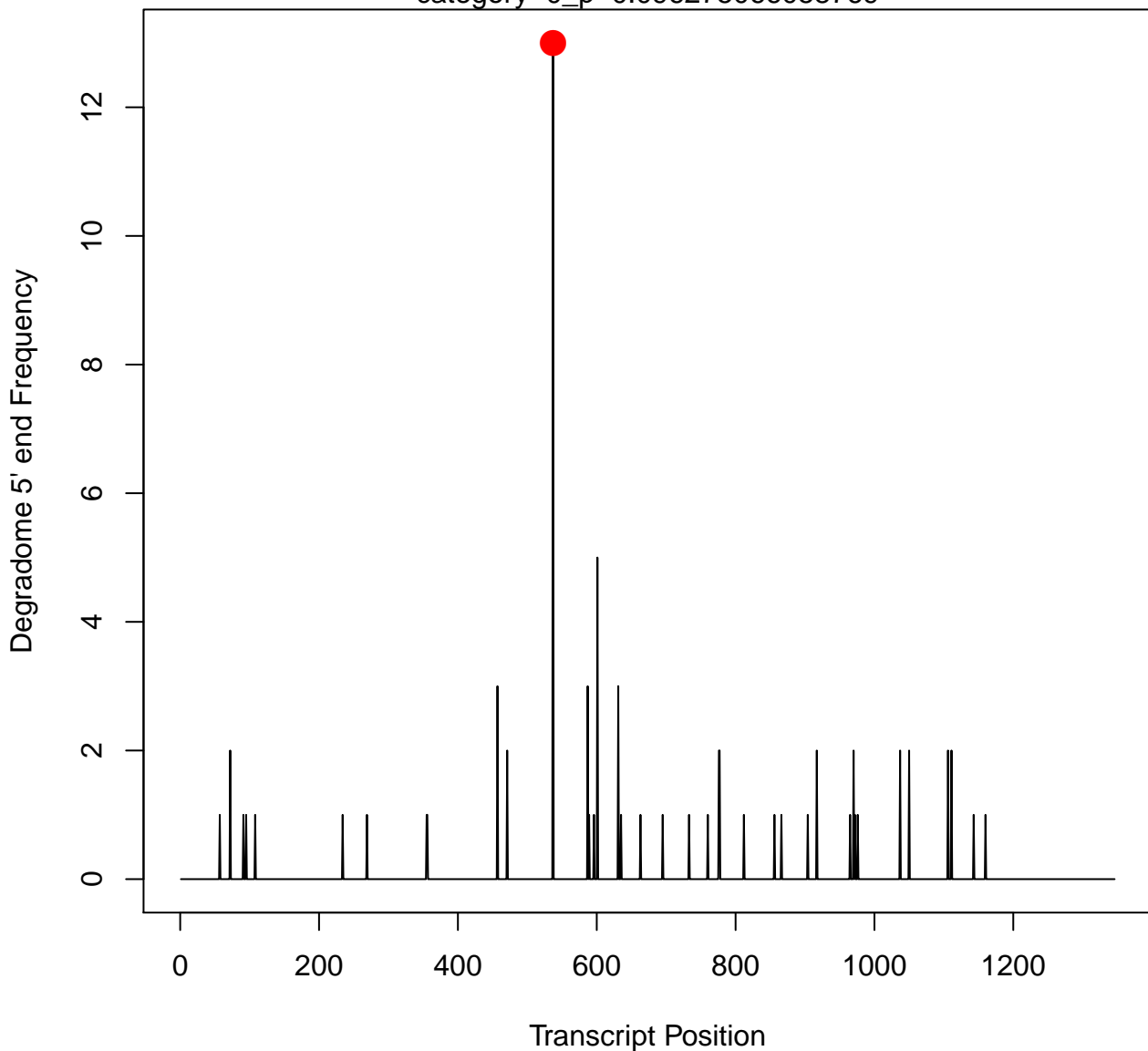

Supplement: Supplementary file 4 [file Data_Sheet_4.zip › Sit-miR168_Seita.5G261900.1_537_TPlot.pdf]

**T=Seita.5G435400.1\_Q=Sit-miR168\_S=595**

category=0\_p=0.00457352039366032

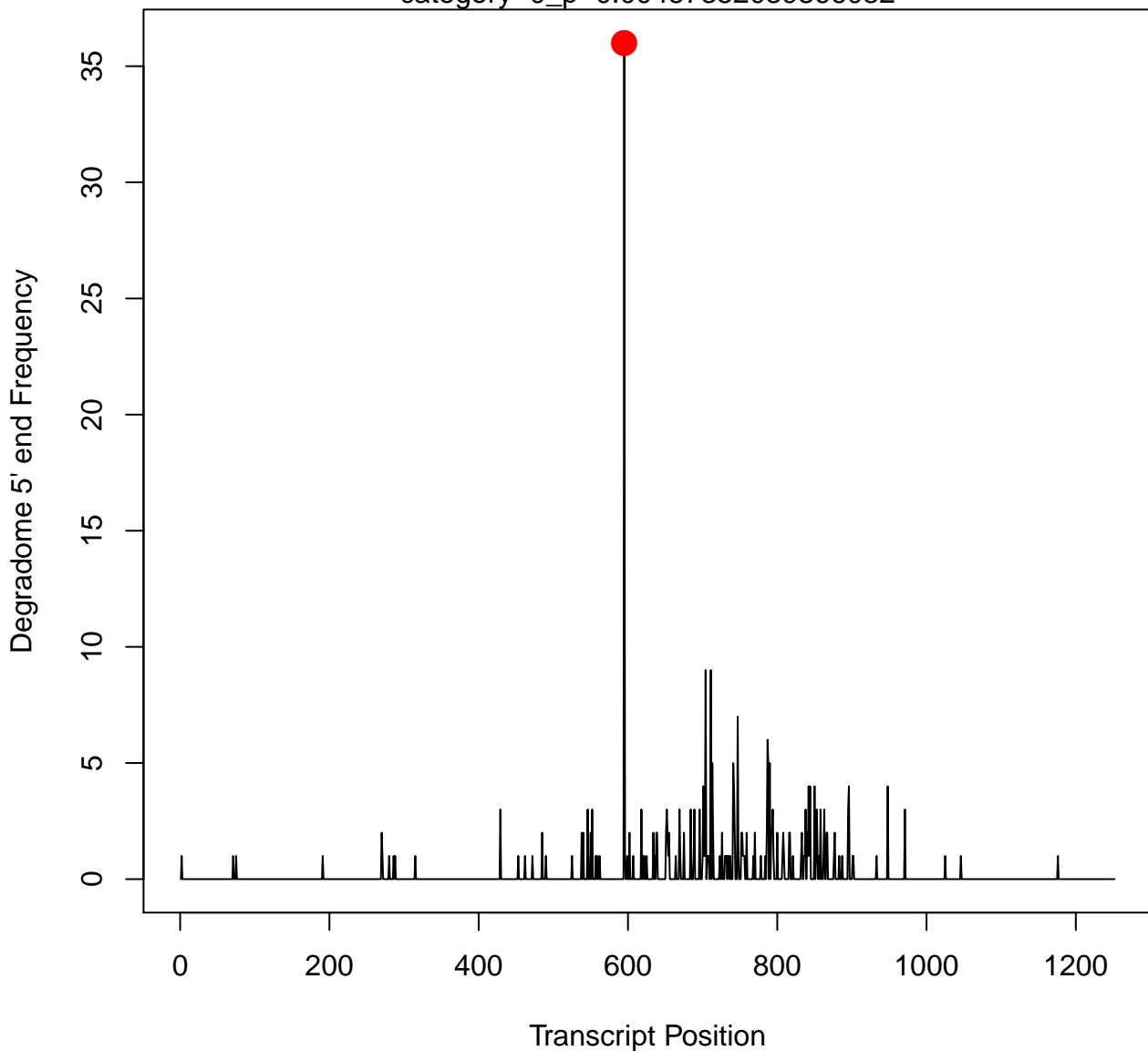

Supplement: Supplementary file 4 [file Data_Sheet_4.zip › Sit-miR168_Seita.5G435400.1_595_TPlot.pdf]

**T=Seita.5G455800.1\_Q=Sit-miR168\_S=851**

category=2\_p=0.971132874121553

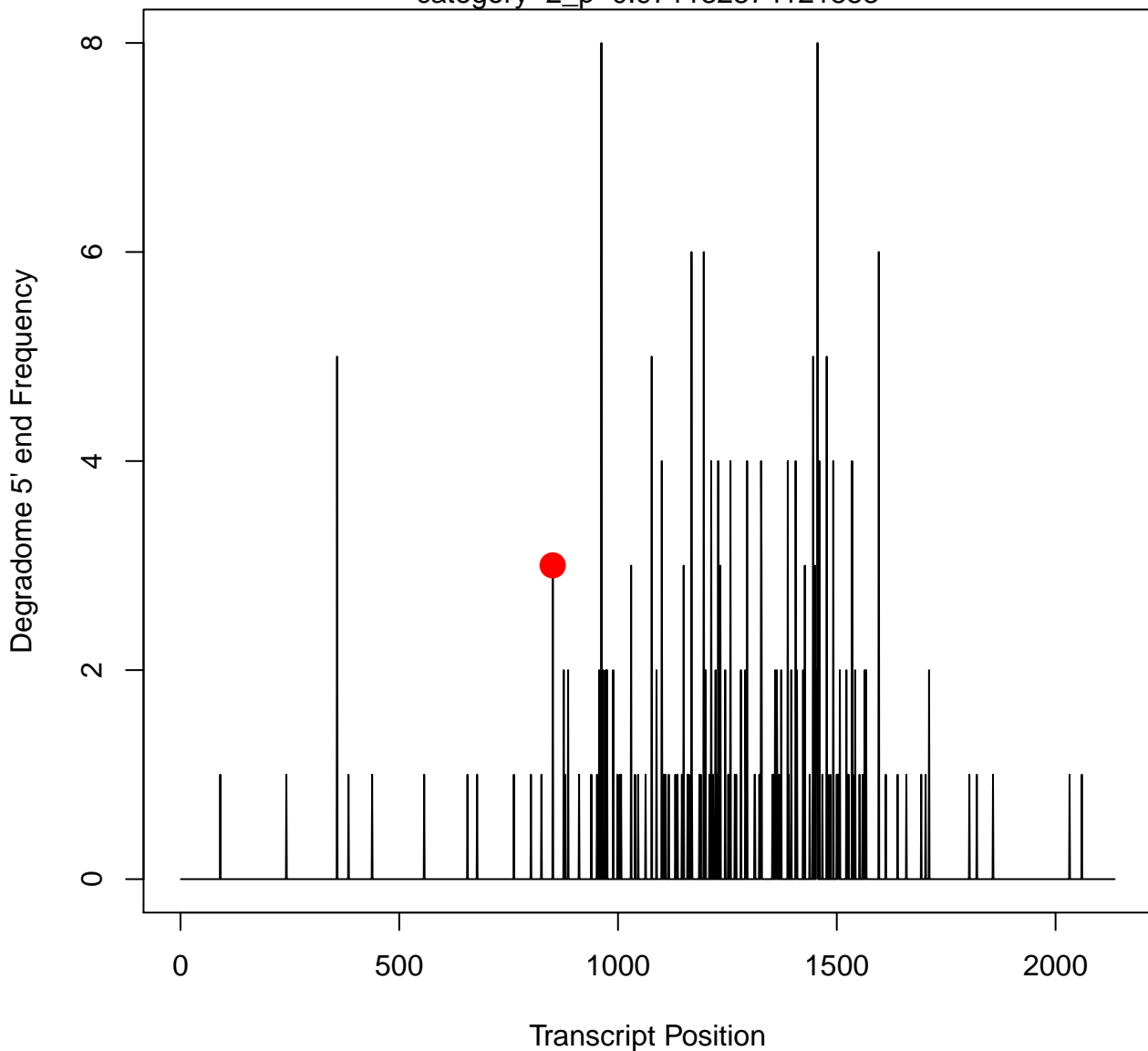

Supplement: Supplementary file 4 [file Data_Sheet_4.zip › Sit-miR168_Seita.5G455800.1_851_TPlot.pdf]

**T=Seita.7G141400.1\_Q=Sit-miR168\_S=1212**

category=1\_p=0.0893824676309303

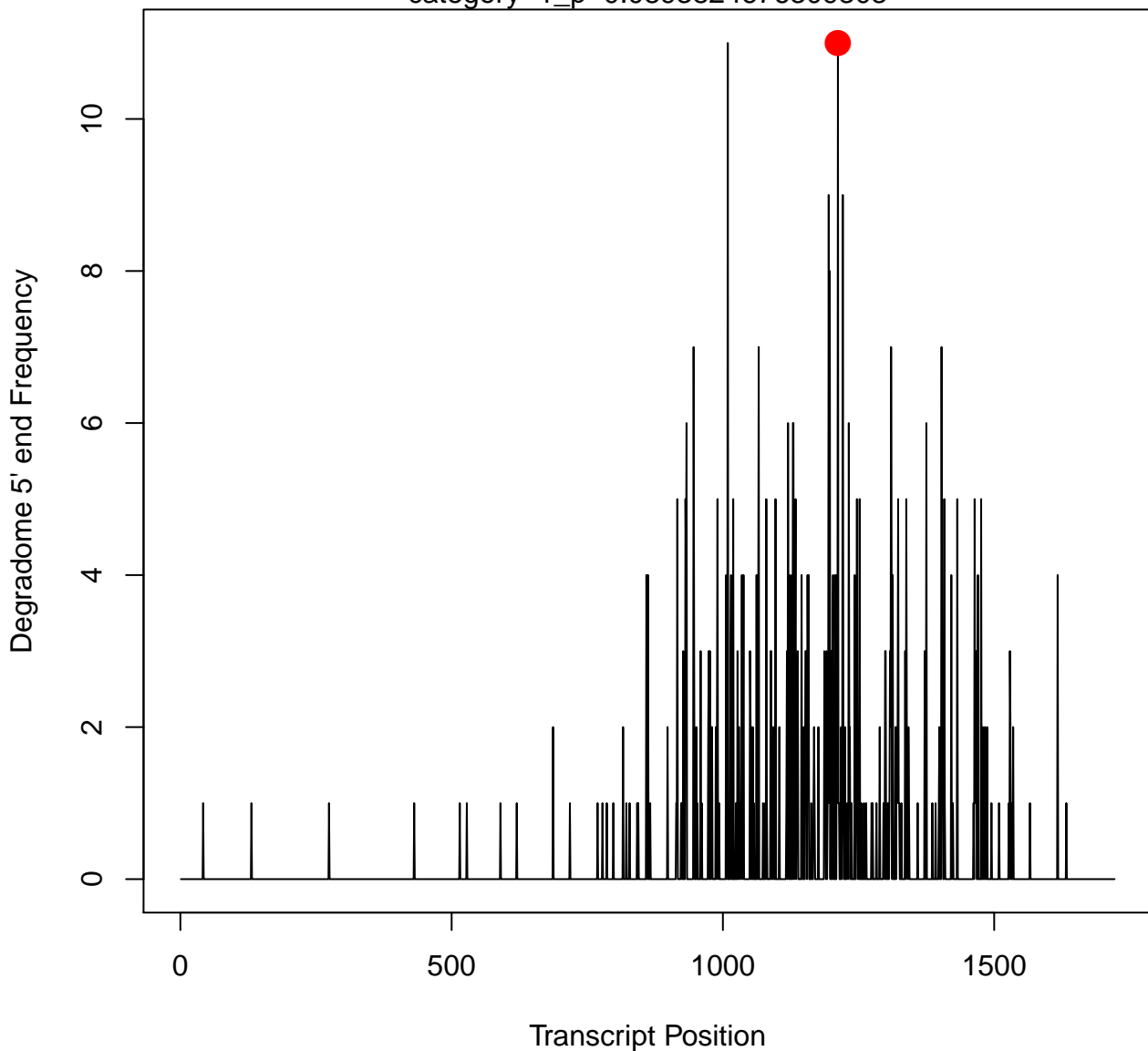

Supplement: Supplementary file 4 [file Data_Sheet_4.zip › Sit-miR168_Seita.7G141400.1_1212_TPlot.pdf]

**T=Seita.7G200400.1\_Q=Sit-miR168\_S=1236**

category=2\_p=0.998550779918306

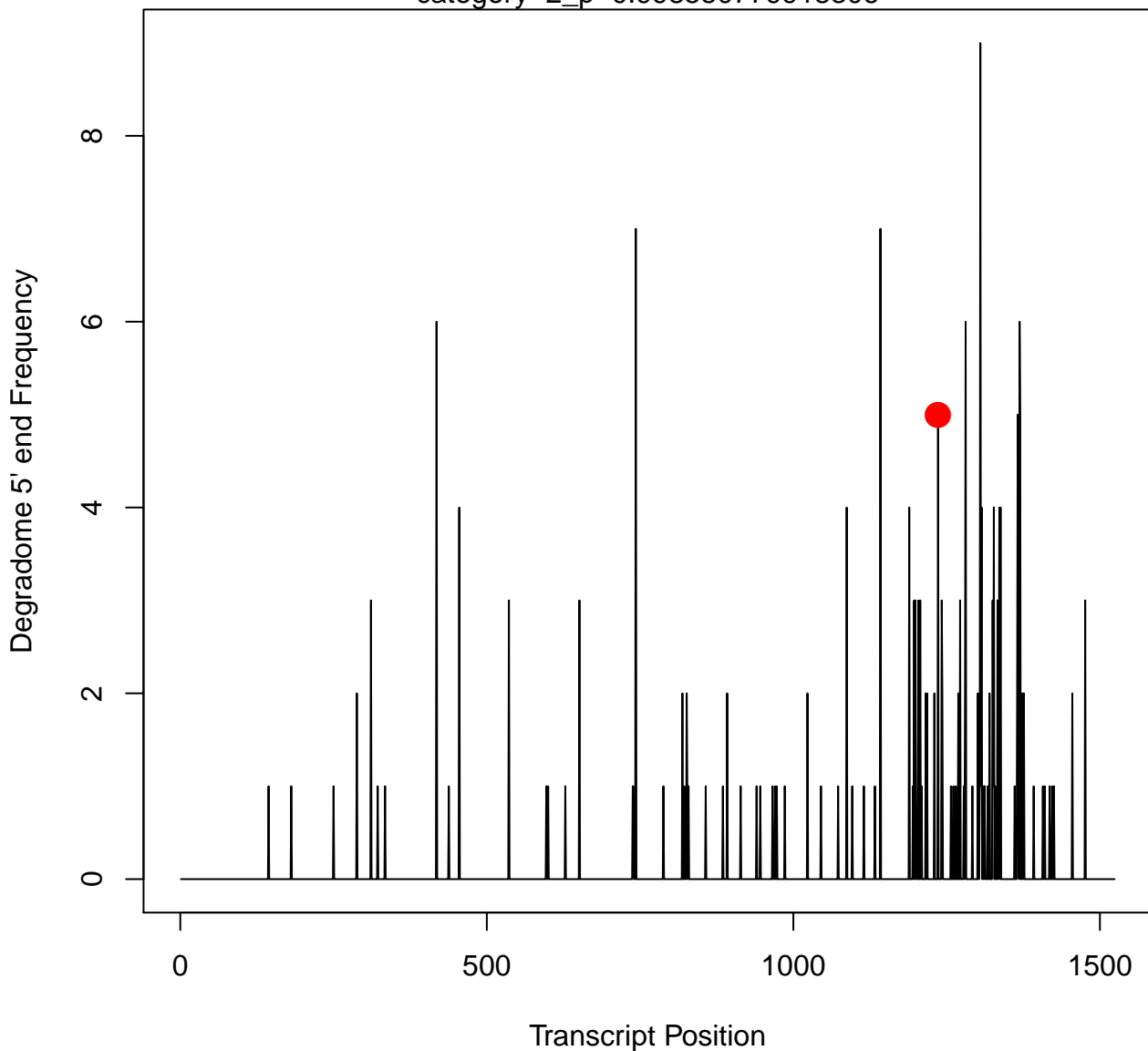

Supplement: Supplementary file 4 [file Data_Sheet_4.zip › Sit-miR168_Seita.7G200400.1_1236_TPlot.pdf]

**T=Seita.7G201100.1\_Q=Sit-miR168\_S=664**

category=0\_p=0.0106390264388001

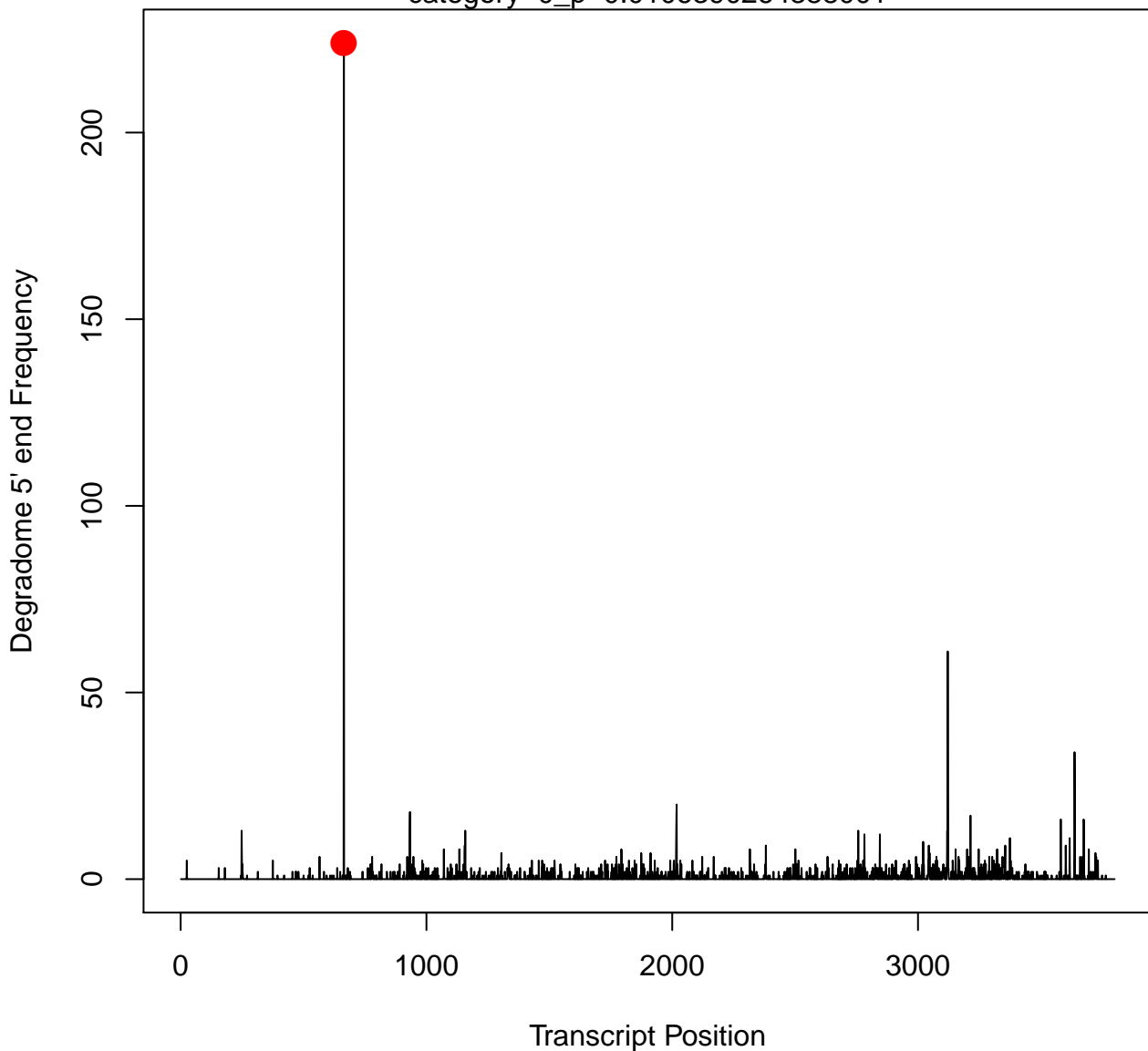

Supplement: Supplementary file 4 [file Data_Sheet_4.zip › Sit-miR168_Seita.7G201100.1_664_TPlot.pdf]

**T=Seita.8G236800.1\_Q=Sit-miR168\_S=1659**

category=2\_p=0.981586444536572

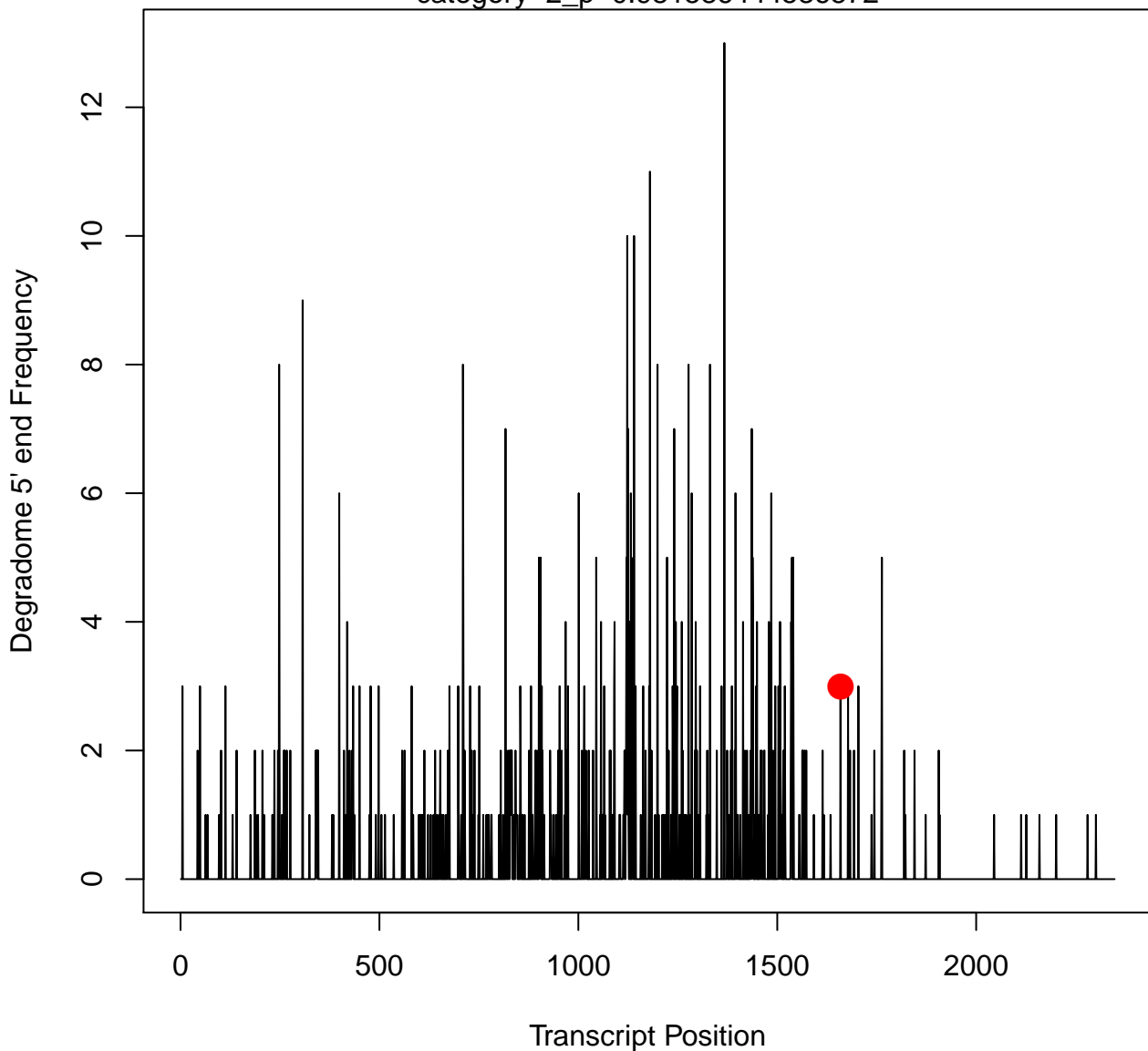

Supplement: Supplementary file 4 [file Data_Sheet_4.zip › Sit-miR168_Seita.8G236800.1_1659_TPlot.pdf]

**T=Seita.9G505300.1\_Q=Sit-miR168\_S=412**

category=2\_p=0.882852803167317

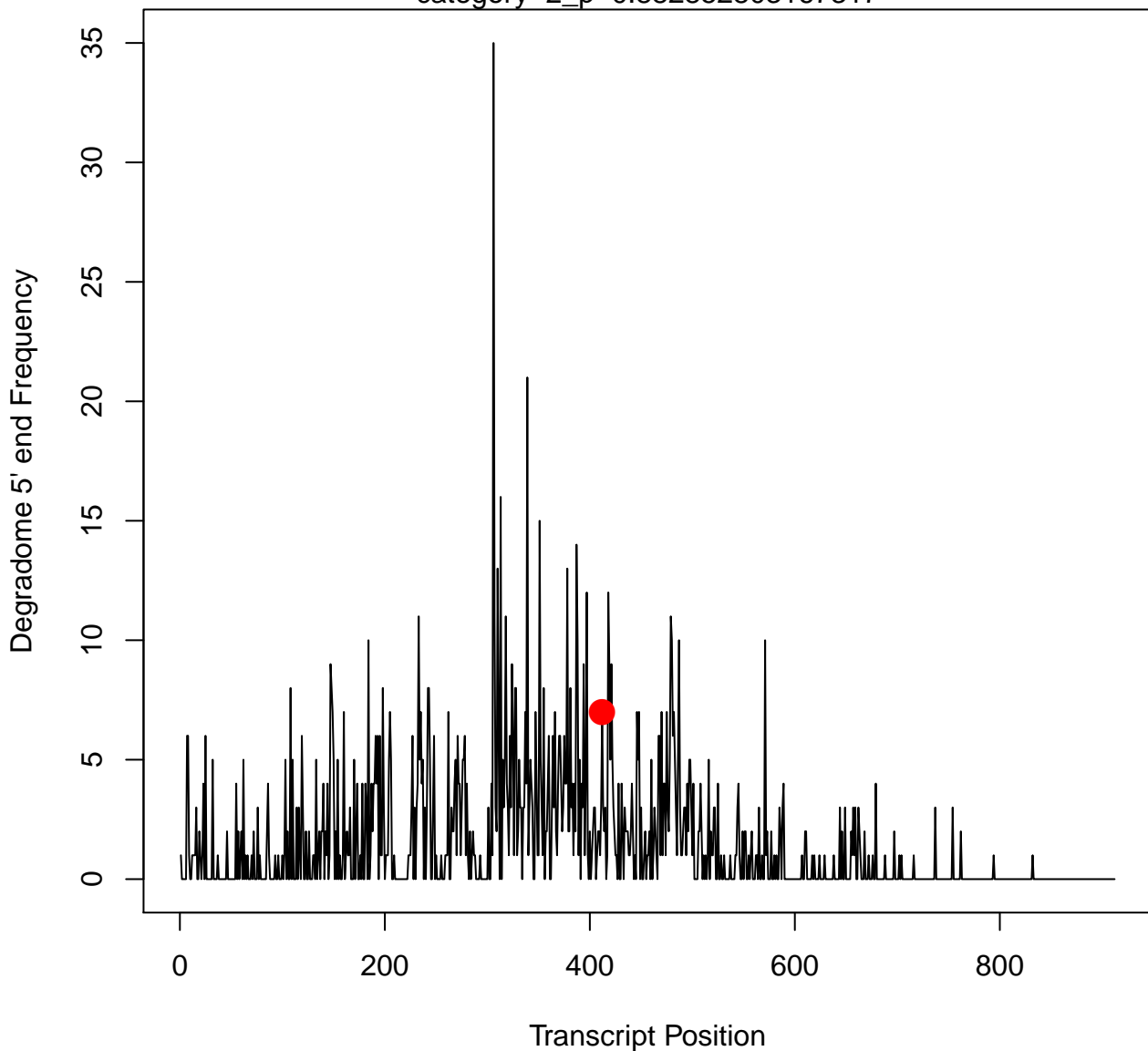

Supplement: Supplementary file 4 [file Data_Sheet_4.zip › Sit-miR168_Seita.9G505300.1_412_TPlot.pdf]

**T=Seita.J030300.1\_Q=Sit-miR168\_S=240**

category=0\_p=0.0959297763641125

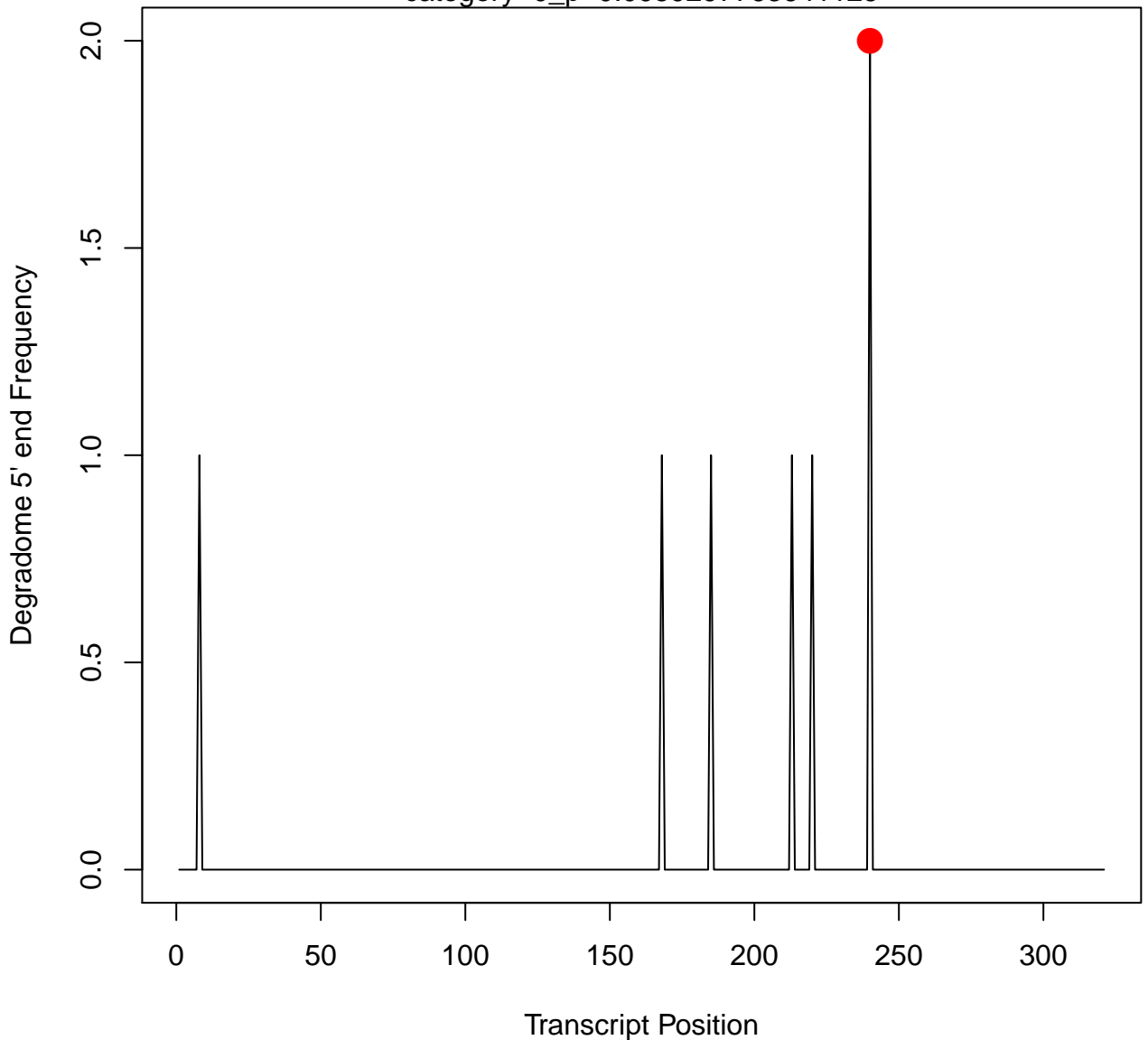

Supplement: Supplementary file 4 [file Data_Sheet_4.zip › Sit-miR168_Seita.J030300.1_240_TPlot.pdf]

**T=Seita.3G184900.1\_Q=Sit-miR169a\_S=536**

category=2\_p=0.964475639773801

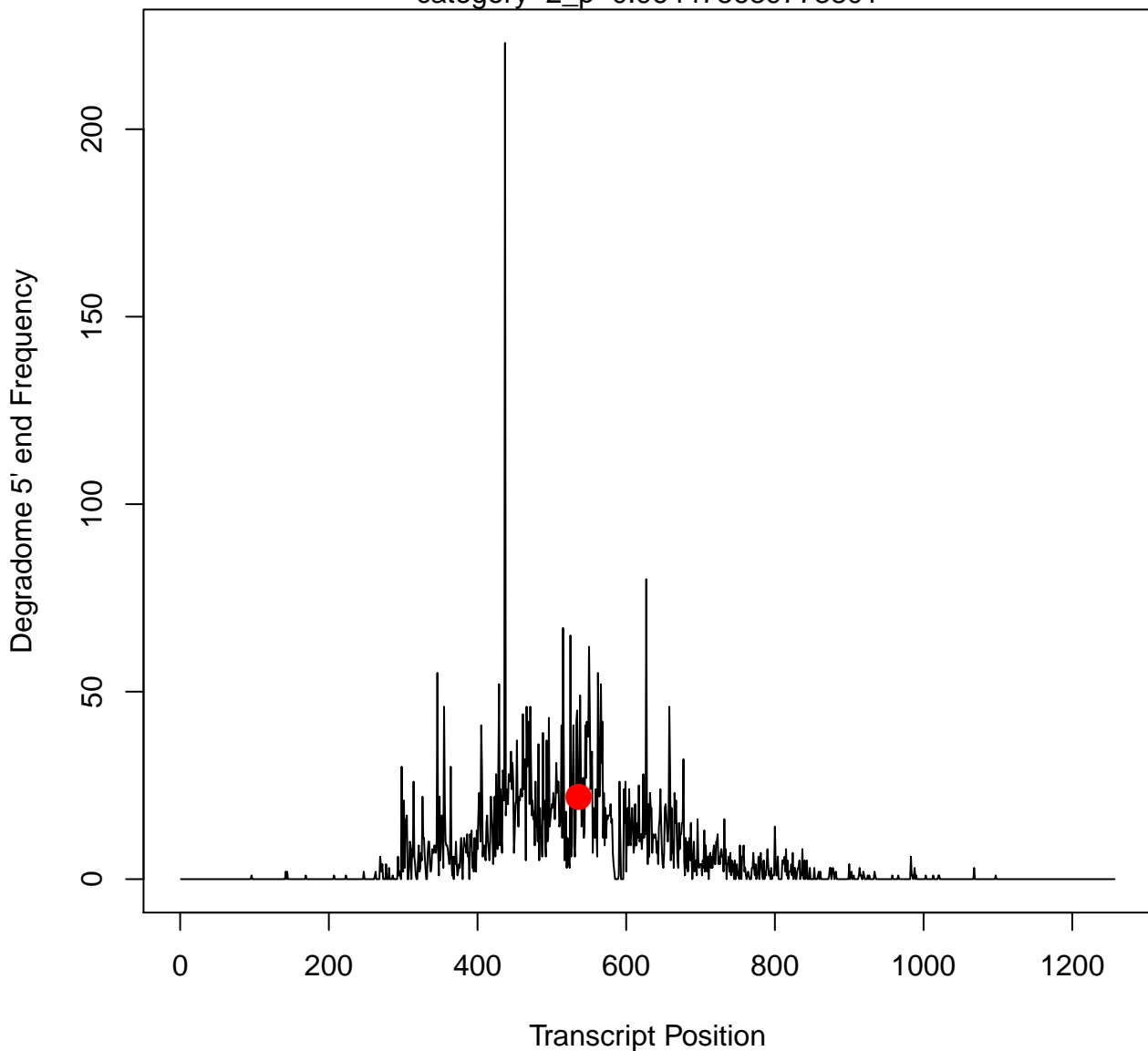

Supplement: Supplementary file 4 [file Data_Sheet_4.zip › Sit-miR169a_Seita.3G184900.1_536_TPlot.pdf]

**T=Seita.1G016500.1\_Q=Sit-miR169e\_S=720**

category=2\_p=0.707063214852899

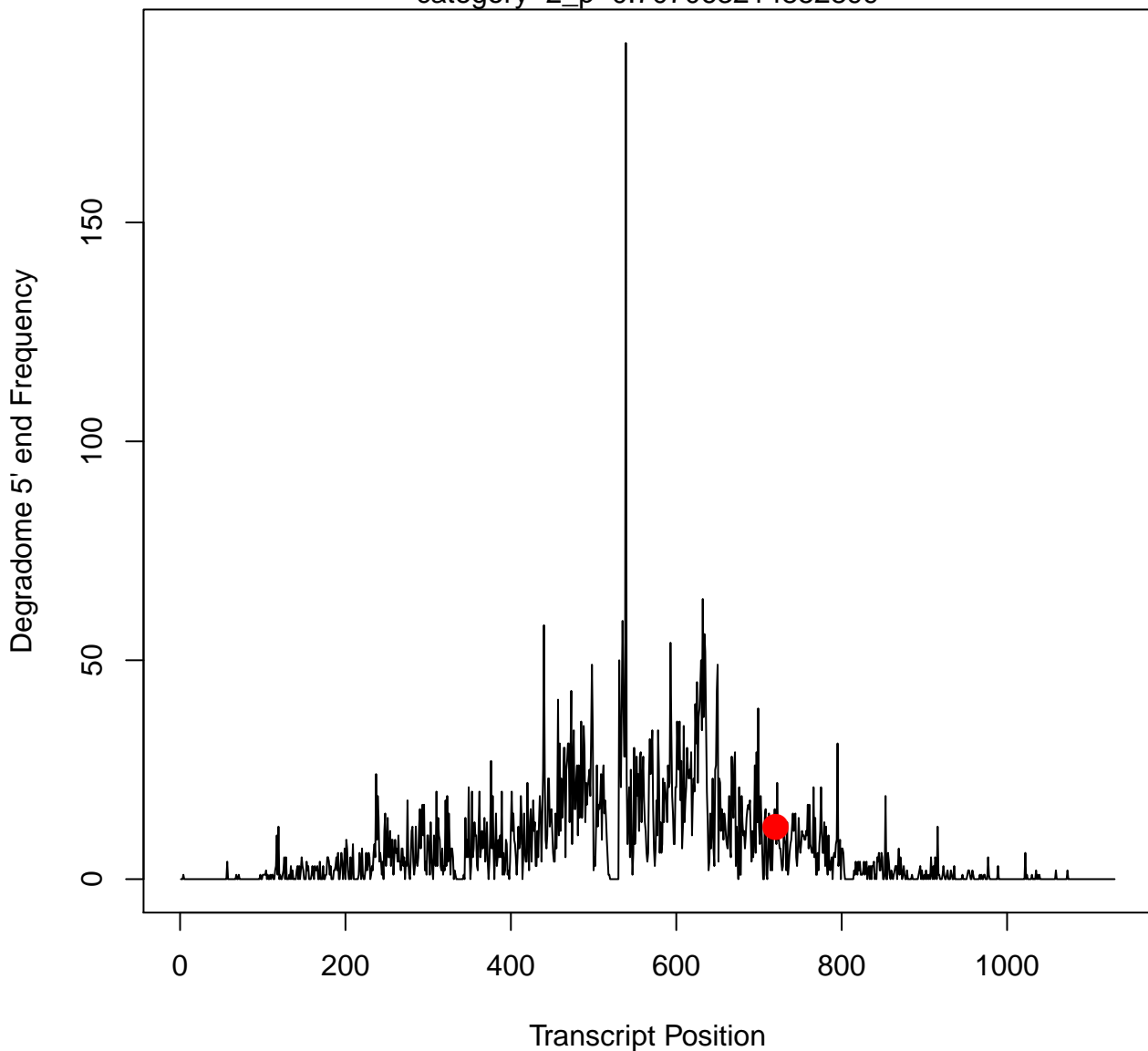

Supplement: Supplementary file 4 [file Data_Sheet_4.zip › Sit-miR169e_Seita.1G016500.1_720_TPlot.pdf]

**T=Seita.2G331100.1\_Q=Sit-miR169e\_S=1441**

category=2\_p=0.991971271403465

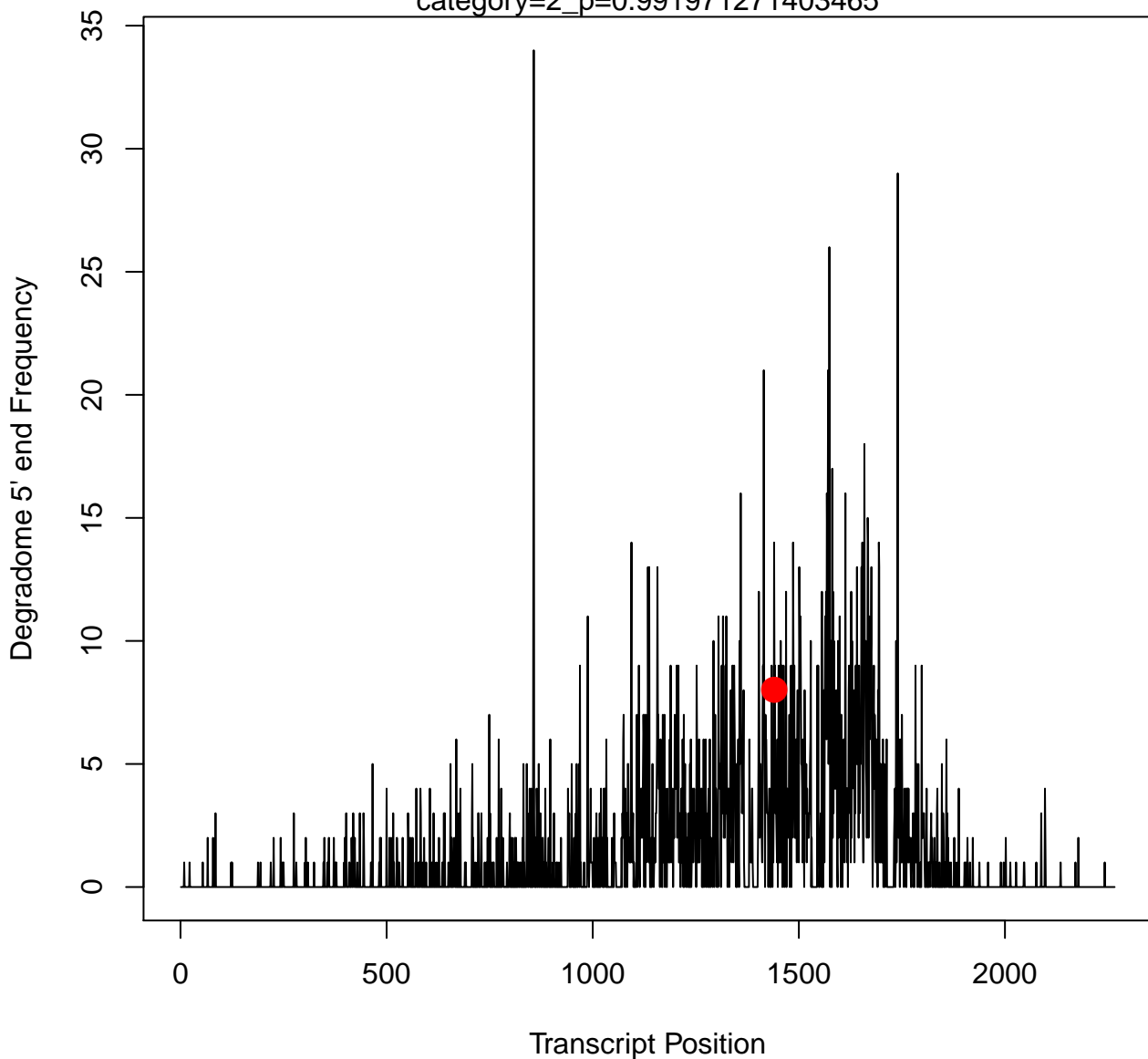

Supplement: Supplementary file 4 [file Data_Sheet_4.zip › Sit-miR169e_Seita.2G331100.1_1441_TPlot.pdf]

**T=Seita.2G383900.1\_Q=Sit-miR169e\_S=2514**

category=2\_p=0.241708174376078

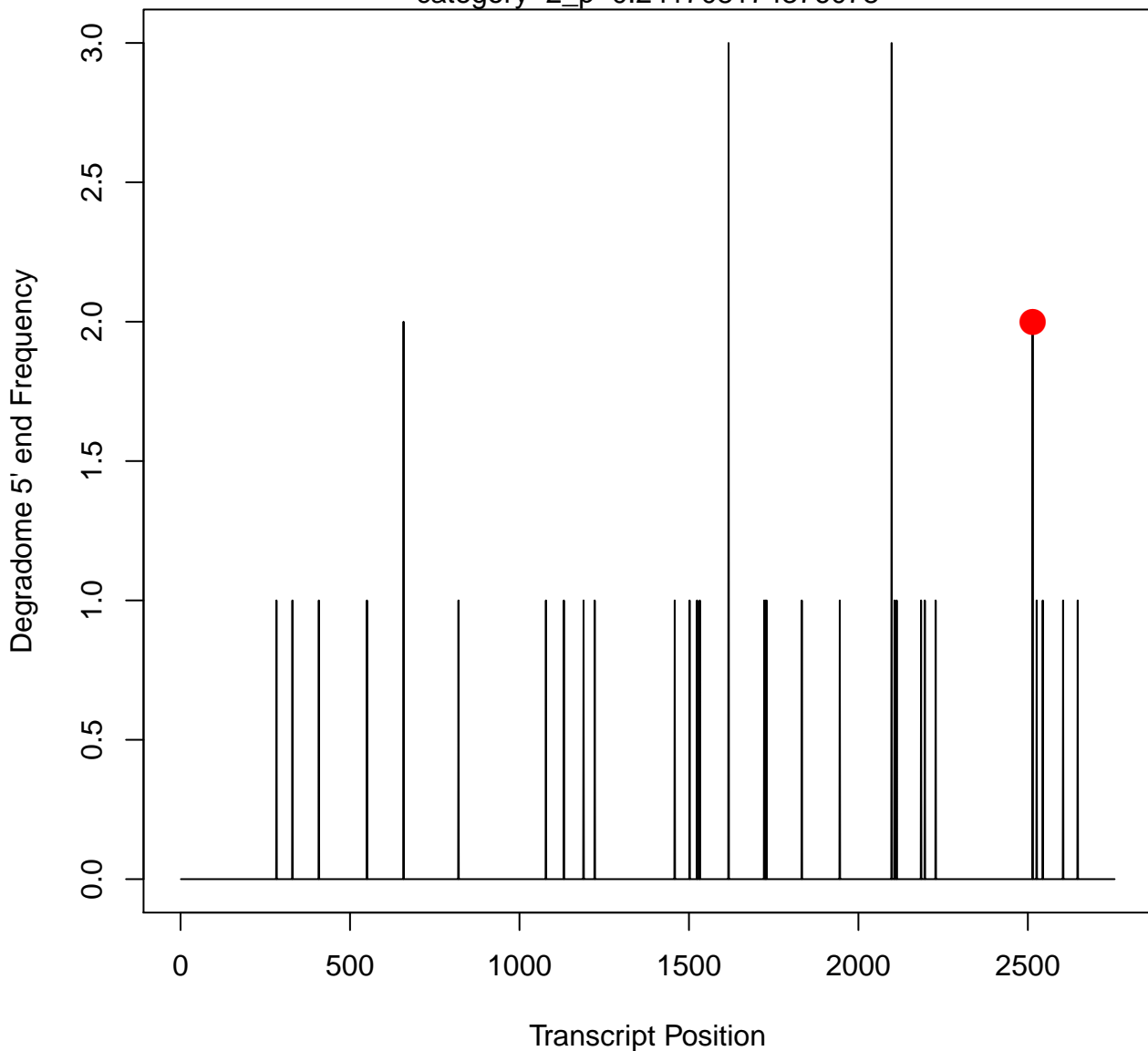

Supplement: Supplementary file 4 [file Data_Sheet_4.zip › Sit-miR169e_Seita.2G383900.1_2514_TPlot.pdf]

**T=Seita.3G115000.1\_Q=Sit-miR169e\_S=715**

category=2\_p=0.992108918103862

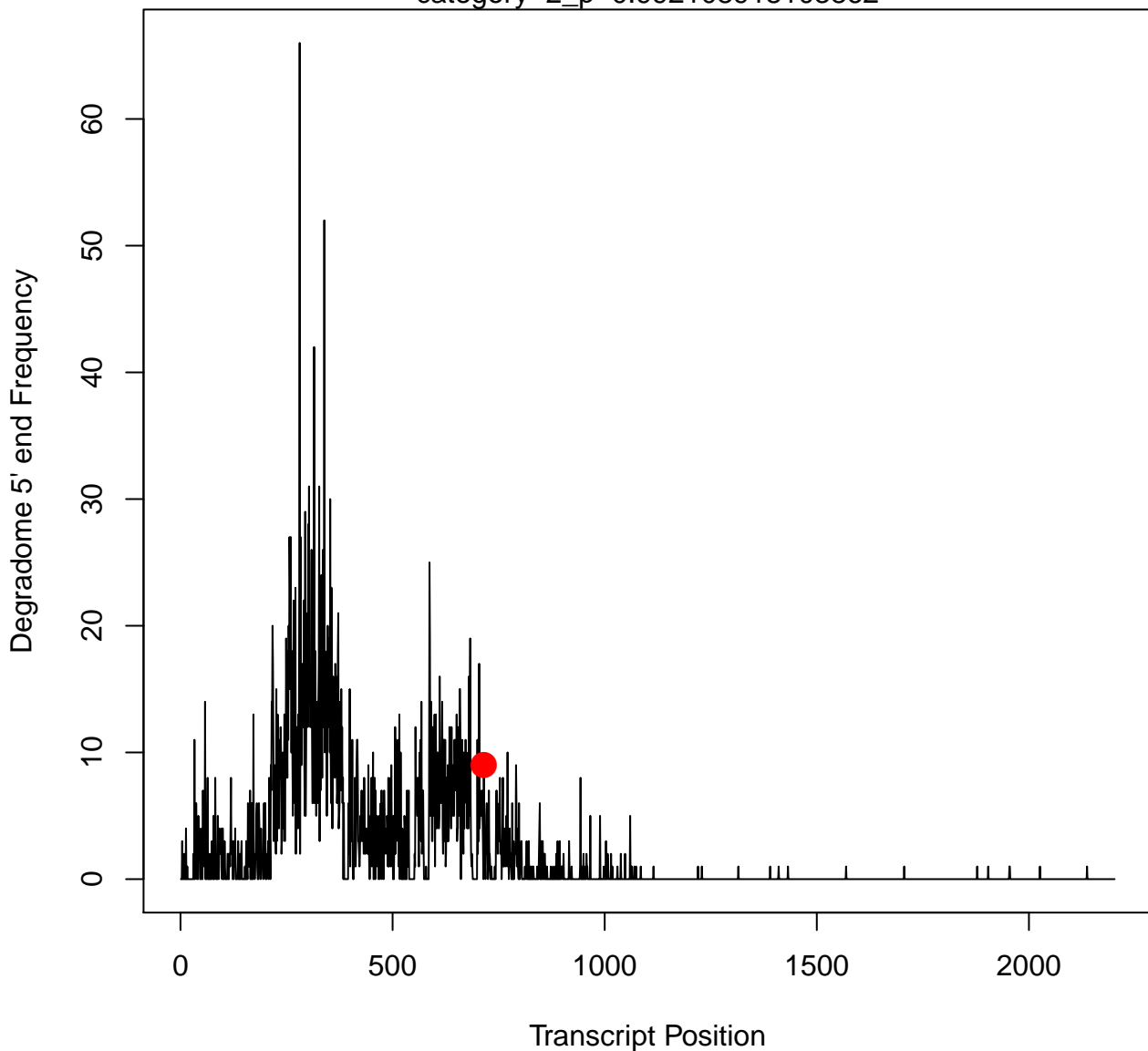

Supplement: Supplementary file 4 [file Data_Sheet_4.zip › Sit-miR169e_Seita.3G115000.1_715_TPlot.pdf]

**T=Seita.9G361700.1\_Q=Sit-miR169e\_S=218**

category=2\_p=0.761959314751217

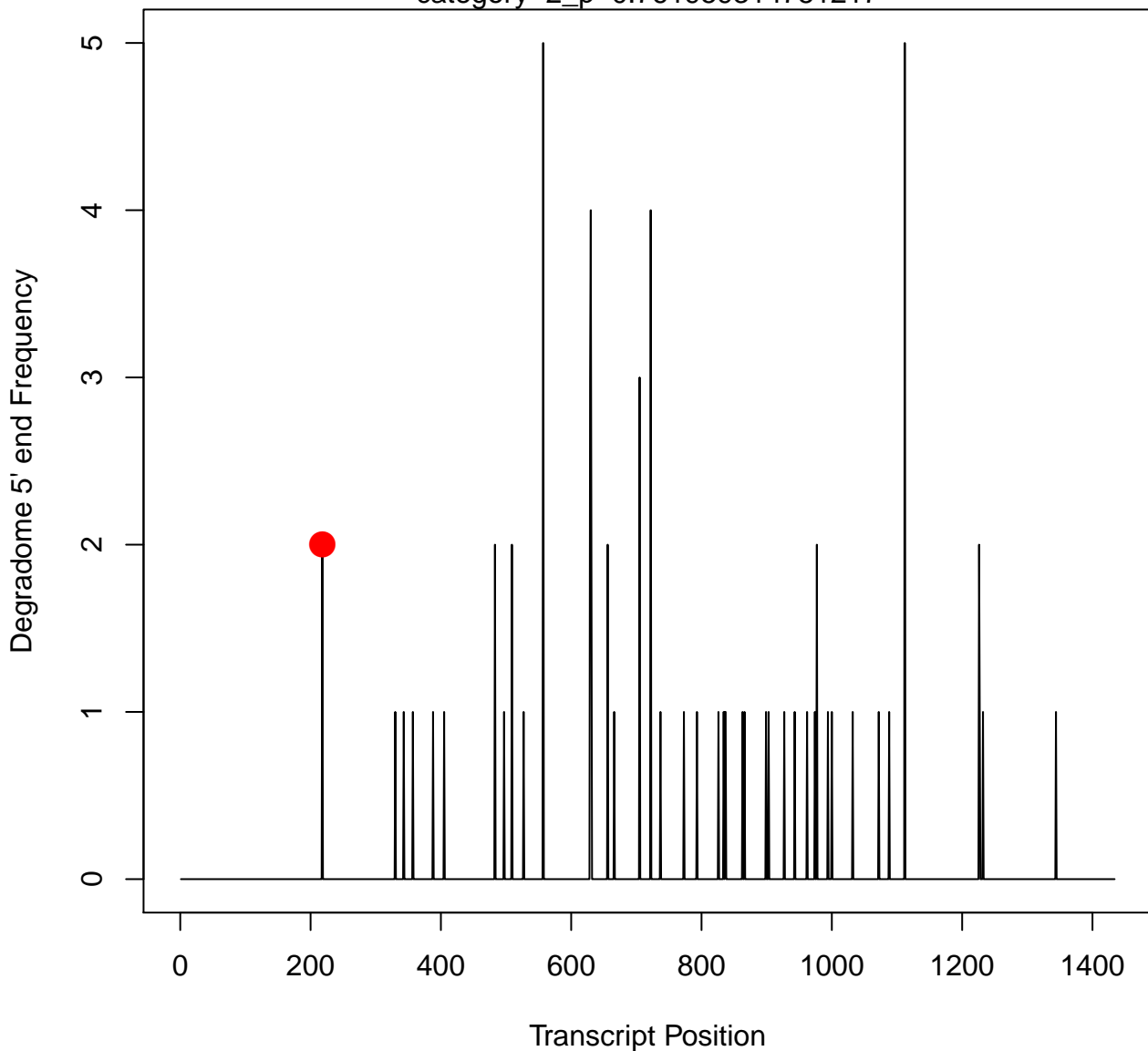

Supplement: Supplementary file 4 [file Data_Sheet_4.zip › Sit-miR169e_Seita.9G361700.1_218_TPlot.pdf]

**T=Seita.9G367200.1\_Q=Sit-miR169e\_S=1285**

category=0\_p=0.00533373759778111

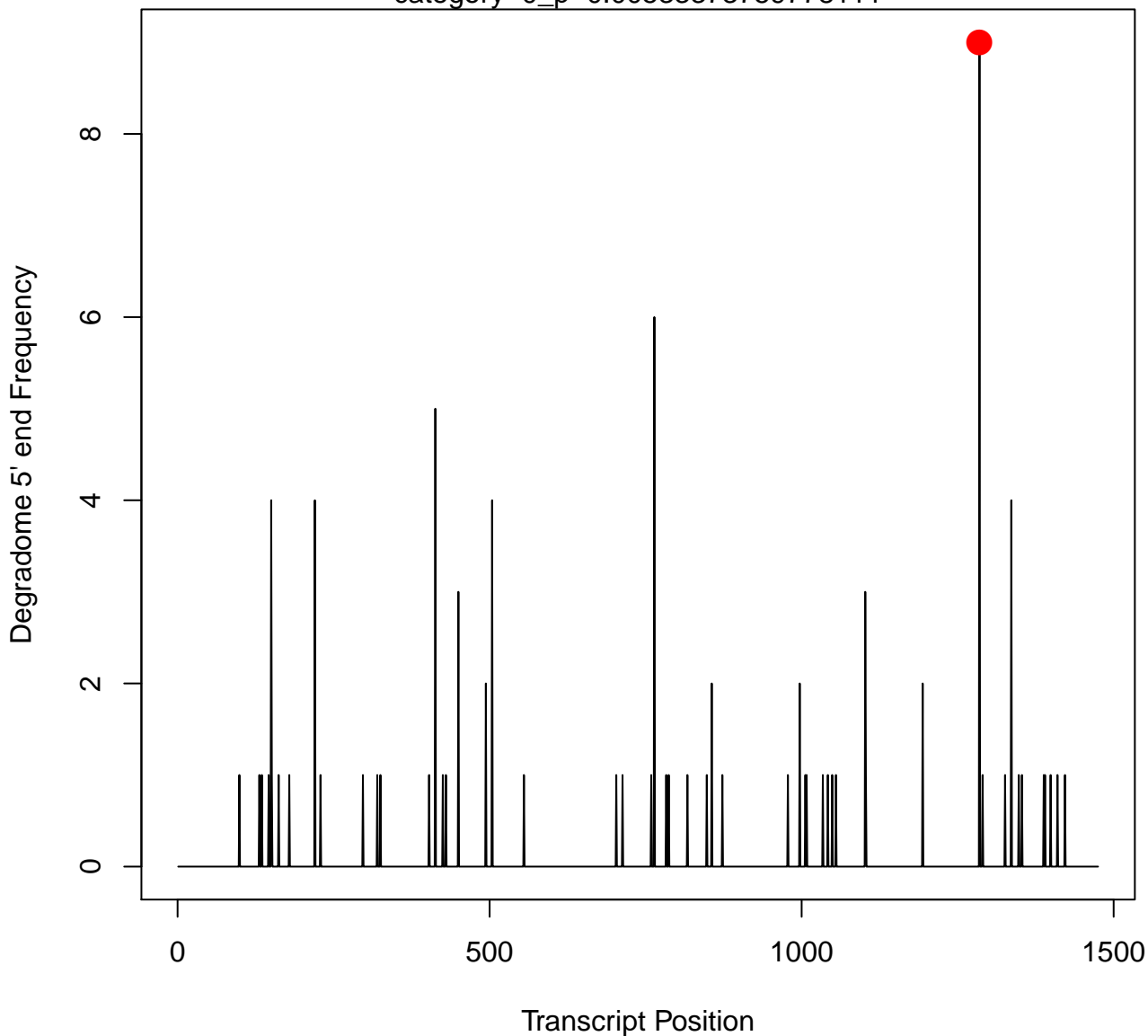

Supplement: Supplementary file 4 [file Data_Sheet_4.zip › Sit-miR169e_Seita.9G367200.1_1285_TPlot.pdf]

**T=Seita.1G041900.1\_Q=Sit-miR169f\_S=955**

category=2\_p=0.978485577465972

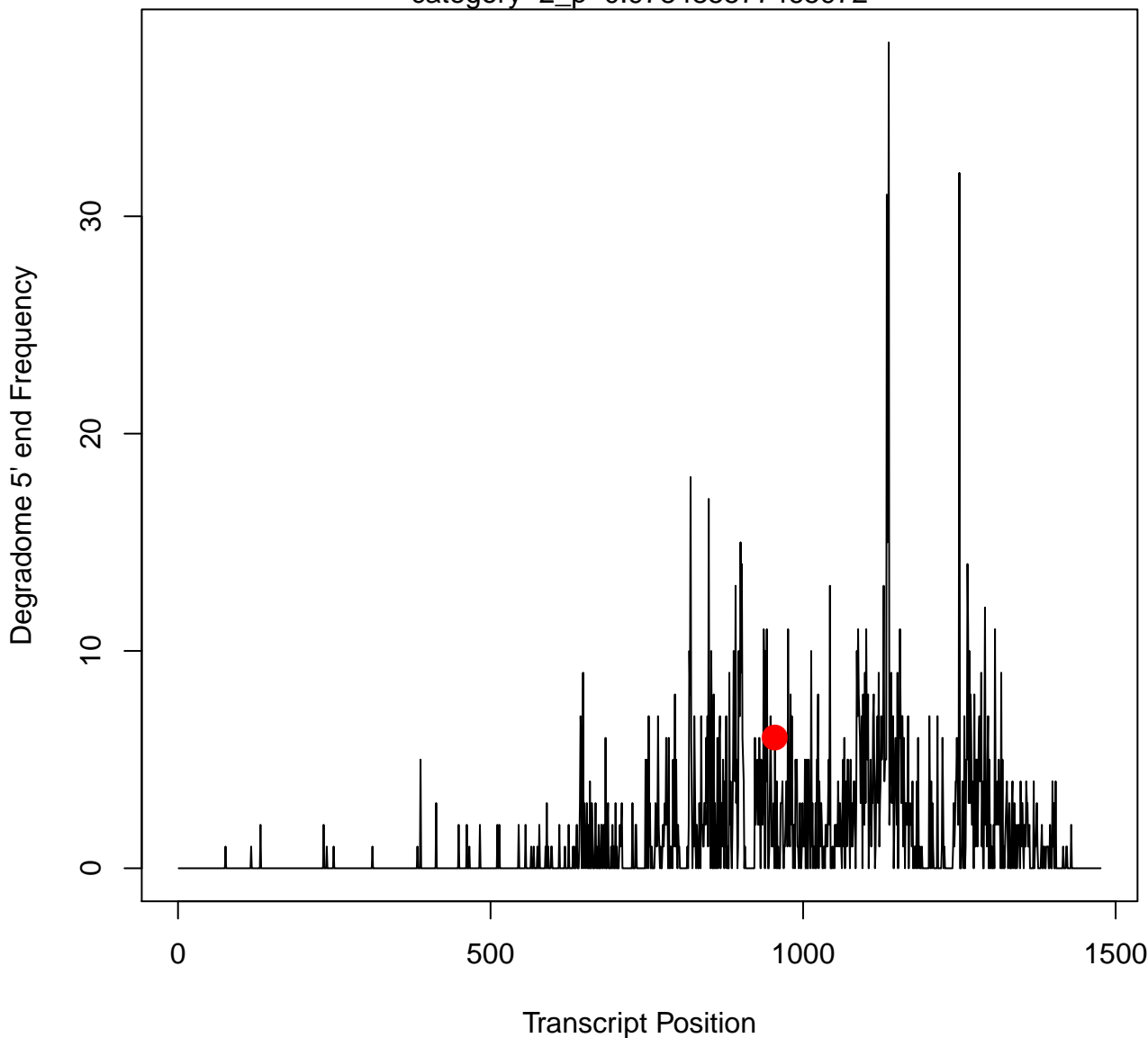

Supplement: Supplementary file 4 [file Data_Sheet_4.zip › Sit-miR169f_Seita.1G041900.1_955_TPlot.pdf]

**T=Seita.1G199800.1\_Q=Sit-miR169f\_S=2032**

category=2\_p=0.796268045147471

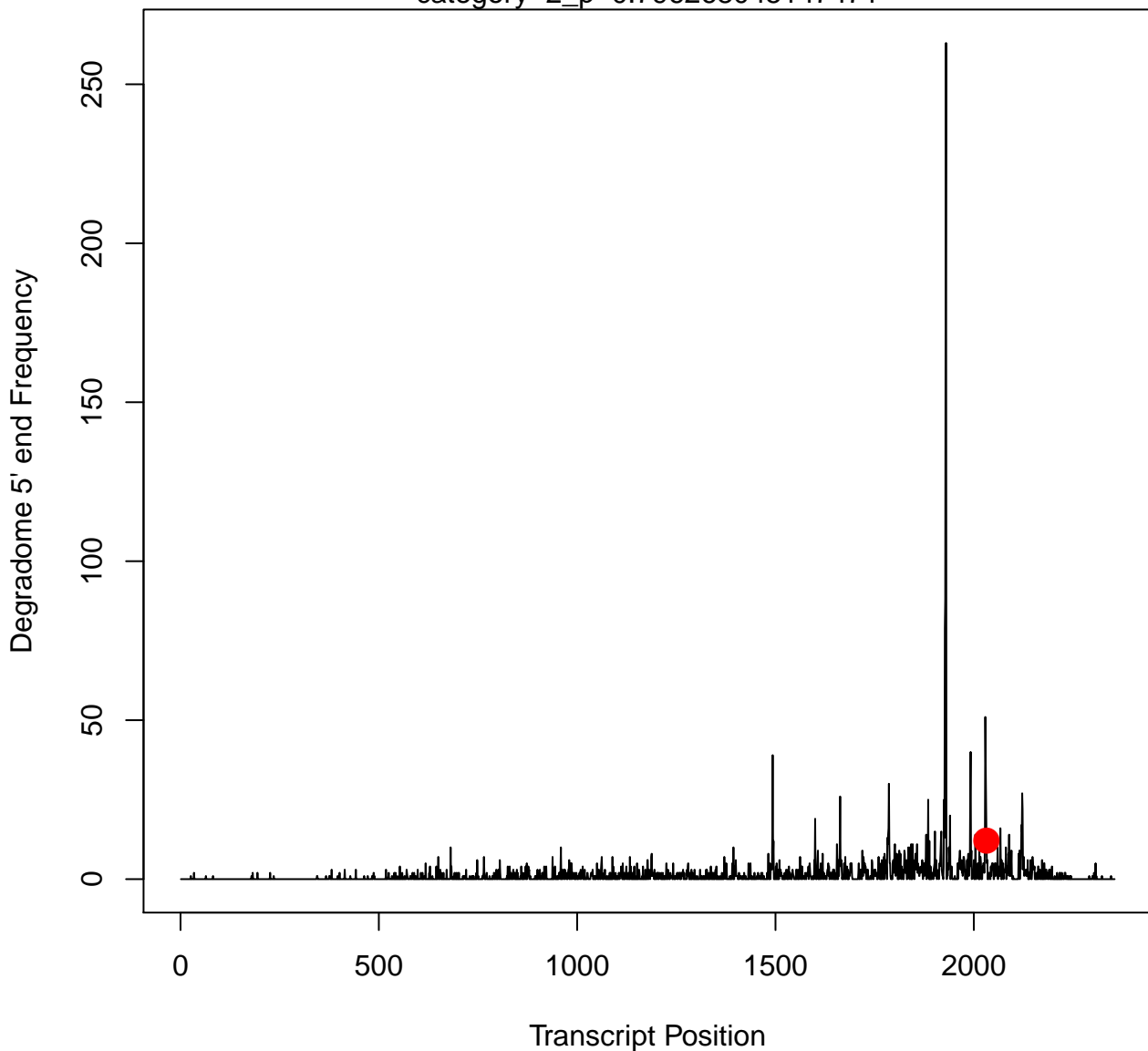

Supplement: Supplementary file 4 [file Data_Sheet_4.zip › Sit-miR169f_Seita.1G199800.1_2032_TPlot.pdf]

**T=Seita.2G108300.1\_Q=Sit-miR169f\_S=919**

category=2\_p=0.923971532614208

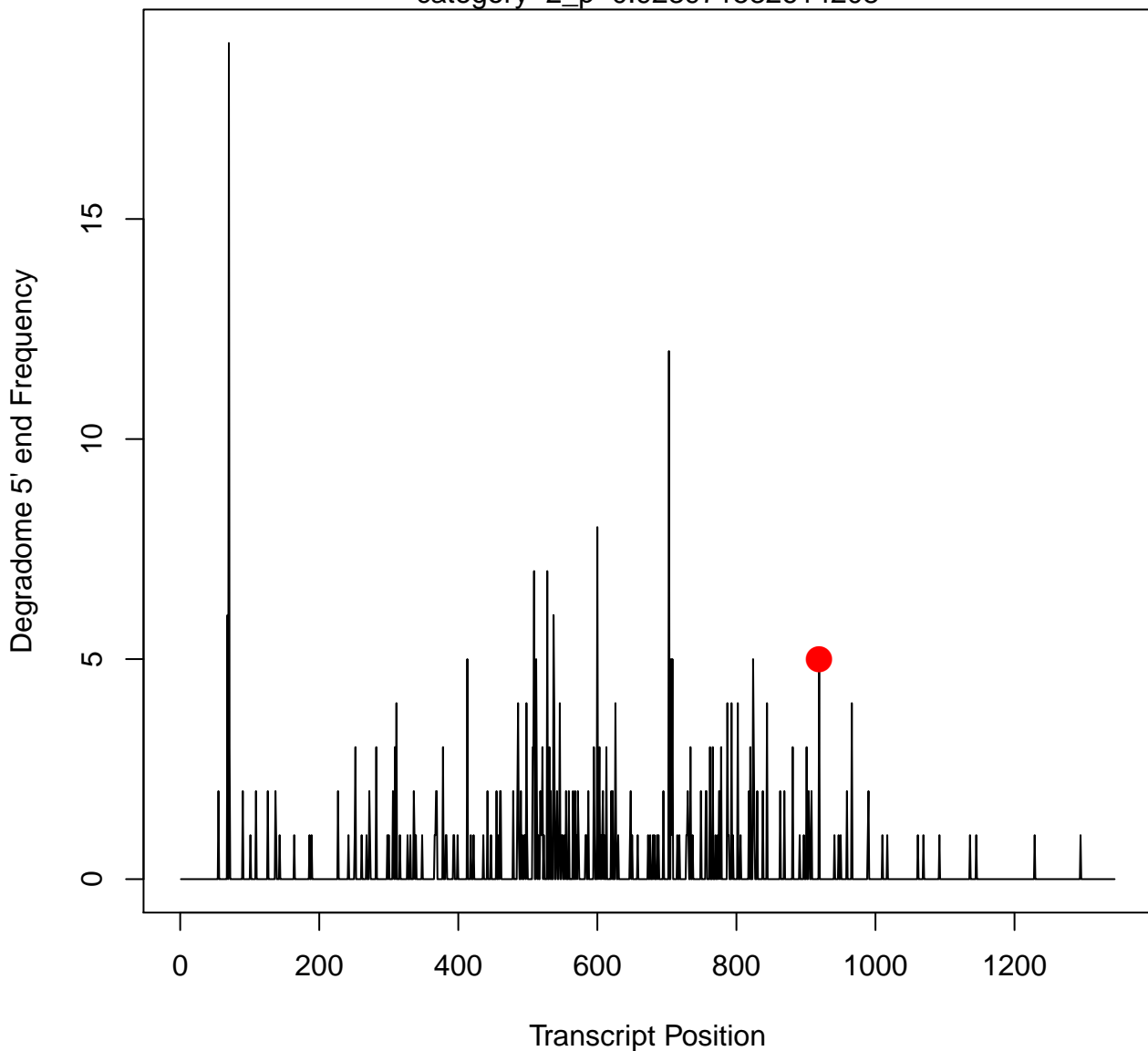

Supplement: Supplementary file 4 [file Data_Sheet_4.zip › Sit-miR169f_Seita.2G108300.1_919_TPlot.pdf]

**T=Seita.3G390000.1\_Q=Sit-miR169f\_S=2048**

category=0\_p=0.00152683684215971

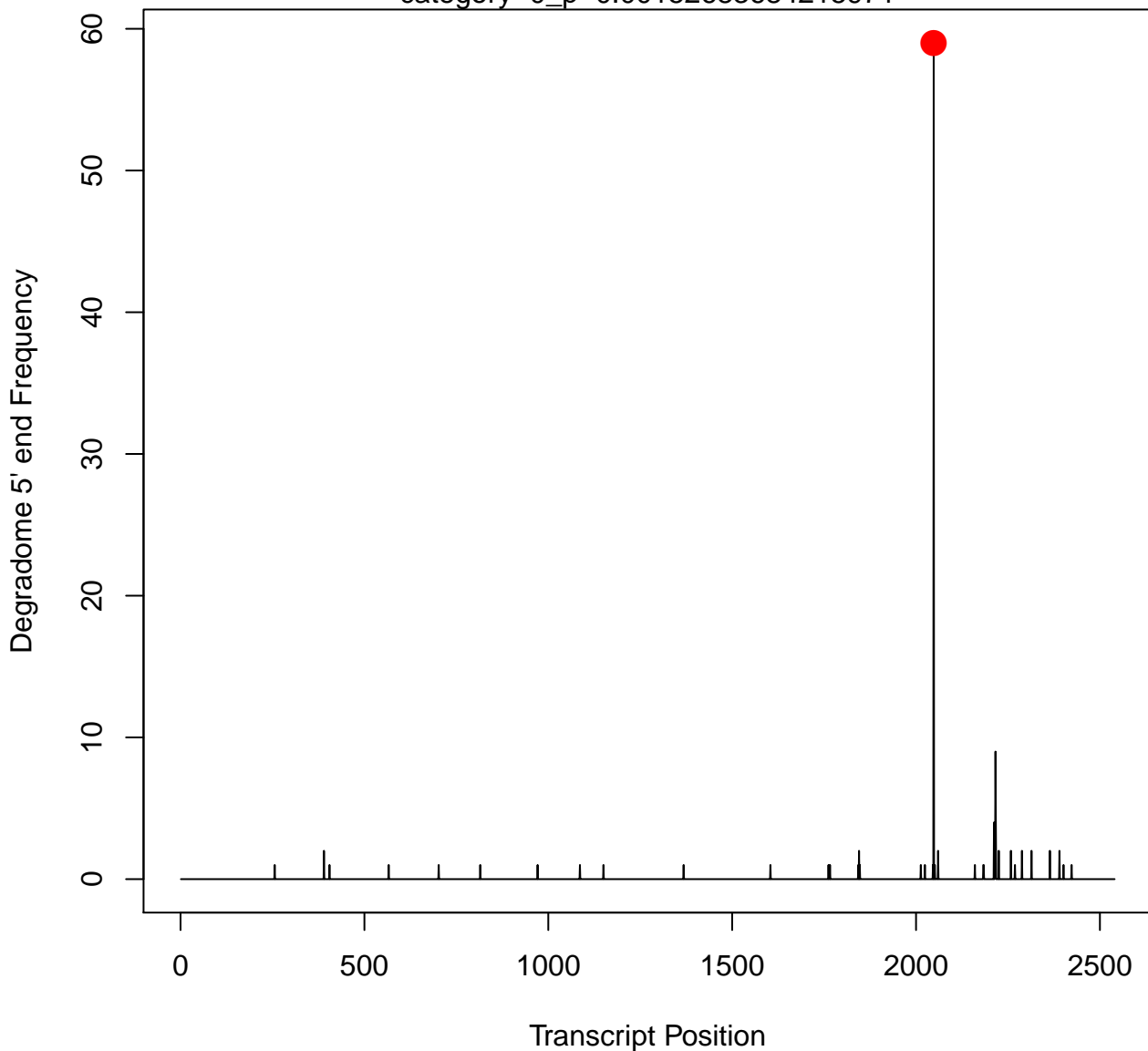

Supplement: Supplementary file 4 [file Data_Sheet_4.zip › Sit-miR169f_Seita.3G390000.1_2048_TPlot.pdf]

**T=Seita.5G374600.1\_Q=Sit-miR169f\_S=205**

category=2\_p=0.985293656407299

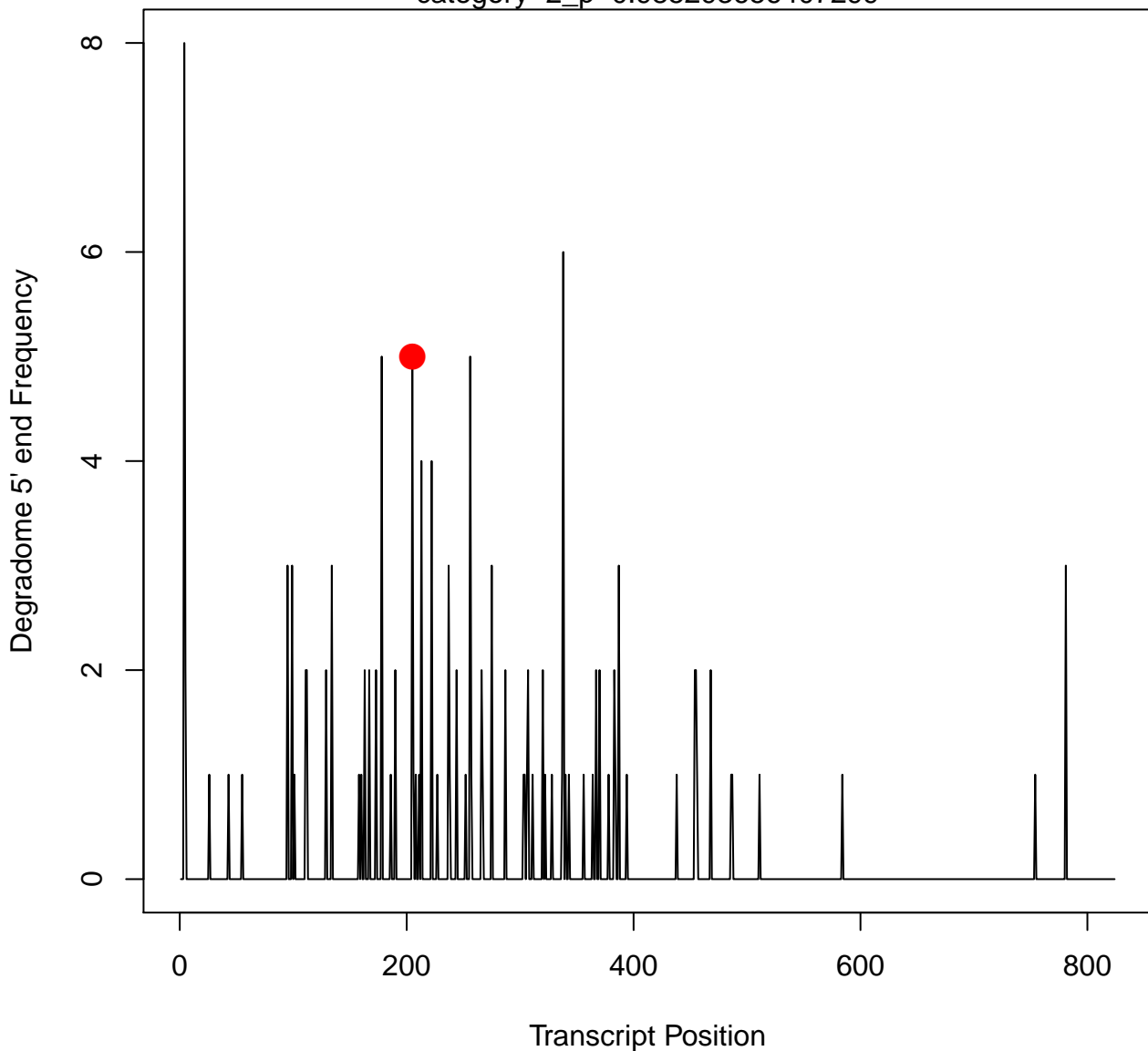

Supplement: Supplementary file 4 [file Data_Sheet_4.zip › Sit-miR169f_Seita.5G374600.1_205_TPlot.pdf]

**T=Seita.7G063700.1\_Q=Sit-miR169f\_S=1128**

category=2\_p=0.995220967553444

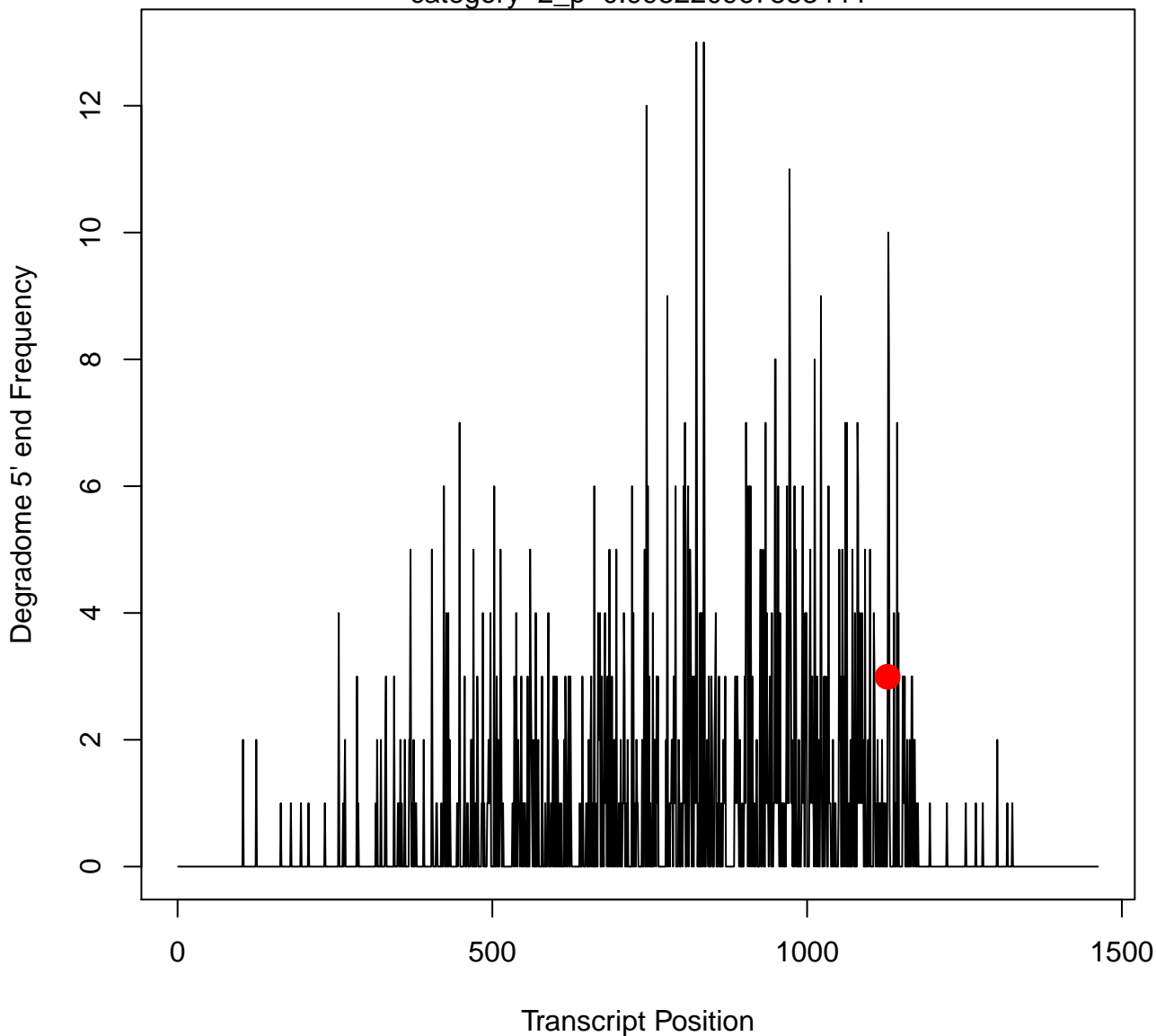

Supplement: Supplementary file 4 [file Data_Sheet_4.zip › Sit-miR169f_Seita.7G063700.1_1128_TPlot.pdf]

**T=Seita.9G129400.1\_Q=Sit-miR169f\_S=2100**

category=0\_p=0.00381272216028461

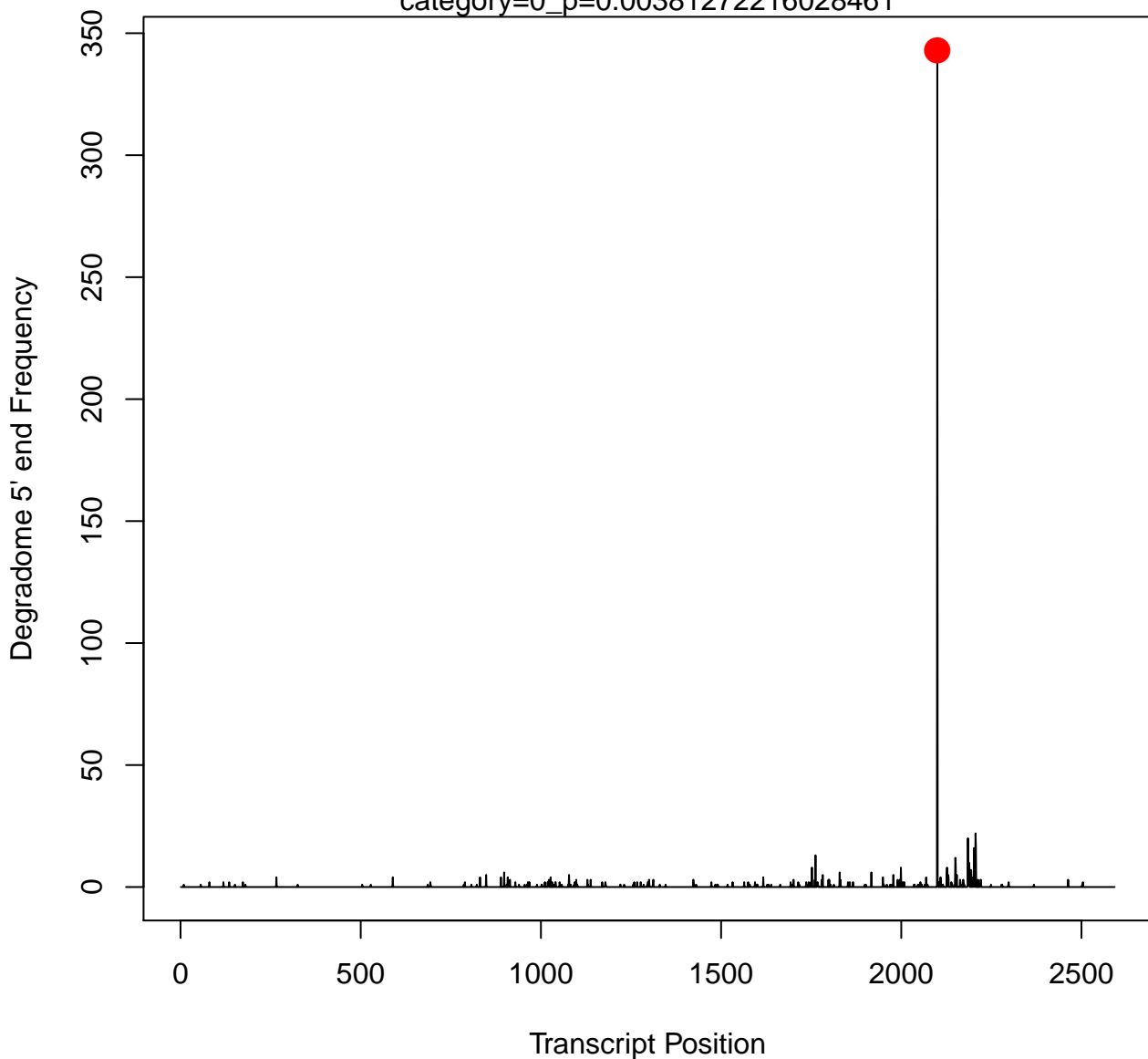

Supplement: Supplementary file 4 [file Data_Sheet_4.zip › Sit-miR169f_Seita.9G129400.1_2100_TPlot.pdf]

**T=Seita.9G155700.1\_Q=Sit-miR169f\_S=1672**

category=0\_p=0.000763710047598232

Degradome 5' end Frequency

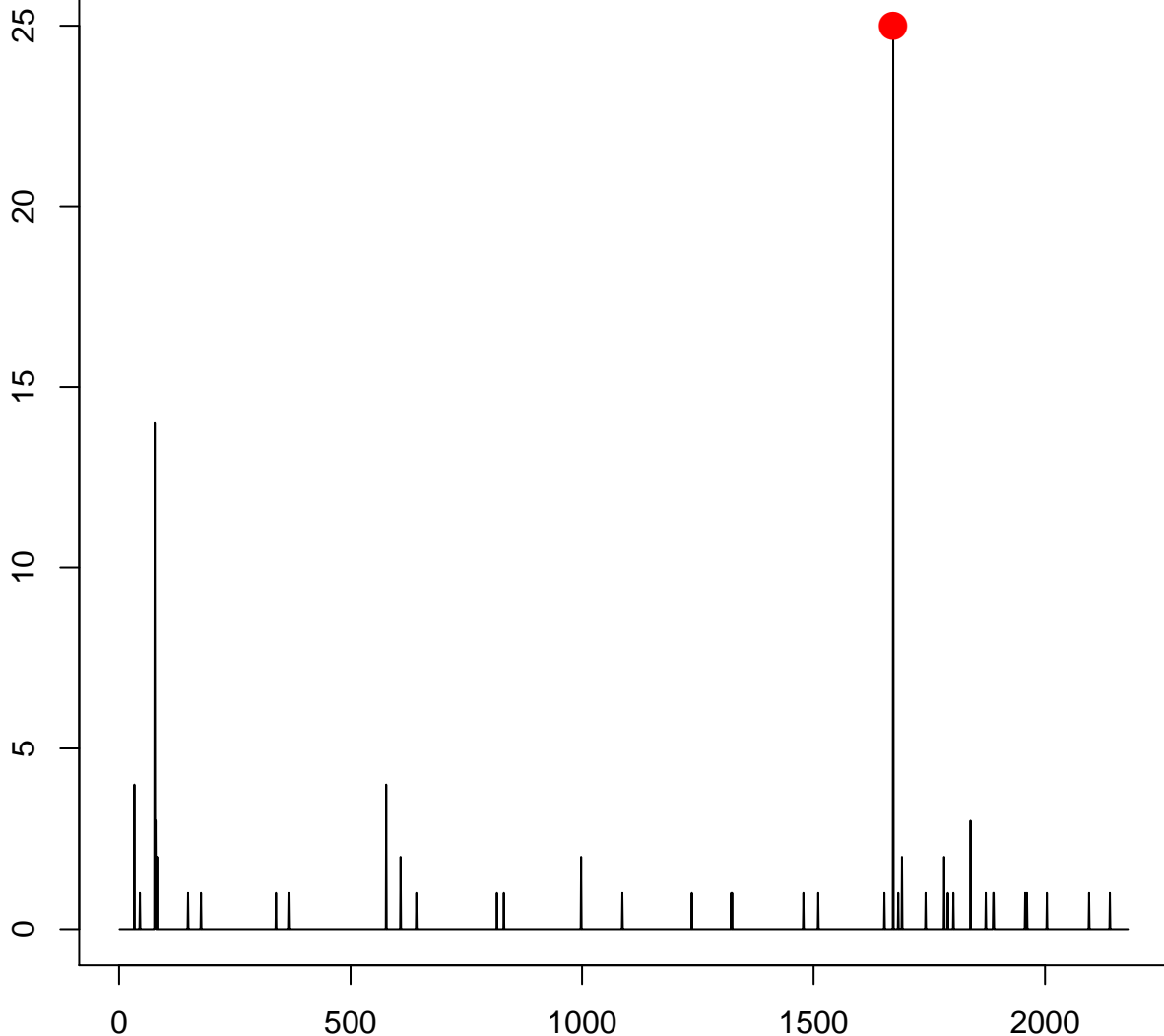

Transcript Position

Supplement: Supplementary file 4 [file Data_Sheet_4.zip › Sit-miR169f_Seita.9G155700.1_1672_TPlot.pdf]

**T=Seita.9G365500.1\_Q=Sit-miR169f\_S=1287**

category=2\_p=0.173224673744341

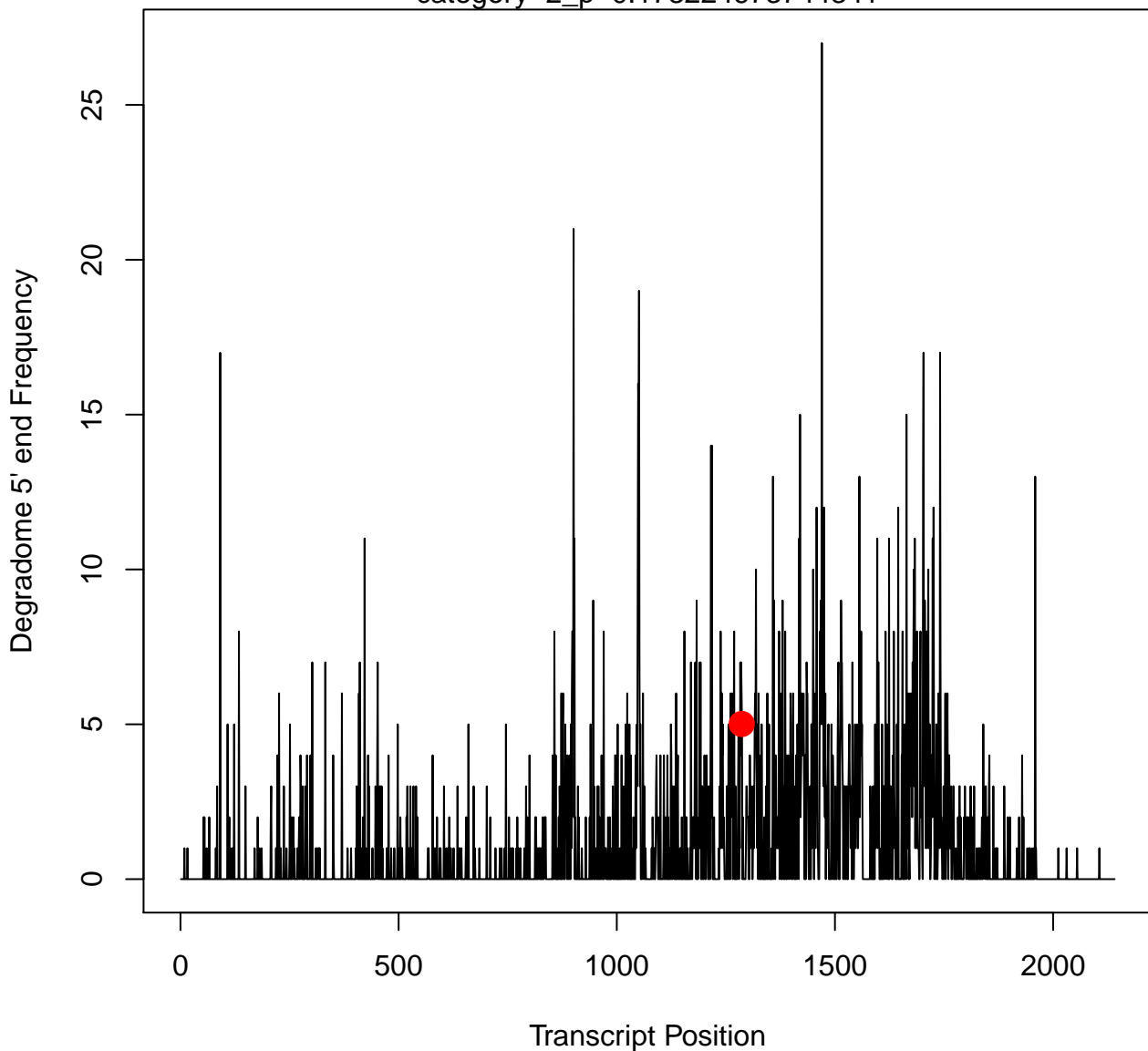

Supplement: Supplementary file 4 [file Data_Sheet_4.zip › Sit-miR169f_Seita.9G365500.1_1287_TPlot.pdf]

**T=Seita.9G559100.1\_Q=Sit-miR169f\_S=811**

category=2\_p=0.997842903593736

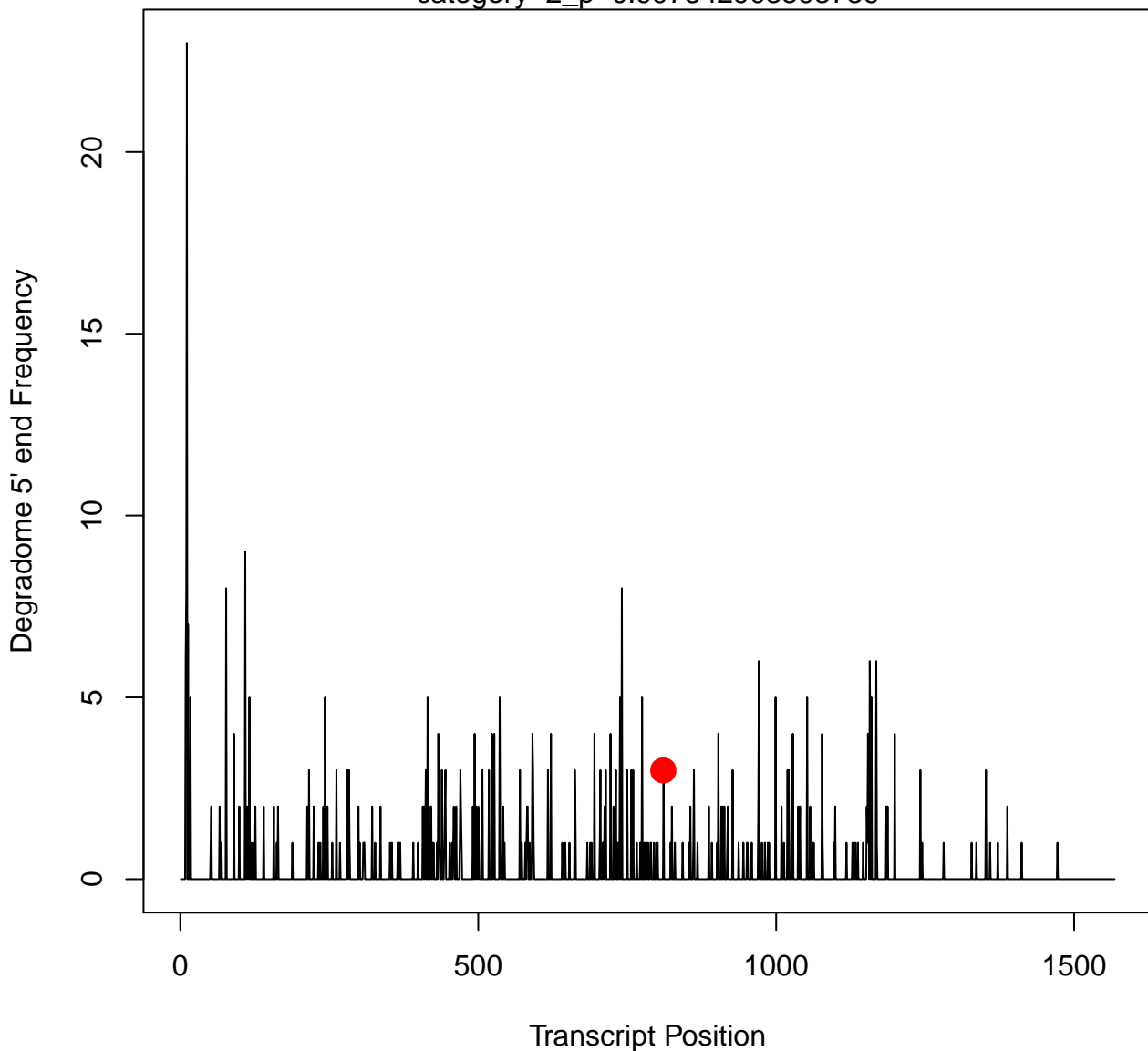

Supplement: Supplementary file 4 [file Data_Sheet_4.zip › Sit-miR169f_Seita.9G559100.1_811_TPlot.pdf]

**T=Seita.2G402500.1\_Q=Sit-miR169g\_S=1831**

category=2\_p=0.999849583784293

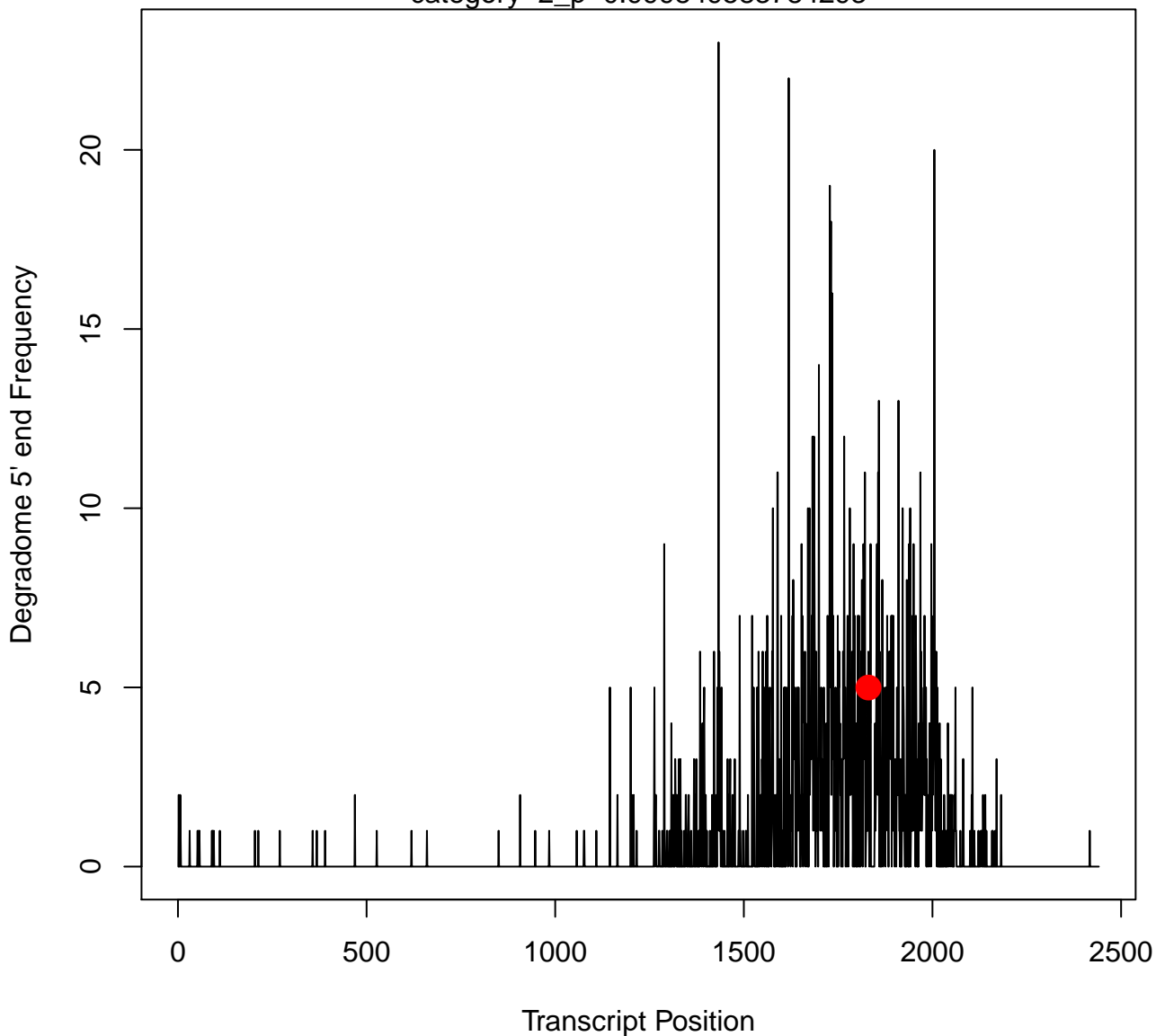

Supplement: Supplementary file 4 [file Data_Sheet_4.zip › Sit-miR169g_Seita.2G402500.1_1831_TPlot.pdf]

**T=Seita.4G268900.1\_Q=Sit-miR169g\_S=574**

category=2\_p=0.999905698513256

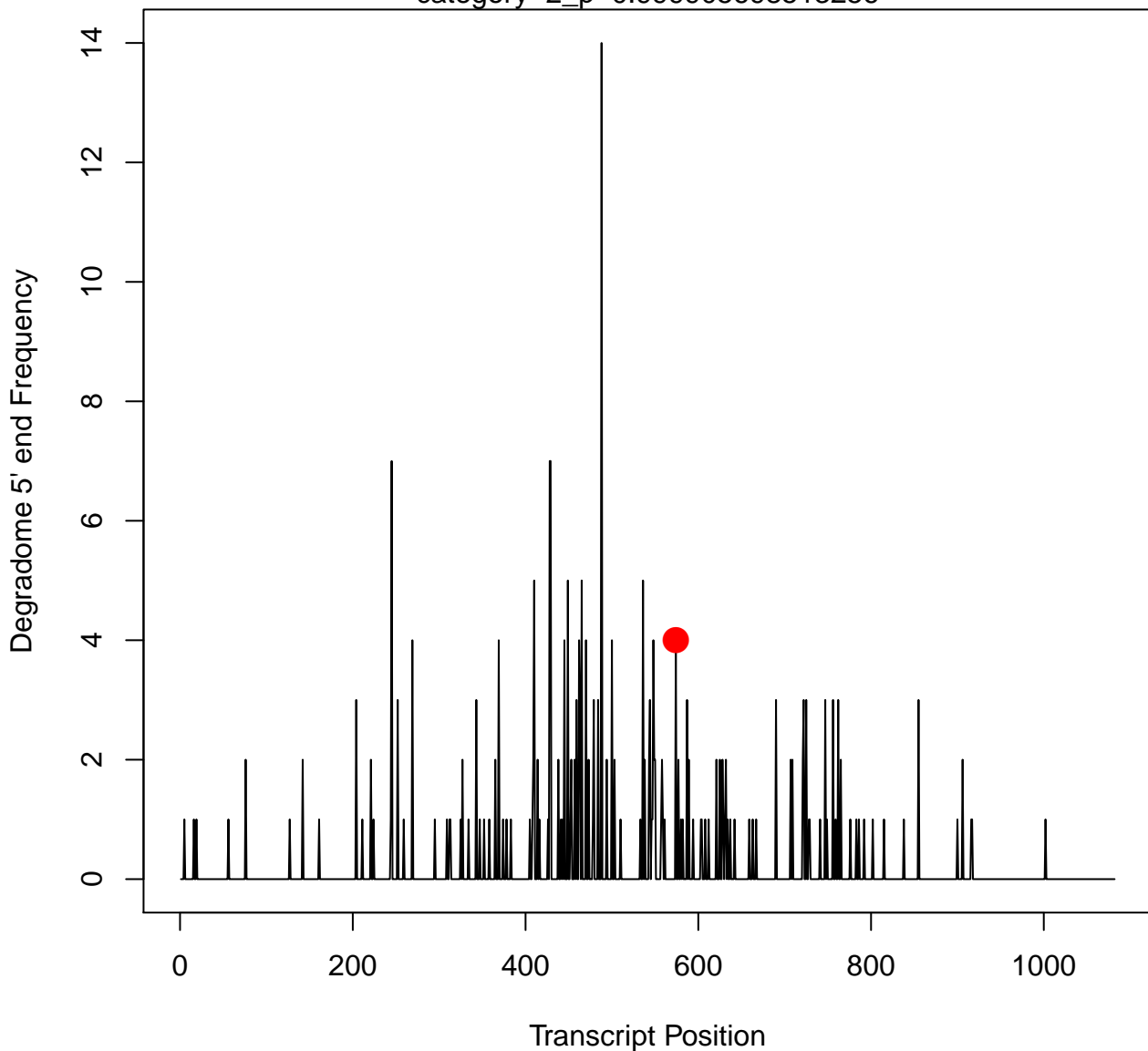

Supplement: Supplementary file 4 [file Data_Sheet_4.zip › Sit-miR169g_Seita.4G268900.1_574_TPlot.pdf]

**T=Seita.5G437000.1\_Q=Sit-miR169g\_S=740**

category=2\_p=0.991688733072879

Degradome 5' end Frequency

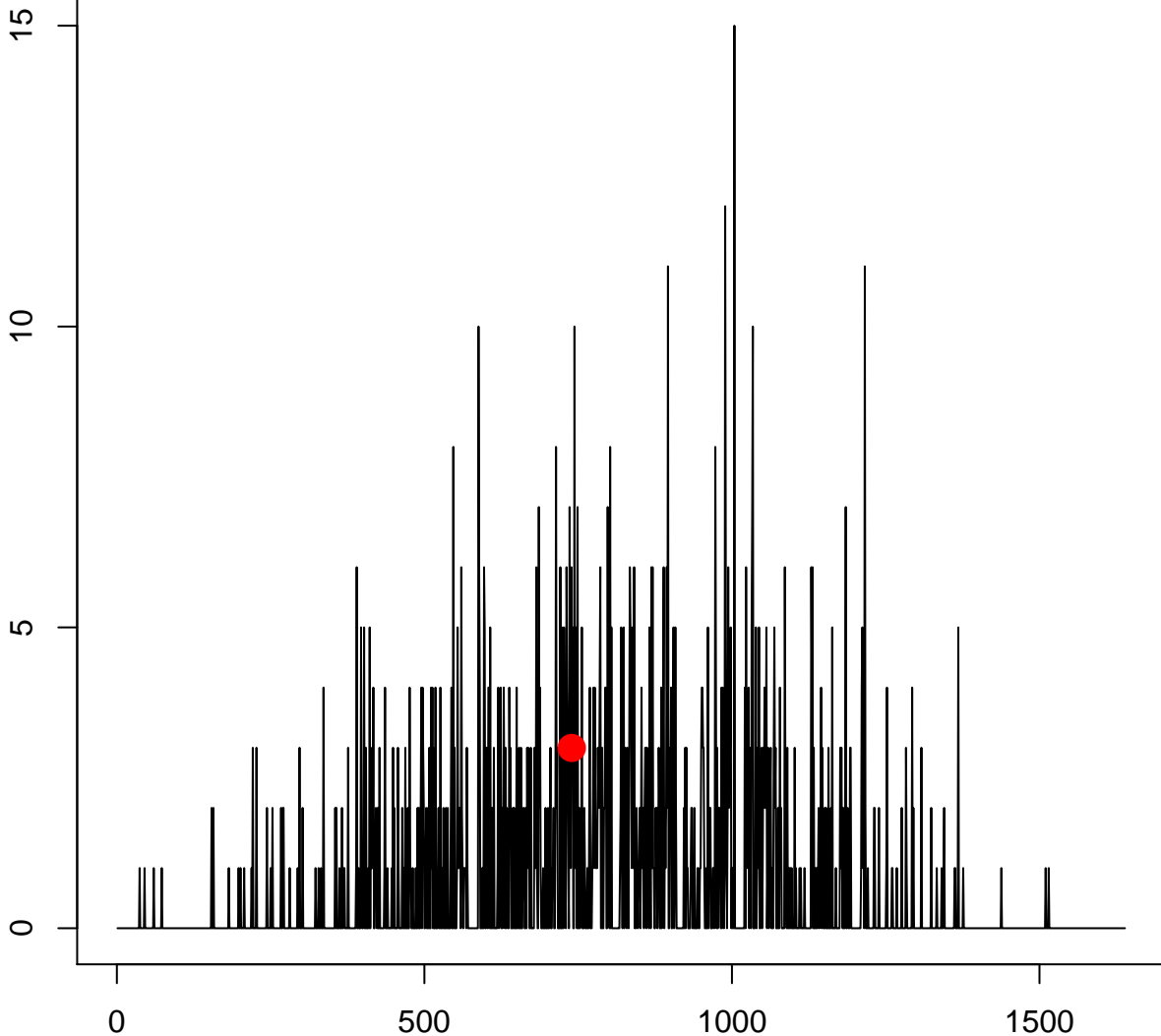

Transcript Position

Supplement: Supplementary file 4 [file Data_Sheet_4.zip › Sit-miR169g_Seita.5G437000.1_740_TPlot.pdf]

**T=Seita.5G438900.1\_Q=Sit-miR169g\_S=506**

category=2\_p=0.999400053137421

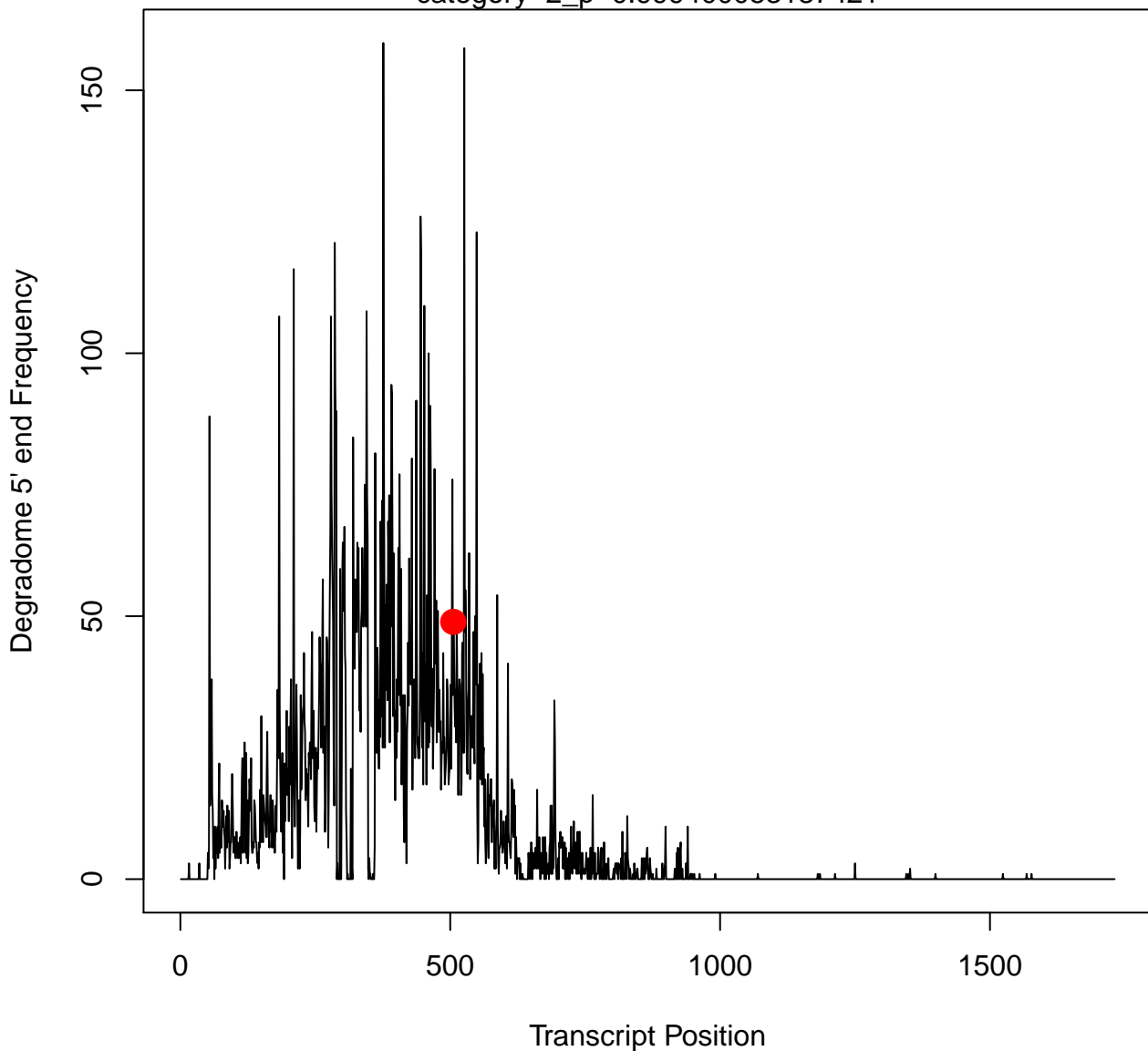

Supplement: Supplementary file 4 [file Data_Sheet_4.zip › Sit-miR169g_Seita.5G438900.1_506_TPlot.pdf]

**T=Seita.6G128600.1\_Q=Sit-miR169g\_S=797**

category=2\_p=0.997345441659089

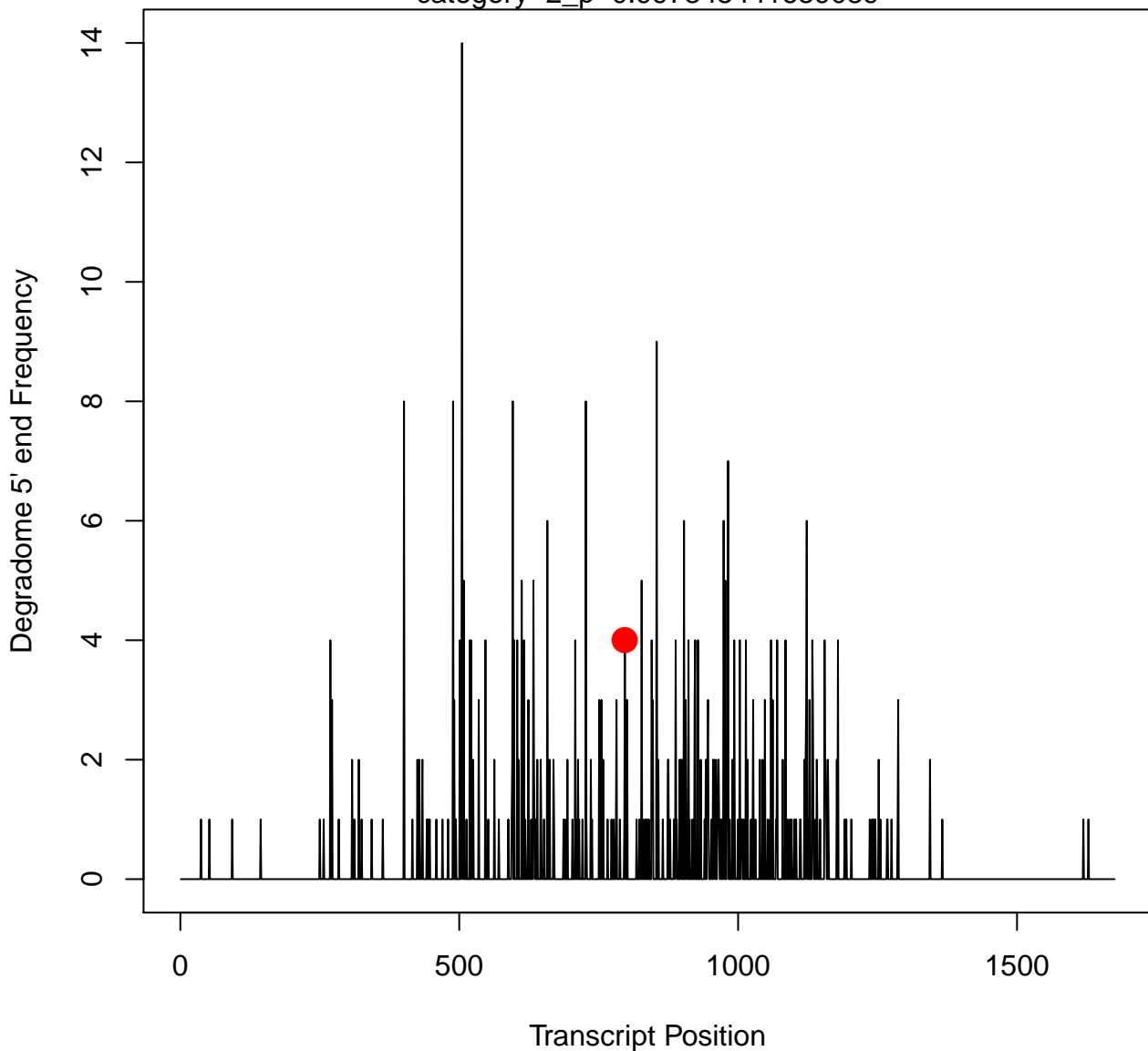

Supplement: Supplementary file 4 [file Data_Sheet_4.zip › Sit-miR169g_Seita.6G128600.1_797_TPlot.pdf]

**T=Seita.6G237000.1\_Q=Sit-miR169g\_S=1581**

category=2\_p=0.961267333335852

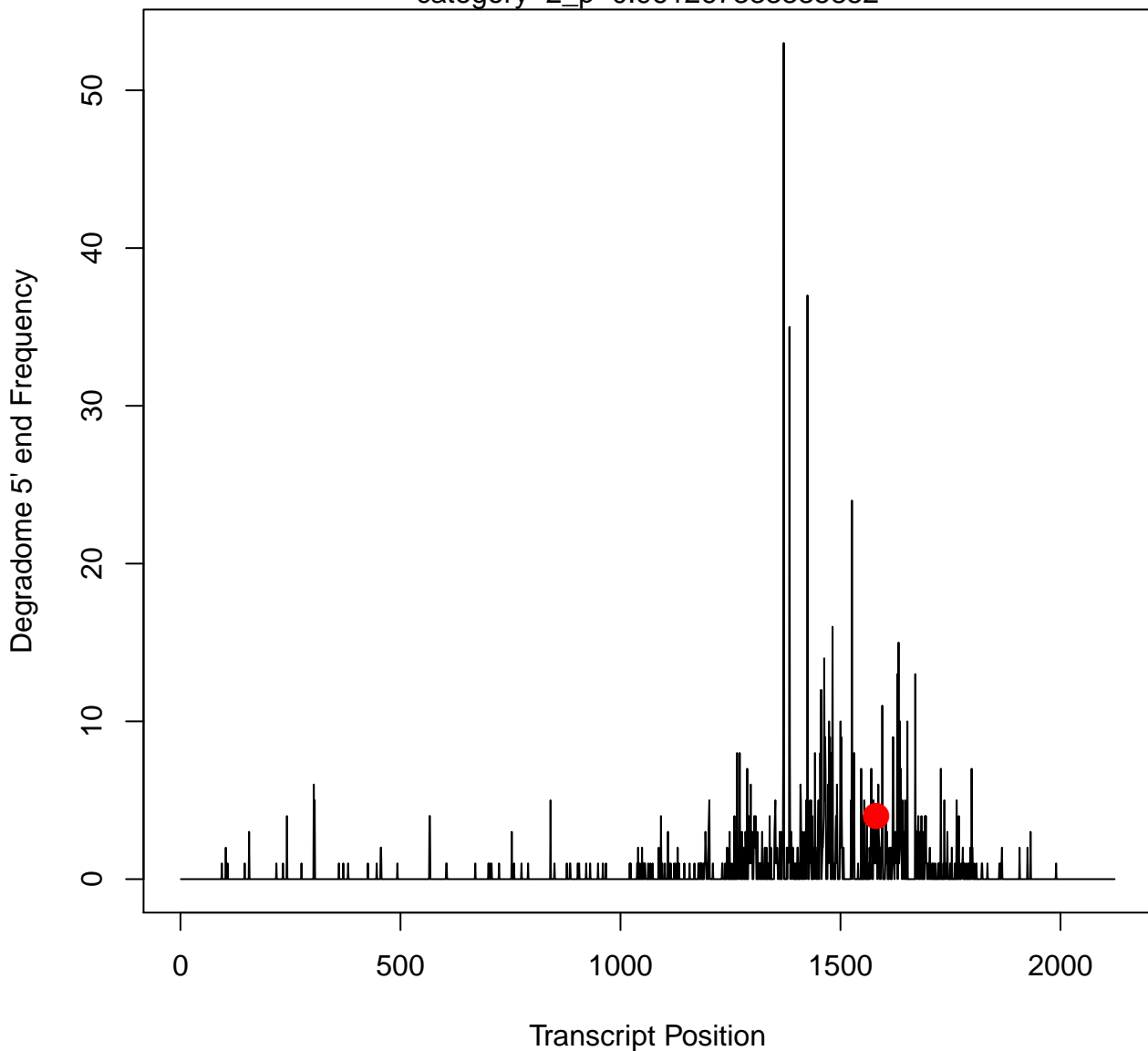

Supplement: Supplementary file 4 [file Data_Sheet_4.zip › Sit-miR169g_Seita.6G237000.1_1581_TPlot.pdf]

**T=Seita.7G104200.1\_Q=Sit-miR169g\_S=1770**

category=2\_p=0.95315212557254

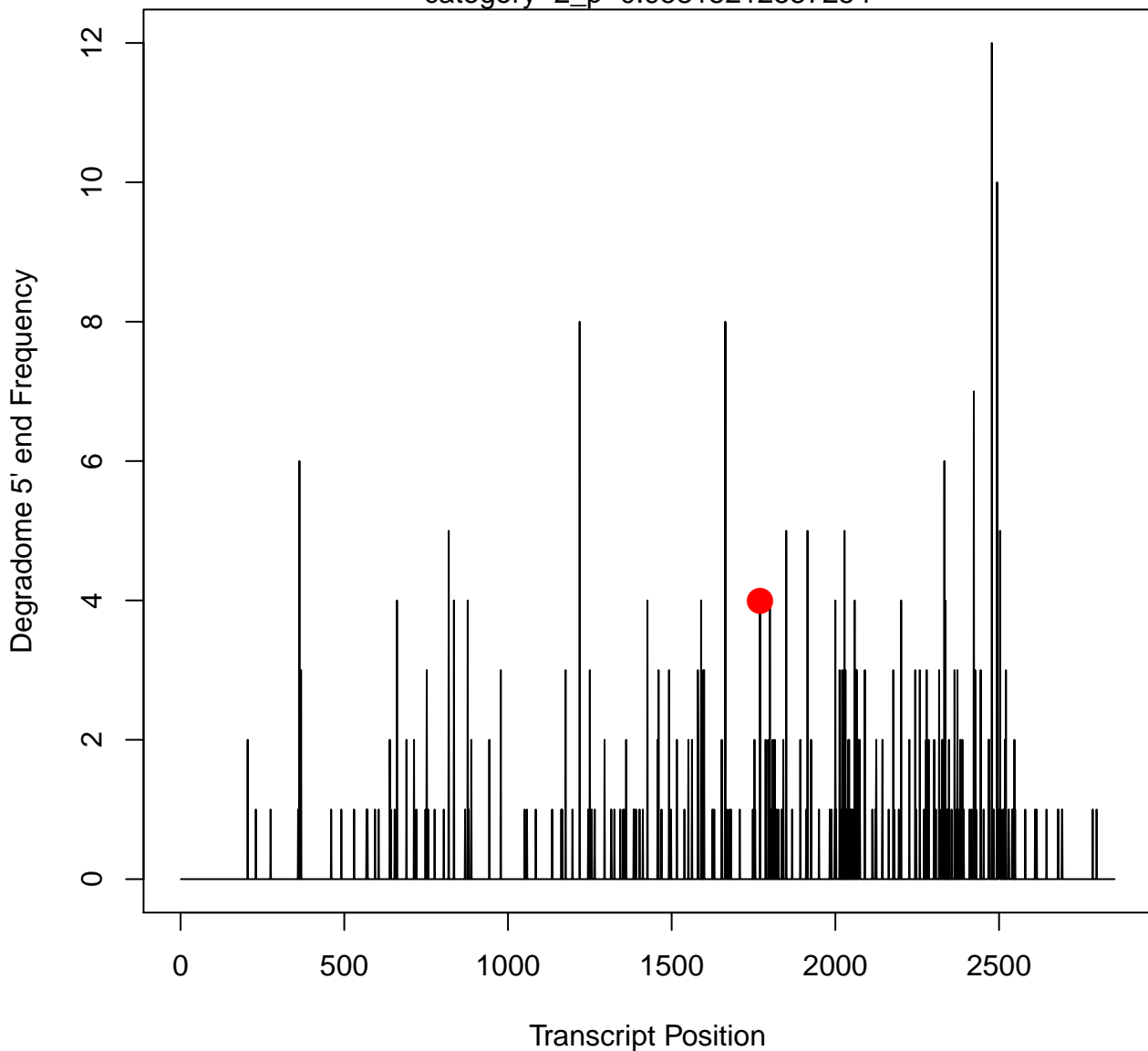

Supplement: Supplementary file 4 [file Data_Sheet_4.zip › Sit-miR169g_Seita.7G104200.1_1770_TPlot.pdf]

**T=Seita.9G137200.1\_Q=Sit-miR169g\_S=445**

category=2\_p=0.761959314751217

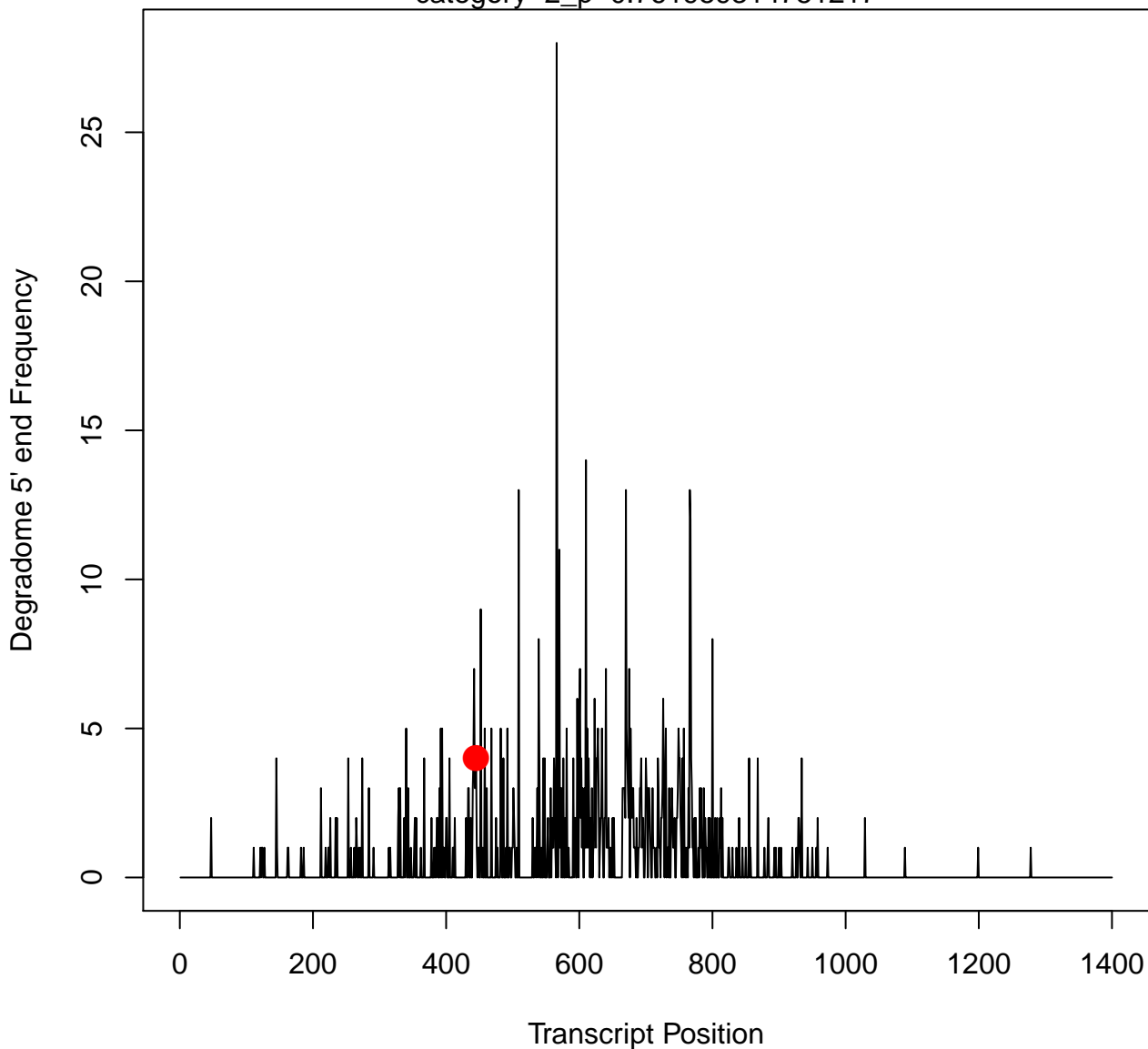

Supplement: Supplementary file 4 [file Data_Sheet_4.zip › Sit-miR169g_Seita.9G137200.1_445_TPlot.pdf]

**T=Seita.9G366600.1\_Q=Sit-miR169g\_S=337**

category=2\_p=0.988049612463137

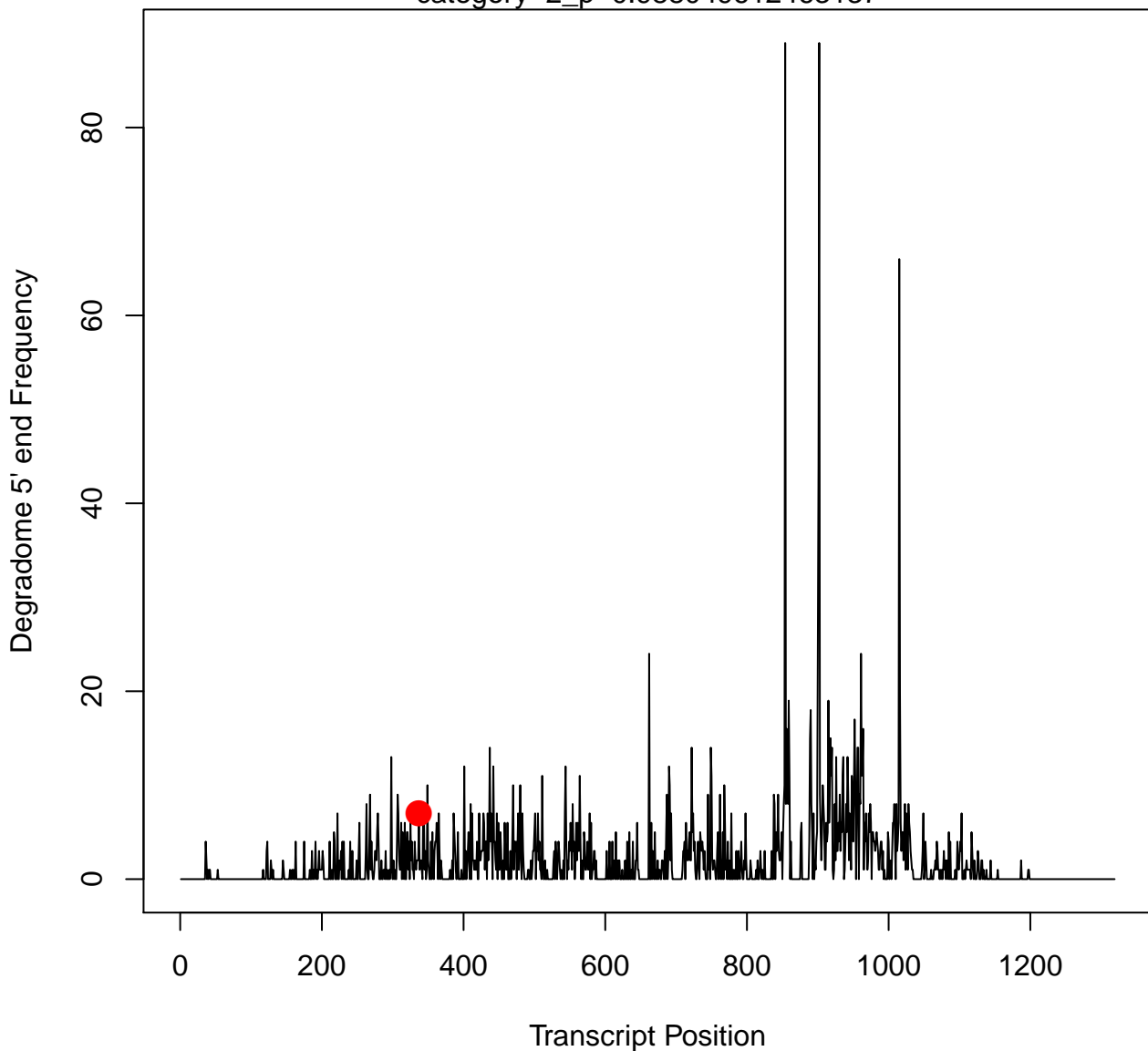

Supplement: Supplementary file 4 [file Data_Sheet_4.zip › Sit-miR169g_Seita.9G366600.1_337_TPlot.pdf]

**T=Seita.9G521100.1\_Q=Sit-miR169g\_S=930**

category=0\_p=0.00228938082912056

Degradome 5' end Frequency

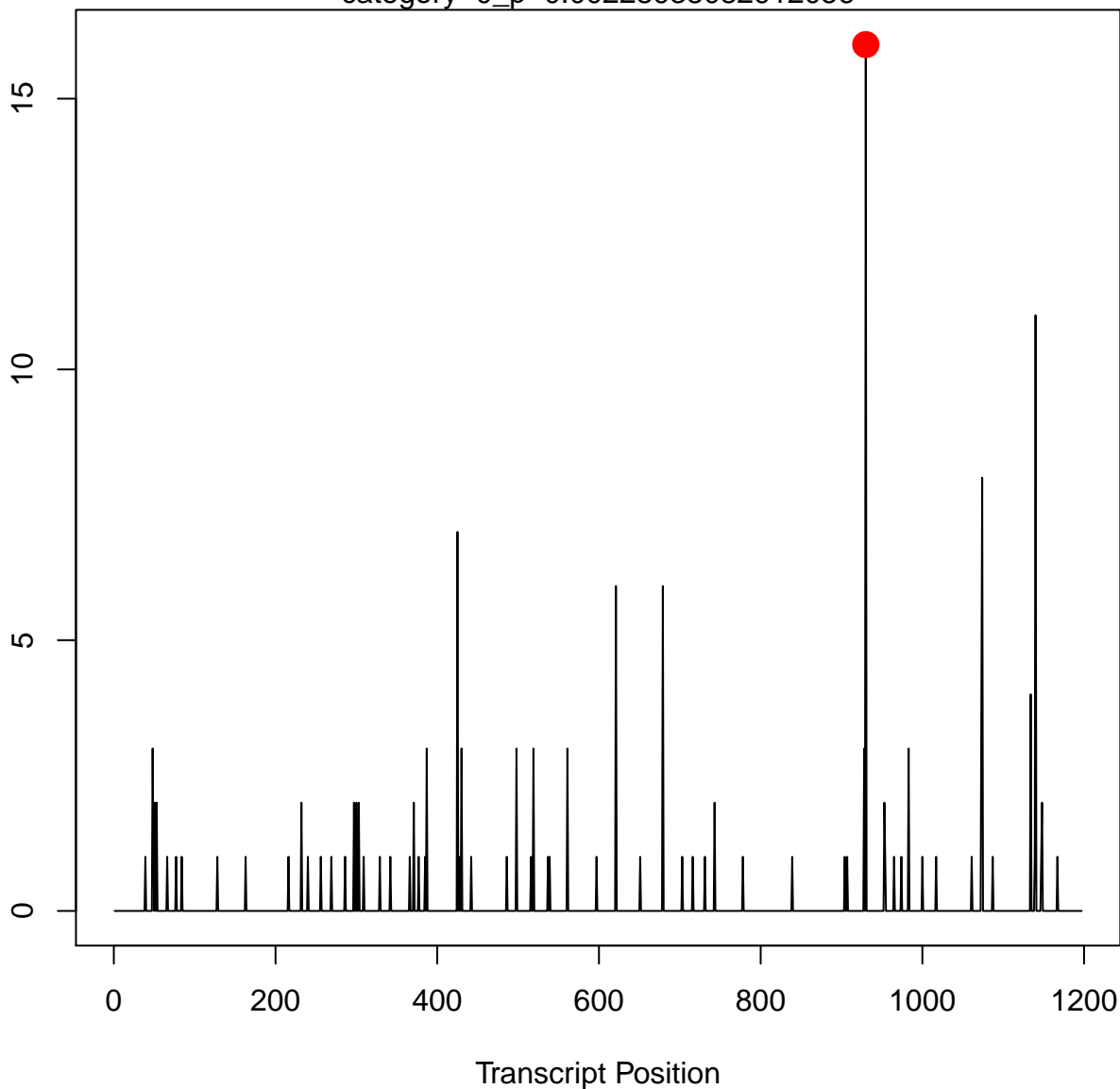

Supplement: Supplementary file 4 [file Data_Sheet_4.zip › Sit-miR169g_Seita.9G521100.1_930_TPlot.pdf]

**T=Seita.9G574700.1\_Q=Sit-miR169g\_S=2609**

category=2\_p=0.999334460024848

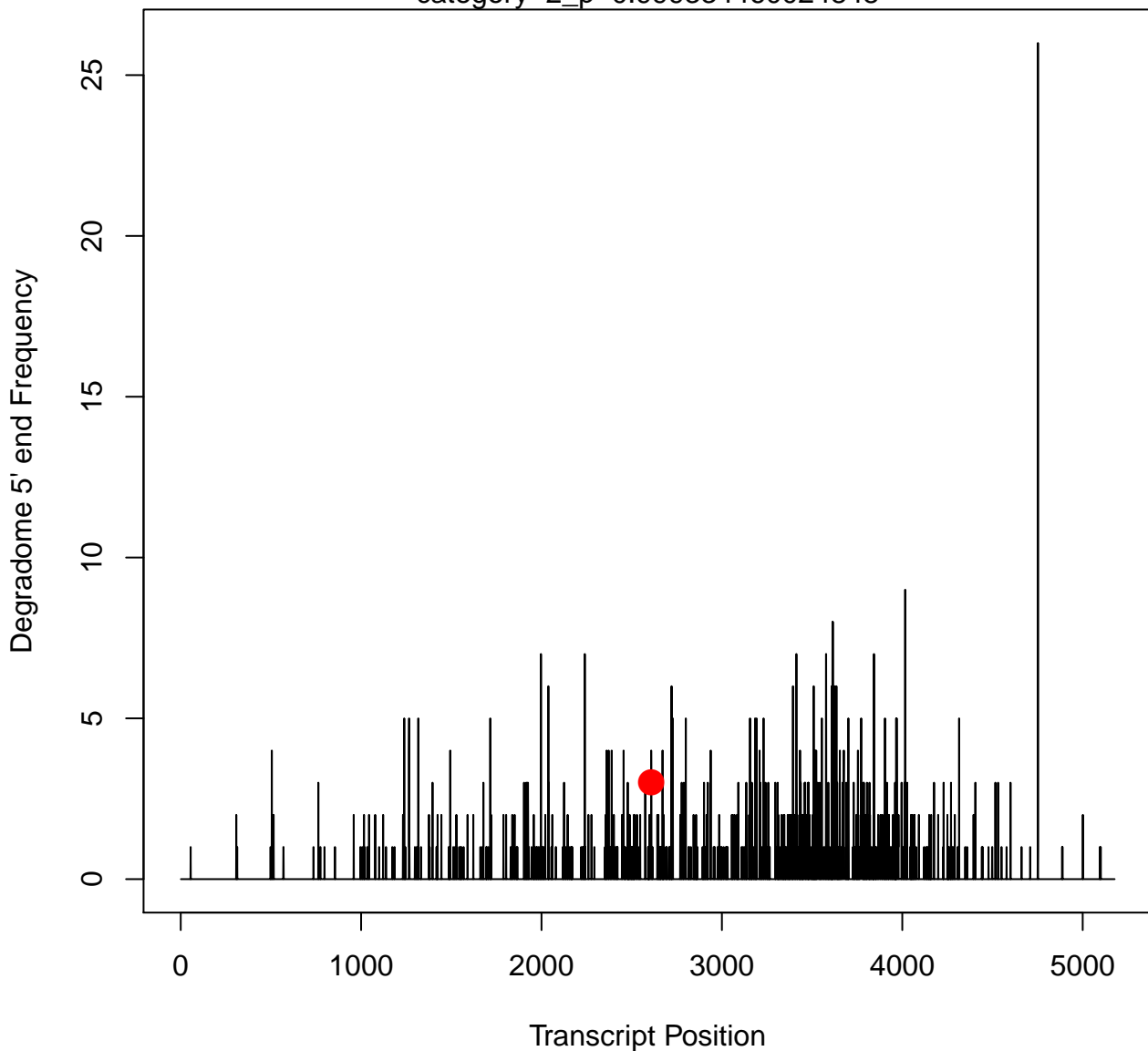

Supplement: Supplementary file 4 [file Data_Sheet_4.zip › Sit-miR169g_Seita.9G574700.1_2609_TPlot.pdf]

**T=Seita.2G170400.1\_Q=Sit-miR171a\_S=1564**

category=2\_p=0.990619222899132

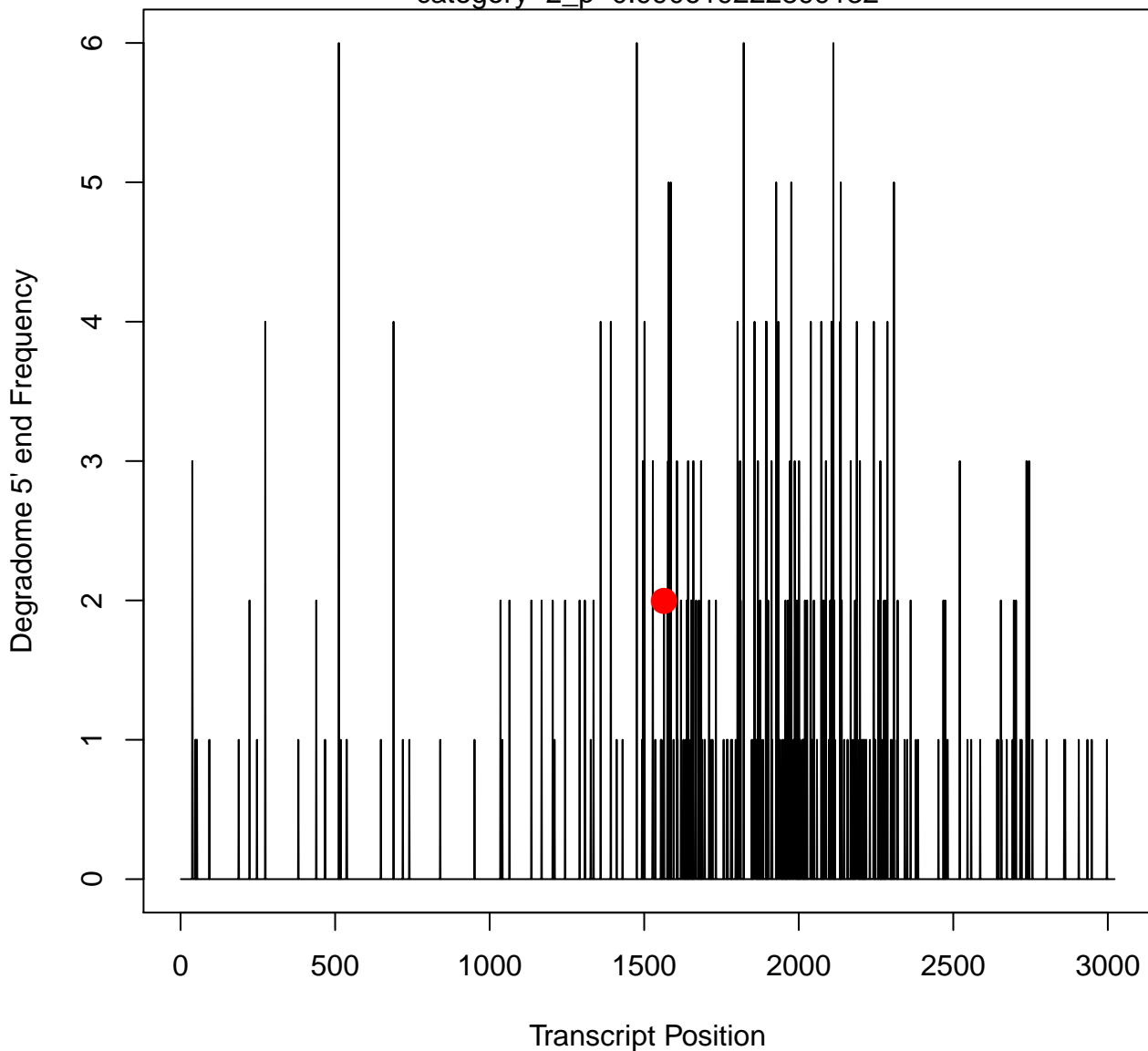

Supplement: Supplementary file 4 [file Data_Sheet_4.zip › Sit-miR171a_Seita.2G170400.1_1564_TPlot.pdf]
