# Supplementary figures and images for: The Construction and Exploration of a Comprehensive MicroRNA Centered Regulatory Network in Foxtail Millet (Setaria italica L.) (part 7 of 14)
Source: Front Plant Sci. 2022 May 6;13:848474. doi: 10.3389/fpls.2022.848474 (PMC9121102; doi:10.3389/fpls.2022.848474)

**T=Seita.1G060100.1\_Q=Sit-miR1133\_S=382**

category=2\_p=0.999999924088919

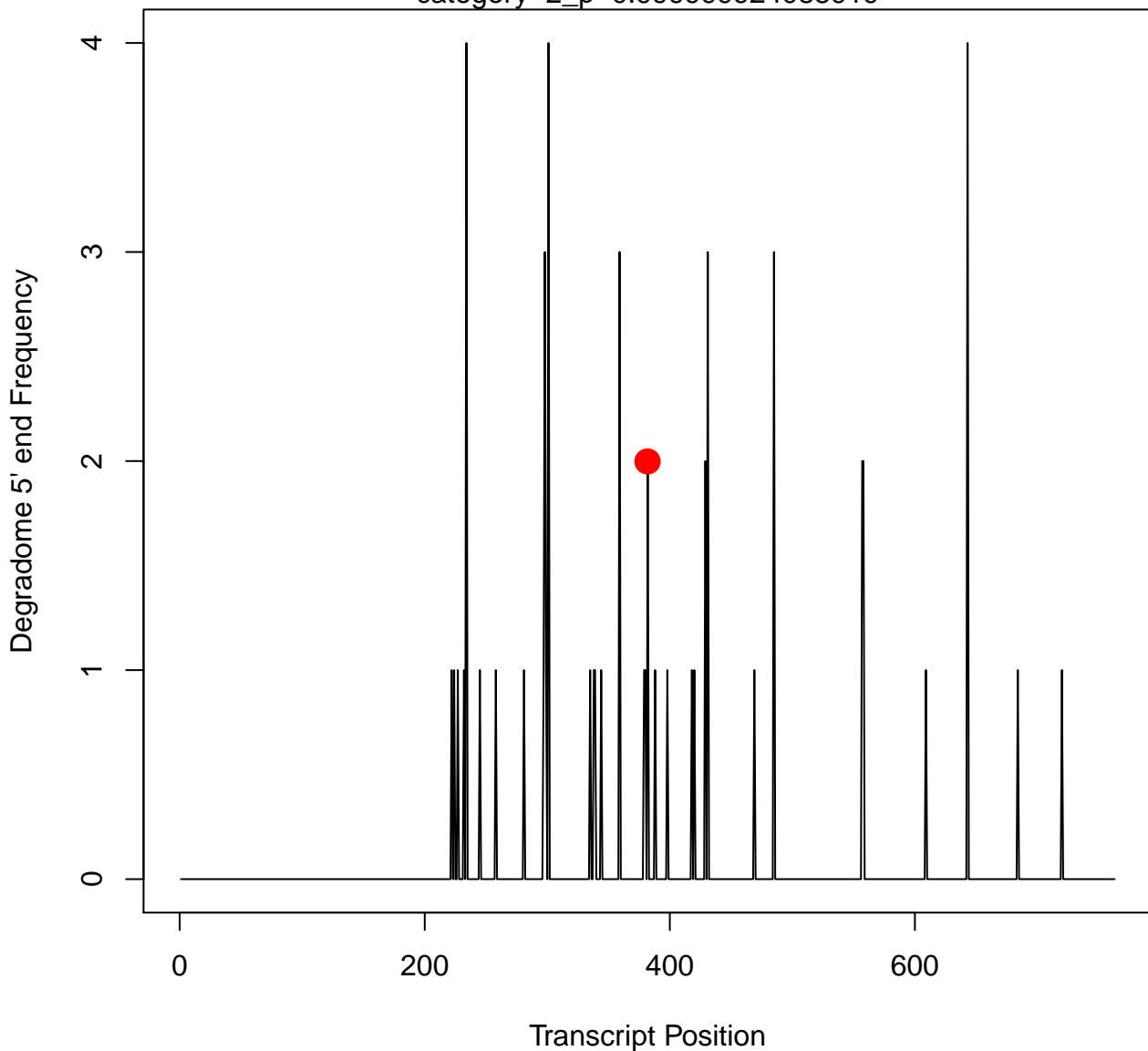

Supplement: Supplementary file 4 [file Data_Sheet_4.zip › Sit-miR1133_Seita.1G060100.1_382_TPlot.pdf]

**T=Seita.2G326100.1\_Q=Sit-miR1133\_S=4134**

category=2\_p=0.999997863126008

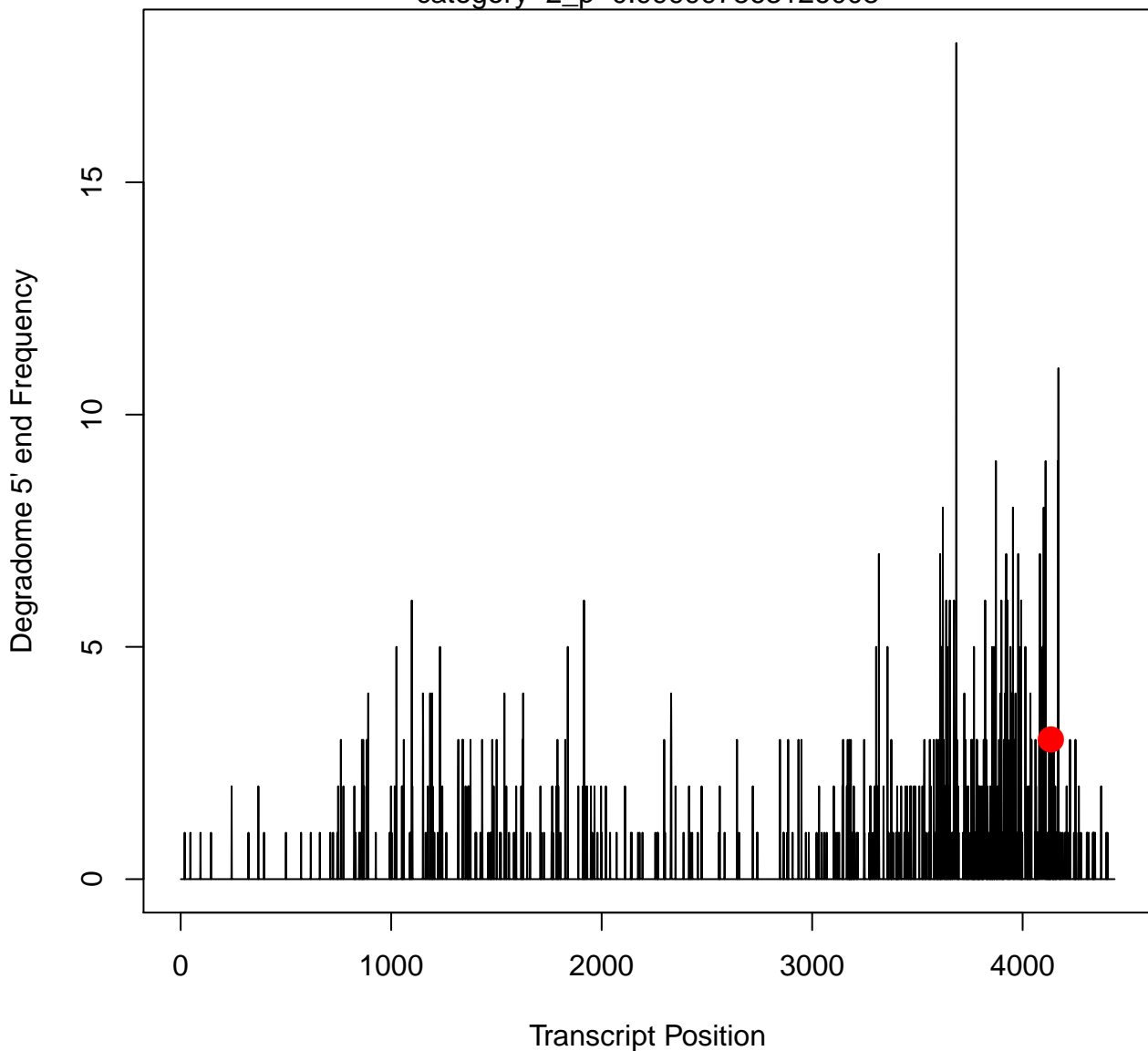

Supplement: Supplementary file 4 [file Data_Sheet_4.zip › Sit-miR1133_Seita.2G326100.1_4134_TPlot.pdf]

**T=Seita.4G129400.1\_Q=Sit-miR1133\_S=457**

category=2\_p=0.999999541468955

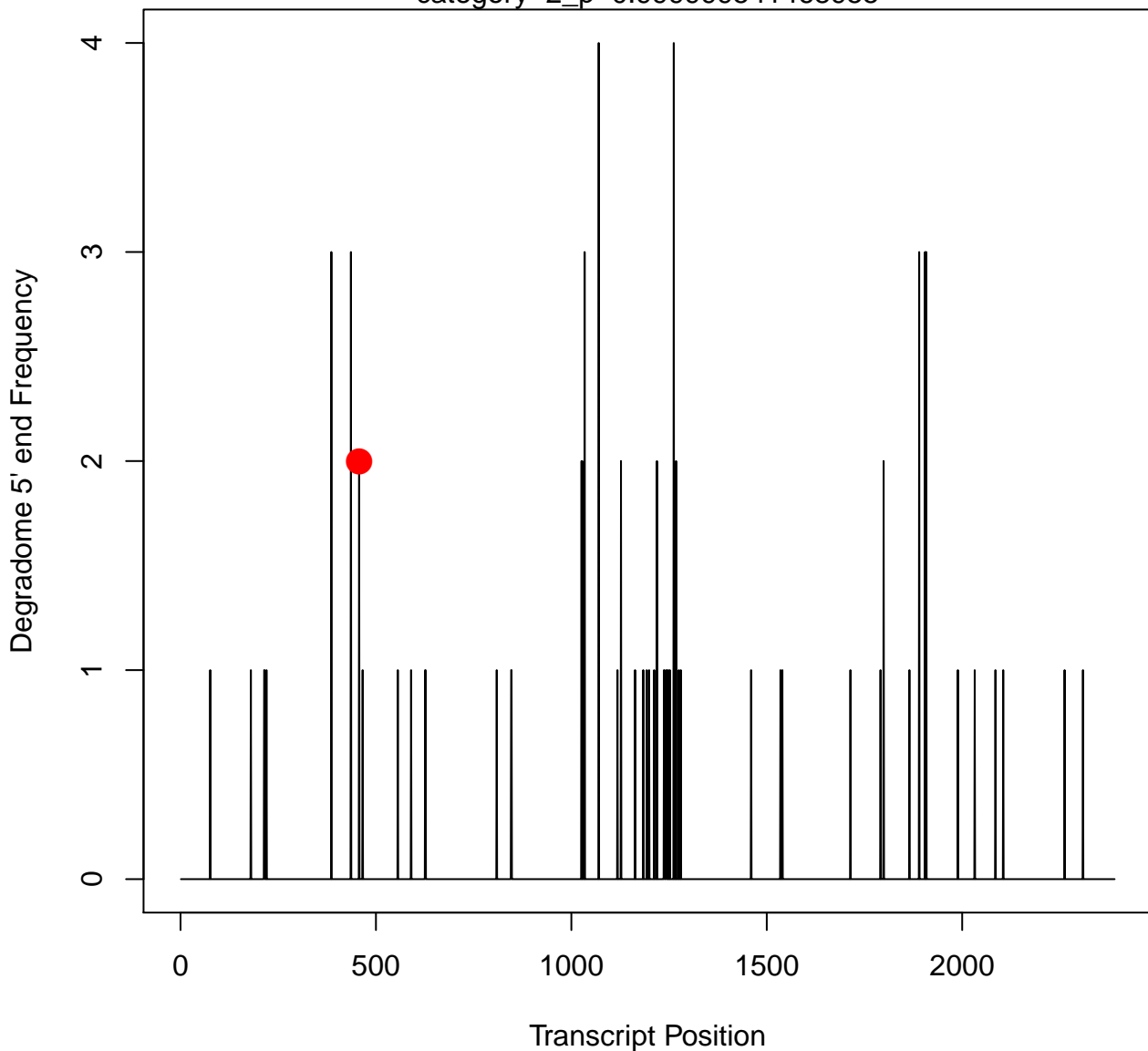

Supplement: Supplementary file 4 [file Data_Sheet_4.zip › Sit-miR1133_Seita.4G129400.1_457_TPlot.pdf]

**T=Seita.6G129100.1\_Q=Sit-miR1133\_S=300**

category=2\_p=0.999998873060688

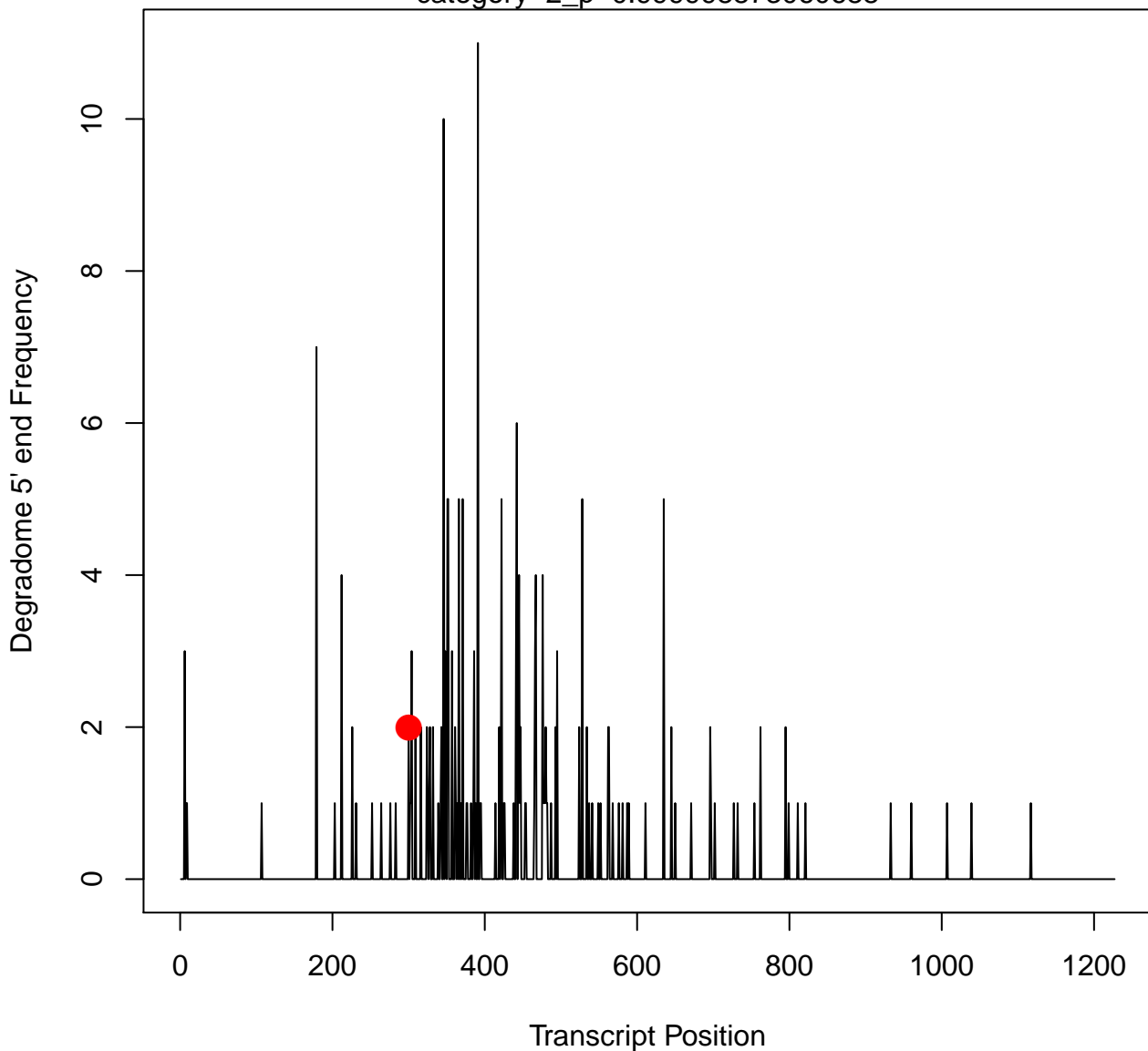

Supplement: Supplementary file 4 [file Data_Sheet_4.zip › Sit-miR1133_Seita.6G129100.1_300_TPlot.pdf]

**T=Seita.9G074400.1\_Q=Sit-miR1133\_S=151**

category=2\_p=0.984240384594638

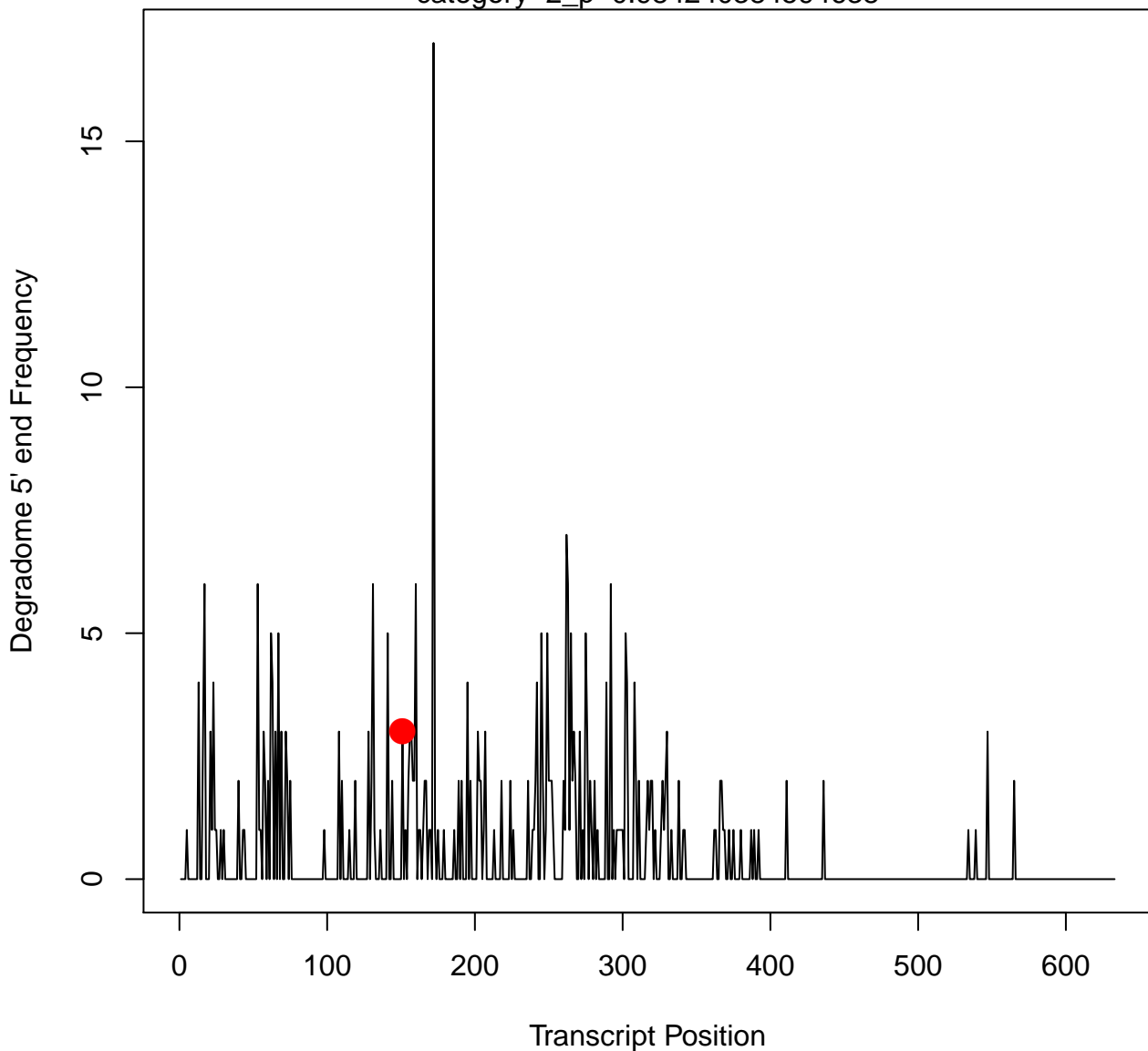

Supplement: Supplementary file 4 [file Data_Sheet_4.zip › Sit-miR1133_Seita.9G074400.1_151_TPlot.pdf]

**T=Seita.9G361400.1\_Q=Sit-miR1133\_S=60**

category=2\_p=0.999999974901332

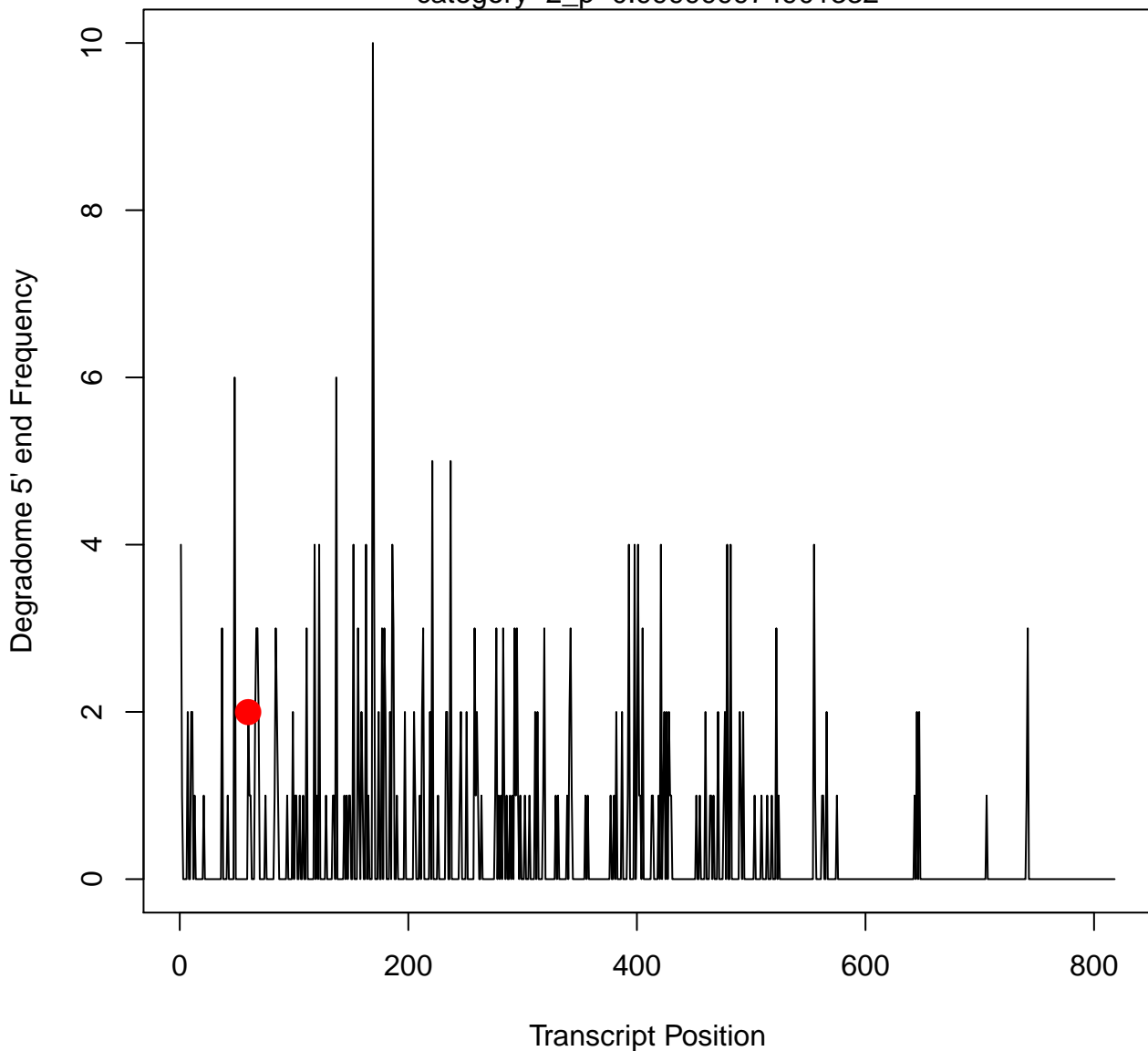

Supplement: Supplementary file 4 [file Data_Sheet_4.zip › Sit-miR1133_Seita.9G361400.1_60_TPlot.pdf]

**T=Seita.9G435700.1\_Q=Sit-miR1133\_S=163**

category=2\_p=0.999990212340451

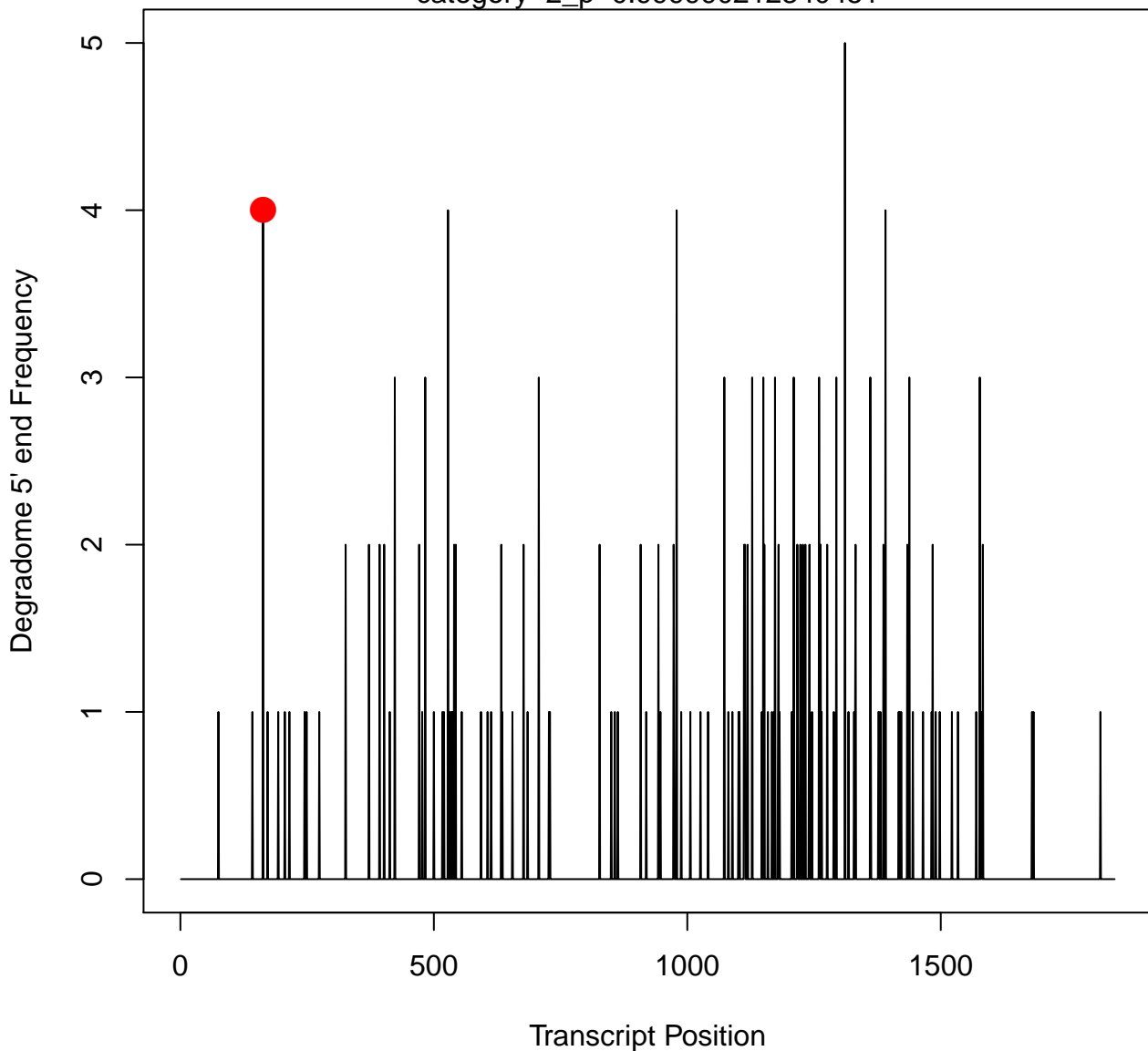

Supplement: Supplementary file 4 [file Data_Sheet_4.zip › Sit-miR1133_Seita.9G435700.1_163_TPlot.pdf]

**T=Seita.1G379300.1\_Q=Sit-miR1432\_S=242**

category=2\_p=0.328161654024246

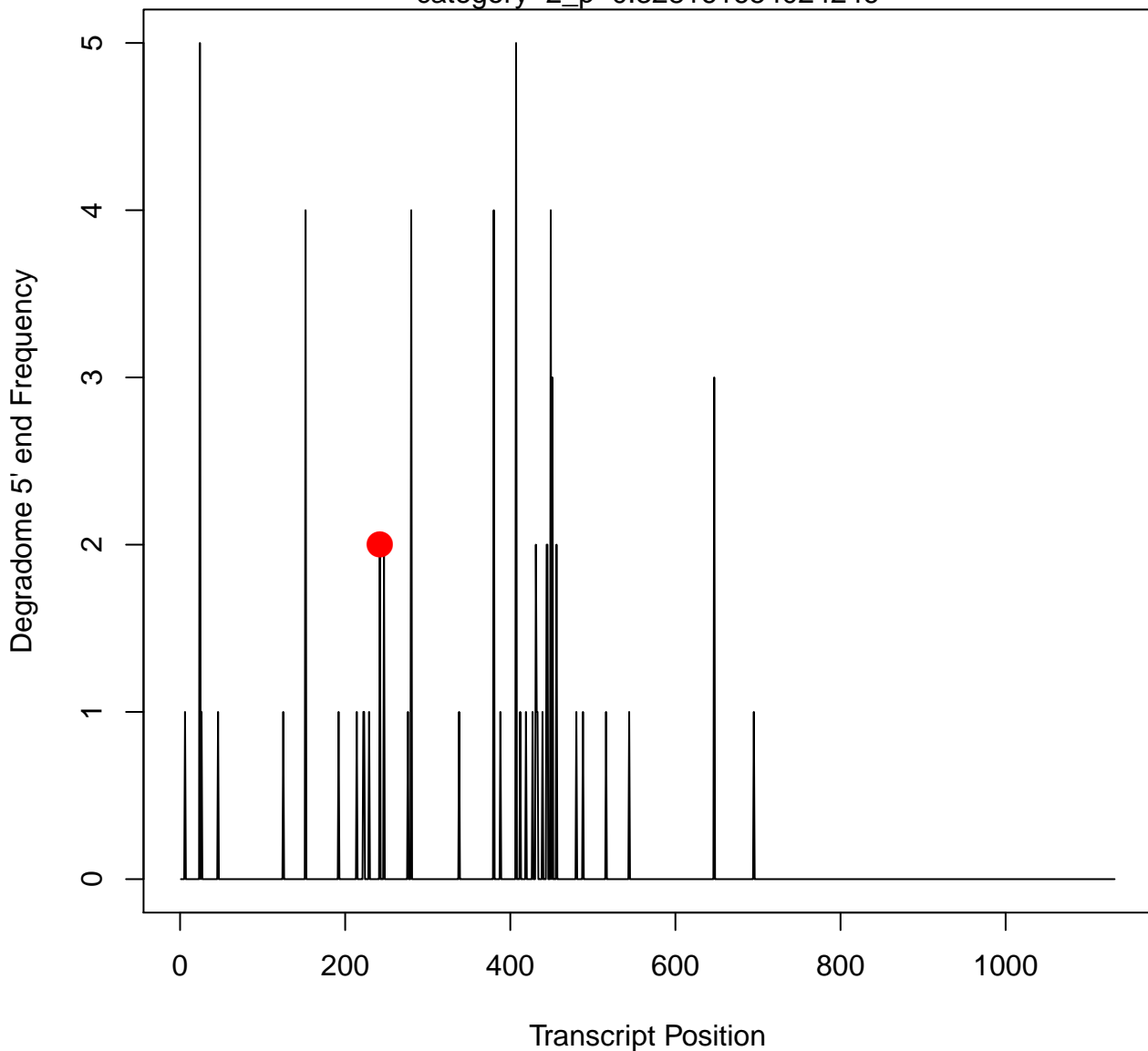

Supplement: Supplementary file 4 [file Data_Sheet_4.zip › Sit-miR1432_Seita.1G379300.1_242_TPlot.pdf]

**T=Seita.2G158400.1\_Q=Sit-miR1432\_S=994**

category=2\_p=0.507869015289345

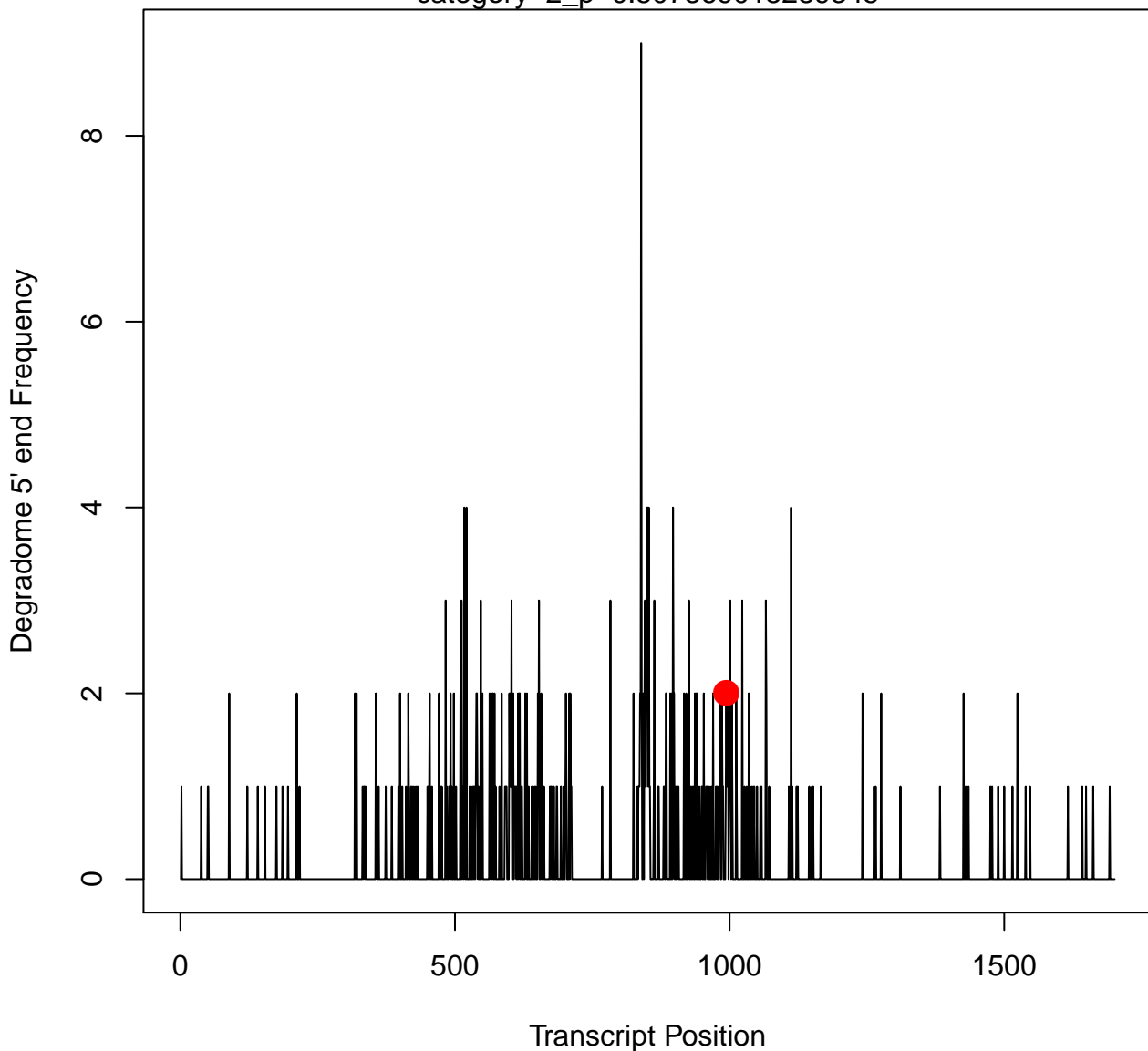

Supplement: Supplementary file 4 [file Data_Sheet_4.zip › Sit-miR1432_Seita.2G158400.1_994_TPlot.pdf]

**T=Seita.2G358600.1 Q=Sit-miR1432 S=138**

category=2\_p=0.999729203926592

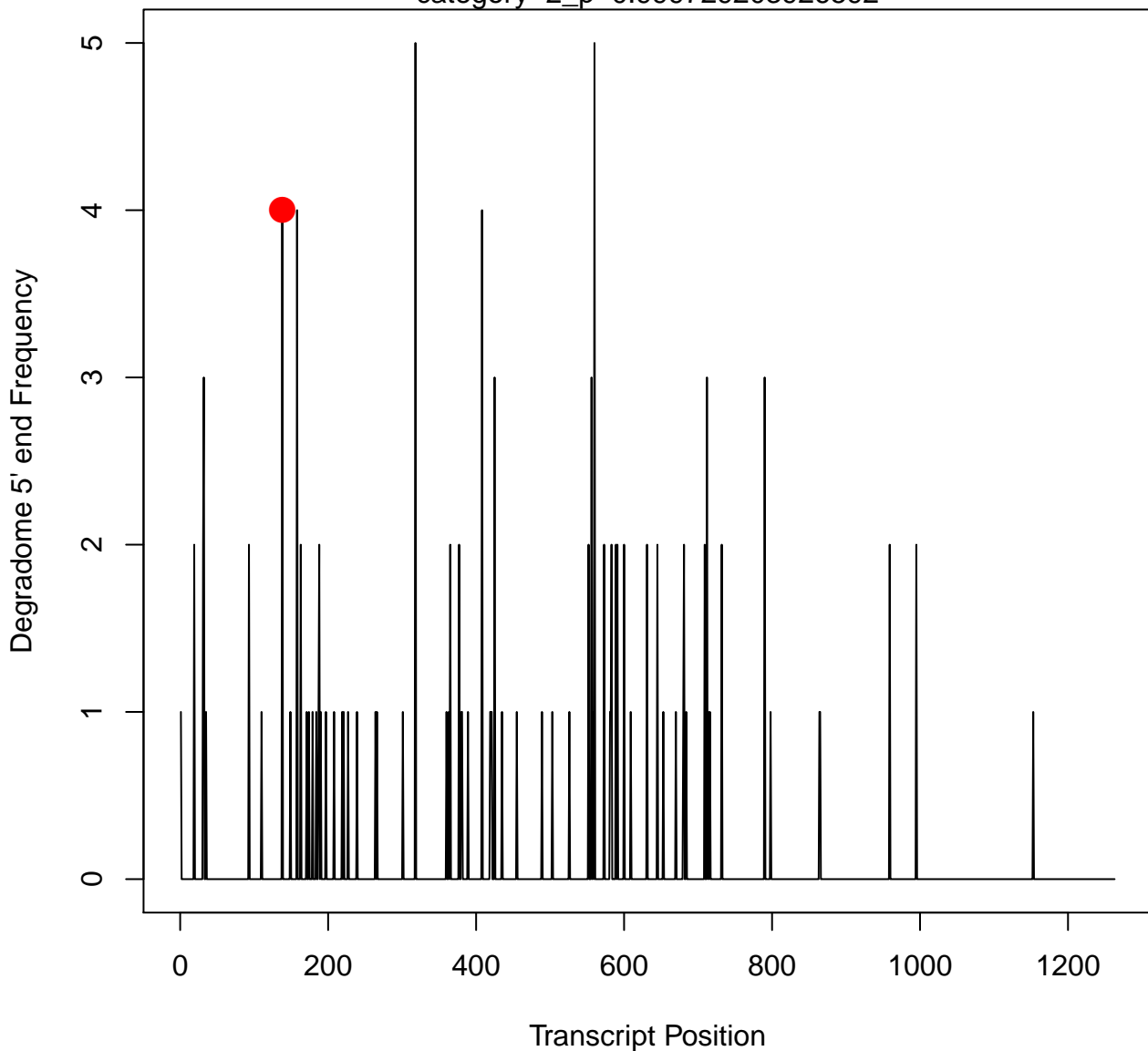

Supplement: Supplementary file 4 [file Data_Sheet_4.zip › Sit-miR1432_Seita.2G358600.1_138_TPlot.pdf]

**T=Seita.2G361800.1\_Q=Sit-miR1432\_S=1740**

category=2\_p=0.267486014070987

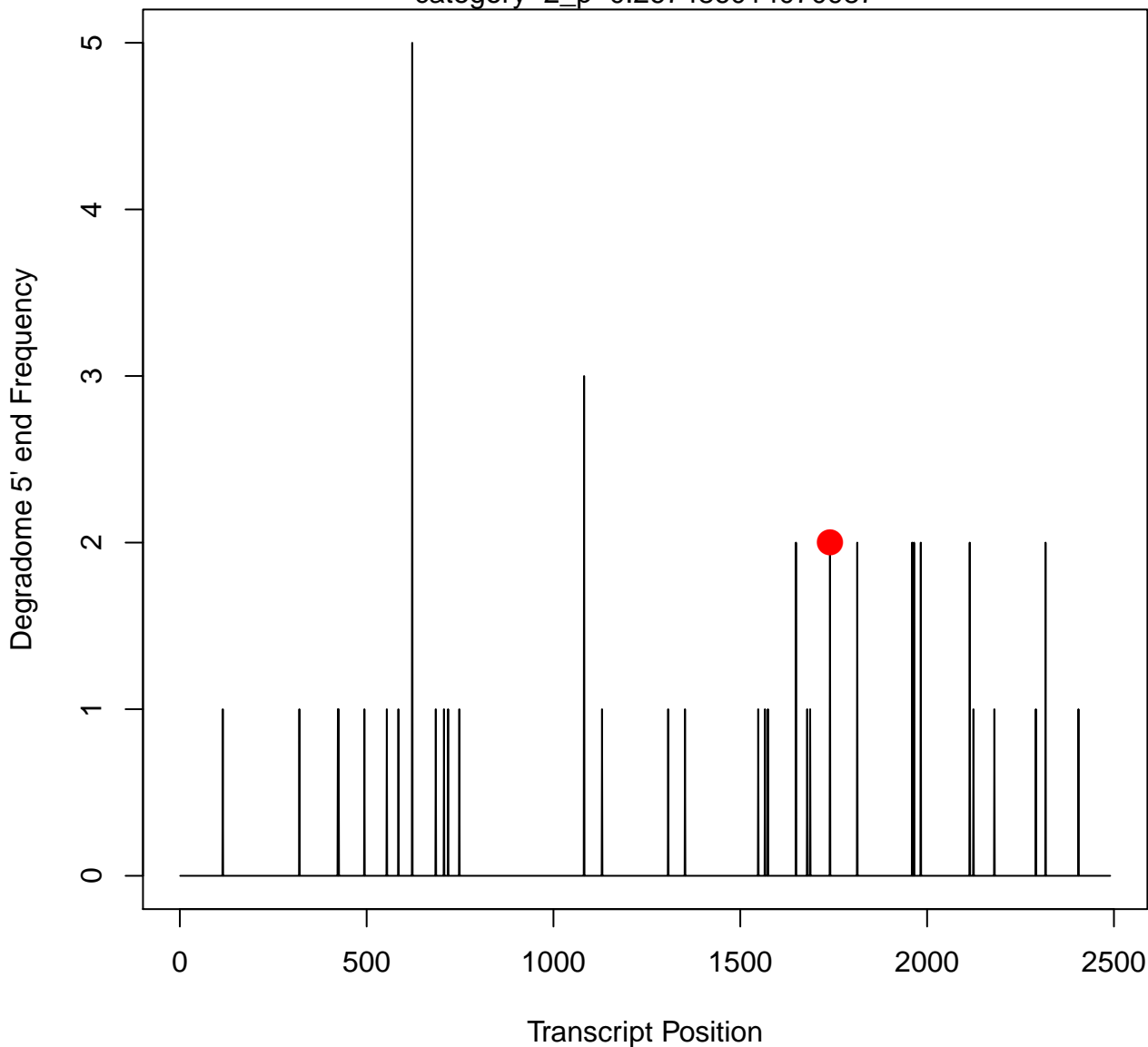

Supplement: Supplementary file 4 [file Data_Sheet_4.zip › Sit-miR1432_Seita.2G361800.1_1740_TPlot.pdf]

**T=Seita.2G405900.1\_Q=Sit-miR1432\_S=124**

category=2\_p=0.993362045295182

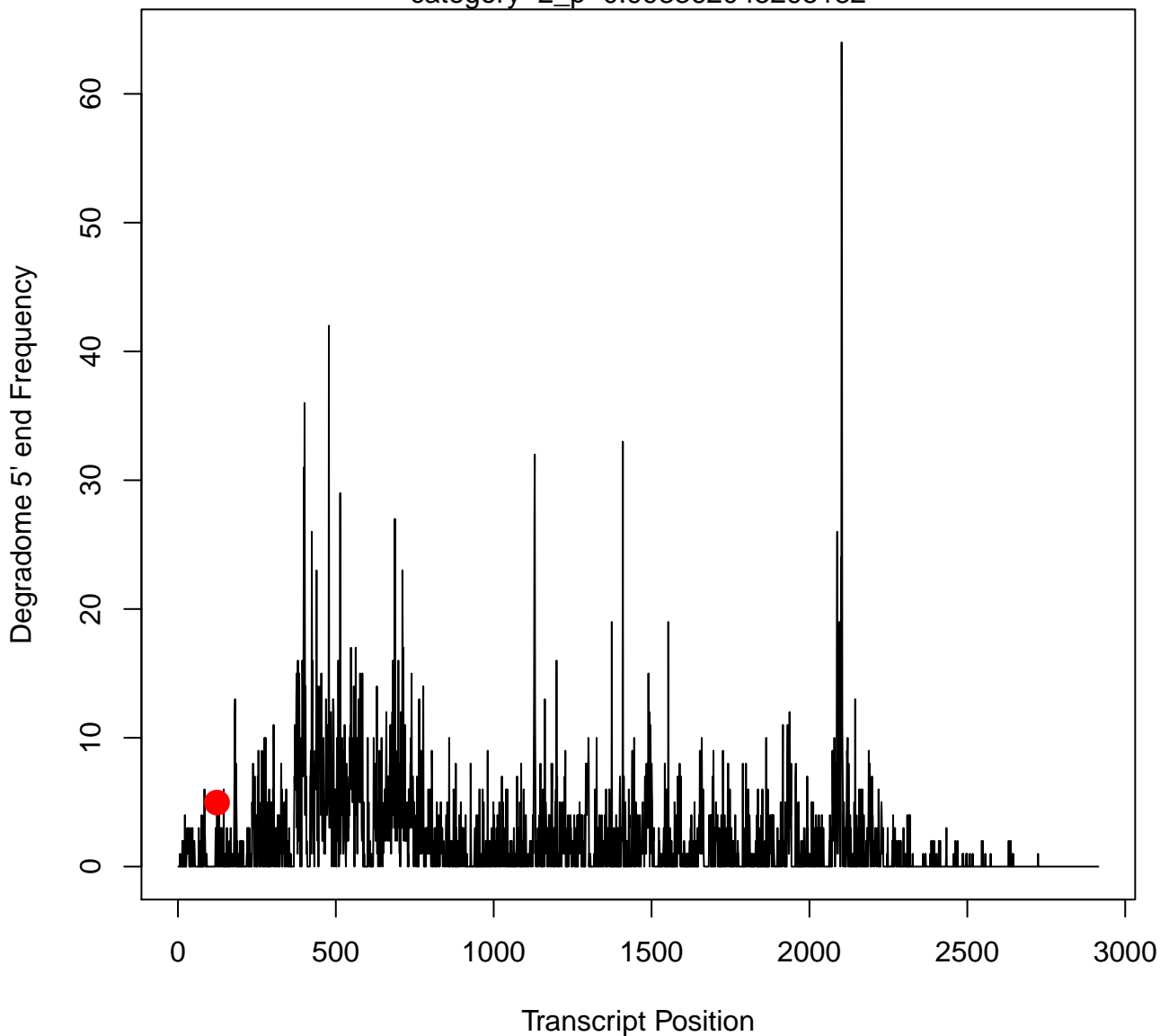

Supplement: Supplementary file 4 [file Data_Sheet_4.zip › Sit-miR1432_Seita.2G405900.1_124_TPlot.pdf]

**T=Seita.3G129700.1\_Q=Sit-miR1432\_S=592**

category=2\_p=0.499284614937144

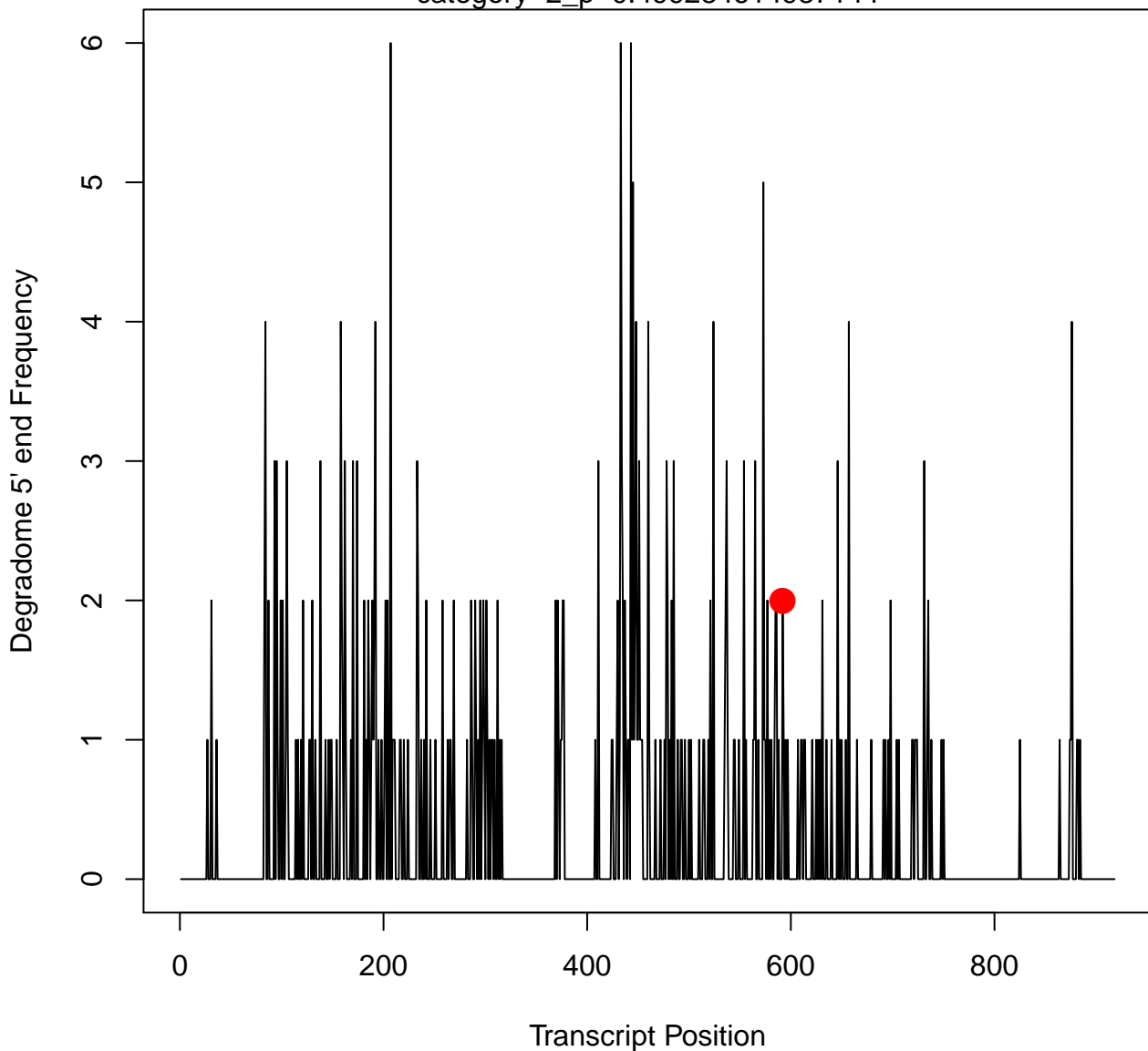

Supplement: Supplementary file 4 [file Data_Sheet_4.zip › Sit-miR1432_Seita.3G129700.1_592_TPlot.pdf]

**T=Seita.3G261200.1\_Q=Sit-miR1432\_S=1434**

category=2\_p=0.999962934803816

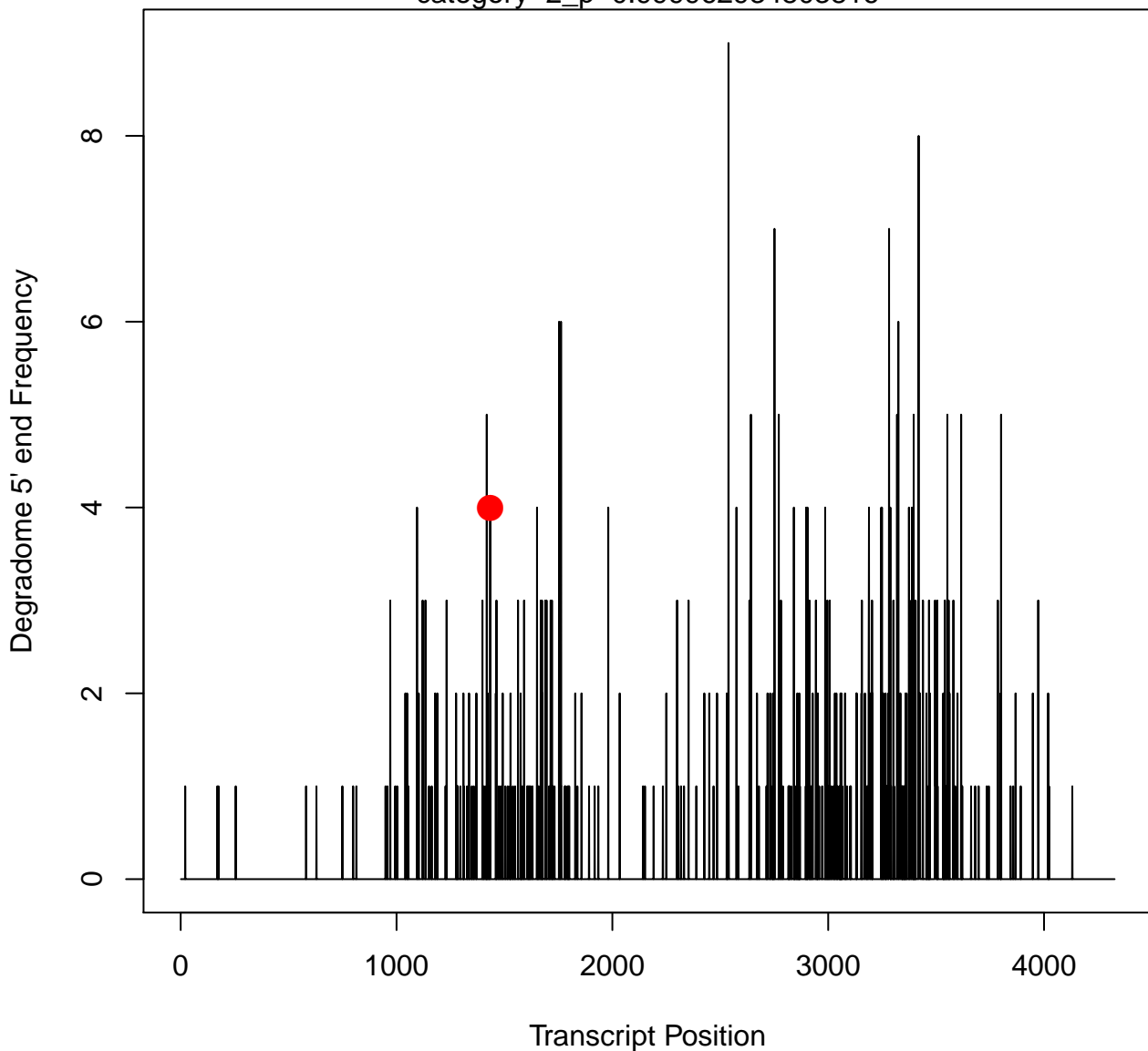

Supplement: Supplementary file 4 [file Data_Sheet_4.zip › Sit-miR1432_Seita.3G261200.1_1434_TPlot.pdf]

**T=Seita.3G395200.1\_Q=Sit-miR1432\_S=629**

category=2\_p=0.999841574408254

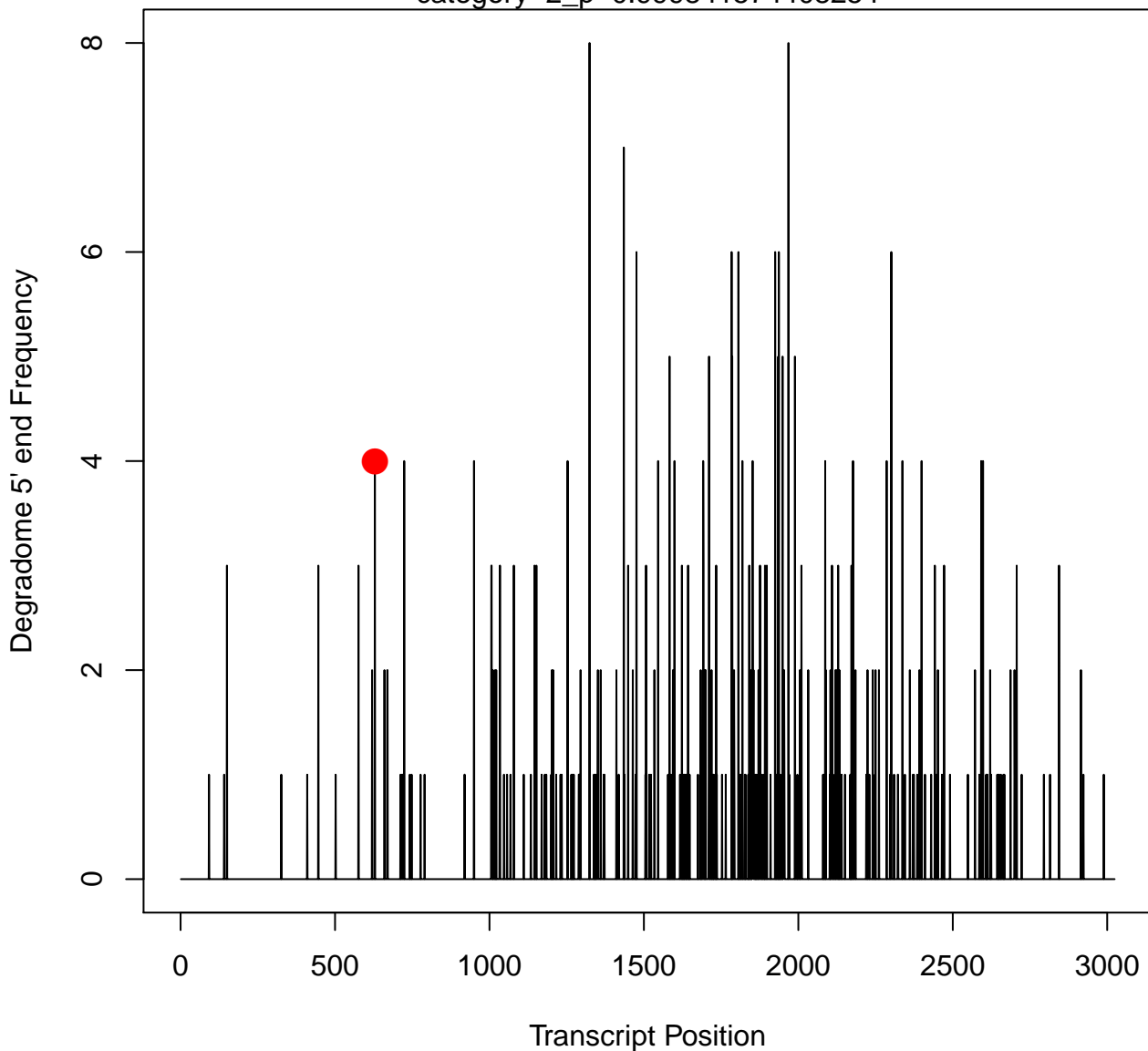

Supplement: Supplementary file 4 [file Data_Sheet_4.zip › Sit-miR1432_Seita.3G395200.1_629_TPlot.pdf]

**T=Seita.6G193400.1\_Q=Sit-miR1432\_S=2270**

category=2\_p=0.999914992512515

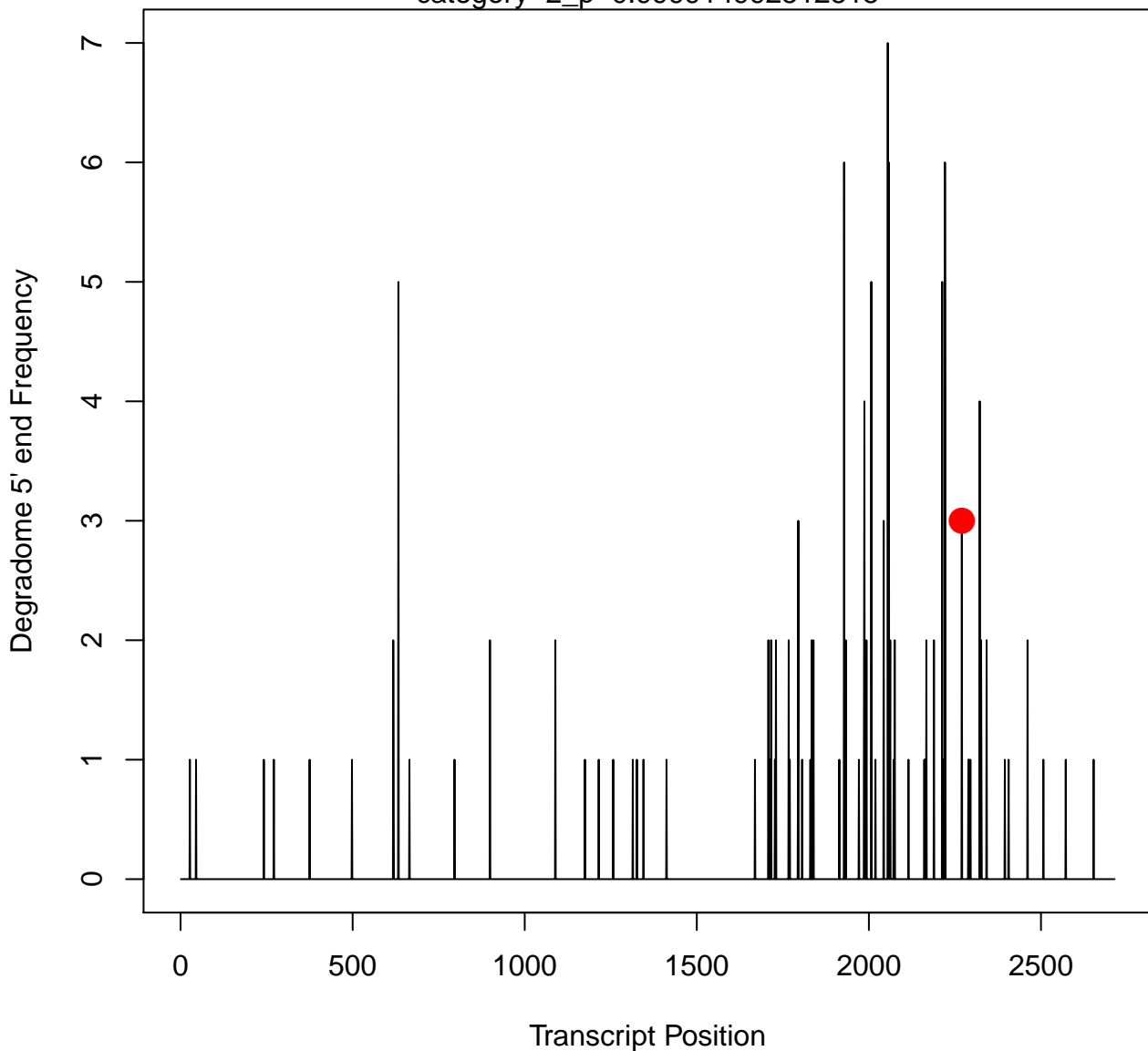

Supplement: Supplementary file 4 [file Data_Sheet_4.zip › Sit-miR1432_Seita.6G193400.1_2270_TPlot.pdf]

**T=Seita.6G254200.1\_Q=Sit-miR1432\_S=1190**

category=2\_p=0.991971271403465

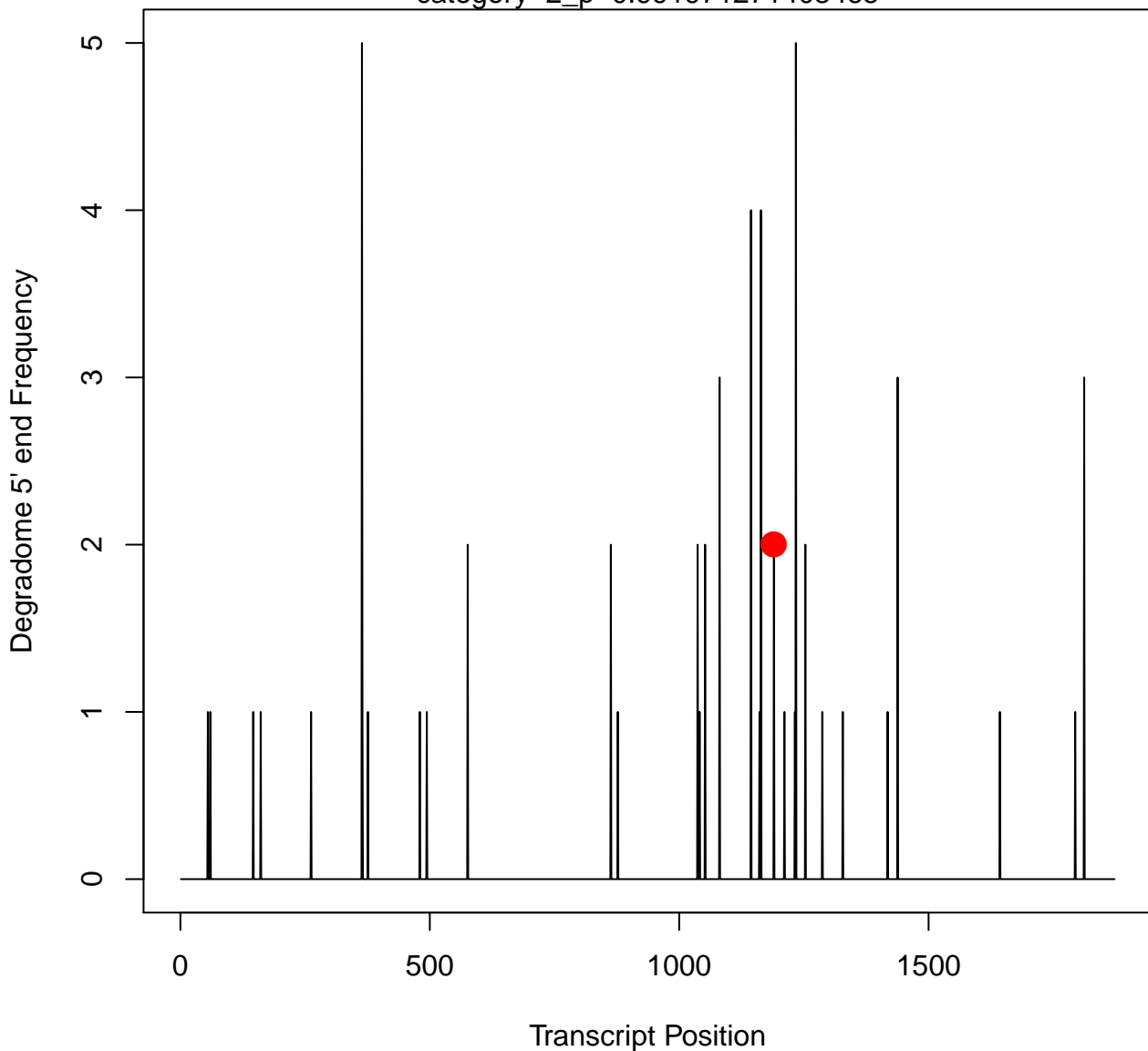

Supplement: Supplementary file 4 [file Data_Sheet_4.zip › Sit-miR1432_Seita.6G254200.1_1190_TPlot.pdf]

**T=Seita.9G141000.1\_Q=Sit-miR1432\_S=443**

category=2\_p=0.999468453576567

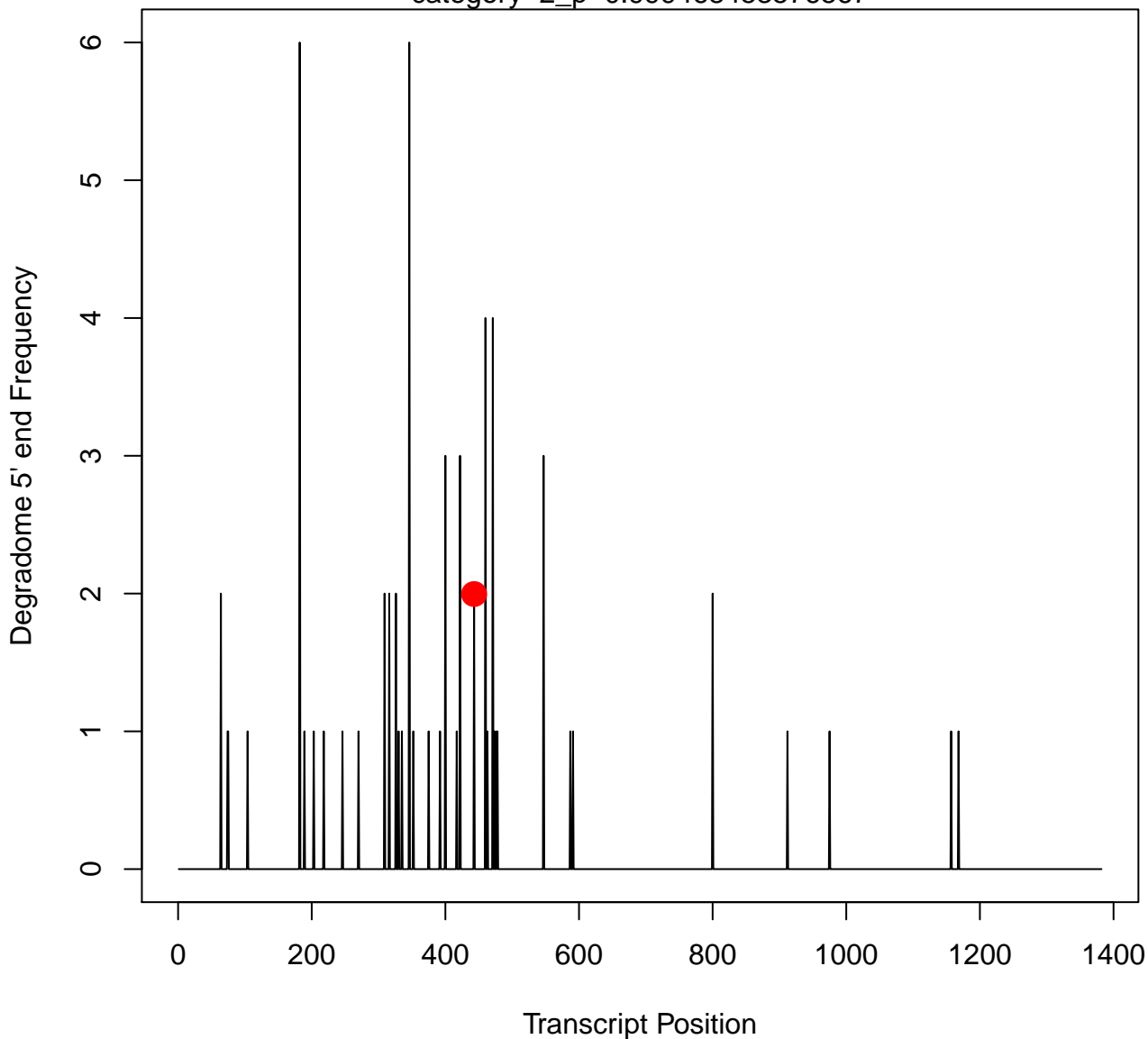

Supplement: Supplementary file 4 [file Data_Sheet_4.zip › Sit-miR1432_Seita.9G141000.1_443_TPlot.pdf]

**T=Seita.9G259600.1\_Q=Sit-miR1432\_S=856**

category=2\_p=0.999827266540563

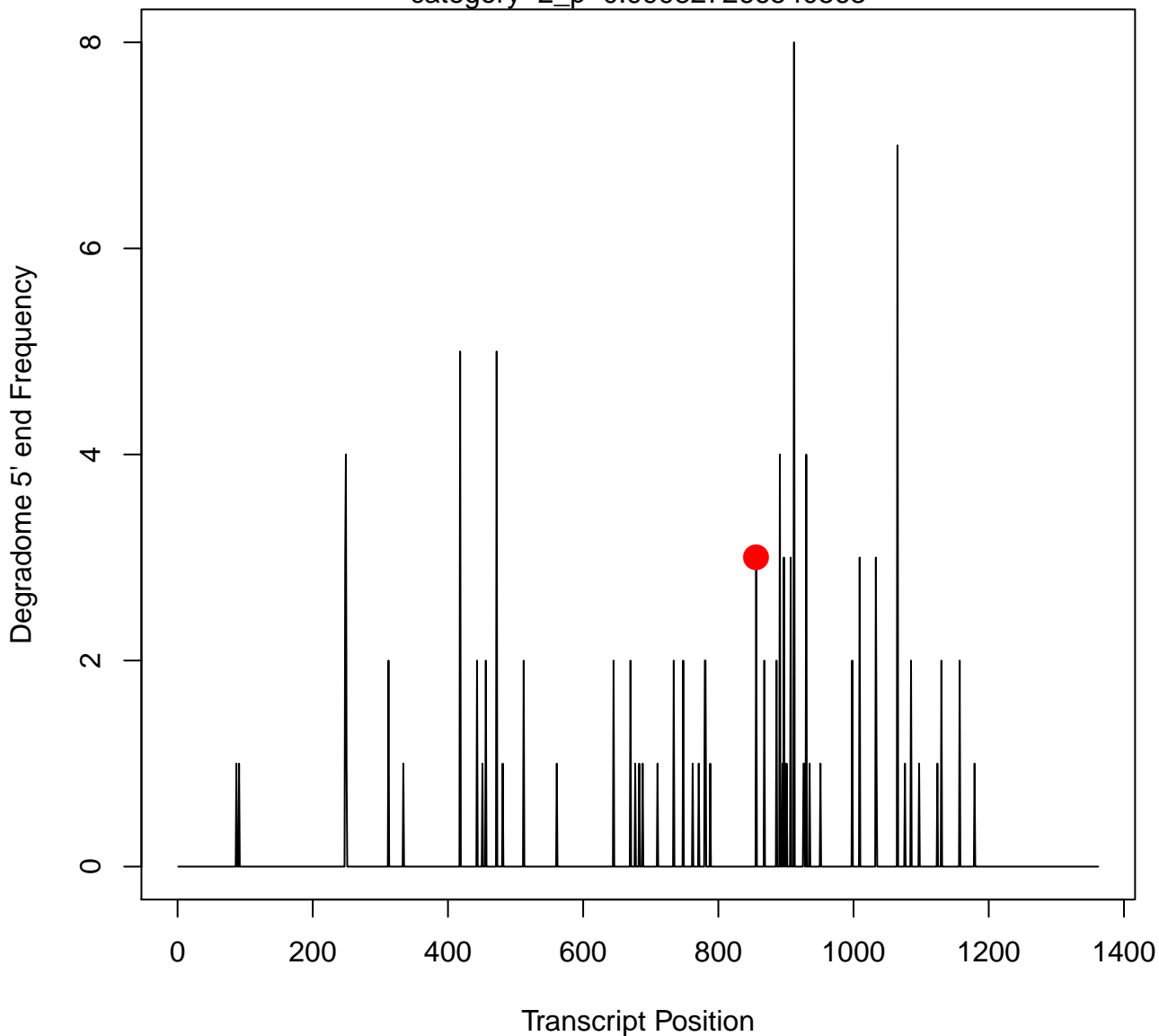

Supplement: Supplementary file 4 [file Data_Sheet_4.zip › Sit-miR1432_Seita.9G259600.1_856_TPlot.pdf]

**T=Seita.9G522200.1\_Q=Sit-miR1432\_S=167**

category=2\_p=0.999912001020984

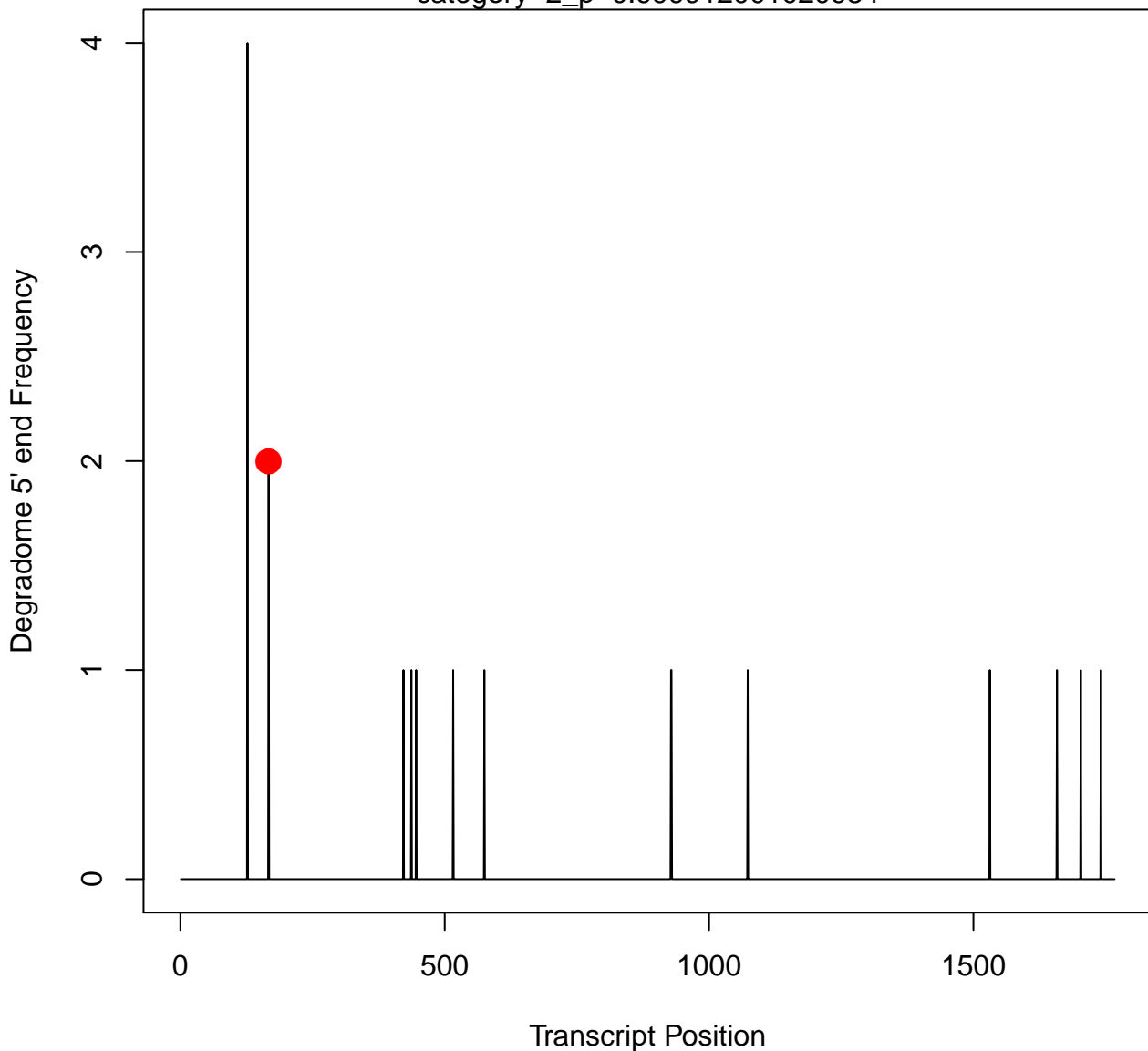

Supplement: Supplementary file 4 [file Data_Sheet_4.zip › Sit-miR1432_Seita.9G522200.1_167_TPlot.pdf]

**T=Seita.2G266500.1\_Q=Sit-miR156a\_S=1896**

category=0\_p=0.00343210501886526

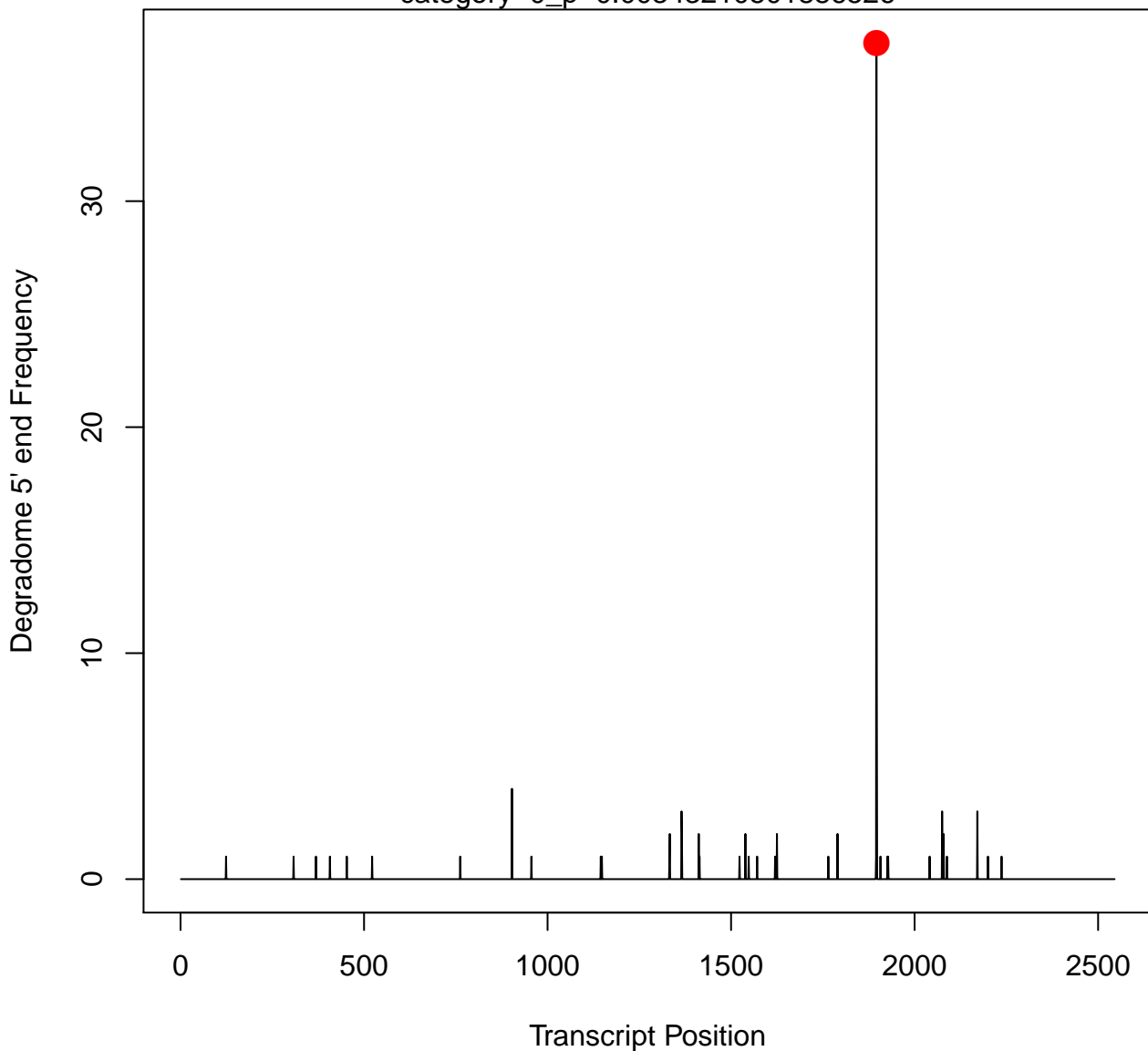

Supplement: Supplementary file 4 [file Data_Sheet_4.zip › Sit-miR156a_Seita.2G266500.1_1896_TPlot.pdf]

**T=Seita.4G270400.1\_Q=Sit-miR156a\_S=2084**

category=1\_p=0.000255096347370198

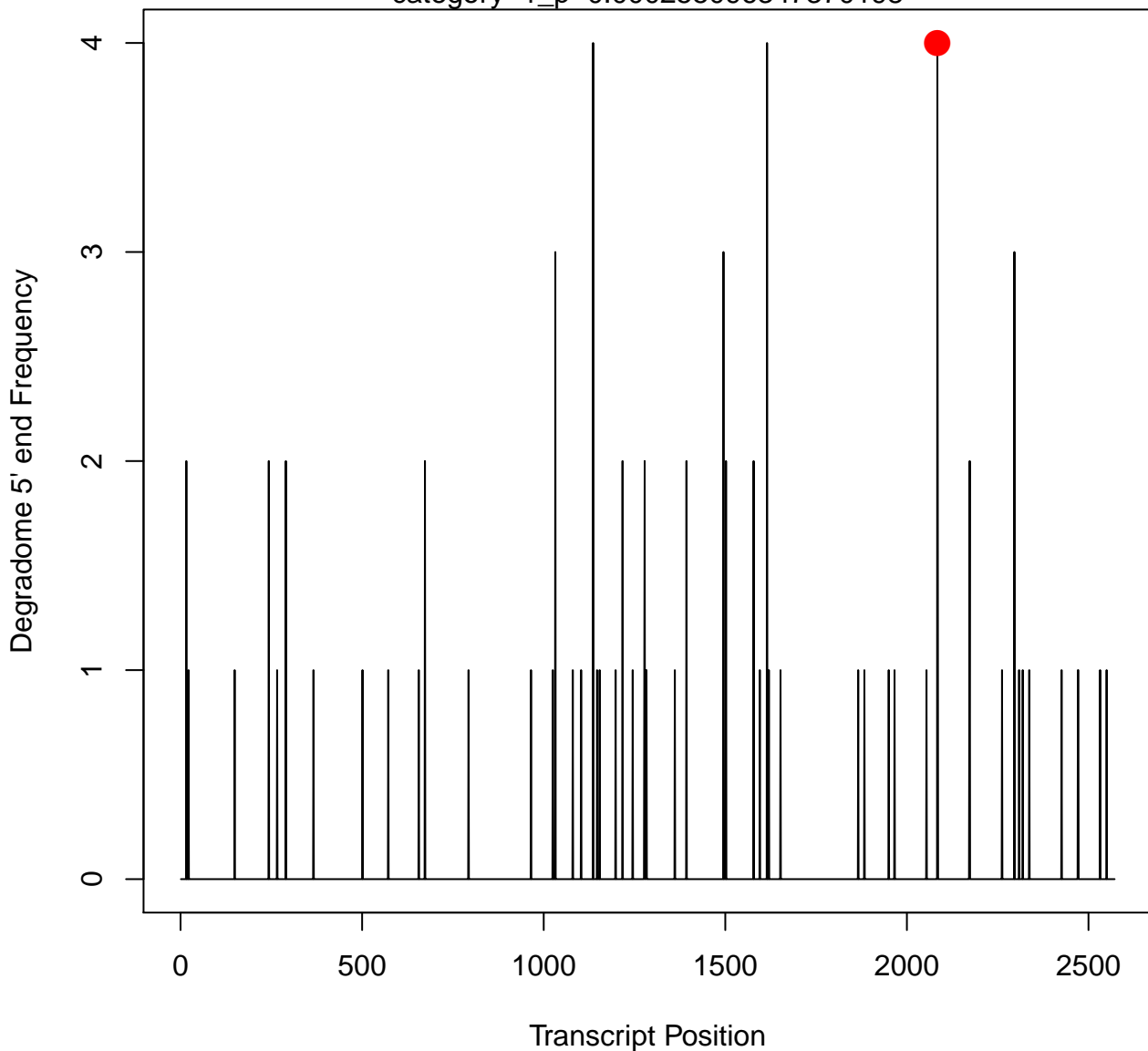

Supplement: Supplementary file 4 [file Data_Sheet_4.zip › Sit-miR156a_Seita.4G270400.1_2084_TPlot.pdf]

**T=Seita.6G223300.1\_Q=Sit-miR156c\_S=981**

category=0\_p=0.00152683684215971

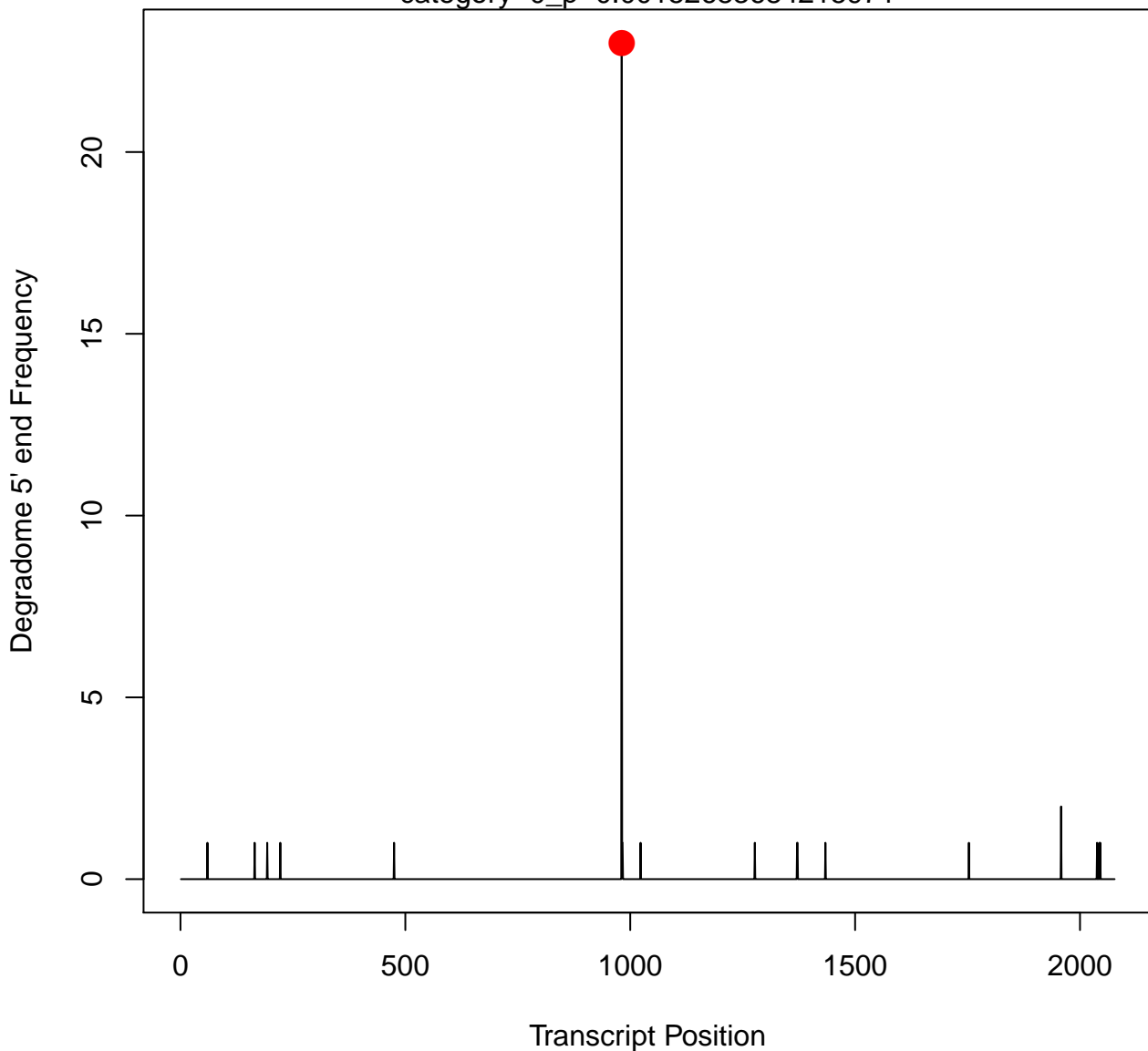

Supplement: Supplementary file 4 [file Data_Sheet_4.zip › Sit-miR156c_Seita.6G223300.1_981_TPlot.pdf]

**T=Seita.1G318200.1\_Q=Sit-miR156d\_S=1439**

category=2\_p=0.964475639773801

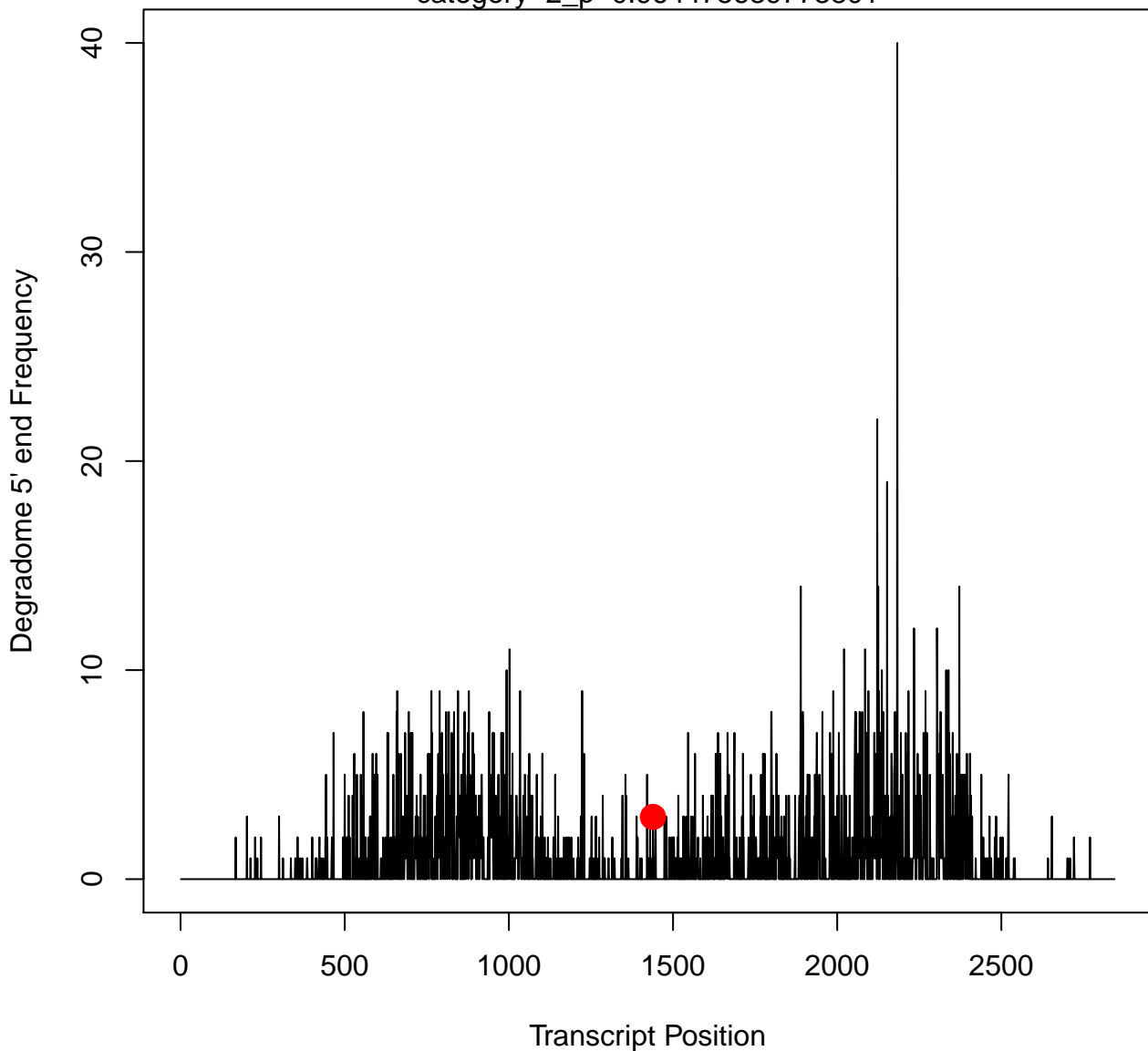

Supplement: Supplementary file 4 [file Data_Sheet_4.zip › Sit-miR156d_Seita.1G318200.1_1439_TPlot.pdf]

**T=Seita.2G254300.1\_Q=Sit-miR156d\_S=1267**

category=2\_p=0.0668335987629942

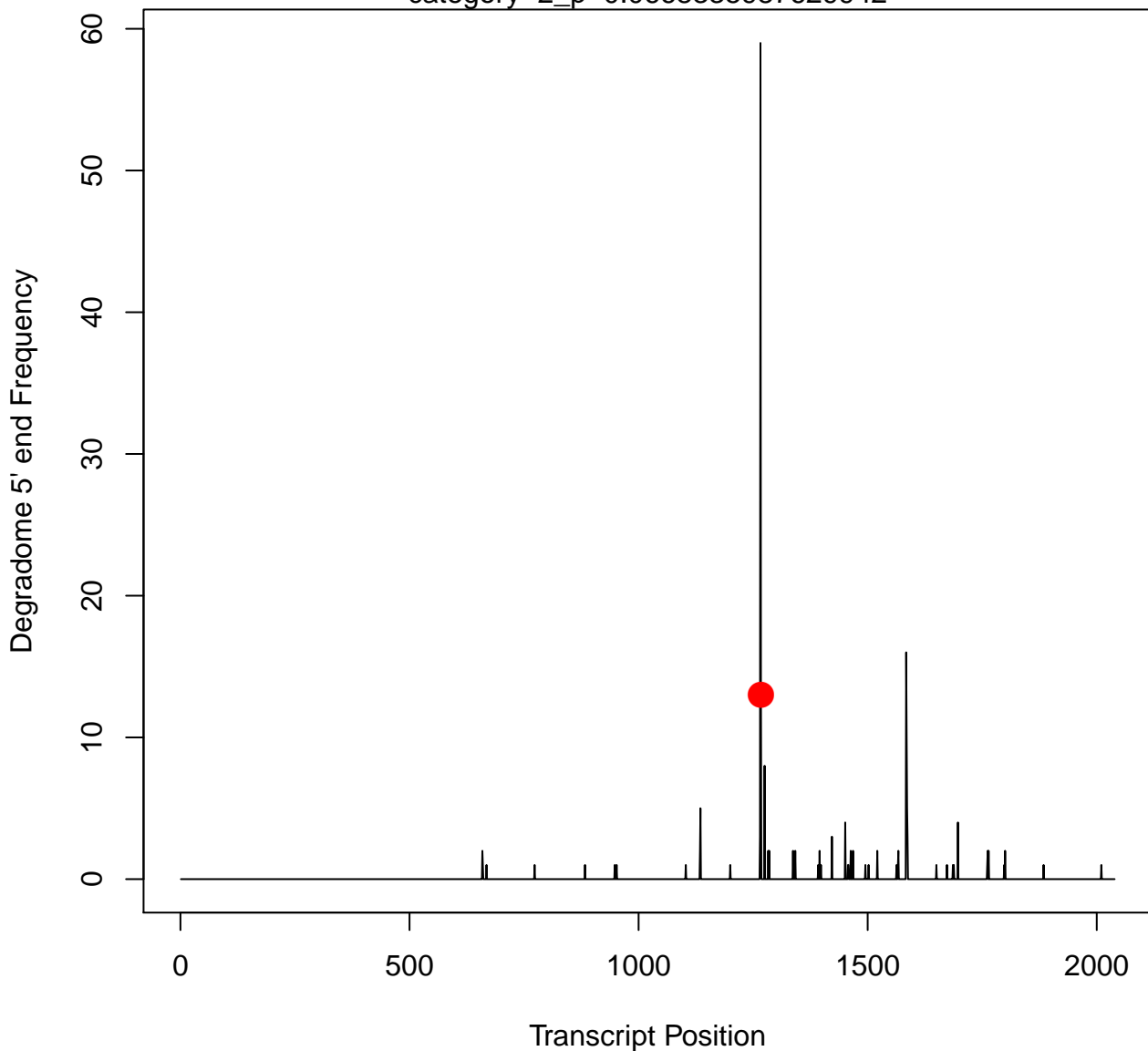

Supplement: Supplementary file 4 [file Data_Sheet_4.zip › Sit-miR156d_Seita.2G254300.1_1267_TPlot.pdf]

**T=Seita.2G266500.1\_Q=Sit-miR156d\_S=1897**

category=2\_p=0.144129690940846

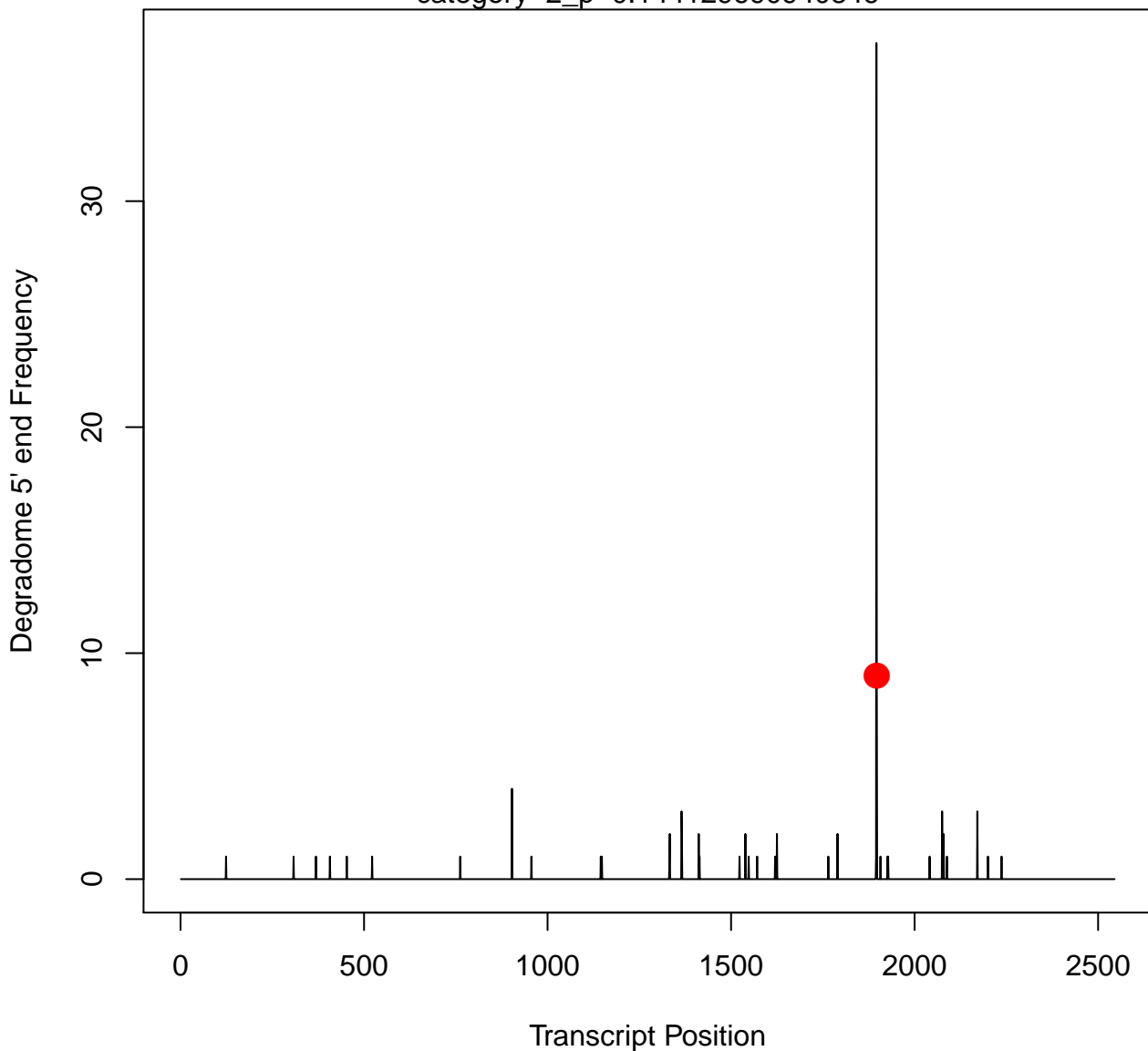

Supplement: Supplementary file 4 [file Data_Sheet_4.zip › Sit-miR156d_Seita.2G266500.1_1897_TPlot.pdf]

**T=Seita.2G324900.1\_Q=Sit-miR156d\_S=834**

category=2\_p=0.187399134153641

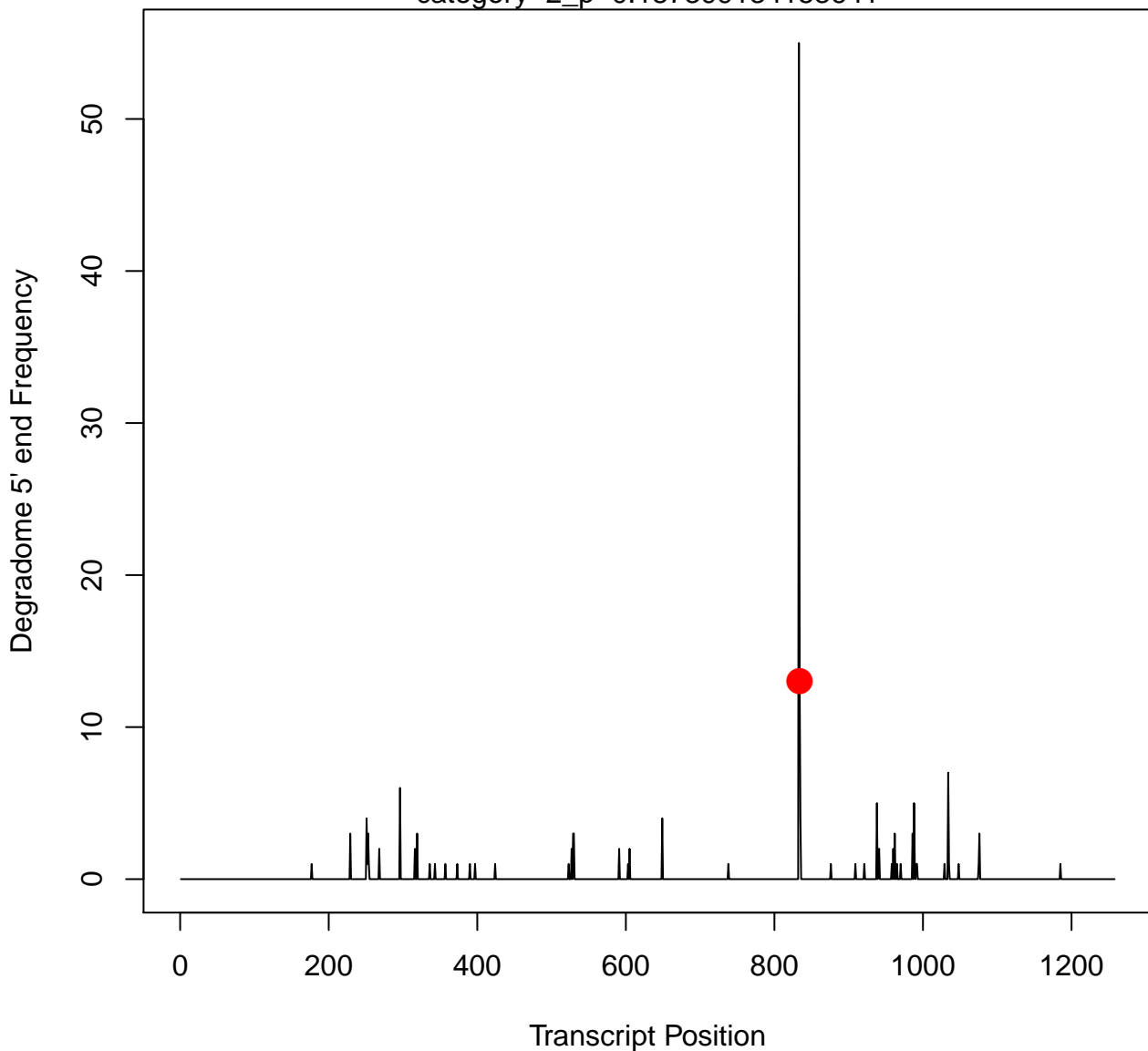

Supplement: Supplementary file 4 [file Data_Sheet_4.zip › Sit-miR156d_Seita.2G324900.1_834_TPlot.pdf]

**T=Seita.3G222000.1\_Q=Sit-miR156d\_S=2038**

category=2\_p=0.744910784471841

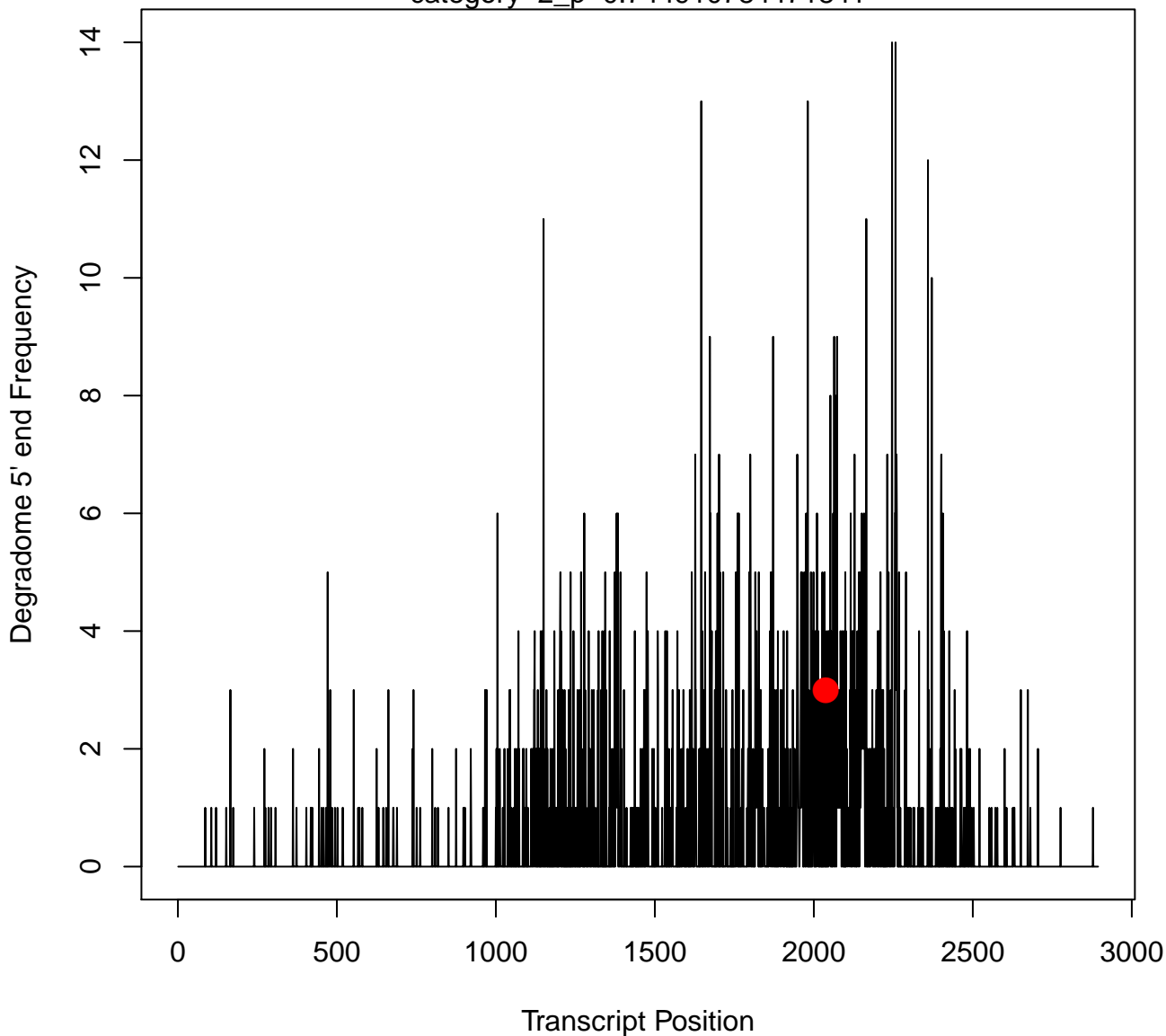

Supplement: Supplementary file 4 [file Data_Sheet_4.zip › Sit-miR156d_Seita.3G222000.1_2038_TPlot.pdf]

**T=Seita.4G270400.1\_Q=Sit-miR156d\_S=2085**

category=2\_p=0.082832056614327

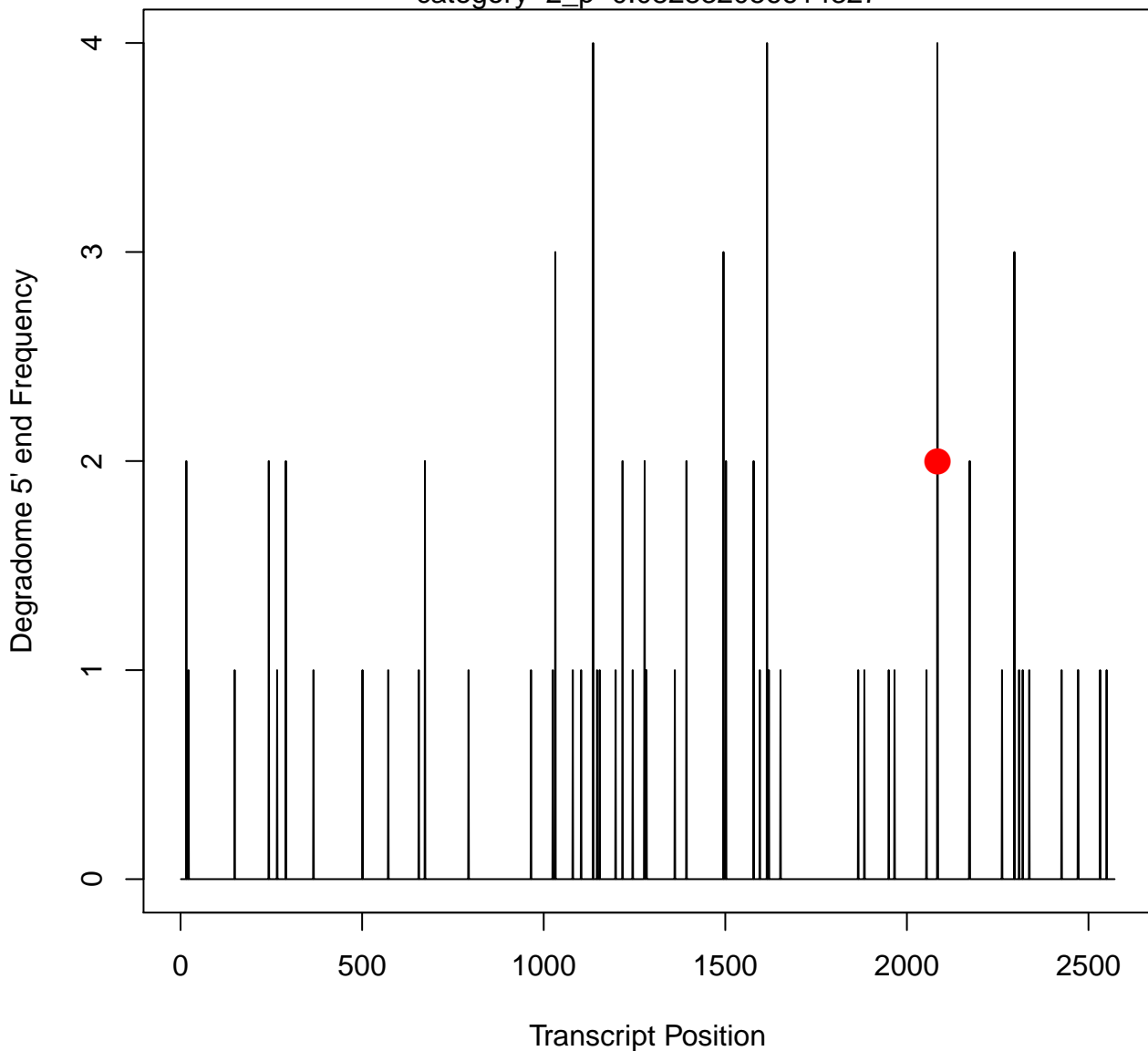

Supplement: Supplementary file 4 [file Data_Sheet_4.zip › Sit-miR156d_Seita.4G270400.1_2085_TPlot.pdf]

**T=Seita.6G176000.1\_Q=Sit-miR156d\_S=189**

category=2\_p=0.686083012891608

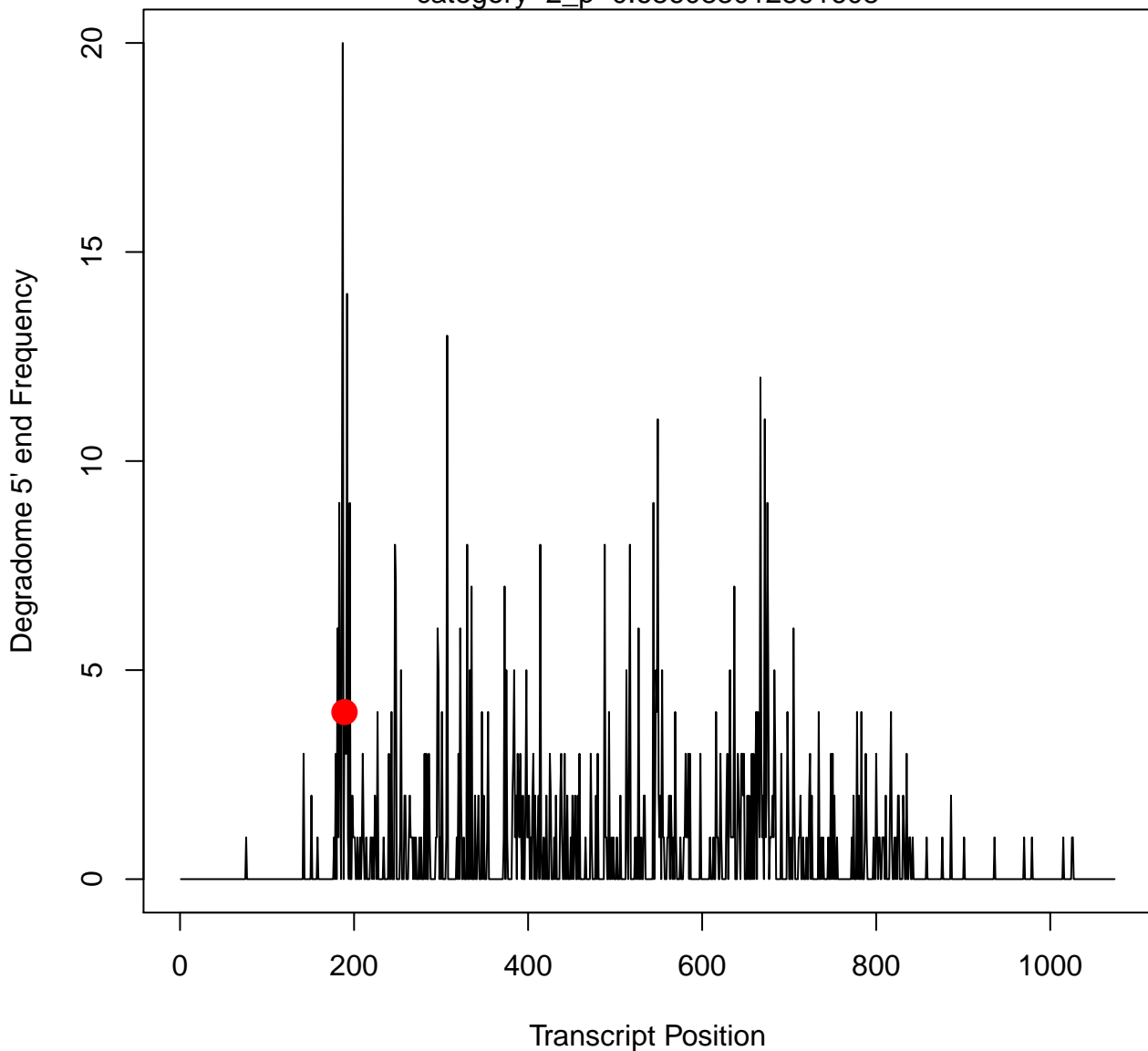

Supplement: Supplementary file 4 [file Data_Sheet_4.zip › Sit-miR156d_Seita.6G176000.1_189_TPlot.pdf]

**T=Seita.6G208700.1\_Q=Sit-miR156d\_S=164**

category=2\_p=0.158802963625695

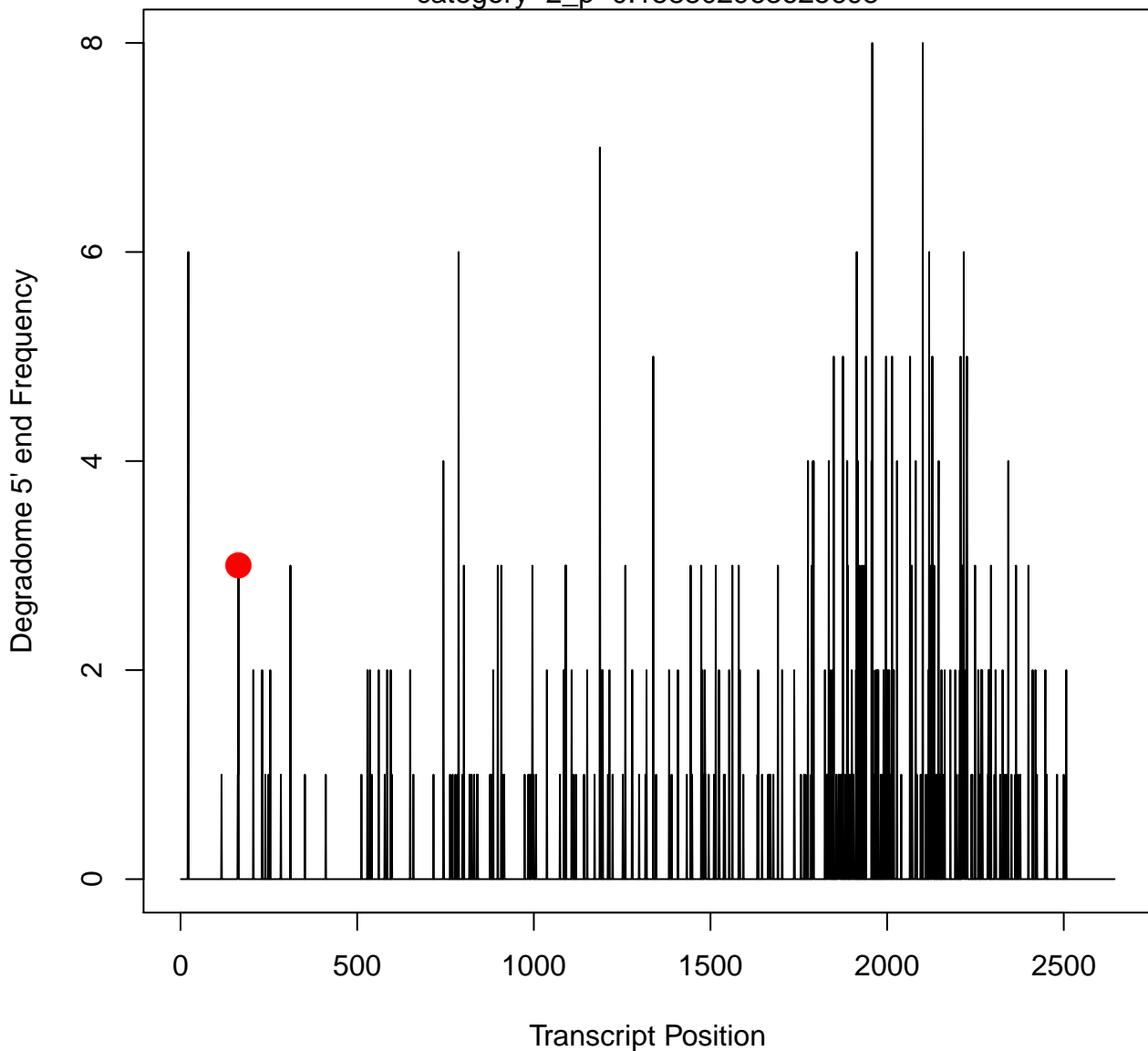

Supplement: Supplementary file 4 [file Data_Sheet_4.zip › Sit-miR156d_Seita.6G208700.1_164_TPlot.pdf]

**T=Seita.8G049200.1\_Q=Sit-miR156d\_S=569**

category=2\_p=0.923971532614208

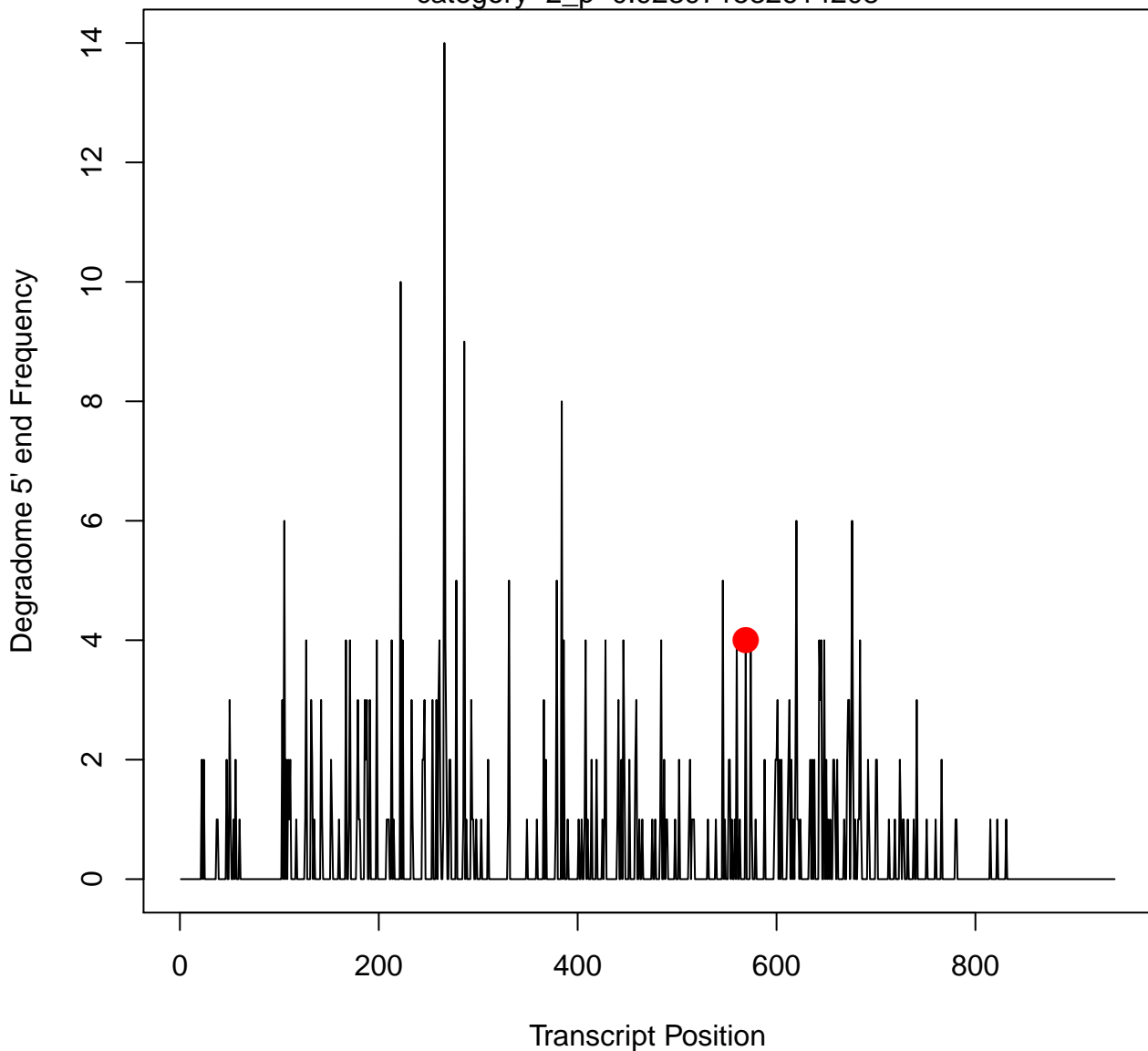

Supplement: Supplementary file 4 [file Data_Sheet_4.zip › Sit-miR156d_Seita.8G049200.1_569_TPlot.pdf]

**T=Seita.2G254300.1\_Q=Sit-miR156f\_S=1266**

category=0\_p=0.00152683684215971

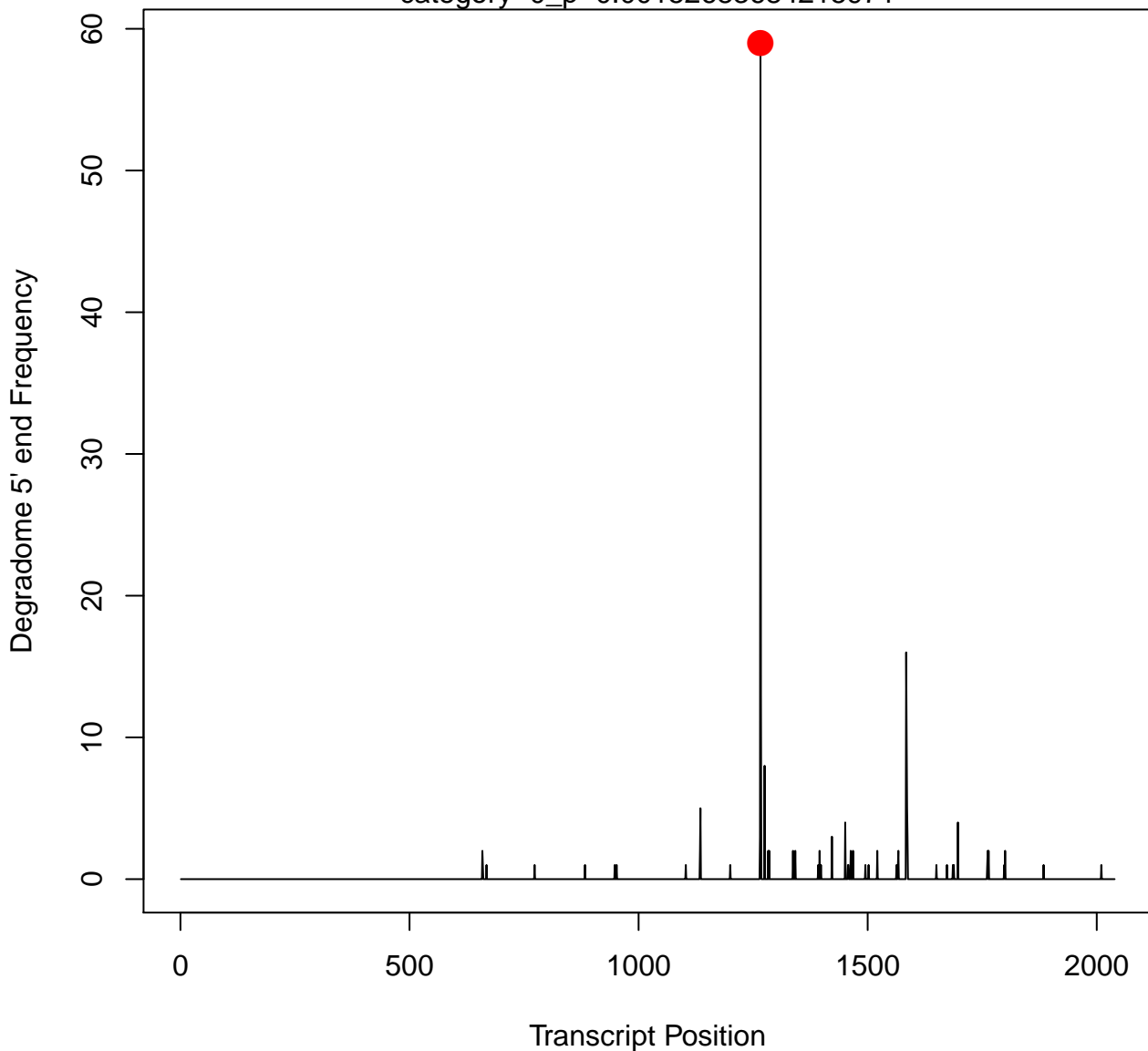

Supplement: Supplementary file 4 [file Data_Sheet_4.zip › Sit-miR156f_Seita.2G254300.1_1266_TPlot.pdf]

**T=Seita.1G091900.1\_Q=Sit-miR156g\_S=1786**

category=2\_p=0.0171442711933534

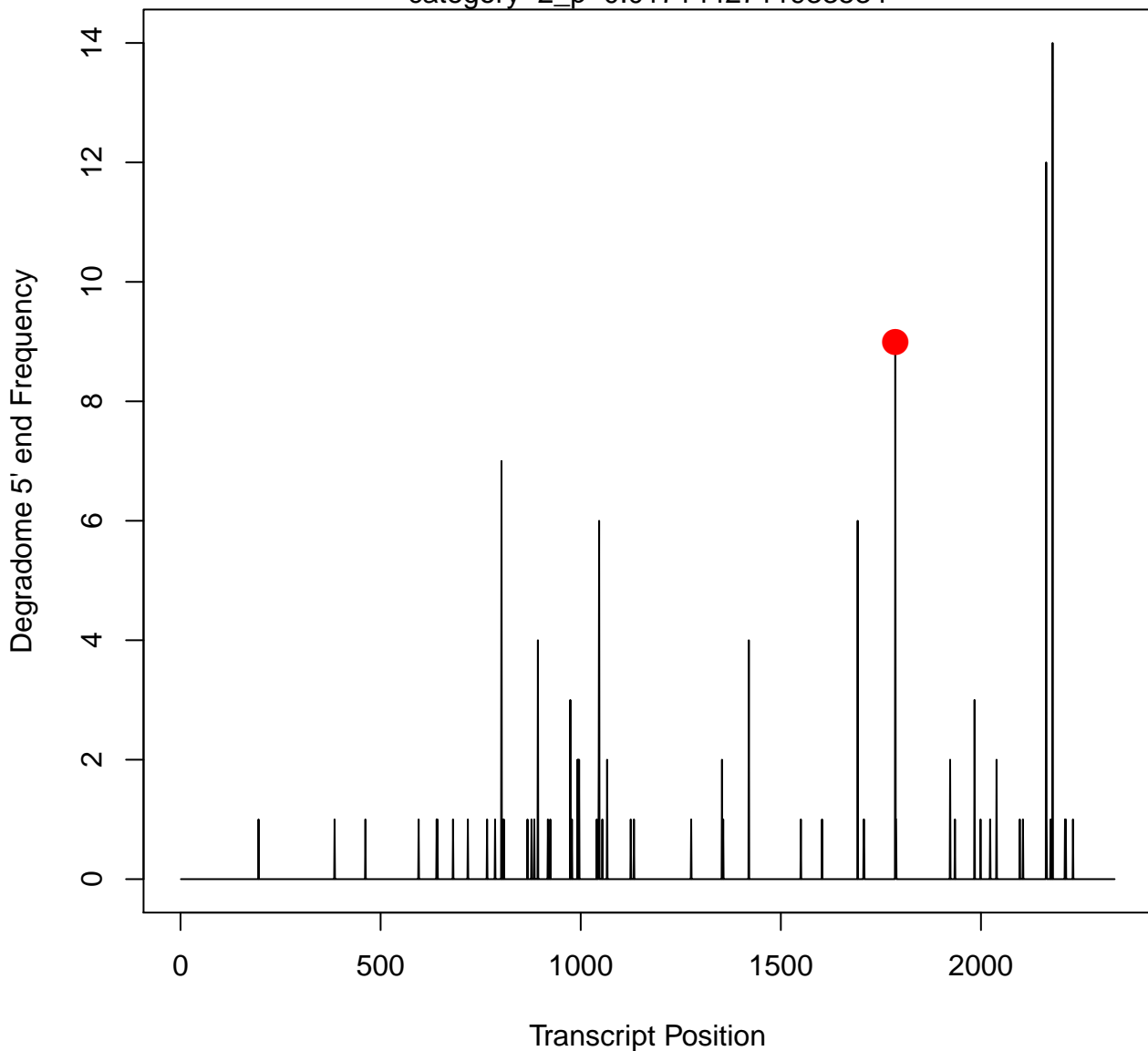

Supplement: Supplementary file 4 [file Data_Sheet_4.zip › Sit-miR156g_Seita.1G091900.1_1786_TPlot.pdf]

**T=Seita.3G179300.1\_Q=Sit-miR156g\_S=2107**

category=2\_p=0.799760881032215

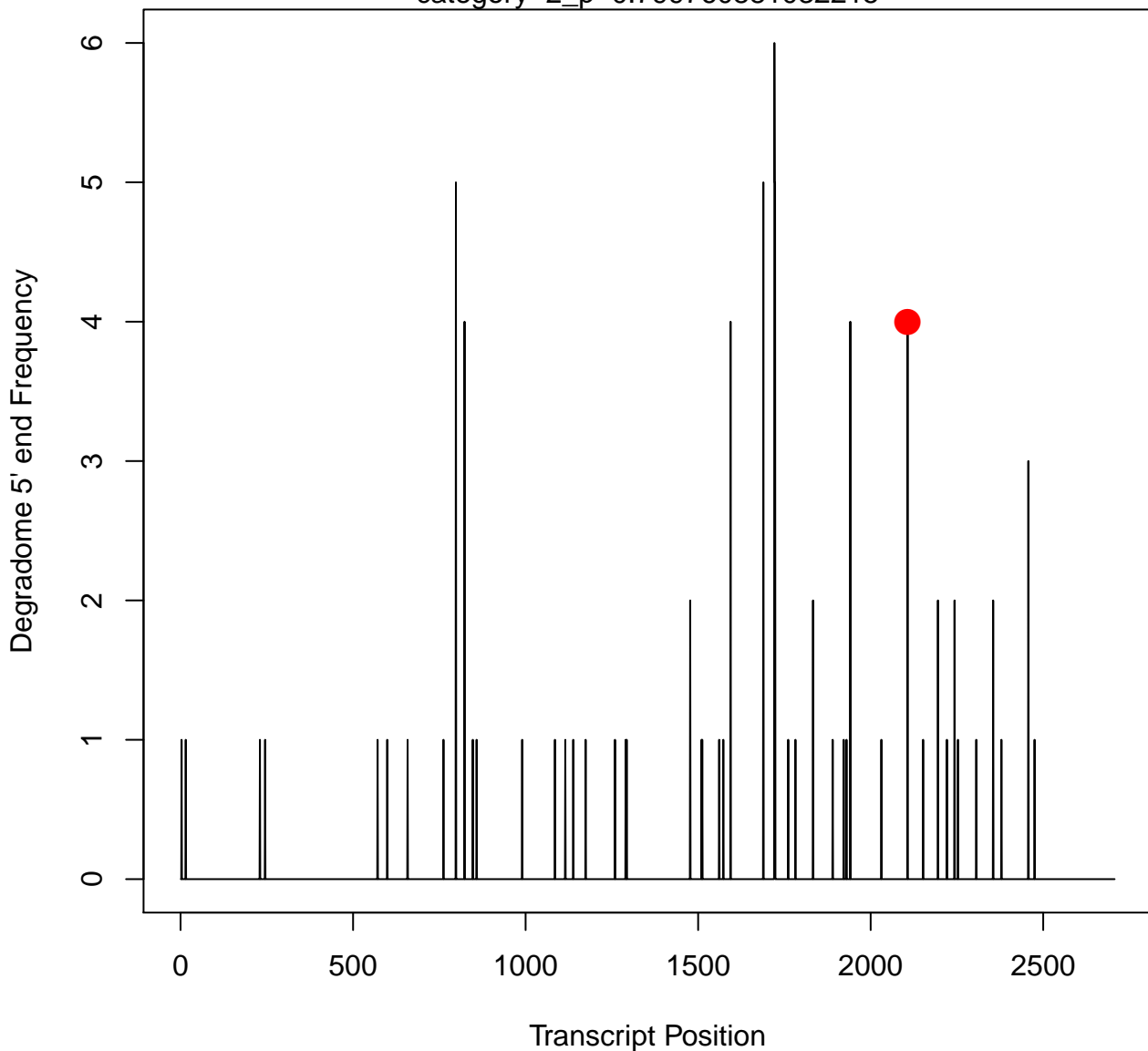

Supplement: Supplementary file 4 [file Data_Sheet_4.zip › Sit-miR156g_Seita.3G179300.1_2107_TPlot.pdf]

**T=Seita.2G324900.1\_Q=Sit-miR156h\_S=833**

category=0\_p=0.00381272216028461

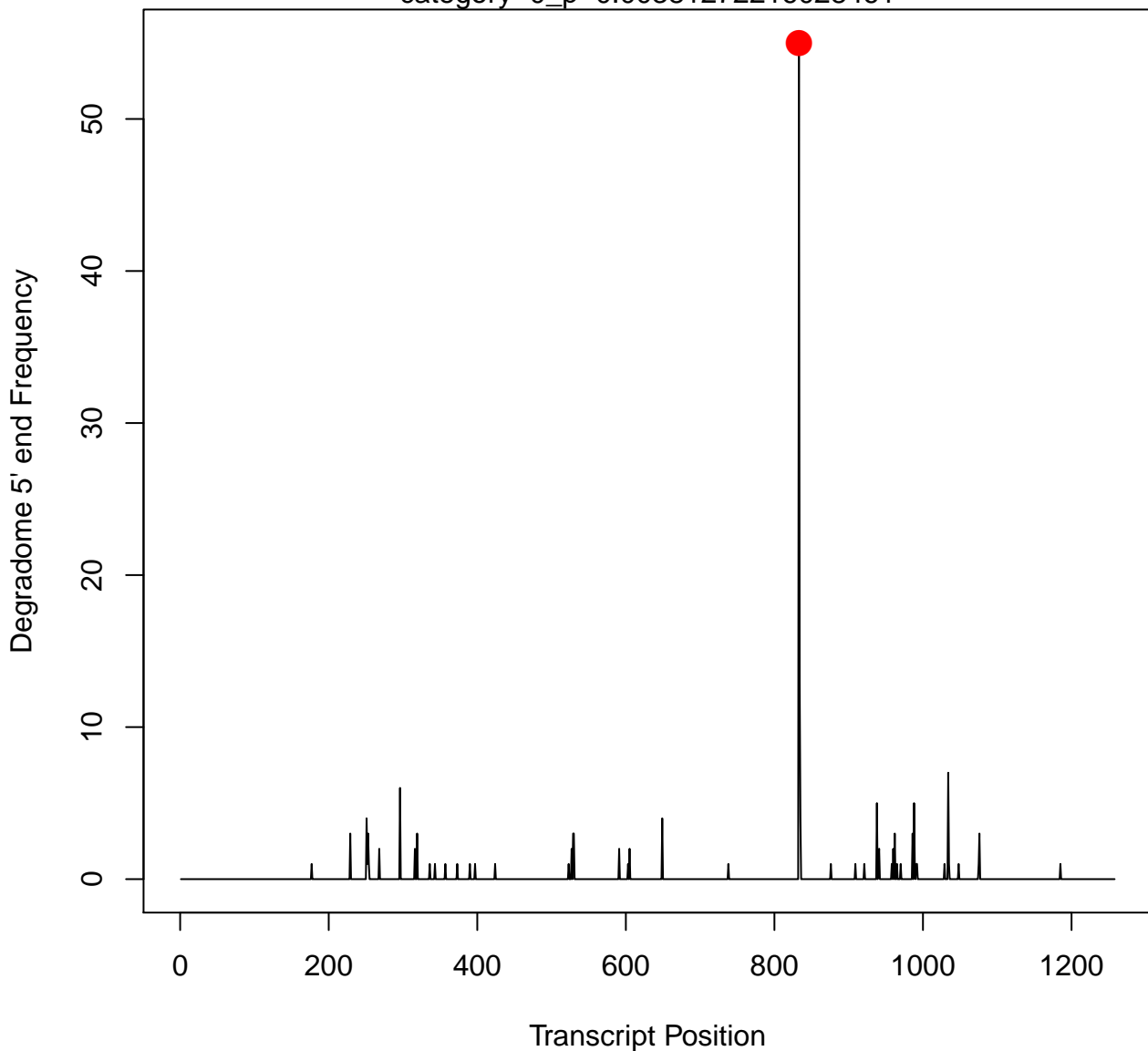

Supplement: Supplementary file 4 [file Data_Sheet_4.zip › Sit-miR156h_Seita.2G324900.1_833_TPlot.pdf]

**T=Seita.1G069300.1\_Q=Sit-miR156i\_S=873**

category=0\_p=0.000381927958281736

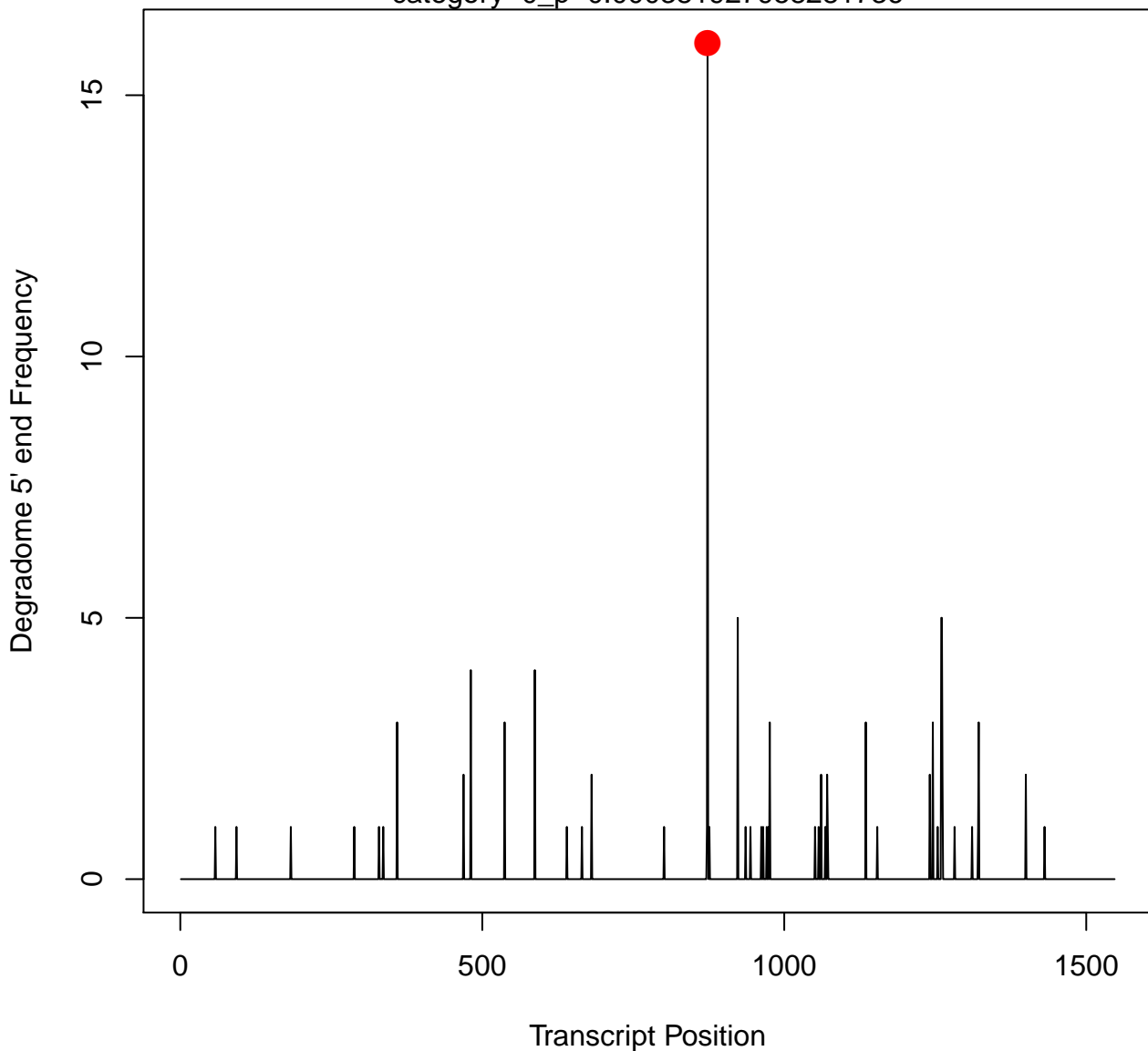

Supplement: Supplementary file 4 [file Data_Sheet_4.zip › Sit-miR156i_Seita.1G069300.1_873_TPlot.pdf]

**T=Seita.8G124900.1\_Q=Sit-miR156i\_S=1295**

category=0\_p=0.00571362845255208

Degradsome 5' end Frequency

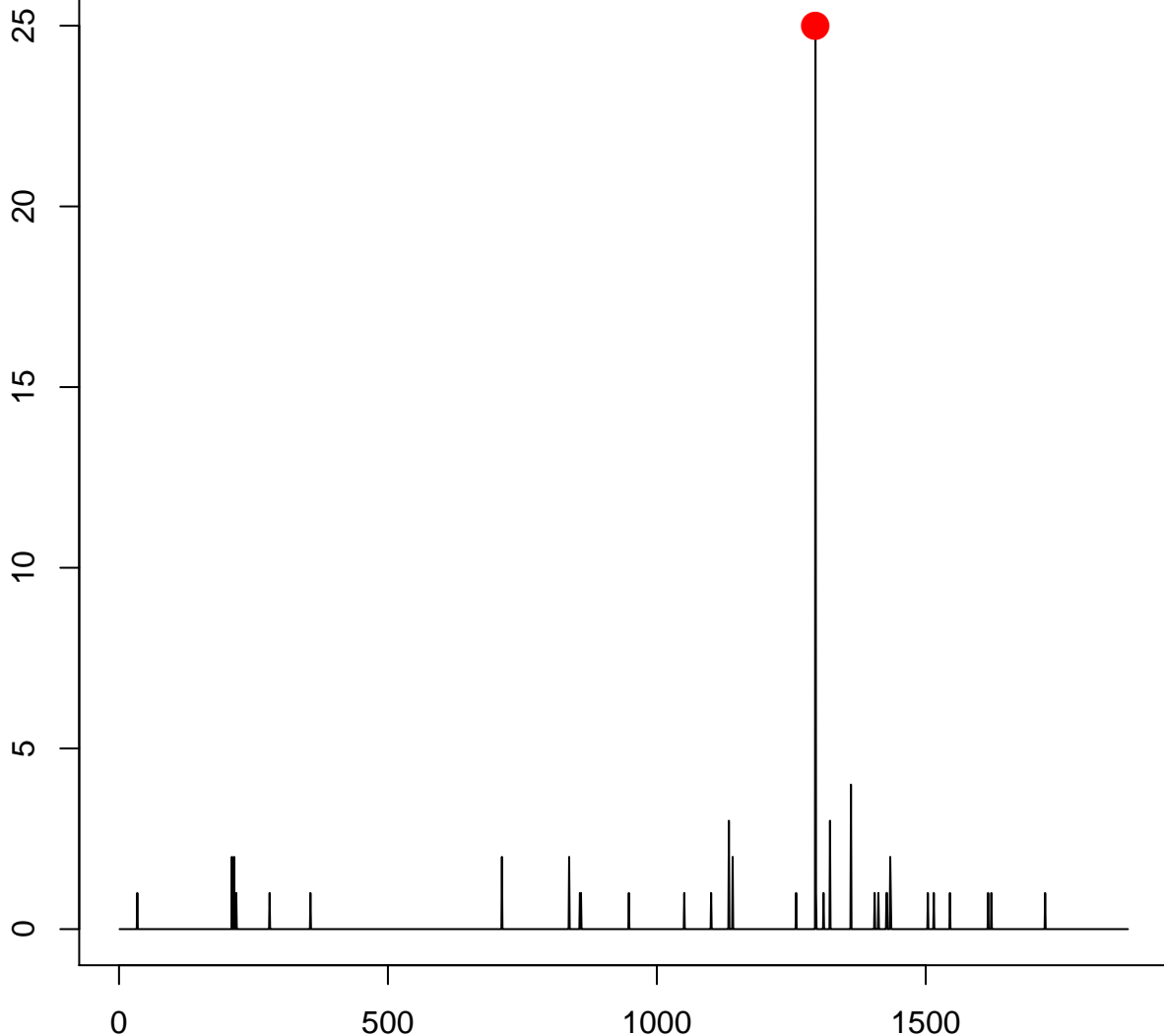

Transcript Position

Supplement: Supplementary file 4 [file Data_Sheet_4.zip › Sit-miR156i_Seita.8G124900.1_1295_TPlot.pdf]

**T=Seita.9G012800.1\_Q=Sit-miR156j\_S=2149**

category=2\_p=0.516306242353877

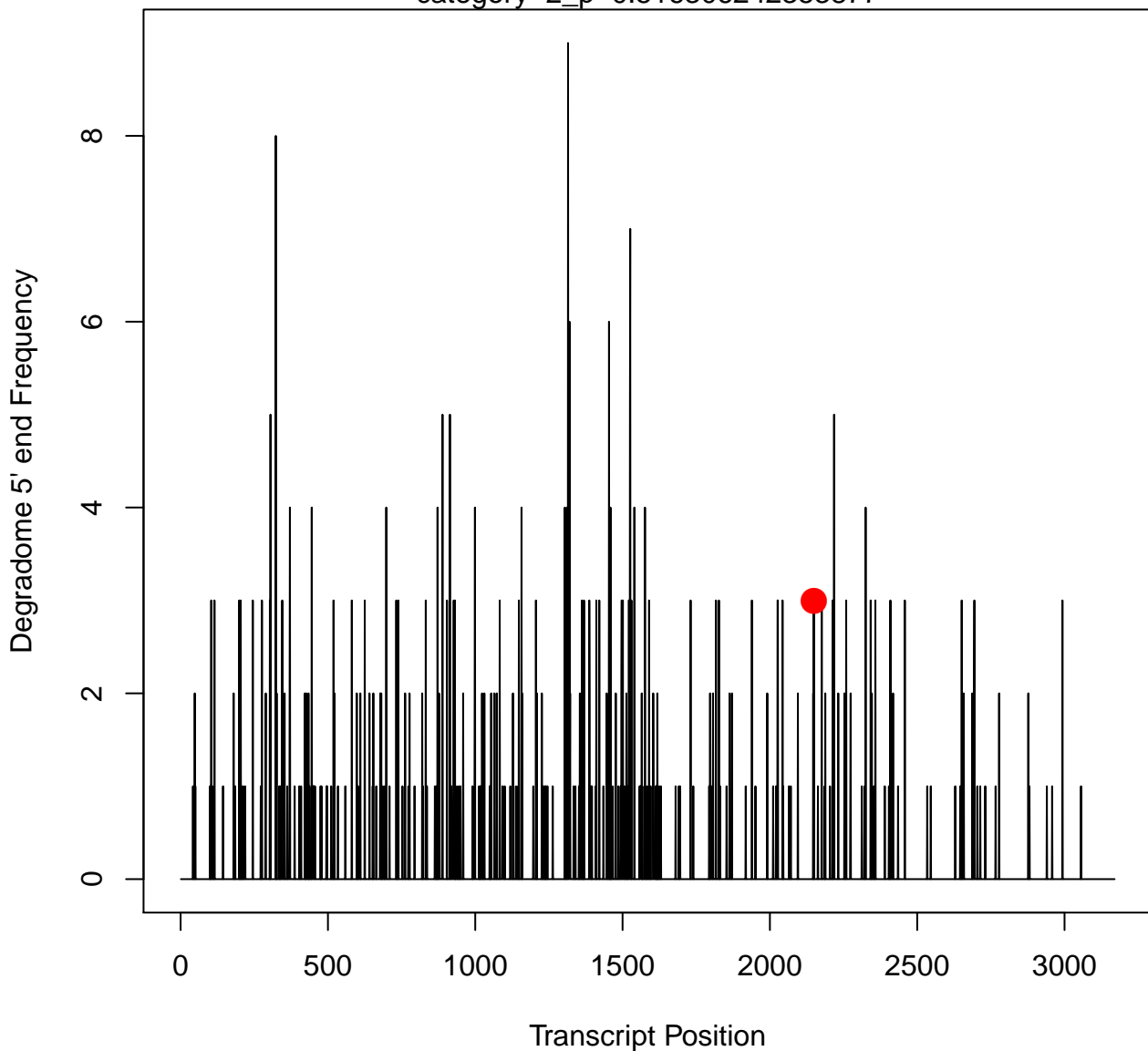

Supplement: Supplementary file 4 [file Data_Sheet_4.zip › Sit-miR156j_Seita.9G012800.1_2149_TPlot.pdf]

**T=Seita.2G014000.1\_Q=Sit-miR159a\_S=1233**

category=2\_p=0.454063578351316

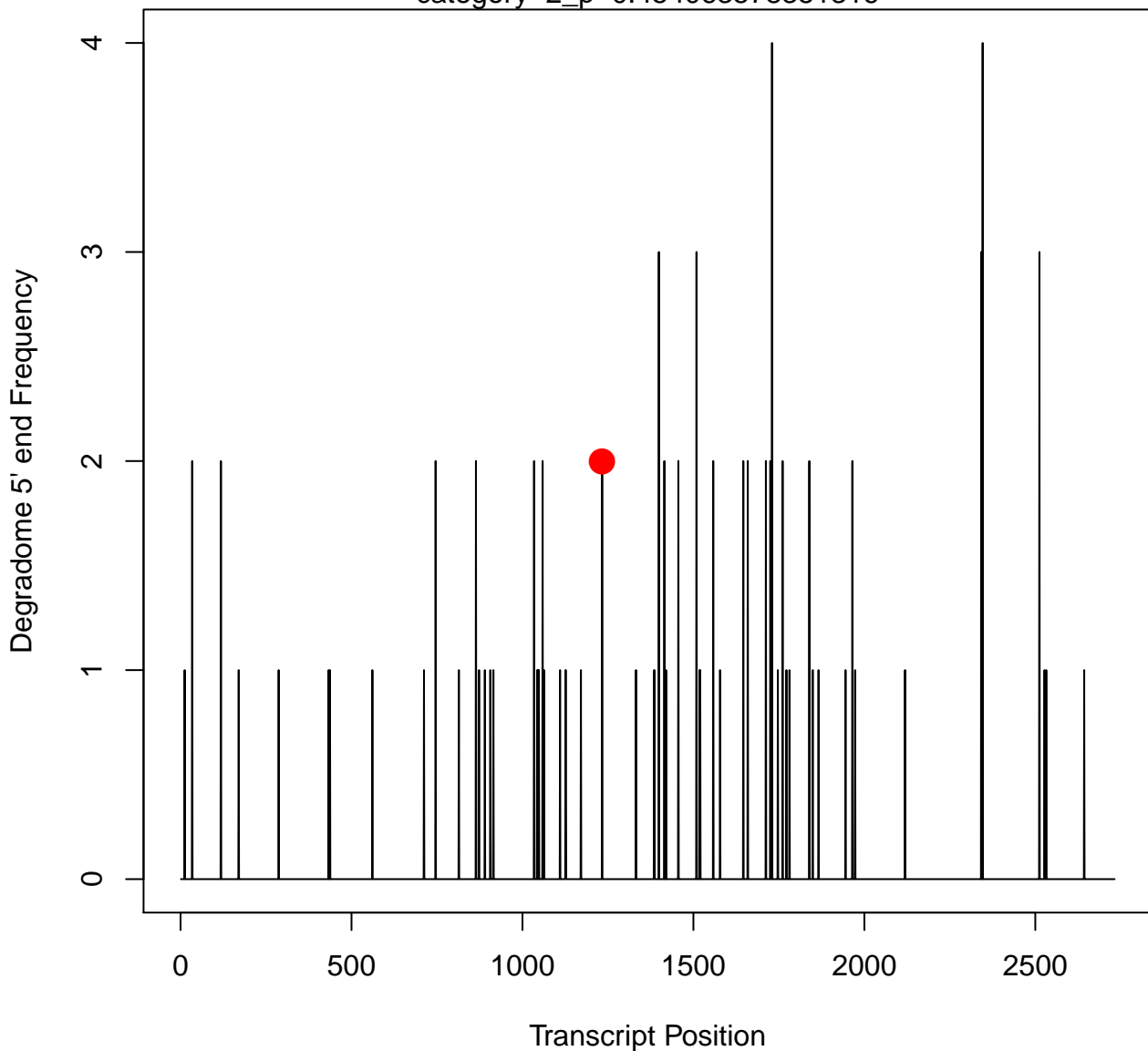

Supplement: Supplementary file 4 [file Data_Sheet_4.zip › Sit-miR159a_Seita.2G014000.1_1233_TPlot.pdf]

**T=Seita.2G193900.1\_Q=Sit-miR159a\_S=1942**

category=2\_p=0.886835177180351

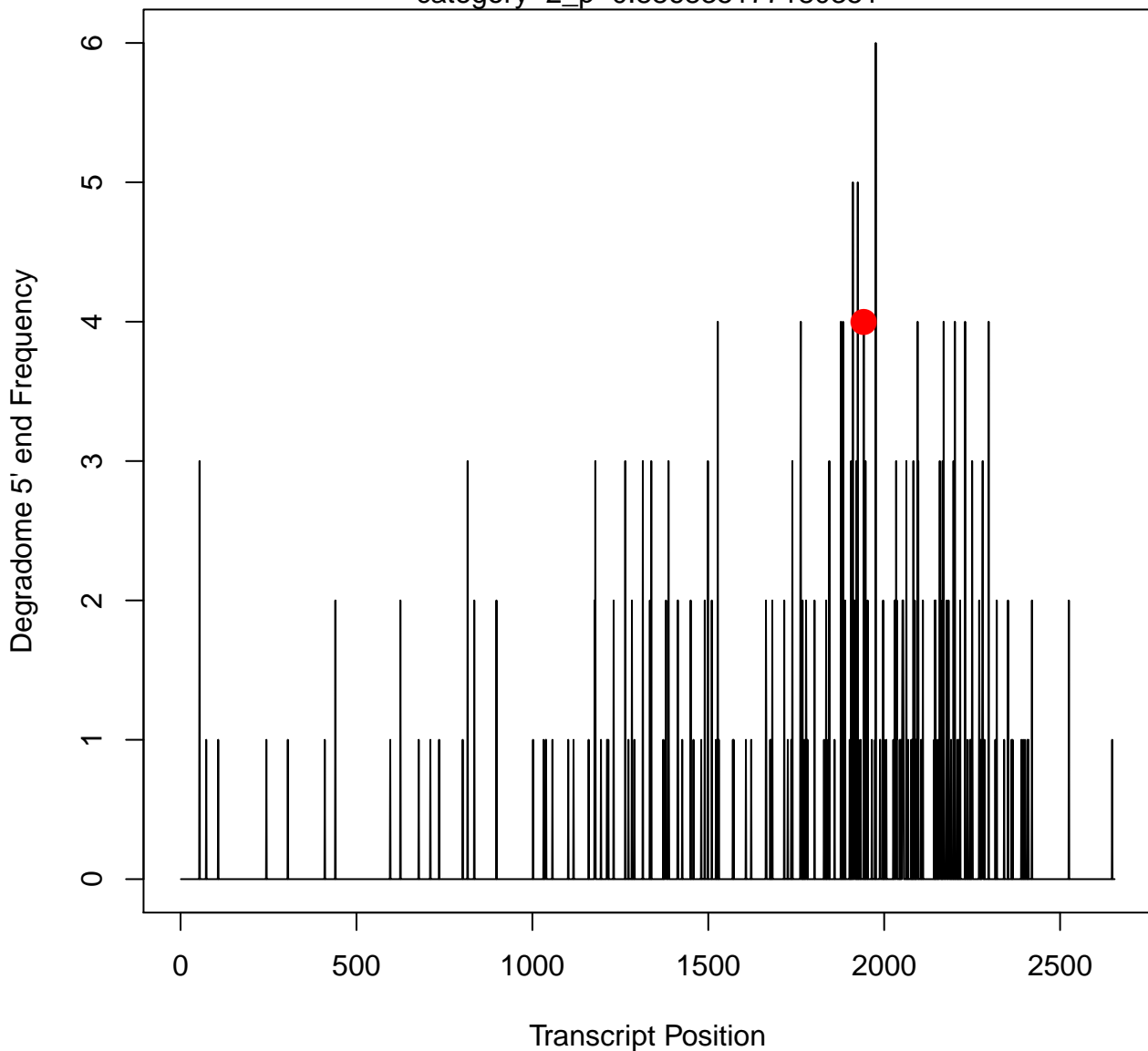

Supplement: Supplementary file 4 [file Data_Sheet_4.zip › Sit-miR159a_Seita.2G193900.1_1942_TPlot.pdf]

**T=Seita.2G258400.1\_Q=Sit-miR159a\_S=480**

category=2\_p=0.999999998058501

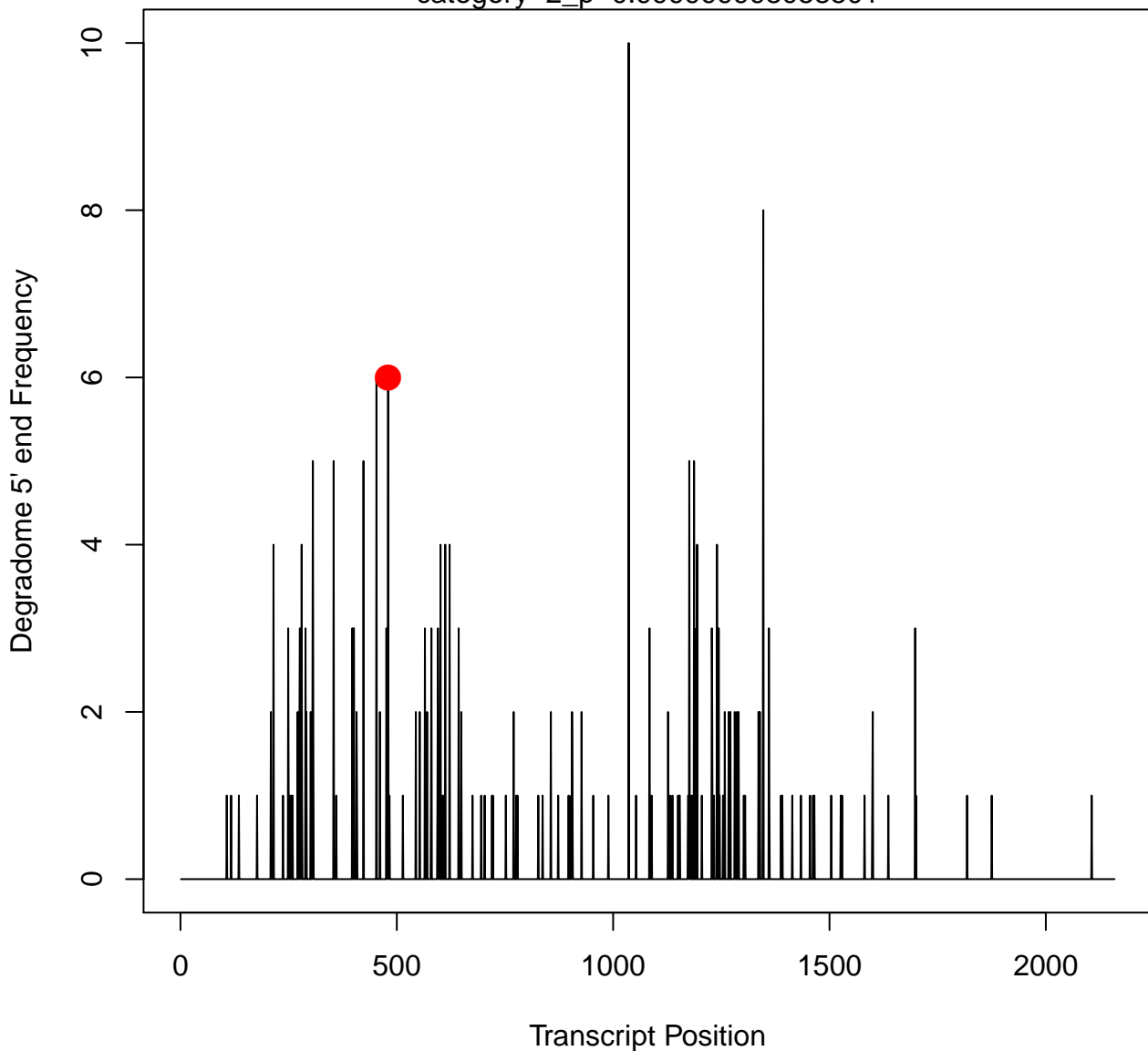

Supplement: Supplementary file 4 [file Data_Sheet_4.zip › Sit-miR159a_Seita.2G258400.1_480_TPlot.pdf]

**T=Seita.2G434400.1\_Q=Sit-miR159a\_S=362**

category=2\_p=0.43485157444258

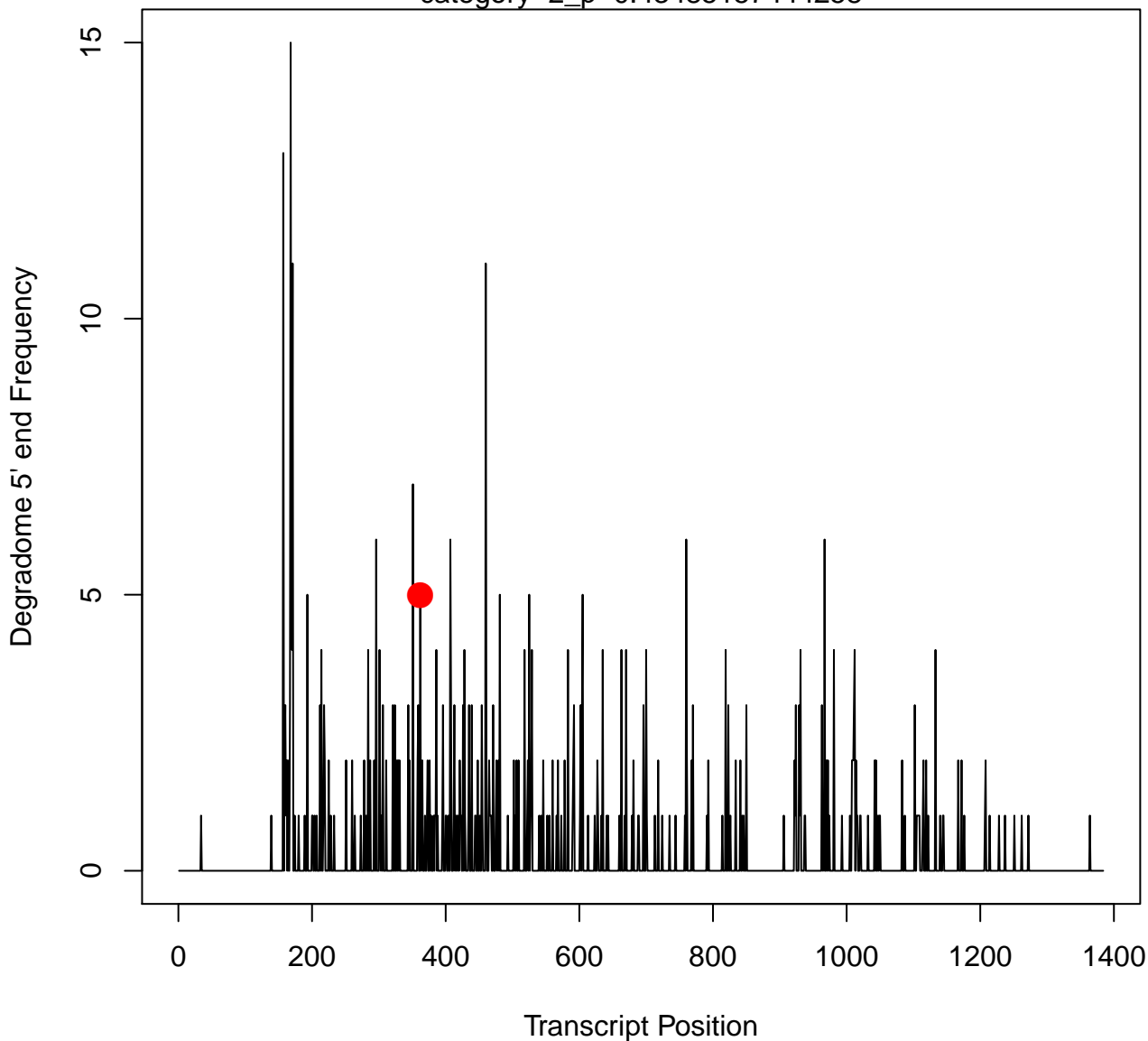

Supplement: Supplementary file 4 [file Data_Sheet_4.zip › Sit-miR159a_Seita.2G434400.1_362_TPlot.pdf]

**T=Seita.3G059500.1\_Q=Sit-miR159a\_S=1421**

category=2\_p=0.999999988865518

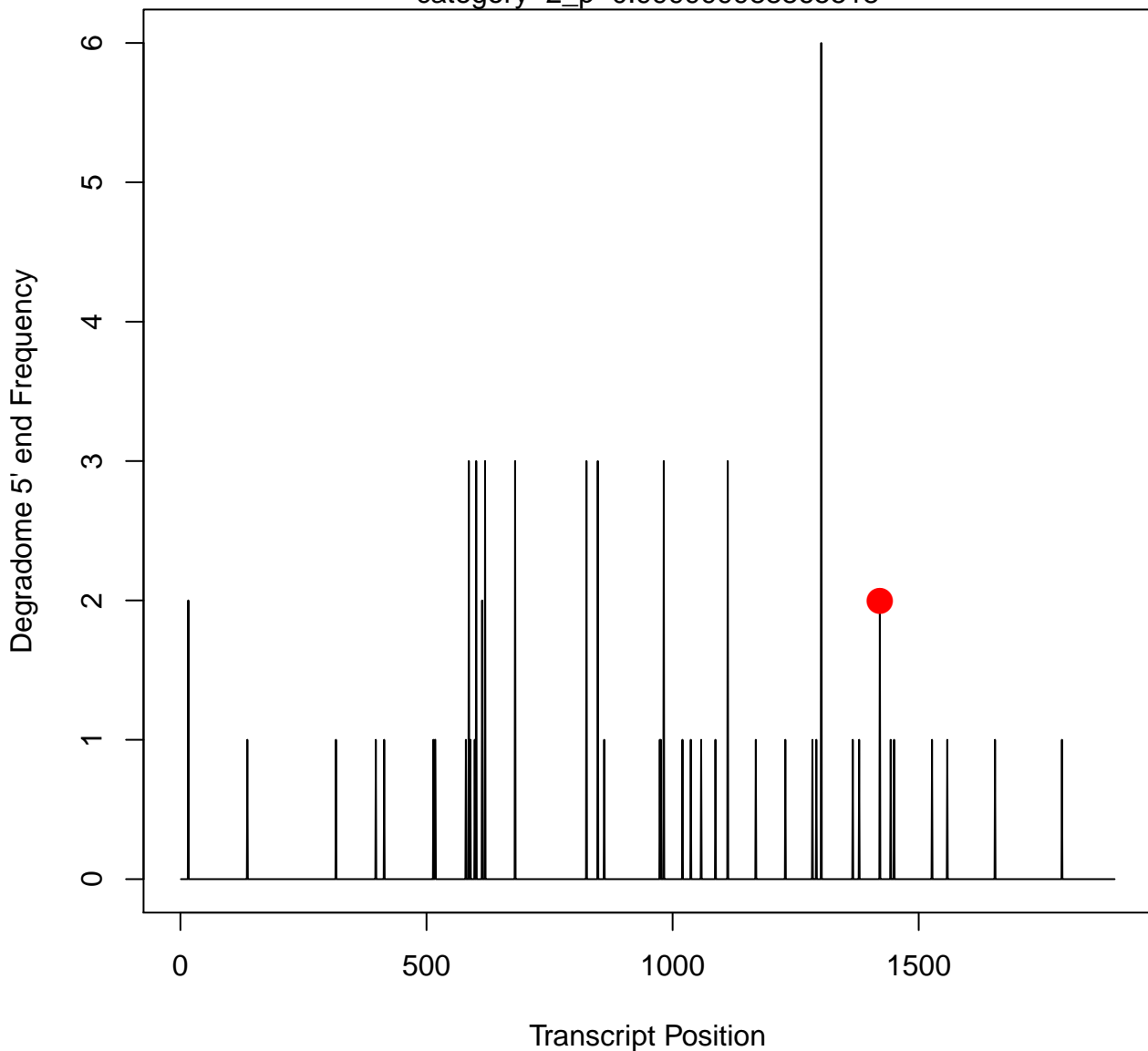

Supplement: Supplementary file 4 [file Data_Sheet_4.zip › Sit-miR159a_Seita.3G059500.1_1421_TPlot.pdf]

**T=Seita.3G096800.1\_Q=Sit-miR159a\_S=1199**

category=2\_p=0.99999998605871

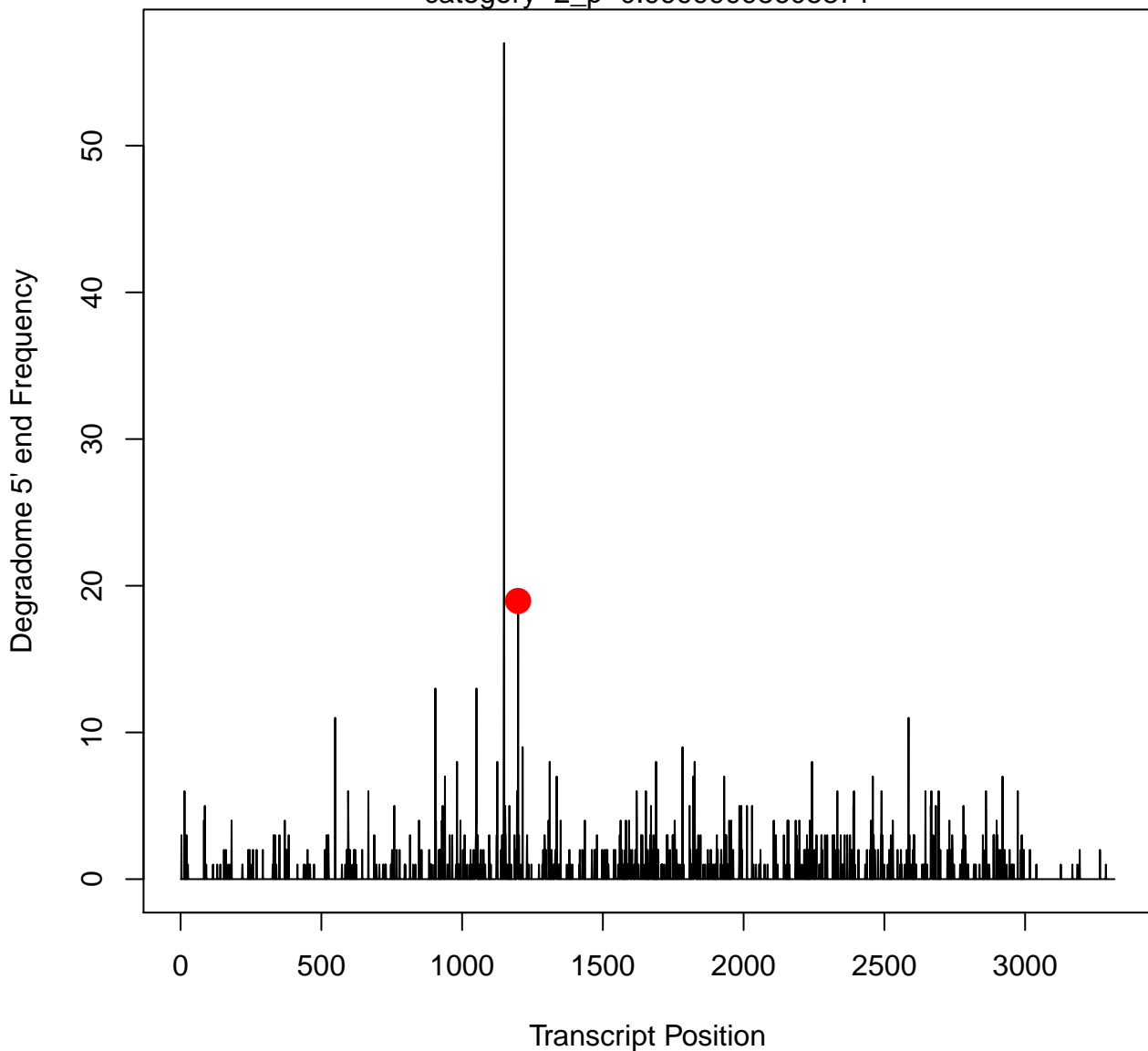

Supplement: Supplementary file 4 [file Data_Sheet_4.zip › Sit-miR159a_Seita.3G096800.1_1199_TPlot.pdf]

**T=Seita.3G134100.1\_Q=Sit-miR159a\_S=32**

category=2\_p=0.999999997808665

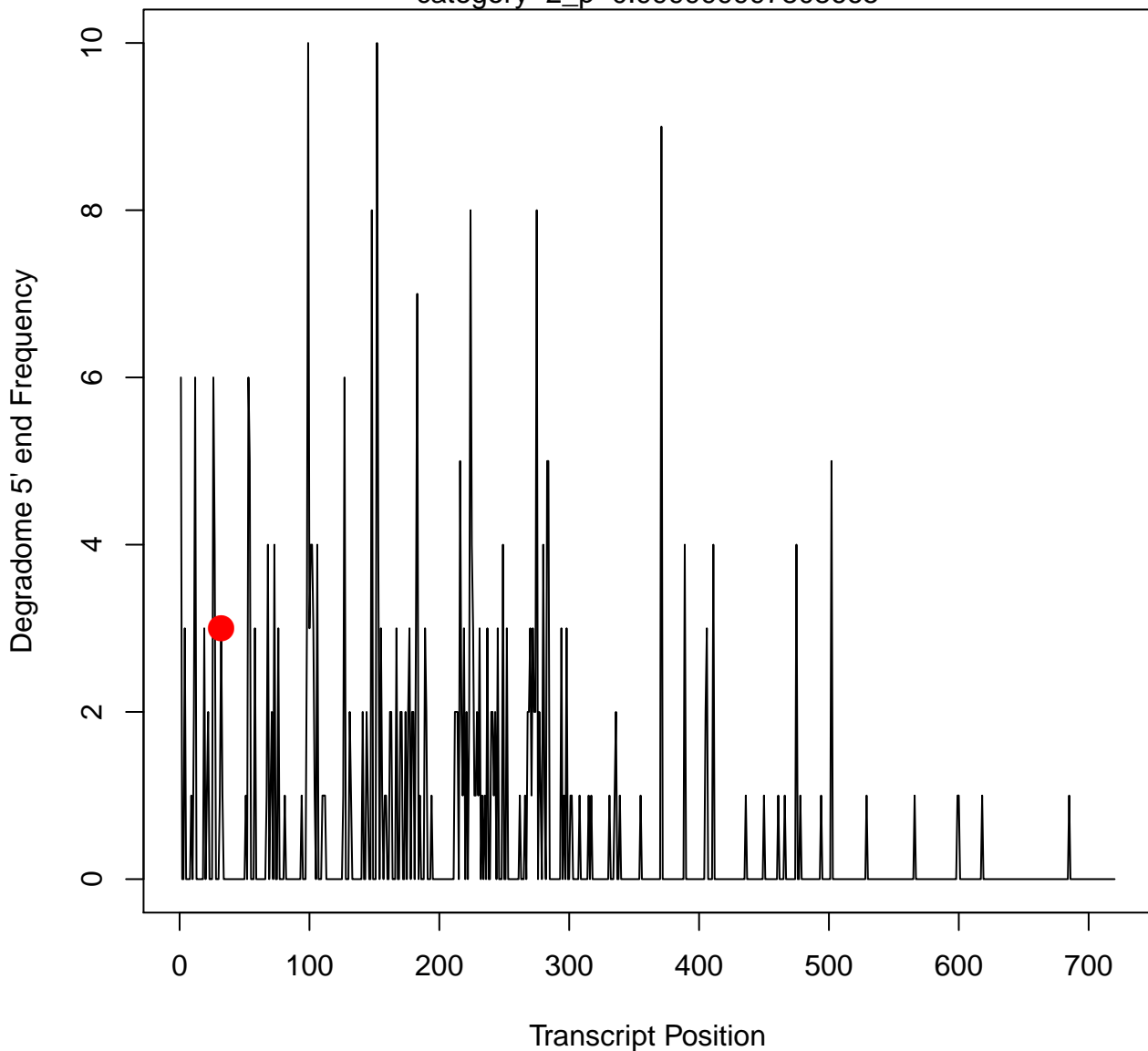

Supplement: Supplementary file 4 [file Data_Sheet_4.zip › Sit-miR159a_Seita.3G134100.1_32_TPlot.pdf]

**T=Seita.3G379300.1\_Q=Sit-miR159a\_S=881**

category=2\_p=0.837285518550466

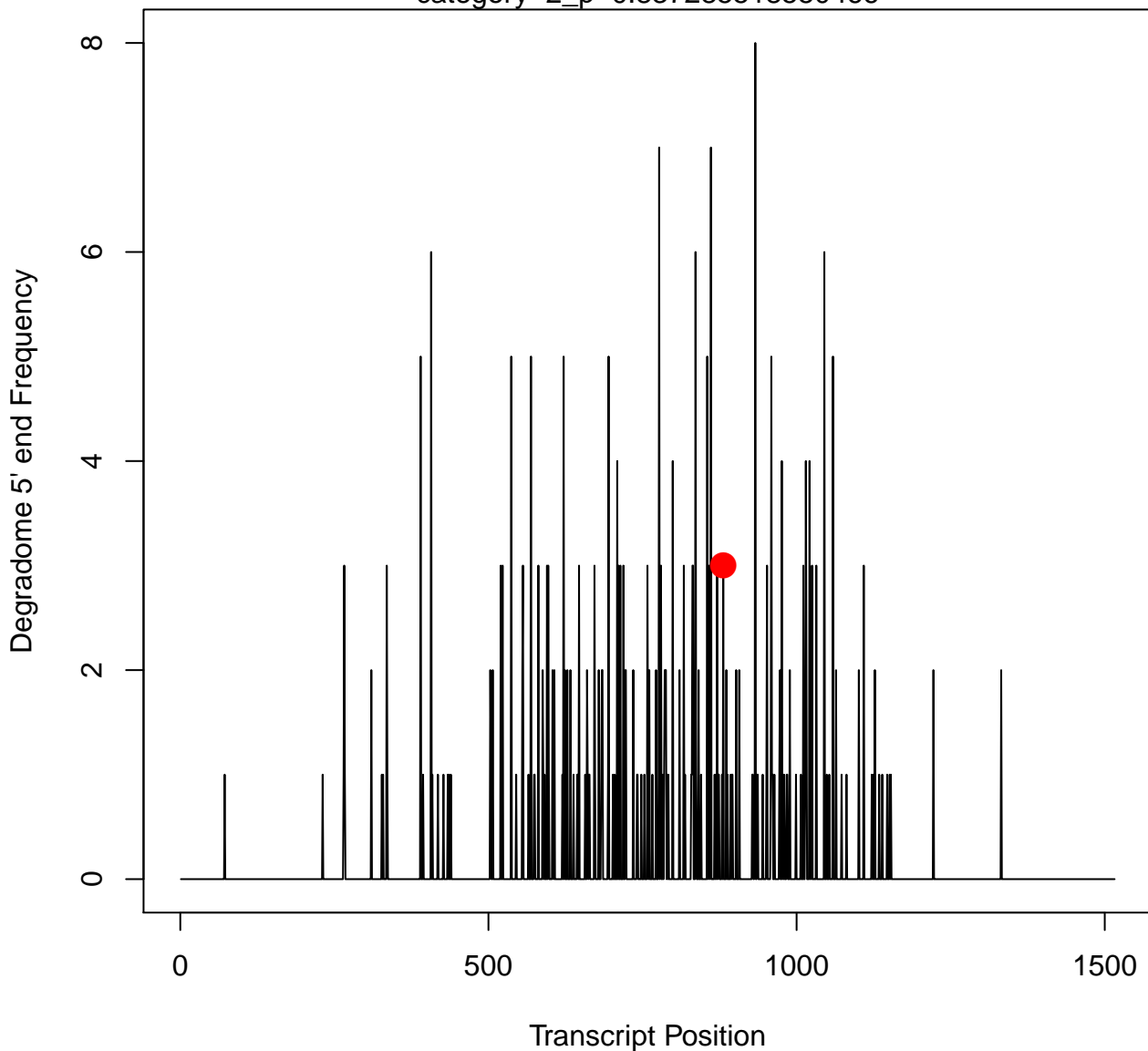

Supplement: Supplementary file 4 [file Data_Sheet_4.zip › Sit-miR159a_Seita.3G379300.1_881_TPlot.pdf]

**T=Seita.4G020800.1\_Q=Sit-miR159a\_S=284**

category=2\_p=0.999999995228346

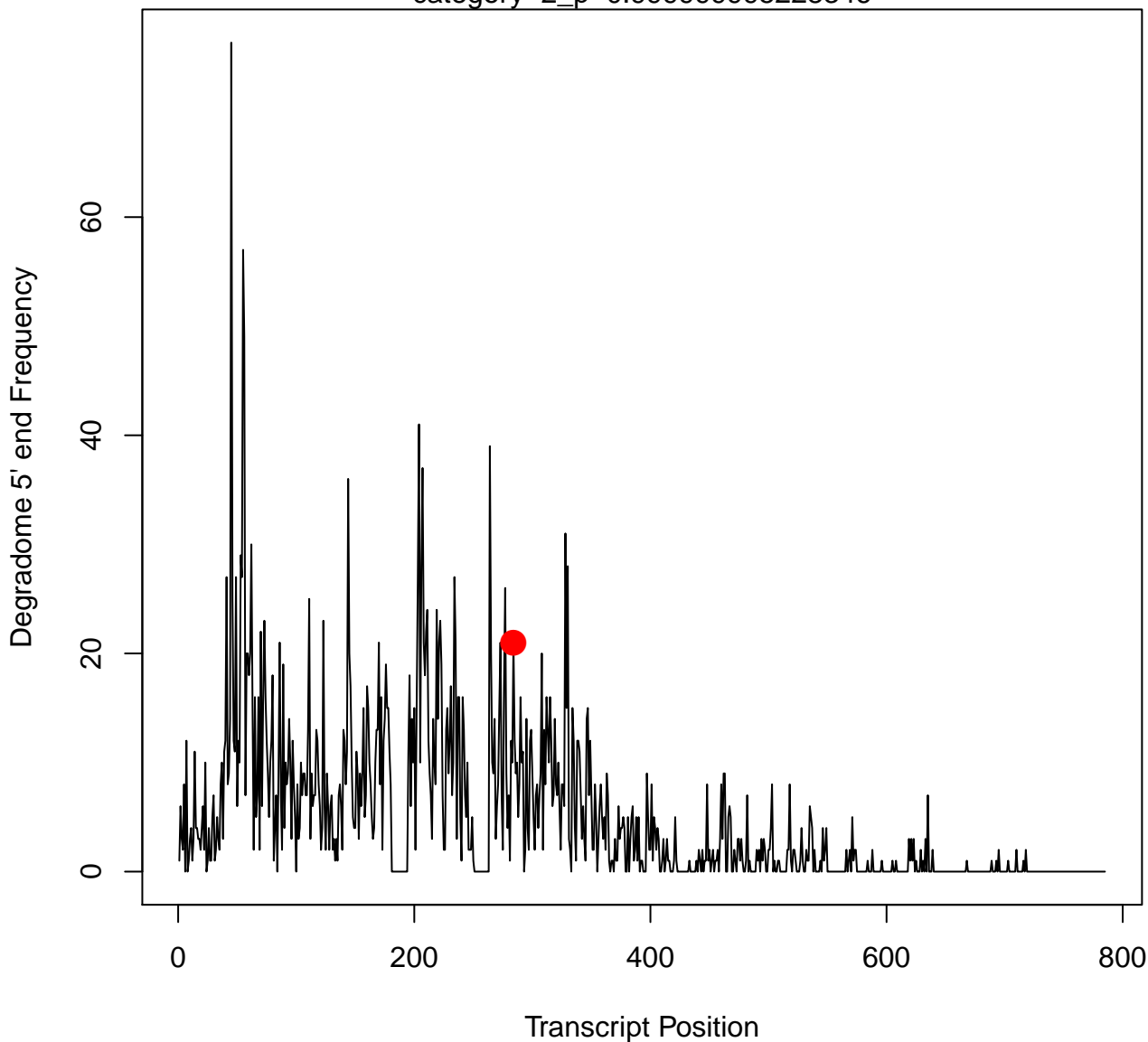

Supplement: Supplementary file 4 [file Data_Sheet_4.zip › Sit-miR159a_Seita.4G020800.1_284_TPlot.pdf]

**T=Seita.4G067800.1\_Q=Sit-miR159a\_S=421**

category=2\_p=0.969065403726301

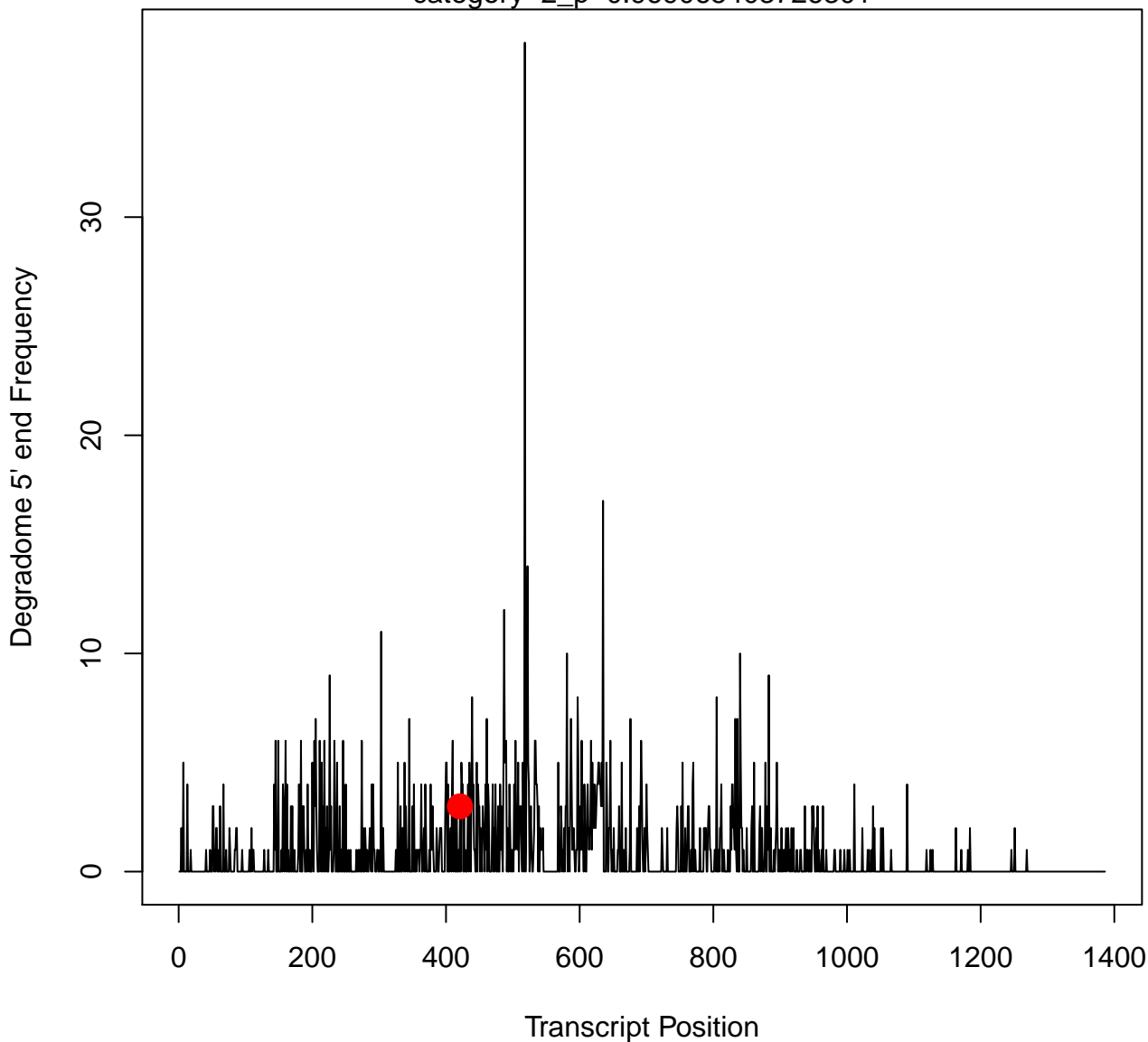

Supplement: Supplementary file 4 [file Data_Sheet_4.zip › Sit-miR159a_Seita.4G067800.1_421_TPlot.pdf]

**T=Seita.4G265500.1\_Q=Sit-miR159a\_S=936**

category=2\_p=0.98603715141141

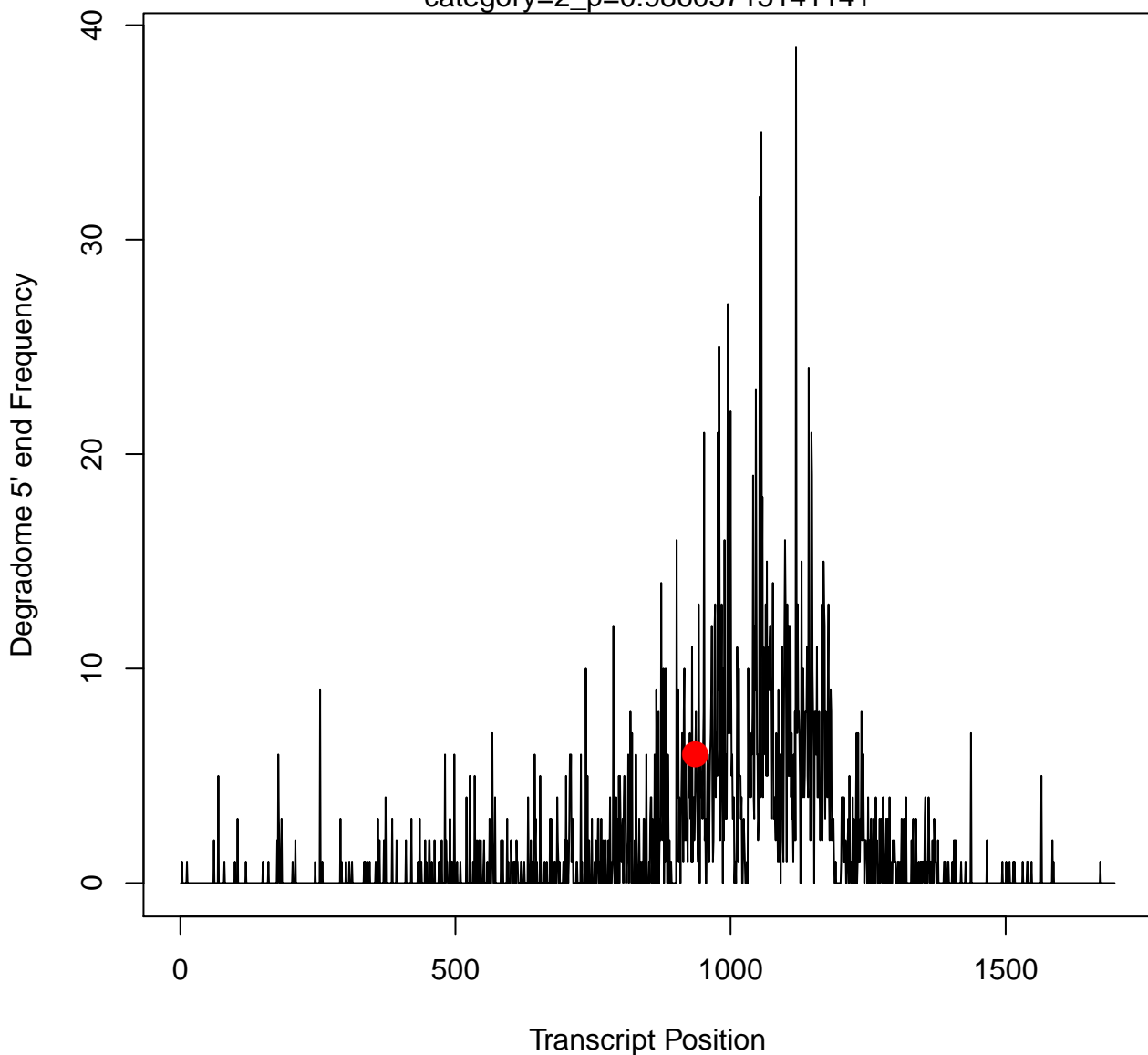

Supplement: Supplementary file 4 [file Data_Sheet_4.zip › Sit-miR159a_Seita.4G265500.1_936_TPlot.pdf]

**T=Seita.6G247500.1\_Q=Sit-miR159a\_S=1051**

category=2\_p=0.999999980298128

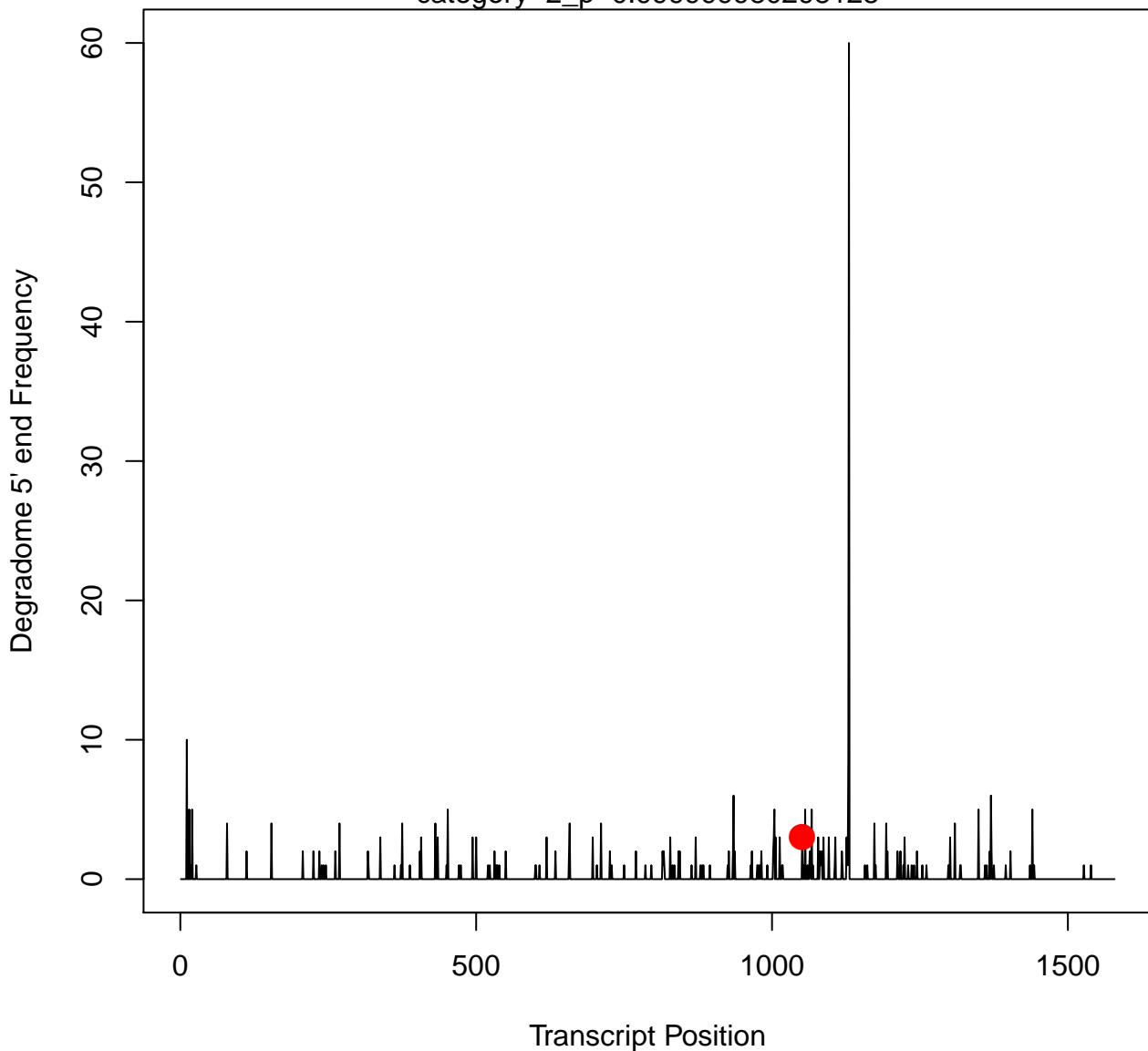

Supplement: Supplementary file 4 [file Data_Sheet_4.zip › Sit-miR159a_Seita.6G247500.1_1051_TPlot.pdf]

**T=Seita.7G085500.1\_Q=Sit-miR159a\_S=2417**

category=2\_p=0.999999999027862

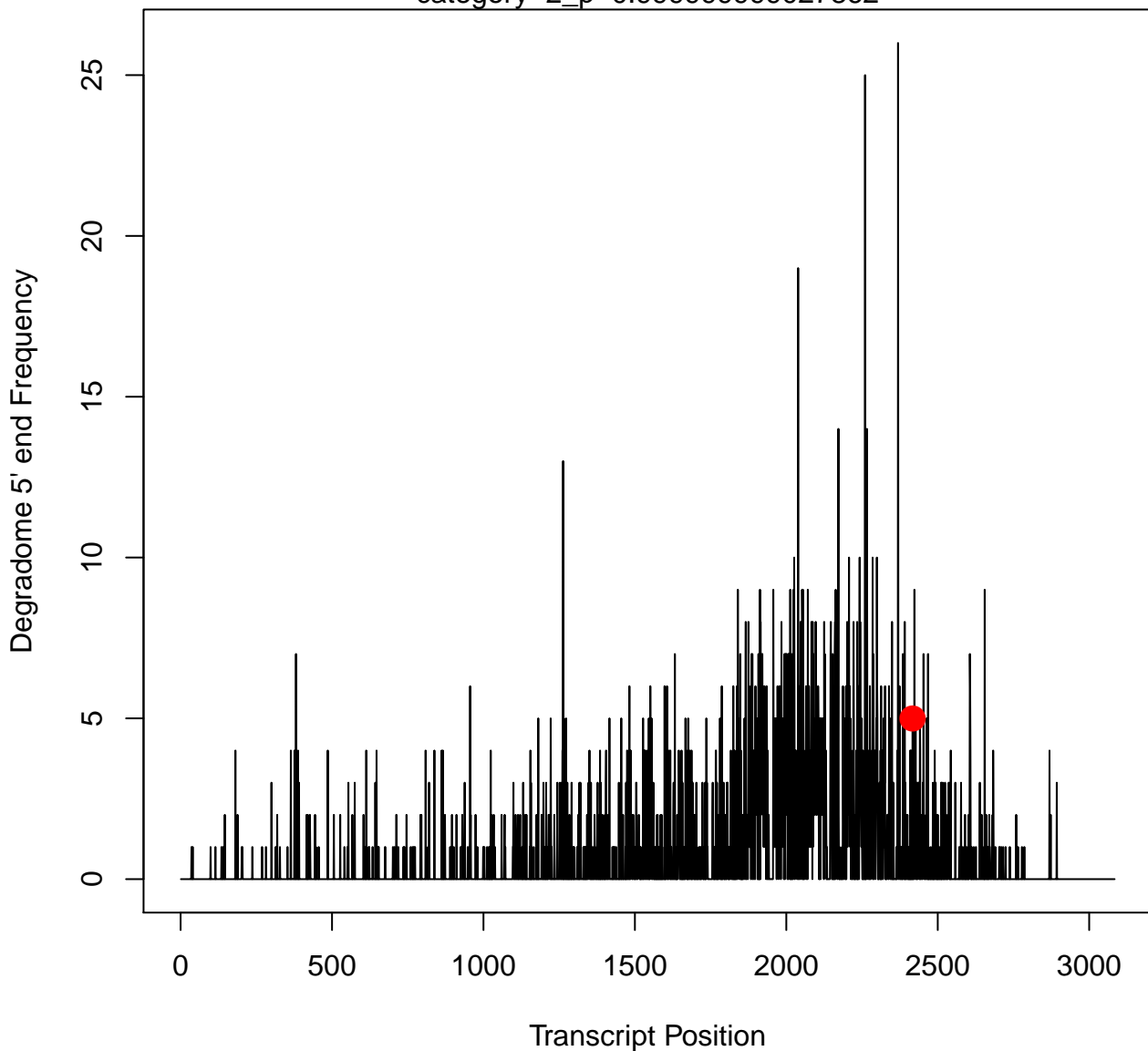

Supplement: Supplementary file 4 [file Data_Sheet_4.zip › Sit-miR159a_Seita.7G085500.1_2417_TPlot.pdf]

**T=Seita.8G199800.1\_Q=Sit-miR159a\_S=3572**

category=2\_p=0.992886633406412

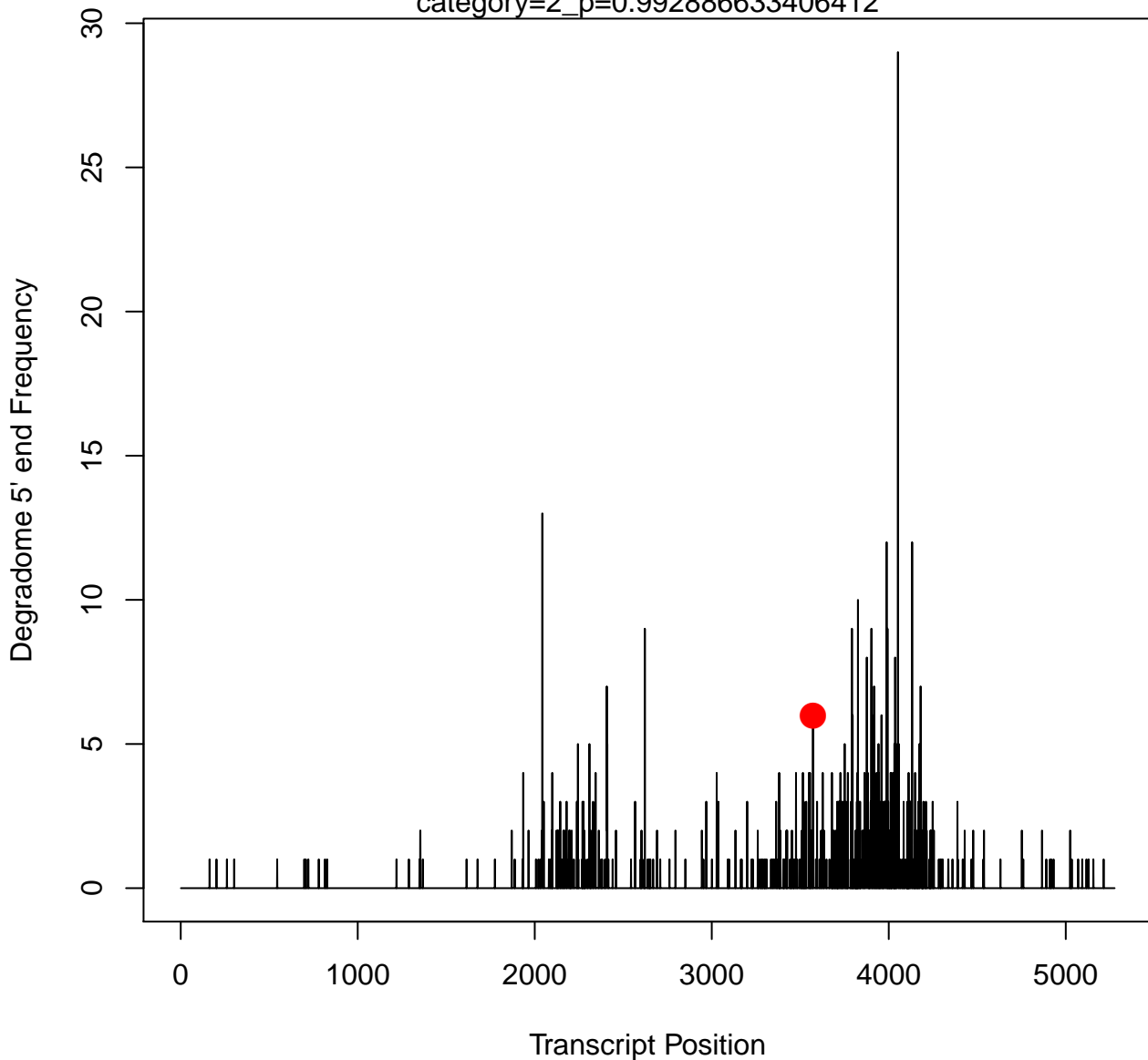

Supplement: Supplementary file 4 [file Data_Sheet_4.zip › Sit-miR159a_Seita.8G199800.1_3572_TPlot.pdf]

**T=Seita.9G294600.1\_Q=Sit-miR159a\_S=1202**

category=2\_p=0.999973314705647

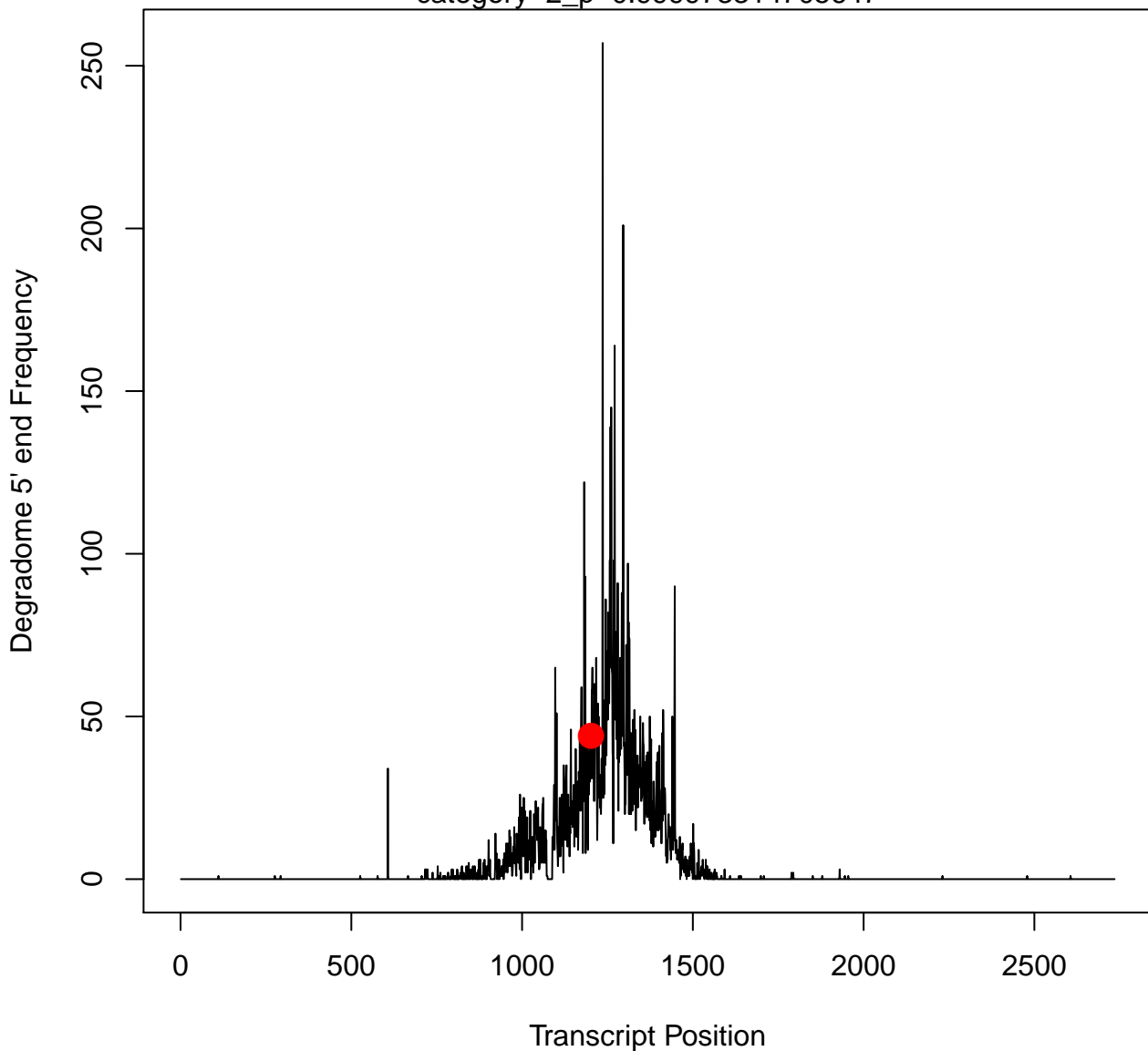

Supplement: Supplementary file 4 [file Data_Sheet_4.zip › Sit-miR159a_Seita.9G294600.1_1202_TPlot.pdf]

**T=Seita.9G319300.1\_Q=Sit-miR159a\_S=840**

category=2\_p=0.999694357355297

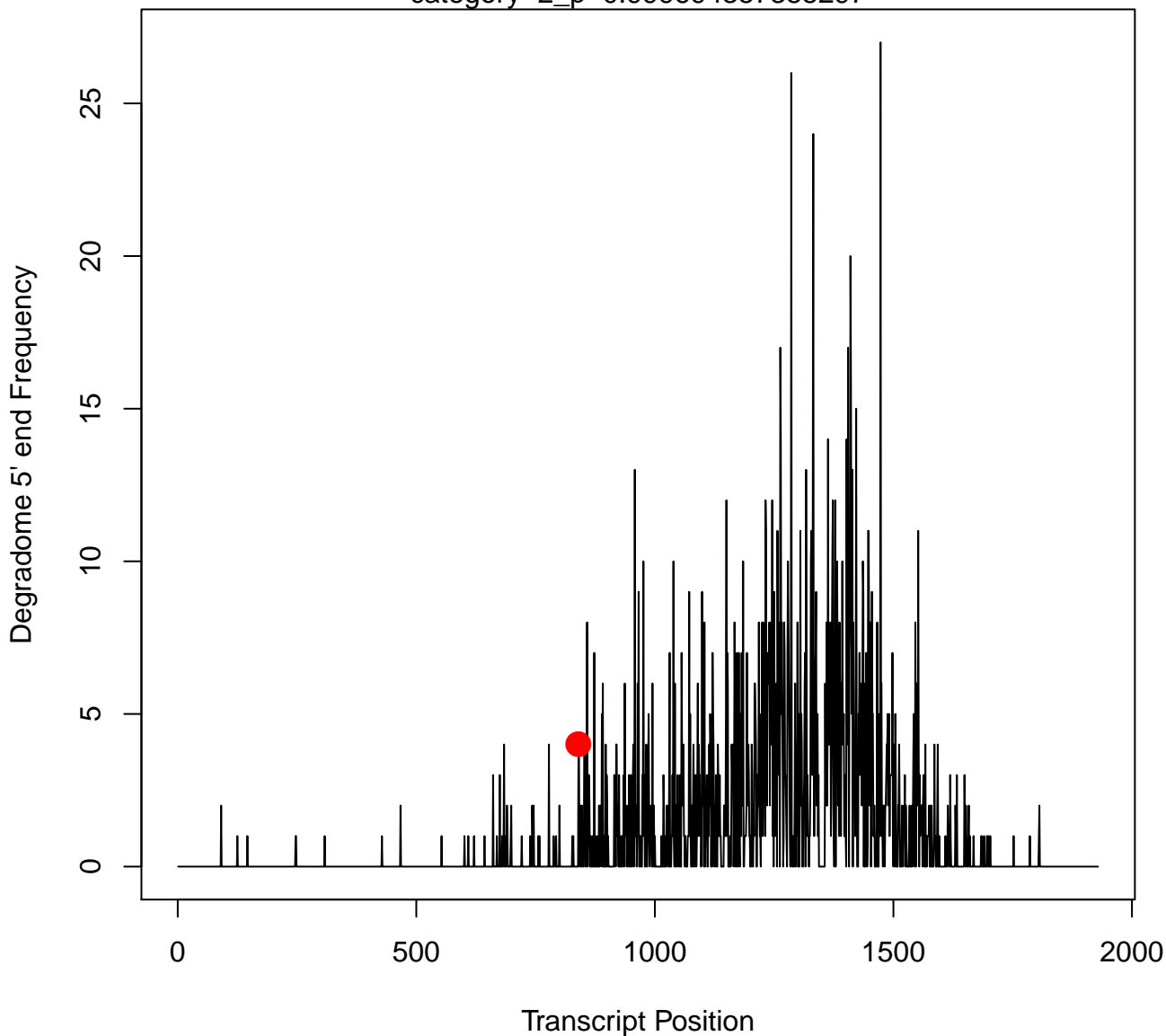

Supplement: Supplementary file 4 [file Data_Sheet_4.zip › Sit-miR159a_Seita.9G319300.1_840_TPlot.pdf]

**T=Seita.9G380100.1\_Q=Sit-miR159a\_S=2377**

category=2\_p=0.999999860952524

Degradome 5' end Frequency

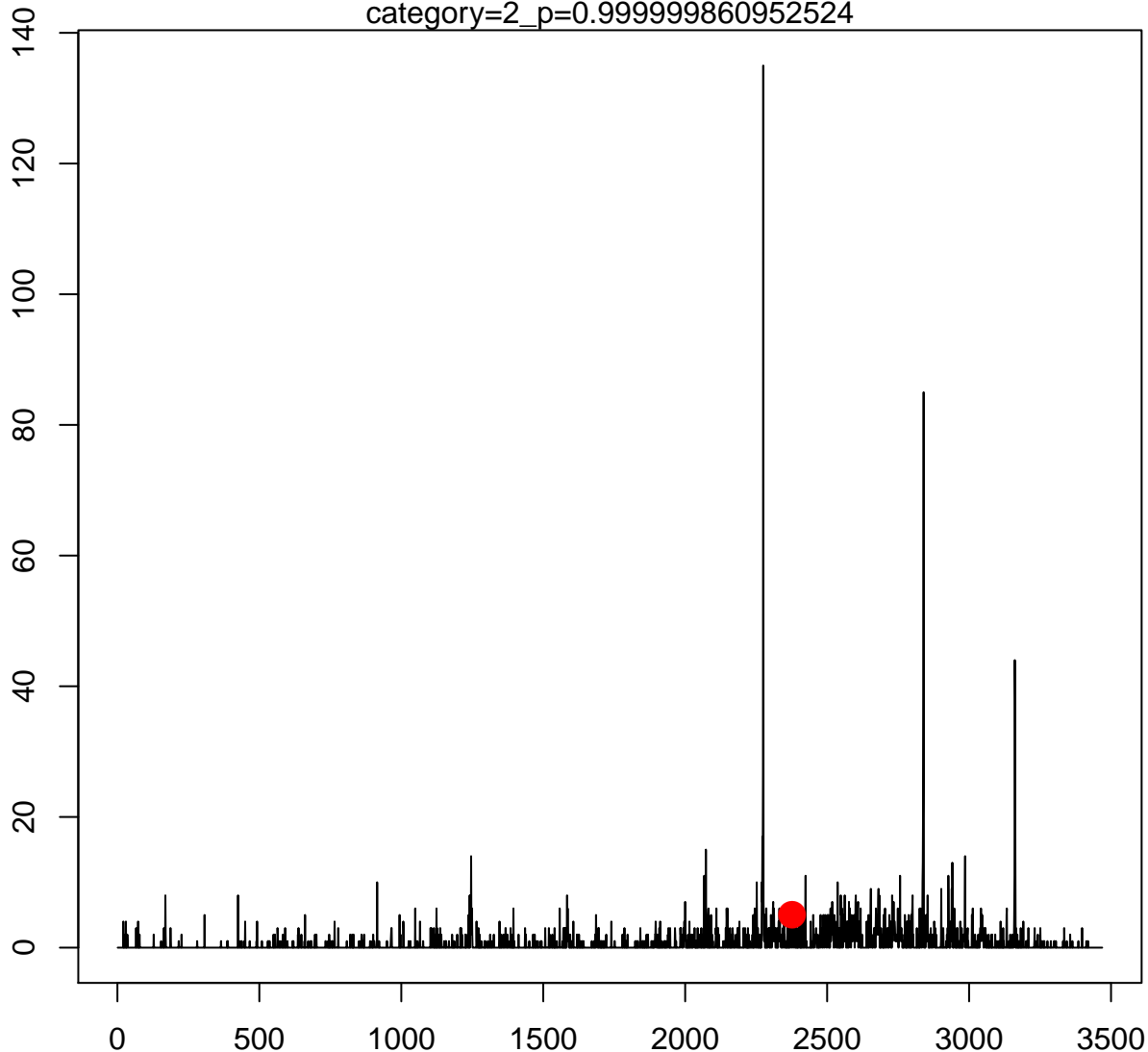

Transcript Position

Supplement: Supplementary file 4 [file Data_Sheet_4.zip › Sit-miR159a_Seita.9G380100.1_2377_TPlot.pdf]

**T=Seita.9G517700.1\_Q=Sit-miR159a\_S=1633**

category=2\_p=0.999999533470649

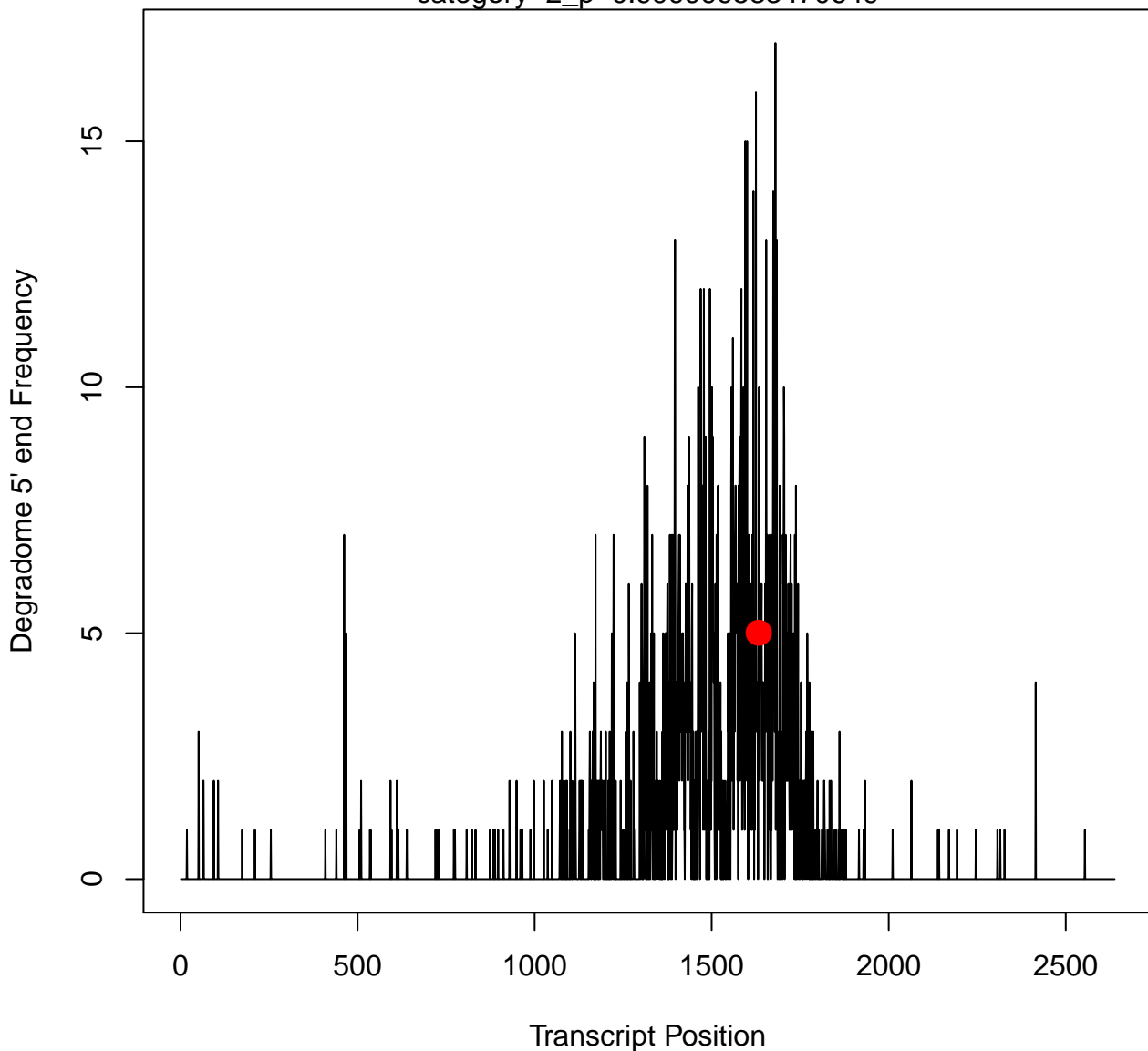

Supplement: Supplementary file 4 [file Data_Sheet_4.zip › Sit-miR159a_Seita.9G517700.1_1633_TPlot.pdf]

**T=Seita.4G083900.1\_Q=Sit-miR159b\_S=4244**

category=2\_p=0.43485157444258

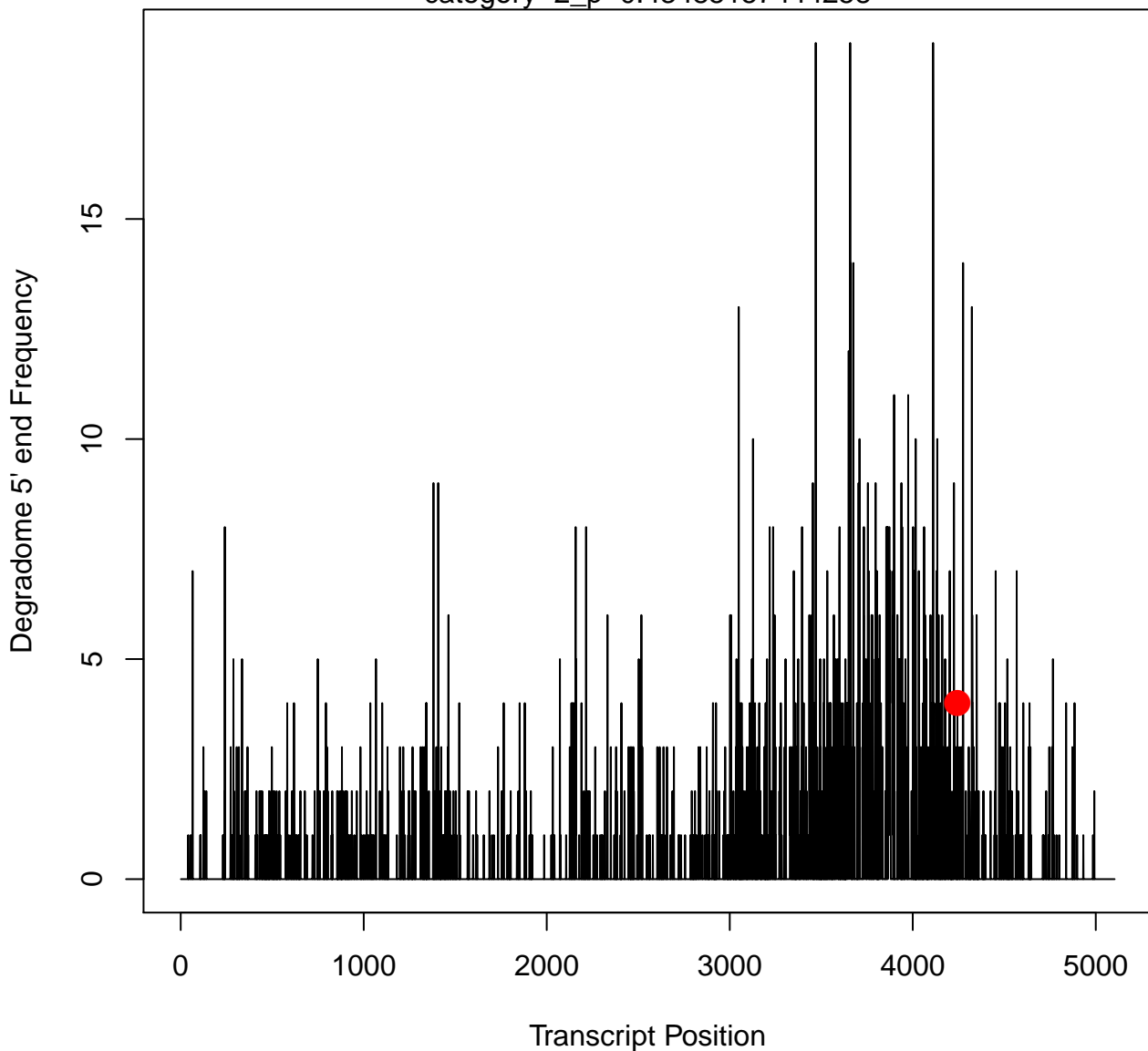

Supplement: Supplementary file 4 [file Data_Sheet_4.zip › Sit-miR159b_Seita.4G083900.1_4244_TPlot.pdf]

**T=Seita.4G221900.1\_Q=Sit-miR159b\_S=1159**

category=2\_p=0.0339946163519554

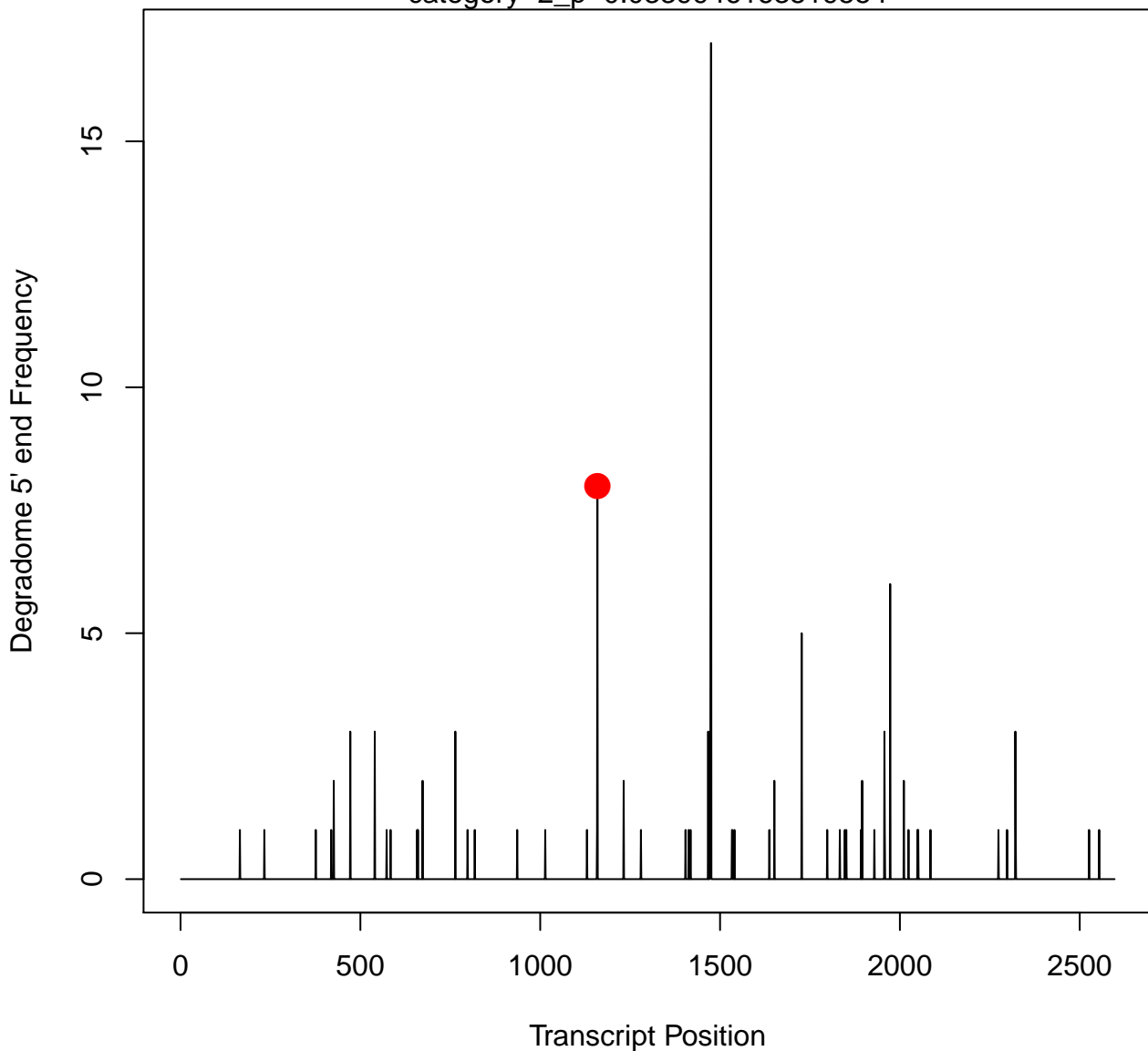

Supplement: Supplementary file 4 [file Data_Sheet_4.zip › Sit-miR159b_Seita.4G221900.1_1159_TPlot.pdf]

**T=Seita.4G265500.1\_Q=Sit-miR159b\_S=937**

category=2\_p=0.98060595789675

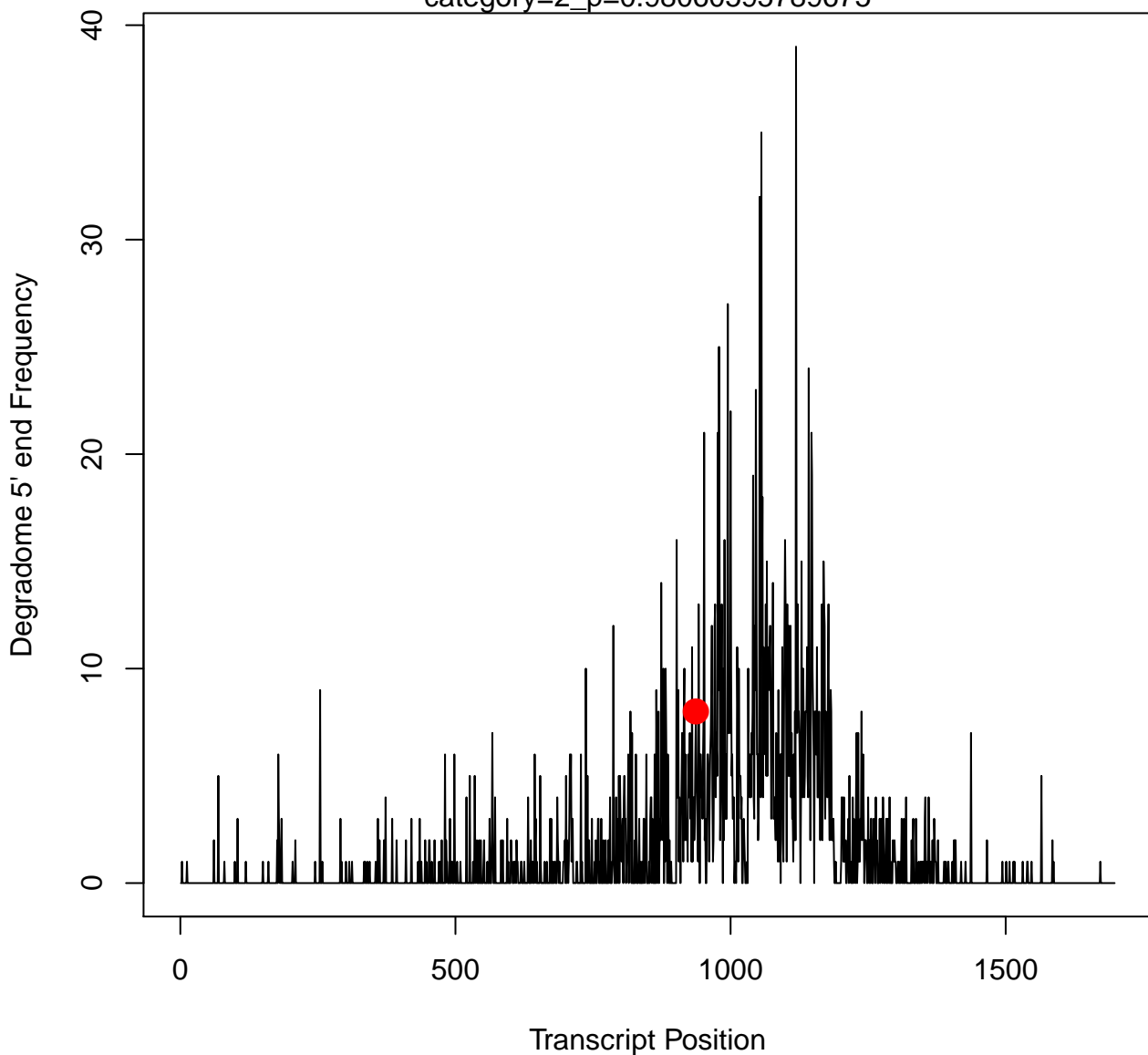

Supplement: Supplementary file 4 [file Data_Sheet_4.zip › Sit-miR159b_Seita.4G265500.1_937_TPlot.pdf]

**T=Seita.5G306500.1\_Q=Sit-miR159c\_S=3489**

category=2\_p=0.950657565786331

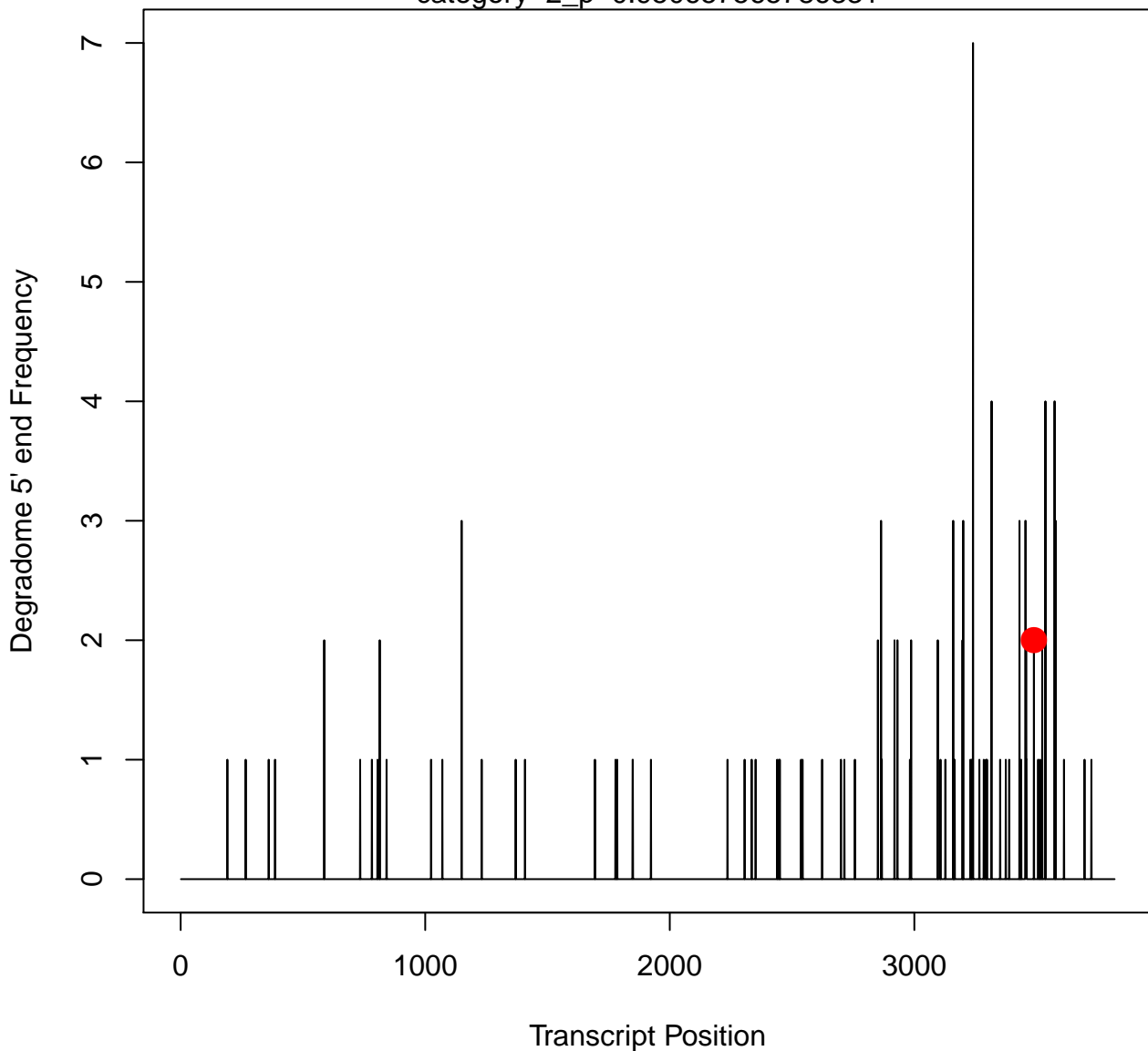

Supplement: Supplementary file 4 [file Data_Sheet_4.zip › Sit-miR159c_Seita.5G306500.1_3489_TPlot.pdf]

**T=Seita.5G355300.1\_Q=Sit-miR159c\_S=1278**

category=0\_p=0.00152683684215971

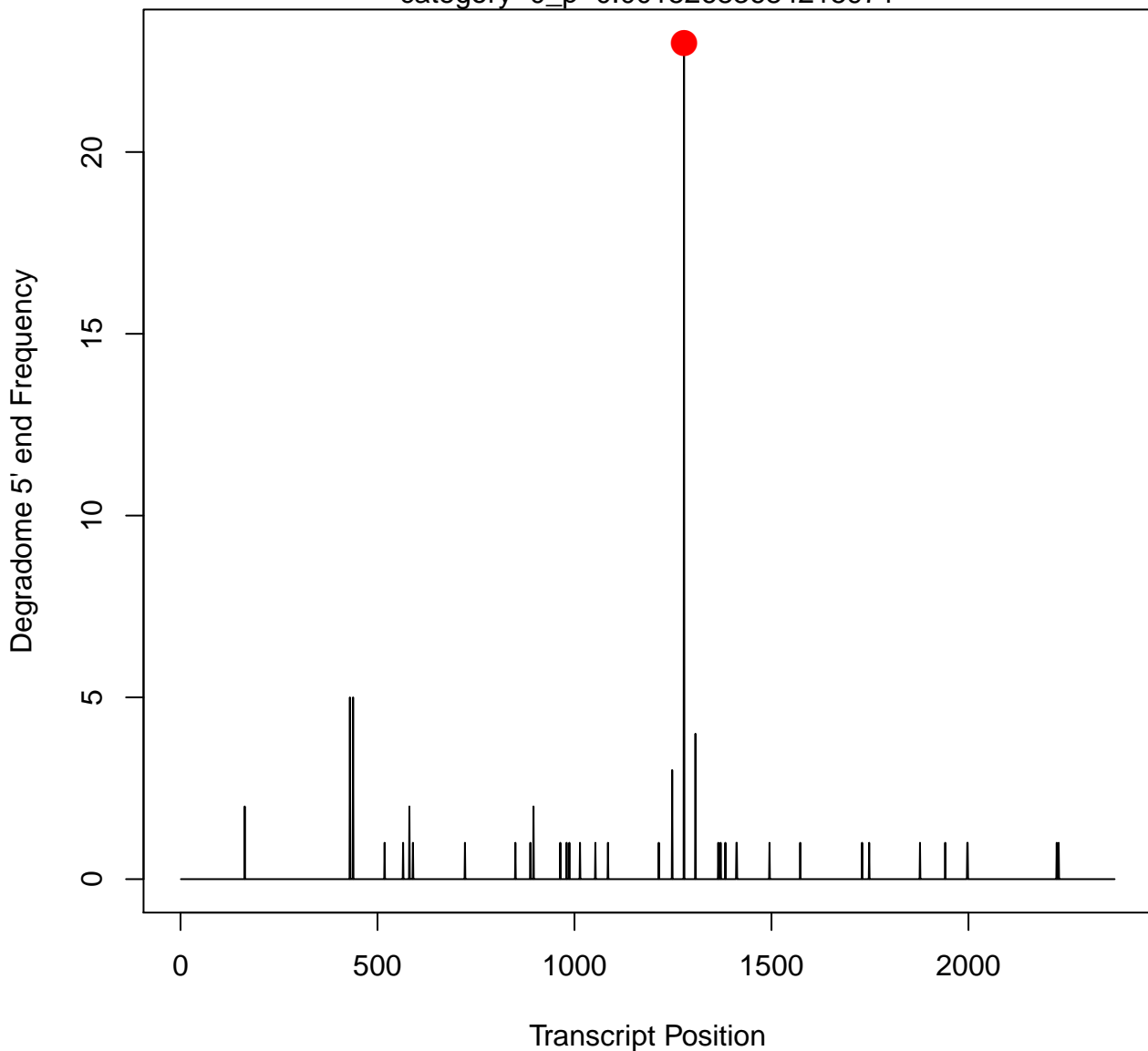

Supplement: Supplementary file 4 [file Data_Sheet_4.zip › Sit-miR159c_Seita.5G355300.1_1278_TPlot.pdf]

**T=Seita.1G185400.1\_Q=Sit-miR160a\_S=1610**

category=2\_p=0.956283137614924

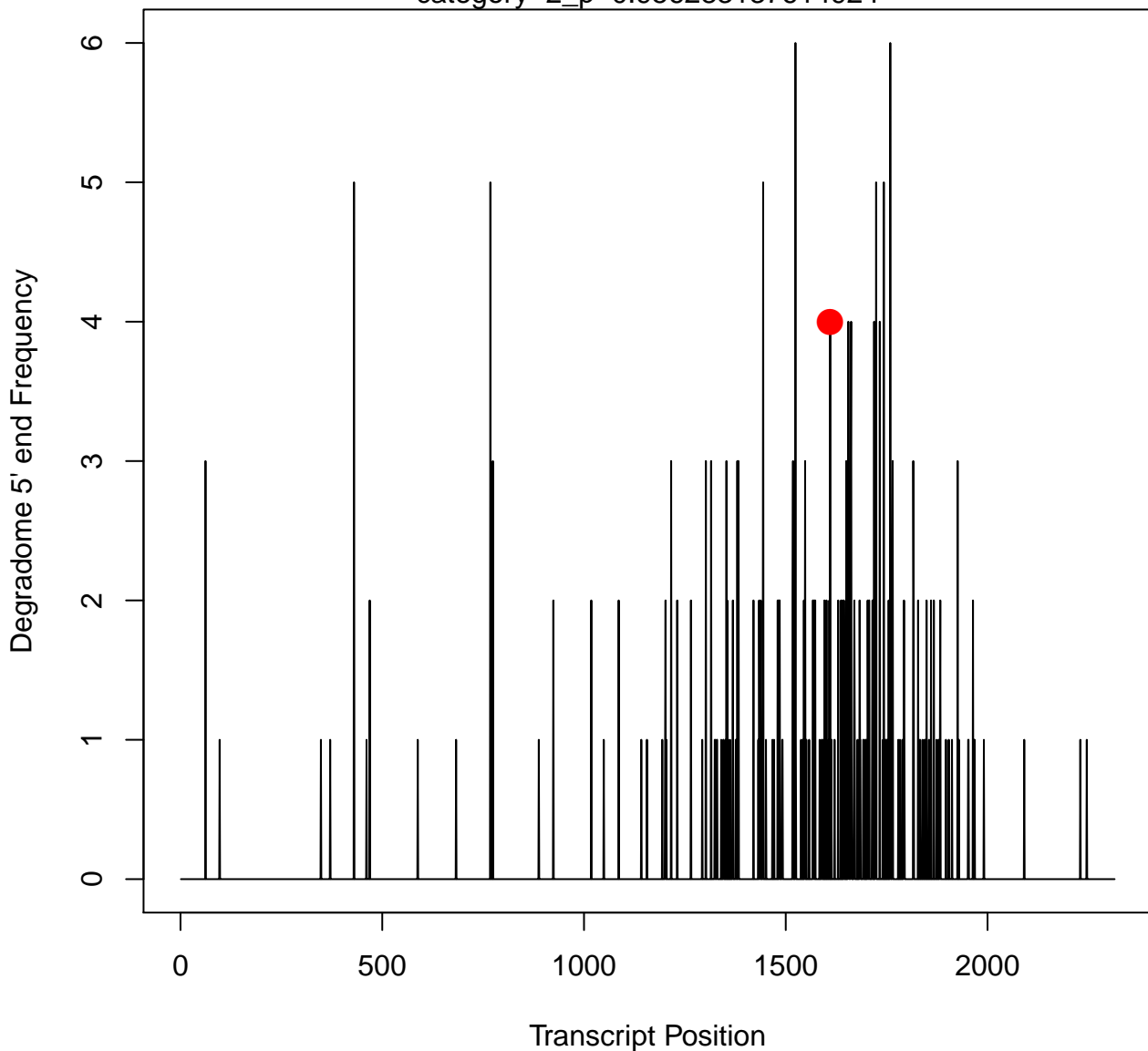

Supplement: Supplementary file 4 [file Data_Sheet_4.zip › Sit-miR160a_Seita.1G185400.1_1610_TPlot.pdf]

**T=Seita.1G331500.1\_Q=Sit-miR160a\_S=450**

category=2\_p=0.563977276822968

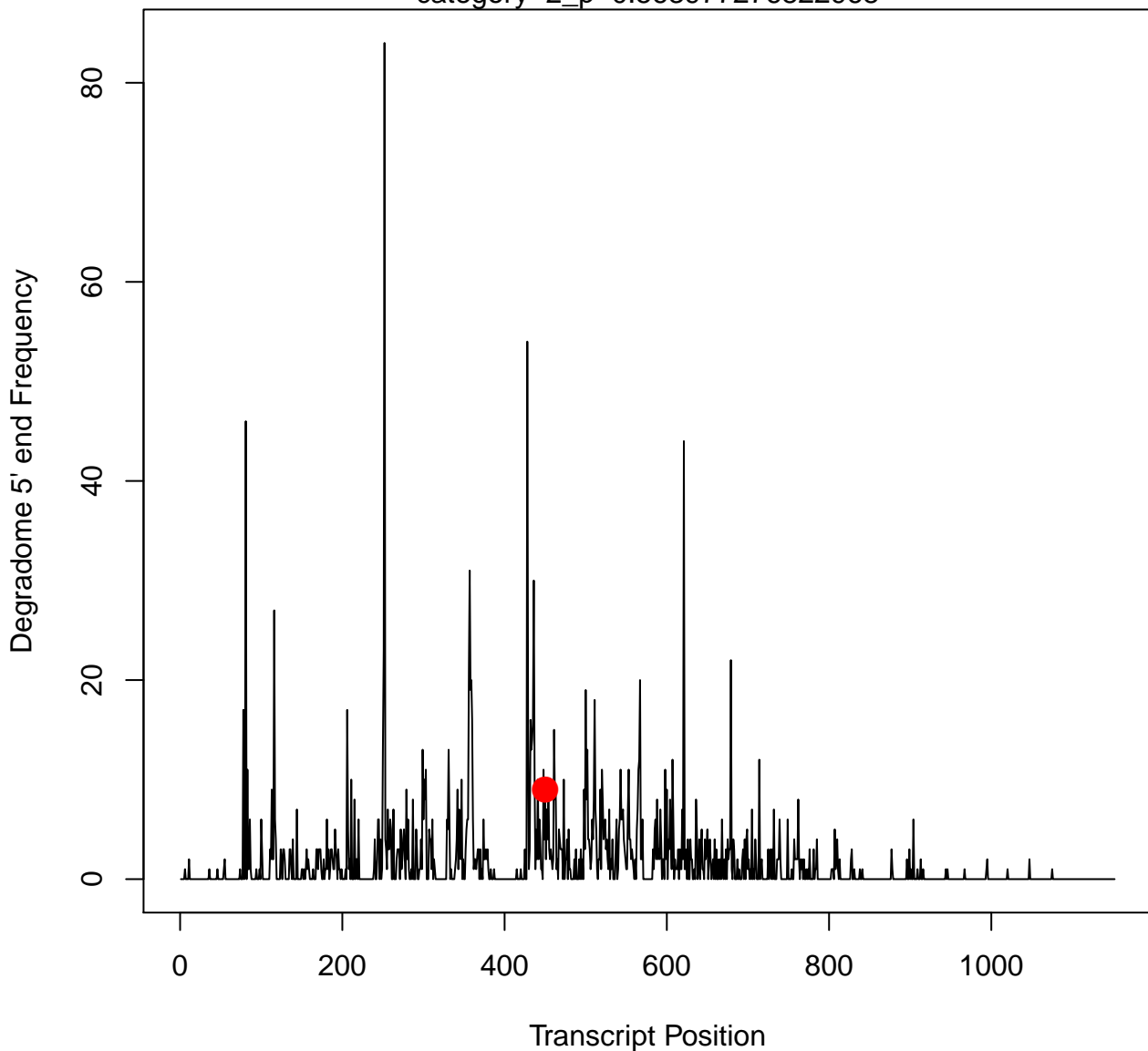

Supplement: Supplementary file 4 [file Data_Sheet_4.zip › Sit-miR160a_Seita.1G331500.1_450_TPlot.pdf]

**T=Seita.5G140500.1\_Q=Sit-miR160a\_S=1934**

category=2\_p=0.9999999999999998

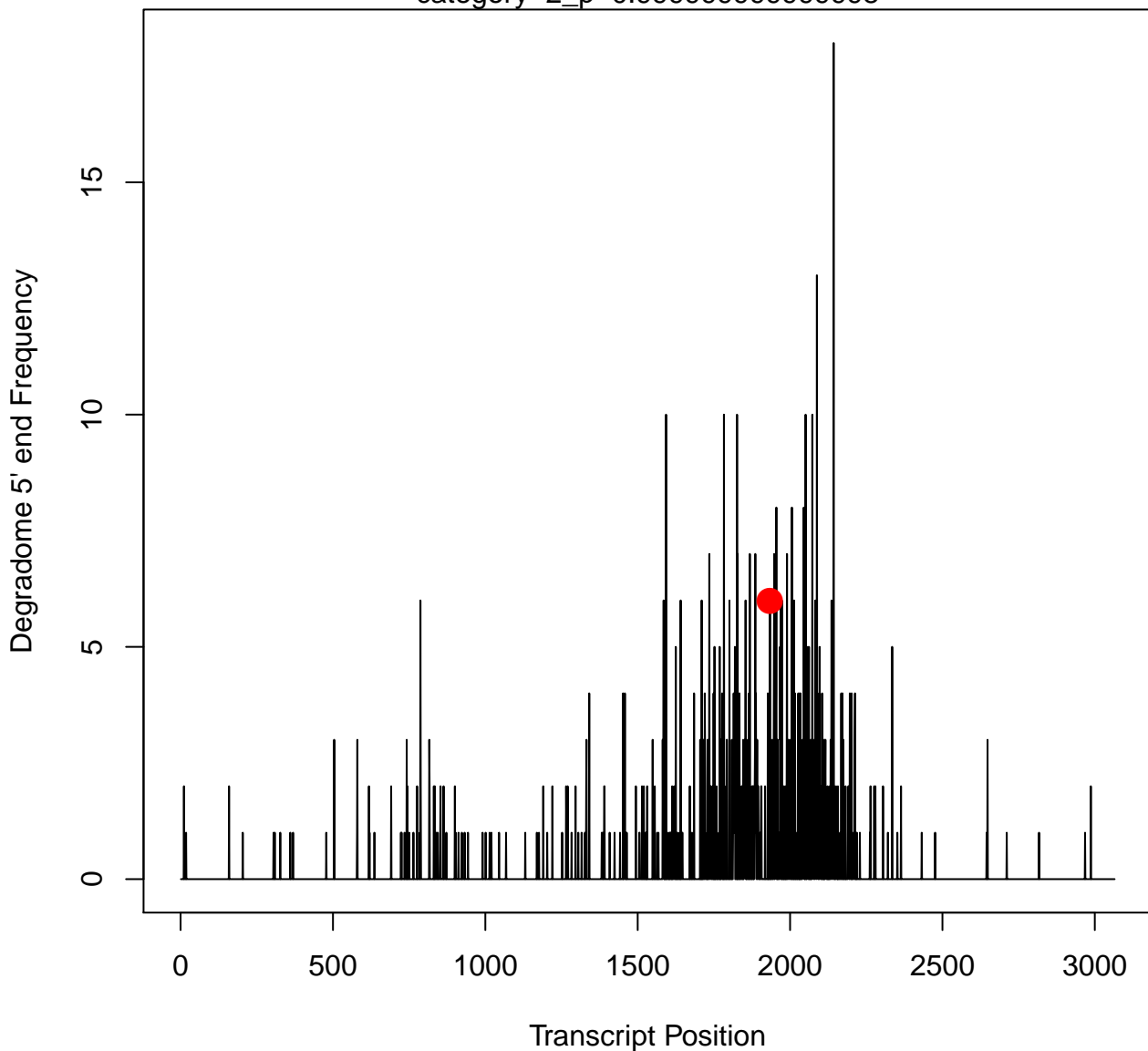

Supplement: Supplementary file 4 [file Data_Sheet_4.zip › Sit-miR160a_Seita.5G140500.1_1934_TPlot.pdf]

**T=Seita.5G183700.1\_Q=Sit-miR160a\_S=2318**

category=2\_p=0.999999999905844

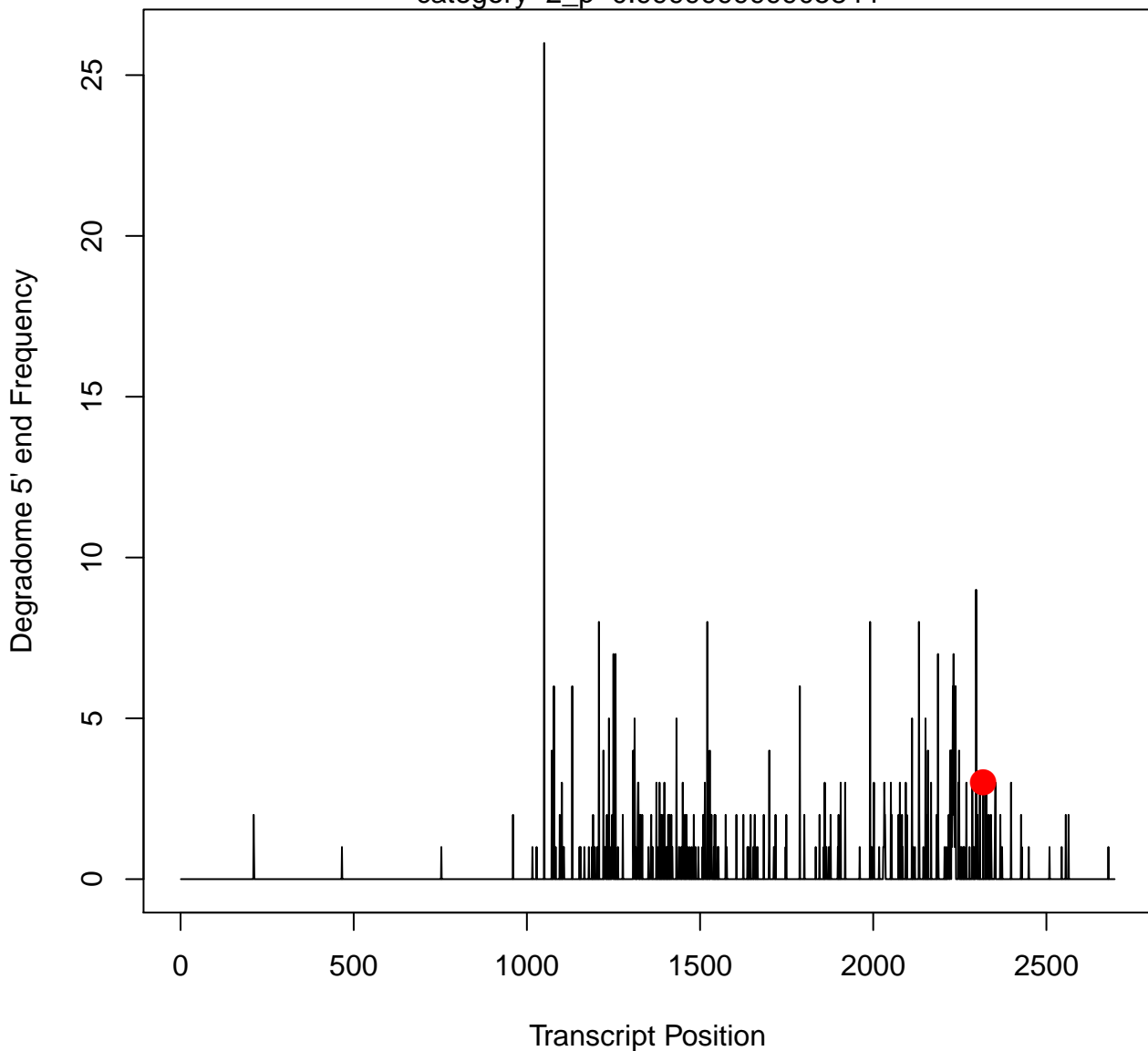

Supplement: Supplementary file 4 [file Data_Sheet_4.zip › Sit-miR160a_Seita.5G183700.1_2318_TPlot.pdf]

**T=Seita.5G257300.1\_Q=Sit-miR160a\_S=995**

category=2\_p=0.740461180566266

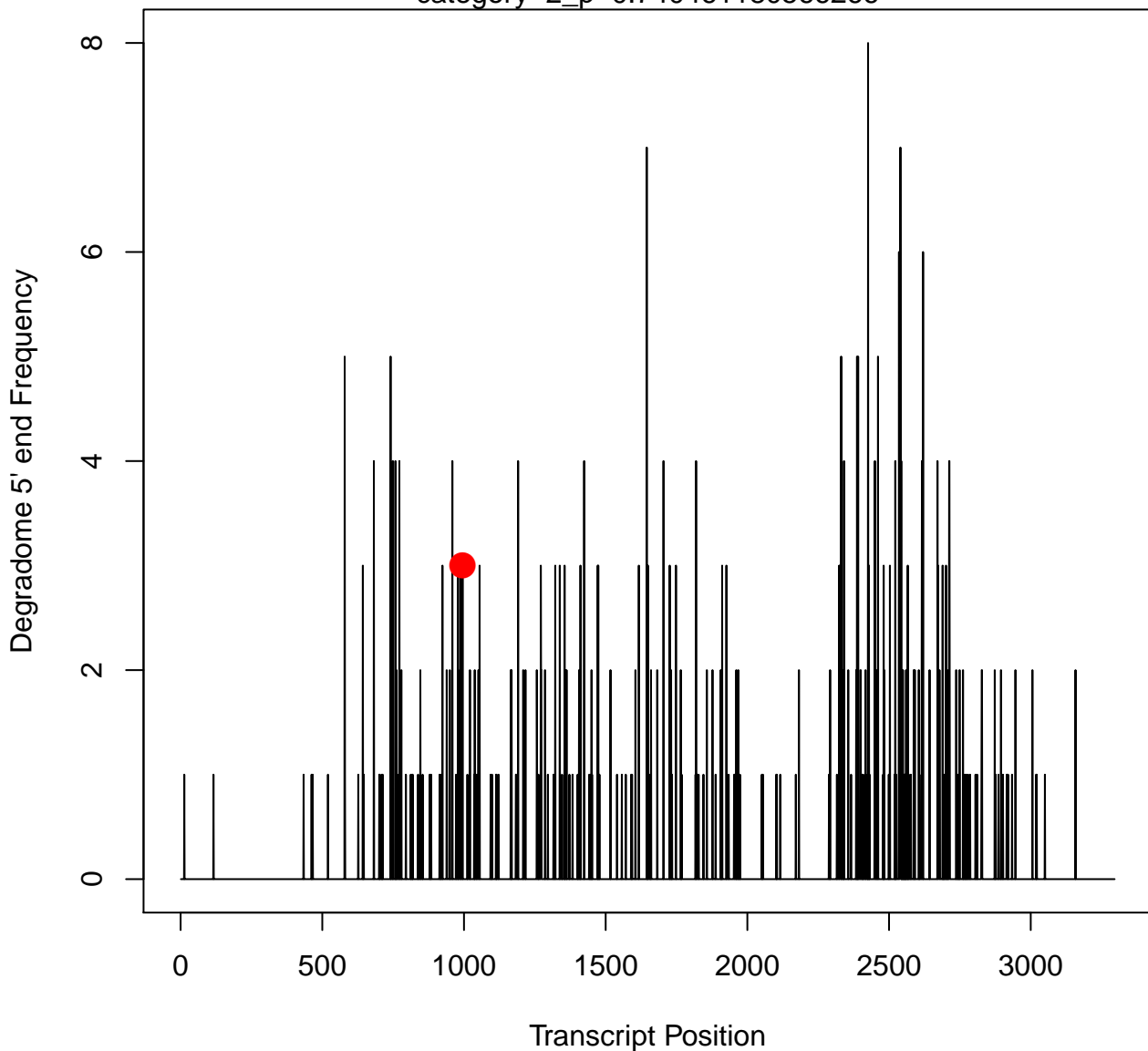

Supplement: Supplementary file 4 [file Data_Sheet_4.zip › Sit-miR160a_Seita.5G257300.1_995_TPlot.pdf]

**T=Seita.9G465800.1\_Q=Sit-miR160a\_S=1303**

category=2\_p=0.42499350719194

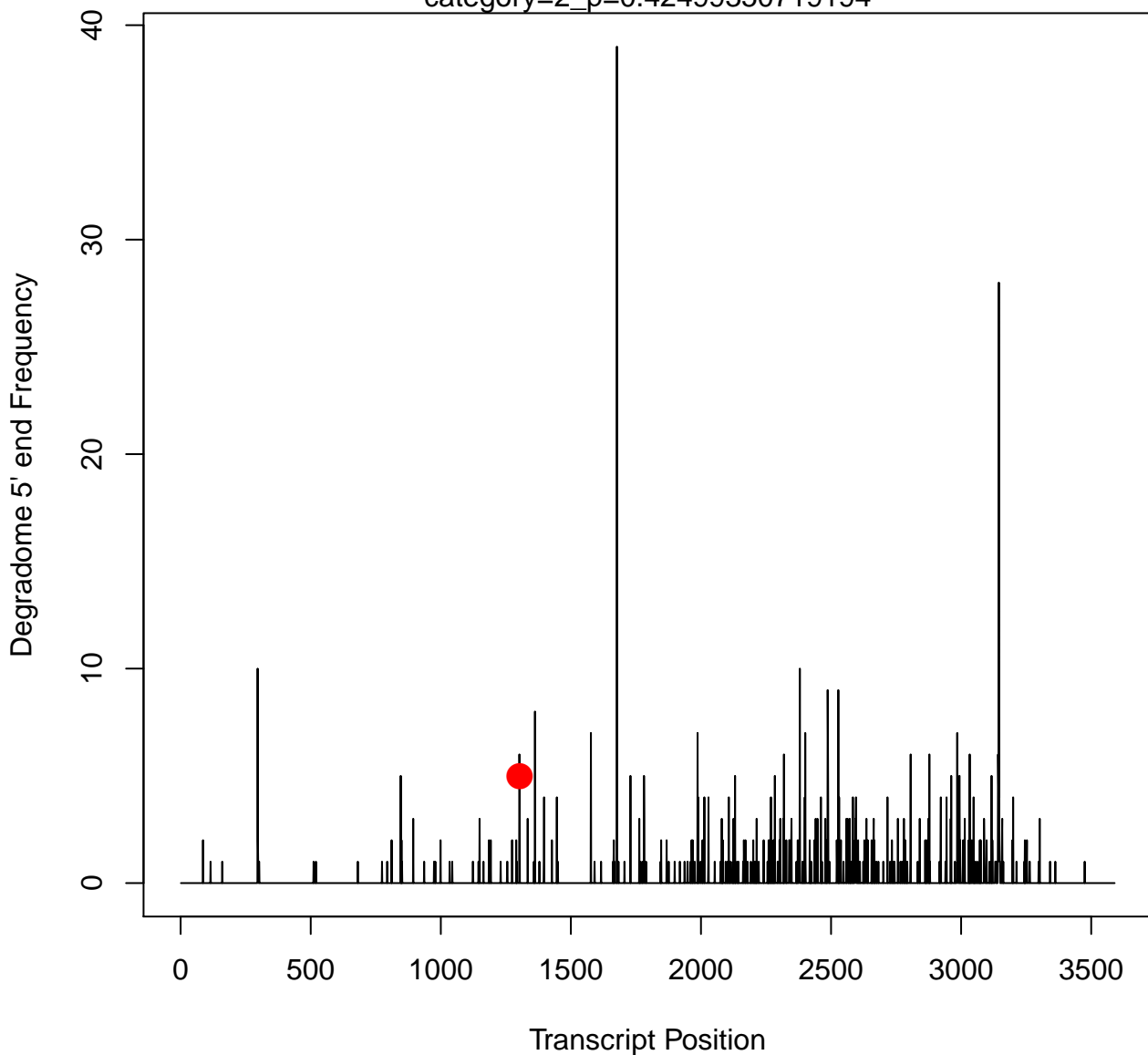

Supplement: Supplementary file 4 [file Data_Sheet_4.zip › Sit-miR160a_Seita.9G465800.1_1303_TPlot.pdf]

**T=Seita.2G068600.1\_Q=Sit-miR160b\_S=3061**

category=2\_p=0.999999991701565

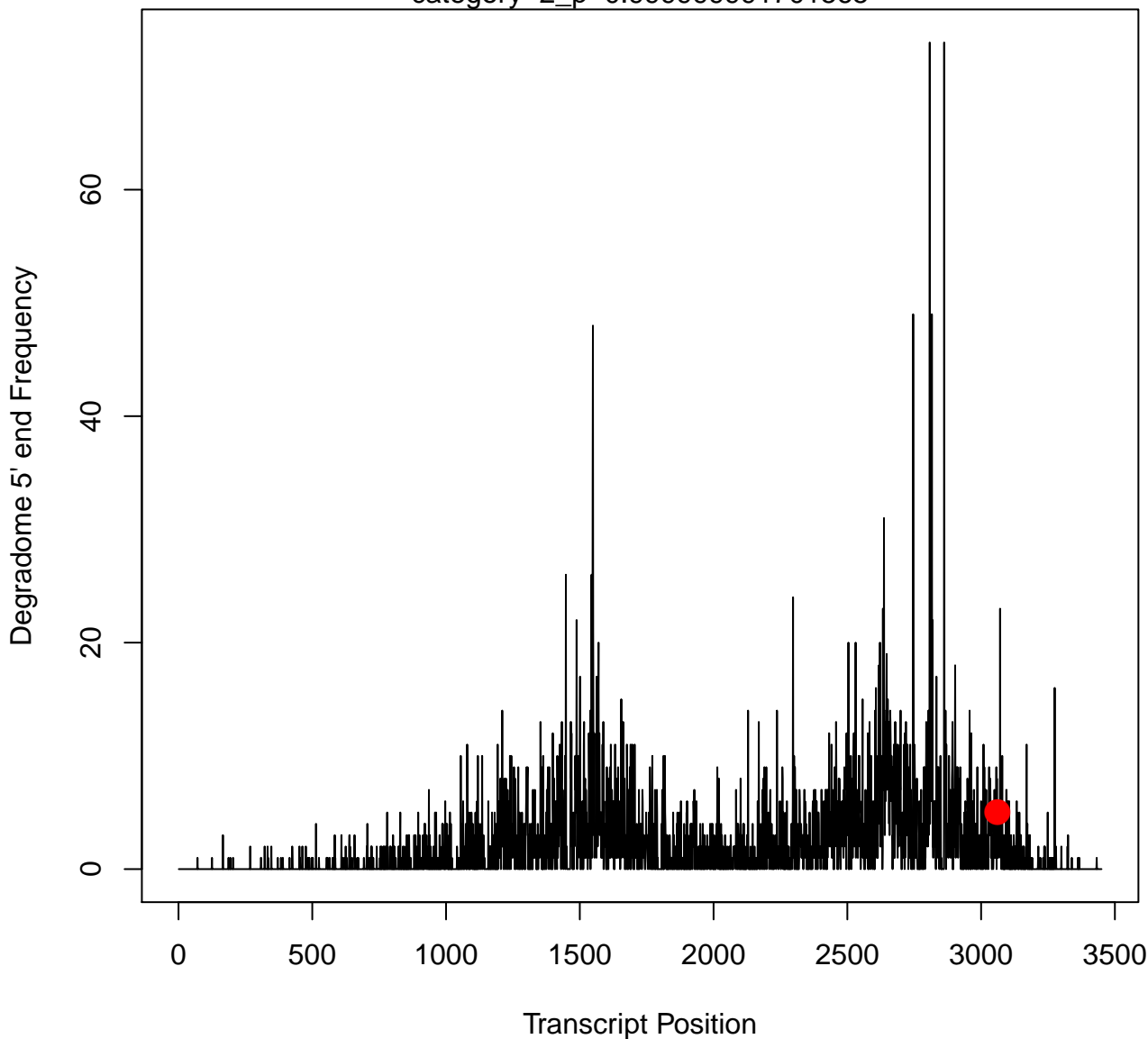

Supplement: Supplementary file 4 [file Data_Sheet_4.zip › Sit-miR160b_Seita.2G068600.1_3061_TPlot.pdf]

**T=Seita.2G249600.1\_Q=Sit-miR160b\_S=971**

category=2\_p=0.99999993037679

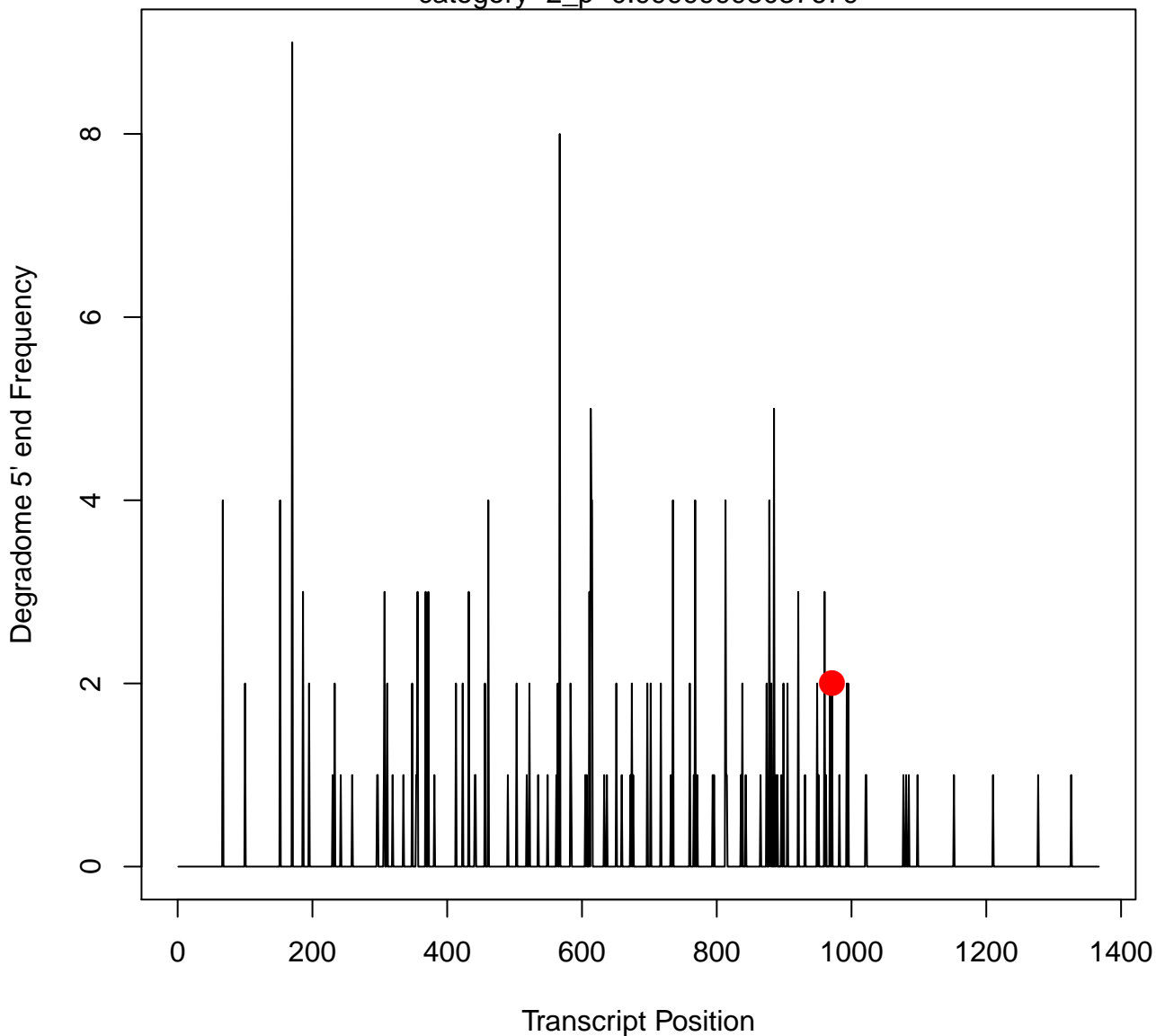

Supplement: Supplementary file 4 [file Data_Sheet_4.zip › Sit-miR160b_Seita.2G249600.1_971_TPlot.pdf]

**T=Seita.2G275500.1\_Q=Sit-miR160b\_S=1524**

category=2\_p=0.99999999938893

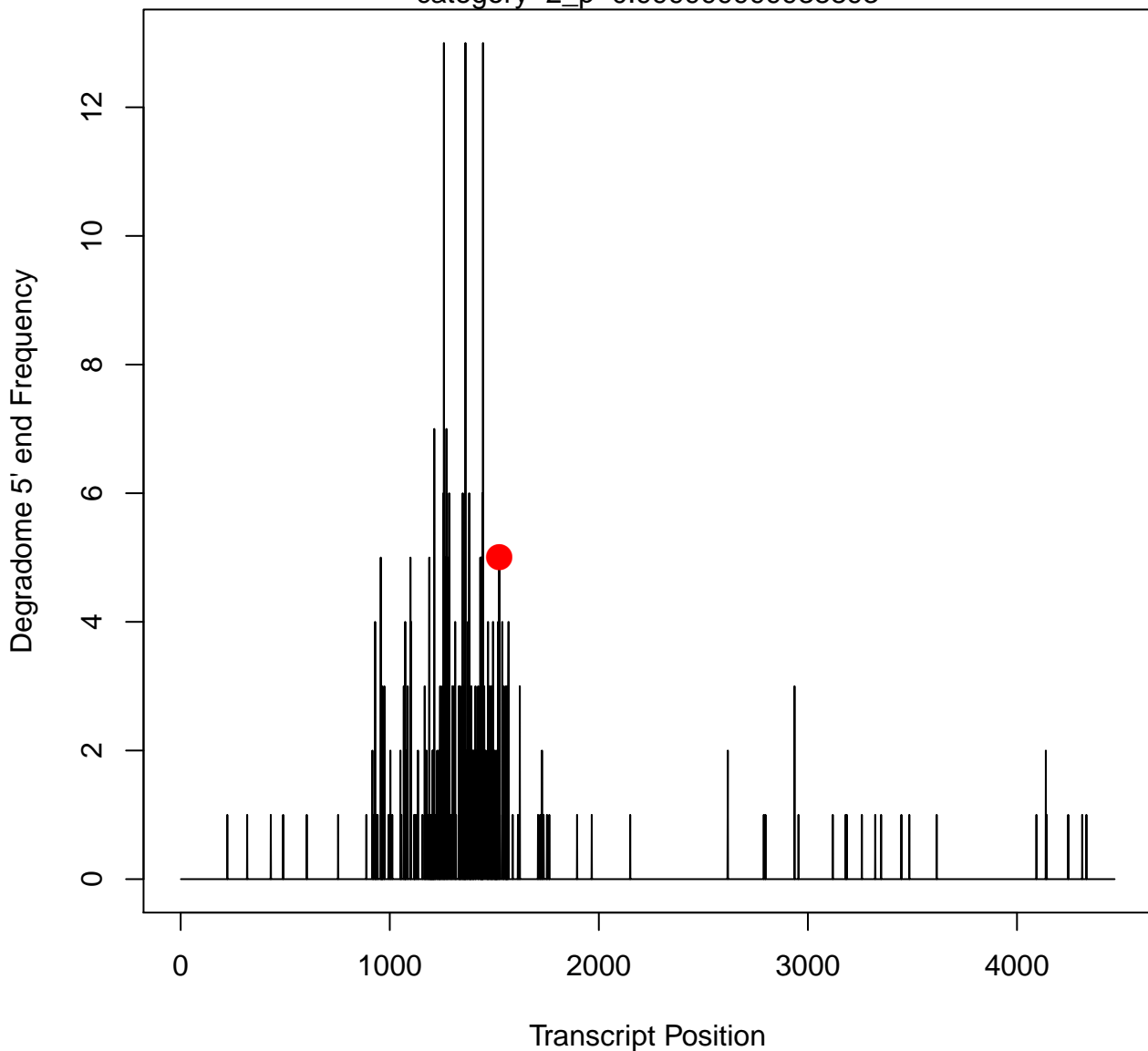

Supplement: Supplementary file 4 [file Data_Sheet_4.zip › Sit-miR160b_Seita.2G275500.1_1524_TPlot.pdf]

**T=Seita.4G090500.1\_Q=Sit-miR160b\_S=945**

category=2\_p=0.999999999700061

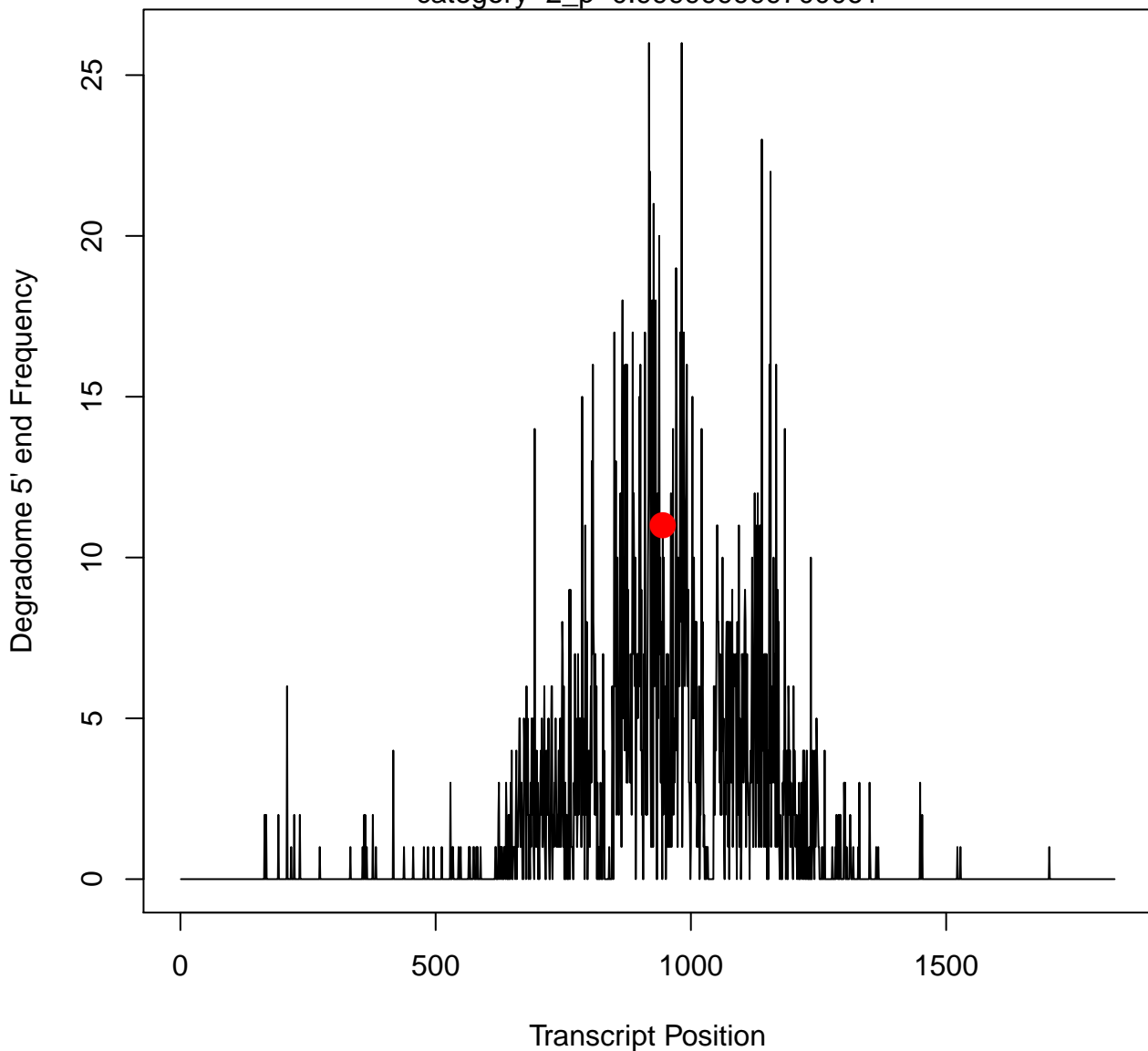

Supplement: Supplementary file 4 [file Data_Sheet_4.zip › Sit-miR160b_Seita.4G090500.1_945_TPlot.pdf]

**T=Seita.4G136400.1\_Q=Sit-miR160b\_S=414**

category=2\_p=0.999999999689506

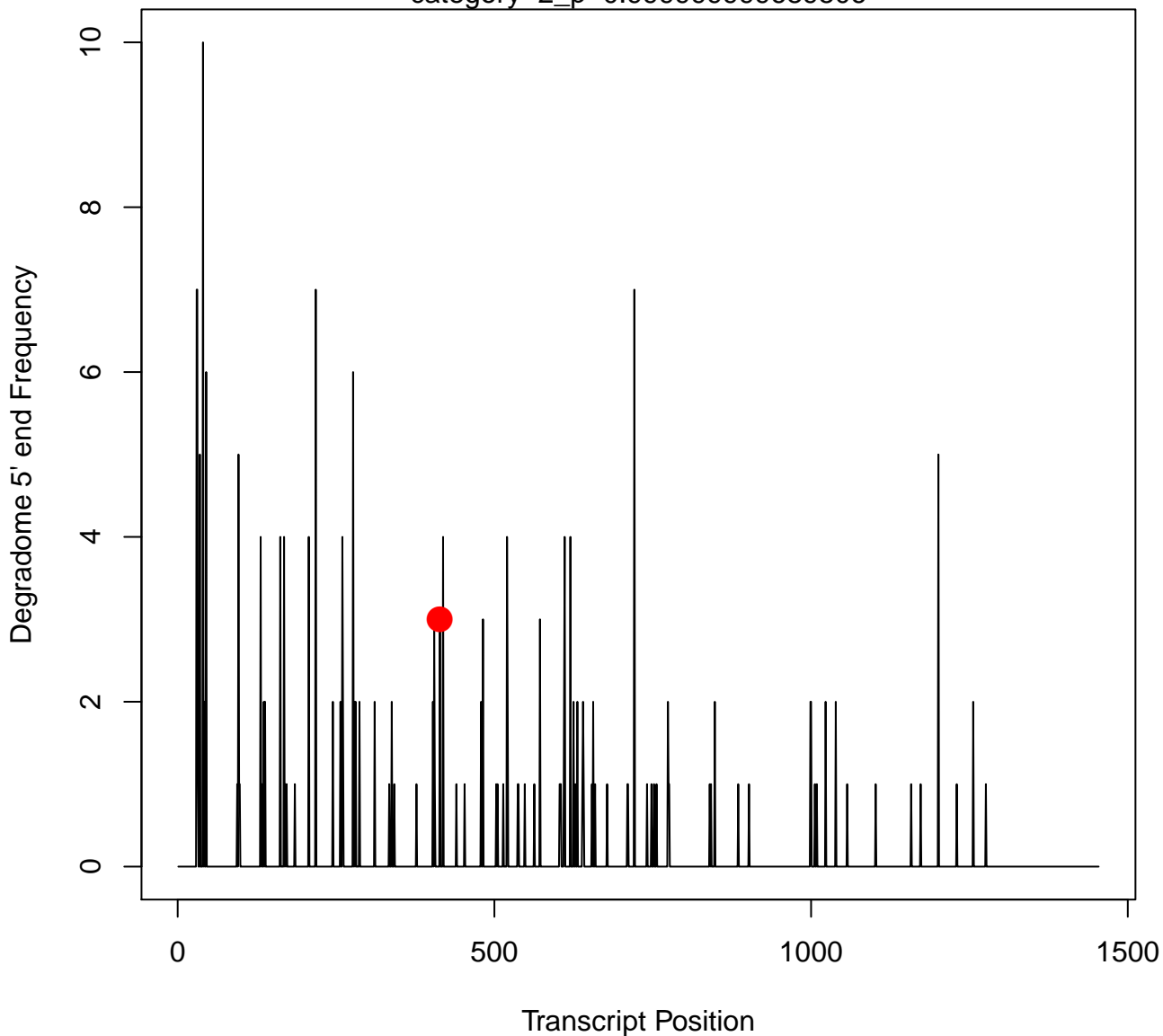

Supplement: Supplementary file 4 [file Data_Sheet_4.zip › Sit-miR160b_Seita.4G136400.1_414_TPlot.pdf]

**T=Seita.5G069000.1\_Q=Sit-miR160b\_S=270**

category=2\_p=0.999999998672874

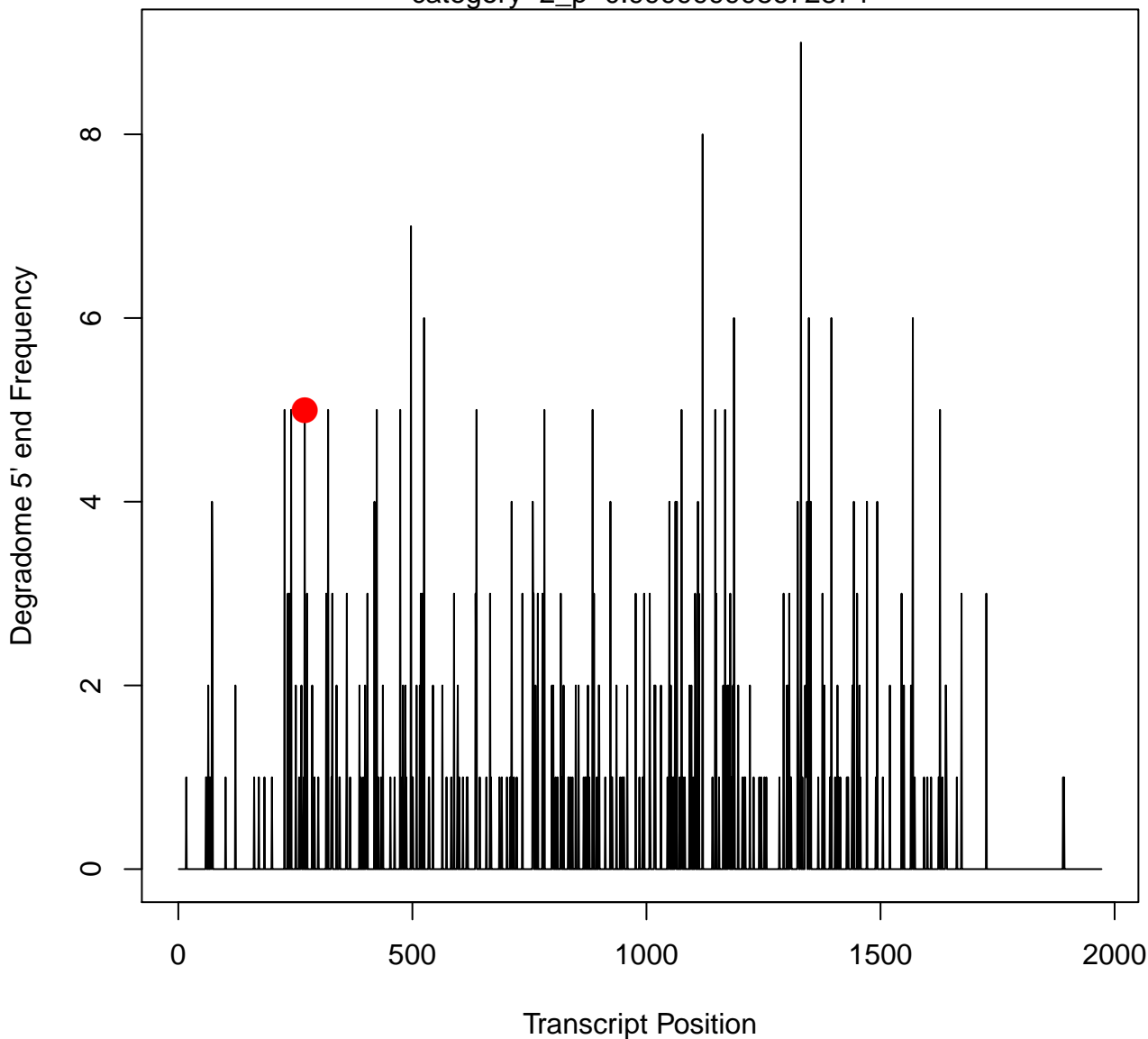

Supplement: Supplementary file 4 [file Data_Sheet_4.zip › Sit-miR160b_Seita.5G069000.1_270_TPlot.pdf]

**T=Seita.5G338300.1\_Q=Sit-miR160b\_S=224**

category=2\_p=0.994318771655266

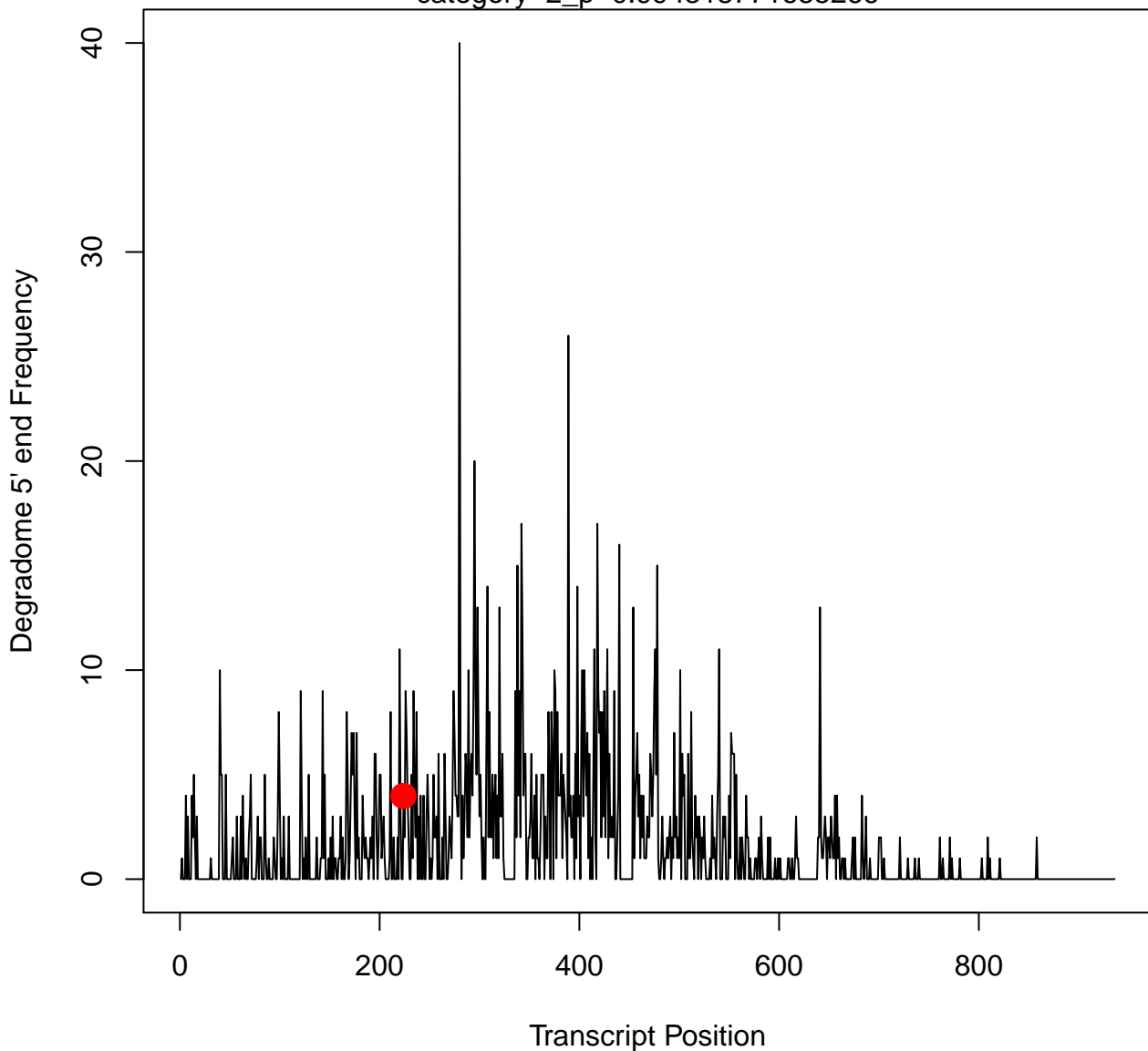

Supplement: Supplementary file 4 [file Data_Sheet_4.zip › Sit-miR160b_Seita.5G338300.1_224_TPlot.pdf]

**T=Seita.7G331800.1\_Q=Sit-miR160b\_S=176**

category=2\_p=0.99999999927357

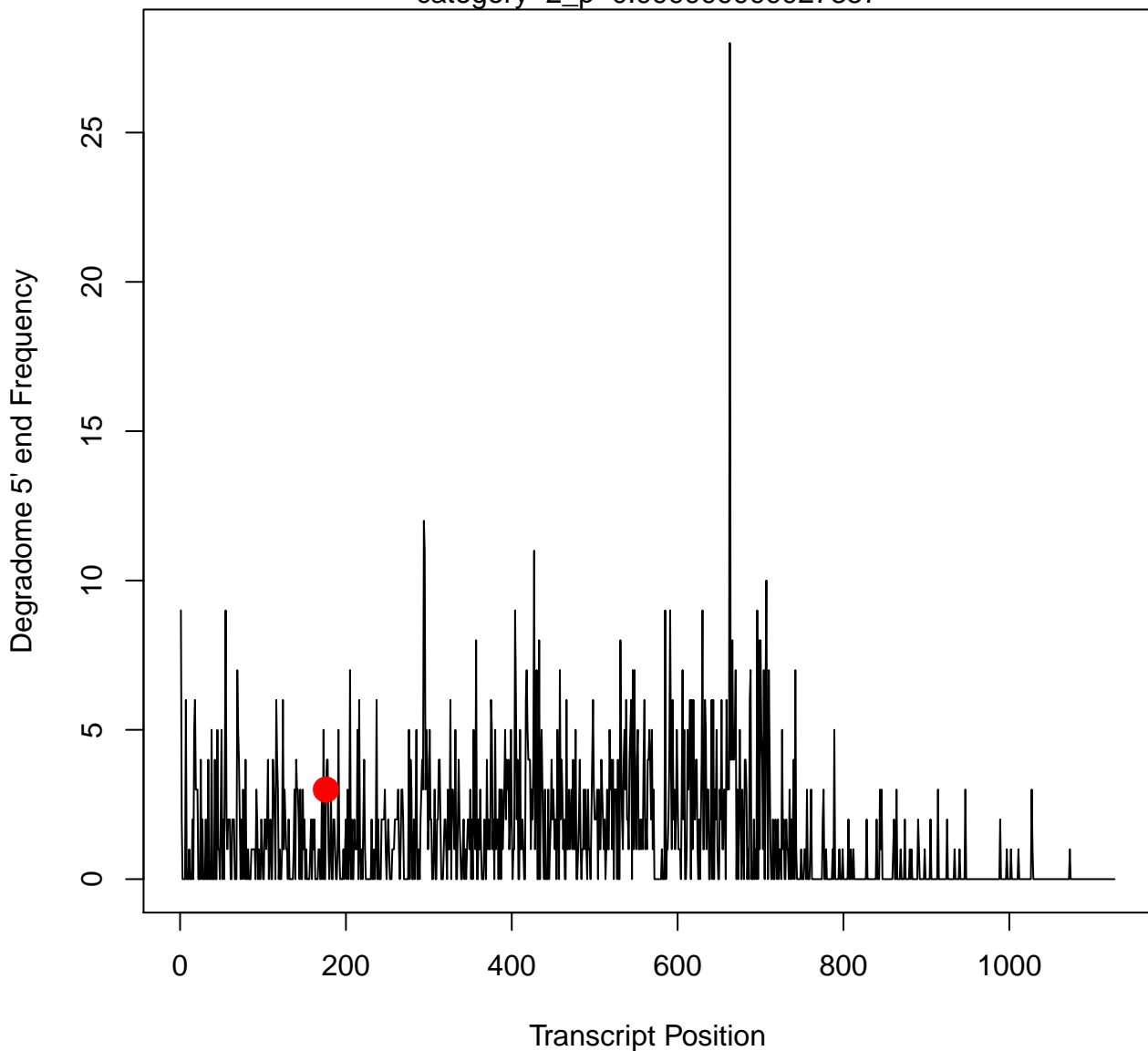

Supplement: Supplementary file 4 [file Data_Sheet_4.zip › Sit-miR160b_Seita.7G331800.1_176_TPlot.pdf]

**T=Seita.9G219800.1\_Q=Sit-miR160b\_S=1729**

category=0\_p=0.000381927958281736

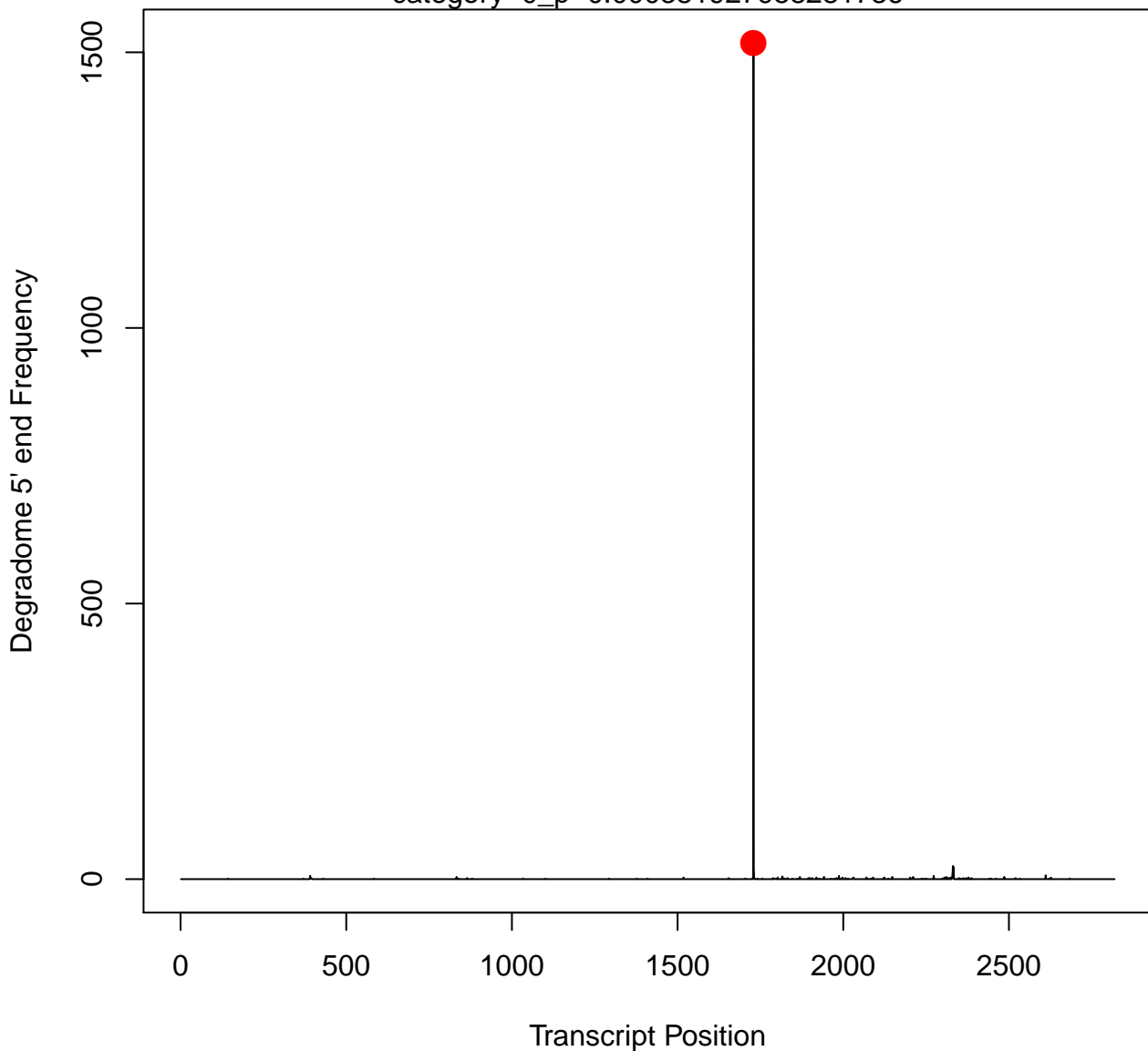

Supplement: Supplementary file 4 [file Data_Sheet_4.zip › Sit-miR160b_Seita.9G219800.1_1729_TPlot.pdf]

**T=Seita.1G125900.1\_Q=Sit-miR160c\_S=678**

category=2\_p=0.99999999110723

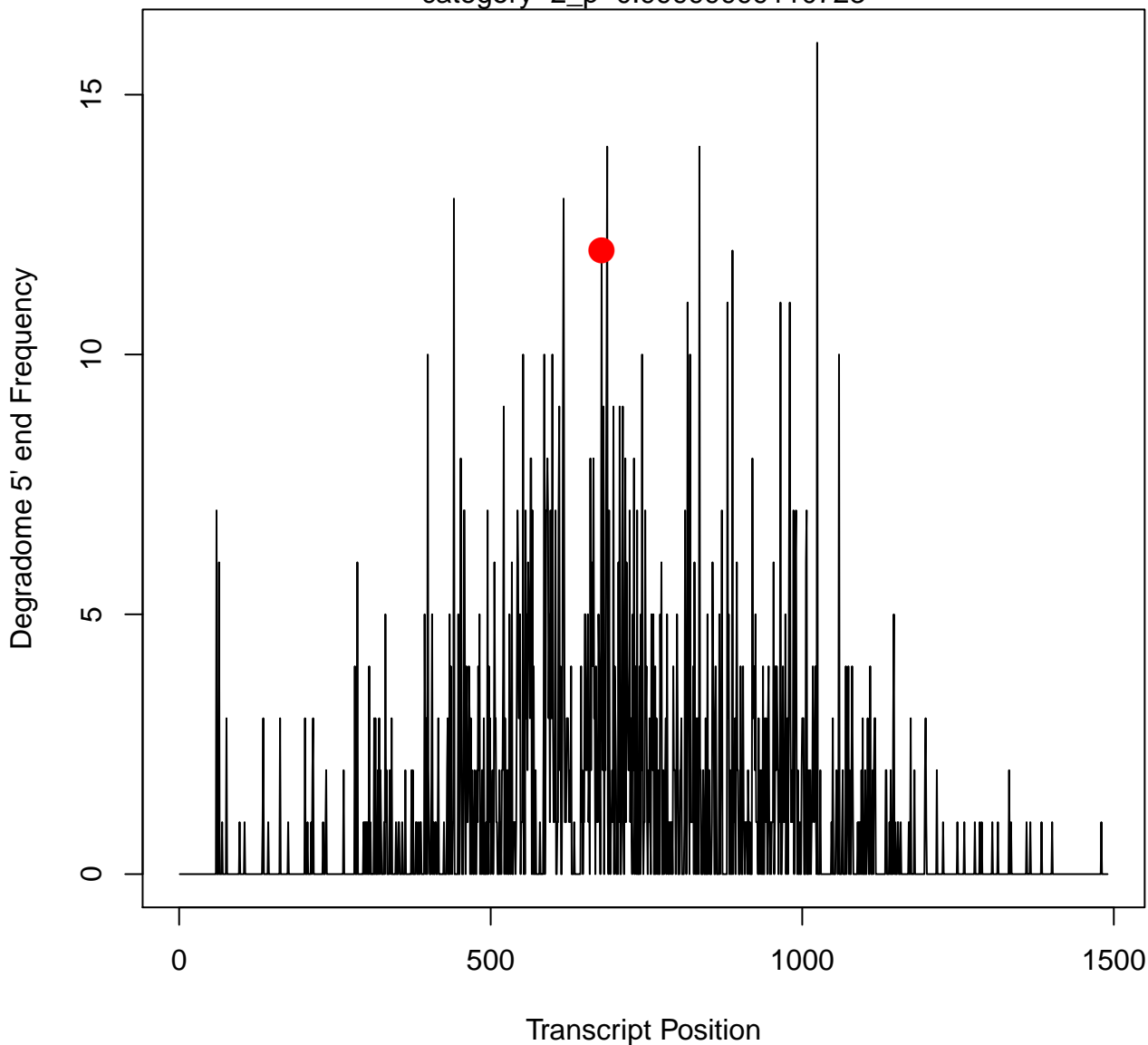

Supplement: Supplementary file 4 [file Data_Sheet_4.zip › Sit-miR160c_Seita.1G125900.1_678_TPlot.pdf]

**T=Seita.2G199300.1\_Q=Sit-miR160c\_S=1175**

category=2\_p=0.994016257102829

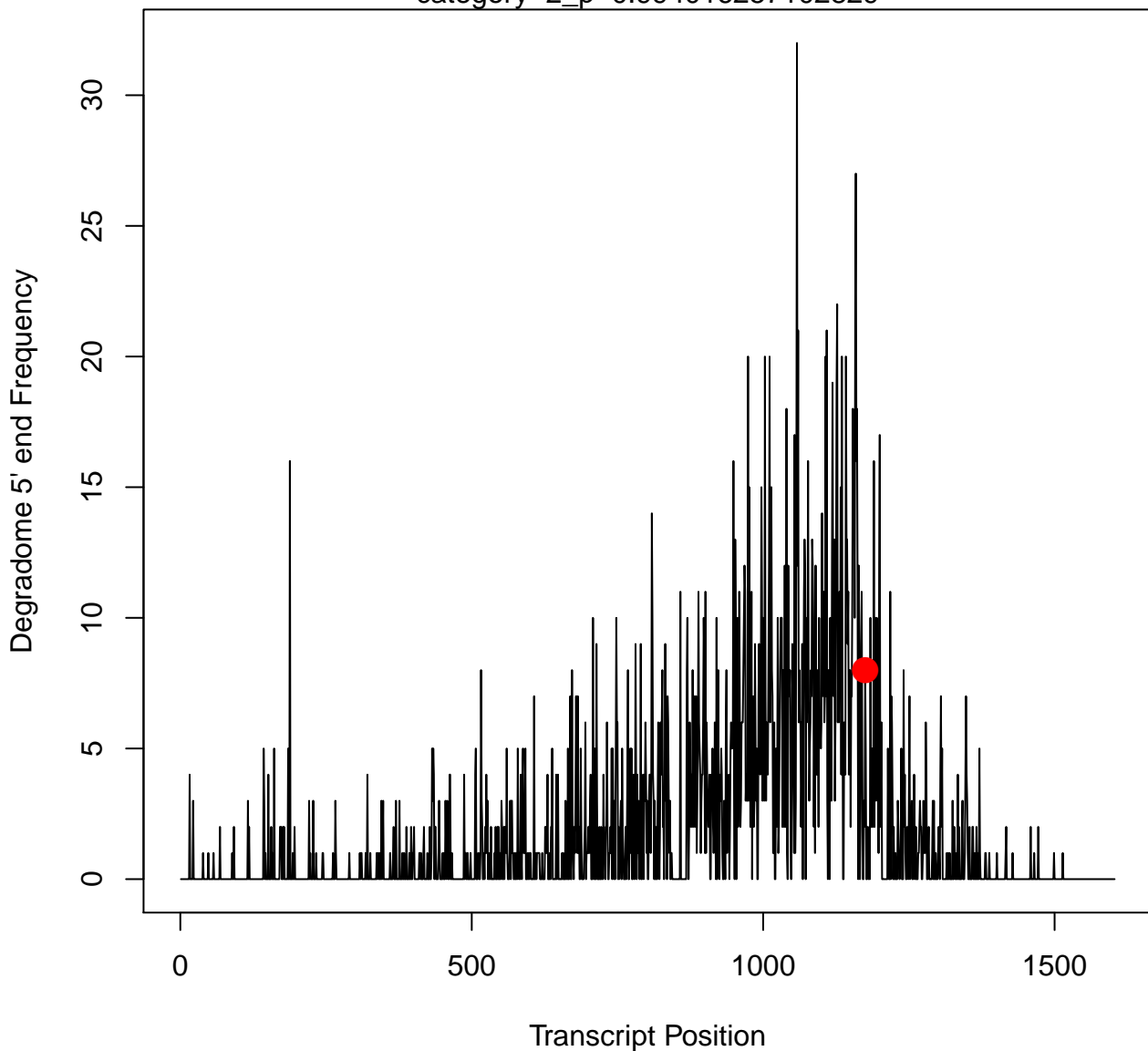

Supplement: Supplementary file 4 [file Data_Sheet_4.zip › Sit-miR160c_Seita.2G199300.1_1175_TPlot.pdf]

**T=Seita.2G283800.1\_Q=Sit-miR160c\_S=1464**

category=2\_p=0.99999573235803

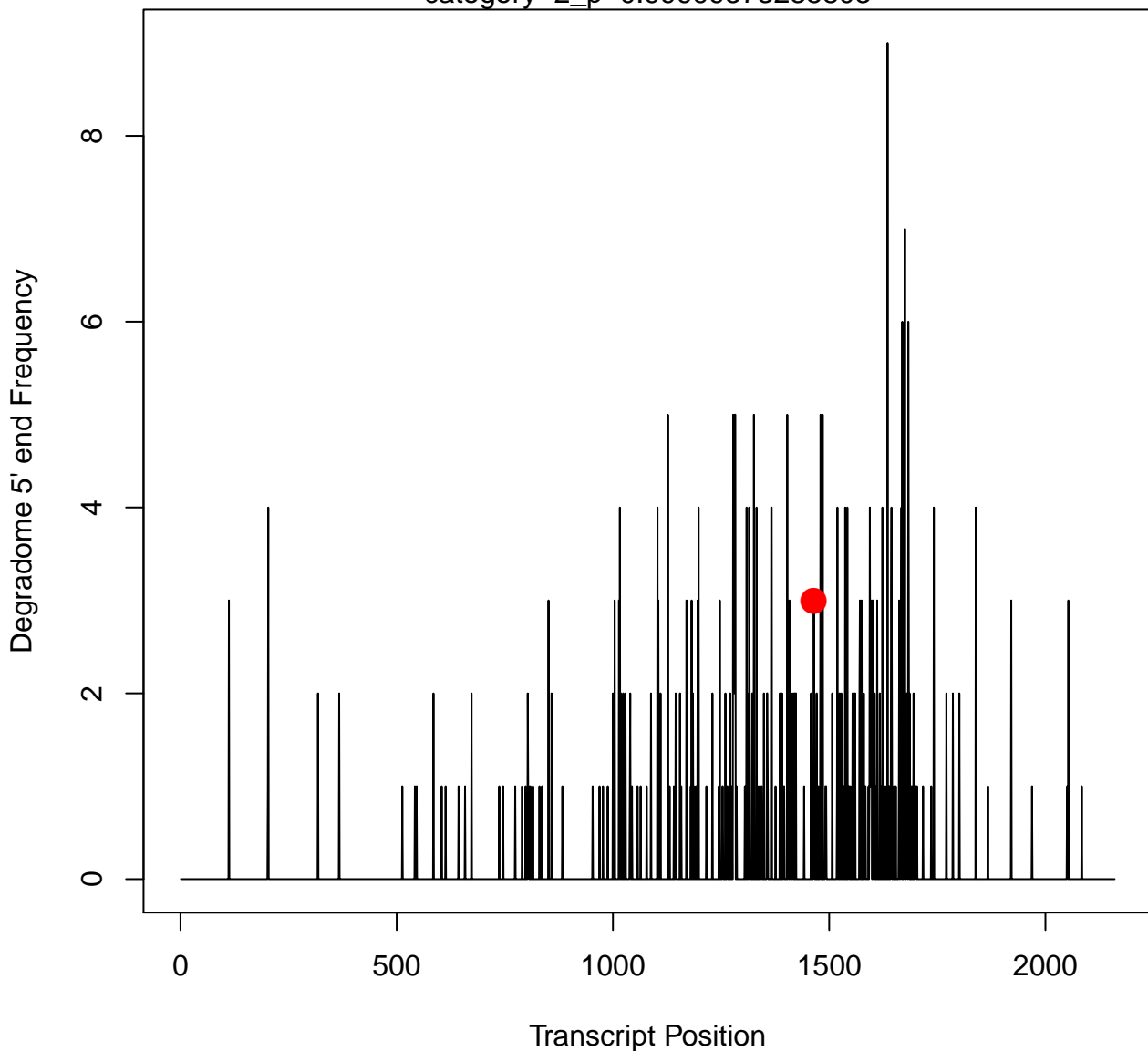

Supplement: Supplementary file 4 [file Data_Sheet_4.zip › Sit-miR160c_Seita.2G283800.1_1464_TPlot.pdf]

**T=Seita.2G444000.1\_Q=Sit-miR160c\_S=581**

category=2\_p=0.999999969642698

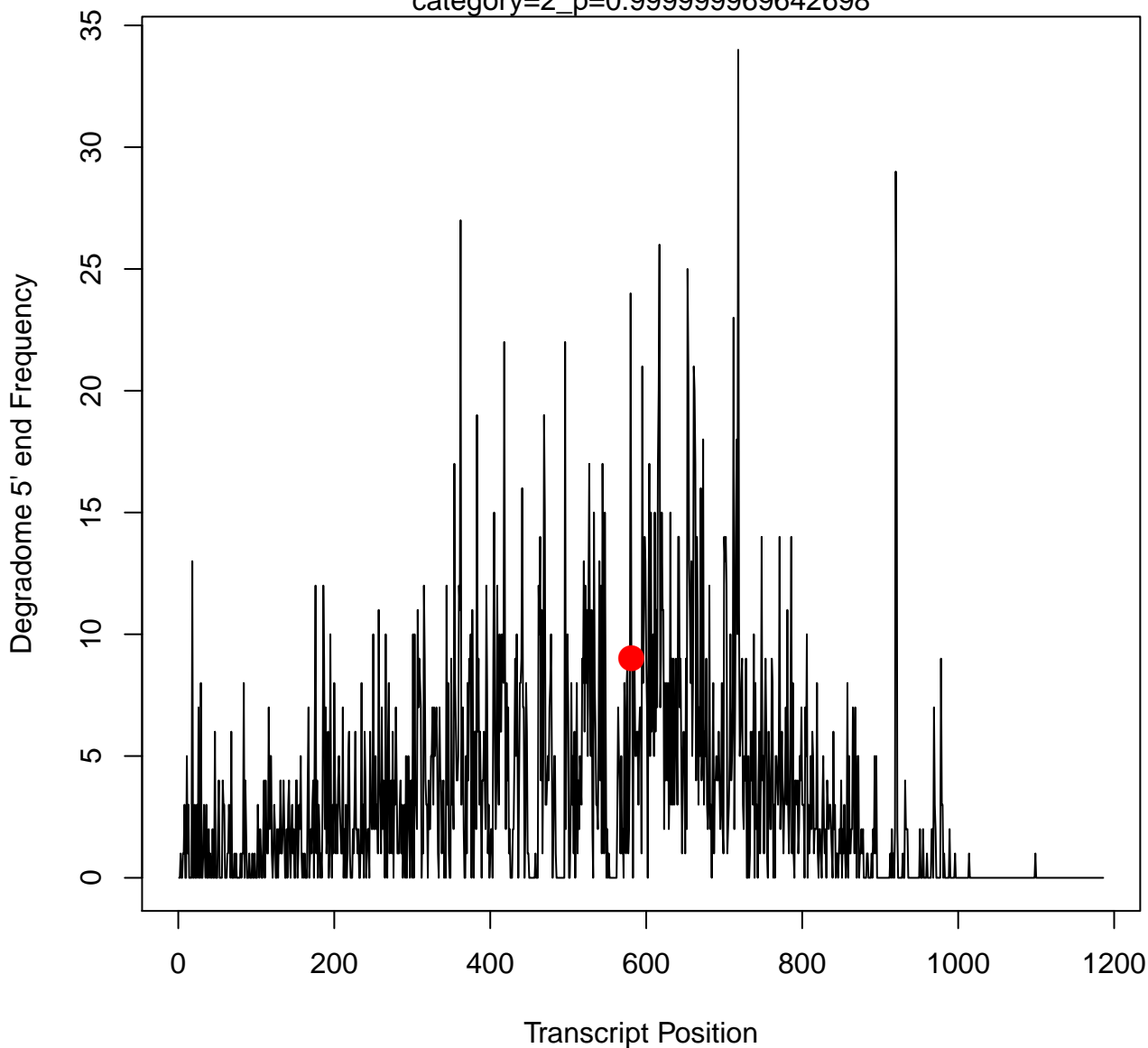

Supplement: Supplementary file 4 [file Data_Sheet_4.zip › Sit-miR160c_Seita.2G444000.1_581_TPlot.pdf]

**T=Seita.3G003300.1\_Q=Sit-miR160c\_S=1363**

category=0\_p=0.000381927958281736

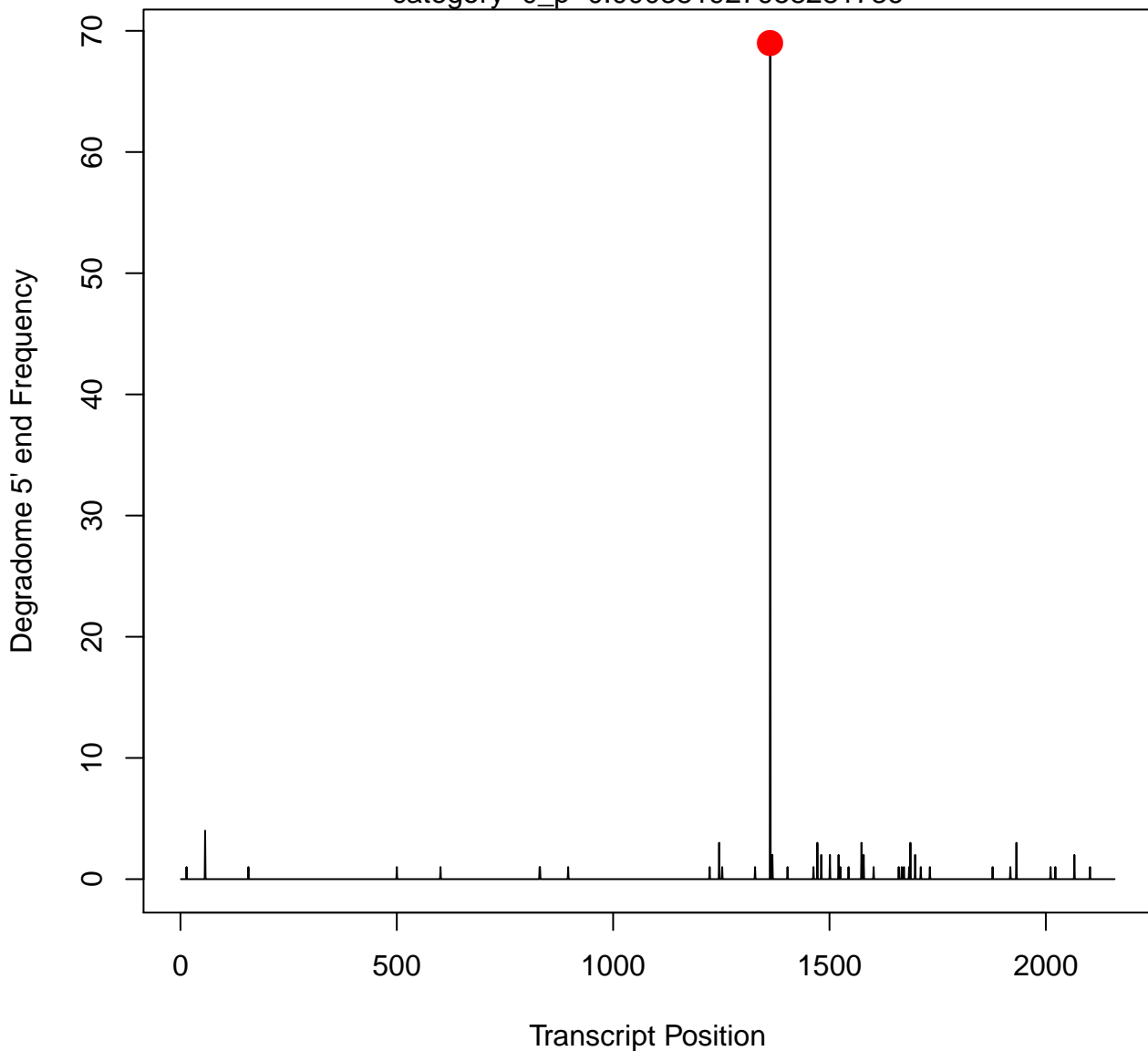

Supplement: Supplementary file 4 [file Data_Sheet_4.zip › Sit-miR160c_Seita.3G003300.1_1363_TPlot.pdf]

**T=Seita.3G014300.1\_Q=Sit-miR160c\_S=744**

category=2\_p=0.999999793034326

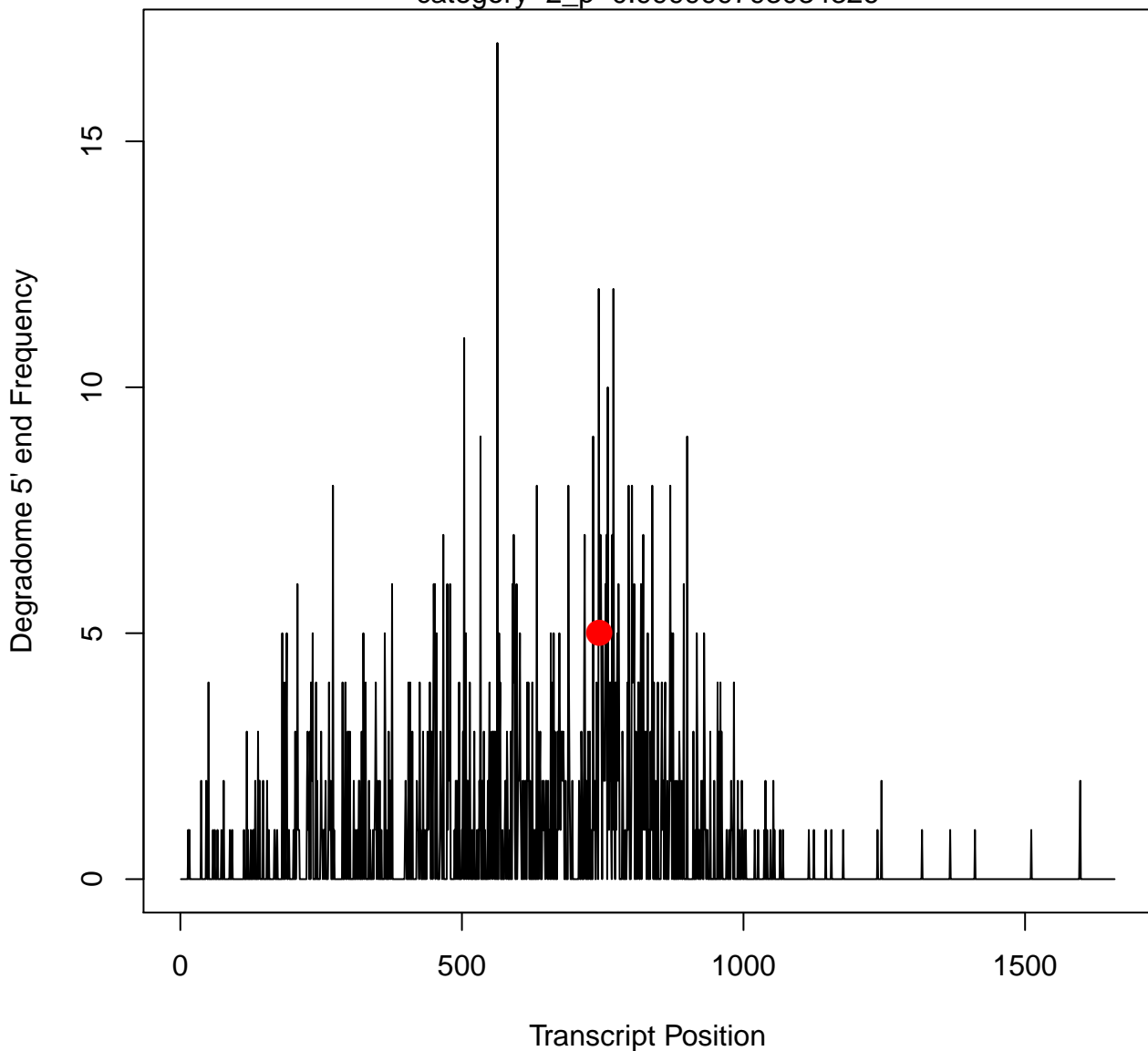

Supplement: Supplementary file 4 [file Data_Sheet_4.zip › Sit-miR160c_Seita.3G014300.1_744_TPlot.pdf]

**T=Seita.3G017100.1\_Q=Sit-miR160c\_S=1483**

category=2\_p=0.999999993921281

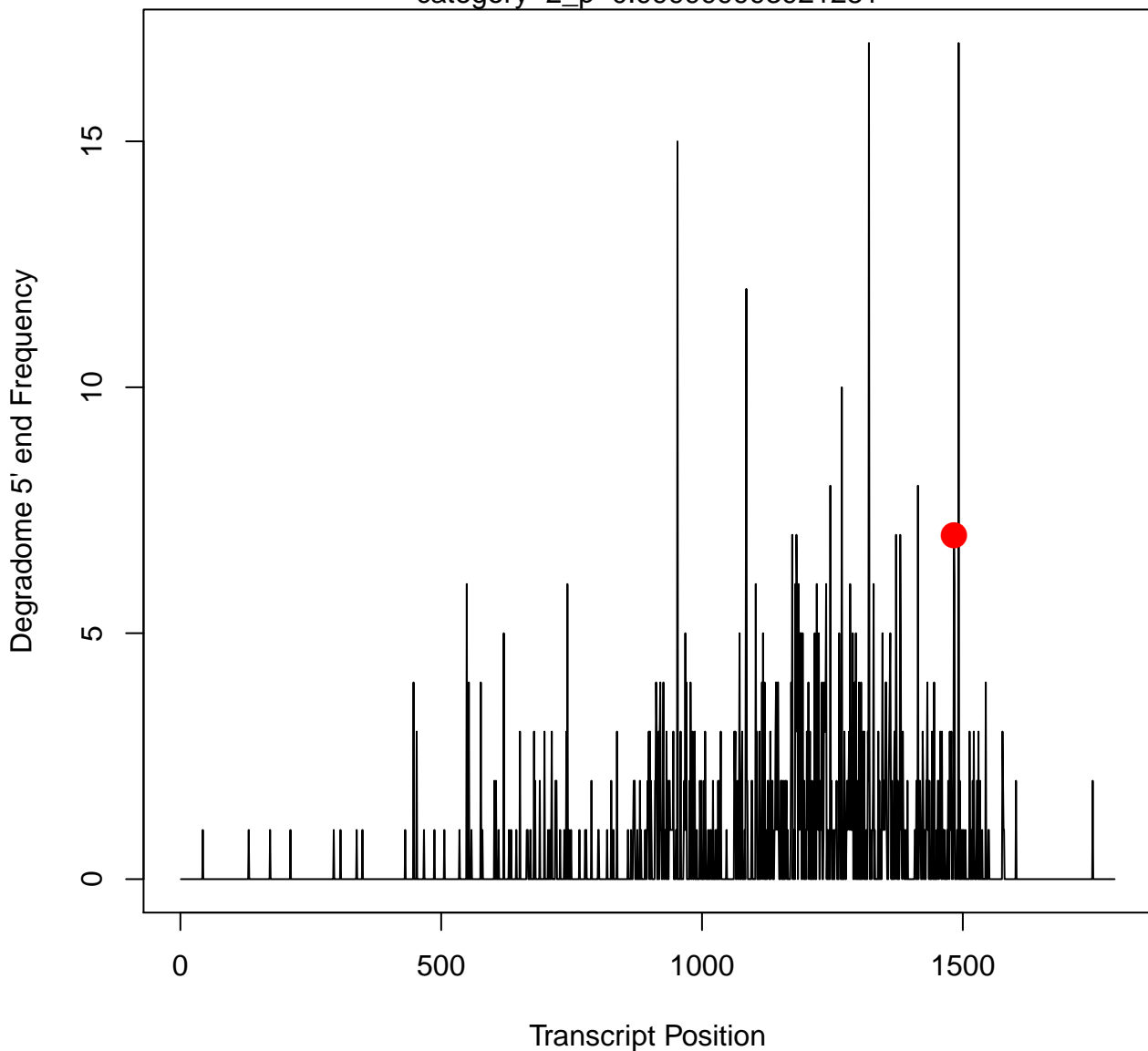

Supplement: Supplementary file 4 [file Data_Sheet_4.zip › Sit-miR160c_Seita.3G017100.1_1483_TPlot.pdf]

**T=Seita.3G025000.1\_Q=Sit-miR160c\_S=957**

category=2\_p=0.999660940975195

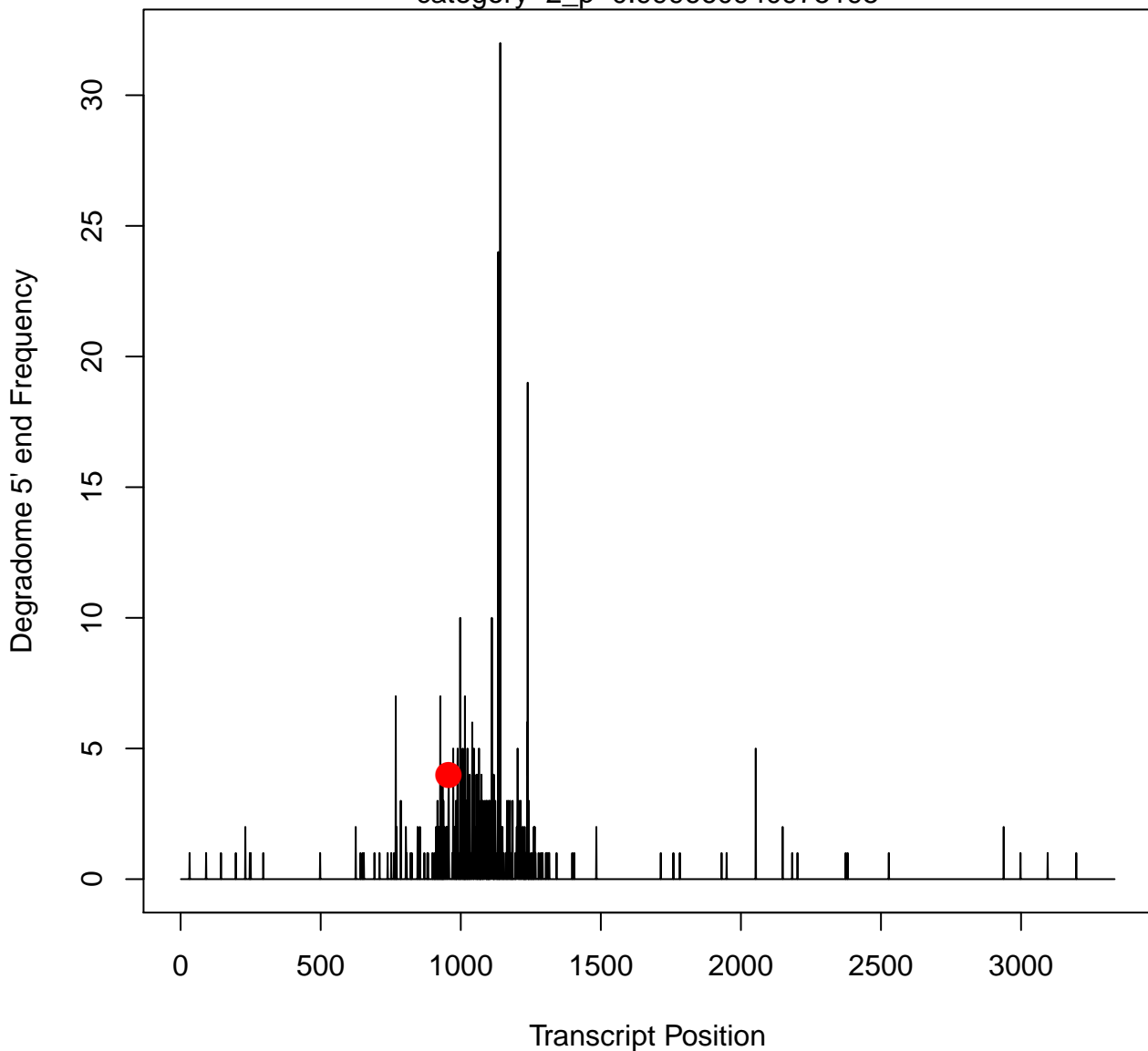

Supplement: Supplementary file 4 [file Data_Sheet_4.zip › Sit-miR160c_Seita.3G025000.1_957_TPlot.pdf]

**T=Seita.4G112500.1\_Q=Sit-miR160c\_S=441**

category=2\_p=0.999948517383771

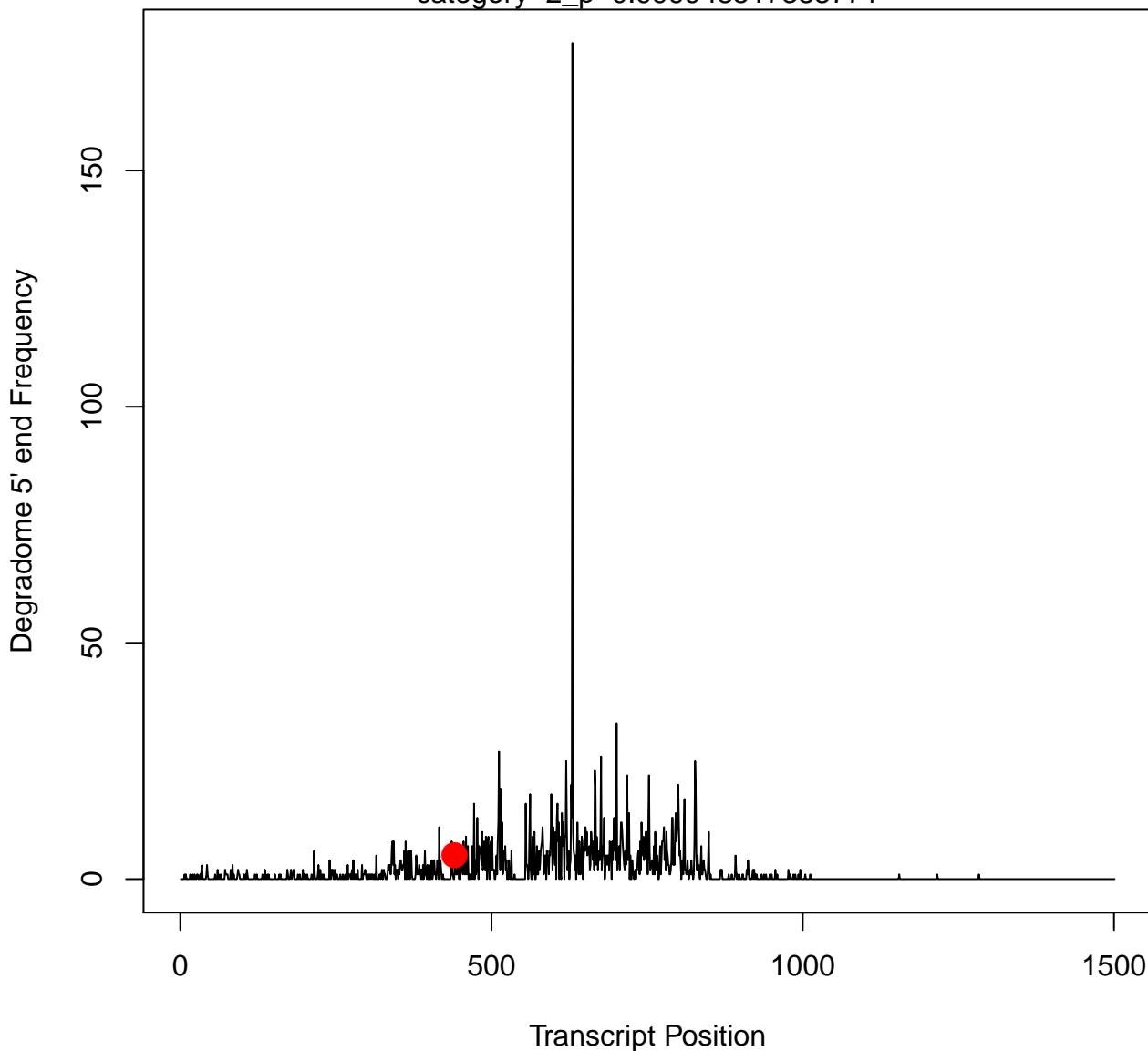

Supplement: Supplementary file 4 [file Data_Sheet_4.zip › Sit-miR160c_Seita.4G112500.1_441_TPlot.pdf]

**T=Seita.5G021300.1\_Q=Sit-miR160c\_S=471**

category=2\_p=0.809884184873285

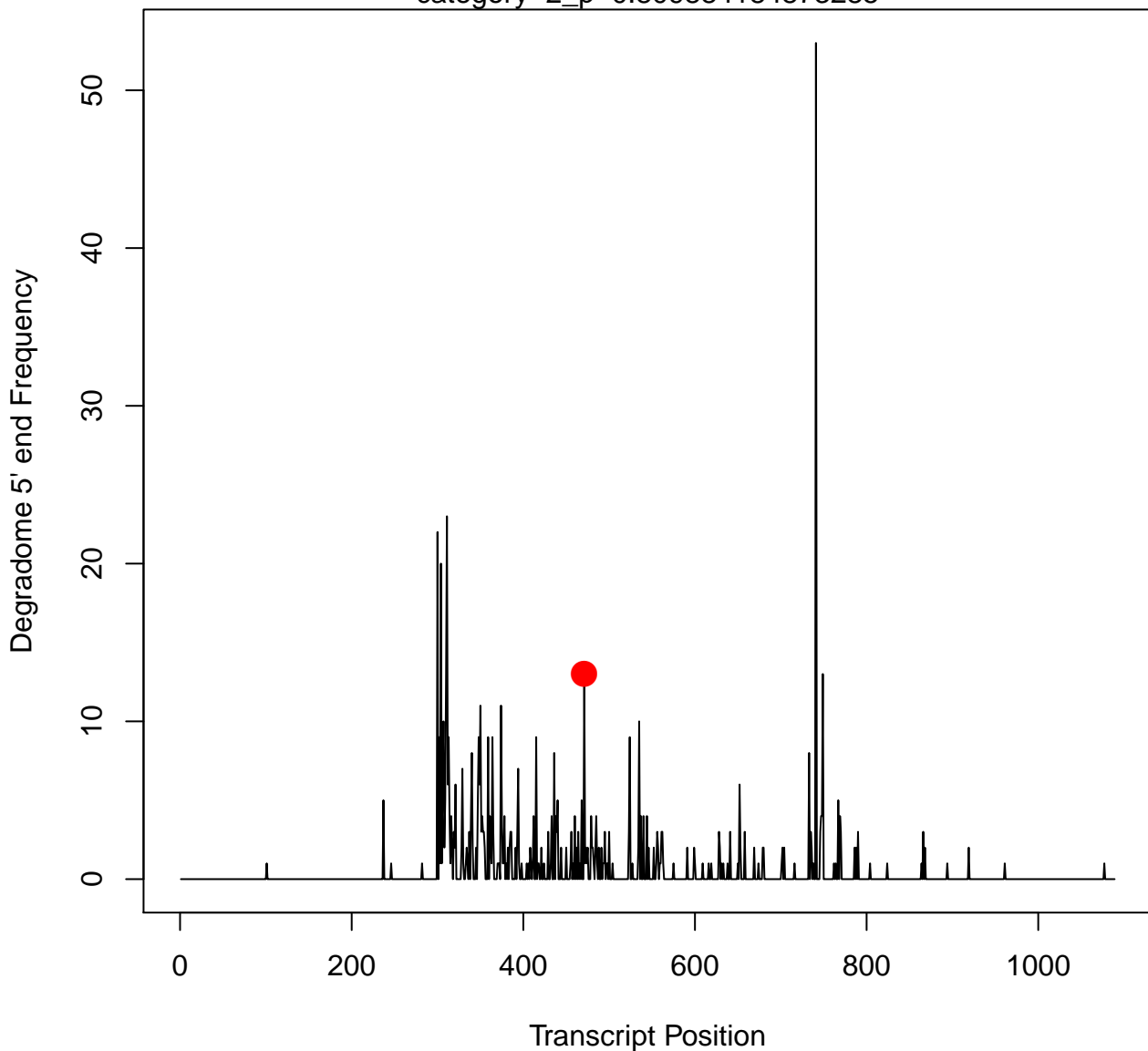

Supplement: Supplementary file 4 [file Data_Sheet_4.zip › Sit-miR160c_Seita.5G021300.1_471_TPlot.pdf]

**T=Seita.5G079900.1\_Q=Sit-miR160c\_S=2436**

category=2\_p=0.999999999847196

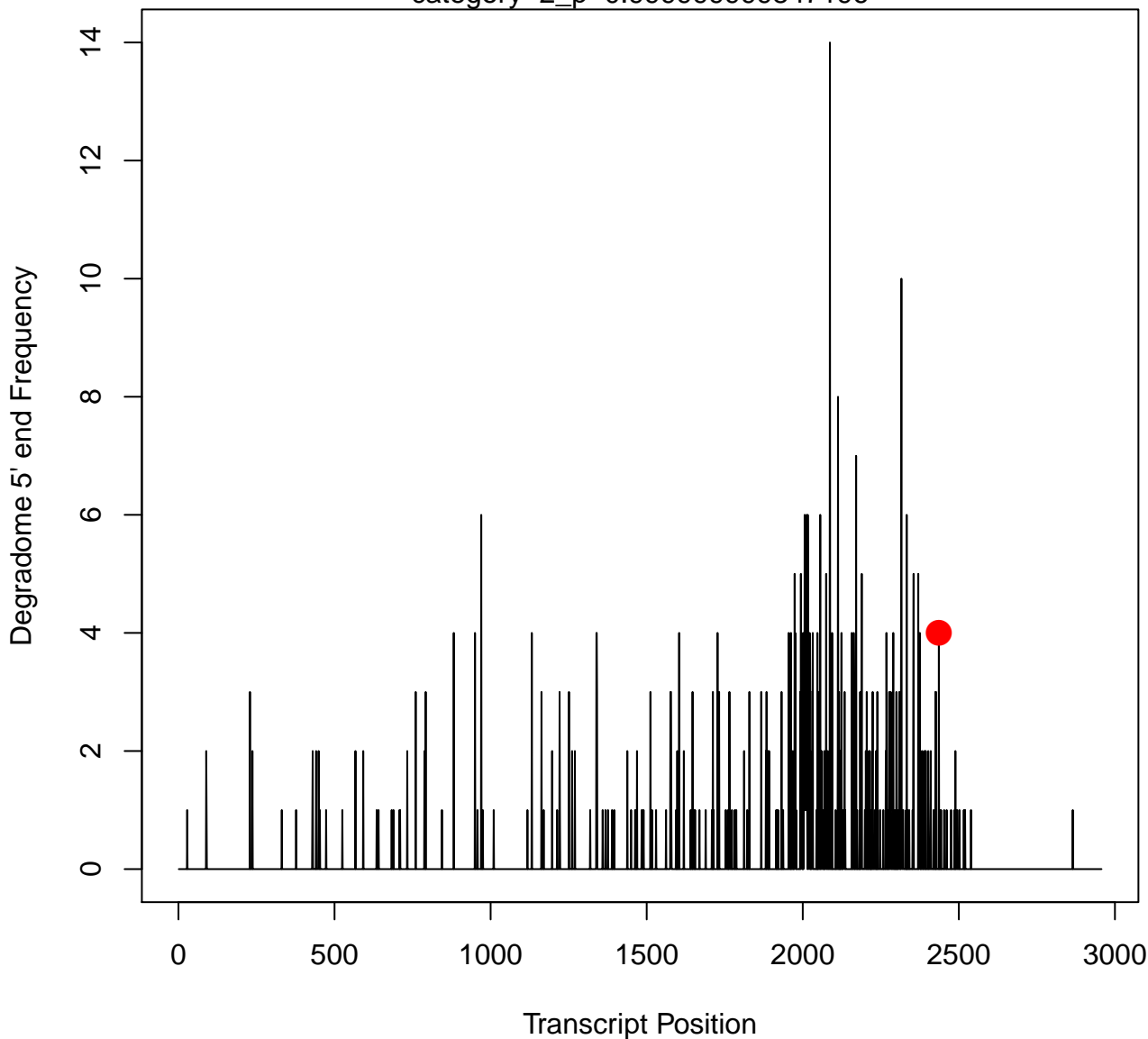

Supplement: Supplementary file 4 [file Data_Sheet_4.zip › Sit-miR160c_Seita.5G079900.1_2436_TPlot.pdf]

**T=Seita.5G153500.1\_Q=Sit-miR160c\_S=1192**

category=2\_p=0.999985926775579

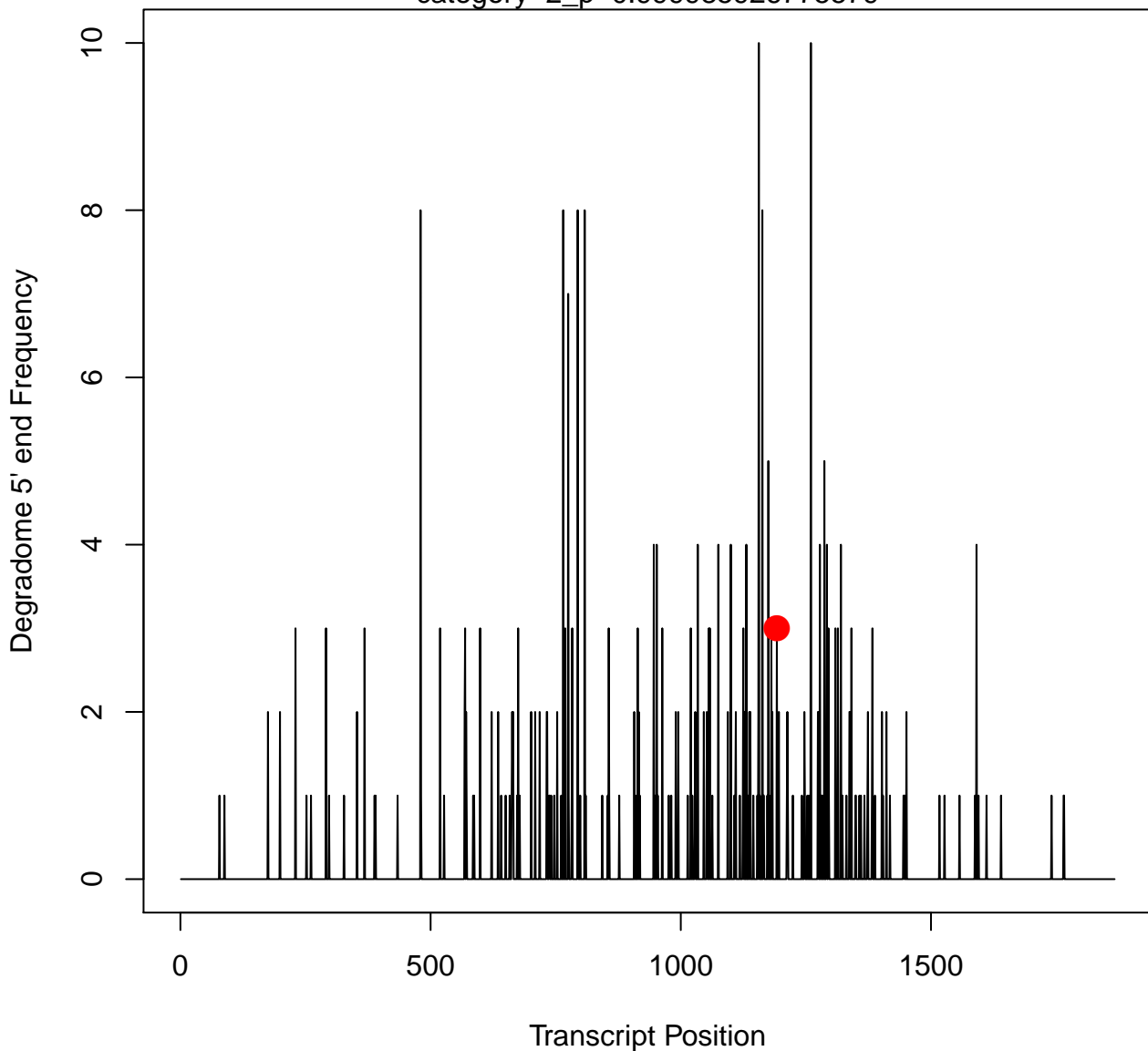

Supplement: Supplementary file 4 [file Data_Sheet_4.zip › Sit-miR160c_Seita.5G153500.1_1192_TPlot.pdf]

**T=Seita.5G165100.1\_Q=Sit-miR160c\_S=762**

category=2\_p=0.822590308979964

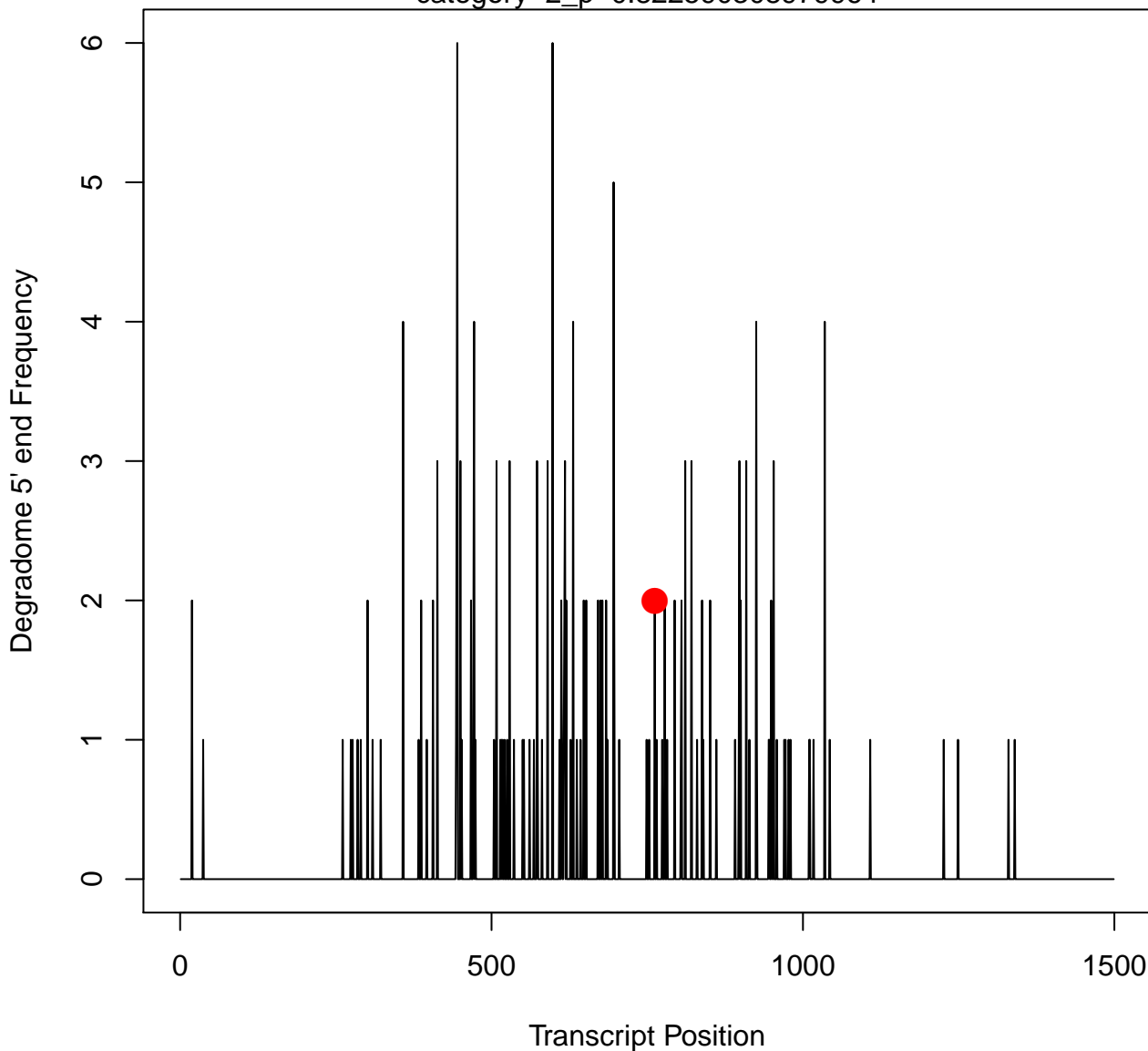

Supplement: Supplementary file 4 [file Data_Sheet_4.zip › Sit-miR160c_Seita.5G165100.1_762_TPlot.pdf]

**T=Seita.5G338300.1\_Q=Sit-miR160c\_S=373**

category=2\_p=0.999999160101749

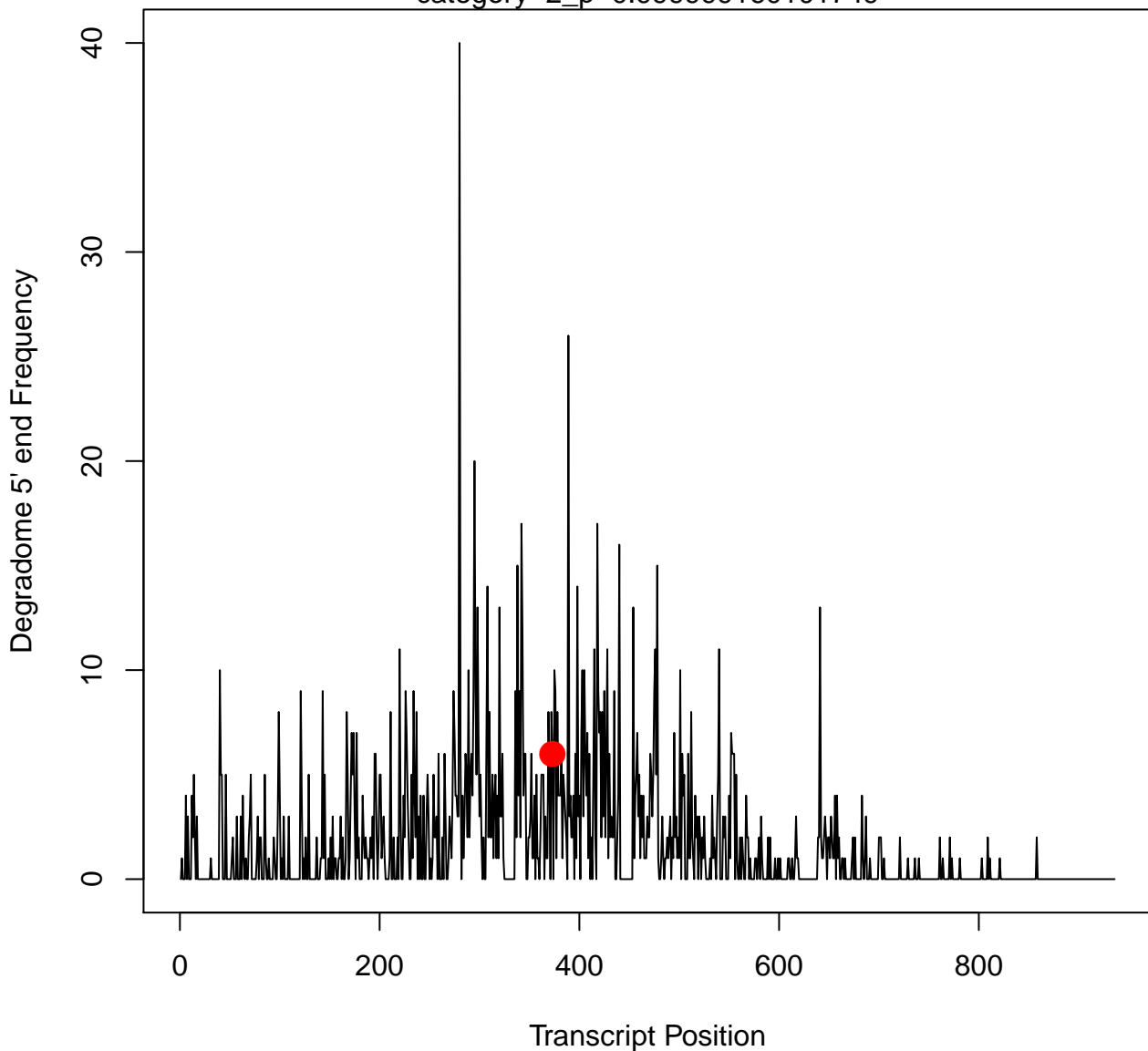

Supplement: Supplementary file 4 [file Data_Sheet_4.zip › Sit-miR160c_Seita.5G338300.1_373_TPlot.pdf]

**T=Seita.5G378000.1\_Q=Sit-miR160c\_S=596**

category=2\_p=0.99999999919626

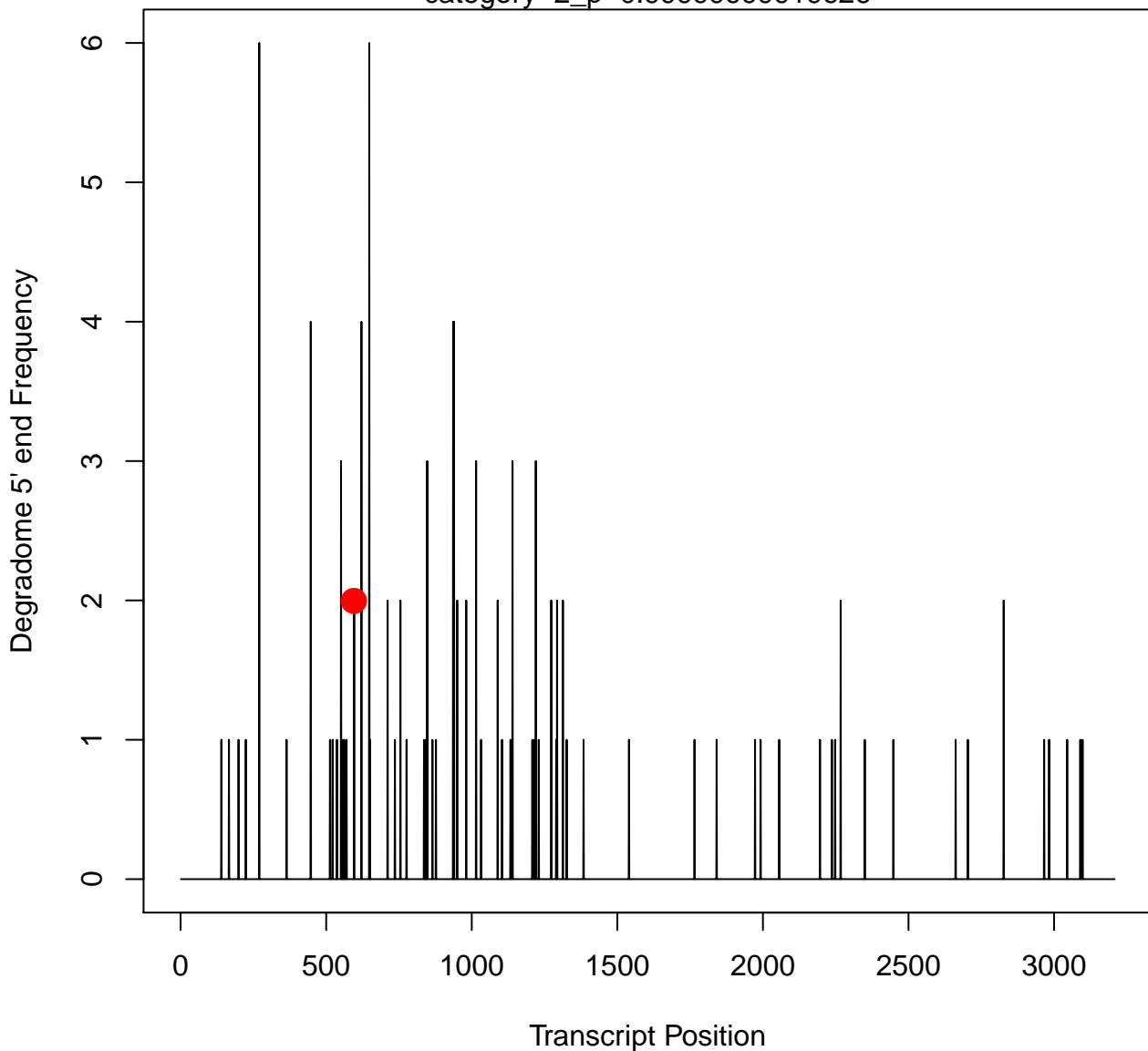

Supplement: Supplementary file 4 [file Data_Sheet_4.zip › Sit-miR160c_Seita.5G378000.1_596_TPlot.pdf]

**T=Seita.6G170500.1\_Q=Sit-miR160c\_S=2580**

category=2\_p=0.0985562325655811

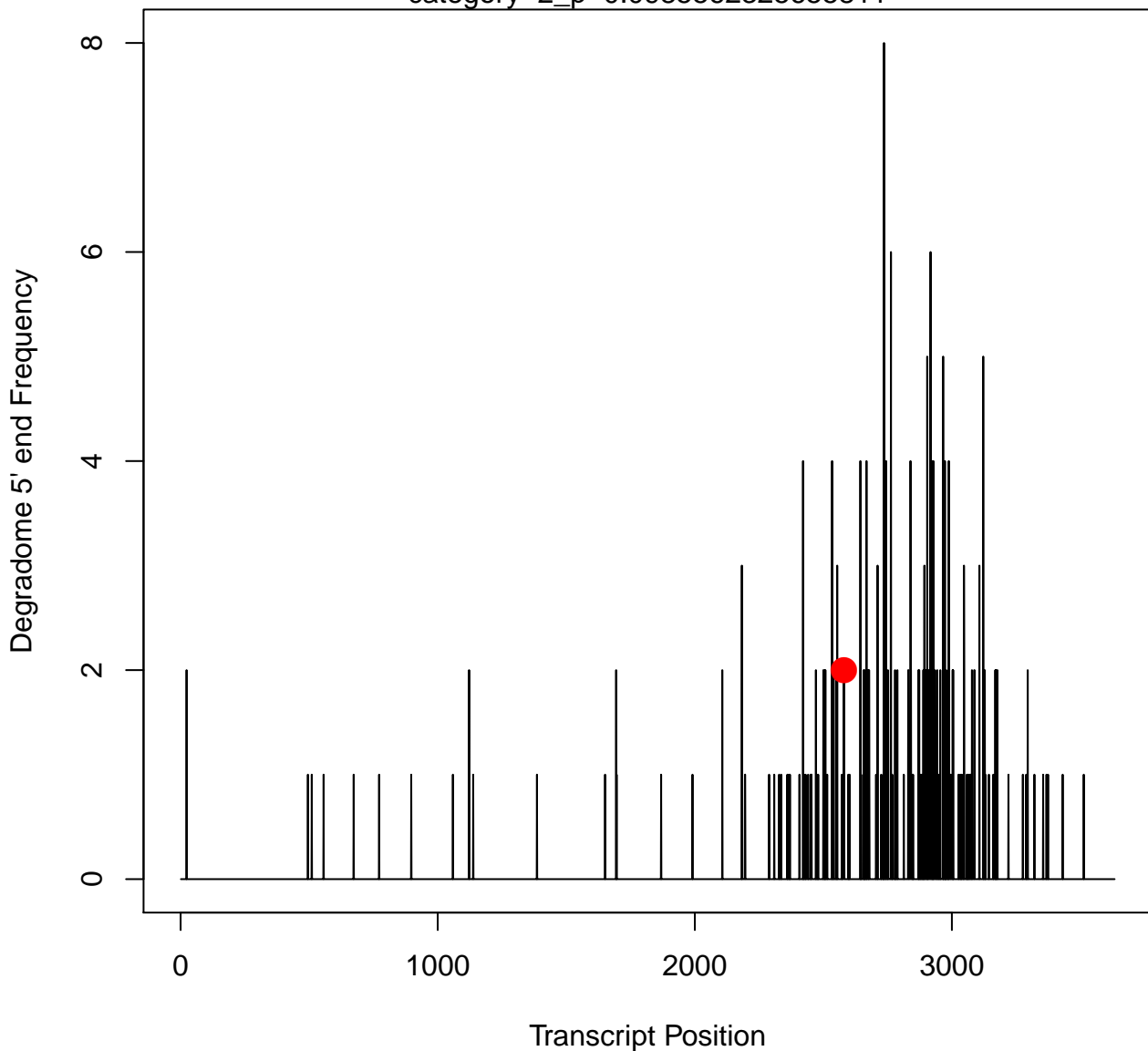

Supplement: Supplementary file 4 [file Data_Sheet_4.zip › Sit-miR160c_Seita.6G170500.1_2580_TPlot.pdf]

**T=Seita.7G216100.1\_Q=Sit-miR160c\_S=932**

category=2\_p=0.999978315506703

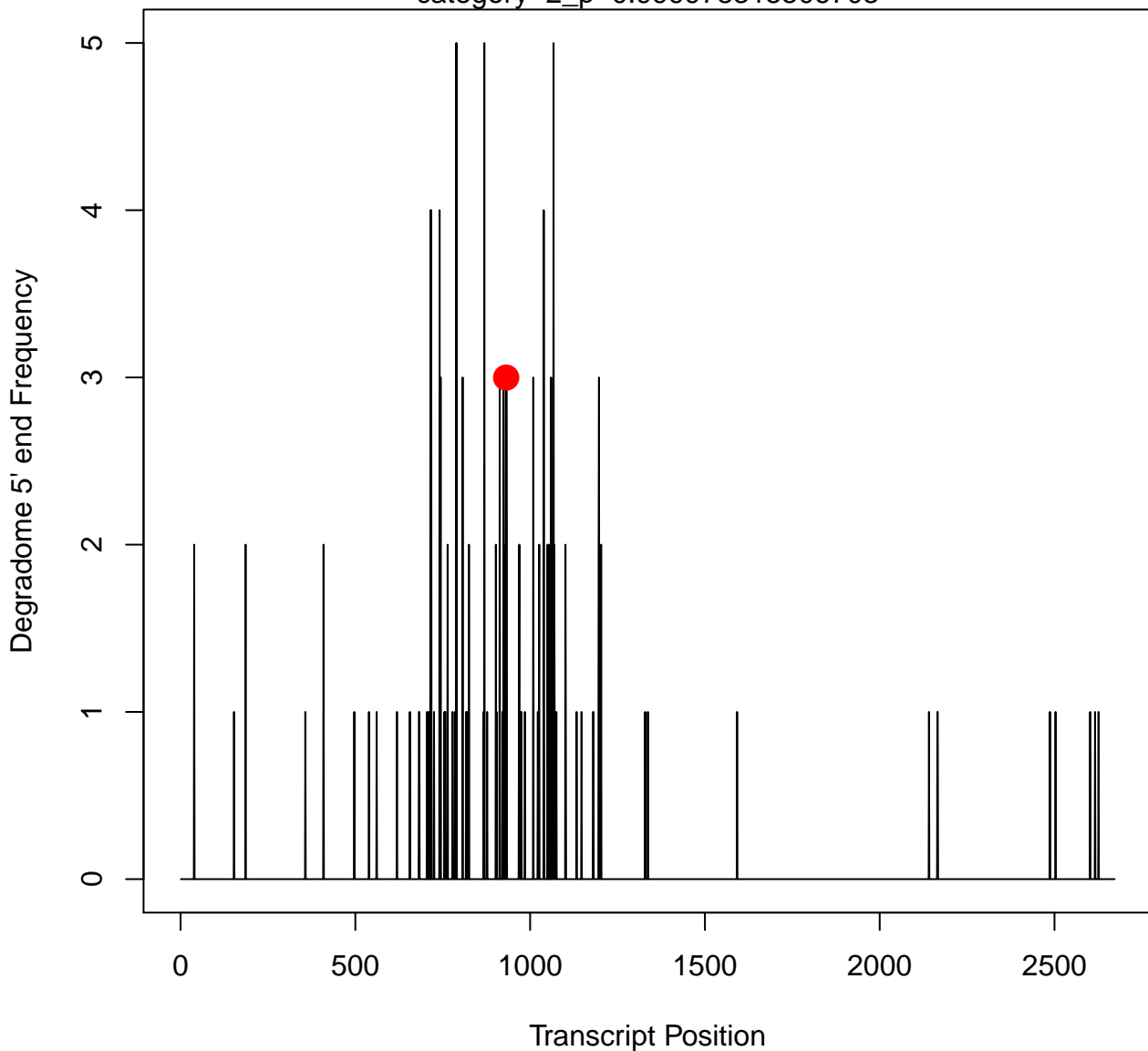

Supplement: Supplementary file 4 [file Data_Sheet_4.zip › Sit-miR160c_Seita.7G216100.1_932_TPlot.pdf]

**T=Seita.9G045100.1\_Q=Sit-miR160c\_S=835**

category=2\_p=0.984510571715051

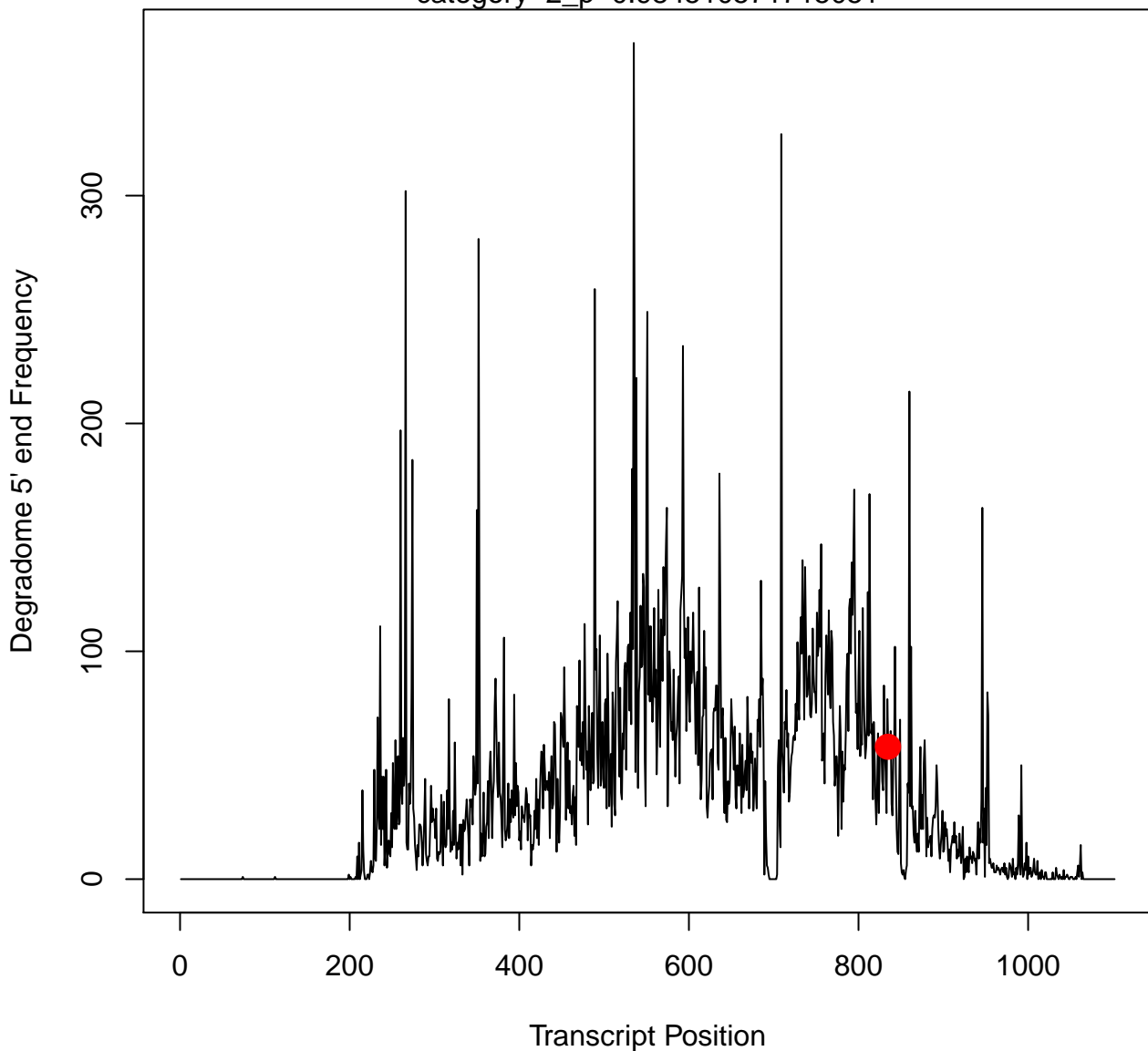

Supplement: Supplementary file 4 [file Data_Sheet_4.zip › Sit-miR160c_Seita.9G045100.1_835_TPlot.pdf]

**T=Seita.9G236900.1\_Q=Sit-miR160c\_S=1291**

category=2\_p=0.979573262217083

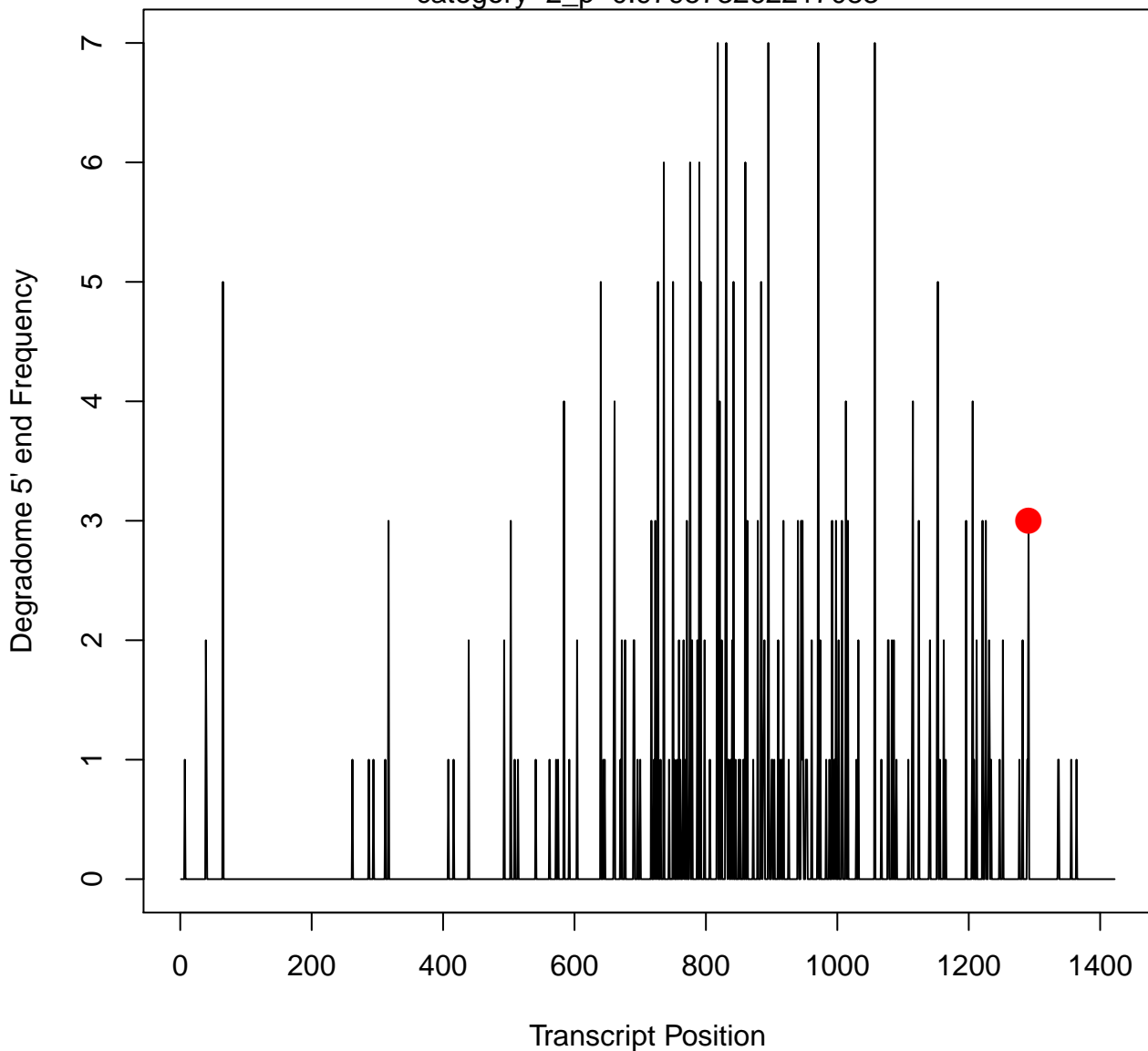

Supplement: Supplementary file 4 [file Data_Sheet_4.zip › Sit-miR160c_Seita.9G236900.1_1291_TPlot.pdf]

**T=Seita.9G250900.1\_Q=Sit-miR160c\_S=327**

category=1\_p=0.0301515596916417

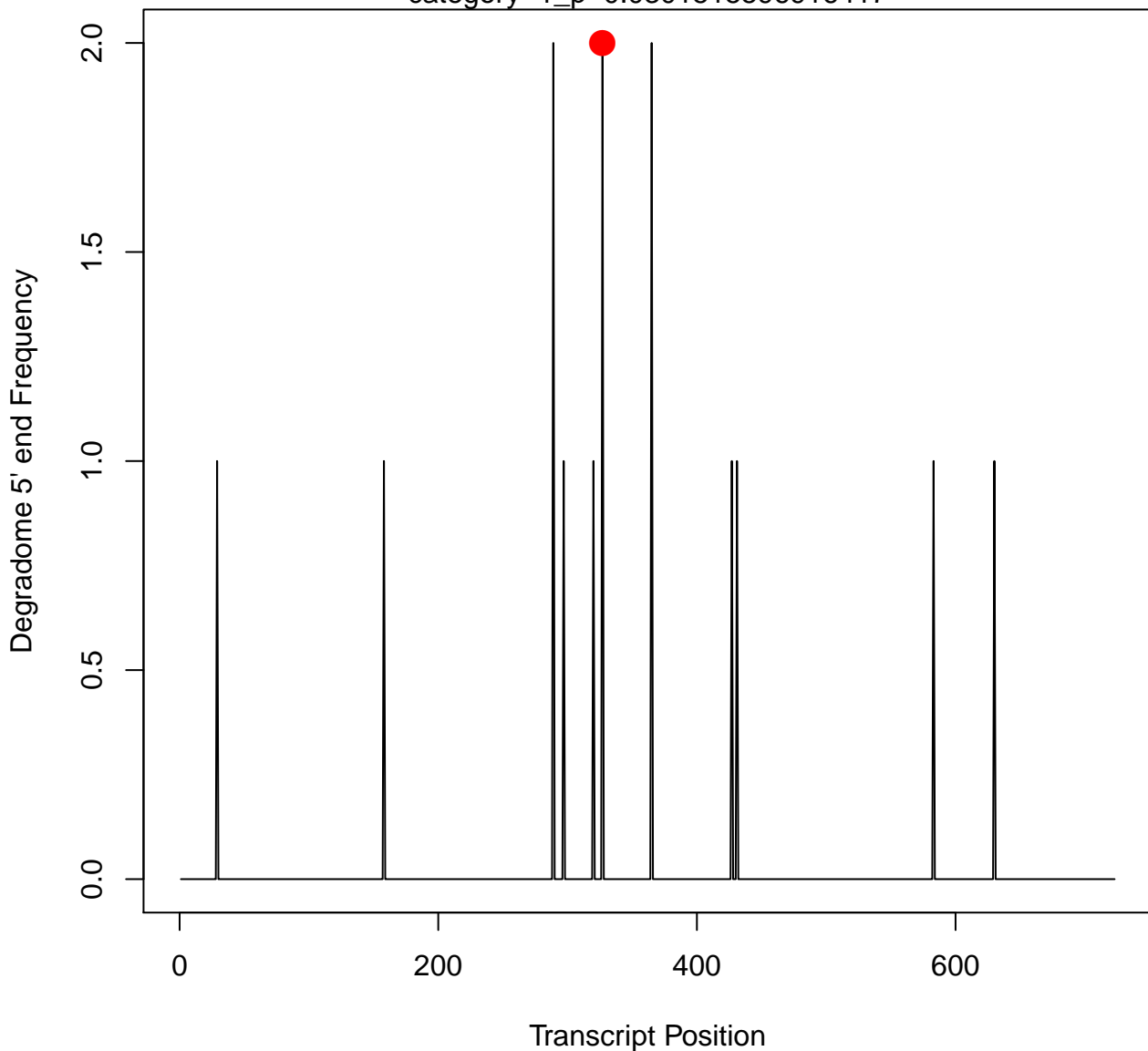

Supplement: Supplementary file 4 [file Data_Sheet_4.zip › Sit-miR160c_Seita.9G250900.1_327_TPlot.pdf]

**T=Seita.9G333200.1\_Q=Sit-miR160c\_S=695**

category=2\_p=0.999999914320531

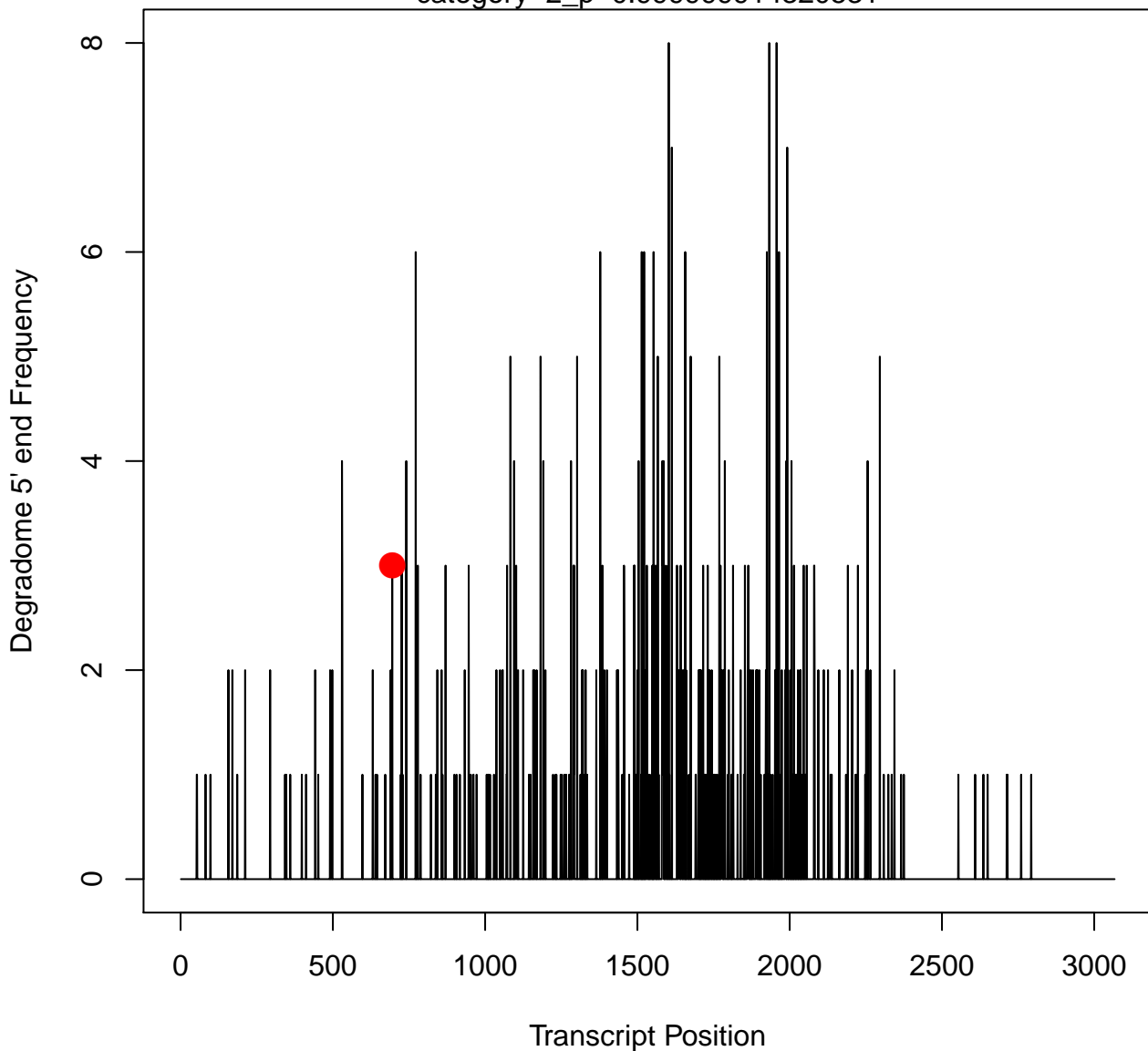

Supplement: Supplementary file 4 [file Data_Sheet_4.zip › Sit-miR160c_Seita.9G333200.1_695_TPlot.pdf]

**T=Seita.1G109500.1\_Q=Sit-miR160d\_S=471**

category=2\_p=0.999999997846234

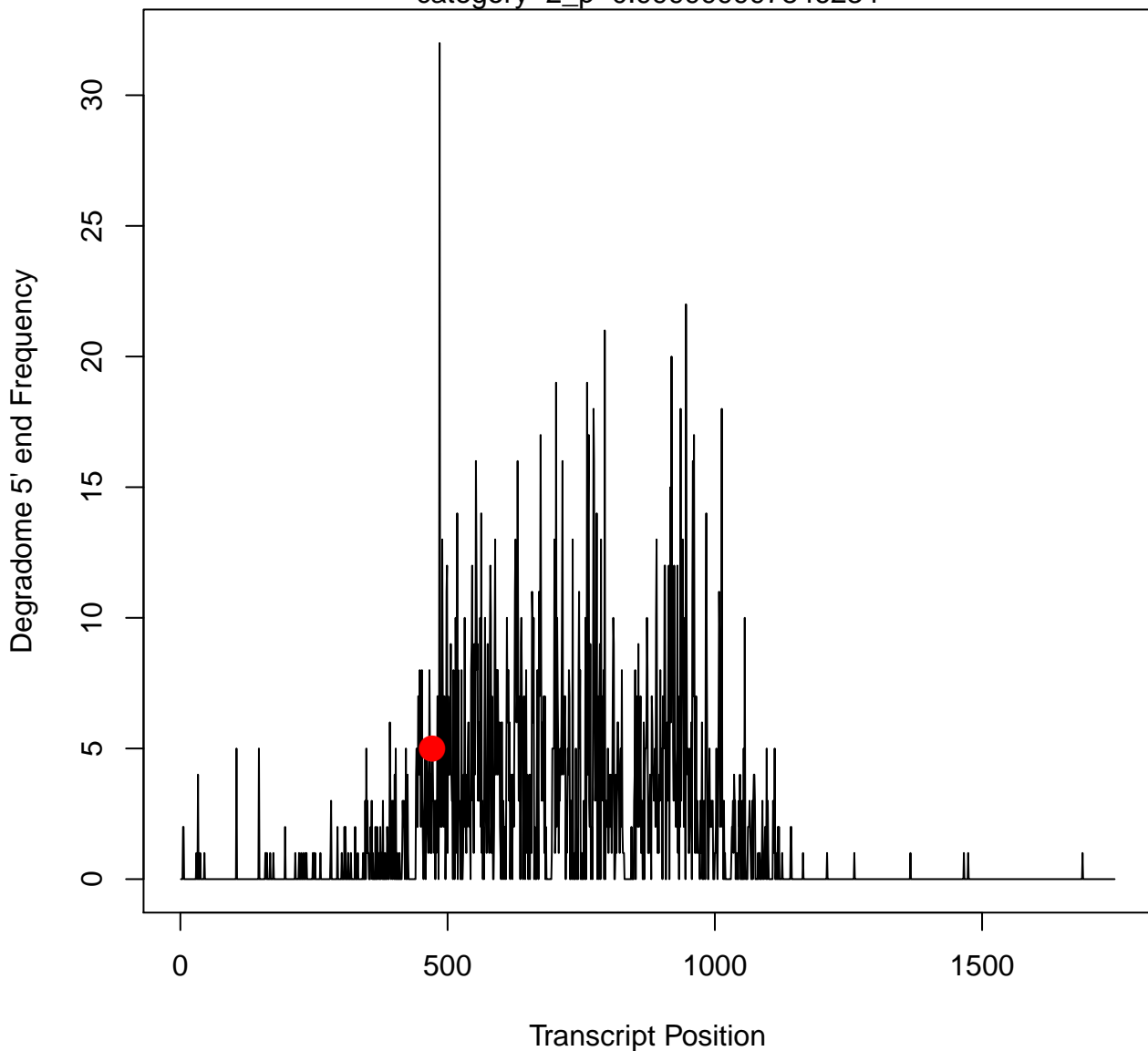

Supplement: Supplementary file 4 [file Data_Sheet_4.zip › Sit-miR160d_Seita.1G109500.1_471_TPlot.pdf]

**T=Seita.1G168000.1\_Q=Sit-miR160d\_S=249**

category=2\_p=0.999994748169551

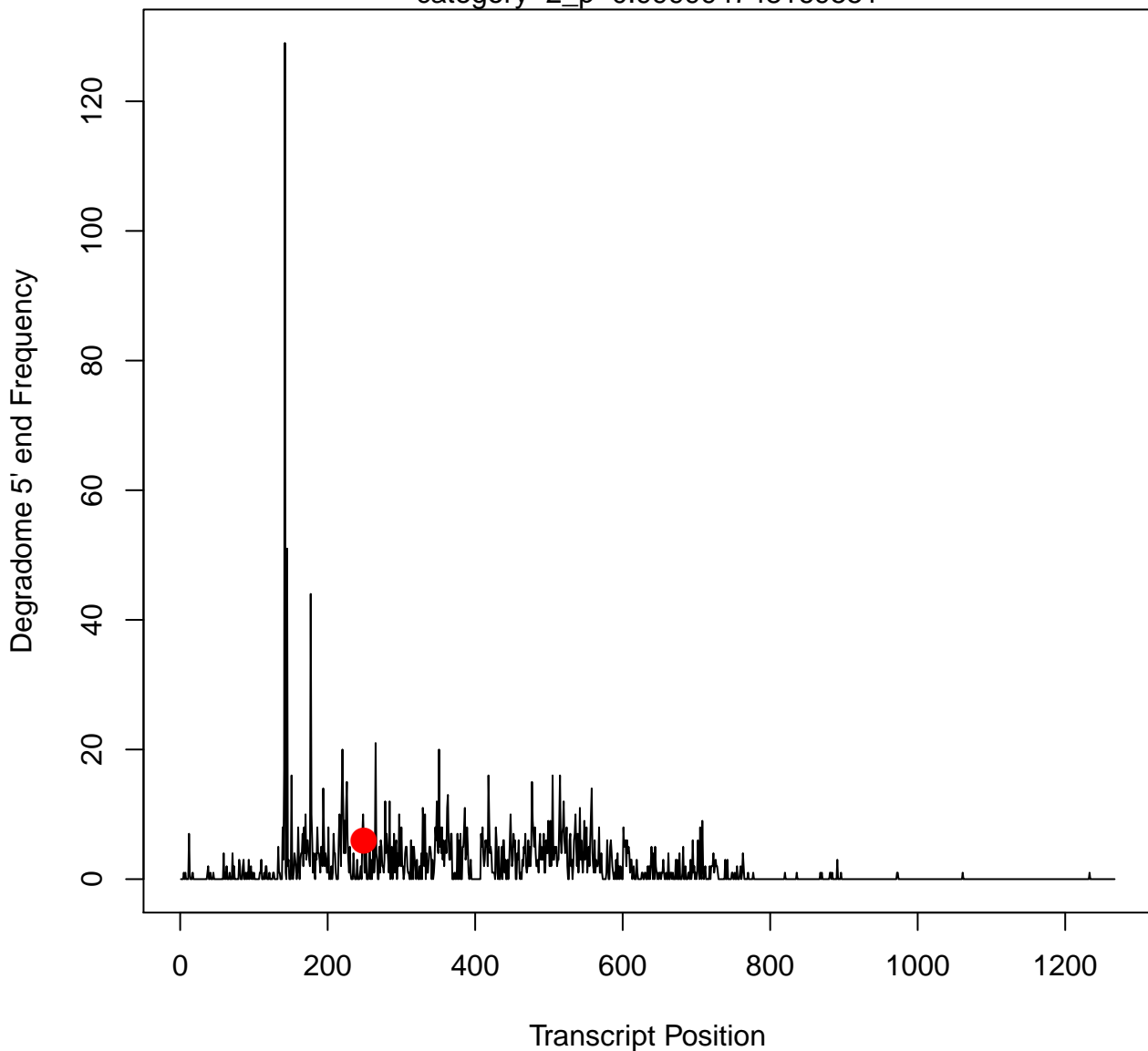

Supplement: Supplementary file 4 [file Data_Sheet_4.zip › Sit-miR160d_Seita.1G168000.1_249_TPlot.pdf]
