# Supplementary figures and images for: The Construction and Exploration of a Comprehensive MicroRNA Centered Regulatory Network in Foxtail Millet (Setaria italica L.) (part 10 of 14)
Source: Front Plant Sci. 2022 May 6;13:848474. doi: 10.3389/fpls.2022.848474 (PMC9121102; doi:10.3389/fpls.2022.848474)

**T=Seita.9G572600.1\_Q=Sit-miR166k\_S=1138**

category=0\_p=0.00084475590689892

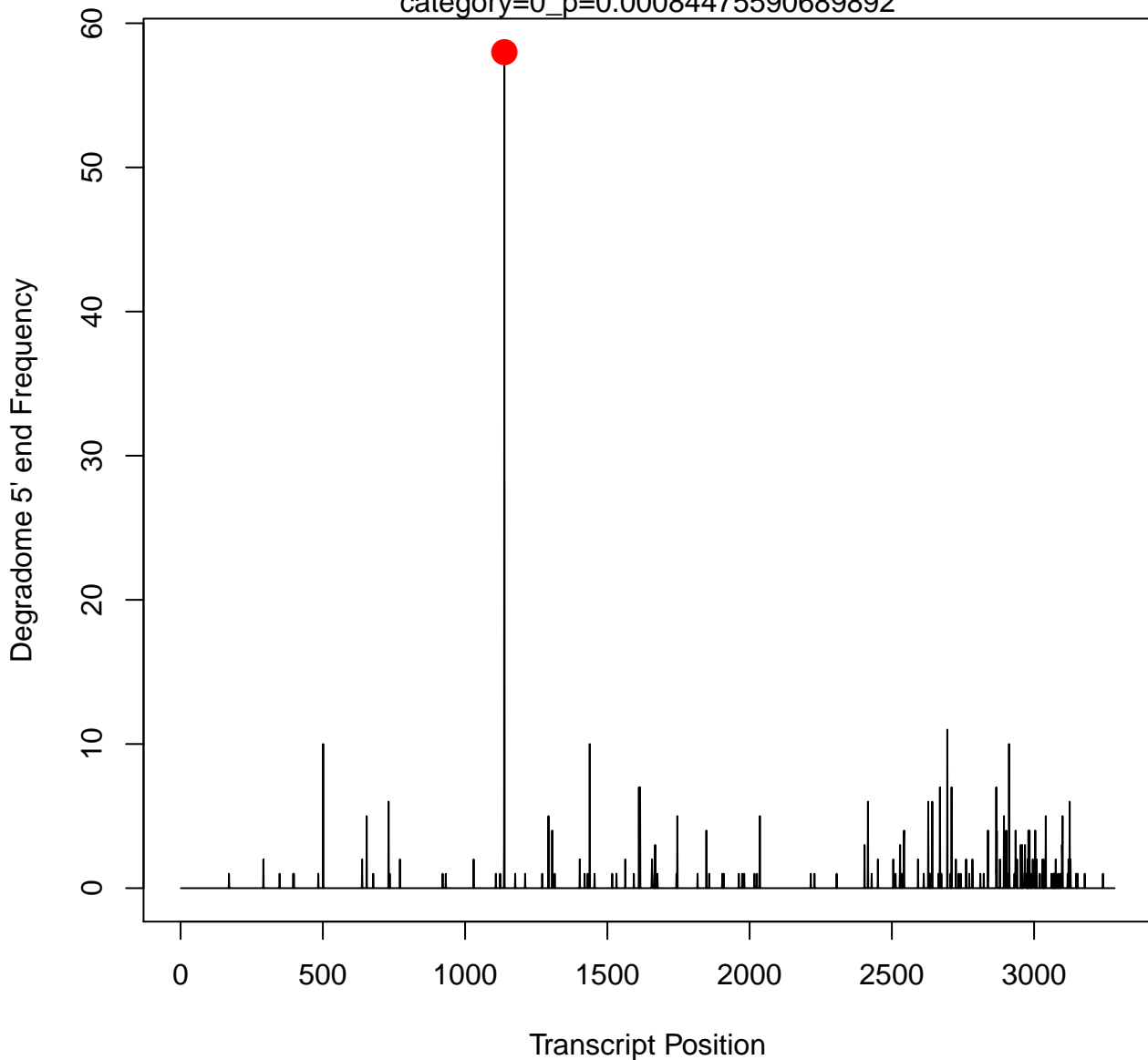

Supplement: Supplementary file 5 [file Data_Sheet_5.zip › Sit-miR166k_Seita.9G572600.1_1138_TPlot.pdf]

**T=Seita.1G035000.1\_Q=Sit-miR167a\_S=1306**

category=2\_p=0.934049776612893

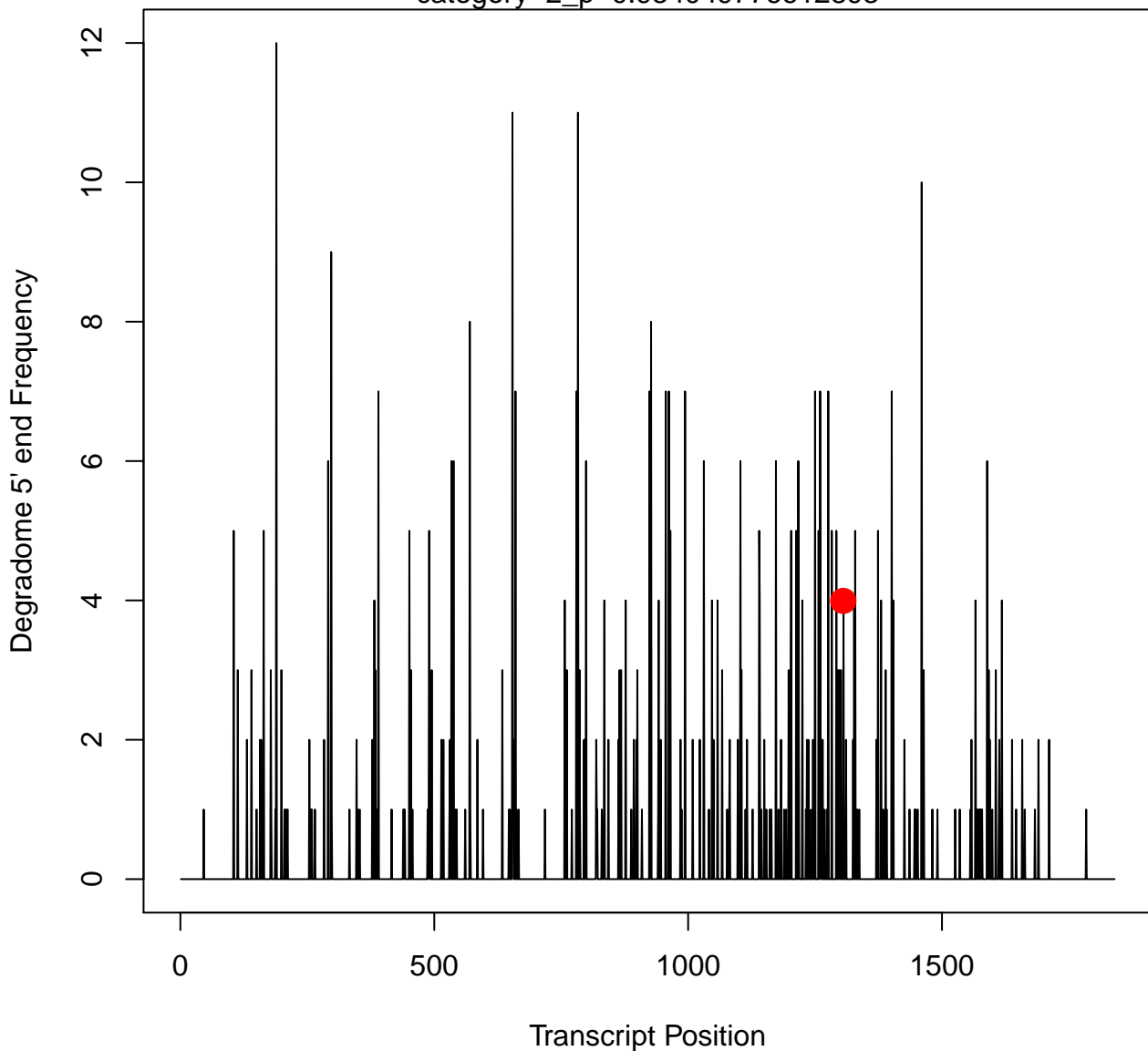

Supplement: Supplementary file 5 [file Data_Sheet_5.zip › Sit-miR167a_Seita.1G035000.1_1306_TPlot.pdf]

**T=Seita.2G255100.1\_Q=Sit-miR167a\_S=1619**

category=2\_p=0.345548011822793

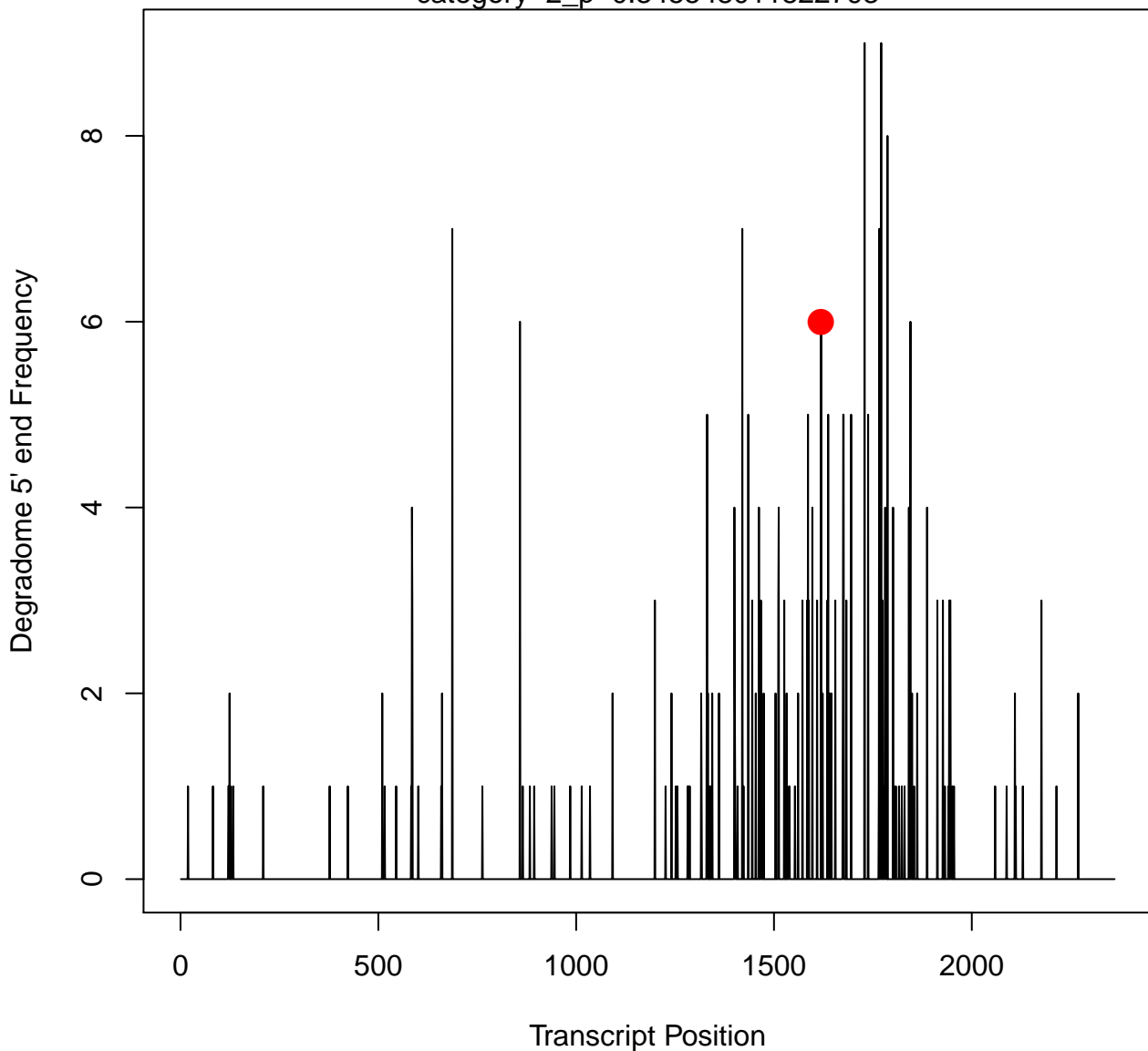

Supplement: Supplementary file 5 [file Data_Sheet_5.zip › Sit-miR167a_Seita.2G255100.1_1619_TPlot.pdf]

**T=Seita.3G020000.1\_Q=Sit-miR167h\_S=2739**

category=0\_p=0.00126686621795646

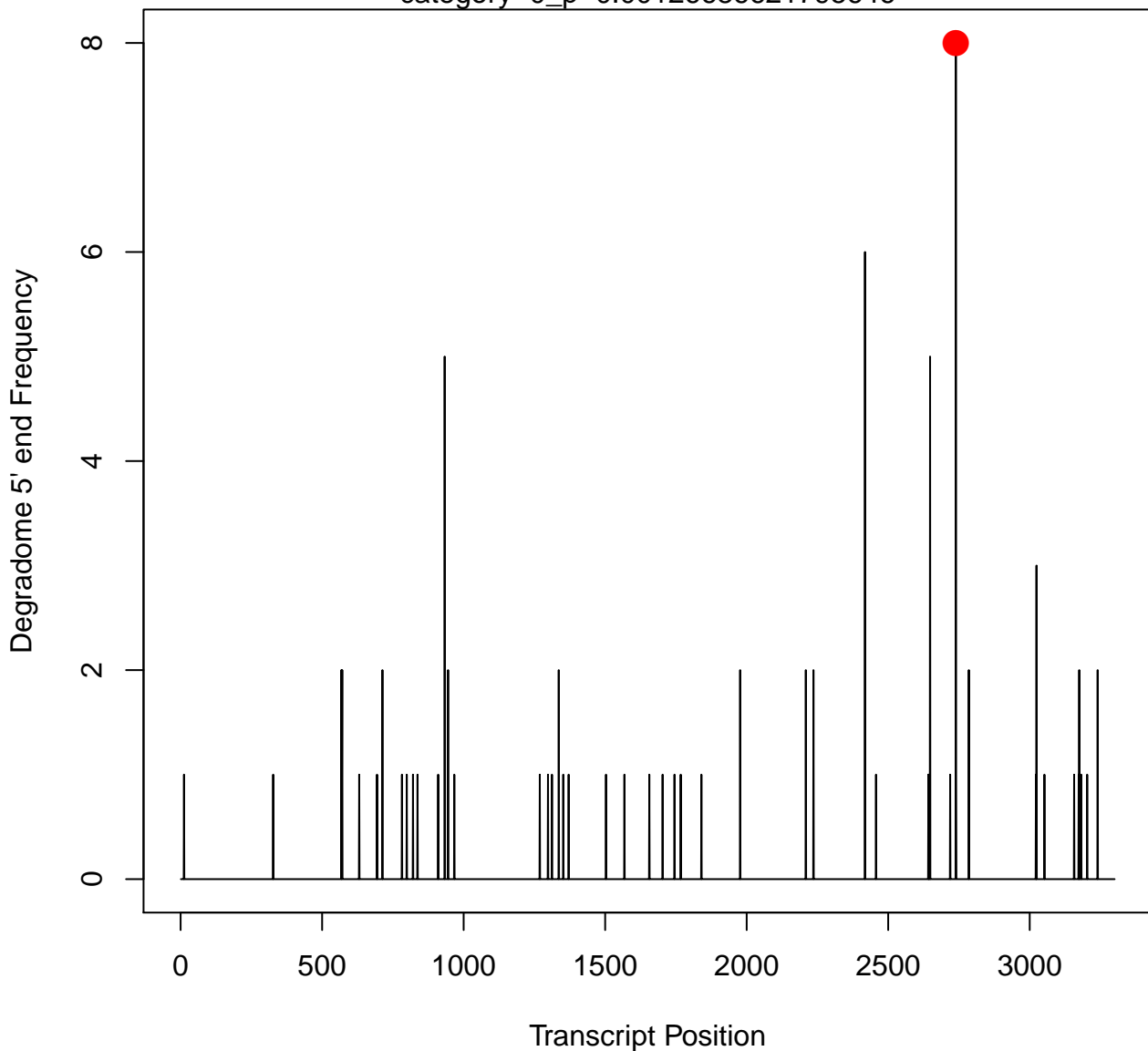

Supplement: Supplementary file 5 [file Data_Sheet_5.zip › Sit-miR167h_Seita.3G020000.1_2739_TPlot.pdf]

**T=Seita.4G262300.1\_Q=Sit-miR167h\_S=3389**

category=0\_p=0.00253212748589871

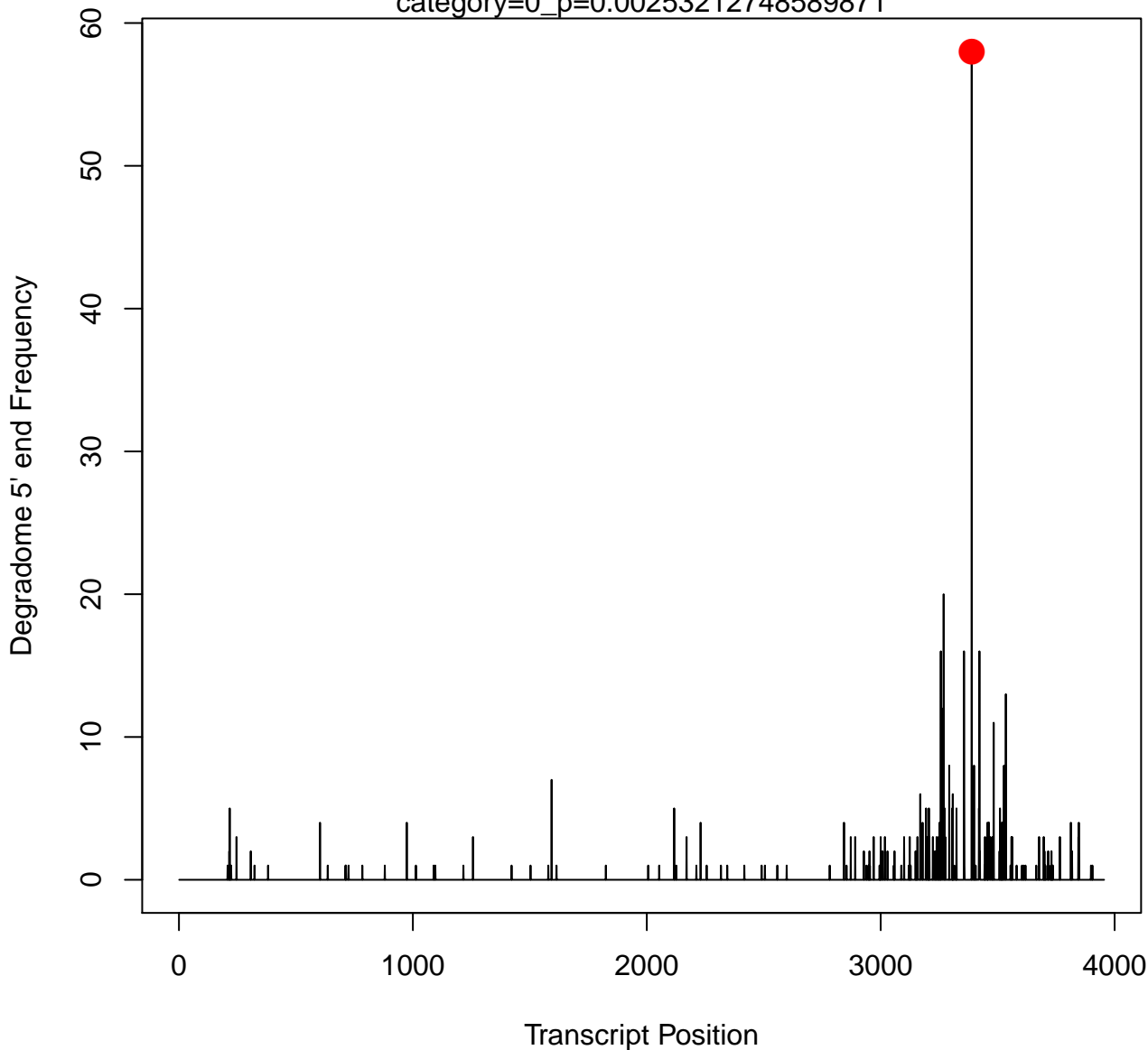

Supplement: Supplementary file 5 [file Data_Sheet_5.zip › Sit-miR167h_Seita.4G262300.1_3389_TPlot.pdf]

**T=Seita.5G393700.1\_Q=Sit-miR167h\_S=1599**

category=2\_p=0.97650806883866

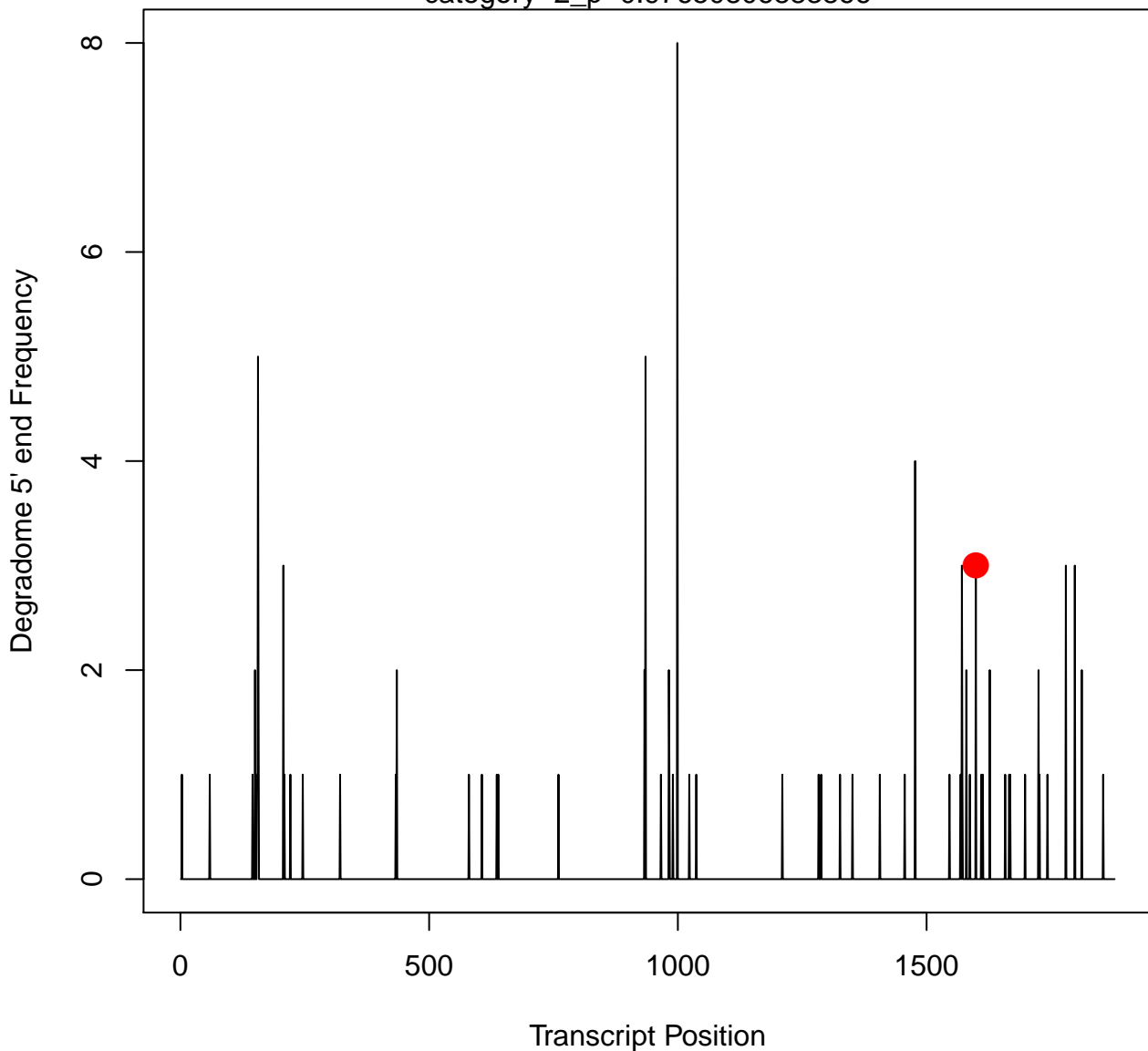

Supplement: Supplementary file 5 [file Data_Sheet_5.zip › Sit-miR167h_Seita.5G393700.1_1599_TPlot.pdf]

**T=Seita.6G097500.1\_Q=Sit-miR167h\_S=1476**

category=2\_p=0.816552766968562

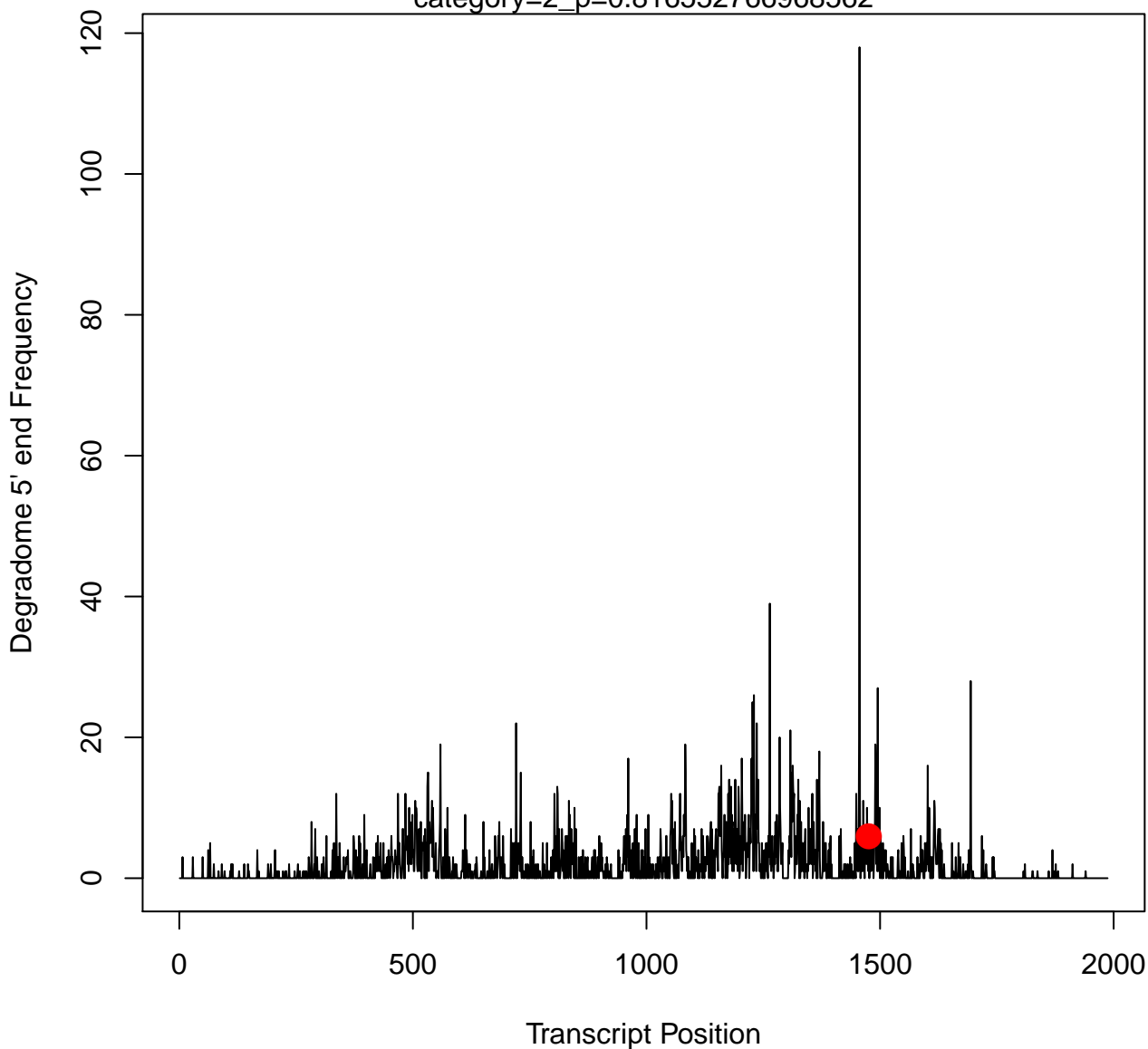

Supplement: Supplementary file 5 [file Data_Sheet_5.zip › Sit-miR167h_Seita.6G097500.1_1476_TPlot.pdf]

**T=Seita.9G210000.1\_Q=Sit-miR167h\_S=1228**

category=2\_p=0.885349573814913

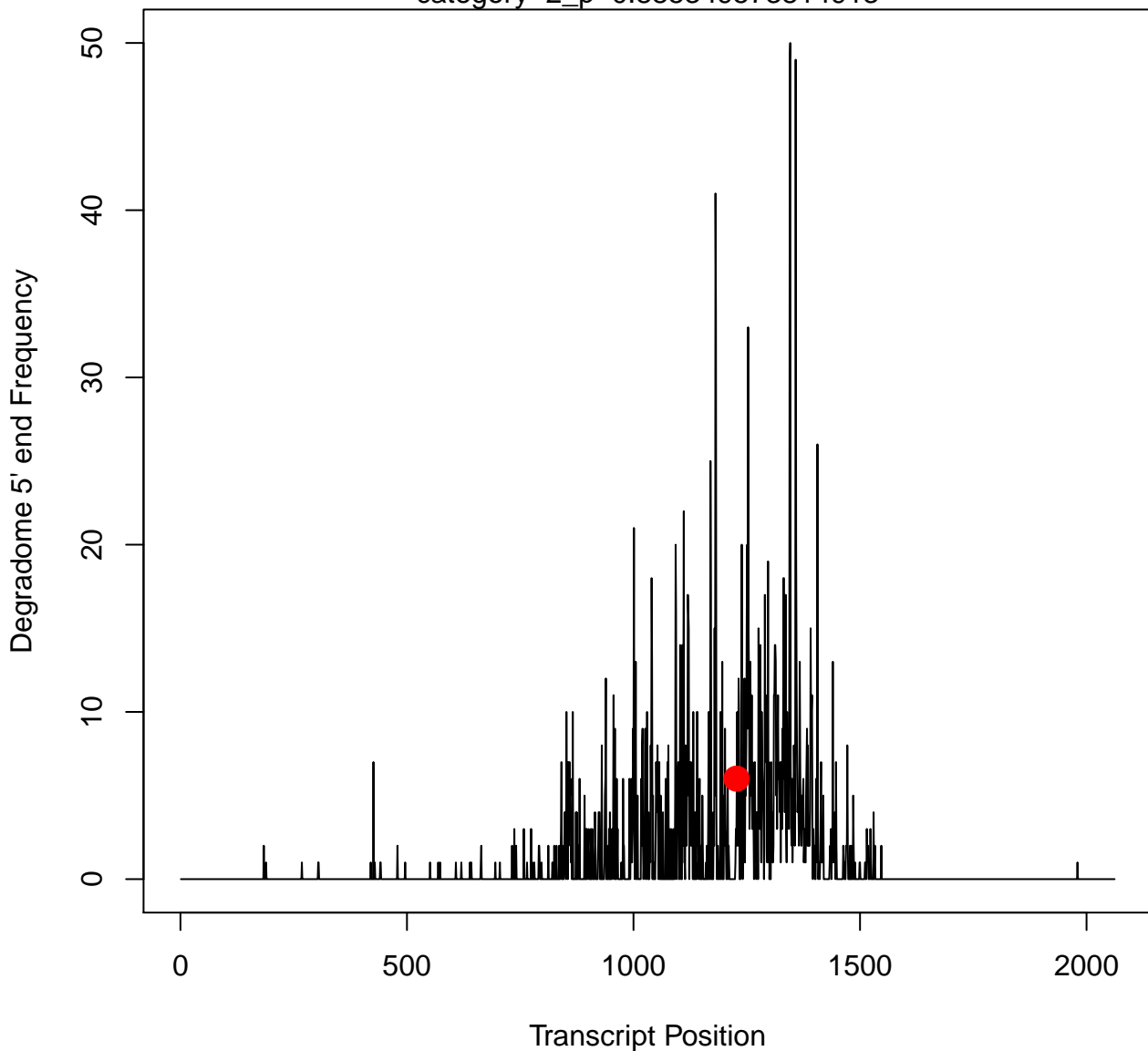

Supplement: Supplementary file 5 [file Data_Sheet_5.zip › Sit-miR167h_Seita.9G210000.1_1228_TPlot.pdf]

**T=Seita.1G077200.1\_Q=Sit-miR167j\_S=3508**

category=0\_p=0.00253212748589871

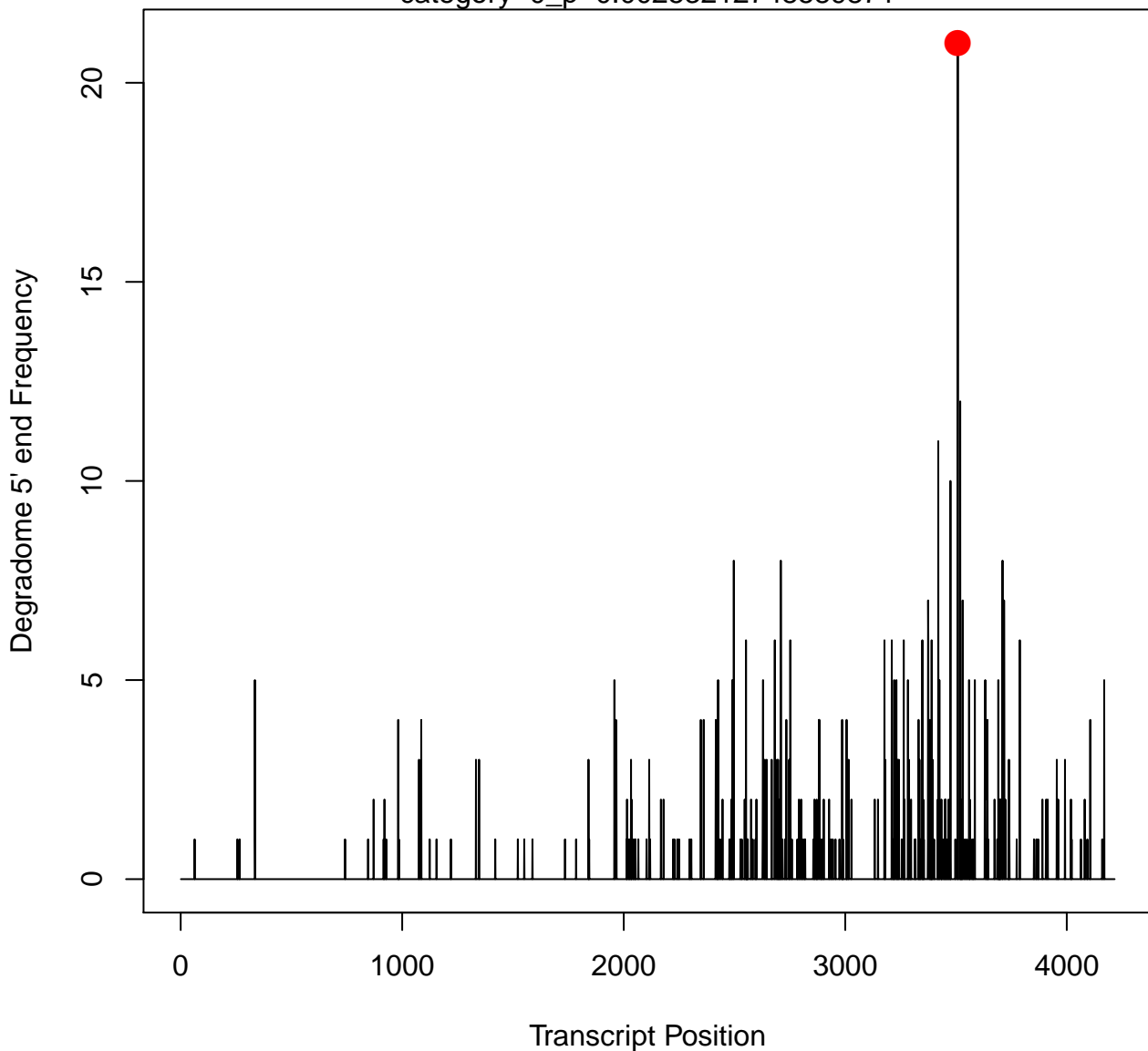

Supplement: Supplementary file 5 [file Data_Sheet_5.zip › Sit-miR167j_Seita.1G077200.1_3508_TPlot.pdf]

**T=Seita.3G394000.1\_Q=Sit-miR167j\_S=3277**

category=0\_p=0.00337474436314689

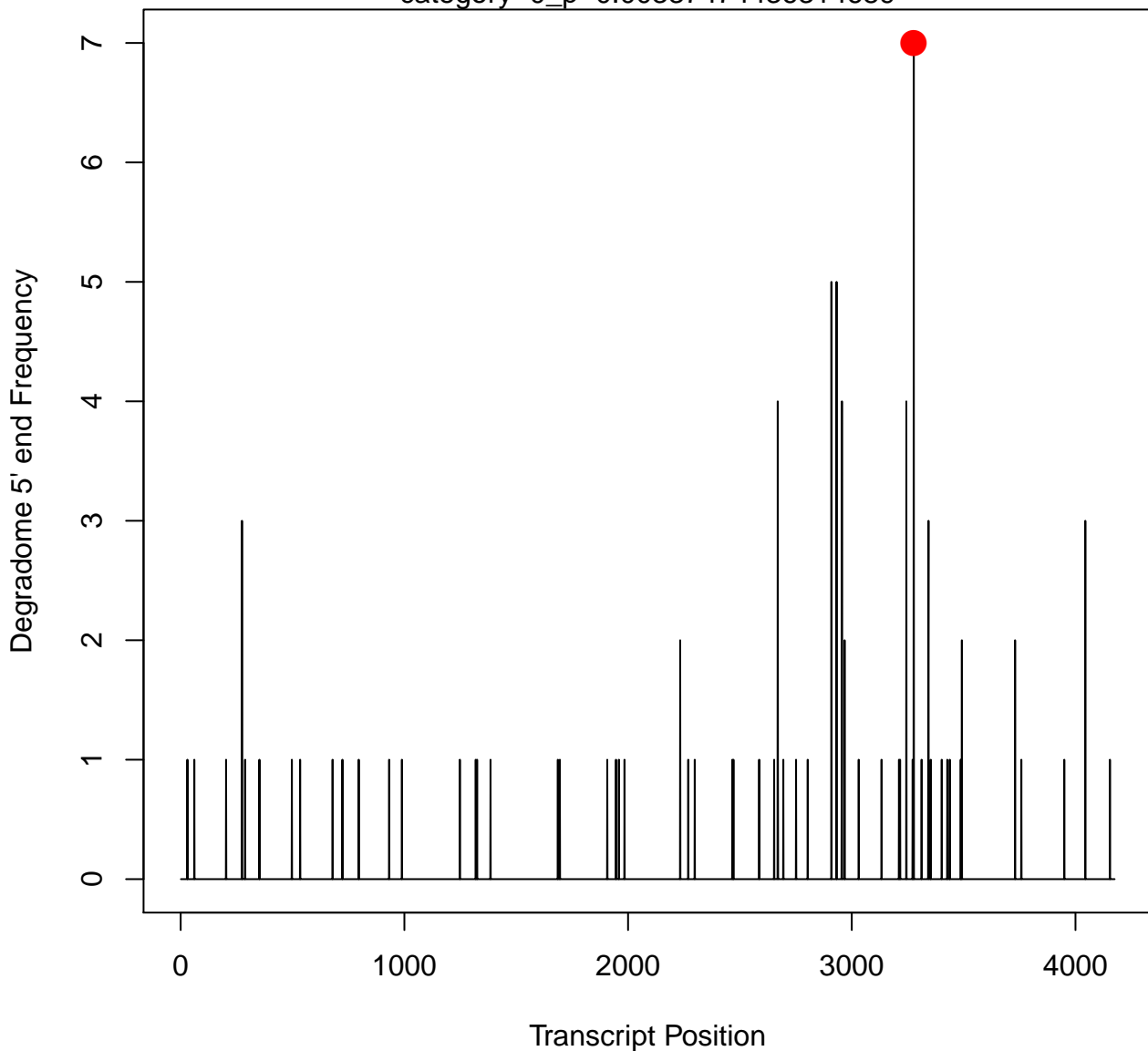

Supplement: Supplementary file 5 [file Data_Sheet_5.zip › Sit-miR167j_Seita.3G394000.1_3277_TPlot.pdf]

**T=Seita.3G070600.1\_Q=Sit-miR168\_S=747**

category=0\_p=0.013430797664279

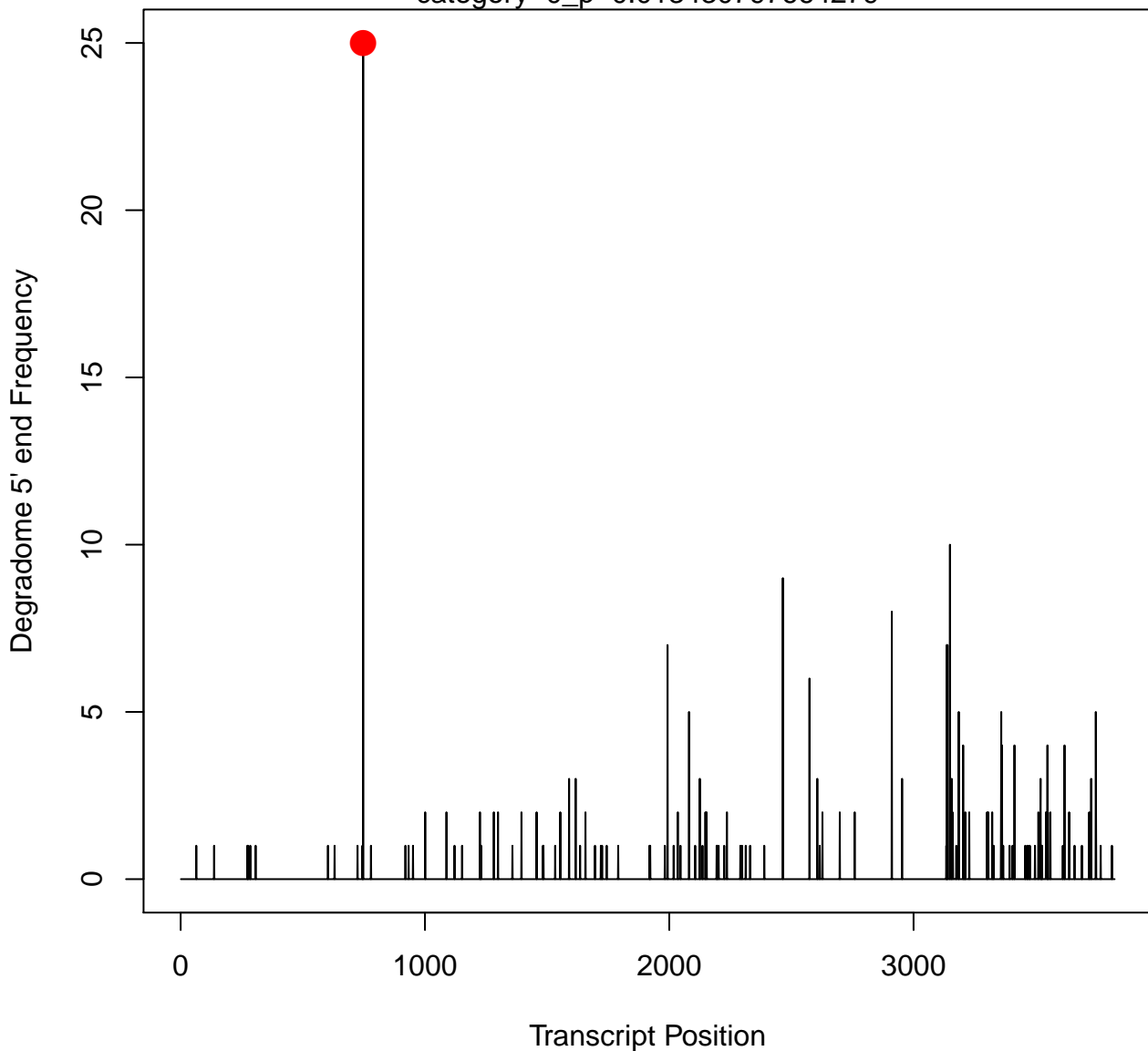

Supplement: Supplementary file 5 [file Data_Sheet_5.zip › Sit-miR168_Seita.3G070600.1_747_TPlot.pdf]

**T=Seita.5G022800.1\_Q=Sit-miR168\_S=2856**

category=2\_p=0.870756053555062

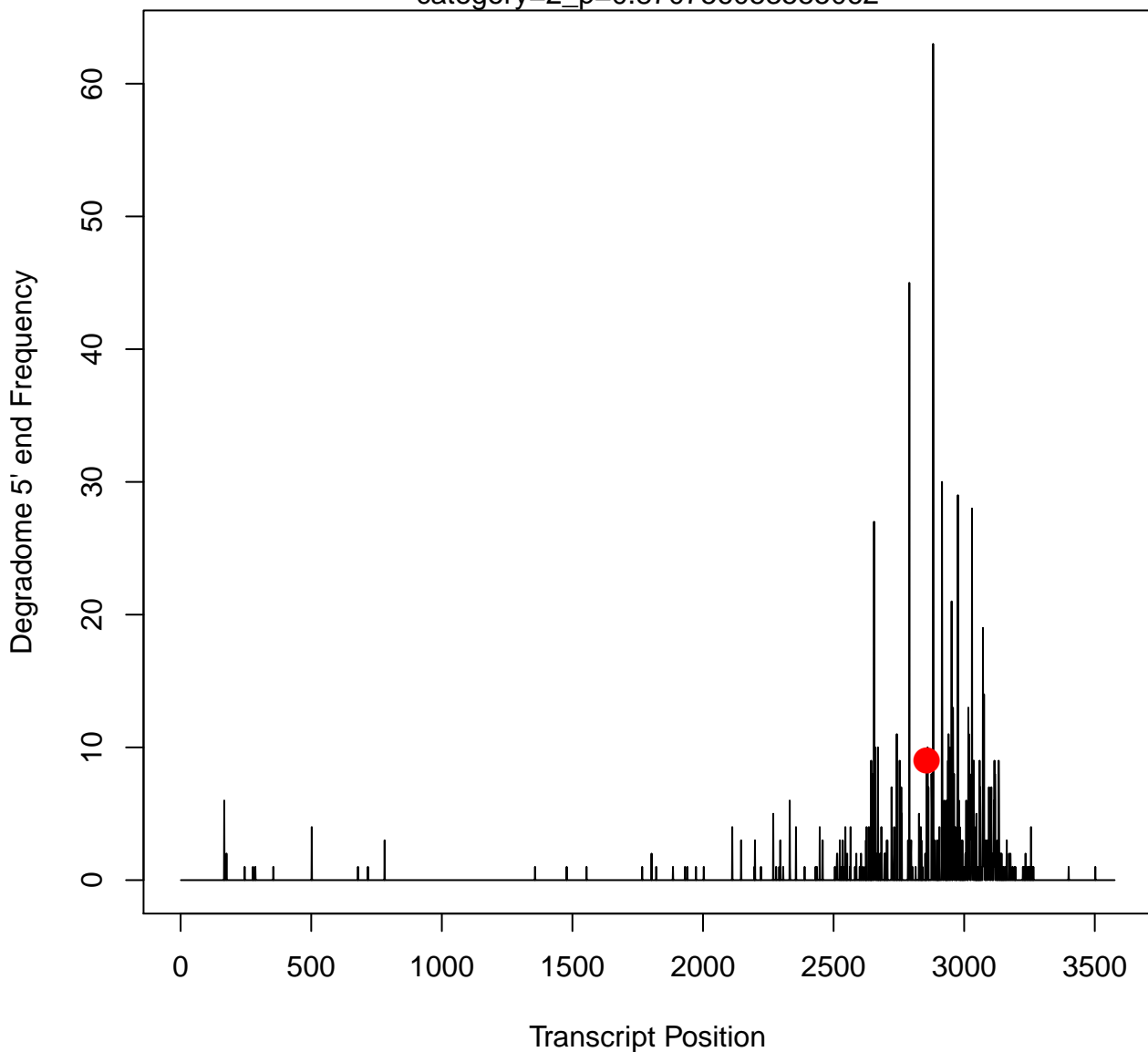

Supplement: Supplementary file 5 [file Data_Sheet_5.zip › Sit-miR168_Seita.5G022800.1_2856_TPlot.pdf]

**T=Seita.5G435400.1\_Q=Sit-miR168\_S=595**

category=2\_p=0.104700958593756

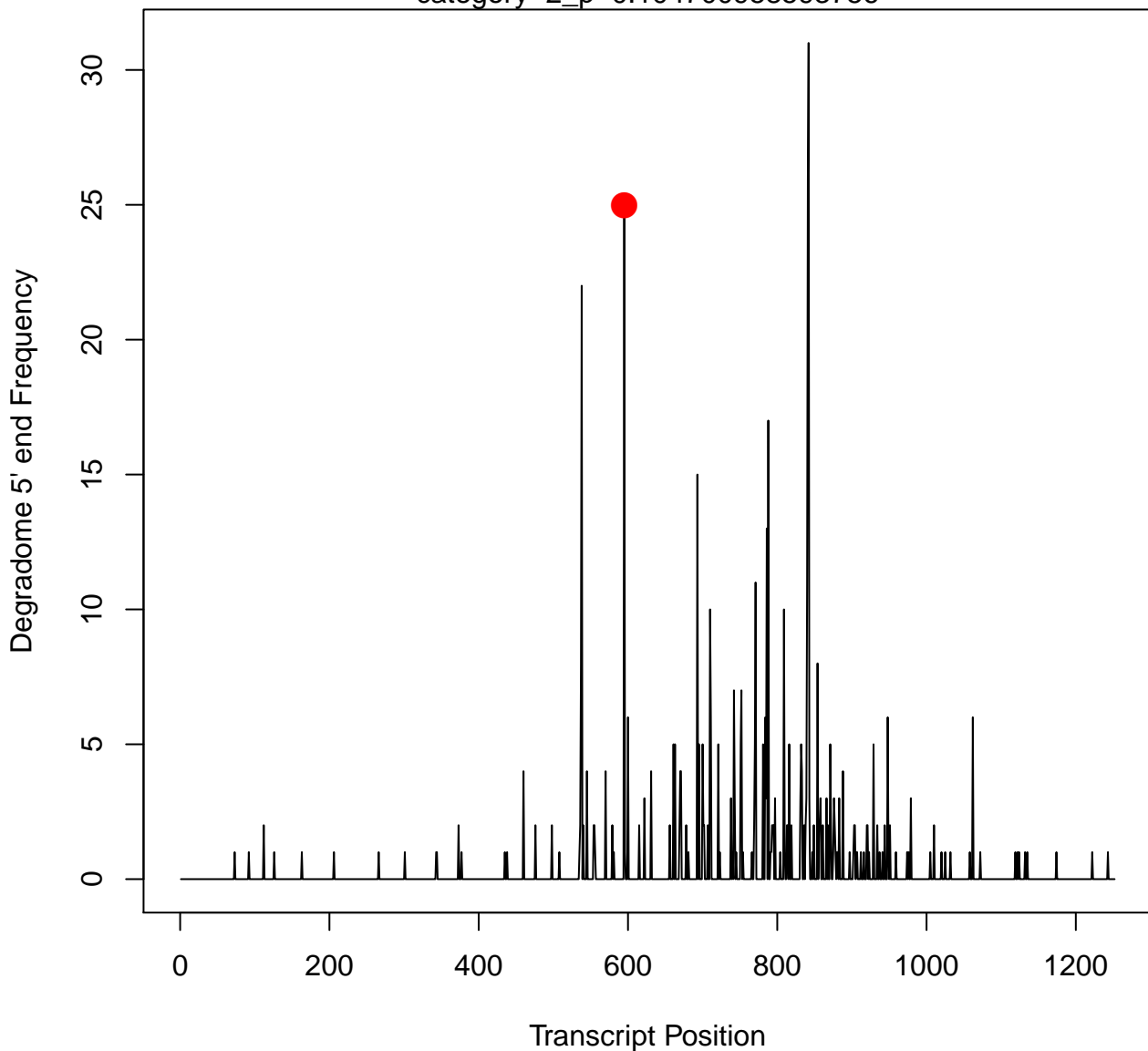

Supplement: Supplementary file 5 [file Data_Sheet_5.zip › Sit-miR168_Seita.5G435400.1_595_TPlot.pdf]

**T=Seita.7G115200.1\_Q=Sit-miR168\_S=418**

category=2\_p=0.875433981355722

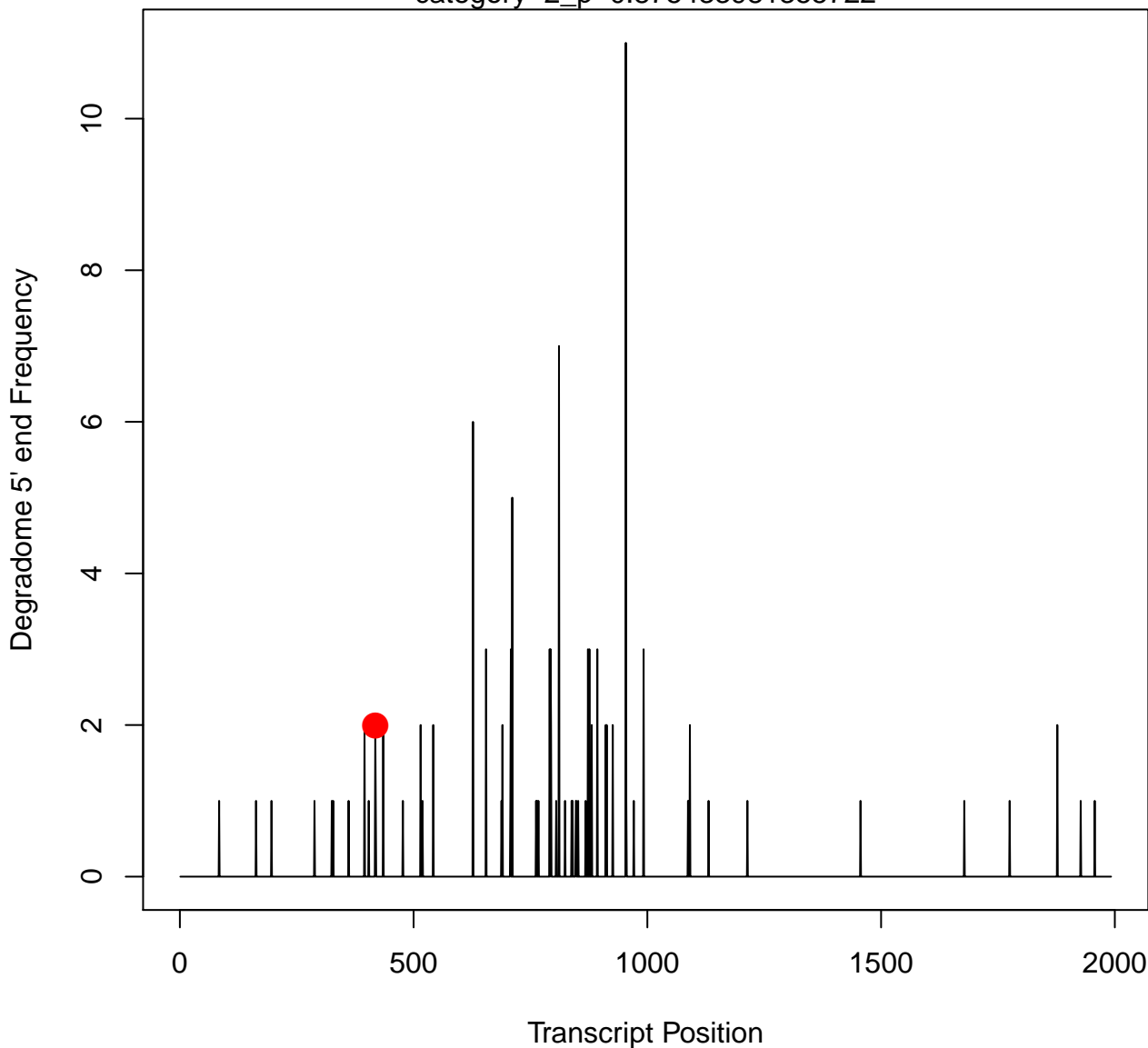

Supplement: Supplementary file 5 [file Data_Sheet_5.zip › Sit-miR168_Seita.7G115200.1_418_TPlot.pdf]

**T=Seita.7G141400.1\_Q=Sit-miR168\_S=1212**

category=2\_p=0.963436627955168

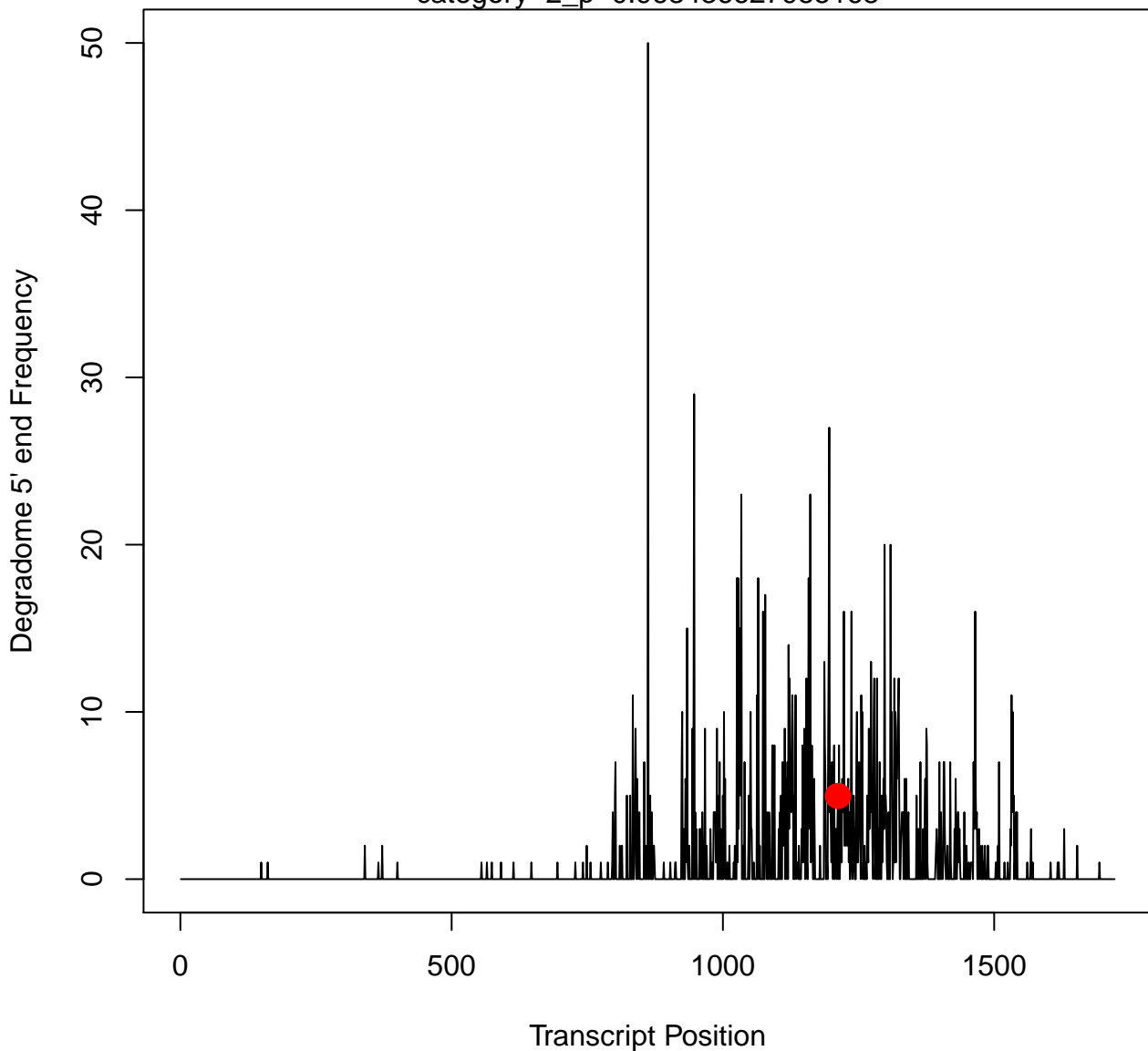

Supplement: Supplementary file 5 [file Data_Sheet_5.zip › Sit-miR168_Seita.7G141400.1_1212_TPlot.pdf]

**T=Seita.7G201100.1\_Q=Sit-miR168\_S=664**

category=2\_p=0.248519627402798

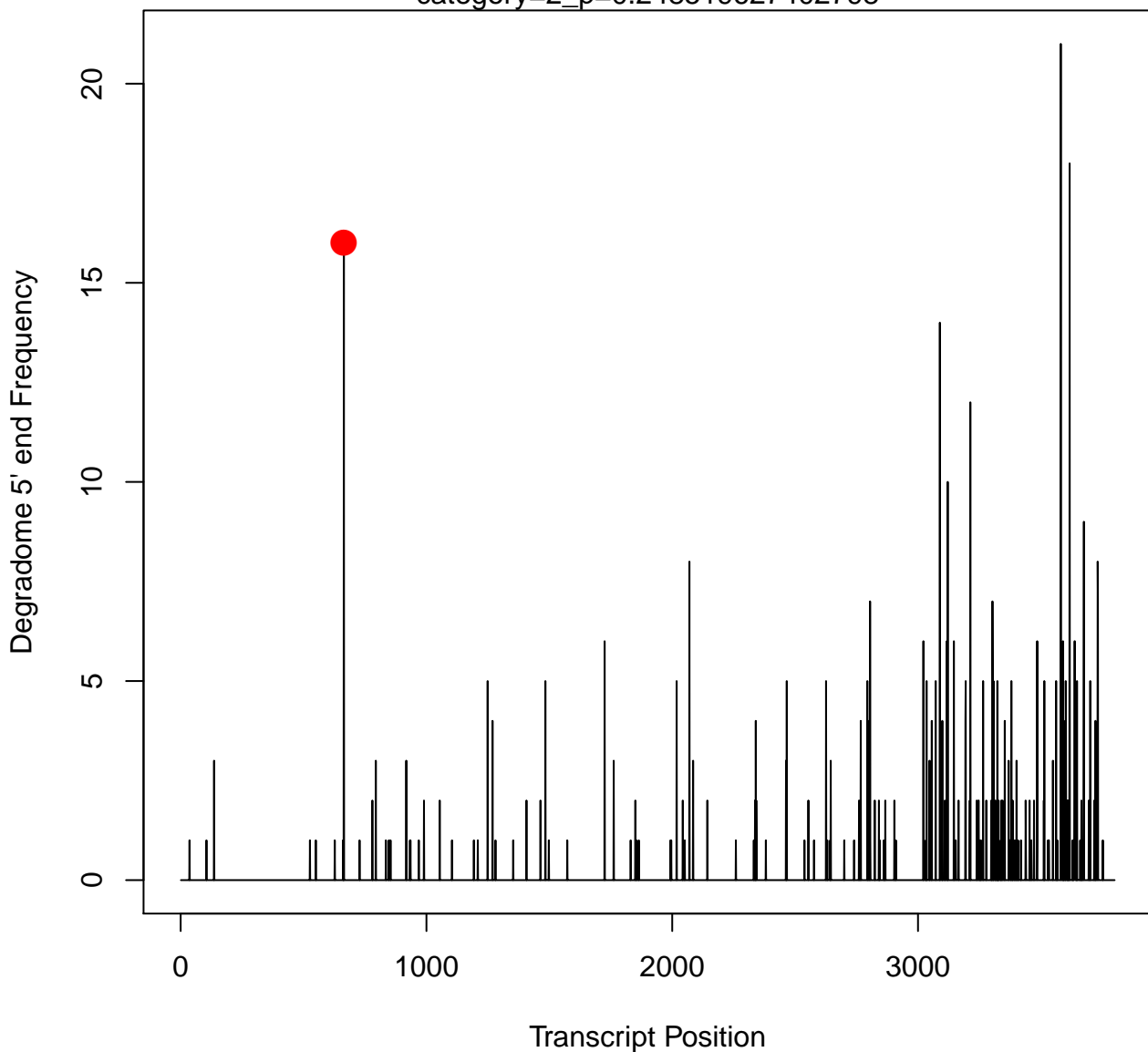

Supplement: Supplementary file 5 [file Data_Sheet_5.zip › Sit-miR168_Seita.7G201100.1_664_TPlot.pdf]

**T=Seita.9G136100.1\_Q=Sit-miR168\_S=1365**

category=2\_p=0.950439701556188

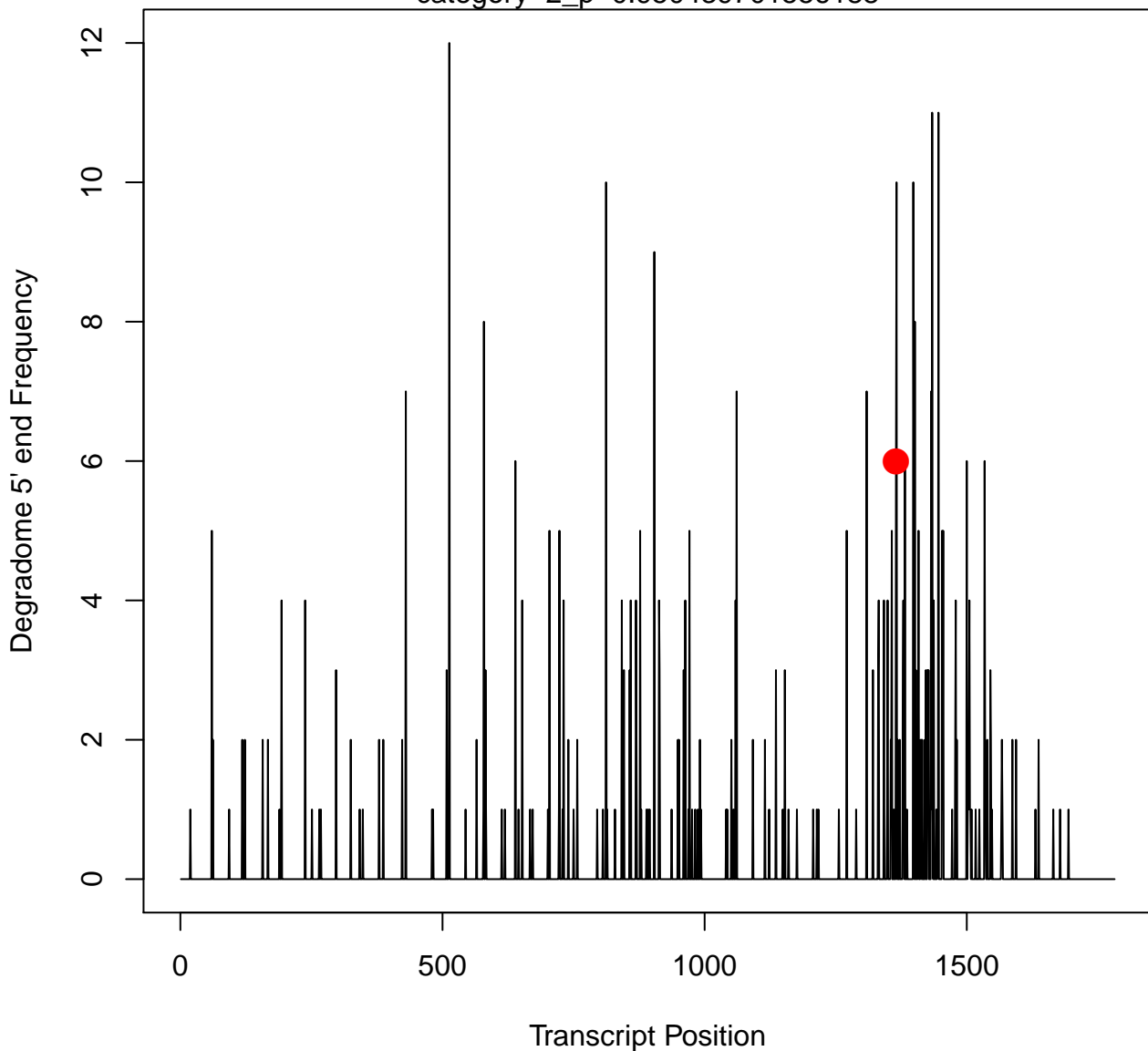

Supplement: Supplementary file 5 [file Data_Sheet_5.zip › Sit-miR168_Seita.9G136100.1_1365_TPlot.pdf]

**T=Seita.1G372100.1\_Q=Sit-miR169a\_S=2170**

category=2\_p=0.112914536125439

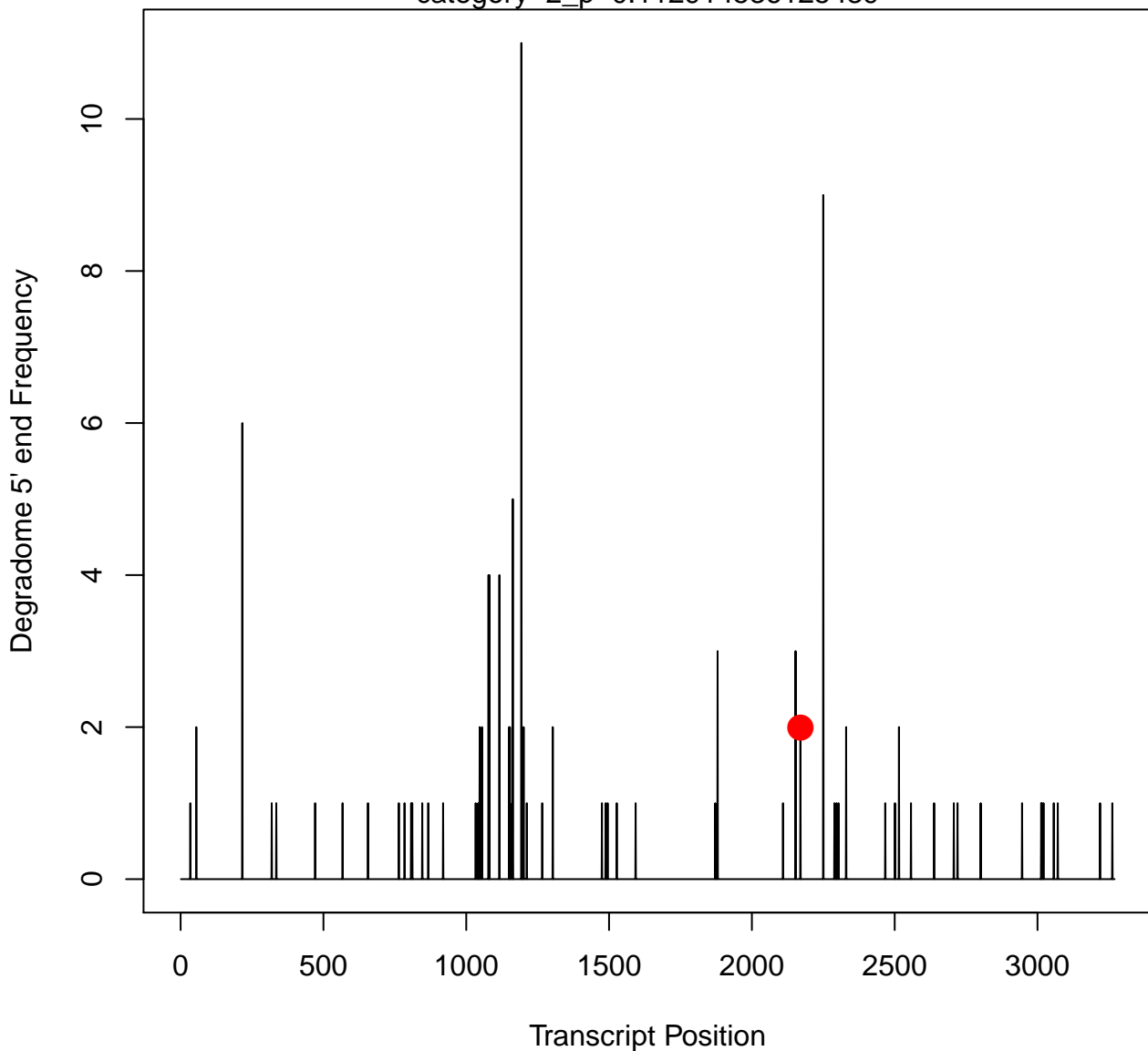

Supplement: Supplementary file 5 [file Data_Sheet_5.zip › Sit-miR169a_Seita.1G372100.1_2170_TPlot.pdf]

**T=Seita.3G038000.1\_Q=Sit-miR169b\_S=685**

category=2\_p=0.598452445017394

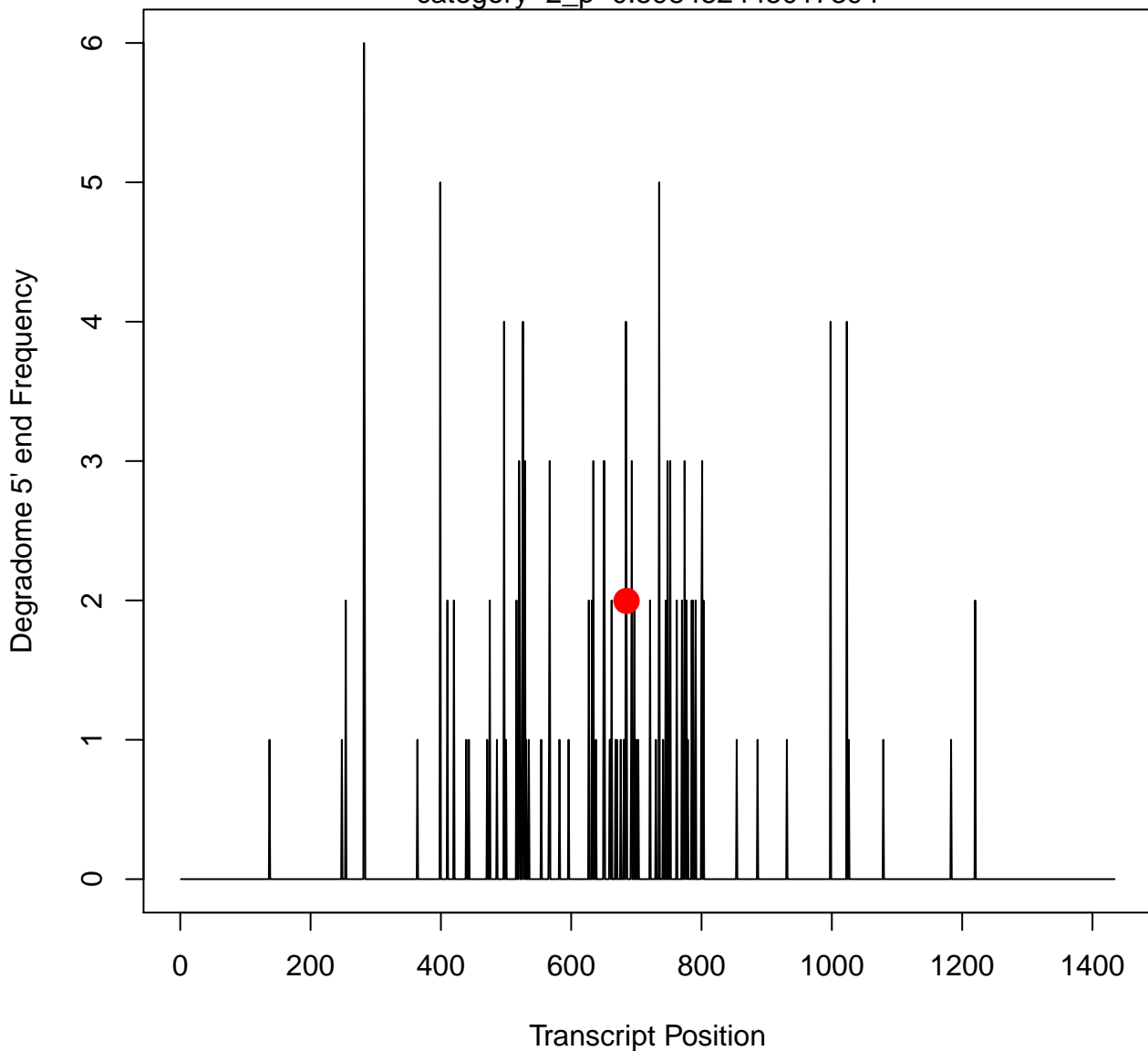

Supplement: Supplementary file 5 [file Data_Sheet_5.zip › Sit-miR169b_Seita.3G038000.1_685_TPlot.pdf]

**T=Seita.2G142500.1\_Q=Sit-miR169e\_S=785**

category=2\_p=0.977975800994102

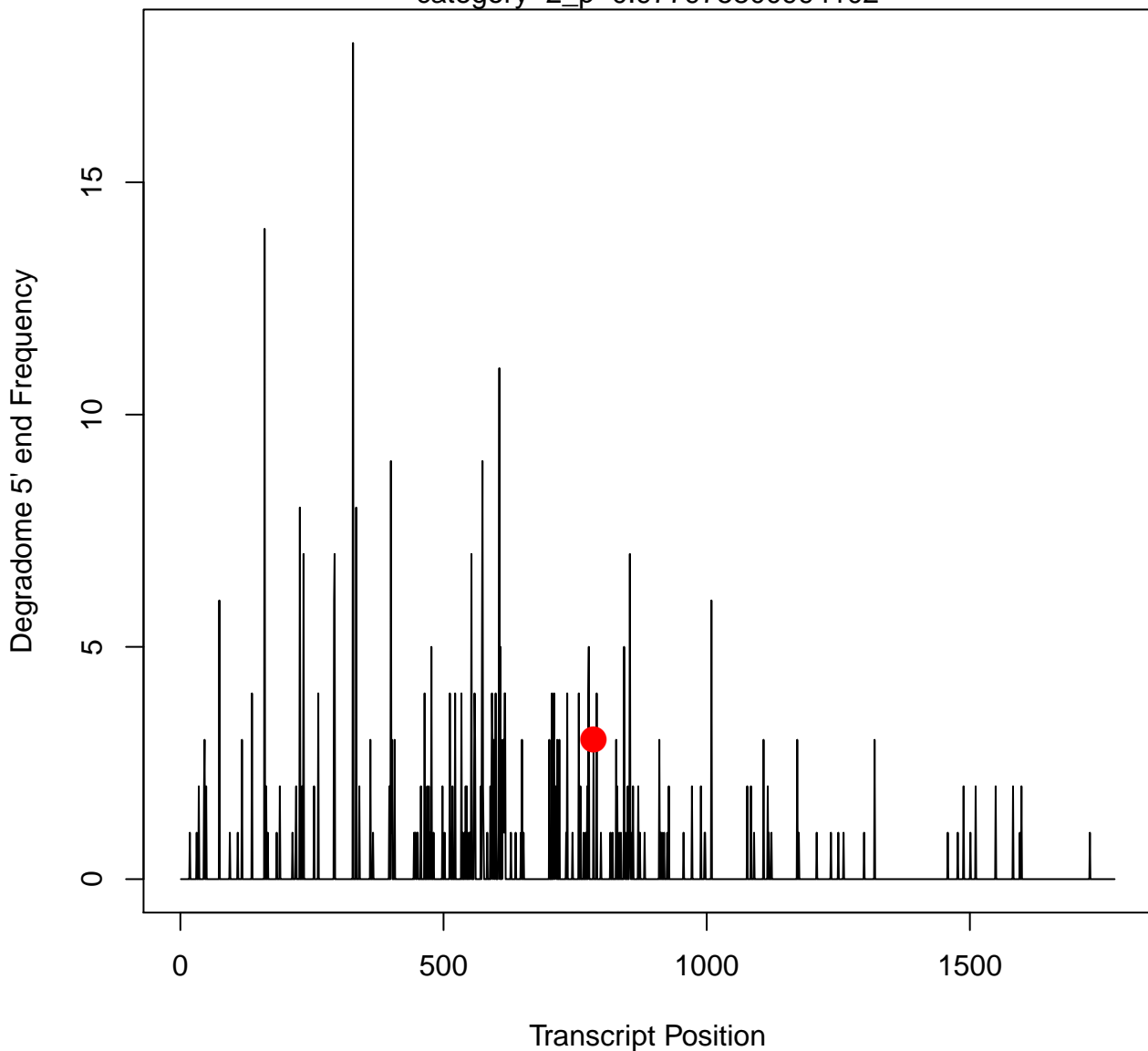

Supplement: Supplementary file 5 [file Data_Sheet_5.zip › Sit-miR169e_Seita.2G142500.1_785_TPlot.pdf]

**T=Seita.4G287200.1\_Q=Sit-miR169e\_S=694**

category=2\_p=0.98584947344857

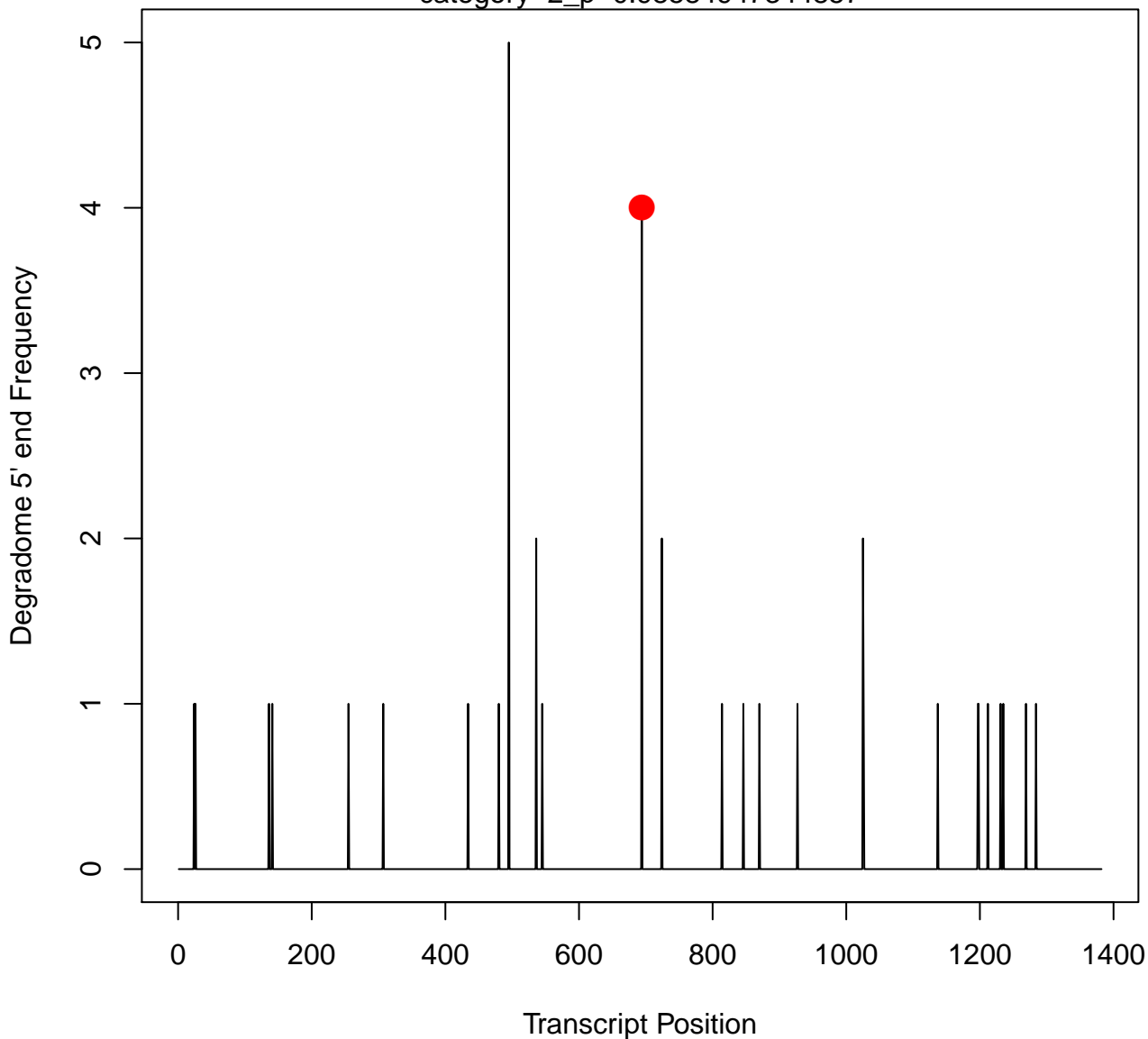

Supplement: Supplementary file 5 [file Data_Sheet_5.zip › Sit-miR169e_Seita.4G287200.1_694_TPlot.pdf]

**T=Seita.7G327200.1\_Q=Sit-miR169e\_S=2250**

category=2\_p=0.227451751654747

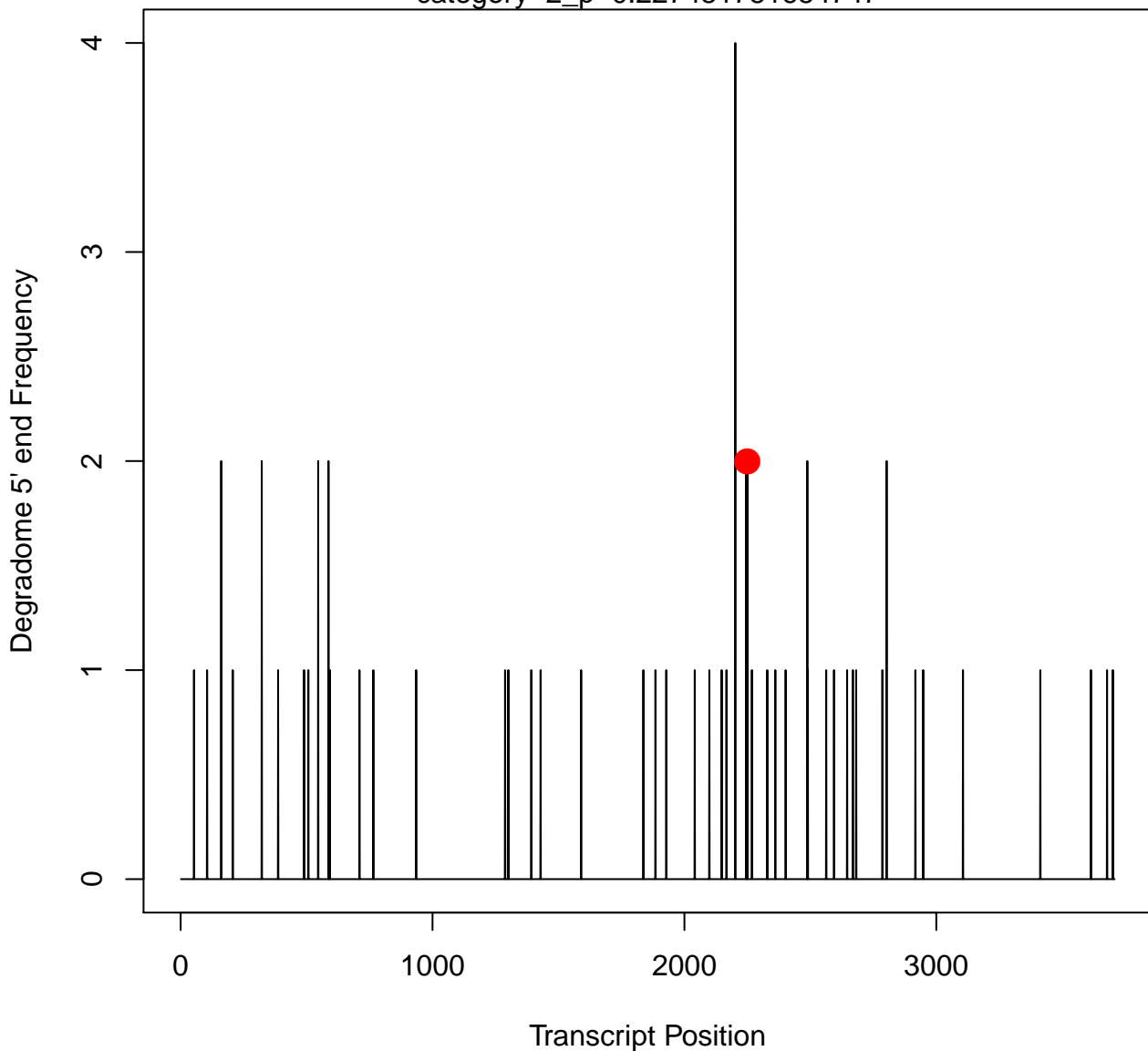

Supplement: Supplementary file 5 [file Data_Sheet_5.zip › Sit-miR169e_Seita.7G327200.1_2250_TPlot.pdf]

**T=Seita.8G014600.1\_Q=Sit-miR169e\_S=2597**

category=2\_p=0.220298681073436

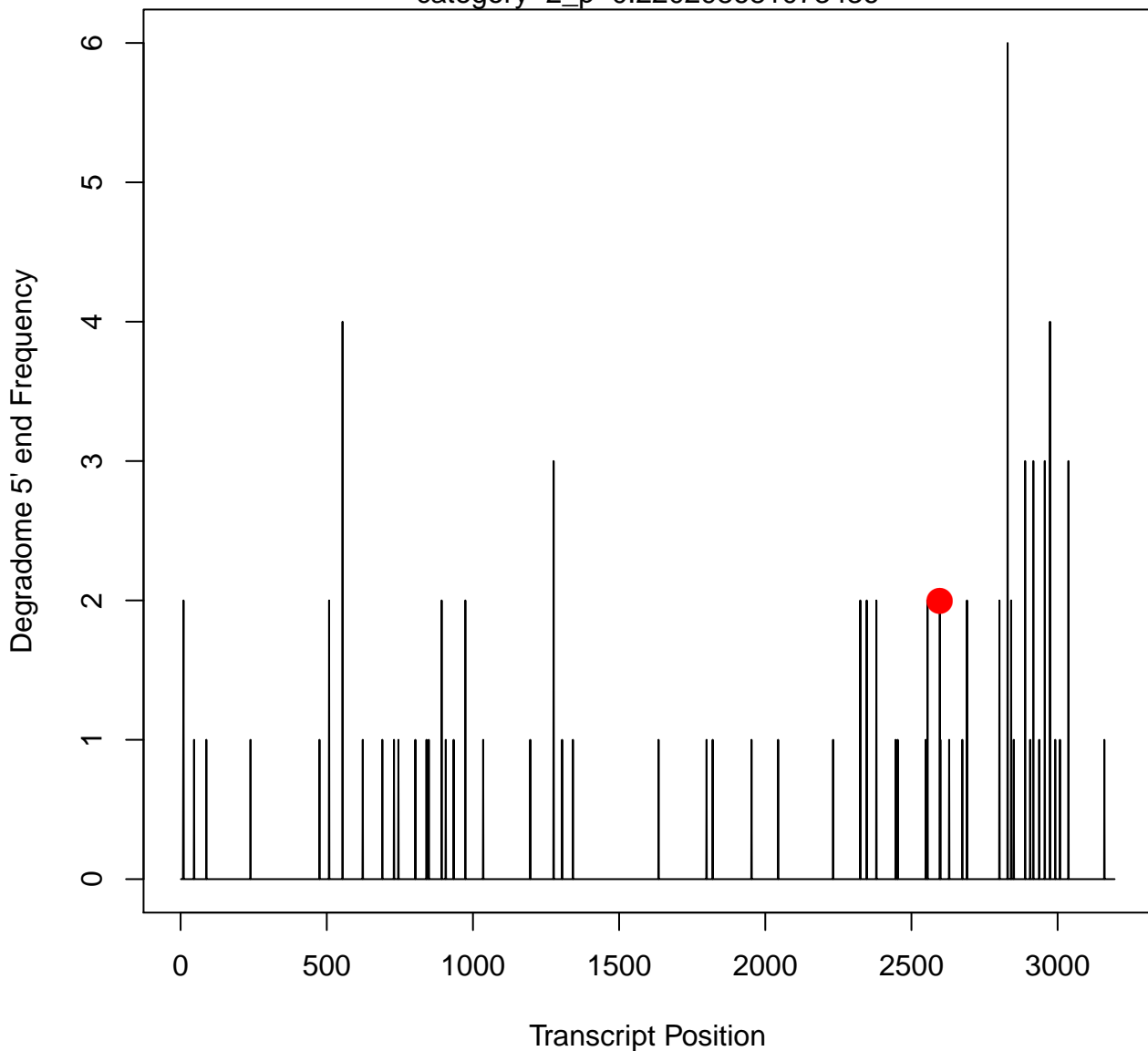

Supplement: Supplementary file 5 [file Data_Sheet_5.zip › Sit-miR169e_Seita.8G014600.1_2597_TPlot.pdf]

**T=Seita.9G367200.1\_Q=Sit-miR169e\_S=1285**

category=2\_p=0.129116325533099

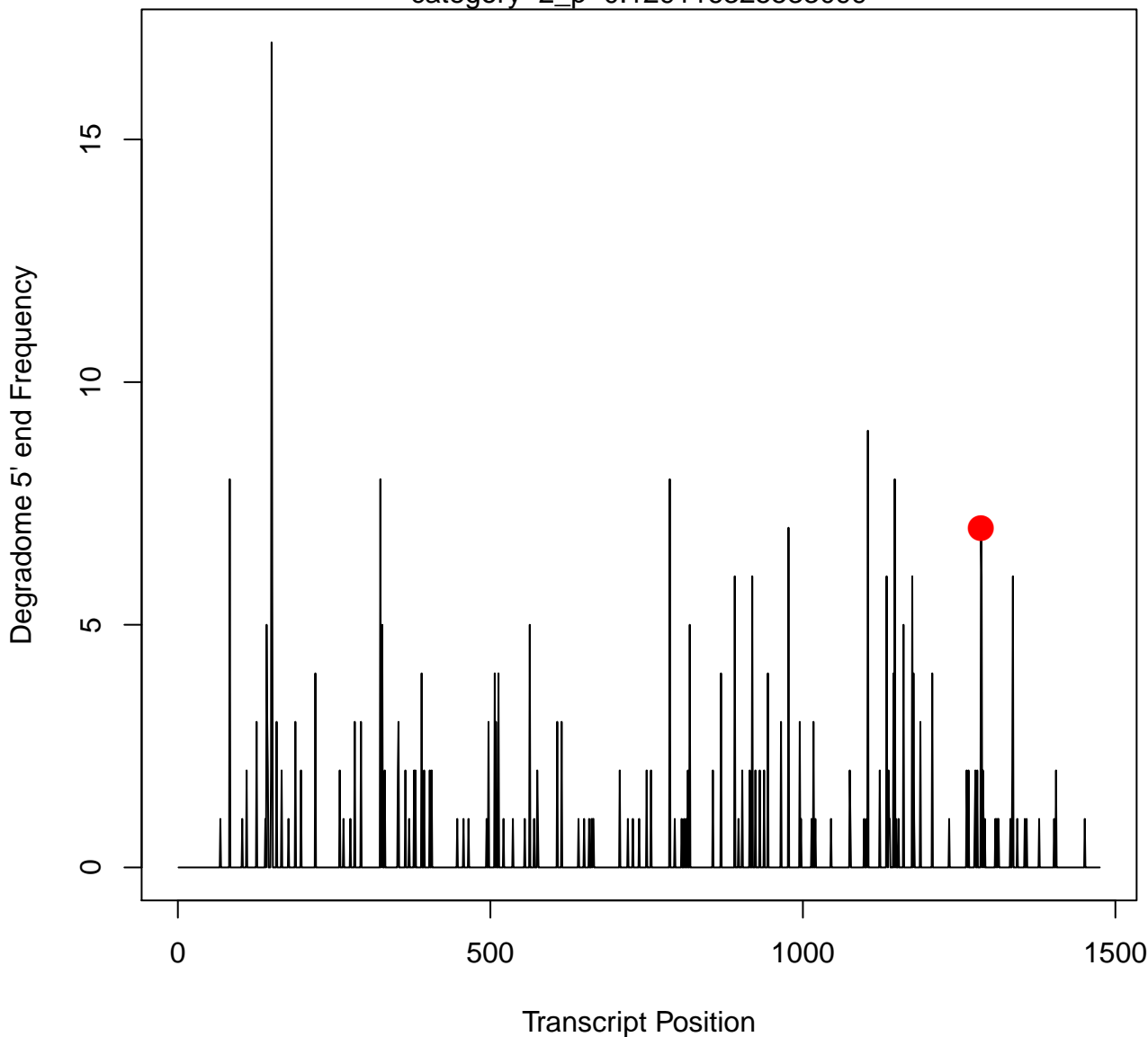

Supplement: Supplementary file 5 [file Data_Sheet_5.zip › Sit-miR169e_Seita.9G367200.1_1285_TPlot.pdf]

**T=Seita.1G199800.1\_Q=Sit-miR169f\_S=2032**

category=2\_p=0.571692595170901

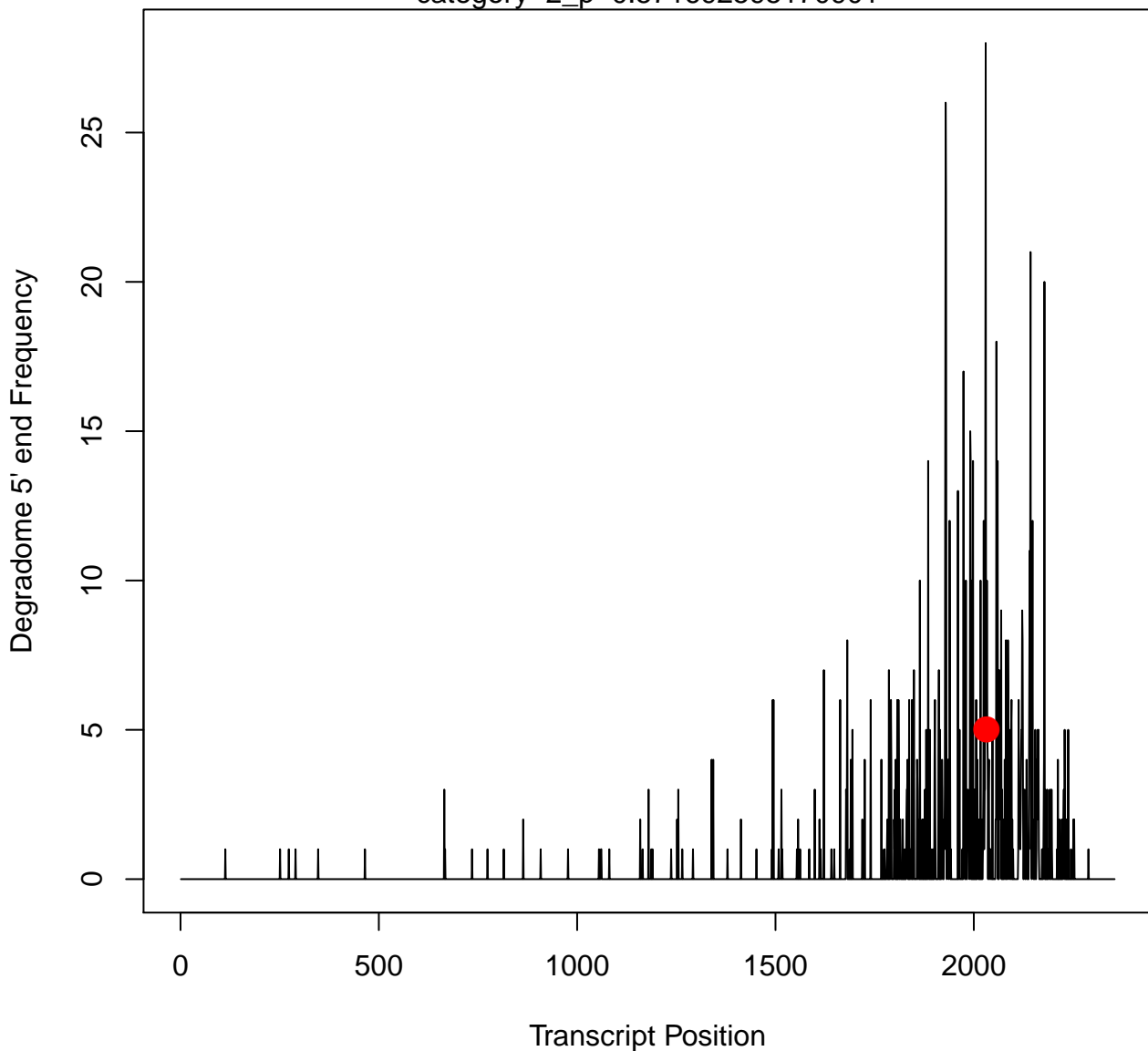

Supplement: Supplementary file 5 [file Data_Sheet_5.zip › Sit-miR169f_Seita.1G199800.1_2032_TPlot.pdf]

**T=Seita.1G234000.1\_Q=Sit-miR169f\_S=739**

category=2\_p=0.809663625606483

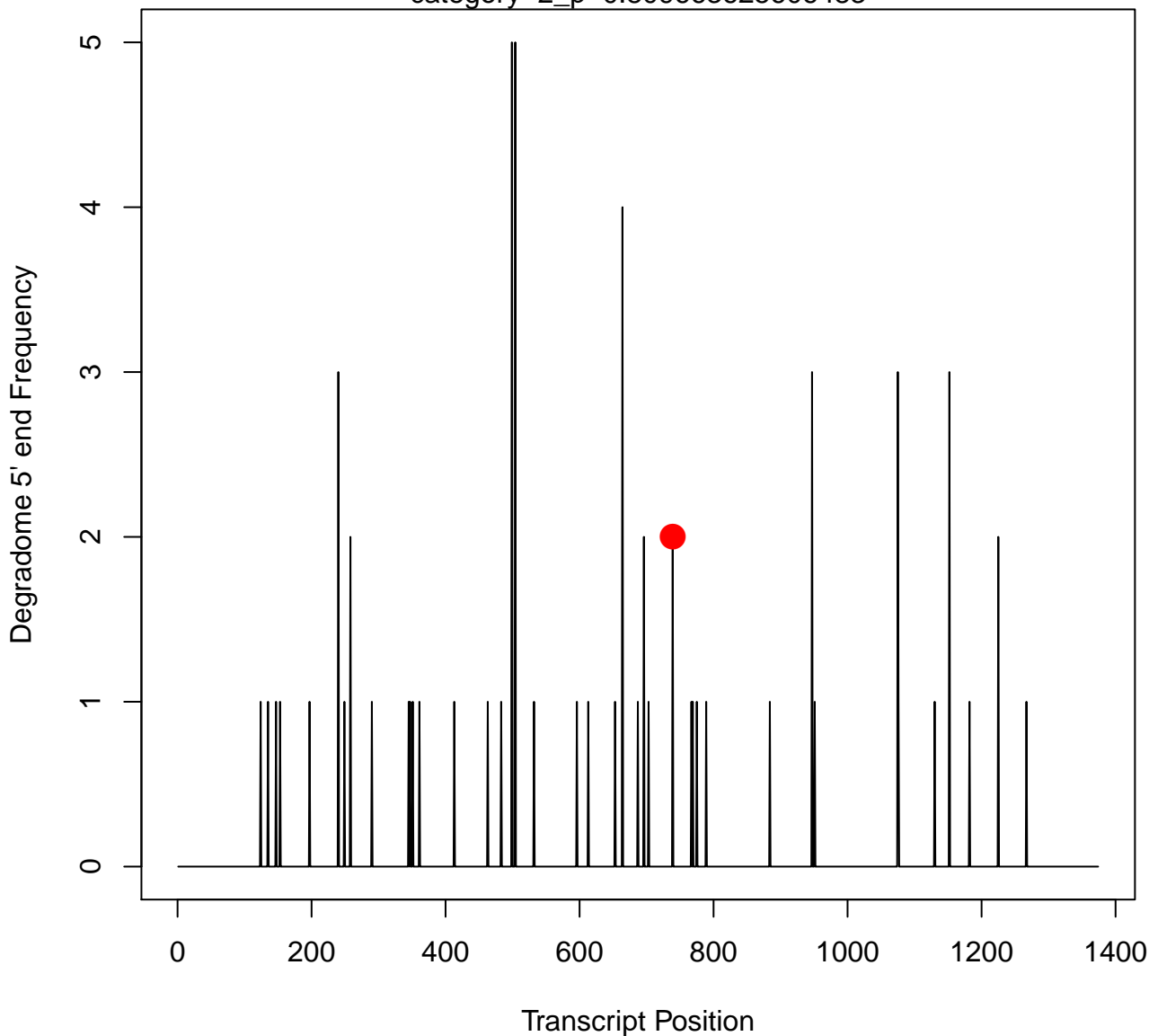

Supplement: Supplementary file 5 [file Data_Sheet_5.zip › Sit-miR169f_Seita.1G234000.1_739_TPlot.pdf]

**T=Seita.3G390000.1\_Q=Sit-miR169f\_S=2048**

category=0\_p=0.00168879820125578

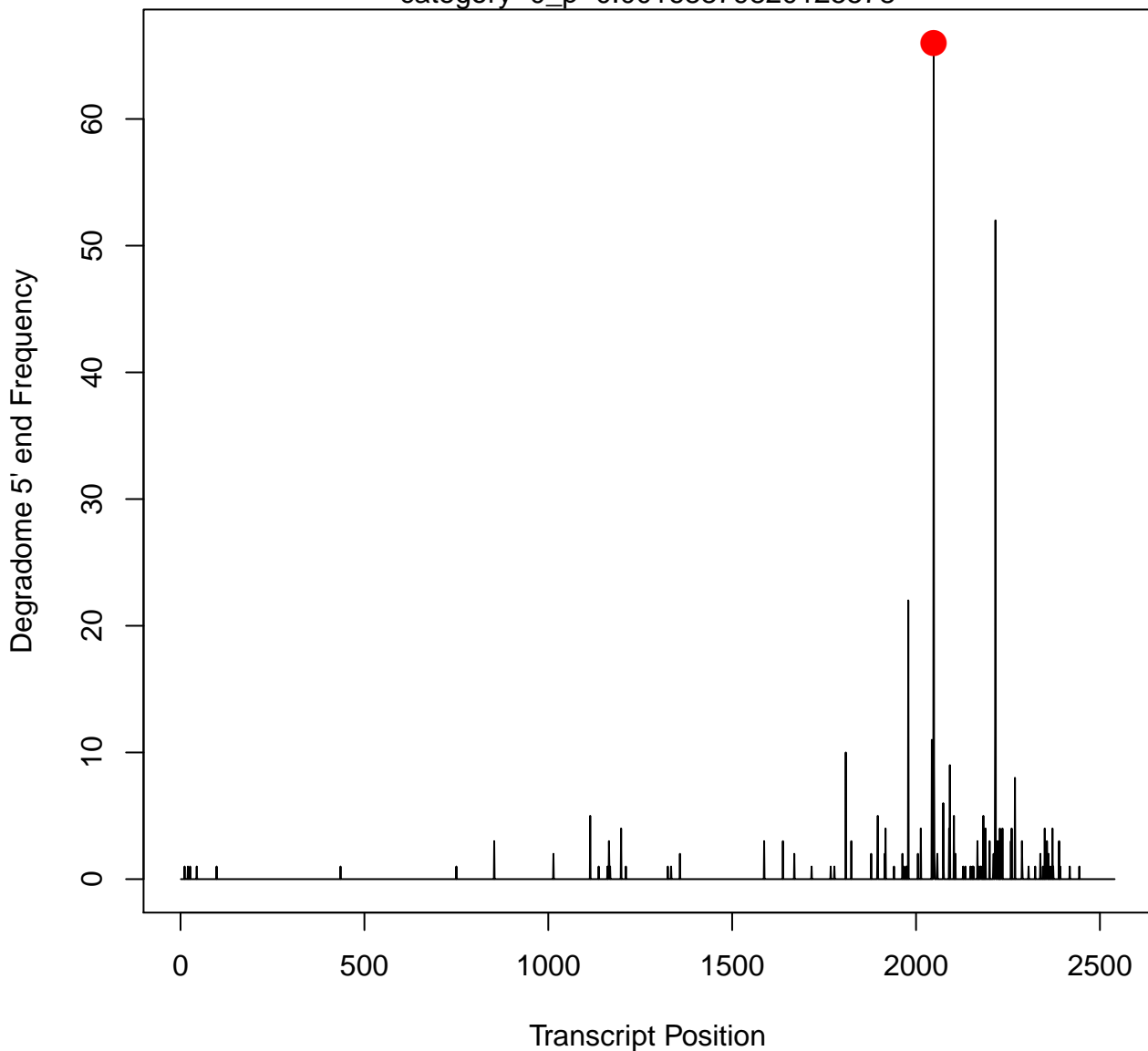

Supplement: Supplementary file 5 [file Data_Sheet_5.zip › Sit-miR169f_Seita.3G390000.1_2048_TPlot.pdf]

**T=Seita.4G197200.1\_Q=Sit-miR169f\_S=299**

category=2\_p=0.744372950634024

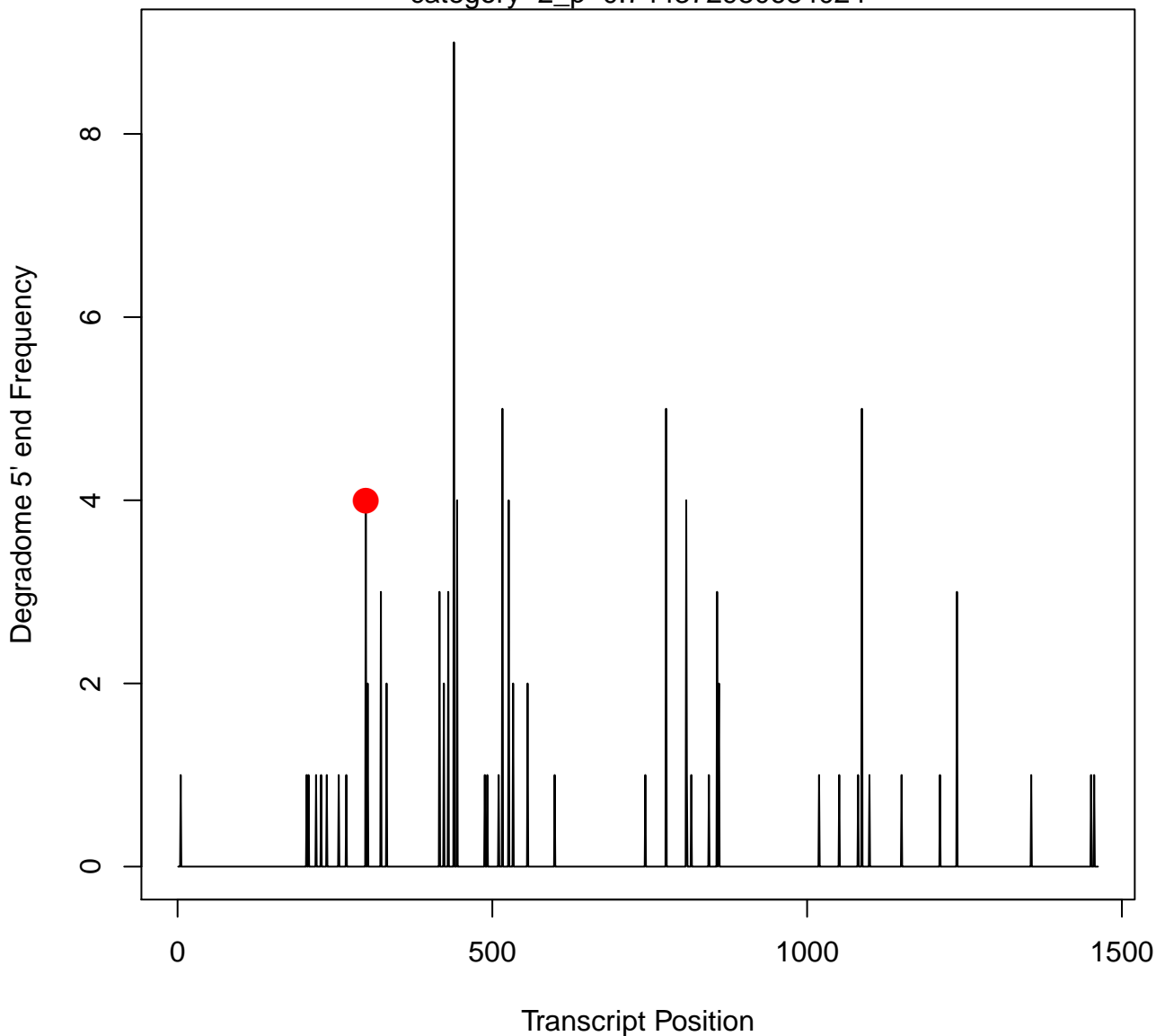

Supplement: Supplementary file 5 [file Data_Sheet_5.zip › Sit-miR169f_Seita.4G197200.1_299_TPlot.pdf]

**T=Seita.5G387000.1\_Q=Sit-miR169f\_S=1594**

category=2\_p=0.791287548372707

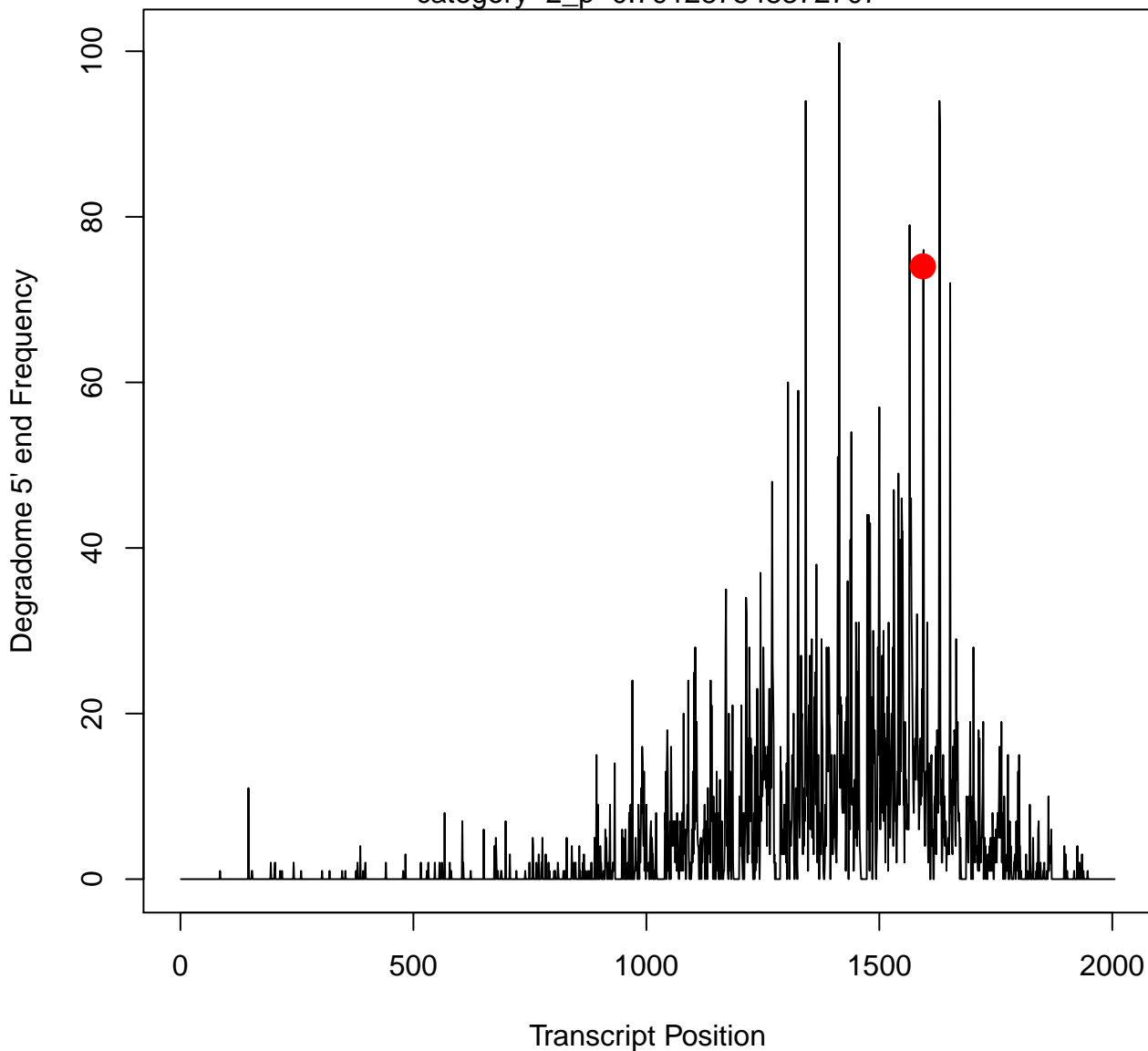

Supplement: Supplementary file 5 [file Data_Sheet_5.zip › Sit-miR169f_Seita.5G387000.1_1594_TPlot.pdf]

**T=Seita.7G063700.1\_Q=Sit-miR169f\_S=1128**

category=2\_p=0.943091942245732

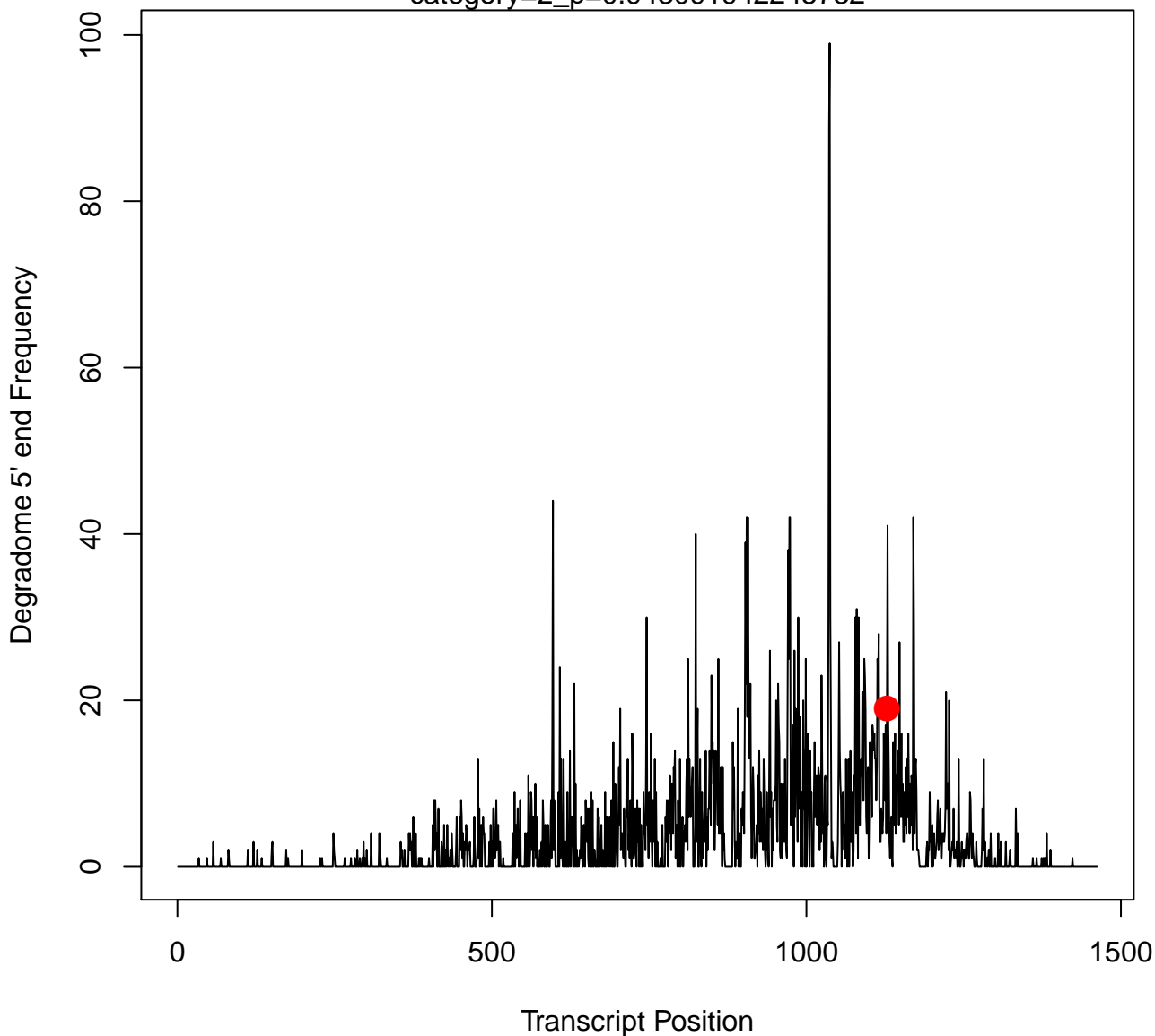

Supplement: Supplementary file 5 [file Data_Sheet_5.zip › Sit-miR169f_Seita.7G063700.1_1128_TPlot.pdf]

**T=Seita.9G129400.1\_Q=Sit-miR169f\_S=2100**

category=0\_p=0.00421664943481093

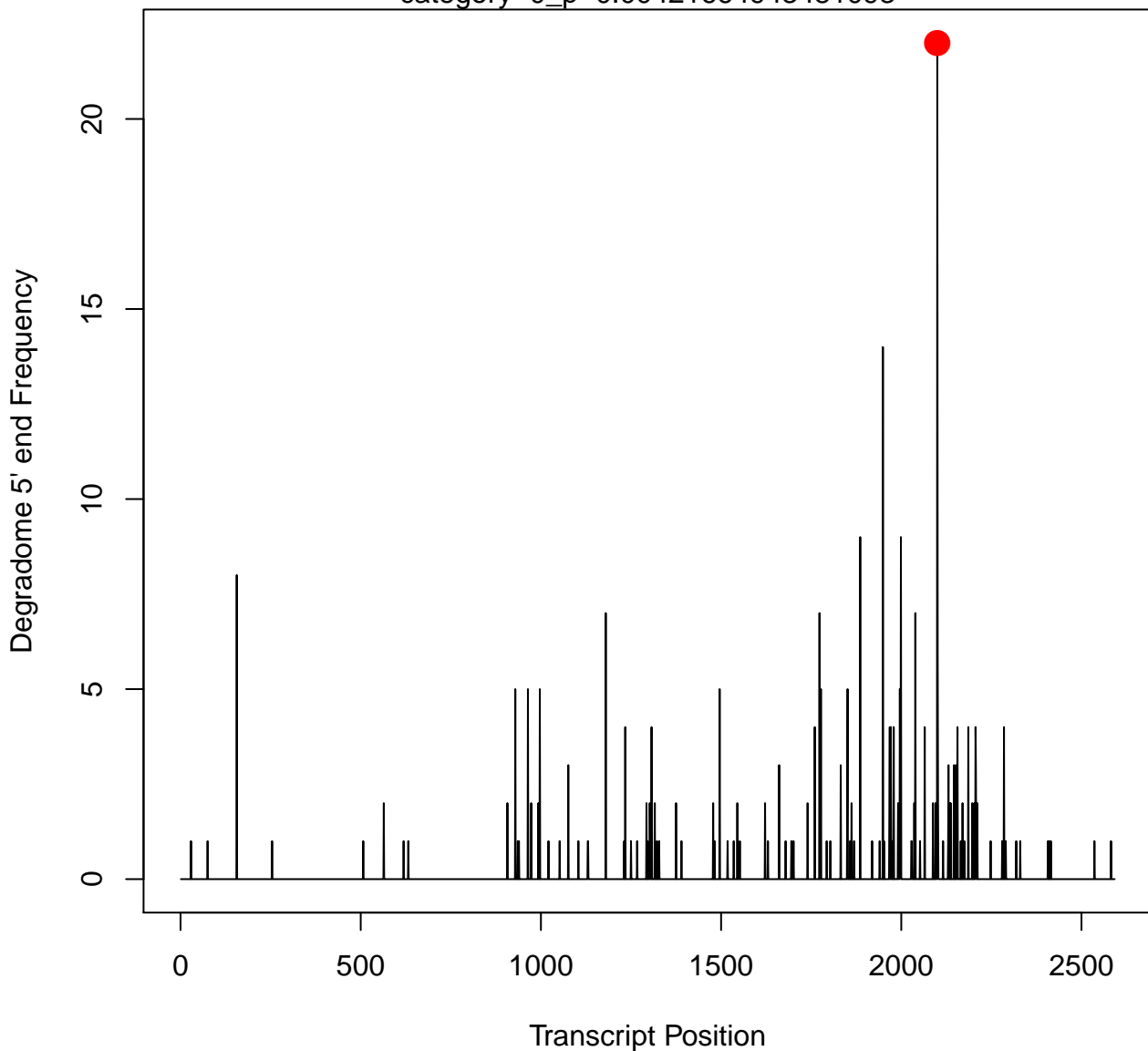

Supplement: Supplementary file 5 [file Data_Sheet_5.zip › Sit-miR169f_Seita.9G129400.1_2100_TPlot.pdf]

**T=Seita.9G155700.1\_Q=Sit-miR169f\_S=1672**

category=2\_p=0.0182640681957454

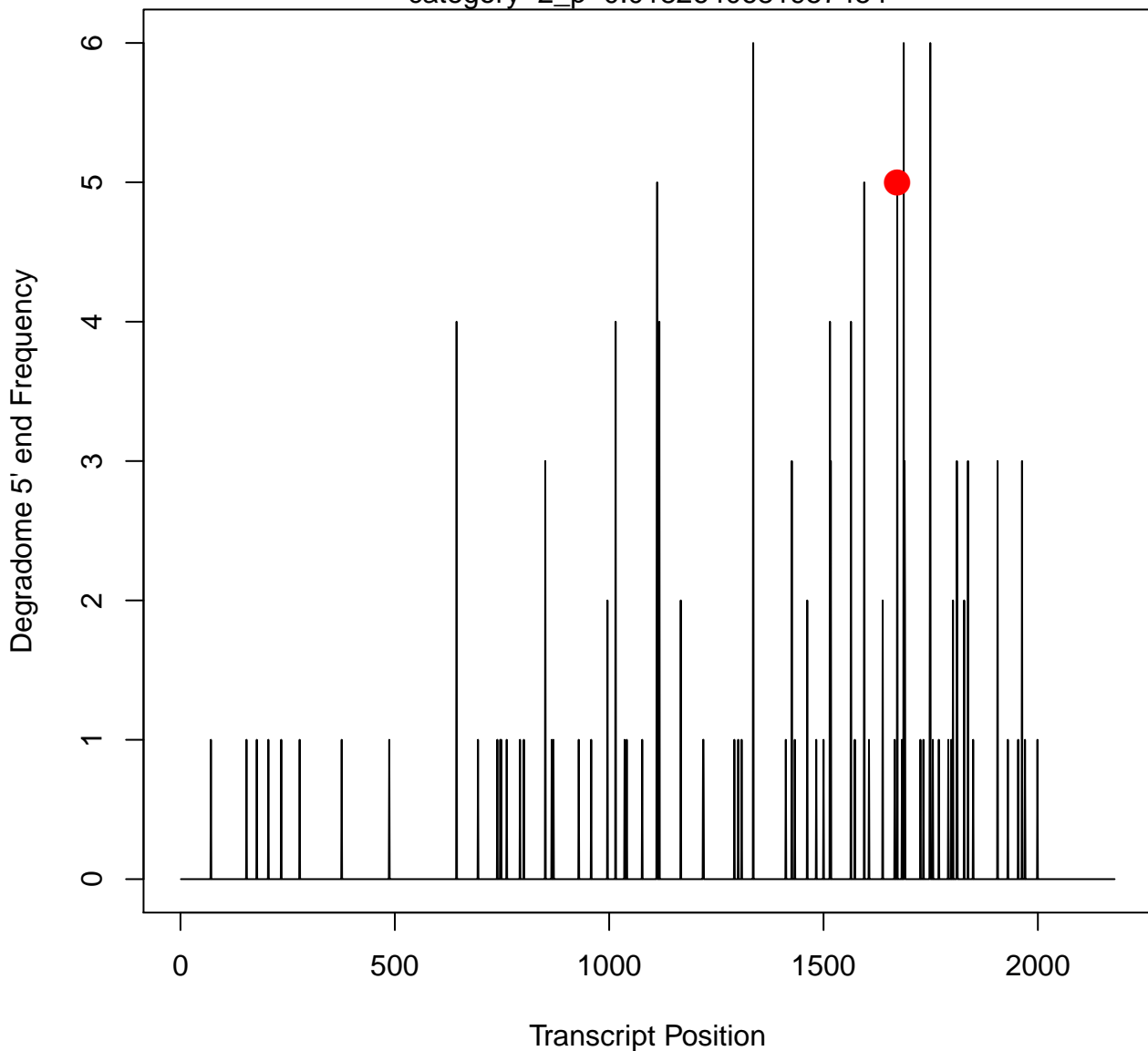

Supplement: Supplementary file 5 [file Data_Sheet_5.zip › Sit-miR169f_Seita.9G155700.1_1672_TPlot.pdf]

**T=Seita.4G209800.1\_Q=Sit-miR169g\_S=2645**

category=2\_p=0.995730006357209

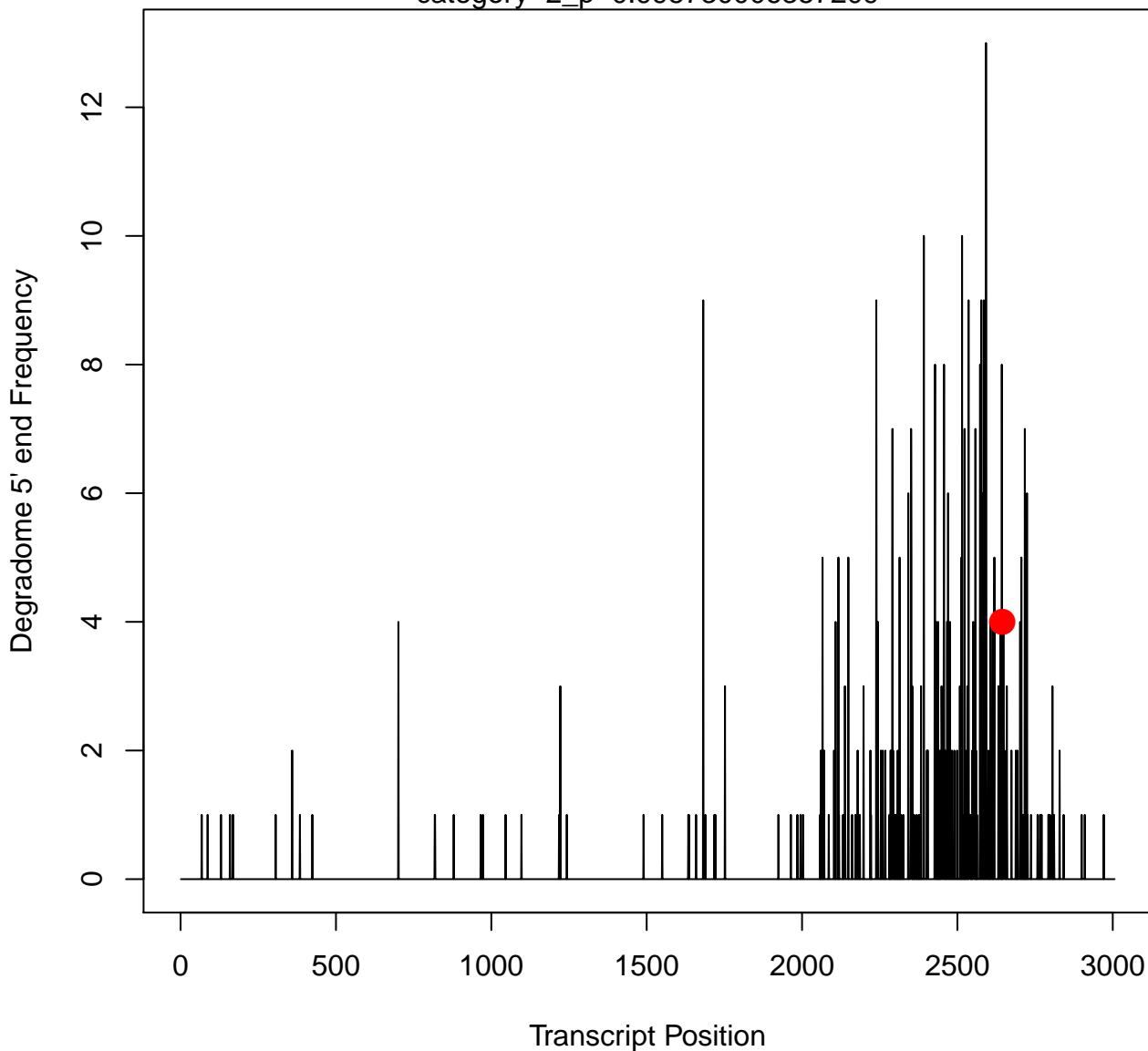

Supplement: Supplementary file 5 [file Data_Sheet_5.zip › Sit-miR169g_Seita.4G209800.1_2645_TPlot.pdf]

**T=Seita.4G268900.1\_Q=Sit-miR169g\_S=574**

category=2\_p=0.992712453858273

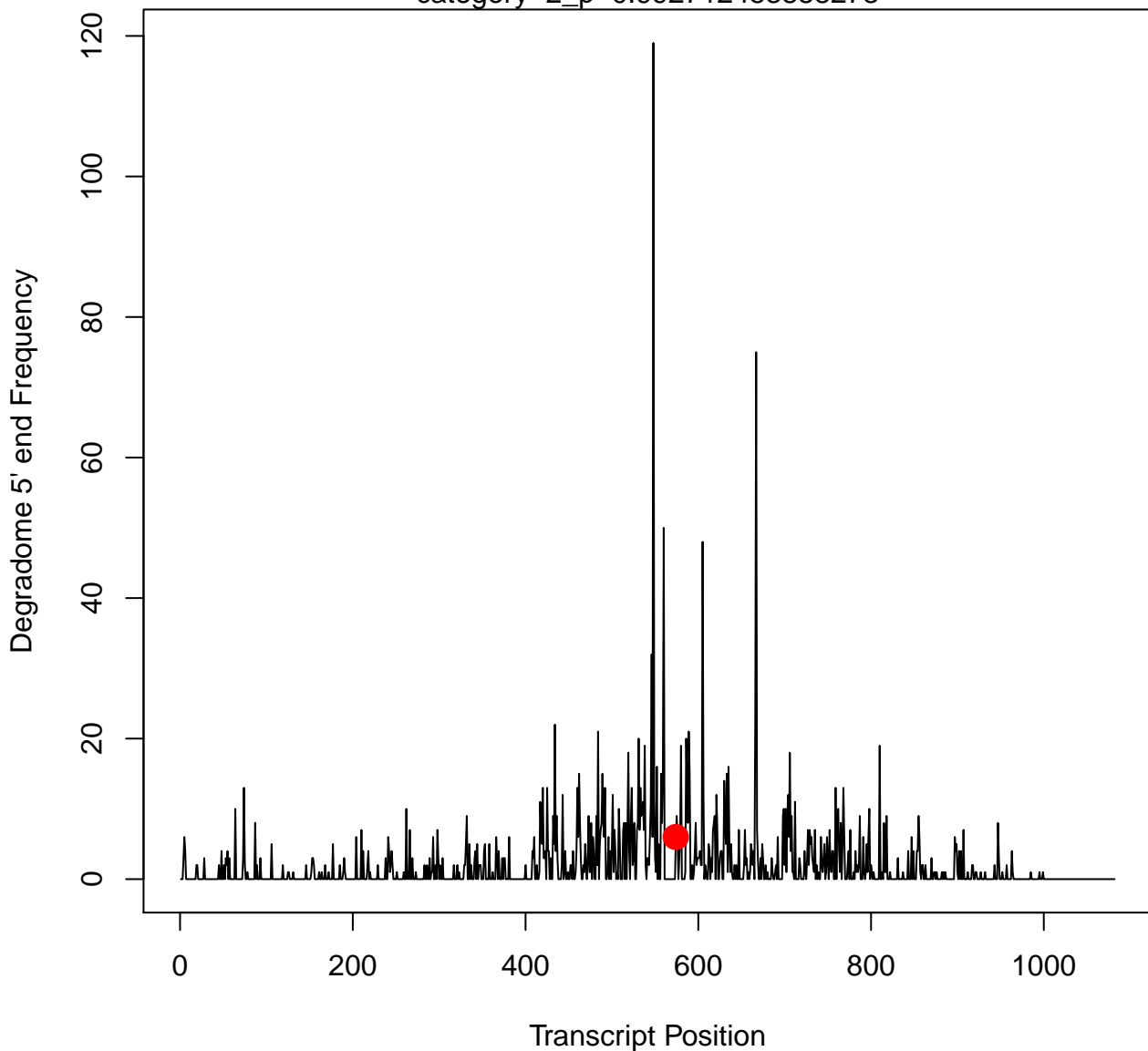

Supplement: Supplementary file 5 [file Data_Sheet_5.zip › Sit-miR169g_Seita.4G268900.1_574_TPlot.pdf]

**T=Seita.5G301600.1\_Q=Sit-miR169g\_S=1032**

category=2\_p=0.191017930867929

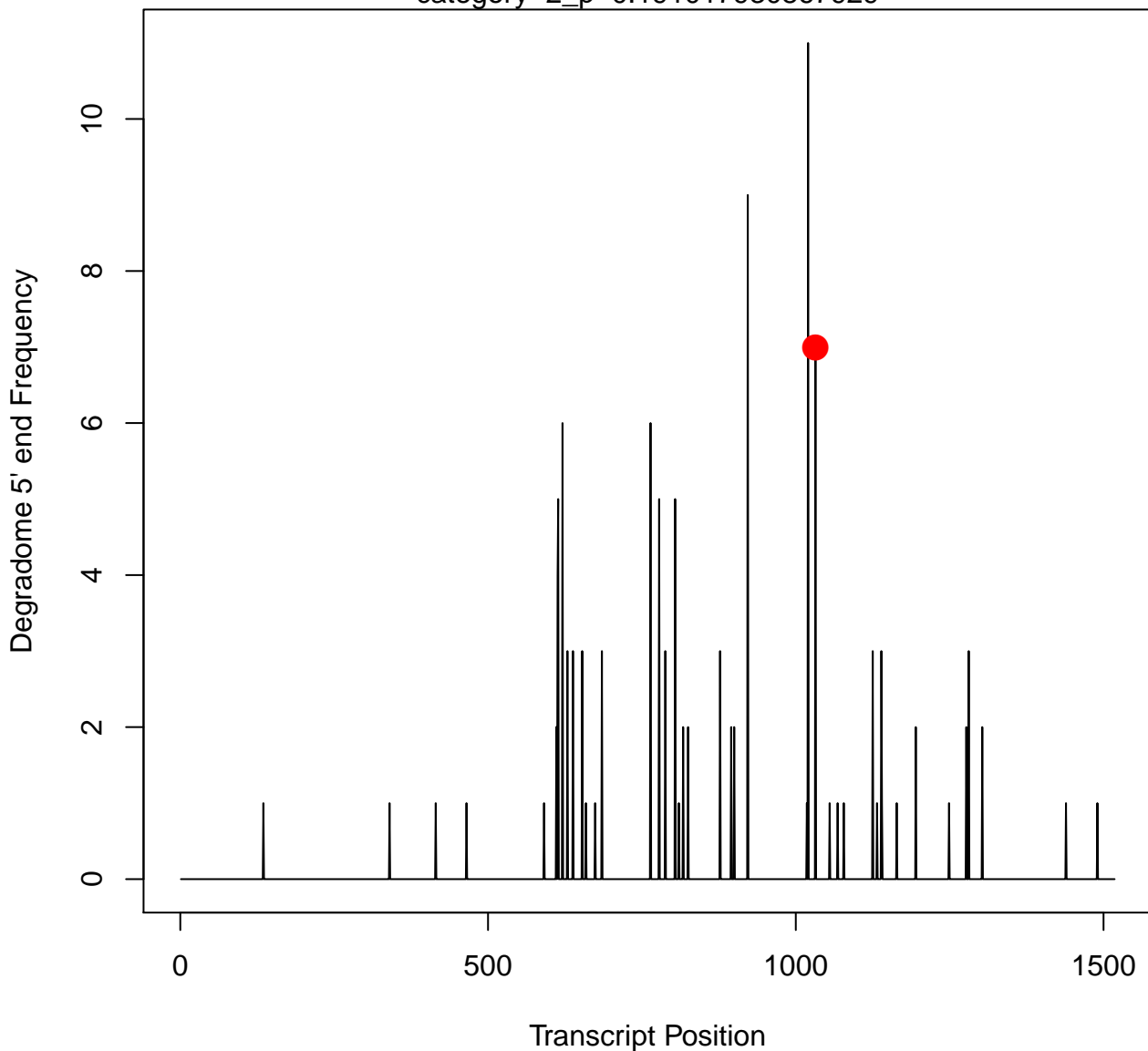

Supplement: Supplementary file 5 [file Data_Sheet_5.zip › Sit-miR169g_Seita.5G301600.1_1032_TPlot.pdf]

**T=Seita.9G467600.1\_Q=Sit-miR169g\_S=1226**

category=2\_p=0.955628712311448

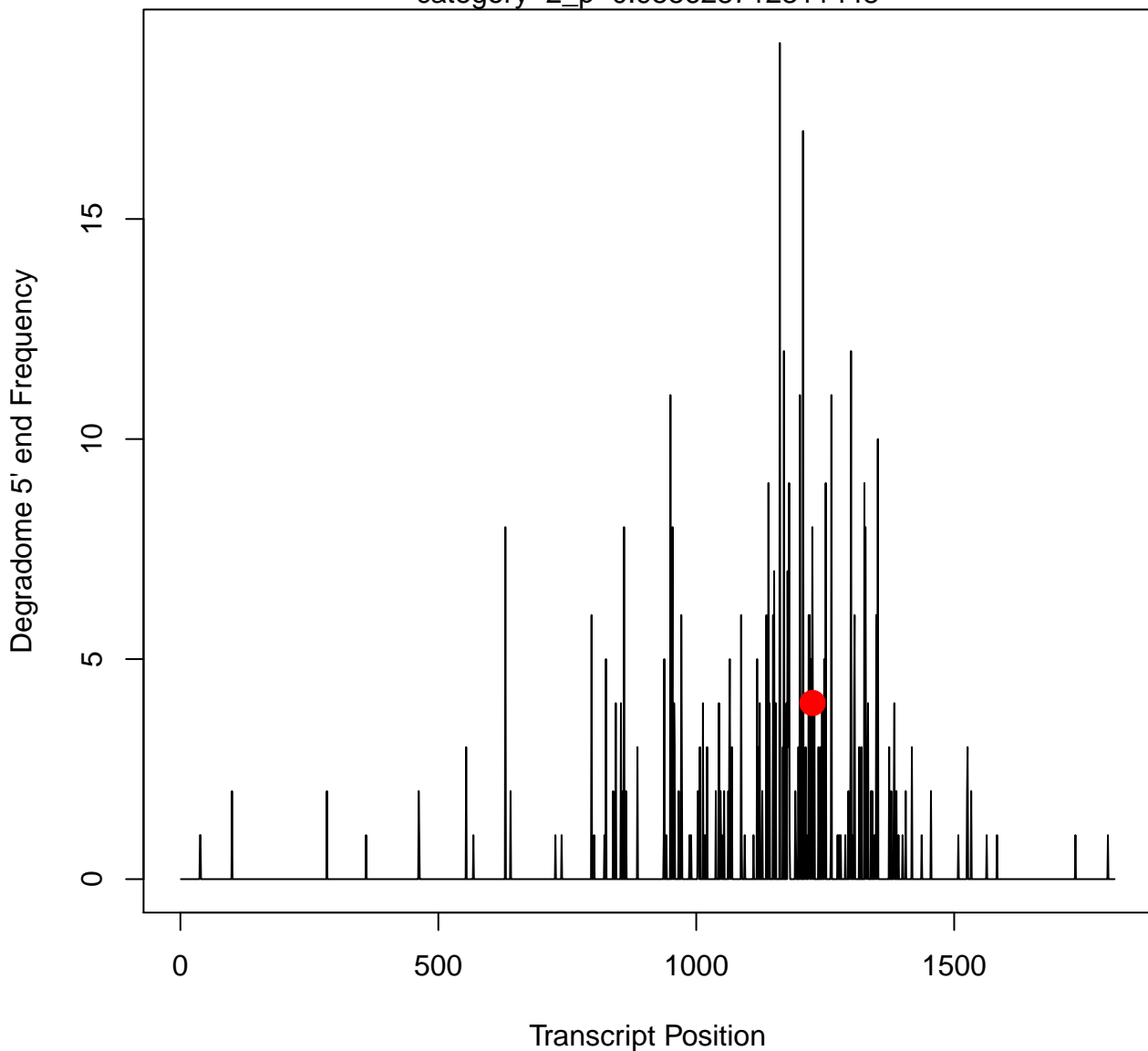

Supplement: Supplementary file 5 [file Data_Sheet_5.zip › Sit-miR169g_Seita.9G467600.1_1226_TPlot.pdf]

**T=Seita.9G521100.1\_Q=Sit-miR169g\_S=930**

category=2\_p=0.0537975684842888

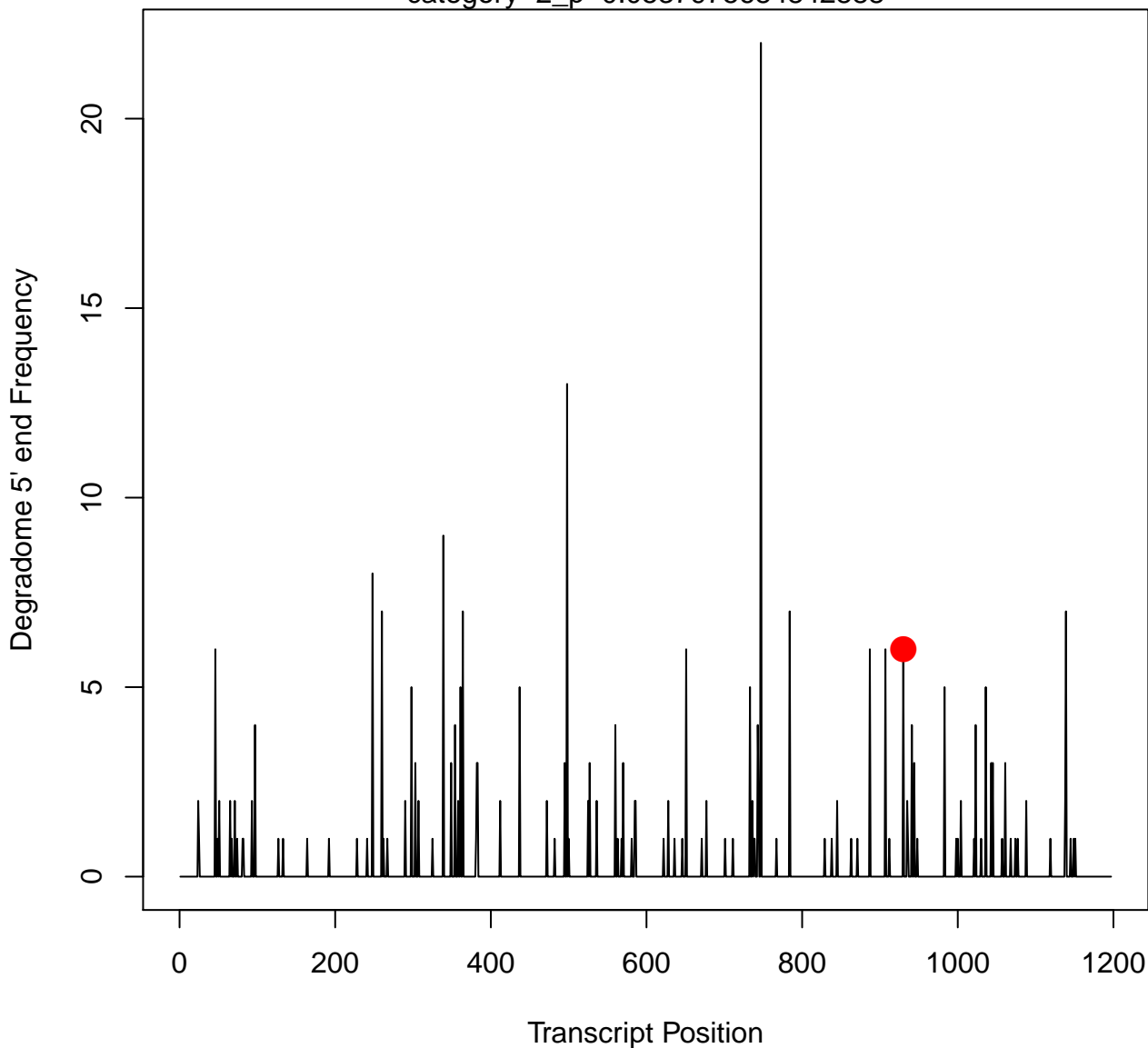

Supplement: Supplementary file 5 [file Data_Sheet_5.zip › Sit-miR169g_Seita.9G521100.1_930_TPlot.pdf]

**T=Seita.5G107300.1\_Q=Sit-miR171a\_S=580**

category=2\_p=0.856965950451959

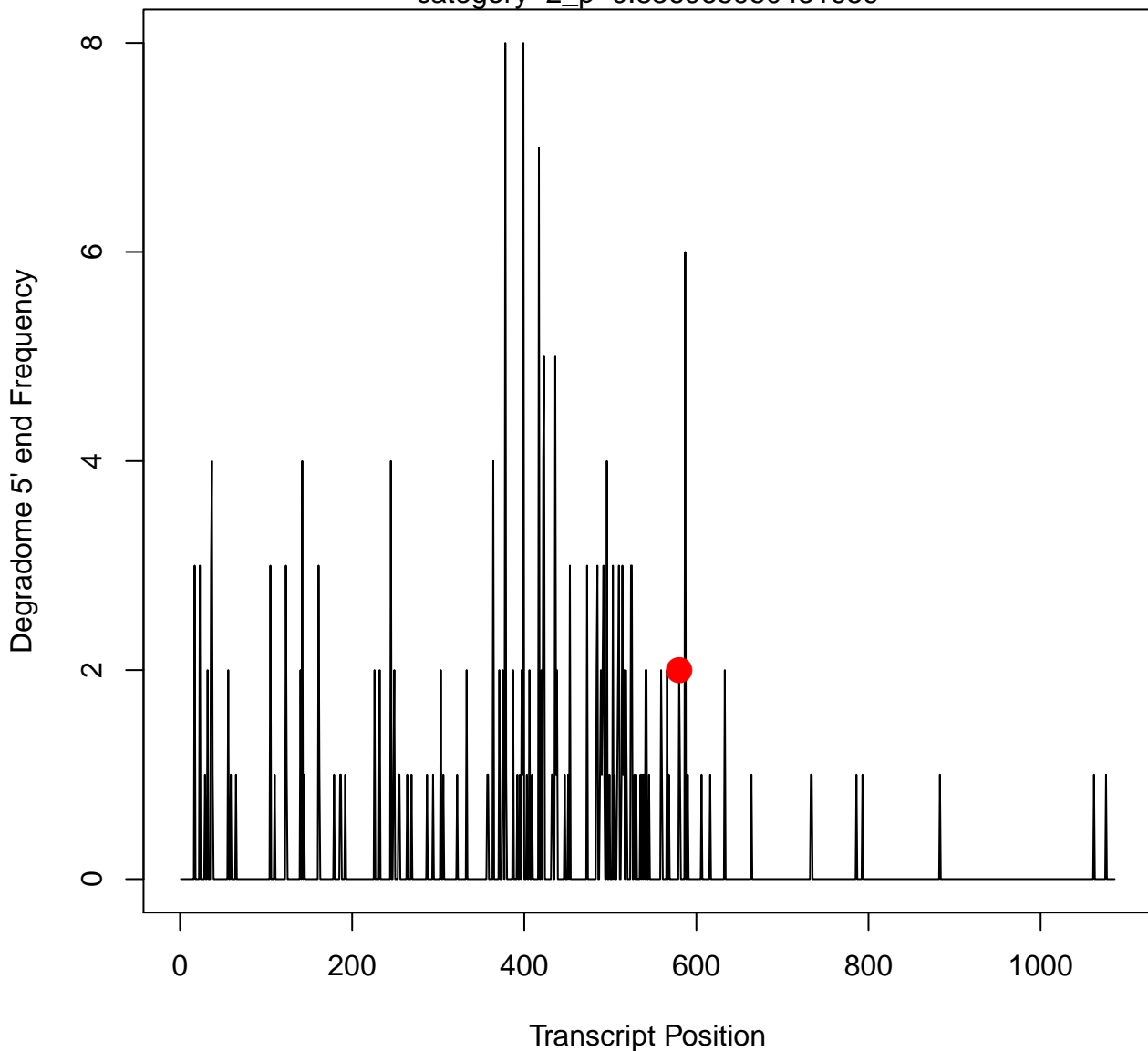

Supplement: Supplementary file 5 [file Data_Sheet_5.zip › Sit-miR171a_Seita.5G107300.1_580_TPlot.pdf]

**T=Seita.1G262900.1\_Q=Sit-miR171d\_S=1085**

category=2\_p=0.00917411630284182

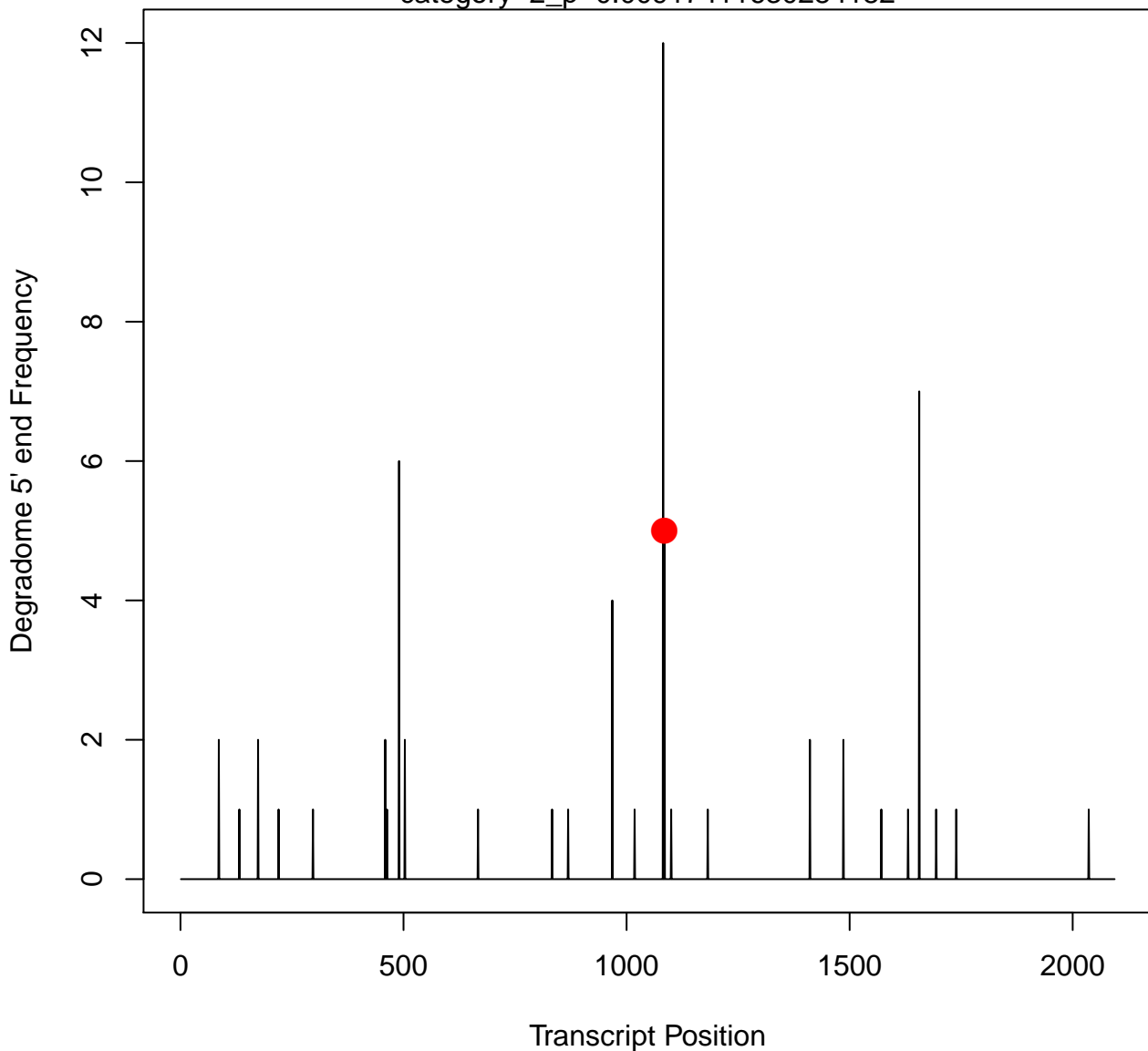

Supplement: Supplementary file 5 [file Data_Sheet_5.zip › Sit-miR171d_Seita.1G262900.1_1085_TPlot.pdf]

**T=Seita.2G335900.1\_Q=Sit-miR171d\_S=143**

category=2\_p=0.971491490592241

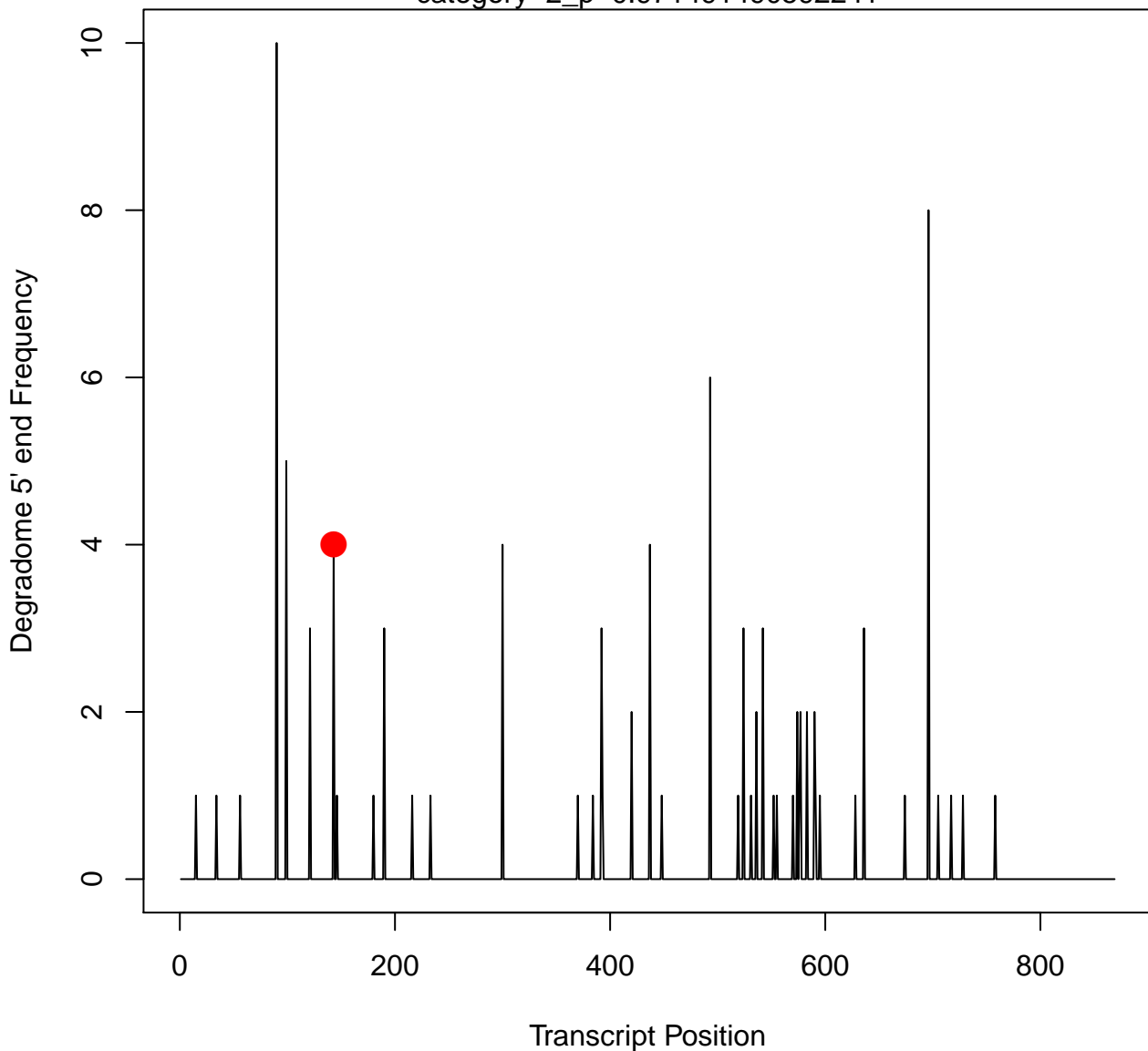

Supplement: Supplementary file 5 [file Data_Sheet_5.zip › Sit-miR171d_Seita.2G335900.1_143_TPlot.pdf]

**T=Seita.3G167200.1\_Q=Sit-miR171d\_S=432**

category=2\_p=0.783449601953312

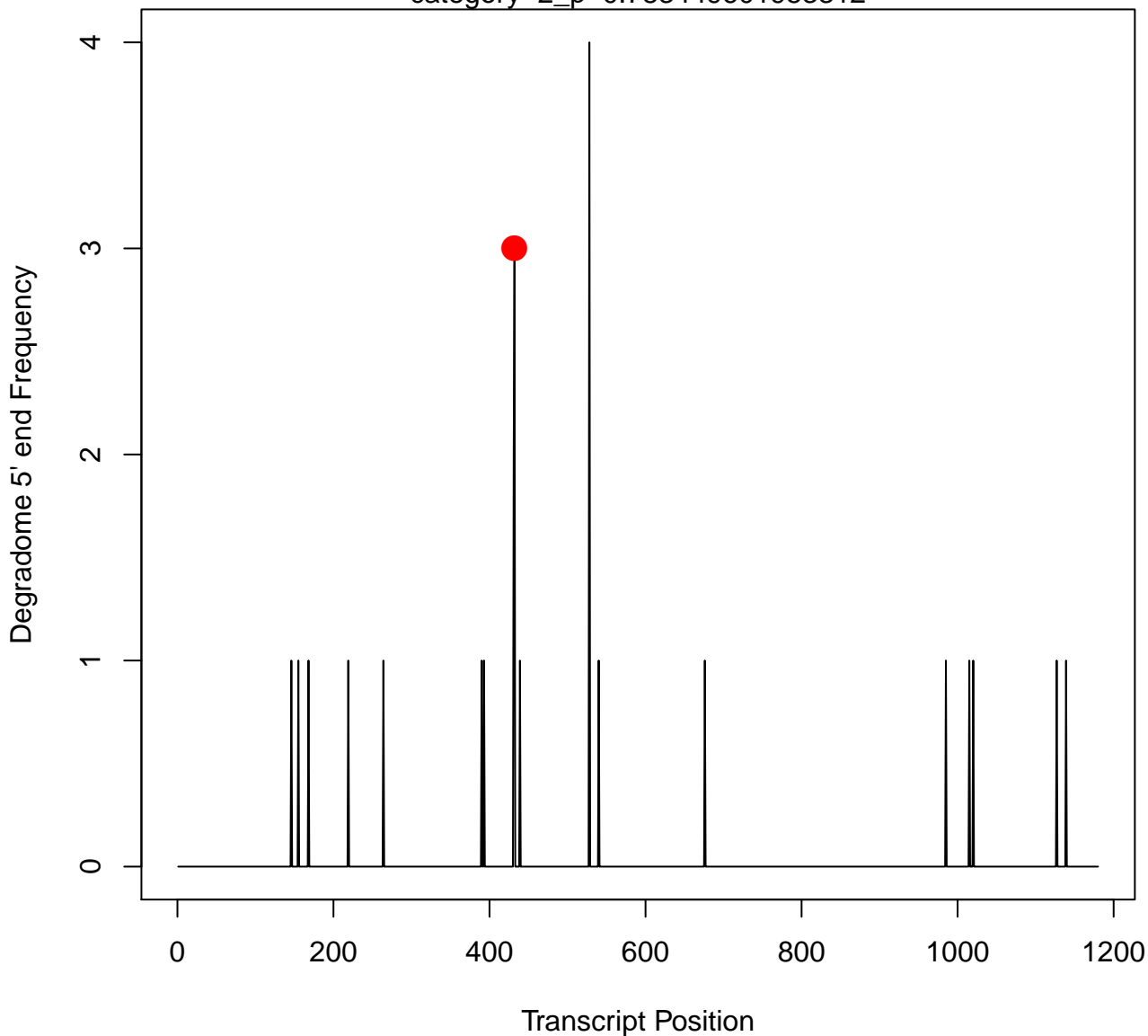

Supplement: Supplementary file 5 [file Data_Sheet_5.zip › Sit-miR171d_Seita.3G167200.1_432_TPlot.pdf]

**T=Seita.6G187200.1\_Q=Sit-miR171d\_S=241**

category=2\_p=0.939856360691118

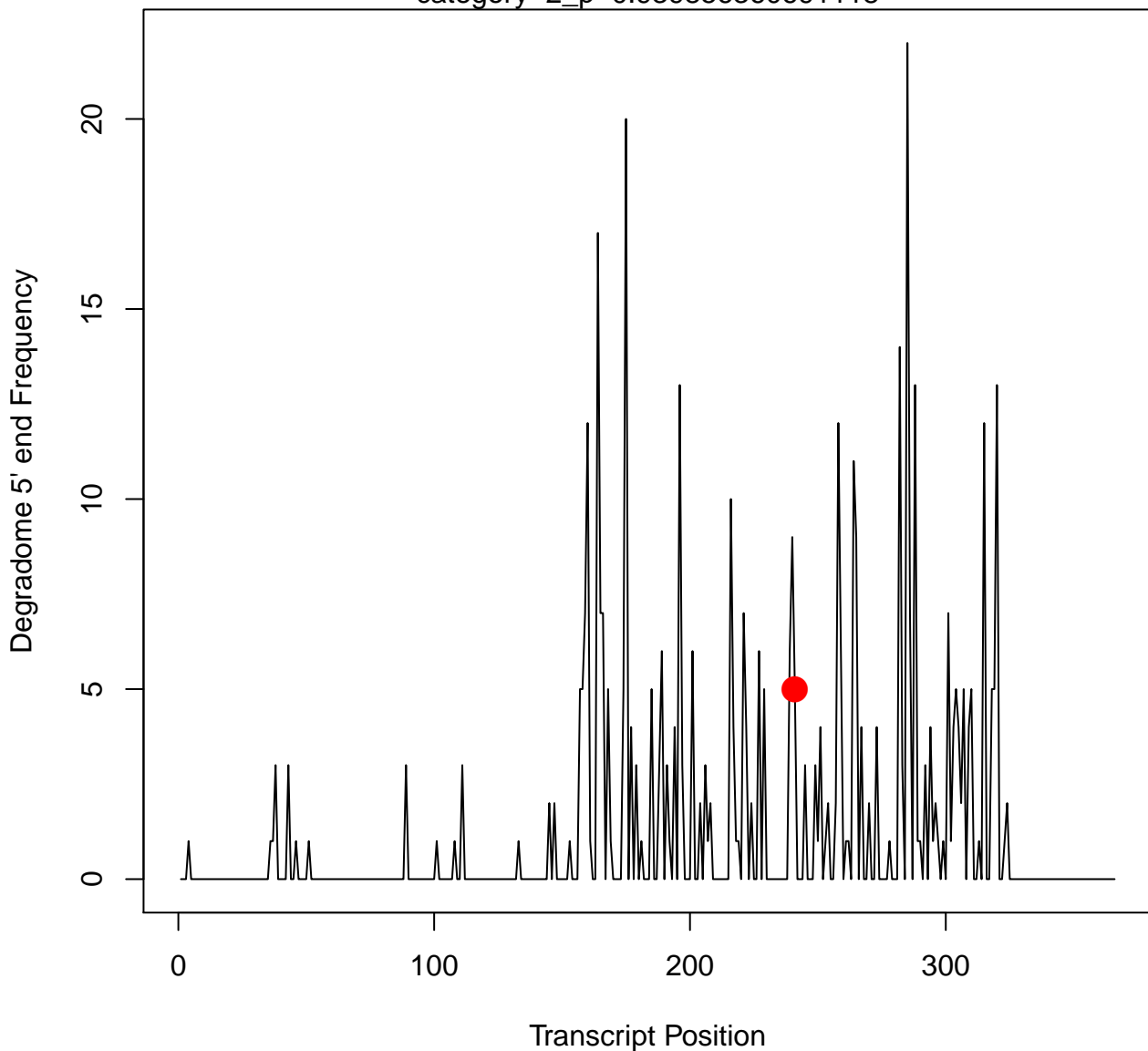

Supplement: Supplementary file 5 [file Data_Sheet_5.zip › Sit-miR171d_Seita.6G187200.1_241_TPlot.pdf]

**T=Seita.4G003600.1\_Q=Sit-miR171e\_S=1532**

category=2\_p=0.00917411630284182

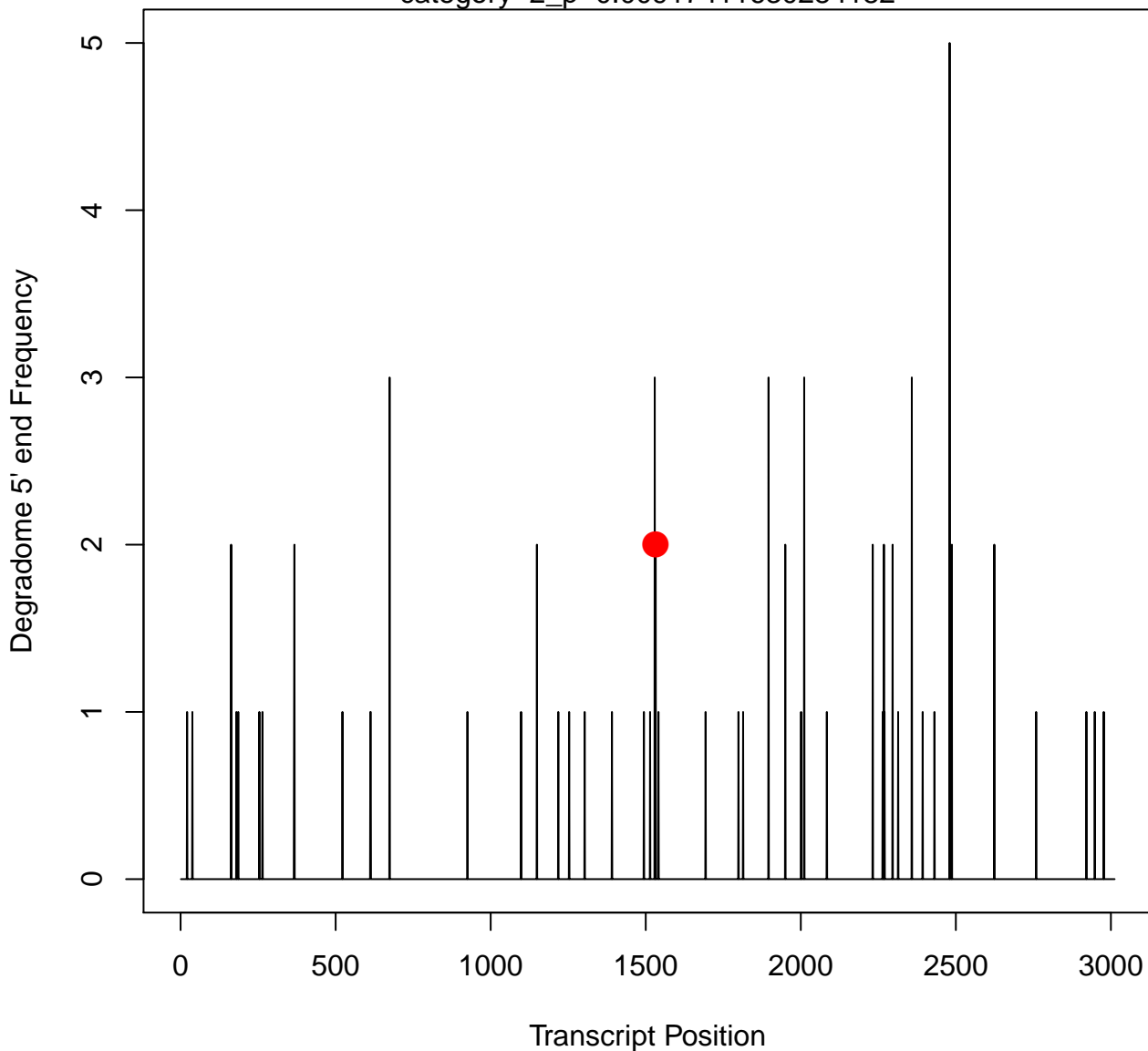

Supplement: Supplementary file 5 [file Data_Sheet_5.zip › Sit-miR171e_Seita.4G003600.1_1532_TPlot.pdf]

**T=Seita.4G018600.1\_Q=Sit-miR171e\_S=1288**

category=2\_p=0.791287548372707

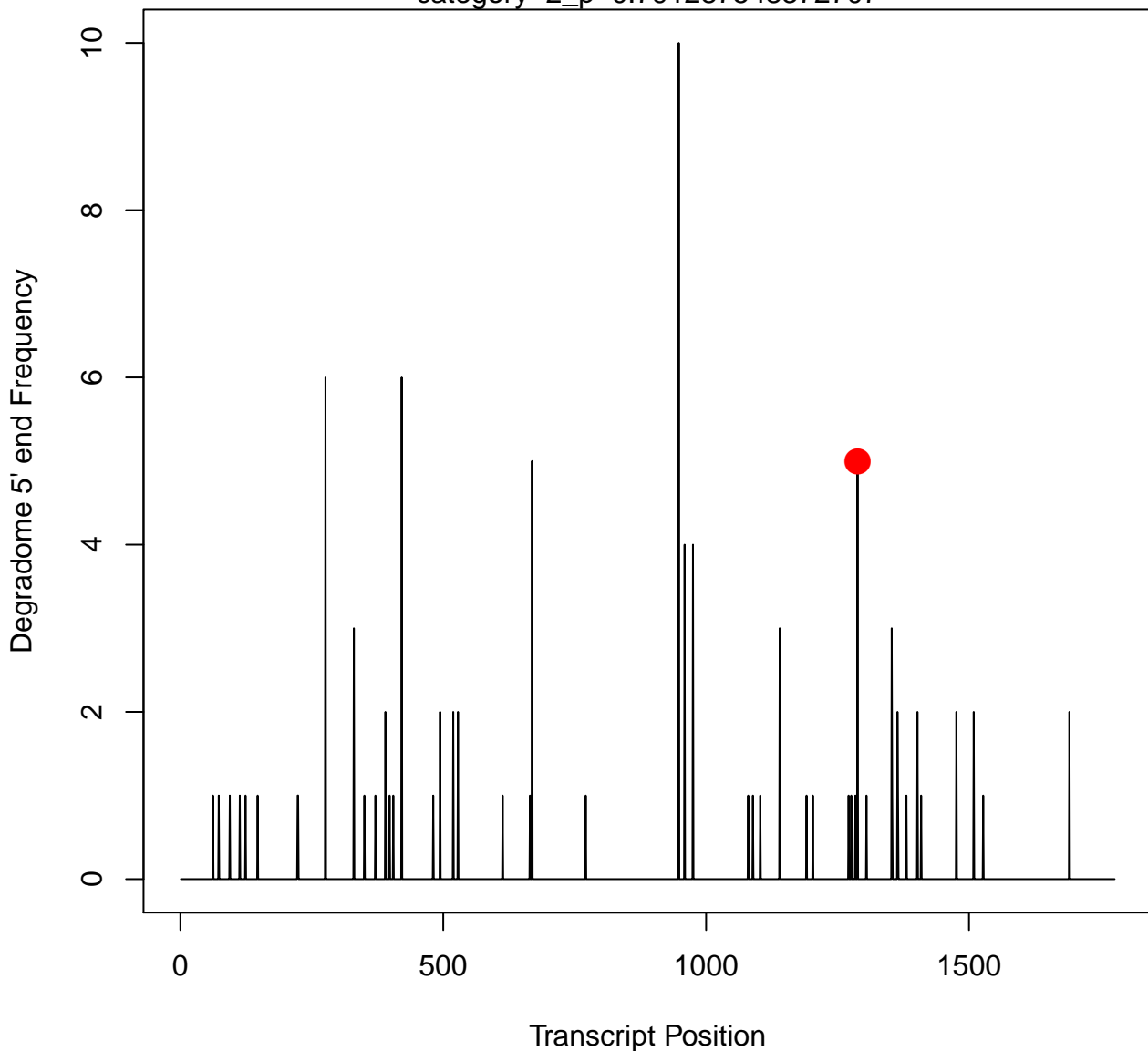

Supplement: Supplementary file 5 [file Data_Sheet_5.zip › Sit-miR171e_Seita.4G018600.1_1288_TPlot.pdf]

**T=Seita.1G262900.1\_Q=Sit-miR171f\_S=1082**

category=0\_p=0.00126686621795646

Degradome 5' end Frequency

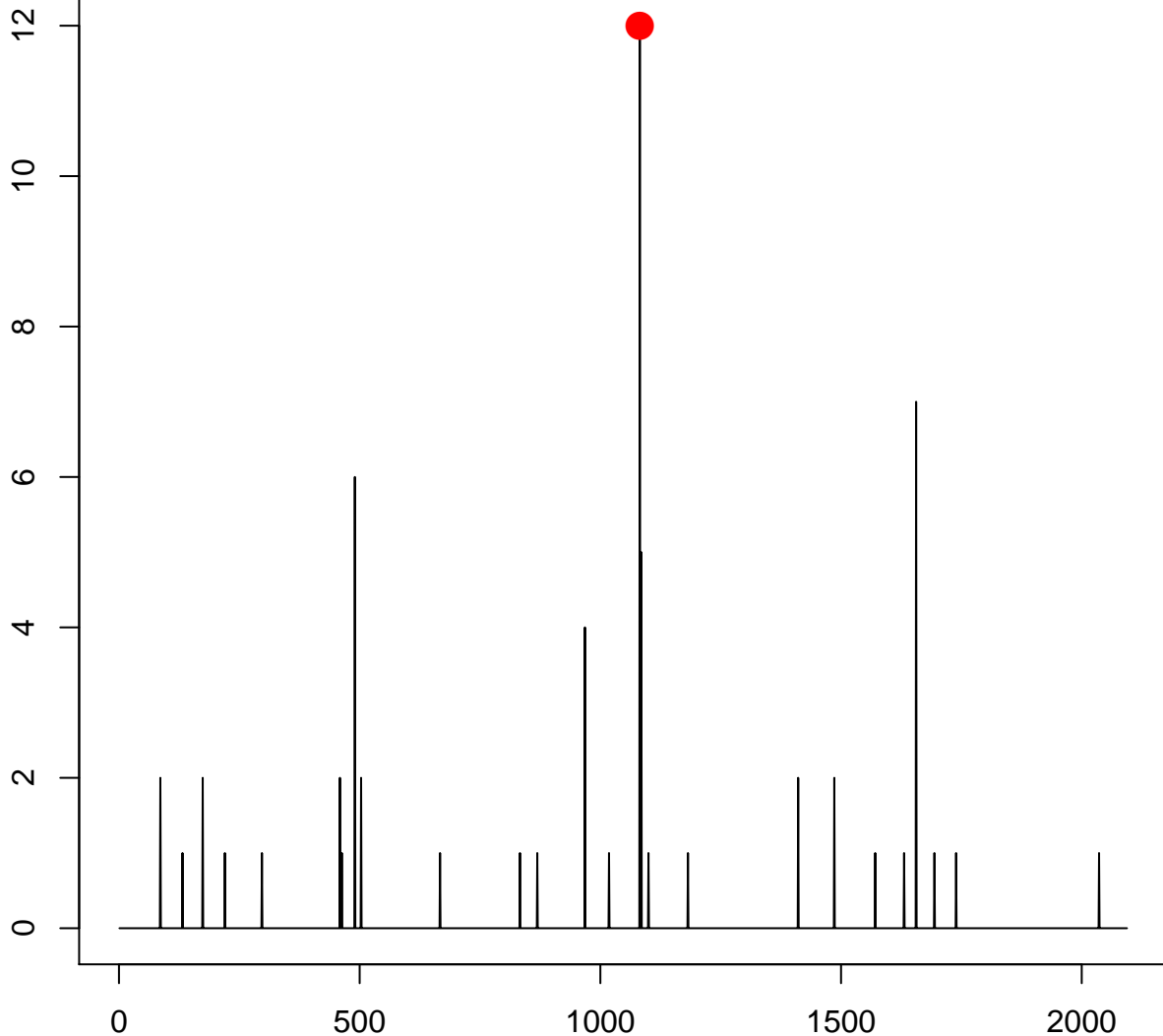

Transcript Position

Supplement: Supplementary file 5 [file Data_Sheet_5.zip › Sit-miR171f_Seita.1G262900.1_1082_TPlot.pdf]

**T=Seita.4G263300.1\_Q=Sit-miR171f\_S=3000**

category=0\_p=0.220660341636088

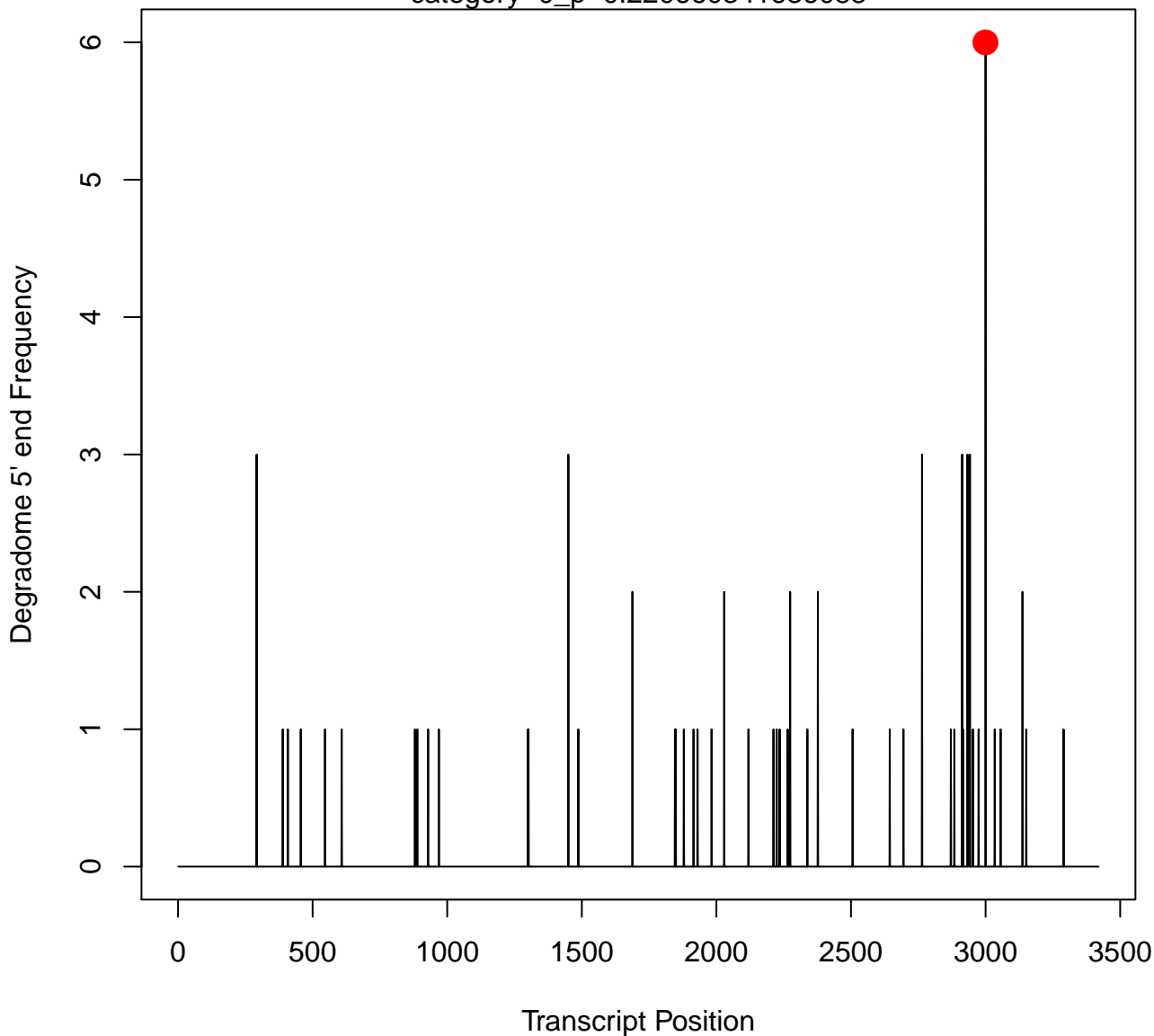

Supplement: Supplementary file 5 [file Data_Sheet_5.zip › Sit-miR171f_Seita.4G263300.1_3000_TPlot.pdf]

**T=Seita.5G367800.1\_Q=Sit-miR171f\_S=399**

category=2\_p=0.82155548818385

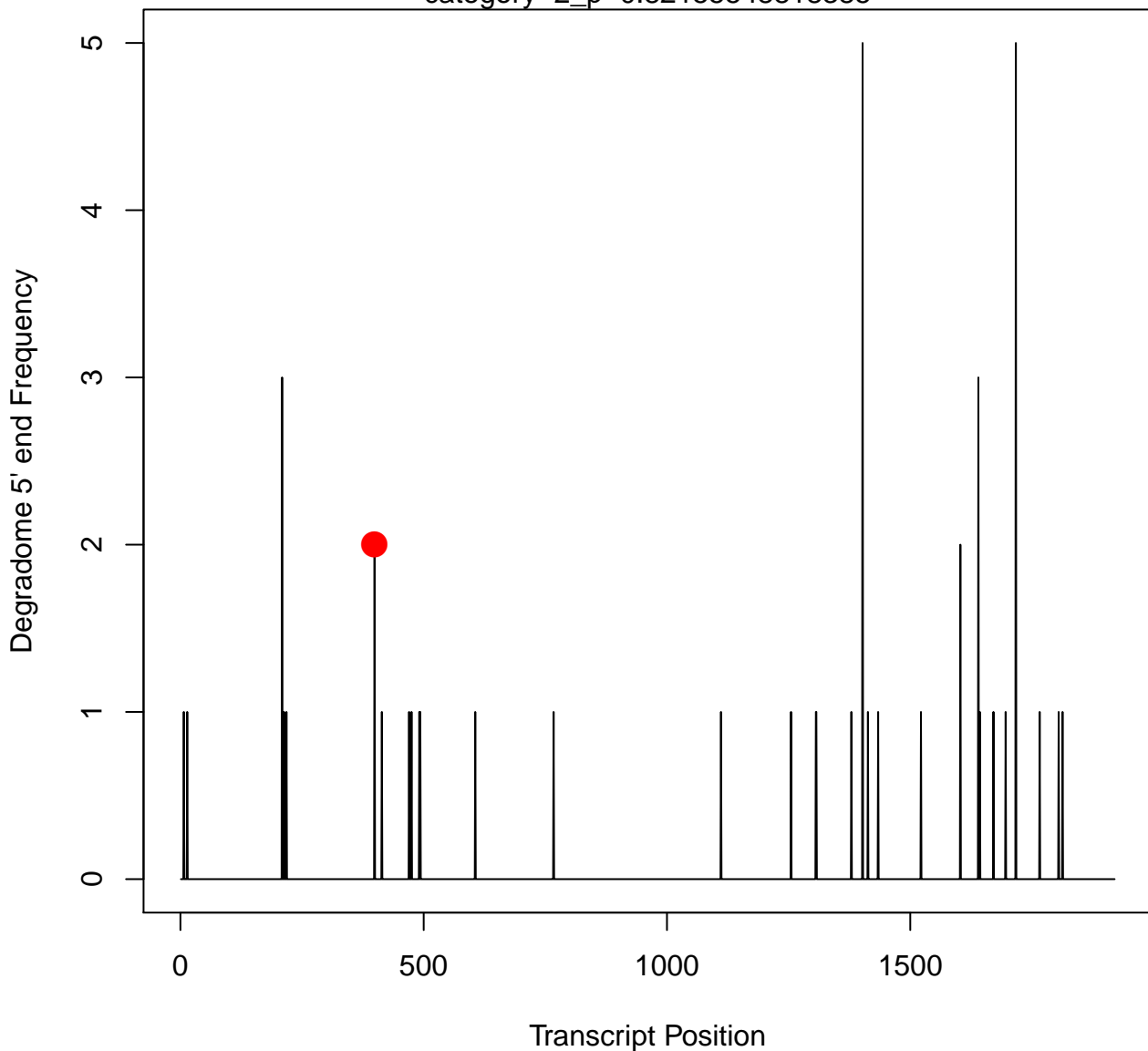

Supplement: Supplementary file 5 [file Data_Sheet_5.zip › Sit-miR171f_Seita.5G367800.1_399_TPlot.pdf]

**T=Seita.1G278900.1\_Q=Sit-miR171g\_S=762**

category=2\_p=0.995884556899185

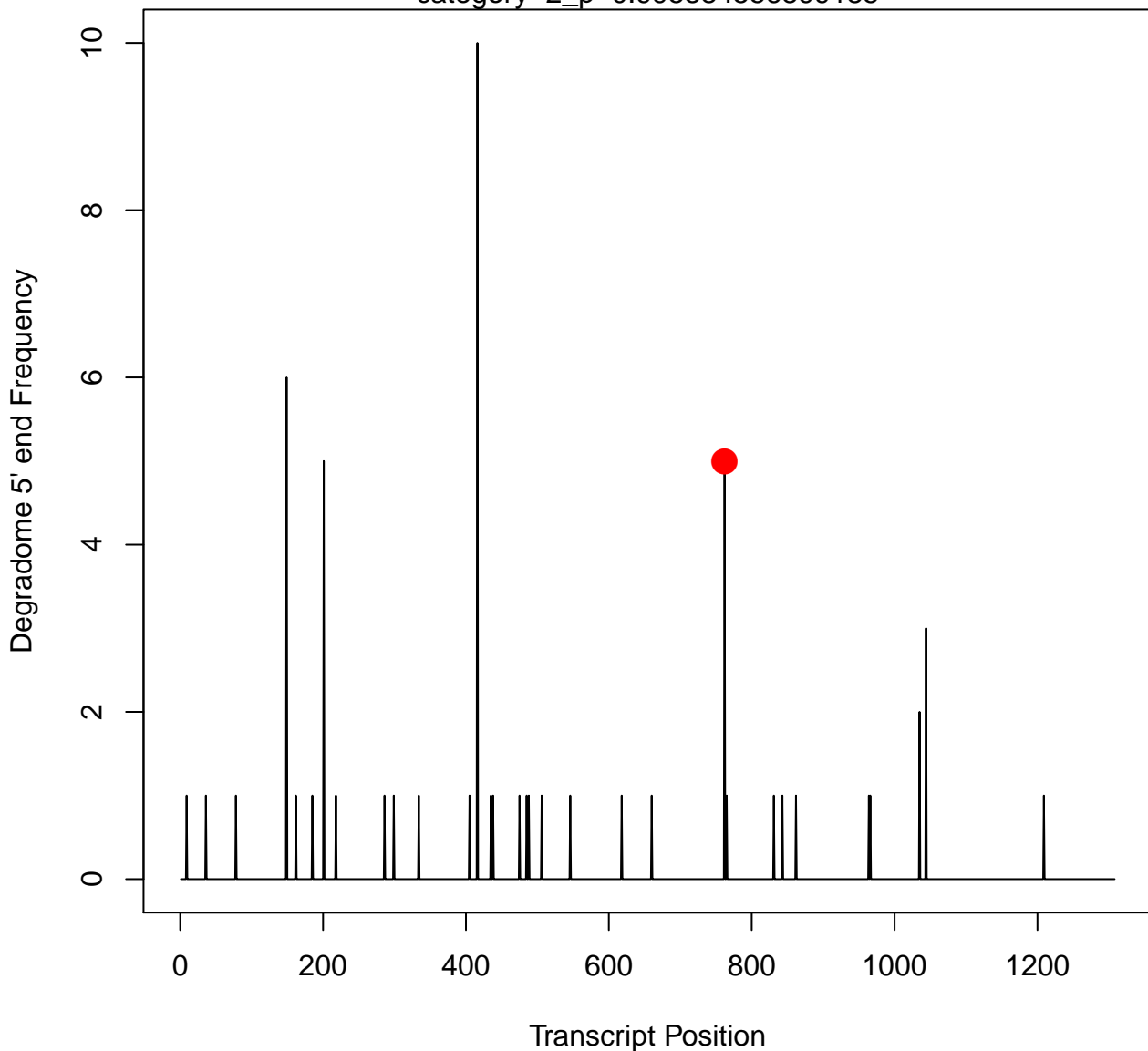

Supplement: Supplementary file 5 [file Data_Sheet_5.zip › Sit-miR171g_Seita.1G278900.1_762_TPlot.pdf]

**T=Seita.2G419600.1\_Q=Sit-miR171g\_S=5912**

category=2\_p=0.751344060756167

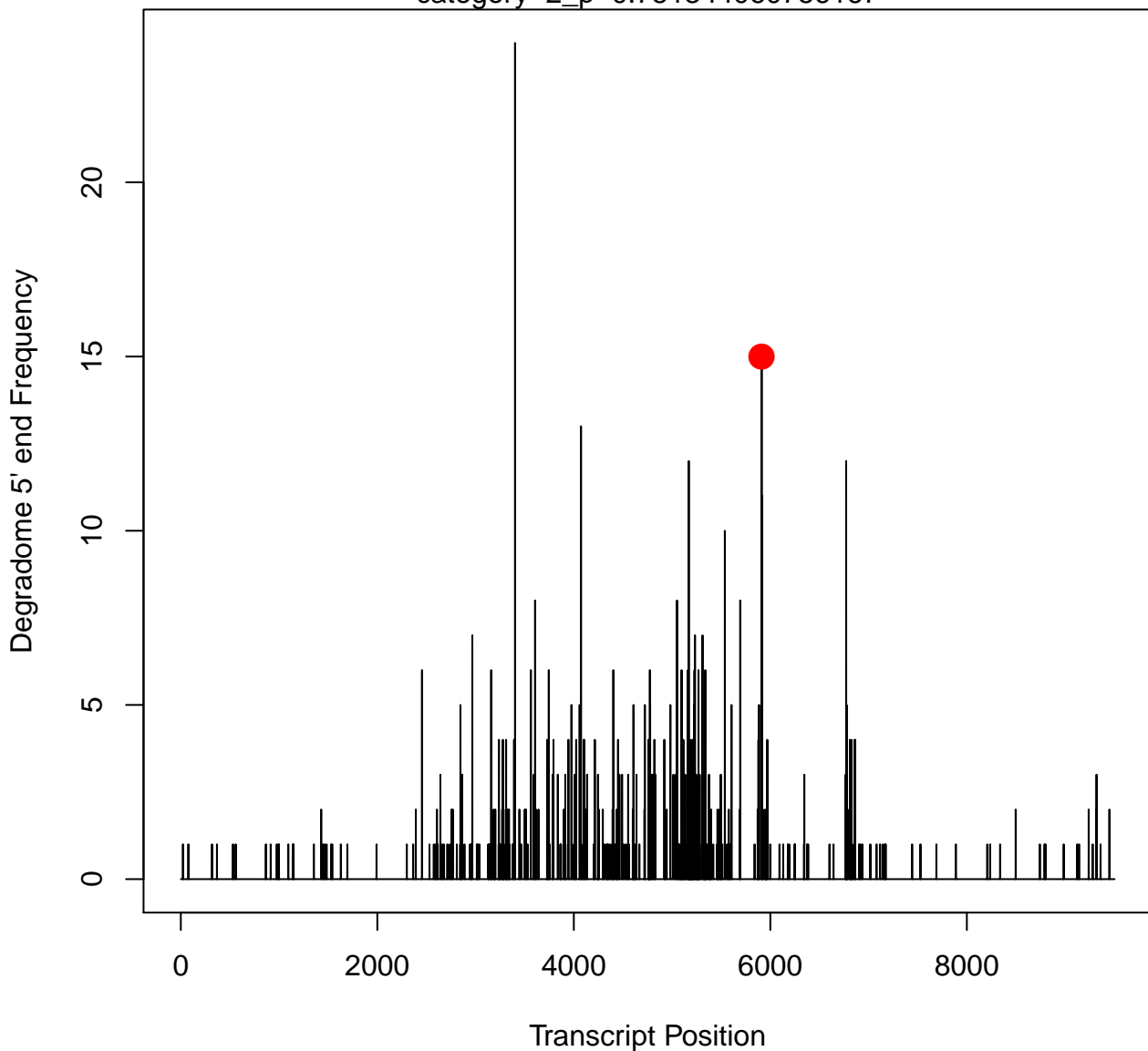

Supplement: Supplementary file 5 [file Data_Sheet_5.zip › Sit-miR171g_Seita.2G419600.1_5912_TPlot.pdf]

**T=Seita.3G155000.1\_Q=Sit-miR171g\_S=663**

category=2\_p=0.816552766968562

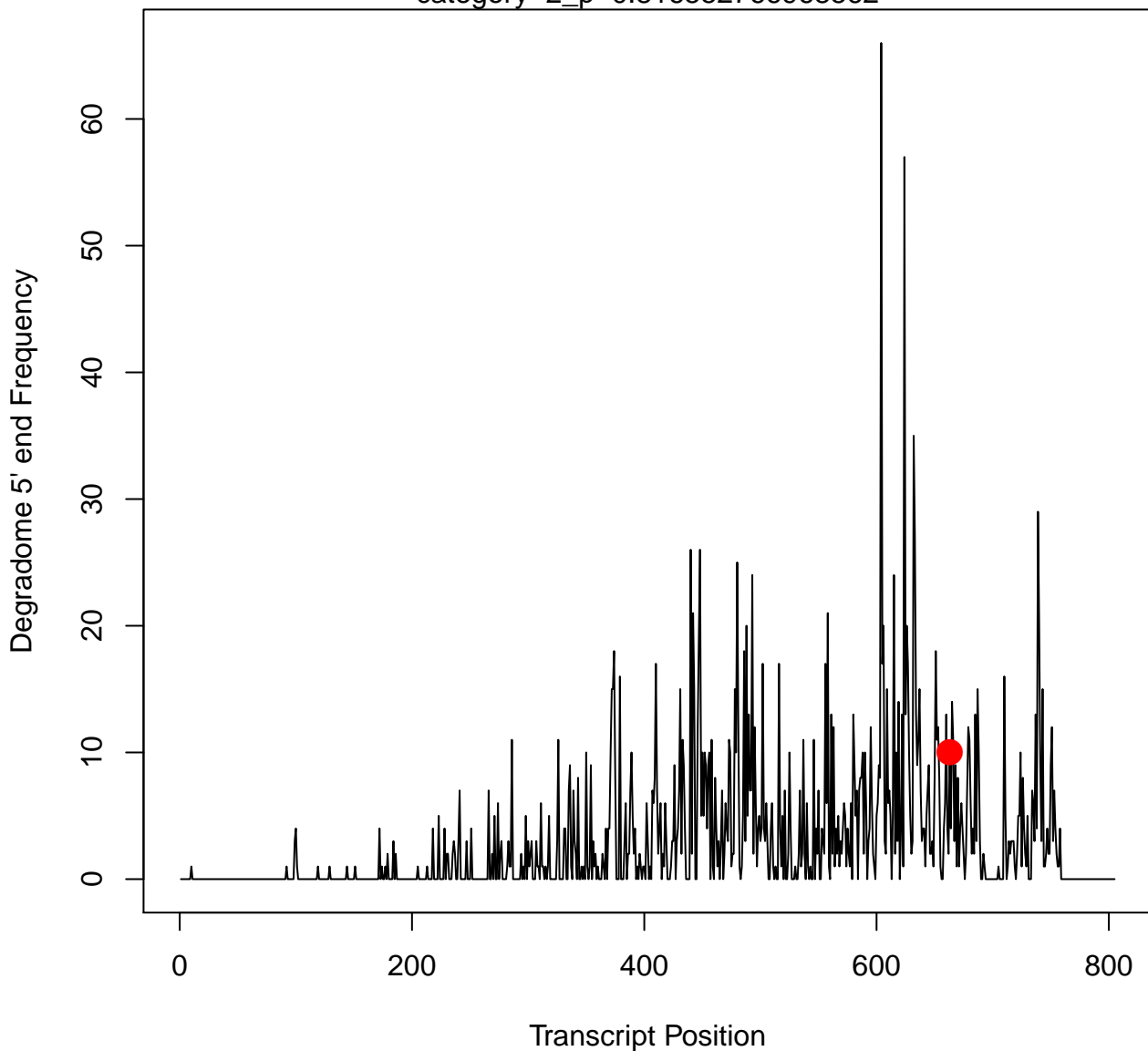

Supplement: Supplementary file 5 [file Data_Sheet_5.zip › Sit-miR171g_Seita.3G155000.1_663_TPlot.pdf]

**T=Seita.4G003600.1\_Q=Sit-miR171g\_S=1529**

category=2\_p=0.00917411630284182

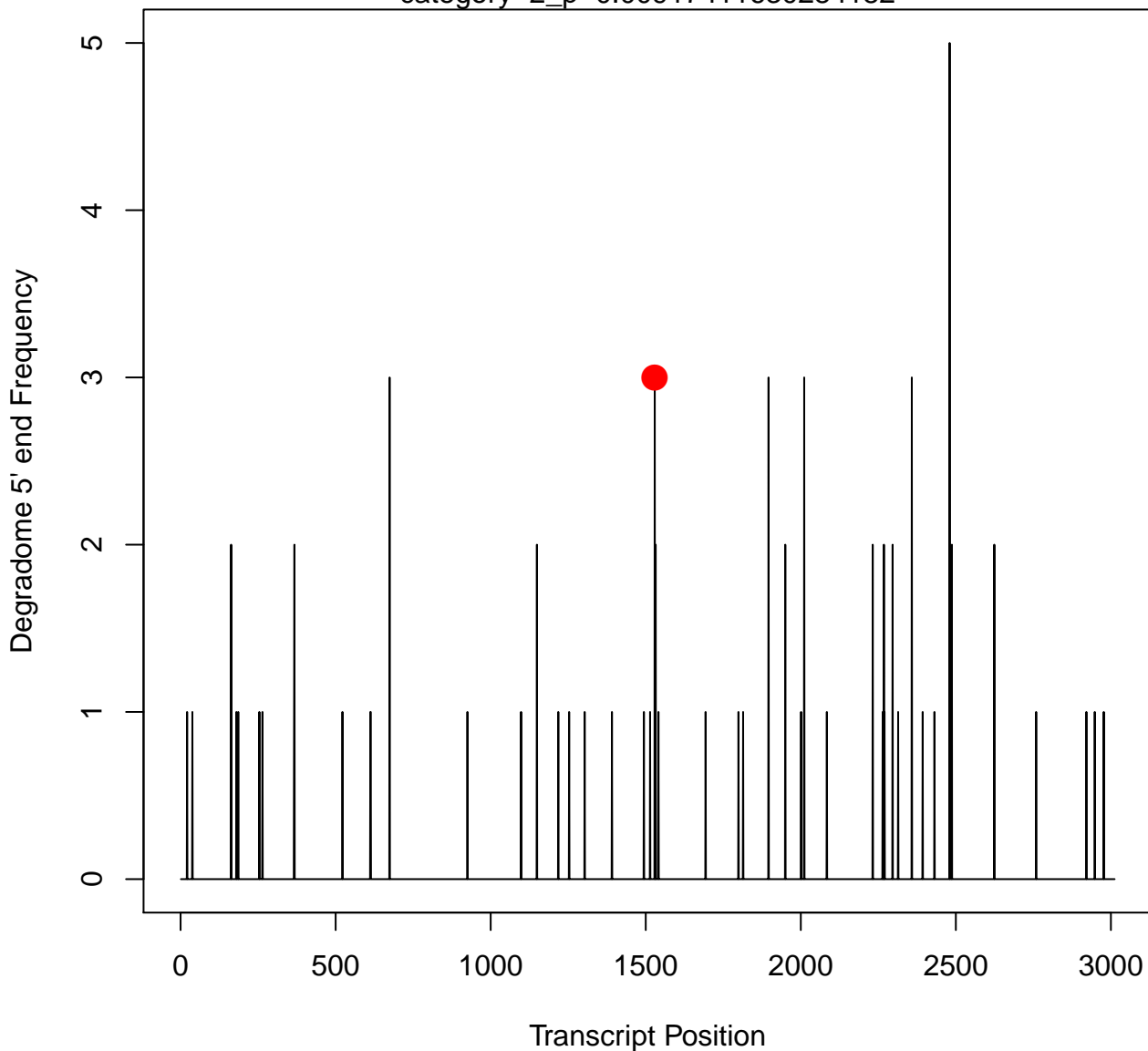

Supplement: Supplementary file 5 [file Data_Sheet_5.zip › Sit-miR171g_Seita.4G003600.1_1529_TPlot.pdf]

**T=Seita.6G252500.1\_Q=Sit-miR171g\_S=1002**

category=2\_p=0.975625857469405

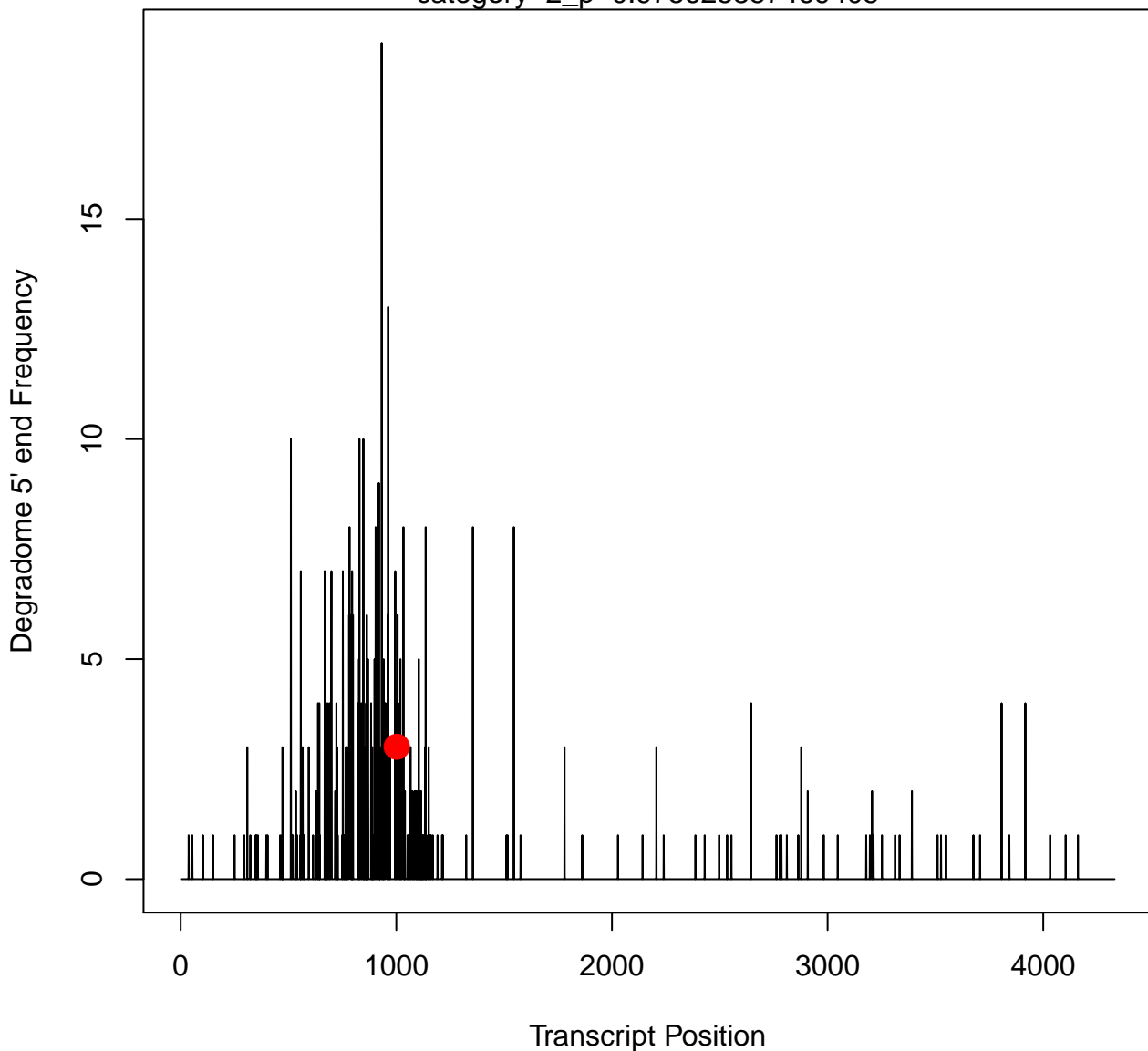

Supplement: Supplementary file 5 [file Data_Sheet_5.zip › Sit-miR171g_Seita.6G252500.1_1002_TPlot.pdf]

**T=Seita.9G078400.1\_Q=Sit-miR171g\_S=565**

category=2\_p=0.98558621917258

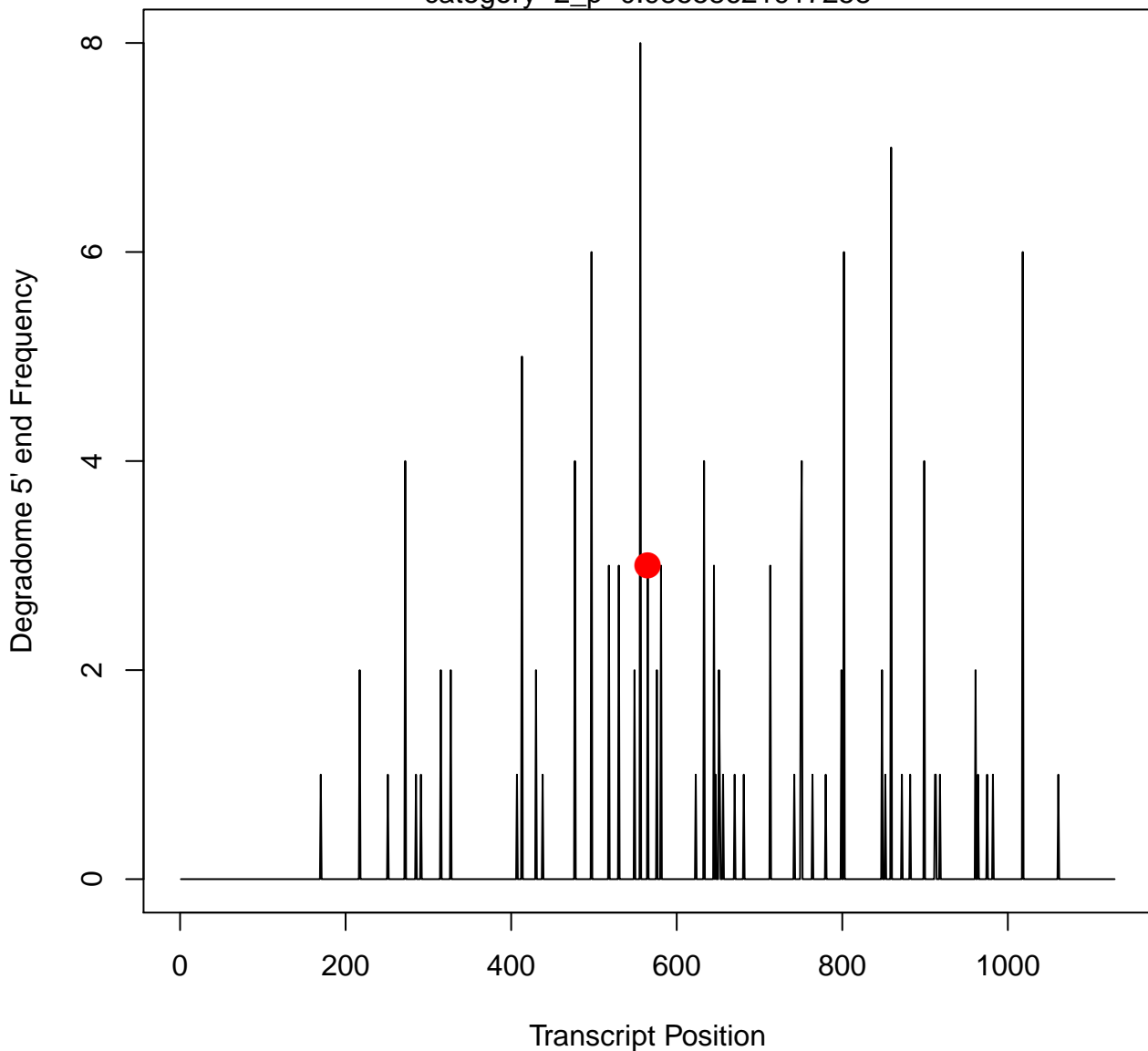

Supplement: Supplementary file 5 [file Data_Sheet_5.zip › Sit-miR171g_Seita.9G078400.1_565_TPlot.pdf]

**T=Seita.9G187400.1\_Q=Sit-miR171g\_S=809**

category=2\_p=0.980995448921191

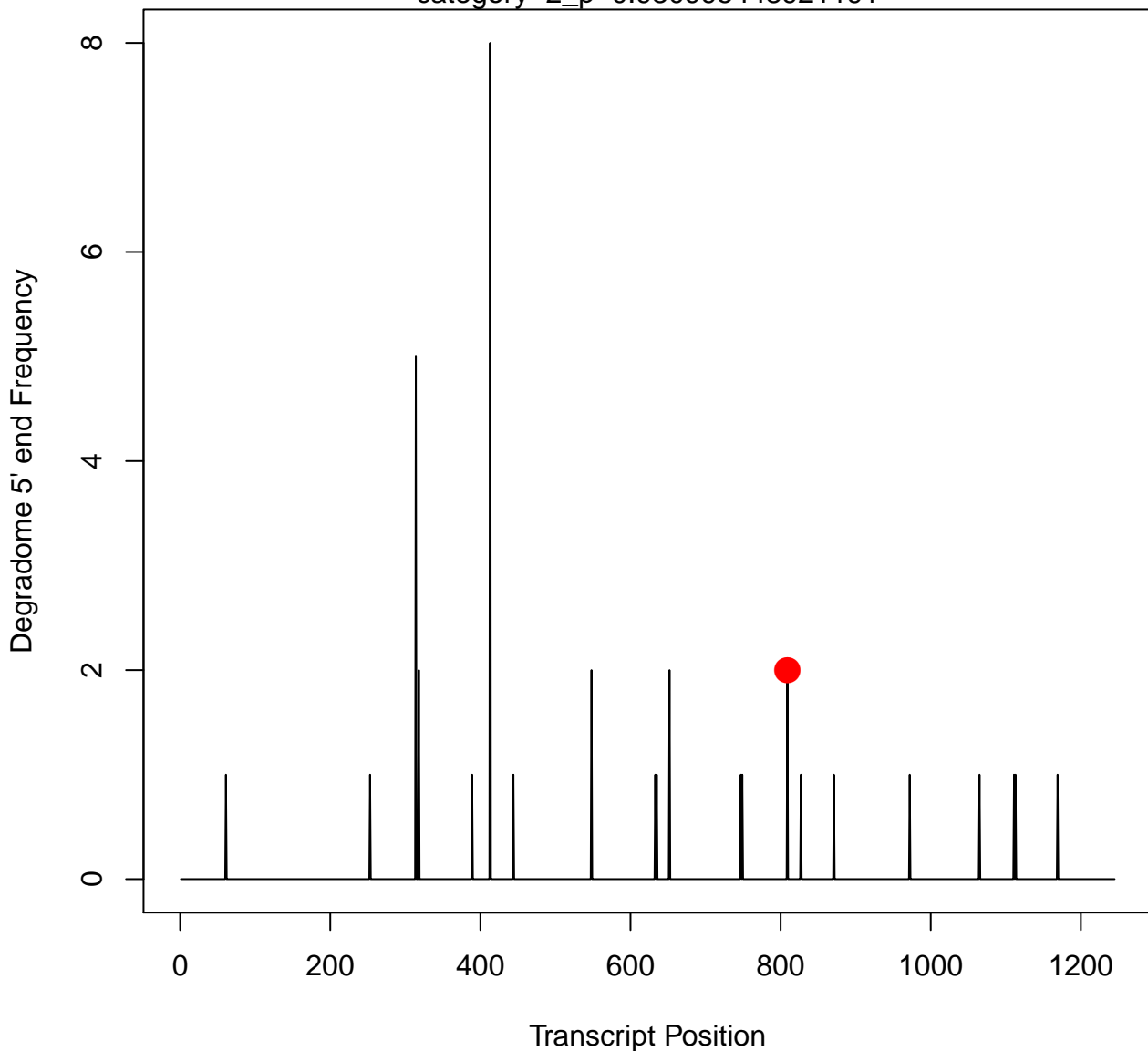

Supplement: Supplementary file 5 [file Data_Sheet_5.zip › Sit-miR171g_Seita.9G187400.1_809_TPlot.pdf]

**T=Seita.9G371700.1\_Q=Sit-miR171g\_S=2577**

category=2\_p=0.999194721017758

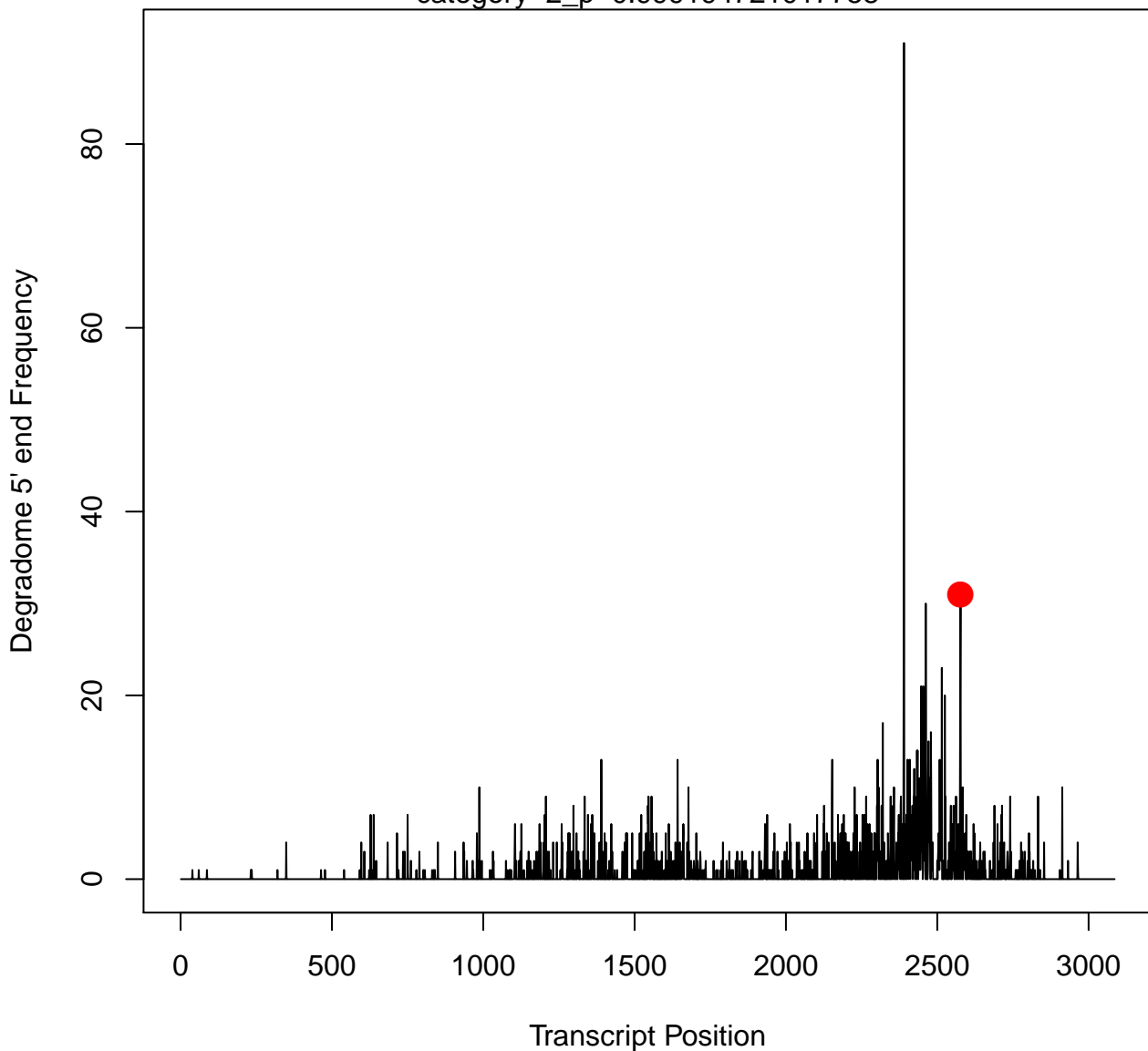

Supplement: Supplementary file 5 [file Data_Sheet_5.zip › Sit-miR171g_Seita.9G371700.1_2577_TPlot.pdf]

**T=Seita.5G438100.1\_Q=Sit-miR171h\_S=1632**

category=2\_p=0.145022204354124

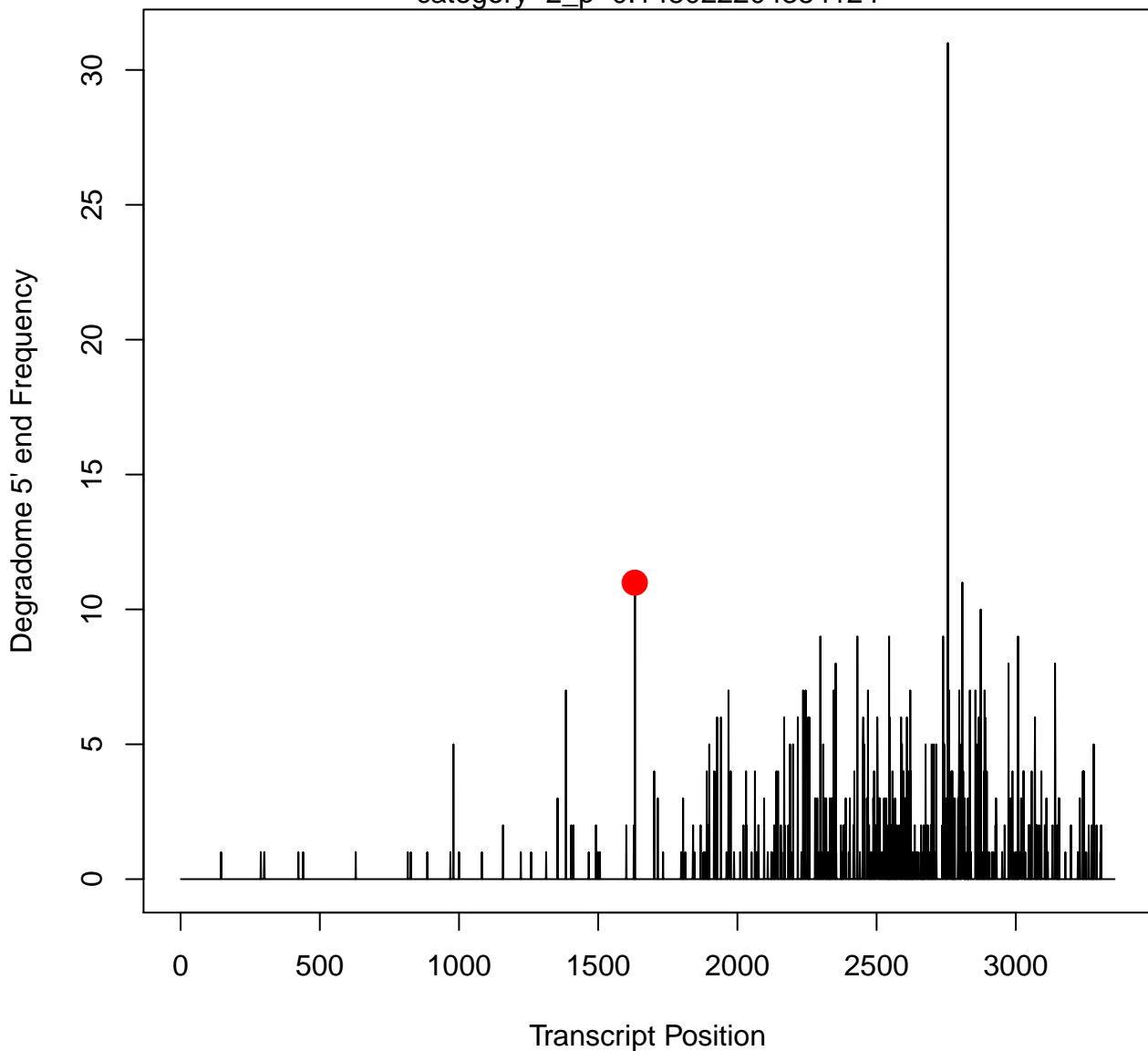

Supplement: Supplementary file 5 [file Data_Sheet_5.zip › Sit-miR171h_Seita.5G438100.1_1632_TPlot.pdf]

**T=Seita.2G031700.1\_Q=Sit-miR171i\_S=851**

category=2\_p=0.999784440853763

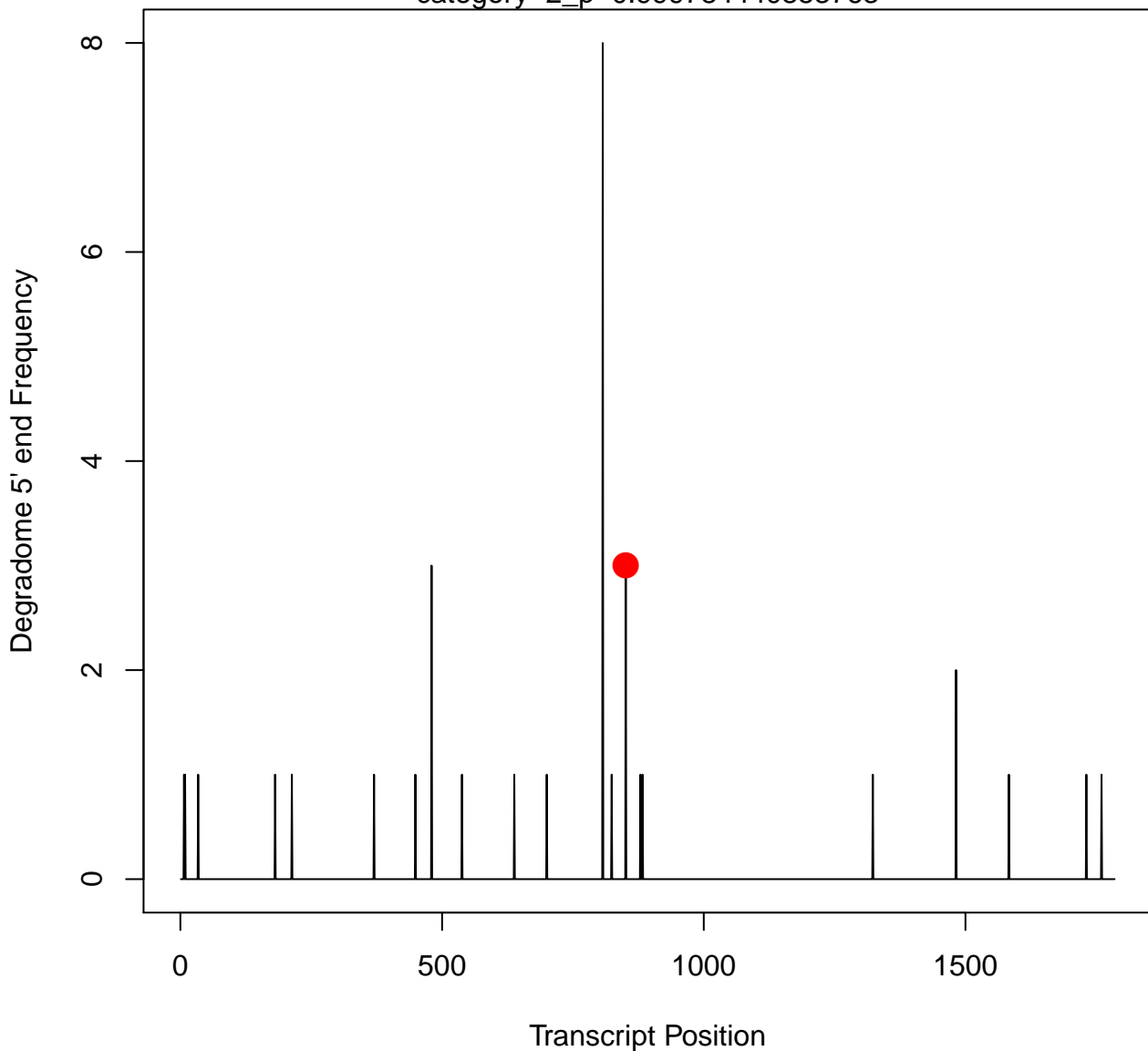

Supplement: Supplementary file 5 [file Data_Sheet_5.zip › Sit-miR171i_Seita.2G031700.1_851_TPlot.pdf]

**T=Seita.9G220200.1\_Q=Sit-miR171i\_S=539**

category=2\_p=0.99958908459368

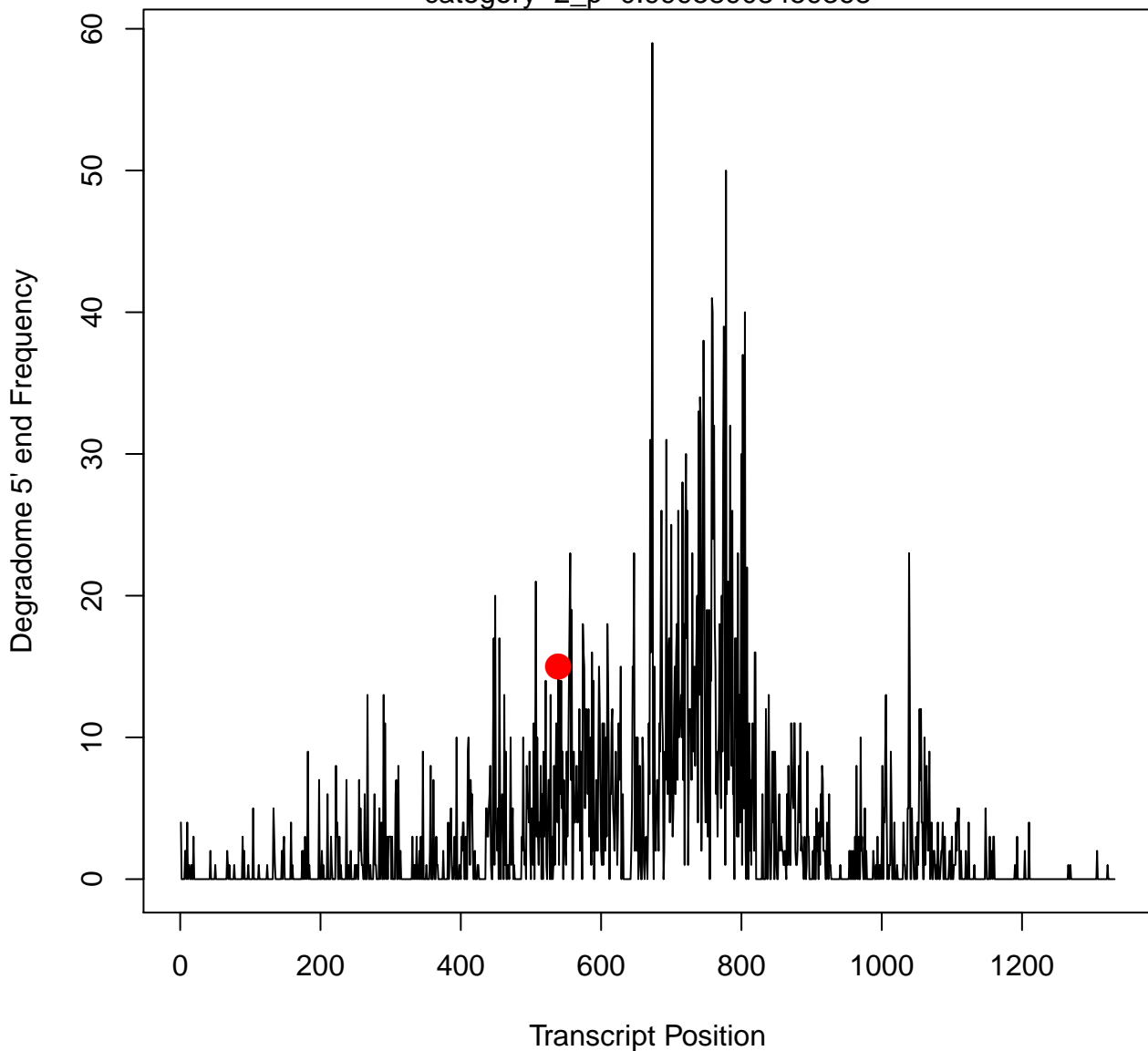

Supplement: Supplementary file 5 [file Data_Sheet_5.zip › Sit-miR171i_Seita.9G220200.1_539_TPlot.pdf]

**T=Seita.9G228200.1\_Q=Sit-miR171i\_S=137**

category=0\_p=0.314898330511524

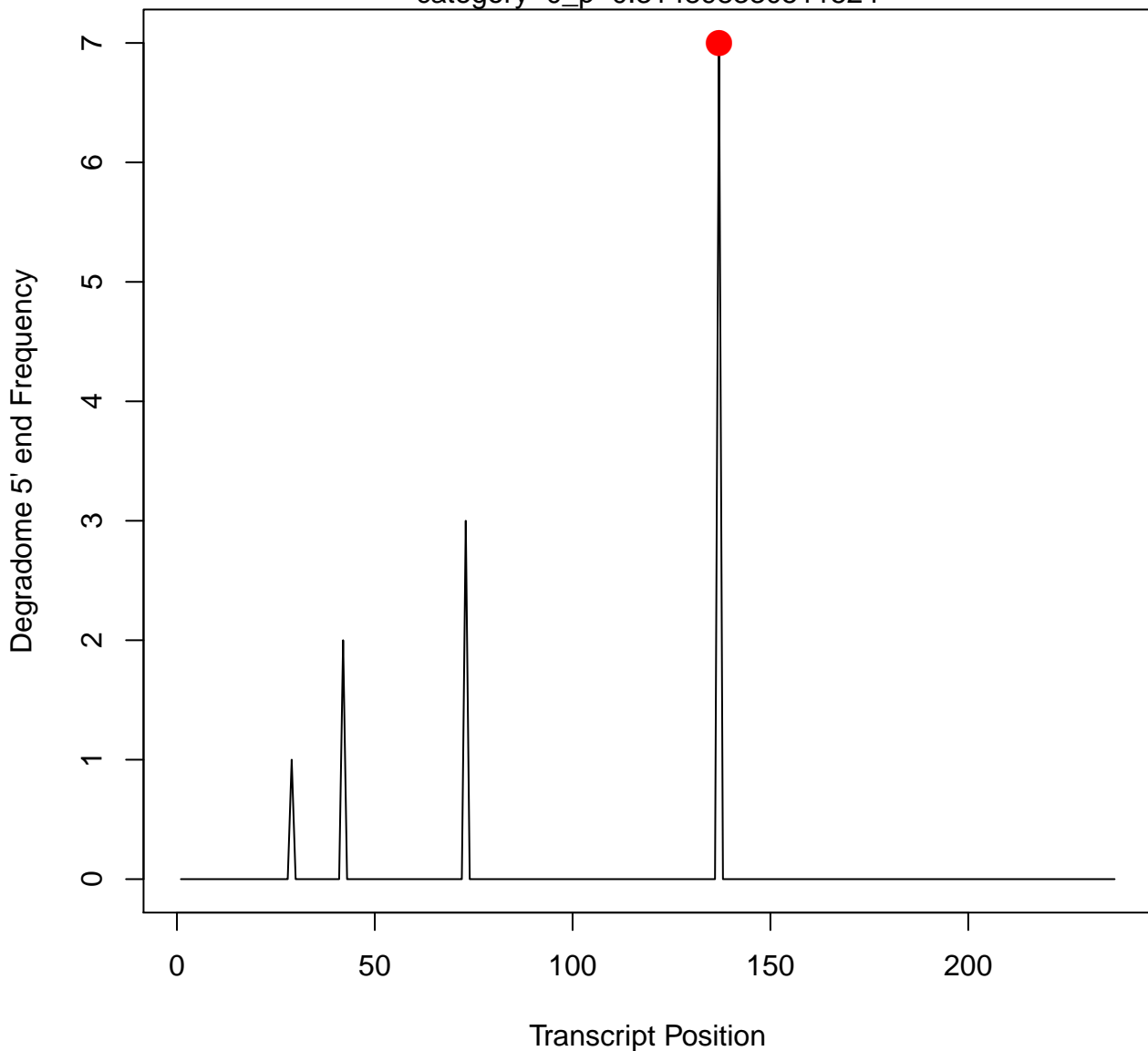

Supplement: Supplementary file 5 [file Data_Sheet_5.zip › Sit-miR171i_Seita.9G228200.1_137_TPlot.pdf]

**T=Seita.9G330300.1\_Q=Sit-miR171i\_S=1241**

category=2\_p=0.00917411630284182

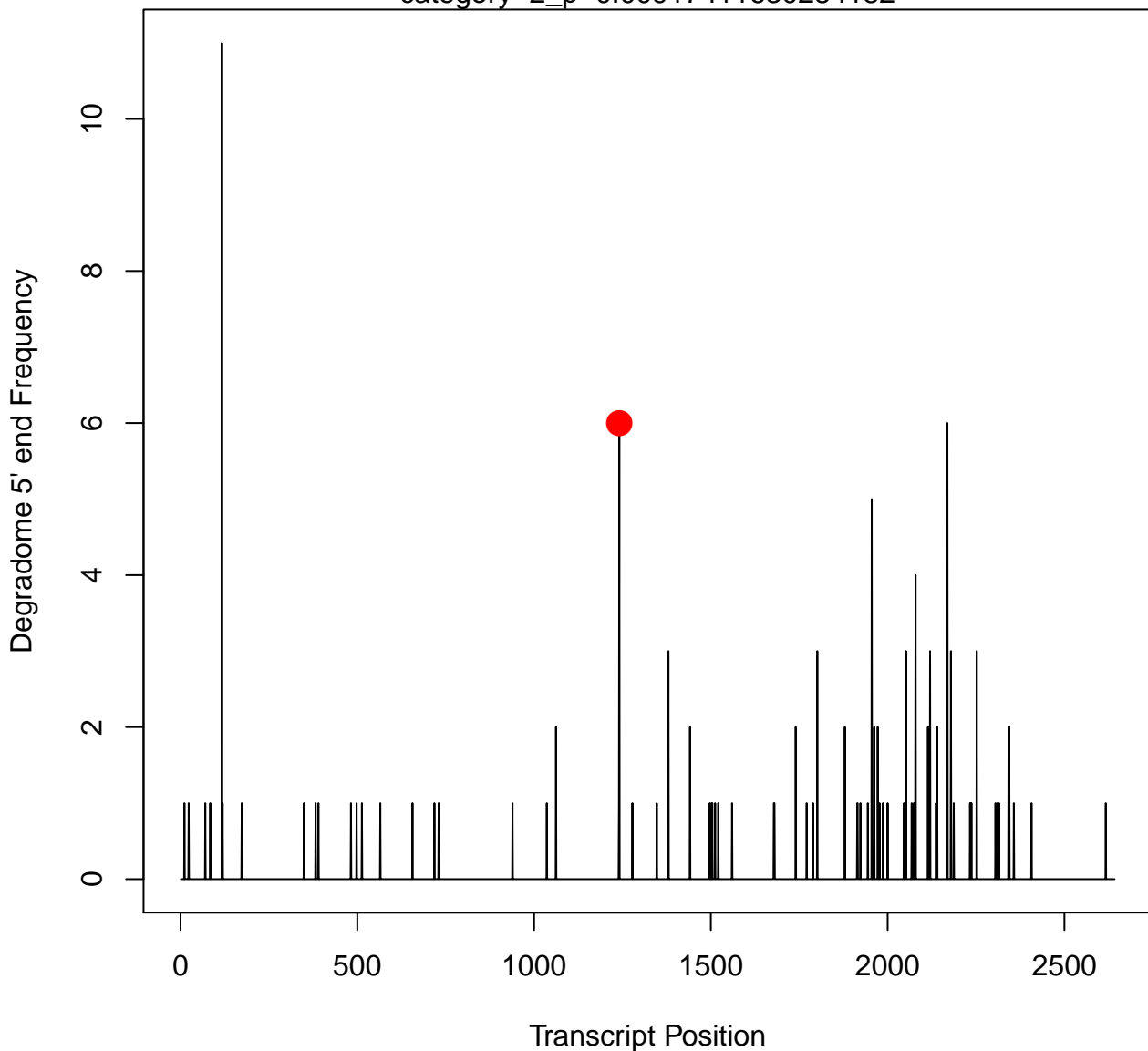

Supplement: Supplementary file 5 [file Data_Sheet_5.zip › Sit-miR171i_Seita.9G330300.1_1241_TPlot.pdf]

**T=Seita.9G528200.1\_Q=Sit-miR171i\_S=1093**

category=1\_p=0.0094011626821765

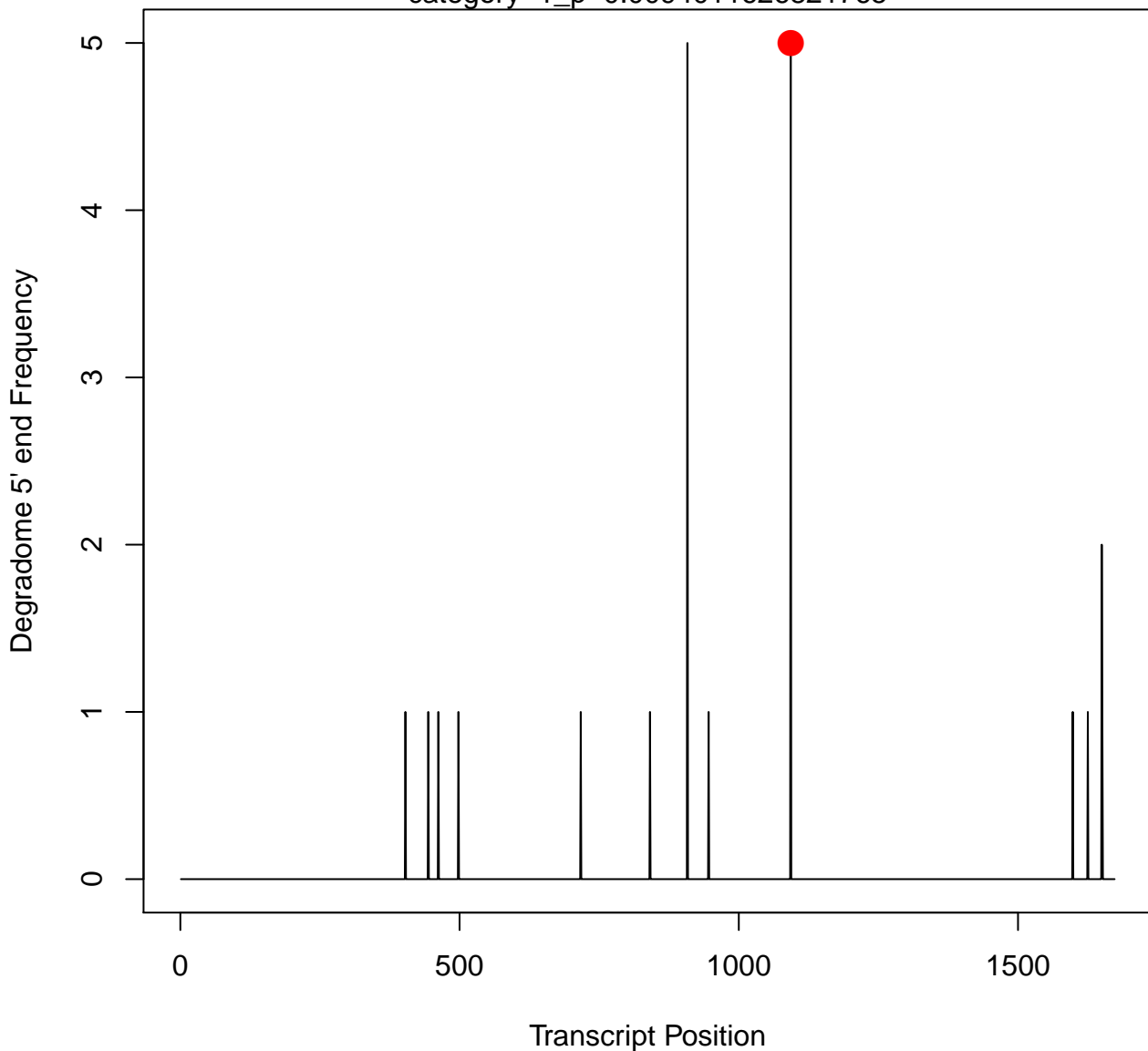

Supplement: Supplementary file 5 [file Data_Sheet_5.zip › Sit-miR171i_Seita.9G528200.1_1093_TPlot.pdf]

**T=Seita.1G373000.1\_Q=Sit-miR172a\_S=390**

category=2\_p=0.991074351804104

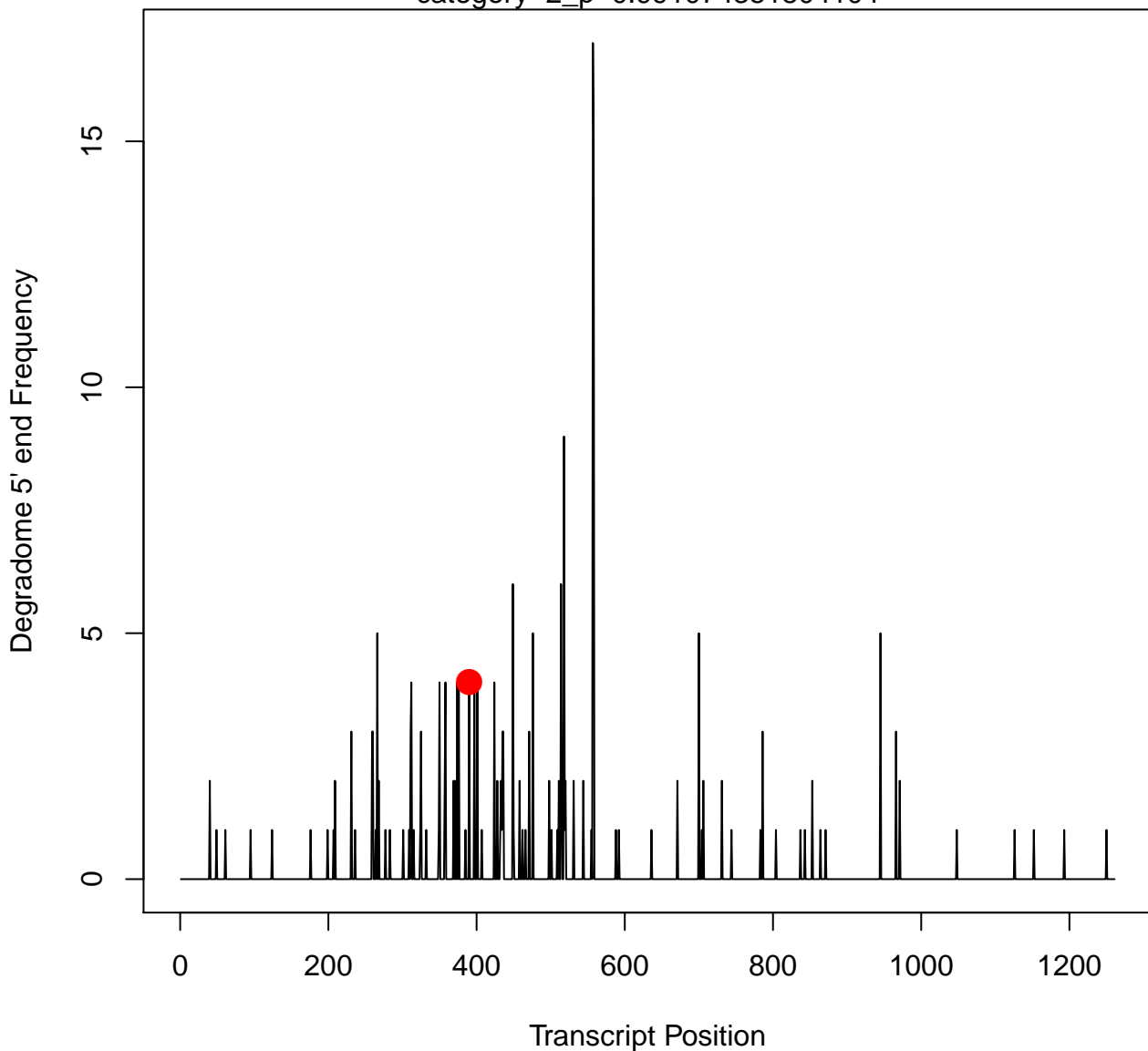

Supplement: Supplementary file 5 [file Data_Sheet_5.zip › Sit-miR172a_Seita.1G373000.1_390_TPlot.pdf]

**T=Seita.2G018200.1\_Q=Sit-miR172a\_S=1960**

category=2\_p=0.998407267065041

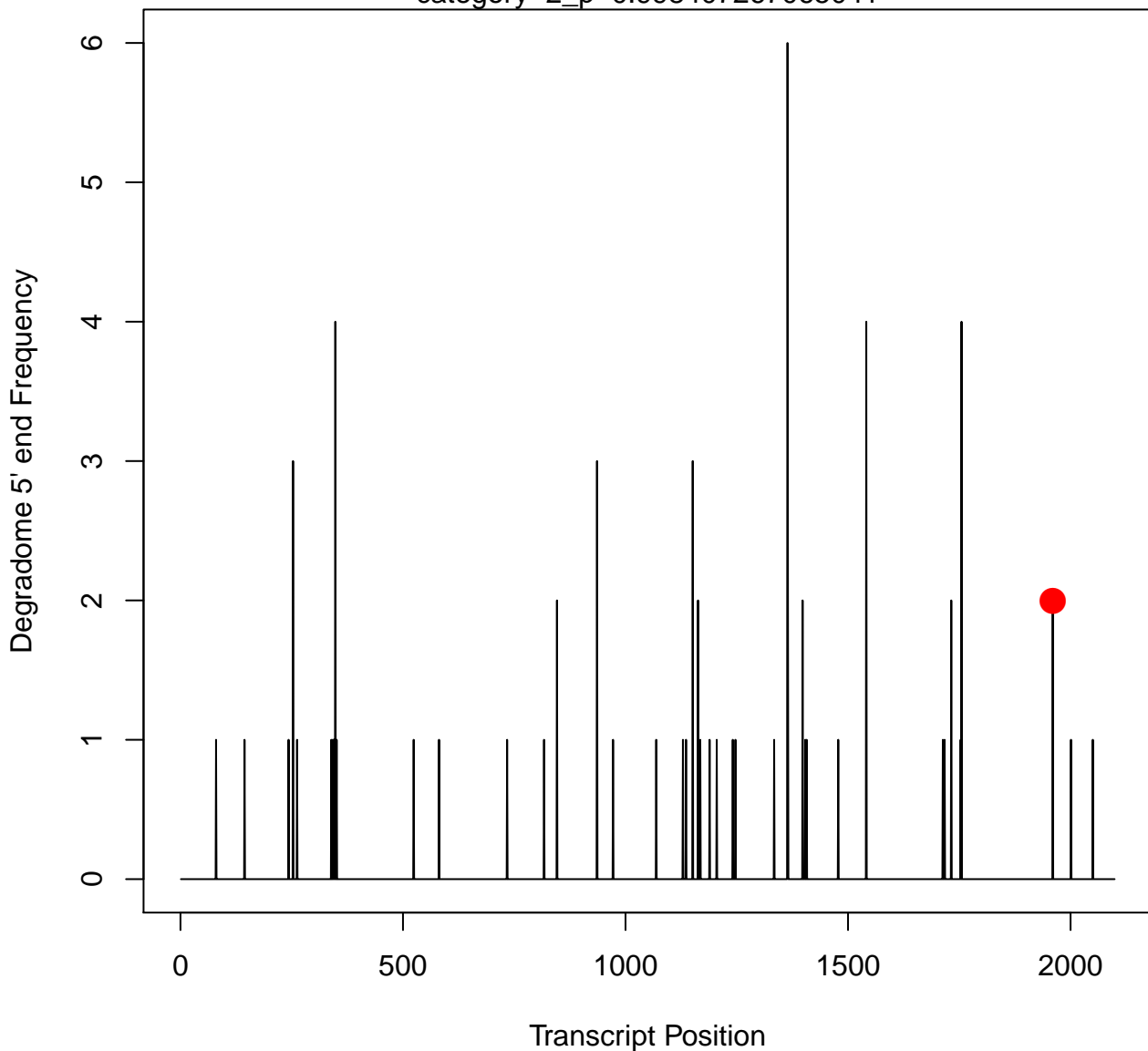

Supplement: Supplementary file 5 [file Data_Sheet_5.zip › Sit-miR172a_Seita.2G018200.1_1960_TPlot.pdf]

**T=Seita.2G091100.1\_Q=Sit-miR172a\_S=1500**

category=0\_p=0.000422467192713993

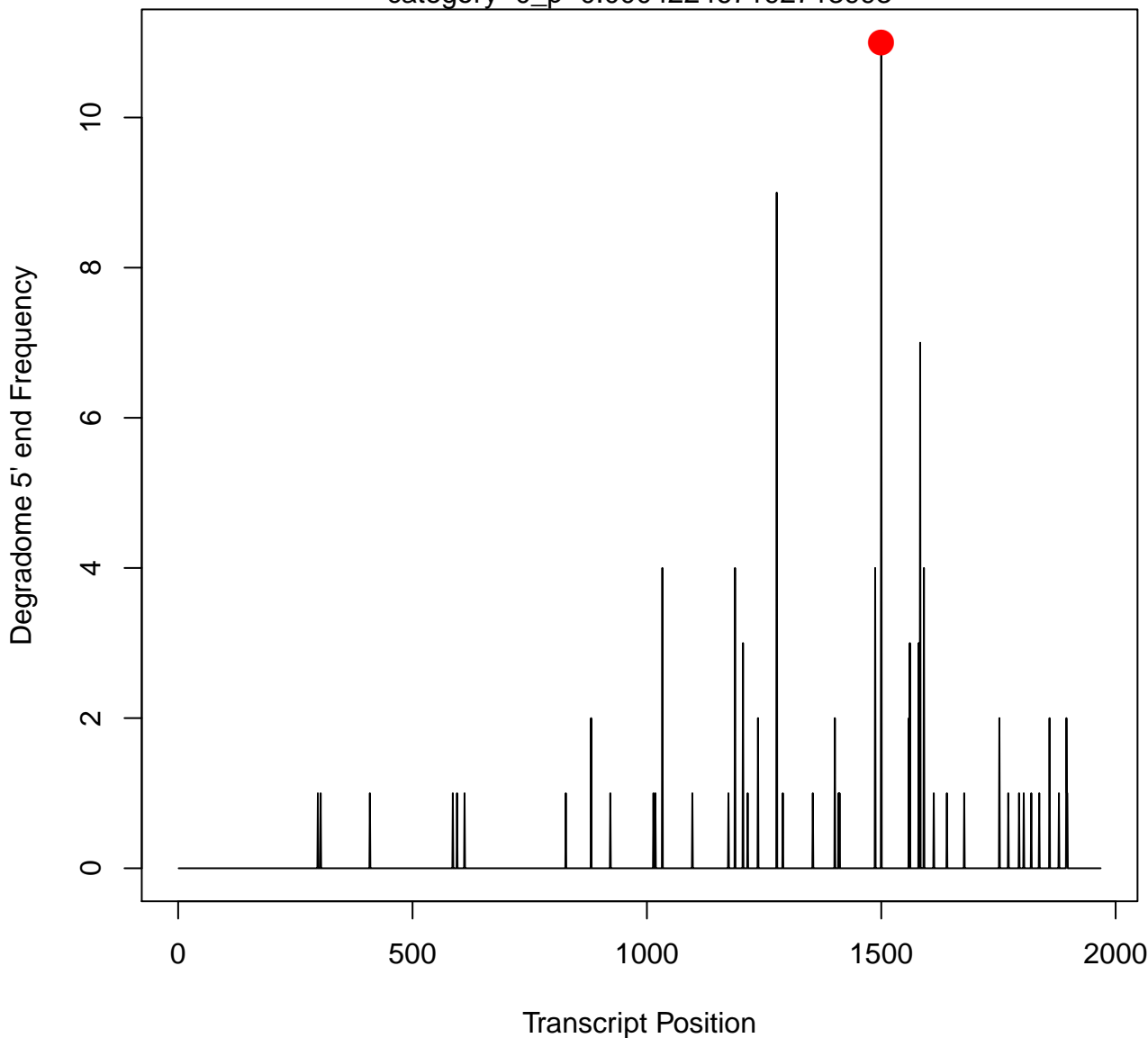

Supplement: Supplementary file 5 [file Data_Sheet_5.zip › Sit-miR172a_Seita.2G091100.1_1500_TPlot.pdf]

**T=Seita.3G044600.1\_Q=Sit-miR172a\_S=1327**

category=0\_p=0.00084475590689892

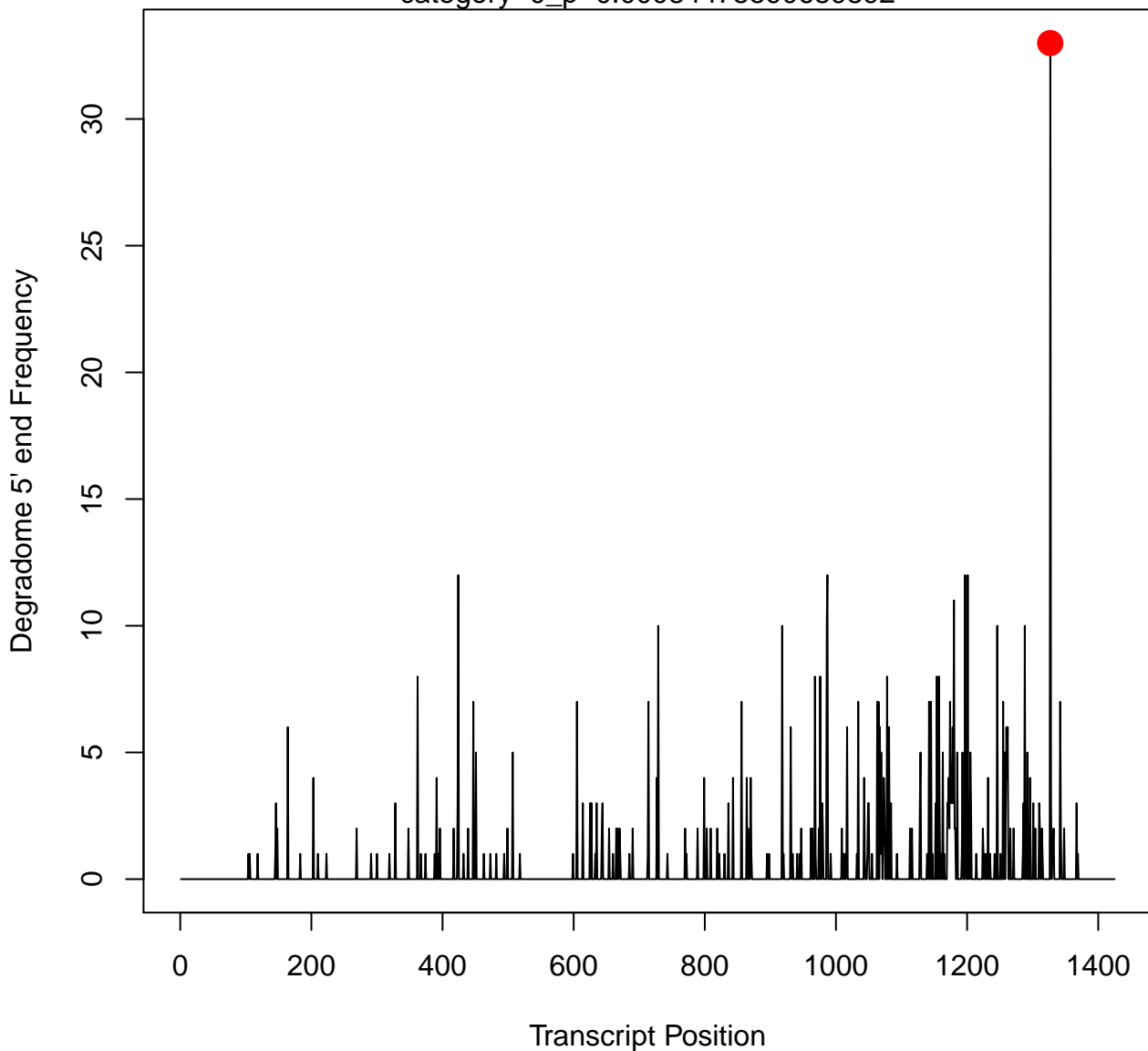

Supplement: Supplementary file 5 [file Data_Sheet_5.zip › Sit-miR172a_Seita.3G044600.1_1327_TPlot.pdf]

**T=Seita.5G073000.1\_Q=Sit-miR172a\_S=1026**

category=2\_p=0.998237325245768

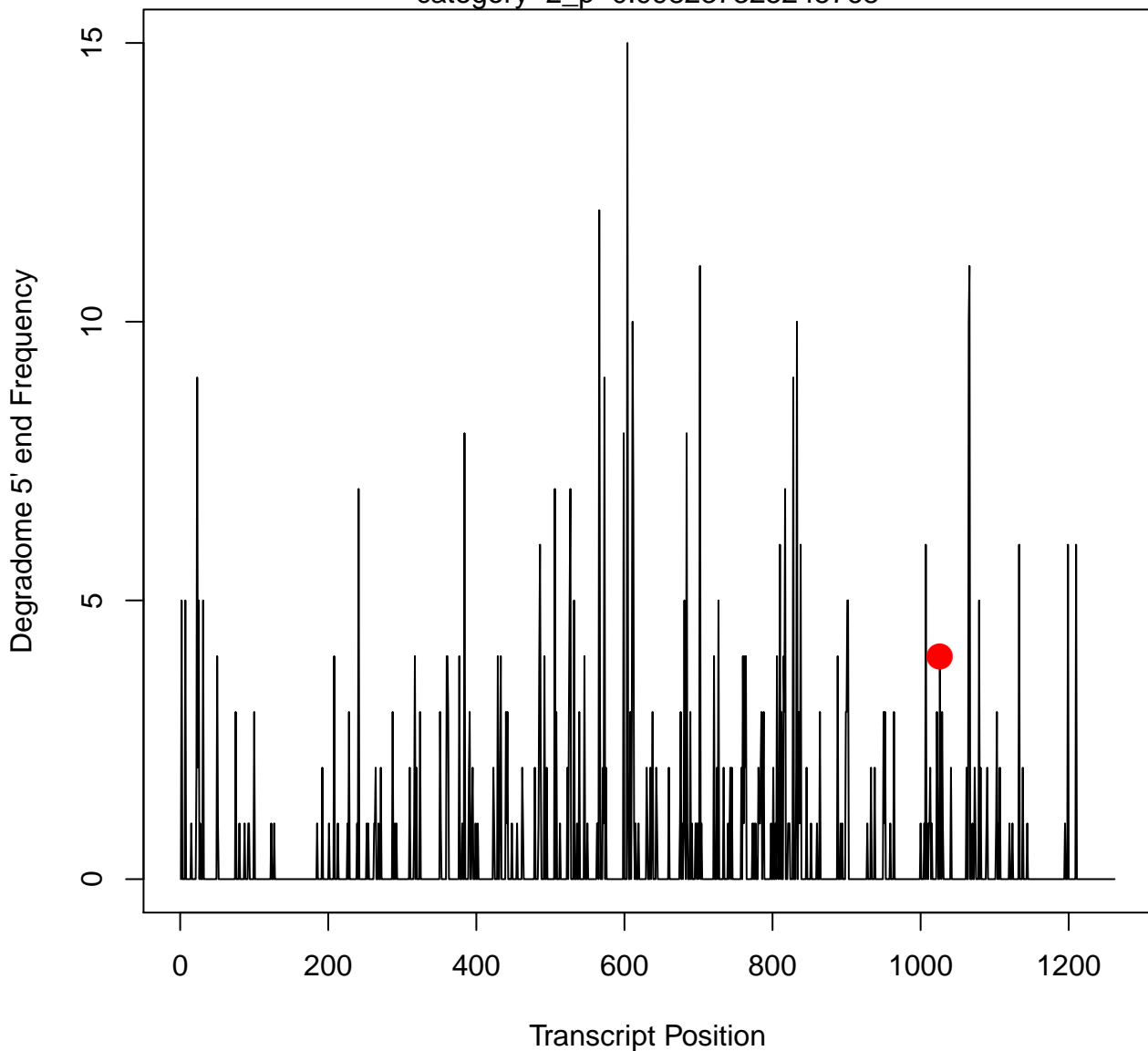

Supplement: Supplementary file 5 [file Data_Sheet_5.zip › Sit-miR172a_Seita.5G073000.1_1026_TPlot.pdf]

**T=Seita.6G204700.1\_Q=Sit-miR172a\_S=1870**

category=2\_p=0.288947444212195

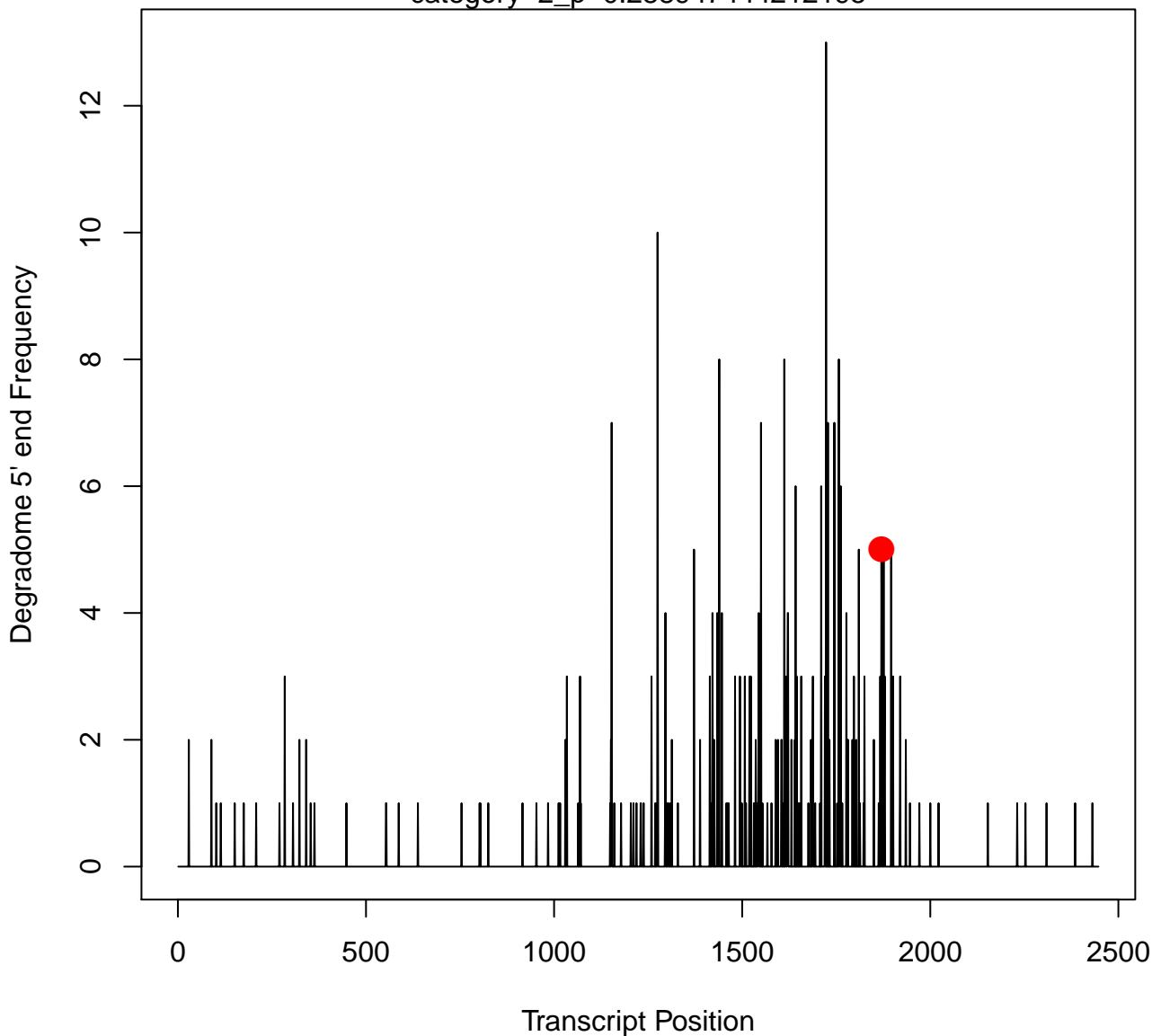

Supplement: Supplementary file 5 [file Data_Sheet_5.zip › Sit-miR172a_Seita.6G204700.1_1870_TPlot.pdf]

**T=Seita.9G098100.1\_Q=Sit-miR172a\_S=862**

category=2\_p=0.992644978031322

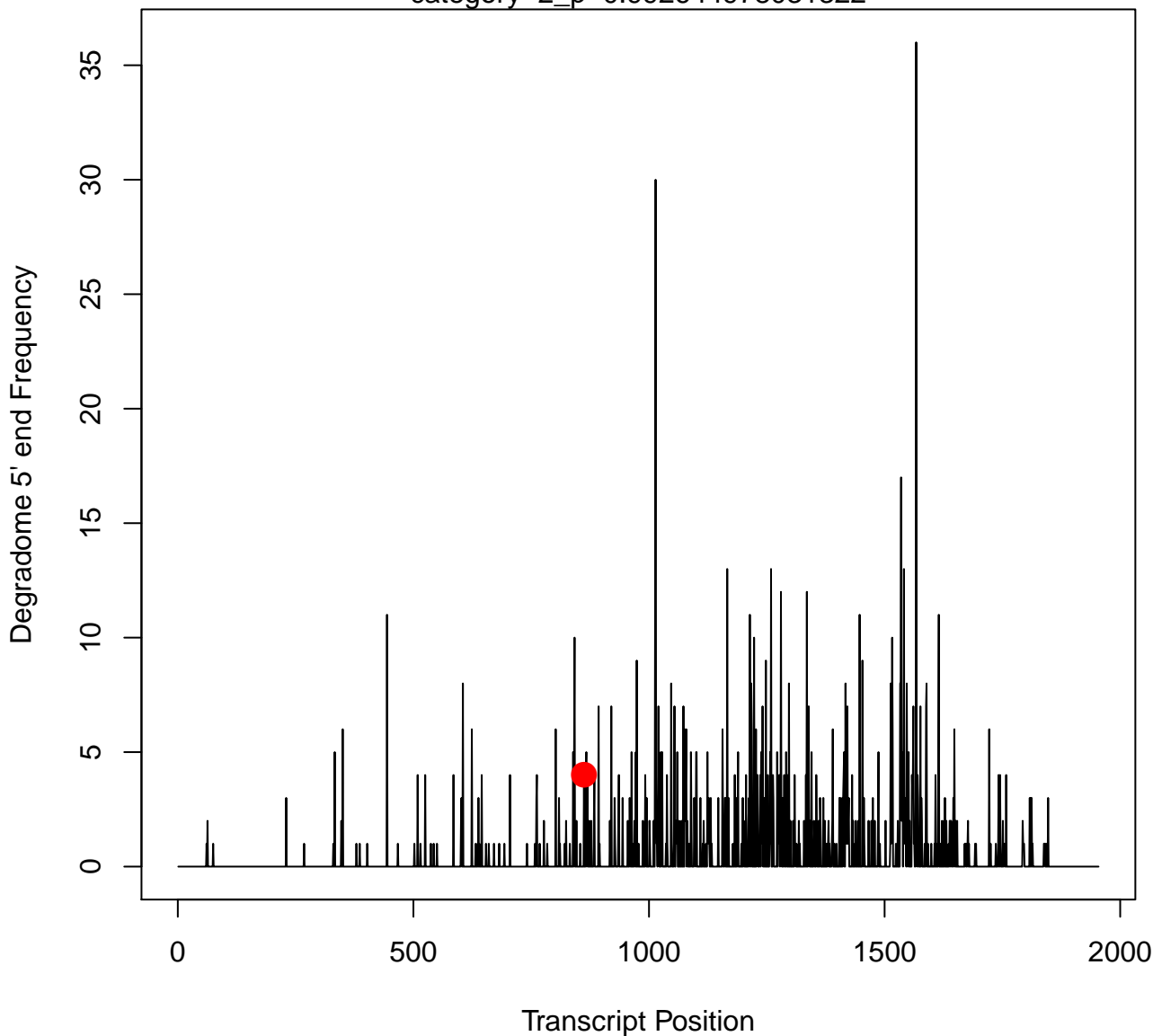

Supplement: Supplementary file 5 [file Data_Sheet_5.zip › Sit-miR172a_Seita.9G098100.1_862_TPlot.pdf]

**T=Seita.9G229600.1\_Q=Sit-miR172a\_S=319**

category=2\_p=0.994672898282237

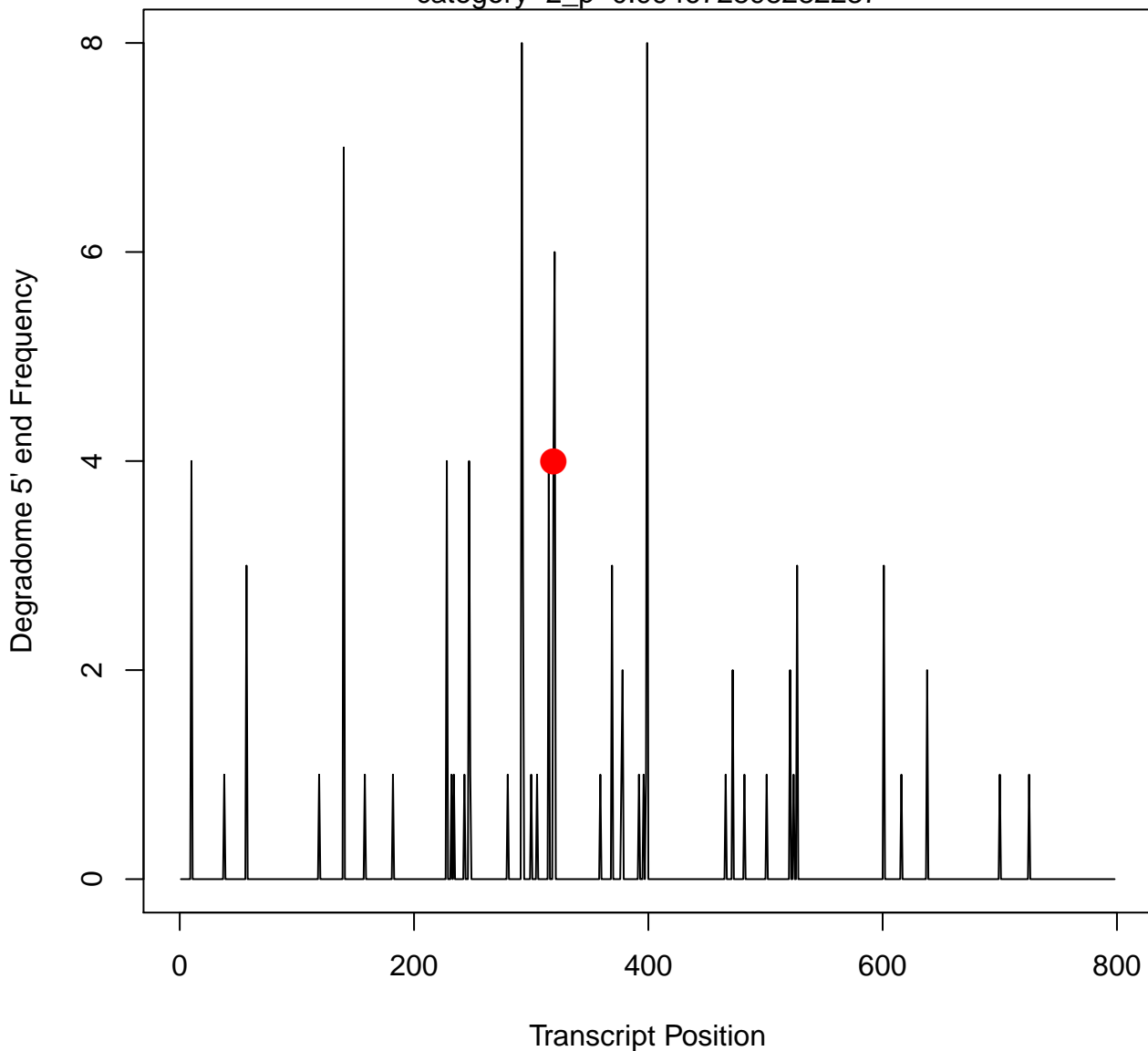

Supplement: Supplementary file 5 [file Data_Sheet_5.zip › Sit-miR172a_Seita.9G229600.1_319_TPlot.pdf]

**T=Seita.1G130300.1\_Q=Sit-miR172b\_S=591**

category=2\_p=0.598452445017394

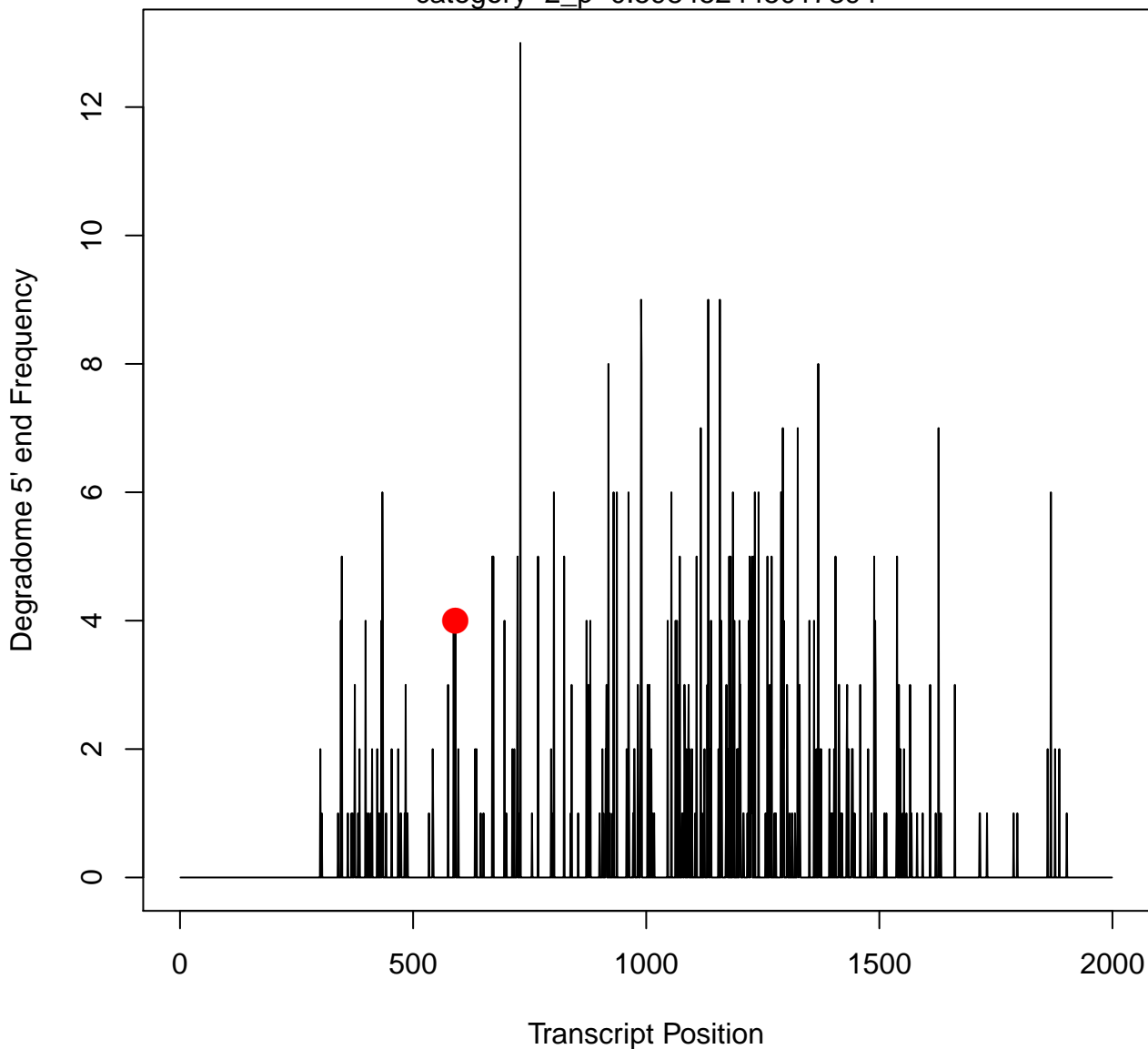

Supplement: Supplementary file 5 [file Data_Sheet_5.zip › Sit-miR172b_Seita.1G130300.1_591_TPlot.pdf]

**T=Seita.1G372300.1\_Q=Sit-miR172b\_S=578**

category=2\_p=0.890513041881939

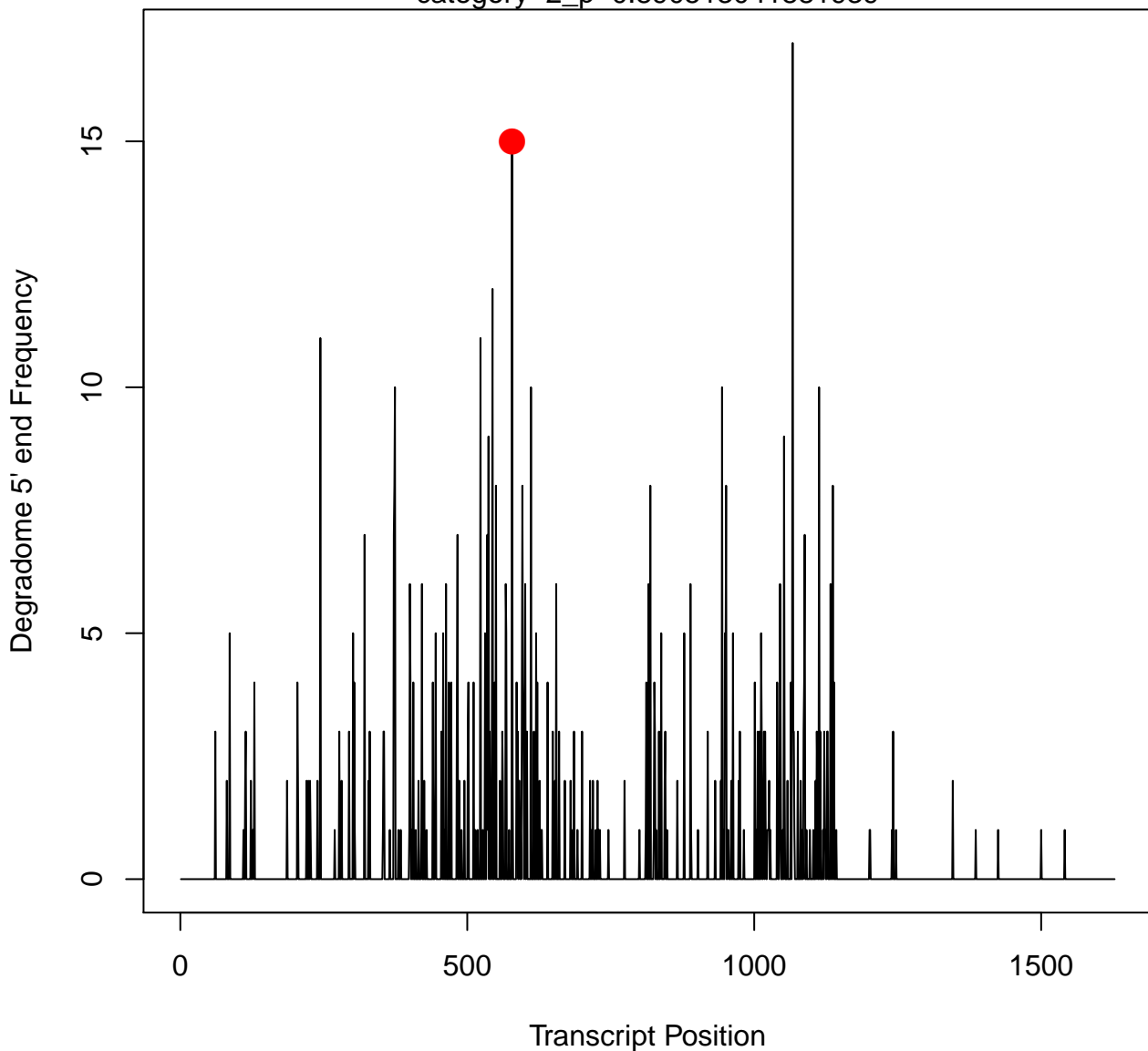

Supplement: Supplementary file 5 [file Data_Sheet_5.zip › Sit-miR172b_Seita.1G372300.1_578_TPlot.pdf]

**T=Seita.2G123000.1\_Q=Sit-miR172b\_S=513**

category=2\_p=0.99592231245283

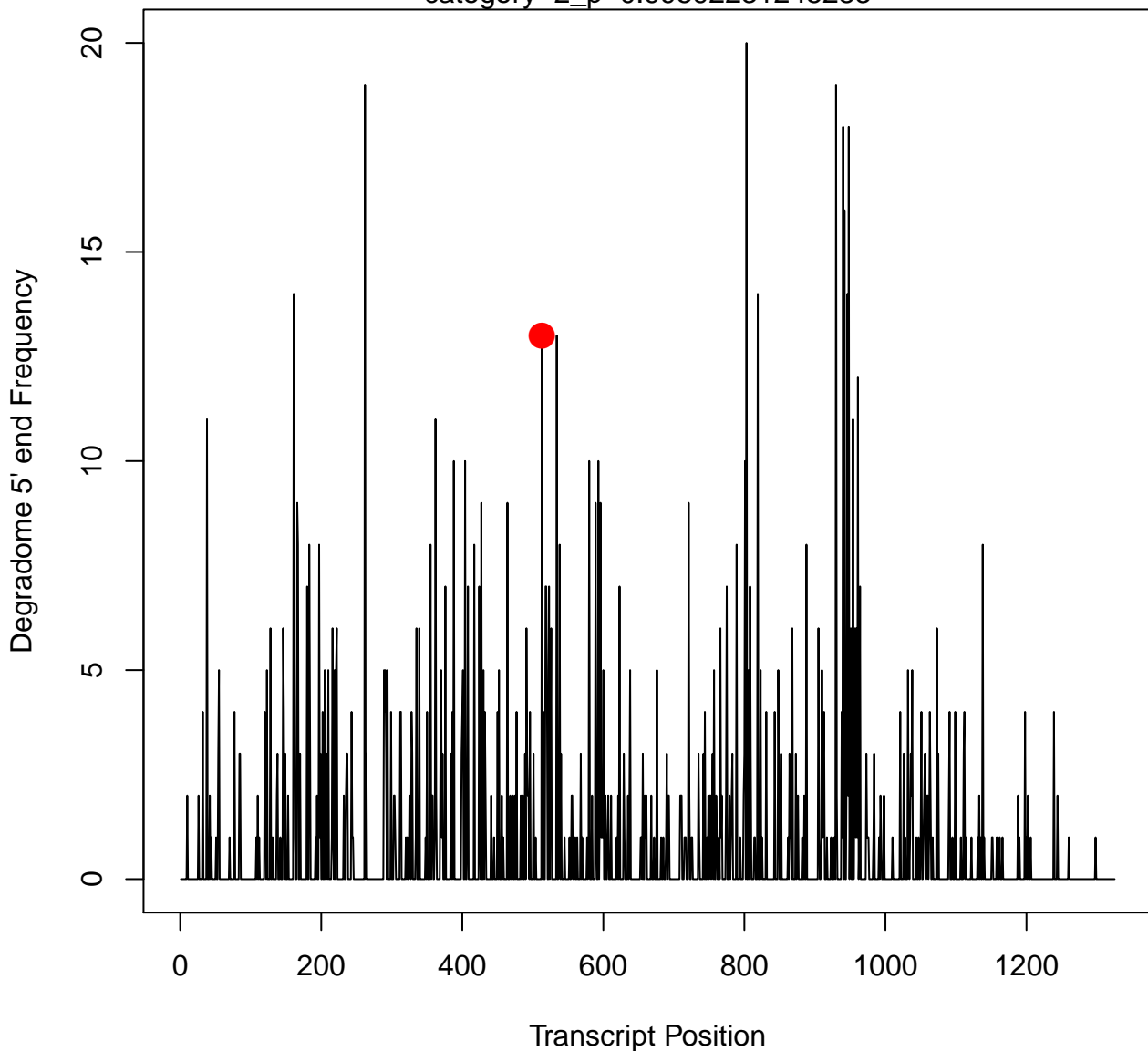

Supplement: Supplementary file 5 [file Data_Sheet_5.zip › Sit-miR172b_Seita.2G123000.1_513_TPlot.pdf]

**T=Seita.2G369800.1\_Q=Sit-miR172b\_S=834**

category=2\_p=0.994623574327827

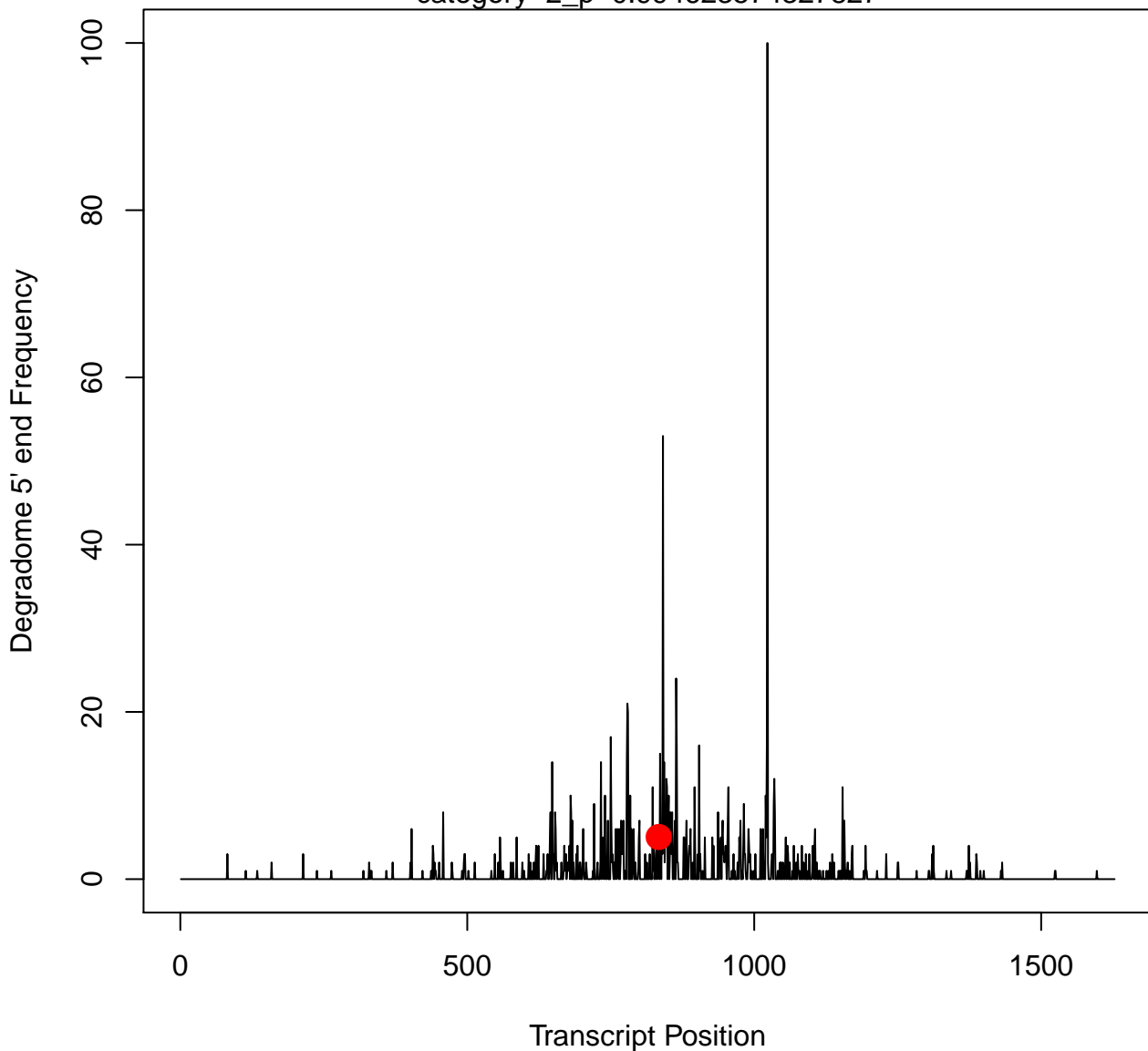

Supplement: Supplementary file 5 [file Data_Sheet_5.zip › Sit-miR172b_Seita.2G369800.1_834_TPlot.pdf]

**T=Seita.3G208400.1\_Q=Sit-miR172b\_S=451**

category=2\_p=0.946647451824576

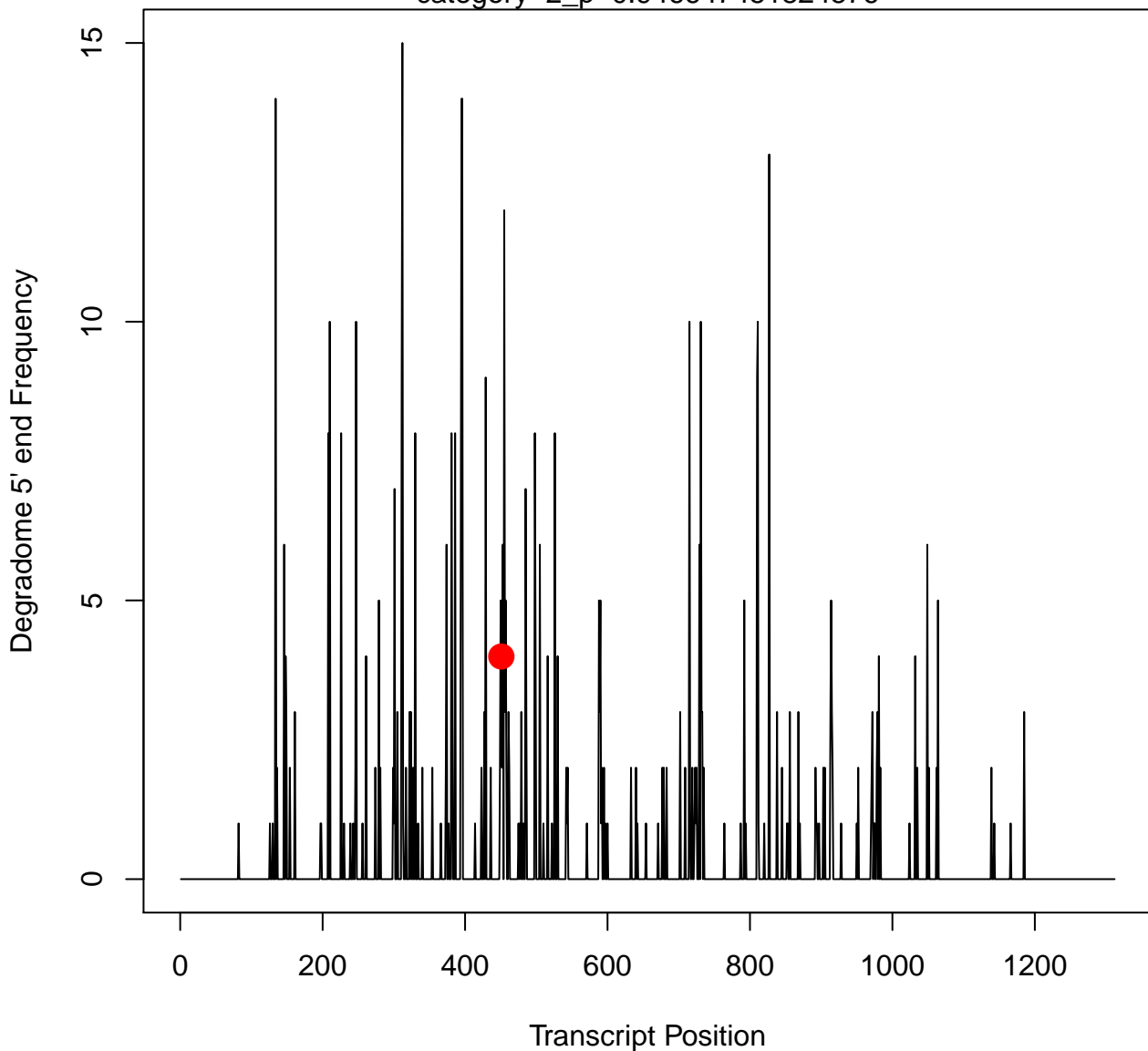

Supplement: Supplementary file 5 [file Data_Sheet_5.zip › Sit-miR172b_Seita.3G208400.1_451_TPlot.pdf]

**T=Seita.5G226400.1\_Q=Sit-miR172b\_S=1097**

category=2\_p=0.998377636100135

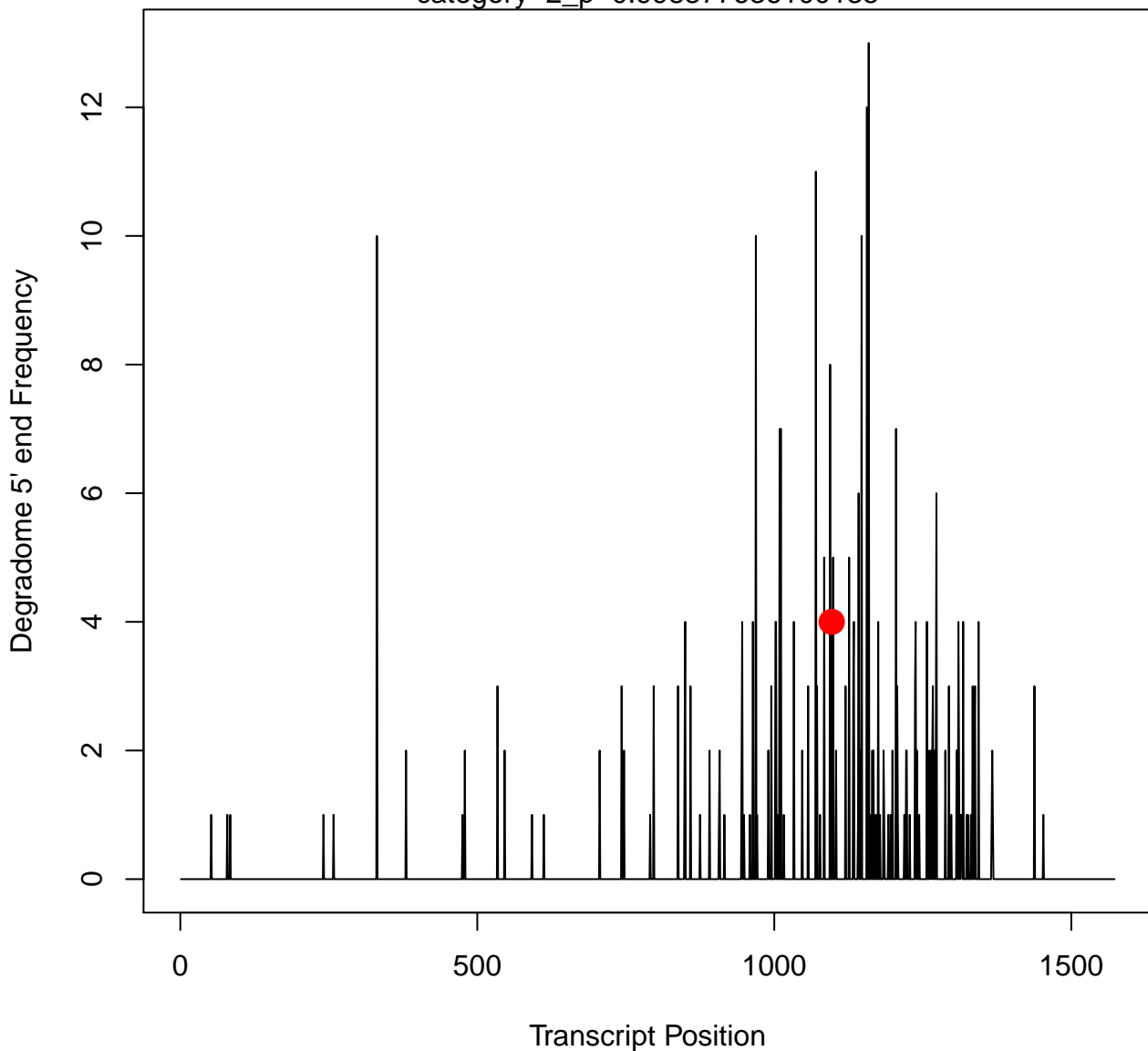

Supplement: Supplementary file 5 [file Data_Sheet_5.zip › Sit-miR172b_Seita.5G226400.1_1097_TPlot.pdf]

**T=Seita.5G371100.1\_Q=Sit-miR172b\_S=2776**

category=2\_p=0.351552030476957

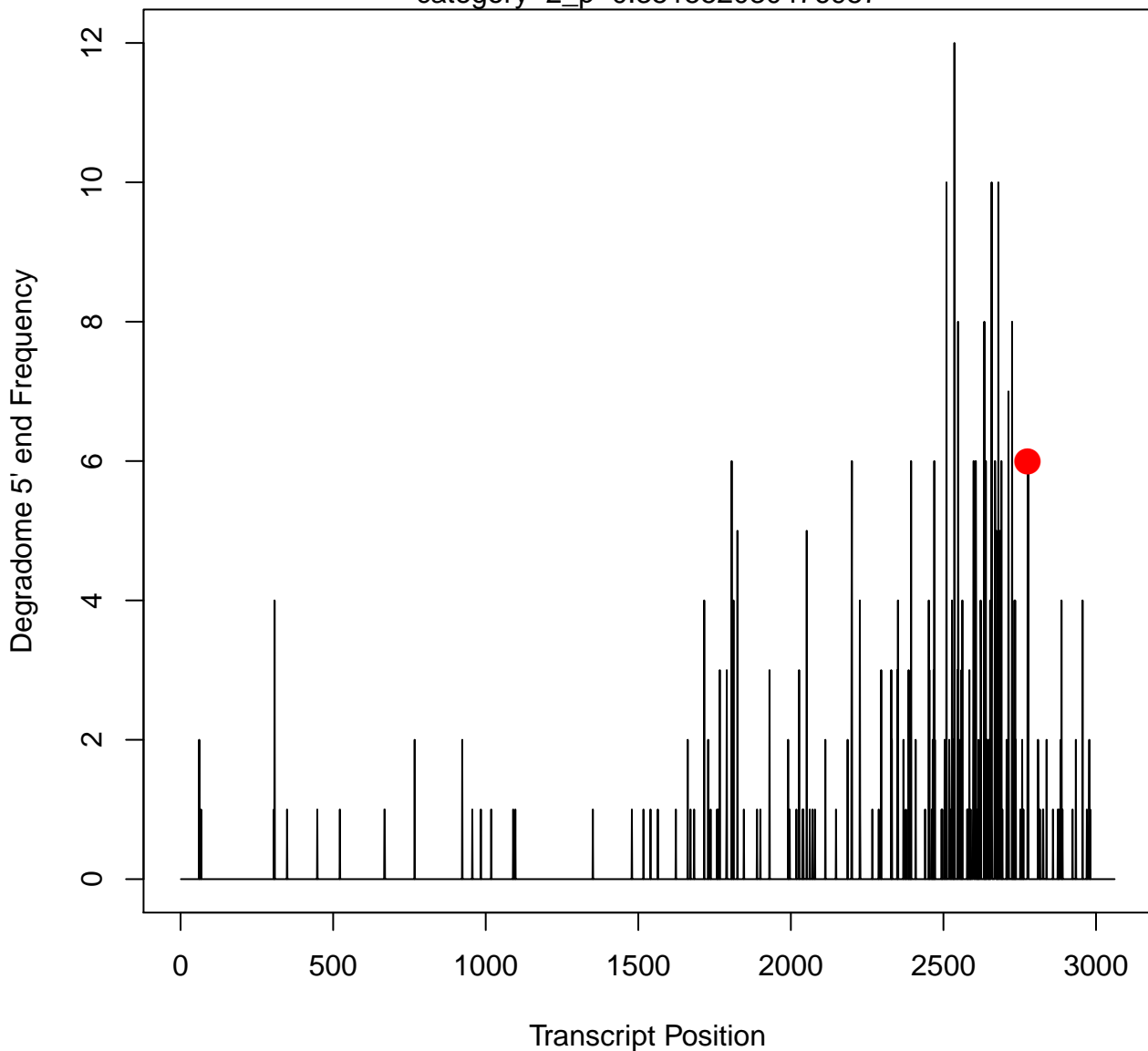

Supplement: Supplementary file 5 [file Data_Sheet_5.zip › Sit-miR172b_Seita.5G371100.1_2776_TPlot.pdf]

**T=Seita.7G104400.1\_Q=Sit-miR172b\_S=323**

category=0\_p=0.250375979206377

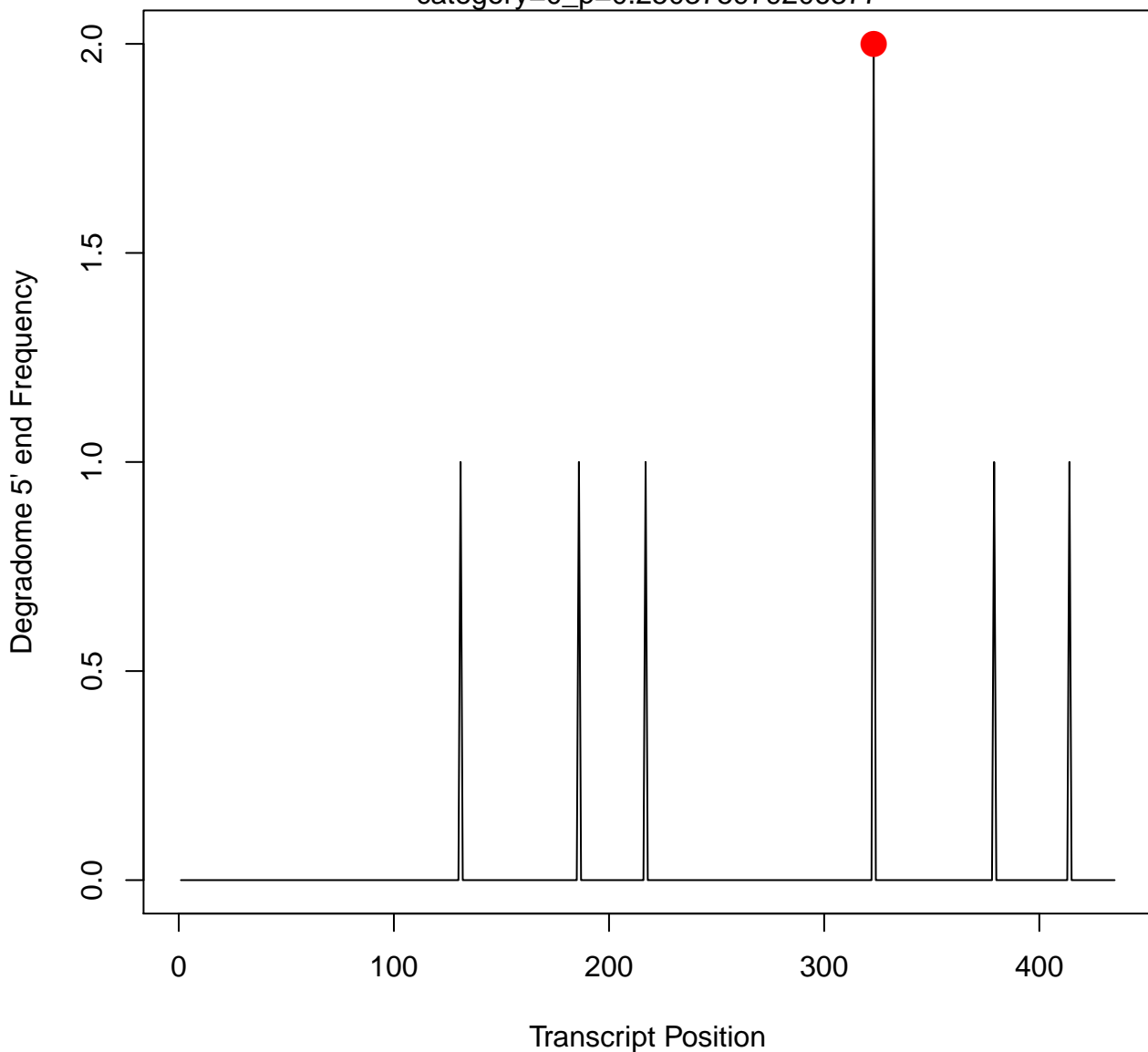

Supplement: Supplementary file 5 [file Data_Sheet_5.zip › Sit-miR172b_Seita.7G104400.1_323_TPlot.pdf]

**T=Seita.7G263000.1\_Q=Sit-miR172b\_S=1947**

category=2\_p=0.0450366234024282

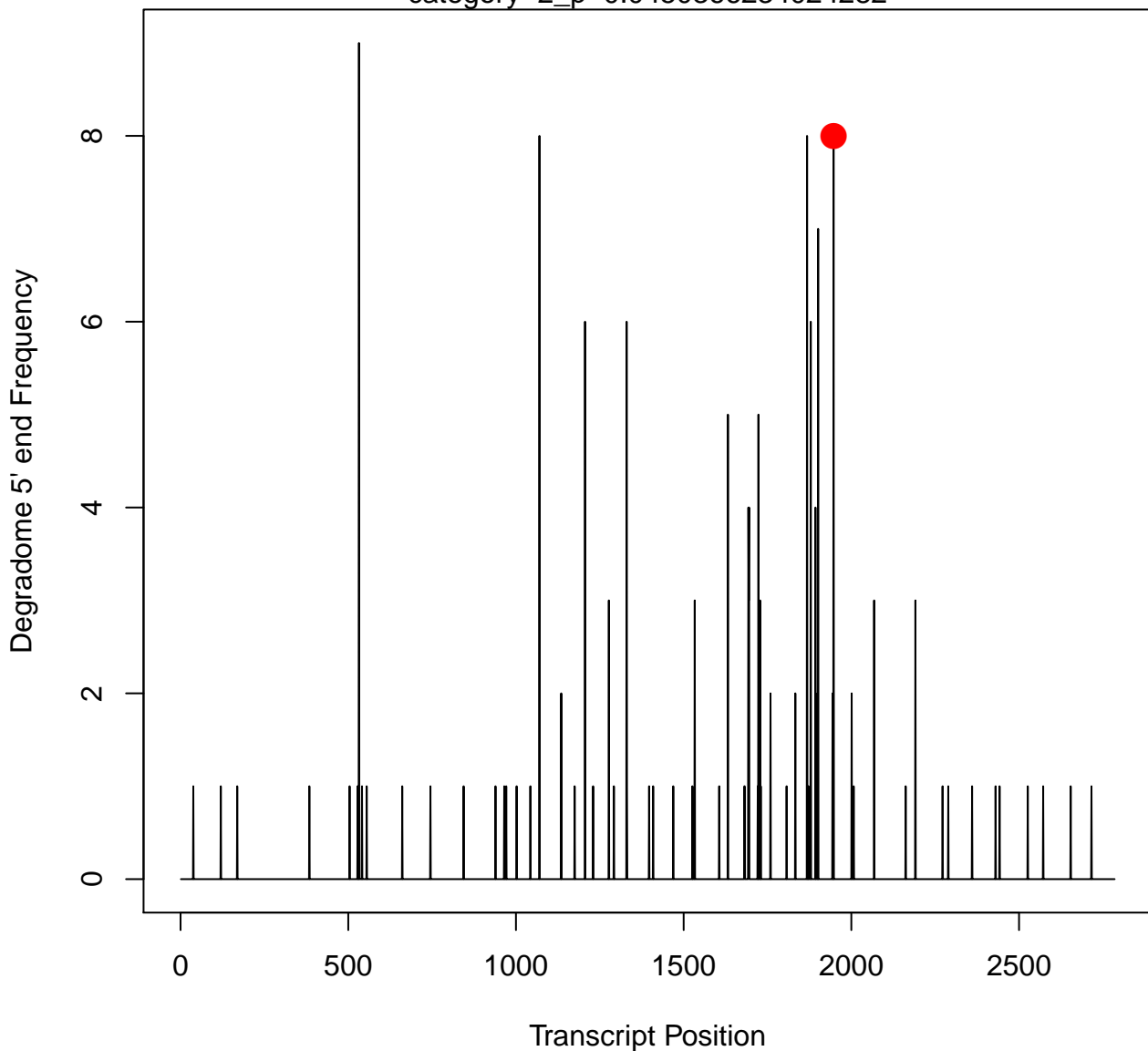

Supplement: Supplementary file 5 [file Data_Sheet_5.zip › Sit-miR172b_Seita.7G263000.1_1947_TPlot.pdf]

**T=Seita.9G103400.1\_Q=Sit-miR172b\_S=186**

category=2\_p=0.999581439985023

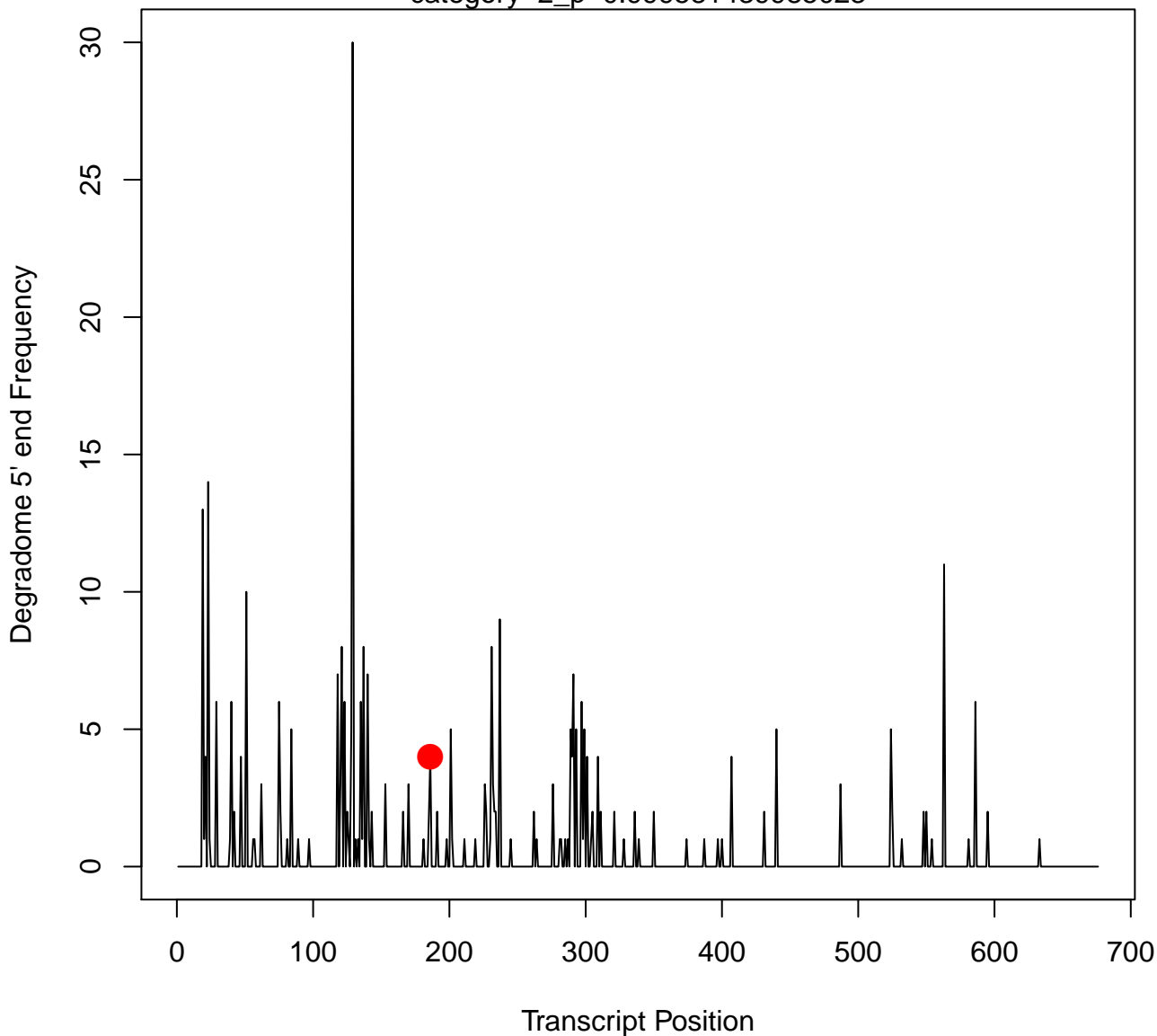

Supplement: Supplementary file 5 [file Data_Sheet_5.zip › Sit-miR172b_Seita.9G103400.1_186_TPlot.pdf]

**T=Seita.2G022100.1\_Q=Sit-miR2118a\_S=386**

category=2\_p=0.999360441984227

Degradome 5' end Frequency

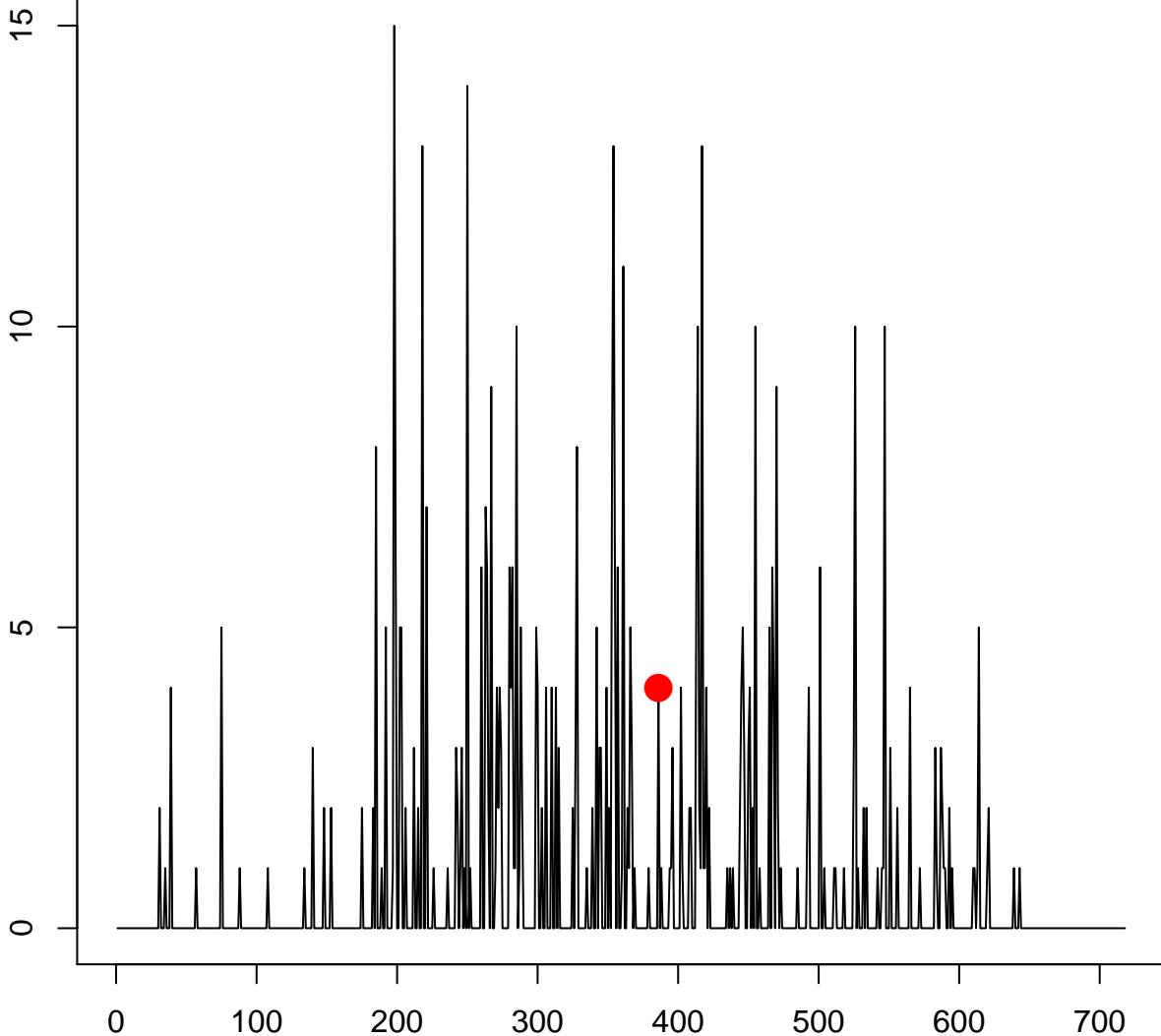

Transcript Position

Supplement: Supplementary file 5 [file Data_Sheet_5.zip › Sit-miR2118a_Seita.2G022100.1_386_TPlot.pdf]

**T=Seita.3G213800.1\_Q=Sit-miR2118a\_S=426**

category=2\_p=0.598452445017394

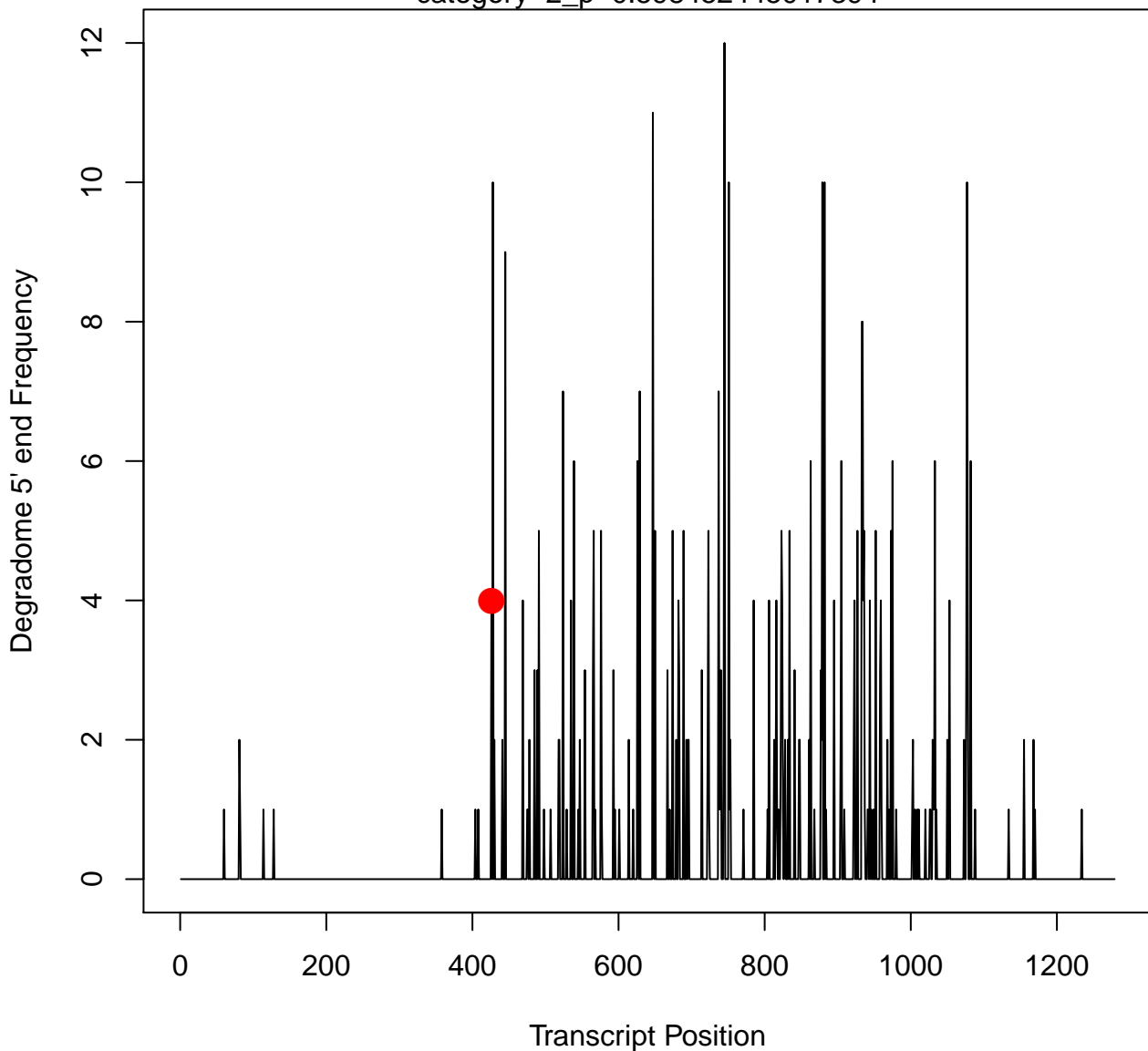

Supplement: Supplementary file 5 [file Data_Sheet_5.zip › Sit-miR2118a_Seita.3G213800.1_426_TPlot.pdf]

**T=Seita.5G353200.1\_Q=Sit-miR2118a\_S=2438**

category=2\_p=0.938170223878767

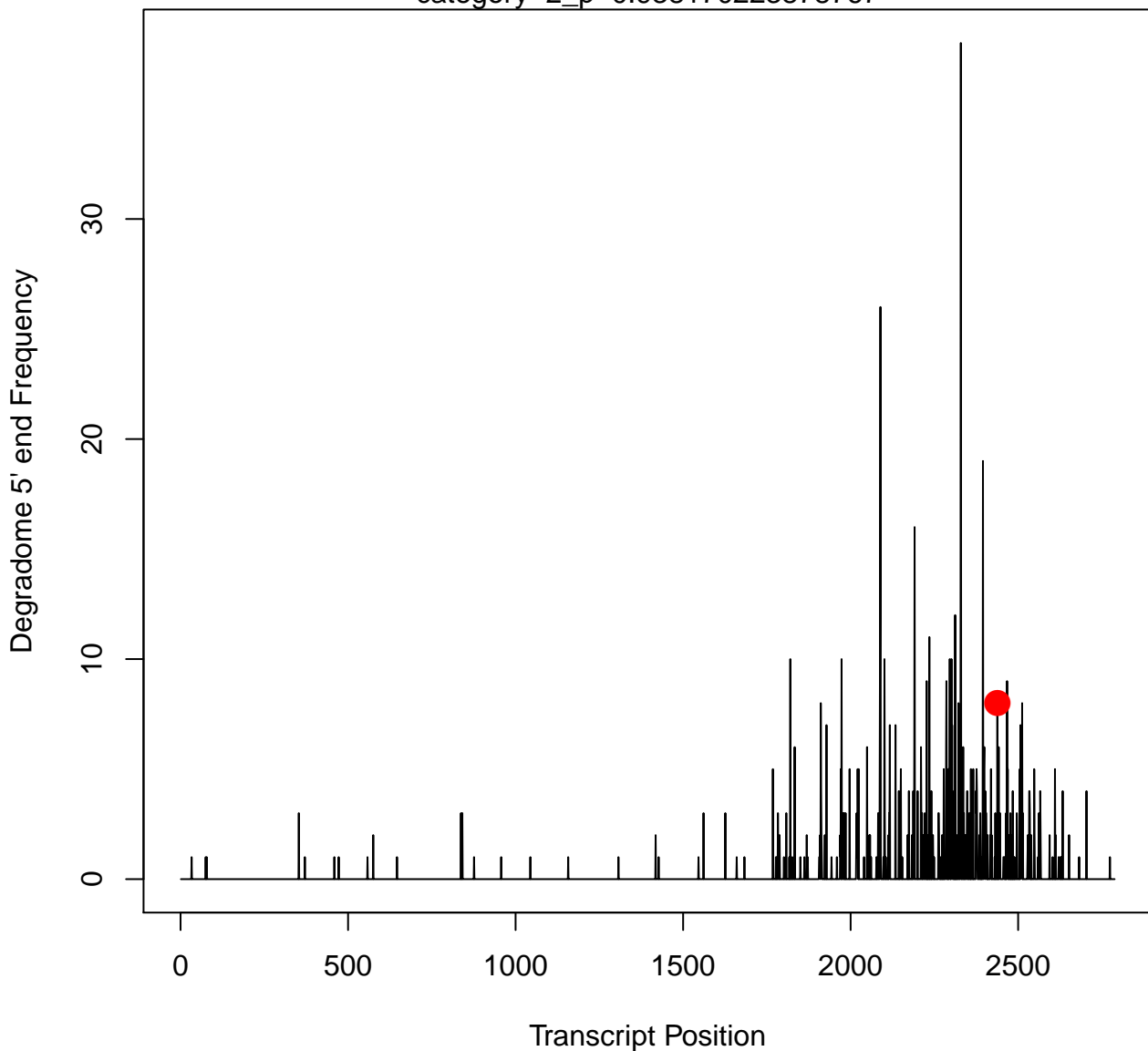

Supplement: Supplementary file 5 [file Data_Sheet_5.zip › Sit-miR2118a_Seita.5G353200.1_2438_TPlot.pdf]

**T=Seita.5G387000.1\_Q=Sit-miR2118a\_S=1489**

category=2\_p=0.567726871212814

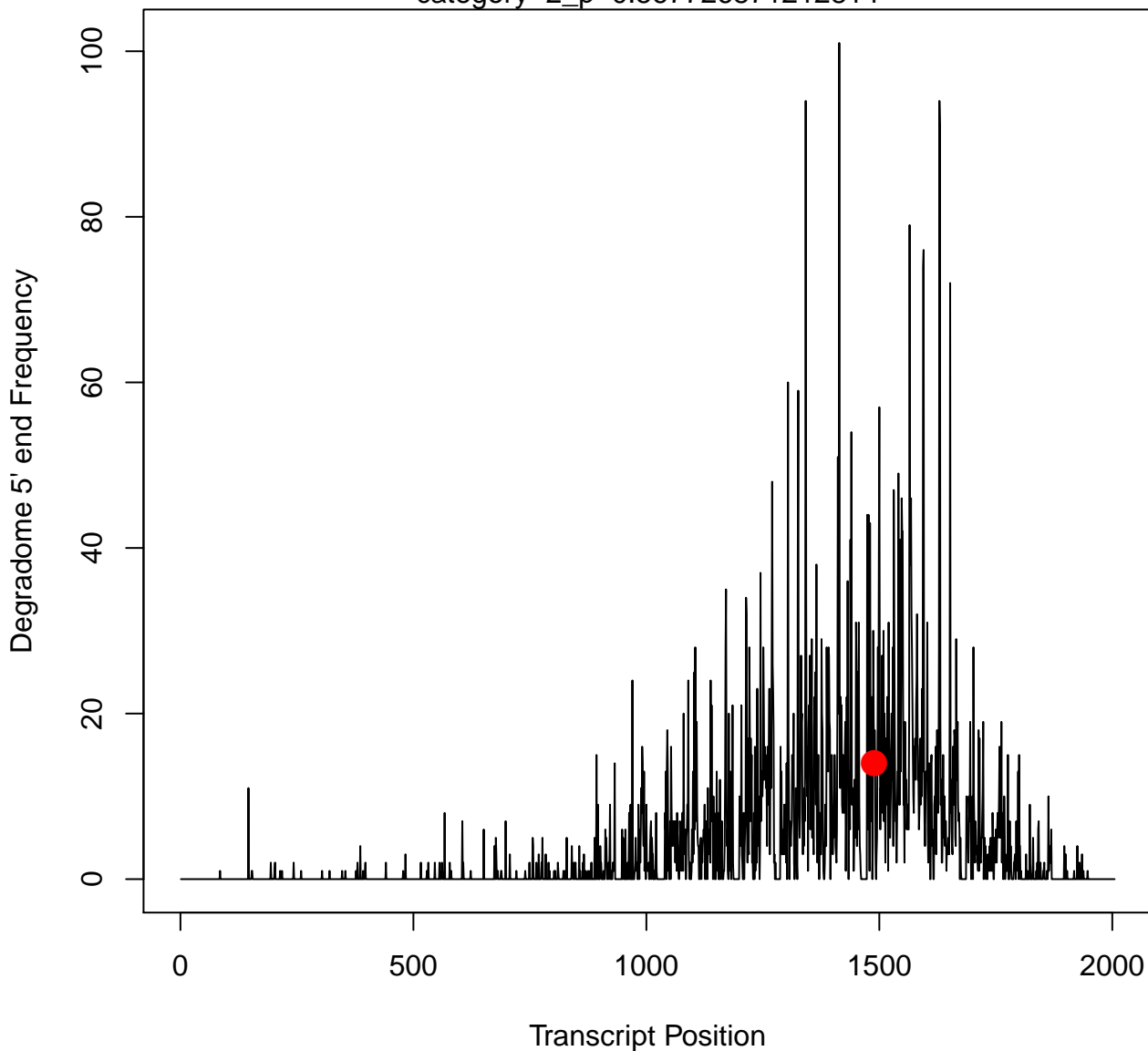

Supplement: Supplementary file 5 [file Data_Sheet_5.zip › Sit-miR2118a_Seita.5G387000.1_1489_TPlot.pdf]

**T=Seita.6G077800.1\_Q=Sit-miR2118a\_S=1437**

category=2\_p=0.987447273590056

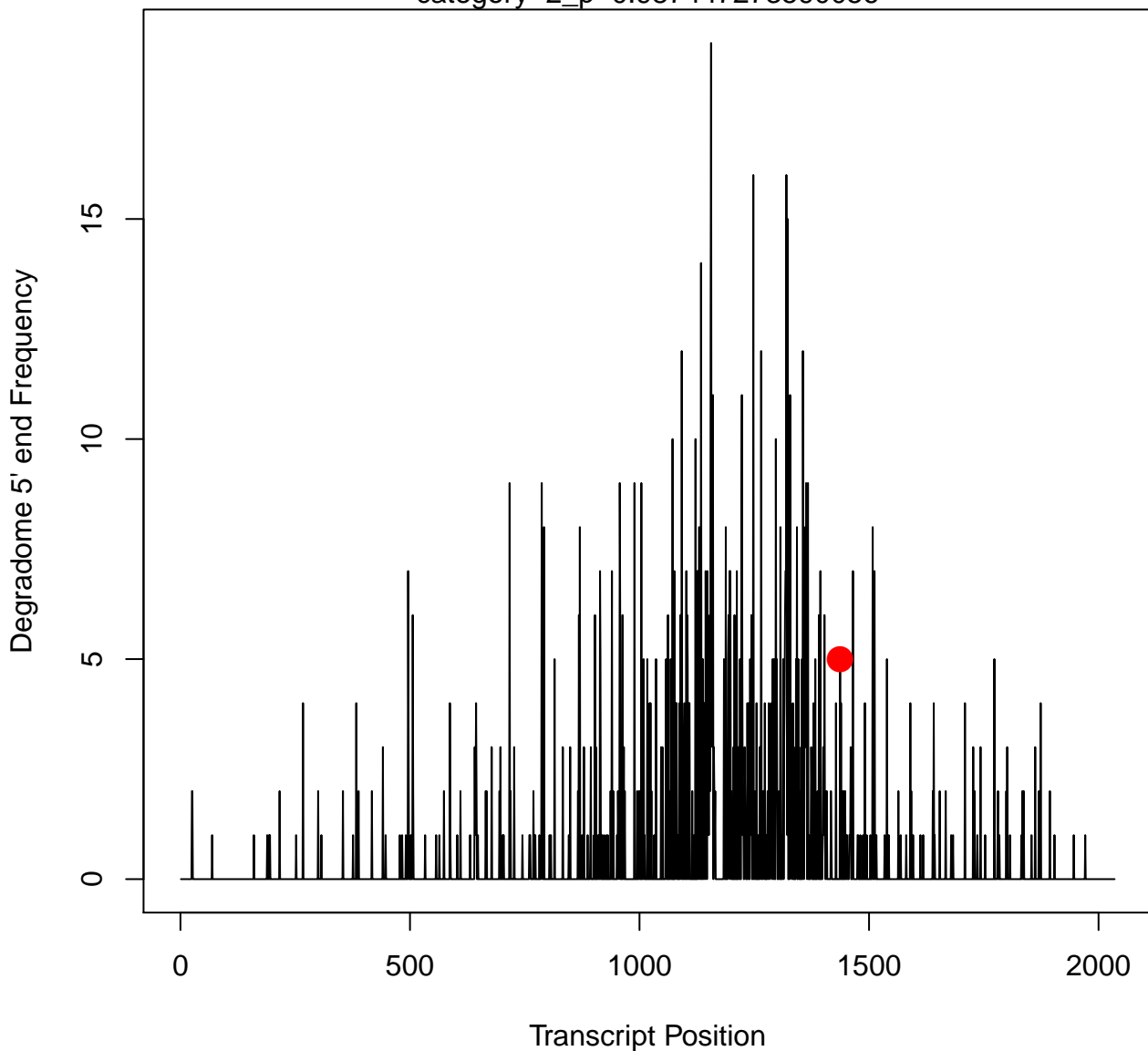

Supplement: Supplementary file 5 [file Data_Sheet_5.zip › Sit-miR2118a_Seita.6G077800.1_1437_TPlot.pdf]

**T=Seita.1G008600.1\_Q=Sit-miR2118b\_S=393**

category=0\_p=0.400280547999873

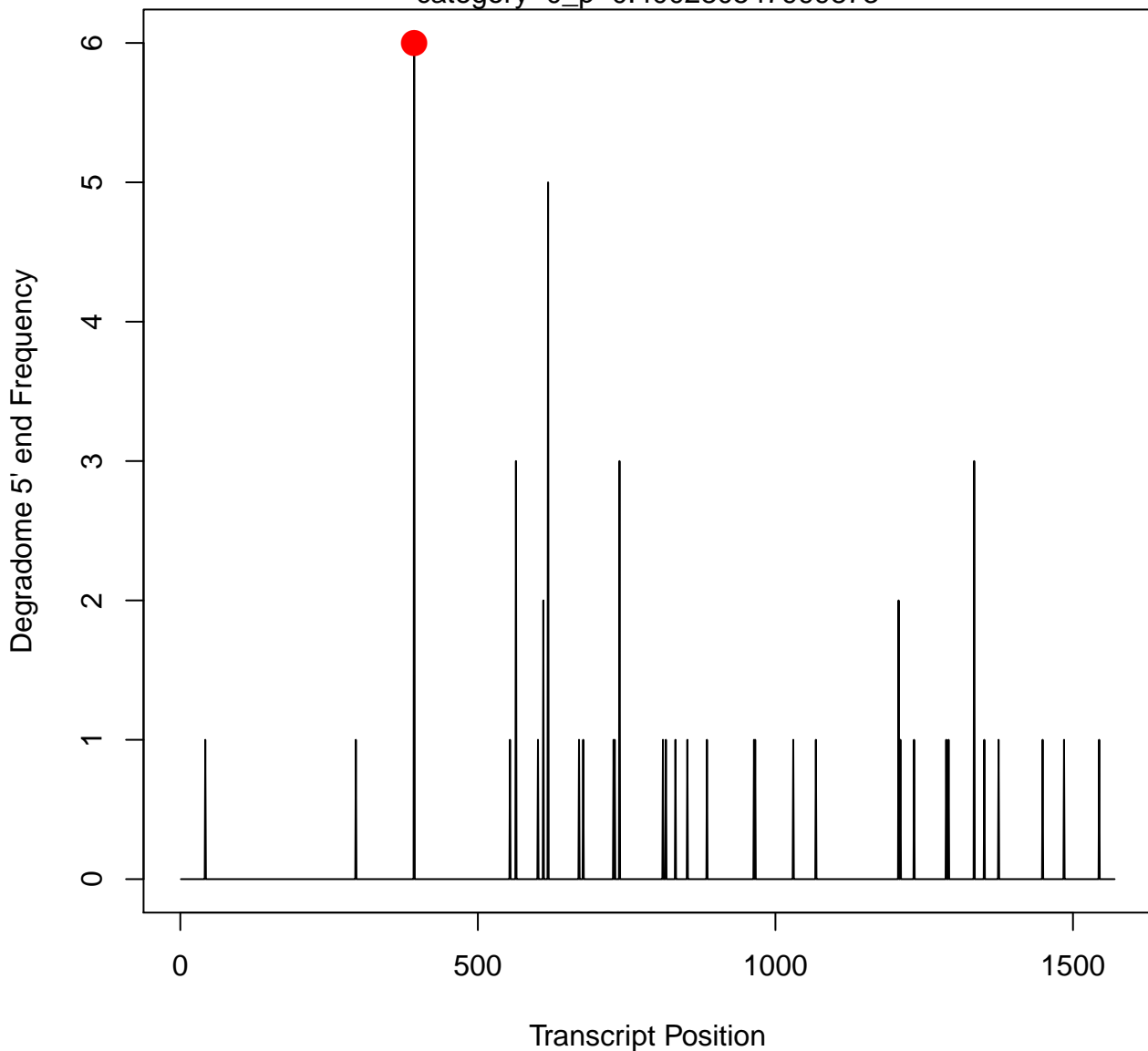

Supplement: Supplementary file 5 [file Data_Sheet_5.zip › Sit-miR2118b_Seita.1G008600.1_393_TPlot.pdf]

**T=Seita.5G348000.1\_Q=Sit-miR2118b\_S=150**

category=2\_p=0.999854971880301

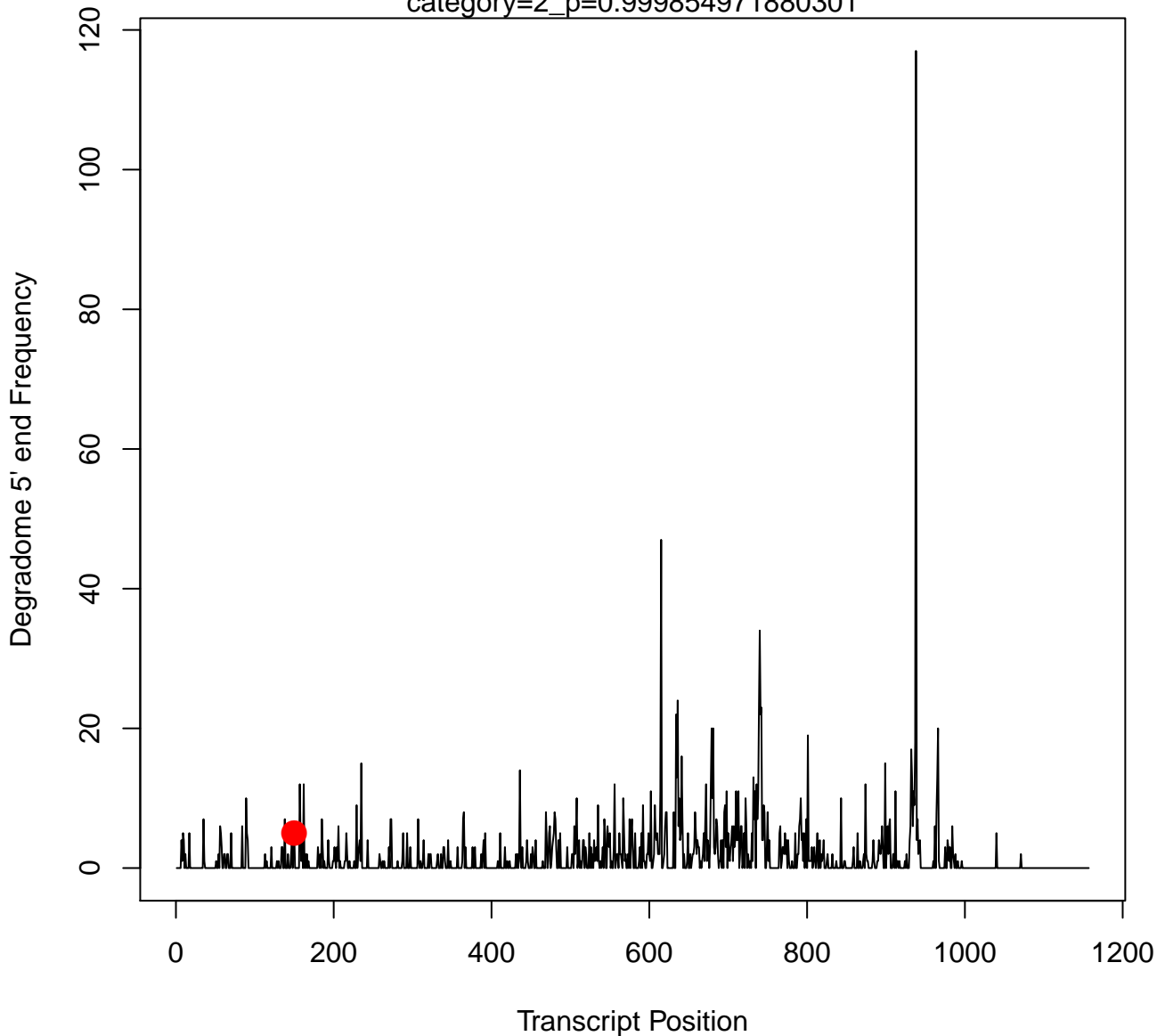

Supplement: Supplementary file 5 [file Data_Sheet_5.zip › Sit-miR2118b_Seita.5G348000.1_150_TPlot.pdf]

**T=Seita.6G047900.1\_Q=Sit-miR2118b\_S=393**

category=2\_p=0.999596589580684

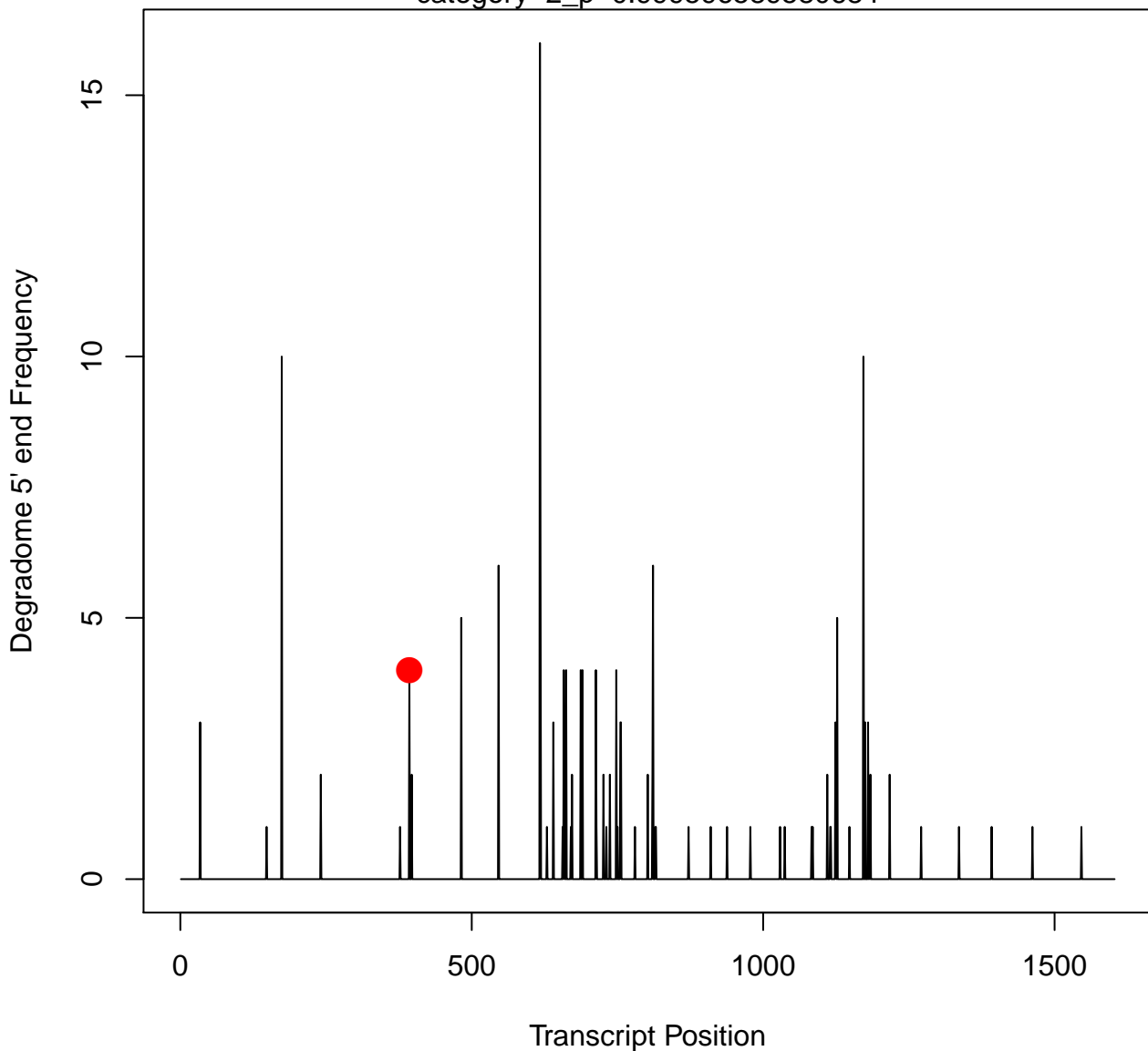

Supplement: Supplementary file 5 [file Data_Sheet_5.zip › Sit-miR2118b_Seita.6G047900.1_393_TPlot.pdf]

**T=Seita.8G034900.1\_Q=Sit-miR2118b\_S=632**

category=2\_p=0.950439701556188

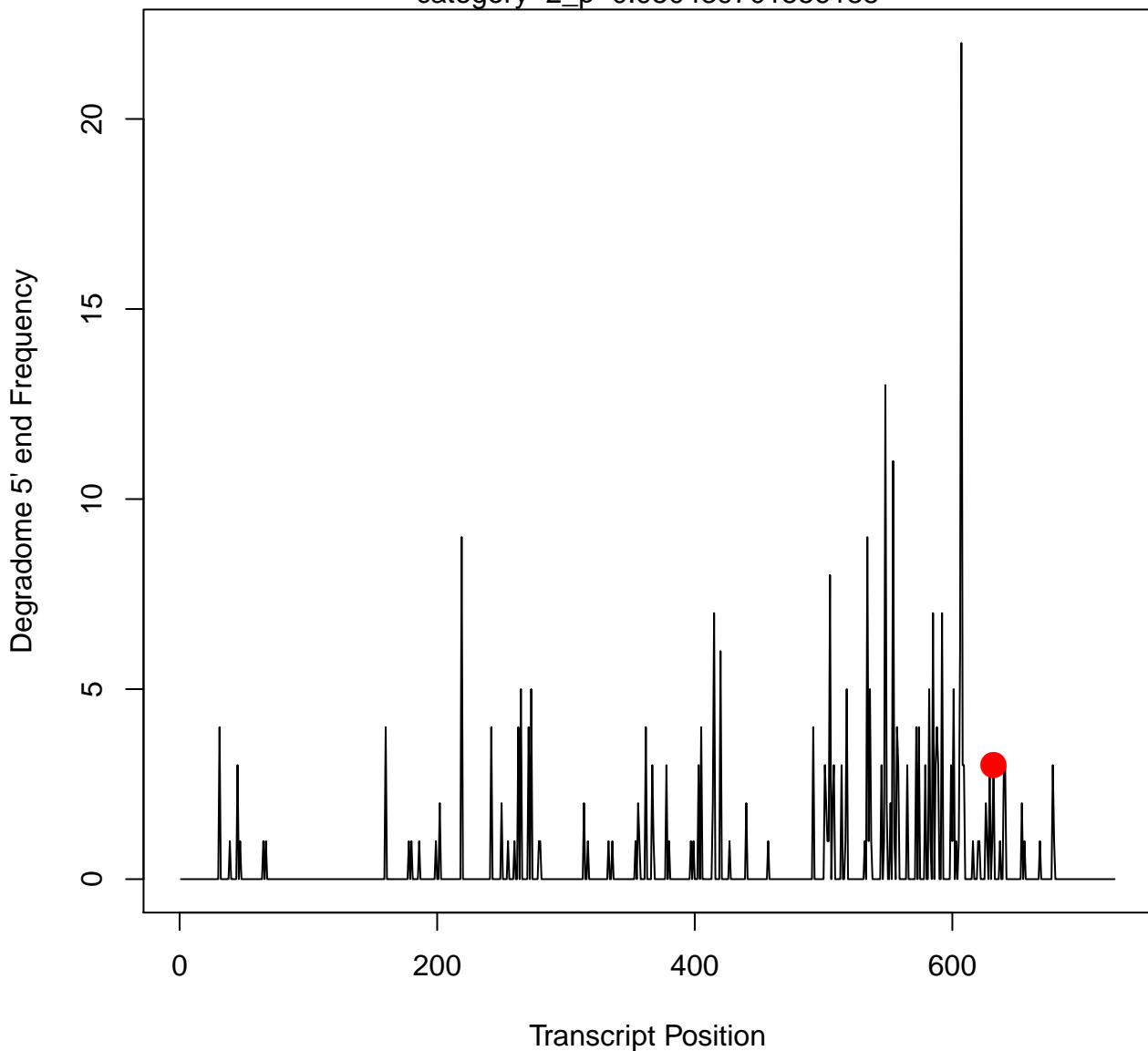

Supplement: Supplementary file 5 [file Data_Sheet_5.zip › Sit-miR2118b_Seita.8G034900.1_632_TPlot.pdf]

**T=Seita.9G134900.1\_Q=Sit-miR2118b\_S=2876**

category=2\_p=0.999075331176997

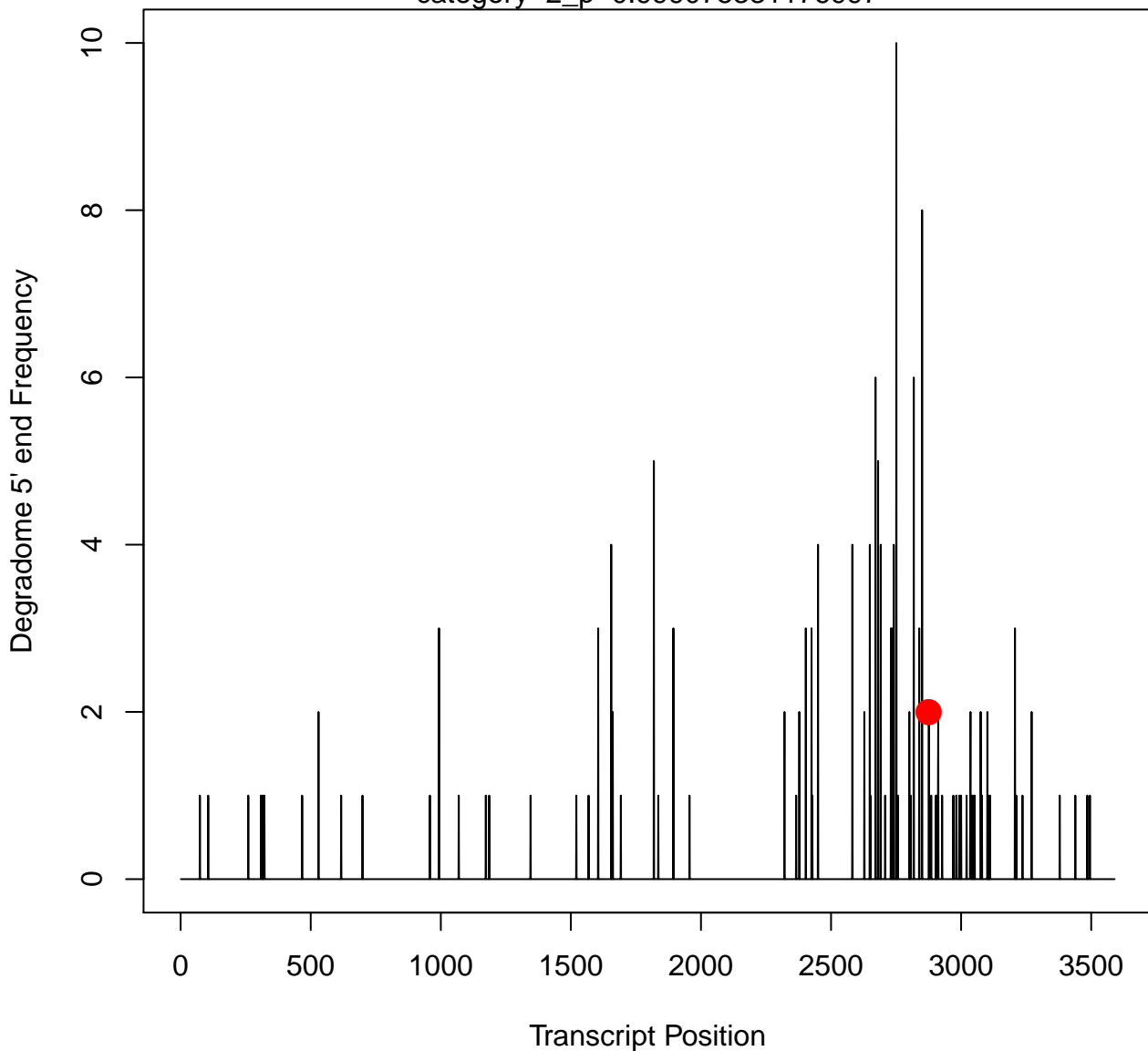

Supplement: Supplementary file 5 [file Data_Sheet_5.zip › Sit-miR2118b_Seita.9G134900.1_2876_TPlot.pdf]

**T=Seita.9G383000.1\_Q=Sit-miR2118b\_S=2552**

category=2\_p=0.91463287435009

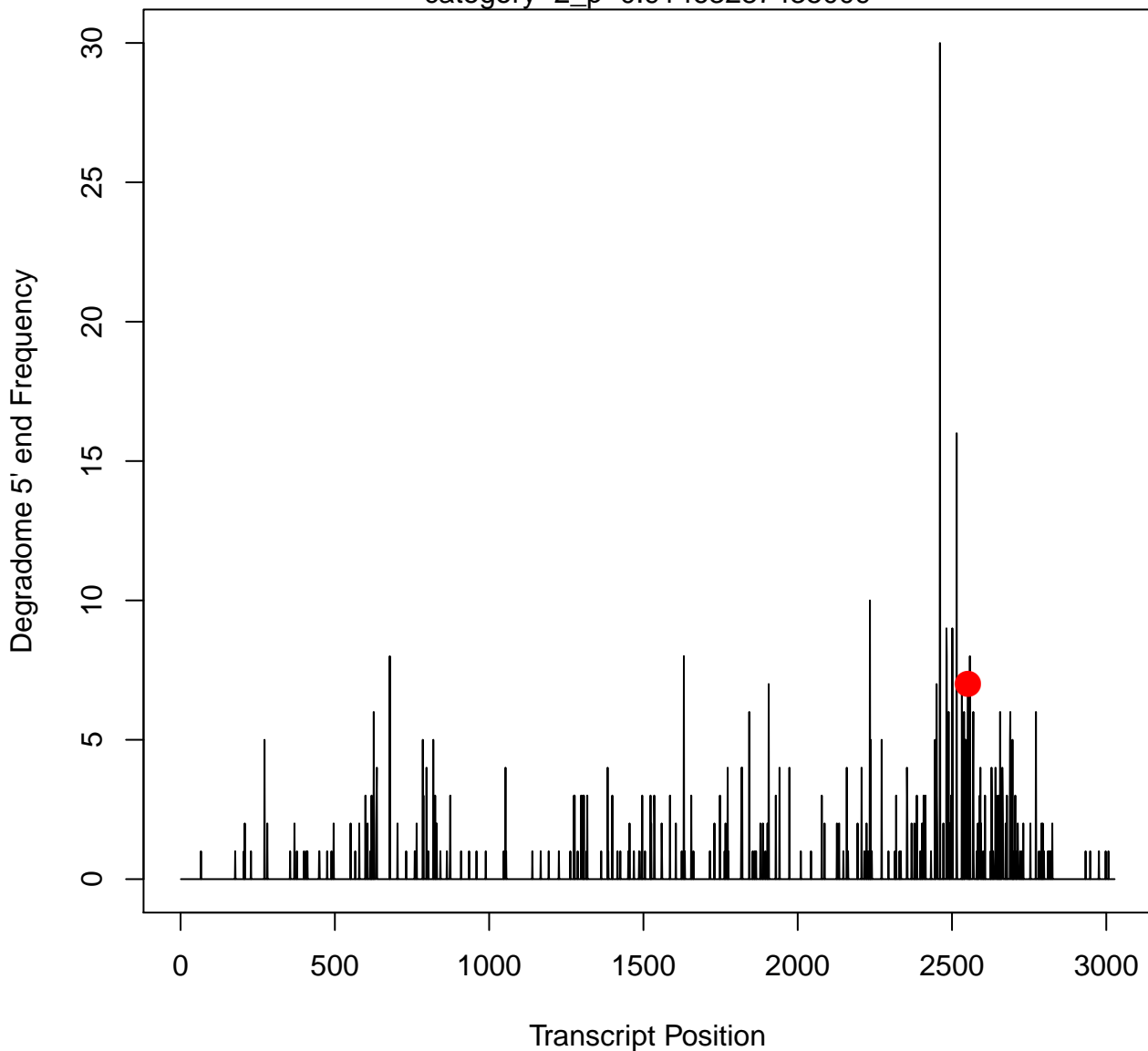

Supplement: Supplementary file 5 [file Data_Sheet_5.zip › Sit-miR2118b_Seita.9G383000.1_2552_TPlot.pdf]

**T=Seita.6G203700.1\_Q=Sit-miR2118c\_S=827**

category=2\_p=0.999565721464421

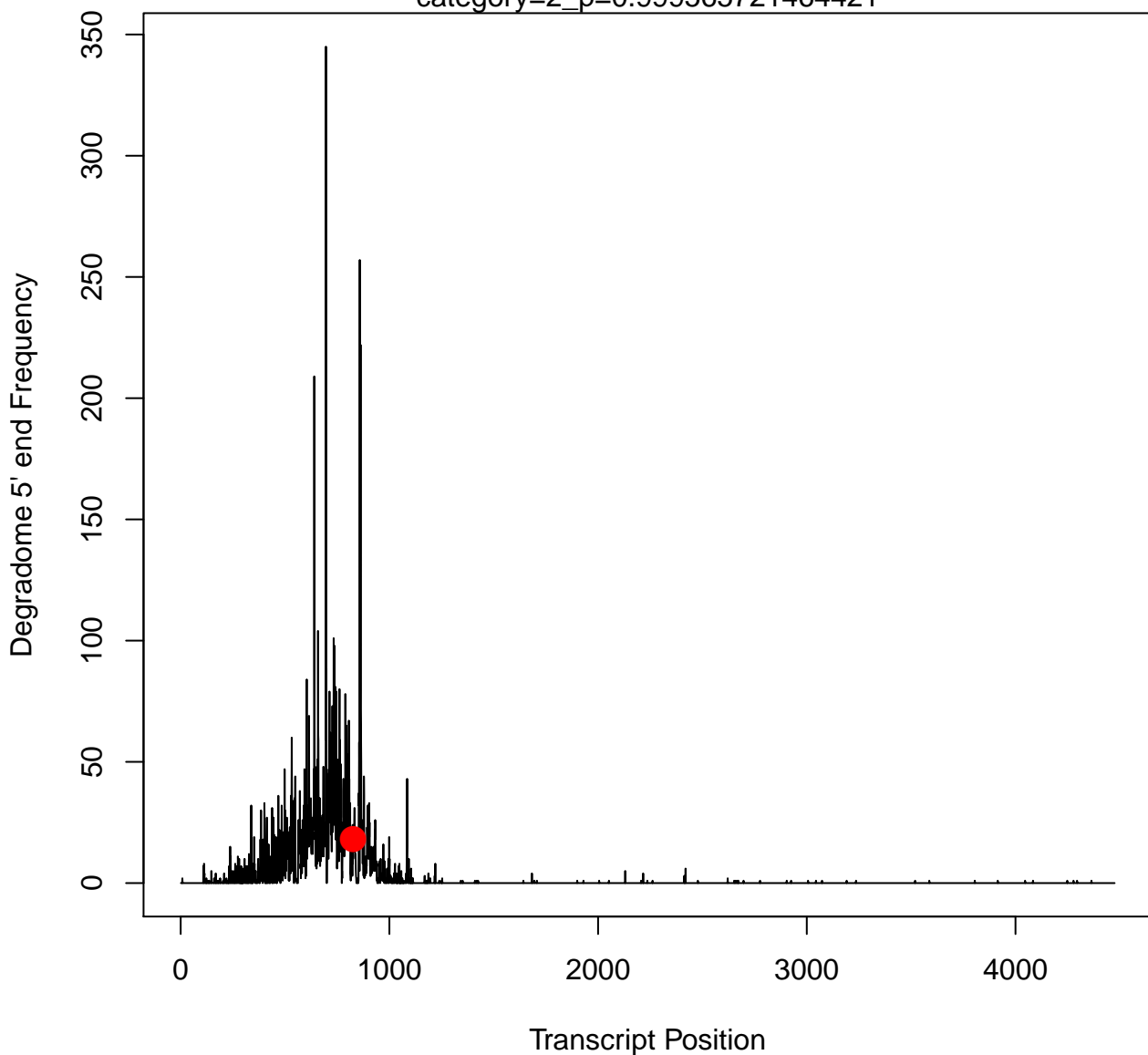

Supplement: Supplementary file 5 [file Data_Sheet_5.zip › Sit-miR2118c_Seita.6G203700.1_827_TPlot.pdf]

**T=Seita.6G209900.1\_Q=Sit-miR2118c\_S=283**

category=2\_p=0.990991708691954

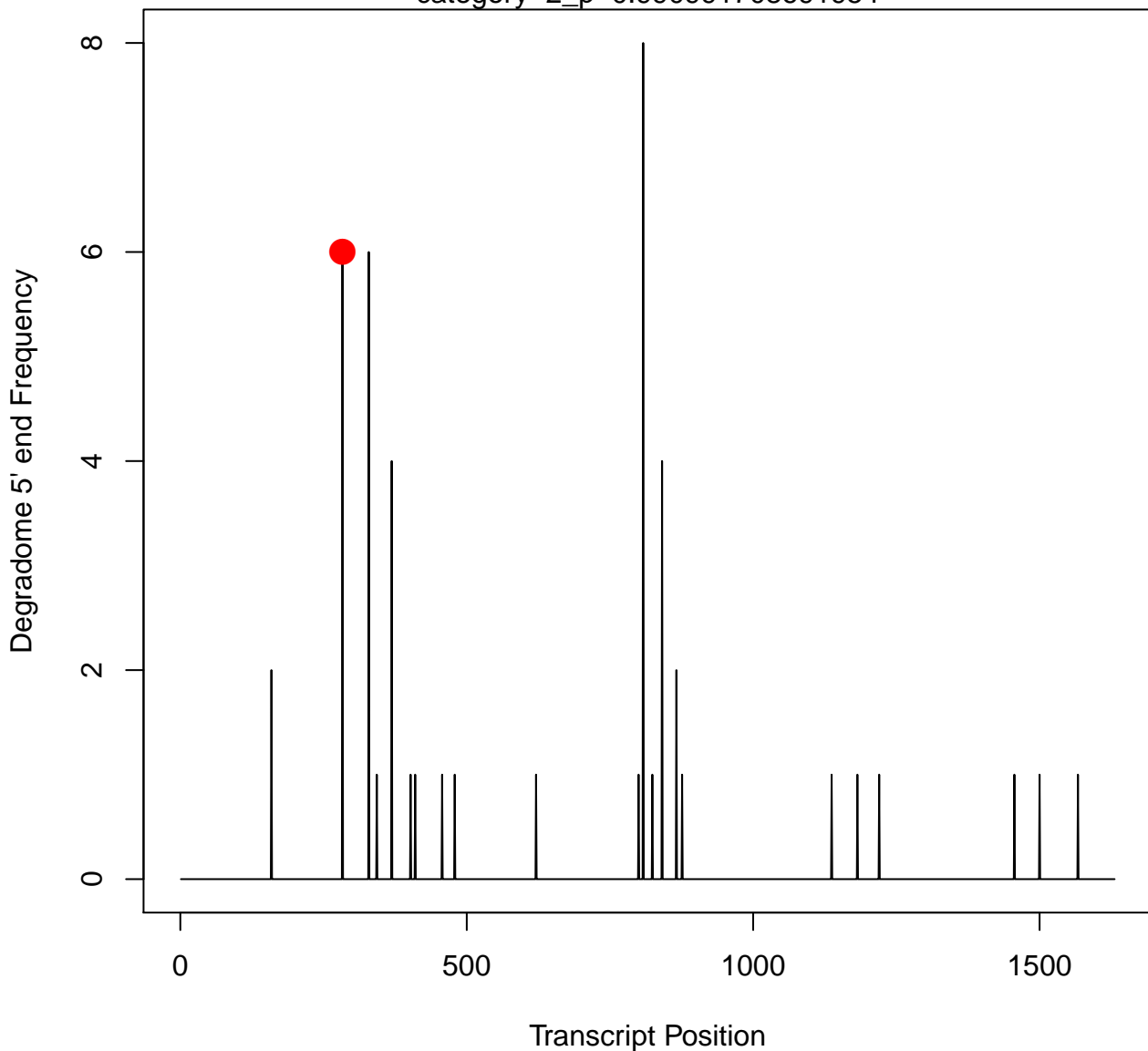

Supplement: Supplementary file 5 [file Data_Sheet_5.zip › Sit-miR2118c_Seita.6G209900.1_283_TPlot.pdf]

**T=Seita.9G339600.1\_Q=Sit-miR2118c\_S=1070**

category=2\_p=0.999949283491034

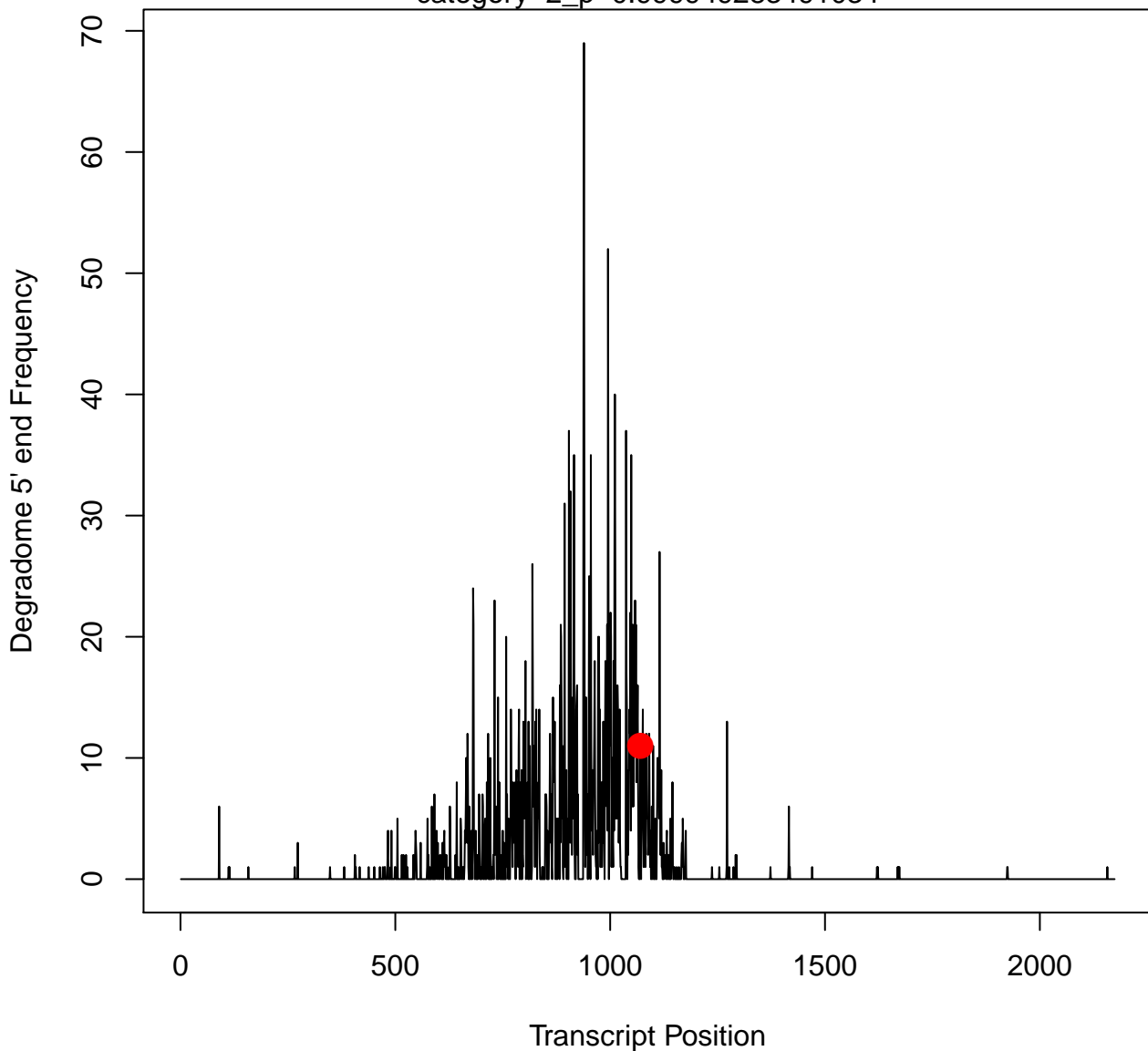

Supplement: Supplementary file 5 [file Data_Sheet_5.zip › Sit-miR2118c_Seita.9G339600.1_1070_TPlot.pdf]

**T=Seita.3G154800.1\_Q=Sit-miR2118d\_S=1712**

category=2\_p=0.974001520859301

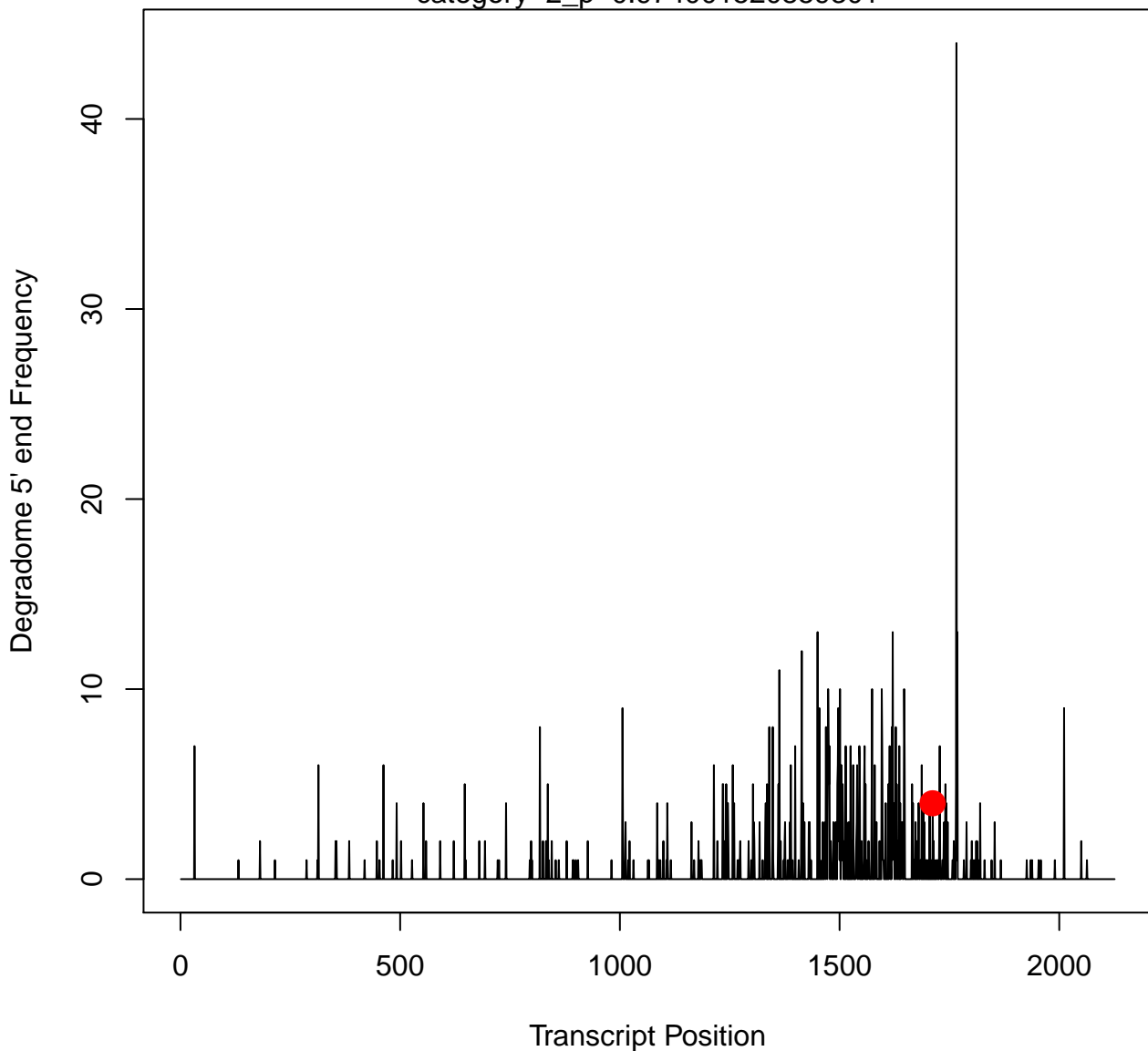

Supplement: Supplementary file 5 [file Data_Sheet_5.zip › Sit-miR2118d_Seita.3G154800.1_1712_TPlot.pdf]

**T=Seita.5G010200.1\_Q=Sit-miR2118d\_S=1437**

category=2\_p=0.994672898282237

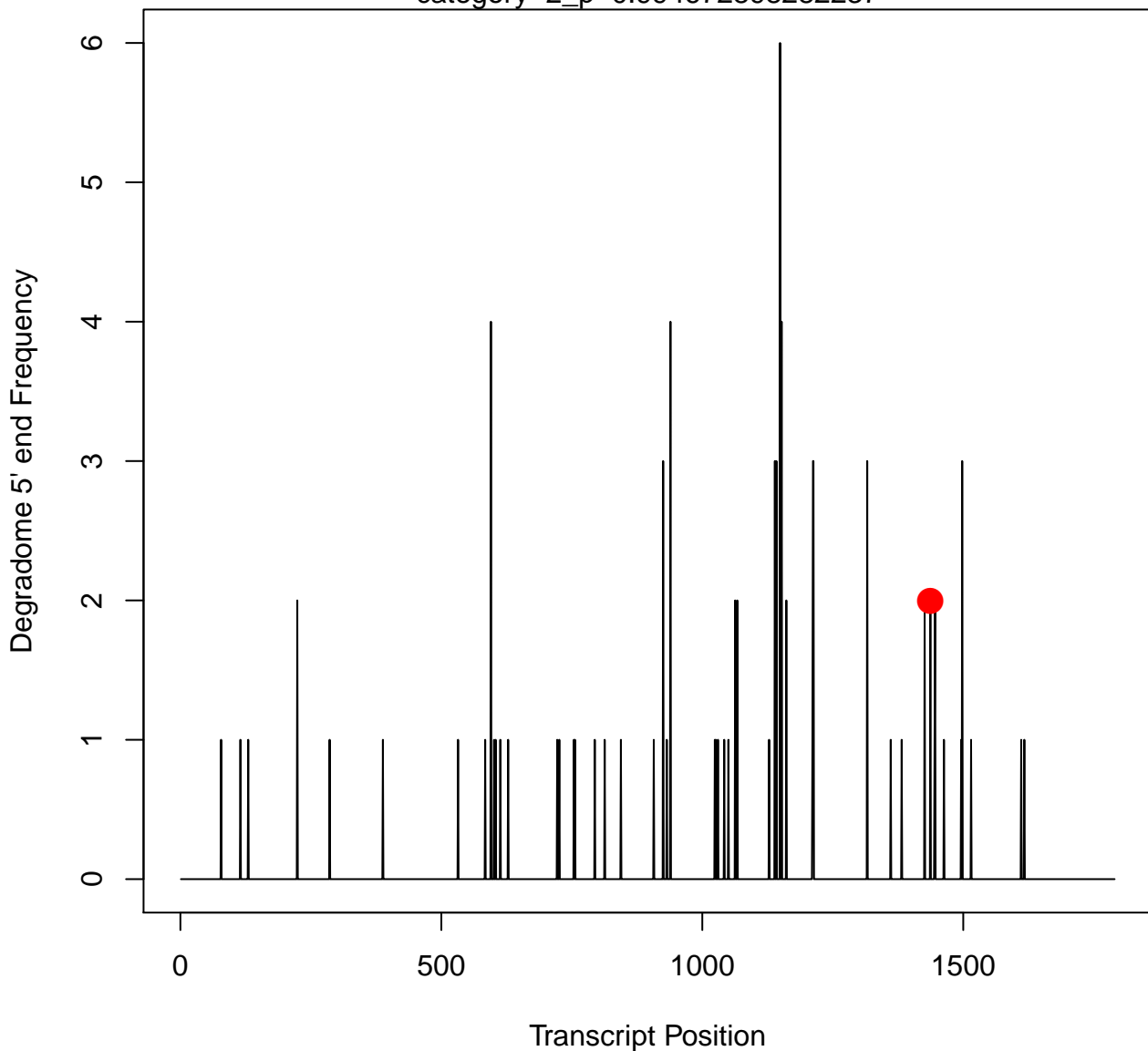

Supplement: Supplementary file 5 [file Data_Sheet_5.zip › Sit-miR2118d_Seita.5G010200.1_1437_TPlot.pdf]

**T=Seita.5G202700.1\_Q=Sit-miR2118d\_S=1725**

category=2\_p=0.974240033930635

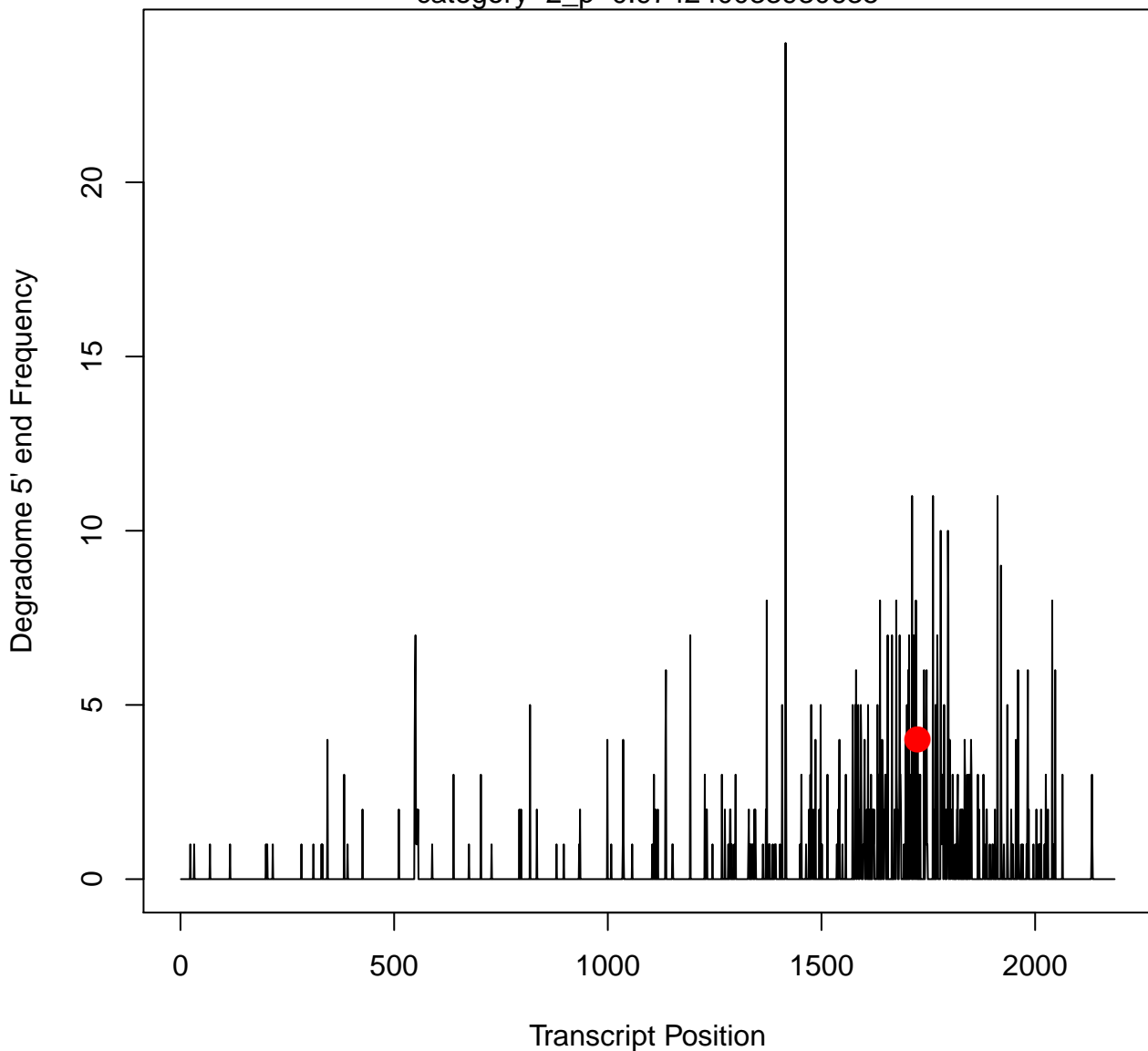

Supplement: Supplementary file 5 [file Data_Sheet_5.zip › Sit-miR2118d_Seita.5G202700.1_1725_TPlot.pdf]

**T=Seita.5G319000.1\_Q=Sit-miR2118d\_S=1396**

category=2\_p=0.999272359036306

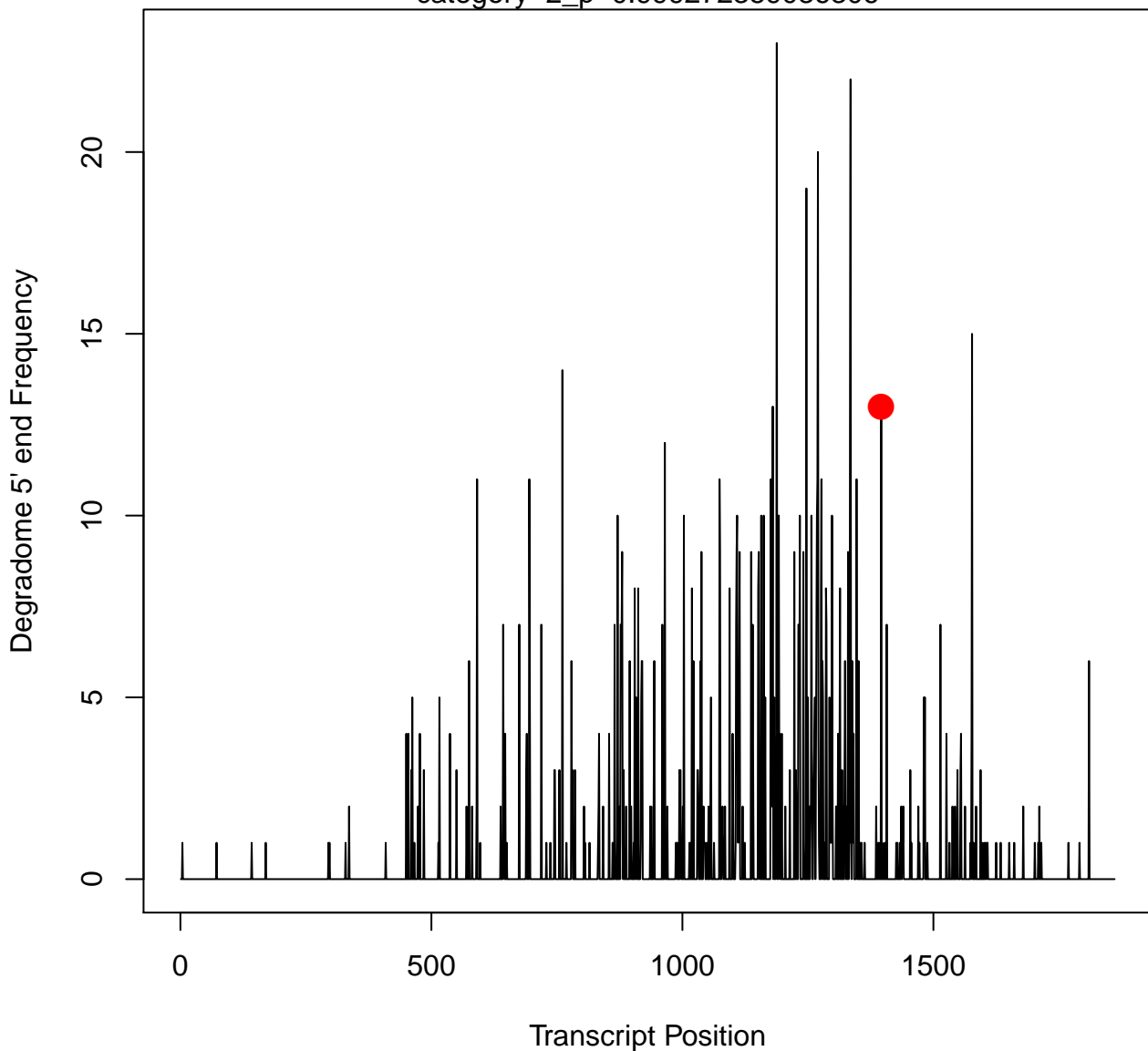

Supplement: Supplementary file 5 [file Data_Sheet_5.zip › Sit-miR2118d_Seita.5G319000.1_1396_TPlot.pdf]

**T=Seita.6G192400.1\_Q=Sit-miR2118d\_S=1588**

category=2\_p=0.975849468687763

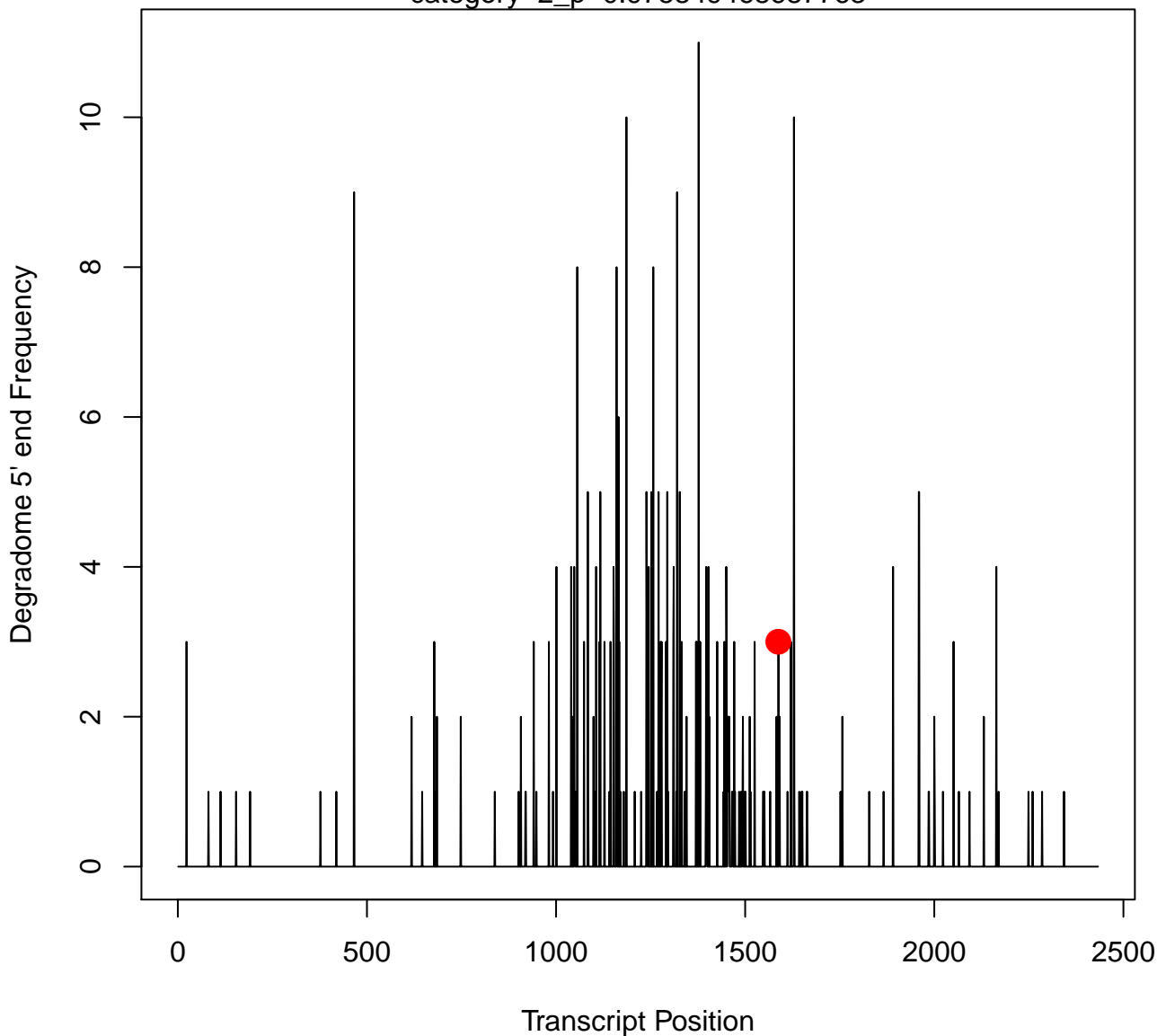

Supplement: Supplementary file 5 [file Data_Sheet_5.zip › Sit-miR2118d_Seita.6G192400.1_1588_TPlot.pdf]

**T=Seita.7G086700.1\_Q=Sit-miR2118d\_S=961**

category=2\_p=0.990566872480344

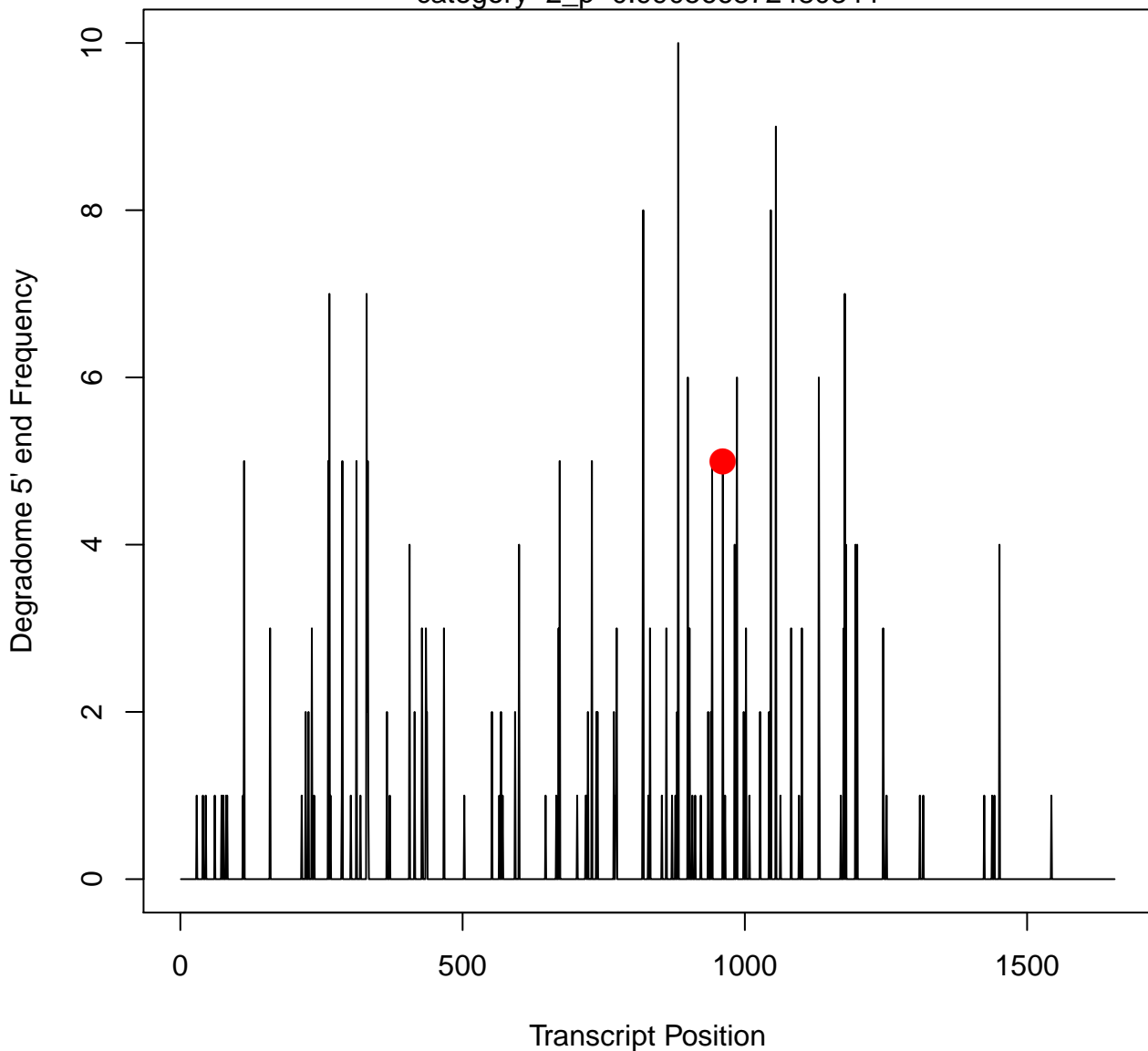

Supplement: Supplementary file 5 [file Data_Sheet_5.zip › Sit-miR2118d_Seita.7G086700.1_961_TPlot.pdf]

**T=Seita.6G192400.1\_Q=Sit-miR2118e\_S=1074**

category=2\_p=0.998347453885198

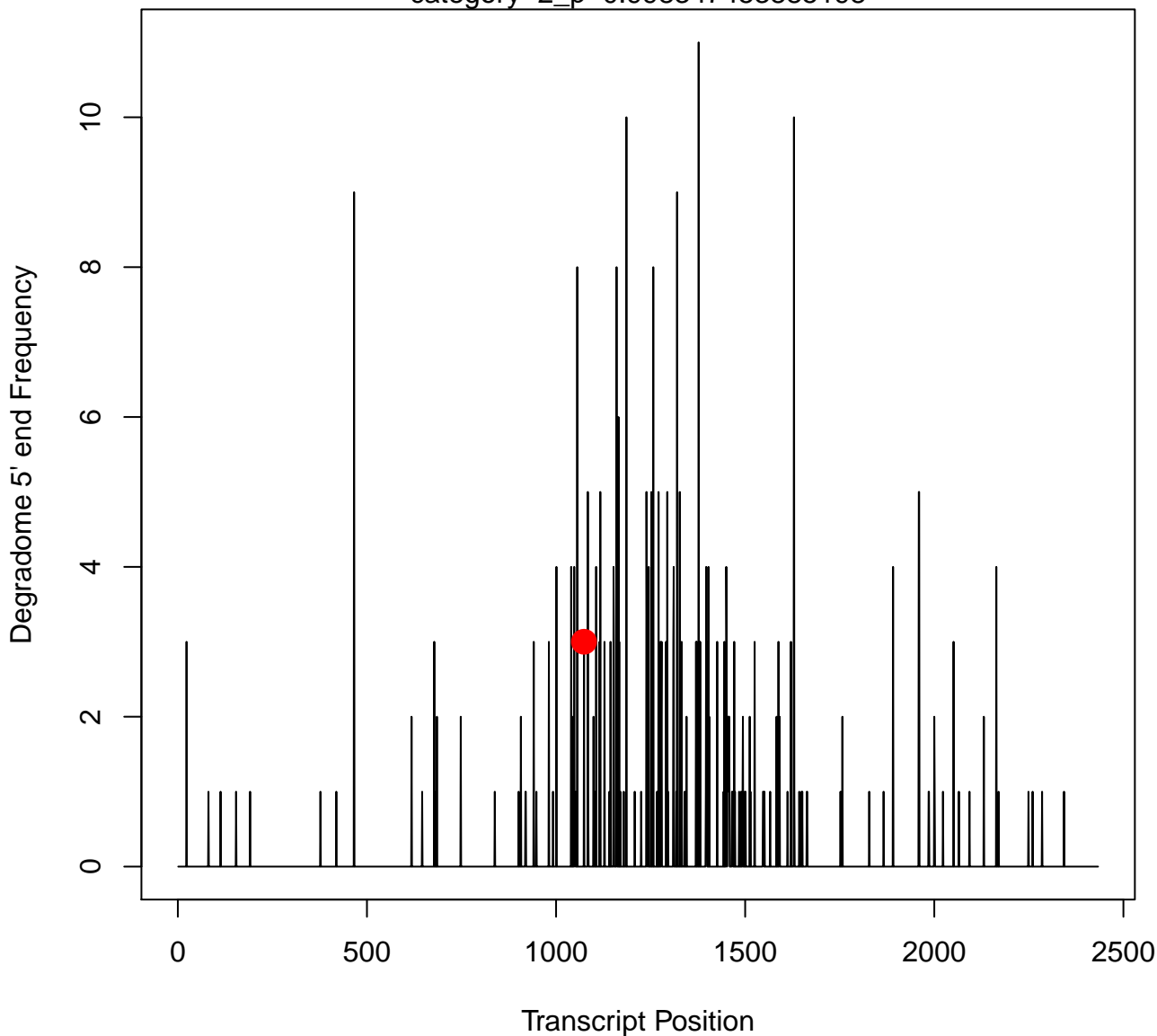

Supplement: Supplementary file 5 [file Data_Sheet_5.zip › Sit-miR2118e_Seita.6G192400.1_1074_TPlot.pdf]
