# Supplementary figures and images for: The Construction and Exploration of a Comprehensive MicroRNA Centered Regulatory Network in Foxtail Millet (Setaria italica L.) (part 9 of 14)
Source: Front Plant Sci. 2022 May 6;13:848474. doi: 10.3389/fpls.2022.848474 (PMC9121102; doi:10.3389/fpls.2022.848474)

**T=Seita.2G144400.1\_Q=Sit-miR1133\_S=1558**

category=2\_p=0.865902451772474

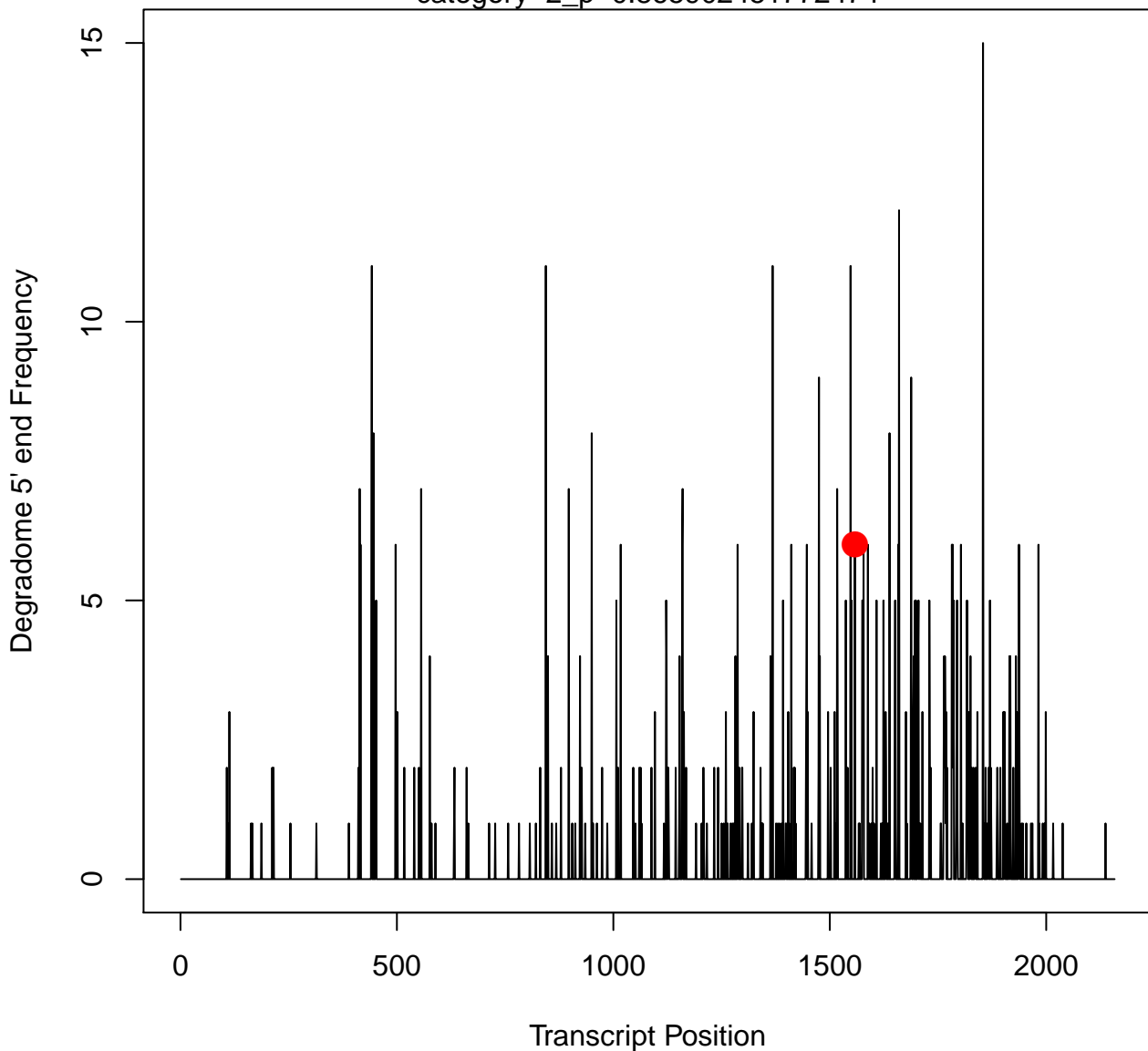

Supplement: Supplementary file 5 [file Data_Sheet_5.zip › Sit-miR1133_Seita.2G144400.1_1558_TPlot.pdf]

**T=Seita.2G387500.1\_Q=Sit-miR1133\_S=21**

category=2\_p=0.818235733219827

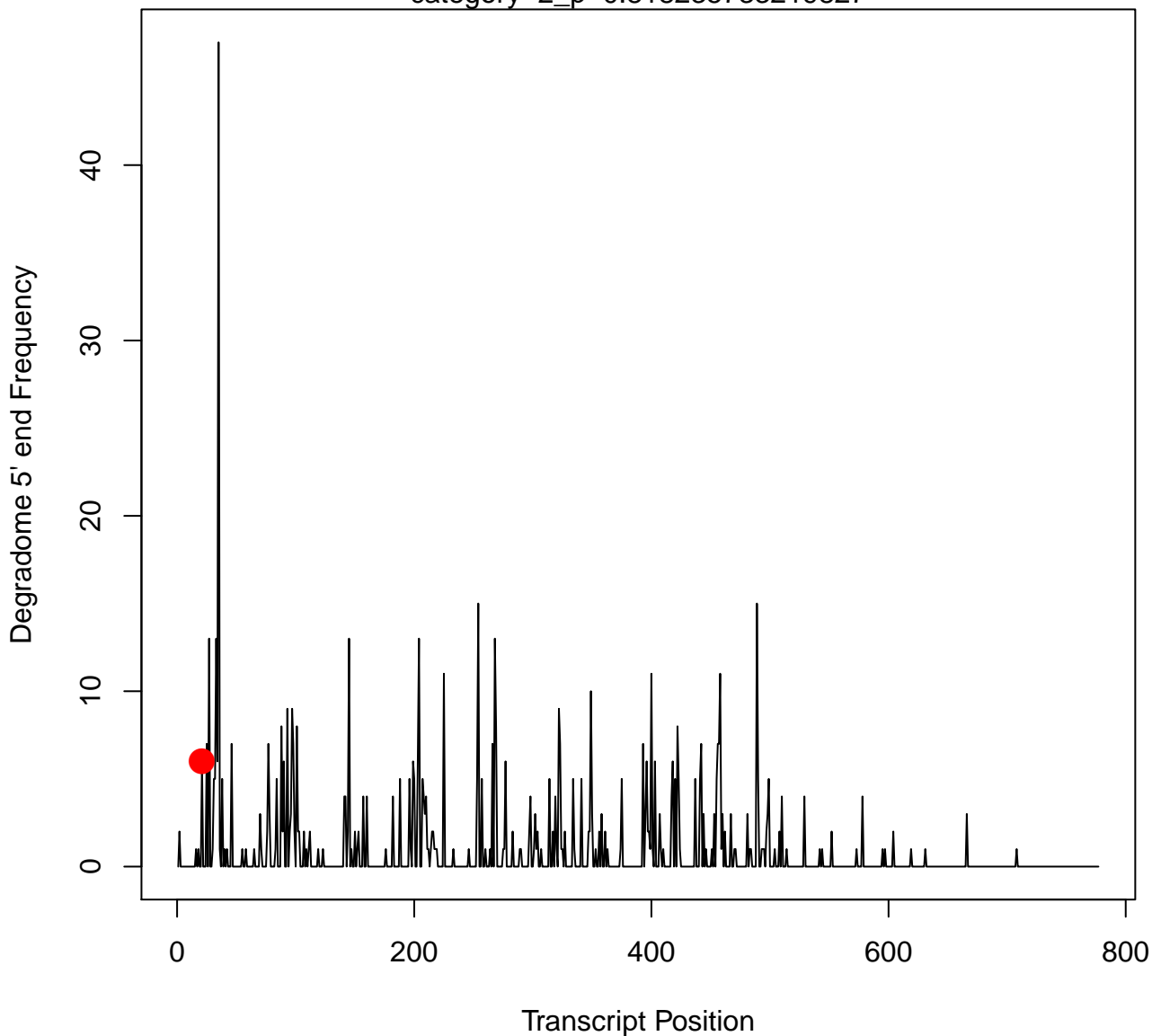

Supplement: Supplementary file 5 [file Data_Sheet_5.zip › Sit-miR1133_Seita.2G387500.1_21_TPlot.pdf]

**T=Seita.2G393200.1\_Q=Sit-miR1133\_S=44**

category=2\_p=0.982668703658484

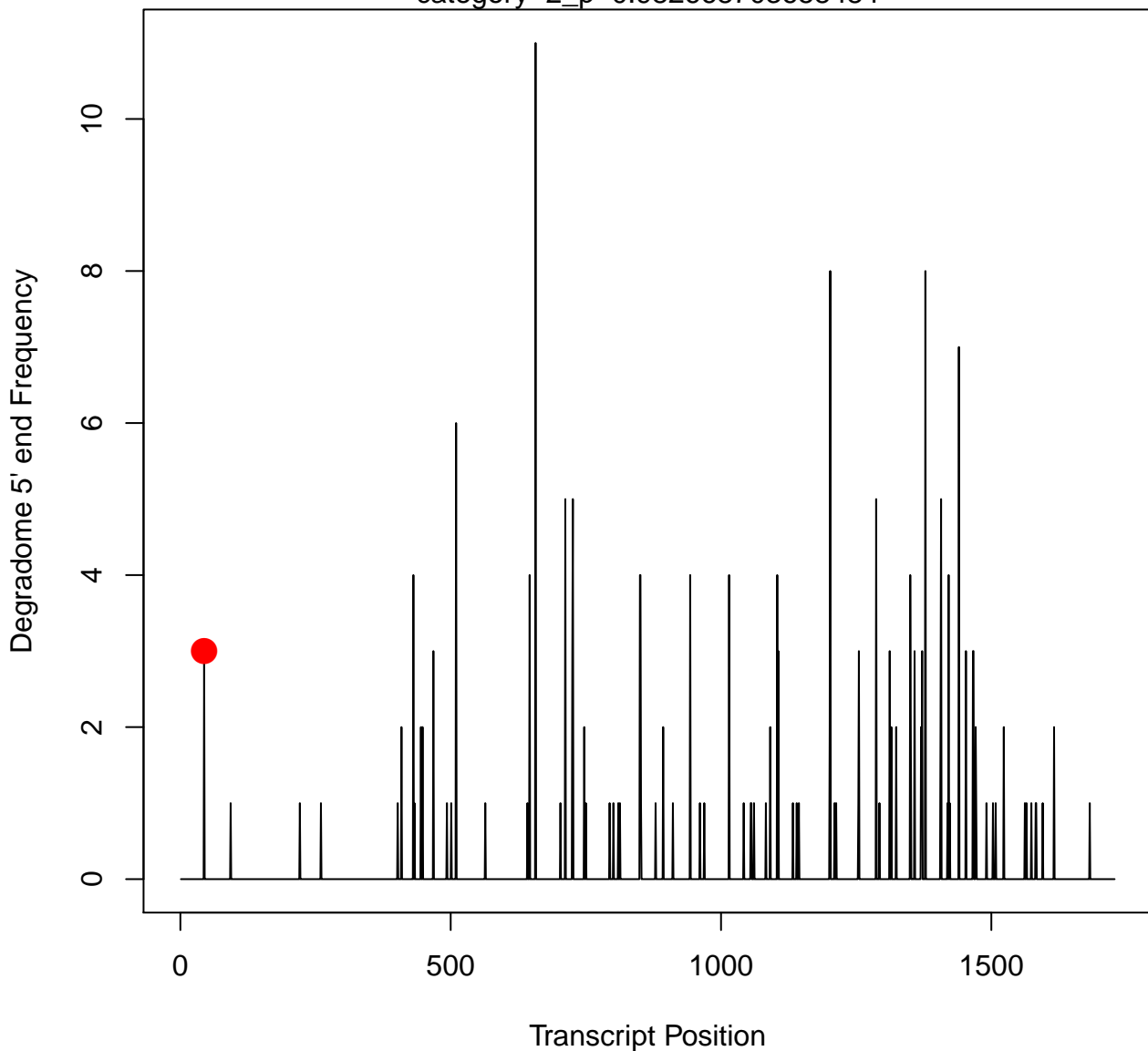

Supplement: Supplementary file 5 [file Data_Sheet_5.zip › Sit-miR1133_Seita.2G393200.1_44_TPlot.pdf]

**T=Seita.5G236300.1\_Q=Sit-miR1133\_S=147**

category=2\_p=0.999031723262207

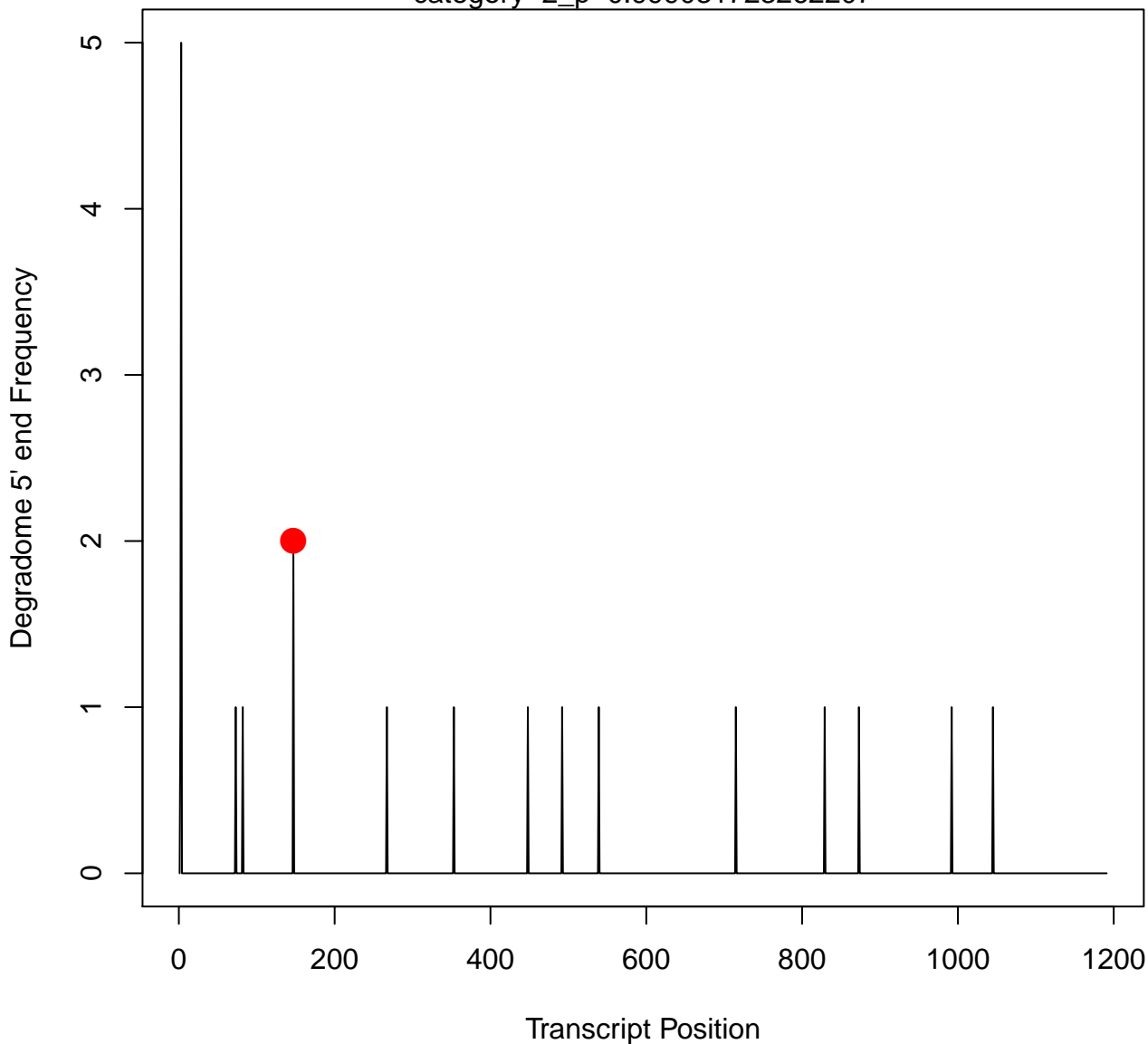

Supplement: Supplementary file 5 [file Data_Sheet_5.zip › Sit-miR1133_Seita.5G236300.1_147_TPlot.pdf]

**T=Seita.7G269200.1\_Q=Sit-miR1133\_S=1315**

category=2\_p=0.930300090984271

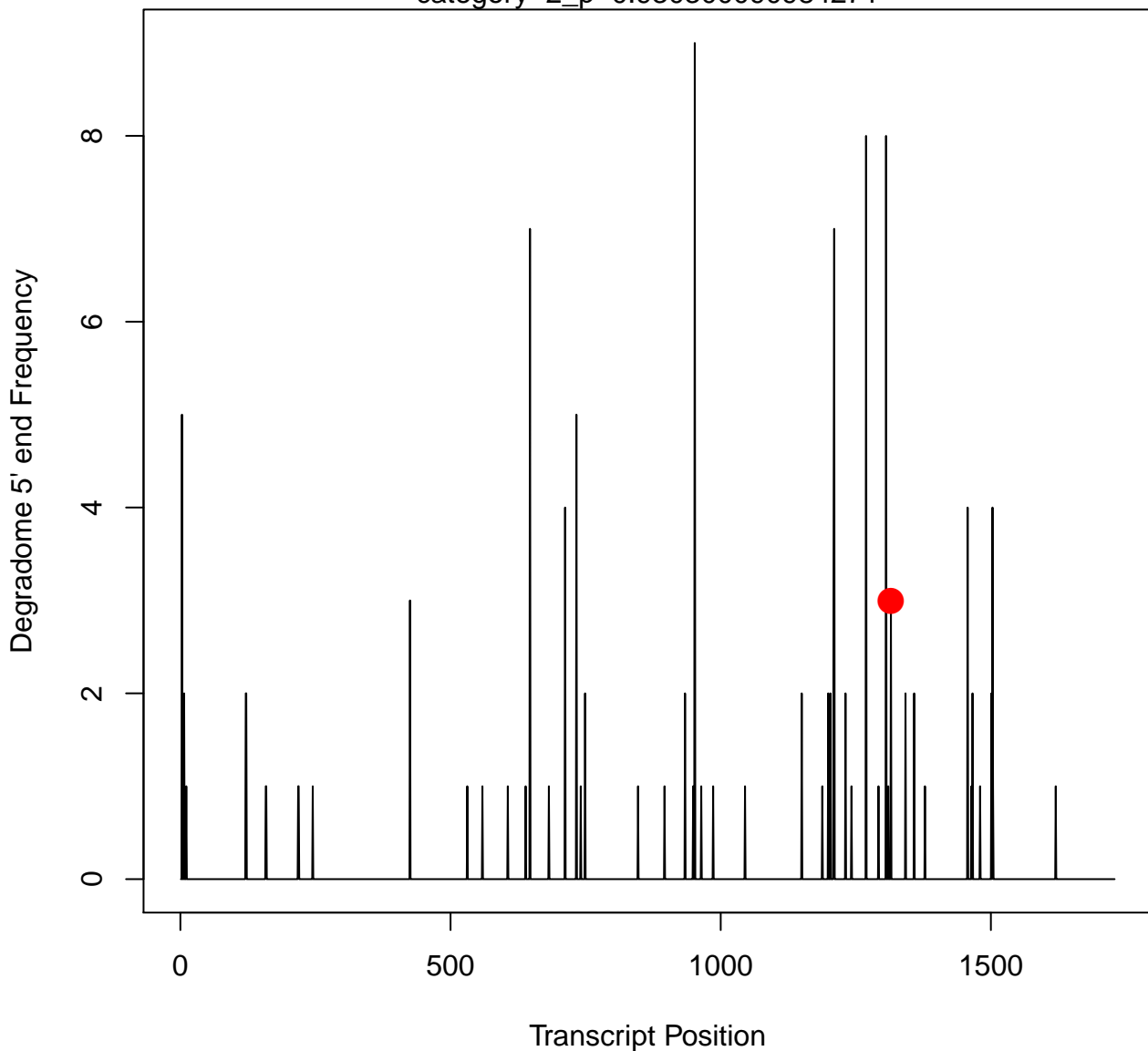

Supplement: Supplementary file 5 [file Data_Sheet_5.zip › Sit-miR1133_Seita.7G269200.1_1315_TPlot.pdf]

**T=Seita.9G150800.1\_Q=Sit-miR1133\_S=573**

category=2\_p=0.995959721632634

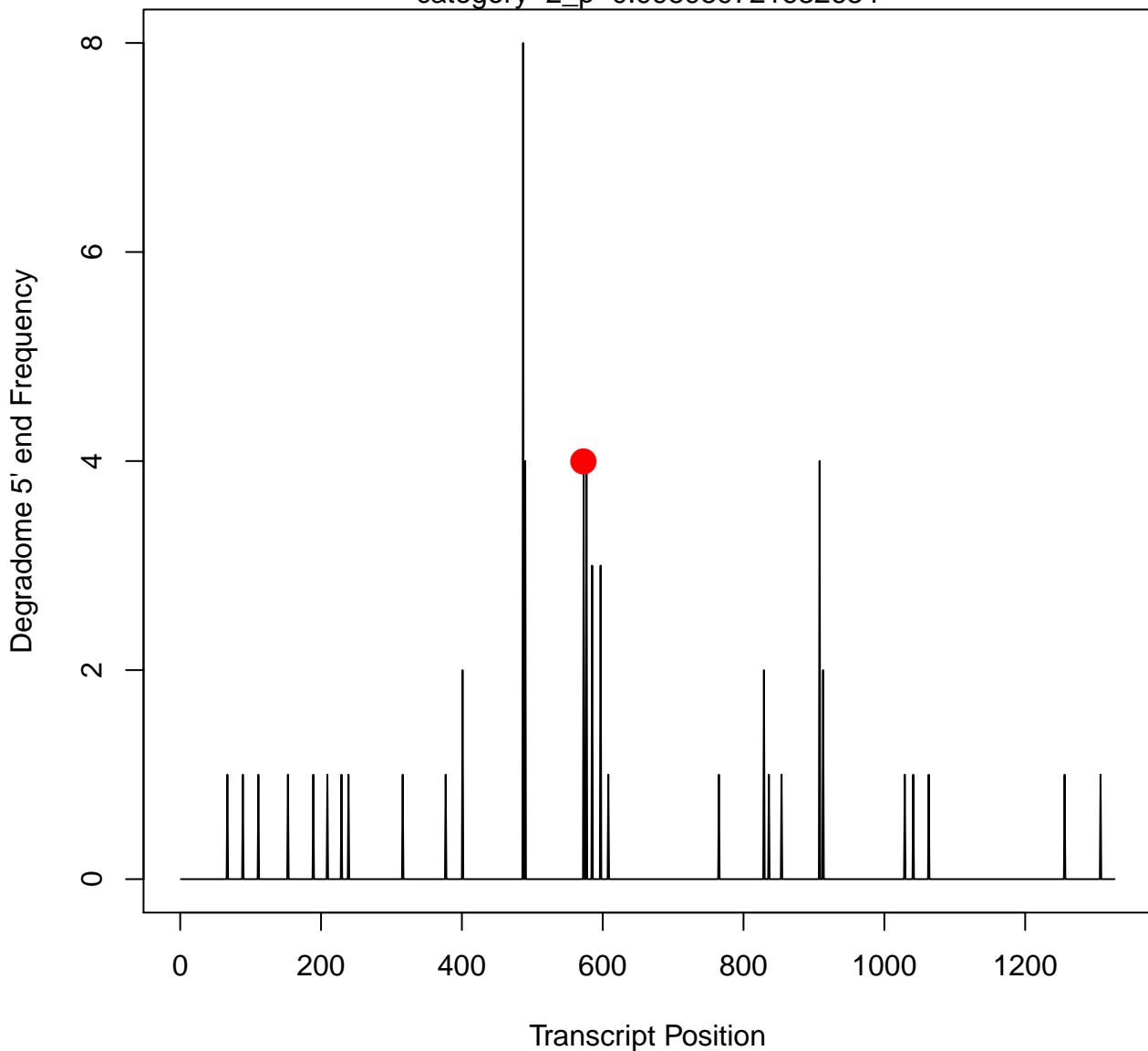

Supplement: Supplementary file 5 [file Data_Sheet_5.zip › Sit-miR1133_Seita.9G150800.1_573_TPlot.pdf]

**T=Seita.9G100400.1\_Q=Sit-miR1432\_S=1145**

category=2\_p=0.701013230205742

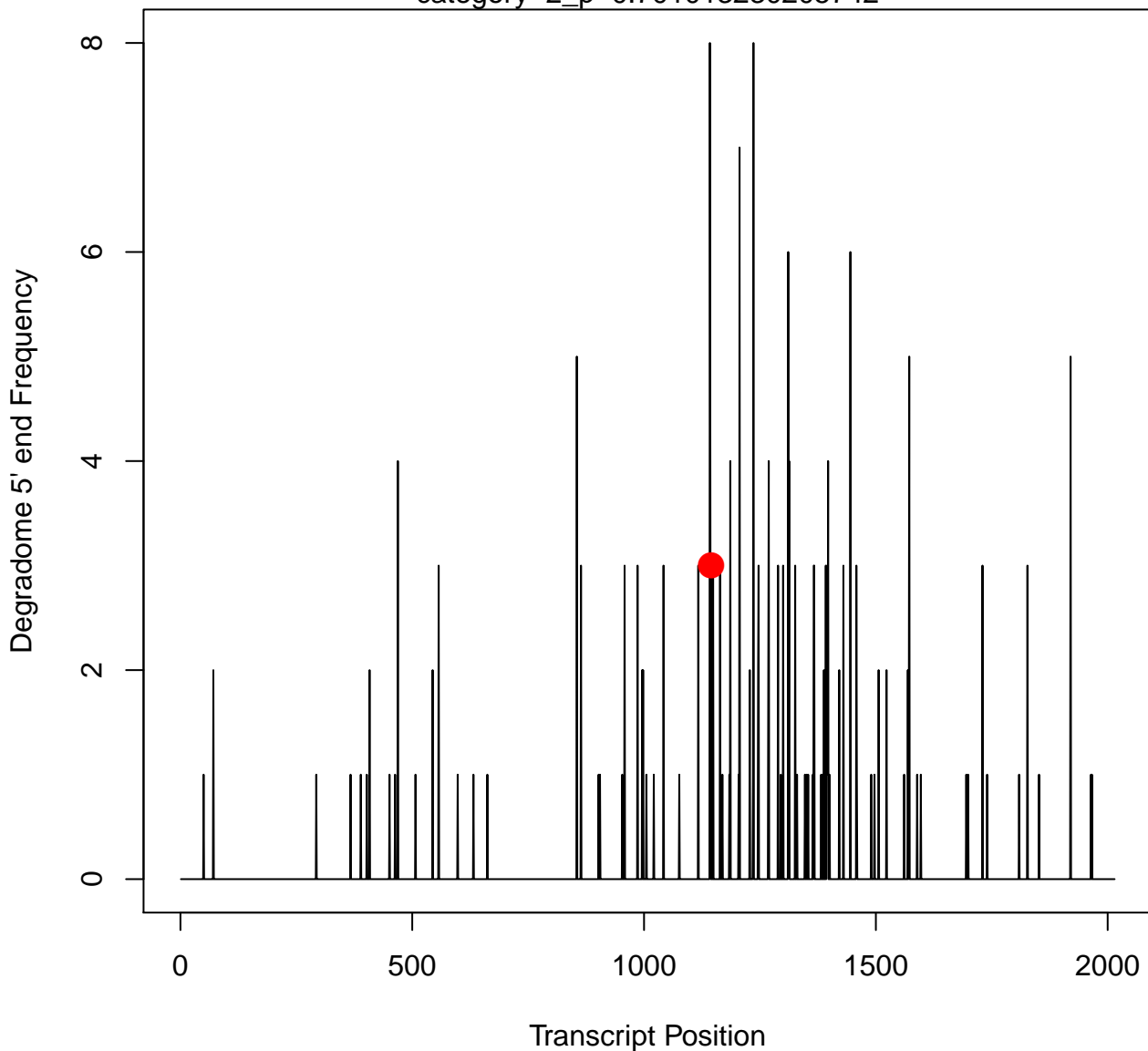

Supplement: Supplementary file 5 [file Data_Sheet_5.zip › Sit-miR1432_Seita.9G100400.1_1145_TPlot.pdf]

**T=Seita.2G254300.1\_Q=Sit-miR156a\_S=1266**

category=2\_p=0.0537975684842888

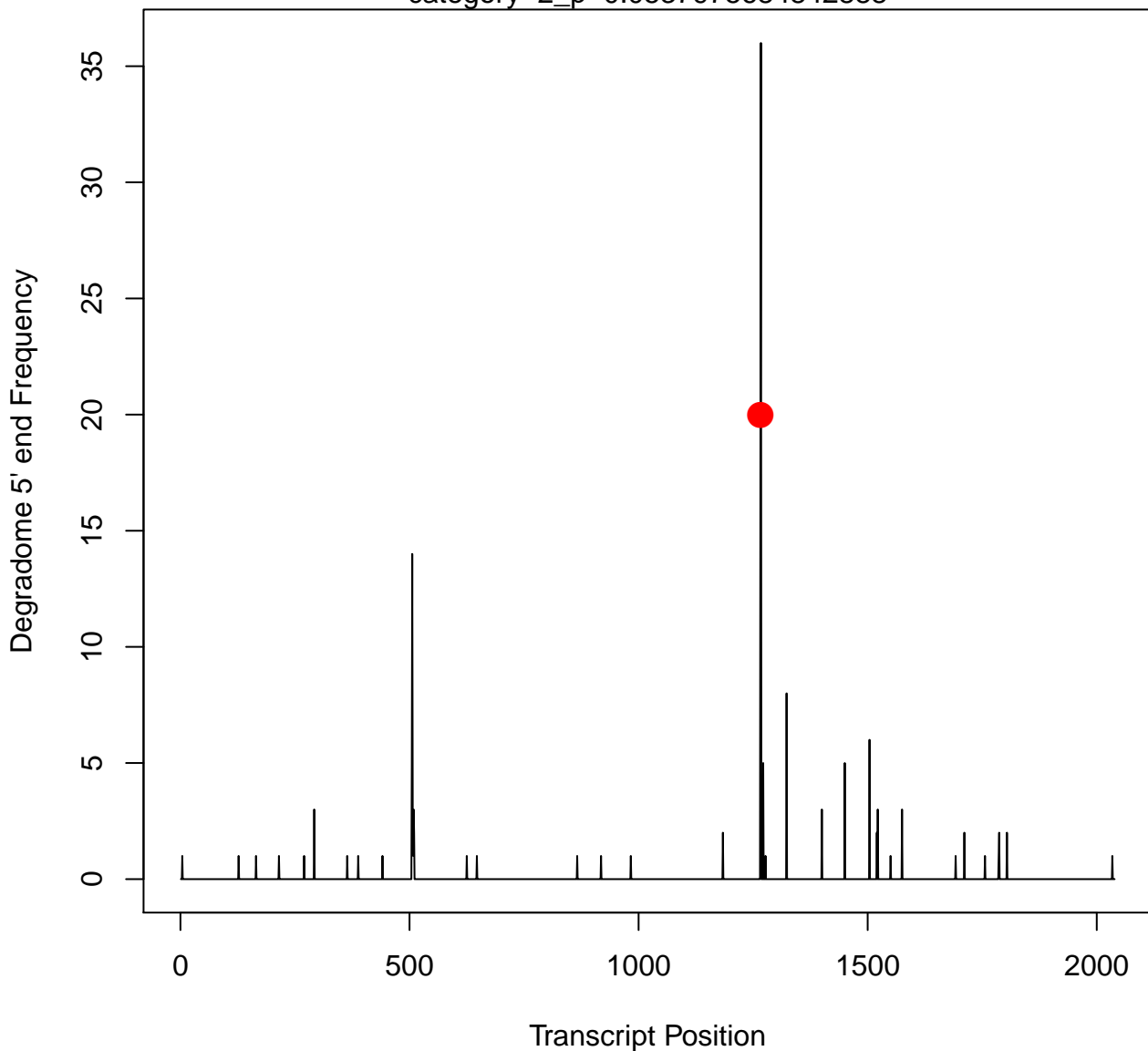

Supplement: Supplementary file 5 [file Data_Sheet_5.zip › Sit-miR156a_Seita.2G254300.1_1266_TPlot.pdf]

**T=Seita.2G266500.1\_Q=Sit-miR156a\_S=1896**

category=0\_p=0.00379578583708362

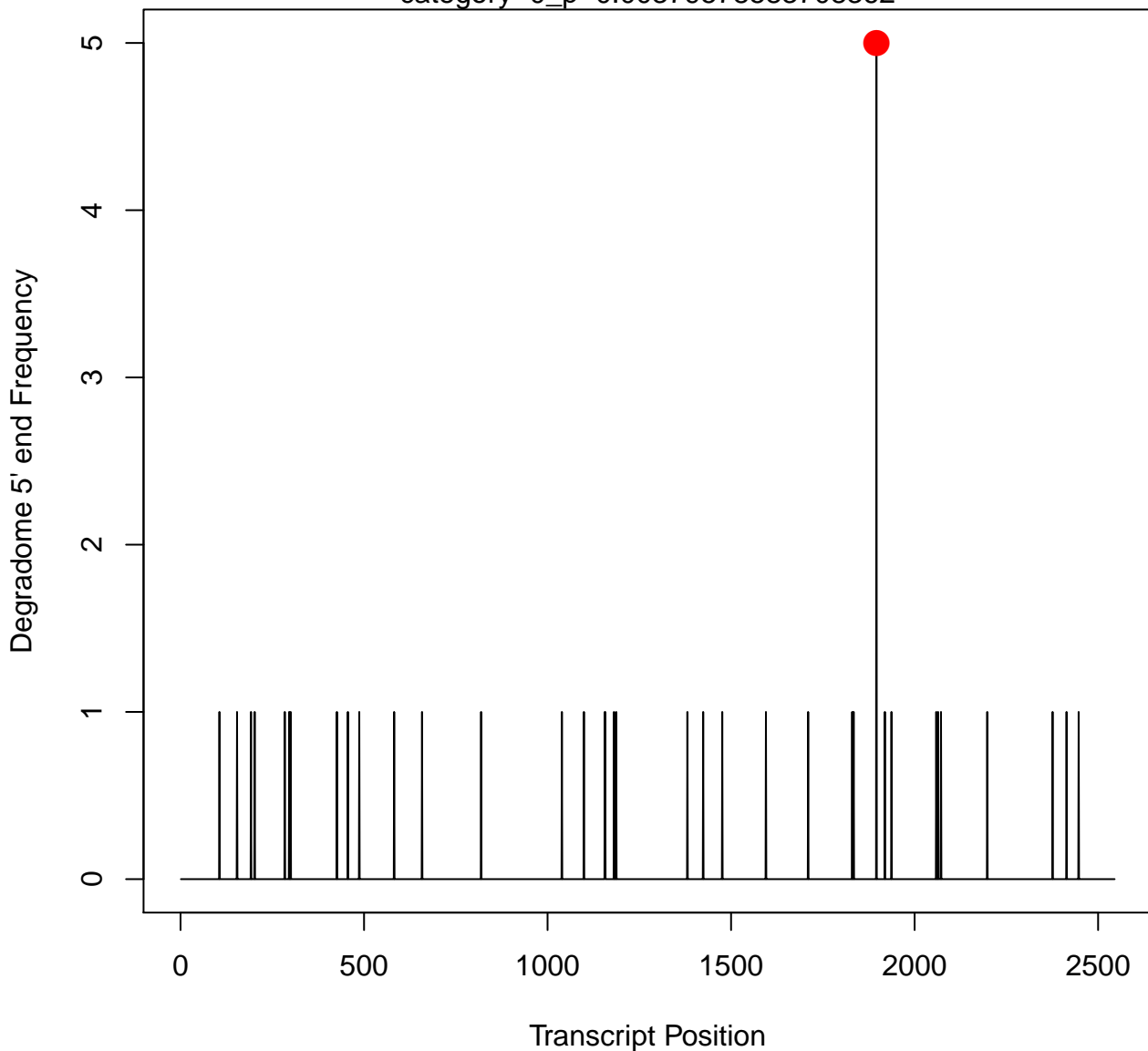

Supplement: Supplementary file 5 [file Data_Sheet_5.zip › Sit-miR156a_Seita.2G266500.1_1896_TPlot.pdf]

**T=Seita.5G432500.1\_Q=Sit-miR156a\_S=2375**

category=2\_p=0.036194560204432

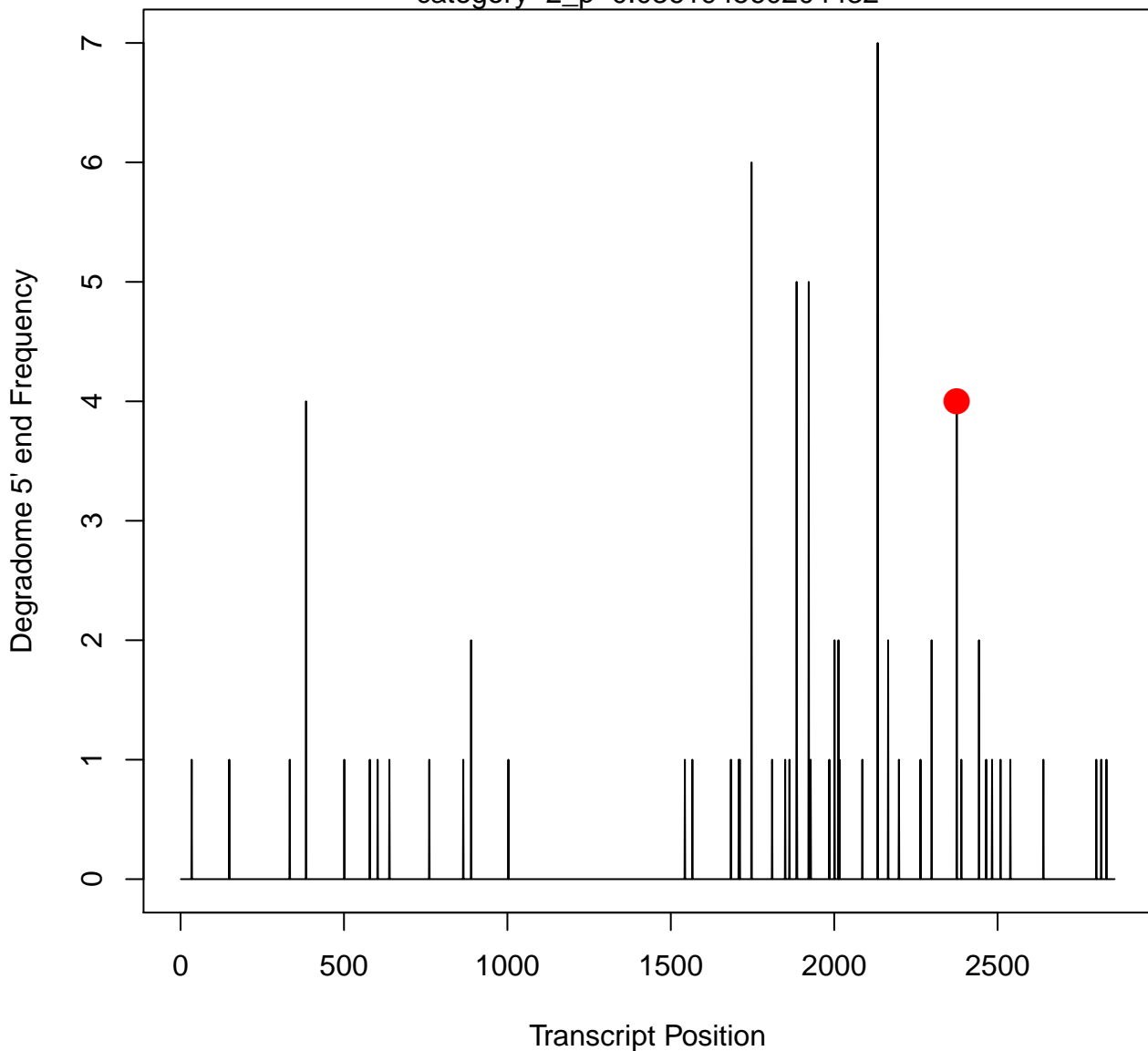

Supplement: Supplementary file 5 [file Data_Sheet_5.zip › Sit-miR156a_Seita.5G432500.1_2375_TPlot.pdf]

**T=Seita.8G124900.1\_Q=Sit-miR156a\_S=1295**

category=0\_p=0.0063183019093338

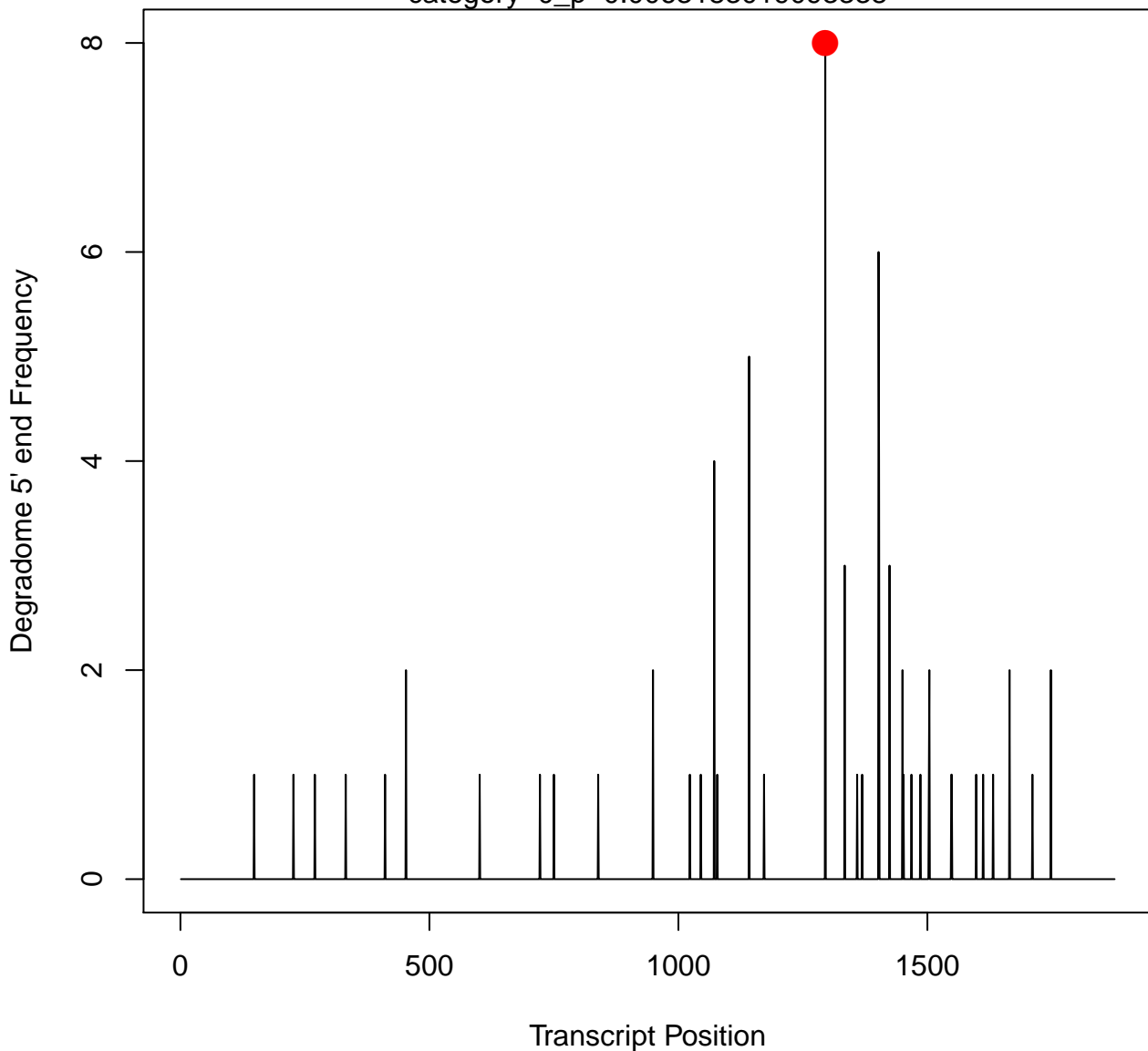

Supplement: Supplementary file 5 [file Data_Sheet_5.zip › Sit-miR156a_Seita.8G124900.1_1295_TPlot.pdf]

**T=Seita.6G223300.1\_Q=Sit-miR156b\_S=981**

category=0\_p=0.00168879820125578

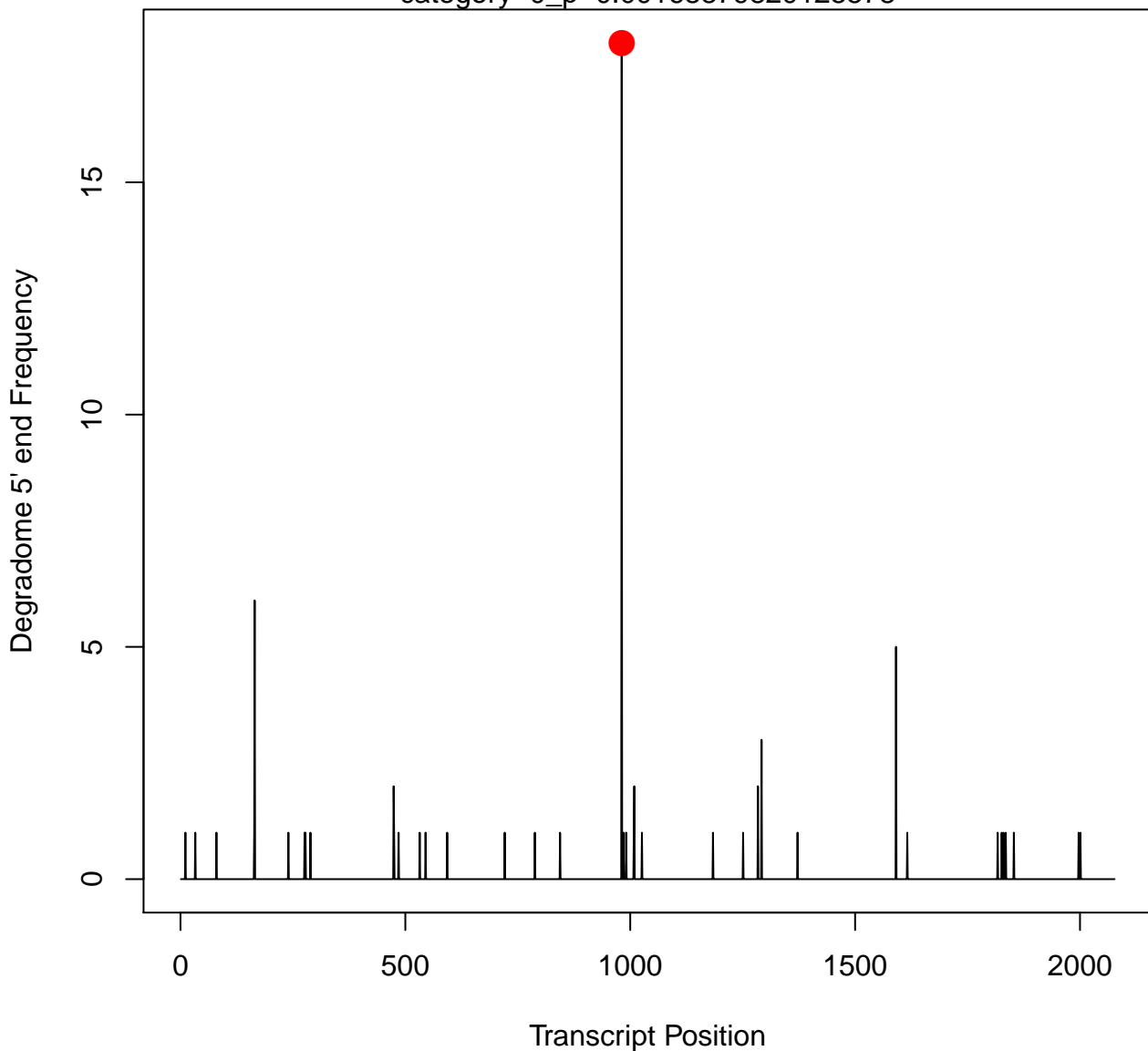

Supplement: Supplementary file 5 [file Data_Sheet_5.zip › Sit-miR156b_Seita.6G223300.1_981_TPlot.pdf]

**T=Seita.1G069300.1\_Q=Sit-miR156d\_S=874**

category=2\_p=0.0182640681957454

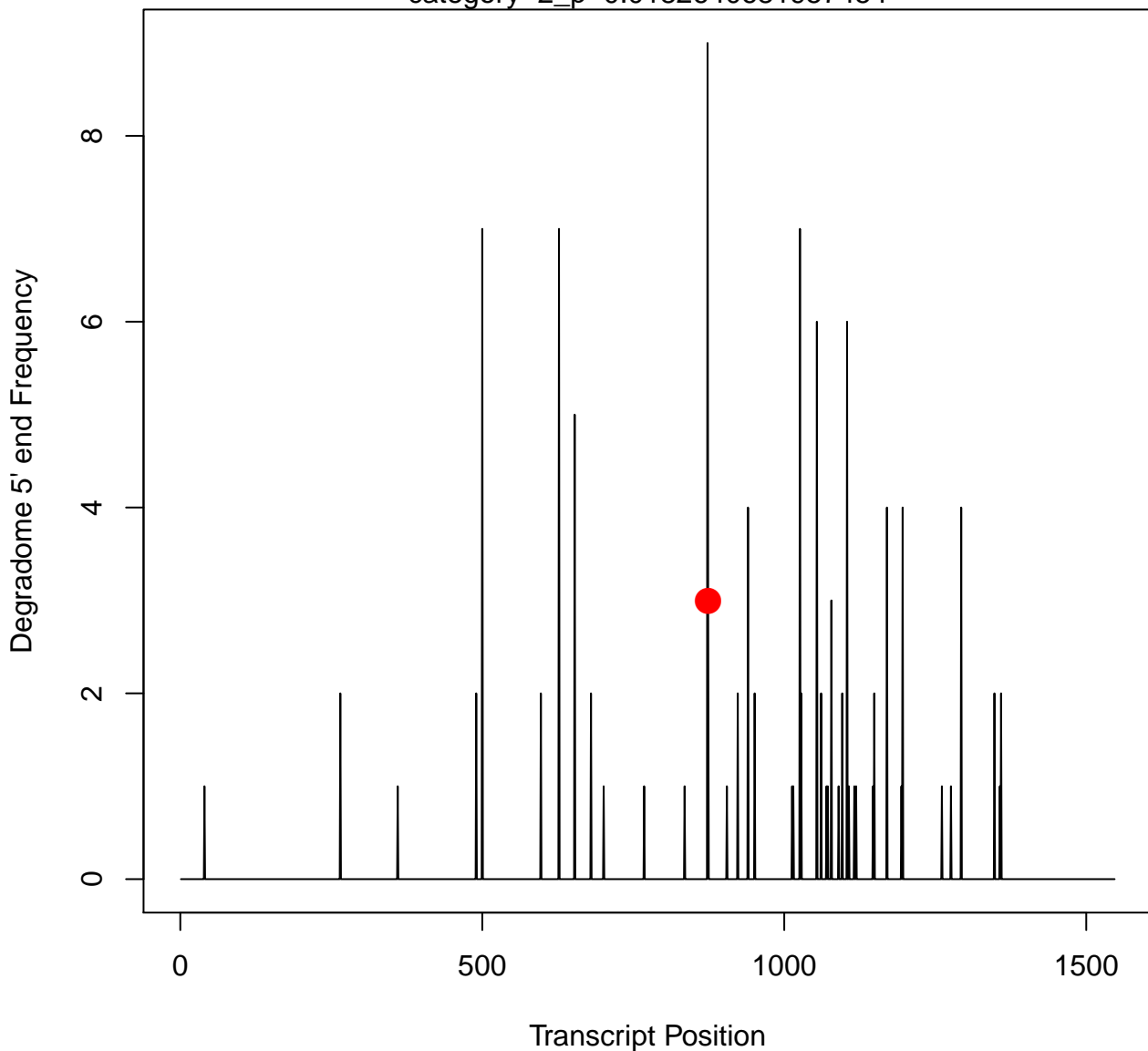

Supplement: Supplementary file 5 [file Data_Sheet_5.zip › Sit-miR156d_Seita.1G069300.1_874_TPlot.pdf]

**T=Seita.2G254300.1\_Q=Sit-miR156d\_S=1267**

category=0\_p=0.00168879820125578

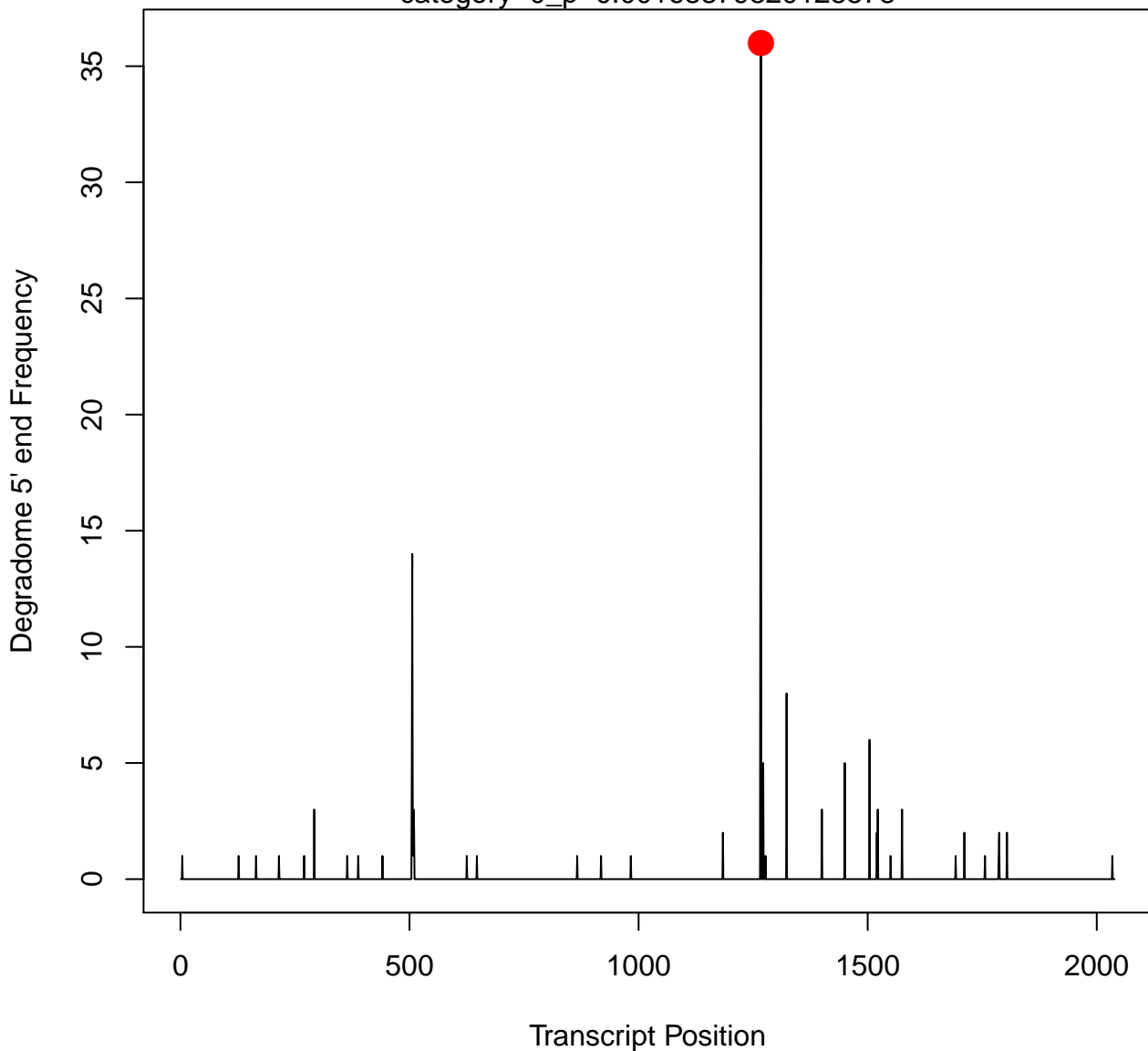

Supplement: Supplementary file 5 [file Data_Sheet_5.zip › Sit-miR156d_Seita.2G254300.1_1267_TPlot.pdf]

**T=Seita.1G069300.1\_Q=Sit-miR156g\_S=873**

category=0\_p=0.000422467192713993

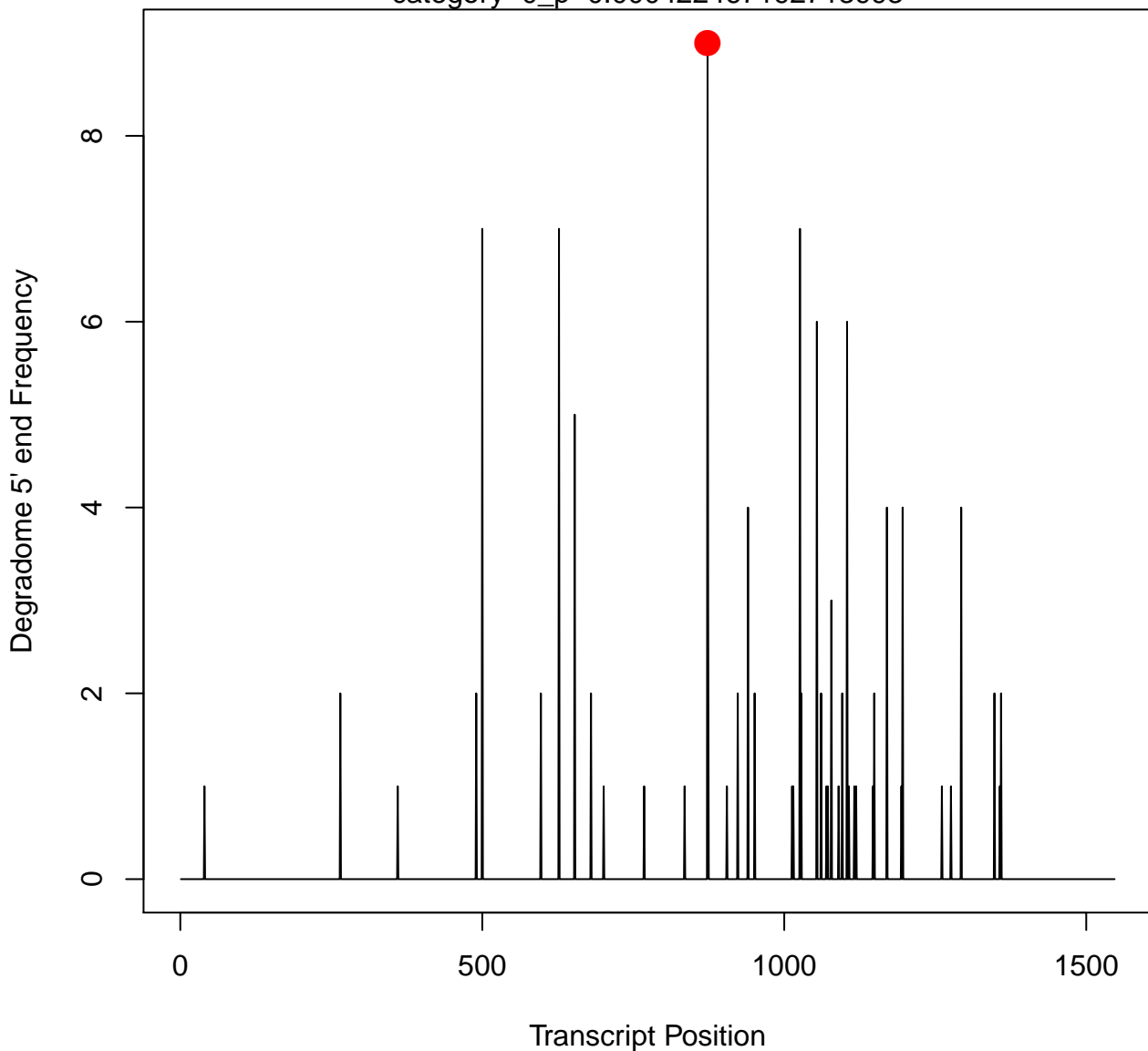

Supplement: Supplementary file 5 [file Data_Sheet_5.zip › Sit-miR156g_Seita.1G069300.1_873_TPlot.pdf]

**T=Seita.6G110400.1\_Q=Sit-miR156h\_S=845**

category=2\_p=0.701013230205742

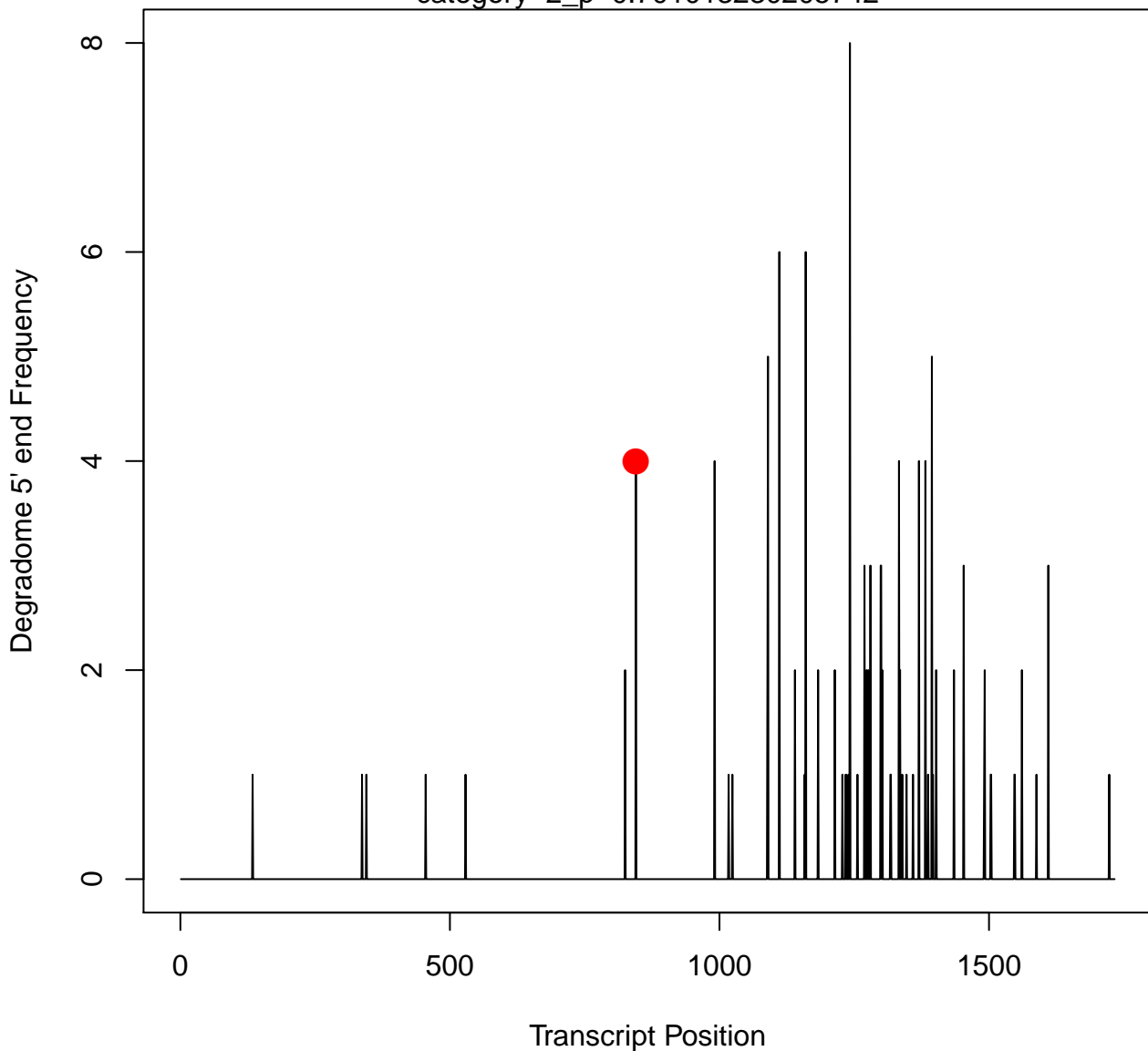

Supplement: Supplementary file 5 [file Data_Sheet_5.zip › Sit-miR156h_Seita.6G110400.1_845_TPlot.pdf]

**T=Seita.8G049200.1\_Q=Sit-miR156i\_S=568**

category=2\_p=0.82801420880993

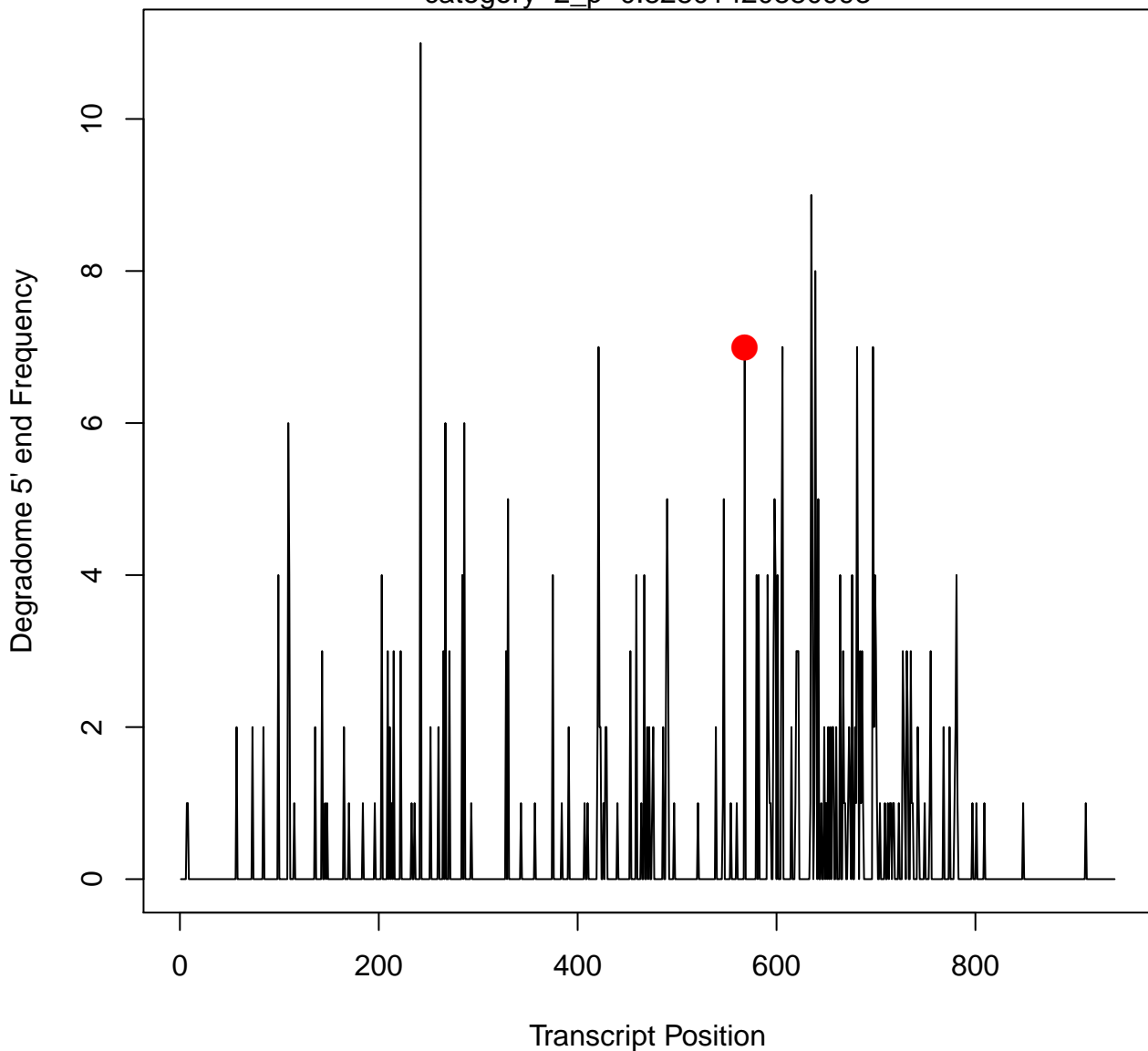

Supplement: Supplementary file 5 [file Data_Sheet_5.zip › Sit-miR156i_Seita.8G049200.1_568_TPlot.pdf]

**T=Seita.1G091900.1\_Q=Sit-miR156j\_S=1786**

category=2\_p=0.00917411630284182

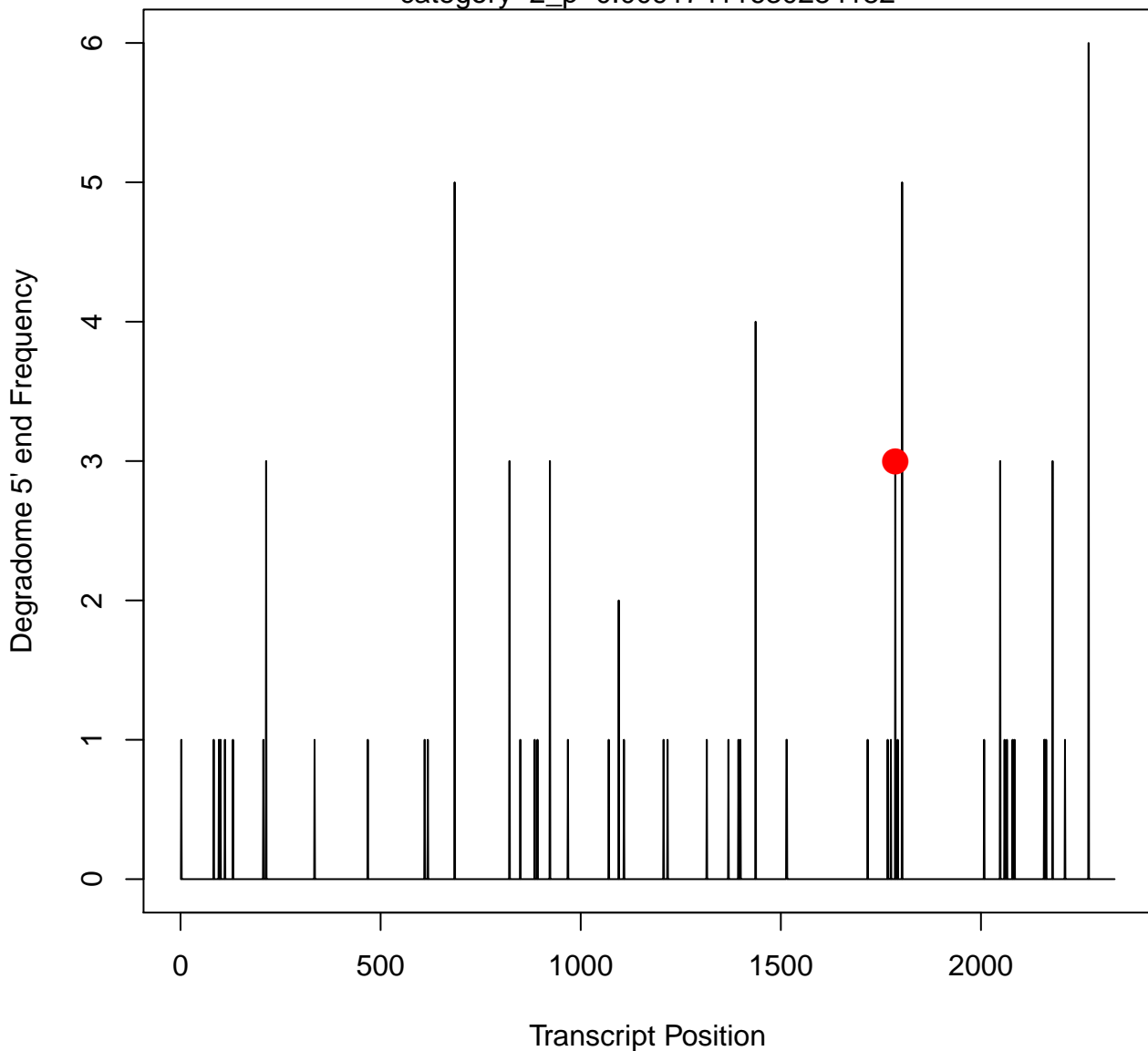

Supplement: Supplementary file 5 [file Data_Sheet_5.zip › Sit-miR156j_Seita.1G091900.1_1786_TPlot.pdf]

**T=Seita.2G324900.1\_Q=Sit-miR156j\_S=833**

category=0\_p=0.00421664943481093

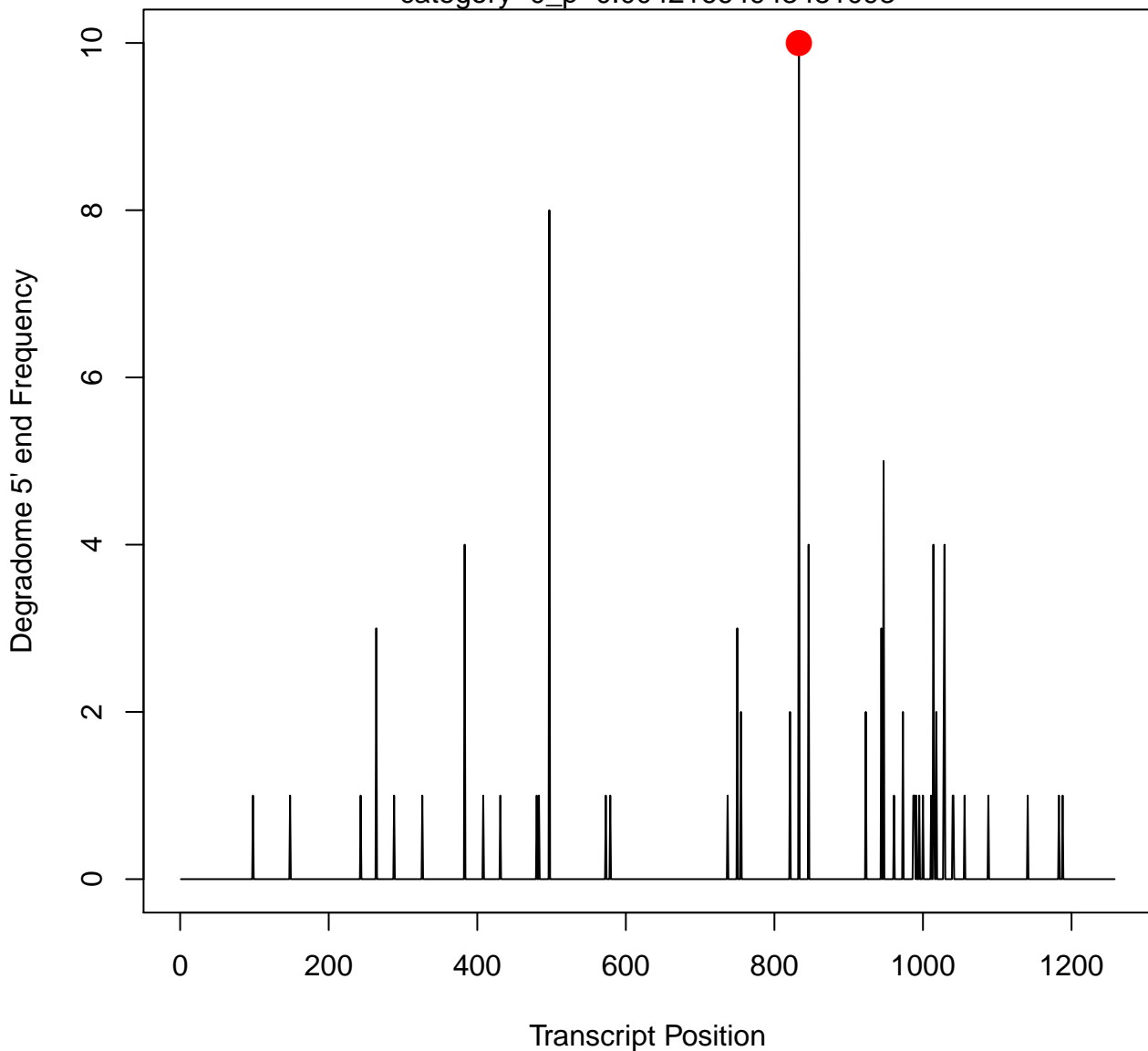

Supplement: Supplementary file 5 [file Data_Sheet_5.zip › Sit-miR156j_Seita.2G324900.1_833_TPlot.pdf]

**T=Seita.1G185100.1\_Q=Sit-miR159a\_S=1132**

category=2\_p=0.997205494170971

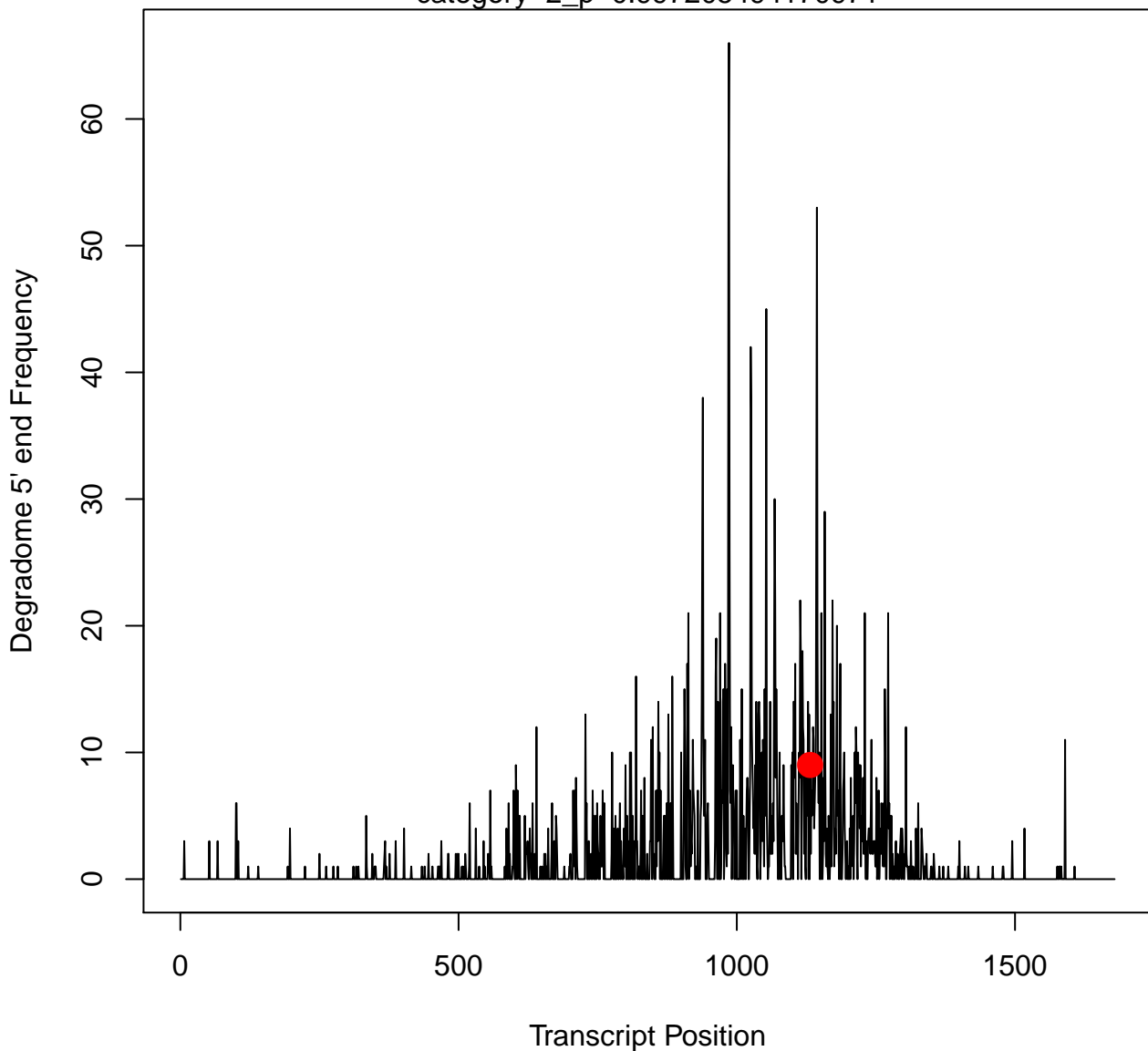

Supplement: Supplementary file 5 [file Data_Sheet_5.zip › Sit-miR159a_Seita.1G185100.1_1132_TPlot.pdf]

**T=Seita.1G230100.1\_Q=Sit-miR159a\_S=1456**

category=2\_p=0.814854218031806

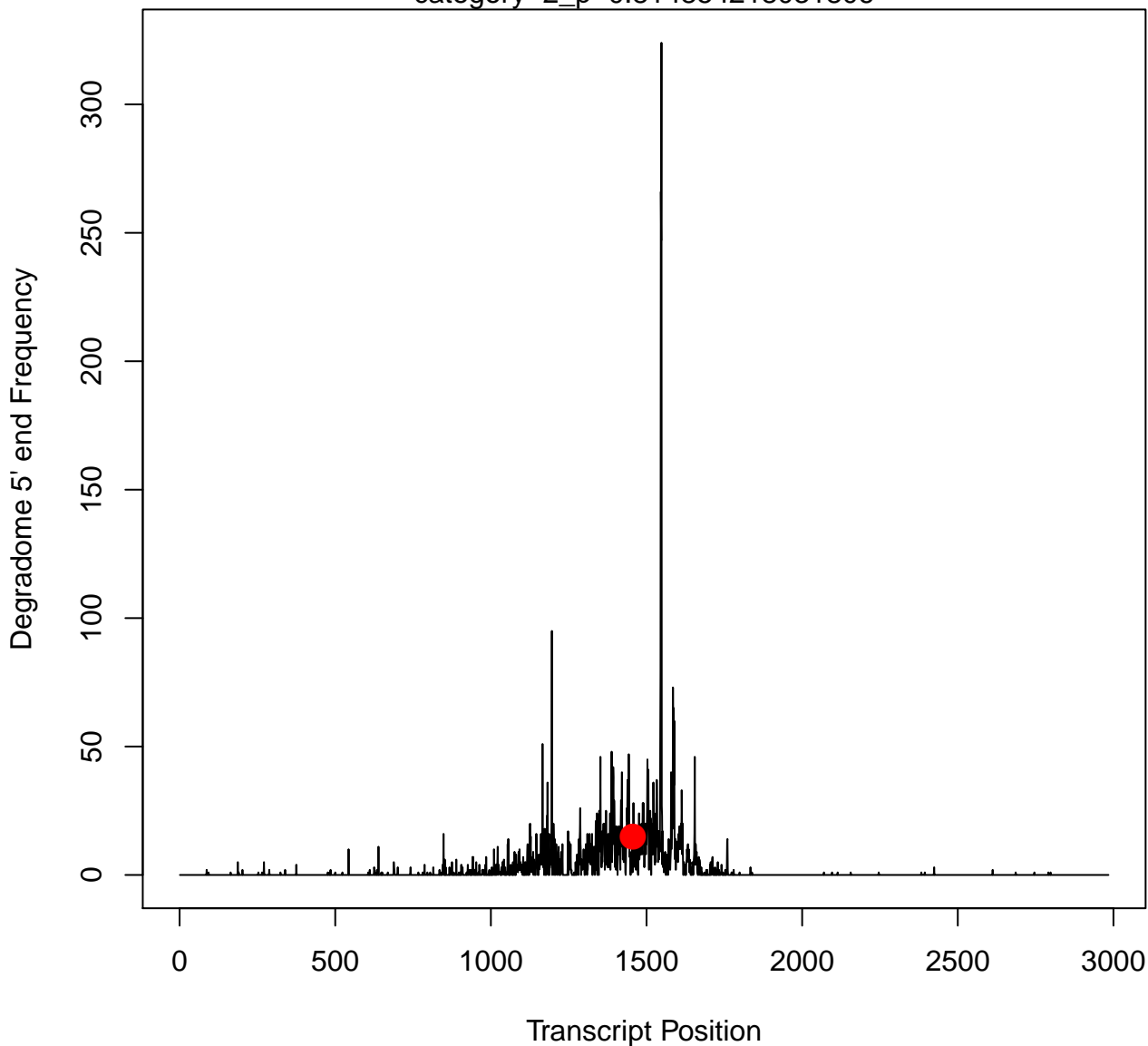

Supplement: Supplementary file 5 [file Data_Sheet_5.zip › Sit-miR159a_Seita.1G230100.1_1456_TPlot.pdf]

**T=Seita.2G319000.1\_Q=Sit-miR159a\_S=794**

category=2\_p=0.885349573814913

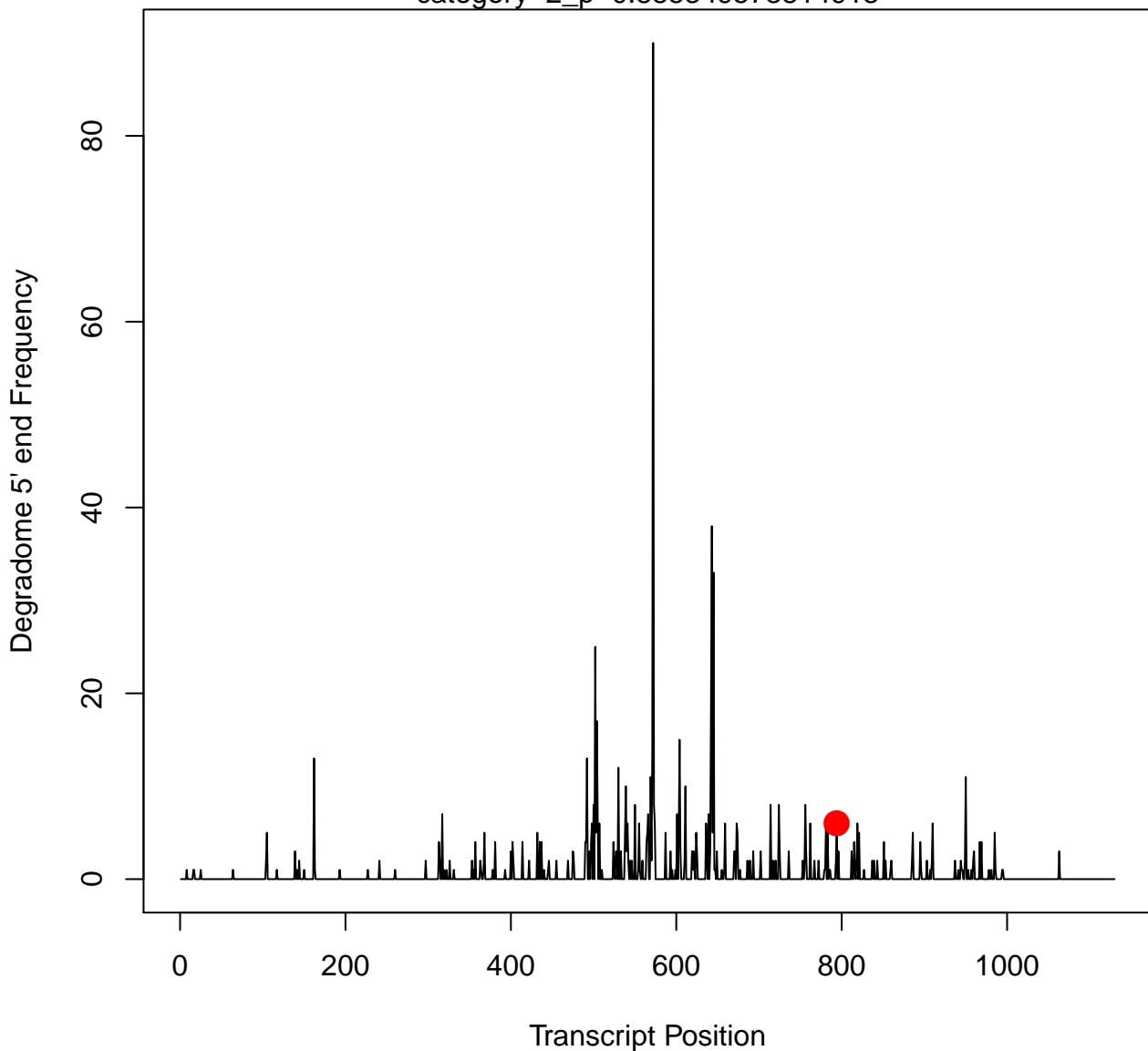

Supplement: Supplementary file 5 [file Data_Sheet_5.zip › Sit-miR159a_Seita.2G319000.1_794_TPlot.pdf]

**T=Seita.3G069700.1\_Q=Sit-miR159a\_S=4218**

category=2\_p=0.99994590365184

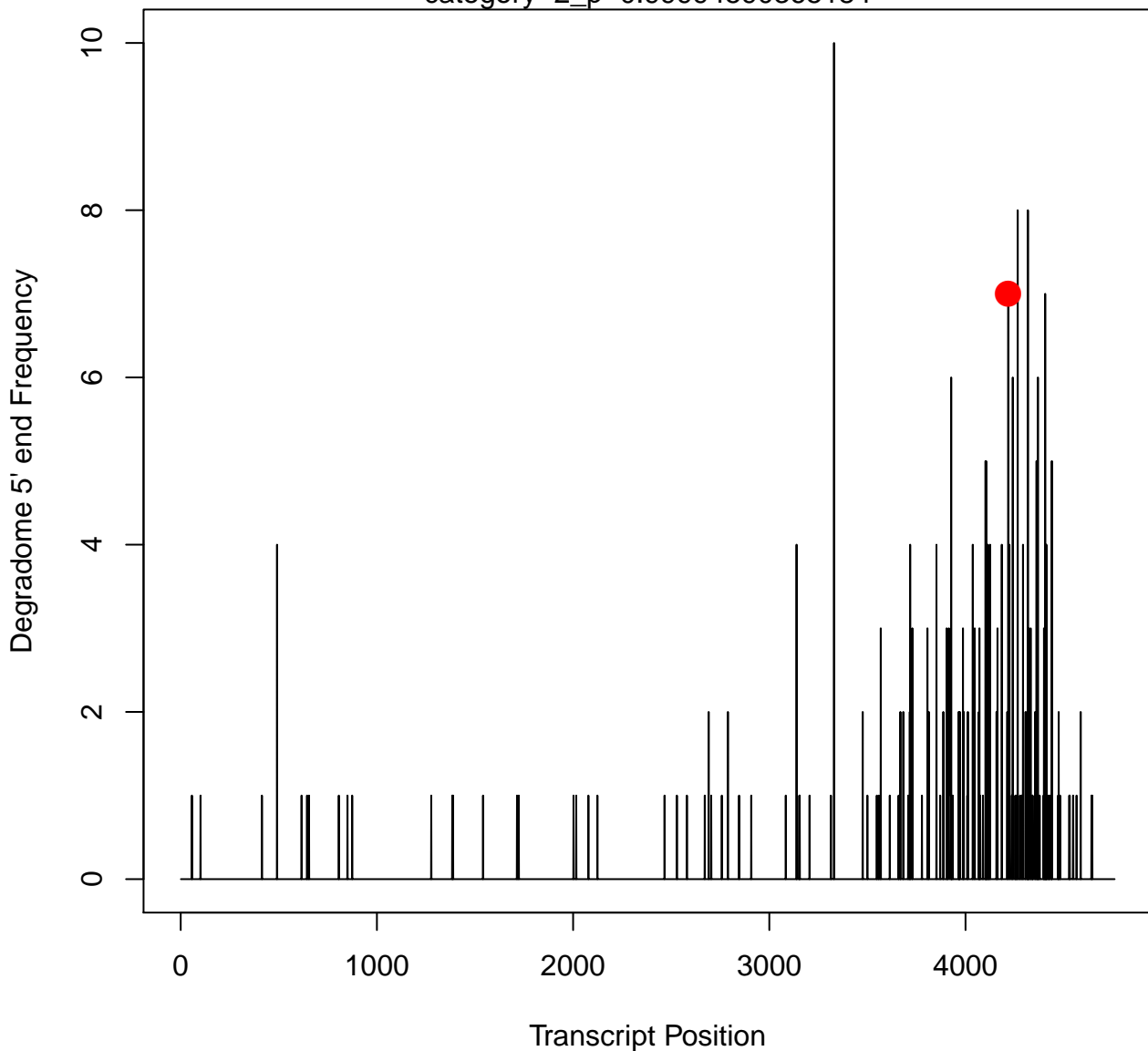

Supplement: Supplementary file 5 [file Data_Sheet_5.zip › Sit-miR159a_Seita.3G069700.1_4218_TPlot.pdf]

**T=Seita.4G020800.1\_Q=Sit-miR159a\_S=284**

category=2\_p=0.999972395873511

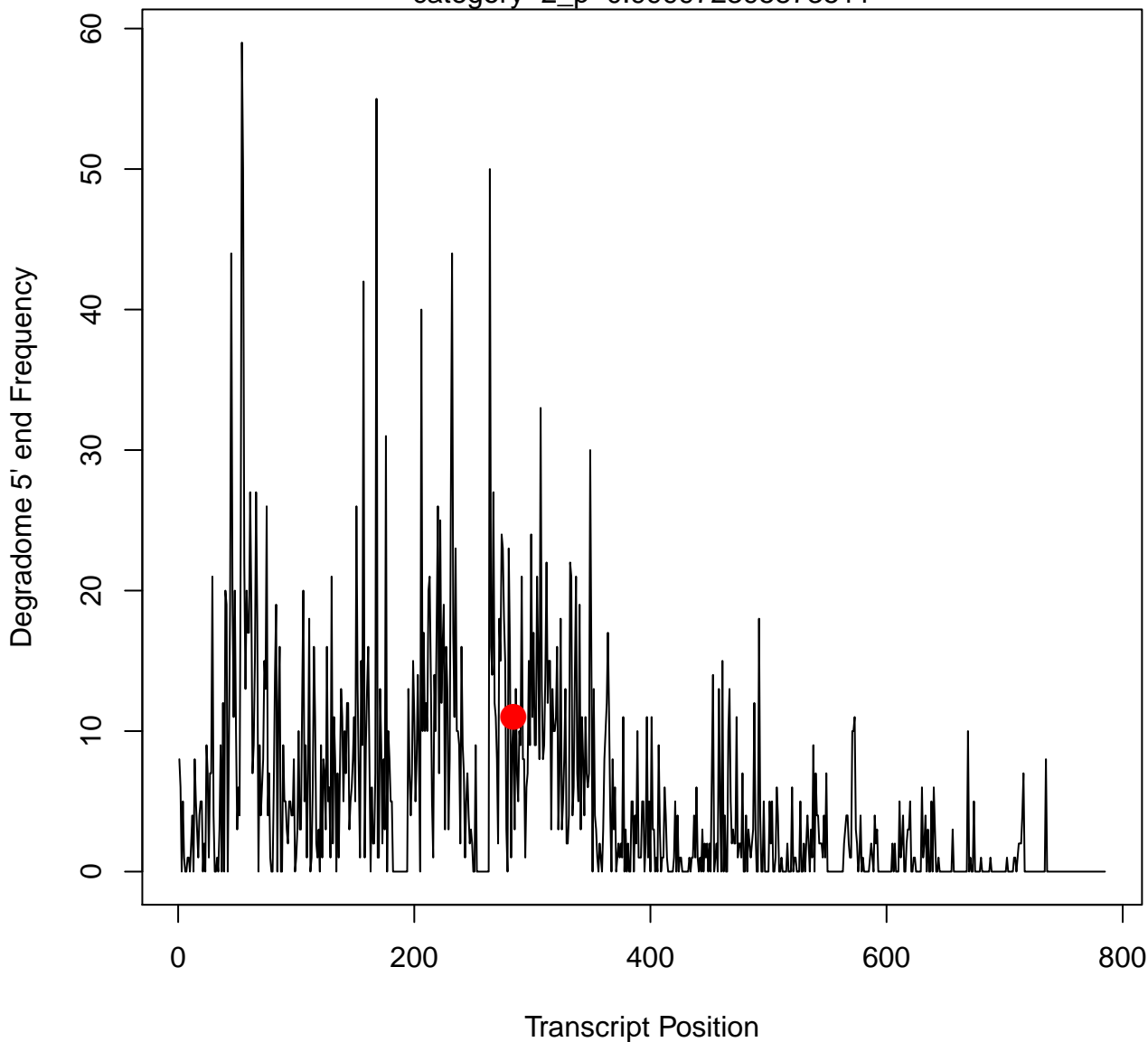

Supplement: Supplementary file 5 [file Data_Sheet_5.zip › Sit-miR159a_Seita.4G020800.1_284_TPlot.pdf]

**T=Seita.5G143900.1\_Q=Sit-miR159a\_S=912**

category=2\_p=0.999976830133509

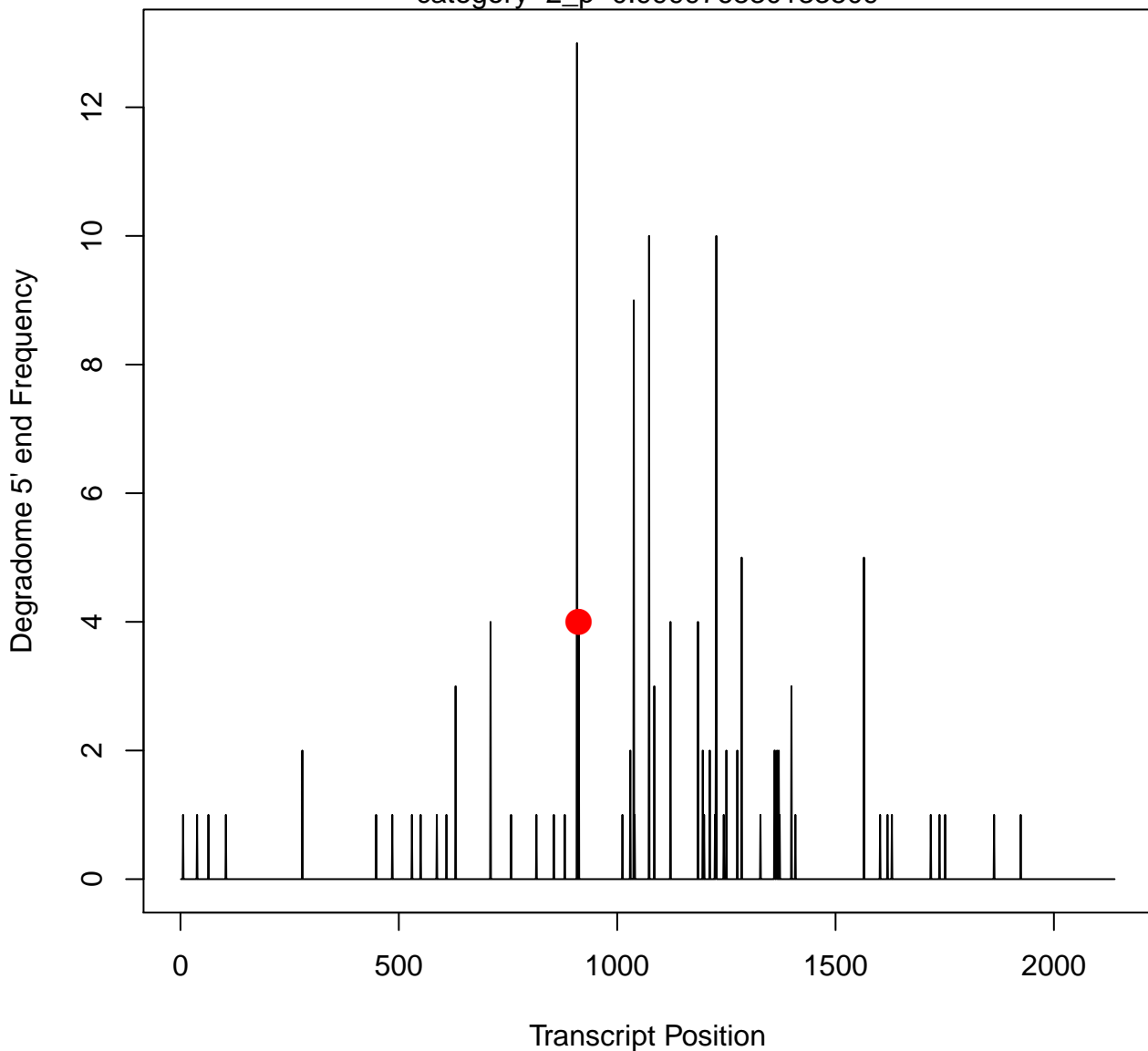

Supplement: Supplementary file 5 [file Data_Sheet_5.zip › Sit-miR159a_Seita.5G143900.1_912_TPlot.pdf]

**T=Seita.5G210200.1\_Q=Sit-miR159a\_S=1203**

category=2\_p=0.999553546394304

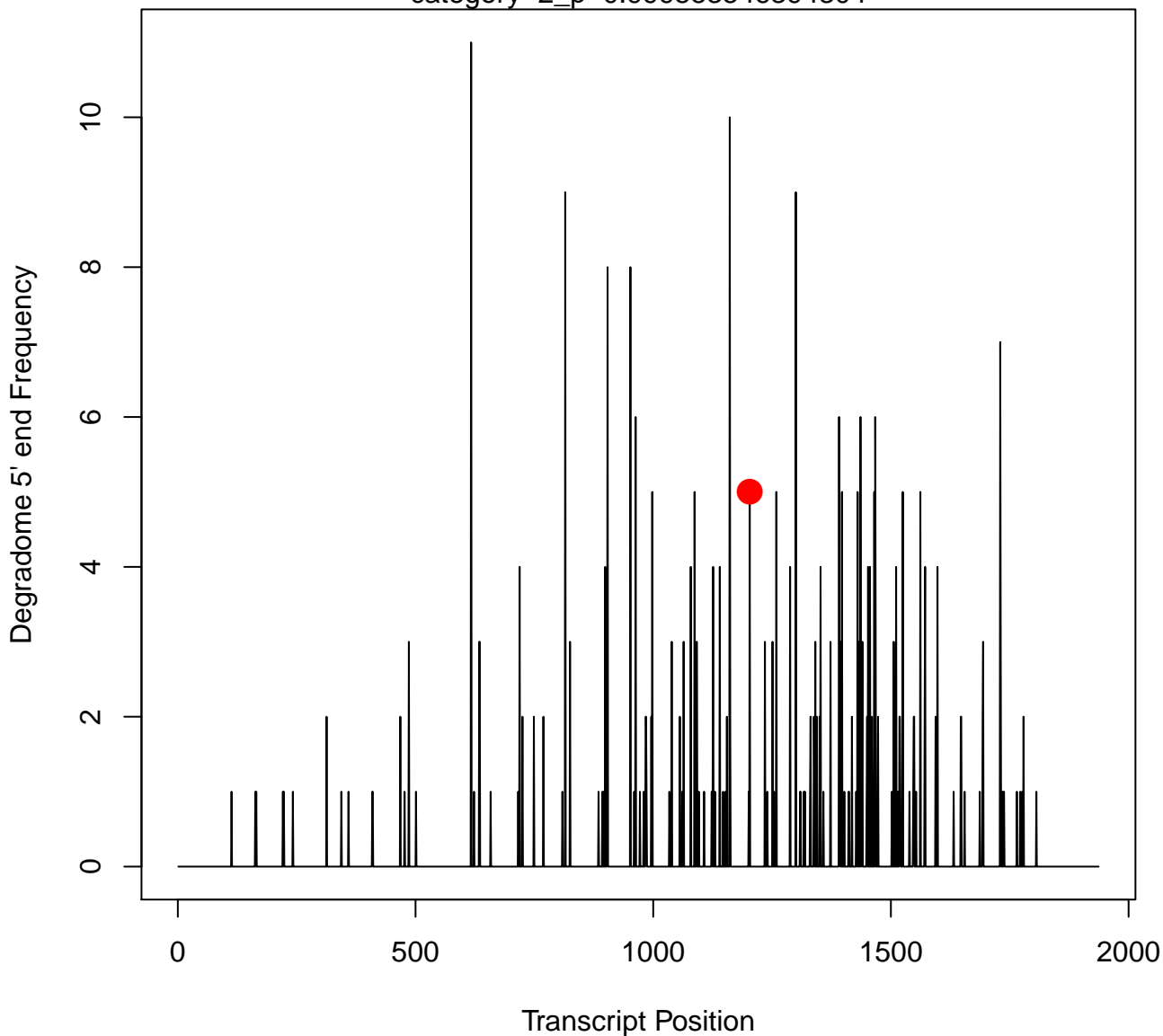

Supplement: Supplementary file 5 [file Data_Sheet_5.zip › Sit-miR159a_Seita.5G210200.1_1203_TPlot.pdf]

**T=Seita.9G098200.1\_Q=Sit-miR159a\_S=453**

category=2\_p=0.99990936066215

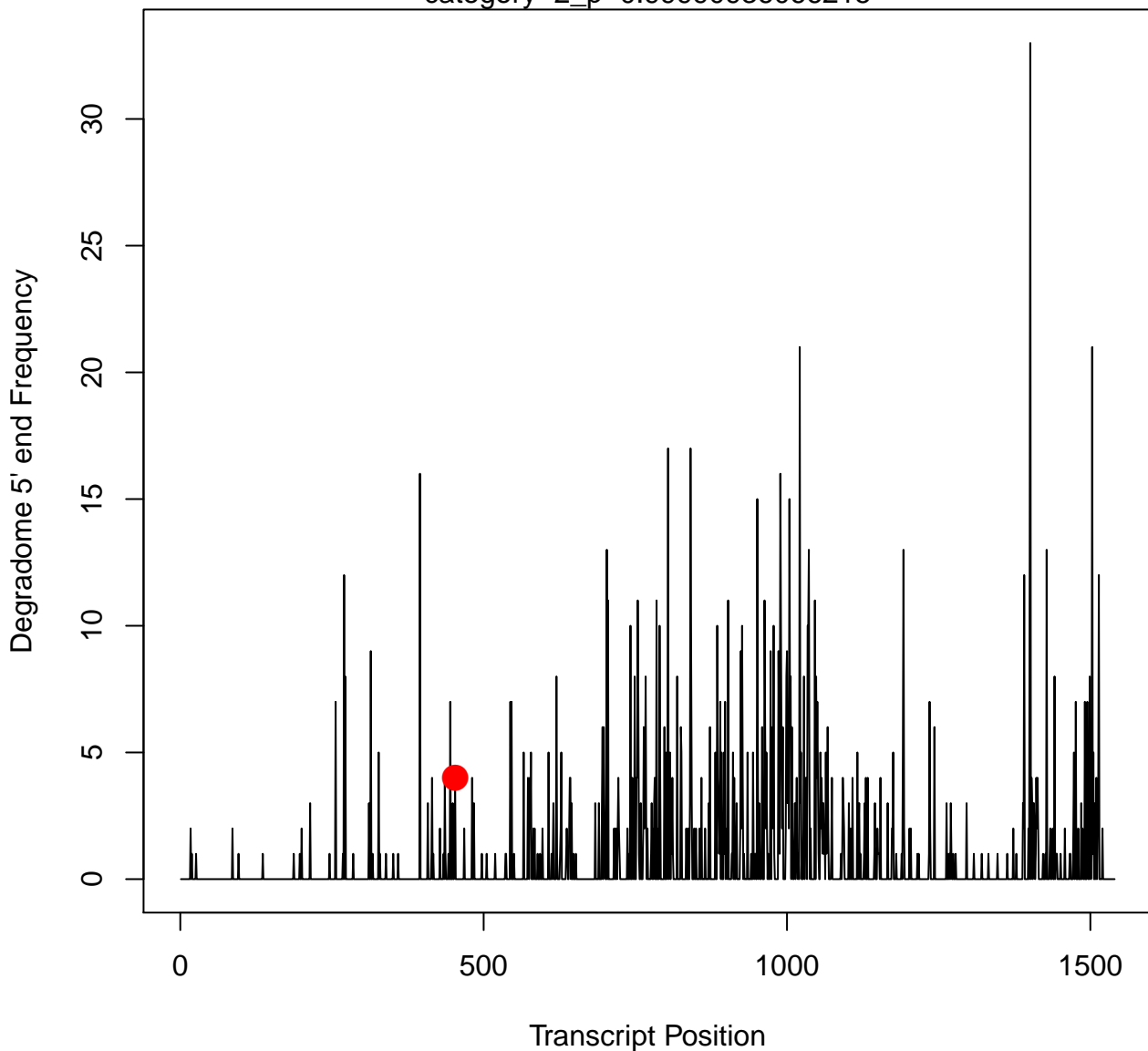

Supplement: Supplementary file 5 [file Data_Sheet_5.zip › Sit-miR159a_Seita.9G098200.1_453_TPlot.pdf]

**T=Seita.2G184400.1\_Q=Sit-miR159b\_S=171**

category=2\_p=0.480229803325949

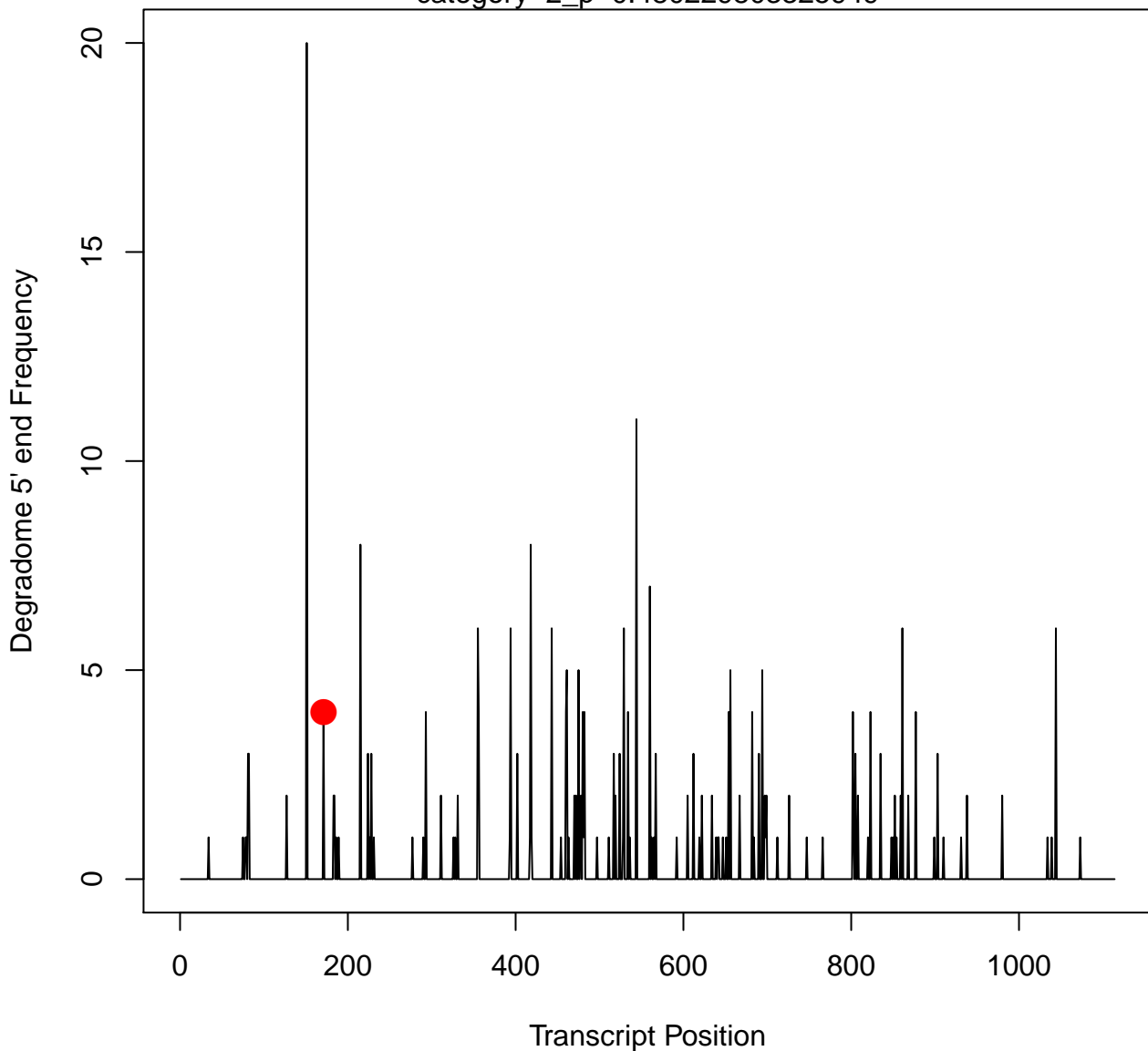

Supplement: Supplementary file 5 [file Data_Sheet_5.zip › Sit-miR159b_Seita.2G184400.1_171_TPlot.pdf]

**T=Seita.5G355300.1\_Q=Sit-miR159b\_S=1278**

category=2\_p=0.036194560204432

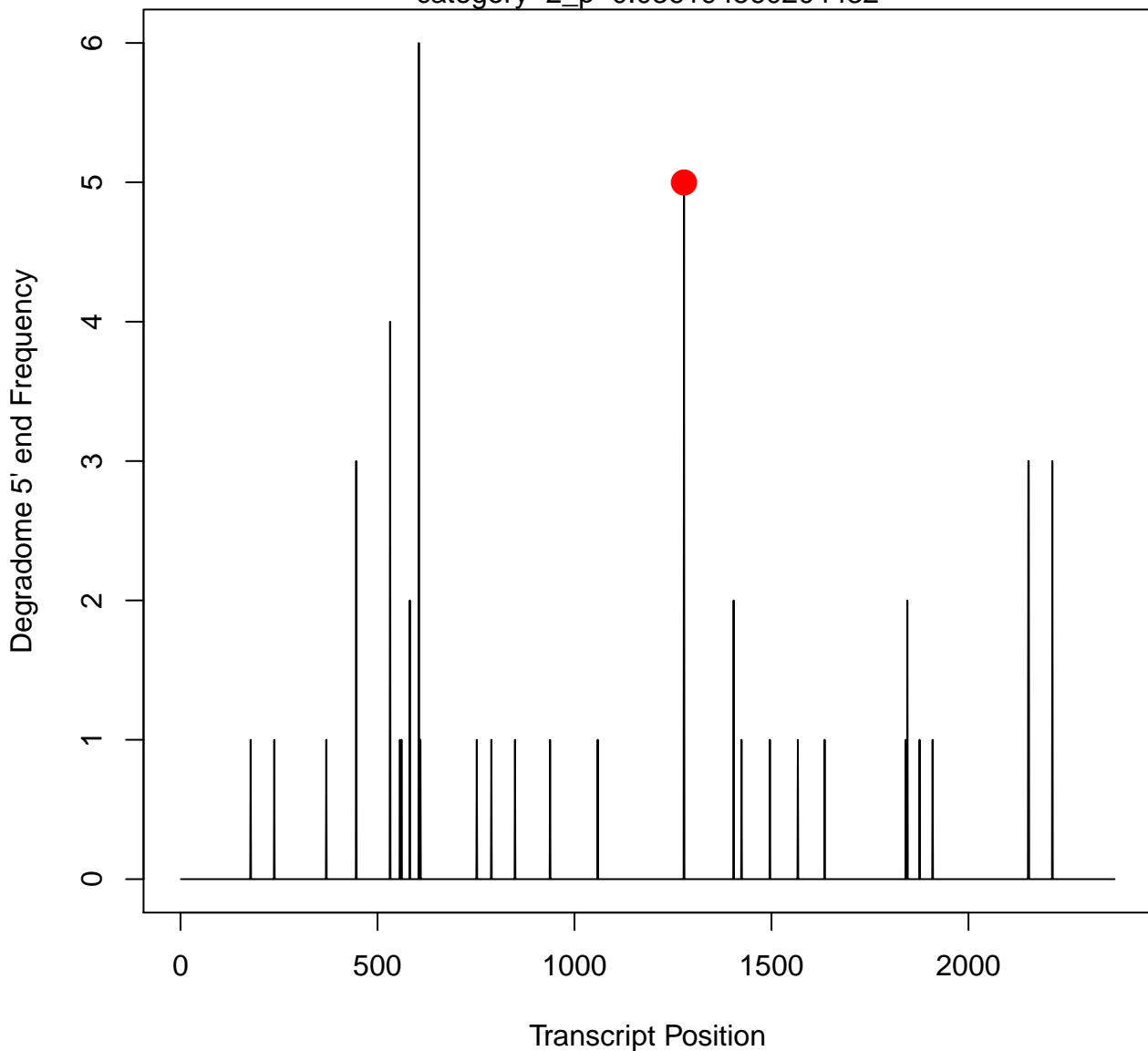

Supplement: Supplementary file 5 [file Data_Sheet_5.zip › Sit-miR159b_Seita.5G355300.1_1278_TPlot.pdf]

**T=Seita.9G313100.1\_Q=Sit-miR159b\_S=4836**

category=2\_p=0.392065853264176

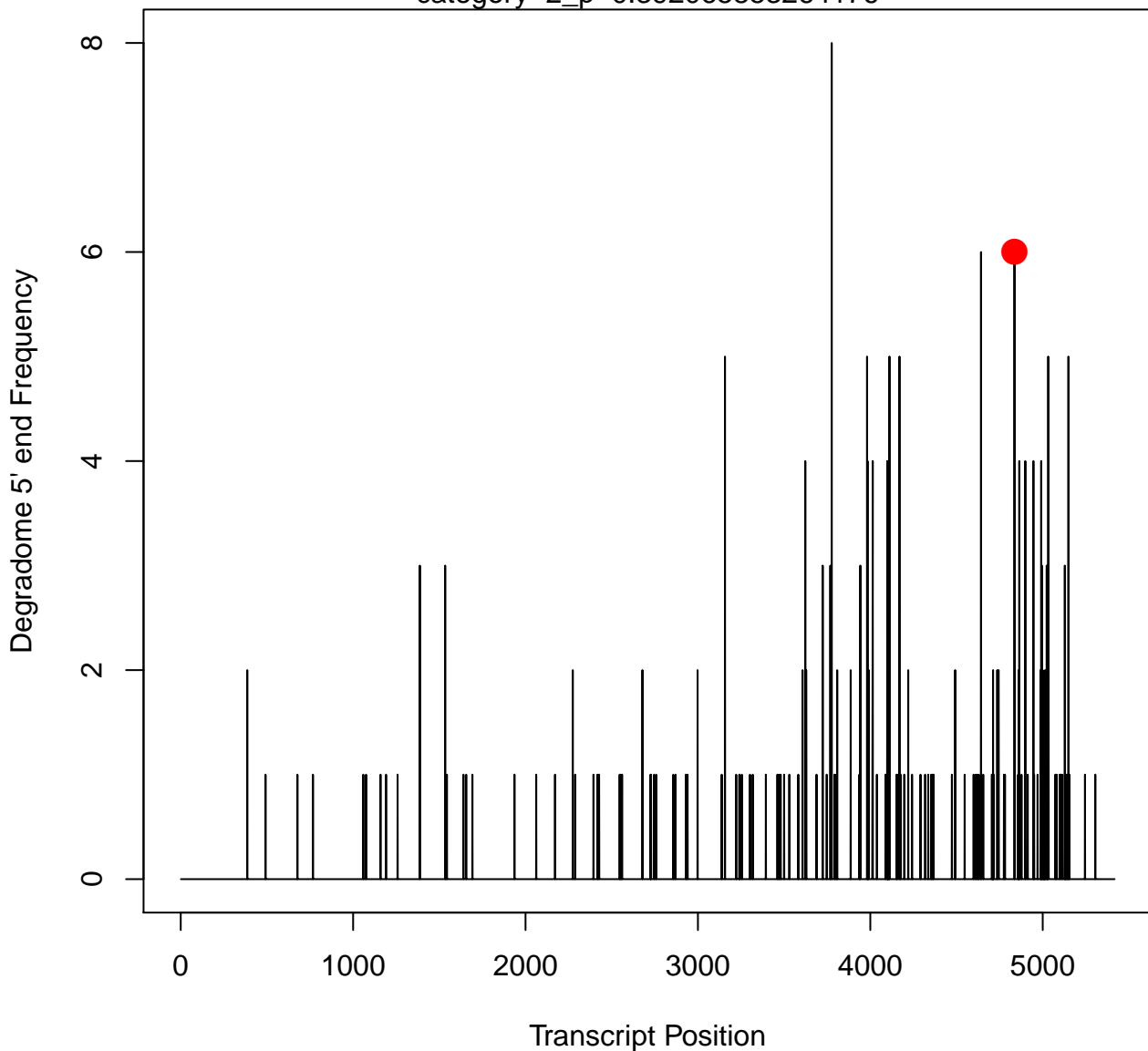

Supplement: Supplementary file 5 [file Data_Sheet_5.zip › Sit-miR159b_Seita.9G313100.1_4836_TPlot.pdf]

**T=Seita.9G409000.1\_Q=Sit-miR159b\_S=1344**

category=2\_p=0.966961751321866

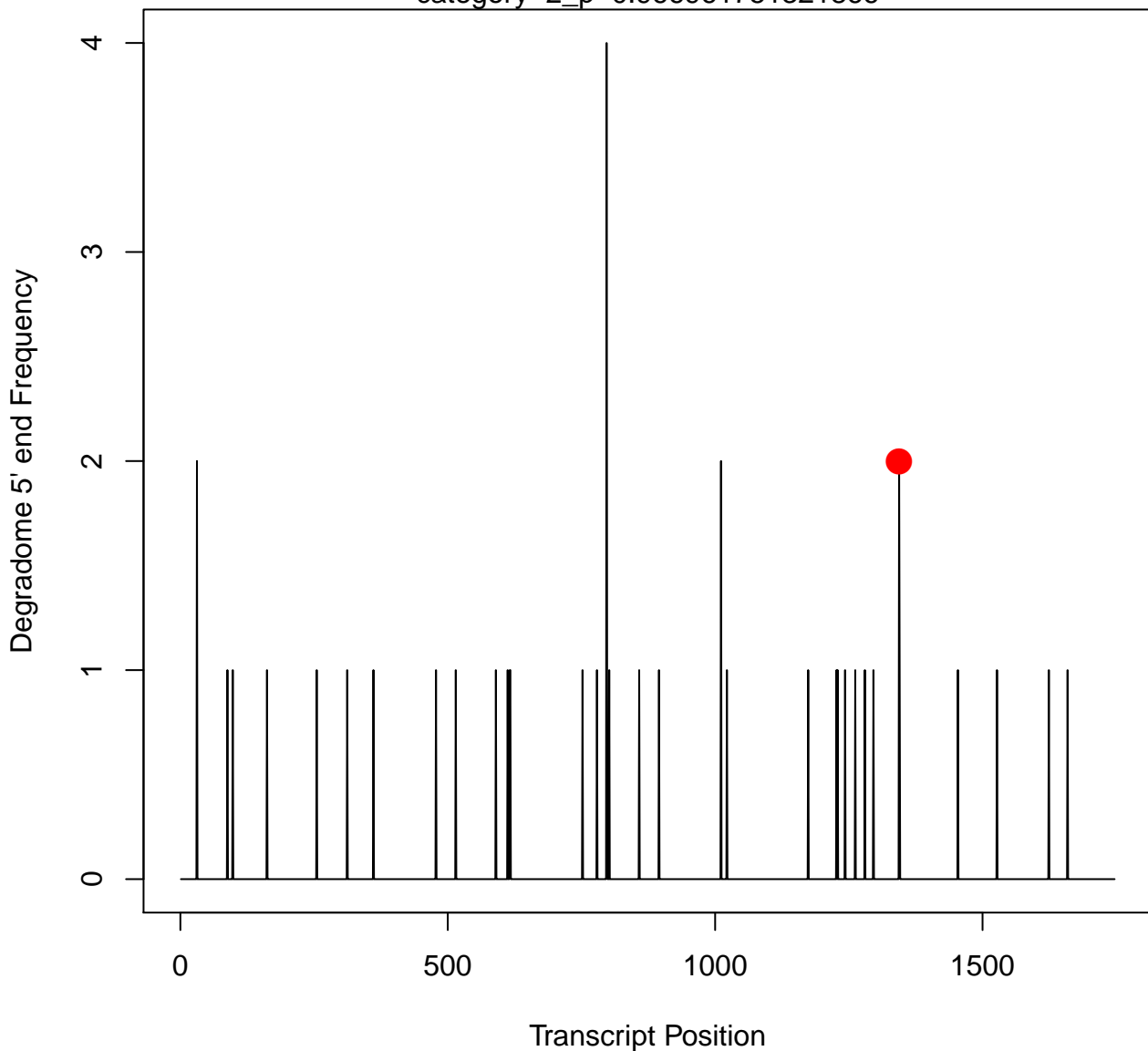

Supplement: Supplementary file 5 [file Data_Sheet_5.zip › Sit-miR159b_Seita.9G409000.1_1344_TPlot.pdf]

**T=Seita.6G163700.1\_Q=Sit-miR159c\_S=854**

category=2\_p=0.966347112693109

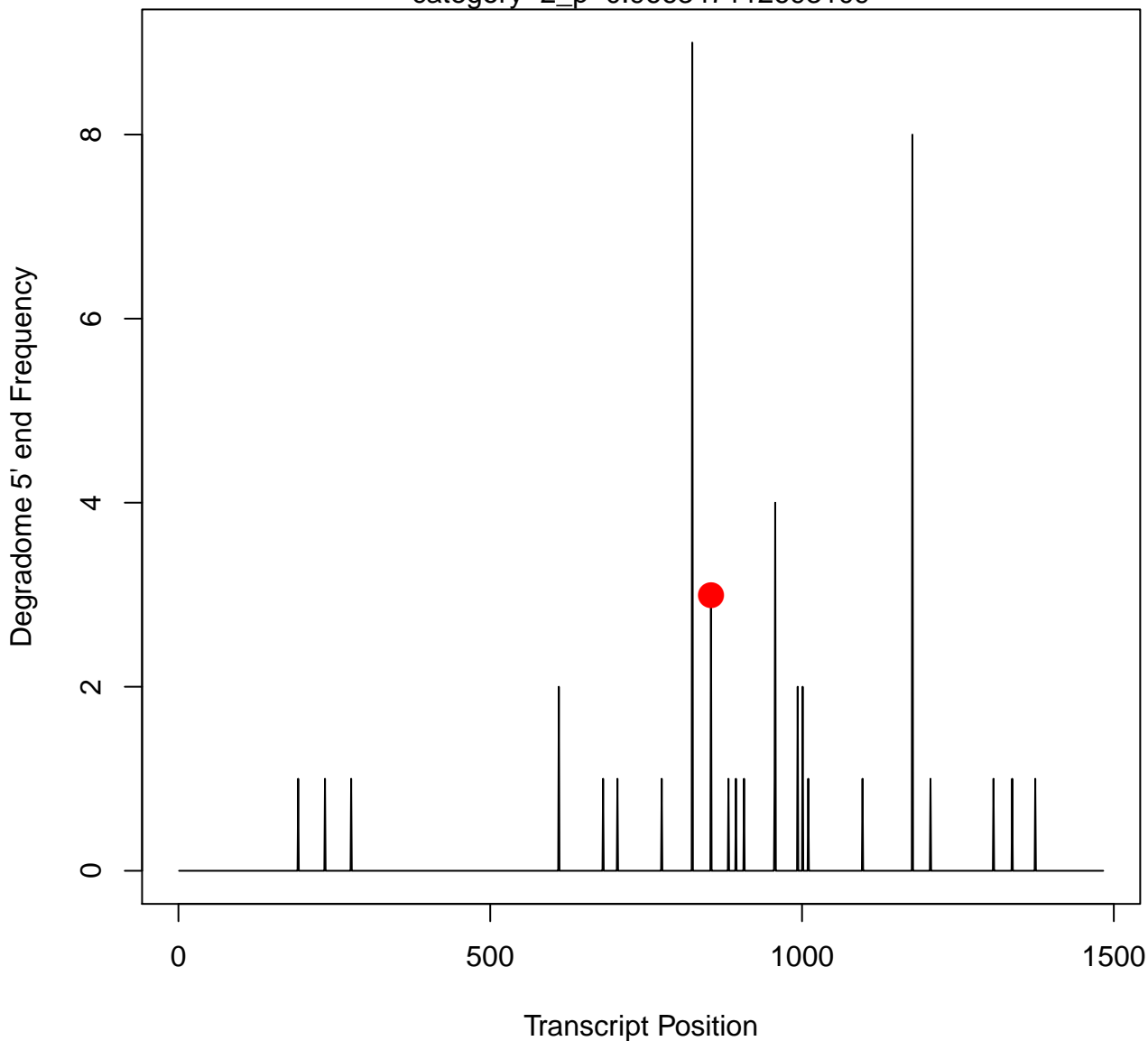

Supplement: Supplementary file 5 [file Data_Sheet_5.zip › Sit-miR159c_Seita.6G163700.1_854_TPlot.pdf]

**T=Seita.9G064600.1\_Q=Sit-miR159c\_S=1006**

category=2\_p=0.525992943969583

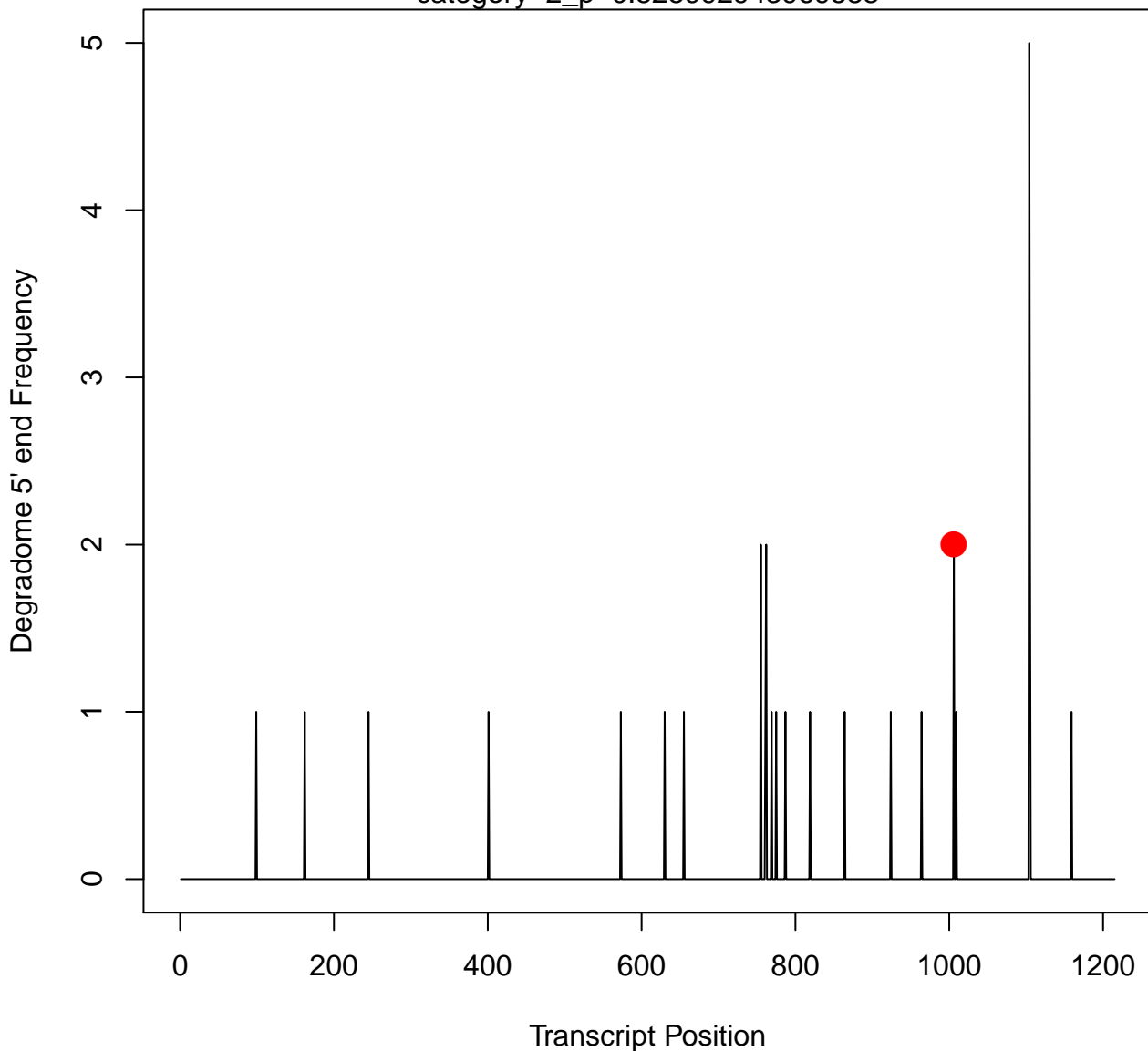

Supplement: Supplementary file 5 [file Data_Sheet_5.zip › Sit-miR159c_Seita.9G064600.1_1006_TPlot.pdf]

**T=Seita.1G241500.1\_Q=Sit-miR160a\_S=1660**

category=2\_p=0.0450366234024282

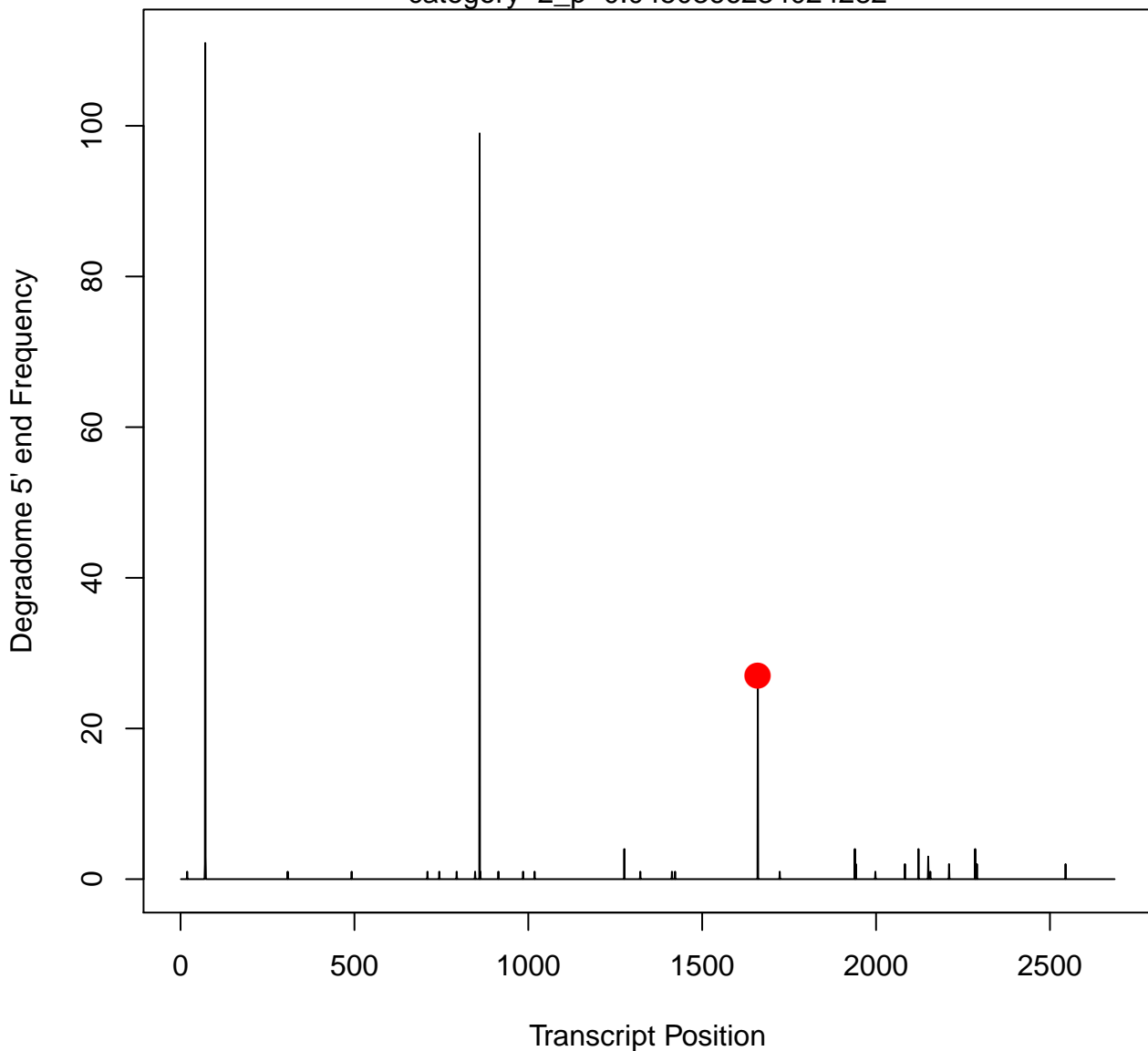

Supplement: Supplementary file 5 [file Data_Sheet_5.zip › Sit-miR160a_Seita.1G241500.1_1660_TPlot.pdf]

**T=Seita.2G058900.1\_Q=Sit-miR160a\_S=570**

category=2\_p=0.998687536695402

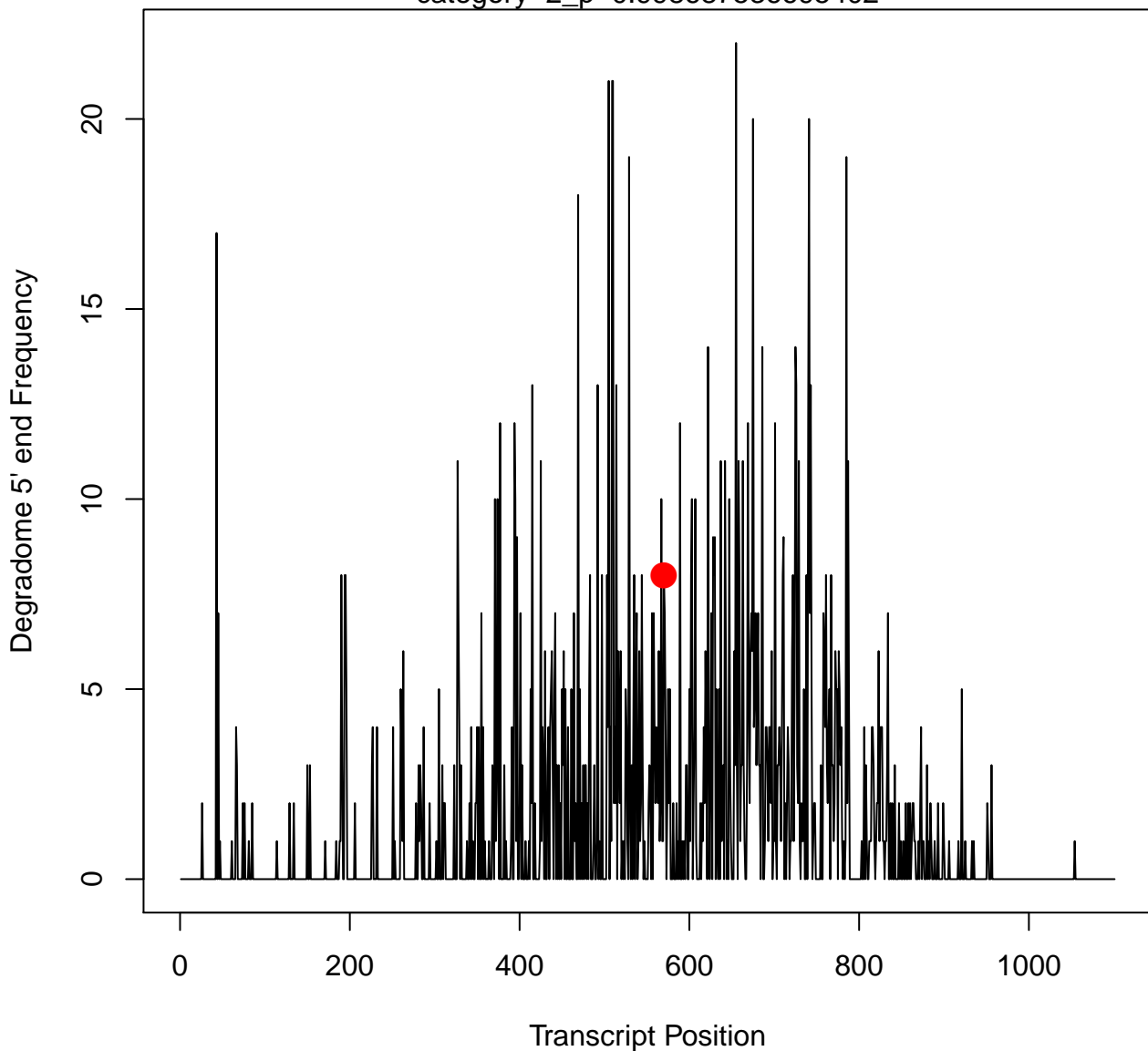

Supplement: Supplementary file 5 [file Data_Sheet_5.zip › Sit-miR160a_Seita.2G058900.1_570_TPlot.pdf]

**T=Seita.3G010200.1\_Q=Sit-miR160a\_S=1143**

category=2\_p=0.998049250931522

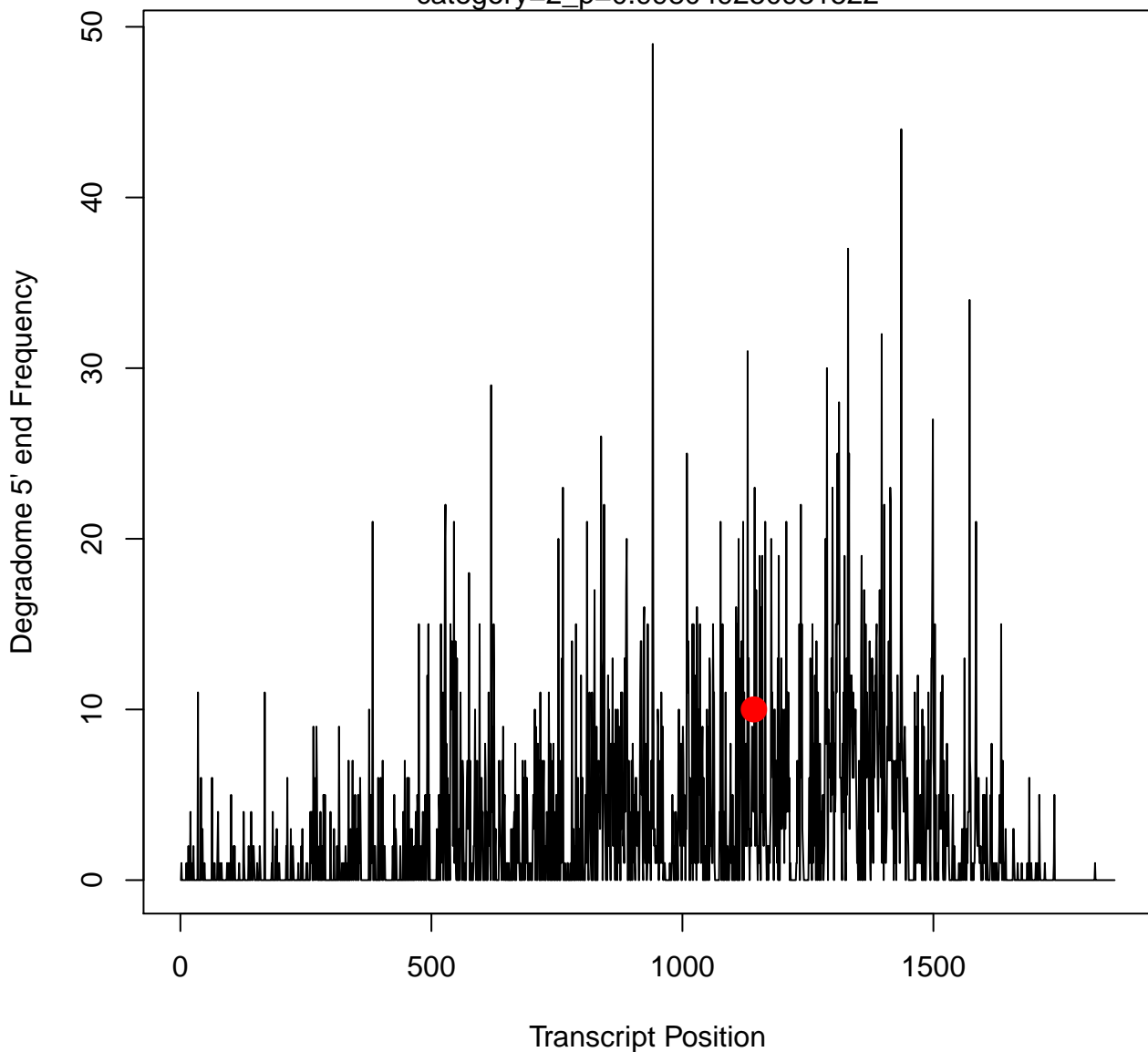

Supplement: Supplementary file 5 [file Data_Sheet_5.zip › Sit-miR160a_Seita.3G010200.1_1143_TPlot.pdf]

**T=Seita.4G090500.1\_Q=Sit-miR160a\_S=945**

category=2\_p=0.999993797830628

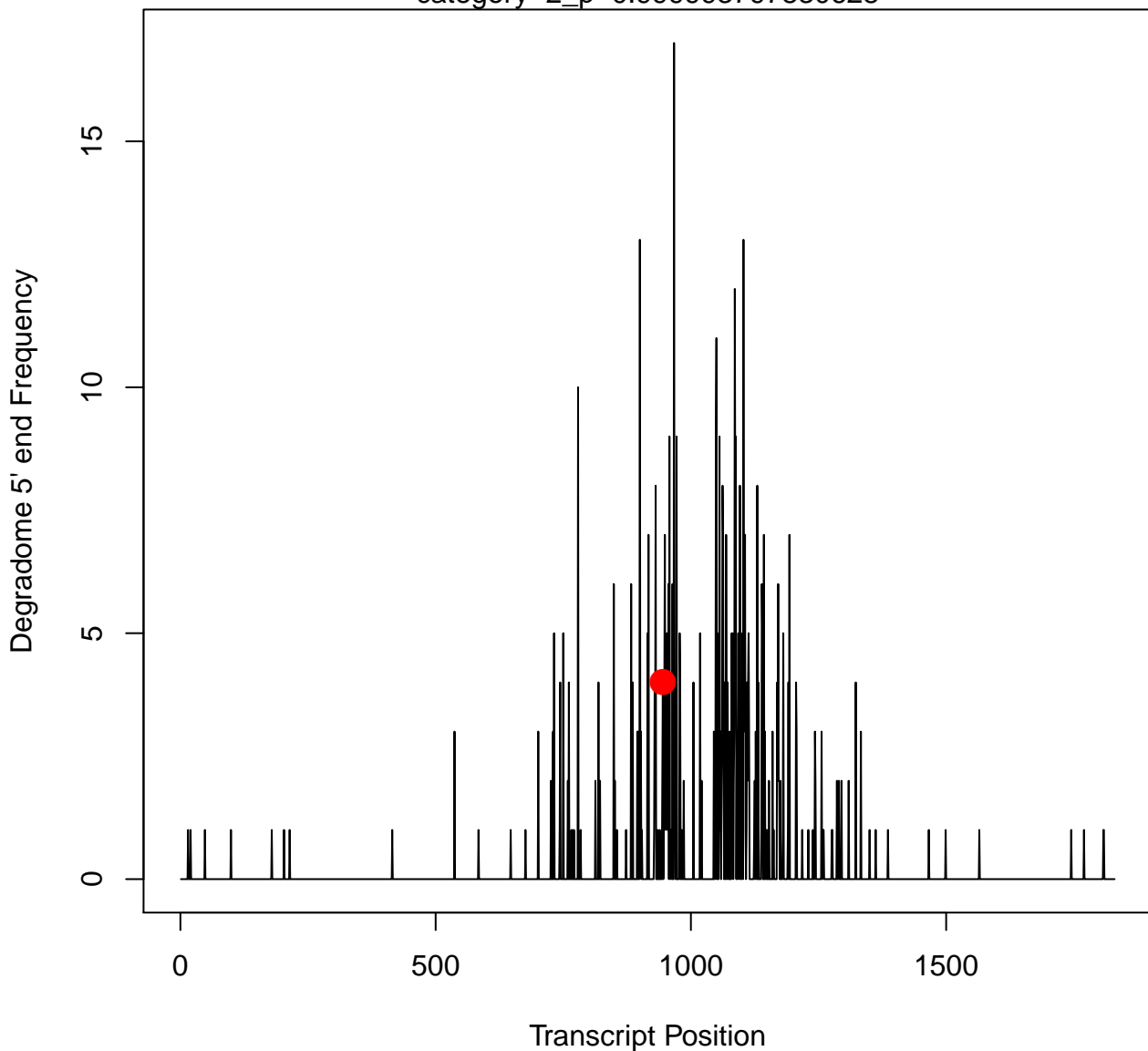

Supplement: Supplementary file 5 [file Data_Sheet_5.zip › Sit-miR160a_Seita.4G090500.1_945_TPlot.pdf]

**T=Seita.4G257800.1\_Q=Sit-miR160a\_S=1974**

category=0\_p=0.000422467192713993

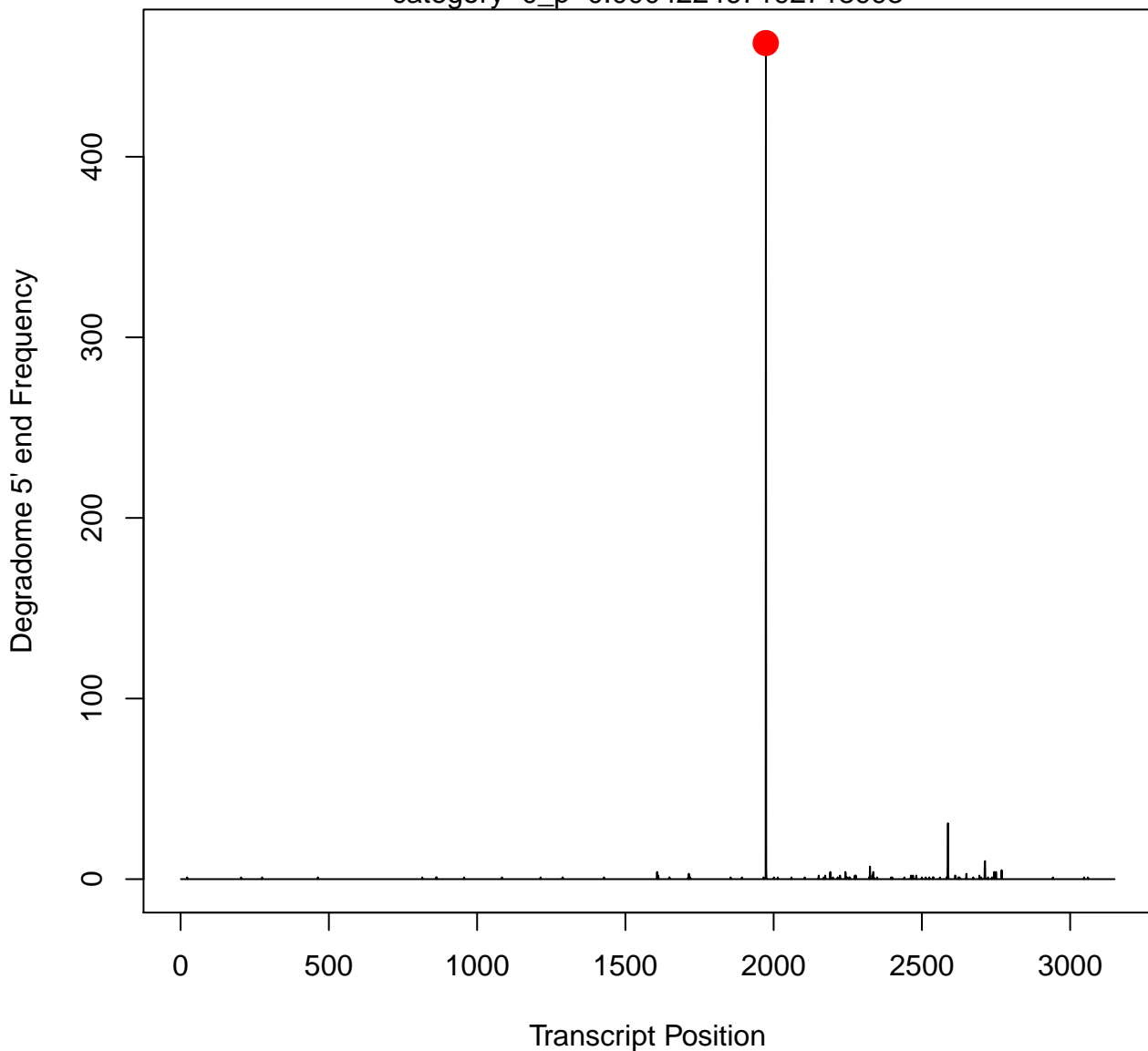

Supplement: Supplementary file 5 [file Data_Sheet_5.zip › Sit-miR160a_Seita.4G257800.1_1974_TPlot.pdf]

**T=Seita.5G273800.1\_Q=Sit-miR160a\_S=477**

category=2\_p=0.999975737429247

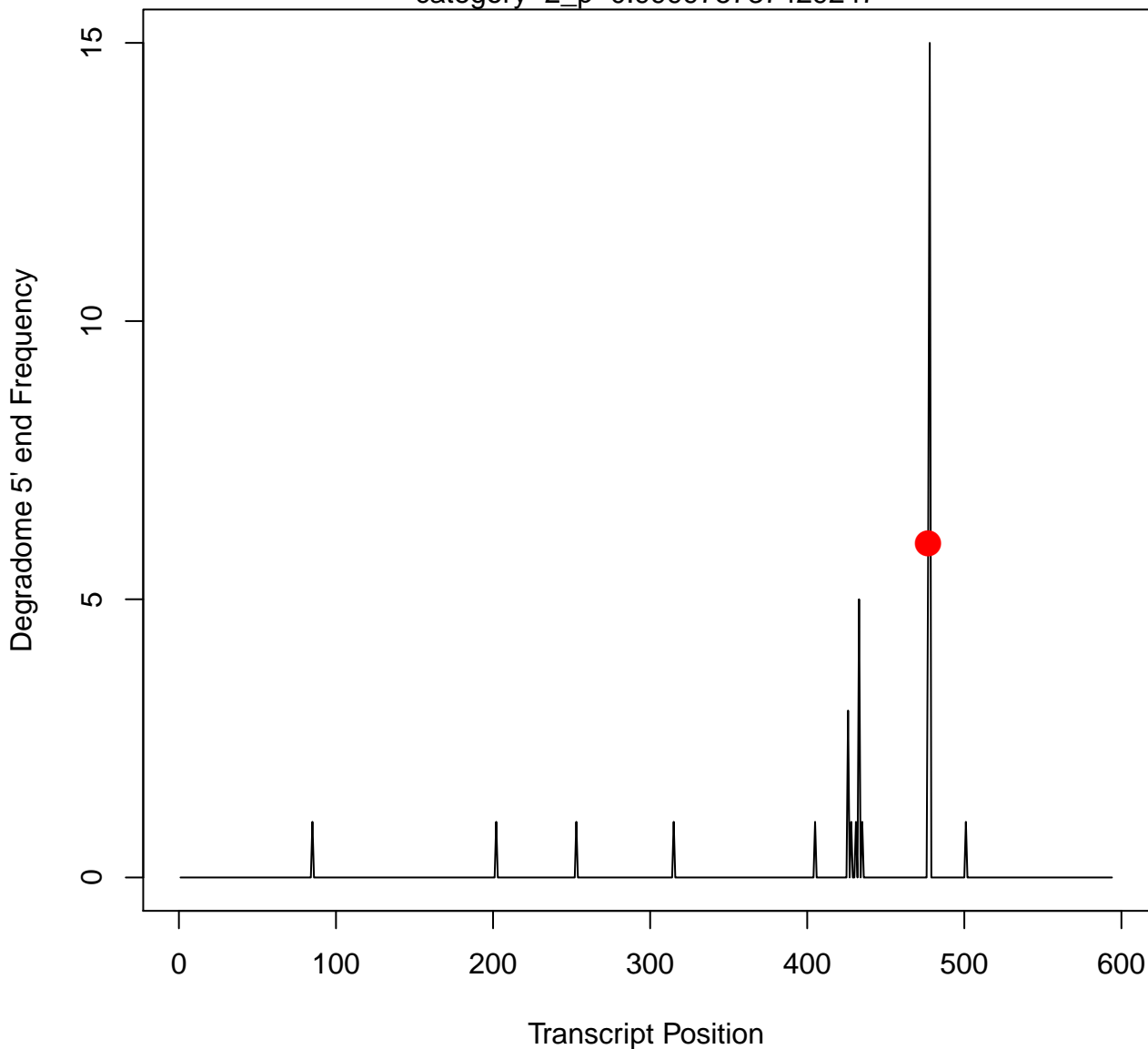

Supplement: Supplementary file 5 [file Data_Sheet_5.zip › Sit-miR160a_Seita.5G273800.1_477_TPlot.pdf]

**T=Seita.9G001700.1\_Q=Sit-miR160a\_S=1263**

category=2\_p=0.999999282334693

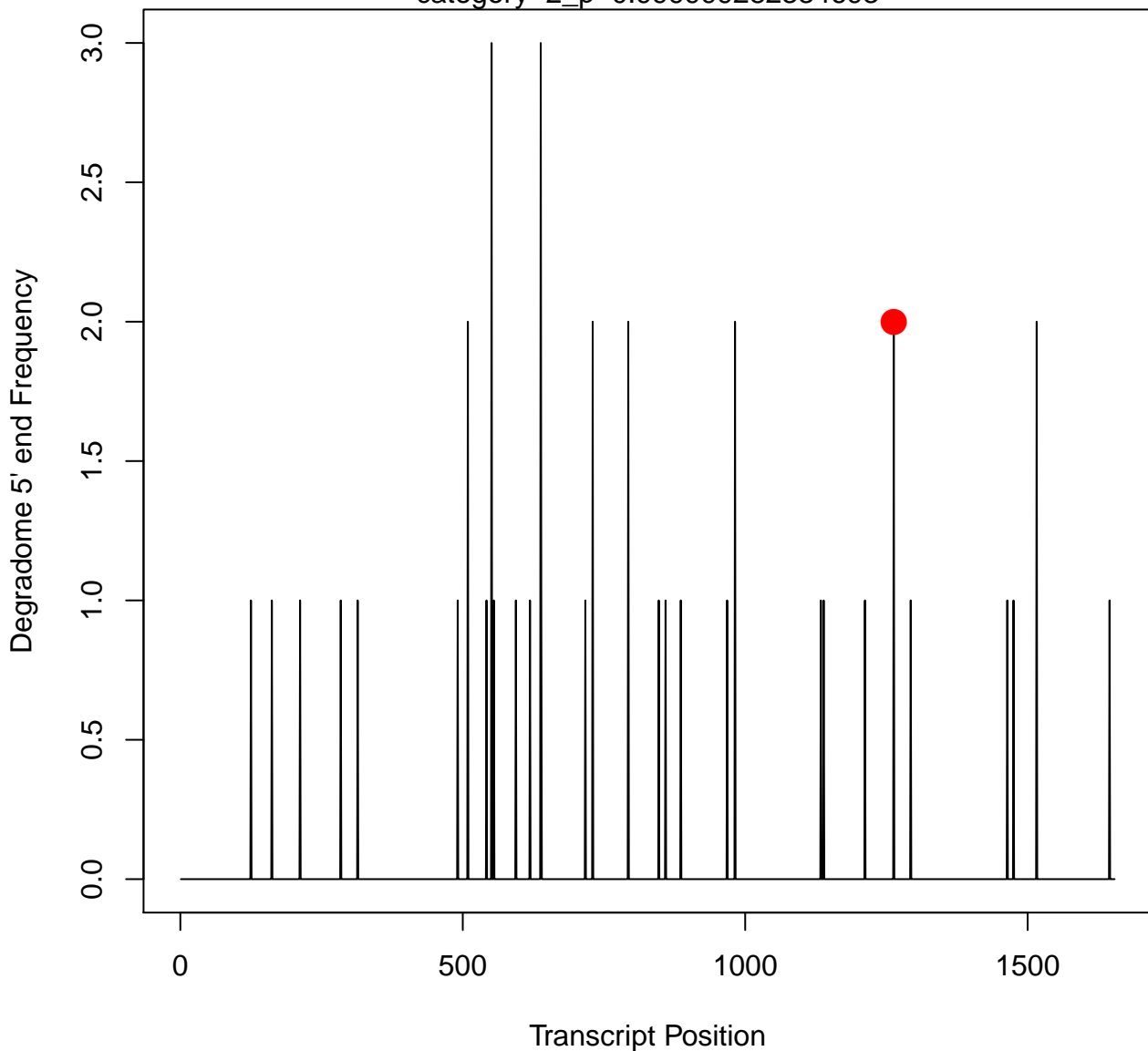

Supplement: Supplementary file 5 [file Data_Sheet_5.zip › Sit-miR160a_Seita.9G001700.1_1263_TPlot.pdf]

**T=Seita.9G219800.1\_Q=Sit-miR160a\_S=1729**

category=0\_p=0.00126686621795646

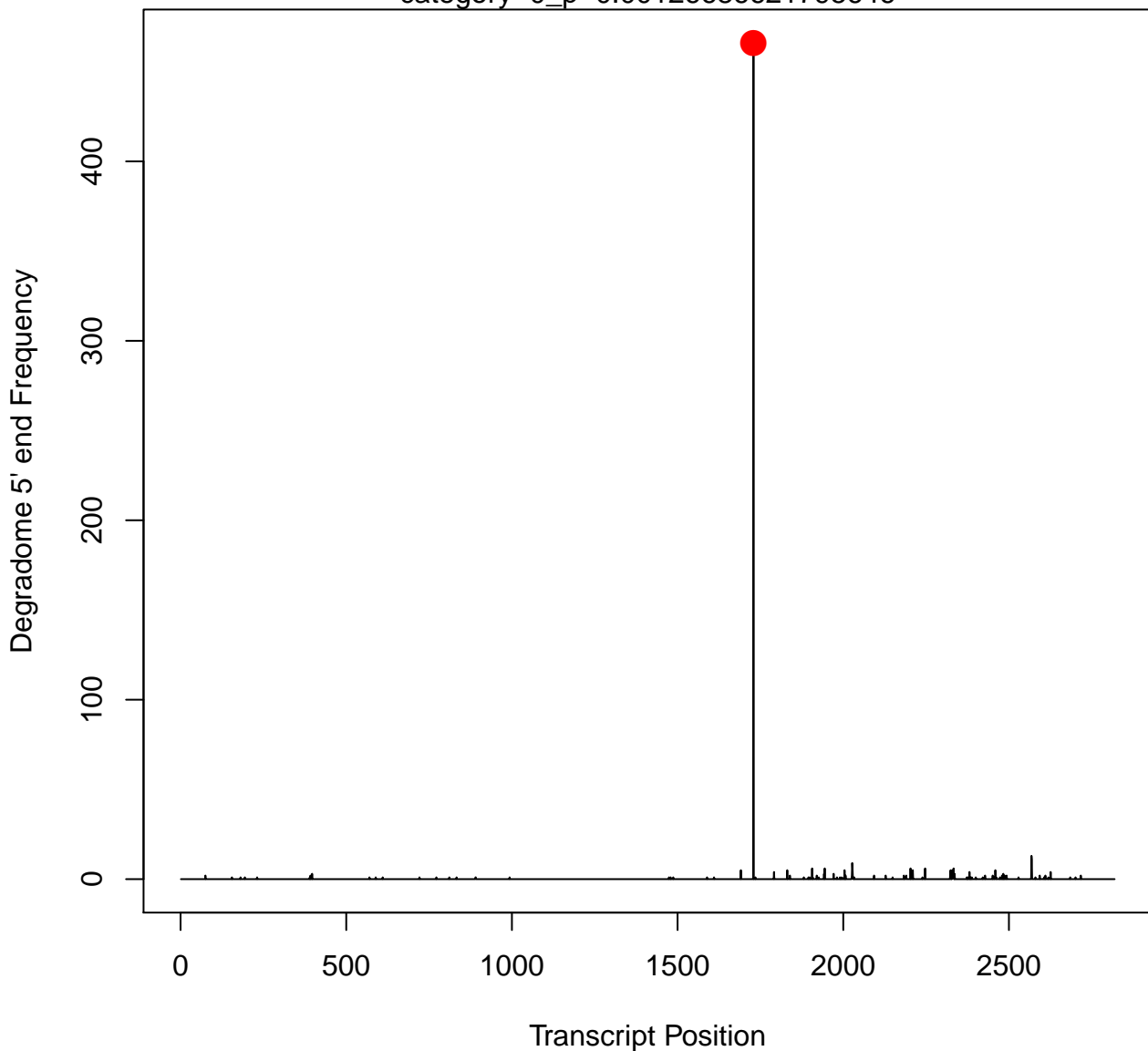

Supplement: Supplementary file 5 [file Data_Sheet_5.zip › Sit-miR160a_Seita.9G219800.1_1729_TPlot.pdf]

**T=Seita.1G173200.1\_Q=Sit-miR160b\_S=372**

category=2\_p=0.999932511199474

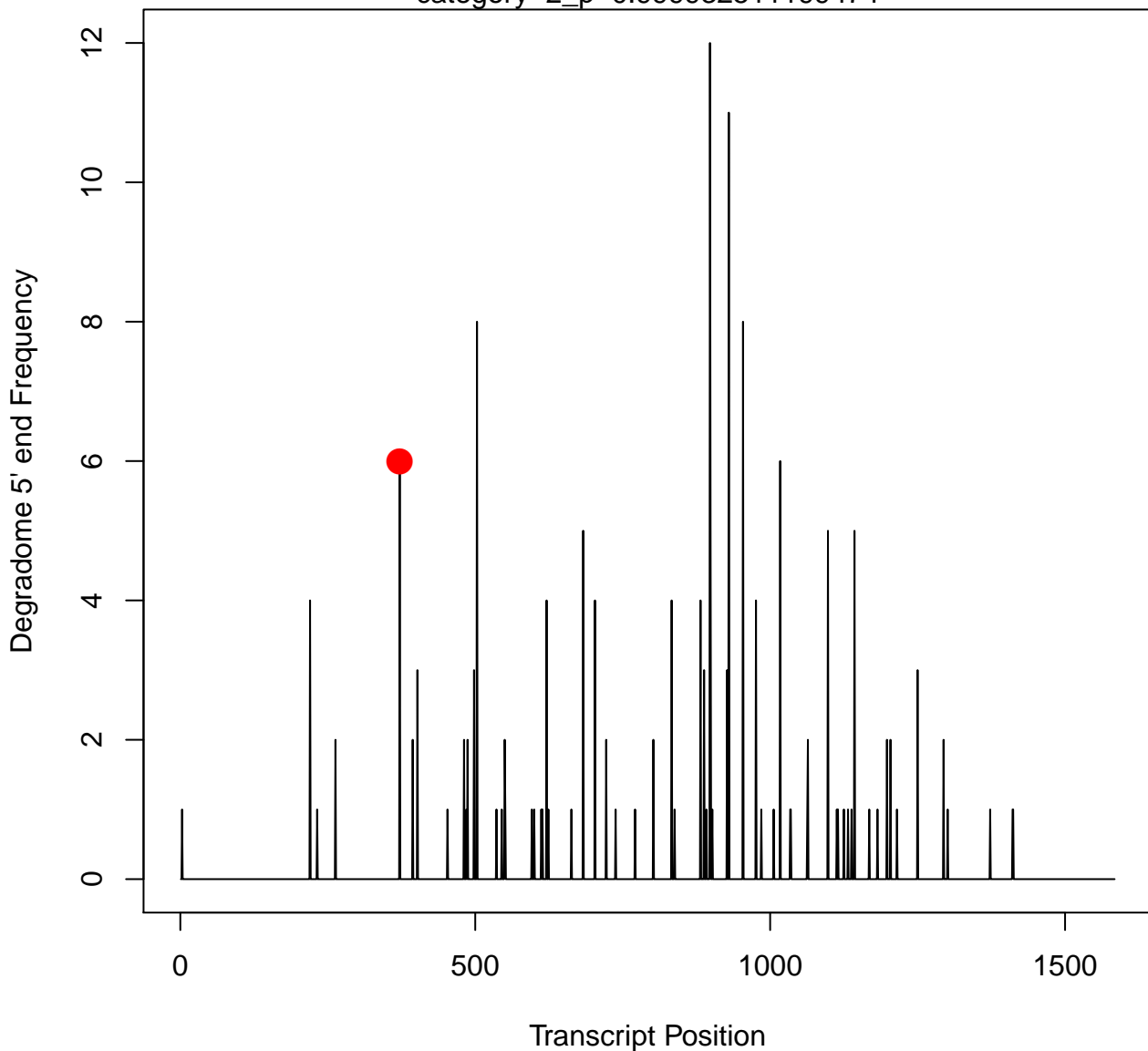

Supplement: Supplementary file 5 [file Data_Sheet_5.zip › Sit-miR160b_Seita.1G173200.1_372_TPlot.pdf]

**T=Seita.1G264900.1\_Q=Sit-miR160b\_S=256**

category=2\_p=0.998464915333145

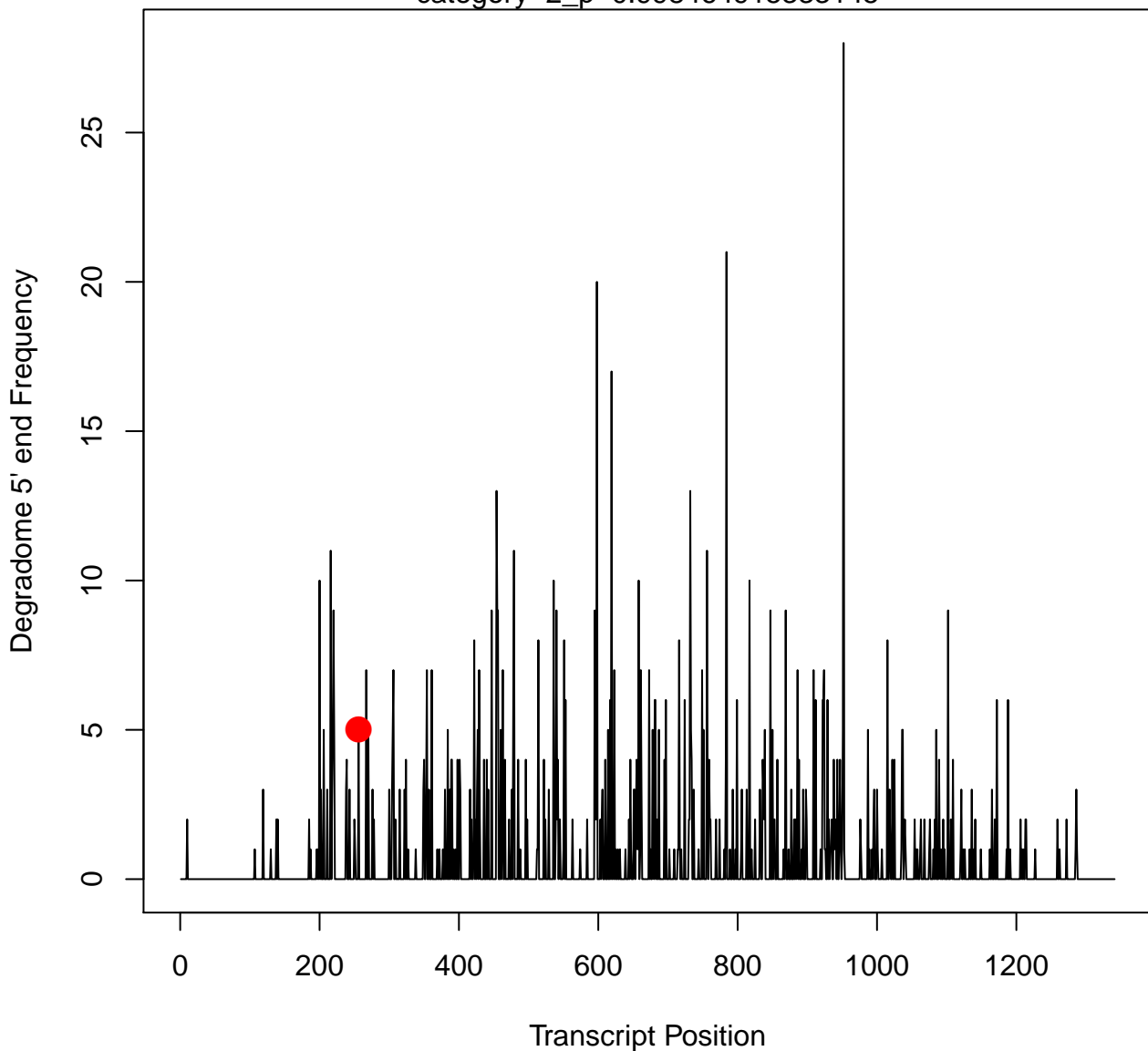

Supplement: Supplementary file 5 [file Data_Sheet_5.zip › Sit-miR160b_Seita.1G264900.1_256_TPlot.pdf]

**T=Seita.2G249600.1\_Q=Sit-miR160b\_S=971**

category=0\_p=0.336269950021032

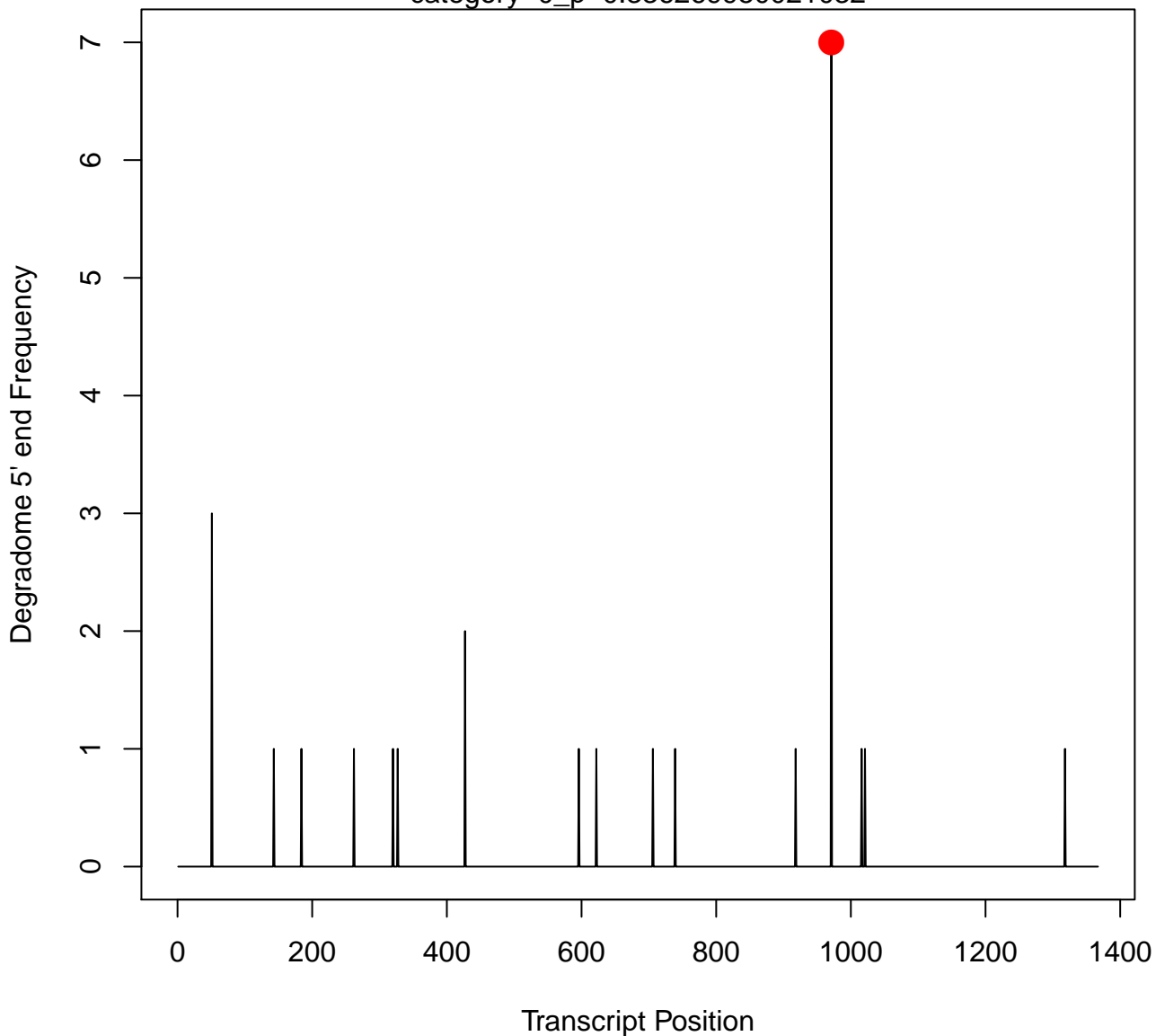

Supplement: Supplementary file 5 [file Data_Sheet_5.zip › Sit-miR160b_Seita.2G249600.1_971_TPlot.pdf]

**T=Seita.3G373100.1\_Q=Sit-miR160b\_S=259**

category=2\_p=0.777378575955027

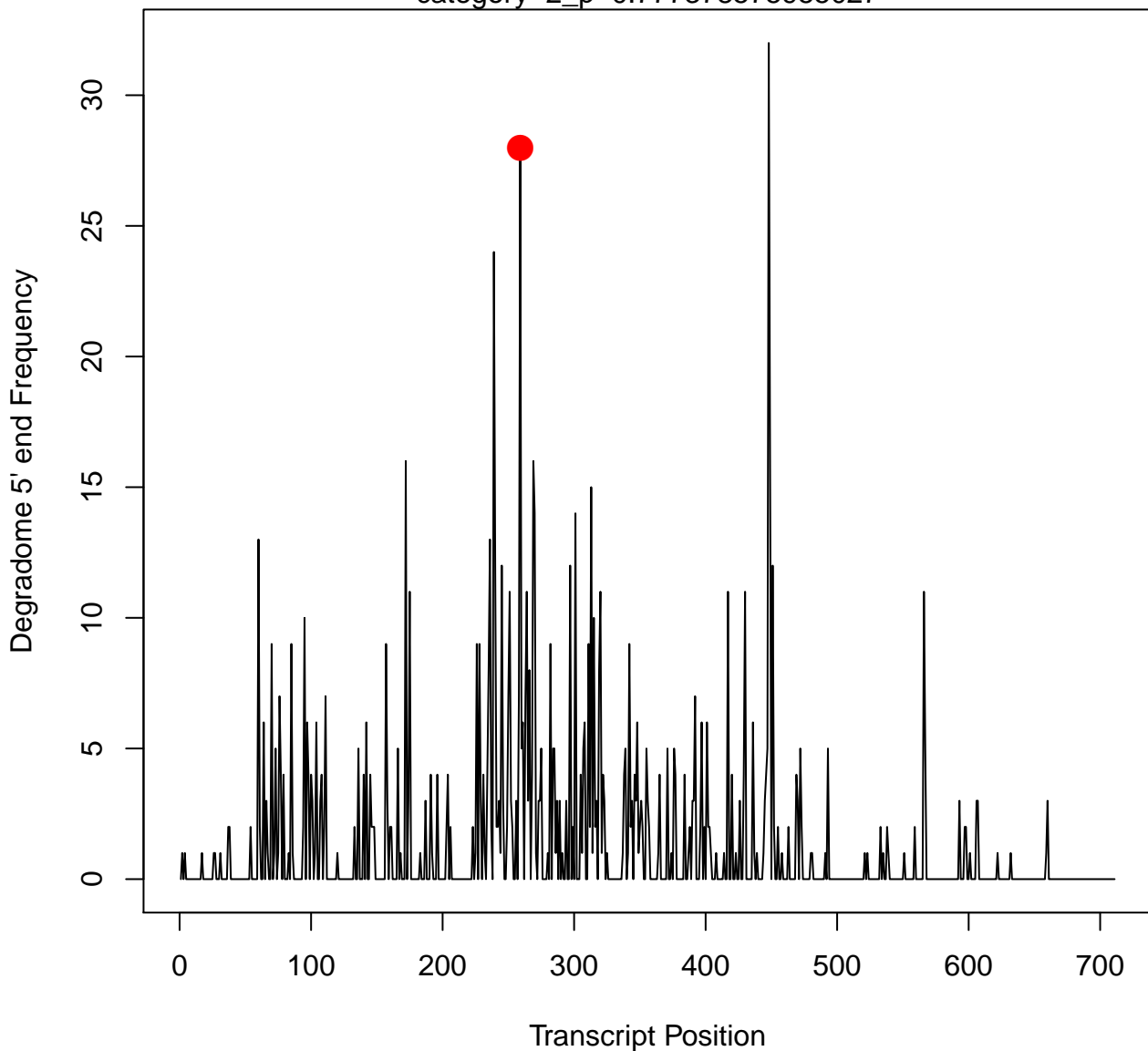

Supplement: Supplementary file 5 [file Data_Sheet_5.zip › Sit-miR160b_Seita.3G373100.1_259_TPlot.pdf]

**T=Seita.5G260500.1\_Q=Sit-miR160b\_S=1065**

category=2\_p=0.996820621641301

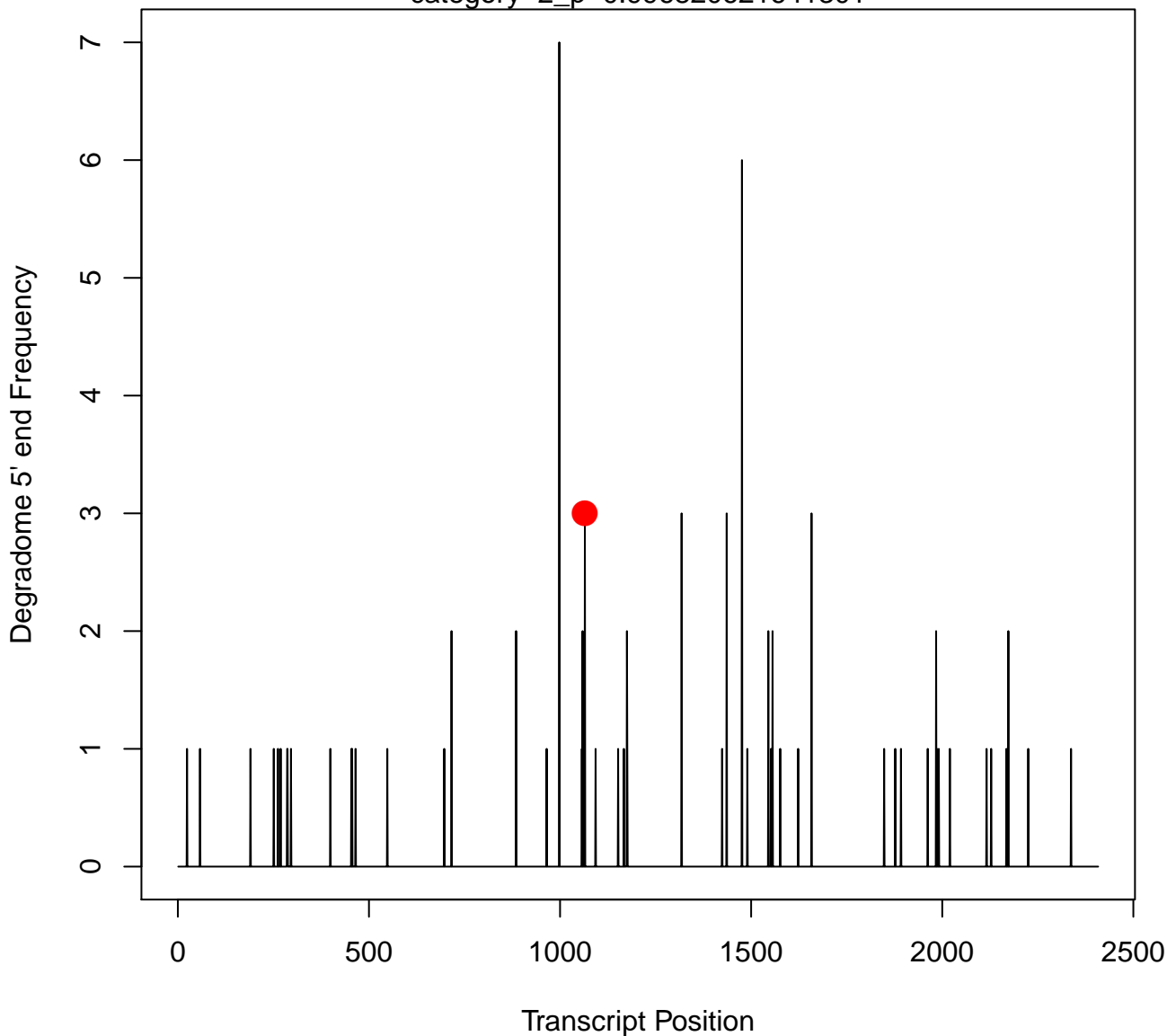

Supplement: Supplementary file 5 [file Data_Sheet_5.zip › Sit-miR160b_Seita.5G260500.1_1065_TPlot.pdf]

**T=Seita.1G095000.1\_Q=Sit-miR160c\_S=1151**

category=2\_p=0.999577564512732

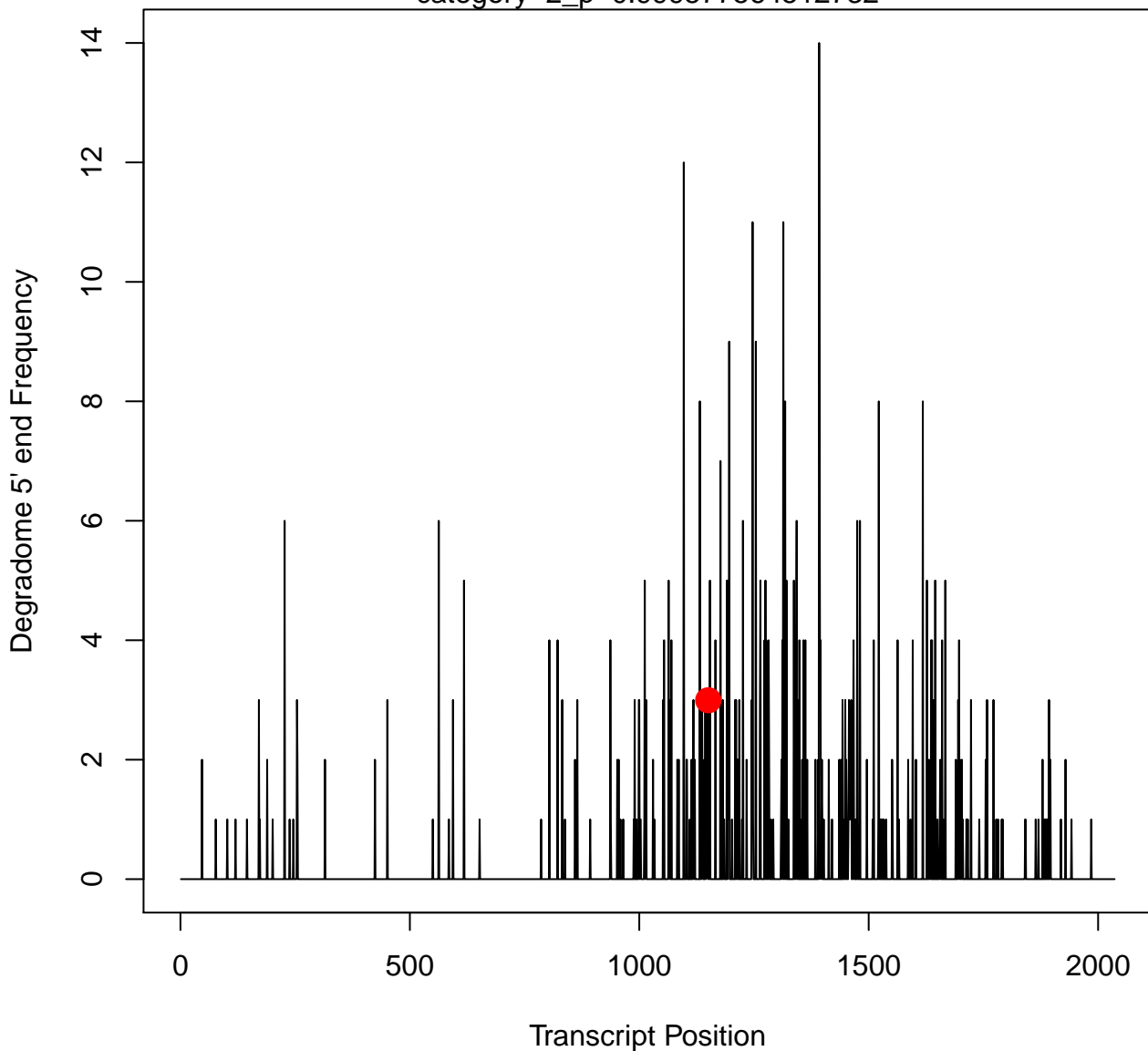

Supplement: Supplementary file 5 [file Data_Sheet_5.zip › Sit-miR160c_Seita.1G095000.1_1151_TPlot.pdf]

**T=Seita.3G373500.1\_Q=Sit-miR160c\_S=1034**

category=2\_p=0.80251577078265

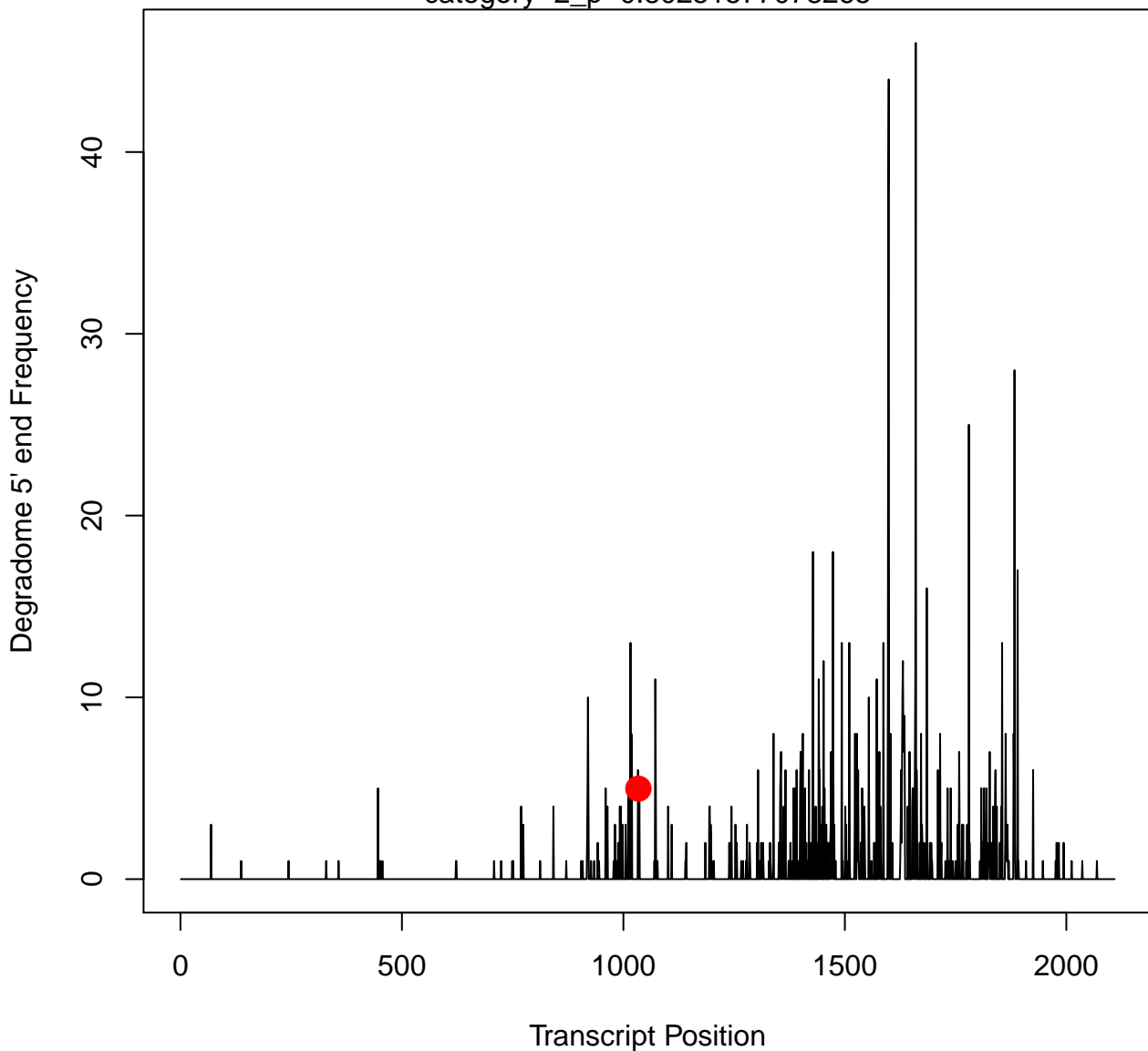

Supplement: Supplementary file 5 [file Data_Sheet_5.zip › Sit-miR160c_Seita.3G373500.1_1034_TPlot.pdf]

**T=Seita.4G100900.1\_Q=Sit-miR160c\_S=657**

category=2\_p=0.191017930867929

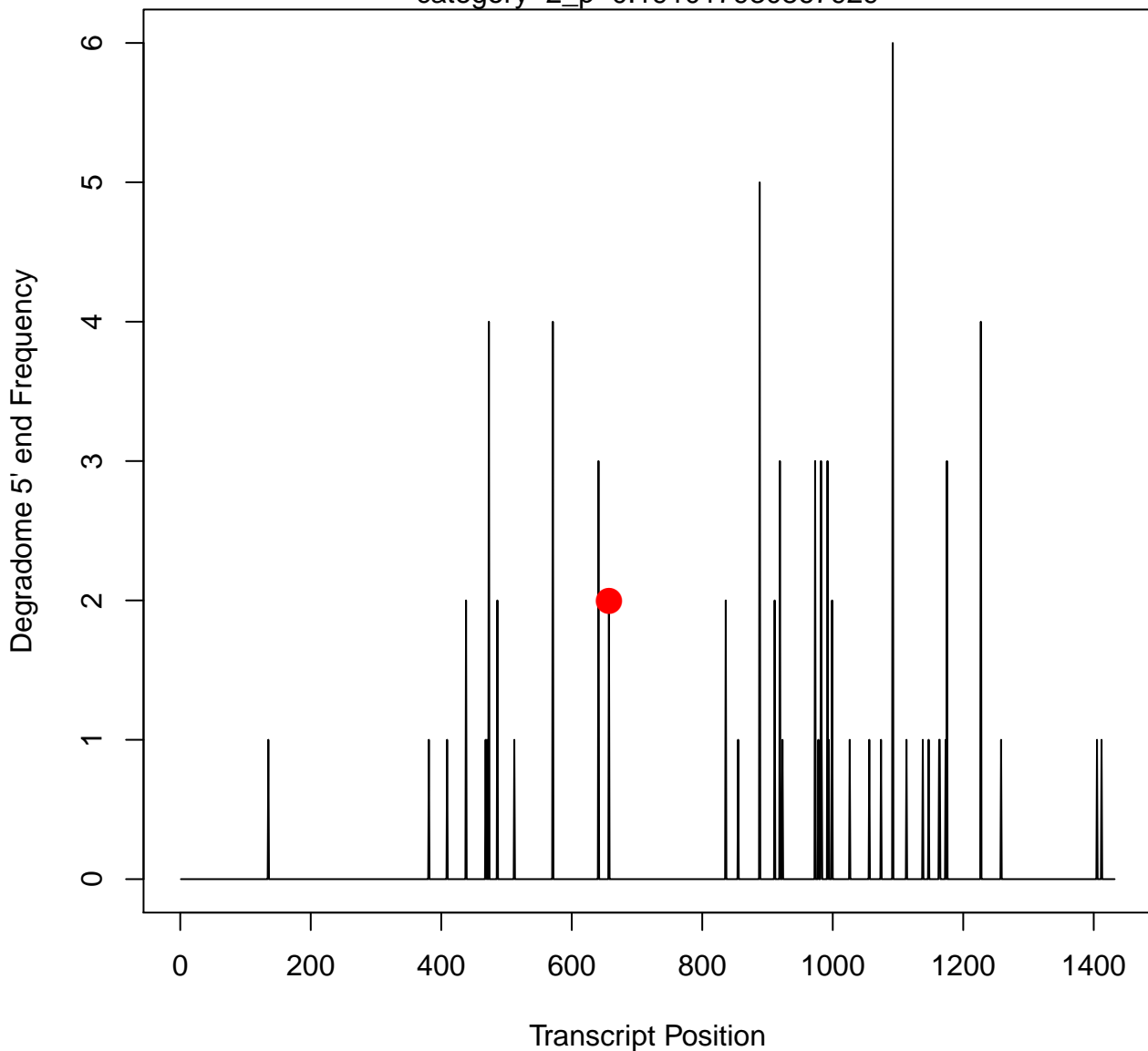

Supplement: Supplementary file 5 [file Data_Sheet_5.zip › Sit-miR160c_Seita.4G100900.1_657_TPlot.pdf]

**T=Seita.4G265400.1\_Q=Sit-miR160c\_S=870**

category=2\_p=0.99999233336608

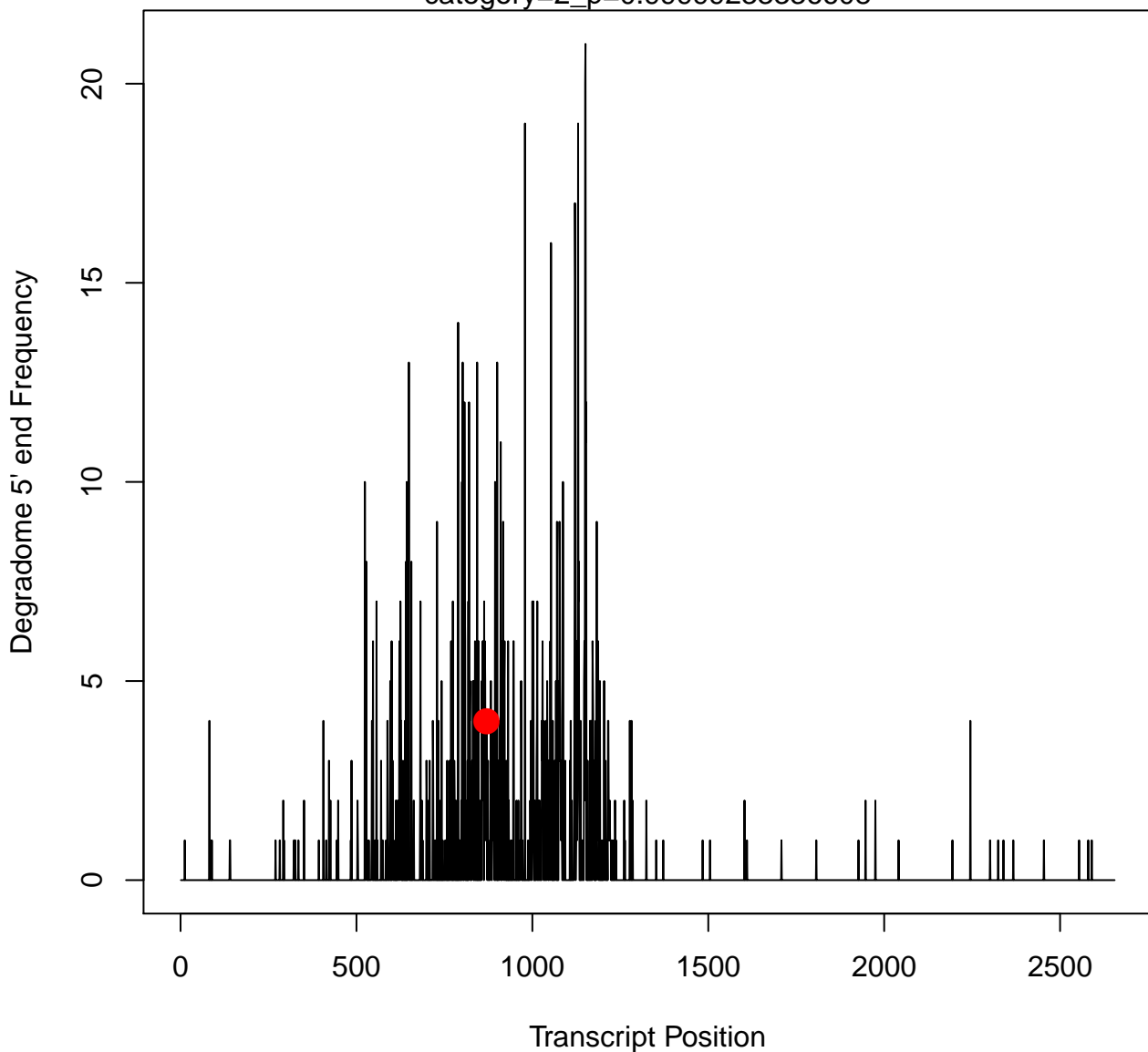

Supplement: Supplementary file 5 [file Data_Sheet_5.zip › Sit-miR160c_Seita.4G265400.1_870_TPlot.pdf]

**T=Seita.5G021300.1\_Q=Sit-miR160c\_S=471**

category=2\_p=0.605786336945431

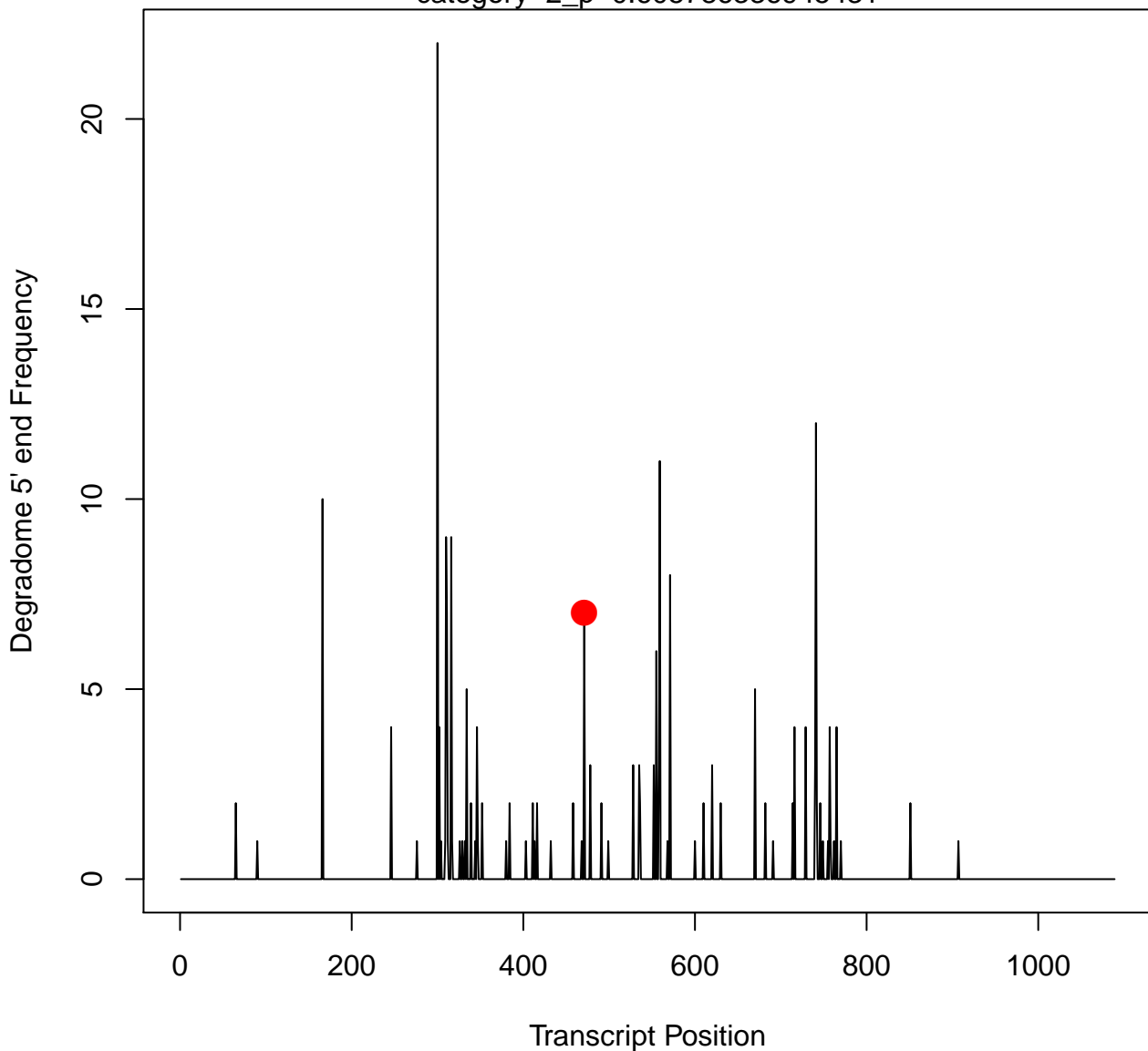

Supplement: Supplementary file 5 [file Data_Sheet_5.zip › Sit-miR160c_Seita.5G021300.1_471_TPlot.pdf]

**T=Seita.5G435000.1\_Q=Sit-miR160c\_S=2985**

category=2\_p=0.0624781396370455

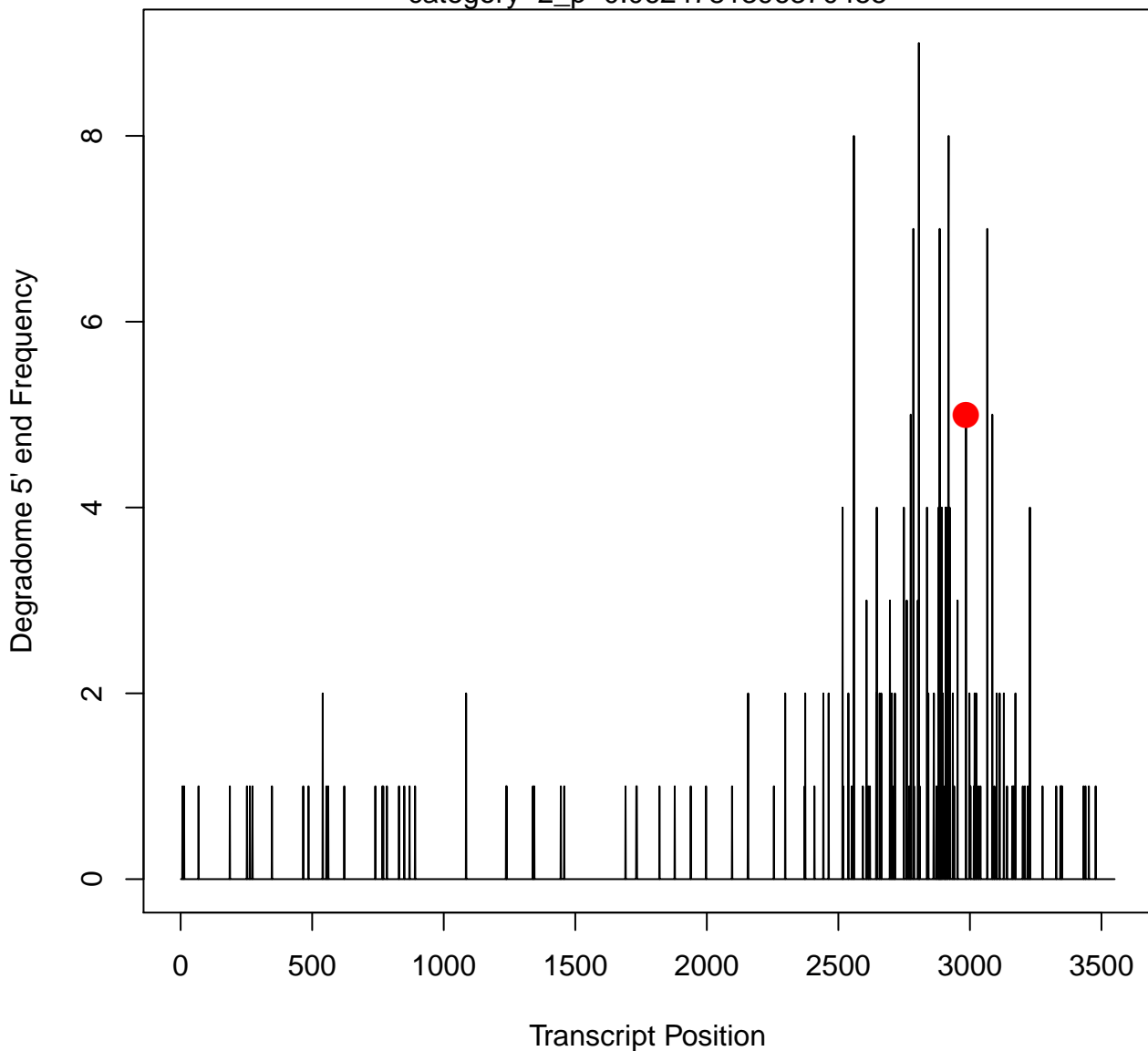

Supplement: Supplementary file 5 [file Data_Sheet_5.zip › Sit-miR160c_Seita.5G435000.1_2985_TPlot.pdf]

**T=Seita.7G155700.1\_Q=Sit-miR160c\_S=1461**

category=0\_p=0.115330696851779

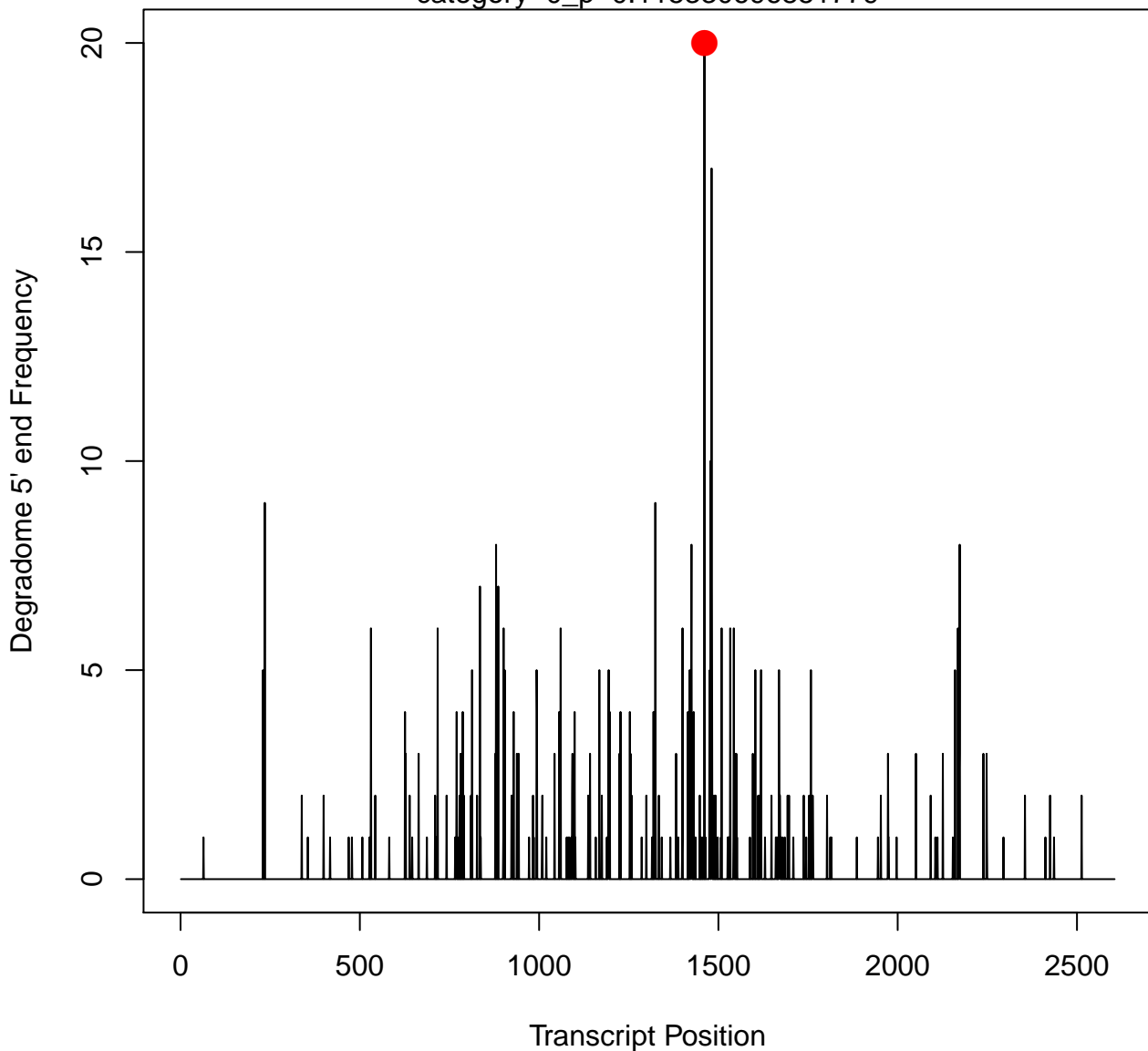

Supplement: Supplementary file 5 [file Data_Sheet_5.zip › Sit-miR160c_Seita.7G155700.1_1461_TPlot.pdf]

**T=Seita.7G169600.1\_Q=Sit-miR160c\_S=1802**

category=0\_p=0.00084475590689892

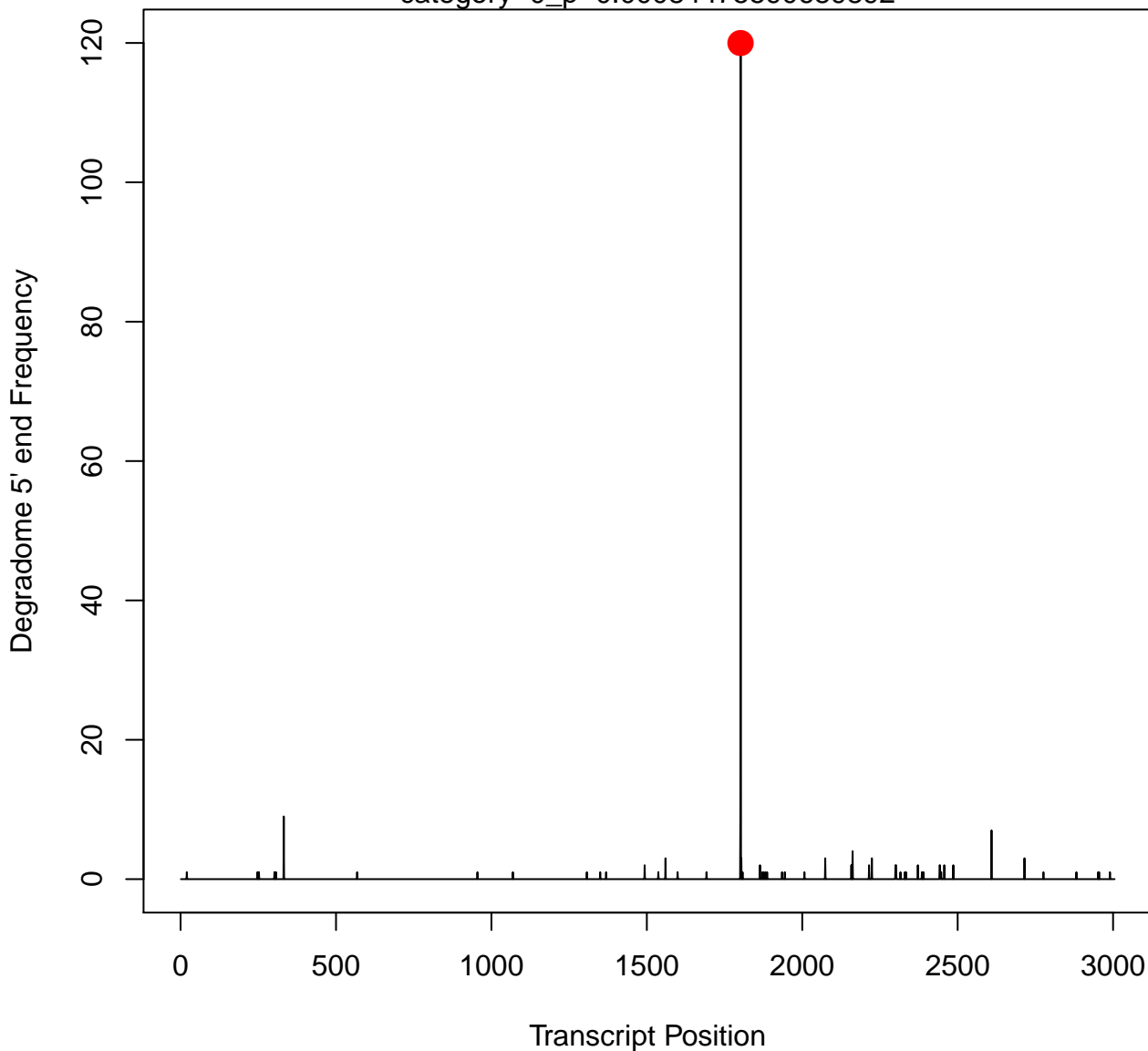

Supplement: Supplementary file 5 [file Data_Sheet_5.zip › Sit-miR160c_Seita.7G169600.1_1802_TPlot.pdf]

**T=Seita.7G171800.1\_Q=Sit-miR160c\_S=1035**

category=1\_p=0.00288200452789211

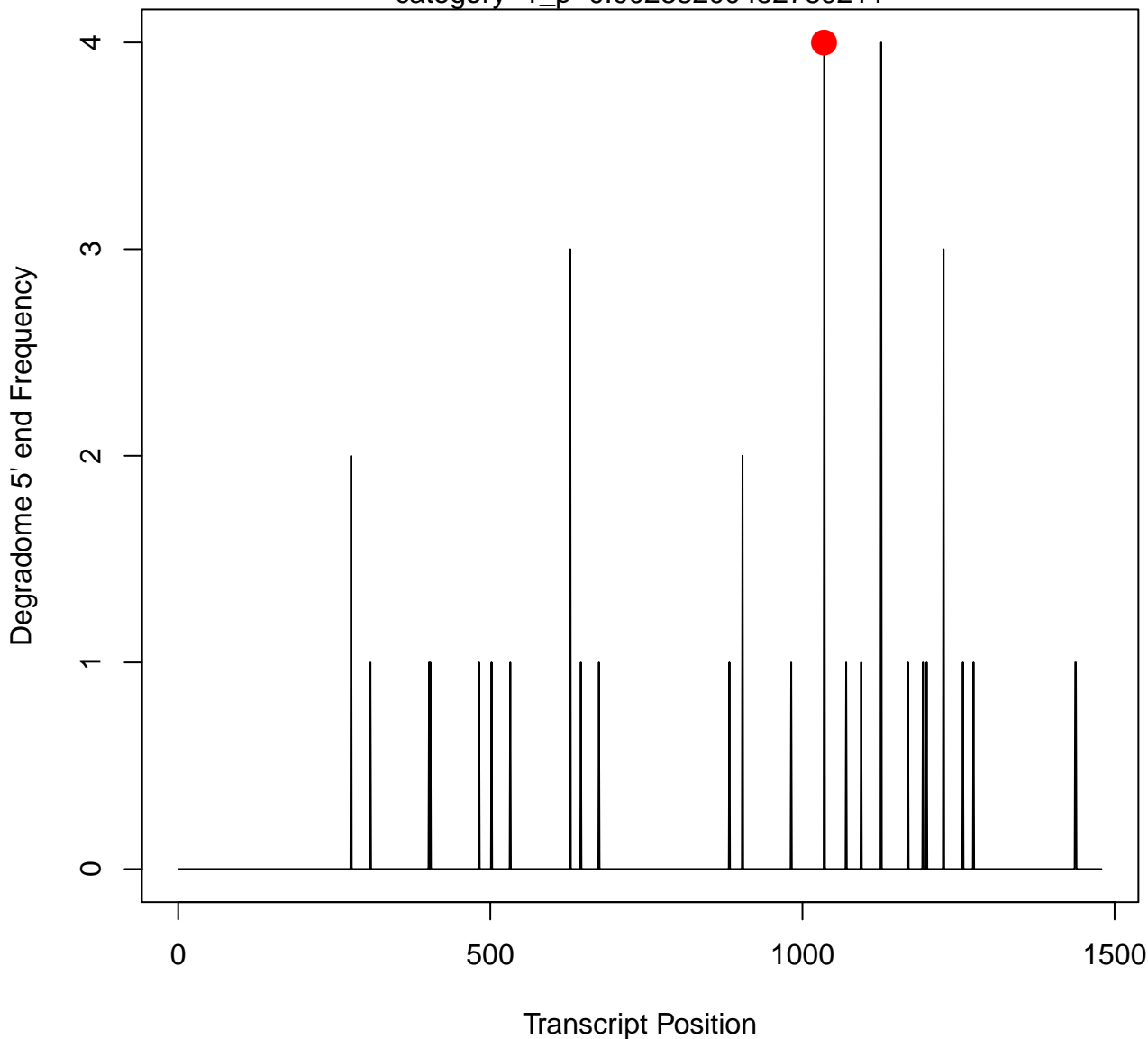

Supplement: Supplementary file 5 [file Data_Sheet_5.zip › Sit-miR160c_Seita.7G171800.1_1035_TPlot.pdf]

**T=Seita.7G260300.1\_Q=Sit-miR160c\_S=1557**

category=2\_p=0.999510443409046

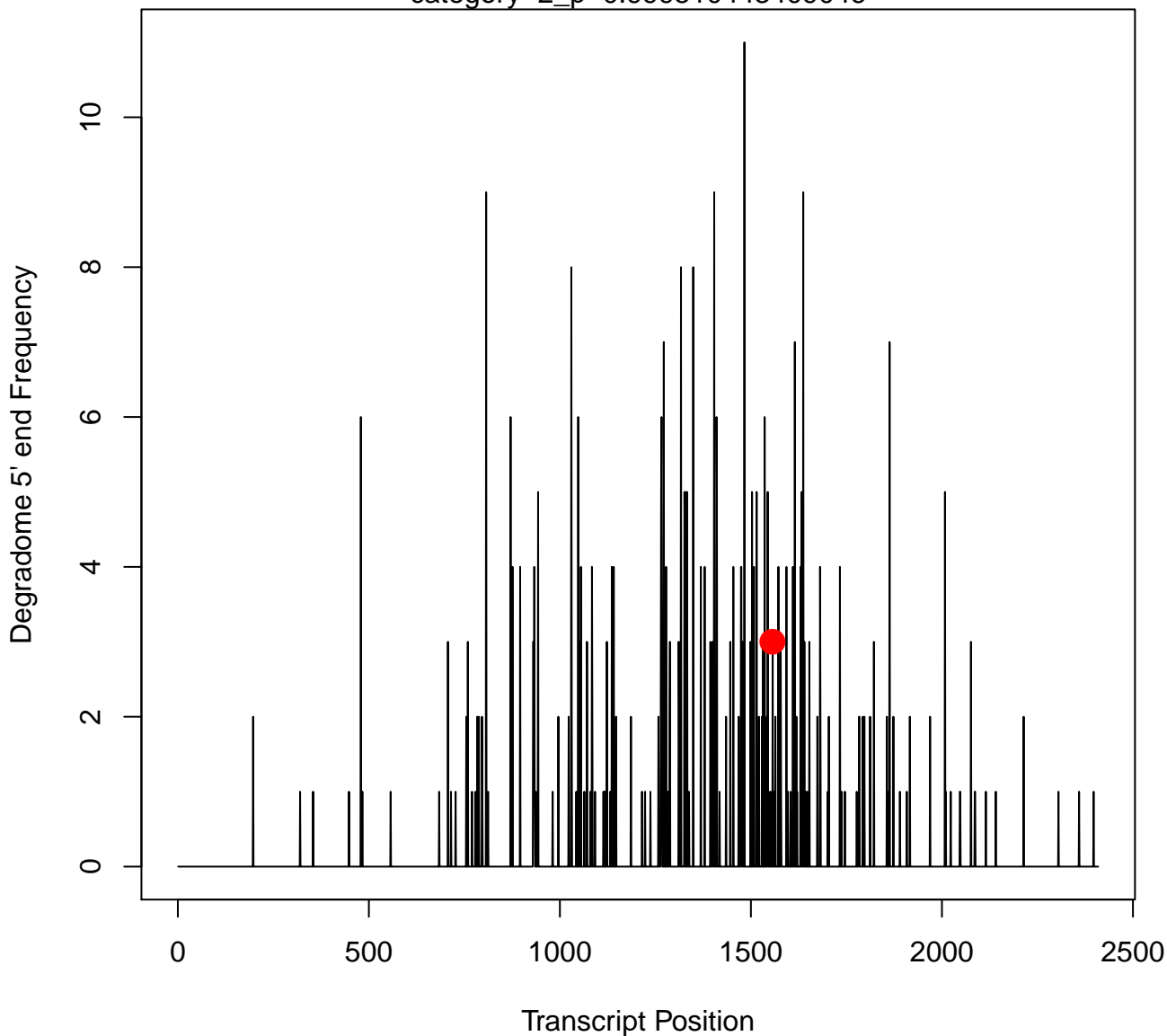

Supplement: Supplementary file 5 [file Data_Sheet_5.zip › Sit-miR160c_Seita.7G260300.1_1557_TPlot.pdf]

**T=Seita.9G204900.1\_Q=Sit-miR160c\_S=1104**

category=2\_p=0.983141338990527

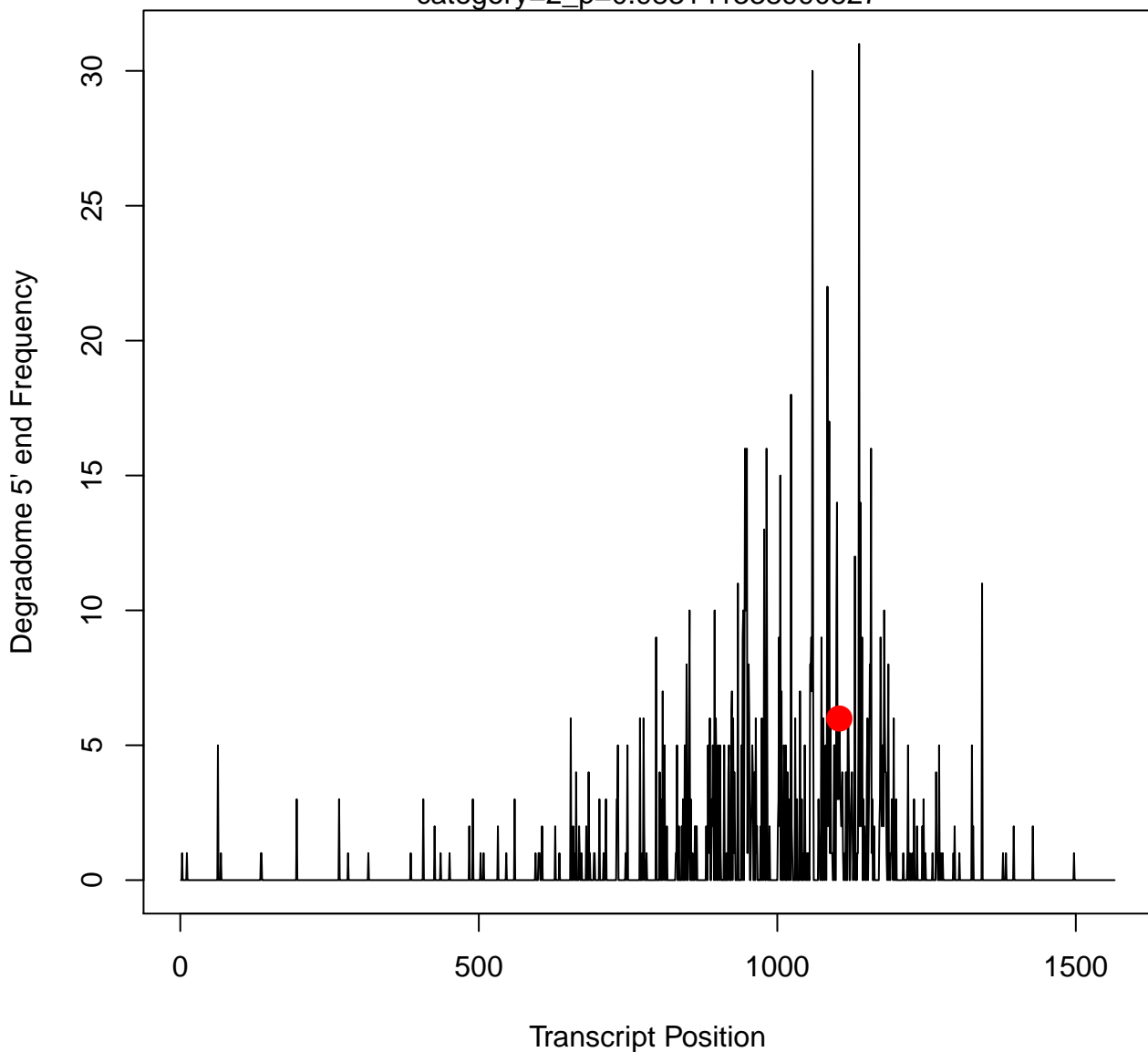

Supplement: Supplementary file 5 [file Data_Sheet_5.zip › Sit-miR160c_Seita.9G204900.1_1104_TPlot.pdf]

**T=Seita.1G291100.1\_Q=Sit-miR160d\_S=289**

category=2\_p=0.320970850386545

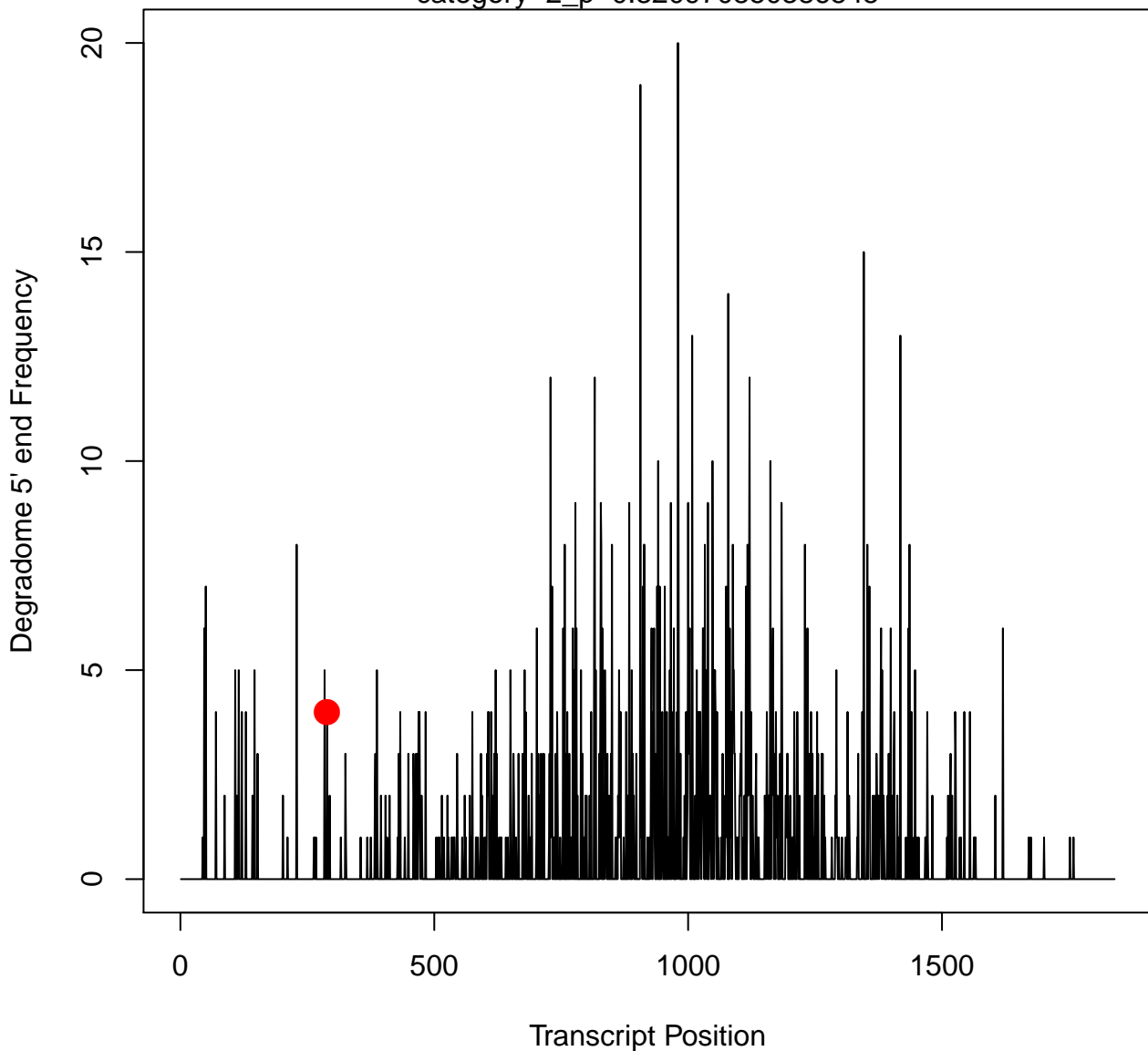

Supplement: Supplementary file 5 [file Data_Sheet_5.zip › Sit-miR160d_Seita.1G291100.1_289_TPlot.pdf]

**T=Seita.2G137400.1\_Q=Sit-miR160d\_S=816**

category=2\_p=0.764721145676662

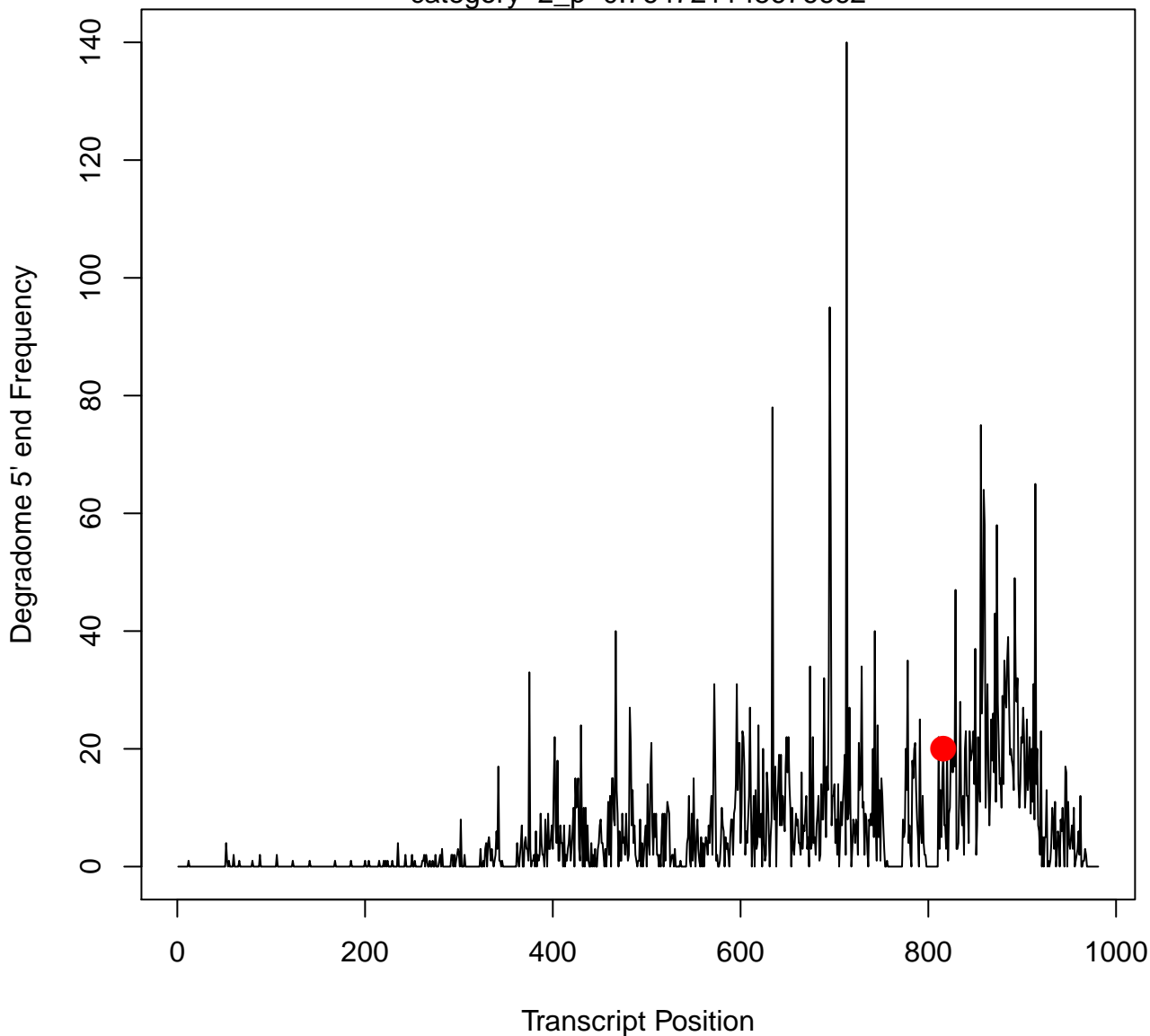

Supplement: Supplementary file 5 [file Data_Sheet_5.zip › Sit-miR160d_Seita.2G137400.1_816_TPlot.pdf]

**T=Seita.3G328100.1\_Q=Sit-miR160d\_S=535**

category=2\_p=0.191017930867929

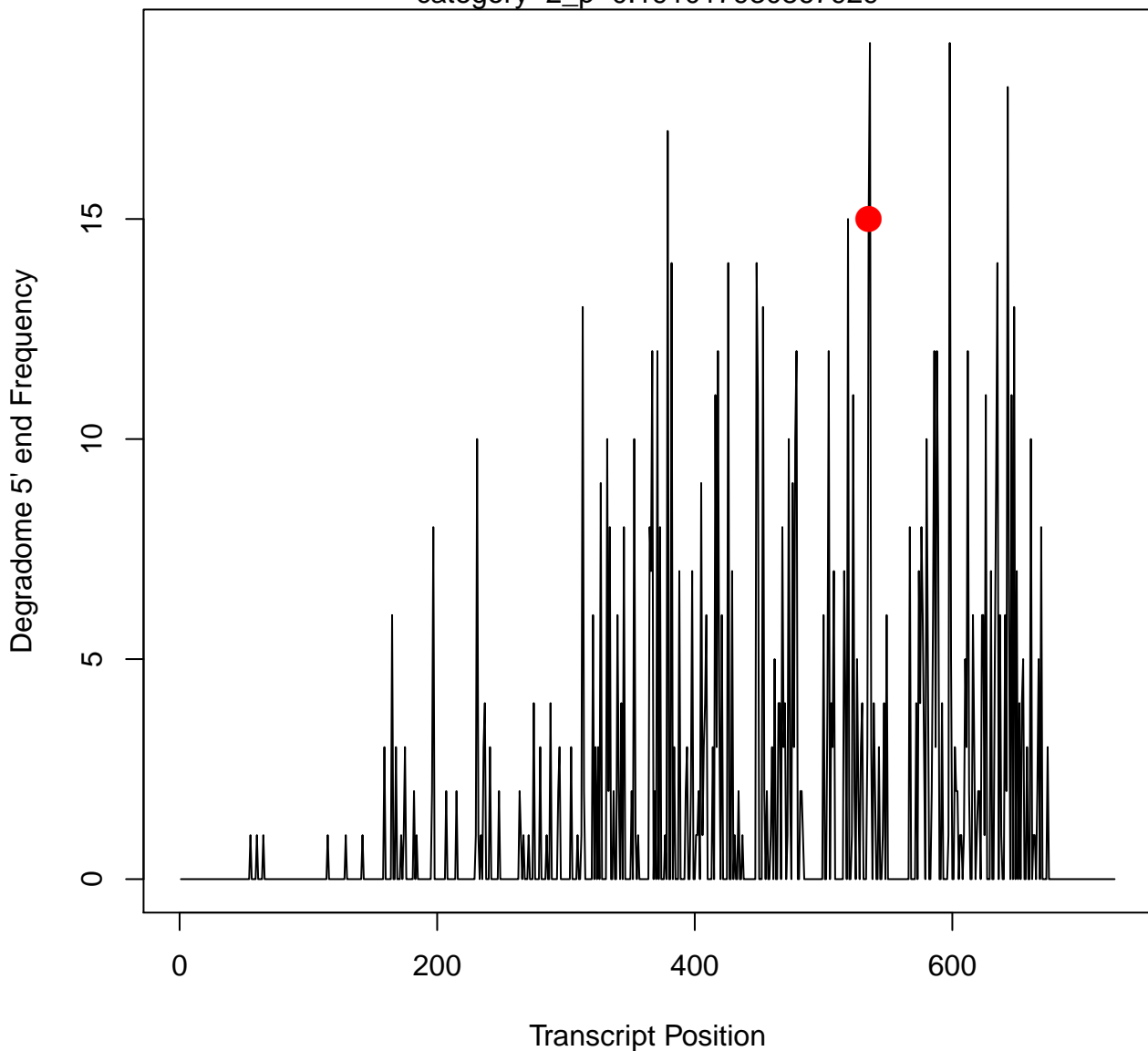

Supplement: Supplementary file 5 [file Data_Sheet_5.zip › Sit-miR160d_Seita.3G328100.1_535_TPlot.pdf]

**T=Seita.5G024400.1\_Q=Sit-miR160d\_S=1642**

category=2\_p=0.999999980994679

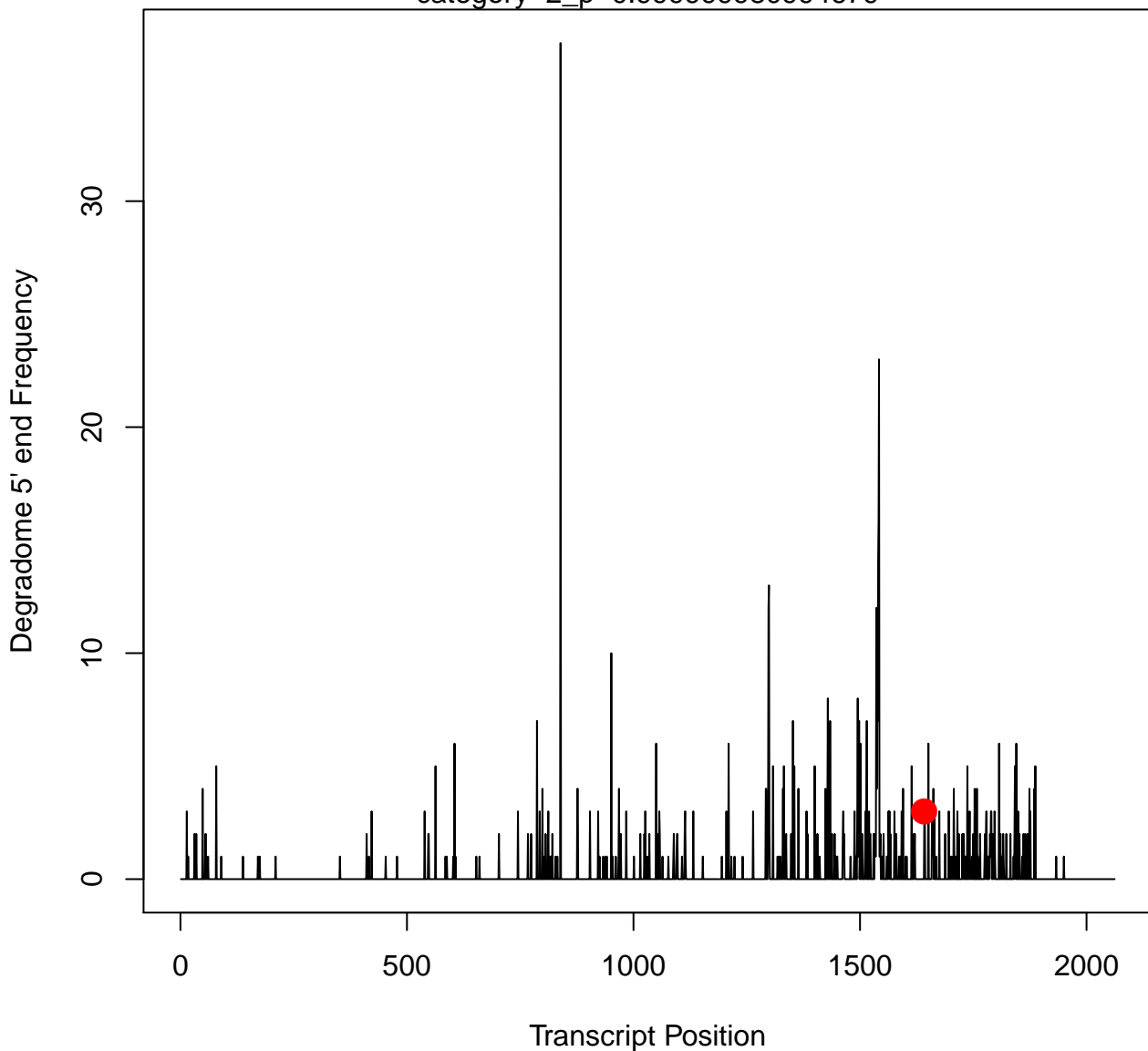

Supplement: Supplementary file 5 [file Data_Sheet_5.zip › Sit-miR160d_Seita.5G024400.1_1642_TPlot.pdf]

**T=Seita.6G069300.1\_Q=Sit-miR160d\_S=129**

category=0\_p=0.219012025958501

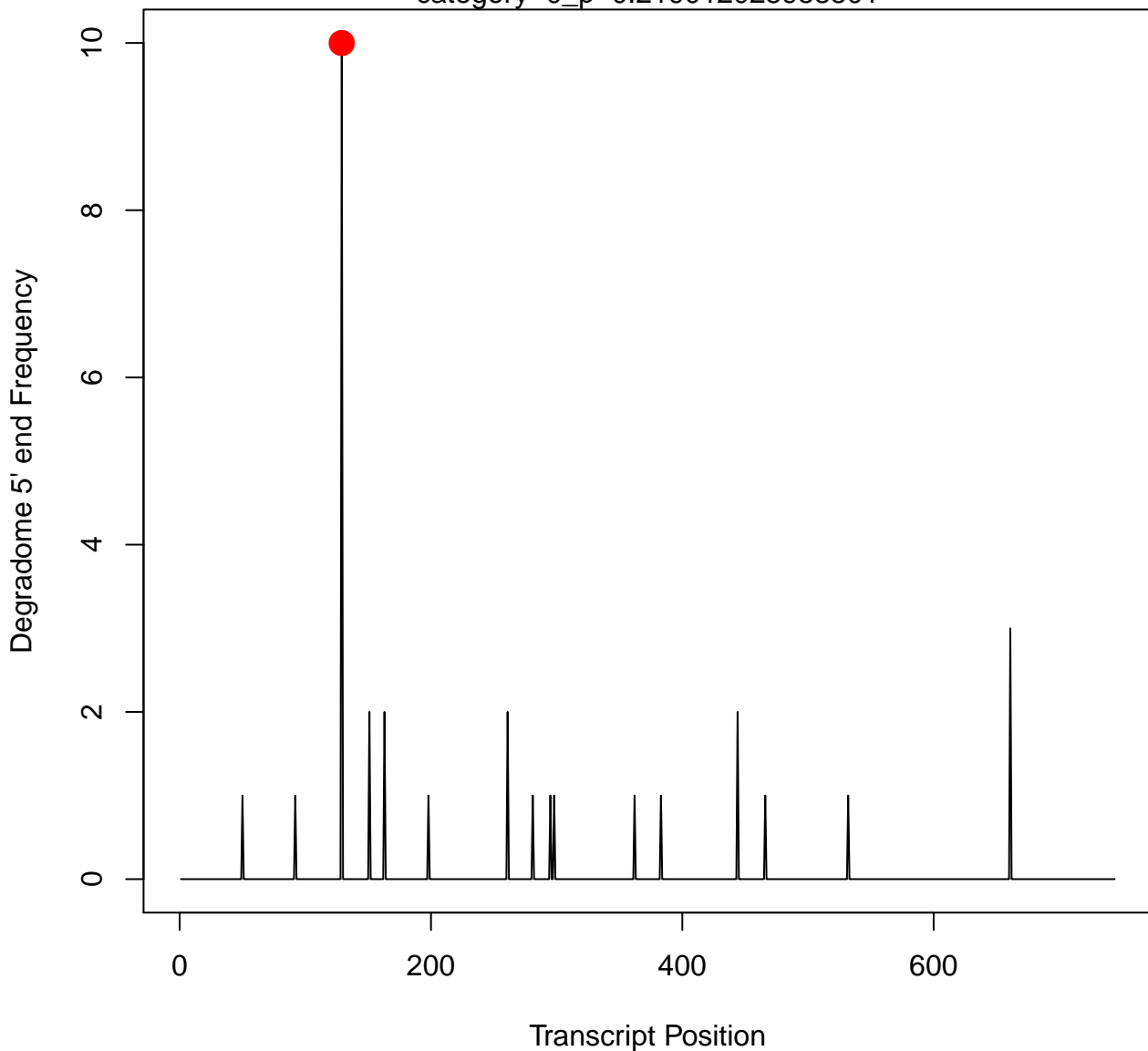

Supplement: Supplementary file 5 [file Data_Sheet_5.zip › Sit-miR160d_Seita.6G069300.1_129_TPlot.pdf]

**T=Seita.7G262800.1\_Q=Sit-miR160d\_S=1155**

category=2\_p=0.999999909775561

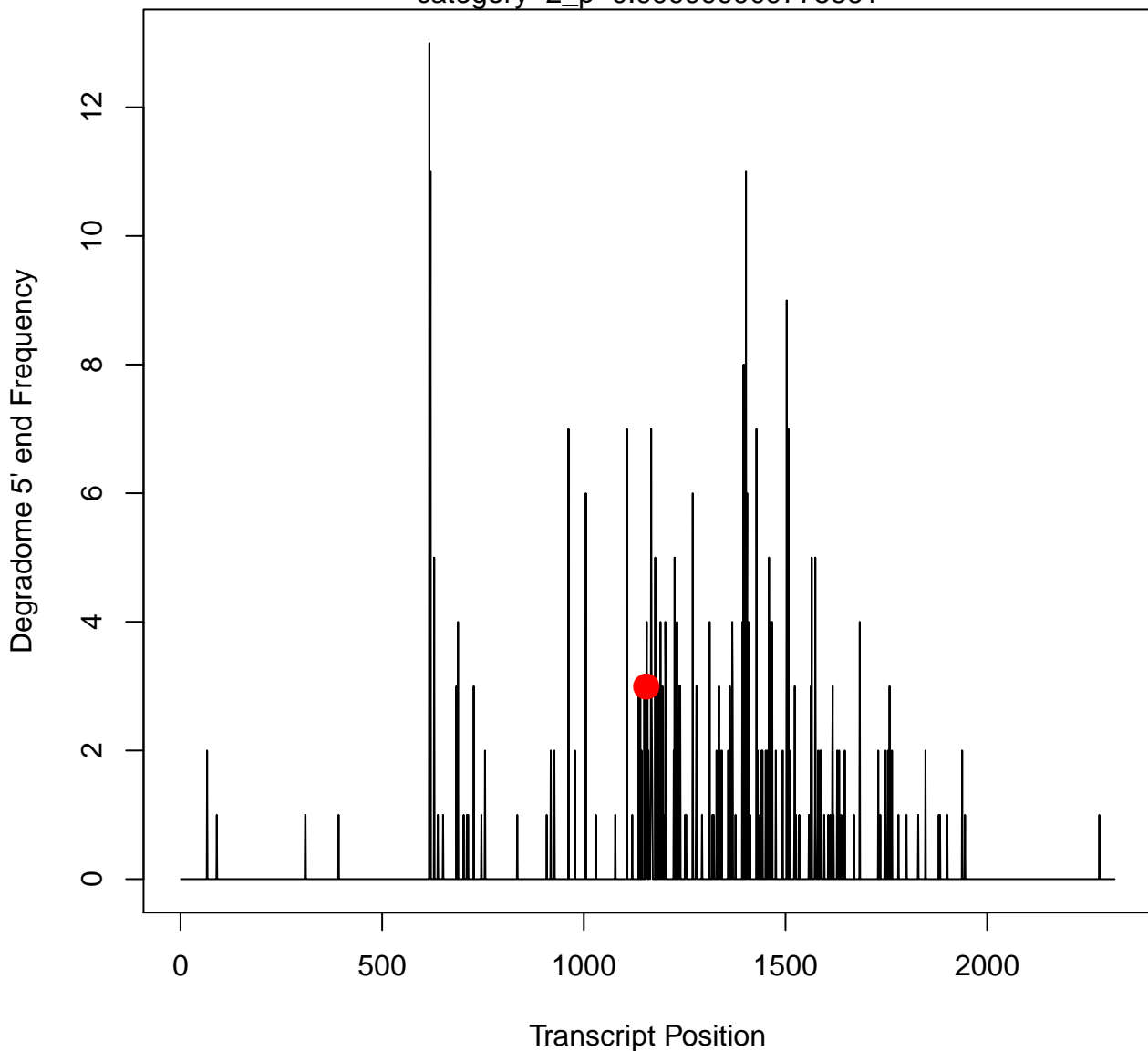

Supplement: Supplementary file 5 [file Data_Sheet_5.zip › Sit-miR160d_Seita.7G262800.1_1155_TPlot.pdf]

**T=Seita.7G295800.1\_Q=Sit-miR160d\_S=368**

category=2\_p=0.999999419421635

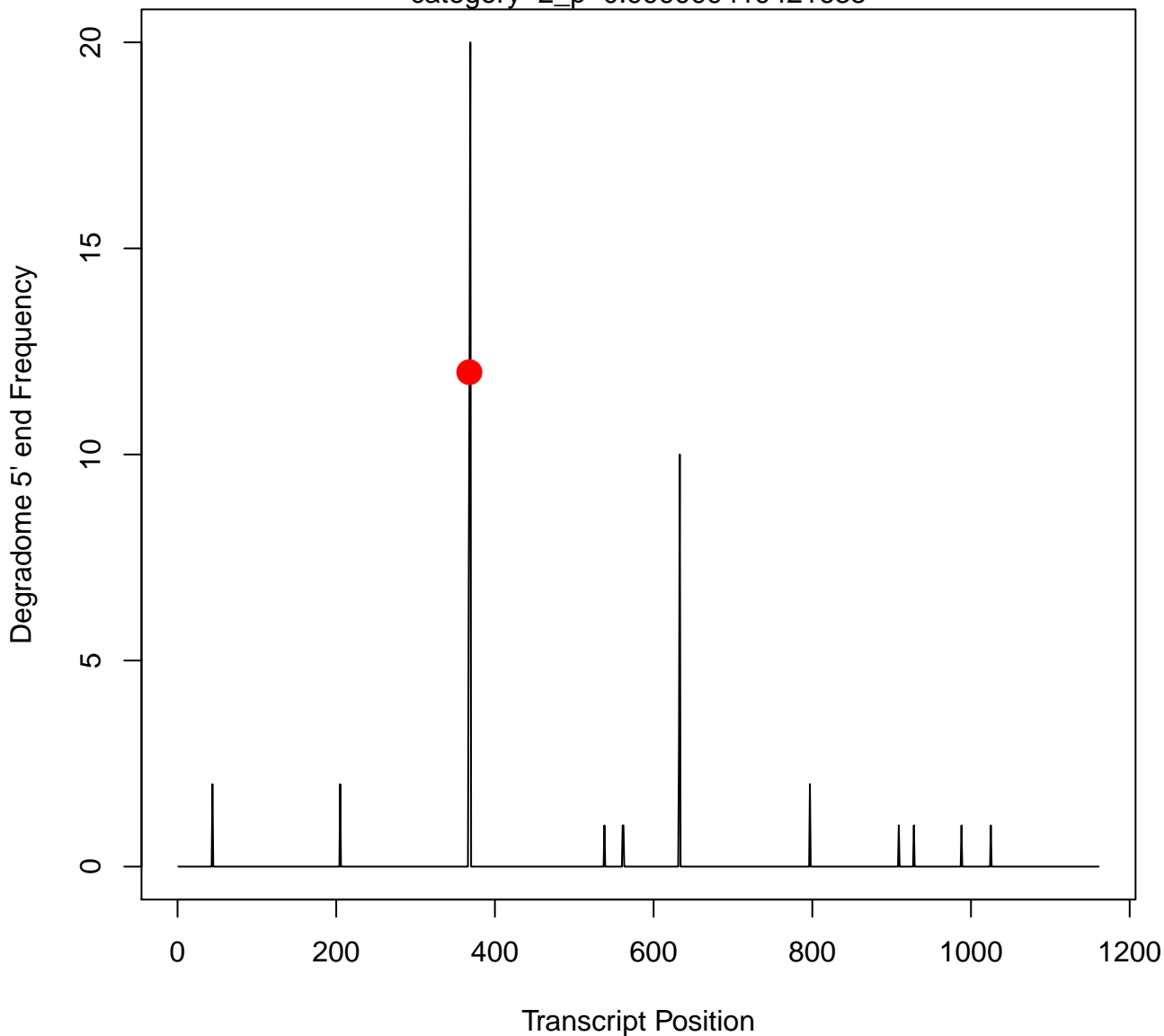

Supplement: Supplementary file 5 [file Data_Sheet_5.zip › Sit-miR160d_Seita.7G295800.1_368_TPlot.pdf]

**T=Seita.8G100700.1\_Q=Sit-miR160d\_S=4526**

category=2\_p=0.974001520859301

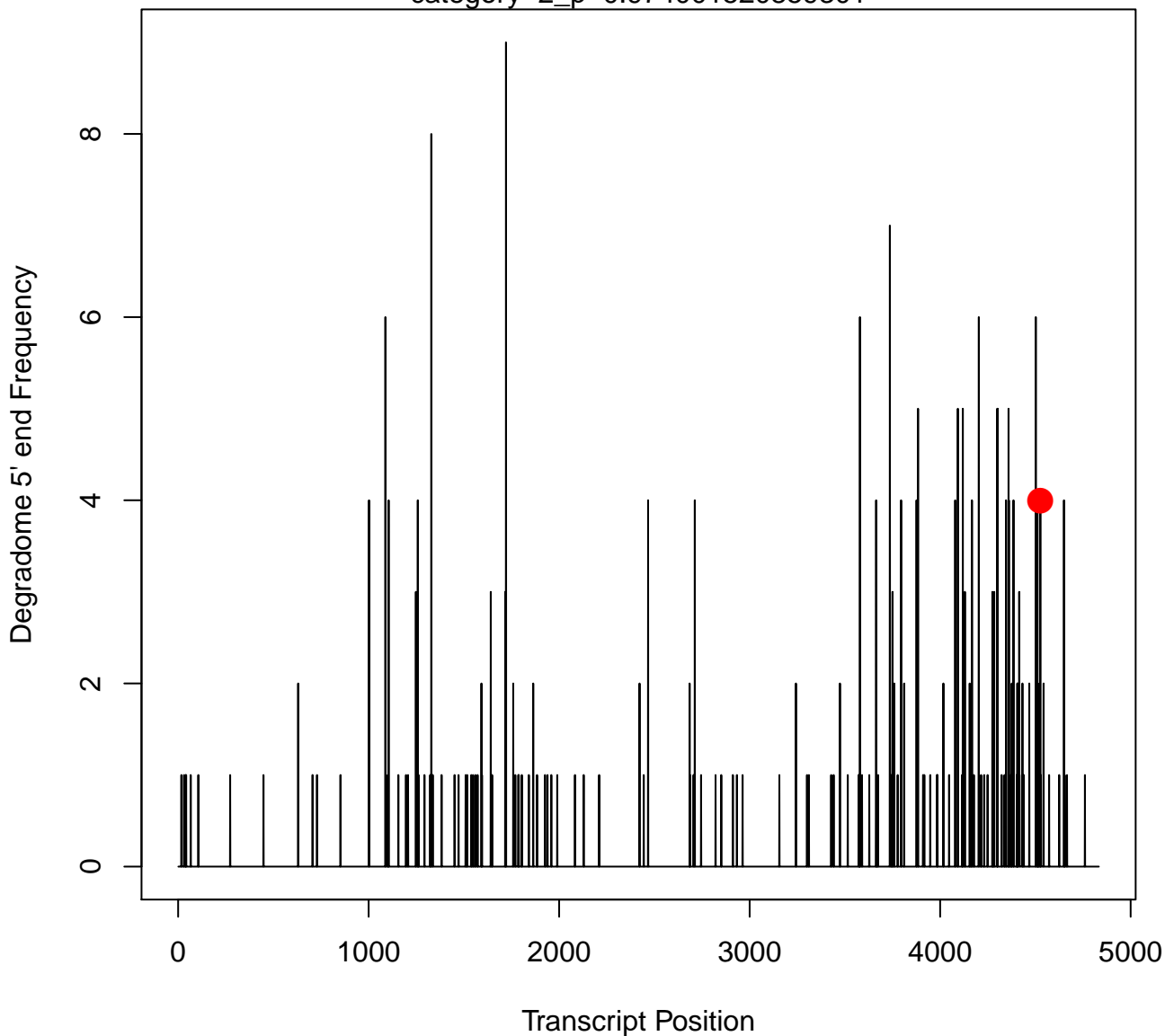

Supplement: Supplementary file 5 [file Data_Sheet_5.zip › Sit-miR160d_Seita.8G100700.1_4526_TPlot.pdf]

**T=Seita.1G231800.1\_Q=Sit-miR162\_S=995**

category=2\_p=0.783449601953312

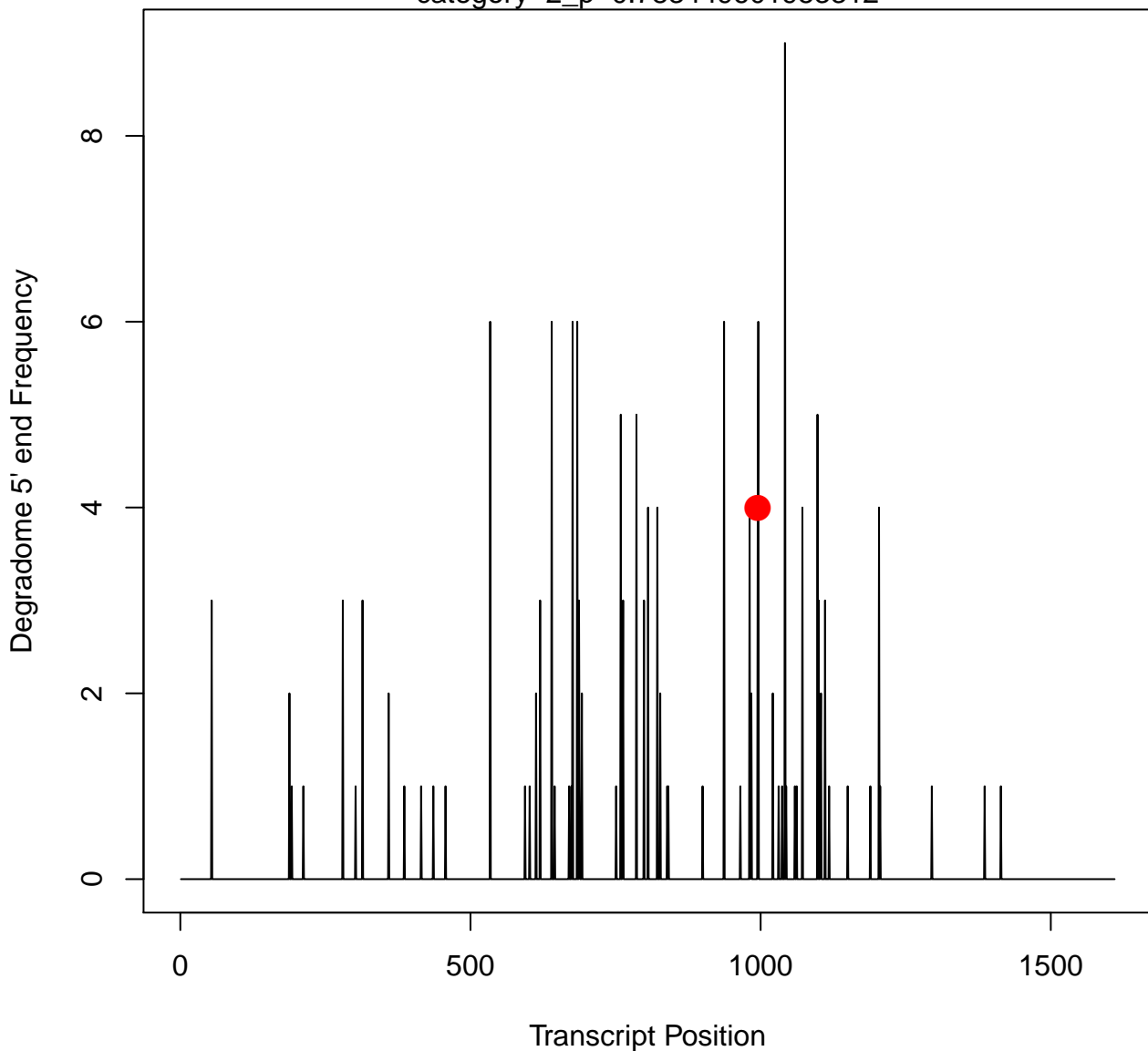

Supplement: Supplementary file 5 [file Data_Sheet_5.zip › Sit-miR162_Seita.1G231800.1_995_TPlot.pdf]

**T=Seita.2G057700.1\_Q=Sit-miR162\_S=1013**

category=2\_p=0.555608023005196

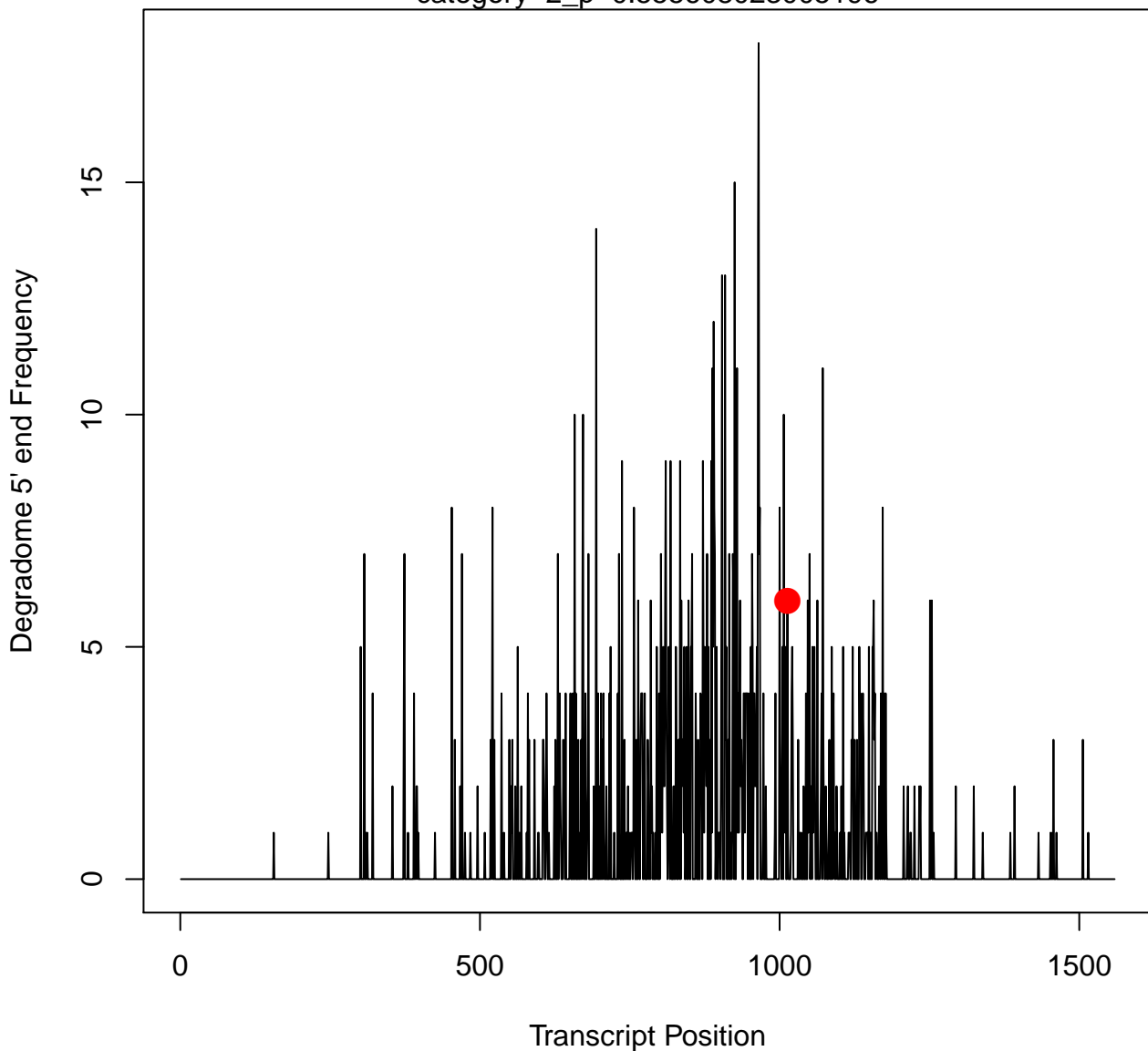

Supplement: Supplementary file 5 [file Data_Sheet_5.zip › Sit-miR162_Seita.2G057700.1_1013_TPlot.pdf]

**T=Seita.9G538200.1\_Q=Sit-miR162\_S=242**

category=0\_p=0.0474321991966474

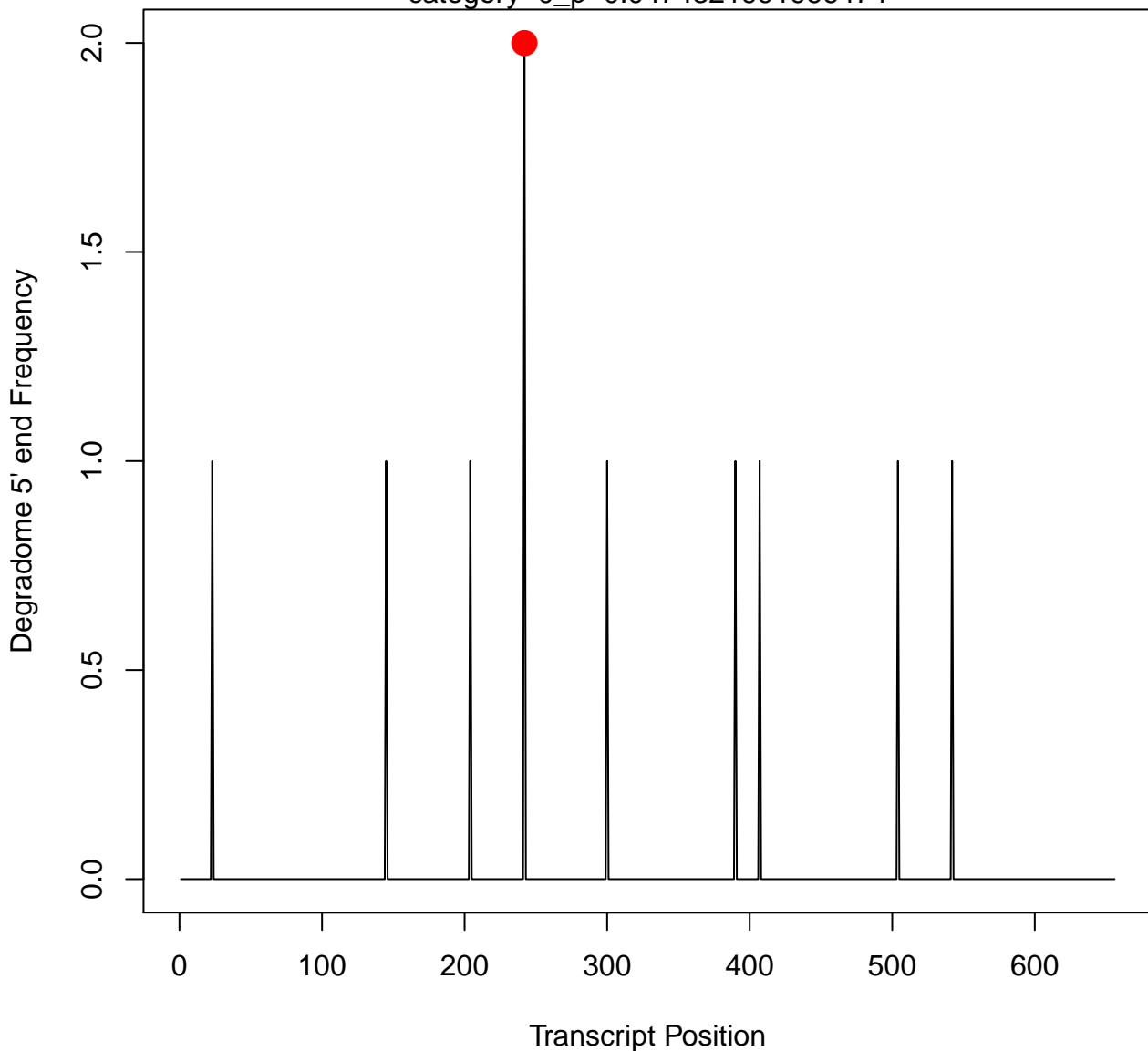

Supplement: Supplementary file 5 [file Data_Sheet_5.zip › Sit-miR162_Seita.9G538200.1_242_TPlot.pdf]

**T=Seita.9G562200.1\_Q=Sit-miR162\_S=3530**

category=2\_p=0.00917411630284182

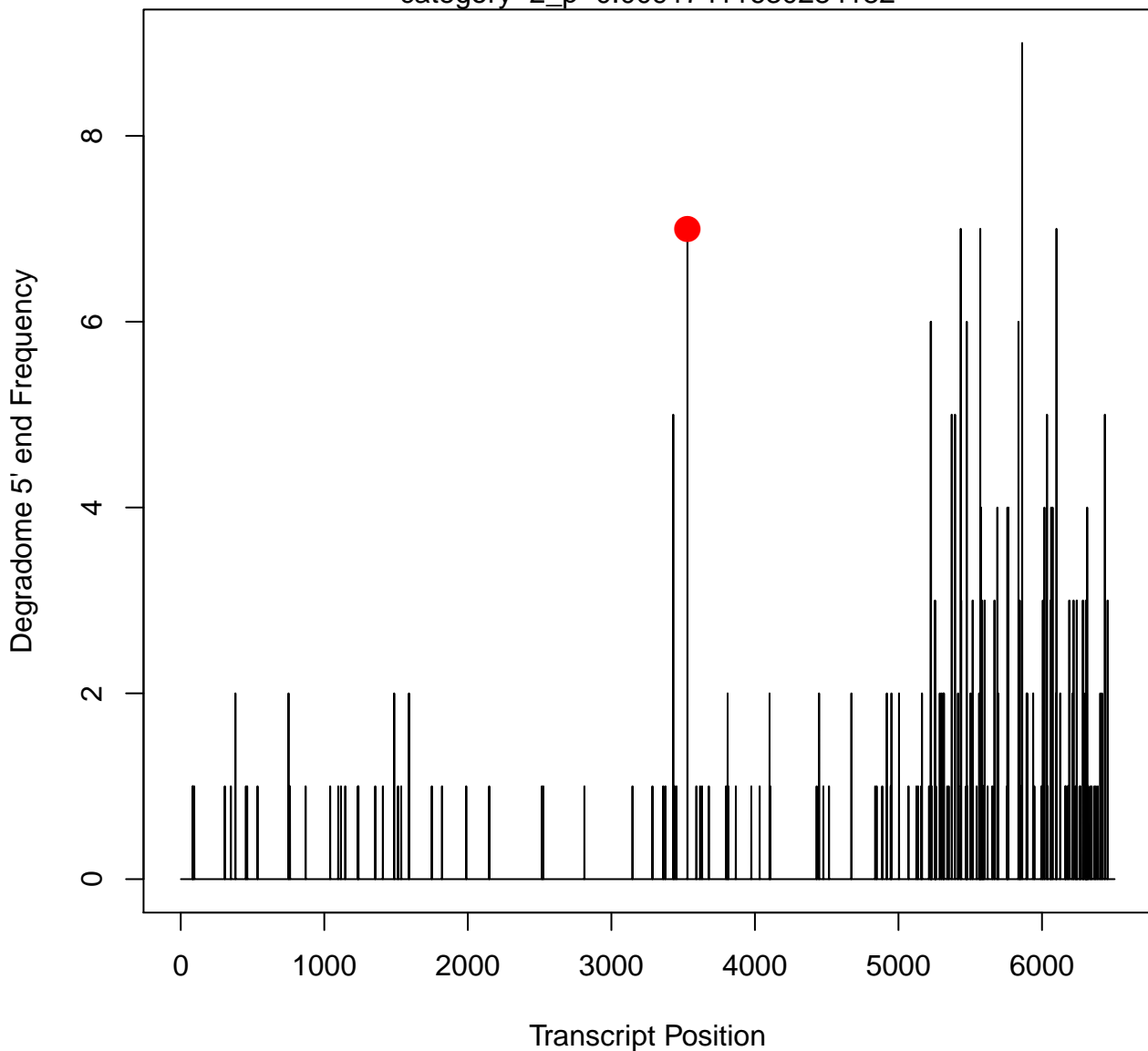

Supplement: Supplementary file 5 [file Data_Sheet_5.zip › Sit-miR162_Seita.9G562200.1_3530_TPlot.pdf]

**T=Seita.1G209000.1\_Q=Sit-miR164a\_S=999**

category=0\_p=0.00168879820125578

Degradome 5' end Frequency

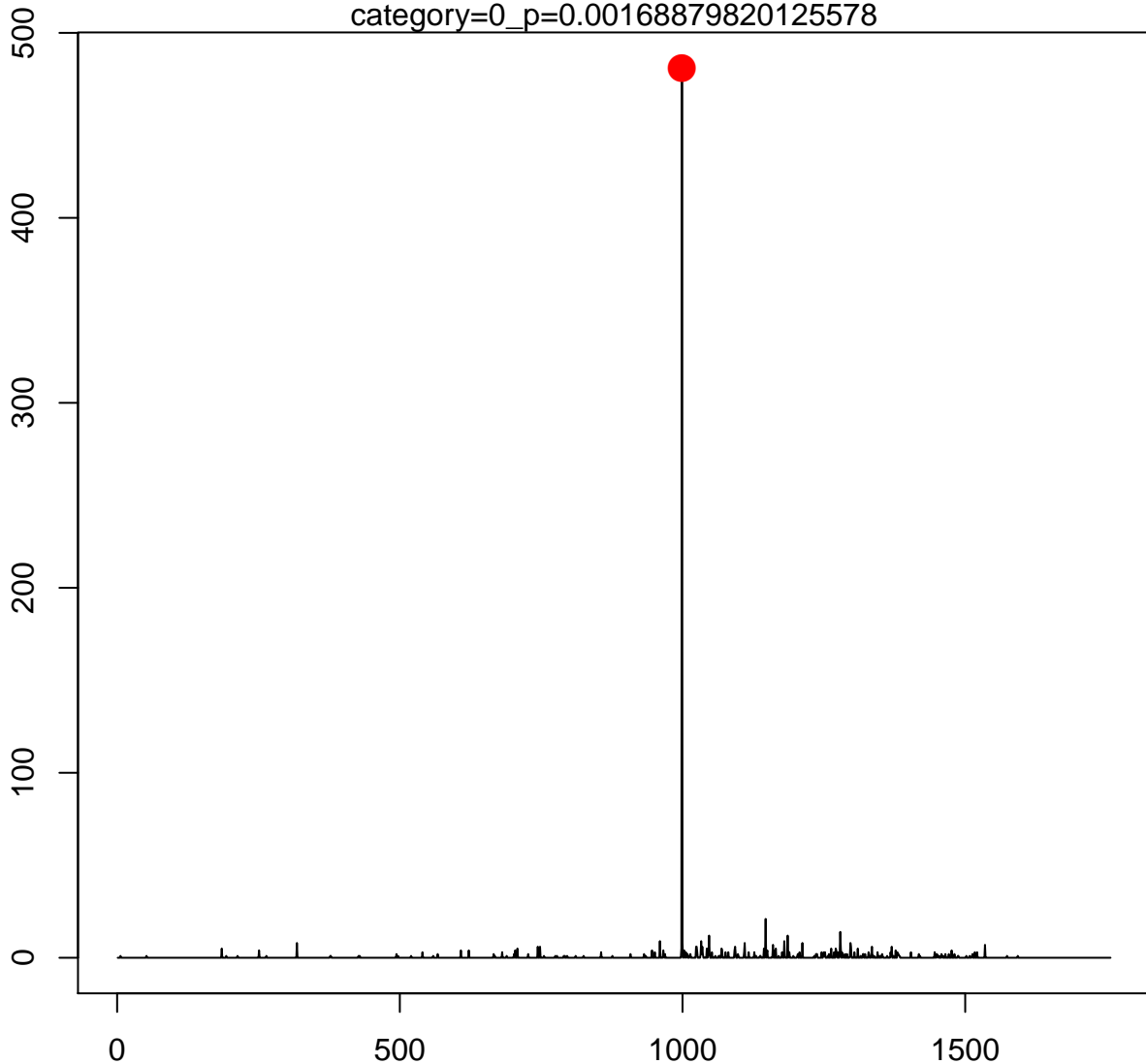

Transcript Position

Supplement: Supplementary file 5 [file Data_Sheet_5.zip › Sit-miR164a_Seita.1G209000.1_999_TPlot.pdf]

**T=Seita.7G124900.1\_Q=Sit-miR164a\_S=859**

category=0\_p=0.0021105519321345

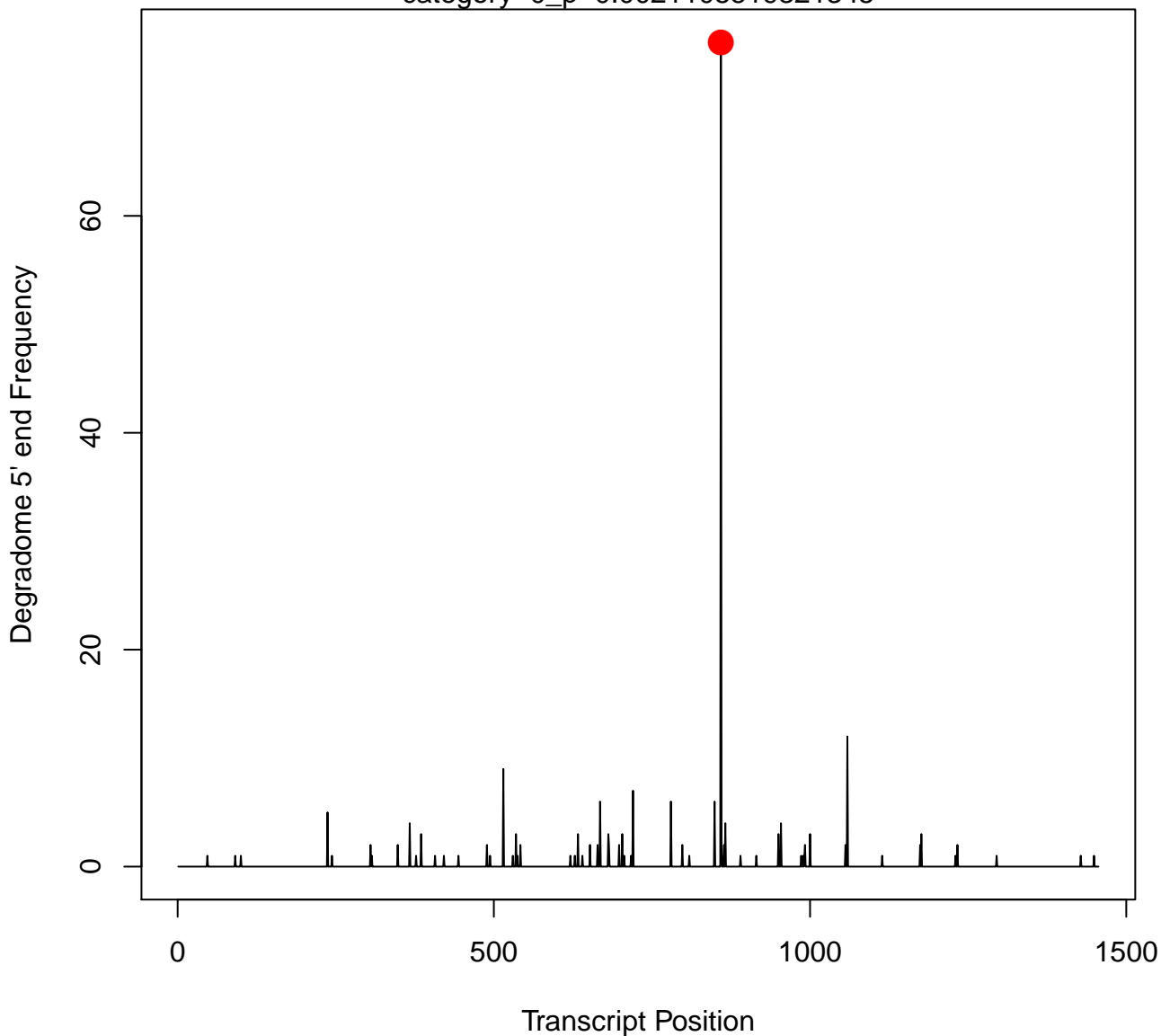

Supplement: Supplementary file 5 [file Data_Sheet_5.zip › Sit-miR164a_Seita.7G124900.1_859_TPlot.pdf]

**T=Seita.3G386200.1\_Q=Sit-miR164b\_S=790**

category=0\_p=0.000422467192713993

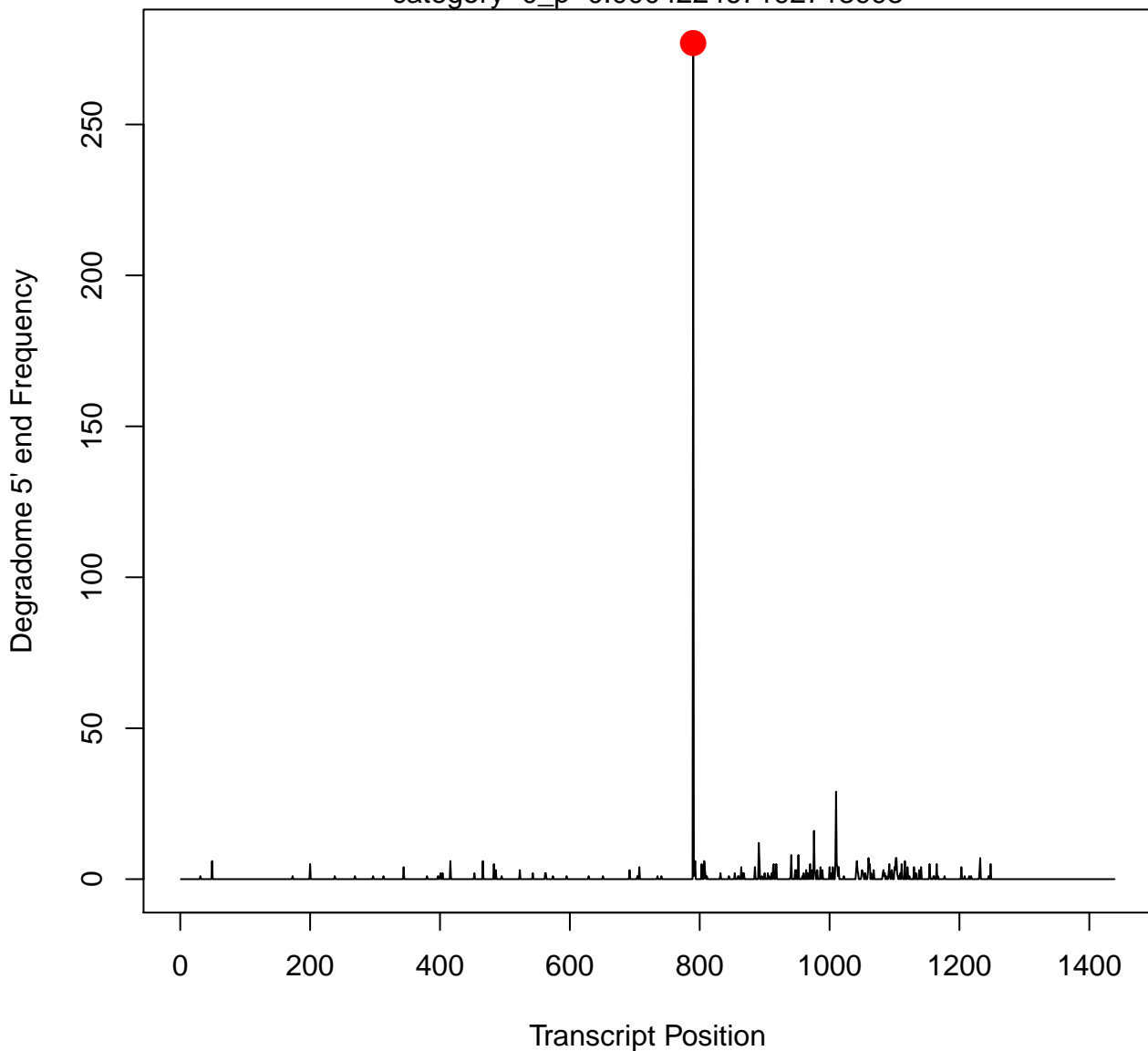

Supplement: Supplementary file 5 [file Data_Sheet_5.zip › Sit-miR164b_Seita.3G386200.1_790_TPlot.pdf]

**T=Seita.4G263400.1\_Q=Sit-miR164b\_S=1216**

category=2\_p=0.036194560204432

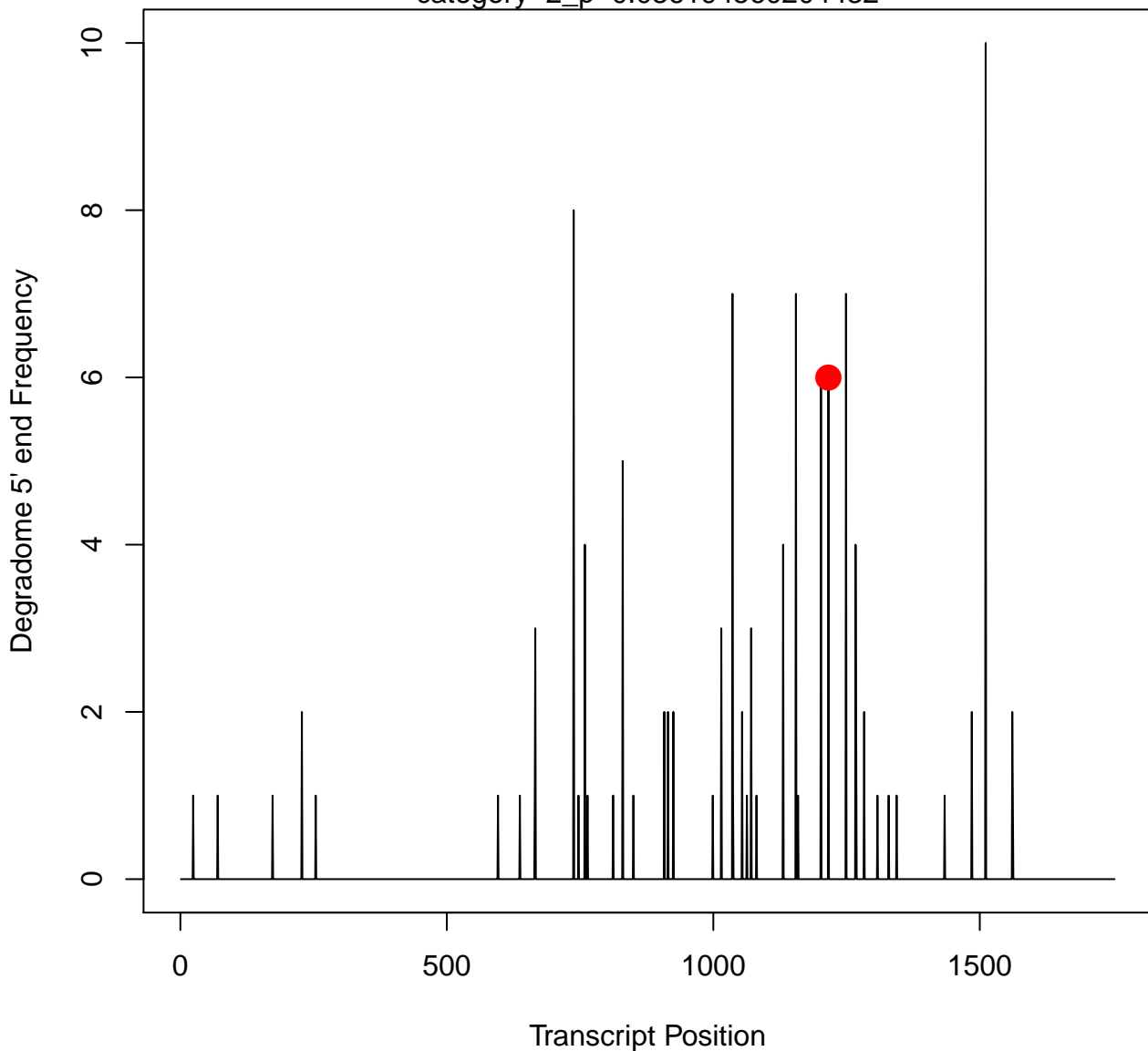

Supplement: Supplementary file 5 [file Data_Sheet_5.zip › Sit-miR164b_Seita.4G263400.1_1216_TPlot.pdf]

**T=Seita.5G001000.1\_Q=Sit-miR164b\_S=1027**

category=2\_p=0.314683678751172

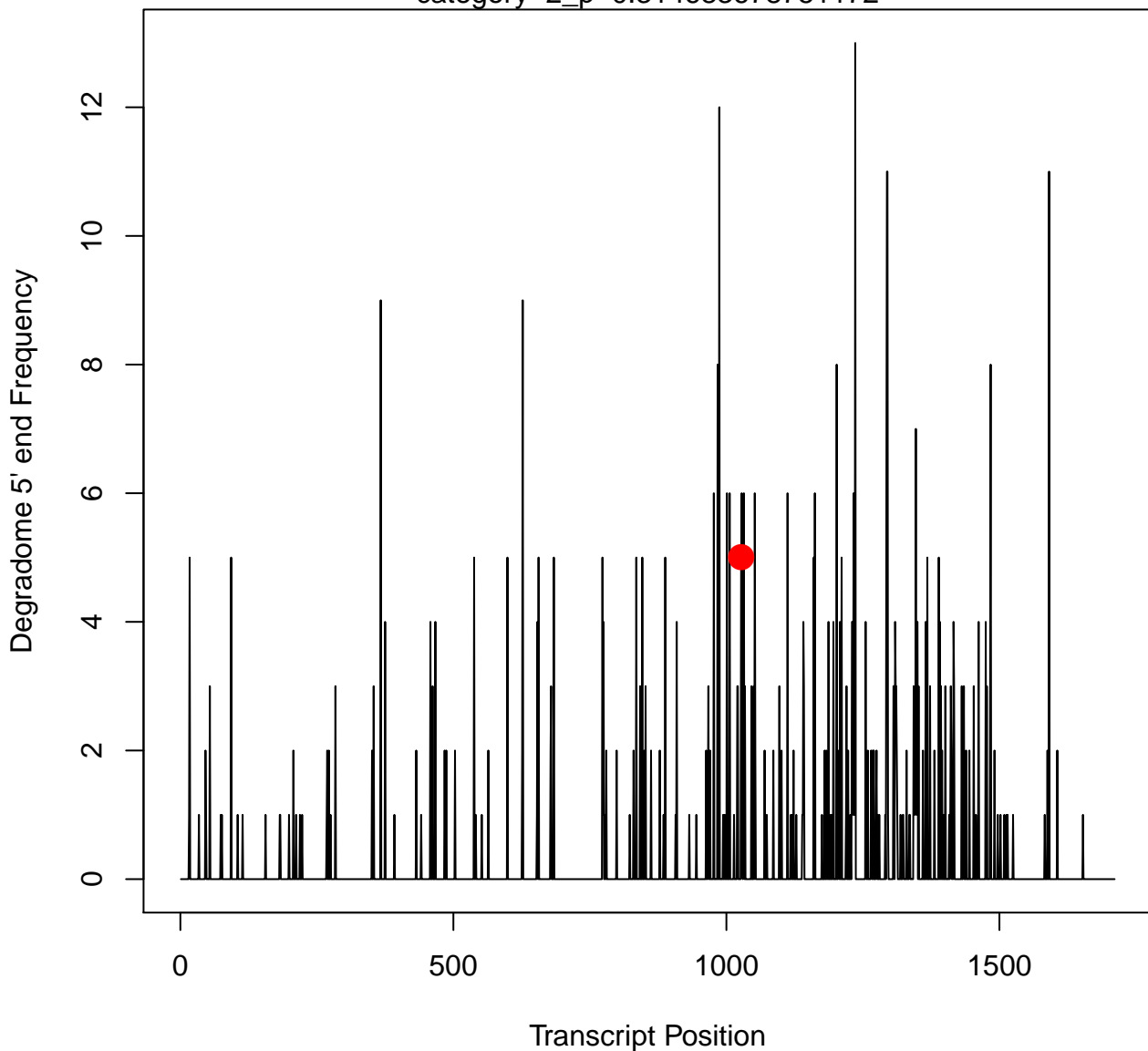

Supplement: Supplementary file 5 [file Data_Sheet_5.zip › Sit-miR164b_Seita.5G001000.1_1027_TPlot.pdf]

**T=Seita.6G171600.1\_Q=Sit-miR164b\_S=302**

category=2\_p=0.997153505603189

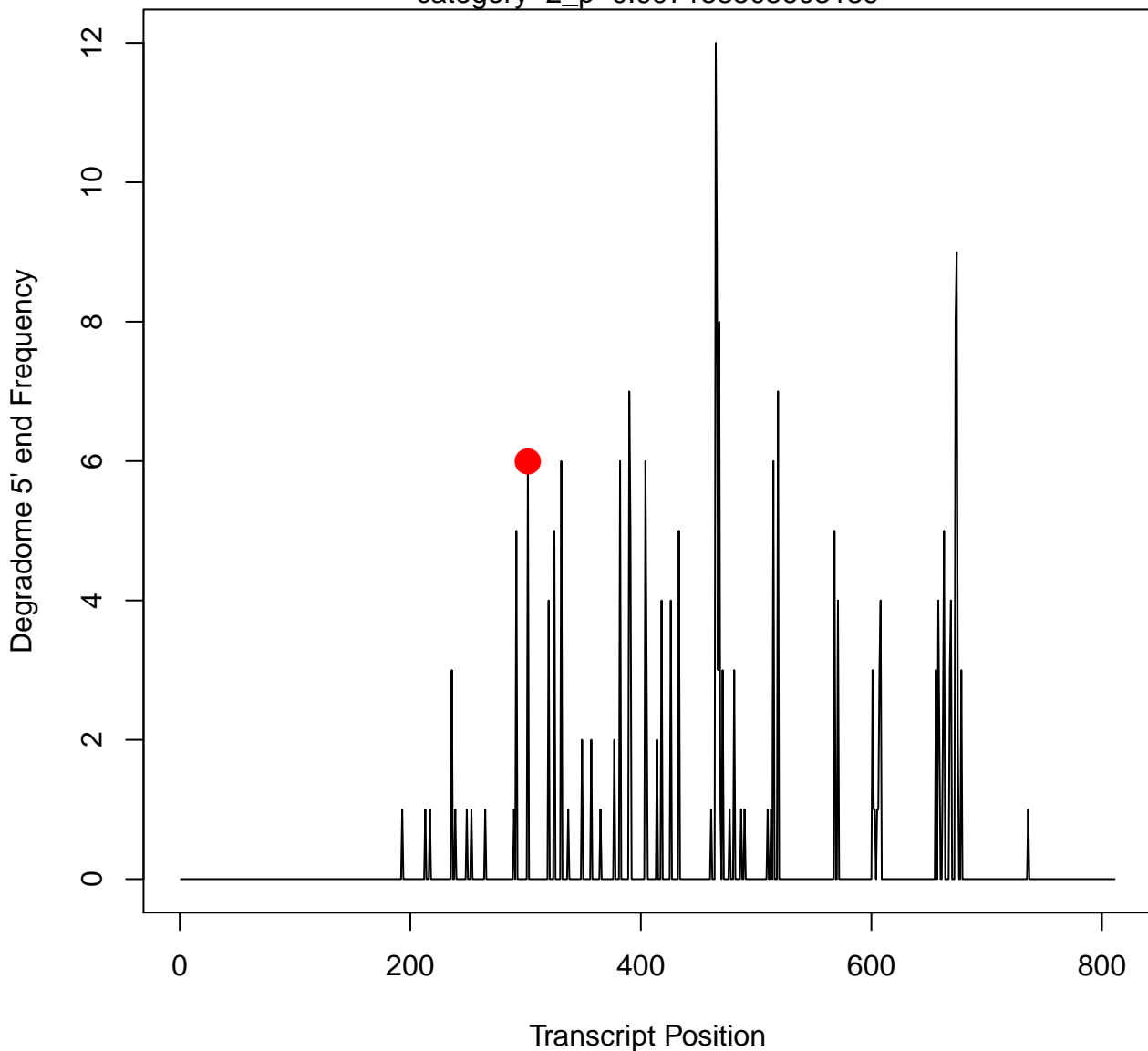

Supplement: Supplementary file 5 [file Data_Sheet_5.zip › Sit-miR164b_Seita.6G171600.1_302_TPlot.pdf]

**T=Seita.9G346700.1\_Q=Sit-miR164b\_S=228**

category=2\_p=0.909779215518226

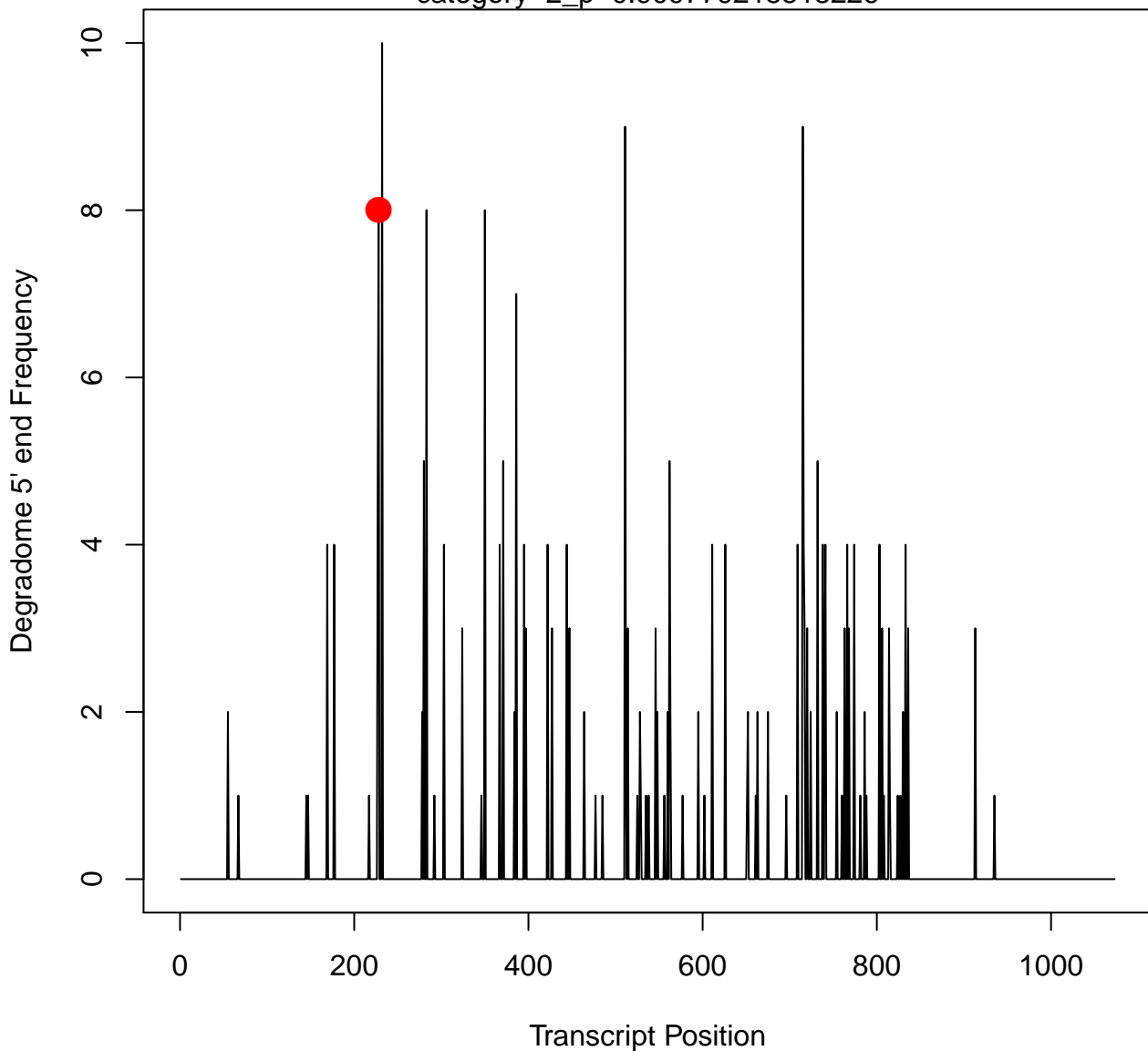

Supplement: Supplementary file 5 [file Data_Sheet_5.zip › Sit-miR164b_Seita.9G346700.1_228_TPlot.pdf]

**T=Seita.1G369500.1\_Q=Sit-miR164c\_S=3079**

category=2\_p=0.999013709586841

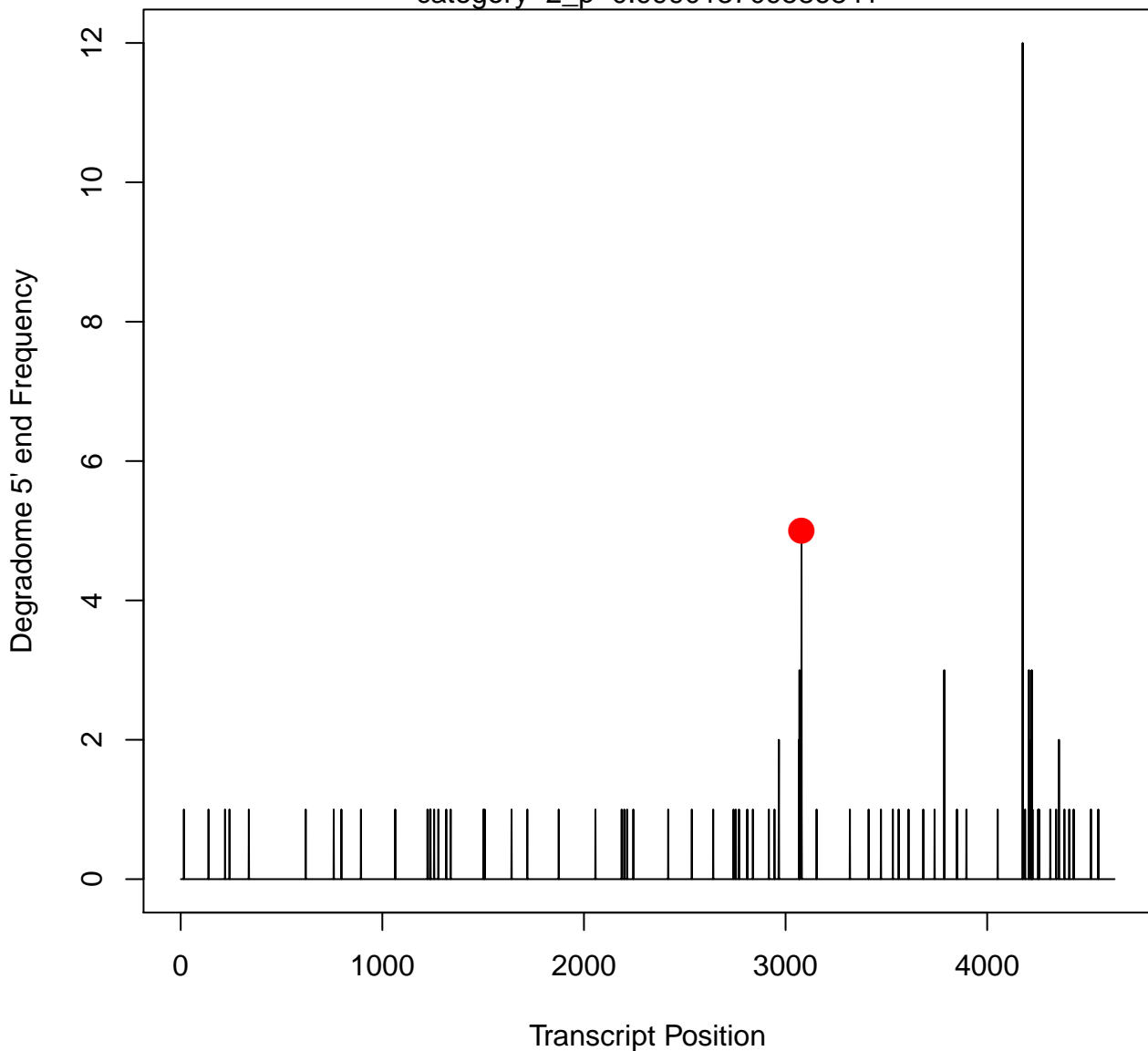

Supplement: Supplementary file 5 [file Data_Sheet_5.zip › Sit-miR164c_Seita.1G369500.1_3079_TPlot.pdf]

**T=Seita.2G386600.1\_Q=Sit-miR164c\_S=180**

category=2\_p=0.999342511870146

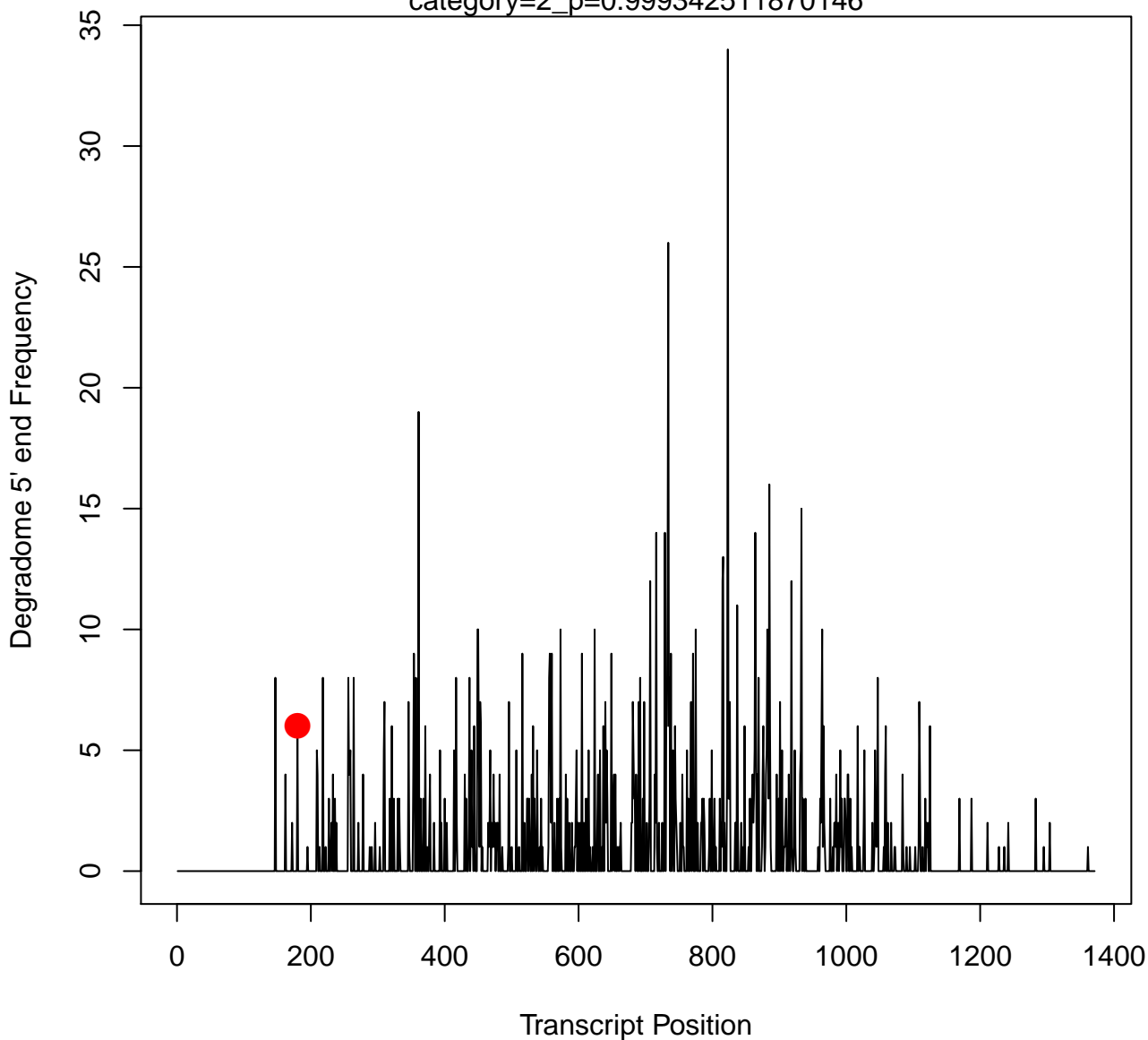

Supplement: Supplementary file 5 [file Data_Sheet_5.zip › Sit-miR164c_Seita.2G386600.1_180_TPlot.pdf]

**T=Seita.5G442800.1\_Q=Sit-miR164c\_S=1129**

category=2\_p=0.980641891100103

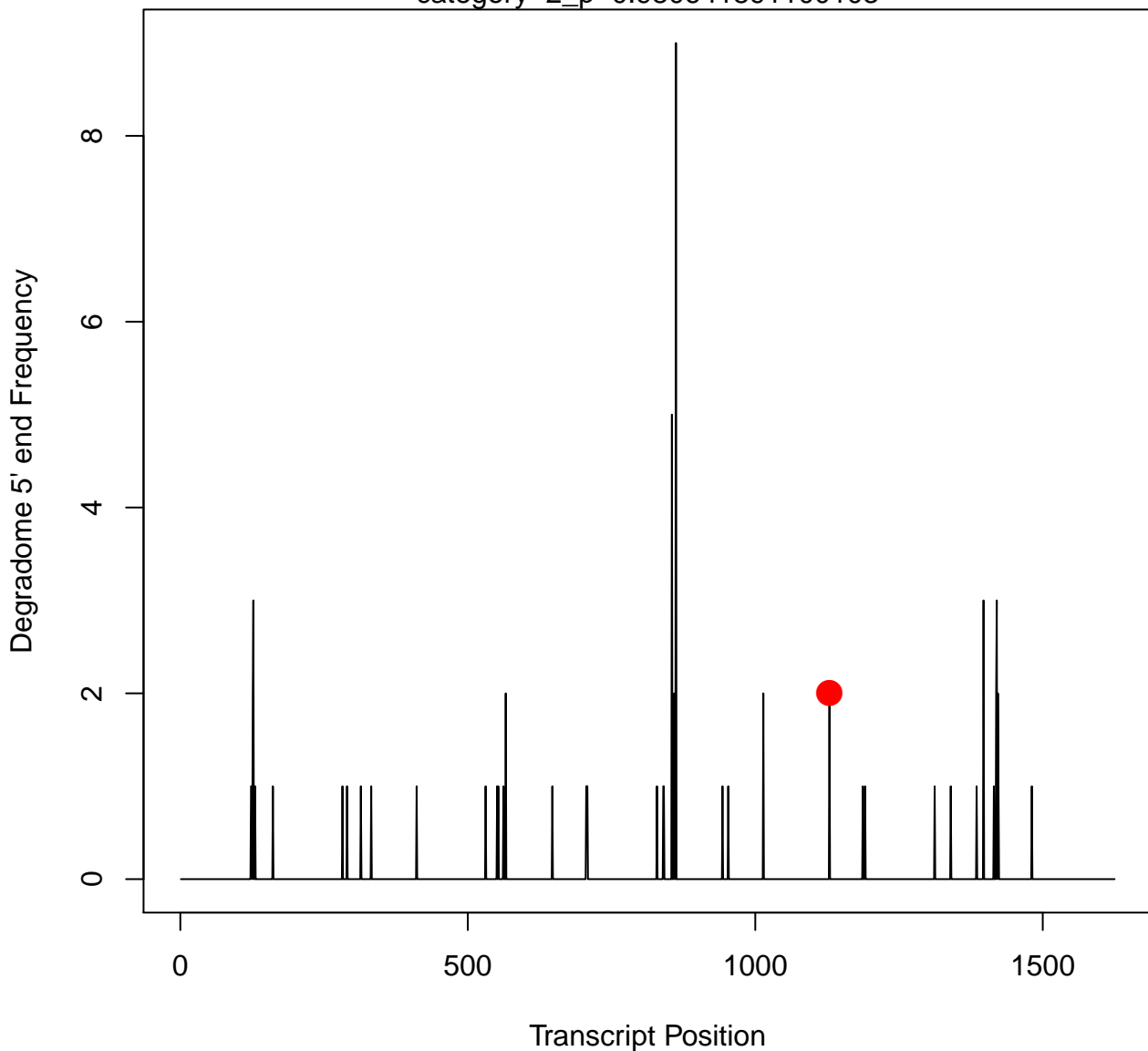

Supplement: Supplementary file 5 [file Data_Sheet_5.zip › Sit-miR164c_Seita.5G442800.1_1129_TPlot.pdf]

**T=Seita.2G368700.1\_Q=Sit-miR164e\_S=1271**

category=2\_p=0.999348543742717

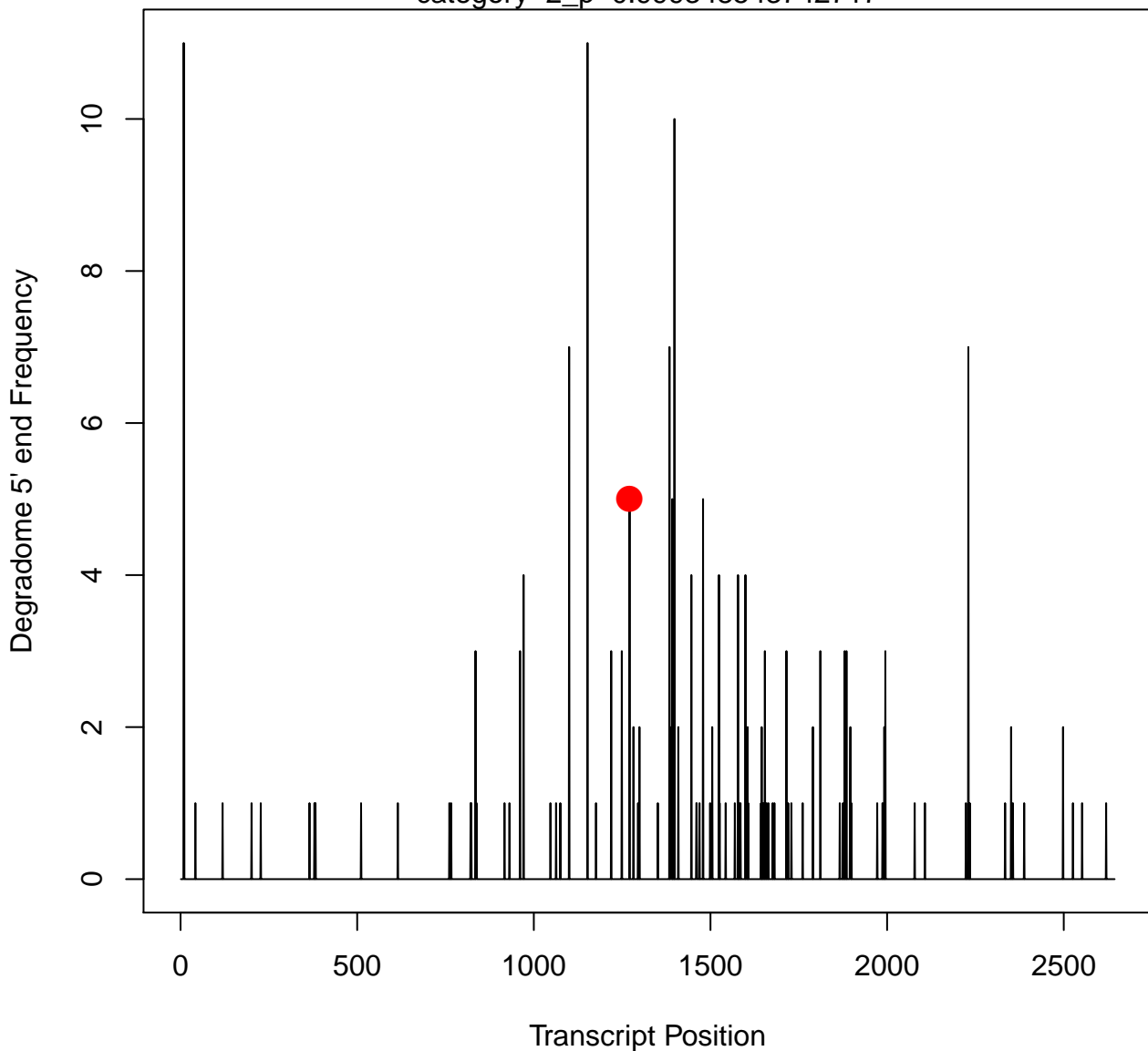

Supplement: Supplementary file 5 [file Data_Sheet_5.zip › Sit-miR164e_Seita.2G368700.1_1271_TPlot.pdf]

**T=Seita.3G098500.1\_Q=Sit-miR164e\_S=201**

category=1\_p=0.172792564839103

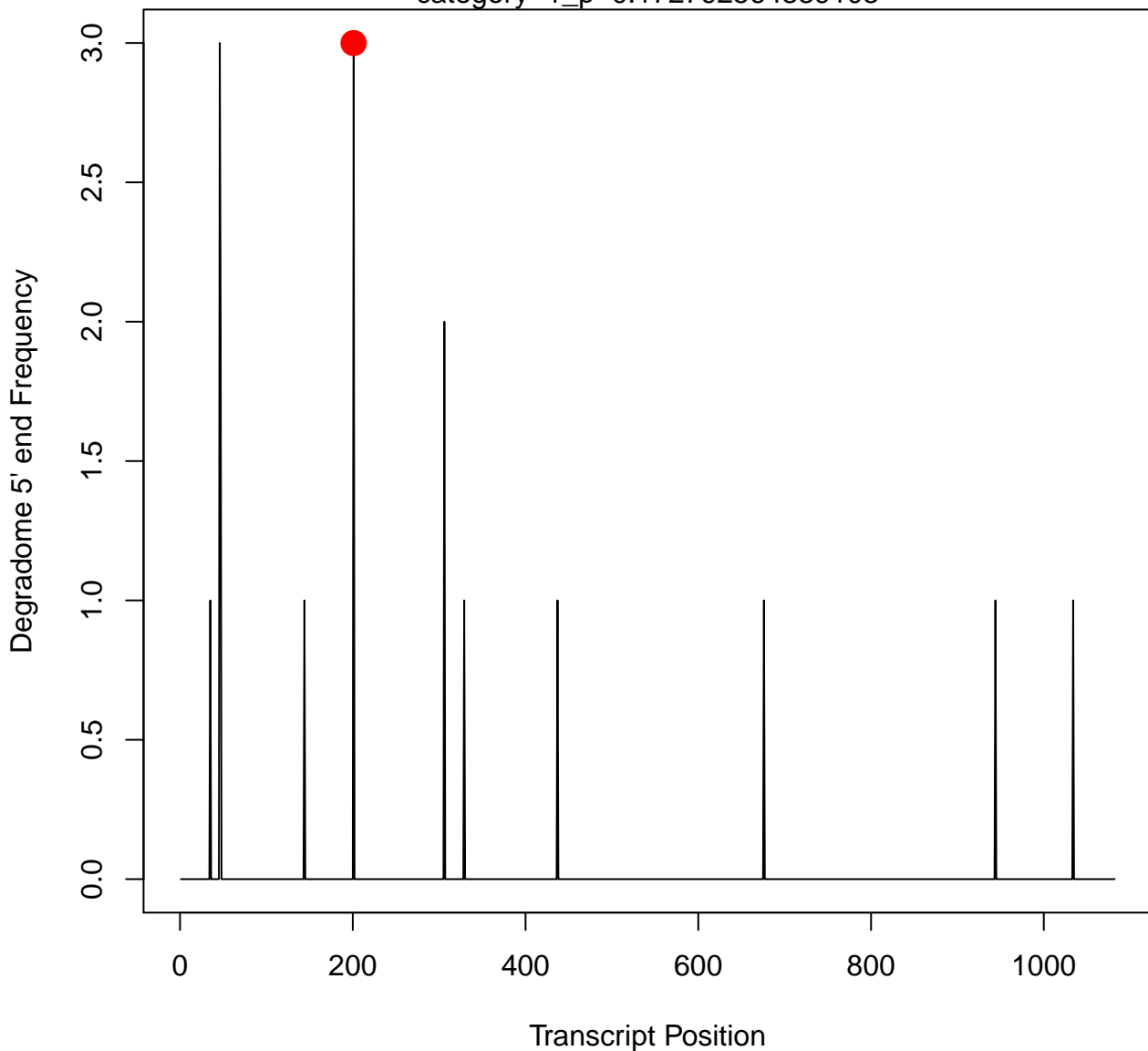

Supplement: Supplementary file 5 [file Data_Sheet_5.zip › Sit-miR164e_Seita.3G098500.1_201_TPlot.pdf]

**T=Seita.4G090500.1\_Q=Sit-miR164e\_S=744**

category=2\_p=0.98558621917258

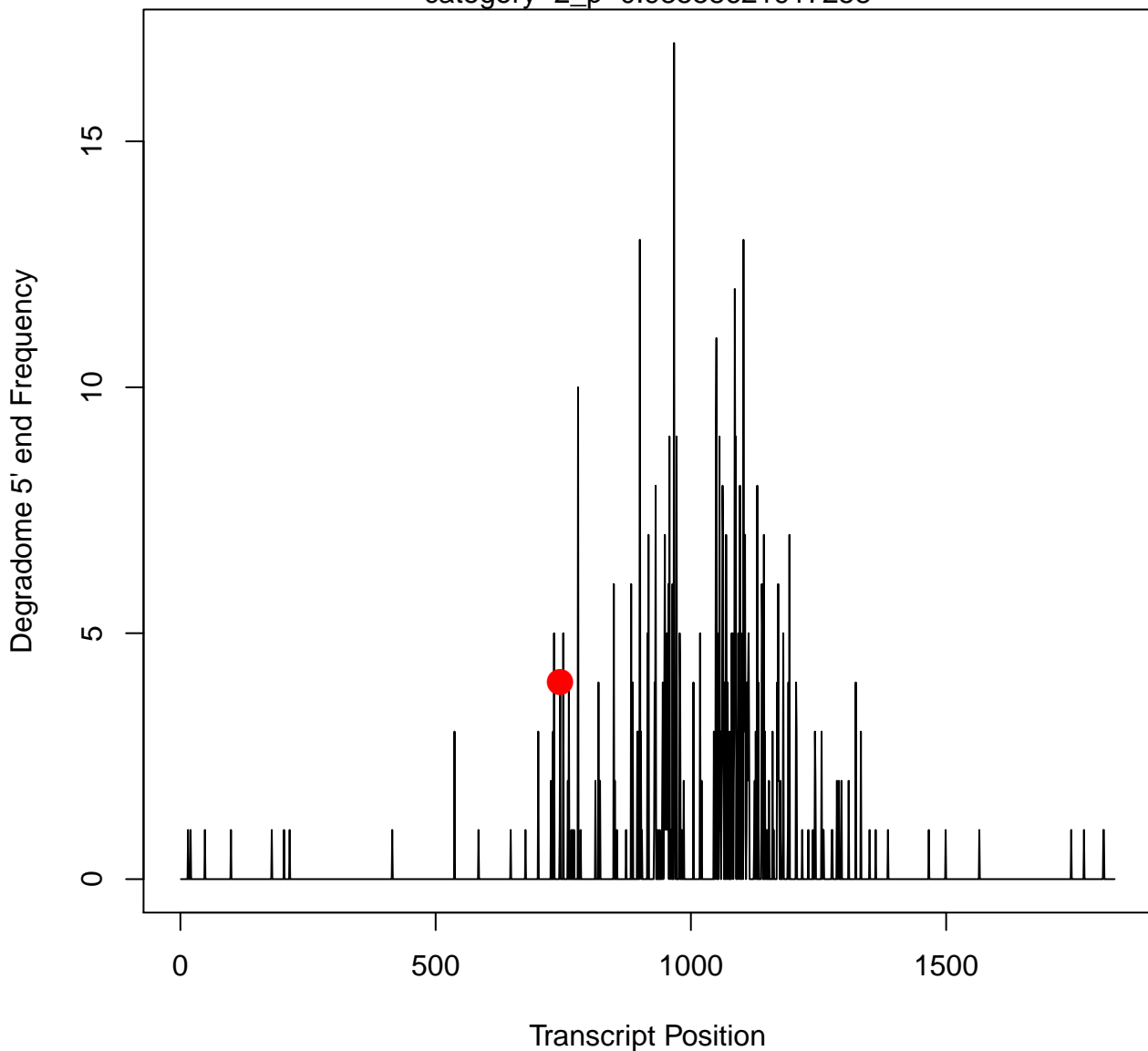

Supplement: Supplementary file 5 [file Data_Sheet_5.zip › Sit-miR164e_Seita.4G090500.1_744_TPlot.pdf]

**T=Seita.4G289900.1\_Q=Sit-miR164e\_S=599**

category=2\_p=0.623540389228512

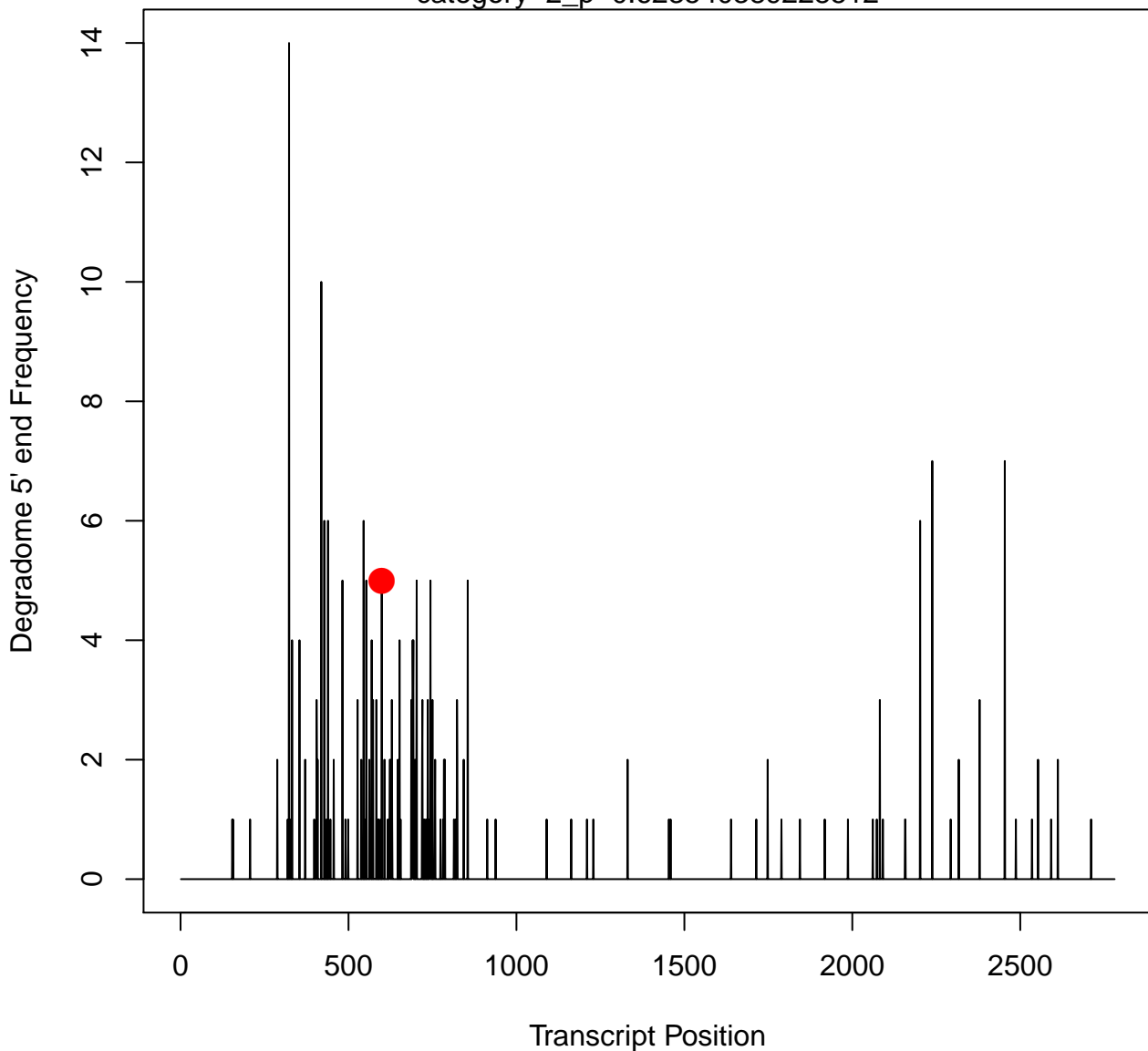

Supplement: Supplementary file 5 [file Data_Sheet_5.zip › Sit-miR164e_Seita.4G289900.1_599_TPlot.pdf]

**T=Seita.7G031200.1\_Q=Sit-miR164e\_S=548**

category=2\_p=0.703756169604845

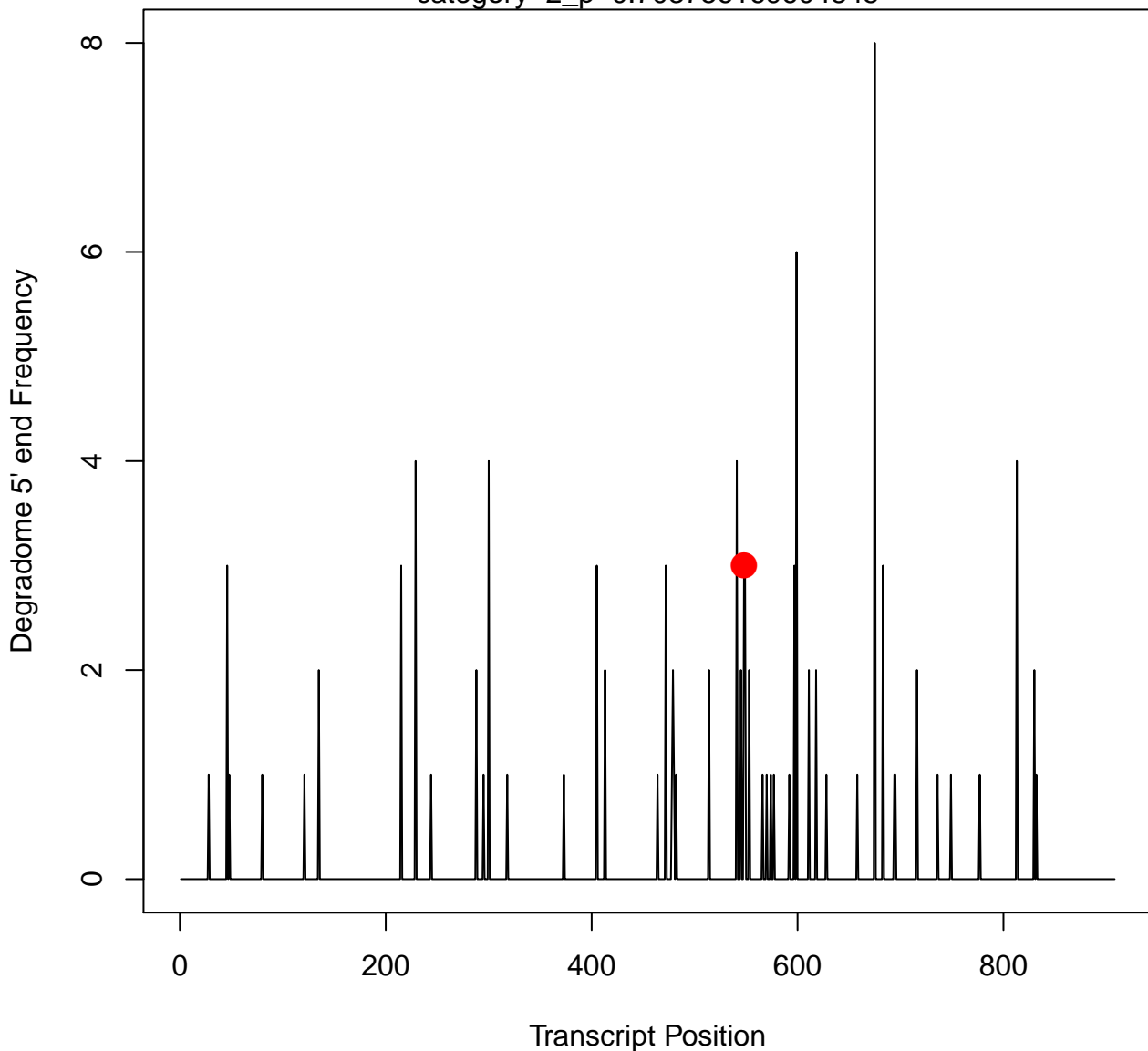

Supplement: Supplementary file 5 [file Data_Sheet_5.zip › Sit-miR164e_Seita.7G031200.1_548_TPlot.pdf]

**T=Seita.9G320000.1\_Q=Sit-miR164e\_S=1141**

category=2\_p=0.984339637007677

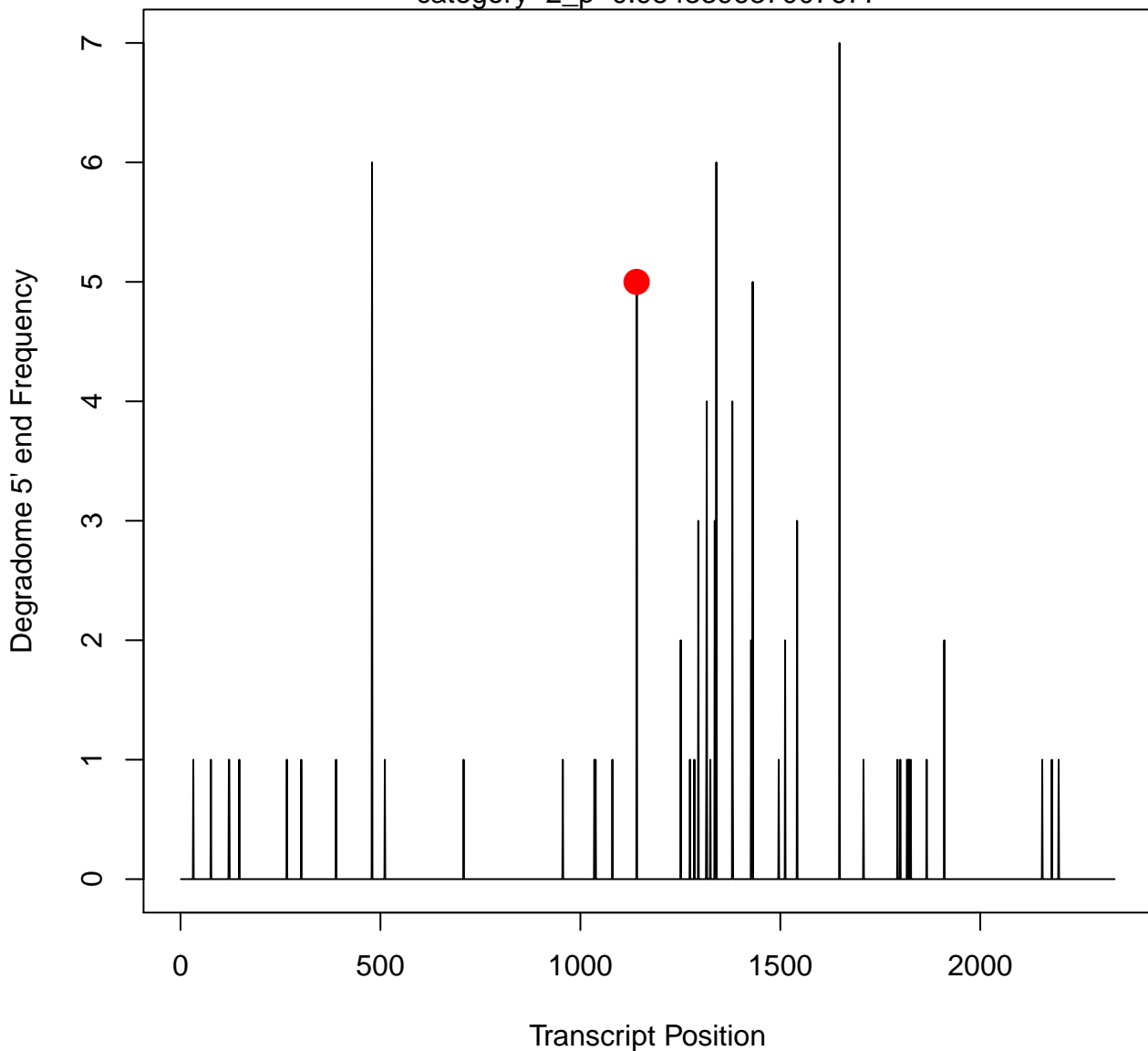

Supplement: Supplementary file 5 [file Data_Sheet_5.zip › Sit-miR164e_Seita.9G320000.1_1141_TPlot.pdf]

**T=Seita.J003600.1\_Q=Sit-miR164e\_S=116**

category=0\_p=0.219341967755437

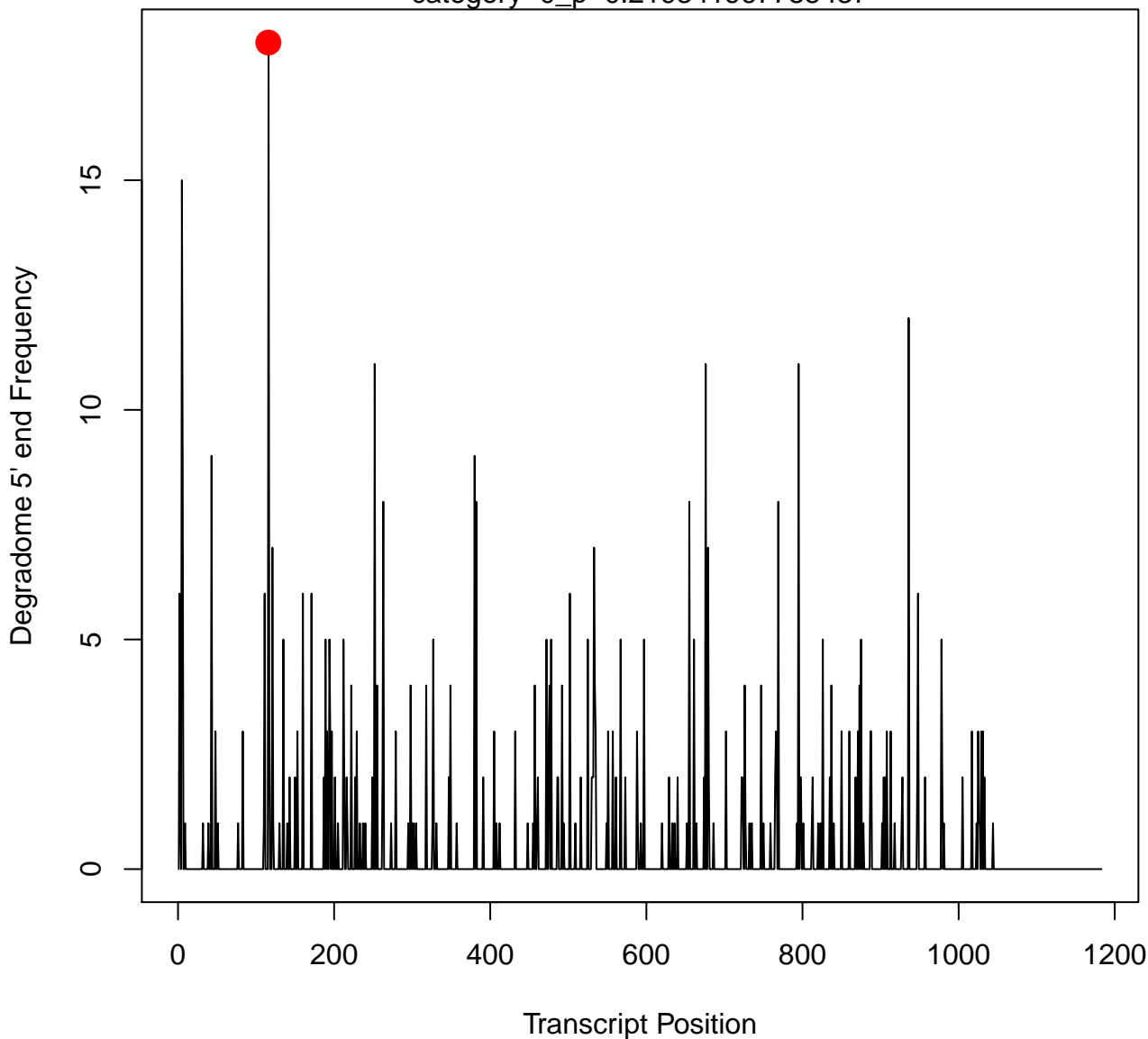

Supplement: Supplementary file 5 [file Data_Sheet_5.zip › Sit-miR164e_Seita.J003600.1_116_TPlot.pdf]

**T=Seita.1G273100.1\_Q=Sit-miR164f\_S=359**

category=2\_p=0.98558621917258

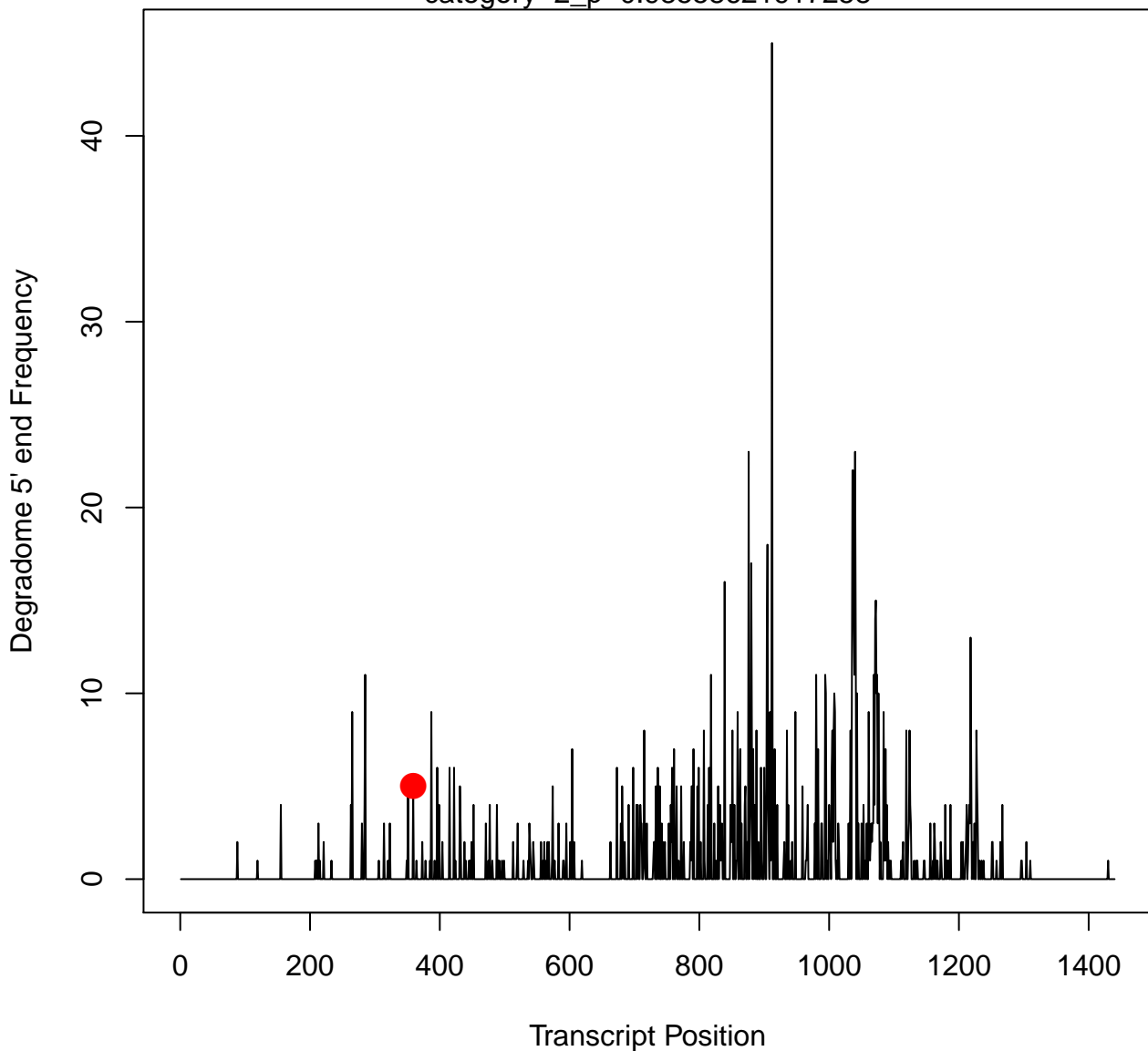

Supplement: Supplementary file 5 [file Data_Sheet_5.zip › Sit-miR164f_Seita.1G273100.1_359_TPlot.pdf]

**T=Seita.2G408400.1\_Q=Sit-miR164f\_S=798**

category=2\_p=0.9957691797755

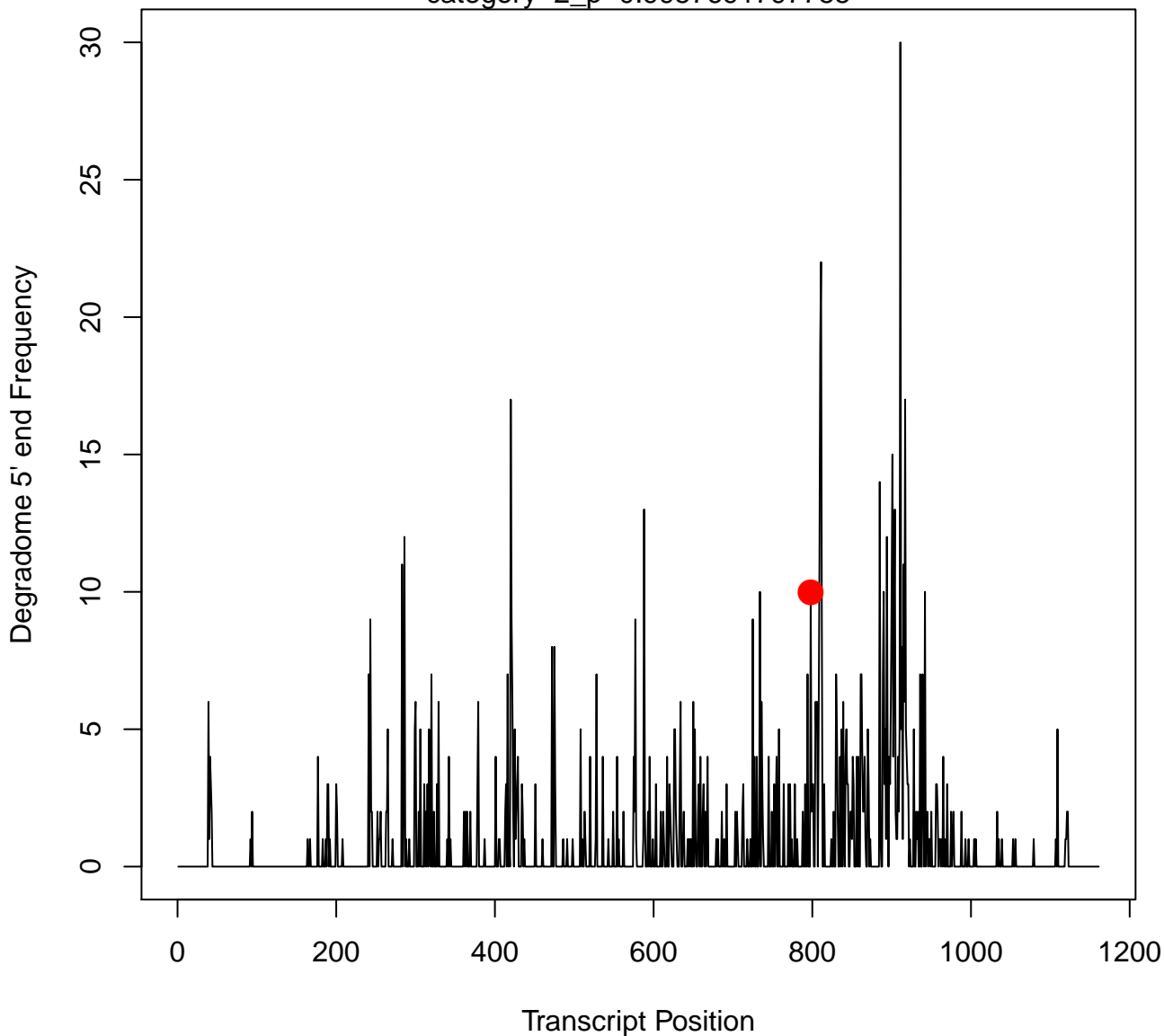

Supplement: Supplementary file 5 [file Data_Sheet_5.zip › Sit-miR164f_Seita.2G408400.1_798_TPlot.pdf]

**T=Seita.6G097500.1\_Q=Sit-miR164f\_S=1205**

category=2\_p=0.980462653208387

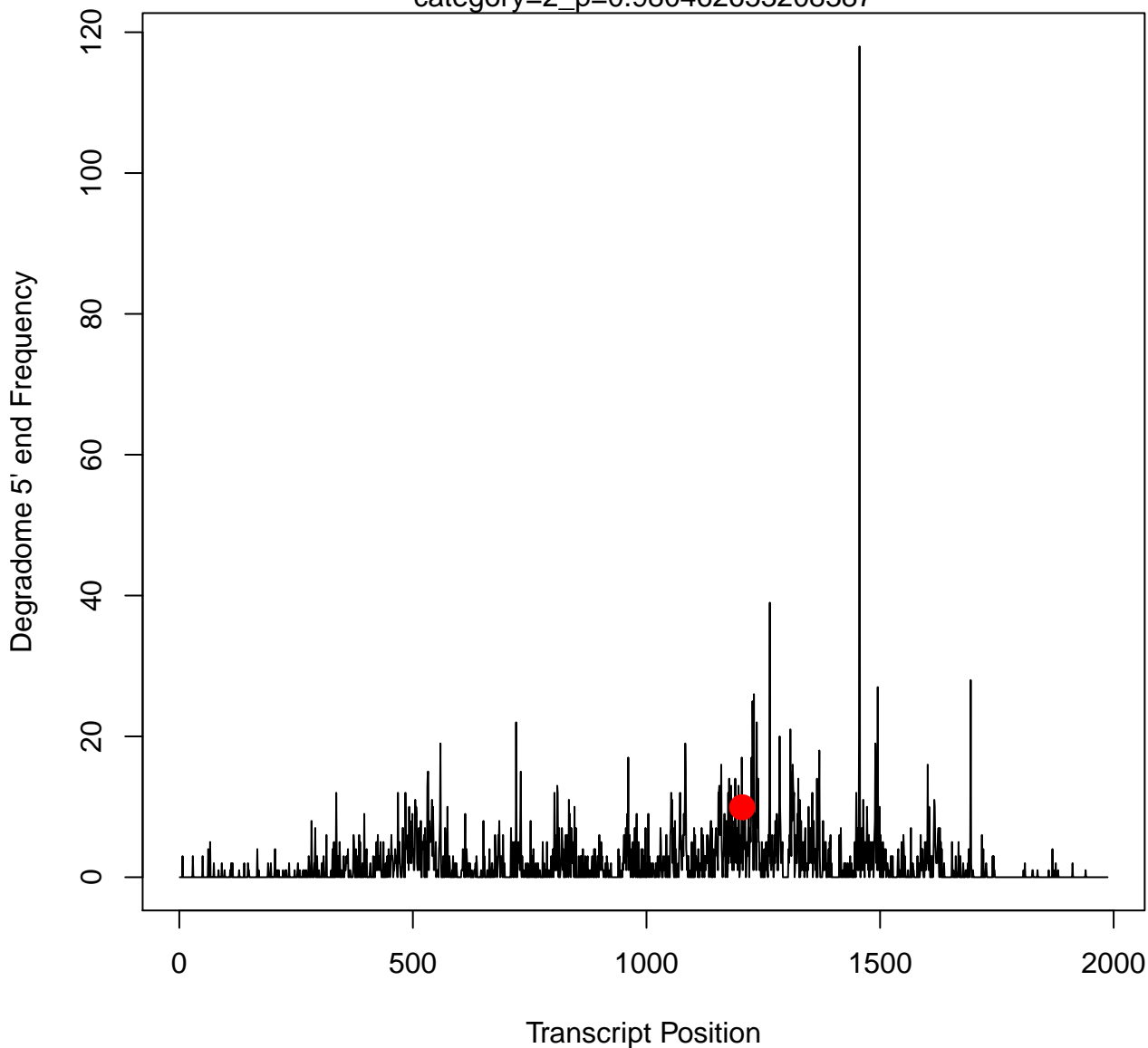

Supplement: Supplementary file 5 [file Data_Sheet_5.zip › Sit-miR164f_Seita.6G097500.1_1205_TPlot.pdf]

**T=Seita.3G395000.1\_Q=Sit-miR166a\_S=774**

category=2\_p=0.0272706278127964

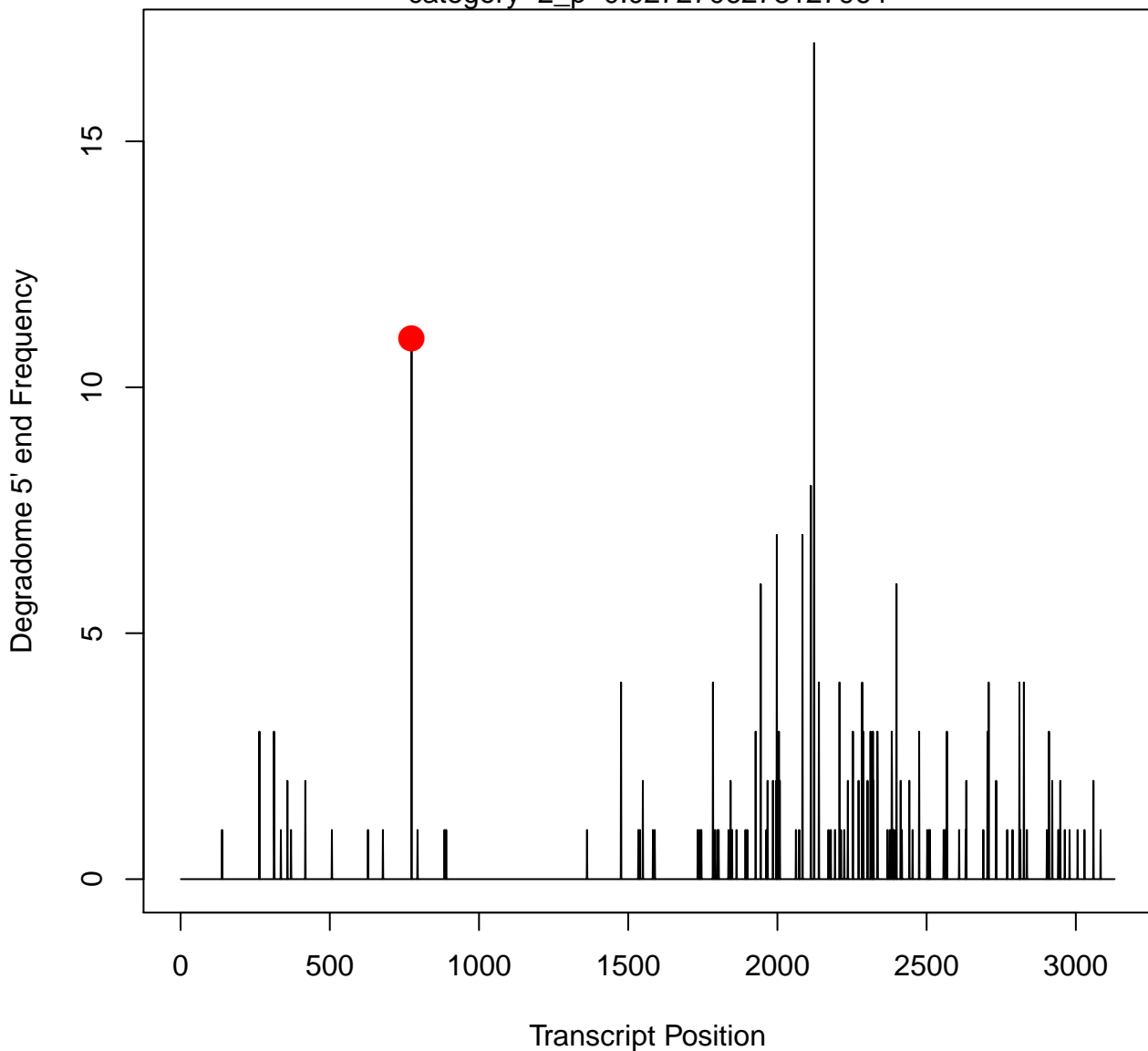

Supplement: Supplementary file 5 [file Data_Sheet_5.zip › Sit-miR166a_Seita.3G395000.1_774_TPlot.pdf]

**T=Seita.7G250800.1\_Q=Sit-miR166a\_S=1446**

category=2\_p=0.605786336945431

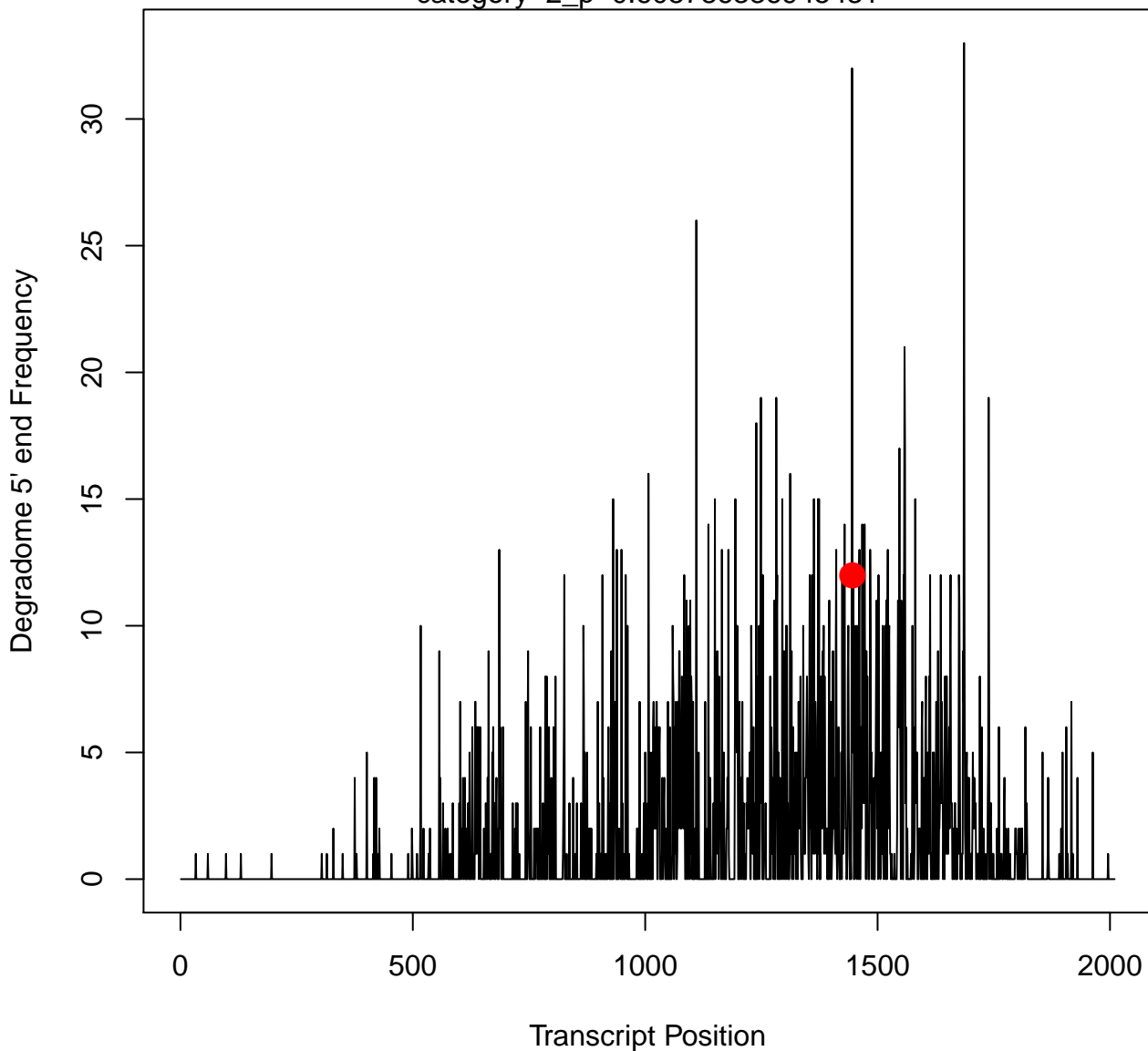

Supplement: Supplementary file 5 [file Data_Sheet_5.zip › Sit-miR166a_Seita.7G250800.1_1446_TPlot.pdf]

**T=Seita.6G173700.1\_Q=Sit-miR166b\_S=1232**

category=2\_p=0.932200851261624

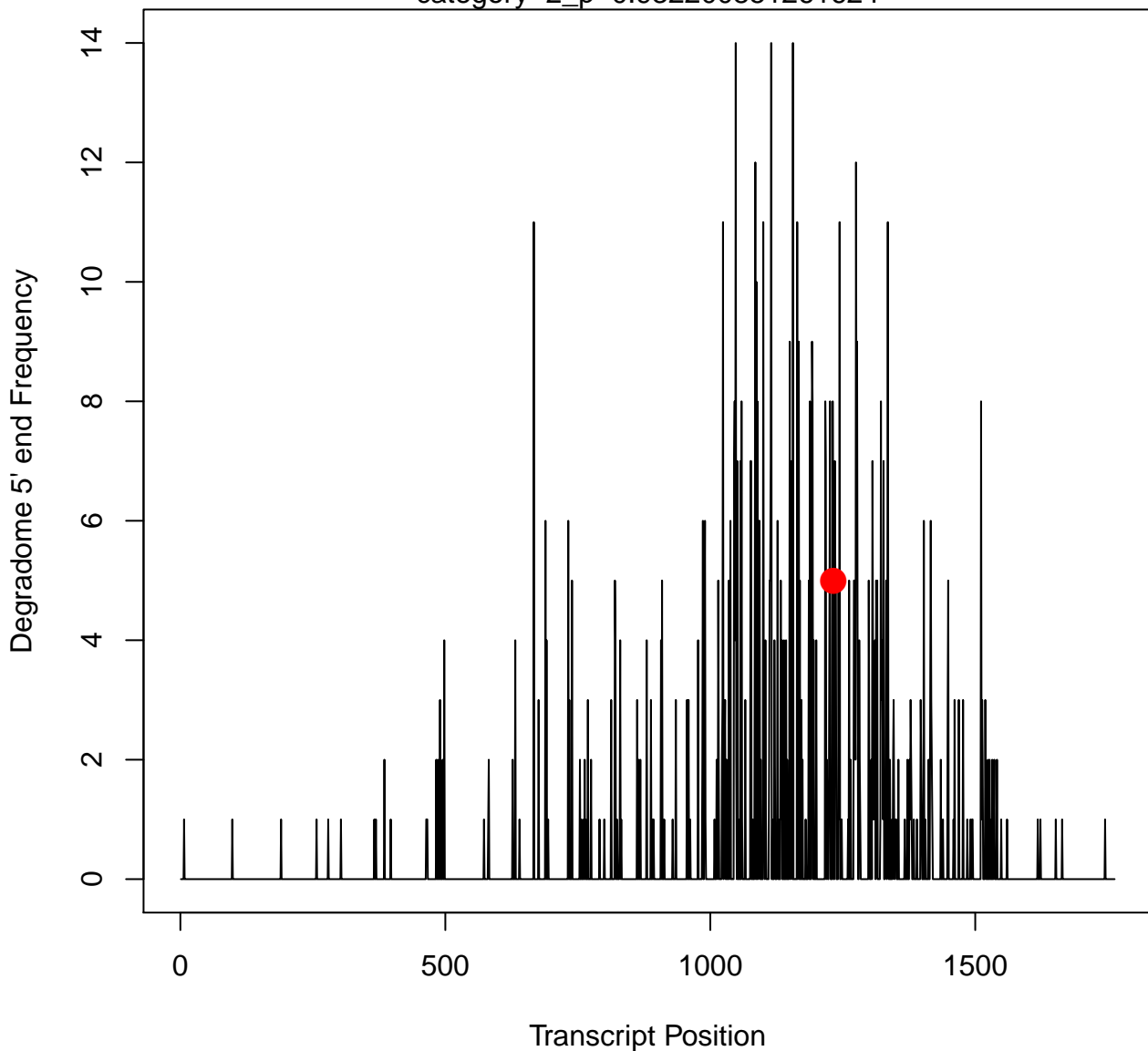

Supplement: Supplementary file 5 [file Data_Sheet_5.zip › Sit-miR166b_Seita.6G173700.1_1232_TPlot.pdf]

**T=Seita.5G381800.1\_Q=Sit-miR166d\_S=188**

category=1\_p=0.0558412232857216

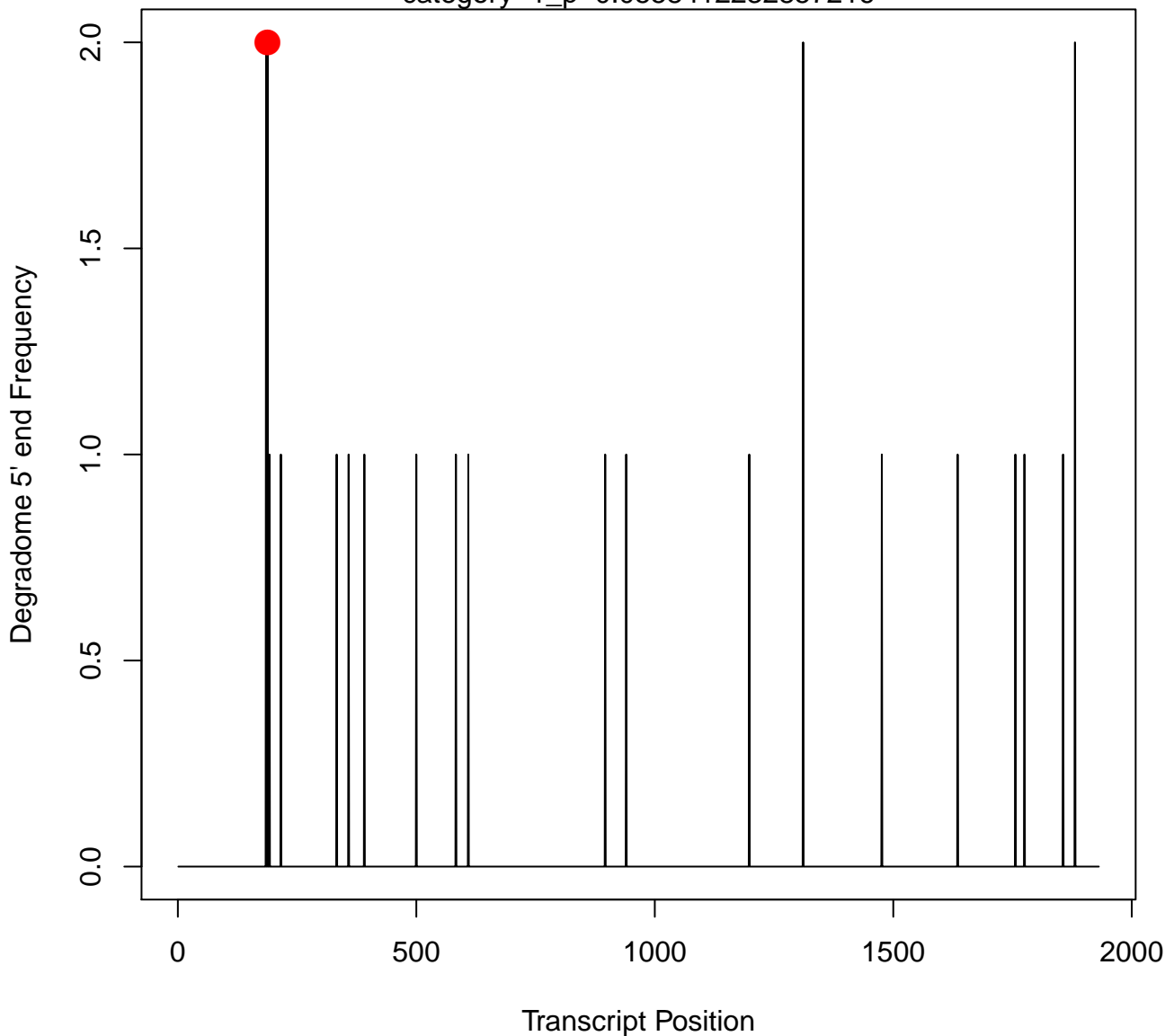

Supplement: Supplementary file 5 [file Data_Sheet_5.zip › Sit-miR166d_Seita.5G381800.1_188_TPlot.pdf]

**T=Seita.8G179500.1\_Q=Sit-miR166f\_S=1695**

category=2\_p=0.919225418138548

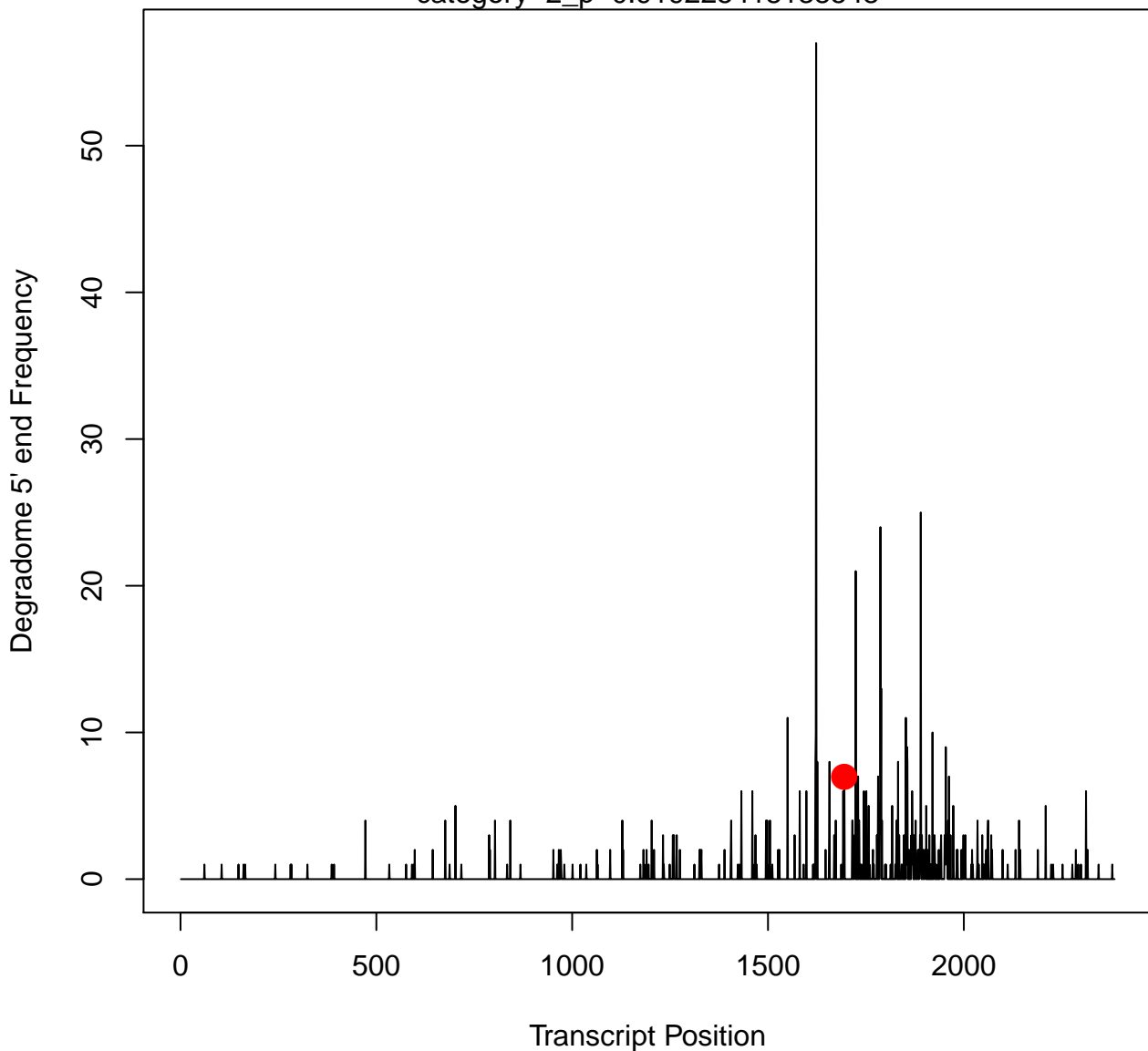

Supplement: Supplementary file 5 [file Data_Sheet_5.zip › Sit-miR166f_Seita.8G179500.1_1695_TPlot.pdf]

**T=Seita.5G390300.1\_Q=Sit-miR166i\_S=1096**

category=2\_p=0.475417219890759

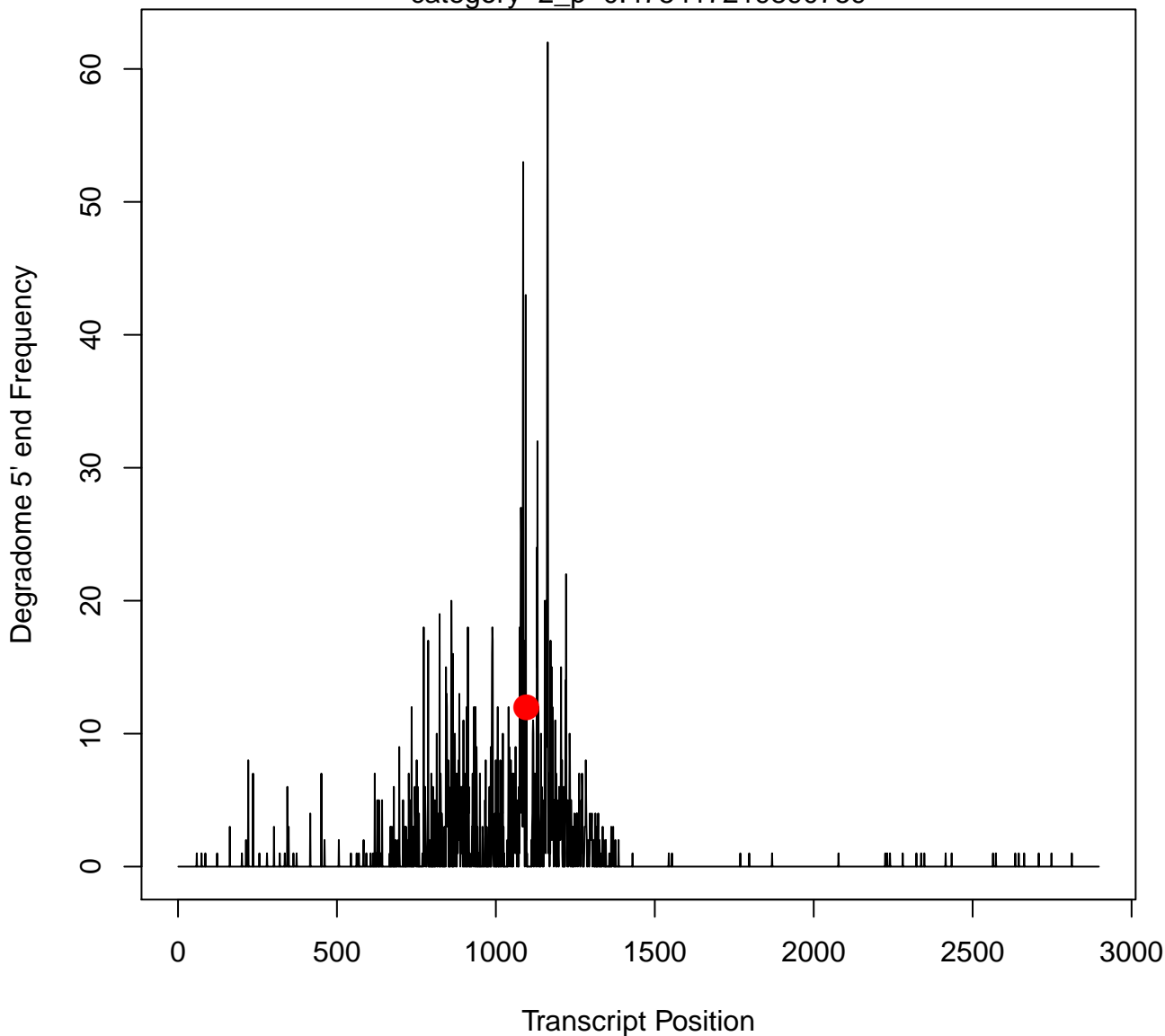

Supplement: Supplementary file 5 [file Data_Sheet_5.zip › Sit-miR166i_Seita.5G390300.1_1096_TPlot.pdf]

**T=Seita.7G130900.1\_Q=Sit-miR166i\_S=386**

category=2\_p=0.551493371028415

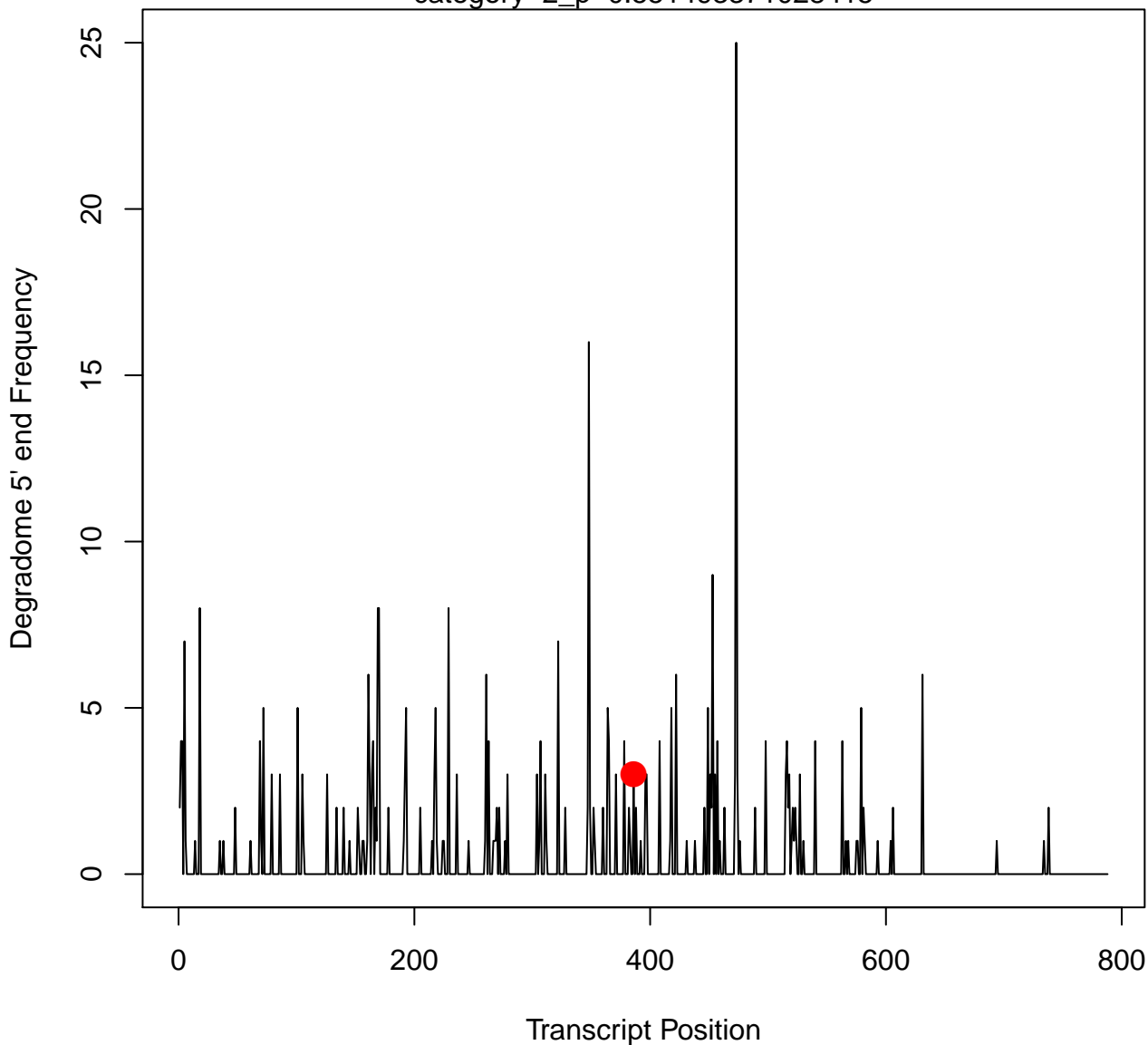

Supplement: Supplementary file 5 [file Data_Sheet_5.zip › Sit-miR166i_Seita.7G130900.1_386_TPlot.pdf]

**T=Seita.9G219700.1\_Q=Sit-miR166i\_S=1105**

category=2\_p=0.0272706278127964

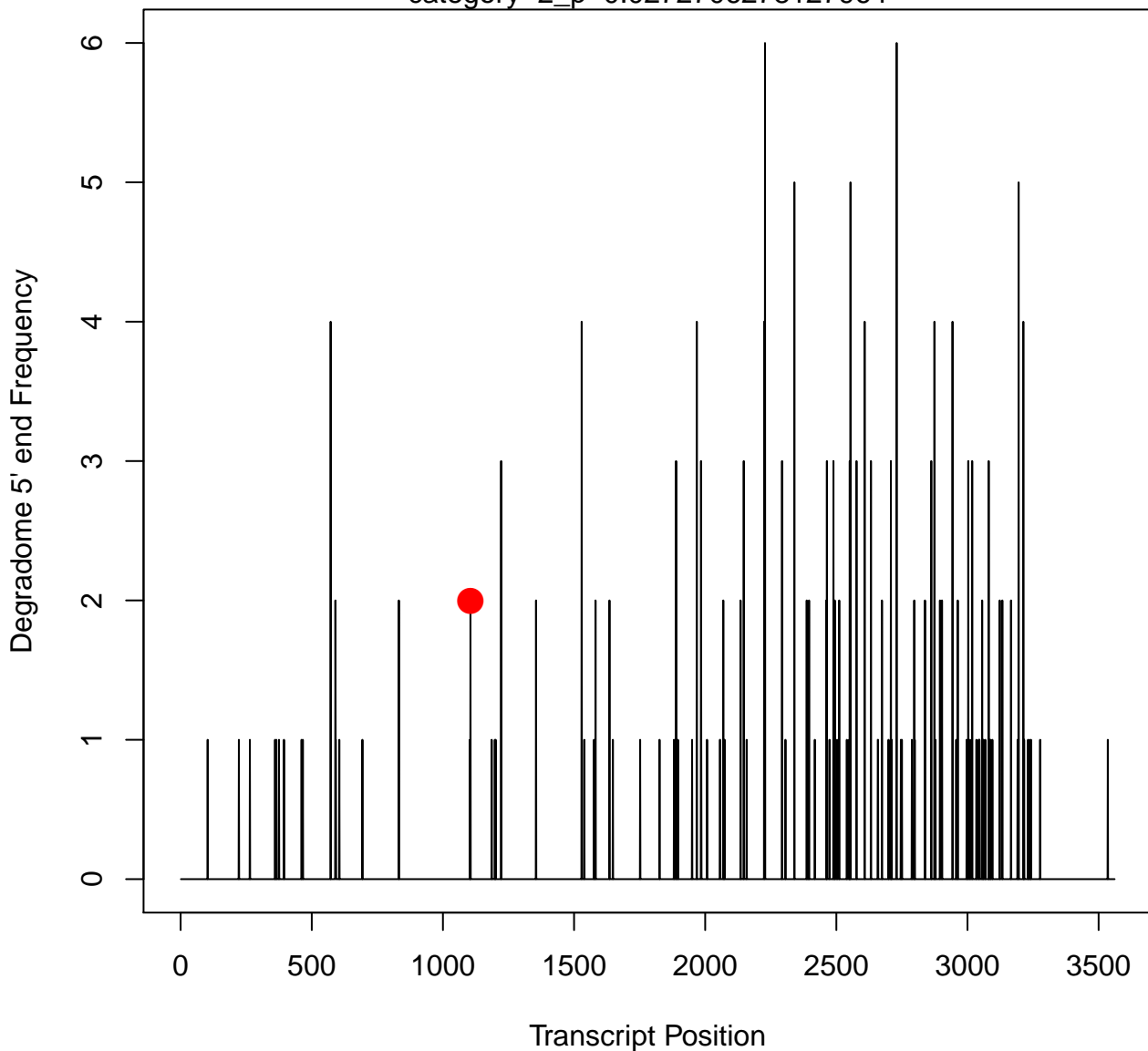

Supplement: Supplementary file 5 [file Data_Sheet_5.zip › Sit-miR166i_Seita.9G219700.1_1105_TPlot.pdf]

**T=Seita.8G135600.1\_Q=Sit-miR166j\_S=2218**

category=2\_p=0.262244716175678

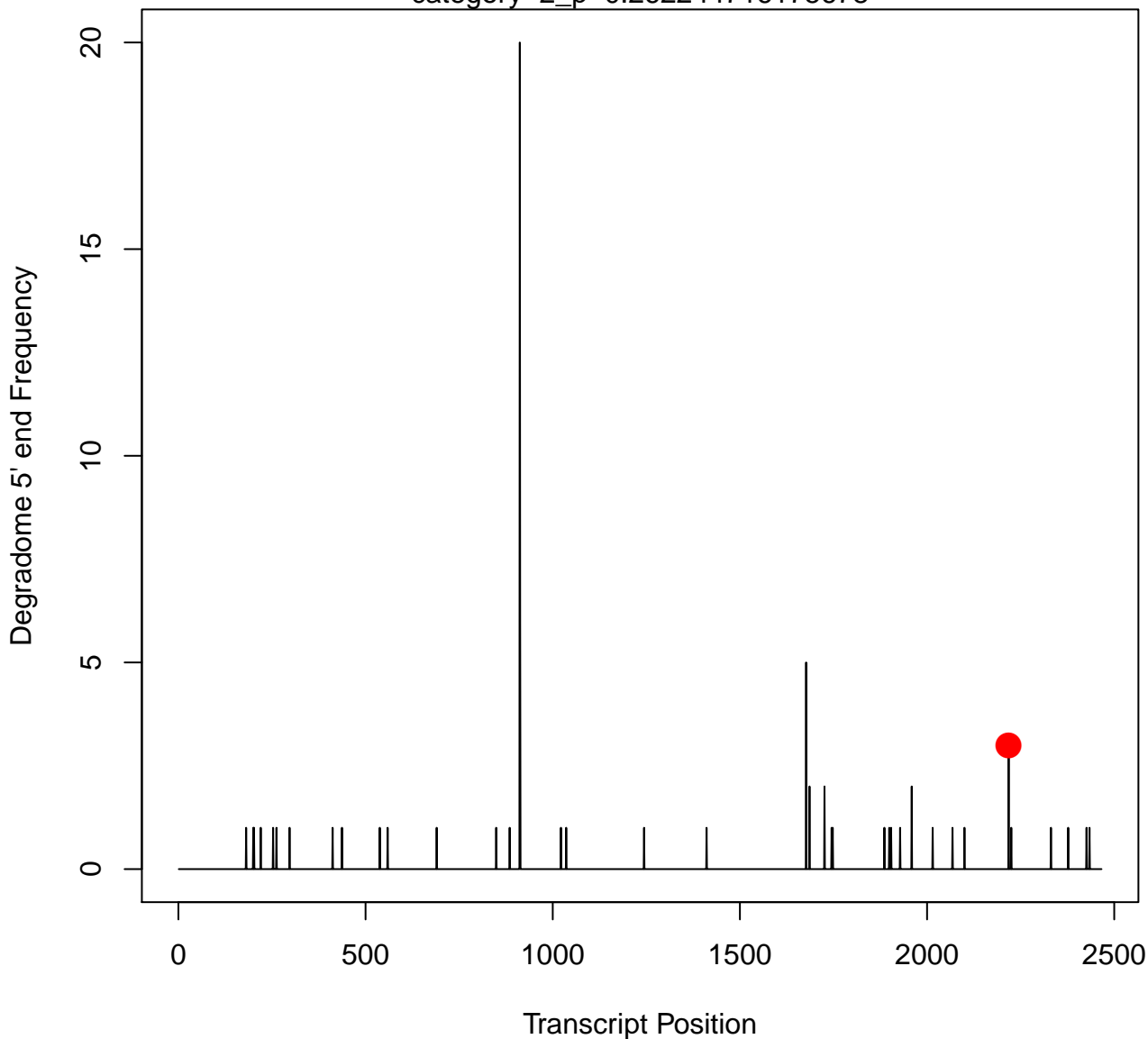

Supplement: Supplementary file 5 [file Data_Sheet_5.zip › Sit-miR166j_Seita.8G135600.1_2218_TPlot.pdf]

**T=Seita.9G056200.1\_Q=Sit-miR166j\_S=1465**

category=2\_p=0.724812906812859

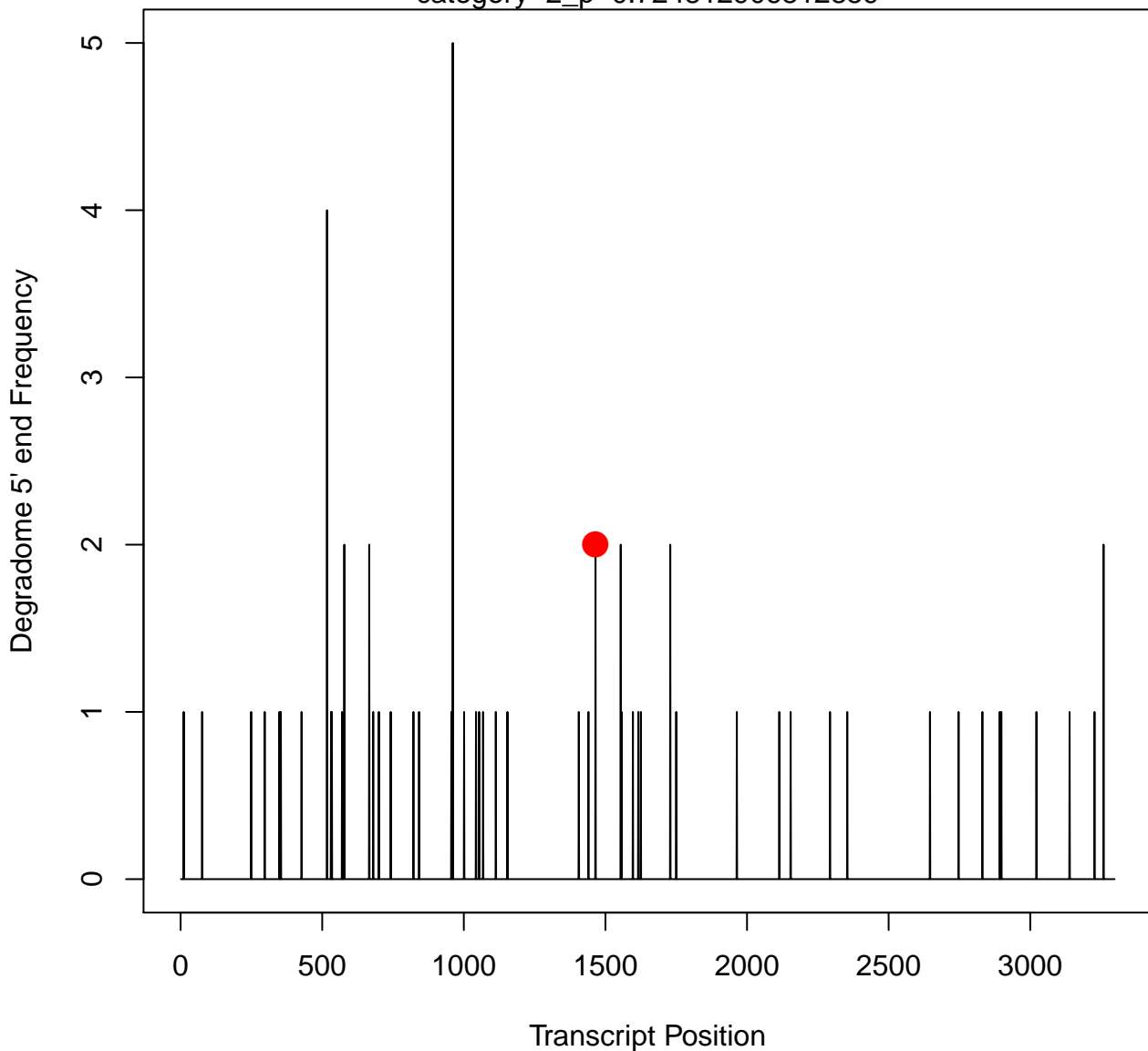

Supplement: Supplementary file 5 [file Data_Sheet_5.zip › Sit-miR166j_Seita.9G056200.1_1465_TPlot.pdf]

**T=Seita.9G560400.1\_Q=Sit-miR166k\_S=917**

category=2\_p=0.881044014226144

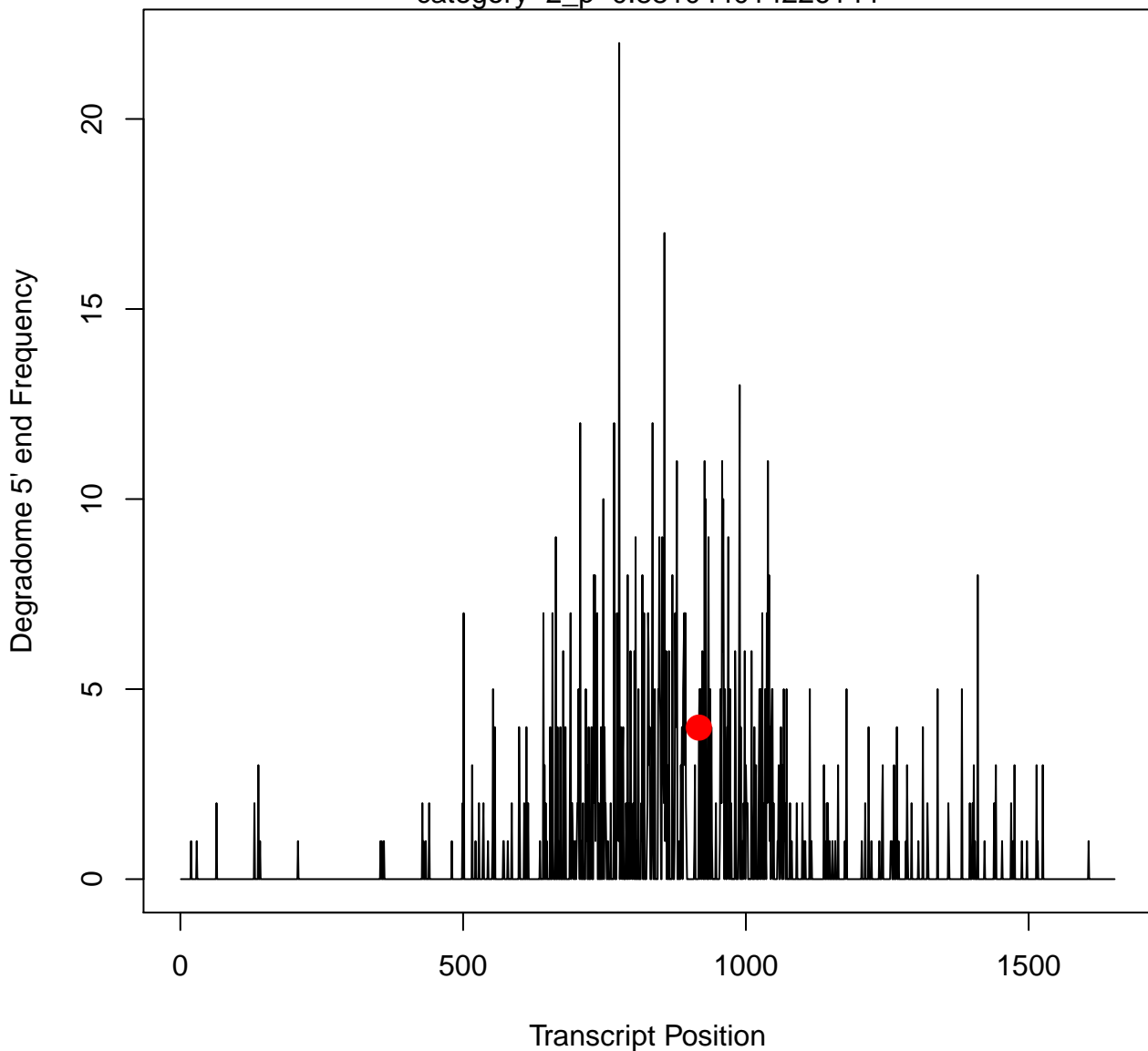

Supplement: Supplementary file 5 [file Data_Sheet_5.zip › Sit-miR166k_Seita.9G560400.1_917_TPlot.pdf]
