# Supplementary figures and images for: The Construction and Exploration of a Comprehensive MicroRNA Centered Regulatory Network in Foxtail Millet (Setaria italica L.) (part 12 of 14)
Source: Front Plant Sci. 2022 May 6;13:848474. doi: 10.3389/fpls.2022.848474 (PMC9121102; doi:10.3389/fpls.2022.848474)

**T=Seita.6G170500.1\_Q=Sit-miR160c\_S=2580**

category=2\_p=0.103158972889376

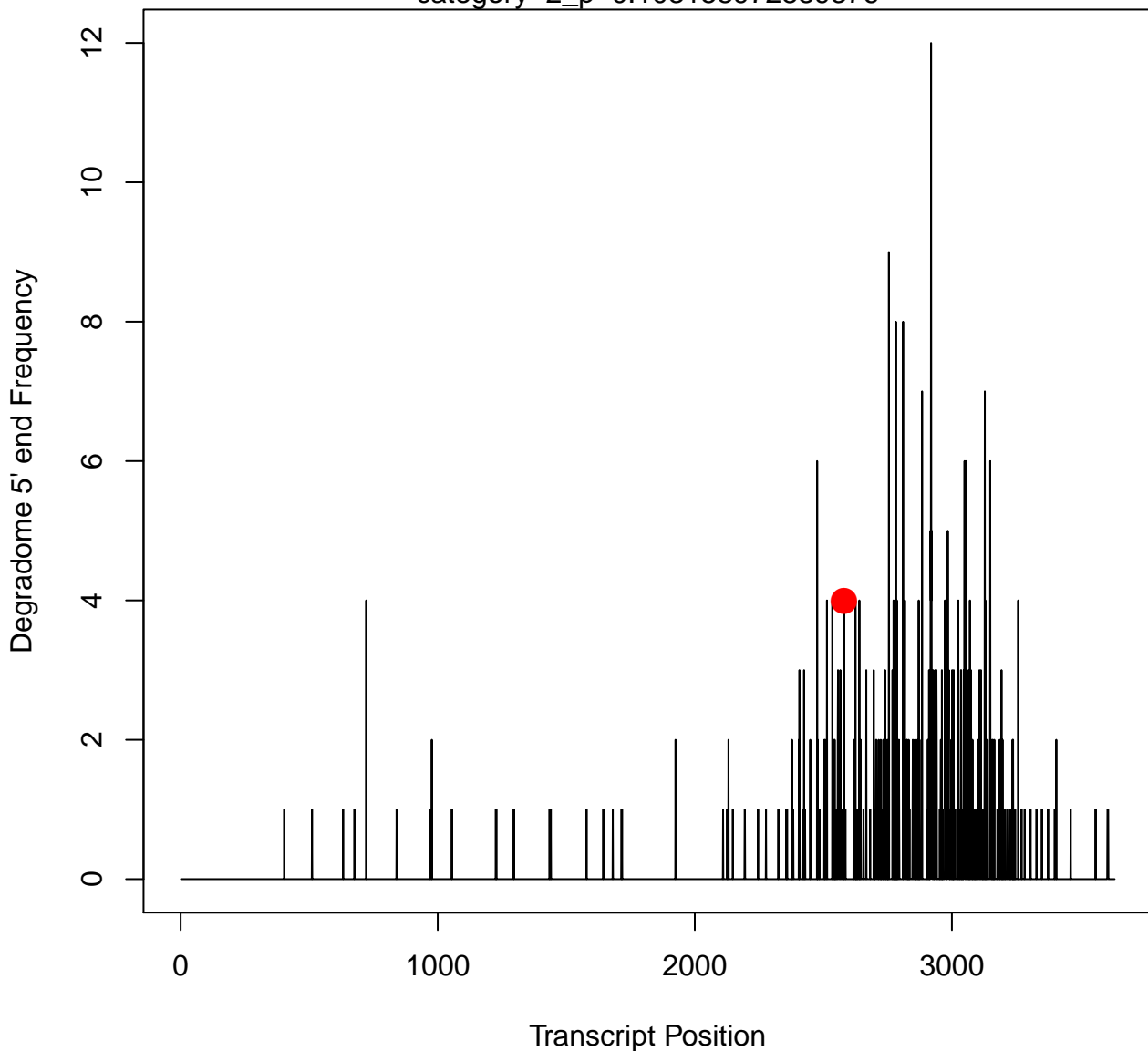

Supplement: Supplementary file 6 [file Data_Sheet_6.zip › Sit-miR160c_Seita.6G170500.1_2580_TPlot.pdf]

**T=Seita.7G155700.1\_Q=Sit-miR160c\_S=1461**

category=2\_p=0.995517349493532

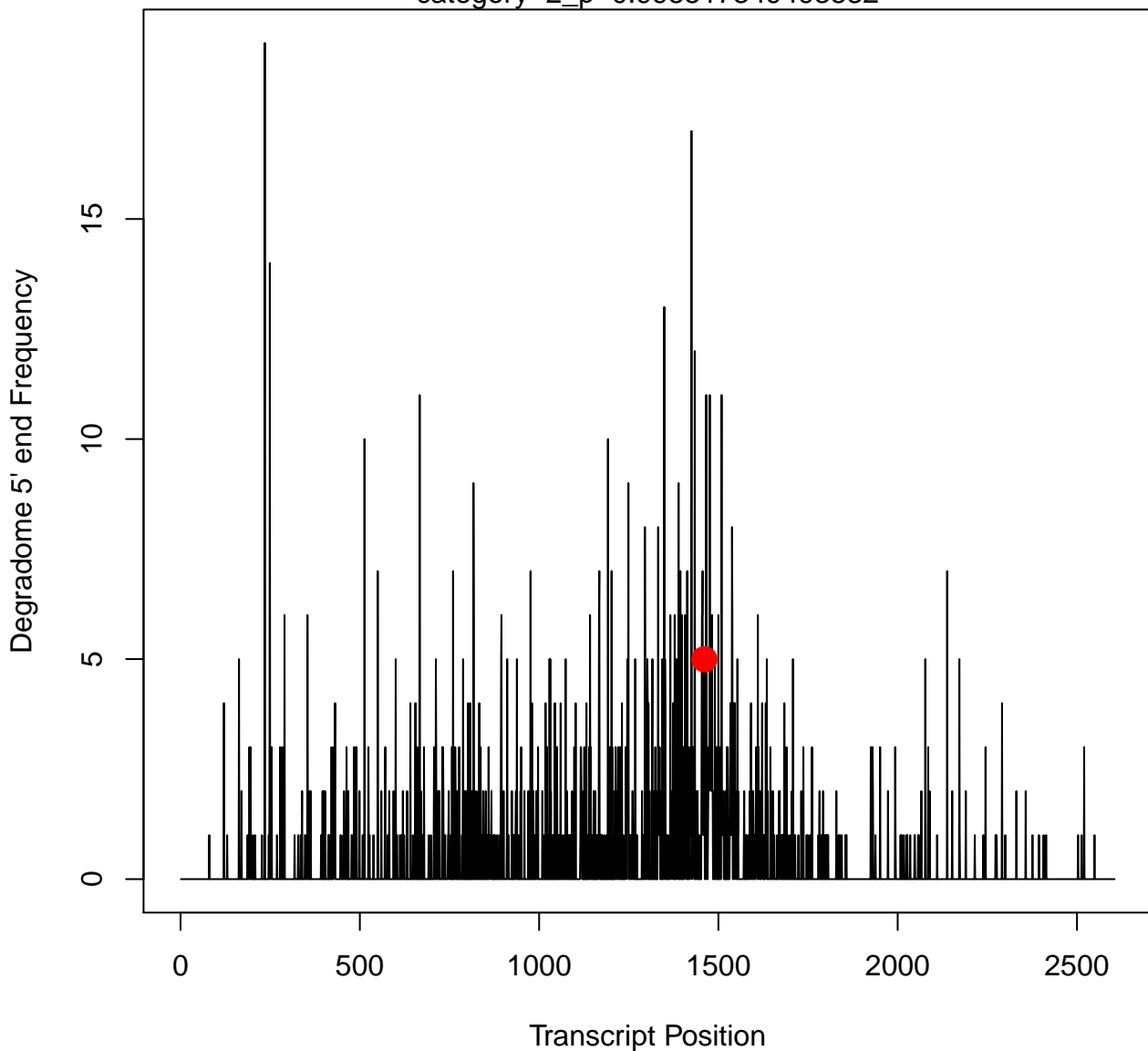

Supplement: Supplementary file 6 [file Data_Sheet_6.zip › Sit-miR160c_Seita.7G155700.1_1461_TPlot.pdf]

**T=Seita.7G194100.1\_Q=Sit-miR160c\_S=435**

category=2\_p=0.999999999419564

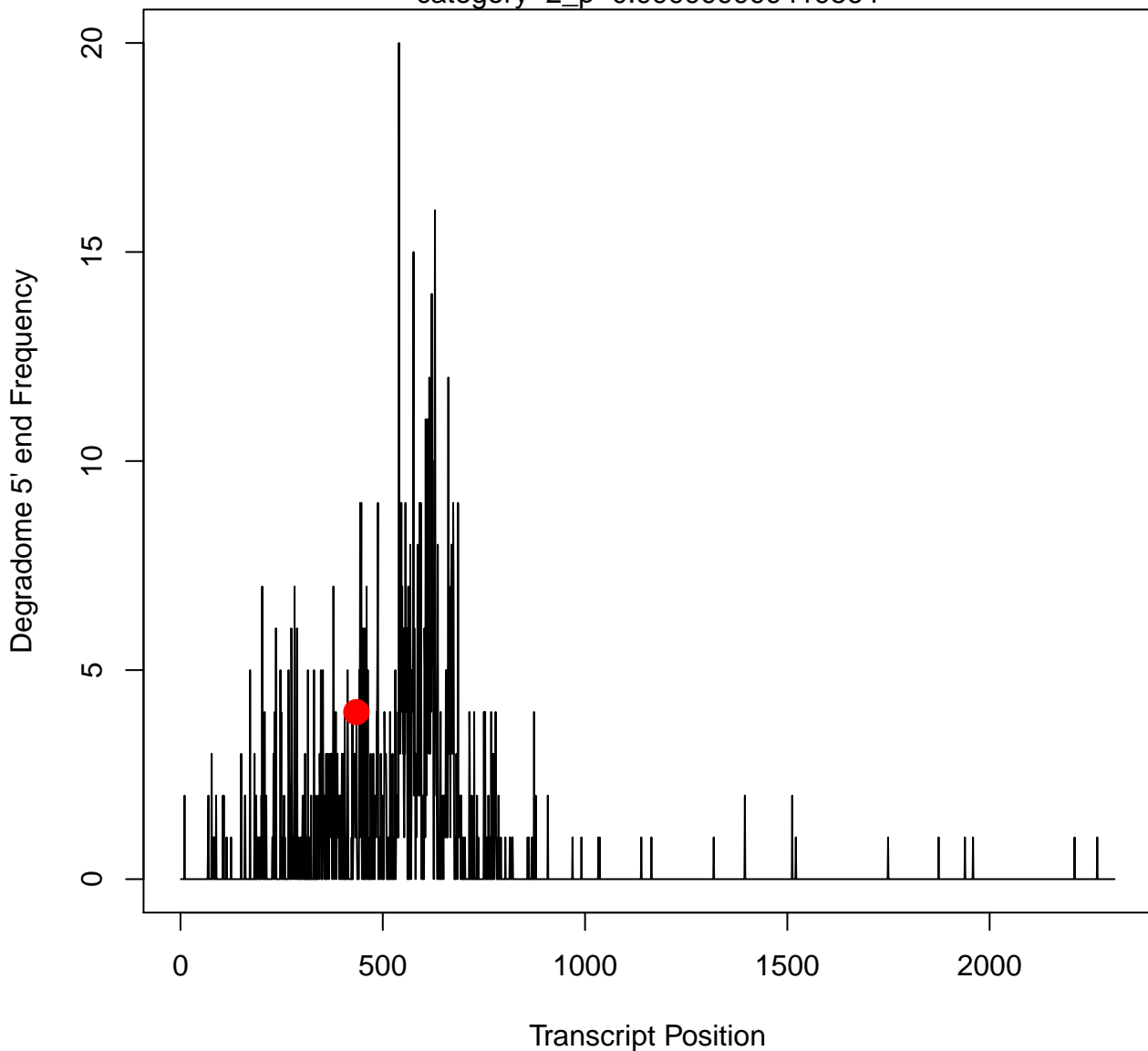

Supplement: Supplementary file 6 [file Data_Sheet_6.zip › Sit-miR160c_Seita.7G194100.1_435_TPlot.pdf]

**T=Seita.9G204900.1\_Q=Sit-miR160c\_S=1104**

category=2\_p=0.999699874898397

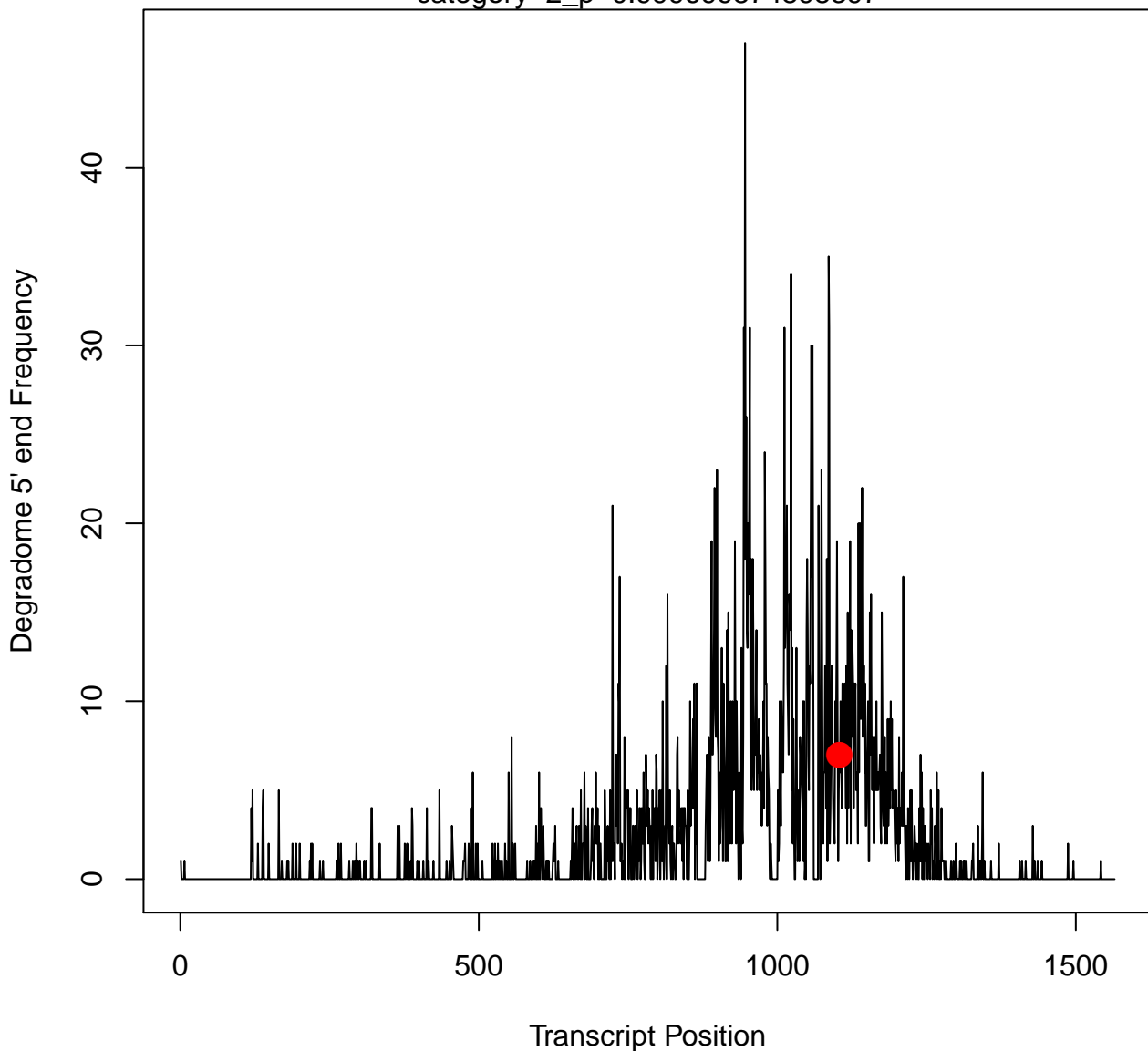

Supplement: Supplementary file 6 [file Data_Sheet_6.zip › Sit-miR160c_Seita.9G204900.1_1104_TPlot.pdf]

**T=Seita.9G524800.1\_Q=Sit-miR160c\_S=277**

category=2\_p=0.999999547110894

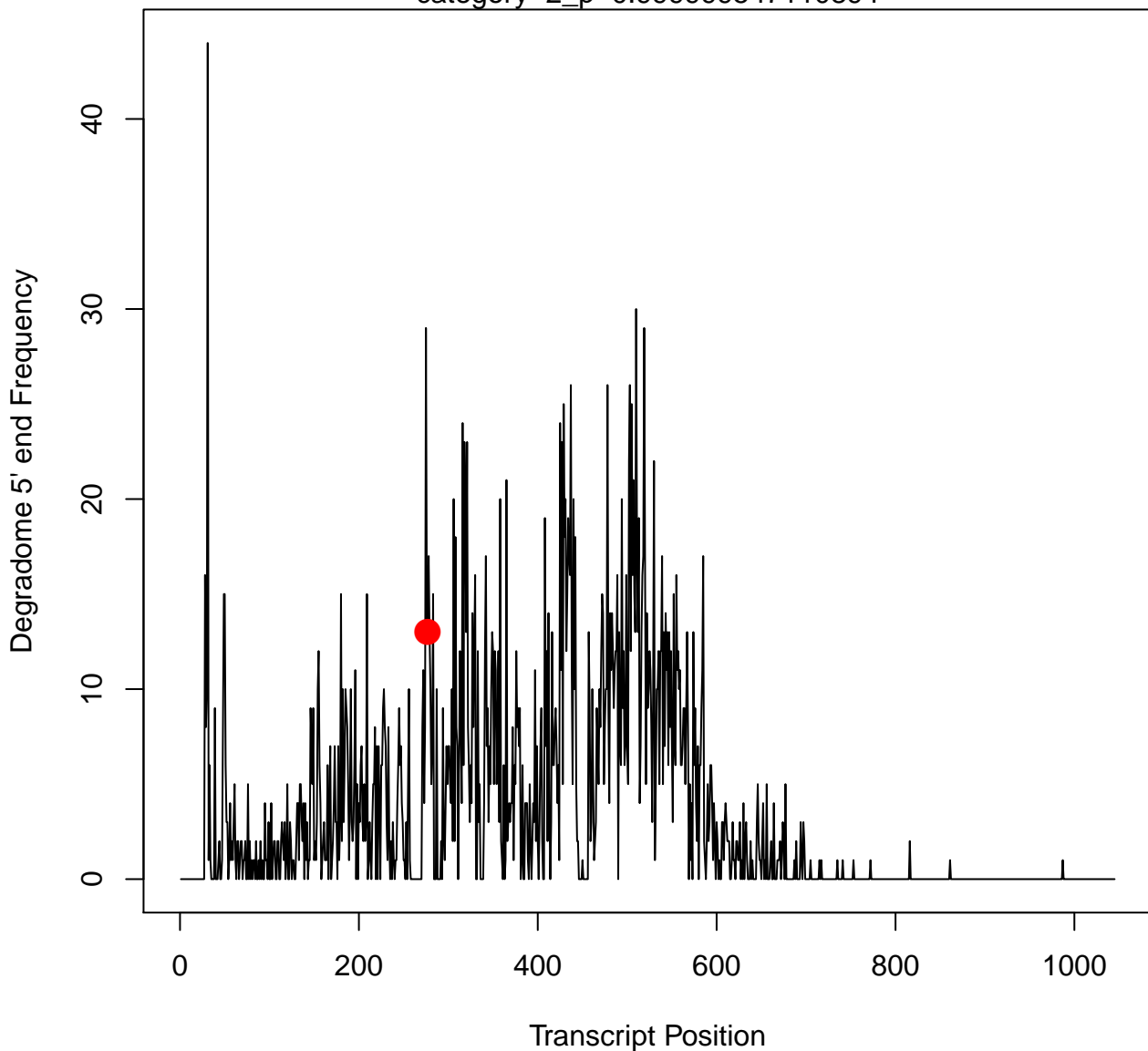

Supplement: Supplementary file 6 [file Data_Sheet_6.zip › Sit-miR160c_Seita.9G524800.1_277_TPlot.pdf]

**T=Seita.1G363500.1\_Q=Sit-miR160d\_S=259**

category=2\_p=0.999453780438229

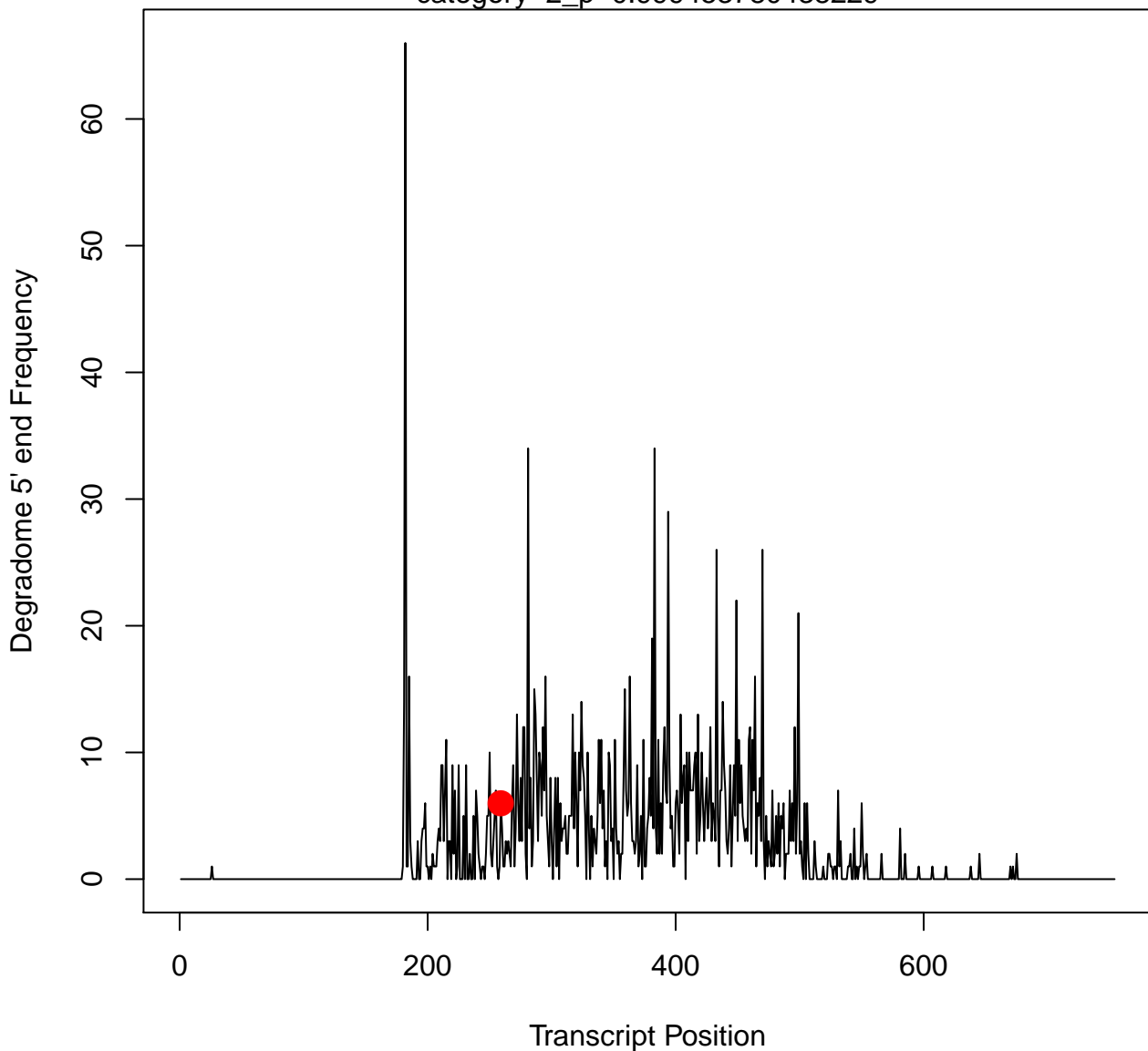

Supplement: Supplementary file 6 [file Data_Sheet_6.zip › Sit-miR160d_Seita.1G363500.1_259_TPlot.pdf]

**T=Seita.2G205400.1\_Q=Sit-miR160d\_S=698**

category=1\_p=0.292177371697852

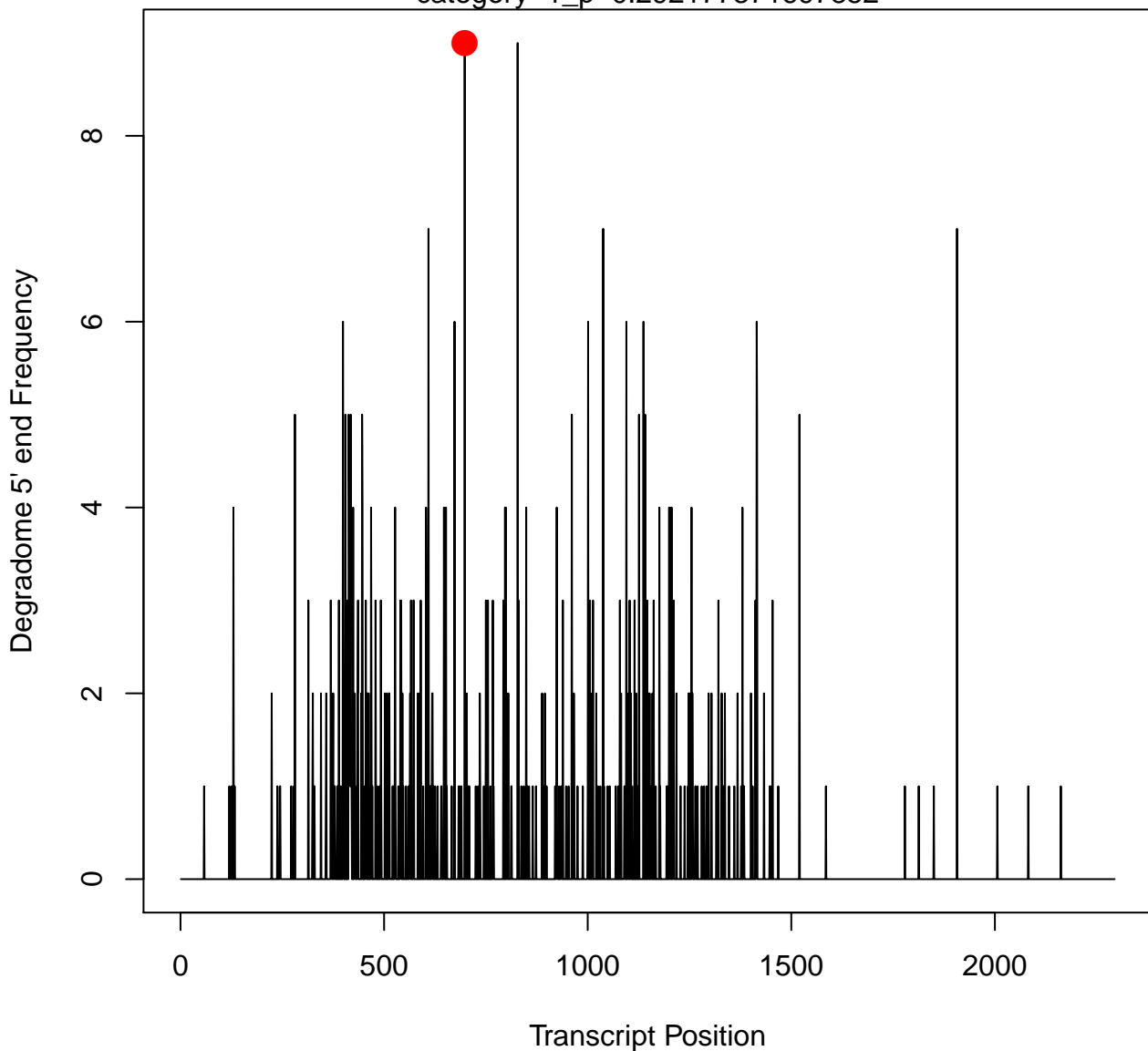

Supplement: Supplementary file 6 [file Data_Sheet_6.zip › Sit-miR160d_Seita.2G205400.1_698_TPlot.pdf]

**T=Seita.2G242400.1\_Q=Sit-miR160d\_S=456**

category=2\_p=0.999999999999689

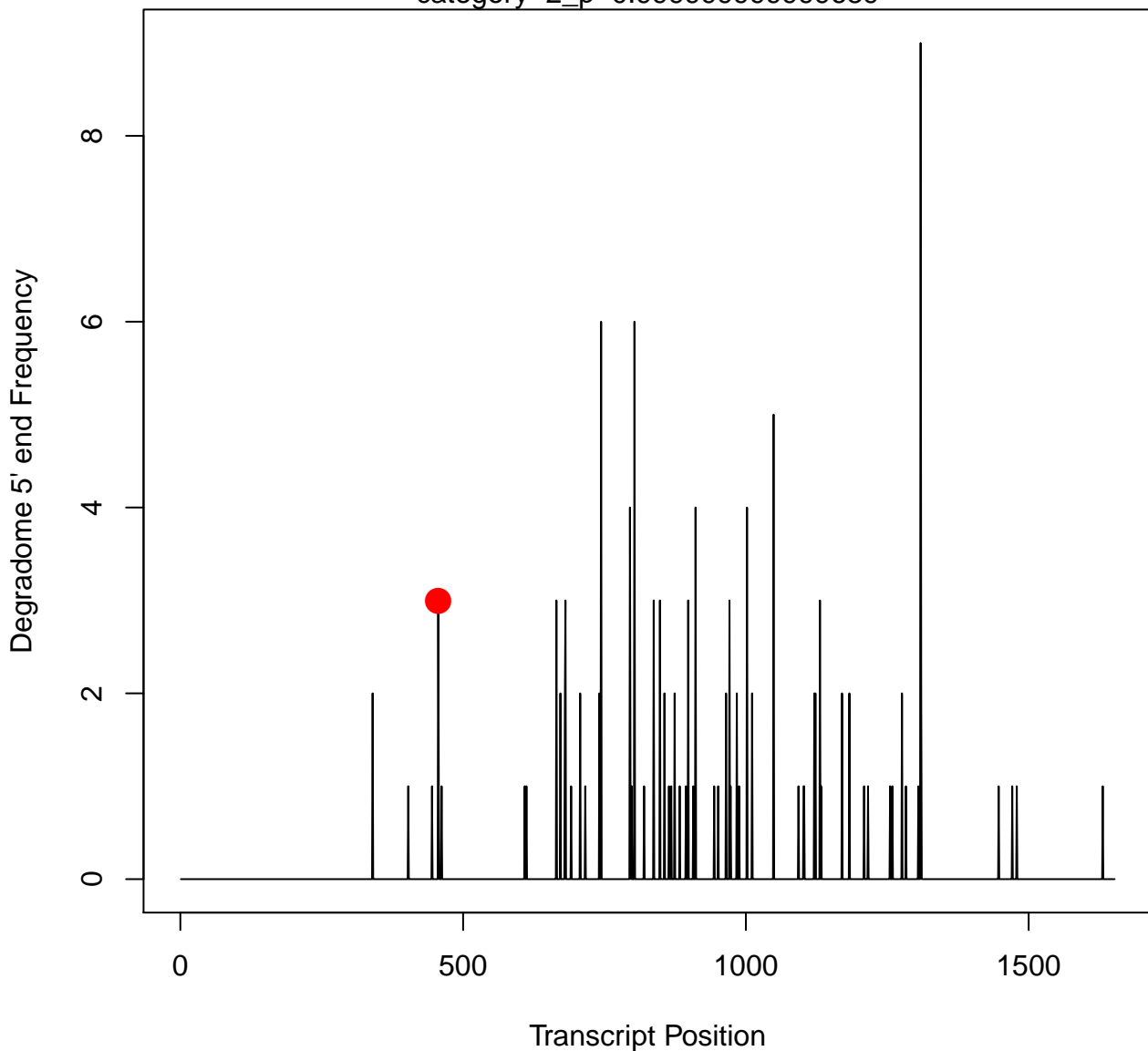

Supplement: Supplementary file 6 [file Data_Sheet_6.zip › Sit-miR160d_Seita.2G242400.1_456_TPlot.pdf]

**T=Seita.4G043900.1\_Q=Sit-miR160d\_S=308**

category=1\_p=0.178796377900209

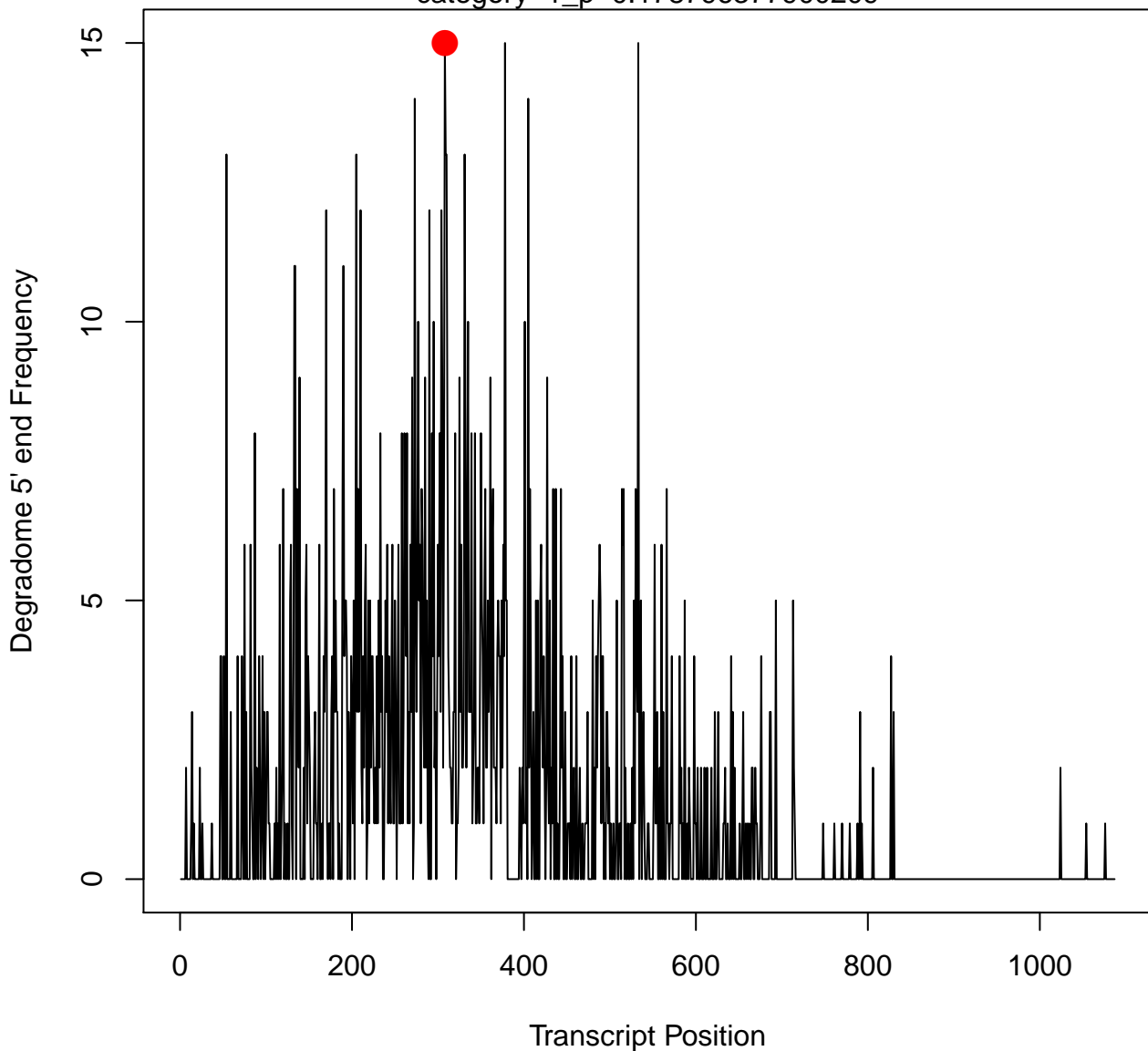

Supplement: Supplementary file 6 [file Data_Sheet_6.zip › Sit-miR160d_Seita.4G043900.1_308_TPlot.pdf]

**T=Seita.4G257800.1\_Q=Sit-miR160d\_S=1974**

category=0\_p=0.000375549221534932

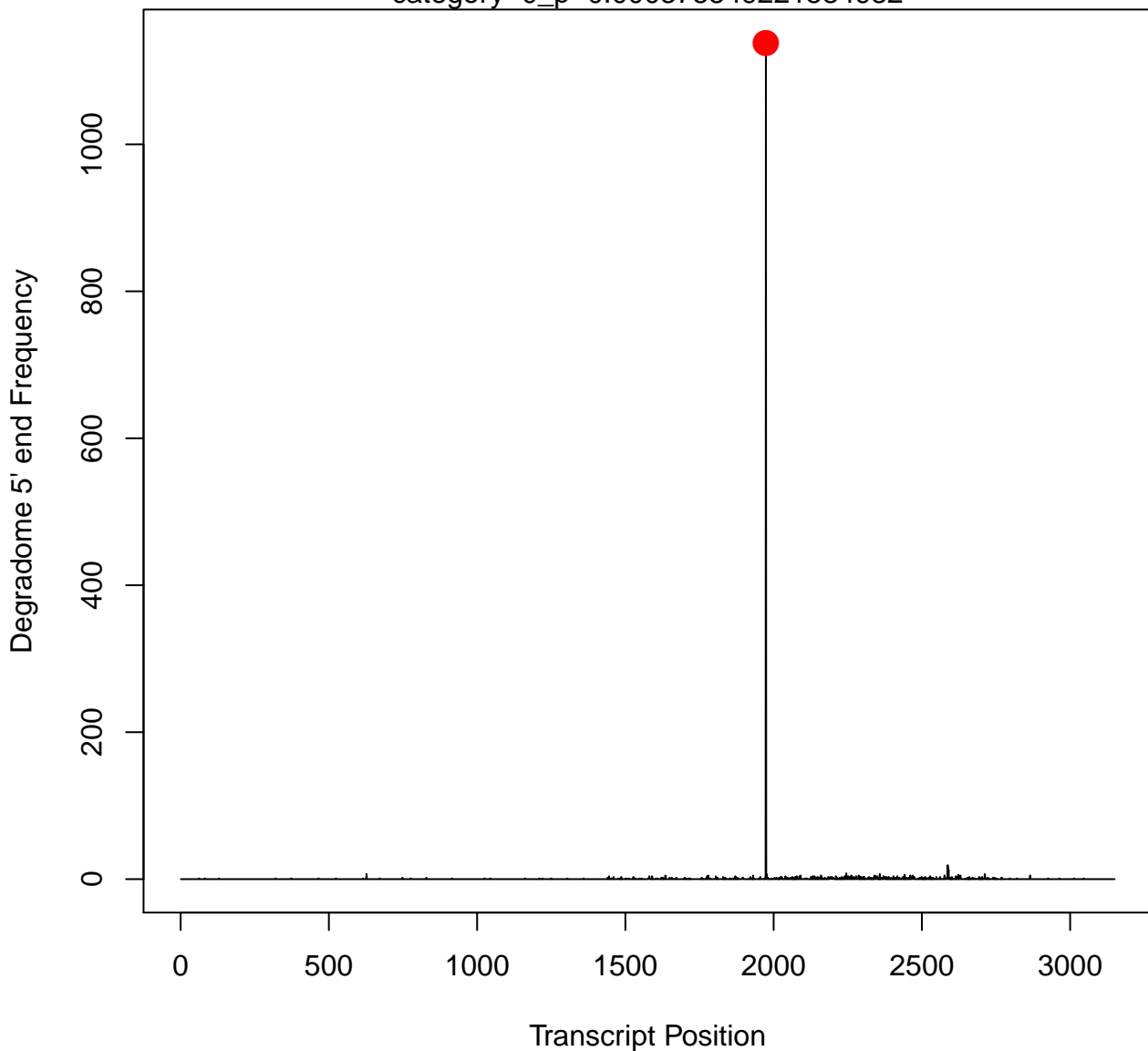

Supplement: Supplementary file 6 [file Data_Sheet_6.zip › Sit-miR160d_Seita.4G257800.1_1974_TPlot.pdf]

**T=Seita.5G079500.1\_Q=Sit-miR160d\_S=3803**

category=2\_p=0.999999999999568

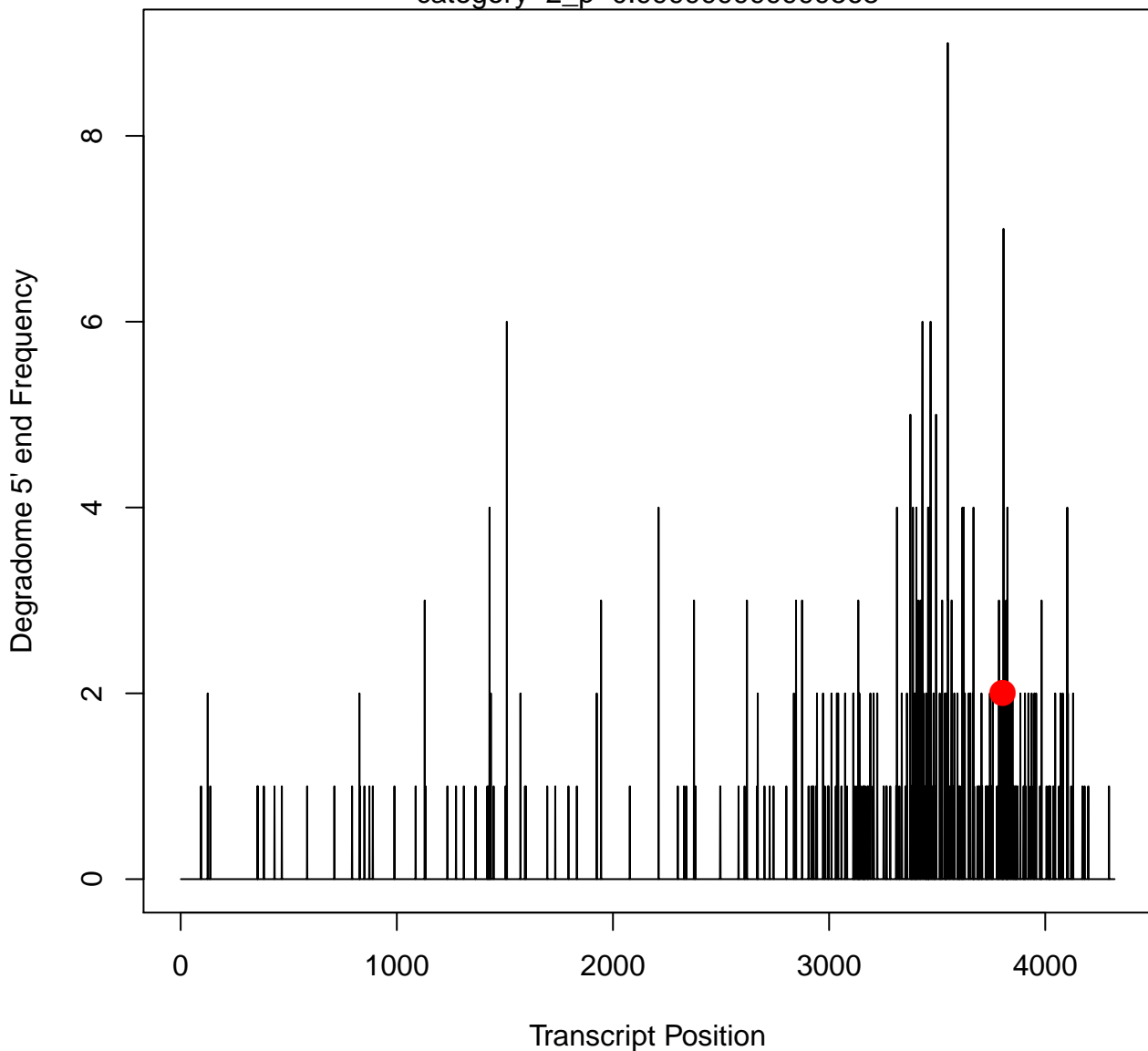

Supplement: Supplementary file 6 [file Data_Sheet_6.zip › Sit-miR160d_Seita.5G079500.1_3803_TPlot.pdf]

**T=Seita.5G080400.1\_Q=Sit-miR160d\_S=1572**

category=2\_p=0.993195562096841

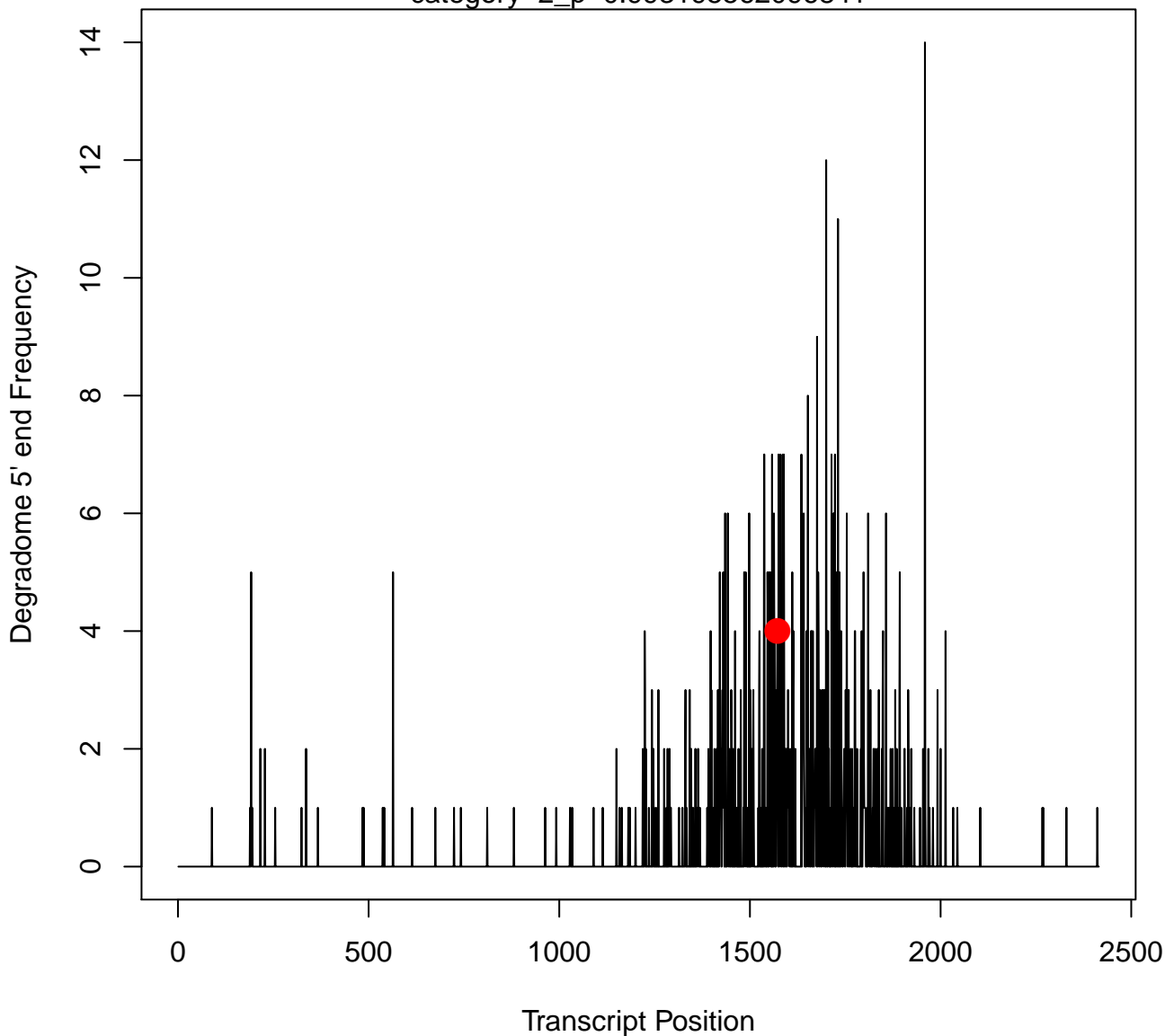

Supplement: Supplementary file 6 [file Data_Sheet_6.zip › Sit-miR160d_Seita.5G080400.1_1572_TPlot.pdf]

**T=Seita.5G140500.1\_Q=Sit-miR160d\_S=1934**

category=2\_p=1

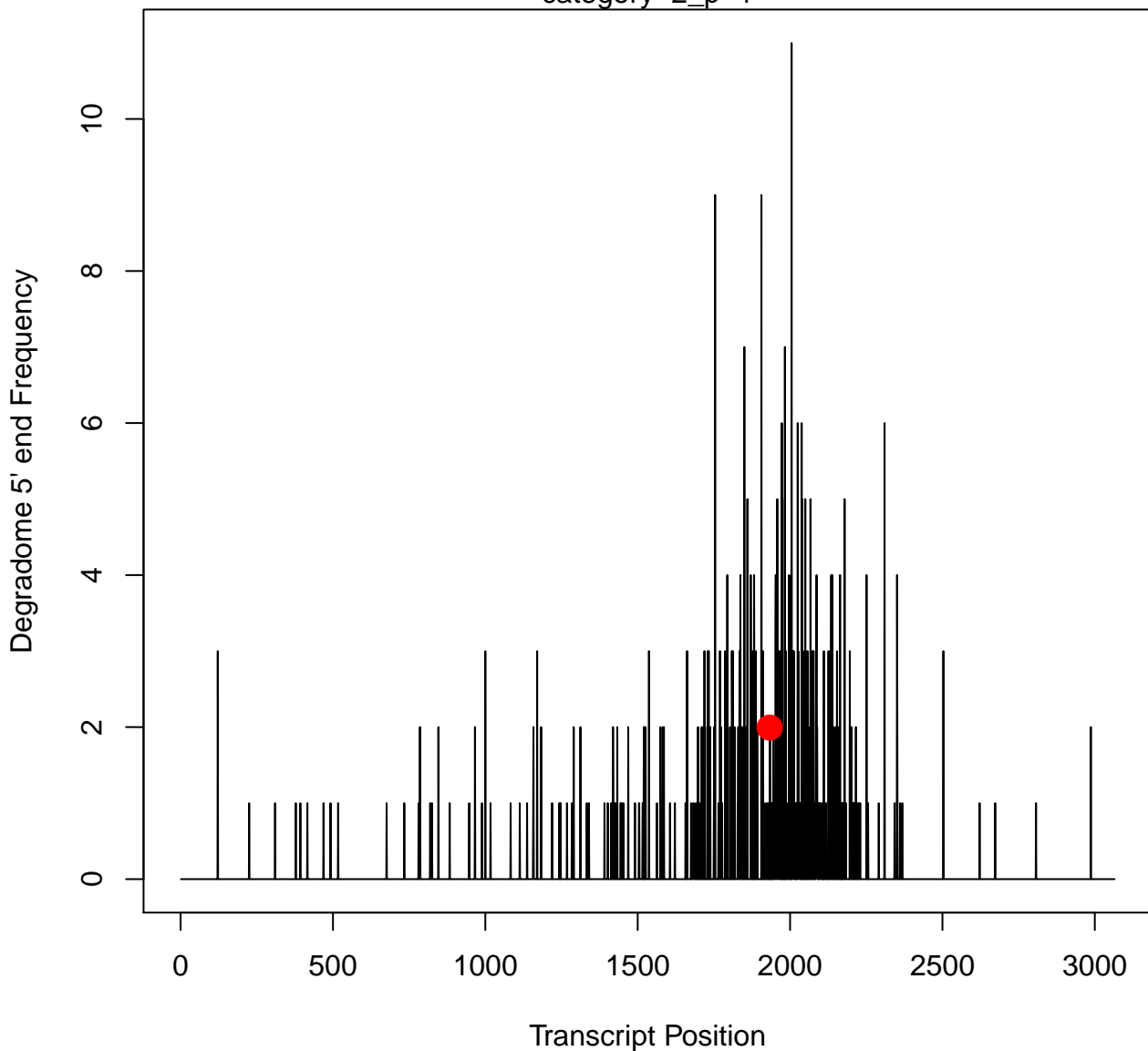

Supplement: Supplementary file 6 [file Data_Sheet_6.zip › Sit-miR160d_Seita.5G140500.1_1934_TPlot.pdf]

**T=Seita.7G107900.1\_Q=Sit-miR160d\_S=1649**

category=2\_p=0.989093336946018

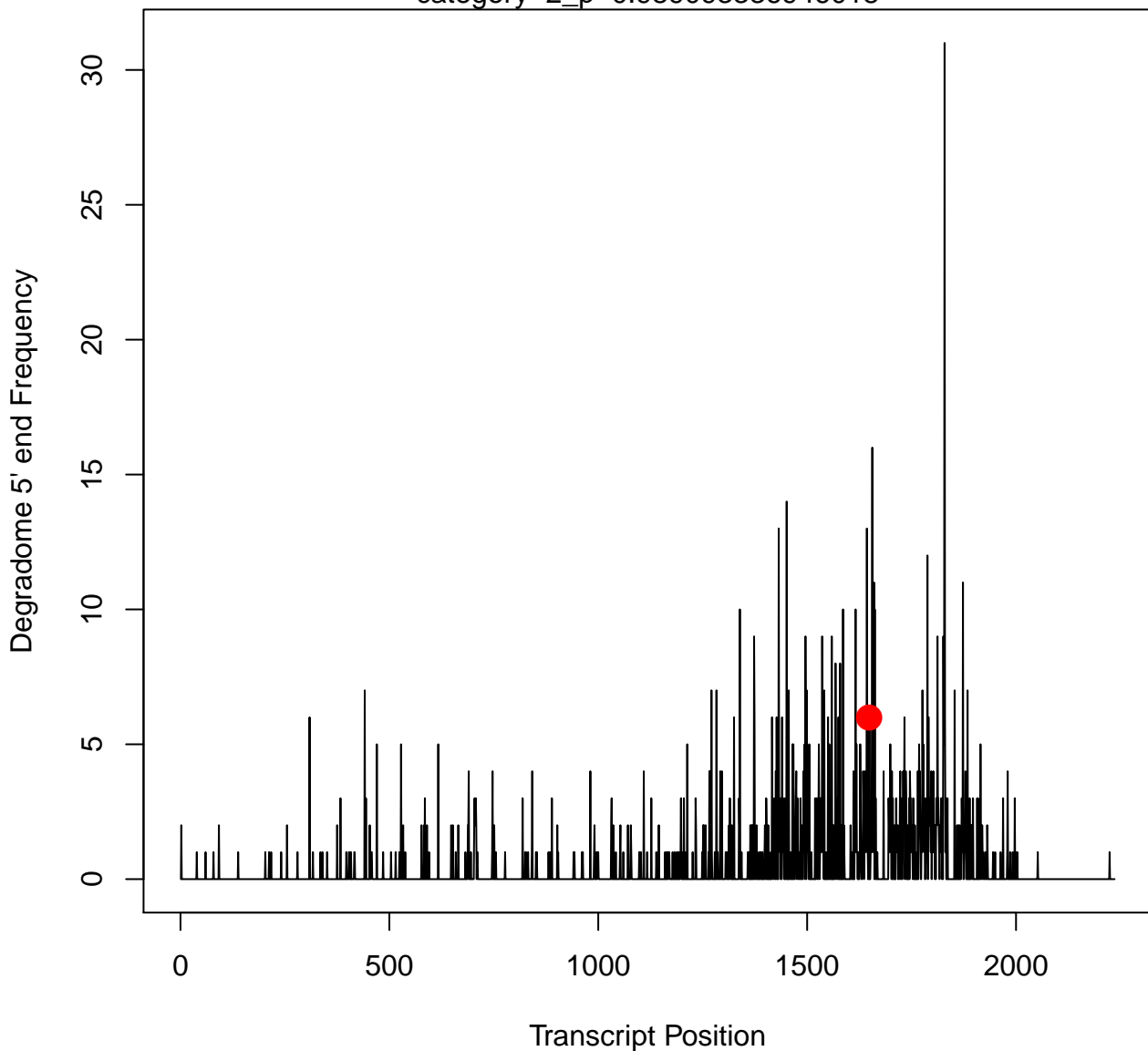

Supplement: Supplementary file 6 [file Data_Sheet_6.zip › Sit-miR160d_Seita.7G107900.1_1649_TPlot.pdf]

**T=Seita.7G295800.1\_Q=Sit-miR160d\_S=368**

category=2\_p=0.999999999998871

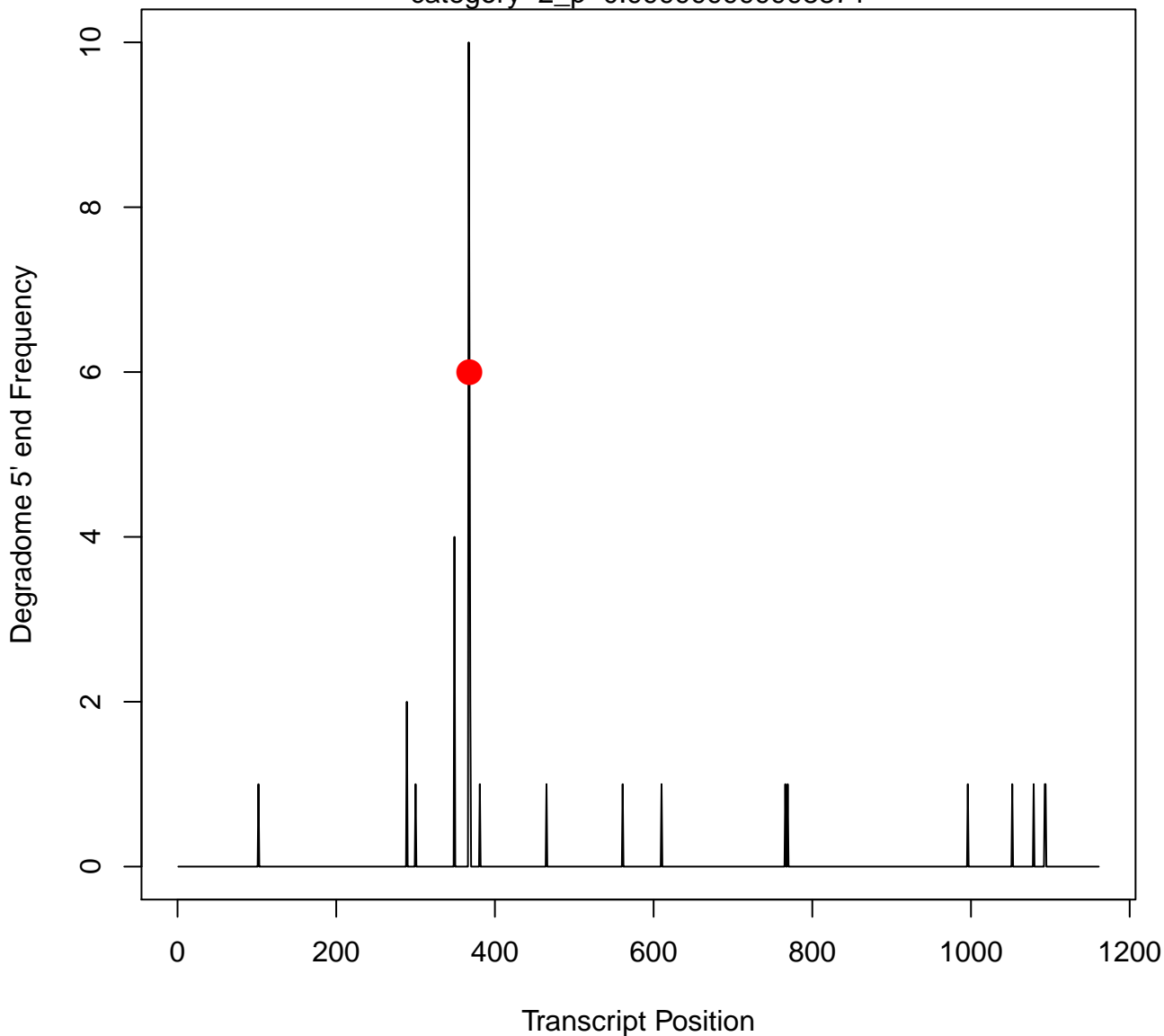

Supplement: Supplementary file 6 [file Data_Sheet_6.zip › Sit-miR160d_Seita.7G295800.1_368_TPlot.pdf]

**T=Seita.9G060800.1\_Q=Sit-miR160d\_S=2103**

category=2\_p=0.99999999999857

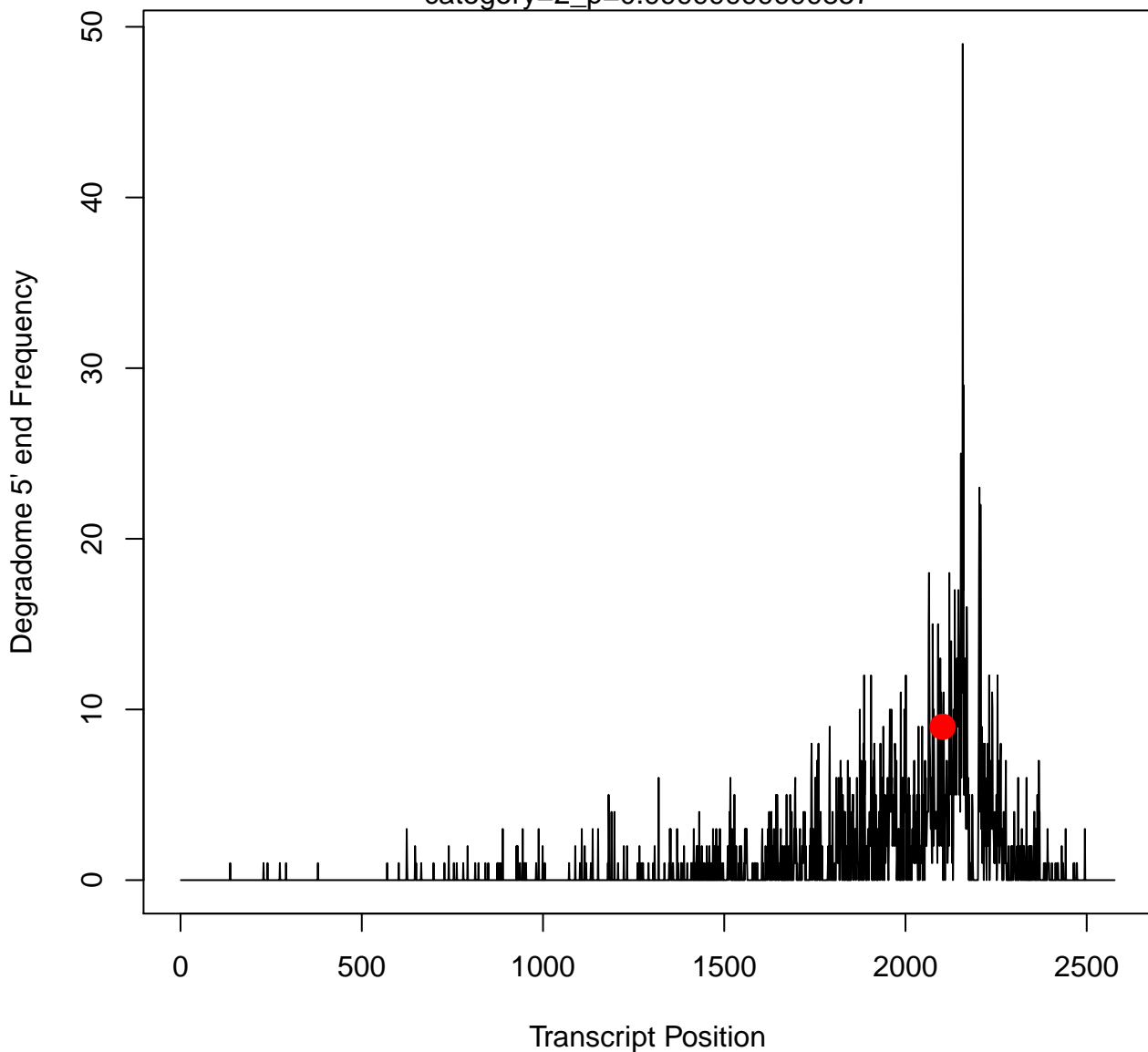

Supplement: Supplementary file 6 [file Data_Sheet_6.zip › Sit-miR160d_Seita.9G060800.1_2103_TPlot.pdf]

**T=Seita.2G271300.1\_Q=Sit-miR162\_S=793**

category=2\_p=0.967600580980398

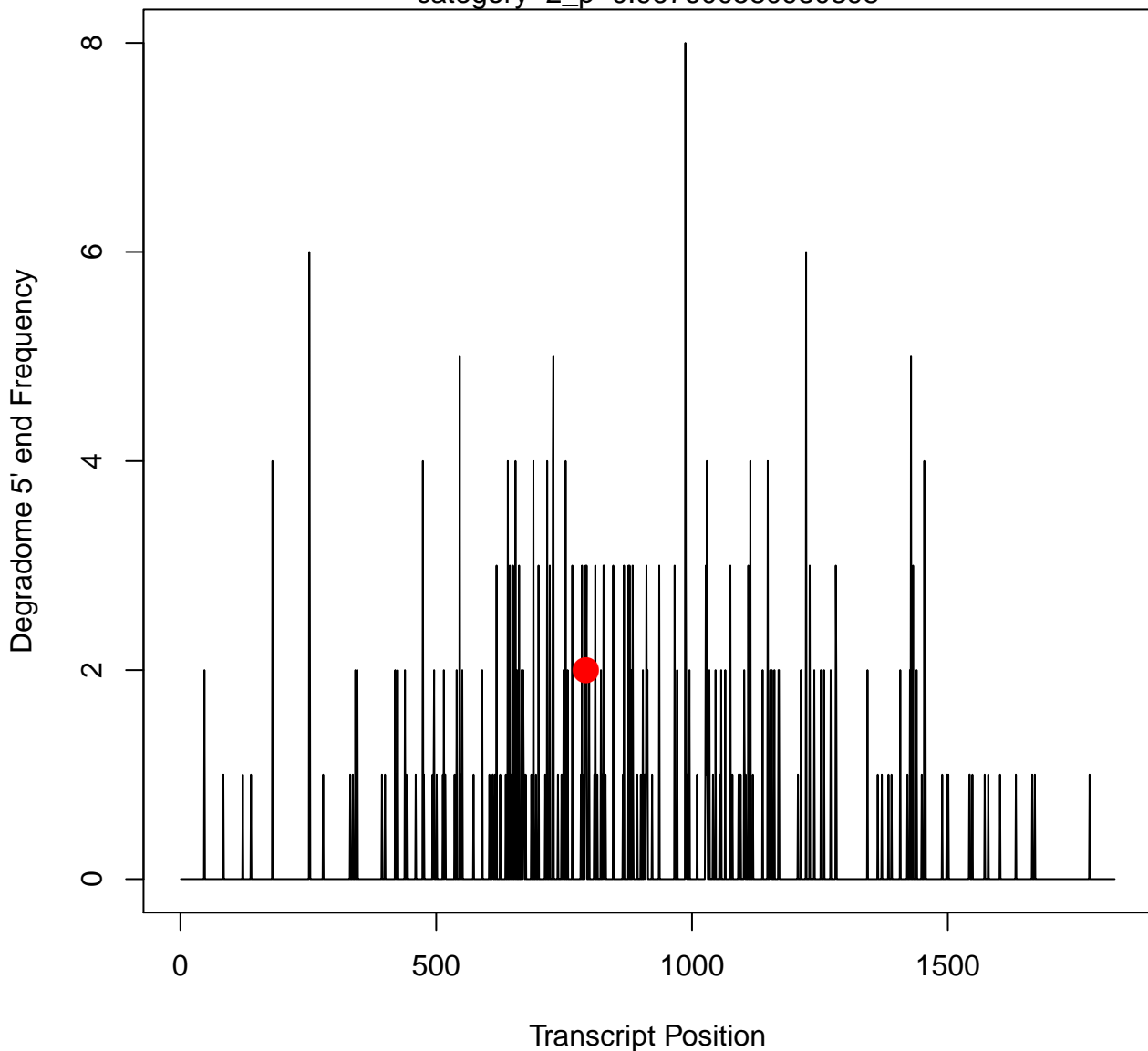

Supplement: Supplementary file 6 [file Data_Sheet_6.zip › Sit-miR162_Seita.2G271300.1_793_TPlot.pdf]

**T=Seita.5G064400.1\_Q=Sit-miR162\_S=878**

category=1\_p=0.000559457601756463

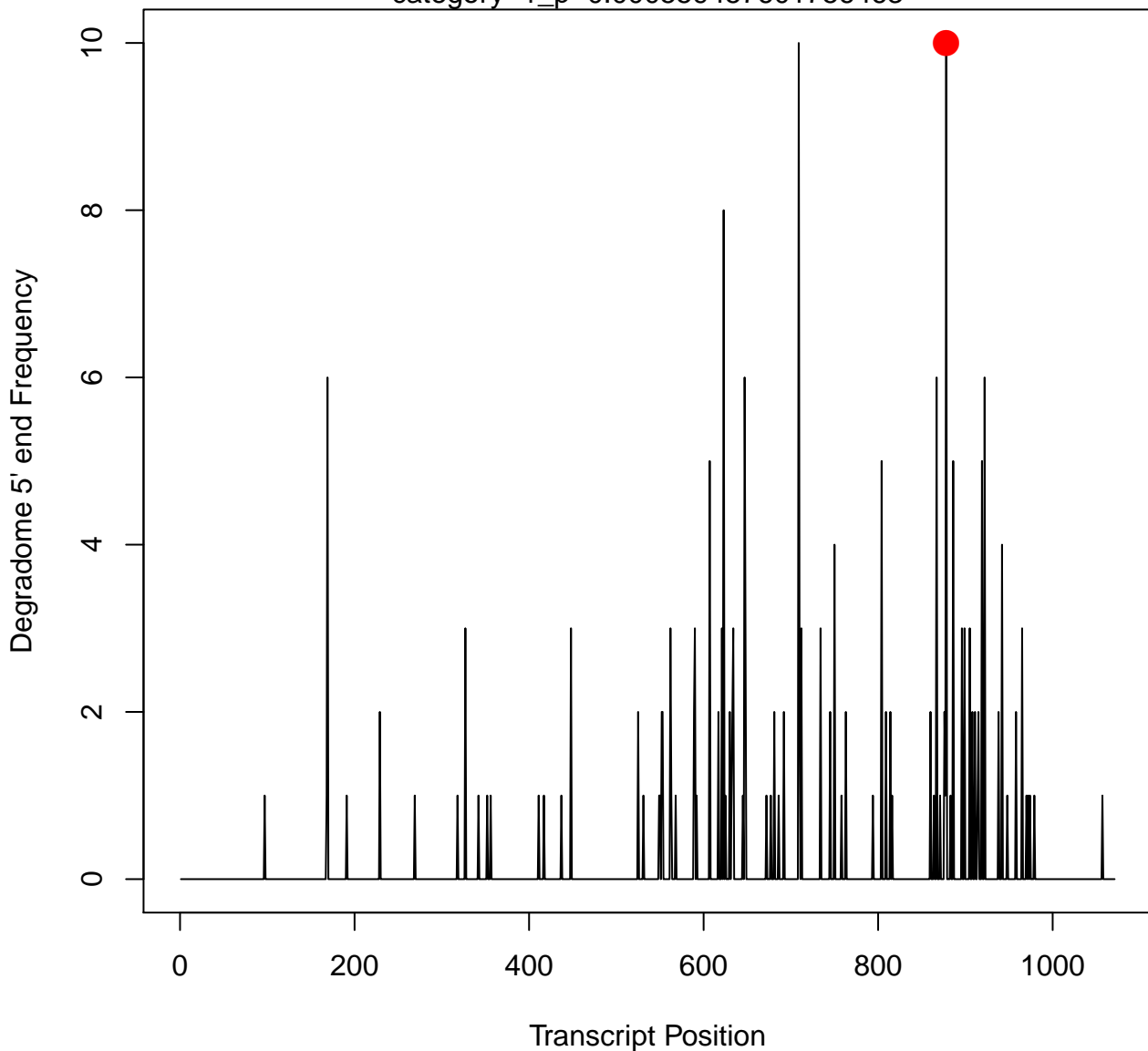

Supplement: Supplementary file 6 [file Data_Sheet_6.zip › Sit-miR162_Seita.5G064400.1_878_TPlot.pdf]

**T=Seita.5G177000.1\_Q=Sit-miR162\_S=1193**

category=2\_p=0.278649392048688

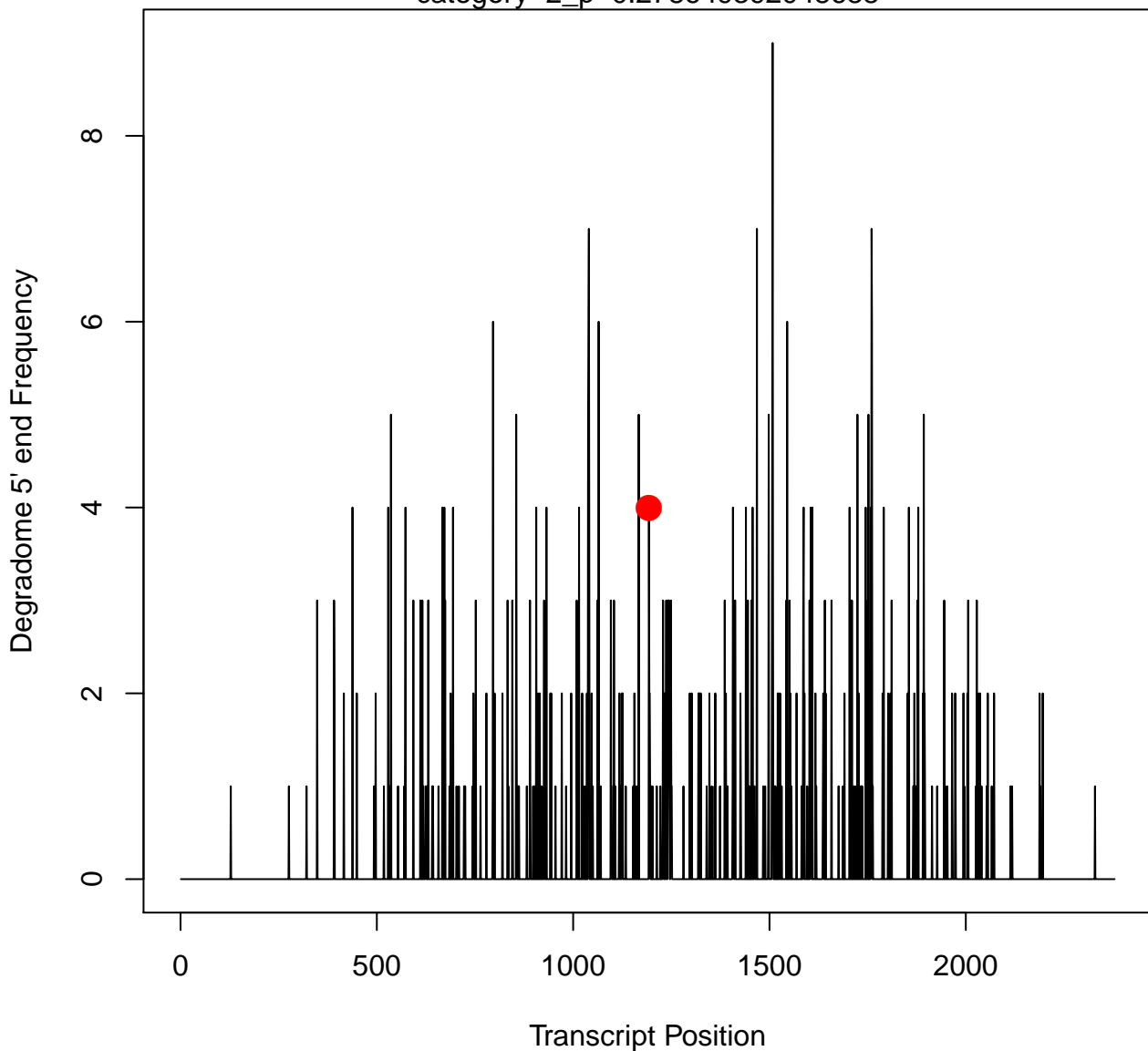

Supplement: Supplementary file 6 [file Data_Sheet_6.zip › Sit-miR162_Seita.5G177000.1_1193_TPlot.pdf]

**T=Seita.7G255700.1\_Q=Sit-miR162\_S=1326**

category=2\_p=0.703524107046325

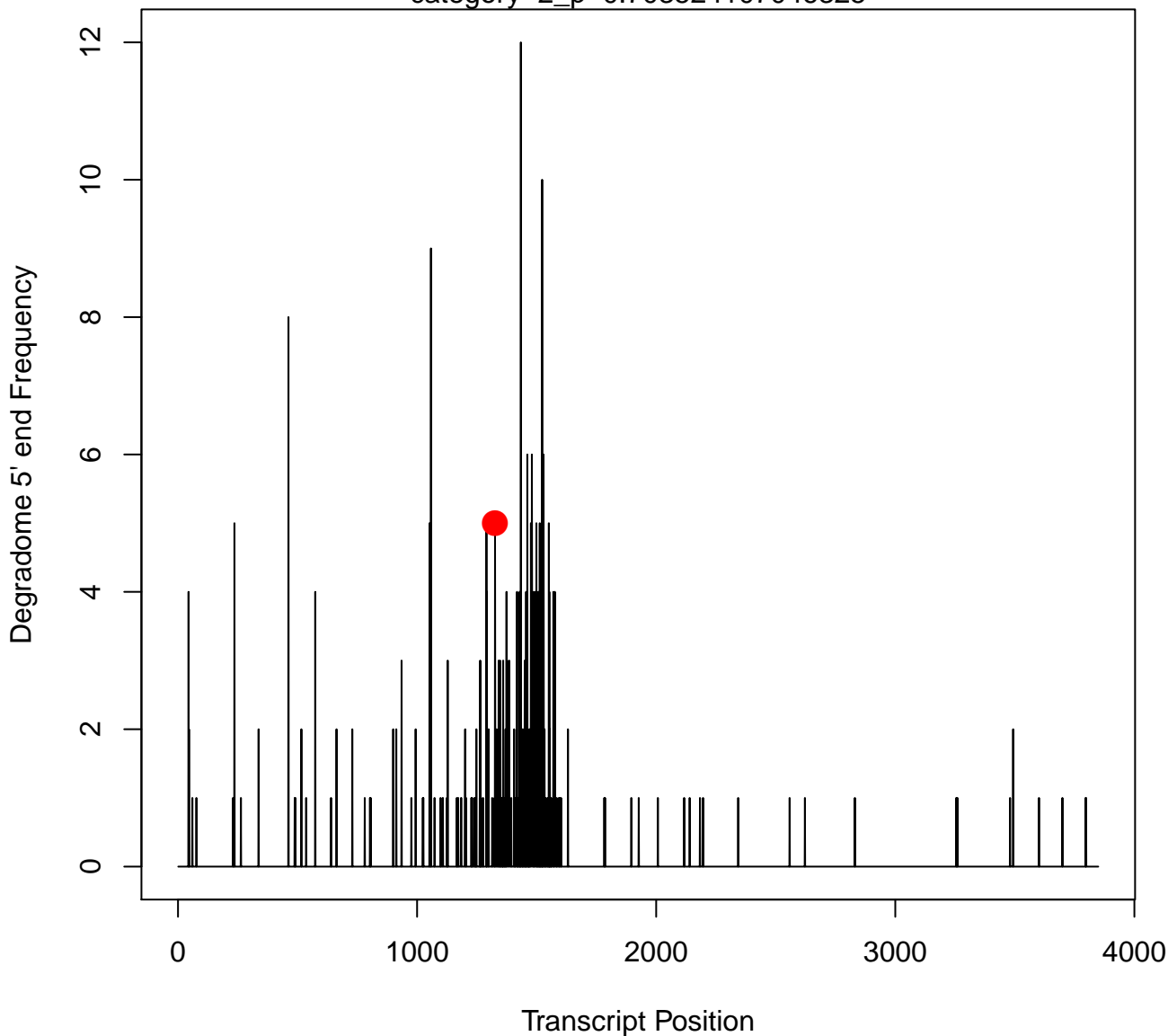

Supplement: Supplementary file 6 [file Data_Sheet_6.zip › Sit-miR162_Seita.7G255700.1_1326_TPlot.pdf]

**T=Seita.9G562200.1\_Q=Sit-miR162\_S=3530**

category=0\_p=0.000375549221534932

Degradome 5' end Frequency

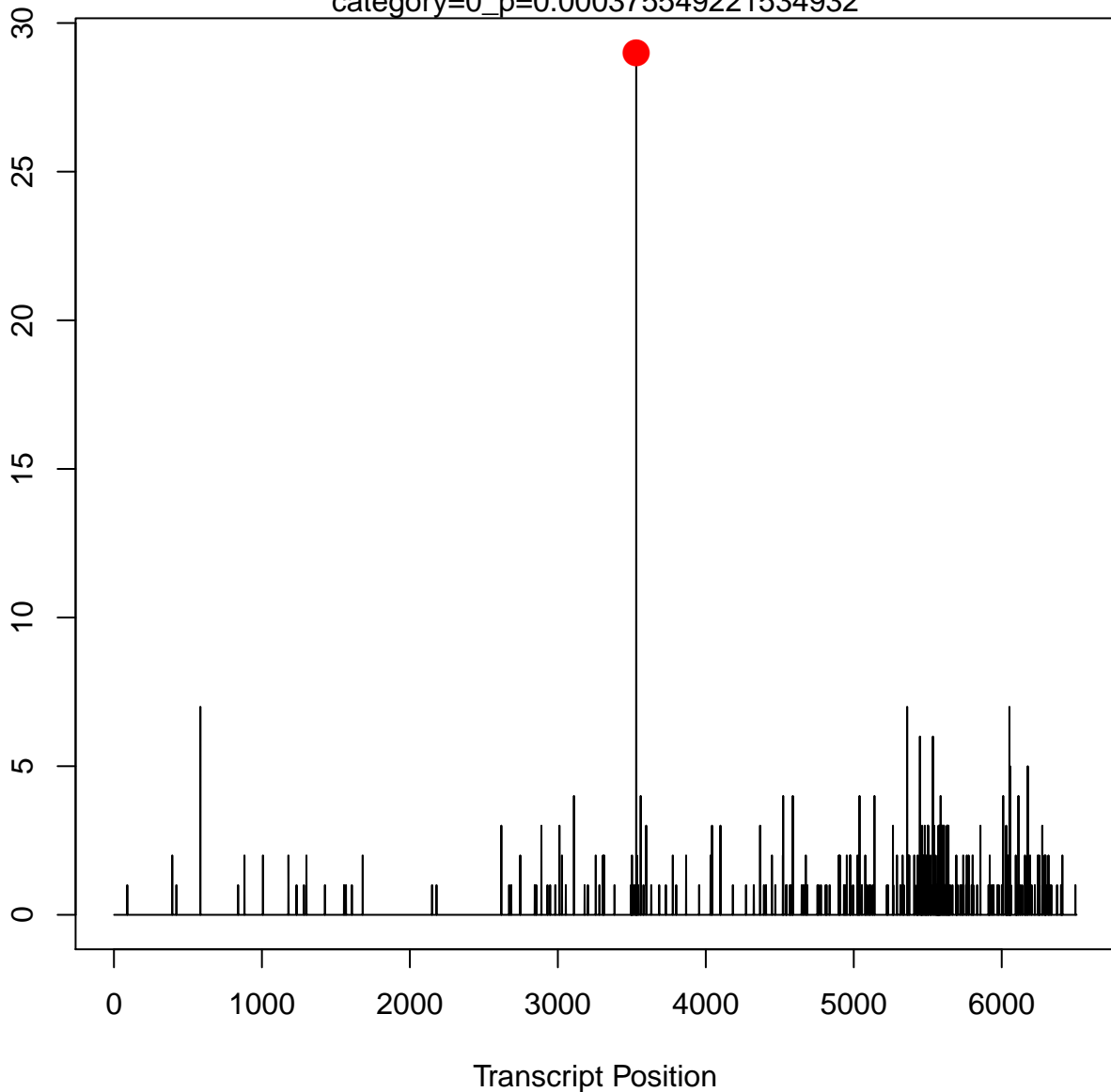

Supplement: Supplementary file 6 [file Data_Sheet_6.zip › Sit-miR162_Seita.9G562200.1_3530_TPlot.pdf]

**T=Seita.1G209000.1\_Q=Sit-miR164a\_S=999**

category=0\_p=0.0015013508746784

Degradsome 5' end Frequency

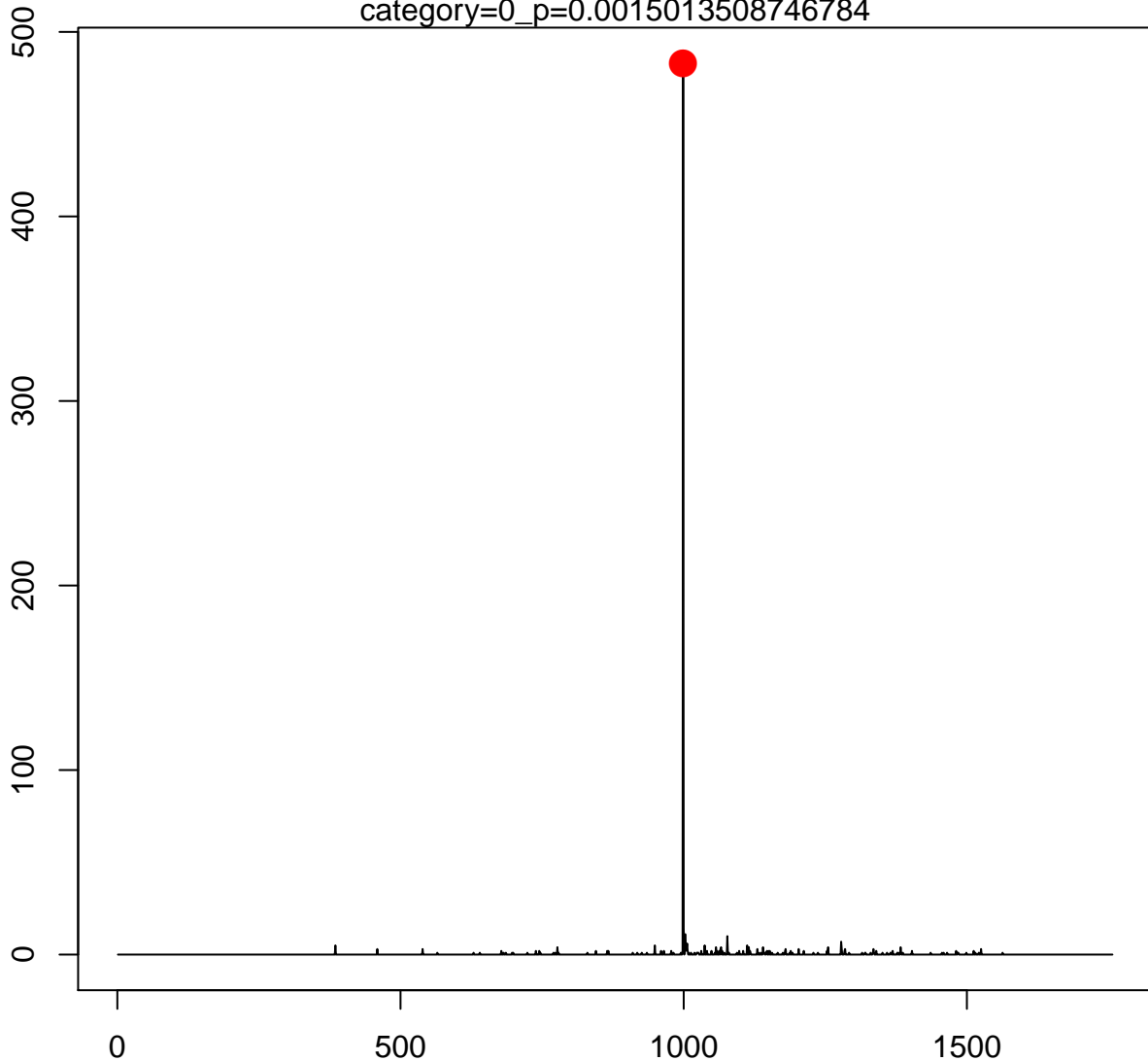

Transcript Position

Supplement: Supplementary file 6 [file Data_Sheet_6.zip › Sit-miR164a_Seita.1G209000.1_999_TPlot.pdf]

**T=Seita.7G124900.1\_Q=Sit-miR164a\_S=859**

category=0\_p=0.00187633626506101

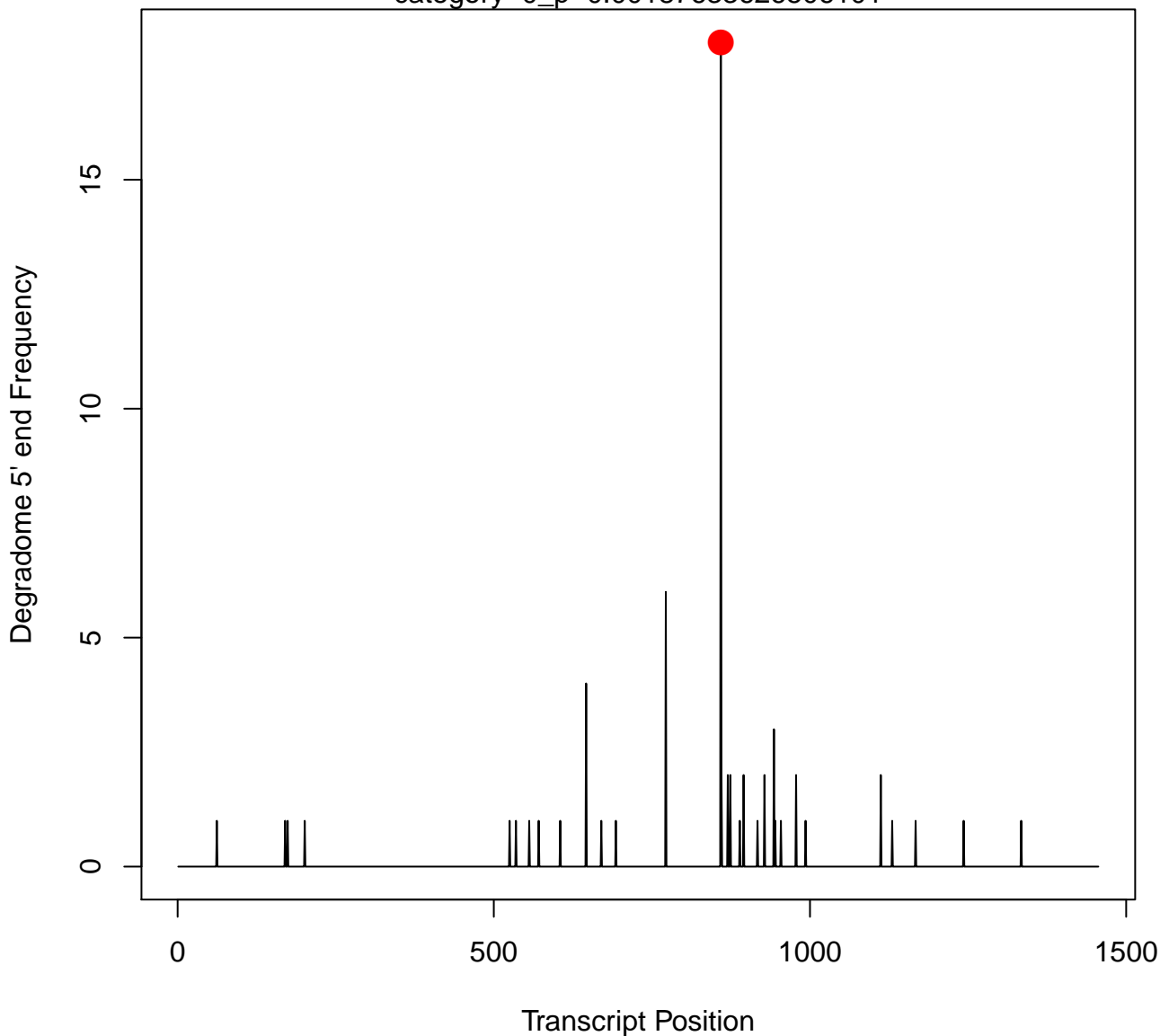

Supplement: Supplementary file 6 [file Data_Sheet_6.zip › Sit-miR164a_Seita.7G124900.1_859_TPlot.pdf]

**T=Seita.J028200.1\_Q=Sit-miR164a\_S=569**

category=2\_p=0.103158972889376

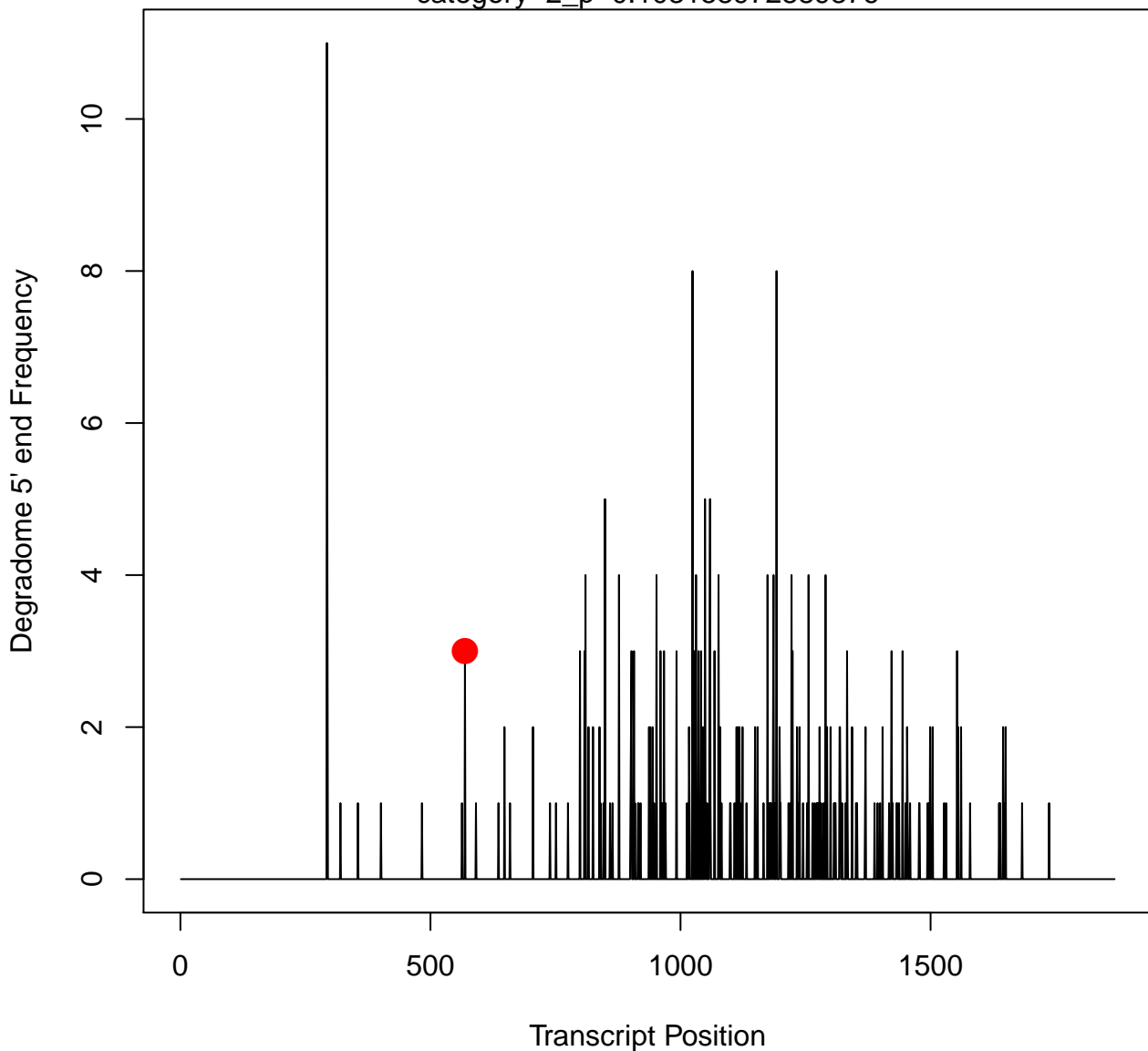

Supplement: Supplementary file 6 [file Data_Sheet_6.zip › Sit-miR164a_Seita.J028200.1_569_TPlot.pdf]

**T=Seita.2G174200.1\_Q=Sit-miR164b\_S=201**

category=0\_p=0.0011262246059176

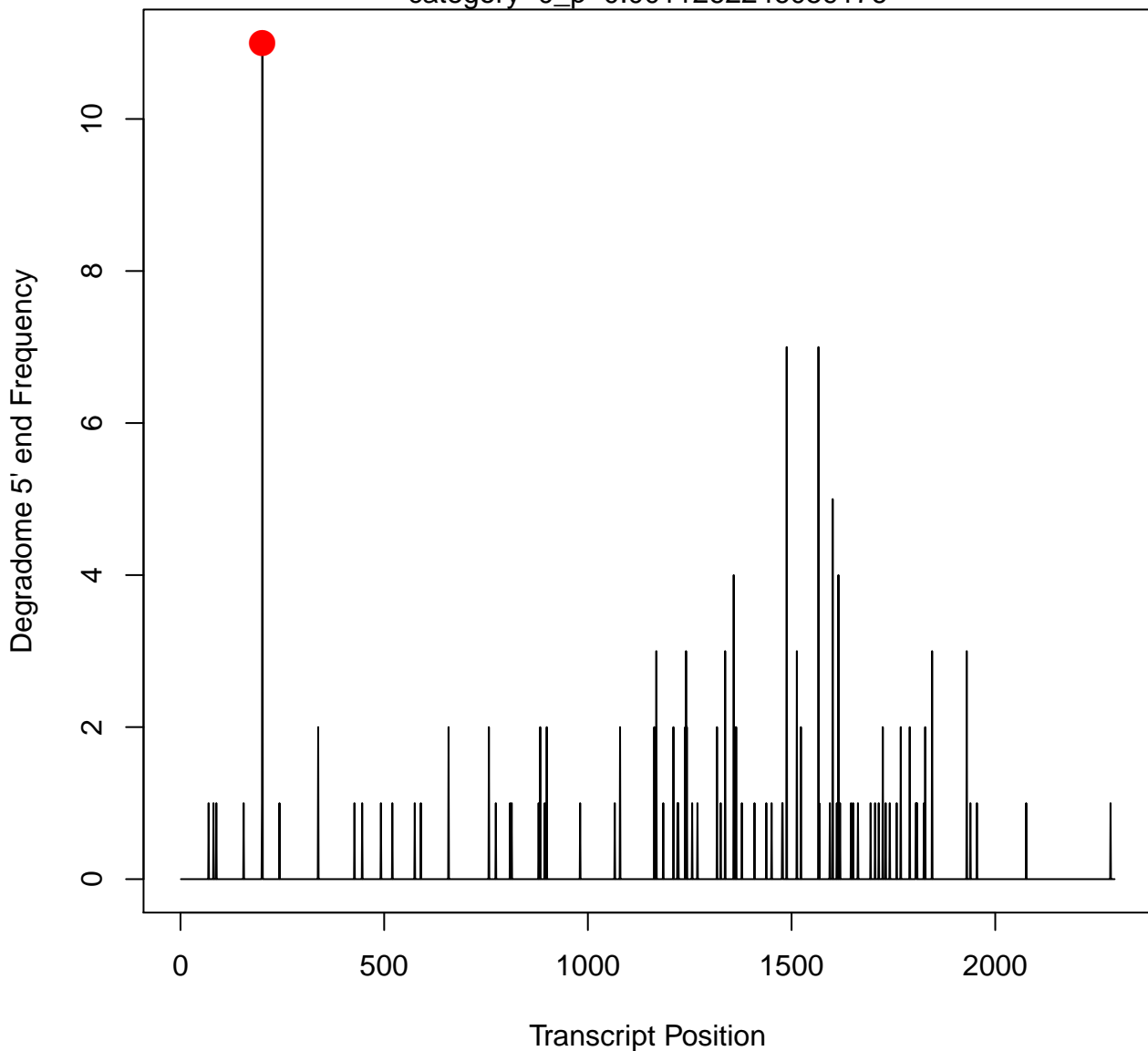

Supplement: Supplementary file 6 [file Data_Sheet_6.zip › Sit-miR164b_Seita.2G174200.1_201_TPlot.pdf]

**T=Seita.2G408400.1\_Q=Sit-miR164b\_S=798**

category=2\_p=0.999976766437687

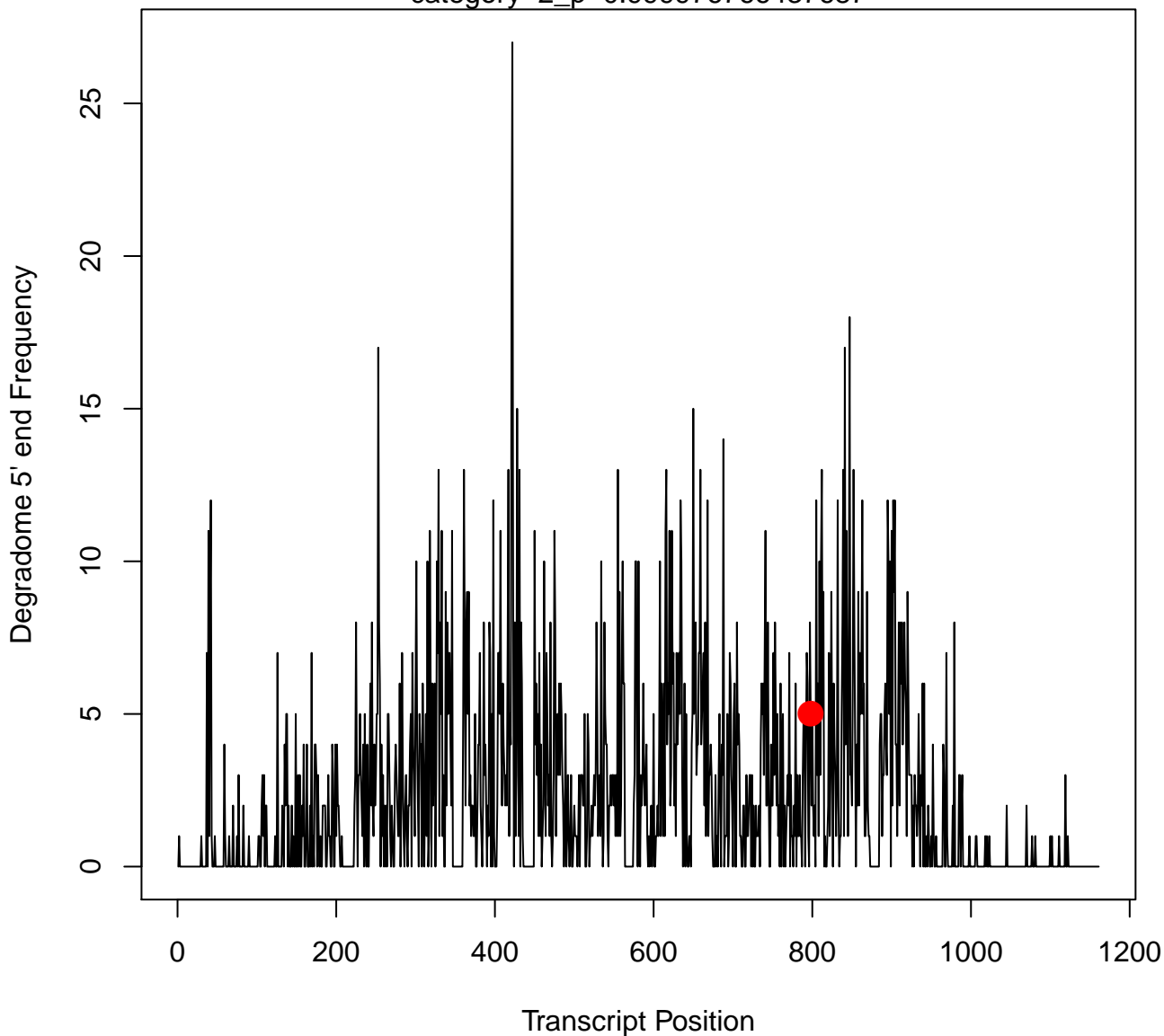

Supplement: Supplementary file 6 [file Data_Sheet_6.zip › Sit-miR164b_Seita.2G408400.1_798_TPlot.pdf]

**T=Seita.3G386200.1\_Q=Sit-miR164b\_S=790**

category=0\_p=0.000375549221534932

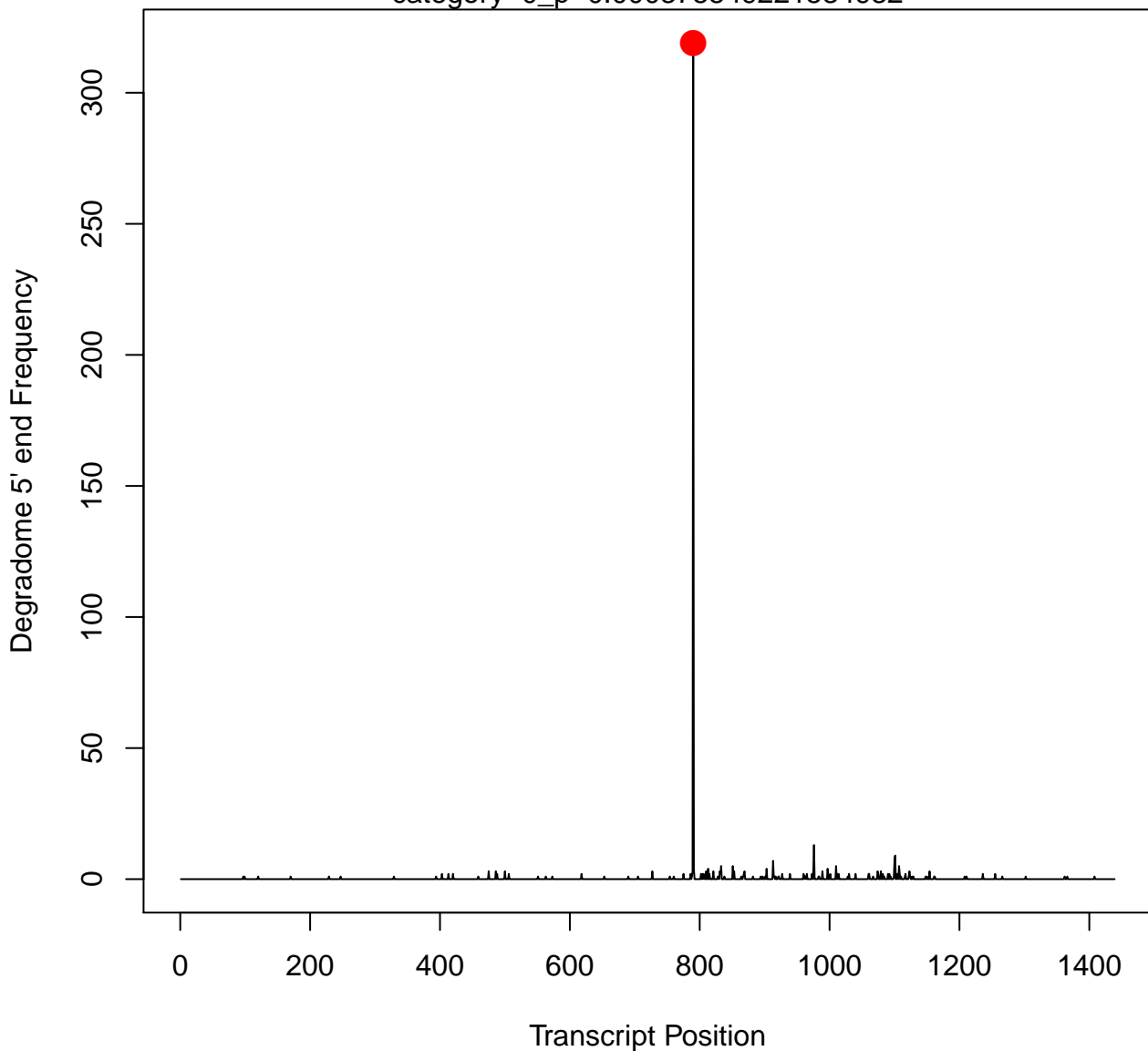

Supplement: Supplementary file 6 [file Data_Sheet_6.zip › Sit-miR164b_Seita.3G386200.1_790_TPlot.pdf]

**T=Seita.5G001000.1\_Q=Sit-miR164b\_S=1027**

category=2\_p=0.52478621833363

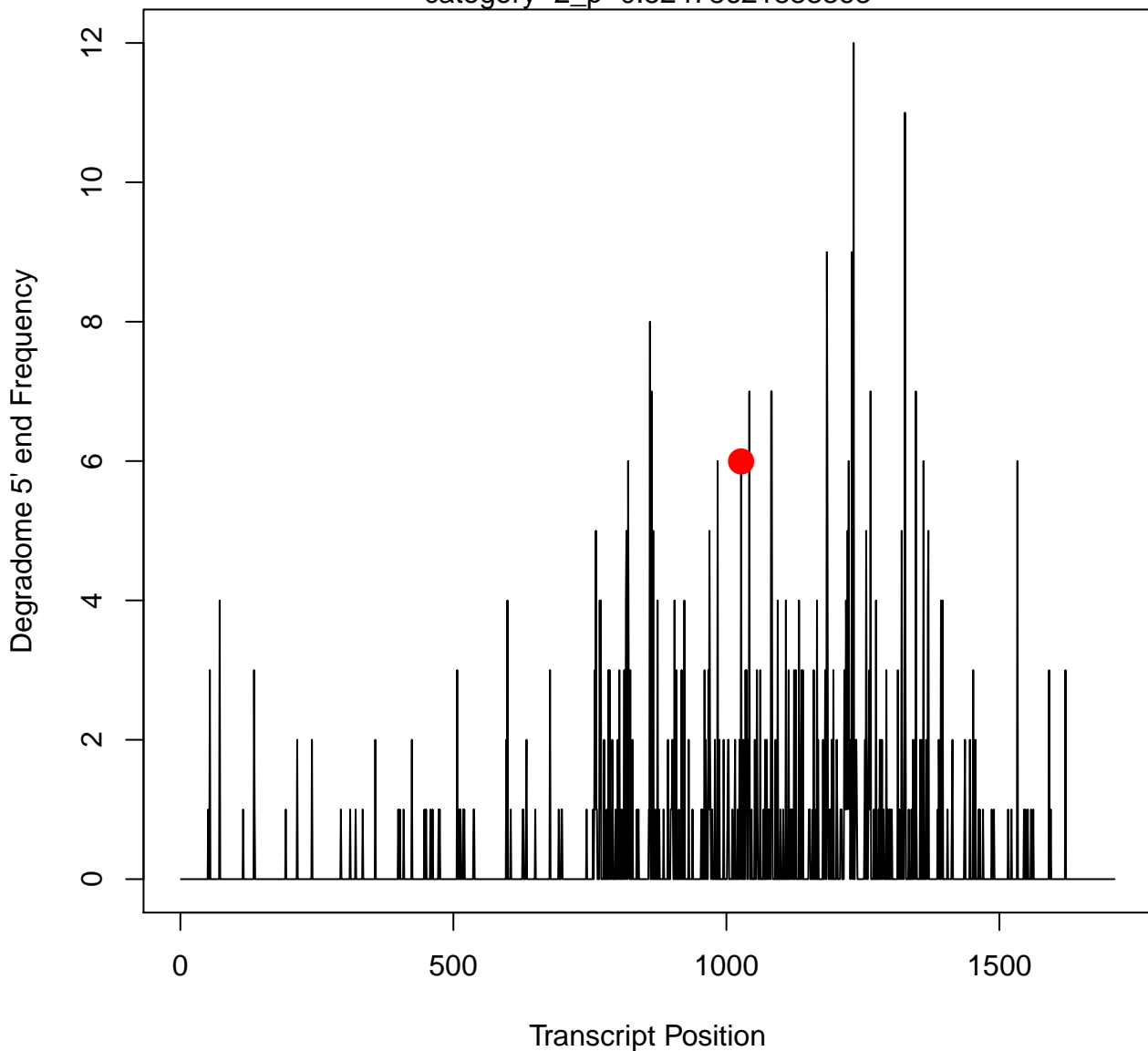

Supplement: Supplementary file 6 [file Data_Sheet_6.zip › Sit-miR164b_Seita.5G001000.1_1027_TPlot.pdf]

**T=Seita.1G229400.1\_Q=Sit-miR164c\_S=1272**

category=2\_p=0.995001733450007

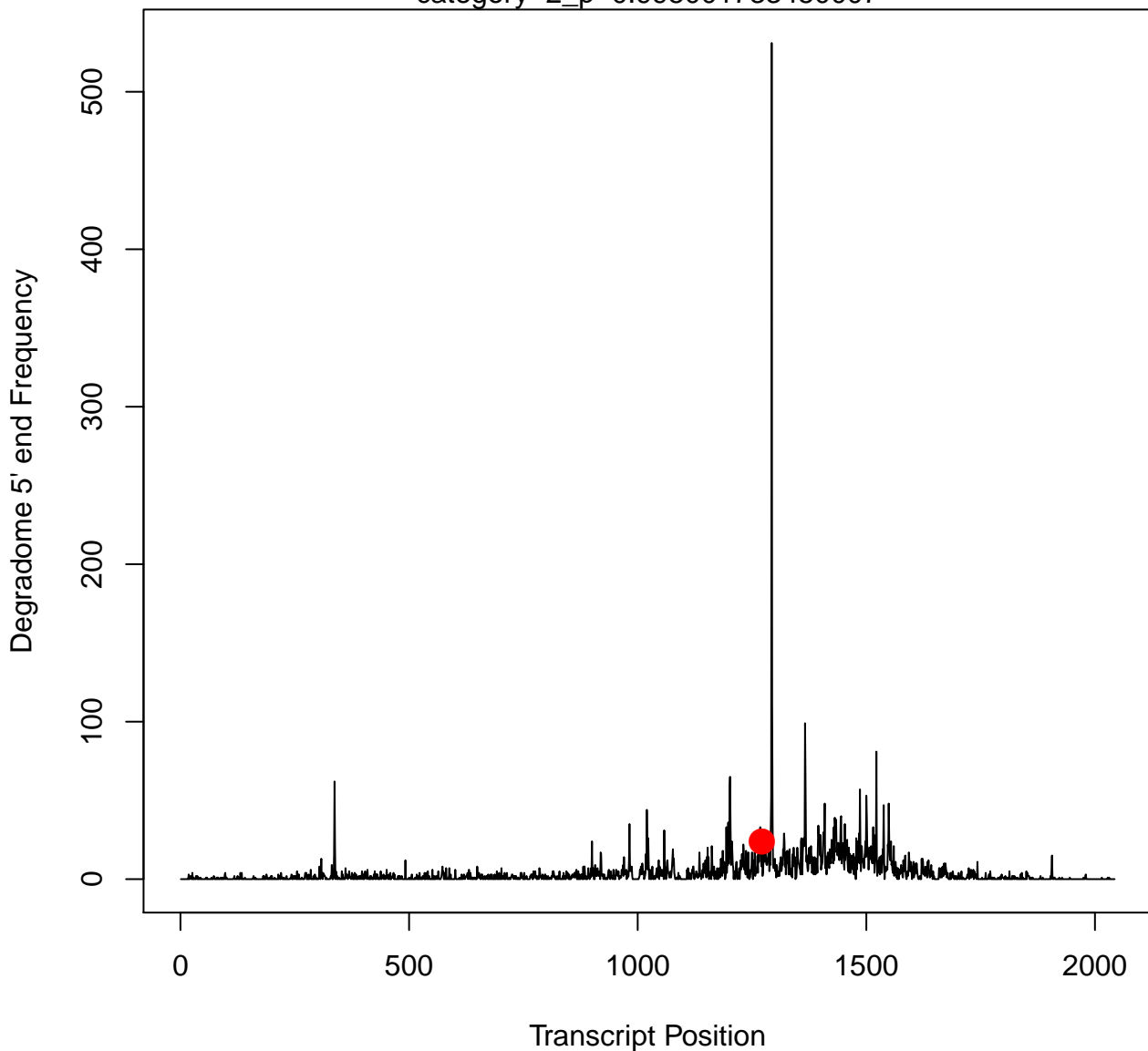

Supplement: Supplementary file 6 [file Data_Sheet_6.zip › Sit-miR164c_Seita.1G229400.1_1272_TPlot.pdf]

**T=Seita.1G273100.1\_Q=Sit-miR164c\_S=359**

category=2\_p=0.999775503159573

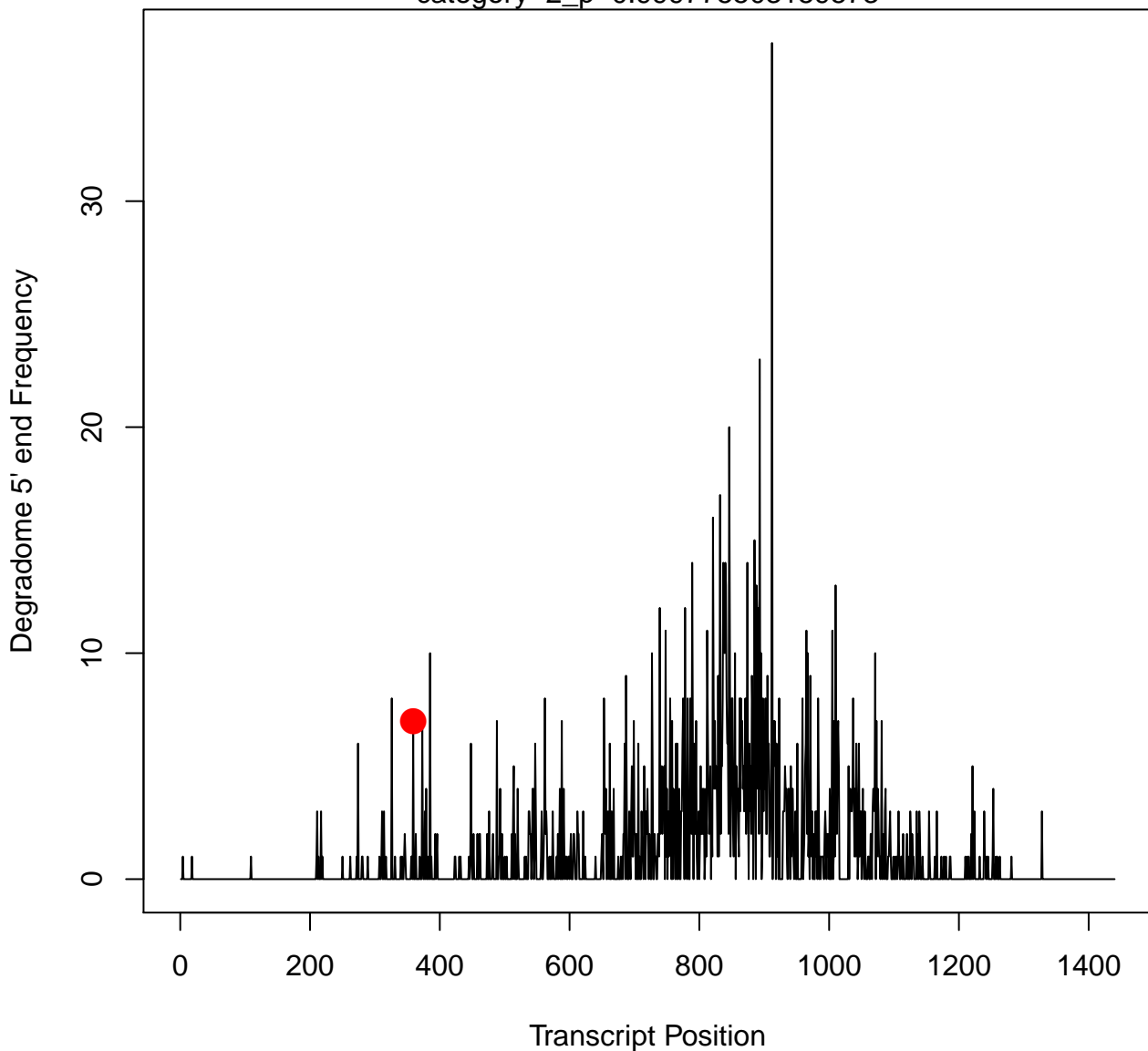

Supplement: Supplementary file 6 [file Data_Sheet_6.zip › Sit-miR164c_Seita.1G273100.1_359_TPlot.pdf]

**T=Seita.5G086100.1\_Q=Sit-miR164d\_S=1397**

category=2\_p=0.999864933053762

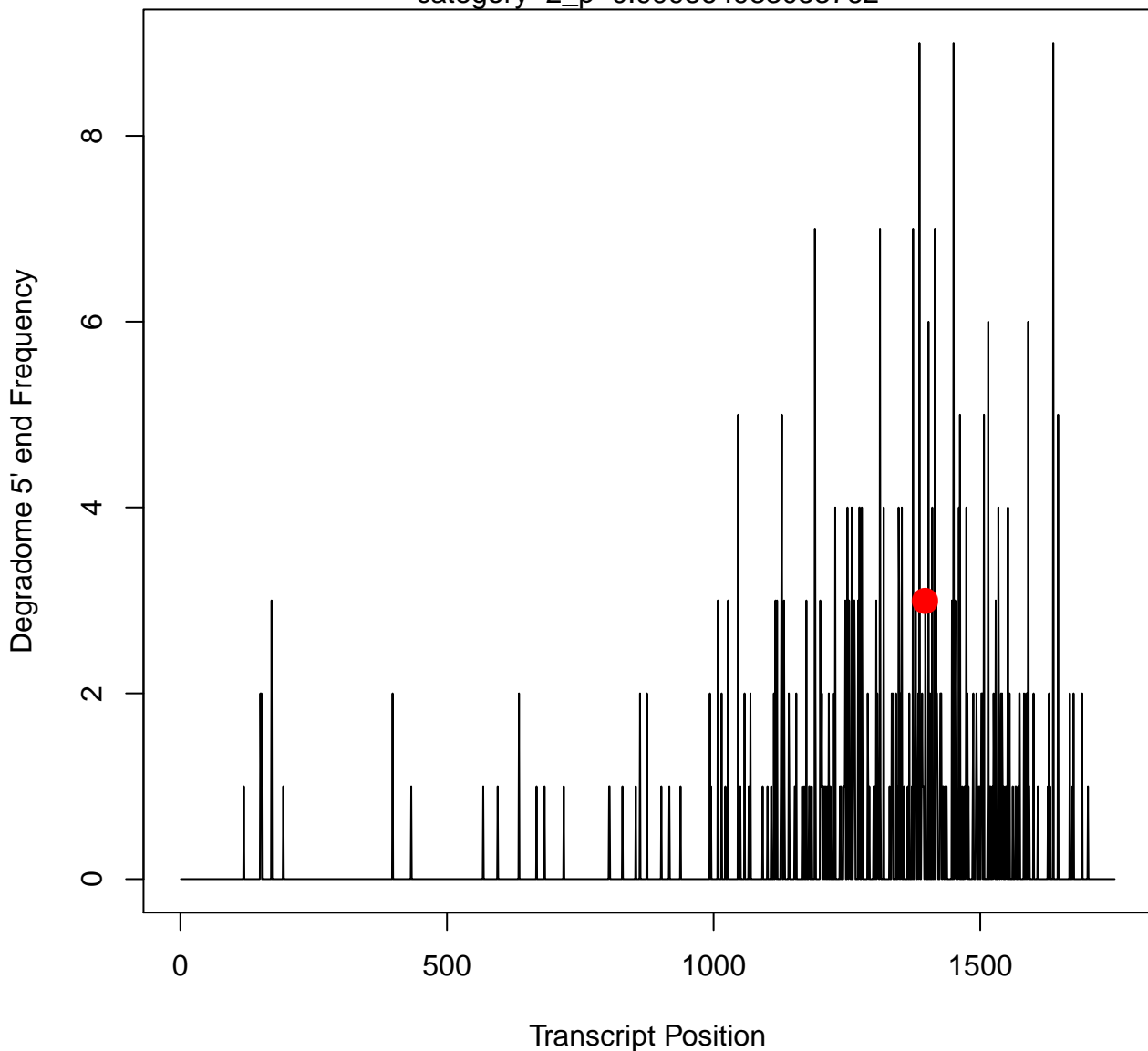

Supplement: Supplementary file 6 [file Data_Sheet_6.zip › Sit-miR164d_Seita.5G086100.1_1397_TPlot.pdf]

**T=Seita.9G078400.1\_Q=Sit-miR164d\_S=622**

category=2\_p=0.99509161458179

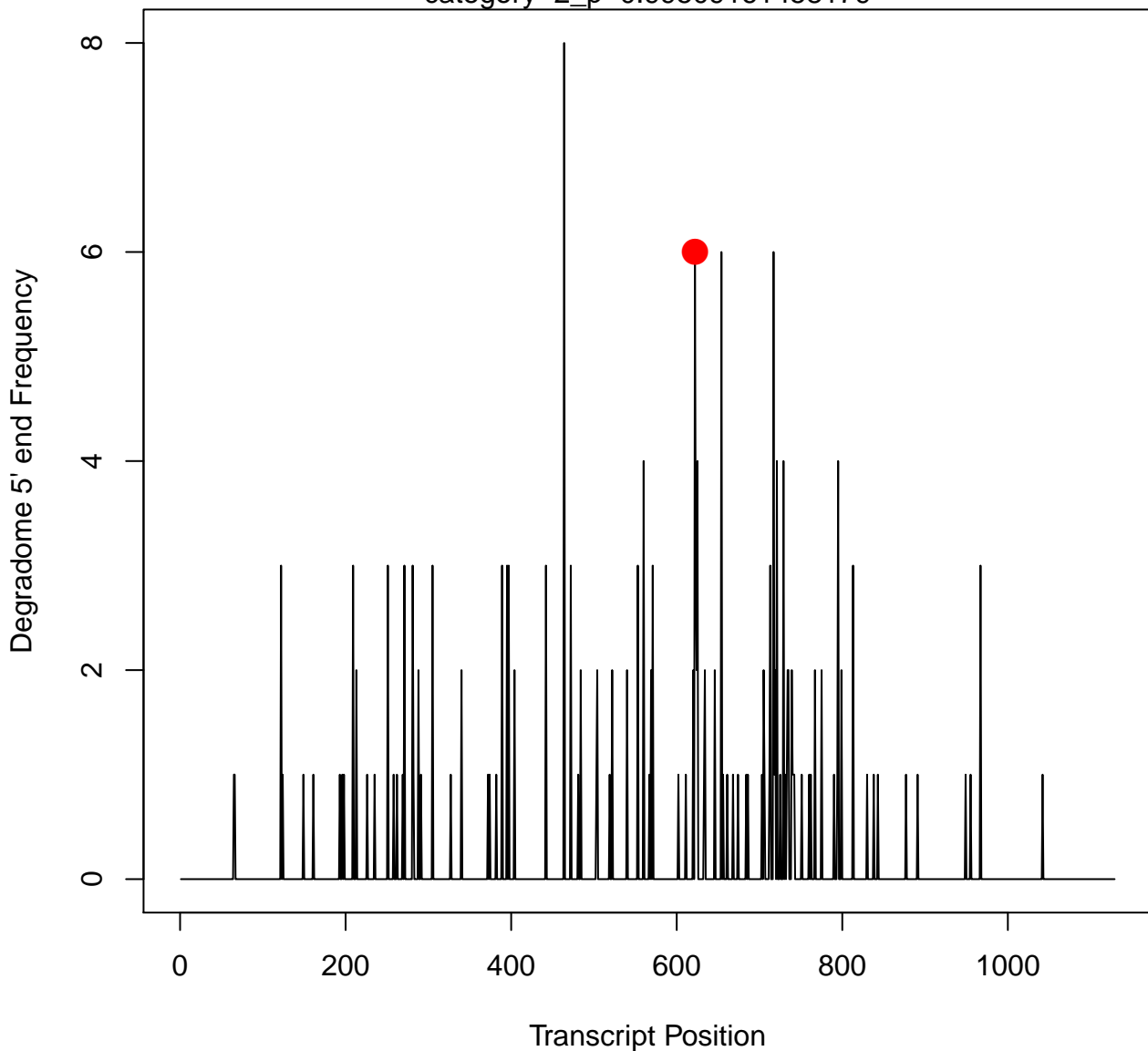

Supplement: Supplementary file 6 [file Data_Sheet_6.zip › Sit-miR164d_Seita.9G078400.1_622_TPlot.pdf]

**T=Seita.1G320200.1\_Q=Sit-miR164e\_S=1595**

category=2\_p=0.999762943155349

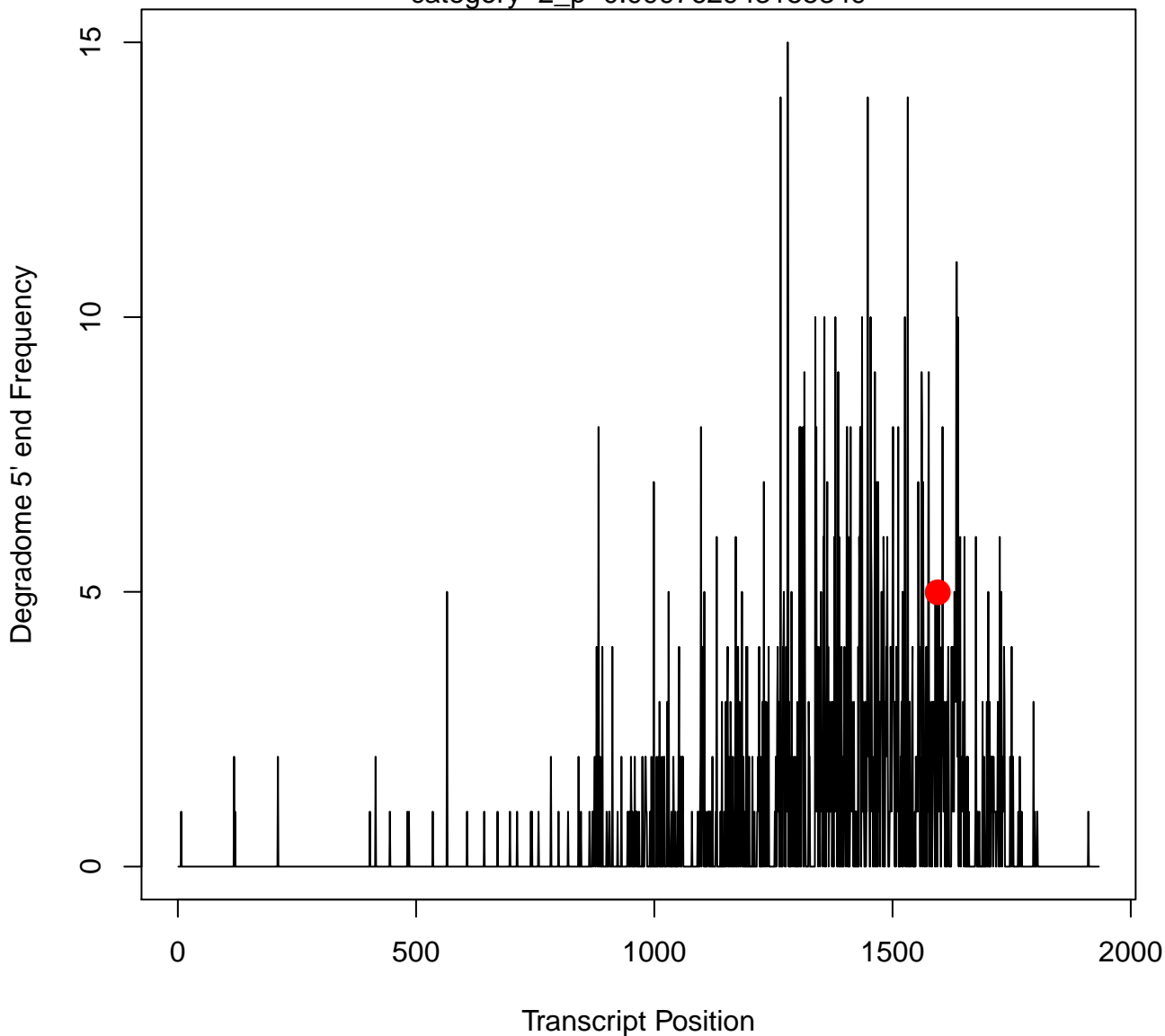

Supplement: Supplementary file 6 [file Data_Sheet_6.zip › Sit-miR164e_Seita.1G320200.1_1595_TPlot.pdf]

**T=Seita.2G324100.1\_Q=Sit-miR164e\_S=1709**

category=2\_p=0.999999615350586

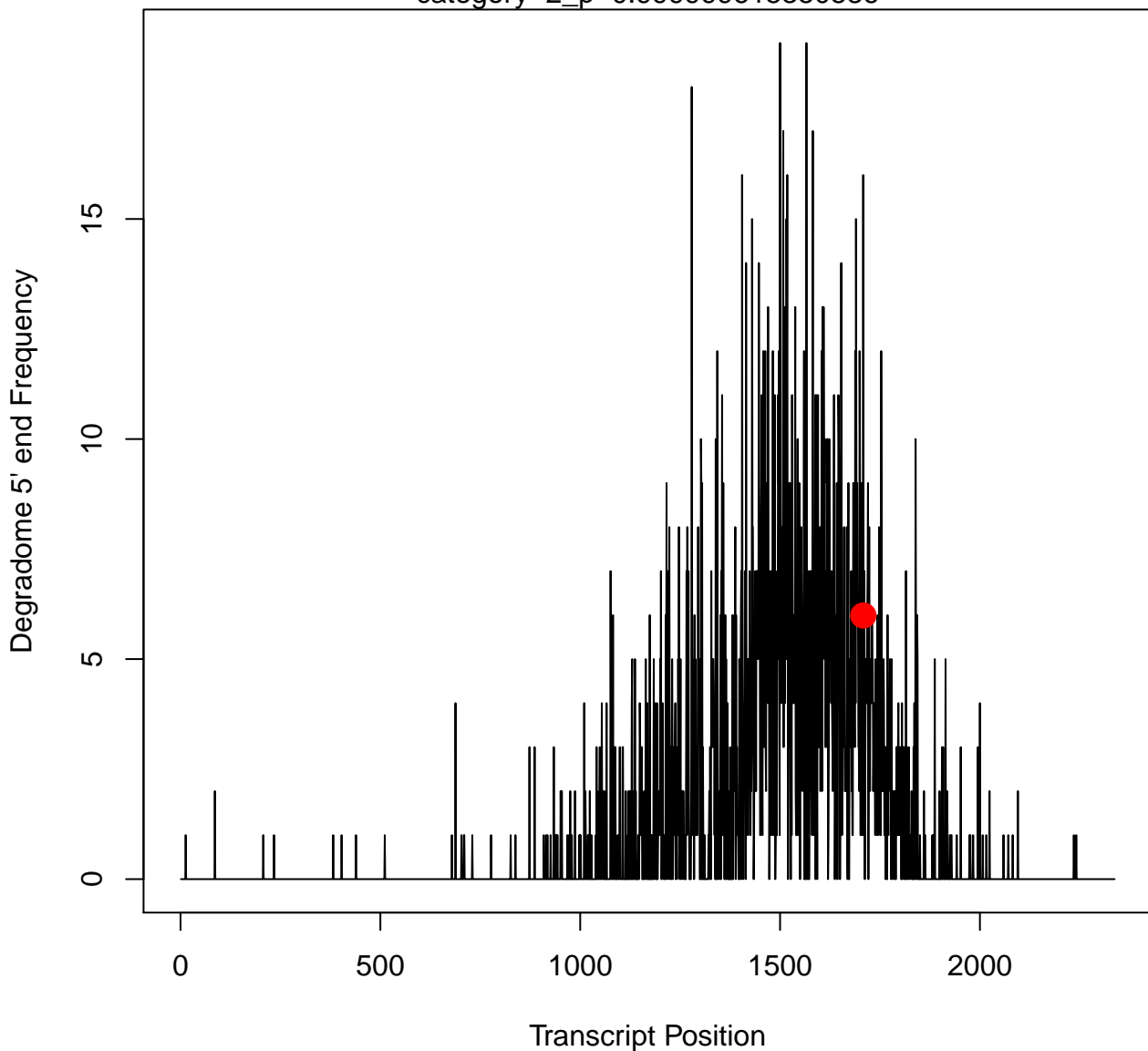

Supplement: Supplementary file 6 [file Data_Sheet_6.zip › Sit-miR164e_Seita.2G324100.1_1709_TPlot.pdf]

**T=Seita.3G201900.1\_Q=Sit-miR164e\_S=239**

category=2\_p=0.894613173724163

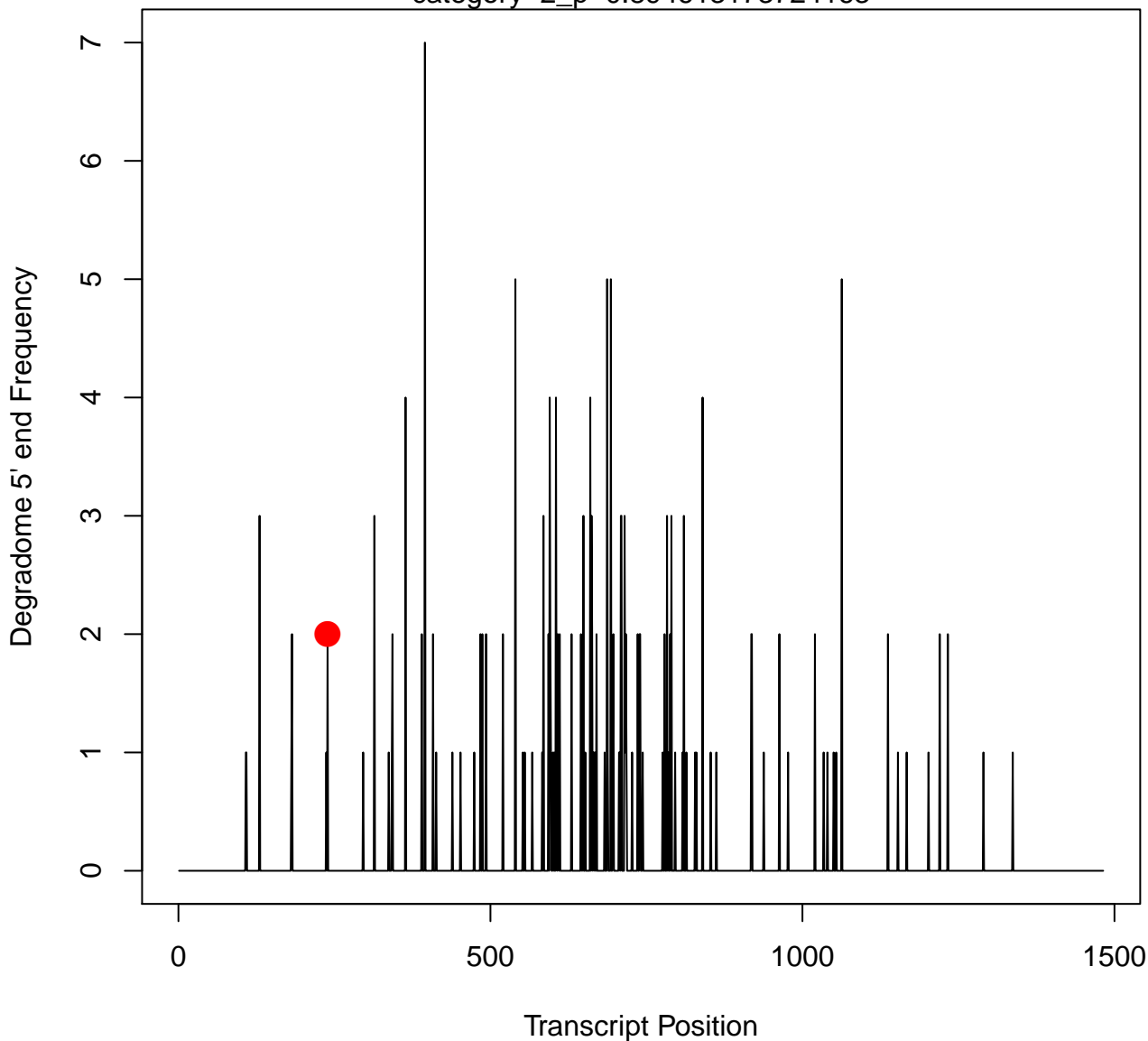

Supplement: Supplementary file 6 [file Data_Sheet_6.zip › Sit-miR164e_Seita.3G201900.1_239_TPlot.pdf]

**T=Seita.4G289900.1\_Q=Sit-miR164e\_S=599**

category=2\_p=0.84290478913823

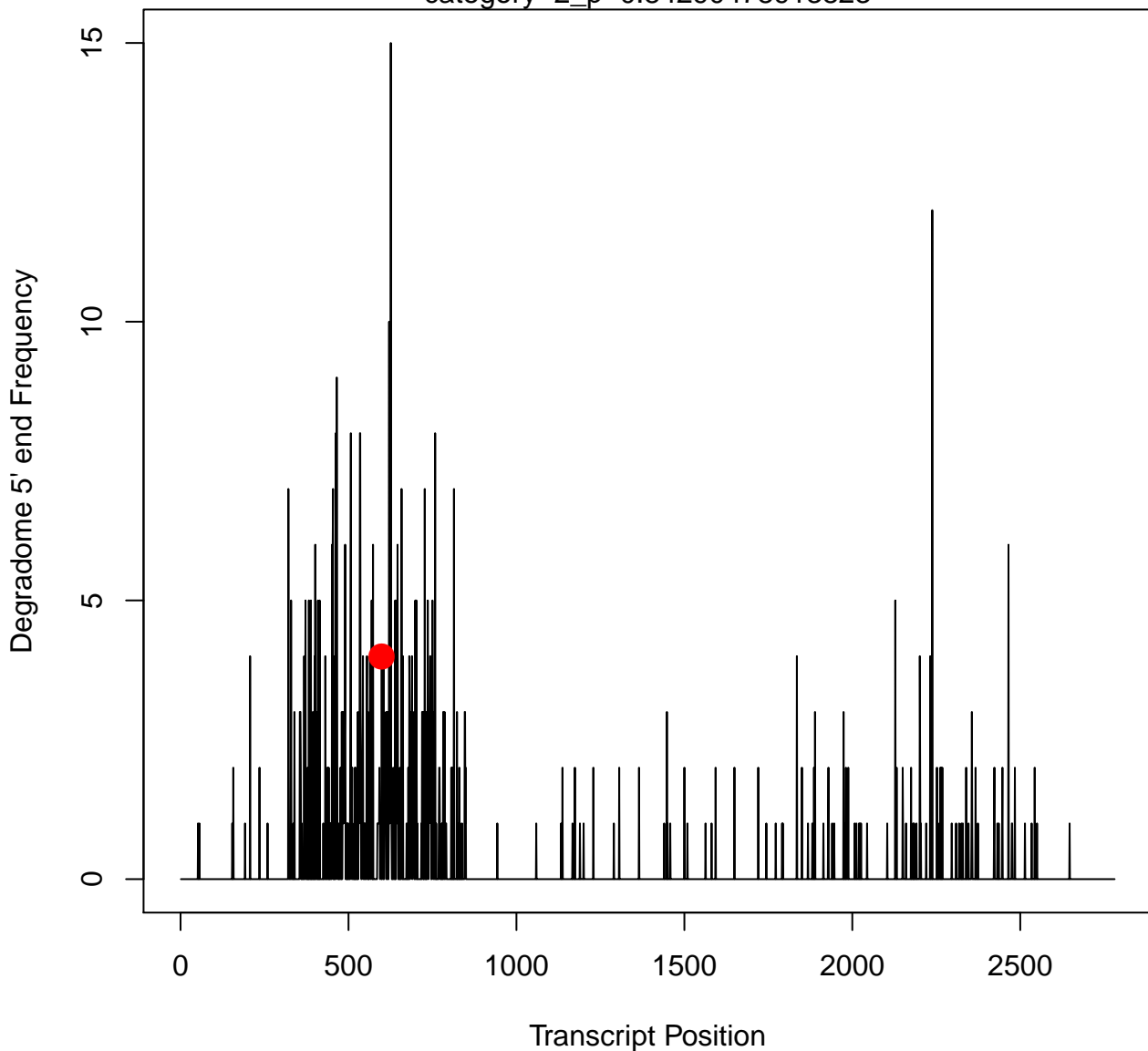

Supplement: Supplementary file 6 [file Data_Sheet_6.zip › Sit-miR164e_Seita.4G289900.1_599_TPlot.pdf]

**T=Seita.7G286800.1\_Q=Sit-miR164e\_S=1119**

category=2\_p=0.999999097472216

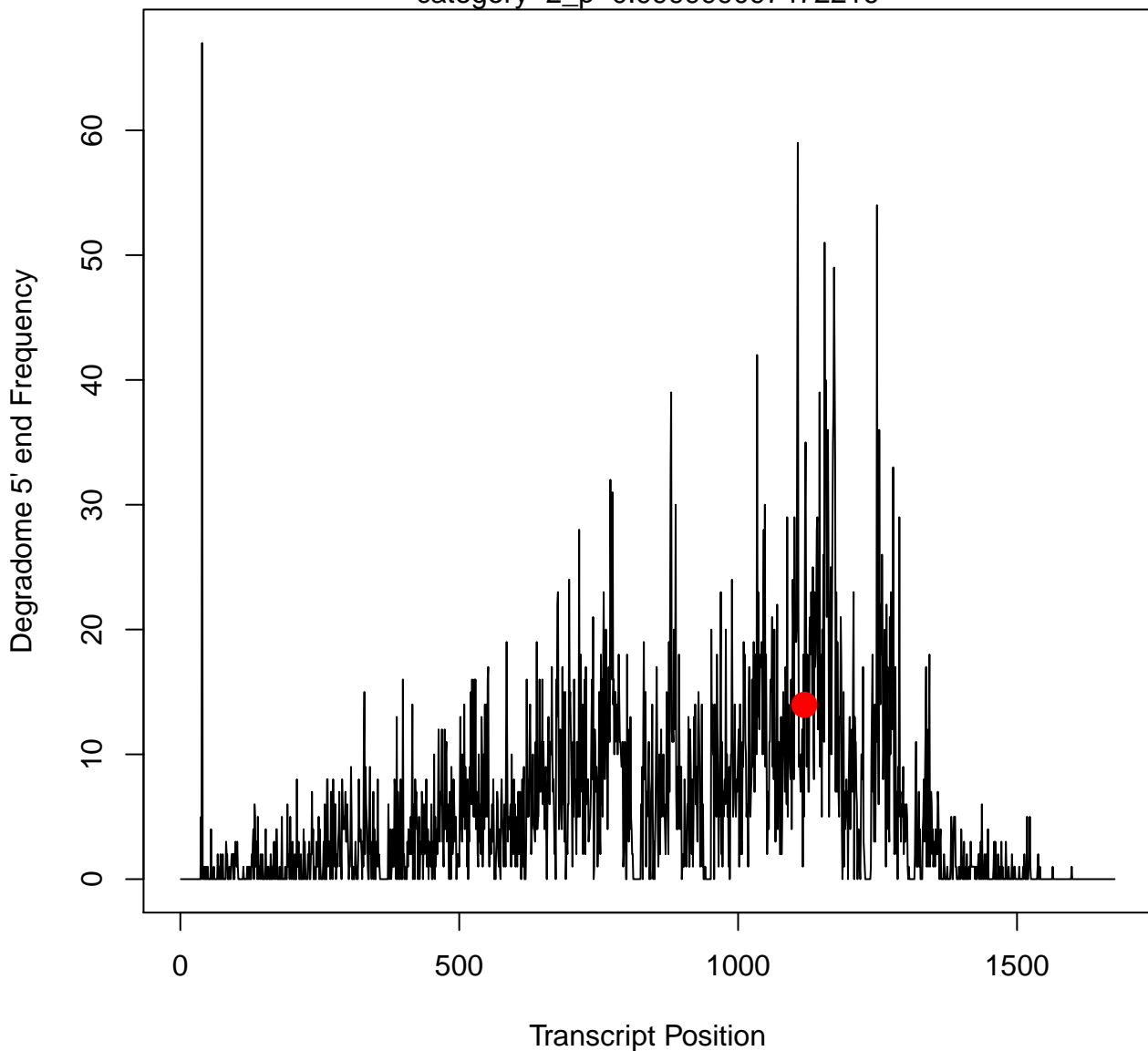

Supplement: Supplementary file 6 [file Data_Sheet_6.zip › Sit-miR164e_Seita.7G286800.1_1119_TPlot.pdf]

**T=Seita.8G030100.1\_Q=Sit-miR164e\_S=133**

category=0\_p=0.135617989427805

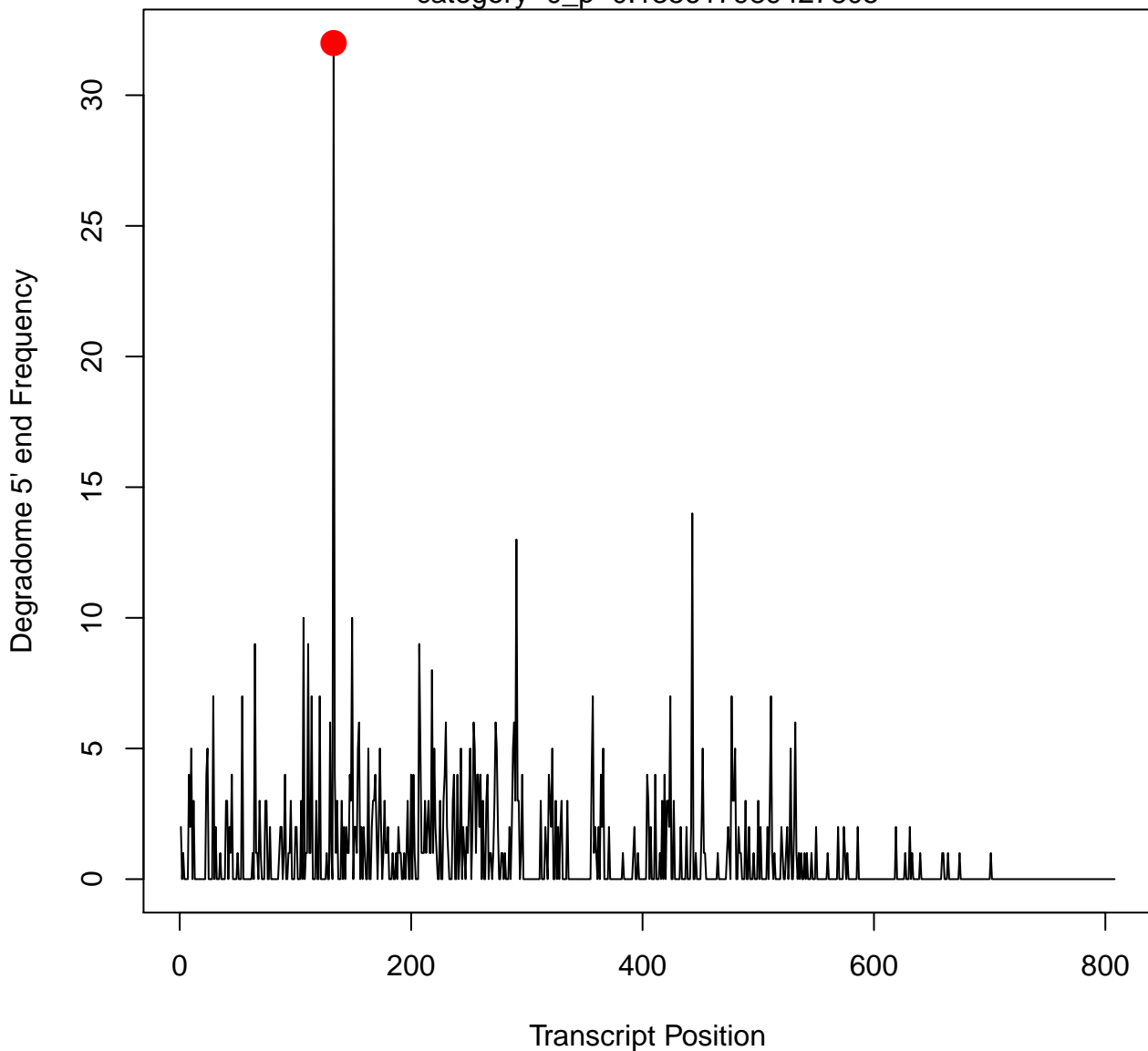

Supplement: Supplementary file 6 [file Data_Sheet_6.zip › Sit-miR164e_Seita.8G030100.1_133_TPlot.pdf]

**T=Seita.8G179000.1\_Q=Sit-miR164e\_S=122**

category=2\_p=0.135123779440266

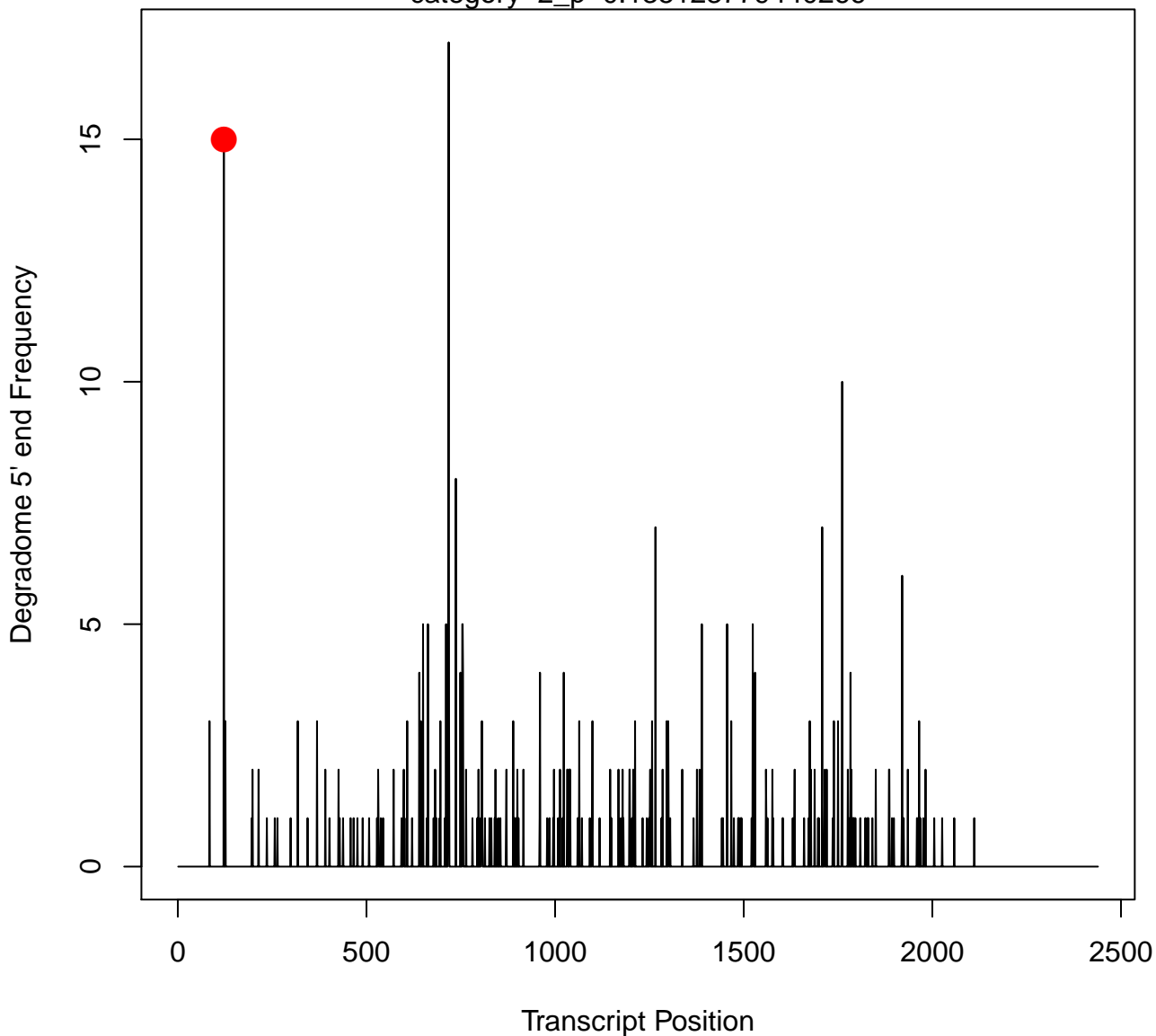

Supplement: Supplementary file 6 [file Data_Sheet_6.zip › Sit-miR164e_Seita.8G179000.1_122_TPlot.pdf]

**T=Seita.9G089800.1\_Q=Sit-miR164e\_S=1145**

category=2\_p=0.692566707201138

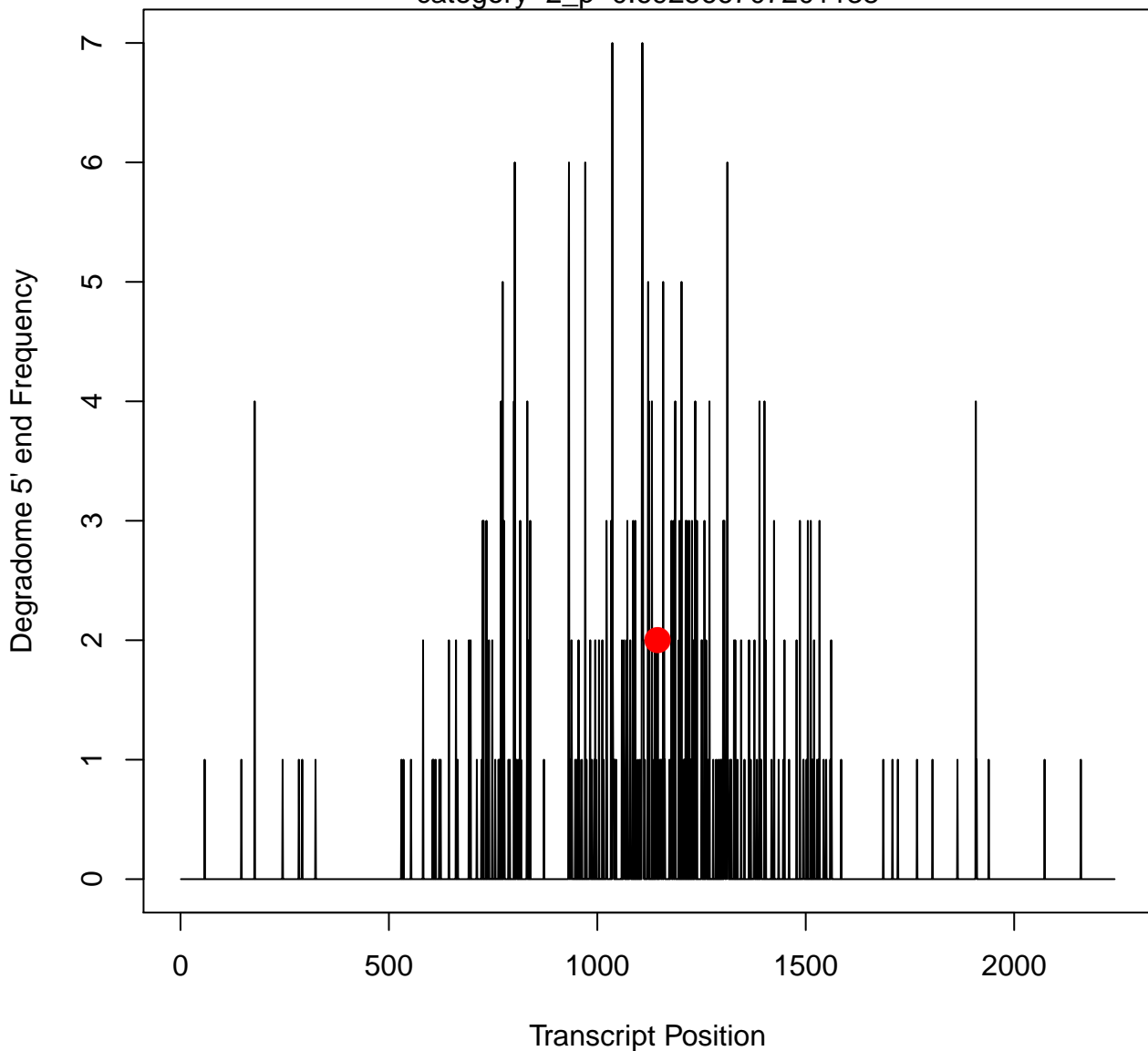

Supplement: Supplementary file 6 [file Data_Sheet_6.zip › Sit-miR164e_Seita.9G089800.1_1145_TPlot.pdf]

**T=Seita.4G263400.1\_Q=Sit-miR164f\_S=1216**

category=0\_p=0.0015013508746784

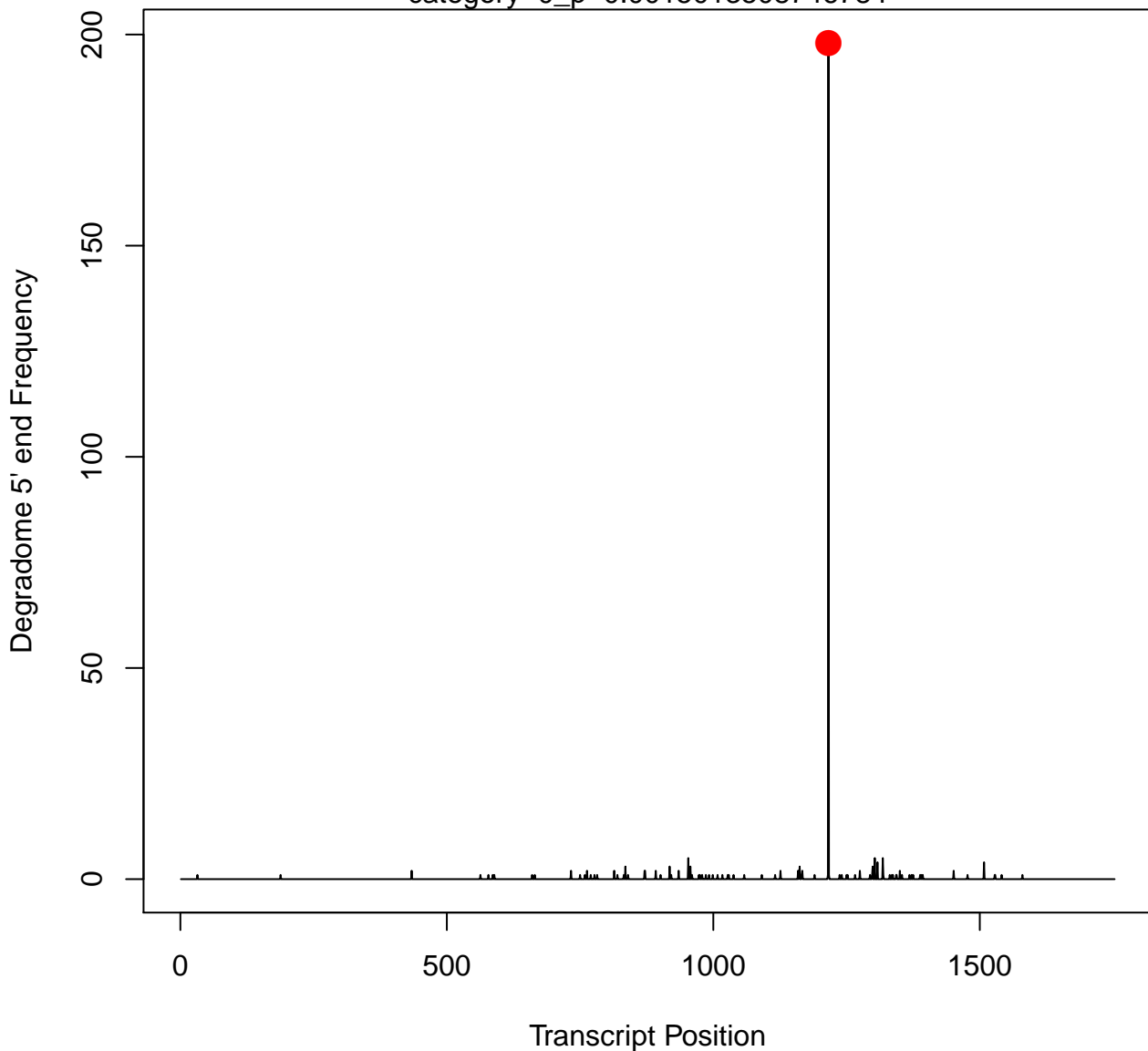

Supplement: Supplementary file 6 [file Data_Sheet_6.zip › Sit-miR164f_Seita.4G263400.1_1216_TPlot.pdf]

**T=Seita.5G312200.1\_Q=Sit-miR166a\_S=1707**

category=2\_p=0.986439960280254

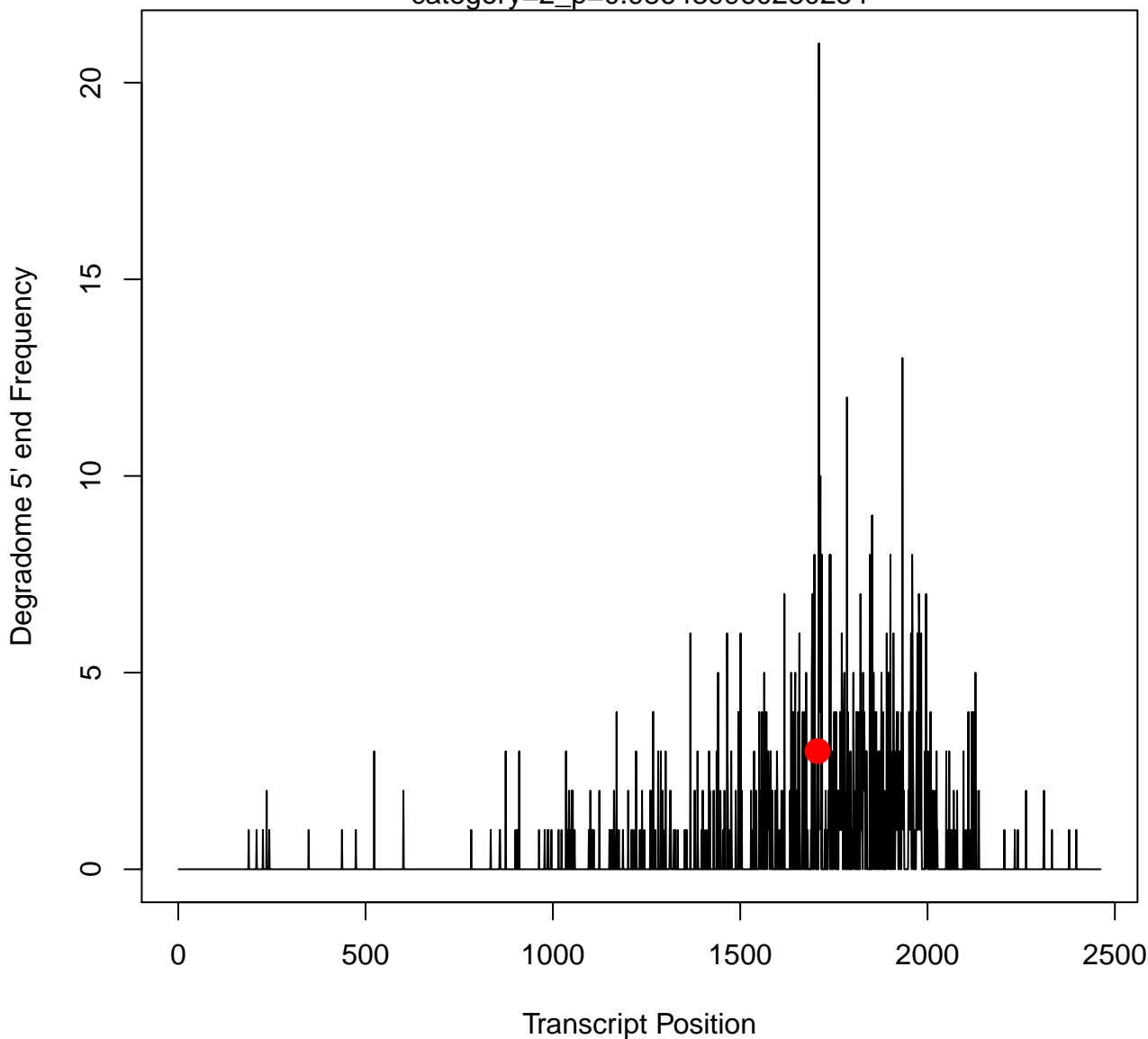

Supplement: Supplementary file 6 [file Data_Sheet_6.zip › Sit-miR166a_Seita.5G312200.1_1707_TPlot.pdf]

**T=Seita.9G158800.1\_Q=Sit-miR166a\_S=868**

category=2\_p=0.0529830903776722

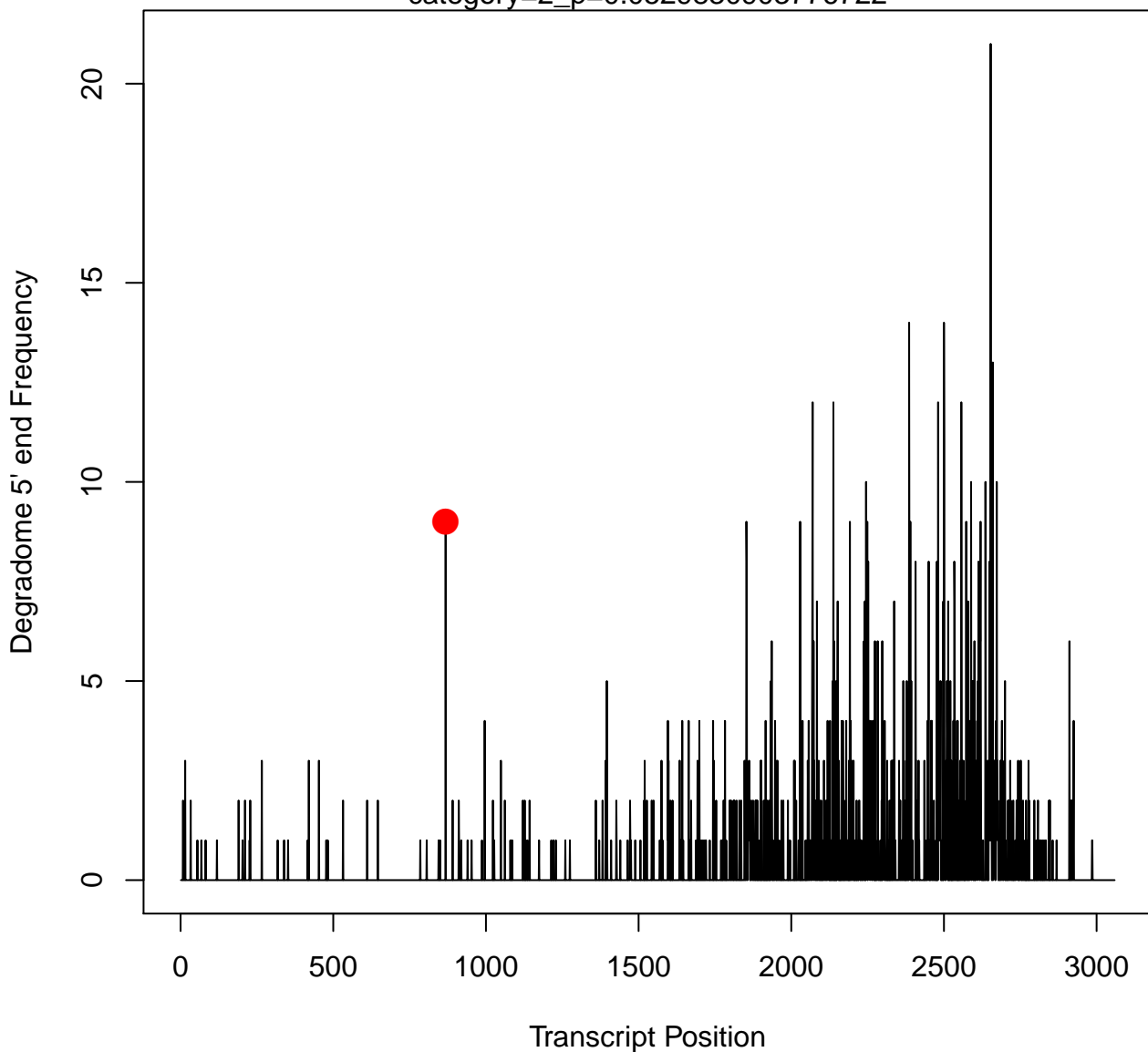

Supplement: Supplementary file 6 [file Data_Sheet_6.zip › Sit-miR166a_Seita.9G158800.1_868_TPlot.pdf]

**T=Seita.1G068500.1\_Q=Sit-miR166b\_S=1734**

category=2\_p=0.992273949488767

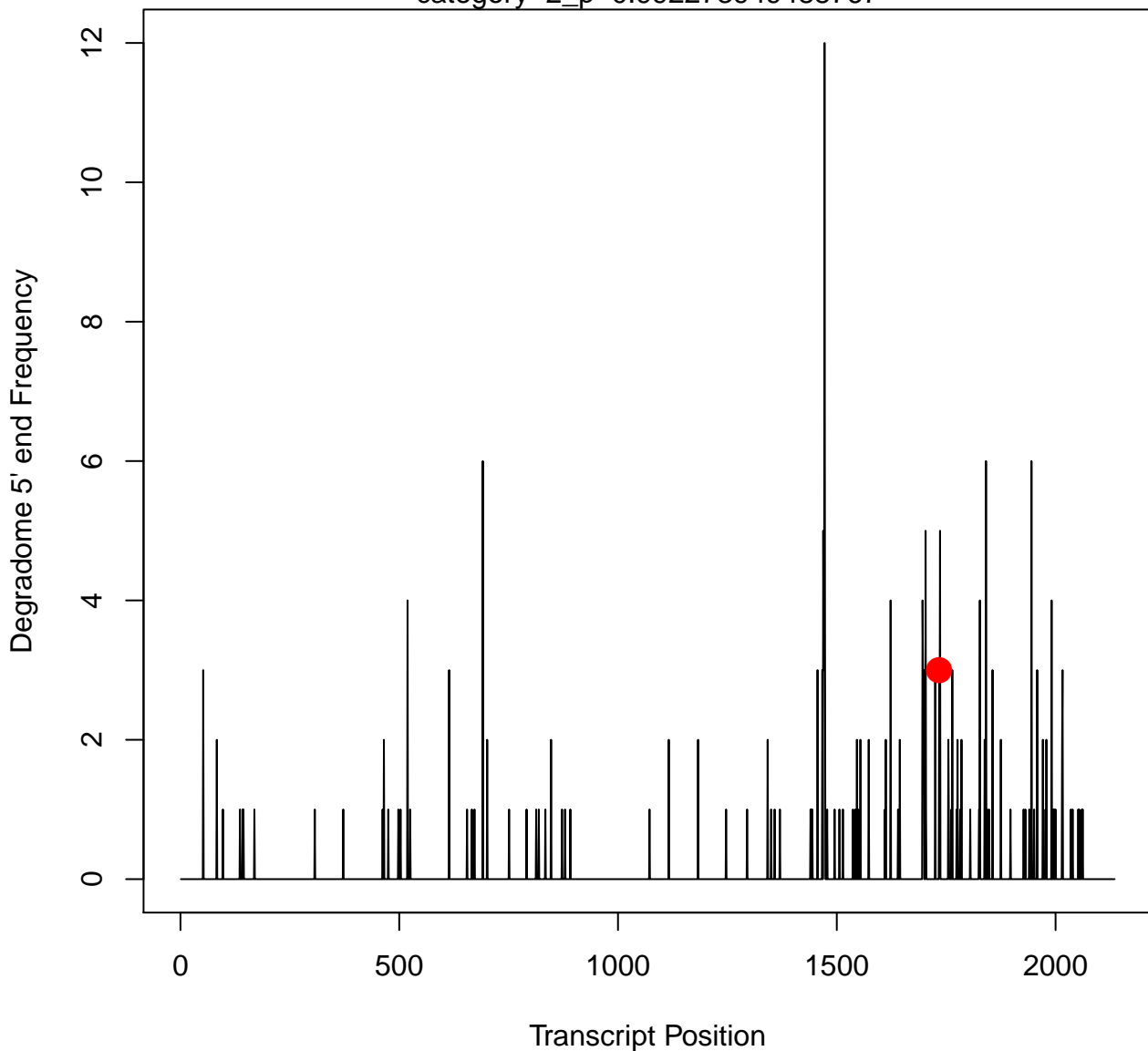

Supplement: Supplementary file 6 [file Data_Sheet_6.zip › Sit-miR166b_Seita.1G068500.1_1734_TPlot.pdf]

**T=Seita.3G035500.1\_Q=Sit-miR166b\_S=2169**

category=2\_p=0.992549317923672

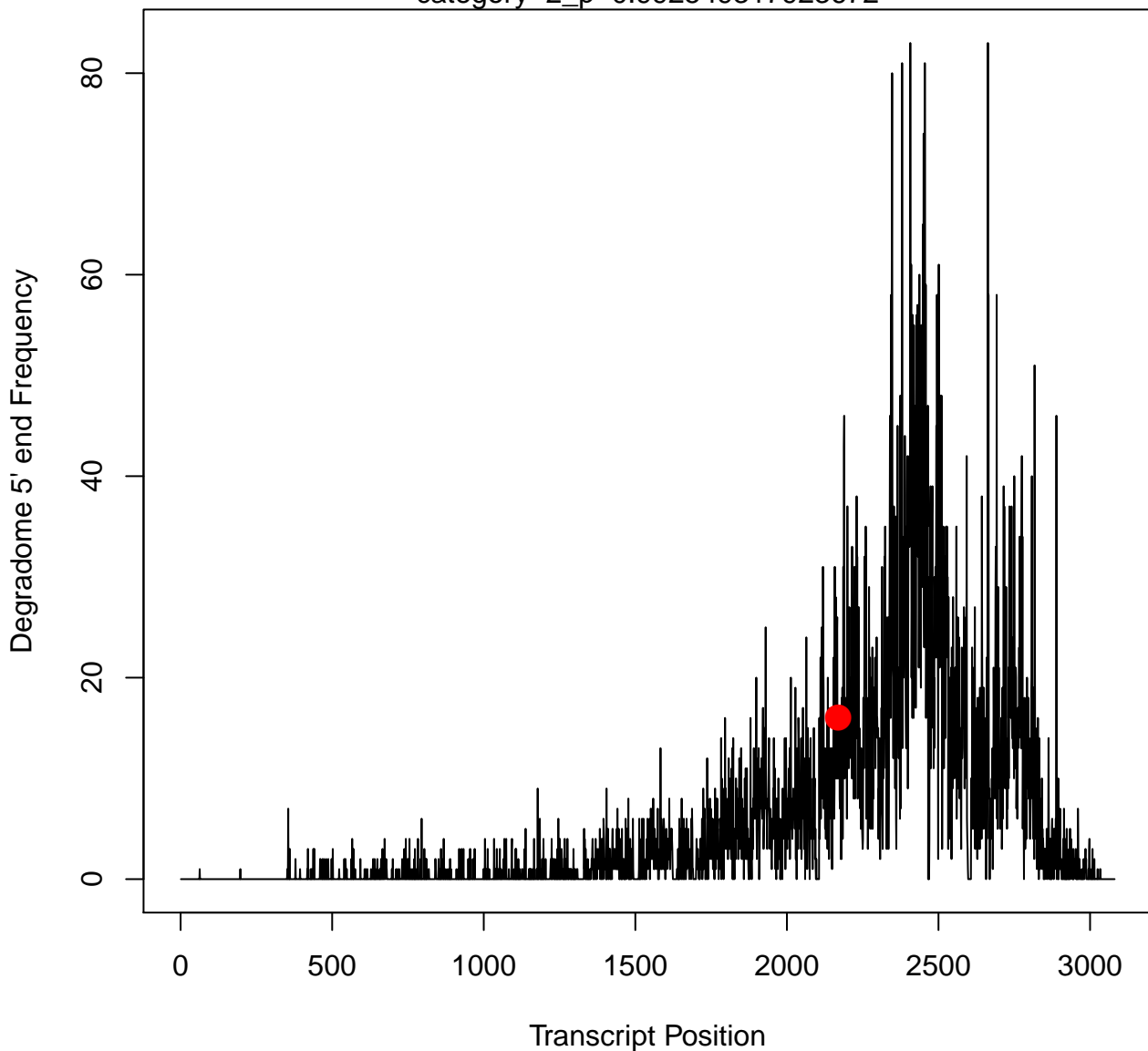

Supplement: Supplementary file 6 [file Data_Sheet_6.zip › Sit-miR166b_Seita.3G035500.1_2169_TPlot.pdf]

**T=Seita.5G402200.1\_Q=Sit-miR166b\_S=1662**

category=2\_p=0.840028100747495

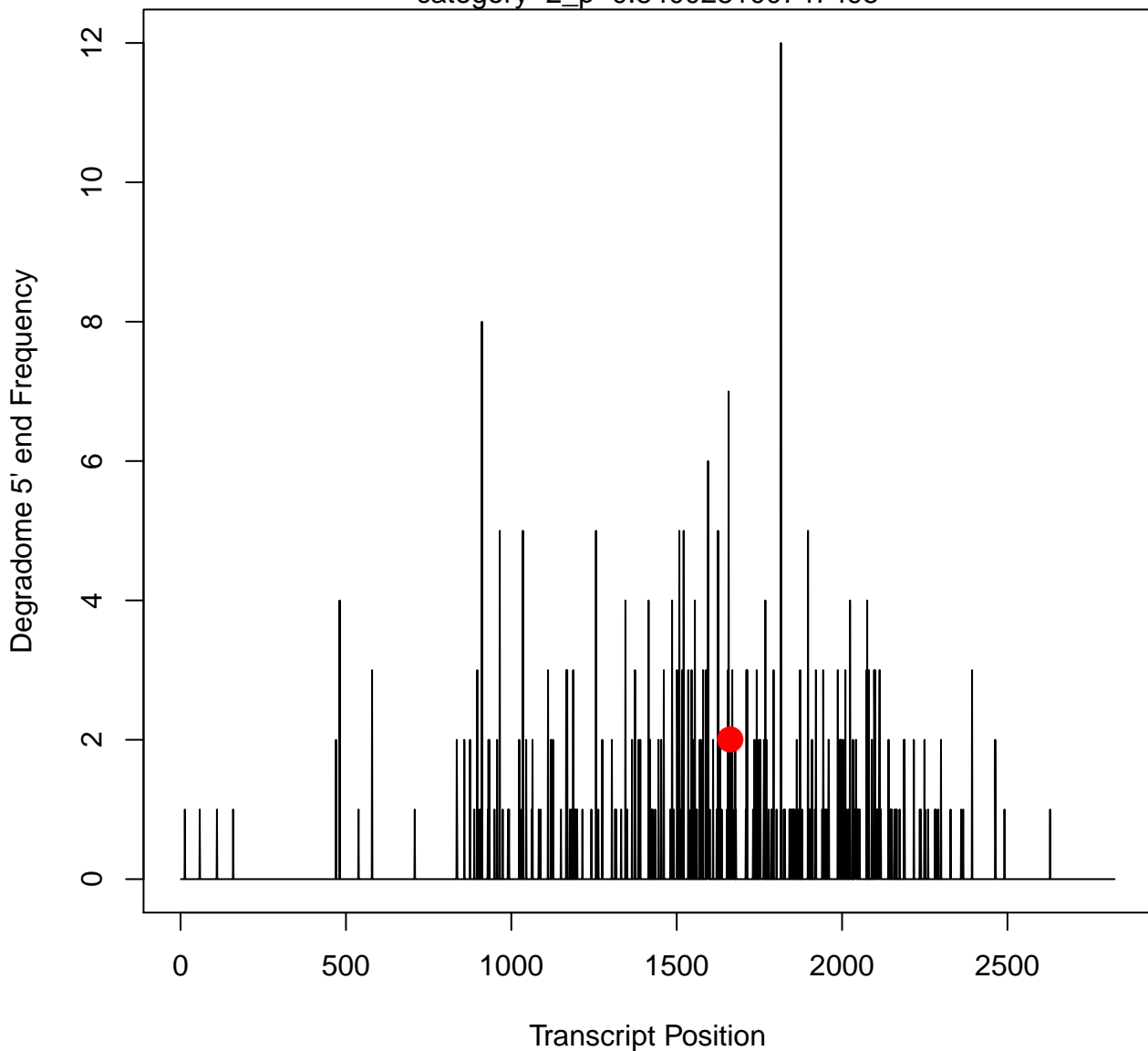

Supplement: Supplementary file 6 [file Data_Sheet_6.zip › Sit-miR166b_Seita.5G402200.1_1662_TPlot.pdf]

**T=Seita.6G091300.1\_Q=Sit-miR166b\_S=2076**

category=2\_p=0.824834941630751

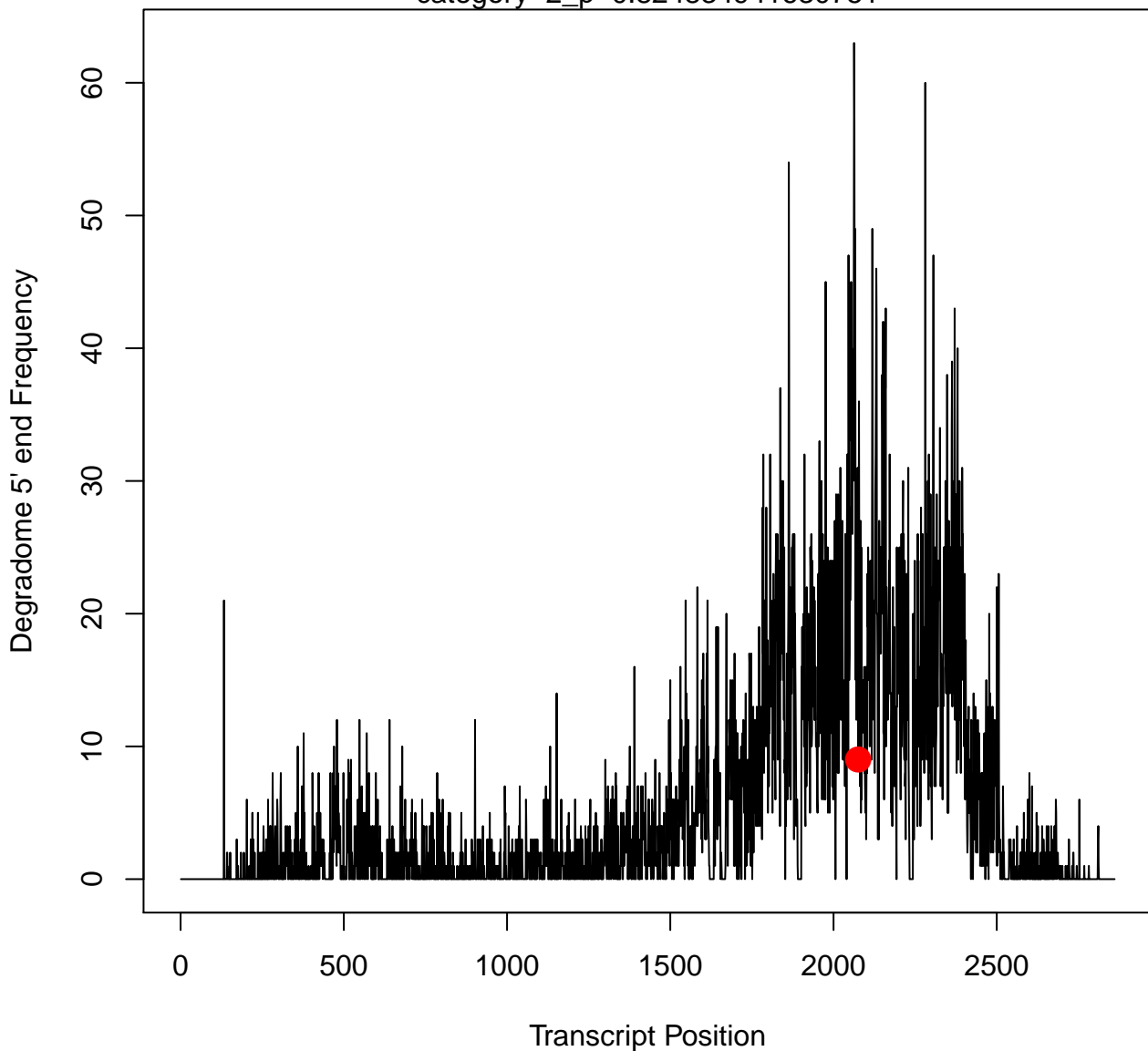

Supplement: Supplementary file 6 [file Data_Sheet_6.zip › Sit-miR166b_Seita.6G091300.1_2076_TPlot.pdf]

**T=Seita.6G173700.1\_Q=Sit-miR166b\_S=1232**

category=2\_p=0.994722093661468

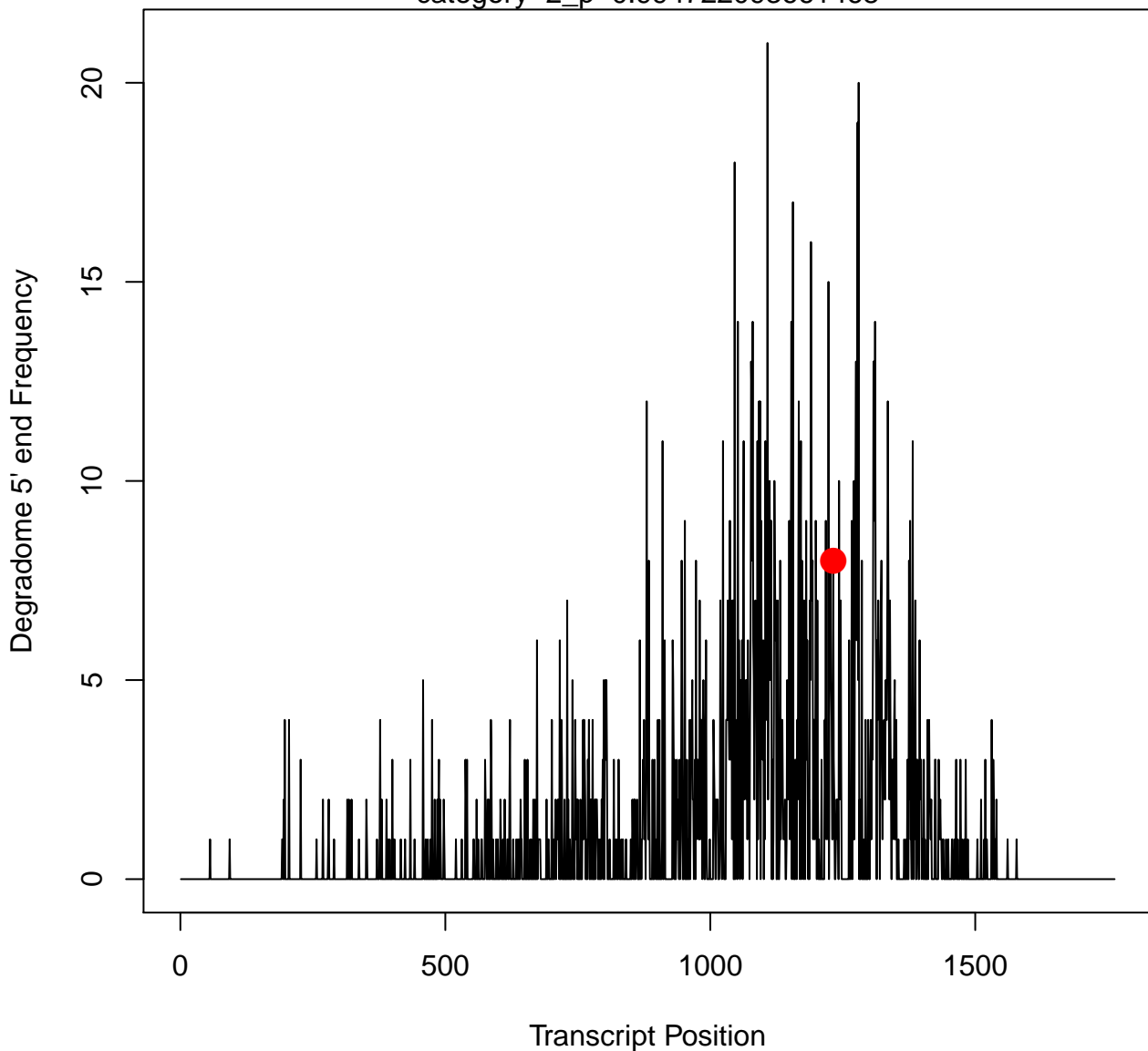

Supplement: Supplementary file 6 [file Data_Sheet_6.zip › Sit-miR166b_Seita.6G173700.1_1232_TPlot.pdf]

**T=Seita.7G130800.1\_Q=Sit-miR166b\_S=381**

category=2\_p=0.748196021971465

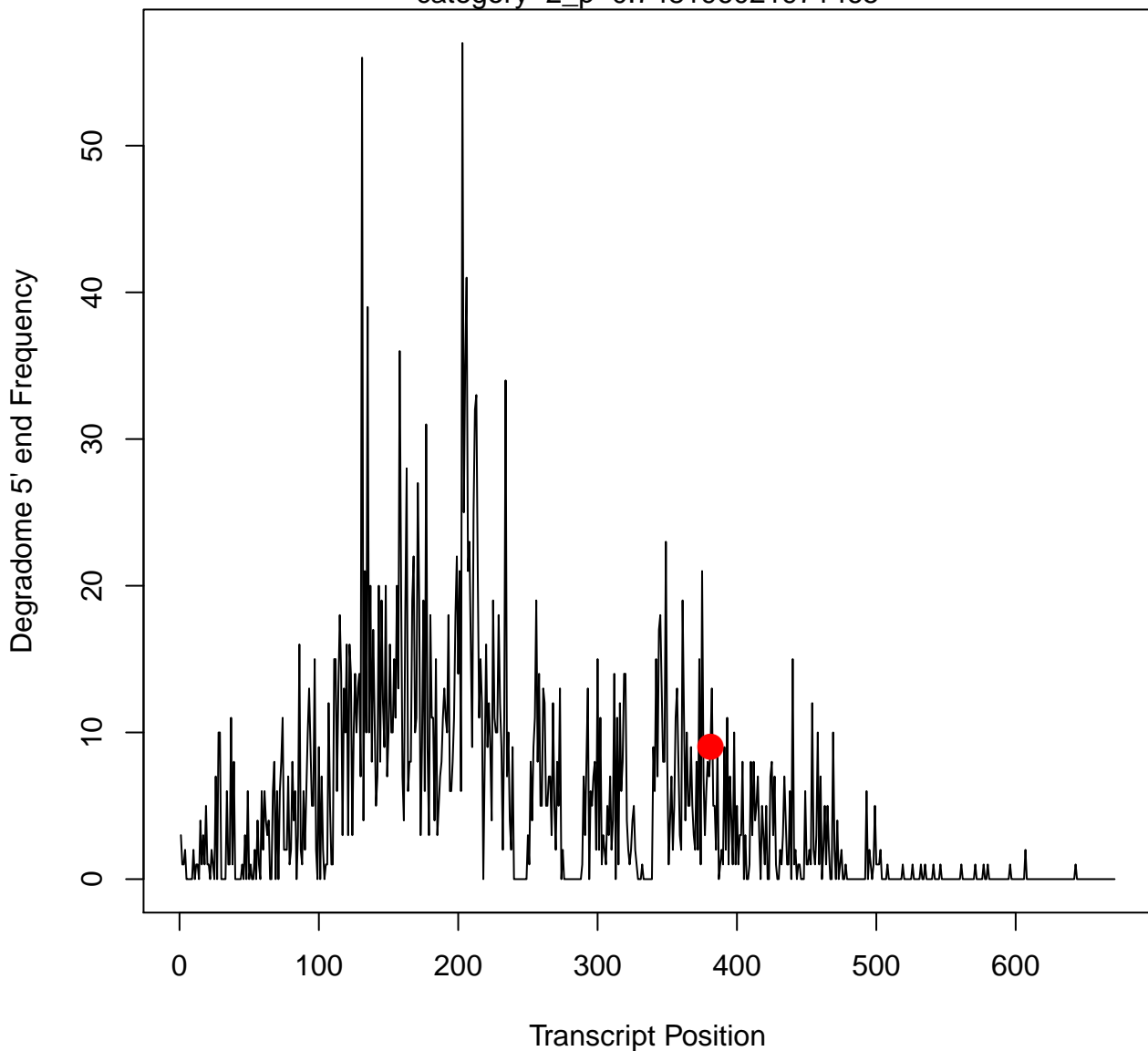

Supplement: Supplementary file 6 [file Data_Sheet_6.zip › Sit-miR166b_Seita.7G130800.1_381_TPlot.pdf]

**T=Seita.7G130900.1\_Q=Sit-miR166b\_S=388**

category=2\_p=0.738889643491991

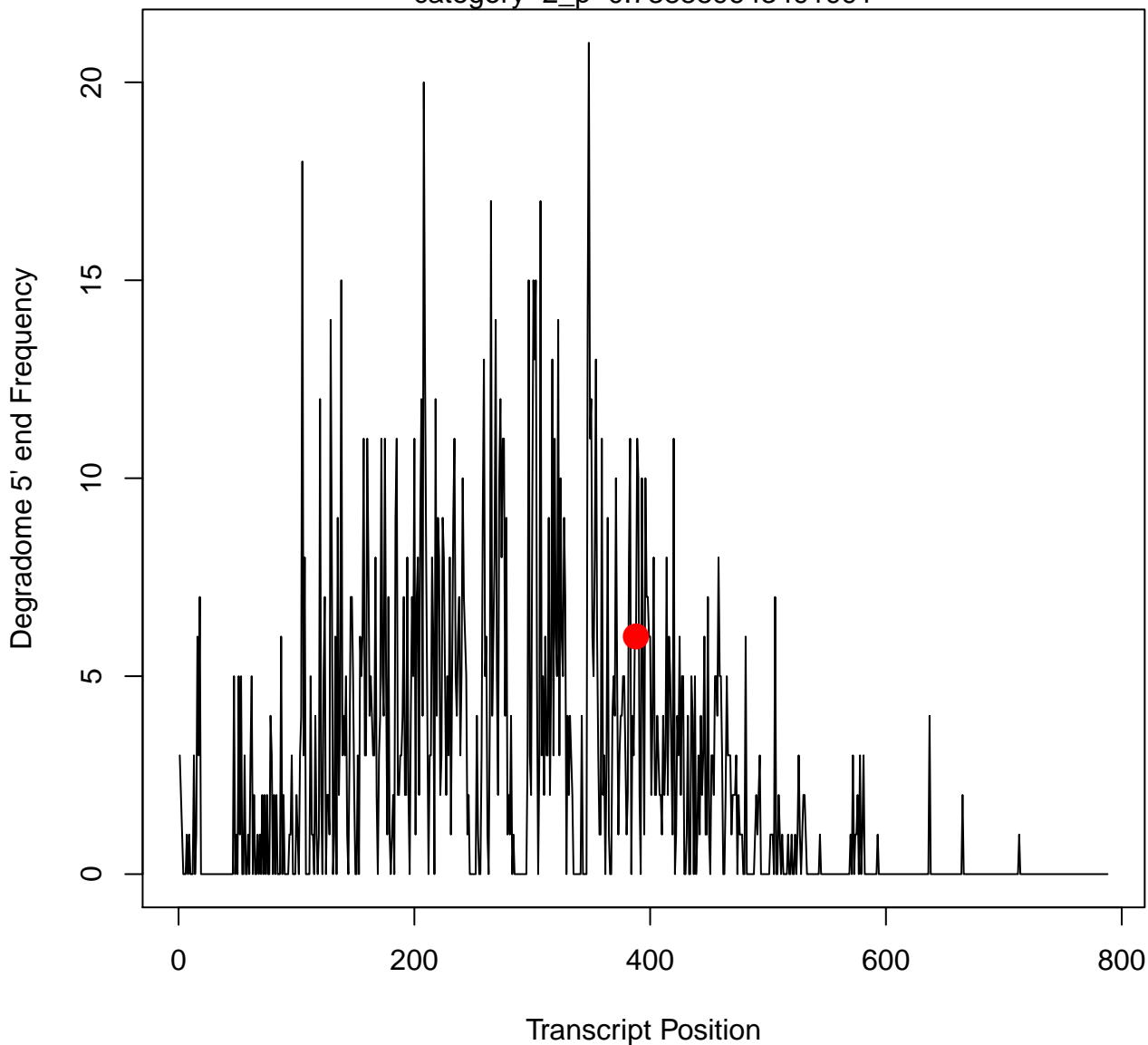

Supplement: Supplementary file 6 [file Data_Sheet_6.zip › Sit-miR166b_Seita.7G130900.1_388_TPlot.pdf]

**T=Seita.7G204000.1\_Q=Sit-miR166b\_S=608**

category=2\_p=0.988893616847507

Degradome 5' end Frequency

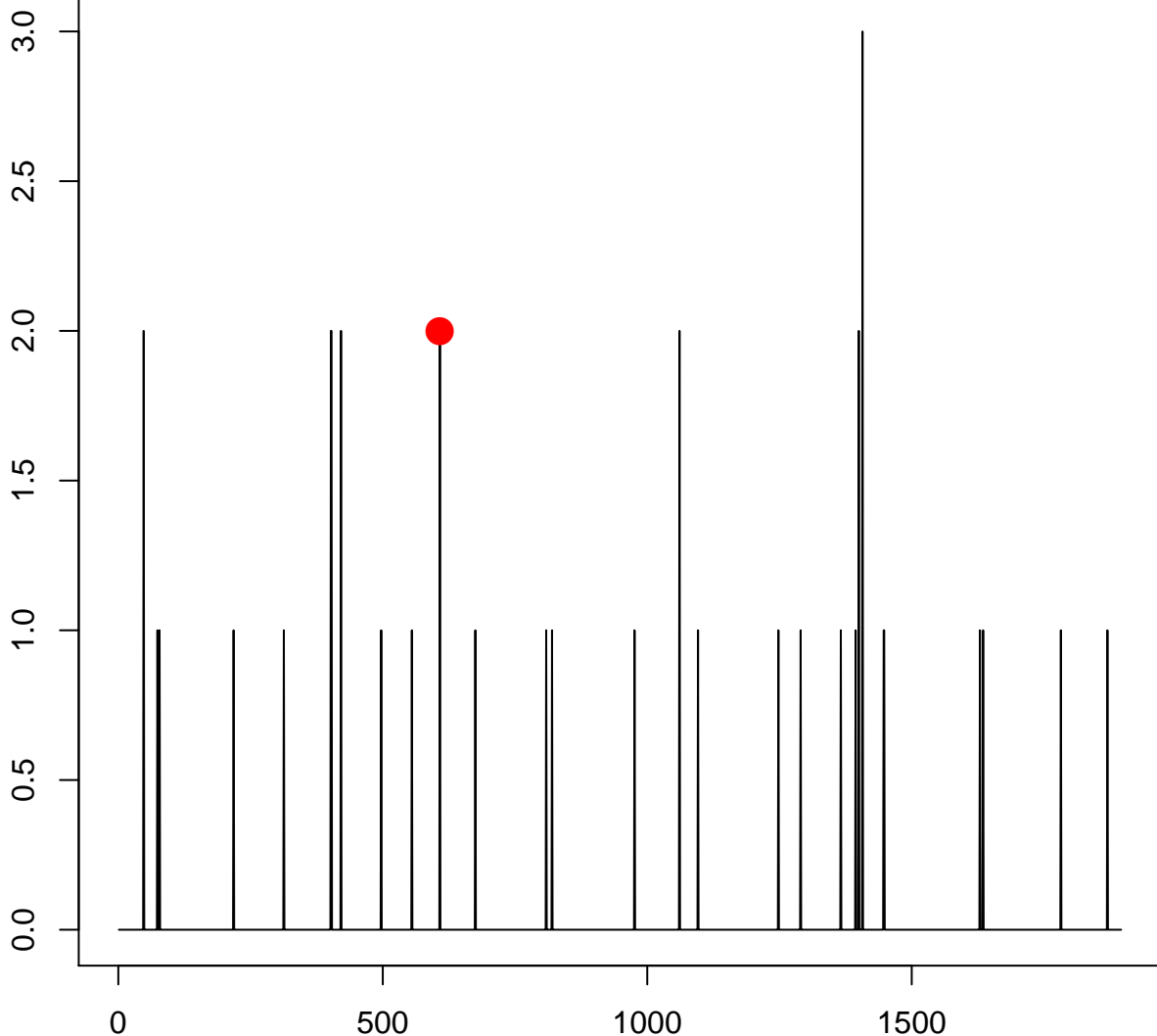

Transcript Position

Supplement: Supplementary file 6 [file Data_Sheet_6.zip › Sit-miR166b_Seita.7G204000.1_608_TPlot.pdf]

**T=Seita.7G243800.1\_Q=Sit-miR166b\_S=1926**

category=2\_p=0.801110083150942

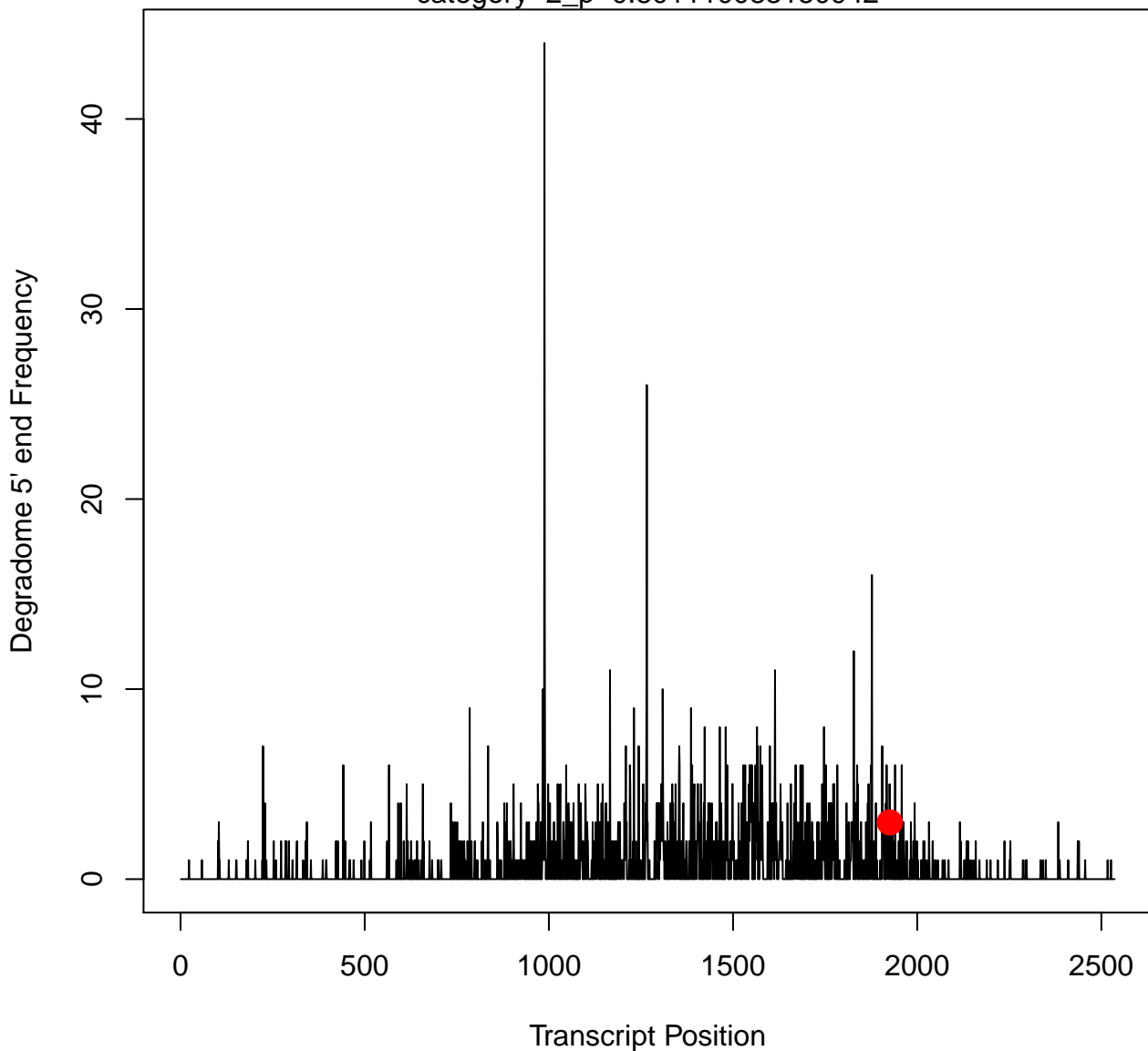

Supplement: Supplementary file 6 [file Data_Sheet_6.zip › Sit-miR166b_Seita.7G243800.1_1926_TPlot.pdf]

**T=Seita.6G190900.1\_Q=Sit-miR166c\_S=657**

category=2\_p=0.969868954529697

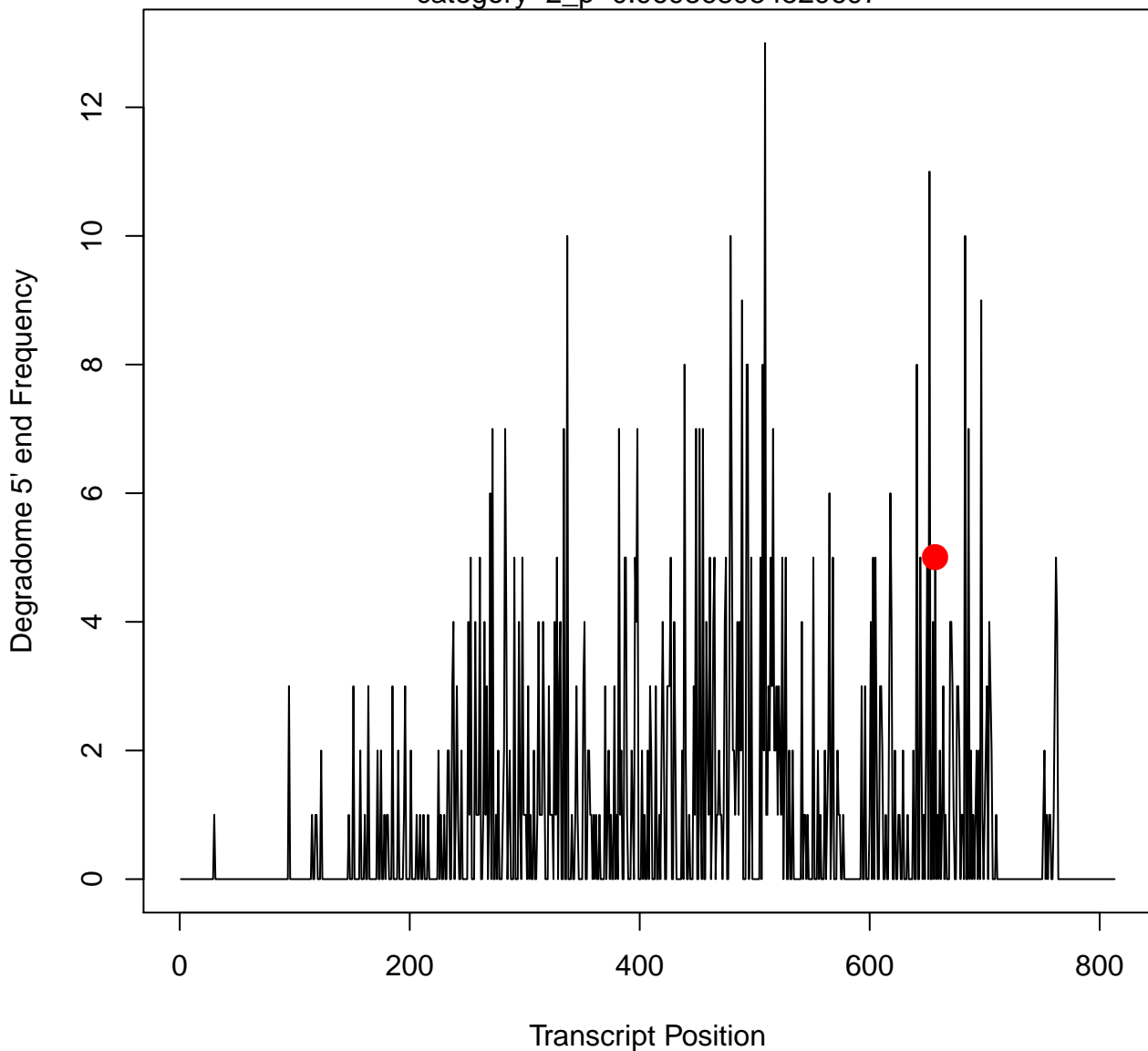

Supplement: Supplementary file 6 [file Data_Sheet_6.zip › Sit-miR166c_Seita.6G190900.1_657_TPlot.pdf]

**T=Seita.7G250800.1\_Q=Sit-miR166d\_S=1446**

category=2\_p=0.848503906343478

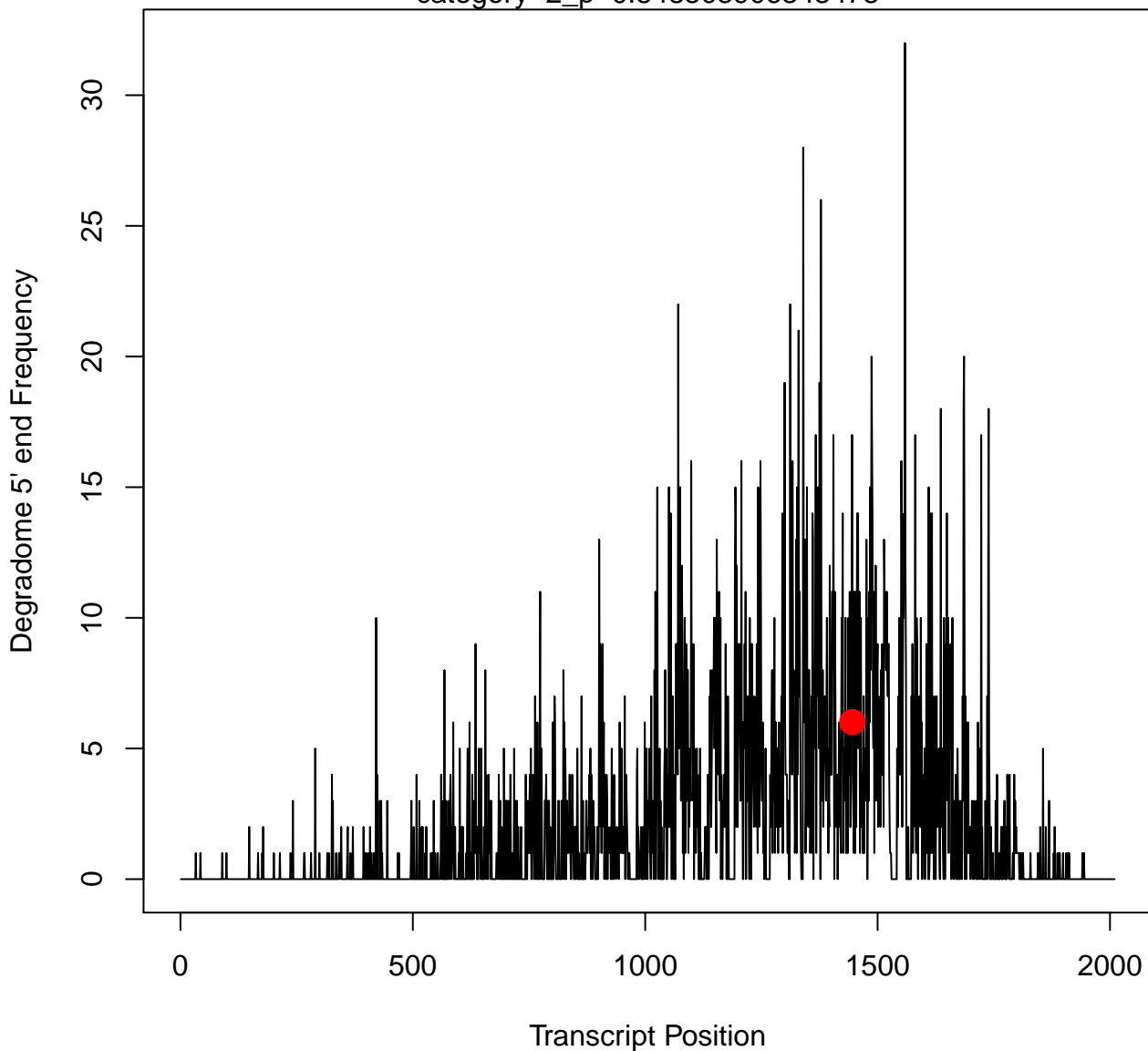

Supplement: Supplementary file 6 [file Data_Sheet_6.zip › Sit-miR166d_Seita.7G250800.1_1446_TPlot.pdf]

**T=Seita.9G300300.1\_Q=Sit-miR166f\_S=2272**

category=2\_p=0.913682666420193

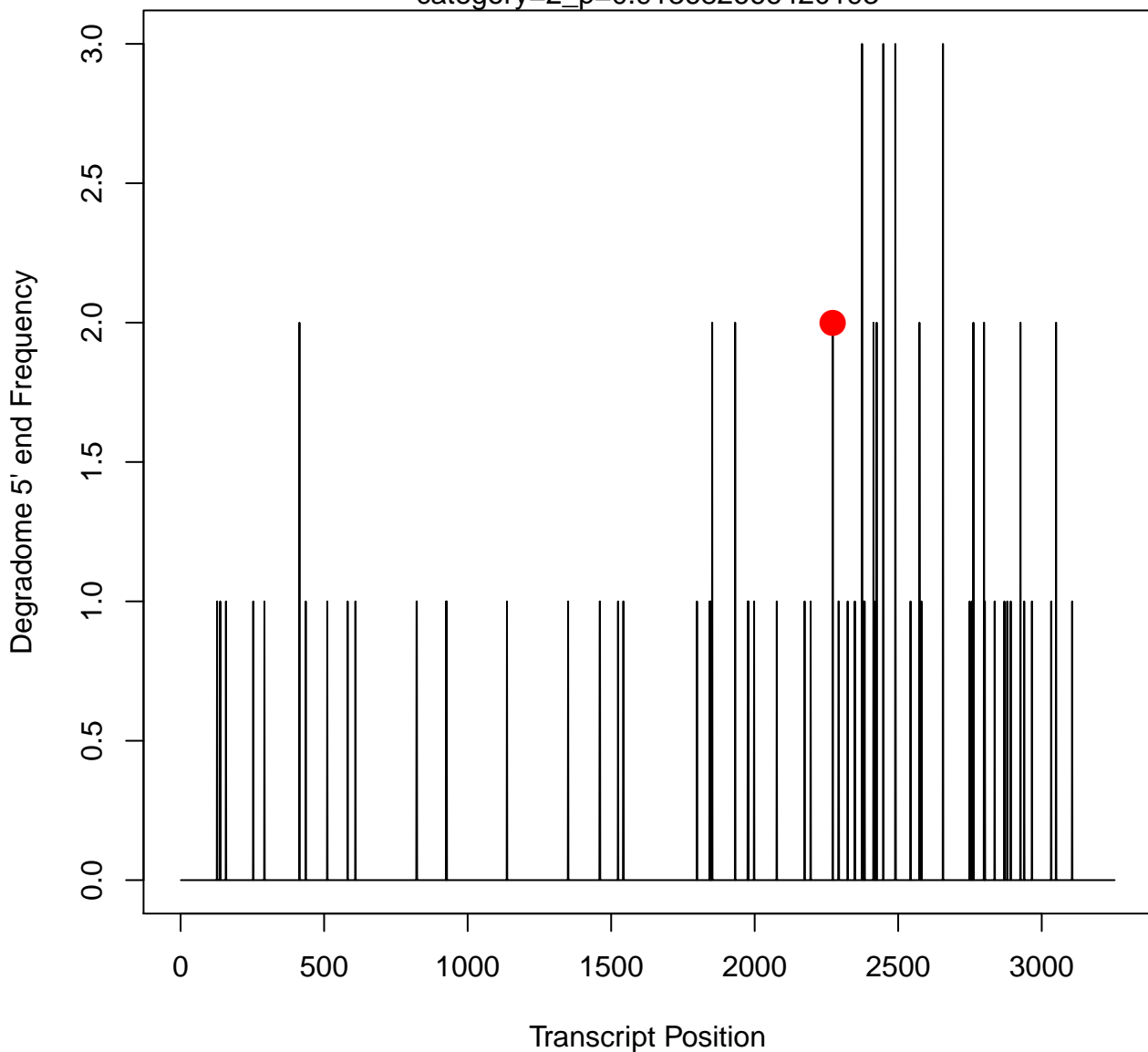

Supplement: Supplementary file 6 [file Data_Sheet_6.zip › Sit-miR166f_Seita.9G300300.1_2272_TPlot.pdf]

**T=Seita.1G013500.1\_Q=Sit-miR166i\_S=507**

category=2\_p=0.998950277647192

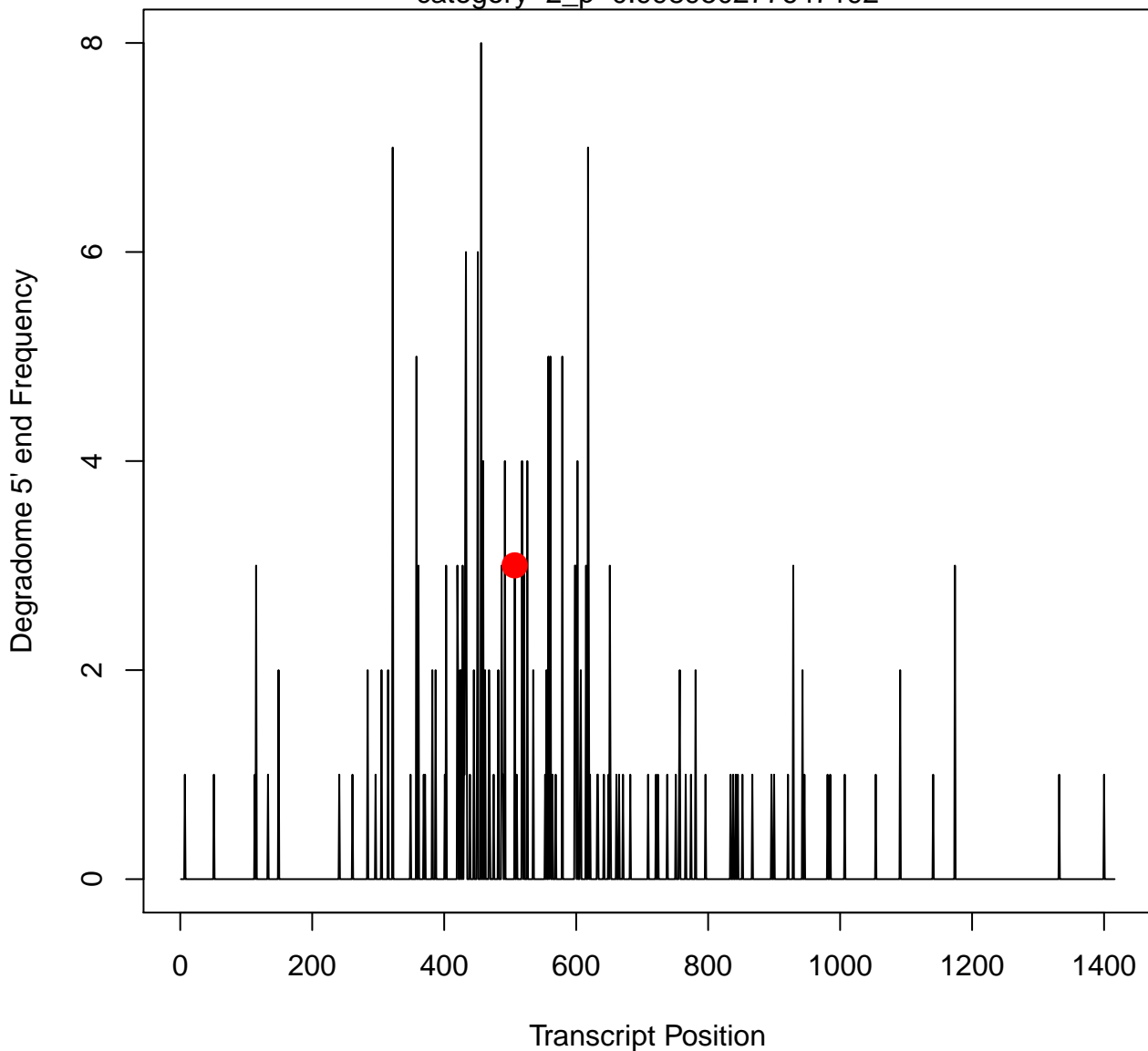

Supplement: Supplementary file 6 [file Data_Sheet_6.zip › Sit-miR166i_Seita.1G013500.1_507_TPlot.pdf]

**T=Seita.1G214100.1\_Q=Sit-miR166i\_S=3966**

category=2\_p=0.989856942798574

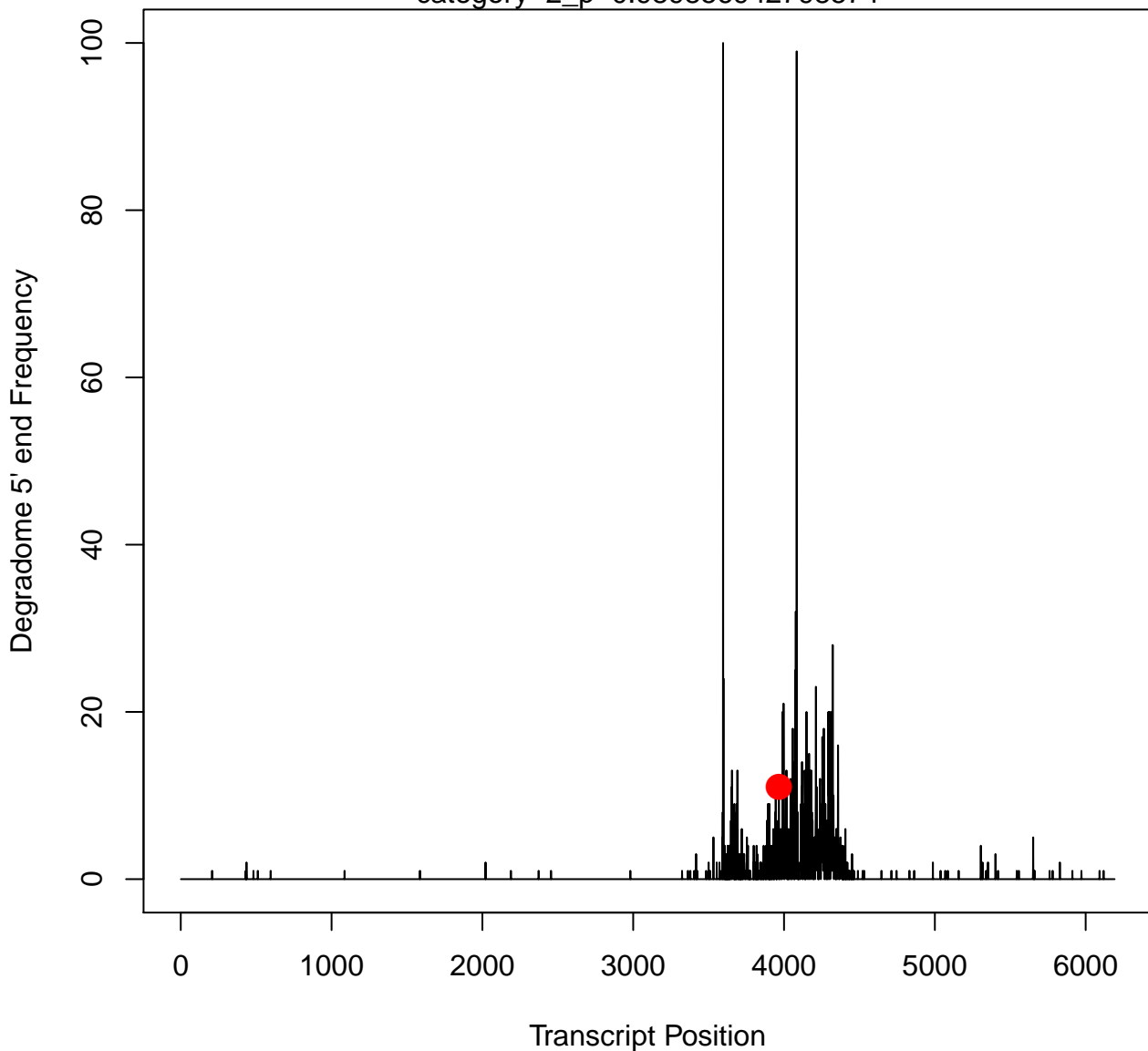

Supplement: Supplementary file 6 [file Data_Sheet_6.zip › Sit-miR166i_Seita.1G214100.1_3966_TPlot.pdf]

**T=Seita.2G180300.1\_Q=Sit-miR166i\_S=829**

category=2\_p=0.939954162387889

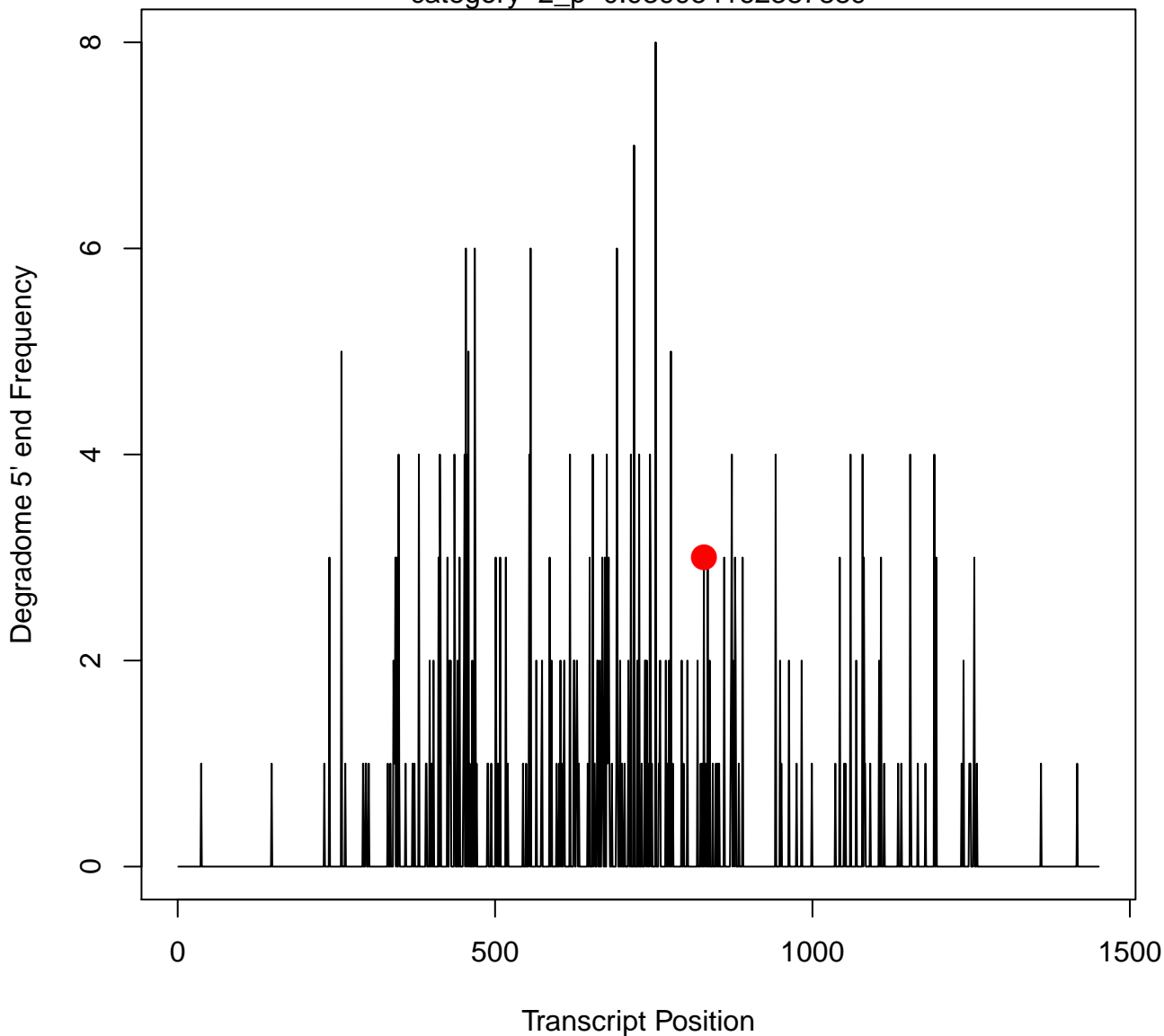

Supplement: Supplementary file 6 [file Data_Sheet_6.zip › Sit-miR166i_Seita.2G180300.1_829_TPlot.pdf]

**T=Seita.5G141300.1\_Q=Sit-miR166i\_S=1222**

category=0\_p=0.0011262246059176

Degradome 5' end Frequency

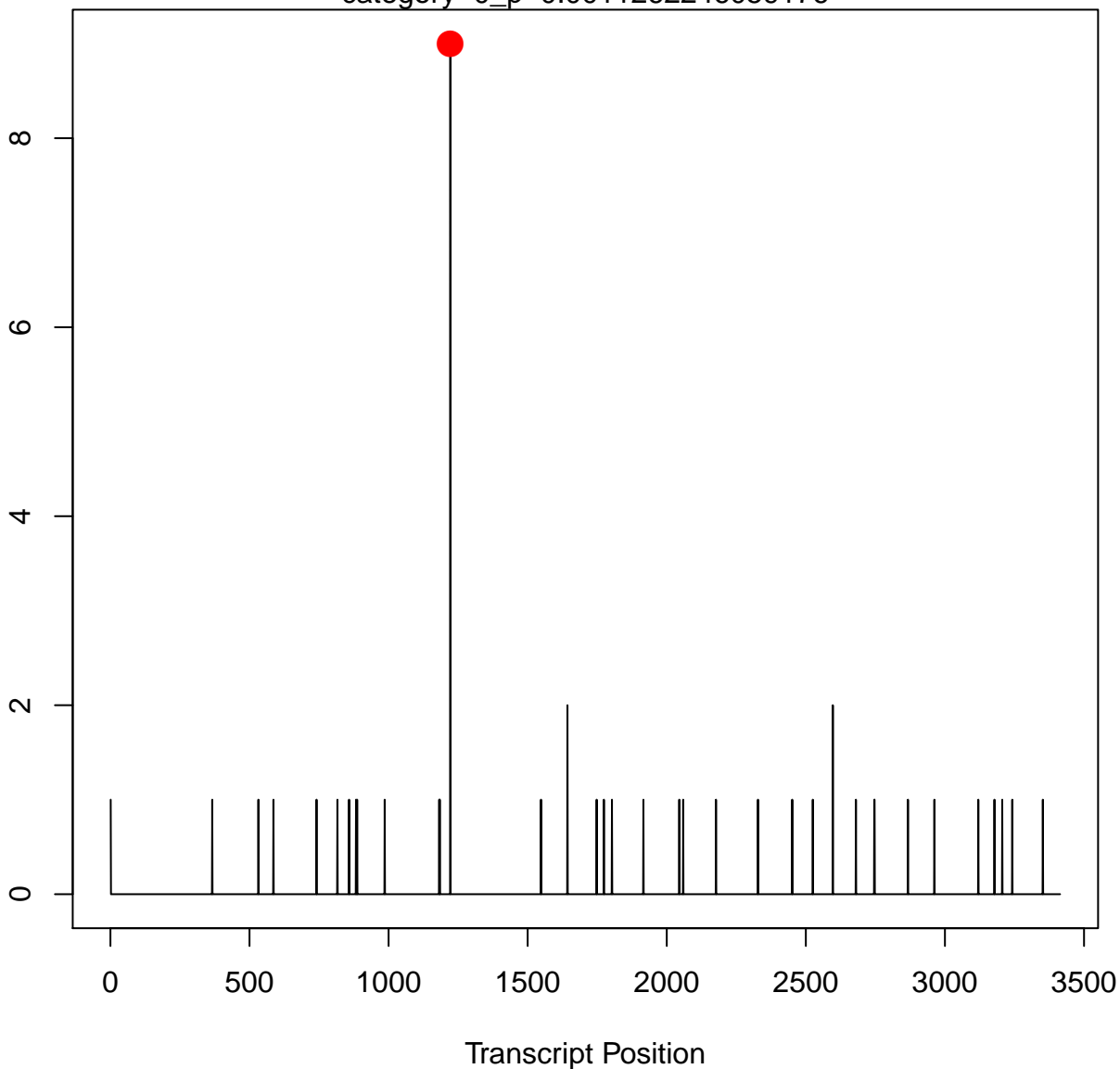

Supplement: Supplementary file 6 [file Data_Sheet_6.zip › Sit-miR166i_Seita.5G141300.1_1222_TPlot.pdf]

**T=Seita.6G091300.1\_Q=Sit-miR166i\_S=2074**

category=2\_p=0.765825519680136

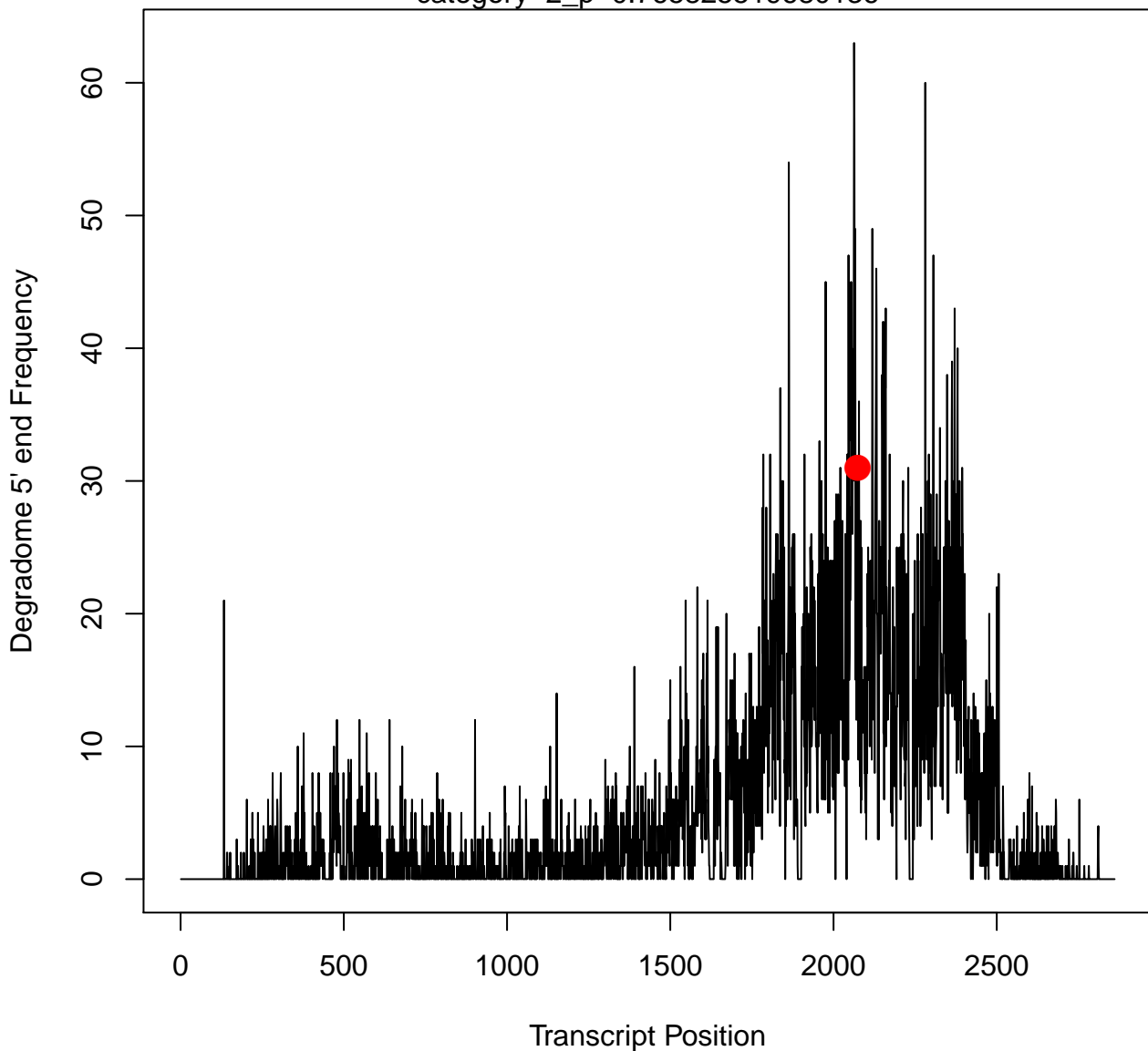

Supplement: Supplementary file 6 [file Data_Sheet_6.zip › Sit-miR166i_Seita.6G091300.1_2074_TPlot.pdf]

**T=Seita.7G130800.1\_Q=Sit-miR166i\_S=379**

category=2\_p=0.793759346055999

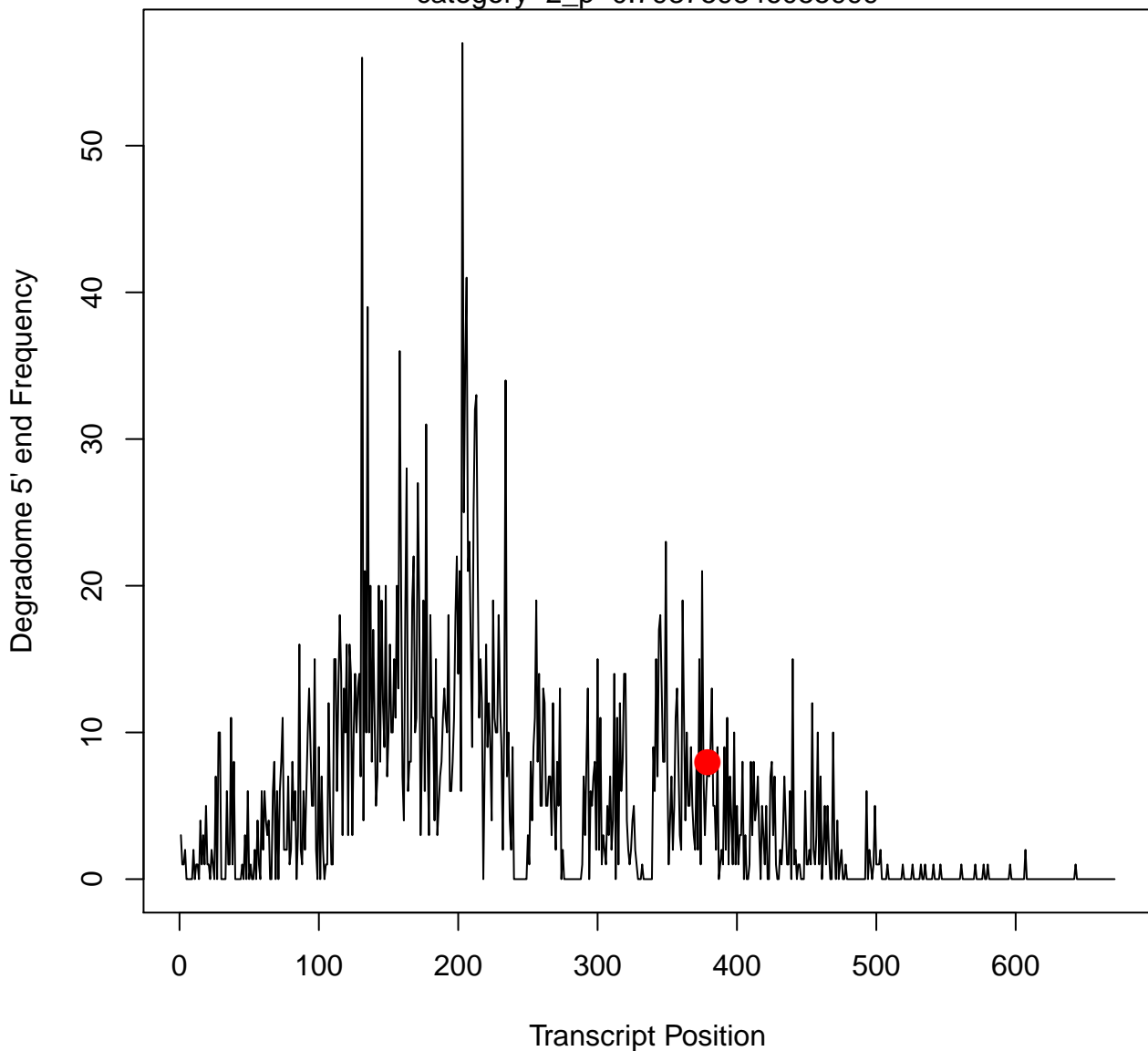

Supplement: Supplementary file 6 [file Data_Sheet_6.zip › Sit-miR166i_Seita.7G130800.1_379_TPlot.pdf]

**T=Seita.9G201400.1\_Q=Sit-miR166i\_S=514**

category=2\_p=0.982197856675798

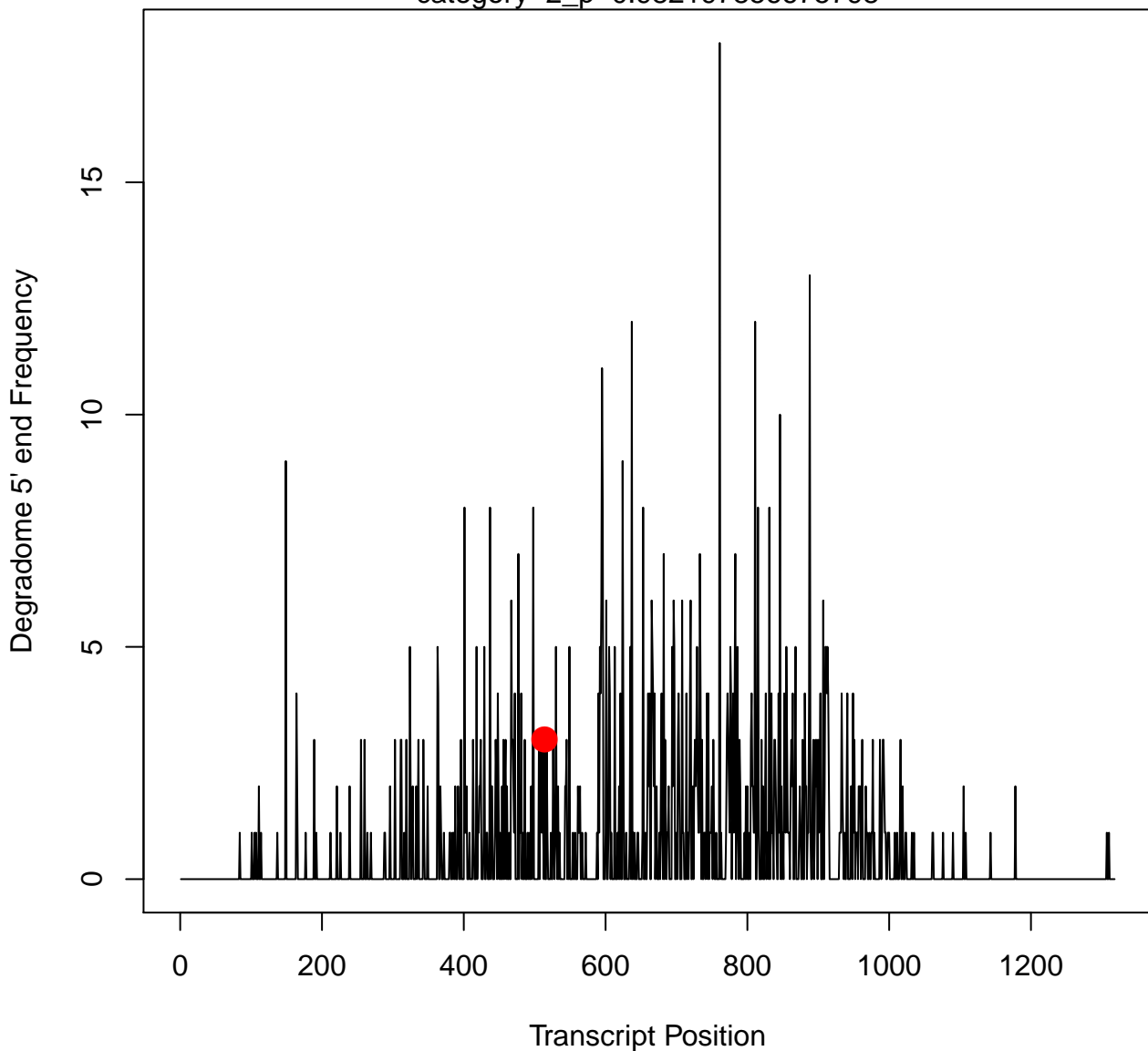

Supplement: Supplementary file 6 [file Data_Sheet_6.zip › Sit-miR166i_Seita.9G201400.1_514_TPlot.pdf]

**T=Seita.9G516200.1\_Q=Sit-miR166i\_S=2503**

category=2\_p=0.972977242232497

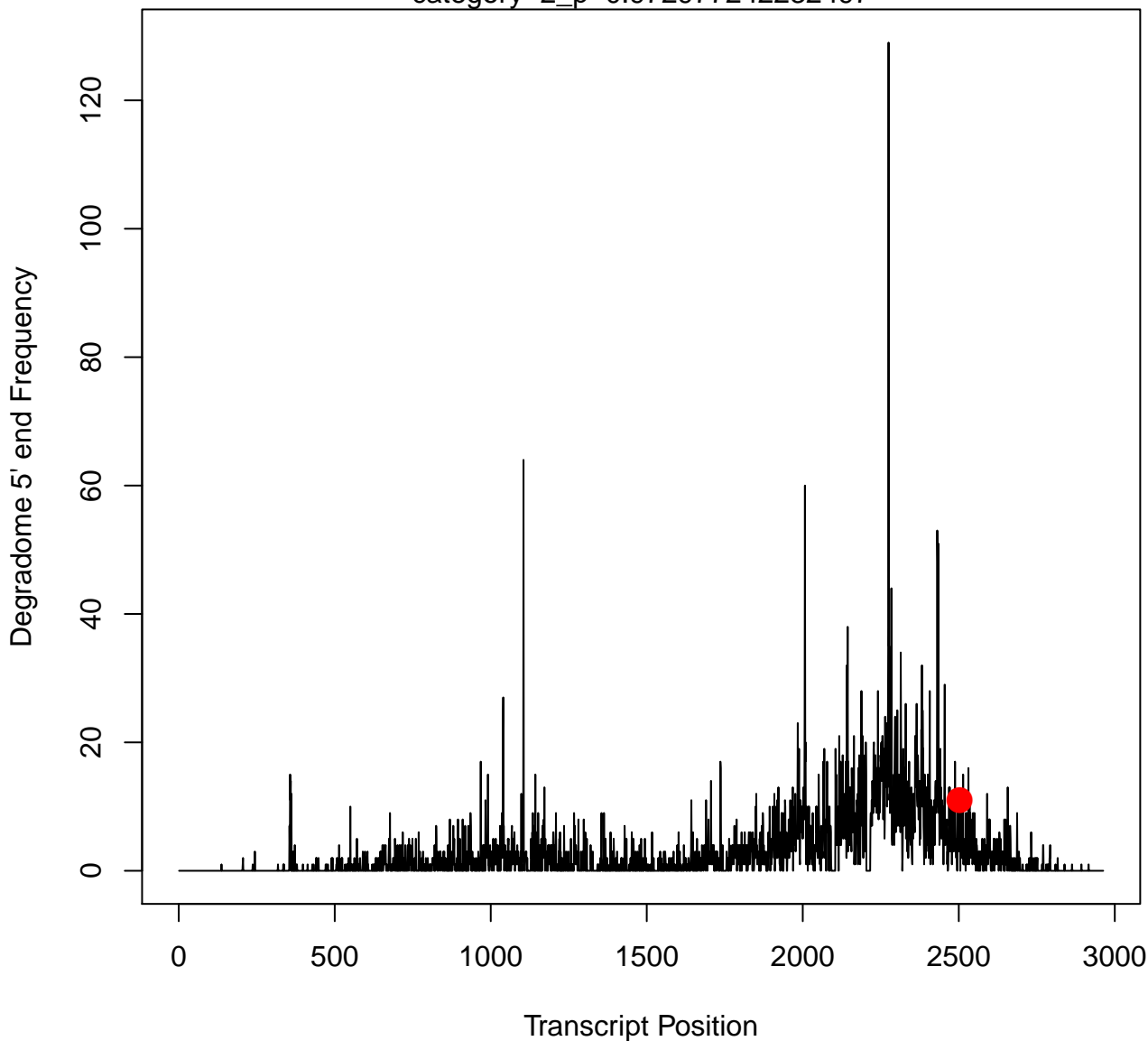

Supplement: Supplementary file 6 [file Data_Sheet_6.zip › Sit-miR166i_Seita.9G516200.1_2503_TPlot.pdf]

**T=Seita.9G572600.1\_Q=Sit-miR166i\_S=1138**

category=0\_p=0.00075095740585196

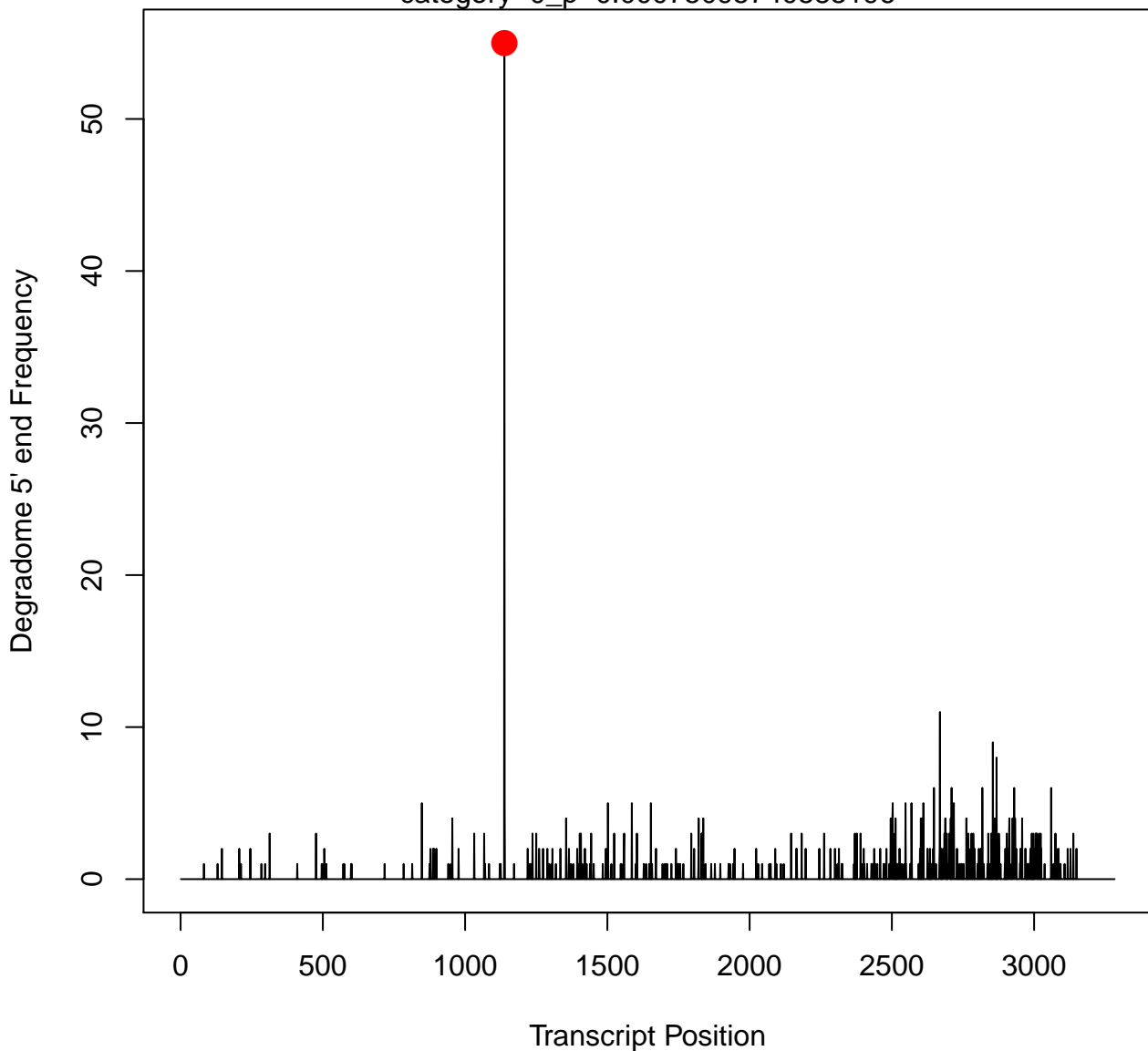

Supplement: Supplementary file 6 [file Data_Sheet_6.zip › Sit-miR166i_Seita.9G572600.1_1138_TPlot.pdf]

**T=Seita.2G373000.1\_Q=Sit-miR166j\_S=891**

category=2\_p=0.638025734462772

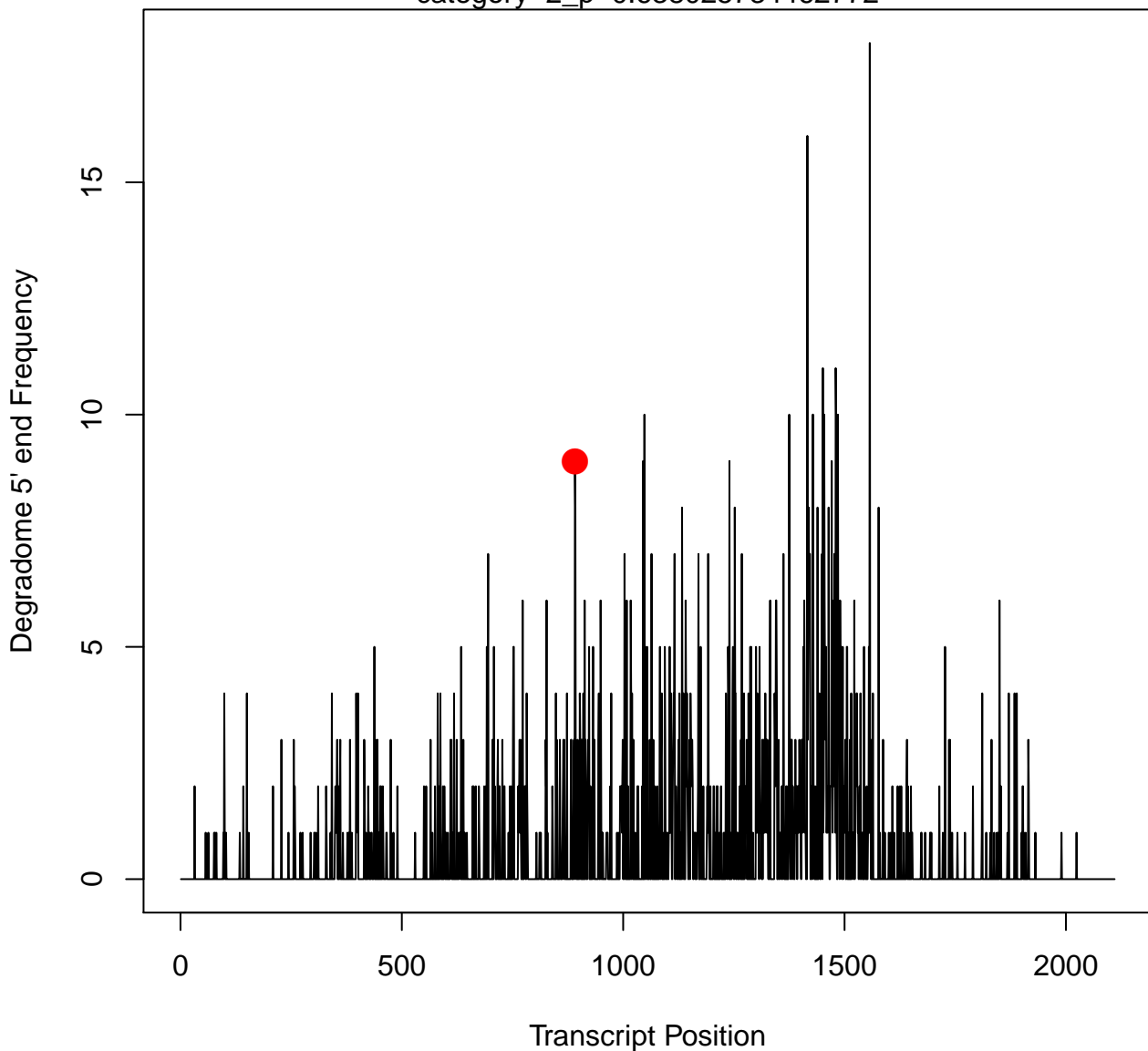

Supplement: Supplementary file 6 [file Data_Sheet_6.zip › Sit-miR166j_Seita.2G373000.1_891_TPlot.pdf]

**T=Seita.9G219700.1\_Q=Sit-miR166j\_S=1105**

category=0\_p=0.0011262246059176

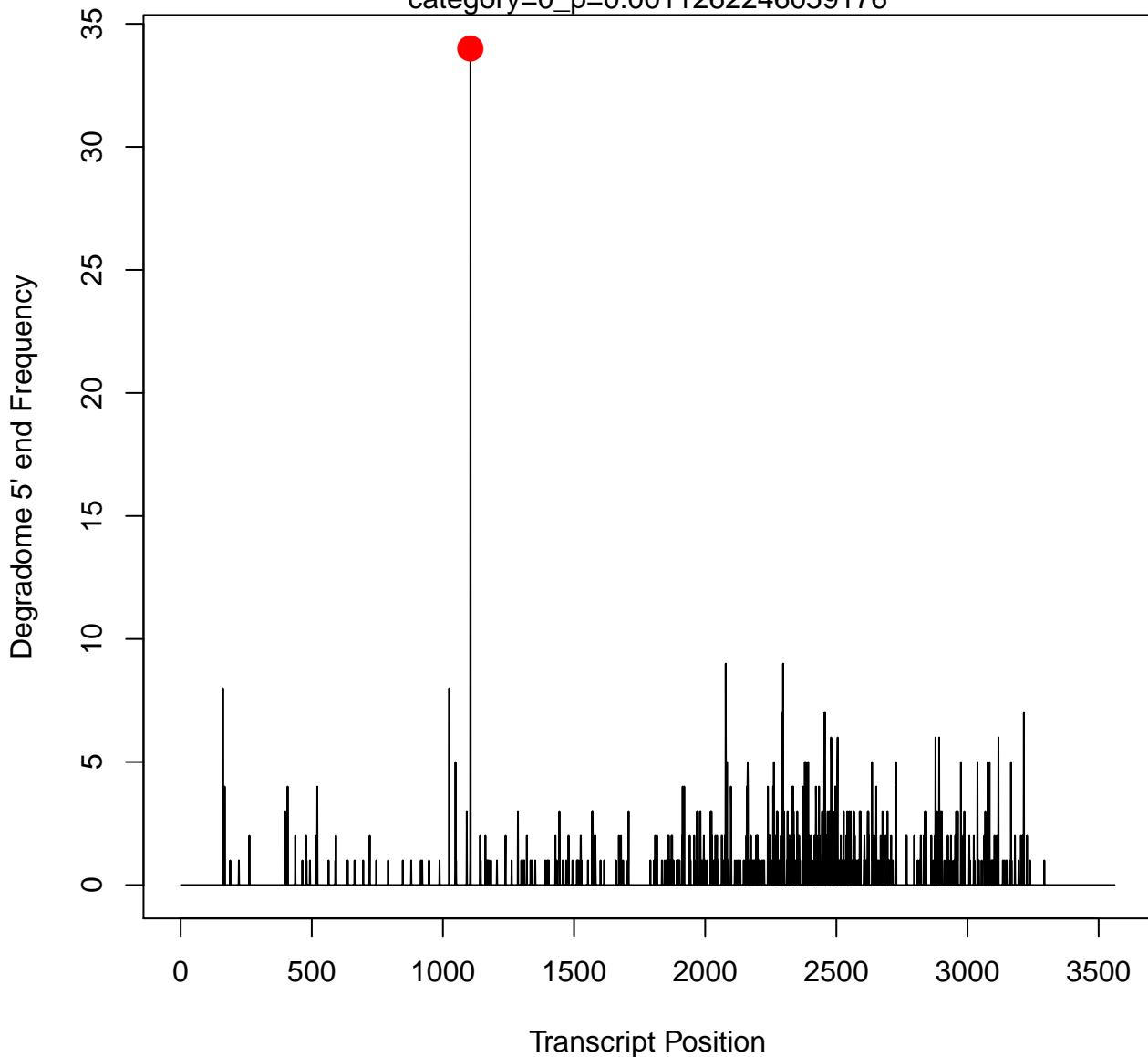

Supplement: Supplementary file 6 [file Data_Sheet_6.zip › Sit-miR166j_Seita.9G219700.1_1105_TPlot.pdf]

**T=Seita.3G185800.1\_Q=Sit-miR166k\_S=1028**

category=2\_p=0.761537374896809

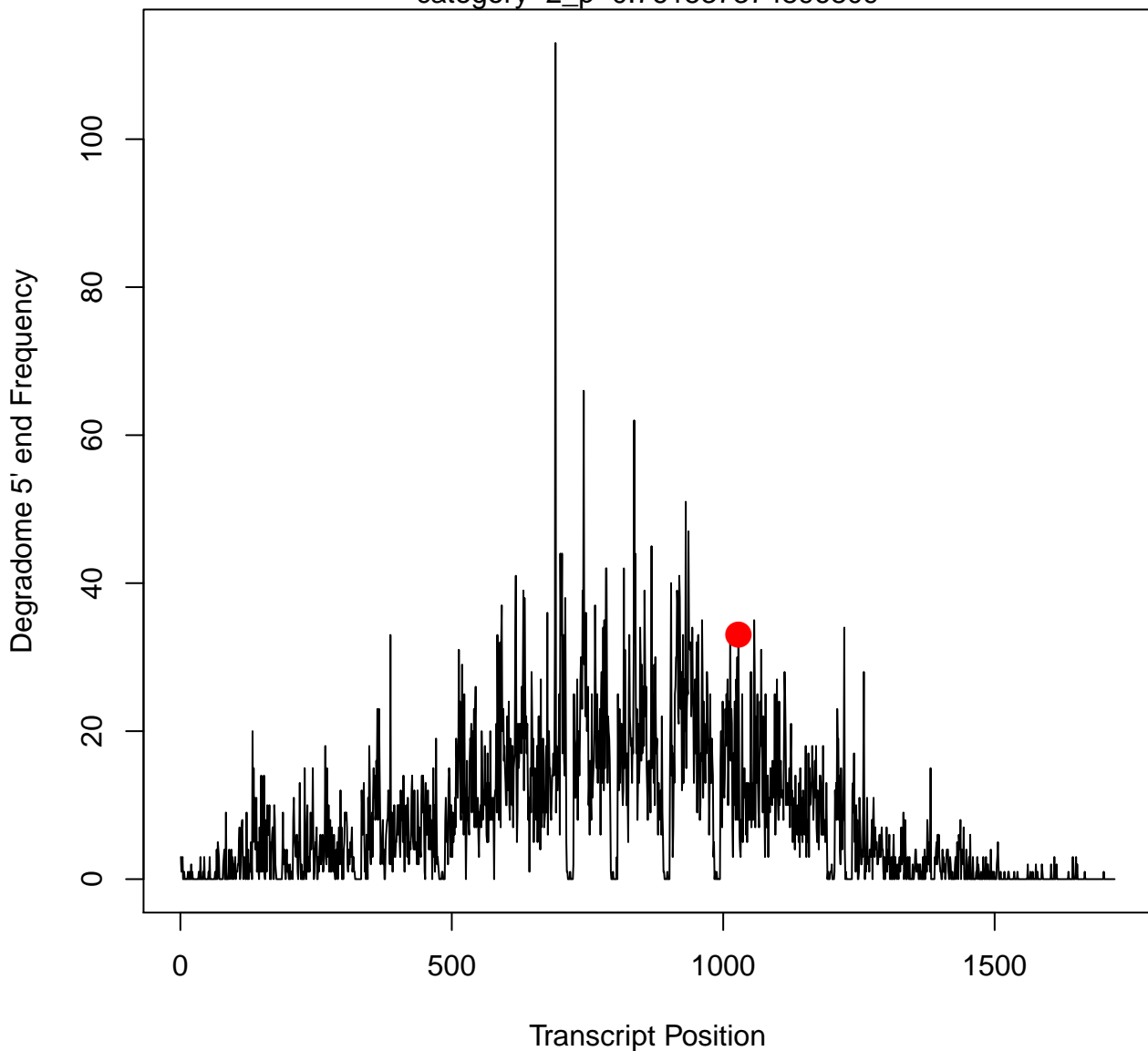

Supplement: Supplementary file 6 [file Data_Sheet_6.zip › Sit-miR166k_Seita.3G185800.1_1028_TPlot.pdf]

**T=Seita.3G395000.1\_Q=Sit-miR166k\_S=774**

category=0\_p=0.00075095740585196

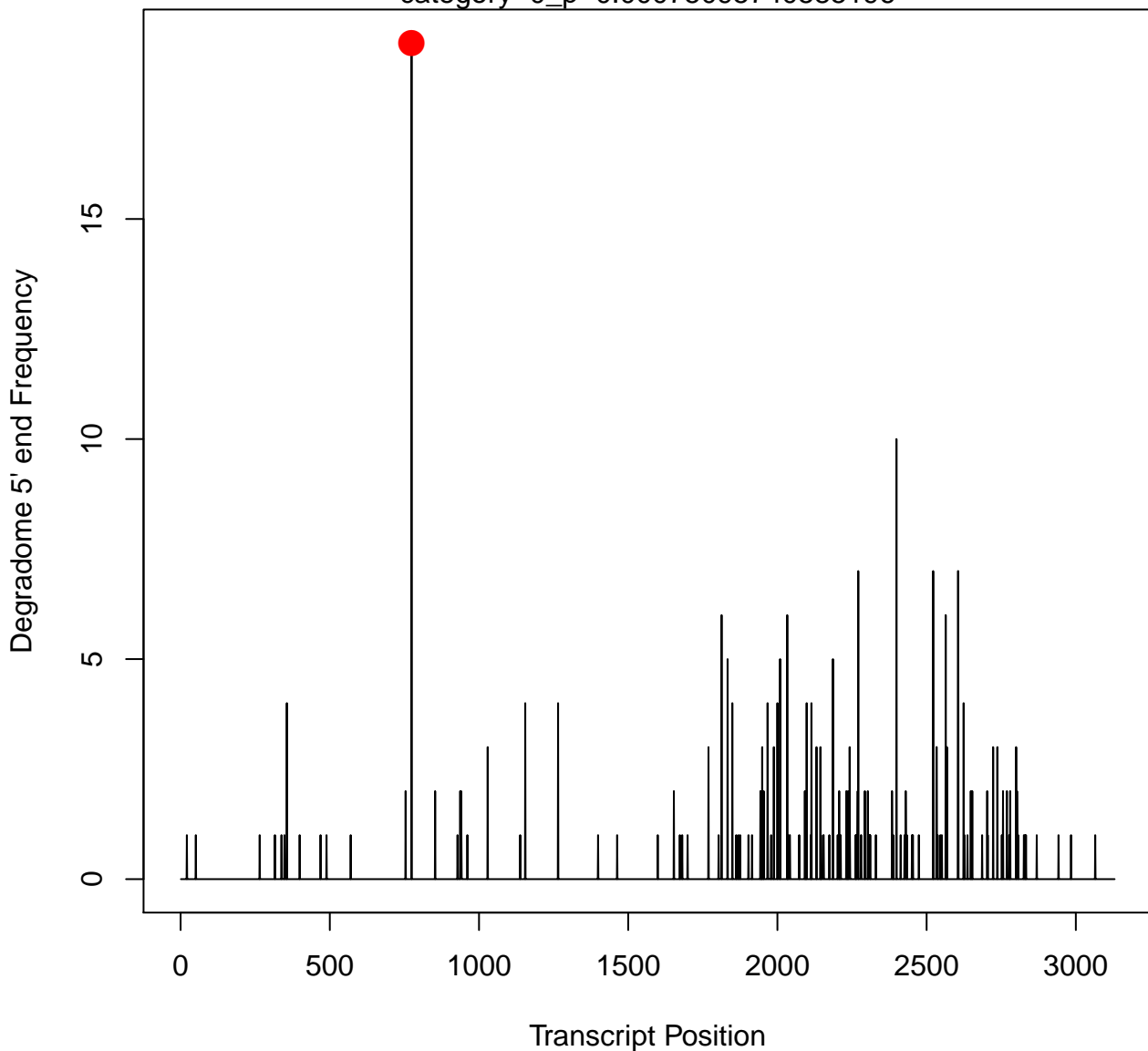

Supplement: Supplementary file 6 [file Data_Sheet_6.zip › Sit-miR166k_Seita.3G395000.1_774_TPlot.pdf]

**T=Seita.4G229600.1\_Q=Sit-miR167a\_S=540**

category=2\_p=0.581472750744651

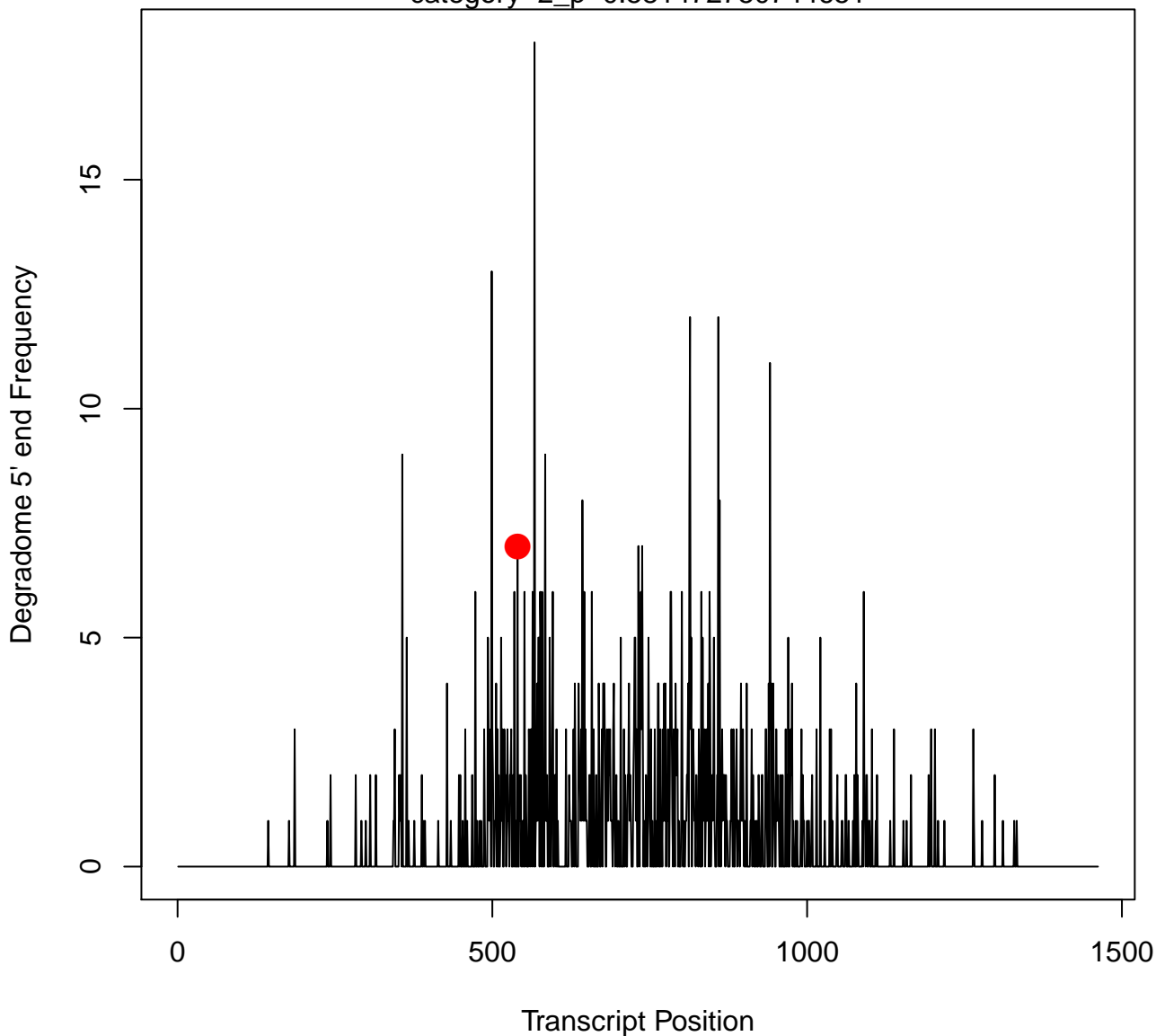

Supplement: Supplementary file 6 [file Data_Sheet_6.zip › Sit-miR167a_Seita.4G229600.1_540_TPlot.pdf]

**T=Seita.5G141100.1\_Q=Sit-miR167a\_S=1956**

category=2\_p=0.610775008951736

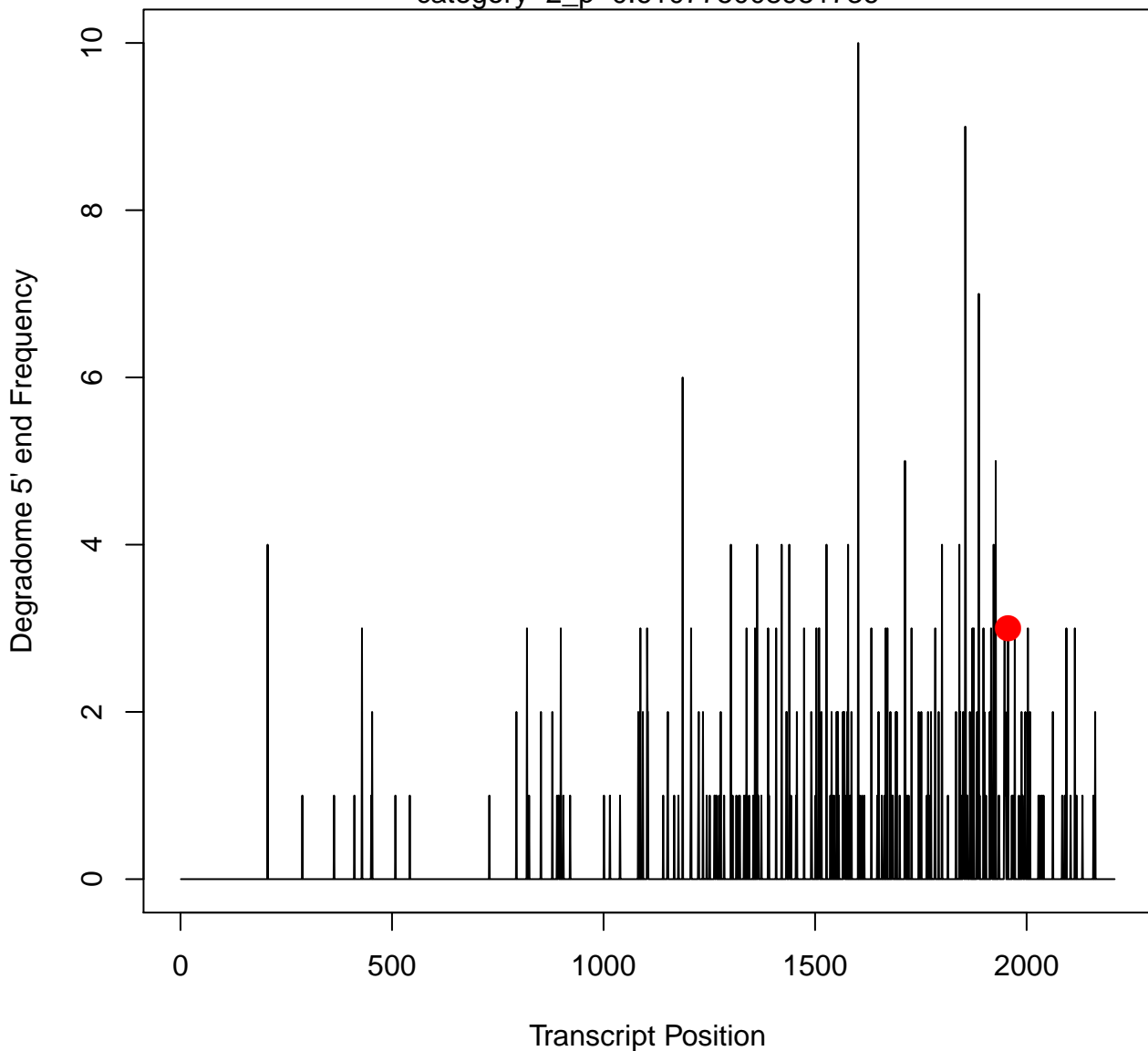

Supplement: Supplementary file 6 [file Data_Sheet_6.zip › Sit-miR167a_Seita.5G141100.1_1956_TPlot.pdf]

**T=Seita.9G532700.1\_Q=Sit-miR167a\_S=1896**

category=2\_p=0.786136934374958

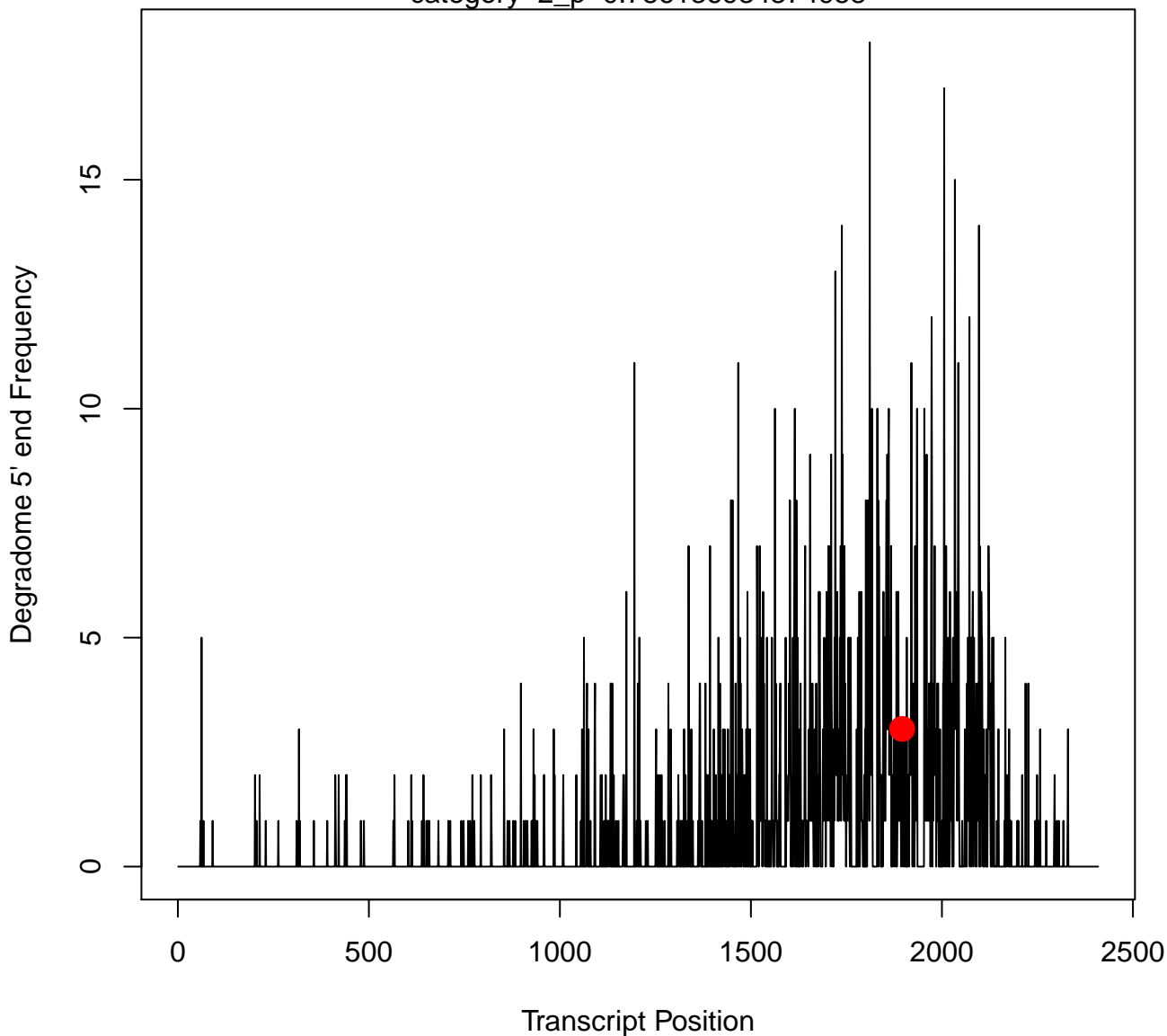

Supplement: Supplementary file 6 [file Data_Sheet_6.zip › Sit-miR167a_Seita.9G532700.1_1896_TPlot.pdf]

**T=Seita.9G044500.1\_Q=Sit-miR167g\_S=216**

category=2\_p=0.969317202326498

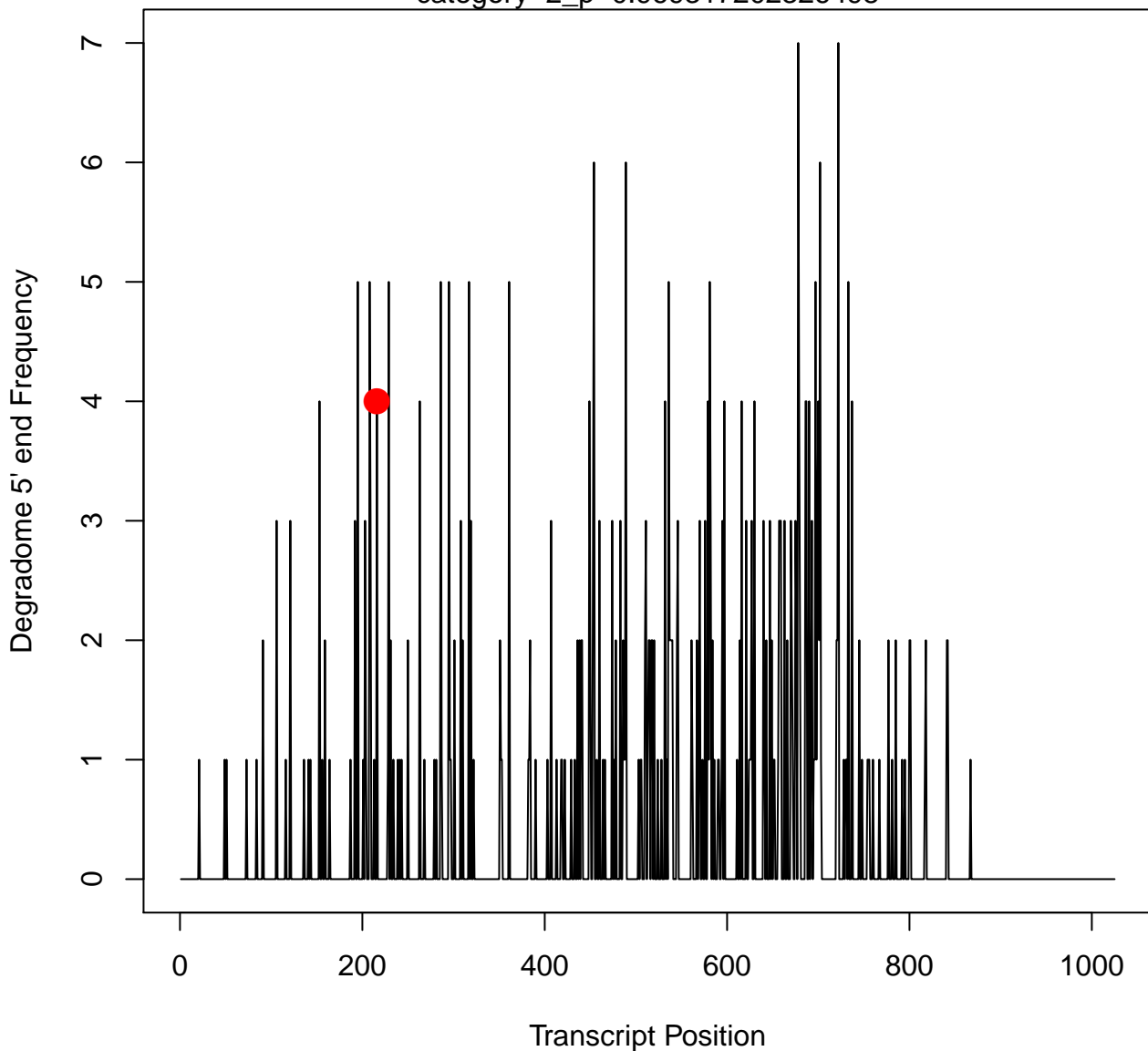

Supplement: Supplementary file 6 [file Data_Sheet_6.zip › Sit-miR167g_Seita.9G044500.1_216_TPlot.pdf]

**T=Seita.3G020000.1\_Q=Sit-miR167h\_S=2739**

category=0\_p=0.0011262246059176

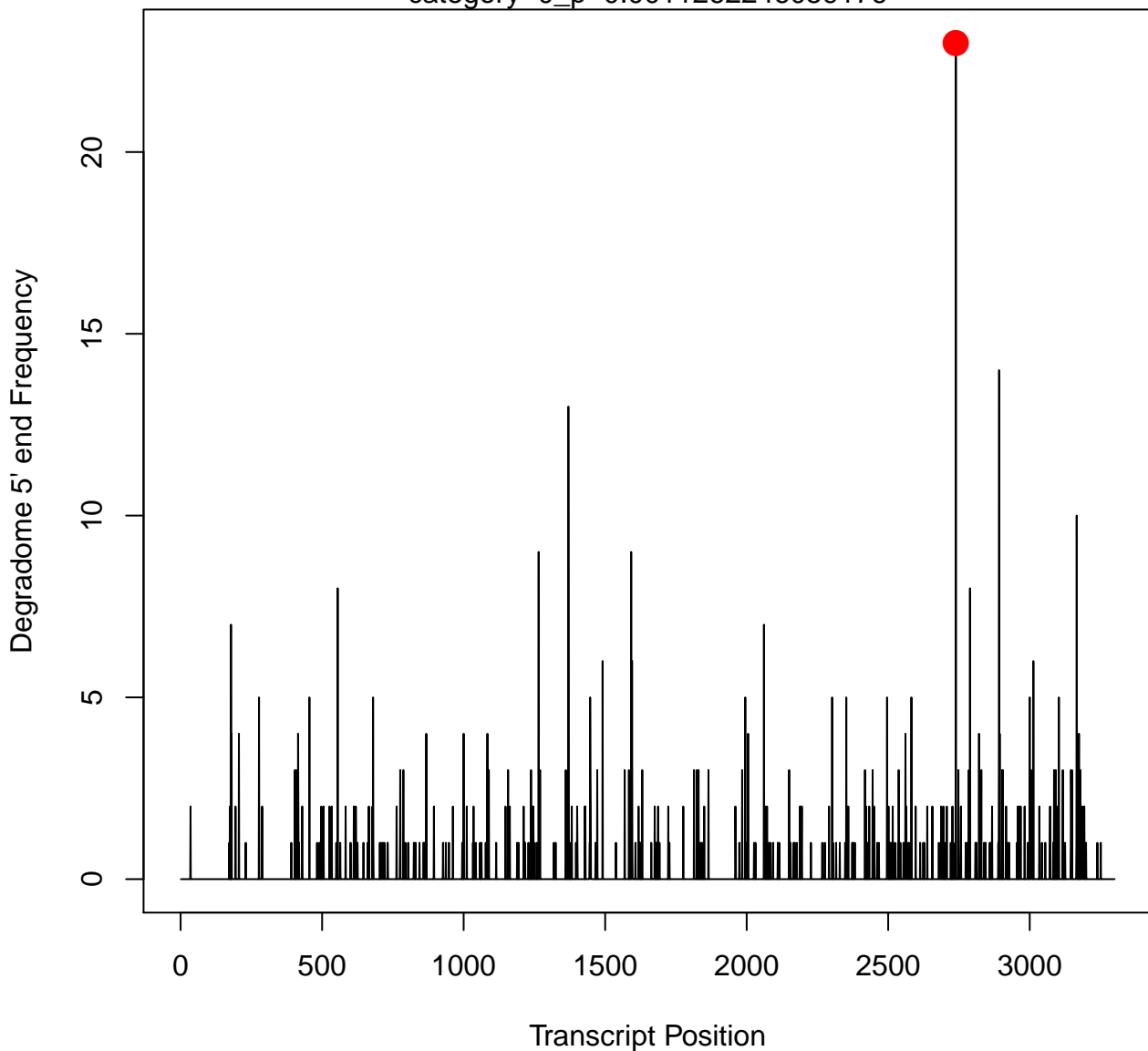

Supplement: Supplementary file 6 [file Data_Sheet_6.zip › Sit-miR167h_Seita.3G020000.1_2739_TPlot.pdf]

**T=Seita.4G163700.1\_Q=Sit-miR167h\_S=1098**

category=2\_p=0.714090968149678

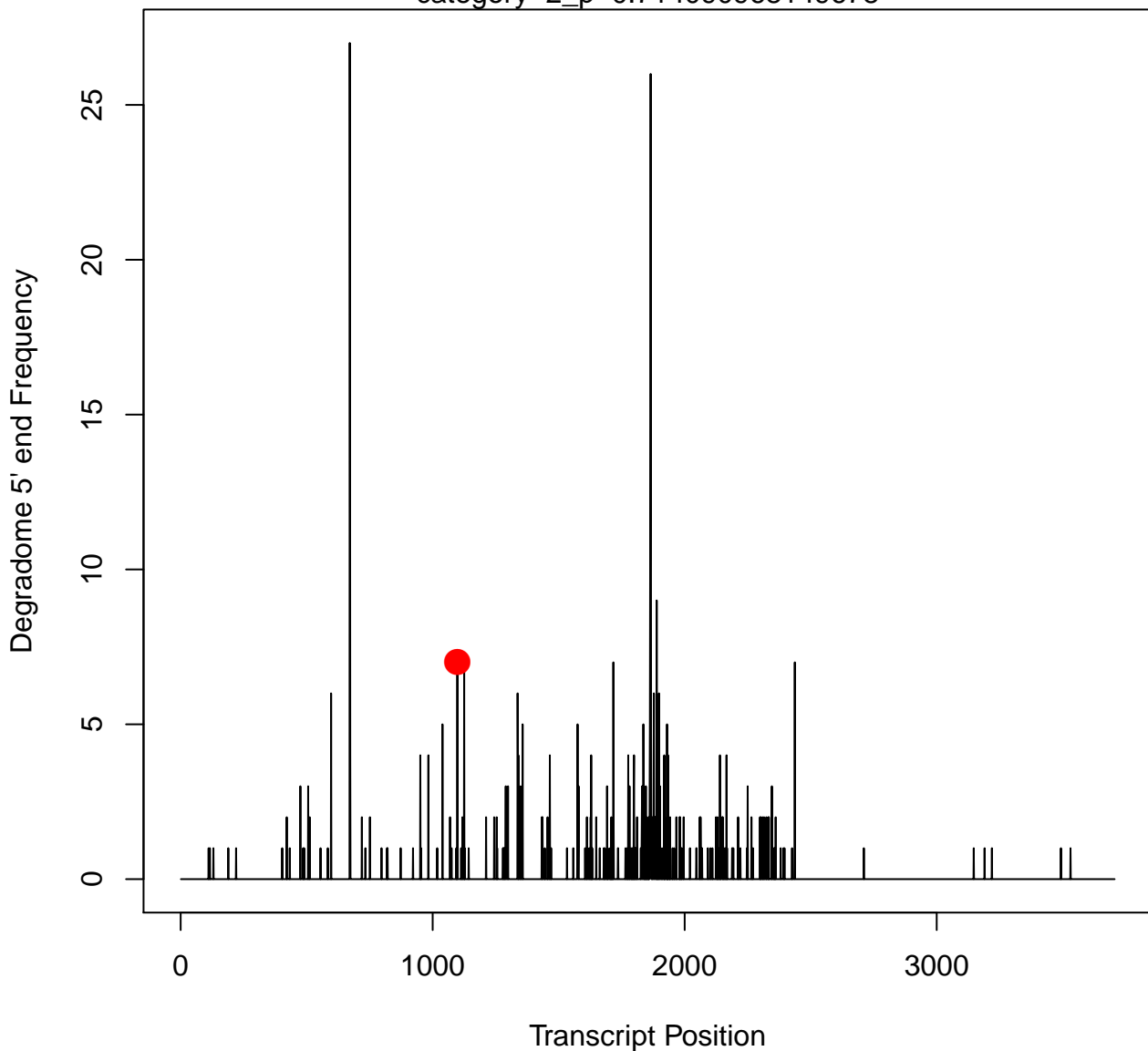

Supplement: Supplementary file 6 [file Data_Sheet_6.zip › Sit-miR167h_Seita.4G163700.1_1098_TPlot.pdf]

**T=Seita.4G262300.1\_Q=Sit-miR167h\_S=3389**

category=0\_p=0.00225118082997222

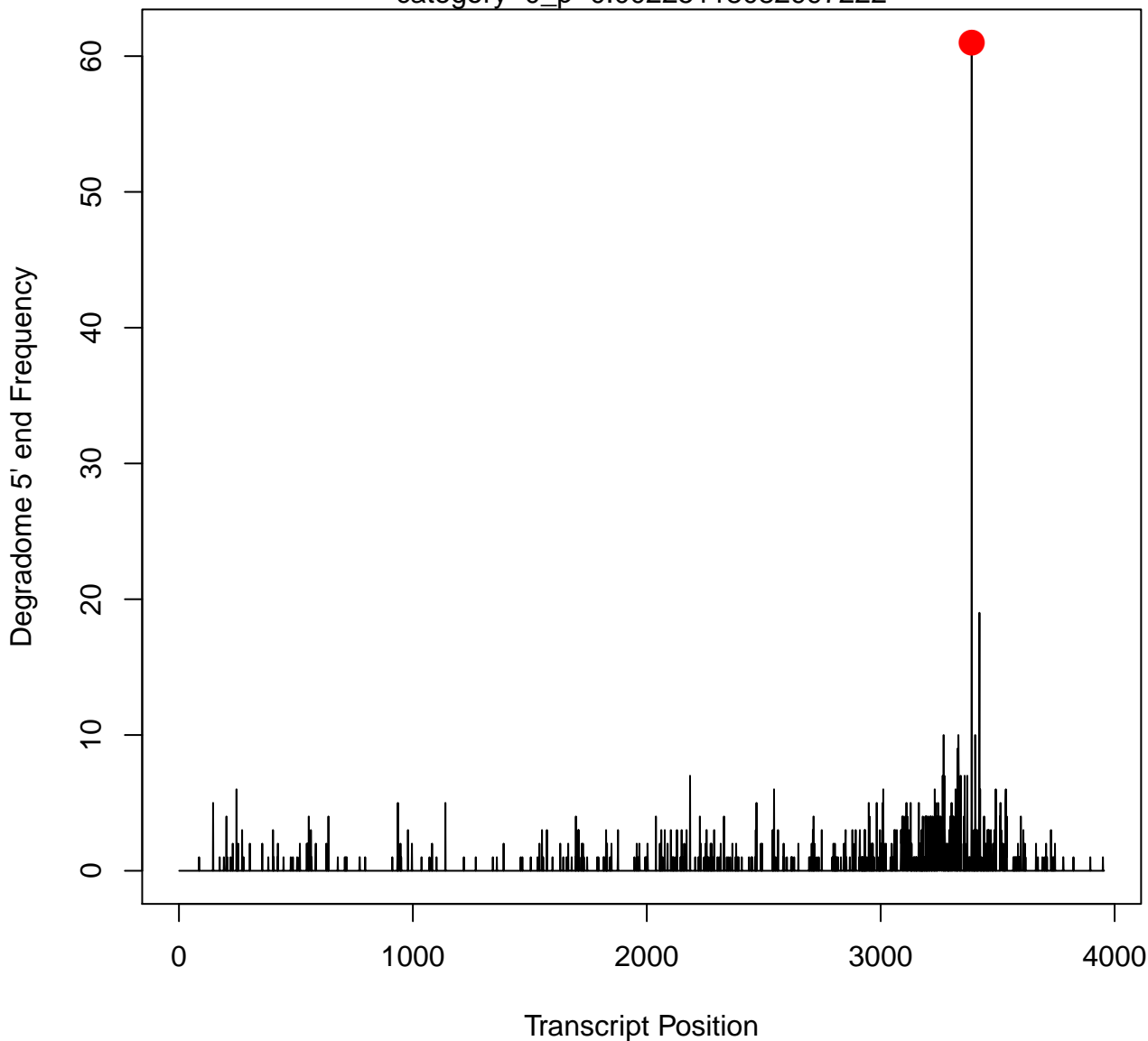

Supplement: Supplementary file 6 [file Data_Sheet_6.zip › Sit-miR167h_Seita.4G262300.1_3389_TPlot.pdf]

**T=Seita.7G229200.1\_Q=Sit-miR167i\_S=1096**

category=2\_p=0.933047401047695

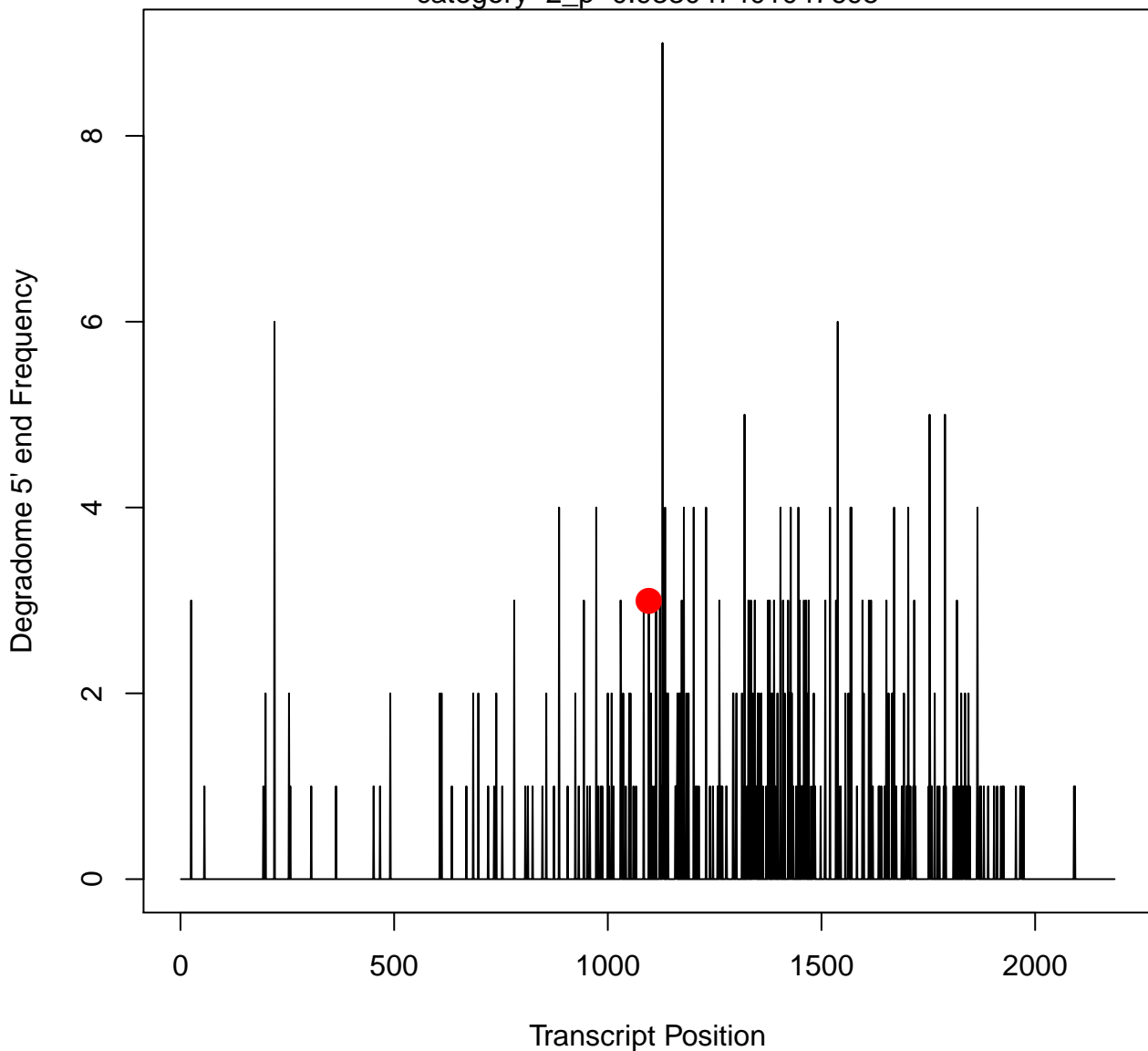

Supplement: Supplementary file 6 [file Data_Sheet_6.zip › Sit-miR167i_Seita.7G229200.1_1096_TPlot.pdf]

**T=Seita.1G272400.1\_Q=Sit-miR167j\_S=963**

category=2\_p=0.9999358143257

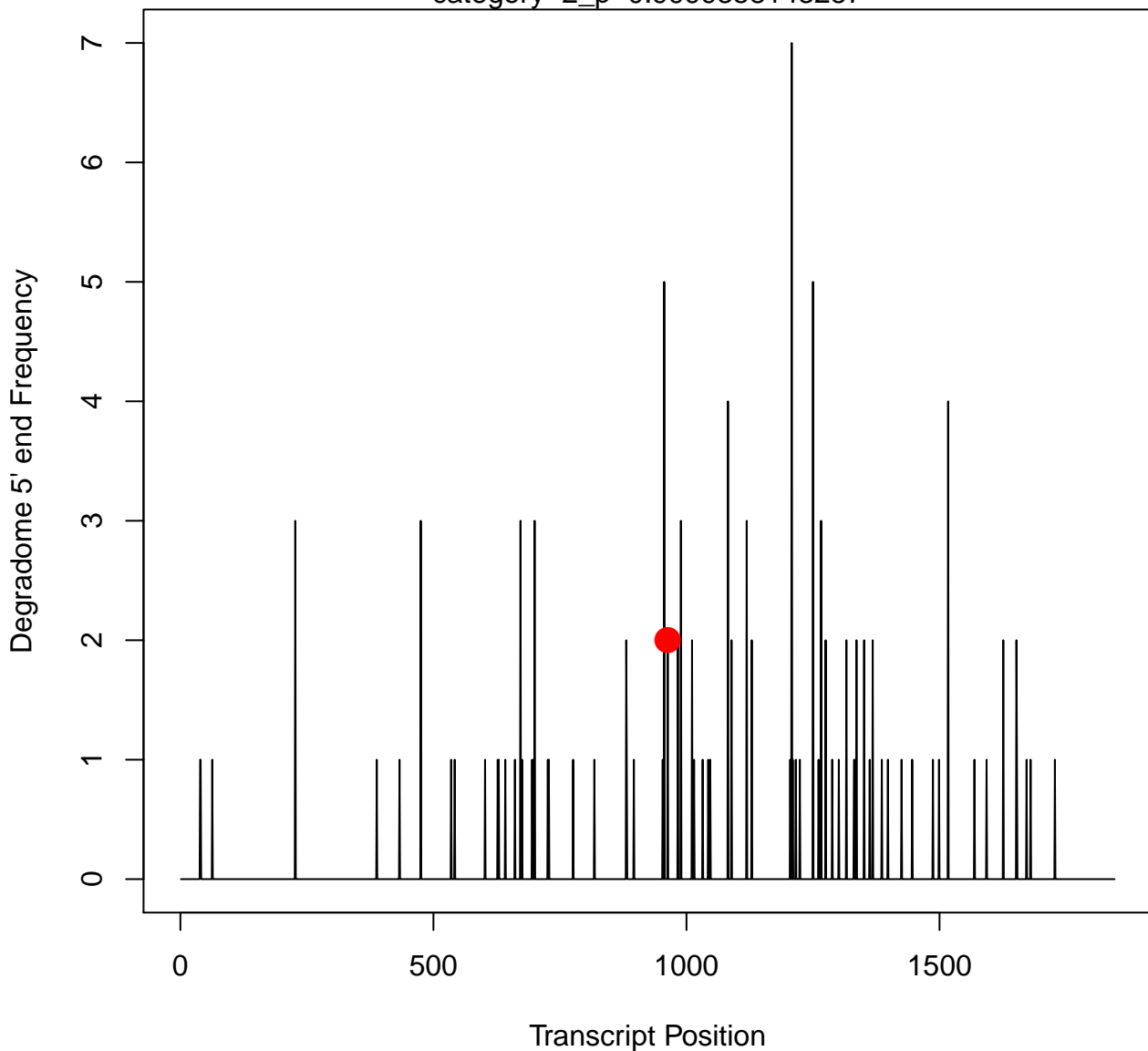

Supplement: Supplementary file 6 [file Data_Sheet_6.zip › Sit-miR167j_Seita.1G272400.1_963_TPlot.pdf]

**T=Seita.3G394000.1\_Q=Sit-miR167j\_S=3277**

category=2\_p=0.135123779440266

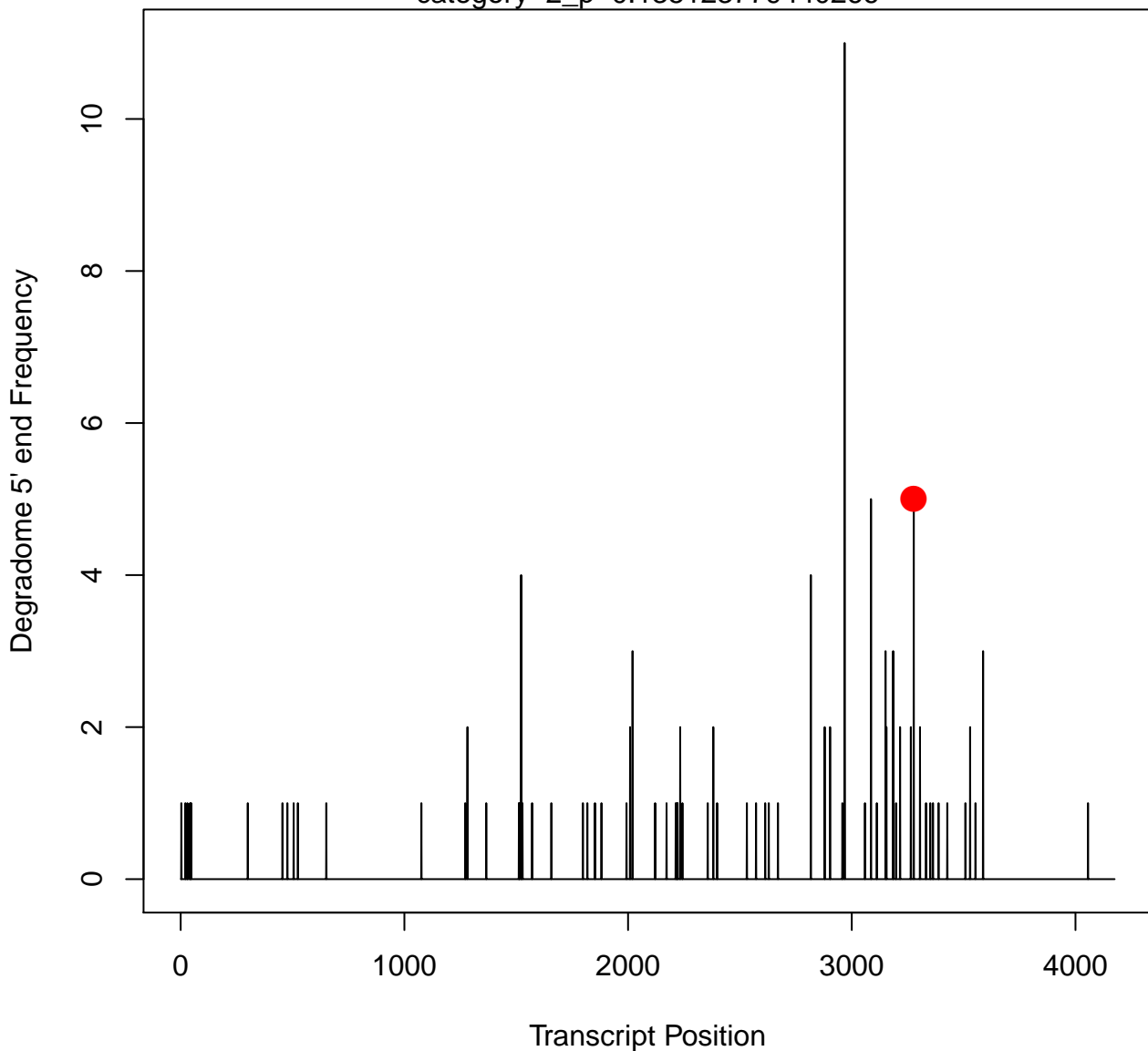

Supplement: Supplementary file 6 [file Data_Sheet_6.zip › Sit-miR167j_Seita.3G394000.1_3277_TPlot.pdf]

**T=Seita.9G403000.1\_Q=Sit-miR167j\_S=1008**

category=2\_p=0.951703702041835

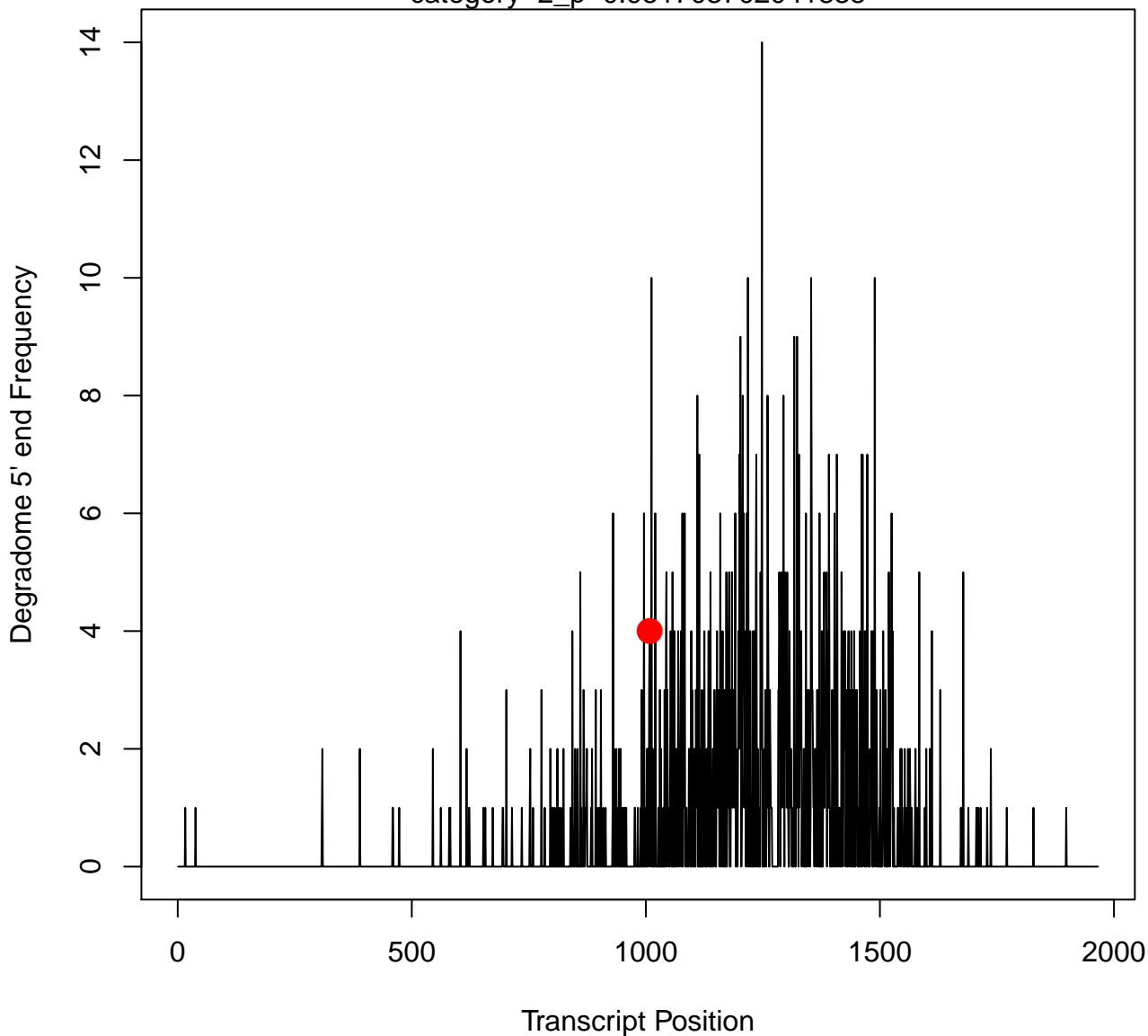

Supplement: Supplementary file 6 [file Data_Sheet_6.zip › Sit-miR167j_Seita.9G403000.1_1008_TPlot.pdf]

**T=Seita.1G323200.1\_Q=Sit-miR168\_S=2230**

category=2\_p=0.989671205660326

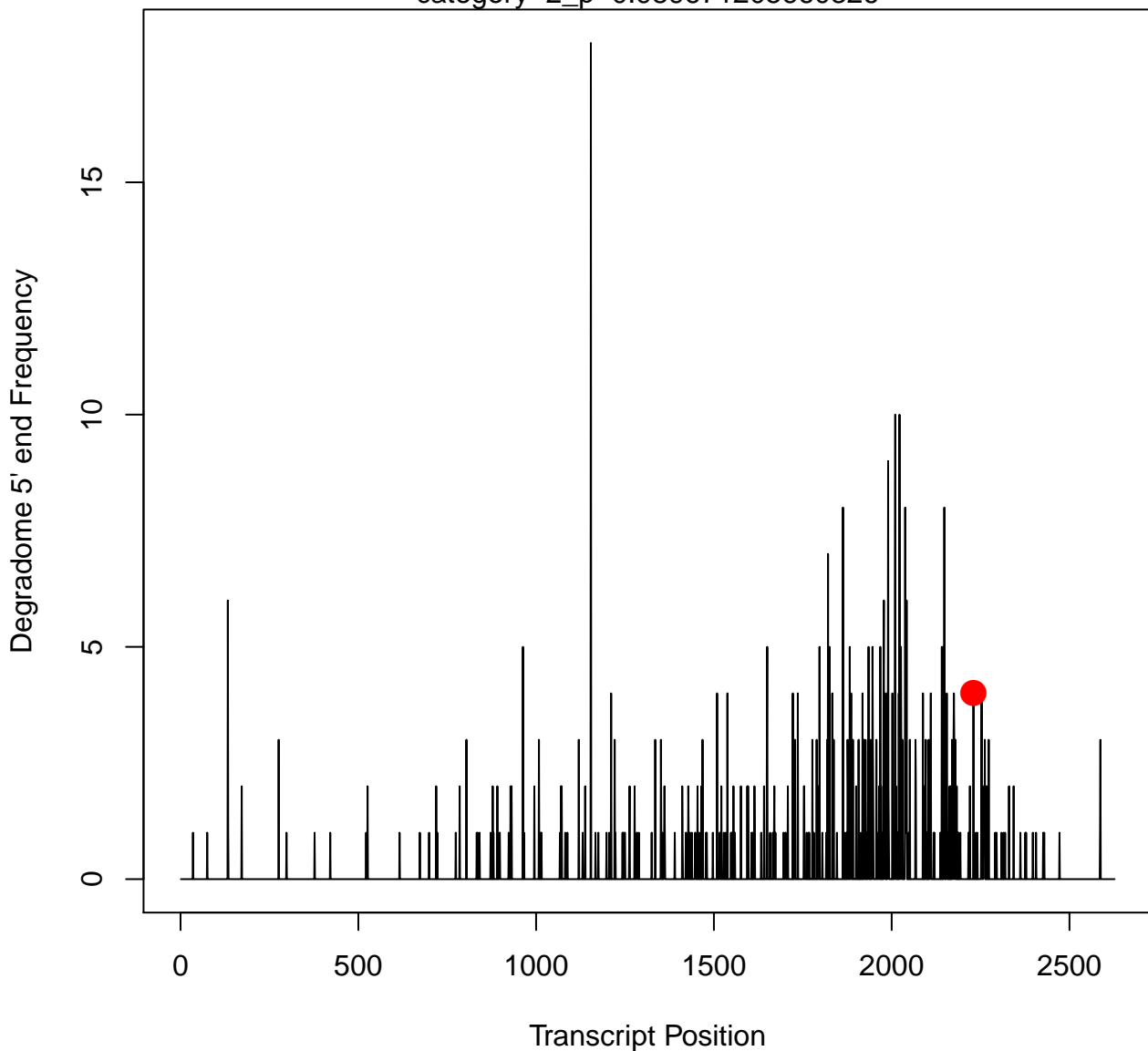

Supplement: Supplementary file 6 [file Data_Sheet_6.zip › Sit-miR168_Seita.1G323200.1_2230_TPlot.pdf]

**T=Seita.1G378700.1\_Q=Sit-miR168\_S=527**

category=0\_p=0.0119478826287224

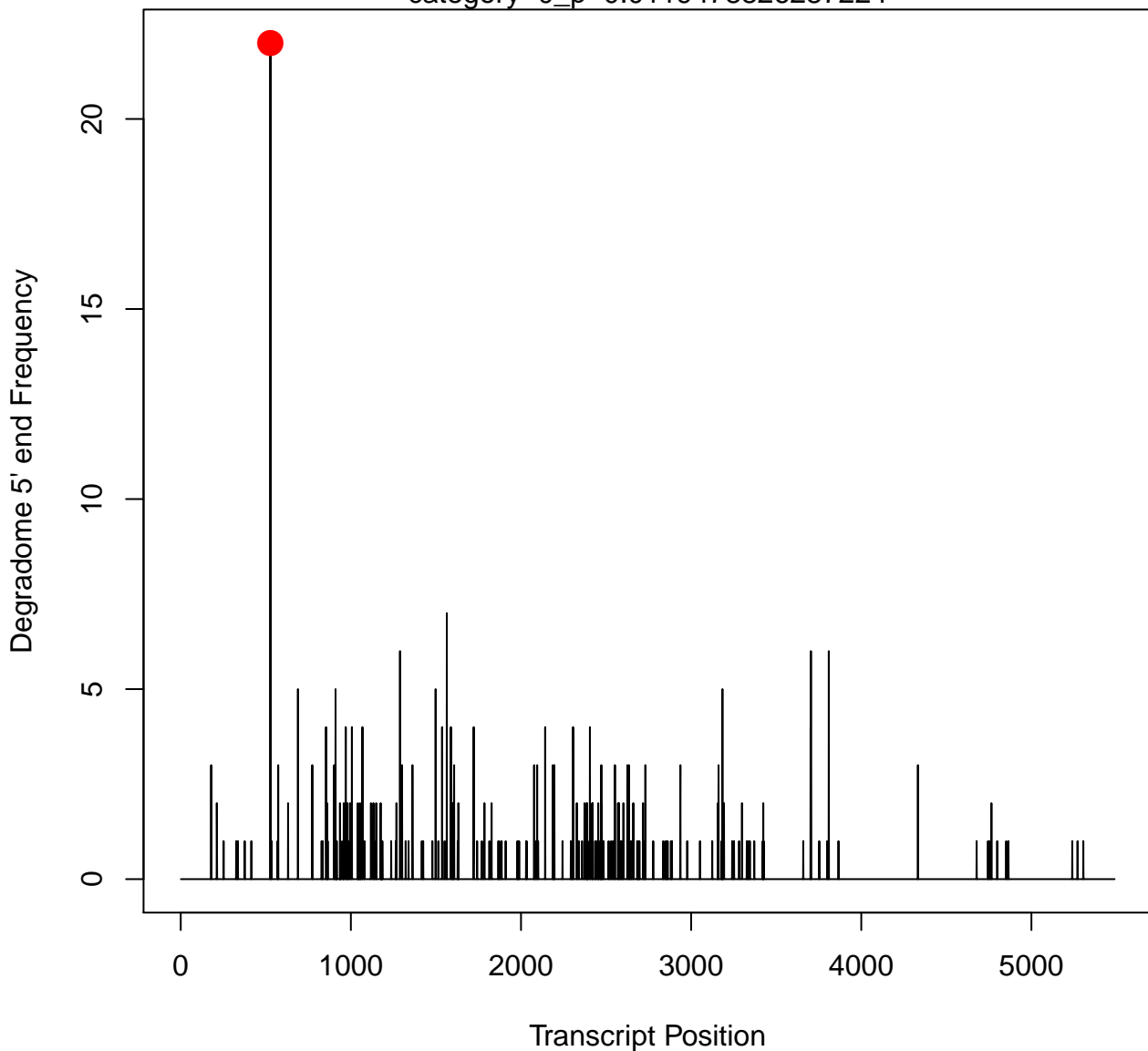

Supplement: Supplementary file 6 [file Data_Sheet_6.zip › Sit-miR168_Seita.1G378700.1_527_TPlot.pdf]

**T=Seita.2G434600.1\_Q=Sit-miR168\_S=2266**

category=2\_p=0.915234864478655

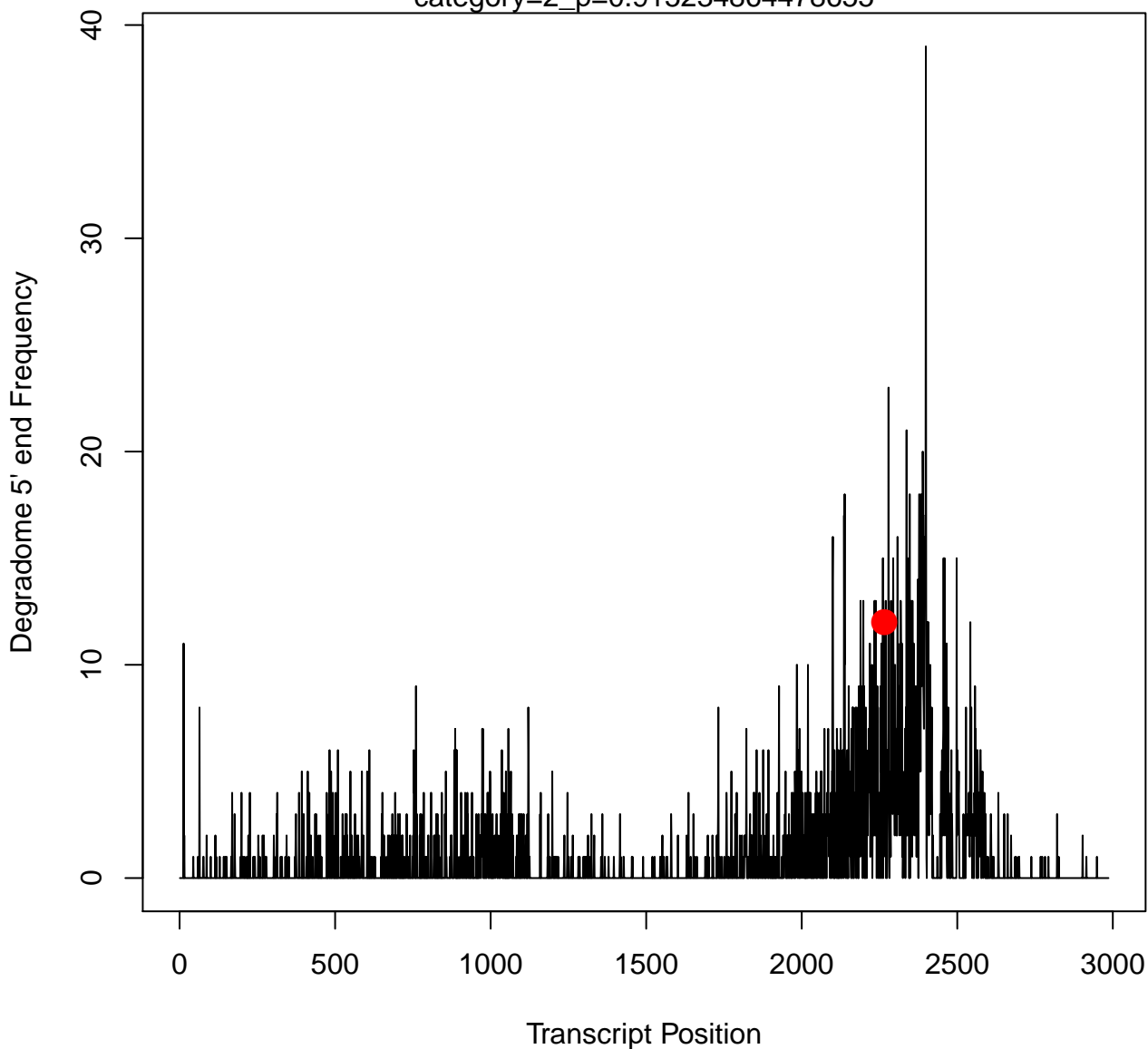

Supplement: Supplementary file 6 [file Data_Sheet_6.zip › Sit-miR168_Seita.2G434600.1_2266_TPlot.pdf]

**T=Seita.3G070600.1\_Q=Sit-miR168\_S=747**

category=0\_p=0.0112053399558953

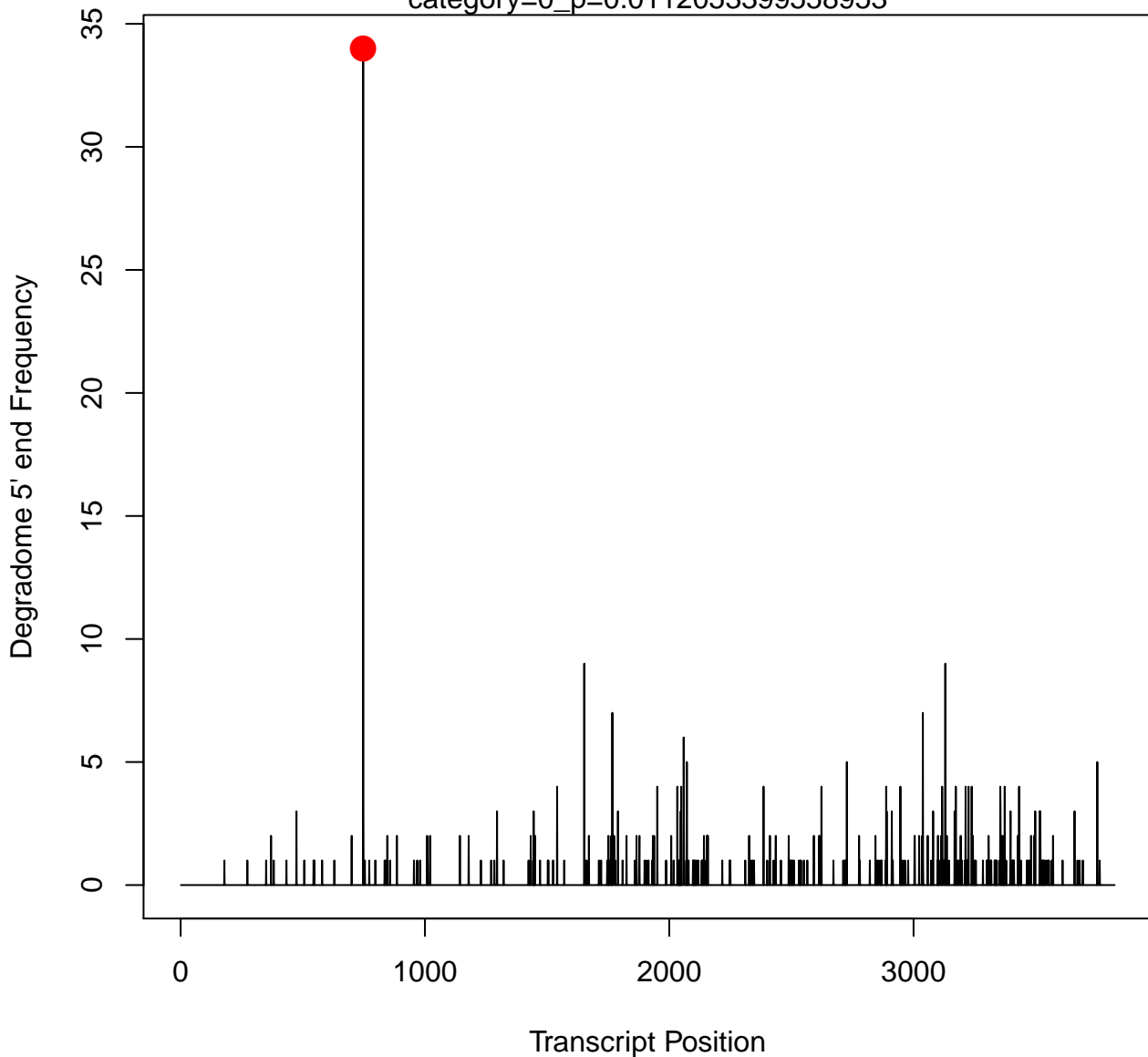

Supplement: Supplementary file 6 [file Data_Sheet_6.zip › Sit-miR168_Seita.3G070600.1_747_TPlot.pdf]

**T=Seita.3G089200.1\_Q=Sit-miR168\_S=125**

category=2\_p=0.669422022419247

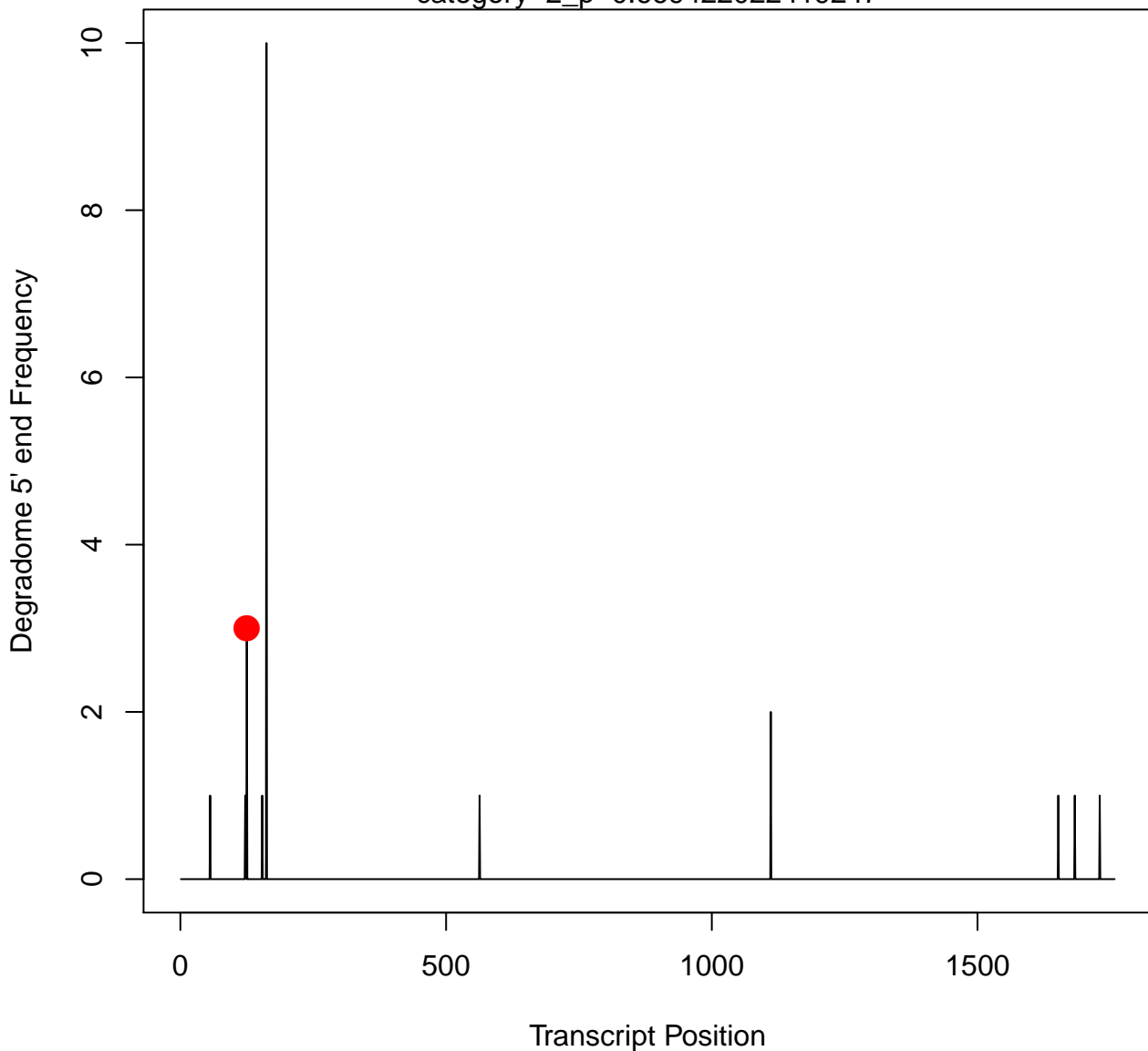

Supplement: Supplementary file 6 [file Data_Sheet_6.zip › Sit-miR168_Seita.3G089200.1_125_TPlot.pdf]

**T=Seita.5G022800.1\_Q=Sit-miR168\_S=2856**

category=2\_p=0.975321094724296

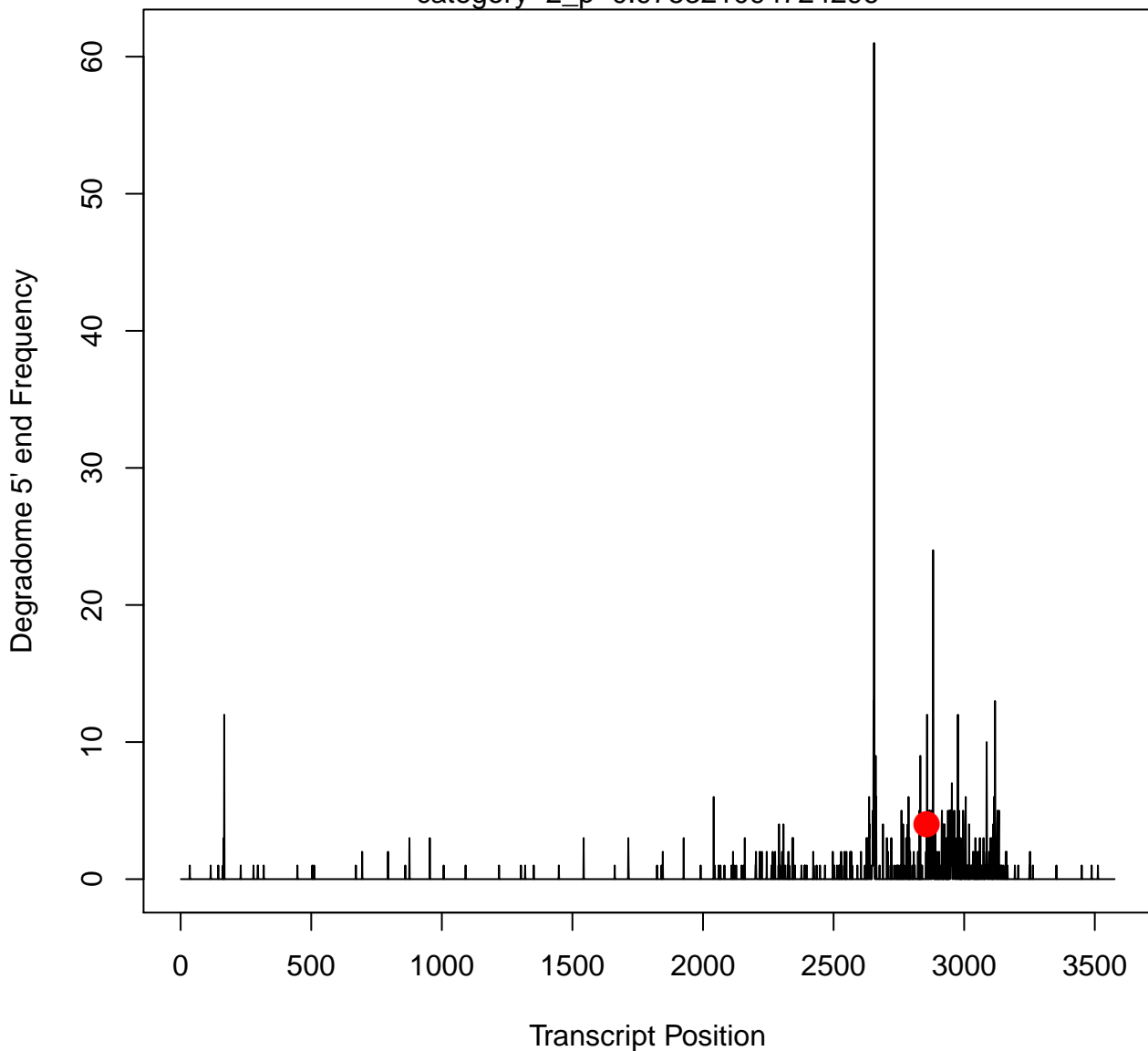

Supplement: Supplementary file 6 [file Data_Sheet_6.zip › Sit-miR168_Seita.5G022800.1_2856_TPlot.pdf]

**T=Seita.5G178200.1\_Q=Sit-miR168\_S=1671**

category=2\_p=0.558057258531678

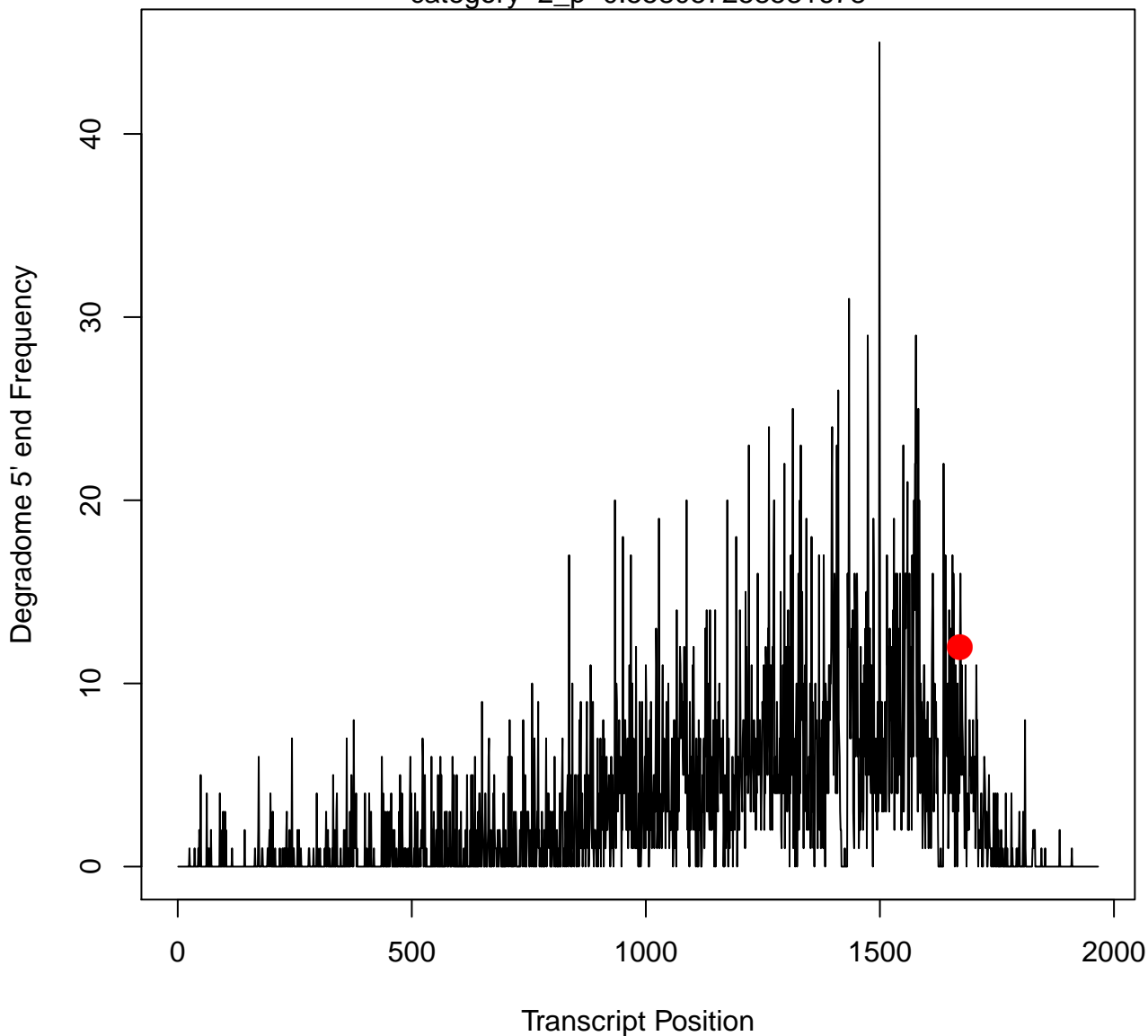

Supplement: Supplementary file 6 [file Data_Sheet_6.zip › Sit-miR168_Seita.5G178200.1_1671_TPlot.pdf]

**T=Seita.5G261900.1\_Q=Sit-miR168\_S=537**

category=0\_p=0.0923622171298326

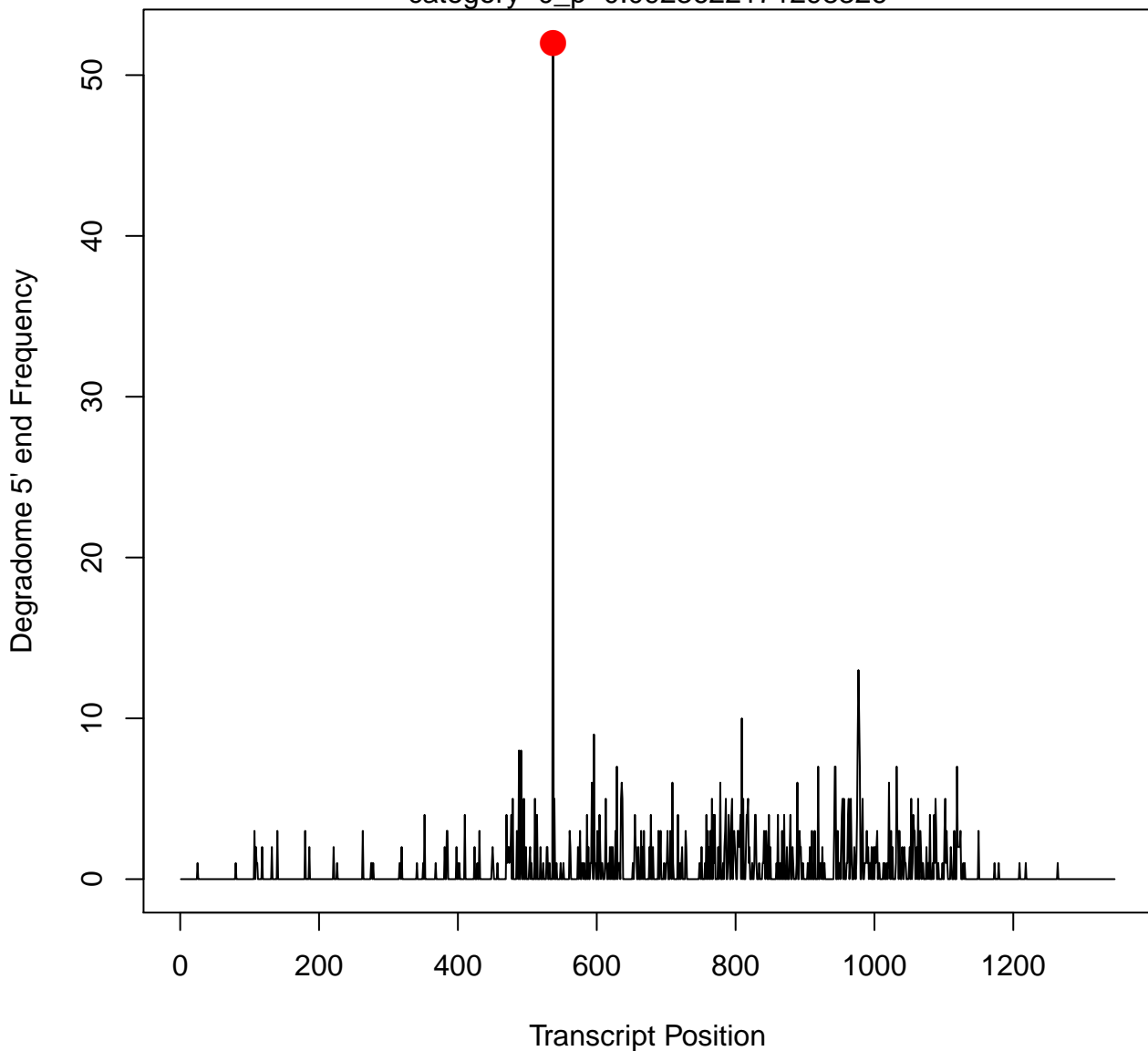

Supplement: Supplementary file 6 [file Data_Sheet_6.zip › Sit-miR168_Seita.5G261900.1_537_TPlot.pdf]

**T=Seita.5G435400.1\_Q=Sit-miR168\_S=595**

category=0\_p=0.00449729384481512

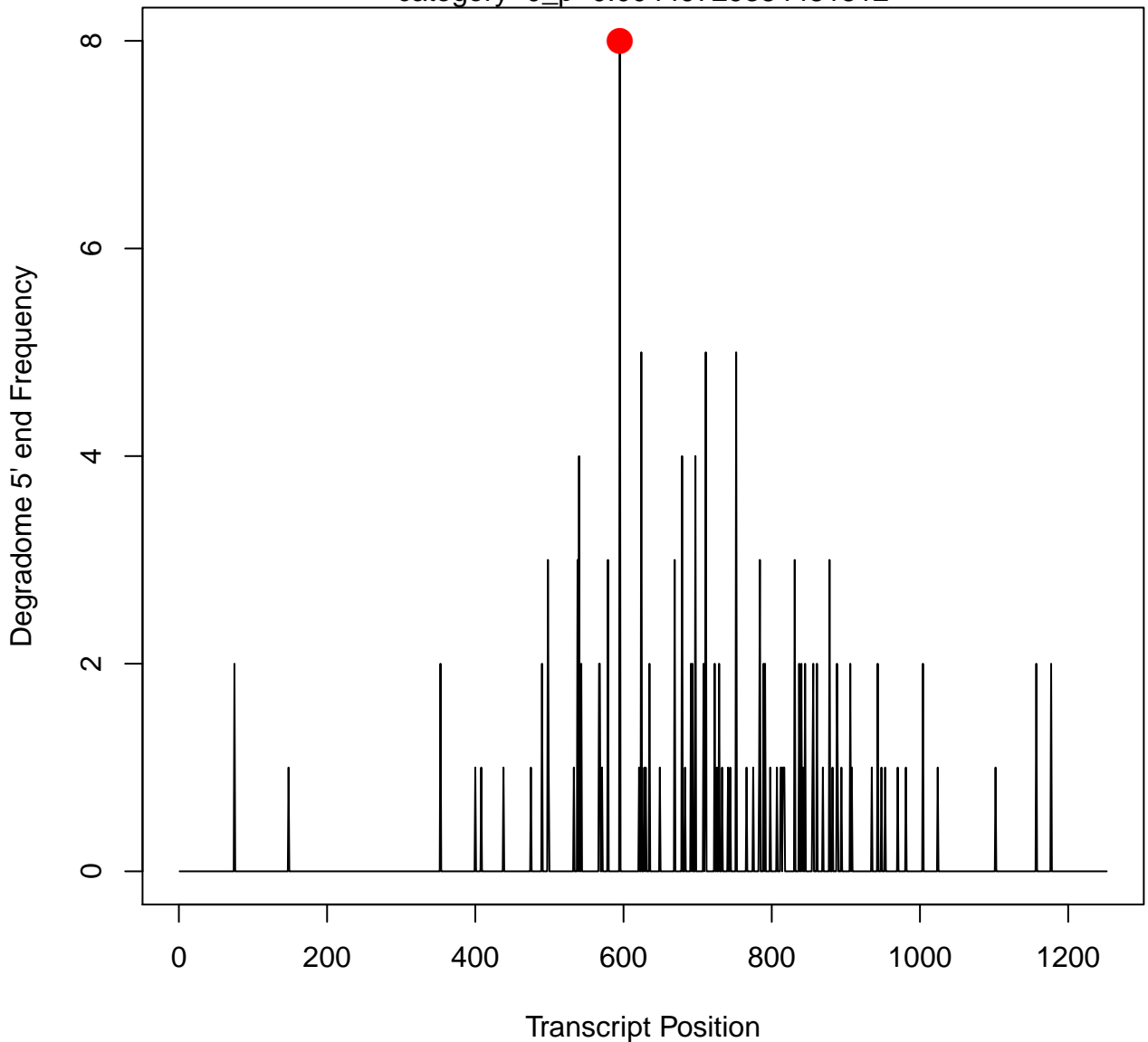

Supplement: Supplementary file 6 [file Data_Sheet_6.zip › Sit-miR168_Seita.5G435400.1_595_TPlot.pdf]

**T=Seita.7G114700.1\_Q=Sit-miR168\_S=402**

category=2\_p=0.981201873859569

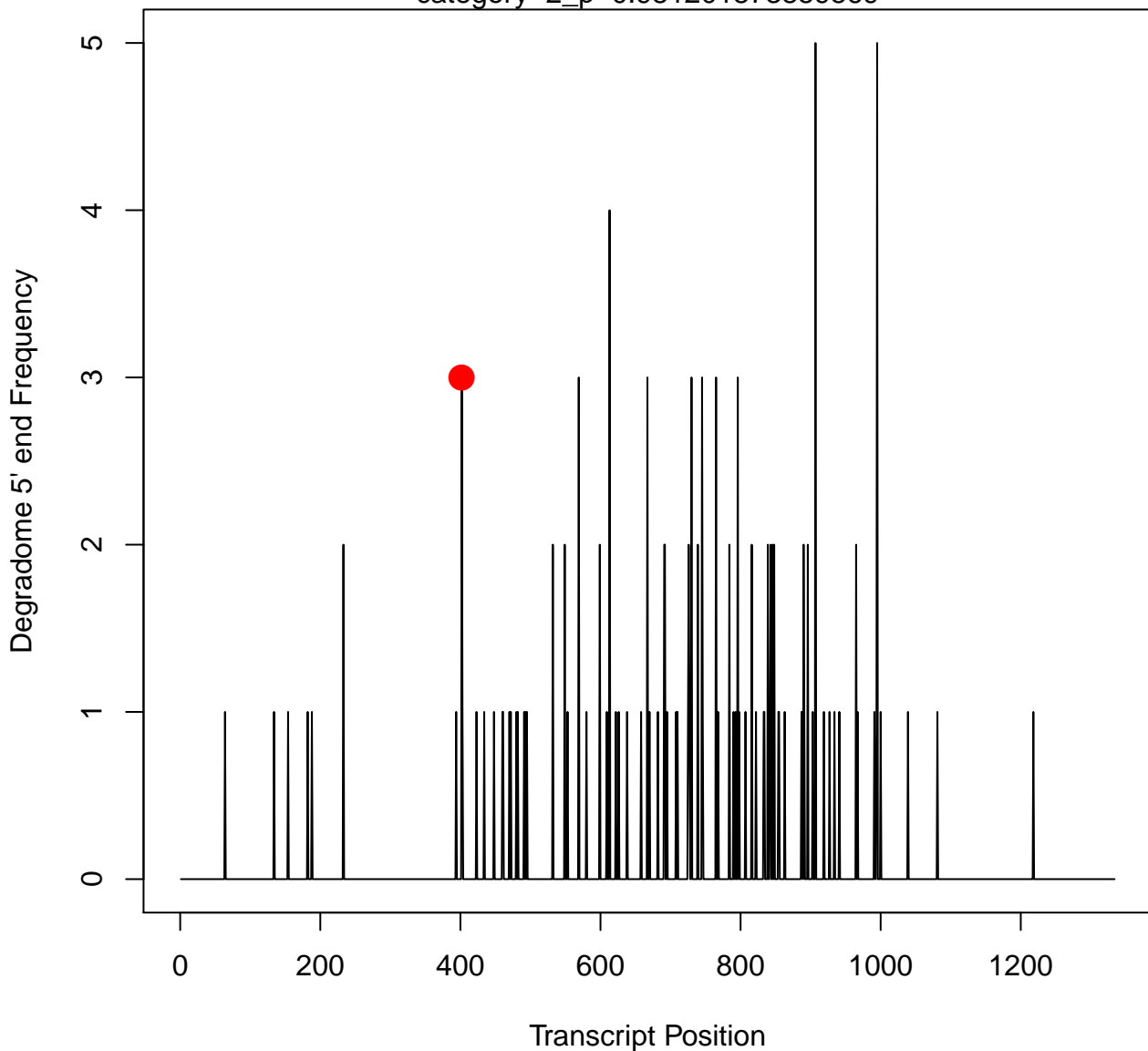

Supplement: Supplementary file 6 [file Data_Sheet_6.zip › Sit-miR168_Seita.7G114700.1_402_TPlot.pdf]

**T=Seita.7G115200.1\_Q=Sit-miR168\_S=418**

category=2\_p=0.976628659392941

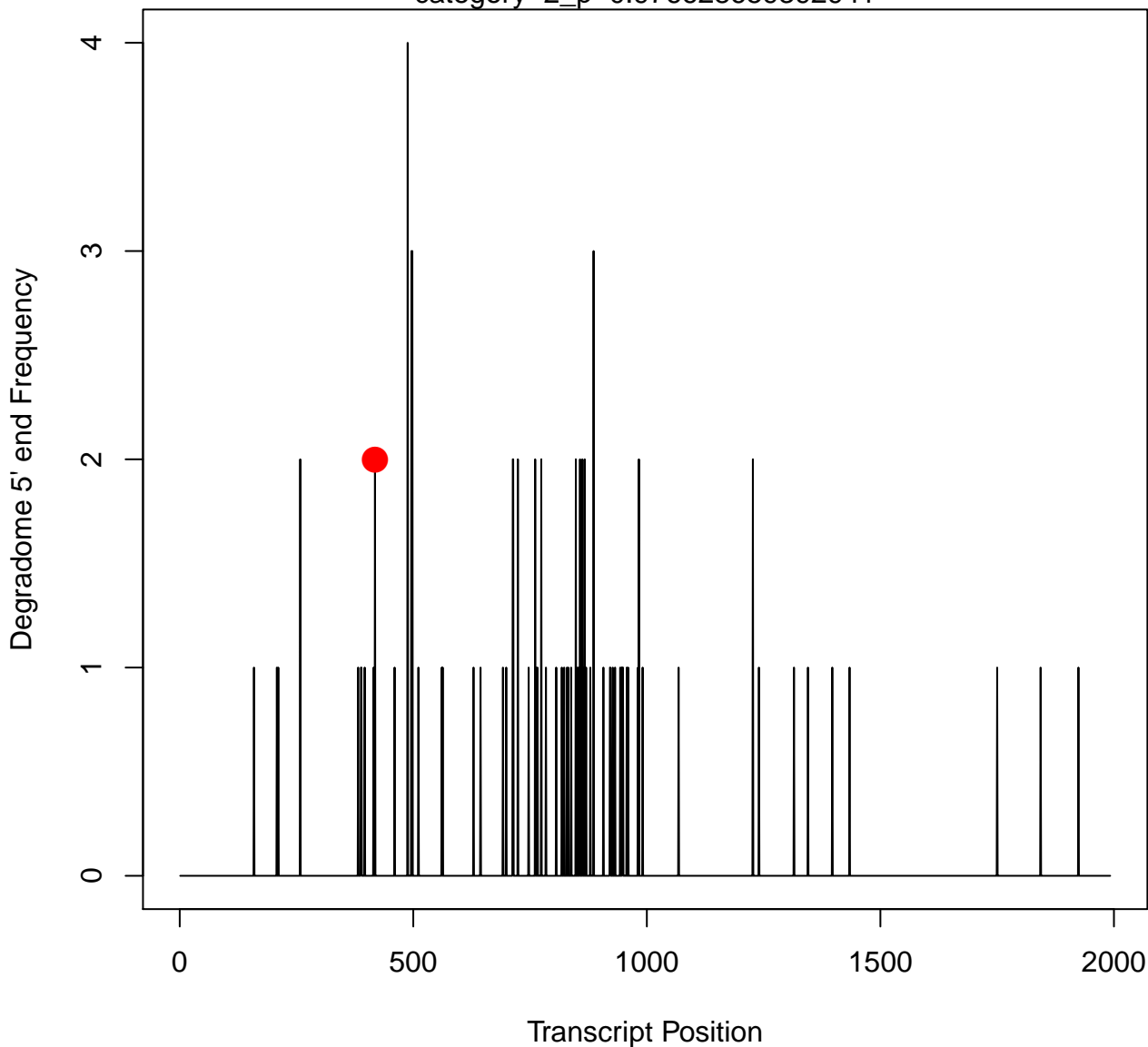

Supplement: Supplementary file 6 [file Data_Sheet_6.zip › Sit-miR168_Seita.7G115200.1_418_TPlot.pdf]

**T=Seita.7G141400.1\_Q=Sit-miR168\_S=1212**

category=2\_p=0.998490998908554

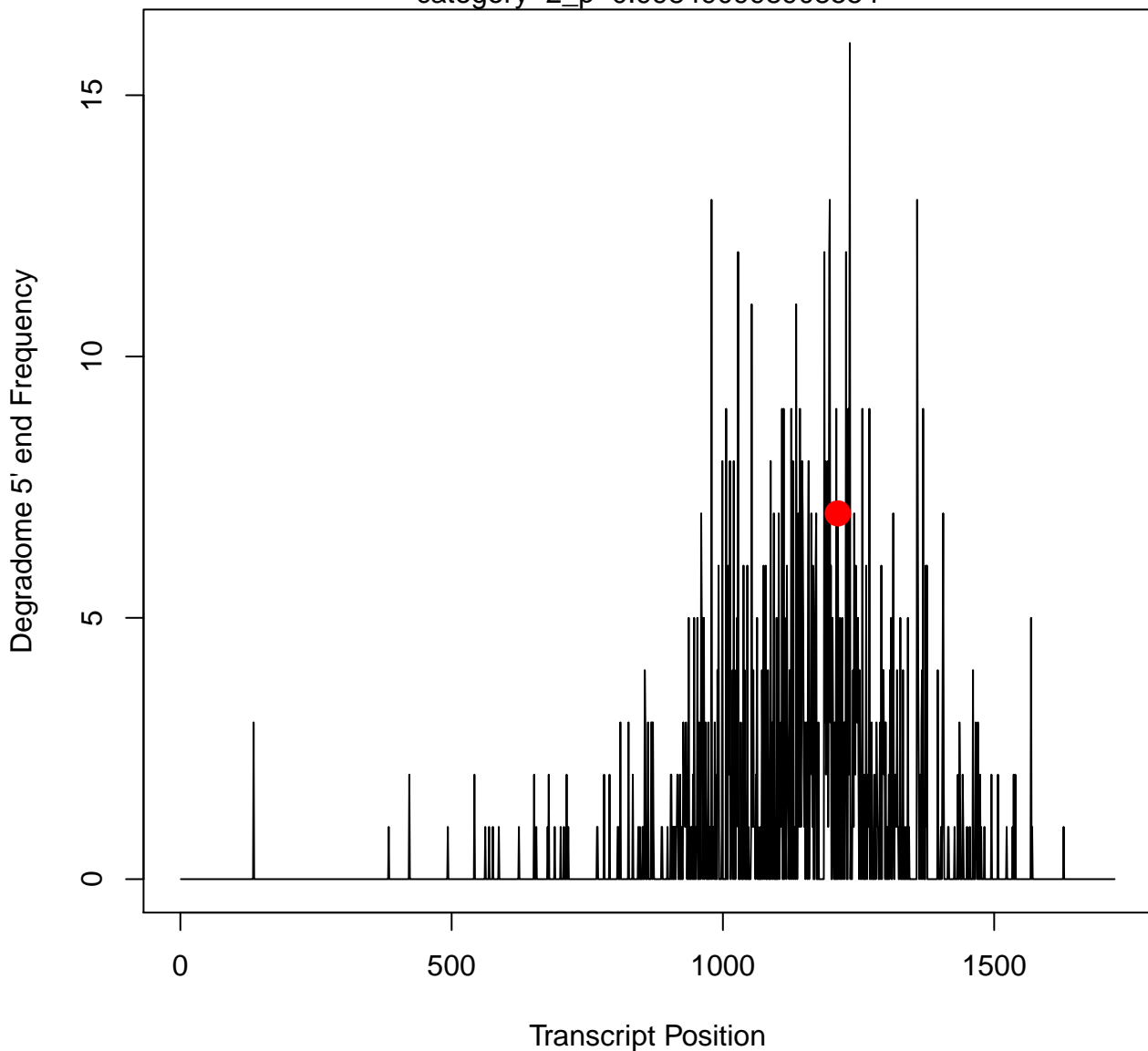

Supplement: Supplementary file 6 [file Data_Sheet_6.zip › Sit-miR168_Seita.7G141400.1_1212_TPlot.pdf]

**T=Seita.7G200400.1\_Q=Sit-miR168\_S=1236**

category=2\_p=0.99887125077036

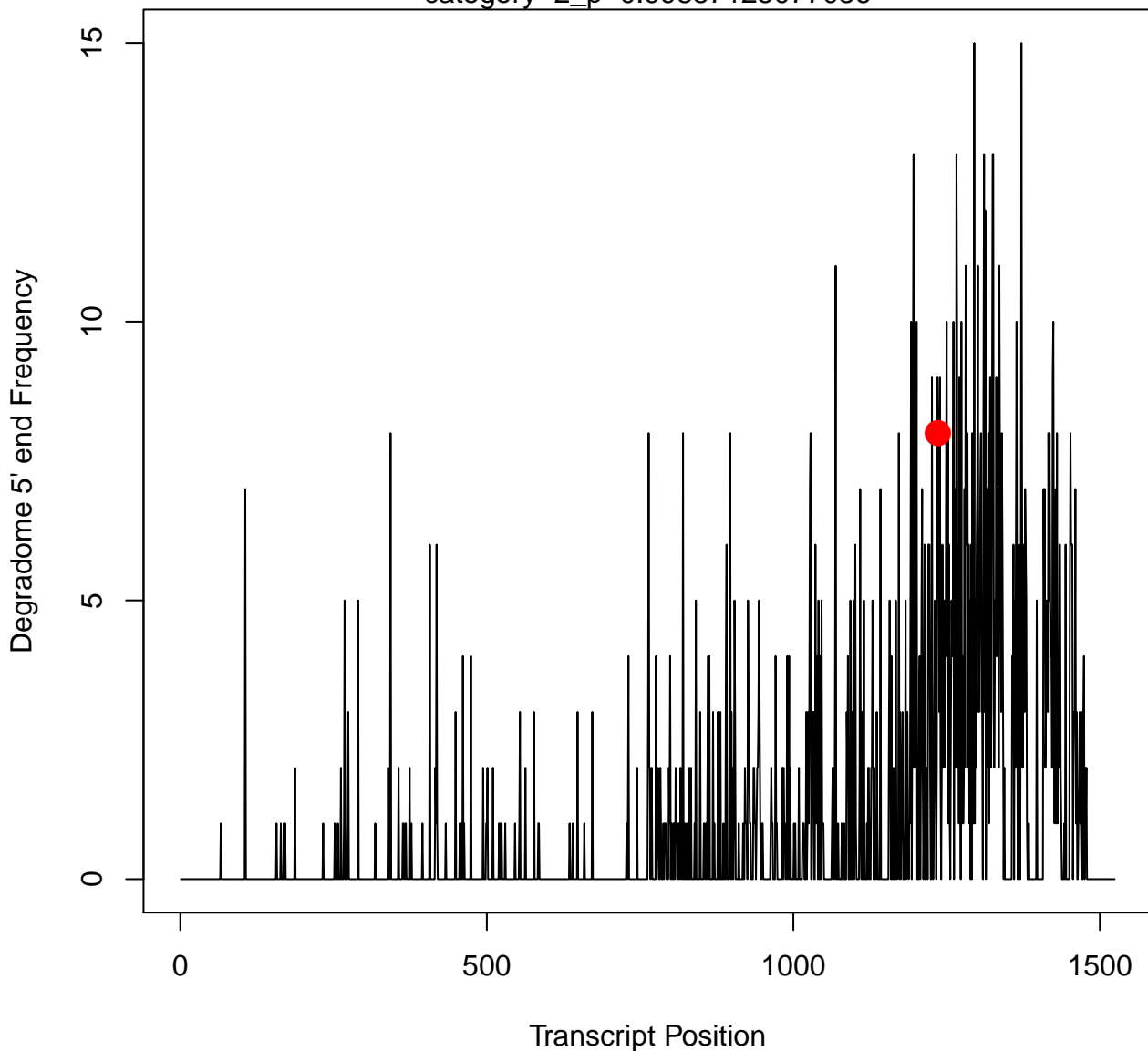

Supplement: Supplementary file 6 [file Data_Sheet_6.zip › Sit-miR168_Seita.7G200400.1_1236_TPlot.pdf]

**T=Seita.7G201100.1\_Q=Sit-miR168\_S=664**

category=0\_p=0.0108338593818176

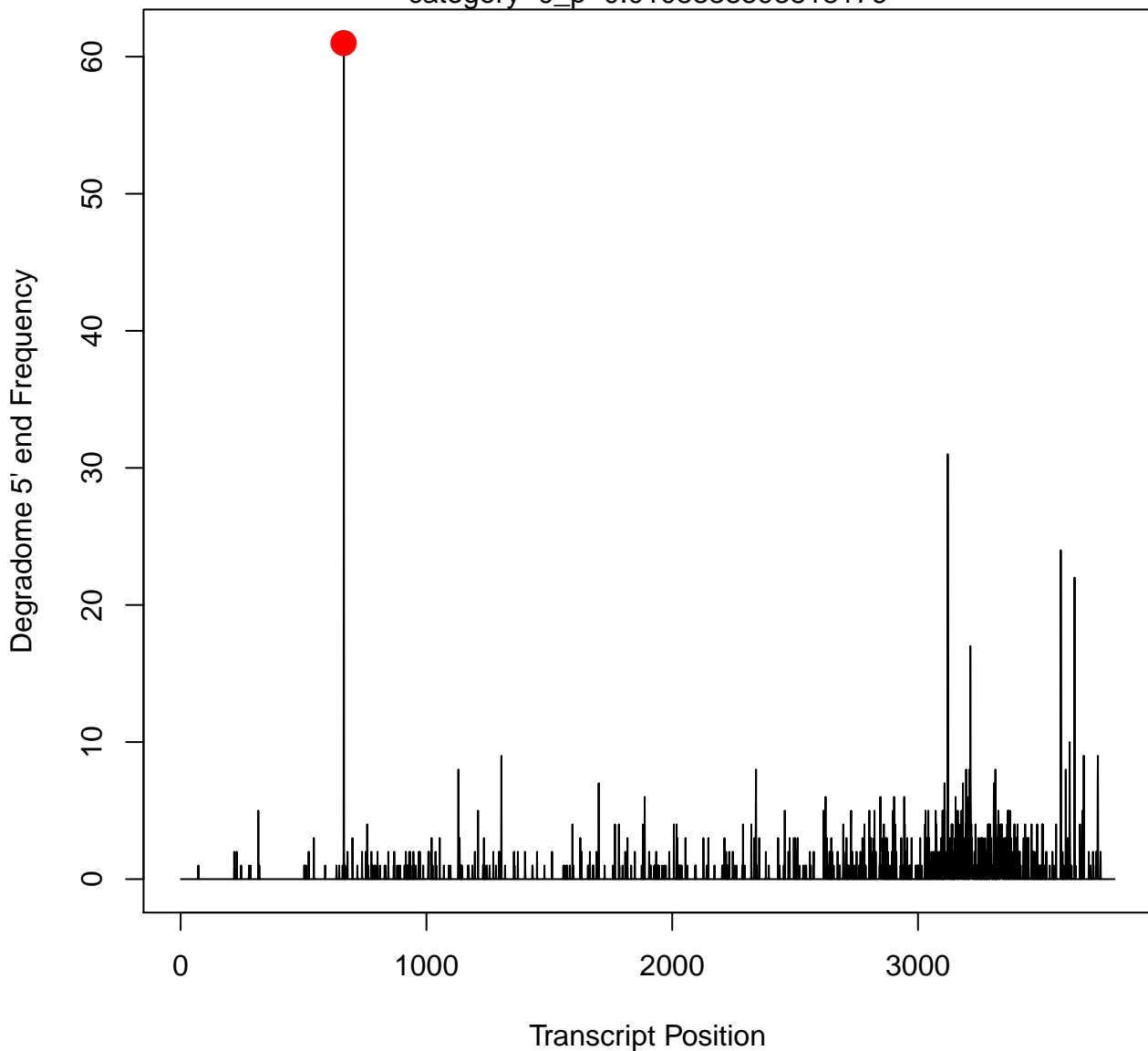

Supplement: Supplementary file 6 [file Data_Sheet_6.zip › Sit-miR168_Seita.7G201100.1_664_TPlot.pdf]

**T=Seita.9G136100.1\_Q=Sit-miR168\_S=1365**

category=2\_p=0.996261242255012

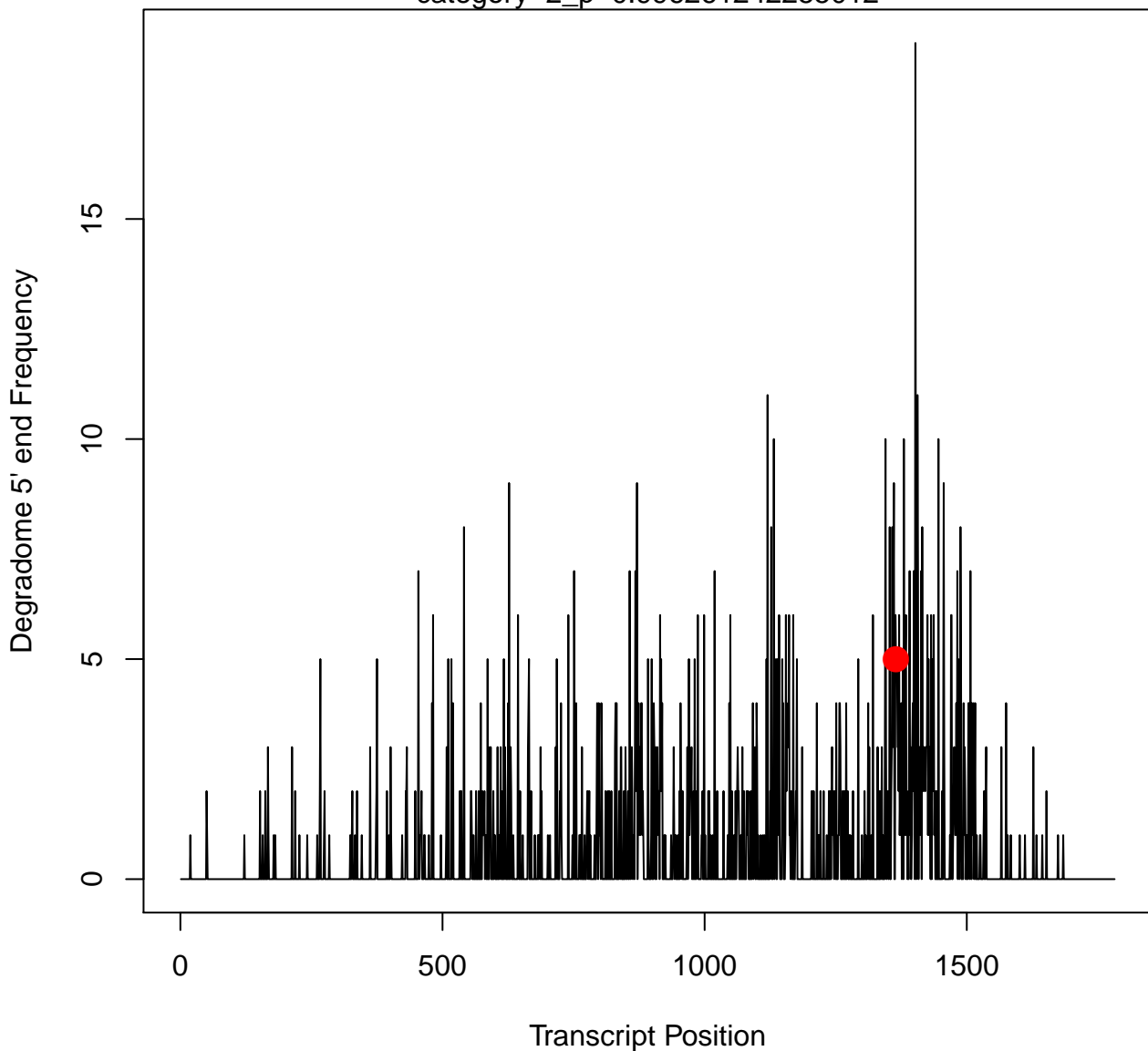

Supplement: Supplementary file 6 [file Data_Sheet_6.zip › Sit-miR168_Seita.9G136100.1_1365_TPlot.pdf]

**T=Seita.9G505300.1\_Q=Sit-miR168\_S=412**

category=2\_p=0.892683356398599

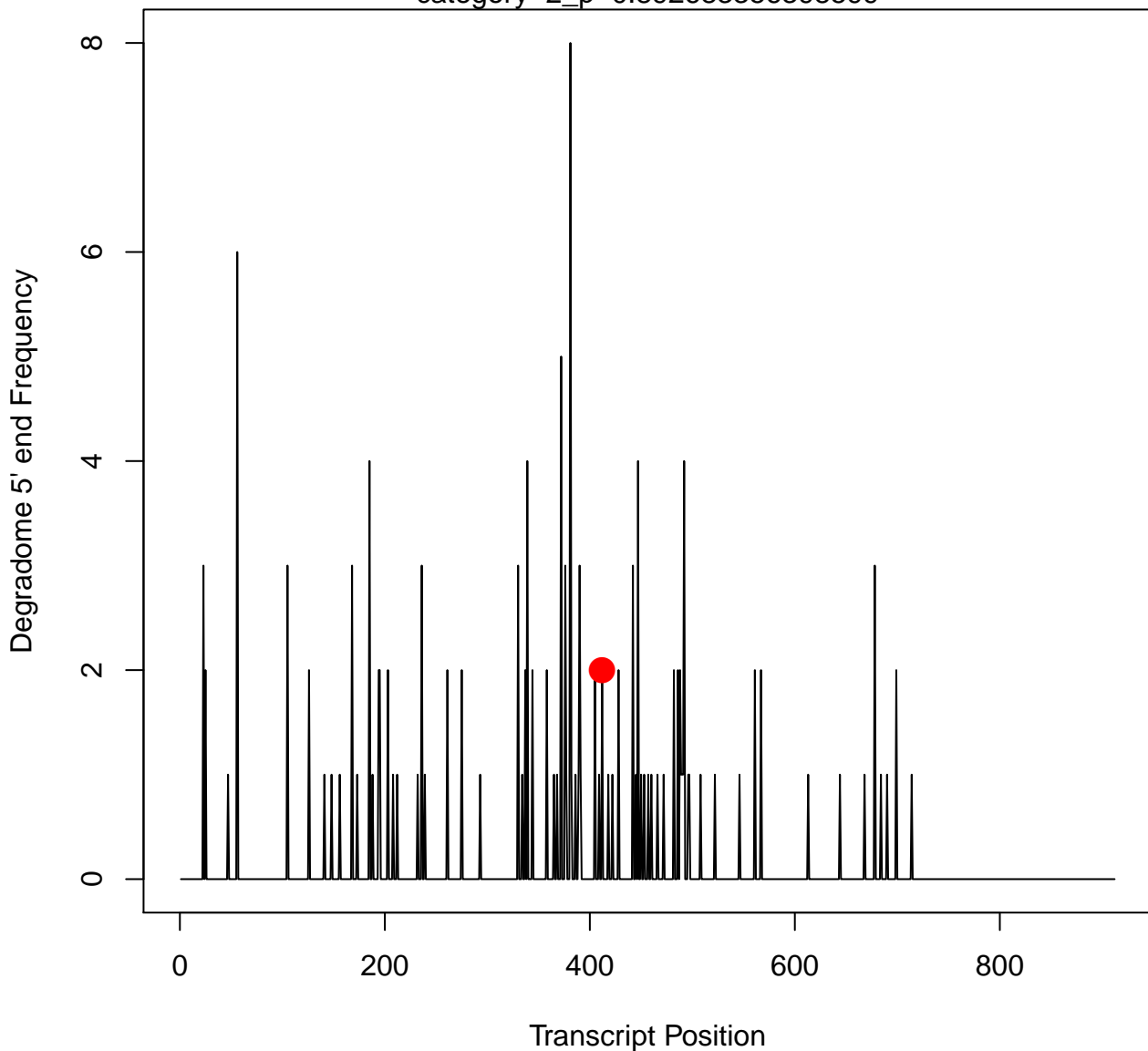

Supplement: Supplementary file 6 [file Data_Sheet_6.zip › Sit-miR168_Seita.9G505300.1_412_TPlot.pdf]

**T=Seita.3G037900.1\_Q=Sit-miR169a\_S=825**

category=2\_p=0.840028100747495

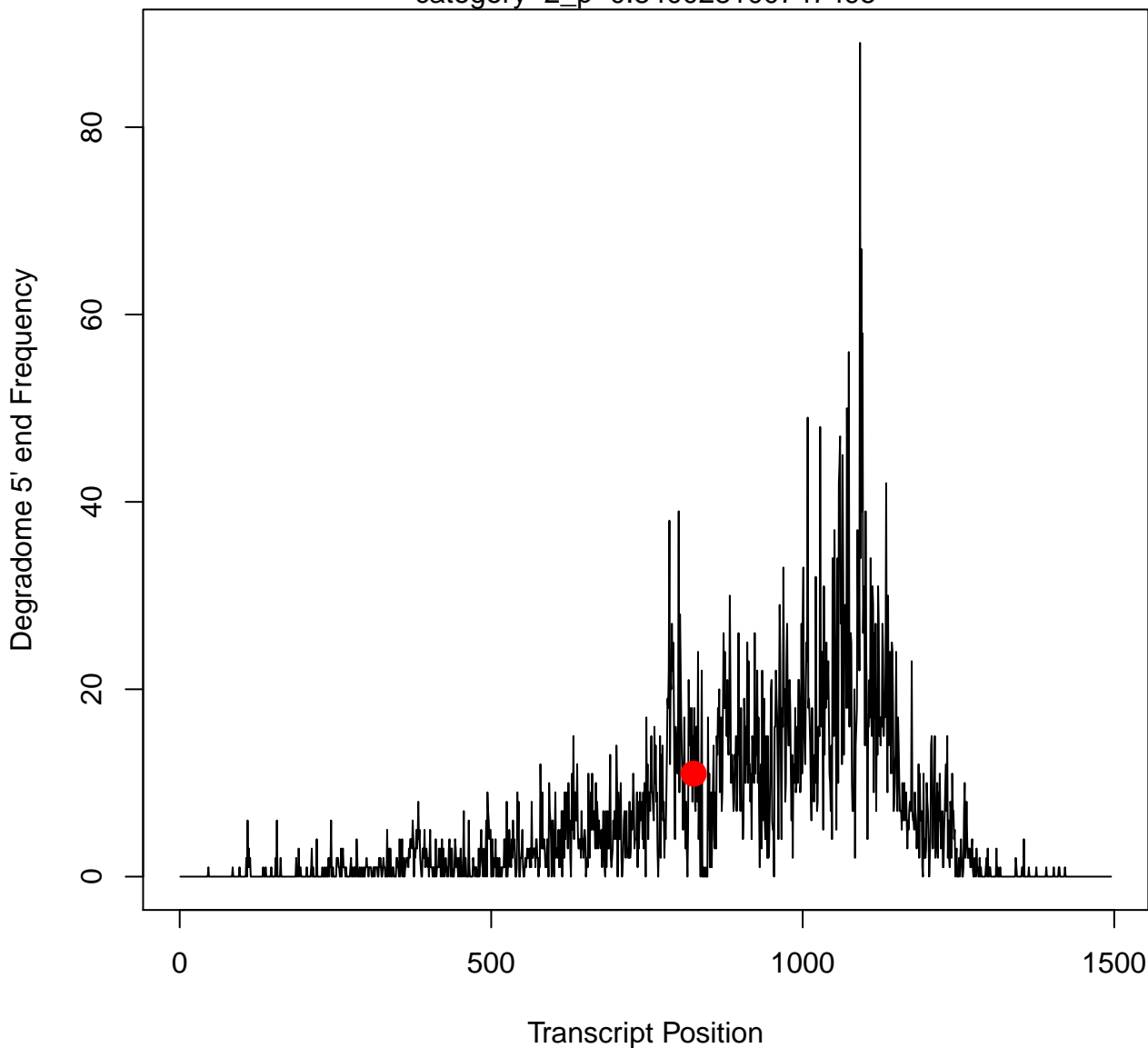

Supplement: Supplementary file 6 [file Data_Sheet_6.zip › Sit-miR169a_Seita.3G037900.1_825_TPlot.pdf]

**T=Seita.7G270500.1\_Q=Sit-miR169a\_S=1480**

category=1\_p=0.0456528473619759

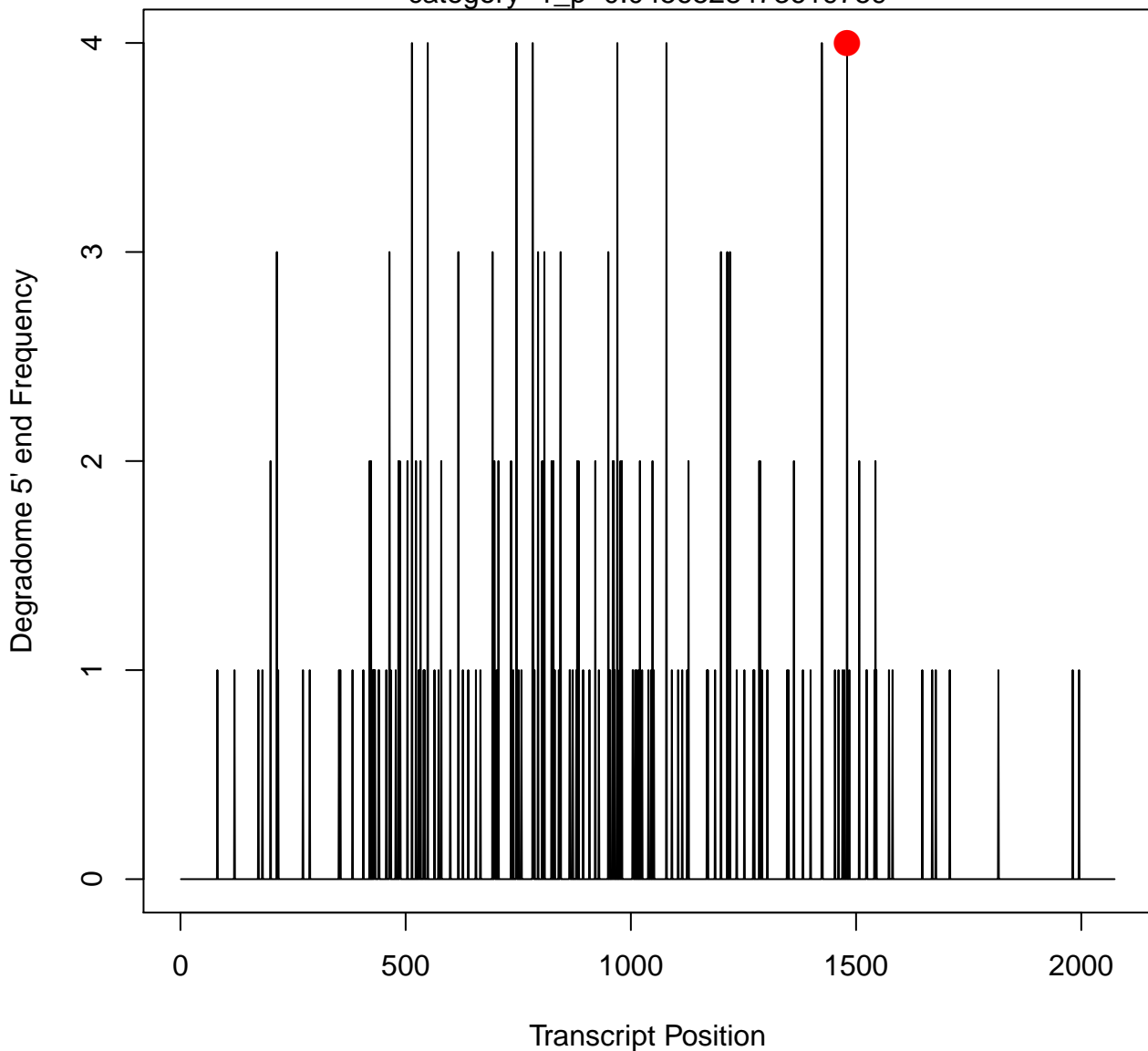

Supplement: Supplementary file 6 [file Data_Sheet_6.zip › Sit-miR169a_Seita.7G270500.1_1480_TPlot.pdf]

**T=Seita.3G038000.1\_Q=Sit-miR169b\_S=685**

category=2\_p=0.837098735155132

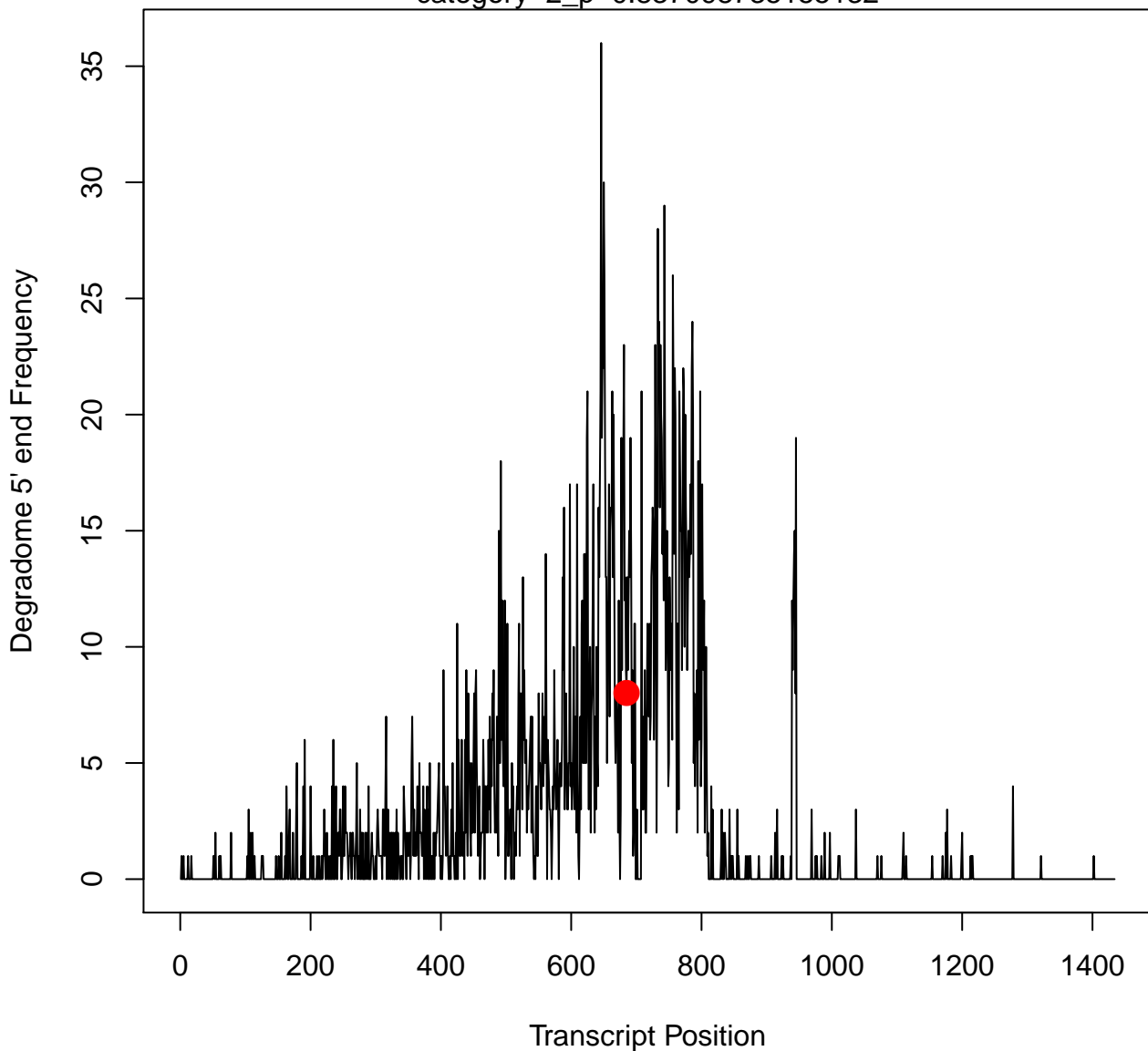

Supplement: Supplementary file 6 [file Data_Sheet_6.zip › Sit-miR169b_Seita.3G038000.1_685_TPlot.pdf]

**T=Seita.9G257700.1\_Q=Sit-miR169c\_S=1133**

category=2\_p=0.824834941630751

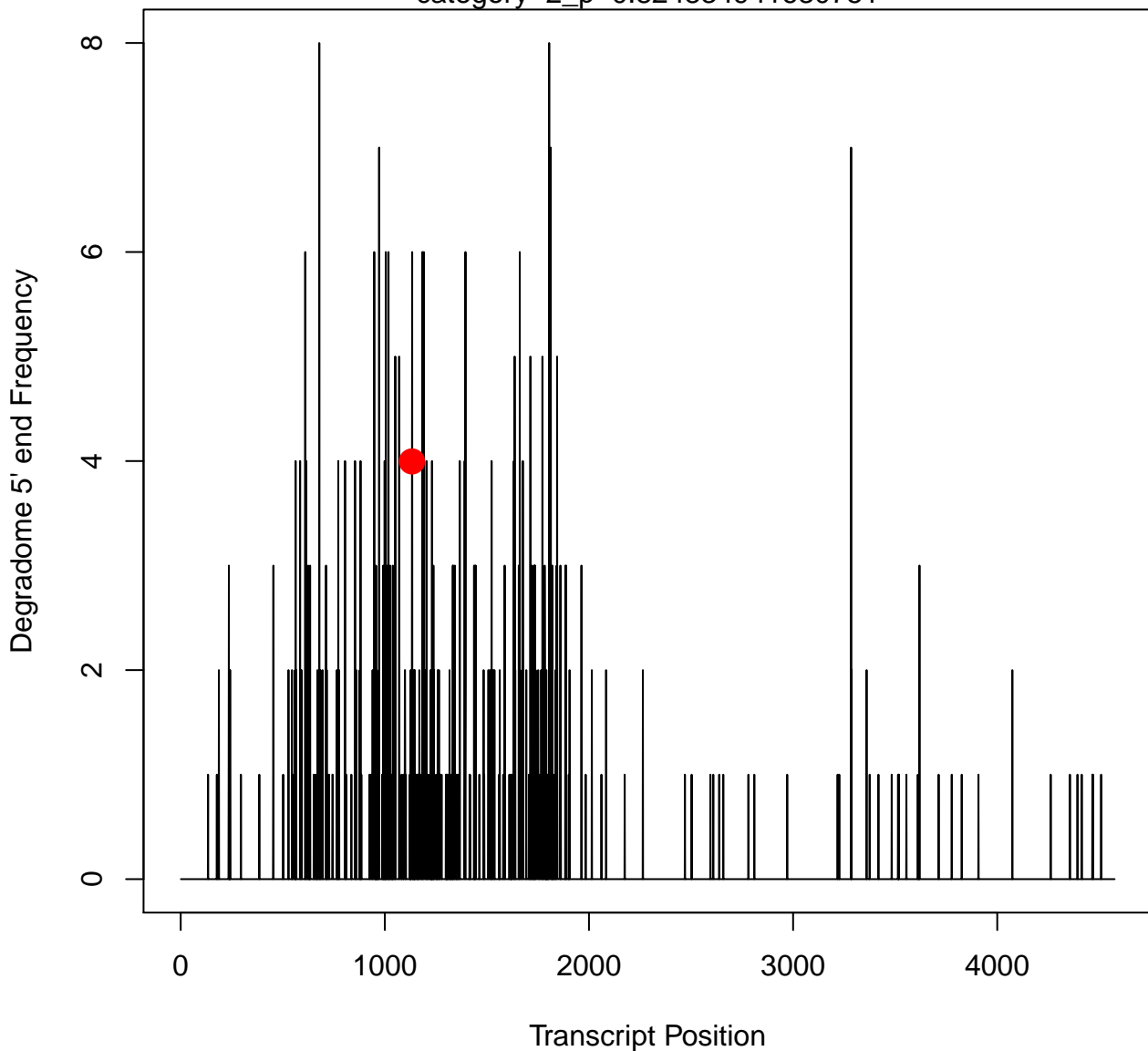

Supplement: Supplementary file 6 [file Data_Sheet_6.zip › Sit-miR169c_Seita.9G257700.1_1133_TPlot.pdf]

**T=Seita.1G016500.1\_Q=Sit-miR169e\_S=720**

category=2\_p=0.703524107046325

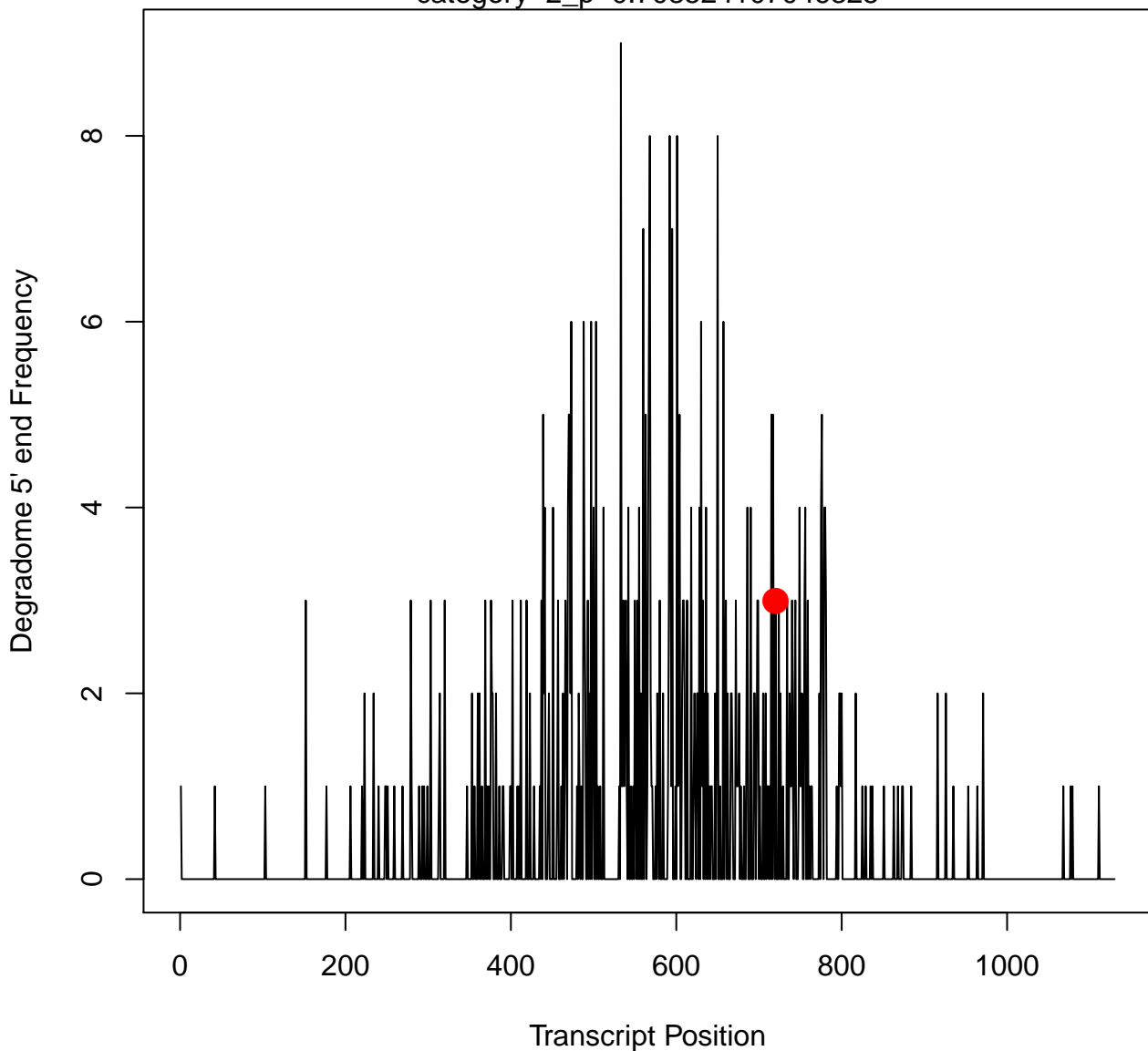

Supplement: Supplementary file 6 [file Data_Sheet_6.zip › Sit-miR169e_Seita.1G016500.1_720_TPlot.pdf]
