# Supplementary figures and images for: The Construction and Exploration of a Comprehensive MicroRNA Centered Regulatory Network in Foxtail Millet (Setaria italica L.) (part 11 of 14)
Source: Front Plant Sci. 2022 May 6;13:848474. doi: 10.3389/fpls.2022.848474 (PMC9121102; doi:10.3389/fpls.2022.848474)

**T=Seita.1G177900.1\_Q=Sit-miR1133\_S=1890**

category=2\_p=0.581472750744651

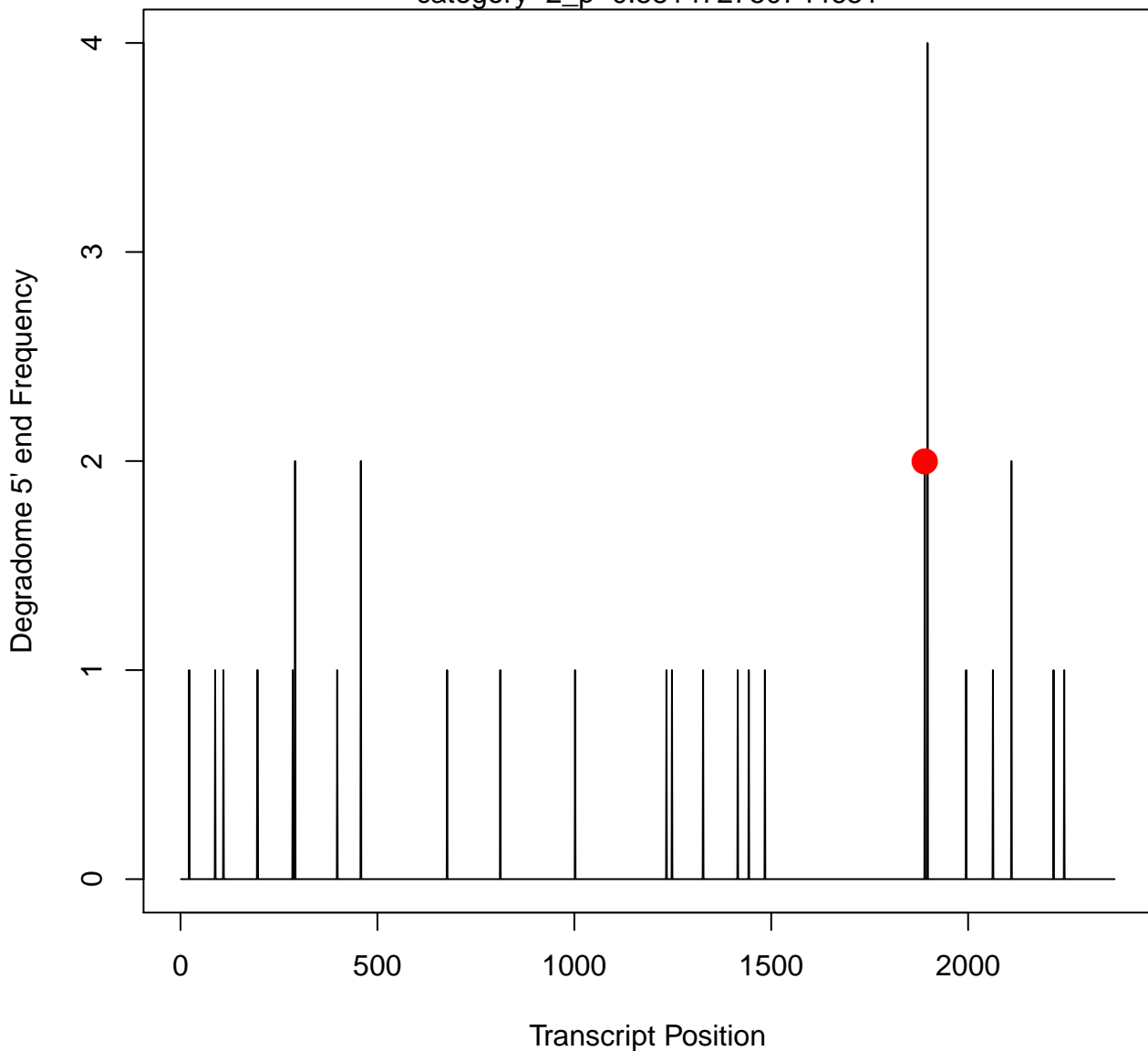

Supplement: Supplementary file 6 [file Data_Sheet_6.zip › Sit-miR1133_Seita.1G177900.1_1890_TPlot.pdf]

**T=Seita.3G120600.1\_Q=Sit-miR1133\_S=1175**

category=2\_p=0.376121012496603

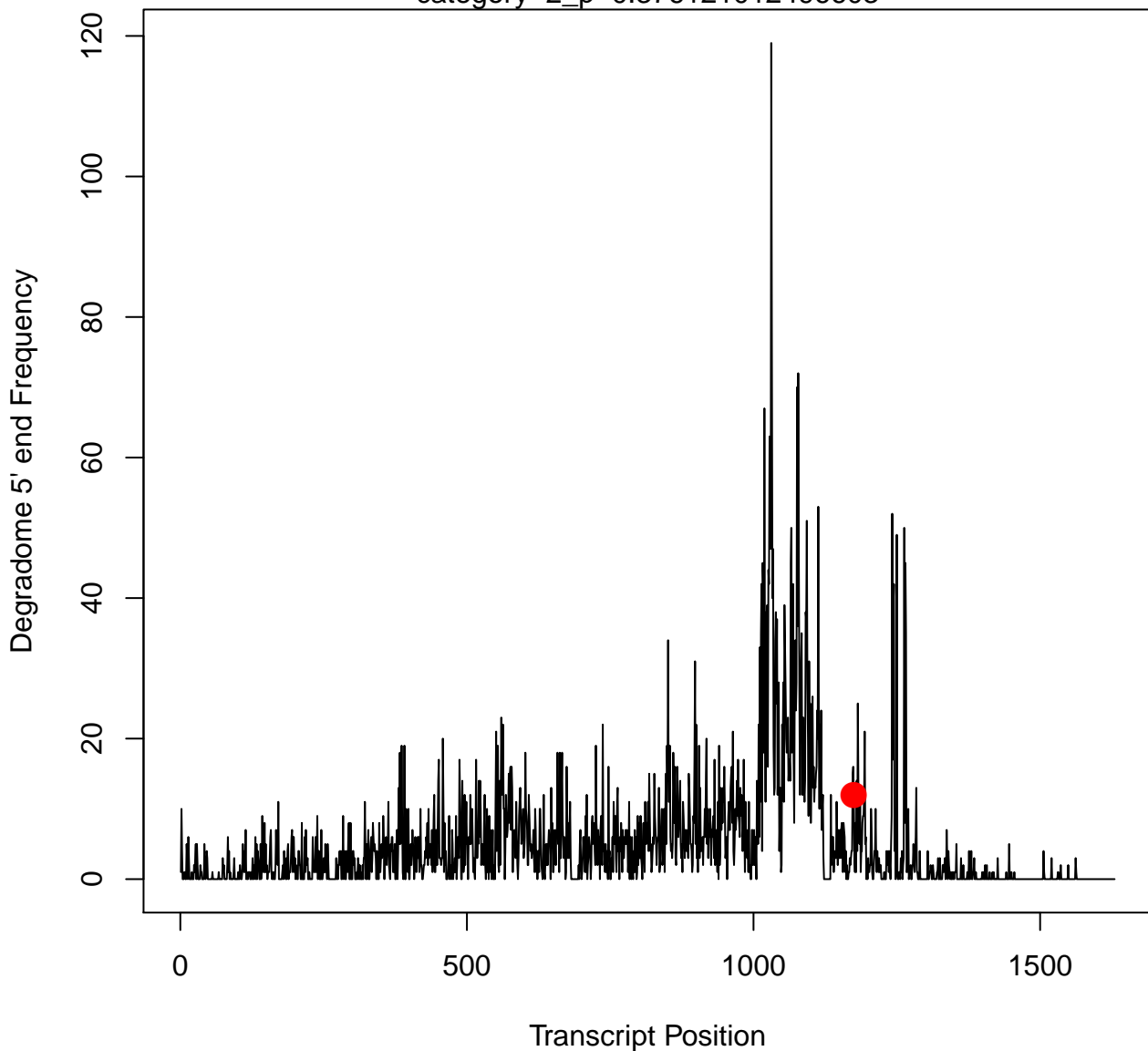

Supplement: Supplementary file 6 [file Data_Sheet_6.zip › Sit-miR1133_Seita.3G120600.1_1175_TPlot.pdf]

**T=Seita.3G163400.1\_Q=Sit-miR1133\_S=876**

category=2\_p=0.999898968455106

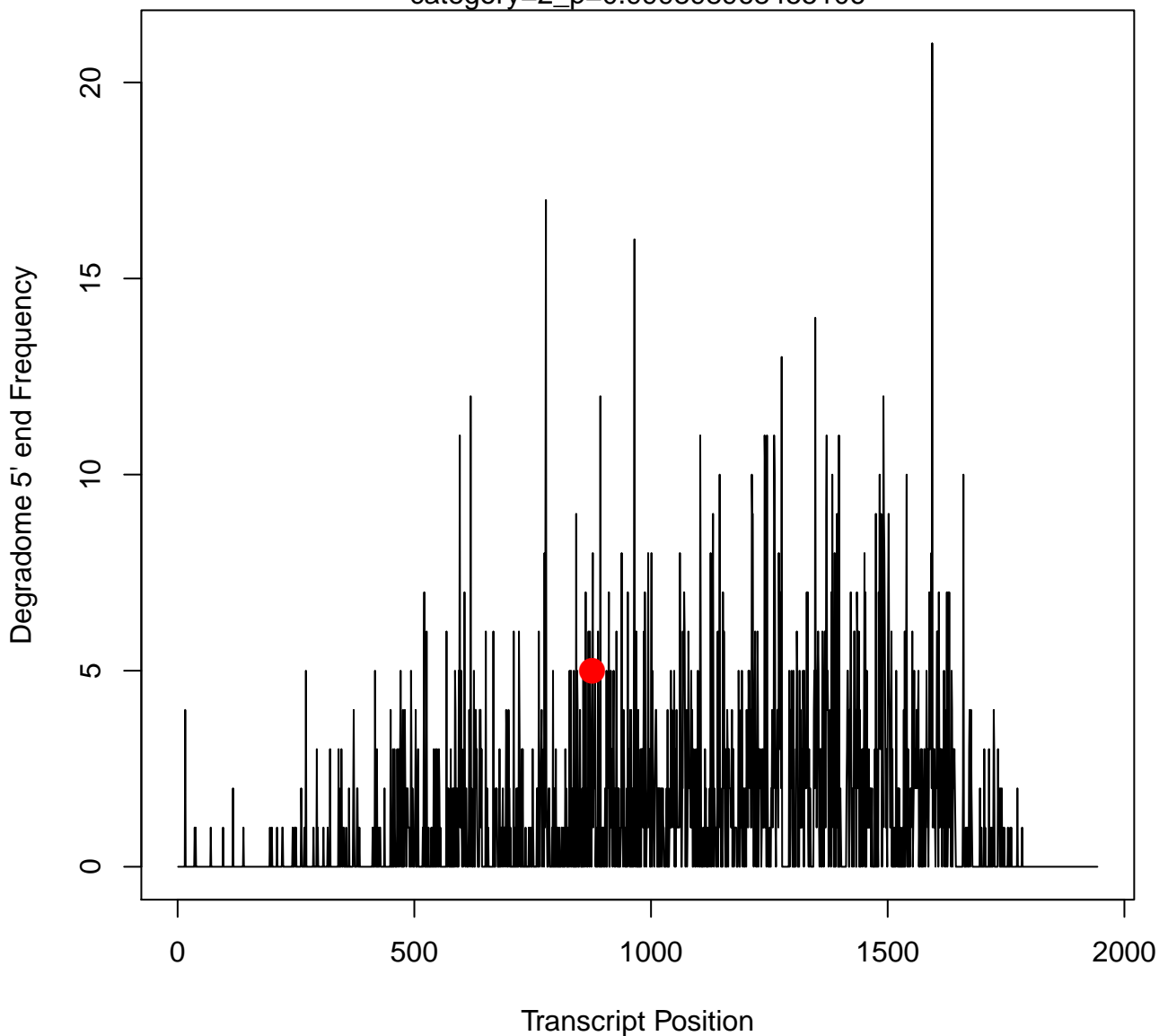

Supplement: Supplementary file 6 [file Data_Sheet_6.zip › Sit-miR1133_Seita.3G163400.1_876_TPlot.pdf]

**T=Seita.7G185200.1\_Q=Sit-miR1133\_S=185**

category=2\_p=0.999591421826625

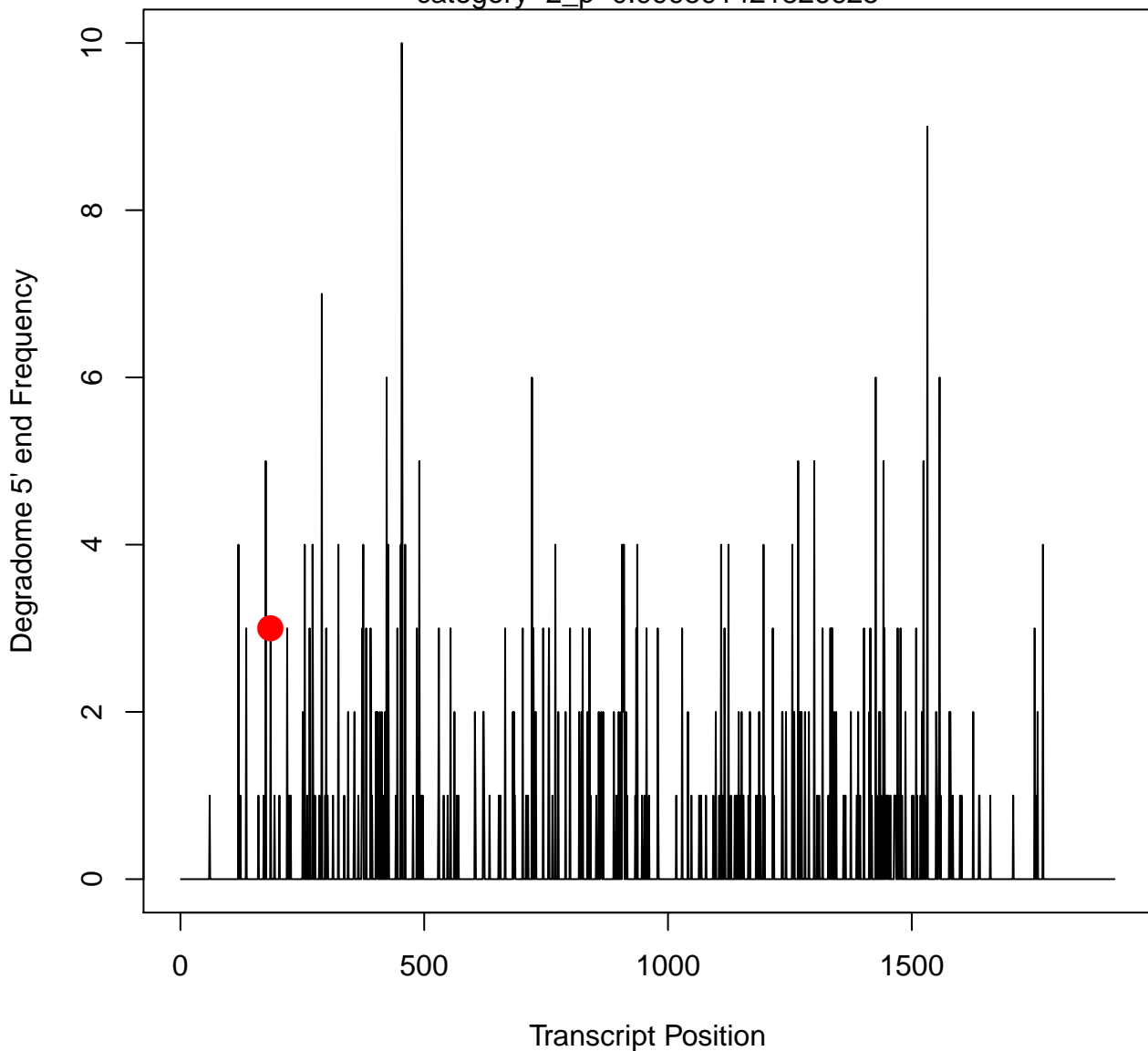

Supplement: Supplementary file 6 [file Data_Sheet_6.zip › Sit-miR1133_Seita.7G185200.1_185_TPlot.pdf]

**T=Seita.2G034500.1\_Q=Sit-miR1432\_S=427**

category=2\_p=0.999909390765507

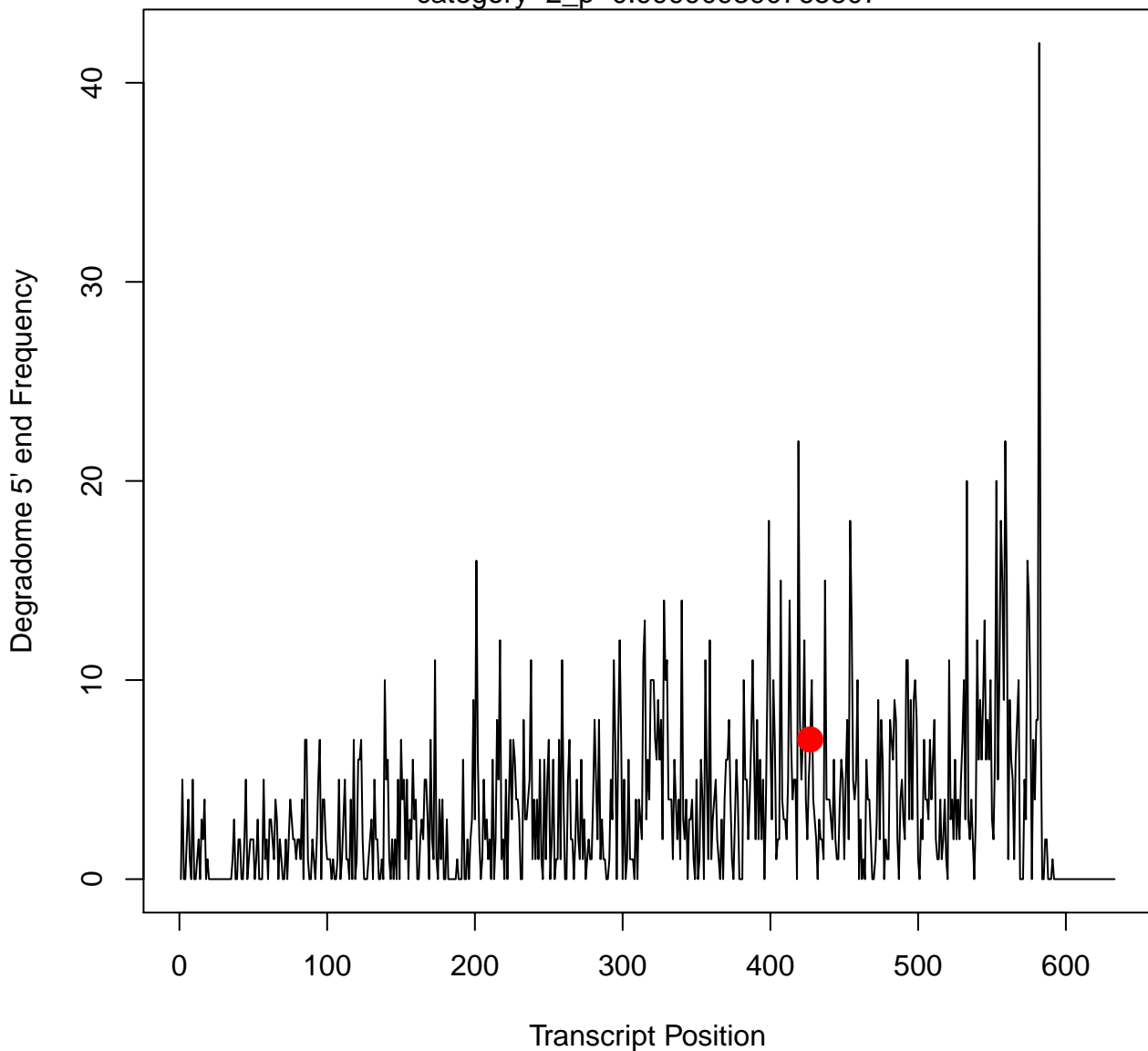

Supplement: Supplementary file 6 [file Data_Sheet_6.zip › Sit-miR1432_Seita.2G034500.1_427_TPlot.pdf]

**T=Seita.2G084300.1\_Q=Sit-miR1432\_S=1096**

category=2\_p=0.999671370839591

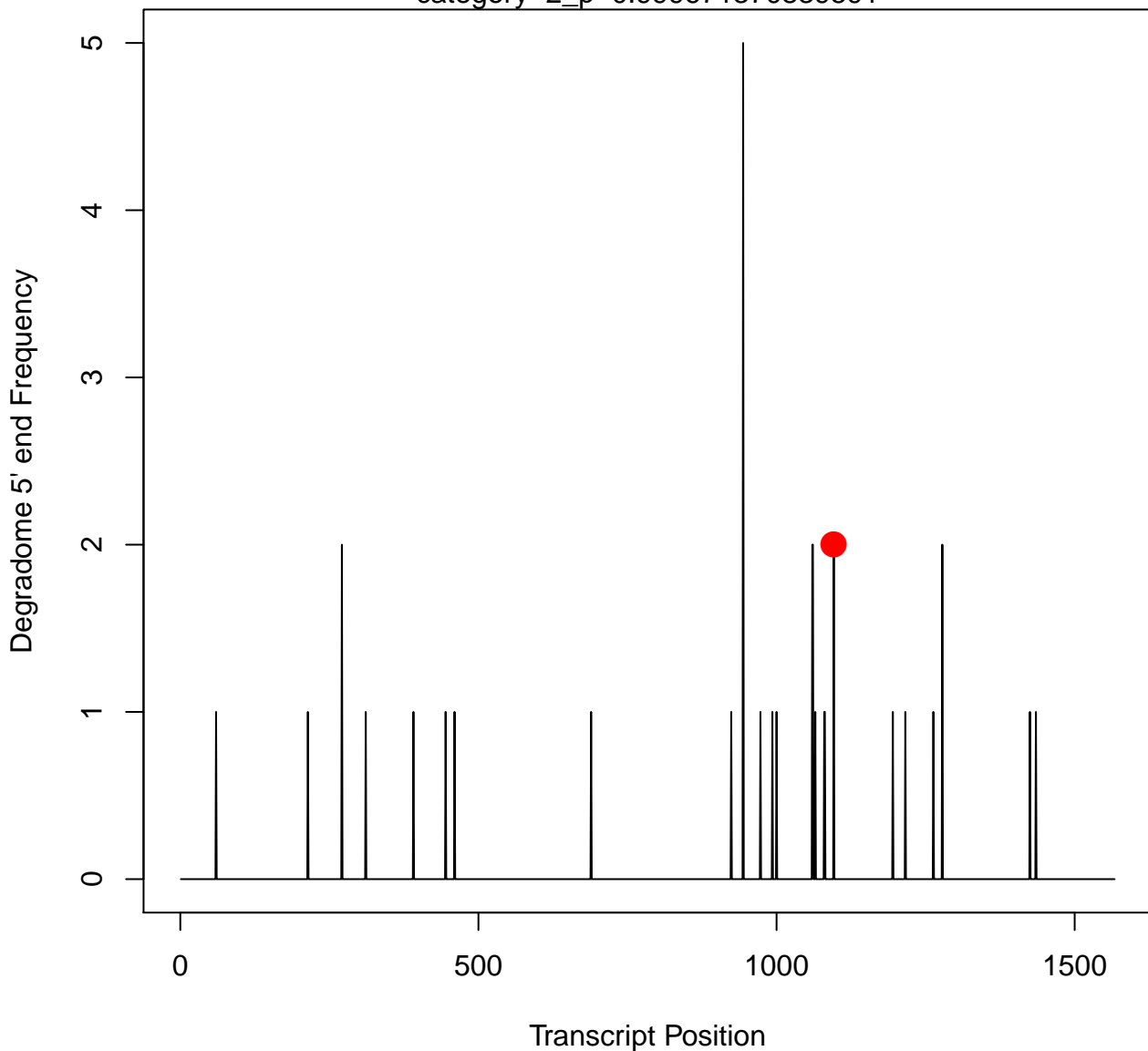

Supplement: Supplementary file 6 [file Data_Sheet_6.zip › Sit-miR1432_Seita.2G084300.1_1096_TPlot.pdf]

**T=Seita.3G129700.1\_Q=Sit-miR1432\_S=592**

category=2\_p=0.541723575281072

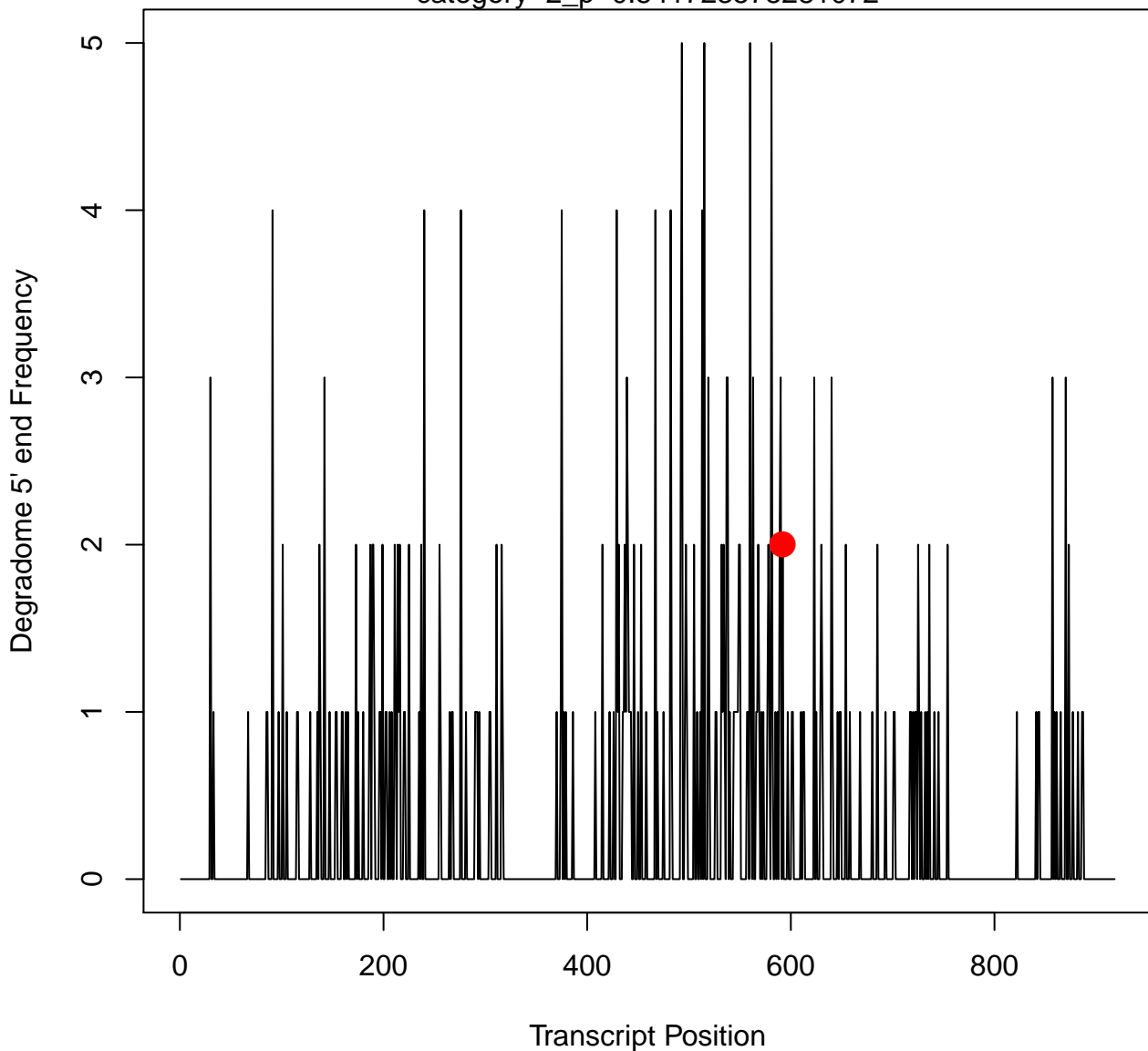

Supplement: Supplementary file 6 [file Data_Sheet_6.zip › Sit-miR1432_Seita.3G129700.1_592_TPlot.pdf]

**T=Seita.4G149100.1\_Q=Sit-miR1432\_S=350**

category=2\_p=0.999955349842075

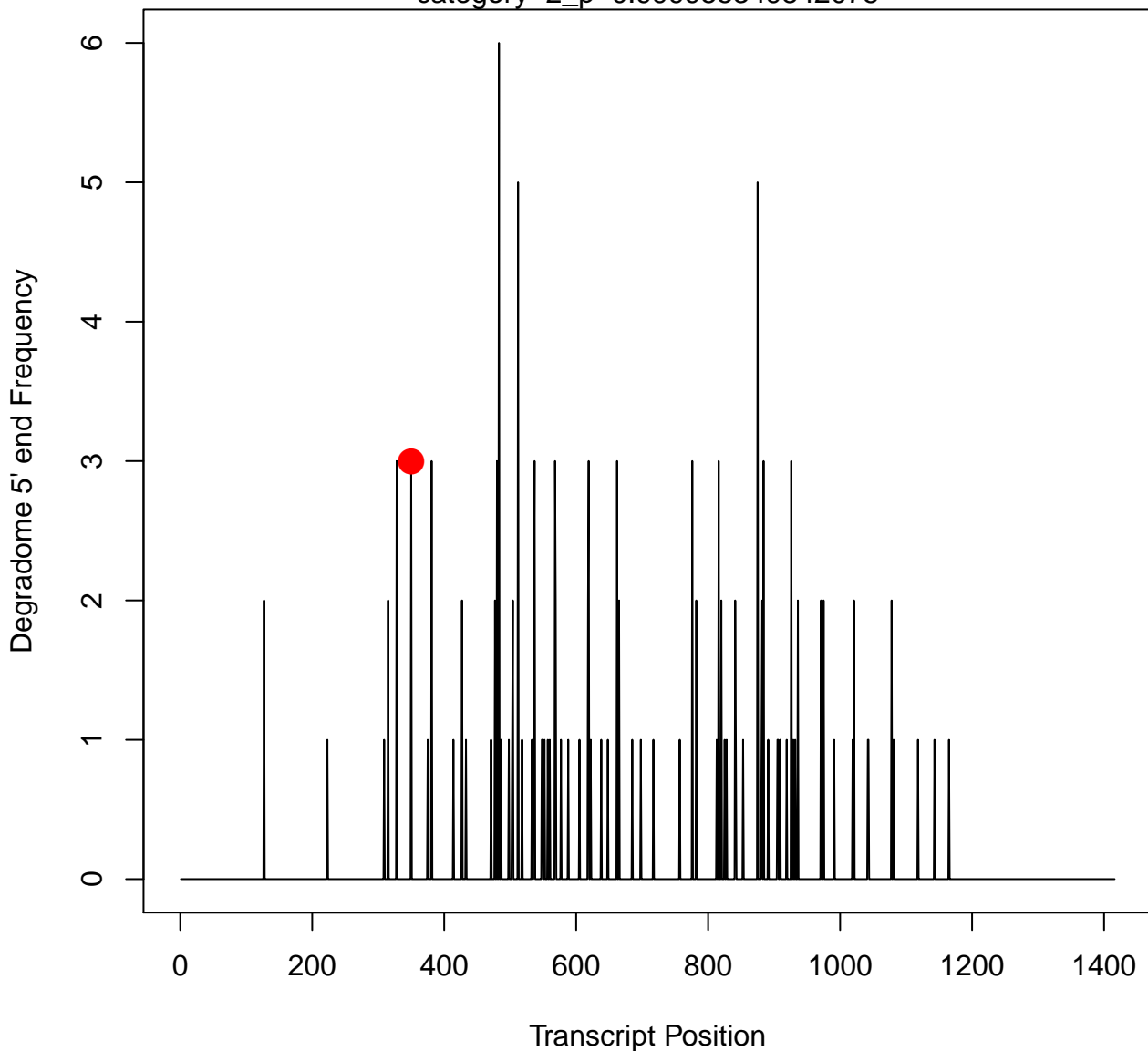

Supplement: Supplementary file 6 [file Data_Sheet_6.zip › Sit-miR1432_Seita.4G149100.1_350_TPlot.pdf]

**T=Seita.5G126800.1\_Q=Sit-miR1432\_S=988**

category=2\_p=0.353063179858072

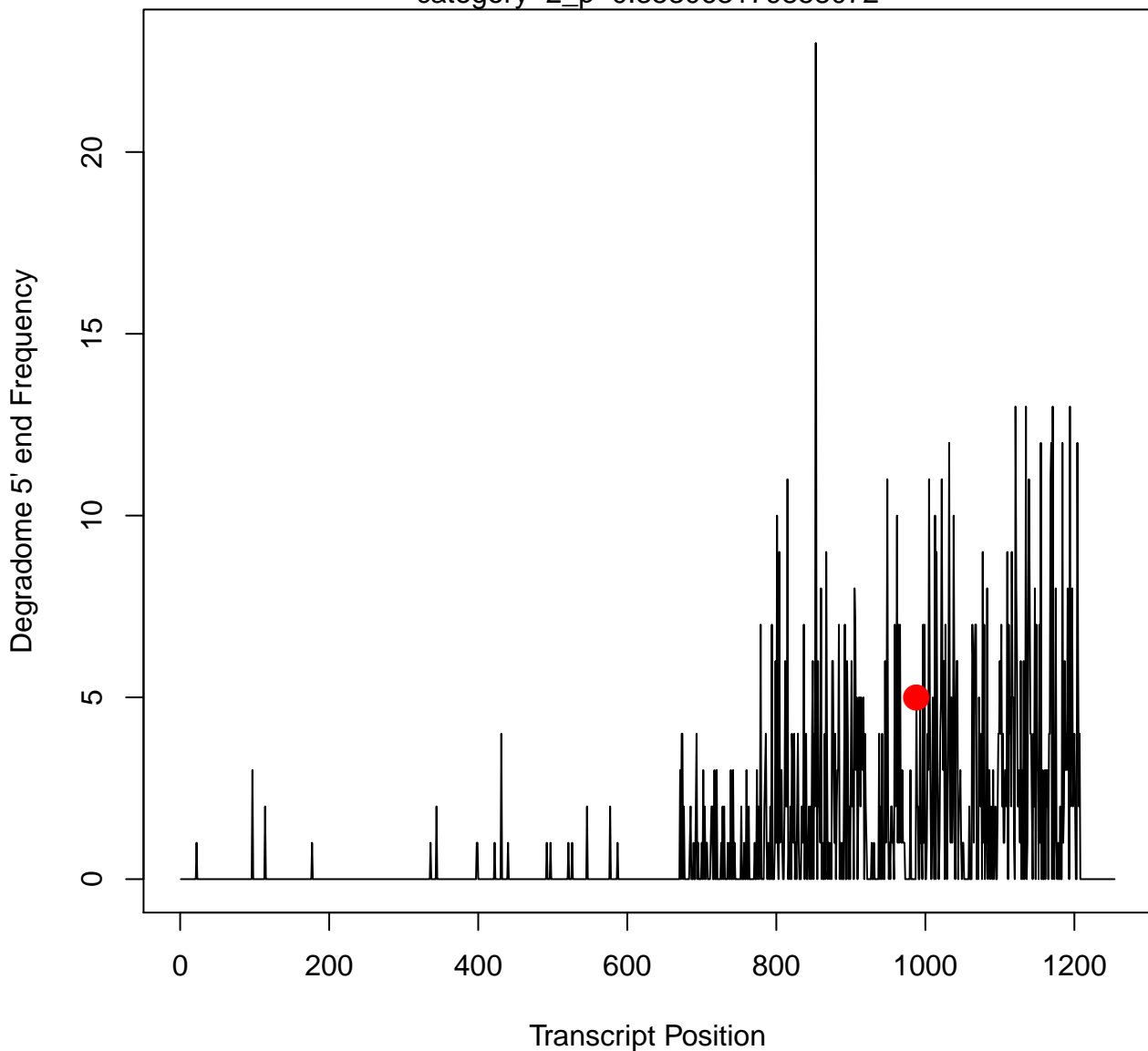

Supplement: Supplementary file 6 [file Data_Sheet_6.zip › Sit-miR1432_Seita.5G126800.1_988_TPlot.pdf]

**T=Seita.5G206500.1\_Q=Sit-miR1432\_S=2119**

category=2\_p=0.93773493894547

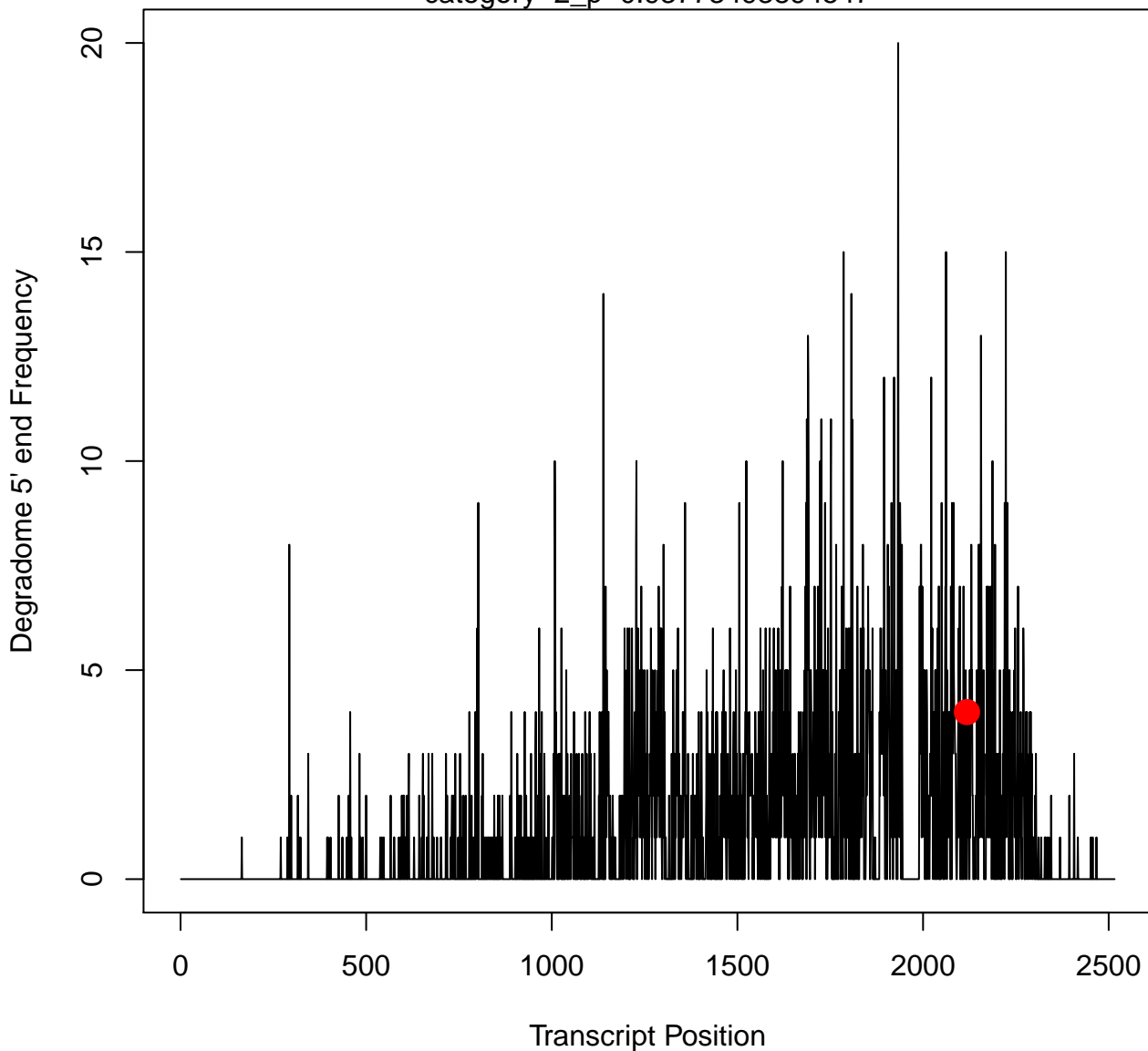

Supplement: Supplementary file 6 [file Data_Sheet_6.zip › Sit-miR1432_Seita.5G206500.1_2119_TPlot.pdf]

**T=Seita.9G067000.1\_Q=Sit-miR1432\_S=834**

category=2\_p=0.998931055392807

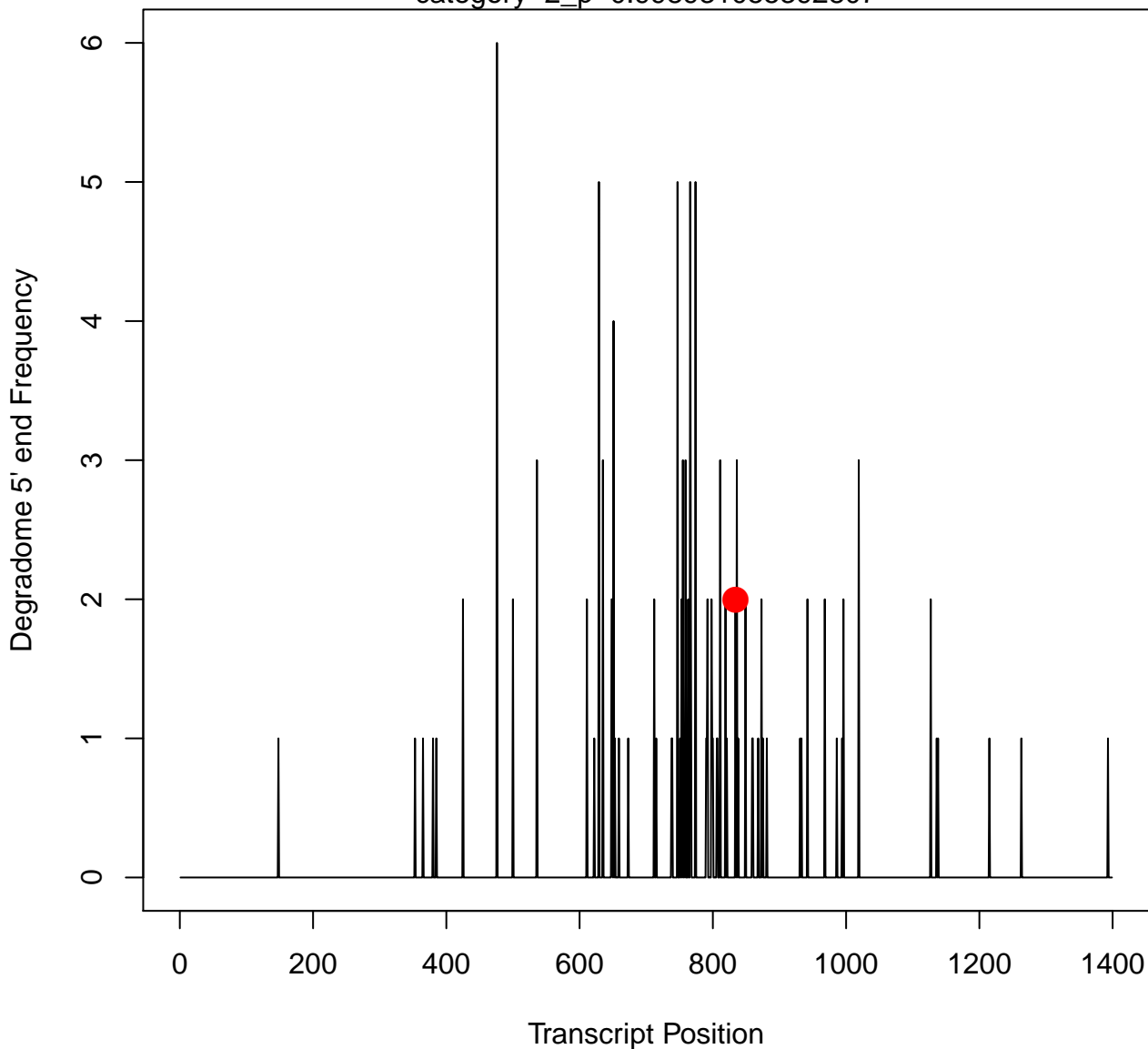

Supplement: Supplementary file 6 [file Data_Sheet_6.zip › Sit-miR1432_Seita.9G067000.1_834_TPlot.pdf]

**T=Seita.3G409000.1\_Q=Sit-miR156a\_S=1545**

category=2\_p=0.942094289261479

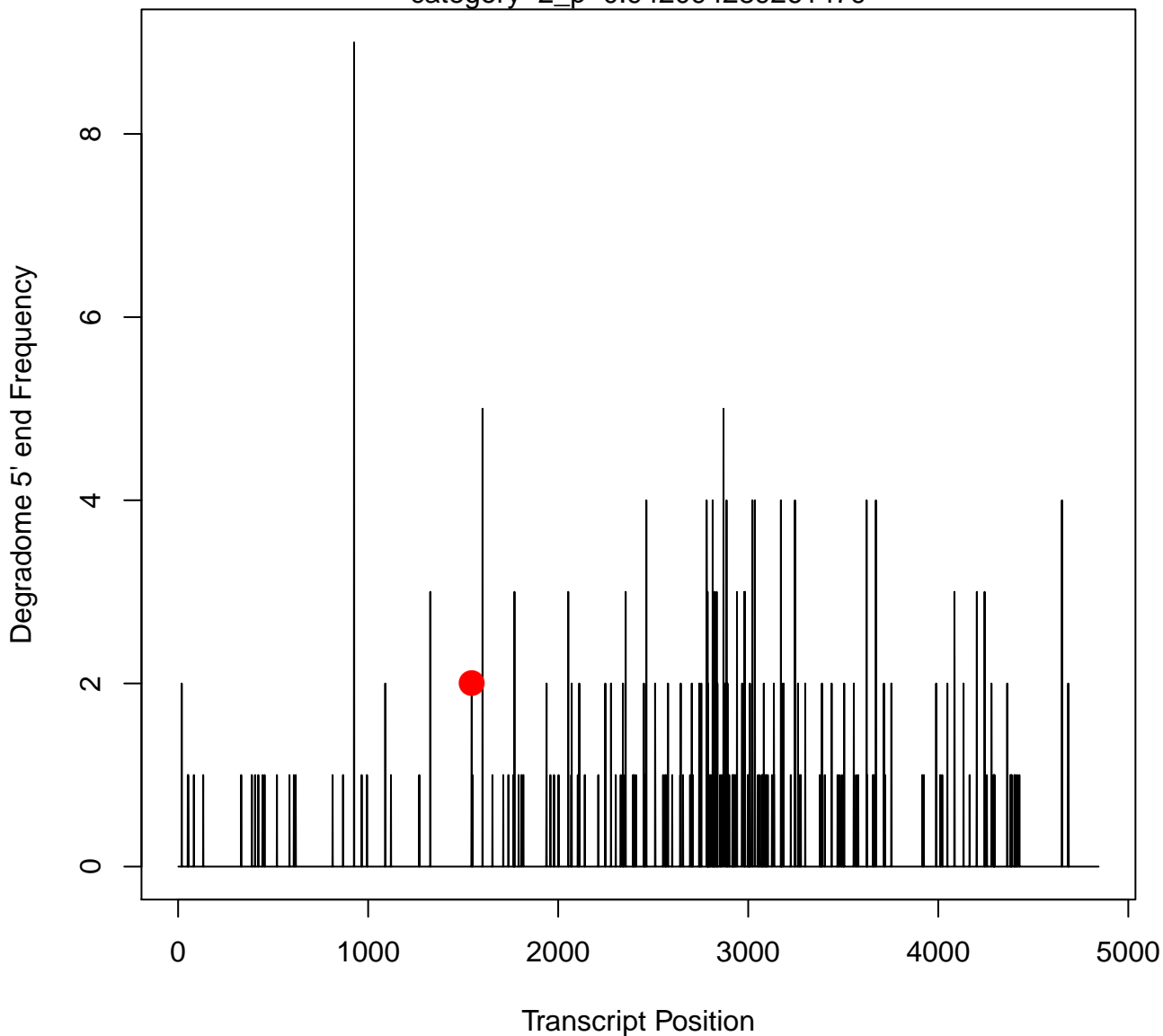

Supplement: Supplementary file 6 [file Data_Sheet_6.zip › Sit-miR156a_Seita.3G409000.1_1545_TPlot.pdf]

**T=Seita.5G424000.1\_Q=Sit-miR156a\_S=1856**

category=2\_p=0.977048933606814

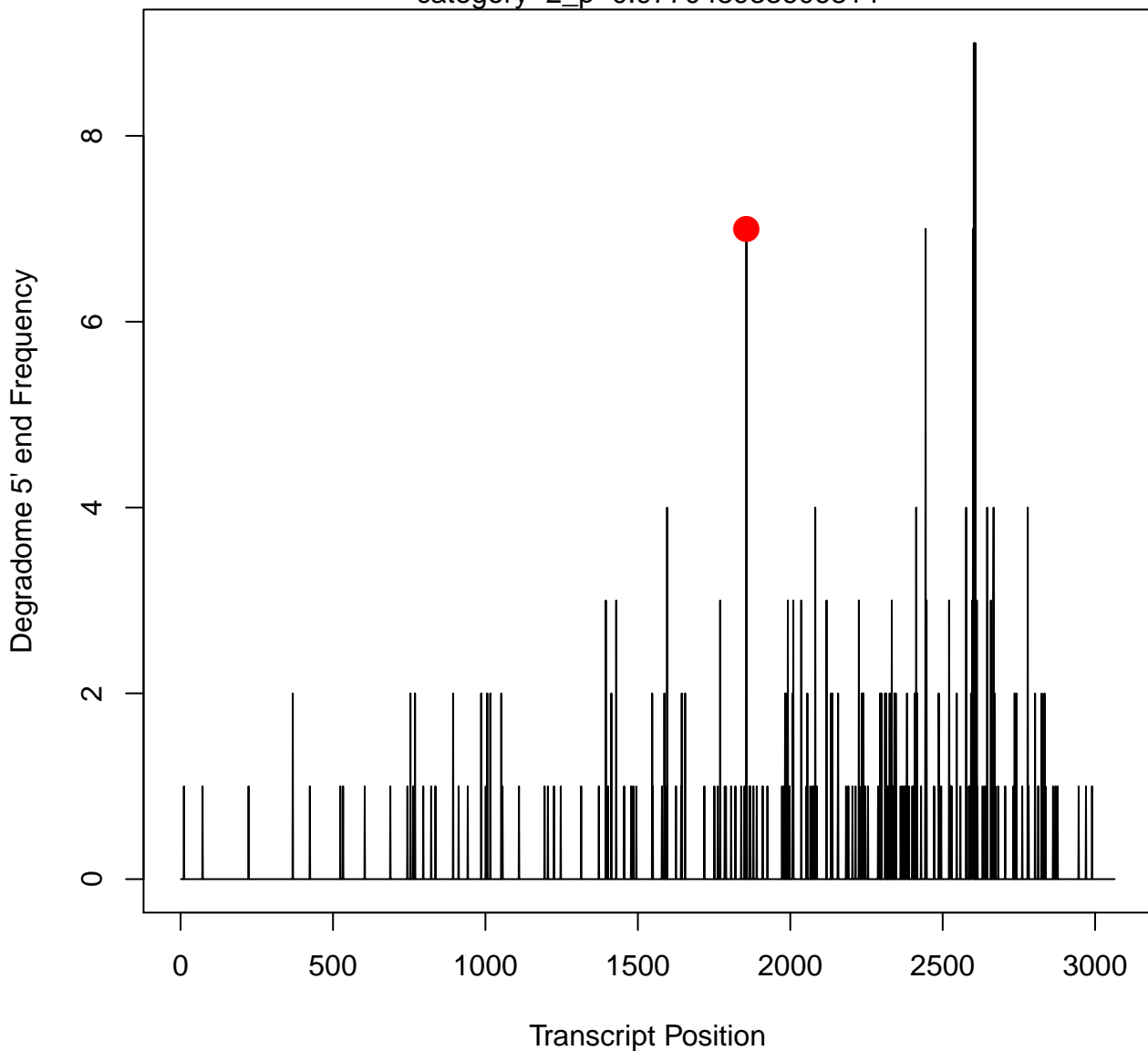

Supplement: Supplementary file 6 [file Data_Sheet_6.zip › Sit-miR156a_Seita.5G424000.1_1856_TPlot.pdf]

**T=Seita.7G162400.1\_Q=Sit-miR156a\_S=1616**

category=2\_p=0.470124841191333

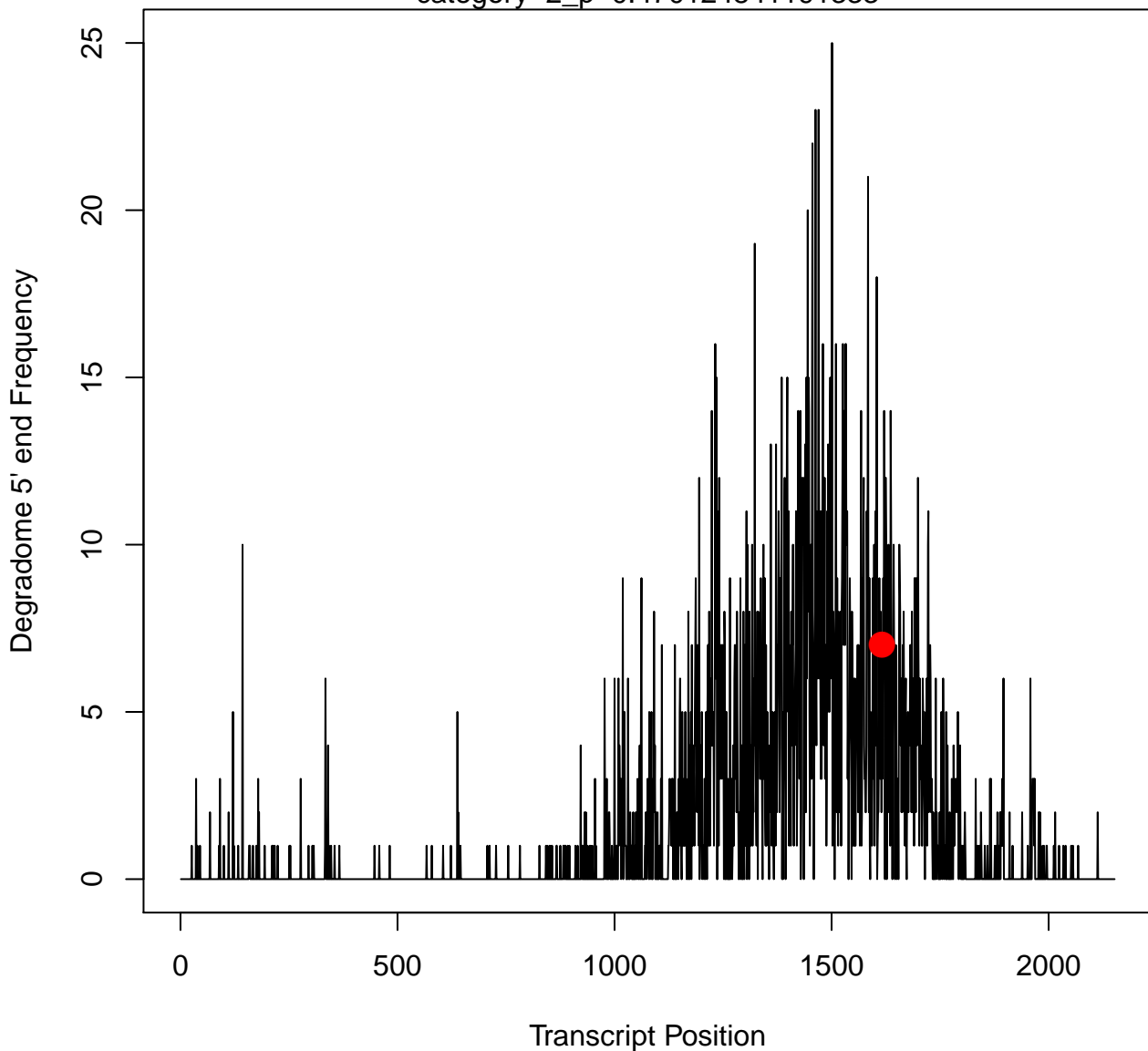

Supplement: Supplementary file 6 [file Data_Sheet_6.zip › Sit-miR156a_Seita.7G162400.1_1616_TPlot.pdf]

**T=Seita.2G065200.1\_Q=Sit-miR156d\_S=1207**

category=2\_p=0.959718439395439

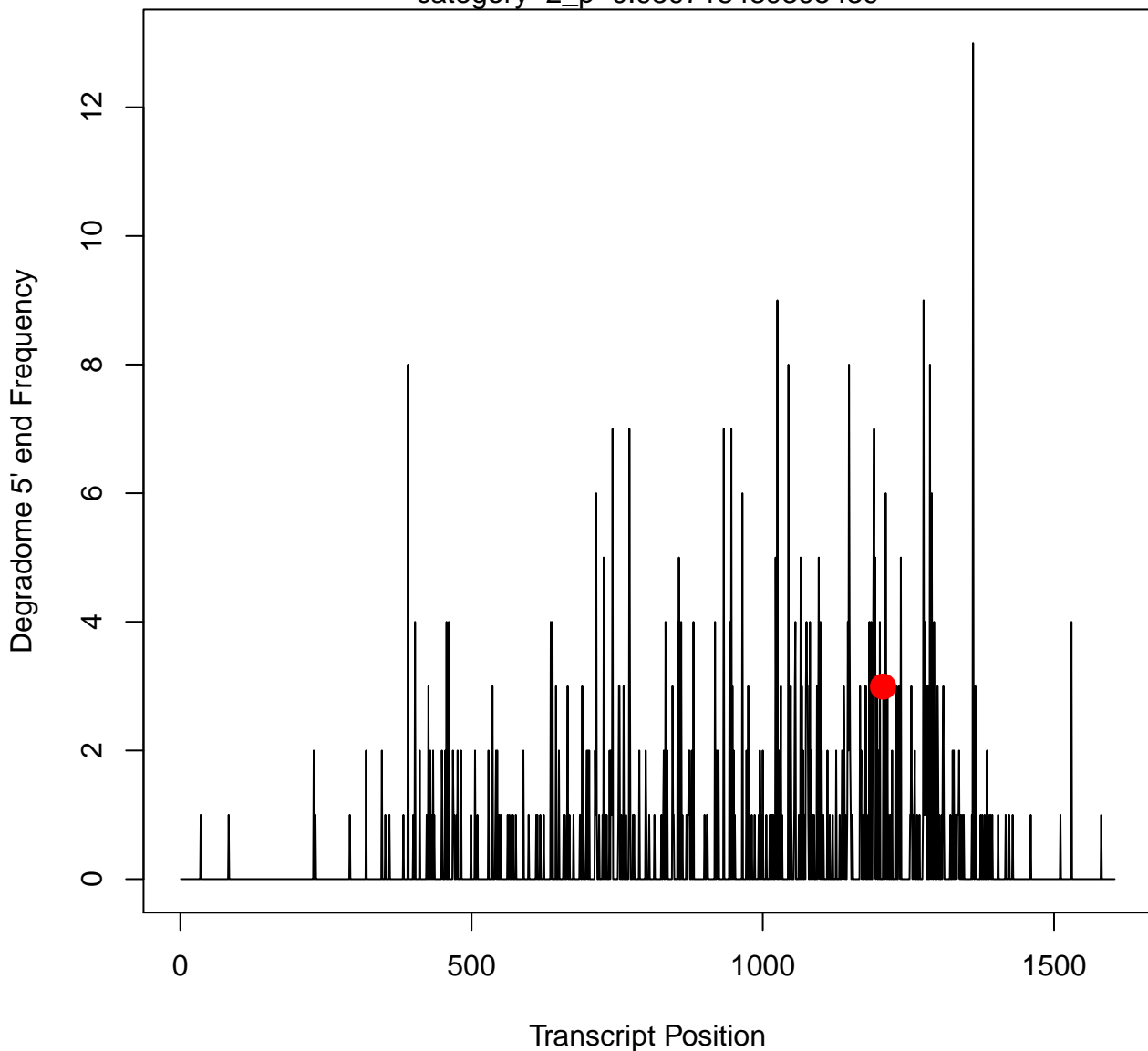

Supplement: Supplementary file 6 [file Data_Sheet_6.zip › Sit-miR156d_Seita.2G065200.1_1207_TPlot.pdf]

**T=Seita.2G254300.1\_Q=Sit-miR156d\_S=1267**

category=2\_p=0.0356415524988544

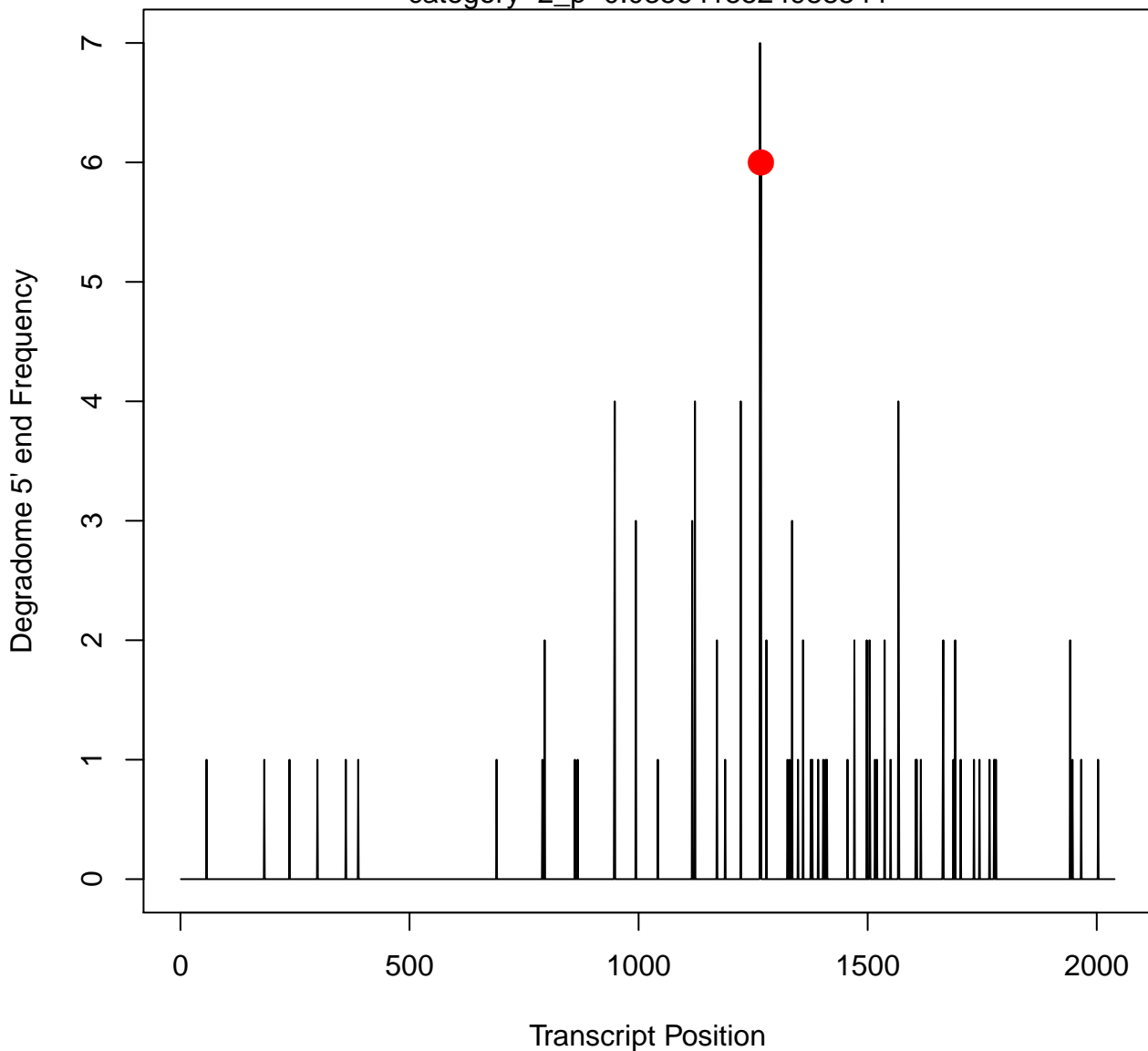

Supplement: Supplementary file 6 [file Data_Sheet_6.zip › Sit-miR156d_Seita.2G254300.1_1267_TPlot.pdf]

**T=Seita.3G205200.1\_Q=Sit-miR156d\_S=48**

category=2\_p=0.968183202259483

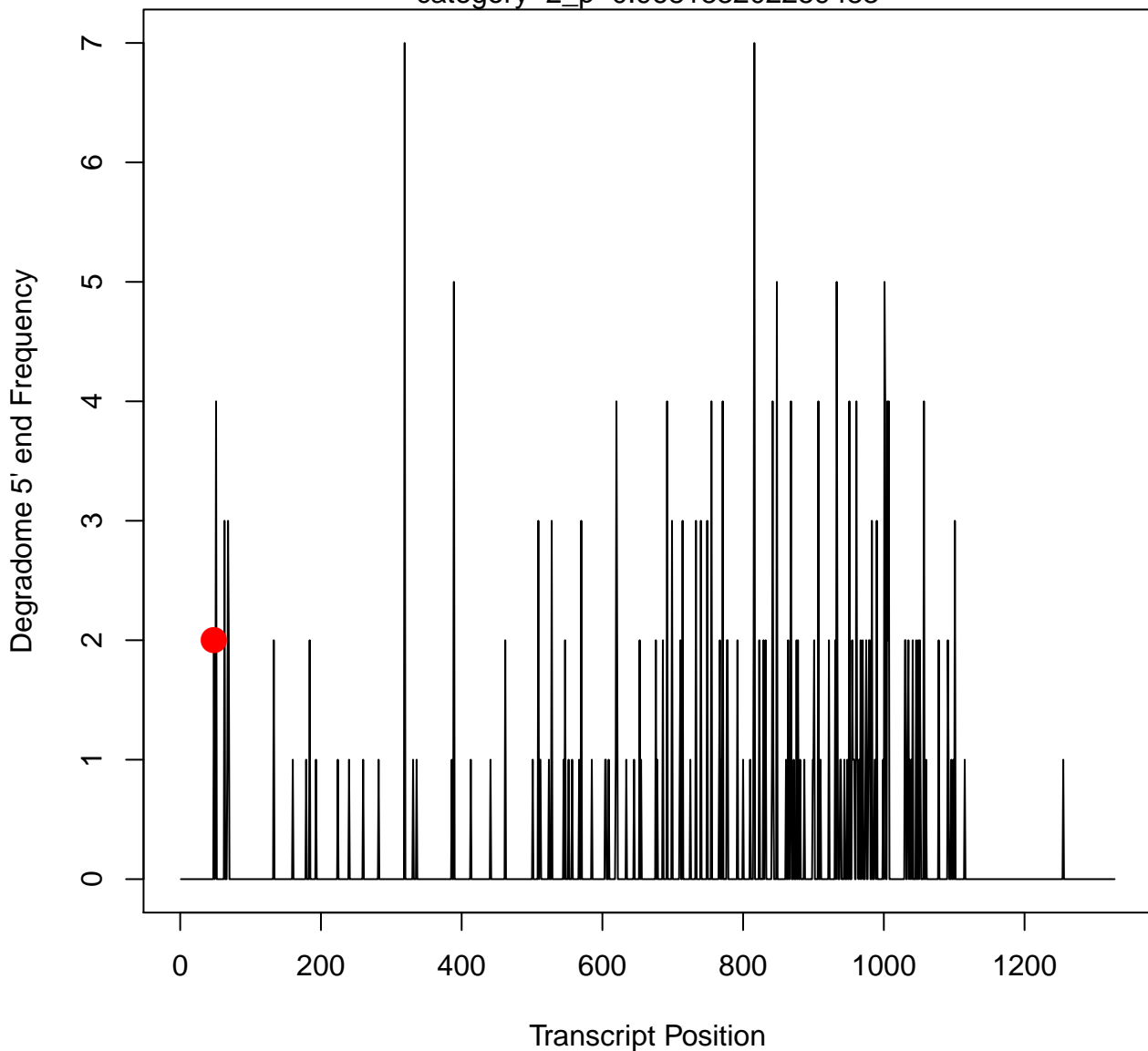

Supplement: Supplementary file 6 [file Data_Sheet_6.zip › Sit-miR156d_Seita.3G205200.1_48_TPlot.pdf]

**T=Seita.3G222000.1\_Q=Sit-miR156d\_S=2038**

category=2\_p=0.761537374896809

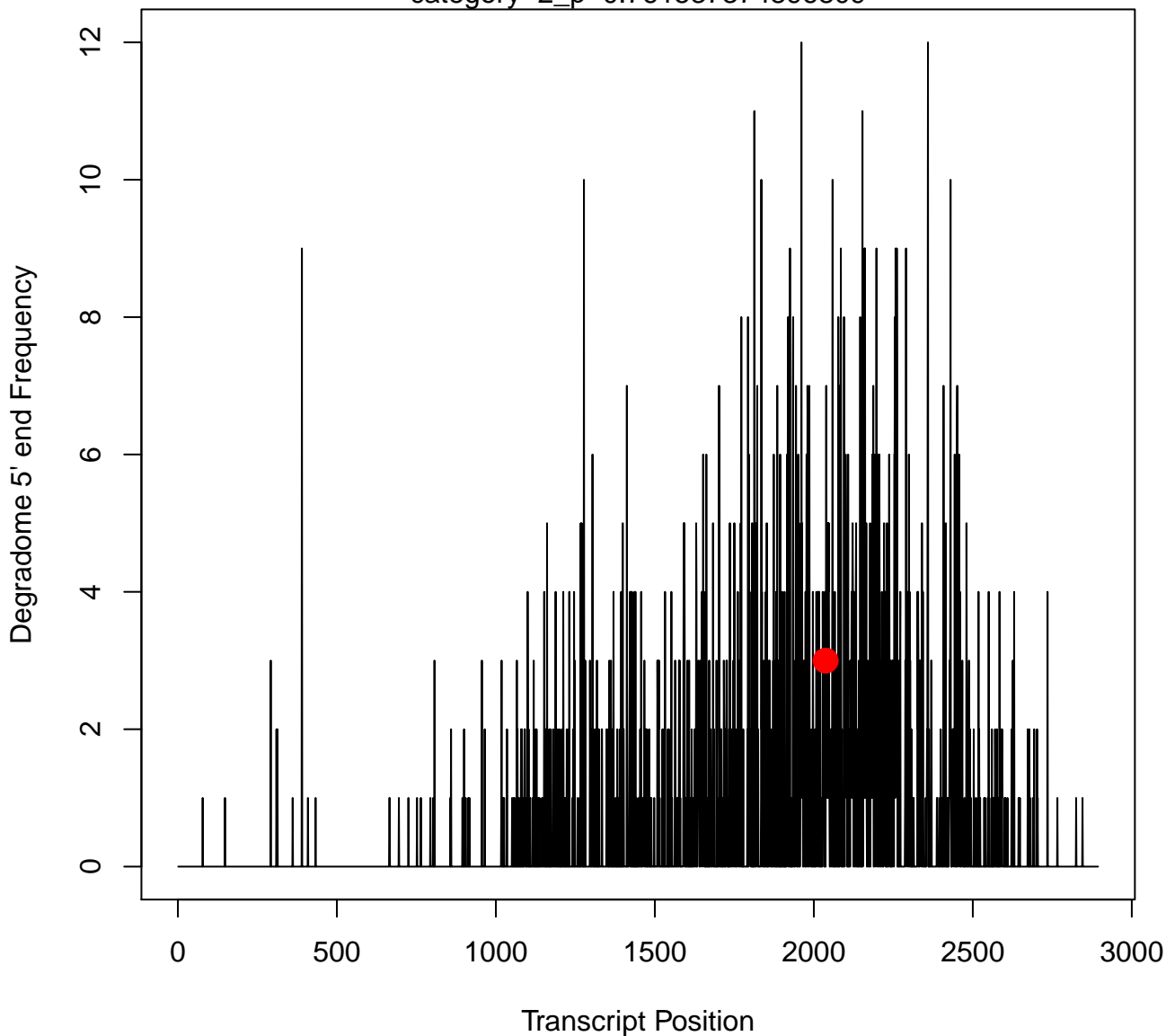

Supplement: Supplementary file 6 [file Data_Sheet_6.zip › Sit-miR156d_Seita.3G222000.1_2038_TPlot.pdf]

**T=Seita.9G485400.1\_Q=Sit-miR156d\_S=1791**

category=2\_p=0.971465390435024

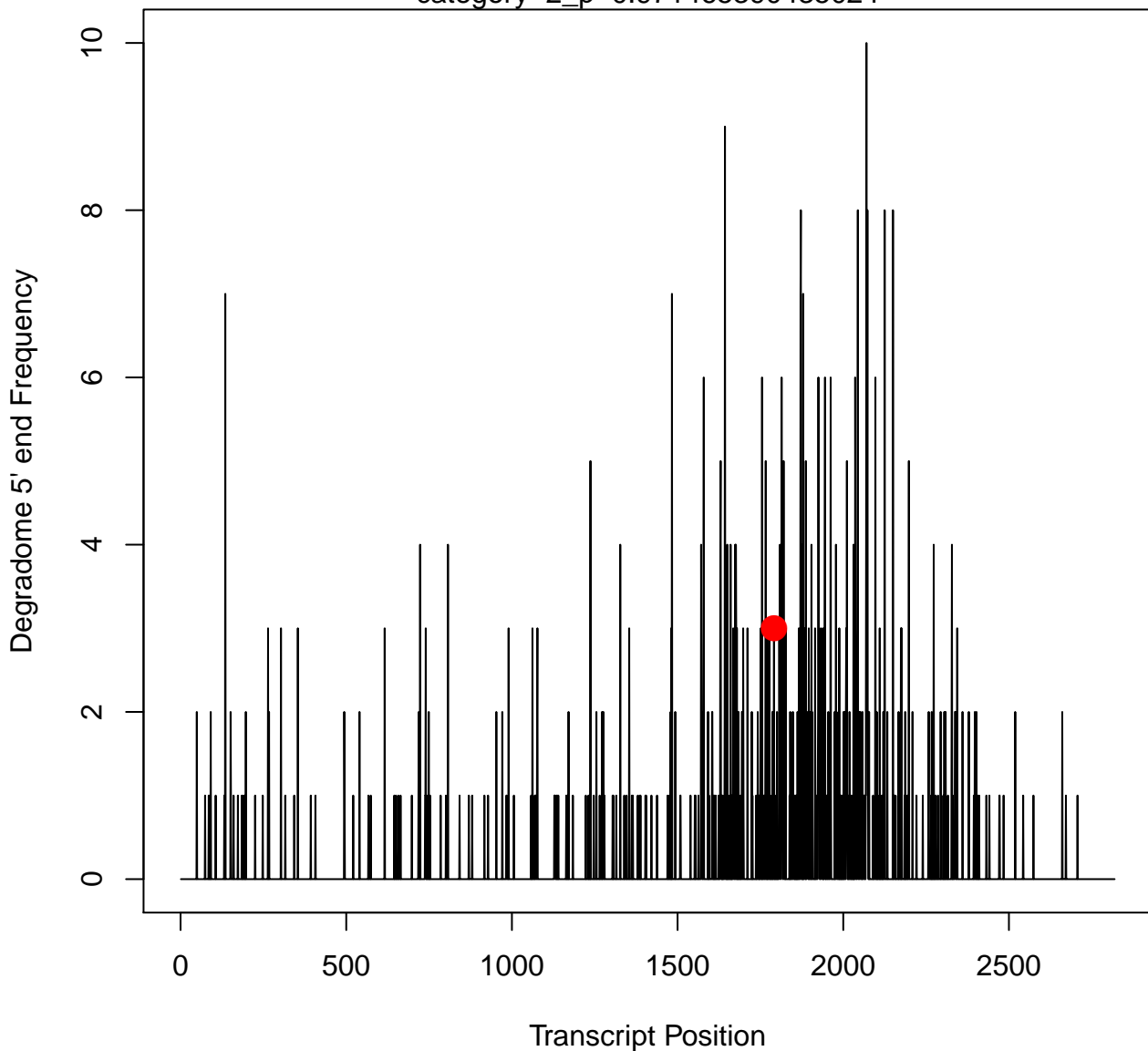

Supplement: Supplementary file 6 [file Data_Sheet_6.zip › Sit-miR156d_Seita.9G485400.1_1791_TPlot.pdf]

**T=Seita.4G270400.1\_Q=Sit-miR156f\_S=2084**

category=2\_p=0.0179824606957646

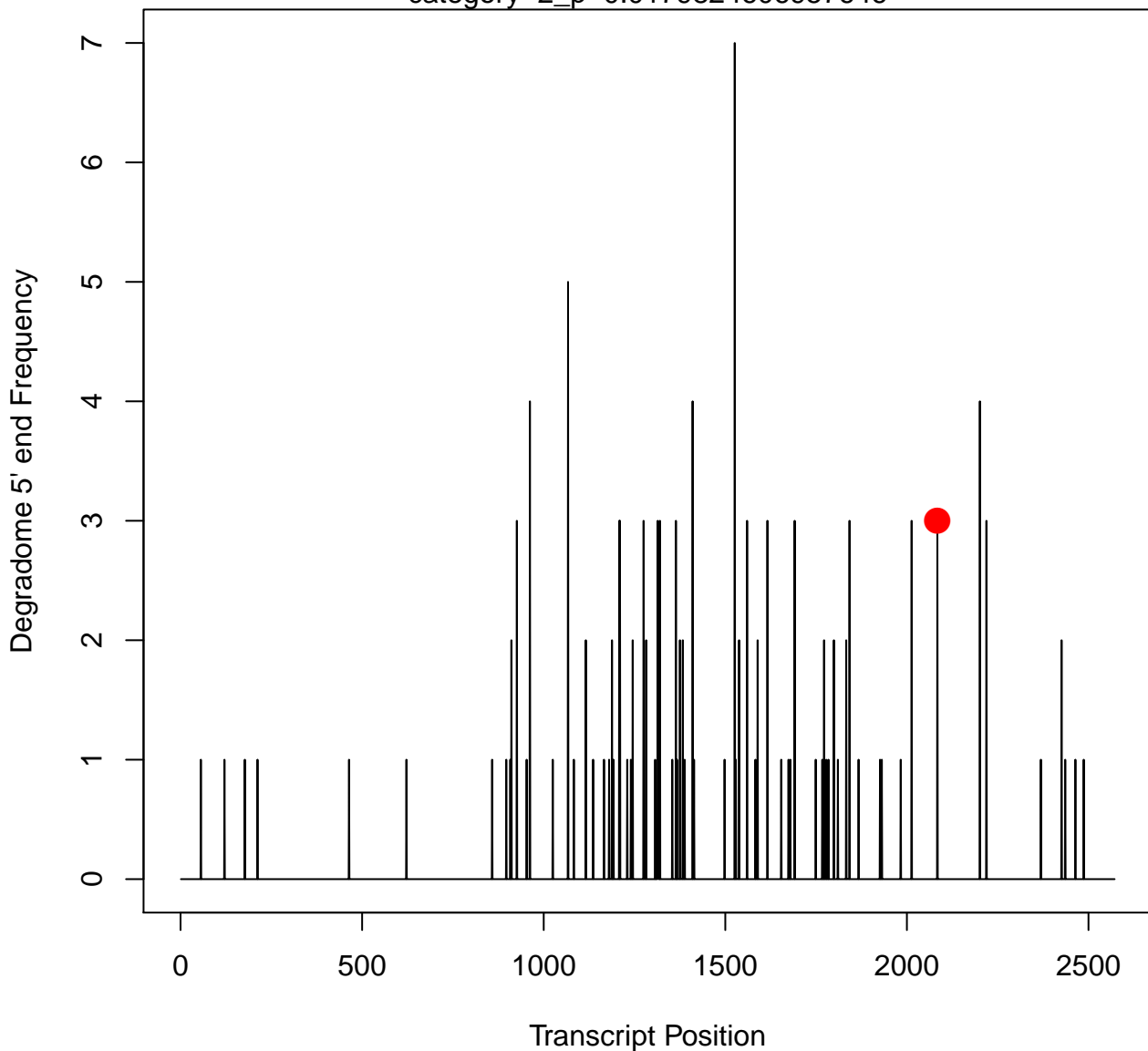

Supplement: Supplementary file 6 [file Data_Sheet_6.zip › Sit-miR156f_Seita.4G270400.1_2084_TPlot.pdf]

**T=Seita.2G254300.1\_Q=Sit-miR156i\_S=1266**

category=2\_p=0.0700127847331802

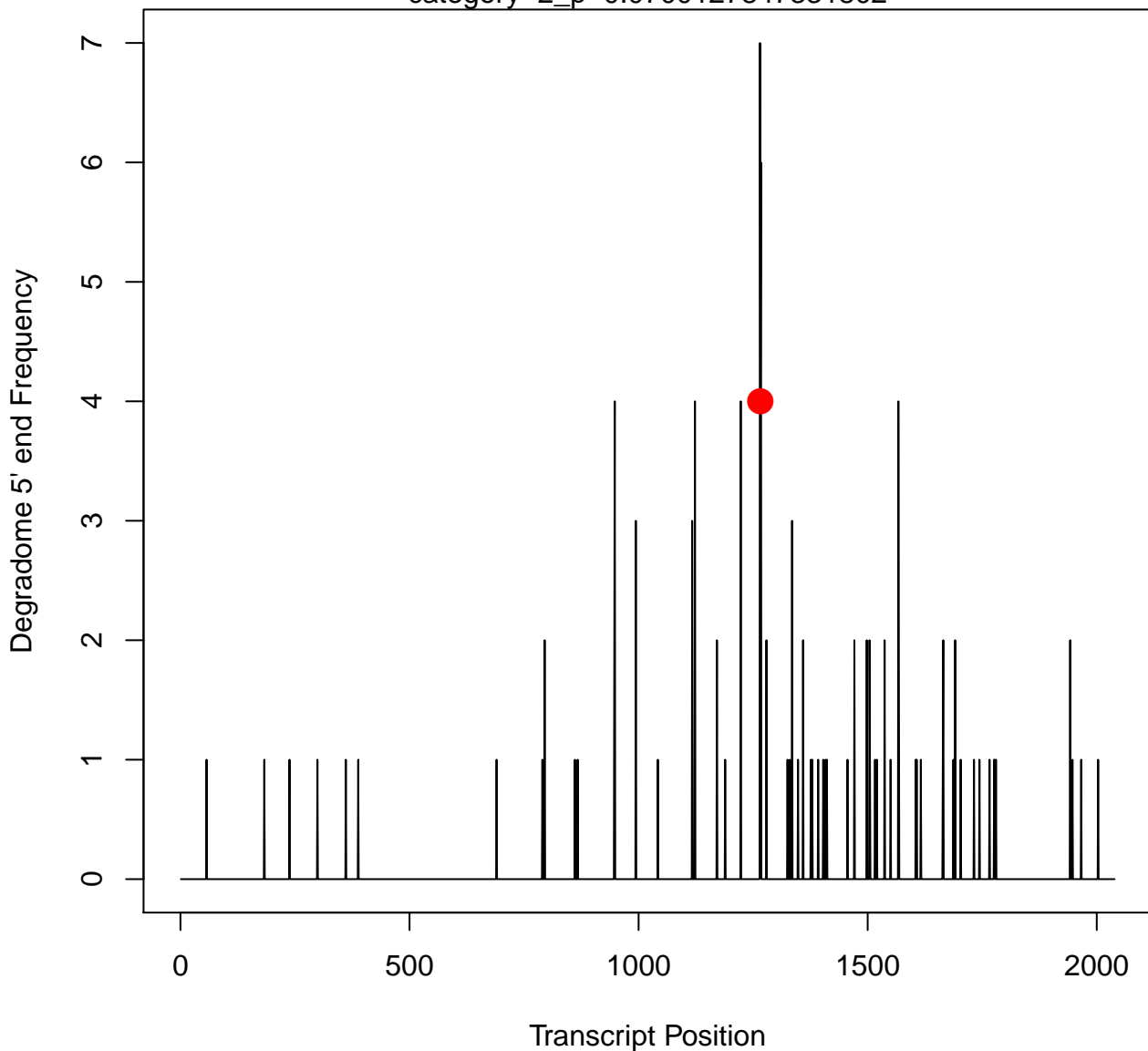

Supplement: Supplementary file 6 [file Data_Sheet_6.zip › Sit-miR156i_Seita.2G254300.1_1266_TPlot.pdf]

**T=Seita.9G510100.1\_Q=Sit-miR156i\_S=2837**

category=2\_p=0.968755346574317

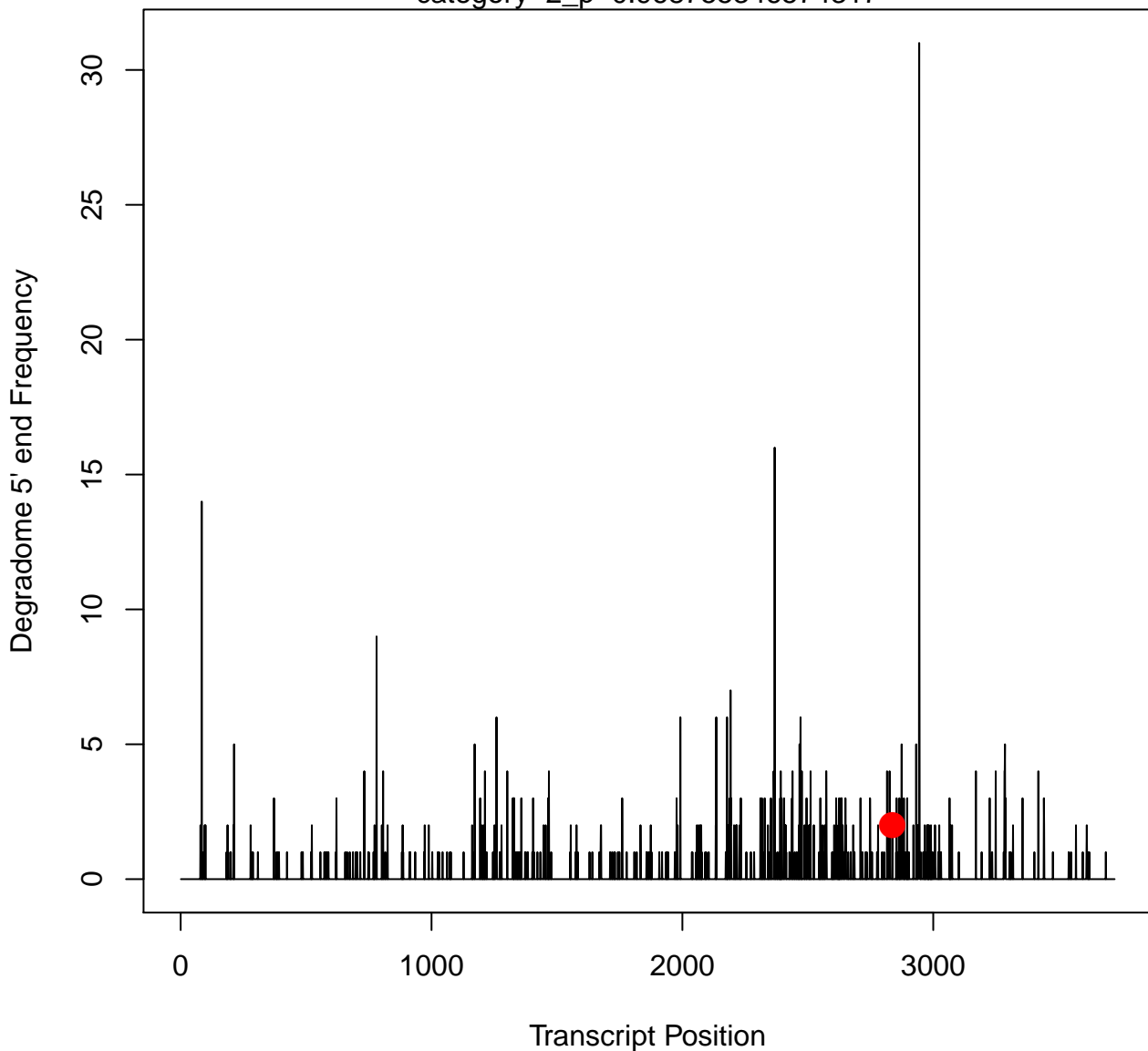

Supplement: Supplementary file 6 [file Data_Sheet_6.zip › Sit-miR156i_Seita.9G510100.1_2837_TPlot.pdf]

**T=Seita.1G069300.1\_Q=Sit-miR156j\_S=873**

category=2\_p=0.0356415524988544

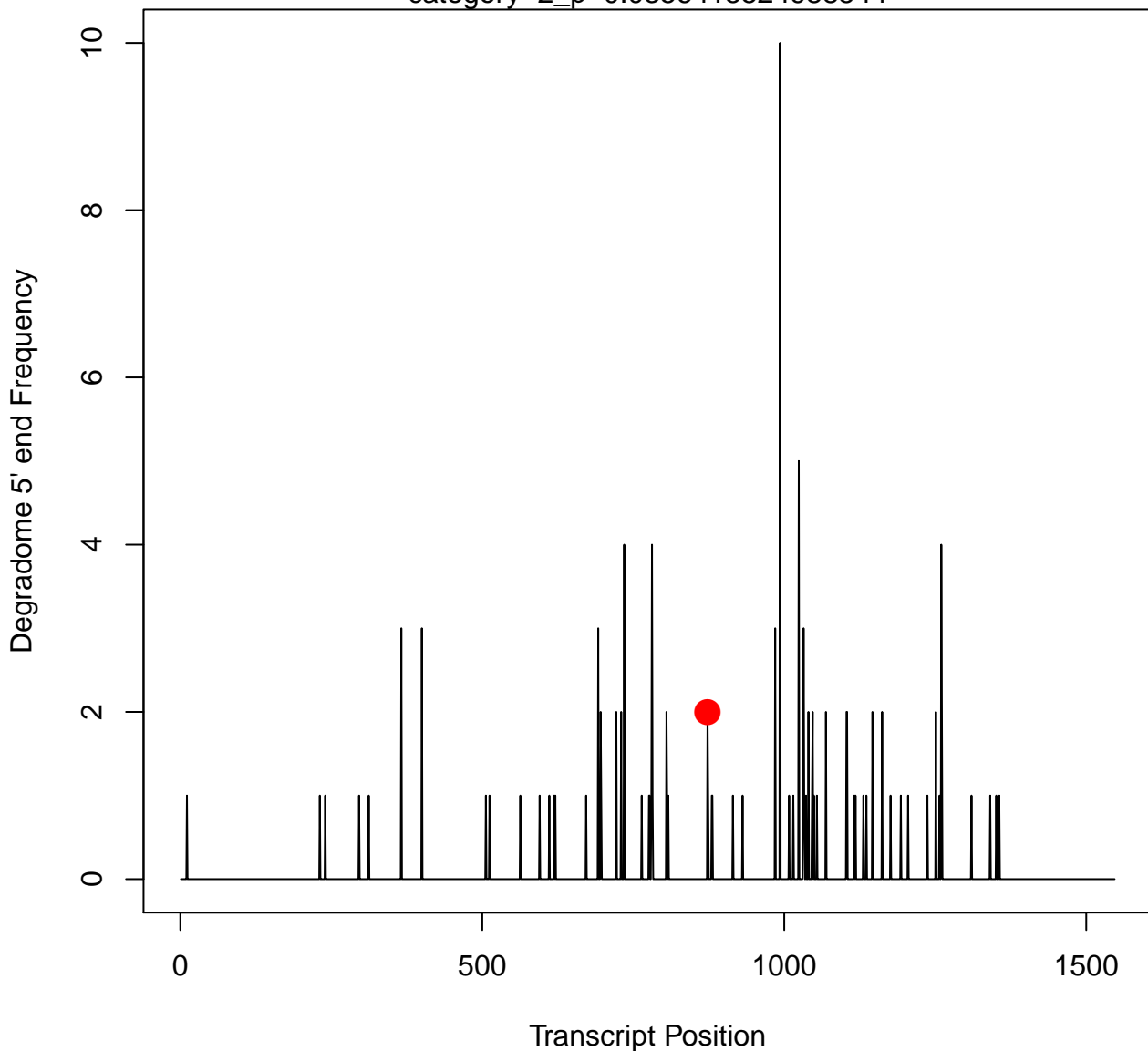

Supplement: Supplementary file 6 [file Data_Sheet_6.zip › Sit-miR156j_Seita.1G069300.1_873_TPlot.pdf]

**T=Seita.1G091900.1\_Q=Sit-miR156j\_S=1786**

category=2\_p=0.0179824606957646

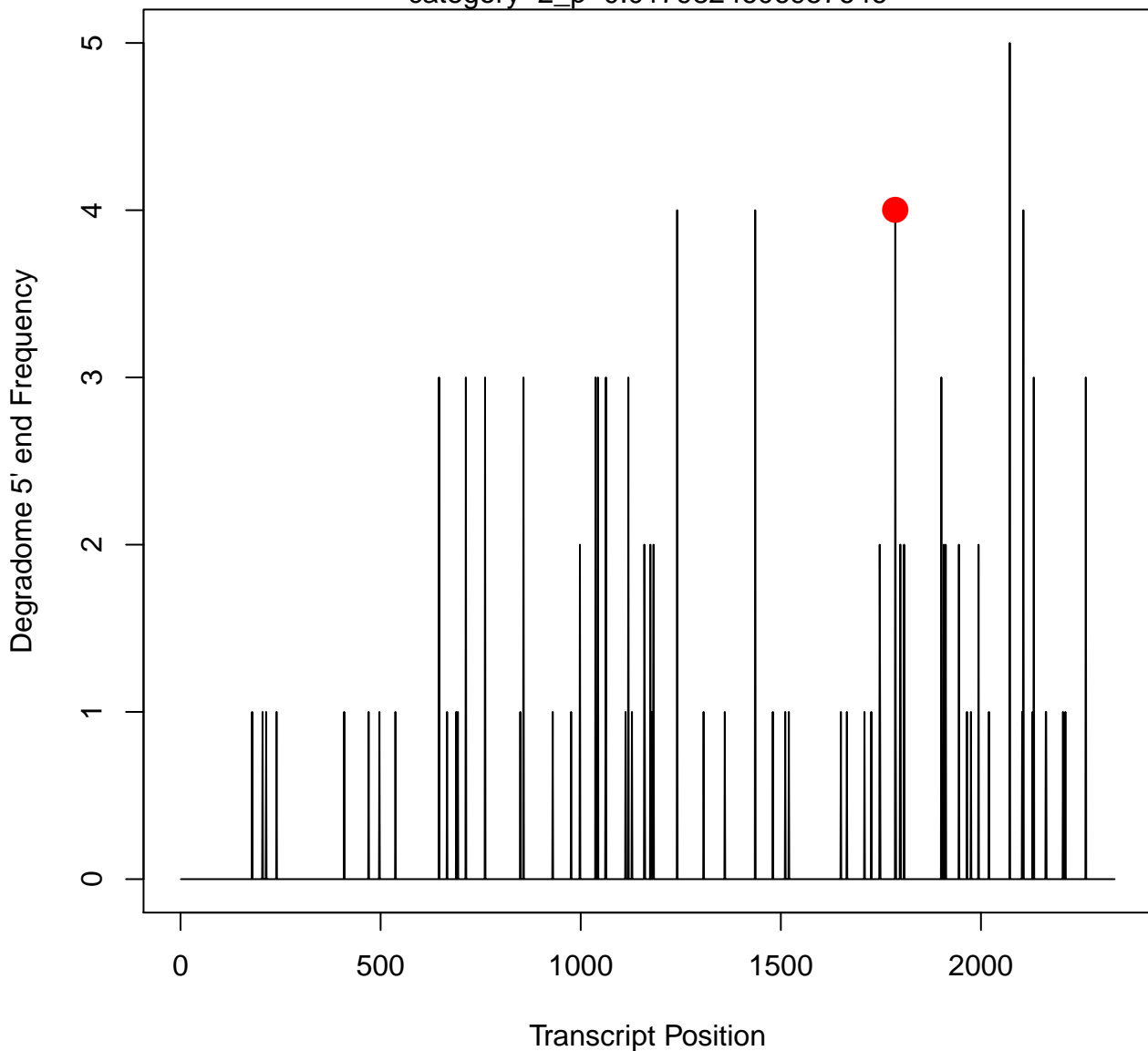

Supplement: Supplementary file 6 [file Data_Sheet_6.zip › Sit-miR156j_Seita.1G091900.1_1786_TPlot.pdf]

**T=Seita.2G324900.1\_Q=Sit-miR156j\_S=833**

category=0\_p=0.00374915189234248

Degradome 5' end Frequency

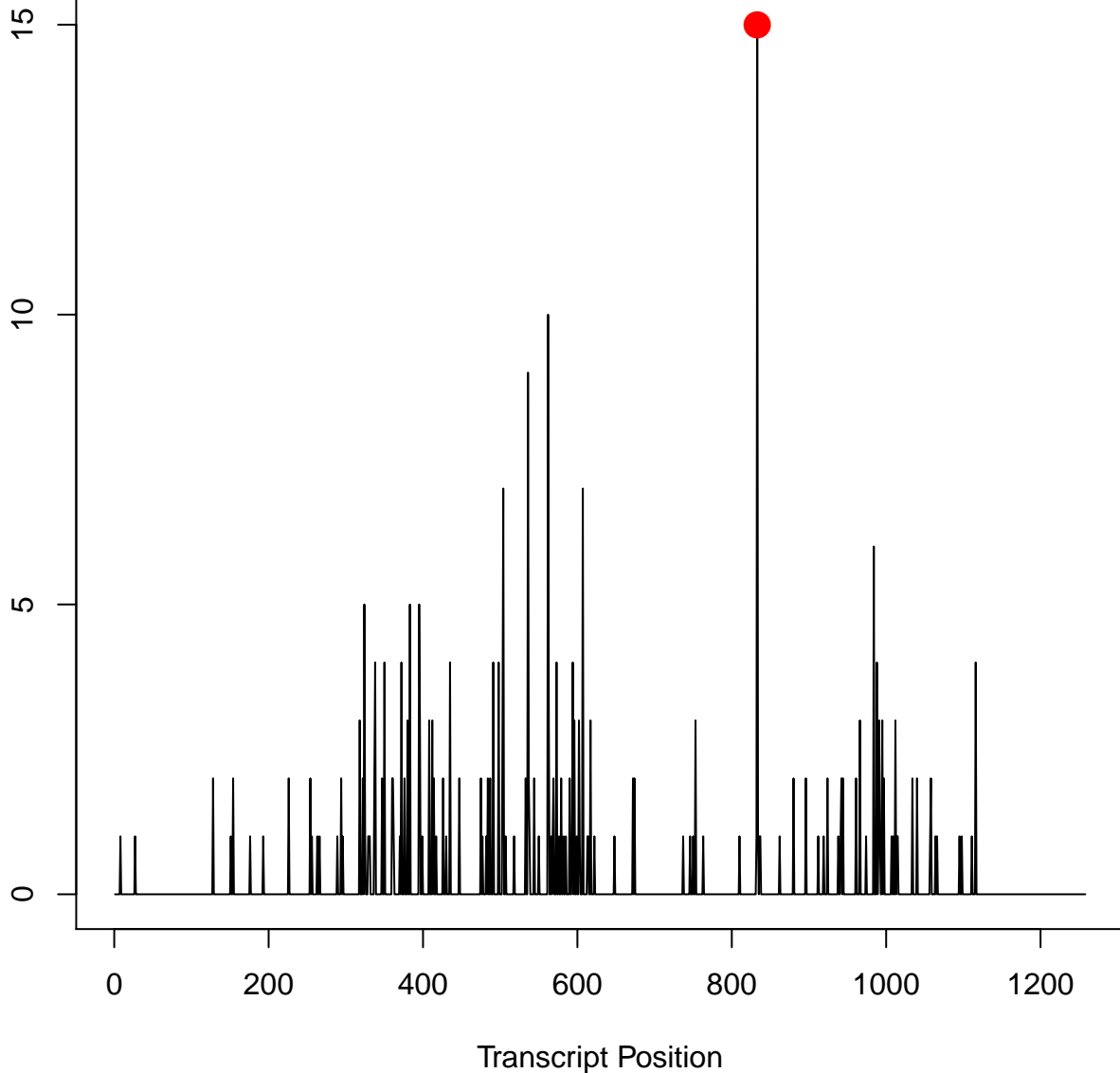

Supplement: Supplementary file 6 [file Data_Sheet_6.zip › Sit-miR156j_Seita.2G324900.1_833_TPlot.pdf]

**T=Seita.9G051300.1\_Q=Sit-miR156j\_S=2276**

category=2\_p=0.907184386878865

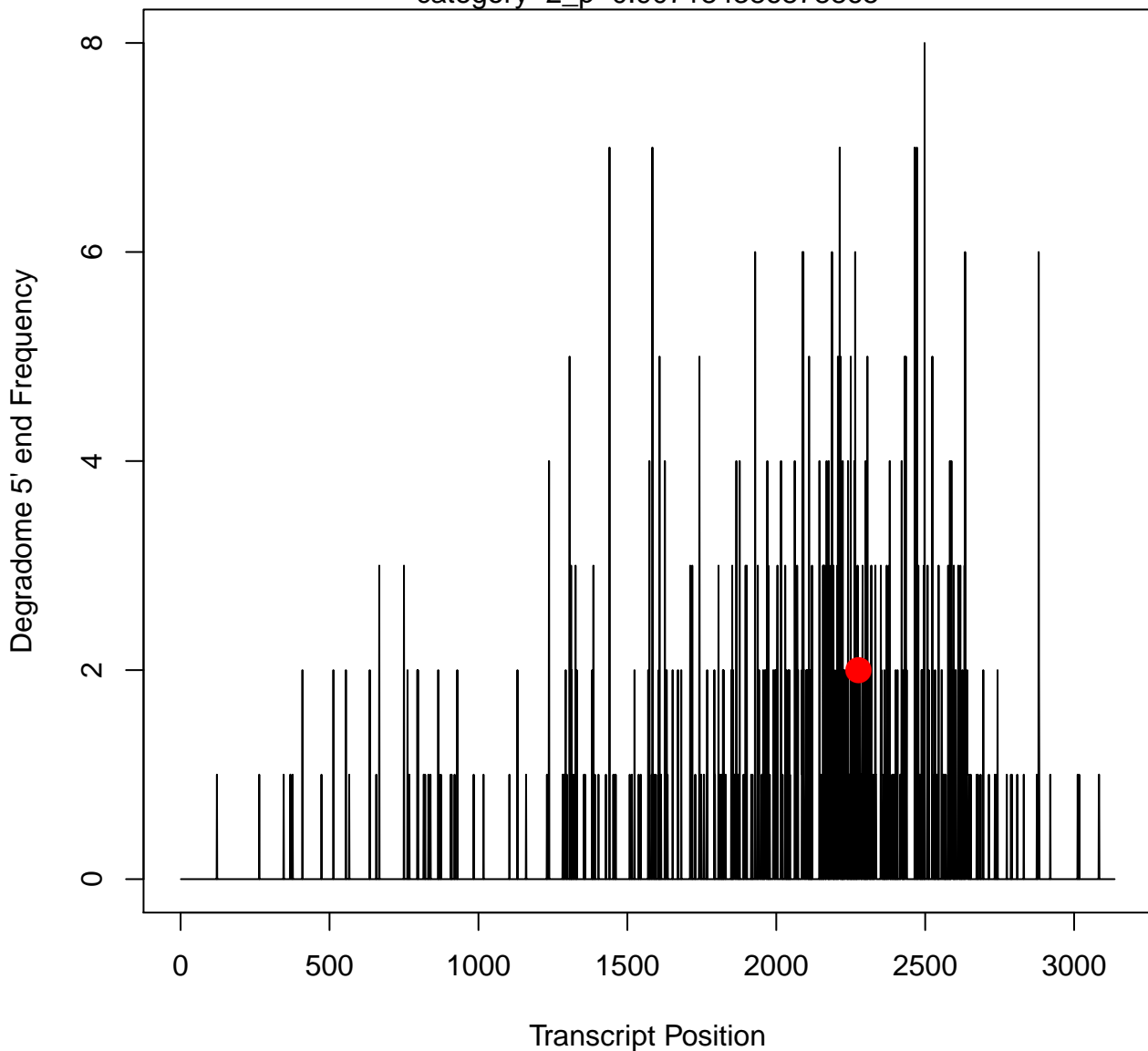

Supplement: Supplementary file 6 [file Data_Sheet_6.zip › Sit-miR156j_Seita.9G051300.1_2276_TPlot.pdf]

**T=Seita.1G185100.1\_Q=Sit-miR159a\_S=1132**

category=2\_p=0.999987234116576

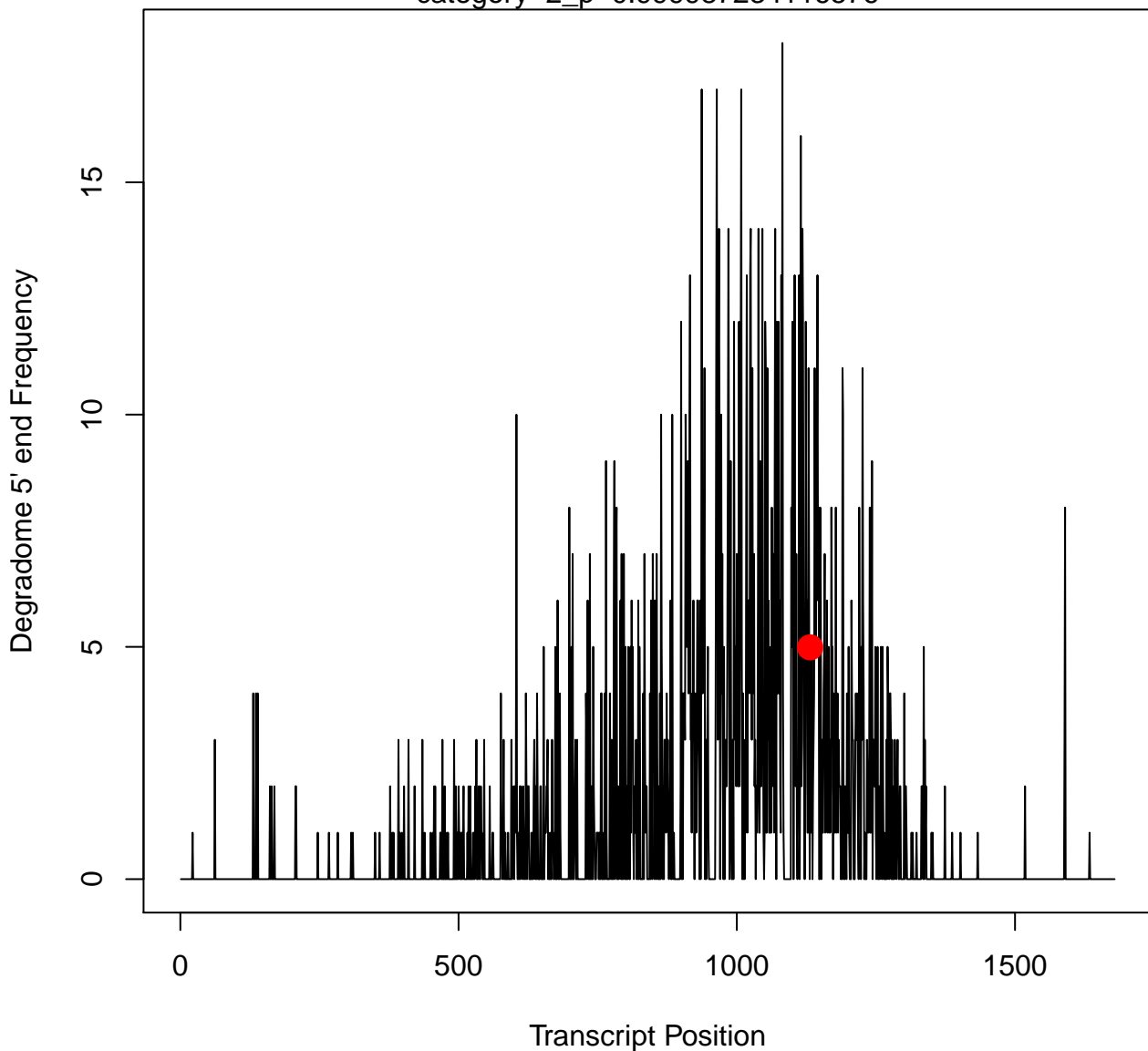

Supplement: Supplementary file 6 [file Data_Sheet_6.zip › Sit-miR159a_Seita.1G185100.1_1132_TPlot.pdf]

**T=Seita.1G348600.1\_Q=Sit-miR159a\_S=4875**

category=2\_p=0.985681311936496

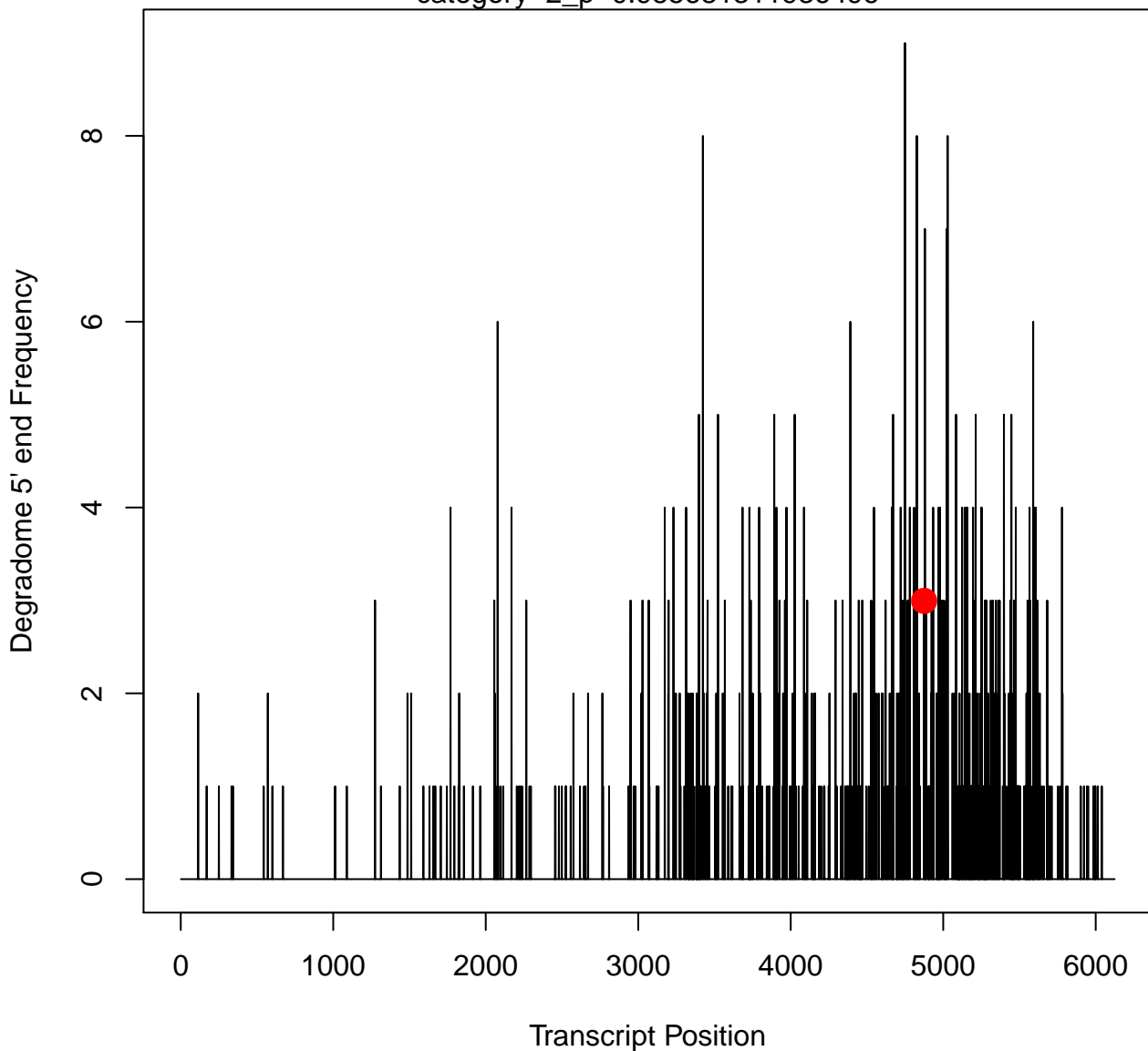

Supplement: Supplementary file 6 [file Data_Sheet_6.zip › Sit-miR159a_Seita.1G348600.1_4875_TPlot.pdf]

**T=Seita.2G184400.1\_Q=Sit-miR159a\_S=170**

category=2\_p=0.23829173415817

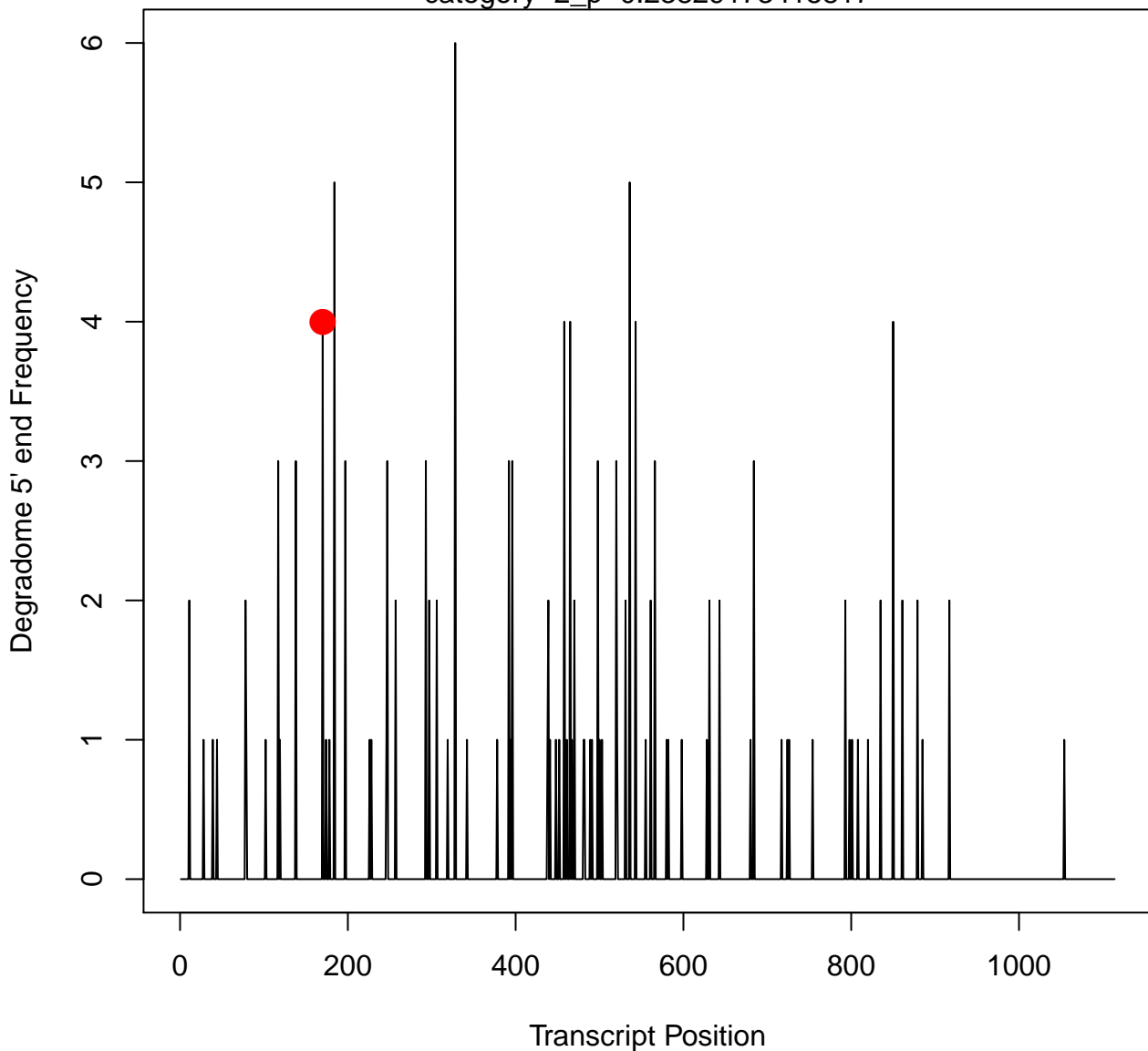

Supplement: Supplementary file 6 [file Data_Sheet_6.zip › Sit-miR159a_Seita.2G184400.1_170_TPlot.pdf]

**T=Seita.2G358600.1\_Q=Sit-miR159a\_S=343**

category=2\_p=0.961154136752463

Degradome 5' end Frequency

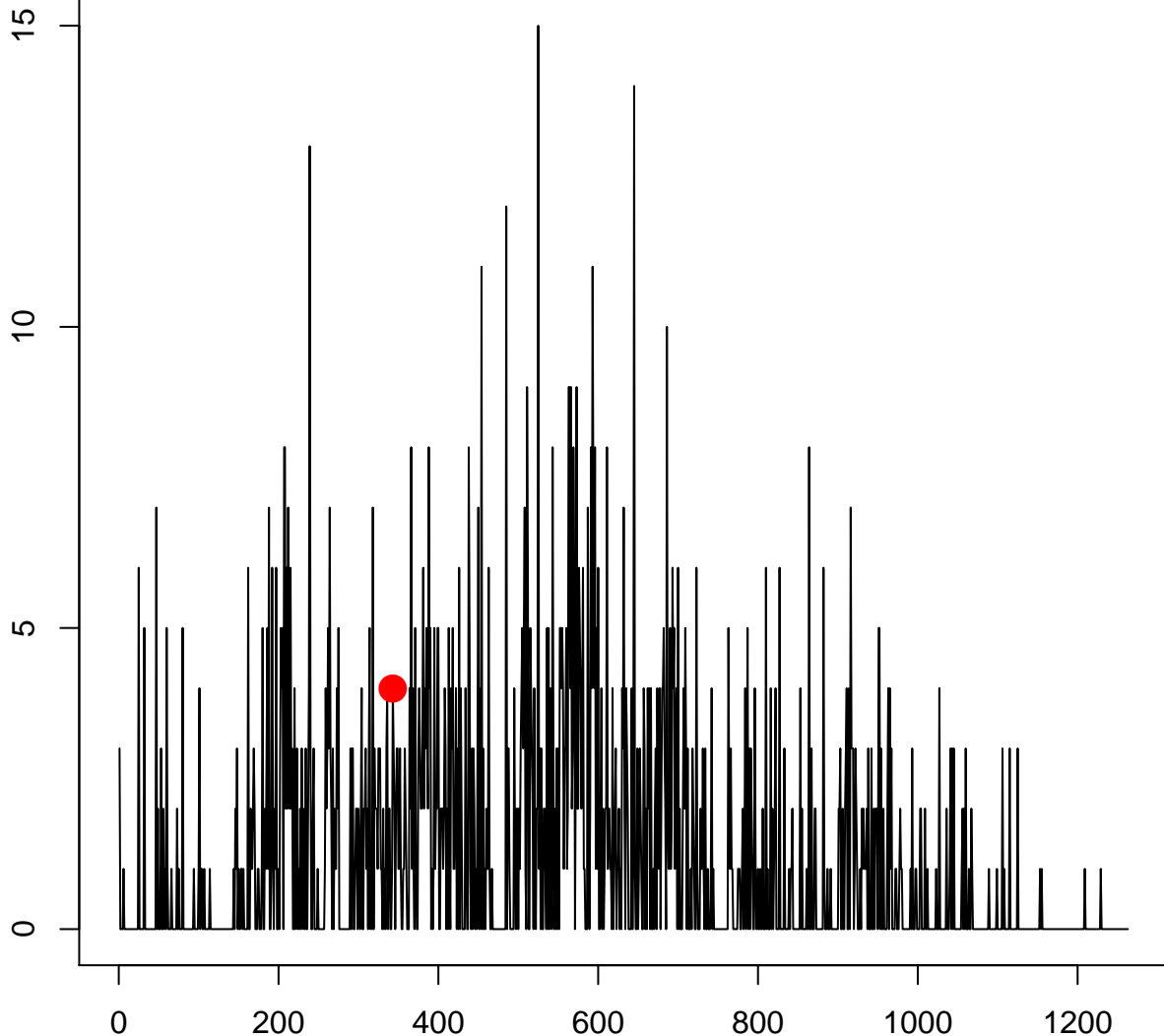

Transcript Position

Supplement: Supplementary file 6 [file Data_Sheet_6.zip › Sit-miR159a_Seita.2G358600.1_343_TPlot.pdf]

**T=Seita.3G096800.1\_Q=Sit-miR159a\_S=1199**

category=2\_p=0.999999990677976

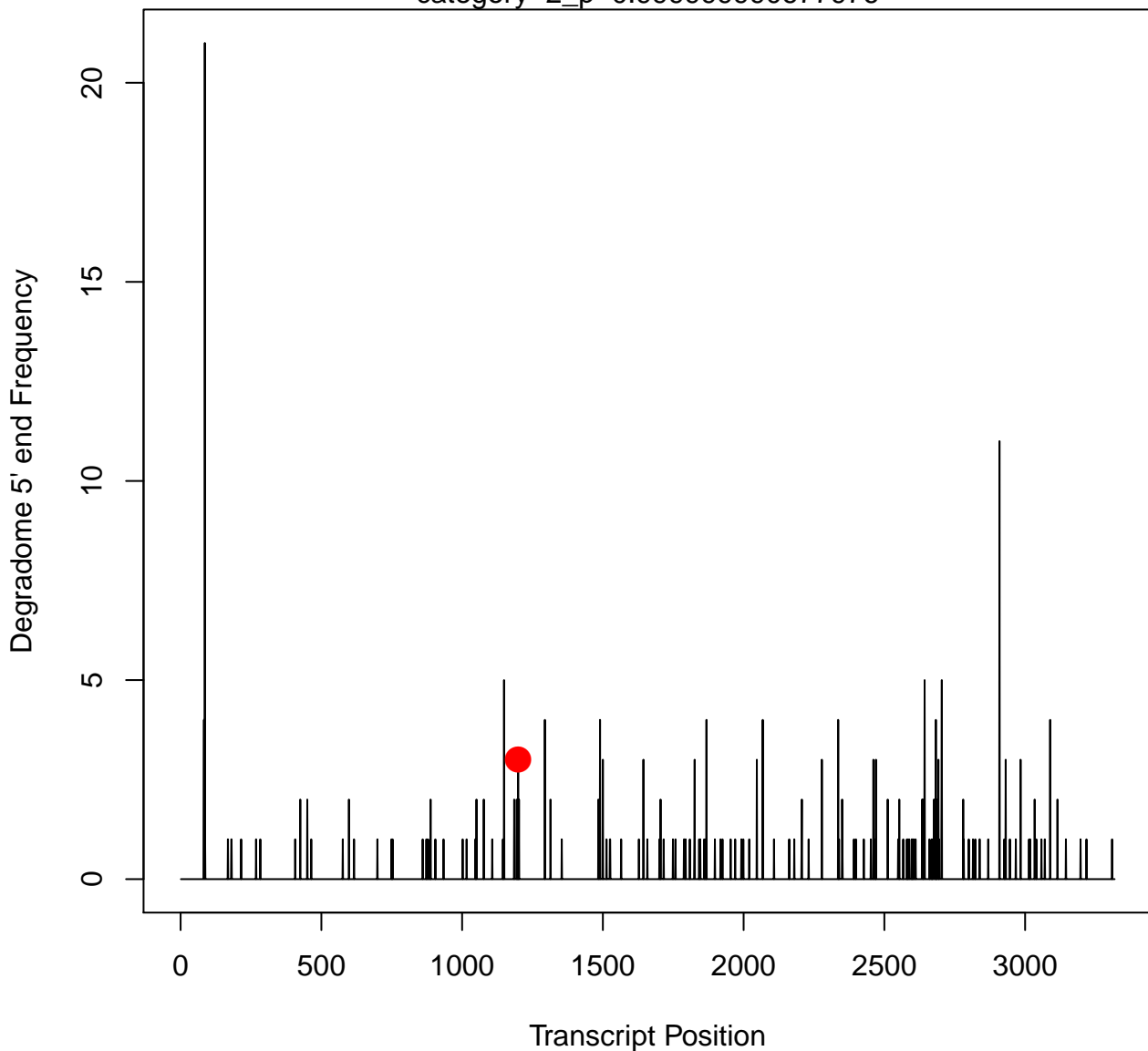

Supplement: Supplementary file 6 [file Data_Sheet_6.zip › Sit-miR159a_Seita.3G096800.1_1199_TPlot.pdf]

**T=Seita.4G020800.1\_Q=Sit-miR159a\_S=284**

category=2\_p=0.999999998962618

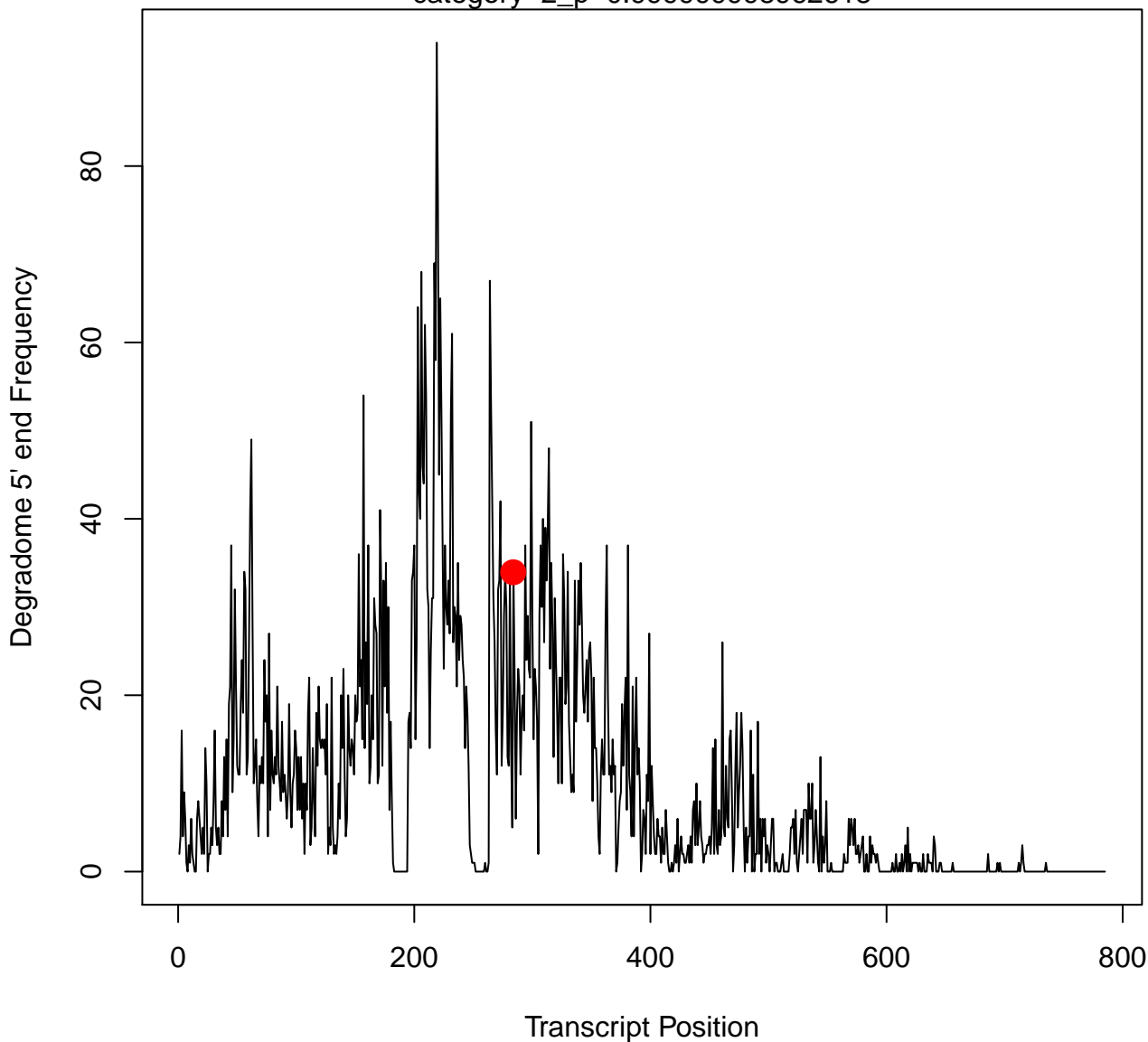

Supplement: Supplementary file 6 [file Data_Sheet_6.zip › Sit-miR159a_Seita.4G020800.1_284_TPlot.pdf]

**T=Seita.4G083900.1\_Q=Sit-miR159a\_S=4243**

category=2\_p=0.558057258531678

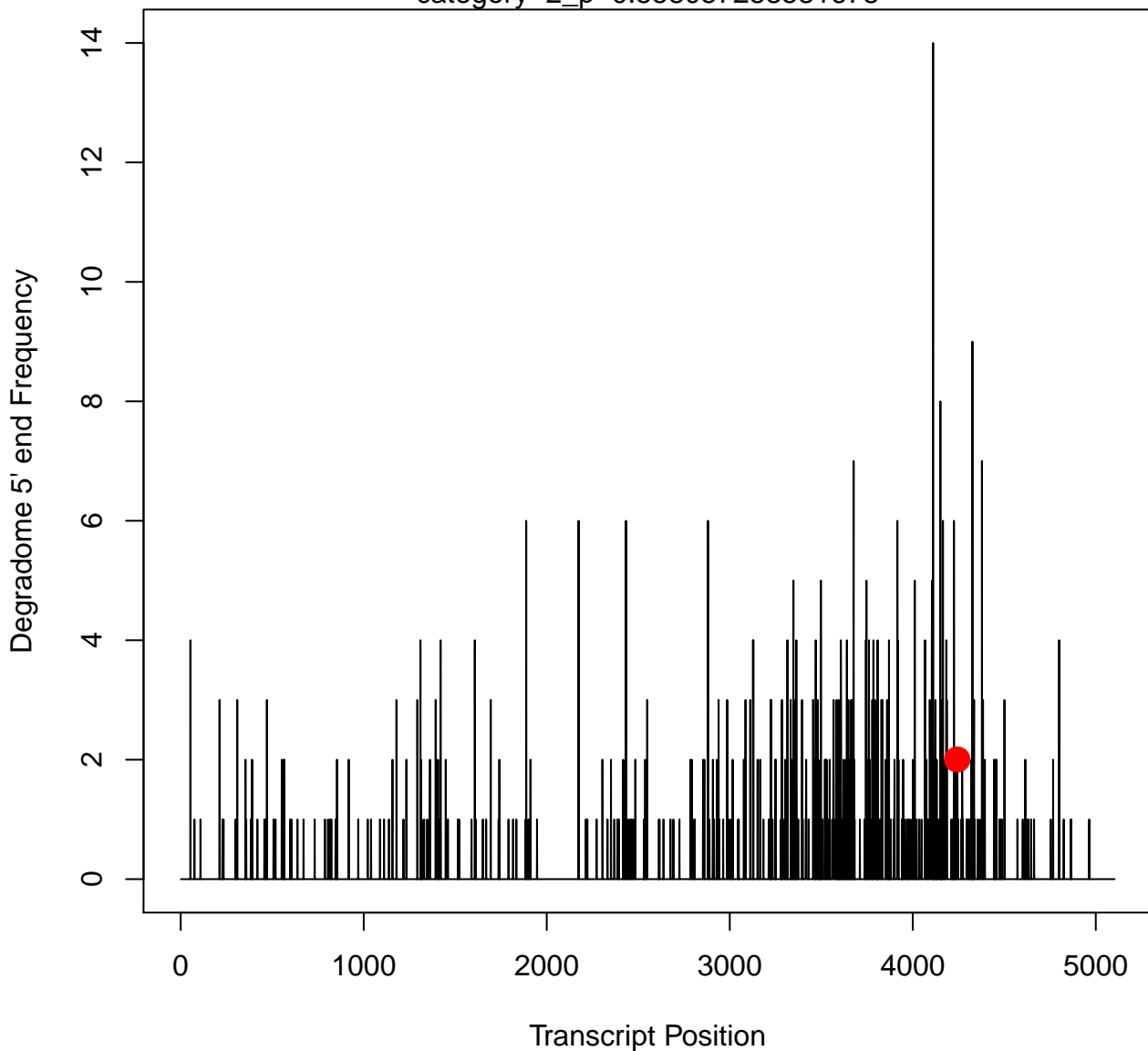

Supplement: Supplementary file 6 [file Data_Sheet_6.zip › Sit-miR159a_Seita.4G083900.1_4243_TPlot.pdf]

**T=Seita.4G221900.1\_Q=Sit-miR159a\_S=1158**

category=2\_p=0.150676382083183

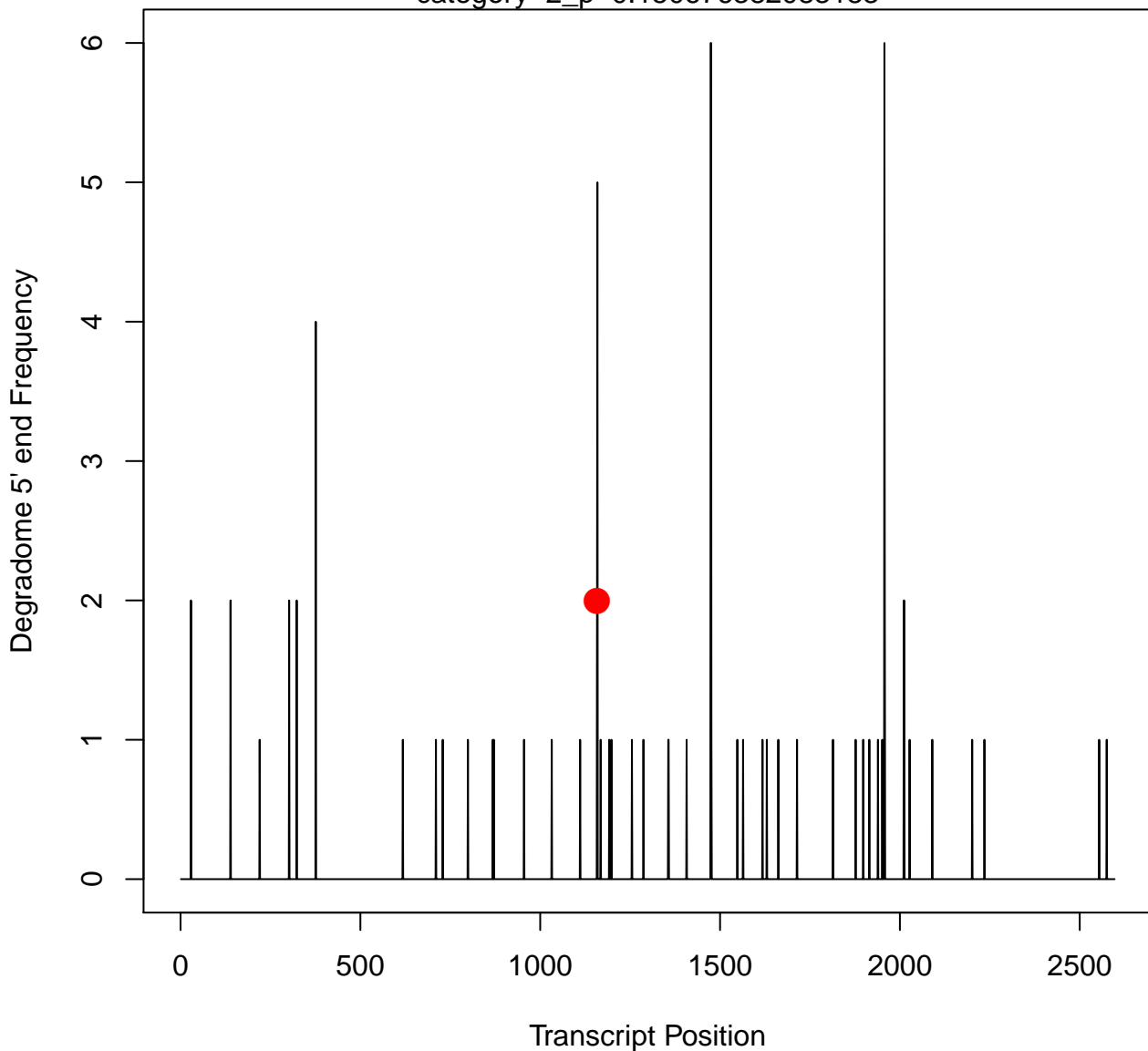

Supplement: Supplementary file 6 [file Data_Sheet_6.zip › Sit-miR159a_Seita.4G221900.1_1158_TPlot.pdf]

**T=Seita.6G018100.1\_Q=Sit-miR159a\_S=2896**

category=2\_p=0.999996215223312

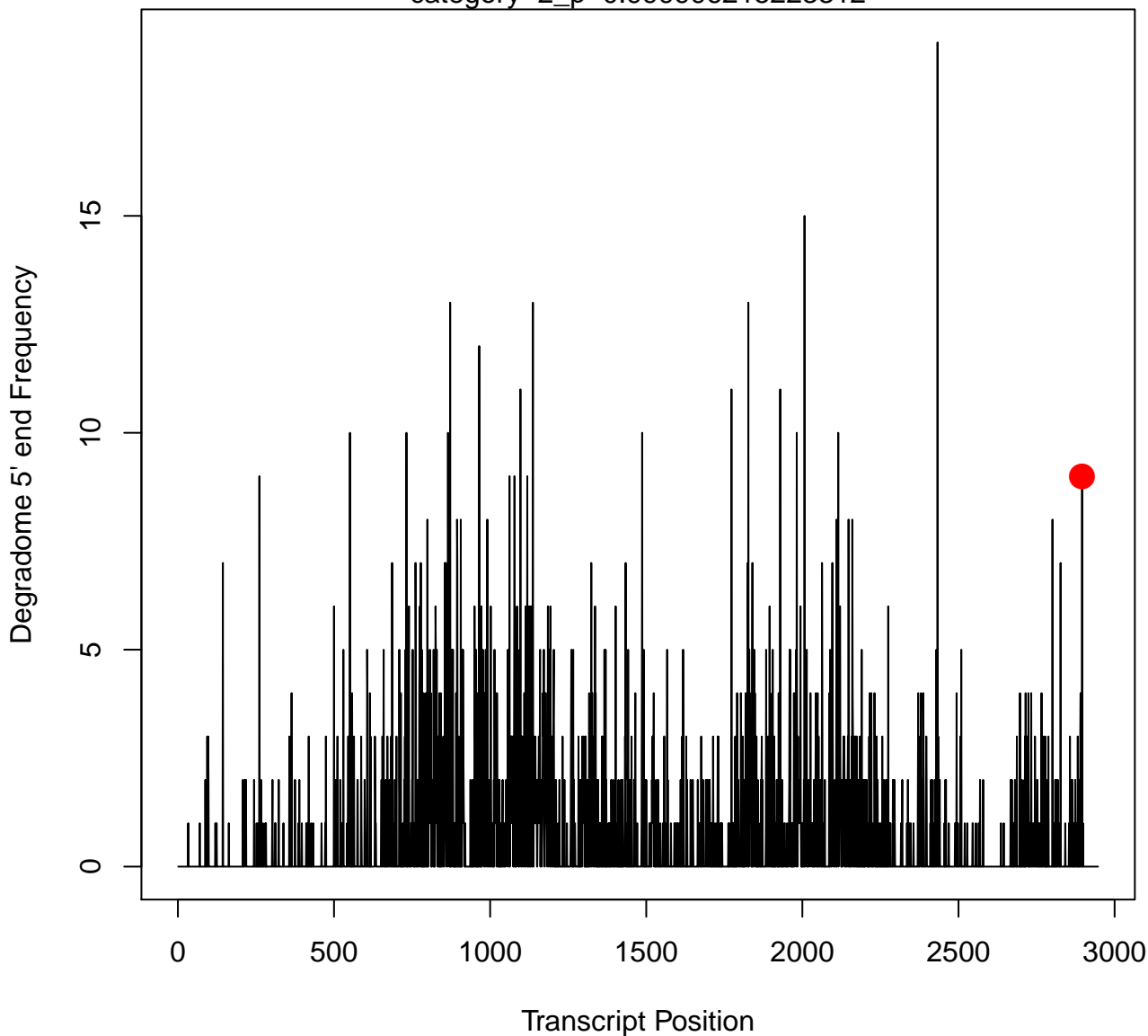

Supplement: Supplementary file 6 [file Data_Sheet_6.zip › Sit-miR159a_Seita.6G018100.1_2896_TPlot.pdf]

**T=Seita.6G243700.1\_Q=Sit-miR159a\_S=691**

category=2\_p=0.999999967981456

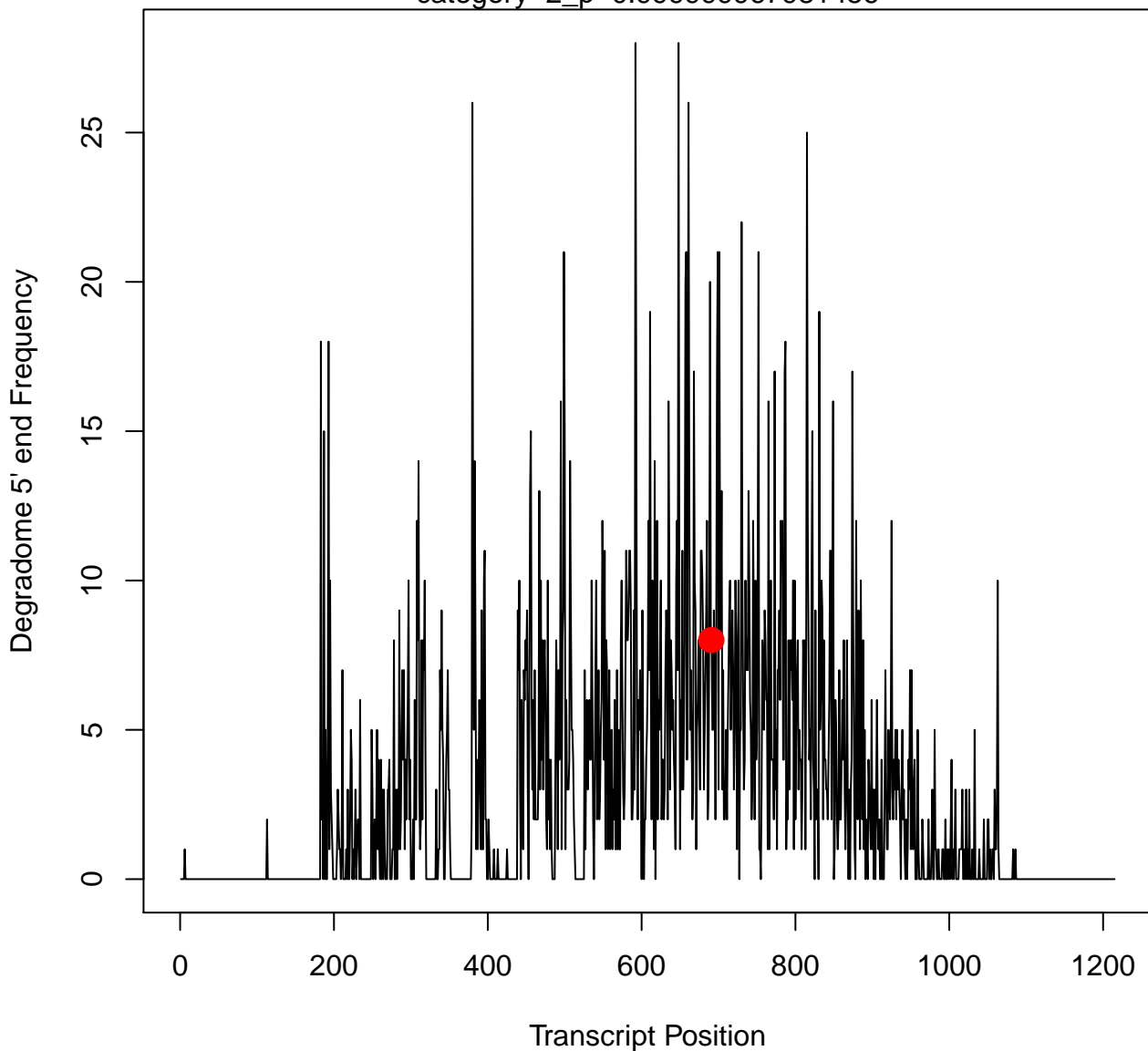

Supplement: Supplementary file 6 [file Data_Sheet_6.zip › Sit-miR159a_Seita.6G243700.1_691_TPlot.pdf]

**T=Seita.7G149200.1\_Q=Sit-miR159a\_S=1252**

category=2\_p=0.884604173219079

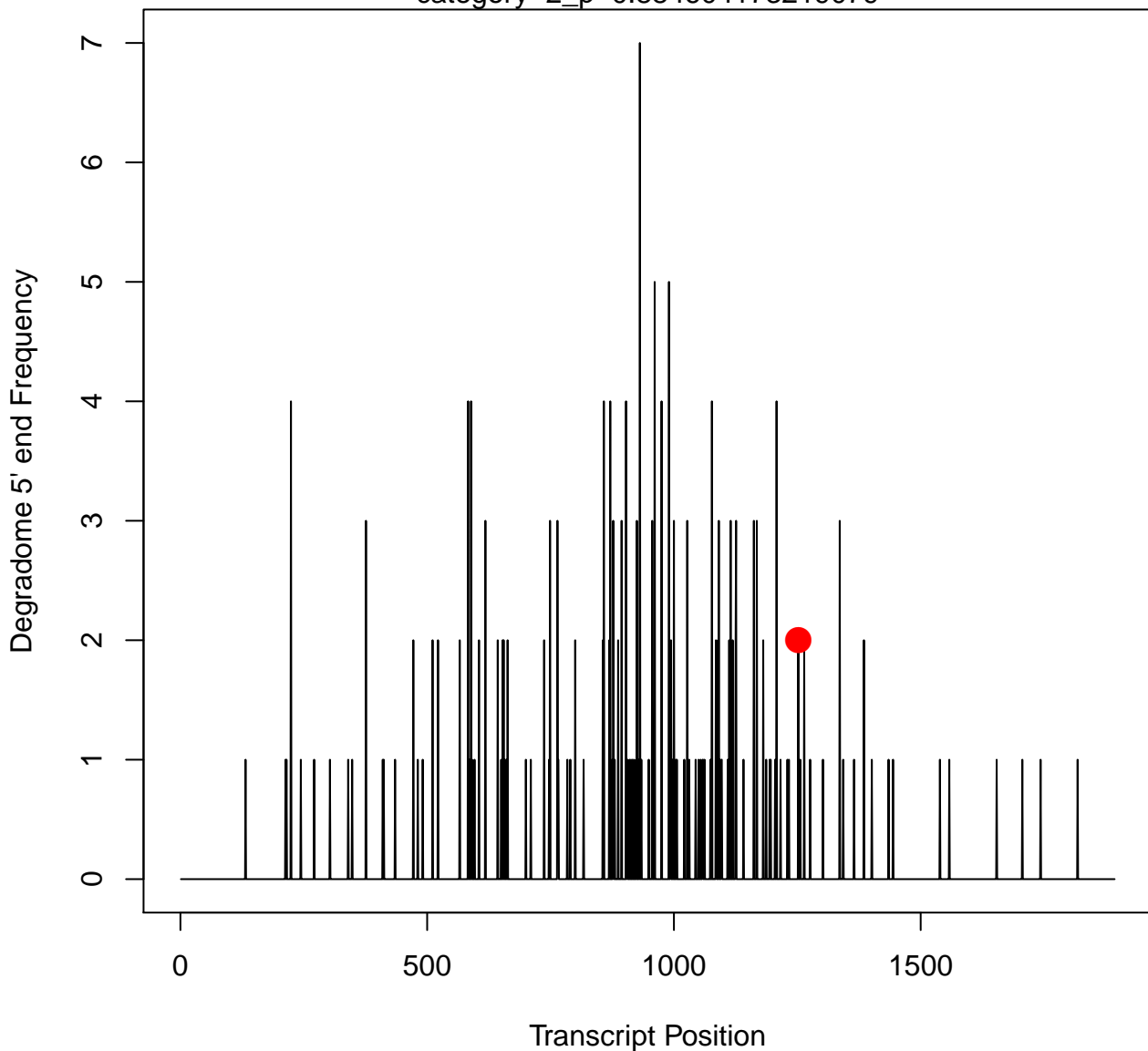

Supplement: Supplementary file 6 [file Data_Sheet_6.zip › Sit-miR159a_Seita.7G149200.1_1252_TPlot.pdf]

**T=Seita.7G229800.1\_Q=Sit-miR159a\_S=122**

category=0\_p=0.328695579470126

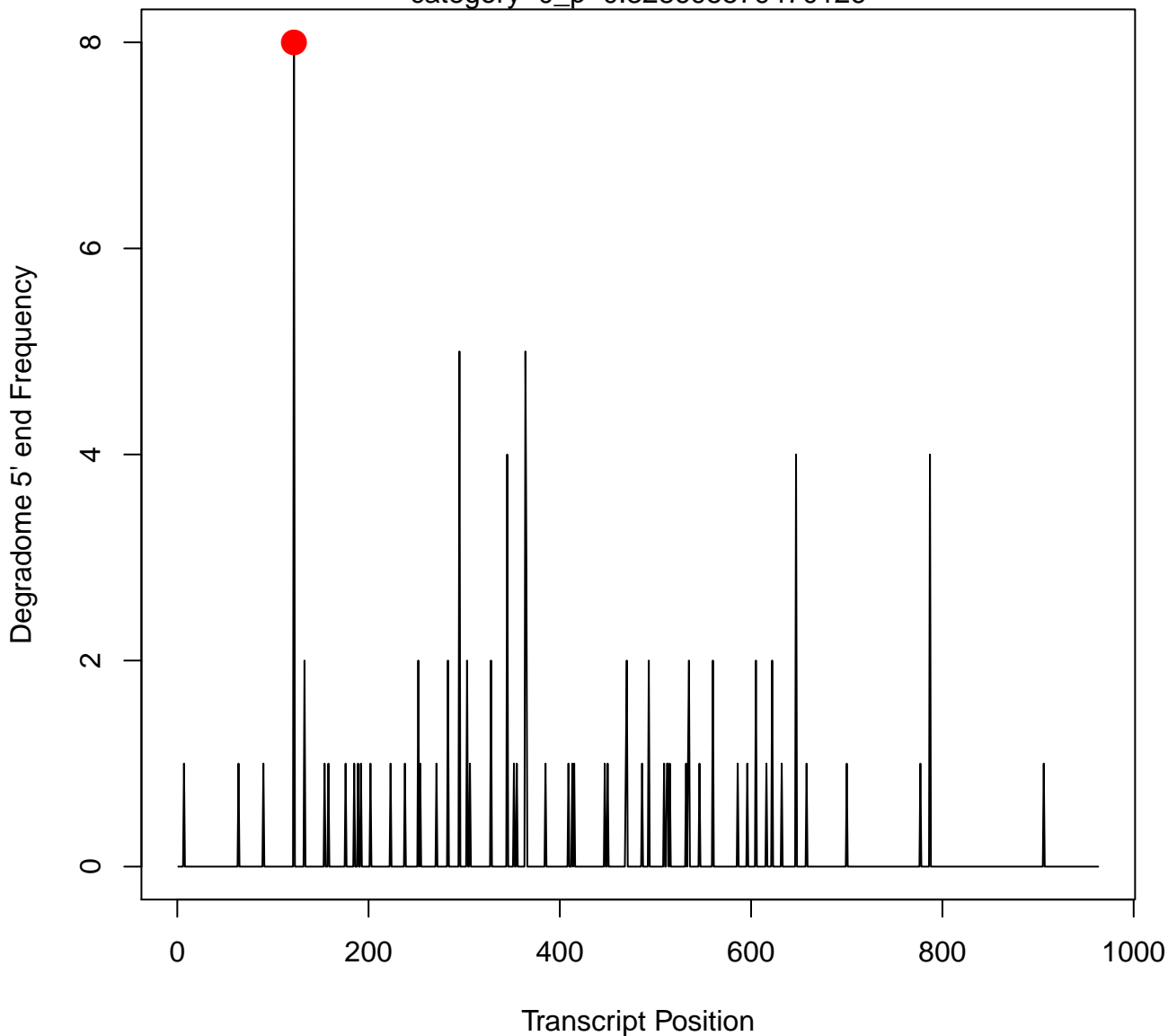

Supplement: Supplementary file 6 [file Data_Sheet_6.zip › Sit-miR159a_Seita.7G229800.1_122_TPlot.pdf]

**T=Seita.7G240000.1\_Q=Sit-miR159a\_S=1128**

category=2\_p=0.999999908275491

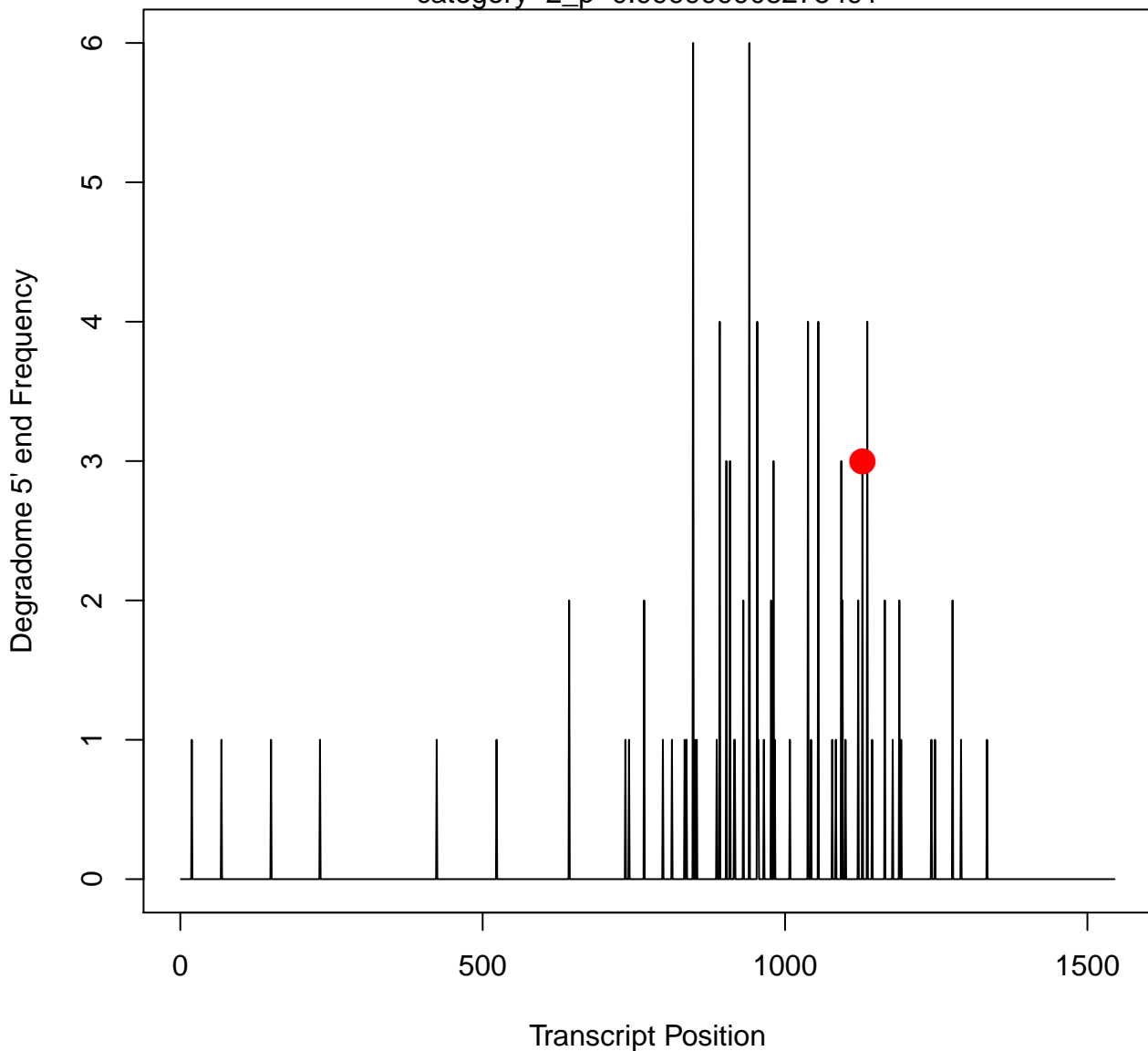

Supplement: Supplementary file 6 [file Data_Sheet_6.zip › Sit-miR159a_Seita.7G240000.1_1128_TPlot.pdf]

**T=Seita.7G297000.1\_Q=Sit-miR159a\_S=741**

category=2\_p=0.999999312537126

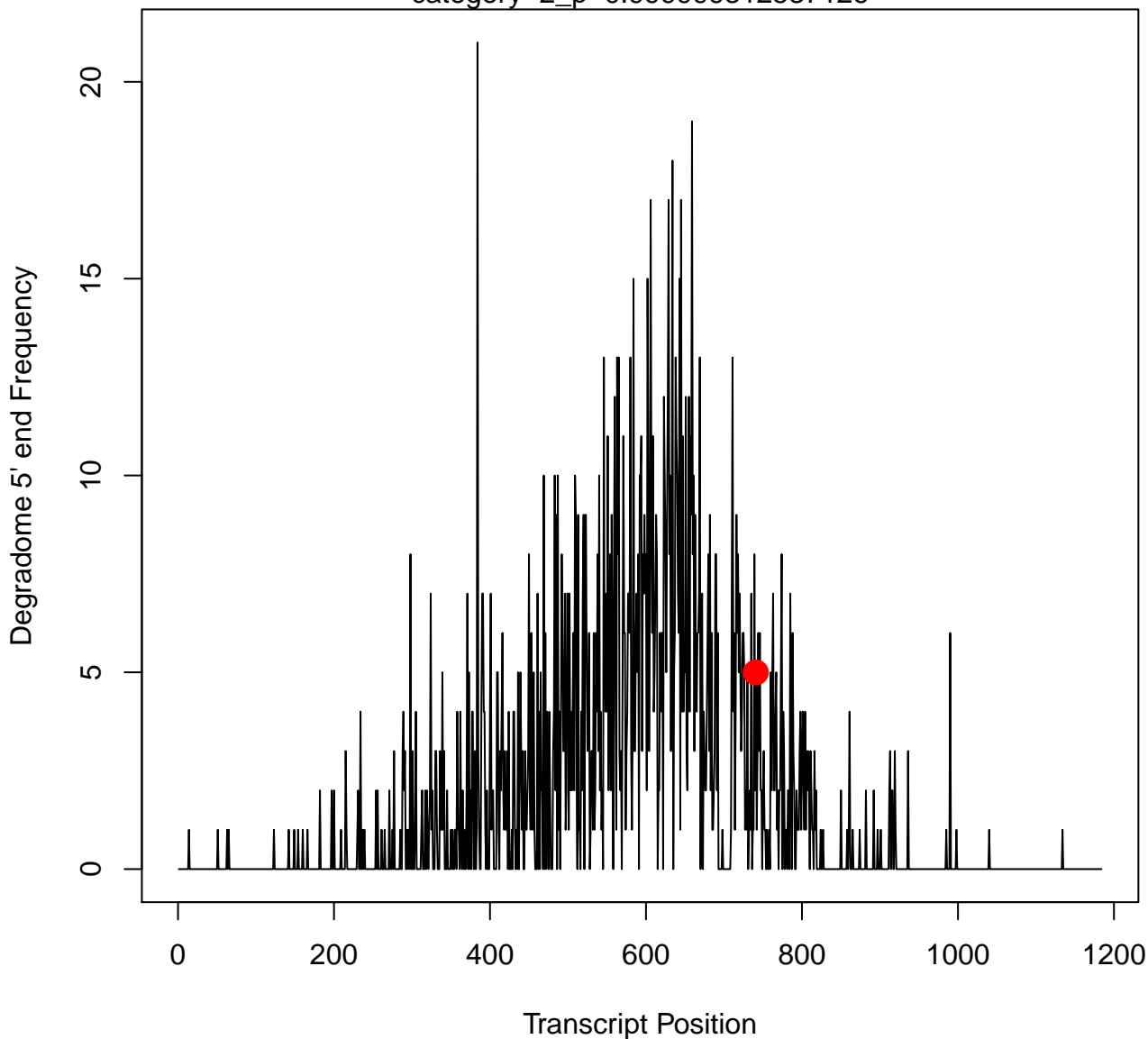

Supplement: Supplementary file 6 [file Data_Sheet_6.zip › Sit-miR159a_Seita.7G297000.1_741_TPlot.pdf]

**T=Seita.8G123100.1\_Q=Sit-miR159a\_S=238**

category=2\_p=0.999819431841955

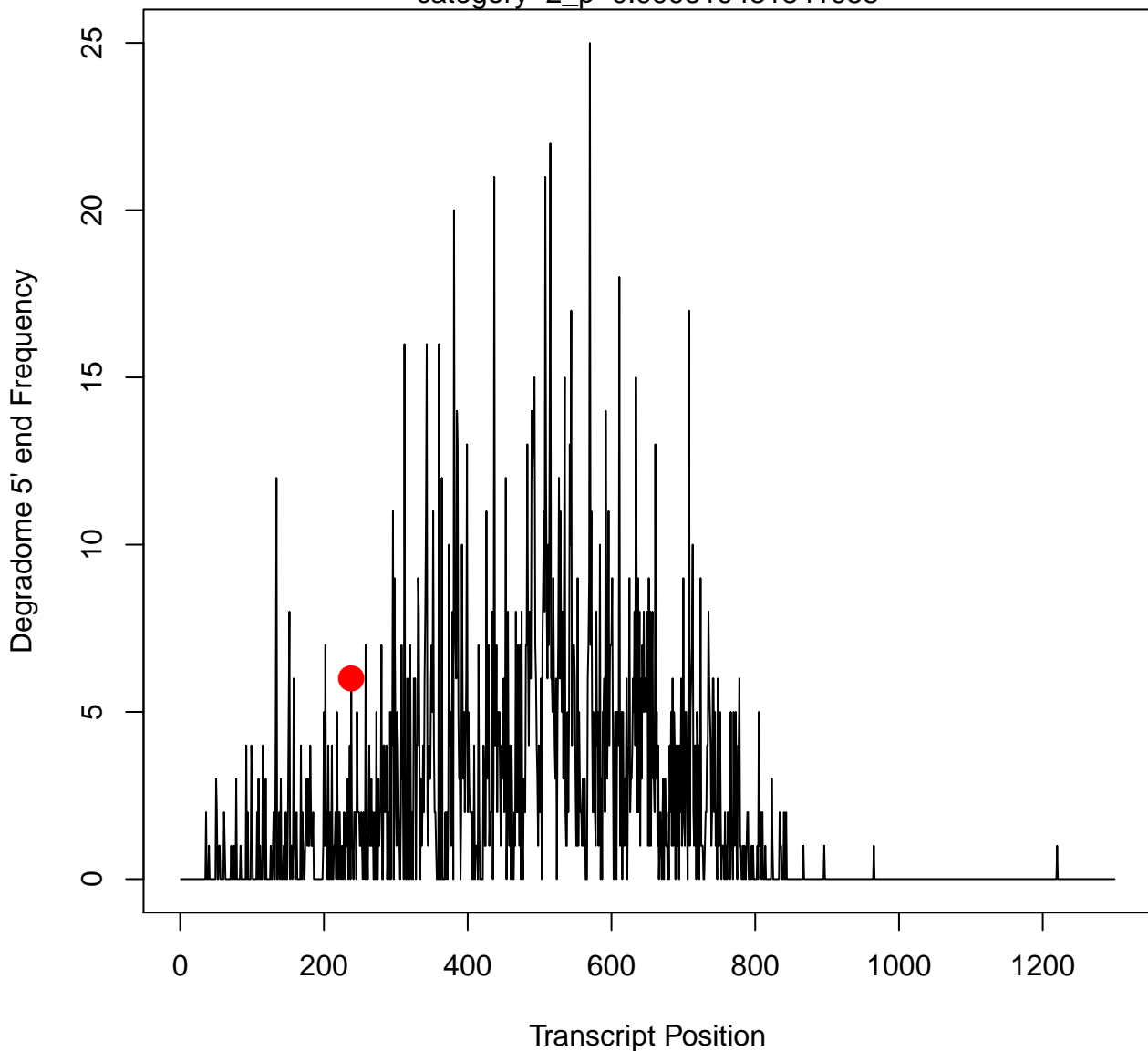

Supplement: Supplementary file 6 [file Data_Sheet_6.zip › Sit-miR159a_Seita.8G123100.1_238_TPlot.pdf]

**T=Seita.9G062800.1\_Q=Sit-miR159a\_S=6173**

category=2\_p=0.969317202326498

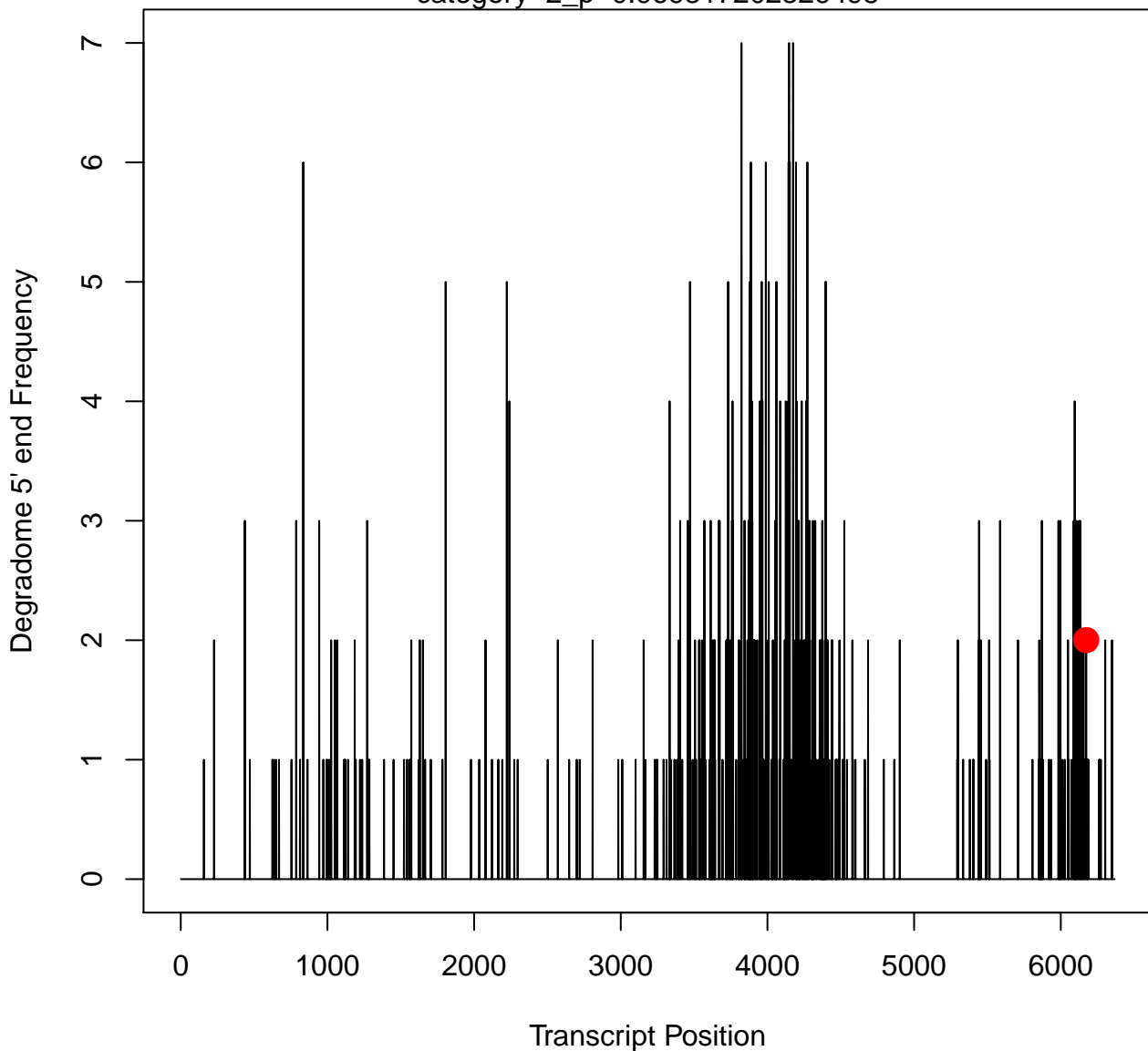

Supplement: Supplementary file 6 [file Data_Sheet_6.zip › Sit-miR159a_Seita.9G062800.1_6173_TPlot.pdf]

**T=Seita.9G134700.1\_Q=Sit-miR159a\_S=4274**

category=0\_p=0.37891408233806

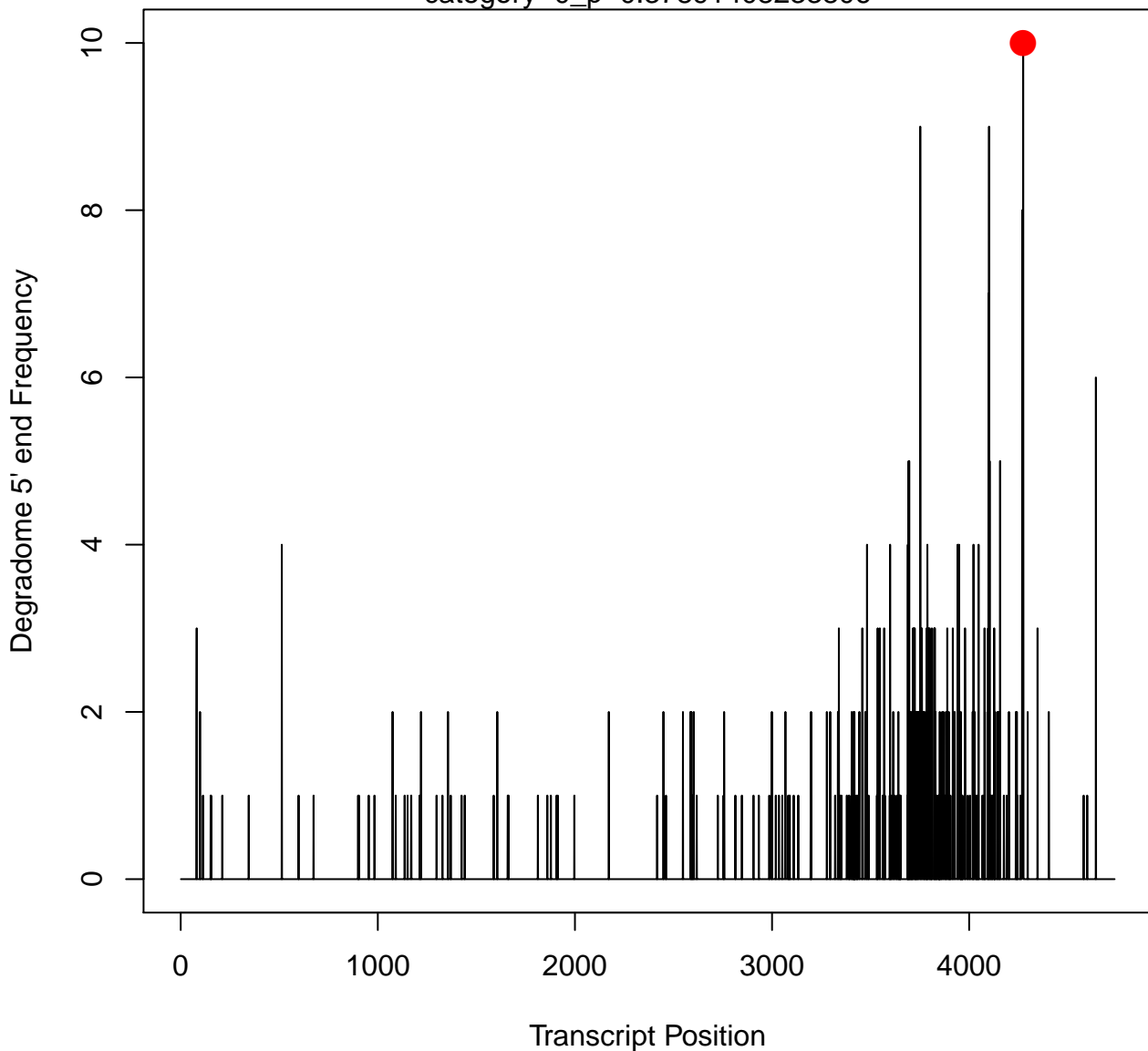

Supplement: Supplementary file 6 [file Data_Sheet_6.zip › Sit-miR159a_Seita.9G134700.1_4274_TPlot.pdf]

**T=Seita.9G380100.1\_Q=Sit-miR159a\_S=2377**

category=2\_p=0.999999952271454

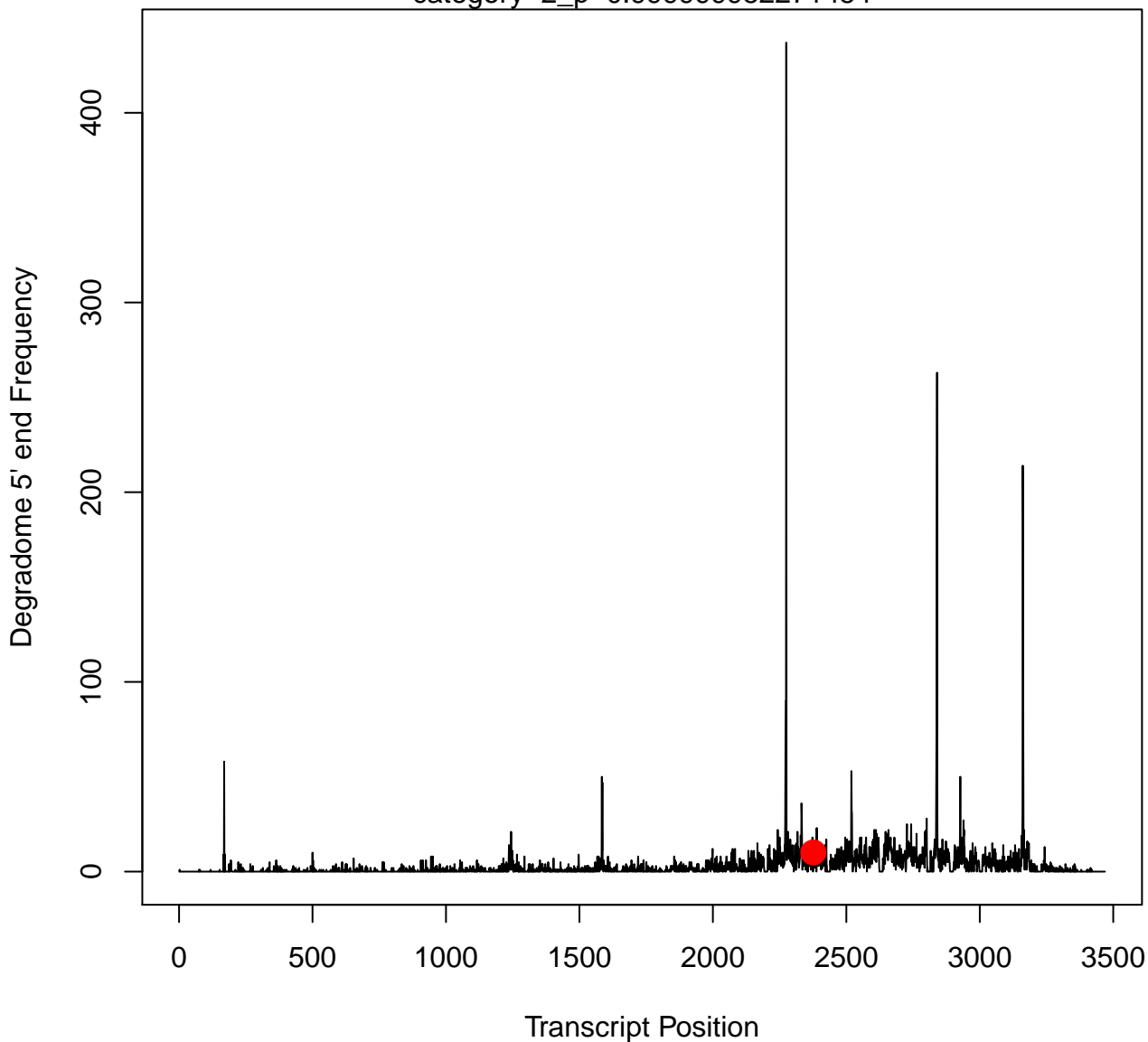

Supplement: Supplementary file 6 [file Data_Sheet_6.zip › Sit-miR159a_Seita.9G380100.1_2377_TPlot.pdf]

**T=Seita.9G444100.1\_Q=Sit-miR159a\_S=1241**

category=2\_p=0.999999985590519

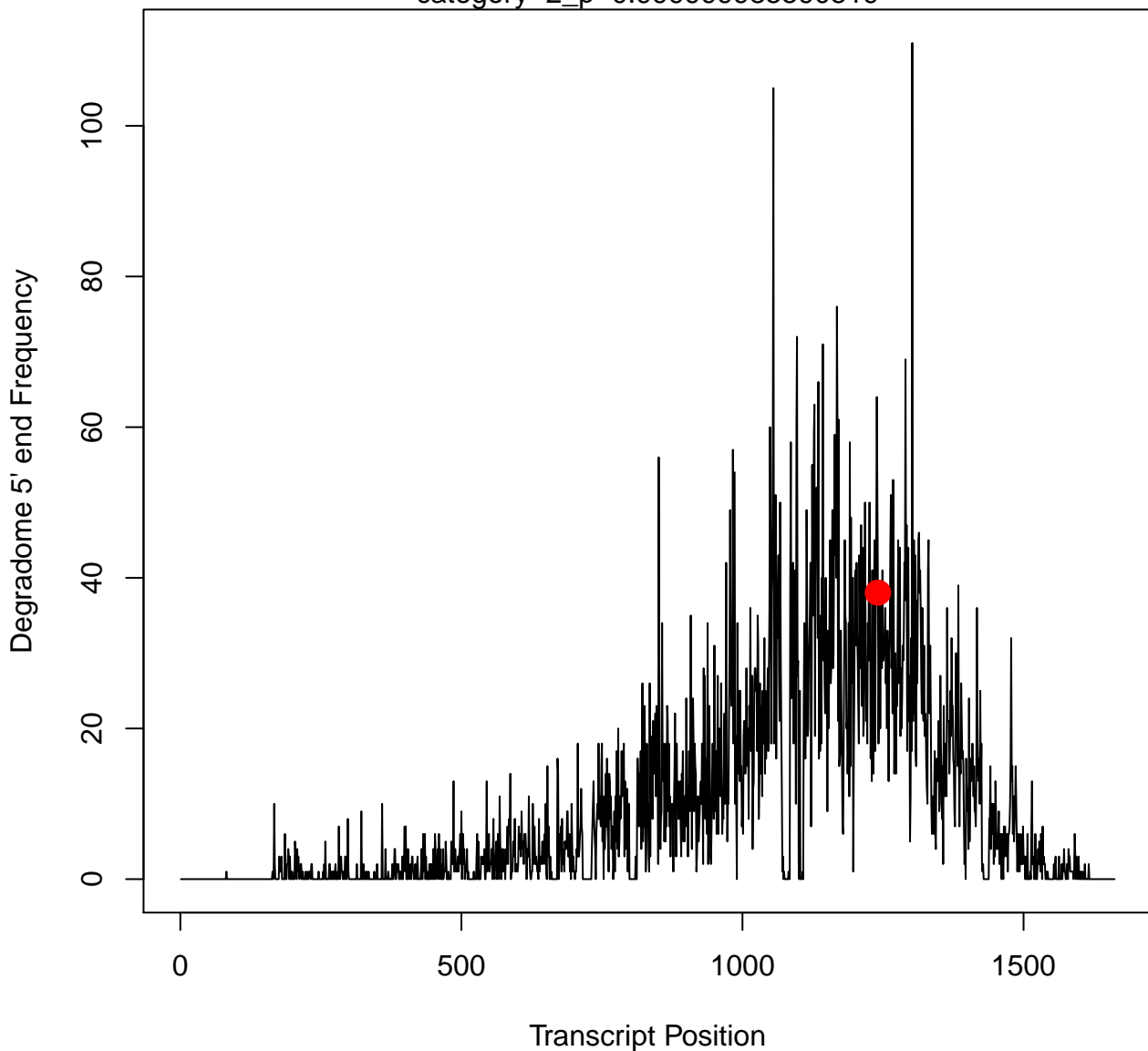

Supplement: Supplementary file 6 [file Data_Sheet_6.zip › Sit-miR159a_Seita.9G444100.1_1241_TPlot.pdf]

**T=Seita.9G569300.1\_Q=Sit-miR159a\_S=1398**

category=2\_p=0.999228916157735

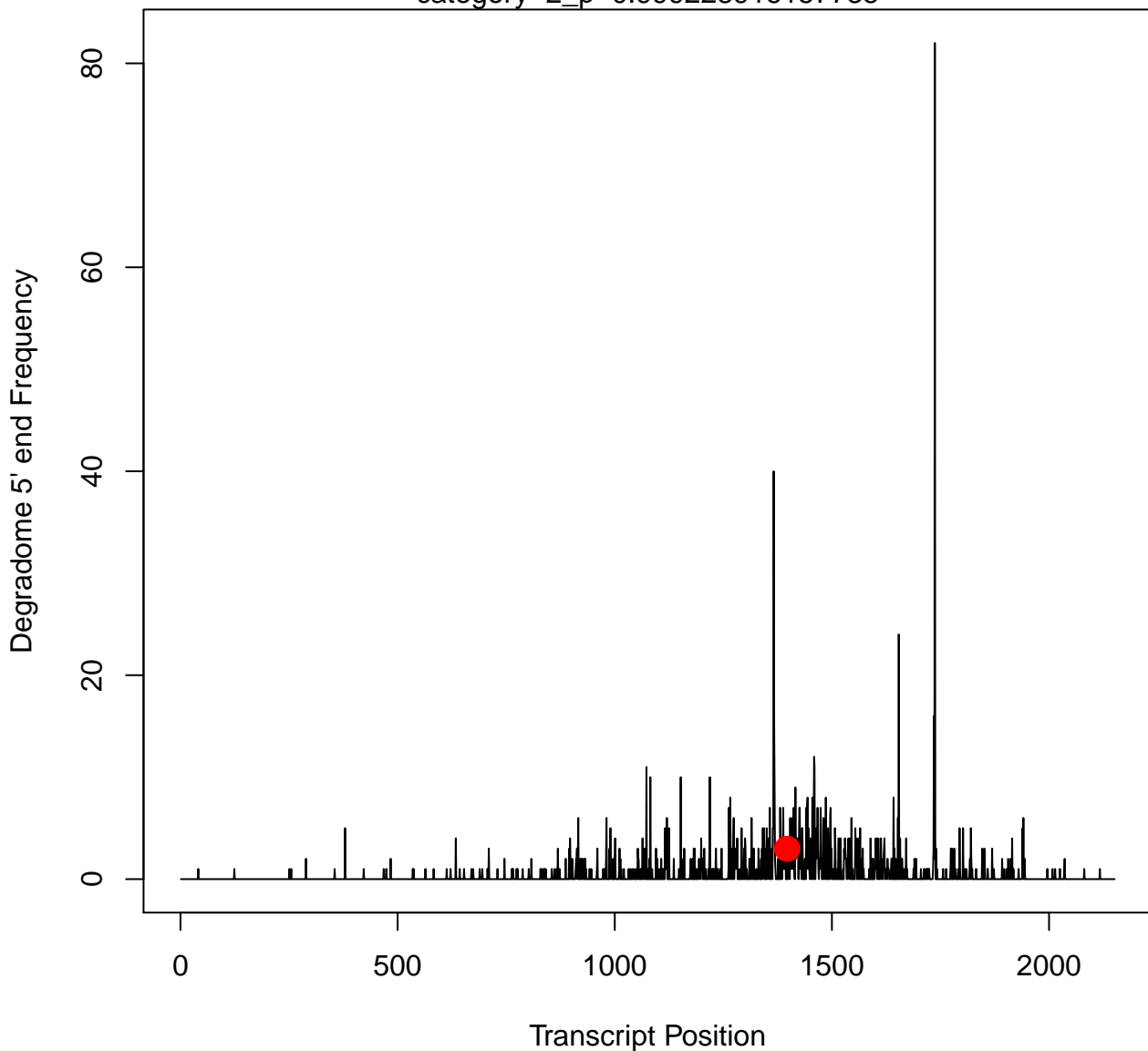

Supplement: Supplementary file 6 [file Data_Sheet_6.zip › Sit-miR159a_Seita.9G569300.1_1398_TPlot.pdf]

**T=Seita.1G185100.1\_Q=Sit-miR159b\_S=1133**

category=2\_p=0.99994742856906

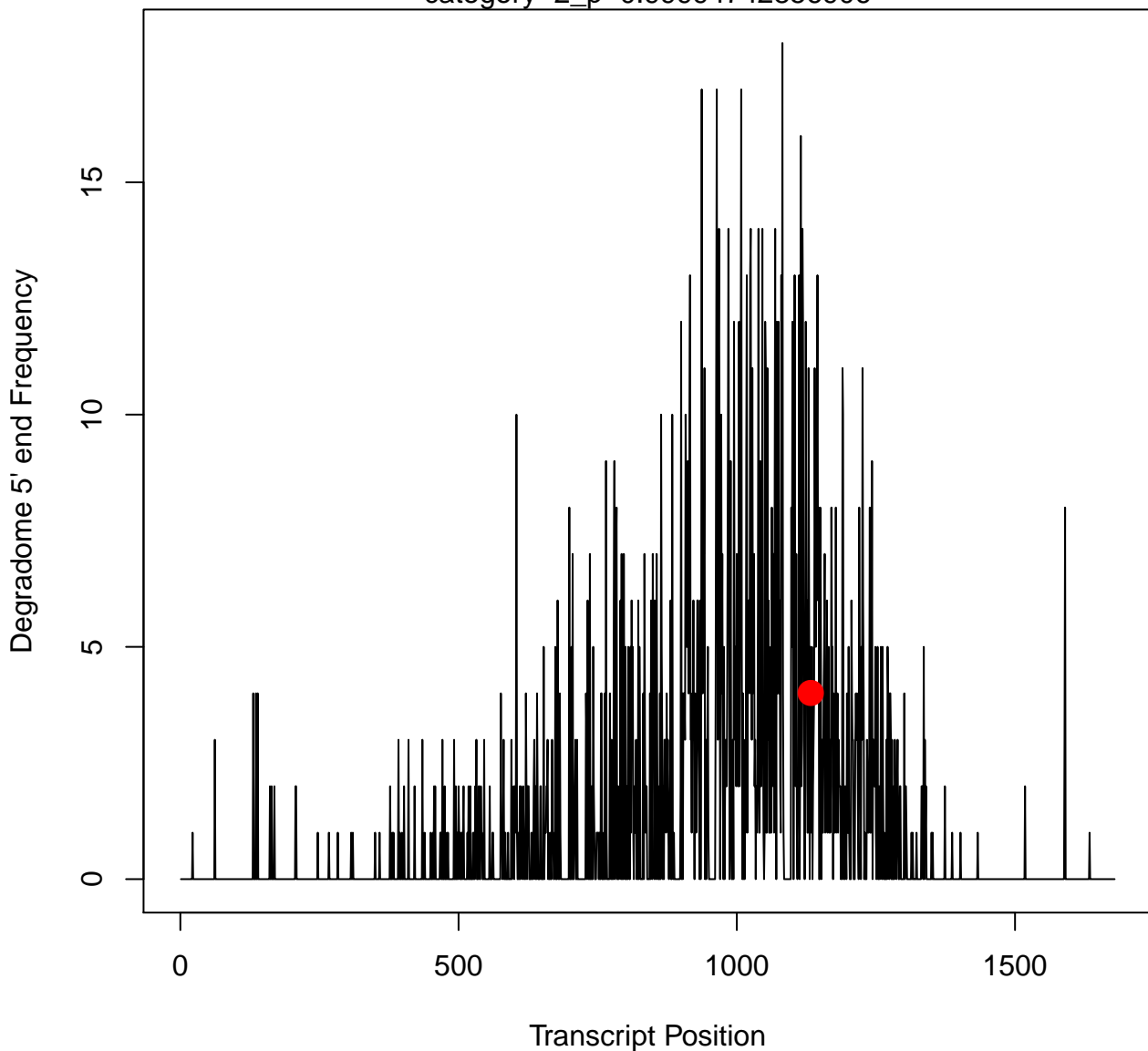

Supplement: Supplementary file 6 [file Data_Sheet_6.zip › Sit-miR159b_Seita.1G185100.1_1133_TPlot.pdf]

**T=Seita.2G353300.1\_Q=Sit-miR159b\_S=1486**

category=2\_p=0.998808100237523

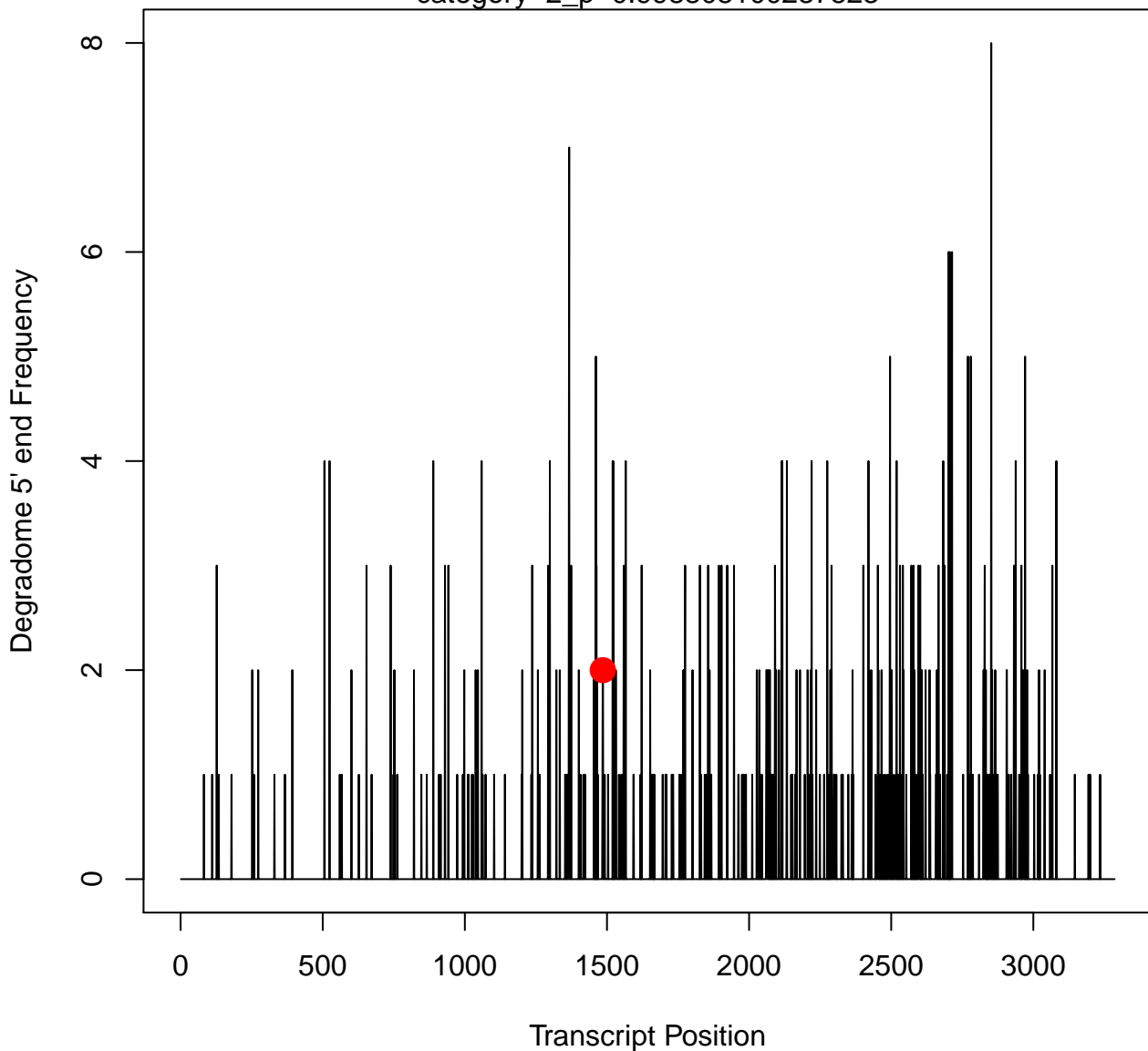

Supplement: Supplementary file 6 [file Data_Sheet_6.zip › Sit-miR159b_Seita.2G353300.1_1486_TPlot.pdf]

**T=Seita.2G370800.1\_Q=Sit-miR159b\_S=463**

category=2\_p=0.985938797181813

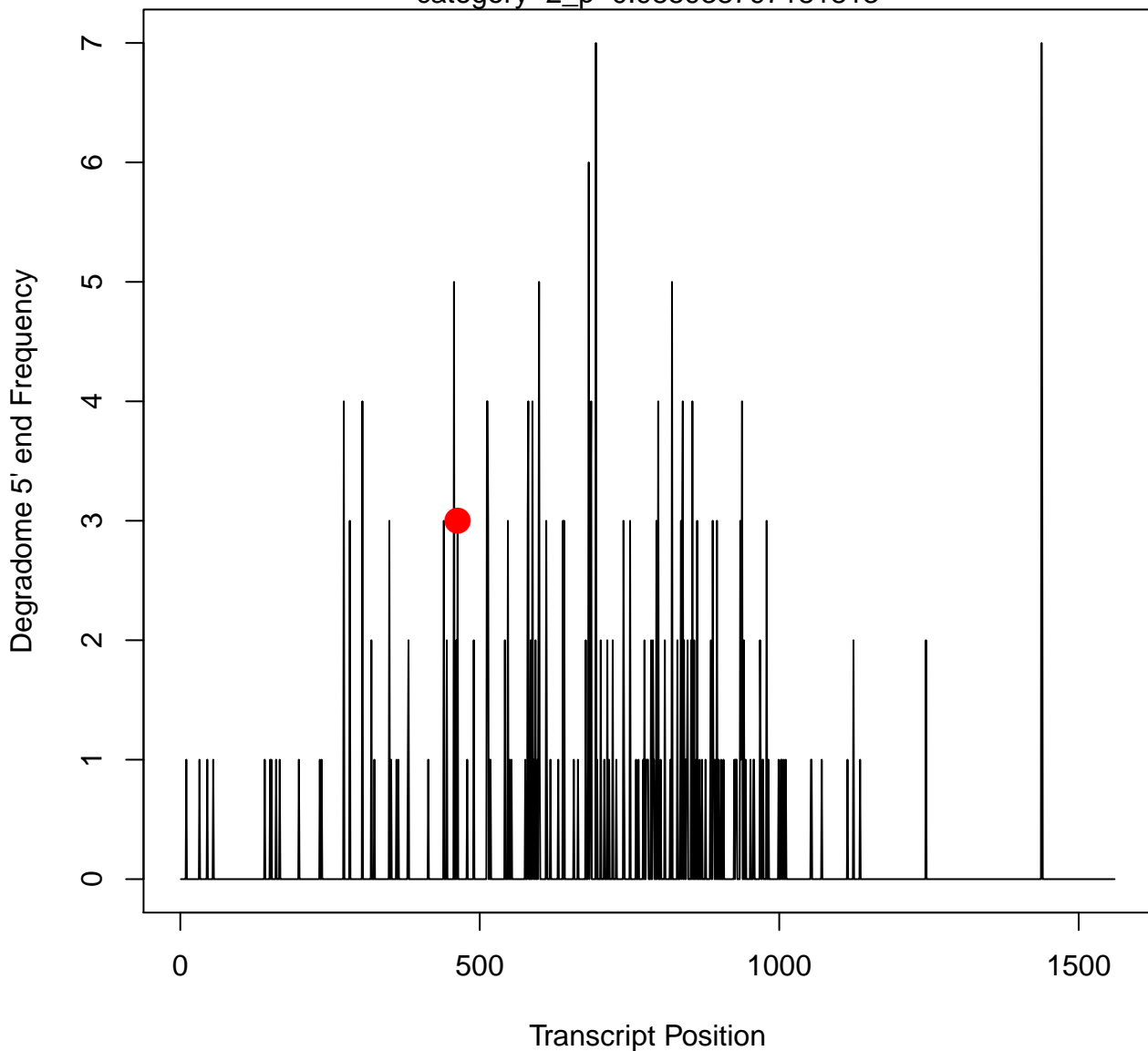

Supplement: Supplementary file 6 [file Data_Sheet_6.zip › Sit-miR159b_Seita.2G370800.1_463_TPlot.pdf]

**T=Seita.2G434400.1\_Q=Sit-miR159b\_S=363**

category=2\_p=0.489010414481851

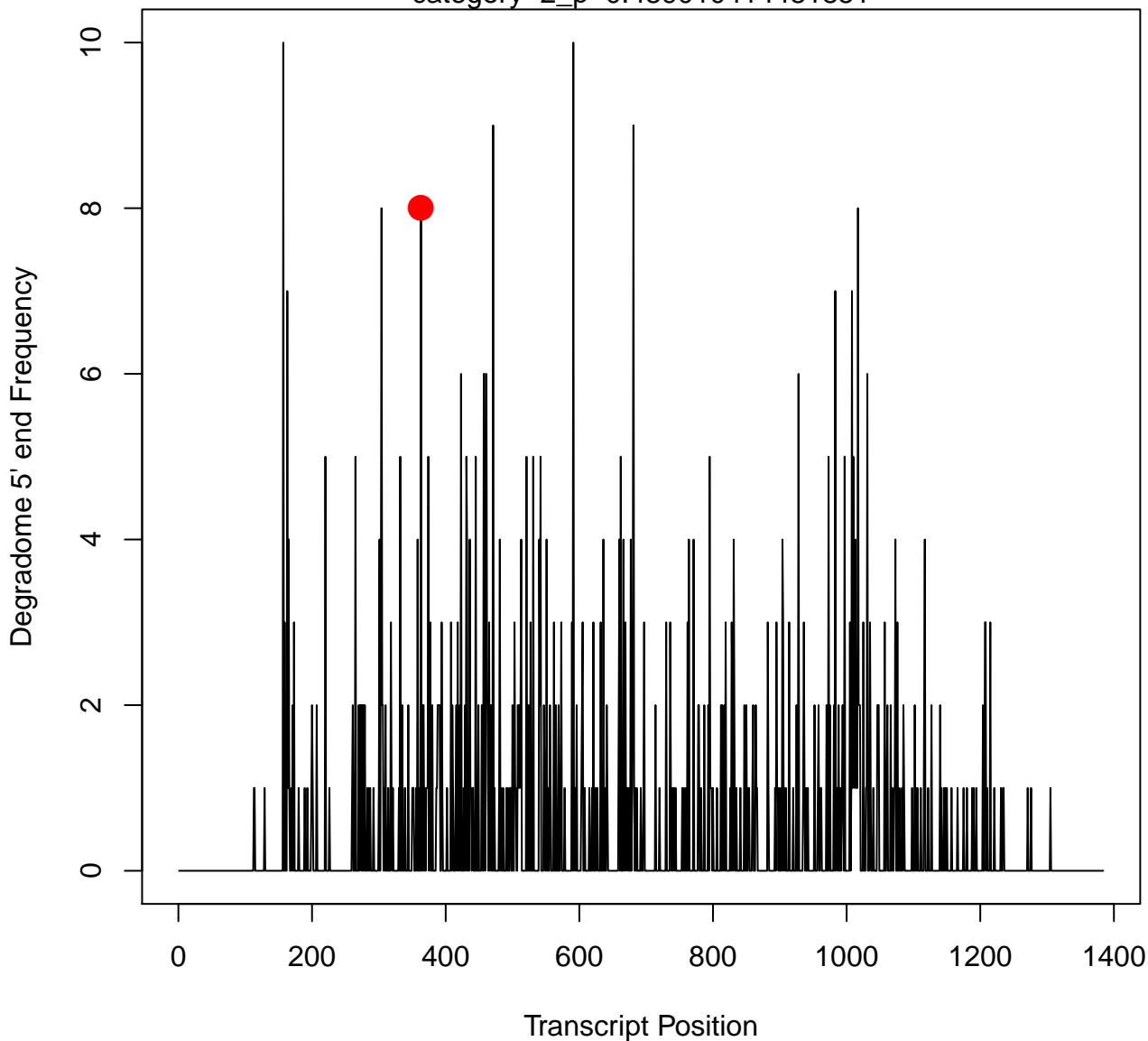

Supplement: Supplementary file 6 [file Data_Sheet_6.zip › Sit-miR159b_Seita.2G434400.1_363_TPlot.pdf]

**T=Seita.7G203700.1\_Q=Sit-miR159b\_S=1002**

category=2\_p=0.999443778201601

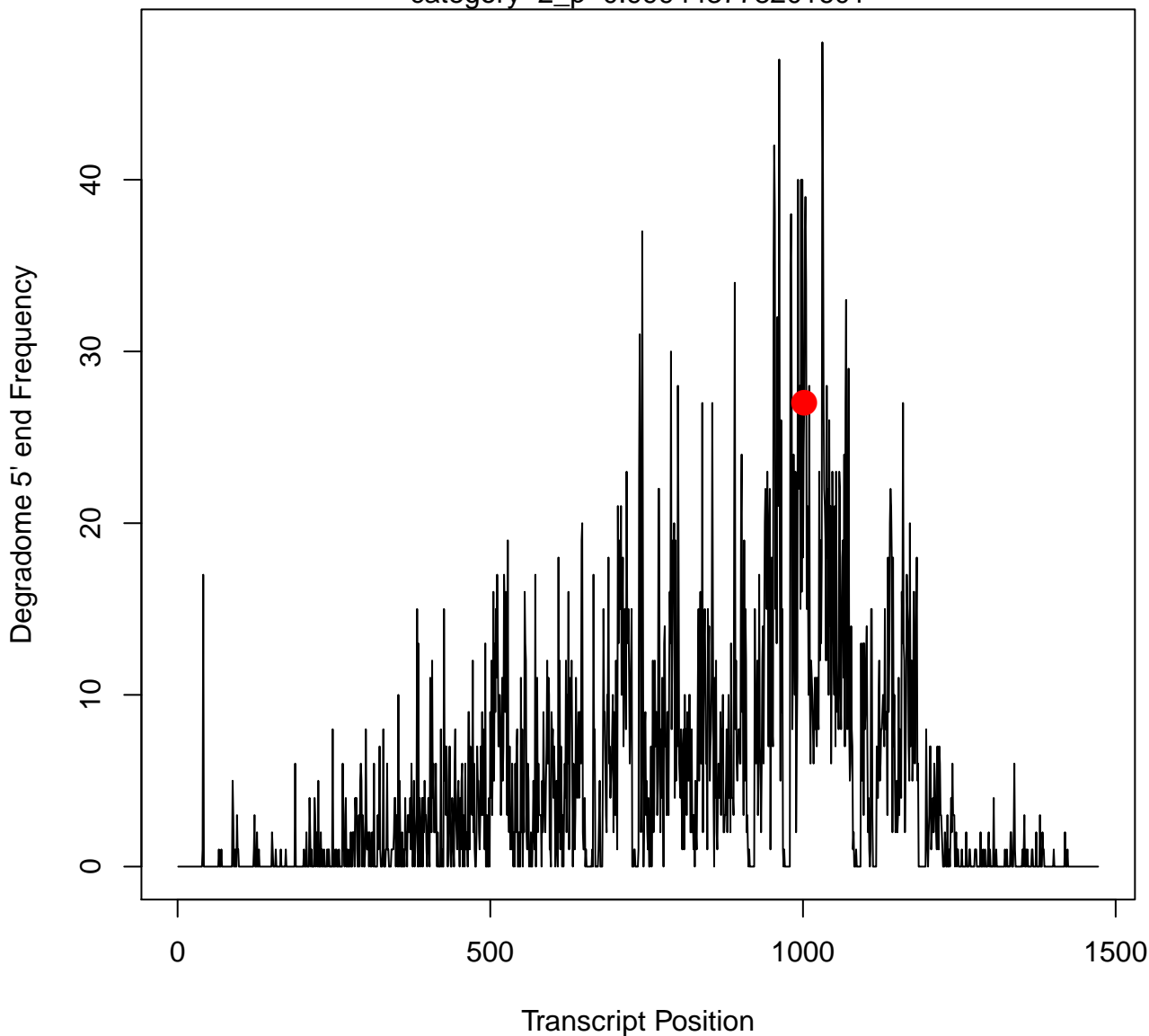

Supplement: Supplementary file 6 [file Data_Sheet_6.zip › Sit-miR159b_Seita.7G203700.1_1002_TPlot.pdf]

**T=Seita.8G008000.1\_Q=Sit-miR159b\_S=589**

category=2\_p=0.970410784870589

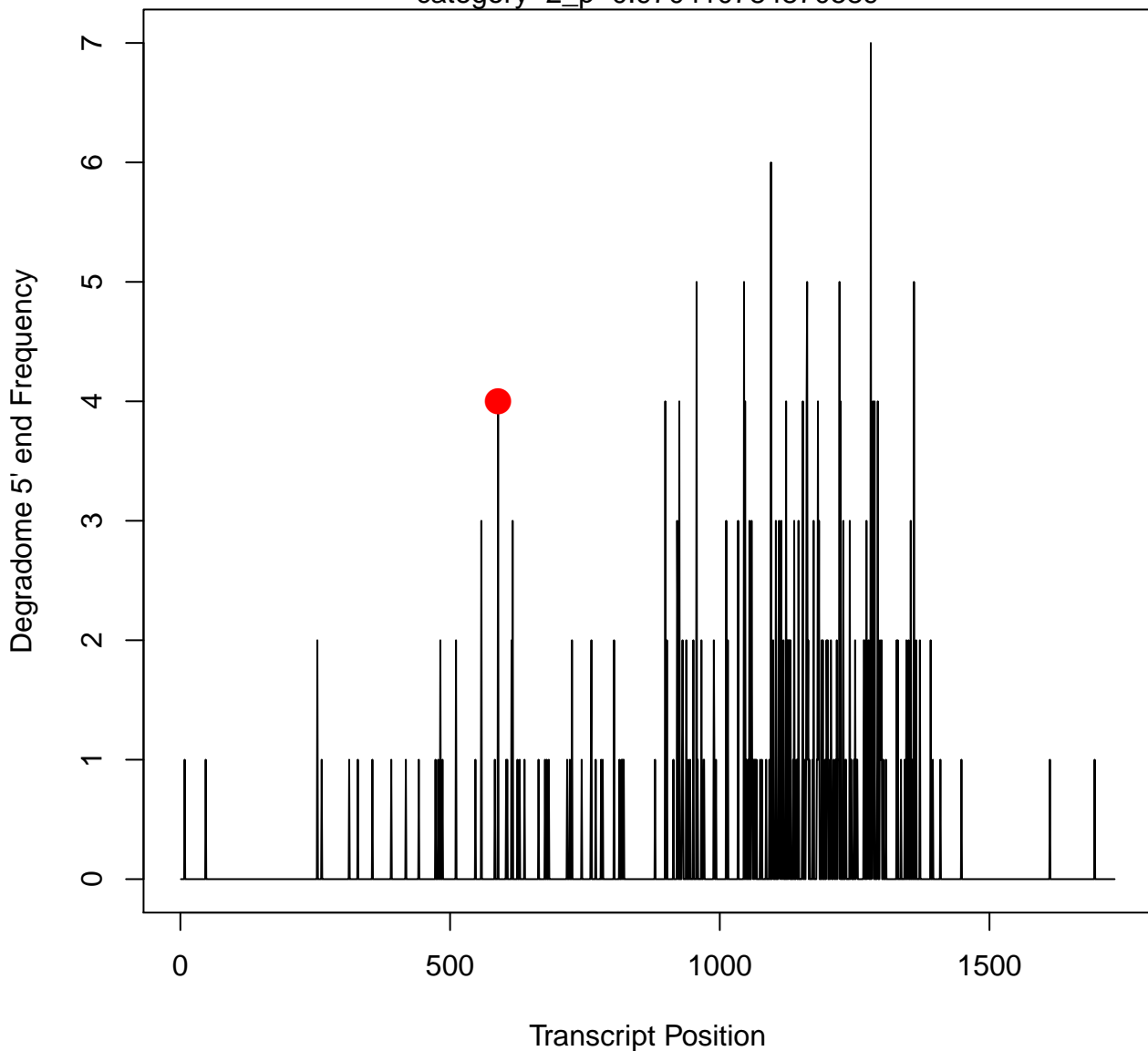

Supplement: Supplementary file 6 [file Data_Sheet_6.zip › Sit-miR159b_Seita.8G008000.1_589_TPlot.pdf]

**T=Seita.8G060100.1\_Q=Sit-miR159b\_S=2349**

category=2\_p=0.99442680876666

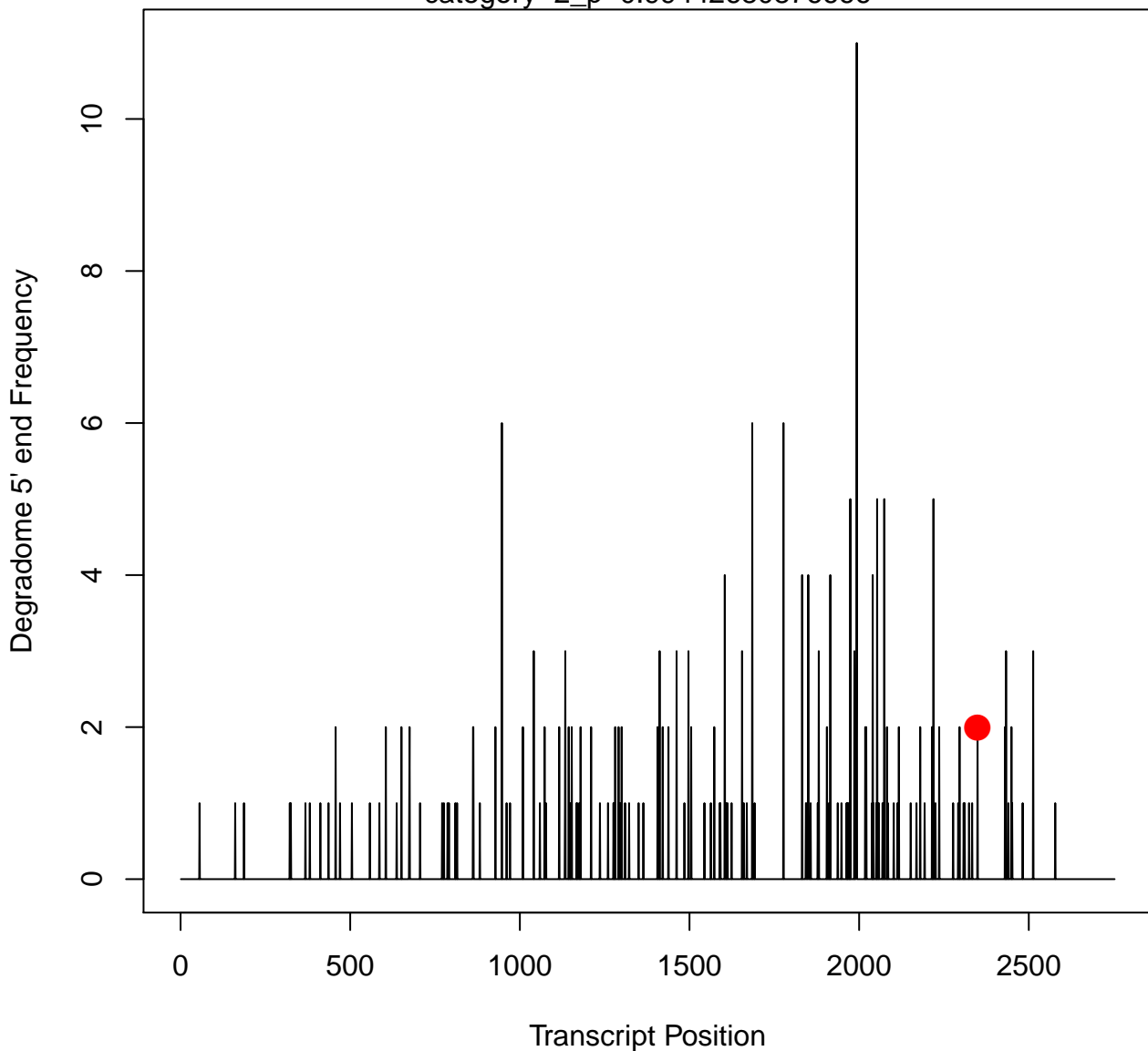

Supplement: Supplementary file 6 [file Data_Sheet_6.zip › Sit-miR159b_Seita.8G060100.1_2349_TPlot.pdf]

**T=Seita.4G083900.1\_Q=Sit-miR159c\_S=4244**

category=2\_p=0.450541279353456

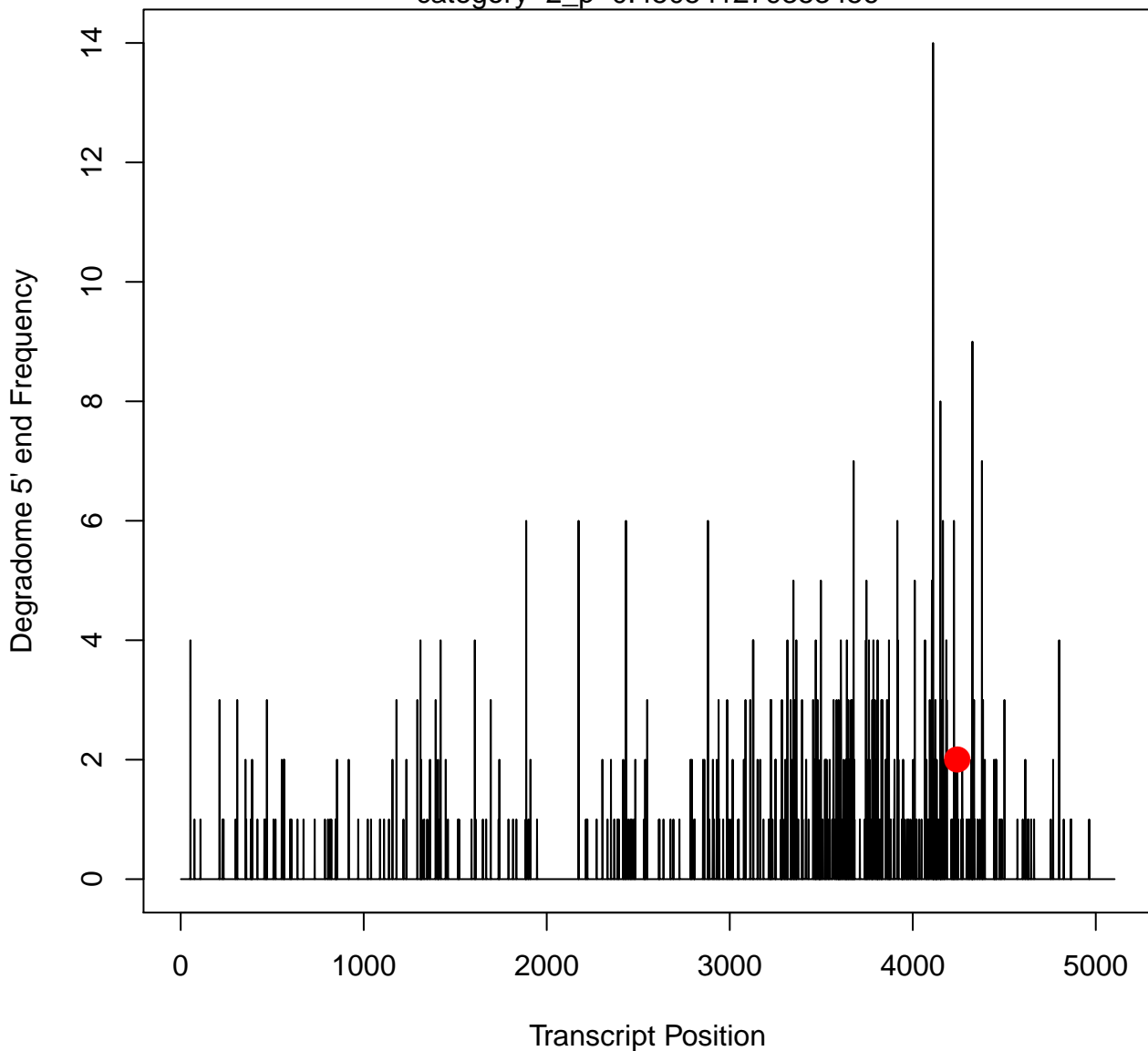

Supplement: Supplementary file 6 [file Data_Sheet_6.zip › Sit-miR159c_Seita.4G083900.1_4244_TPlot.pdf]

**T=Seita.4G221900.1\_Q=Sit-miR159c\_S=1159**

category=2\_p=0.0356415524988544

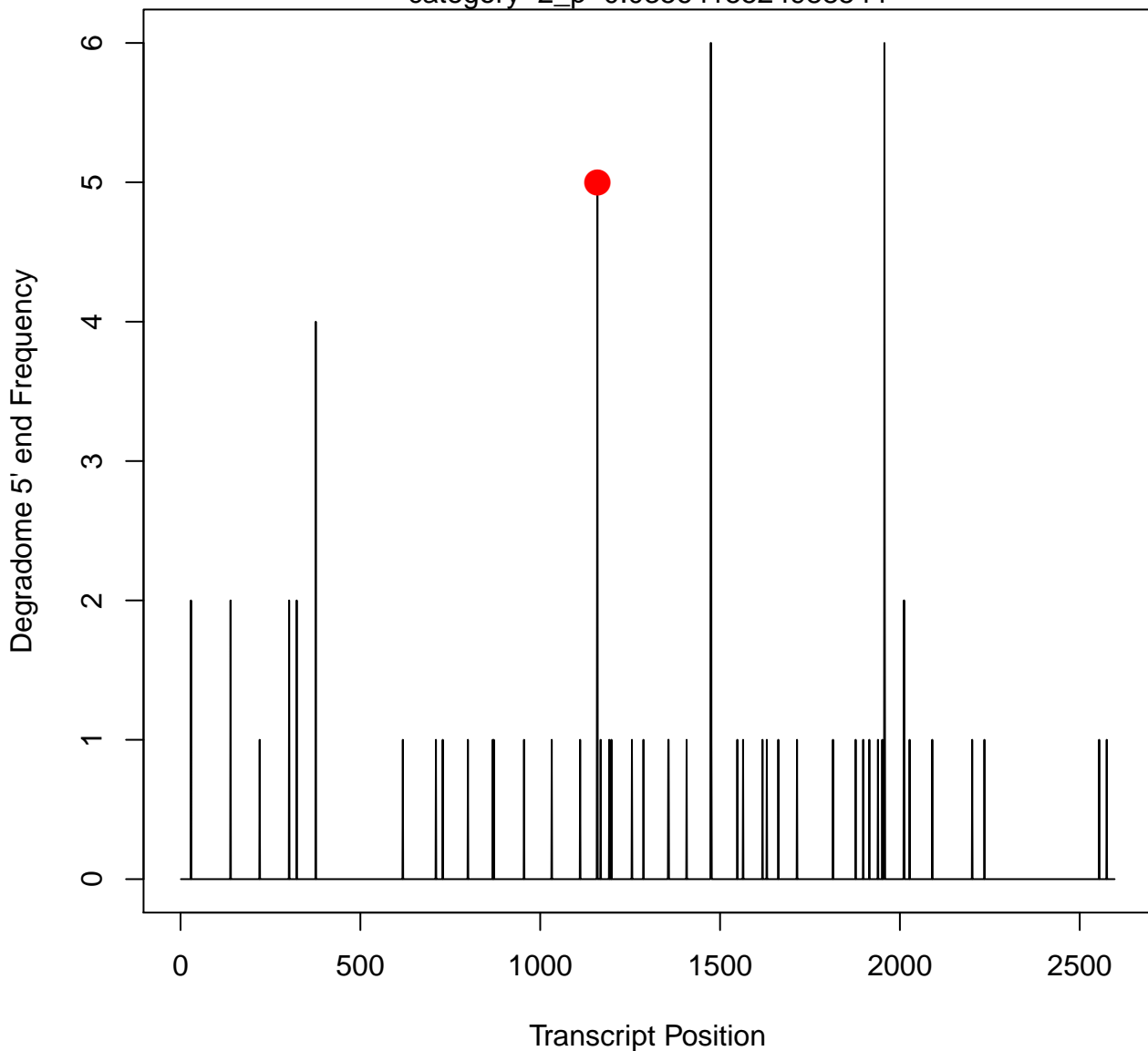

Supplement: Supplementary file 6 [file Data_Sheet_6.zip › Sit-miR159c_Seita.4G221900.1_1159_TPlot.pdf]

**T=Seita.5G318300.1\_Q=Sit-miR159c\_S=1126**

category=2\_p=0.999941381549961

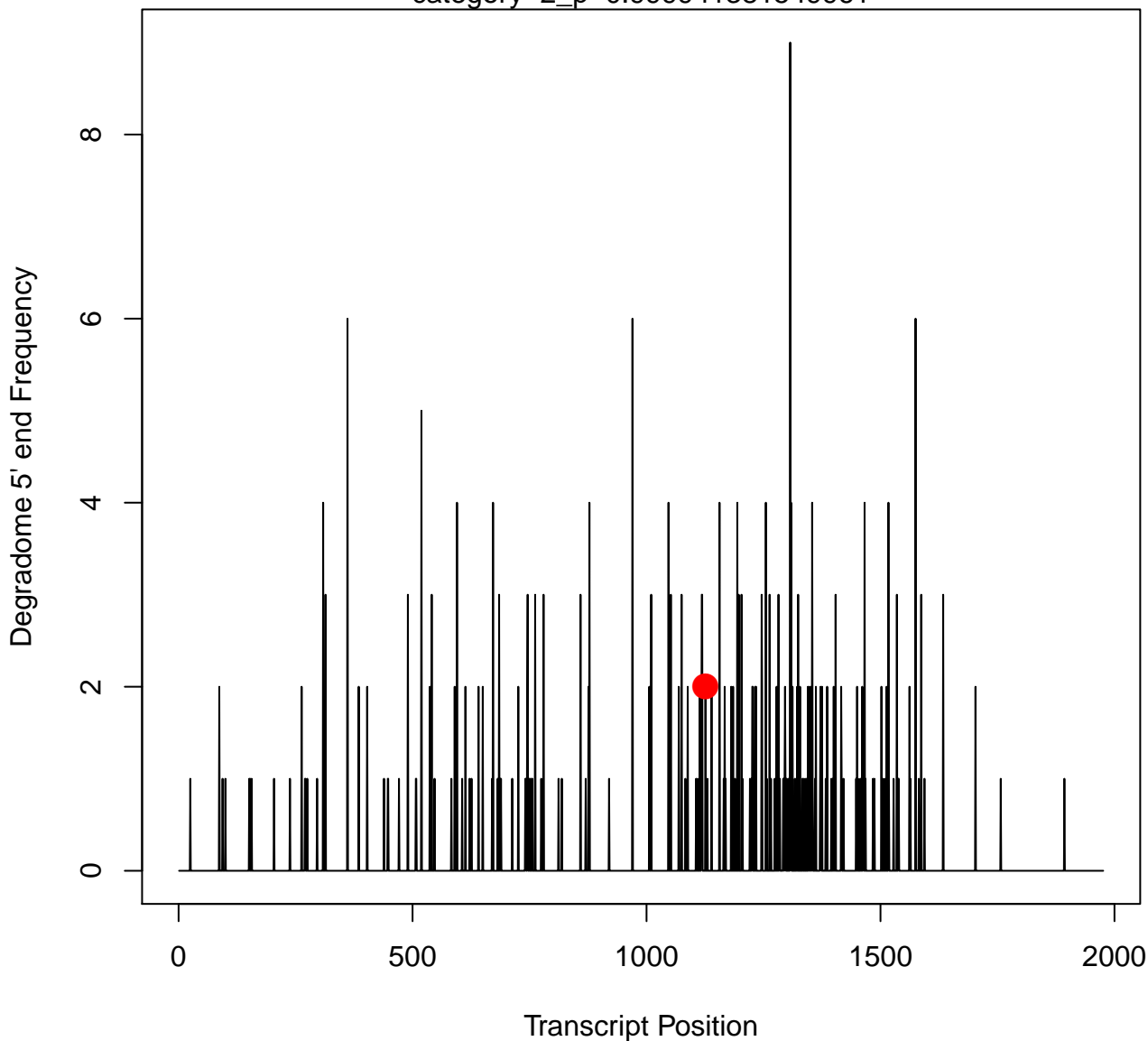

Supplement: Supplementary file 6 [file Data_Sheet_6.zip › Sit-miR159c_Seita.5G318300.1_1126_TPlot.pdf]

**T=Seita.5G355300.1\_Q=Sit-miR159c\_S=1278**

category=0\_p=0.0015013508746784

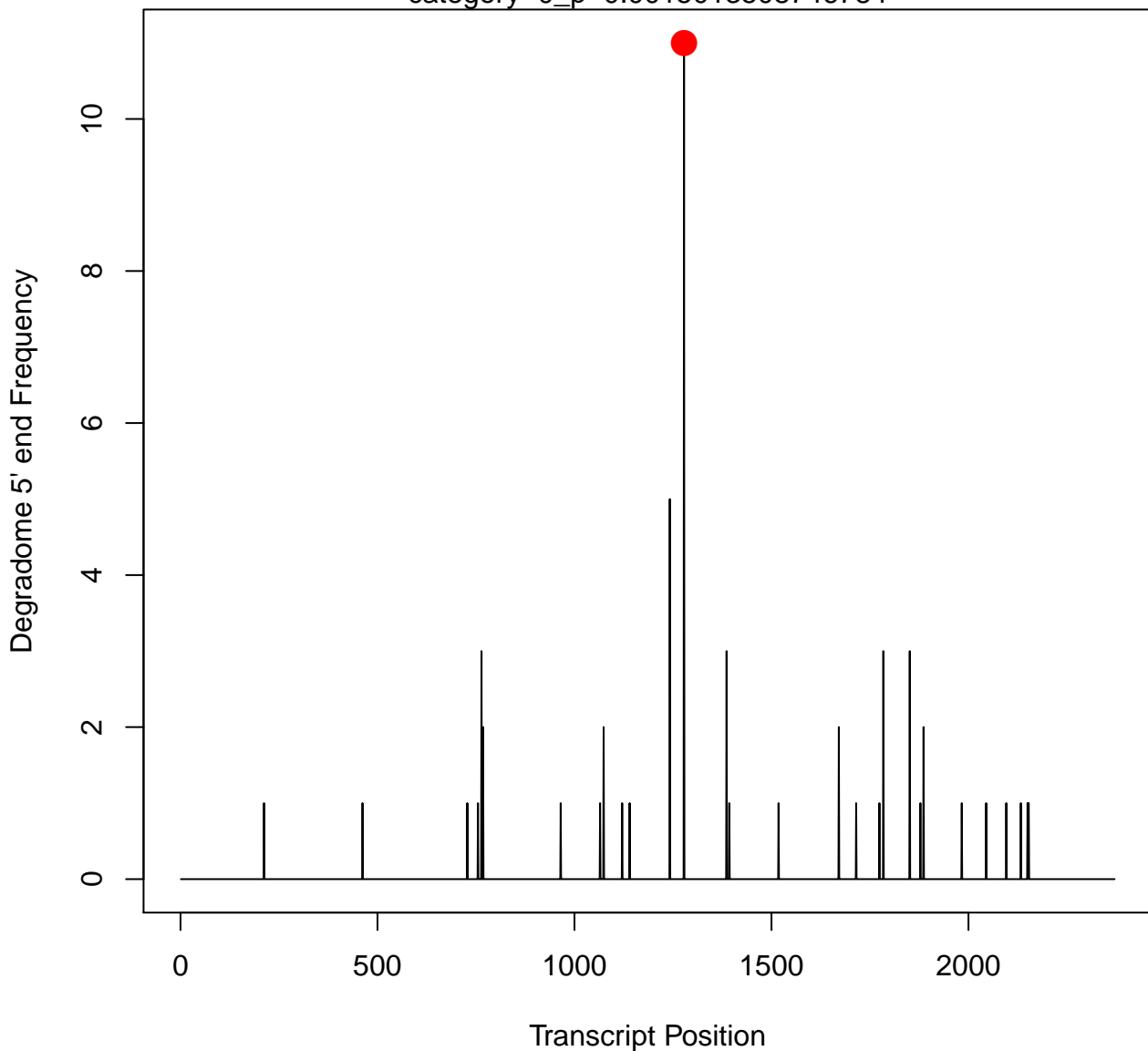

Supplement: Supplementary file 6 [file Data_Sheet_6.zip › Sit-miR159c_Seita.5G355300.1_1278_TPlot.pdf]

**T=Seita.7G095500.1\_Q=Sit-miR159c\_S=267**

category=2\_p=0.985419111685471

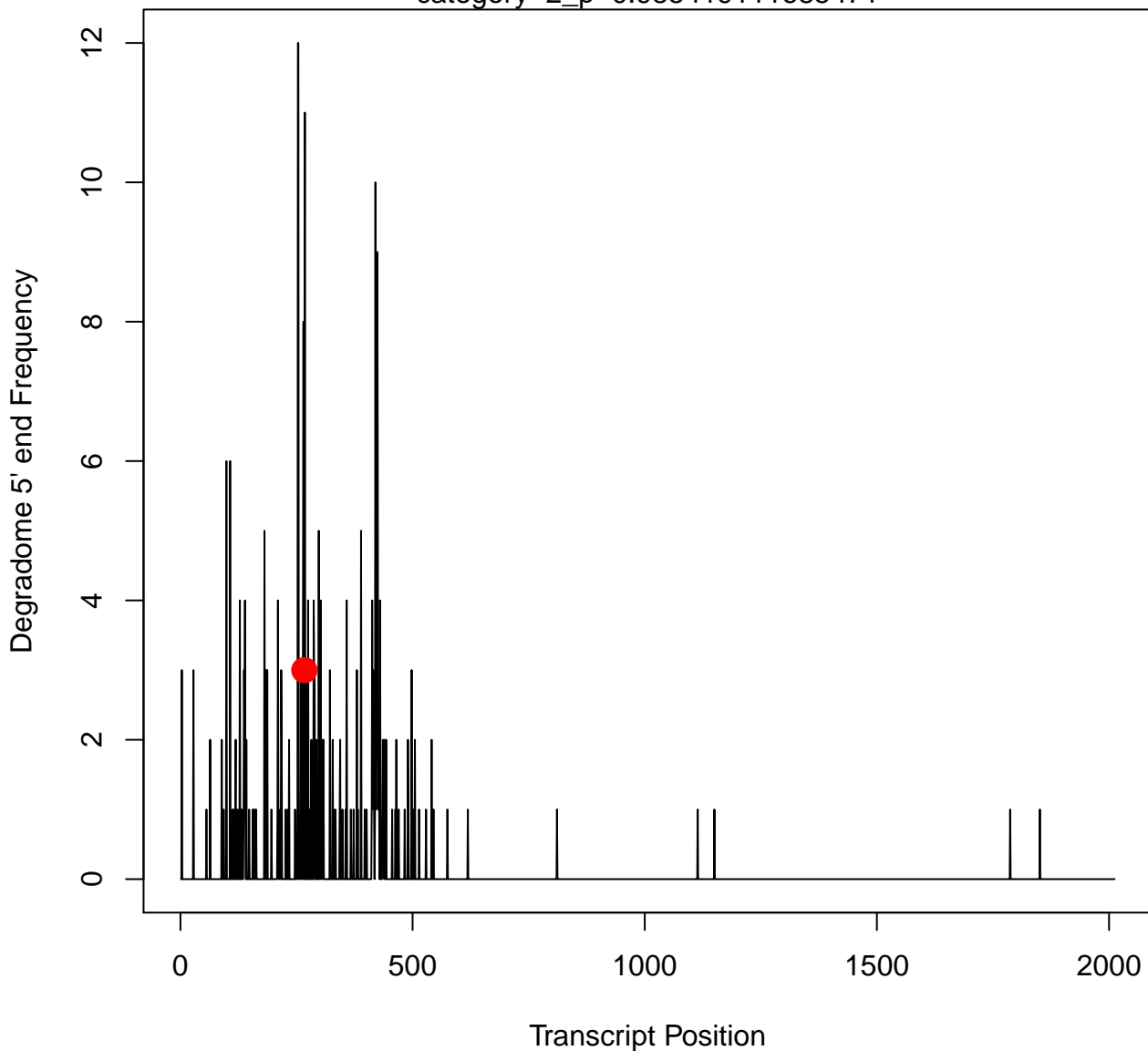

Supplement: Supplementary file 6 [file Data_Sheet_6.zip › Sit-miR159c_Seita.7G095500.1_267_TPlot.pdf]

**T=Seita.9G018400.1\_Q=Sit-miR159c\_S=369**

category=2\_p=0.990394353314983

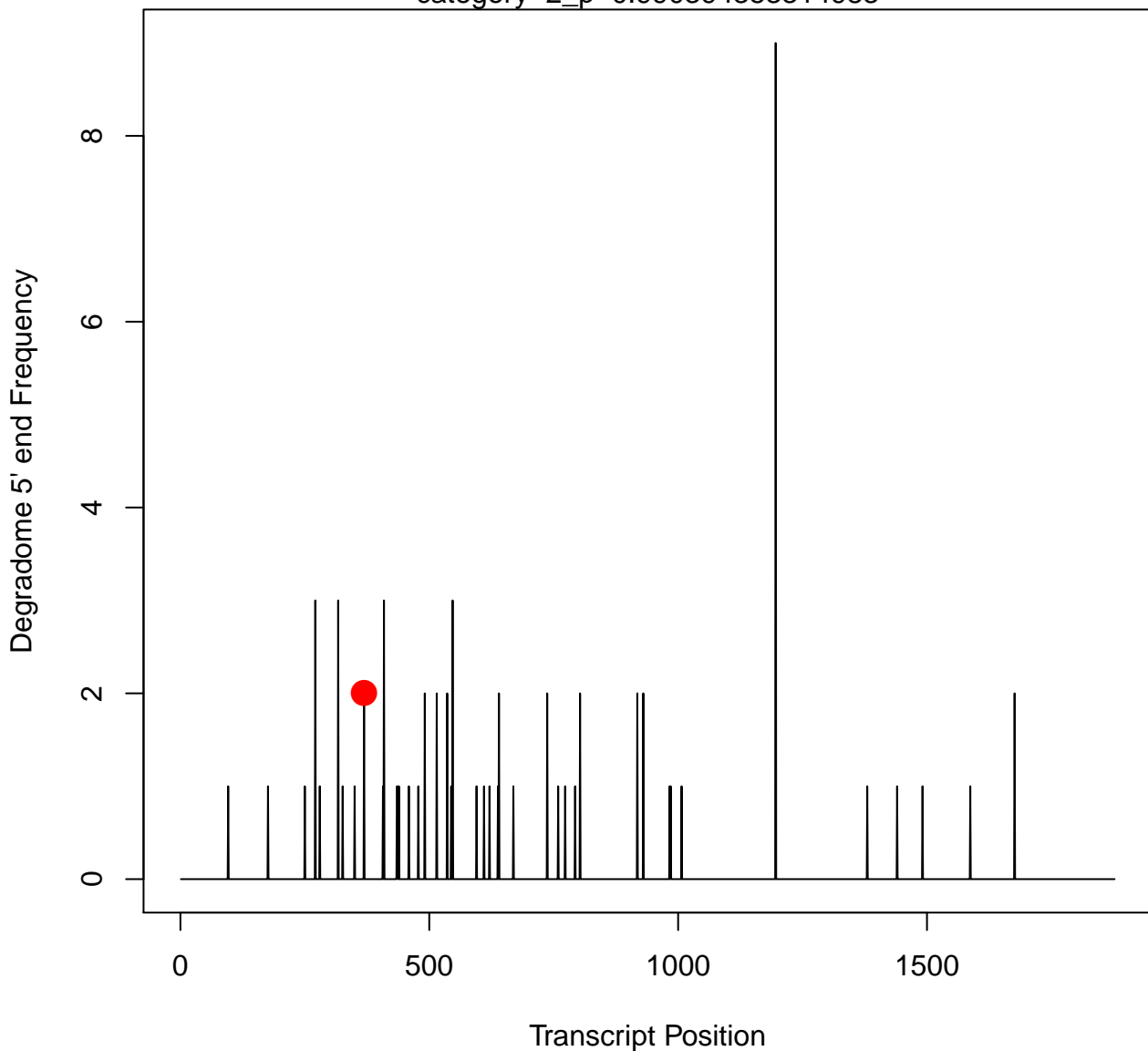

Supplement: Supplementary file 6 [file Data_Sheet_6.zip › Sit-miR159c_Seita.9G018400.1_369_TPlot.pdf]

**T=Seita.9G570600.1\_Q=Sit-miR159c\_S=2804**

category=2\_p=0.990039339926034

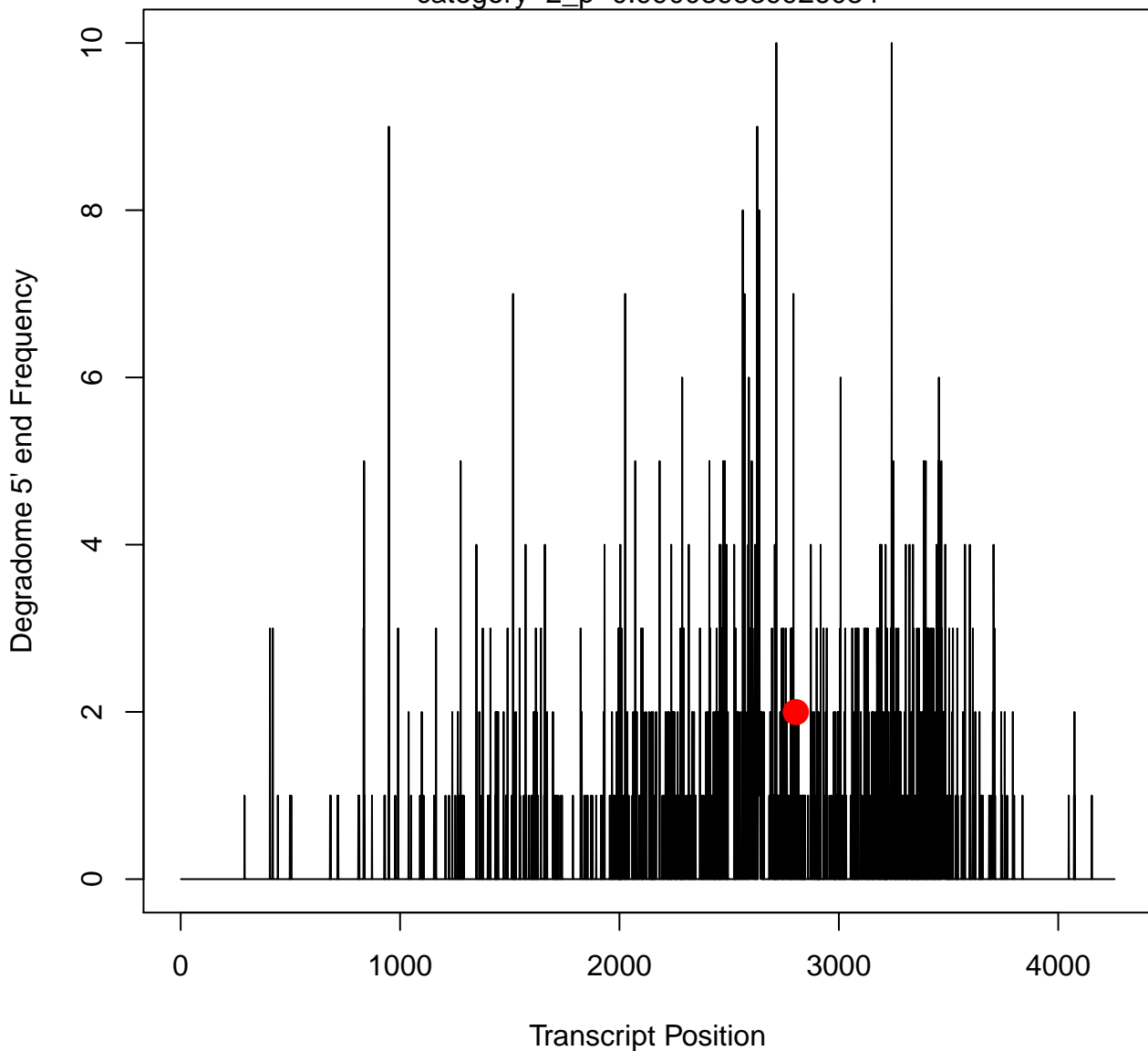

Supplement: Supplementary file 6 [file Data_Sheet_6.zip › Sit-miR159c_Seita.9G570600.1_2804_TPlot.pdf]

**T=Seita.1G241500.1\_Q=Sit-miR160a\_S=1660**

category=2\_p=0.0867362432792793

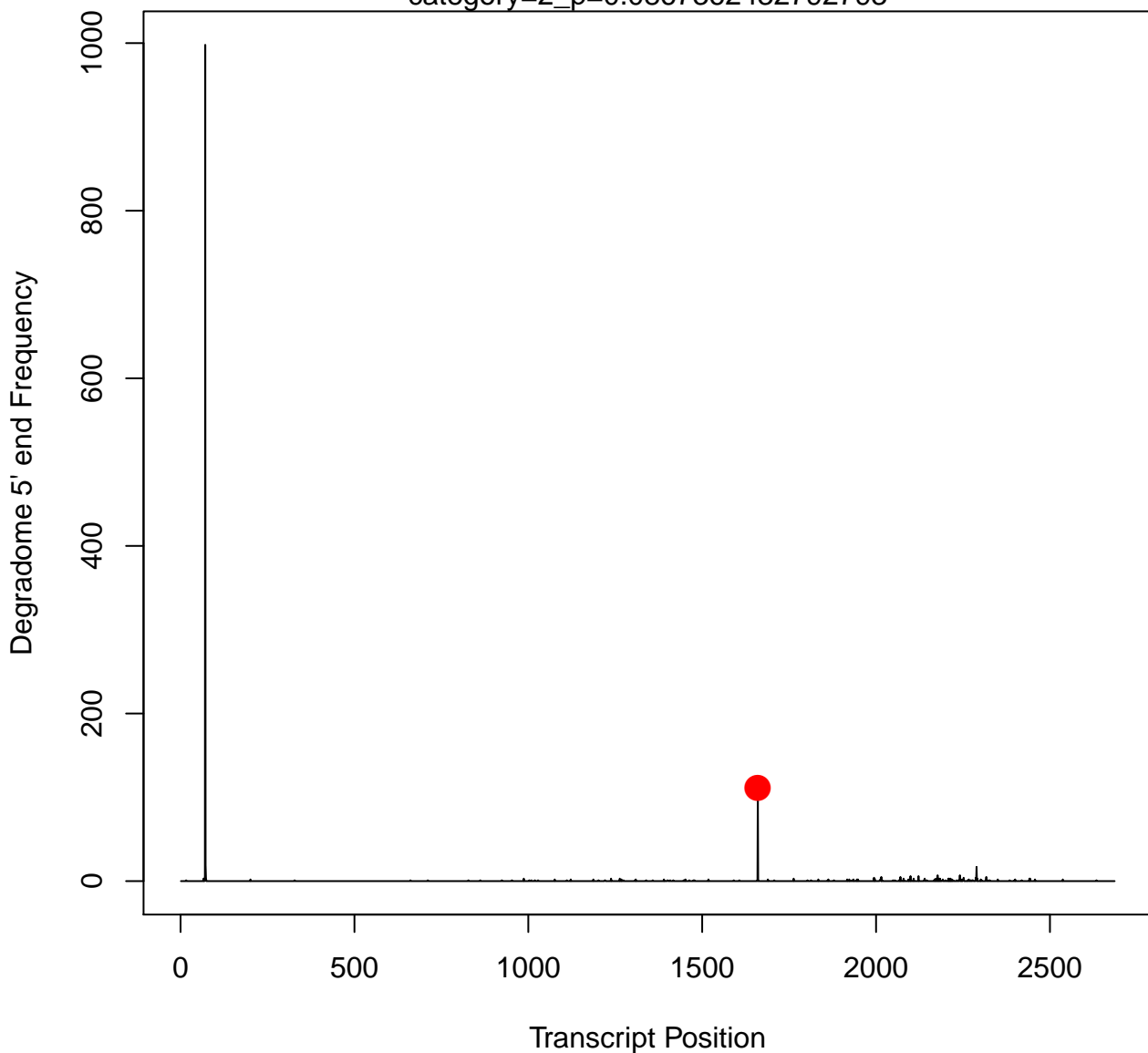

Supplement: Supplementary file 6 [file Data_Sheet_6.zip › Sit-miR160a_Seita.1G241500.1_1660_TPlot.pdf]

**T=Seita.1G327600.1\_Q=Sit-miR160a\_S=1165**

category=2\_p=0.999999995880199

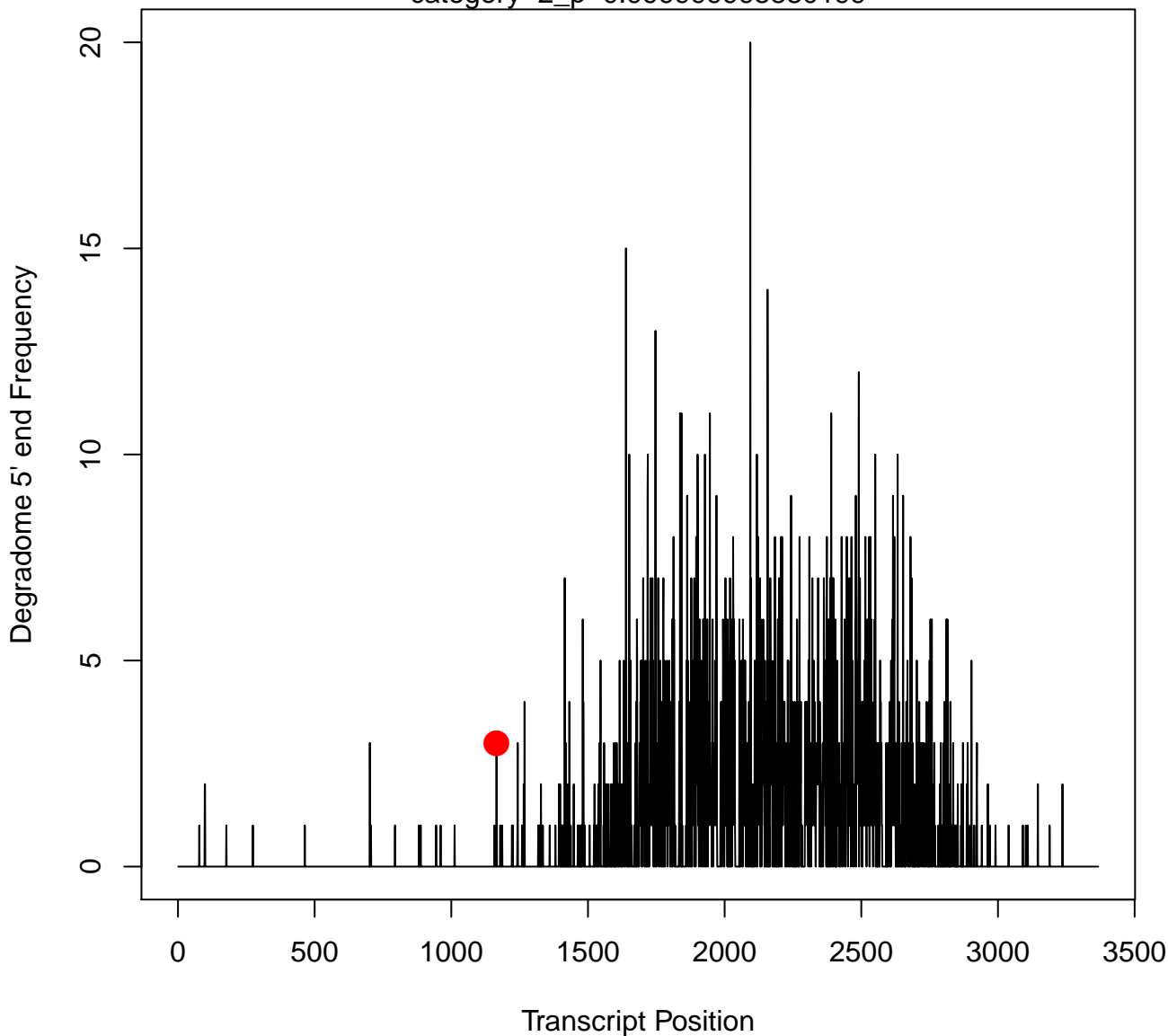

Supplement: Supplementary file 6 [file Data_Sheet_6.zip › Sit-miR160a_Seita.1G327600.1_1165_TPlot.pdf]

**T=Seita.2G278900.1\_Q=Sit-miR160a\_S=1439**

category=2\_p=0.999997506652074

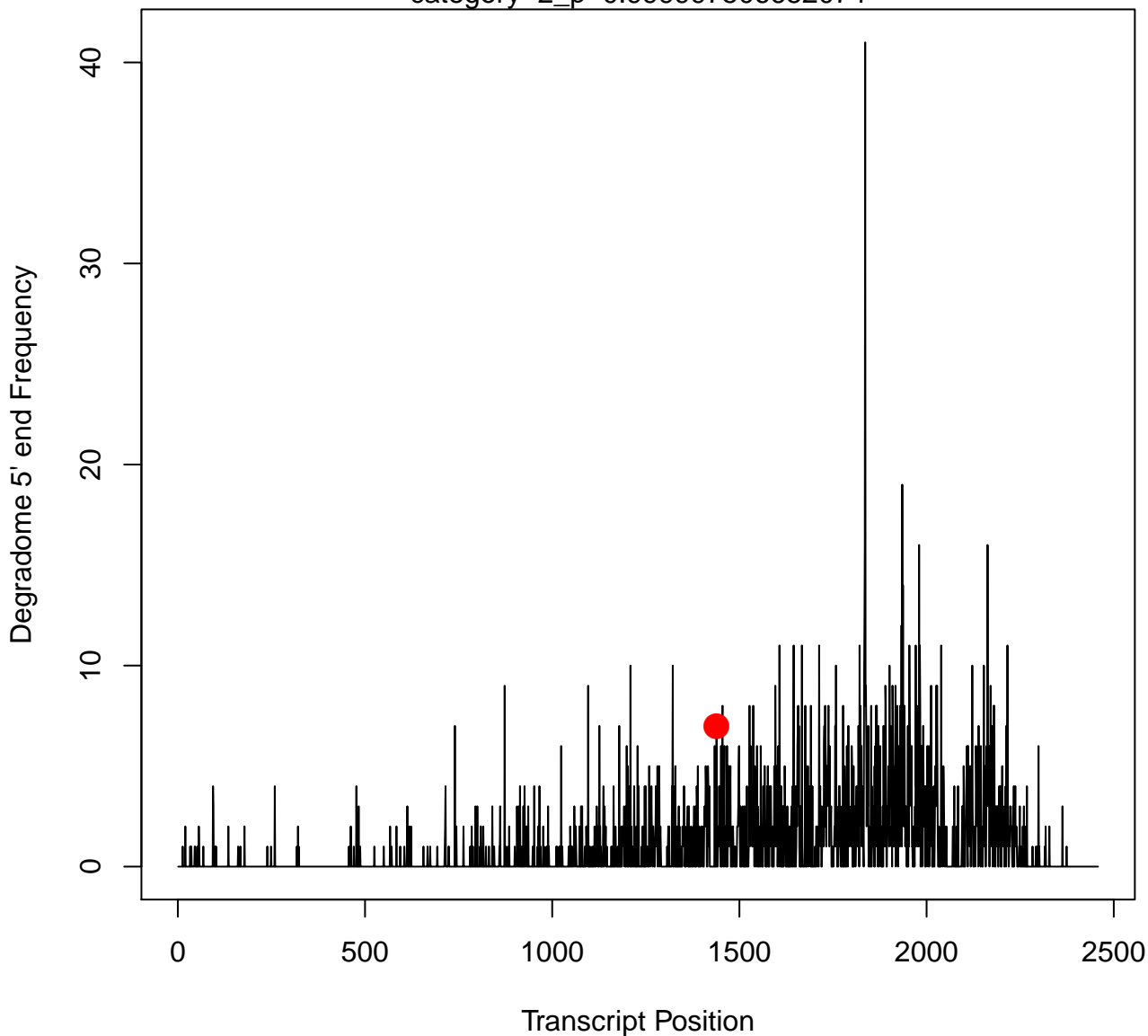

Supplement: Supplementary file 6 [file Data_Sheet_6.zip › Sit-miR160a_Seita.2G278900.1_1439_TPlot.pdf]

**T=Seita.3G010200.1\_Q=Sit-miR160a\_S=1143**

category=2\_p=0.999994657129926

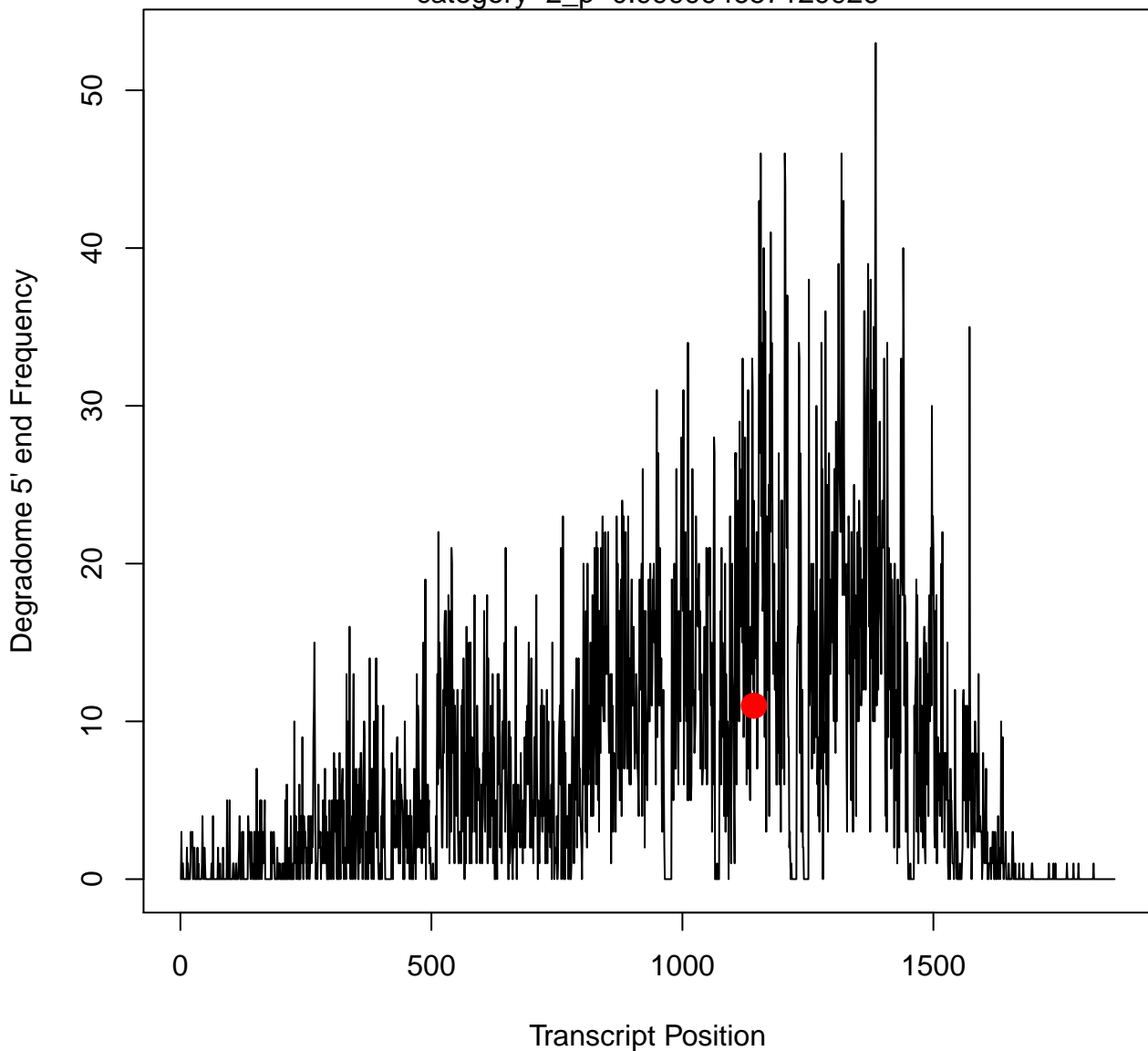

Supplement: Supplementary file 6 [file Data_Sheet_6.zip › Sit-miR160a_Seita.3G010200.1_1143_TPlot.pdf]

**T=Seita.3G163700.1\_Q=Sit-miR160a\_S=147**

category=2\_p=0.999997882340719

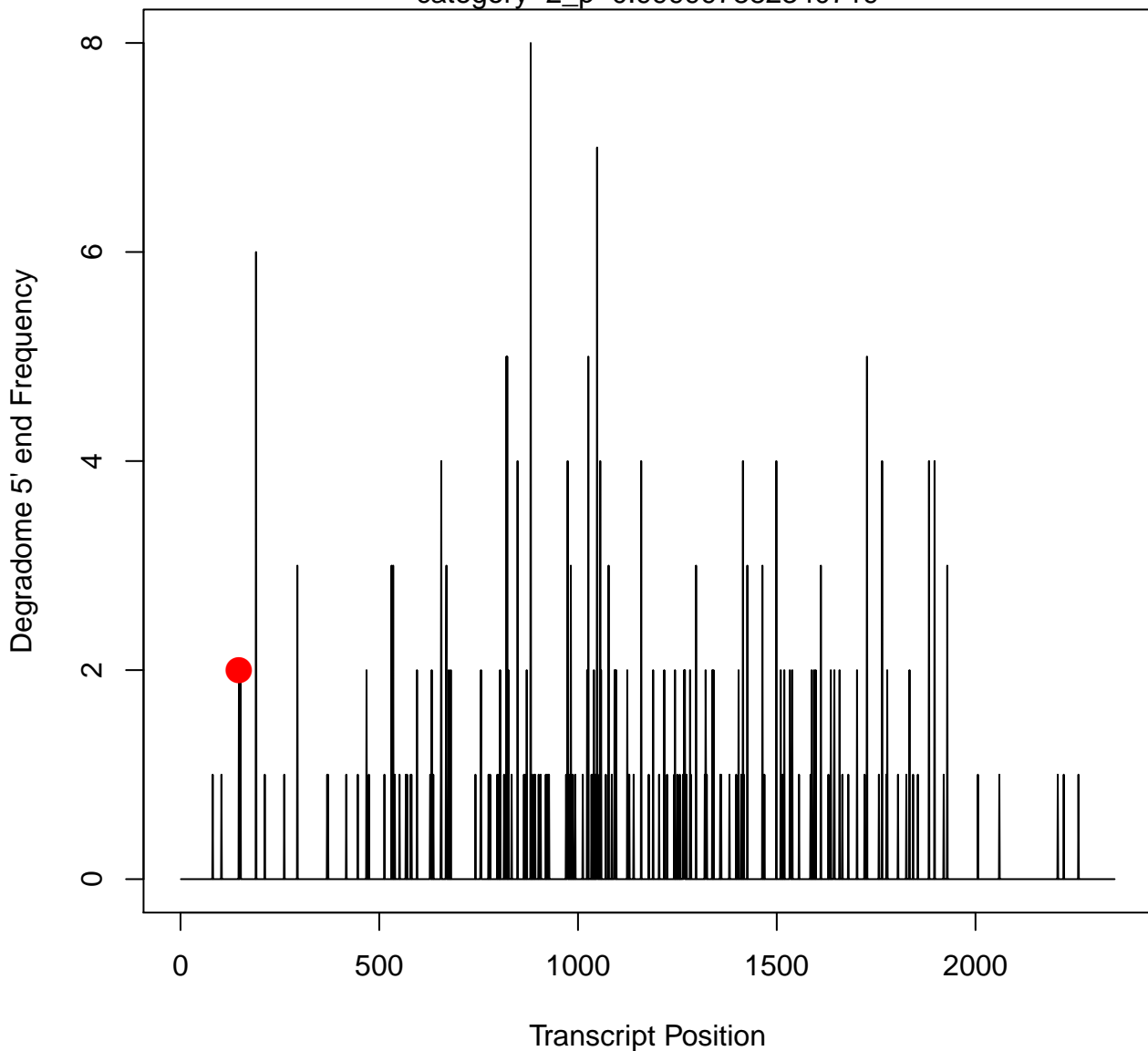

Supplement: Supplementary file 6 [file Data_Sheet_6.zip › Sit-miR160a_Seita.3G163700.1_147_TPlot.pdf]

**T=Seita.4G090500.1\_Q=Sit-miR160a\_S=945**

category=2\_p=0.999999999834048

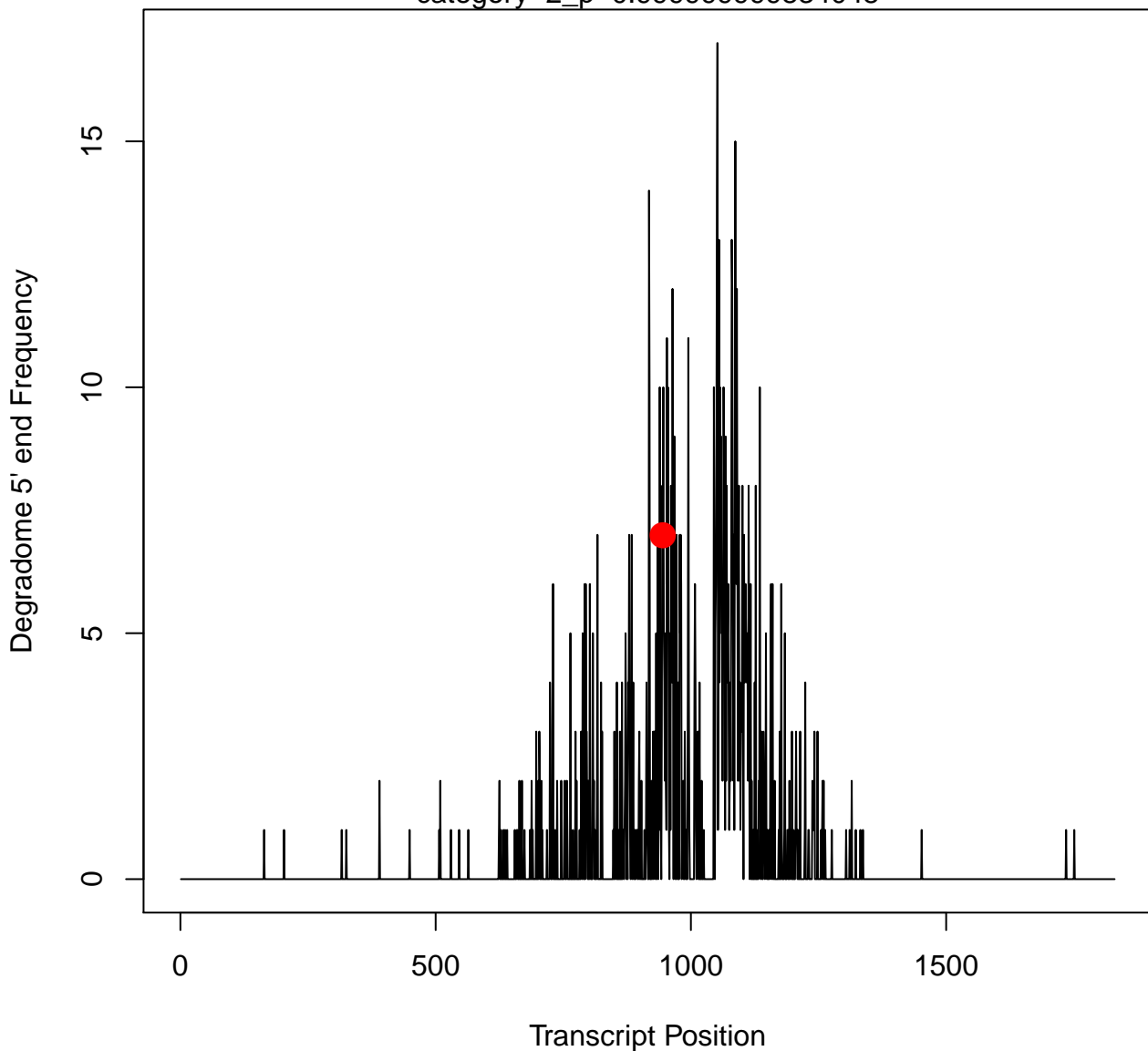

Supplement: Supplementary file 6 [file Data_Sheet_6.zip › Sit-miR160a_Seita.4G090500.1_945_TPlot.pdf]

**T=Seita.4G258300.1\_Q=Sit-miR160a\_S=212**

category=2\_p=0.99999999866521

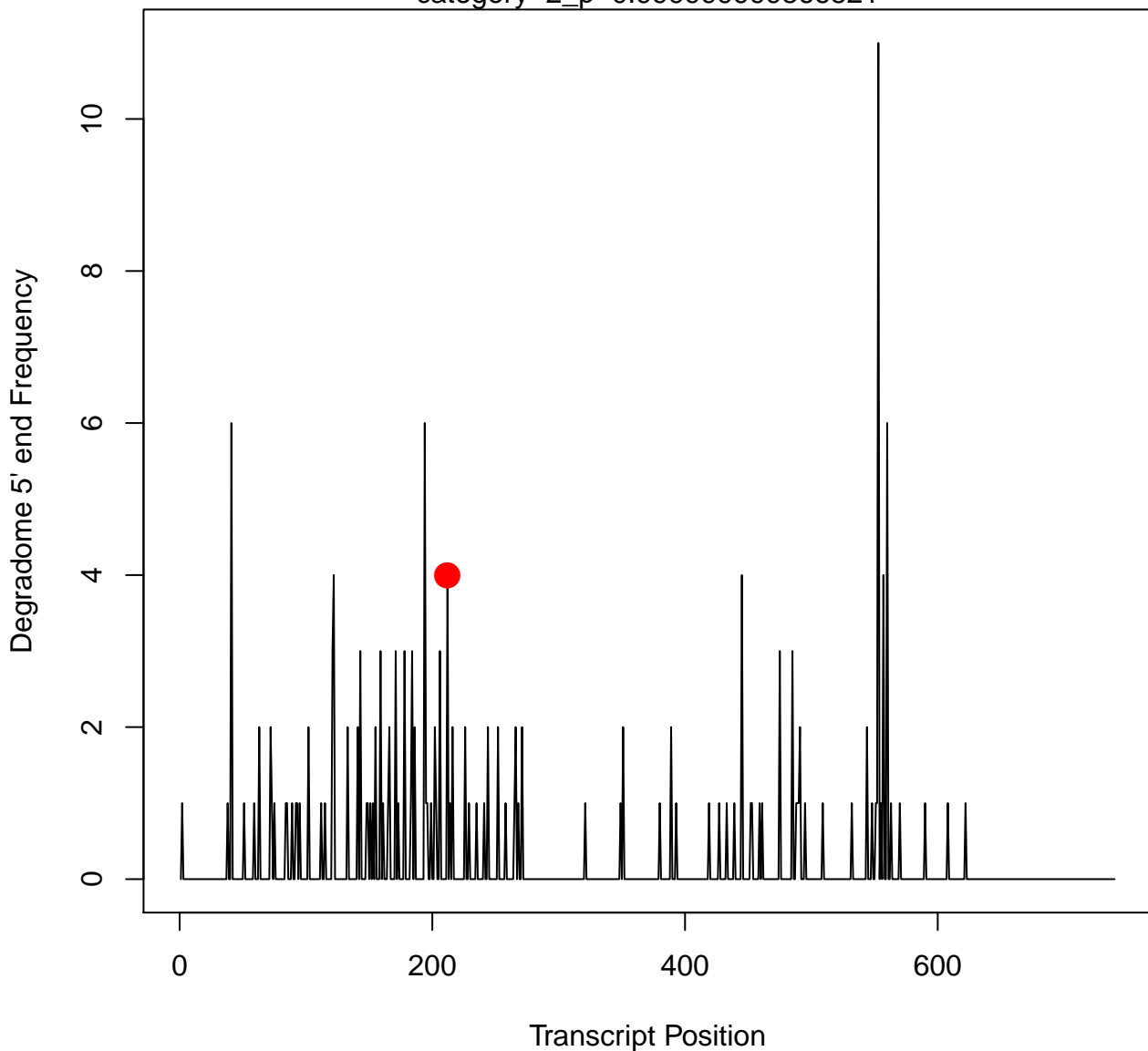

Supplement: Supplementary file 6 [file Data_Sheet_6.zip › Sit-miR160a_Seita.4G258300.1_212_TPlot.pdf]

**T=Seita.5G273800.1\_Q=Sit-miR160a\_S=477**

category=0\_p=0.359005109505183

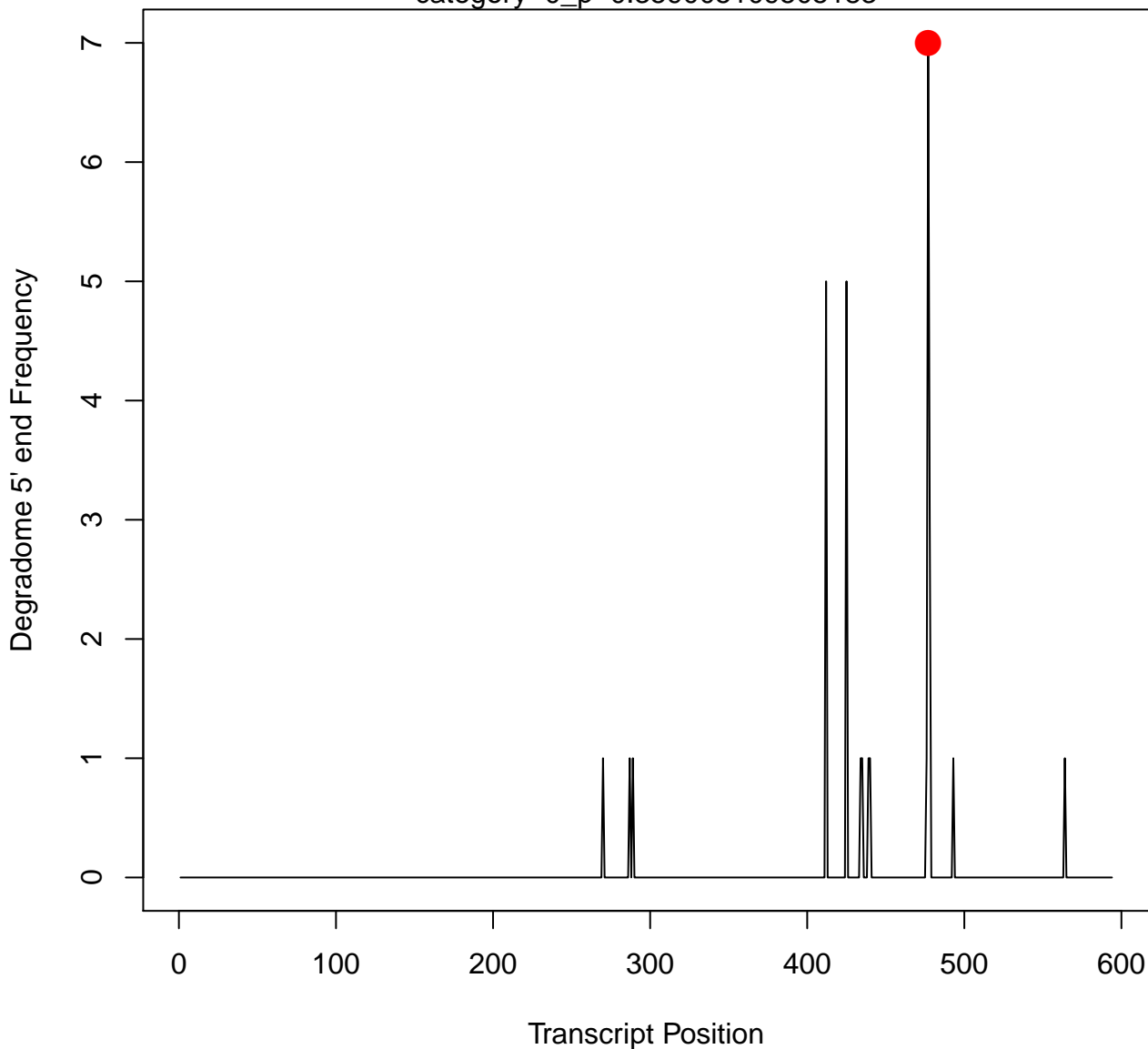

Supplement: Supplementary file 6 [file Data_Sheet_6.zip › Sit-miR160a_Seita.5G273800.1_477_TPlot.pdf]

**T=Seita.6G173700.1\_Q=Sit-miR160a\_S=1111**

category=2\_p=0.999999999996191

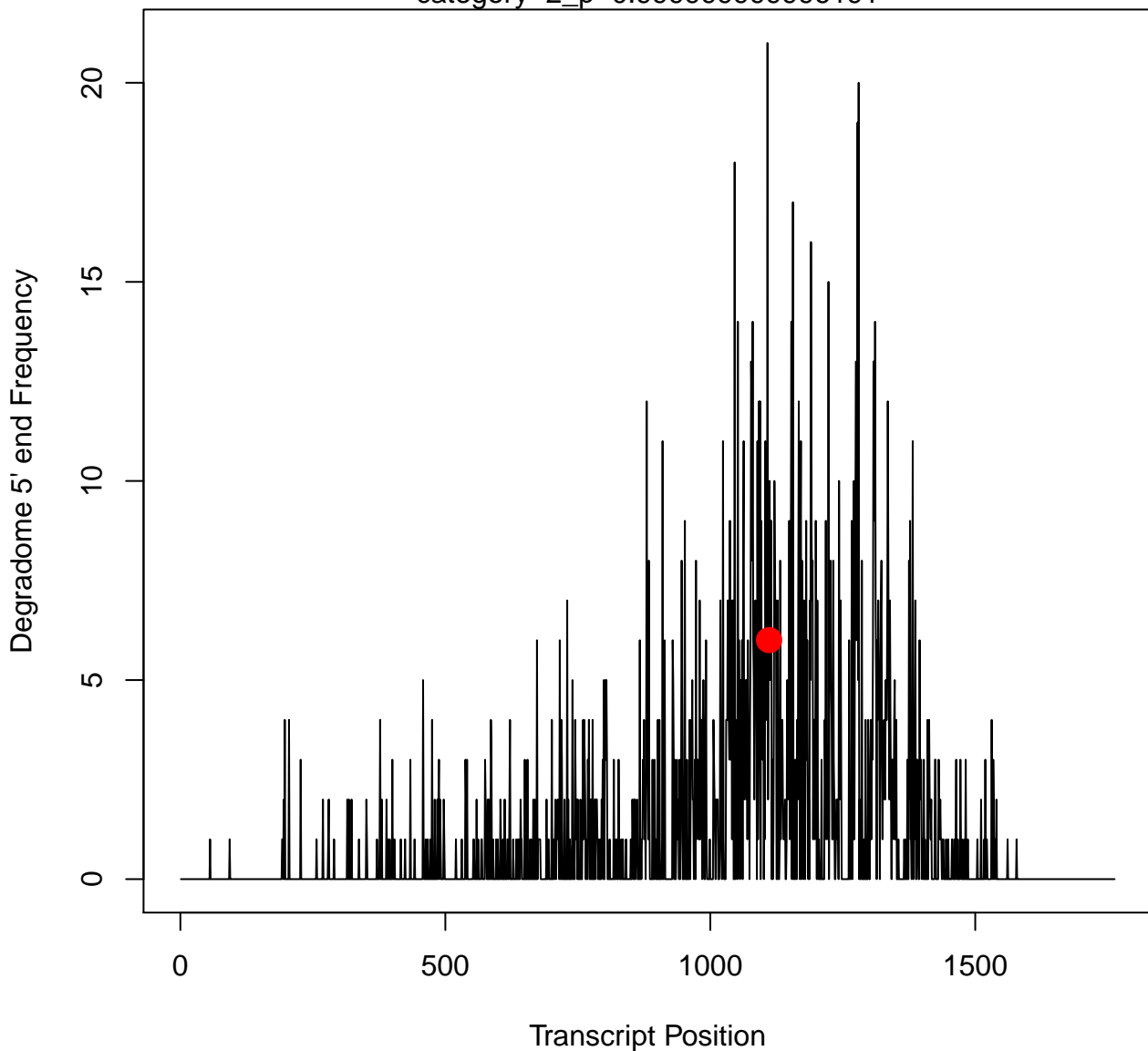

Supplement: Supplementary file 6 [file Data_Sheet_6.zip › Sit-miR160a_Seita.6G173700.1_1111_TPlot.pdf]

**T=Seita.7G162100.1\_Q=Sit-miR160a\_S=219**

category=2\_p=0.99999999814959

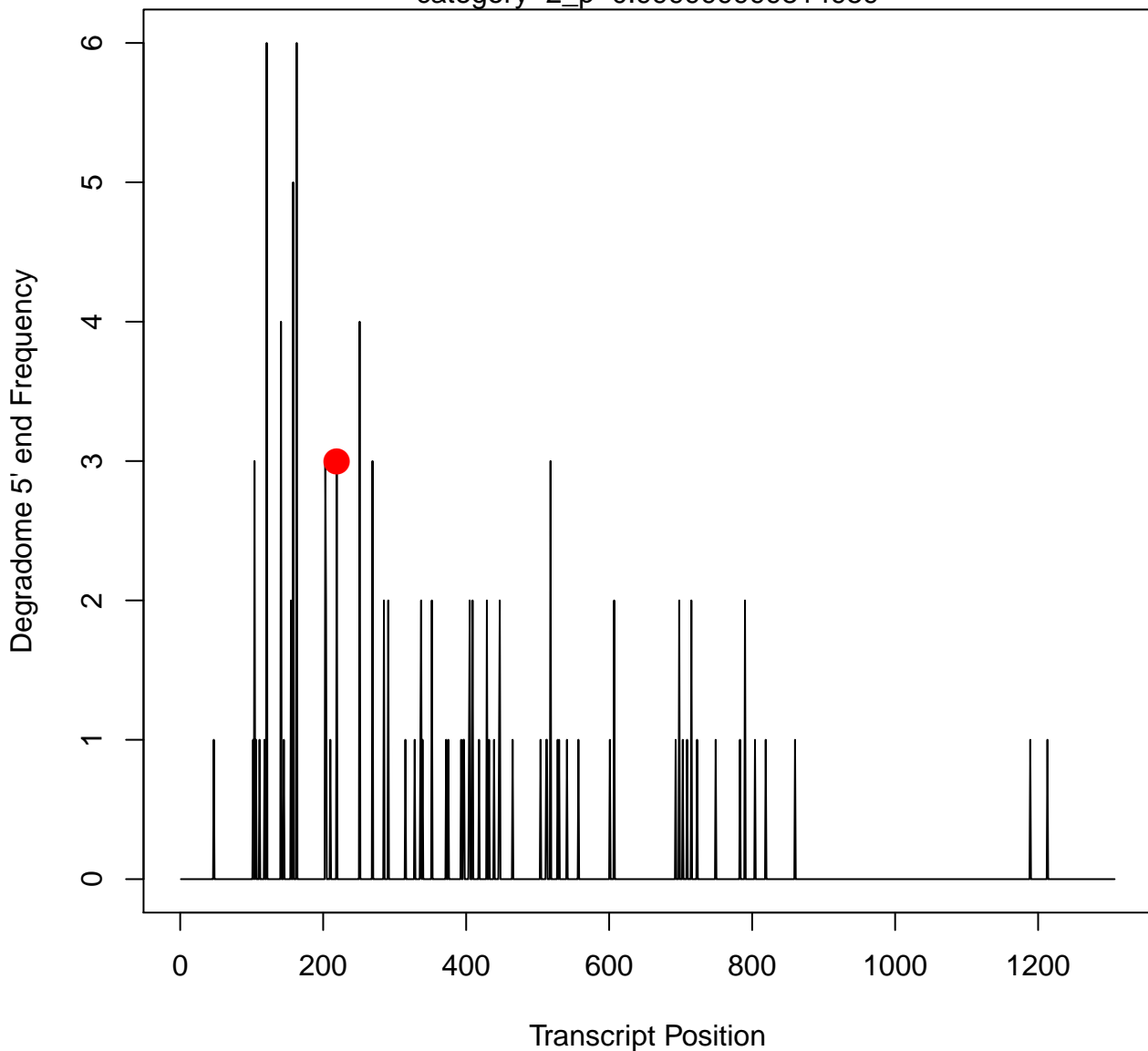

Supplement: Supplementary file 6 [file Data_Sheet_6.zip › Sit-miR160a_Seita.7G162100.1_219_TPlot.pdf]

**T=Seita.7G169600.1\_Q=Sit-miR160a\_S=1802**

category=0\_p=0.000375549221534932

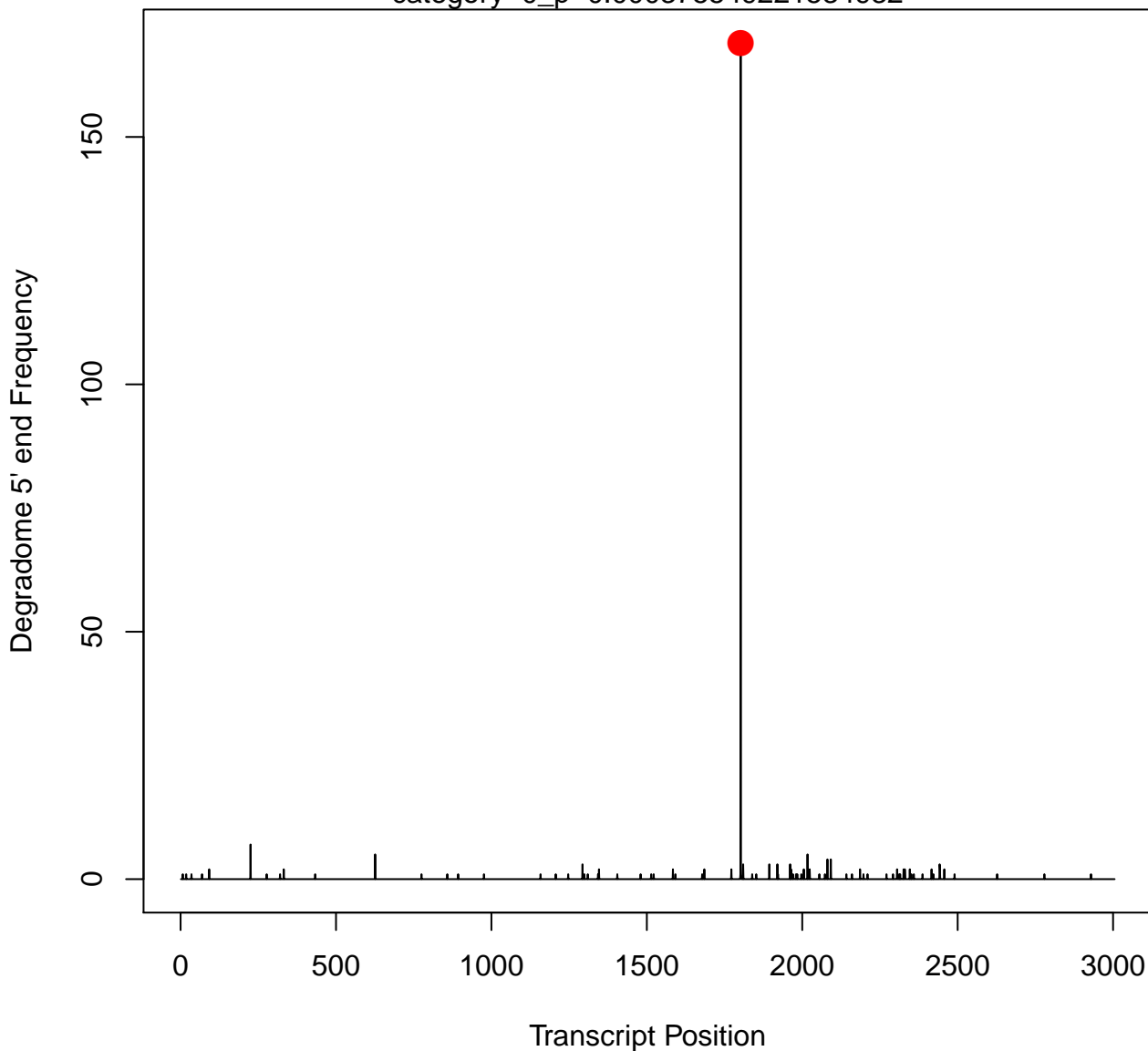

Supplement: Supplementary file 6 [file Data_Sheet_6.zip › Sit-miR160a_Seita.7G169600.1_1802_TPlot.pdf]

**T=Seita.7G178800.1\_Q=Sit-miR160a\_S=792**

category=2\_p=0.999999967981456

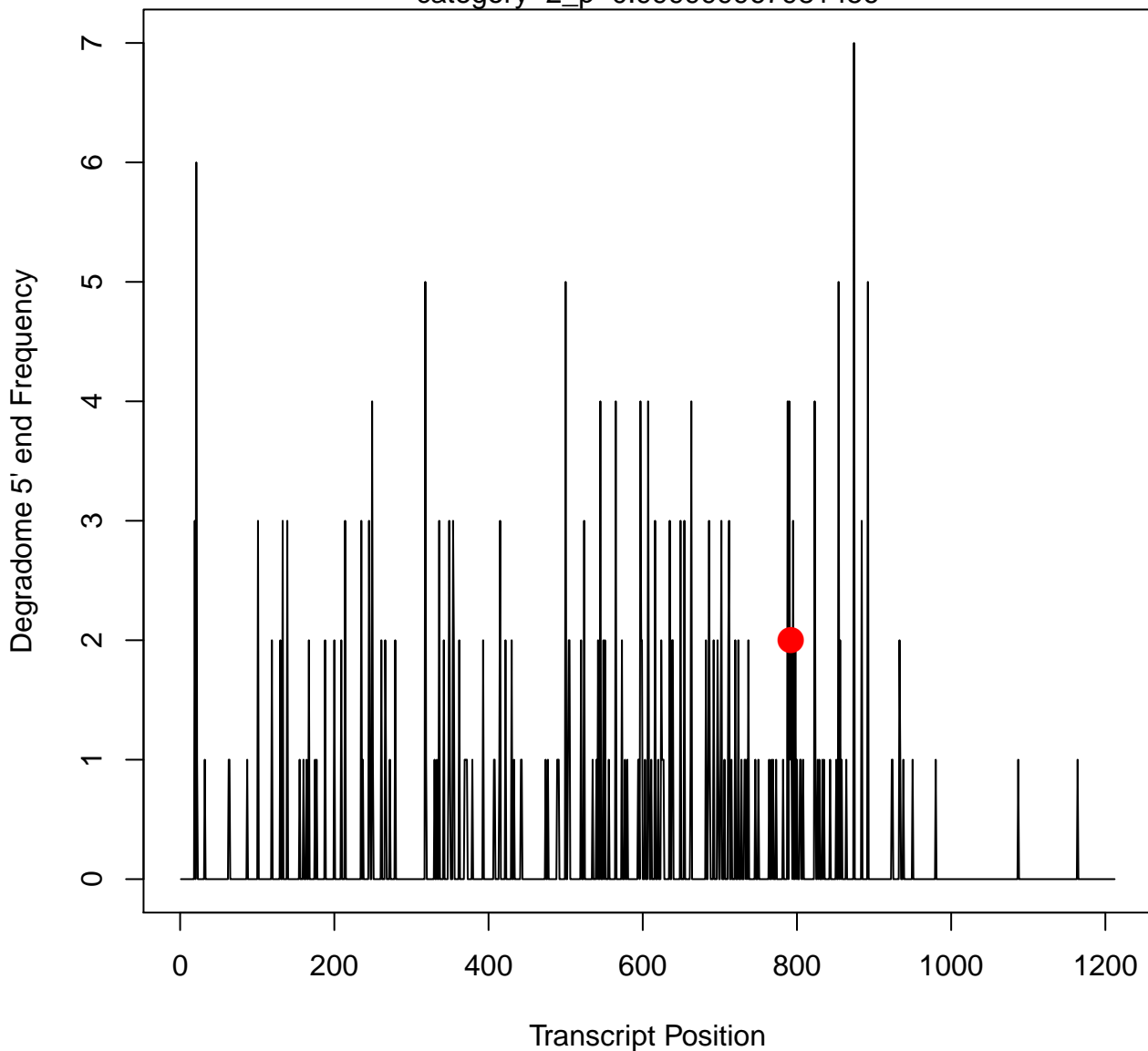

Supplement: Supplementary file 6 [file Data_Sheet_6.zip › Sit-miR160a_Seita.7G178800.1_792_TPlot.pdf]

**T=Seita.7G182200.1\_Q=Sit-miR160a\_S=1026**

category=2\_p=0.99988528445256

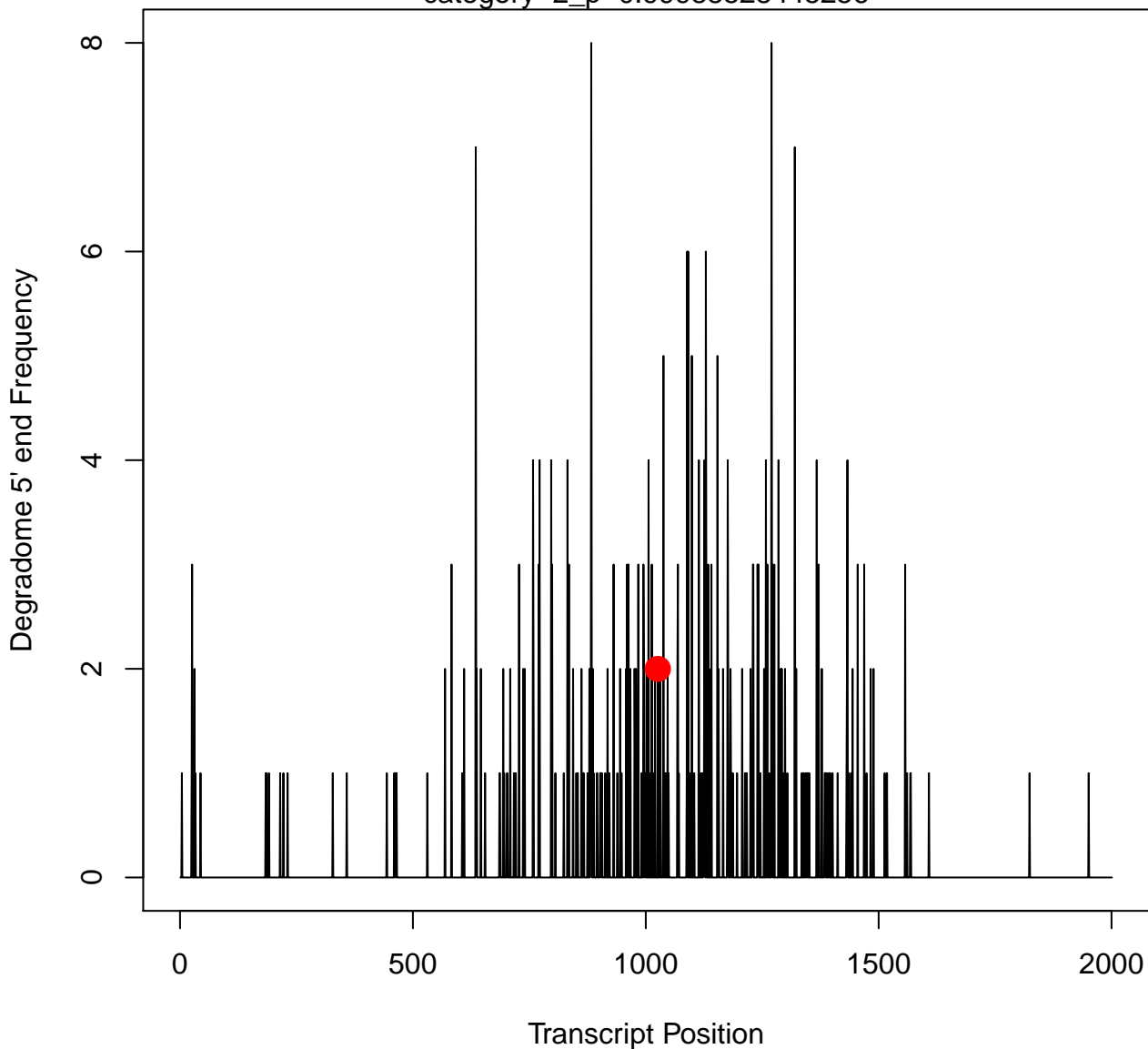

Supplement: Supplementary file 6 [file Data_Sheet_6.zip › Sit-miR160a_Seita.7G182200.1_1026_TPlot.pdf]

**T=Seita.7G262800.1\_Q=Sit-miR160a\_S=1155**

category=2\_p=0.99999999999998

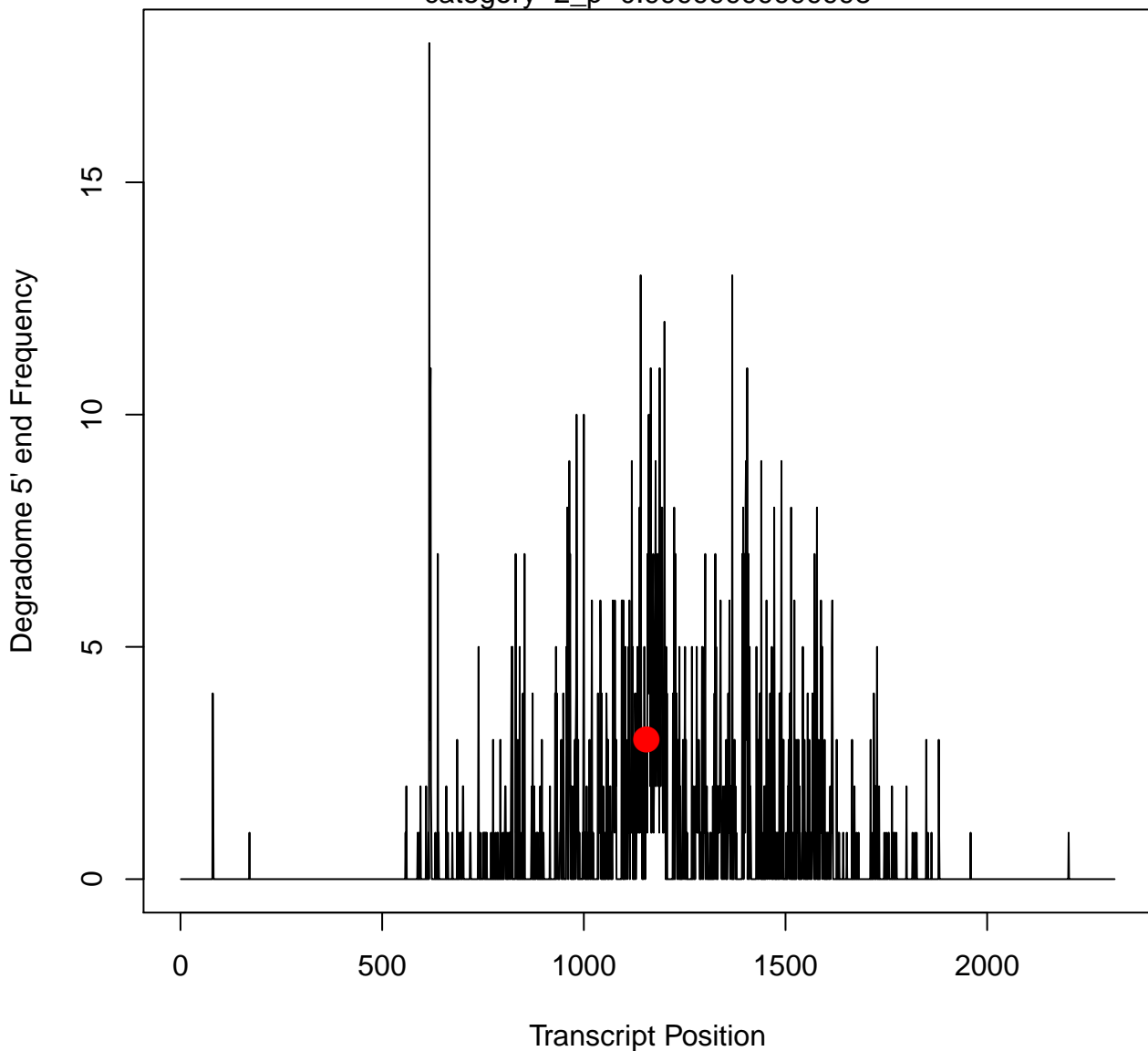

Supplement: Supplementary file 6 [file Data_Sheet_6.zip › Sit-miR160a_Seita.7G262800.1_1155_TPlot.pdf]

**T=Seita.8G100700.1\_Q=Sit-miR160a\_S=4526**

category=2\_p=0.999058565926927

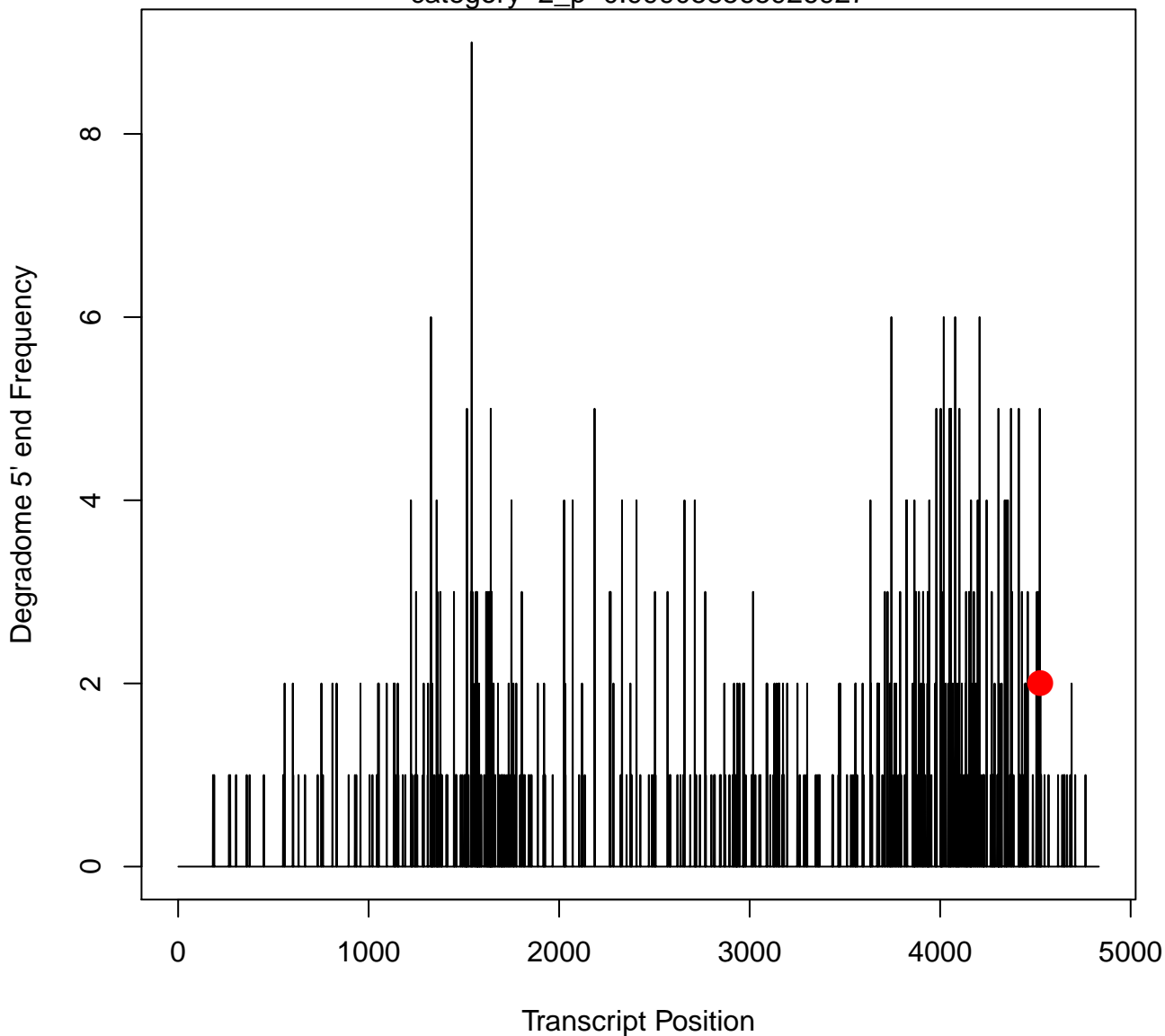

Supplement: Supplementary file 6 [file Data_Sheet_6.zip › Sit-miR160a_Seita.8G100700.1_4526_TPlot.pdf]

**T=Seita.9G270700.1\_Q=Sit-miR160a\_S=69**

category=2\_p=0.999999999993193

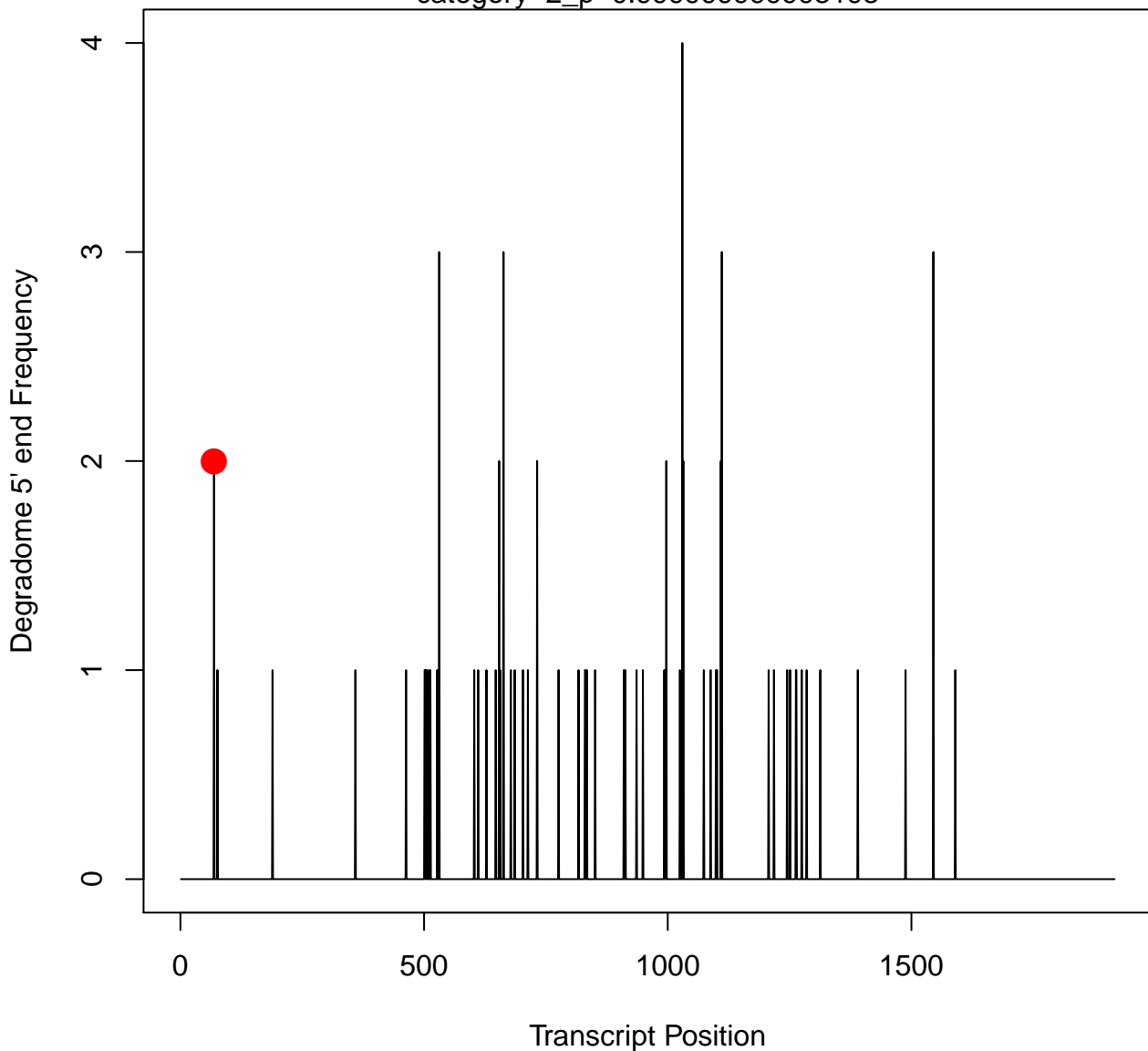

Supplement: Supplementary file 6 [file Data_Sheet_6.zip › Sit-miR160a_Seita.9G270700.1_69_TPlot.pdf]

**T=Seita.9G318500.1\_Q=Sit-miR160a\_S=1198**

category=2\_p=0.9999999997393

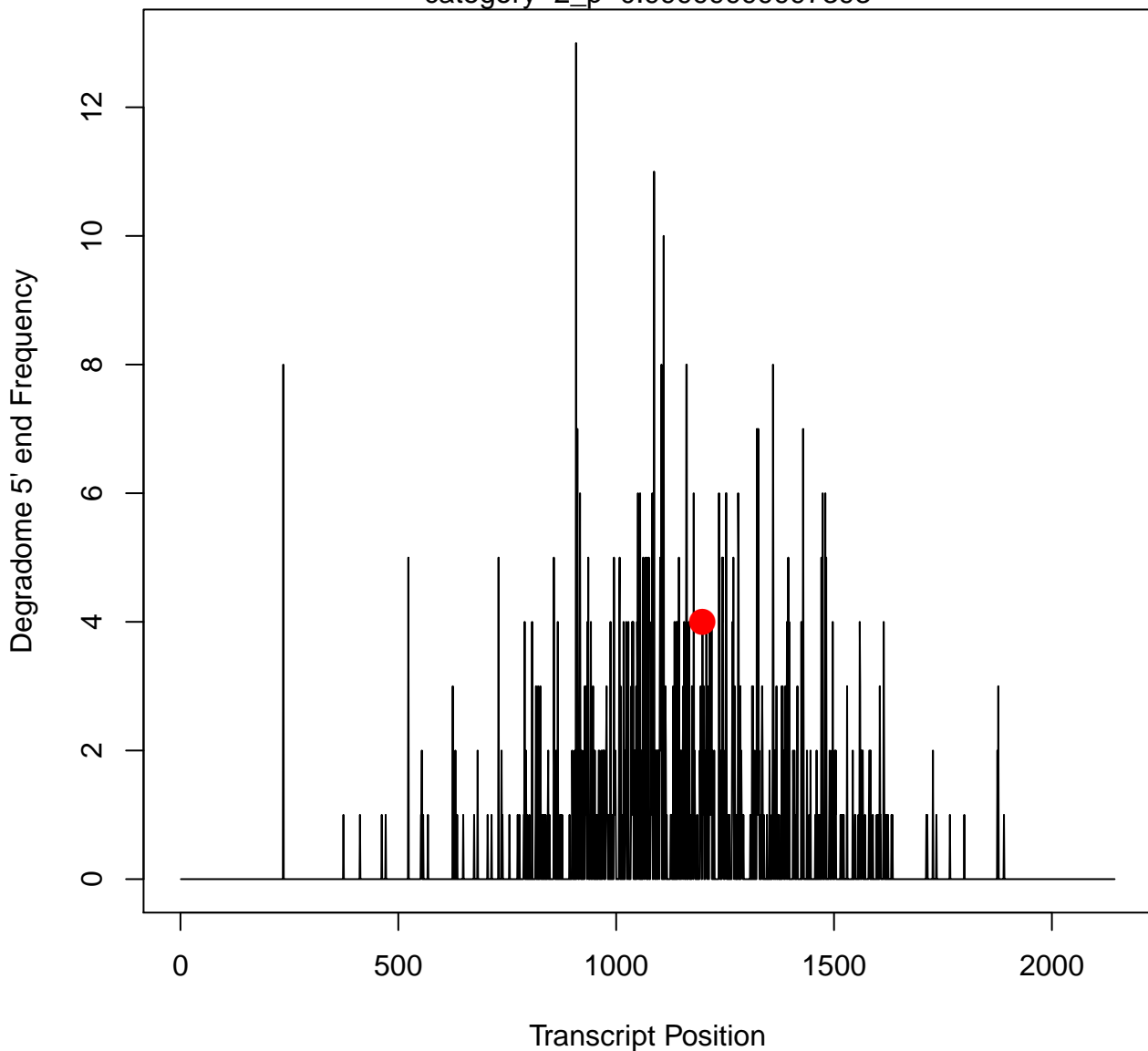

Supplement: Supplementary file 6 [file Data_Sheet_6.zip › Sit-miR160a_Seita.9G318500.1_1198_TPlot.pdf]

**T=Seita.J002600.1\_Q=Sit-miR160a\_S=1009**

category=2\_p=0.951703702041835

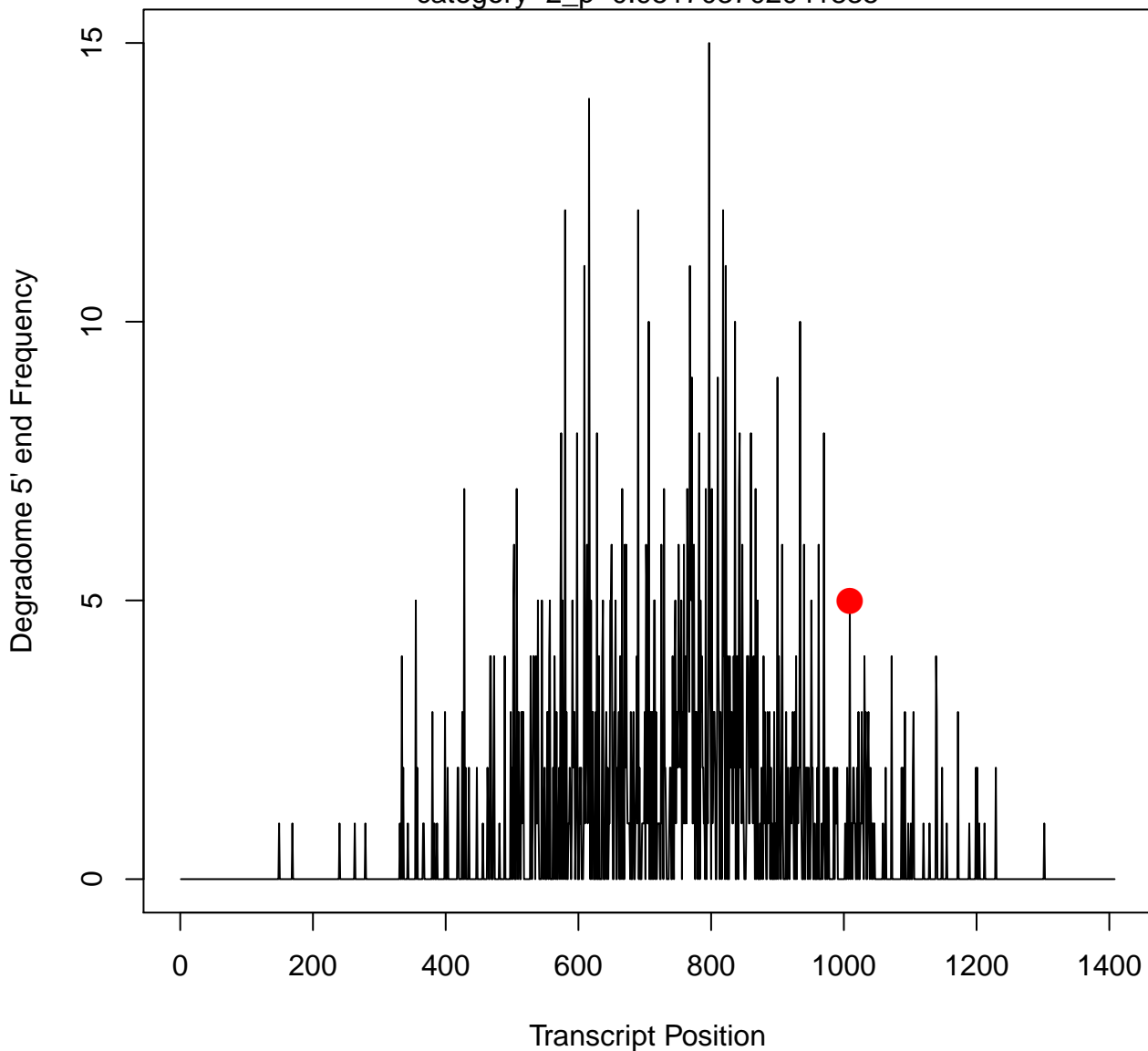

Supplement: Supplementary file 6 [file Data_Sheet_6.zip › Sit-miR160a_Seita.J002600.1_1009_TPlot.pdf]

**T=Seita.1G185400.1\_Q=Sit-miR160b\_S=1610**

category=2\_p=0.960442800975775

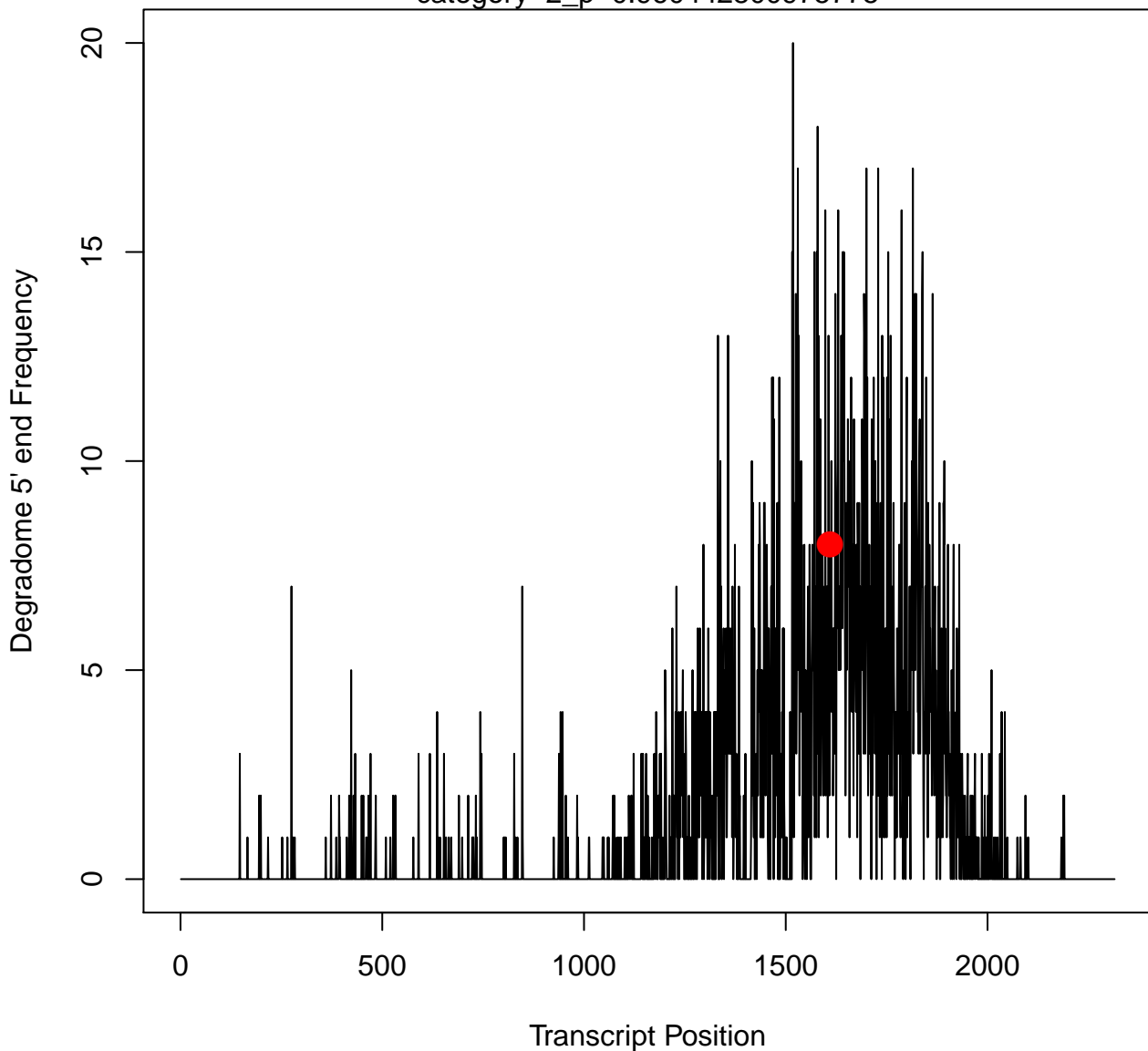

Supplement: Supplementary file 6 [file Data_Sheet_6.zip › Sit-miR160b_Seita.1G185400.1_1610_TPlot.pdf]

**T=Seita.2G137400.1\_Q=Sit-miR160b\_S=816**

category=2\_p=0.938854617958602

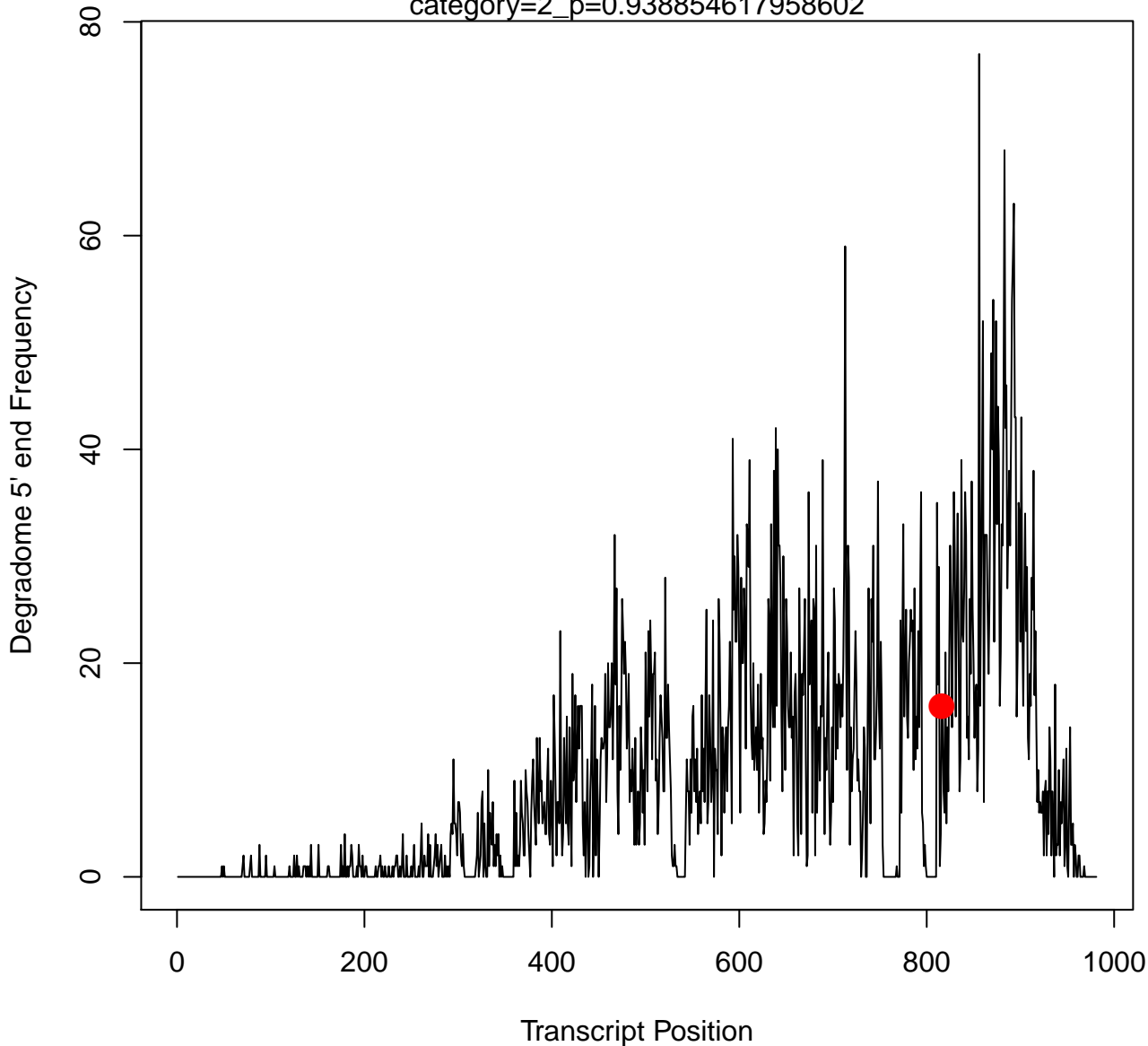

Supplement: Supplementary file 6 [file Data_Sheet_6.zip › Sit-miR160b_Seita.2G137400.1_816_TPlot.pdf]

**T=Seita.4G152100.1\_Q=Sit-miR160b\_S=1570**

category=2\_p=0.9999999999999973

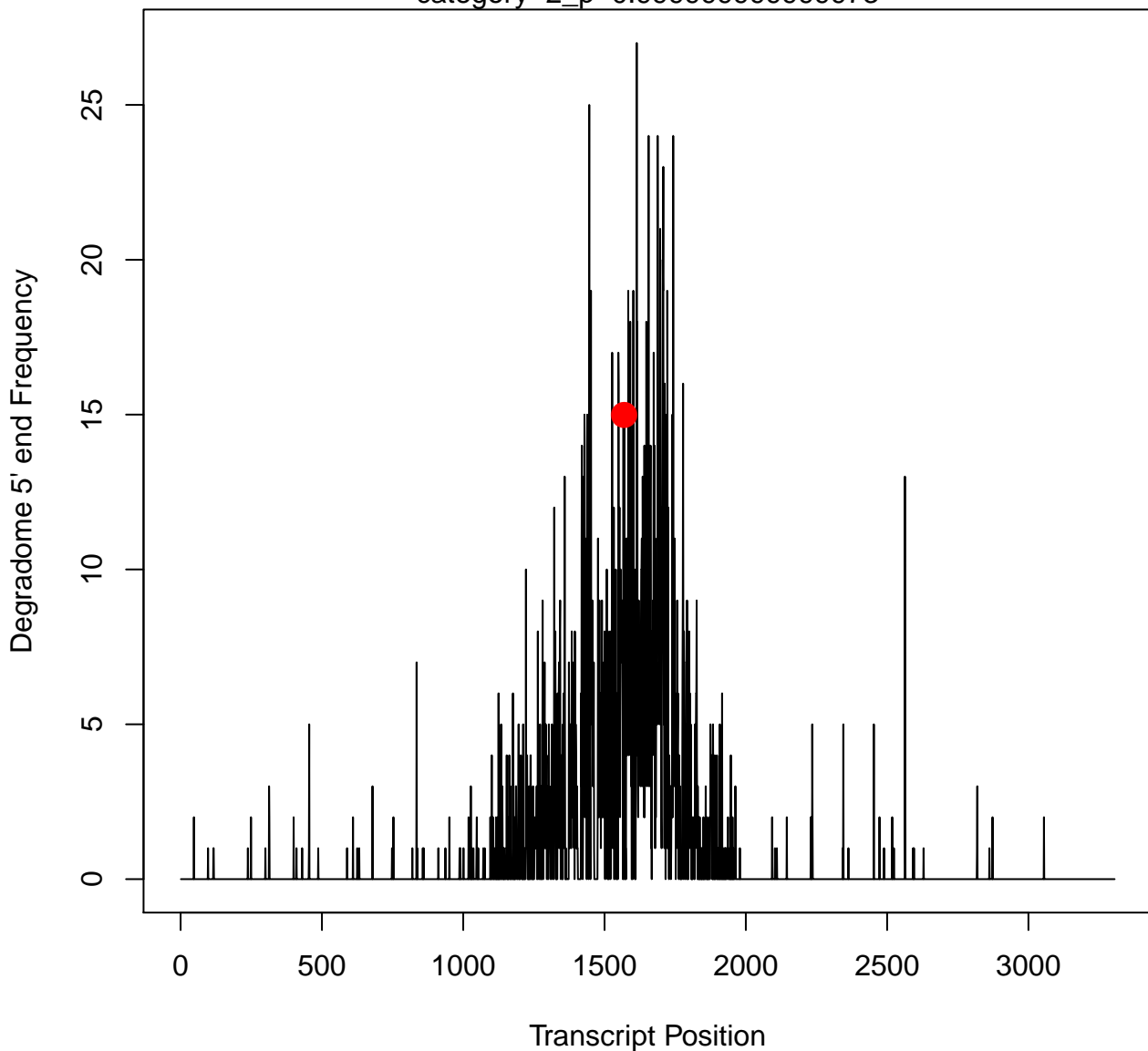

Supplement: Supplementary file 6 [file Data_Sheet_6.zip › Sit-miR160b_Seita.4G152100.1_1570_TPlot.pdf]

**T=Seita.4G202900.1\_Q=Sit-miR160b\_S=204**

category=2\_p=0.999999999969857

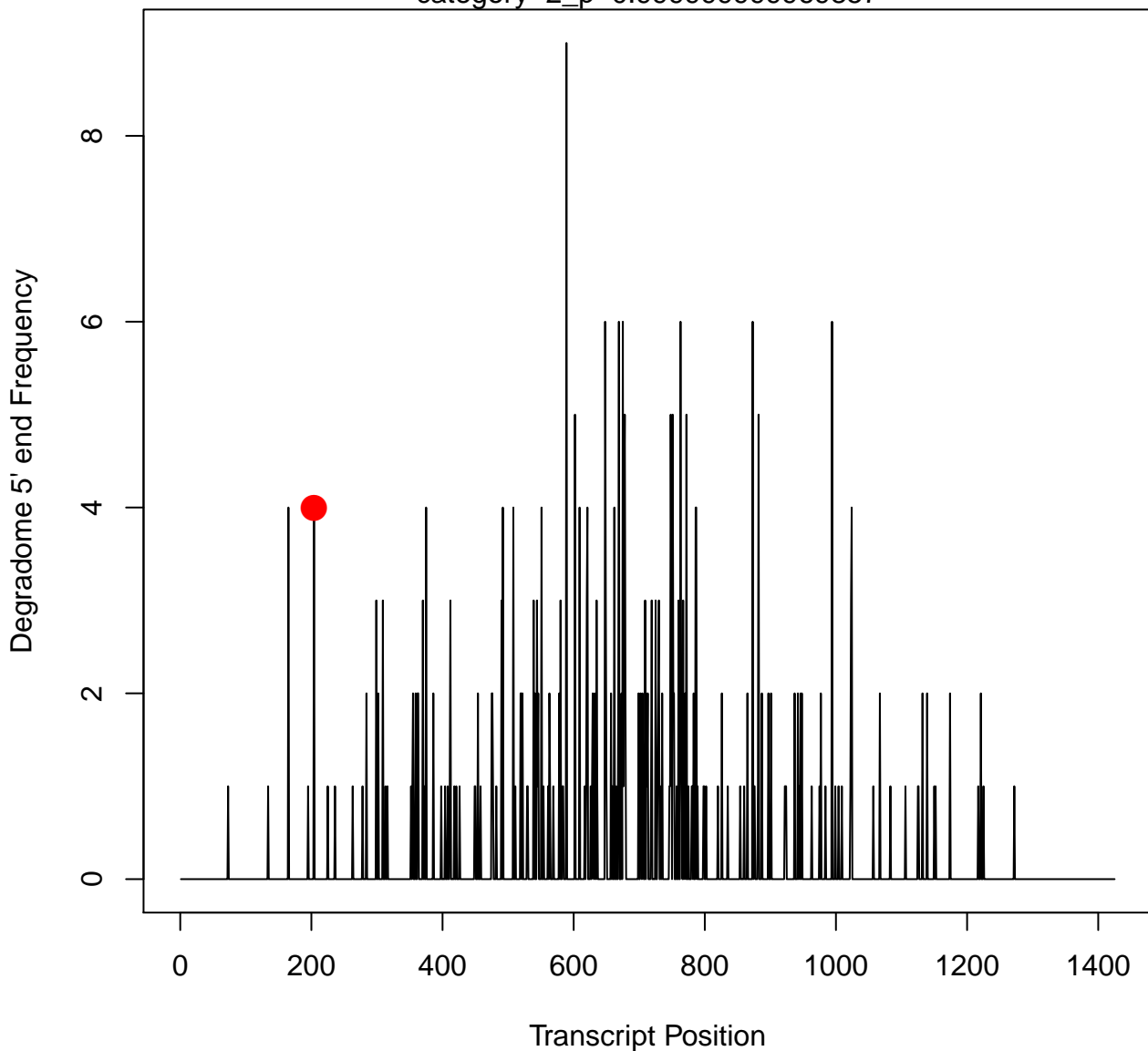

Supplement: Supplementary file 6 [file Data_Sheet_6.zip › Sit-miR160b_Seita.4G202900.1_204_TPlot.pdf]

**T=Seita.9G127600.1\_Q=Sit-miR160b\_S=2497**

category=2\_p=0.999999968557228

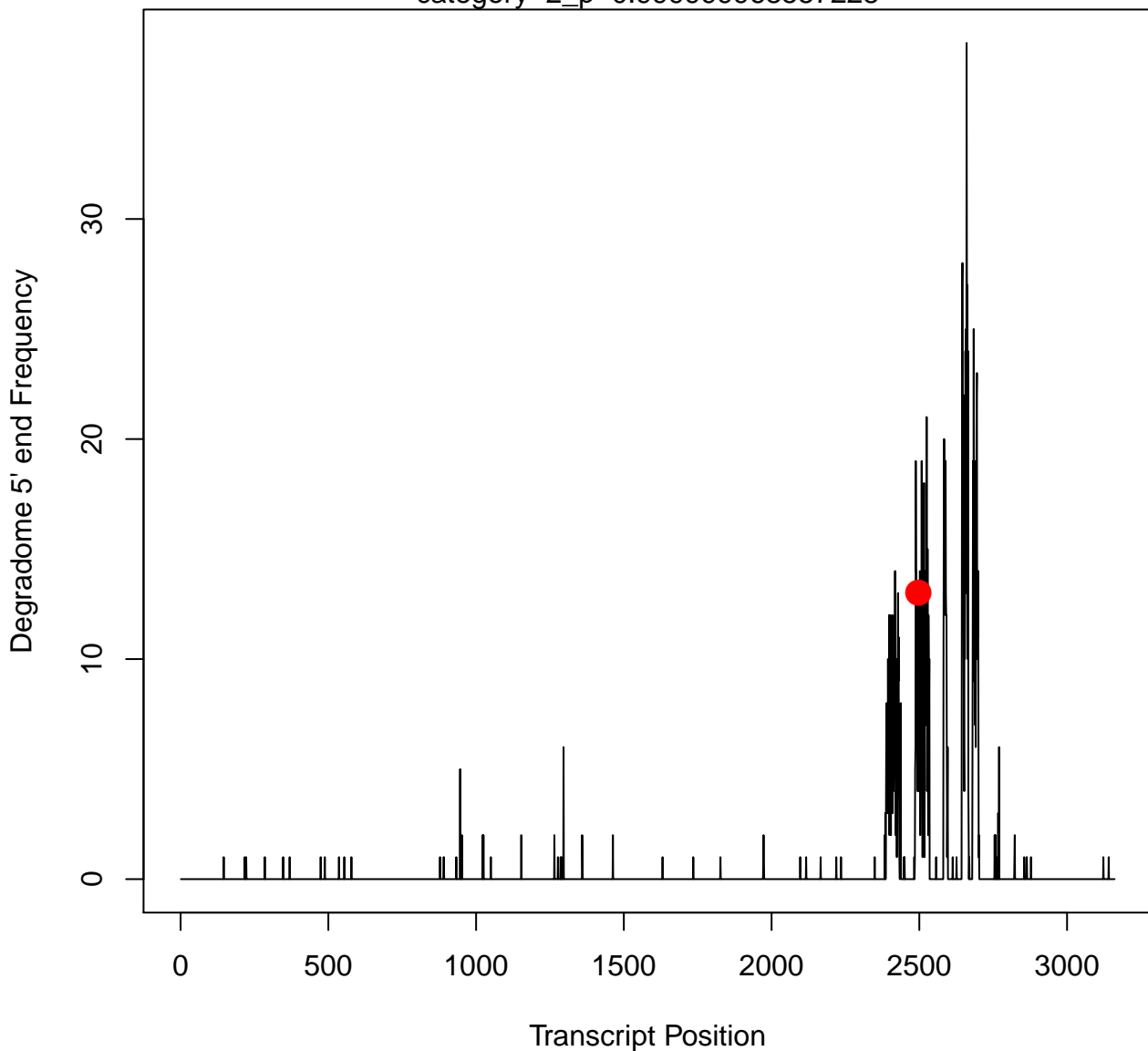

Supplement: Supplementary file 6 [file Data_Sheet_6.zip › Sit-miR160b_Seita.9G127600.1_2497_TPlot.pdf]

**T=Seita.9G219800.1\_Q=Sit-miR160b\_S=1729**

category=0\_p=0.000375549221534932

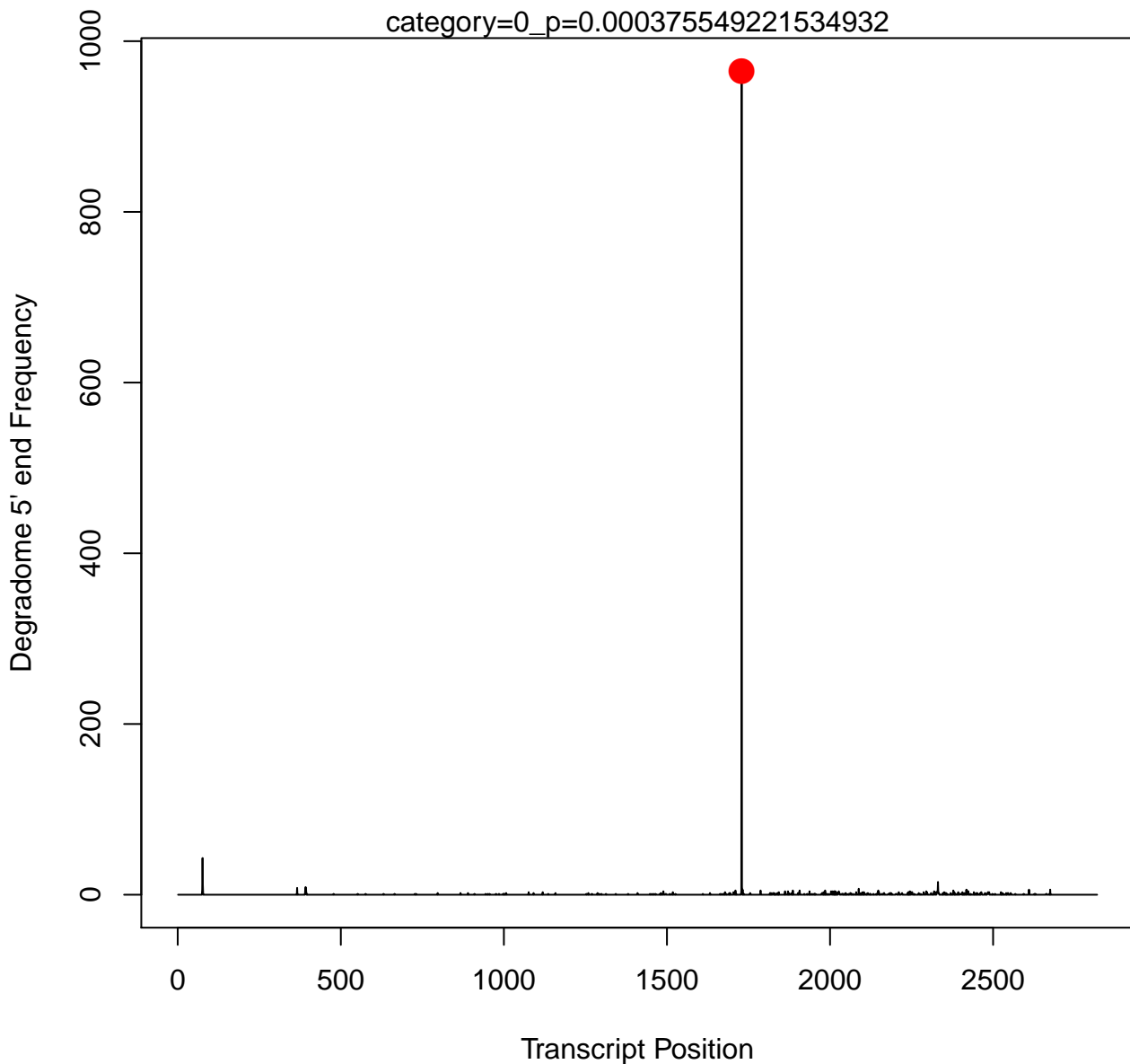

Supplement: Supplementary file 6 [file Data_Sheet_6.zip › Sit-miR160b_Seita.9G219800.1_1729_TPlot.pdf]

**T=Seita.2G045800.1\_Q=Sit-miR160c\_S=608**

category=2\_p=0.999999990845609

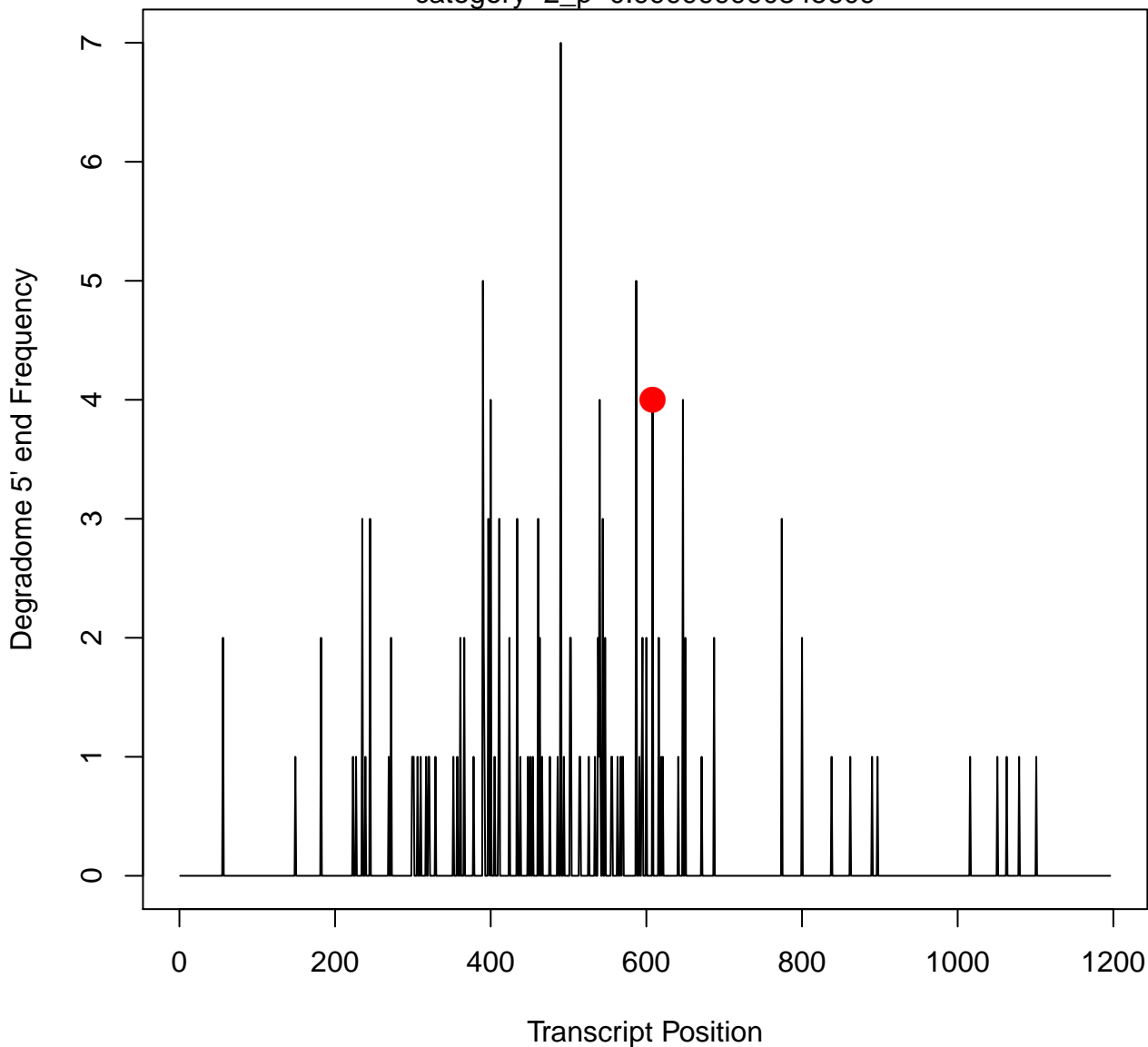

Supplement: Supplementary file 6 [file Data_Sheet_6.zip › Sit-miR160c_Seita.2G045800.1_608_TPlot.pdf]

**T=Seita.2G137400.1\_Q=Sit-miR160c\_S=817**

category=2\_p=0.999999999631247

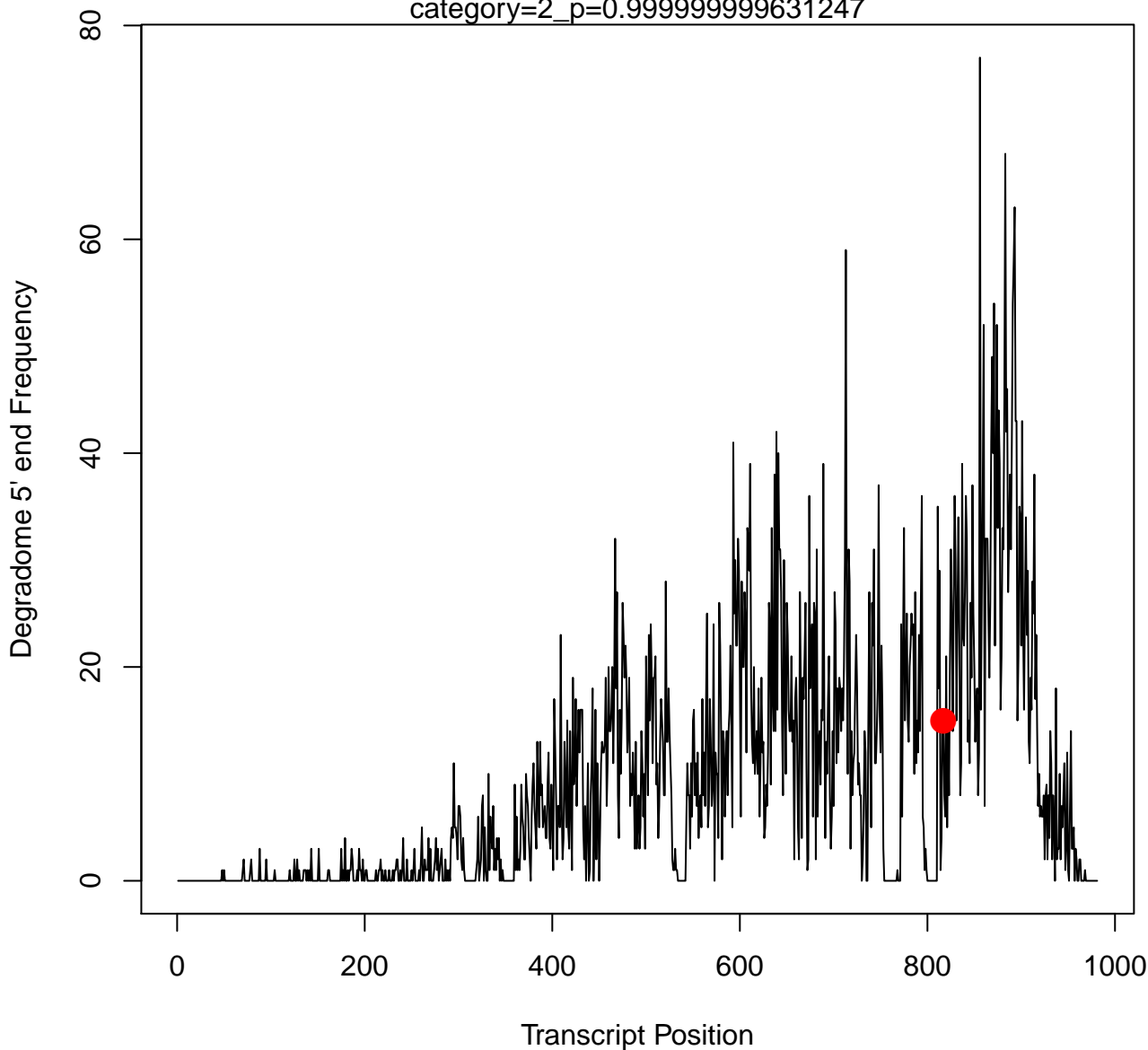

Supplement: Supplementary file 6 [file Data_Sheet_6.zip › Sit-miR160c_Seita.2G137400.1_817_TPlot.pdf]

**T=Seita.2G185600.1\_Q=Sit-miR160c\_S=3070**

category=2\_p=0.999999998962618

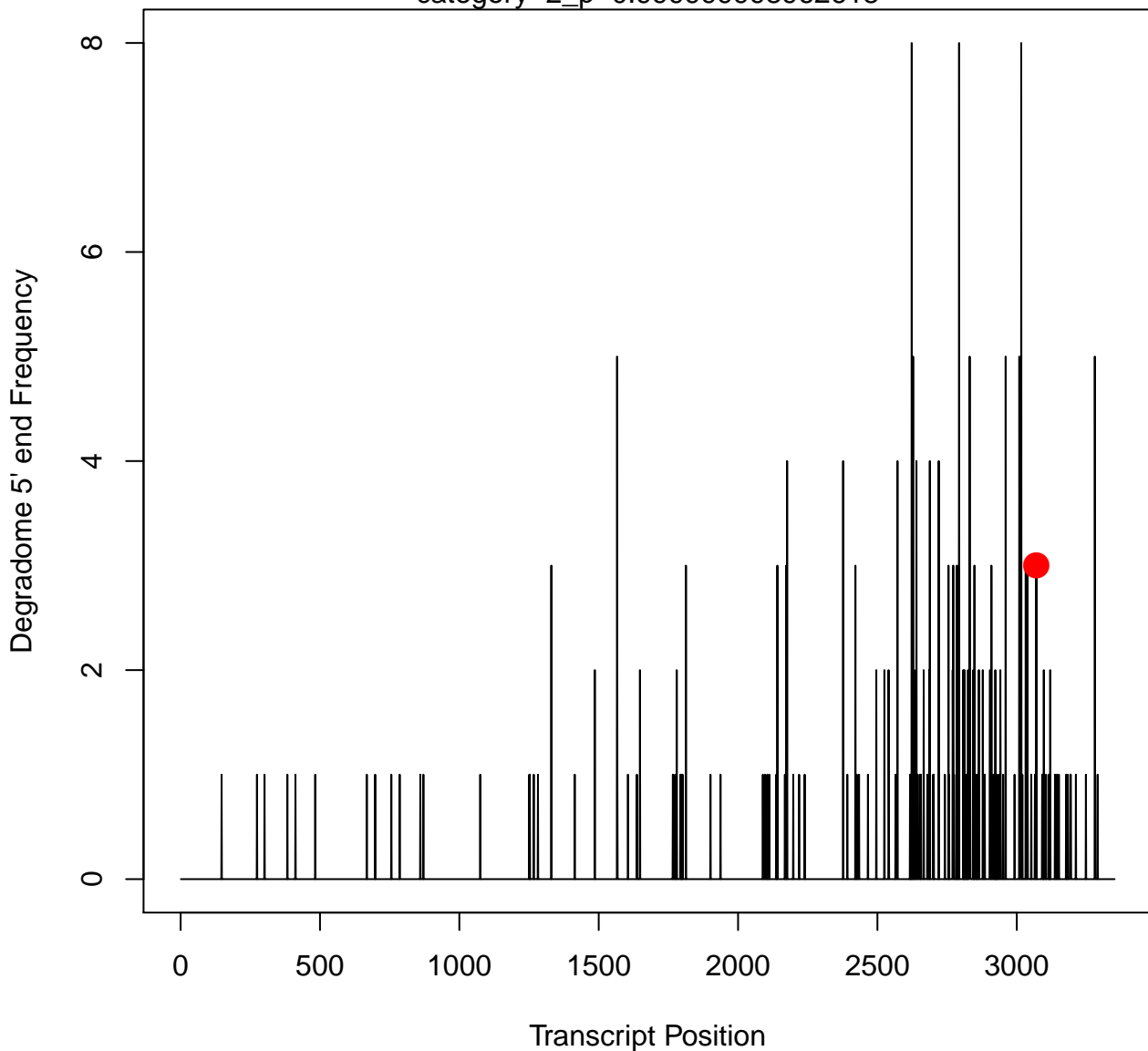

Supplement: Supplementary file 6 [file Data_Sheet_6.zip › Sit-miR160c_Seita.2G185600.1_3070_TPlot.pdf]

**T=Seita.2G377800.1\_Q=Sit-miR160c\_S=3176**

category=2\_p=0.987158413090251

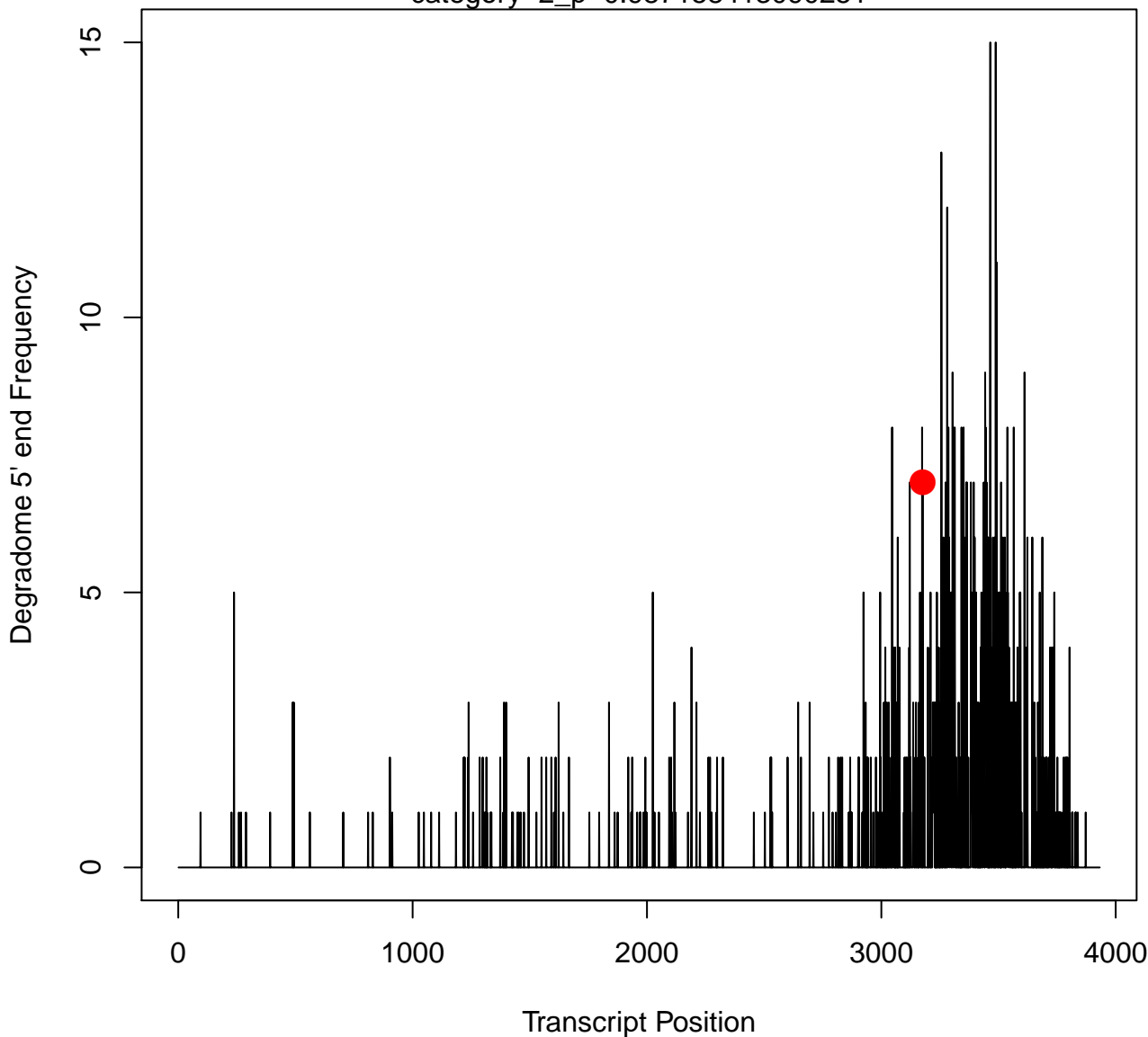

Supplement: Supplementary file 6 [file Data_Sheet_6.zip › Sit-miR160c_Seita.2G377800.1_3176_TPlot.pdf]

**T=Seita.3G122800.1\_Q=Sit-miR160c\_S=280**

category=2\_p=0.999965366815763

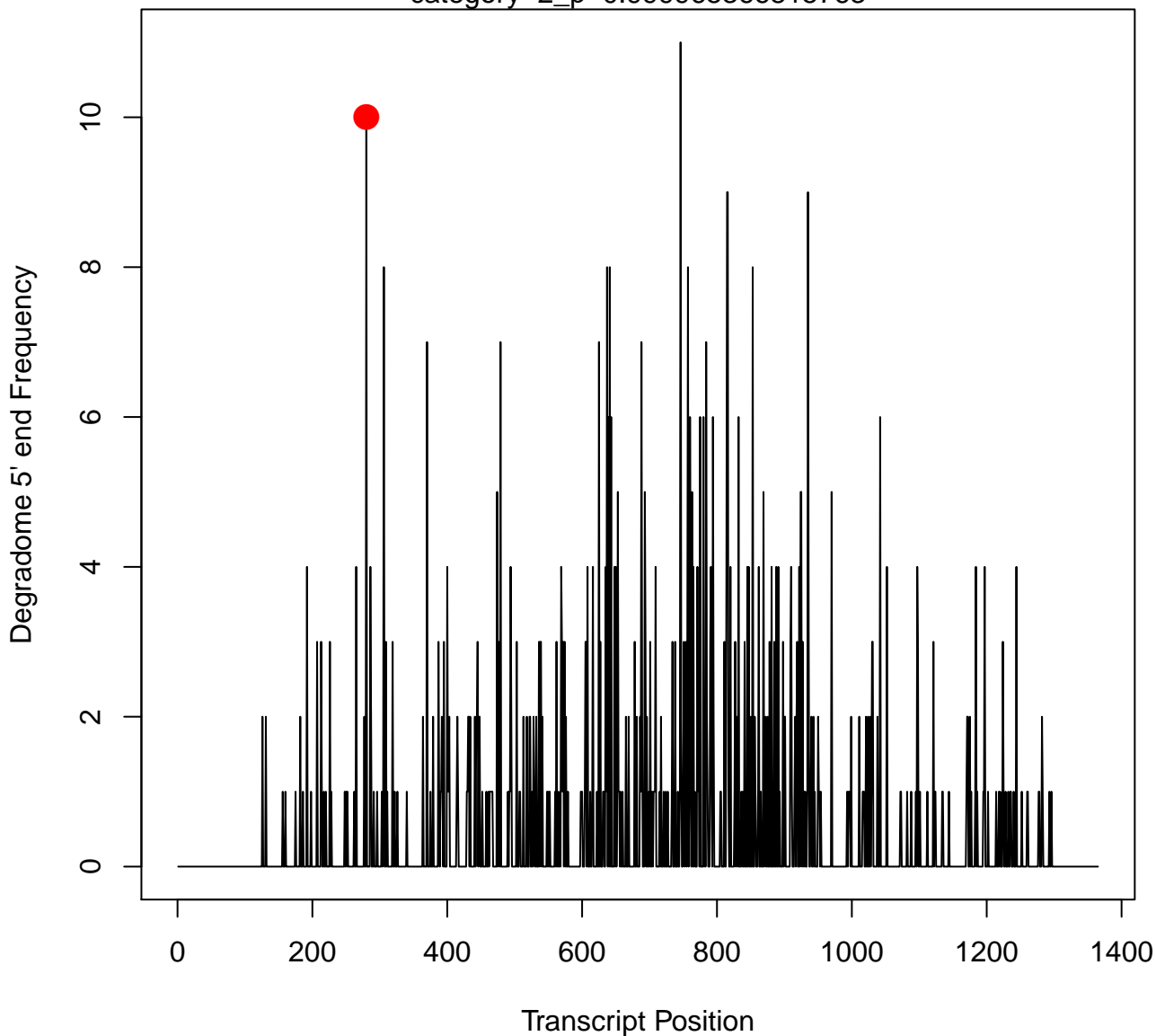

Supplement: Supplementary file 6 [file Data_Sheet_6.zip › Sit-miR160c_Seita.3G122800.1_280_TPlot.pdf]

**T=Seita.4G112500.1\_Q=Sit-miR160c\_S=441**

category=2\_p=0.999972143704682

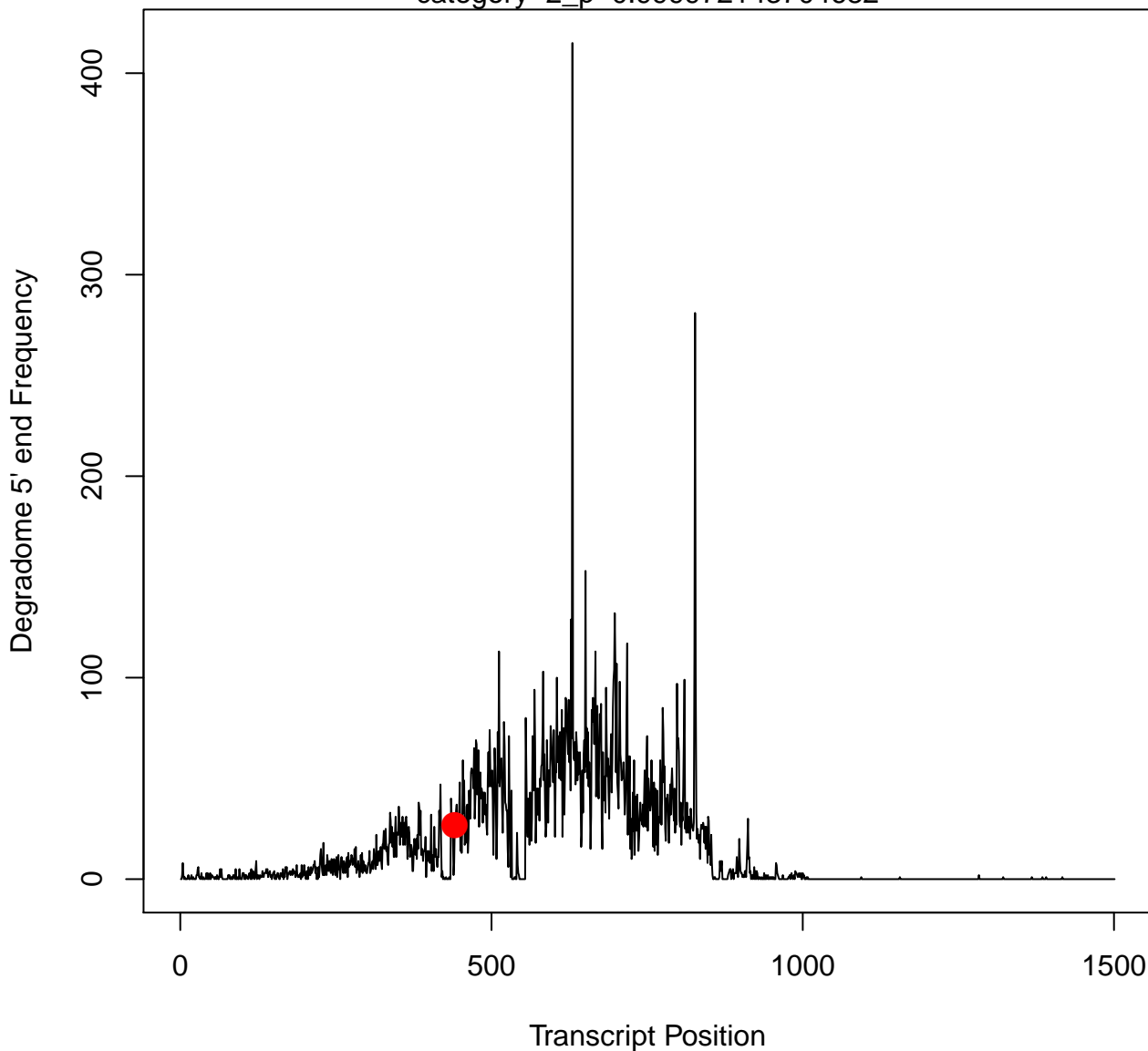

Supplement: Supplementary file 6 [file Data_Sheet_6.zip › Sit-miR160c_Seita.4G112500.1_441_TPlot.pdf]

**T=Seita.4G173800.1\_Q=Sit-miR160c\_S=259**

category=1\_p=0.0367999461200628

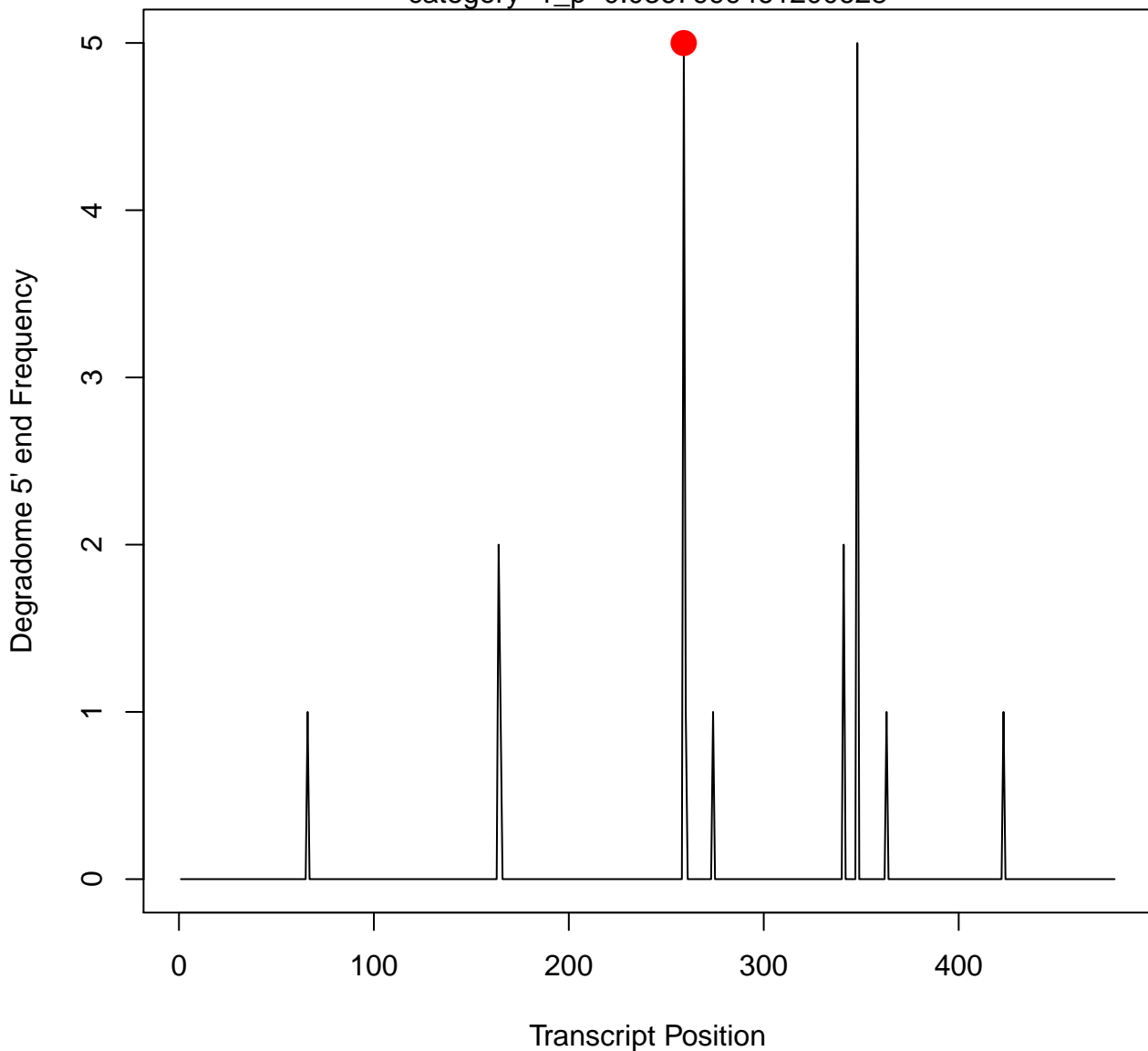

Supplement: Supplementary file 6 [file Data_Sheet_6.zip › Sit-miR160c_Seita.4G173800.1_259_TPlot.pdf]

**T=Seita.4G217600.1\_Q=Sit-miR160c\_S=1085**

category=2\_p=0.999999999603486

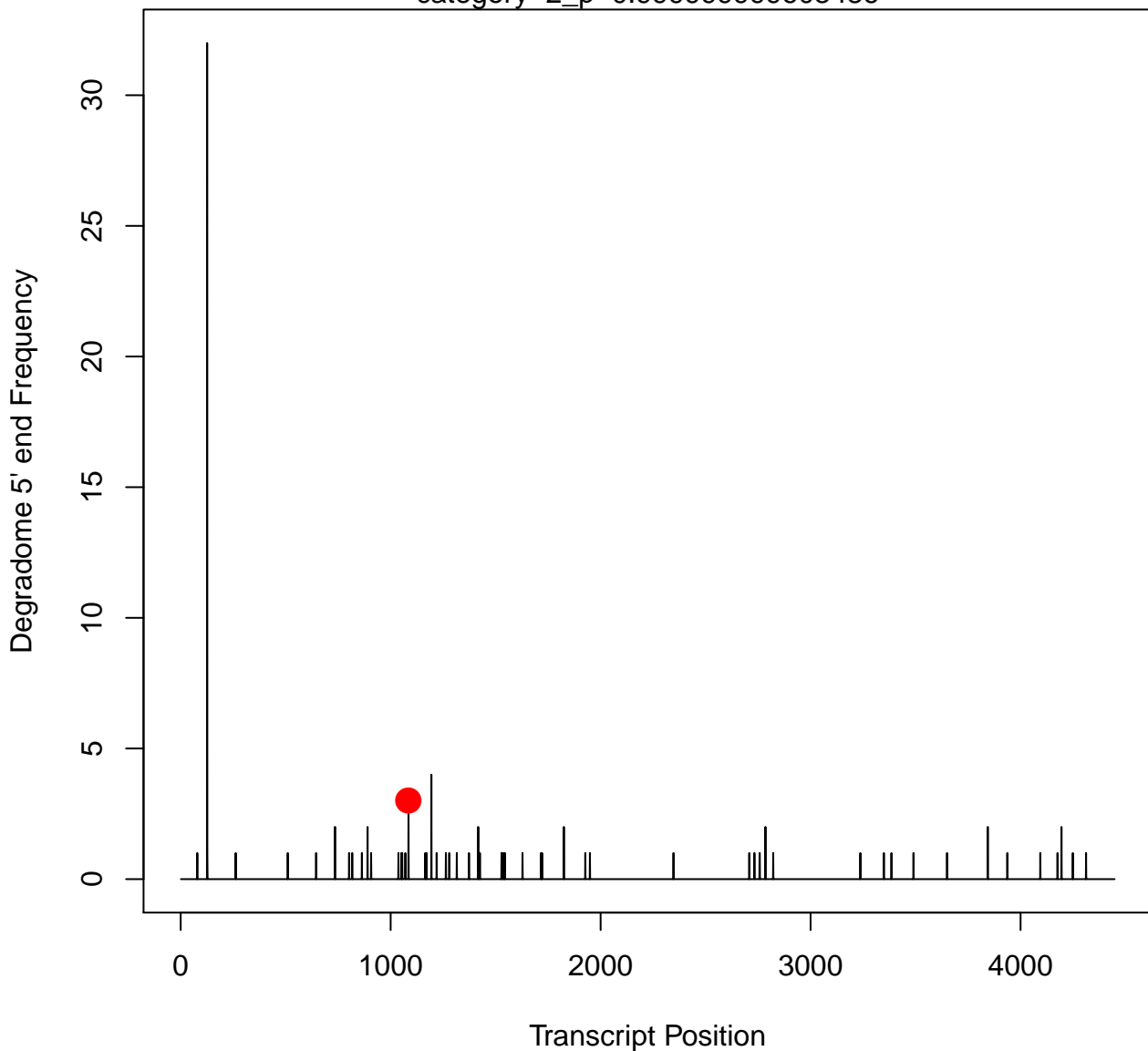

Supplement: Supplementary file 6 [file Data_Sheet_6.zip › Sit-miR160c_Seita.4G217600.1_1085_TPlot.pdf]

**T=Seita.4G265400.1\_Q=Sit-miR160c\_S=870**

category=2\_p=0.999999999930545

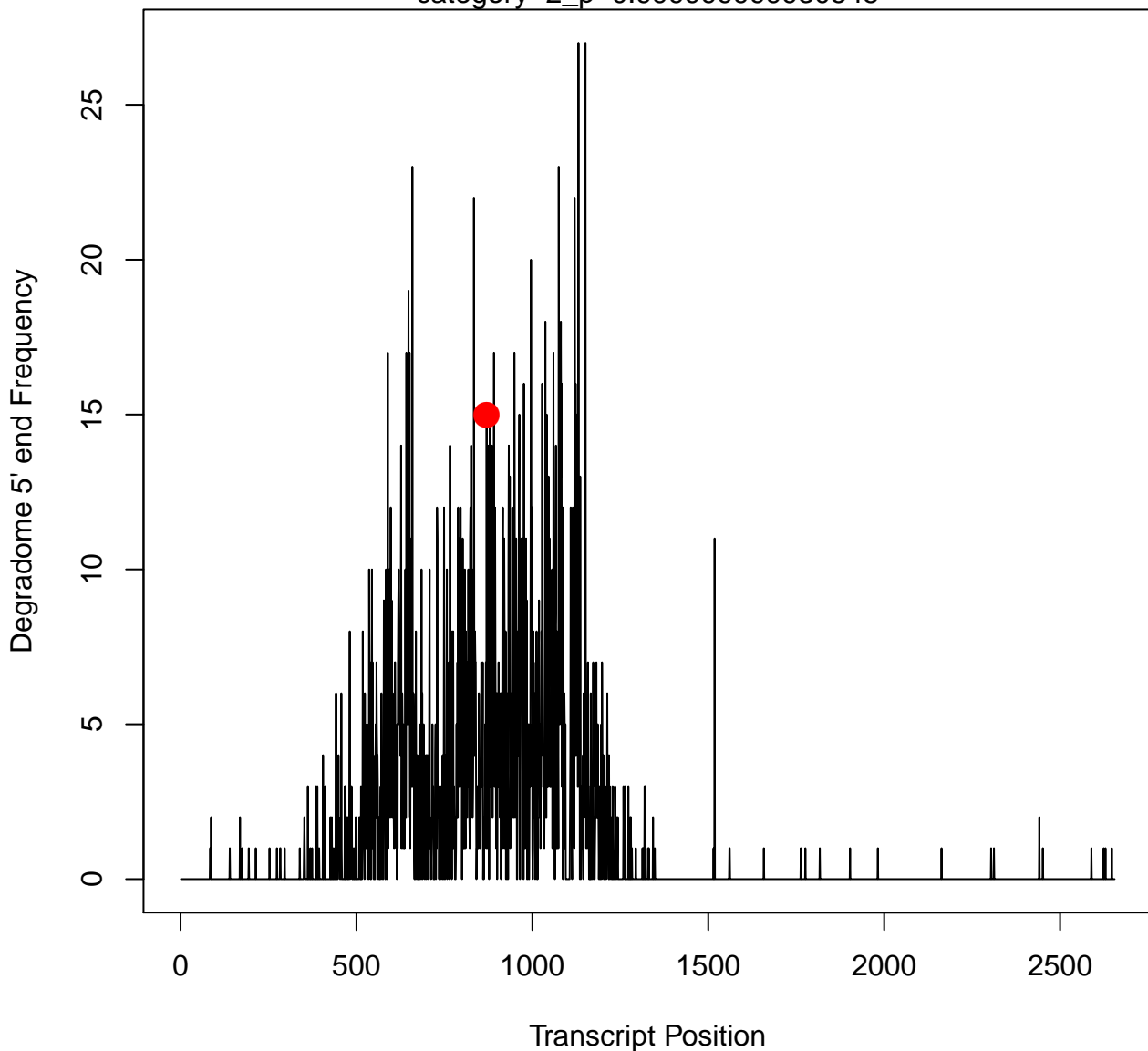

Supplement: Supplementary file 6 [file Data_Sheet_6.zip › Sit-miR160c_Seita.4G265400.1_870_TPlot.pdf]

**T=Seita.5G022000.1\_Q=Sit-miR160c\_S=417**

category=2\_p=0.99999999950799

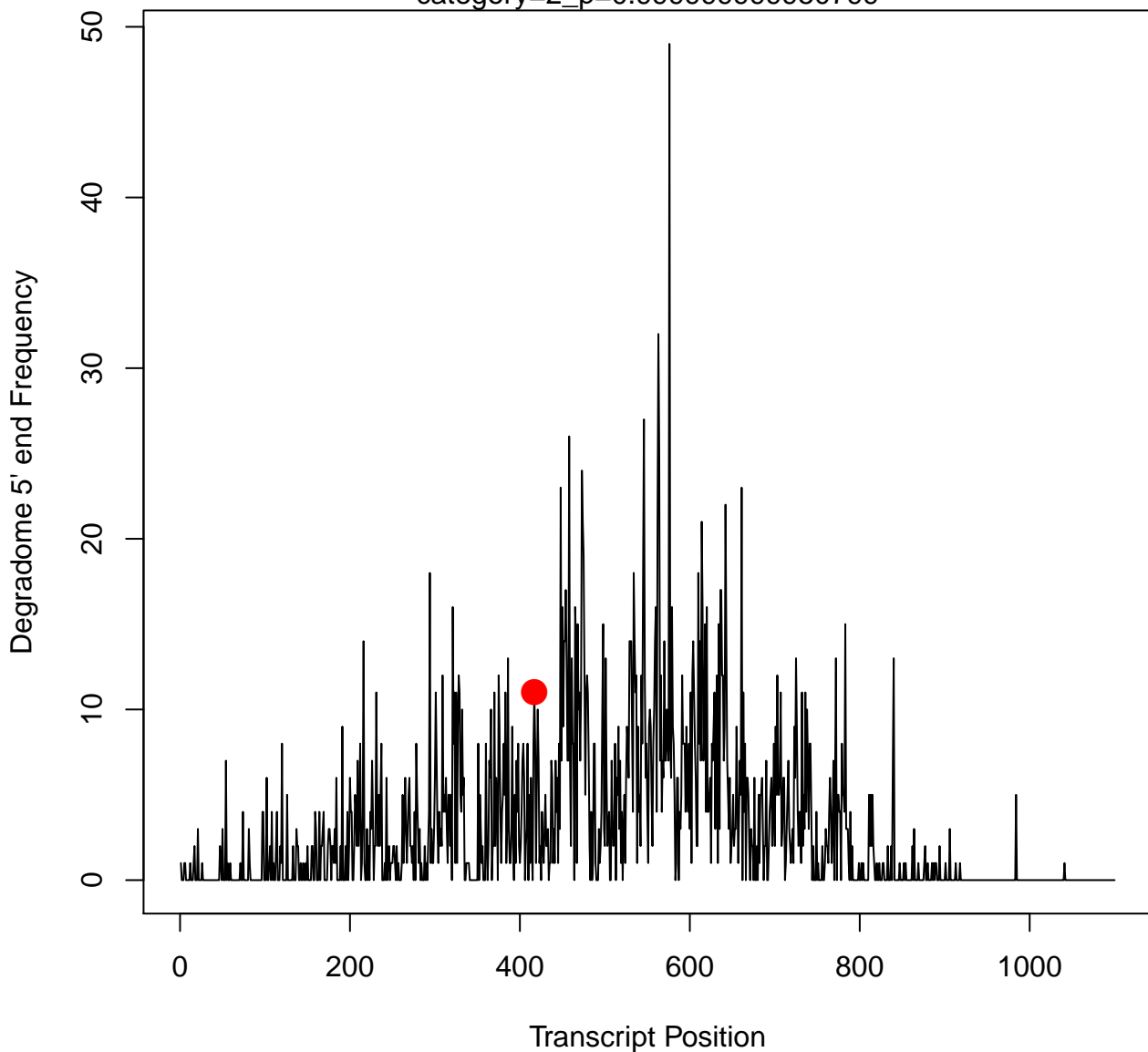

Supplement: Supplementary file 6 [file Data_Sheet_6.zip › Sit-miR160c_Seita.5G022000.1_417_TPlot.pdf]

**T=Seita.5G070500.1\_Q=Sit-miR160c\_S=709**

category=2\_p=0.868974631088784

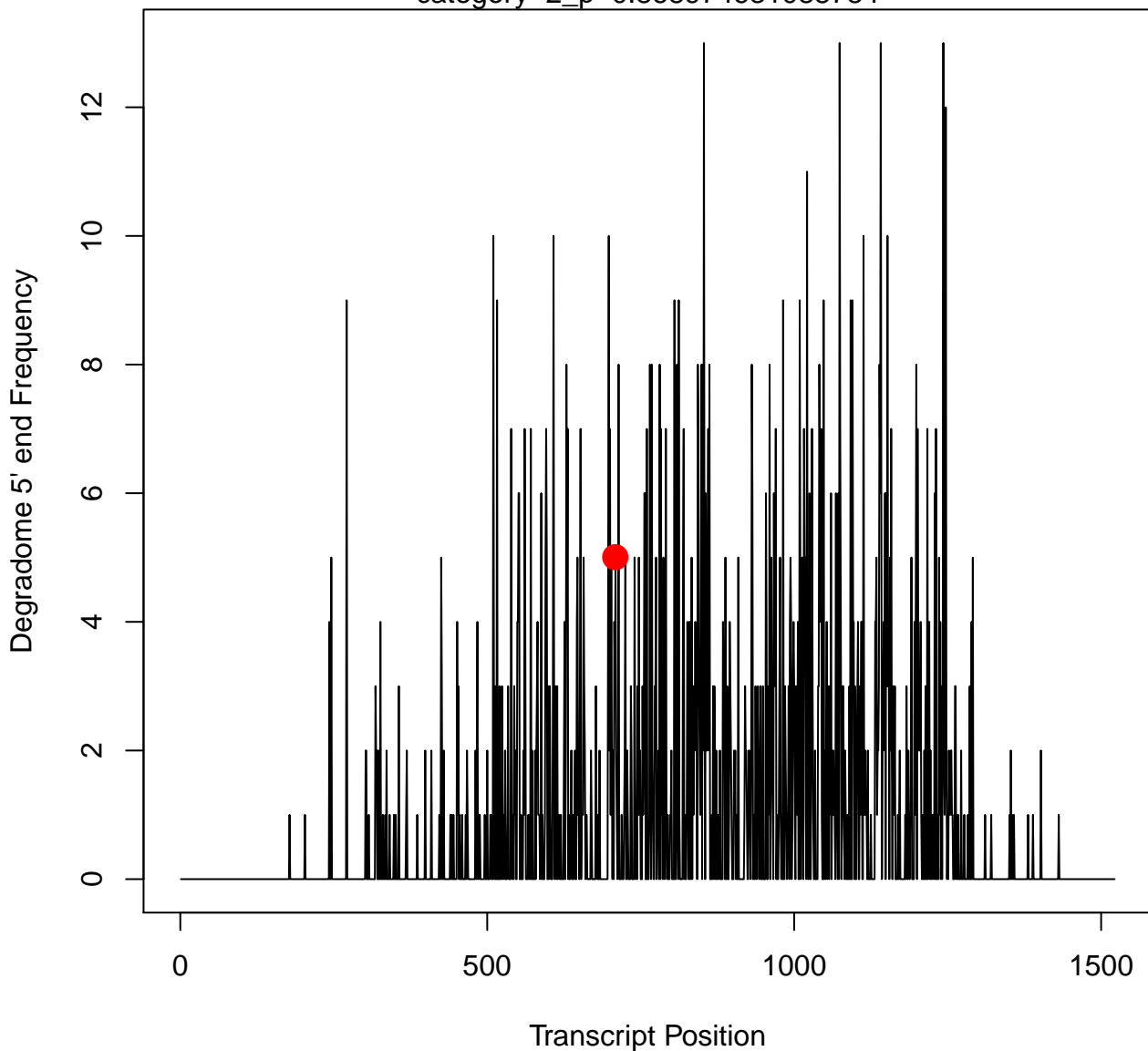

Supplement: Supplementary file 6 [file Data_Sheet_6.zip › Sit-miR160c_Seita.5G070500.1_709_TPlot.pdf]

**T=Seita.5G135500.1\_Q=Sit-miR160c\_S=1290**

category=2\_p=0.999992457610321

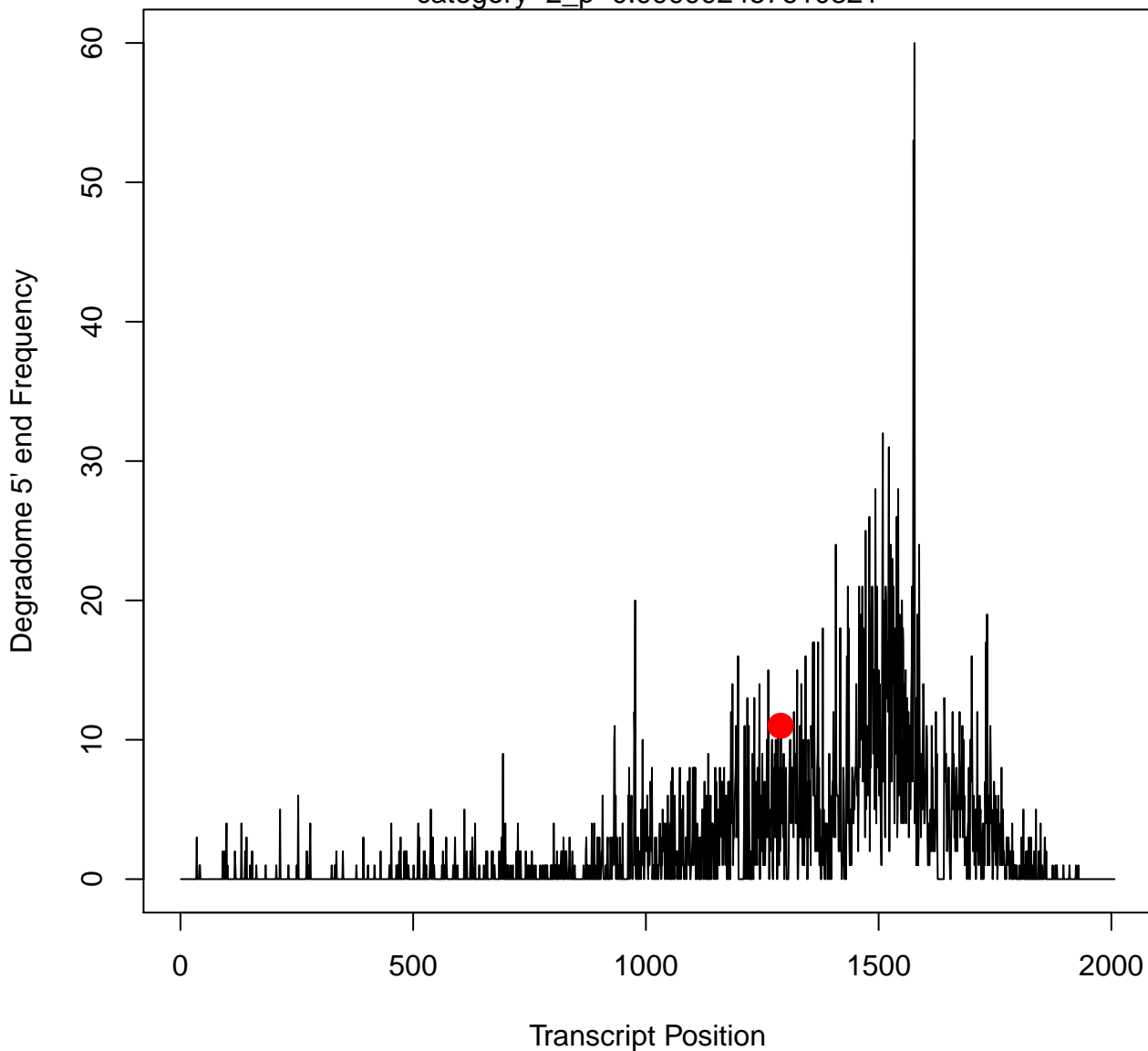

Supplement: Supplementary file 6 [file Data_Sheet_6.zip › Sit-miR160c_Seita.5G135500.1_1290_TPlot.pdf]

**T=Seita.5G165100.1\_Q=Sit-miR160c\_S=762**

category=2\_p=0.84290478913823

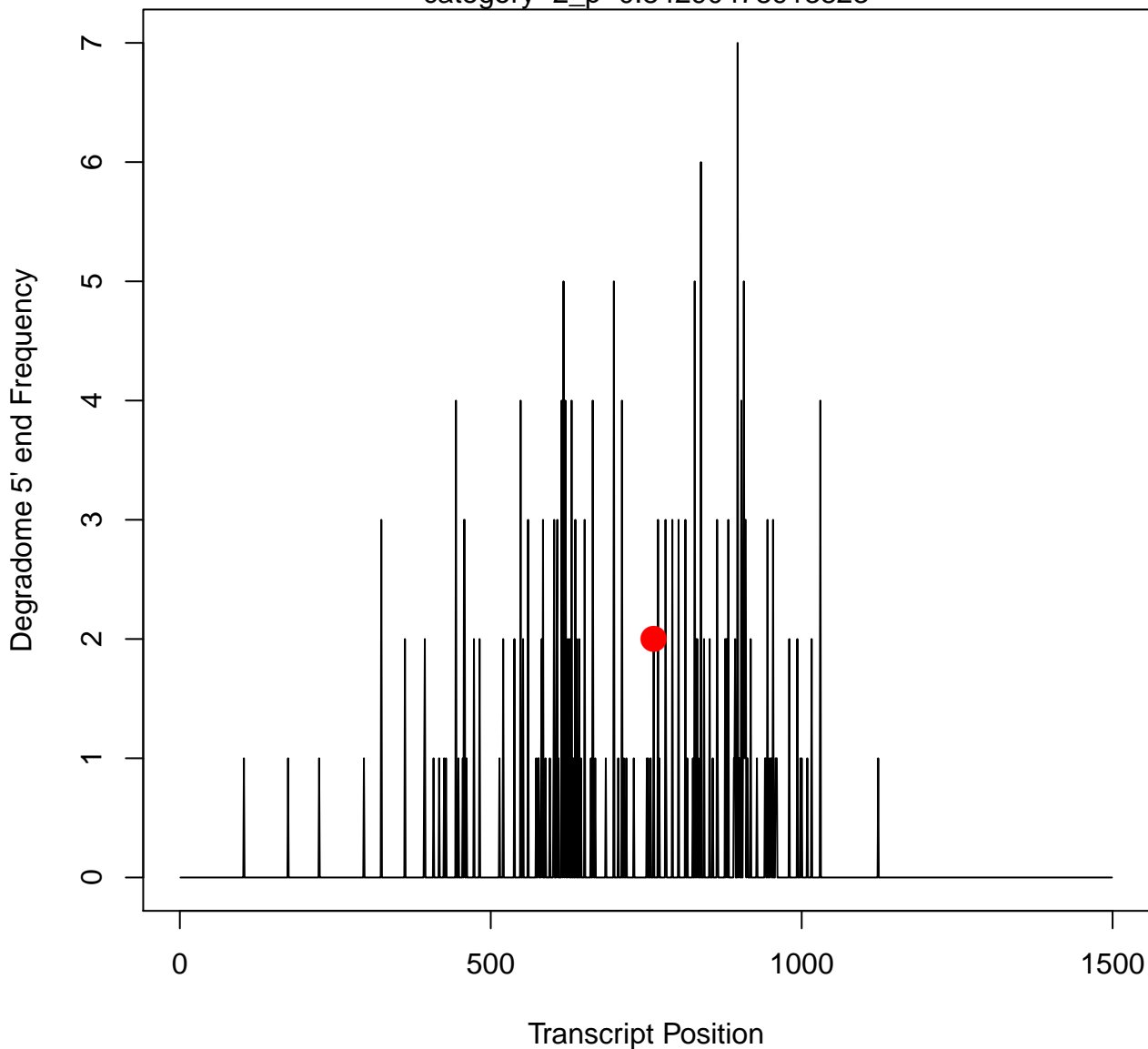

Supplement: Supplementary file 6 [file Data_Sheet_6.zip › Sit-miR160c_Seita.5G165100.1_762_TPlot.pdf]

**T=Seita.5G267100.1\_Q=Sit-miR160c\_S=1208**

category=2\_p=0.999576321258518

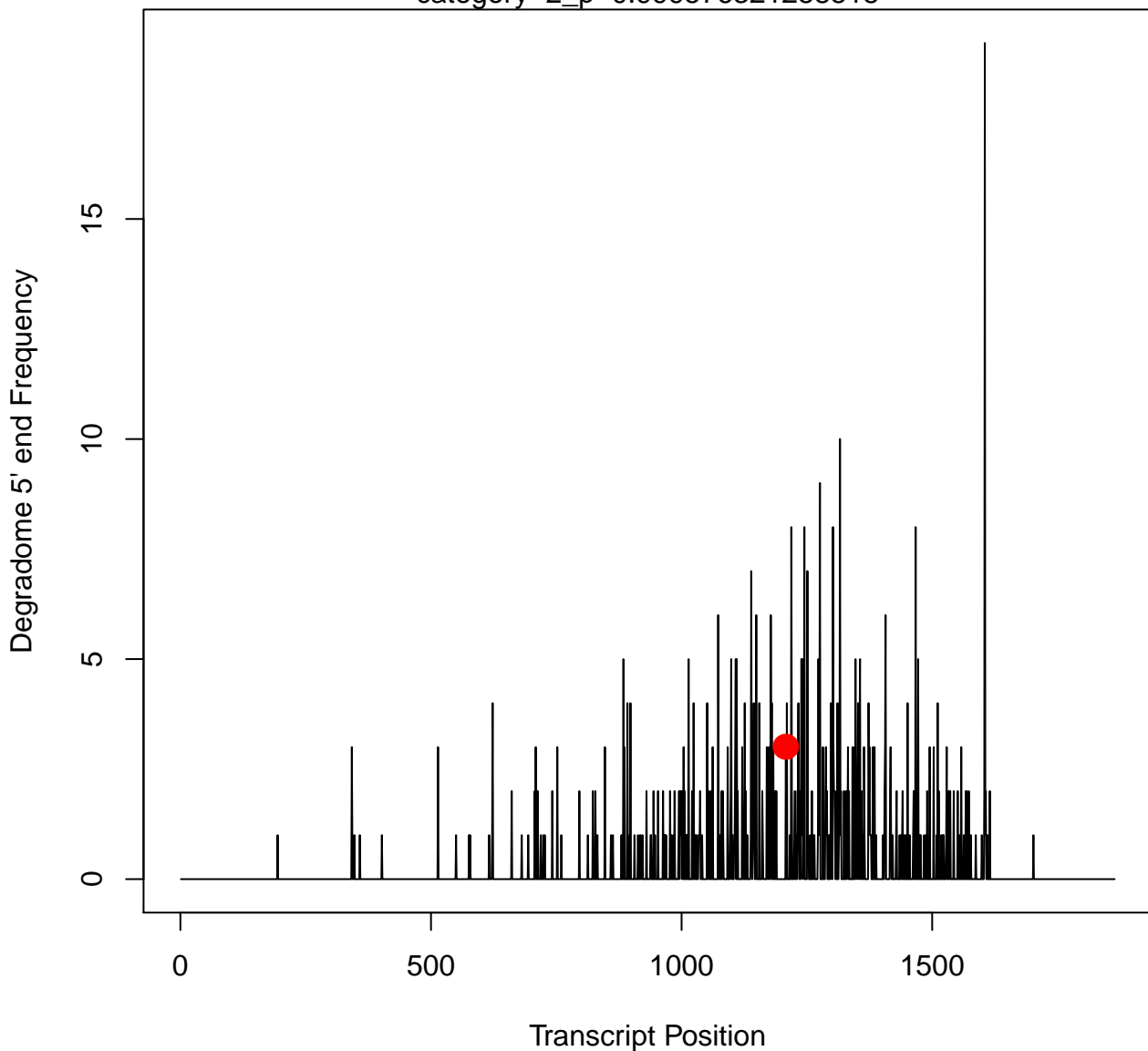

Supplement: Supplementary file 6 [file Data_Sheet_6.zip › Sit-miR160c_Seita.5G267100.1_1208_TPlot.pdf]

**T=Seita.5G322600.1\_Q=Sit-miR160c\_S=463**

category=2\_p=0.999155683298948

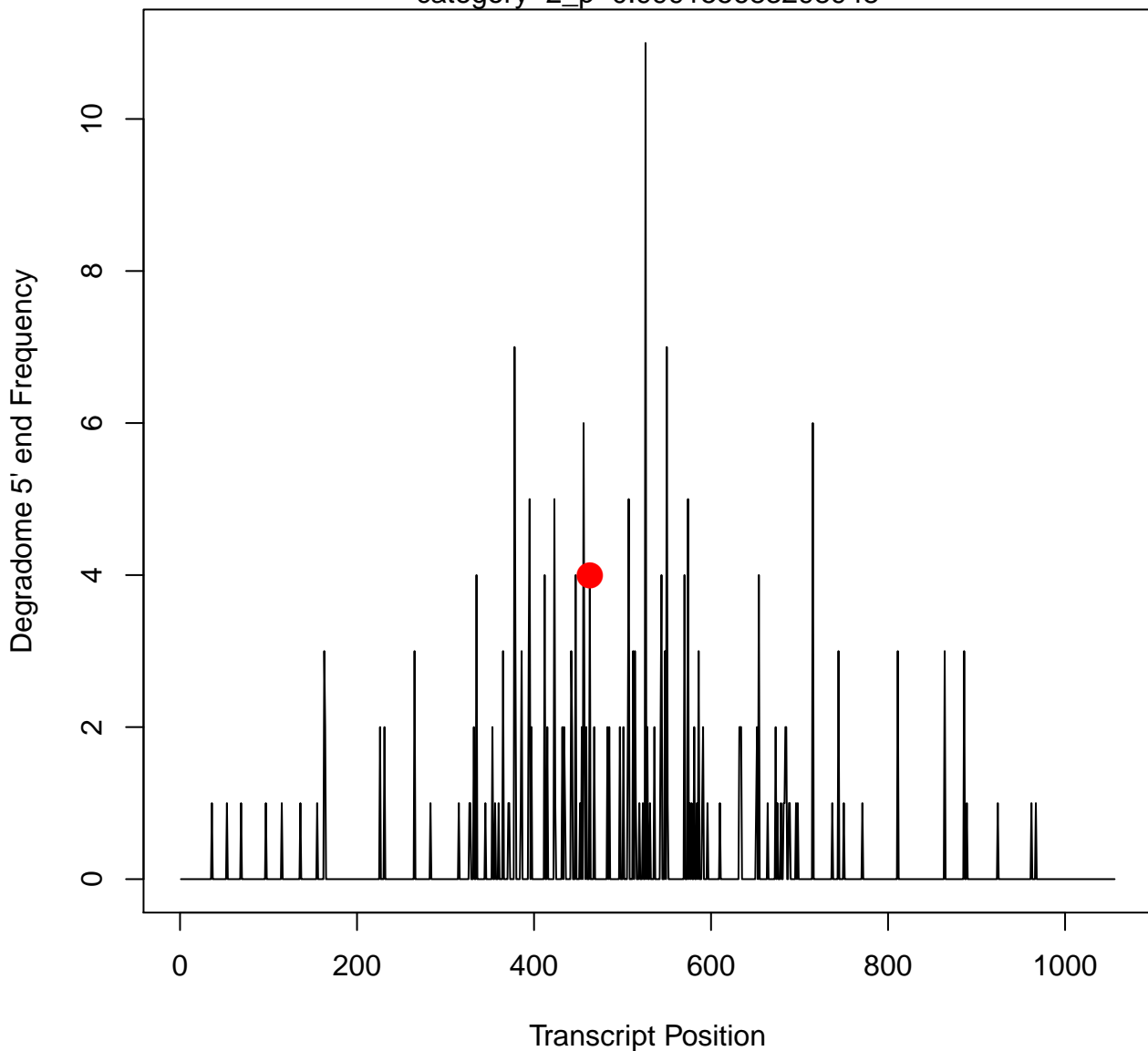

Supplement: Supplementary file 6 [file Data_Sheet_6.zip › Sit-miR160c_Seita.5G322600.1_463_TPlot.pdf]

**T=Seita.5G338300.1\_Q=Sit-miR160c\_S=373**

category=2\_p=0.999999722532911

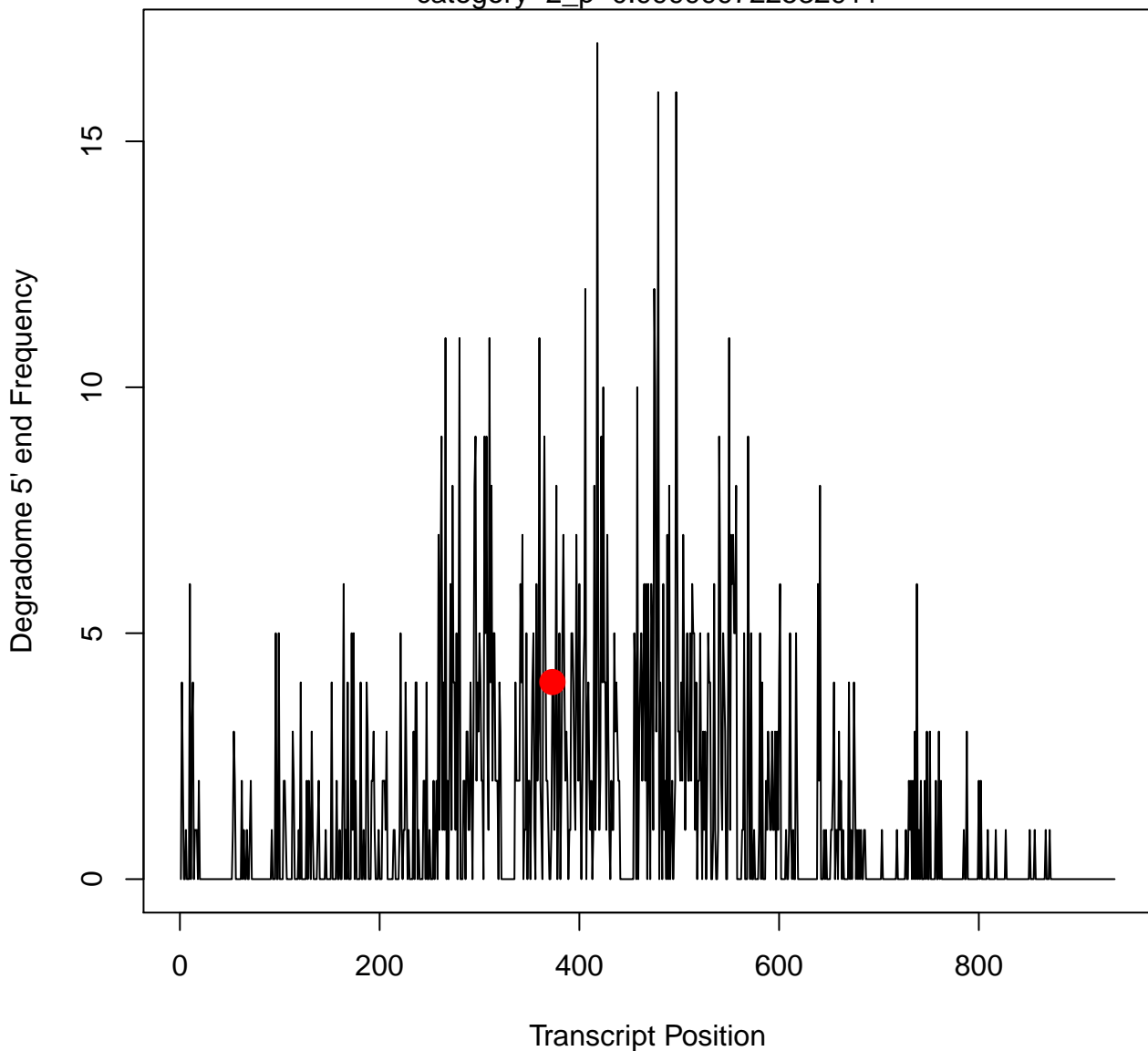

Supplement: Supplementary file 6 [file Data_Sheet_6.zip › Sit-miR160c_Seita.5G338300.1_373_TPlot.pdf]
