# Supplementary figures and images for: The Construction and Exploration of a Comprehensive MicroRNA Centered Regulatory Network in Foxtail Millet (Setaria italica L.) (part 14 of 14)
Source: Front Plant Sci. 2022 May 6;13:848474. doi: 10.3389/fpls.2022.848474 (PMC9121102; doi:10.3389/fpls.2022.848474)

**T=Seita.6G215000.1\_Q=Sit-miR171h\_S=301**

category=2\_p=0.207913115291402

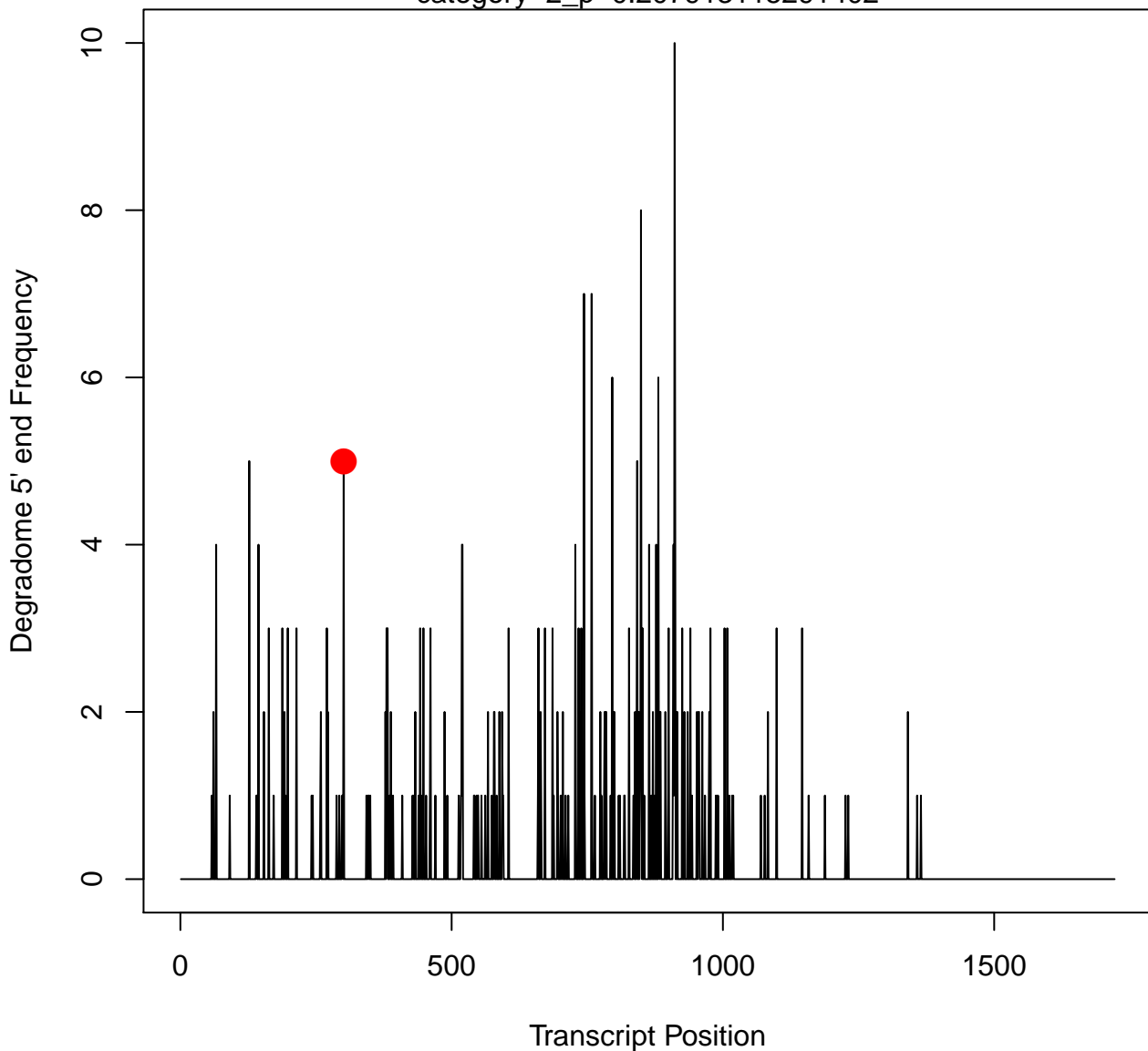

Supplement: Supplementary file 7 [file Data_Sheet_7.zip › Sit-miR171h_Seita.6G215000.1_301_TPlot.pdf]

**T=Seita.4G192300.1\_Q=Sit-miR171i\_S=1041**

category=2\_p=0.999251438556956

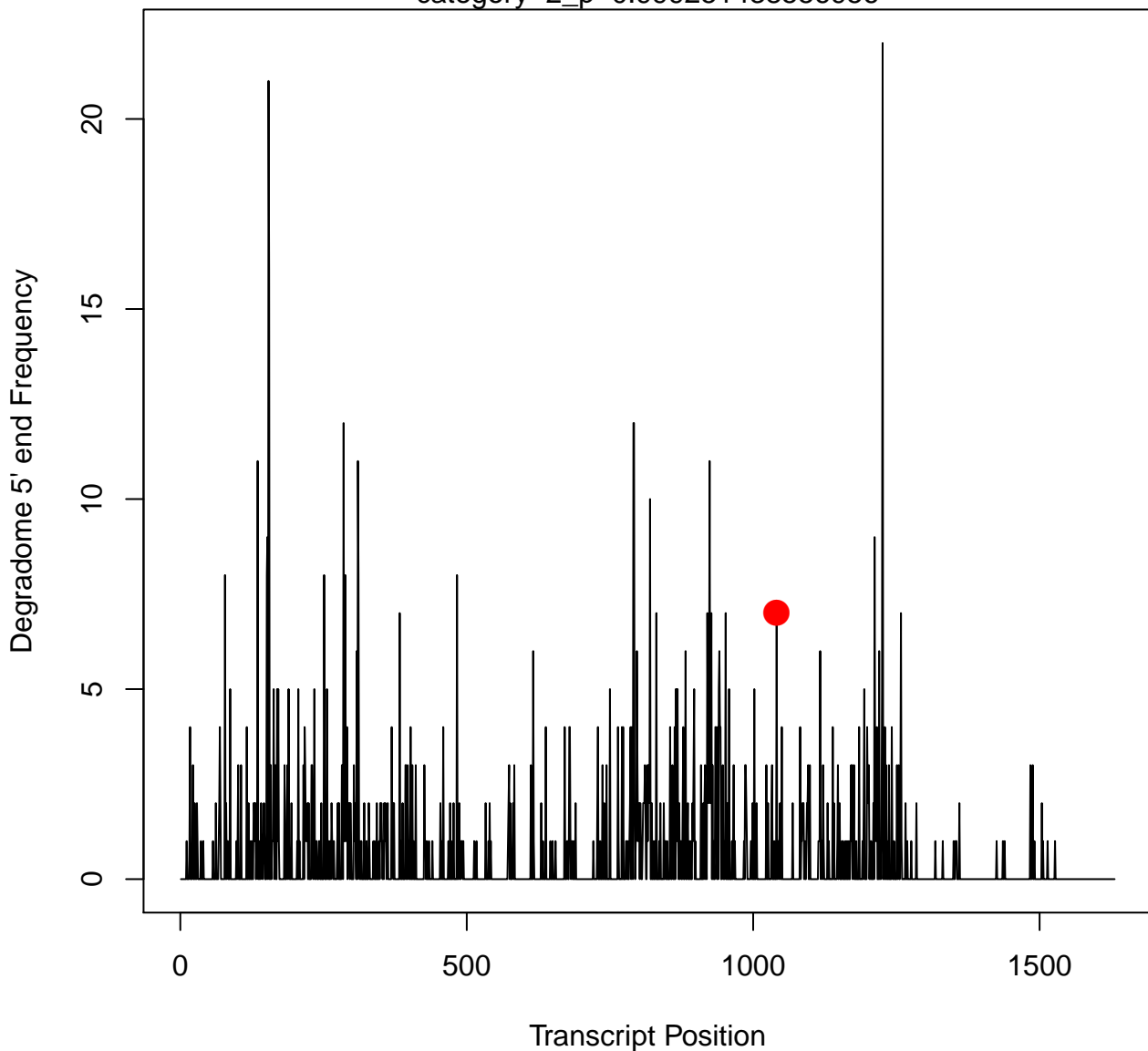

Supplement: Supplementary file 7 [file Data_Sheet_7.zip › Sit-miR171i_Seita.4G192300.1_1041_TPlot.pdf]

**T=Seita.6G208600.1\_Q=Sit-miR171i\_S=3603**

category=2\_p=0.993726036128558

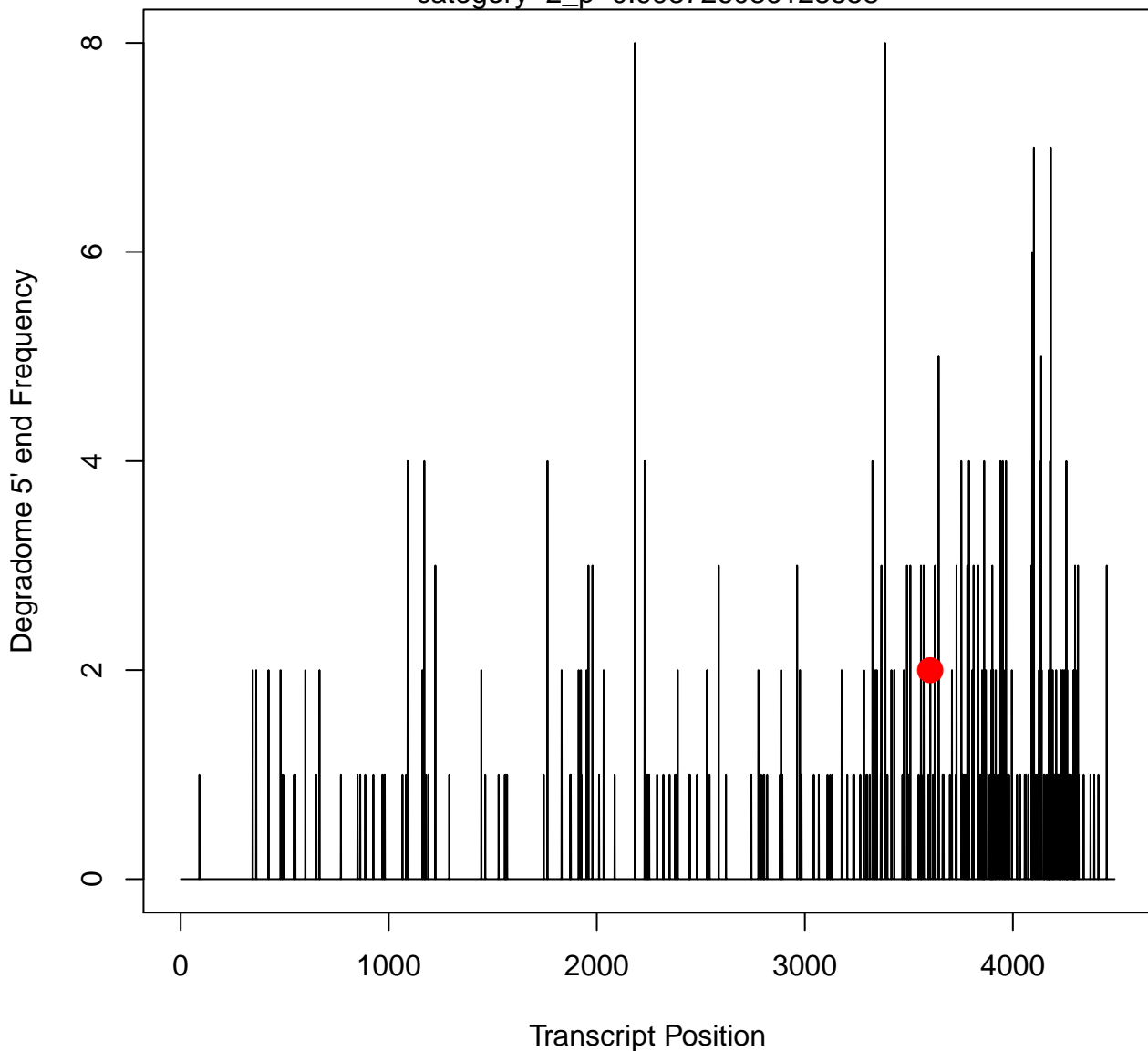

Supplement: Supplementary file 7 [file Data_Sheet_7.zip › Sit-miR171i_Seita.6G208600.1_3603_TPlot.pdf]

**T=Seita.1G053900.1\_Q=Sit-miR172a\_S=692**

category=2\_p=0.928754300749882

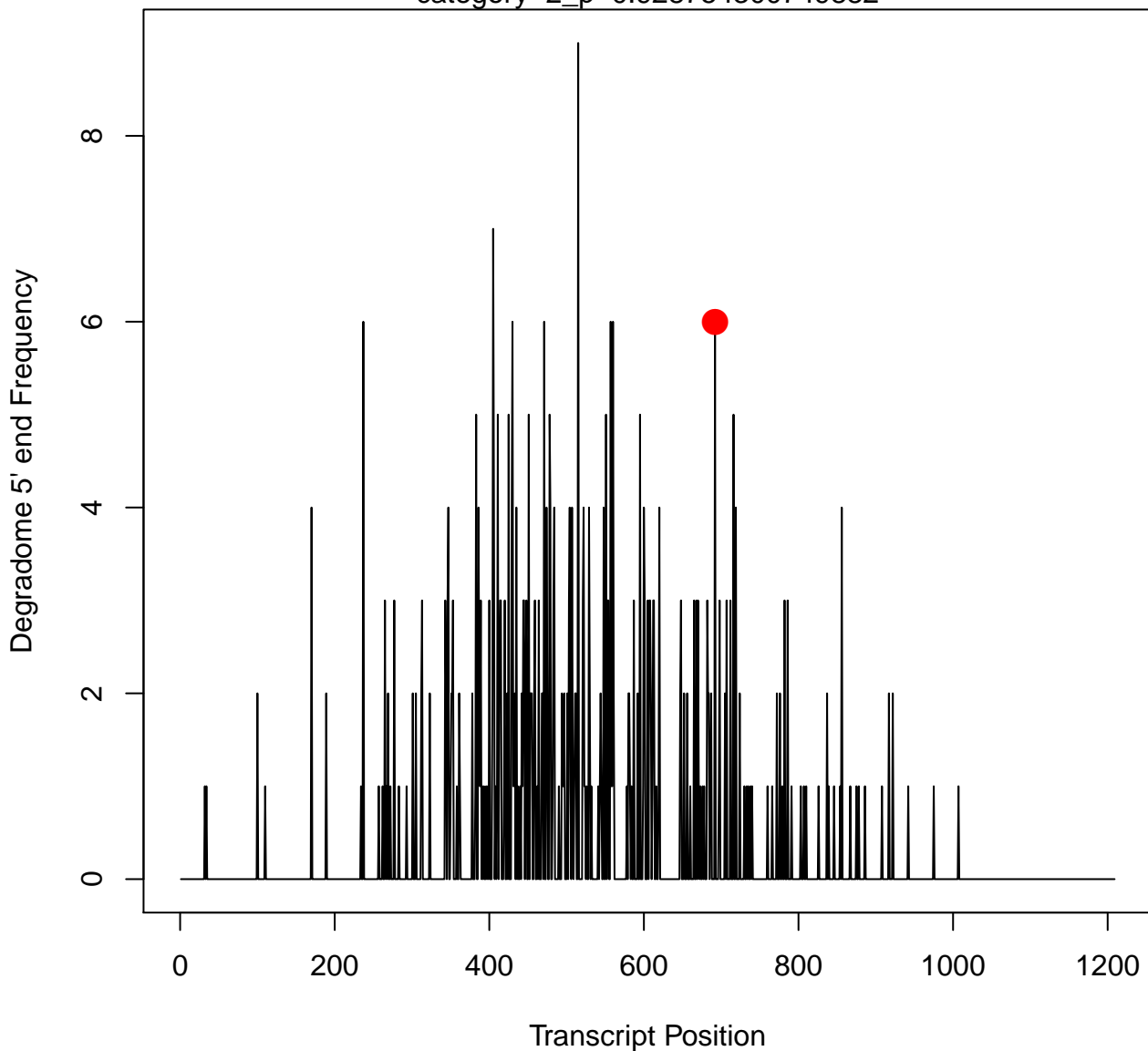

Supplement: Supplementary file 7 [file Data_Sheet_7.zip › Sit-miR172a_Seita.1G053900.1_692_TPlot.pdf]

**T=Seita.2G091100.1\_Q=Sit-miR172a\_S=1500**

category=0\_p=0.000897462201815302

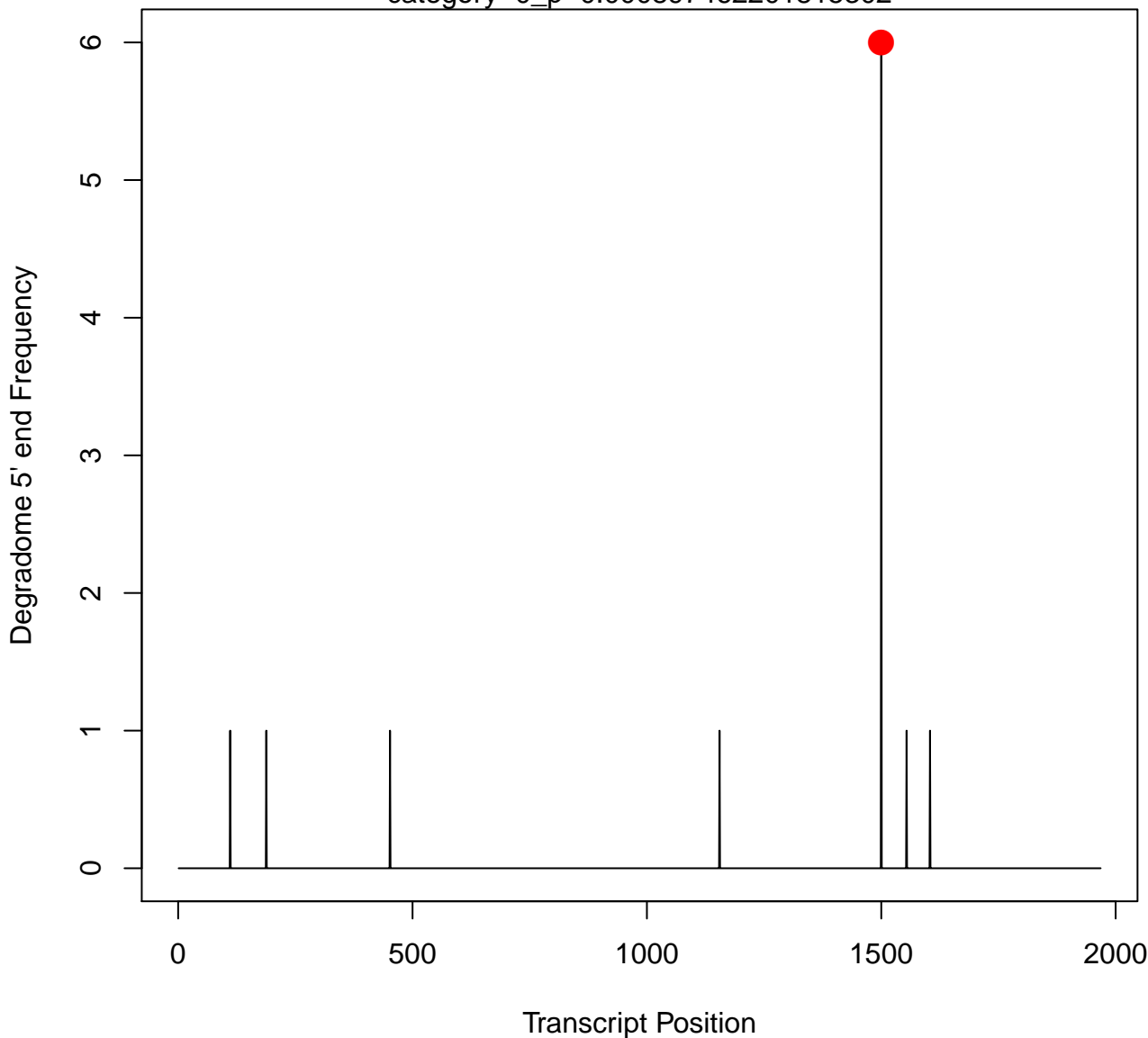

Supplement: Supplementary file 7 [file Data_Sheet_7.zip › Sit-miR172a_Seita.2G091100.1_1500_TPlot.pdf]

**T=Seita.3G044600.1\_Q=Sit-miR172a\_S=1327**

category=0\_p=0.000598397663414518

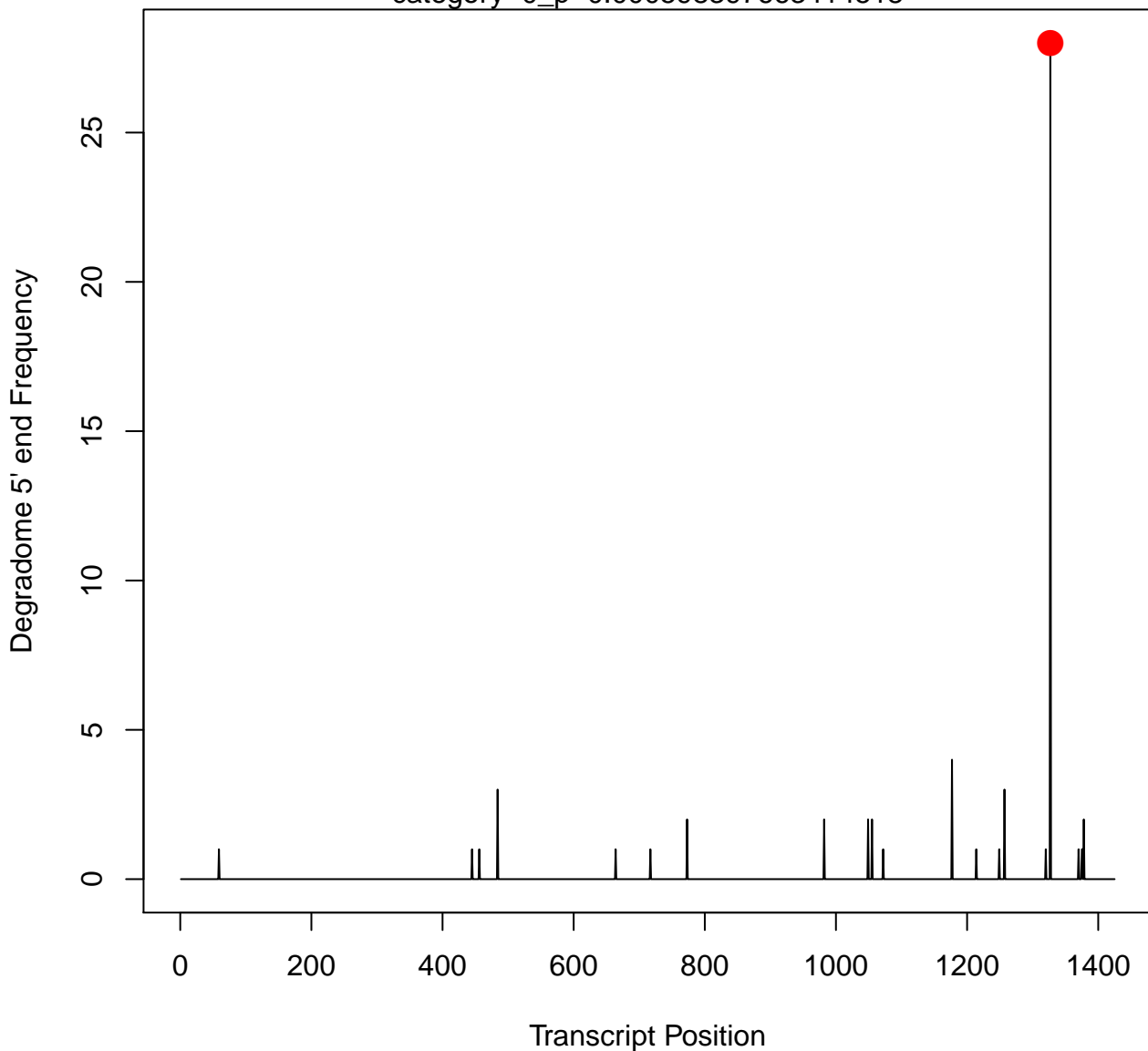

Supplement: Supplementary file 7 [file Data_Sheet_7.zip › Sit-miR172a_Seita.3G044600.1_1327_TPlot.pdf]

**T=Seita.3G404200.1\_Q=Sit-miR172a\_S=231**

category=2\_p=0.423584352287658

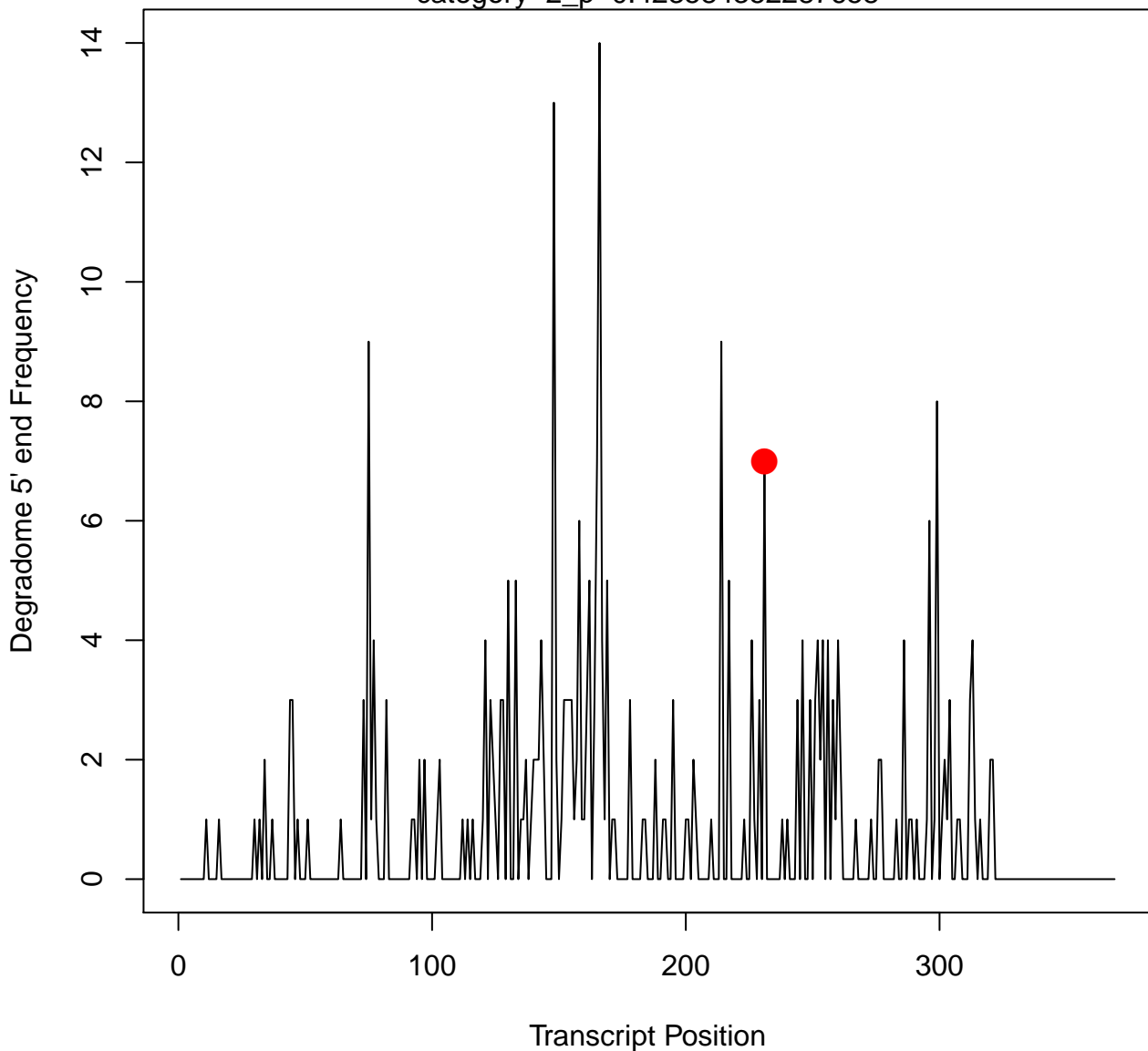

Supplement: Supplementary file 7 [file Data_Sheet_7.zip › Sit-miR172a_Seita.3G404200.1_231_TPlot.pdf]

**T=Seita.5G302800.1\_Q=Sit-miR172a\_S=2750**

category=2\_p=0.868286496025829

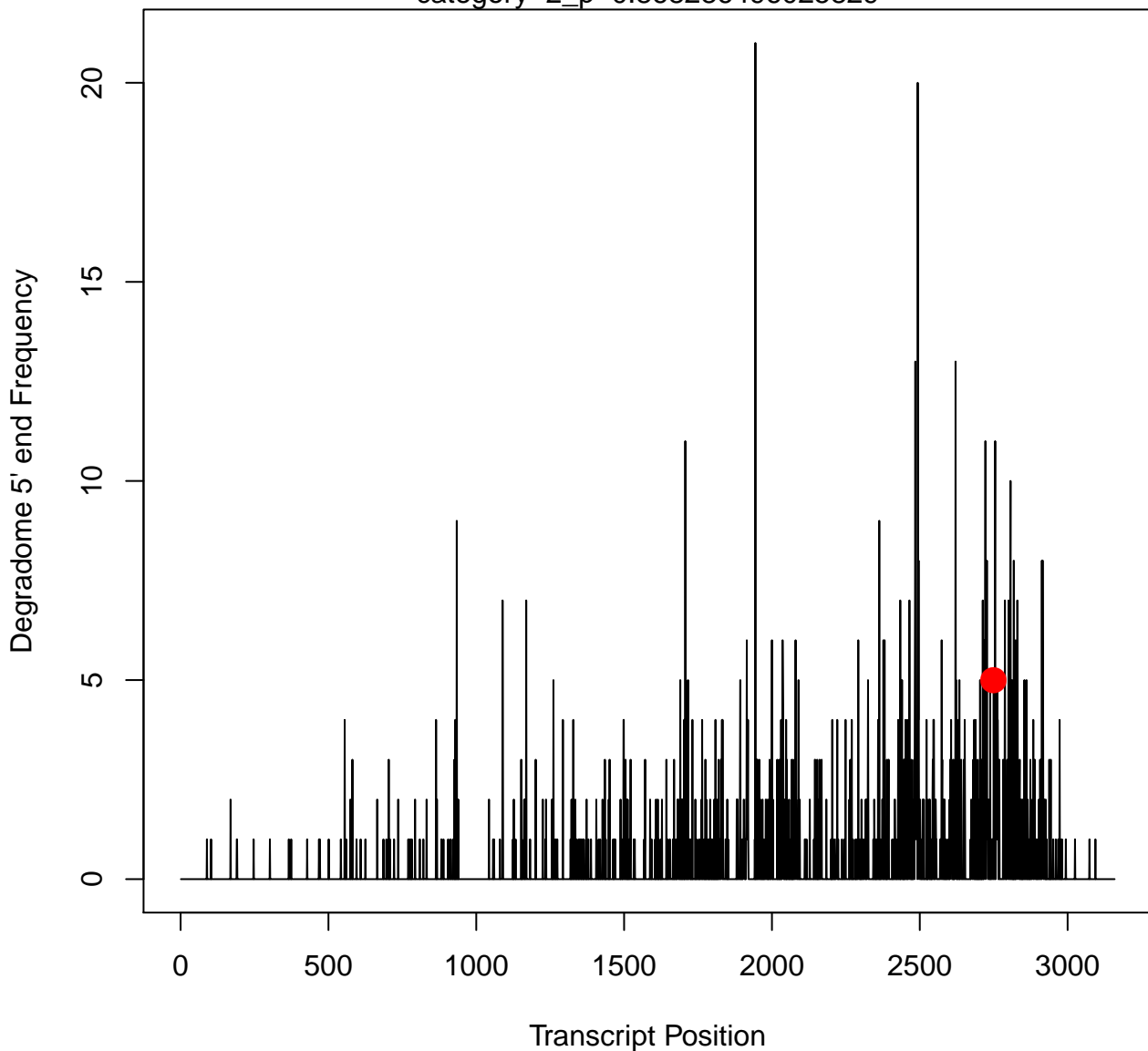

Supplement: Supplementary file 7 [file Data_Sheet_7.zip › Sit-miR172a_Seita.5G302800.1_2750_TPlot.pdf]

**T=Seita.6G204700.1\_Q=Sit-miR172a\_S=1870**

category=0\_p=0.0113085311008065

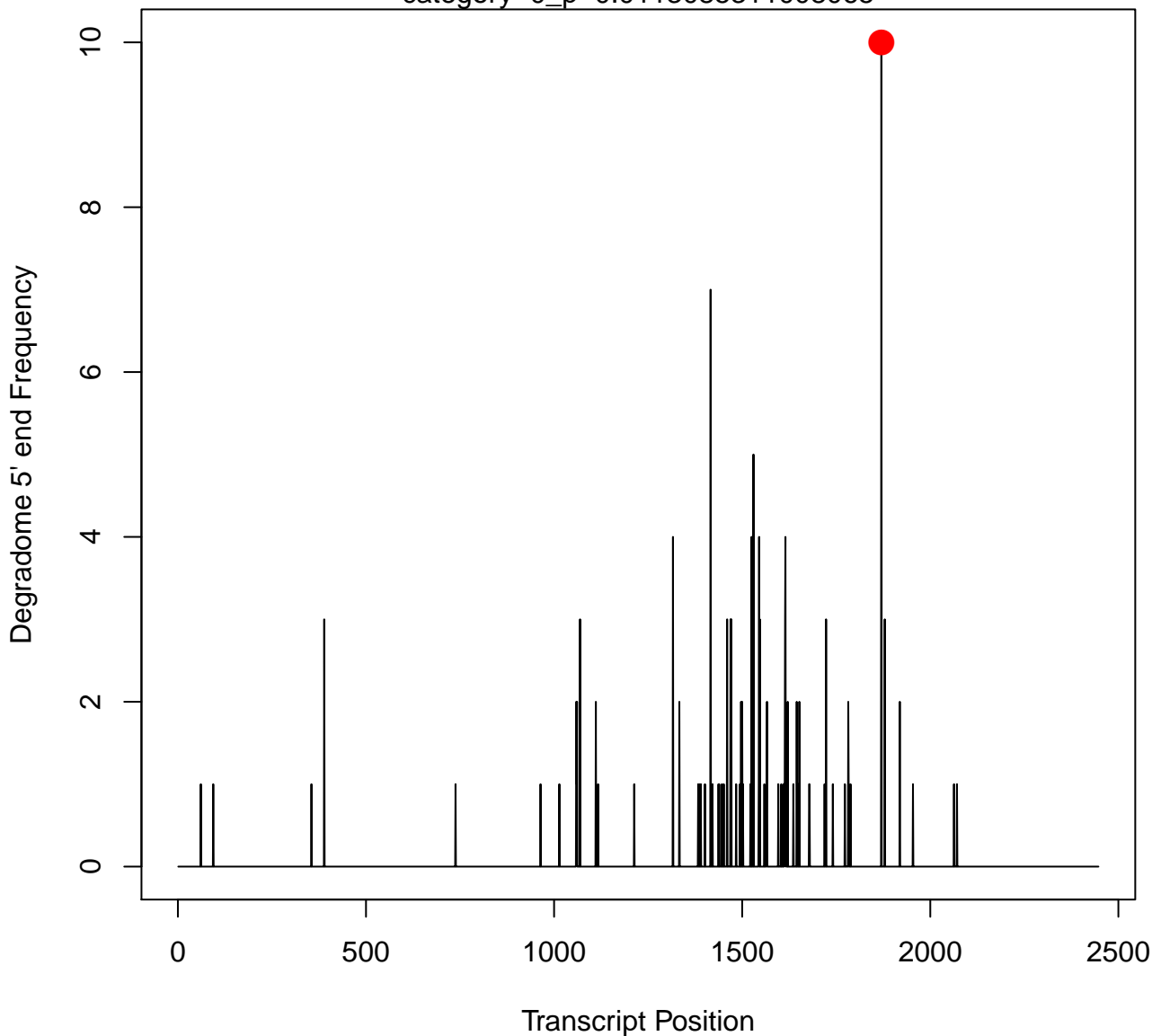

Supplement: Supplementary file 7 [file Data_Sheet_7.zip › Sit-miR172a_Seita.6G204700.1_1870_TPlot.pdf]

**T=Seita.7G057600.1\_Q=Sit-miR172a\_S=993**

category=2\_p=0.947785745330705

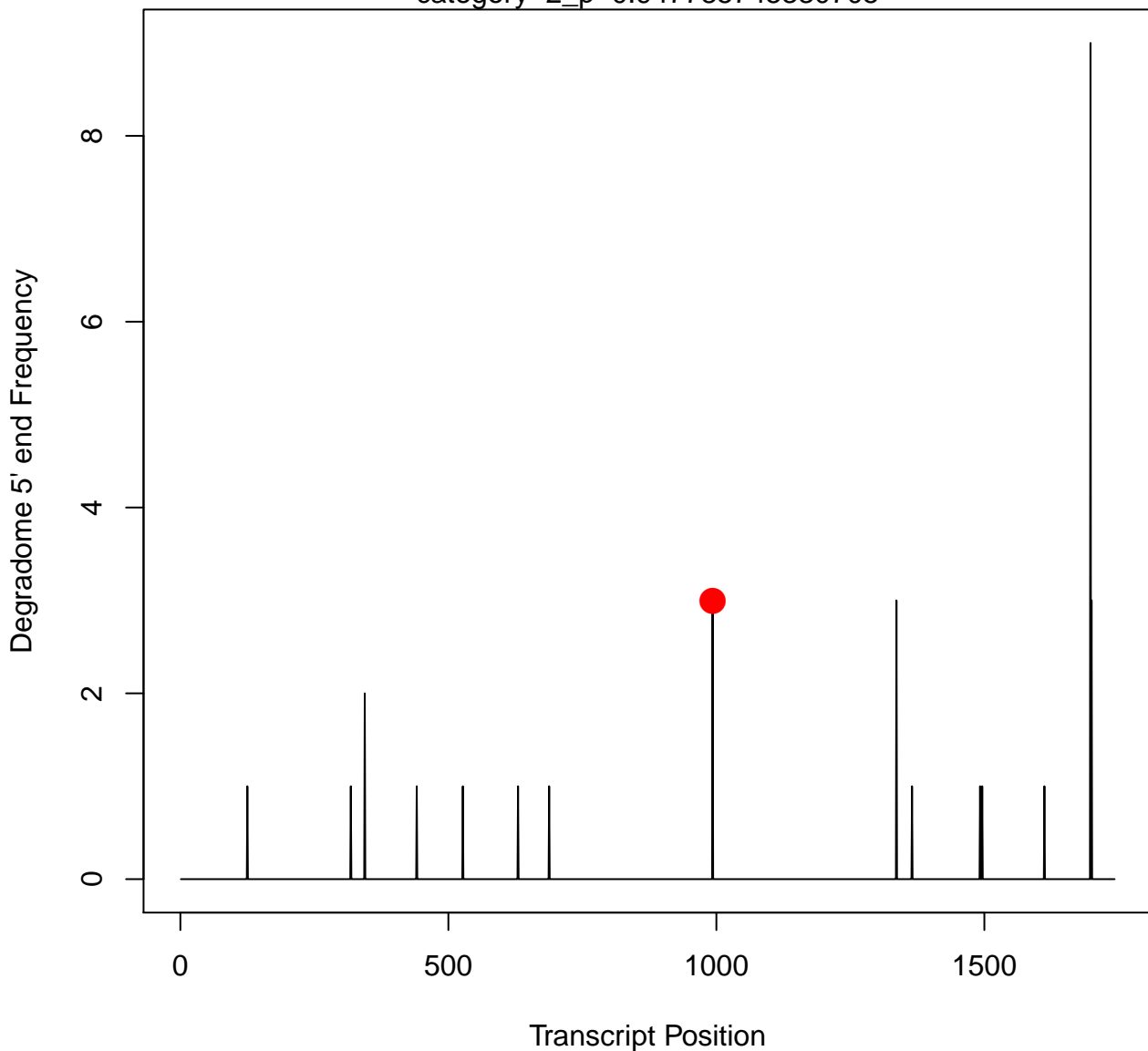

Supplement: Supplementary file 7 [file Data_Sheet_7.zip › Sit-miR172a_Seita.7G057600.1_993_TPlot.pdf]

**T=Seita.7G074500.1\_Q=Sit-miR172a\_S=3253**

category=2\_p=0.995466459886192

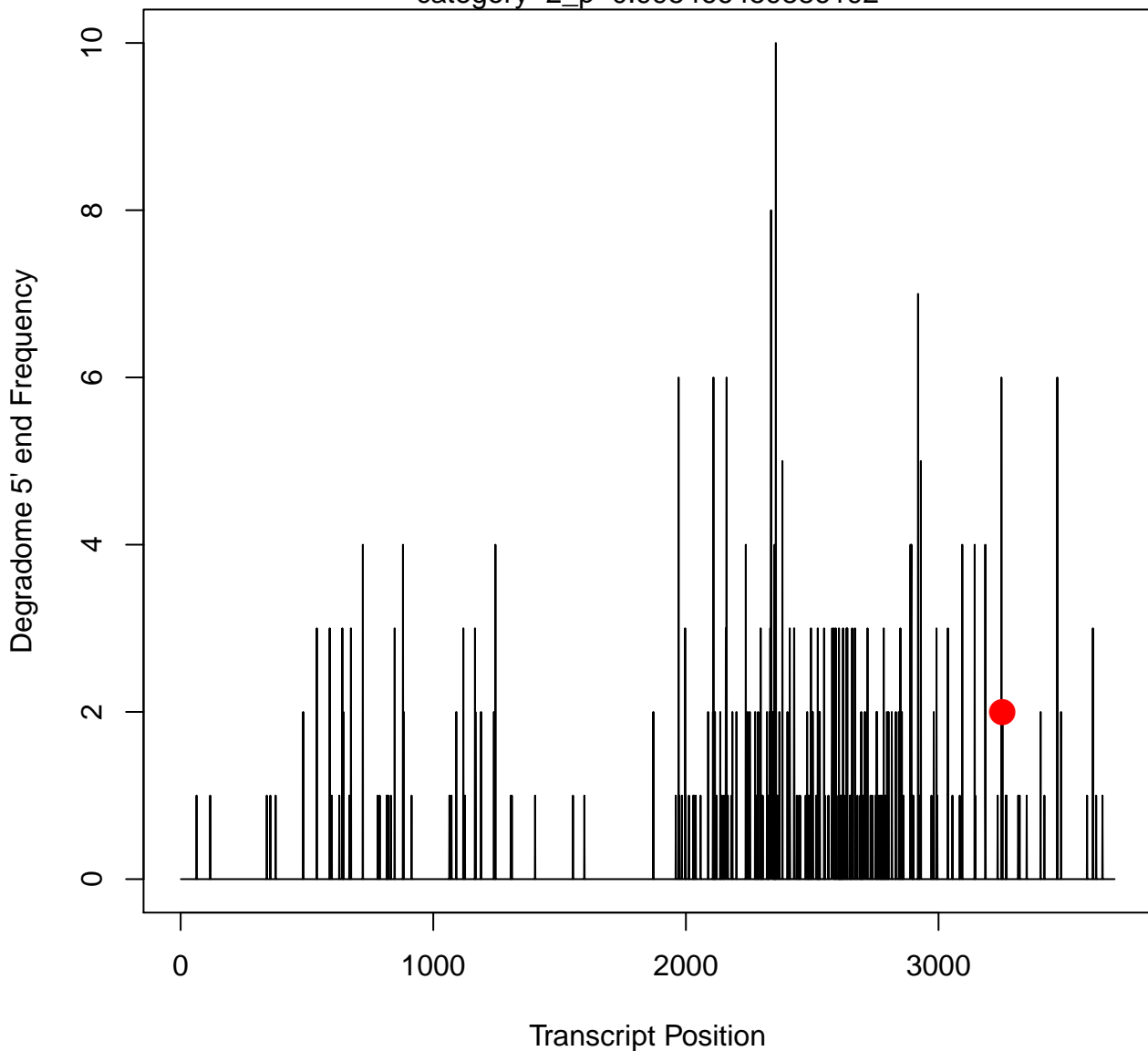

Supplement: Supplementary file 7 [file Data_Sheet_7.zip › Sit-miR172a_Seita.7G074500.1_3253_TPlot.pdf]

**T=Seita.7G079600.1\_Q=Sit-miR172a\_S=2478**

category=2\_p=0.725432399997792

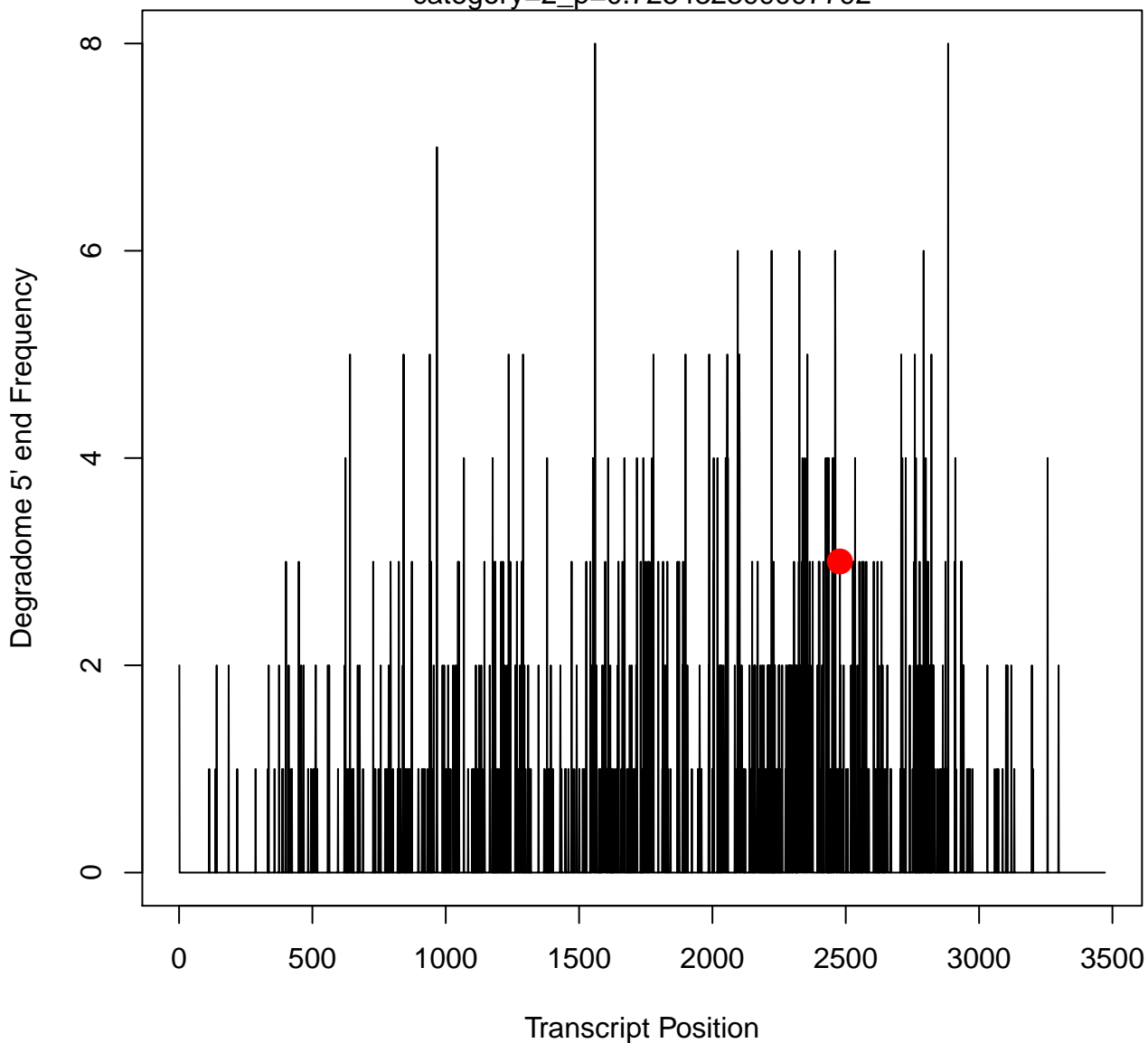

Supplement: Supplementary file 7 [file Data_Sheet_7.zip › Sit-miR172a_Seita.7G079600.1_2478_TPlot.pdf]

**T=Seita.4G192300.1\_Q=Sit-miR172b\_S=771**

category=2\_p=0.978557011442308

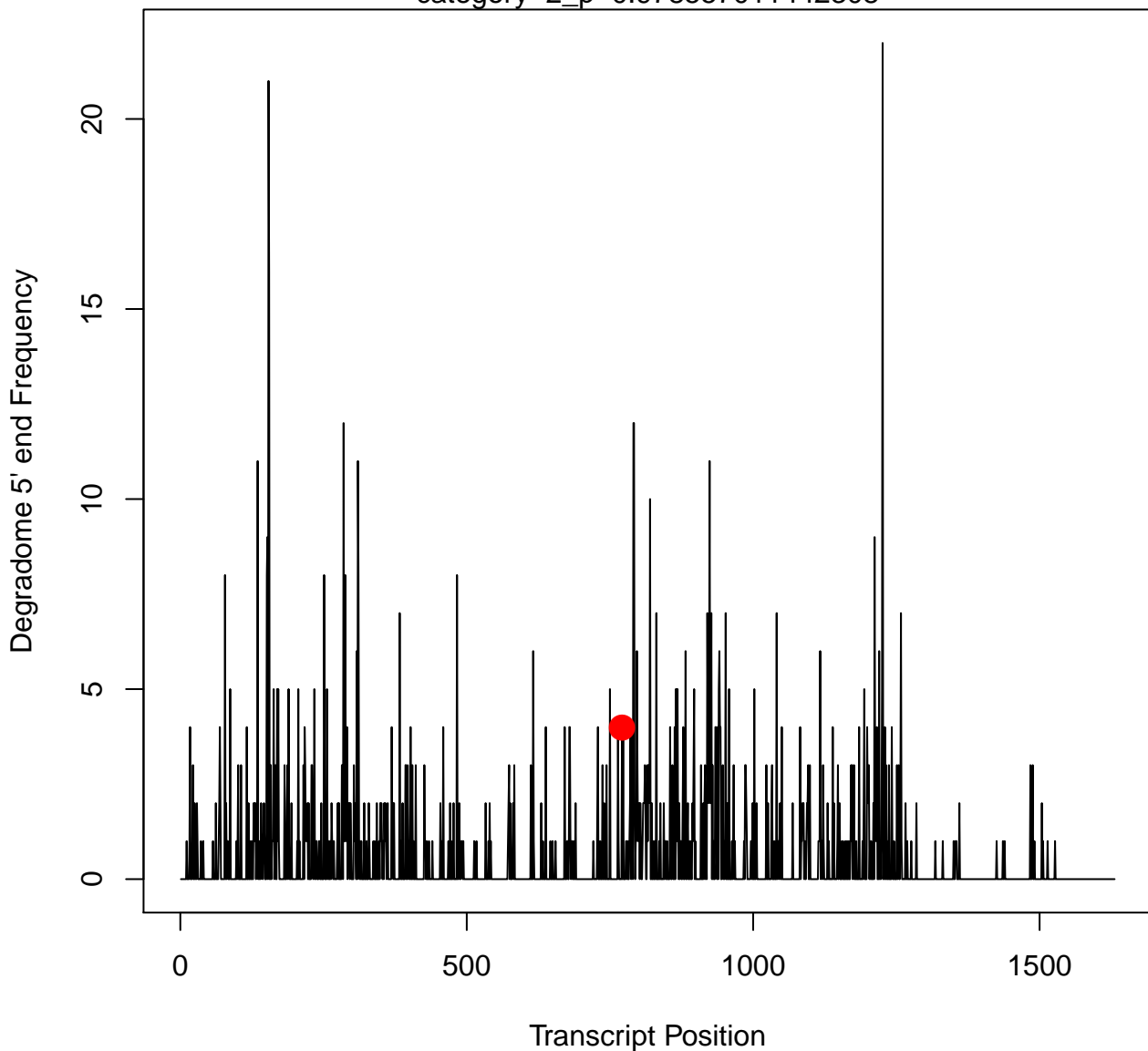

Supplement: Supplementary file 7 [file Data_Sheet_7.zip › Sit-miR172b_Seita.4G192300.1_771_TPlot.pdf]

**T=Seita.7G263000.1\_Q=Sit-miR172b\_S=1947**

category=0\_p=0.00149532282594533

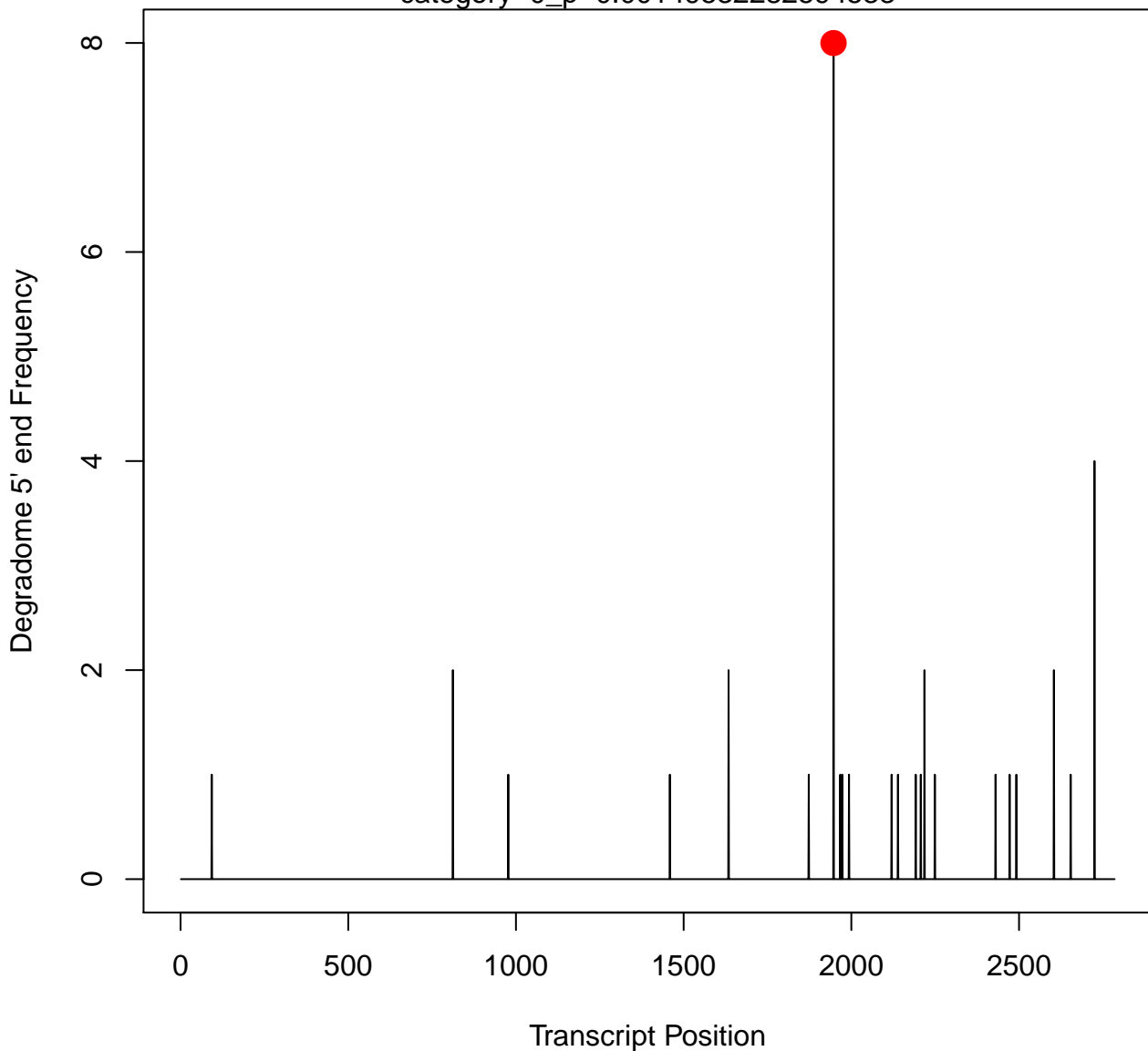

Supplement: Supplementary file 7 [file Data_Sheet_7.zip › Sit-miR172b_Seita.7G263000.1_1947_TPlot.pdf]

**T=Seita.9G103500.1\_Q=Sit-miR172b\_S=44**

category=2\_p=0.987726919423007

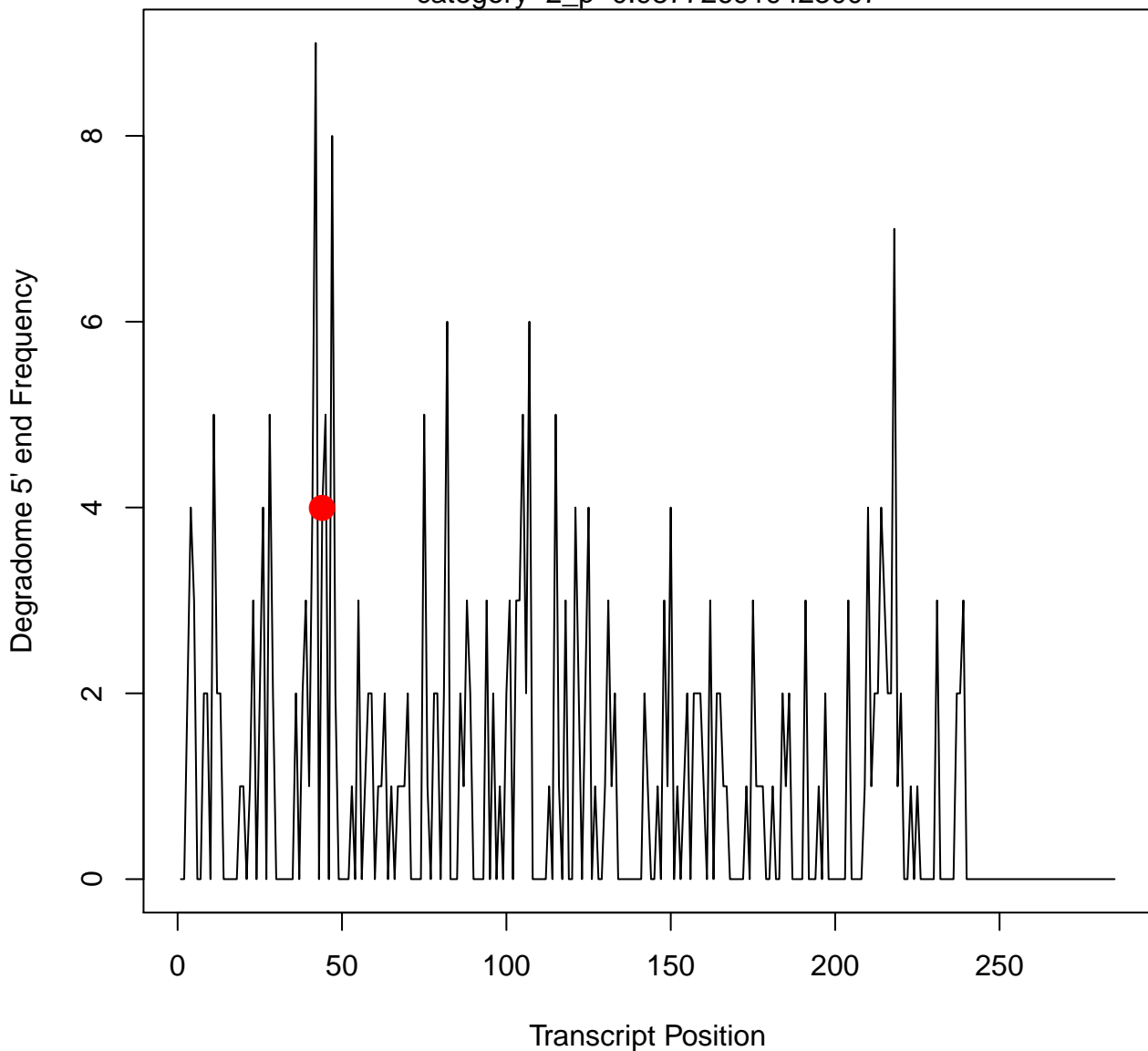

Supplement: Supplementary file 7 [file Data_Sheet_7.zip › Sit-miR172b_Seita.9G103500.1_44_TPlot.pdf]

**T=Seita.1G146800.1\_Q=Sit-miR2118a\_S=1189**

category=2\_p=0.811169429487821

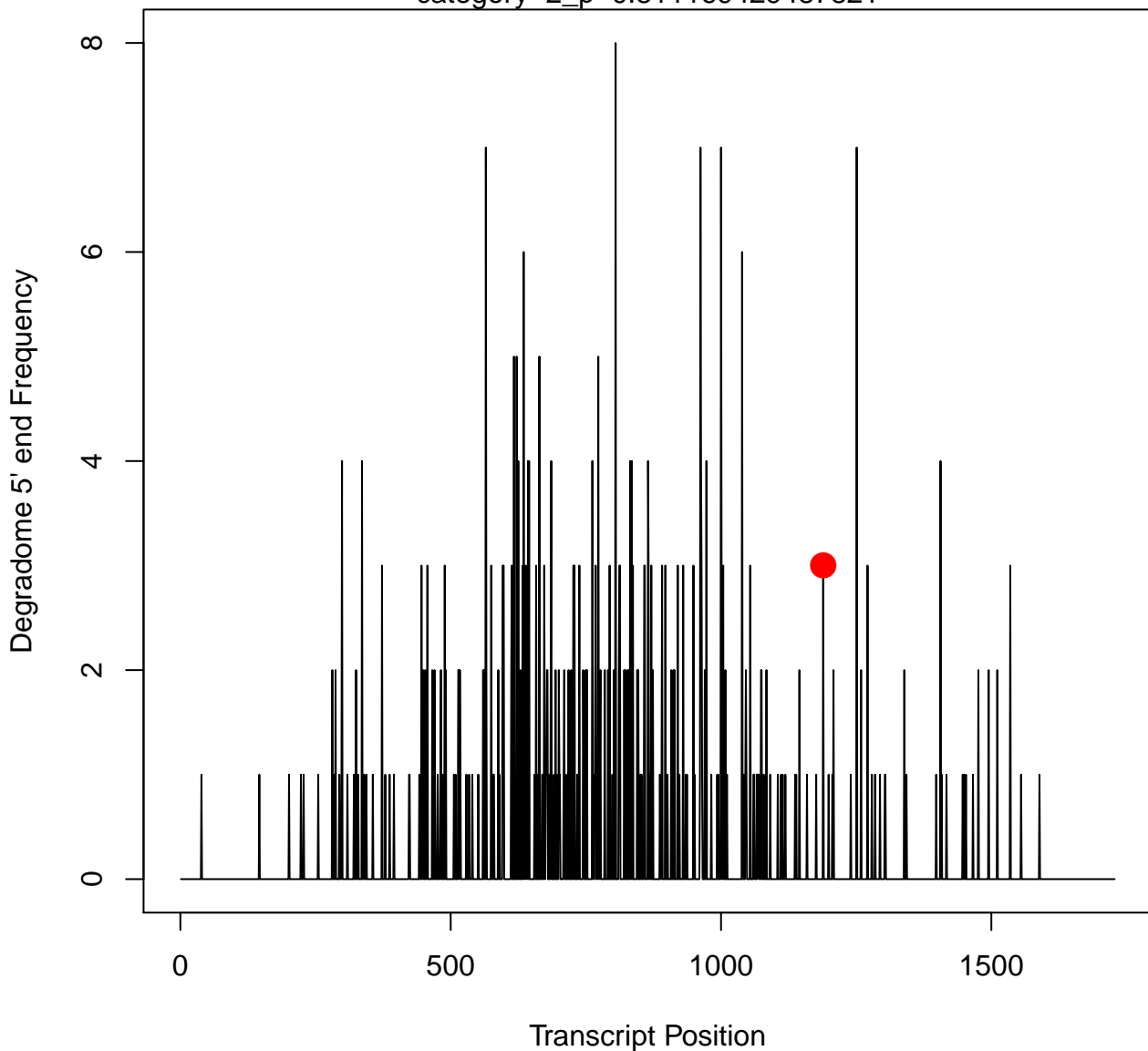

Supplement: Supplementary file 7 [file Data_Sheet_7.zip › Sit-miR2118a_Seita.1G146800.1_1189_TPlot.pdf]

**T=Seita.9G068800.1\_Q=Sit-miR2118a\_S=218**

category=2\_p=0.987552315675952

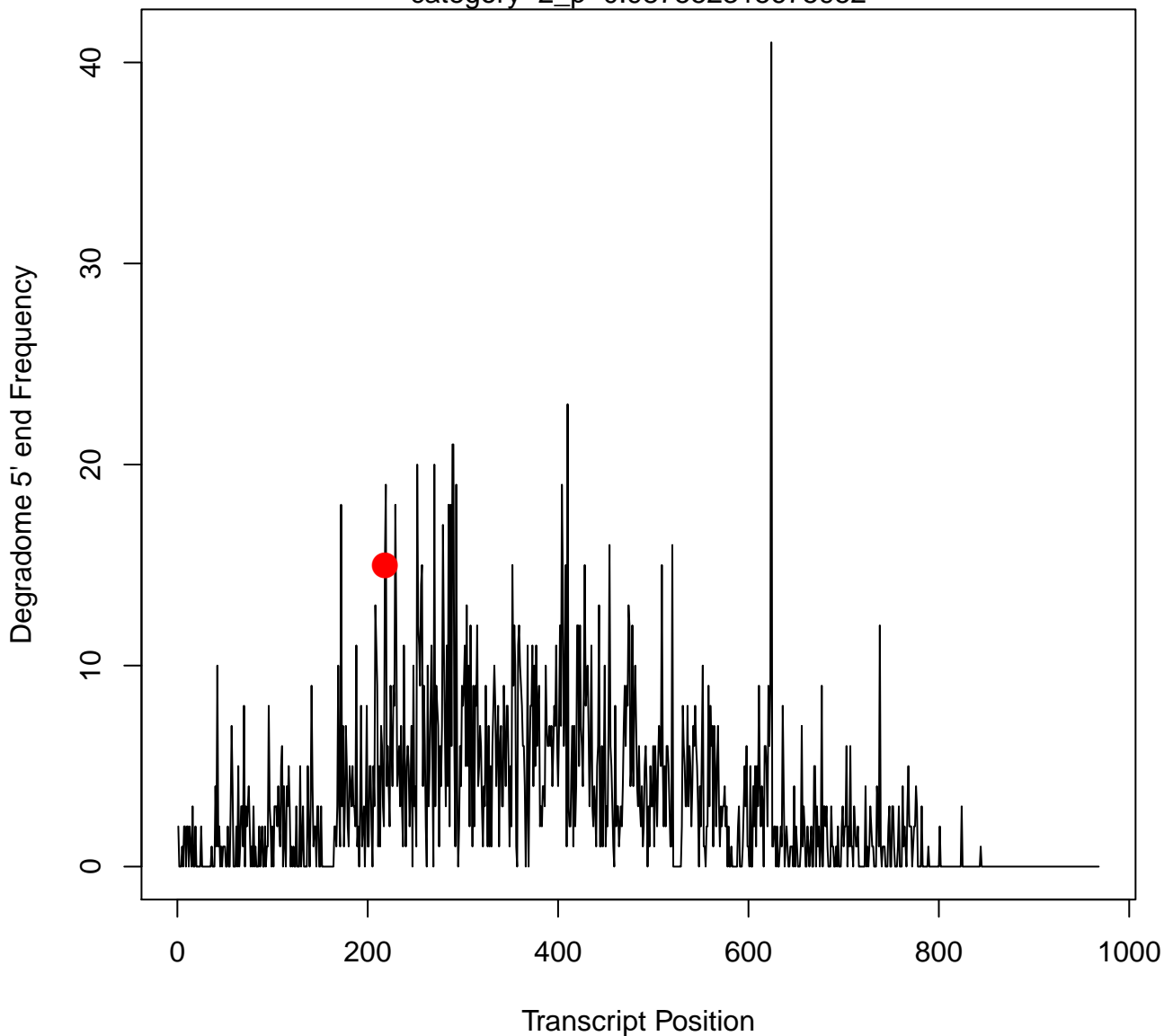

Supplement: Supplementary file 7 [file Data_Sheet_7.zip › Sit-miR2118a_Seita.9G068800.1_218_TPlot.pdf]

**T=Seita.3G350700.1\_Q=Sit-miR2118b\_S=1541**

category=2\_p=0.326681500356753

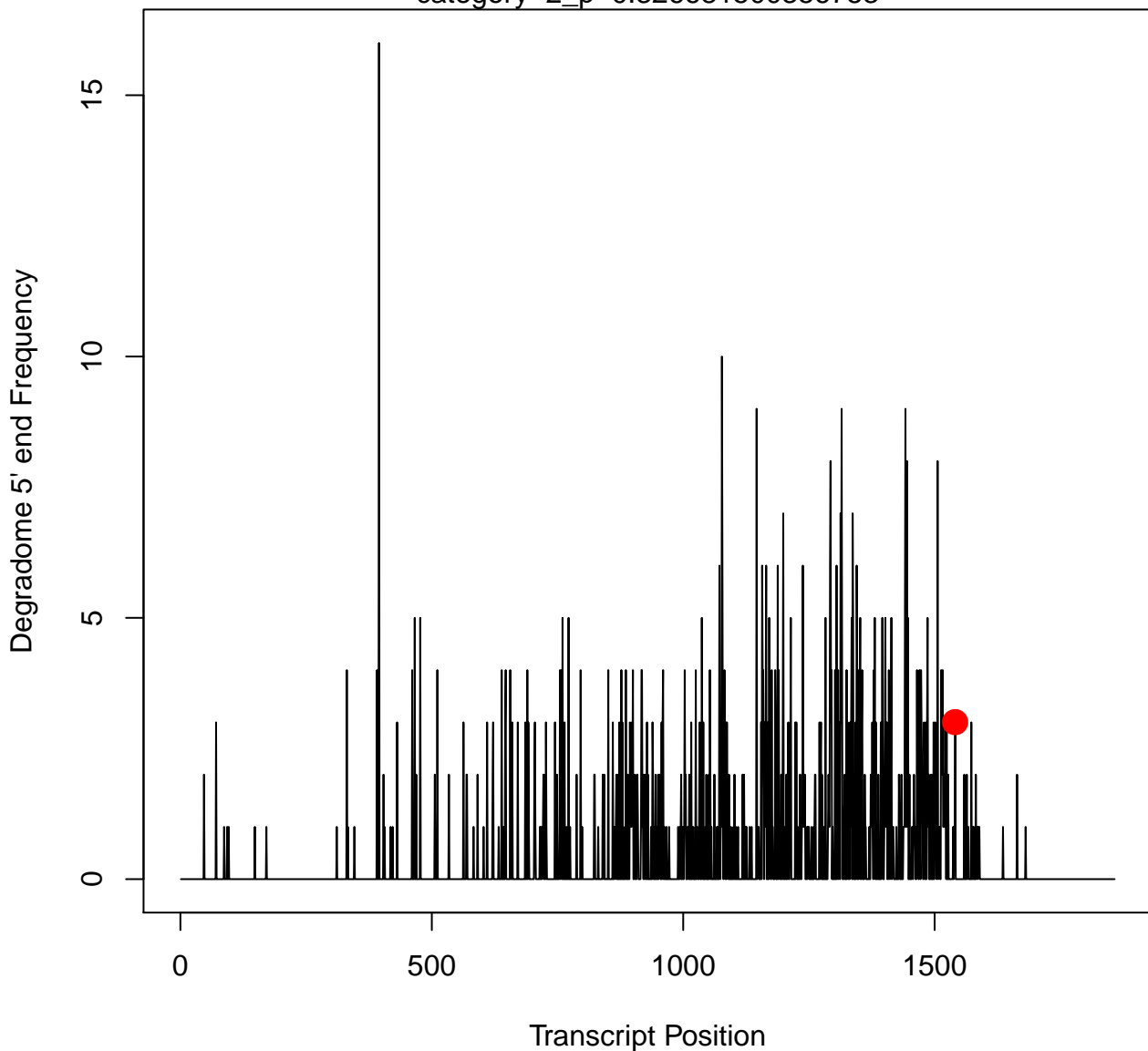

Supplement: Supplementary file 7 [file Data_Sheet_7.zip › Sit-miR2118b_Seita.3G350700.1_1541_TPlot.pdf]

**T=Seita.6G047900.1\_Q=Sit-miR2118b\_S=393**

category=2\_p=0.997565003605941

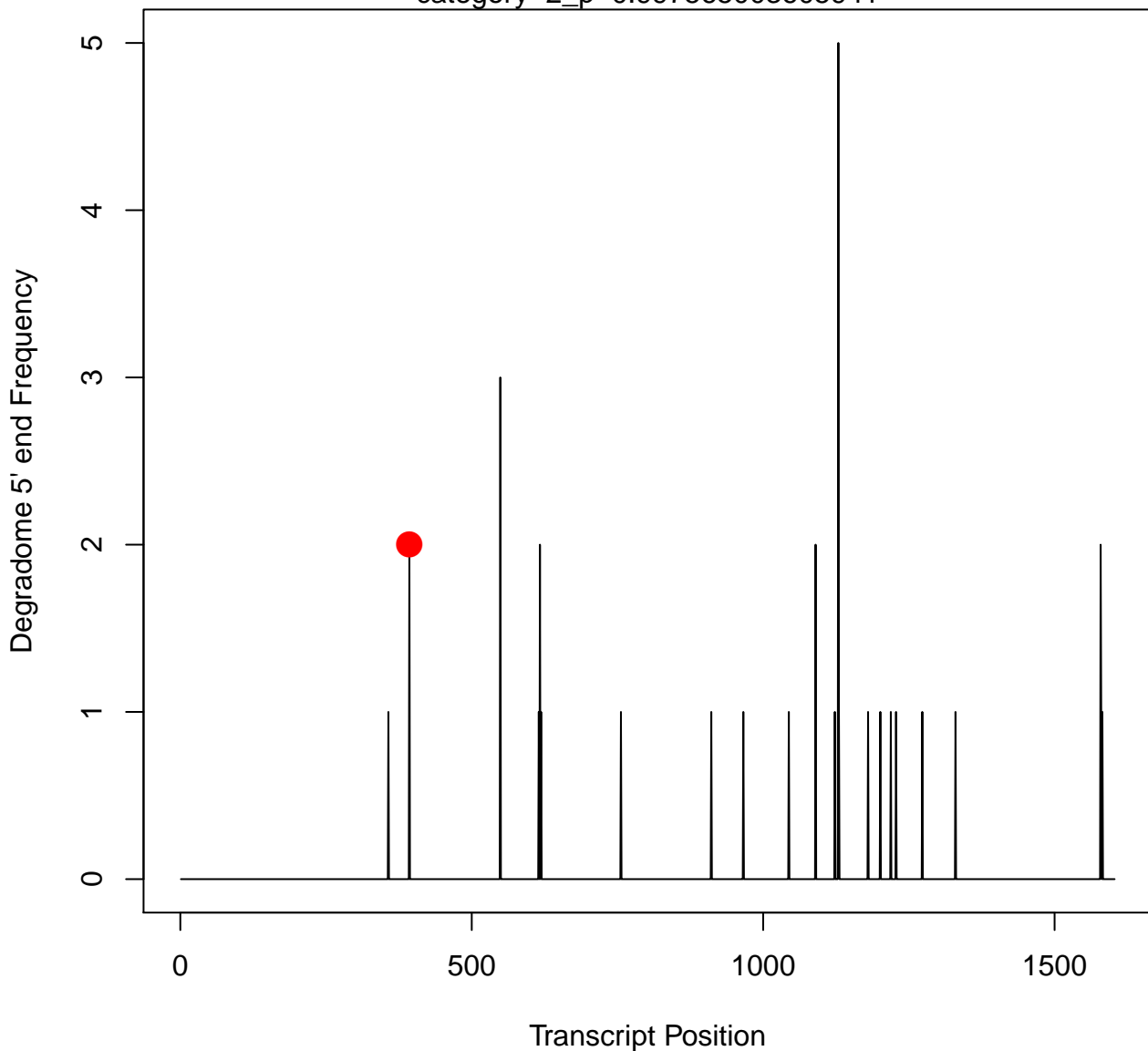

Supplement: Supplementary file 7 [file Data_Sheet_7.zip › Sit-miR2118b_Seita.6G047900.1_393_TPlot.pdf]

**T=Seita.6G204200.1\_Q=Sit-miR2118b\_S=190**

category=1\_p=0.0676492921494836

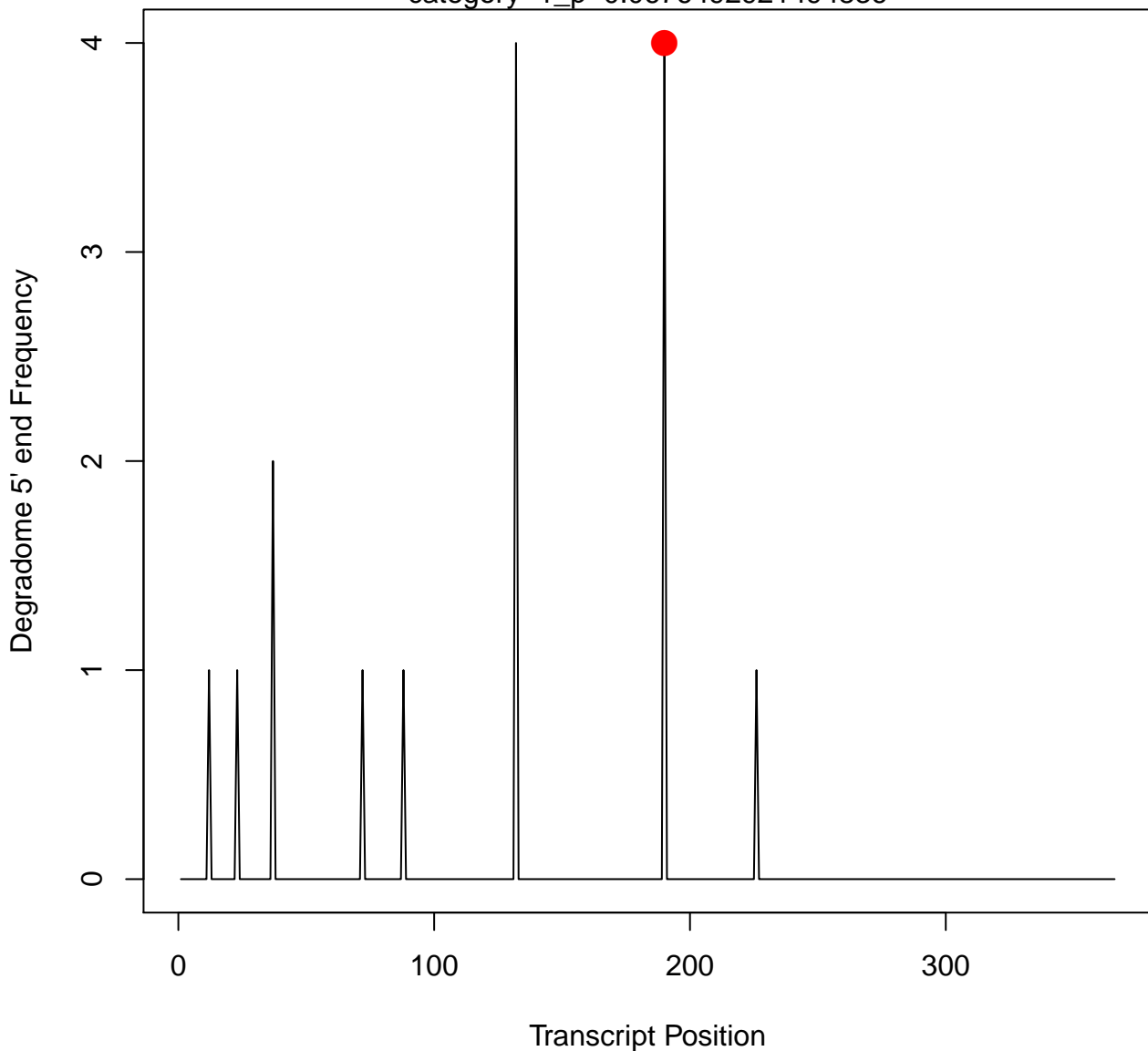

Supplement: Supplementary file 7 [file Data_Sheet_7.zip › Sit-miR2118b_Seita.6G204200.1_190_TPlot.pdf]

**T=Seita.9G517500.1\_Q=Sit-miR2118b\_S=2071**

category=2\_p=0.990209747380971

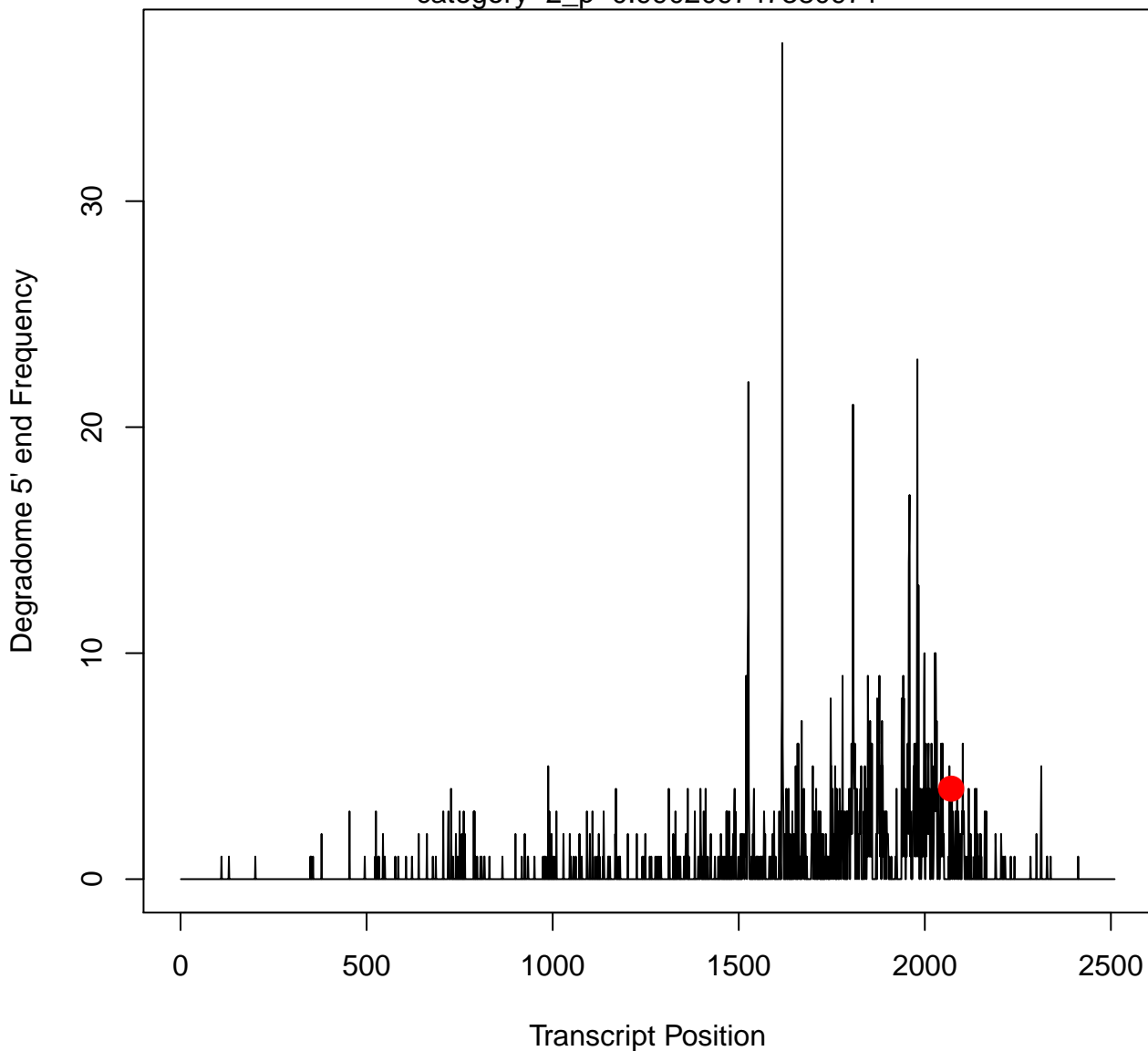

Supplement: Supplementary file 7 [file Data_Sheet_7.zip › Sit-miR2118b_Seita.9G517500.1_2071_TPlot.pdf]

**T=Seita.2G418200.1\_Q=Sit-miR2118c\_S=4729**

category=2\_p=0.727364881396822

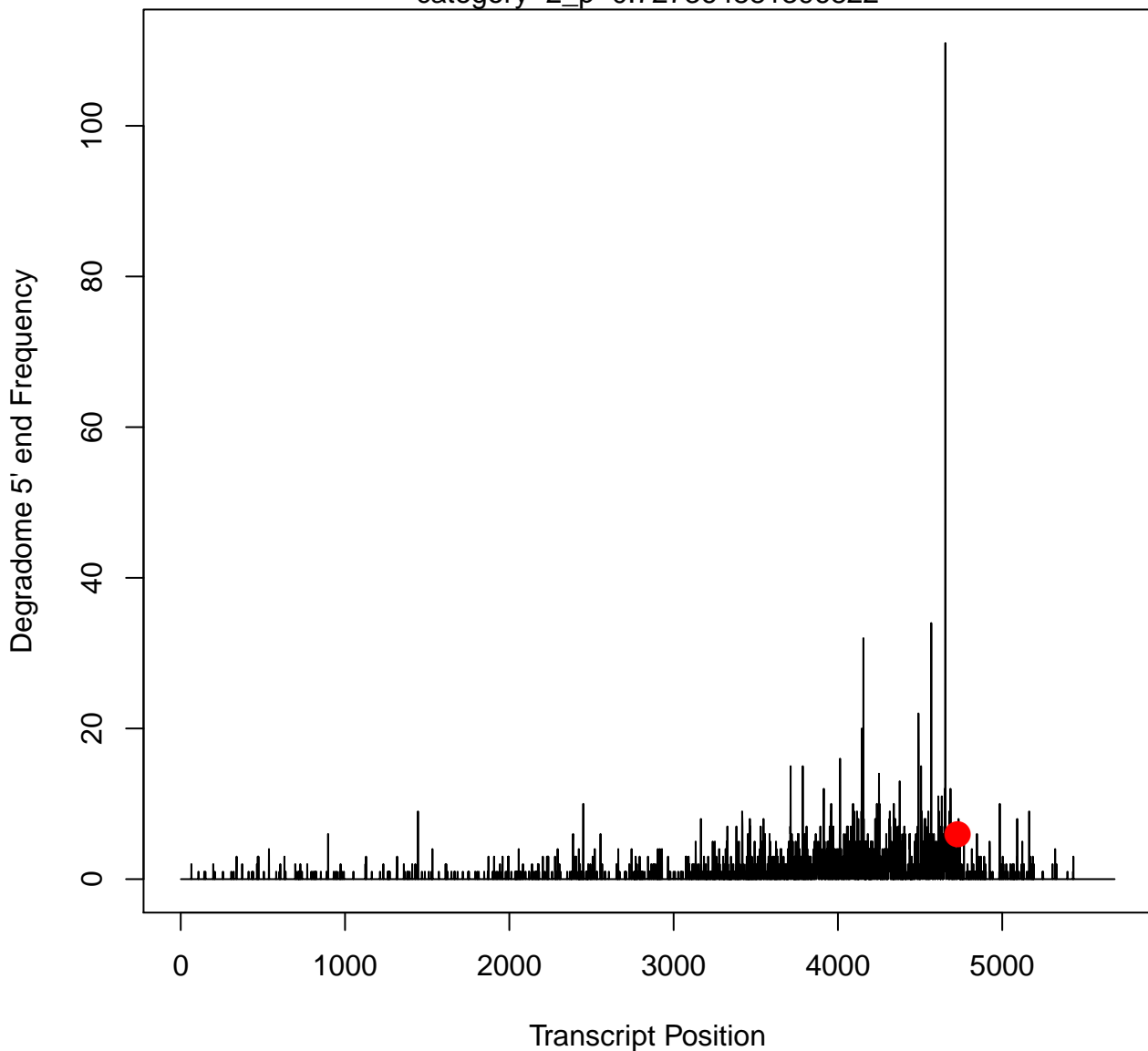

Supplement: Supplementary file 7 [file Data_Sheet_7.zip › Sit-miR2118c_Seita.2G418200.1_4729_TPlot.pdf]

**T=Seita.3G213900.1\_Q=Sit-miR2118d\_S=688**

category=2\_p=0.917362732528019

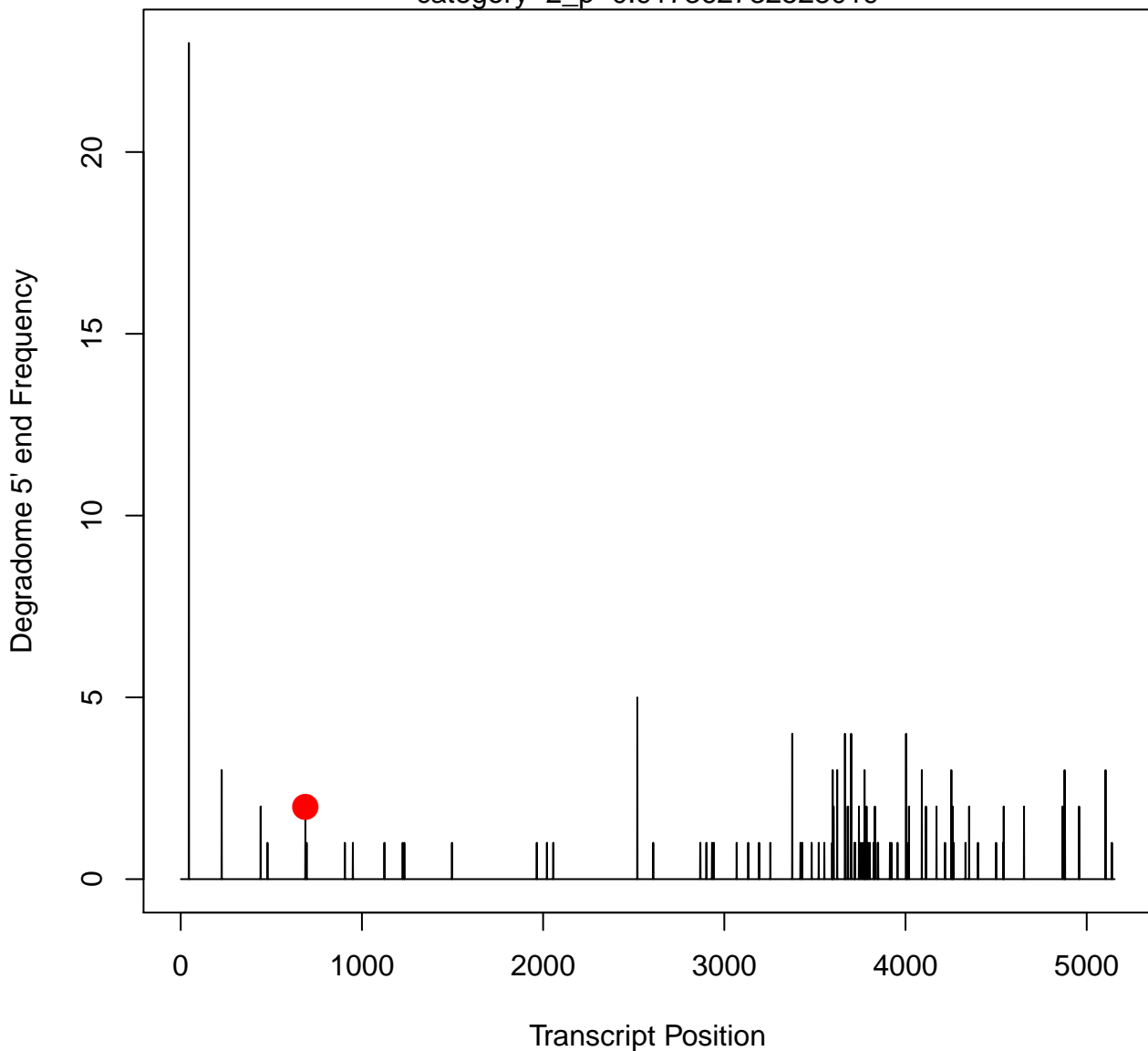

Supplement: Supplementary file 7 [file Data_Sheet_7.zip › Sit-miR2118d_Seita.3G213900.1_688_TPlot.pdf]

**T=Seita.7G072900.1\_Q=Sit-miR2118d\_S=1010**

category=2\_p=0.910053176456357

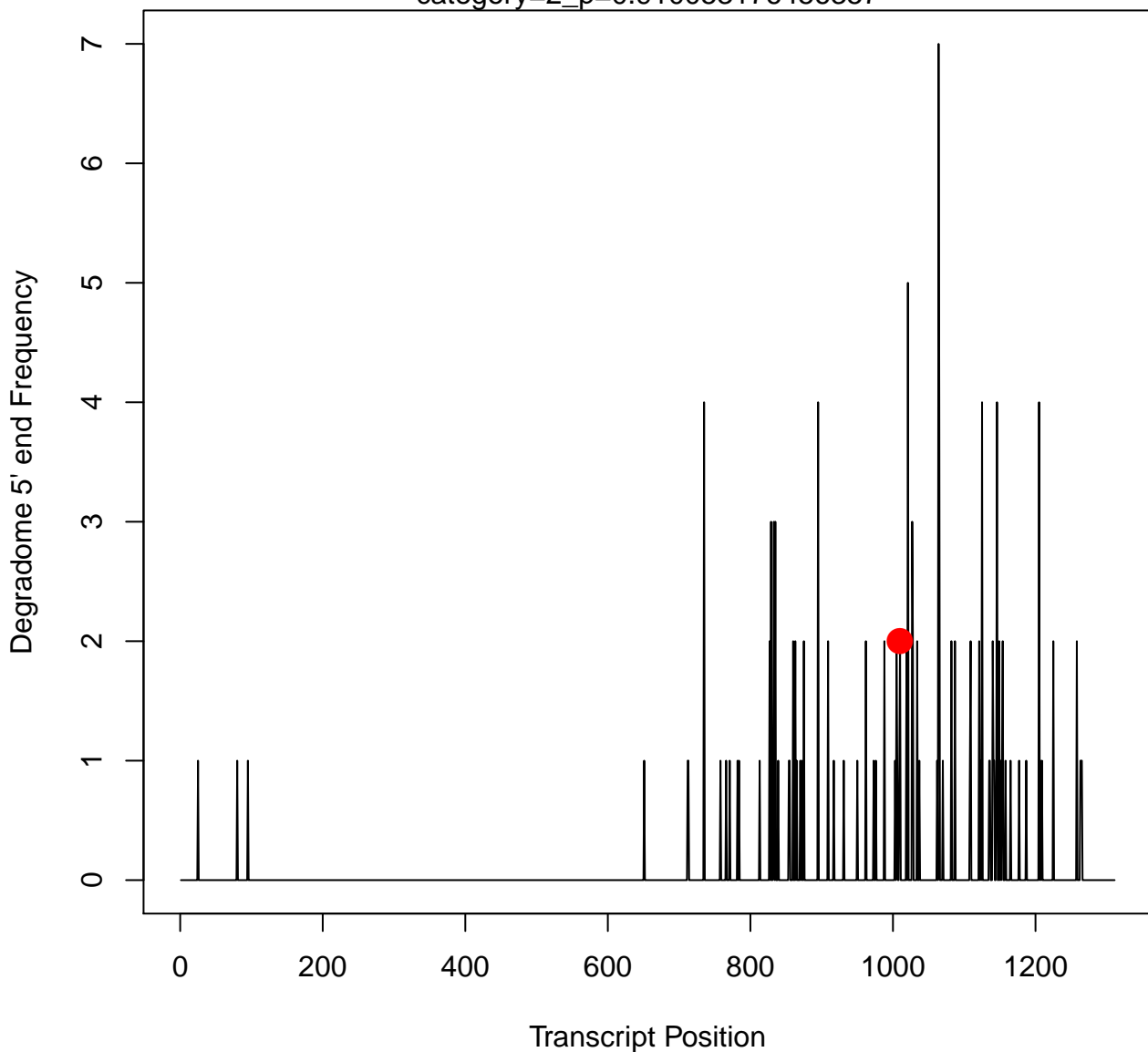

Supplement: Supplementary file 7 [file Data_Sheet_7.zip › Sit-miR2118d_Seita.7G072900.1_1010_TPlot.pdf]

**T=Seita.2G384200.1\_Q=Sit-miR2118e\_S=641**

category=2\_p=0.994959776251774

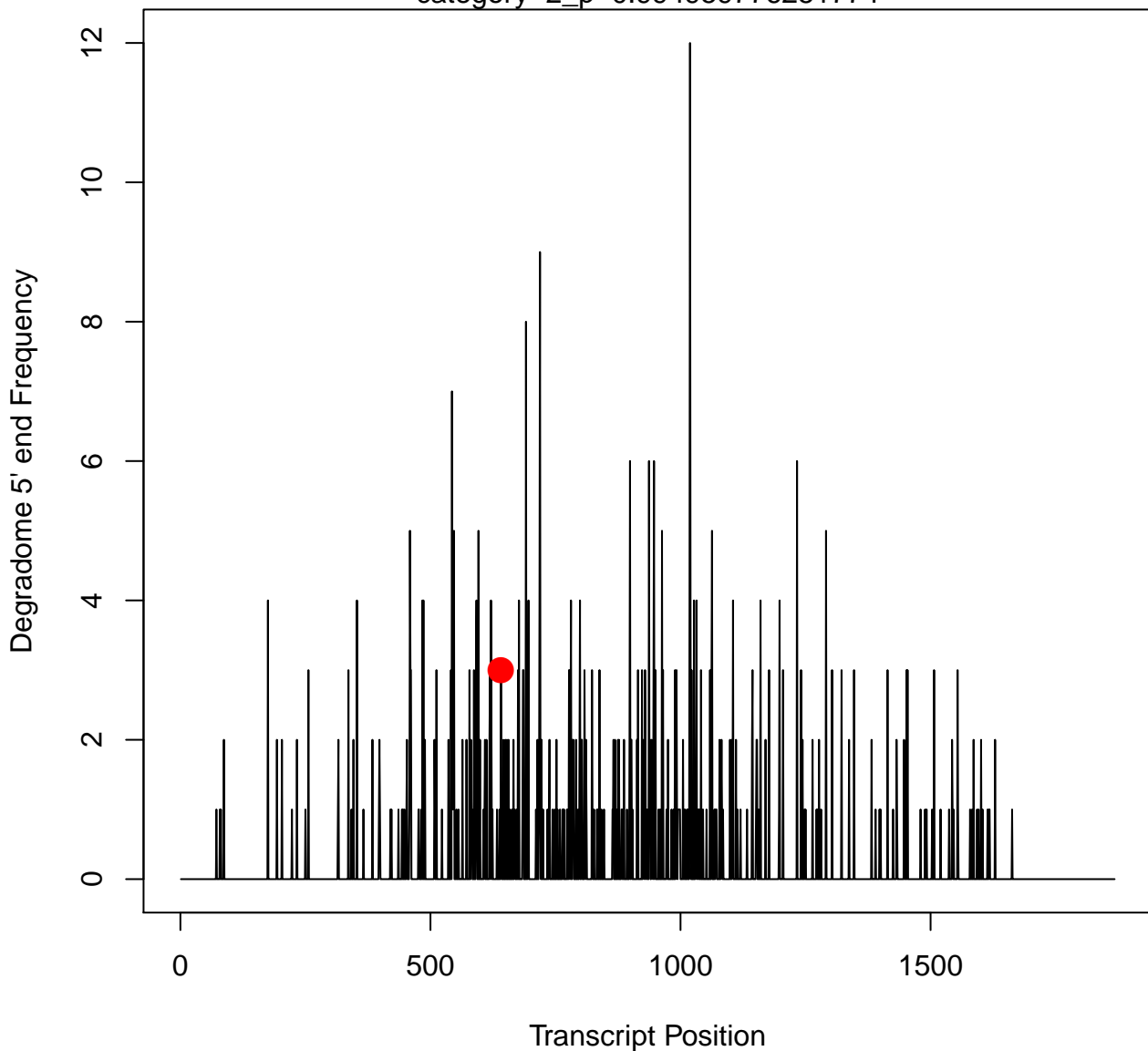

Supplement: Supplementary file 7 [file Data_Sheet_7.zip › Sit-miR2118e_Seita.2G384200.1_641_TPlot.pdf]

**T=Seita.7G212200.1\_Q=Sit-miR2118e\_S=1747**

category=2\_p=0.996484332336168

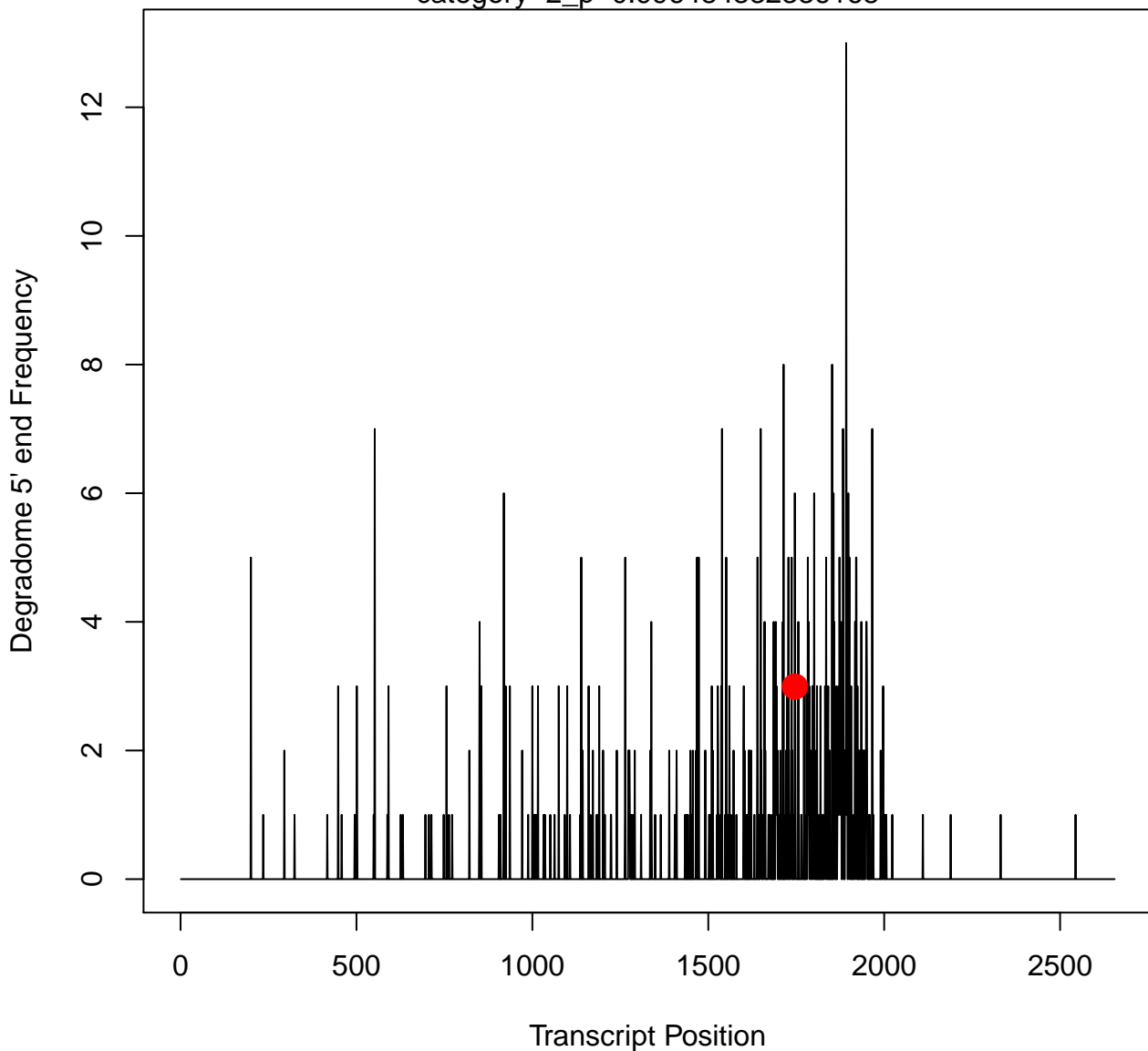

Supplement: Supplementary file 7 [file Data_Sheet_7.zip › Sit-miR2118e_Seita.7G212200.1_1747_TPlot.pdf]

**T=Seita.9G230400.1\_Q=Sit-miR2118e\_S=551**

category=0\_p=0.0297758261510233

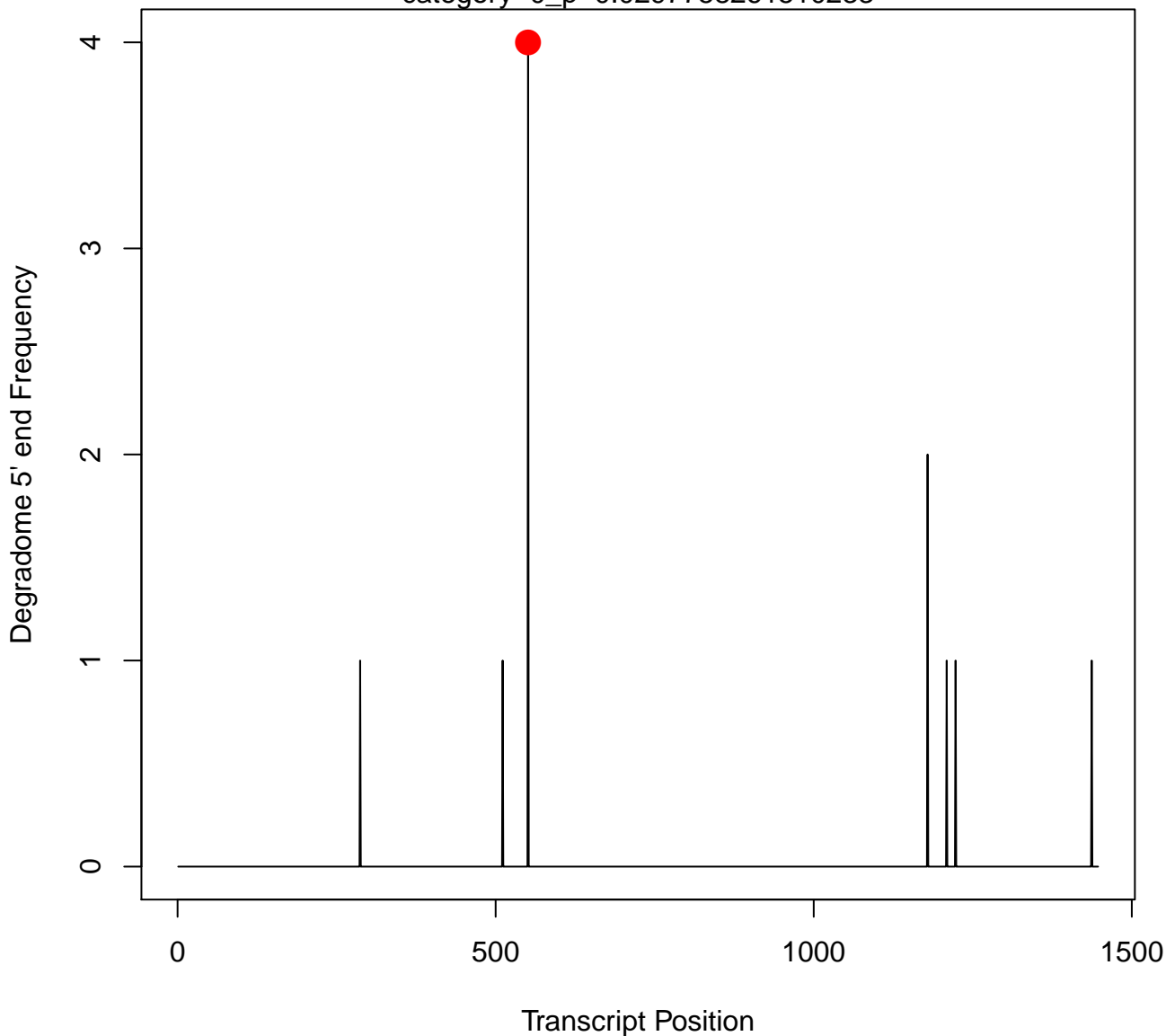

Supplement: Supplementary file 7 [file Data_Sheet_7.zip › Sit-miR2118e_Seita.9G230400.1_551_TPlot.pdf]

**T=Seita.3G247900.1\_Q=Sit-miR2275a\_S=2781**

category=2\_p=0.936367525878408

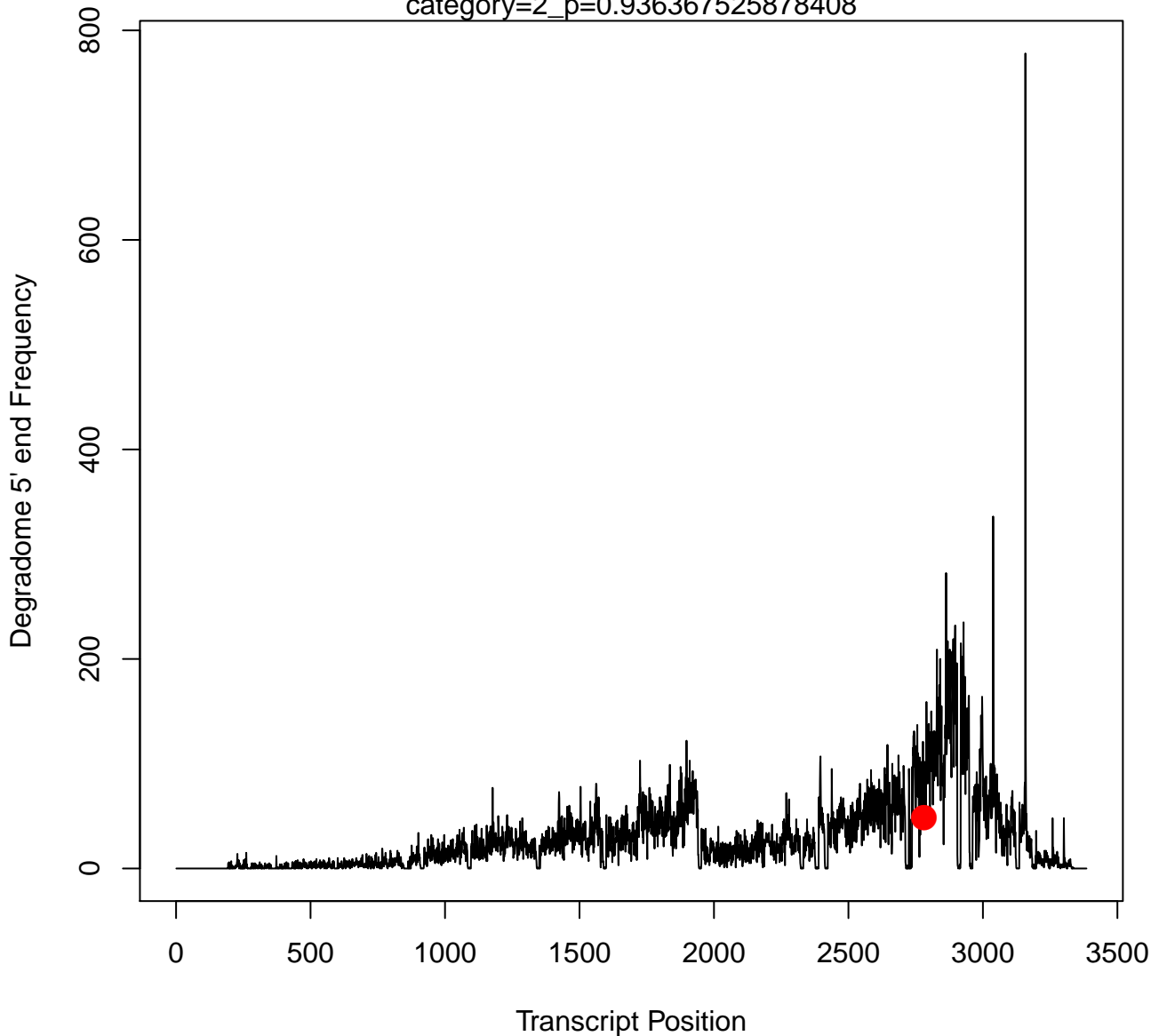

Supplement: Supplementary file 7 [file Data_Sheet_7.zip › Sit-miR2275a_Seita.3G247900.1_2781_TPlot.pdf]

**T=Seita.5G137400.1\_Q=Sit-miR2275a\_S=1325**

category=2\_p=0.506541110776428

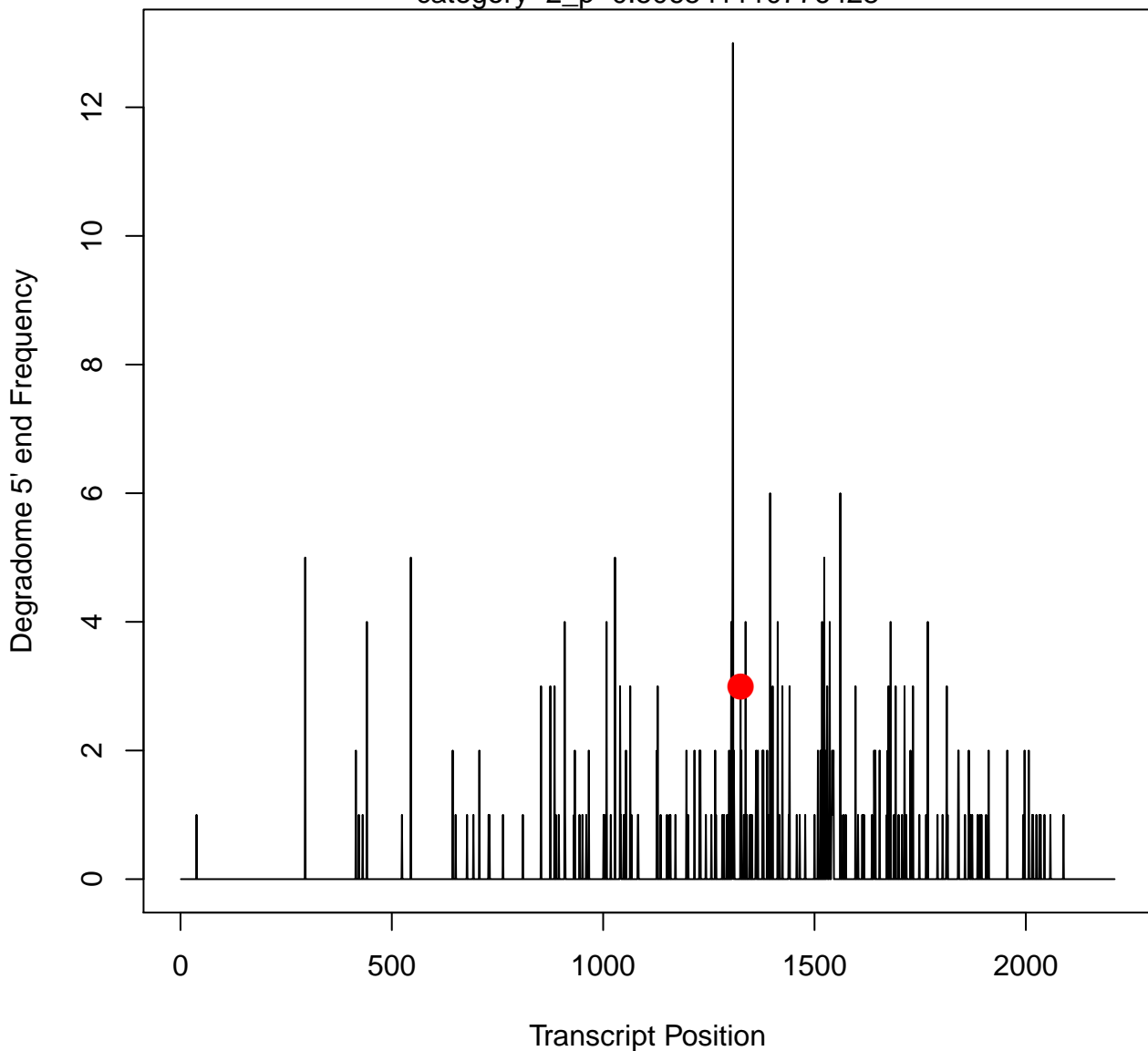

Supplement: Supplementary file 7 [file Data_Sheet_7.zip › Sit-miR2275a_Seita.5G137400.1_1325_TPlot.pdf]

**T=Seita.7G186500.1\_Q=Sit-miR2275a\_S=916**

category=2\_p=0.326681500356753

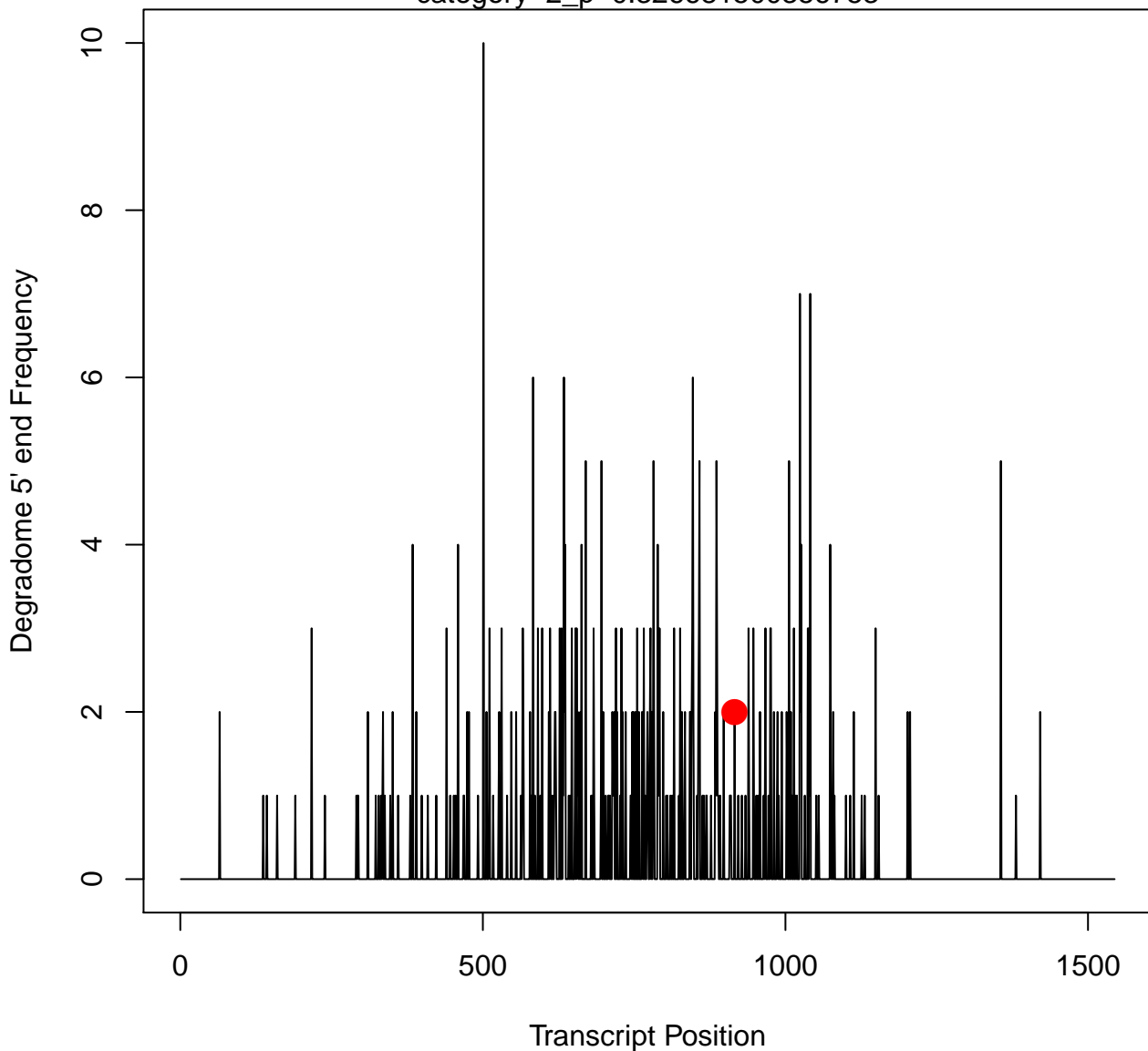

Supplement: Supplementary file 7 [file Data_Sheet_7.zip › Sit-miR2275a_Seita.7G186500.1_916_TPlot.pdf]

**T=Seita.1G117800.1\_Q=Sit-miR2275b\_S=845**

category=2\_p=0.277404172752107

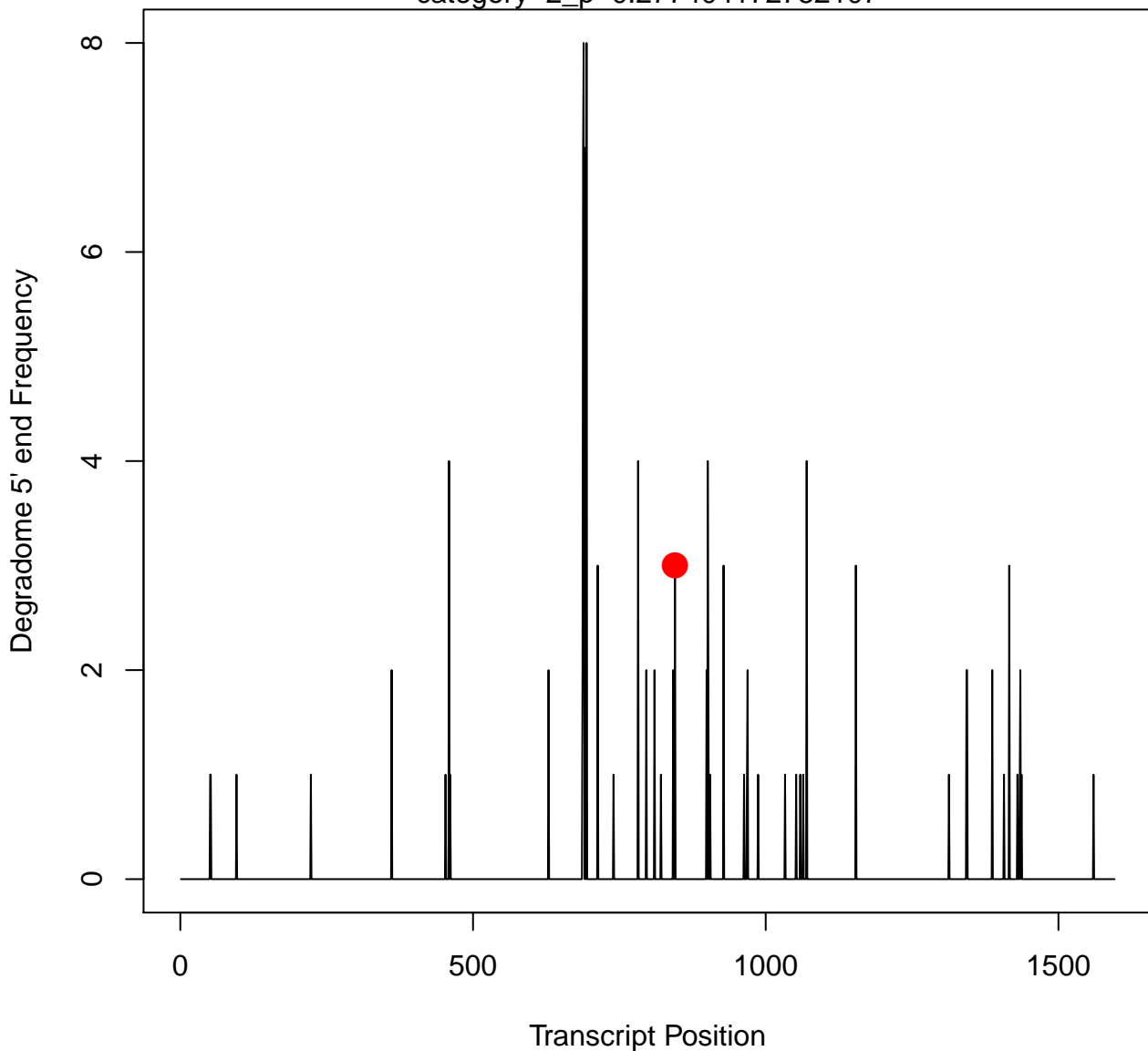

Supplement: Supplementary file 7 [file Data_Sheet_7.zip › Sit-miR2275b_Seita.1G117800.1_845_TPlot.pdf]

**T=Seita.1G138300.1\_Q=Sit-miR2275b\_S=224**

category=2\_p=0.492400789735958

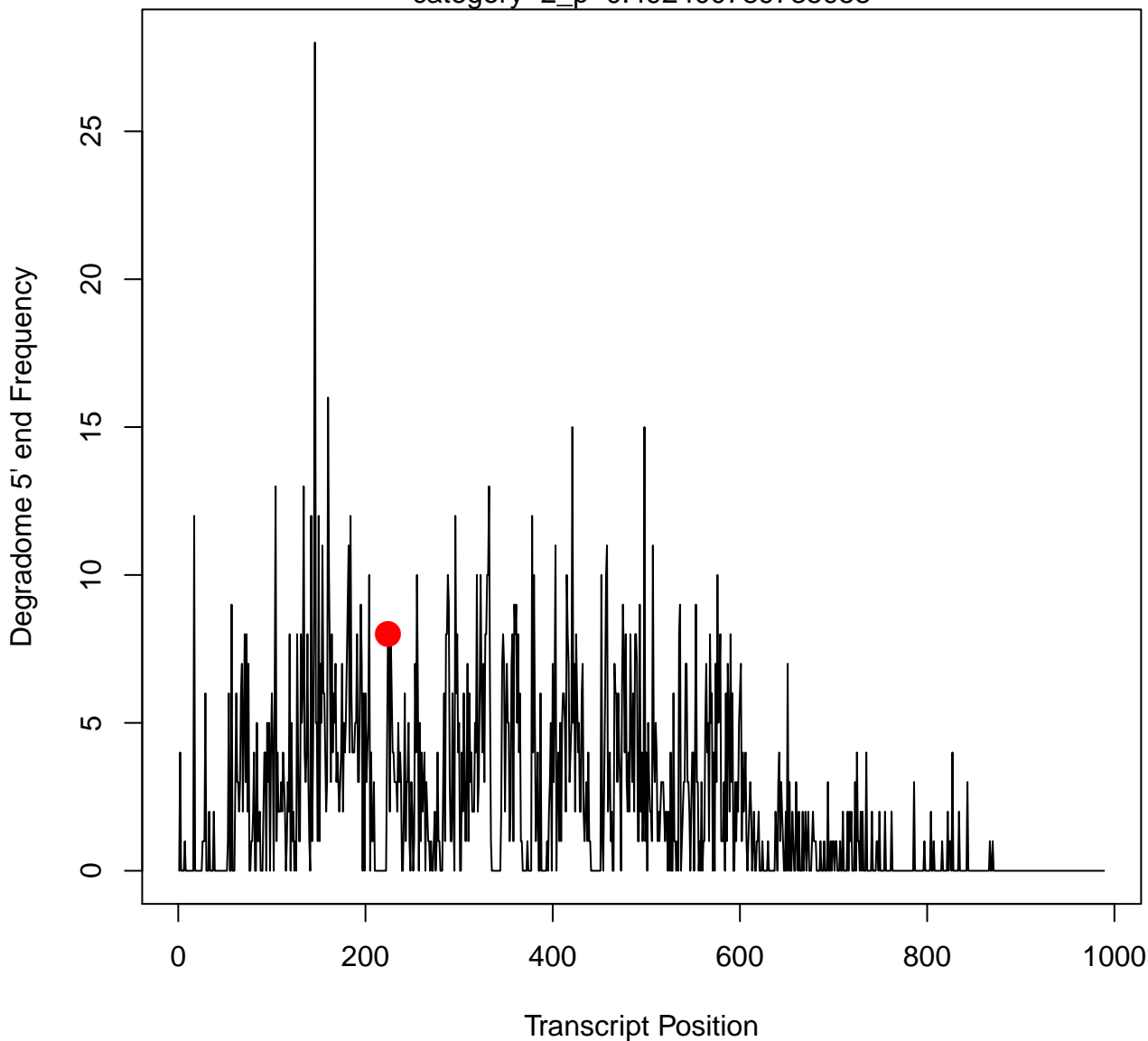

Supplement: Supplementary file 7 [file Data_Sheet_7.zip › Sit-miR2275b_Seita.1G138300.1_224_TPlot.pdf]

**T=Seita.2G046600.1\_Q=Sit-miR2275b\_S=896**

category=2\_p=0.999207923049376

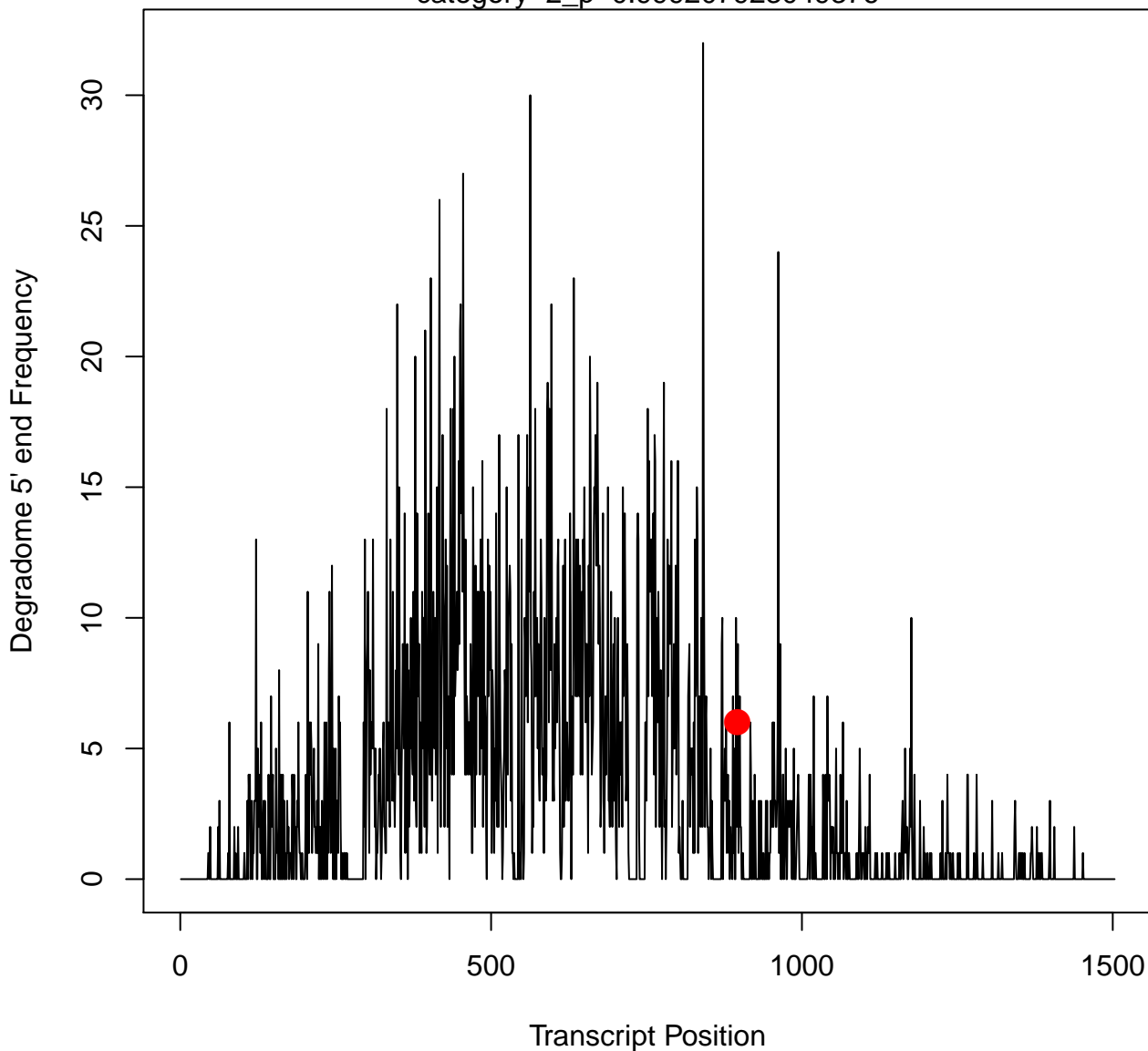

Supplement: Supplementary file 7 [file Data_Sheet_7.zip › Sit-miR2275b_Seita.2G046600.1_896_TPlot.pdf]

**T=Seita.3G022000.1\_Q=Sit-miR2275b\_S=77**

category=2\_p=0.9754773370952

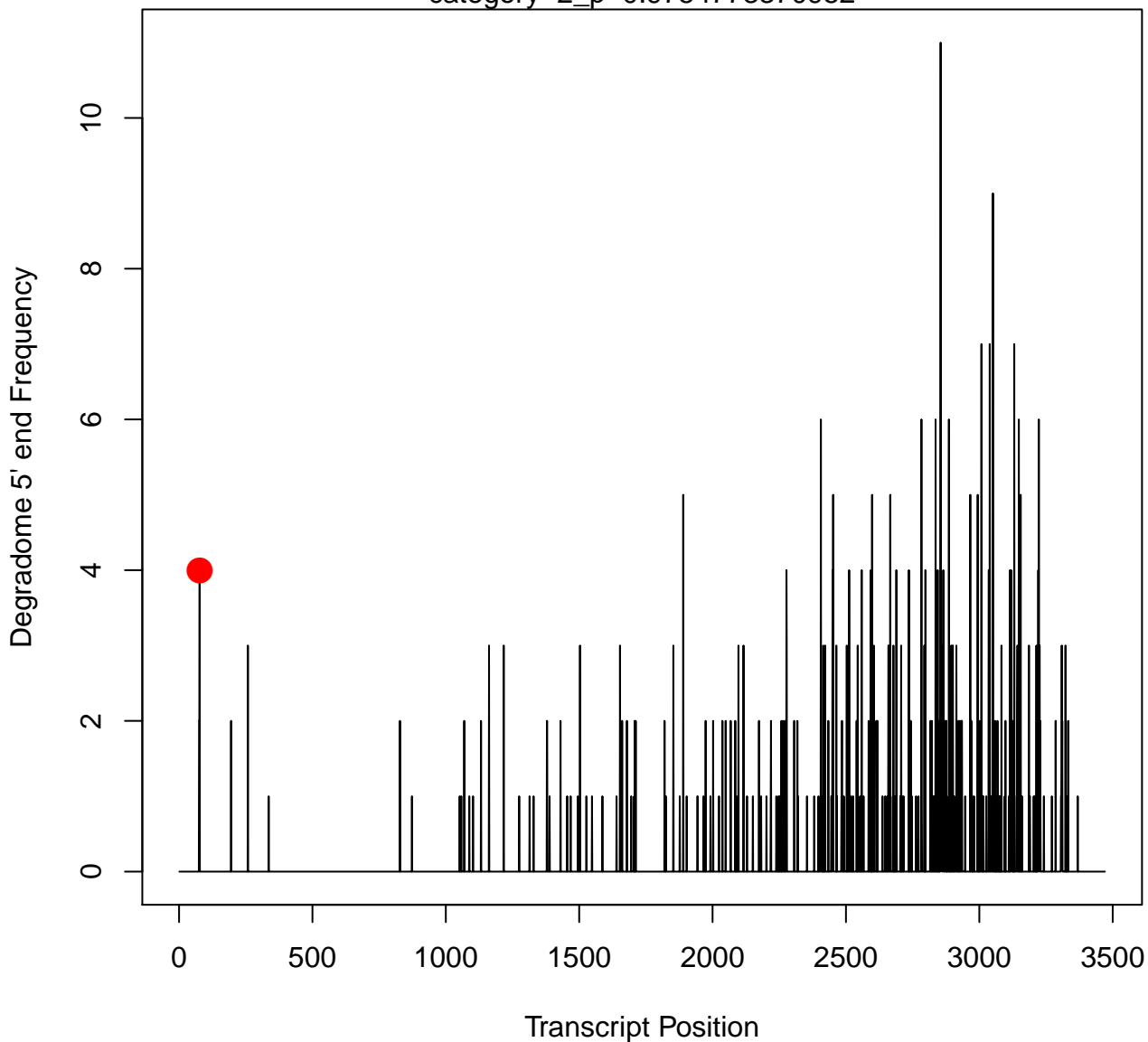

Supplement: Supplementary file 7 [file Data_Sheet_7.zip › Sit-miR2275b_Seita.3G022000.1_77_TPlot.pdf]

**T=Seita.3G171200.1\_Q=Sit-miR2275b\_S=1198**

category=2\_p=0.999758215188045

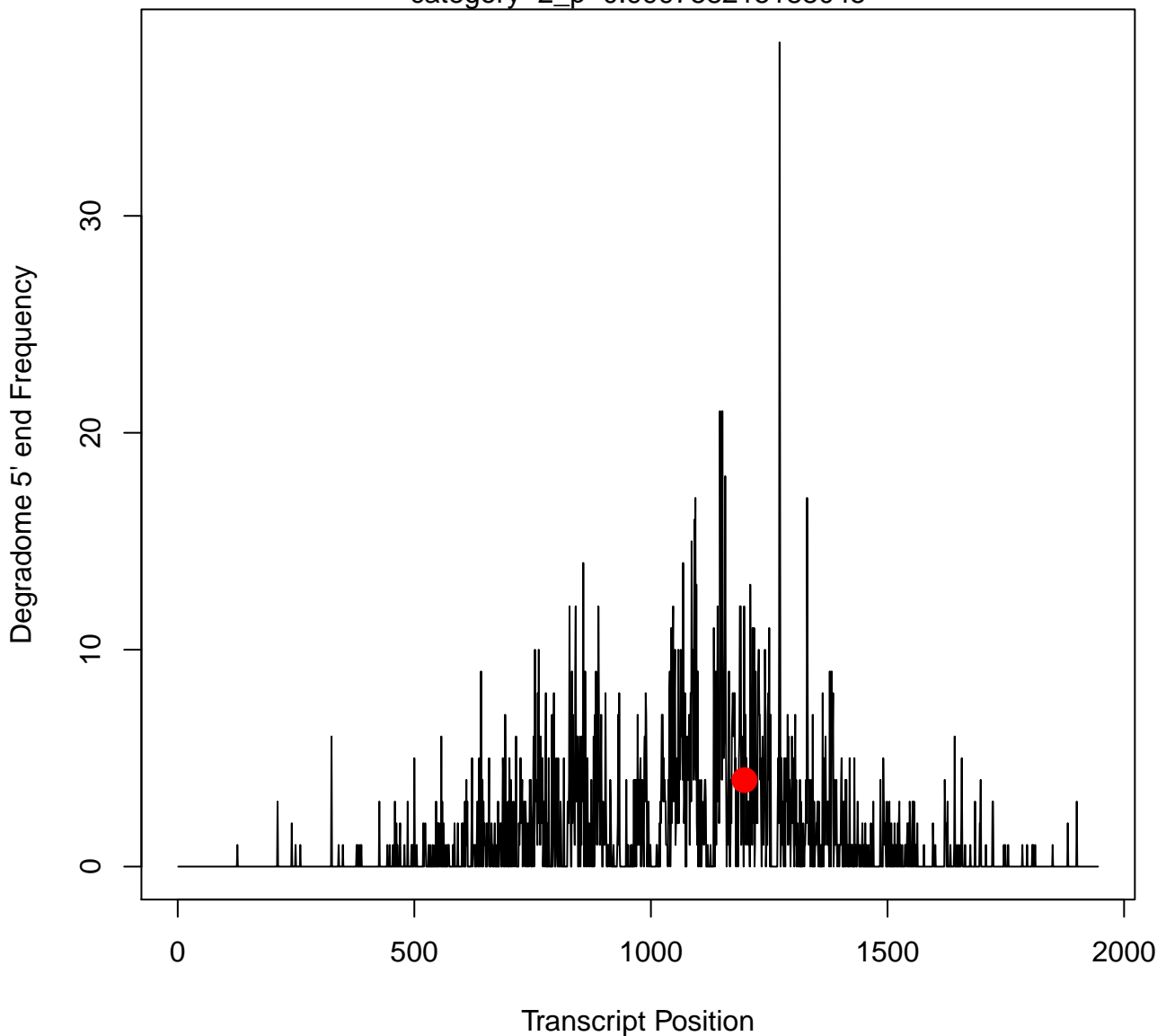

Supplement: Supplementary file 7 [file Data_Sheet_7.zip › Sit-miR2275b_Seita.3G171200.1_1198_TPlot.pdf]

**T=Seita.4G096600.1\_Q=Sit-miR2275b\_S=2210**

category=2\_p=0.969689553413221

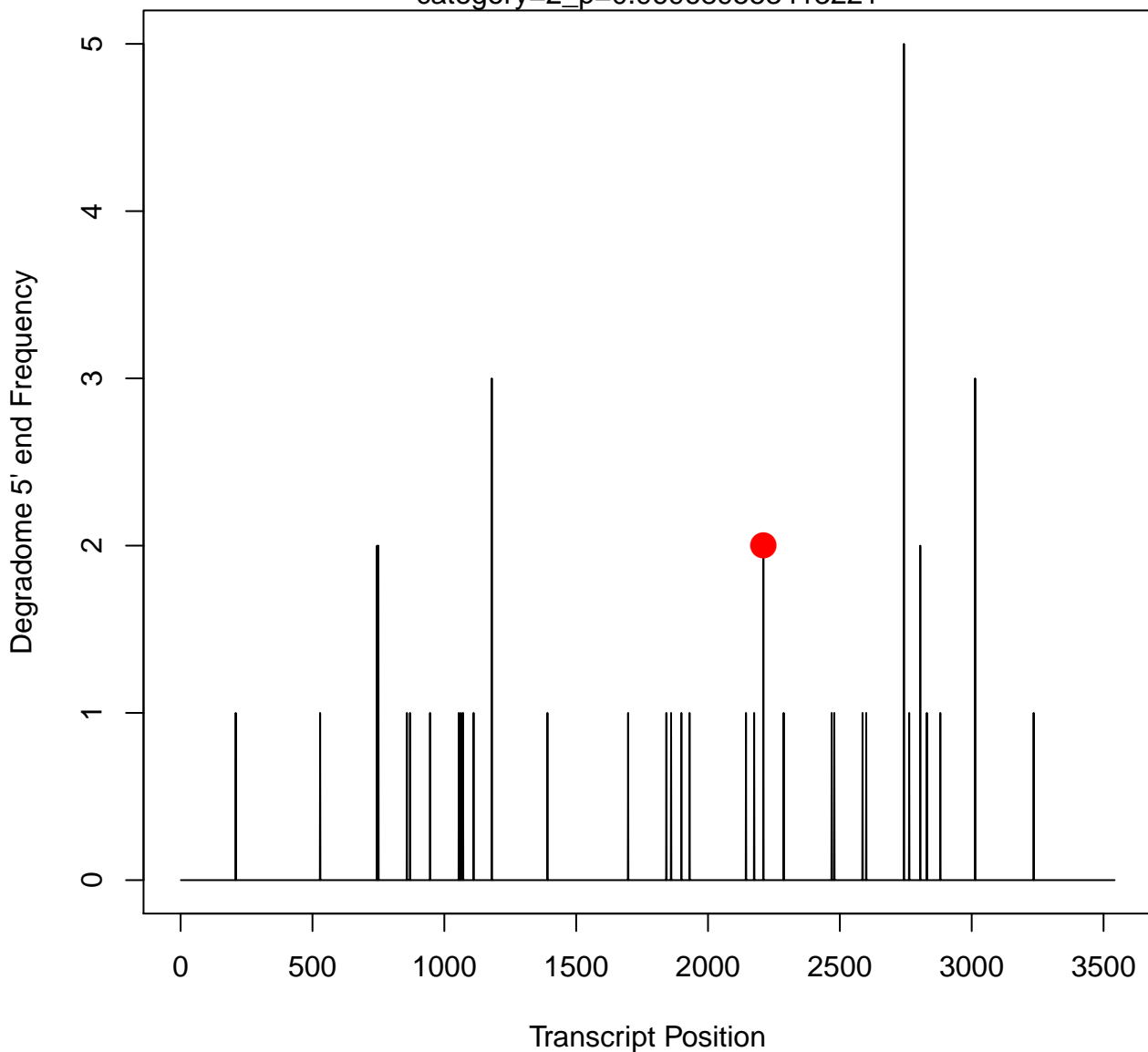

Supplement: Supplementary file 7 [file Data_Sheet_7.zip › Sit-miR2275b_Seita.4G096600.1_2210_TPlot.pdf]

**T=Seita.5G308400.1\_Q=Sit-miR2275b\_S=1280**

category=2\_p=0.998265162539726

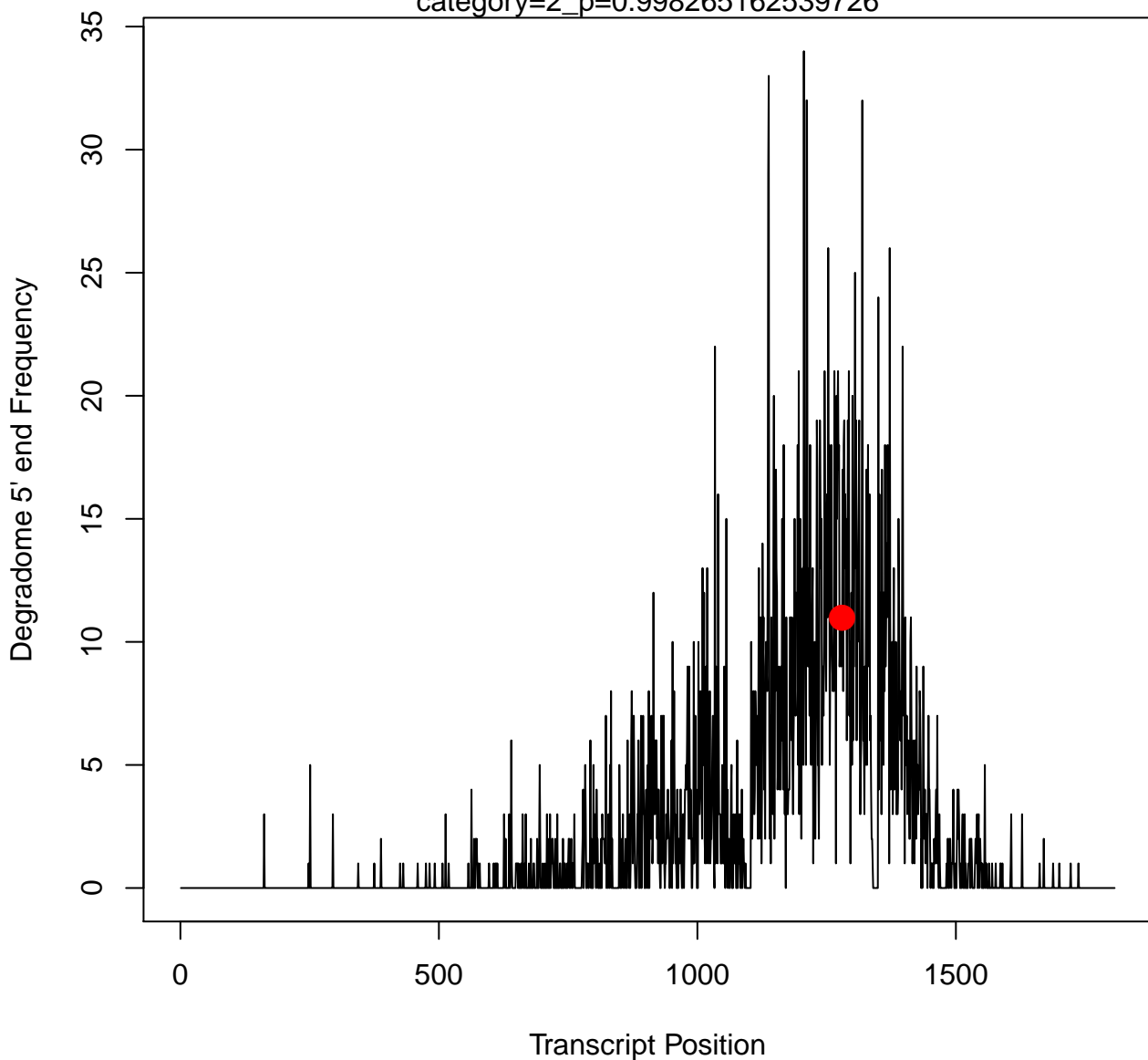

Supplement: Supplementary file 7 [file Data_Sheet_7.zip › Sit-miR2275b_Seita.5G308400.1_1280_TPlot.pdf]

**T=Seita.5G319300.1\_Q=Sit-miR2275b\_S=751**

category=2\_p=0.999443592642742

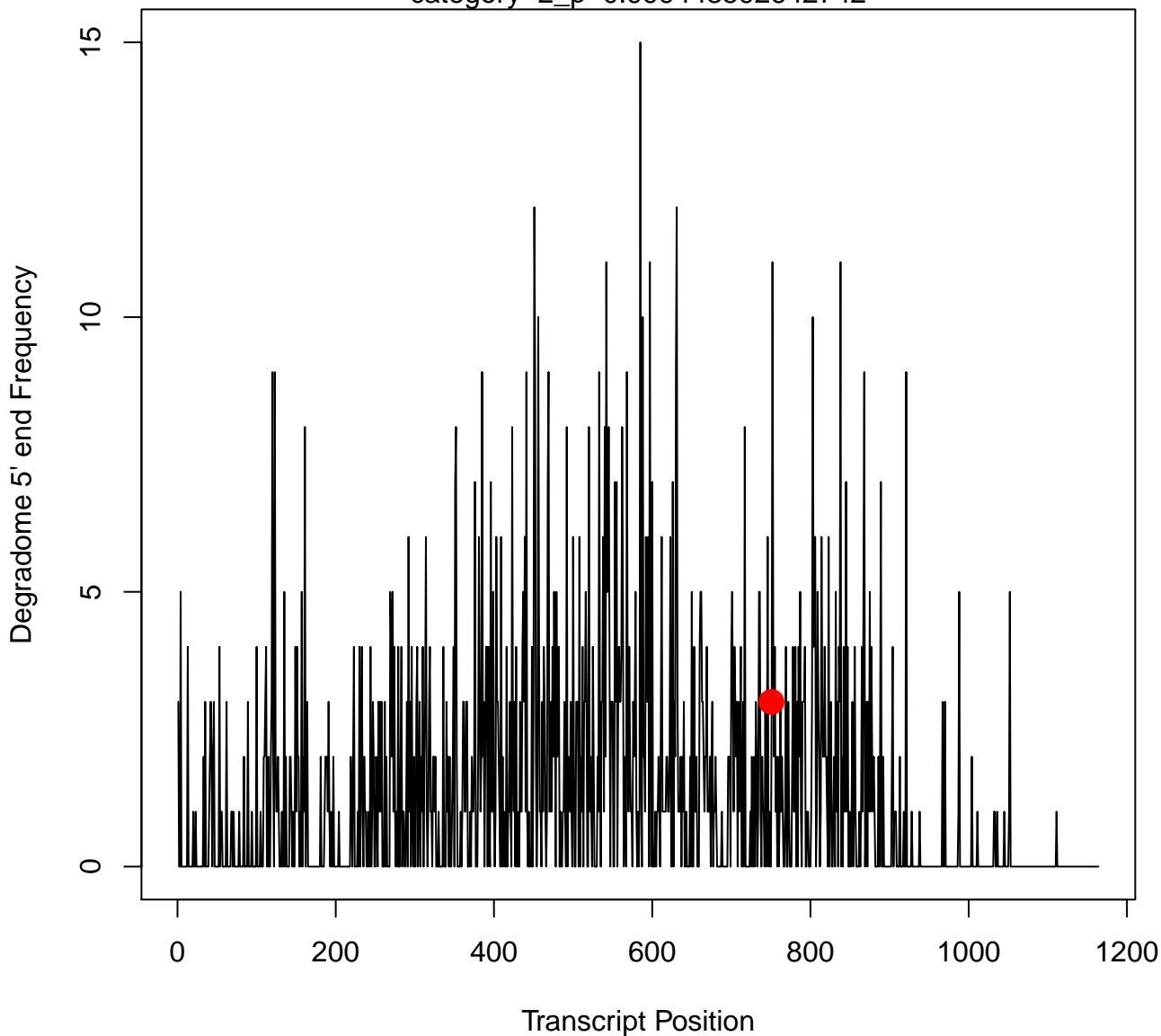

Supplement: Supplementary file 7 [file Data_Sheet_7.zip › Sit-miR2275b_Seita.5G319300.1_751_TPlot.pdf]

**T=Seita.1G004300.1\_Q=Sit-miR2275c\_S=822**

category=1\_p=0.00514800519603853

Degradome 5' end Frequency

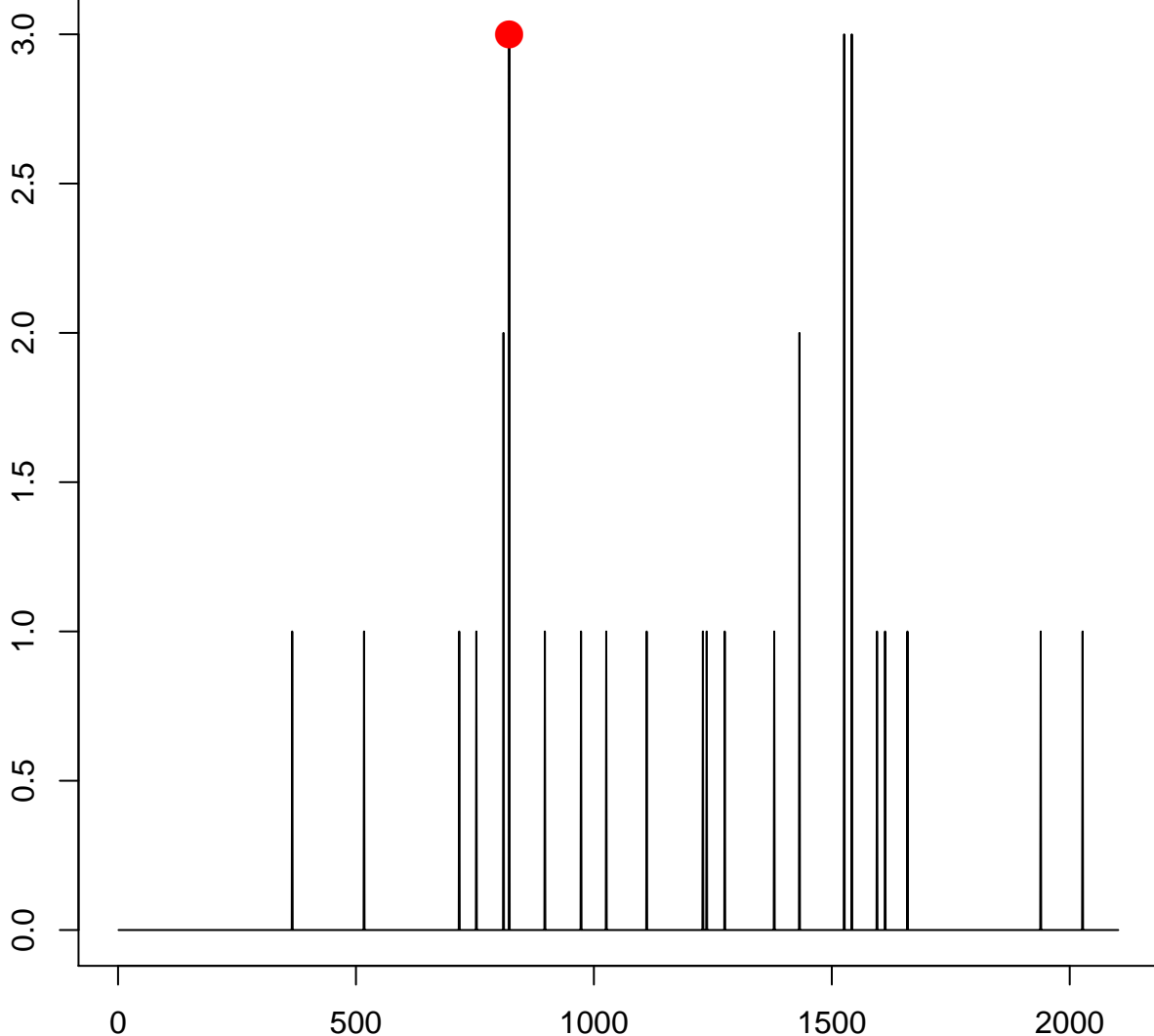

Transcript Position

Supplement: Supplementary file 7 [file Data_Sheet_7.zip › Sit-miR2275c_Seita.1G004300.1_822_TPlot.pdf]

**T=Seita.2G207500.1\_Q=Sit-miR2275c\_S=112**

category=2\_p=0.620026037172209

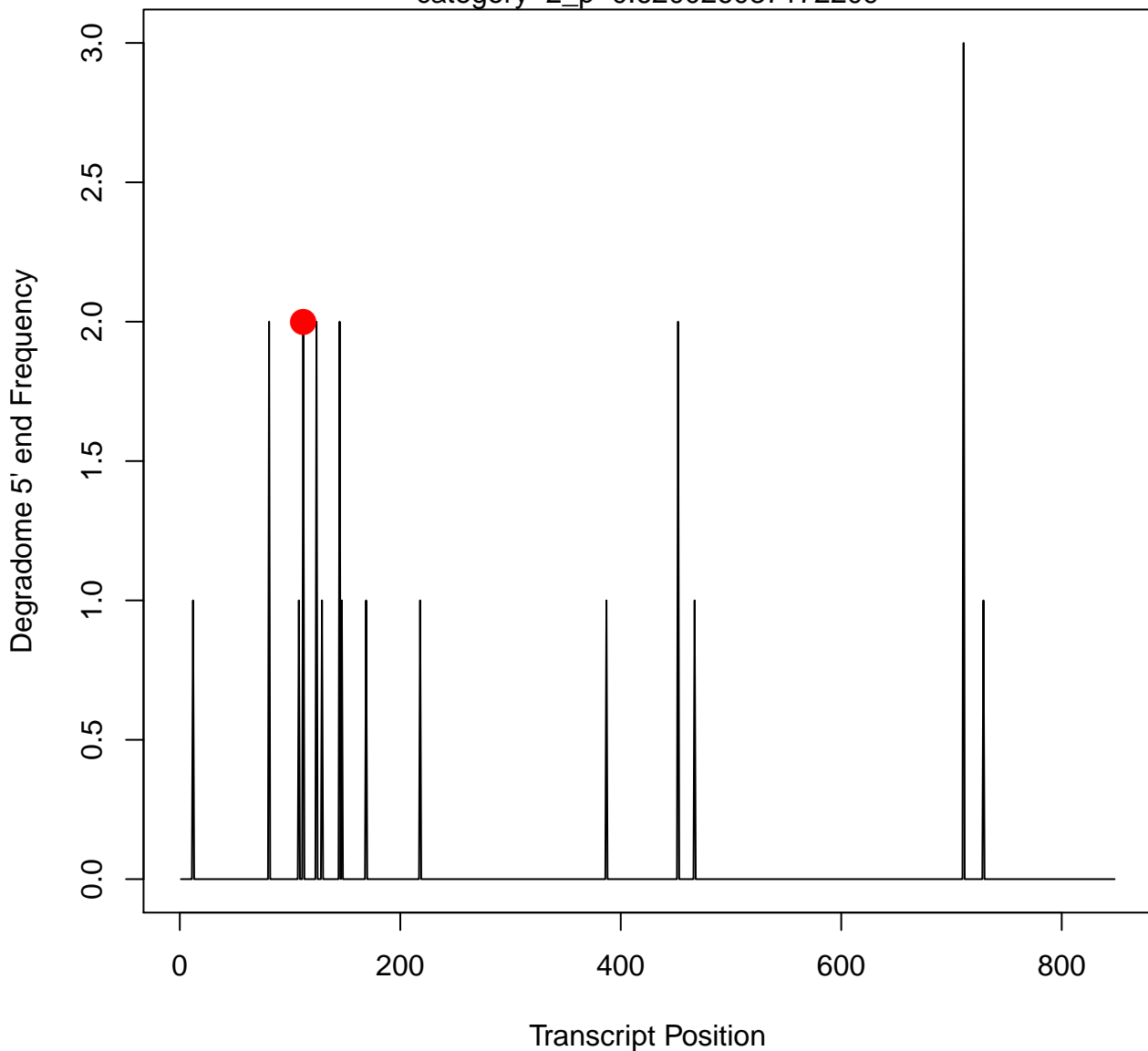

Supplement: Supplementary file 7 [file Data_Sheet_7.zip › Sit-miR2275c_Seita.2G207500.1_112_TPlot.pdf]

**T=Seita.5G464000.1\_Q=Sit-miR2275c\_S=943**

category=2\_p=0.106858732648528

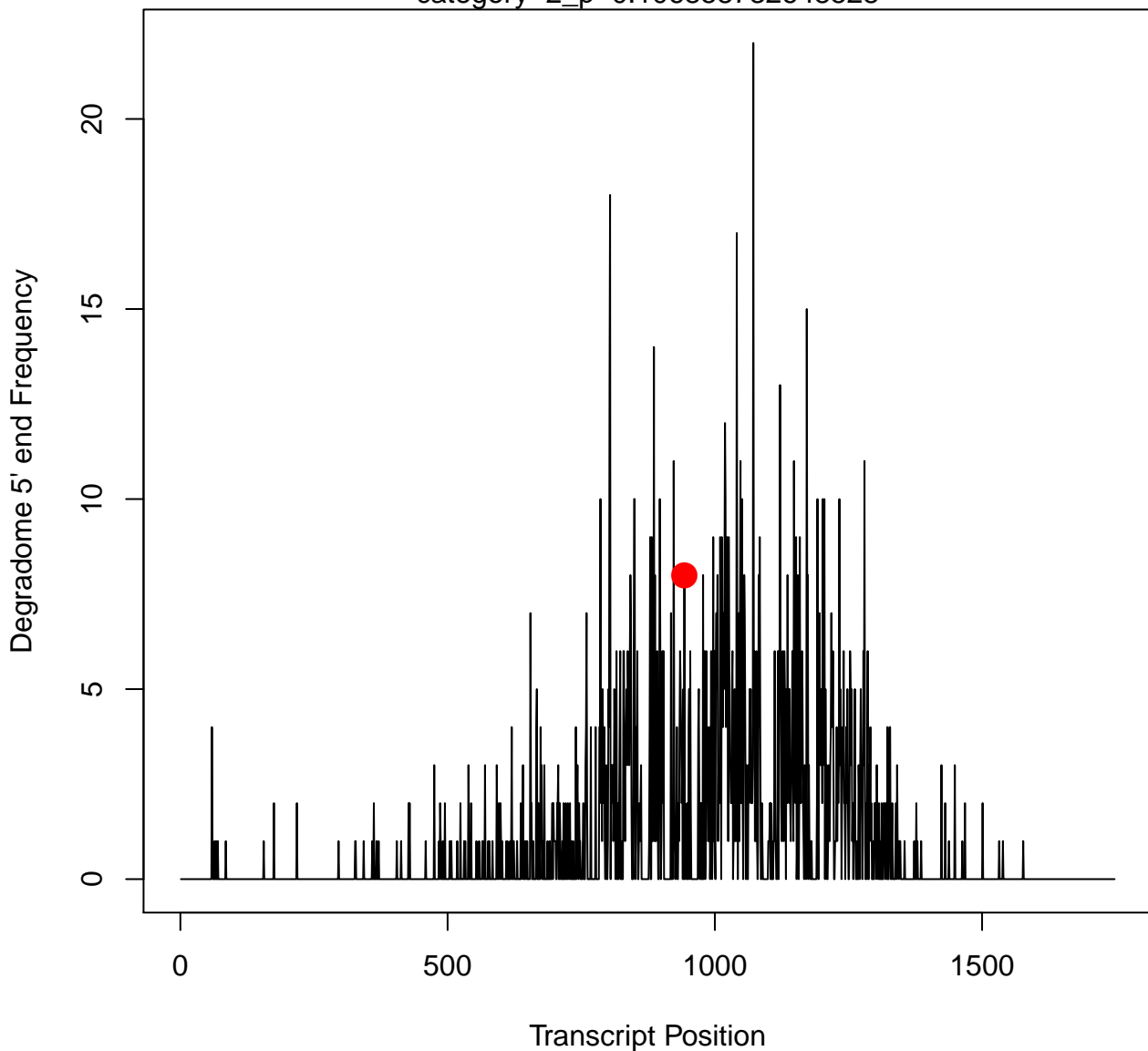

Supplement: Supplementary file 7 [file Data_Sheet_7.zip › Sit-miR2275c_Seita.5G464000.1_943_TPlot.pdf]

**T=Seita.1G095000.1\_Q=Sit-miR2275d\_S=1528**

category=2\_p=0.999983720249377

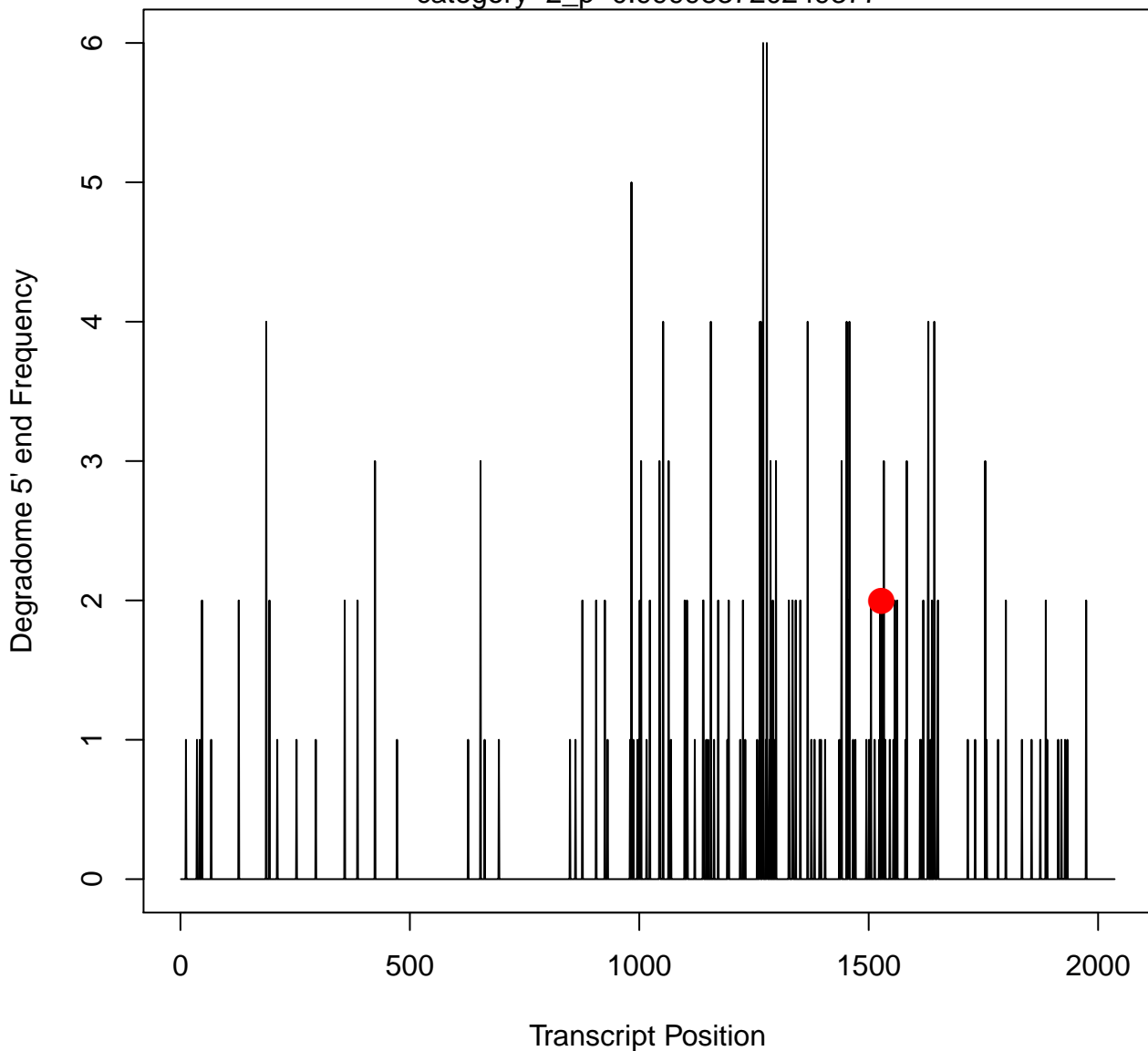

Supplement: Supplementary file 7 [file Data_Sheet_7.zip › Sit-miR2275d_Seita.1G095000.1_1528_TPlot.pdf]

**T=Seita.1G100400.1\_Q=Sit-miR2275d\_S=955**

category=0\_p=0.383286524111916

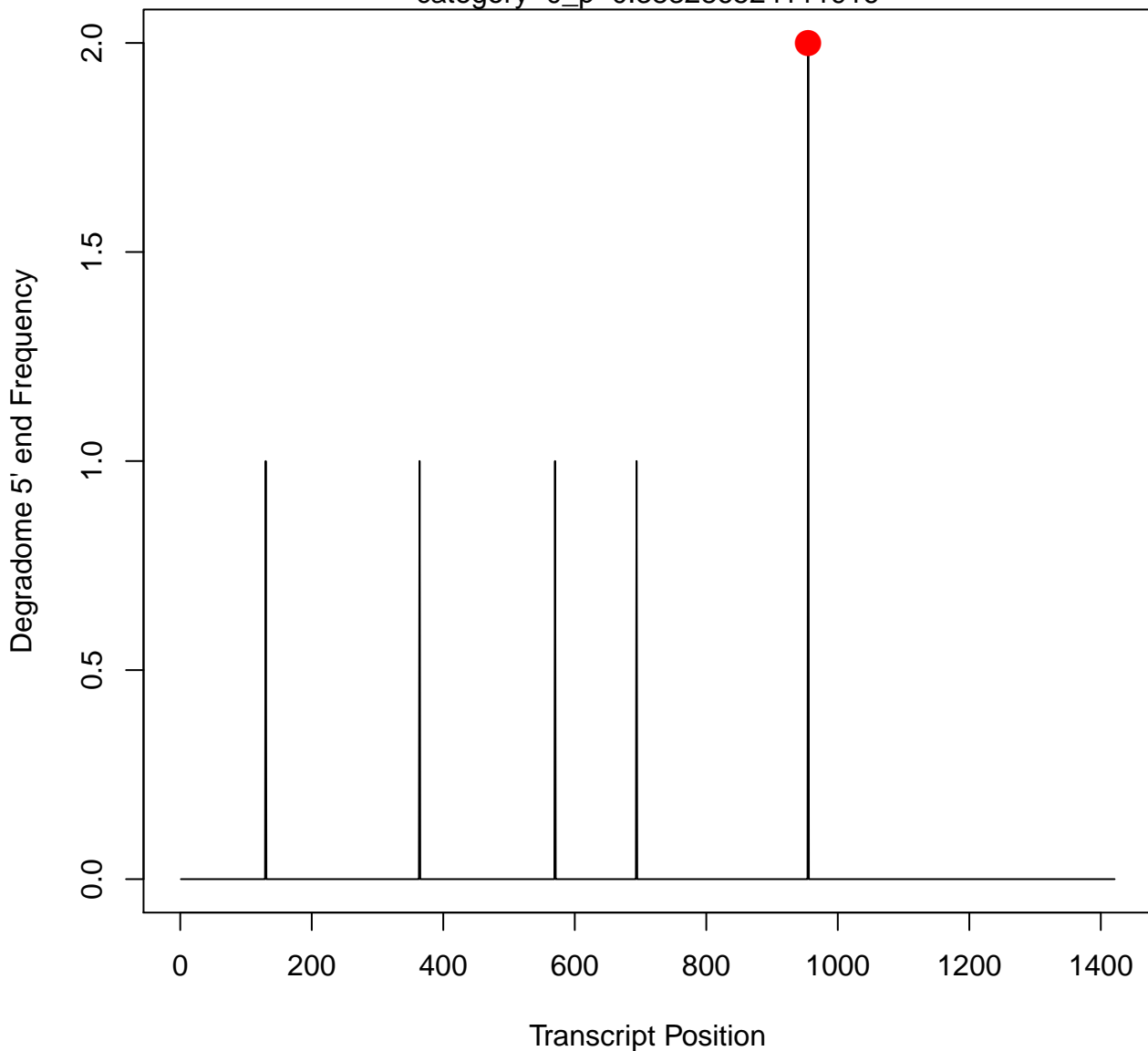

Supplement: Supplementary file 7 [file Data_Sheet_7.zip › Sit-miR2275d_Seita.1G100400.1_955_TPlot.pdf]

**T=Seita.2G236800.1\_Q=Sit-miR2275d\_S=1057**

category=2\_p=0.999721529739418

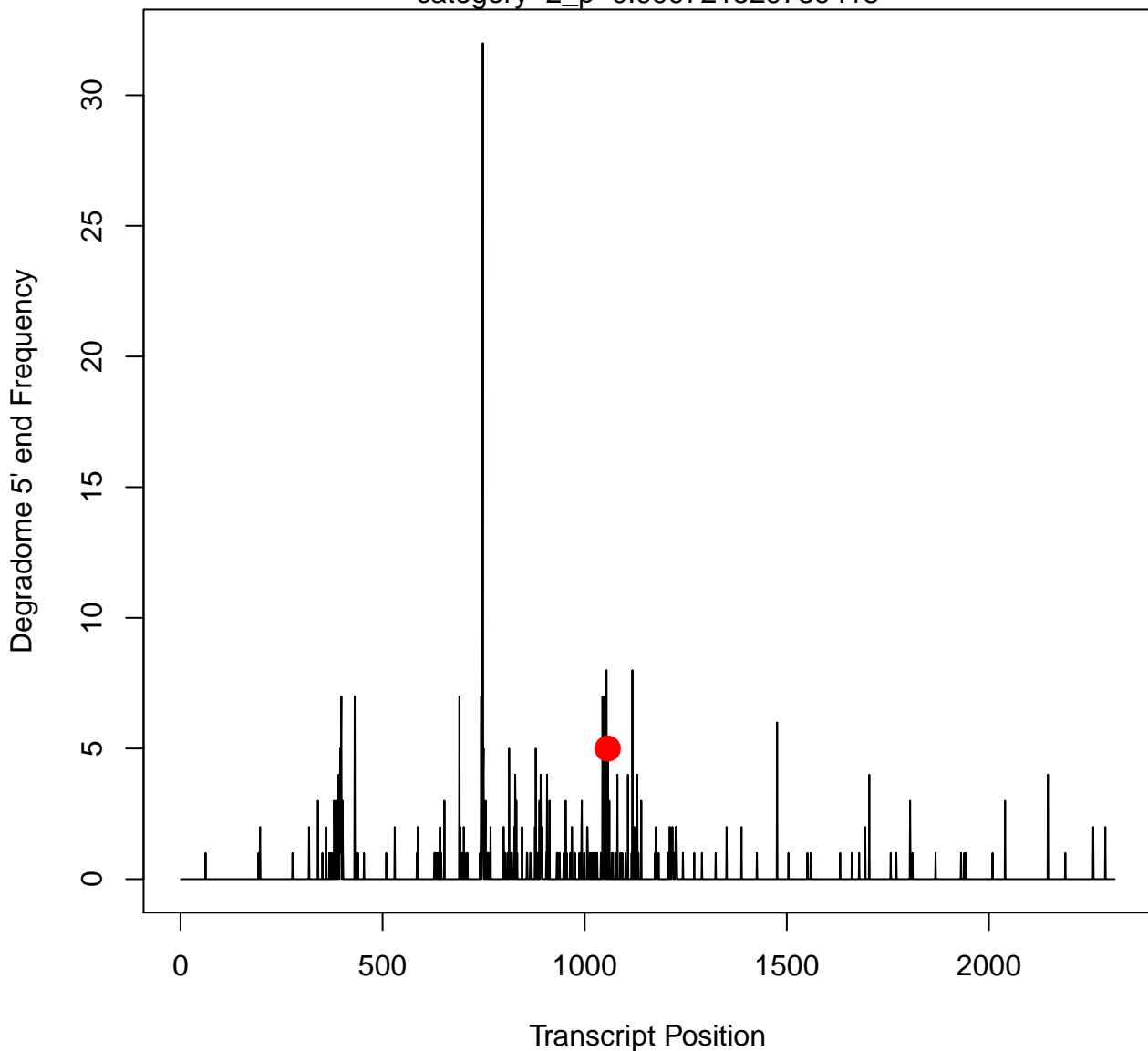

Supplement: Supplementary file 7 [file Data_Sheet_7.zip › Sit-miR2275d_Seita.2G236800.1_1057_TPlot.pdf]

**T=Seita.4G277800.1\_Q=Sit-miR2275d\_S=1177**

category=2\_p=0.99942764850062

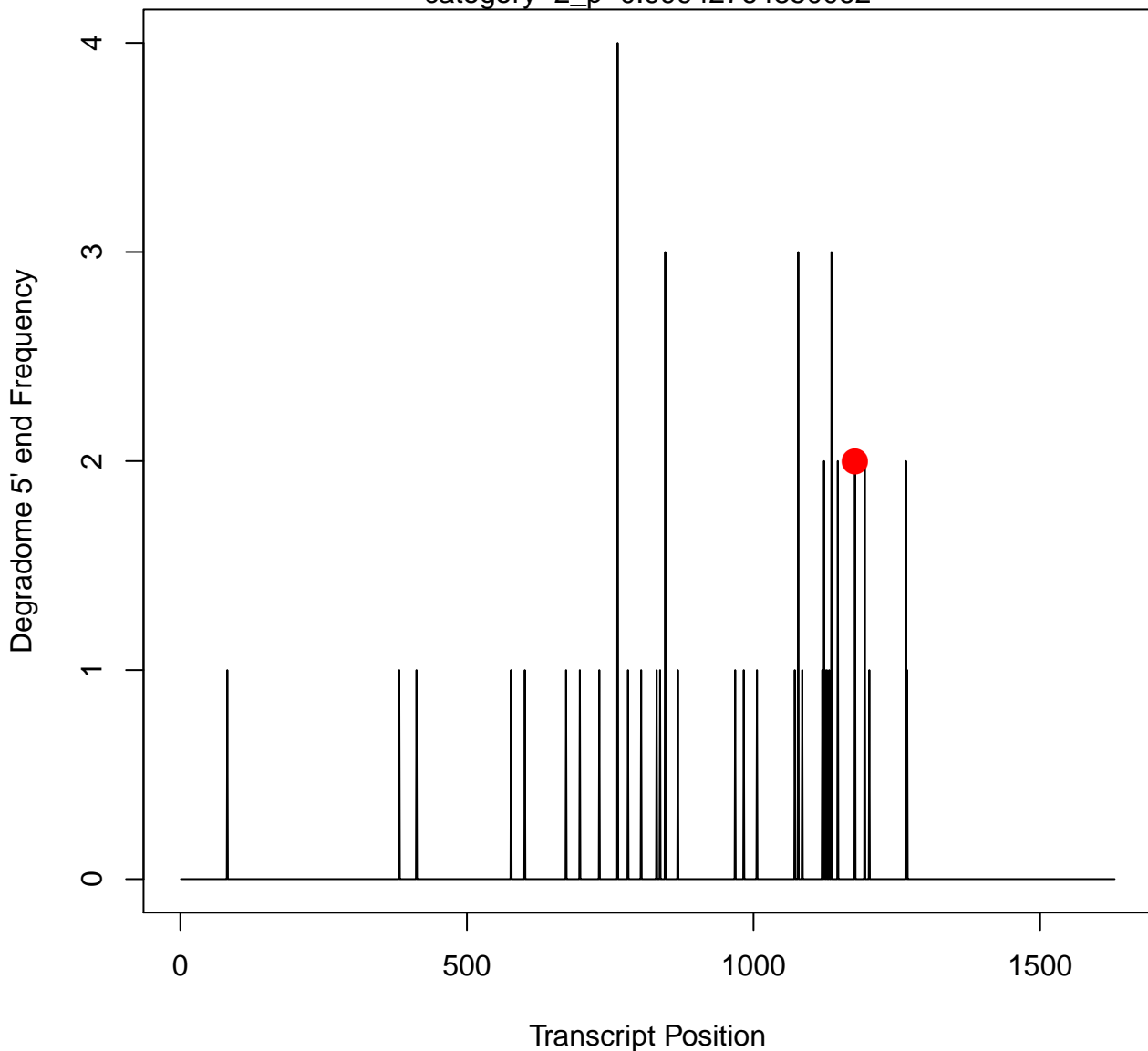

Supplement: Supplementary file 7 [file Data_Sheet_7.zip › Sit-miR2275d_Seita.4G277800.1_1177_TPlot.pdf]

**T=Seita.9G040500.1\_Q=Sit-miR2275d\_S=459**

category=2\_p=0.949952297937

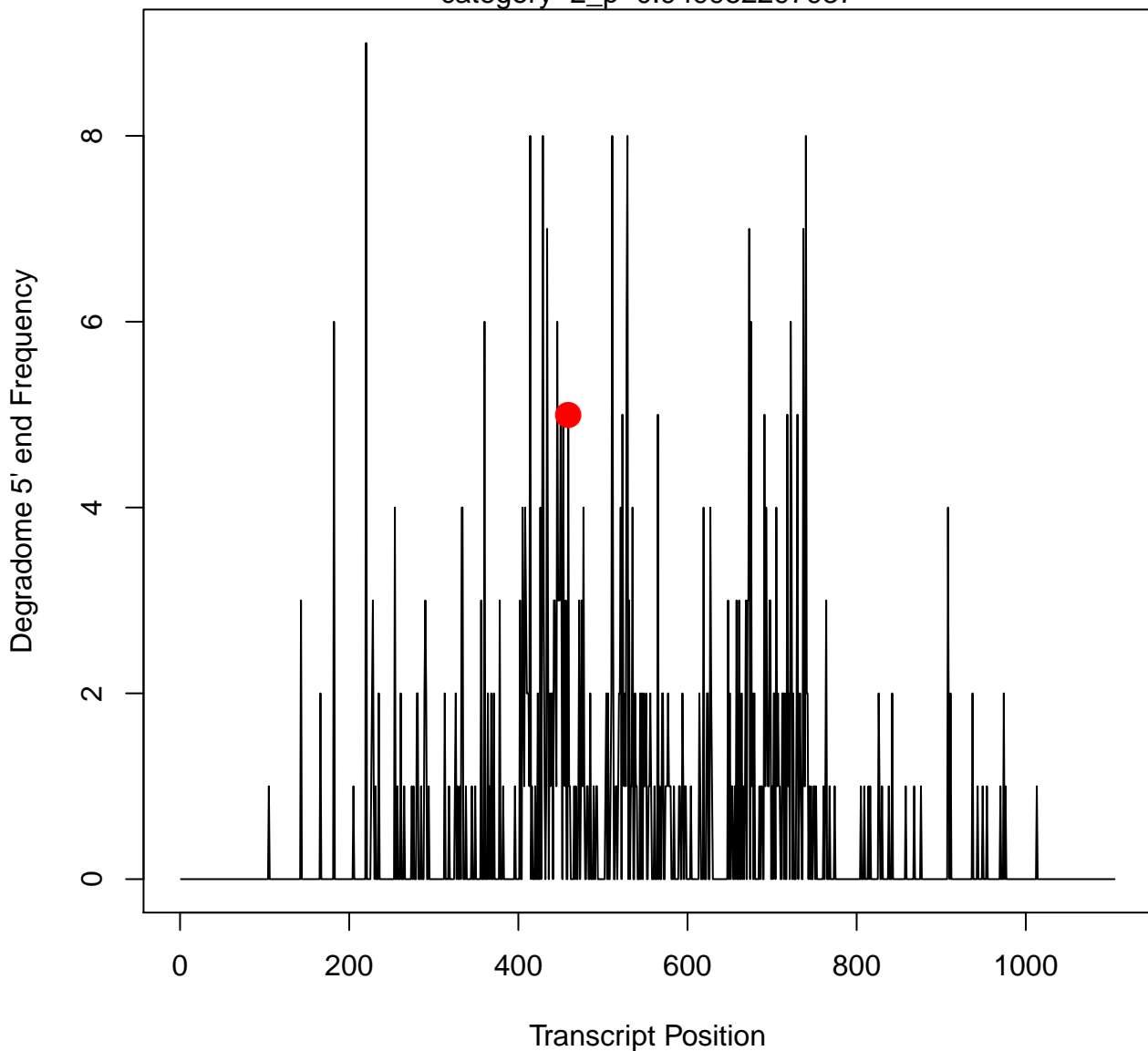

Supplement: Supplementary file 7 [file Data_Sheet_7.zip › Sit-miR2275d_Seita.9G040500.1_459_TPlot.pdf]

**T=Seita.9G458800.1\_Q=Sit-miR2275d\_S=1295**

category=2\_p=0.893437206584162

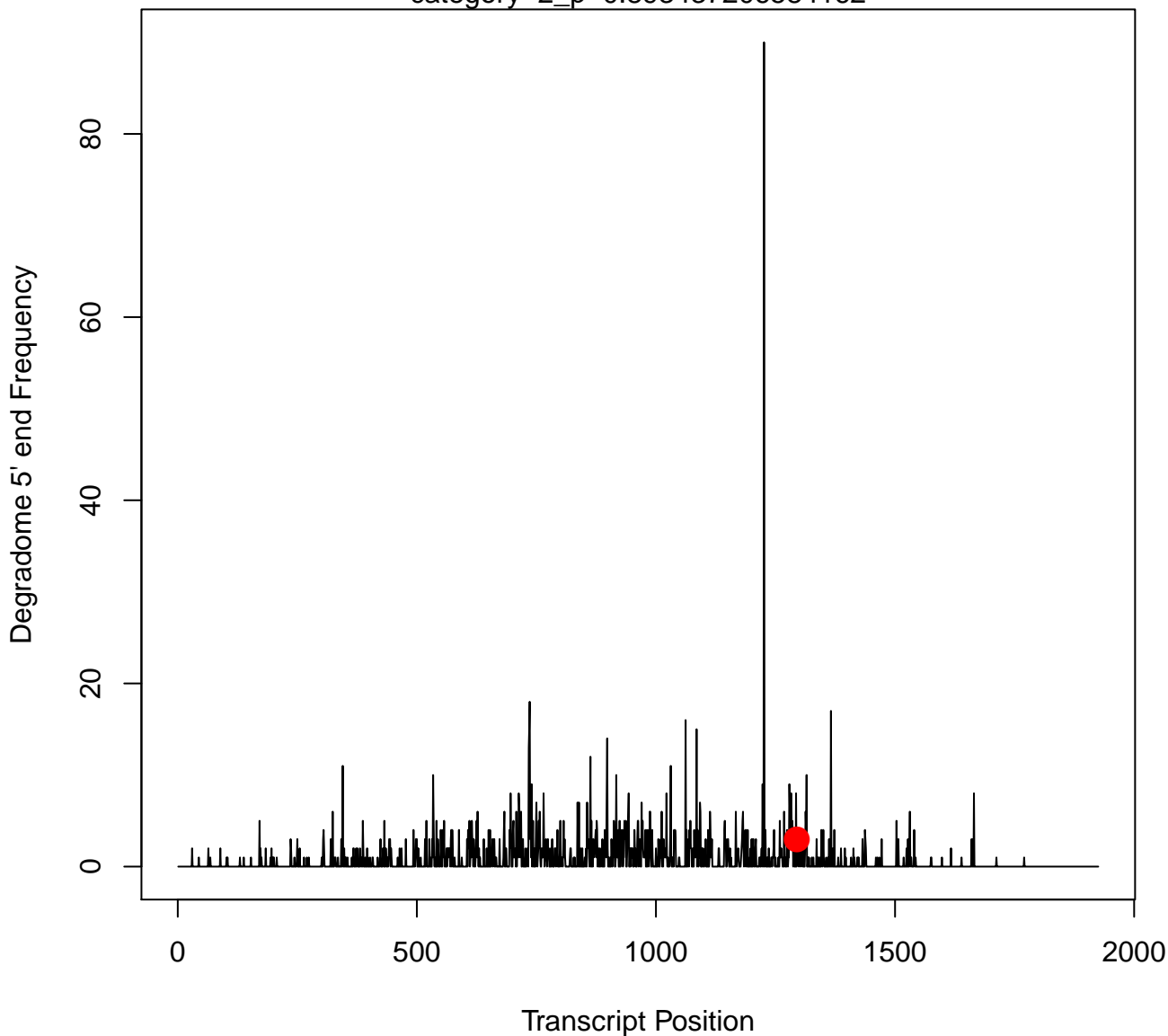

Supplement: Supplementary file 7 [file Data_Sheet_7.zip › Sit-miR2275d_Seita.9G458800.1_1295_TPlot.pdf]

**T=Seita.6G013100.1\_Q=Sit-miR2275e\_S=2767**

category=2\_p=0.0877316097542381

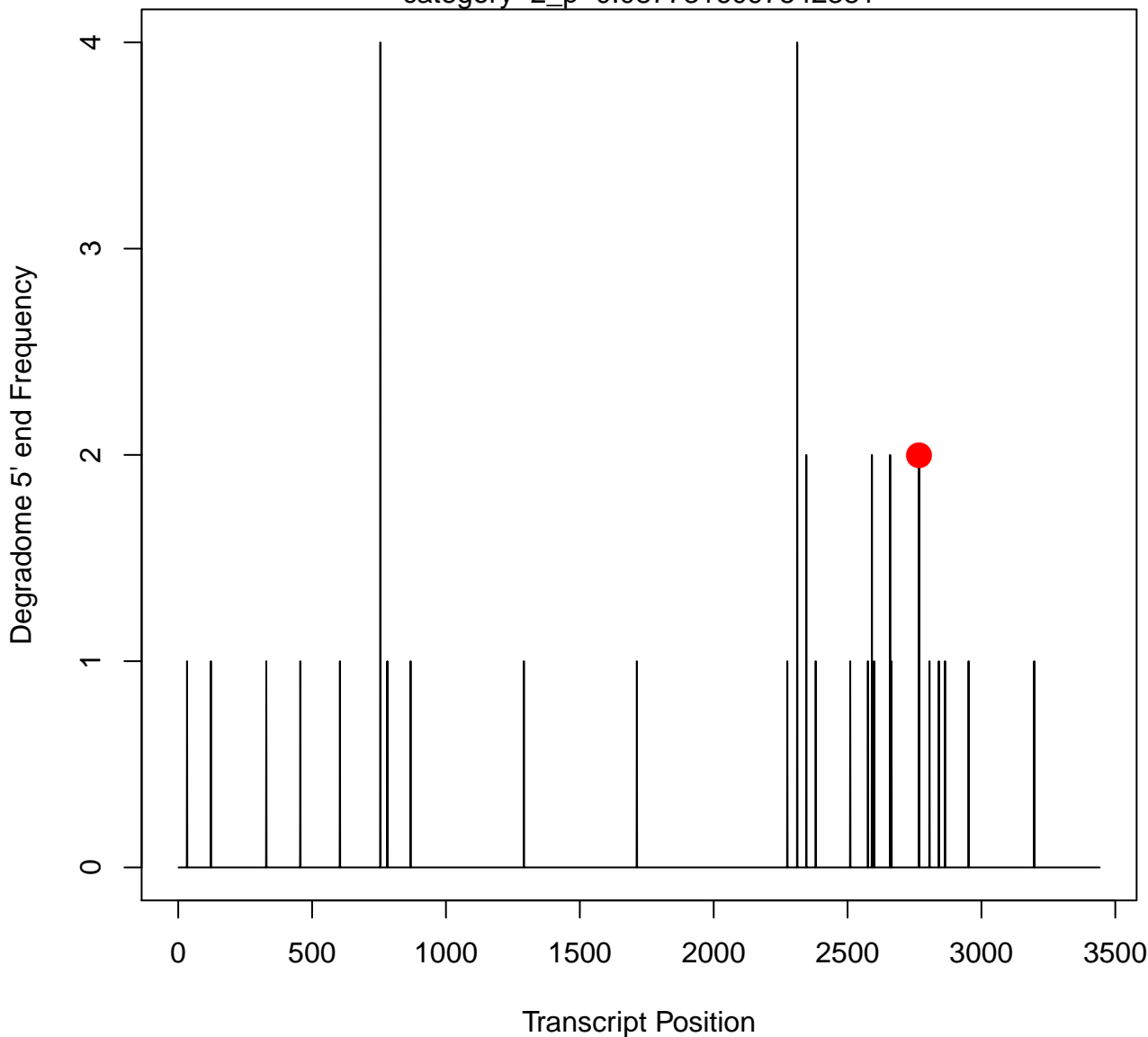

Supplement: Supplementary file 7 [file Data_Sheet_7.zip › Sit-miR2275e_Seita.6G013100.1_2767_TPlot.pdf]

**T=Seita.7G222300.1\_Q=Sit-miR319\_S=386**

category=0\_p=0.104021478797898

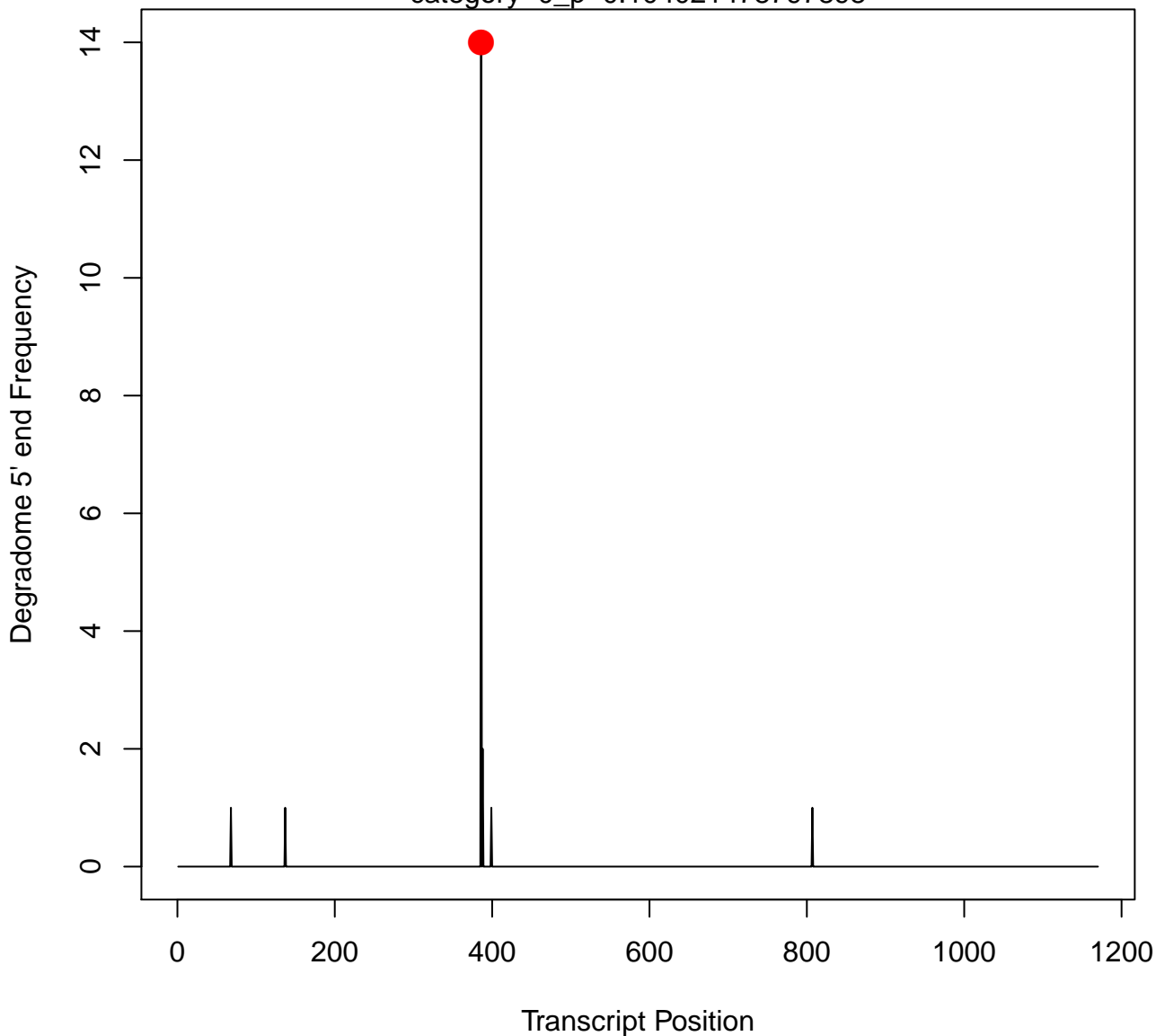

Supplement: Supplementary file 7 [file Data_Sheet_7.zip › Sit-miR319_Seita.7G222300.1_386_TPlot.pdf]

**T=Seita.2G188000.1\_Q=Sit-miR390\_S=768**

category=2\_p=0.935462254512649

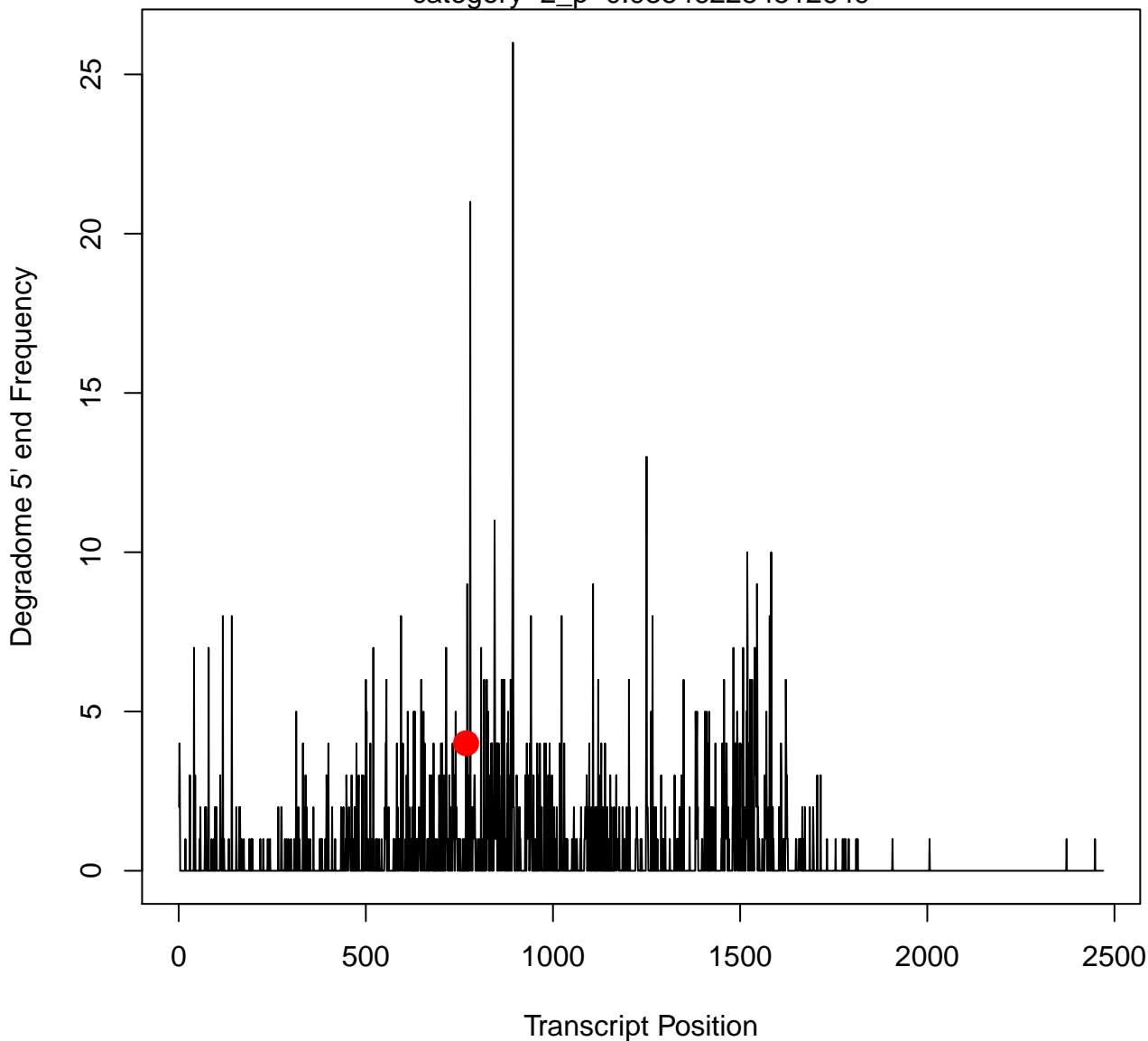

Supplement: Supplementary file 7 [file Data_Sheet_7.zip › Sit-miR390_Seita.2G188000.1_768_TPlot.pdf]

**T=Seita.4G008800.1\_Q=Sit-miR390\_S=3392**

category=2\_p=0.903470349617193

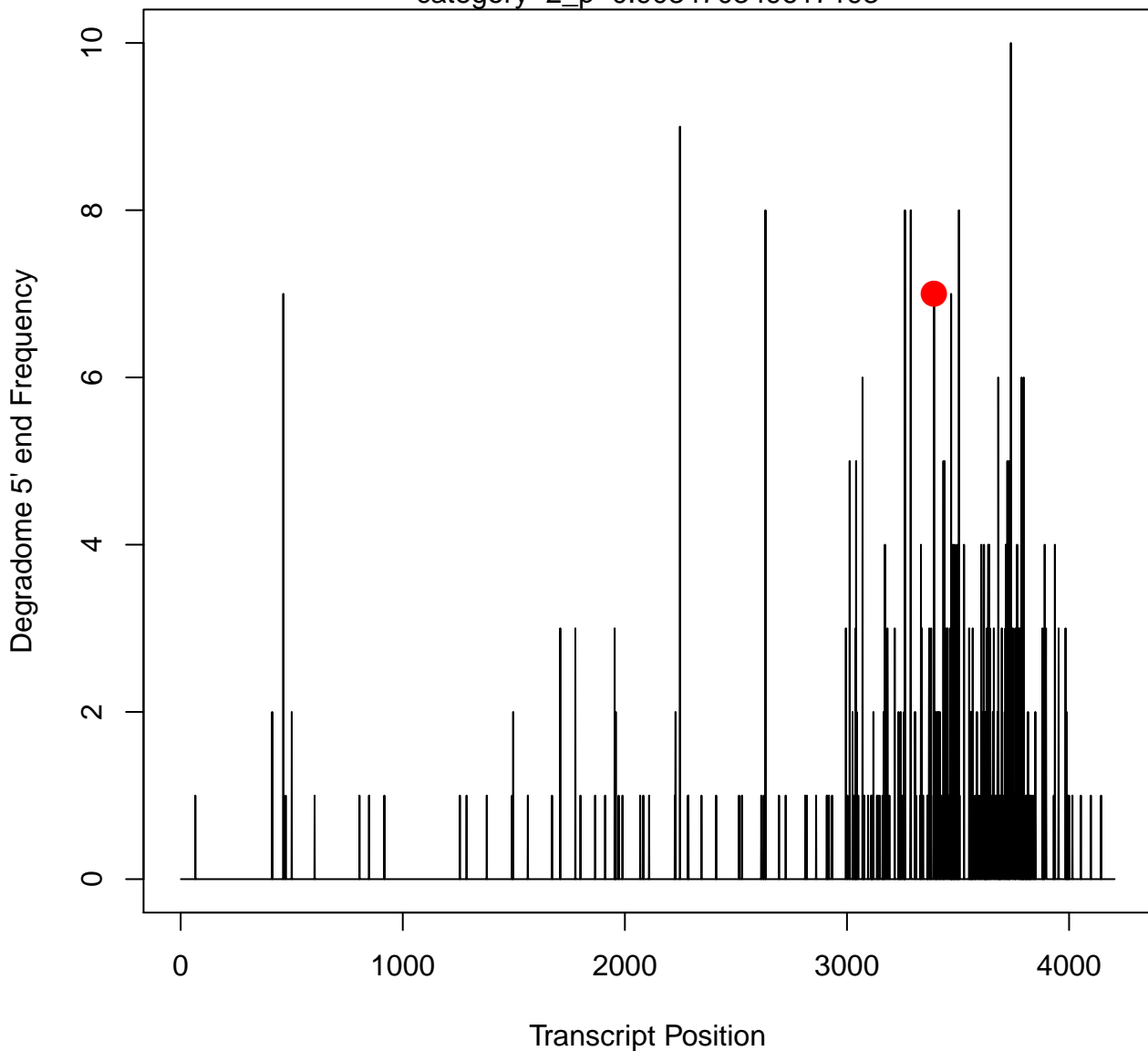

Supplement: Supplementary file 7 [file Data_Sheet_7.zip › Sit-miR390_Seita.4G008800.1_3392_TPlot.pdf]

**T=Seita.5G251600.1\_Q=Sit-miR390\_S=336**

category=2\_p=0.580532129313223

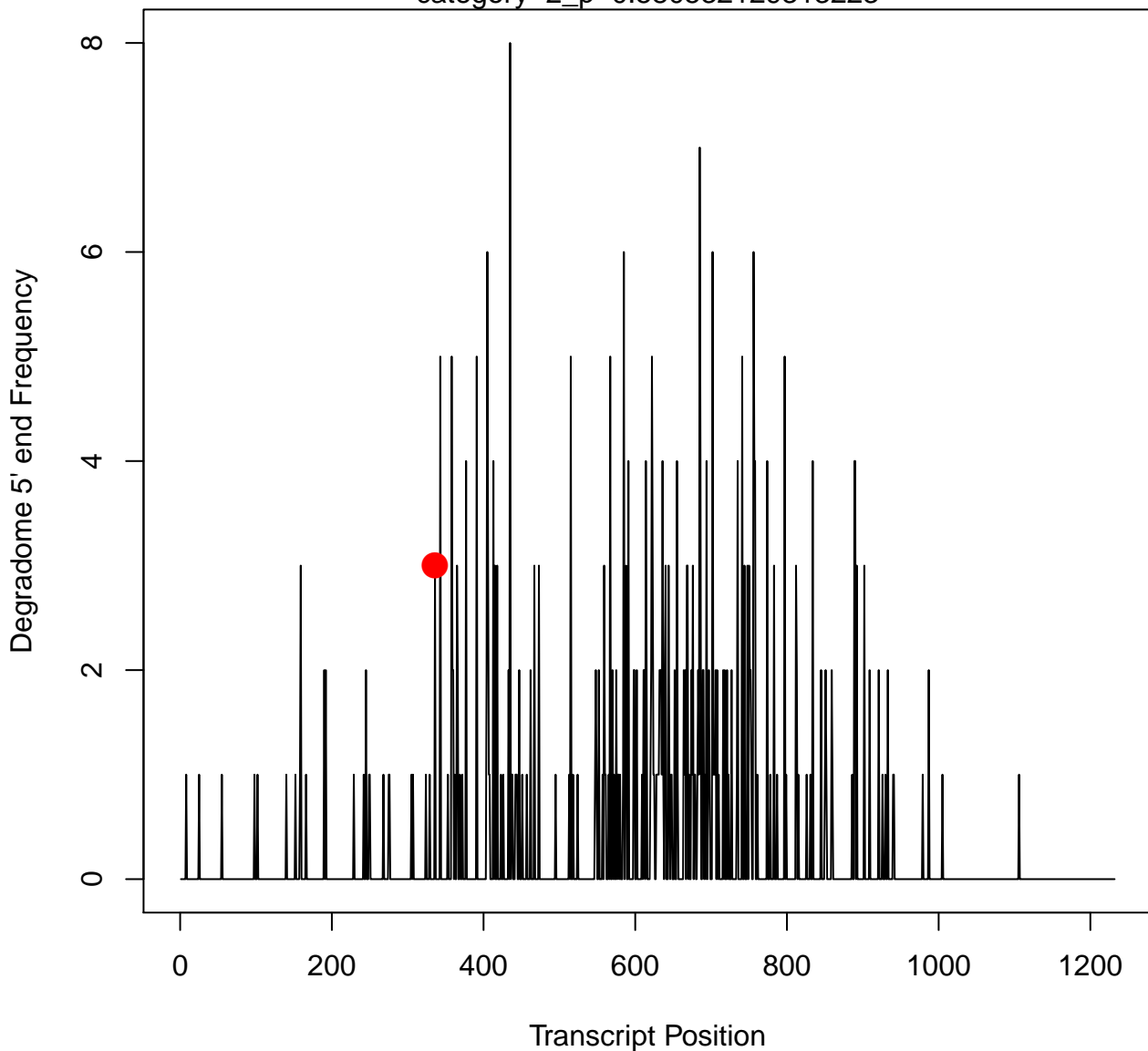

Supplement: Supplementary file 7 [file Data_Sheet_7.zip › Sit-miR390_Seita.5G251600.1_336_TPlot.pdf]

**T=Seita.5G271700.1\_Q=Sit-miR390\_S=883**

category=0\_p=0.176538237546587

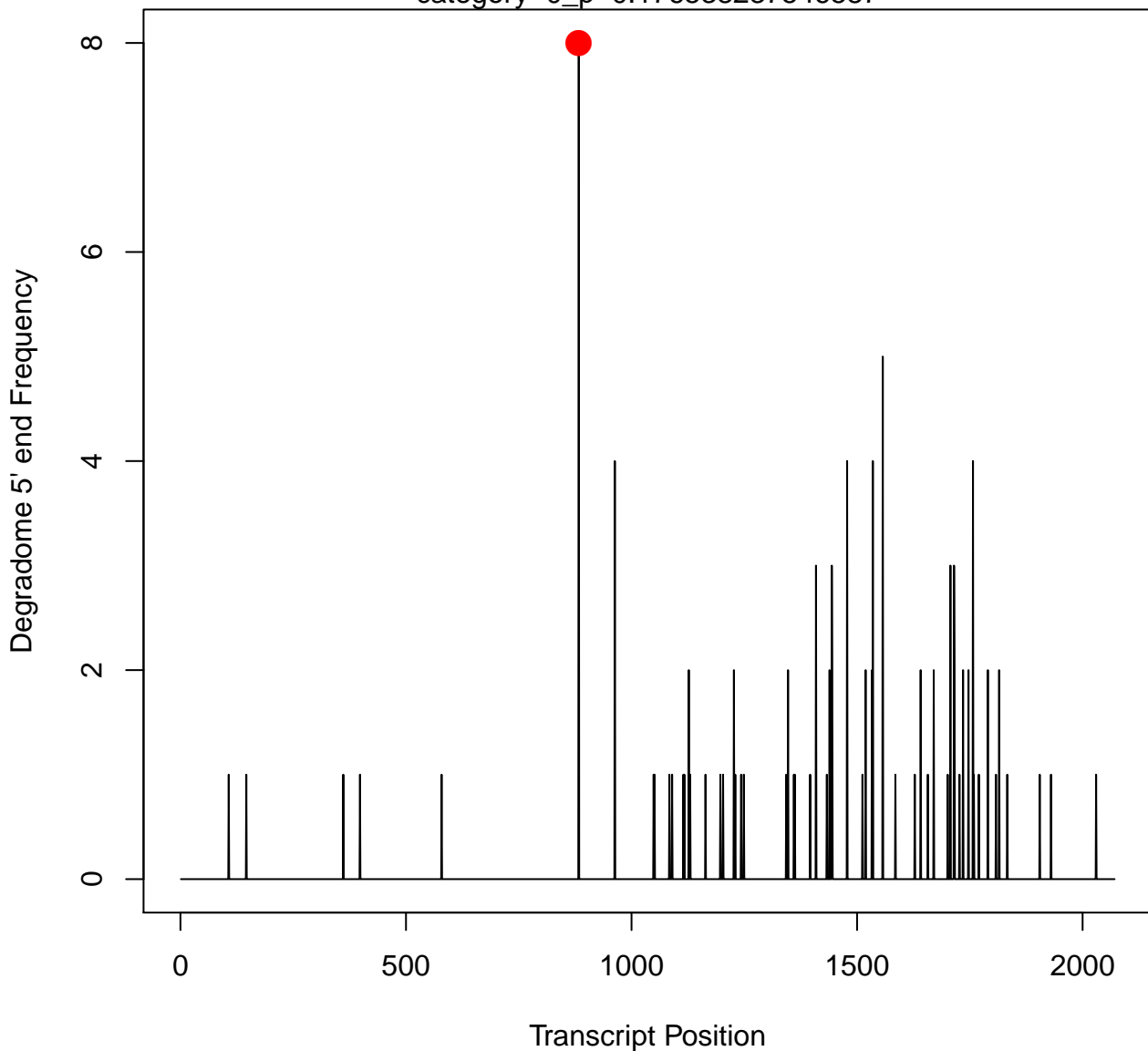

Supplement: Supplementary file 7 [file Data_Sheet_7.zip › Sit-miR390_Seita.5G271700.1_883_TPlot.pdf]

**T=Seita.9G074100.1\_Q=Sit-miR390\_S=1002**

category=2\_p=0.985562039116467

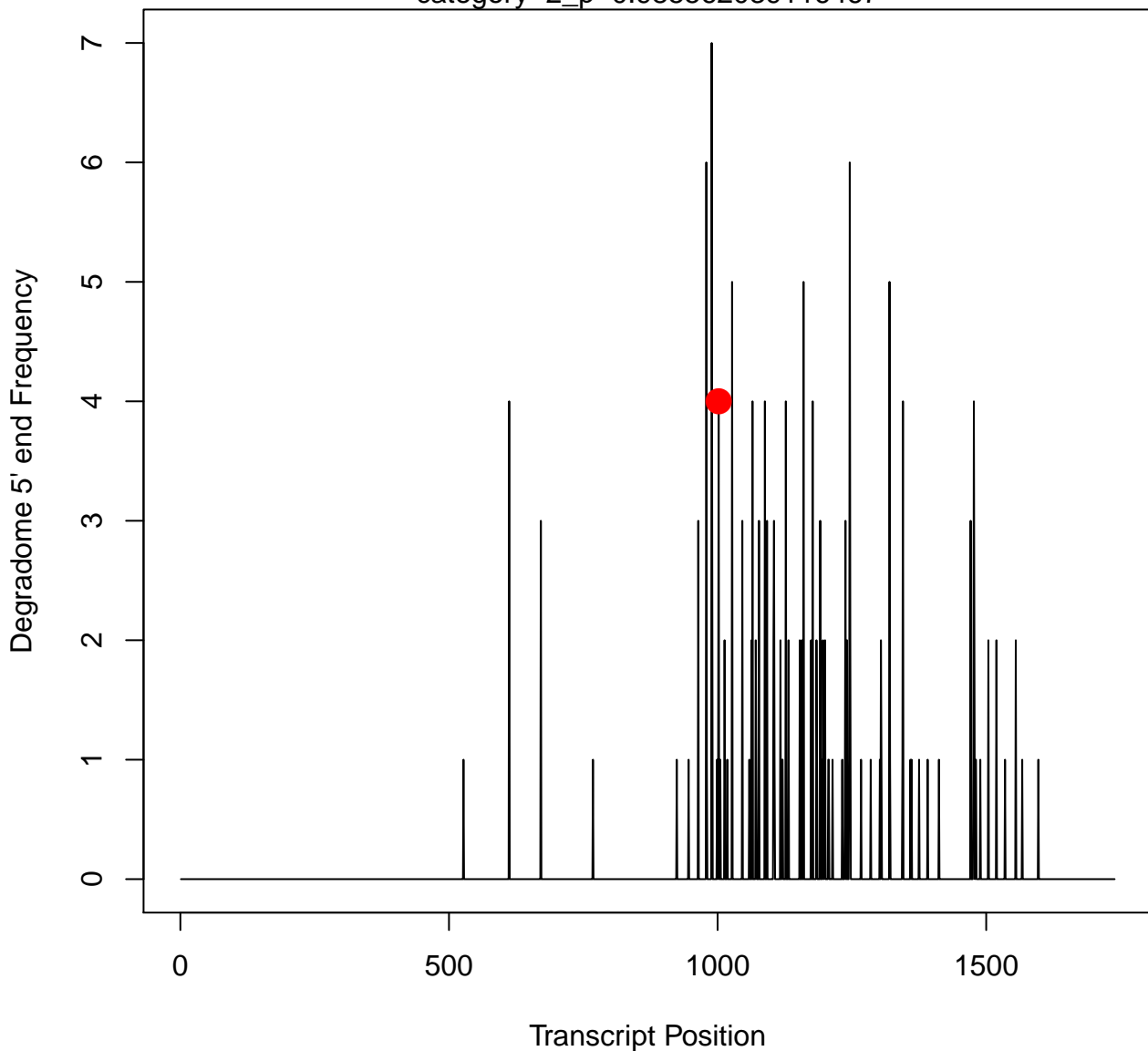

Supplement: Supplementary file 7 [file Data_Sheet_7.zip › Sit-miR390_Seita.9G074100.1_1002_TPlot.pdf]

**T=Seita.9G345300.1\_Q=Sit-miR390\_S=137**

category=2\_p=0.943567216032088

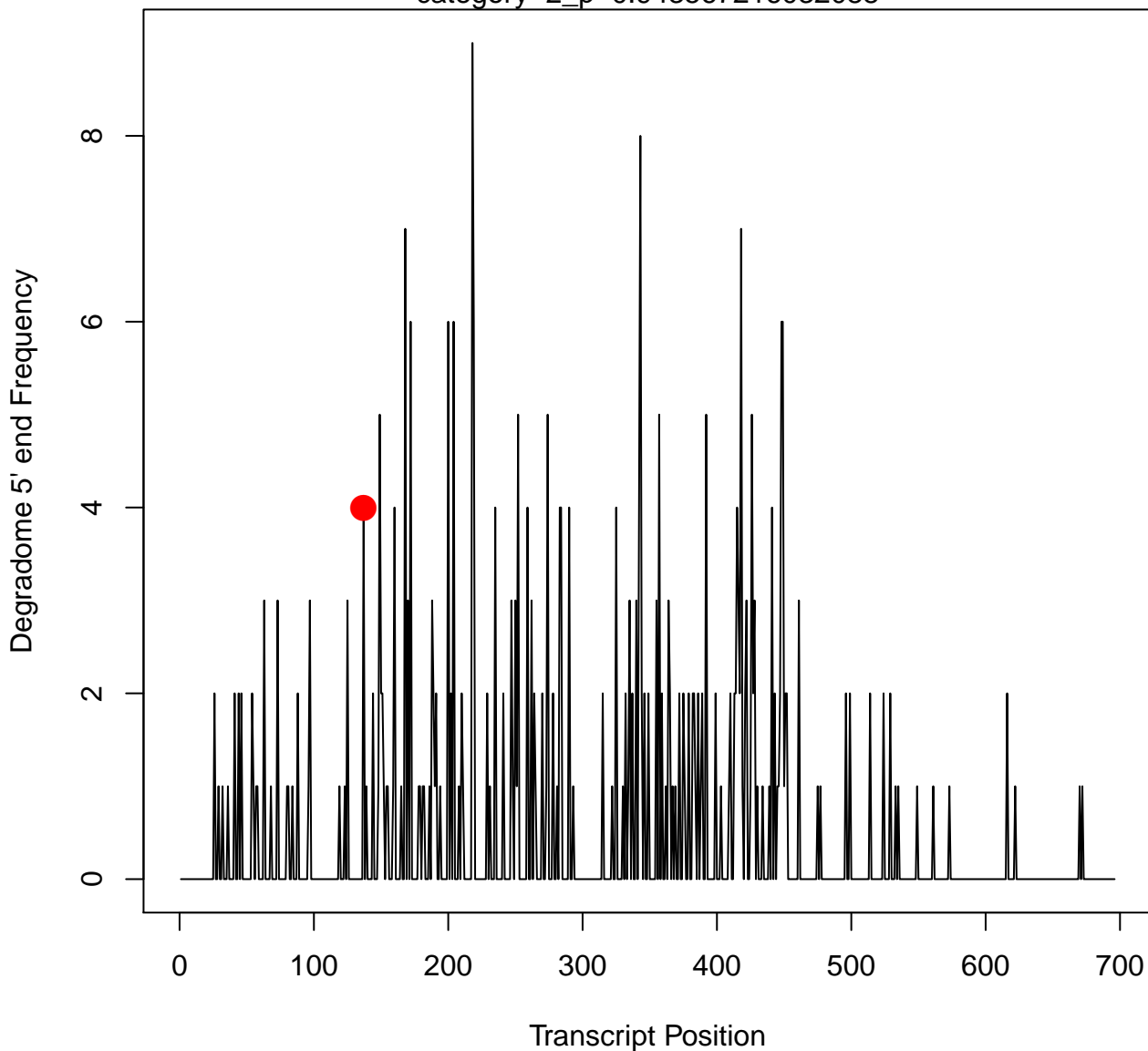

Supplement: Supplementary file 7 [file Data_Sheet_7.zip › Sit-miR390_Seita.9G345300.1_137_TPlot.pdf]

**T=Seita.9G414000.1\_Q=Sit-miR390\_S=765**

category=2\_p=0.997349619636684

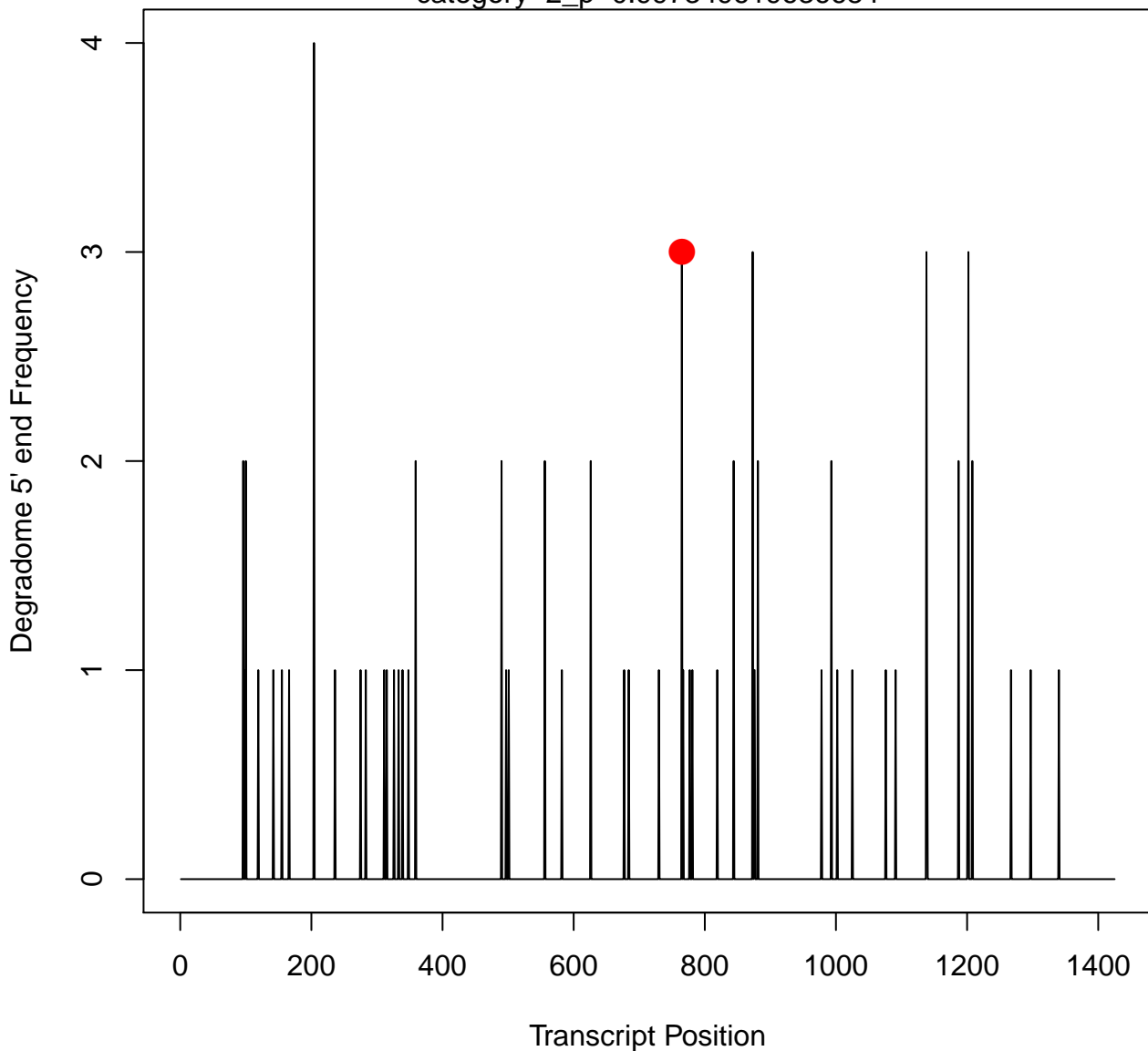

Supplement: Supplementary file 7 [file Data_Sheet_7.zip › Sit-miR390_Seita.9G414000.1_765_TPlot.pdf]

**T=Seita.9G505400.1\_Q=Sit-miR390\_S=1079**

category=2\_p=0.988318969607858

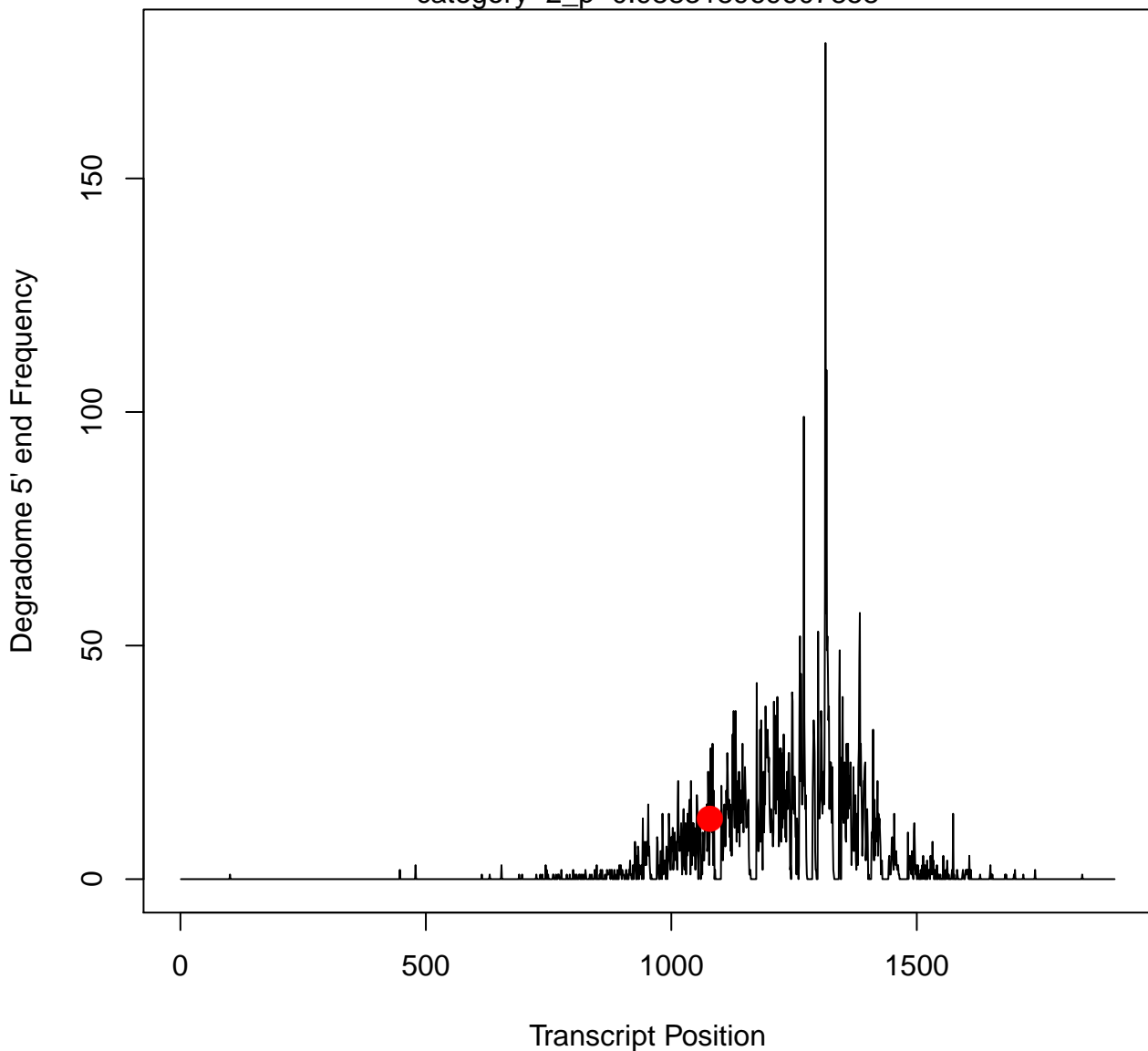

Supplement: Supplementary file 7 [file Data_Sheet_7.zip › Sit-miR390_Seita.9G505400.1_1079_TPlot.pdf]

**T=Seita.3G037200.1\_Q=Sit-miR393a\_S=1766**

category=2\_p=0.0140270063498924

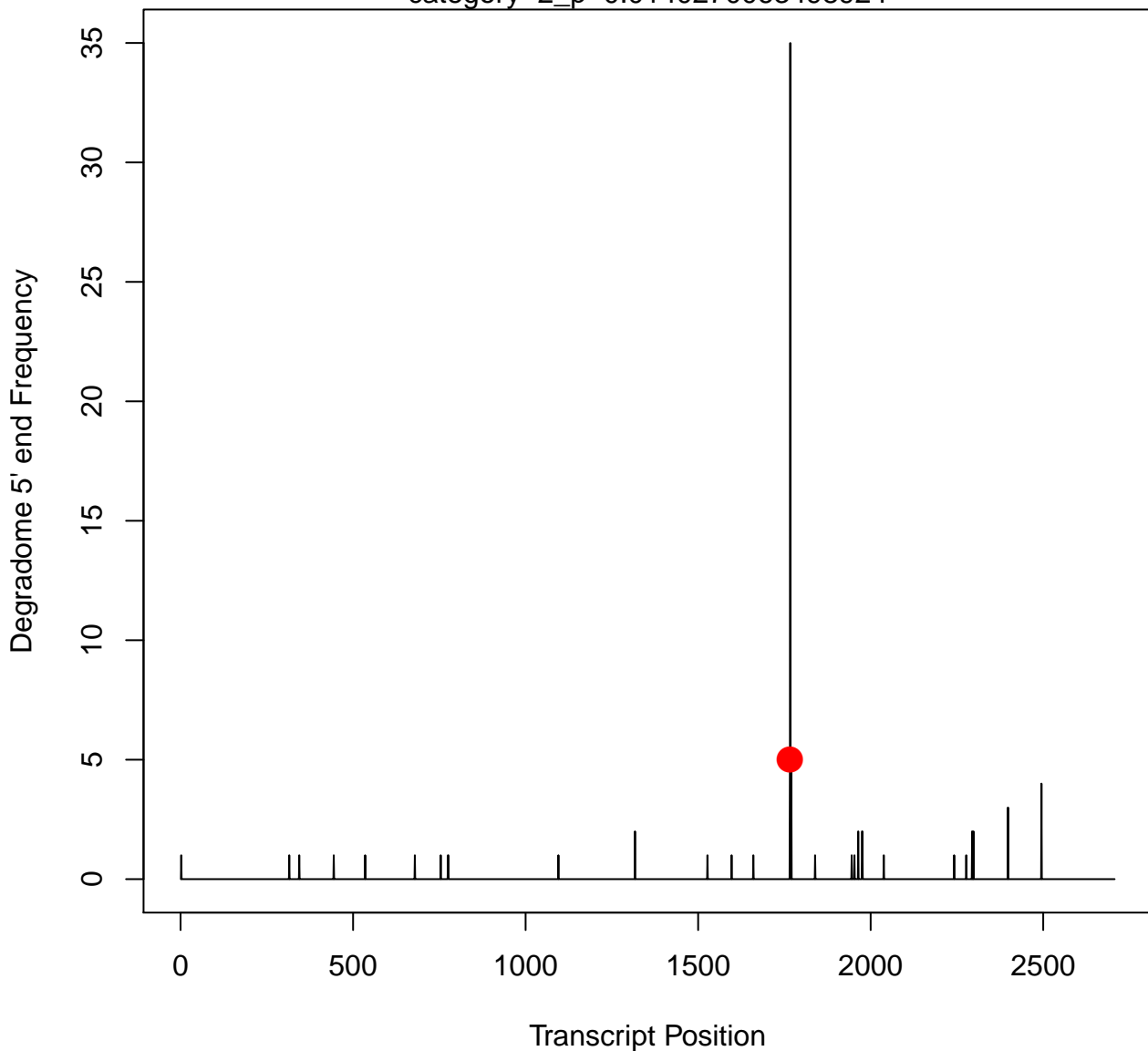

Supplement: Supplementary file 7 [file Data_Sheet_7.zip › Sit-miR393a_Seita.3G037200.1_1766_TPlot.pdf]

**T=Seita.7G078300.1\_Q=Sit-miR393a\_S=2366**

category=2\_p=0.00703827180998173

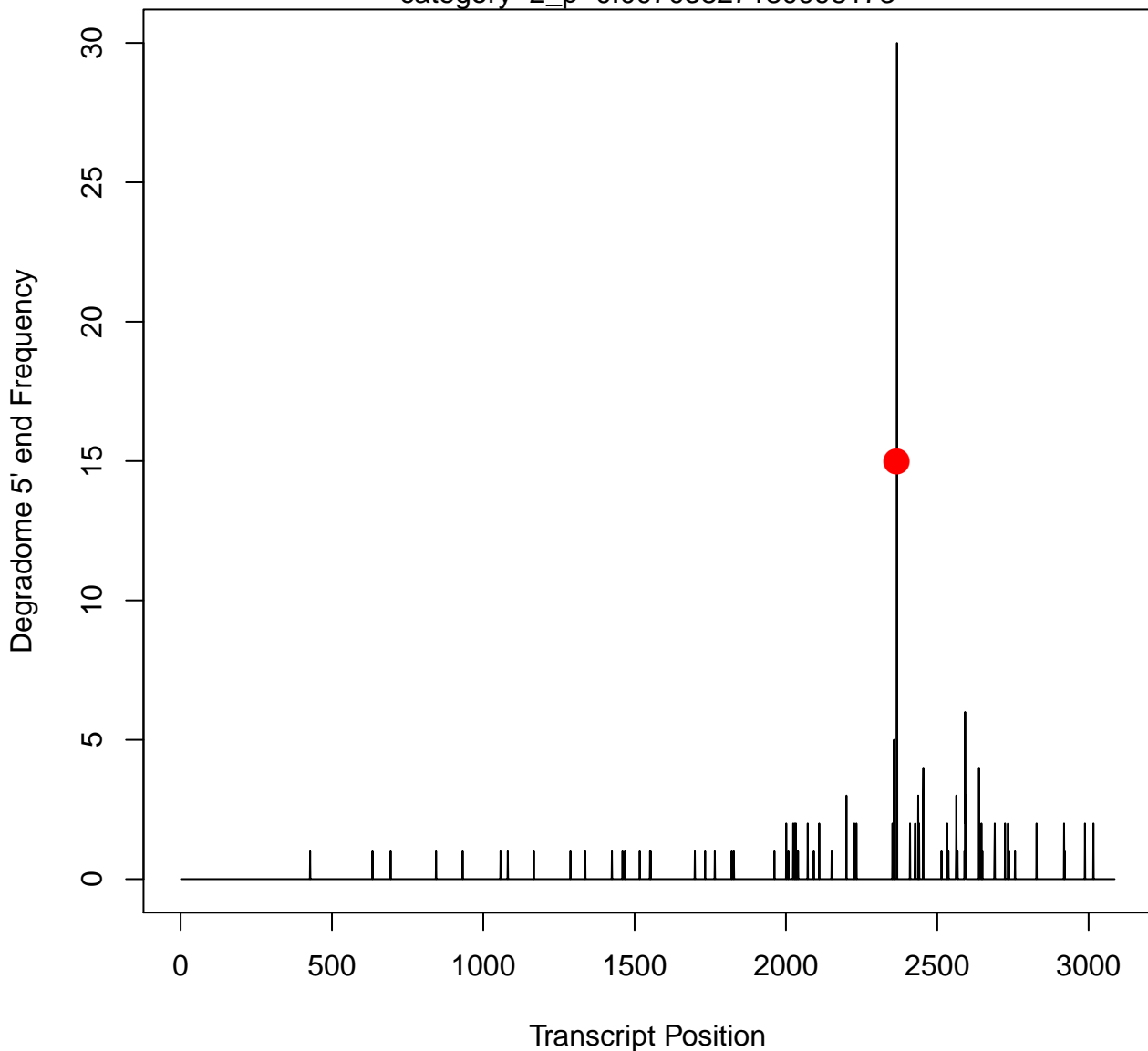

Supplement: Supplementary file 7 [file Data_Sheet_7.zip › Sit-miR393a_Seita.7G078300.1_2366_TPlot.pdf]

**T=Seita.3G009800.1\_Q=Sit-miR393b\_S=470**

category=0\_p=0.00149532282594533

Degradome 5' end Frequency

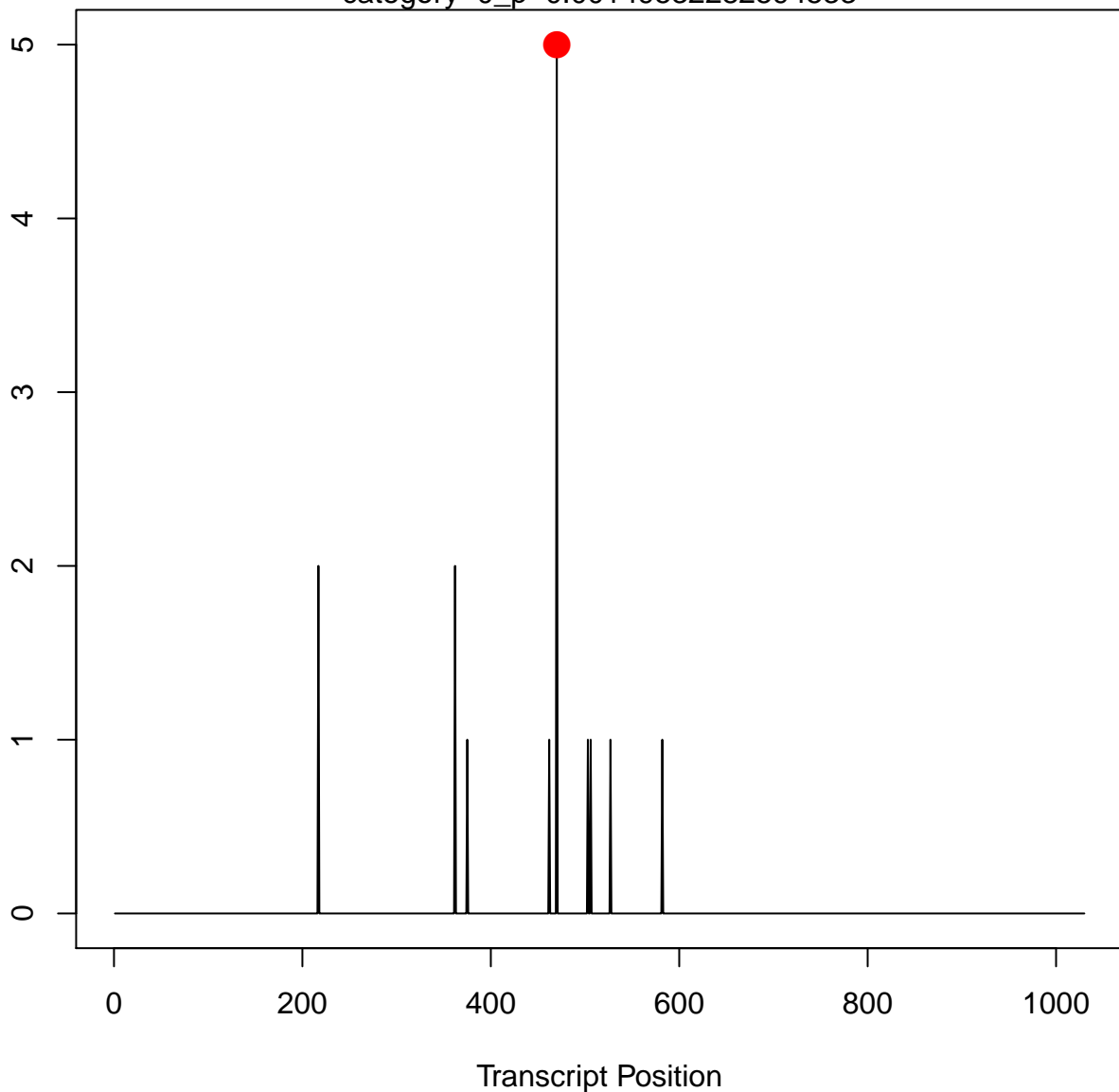

Supplement: Supplementary file 7 [file Data_Sheet_7.zip › Sit-miR393b_Seita.3G009800.1_470_TPlot.pdf]

**T=Seita.3G037200.1\_Q=Sit-miR393b\_S=1767**

category=0\_p=0.000299243605074828

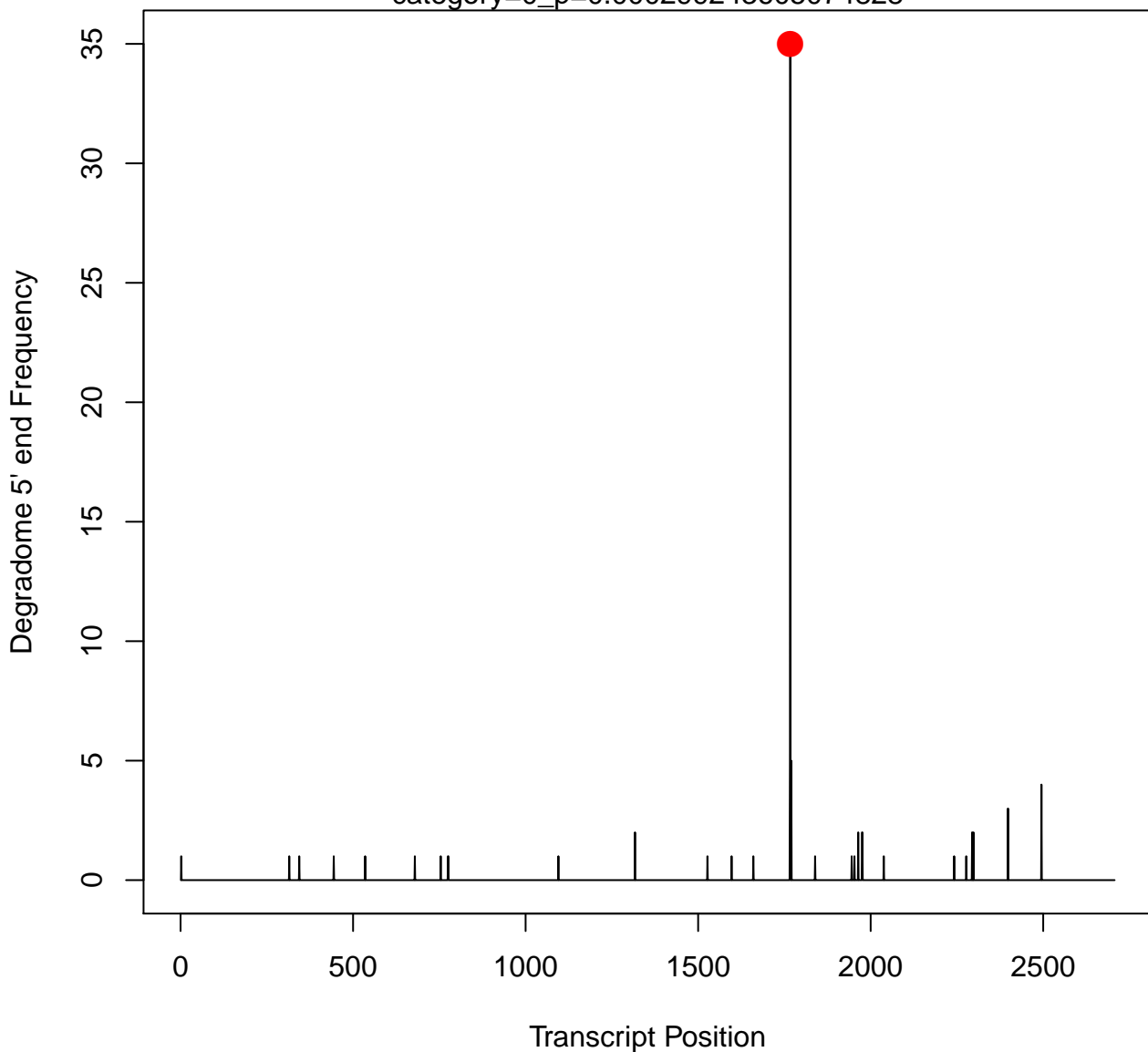

Supplement: Supplementary file 7 [file Data_Sheet_7.zip › Sit-miR393b_Seita.3G037200.1_1767_TPlot.pdf]

**T=Seita.5G373000.1\_Q=Sit-miR393b\_S=624**

category=2\_p=0.402864057126576

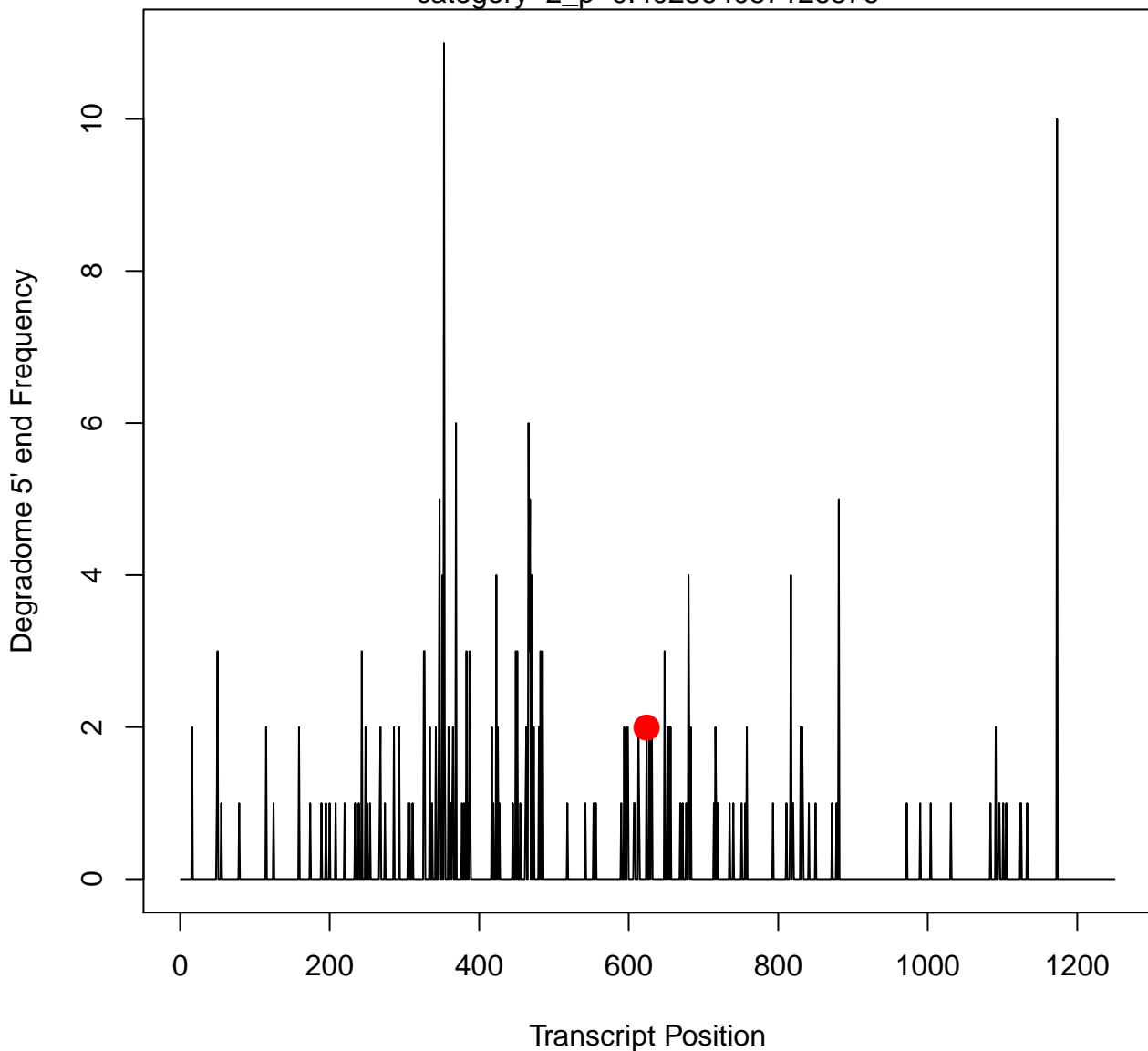

Supplement: Supplementary file 7 [file Data_Sheet_7.zip › Sit-miR393b_Seita.5G373000.1_624_TPlot.pdf]

**T=Seita.7G078300.1\_Q=Sit-miR393b\_S=2367**

category=0\_p=0.000598397663414518

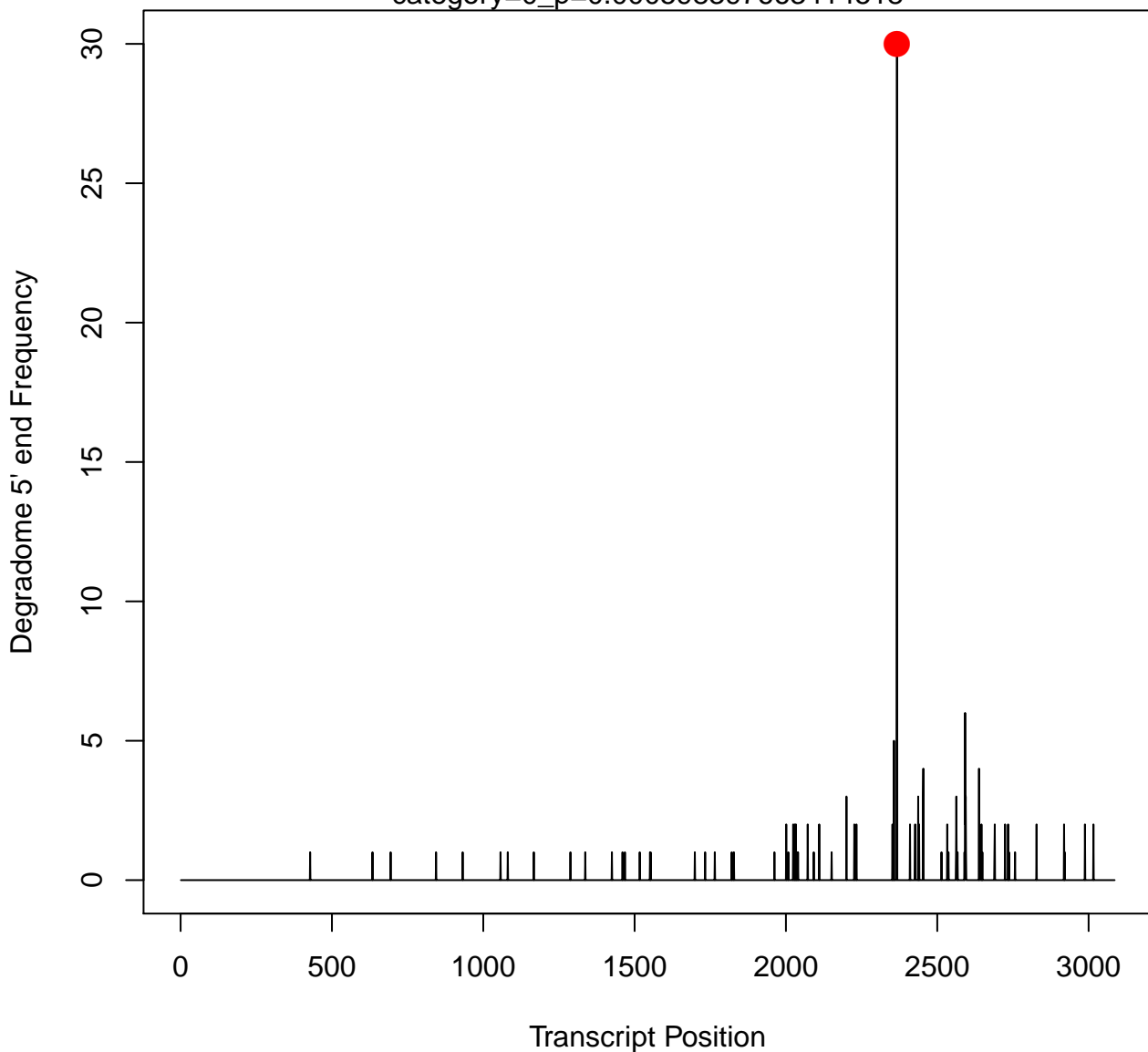

Supplement: Supplementary file 7 [file Data_Sheet_7.zip › Sit-miR393b_Seita.7G078300.1_2367_TPlot.pdf]

**T=Seita.8G222300.1\_Q=Sit-miR393b\_S=998**

category=2\_p=0.681531426607696

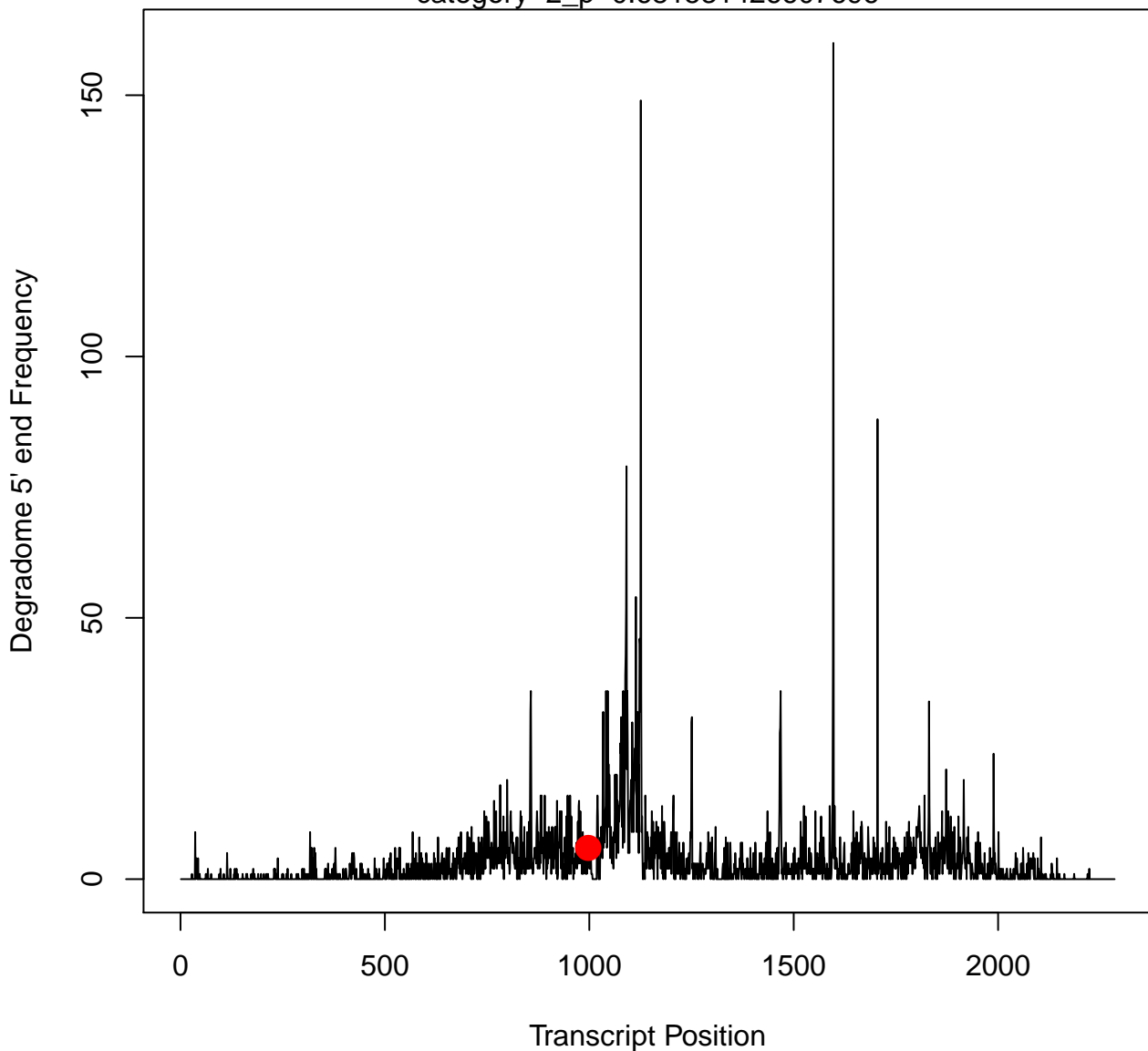

Supplement: Supplementary file 7 [file Data_Sheet_7.zip › Sit-miR393b_Seita.8G222300.1_998_TPlot.pdf]

**T=Seita.2G373600.1\_Q=Sit-miR394\_S=746**

category=1\_p=0.0978552186202288

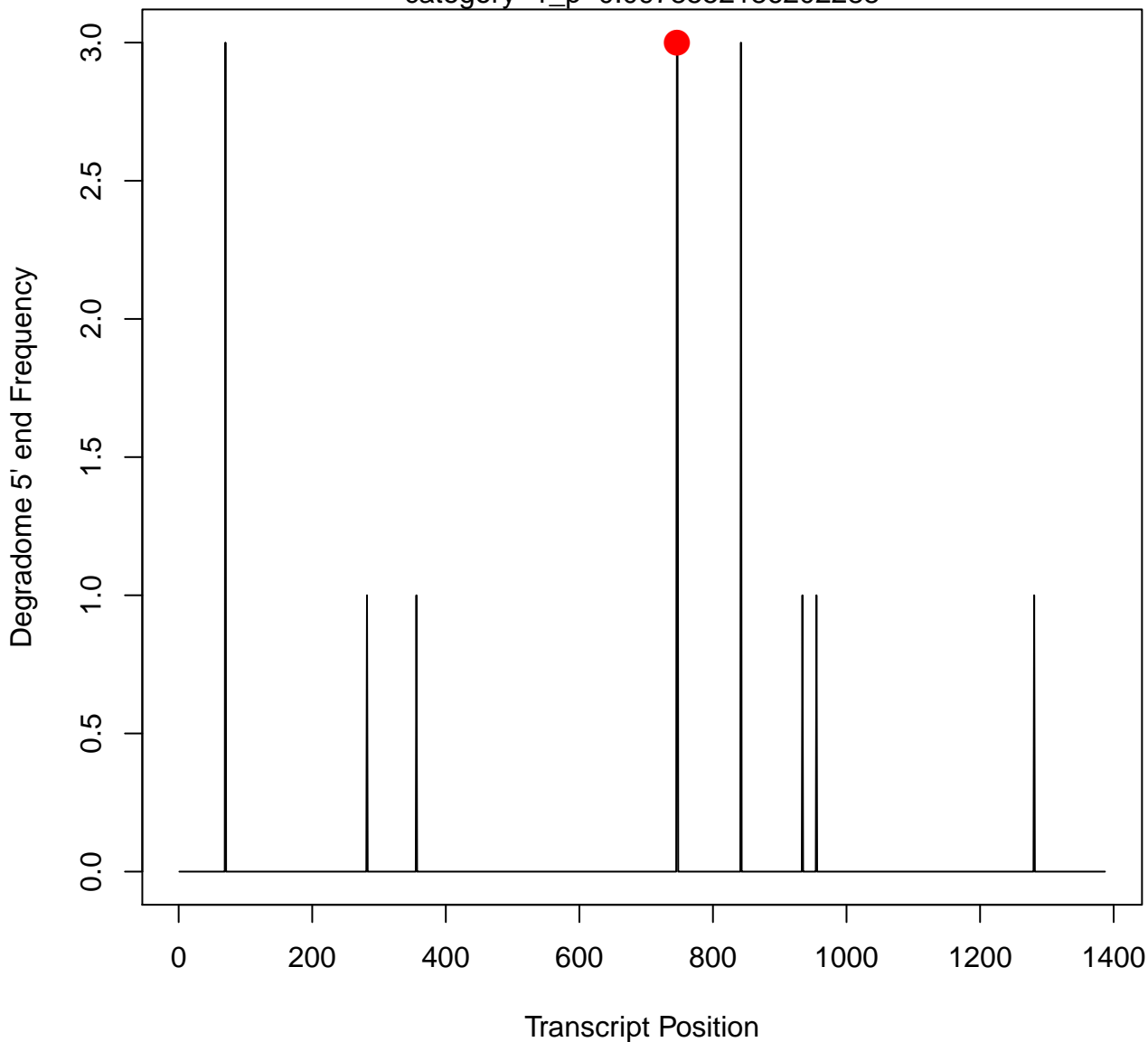

Supplement: Supplementary file 7 [file Data_Sheet_7.zip › Sit-miR394_Seita.2G373600.1_746_TPlot.pdf]

**T=Seita.2G413500.1\_Q=Sit-miR394\_S=595**

category=2\_p=0.999094163763482

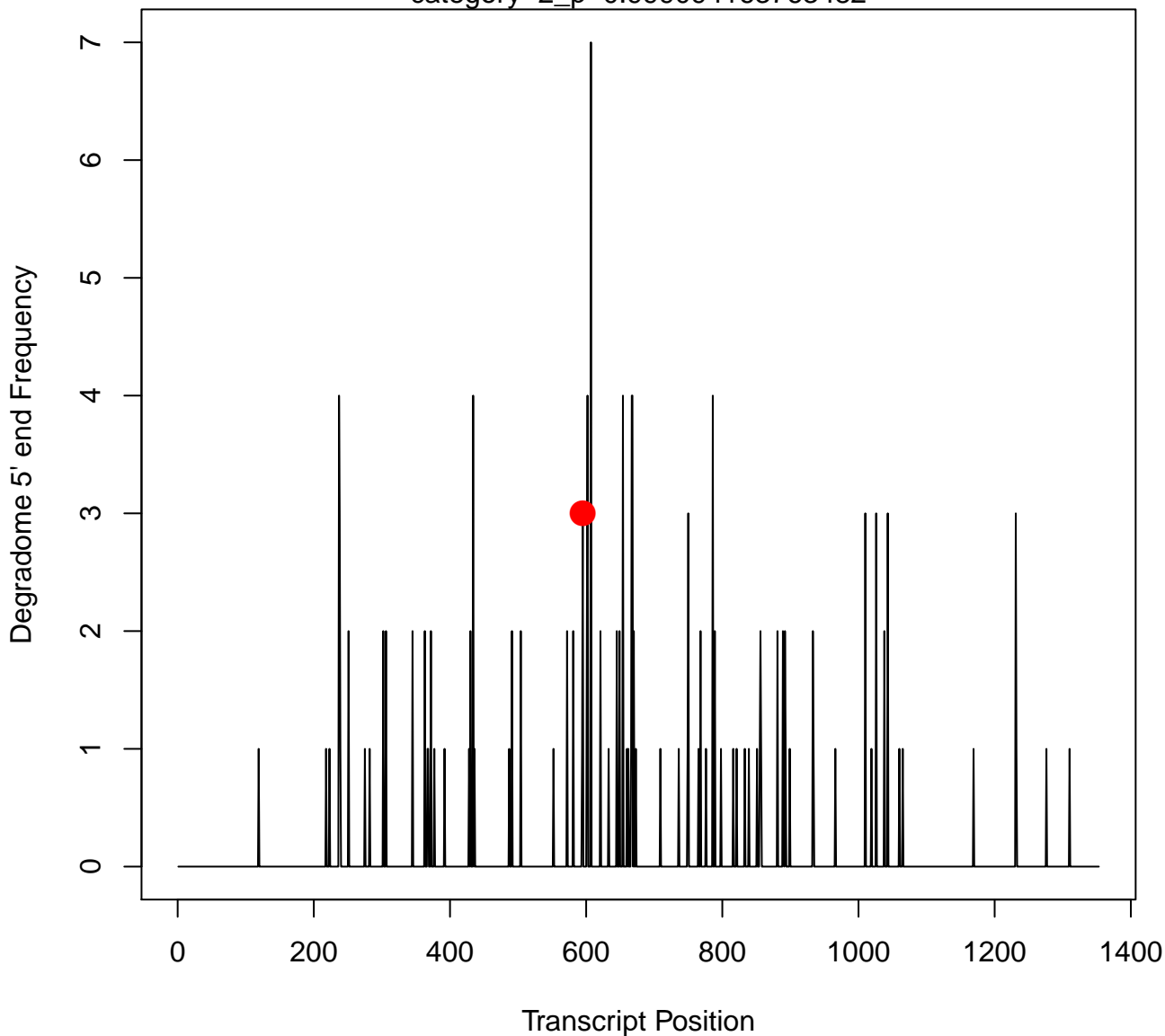

Supplement: Supplementary file 7 [file Data_Sheet_7.zip › Sit-miR394_Seita.2G413500.1_595_TPlot.pdf]

**T=Seita.3G339200.1\_Q=Sit-miR394\_S=287**

category=2\_p=0.979591415580674

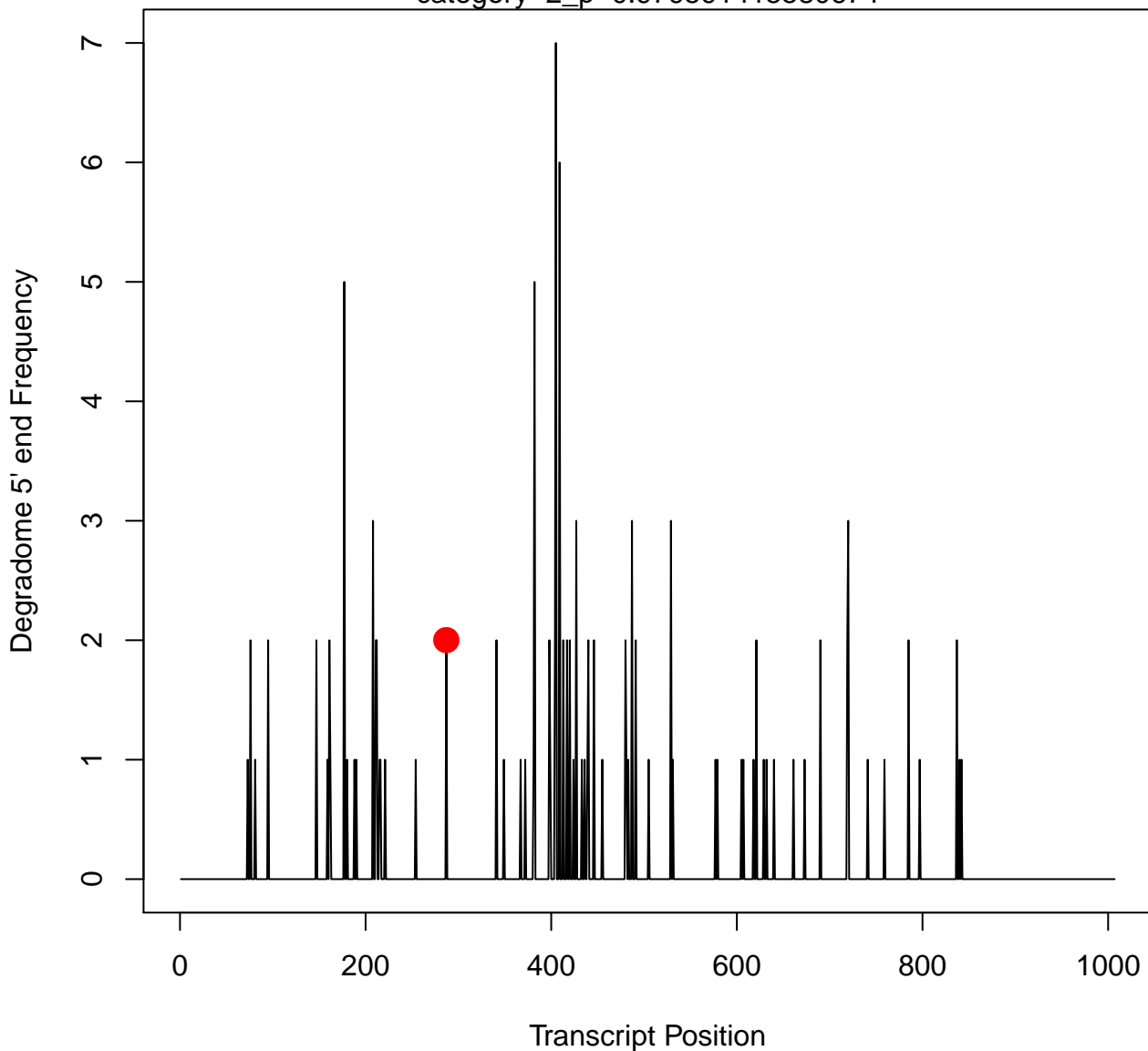

Supplement: Supplementary file 7 [file Data_Sheet_7.zip › Sit-miR394_Seita.3G339200.1_287_TPlot.pdf]

**T=Seita.4G207000.1\_Q=Sit-miR394\_S=730**

category=2\_p=0.99999597945727

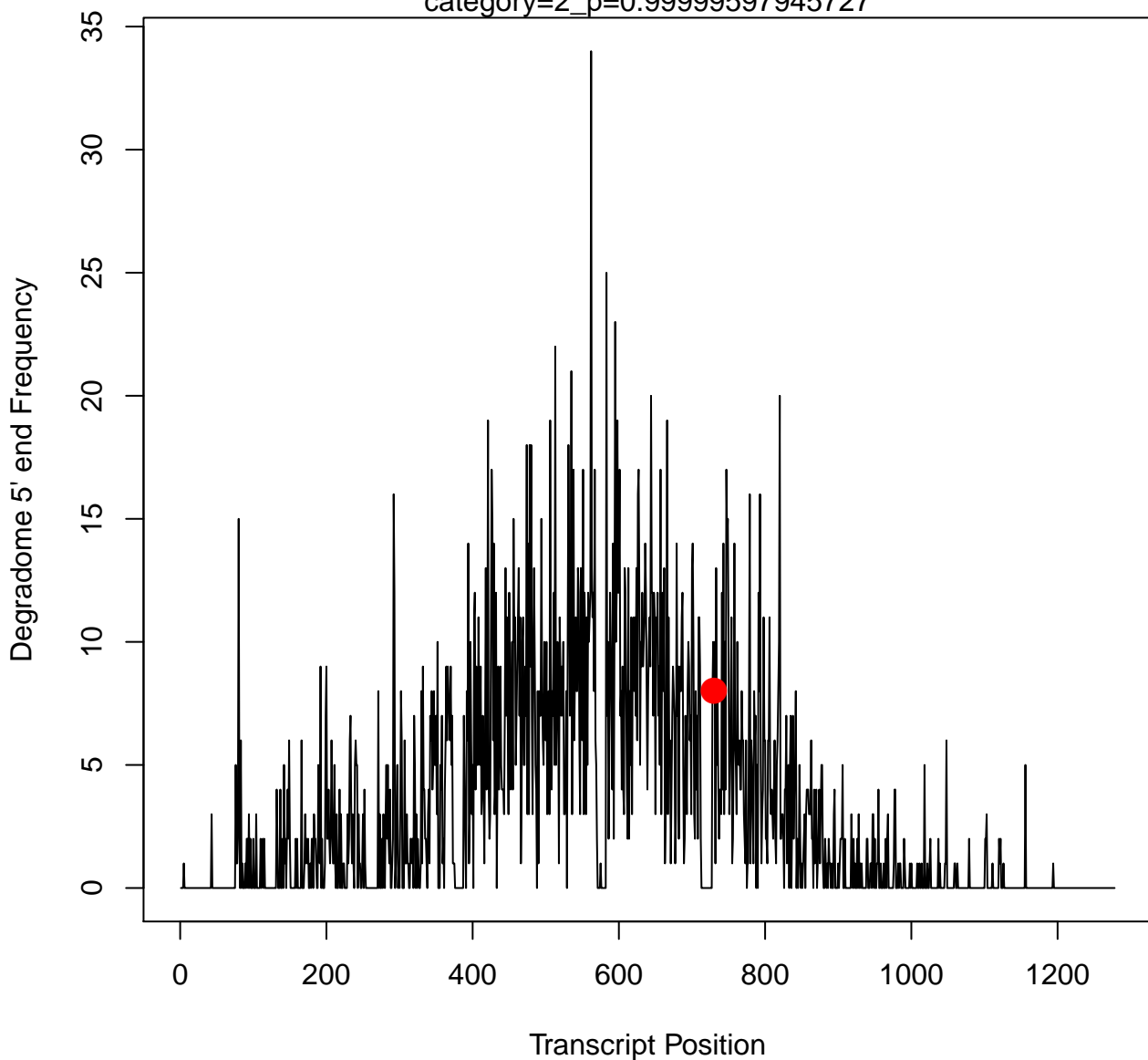

Supplement: Supplementary file 7 [file Data_Sheet_7.zip › Sit-miR394_Seita.4G207000.1_730_TPlot.pdf]

**T=Seita.5G231900.1\_Q=Sit-miR394\_S=2033**

category=2\_p=0.933142329401601

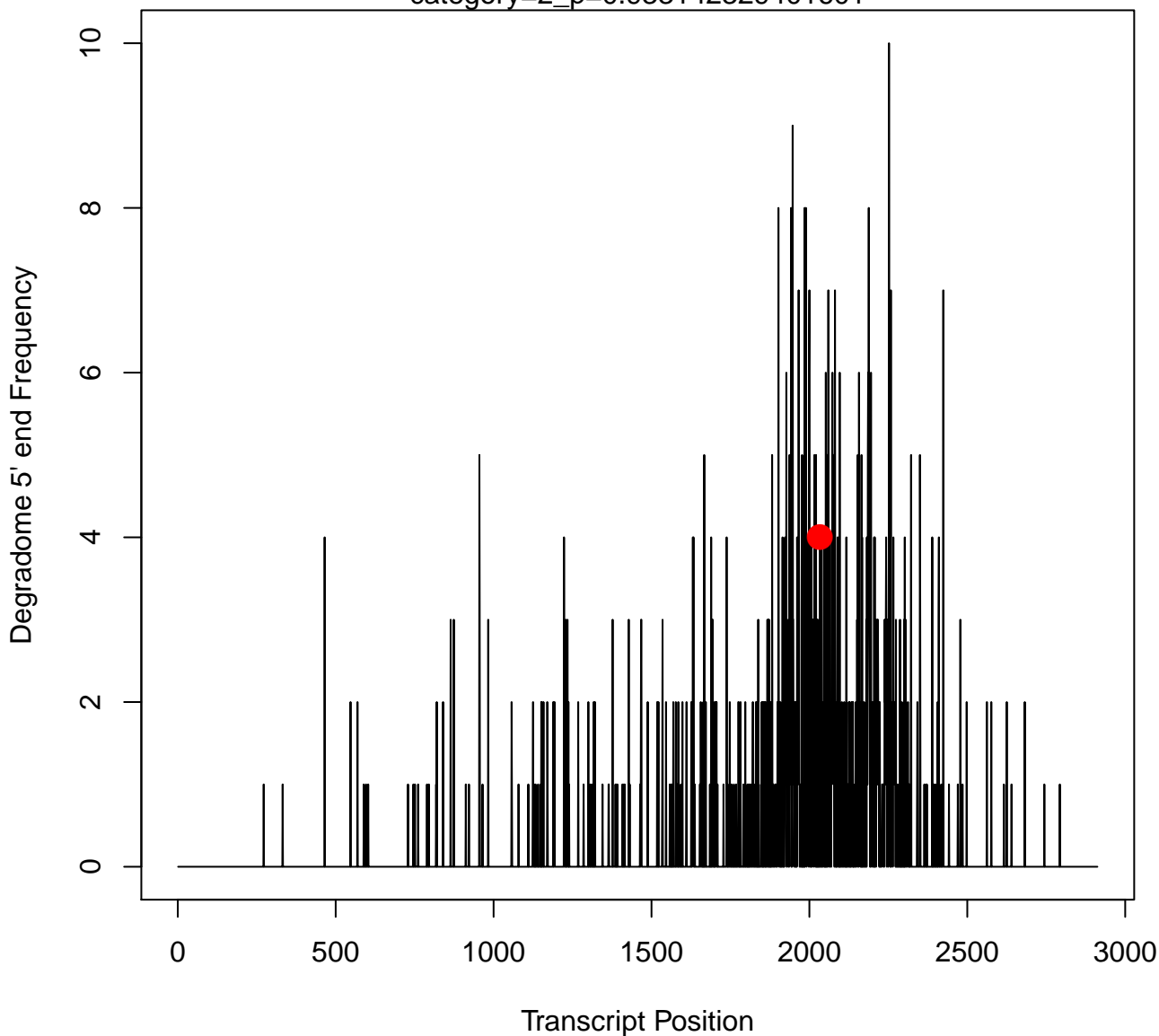

Supplement: Supplementary file 7 [file Data_Sheet_7.zip › Sit-miR394_Seita.5G231900.1_2033_TPlot.pdf]

**T=Seita.5G433500.1\_Q=Sit-miR394\_S=1382**

category=0\_p=0.000299243605074828

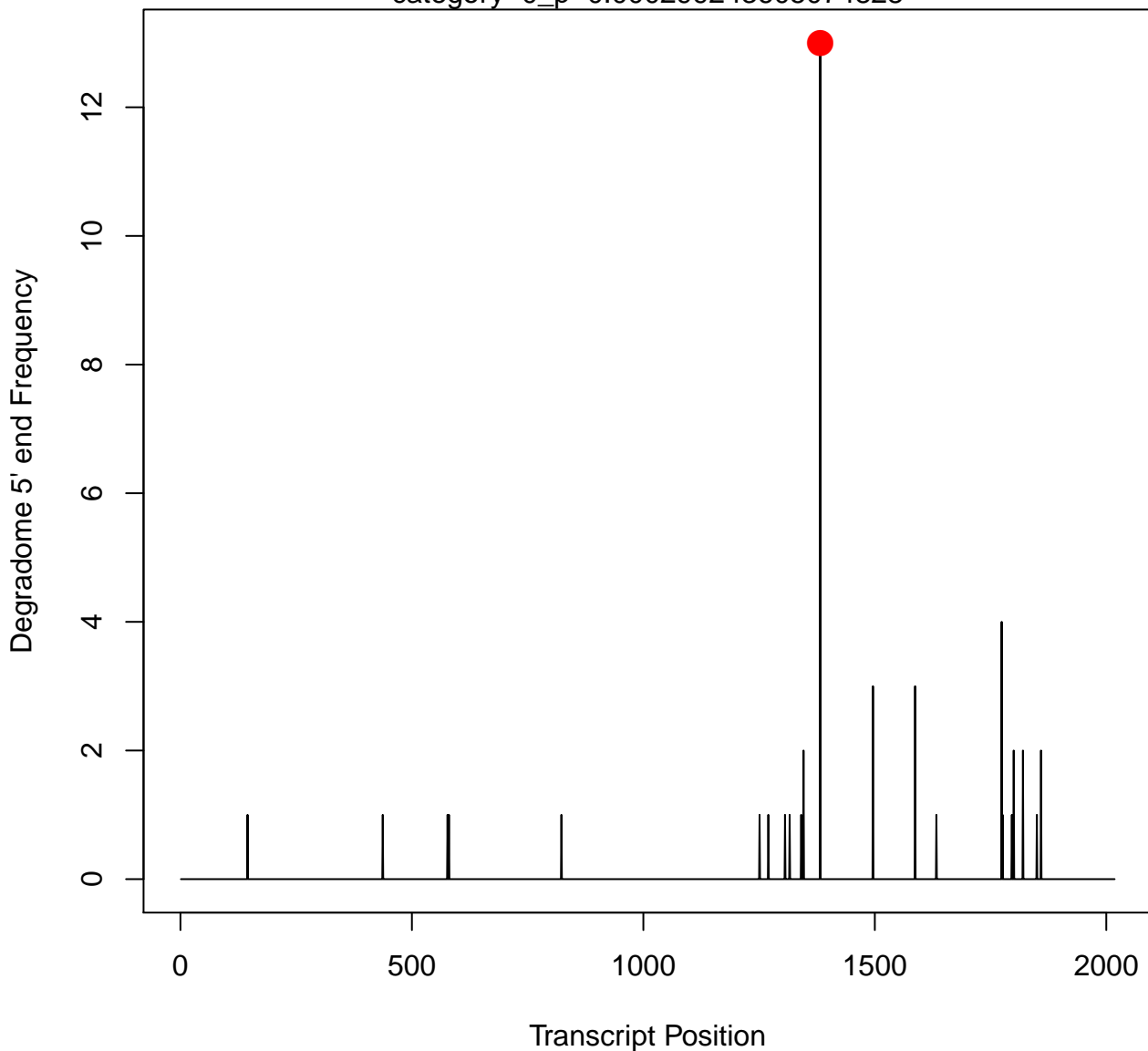

Supplement: Supplementary file 7 [file Data_Sheet_7.zip › Sit-miR394_Seita.5G433500.1_1382_TPlot.pdf]

**T=Seita.8G081400.1\_Q=Sit-miR394\_S=2312**

category=2\_p=0.946667547681127

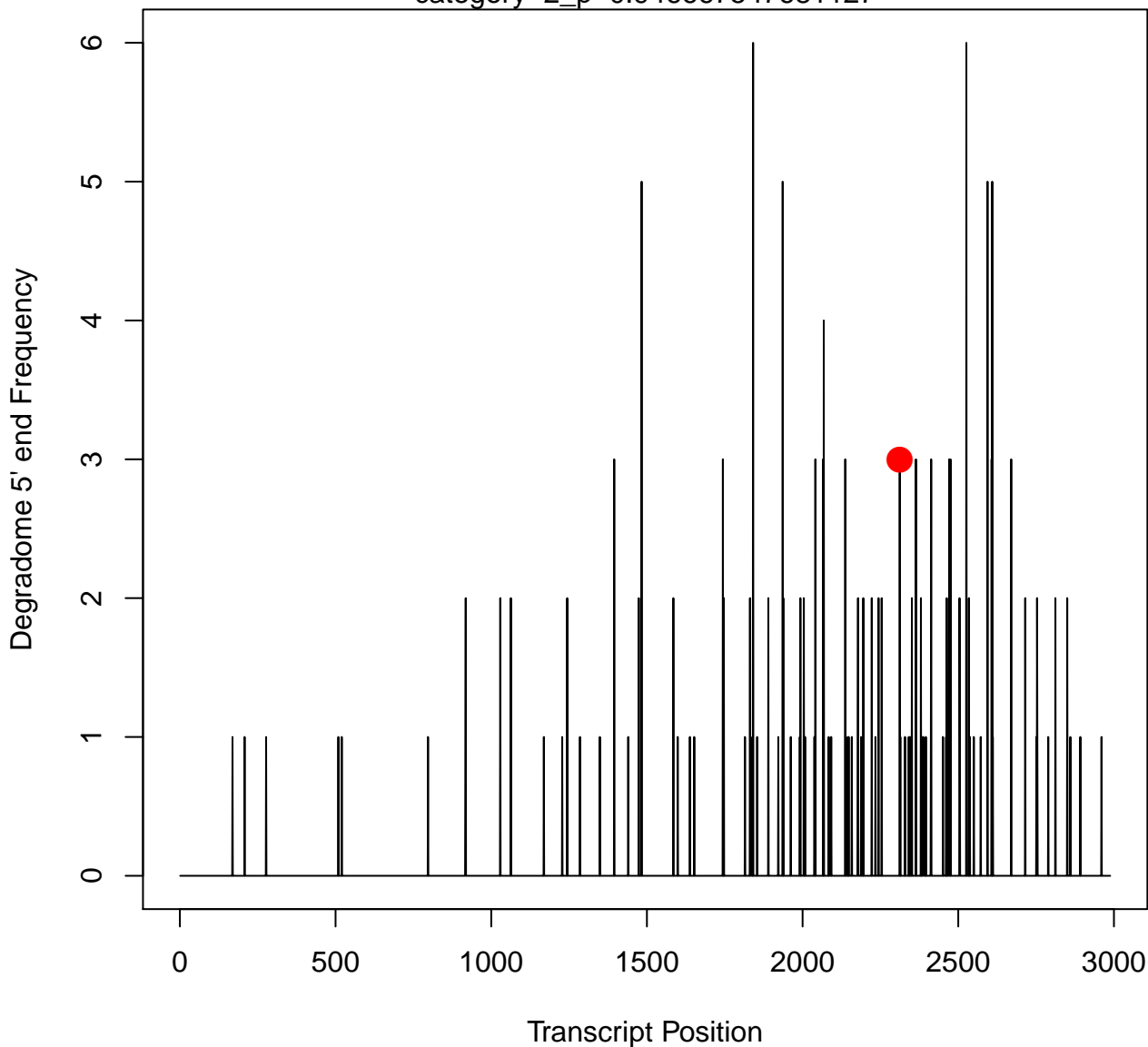

Supplement: Supplementary file 7 [file Data_Sheet_7.zip › Sit-miR394_Seita.8G081400.1_2312_TPlot.pdf]

**T=Seita.8G123100.1\_Q=Sit-miR394\_S=335**

category=2\_p=0.999988803796959

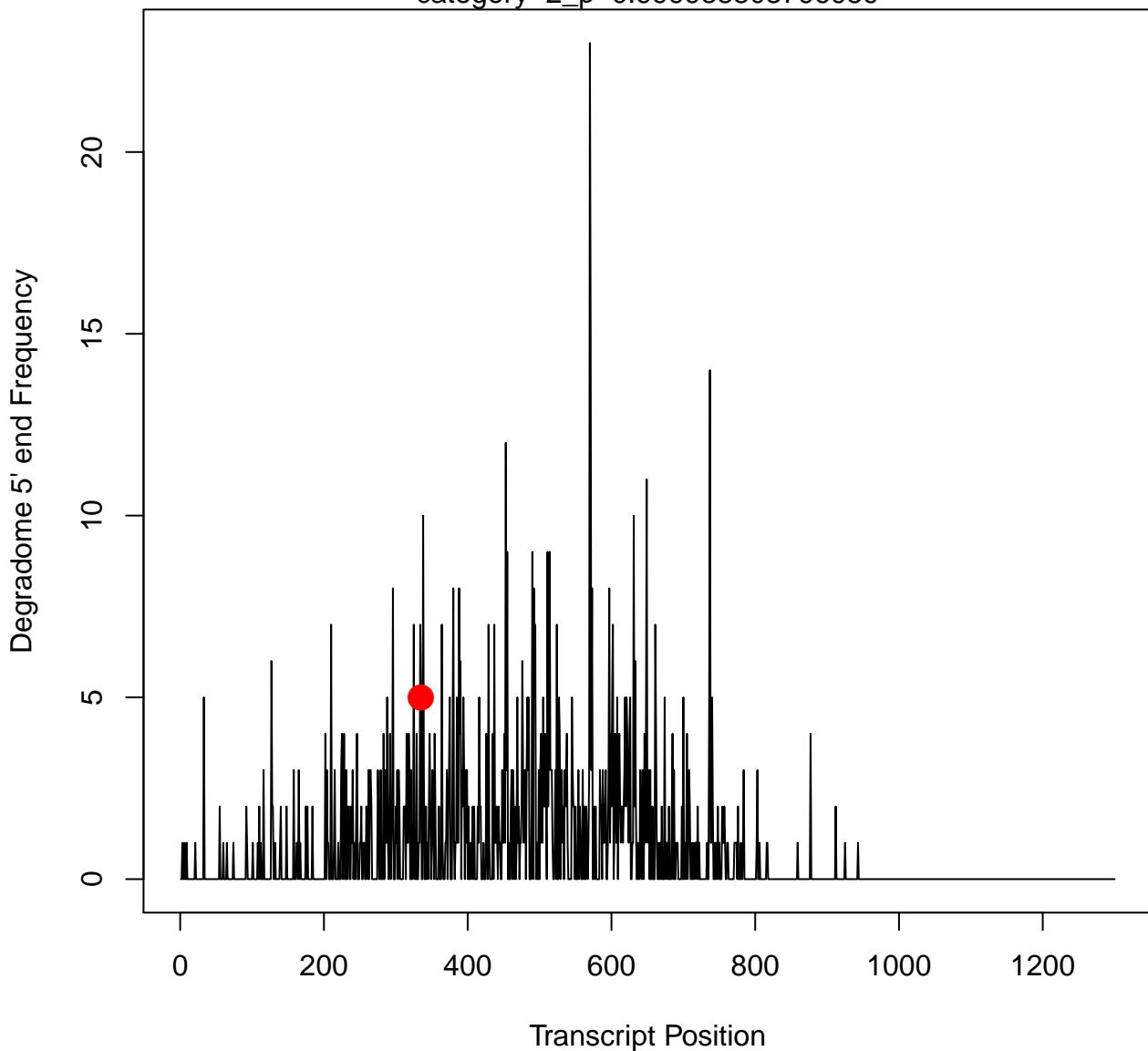

Supplement: Supplementary file 7 [file Data_Sheet_7.zip › Sit-miR394_Seita.8G123100.1_335_TPlot.pdf]

**T=Seita.8G175400.1\_Q=Sit-miR394\_S=844**

category=2\_p=0.994704313736769

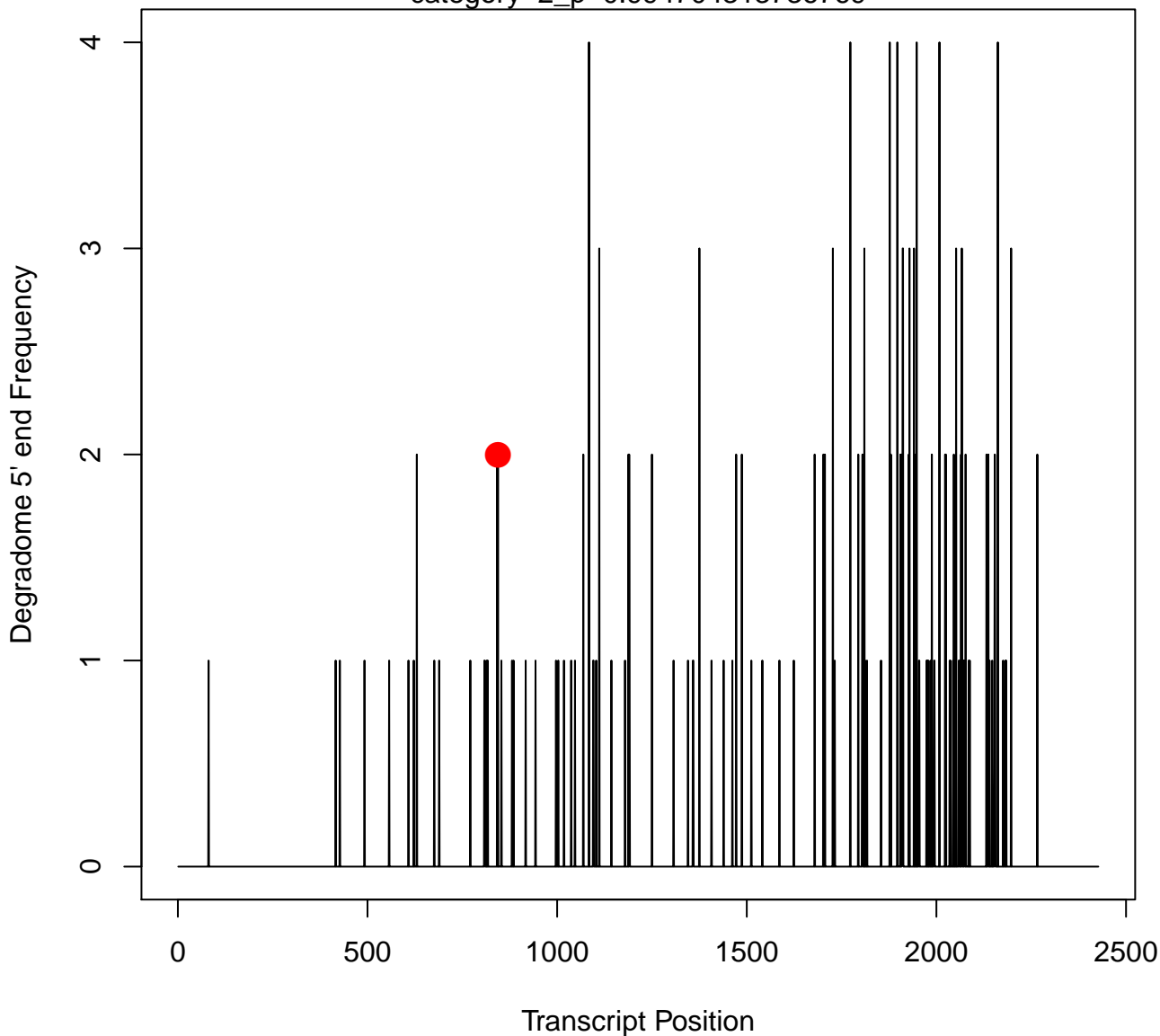

Supplement: Supplementary file 7 [file Data_Sheet_7.zip › Sit-miR394_Seita.8G175400.1_844_TPlot.pdf]

**T=Seita.8G202200.1\_Q=Sit-miR394\_S=223**

category=2\_p=0.999998029991189

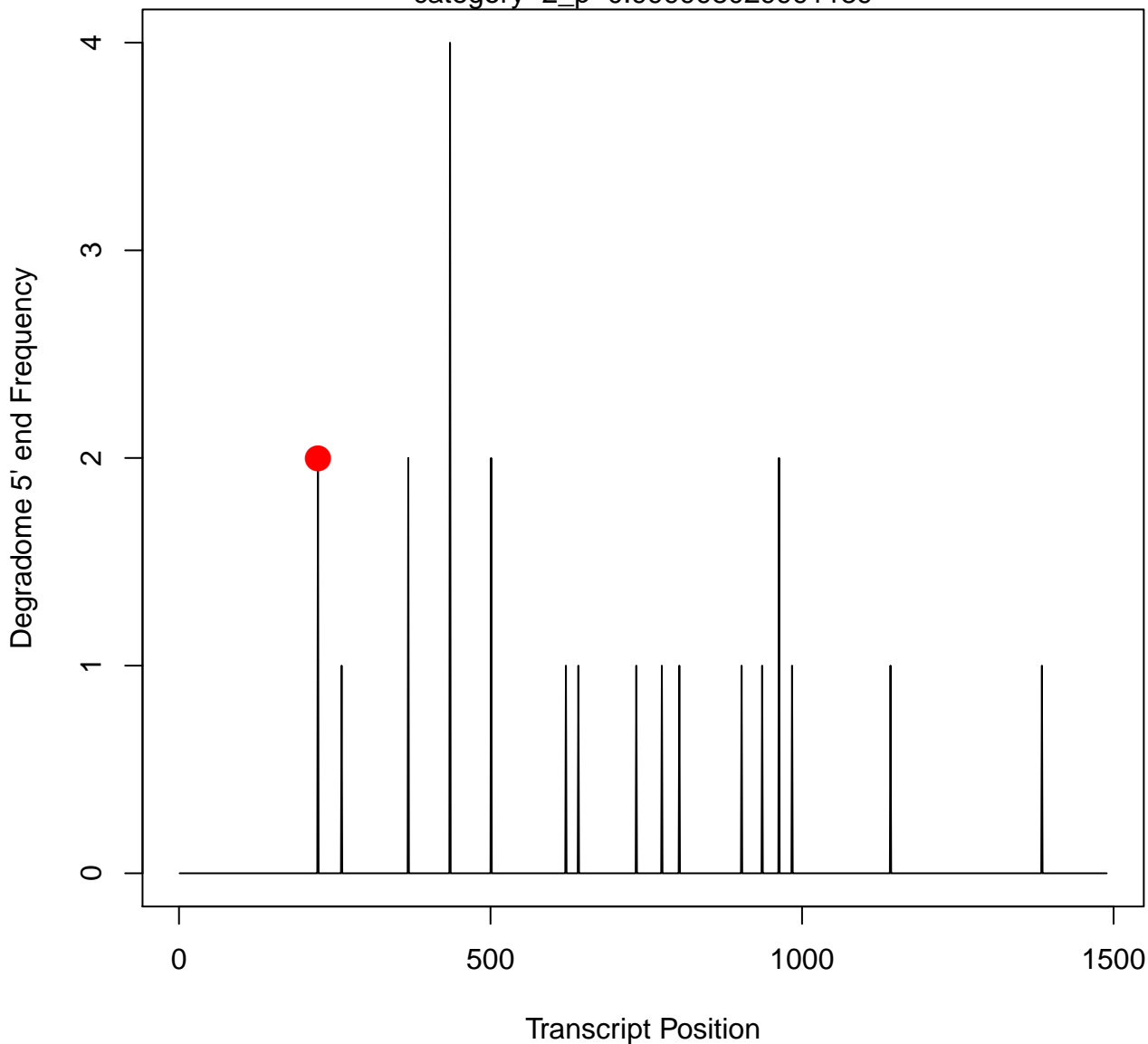

Supplement: Supplementary file 7 [file Data_Sheet_7.zip › Sit-miR394_Seita.8G202200.1_223_TPlot.pdf]

**T=Seita.9G499500.1\_Q=Sit-miR394\_S=2236**

category=2\_p=0.999970741676515

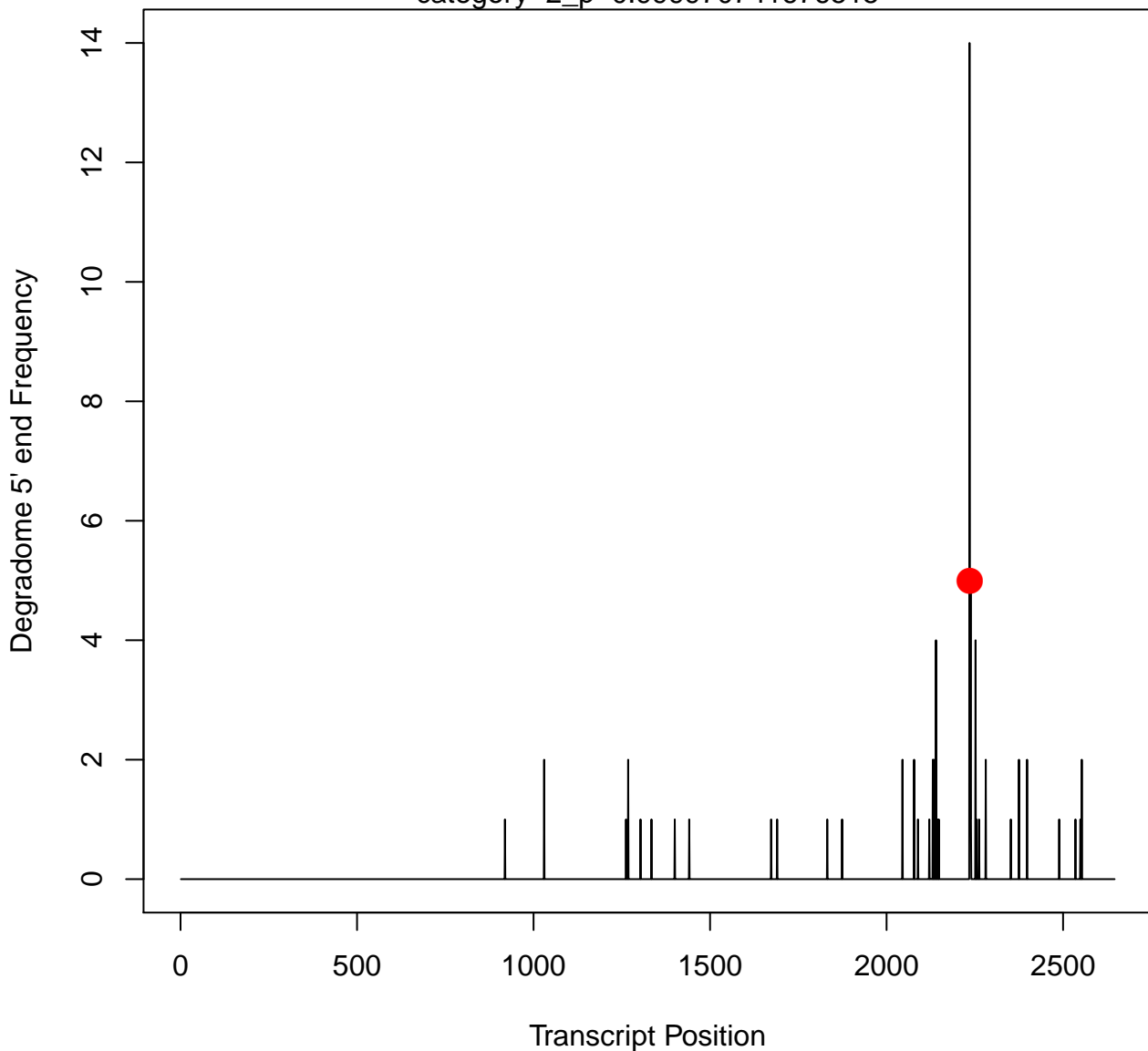

Supplement: Supplementary file 7 [file Data_Sheet_7.zip › Sit-miR394_Seita.9G499500.1_2236_TPlot.pdf]

**T=Seita.9G537700.1\_Q=Sit-miR394\_S=1887**

category=2\_p=0.999995100244506

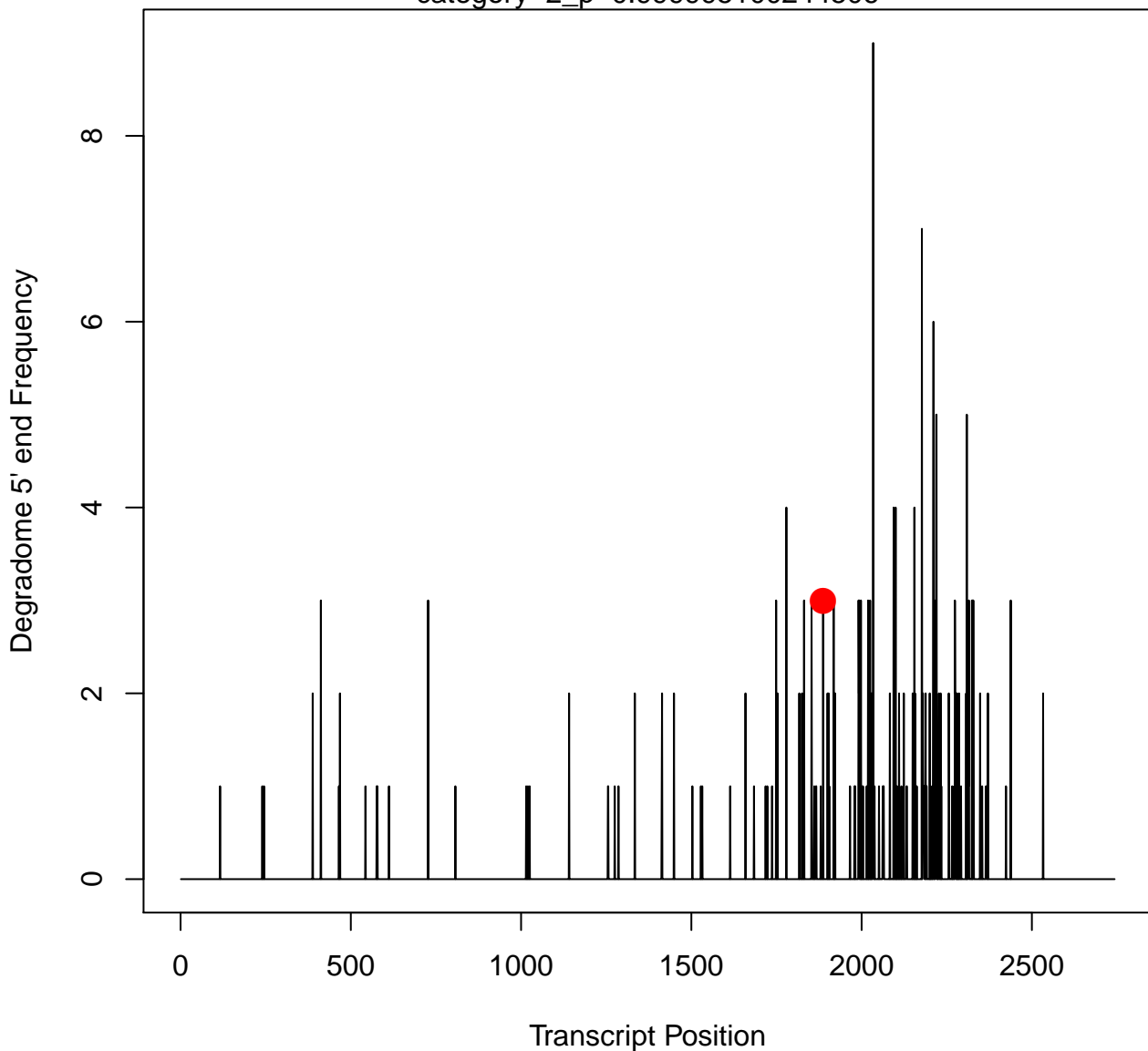

Supplement: Supplementary file 7 [file Data_Sheet_7.zip › Sit-miR394_Seita.9G537700.1_1887_TPlot.pdf]

**T=Seita.1G217200.1\_Q=Sit-miR395e\_S=886**

category=2\_p=0.721526246894706

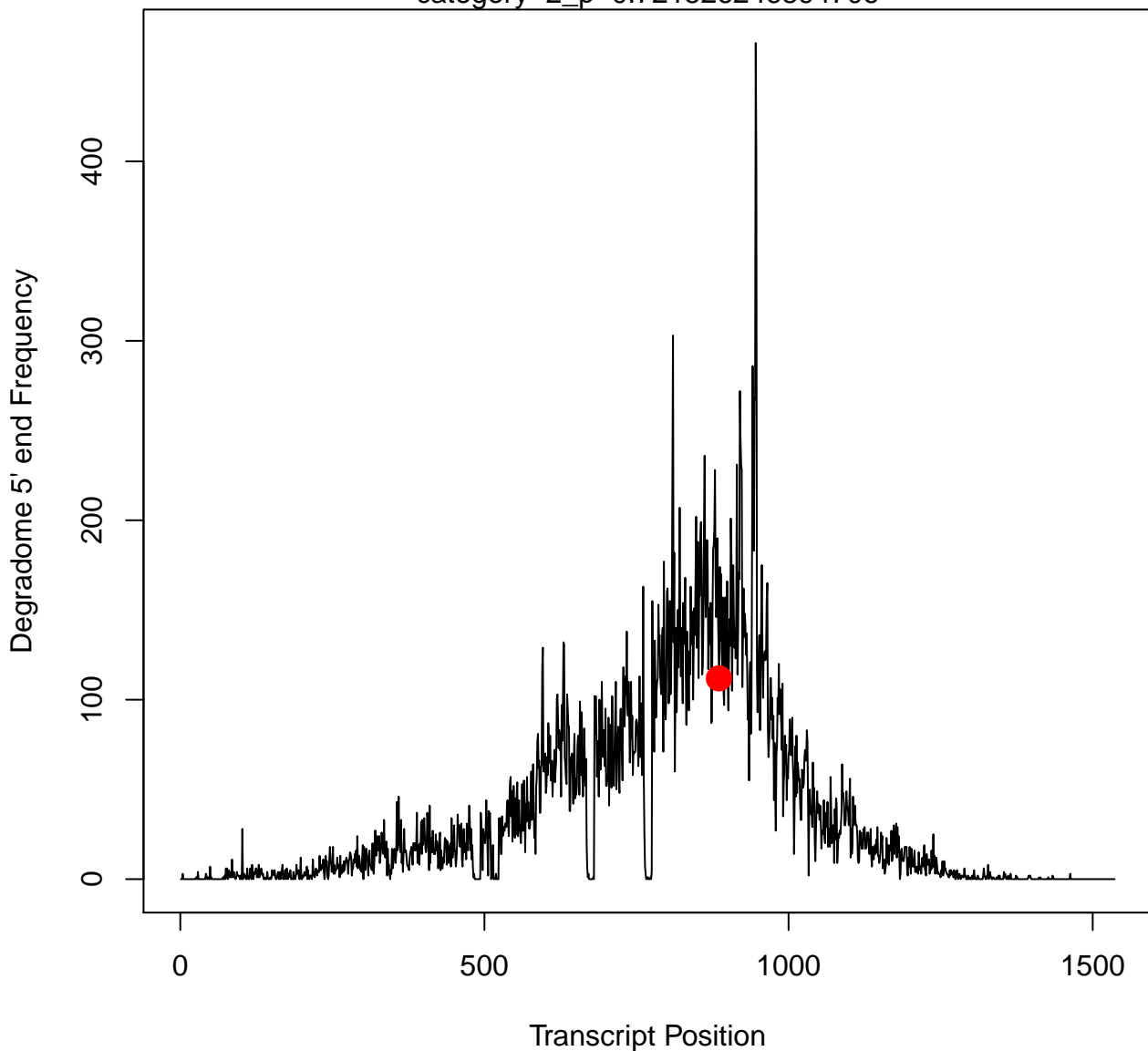

Supplement: Supplementary file 7 [file Data_Sheet_7.zip › Sit-miR395e_Seita.1G217200.1_886_TPlot.pdf]

**T=Seita.9G397100.1\_Q=Sit-miR395e\_S=197**

category=2\_p=0.846143769291111

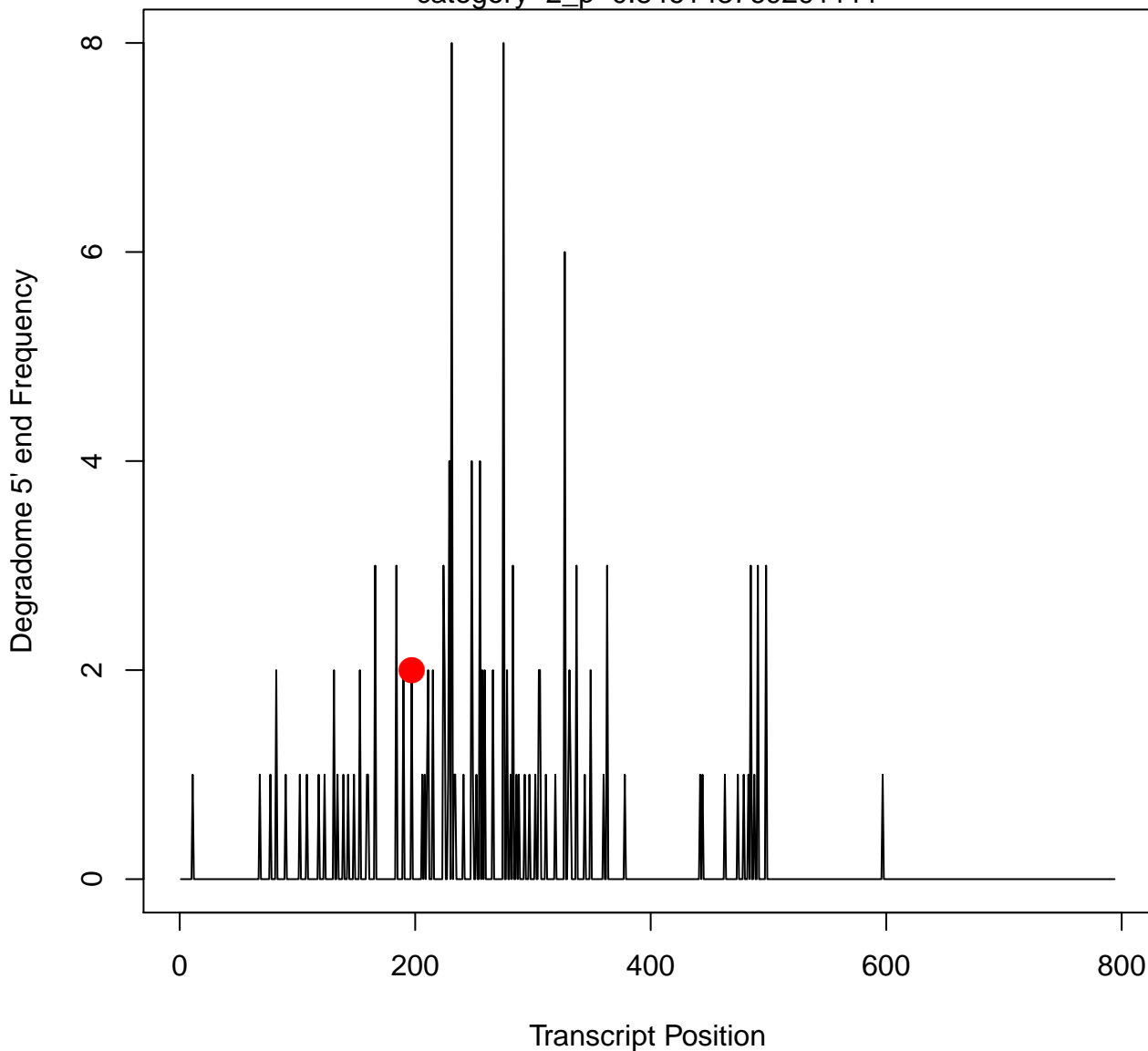

Supplement: Supplementary file 7 [file Data_Sheet_7.zip › Sit-miR395e_Seita.9G397100.1_197_TPlot.pdf]

**T=Seita.4G158600.1\_Q=Sit-miR395g\_S=553**

category=2\_p=0.768244781536202

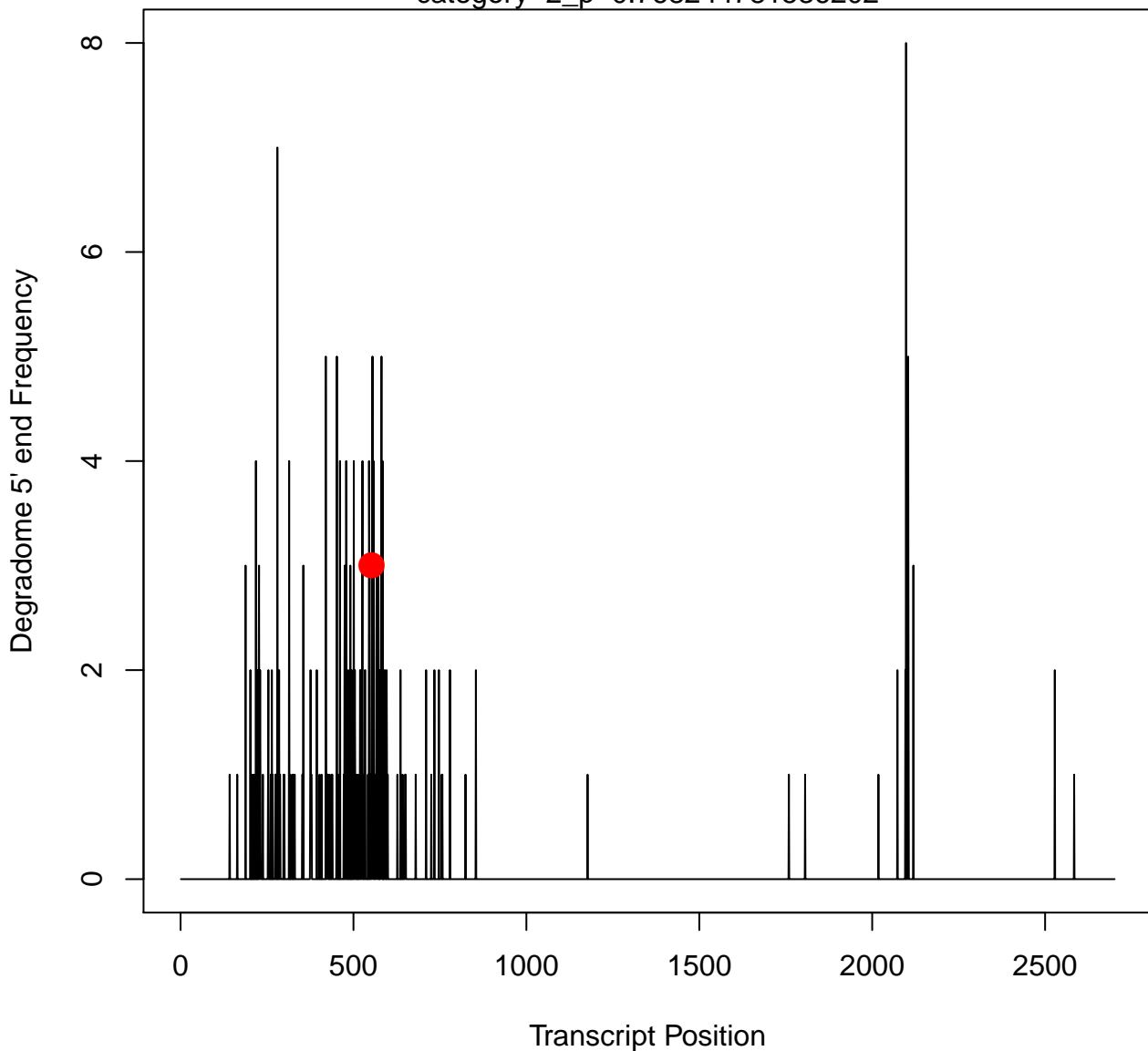

Supplement: Supplementary file 7 [file Data_Sheet_7.zip › Sit-miR395g_Seita.4G158600.1_553_TPlot.pdf]

**T=Seita.6G213700.1\_Q=Sit-miR395j\_S=2545**

category=2\_p=0.261929376439474

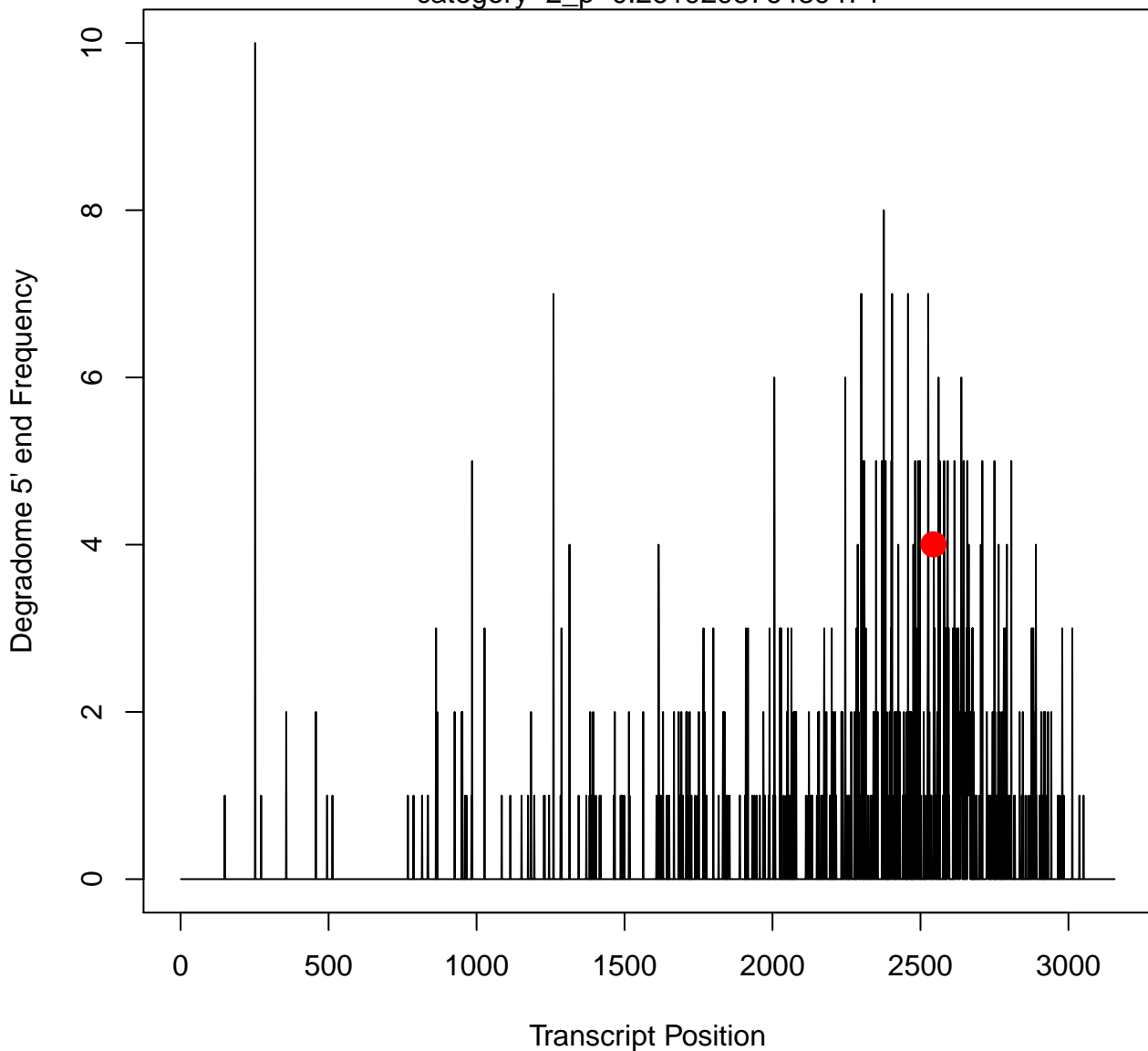

Supplement: Supplementary file 7 [file Data_Sheet_7.zip › Sit-miR395j_Seita.6G213700.1_2545_TPlot.pdf]

**T=Seita.9G096900.1\_Q=Sit-miR395j\_S=1005**

category=1\_p=0.000245746077032871

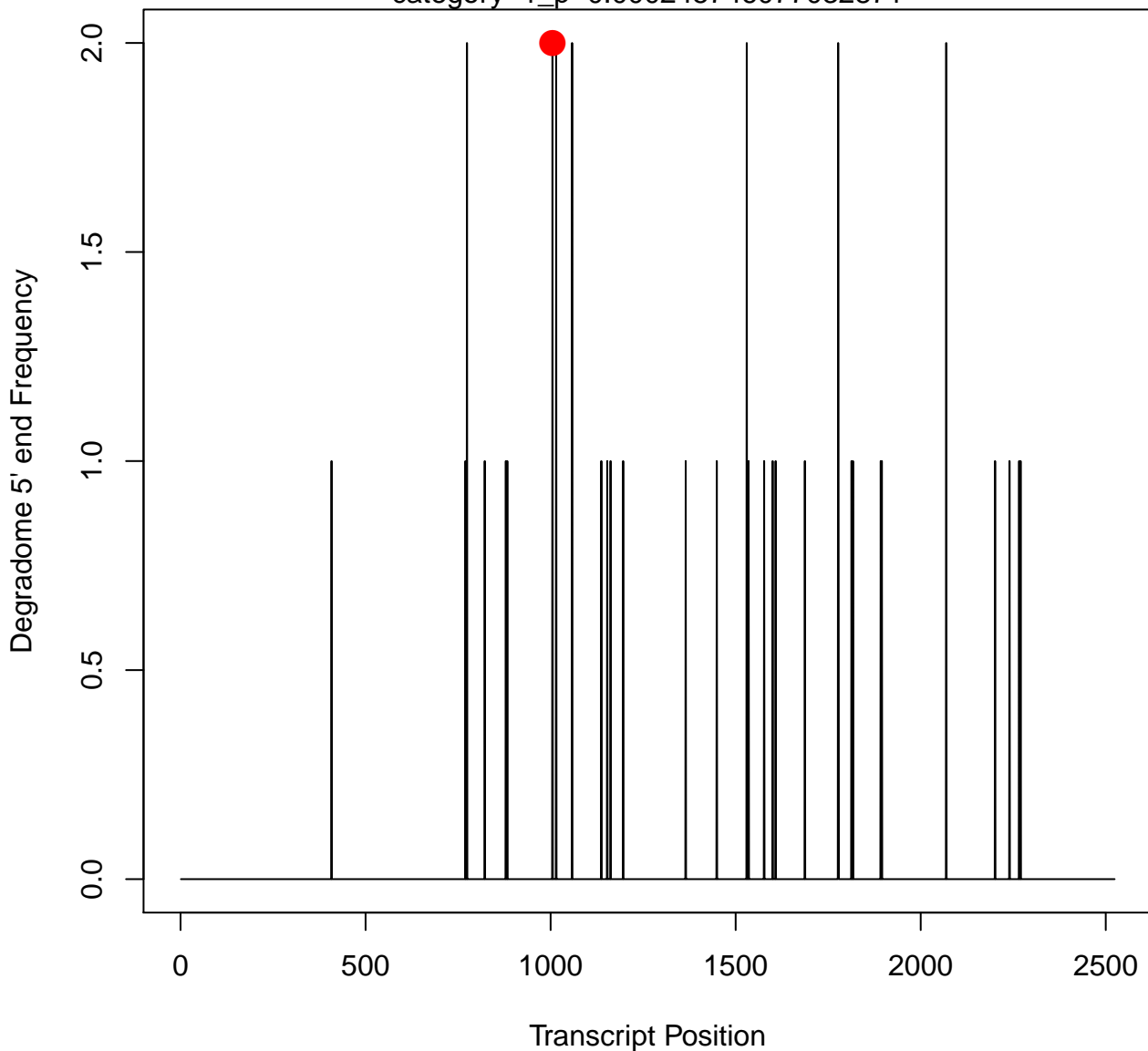

Supplement: Supplementary file 7 [file Data_Sheet_7.zip › Sit-miR395j_Seita.9G096900.1_1005_TPlot.pdf]

**T=Seita.2G112500.1\_Q=Sit-miR396a\_S=1522**

category=2\_p=0.988068813768004

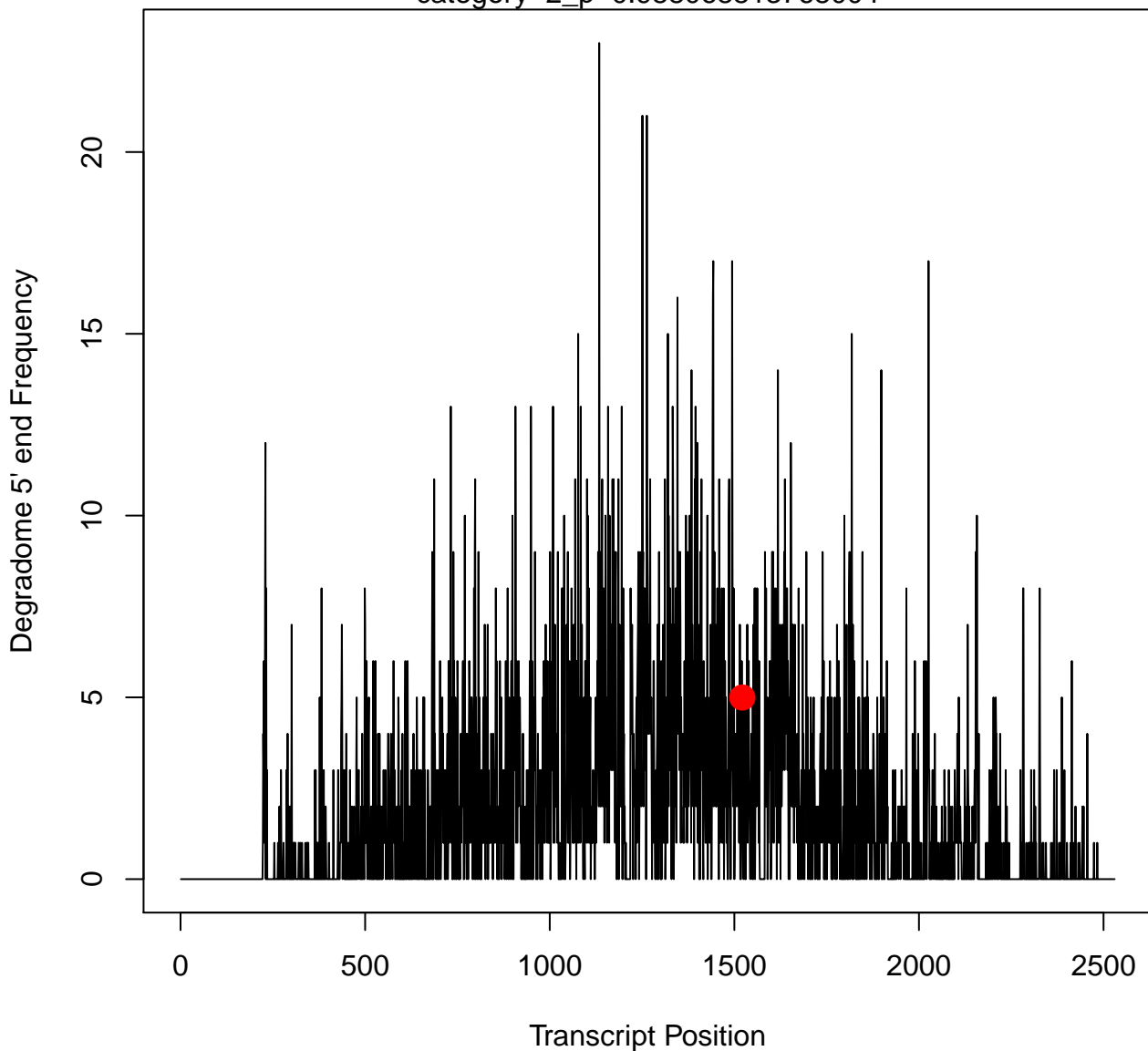

Supplement: Supplementary file 7 [file Data_Sheet_7.zip › Sit-miR396a_Seita.2G112500.1_1522_TPlot.pdf]

**T=Seita.3G187400.1\_Q=Sit-miR396a\_S=1138**

category=2\_p=0.933142329401601

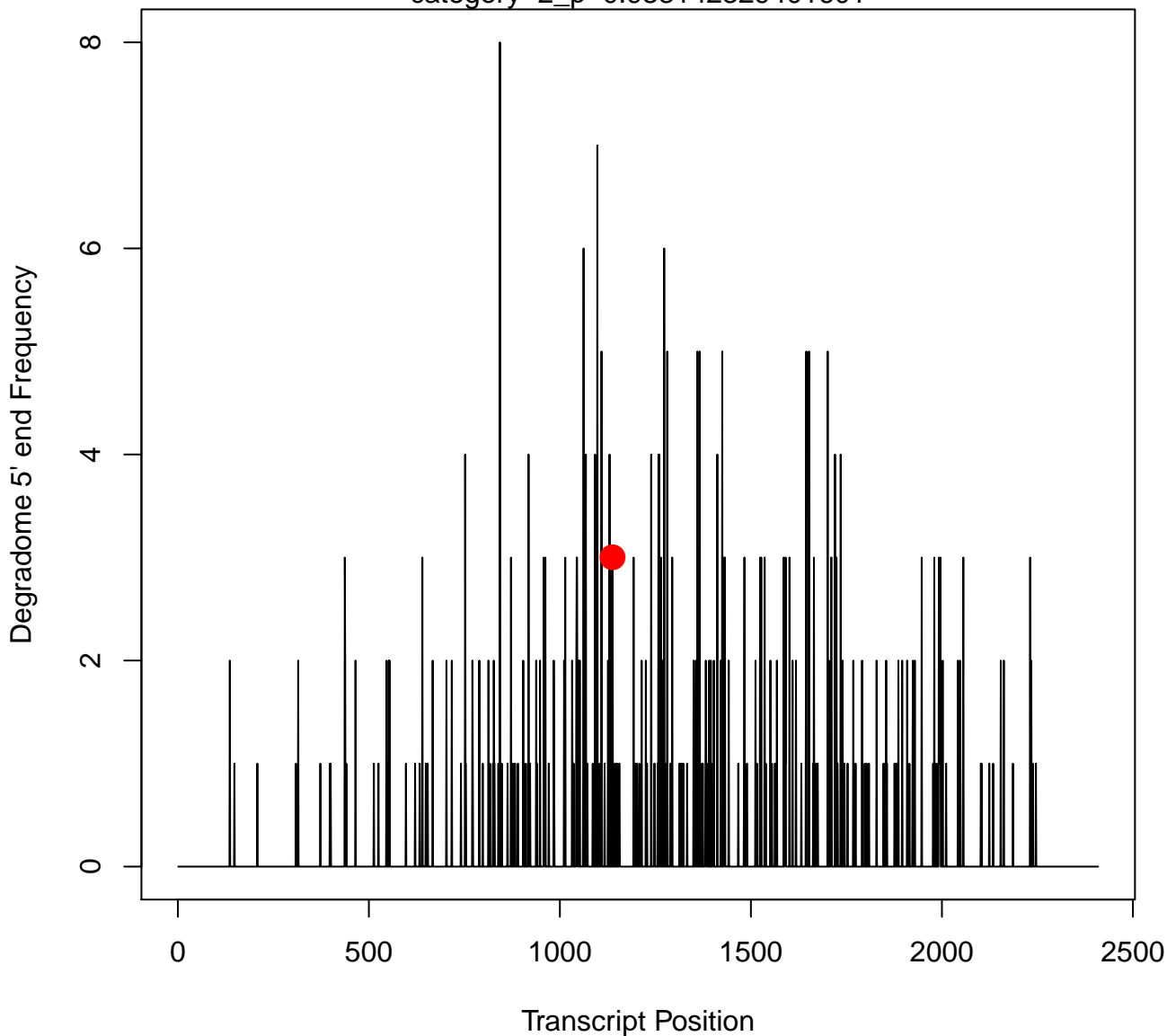

Supplement: Supplementary file 7 [file Data_Sheet_7.zip › Sit-miR396a_Seita.3G187400.1_1138_TPlot.pdf]

**T=Seita.4G012400.1\_Q=Sit-miR396a\_S=809**

category=0\_p=0.00209282569167468

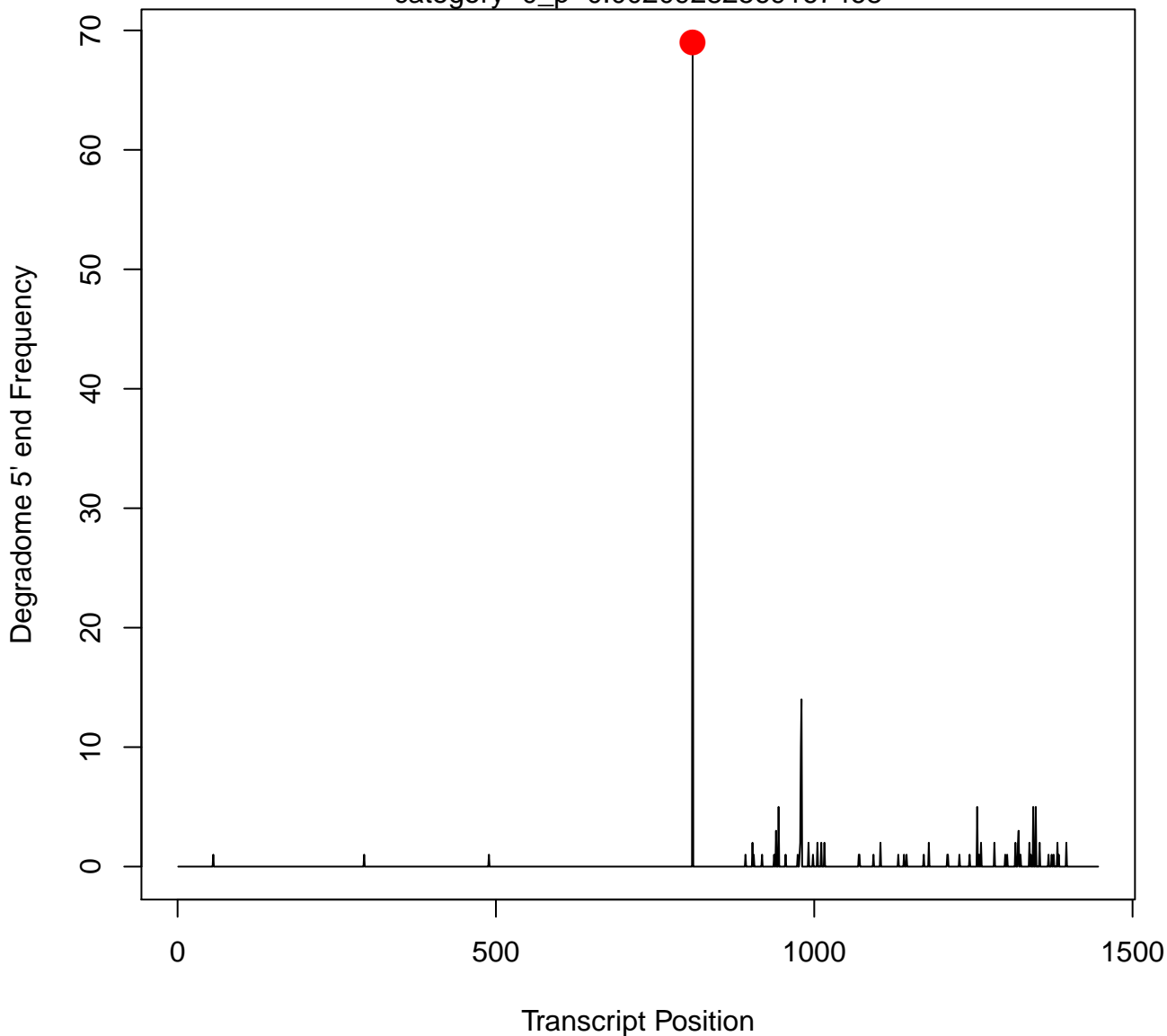

Supplement: Supplementary file 7 [file Data_Sheet_7.zip › Sit-miR396a_Seita.4G012400.1_809_TPlot.pdf]

**T=Seita.4G086400.1\_Q=Sit-miR396a\_S=765**

category=0\_p=0.00119643724706542

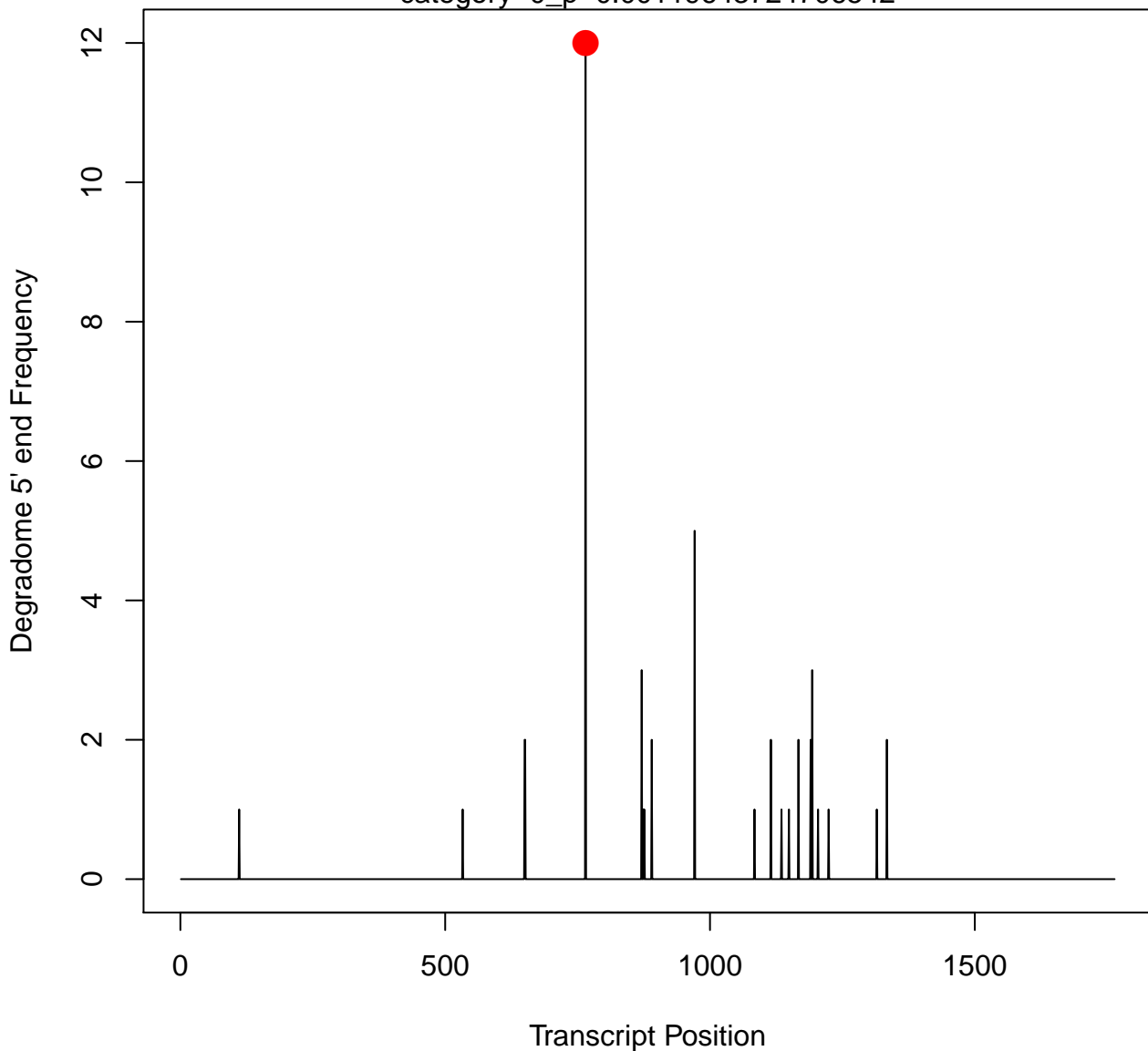

Supplement: Supplementary file 7 [file Data_Sheet_7.zip › Sit-miR396a_Seita.4G086400.1_765_TPlot.pdf]

**T=Seita.4G280300.1\_Q=Sit-miR396a\_S=459**

category=2\_p=0.891155104390331

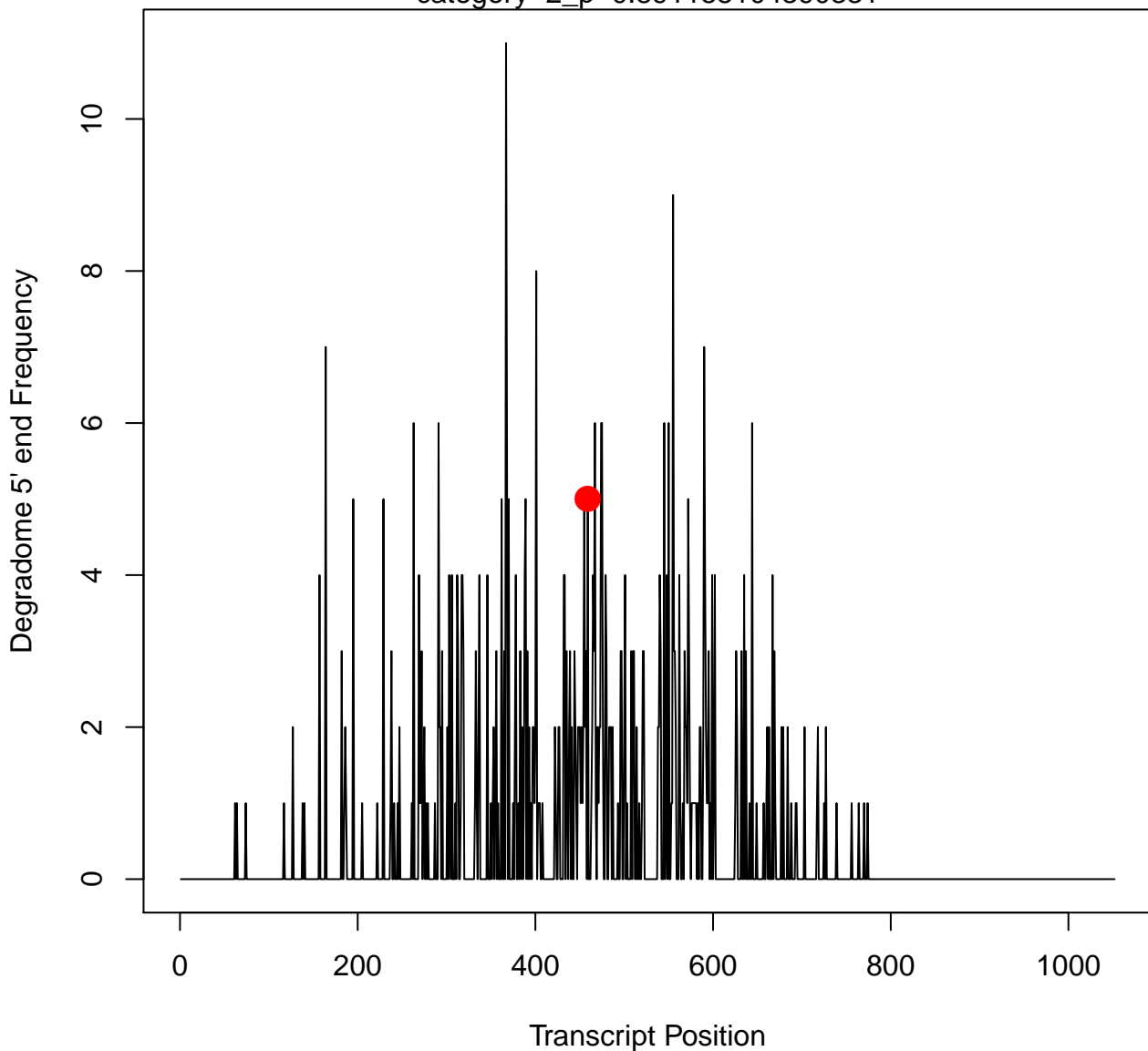

Supplement: Supplementary file 7 [file Data_Sheet_7.zip › Sit-miR396a_Seita.4G280300.1_459_TPlot.pdf]

**T=Seita.5G027200.1\_Q=Sit-miR396a\_S=119**

category=2\_p=0.562373469254276

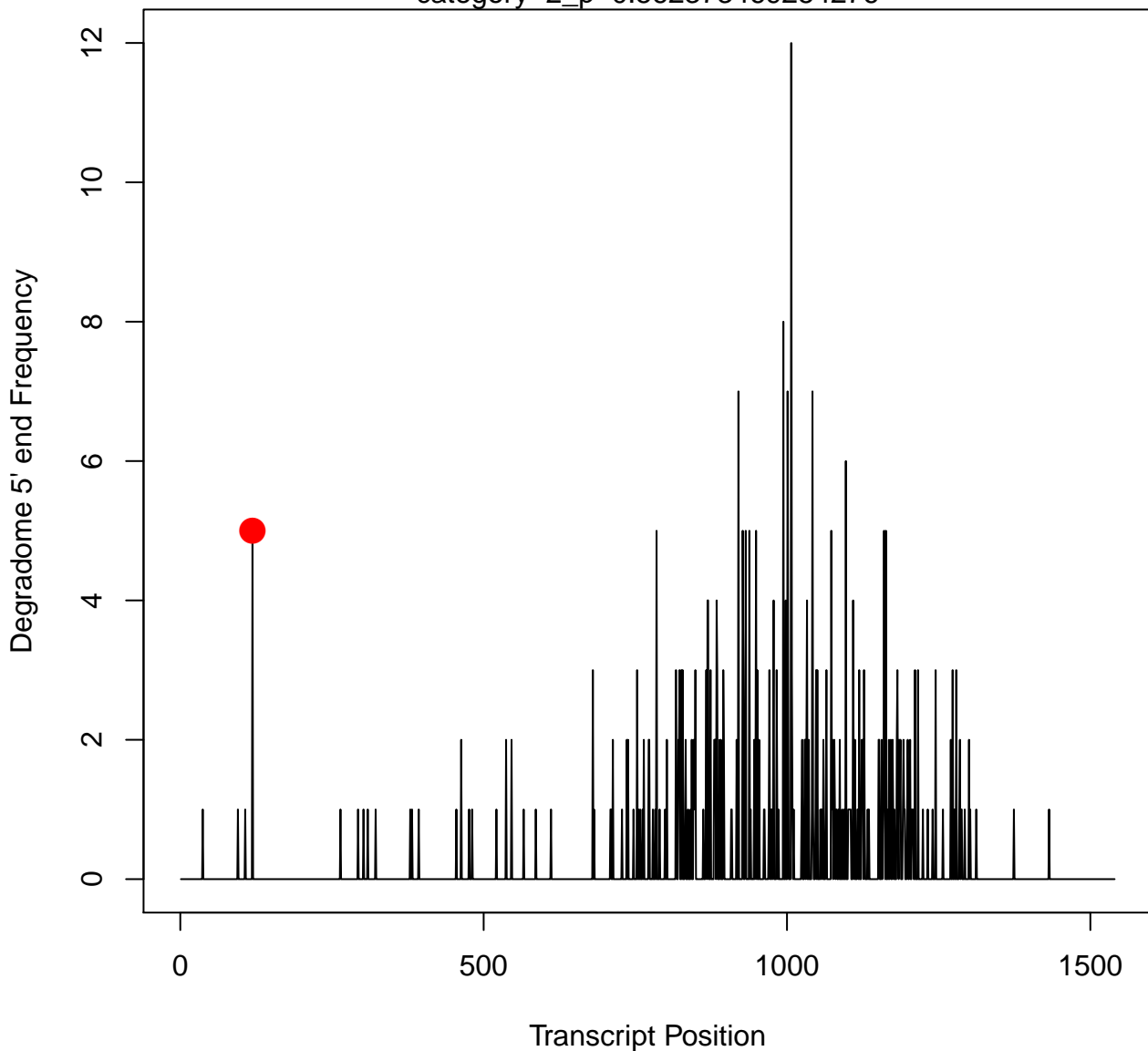

Supplement: Supplementary file 7 [file Data_Sheet_7.zip › Sit-miR396a_Seita.5G027200.1_119_TPlot.pdf]

**T=Seita.6G141500.1\_Q=Sit-miR396a\_S=1360**

category=2\_p=0.994153888271367

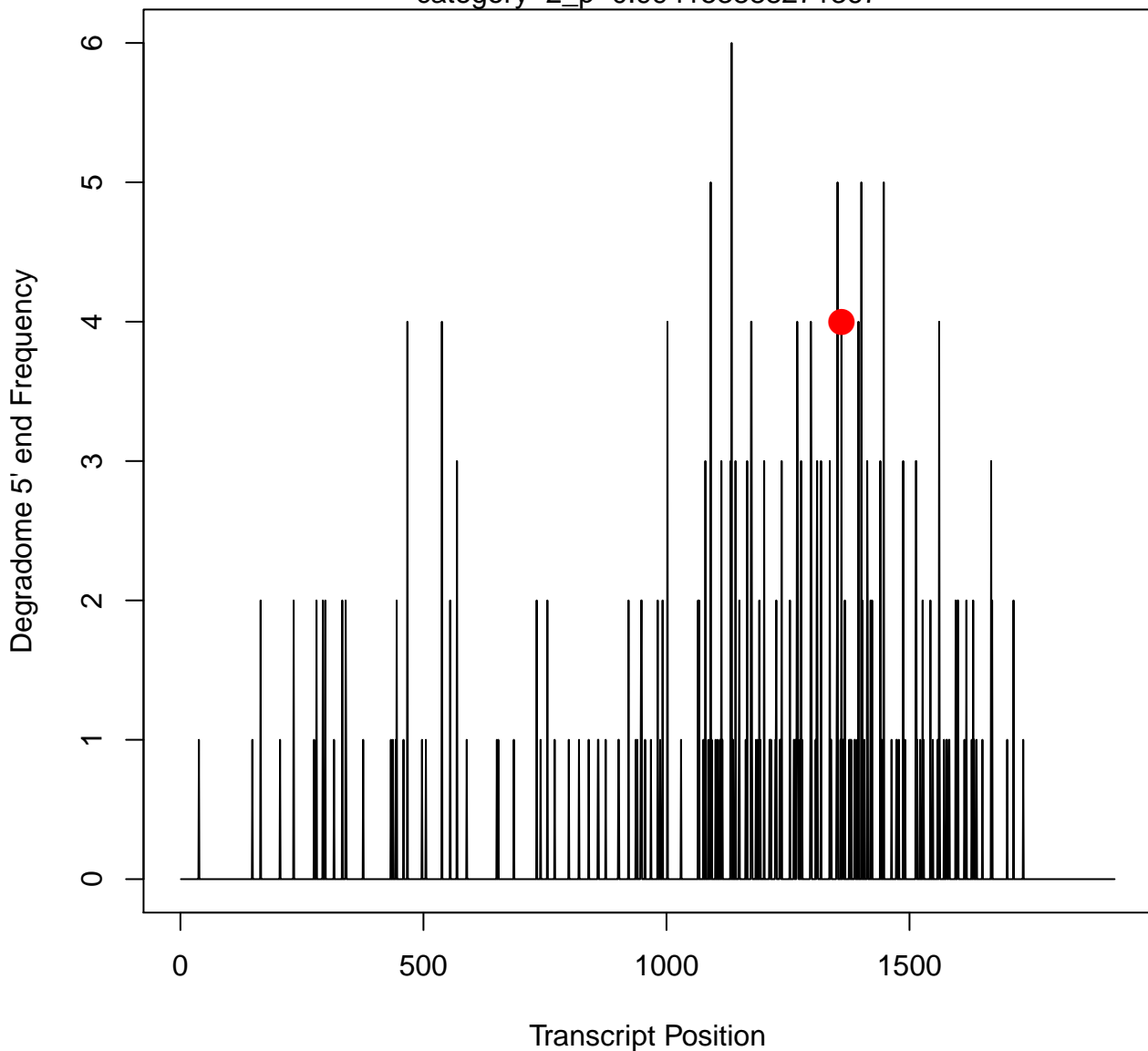

Supplement: Supplementary file 7 [file Data_Sheet_7.zip › Sit-miR396a_Seita.6G141500.1_1360_TPlot.pdf]

**T=Seita.7G212200.1\_Q=Sit-miR396a\_S=1746**

category=2\_p=0.29753370954645

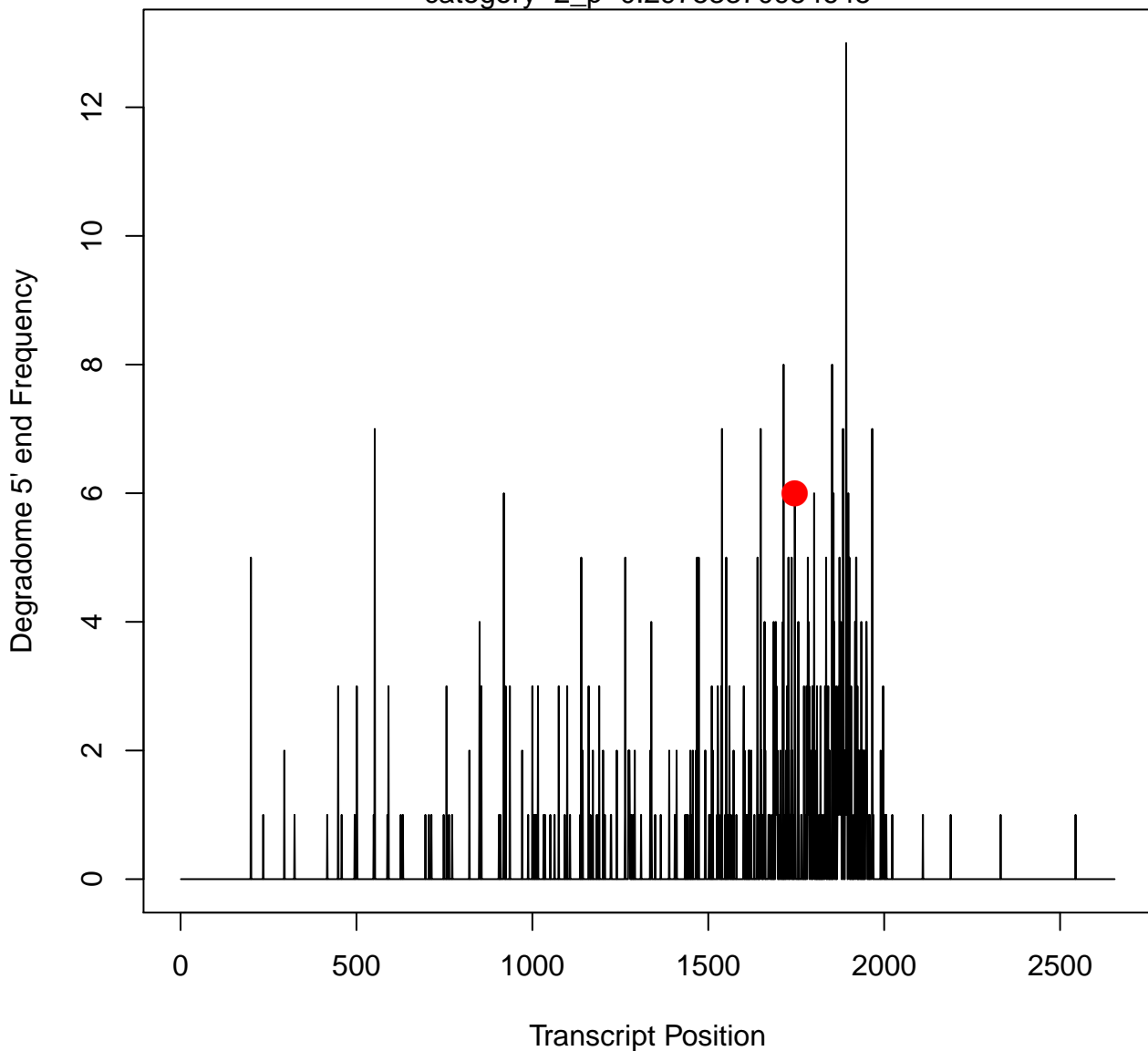

Supplement: Supplementary file 7 [file Data_Sheet_7.zip › Sit-miR396a_Seita.7G212200.1_1746_TPlot.pdf]

**T=Seita.7G224500.1\_Q=Sit-miR396a\_S=868**

category=2\_p=0.0140270063498924

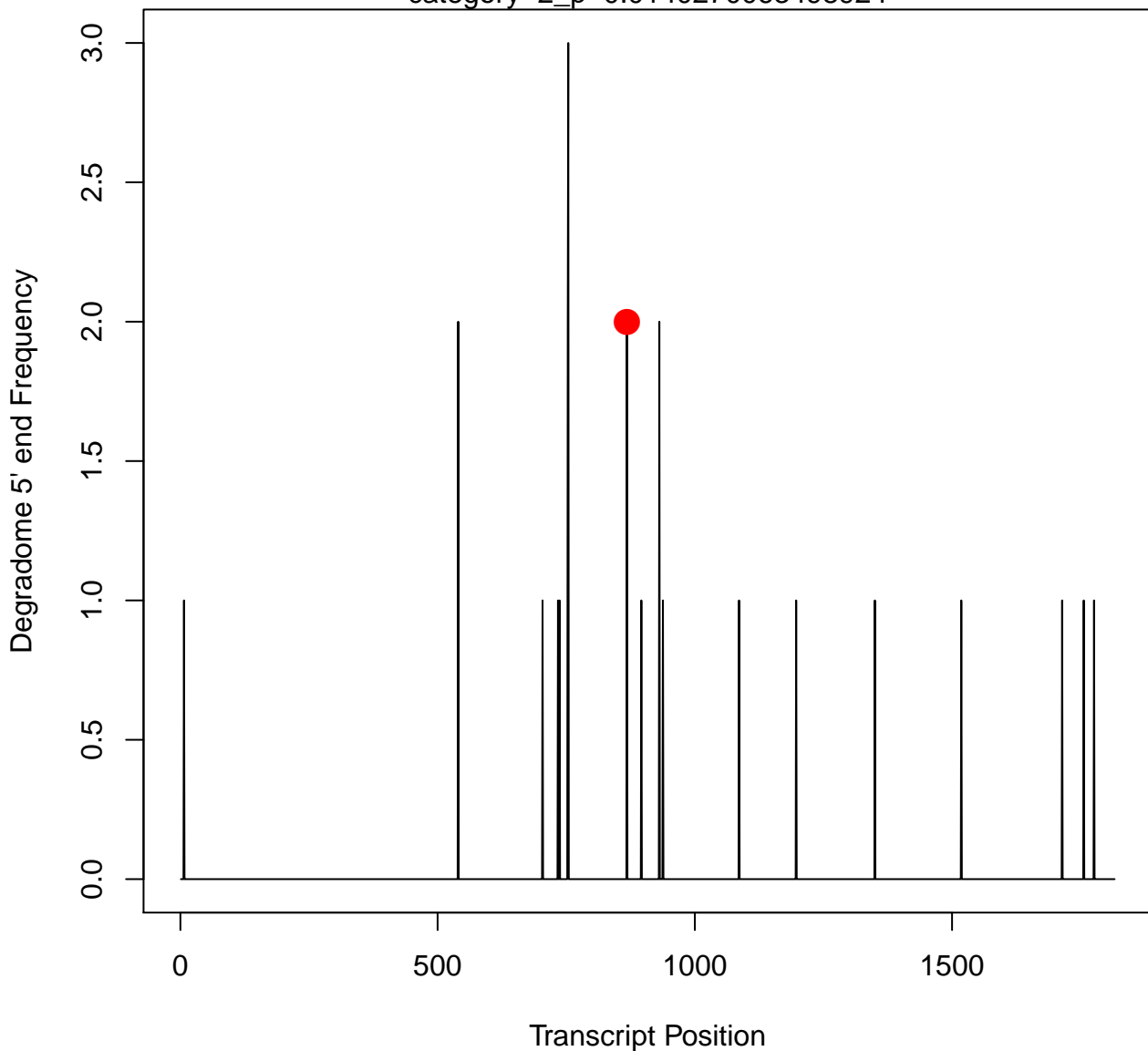

Supplement: Supplementary file 7 [file Data_Sheet_7.zip › Sit-miR396a_Seita.7G224500.1_868_TPlot.pdf]

**T=Seita.9G033500.1\_Q=Sit-miR396a\_S=1068**

category=2\_p=0.643428665728667

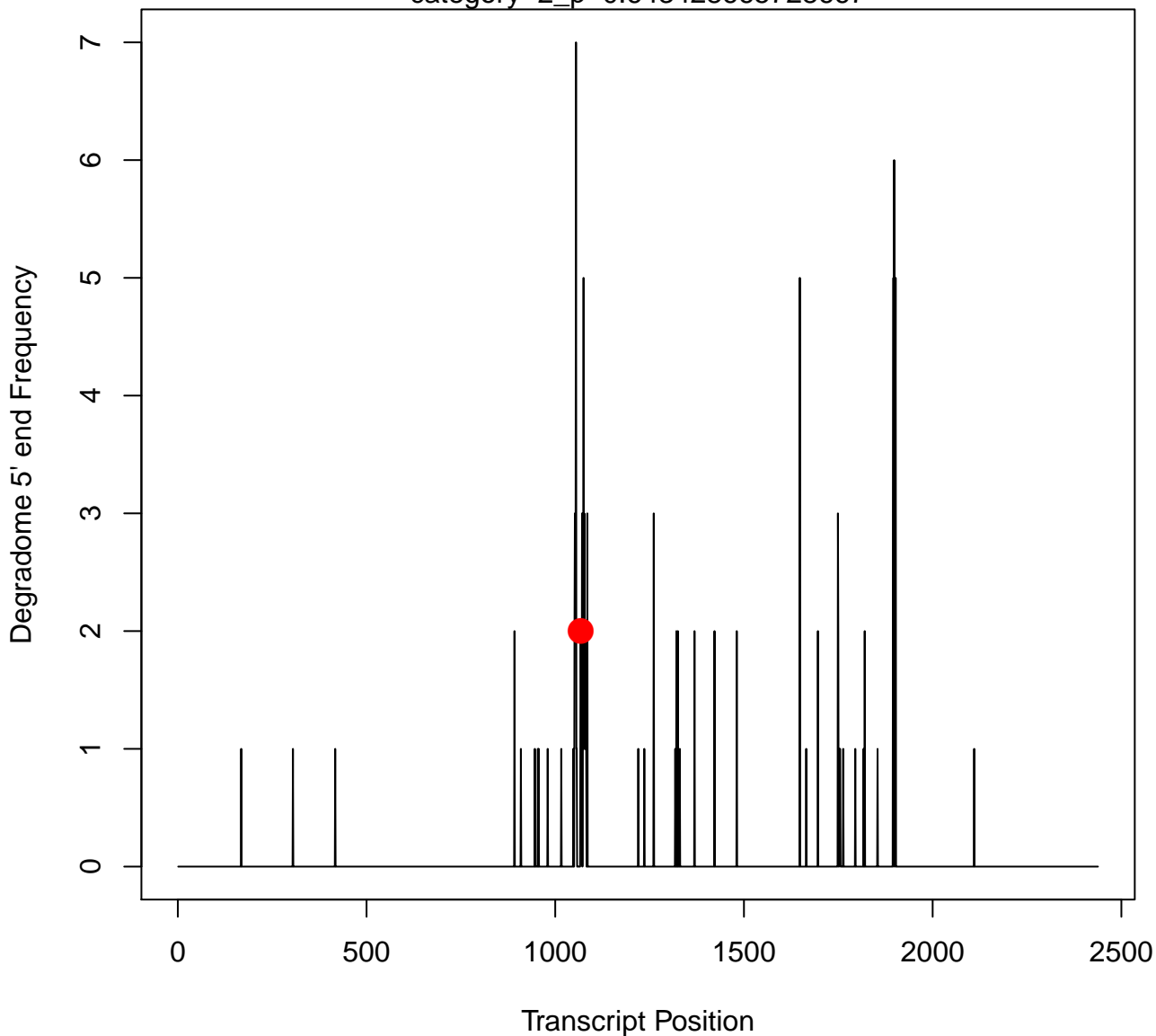

Supplement: Supplementary file 7 [file Data_Sheet_7.zip › Sit-miR396a_Seita.9G033500.1_1068_TPlot.pdf]

**T=Seita.9G127700.1\_Q=Sit-miR396a\_S=2748**

category=2\_p=0.131739197392104

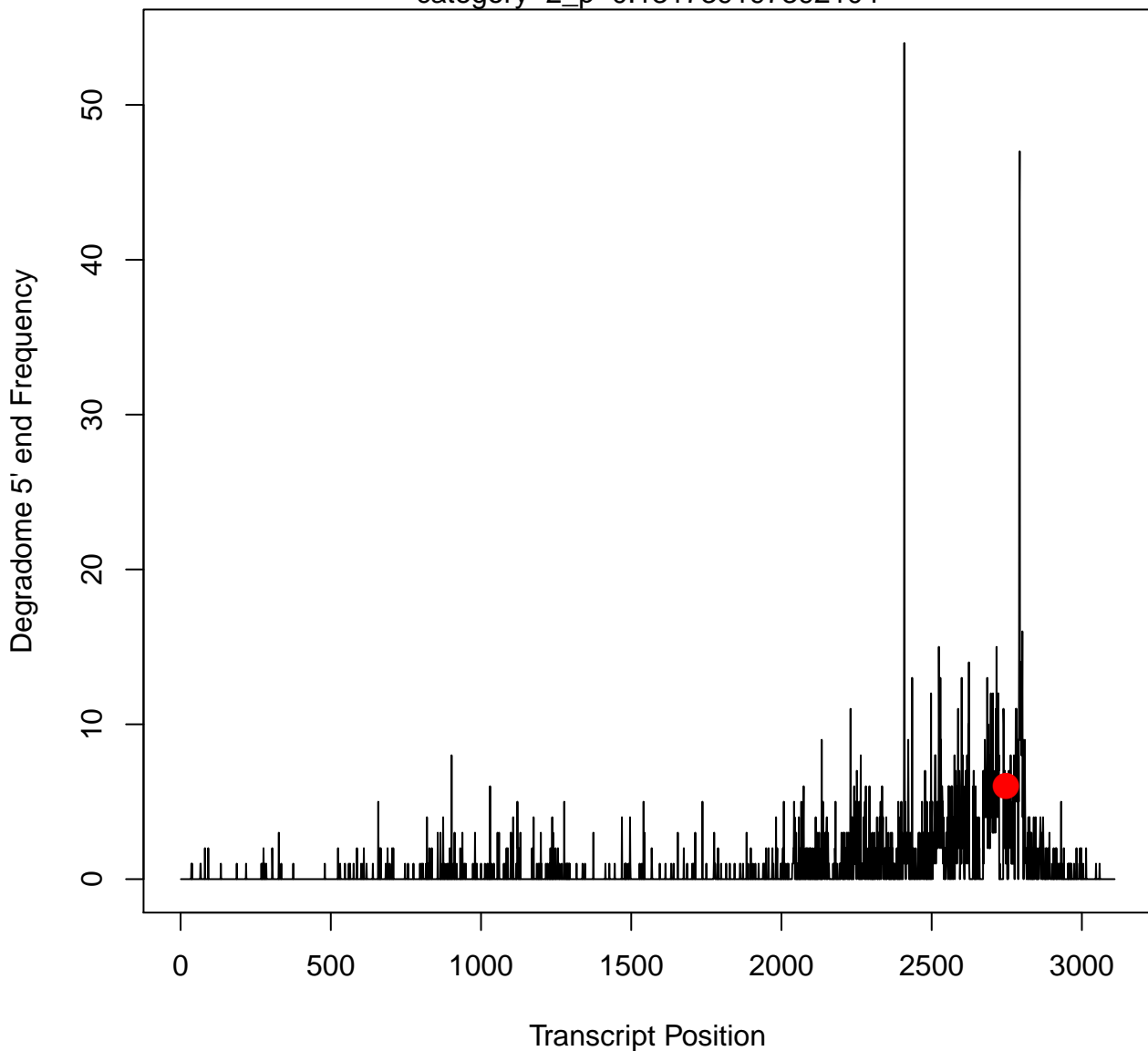

Supplement: Supplementary file 7 [file Data_Sheet_7.zip › Sit-miR396a_Seita.9G127700.1_2748_TPlot.pdf]

**T=Seita.2G226800.1\_Q=Sit-miR396b\_S=1233**

category=2\_p=0.763281613102506

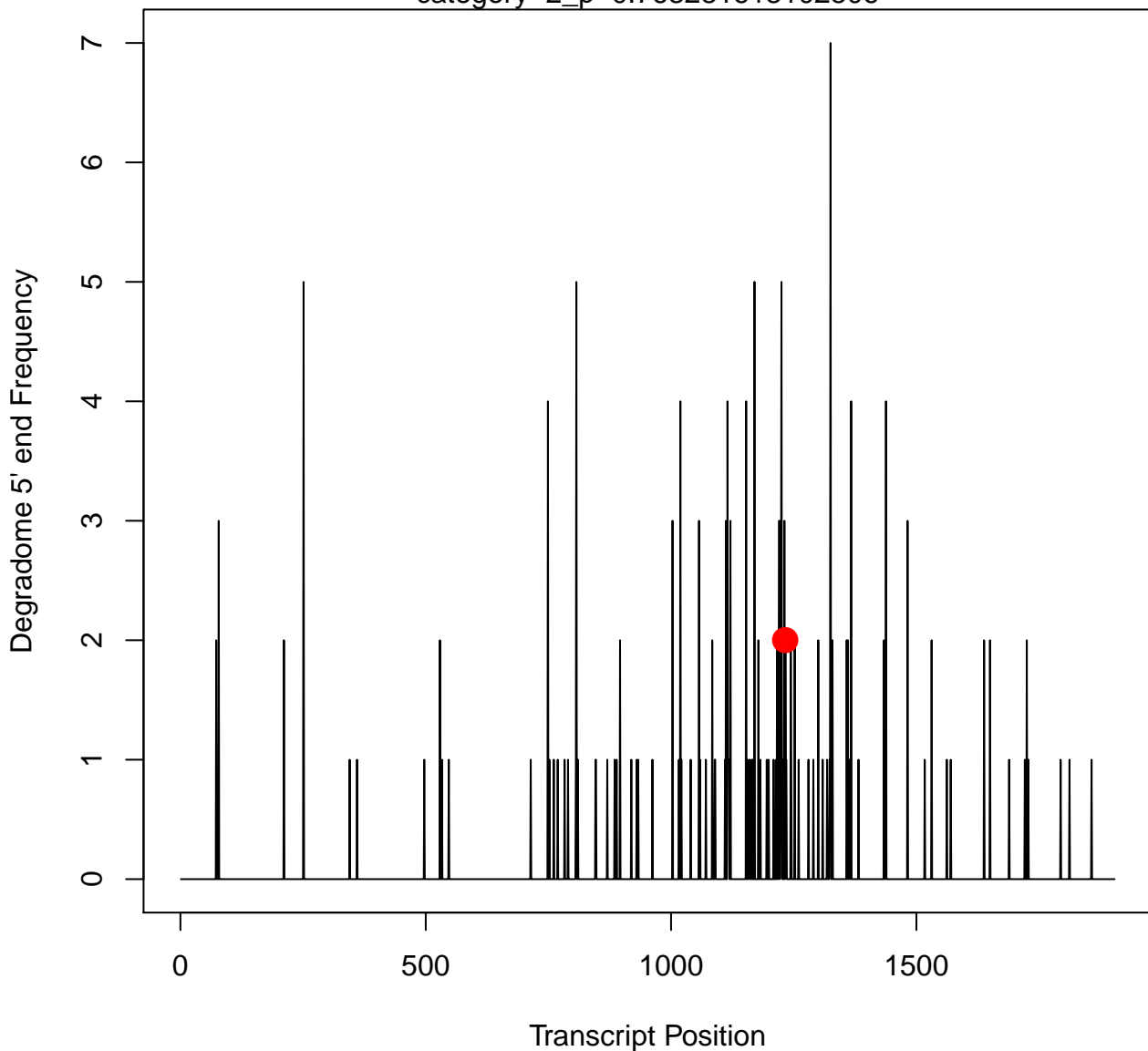

Supplement: Supplementary file 7 [file Data_Sheet_7.zip › Sit-miR396b_Seita.2G226800.1_1233_TPlot.pdf]

**T=Seita.3G207400.1\_Q=Sit-miR396b\_S=2413**

category=2\_p=0.998941881092028

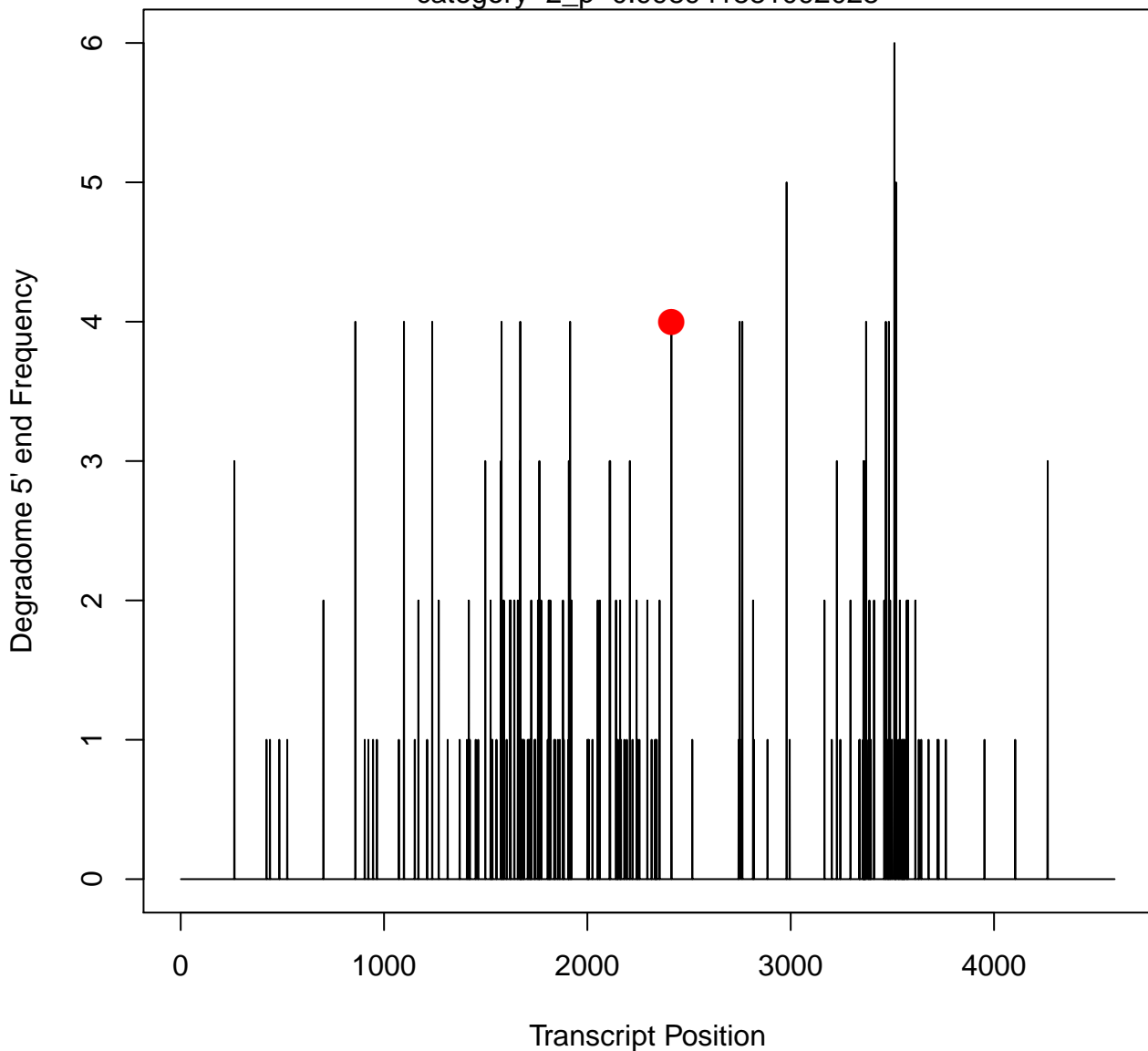

Supplement: Supplementary file 7 [file Data_Sheet_7.zip › Sit-miR396b_Seita.3G207400.1_2413_TPlot.pdf]

**T=Seita.7G267500.1\_Q=Sit-miR396b\_S=720**

category=2\_p=0.17944013037241

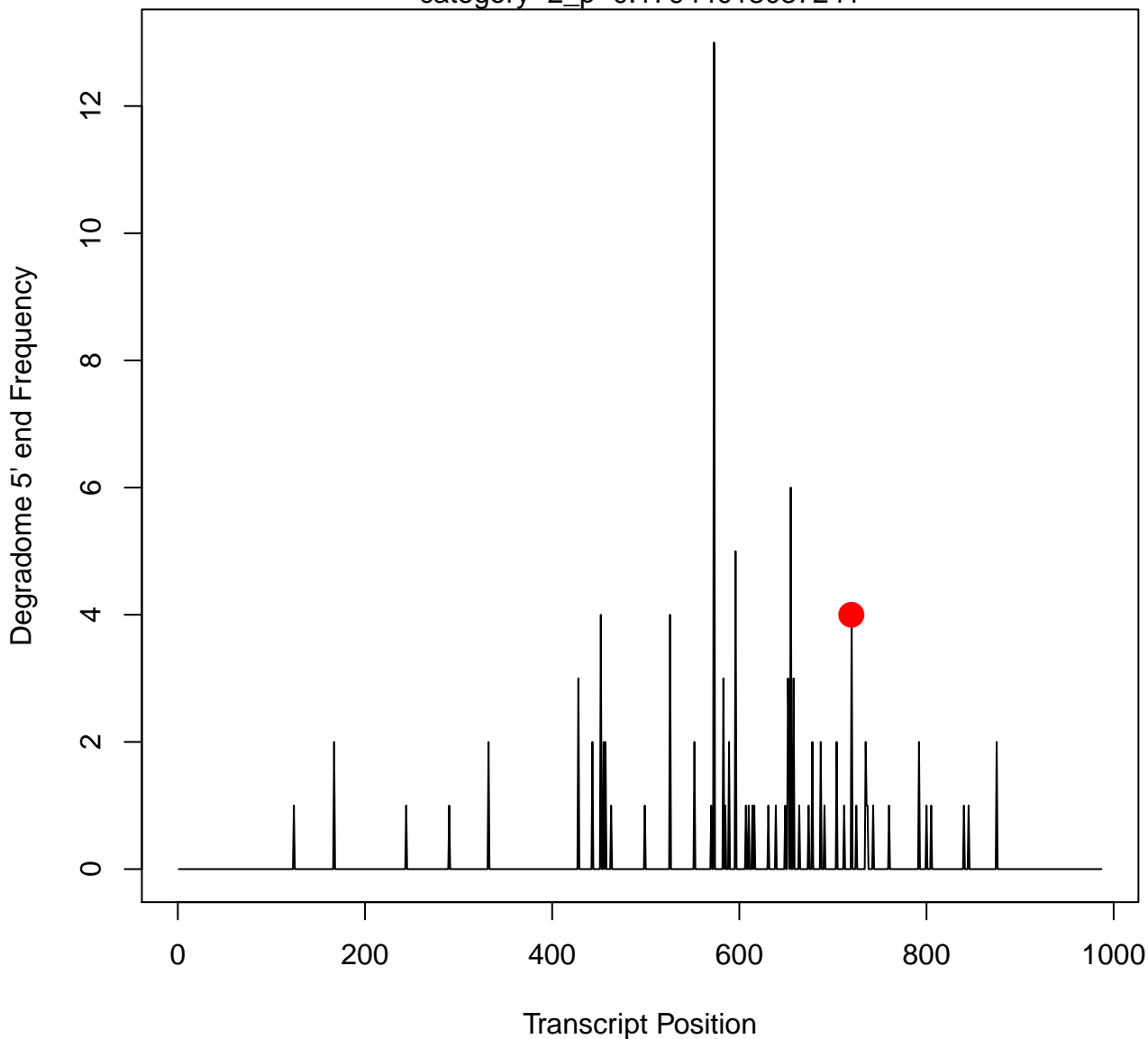

Supplement: Supplementary file 7 [file Data_Sheet_7.zip › Sit-miR396b_Seita.7G267500.1_720_TPlot.pdf]

**T=Seita.8G116700.1\_Q=Sit-miR396b\_S=382**

category=2\_p=0.933612891859855

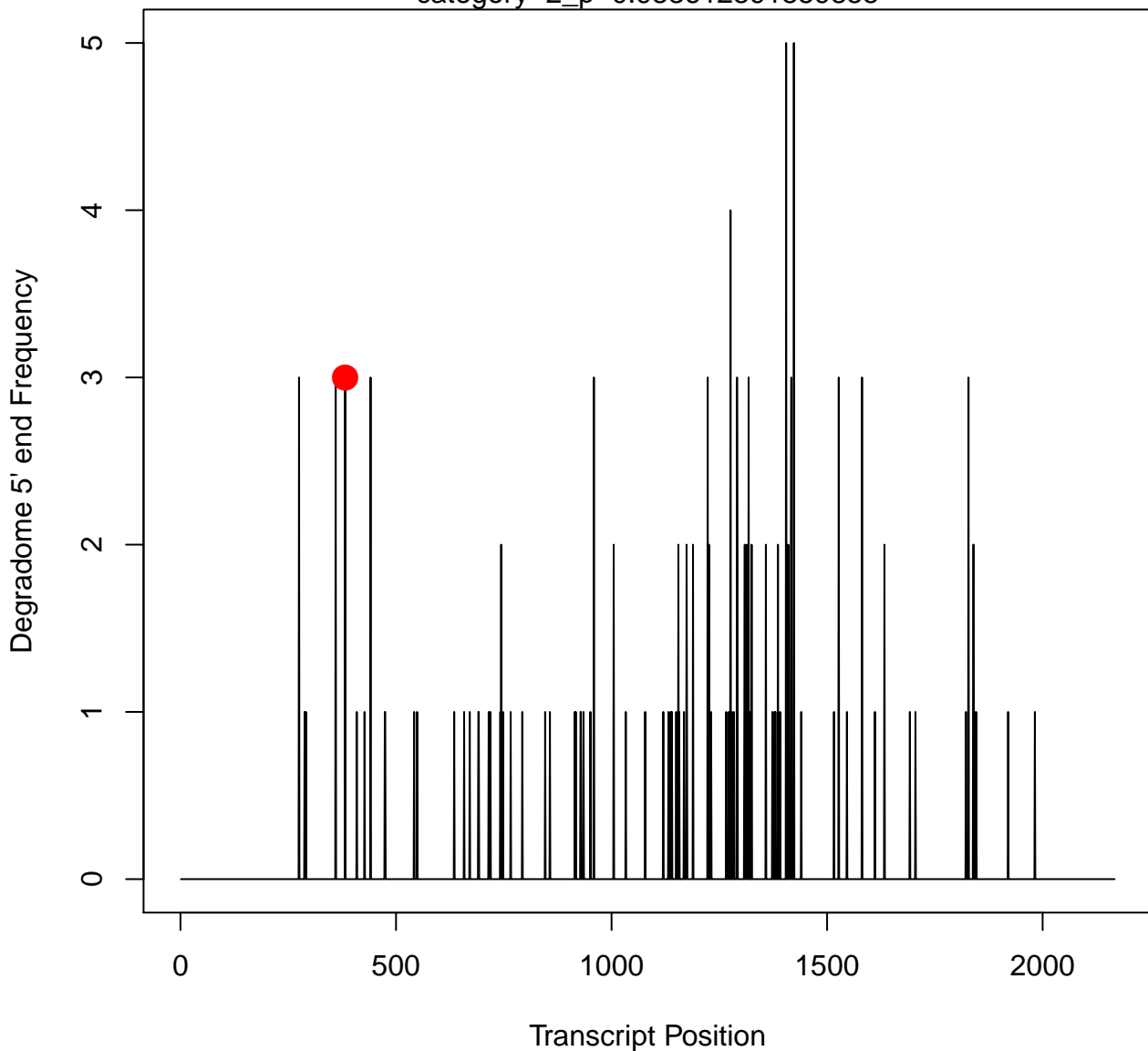

Supplement: Supplementary file 7 [file Data_Sheet_7.zip › Sit-miR396b_Seita.8G116700.1_382_TPlot.pdf]

**T=Seita.8G149500.1\_Q=Sit-miR396b\_S=729**

category=2\_p=0.0346994606644779

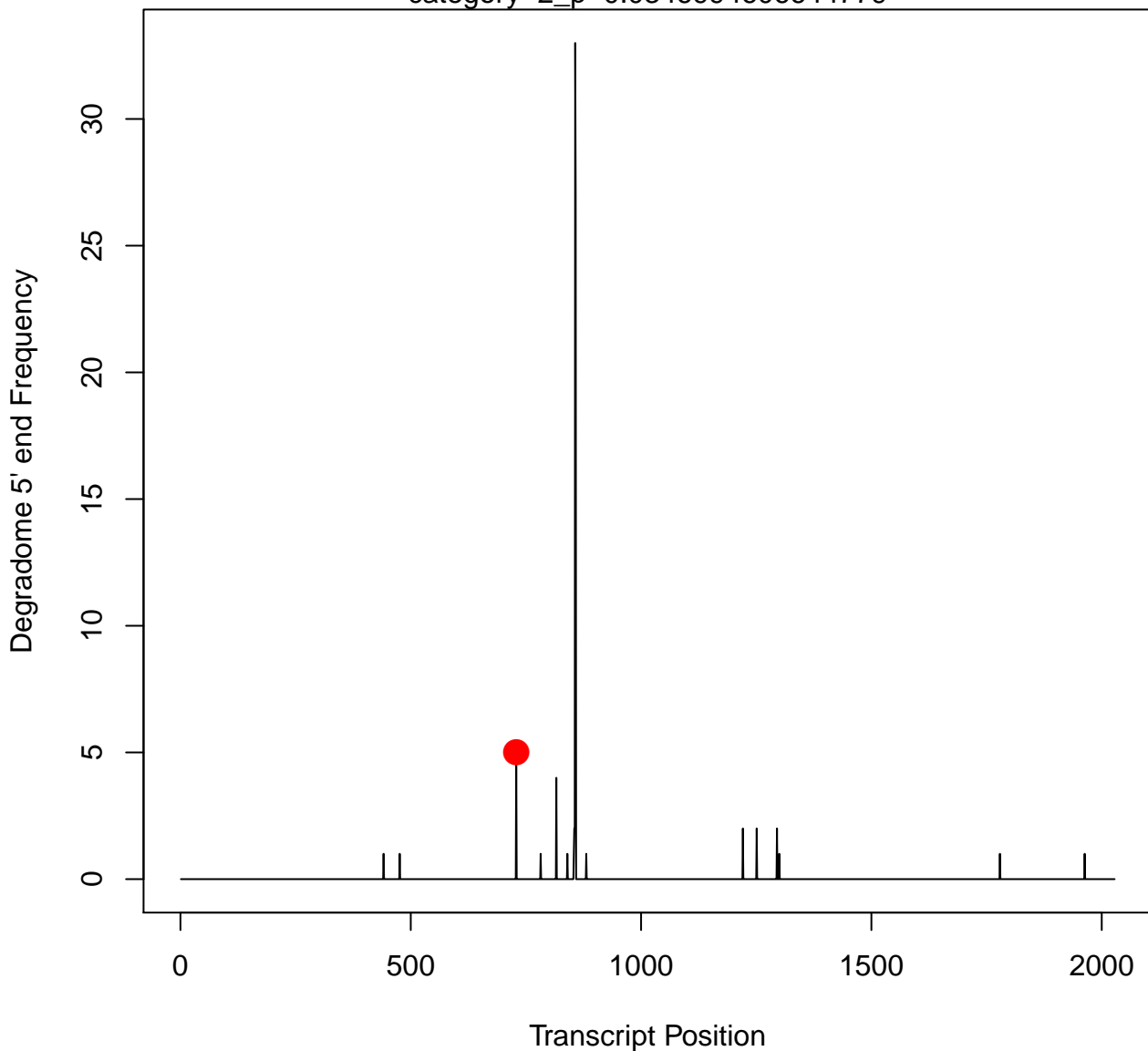

Supplement: Supplementary file 7 [file Data_Sheet_7.zip › Sit-miR396b_Seita.8G149500.1_729_TPlot.pdf]

**T=Seita.9G566700.1\_Q=Sit-miR396b\_S=939**

category=2\_p=0.996837755390044

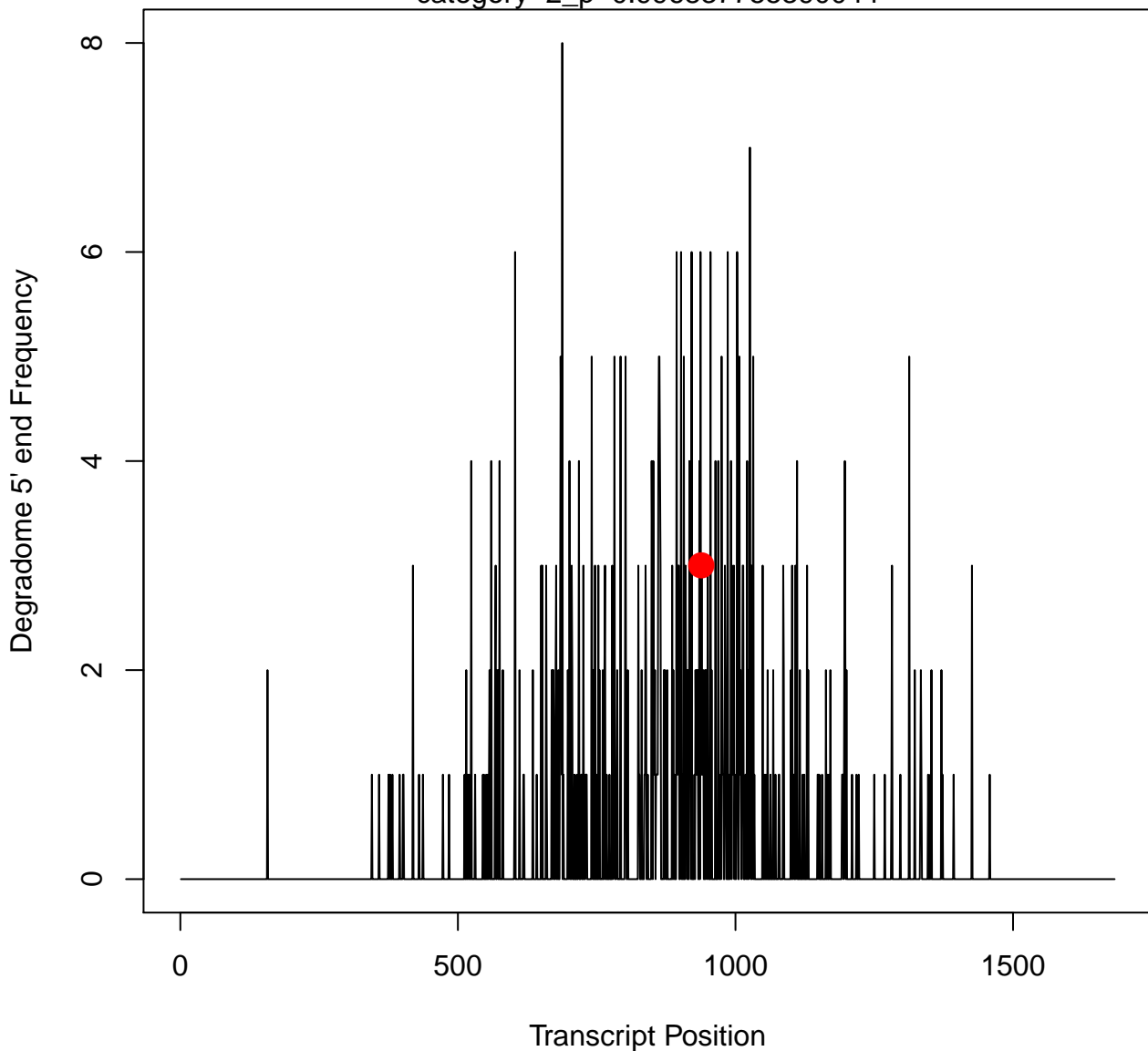

Supplement: Supplementary file 7 [file Data_Sheet_7.zip › Sit-miR396b_Seita.9G566700.1_939_TPlot.pdf]

**T=Seita.3G400500.1\_Q=Sit-miR396d\_S=476**

category=2\_p=0.98763992586163

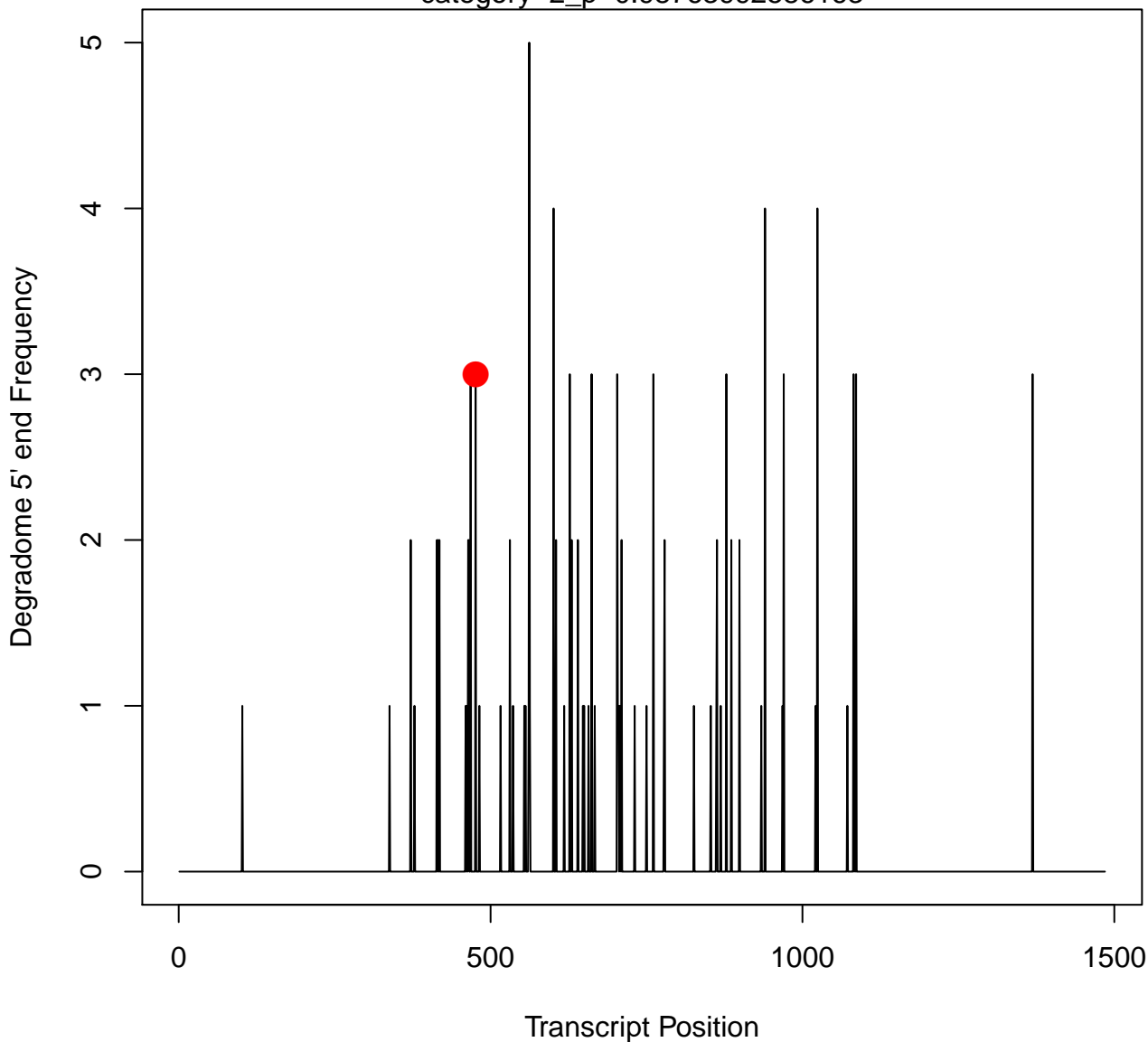

Supplement: Supplementary file 7 [file Data_Sheet_7.zip › Sit-miR396d_Seita.3G400500.1_476_TPlot.pdf]

**T=Seita.5G127600.1\_Q=Sit-miR396d\_S=1520**

category=2\_p=0.845053211678823

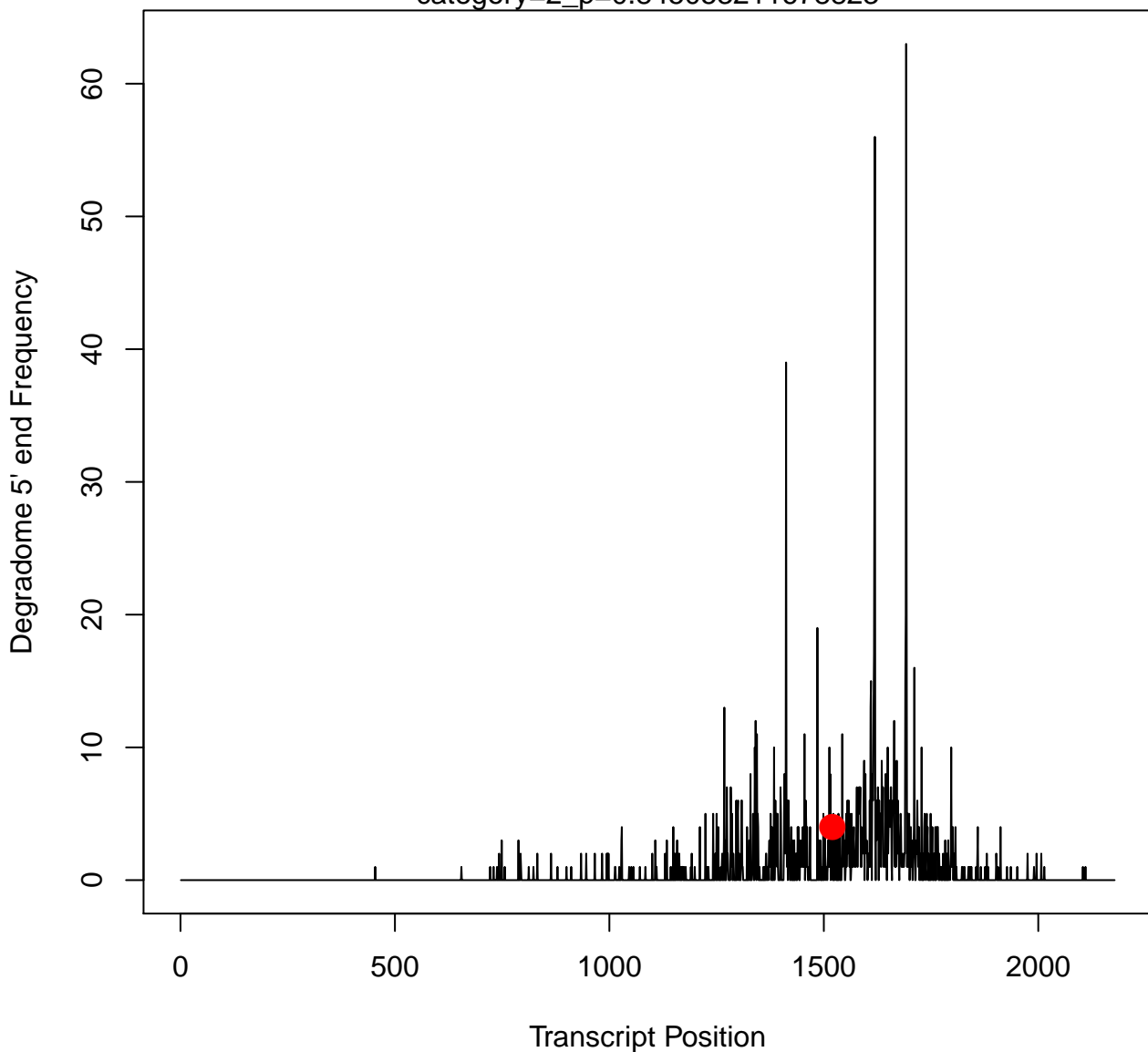

Supplement: Supplementary file 7 [file Data_Sheet_7.zip › Sit-miR396d_Seita.5G127600.1_1520_TPlot.pdf]
