# Supplementary figures and images for: The Construction and Exploration of a Comprehensive MicroRNA Centered Regulatory Network in Foxtail Millet (Setaria italica L.) (part 13 of 14)
Source: Front Plant Sci. 2022 May 6;13:848474. doi: 10.3389/fpls.2022.848474 (PMC9121102; doi:10.3389/fpls.2022.848474)

**T=Seita.2G293100.1\_Q=Sit-miR1133\_S=567**

category=2\_p=0.998645133099167

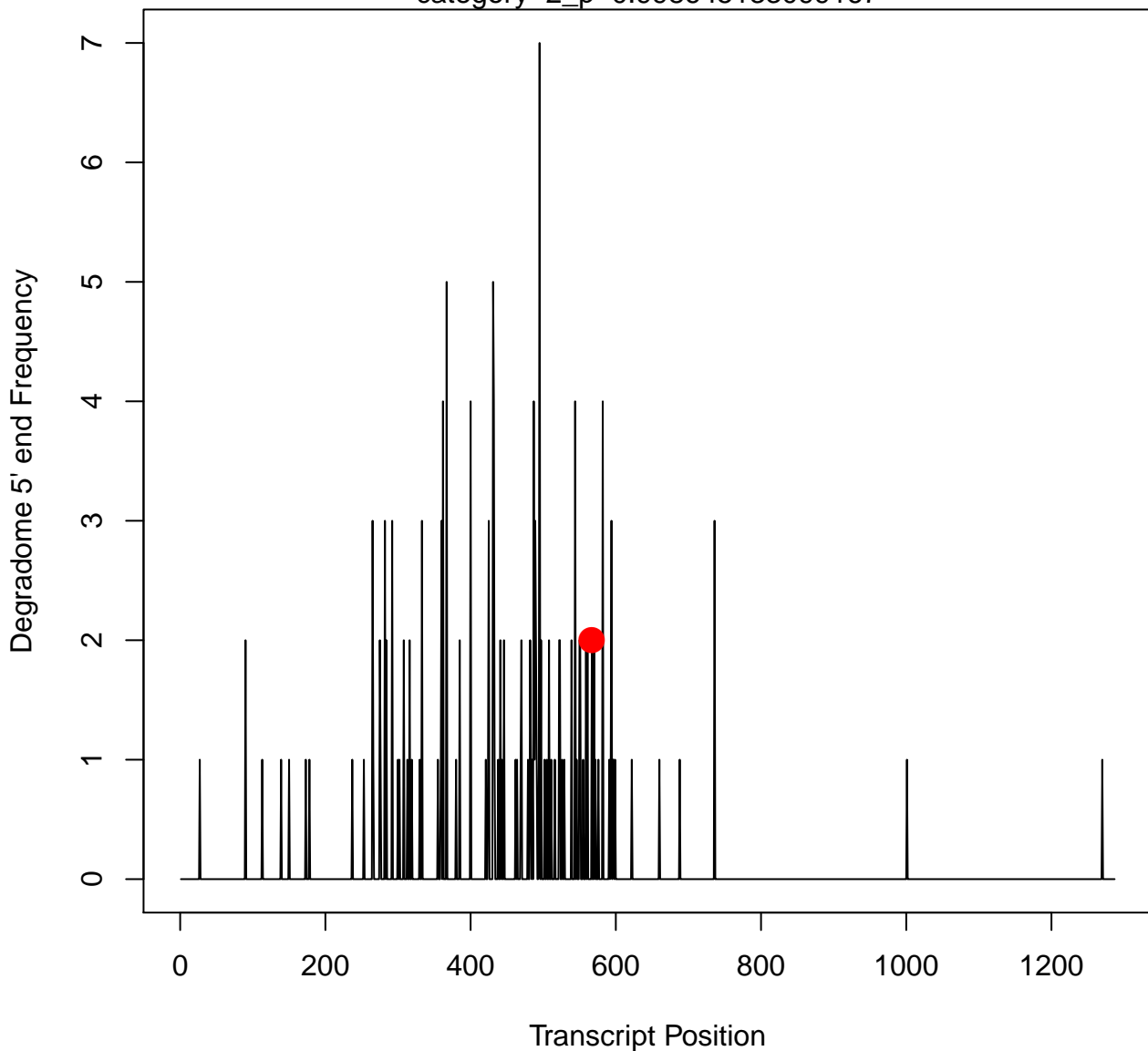

Supplement: Supplementary file 7 [file Data_Sheet_7.zip › Sit-miR1133_Seita.2G293100.1_567_TPlot.pdf]

**T=Seita.3G120600.1\_Q=Sit-miR1133\_S=1175**

category=2\_p=0.167766384158407

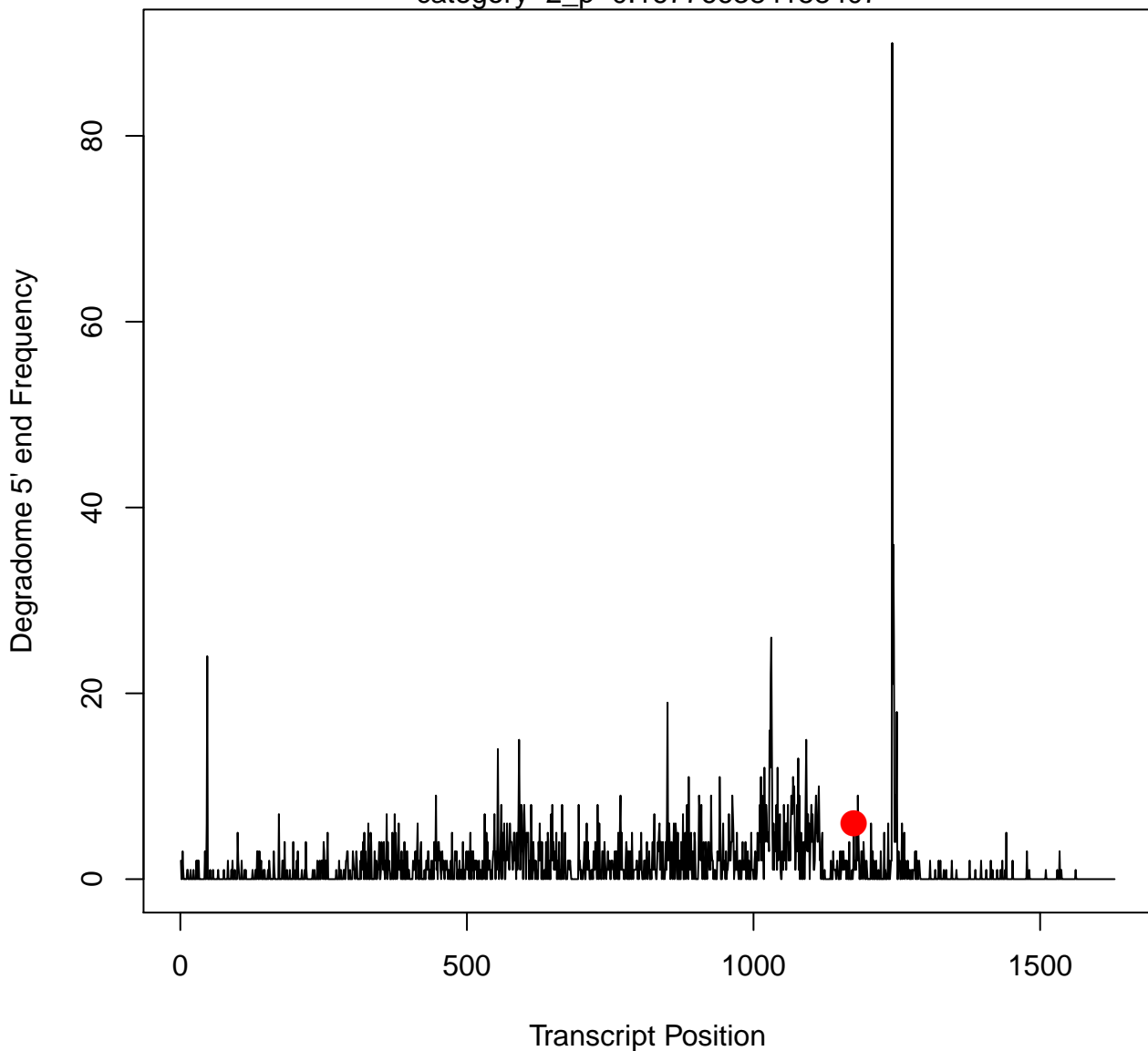

Supplement: Supplementary file 7 [file Data_Sheet_7.zip › Sit-miR1133_Seita.3G120600.1_1175_TPlot.pdf]

**T=Seita.9G173800.1\_Q=Sit-miR1133\_S=1339**

category=2\_p=0.996653927221265

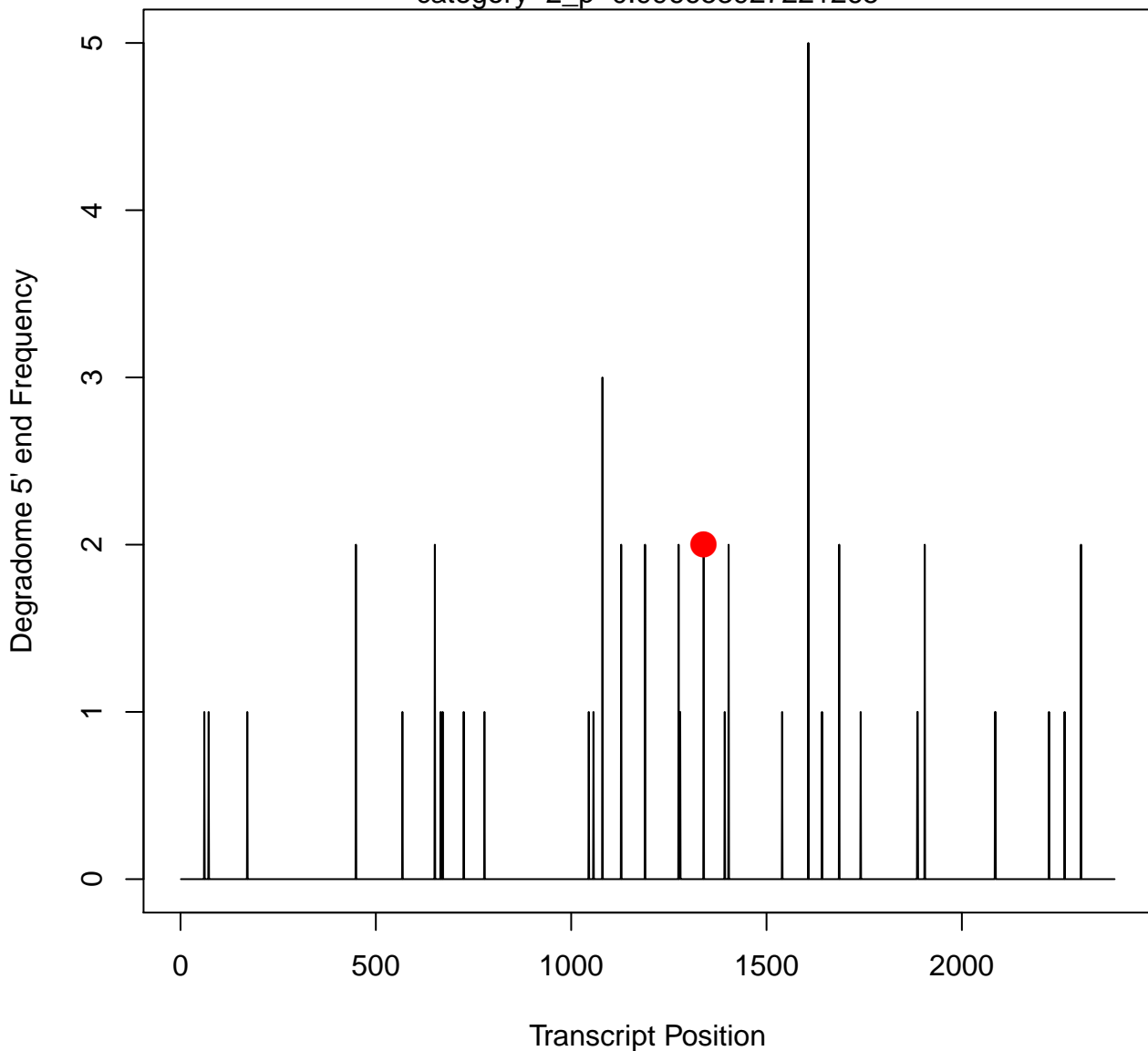

Supplement: Supplementary file 7 [file Data_Sheet_7.zip › Sit-miR1133_Seita.9G173800.1_1339_TPlot.pdf]

**T=Seita.9G480400.1\_Q=Sit-miR1133\_S=630**

category=2\_p=0.985356636564575

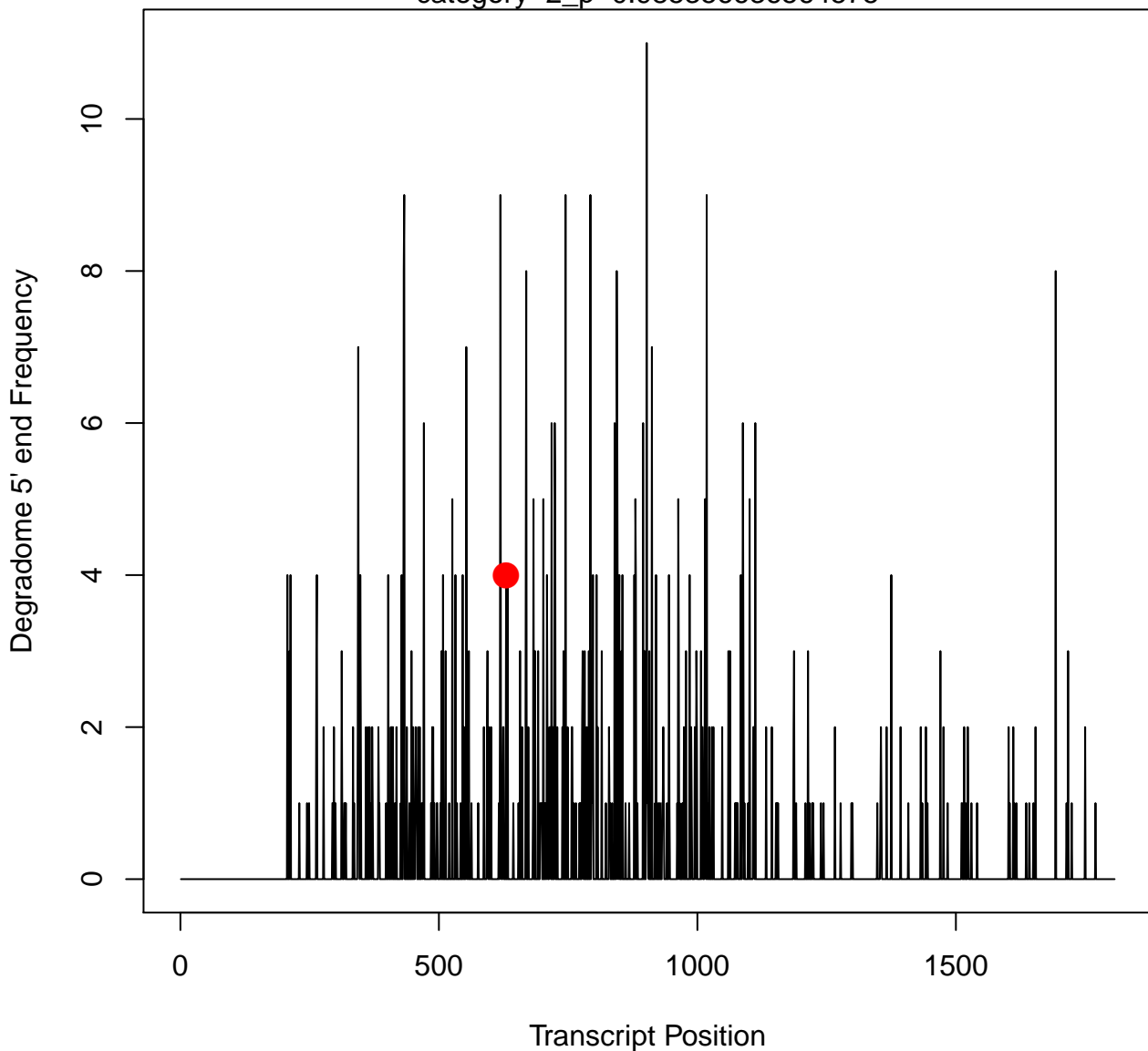

Supplement: Supplementary file 7 [file Data_Sheet_7.zip › Sit-miR1133_Seita.9G480400.1_630_TPlot.pdf]

**T=Seita.9G141000.1\_Q=Sit-miR1432\_S=443**

category=2\_p=0.958932864120024

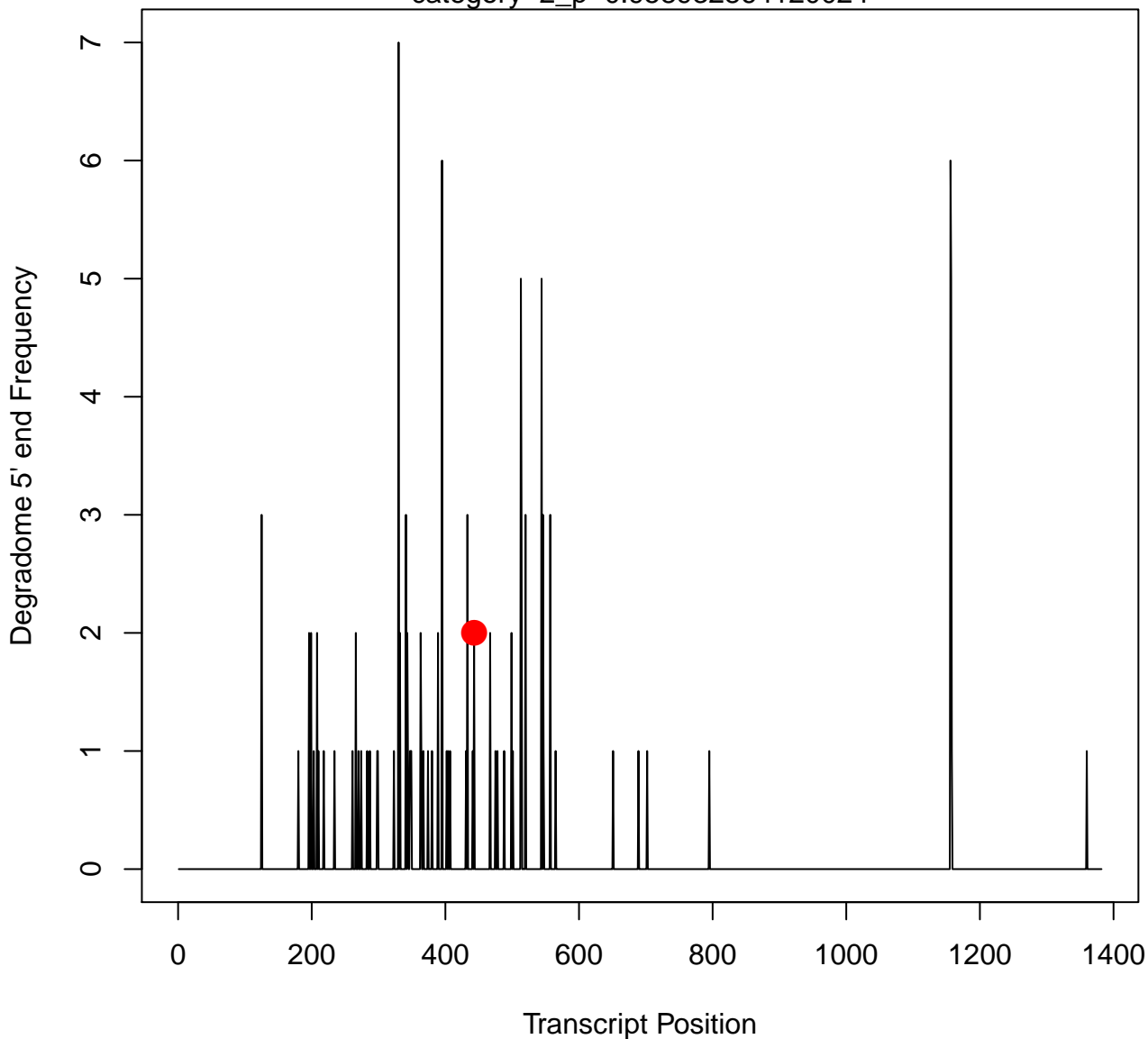

Supplement: Supplementary file 7 [file Data_Sheet_7.zip › Sit-miR1432_Seita.9G141000.1_443_TPlot.pdf]

**T=Seita.2G324900.1\_Q=Sit-miR156a\_S=833**

category=0\_p=0.00298840966153668

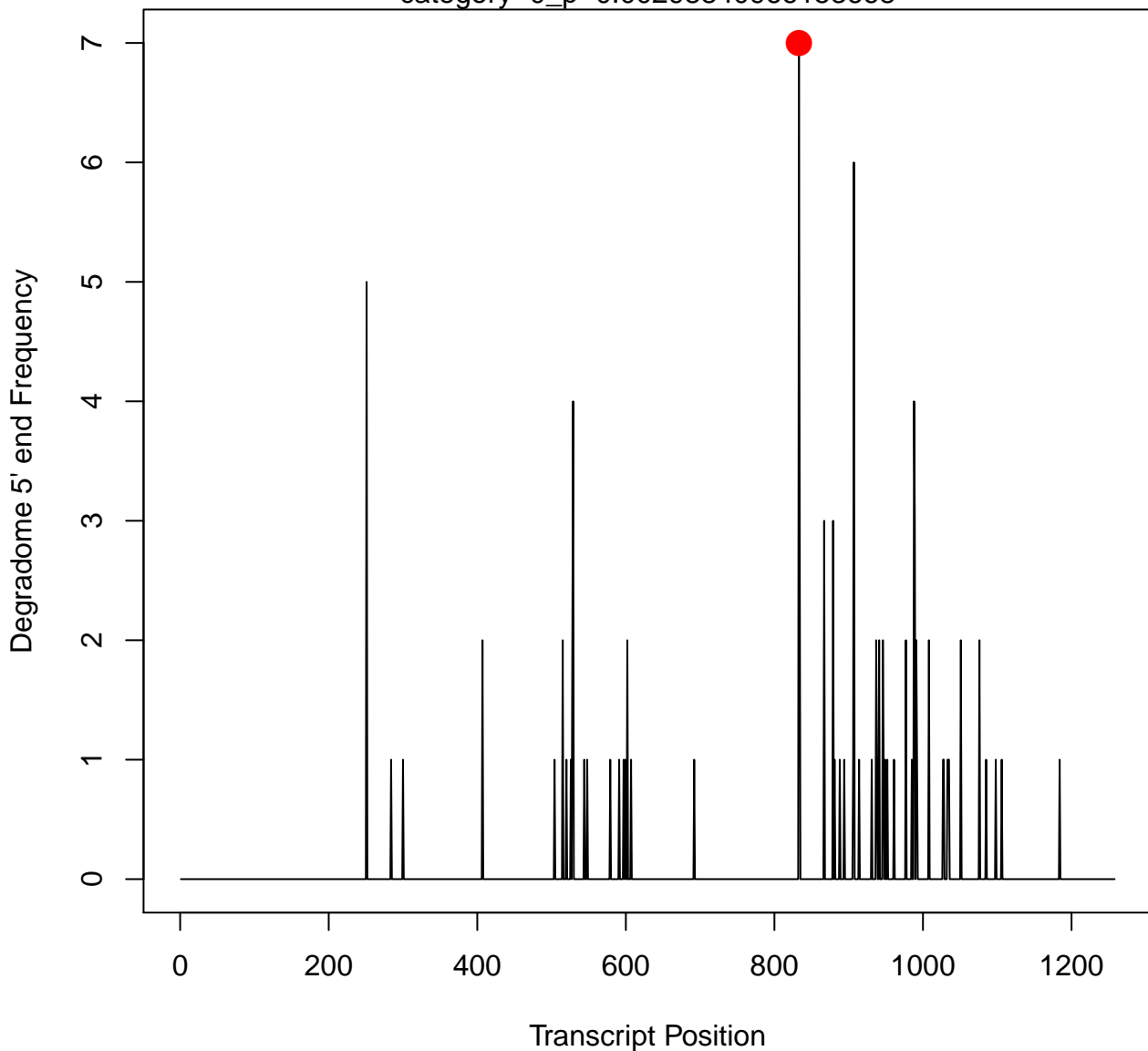

Supplement: Supplementary file 7 [file Data_Sheet_7.zip › Sit-miR156a_Seita.2G324900.1_833_TPlot.pdf]

**T=Seita.1G091900.1\_Q=Sit-miR156c\_S=1786**

category=0\_p=0.000299243605074828

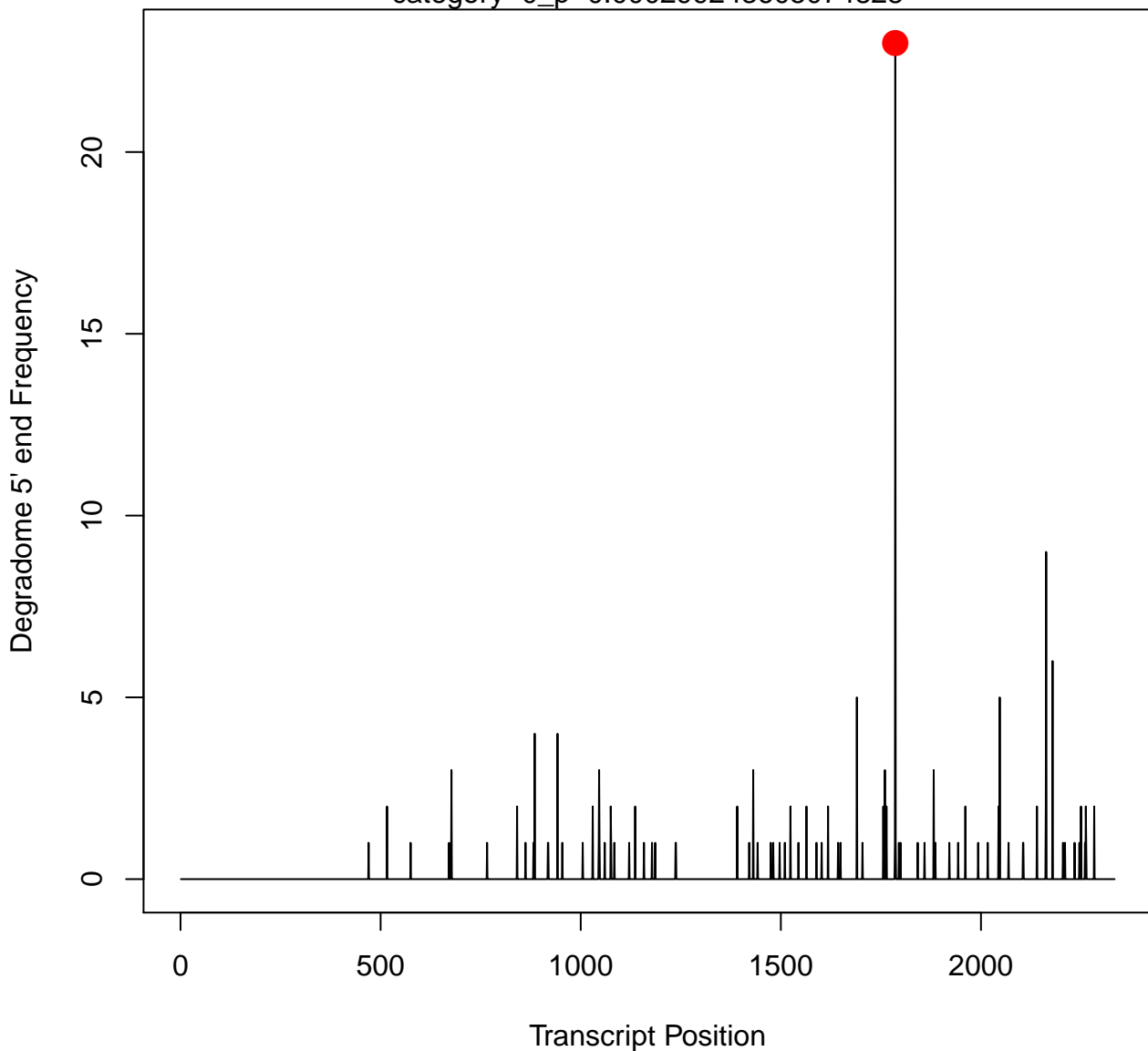

Supplement: Supplementary file 7 [file Data_Sheet_7.zip › Sit-miR156c_Seita.1G091900.1_1786_TPlot.pdf]

**T=Seita.2G324900.1\_Q=Sit-miR156d\_S=834**

category=2\_p=0.0747531665461634

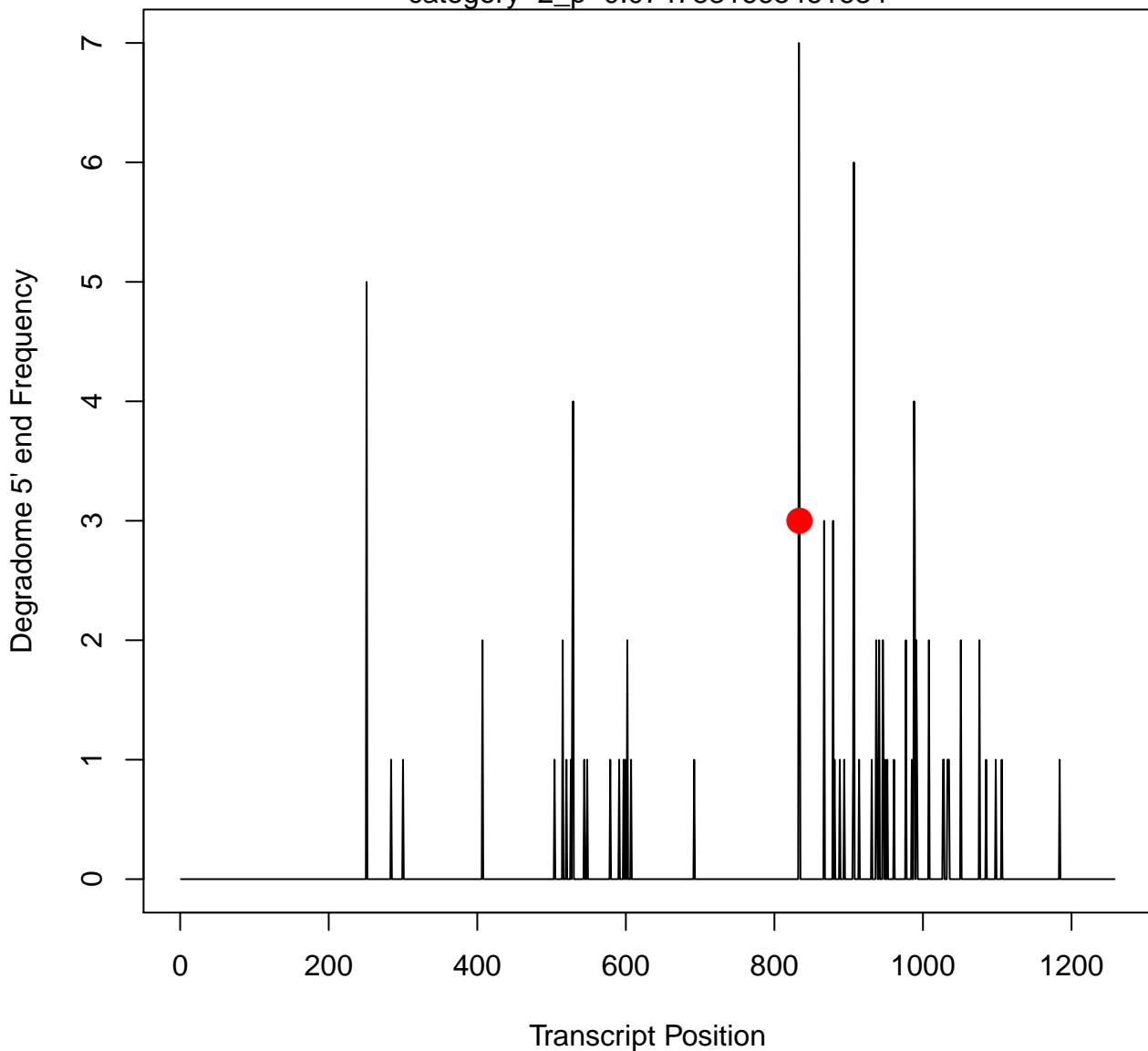

Supplement: Supplementary file 7 [file Data_Sheet_7.zip › Sit-miR156d_Seita.2G324900.1_834_TPlot.pdf]

**T=Seita.2G254300.1\_Q=Sit-miR156f\_S=1266**

category=0\_p=0.00119643724706542

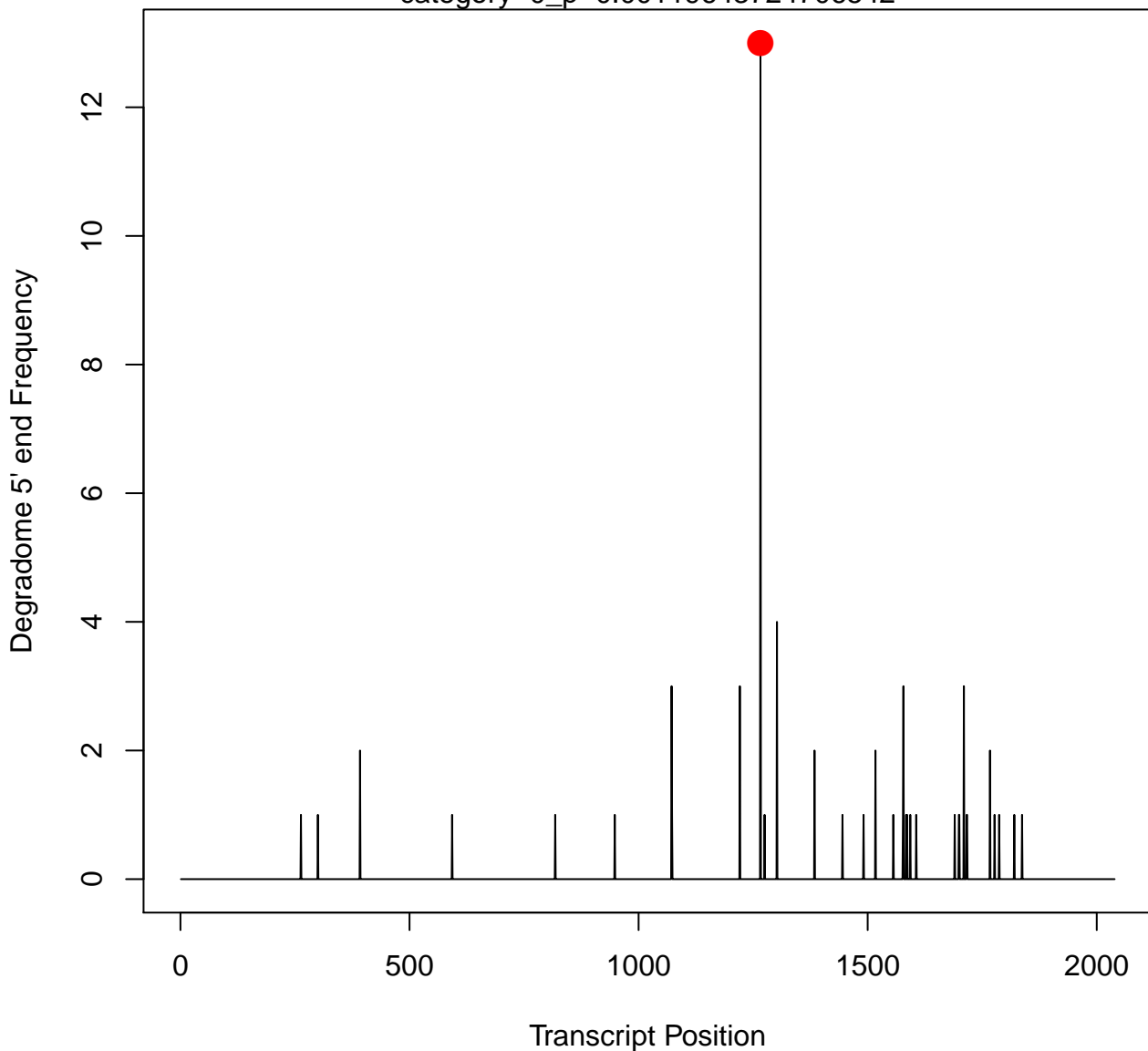

Supplement: Supplementary file 7 [file Data_Sheet_7.zip › Sit-miR156f_Seita.2G254300.1_1266_TPlot.pdf]

**T=Seita.8G124900.1\_Q=Sit-miR156j\_S=1295**

category=2\_p=0.100528004257022

Degradome 5' end Frequency

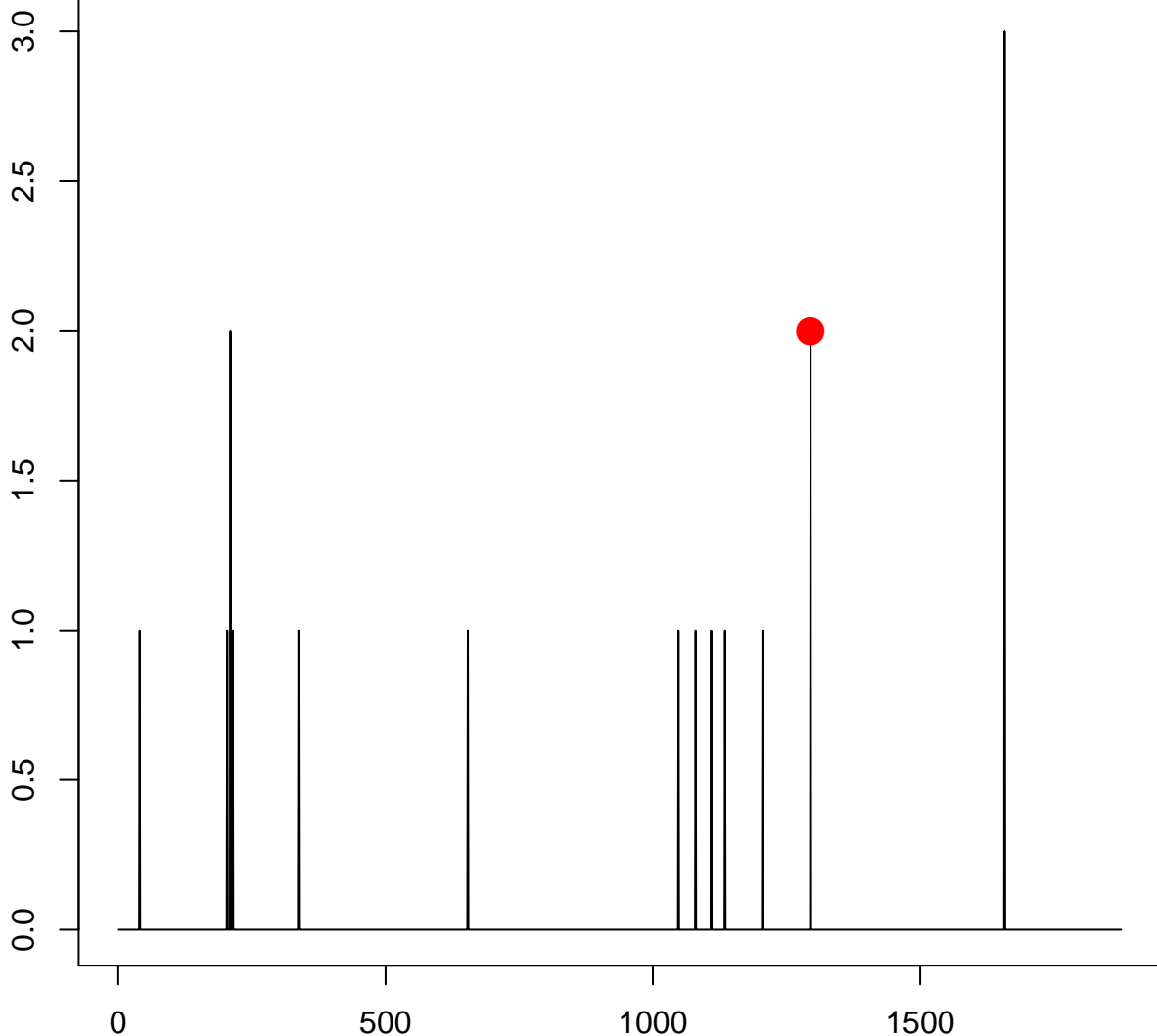

Transcript Position

Supplement: Supplementary file 7 [file Data_Sheet_7.zip › Sit-miR156j_Seita.8G124900.1_1295_TPlot.pdf]

**T=Seita.3G096800.1\_Q=Sit-miR159a\_S=1199**

category=2\_p=0.999377022003576

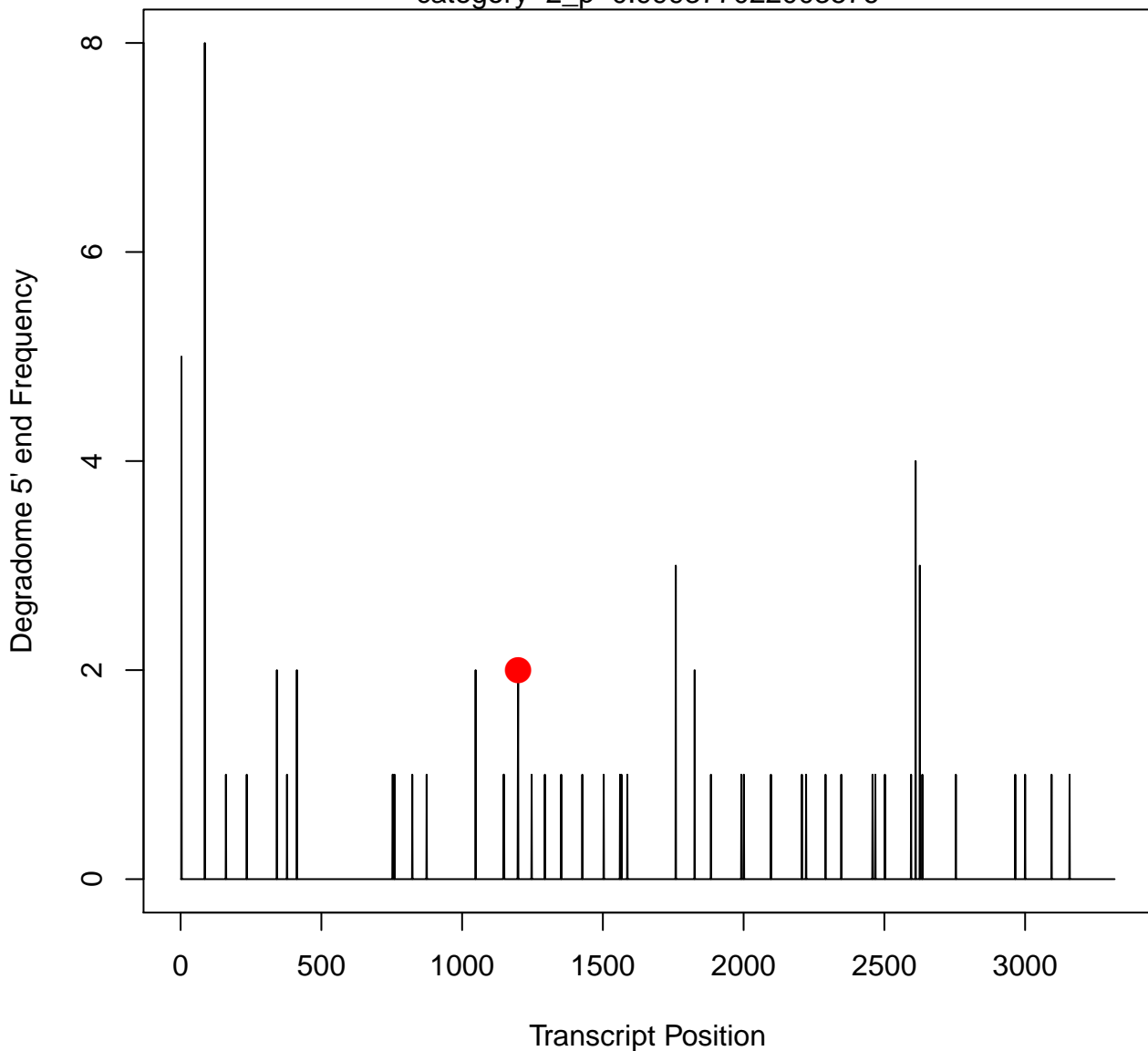

Supplement: Supplementary file 7 [file Data_Sheet_7.zip › Sit-miR159a_Seita.3G096800.1_1199_TPlot.pdf]

**T=Seita.4G020800.1\_Q=Sit-miR159a\_S=284**

category=2\_p=0.999655805815537

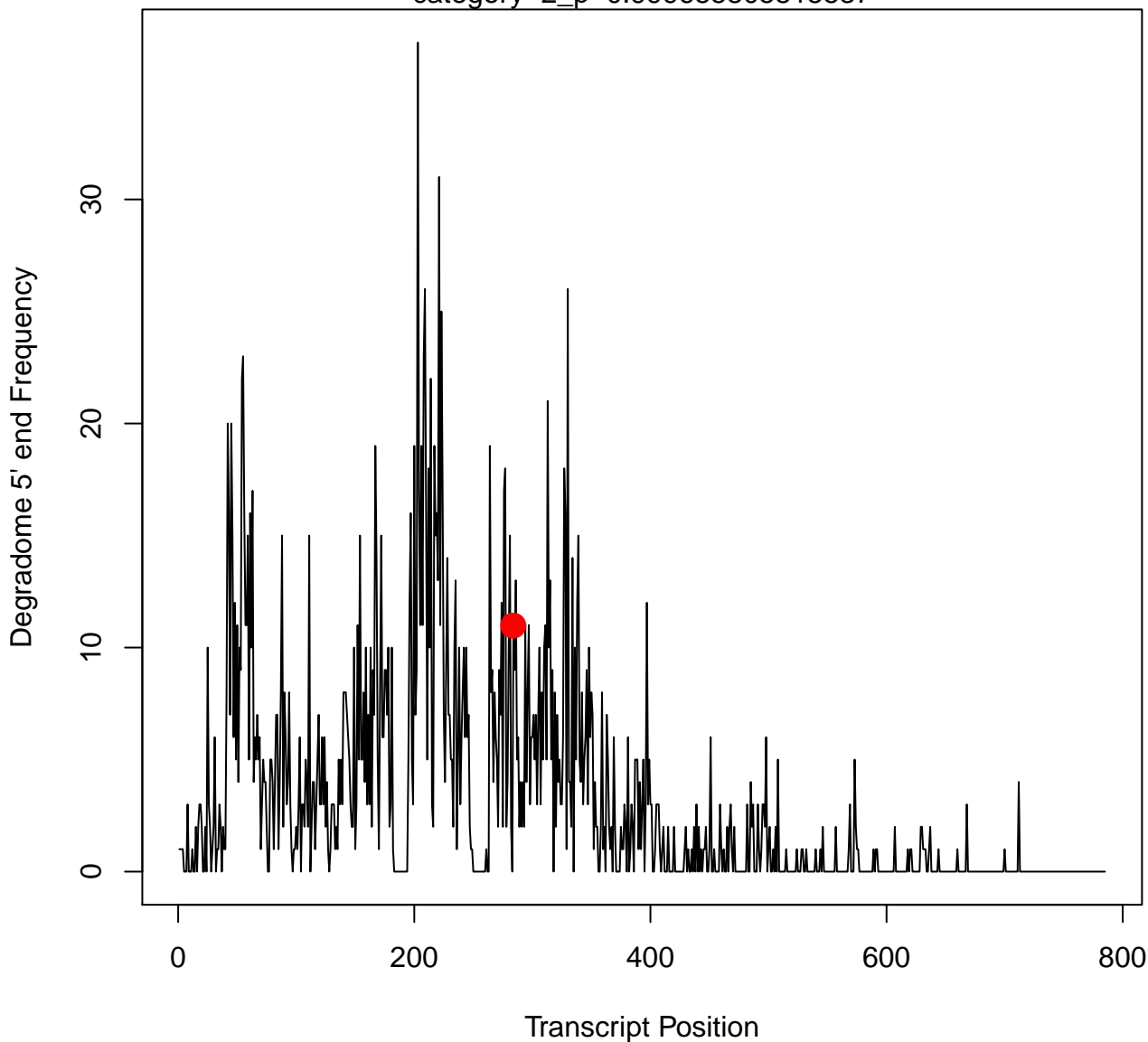

Supplement: Supplementary file 7 [file Data_Sheet_7.zip › Sit-miR159a_Seita.4G020800.1_284_TPlot.pdf]

**T=Seita.5G046700.1\_Q=Sit-miR159a\_S=479**

category=2\_p=0.959793900371298

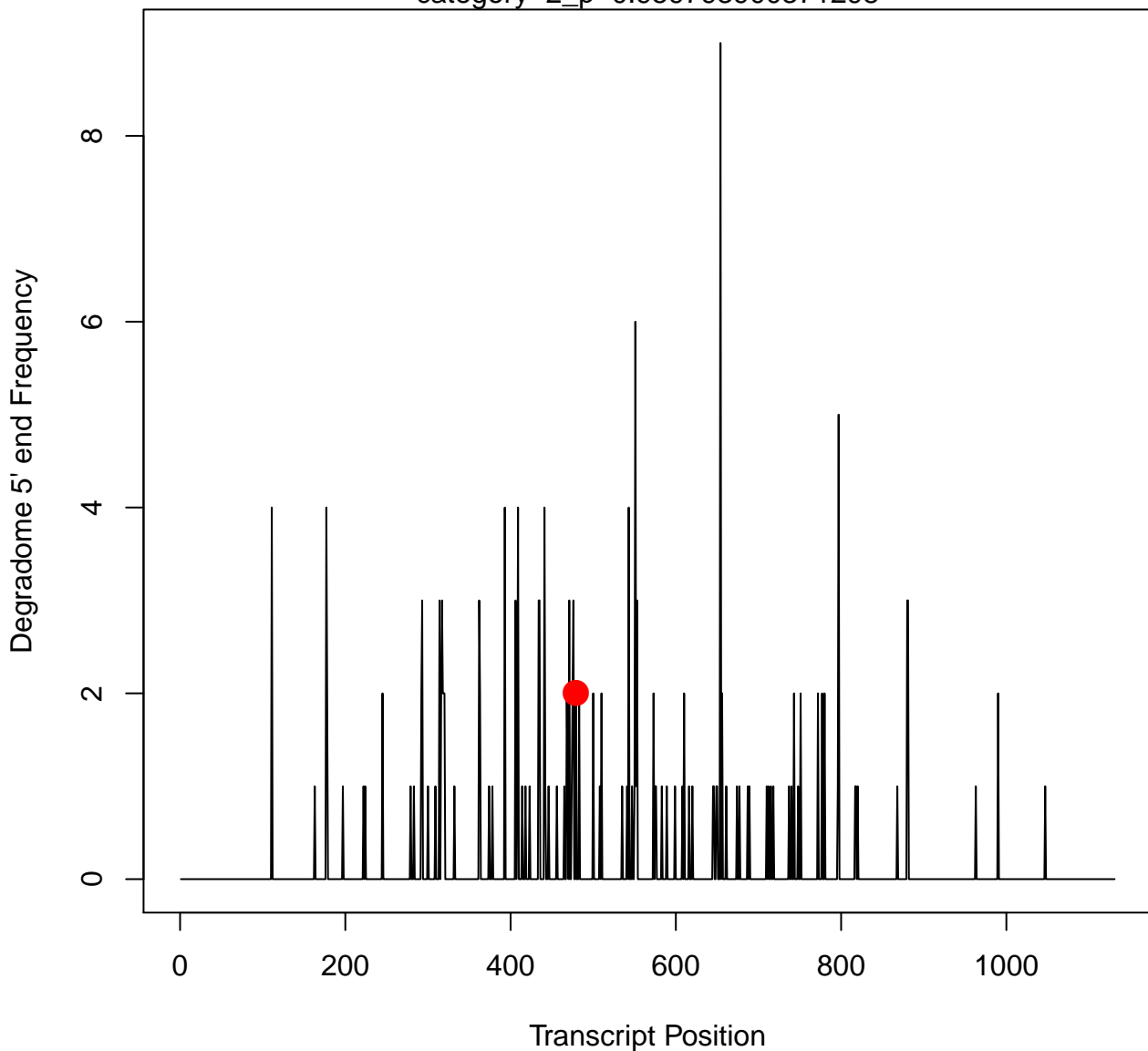

Supplement: Supplementary file 7 [file Data_Sheet_7.zip › Sit-miR159a_Seita.5G046700.1_479_TPlot.pdf]

**T=Seita.7G085500.1\_Q=Sit-miR159a\_S=2417**

category=2\_p=0.999862586374531

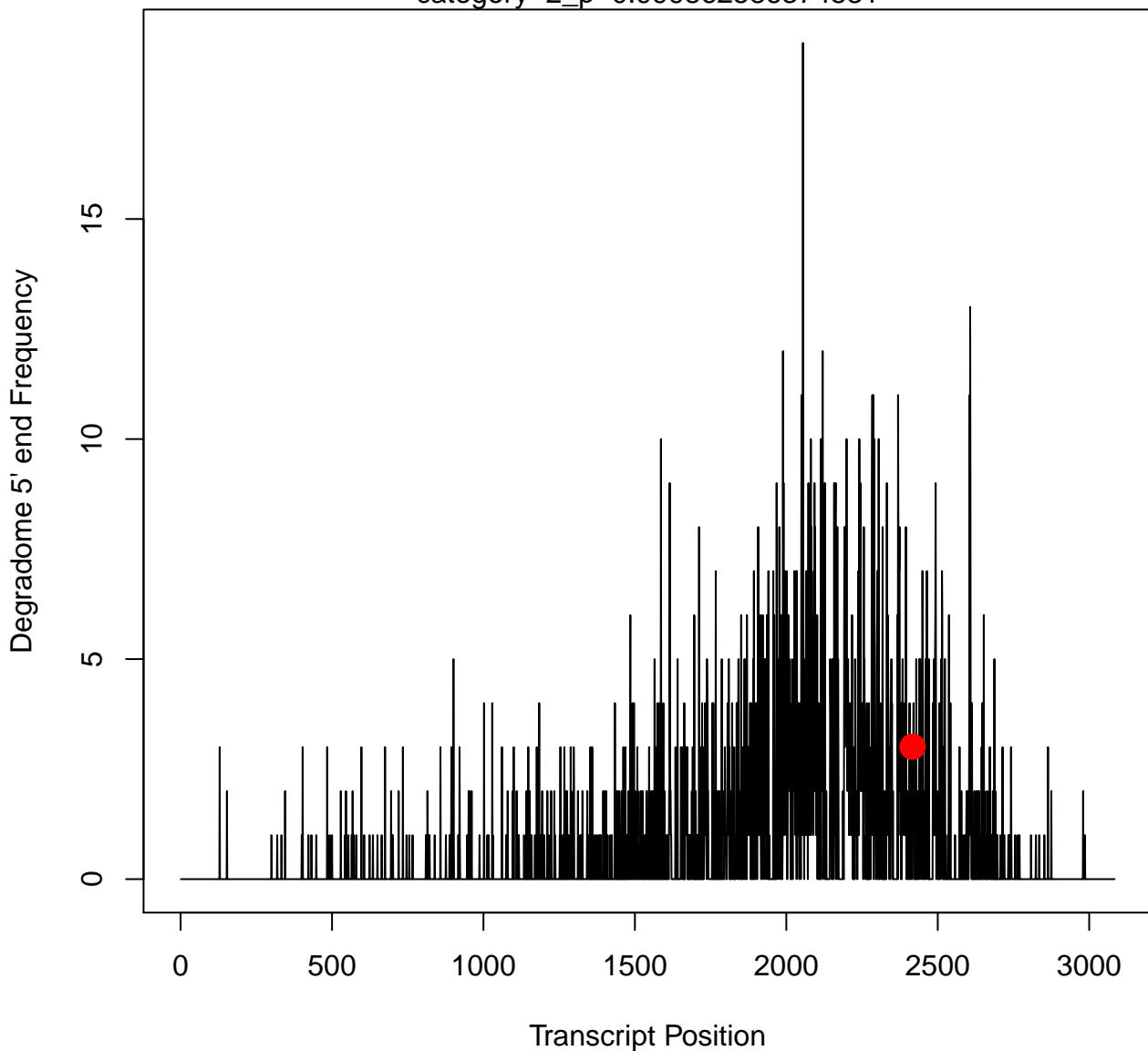

Supplement: Supplementary file 7 [file Data_Sheet_7.zip › Sit-miR159a_Seita.7G085500.1_2417_TPlot.pdf]

**T=Seita.9G062800.1\_Q=Sit-miR159a\_S=4205**

category=2\_p=0.994235891717707

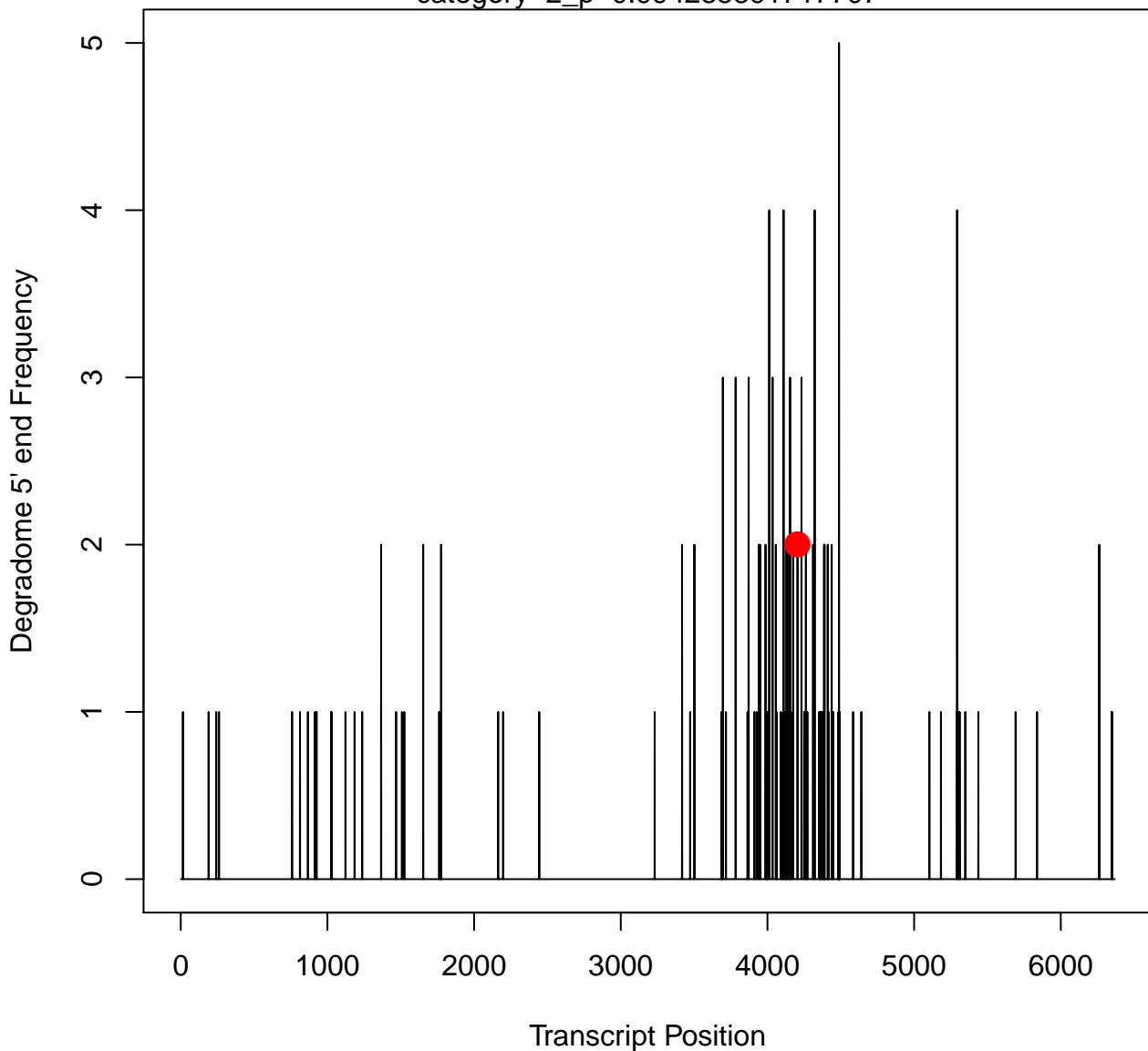

Supplement: Supplementary file 7 [file Data_Sheet_7.zip › Sit-miR159a_Seita.9G062800.1_4205_TPlot.pdf]

**T=Seita.8G006100.1\_Q=Sit-miR159b\_S=1440**

category=2\_p=0.492400789735958

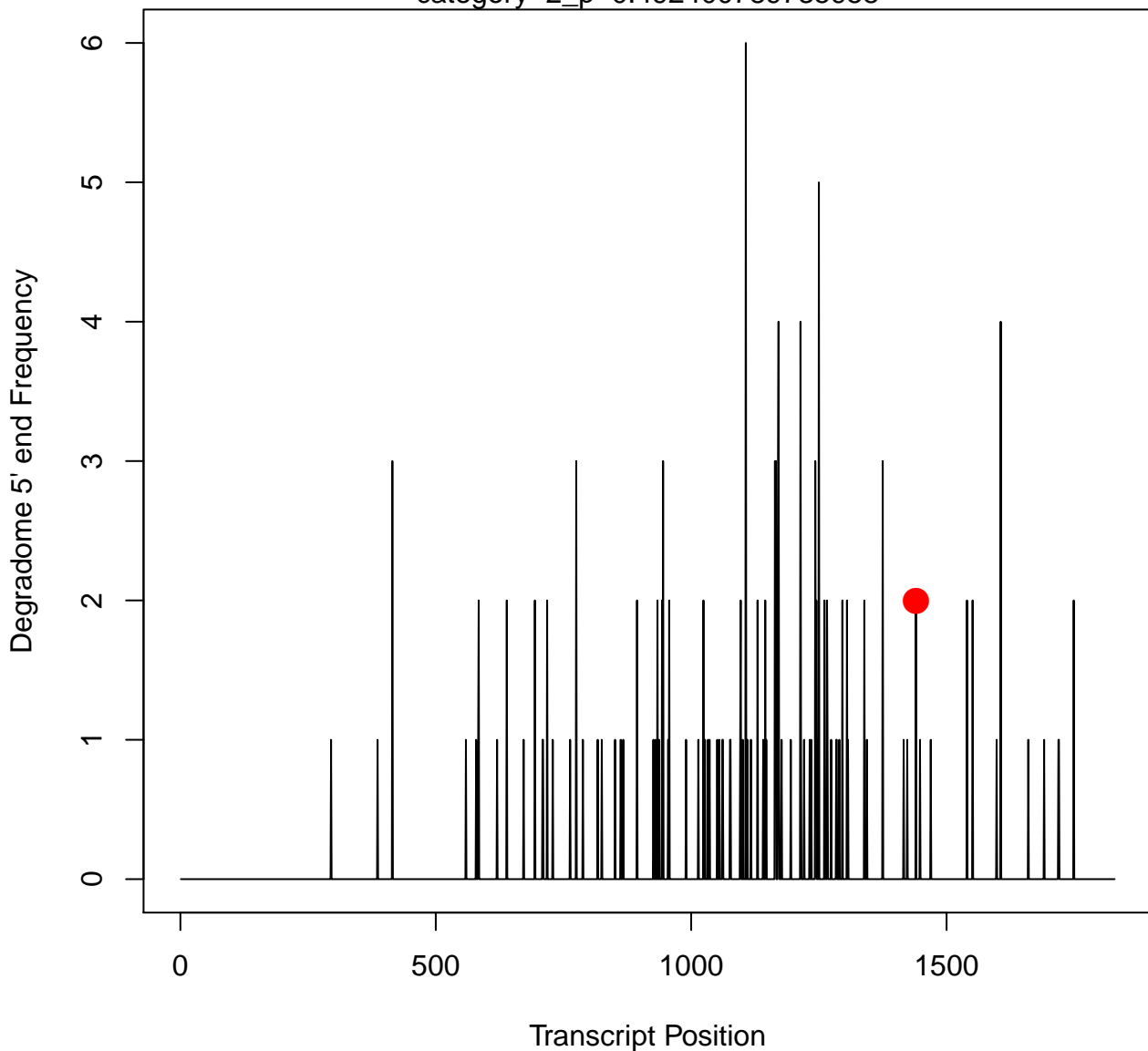

Supplement: Supplementary file 7 [file Data_Sheet_7.zip › Sit-miR159b_Seita.8G006100.1_1440_TPlot.pdf]

**T=Seita.9G451100.1\_Q=Sit-miR159b\_S=1813**

category=2\_p=0.523663868092578

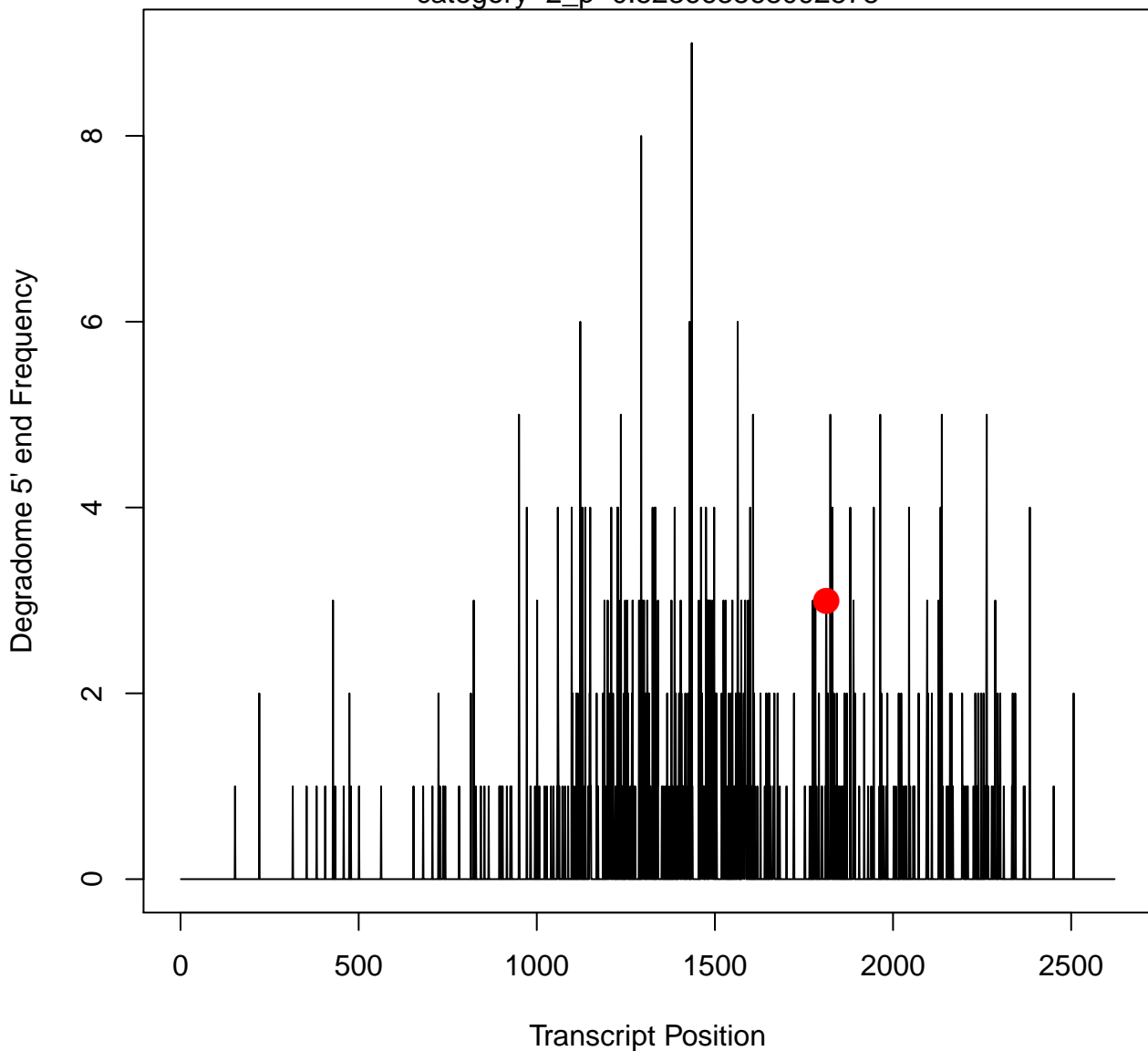

Supplement: Supplementary file 7 [file Data_Sheet_7.zip › Sit-miR159b_Seita.9G451100.1_1813_TPlot.pdf]

**T=Seita.7G203700.1\_Q=Sit-miR159c\_S=1002**

category=2\_p=0.94741564232846

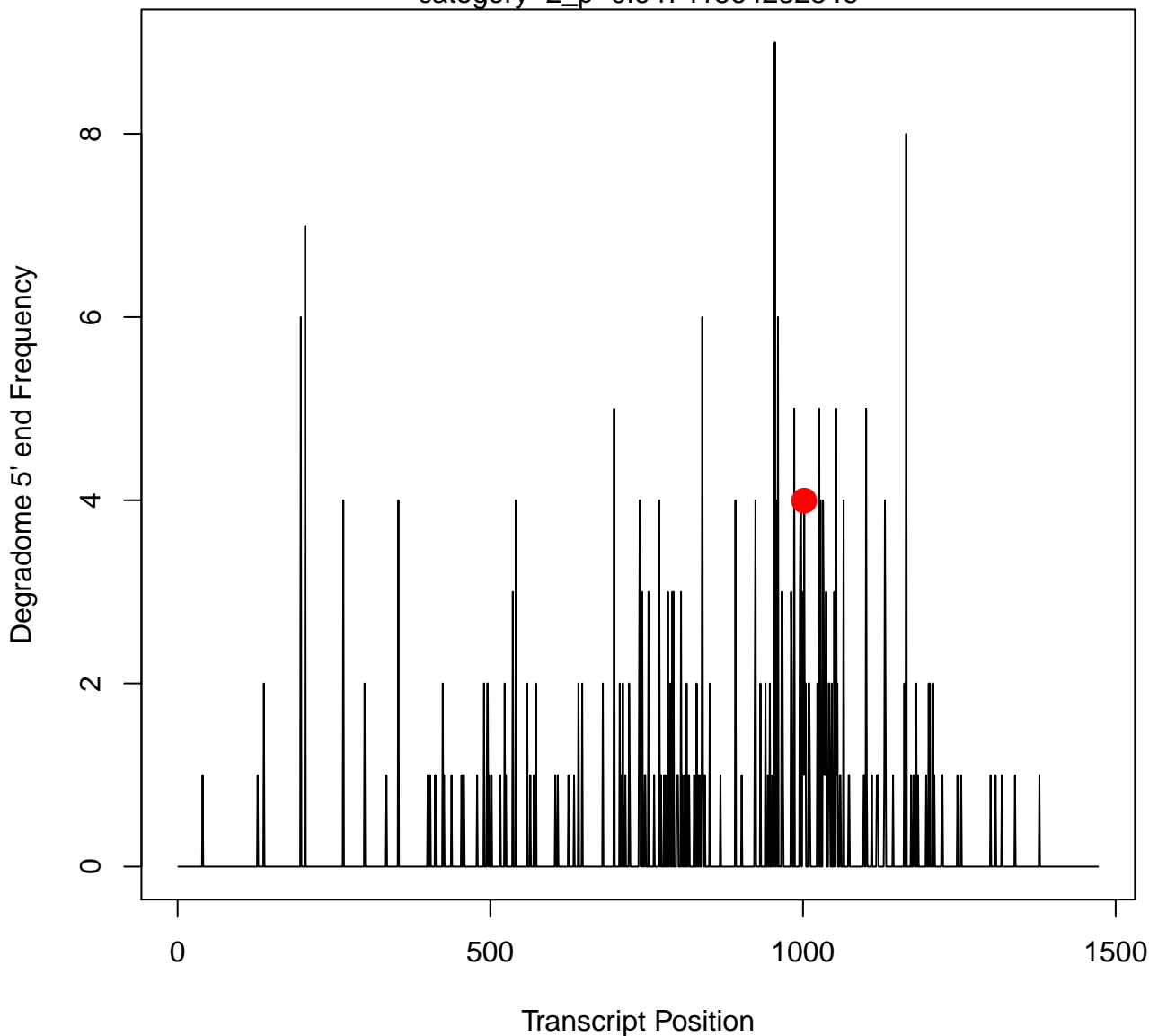

Supplement: Supplementary file 7 [file Data_Sheet_7.zip › Sit-miR159c_Seita.7G203700.1_1002_TPlot.pdf]

**T=Seita.2G324800.1\_Q=Sit-miR160a\_S=712**

category=2\_p=0.999960077382531

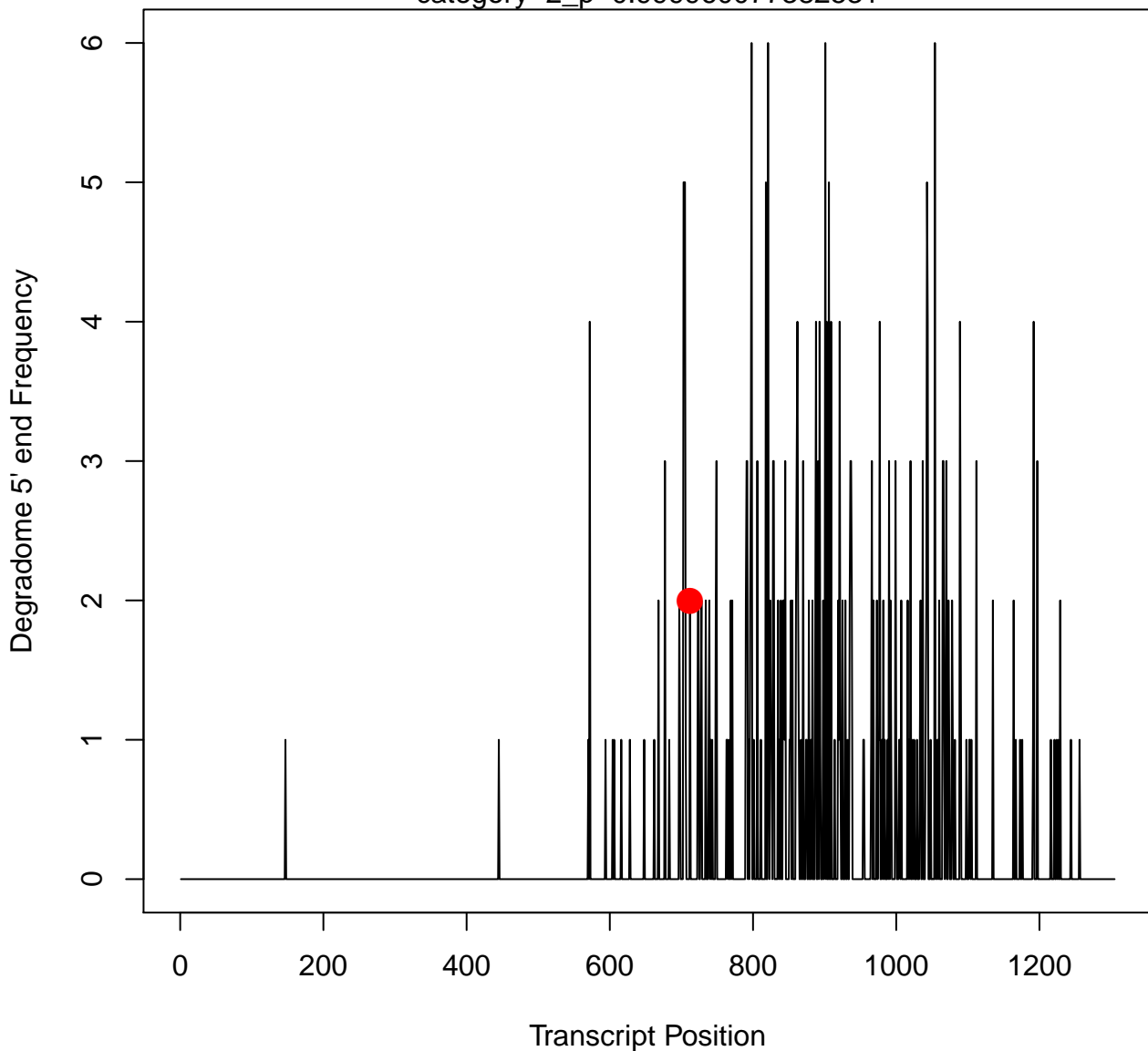

Supplement: Supplementary file 7 [file Data_Sheet_7.zip › Sit-miR160a_Seita.2G324800.1_712_TPlot.pdf]

**T=Seita.3G010200.1\_Q=Sit-miR160a\_S=1143**

category=2\_p=0.990482476952454

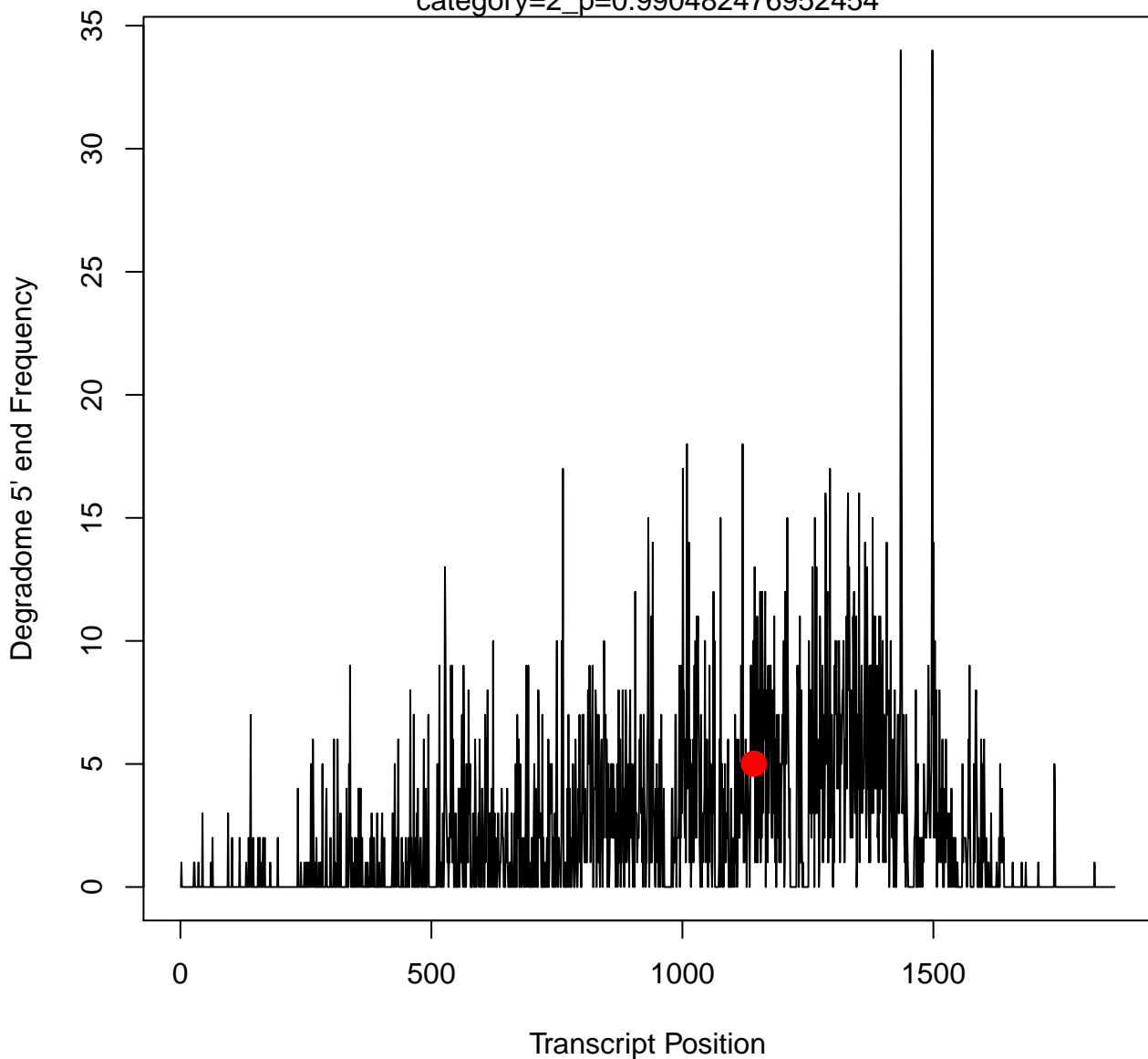

Supplement: Supplementary file 7 [file Data_Sheet_7.zip › Sit-miR160a_Seita.3G010200.1_1143_TPlot.pdf]

**T=Seita.4G006200.1\_Q=Sit-miR160a\_S=649**

category=2\_p=0.742343041739321

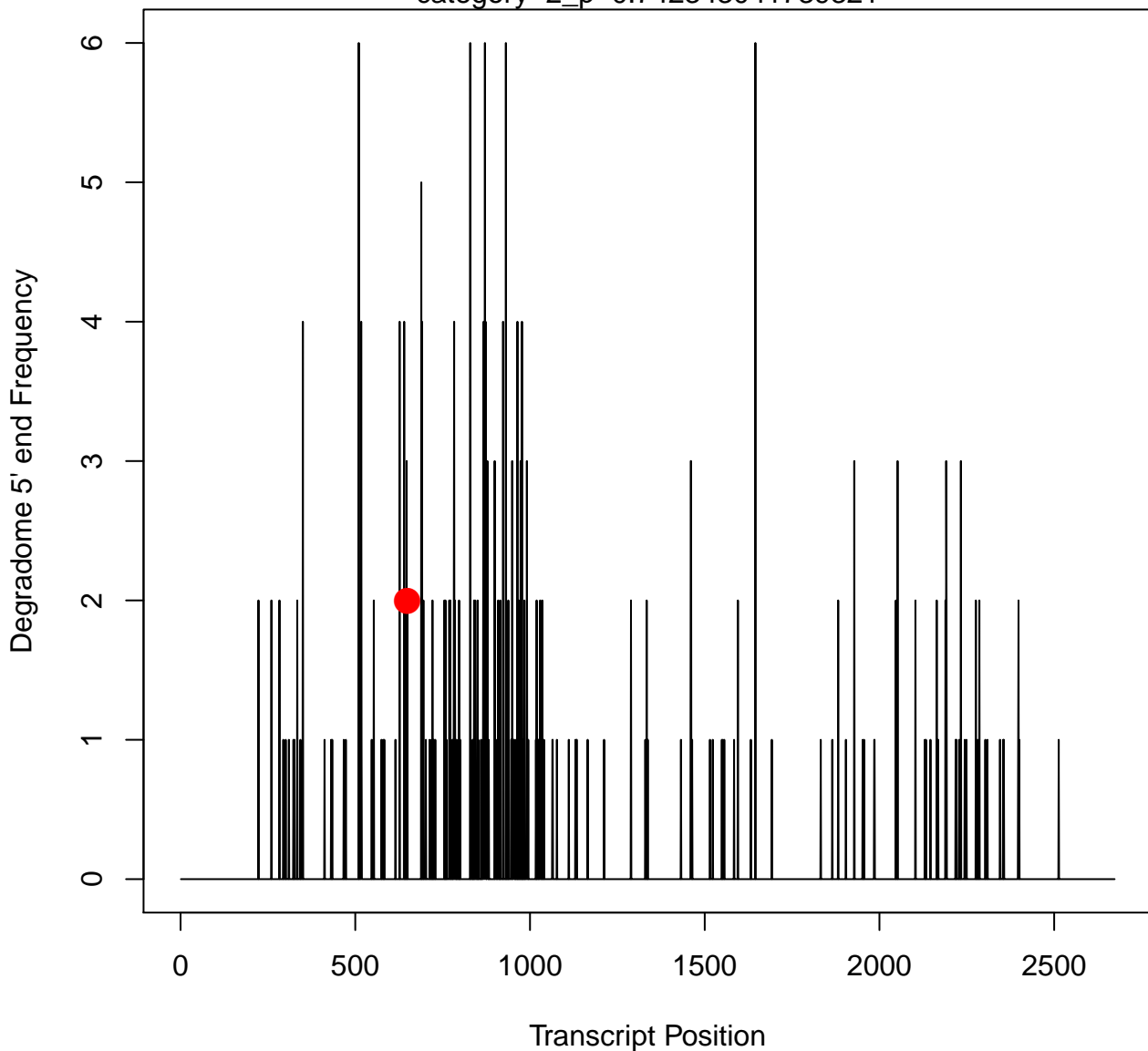

Supplement: Supplementary file 7 [file Data_Sheet_7.zip › Sit-miR160a_Seita.4G006200.1_649_TPlot.pdf]

**T=Seita.5G079000.1\_Q=Sit-miR160a\_S=2146**

category=2\_p=0.99991317537166

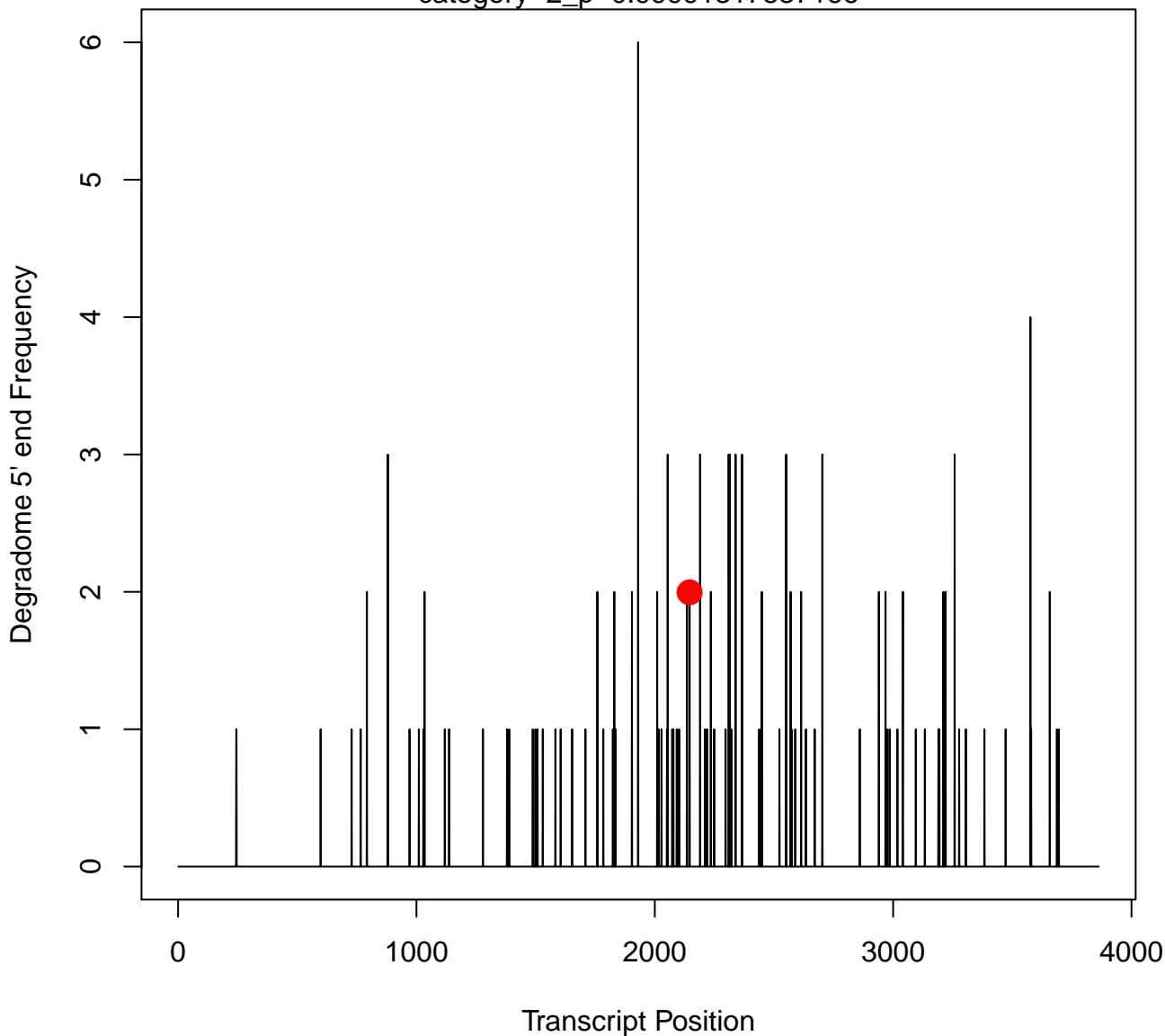

Supplement: Supplementary file 7 [file Data_Sheet_7.zip › Sit-miR160a_Seita.5G079000.1_2146_TPlot.pdf]

**T=Seita.6G214100.1\_Q=Sit-miR160a\_S=912**

category=2\_p=0.998461450557022

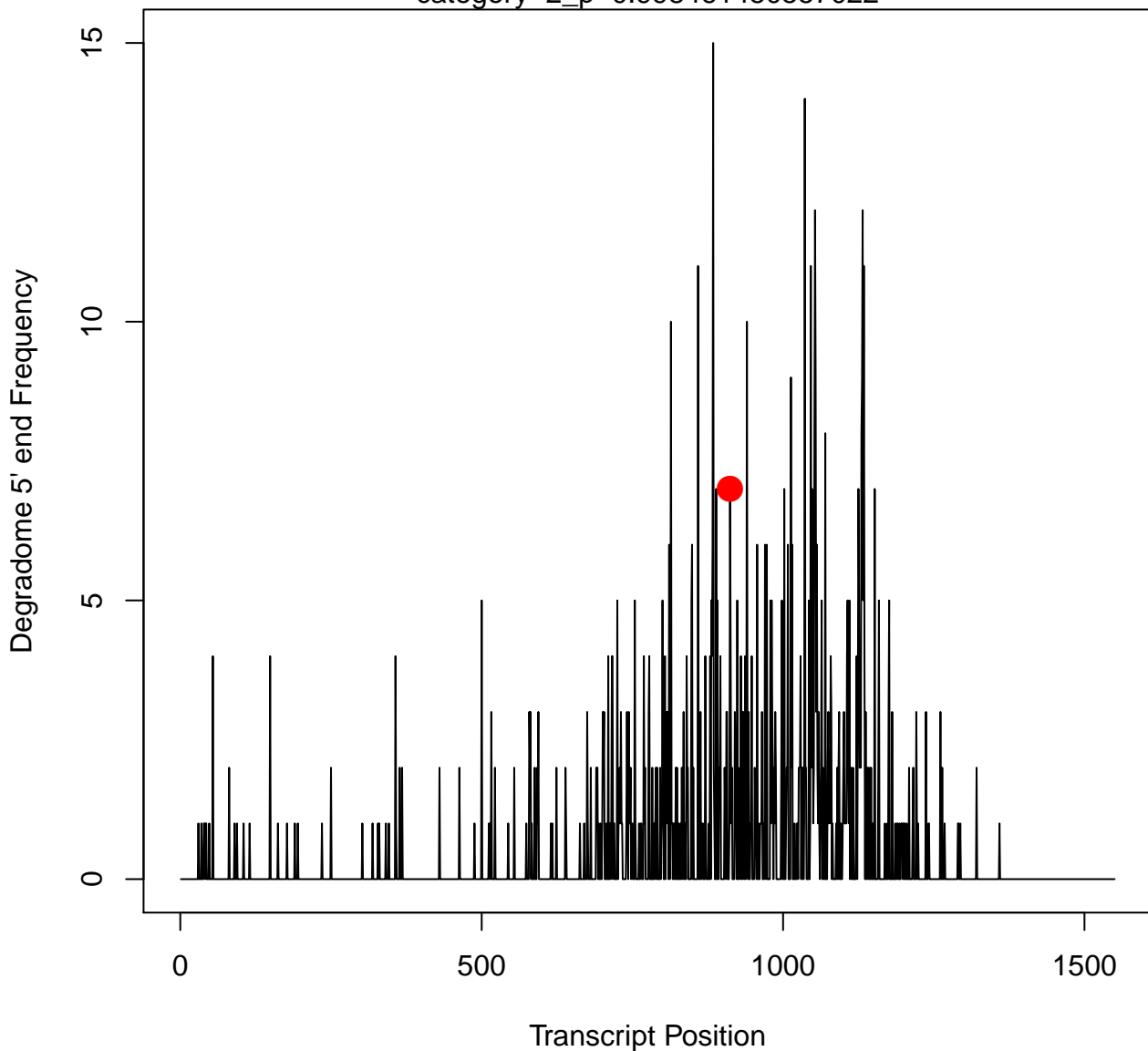

Supplement: Supplementary file 7 [file Data_Sheet_7.zip › Sit-miR160a_Seita.6G214100.1_912_TPlot.pdf]

**T=Seita.9G219800.1\_Q=Sit-miR160a\_S=1729**

category=0\_p=0.000299243605074828

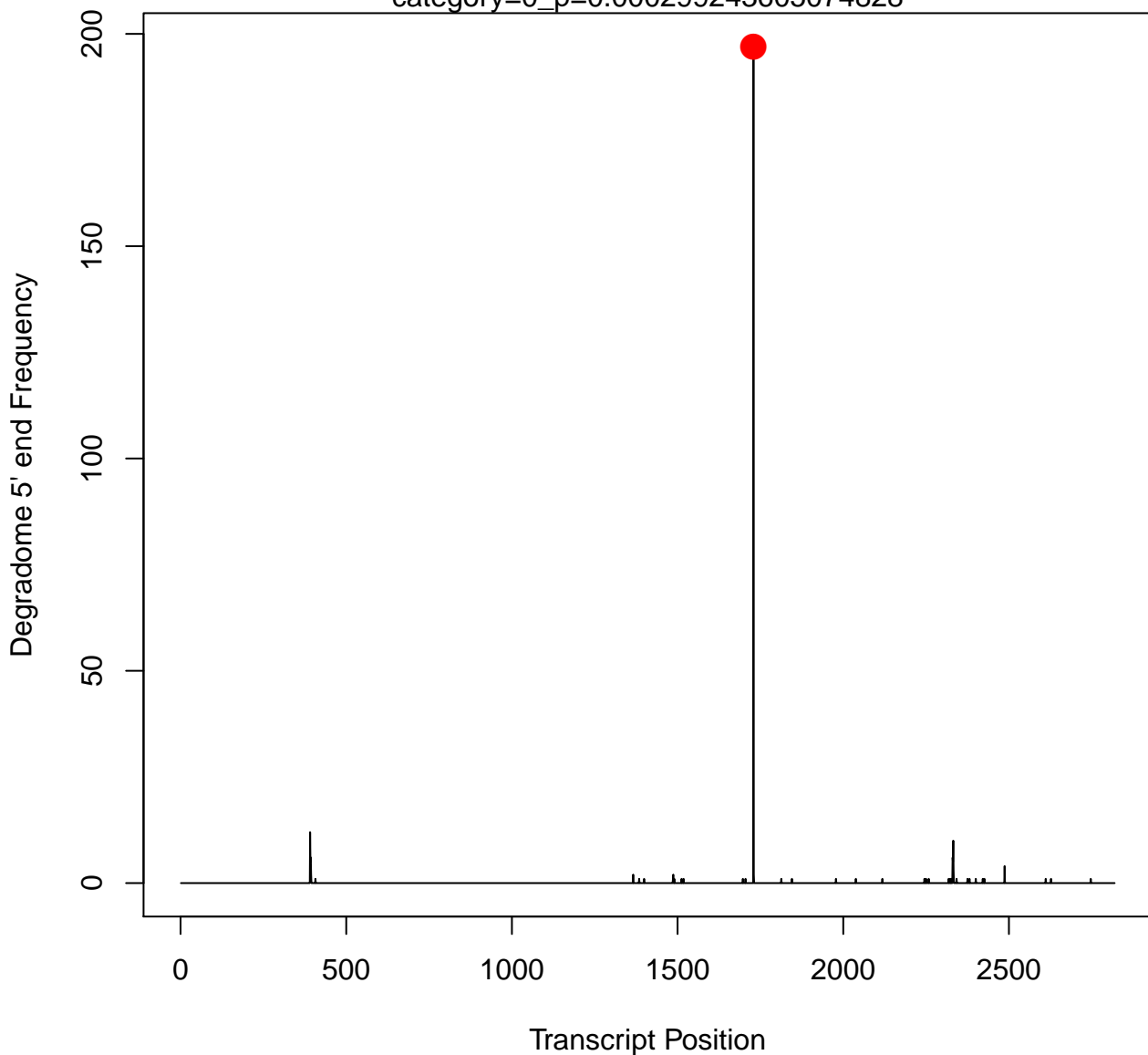

Supplement: Supplementary file 7 [file Data_Sheet_7.zip › Sit-miR160a_Seita.9G219800.1_1729_TPlot.pdf]

**T=Seita.2G412300.1\_Q=Sit-miR160b\_S=1580**

category=2\_p=0.999998764013255

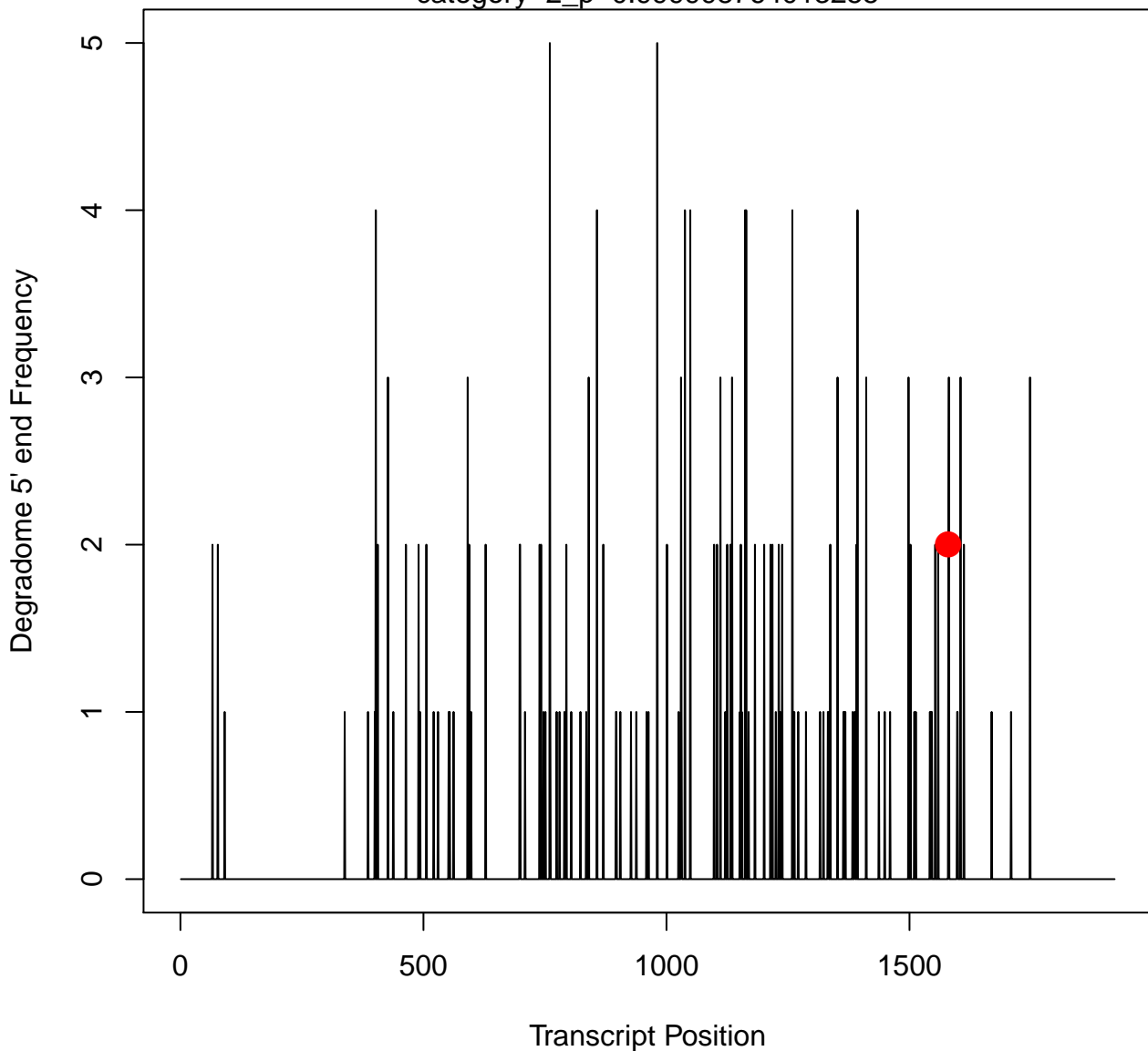

Supplement: Supplementary file 7 [file Data_Sheet_7.zip › Sit-miR160b_Seita.2G412300.1_1580_TPlot.pdf]

**T=Seita.4G043900.1\_Q=Sit-miR160b\_S=308**

category=0\_p=0.191919285940511

Degradsome 5' end Frequency

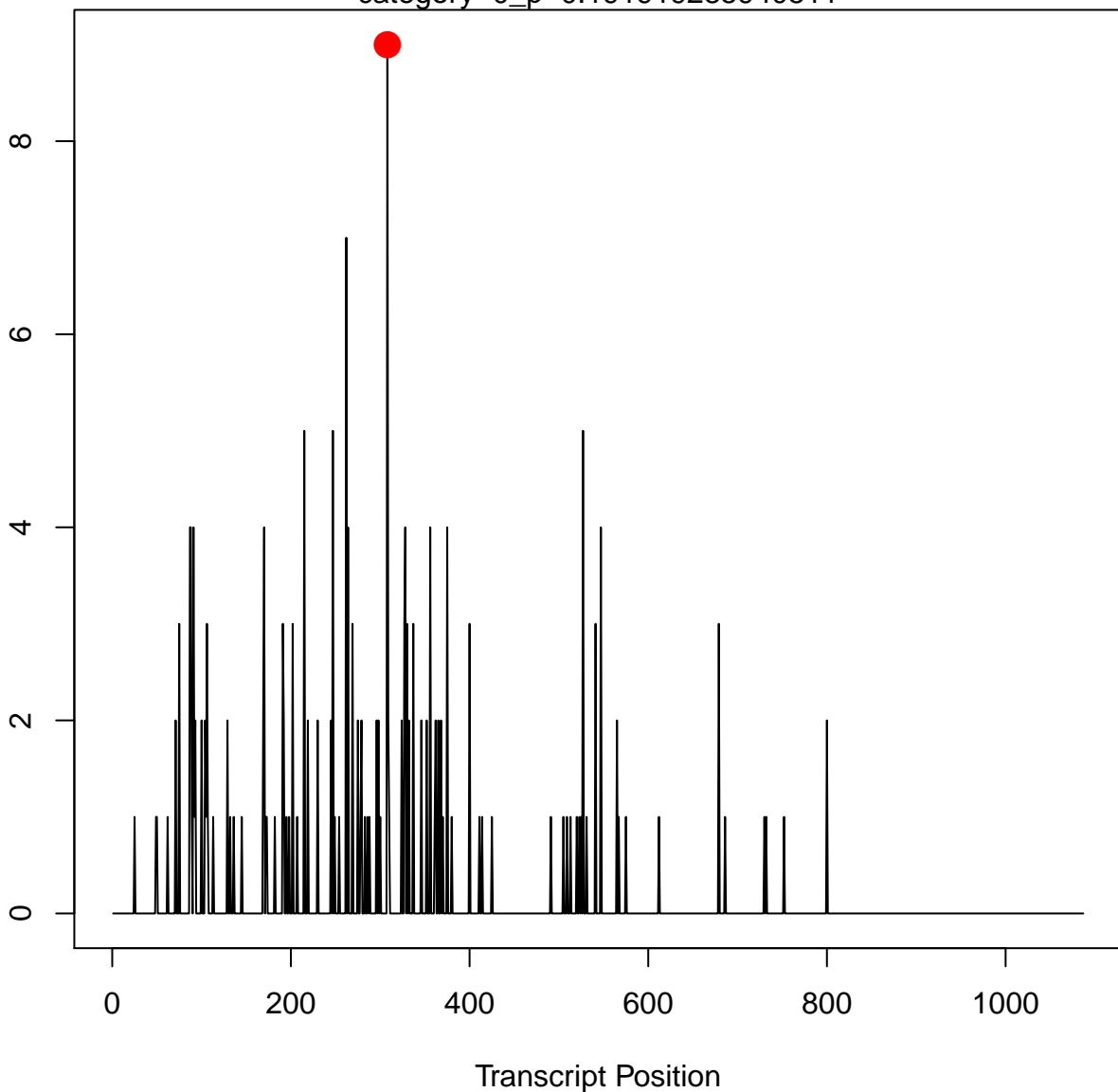

Supplement: Supplementary file 7 [file Data_Sheet_7.zip › Sit-miR160b_Seita.4G043900.1_308_TPlot.pdf]

**T=Seita.6G112700.1\_Q=Sit-miR160b\_S=2260**

category=2\_p=0.994628974325528

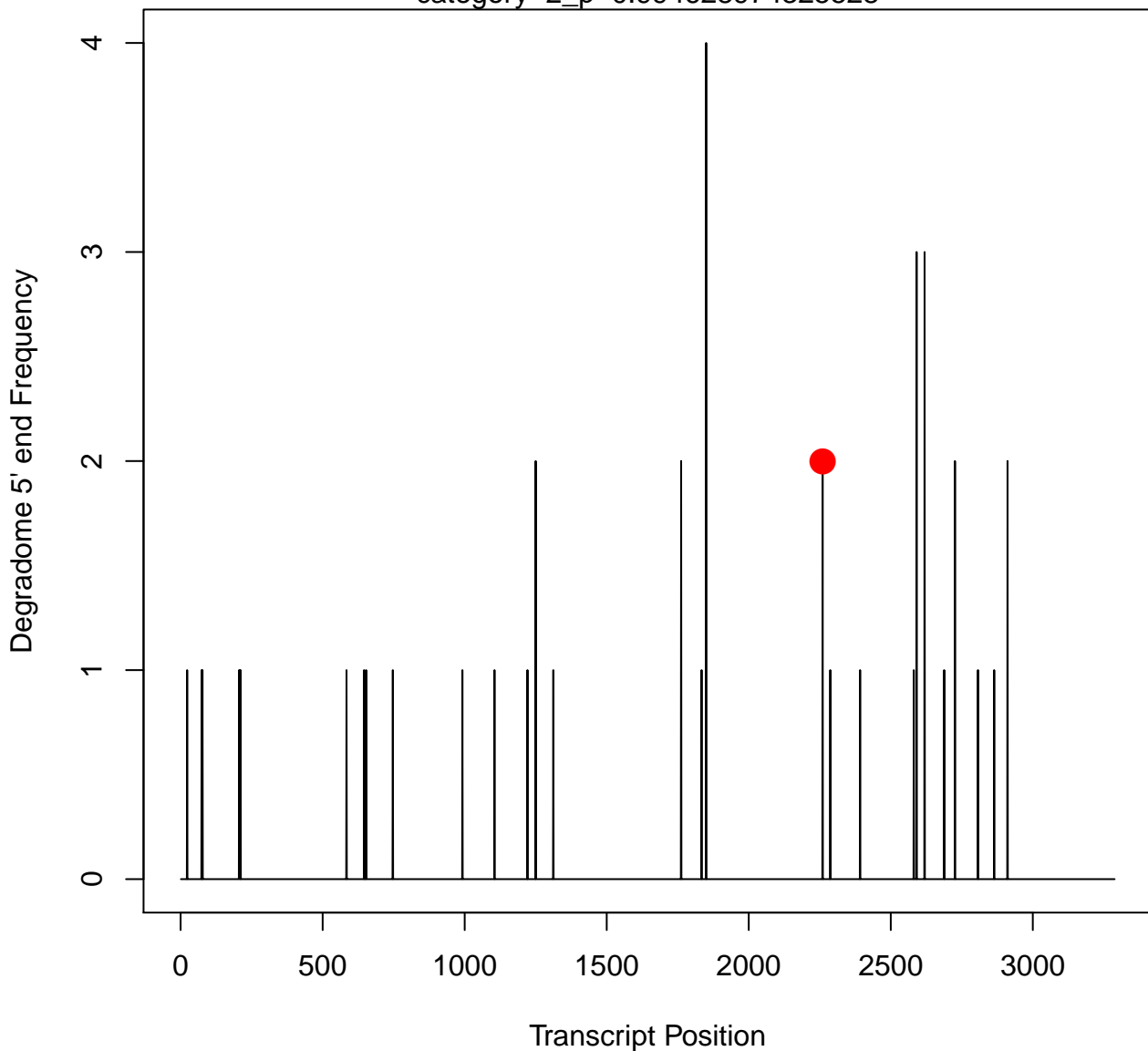

Supplement: Supplementary file 7 [file Data_Sheet_7.zip › Sit-miR160b_Seita.6G112700.1_2260_TPlot.pdf]

**T=Seita.9G108700.1\_Q=Sit-miR160b\_S=370**

category=2\_p=0.999988724436479

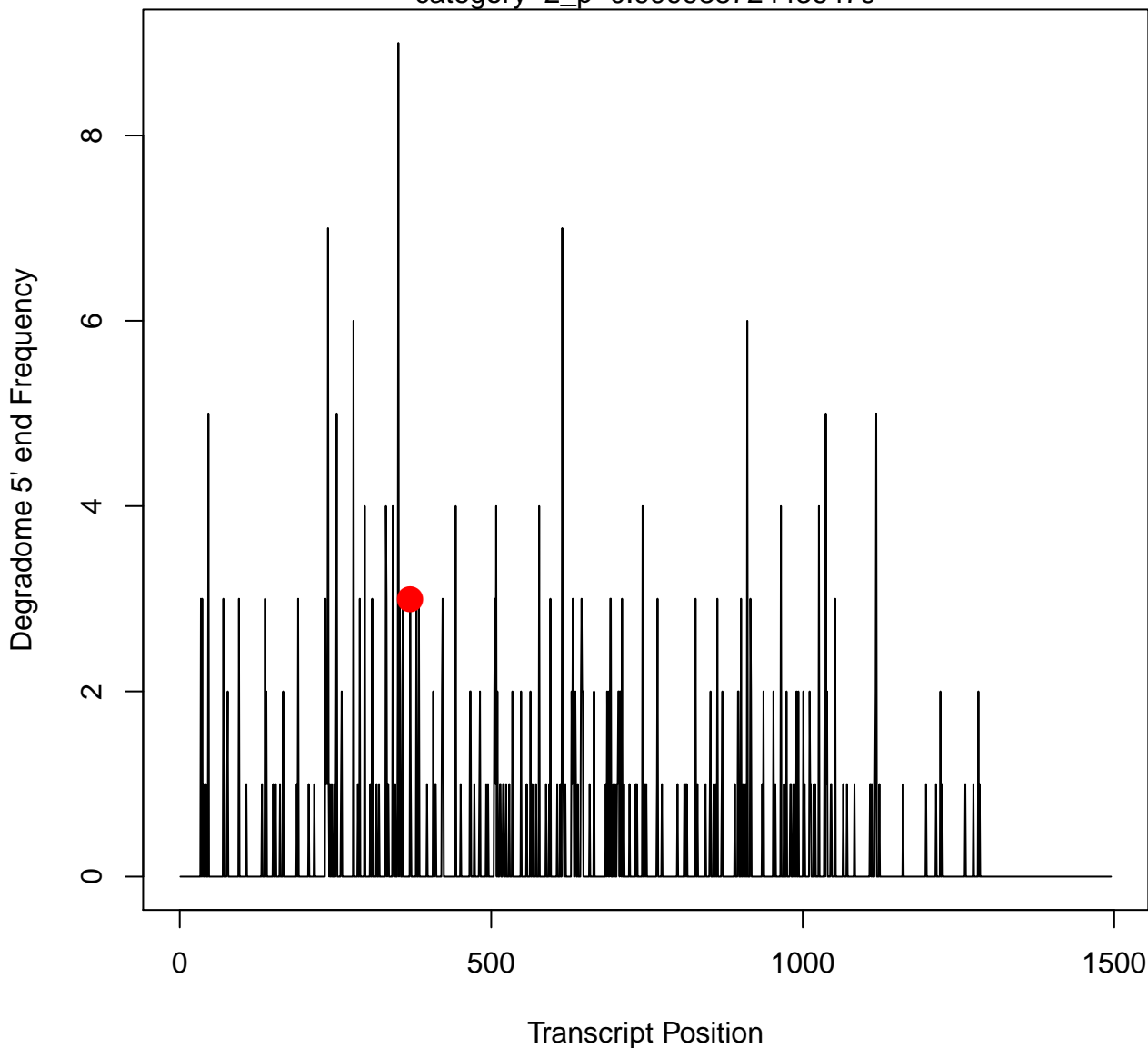

Supplement: Supplementary file 7 [file Data_Sheet_7.zip › Sit-miR160b_Seita.9G108700.1_370_TPlot.pdf]

**T=Seita.1G057100.1\_Q=Sit-miR160c\_S=1565**

category=2\_p=0.996653927221265

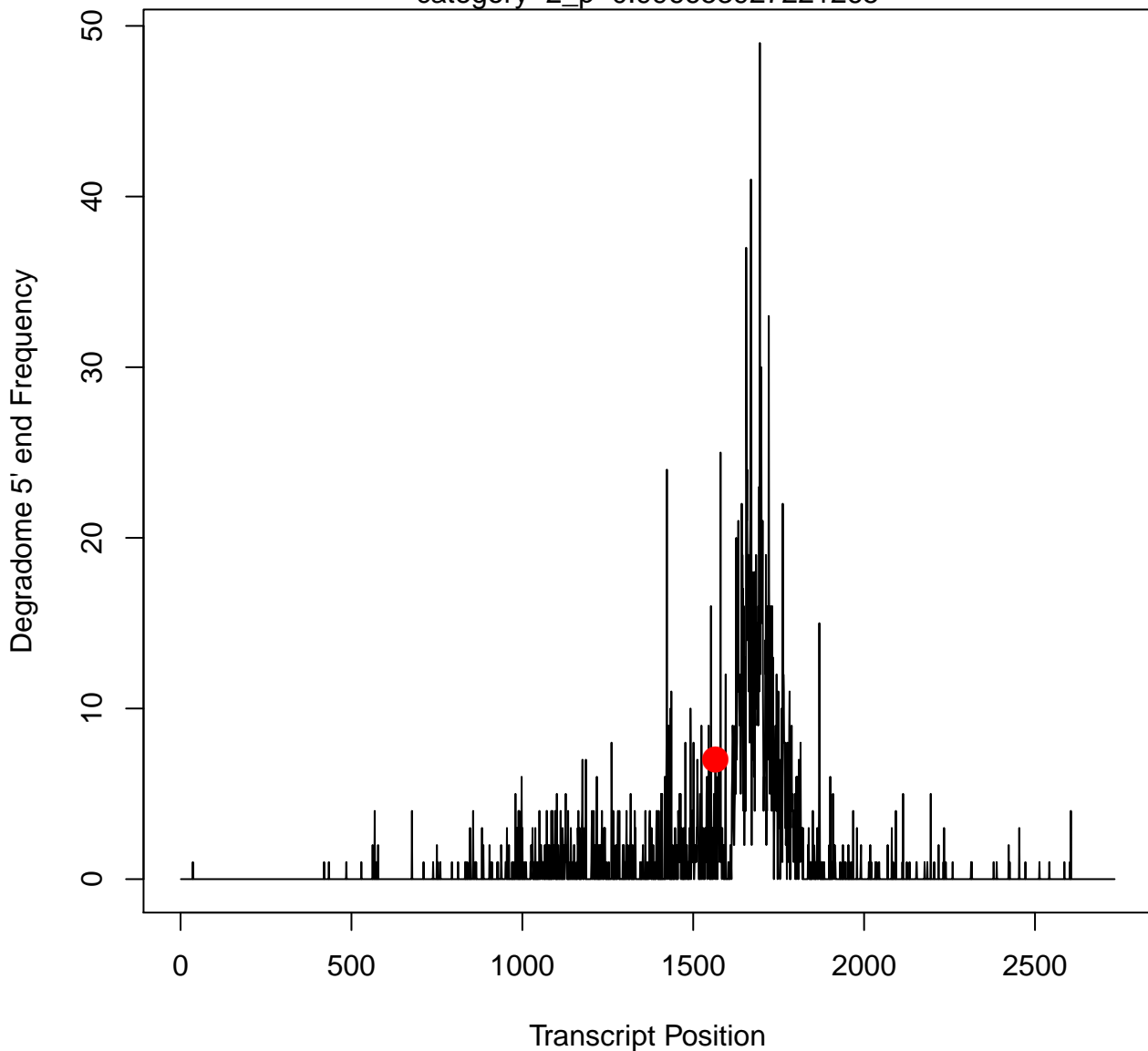

Supplement: Supplementary file 7 [file Data_Sheet_7.zip › Sit-miR160c_Seita.1G057100.1_1565_TPlot.pdf]

**T=Seita.1G099400.1\_Q=Sit-miR160c\_S=538**

category=2\_p=0.997311913834978

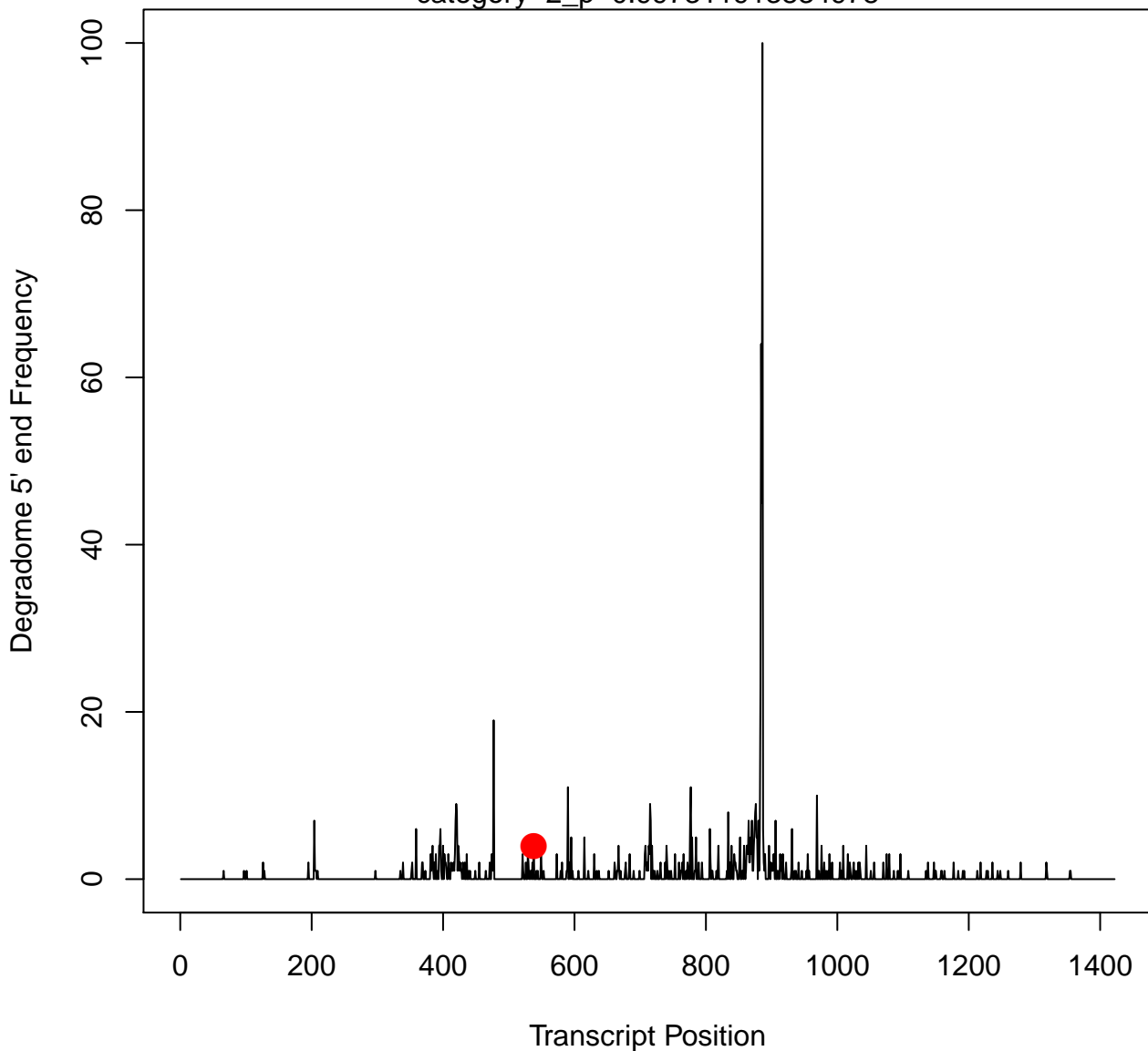

Supplement: Supplementary file 7 [file Data_Sheet_7.zip › Sit-miR160c_Seita.1G099400.1_538_TPlot.pdf]

**T=Seita.1G141900.1\_Q=Sit-miR160c\_S=1482**

category=2\_p=0.999655805815537

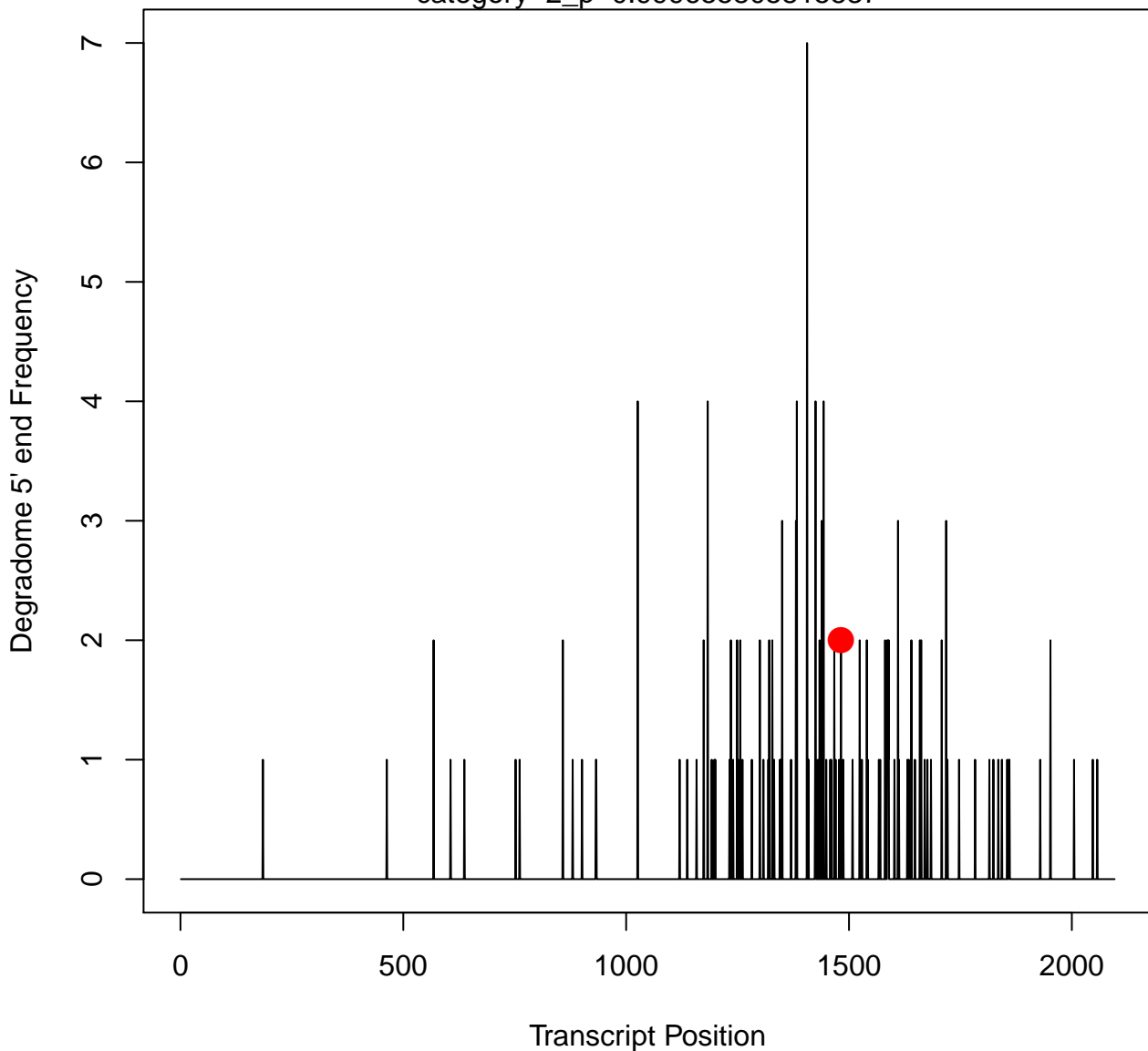

Supplement: Supplementary file 7 [file Data_Sheet_7.zip › Sit-miR160c_Seita.1G141900.1_1482_TPlot.pdf]

**T=Seita.2G112500.1\_Q=Sit-miR160c\_S=2104**

category=2\_p=0.9998872438934

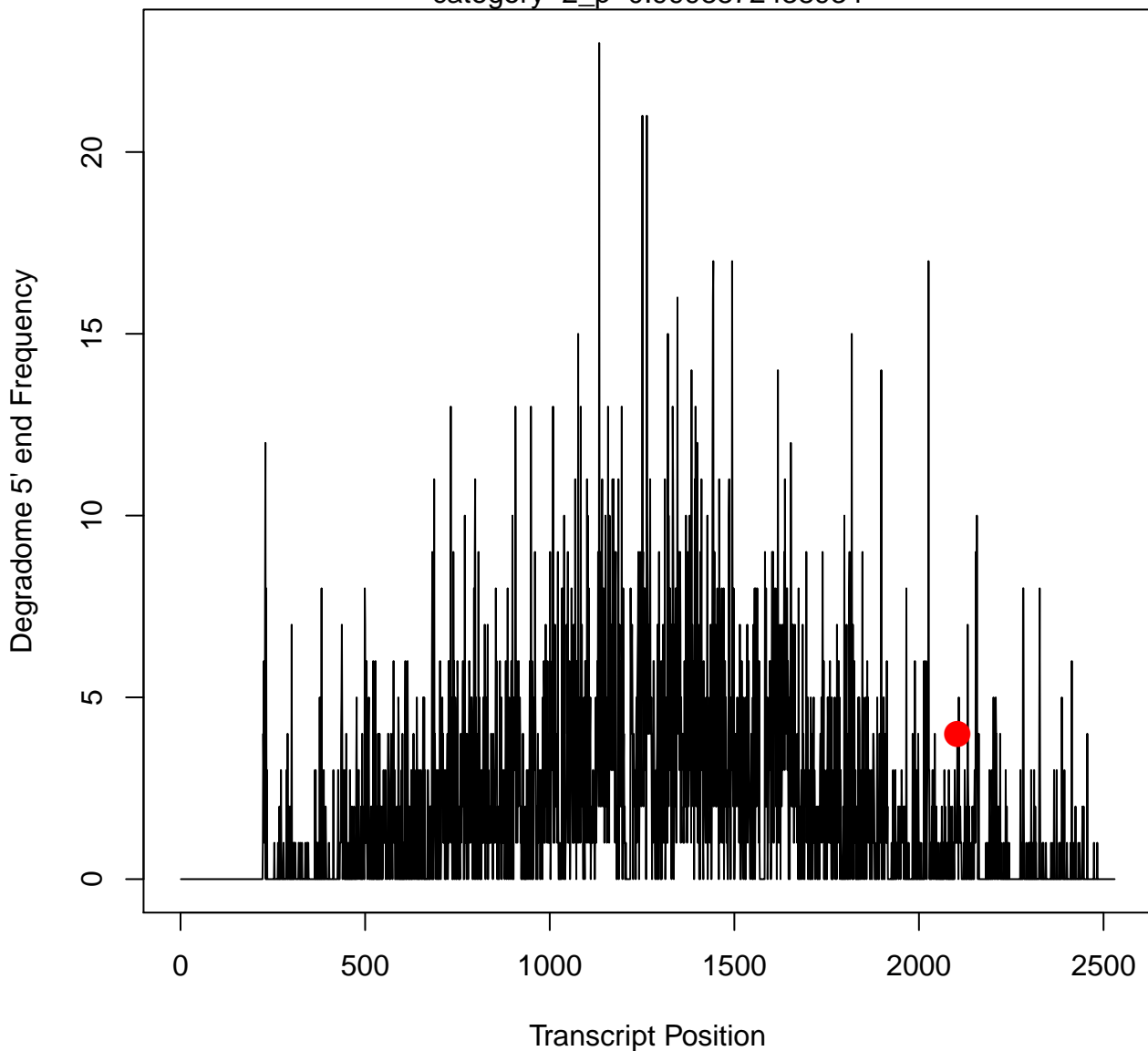

Supplement: Supplementary file 7 [file Data_Sheet_7.zip › Sit-miR160c_Seita.2G112500.1_2104_TPlot.pdf]

**T=Seita.3G003300.1\_Q=Sit-miR160c\_S=1363**

category=0\_p=0.000299243605074828

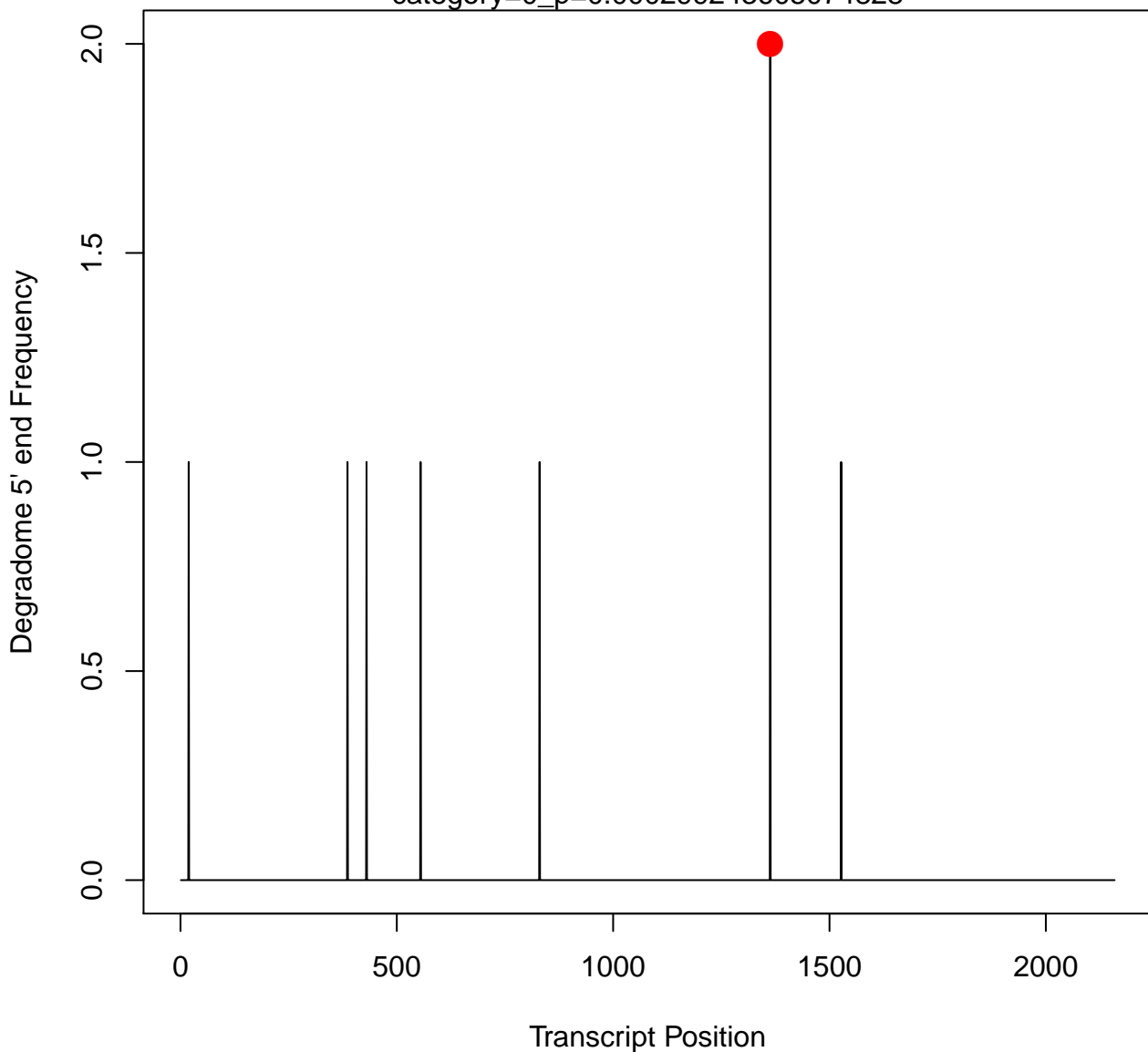

Supplement: Supplementary file 7 [file Data_Sheet_7.zip › Sit-miR160c_Seita.3G003300.1_1363_TPlot.pdf]

**T=Seita.4G127200.1\_Q=Sit-miR160c\_S=1448**

category=2\_p=0.954664409333265

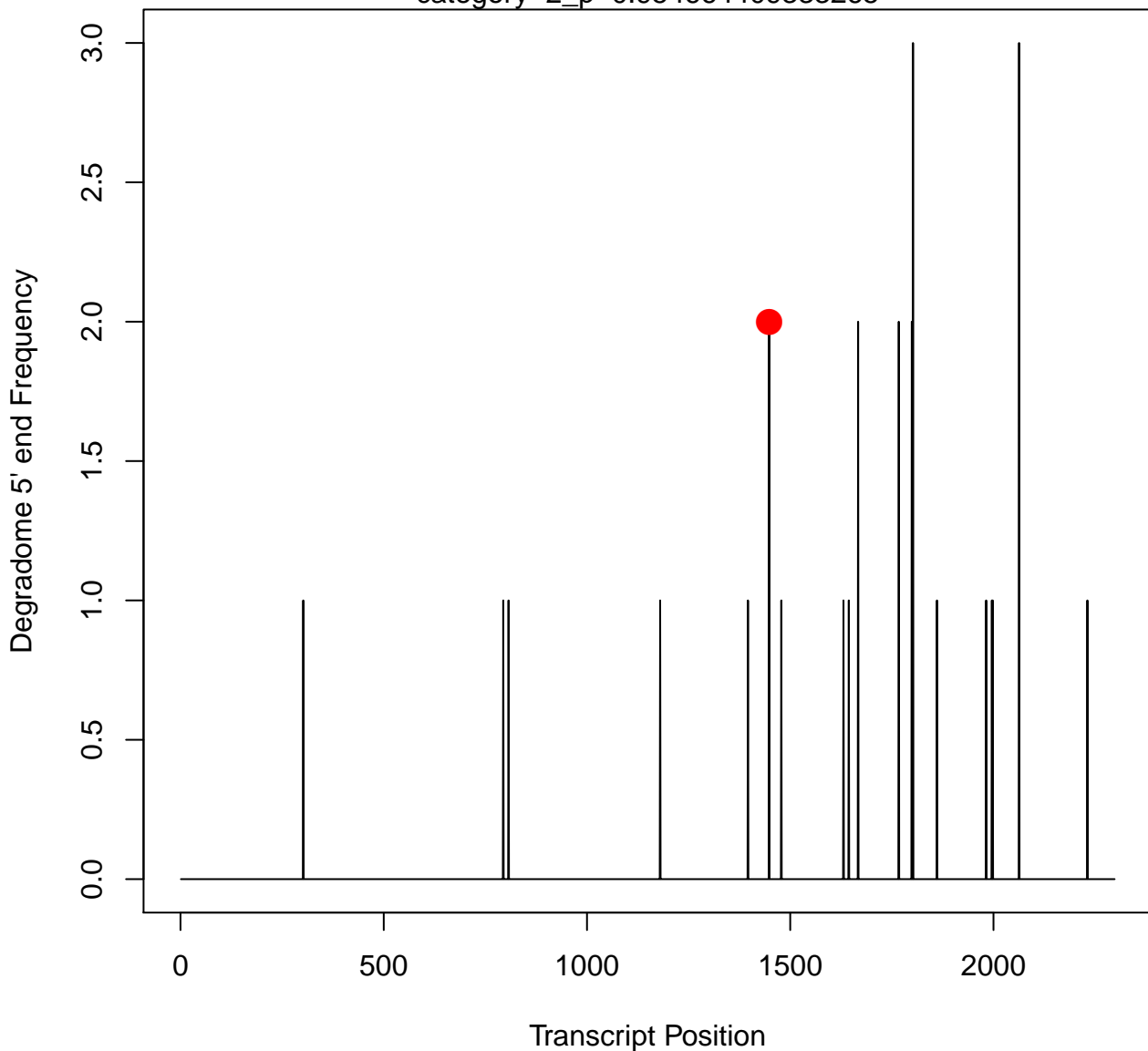

Supplement: Supplementary file 7 [file Data_Sheet_7.zip › Sit-miR160c_Seita.4G127200.1_1448_TPlot.pdf]

**T=Seita.4G173800.1\_Q=Sit-miR160c\_S=259**

category=2\_p=0.611888680911436

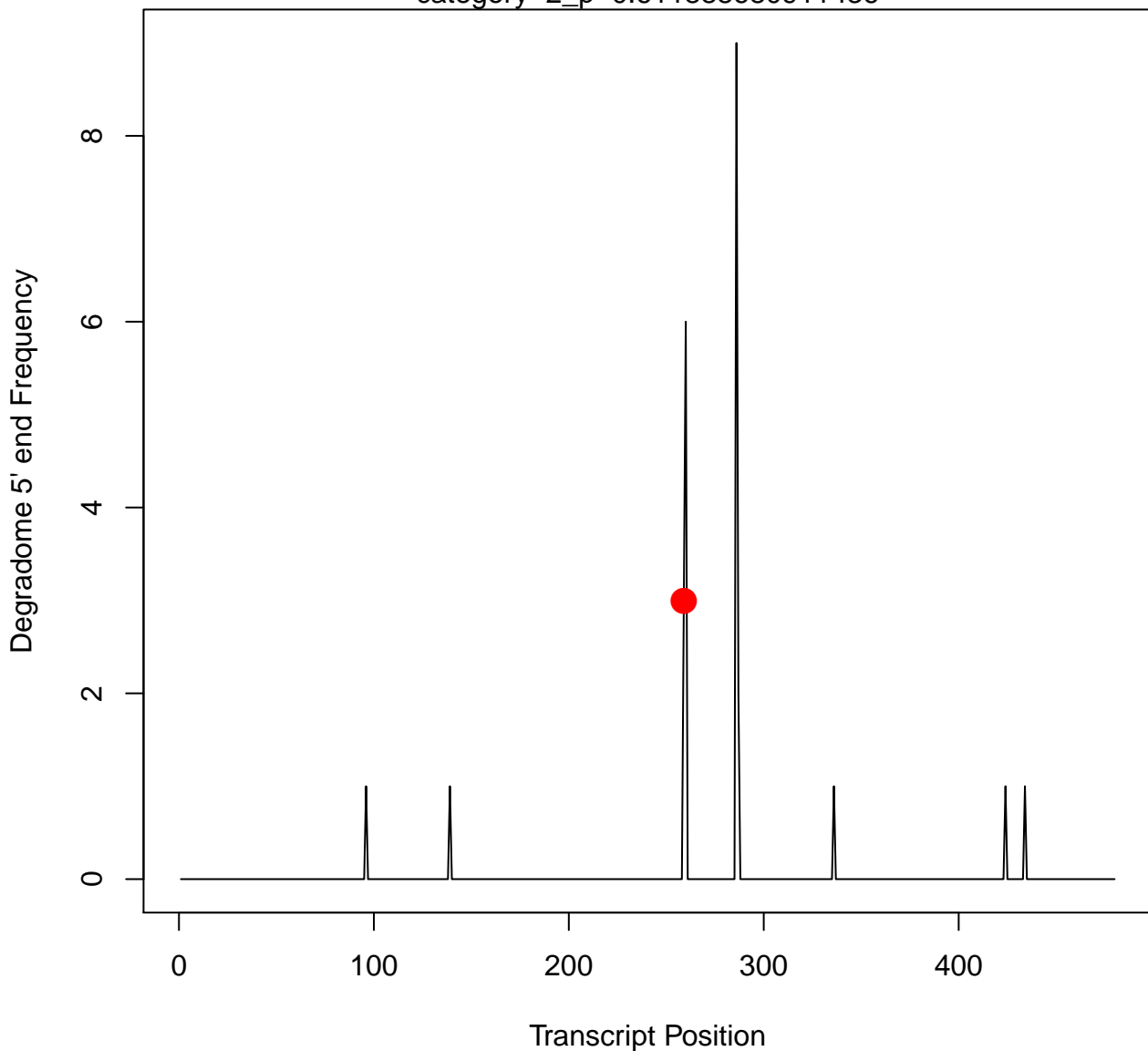

Supplement: Supplementary file 7 [file Data_Sheet_7.zip › Sit-miR160c_Seita.4G173800.1_259_TPlot.pdf]

**T=Seita.5G021300.1\_Q=Sit-miR160c\_S=471**

category=2\_p=0.492400789735958

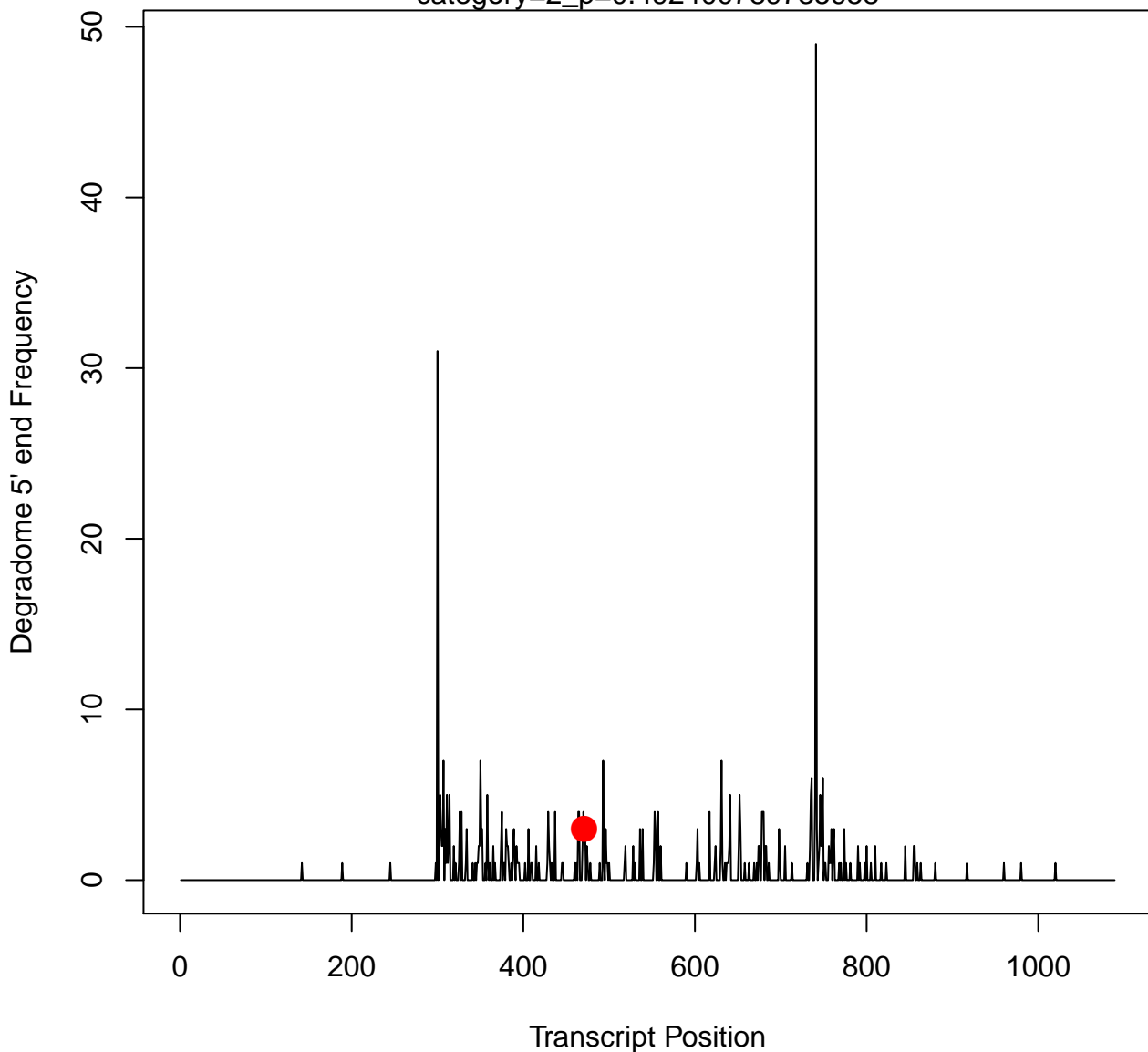

Supplement: Supplementary file 7 [file Data_Sheet_7.zip › Sit-miR160c_Seita.5G021300.1_471_TPlot.pdf]

**T=Seita.5G392600.1\_Q=Sit-miR160c\_S=931**

category=2\_p=0.999640905734681

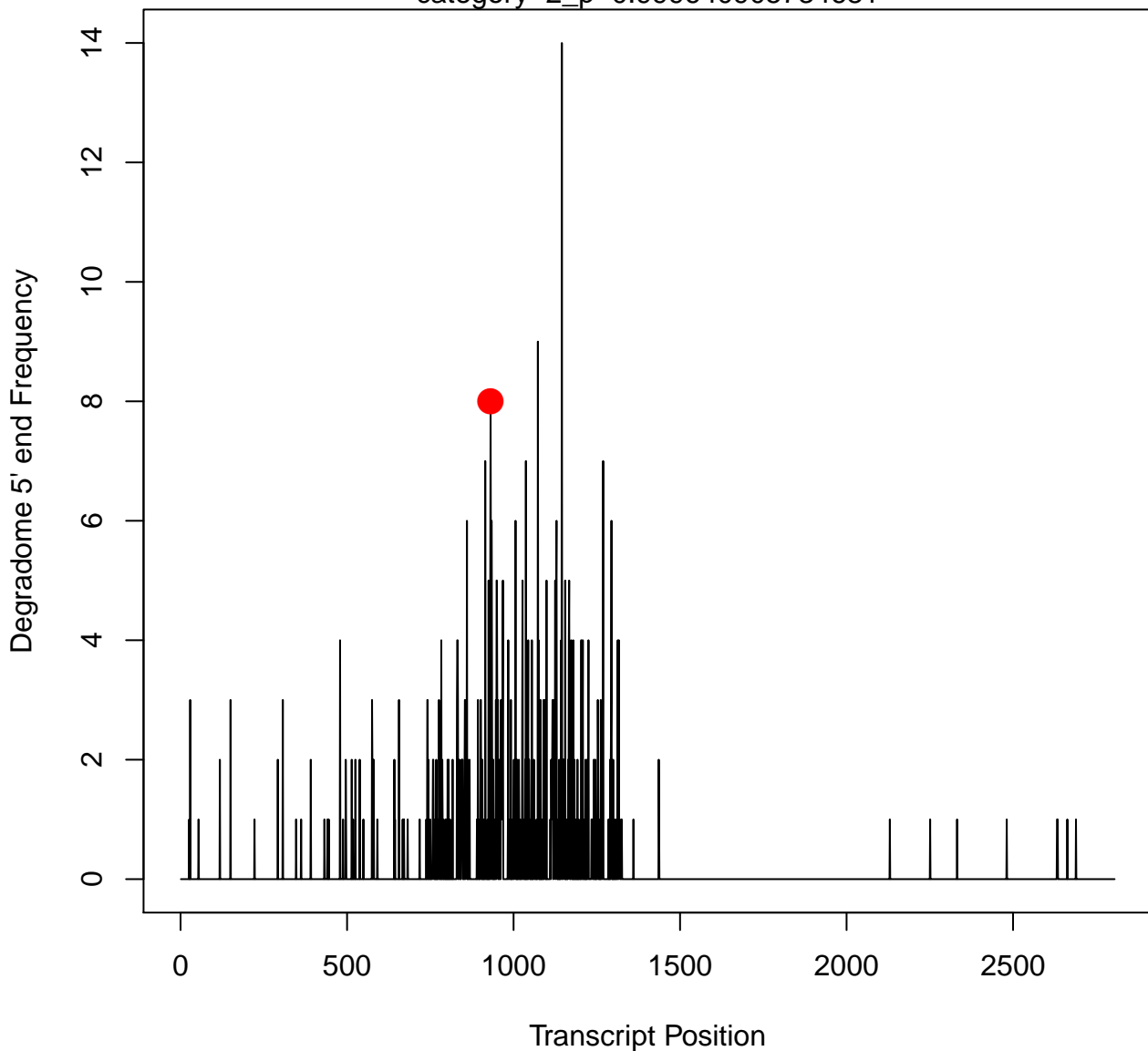

Supplement: Supplementary file 7 [file Data_Sheet_7.zip › Sit-miR160c_Seita.5G392600.1_931_TPlot.pdf]

**T=Seita.7G117100.1\_Q=Sit-miR160c\_S=894**

category=2\_p=0.998417362622778

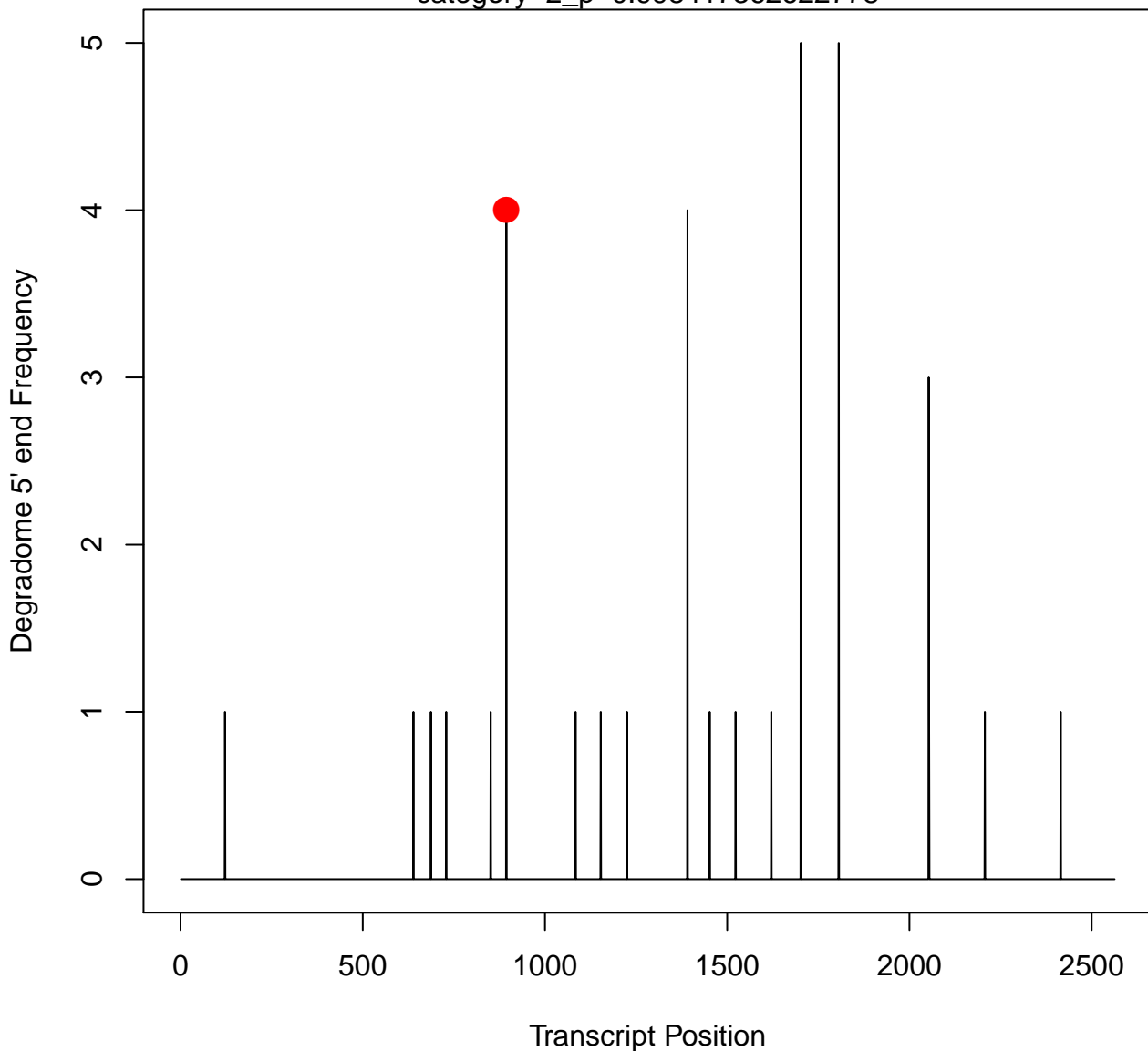

Supplement: Supplementary file 7 [file Data_Sheet_7.zip › Sit-miR160c_Seita.7G117100.1_894_TPlot.pdf]

**T=Seita.7G155700.1\_Q=Sit-miR160c\_S=1461**

category=2\_p=0.884827713467495

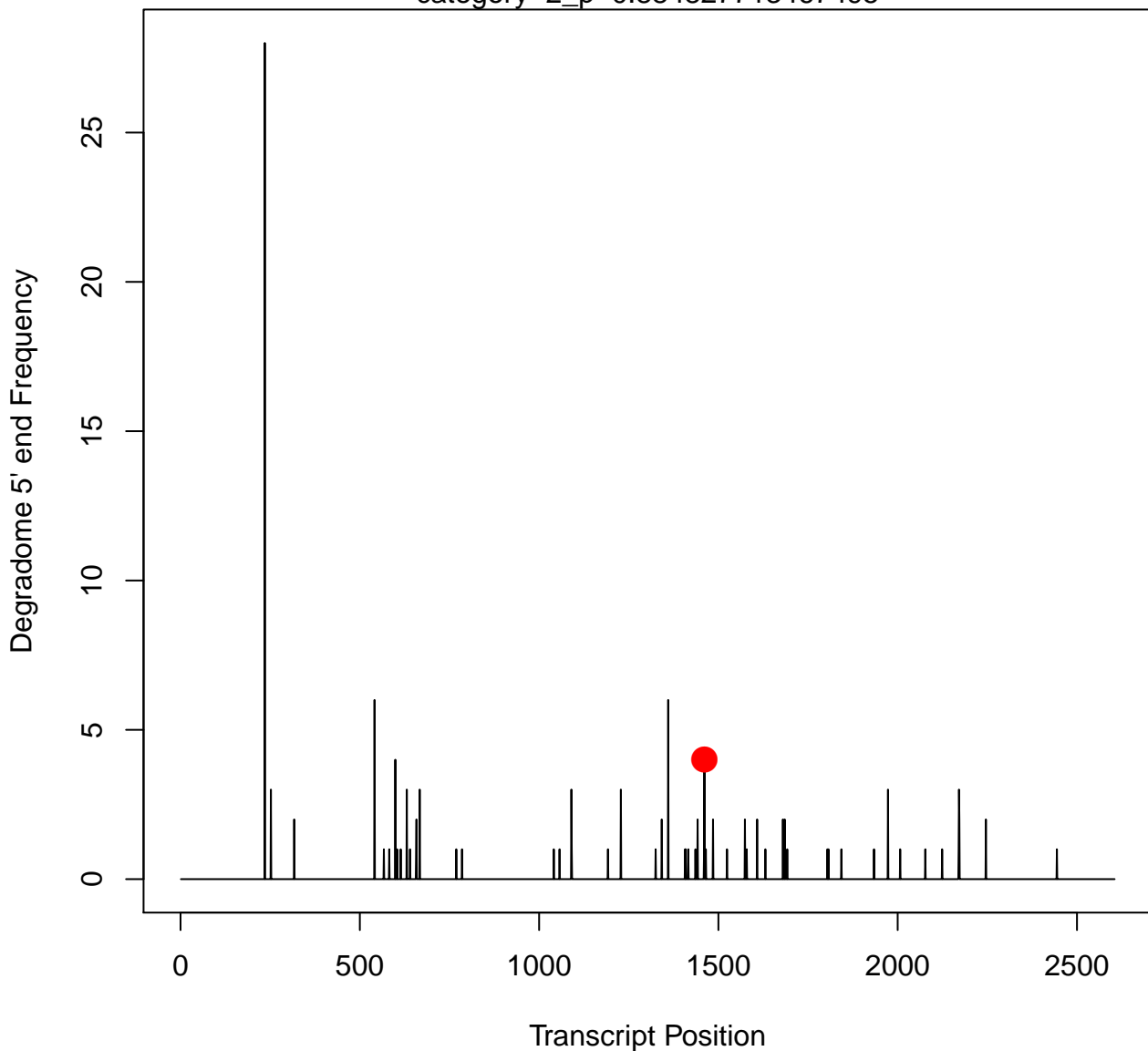

Supplement: Supplementary file 7 [file Data_Sheet_7.zip › Sit-miR160c_Seita.7G155700.1_1461_TPlot.pdf]

**T=Seita.7G295400.1\_Q=Sit-miR160c\_S=1097**

category=1\_p=0.255783346510205

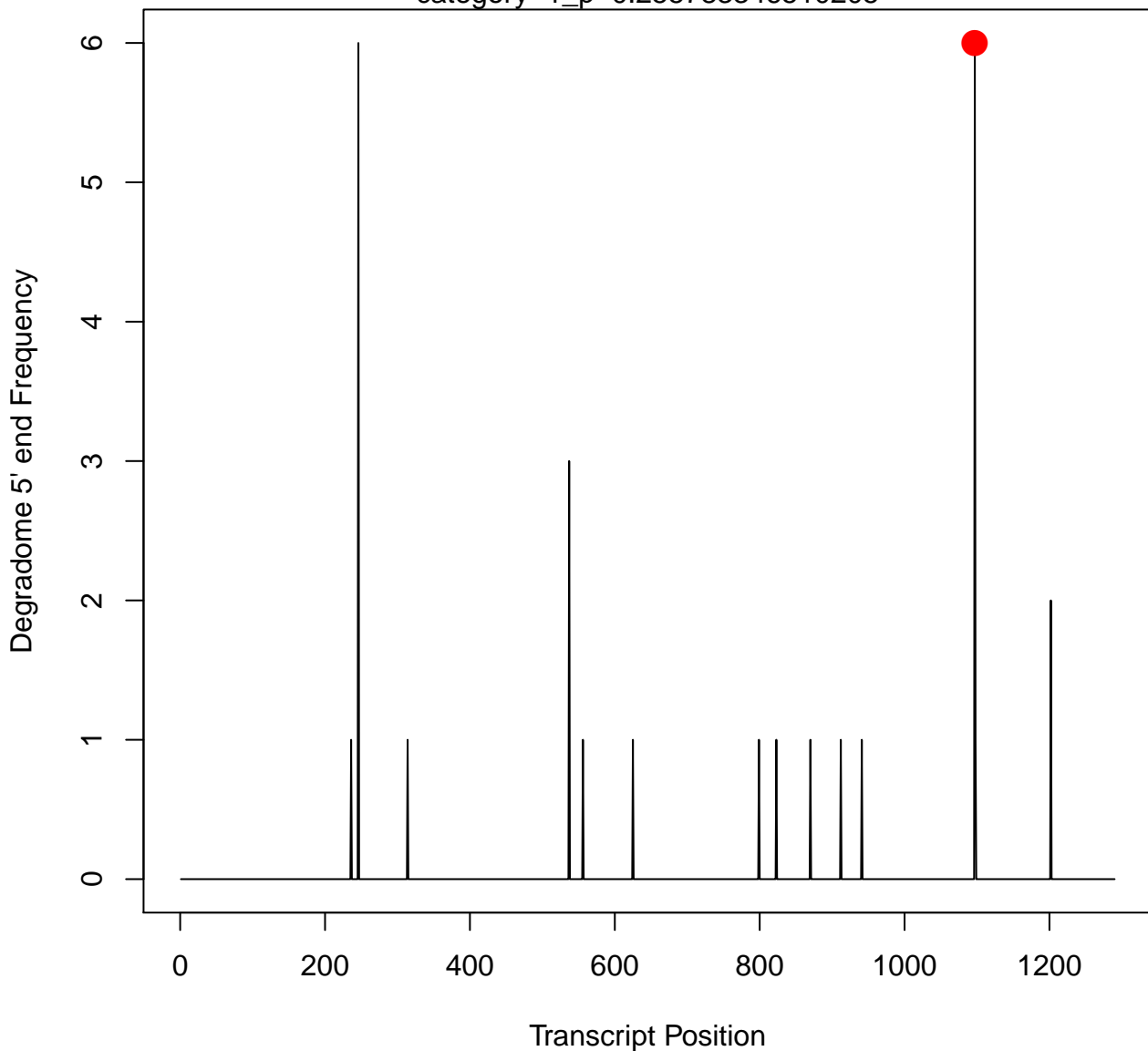

Supplement: Supplementary file 7 [file Data_Sheet_7.zip › Sit-miR160c_Seita.7G295400.1_1097_TPlot.pdf]

**T=Seita.9G045100.1\_Q=Sit-miR160c\_S=835**

category=2\_p=0.819004672321076

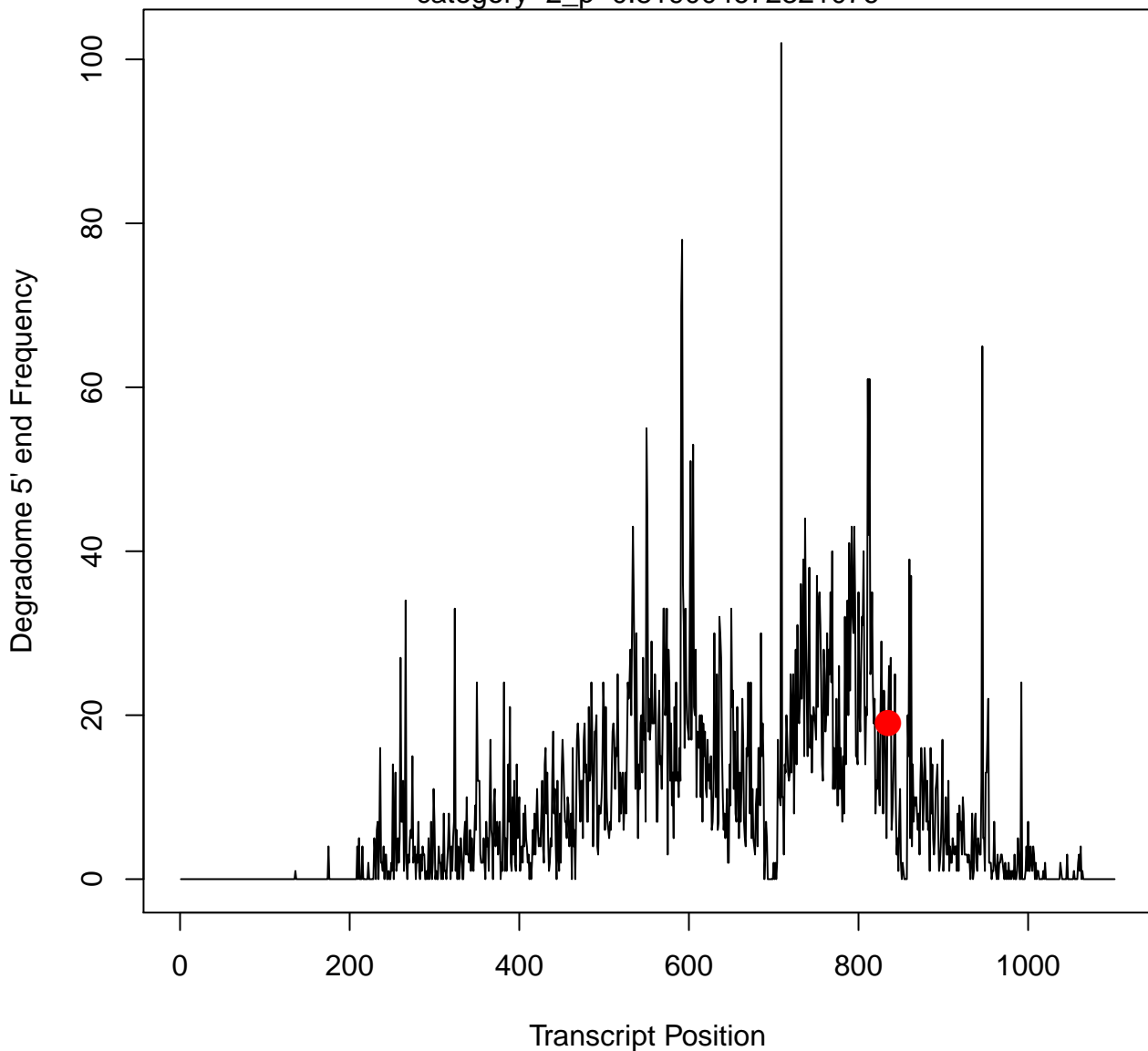

Supplement: Supplementary file 7 [file Data_Sheet_7.zip › Sit-miR160c_Seita.9G045100.1_835_TPlot.pdf]

**T=Seita.9G427100.1\_Q=Sit-miR160c\_S=386**

category=2\_p=0.99908127682771

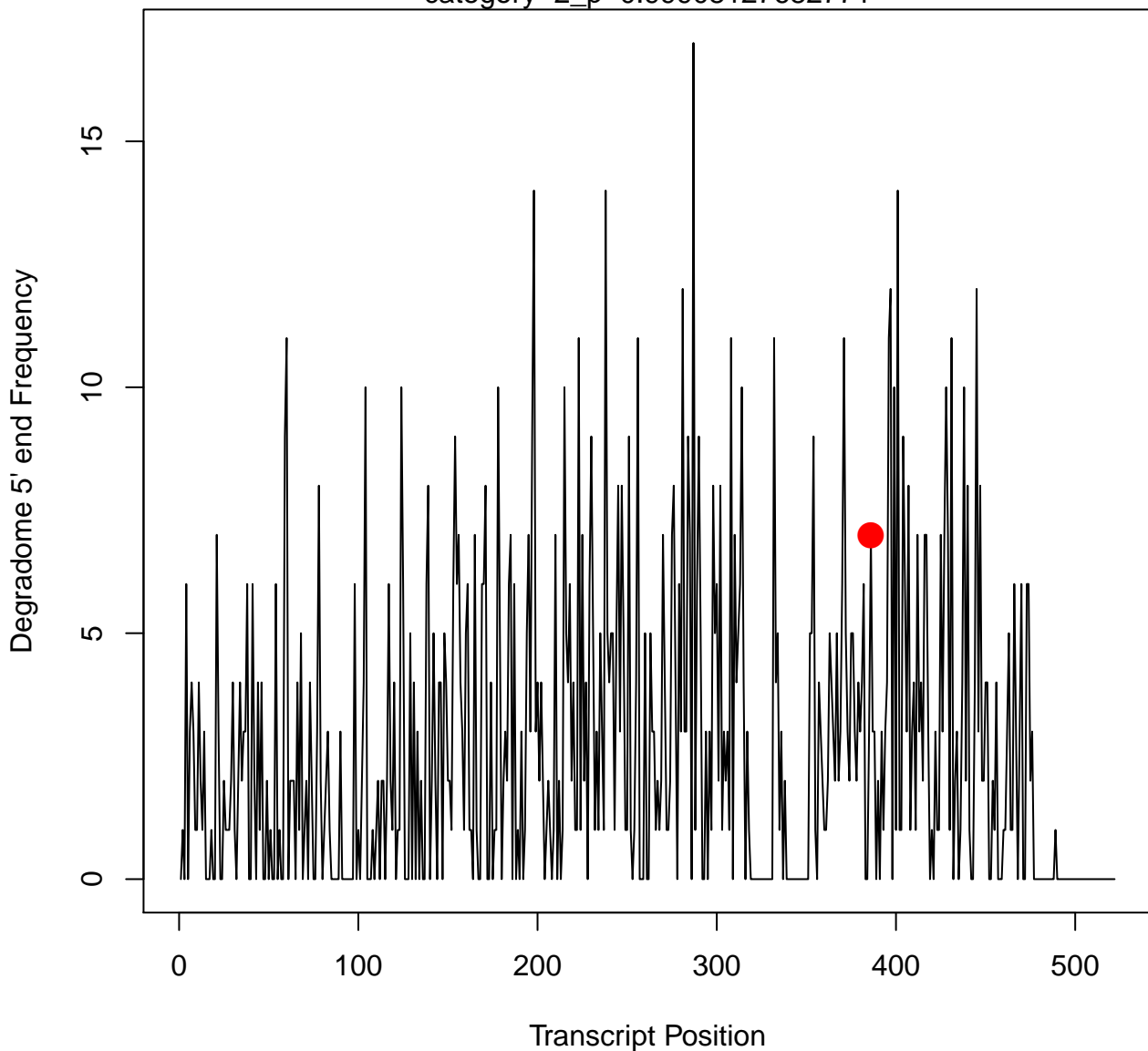

Supplement: Supplementary file 7 [file Data_Sheet_7.zip › Sit-miR160c_Seita.9G427100.1_386_TPlot.pdf]

**T=Seita.1G099500.1\_Q=Sit-miR160d\_S=838**

category=2\_p=0.999240789100853

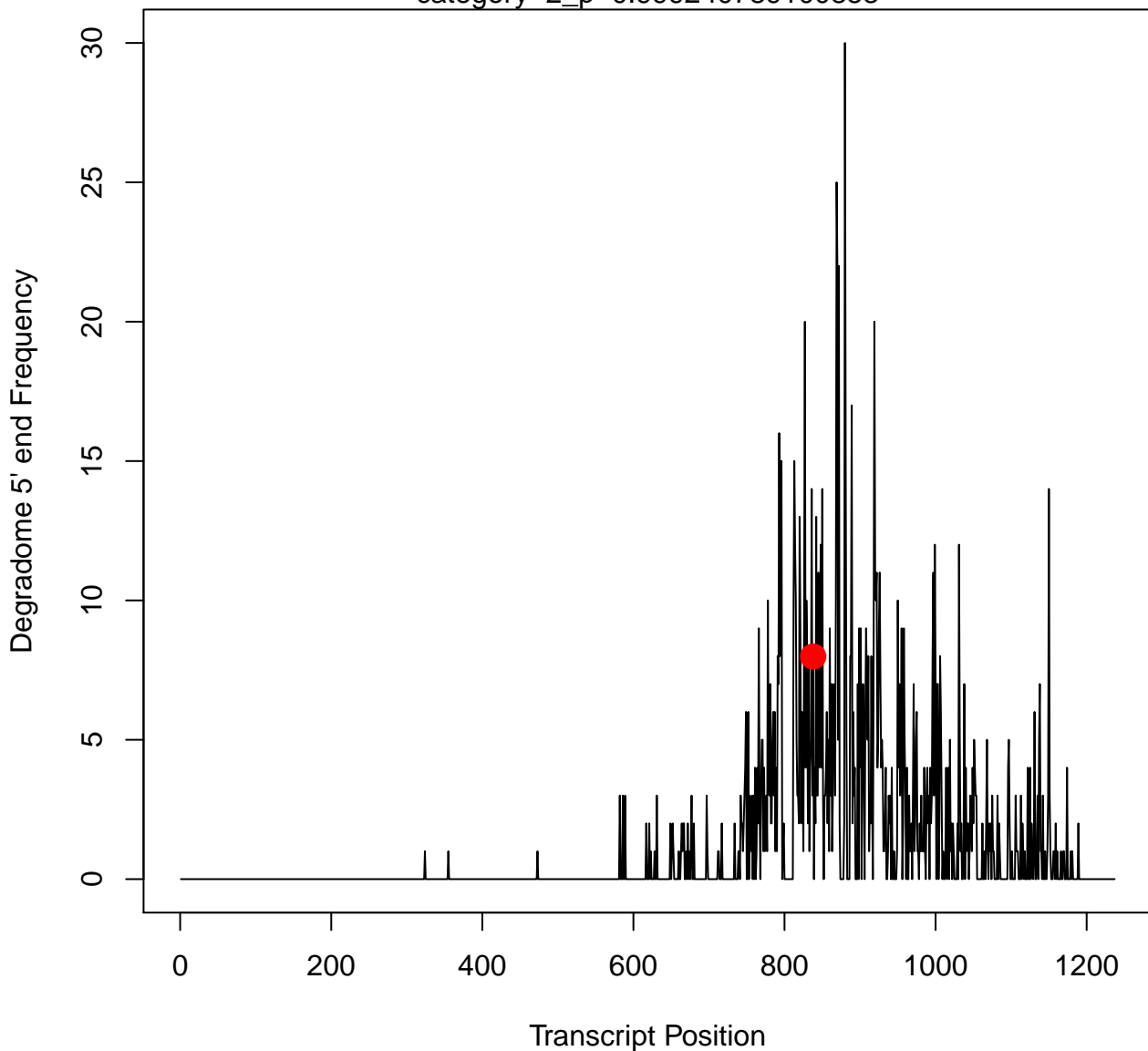

Supplement: Supplementary file 7 [file Data_Sheet_7.zip › Sit-miR160d_Seita.1G099500.1_838_TPlot.pdf]

**T=Seita.1G241500.1\_Q=Sit-miR160d\_S=1660**

category=2\_p=0.0346994606644779

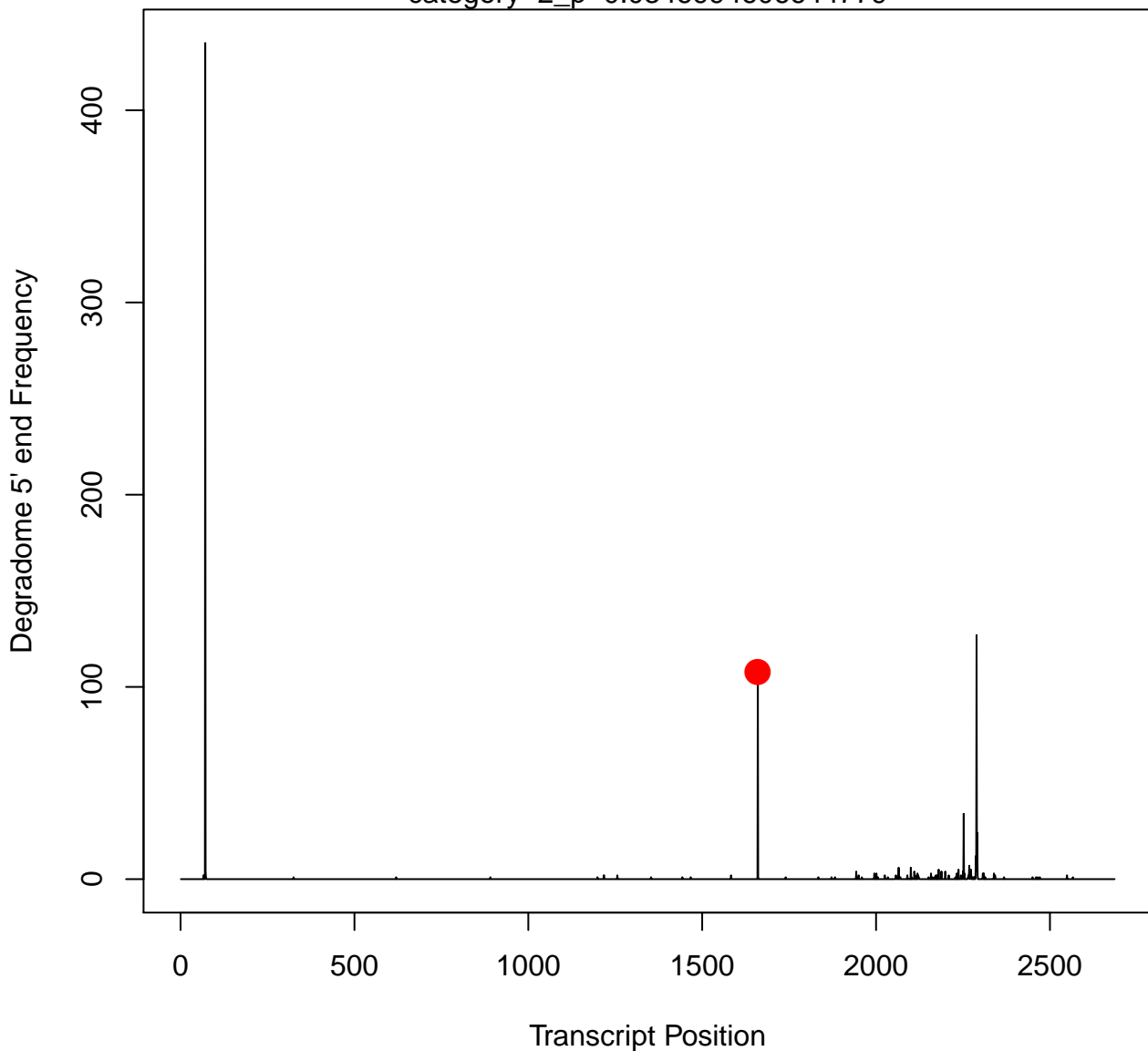

Supplement: Supplementary file 7 [file Data_Sheet_7.zip › Sit-miR160d_Seita.1G241500.1_1660_TPlot.pdf]

**T=Seita.4G257800.1\_Q=Sit-miR160d\_S=1974**

category=0\_p=0.000299243605074828

Degradome 5' end Frequency

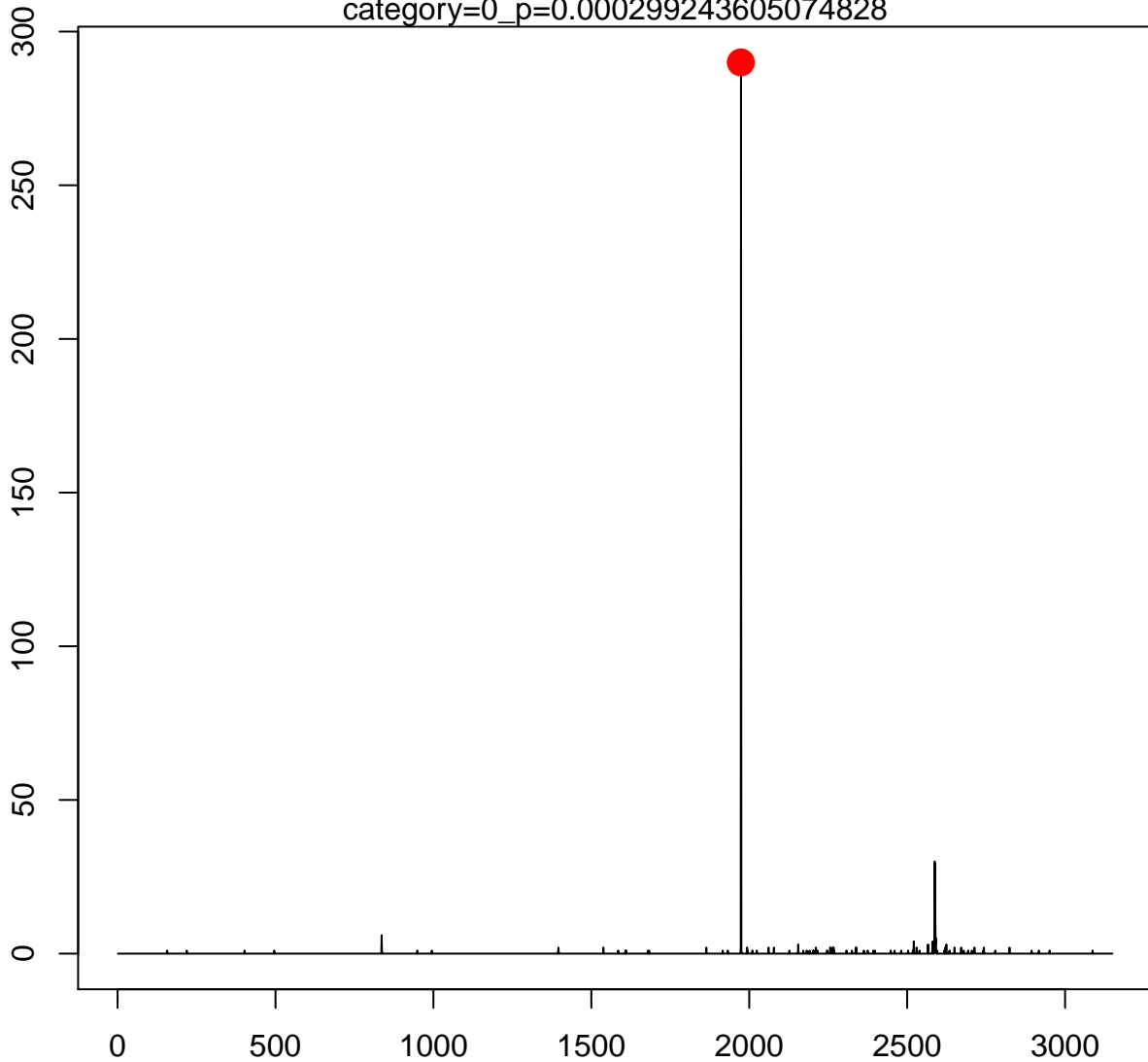

Transcript Position

Supplement: Supplementary file 7 [file Data_Sheet_7.zip › Sit-miR160d_Seita.4G257800.1_1974_TPlot.pdf]

**T=Seita.7G295800.1\_Q=Sit-miR160d\_S=368**

category=1\_p=0.309870765685019

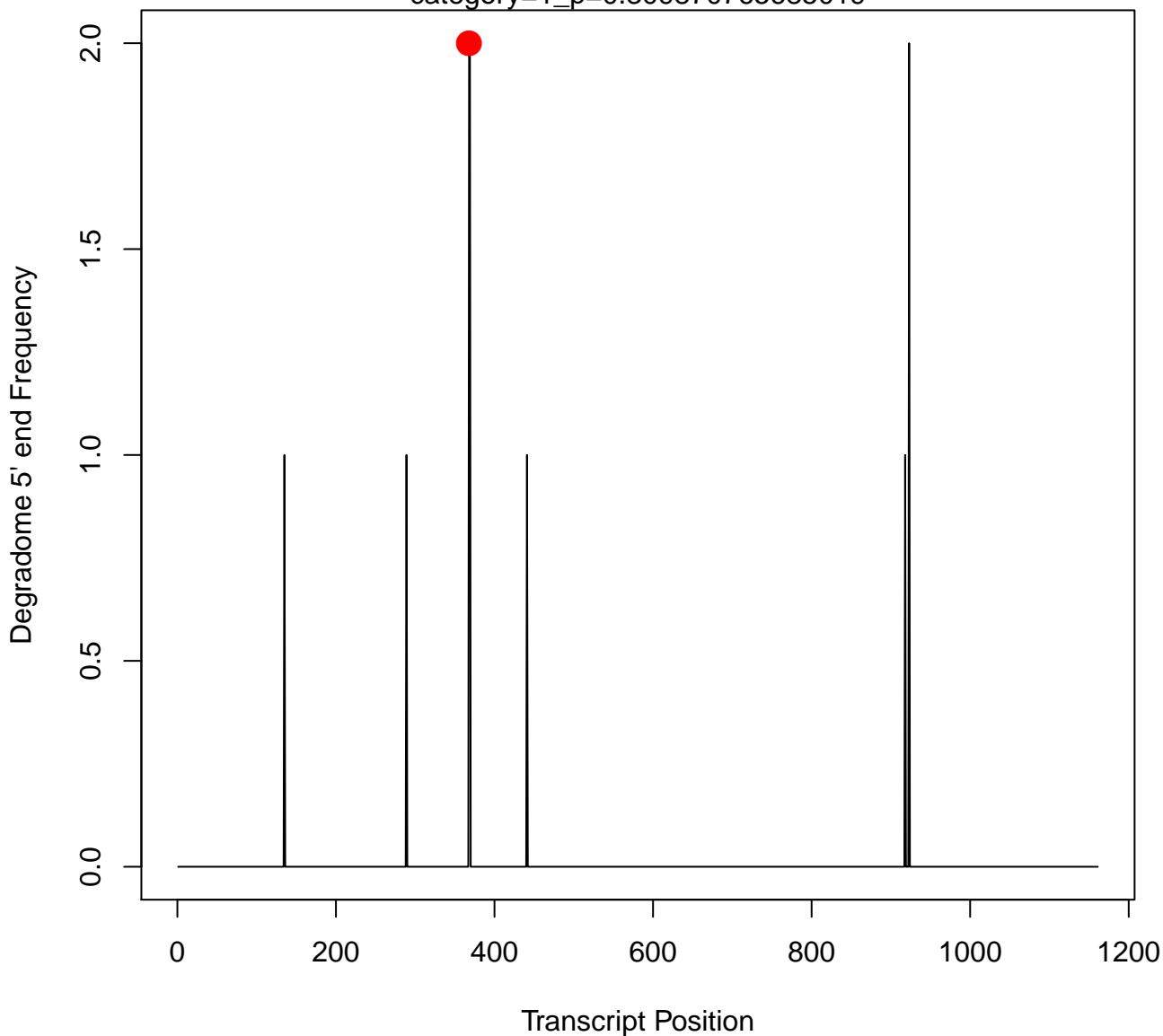

Supplement: Supplementary file 7 [file Data_Sheet_7.zip › Sit-miR160d_Seita.7G295800.1_368_TPlot.pdf]

**T=Seita.8G248200.1\_Q=Sit-miR160d\_S=820**

category=0\_p=0.0341217162196339

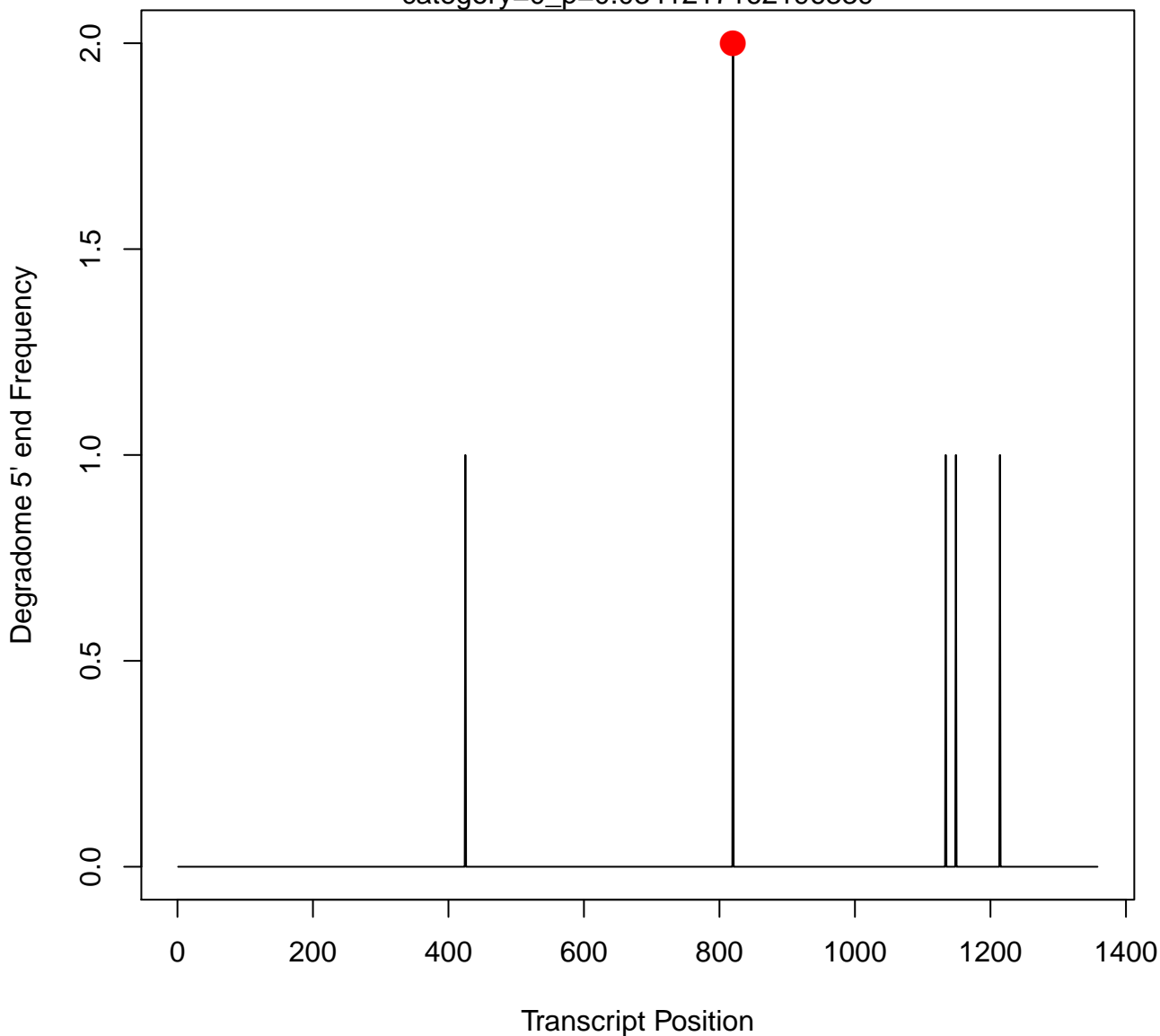

Supplement: Supplementary file 7 [file Data_Sheet_7.zip › Sit-miR160d_Seita.8G248200.1_820_TPlot.pdf]

**T=Seita.1G229400.1\_Q=Sit-miR162\_S=1479**

category=2\_p=0.571548988788741

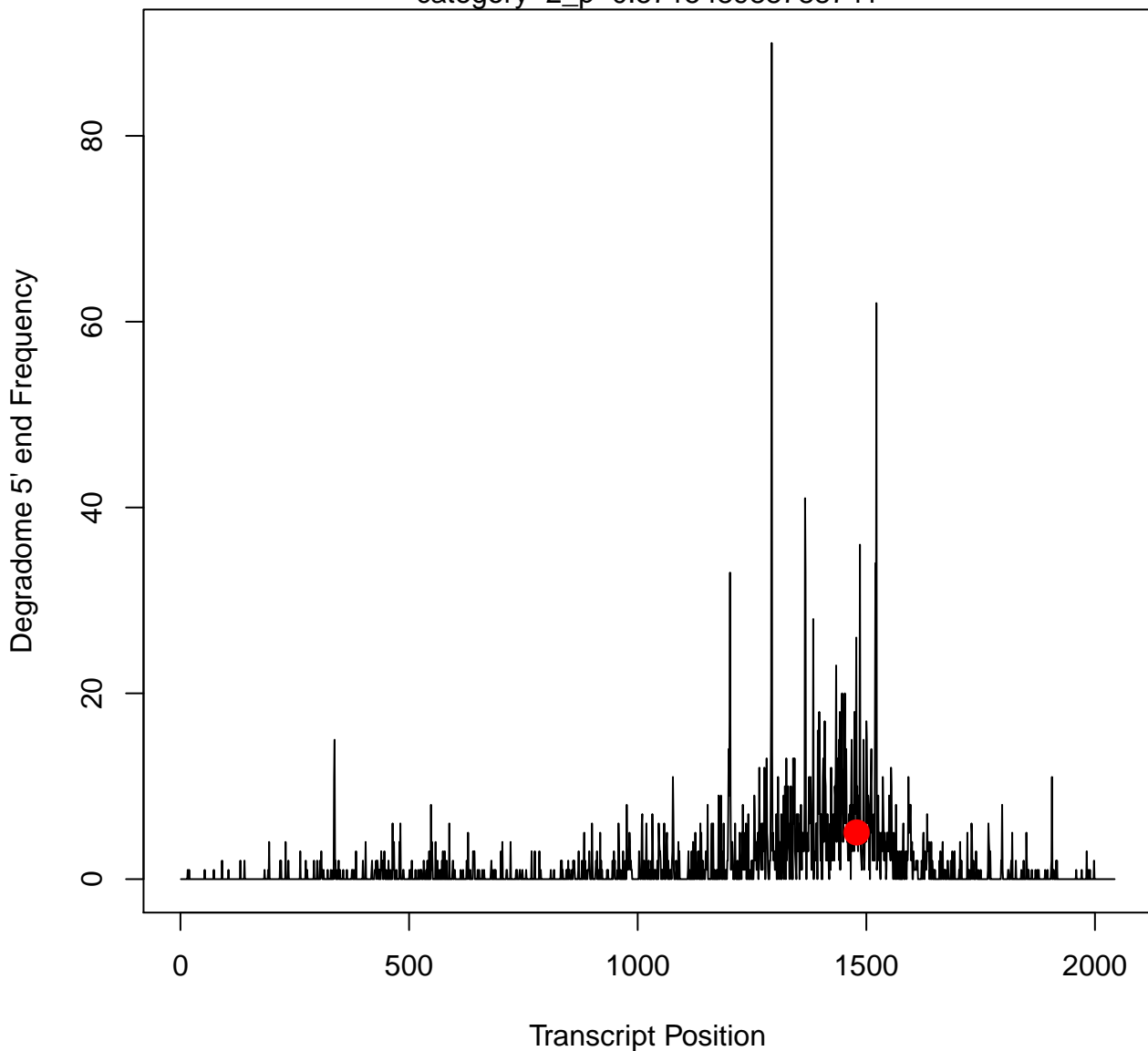

Supplement: Supplementary file 7 [file Data_Sheet_7.zip › Sit-miR162_Seita.1G229400.1_1479_TPlot.pdf]

**T=Seita.2G116400.1\_Q=Sit-miR162\_S=761**

category=2\_p=0.131739197392104

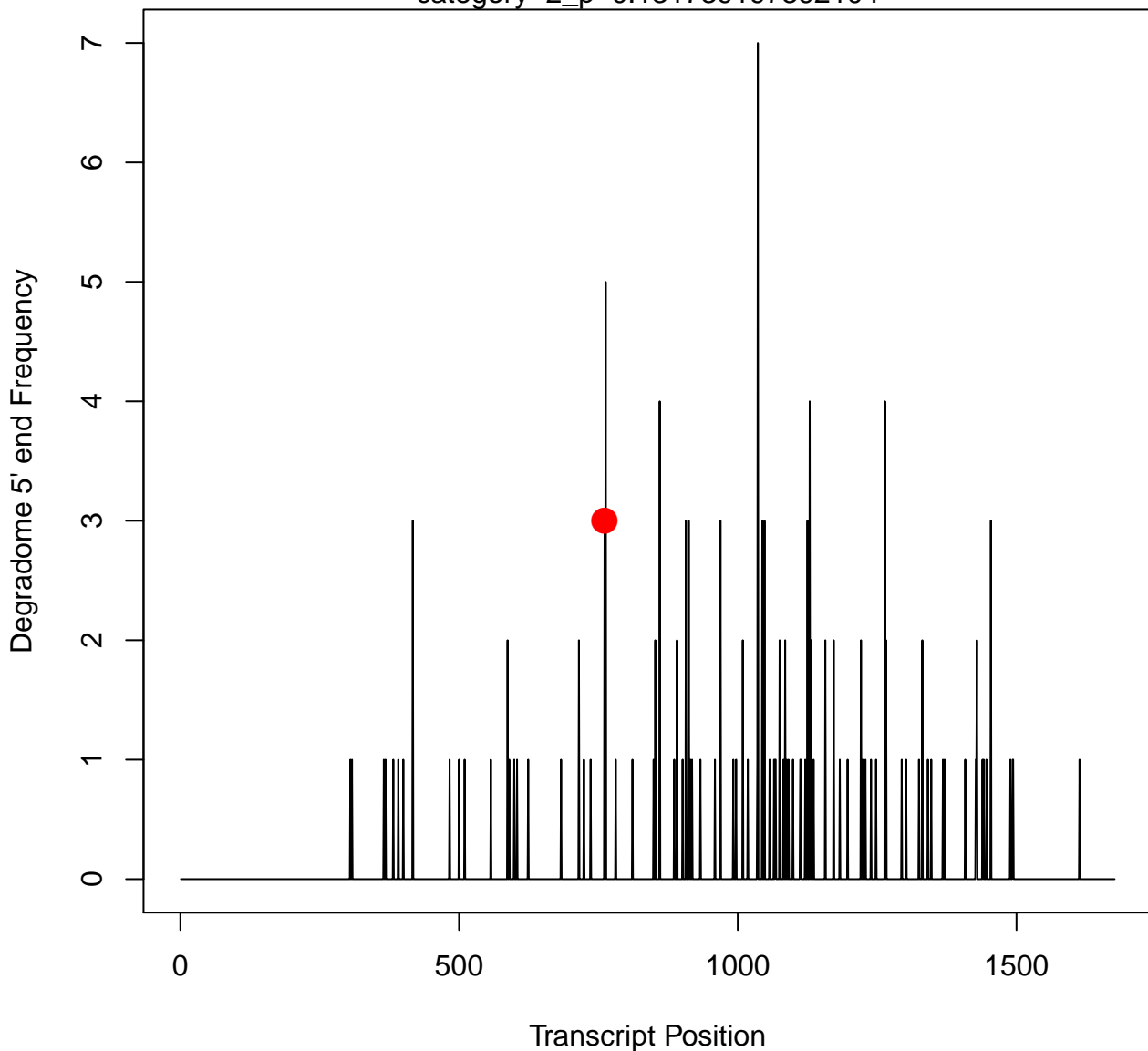

Supplement: Supplementary file 7 [file Data_Sheet_7.zip › Sit-miR162_Seita.2G116400.1_761_TPlot.pdf]

**T=Seita.9G562200.1\_Q=Sit-miR162\_S=3530**

category=2\_p=0.00703827180998173

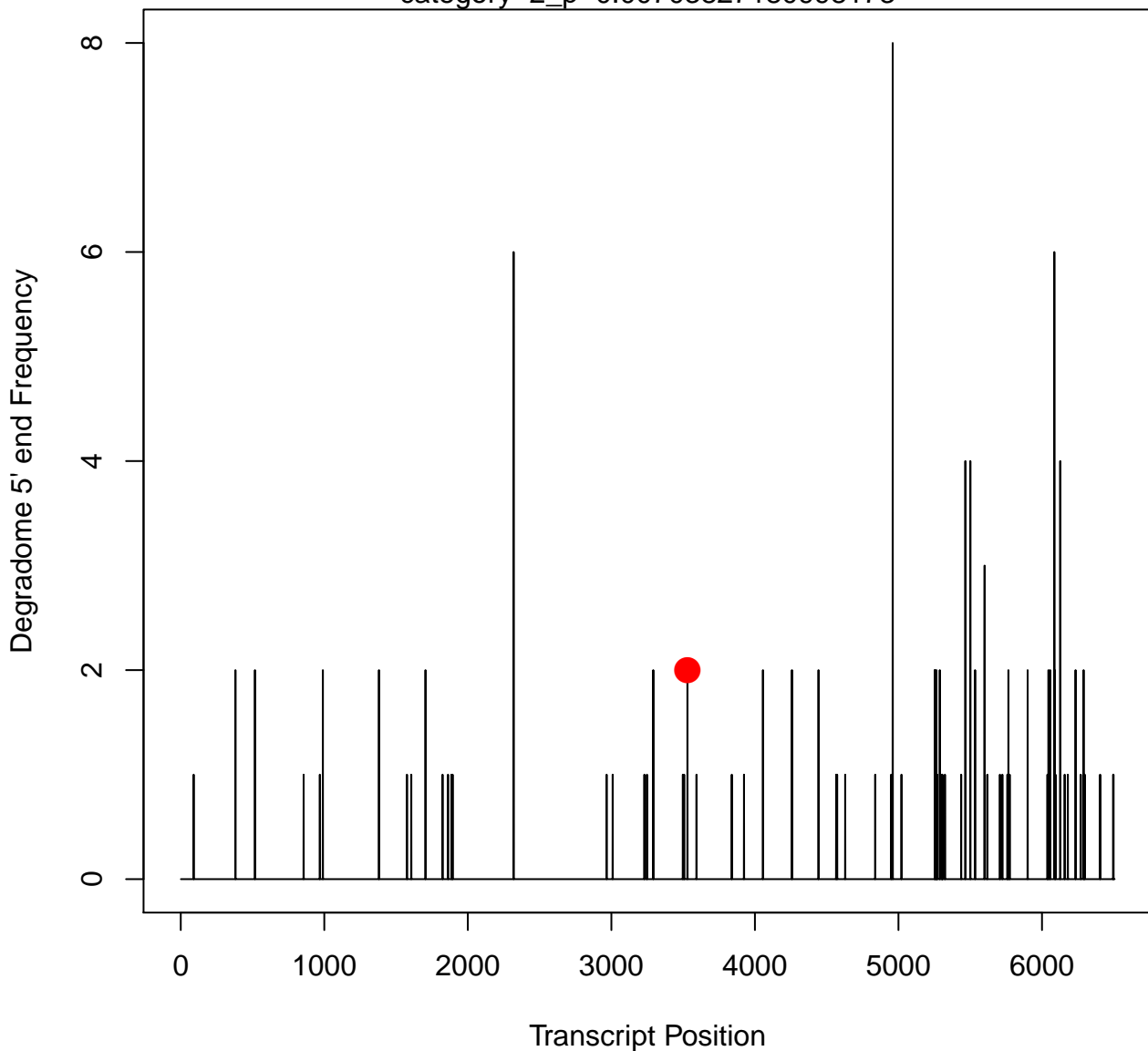

Supplement: Supplementary file 7 [file Data_Sheet_7.zip › Sit-miR162_Seita.9G562200.1_3530_TPlot.pdf]

**T=Seita.7G280600.1\_Q=Sit-miR164a\_S=215**

category=2\_p=0.261929376439474

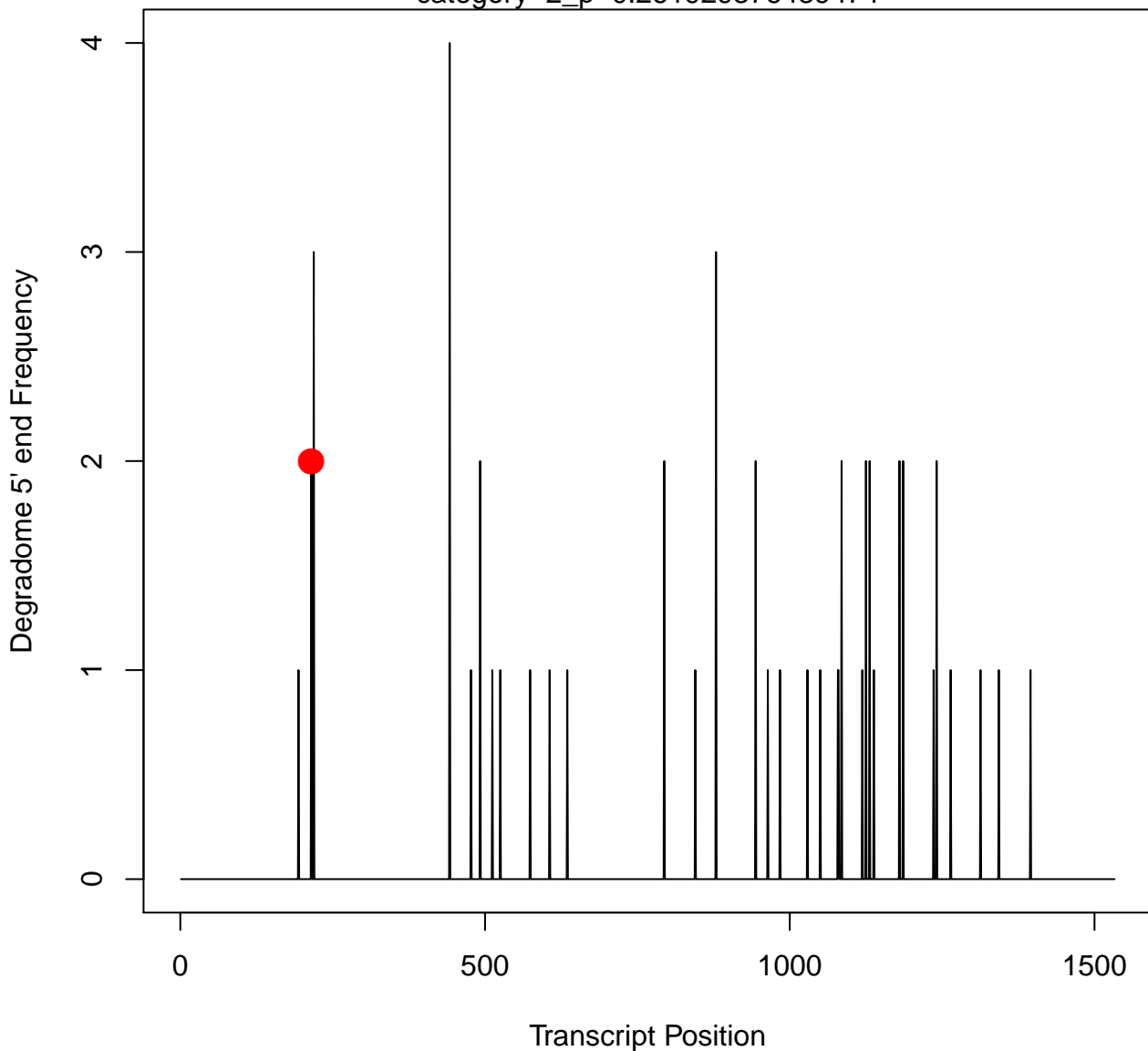

Supplement: Supplementary file 7 [file Data_Sheet_7.zip › Sit-miR164a_Seita.7G280600.1_215_TPlot.pdf]

**T=Seita.7G322100.1\_Q=Sit-miR164a\_S=1693**

category=2\_p=0.677000713565874

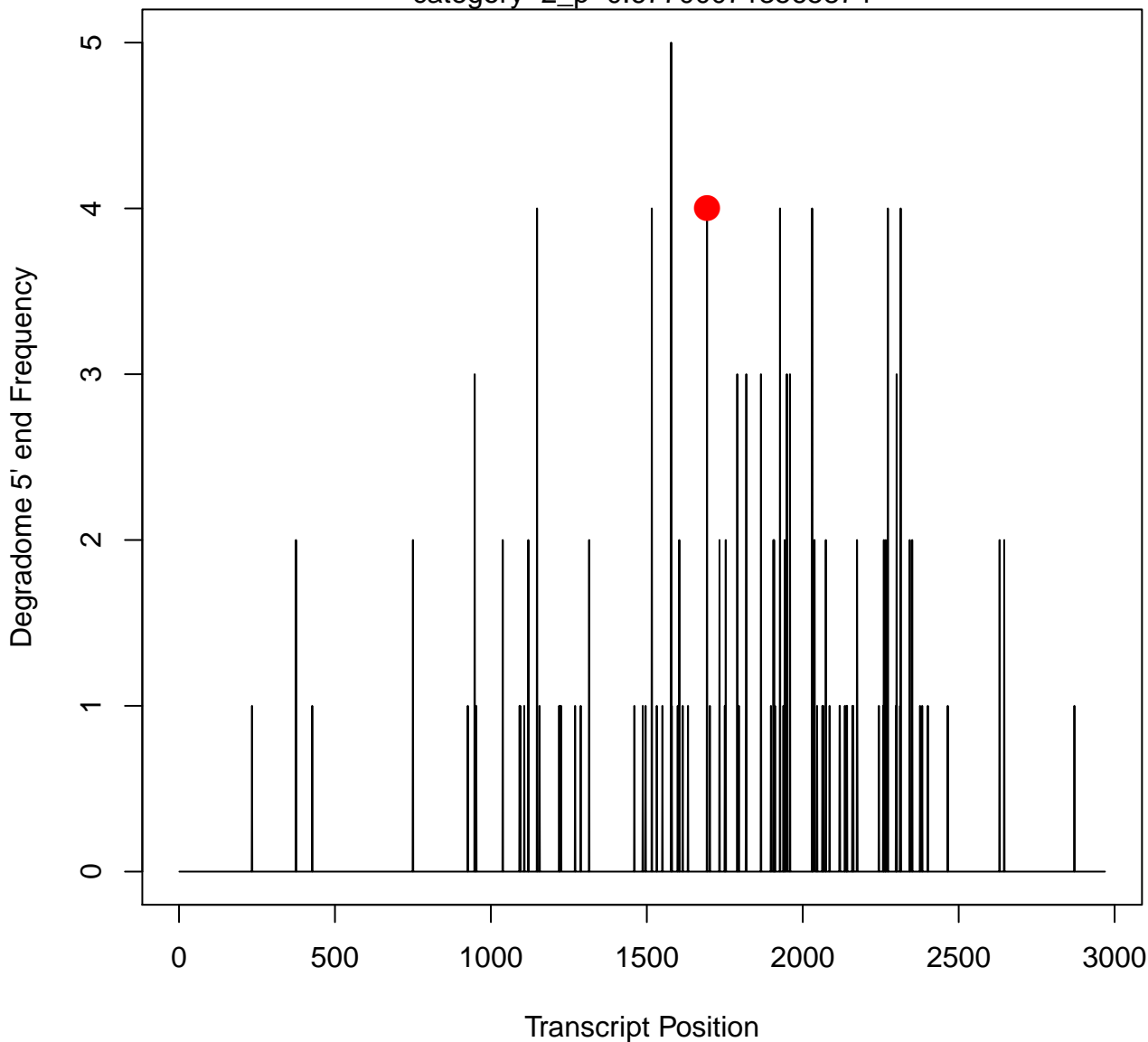

Supplement: Supplementary file 7 [file Data_Sheet_7.zip › Sit-miR164a_Seita.7G322100.1_1693_TPlot.pdf]

**T=Seita.9G579100.1\_Q=Sit-miR164a\_S=819**

category=2\_p=0.55614754809296

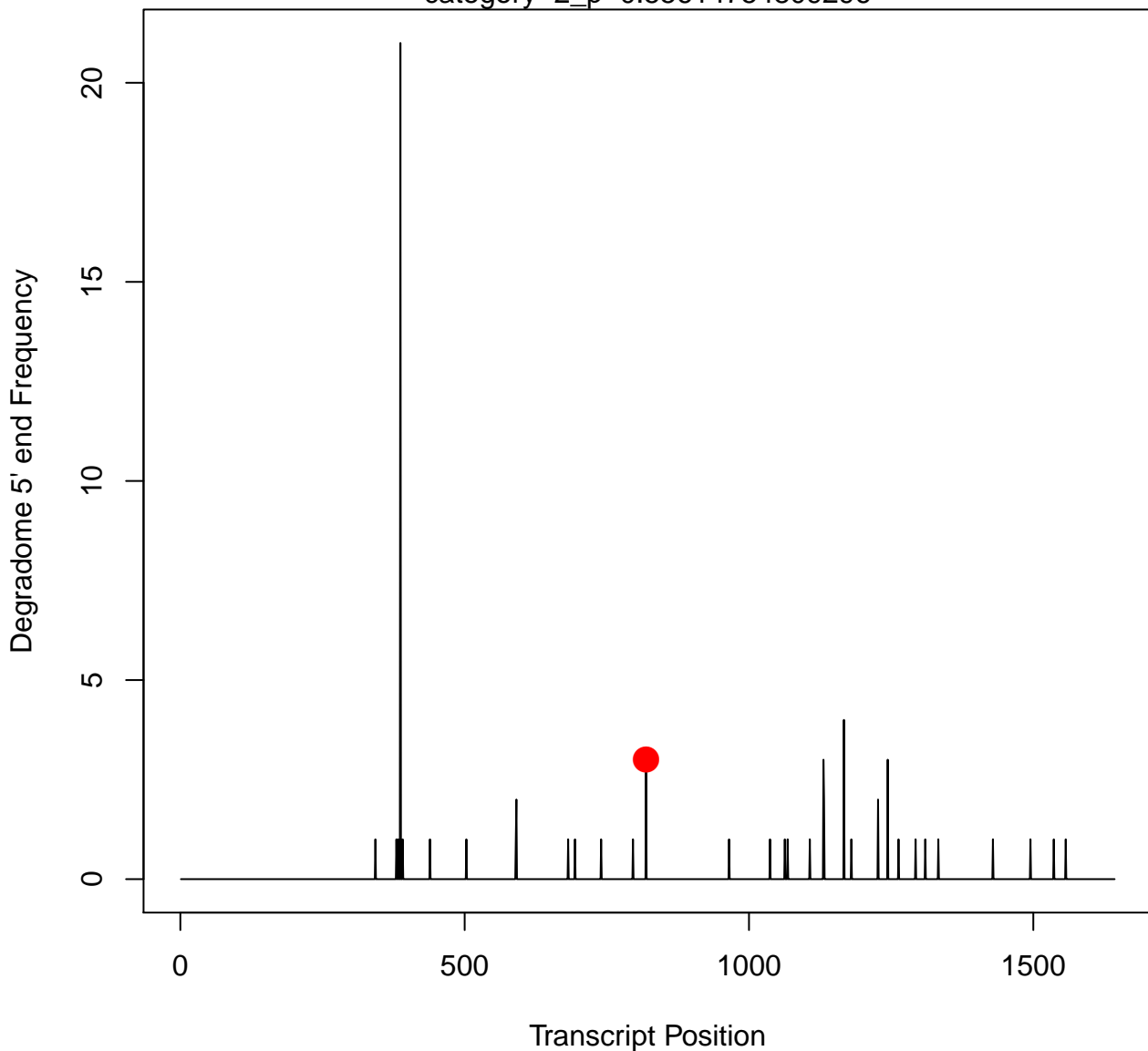

Supplement: Supplementary file 7 [file Data_Sheet_7.zip › Sit-miR164a_Seita.9G579100.1_819_TPlot.pdf]

**T=Seita.2G174200.1\_Q=Sit-miR164b\_S=201**

category=2\_p=0.0209665522765033

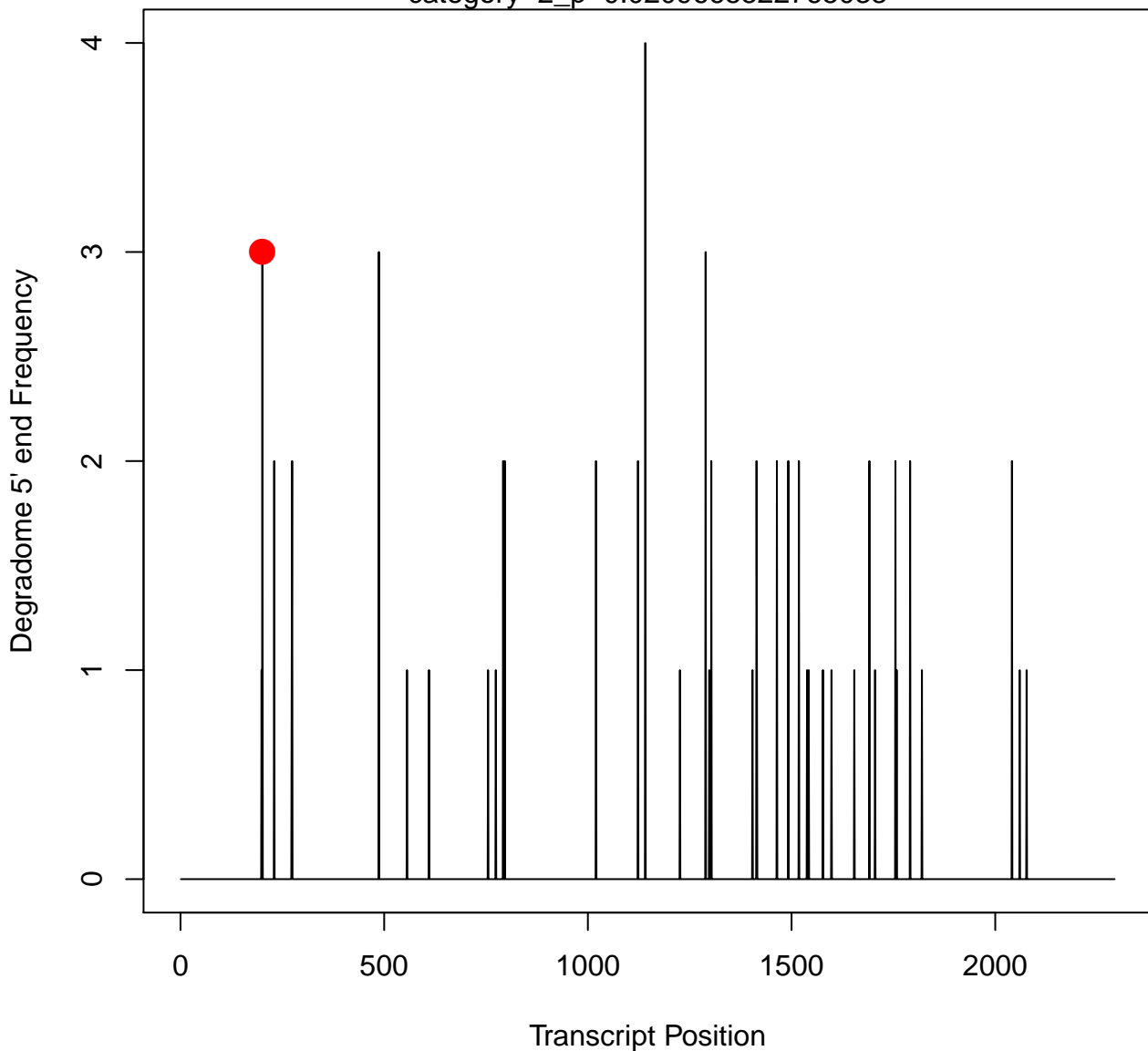

Supplement: Supplementary file 7 [file Data_Sheet_7.zip › Sit-miR164b_Seita.2G174200.1_201_TPlot.pdf]

**T=Seita.3G001500.1\_Q=Sit-miR164b\_S=686**

category=2\_p=0.834883683776343

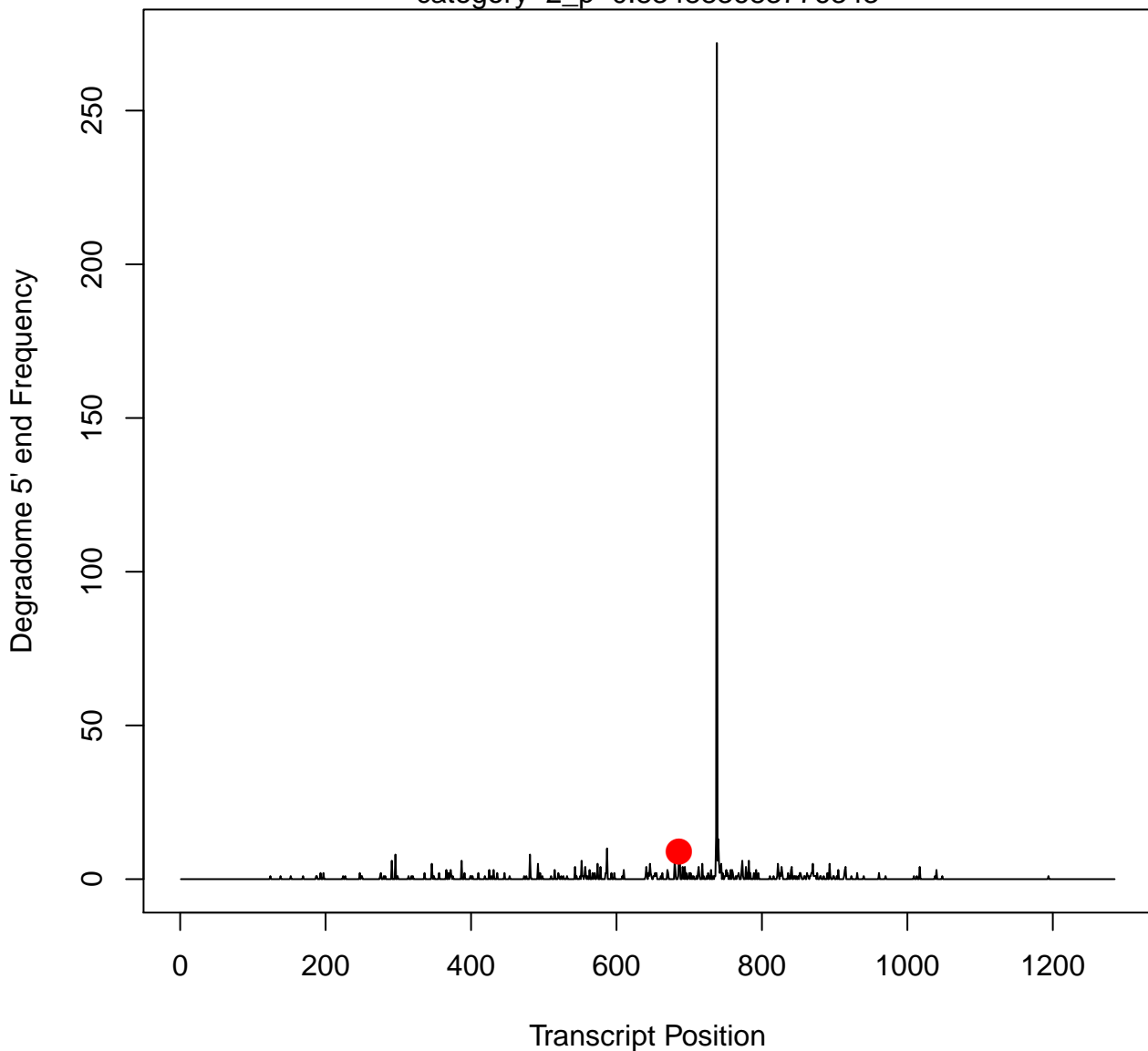

Supplement: Supplementary file 7 [file Data_Sheet_7.zip › Sit-miR164b_Seita.3G001500.1_686_TPlot.pdf]

**T=Seita.3G386200.1\_Q=Sit-miR164b\_S=790**

category=0\_p=0.000299243605074828

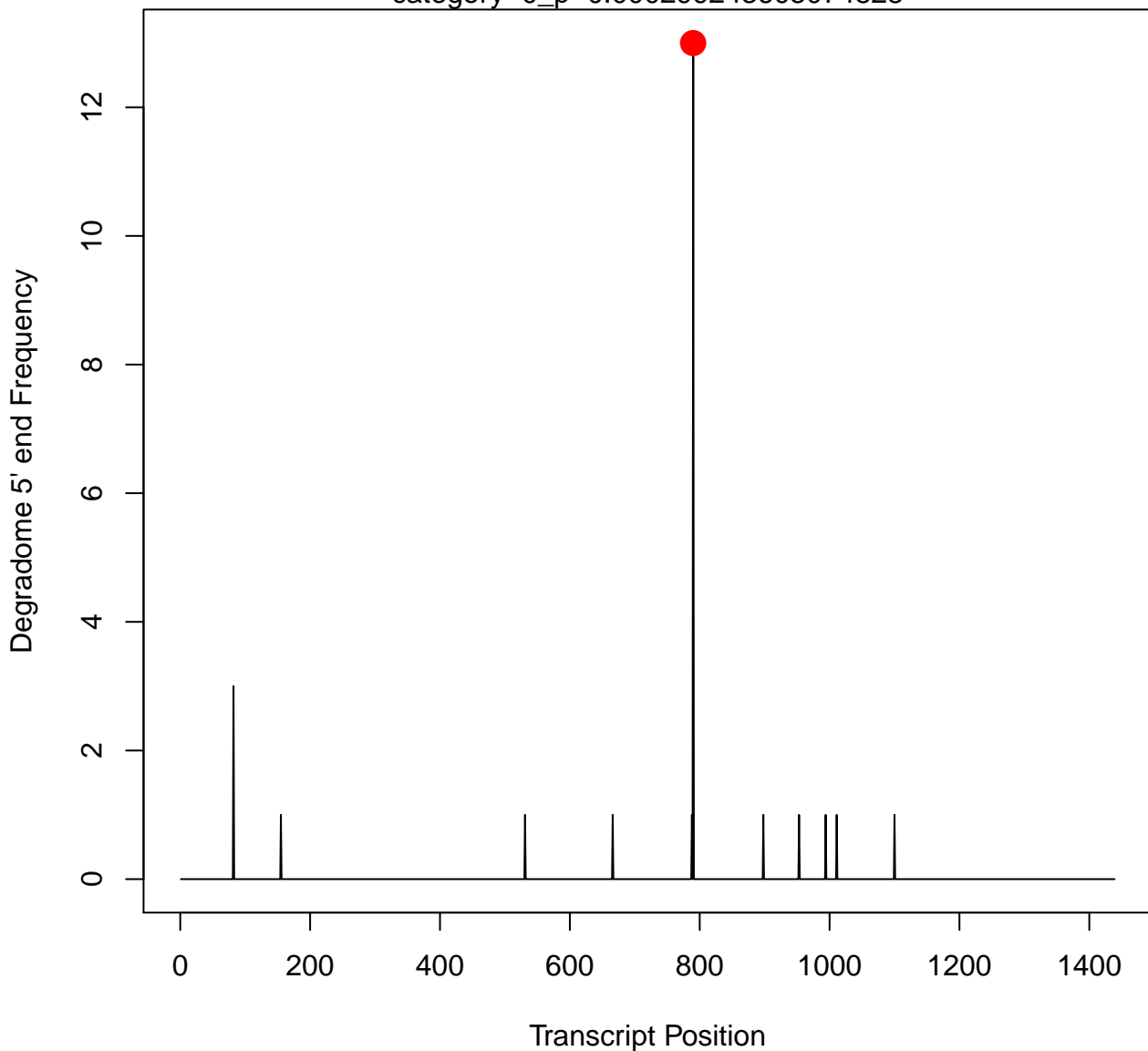

Supplement: Supplementary file 7 [file Data_Sheet_7.zip › Sit-miR164b_Seita.3G386200.1_790_TPlot.pdf]

**T=Seita.2G037200.1\_Q=Sit-miR164c\_S=1419**

category=2\_p=0.988960707719498

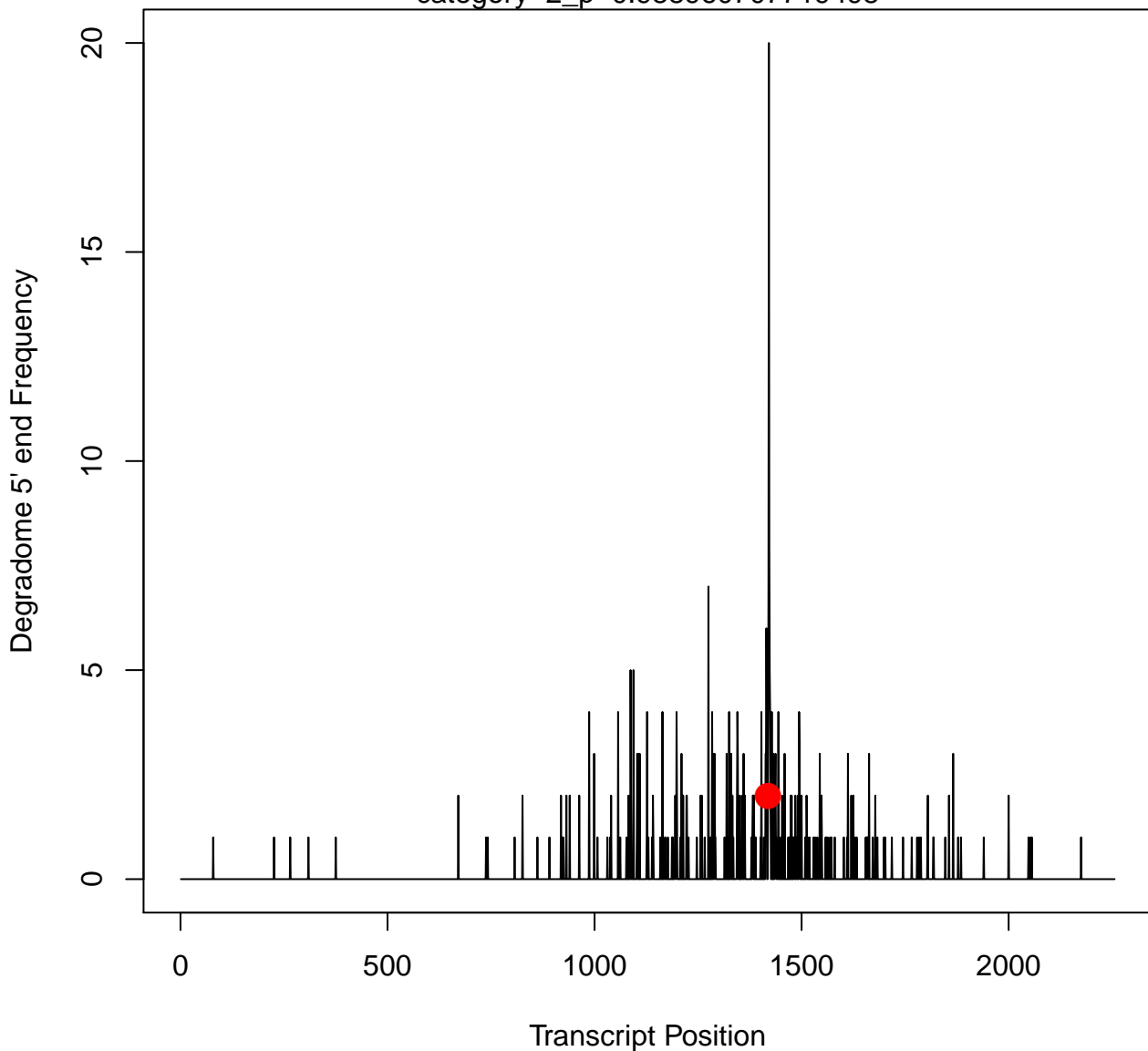

Supplement: Supplementary file 7 [file Data_Sheet_7.zip › Sit-miR164c_Seita.2G037200.1_1419_TPlot.pdf]

**T=Seita.6G069100.1\_Q=Sit-miR164c\_S=415**

category=2\_p=0.967471260276925

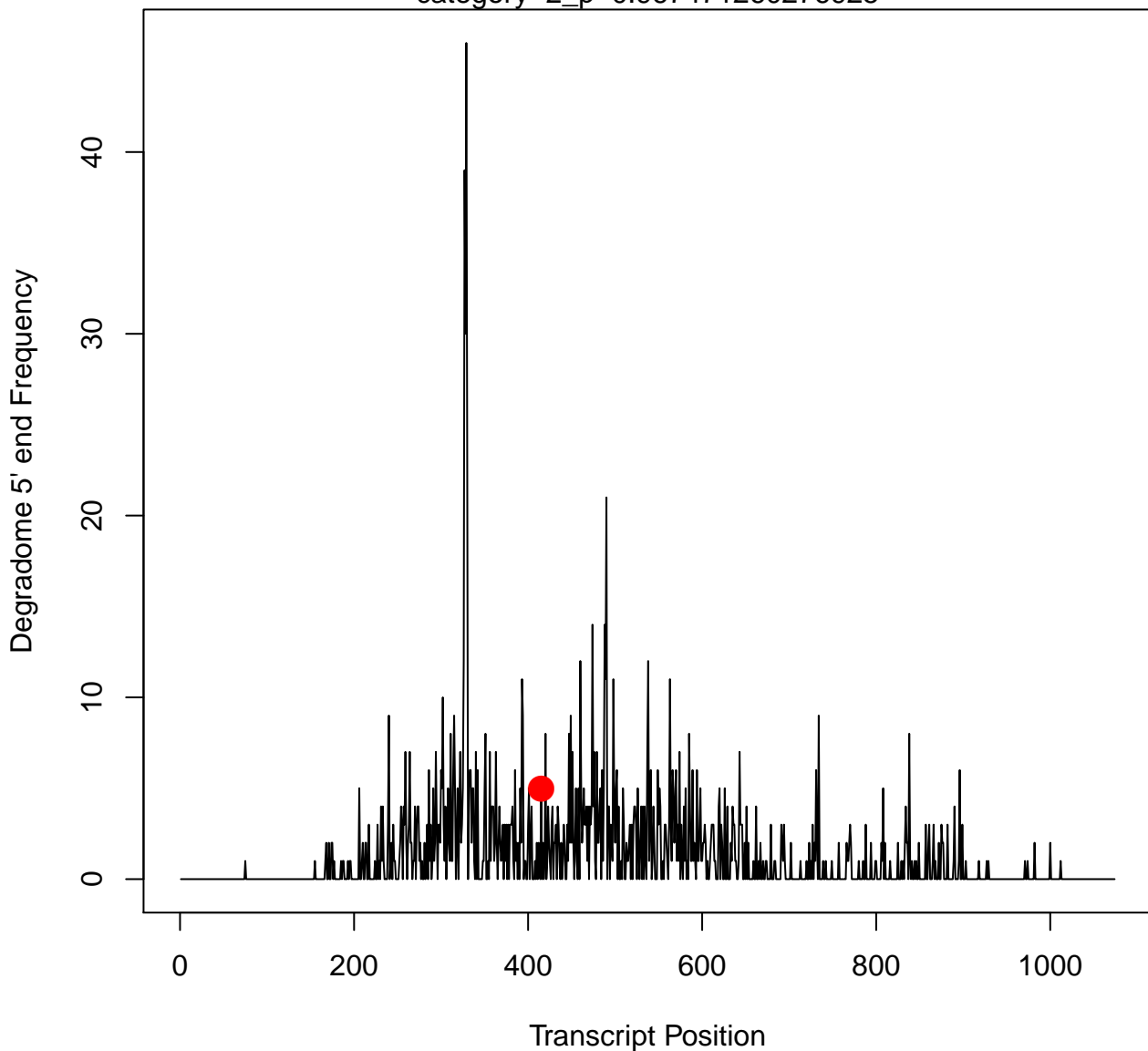

Supplement: Supplementary file 7 [file Data_Sheet_7.zip › Sit-miR164c_Seita.6G069100.1_415_TPlot.pdf]

**T=Seita.4G285900.1\_Q=Sit-miR164e\_S=994**

category=2\_p=0.94475041694726

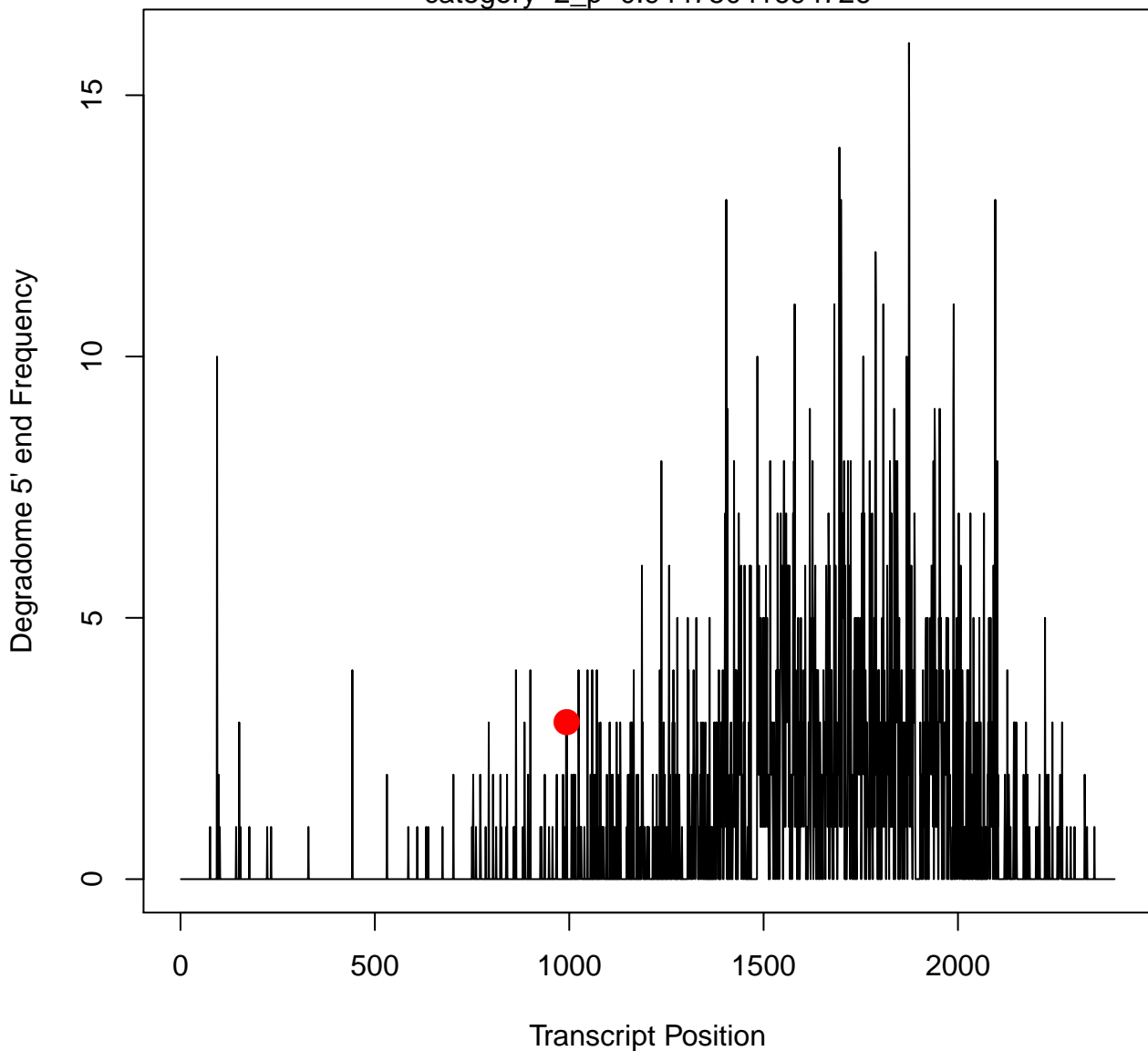

Supplement: Supplementary file 7 [file Data_Sheet_7.zip › Sit-miR164e_Seita.4G285900.1_994_TPlot.pdf]

**T=Seita.7G256100.1\_Q=Sit-miR164e\_S=1791**

category=2\_p=0.993171082024764

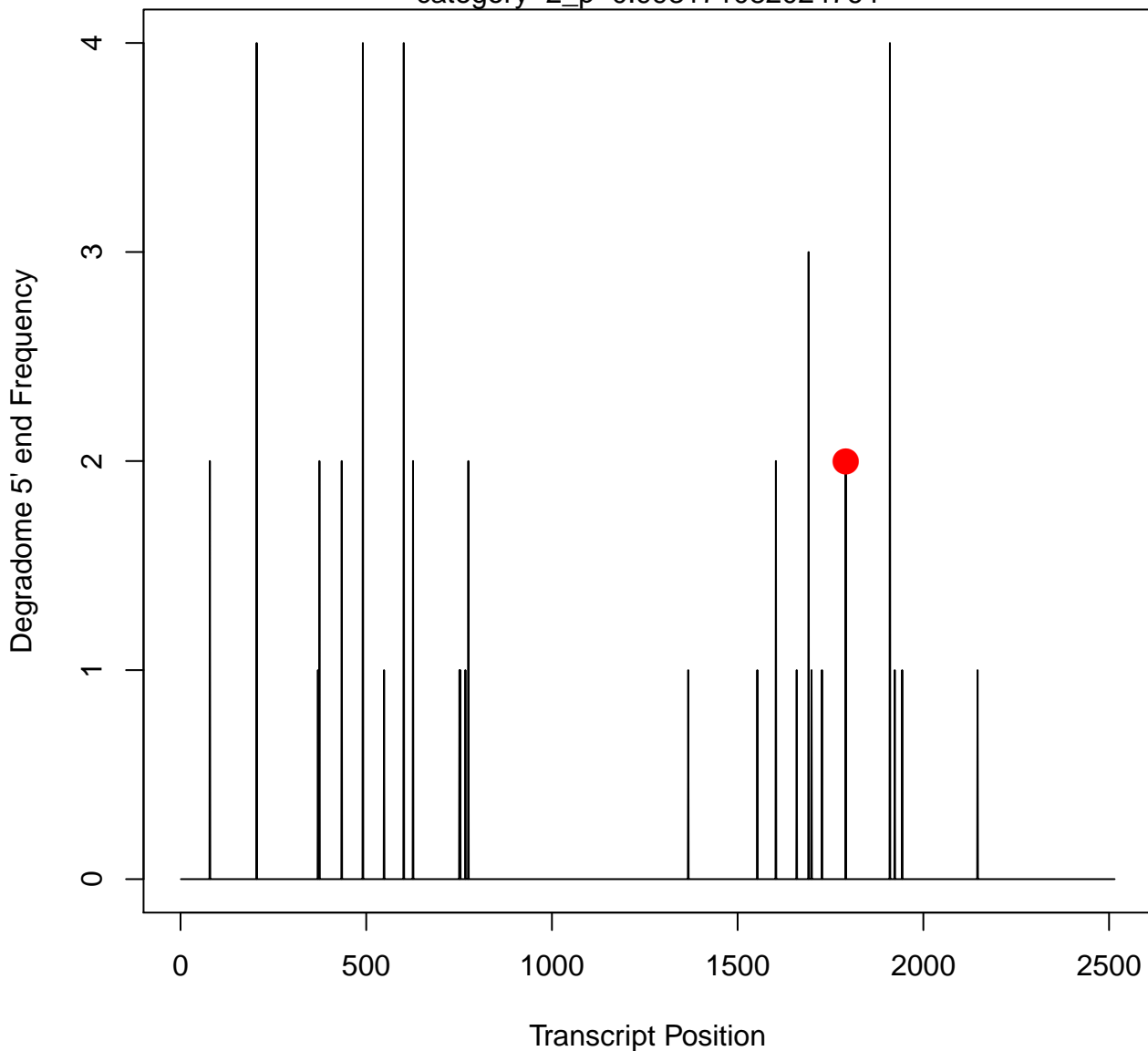

Supplement: Supplementary file 7 [file Data_Sheet_7.zip › Sit-miR164e_Seita.7G256100.1_1791_TPlot.pdf]

**T=Seita.4G263400.1\_Q=Sit-miR164f\_S=1216**

category=0\_p=0.00119643724706542

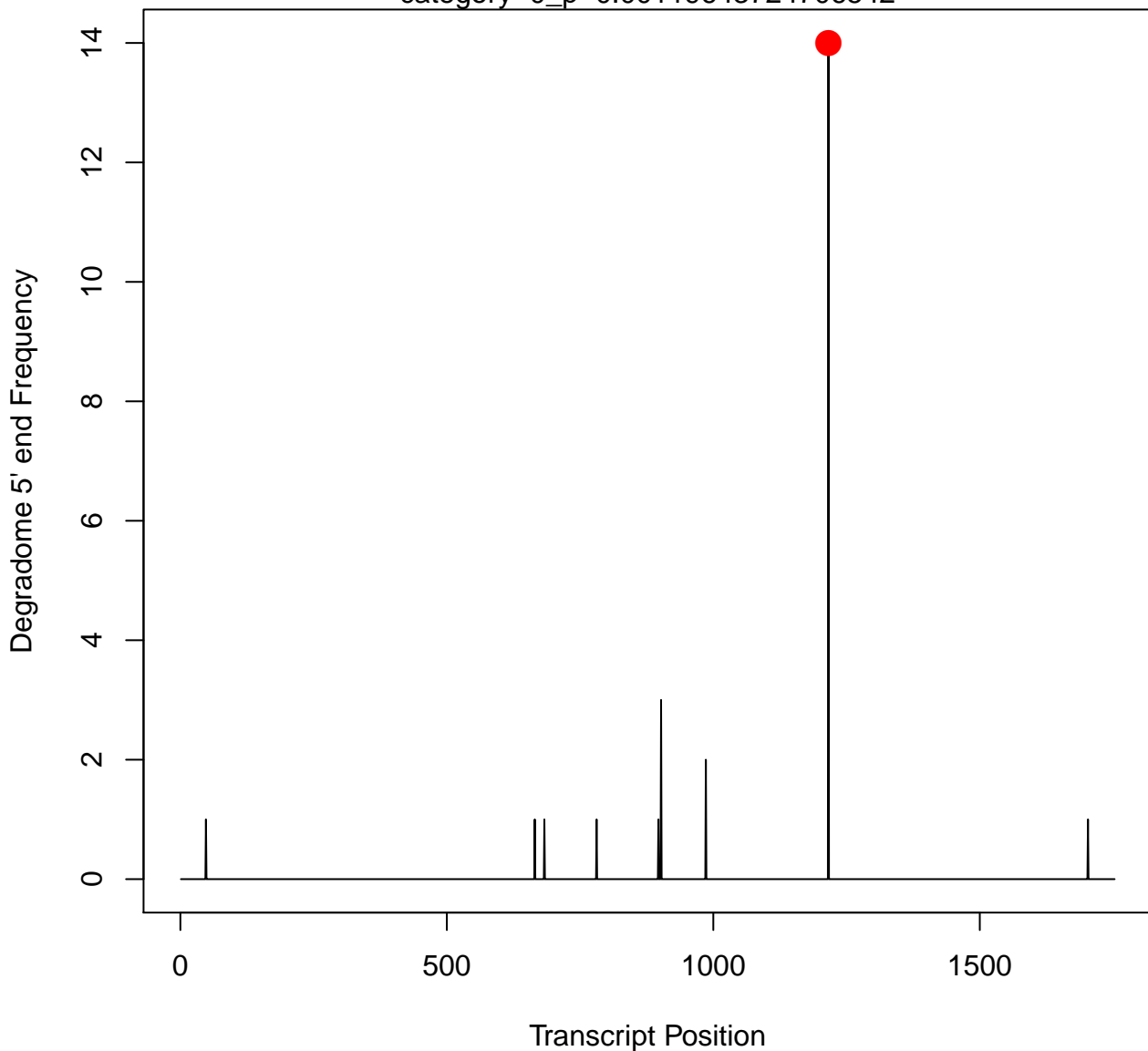

Supplement: Supplementary file 7 [file Data_Sheet_7.zip › Sit-miR164f_Seita.4G263400.1_1216_TPlot.pdf]

**T=Seita.4G110900.1\_Q=Sit-miR166b\_S=2613**

category=2\_p=0.790065092180883

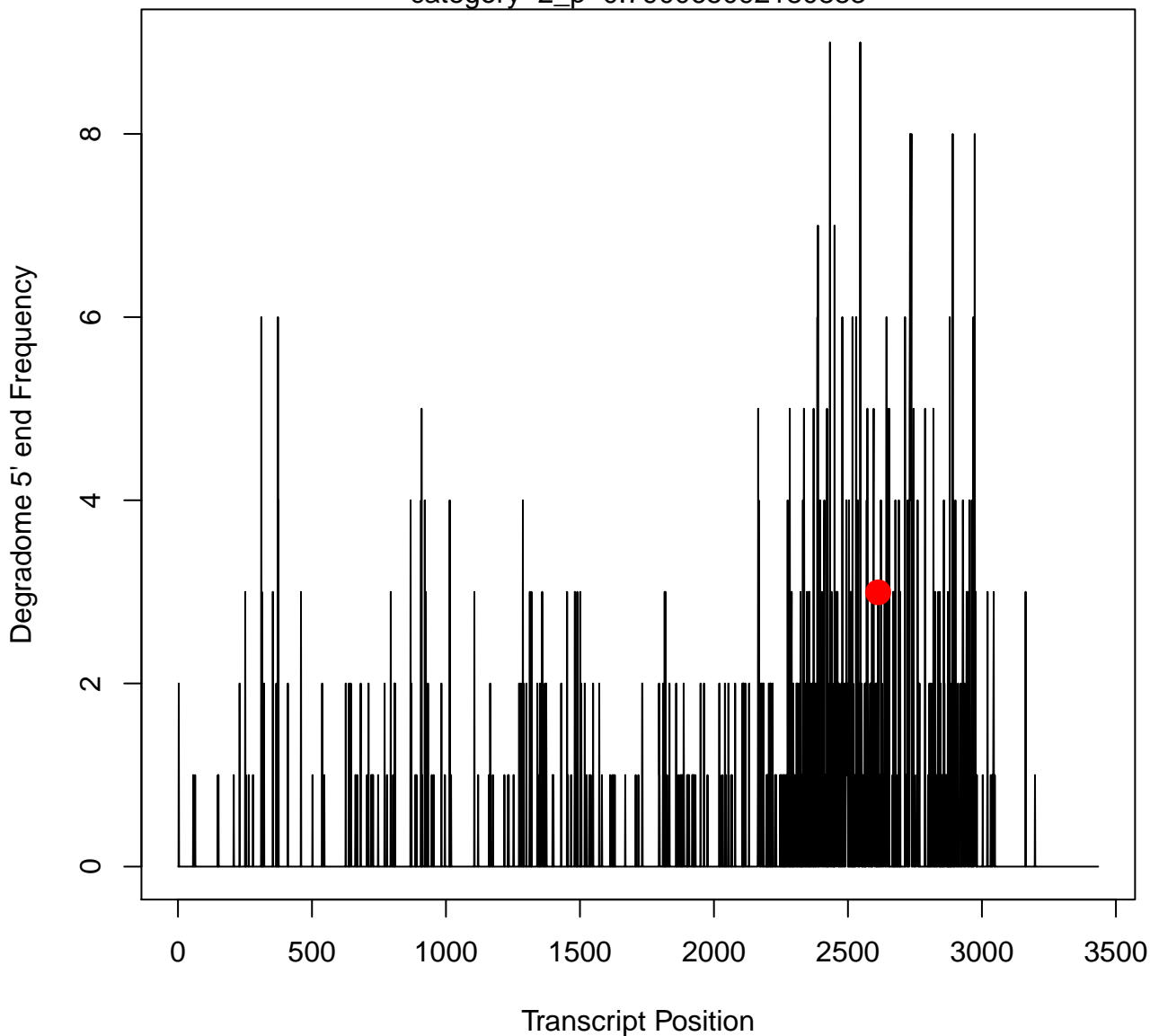

Supplement: Supplementary file 7 [file Data_Sheet_7.zip › Sit-miR166b_Seita.4G110900.1_2613_TPlot.pdf]

**T=Seita.4G049700.1\_Q=Sit-miR166g\_S=680**

category=2\_p=0.520287521276684

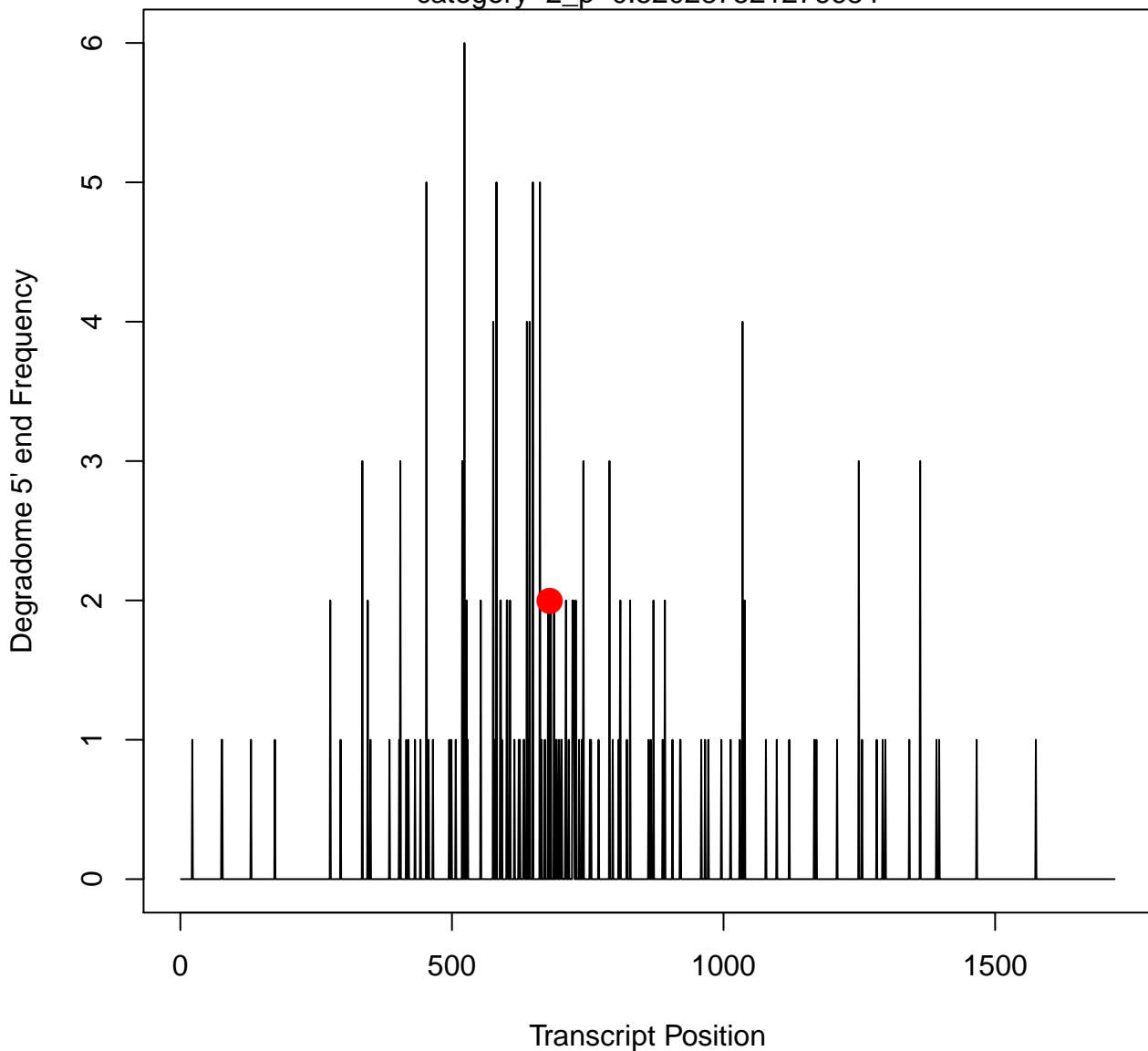

Supplement: Supplementary file 7 [file Data_Sheet_7.zip › Sit-miR166g_Seita.4G049700.1_680_TPlot.pdf]

**T=Seita.7G130800.1\_Q=Sit-miR166i\_S=379**

category=2\_p=0.462892941733776

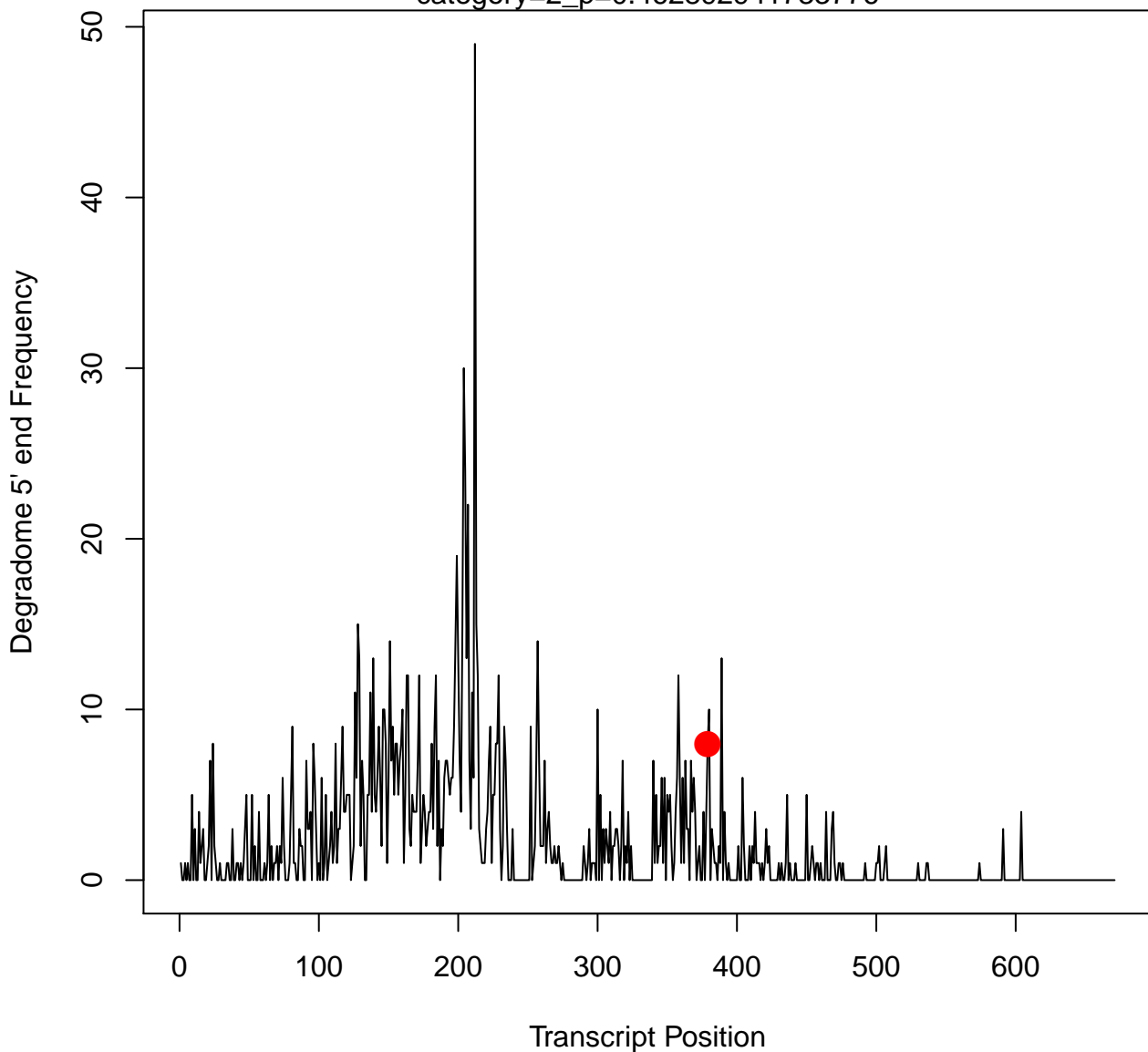

Supplement: Supplementary file 7 [file Data_Sheet_7.zip › Sit-miR166i_Seita.7G130800.1_379_TPlot.pdf]

**T=Seita.9G572600.1\_Q=Sit-miR166i\_S=1138**

category=0\_p=0.00119643724706542

Degradome 5' end Frequency

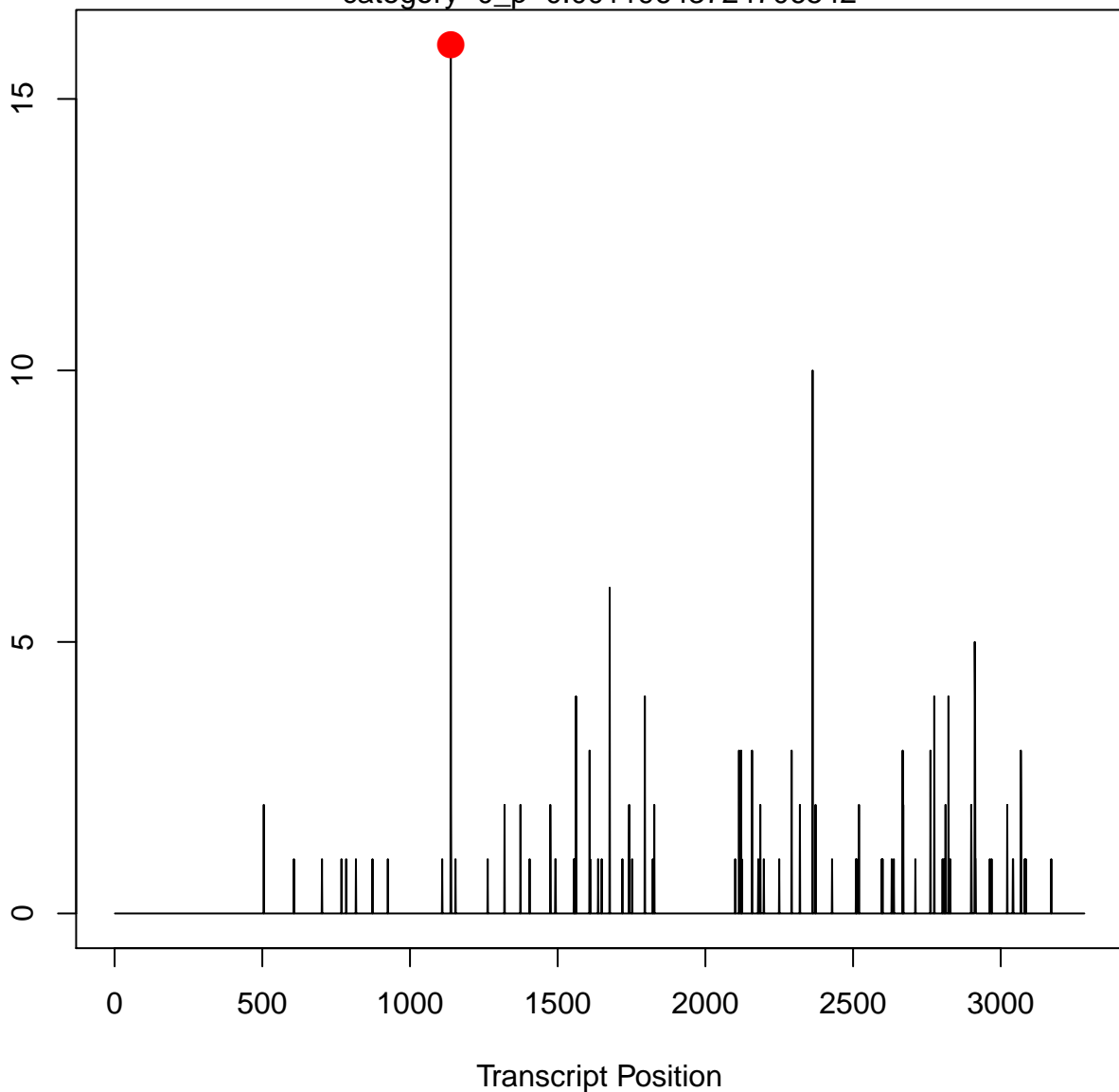

Supplement: Supplementary file 7 [file Data_Sheet_7.zip › Sit-miR166i_Seita.9G572600.1_1138_TPlot.pdf]

**T=Seita.5G069000.1\_Q=Sit-miR166j\_S=821**

category=2\_p=0.577558873843619

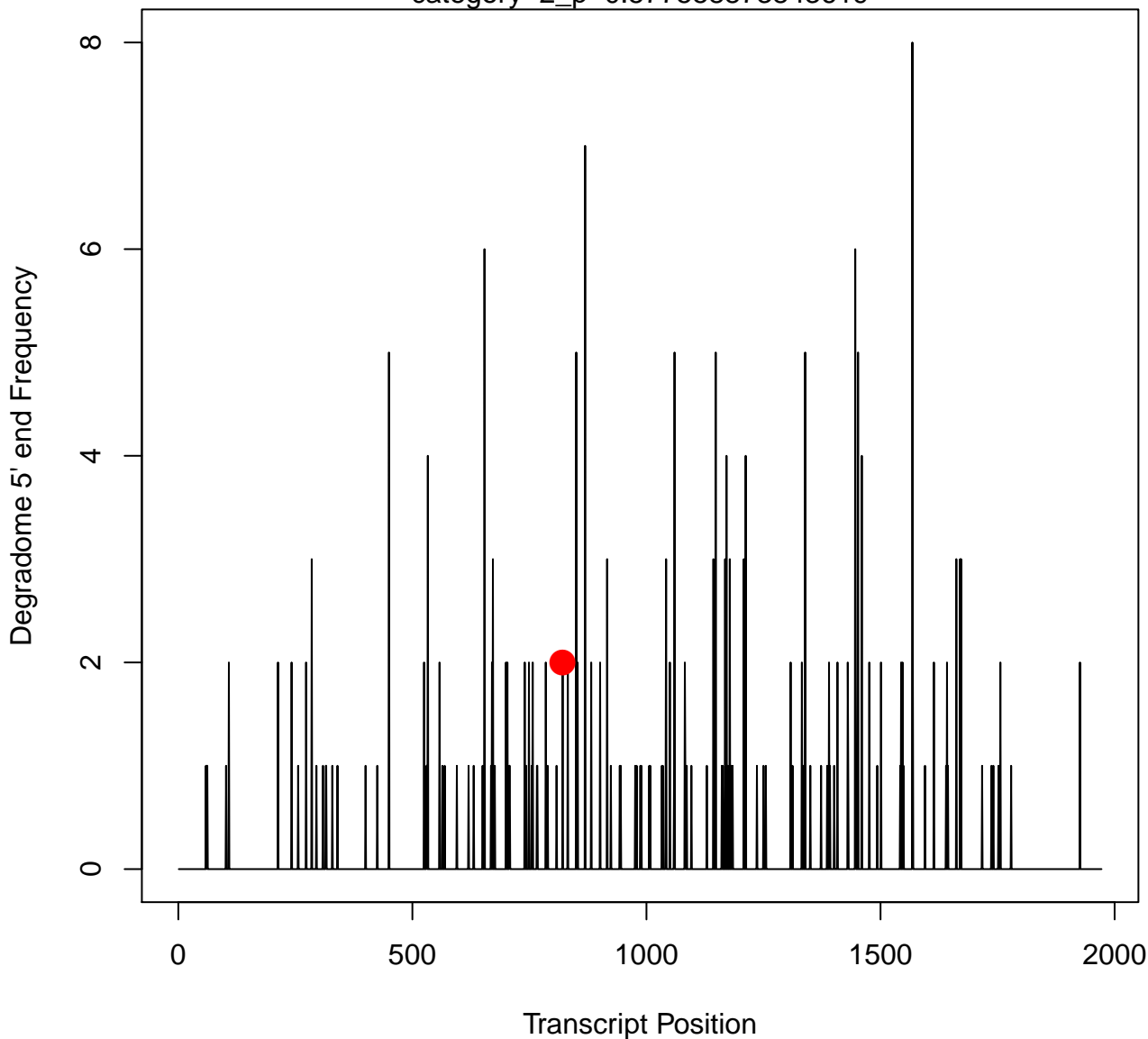

Supplement: Supplementary file 7 [file Data_Sheet_7.zip › Sit-miR166j_Seita.5G069000.1_821_TPlot.pdf]

**T=Seita.9G219700.1\_Q=Sit-miR166j\_S=1105**

category=0\_p=0.000897462201815302

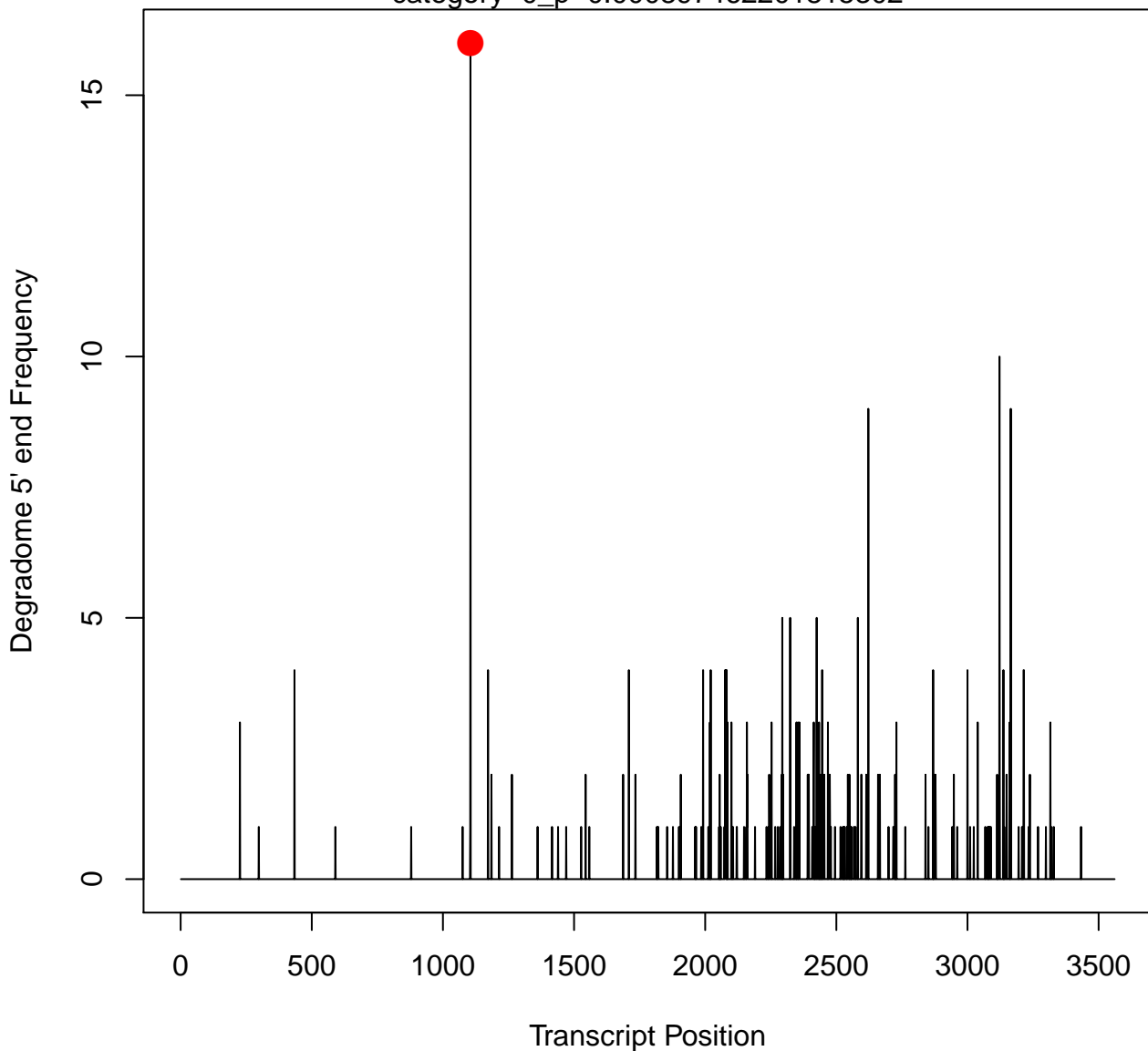

Supplement: Supplementary file 7 [file Data_Sheet_7.zip › Sit-miR166j_Seita.9G219700.1_1105_TPlot.pdf]

**T=Seita.1G270500.1\_Q=Sit-miR166k\_S=185**

category=2\_p=0.119386830877018

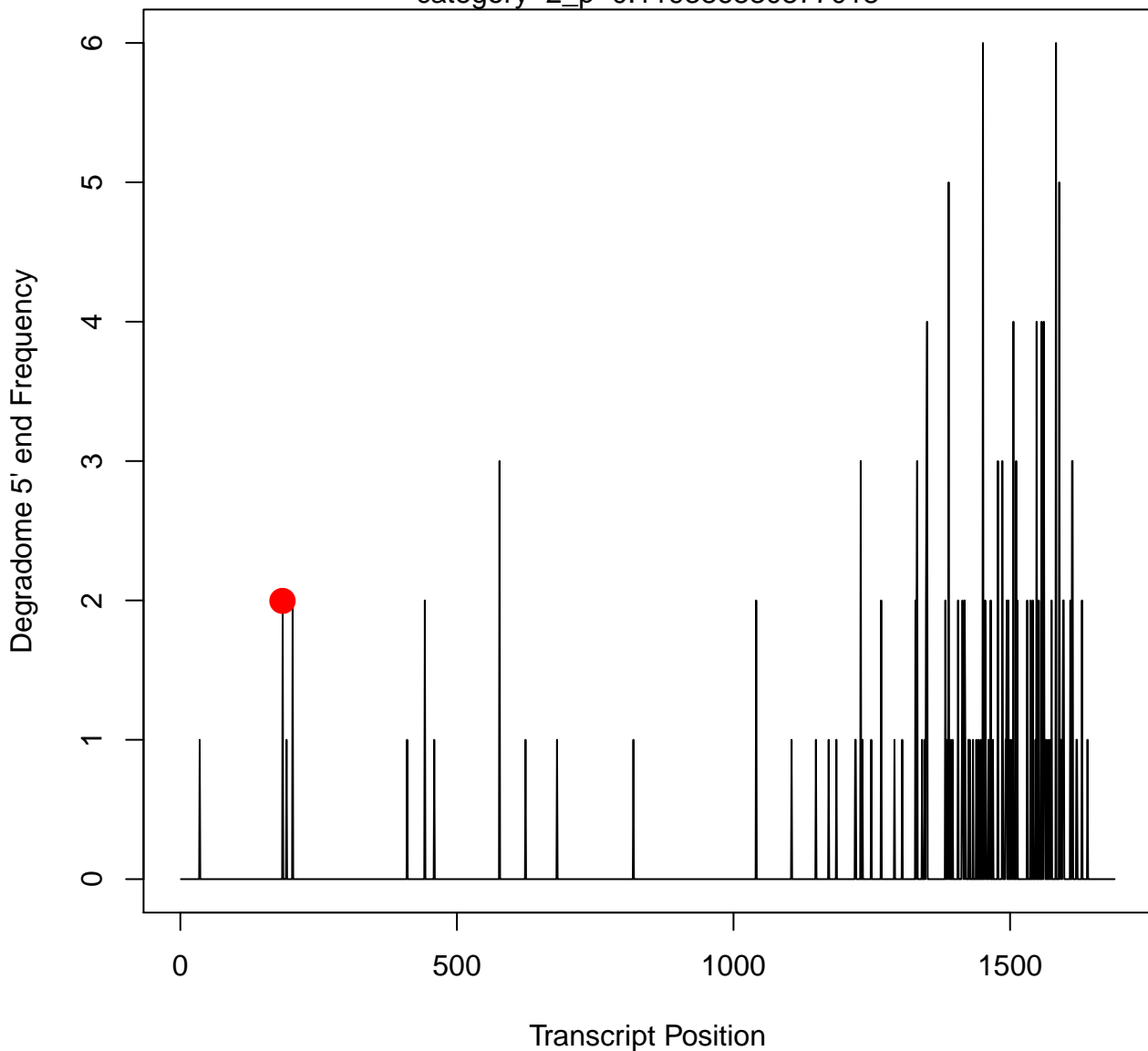

Supplement: Supplementary file 7 [file Data_Sheet_7.zip › Sit-miR166k_Seita.1G270500.1_185_TPlot.pdf]

**T=Seita.9G544600.1\_Q=Sit-miR167a\_S=1287**

category=2\_p=0.149943632899622

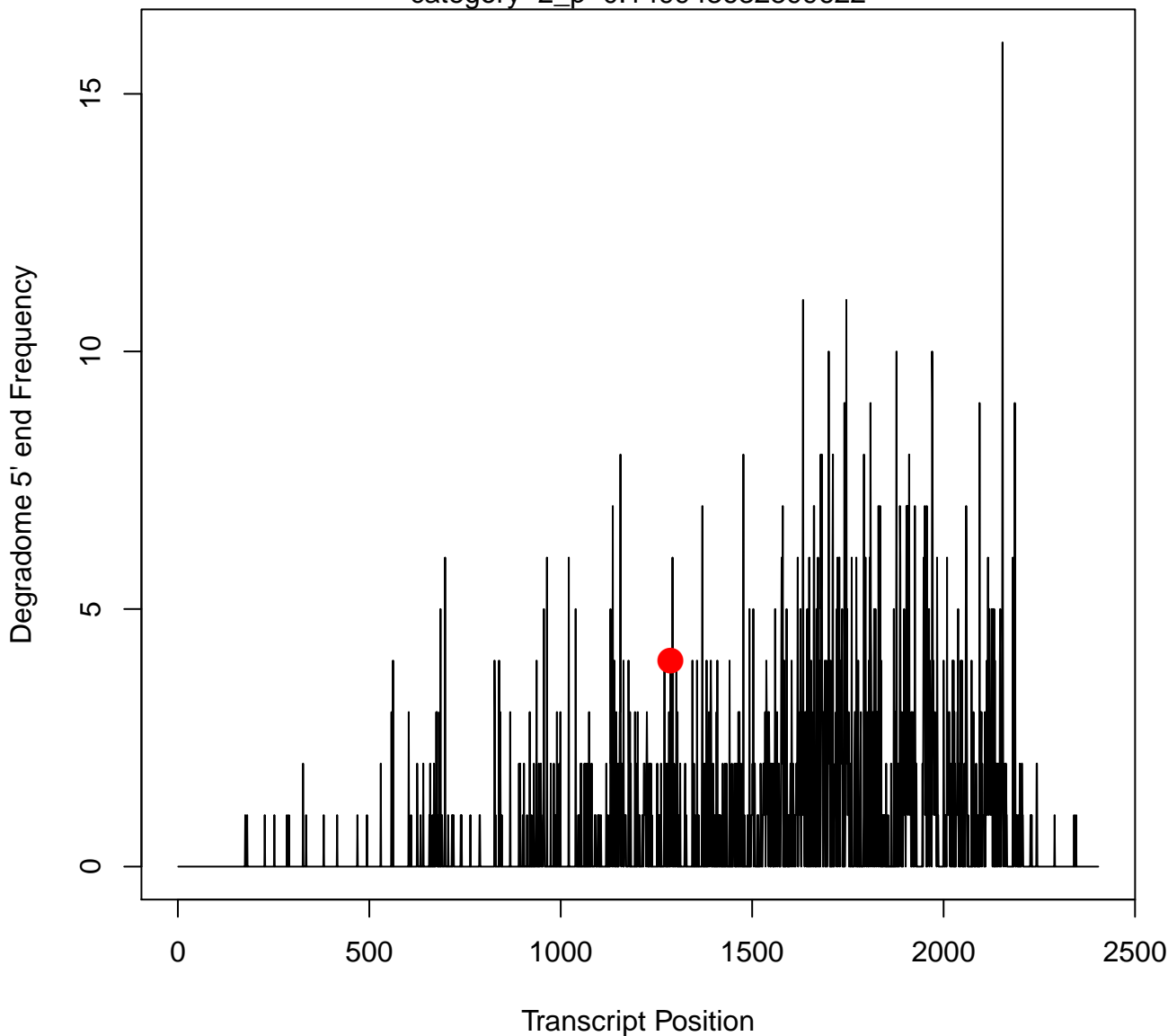

Supplement: Supplementary file 7 [file Data_Sheet_7.zip › Sit-miR167a_Seita.9G544600.1_1287_TPlot.pdf]

**T=Seita.3G020000.1\_Q=Sit-miR167h\_S=2739**

category=0\_p=0.000897462201815302

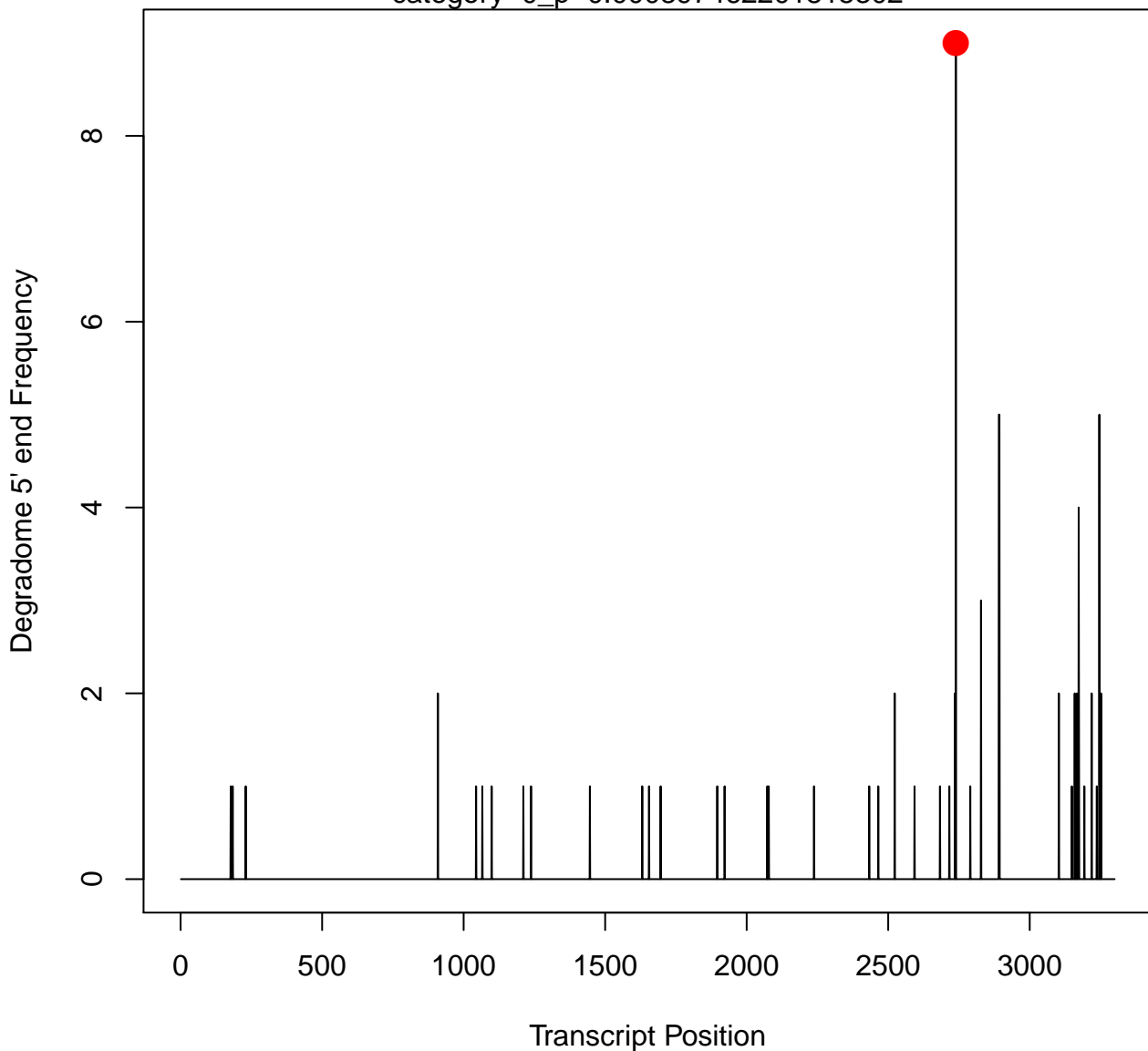

Supplement: Supplementary file 7 [file Data_Sheet_7.zip › Sit-miR167h_Seita.3G020000.1_2739_TPlot.pdf]

**T=Seita.4G262300.1\_Q=Sit-miR167h\_S=3389**

category=0\_p=0.00179411896522685

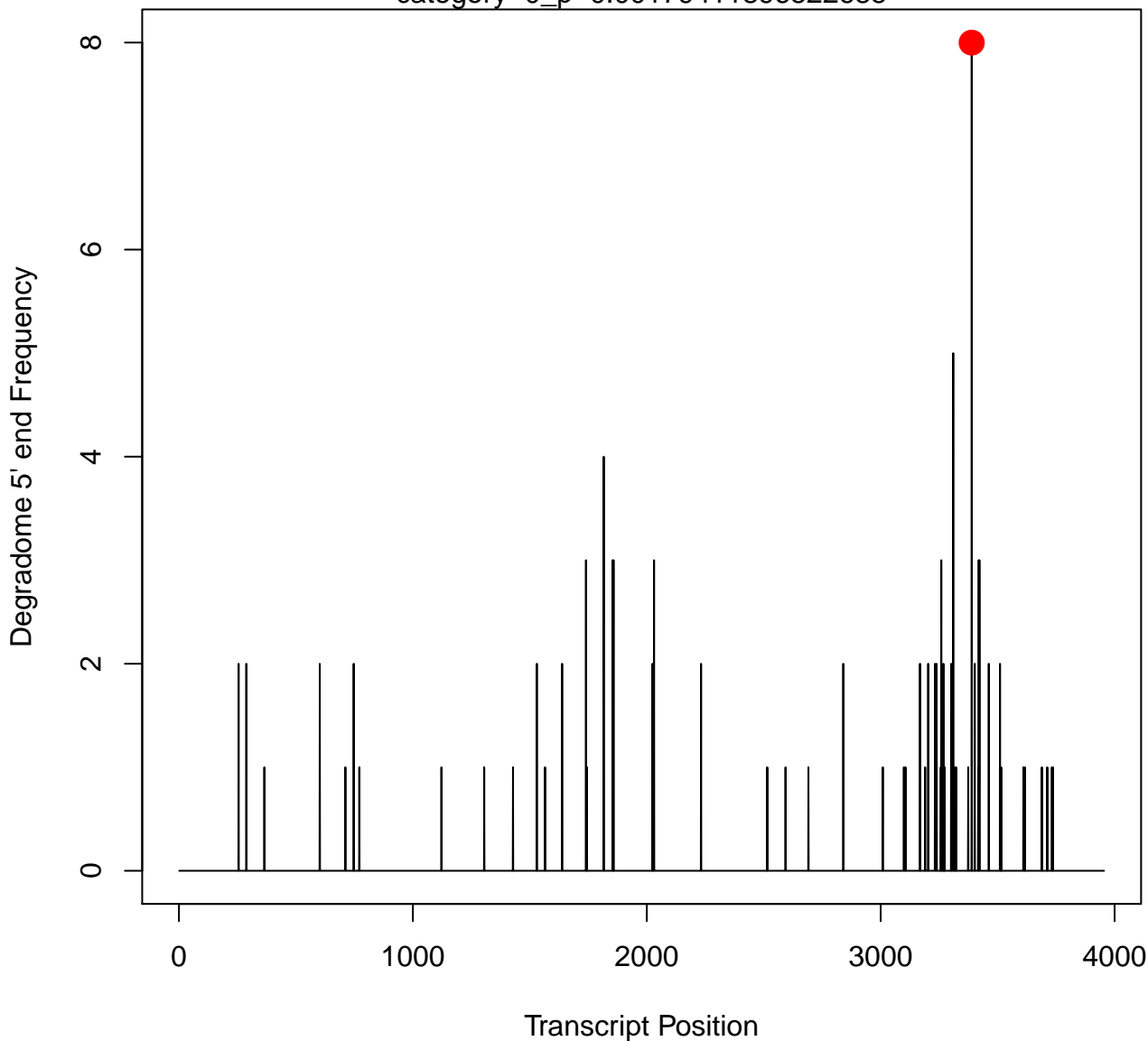

Supplement: Supplementary file 7 [file Data_Sheet_7.zip › Sit-miR167h_Seita.4G262300.1_3389_TPlot.pdf]

**T=Seita.9G320900.1\_Q=Sit-miR167h\_S=1109**

category=2\_p=0.466673247200913

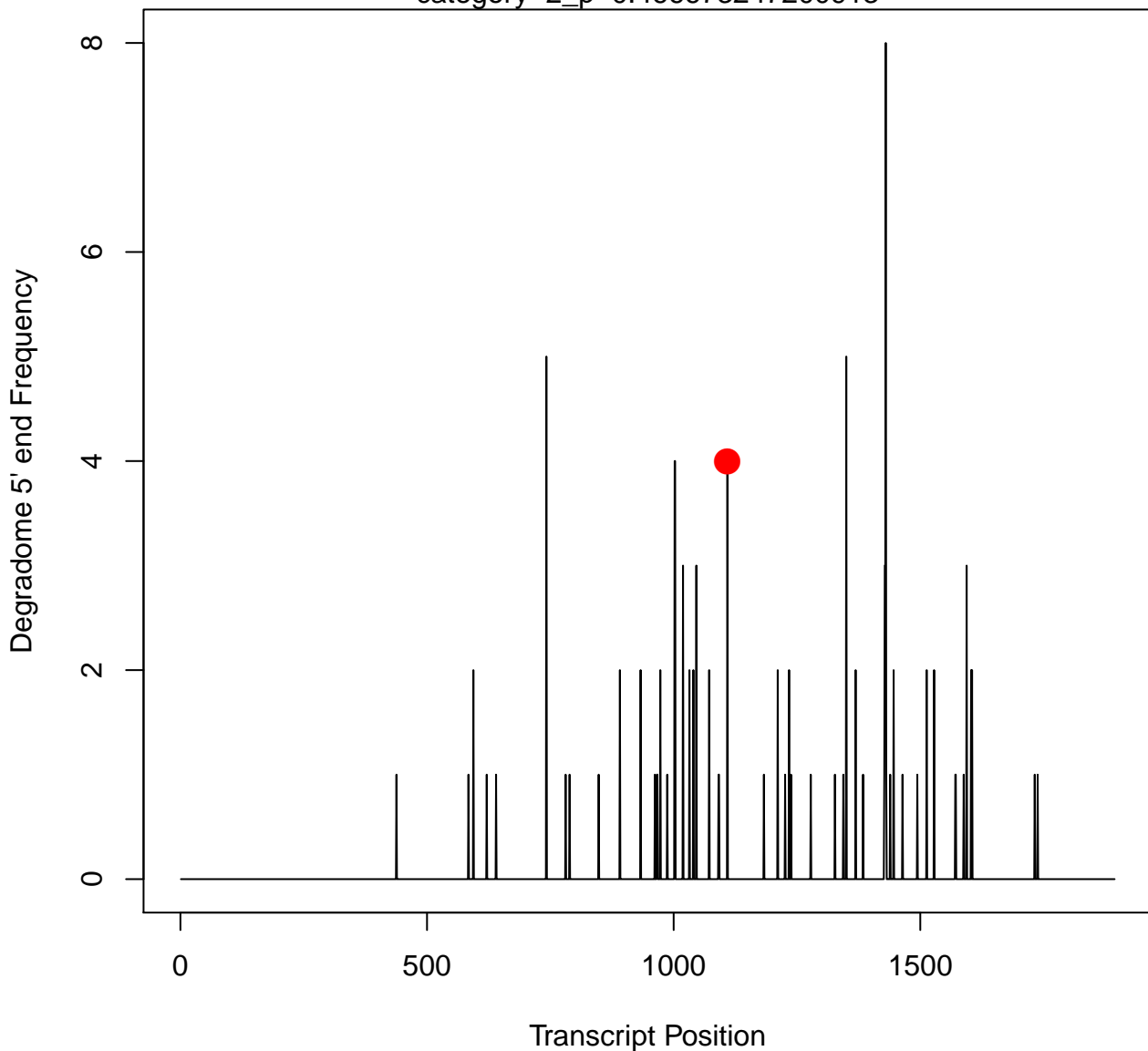

Supplement: Supplementary file 7 [file Data_Sheet_7.zip › Sit-miR167h_Seita.9G320900.1_1109_TPlot.pdf]

**T=Seita.5G376700.1\_Q=Sit-miR167j\_S=962**

category=2\_p=0.603577058586688

Degradome 5' end Frequency

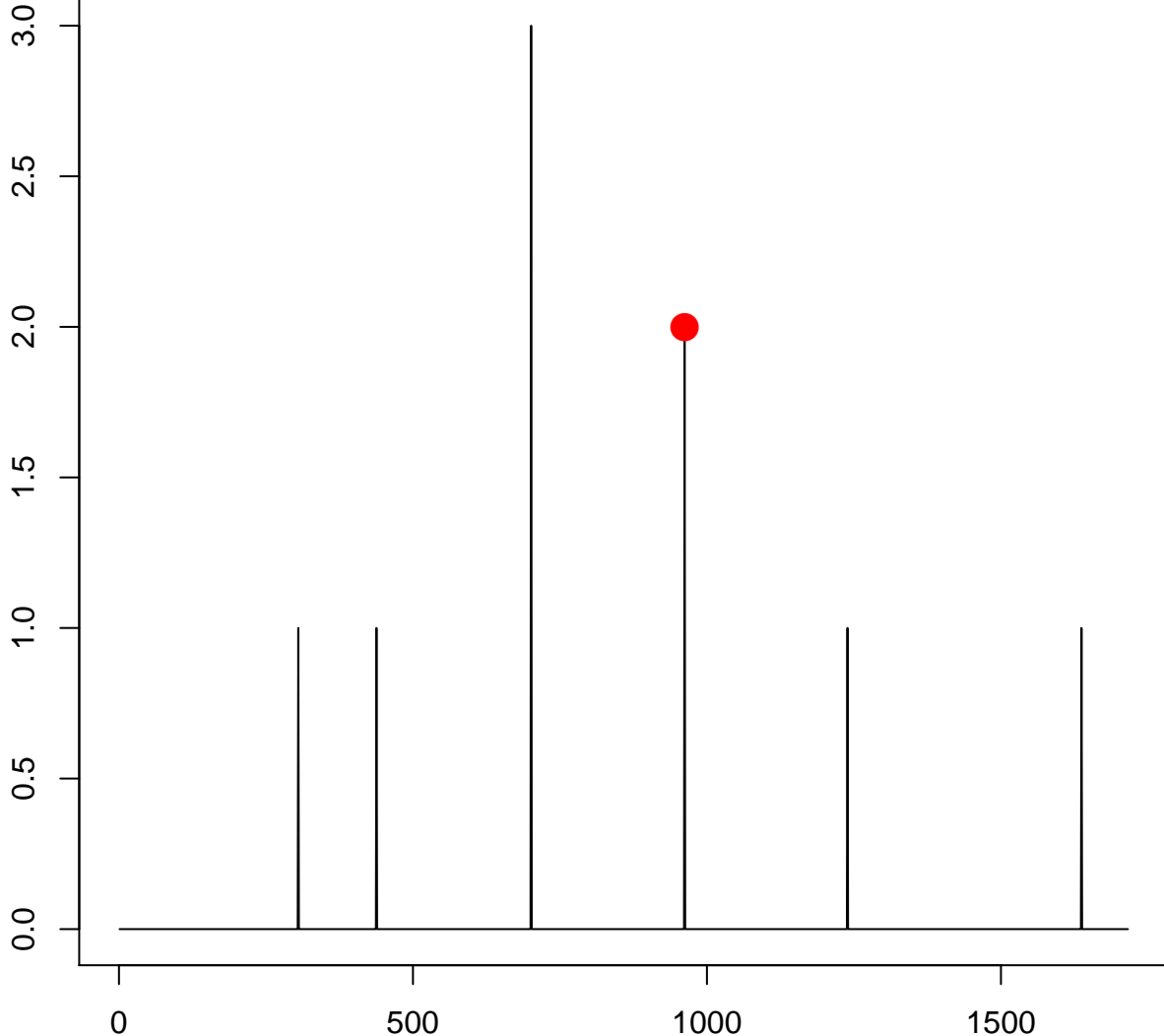

Transcript Position

Supplement: Supplementary file 7 [file Data_Sheet_7.zip › Sit-miR167j_Seita.5G376700.1_962_TPlot.pdf]

**T=Seita.3G070600.1\_Q=Sit-miR168\_S=747**

category=2\_p=0.17944013037241

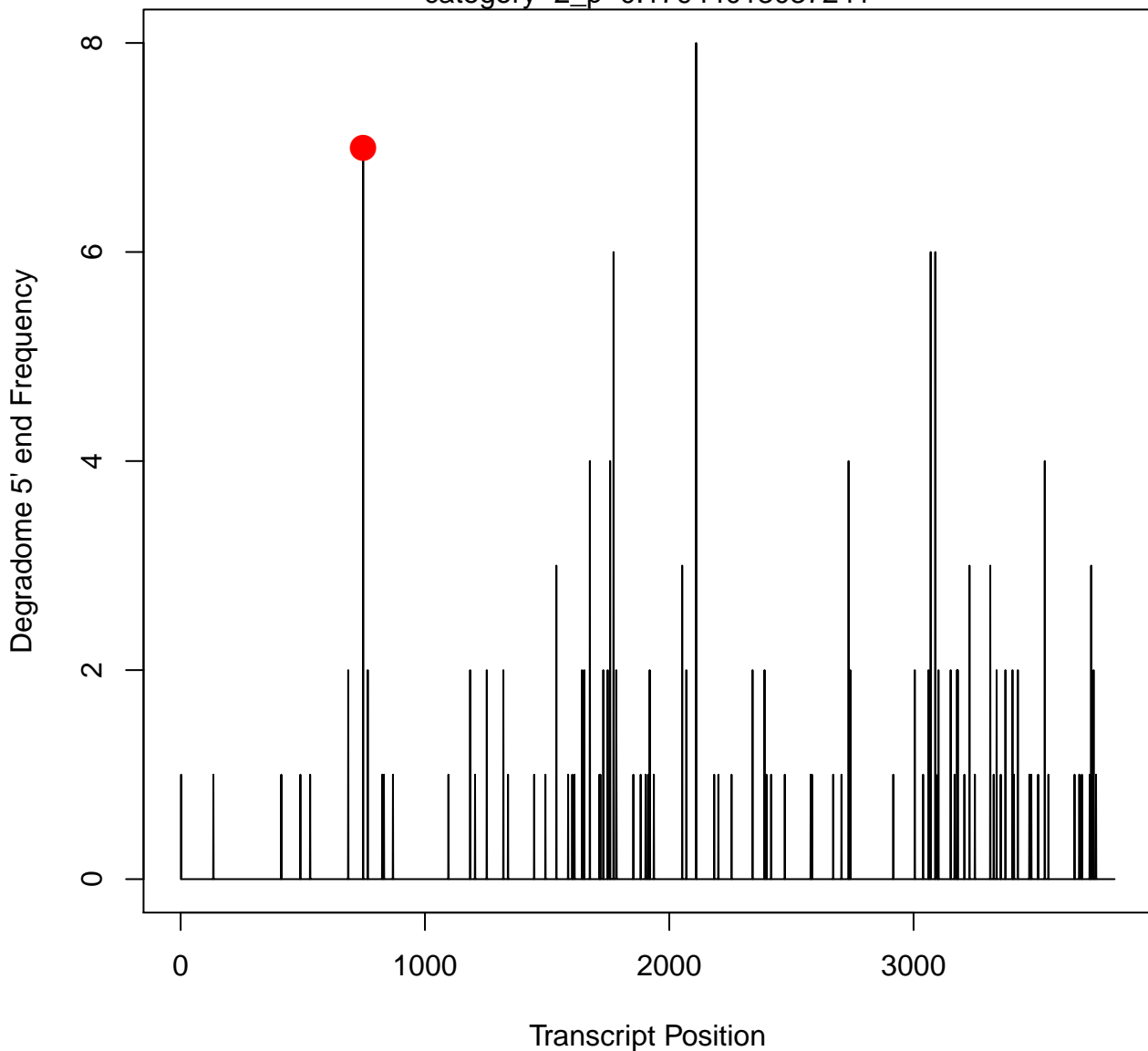

Supplement: Supplementary file 7 [file Data_Sheet_7.zip › Sit-miR168_Seita.3G070600.1_747_TPlot.pdf]

**T=Seita.3G089200.1\_Q=Sit-miR168\_S=125**

category=2\_p=0.377014190299197

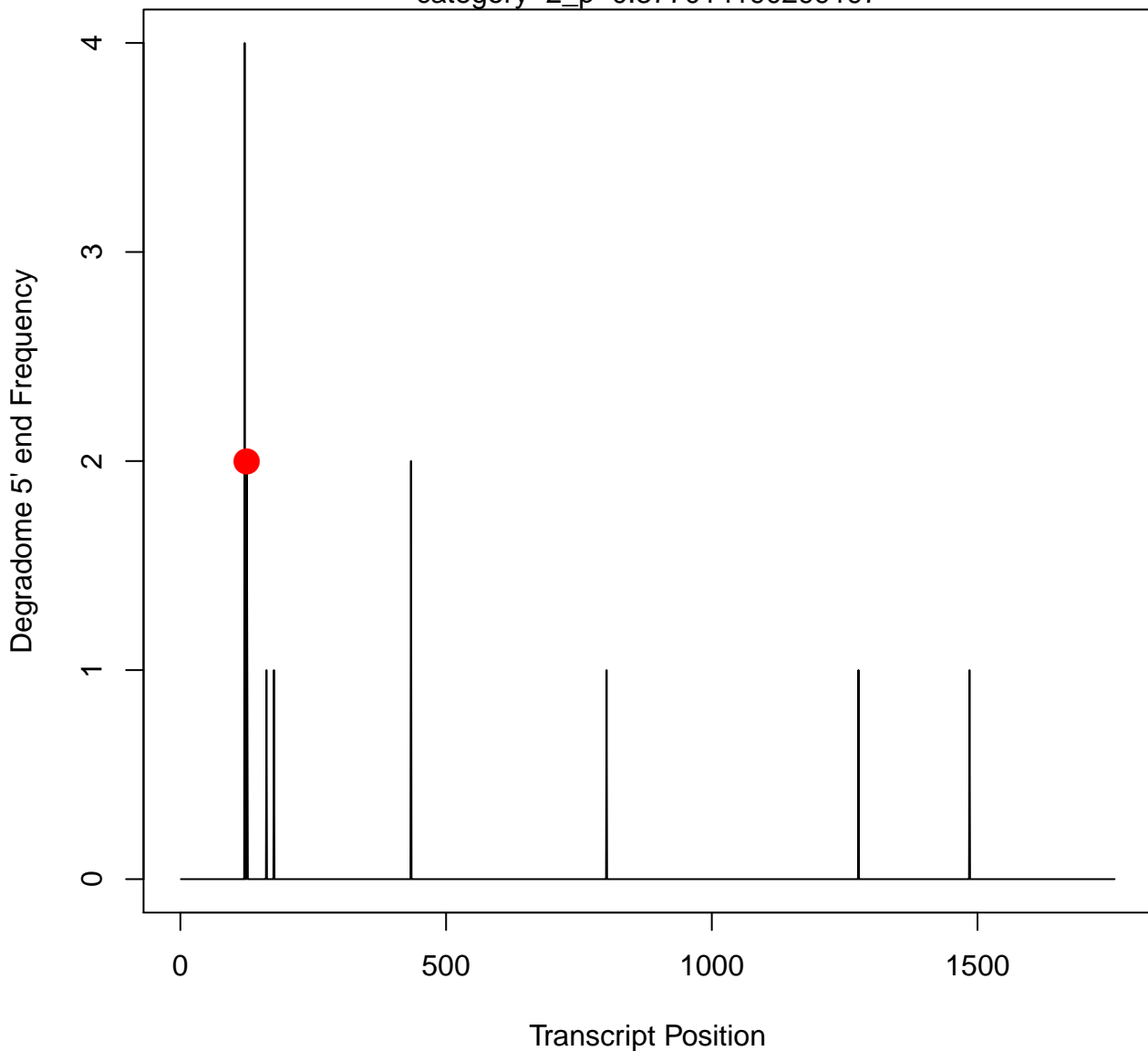

Supplement: Supplementary file 7 [file Data_Sheet_7.zip › Sit-miR168_Seita.3G089200.1_125_TPlot.pdf]

**T=Seita.5G261900.1\_Q=Sit-miR168\_S=537**

category=0\_p=0.0745874975961638

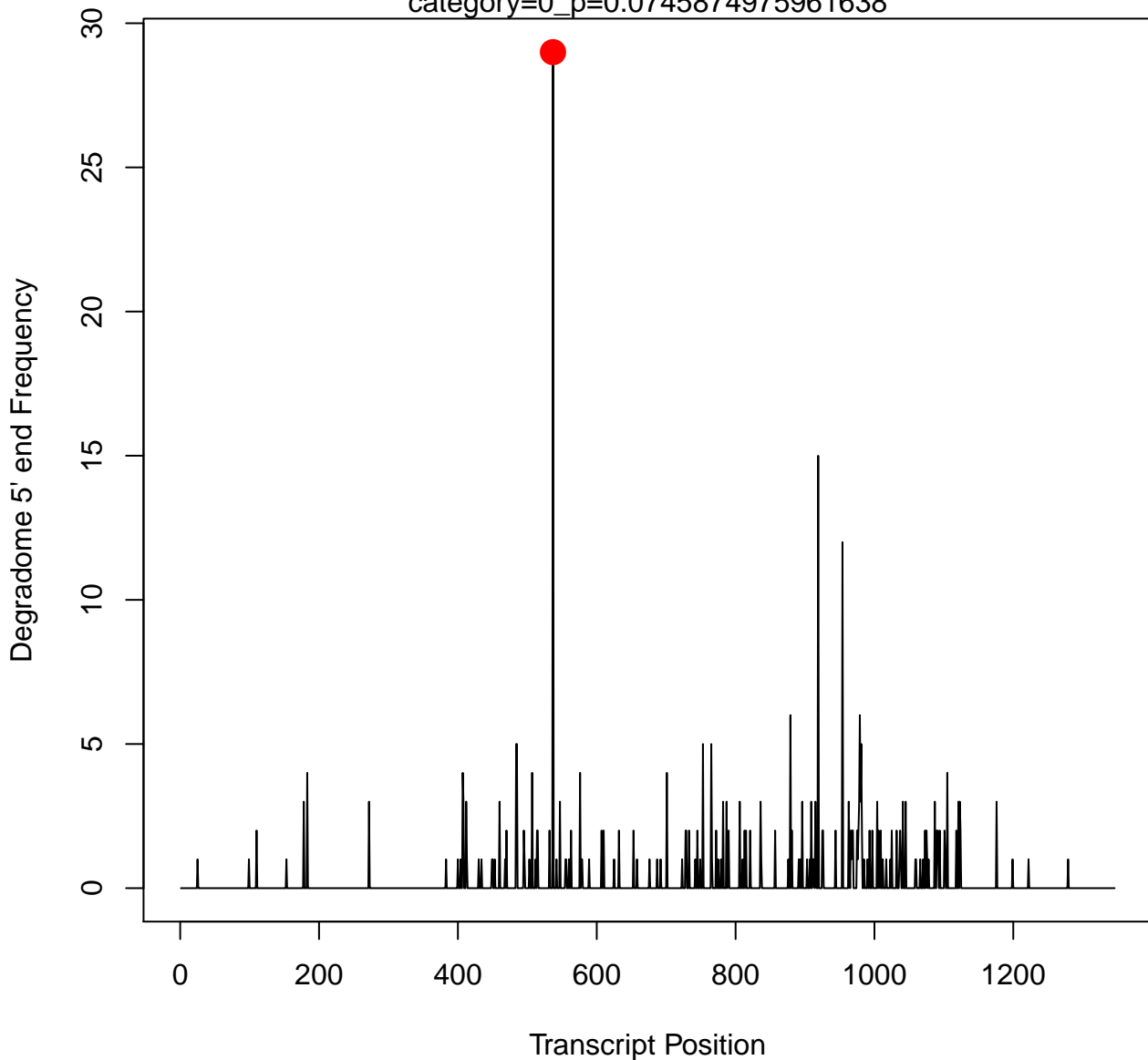

Supplement: Supplementary file 7 [file Data_Sheet_7.zip › Sit-miR168_Seita.5G261900.1_537_TPlot.pdf]

**T=Seita.5G435400.1\_Q=Sit-miR168\_S=595**

category=1\_p=0.00269988778251407

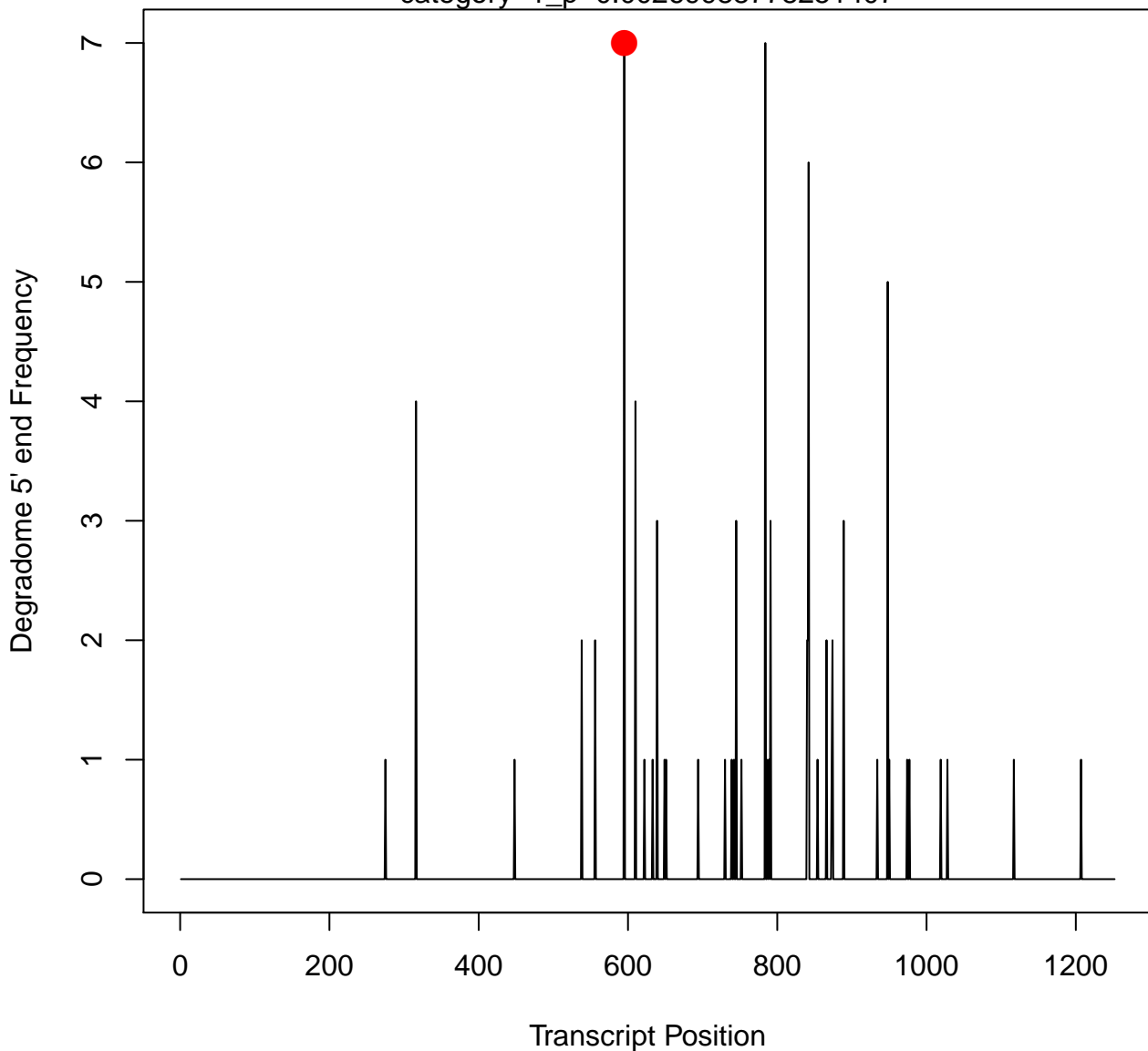

Supplement: Supplementary file 7 [file Data_Sheet_7.zip › Sit-miR168_Seita.5G435400.1_595_TPlot.pdf]

**T=Seita.7G200400.1\_Q=Sit-miR168\_S=1236**

category=2\_p=0.930739010418018

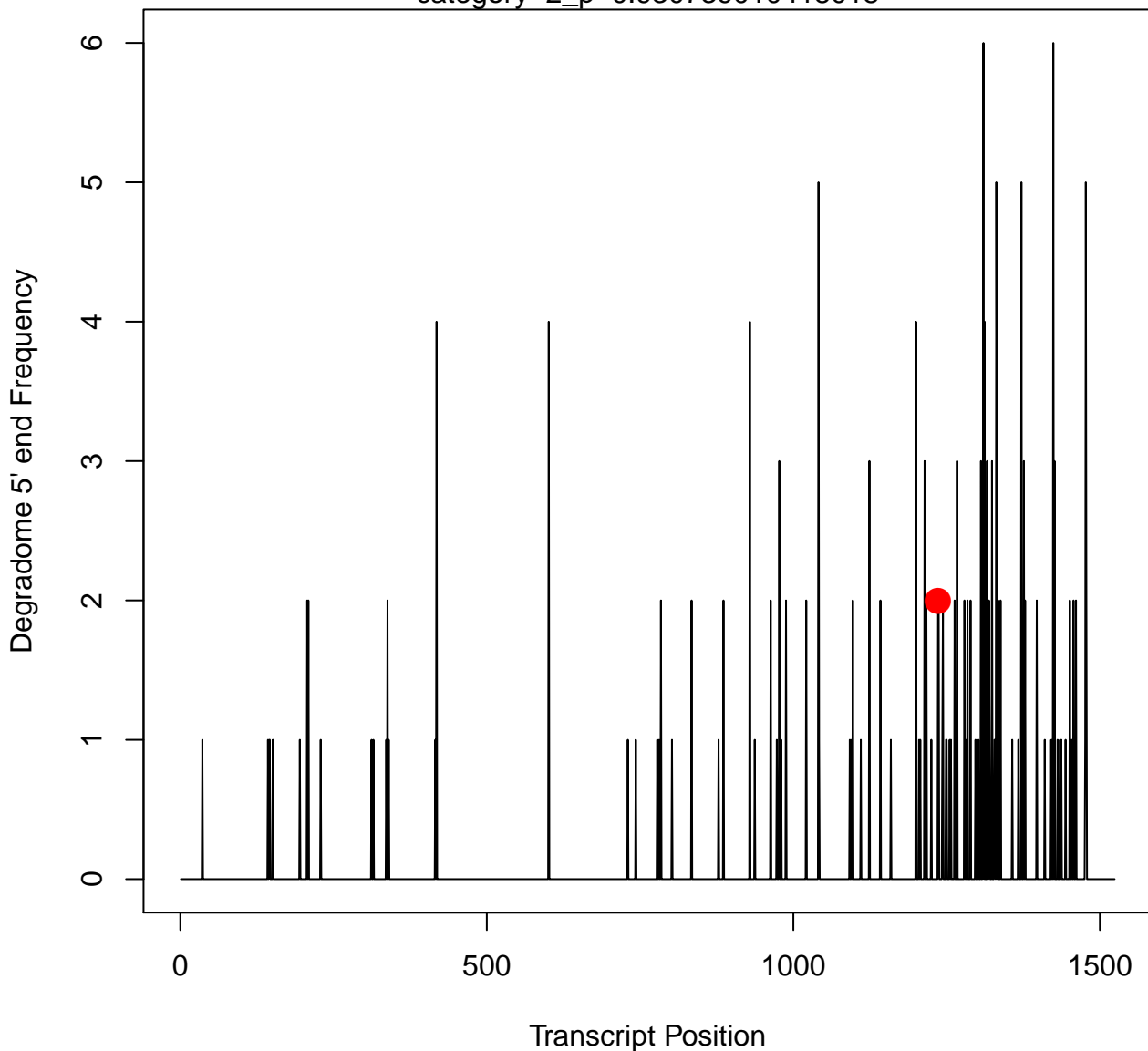

Supplement: Supplementary file 7 [file Data_Sheet_7.zip › Sit-miR168_Seita.7G200400.1_1236_TPlot.pdf]

**T=Seita.7G201100.1\_Q=Sit-miR168\_S=664**

category=2\_p=0.167766384158407

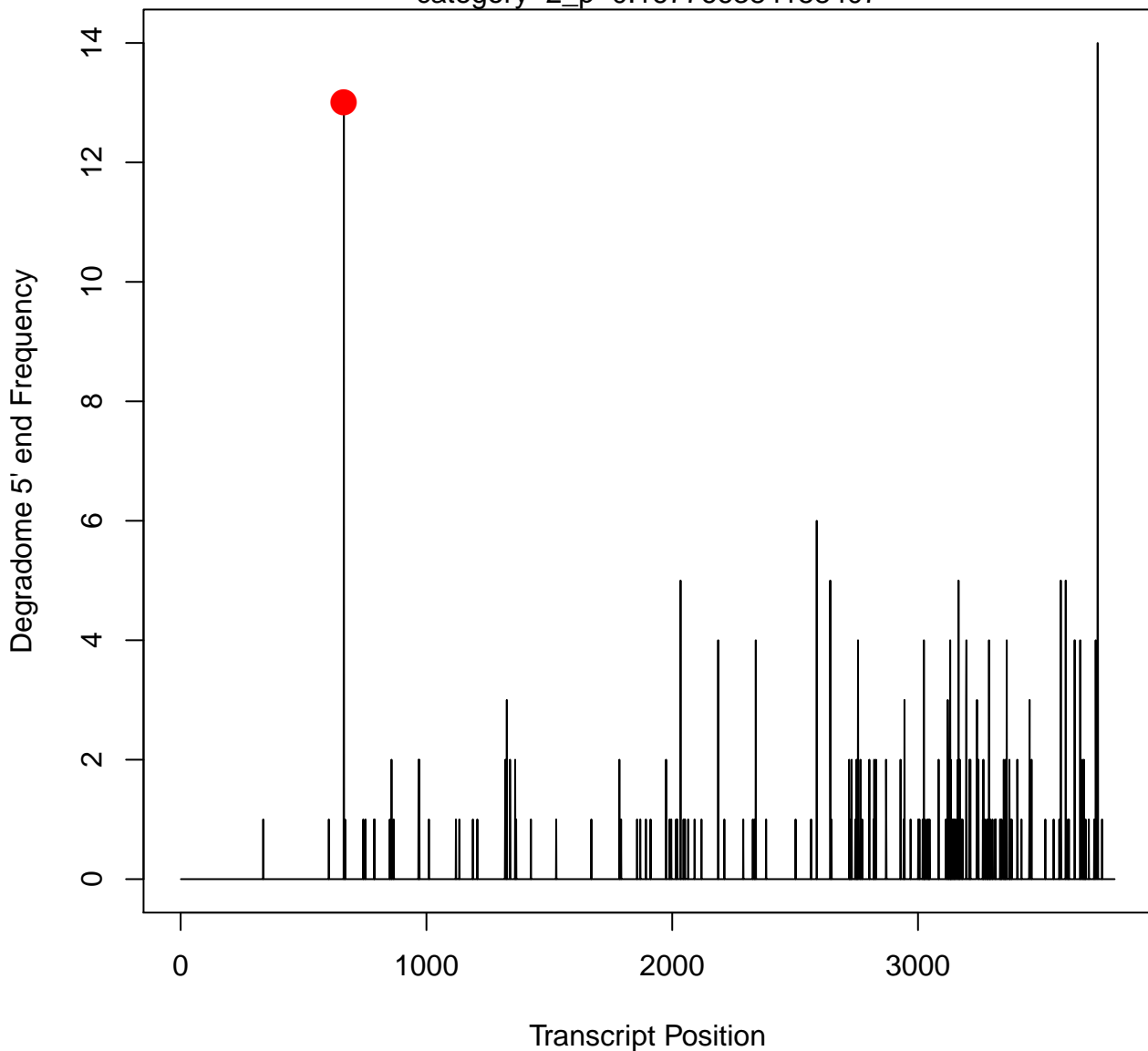

Supplement: Supplementary file 7 [file Data_Sheet_7.zip › Sit-miR168_Seita.7G201100.1_664_TPlot.pdf]

**T=Seita.9G431700.1\_Q=Sit-miR168\_S=1457**

category=2\_p=0.934080142371624

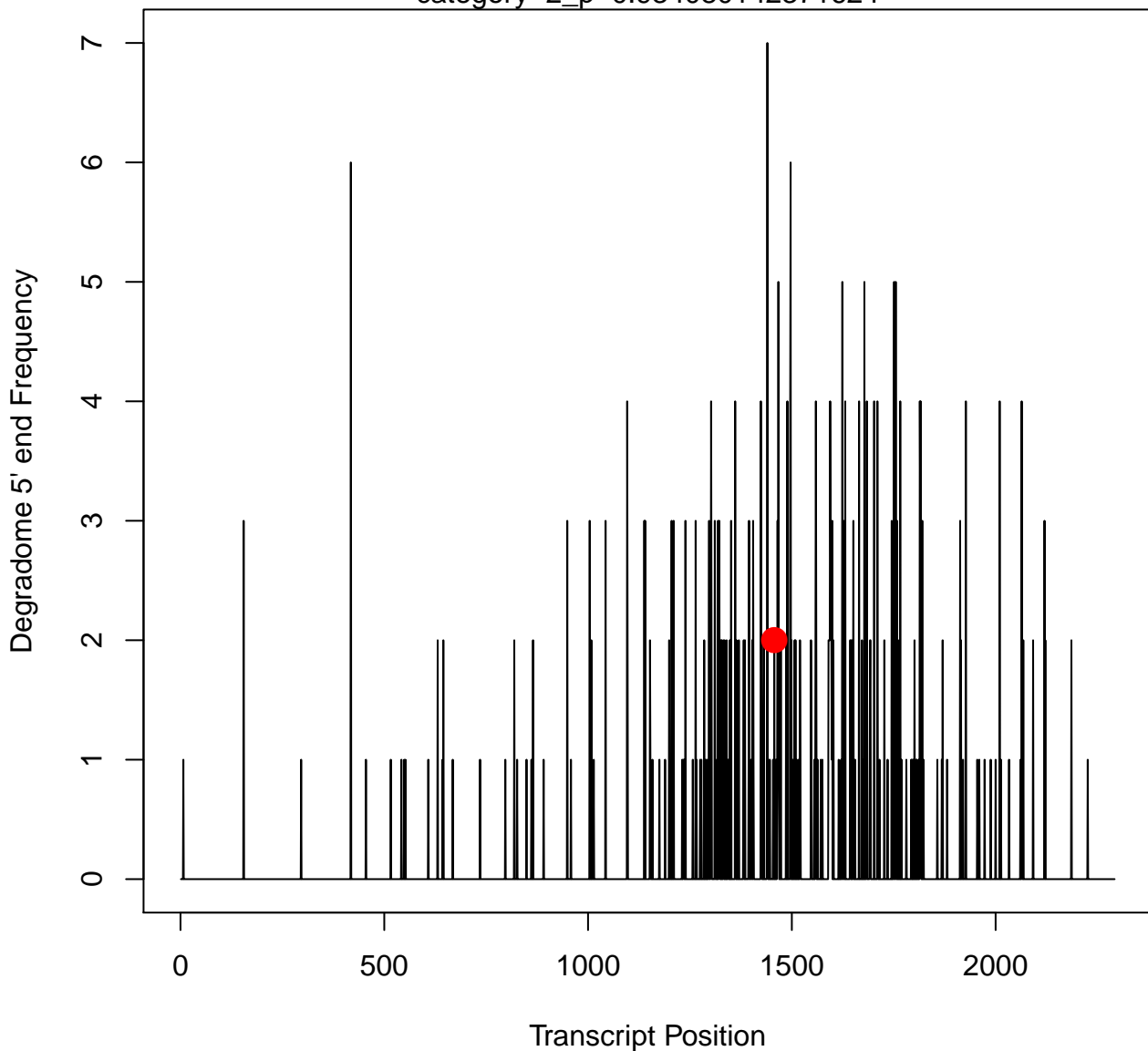

Supplement: Supplementary file 7 [file Data_Sheet_7.zip › Sit-miR168_Seita.9G431700.1_1457_TPlot.pdf]

**T=Seita.9G505300.1\_Q=Sit-miR168\_S=412**

category=2\_p=0.58348445820267

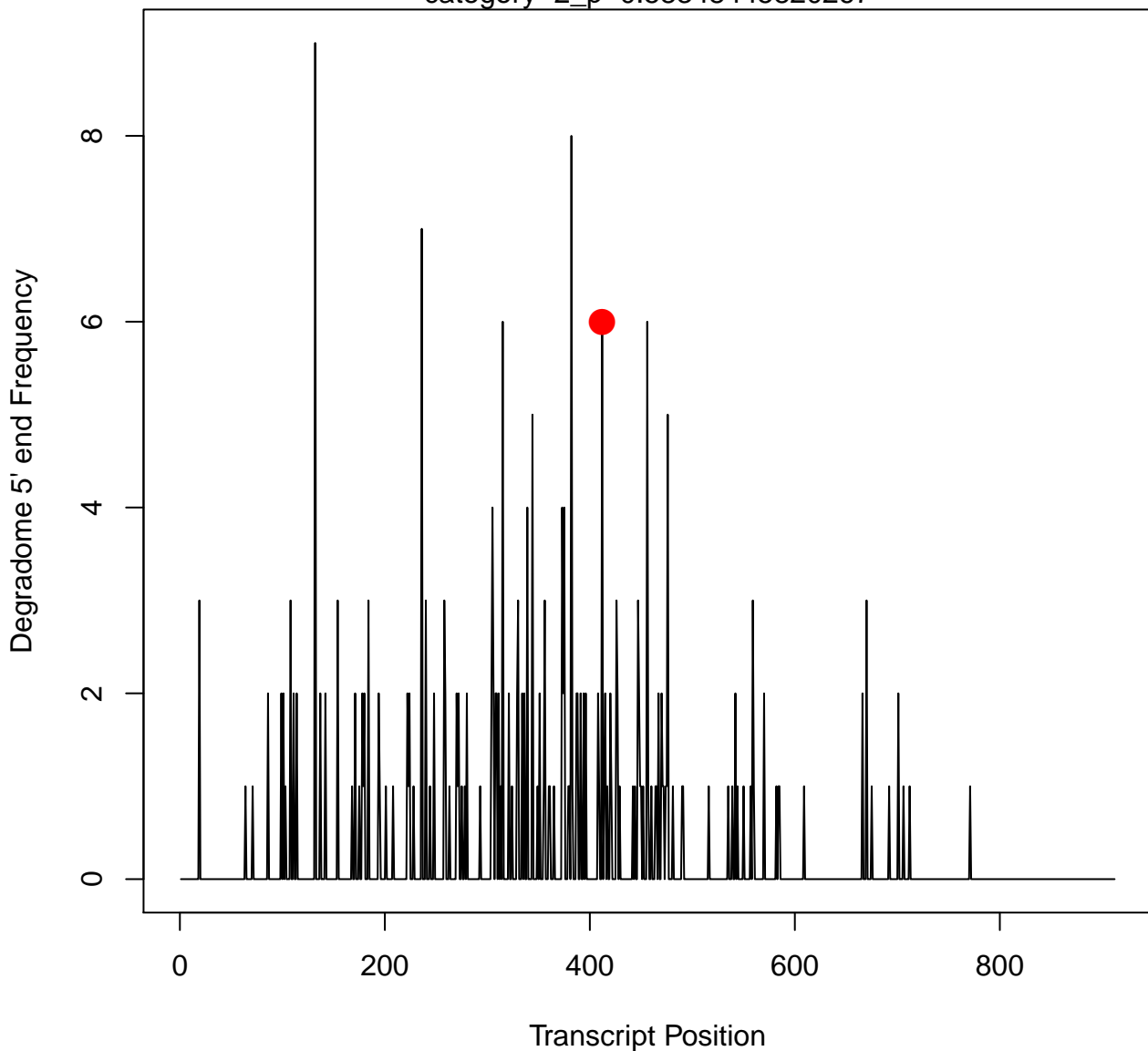

Supplement: Supplementary file 7 [file Data_Sheet_7.zip › Sit-miR168_Seita.9G505300.1_412_TPlot.pdf]

**T=Seita.3G184900.1\_Q=Sit-miR169d\_S=536**

category=2\_p=0.740516727940422

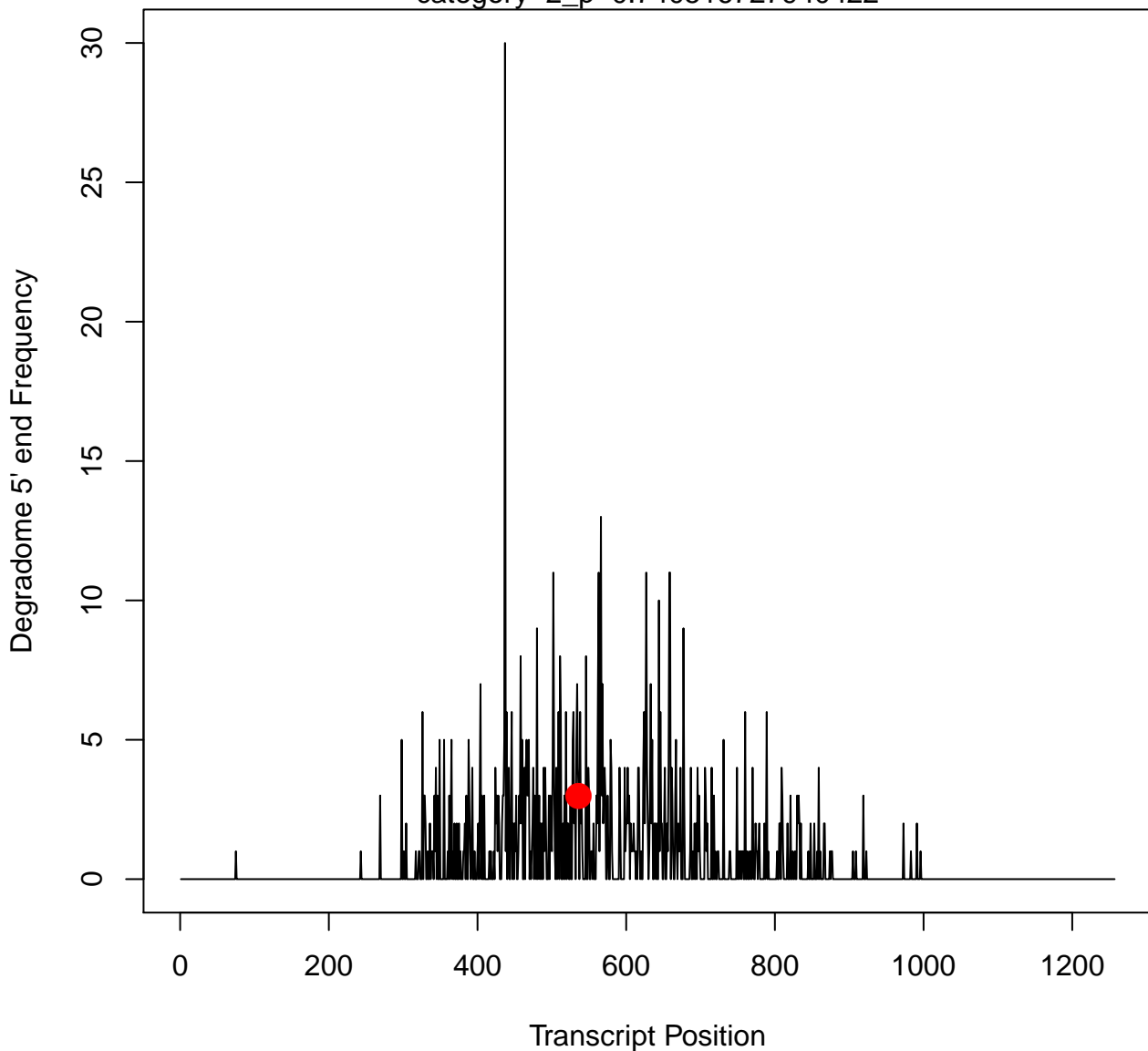

Supplement: Supplementary file 7 [file Data_Sheet_7.zip › Sit-miR169d_Seita.3G184900.1_536_TPlot.pdf]

**T=Seita.3G390000.1\_Q=Sit-miR169f\_S=2048**

category=2\_p=0.027857255792645

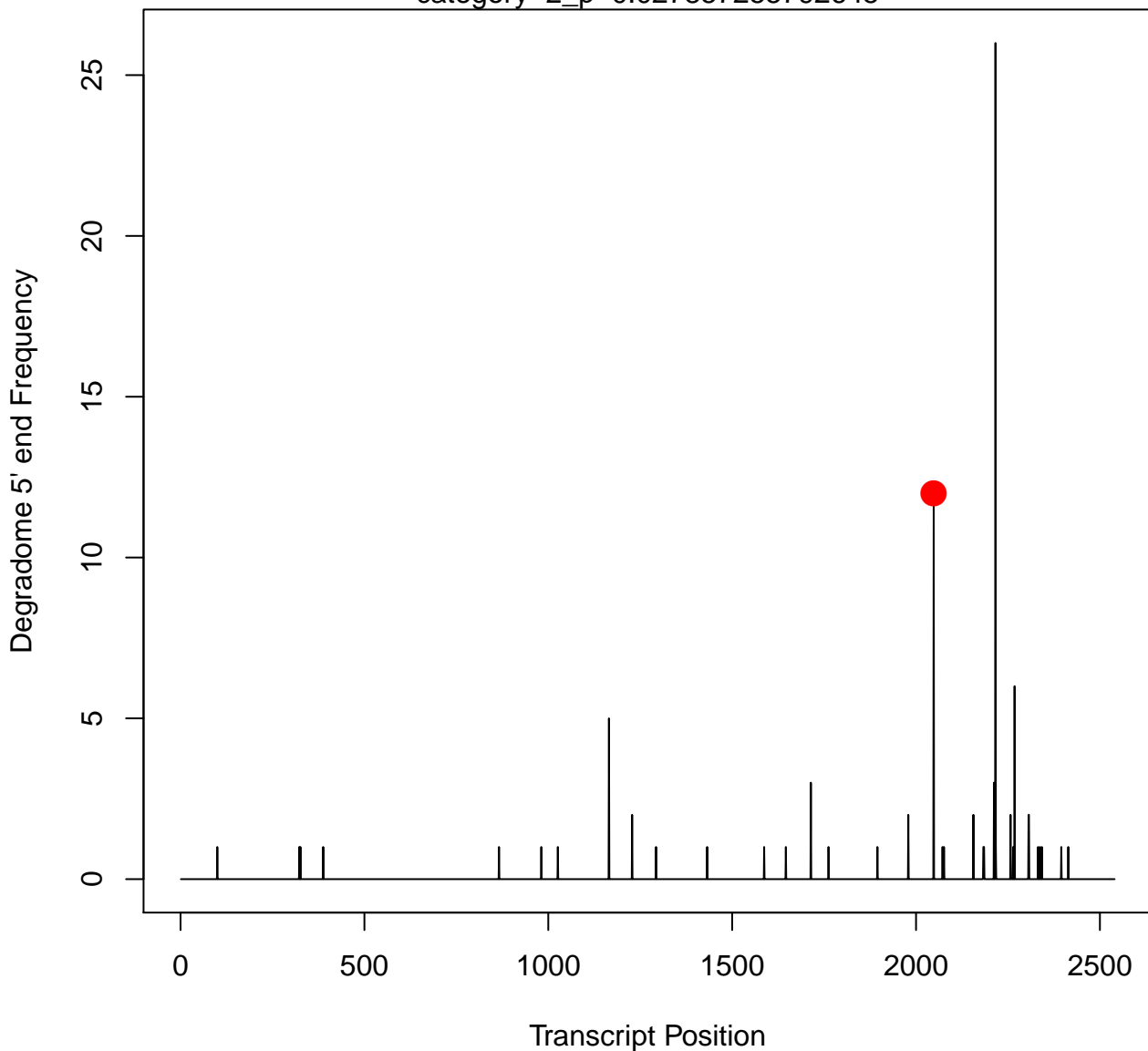

Supplement: Supplementary file 7 [file Data_Sheet_7.zip › Sit-miR169f_Seita.3G390000.1_2048_TPlot.pdf]

**T=Seita.7G063700.1\_Q=Sit-miR169f\_S=1128**

category=2\_p=0.891921184350761

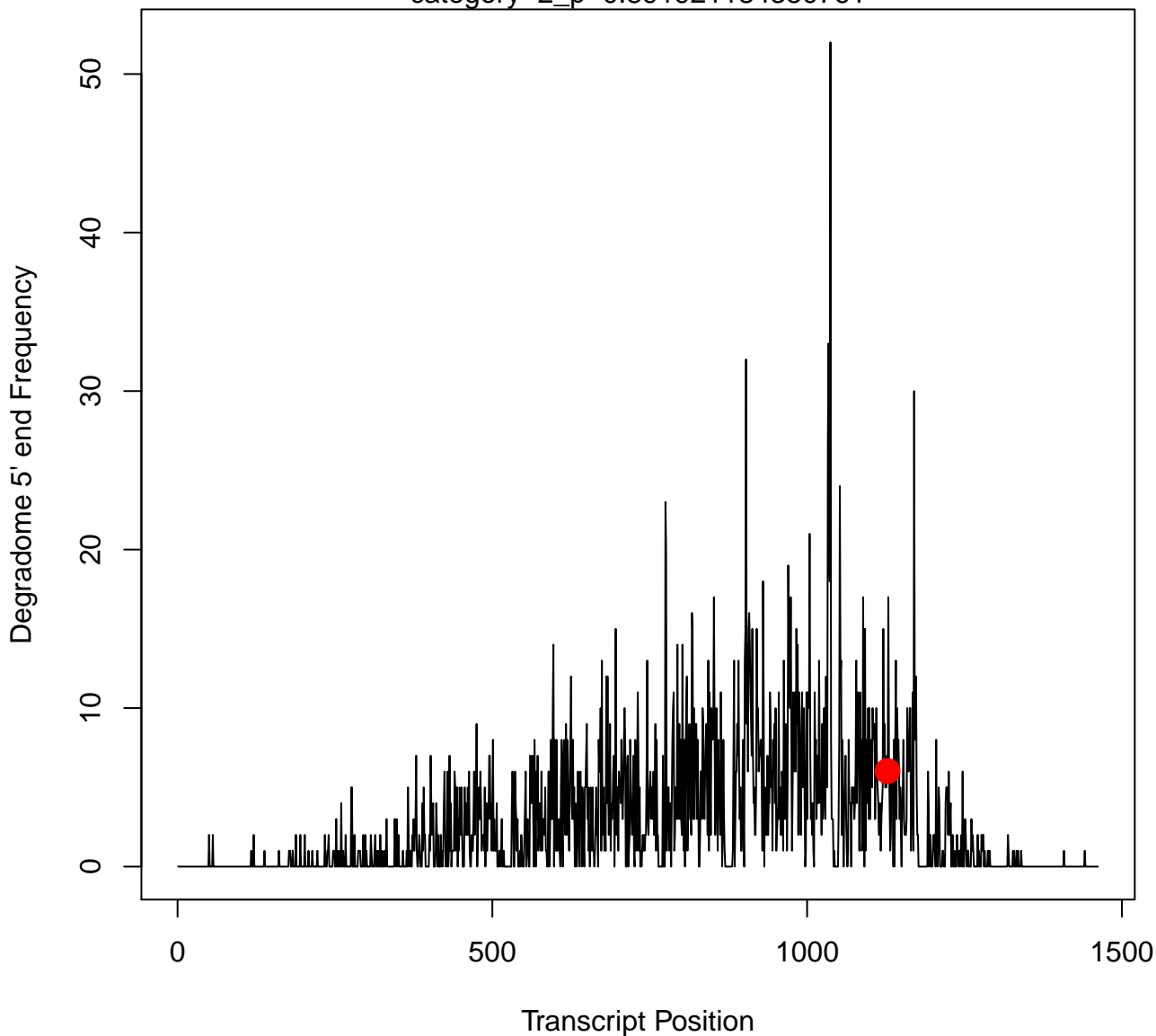

Supplement: Supplementary file 7 [file Data_Sheet_7.zip › Sit-miR169f_Seita.7G063700.1_1128_TPlot.pdf]

**T=Seita.7G160400.1\_Q=Sit-miR169f\_S=1070**

category=2\_p=0.747745219824098

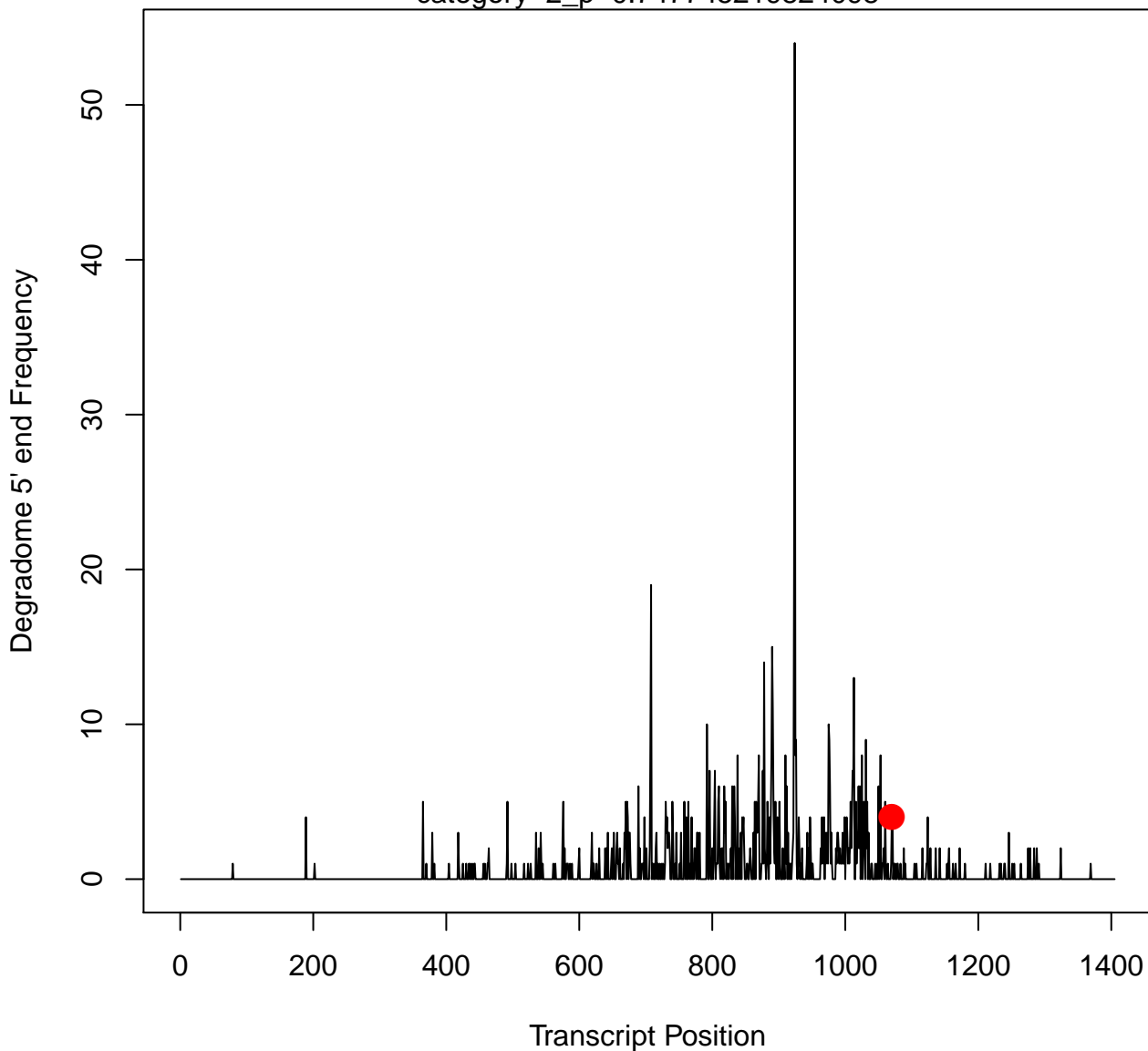

Supplement: Supplementary file 7 [file Data_Sheet_7.zip › Sit-miR169f_Seita.7G160400.1_1070_TPlot.pdf]

**T=Seita.9G129400.1\_Q=Sit-miR169f\_S=2100**

category=0\_p=0.00298840966153668

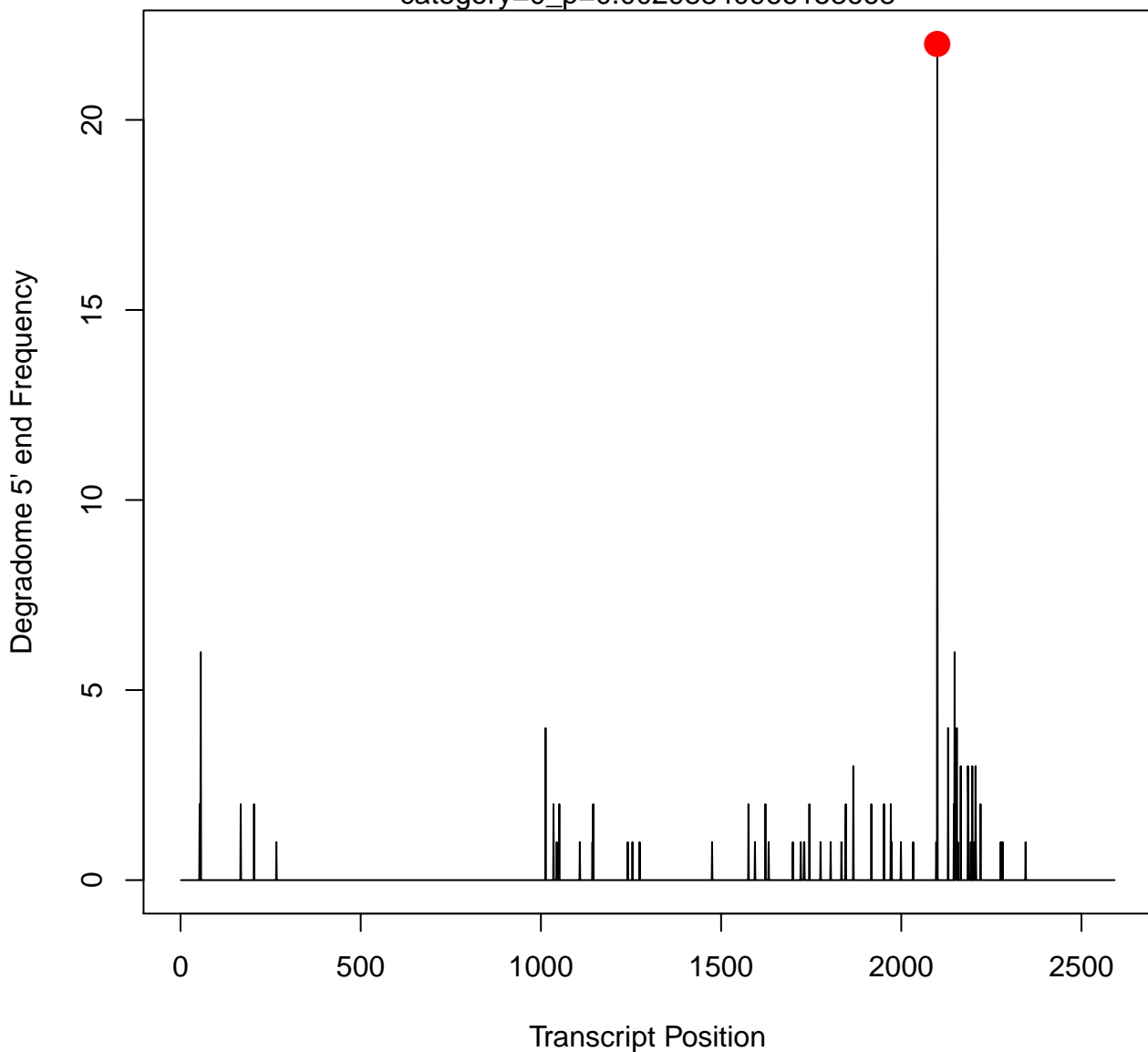

Supplement: Supplementary file 7 [file Data_Sheet_7.zip › Sit-miR169f_Seita.9G129400.1_2100_TPlot.pdf]

**T=Seita.9G155700.1\_Q=Sit-miR169f\_S=1672**

category=0\_p=0.000598397663414518

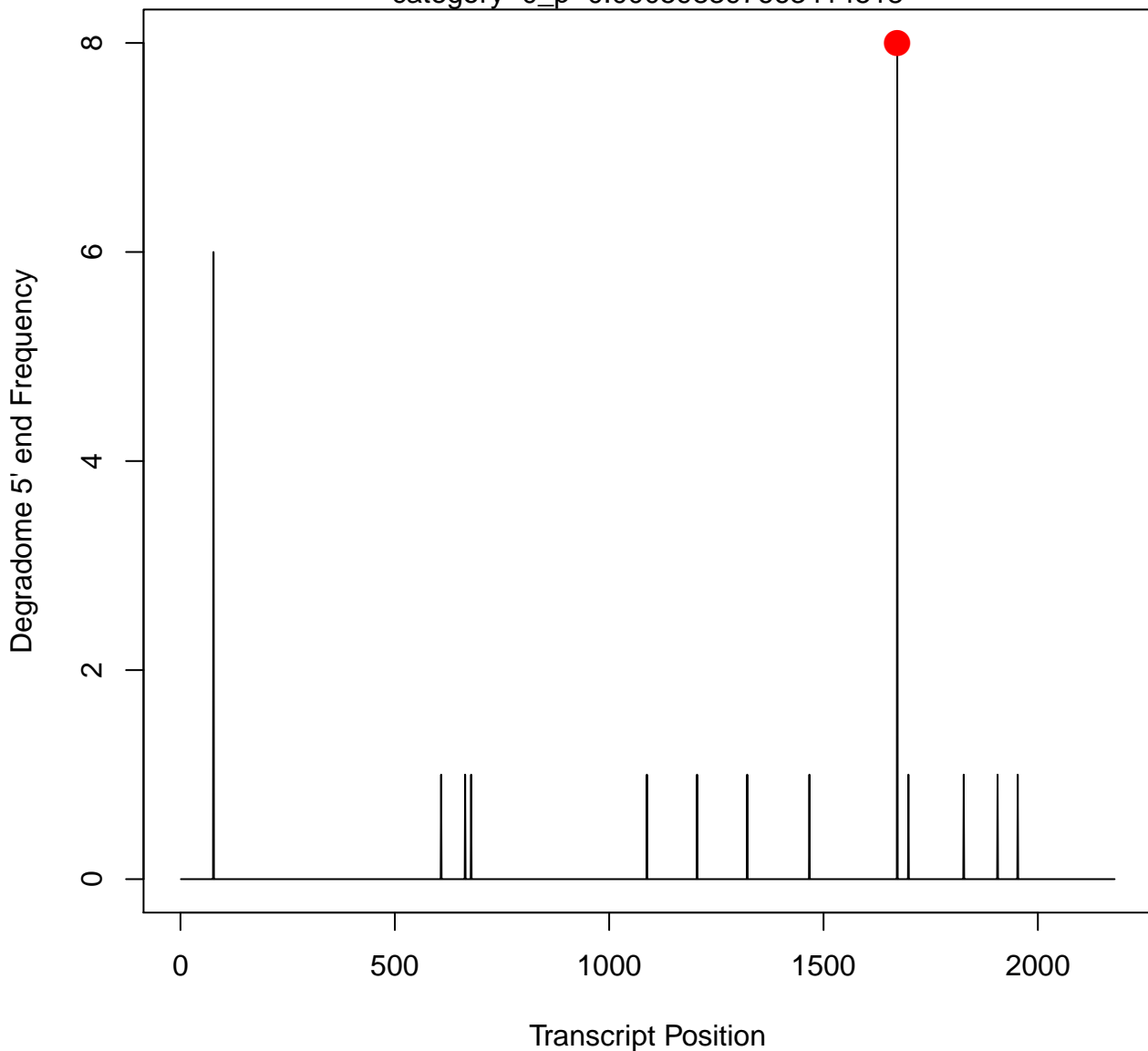

Supplement: Supplementary file 7 [file Data_Sheet_7.zip › Sit-miR169f_Seita.9G155700.1_1672_TPlot.pdf]

**T=Seita.2G066700.1\_Q=Sit-miR169g\_S=1865**

category=2\_p=0.979006596982867

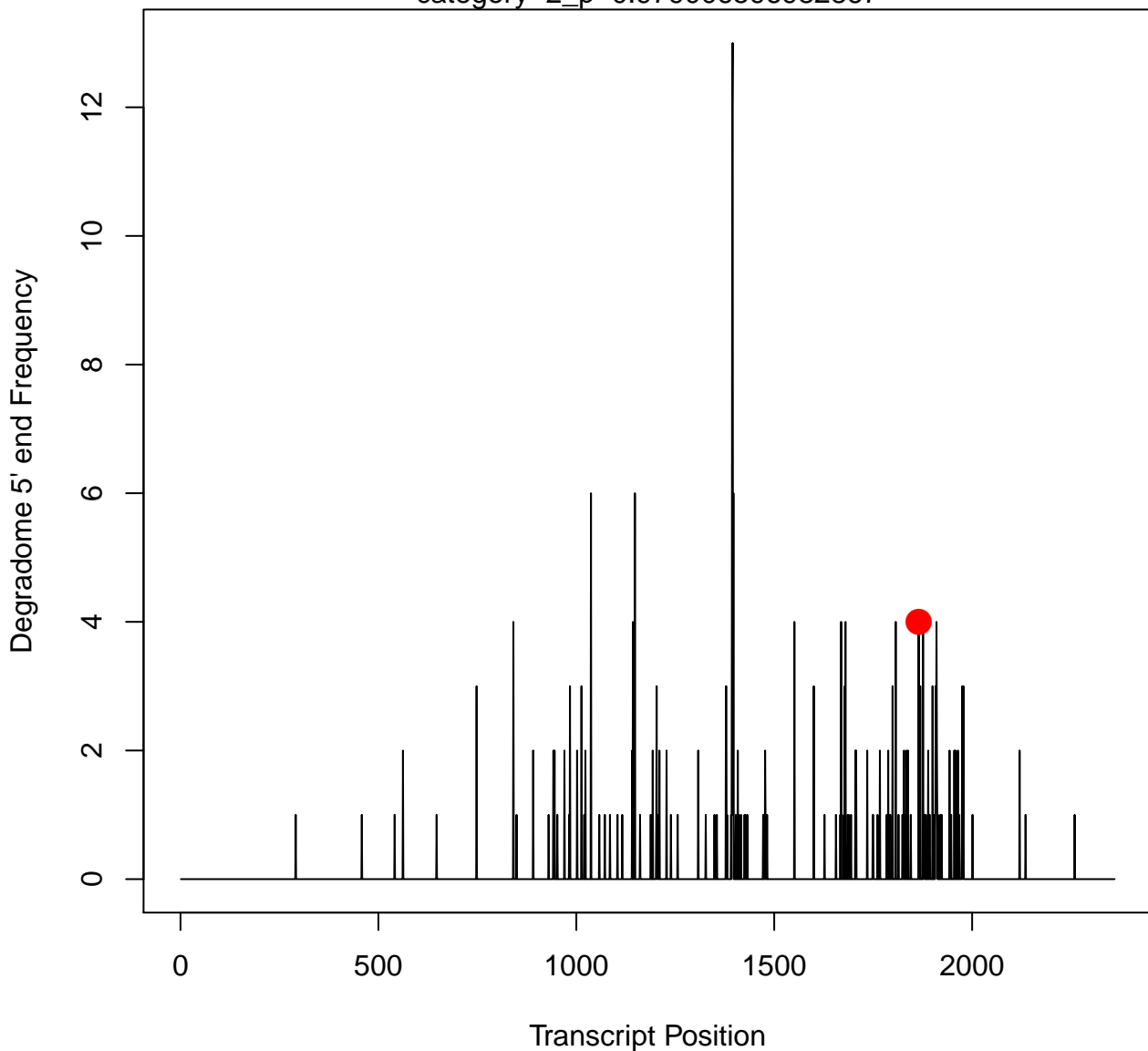

Supplement: Supplementary file 7 [file Data_Sheet_7.zip › Sit-miR169g_Seita.2G066700.1_1865_TPlot.pdf]

**T=Seita.4G131600.1\_Q=Sit-miR169g\_S=1407**

category=2\_p=0.94475041694726

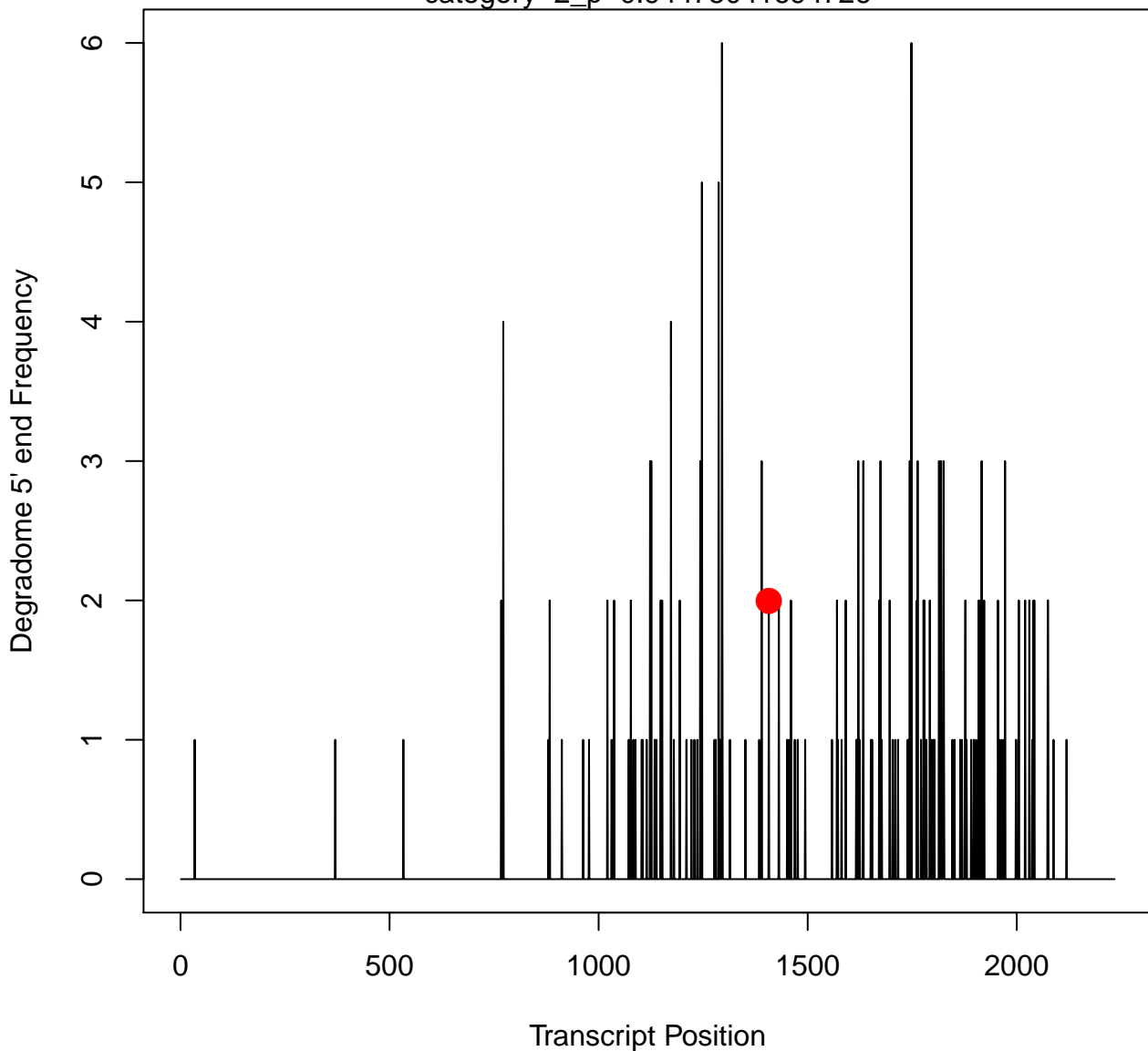

Supplement: Supplementary file 7 [file Data_Sheet_7.zip › Sit-miR169g_Seita.4G131600.1_1407_TPlot.pdf]

**T=Seita.5G438900.1\_Q=Sit-miR169g\_S=506**

category=2\_p=0.948518155012506

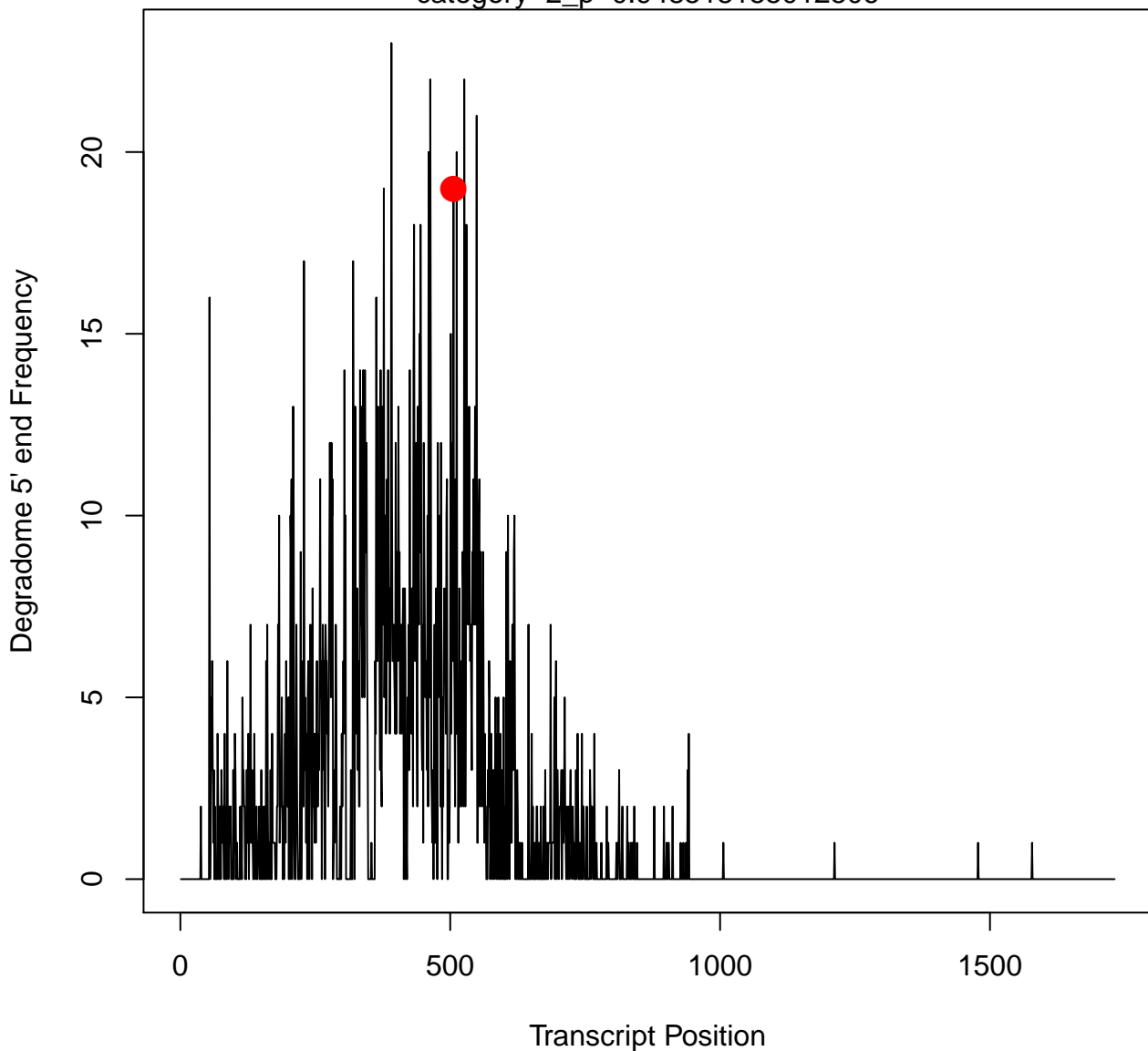

Supplement: Supplementary file 7 [file Data_Sheet_7.zip › Sit-miR169g_Seita.5G438900.1_506_TPlot.pdf]

**T=Seita.7G226200.1\_Q=Sit-miR169g\_S=1440**

category=2\_p=0.58348445820267

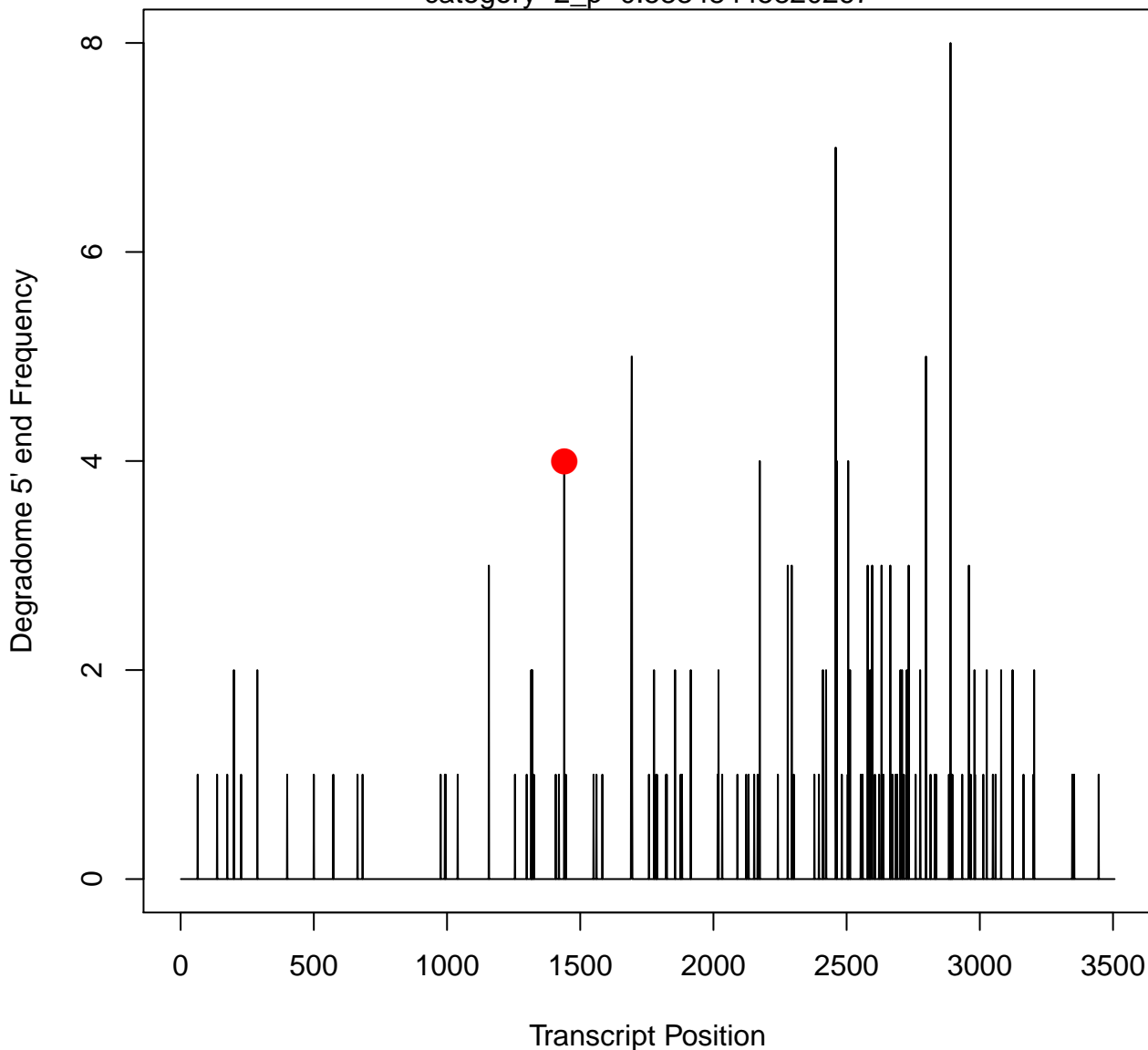

Supplement: Supplementary file 7 [file Data_Sheet_7.zip › Sit-miR169g_Seita.7G226200.1_1440_TPlot.pdf]

**T=Seita.9G521100.1\_Q=Sit-miR169g\_S=930**

category=2\_p=0.0414935082386434

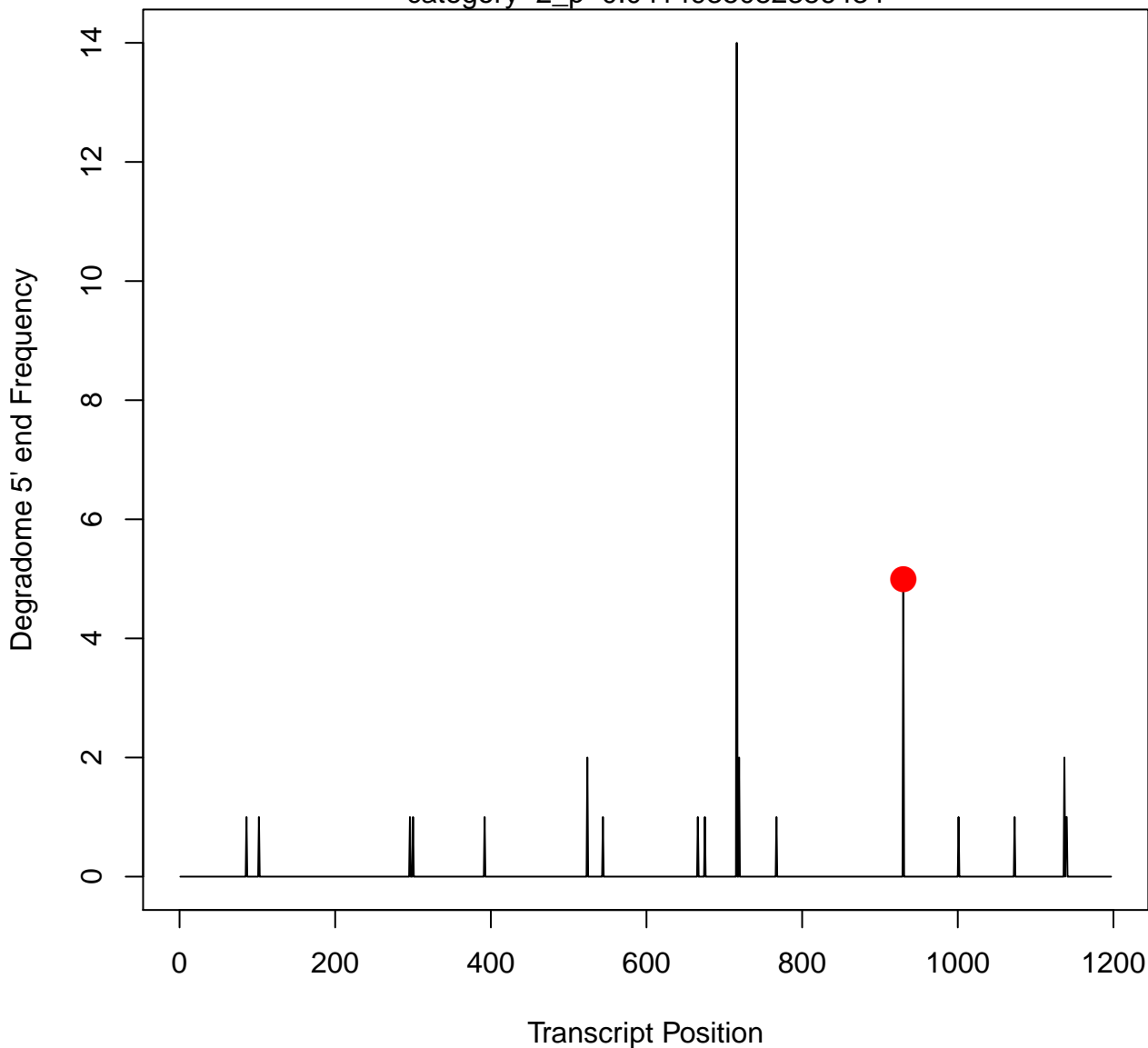

Supplement: Supplementary file 7 [file Data_Sheet_7.zip › Sit-miR169g_Seita.9G521100.1_930_TPlot.pdf]

**T=Seita.5G175600.1\_Q=Sit-miR171a\_S=1529**

category=2\_p=0.207913115291402

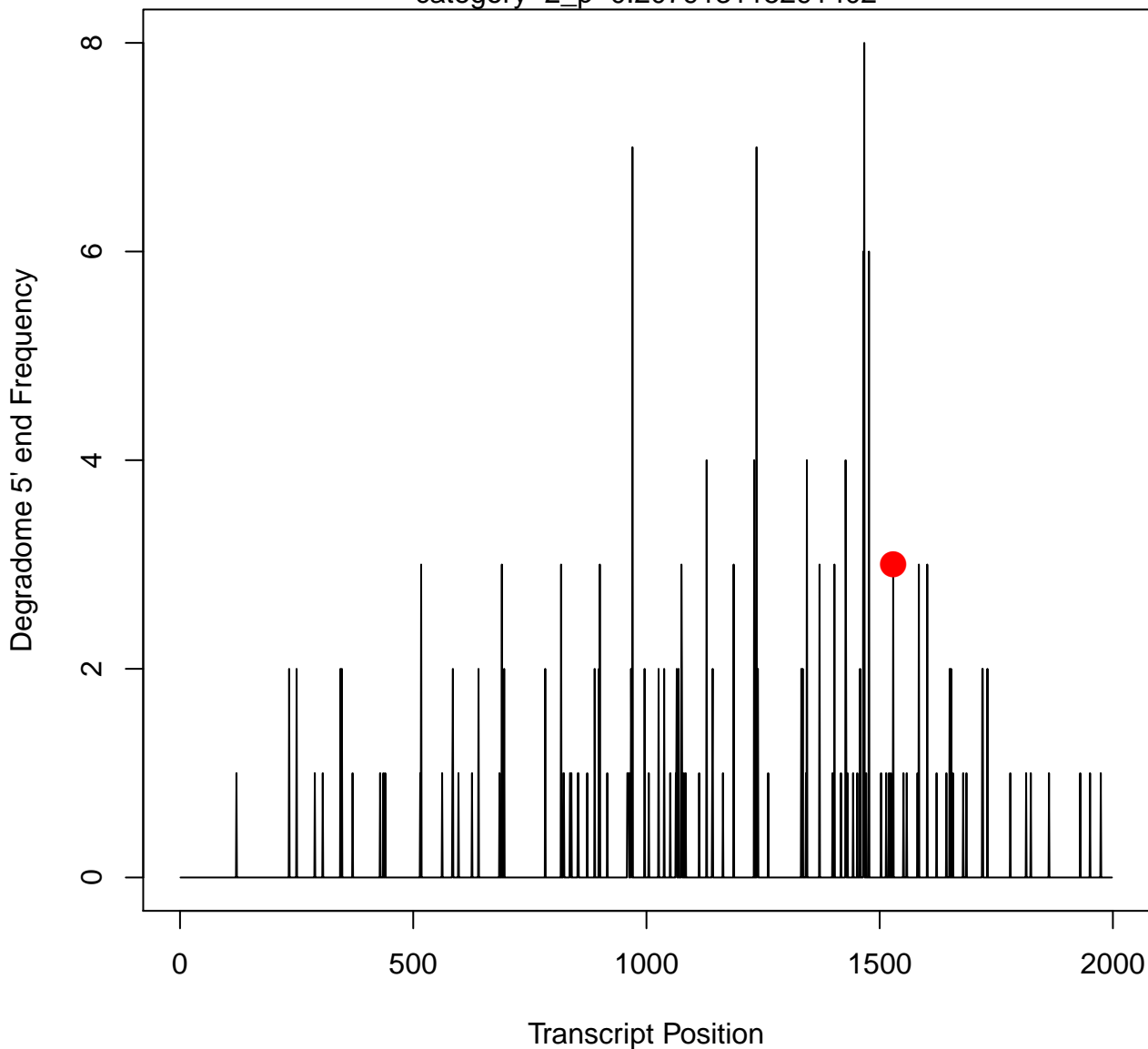

Supplement: Supplementary file 7 [file Data_Sheet_7.zip › Sit-miR171a_Seita.5G175600.1_1529_TPlot.pdf]

**T=Seita.9G414200.1\_Q=Sit-miR171a\_S=182**

category=2\_p=0.485179397880979

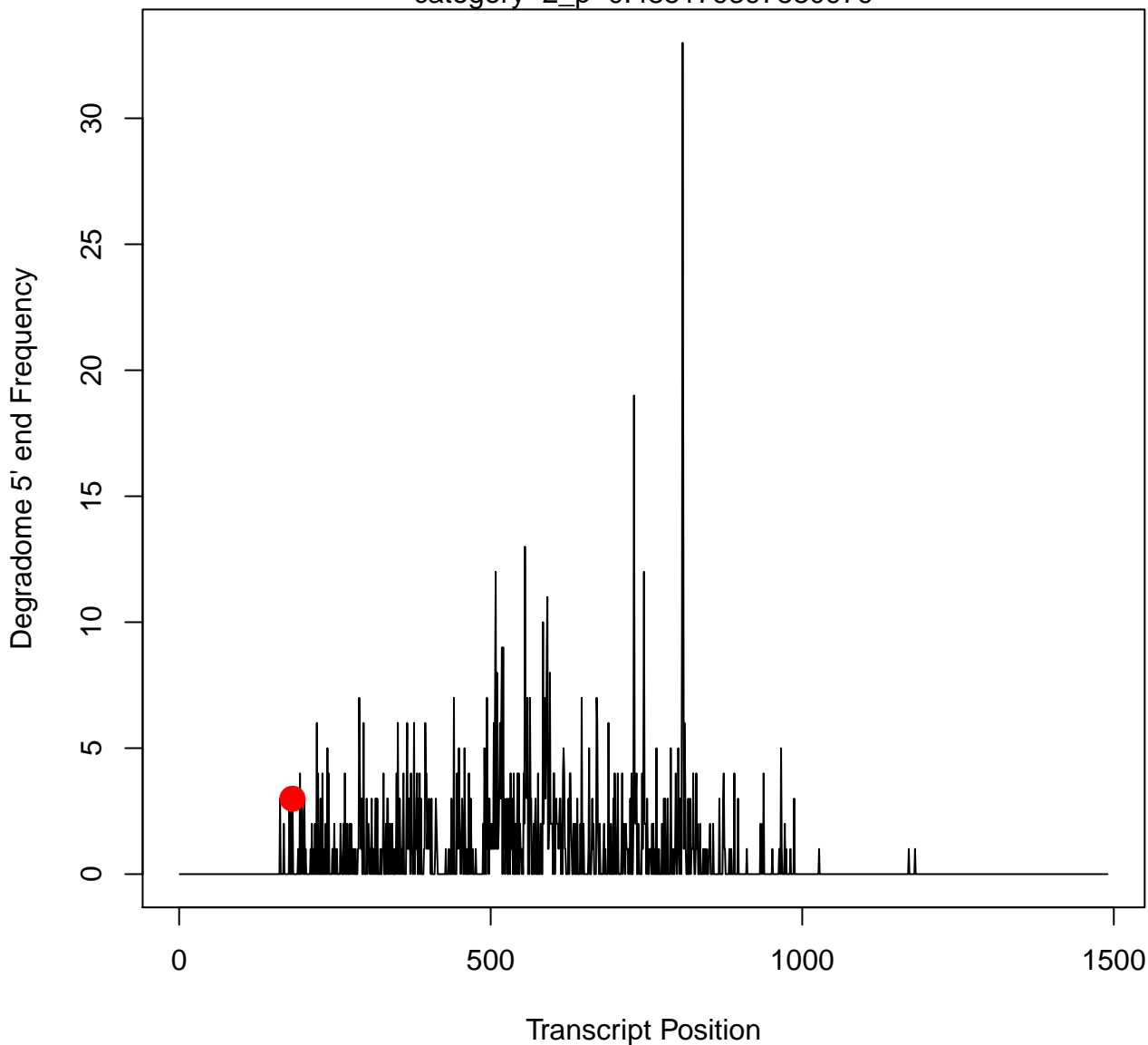

Supplement: Supplementary file 7 [file Data_Sheet_7.zip › Sit-miR171a_Seita.9G414200.1_182_TPlot.pdf]

**T=Seita.1G262900.1\_Q=Sit-miR171f\_S=1082**

category=0\_p=0.000598397663414518

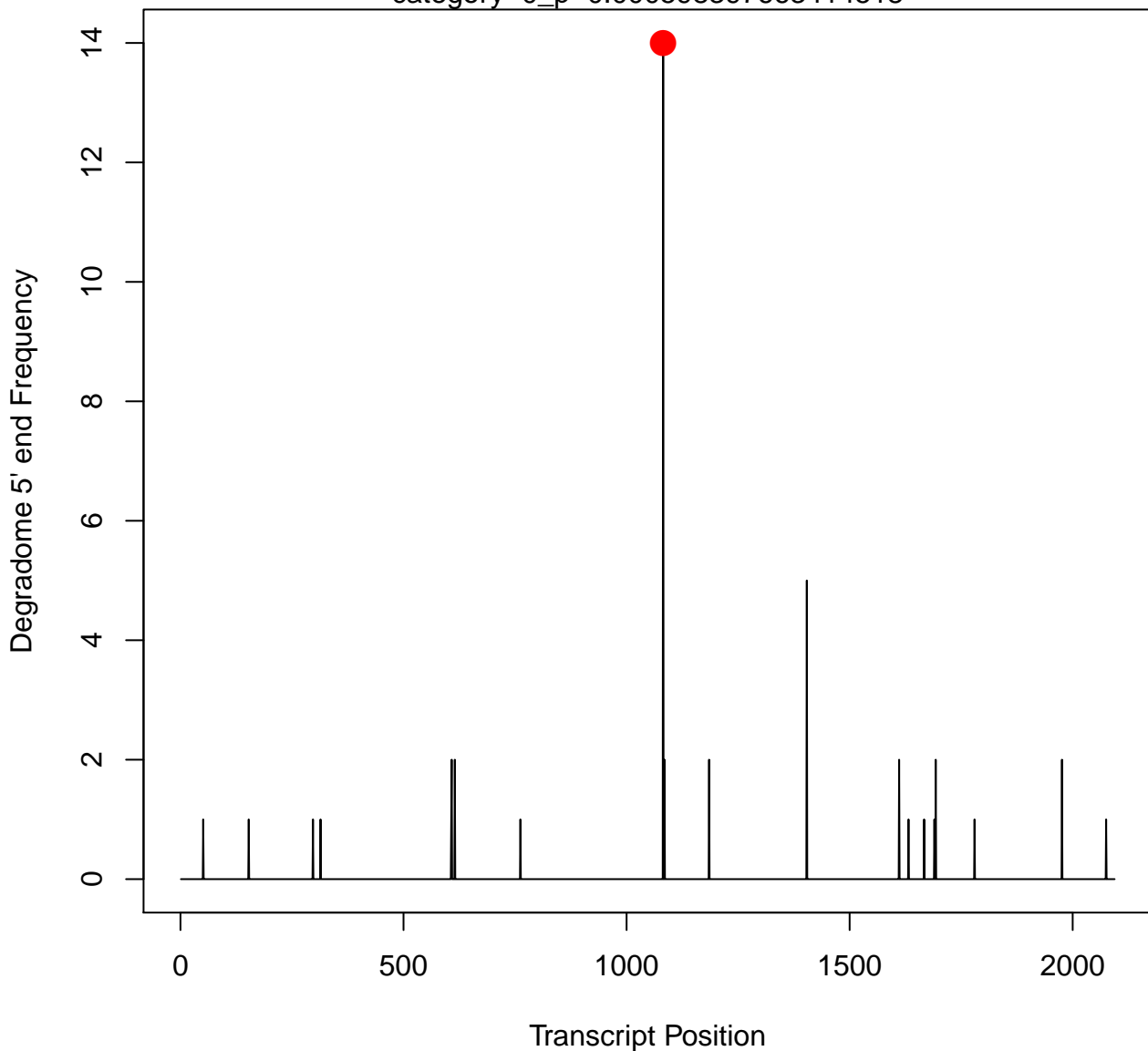

Supplement: Supplementary file 7 [file Data_Sheet_7.zip › Sit-miR171f_Seita.1G262900.1_1082_TPlot.pdf]

**T=Seita.3G218500.1\_Q=Sit-miR171f\_S=576**

category=0\_p=0.122071785121309

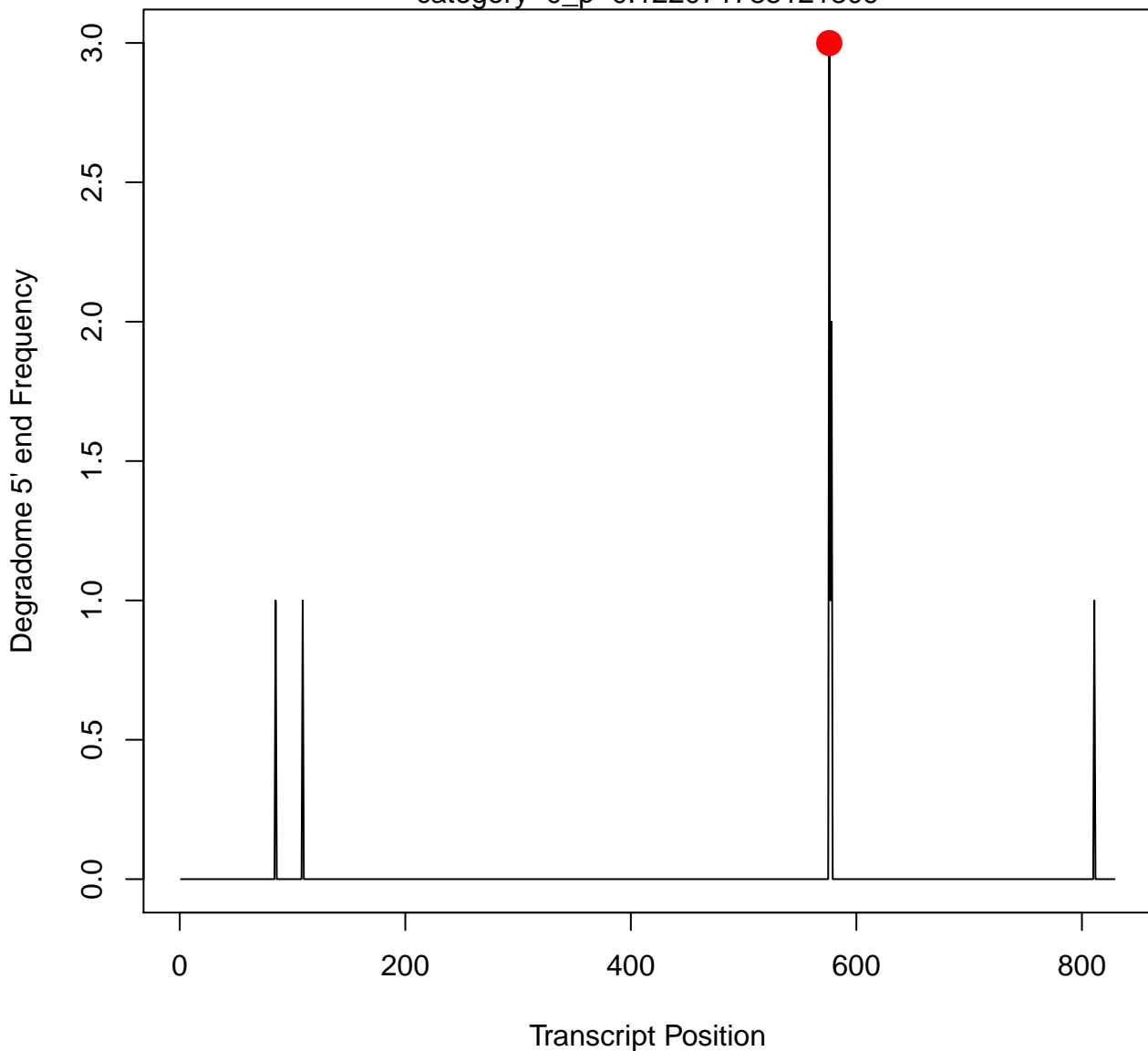

Supplement: Supplementary file 7 [file Data_Sheet_7.zip › Sit-miR171f_Seita.3G218500.1_576_TPlot.pdf]

**T=Seita.5G413000.1\_Q=Sit-miR171f\_S=965**

category=2\_p=0.972736602116729

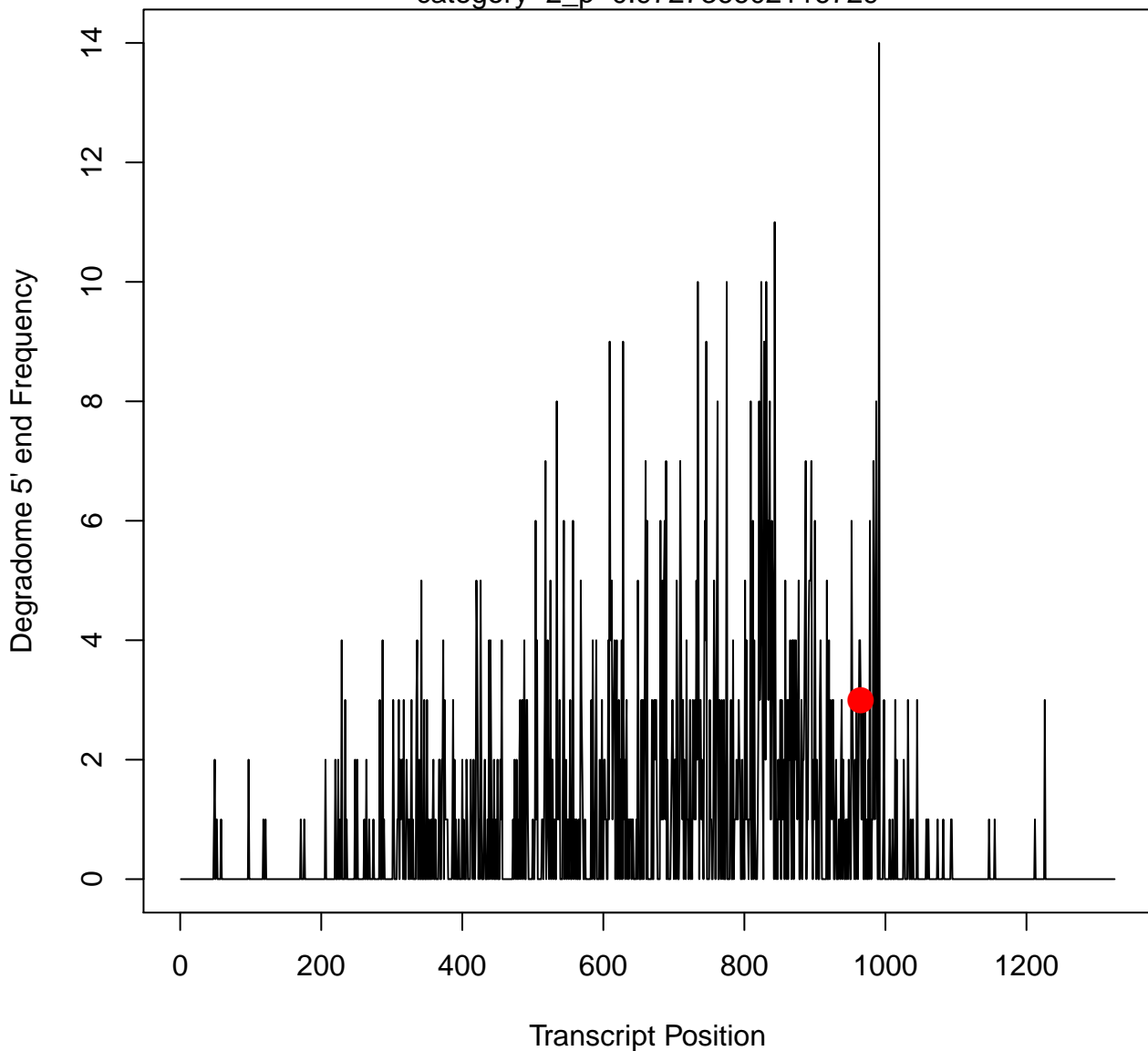

Supplement: Supplementary file 7 [file Data_Sheet_7.zip › Sit-miR171f_Seita.5G413000.1_965_TPlot.pdf]

**T=Seita.9G254900.1\_Q=Sit-miR171f\_S=1010**

category=1\_p=0.0731326964343855

Degradome 5' end Frequency

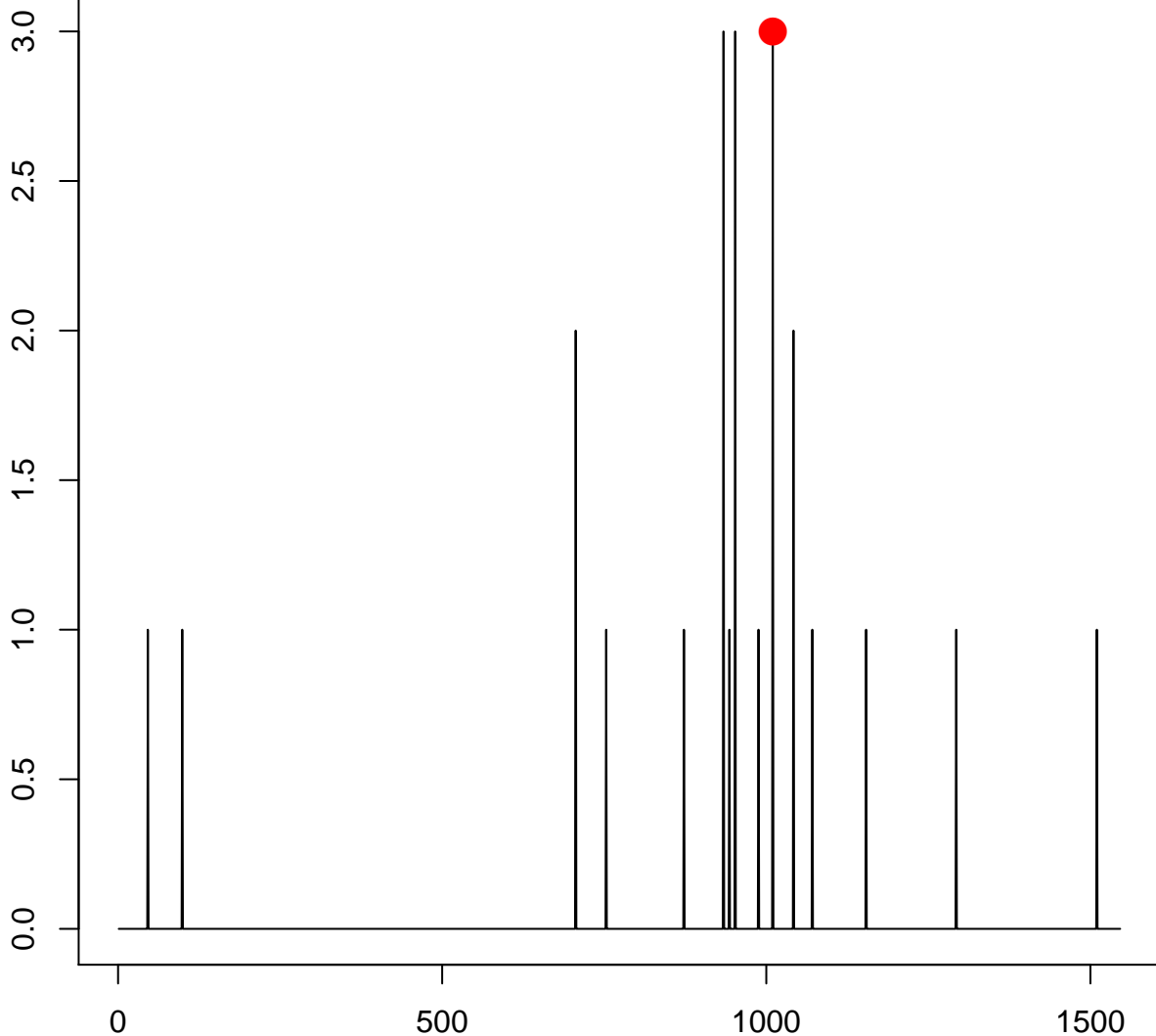

Transcript Position

Supplement: Supplementary file 7 [file Data_Sheet_7.zip › Sit-miR171f_Seita.9G254900.1_1010_TPlot.pdf]

**T=Seita.2G419600.1\_Q=Sit-miR171g\_S=5912**

category=2\_p=0.653361764595788

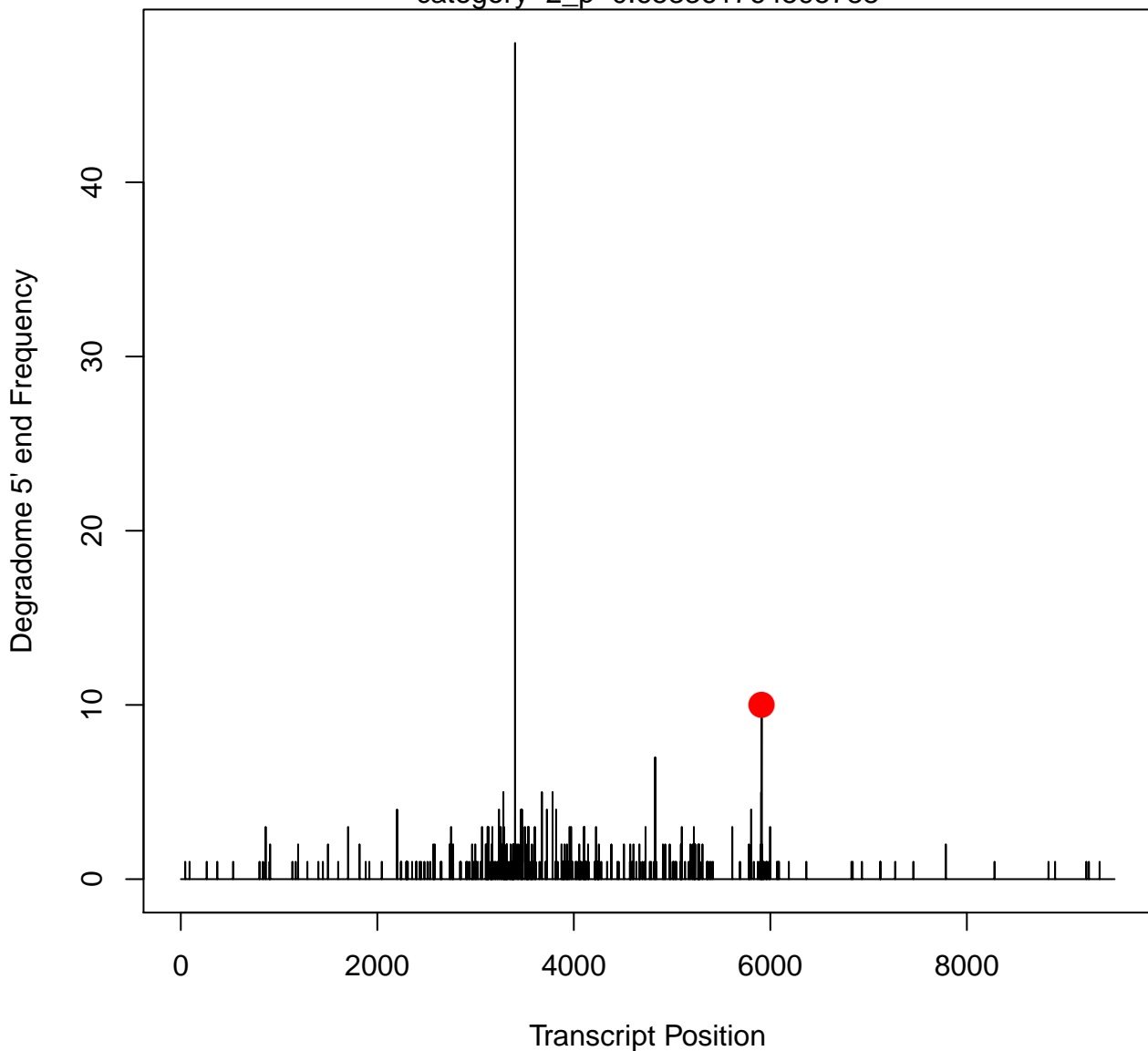

Supplement: Supplementary file 7 [file Data_Sheet_7.zip › Sit-miR171g_Seita.2G419600.1_5912_TPlot.pdf]

**T=Seita.9G460900.1\_Q=Sit-miR171g\_S=946**

category=2\_p=0.99606370482213

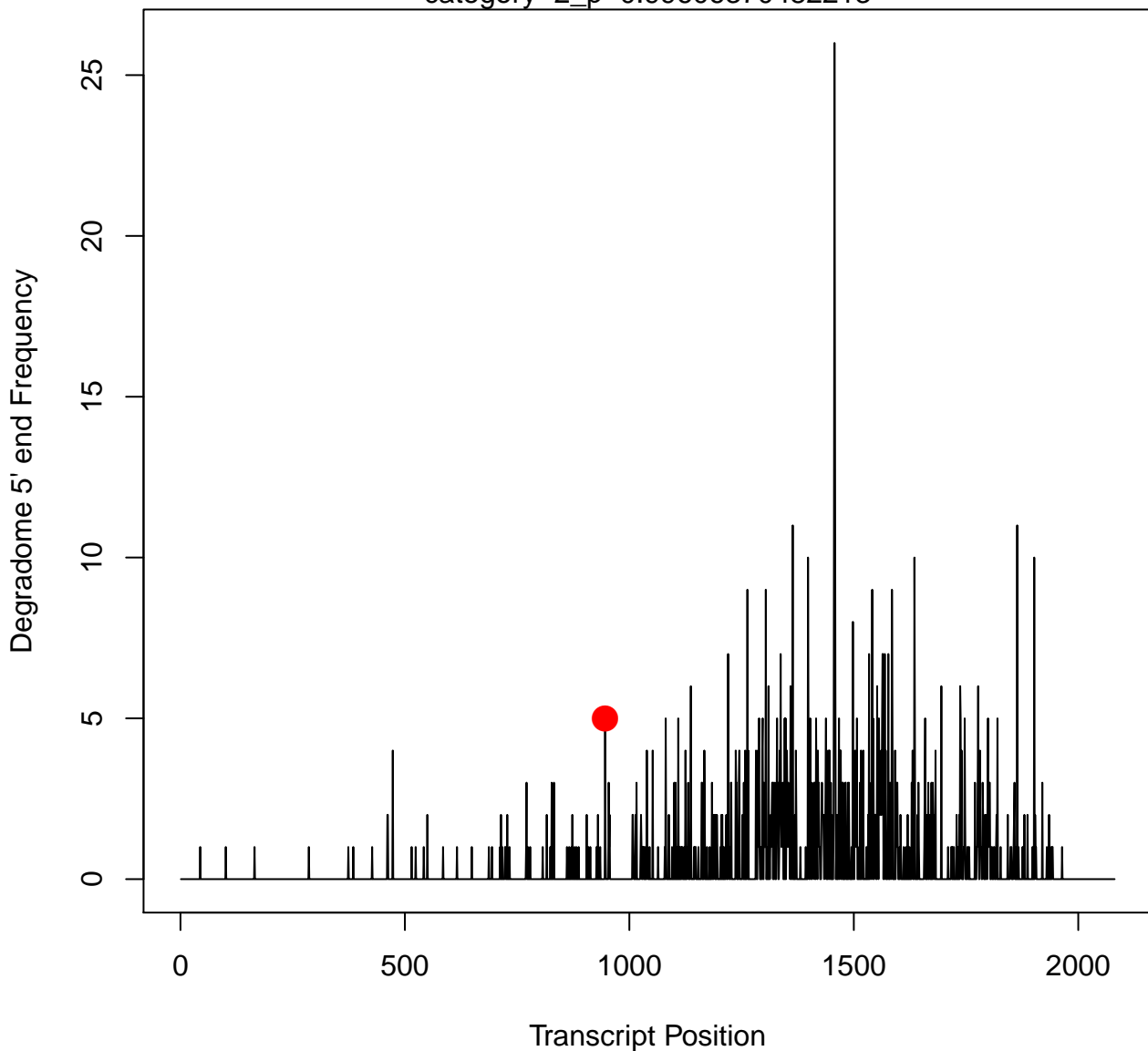

Supplement: Supplementary file 7 [file Data_Sheet_7.zip › Sit-miR171g_Seita.9G460900.1_946_TPlot.pdf]
