# Supplementary material for: The Construction and Exploration of a Comprehensive MicroRNA Centered Regulatory Network in Foxtail Millet (Setaria italica L.)
Source: Front Plant Sci. 2022 May 6;13:848474. doi: 10.3389/fpls.2022.848474 (PMC9121102; doi:10.3389/fpls.2022.848474)
Supplement: Supplementary file 8 [file Data_Sheet_8.PDF]

The construction and exploration of a comprehensive microRNA centered regulatory network in foxtail millet (*Setaria italica* L.)

Supplementary Materials

Supplementary Figures

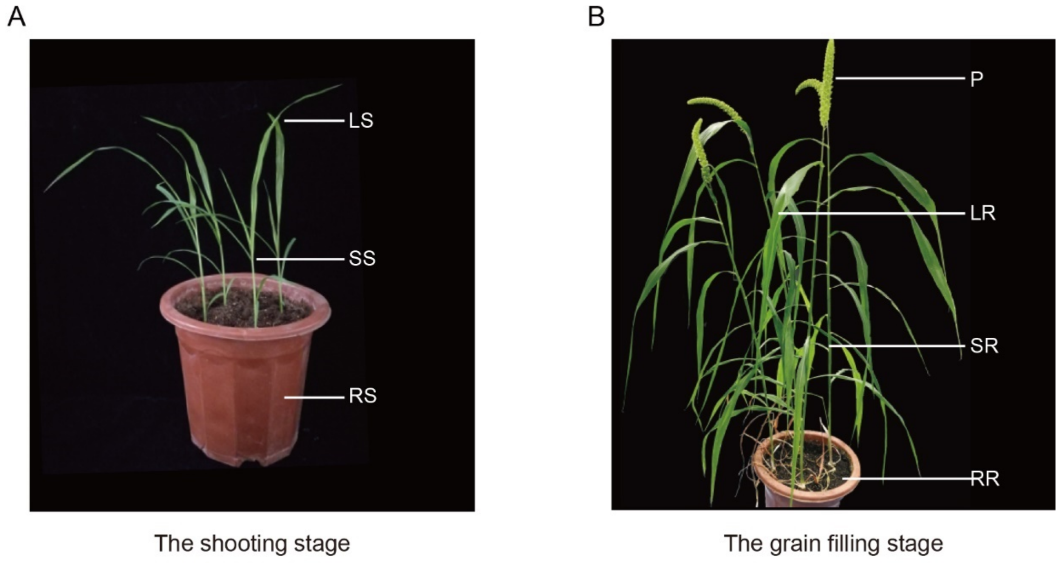

Figure S1. The samples collected from various tissues at the shooting and grain filling stages.

(A) Various tissues at the shooting stage. (B) Various tissues at the grain filling stage. RS, SS, and LS indicate samples from the roots, stems, and leaves at the shooting stage, respectively, while RG, SG, LG, and P indicate samples from the roots, stems, flag leaves, and panicle at the grain filling stage, respectively.

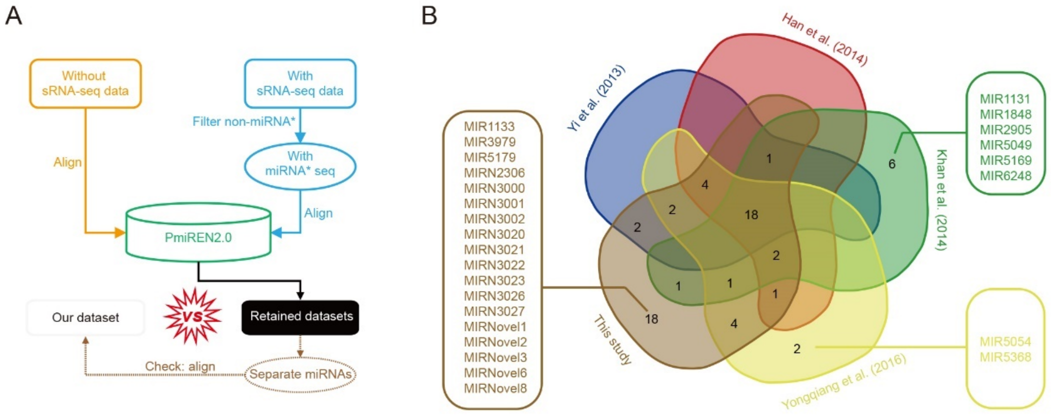

Figure S2. A comparison of miRNA identification and annotation between our study and previous studies.

(A) The detailed process of how the miRNAs were compared. The previously identified miRNAs were filtered out by either their alignment to PmiREN or supported by sRNA-seq datasets. (B) Comparison results displayed by Venn diagram. Our study identified most of the miRNA families, including 18 that were previously unknown.

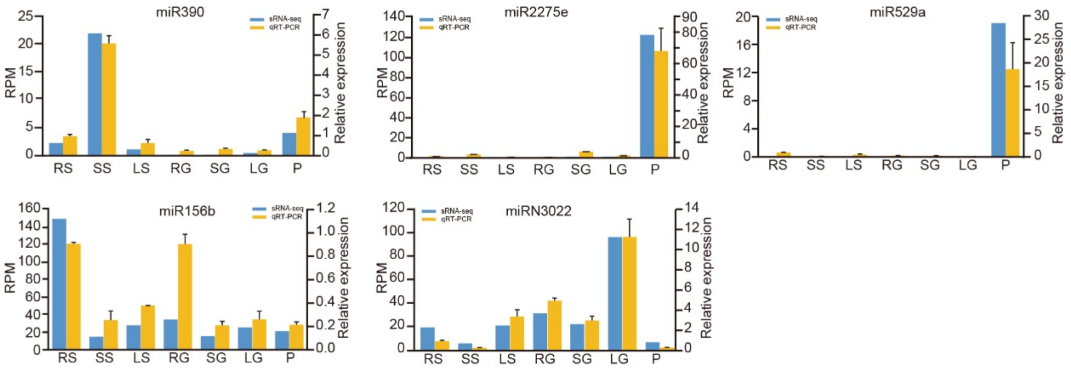

Figure S3. Comparison of sRNA-seq results and the qRT-PCR results of five other randomly selected miRNAs.

RS, SS and LS indicate samples from root, stem, and leaf tissues at the shooting stage, respectively. RG, SG, LG, and P indicate samples from the roots, stems, flag leaves, and panicle tissues at the grain filling stage. The blue and yellow bar plot is the quantitative result from sRNA-seq and the relative expression from qRT-PCR, respectively. qRT-PCR, real-time quantitative reverse transcription PCR; sRNA, small RNA.

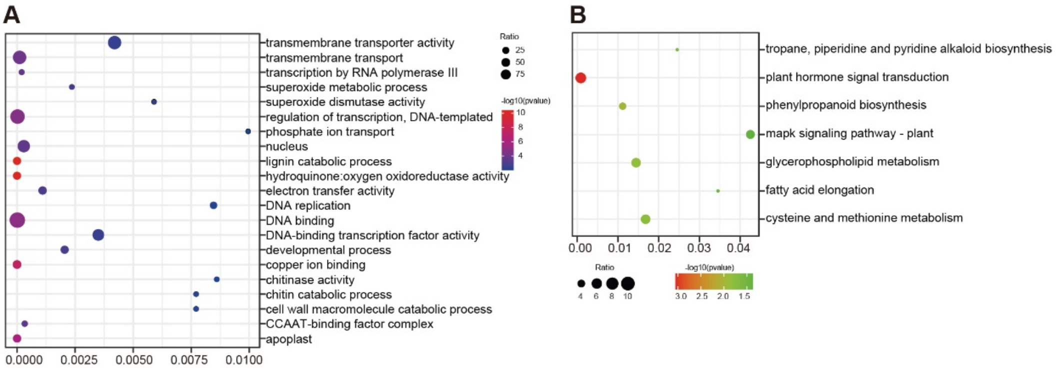

Figure S4. GO and KEGG enrichment results of the overlap target genes dataset.

(A) The GO enrichment result of the overlap target genes dataset. (B) The result of KEGG enrichment of the overlap target genes dataset. GO, Gene Ontology; KEGG, the Kyoto Encyclopedia of Genes and Genomes.

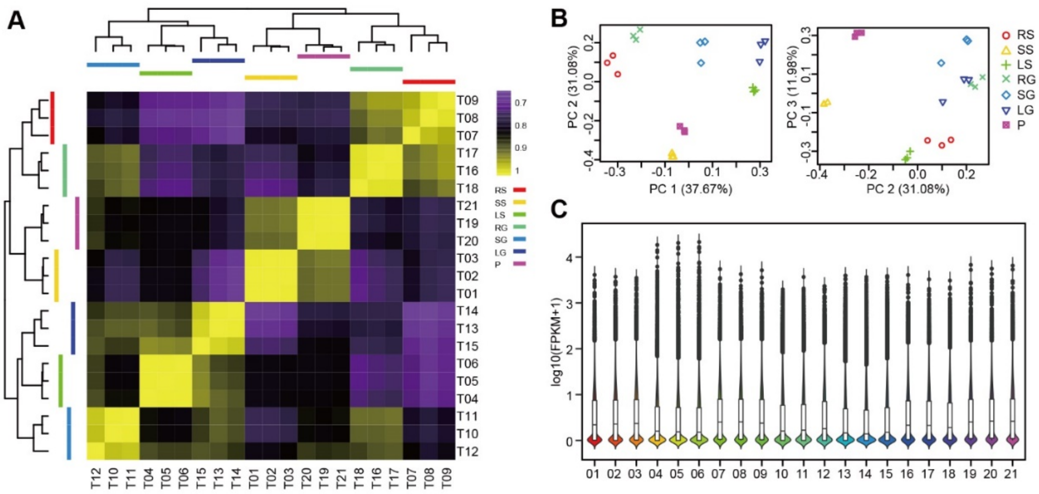

Figure S5. Evaluation of the mRNA libraries via expression.

(A) A correlation analysis of RNA samples via the mRNA transcript expression values. (B) Principal component analysis (PCA) of RNA samples via the mRNA transcript expression values. (C) Distribution of the expression of the mRNA transcripts in each sample.

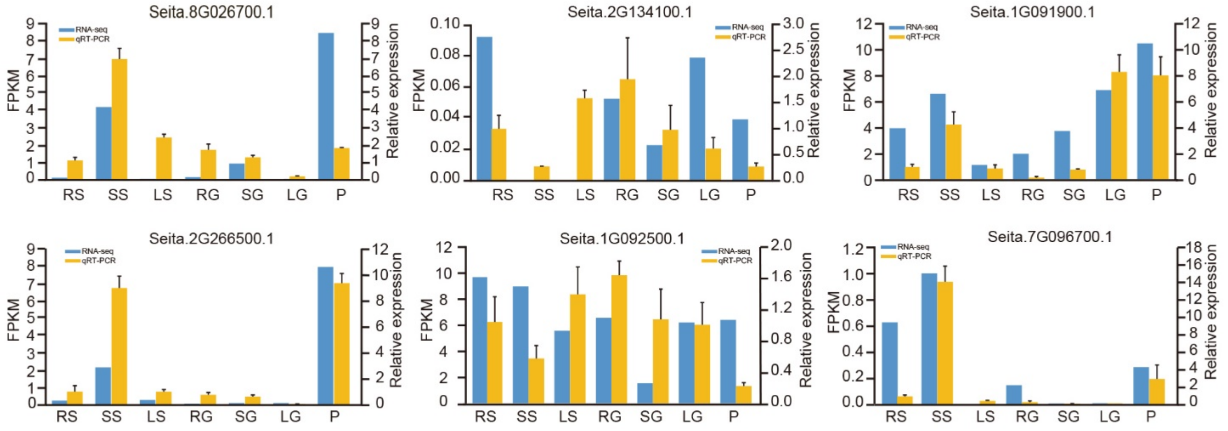

Figure S6. Comparison of the RNA-Seq results and the qRT-PCR results of six other randomly selected transcripts.

RS, SS and LS indicate samples from the root, stem, and leaf tissues at the shooting stage, respectively. RG, SG, LG, and P indicate samples from the root, stem, flag leaf, and panicle tissues at the grain filling stage. The blue and yellow bar plot is the quantitative result from RNA-Seq and the relative expression from qRT-PCR, respectively. qRT-PCR, real-time quantitative reverse transcription PCR.

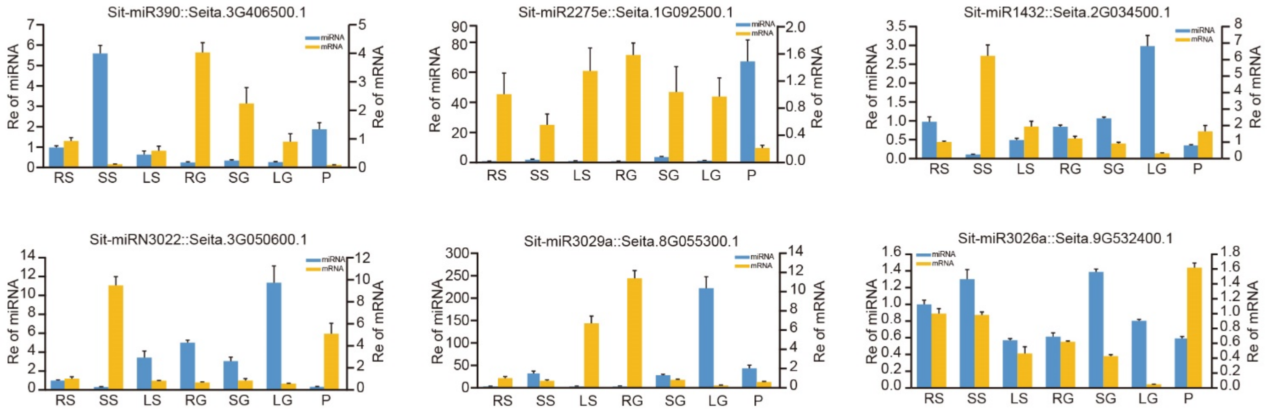

Figure S7. Comparative qRT-PCR of the selected miRNA and its target.

RS, SS and LS indicate samples from the root, stem, and leaf tissues at the shooting stage, respectively. RG, SG, LG, and P indicate samples from the root, stem, flag leaf, and panicle tissues at the grain filling stage. The blue and yellow bar plot is the qRT-PCR results of miRNA and their targets, respectively. Sit-miR390 and Sit-miR2275e belong to conserved miRNAs, Sit-miR1432 belongs to Poaceae-specific miRNA, and Sit-miRN3026a, miRN3022 and miRNA3029a belong to foxtail millet specific miRNAs qRT-PCR, real-time quantitative reverse transcription PCR. Re, relative expression.

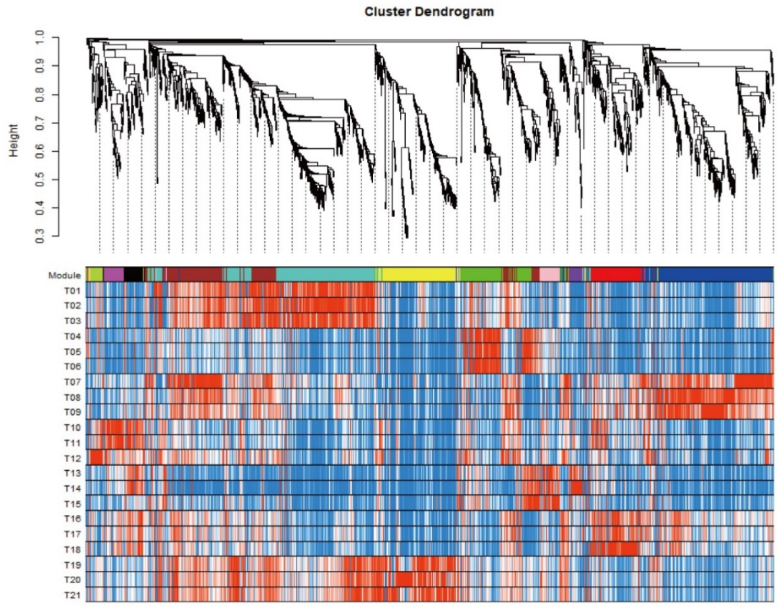

Figure S8. The cluster dendrogram and expression clustering results of a WGCNA of target genes. WGCNA, weighted gene co-expression network analysis.

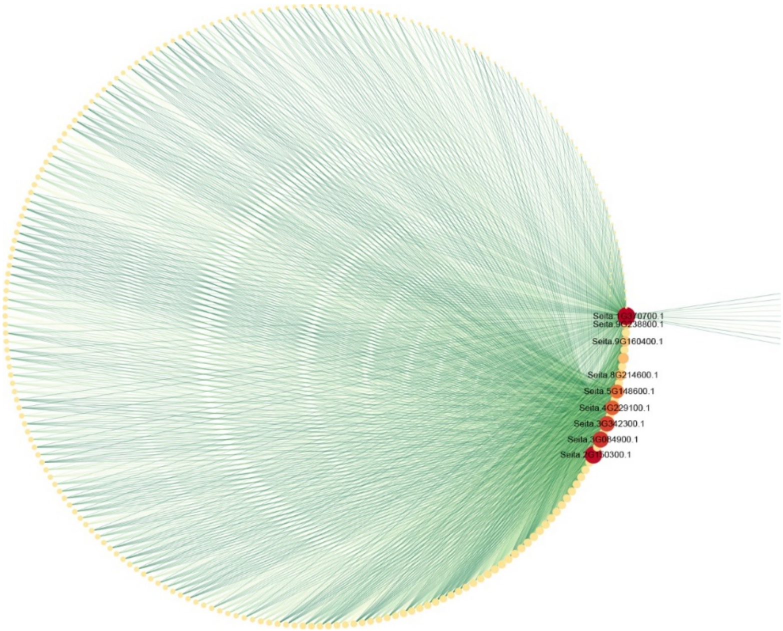

Figure S9. The regulatory network of the nine highest-connectivity target genes with other target genes in the yellow module.

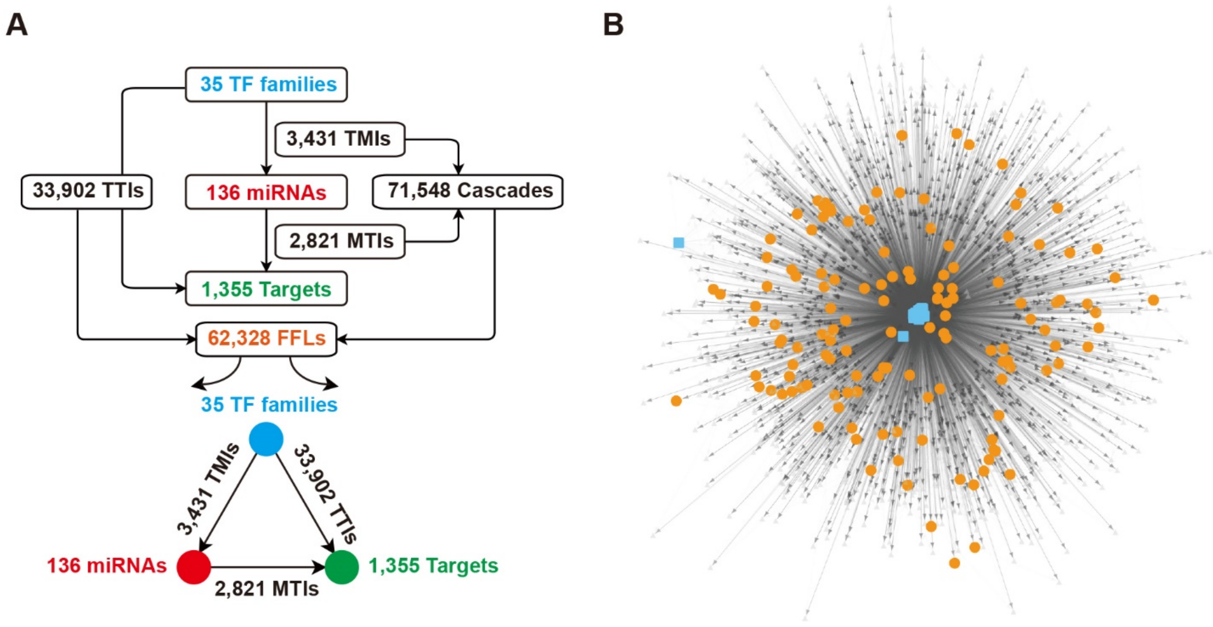

Figure S10. FFLs network based on overlap datasets.

(A) The analytical process and detailed information of the FFLs network. (B) The FFLs regulatory network where the blue, orange, and gray nodes are transcription factors, miRNAs, and their targets, respectively. FFLs, feed forward loops.

Supplementary Tables

Supplementary Table 1. The number of reads produced in sRNA libraries. sRNA, small RNA.

Supplementary Table 2. The proportion of different length reads in clean reads from sRNA libraries. sRNA, small RNA.

Supplementary Table 3. The proportion of different length reads in unique reads from sRNA libraries. sRNA, small RNA.

Supplementary Table 4. The detailed genomic information of 136 high confidence miRNAs in foxtail millet.

Supplementary Table 5. Details of the comparison between the previous four studies and our research.

Supplementary Table 6. A comparison of the concrete information of miRNA families contained in the five studies.

Supplementary Table 7. The list consists of miRNA clusters less than 10 kb in genomic locations.

Supplementary Table 8. The synteny blocks contain the list of single miRNAs.

Supplementary Table 9. The synteny blocks contain the list of paired miRNAs.

Supplementary Table 10. Average expression profile of 136 miRNAs in millet.

Supplementary Table11. Detailed annotation information and profile of expression of 5,033 miRNA-Target regulatory pattern.

Supplementary Table 12. The result of significantly enriched GO terms of 2,417 candidate target genes.

Supplementary Table 13. The result of significant enriched KEGG pathways of 2,417 candidate targets. KEGG, Kyoto Encyclopedia of Genes and Genomes.

Supplementary Table 14. Detailed information of tissue-specific regulatory pairs in the seven tissues at two stages.

Supplementary Table 15. The correlation profile of the dynamic expression in seven tissues between 136 miRNAs and 2,417 targets among 5,033 regulatory pairs.

Supplementary Table 16. The GO enrichment analysis of targets in the yellow module. GO, gene ontology.

Supplementary Table 17. Detailed information of 127,645 cascade motifs and 110,922 FFLs motifs based on the overall dataset. FFLs, feed forward loops.

Supplementary Table 18. Detailed information of 71,548 cascade motifs and 62,328 FFLs motifs based on the overlap datasets. FFLs, feed forward loops.

Supplementary Table 19. Primers of all the qRT-PCR experiments in this study.

Supplementary Data

Supplementary Data 1. The results from the PARE-seq analysis.
